# A ferroptosis-associated gene signature for the prediction of prognosis and therapeutic response in luminal-type breast carcinoma

**DOI:** 10.1038/s41598-021-97102-z

**Published:** 2021-09-02

**Authors:** Yang Peng, Haochen Yu, Yingzi Zhang, Fanli Qu, Zhenrong Tang, Chi Qu, Jiao Tian, Beige Zong, Yu Wang, Haoyu Ren, Shengchun Liu

**Affiliations:** 1 Department of Endocrine and Breast Surgery, The First Affiliated Hospital of Chongqing Medical University, Chongqing, China; 2 Medical Faculty of Ludwig-Maximilians-University of Munich, University Hospital of LMU Munich, Munich, Germany; 3 Department of Breast Surgery, The Sixth Affiliated Hospital, Sun Yat-sen University, Guangzhou, China

**Keywords:** Cancer, Breast cancer

## Abstract

Ferroptosis is a new form of regulated cell death (RCD), and its emergence has provided a new approach to the progression and drug resistance of breast cancer (BRCA). However, there is still a great gap in the study of ferroptosis-related genes in BRCA, especially luminal-type BRCA patients. We downloaded the mRNA expression profiles and corresponding clinical data of BRCA patients from the Molecular Taxonomy of Breast Cancer International Consortium (METABRIC) and The Cancer Genome Atlas (TCGA) databases. Then, we built a prognostic multigene signature with ferroptosis-related differentially expressed genes (DEGs) in the METABRIC cohort and validated it in the TCGA cohort. The predictive value of this signature was investigated in terms of the immune microenvironment and the probability of a response to immunotherapy and chemotherapy. The patients were divided into a high-risk group and a low-risk group according to the ferroptosis-associated gene signature, and the high-risk group had a worse overall survival (OS). The risk score based on the 10 ferroptosis-related gene-based signature was determined to be an independent prognostic predictor in both the METABRIC and TCGA cohorts (HR, 1.41, 95% CI, 1.14–1.76, *P* = 0.002; HR, 2.19, 95% CI, 1.13–4.26, *P* = 0.02). Gene set enrichment analysis indicated that the term “cytokine-cytokine receptor interaction” was enriched in the high-risk score subgroup. Moreover, the immune infiltration scores of most immune cells were significantly different between the two groups, the low-risk group was much more sensitive to immunotherapy, and six drugs might have potential therapeutic implications in the high-risk group. Finally, a nomogram incorporating a classifier based on the 10 ferroptosis-related genes, tumor stage, age and histologic grade was established. This nomogram showed favorable discriminative ability and could help guide clinical decision-making for luminal-type breast carcinoma.

## Introduction

Breast cancer (BRCA) is the most common type of malignant tumor in females and includes numerous subtypes with high heterogeneity^1,2^. Additionally, BRCA is the leading cause of cancer-related deaths among females worldwide^3^. With the growing understanding of BRCA at the molecular level, there is a growing focus on the precision treatment of BRCA. Four molecular features can be used to group breast tumors based on the following: expression of the estrogen receptor (ER), the progesterone receptor (PR), Ki-67 (a proliferation index marker), and the HER2 receptor tyrosine kinase (HER2)^4^. Therefore, substantial heterogeneity exists within and between well-established BRCA subtypes and therapies. Luminal-type BRCAs have better potential efficacy than TNBCs because of their positive expression of hormone receptors and the location of the target sites of Ki67 and HER2. Additionally, in clinical practice, we have also found that the luminal subtype of BRCA accounts for almost 70% of new cases of BRCA. Since luminal-type BRCA has high ER and PR expression, inhibitors of hormone receptors, such as tamoxifen, are still used in the treatment of this type of BRCA. However, the emergence of drug resistance has also made the search for new treatment options imminent.

Ferroptosis is a new form of regulated cell death (RCD)^5^. The emergence of ferroptosis has provided a new approach to the progression and drug resistance of BRCA. Ferroptosis can be induced by experimental compounds (e.g., erastin) or clinical drugs (e.g., sorafenib and artemisinin) in cancer cells and normal cells (e.g., kidney tubule cells, fibroblasts and T cells)^5–7^. Ferroptosis is triggered by the accumulation of lipid peroxidation products and toxic reactive oxygen species (ROS) derived from iron metabolism^8^. Iron metabolism and lipid peroxidation signaling are recognized as the main mediators of ferroptosis^5^. Previous studies have reported that ferroptosis plays a vital role in BRCA, and some genes, such as ACSL4^9^ and P53RRA^10^, are known to positively regulate ferroptosis. On the other hand, other ferroptosis-related genes, such as ATF2^11^, NRF2^12^ and GPX4^13^, might inhibit ferroptosis in BRCA.

For better treatment decision-making in early BRCA patients, it is important to accurately predict the risk of recurrence and response to therapy. Currently, prediction at the molecular level still relies heavily on ER, PR and HER2. However, these traditional factors alone are not sufficient for optimal treatment decisions, and consequently, several molecular assays based on multiple gene expression signatures have been developed to better predict the prognosis and treatment responses of BRCA patients. Since the introduction of first-generation multigene assays, several prognostic assays for early BRCA, such as Prosigna^14^ and EndoPredict^15^, have subsequently been developed. The most commonly used signature in clinical work is a 21-gene signature assay based on quantitative real-time reverse transcription-PCR (qRT-PCR). Patients are classified into low-, intermediate-, and high-risk categories according to the recurrence score (RS) calculated from the expression of 21 genes, comprising 16 cancer-related genes and 5 reference genes^16^. Moreover, many immune gene signatures have been reported as prognostic or predictive biomarkers in BRCA. Finak et al.^17^ identified a new 26-gene stroma-derived prognostic predictor (SDPP) associated with the clinical outcome of BRCA patients. These 26 genes comprise CD48, TRBV5-4, and other genes that are closely related to the immune response. Thus, the exploration of BRCA-associated gene signatures is important for precise BRCA treatment.

Ferroptosis has emerged as a new form of death with a growing number of studies on the genes associated with it. However, there is still a great gap in the study of ferroptosis-related genes in BRCA patients, especially luminal-type BRCA patients. In our study, we downloaded the mRNA expression profiles and corresponding clinical data of BRCA patients from different databases. Then, we built a prognostic multigene signature with ferroptosis-related differentially expressed genes (DEGs) in the Molecular Taxonomy of Breast Cancer International Consortium (METABRIC) cohort and validated it in The Cancer Genome Atlas (TCGA) cohort. Then, we further explored the underlying mechanisms. Finally, a quantifiable and clinically usable gene signature was obtained.

## Methods

### Data collection

The RNA sequencing (RNA-seq) data and clinical information of 1139 luminal subtype BRCA patients were downloaded from the METABRIC database (https://www.cbioportal.org/study?id=brca_metabric). The mRNA expression profiles and clinical information of another 754 tumor samples were obtained from the TCGA database (https://gdc-portal.nci.nih.gov/). All METABRIC and TCGA data are publicly available. Ferroptosis-related genes were identified from the latest literature and are provided in Supplementary Table S1. Six immune infiltration cell scores and 28 immune infiltration cell scores were downloaded from the Tumor IMmune Estimation Resource (TIMER, available at http://cistrome.org/TIMER) ^18^ and The Cancer Immunome Atlas (https://tcia.at/) ^19^. The TCGAbiolinks package (version 2.18.0; https://github.com/BioinformaticsFMRP/TCGAbiolinks) was used to download the somatic mutation profiles of BRCA patients from the TCGA database^20^, and the maftools package (version 2.6.05; https://github.com/PoisonAlien/maftools) was used to summarize and analyze the data^21^. The copy number variation (CNV) profiles of BRCA patients were also obtained from the TCGAbiolinks package, and GISTIC 2.0 was used to generate discrete copy number data files (https://cloud.genepattern.org/) ^22^. Drug sensitivity data of cancer cell-lines (CCLs) were obtained from the Cancer Therapeutics Response Portal (CTRP v.2.0, released October 2015, https://portals.broadinstitute.org/ctrp) and PRISM Repurposing dataset (19Q4, released December 2019, https://depmap.org/portal/prism/).

### Study design

The METABRIC cohort was used as the training set, and the TCGA cohort was used as the validation set. The R package *limm*a (version: 3.46.0; http://bioinf.wehi.edu.au/limma) was utilized to carry out normalization and compare DEGs between tumor and adjacent samples with false discovery rate (FDR) < 0.05 in the METABRIC cohort^23^. Then, univariate Cox analysis was used to select DEGs significantly associated with overall survival (OS). Finally, the 12 DEGs in the METABRIC cohort most related to OS with *P* < 0.05 were selected for least absolute shrinkage and selector operator (LASSO) Cox regression to narrow down the candidate ferroptosis-related genes using the R package *glmnet* (version: 4.0-2; https://cran.r-project.org/web/packages/glmnet/index.html). Ten ferroptosis-related genes were identified to have nonzero coefficients in the model, and the samples were divided into high-risk and low-risk groups based on the optimal cutoff value of 0.22, which was derived from the *surv_cutpoint* function of the *survminer* R package (Version: 0.4.3, https://CRAN.R-project.org/package=survminer). The formula of the risk score was as follows:

$$ Risk\;score = sum\;of\;coefficients \times normalized\;expression\;level\;of\;ferroptosis{ - }related\;genes $$

The coefficients of the normalized expression level of each ferroptosis-related gene are supplied in Table S2. Finally, independent risk factors identified through multivariate Cox regression analysis were chosen to develop a nomogram for predicting OS likelihood. In addition, we plotted calibration plots to investigate the performance of the nomogram. The concordance index (C-index) was used to assess the agreement between the actual outcomes and the probabilities predicted by the model. The R package *rms* was used to plot the nomogram and calibration plots (Version: 4.0.2, https://cran.r-project.org/web/packages/rms/).

### Gene set enrichment analysis (GSEA) and functional enrichment analysis

To assess the potential mechanisms of the ferroptosis-related genes included in the risk score, GSEA was performed to identify the differences in the pathways in luminal subtype BRCA patients. The annotated gene set file (c2.cp.kegg.v7.1.entrez.gmt) was used as a reference^24–26^. A *P* value less than 0.05 was set as the significance threshold. Genes with *P* < 0.05 and |log2-fold change (FC)|≥ 0.2 were recognized as significantly differentially expressed between the high-risk and low-risk groups based on the risk score. Gene Ontology (GO) and Kyoto Encyclopedia of Genes and Genomes (KEGG) analyses of the DEGs were performed using the *clusterProfiler* R package (version 3.18.1; https://yulab-smu.top/biomedical-knowledge-mining-book/) ^27^.

### Estimation of immunotherapy and chemotherapy response

The normalized gene expression data with standard annotation files from the TCGA cohort were uploaded to the Immune Cell Abundance Identifier (ImmuCellAI) (http://bioinfo.life.hust.edu.cn/web/ImmuCellAI/), which uses a gene set signature-based method to precisely estimate the infiltration score of 24 immune cell types, including 18T-cell subsets, and to predict the immunotherapy response (anti-PD1 or anti-CTLA4 therapy) with high accuracy^28^. The R package *pRRophetic* (*version 0.5; *https://doi.org/10.1371/journal.pone.0107468) was used to predict chemotherapy response as determined by the half maximal inhibitory concentration (IC50) of each BRCA patient in the TCGA cohort^29^. The methods used to assess the drug sensitivity of each sample from TCGA were referenced from the published literature^30^.

### Statistical analysis

All statistical analyses were conducted using R version 4.0.0 (2020-04-24). The Mann–Whitney U-test and the Pearson chi-square test were used for comparisons of continuous and categorical variables, respectively, between the training set and testing set. Univariate and multivariate Cox regression analyses were used to identify the predominant prognostic factors of OS (*P* < 0.05). Kaplan–Meier survival curves were compared using the log-rank test. The ggplot2 R package (version 3.3.3; https://ggplot2.tidyverse.org) was used to plot the volcano plot and heatmap^31^. Correlations between two continuous variables were measured by either Pearson’s *r* correlation or Spearman’s rank-order correlation. The estimated scores, immune scores and stromal scores of BRCA were analyzed by the estimate R package^32^. K-nearest neighbor (k-NN) imputation was applied to impute the missing AUC values. *P* < 0.05 (two-sided) was considered statistically significant.

## Results

### Patient characteristics

**Figure 1:**
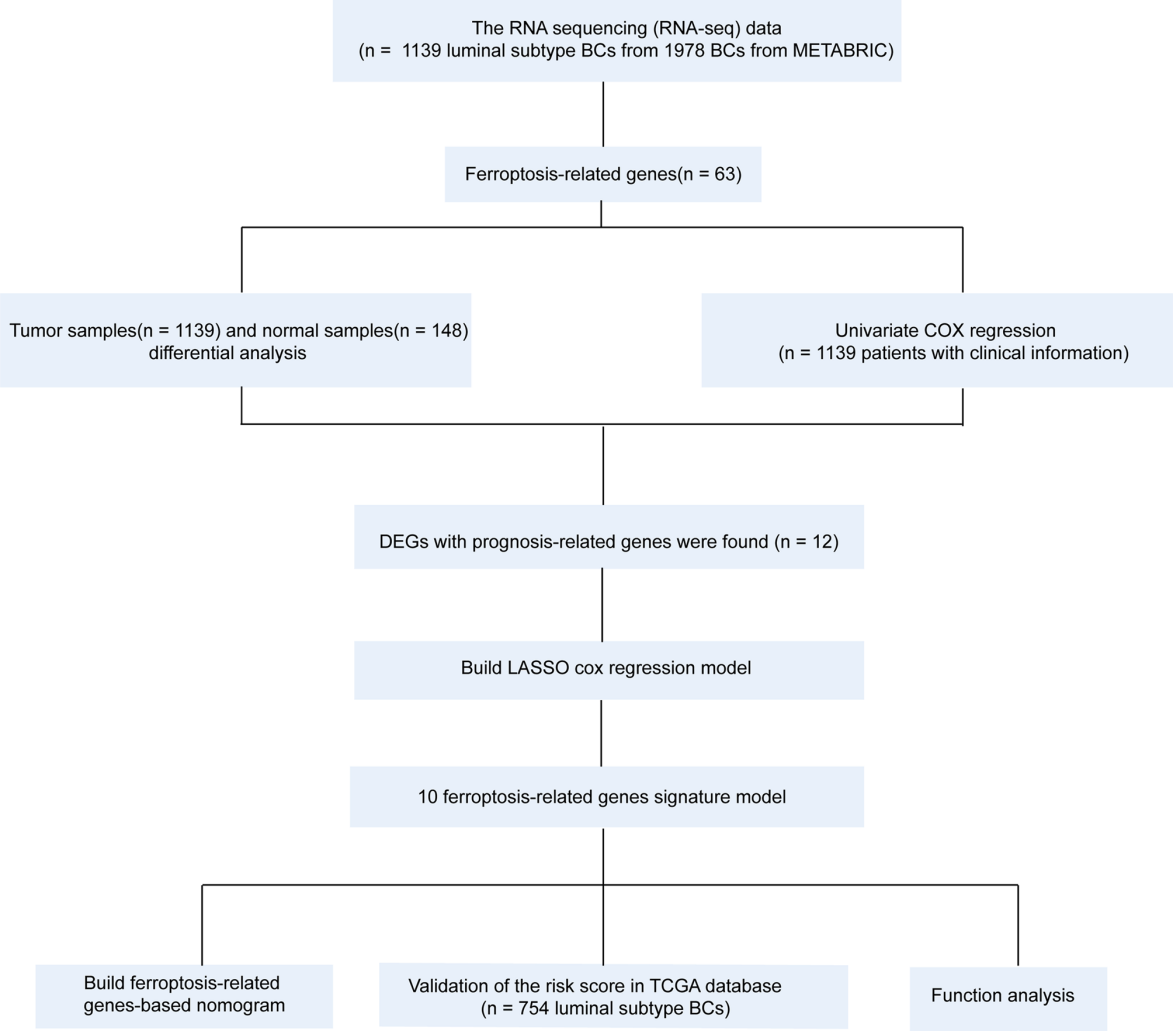
Study flow chart of the collection and analysis of data from the METABRIC and TCGA databases.

The flow chart of our research is shown in Fig. 1. A total of 1139 luminal subtype BRCA patients from the METABRIC cohort were included as a training set, and 754 patients from the TCGA cohort were enrolled as a testing set. The detailed clinical features of these patients are shown in Table 1.

**Table 1:** Clinical characteristics of the BRCA patients used in this study.

	METABRIC cohort	TCGA cohort
**No of patients**	1139	754
**Age**		
Mean (SD)	63.7 (12.2)	59.3 (13.4)
Median [Min, Max]	64.3 [26.4, 92.1]	60.0 [26.0, 90.0]
**Menopausal state**		
Post	964 (84.6%)	490 (65.0%)
Pre	175 (15.4%)	264 (35.0%)
**Histologic subtype**		
Ductal/NST	839 (73.7%)	500 (66.3%)
Other	293 (25.7%)	254 (33.7%)
Missing	7 (0.6%)	0 (0%)
**Histologic grade**		
1	134 (11.8%)	NA
2	543 (47.7%)	NA
3	416 (36.5%)	NA
Missing	46 (4.0%)	NA
**Tumor stage**		
0	1 (0.1%)	0 (0%)
I	312 (27.4%)	140 (18.6%)
II	483 (42.4%)	396 (52.5%)
III	56 (4.9%)	177 (23.5%)
IV	7 (0.6%)	11 (1.5%)
Missing	280 (24.6%)	30 (4.0%)
**OS time (month)**		
Mean (SD)	131 (73.3)	42.9 (38.5)
Median [Min, Max]	122 [1.23, 337]	29.8 [1.03, 285]
**OS**		
Alive	473 (41.5%)	654 (86.7%)
dead	666 (58.5%)	100 (13.3%)

### Candidate prognostic ferroptosis-related DEGs were identified in the METABRIC cohort

**Figure 2:**
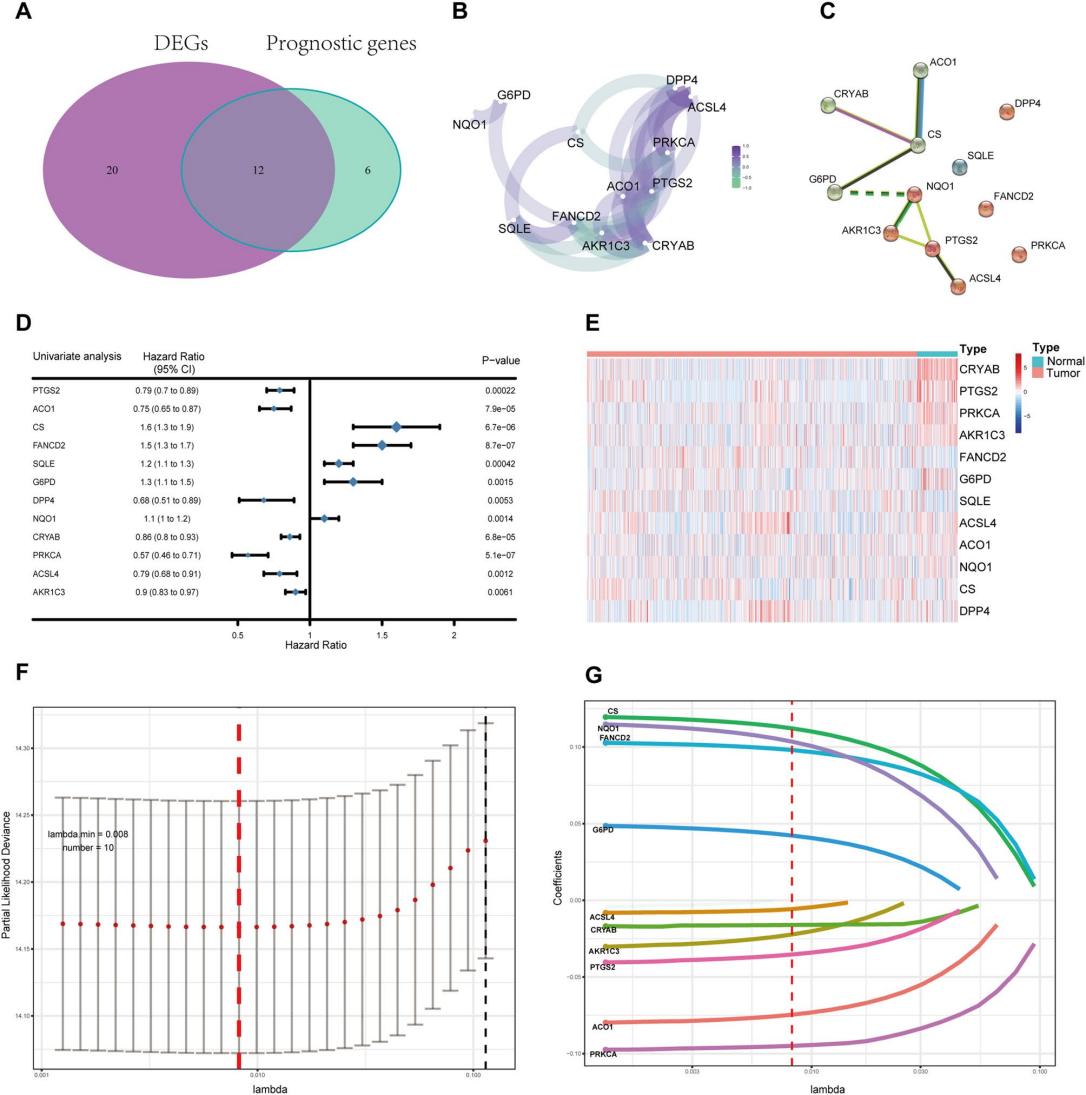
Identification of candidate genes associated with ferroptosis in the METABRIC cohort. (**A**) Venn diagram to identify differentially expressed genes associated with OS between tumor tissue and adjacent normal tissue. (**B**) The correlation network of candidate genes. Correlation coefficients are shown in different colors. (**C**) The PPI network downloaded from the STRING database shows the interactions between candidate genes. (**D**) The forest plot displays the results of the univariate Cox regression analysis of the relationship between the gene expression of candidate genes and OS. (**E**) Heatmap showing differences in the expression of candidate genes between tumor tissue and adjacent normal tissue. (**F**) The partial likelihood deviation curve was plotted versus lambda. Dotted vertical lines were drawn at the optimal values by using the minimum criteria and the 1 standard error of the minimum criteria (the 1-SE criteria). (**G**) The optimal lambda resulted in 10 nonzero coefficients.

More than half of the ferroptosis-related genes (32/63) were differentially expressed between 140 adjacent normal breast tissues and 1139 luminal subtype BRCA samples, and in the univariate Cox regression analysis, twelve of them were associated with OS (Fig. 2A). Some ferroptosis-related genes showed correlations with each other (Fig. 2B). The protein–protein interaction (PPI) network showing interactions between candidate genes is presented in Fig. 2C. A forest plot was used to display the results of the univariate Cox regression analysis of the relationship between the expression of candidate genes and OS (Fig. 2D). The heatmap showed that more than half of the genes were downregulated in tumor tissue, and consistent with the univariate Cox regression analysis, they represented a better prognosis, including PTGS2, ACO1, DPP4, CRYAB, PRKCA, ACSL4 and AKR1C3 (Fig. 2E).

### Constructing a prognostic model based on the METABRIC cohort

**Figure 3:**
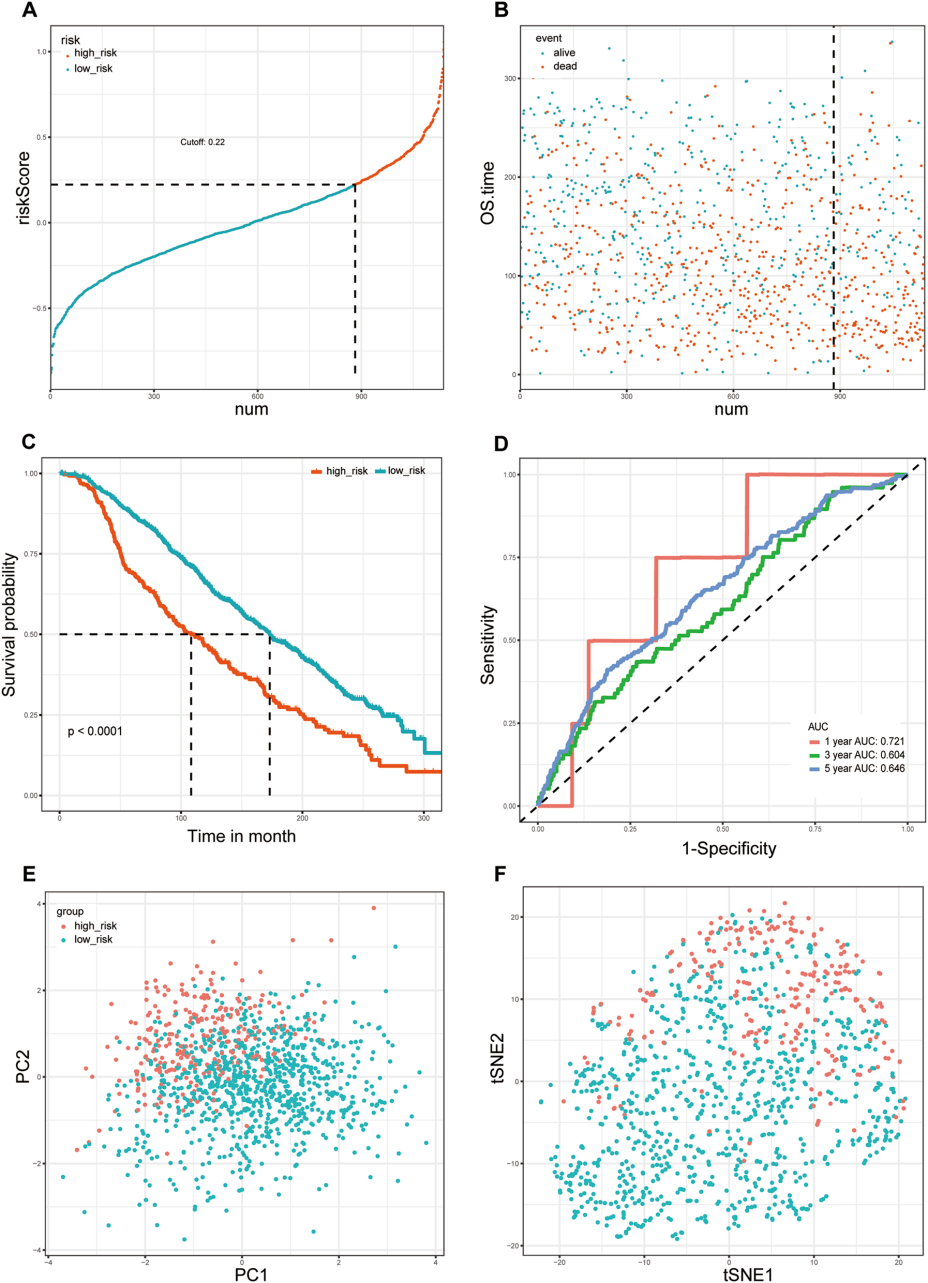
Prognostic analysis of the 10 ferroptosis-related gene signature model in the METABRIC cohort. (**A**) The distribution and median value of the risk score in the METABRIC cohort. (**B**) The distributions of OS status, OS and the risk score in the METABRIC cohort. (**C**) Kaplan–Meier curves for the OS of patients in the high-risk group and low-risk group in the METABRIC cohort. (**D**) The AUCs of time-dependent ROC curves verified the prognostic performance of the risk score in the METABRIC cohort. (**E**) PCA plot of the METABRIC cohort. (**F**) t-SNE analysis of the METABRIC cohort.

To further identify the best candidate genes for building a predictive model, LASSO Cox regression was performed in the METABRIC cohort. Finally, 10 candidate gene signatures were found to have the optimal value of lambda (Fig. 2F, G). A risk score was established to identify the predictive performance of the 10 ferroptosis-related gene-based signature in the METABRIC cohort (Table S3). Patients with a risk score greater than 0.22 were categorized into the high-risk group, and the remaining patients were stratified into the low-risk group. The distributions of the risk scores, survival time, and survival status are displayed in Fig. 3A, B. The high-risk group was found to be significantly associated with higher age, postmenopausal status and histologic grade (Table 2). Kaplan–Meier curves were constructed and indicated that patients with low-risk scores were significantly correlated with better prognosis in the METABRIC cohort (Fig. 3C, P < 0.001). Then, time-dependent receiver operating characteristic (ROC) curve analysis was performed to evaluate the area under the curve (AUC). The AUCs of the 10 ferroptosis-related gene-based signature for predicting OS at 1, 3 and 5 years reached 0.721, 0.604 and 0.646, respectively (Fig. 3D). As shown in Fig. 3E, F, principal component analysis (PCA) and t-distributed stochastic neighbor embedding (t-SNE) analyses showed that patients in different risk groups were spread out in two directions. In summary, we believe that increasing the level of ferroptosis in the tumor tissues of BRCA patients would be beneficial to these patients. In addition, ferroptosis was found to be increased in the low-risk group. In other words, low-risk patients are more prone to ferroptosis, or ferroptosis activity is greater in the low-risk group; that is, low-risk patients survive longer because the cancer tissue in their bodies exhibits greater levels of ferroptosis, and more cancer cells die.

**Table 2:** Baseline characteristics of the patients in different risk groups.

Characteristics	METABRIC cohort			TCGA cohort		
	High risk (N = 257)	Low risk (N = 882)	*P*-value	High risk (N = 182)	Low risk (N = 572)	*P*-value
**Age**			< 0.001			0.528
< 60	68 (26.5)	342 (38.8)		94 (51.6)	278 (48.6)	
≥ 60	189 (73.5)	540 (61.2)		88 (48.4)	294 (51.4)	
**Menopausal state**			0.018			0.692
Post	230 (89.5)	734 (83.2)		121 (66.5)	369 (64.5)	
Pre	27 (10.5)	148 (16.8)		61 (33.5)	203 (35.5)	
**Histologic subtype**			0.239			< 0.001
Ductal/NST	196 (76.3)	643 (72.9)		153 (84.1)	347 (60.7)	
Other	58 (22.6)	235 (26.6)		29 (15.9)	225 (39.3)	
Missing	3 (1.2)	4 (0.5)		–	–	
**Histologic grade**			< 0.001			
1	41 (16.0)	93 (10.5)		–	–	–
2	141 (54.9)	402 (45.6)		–	–	–
3	67 (26.1)	349 (39.6)		–	–	–
Missing	8 (3.1)	38 (4.3)		–	–	–
**Tumor stage**			1			0.023
I + II	37 (14.4)	758 (85.9)		114 (62.6%)	422 (73.8%)	
III + IV	3 (1.2)	61 (6.9)		56 (30.8%)	132 (23.1%)	
Missing	217 (84.4)	63 (7.1)		12 (6.6%)	18 (3.1%)	

### Validation of the 10 ferroptosis-related gene-based signature in the TCGA cohort

The baseline characteristics of the patients in different risk groups in the METABRIC and TCGA cohorts are shown in Table 2. To examine the soundness of the model constructed based on the METABRIC cohort, patients in the TCGA cohort were also assigned to either the high-risk or low-risk group with the same calculation formula as that for the METABRIC cohort. The high-risk group was also associated with higher tumor stage in the TCGA cohort (Table 2). Similar outcomes as those in the METABRIC cohort were obtained, and patients in the low-risk group had a longer survival time than those in the high-risk group (Fig. S1C, *P* = 0.0029). In addition, the AUC values of the 10 ferroptosis-related gene-based signature were 0.628 at 1 year, 0.593 at 3 years, and 0.649 at 5 years in the TCGA cohort (Fig. S1D). PCA and t-SNE analyses also confirmed that patients were distributed in two subgroups in a discrete direction (Fig. S1E, F). The complete list of the 10 candidate genes in the TCGA cohort is provided in Table S4.

### Estimation of the independent prognostic value of the 10 ferroptosis-related gene-based signature

**Figure 4:**
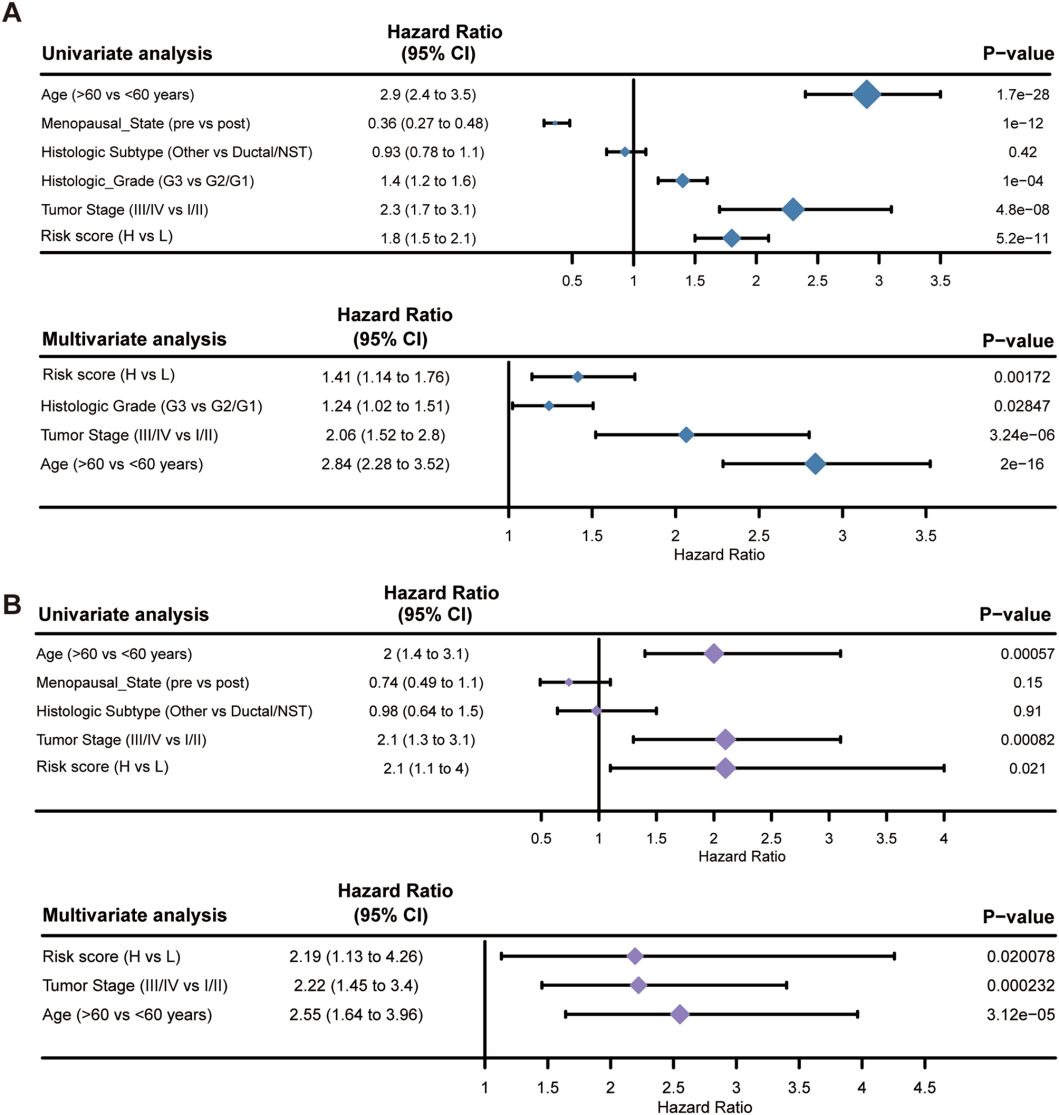
Results of the univariate and multivariate Cox regression analyses of OS in the METABRIC derivation cohort (**A**) and the TCGA validation cohort (**B**).

The outcomes of univariate and multivariate Cox regression analyses are illustrated with forest plots (Fig. 4), and the complete data are shown in Tables S5 and S6. The risk score based on the 10 ferroptosis-related gene-based signature was determined to be an independent prognostic predictor in both the METABRIC (Fig. 4A) and TCGA (Fig. 4B) cohorts (hazard ratio (HR), 1.41, 95% confidence interval (CI), 1.14–1.76, *P* = 0.002; HR, 2.19, 95% CI, 1.13–4.26, *P* = 0.02). In addition, tumor stage and age were also independent prognostic predictors in both cohorts (*P* < 0.01).

### Gene expression differences and functional analyses between the high- and low-risk score groups in the METABRIC and TCGA cohorts

**Figure 5:**
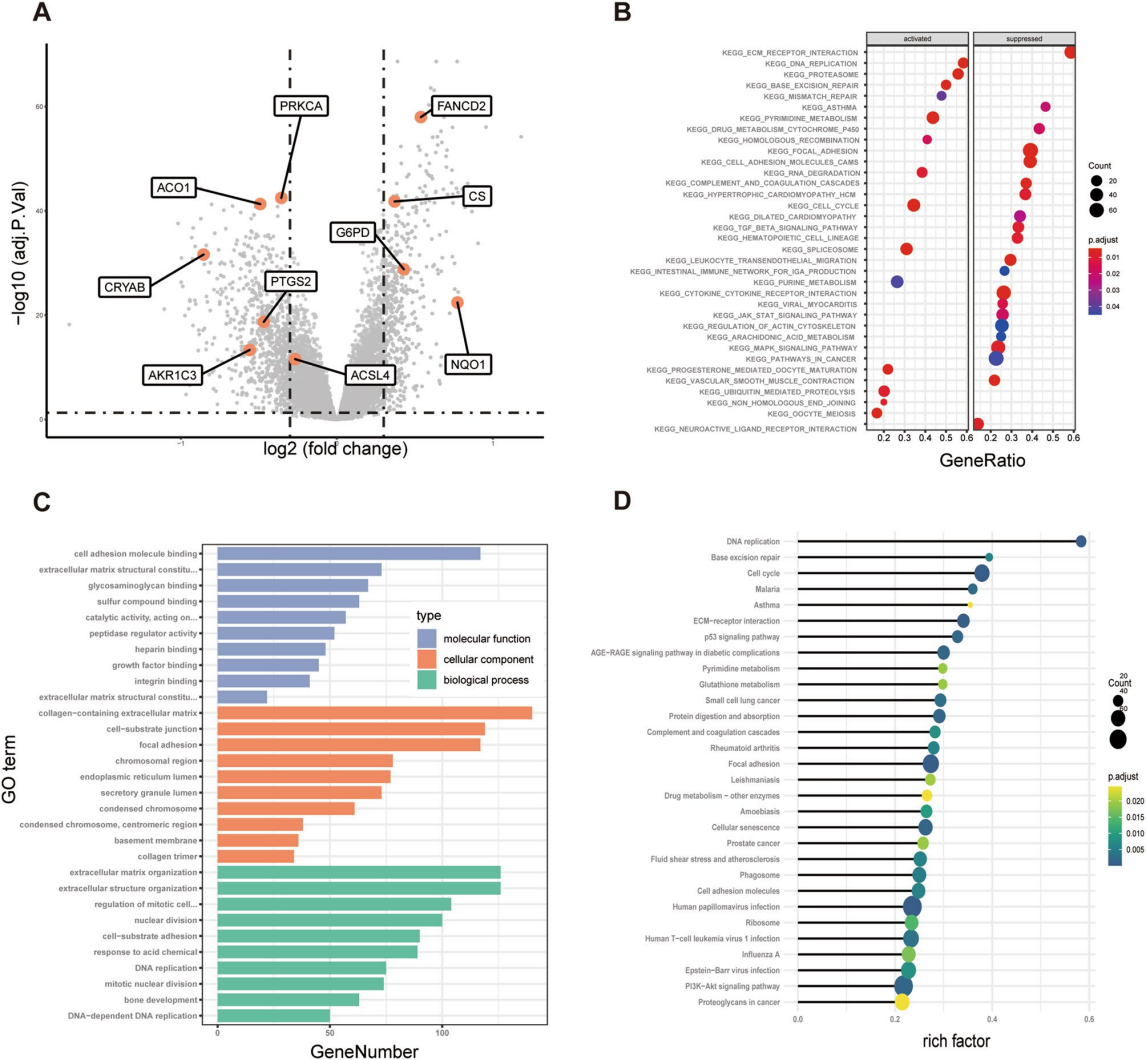
Functional annotation of genes differentially expressed between the low- and high-risk groups in the METABRIC derivation cohort. (**A**) Volcano plot of differentially expressed genes between the low- and high-risk groups. Orange indicates the 10 ferroptosis-related gene signature. (**B**) Enrichment plots from gene set enrichment analysis (GSEA) in the METABRIC cohort. (**C**) The most significant or shared GO enrichment terms in the METABRIC cohort. (**D**) The most significant or shared KEGG pathways in the METABRIC cohort.

To better explore the biological functions of the genes in the risk score, the DEGs between the high- and low-risk groups were identified and were consistent with the results of previous univariate Cox regression analysis (Fig. 2D). High expression of ferroptosis-related genes, including FANCD2, CS, G6PD and NQO1, in the high-risk group represented a higher risk of survival (Figs. 5A and S2A). GSEA using the KEGG pathway database (c2.cp.kegg.v7.1.entrez.gmt) showed that cytokine-cytokine receptor interactions were enriched in the METABRIC and TCGA cohorts (Figs. 5B and S2B). GO and KEGG pathway analyses were also used to explore the potential functions of the DEGs between the two groups. Interestingly, the DEGs from the METABRIC and TCGA cohorts showed enrichment of several cancer-related molecular pathways, including the PI3K-Akt signaling pathway, proteoglycans in cancer and the cell cycle (Figs. 5C, D and S2C, D).

### Immune cell infiltration landscapes of high- and low-risk patients with luminal subtype BRCA

**Figure 6:**
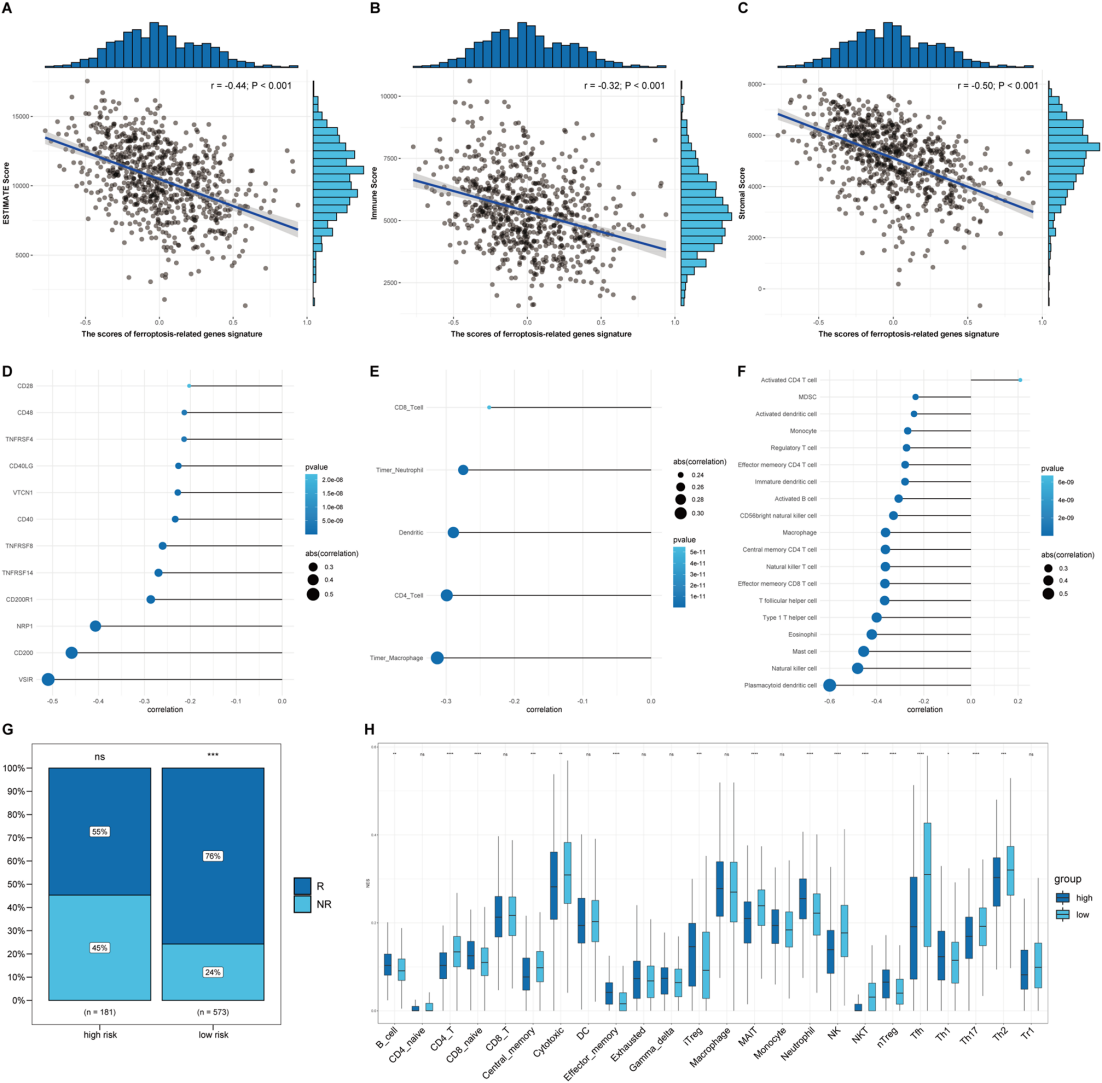
Correlation analysis of immune-related scores and ferroptosis-related gene signature scores. (**A**–**C**) The correlation analysis showed that the estimated score (*r* =  − 0.44; *P* < 0.001) (**A**), immune score (*r* =  − 0.32; *P* < 0.001) (**B**) and stromal score (*r* =  − 0.50; *P* < 0.001) (**C**) were negatively correlated with the risk score of the ferroptosis-related gene signature. (**D**–**F**) The results of Spearman’s correlation analysis of common immune checkpoint genes (**D**), infiltration cell scores from TIMER (**E**) and immune infiltration cell scores from TCIA (**F**) (*r* > 0.2 and *P* < 0.05). (**G**) The ratio of immunotherapy response is greatly elevated in the low ferroptosis-related gene signature score group. (**H**) The immune infiltration scores of high- and low- ferroptosis-related gene signature score groups in the TCGA cohort.

The latest literature reported that CD8 + T cells downregulated the expression of SLC3A2 and SLC7A11 to promote tumor cell lipid peroxidation and ferroptosis^33^. The use of immunotherapy with immune checkpoint blockade targeting CTLA-4 and PD-1 has emerged as a promising strategy for the treatment of various malignancies^34^. Therefore, the immune microenvironment may have a strong link to ferroptosis. To identify immunotherapy targets and assess immunotherapy response in patients in high- and low-risk groups, a correlation analysis was performed, and the results showed that the estimated score (*r* =  − 0.44; *P* < 0.001, Fig. 6A), immune score (*r* =  − 0.32; *P* < 0.001, Fig. 6B) and stromal score (*r* =  − 0.50; *P* < 0.001 Fig. 6C) were negatively correlated with the gene signature score. Twelve common immune checkpoint genes were negatively correlated with the gene signature score (*r* > 0.2; *P* < 0.005, Fig. 6D). The correlation analysis bubble diagram shows the relationships among the 5 immune infiltration cell scores from TIMER (*r* > 0.2; *P* < 0.005, Fig. 6E). Similarly, 18 and 1 immune infiltrating cell scores from TCIA (*r* > 0.2; *P* < 0.005, Fig. 6F) were negatively and positively correlated with the gene signature score, respectively. This suggests that low risk patients may have more options for immune targets when faced with immunotherapy. As shown in Fig. 6G, the percentage of immunotherapy response in the low-risk group was much higher than that in the high-risk group (*P* < 0.001). This demonstrates that the low-risk group had a better response to immunotherapy. To better explore the association between the risk score and immune status, ImmuCellAI, which is used for precisely estimating the abundance of 24 immune cell types, including 18 T-cell subsets, was used to calculate the immune infiltration scores in and TCGA cohorts (Fig. 6H). Interestingly, several T-cell subsets, including Th2, Th17, Tgd, Tfh, Tem, Tcm and Tc, were significantly different between the two groups in TCGA cohorts (adjusted *P* < 0.05) (Table S7).

### Chemotherapy response and chemotherapeutic agent prediction based on the gene signature in high- and low-risk patients with luminal subtype BRCA

Chemotherapeutic agents with higher drug sensitivity are urgently needed for these patients, so two chemotherapy response datasets (CTRP2.0 and PRISM) were analyzed. CTRP2.0 contains sensitivity data for 481 compounds over 835 CCLs, and PRISM contains sensitivity data for 1448 compounds over 482 CCLs. Both of these datasets provide area under the dose–response curve (area under the curve, AUC) values as a measure of drug sensitivity, with lower AUC values indicating increased sensitivity to treatment.

**Figure 7:**
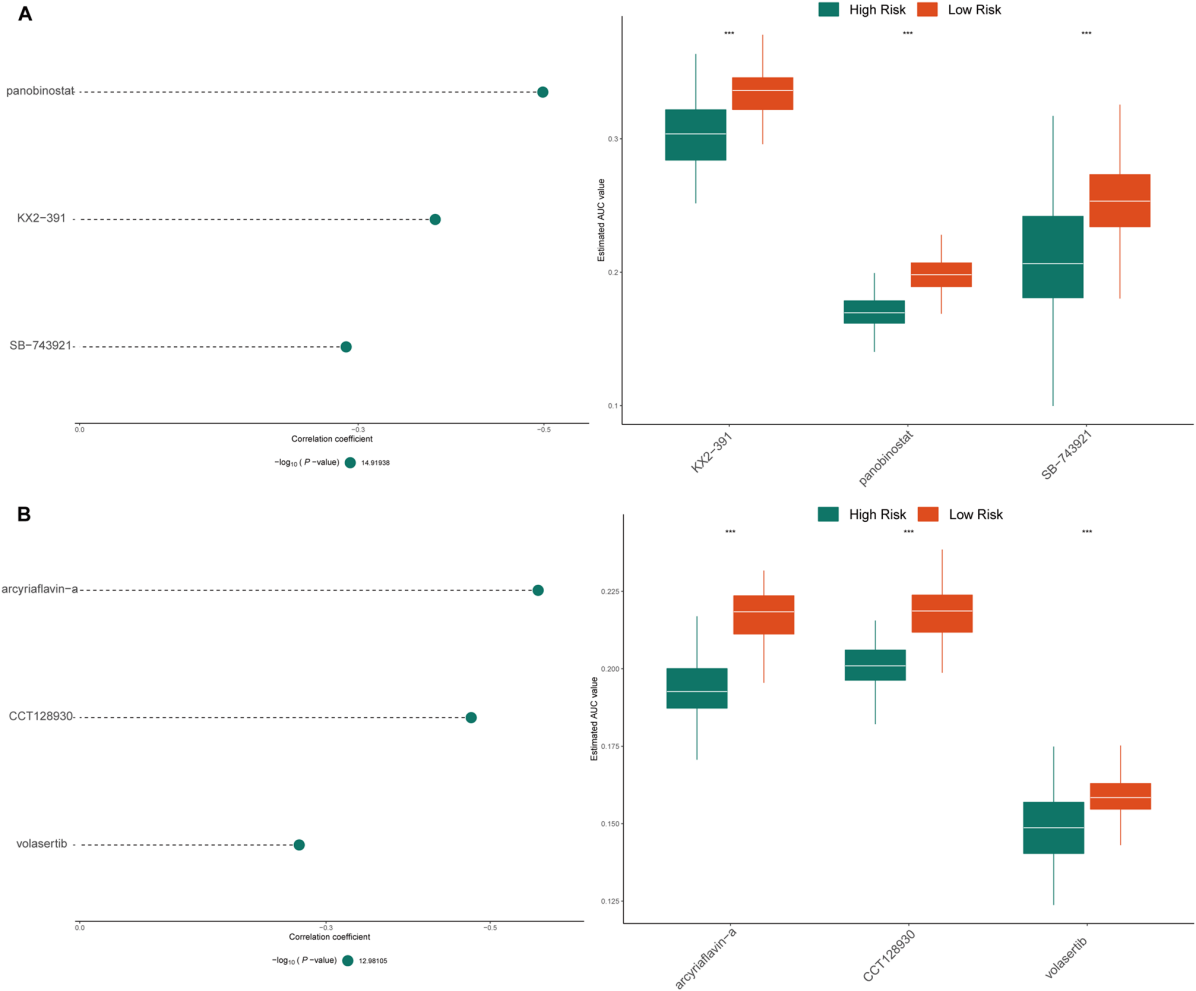
Identification of candidate agents with higher drug sensitivity in high- ferroptosis-related gene signature score patients. (**A**) The results of Spearman’s correlation analysis and differential drug response analysis of three CTRP-derived compounds. (**B**) The results of Spearman’s correlation analysis and differential drug response analysis of three PRISM-derived compounds. *Note* that lower values on the y-axis of boxplots imply greater drug sensitivity.

The pRRophetic package of R was used to estimate the drug response for each sample in the TCGA database based on CTRP and PRISM, respectively. Then, we used two strategies to identify chemotherapy candidates. First, we searched for agents with lower AUC values in the high-risk group (Log2FC > 0.10). Second, we searched for agents that had negative Spearman correlation coefficients (*r* <  − 0.20). These analyses yielded three CTRP-derived compounds (including panobinostat, SB-743921 and KX2-391) and three PRISM-derived compounds (including volasertib, arcyriaflavin-a and CCT128930). All these compounds had lower estimated AUC values in the high-risk group and a negative correlation with the risk score (Fig. 7A, B).

We also selected several common ferroptosis inducers (erastin, 1S, 3R-RSL3, ML162, ML210) as positive controls for the analysis. The results showed that patients in the low-risk group were more sensitive to these drugs (Fig. S3 and Table S11). This result is consistent with our initial hypothesis. The patients in the low-risk group survived longer and were more sensitive to known inducers of ferroptosis, illustrating how they could benefit from ferroptosis-targeted treatment.

### The heterogeneity between high- and low-risk patients

**Figure 8:**
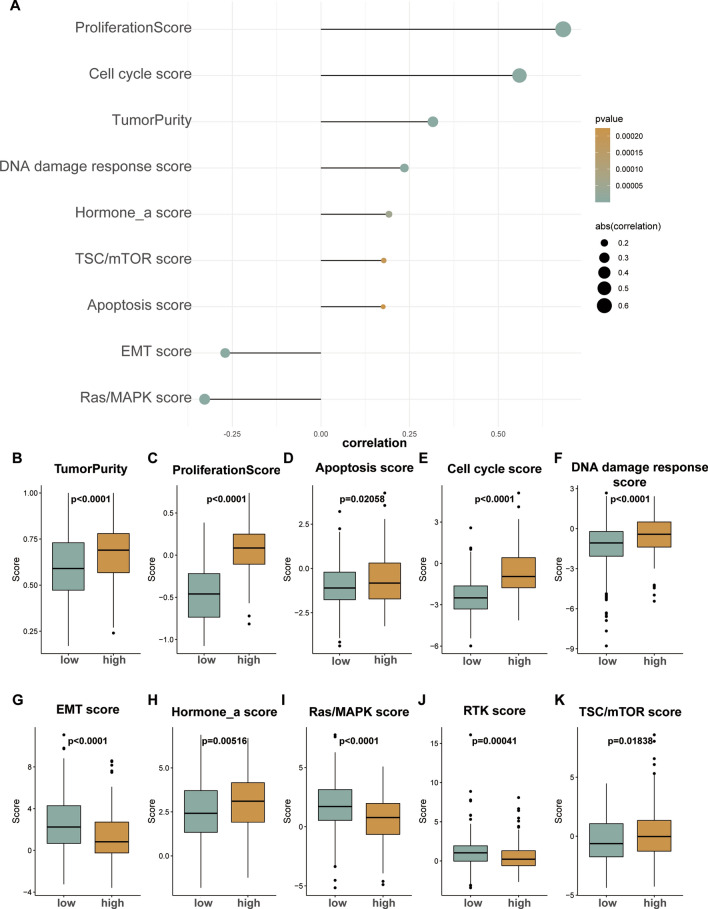
Phenotype heterogeneity among the network-based subtypes. The bubble map shows the correlation between the ferroptosis-related risk score and RPPA data-based scores (**A**). Boxplots show differences in (**B**) tumor purity, (**C**) proliferation, (**D**) apoptosis, (**E**) cell cycle, (**F**) DNA damage response, (**G**) EMT, (**H**) hormone a, (**I**) Ras/MAPK, (**J**) RTK, (**K**) TSC-mTOR scores from TCGA between low- and high-risk groups. The Kruskal–Wallis test was performed to calculate the *P*-value, and those associations with *P*-value < 0.01 were considered significant.

To further explore the heterogeneity of the two patient groups, a reverse-phase protein microarray (RPPA) was obtained from prior work in the literature^35^. Our analysis revealed that the risk score obtained from the ferroptosis-related gene-based signature was significantly correlated with tumor purity scores (*r* = 0.3, *P* < 0.001) and most pathway scores (Fig. 8A and Table S8). In addition, we sought to investigate whether pathway scores exhibit differences between the high- and low-risk patients with luminal subtype BRCA (Fig. 8B–K). The results of our analysis suggest that the pathway scores, except for EMT, RAS/MAPK and RTK, were all significantly higher in the high-risk group. These results suggest that the ferroptosis-related gene-based signature shows differences across most BRCA-associated phenotypes.

### Building a predictive nomogram for luminal subtype BRCA patients

**Figure 9:**
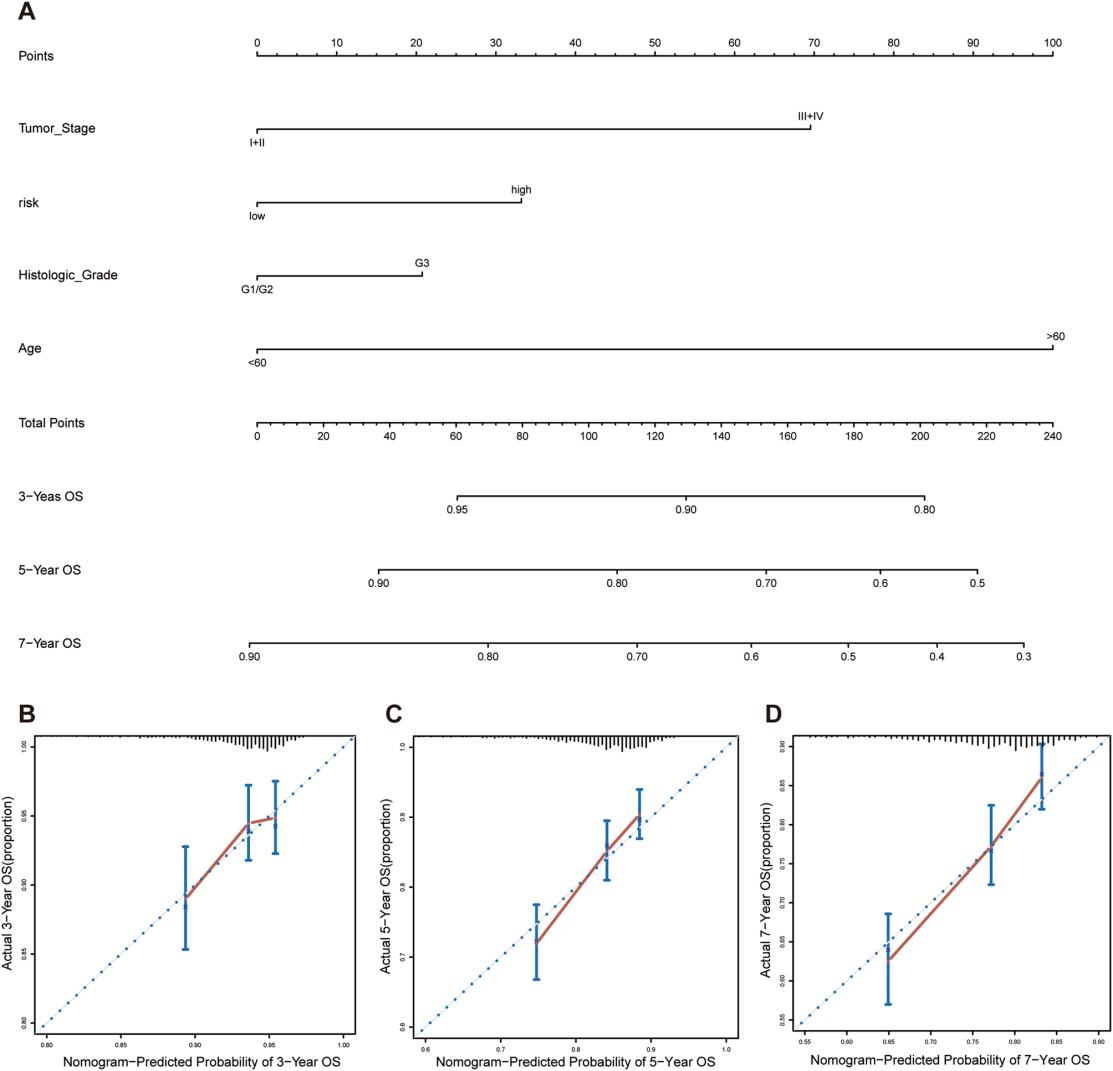
The 10 ferroptosis-related prognostic gene signature model for predicting 3-, 5-, and 7-year OS in luminal-type BRCA patients. (**A**) The independent risk factors were used to build a risk estimation nomogram to predict the probability of OS in luminal subtype BRCA patients. (**B**) The calibration plots for 3-year survival probabilities in the METABRIC cohort. (**C**) The calibration plots for 5-year survival probabilities in the METABRIC cohort. (**D**) The calibration plots for 7-year survival probabilities in the METABRIC cohort.

To provide a clinically appropriate approach for predicting the probability of OS in luminal subtype BRCA patients, the independent risk factors were used to build a risk estimation nomogram (Fig. 9A). These predictors included tumor stage, risk score related to ferroptosis, age and histologic stage. The C-index of our nomogram was 0.66 in the METABRIC cohort. The calibration plots for 3-, 5- and 7-year survival probabilities in the METABRIC cohort are presented in Fig. 9B–D, respectively. Importantly, there was good agreement between the predicted survival rate and the actual observed survival rate. This means that our nomogram has good predictive value.

## Discussion

In our study, we systematically investigated the potential mechanisms of 63 ferroptosis-related genes in luminal-type BRCA tumor tissues. A new prognostic model comprising 10 ferroptosis-related genes was constructed and validated in an external cohort. These genes were also explored for their associations with OS. The functional analyses revealed some potential mechanisms for these genes. Additionally, immune-related pathways, especially the T-cell pathway, were enriched in our study. Although a few previous studies have suggested that some genes might regulate drug-induced ferroptosis in several cancers, especially luminal-type BRCA^36,37^, the relationships among these genes and their correlation with BRCA remain largely unknown. To our surprise, more than half of the ferroptosis-related genes (32/63) were differentially expressed between 140 adjacent normal breast tissues and 1139 luminal subtype BRCA tissues, and in the univariate Cox regression analysis, 12 of them were associated with OS. LASSO Cox regression was performed to find the optimal candidate genes for building a predictive model. Finally, DPP4 and SQLE were removed from the model, and a 10-gene signature was obtained. These results significantly suggested the vital role of ferroptosis in luminal subtype BRCA and the possibility of constructing a prognostic signature with ferroptosis-related genes.

The prognostic model proposed in the present study included 10 ferroptosis-related genes (ACO1, ACSL4, PTGS2, CRYAB, G6PD, PRKCA, NQO1, FANCD2, CS, and AKR1C3). Previous studies^38^ indicated that iron metabolism, lipid metabolism and (anti)oxidant metabolism are the three main pathways that regulate ferroptosis. Moreover, energy metabolism has crosstalk with ferroptosis^39^. To our surprise, of the genes in the 10-gene prognostic model, only ACO1 was reported to be related to iron metabolism. The remaining genes could be roughly classified into other categories. CRYAB, FANCD2 and G6PD are related to energy metabolism. PRKCA, CS, and ACSL4 are included in the lipid metabolism category. The (anti)oxidant metabolism category includes NQO1, AKR1C3 and PTGS2. The analysis of these ferroptosis-related genes led to an interesting finding that most of the genes significantly related to prognosis in luminal-type BRCA are associated with the metabolism of the three major nutrients (glucose, lipids and amino acids). They are closely related to the tricarboxylic acid cycle (TAC). In contrast, there is less regulation of iron metabolism. ACO1, as an iron homeostasis-regulating gene, has been reported to regulate ferroptosis^5^. Genetic knockdown of PRKCA significantly protected rhabdomyosarcoma (RMS) cells from erastin-induced cell death^40^. CRYAB, a member of the small heat shock protein (sHSP) family, has been shown to be significantly differentially expressed in BRCA^41^. sHSP has an inhibitory effect on erastin-induced ferroptosis^42^. AKR1C3 has been proven to inhibit lipid peroxidation to promote ferroptosis and has increased expression in zero-valent iron (ZVI)-induced ferroptosis^43^. The increase in PTGS2 has been confirmed by many studies as one of the hallmarks of the occurrence of ferroptosis^44,45^. G6PD is involved in the pentose phosphate pathway^46^. Dixon SJ et al. reported that G6PD, when knocked down in non-small cell lung cancer cells, prevents erastin-induced ferroptosis^5^. Ferroptosis caused by lipid peroxidation is controlled by integrated oxidation and antioxidant systems. The iron-containing enzyme lipoxygenase is the main promoter of ferroptosis by producing lipid hydroperoxides, and its function relies on the activation of ACSL4-dependent lipid biosynthesis^47^. In hepatocellular carcinoma (HCC) cells, knockdown of NQO1 enhances erastin and sorafenib-induced ferroptosis^48^. FANCD2, a nuclear protein involved in DNA damage repair, protects against ferroptosis-mediated injury in bone marrow stroma^49^. Dixon SJ et al. found that cell viability was rescued from erastin-induced ferroptosis by silencing CS^5^. According to the differential expression of 10 genes in BRCA tissues and normal tissues, six of the genes (PRKCA, ACO1, CRYAB, AKR1C3, PTGS2, and ACSL4) in the prognostic model were proven to protect cells from ferroptosis, while the remaining four genes (NQO1, FANCD2, G6PD, and CS) had the opposite effects. The role these genes play in BRCA patient prognosis by influencing the process of ferroptosis remains to be further investigated since few related studies on these genes have been reported, especially in luminal-type BRCA.

In recent years, cancer immunotherapy based on immune checkpoint inhibitors (ICIs) has achieved great success in basic medical research and clinical practice. However, ICIs are significantly limited by the fact that only one-third of patients with most types of cancer respond to these agents^50^. Tumor cells undergoing ferroptosis can trigger robust antitumor immunity in vivo and in vitro, and their efficacy can be synergistically improved by ICIs, even in ICI resistance. In 2019, direct evidence for a link between ferroptosis and antitumor immunity emerged with the discovery by Wang et al. that CD8 + T cells induce tumor cells to undergo ferroptosis in vivo^51^. The paper reported that CD8 + T cells downregulated the expression of SLC3A2 and SLC7A11 to promote tumor cell lipid peroxidation and ferroptosis^51^. Subsequently, the same team reported that IFN-γ derived from immunotherapy-activated CD8 + T cells synergizes with radiotherapy-activated ataxia-telangiectasia mutated (ATM) to induce ferroptosis in human fibrosarcoma cells and melanoma cells^52^. Although these findings suggest that ferroptosis has a synergistic effect on antitumor immunity, scientific hypotheses still need to be validated by more evidence. The immunoediting hypothesis was proposed in 2009 by Dunn et al. Less immunogenic cancer cells are selected during tumor development in immune-competent hosts to evade antitumor immune responses^53^. Thus, we hypothesized that patients in different groups would have different immunotherapeutic responses. As expected, we found that patients with low risk scores would generally be more sensitive to tumor immune responses than patients with high risk scores in both the METABRIC and TCGA cohorts. To better explore the relationship between our prognostic model and immune status, ImmuCellAI was used to determine the immune infiltration scores of our two cohorts. Interestingly, several T-cell subsets, including Th2, Th17, Tgd, Tfh, Tem, Tcm and Tc, were significantly different between the two groups in both the METABRIC and TCGA cohorts. The interpretation of the results reinforces our belief that there is some relationship between ferroptosis and tumor immunity, particularly with the T-cell family.

Chemotherapy is the foundation of comprehensive BRCA treatment. Many studies have reported that chemotherapeutic agents can induce ferroptosis in different tumors. Recent studies have shown that ferroptosis inhibitors can reduce the ototoxicity caused by cisplatin^54^. Doxorubicin is a chemotherapy drug commonly used for BRCA, but a common side effect is cardiotoxicity. Studies have suggested that this cardiotoxicity is most likely caused by ferroptosis induced by doxorubicin^55^.

In modern clinical practice, clinicians have more targeted therapy options to offer patients for whom first-line treatment has failed. Lapatinib is one such option; lapatinib is a small molecule tyrosine kinase inhibitor that has been approved by the U.S. Food and Drug Administration (FDA) for the treatment of anthracycline-, paclitaxel- and trastuzumab-resistant HER2-positive progressive or metastatic BRCA. S Ma et al. found that ferroptosis is induced following siramesine and lapatinib treatment of BRCA cells^56^. Based on these studies, we can speculate that the ferroptosis signature plays an important role in predicting chemotherapy and targeted therapy drug sensitivity in luminal BRCA patients.

As expected, multiple therapeutic agents that exhibit significant differences in drug sensitivity between the high- and low-risk groups were predicted by our signature. We chose the drugs that have been commonly used as ferroptosis inducers in clinical treatment to date as positive controls. Interestingly, the low-risk group had greater sensitivity to these drugs. These results suggest that known ferroptosis inducers are more effective in patients in the low-risk group, whereas the drugs predicted using our prediction model are more effective in patients in the high-risk group. This result is consistent with our initial hypothesis. The patients in the low-risk group survived longer and more sensitive to known inducers of ferroptosis, illustrating the benefit they could receive from ferroptosis-targeted treatment. This also explains why low-risk patients are more sensitive to known inducers of ferroptosis (such as erastin). The new drugs we predicted through our gene model are intended for the high-risk group, and we continue to look for a potential drug target for high-risk patients with a poor prognosis.

Recently, we found three studies that have reported ferroptosis gene signatures. These three studies focused on HCC^57^, clear cell renal cell carcinoma^58^, and glioma^59^. There are some similarities between our research and these three articles. Univariate and multivariate Cox regression analyses were performed to identify independent predictors of OS. To clarify the functional characteristics of the ferroptosis-related genes in different tumors, GO analysis and KEGG analysis were used in several datasets. Although some similarities exist between our study and the three studies, several innovations still exist in our study. First, the ferroptosis gene signature has never been reported in BRCA, especially luminal-type BRCA. Our team believes that the most important aspect of a good signature is that it should provide valuable suggestions on options in clinical work. Therefore, we provide more predictions on the choice of treatment, including immunotherapy and chemotherapy. Our research learned from the advantages of all three articles. We made two sets, a training set and a validation set, from different public databases to clarify the effectiveness of our signature. We built a predictive nomogram in luminal subtype BRCA patients and investigated the link between ferroptosis and tumor immunity. It is worth noting that our predictive nomogram could predict 3-, 5-, and 7-year OS. Our research went above and beyond what the three previous studies did. By comparing the three prediction models associated with ferroptosis, we found that only a few genes recurred in the different signatures. This may be due to the heterogeneity of the tumors. In addition, due to the physiological characteristics of the breast tissue itself, the development of BRCA may be more related to lipid metabolism and energy metabolism than to iron metabolism, which is more widespread in other carcinomas.

here are still some limitations in our study. First, our prognostic model was established by bioinformatics analyses of data from public databases. More real-world data are needed to verify its clinical utility. Second, we considered only the single hallmark (ferroptosis) genes and excluded genes that are themselves highly correlated with BRCA. In addition, we identified the potential relationship between the ferroptosis gene signature and tumor immunity, but we did not have a validated risk score to evaluate BRCA patients. Indeed, the most malignant molecular type of breast cancer seems to be triple negative breast cancer, not the luminal type. Our initial goal was to identify potential therapeutic targets for TNBC. Although we exhausted all available analysis methods, we did not obtain a positive result. However, in clinical practice, the luminal subtype of BRCA accounts for almost 70% of new cases. Therefore, we think it is also meaningful to analyze and predict targets for the luminal subtype. Finally, we analyzed the single mutations in luminal-type BRCA to find new potential targets. Although we obtained some results from TCGA, these genes with higher mutation rates were not significantly related to ferroptosis.

We think there is a common issue to all predictive modeling research; that is, we may overinterpret the relationship between these genes and ferroptosis. In our logic, with a gene set, it is possible to divide the luminal type BRCA patients into two subgroups. These two subgroups do have some differences in biological function, especially in terms of prognosis and survival. Thus, our gene set is of some significance. In addition, regarding evidence on ferroptosis-related genes, we only have the studies reported in the literature. Therefore, we really cannot definitely say gene expression is related to ferroptosis. To prove that gene expression is relevant to the occurrence of ferroptosis, the transcriptome of samples that are sensitive to ferroptosis must be compared with those resistant to it.

## Conclusion

Our study systematically developed a novel prognostic model of 10 ferroptosis-related genes. The prognostic model was established in the derivation cohort and validated in the validation cohort and exhibited potential as a biomarker of OS in luminal-type BRCA patients. These 10 genes can provide insights into the identification of therapeutic targets for luminal-type BRCA, especially immunotherapy. This study provides a new reference for further study of the mechanisms among ferroptosis, tumor immunity and the choice of chemotherapy drugs.

## Supplementary Information

Supplementary Legends.

Supplementary Figure S1.

Supplementary Figure S2.

Supplementary Figure S3.

Supplementary Table S1.

Supplementary Table S2.

Supplementary Table S3.

Supplementary Table S4.

Supplementary Table S5.

Supplementary Table S6.

Supplementary Table S7.

Supplementary Table S8.

Supplementary Table S9.

Supplementary Table S10.

Supplementary Table S11.
