# Supplementary material for: A ferroptosis-associated gene signature for the prediction of prognosis and therapeutic response in luminal-type breast carcinoma
Source: Sci Rep. 2021 Sep 2;11:17610. doi: 10.1038/s41598-021-97102-z (PMC8413464; doi:10.1038/s41598-021-97102-z)
Supplement: Supplementary file 1 — Supplementary Legends. [file 41598_2021_97102_MOESM1_ESM.docx]

**legends for the Supplementary Figures and Tables**

**Figure S1**. **Prognostic analysis of the 10 ferroptosis-related gene signature model in the TCGA cohort.**

1. The distribution and median value of the risk score in the TCGA cohort.
2. The distributions of OS status, OS and risk score in the TCGA cohort.
3. Kaplan-Meier curves for the OS of patients in the high-risk group and low-risk group in the TCGA cohort.
4. The AUCs of time-dependent ROC curves verified the prognostic performance of the risk score in the TCGA cohort.
5. PCA plot of the TCGA cohort.
6. t-SNE analysis of the TCGA cohort.

**Figure S2.** **Functional annotation of genes differentially expressed between the low- and high-risk groups in the TCGA validation cohort.**

1. Volcano plot of differentially expressed genes between the low- and high-risk groups. Blue indicates the 10 ferroptosis-related genes signature.
2. Enrichment plots from gene set enrichment analysis (GSEA) in the TCGA cohort.
3. The most significant or shared GO enrichment terms in the TCGA cohort.
4. The most significant or shared KEGG pathways in the TCGA cohort.

**Figure S3.** **Validation of drug sensitivity of commonly used ferroptosis inducers in different risk groups.**

The results of Spearman’s correlation analysis and differential drug response analysis of three CTRP-derived compounds.

**Table S1: The complete list of ferroptosis-related genes**

**Table S2: The coefficients of each normalized expression level of ferroptosis-related genes**

**Table S3: Complete list of 10 candidate gene in the METABRIC cohort**

**Table S4:** **Complete list of 10 candidate gene in the TCGA cohort**

**Table S5:** **Clinical-related data with ferroptosis-related riskscore in METABRIC cohort**

**Table S6:** **Clinical-related data with ferroptosis-related riskscore in TCGA cohort**

**Table S7:** **Immune infiltration score in the TCGA cohort**

**Table S8:** **The correlation between risk score and pathway score**

**Table S9:** **Gistic scores data of high risk subgroup**

**Table S10:** **Gistic scores data of low risk subgroup**

**Table S11: Estimated AUC value of commonly used ferroptosis inducer**
