# Supplementary material for: A ferroptosis-associated gene signature for the prediction of prognosis and therapeutic response in luminal-type breast carcinoma
Source: Sci Rep. 2021 Sep 2;11:17610. doi: 10.1038/s41598-021-97102-z (PMC8413464; doi:10.1038/s41598-021-97102-z)
Supplement: Supplementary file 5 — Supplementary Table S1. [file 41598_2021_97102_MOESM5_ESM.pdf]

TableS1 the complete list of ferroptosis-related genes

Ferrotosis-related genes	Name
ACSL4	acyl-CoA synthetase long-chain family member 4
AKR1C1	aldo-keto reductase family 1 member C1
AKR1C2	aldo-keto reductase family 1 member C2
AKR1C3	aldo-keto reductase family 1 member C3
ALOX15	arachidonate 15-lipoxygenase
ALOX5	arachidonate 5-lipoxygenase
ALOX12	arachidonate 12-lipoxygenase
ATP5G3	ATP synthase membrane subunit c locus 3
CARS	cysteinyl tRNA synthetase
CD44	CD44 molecule
CHAC1	ChaC glutathione- specific gamma-glutamyl cyclotransferase 1
CISD1	CDGSH iron sulfur domain 1
CS	citrate synthase
DPP4	dipeptidyl-dippeptidase-4
FANCD2	Fanconi anemia comple mentation group D2
GCLC	glutamate-cysteine ligase catalytic subunit
GCLM	glutamate-cysteine ligase modifier subunit
GLS2	glutaminase 2
GPX4	glutathio ne peroxidase 4
GSS	glutathione synthetase
HSPB1	heat shock protein beta 1
CRYAB	heat shock protein beta 5
LPCAT3	lysophosp hatidylcholine acyltransferase 3
MT1G	metallothionein-1G
NCOA4	nuclear receptor coactiva tor 4
PTGS2	prostagla ndin-endoperoxide synthase 2
RPL8	ribosomal protein L8
SAT1	spermidine/spermine N1-acetyltra nsferase 1
SLC7A11	solute carrier family 7 member 11
FDFT1	farnesyl-diphosphate farnesyltransferase 1
TFRC	transferrin receptor
TP53	tumor protein 53
EMC2	ER membrane protein complex subunit 2
AIFM2	apoptosis inducing factor mitochondria associated 2
PHKG2	phospho rylase kinase ,g2
HSBP1	heat-shock 27-k Da protein 1
ACO1	aconitase 1
FTH1	ferritin heavy chain 1
STEAP3	six-transm embrane epithelial antigen of prostate 3
NFS1	cysteine desulfurase
ACSL3	acyl-CoA synthetase long-chain family member 3
ACACA	Acetyl-CoA carboxylase alpha
PEBP1	phosphatidy lethanolamine-binding protein 1
ZEB1	zinc finger E-box-binding homeobox 1
SQLE	squalene monooxygenase
FADS2	fatty acid desaturase 2/acyl-CoA 6-desaturase
NFE2L2	nuclear factor, erythroid 2 like 2
KEAP1	kelch-like ECH- associated protein 1
NQO1	quinone oxidoreductas e-1
NOX1	NADPH oxidase 1
ABCC1	ATP binding cassette subfamily C member 1
SLC1A5	solute carrier family 1 member 5

GOT1	glutamic-oxa loacetic transaminase 1
G6PD	glucose-6-phosphate dehydrogenas e
PGD	phosphoglycerate dehydrogenas e
IREB2	iron response element-binding protein 2
HMOX1	heme oxygenase 1
SLC11A2	Solute Carrier Family 11 Member 2
PRKCA	Protein Kinase C Alpha
FTL	Ferritin Light Chain
LOX	Lysyl Oxidase
VDAC2	Voltage Dependent Anion Channel 2
VDAC3	Voltage Dependent Anion Channel 3
