# Supplementary material for: A ferroptosis-associated gene signature for the prediction of prognosis and therapeutic response in luminal-type breast carcinoma
Source: Sci Rep. 2021 Sep 2;11:17610. doi: 10.1038/s41598-021-97102-z (PMC8413464; doi:10.1038/s41598-021-97102-z)
Supplement: Supplementary file 6 — Supplementary Table S2. [file 41598_2021_97102_MOESM6_ESM.pdf]

TableS2 the coefficients of each normalized expression level of ferroptosis-related genes

Genes	Coefficients
CRYAB	0.983799336
PTGS2	0.959648823
PRKCA	0.907204062
AKR1C3	0.968377806
FANCD2	1.109649176
G6PD	1.050621819
ACSL4	0.988587942
ACO1	0.921905453
NQO1	1.123790804
CS	1.12909727
