# Supplementary material for: A ferroptosis-associated gene signature for the prediction of prognosis and therapeutic response in luminal-type breast carcinoma
Source: Sci Rep. 2021 Sep 2;11:17610. doi: 10.1038/s41598-021-97102-z (PMC8413464; doi:10.1038/s41598-021-97102-z)
Supplement: Supplementary file 7 — Supplementary Table S3. [file 41598_2021_97102_MOESM7_ESM.pdf]

TableS3 Complete list of 10 candidate gene in the METABRIC cohort

	OS.time	OS	CRYAB	PTGS2	PRKCA	AKR1C3	FANCD2
MB-0002	84.63333333	0	6.907372846	5.754286058	5.860844768	7.562530582	6.725265946
MB-0005	163.7	1	8.644812412	7.404578153	6.105563475	7.78853005	6.778085241
MB-0006	164.9333333	0	7.92320109	6.038329602	6.654168271	7.611662442	6.960574097
MB-0008	41.36666667	1	9.026136374	6.716833943	5.885327969	8.322077602	6.796827192
MB-0010	7.8	1	7.560317162	6.169141659	6.50126818	8.415016269	6.998417681
MB-0014	164.3333333	0	8.945449298	7.925600849	6.373077473	9.619248934	6.671650237
MB-0028	36.56666667	1	6.965302648	5.865020461	5.784725767	7.945142773	7.558923274
MB-0036	132.0333333	1	9.612482224	6.788706102	6.256813088	8.929415466	6.752797857
MB-0039	163.5333333	0	5.257978001	5.8447905	5.675203228	10.12718117	6.606781229
MB-0046	14.13333333	1	8.582921766	6.674774997	6.066494859	8.950778648	7.238893409
MB-0053	161.0666667	0	7.29740766	6.899224394	6.366296492	10.5068791	6.844362908
MB-0054	160.3	0	7.553058442	6.735961315	6.573325528	8.497650915	7.124936628
MB-0056	62.86666667	0	6.742033756	7.588093635	6.062184164	9.95308368	6.495244152
MB-0060	140.8666667	0	6.009638762	6.868691131	5.820141549	7.088788611	7.721604504
MB-0064	108.9333333	0	7.499895472	6.557222902	6.024826774	9.374590225	6.996529453
MB-0066	157.4333333	0	7.41295206	6.793211213	6.128010818	9.371040947	6.997305225
MB-0068	103.1333333	0	9.522032677	6.461100491	6.553789156	9.232774567	6.808083943
MB-0083	86.06666667	1	8.101688377	8.85115904	6.44308262	9.765207302	7.237655753
MB-0093	153.2	0	6.489780674	8.489576883	6.741674231	8.262820392	6.38571
MB-0095	49.76666667	1	6.040348046	6.256278533	6.08812506	7.174527208	7.649509165
MB-0097	98.7	0	6.896602986	6.630726266	5.914380732	7.292255221	6.606655313
MB-0102	140.7666667	1	7.095306334	6.037154835	6.273512012	7.61842528	6.916258651
MB-0106	85.33333333	0	9.779914196	7.621199586	6.719096457	7.703120468	6.677960495
MB-0107	158.0333333	0	7.273883645	6.032967585	6.176498632	7.721951992	8.08906458
MB-0111	127.1	0	8.153666118	7.128819242	7.075965034	8.362949534	6.927266329
MB-0112	39.16666667	1	8.876832899	6.275397004	6.452671117	8.730560259	6.477389031
MB-0114	13.4	0	7.72787379	5.616743698	5.947403679	7.456728625	6.9752009
MB-0117	2.4	0	9.726590387	8.099975878	6.647113492	9.361347108	6.82666656
MB-0119	95.86666667	1	7.124938755	5.853062855	5.772581089	7.623422295	6.709233733
MB-0120	29.06666667	1	7.787718295	6.548733967	5.902654562	7.980026988	7.267387483
MB-0121	152.2	0	7.964192774	6.082326893	5.826264028	7.620452644	7.055282583
MB-0122	138.9	0	9.672354087	7.004535045	6.106432808	8.395613416	6.522523298
MB-0123	114.2333333	1	8.30698042	8.372891763	5.877954935	8.694290101	7.363222198
MB-0124	118.2	0	8.490774514	9.153118458	6.322179909	9.744106162	6.611510347
MB-0125	1.266666667	0	6.648916205	5.898018839	5.742430373	9.189919852	6.45645174
MB-0126	127.6333333	0	8.45011259	6.013033747	6.834754396	7.069625913	6.673826888
MB-0130	153.5666667	0	9.281744822	7.099205923	6.727767795	8.575720674	6.755015034
MB-0131	66.63333333	1	7.109705859	6.172958271	6.640763822	6.326203973	6.743066457
MB-0133	151	0	9.712312512	7.165499938	6.004199986	9.32318342	6.590125827
MB-0134	12.93333333	1	7.546962057	5.879524372	6.094823477	8.451349765	6.645520511
MB-0135	116.6333333	0	7.277735156	5.745076475	6.024832042	8.582680277	7.513172041
MB-0136	88.2	0	7.880720775	7.042810148	6.161919505	8.782253264	6.678017812
MB-0138	150.5666667	0	8.788957447	7.796639387	6.510973401	9.757367448	6.780966649
MB-0139	109.2	0	11.4706325	7.526544713	6.630832687	9.383487505	6.367377306
MB-0140	147.9333333	0	8.22705974	6.6492529	5.700163128	7.980749862	7.082164121
MB-0142	12.4	0	8.417875605	6.821712826	5.782267945	8.102426032	5.705721425
MB-0143	54.33333333	1	7.196437528	5.704735174	5.948490291	7.896715966	7.369218498
MB-0144	152.0666667	1	8.131362194	6.443100394	6.497791389	7.679343782	7.084597765
MB-0145	147.6666667	0	10.49277101	6.839365026	6.466934931	9.334611098	6.090331502
MB-0146	122.1666667	1	6.234045588	5.665367886	6.008241569	6.333510538	7.076770013
MB-0147	51.76666667	1	8.041666091	5.496078639	6.354127609	7.861924672	6.977011707
MB-0151	49.23333333	1	8.200407417	5.948466353	5.957491232	8.038537114	6.957960238

MB-0154	114.7666667	0	8.823575761	8.896079676	6.630506064	8.831709803	6.089443061
MB-0162	55.76666667	0	8.027582426	7.863133252	6.456840989	8.102048983	6.894847285
MB-0165	47.63333333	1	8.453344739	6.074209886	6.776015697	8.453969366	7.110313956
MB-0166	104.4	0	8.973794644	6.580536011	6.101724297	8.994090006	7.028038486
MB-0167	43.1	1	7.147932198	5.703494953	5.723439234	7.862995513	7.496951746
MB-0170	93.36666667	1	8.936551248	9.665950639	6.218567277	8.99834803	6.531097474
MB-0172	138.1	0	8.278778986	6.608454641	6.227030889	8.822194075	6.511088183
MB-0173	3.766666667	0	5.837761873	6.437628814	5.599302434	6.60726789	6.782723241
MB-0175	72.36666667	0	6.85417761	6.958709809	6.136468863	13.47609347	5.984448209
MB-0176	113.4333333	0	9.748572458	8.351577455	6.456968416	8.570300772	6.877372204
MB-0177	45.6	1	8.591595513	7.063873779	6.461625629	9.310151871	6.352052007
MB-0178	104.4666667	1	6.856913273	6.270272374	5.91842615	8.310073328	7.620937038
MB-0180	62.33333333	0	7.304909725	6.629970842	5.872995729	8.628063563	6.254987238
MB-0181	85.96666667	0	9.83196732	8.719879487	6.259587326	9.565702215	6.518820918
MB-0184	109.0333333	0	9.271764644	5.923952978	6.053316188	7.632615398	6.262132306
MB-0193	19	0	7.38520847	5.449549644	6.304016382	9.66788337	6.85560032
MB-0194	91.6	0	9.692348049	7.846646522	6.260794477	8.761458893	6.16297361
MB-0195	146	0	6.441238867	5.92689094	5.878029876	8.679407673	7.381391333
MB-0197	70.73333333	0	7.951421844	6.180676997	5.812294749	7.055953955	7.448996659
MB-0198	144.7666667	0	7.860093002	6.701603951	6.532923601	8.245576153	7.2555524
MB-0199	144.4	0	8.519796433	6.262814931	6.189639041	10.05136329	6.523982974
MB-0202	128.3666667	0	8.71577165	6.351149721	5.910333862	7.551251444	7.041658609
MB-0203	58.76666667	1	9.773498546	7.523318057	5.955346718	9.079423914	6.672381641
MB-0204	24.3	1	9.552829218	8.172470094	6.76004629	8.871749362	6.555509104
MB-0207	173.6333333	0	8.329199289	6.975433296	6.092837582	8.204280005	7.02115826
MB-0215	122.2	0	8.191940122	6.189805288	5.981916396	8.962652337	7.127328836
MB-0218	131.0666667	0	7.555091499	6.971866864	6.095088257	8.196917642	7.054483989
MB-0223	81.33333333	1	8.875988972	6.173080161	6.080165283	8.308949462	6.706065356
MB-0224	176.7666667	0	10.07453343	7.217158578	6.308259939	8.773661601	6.518394974
MB-0225	212.2	0	6.790854103	7.150574785	5.578233761	7.675114185	7.33614338
MB-0226	57.63333333	0	9.361826407	7.665001319	6.533318478	9.689527264	6.889383633
MB-0227	111.8666667	0	9.122583828	9.624197815	6.576029776	9.66079746	6.406309983
MB-0229	71.5	0	8.053116917	5.79406647	6.141860549	7.726988575	6.695202093
MB-0231	19.6	0	7.822472232	6.936114901	6.364493091	7.628338973	5.912275666
MB-0232	207.6333333	0	6.84429685	7.006734771	6.125098834	7.588390899	6.956097074
MB-0233	72.43333333	1	7.40489968	7.423107888	6.522951988	8.346205302	6.303870762
MB-0234	94.23333333	1	7.633726358	6.578436883	5.979392865	7.973837269	6.948158342
MB-0235	142.5666667	1	8.010657297	7.277523815	6.018398998	9.199190352	5.974273091
MB-0236	205.0333333	0	7.872372353	6.159624962	6.445691087	8.51727967	7.114192554
MB-0239	85.56666667	1	10.70940031	6.752893509	6.111507644	10.0308897	6.21146383
MB-0242	199.1	0	8.484651052	6.342306529	6.440180324	7.875951999	7.019100875
MB-0243	149.7666667	0	8.919953781	7.215641749	6.533183754	10.36400488	6.466750617
MB-0245	164.7	0	8.308783679	7.391282516	6.671294372	8.748108386	6.807473496
MB-0247	185.6	0	9.435853453	6.803627595	7.125264854	8.402801121	6.67010533
MB-0248	71	0	6.482962271	5.560058846	5.991146813	9.924380071	6.836715637
MB-0256	200.7	0	7.33649432	6.188186534	6.426883315	8.464108333	6.238027216
MB-0257	91.73333333	1	8.941838355	6.568743721	5.979337699	8.24980017	7.192336535
MB-0258	86.13333333	0	10.09119625	6.53596128	6.073398136	9.832526143	7.229086774
MB-0260	169.8333333	1	9.72319596	8.275302482	6.295875853	8.972949841	6.552448139
MB-0261	90.23333333	1	7.268868312	6.330621368	5.881943575	8.055216672	6.8267769
MB-0263	176.0333333	1	9.262217671	8.789725691	6.29788758	8.973842767	6.513476843
MB-0266	90.66666667	1	8.841133996	6.999306726	6.646806301	9.571953398	6.00529946
MB-0268	28.5	1	7.404428382	5.73153096	6.143062305	7.36692629	7.68404382

MB-0270	337.0333333	0	7.64777017	5.970345152	5.847039306	7.440183168	7.590389429
MB-0272	122	1	7.719198372	6.330380631	6.366651619	9.099443517	6.98271442
MB-0273	186.5333333	0	8.624417743	6.482394009	5.801076958	9.115904799	6.40220992
MB-0287	94.73333333	1	7.264813064	5.979095287	6.13411489	6.798046134	7.154137077
MB-0290	199.5333333	0	8.257886745	6.665043515	6.346357509	7.962407212	6.578330737
MB-0291	39.2	1	8.75225912	6.177834649	6.282867309	8.26564114	7.095634563
MB-0295	164.5	0	9.145622475	6.932608283	6.378340548	8.683113847	6.889092972
MB-0301	122.7	1	8.186968842	6.633368751	6.251027771	9.74954275	6.773014834
MB-0302	84.2	1	8.710999327	7.412238056	6.593874833	9.615887712	6.498621493
MB-0304	111.5333333	1	8.567114298	5.613692579	5.549116023	6.662735746	7.444420648
MB-0306	42.96666667	1	8.897345035	8.070403667	6.366232309	7.891017102	6.65224465
MB-0308	183.2666667	0	10.2738317	7.975422431	6.894645936	8.6845598	6.791160859
MB-0309	42.33333333	1	8.275683255	8.505619502	6.09577801	7.530612195	6.536833191
MB-0310	185.1666667	0	10.44483785	8.147550686	6.375611884	9.660545198	6.768269377
MB-0311	151.1666667	1	7.751462979	5.951415235	6.454590809	7.334273882	7.687212008
MB-0312	169.6	1	7.823982179	6.966160843	5.967707458	8.470978479	7.112422856
MB-0313	184.7666667	0	7.365948538	6.773138613	6.00478536	6.944170247	7.829160638
MB-0315	186.2	0	8.956022007	6.426947017	6.780577068	8.621521617	6.733276494
MB-0317	151.6666667	1	8.310147418	6.136293327	6.515156016	9.1722463	6.728699284
MB-0319	40.7	1	7.102130249	6.267829714	5.702712191	6.968394806	7.245754604
MB-0320	63.73333333	0	9.993502915	6.475234896	6.719481178	9.794221032	6.47927033
MB-0321	174.6333333	0	6.200332012	6.639793849	5.525746614	7.19341736	6.388939843
MB-0322	50.53333333	1	6.950094005	5.732728112	5.575157841	8.280516215	6.99606459
MB-0324	144.9666667	1	7.532446579	7.219349468	6.220444659	7.855300102	7.111683404
MB-0325	177.5333333	0	5.873878721	5.474279709	6.260305908	6.672756324	7.535505993
MB-0328	125.6	1	8.477185453	7.186974294	5.973339386	7.970444201	7.601268827
MB-0336	170.6333333	1	8.368970098	6.217806176	5.964399704	8.368116987	7.814653685
MB-0339	26.73333333	1	8.733871732	6.733533821	6.040116226	6.963368792	6.271450066
MB-0341	173.9	1	9.218694609	7.763266089	6.152705733	8.789880414	6.843445438
MB-0345	72.66666667	0	8.922460259	7.669370514	6.274953678	8.808179065	6.264937792
MB-0348	122.7	1	7.105142371	6.088888182	5.760463423	8.229132133	6.5155572
MB-0349	146.0333333	1	8.325232719	6.13713117	5.819870467	7.785585102	6.752989146
MB-0353	41.83333333	1	6.976017248	5.709835469	5.963488367	11.01772519	6.900335932
MB-0356	213.5	0	8.643870165	6.972562814	6.075128753	8.633230297	6.80470868
MB-0358	32.86666667	0	9.737715259	6.688299837	6.33408232	8.552813596	7.366196924
MB-0359	21.6	0	9.474444609	8.990532344	6.322713815	9.441914895	6.634650114
MB-0360	132.5666667	1	6.55750152	5.813741993	5.719991226	7.866158314	6.974340474
MB-0362	47.03333333	1	8.41453381	7.488569504	5.934667127	8.611163666	7.116153727
MB-0363	89.9	1	7.242108053	6.886928271	6.426975441	7.339715569	7.485510039
MB-0364	176.6	0	8.926513947	7.70756019	6.235632561	9.188521188	5.943379471
MB-0366	64.23333333	0	8.551655592	6.617943194	6.214234947	8.762333595	7.040518665
MB-0368	61.9	1	6.299041145	6.592460105	5.447277681	7.30004054	7.248250616
MB-0369	144.3333333	0	7.172012999	5.839128803	5.718391686	8.251116834	6.779379674
MB-0370	93.5	1	7.61724155	6.663654151	5.636203319	7.345468135	7.806252365
MB-0371	131.1333333	0	10.31196596	7.883155656	6.161107945	9.519779008	7.13204731
MB-0373	27	1	7.420592409	6.0775707	6.200791111	7.358261998	7.457902216
MB-0374	1.433333333	0	8.657988336	6.357939902	6.68202652	7.517390746	7.252933154
MB-0377	134.3666667	0	9.740833951	11.29193544	6.67222236	9.594982433	6.306486422
MB-0379	135.6666667	0	9.579401399	6.201249134	6.702922815	9.451246029	6.946401293
MB-0380	69.33333333	1	6.296901586	5.717391253	5.871540202	5.777236948	7.862859595
MB-0382	136.4666667	0	8.8111475	7.833714266	6.621532819	8.832136178	6.86060587
MB-0383	85.73333333	1	9.792591059	7.020072578	6.502497483	9.299651847	6.315016552
MB-0384	61.1	1	8.970204003	6.153235642	6.810113543	7.507748945	7.187916622

MB-0385	36.43333333	1	8.267875568	5.830468774	6.086376468	7.919037323	7.194309819
MB-0386	138.1333333	0	10.74387302	6.971766553	6.116906996	9.517136295	6.887792286
MB-0389	96.96666667	0	7.714460473	6.239731806	6.414014087	6.573711692	7.510467546
MB-0394	64.23333333	0	7.720832277	7.591530843	6.107603845	8.899679974	6.506834244
MB-0397	57.23333333	0	8.634002844	8.900561392	6.398238676	7.688950771	6.480225594
MB-0398	43.2	1	7.590486886	5.966196228	6.403173558	7.820550997	6.995121246
MB-0404	63.5	0	9.234853672	6.809695737	5.919282022	8.203262461	6.983909482
MB-0406	96.9	1	8.031464218	5.761497048	6.341351362	8.335895487	6.847116645
MB-0410	63	0	9.324512142	7.773083082	6.582911534	9.355190388	6.883868289
MB-0411	139.1666667	0	9.154535506	6.15689128	6.194997019	8.308468103	7.196986946
MB-0412	136.1666667	0	7.852258708	5.701835026	6.094021347	7.337797066	7.017313132
MB-0413	140.0666667	0	8.168465969	6.020804736	6.341127413	8.267395376	7.119524185
MB-0419	104.3	1	8.169137935	6.304626243	6.110517562	8.385173611	6.73336358
MB-0422	33.8	1	8.473570141	7.804751381	5.885301732	8.432906578	6.772996563
MB-0425	24.4	1	7.470711488	7.027151183	6.355598762	9.263636426	7.205753381
MB-0427	116.1	0	7.03292203	8.073770142	6.699789481	7.645476731	7.353026834
MB-0428	55.93333333	0	8.090572116	6.863215356	6.506013692	7.959284617	6.98064055
MB-0429	74.46666667	1	7.481584819	6.291781985	5.86938262	8.189181783	6.945230522
MB-0431	85.13333333	0	9.607843934	7.36565905	6.290097174	9.667769753	6.251159413
MB-0434	45.5	1	6.995744298	6.098052054	5.769031039	7.368741622	7.729395706
MB-0437	90.56666667	0	7.901312531	5.928446382	6.225392846	8.641837962	7.410290534
MB-0439	112.1333333	0	6.14363981	5.627728283	5.745814088	7.658692714	7.559162428
MB-0440	100.8333333	0	7.671979693	6.722525742	5.717794663	8.395153727	7.980447791
MB-0444	55.46666667	1	8.246680707	6.160158708	6.35561253	8.359789203	6.962334821
MB-0445	132.5333333	0	8.588268974	6.659159769	6.436354042	7.036883383	6.43895001
MB-0449	112.5666667	0	9.432210784	6.143643546	6.30806422	8.305956051	6.504397776
MB-0451	50.06666667	0	9.324250175	5.839403	6.540363289	7.998445619	7.43607128
MB-0452	62.8	0	8.526640011	6.521782536	6.137837736	8.052827376	7.083838755
MB-0454	46.83333333	1	8.386433649	6.738903254	5.960477094	8.131552108	6.366340716
MB-0455	99.33333333	0	7.940283293	6.560845444	6.731936717	8.231414276	7.693618119
MB-0459	35.6	1	8.386875871	5.956857132	6.233579679	7.99574558	7.635900726
MB-0463	120.5666667	1	8.249946205	5.755779614	6.577023122	7.306885459	6.918550858
MB-0465	28.6	1	8.058332496	5.697444253	5.945887839	7.440428996	7.389469634
MB-0466	43.86666667	0	8.888319758	7.305194067	6.280473756	9.518493612	6.921421655
MB-0468	129.6	1	8.200882078	6.829263496	5.950942716	7.991321949	6.916985821
MB-0469	131.2666667	0	8.725354029	6.83344009	5.738578035	7.718525972	6.797204065
MB-0471	107.4666667	0	6.275599262	6.135200133	5.672293413	8.34465482	6.778173441
MB-0472	27.4	0	6.452331058	5.566536486	5.557839827	6.938406732	6.950370785
MB-0474	111.1	1	7.481411914	5.78930336	5.710901261	8.162269451	8.217356113
MB-0475	130.7	0	7.977073163	7.141667625	6.18957255	8.232146097	6.829210829
MB-0480	94.56666667	0	7.779039362	6.28989448	6.39349012	7.385389926	6.092683689
MB-0483	74.46666667	1	8.294451488	7.483144841	5.817373891	8.305282623	7.588711206
MB-0485	36.76666667	1	9.032368972	7.221803103	6.252661394	10.80911604	6.58834698
MB-0486	89.96666667	0	9.075016527	7.214013349	6.08189181	9.551526021	6.938397794
MB-0490	101.9666667	0	8.9603888	5.97109144	6.45071622	7.980880665	6.877893223
MB-0491	125.8	0	8.234691183	5.808406672	5.943344316	8.213512447	6.938683667
MB-0492	81.13333333	1	9.307942383	5.527378406	5.736938553	6.70425592	6.780048636
MB-0497	73.7	0	7.911227353	7.179281943	6.298467201	8.374875168	6.991138066
MB-0501	71.5	0	7.552203351	6.227445856	6.752501815	7.038241437	6.202856493
MB-0503	101.2333333	0	9.148085817	8.140168039	5.647035859	8.998663138	6.36011464
MB-0504	130.4666667	0	8.363245553	7.465758059	6.631173428	8.923012164	6.728367661
MB-0505	60.7	0	9.871772346	7.459308725	7.128182027	9.667045234	6.303235638
MB-0507	23.8	0	8.721614263	8.099197044	6.544794572	9.588569664	6.862612416

MB-0511	61.7	0	10.64408936	6.61180193	6.27089842	9.357761301	7.055210894
MB-0512	121.5333333	0	7.266980275	6.267510903	6.797317398	8.086761837	6.654188508
MB-0513	117.0333333	0	8.022933833	6.266880698	5.989446116	7.037676124	7.021610888
MB-0514	13.4	0	7.572639405	5.766068553	5.981332265	7.42449412	7.242025563
MB-0517	61.9	0	8.659681547	6.303837671	6.31947737	9.054591458	7.001419531
MB-0521	212.2	0	10.09641151	6.545355383	6.666256759	8.913988039	6.927198704
MB-0526	139.6333333	1	8.595992847	5.906804282	5.678367369	7.820392445	7.595662387
MB-0528	193.9666667	0	8.635469136	6.385489247	5.910775068	6.557310331	6.293081551
MB-0529	48.53333333	1	8.4251322	6.307699517	6.224165449	8.319563981	7.218774528
MB-0532	143.1333333	1	7.144539644	5.621855375	5.90962707	8.717190622	6.826825418
MB-0535	199.1333333	0	8.506500992	6.347206342	5.798646798	8.799351052	6.394384141
MB-0536	147.3666667	1	8.846953055	6.409957562	6.837755385	8.915082843	7.907587402
MB-0538	24.1	1	9.540536557	7.346443152	6.226651463	9.362305216	6.604212864
MB-0541	174.2666667	0	7.620508468	6.079799015	6.051624069	8.081581424	6.783693782
MB-0544	79.33333333	1	7.721951992	6.390798335	6.005078708	8.355286466	6.715541651
MB-0550	132.3333333	1	8.360033587	7.928389651	6.017910392	8.297951043	7.443014219
MB-0553	85.73333333	1	7.69628639	6.274585936	5.95409892	7.41661248	6.502339866
MB-0554	111.1666667	1	8.495574115	6.833368816	6.109511759	7.299171481	6.798838254
MB-0559	161.7666667	0	7.064401446	6.087493637	5.978523822	6.881927576	6.574680619
MB-0568	181.4666667	0	8.990819693	8.273525748	6.32687143	10.19933395	6.81032483
MB-0569	59.5	1	7.950171982	7.862632571	6.182625741	8.052571051	7.078293427
MB-0570	272.2	0	6.589023306	5.991254412	5.692709083	6.751920598	7.580753054
MB-0571	149.8666667	0	8.383588137	7.225366258	6.247150844	7.703260524	7.059016488
MB-0574	119.8	0	8.322215878	5.374559464	5.99381447	8.715112403	6.780164238
MB-0575	128.5666667	0	9.041374189	6.38221596	6.179001332	8.943963712	6.64026893
MB-0576	31	1	8.660237736	5.920572427	6.714071571	7.744251841	7.263622525
MB-0577	65.4	0	6.772254641	6.379238967	6.295010689	6.224016586	6.839600538
MB-0579	92.76666667	1	7.734391375	5.738358365	6.001440459	8.716617023	6.222309352
MB-0580	79.36666667	1	8.788379641	6.55946664	6.178373983	7.806949882	7.250240881
MB-0583	62.63333333	0	7.89846852	5.91154426	5.977901822	8.124820019	6.443712468
MB-0584	118.2	0	7.125356284	7.13657401	6.662567946	8.113352852	7.064491854
MB-0585	115.7	1	7.85510703	6.173136208	6.375146092	10.15661082	6.345266106
MB-0586	77.23333333	1	8.332105513	6.802805317	5.935022834	8.962160417	6.342453683
MB-0587	91.26666667	0	7.144407125	5.474090042	6.382908289	7.645410356	7.380108206
MB-0589	125.8	0	8.641286459	6.014263385	5.943004555	10.15740458	6.940746983
MB-0590	72.46666667	1	7.69301156	5.642007104	5.712182889	7.009806668	7.695013324
MB-0591	119.4666667	1	7.063259682	5.926174535	5.997858831	7.862719436	7.5374171
MB-0594	44.23333333	0	7.908046568	6.08639197	5.807063546	8.407728251	6.790409711
MB-0596	107.1	1	8.59367505	6.705374578	6.815556755	8.713604516	6.627114124
MB-0598	89.36666667	1	9.126572145	6.011772555	6.419896407	9.556056921	6.888118855
MB-0599	123.2666667	0	8.215142356	6.708640744	6.802317224	8.951995254	6.016557364
MB-0600	111	0	7.354749485	5.644351416	6.141570813	8.339264446	6.708108429
MB-0601	29.66666667	1	8.567030946	6.483085322	6.053977462	7.372076538	6.956589475
MB-0603	121.9666667	0	8.336343169	5.871738405	6.000347598	7.994222083	6.336588761
MB-0605	114.6	0	9.414591636	7.079739965	6.732990431	8.741202539	6.289765137
MB-0606	27.46666667	1	7.039627408	5.523957049	6.102769673	6.381741704	6.77868869
MB-0607	87.83333333	0	9.657457052	5.868243513	6.515334122	8.737490216	6.847190327
MB-0609	98.7	1	7.596101858	7.173562236	6.098491442	7.575949558	6.571632459
MB-0611	104.5333333	0	8.526767663	5.933182256	6.16836085	8.405290792	6.566676296
MB-0614	119.3333333	0	8.601539115	7.27925896	6.455765253	9.323908368	7.362591439
MB-0616	29.03333333	1	7.832895823	5.980072983	6.147374043	7.581158827	7.445825529
MB-0618	101.9	0	8.095116478	6.374204452	6.235902119	8.722535035	6.99393319
MB-0621	33.56666667	0	10.80700412	6.374026675	6.899211453	9.524179989	6.288390157

MB-0624	64.03333333	0	10.31419538	8.68478286	6.759745699	9.646626702	5.483602018
MB-0626	35.53333333	1	6.917986557	6.218579527	5.808295635	6.773738492	8.05053484
MB-0630	86.8	0	6.933397039	6.65443495	5.486045848	7.019723436	7.343184035
MB-0631	47.43333333	1	9.562849122	8.795071095	6.824191392	8.943168471	6.976115885
MB-0632	56.5	0	10.09082921	6.404238211	7.119196211	8.986466193	6.529144533
MB-0636	32.63333333	1	9.954824001	7.581346138	6.161178875	8.924905659	6.819185206
MB-0637	80.66666667	0	8.880133002	6.731384877	5.914747509	7.961728035	8.271445026
MB-0642	84.2	1	6.324239826	5.549269633	6.070296095	10.30897421	7.089872421
MB-0646	15.16666667	1	7.500314172	6.402653786	6.058954443	8.67400558	6.992242474
MB-0649	61.43333333	0	8.327756139	7.298491064	6.576736309	9.16960705	6.902591207
MB-0650	25.53333333	0	8.977619665	6.297021171	5.965874306	8.304293769	6.966052307
MB-0654	69.4	0	7.303332572	5.80627389	6.175269465	8.297041642	6.711961772
MB-0661	20	1	8.123007748	5.806813725	5.837320476	10.01712354	6.572948887
MB-0666	24.33333333	1	7.577368766	5.784411899	5.825460885	10.66530215	6.726713168
MB-0877	14.43333333	1	6.769603161	5.637797974	5.548749225	8.337309129	8.17018431
MB-0880	1.233333333	0	6.623586988	7.193234682	6.612324736	9.824859934	6.530984706
MB-0882	42.36666667	1	7.465044867	5.944255849	5.9498847	6.892041067	7.867288329
MB-0884	93.66666667	1	6.58843943	6.396907849	5.86990042	6.969718689	7.981700053
MB-0891	149.4	0	9.800324621	7.122870907	6.66412583	10.05551464	6.004499793
MB-0895	43.1	1	7.692022138	6.579300597	7.683682455	7.852723416	7.540718642
MB-0899	175.8	0	8.940805289	6.992492076	6.438421989	9.794006045	7.105652372
MB-0904	144.7	1	8.761132312	6.271845111	6.578596838	9.330264838	6.799275649
MB-2513	59.7	1	8.533401348	8.008674372	6.419743792	9.172377178	9.063781501
MB-2536	47.9	1	8.523309558	8.228940094	7.11697619	9.326973359	6.662306724
MB-2564	285.4333333	0	8.599435966	7.46705661	6.610466063	9.645187708	6.937980292
MB-2610	280.7	0	9.739584437	7.077254926	6.470329698	9.190941031	6.748657183
MB-2613	163.4	0	7.165113347	6.824727339	6.560229933	7.450592947	7.420620945
MB-2614	64.93333333	1	8.625049405	6.998578175	6.191728957	9.477092825	6.765042462
MB-2617	89.1	1	8.145389961	7.278212742	6.287575276	8.328034497	7.266389201
MB-2618	89.1	1	7.49571745	6.555342188	6.305777751	8.399332743	7.627826711
MB-2624	128.5333333	0	9.554707115	7.288898957	7.390277426	9.755985954	6.786853917
MB-2626	193.7	0	9.153355522	6.381694176	6.608389169	9.215702913	6.981045894
MB-2629	279.1	0	7.012824904	7.600320998	5.884270077	7.99355691	7.26705431
MB-2634	274.0333333	0	8.007689953	5.794341743	6.053602694	7.959879373	7.855913572
MB-2642	45.16666667	1	7.776709528	5.780865234	5.960649152	7.2510977	7.437514761
MB-2669	73.7	1	8.57135354	6.215937927	6.823858237	7.662931726	6.750759159
MB-2686	177.2666667	0	7.118060209	6.233659383	6.017449138	7.281107556	7.798250022
MB-2705	163.7333333	0	7.194812469	5.88177462	5.776161858	7.520156524	7.170078055
MB-2708	133.2333333	1	8.466495034	8.167377558	6.278434575	9.045387053	6.982778155
MB-2711	201.4666667	0	8.229274582	6.008399421	6.209012123	9.056679144	6.496718862
MB-2712	234.4333333	1	8.315121435	6.666550521	6.299926481	8.23939792	6.613231505
MB-2721	251.6333333	0	8.827095265	6.285079028	6.164686946	8.823828573	6.757355614
MB-2725	236.9333333	0	10.69984189	8.949068268	6.358016434	9.575944499	6.126940148
MB-2728	51.4	1	7.997643535	6.253708741	6.045807784	7.695178453	6.61184994
MB-2730	133.7333333	0	7.244210927	5.934637386	6.302240092	7.374775871	7.716457299
MB-2735	274.5	0	7.780874194	6.293672856	6.539872681	7.63885988	7.142287477
MB-2745	168.9666667	1	7.82971233	6.845597031	6.021294603	8.498075921	7.539604503
MB-2747	234.3333333	1	6.968981433	6.119775896	6.398891264	8.155927009	6.905304643
MB-2749	97.76666667	1	10.21200144	7.755222627	6.421417106	9.748465694	6.286149419
MB-2750	145.4333333	1	8.77006178	7.79439915	6.708839752	8.456399873	6.867213084
MB-2752	81.93333333	1	8.702612783	6.85386693	6.325256581	8.655335613	6.822906874
MB-2760	118.0333333	1	9.190814085	7.370428532	6.058012952	9.453493293	6.803647929
MB-2763	275.6	0	7.144265486	6.800810729	6.117154404	8.374719352	6.949531306

MB-2767	271.3333333	0	7.875839178	6.165106964	6.428403429	9.313540919	7.343576635
MB-2769	160	0	7.388536223	6.845655123	6.279257158	8.734725251	7.007655535
MB-2770	274.3666667	0	8.421216678	6.031205906	6.462278387	9.325627525	6.309306426
MB-2772	150.7333333	1	9.0930846	6.717063817	6.707158667	9.187087026	7.270862138
MB-2774	131.6666667	1	7.955795325	6.375748614	6.61451489	8.624694238	7.068293393
MB-2779	275.2333333	0	7.685489236	6.130294416	6.242927409	9.57579136	6.765625647
MB-2781	40.43333333	1	8.158652703	7.357437803	5.794503246	9.779154185	7.407219417
MB-2786	68.26666667	1	8.550147774	6.412861319	6.436571484	9.708118714	6.766896813
MB-2790	116.9333333	0	7.43846472	6.256190066	6.501325416	8.205702344	6.99208095
MB-2791	271.9333333	0	9.37966125	6.053535219	6.158745497	8.995972135	6.58024613
MB-2793	267.2333333	0	6.602255366	5.776153624	6.132260138	6.989564029	7.525463167
MB-2797	252.9666667	0	9.187517399	6.640108995	6.322466412	8.480928745	7.431201656
MB-2801	271.2666667	0	9.006487441	6.452374401	6.44647006	9.056127348	6.900941264
MB-2803	85.86666667	1	7.763219526	6.293840408	5.964176517	9.908845181	6.981866693
MB-2814	231.0333333	1	7.400456463	6.215150453	6.376837081	7.779392031	7.185983184
MB-2819	270.5666667	0	8.175639818	6.319111358	6.520384153	8.020744286	7.011227761
MB-2820	254.6333333	0	10.26447498	6.442209234	6.501935413	9.962144001	6.770271299
MB-2835	55.03333333	1	9.689894689	6.122901987	6.595476027	8.084812139	6.816721839
MB-2838	270.3	0	9.019674002	7.029525025	6.504036684	9.069424196	7.501190446
MB-2840	269	0	9.212701441	7.857677649	6.441890726	9.139231693	7.01512731
MB-2843	231.5333333	1	9.400624576	8.513550733	6.550919049	10.04205555	7.171363799
MB-2844	146.7333333	0	7.270305829	6.111487754	6.181497162	8.518057009	7.854927271
MB-2848	227.7333333	1	8.882946859	6.527177628	6.639773831	8.434972356	6.466930582
MB-2851	159	1	9.096458619	6.45247079	6.925102431	9.563848538	6.524651839
MB-2853	269.6333333	0	8.535588025	7.305778339	6.644400117	9.114153832	7.208815097
MB-2854	270.4333333	0	8.781353454	6.768660205	6.414604657	9.310173093	7.122588765
MB-2858	141.5666667	1	7.187162194	5.839652472	6.342544111	7.271850952	7.541837606
MB-2863	258.3333333	0	7.998138651	6.19210043	6.514125122	9.333276327	7.172409626
MB-2867	269.3333333	0	9.922693653	6.956299118	6.705772239	9.769939934	6.862352182
MB-2901	218.3	1	5.904705754	5.914844452	5.787716757	9.468802491	7.913791581
MB-2916	266.9333333	0	9.143341541	6.637991245	6.922629027	9.791135233	6.538950481
MB-2919	240.0333333	1	7.811115882	6.208555836	6.052685412	7.240568122	6.349603279
MB-2927	263.7	1	8.926925061	6.248246714	5.95409892	8.55352477	6.649006652
MB-2931	228.1	1	8.129912796	6.490478806	6.181493311	8.978474431	6.409641962
MB-2932	108.4333333	0	6.776693398	6.657650234	6.124137024	8.480481294	7.25015595
MB-2933	161.9333333	1	7.929186975	6.683639446	6.365651397	9.024083224	6.689509581
MB-2939	101.2666667	1	9.010096909	6.062366753	6.42374203	8.866161813	7.9883148
MB-2944	27.2	1	9.240143877	6.626393137	6.224016586	8.575720674	6.951869614
MB-2947	112	0	8.902358489	8.426585496	6.888359237	10.51173789	6.802086452
MB-2951	263.3666667	0	9.900906524	6.830827654	7.059030553	9.021075648	6.953674115
MB-2952	189.1	1	9.64263221	6.38172796	6.468386493	8.047801613	7.349415848
MB-2953	30.13333333	1	8.500945753	7.104613616	6.285463297	9.204159443	7.436195731
MB-2954	5.5	1	6.815667418	5.824901896	5.695680862	7.765310536	6.733711699
MB-2960	222.2	1	8.31679131	8.36250241	6.362336856	9.165530145	7.08666394
MB-2966	265	0	8.880406302	6.237980893	6.754605588	9.318532173	6.749504427
MB-2969	172.9	1	8.650880715	7.443761992	6.904489044	9.186425548	7.14345137
MB-2970	252.8666667	1	9.435013758	7.081083464	6.462607707	9.227033679	7.096230808
MB-2971	264.2333333	0	8.238076795	8.640943416	6.606713783	8.520175982	6.89942957
MB-2977	110.6	1	8.739774631	7.943268872	6.695648192	9.217963322	6.112972456
MB-2983	180.8333333	1	7.393001177	6.249243068	6.245215488	9.296347192	8.13436631
MB-2984	261.2	0	6.793553128	6.20051947	7.162274215	8.028225712	6.886053536
MB-2990	260	0	8.799351052	6.300510938	6.290975969	9.028648477	6.697663913
MB-2994	254.5	0	10.17825604	7.984651046	6.633318031	9.639565656	7.189670525

MB-2996	191.2333333	0	7.740878978	7.633323252	6.633794993	8.151760515	6.531590013
MB-2999	262	0	8.771100321	5.981550185	6.011427467	9.314972317	7.28298288
MB-3002	140.5666667	1	9.317492433	7.176814219	6.463582271	9.561385989	7.075870551
MB-3005	253.0666667	0	8.11706624	6.155956528	6.193226701	7.742936325	6.414591903
MB-3007	114.9	1	7.412380344	6.117123914	6.514706663	9.684161444	7.133767519
MB-3008	149.6	0	9.724046203	7.393078146	6.576023756	9.233333758	6.240961842
MB-3016	258.8666667	0	8.002093148	6.781713821	6.098386477	8.596623099	7.189483324
MB-3021	217.5666667	1	8.810907283	6.439972542	6.426620114	8.411910617	6.947081726
MB-3026	261.2	0	7.906330512	6.322205545	6.12108583	7.727590871	6.726135879
MB-3028	42.56666667	1	8.137173324	6.264464749	6.306548308	7.839419624	6.961439092
MB-3032	250.1333333	0	9.734671673	7.029490502	6.860565127	9.496700979	6.579424942
MB-3033	70.4	1	8.706782949	7.553067395	6.490260174	8.970638906	6.582775102
MB-3035	260.7333333	0	9.415451172	5.943158958	6.306060839	8.35144919	6.621872314
MB-3037	98.83333333	0	9.214537142	6.810468077	6.197078122	8.486291795	6.517279676
MB-3049	259.9666667	0	10.54914099	6.674445779	6.459280496	9.399233153	6.696438553
MB-3050	255.1	1	7.456728625	5.808737895	5.958272653	7.021257382	6.478849063
MB-3060	44.6	1	8.03289435	5.667418225	6.341177149	6.063472808	7.338105042
MB-3064	165.1666667	1	8.691772501	7.294420575	6.996625223	8.726112712	6.721954319
MB-3079	149.6	0	8.146343573	6.541764279	6.171745645	7.291777643	6.873188122
MB-3083	81.1	1	6.346766874	6.337871942	5.892876982	6.748748724	7.654733032
MB-3088	256.5	0	8.605555942	6.368674283	6.083356822	7.19916364	6.798222405
MB-3092	152.3333333	1	6.786792778	5.783950357	6.19054037	6.883467466	7.160146912
MB-3102	258.3333333	0	6.826597986	5.917902452	6.403173558	8.40768758	7.062278721
MB-3103	28.73333333	1	7.731497471	6.025646776	6.866215785	7.555122265	7.099600773
MB-3104	75.36666667	1	9.12671529	8.492178682	6.125652921	8.455346457	7.009825421
MB-3105	252.3333333	0	7.013007649	5.742319551	6.663138894	7.757443506	6.50566302
MB-3110	100.7333333	0	9.260584537	6.250572156	7.612101197	8.67614504	6.587007196
MB-3121	131.3333333	1	9.128948002	6.643829066	6.452023857	9.183113651	6.944322211
MB-3167	136.9333333	0	7.003370982	5.790881603	6.157198401	6.709406011	7.118796369
MB-3171	219.6666667	1	8.0481714	7.867819272	6.49619743	8.573618268	6.615406397
MB-3181	178.1666667	1	7.670714312	5.603439666	5.709400208	9.67073298	6.797486752
MB-3222	23.93333333	1	8.170356956	6.01734258	6.475135709	8.6038218	6.415061901
MB-3228	252	0	8.23345388	6.926715079	6.659618036	8.550901717	6.492707476
MB-3235	236.1333333	0	8.384283785	6.368563545	6.606797268	8.521366069	7.584127078
MB-3252	99.7	0	9.834633581	6.581723738	6.795099227	9.946123032	6.179304254
MB-3253	55.83333333	1	7.395034346	6.041825885	6.073539459	5.836708679	6.850323746
MB-3254	123.9	0	9.099267683	6.214512539	6.510610886	8.913806396	5.865403555
MB-3266	51.2	1	9.264361322	6.218628076	6.710037819	8.092446677	6.783604629
MB-3272	22.66666667	1	6.803555457	6.030492798	5.866980982	8.286260655	6.838105958
MB-3275	41.53333333	1	9.205583562	6.162054596	6.76600754	8.334012529	6.863474629
MB-3298	242.5666667	0	9.699877623	8.44923276	6.846993963	9.872900303	6.677496098
MB-3300	247	0	7.709951747	6.156604635	5.781251138	8.541268058	7.153672277
MB-3301	248.7666667	0	7.44079759	6.068628882	6.654168271	9.245420097	7.078921273
MB-3303	243.9	0	7.416371688	6.603767421	5.975965496	8.93689147	7.199053064
MB-3328	191.1333333	1	7.854411732	5.896624434	6.458483924	8.846692862	6.442772766
MB-3341	148.7666667	1	7.689679353	6.037380837	6.210414148	8.487677791	7.085365575
MB-3344	241.2666667	0	9.368818137	6.893750401	6.458757599	8.730748033	6.667583385
MB-3350	227.8	1	6.316626267	6.010805372	6.35806709	6.960143173	7.078060577
MB-3351	241.8666667	0	6.102606975	6.071287328	6.182740898	7.43645473	6.861242109
MB-3357	38.03333333	1	7.579473435	5.613798117	6.061344941	7.696682838	8.350463617
MB-3360	26.76666667	1	7.806569194	5.792543559	6.366078348	7.86233739	8.310226111
MB-3361	68.2	1	8.331453859	5.740712172	6.211951586	7.382713635	7.419464752
MB-3365	241.6	0	6.498559901	6.499869071	6.345214475	7.609466298	6.304511233

MB-3371	241.2666667	0	8.336074334	5.709653973	6.065482942	7.915641142	7.112096587
MB-3378	87.53333333	1	8.383138144	7.861027925	6.844066343	9.888260634	6.646373707
MB-3379	113.8333333	1	7.912188477	6.215337174	6.266914495	7.982367987	7.02702009
MB-3381	236.6333333	0	9.998518586	6.457534402	6.812410158	9.518144927	7.004699591
MB-3382	136.2333333	0	8.059489466	5.703640197	6.559350995	8.455943957	7.623923355
MB-3388	228.6	1	6.886830249	6.426739124	6.095365313	7.093162519	7.073961154
MB-3389	240.7	0	6.371519053	6.140081059	6.398980596	6.237122342	8.100142888
MB-3402	50.03333333	1	7.82556222	5.652419751	6.185497349	8.088452386	7.098574043
MB-3403	138.3333333	0	8.235590619	5.959489313	6.43210925	8.12977978	7.056573605
MB-3412	228.8	0	8.597321471	6.155778251	6.515768822	8.345318567	6.513438195
MB-3417	103.4666667	0	6.647893353	5.674901908	5.945448416	7.188037498	7.251443707
MB-3430	219.7666667	1	8.471810631	6.451933781	6.936988994	8.070052913	6.852231634
MB-3436	50.76666667	1	7.056264322	6.363388858	6.241713589	7.657950231	7.174164186
MB-3437	108.7666667	0	8.09099562	7.032356206	6.149394444	6.392785353	6.881758784
MB-3439	188.7333333	0	8.693454238	6.116580298	6.651861027	8.798523869	7.035011872
MB-3450	240.4666667	0	7.961519936	5.941364977	5.528721413	7.31321225	7.209850889
MB-3462	234.2333333	0	9.055021451	7.013721354	7.044858223	8.934785351	6.647736628
MB-3487	237.1333333	0	7.599686267	5.728599922	6.095163191	8.47144018	7.185352987
MB-3490	102.4	1	7.313200164	5.8543322	6.488910612	7.988788685	7.533301059
MB-3492	118.1333333	1	8.498598009	6.306923495	6.007768035	8.340200111	6.618302442
MB-3506	240.8333333	0	7.46778568	6.18264821	6.510628801	7.800652525	7.961488761
MB-3510	59.6	1	8.415202541	5.988587114	6.742395058	9.227404193	7.076068345
MB-3525	236.7	0	9.208017803	7.3781352	6.167221221	8.568509484	7.550012811
MB-3545	232.7333333	1	10.47199434	7.28689716	6.87670572	9.577050176	6.682612513
MB-3548	234.5333333	1	7.063622437	5.912329632	5.90878129	7.798247231	7.727400755
MB-3600	214.7	0	6.015118044	6.149256949	5.606529689	6.507519761	7.747741575
MB-3614	235.4	0	8.293013721	6.033779955	6.82373595	7.517945701	7.132449691
MB-3711	222.1	0	8.082642895	6.051370502	6.557784303	9.135779009	6.963689977
MB-3748	228	0	9.624679387	6.674215738	6.494675864	8.756787008	6.672927375
MB-3754	92.73333333	1	9.423876902	5.845182759	6.855359778	7.880688421	7.156572096
MB-3781	224.5666667	0	7.998709915	7.022601842	6.231914985	8.770976868	6.50609767
MB-3797	228.3333333	0	7.089501061	5.679681743	5.599970266	7.169934084	6.220633402
MB-3824	126.4666667	1	6.835350027	5.853848425	5.762700096	10.06784526	7.443619358
MB-3838	134.4666667	1	6.640336094	6.253633339	6.033344777	7.567399462	6.896001201
MB-3840	287.2333333	0	7.76944926	7.053105121	6.051794885	7.637089558	7.724217532
MB-3842	226.0666667	0	8.035093741	6.244717126	6.360047173	8.0028502	6.702482052
MB-3850	72.3	0	9.388549535	6.418910542	6.901819981	8.644834149	6.347912291
MB-3852	225.5	0	7.649795132	6.150729811	6.564641162	7.554655525	7.163959964
MB-3854	99.23333333	0	10.23314706	8.505391061	6.68899285	9.263341608	6.311032385
MB-3871	172.8	1	10.37184671	9.191798657	6.818169956	9.353568993	7.086525034
MB-3874	186.4333333	0	7.993319612	6.081055954	6.57937261	8.304422349	6.726616739
MB-4000	28.06666667	1	10.24426351	5.666002572	5.646809314	9.109961884	6.677108297
MB-4001	49.66666667	1	7.516538161	5.84661925	5.914557582	9.287225159	7.333887651
MB-4004	256.8666667	1	6.334725521	5.662507149	5.759082607	8.494721346	7.285433133
MB-4008	44.73333333	1	7.89863958	5.620705827	5.991081453	7.640510018	6.624044154
MB-4011	193.1666667	0	7.0602447	5.895761732	6.23817502	7.66873127	7.028805974
MB-4017	139.3	1	6.116461457	5.579942928	5.804489185	7.517284454	8.303232689
MB-4018	105.6666667	1	9.1265386	5.971118289	6.321653176	9.863306106	6.865298403
MB-4033	15.2	1	8.742547976	6.540753659	6.706086666	9.055021451	7.114472862
MB-4091	281.3666667	1	8.090720617	6.075096889	5.948956719	12.43176215	6.541731463
MB-4120	217.1	1	6.850384471	5.525329757	5.659853926	8.240956077	7.169214841
MB-4139	165.4	1	6.525926857	5.684313604	5.961512568	6.968467798	7.626250684
MB-4140	114.5	1	6.302424694	6.034888656	6.657076552	8.289997116	6.7579513

MB-4148	63.03333333	1	6.974613644	5.843731573	6.124031087	8.7481865	7.490040581
MB-4154	49.56666667	1	7.126261923	5.993311894	6.980776702	6.910363611	7.173643106
MB-4173	160.3333333	1	7.069886722	6.180866478	6.100952318	8.180887168	6.886665251
MB-4212	330.3666667	0	8.9603888	5.904036931	6.728138905	8.170096297	6.174837178
MB-4213	84.5	1	9.980146695	6.114608149	6.65840572	6.957803162	6.469452543
MB-4230	115.6	1	8.205058512	6.172758327	6.433918704	8.277839971	6.807829628
MB-4233	36.36666667	1	7.363048687	5.709530856	6.044990146	6.943275106	7.917018195
MB-4234	253.5333333	1	7.79315526	6.036268027	6.177820113	8.96664425	7.115375239
MB-4250	100.1666667	1	8.492129007	6.216523442	6.496992784	8.334078787	6.510992004
MB-4264	163.2	1	8.723850179	5.754847582	6.37918506	8.114907201	7.586866566
MB-4266	33.9	1	7.50767692	6.611024084	6.572897548	10.90684245	7.504543348
MB-4274	59.93333333	1	7.152758574	5.75337489	6.226226578	8.143224879	6.859327264
MB-4282	34.1	0	8.080483019	6.024589527	6.048744593	8.021608457	6.715353858
MB-4283	174.8333333	0	8.494219048	6.162103723	7.086601912	7.256554671	6.696117175
MB-4292	335.6	1	7.023318138	5.845185604	5.533670048	7.218353013	6.86279388
MB-4293	219.1666667	1	7.760153726	6.145252561	6.975089692	7.957782378	6.707443311
MB-4306	234.7	1	6.059898434	5.669762757	5.840405437	6.910196229	6.689071254
MB-4310	265.5666667	1	6.098441321	5.507474196	5.598751166	6.606988948	7.39643042
MB-4313	300.7	1	7.777717273	6.470280002	5.953109324	8.867903822	6.284979438
MB-4318	17.13333333	1	6.737636627	6.132688305	6.261344764	7.49709247	8.014970577
MB-4322	196.4666667	1	6.817273596	5.667592448	6.448853562	8.007910818	6.827322191
MB-4323	126.6333333	1	6.735786644	5.955040452	6.016201409	8.411928298	7.151477713
MB-4328	199.2666667	1	8.883161341	5.836638867	6.015980472	9.550833718	6.524708741
MB-4329	168.2666667	1	8.078230098	6.34270005	6.552439326	10.80238606	7.175243457
MB-4333	207.9666667	1	9.608338027	6.522273554	6.40581877	9.174662208	6.190818642
MB-4339	173.6	1	8.081641374	5.662019133	6.107542653	6.873077309	7.176479245
MB-4342	232.7666667	1	8.872098022	6.072780366	6.623707598	8.31574731	6.37817782
MB-4353	128.5333333	1	7.973298926	6.269208659	6.085763403	7.99407682	8.179281979
MB-4357	114.0333333	1	7.194661519	6.562873258	6.786763434	6.900750074	7.20246889
MB-4368	119.4666667	1	7.172850115	5.924395931	6.550115558	8.084512399	6.972731461
MB-4374	42.56666667	1	7.253072067	5.714745004	5.585173079	7.259564011	7.079735053
MB-4390	164.3333333	1	7.230296683	5.893006907	6.332274875	7.676130921	6.706983877
MB-4418	307.6333333	0	6.873466347	5.89643423	5.525184131	6.90514988	7.538173256
MB-4421	264.7666667	1	6.6530457	5.633527904	5.78821041	6.986240524	7.532699787
MB-4442	296.8666667	0	7.714035668	6.174446812	5.829142381	8.576447998	7.897587149
MB-4529	126.4666667	1	9.55729866	5.746937082	6.449166931	8.261616185	6.142929978
MB-4557	279.8	0	8.064763694	6.425207953	6.40581877	8.181260244	7.053268416
MB-4564	210.4333333	1	7.427494191	5.796899805	6.119850646	7.137195754	7.039609931
MB-4578	197.3333333	1	7.146051986	5.662049835	6.159734812	7.316661186	7.144360021
MB-4591	41.16666667	1	7.014852729	5.7636977	6.186549726	6.583190548	7.727923204
MB-4593	140.2333333	0	8.528205088	6.444193818	6.290403452	8.282992264	7.130363863
MB-4598	119.3666667	1	6.578428051	5.657935857	5.978760773	7.878062666	7.380130775
MB-4599	191.1666667	0	6.382487088	5.661687887	5.761831836	8.077032433	7.56170376
MB-4602	122.8	1	6.540015089	5.821399246	5.688080516	6.952951743	8.321434764
MB-4607	121.7333333	0	8.15897502	6.286535321	6.451043234	8.572178453	7.293170482
MB-4616	90.13333333	1	6.472714638	5.981690857	6.43606813	8.744415682	6.90017101
MB-4618	267.4	0	7.158754231	5.758041033	8.42529374	9.780613411	6.966399683
MB-4623	292.0333333	1	7.789820449	6.116630419	6.53248081	8.940804482	7.757431049
MB-4627	186.6333333	1	8.040481266	5.549878144	6.244042201	8.180217043	7.122237788
MB-4630	95.83333333	1	6.276062175	6.189103139	5.750092467	7.244420616	8.216159253
MB-4633	318.2	0	9.371665435	6.099991208	6.40496603	8.838085363	6.624555362
MB-4634	109.6	1	8.2034653	6.223250789	6.396526332	7.729709648	7.459178585
MB-4639	119	1	7.467701873	5.910178996	5.81109497	8.039461235	6.731350077

MB-4641	148.5666667	1	8.997106247	5.959715807	6.259860808	9.229696073	6.33873425
MB-4642	285.7	1	7.510474667	5.677385852	6.210340503	8.85427694	7.154853926
MB-4644	90	1	7.135426579	5.671139405	6.096104717	7.010729293	7.921816344
MB-4648	168.5333333	1	6.740400902	6.765733612	6.466653884	8.904396579	7.818657984
MB-4649	16.83333333	1	7.39729588	5.899244925	6.177916883	7.441211792	7.427303583
MB-4651	30.3	1	7.475187959	6.046956553	6.092409998	7.283064159	7.539327884
MB-4654	64.3	1	8.69224227	5.813230488	5.896843776	8.680809165	6.981454121
MB-4655	99	1	7.772576462	6.206750714	6.442202788	8.534286551	7.415359936
MB-4661	234.2333333	0	7.760484267	6.499413858	6.218654915	7.963570266	7.115179429
MB-4665	260.2	0	6.433820263	6.022173614	6.199647618	8.794770643	6.82370372
MB-4666	111.5666667	1	8.776640479	5.976757792	6.296766674	9.140528734	7.211605854
MB-4669	119.4666667	1	6.846969846	6.07129715	6.289808421	8.164240629	7.221086608
MB-4670	67.8	1	6.85150376	5.667948172	6.417232358	6.563884585	6.862405735
MB-4671	34.7	1	7.837750669	5.625567798	6.094347857	9.780613411	7.362875766
MB-4672	220.9333333	1	6.945432315	5.849544215	5.737910101	7.610898038	6.911390427
MB-4673	87	1	7.413039025	6.111965264	6.448189408	7.704173281	6.925023602
MB-4674	81.06666667	1	6.049912089	5.466453015	6.068828346	6.883729029	6.702331172
MB-4675	263.0333333	0	8.015040424	6.249692333	5.873906467	6.877660287	6.884673895
MB-4681	251.8	1	8.78413646	5.960449967	6.738368933	7.785485576	6.975651059
MB-4682	139.5333333	1	7.000665182	5.601523302	6.048343256	6.576648902	7.160246471
MB-4685	43.3	1	9.63050505	5.735219431	5.586804086	8.323101989	7.287766128
MB-4686	48.43333333	1	8.826067111	5.982458589	6.76808957	9.350694454	6.810810225
MB-4687	74.1	1	7.974622482	7.311095517	6.90957431	7.55108639	7.248832099
MB-4688	85.3	1	6.493097936	6.52053907	6.237298114	6.838990501	7.728228396
MB-4691	282.5666667	1	6.994129096	5.47690719	6.603896143	7.554835464	7.089829178
MB-4692	107.2666667	1	6.97121946	5.899204767	5.661677983	8.750435903	7.145879862
MB-4698	170.6666667	1	9.530572219	6.08568314	6.686836177	8.70640653	6.511476746
MB-4701	127.5333333	0	7.897370355	5.790556078	6.153124651	8.240629155	6.783180728
MB-4702	300.8666667	0	6.661447185	6.714327677	5.948303375	6.943003457	7.876110179
MB-4704	64	1	9.341120624	6.472748636	6.809127963	8.868116248	6.719554469
MB-4705	213.1	1	10.64428604	6.031517938	6.284074367	8.98472024	6.357762988
MB-4706	55.66666667	1	7.637726644	5.964792645	6.23422482	8.097397602	6.531167058
MB-4708	227.9	1	9.152471225	6.247501502	6.511534613	8.693305965	6.981318891
MB-4709	146.7666667	0	8.714236798	6.306787913	6.354876655	8.69555707	6.485156993
MB-4710	57.66666667	0	9.6312386	6.205916394	6.568662057	8.654560617	6.087125106
MB-4716	76.13333333	1	8.746239208	6.302208785	6.617287194	8.905928904	6.95858205
MB-4719	81.56666667	1	7.335742992	5.789113566	6.254968879	7.031736927	6.671576821
MB-4721	23.73333333	1	8.092281506	5.859320682	6.234752316	8.26181984	6.708771928
MB-4722	86.1	0	7.388783331	6.170386609	5.818378383	9.133538848	6.702124013
MB-4723	211.9666667	0	7.813245122	6.934297682	6.060164222	7.784617541	7.180734833
MB-4729	28	1	8.202986227	6.222414418	6.099998277	8.184204545	7.706983923
MB-4730	131.3	1	7.625498333	5.653256578	6.03602729	7.080161407	6.626997309
MB-4735	297.8	0	9.882704885	6.326024435	6.092340638	7.602845605	6.060165296
MB-4737	122.2	0	6.779752175	5.755506728	6.276554735	7.488191864	7.293897197
MB-4739	161.6666667	1	7.864129026	5.89208193	6.266442585	7.585663774	6.694217345
MB-4741	56.5	1	7.846298851	6.123045592	6.096540458	9.777545665	7.167478795
MB-4742	83.13333333	1	8.413888917	5.568713978	6.321002209	8.664306956	6.714736204
MB-4744	153	1	6.648579255	5.840609733	5.931377374	7.649721941	6.887694581
MB-4749	51.46666667	1	9.102985949	5.766364196	6.007194902	8.618068326	7.132456906
MB-4750	37.73333333	1	8.861143767	5.805901093	6.15689475	8.672517312	7.495967503
MB-4752	125.2666667	1	7.719079754	5.656740556	6.336930928	7.013634483	6.338549266
MB-4760	124.8	1	9.460065155	6.154729294	6.712561787	8.375248724	6.908268214
MB-4762	252.1	0	8.034219797	5.763426116	6.66548179	7.778987157	6.745973652

MB-4764	245.5	0	8.880146422	5.932651455	6.75014238	8.403517804	6.798945774
MB-4767	47.13333333	1	8.249879106	5.527475363	6.368531803	7.199842804	7.035074149
MB-4771	297.2333333	0	8.062032615	5.794119921	6.216038324	8.347588269	6.446751334
MB-4778	206.1333333	1	8.576998523	6.73257973	6.695700753	9.526624364	6.631773641
MB-4779	101.5666667	0	8.356126882	6.7412209	7.052937639	9.091237448	6.621257814
MB-4785	121.6666667	1	7.572270689	6.39313948	6.398864253	7.095688913	7.456528964
MB-4787	81.06666667	1	9.165285155	5.867075155	6.062352897	9.139956953	7.197825734
MB-4790	70.46666667	1	9.13429034	6.267511533	6.556358937	8.956520348	7.288759945
MB-4791	173.8333333	1	6.983061548	6.040945082	6.476458396	6.685794804	7.167207302
MB-4794	45.5	1	7.391050158	5.876086605	6.104305618	9.04460851	7.292040414
MB-4796	128.2666667	0	6.961487873	5.413480763	5.68297143	7.819865998	7.524682861
MB-4797	99.36666667	1	8.741470322	6.576987996	6.229364075	8.693275343	7.408408635
MB-4800	110.9333333	1	7.264924388	6.02221829	6.307521103	7.426480005	6.72485013
MB-4801	130.3666667	1	7.358311932	6.109597706	6.133191223	7.183819021	7.278678378
MB-4802	43.9	1	7.148995528	5.488534233	5.668292703	6.54336724	7.395910723
MB-4805	129.8	1	10.65631663	6.034013974	6.52291539	8.625026967	6.48342825
MB-4806	201.7666667	1	7.936136613	6.009777283	6.516233574	8.279602049	7.160305196
MB-4818	76.23333333	1	8.835828162	5.603168314	6.445053114	6.651256179	7.178340443
MB-4820	57.3	1	6.212619659	5.979548564	6.272046871	6.223733439	6.368724393
MB-4822	91.63333333	1	7.84805618	5.873073697	6.135734868	8.891117393	6.747631091
MB-4825	214.4333333	1	9.234798485	5.652524833	5.944364932	7.064694964	7.047889219
MB-4827	45.7	1	8.221729548	6.061519562	6.439158136	7.978853993	6.461583376
MB-4828	42.5	1	6.802958566	5.658057068	7.282206423	7.696757347	7.799272659
MB-4829	176.2666667	1	9.967590094	6.113199174	6.001410352	8.917449862	7.18911832
MB-4832	141.7333333	1	9.248298031	5.824452907	6.373405515	7.554094974	6.727004954
MB-4834	36.16666667	1	7.063447166	5.629674989	5.975851601	7.051503175	8.047633134
MB-4838	200.7666667	1	6.157408595	5.802489638	5.57619354	9.239081033	7.982452254
MB-4839	299.4	0	9.375749391	5.961687353	6.47002043	7.771038433	6.979243526
MB-4843	265.1333333	0	7.810600372	6.223191676	6.638296061	7.743962042	6.934482344
MB-4845	25.46666667	1	7.474525466	6.738053415	6.012681465	10.5010092	6.210715491
MB-4846	101.6333333	1	7.294387046	5.905378994	6.077364915	7.732430293	7.761770364
MB-4849	45.33333333	1	6.955514761	5.632575565	5.753750295	6.970588882	7.714638839
MB-4851	90.26666667	1	7.617112259	5.69497913	5.8239202	8.209669441	7.243889604
MB-4853	265.9333333	0	9.963947749	5.994501714	6.204716172	8.848757964	6.919474397
MB-4855	41.46666667	1	7.568581328	5.897115402	5.660869334	11.62335573	6.558313202
MB-4858	129.3333333	1	8.24226908	5.695637252	5.803065423	7.754435164	7.464870354
MB-4860	234.6	0	8.716163319	5.766475853	6.103069123	8.813914785	7.541027667
MB-4862	187.3	0	7.992935049	5.650912714	6.489791159	7.669535269	7.609250358
MB-4866	224.3	0	8.562656172	6.25349742	6.450732529	8.112530197	7.194868083
MB-4867	221.9	0	8.637394202	6.219930164	6.526045599	7.417396787	7.152546998
MB-4869	29.3	1	7.41385197	5.942729002	6.143909985	8.514984926	6.699224579
MB-4870	229.0666667	0	9.940854181	5.901128312	6.415230913	8.853636976	6.432143767
MB-4872	157.1	0	9.76260695	6.040026273	6.485076547	8.699971761	6.857142442
MB-4873	117.6666667	1	8.31574731	6.121316803	5.875080384	8.248024388	7.174185026
MB-4878	22.46666667	1	10.15692524	8.153346849	6.599712871	8.556815928	7.115481624
MB-4882	266.1	1	8.181807205	6.450025778	6.815007366	7.095661185	6.347640411
MB-4883	268.9	0	8.589374229	6.285395637	7.06655477	8.124179226	6.895064506
MB-4894	232.4	0	9.272135177	5.85950147	6.098144125	8.430214627	7.87243469
MB-4898	117.9	1	7.135266546	5.96800202	5.675065274	7.972479656	7.344133097
MB-4899	275.7333333	0	8.898724702	6.225405091	6.149394444	8.495019843	6.881022281
MB-4900	224.6	0	8.953149524	5.810250874	7.129798804	8.007644844	9.19783304
MB-4906	233.8666667	1	8.140943726	5.905344742	6.178772166	6.689930313	6.775681671
MB-4908	47.9	1	7.650233612	5.409147304	5.977861425	8.191703047	7.512584289

MB-4912	50	1	7.590486886	5.774372517	5.993961724	7.129939049	7.823876741
MB-4925	102.7666667	1	9.154340403	5.980046397	6.373393153	8.889111704	6.791881373
MB-4930	58.43333333	0	6.878880684	5.628031253	6.047058038	7.690572068	8.160144418
MB-4933	272.9	0	7.566964594	6.001954227	6.475829086	7.039340328	7.821956048
MB-4934	70.23333333	0	7.709043906	5.656653306	6.026768686	8.125987897	7.343232812
MB-4937	77.23333333	1	7.992690441	5.907965088	6.548916366	6.693451456	8.778683699
MB-4941	58.63333333	1	9.487120656	7.234370698	6.654238083	8.564309118	6.608135001
MB-4944	237.2666667	1	6.066283784	5.709289255	5.902178316	7.348792356	7.546340568
MB-4949	216.9666667	0	6.795482292	5.897678497	6.723780456	7.875951999	7.109345154
MB-4950	150.6	1	7.848133682	5.875871075	6.158232101	7.789677792	6.530072206
MB-4956	69.3	1	8.273251487	5.698266215	6.067119461	7.660871237	6.840439532
MB-4957	197.6666667	1	6.485806648	5.943187953	5.840560553	6.985646539	7.642552247
MB-4959	117.5666667	1	8.500974633	5.98085528	6.627263625	9.002415432	6.750626724
MB-4961	219.4666667	0	7.865030779	5.967965272	6.234872081	7.489130103	7.300864082
MB-4962	197.8333333	1	7.14784719	5.642010401	5.926202983	8.222715441	7.182783194
MB-4965	263.6	1	8.074471279	5.993303991	6.020517326	7.599882718	7.252530211
MB-4966	49.3	1	7.780206308	6.378844474	6.406651233	8.478275886	7.032404385
MB-4967	130.7	1	9.511604293	6.177540552	6.503736725	9.022647572	6.537993717
MB-4968	78.7	0	8.015782995	7.232454281	6.155870864	8.387026671	7.303272233
MB-4969	198.1	1	6.774477232	5.980859172	5.419229896	7.163389848	7.068084497
MB-4970	88.8	1	7.371960768	5.69029813	5.513668029	6.547300533	7.890193612
MB-4976	224.1	1	7.348607027	6.477192349	6.256870762	8.653760831	6.248259653
MB-4977	216.7333333	1	8.127816408	5.993728497	6.060425686	9.861339515	6.764545465
MB-4981	180.7666667	1	8.246699125	8.357902833	6.521283342	8.915994081	5.83990174
MB-4986	79.8	1	8.999168469	6.860726729	6.568593126	8.21292423	7.339435922
MB-4987	221.7666667	1	11.17538031	5.872329675	6.21693143	10.56087924	6.508122118
MB-4991	126.8666667	0	8.029303798	6.088440911	6.383522771	8.507727434	8.017836361
MB-4992	165.3666667	0	7.791258025	7.5836673	6.238961982	8.006015949	7.087157033
MB-4994	174.1333333	1	7.369485885	6.0053109	5.991337493	7.789155432	7.685408647
MB-4996	99.76666667	1	9.527294322	6.128298529	6.009201263	8.539422265	6.46664161
MB-4998	65.56666667	1	8.116150195	6.761299216	5.929568879	10.11888514	7.547632554
MB-4999	187.9333333	0	9.73565871	6.461495101	6.631911909	8.10794285	6.690813911
MB-5001	81.8	1	8.169581516	5.88559046	5.972709278	8.286840777	6.828390846
MB-5011	80.5	1	7.637979473	6.133330196	6.34304379	9.976275649	6.375477258
MB-5013	251.2	1	7.745291776	6.479382154	5.924742645	9.621067905	6.758751621
MB-5014	213.3666667	0	7.086596058	5.631758932	6.511610852	7.466811186	7.970481159
MB-5015	252.3	1	7.465367506	5.365146068	6.018963327	7.231233333	7.050566087
MB-5018	108.6	1	8.012902984	6.792277206	6.165985683	8.633301205	7.628450383
MB-5020	84.83333333	1	7.771826301	6.228412983	6.191988235	7.6424744	6.808954726
MB-5027	189.8666667	0	8.647519285	6.735712466	6.672934649	8.400486353	6.786984854
MB-5033	52.73333333	1	6.74019055	5.670073606	5.646966392	8.826513799	7.070917821
MB-5035	213.2	1	9.18702101	6.034260111	6.34304379	7.648153556	7.498901282
MB-5039	62.13333333	1	6.90668033	5.899740083	6.011817577	9.897776948	7.10701864
MB-5040	79.16666667	1	7.235751702	5.768459937	6.027853058	7.736106674	8.122295601
MB-5043	128.3666667	1	7.379783527	5.768929931	6.051807571	8.266322113	6.723463866
MB-5044	139.4333333	0	7.331423477	5.709956104	5.886640819	6.947715747	7.002119077
MB-5045	168.3	1	7.373859961	5.903053936	5.864581713	10.32239154	7.745072815
MB-5048	86.36666667	1	7.871718542	6.169306488	6.389426081	9.091550293	7.717503566
MB-5049	255.3	0	7.329126514	5.782705207	6.272631109	7.296745028	7.161001663
MB-5050	184.3333333	0	8.281297663	5.91409801	5.992645754	9.287193156	6.569307754
MB-5053	150.6	1	7.365333661	6.429547941	6.340613355	8.678959579	6.780635184
MB-5059	257.6333333	0	8.475685834	6.30103961	6.636805368	7.782289232	6.518108253
MB-5060	150.1	1	7.429956459	5.760256756	6.134613121	7.702909504	7.25813146

MB-5061	39.43333333	0	6.612101588	5.967964204	6.813461055	8.000863775	7.206679499
MB-5062	168.2666667	0	7.091247412	6.005844044	5.994875038	8.492466921	8.133140893
MB-5064	91.5	0	9.645175675	6.455557219	7.35922713	7.325133702	6.647285304
MB-5066	94.7	0	7.967161082	6.206157501	6.24703812	7.855960455	6.550524334
MB-5068	182.5	0	9.499433752	5.994554864	5.806496224	6.759525123	6.303319748
MB-5074	221.6	1	7.398501161	6.003527444	6.088354791	8.470735585	7.430270603
MB-5078	87.1	1	7.166067897	5.856604491	5.643246978	7.428274128	7.678533289
MB-5079	199.9333333	0	9.119662348	6.00557787	5.684594723	9.199190352	6.862122482
MB-5084	198.1	1	9.247448357	7.240307204	6.215128458	9.194391414	6.614341433
MB-5086	84.23333333	1	7.324630202	5.675316211	5.567565976	7.001216942	6.757445199
MB-5088	199.3666667	1	6.944983596	5.922658272	6.456187443	7.528716037	6.960167364
MB-5092	174.5666667	1	10.33220106	6.817675772	6.79767224	9.901092263	6.009213887
MB-5097	61.6	0	7.692036435	6.331153721	6.78319072	7.919037323	7.41828983
MB-5098	186.4	1	8.609260322	6.209682754	6.112901392	8.045019301	7.061884308
MB-5101	34.33333333	1	7.410744602	5.839519713	6.121466417	7.283064159	7.203426326
MB-5105	204.4333333	1	9.304679572	6.112663323	5.969769362	9.408490879	7.212268096
MB-5107	82.63333333	1	6.552316003	5.867357682	5.410456542	7.456958589	7.65245459
MB-5116	71.76666667	1	6.618761568	5.681565436	5.876347449	8.609263939	7.444561984
MB-5117	209.2666667	0	8.159271423	6.082955052	6.178859689	8.608128294	7.347252518
MB-5118	211.7333333	0	8.279275344	6.575327761	6.286794721	8.548688588	7.034561735
MB-5119	59.76666667	0	8.45803716	6.228257059	6.173751772	9.101402294	6.423007887
MB-5121	114.9	1	7.063635809	5.602192389	5.79762447	7.605934663	7.392625251
MB-5122	125.7	1	7.74945798	6.920726599	6.169931122	9.172639839	6.654214471
MB-5123	90.3	1	7.873165293	6.167637887	6.625122729	8.182519832	6.608137628
MB-5124	124.2	1	7.639799874	5.865721049	6.317717247	8.058561046	7.094764653
MB-5127	191.4666667	1	6.963753737	6.181531948	5.693339154	7.481834596	7.132069843
MB-5130	255	1	8.512796951	5.801797217	6.243364202	8.200882078	6.929187044
MB-5134	196.5333333	0	7.99355691	5.863443733	6.670576869	7.517012576	6.001610243
MB-5139	89.96666667	1	7.071574782	6.060908146	6.366566624	7.841086496	7.14348985
MB-5143	127.9333333	1	9.08504924	6.496025358	6.160920657	9.334950483	6.830373496
MB-5144	213.0333333	0	8.222278197	5.933799742	5.937966592	7.51447265	7.876150007
MB-5147	20.26666667	0	7.147046418	5.903684377	6.683097643	7.33923749	6.263993956
MB-5152	115.9333333	1	6.42315414	5.868809052	6.482843826	7.504866524	6.836221933
MB-5160	87.73333333	1	6.790886646	6.083538643	6.048230104	6.868316478	7.503017337
MB-5163	212.2	1	7.058756643	6.057976324	6.825804046	7.760267748	7.109756674
MB-5166	80.73333333	1	7.705162487	5.845529519	6.149878782	6.881268806	8.409016078
MB-5167	208.4	0	6.764797295	5.756546876	6.172299339	7.442612768	7.49631402
MB-5169	51.96666667	1	8.14230818	6.868186983	6.234550312	7.169117666	7.390895963
MB-5179	246.6	1	9.495896511	5.944927124	5.929198788	9.322548514	7.038992056
MB-5182	134.2666667	1	8.225325552	6.123447961	6.268867675	7.966935551	6.841108353
MB-5183	118.5333333	1	8.506983573	8.290235632	6.21002969	8.390017977	6.895466346
MB-5184	89.33333333	1	9.344090435	7.528880205	7.160682767	9.75568975	6.484514285
MB-5185	77.46666667	1	8.020727095	6.43331635	6.876440649	6.635044188	7.325068654
MB-5186	203.2666667	0	8.766414905	6.015810642	6.215872234	8.36423566	6.452088212
MB-5189	111.9666667	1	9.075908956	6.043687407	5.94683011	10.73396537	6.793124483
MB-5191	142.1666667	1	9.256919587	7.621577374	6.760755153	9.048328265	6.887386806
MB-5193	54.26666667	1	6.707410044	6.490191778	6.097305361	8.987699684	7.172211859
MB-5195	196.8666667	1	7.694911646	6.125603301	6.296926653	7.90715725	7.031652104
MB-5196	94.03333333	1	8.304695055	6.21310075	5.99508195	8.043711205	7.385830858
MB-5197	223.8333333	1	7.940339388	6.279411566	6.477745663	9.116061139	6.938667608
MB-5200	52.3	1	7.288205724	5.773549025	5.946736791	7.189125907	8.135758967
MB-5201	96.96666667	1	8.124106828	5.948475445	5.602931739	9.645101786	6.967477328
MB-5204	191.9333333	1	8.394756916	6.334557621	6.06907192	9.559905947	6.711734418

MB-5206	205.7333333	0	8.419646735	5.97431995	6.353142525	7.380428337	6.999080104
MB-5211	247.8333333	1	6.875417055	5.750830384	5.902371334	9.391326502	7.280394427
MB-5212	179.1	1	6.306241917	5.992277152	6.023654882	10.83178876	7.259406905
MB-5215	118.7	1	7.211622677	5.788508092	5.537704471	10.20872052	6.701793294
MB-5218	254.9666667	0	7.175592216	5.853636351	5.947019969	9.009346124	6.831353288
MB-5221	131.3	1	6.997338792	5.809671367	6.384406895	7.225563158	6.908338515
MB-5224	99.4	1	9.019857811	5.784469986	6.265299814	8.367975445	6.978247683
MB-5226	200.6	0	8.358058431	5.607311925	6.488618471	9.700340793	7.219494922
MB-5227	187.0333333	1	8.603796369	5.767509622	6.216869896	8.833165334	7.14915216
MB-5228	180.7333333	1	7.862956625	6.077595021	5.888869265	7.608069862	7.556779491
MB-5230	176.5	0	9.092251608	6.136438857	6.914996051	8.445375291	6.710787066
MB-5233	209.0333333	1	5.873564337	5.761832196	5.691336501	7.590039068	7.874232768
MB-5239	192.1333333	0	8.329625317	5.954579923	5.860474281	9.518144927	7.367591713
MB-5240	103.8333333	1	9.379370805	7.4995231	6.322466412	8.793523456	6.218348467
MB-5243	38.43333333	1	7.790125131	5.915120775	6.189995492	9.365072636	7.356657584
MB-5244	216.8666667	1	6.224943947	5.565495268	6.155351241	7.663791355	7.929956804
MB-5251	112.4666667	1	8.67400558	6.049353664	6.319536272	8.498956482	7.023131739
MB-5253	102.0666667	1	8.789942244	7.312571754	6.571940857	9.230711346	7.039751229
MB-5256	108.0666667	1	8.907494197	6.472690411	6.155620528	8.299387472	7.138609107
MB-5260	202.1	1	8.542217949	6.500743229	5.959620154	8.737491954	7.728907663
MB-5261	116.4666667	1	7.373434202	5.986947792	6.076105258	9.031667145	7.69385676
MB-5264	199.3	0	8.753934042	6.732424012	6.531193215	8.325776827	6.538874288
MB-5266	83.63333333	1	6.373903147	5.761961992	6.77843974	6.841242931	7.287285037
MB-5267	71.06666667	1	9.717078971	6.579385089	6.659618036	8.238314	6.812542455
MB-5268	186.1333333	0	9.122644962	6.116132703	6.511394199	9.529510438	6.167887172
MB-5270	187.8333333	1	7.461021398	6.035718639	6.058193277	7.121769909	7.600873744
MB-5271	167.5	0	6.085515979	6.090001159	6.070704072	5.846569834	7.257171724
MB-5273	60.86666667	1	7.201545166	6.040786931	6.789953202	6.909144264	7.605934395
MB-5279	68.76666667	1	7.019785971	5.90246024	5.814598305	6.494263352	7.170464807
MB-5281	79.3	0	9.450974737	5.78127193	6.544553838	9.954430982	6.770374949
MB-5284	65.33333333	1	9.160360704	6.442369728	6.464200556	8.725414884	6.64023595
MB-5287	124	1	8.883546926	6.617438644	5.928251776	8.777708979	7.048842833
MB-5288	128.4	1	6.636447415	5.588276898	6.623311443	6.091577156	7.61077099
MB-5290	36.93333333	1	7.392302826	5.607052837	5.808643562	6.737317834	6.70871566
MB-5291	153.5333333	0	6.56419933	6.310828324	6.069505357	7.019477937	7.226102766
MB-5292	48.43333333	1	7.603180981	5.719220335	6.326735313	9.408359742	6.827166609
MB-5293	208.9666667	1	8.097404941	5.775307962	6.627005931	6.838391111	7.08452764
MB-5300	190.1666667	0	8.33472274	5.941577159	6.46043887	8.891555808	7.201089592
MB-5305	149.4333333	1	7.389838785	5.870592365	5.858131171	7.360239744	7.234365616
MB-5306	159.7333333	0	6.930074031	6.037839808	6.172779392	7.335160046	7.513751944
MB-5310	153.3	1	7.959345773	7.228022806	6.181063297	10.77618699	6.580117018
MB-5317	53.63333333	1	7.582291016	5.968234344	6.453631444	7.648269233	7.037377649
MB-5318	14.16666667	1	10.21354465	6.30335726	6.056712306	10.02359342	7.566459544
MB-5322	102.3	1	8.857218231	5.802661658	5.986426157	8.679003559	7.084473035
MB-5324	190.1	1	9.329678698	6.314338894	6.525590624	8.367509019	6.900011198
MB-5328	14.2	1	6.895941582	6.096686923	6.461207397	8.220224754	6.695163861
MB-5329	45.73333333	1	9.230882464	6.183367121	6.231160244	8.59692051	7.090144941
MB-5330	114.5333333	1	7.66984998	5.920732045	5.999286704	7.533274852	7.064116786
MB-5331	124.1333333	1	9.012815285	5.927894017	6.175814462	8.368132947	7.186395654
MB-5334	85.4	1	7.842689676	6.680132016	6.092912923	7.671719158	6.949374996
MB-5338	51.66666667	1	7.538949649	6.171955794	6.311796437	7.437859245	7.972715042
MB-5341	250.8333333	0	7.212487911	6.560038462	5.902908353	7.100649207	7.162342381
MB-5347	211.9	1	8.470420836	5.967911295	5.993647953	6.843595409	7.000842213

MB-5358	28.83333333	1	8.830101391	6.70010222	6.350663618	8.318039957	6.879514313
MB-5360	161.1333333	1	6.560233054	5.706760087	6.433918704	6.973408237	6.756210644
MB-5361	15.36666667	1	7.197842115	6.162543519	6.060762895	9.604677261	7.349205786
MB-5365	23.03333333	1	8.004464484	6.279461766	6.154197907	8.089710988	6.983021337
MB-5369	39.3	1	6.875415037	5.590819121	6.231265866	6.759120466	6.632999917
MB-5370	119.3	1	6.176505498	5.825577286	6.206992471	6.245110193	7.147455922
MB-5373	2.533333333	0	7.664365391	5.988062484	6.328411976	8.763122875	6.657582705
MB-5377	102	1	7.544450164	5.9350513	6.123207032	7.507849906	6.895666099
MB-5382	116.2333333	1	7.003311262	5.910962086	6.240543695	8.89416759	6.846154418
MB-5383	113.6666667	1	8.06291371	6.2434139	7.093581491	8.253555768	6.758591375
MB-5384	71.16666667	1	9.058322668	6.84437998	6.460680228	8.596311189	7.485959063
MB-5386	192.2	0	9.426378222	5.812776612	6.184959226	9.92678187	6.959390723
MB-5388	146.9333333	1	8.011223323	5.925139281	6.290720997	8.130341865	6.962405301
MB-5389	124.7666667	1	6.271113094	5.584328706	6.471725741	7.283751673	6.511904118
MB-5393	154	1	9.23686971	5.804395431	6.323679564	10.39659223	6.679831279
MB-5395	165.1666667	0	8.475152552	7.639321218	7.011160043	7.918506697	6.970330067
MB-5396	165.6666667	0	9.127565417	5.769925231	6.204378475	8.962918551	7.660080036
MB-5397	172.8666667	1	8.572192188	6.486177651	6.357724962	9.509989746	6.640471806
MB-5398	213	0	8.254529561	6.748110797	6.002905549	9.575944499	6.613289902
MB-5399	106.8	1	7.517484117	5.679778744	6.033319044	8.620253958	6.069384399
MB-5401	70.16666667	0	7.758466771	5.916944311	6.350649153	7.871713923	7.02273212
MB-5402	107.7666667	1	7.698523701	5.882125688	6.205334872	8.386721879	7.108631598
MB-5403	95.73333333	1	6.25995366	6.215609832	6.251605054	7.997157458	6.636702712
MB-5404	74.73333333	1	7.833710828	5.66616656	6.71636834	7.259810697	7.231589241
MB-5406	97.43333333	1	6.682597925	5.708175369	6.27148568	7.279604334	7.180994332
MB-5407	205.6	0	9.182798537	6.975119659	6.751203357	7.940279483	6.904596624
MB-5410	202.7666667	0	7.807526973	6.130825458	5.567565976	6.954940478	6.949442407
MB-5412	220.2333333	0	8.593496484	6.475492264	7.161889498	7.93579804	6.481020249
MB-5414	140.6	1	8.100627647	6.860169561	5.843783314	8.206611994	7.684402848
MB-5418	207.1666667	0	7.630285297	5.934001743	5.975081185	8.758879649	7.203043335
MB-5422	46.06666667	1	8.228473539	6.621336179	6.340289478	8.614804384	7.386970308
MB-5424	83.53333333	1	8.350822222	6.151131794	6.625904023	9.166461571	7.161858553
MB-5425	49.46666667	1	8.222381301	6.198960833	6.426883315	9.013762297	6.930098914
MB-5428	150.4666667	0	9.082551472	7.034293611	6.757800081	7.55350223	6.603206621
MB-5429	30.7	1	8.615410969	5.889594273	6.42898065	7.999174057	6.723843067
MB-5432	98.5	1	7.501888572	6.392990774	6.105986642	7.280076148	7.409499908
MB-5433	176.3666667	1	7.421809084	5.756548399	6.004497588	8.868539917	6.85181785
MB-5434	45.16666667	1	7.179603244	6.060497278	6.059198318	8.90702794	6.851382962
MB-5435	104	1	7.472686758	5.755624453	5.81533866	8.342377686	7.134890019
MB-5444	240.4333333	0	8.347529992	6.692593878	7.099822806	7.107327259	6.463781546
MB-5447	148.8666667	1	8.204444624	5.894056608	6.402368606	7.588018116	7.058165424
MB-5451	189.9	0	8.519835628	6.242646181	6.494930239	8.497319186	7.025887022
MB-5452	16.16666667	1	8.179471694	6.275610354	5.783438989	8.357188366	6.633845249
MB-5454	50.66666667	1	8.597278816	5.890769649	6.604457811	8.427433319	8.279910086
MB-5455	61.8	1	8.587384825	6.275612885	6.830679513	8.306160054	7.282192972
MB-5457	164.5666667	1	8.259737489	5.926208778	6.492389141	8.595844353	6.529653706
MB-5459	90.8	1	7.476820342	5.874392048	9.626605953	8.039544393	7.767489692
MB-5463	58.13333333	1	6.917227111	6.602129416	6.737636627	8.685335506	7.221768325
MB-5464	116.5333333	1	9.20745274	6.157696636	6.098908782	7.567109794	7.007603438
MB-5471	185.7666667	0	8.210389097	6.781526851	6.602511627	8.656084388	6.902553733
MB-5472	19.56666667	1	6.613008794	6.218848161	6.034339461	9.447005951	7.679935609
MB-5473	125.9	1	7.914427528	5.800102955	6.024277877	8.756084665	7.305438001
MB-5475	2.533333333	1	6.652522186	5.648813564	6.241008108	7.146420158	6.867694609

MB-5477	110.4666667	0	6.728038884	6.249506906	5.924605692	6.668243847	7.618463989
MB-5478	34.3	1	7.723416446	6.128217591	6.797064892	9.119118616	6.869476414
MB-5481	46.36666667	1	9.881208462	6.007931454	6.718707473	7.097759773	6.796890344
MB-5484	238.5	0	7.888308631	6.110085903	6.339492526	7.491027538	7.34844093
MB-5485	31.8	1	7.087501003	6.71139123	5.752381682	6.763824867	8.141752557
MB-5486	123.3	1	7.649242609	5.865350929	5.909982112	7.517298178	7.738813964
MB-5489	216.9666667	0	8.269923734	6.147500594	6.573043919	9.354726801	6.821435122
MB-5490	51.2	1	8.177702877	6.091851082	6.276283708	8.159060996	6.697363409
MB-5491	65.83333333	1	7.401636074	5.719207287	5.690552723	7.365678448	7.523545429
MB-5492	23.36666667	1	10.11087489	6.614722979	7.470440616	9.967590094	6.764458868
MB-5493	57.23333333	1	6.820240705	6.142638766	5.804927769	7.206814208	7.561055215
MB-5495	228.8	0	8.710201759	7.221610223	6.727491605	8.5576222	7.329152644
MB-5497	237.5	0	8.23603279	5.8887332	6.27767016	8.902241048	7.422493825
MB-5499	123.7333333	0	8.407461774	5.903340918	6.184889547	8.740862974	6.609340864
MB-5502	189.7333333	0	7.408654442	5.651275124	5.874242334	7.755970152	7.141940618
MB-5505	164.6	1	7.243796149	5.7197213	6.168867123	8.681725124	7.160408098
MB-5510	103.1	1	7.497871822	5.885695997	6.127092458	8.041858015	7.335120876
MB-5514	182.9	0	10.05811501	5.892765008	6.075938309	7.9043289	6.372887492
MB-5518	30.86666667	1	7.779275774	5.891848661	6.248404296	8.347382827	9.122631657
MB-5519	169.2333333	1	8.17191406	5.758514982	6.460199756	7.962000433	7.941352995
MB-5520	16.3	1	8.28311468	5.796691009	6.267519632	7.508294667	7.060769166
MB-5521	124.8	0	6.797294412	5.954529279	6.533772827	7.498771727	7.814403456
MB-5525	2	0	6.741881381	6.007080233	5.622494651	7.678432073	7.148062522
MB-5532	79.86666667	1	7.645749221	6.03503289	6.07026318	8.66755054	7.390397464
MB-5540	48.43333333	1	7.162001532	5.901802107	6.179019599	7.037551684	7.816509413
MB-5541	200.1333333	0	9.902117485	7.937789603	7.088538696	8.961218673	6.210078543
MB-5543	34.43333333	1	9.244408341	6.020979637	6.38074009	8.3648883	6.595673435
MB-5550	171.3	1	6.857141138	6.115493535	5.87504026	8.528002556	7.611838711
MB-5552	34.7	1	9.114767965	6.004527047	6.729960127	8.706819335	8.739720399
MB-5553	154.5	1	9.54158038	5.978234903	6.020593429	8.570711894	6.887601651
MB-5554	229.8333333	0	8.327191059	6.918534863	6.302168454	8.547993263	7.904206898
MB-5556	225.4	0	6.768775656	5.601346116	5.643290355	10.09464192	6.888422861
MB-5562	102.9666667	1	7.464880572	5.774911336	6.027655002	9.104622945	7.723226733
MB-5563	117.6666667	1	8.158379571	6.862366038	6.156641825	9.027774772	7.547309454
MB-5567	178.5666667	1	7.72754151	6.248404126	6.040879274	8.114161061	6.89437783
MB-5571	153.8666667	1	7.435252166	6.009063882	6.05874405	8.45332538	6.840123795
MB-5575	117.6666667	1	7.065601049	6.01353333	6.15805618	6.341502381	6.875681338
MB-5576	194	0	8.10351131	5.896496739	6.365172923	7.367994602	7.231844216
MB-5579	90.6	1	8.753100678	6.758454482	6.57432398	7.942332346	6.33329035
MB-5580	71.63333333	1	9.809906878	6.035686986	6.647468665	9.852353847	6.438410424
MB-5582	222.3333333	0	8.657427573	7.005660277	6.568229435	8.403323761	6.322359403
MB-5583	111.3666667	0	9.276854291	6.144046372	6.494279777	9.494058901	6.793479943
MB-5589	185.1333333	0	7.937442219	5.924897272	6.551258553	7.769137666	6.566956566
MB-5590	17.83333333	0	6.1314288	5.758044043	6.295255948	6.958931342	6.769485987
MB-5591	155.3666667	1	8.220572529	5.988201711	6.273997717	8.362713386	6.453796687
MB-5592	26.33333333	1	7.749987635	6.011379601	5.803990278	8.001786068	7.141406825
MB-5596	167.9333333	1	8.309886609	7.664067361	6.951374874	7.669078246	6.443874716
MB-5597	32.73333333	1	7.947933199	5.777385257	6.049407672	7.950259331	6.548992705
MB-5599	224.8666667	0	9.218618875	6.926736452	6.620766232	9.065255936	6.733491686
MB-5601	214.8	1	9.108581663	6.302000469	6.60702252	6.586357201	7.014185131
MB-5603	171.6333333	1	8.988251146	5.938354413	6.909200702	8.439824416	6.512767012
MB-5604	145.6333333	0	6.681054525	5.902406314	5.989894581	7.033110671	7.200747589
MB-5605	123.7	0	6.35174311	5.853149865	6.414285964	6.433544849	7.508257661

MB-5613	212.3666667	1	9.060906249	6.271622699	6.35714015	8.397444453	7.541723298
MB-5614	116.4333333	1	9.298073819	6.709428787	6.840829564	8.846237726	6.520411372
MB-5617	128.2	1	9.2303856	5.763103821	5.759804049	6.940782538	6.544119059
MB-5620	42.06666667	1	7.590432117	6.715197902	6.2515149	9.66788337	6.635163803
MB-5622	182.9333333	0	7.506812842	6.003931063	6.016492098	7.726435673	7.092379381
MB-5623	137.8	1	6.934959328	6.073771497	6.23259784	6.705877614	7.418096638
MB-5626	82.96666667	0	8.126566587	6.275850276	6.379134416	8.390319709	6.992179362
MB-5628	42.6	1	6.176236918	6.074970415	6.070704072	6.723069745	6.906857244
MB-5629	103.8	1	7.441827067	5.938479563	6.331777434	8.470078678	7.186004685
MB-5632	38.16666667	1	7.810682473	5.823812602	6.236429456	8.047863767	7.400798303
MB-5635	21.06666667	1	7.860181812	6.147208003	5.990029649	7.357935481	6.432329694
MB-5636	108.3	1	7.167025184	5.875804182	5.997178827	7.493843568	7.669308265
MB-5638	195.7	1	7.319180125	6.168862102	6.501543902	7.682275753	7.50729004
MB-5641	181.8666667	0	7.536660808	5.649650513	6.176236918	8.531750969	7.19475568
MB-5642	156.8	1	7.118356748	5.936178937	6.262289096	6.339742122	7.046666043
MB-5645	22.23333333	1	6.750469828	5.894025141	6.327254664	6.816620011	6.794133239
MB-5646	167.1	1	6.865875872	5.686869711	6.070051426	7.977193945	7.15788982
MB-5647	30.8	1	7.155681266	5.635782175	5.976599747	7.025825054	6.85919448
MB-5653	194.6	0	7.956213601	6.046639984	6.714822689	7.207910873	6.540552134
MB-5654	173.0333333	1	6.566007551	5.596988574	6.013396041	6.115454465	6.883823317
MB-6001	135.3	1	7.937045684	7.253330792	6.127631052	9.055021451	7.063452529
MB-6006	63.56666667	1	8.719961128	5.840894278	6.192398702	8.453366585	6.675336466
MB-6008	69.1	1	7.474525466	5.440967036	5.841925824	6.939562142	7.864215716
MB-6010	218.2333333	0	7.132557332	5.993352821	6.392132158	7.480481997	7.304901213
MB-6011	211.1333333	1	6.560866513	6.094398705	5.500858875	7.019667354	7.684762445
MB-6012	144.4333333	1	6.170465523	5.53896137	6.065769124	8.596734081	7.234239414
MB-6016	143.5333333	1	9.072978523	6.564651421	6.23384194	7.850540293	6.790820713
MB-6017	70.9	1	7.536660808	5.872087883	6.623707598	9.706965011	6.33418022
MB-6018	143.1666667	1	9.46129365	5.92647995	6.163929451	8.761573539	6.887231688
MB-6021	301.2333333	0	8.710668726	6.048729034	7.025792201	8.828744346	6.411881711
MB-6022	52.06666667	1	8.238172233	5.826130418	5.767520602	7.956439321	7.306490469
MB-6024	252.2666667	1	9.550833718	6.183850984	6.850387269	9.001051172	6.877963856
MB-6026	49.73333333	1	7.815210511	5.557635368	6.07950352	8.268981831	7.308741612
MB-6029	278.3666667	1	7.946165729	6.602952208	6.169404397	7.168683198	6.72111566
MB-6030	145.3666667	1	9.20434481	6.831186459	6.368770077	8.155988117	7.393793953
MB-6050	192.2	0	7.675626039	6.11886101	6.726316635	8.032784313	6.822012874
MB-6051	186.8333333	1	11.0984257	5.989117176	6.721277002	9.751724953	6.629246416
MB-6053	250.8	0	8.023323527	6.185535726	6.675528676	10.23047638	7.150811784
MB-6060	38.03333333	1	7.613074924	6.107239571	5.955655595	7.324189646	8.017209073
MB-6065	188.5333333	1	8.577877032	6.062583789	6.002701563	10.09078719	6.702860068
MB-6069	258.1333333	1	7.520741014	6.35876943	6.51997584	7.3472697	6.516367871
MB-6071	57.4	1	8.015598243	5.887116242	6.514629073	7.592850209	7.077773525
MB-6075	182.3333333	1	7.255700255	5.988877253	6.2515149	8.181807205	8.163388786
MB-6079	239.1666667	1	8.362949534	6.503690872	5.748362093	8.241262109	7.019494329
MB-6080	54.1	1	6.917606623	6.05121431	6.851895488	7.566647023	7.881772329
MB-6083	164.0333333	1	8.0167114	6.732981749	6.170465523	8.102825583	6.865900729
MB-6097	159.7	1	7.022908987	5.75496742	6.083593787	8.227758372	7.426971264
MB-6108	193.9666667	0	9.135515203	6.085527334	6.70645319	8.635141519	6.860646638
MB-6118	264.6	0	8.72994463	5.921737033	6.512643284	8.702133132	6.355036081
MB-6124	115.6333333	1	7.753446754	6.170755838	5.870692669	11.33617166	6.757483635
MB-6133	61.8	1	8.580730354	7.979298274	6.339761684	8.392142723	7.749604431
MB-6135	78.6	1	8.0653723	5.950376655	5.933089116	8.106862505	7.662247413
MB-6138	25.63333333	1	9.071053926	5.8110743	6.425934625	9.232774567	6.751701277

MB-6141	78.86666667	1	8.883161341	5.861965519	5.860141615	9.273088963	7.744017367
MB-6145	34.33333333	1	7.354856945	6.814475051	5.904526334	7.940836676	7.743912333
MB-6149	74.93333333	1	9.246479116	6.336655947	5.562138351	8.818048062	7.11304547
MB-6150	77.66666667	1	8.434701464	8.36417873	6.284653164	9.273088963	6.903097177
MB-6154	195.3	0	7.933876402	5.945731036	5.76789258	9.162533956	7.770251108
MB-6163	88.66666667	1	7.531068974	6.056913637	6.179152087	7.978673885	7.433004384
MB-6164	205.9	0	8.069827792	5.70791894	6.290403452	8.473674801	7.78125084
MB-6167	122.8	1	8.39146412	6.203408658	6.727767795	9.575953078	7.002807134
MB-6168	225.7	1	8.493499191	7.565957527	6.80184653	8.656853243	6.643348652
MB-6171	220.3	0	8.379065814	5.712869893	6.047147063	8.653012305	6.755526753
MB-6179	58.46666667	1	8.604885548	5.853079757	5.727804522	9.297022759	6.848567882
MB-6181	54.93333333	1	6.605623421	5.673939349	5.507453521	6.202579907	7.115745654
MB-6183	79.13333333	1	6.932910725	7.334796565	6.042401985	7.132138692	7.201404767
MB-6185	197.7333333	1	9.351526138	6.094941945	6.569943391	9.69684626	6.713907629
MB-6189	240.2	0	7.076366962	5.992055755	5.912161928	10.11695527	7.705824151
MB-6190	24.63333333	1	7.816240844	5.919724832	6.089554889	7.192306552	6.693368975
MB-6192	42.63333333	1	8.174221391	6.059218714	5.7377013	7.761471523	7.455947217
MB-6195	202.2333333	0	9.137489172	7.76317437	6.947003171	9.727652481	6.893868457
MB-6200	30.06666667	1	8.775296295	6.256488801	5.642035174	8.470224475	7.820249143
MB-6201	222.7	1	7.663419357	5.965970628	6.105809934	7.932262725	6.936311836
MB-6207	118.3	1	8.080483019	6.874643169	6.412721124	7.895470885	6.933318553
MB-6211	42.3	1	7.809588248	6.217554596	6.264702017	8.849585961	7.142618433
MB-6212	224.2333333	0	8.564235932	8.187754175	6.646465561	8.569991171	6.625807213
MB-6214	174.5	1	8.332767938	6.578464364	6.316896916	8.502374985	6.524668528
MB-6217	211.5333333	1	7.958096929	6.172409072	5.505692978	7.490105023	7.703072935
MB-6218	147.7666667	1	8.181807205	6.029626415	6.062905682	8.015040424	6.588241973
MB-6225	117.8666667	0	8.202644693	6.671316702	6.540354921	8.332105513	6.682944716
MB-6232	85	1	8.223598726	6.39831332	6.015980472	7.892319227	6.602235711
MB-6233	201.1666667	0	8.88739025	6.342080177	6.383222253	9.665017994	6.934478536
MB-6238	172.9666667	1	8.042216515	7.988562349	6.228099526	8.175970165	7.015637654
MB-6253	194.3666667	0	8.16661349	6.230801646	6.199354622	8.188344712	6.581327924
MB-6254	60.9	1	9.593148489	5.649664943	5.688958867	9.306837319	6.937661793
MB-6256	127.8333333	1	9.593148489	5.997884944	6.476823016	7.670217031	6.229160868
MB-6257	91	0	6.92071605	5.861578657	6.279206792	8.342377686	7.22168825
MB-6263	75.86666667	0	9.726206725	6.839775568	6.261914831	9.853022727	6.478854823
MB-6271	157.7333333	0	7.269551696	7.421795308	5.663464487	8.490774514	7.208134238
MB-6273	189.4333333	0	8.219365355	6.216733122	6.900150312	7.771038433	6.428658343
MB-6281	3.5	1	6.246759534	5.566967248	6.087189389	10.14803205	6.072355196
MB-6283	92.4	1	9.426378222	7.779559042	6.121605285	9.537608851	7.129785216
MB-6284	88.33333333	1	8.052868145	5.873299851	6.369690778	7.122697244	6.646822011
MB-6286	49.53333333	1	9.065314107	6.185496604	6.03264956	9.58258284	6.502644227
MB-6287	35.2	1	7.91300519	5.964379969	6.205538773	7.069023792	6.841966155
MB-6288	100.3	1	7.636600725	7.430038208	6.352941299	7.277812697	6.997466622
MB-6297	170.8666667	0	8.405950265	5.791980113	6.120598565	7.86964067	7.183813962
MB-6300	63.83333333	1	7.888089313	6.311140889	6.549076216	8.174221391	6.411112616
MB-6302	125.8666667	1	8.004575505	6.318601517	6.047823645	8.140877579	6.945897831
MB-6306	64.6	1	8.639553747	6.195865782	5.965711604	8.077677099	7.13042622
MB-6308	151.9333333	1	8.139710053	5.943309082	6.448853562	8.386211759	6.988419618
MB-6312	162.7666667	0	7.826109755	5.834058824	6.058493579	10.10886483	7.193698285
MB-6317	29.23333333	1	7.29180685	6.597314147	5.892996966	8.398653147	7.499962206
MB-6322	80.36666667	1	7.819358022	6.173203369	6.693404522	8.419180871	6.849187497
MB-6327	18.26666667	1	6.873848433	6.286660474	5.988536964	6.425307214	7.243304516
MB-6328	126.6666667	0	8.569991171	5.956427228	5.976493097	8.73624643	7.395143197

MB-6329	81.5	1	6.584548984	6.186008321	5.978160452	6.783438396	7.659697127
MB-6344	152.9333333	1	7.738420164	5.703173876	6.289808421	7.493367962	7.249292843
MB-6346	281.5	0	7.210734889	5.7884925	5.991938594	6.642900036	6.694939972
MB-7000	97.4	0	7.646813928	6.727235791	6.0223794	7.422012902	6.777106534
MB-7001	102.7	0	8.805740355	6.511921468	6.409281097	9.235921127	7.272190812
MB-7002	57.93333333	0	9.131500929	5.8887437	6.467731636	9.138473639	7.024109847
MB-7004	50.46666667	0	9.808889823	7.579766612	6.189205554	8.858621675	7.011312423
MB-7005	45.33333333	0	7.390130753	5.726145474	6.610147846	8.55565509	6.575904029
MB-7006	109.9333333	0	9.23059958	5.945089057	6.279485488	9.258968994	6.219021514
MB-7010	74.03333333	0	7.049730818	5.938723095	6.301095612	7.802383102	6.596176367
MB-7011	70.06666667	1	7.351695589	5.633965521	5.587004587	7.928521164	7.390443765
MB-7013	46.43333333	0	6.584203952	5.482943922	6.314252832	8.883161341	6.818406068
MB-7015	78.16666667	0	6.960175867	6.143188836	5.838414805	6.956978145	7.659955104
MB-7019	80.73333333	1	8.481158874	5.769171911	6.292407153	7.809064678	6.974396231
MB-7022	85.5	0	8.00401381	5.809119841	6.416474382	7.989651297	7.173644743
MB-7024	70.26666667	0	6.85998445	5.736200712	5.739463555	7.542710281	7.567256407
MB-7026	111.7	1	7.897586504	5.75644533	6.512643284	8.880576609	6.541593589
MB-7028	89.8	0	7.996271402	5.859993515	6.234964991	7.159463407	6.821299565
MB-7029	76.86666667	0	9.720194288	6.831946751	6.566216349	9.465000738	6.946884648
MB-7032	71.46666667	0	7.618851201	6.17105912	6.328127991	8.212150892	6.84274736
MB-7034	75.1	0	6.563193046	5.990328467	6.054793226	6.022619108	7.018745734
MB-7037	86.4	0	7.860912458	5.854055165	6.079025113	8.113765166	6.749292043
MB-7040	71.83333333	0	8.51350796	6.681807912	6.251790273	12.01570215	6.710919805
MB-7041	101.9666667	1	9.55472581	6.796164399	6.287254079	9.244408341	6.802653572
MB-7042	88.5	0	8.454040371	6.082418201	6.225945595	7.391910944	7.443247769
MB-7053	122.1333333	0	8.015040424	5.80071633	6.065052469	8.035026309	6.508610763
MB-7056	117	0	6.517303065	6.008870814	6.15404632	7.126358472	6.87985785
MB-7060	112.6666667	1	8.339225486	6.26251168	6.442751677	8.617280147	7.026242197
MB-7062	95.86666667	0	8.477023247	6.16712928	6.008552524	9.501592824	6.649117262
MB-7063	86.6	0	8.926925061	7.44100515	6.576029776	8.678518159	6.949393673
MB-7065	107.8666667	0	7.048093491	5.606797954	6.084065085	6.601801669	6.90835007
MB-7072	52.33333333	1	7.652642531	5.646196391	5.761141593	7.822977504	7.209130576
MB-7075	111.6333333	0	6.698845119	5.95827069	6.020160572	7.547440942	7.396131977
MB-7076	142.6666667	1	9.080608147	5.435554449	6.507918674	8.182397095	6.299856327
MB-7077	101.2666667	0	8.456724789	6.331937966	6.67659912	8.15897502	6.341754624
MB-7080	91.23333333	0	8.435351589	5.95786744	5.996120014	8.436052659	6.711056269
MB-7083	105.4666667	0	7.936506647	7.372168132	5.792729162	8.268981831	6.67578591
MB-7086	123.5333333	0	6.725213458	6.228606465	6.597671504	8.733871732	6.959389026
MB-7091	110.2666667	0	8.496236187	7.124560595	7.1914345	7.683560446	6.426333991
MB-7093	125.8333333	1	8.40265997	6.58558039	6.976798543	8.916596254	6.140148671
MB-7094	113.3666667	0	6.442429028	5.652154945	6.113879199	8.557096004	7.405816012
MB-7095	115.6333333	0	7.322426913	5.539742643	6.165211763	7.179187225	6.992997267
MB-7096	124	0	9.132517866	6.272103949	6.689099107	8.705993958	6.992136305
MB-7097	65.16666667	1	7.943553359	6.539293631	6.034909039	8.717658278	7.347081433
MB-7099	163.1666667	1	7.113106537	6.020220528	5.912542611	6.520964742	7.219457219
MB-7100	156.3333333	1	6.731066912	6.095503042	5.96652848	6.969254734	6.921265854
MB-7101	124.2666667	0	9.824895328	6.49873417	6.201500187	8.878890353	6.465284913
MB-7102	104.1	1	6.776323075	7.130420923	5.874243214	7.308721486	7.117617716
MB-7106	37.5	1	9.62746027	7.300330034	6.686198258	9.480148757	6.140993688
MB-7107	56.93333333	1	7.38833935	5.750852692	5.715682829	7.04685423	7.380267559
MB-7109	117.5333333	0	6.906296645	5.683119613	6.167556225	7.927405729	8.08373846
MB-7112	64.7	1	8.035587531	7.060962306	6.384469426	10.10079568	7.916498642
MB-7113	136.7	1	8.896717806	6.33809155	5.911798674	8.478425387	7.138341745

MB-7116	33.36666667	1	8.012315267	6.133969482	6.024386445	7.019667354	7.389228771
MB-7118	140.5666667	0	8.572880416	6.010981731	6.093856729	8.861953254	6.793349671
MB-7123	137.6666667	0	8.841996963	8.239975595	6.327225149	8.303034329	6.71018672
MB-7124	139.6	0	7.057514105	5.588875303	5.789563435	7.679596596	6.964428439
MB-7128	60.26666667	1	9.122583828	6.252707262	6.55447529	8.662784894	6.913537827
MB-7130	102.9666667	1	6.941530285	5.711376535	5.846569834	7.019246383	7.518983253
MB-7131	134.7333333	0	5.861783483	5.810542625	6.196652302	8.306238205	7.331231599
MB-7132	131.6	0	8.638828787	6.370261854	6.822209841	9.036159953	6.771187239
MB-7133	23.83333333	1	8.009559131	6.095315194	6.653108714	8.488051463	7.523495502
MB-7137	179.8	0	7.88227922	6.569607909	6.259395373	12.30013786	6.407828211
MB-7138	123	1	6.192398702	8.084833713	6.1374867	7.17831762	7.618516563
MB-7140	90.8	1	8.546532887	6.255630077	5.667024478	10.31983034	7.338194146
MB-7141	138.5666667	0	9.145344091	7.667270928	6.225392846	9.084423032	7.030030892
MB-7142	175.9	1	7.657950231	6.05459097	6.260807182	7.826109755	7.3651144
MB-7144	102.6333333	1	7.60736891	5.886955388	5.710079669	12.96641973	7.793457762
MB-7147	168.6	1	6.207162867	5.686454795	6.314537301	6.074249132	7.690296697
MB-7149	44.63333333	1	7.835449298	7.461316466	6.179152087	10.87385021	8.15145985
MB-7150	128.9666667	0	7.815210511	5.949935279	6.228099526	8.354661507	7.706188961
MB-7157	219.6333333	0	7.009990205	5.877056581	5.809963894	7.454005849	7.315714277
MB-7161	121.5333333	0	7.581823586	7.129088287	6.075436521	7.510924963	6.384950007
MB-7162	140.2	0	8.757571736	6.945280775	6.286385212	8.856987001	6.998720793
MB-7163	52.63333333	1	10.17804917	7.875133205	6.15249541	9.482621742	6.443351979
MB-7164	211.4	0	9.820046973	6.306582874	6.170197166	8.448715244	6.793314242
MB-7168	150.5	0	9.866437045	6.574253043	6.433544849	9.140438578	7.02397939
MB-7170	155.4	0	7.247473152	5.776699962	6.4262533	11.28815274	6.453280372
MB-7171	160.4	1	7.80395997	6.274750887	6.163670207	7.936506647	7.224701812
MB-7172	149.3	0	7.795704977	7.617508692	5.996331877	8.672517312	6.58857593
MB-7173	63.53333333	1	8.304967751	6.46335222	6.228650233	8.727621616	7.53409061
MB-7174	140.7666667	0	7.240997406	6.348505666	6.456820642	7.723885622	7.48282736
MB-7176	194.4	1	8.144387543	5.875290216	6.330485695	8.419180871	6.842243163
MB-7181	157.5333333	0	6.226226578	5.9057702	6.188941056	7.509111766	6.661796198
MB-7185	125.0333333	1	6.342212141	6.143730136	5.512735861	6.764797295	6.980983529
MB-7186	4.866666667	0	6.589362217	6.480449102	6.241433734	6.780786329	7.869136819
MB-7189	143.6	1	7.79161569	6.117902544	6.425934625	7.0936013	6.977027321
MB-7193	87.46666667	1	7.316246435	6.350257425	5.957868627	9.796397318	7.611968881
MB-7194	46.66666667	1	6.953343702	5.643061058	5.889493461	9.456524366	7.841320817
MB-7195	80.43333333	1	7.04607268	6.580915618	5.832735785	7.589484023	6.380260321
MB-7197	187.8666667	1	7.219604042	5.883019126	5.973435167	7.170336626	7.291501826
MB-7199	76	1	6.424366973	7.012591848	5.704638369	6.642900036	7.297908244
MB-7200	97.6	1	7.69969648	6.271649381	6.35654056	7.614007844	6.969596079
MB-7212	52.5	1	10.54841106	6.676844175	6.238631829	8.858621675	6.632160735
MB-7216	207.4666667	1	7.783416302	6.041470856	5.899676483	6.128619669	6.994107226
MB-7217	90.4	1	10.99476344	7.985884807	6.836277061	9.365285393	6.704631533
MB-7218	165.4333333	0	8.909625572	7.332363649	6.048987249	7.97652345	6.413396062
MB-7219	165.2	0	9.218785489	7.819437879	6.226765196	8.919217327	6.792907955
MB-7220	183.2	1	6.700314348	6.578797506	6.345162654	7.121438822	6.956812677
MB-7226	53.36666667	1	7.180001103	5.818008275	6.433212429	7.026205939	7.77116315
MB-7229	111.6	1	6.84237763	5.620751741	6.143062305	7.217940953	6.973427913
MB-7230	182.6	0	7.929016872	6.642264648	6.243099935	7.720948932	7.321829262
MB-7231	199.0333333	1	8.112043322	5.316921829	5.927408644	7.617388727	6.360137911
MB-7232	177.6	0	6.863819059	5.672144563	6.268077757	7.253072067	6.670785167
MB-7234	222.2	0	7.565238792	5.61576311	6.561195977	8.836181104	7.946591714
MB-7235	170.8	0	9.523348923	7.719759245	6.105563475	8.490774514	7.46559097

MB-7236	201.4666667	1	7.002785641	5.809907134	5.814341862	6.997613476	6.320617822
MB-7241	171.1	0	7.487321488	6.104063115	6.180447843	10.9641142	6.912531943
MB-7243	148.8	1	7.362900028	7.624210395	6.779308176	7.972179016	6.962869421
MB-7244	184.1666667	0	8.097044899	6.168427276	6.635886295	7.752973028	6.503448096
MB-7249	58.66666667	1	8.865289454	6.82950675	5.911227809	6.883093947	6.399339524
MB-7253	59.96666667	1	6.679434324	5.575049077	6.113150783	9.012862121	6.913999373
MB-7254	192.2	0	9.766704544	6.502032825	6.567238033	9.72319596	7.470792596
MB-7263	164.9666667	1	7.089911641	6.258587199	6.06719714	7.114799152	8.20827606
MB-7266	68.16666667	1	7.00879786	5.647328352	5.843014093	7.168273208	6.460352419
MB-7268	227.4666667	0	9.395520627	6.264721573	6.213168484	8.638072536	7.016565896
MB-7276	185.3333333	0	7.724374836	6.712962754	6.406445533	8.348152189	6.785976832
MB-7277	187.0333333	0	8.249279301	5.732736902	5.786935045	11.75251707	7.07991302
MB-7278	241.3	1	8.361019017	7.860510441	6.769959503	8.105142567	6.175984579
MB-7280	195.5333333	0	9.159409407	8.60992057	6.127631052	8.833713024	7.114021544
MB-7283	28.86666667	1	7.977584376	5.945858328	5.783825788	7.589484023	8.003102667
MB-7284	203	1	8.971176229	6.146515896	6.423099987	8.547228763	7.179245666
MB-7286	158.6333333	1	8.063599427	5.963548474	6.139510087	9.266545191	7.181867606
MB-7287	96.96666667	1	7.16743385	5.73033649	6.054326292	8.074272147	8.095142525
MB-7288	27.06666667	1	7.07555742	5.907525636	6.146858302	8.501000445	6.961166077
MB-7292	78.46666667	1	8.108014435	5.727921446	6.178615002	7.44079759	7.730026805
MB-7293	199.2333333	0	9.537608851	6.796690074	6.572973345	9.327830183	6.429305351
MB-7294	82.73333333	1	6.938009166	5.704964867	6.192398702	11.29401764	7.281481455
MB-7295	196.8666667	0	8.696054228	5.98428069	6.589023306	8.359741797	6.984462838
MB-7296	44.73333333	1	7.656987047	5.772020584	6.09795913	7.501218613	8.697668386
MB-7297	175.9666667	1	7.97652345	6.350892267	5.888597304	6.694502941	7.165923041
MB-7298	86.23333333	1	6.829057013	5.666672097	6.315422206	8.114318634	6.689575989
MB-7299	201.9	1	8.009038859	5.741076029	6.308402514	6.664180568	6.656057181

G6PD	ACSL4	ACO1	NQO1	CS
8.76389733	6.580079287	9.728624716	10.9415924	7.733852864
8.129927467	8.561804754	9.633105012	9.076757856	7.257595107
8.469311588	8.236440748	8.788133595	11.20280706	7.463015606
8.986709689	7.149215988	9.436694845	12.25632061	7.562216133
8.843560911	7.336077871	8.7296657	11.5004318	8.026297721
7.421128677	8.299278654	9.666427355	9.570582128	6.893359146
8.638876989	6.115421449	8.570711894	9.702629103	8.244554003
8.305487072	6.131930446	9.138918196	8.544771736	7.098479423
6.987952586	6.512283579	8.926925061	8.982963133	5.709365653
9.015240736	6.391328807	9.810547853	10.3222849	7.641607428
7.513778924	8.450670158	9.090256463	11.51130754	7.089635766
7.941522993	8.730763539	8.648522785	9.371092462	5.988465011
8.462117733	8.053759702	9.017441445	10.74574764	6.591972636
10.18762719	7.059133999	9.567036586	9.409471977	7.615463994
8.574584126	7.610469393	8.927808489	8.949880786	7.176247056
7.811386203	6.799655228	9.038403197	11.1373501	7.552160131
8.109280688	8.25586935	8.983844673	10.50382652	7.185744451
8.088179082	8.134010101	9.159409407	10.09078719	7.202452753
7.907565938	8.909912116	8.528916879	9.019288141	7.515003317
9.377408421	6.405088743	8.627760298	8.940135454	7.354892077
8.682998641	6.174256272	9.913083989	13.16680437	7.49864061
9.415529016	7.27269162	9.525599119	10.51542861	7.865232133
8.42128081	6.746647079	8.738623594	10.05456541	6.620851038
9.306029726	7.657584	9.389527825	11.61035205	8.196620266
8.760011939	7.282598413	10.04582883	10.32469079	7.389904979
8.106557615	6.67429062	9.733823644	11.04784345	7.618898918
8.840884329	6.457033255	9.53245947	9.546839147	7.887104523
8.68196211	7.105072888	9.891377391	11.25954676	7.520025213
9.756864493	6.576358843	9.698324474	10.97137457	8.704774015
9.429943456	7.498397665	8.649531094	9.867090254	7.309962862
8.845701225	6.898830997	8.635635859	7.666217939	7.42407169
8.081235577	6.696805496	9.0571585	10.2989693	7.140777837
8.193669512	7.32182391	8.711249898	10.60997825	7.43366091
8.172846514	6.987181016	9.365285393	9.705520108	7.103994919
8.212168648	6.295611649	9.061564271	9.831362477	7.095583446
8.556109955	6.937247542	9.660764418	10.45004053	7.660870541
9.326216298	7.146588943	9.49518404	11.83133169	7.425647822
8.677357383	6.803296672	8.666511426	9.837304972	7.762524854
8.595023981	6.623251652	8.572501467	9.884586202	8.023162499
8.480823618	6.681915752	9.783853043	8.342377686	7.649380378
7.896984472	6.662477703	9.045872354	9.425634504	7.767929489
8.440919072	7.797451991	9.729979684	8.746796992	7.18533799
8.487905448	6.731421141	9.724360601	10.5326533	7.308404324
8.284870581	6.747193292	9.866207452	9.882332543	7.809881806
7.860234778	6.522233301	8.935194601	10.90018533	7.415967205
8.921683049	5.916761923	8.649487332	12.20312555	7.499724025
8.572116845	6.413935642	9.234754591	10.15134506	7.964788153
8.830324459	7.072416387	9.70589944	10.14043813	7.591531151
8.179724121	6.603221862	10.12223085	9.620310093	7.41383376
8.713237466	6.447921919	8.593055095	10.22257771	8.573362868
8.943640243	5.462326488	9.047396353	10.78333093	7.304885244
8.683514566	6.237657851	9.614790083	9.21195825	7.938166258

8.875290658	7.218760912	9.59425161	10.95999004	7.33993749
8.486982328	7.383249477	9.164440878	10.09564331	6.981458703
8.743467543	7.369913653	9.073307374	9.939432002	6.940264478
8.815896308	6.362292326	9.712545862	8.29195861	7.263433038
8.864258935	6.572073351	9.222787972	10.24405132	7.64717662
8.412660998	7.072489456	9.211611241	9.972936878	6.980021851
8.402739715	6.945015028	9.081552428	9.853022727	7.139737837
9.16321863	6.249092411	8.501021168	7.830167682	8.101307399
8.924576548	7.01778055	8.582921766	12.75241716	7.227294671
8.693753788	6.499128431	9.458961598	9.63242034	8.036436796
8.605559476	6.824074206	9.408490879	10.4095795	7.151693304
9.369830184	6.348395498	8.946296265	11.48047936	7.693913513
9.078028105	6.286015392	9.904503053	10.13706619	7.226912928
8.931198049	6.700471586	9.864175754	9.821965925	7.748492129
8.647064476	6.159646435	9.634585667	7.010103925	7.800406348
8.733693187	6.633693468	9.290527406	9.66079746	7.074073718
9.009584994	7.362512483	9.506786669	7.891884567	7.155899878
9.355532593	5.932384663	9.080756655	10.87819683	8.211094148
8.413305888	7.405405259	8.942660912	9.449090863	7.540268543
8.054990016	7.314054288	10.15512195	9.861217352	7.502531765
8.664135343	6.712722269	9.290933736	9.908743421	7.161737693
9.374731909	6.80747059	9.433363435	11.1599498	7.543315978
8.52328841	6.990123588	9.240384976	9.68698734	8.105784363
8.221521634	7.042531551	9.884737403	8.999976269	7.496519013
8.448350544	6.876272866	9.57530858	8.96059487	7.189127583
8.563083016	7.034877065	9.112502762	11.05217728	7.405867696
9.515461474	6.941417529	9.206621277	7.352716538	7.938121622
9.3326644	6.712517136	9.45369184	9.554619814	7.330922663
8.756974071	7.472540653	10.30347291	9.86215275	7.314398365
9.429781092	6.726254913	7.500754353	8.701710043	7.659344853
9.262293325	6.950882835	9.714250162	11.05977372	7.612928789
8.769004355	7.971728146	9.240143877	9.039898131	7.099670775
8.650275464	7.217571964	8.826765511	9.425786654	7.436824493
8.497561363	6.880101412	9.805741442	10.34512536	7.553296108
8.757563192	6.893401633	9.534069336	9.112994061	7.838519602
9.745867362	7.308655288	10.01898351	7.293534842	7.554109293
8.472170095	6.707394101	9.427588974	10.06514747	7.806831923
8.841381111	7.285856946	9.108812156	10.50382652	7.210160694
8.968062755	7.105637504	9.857588949	10.58069171	7.247374583
8.849211159	7.317223599	9.317889957	8.709070389	7.002950317
8.486302387	6.972611218	9.478886469	9.129551752	7.525252281
8.693140829	7.010696705	9.429012939	8.83729887	7.552020538
8.215752247	7.061951261	9.552107441	10.74946685	7.549198239
8.413195343	7.698285406	9.989051052	8.930328907	7.350156098
8.353724166	5.933406077	8.826066137	10.24623017	7.611017421
8.298966627	7.174321929	10.27621932	10.54841106	7.084764832
8.363536003	6.536447269	9.655829194	11.43643363	7.732785184
8.622852176	6.60903676	9.263498004	10.59214151	7.452335518
8.67180039	7.352392507	9.498942742	9.213641222	7.568581006
8.201971595	6.507871782	9.449186097	10.11576008	7.764039769
8.358138439	7.085239443	9.946123032	11.12345673	7.382703447
8.120274366	7.167939757	9.216737614	9.335534721	7.92615419
8.975063675	6.877299279	9.544203668	11.04081993	7.119405686

8.813197885	6.053308929	10.04491855	11.7211064	7.796034015
8.258818018	7.141004508	9.511496949	7.863518822	7.3975761
8.223047859	6.198502318	9.149202088	9.099380774	7.764712662
8.992666341	6.878606519	8.874681441	8.039989658	7.448290537
8.151209529	7.042206326	9.3919056	9.689527264	7.547926792
9.751446791	7.250829483	9.615892956	11.77087993	7.573595466
8.867880557	6.836478884	9.832191505	9.34763055	7.68092101
8.771474863	7.346761052	9.652417606	10.05964628	7.240709072
8.53346074	7.156348063	9.489234425	10.59273092	7.161084397
9.593092245	6.33652894	9.265419591	11.31180683	8.560683219
9.317533119	7.186242269	9.421385204	10.69236519	8.215952108
7.9448554	6.449703932	9.135515203	10.38547722	7.682715892
8.599889094	6.866300085	9.345824735	8.482559826	7.652252374
8.332687801	6.806319248	9.861771152	10.50346151	7.220946311
8.927848156	7.217623271	9.409340224	10.17035367	7.235436785
8.248476663	7.061439579	9.130704225	9.786959628	7.630355455
9.372685572	6.947720266	8.938984507	10.30155796	8.080911017
8.806278484	7.405416935	9.236943308	10.96226596	7.589142672
8.676340935	6.591649454	9.42587946	8.397344229	7.903417896
8.02269546	6.404219545	9.36697781	9.150841991	7.608401587
8.37669096	6.854631896	9.81303762	9.660731828	6.920629634
9.277882719	5.888643367	9.393016347	10.99895437	7.999757668
9.395481203	6.654204622	9.097047104	10.84137812	7.415180405
8.883629634	6.80398906	8.503532365	10.22492176	7.415871295
9.347789644	7.206721028	9.51131904	7.667801172	7.670189436
8.327309817	6.333219164	9.045120924	9.631780462	7.585079622
9.306004499	6.718524929	9.462612989	9.535644106	7.305307927
9.00924162	6.196576535	9.154340403	8.149558294	7.395329917
9.032191771	7.589801475	9.459085248	10.74683709	7.331365212
8.645385707	6.365965638	9.148591019	9.998981684	7.704343066
8.31959417	6.079268171	9.152515928	7.145742442	7.91658929
8.784764289	6.649266745	9.33039461	10.50808328	7.914237774
9.164134894	6.483656451	9.466304967	10.56715012	7.269037393
8.68029982	7.136872987	9.529340884	10.14138407	7.177876694
9.093455424	7.122012085	9.231730125	9.374176071	7.552291602
8.663274578	6.703308207	9.312555133	10.56716625	7.501957755
8.994832905	6.522114833	9.28177735	9.30693078	8.056663869
8.897424143	6.264875087	9.324151354	9.560937011	7.998483752
8.684064403	6.814120482	8.779097295	10.36850575	7.537786791
9.255243069	7.131275334	9.205662227	10.68483773	7.144860972
9.190773816	6.496368854	9.153208993	9.277107553	7.867710844
9.350199923	5.893877042	8.630741341	9.549423037	8.293873402
8.041950219	6.037903871	8.888285778	10.49537146	8.073630302
8.504612715	6.640817293	8.55416107	9.850242646	8.14565702
9.793439255	7.000349299	9.687079923	11.49423953	7.919568956
9.394187001	7.042582729	8.475365775	10.13556951	7.618828609
8.840316253	7.16579057	8.480755129	10.90192634	7.186110039
8.338240954	8.297113823	9.770099849	9.704359199	6.844225482
9.162622218	6.952635271	9.549455941	10.2228464	7.604863387
8.708571544	6.585765431	10.05165973	11.32575554	8.281662841
8.834093594	7.049433368	9.563111547	10.80942305	7.31141425
9.09722575	8.01930605	9.927802249	9.785286225	7.899947827
8.393525321	7.085374483	9.104016762	10.29098293	7.802805906

8.367955358	6.634227317	8.061214277	11.42451925	7.762703078
8.62284919	6.718649964	10.27162989	10.00223111	7.720920136
8.71498488	7.52262756	9.372876182	10.51359813	7.850515778
8.430915981	6.93380055	9.301202497	10.11090173	6.965051682
9.037949236	8.255861542	9.498545411	9.805336784	7.344388923
9.54447521	7.904409691	9.100338122	10.12010173	7.295684465
9.905087858	6.667633027	9.227206427	11.21741704	7.511035749
8.061334467	6.868285699	9.58360121	11.09710789	7.079779715
8.224148207	7.2142736	9.867798916	7.96368509	6.859864577
8.107235069	6.567431502	9.301215966	9.952585828	7.134144817
9.77506859	7.208621583	8.556660199	11.12867507	7.625023054
8.03900554	6.481089404	9.149579348	9.85680979	7.278427818
9.052598707	6.576288912	9.694919854	11.04725386	8.018497655
9.184787364	5.877591098	9.567483485	9.984721534	7.76819052
8.726395552	7.315110556	9.474786134	8.867701059	7.20750304
8.590709953	6.548166485	9.050645953	9.91323105	7.491268596
8.791160545	7.137549316	9.241486599	10.16515422	7.264346267
8.547340862	6.628465789	8.049608791	10.52802023	8.400697956
7.986289303	6.808225624	9.108273589	9.829746797	8.027158511
9.408323248	5.904538484	8.468001238	10.25035782	8.097692493
9.195828261	6.766126358	9.386073922	9.747157981	7.77155524
8.29680243	5.76704564	9.09328778	8.140529556	7.696551475
8.218983113	6.640523852	9.136394827	8.051340964	7.494051817
8.400912655	7.200215409	9.456760059	10.24535517	7.552499671
8.403662501	6.793911999	9.569478803	10.35498571	7.492518318
9.252839002	6.827178149	9.950078108	10.55162845	7.52068918
8.090669057	6.007086903	9.487367356	9.668130298	8.125723152
9.04130858	7.510609428	10.03731985	10.53331428	7.647951488
8.285198409	5.938119894	8.896987265	9.331899434	7.590241065
9.246368473	7.348152119	8.883043434	9.783933014	7.63020263
8.594986068	6.793442911	9.195065286	11.28815274	7.238054431
8.490809422	6.989069802	9.606178704	9.313574272	7.610848217
9.185718968	6.694399094	8.506349466	11.21308623	7.156782767
8.947782745	7.324187127	9.753957164	11.11609632	7.020475806
8.604843096	7.274394631	8.890810132	10.23047638	8.037759847
9.054578951	6.545214661	8.761573539	11.71129612	8.229665252
8.875105571	5.934136798	9.584756959	9.723023958	7.355426396
8.884681931	5.894682209	9.228473232	8.615801482	8.188640941
8.806455945	7.133865906	9.229217365	9.290258753	7.693552313
8.504028208	7.199208932	9.200289956	10.13192188	7.69699143
8.668900893	6.397997495	9.443185789	11.18270481	7.872066735
8.752448547	6.308126494	9.496159572	10.52458763	7.885396693
8.872618165	6.627272811	9.661045786	12.32509571	7.425333046
8.310832892	6.137970121	9.294922649	8.950011077	7.706897864
8.708380728	6.110003331	9.573283758	7.816746814	7.616246578
9.102107843	6.64556605	8.92749319	11.08536813	7.763549604
8.320323187	5.932520136	9.470025359	9.232774567	7.586843557
8.215822104	6.648511618	9.137672508	8.506805499	7.241420181
8.723966288	6.62362955	8.43117225	8.782649704	7.484566401
8.58290925	6.350481484	9.562933478	10.33544292	7.587185998
9.535099581	7.498622818	9.645678172	8.992980071	7.110346694
8.41642257	7.183743688	9.525102786	10.28297008	7.144596416
8.735467904	7.341948753	9.966895748	11.27121074	7.132396233

8.338058963	6.937972006	10.26371526	8.812239466	7.611366028
8.733973409	7.150104509	9.591889319	9.748659873	7.402623557
8.198904011	6.100955215	8.835311771	6.40810887	8.282026068
9.115899801	6.639787573	9.091337824	9.224119195	7.631486896
8.460369226	6.472770284	9.275463594	11.0543857	7.798707451
8.496707828	7.351184578	9.506214972	9.062519658	7.337450191
8.825651709	6.029599276	9.456524366	9.093160196	7.950220534
9.1466105	7.089404453	9.578489581	10.9882781	7.200594837
8.483165519	6.30422674	9.554465498	10.51067122	7.472456371
8.547159944	6.602294879	9.317491108	10.83156086	7.929872323
8.558623642	6.344566958	9.502797671	8.598981264	8.466884025
10.38511879	7.001102096	9.264795148	9.295480982	7.549459894
8.909896664	6.929550879	9.393610571	11.77911857	7.498429782
8.366569939	6.746675314	9.294922649	9.75055331	7.351861236
8.359124664	6.807998607	9.686657161	10.22829588	7.548701971
8.482162678	7.431680426	9.236943308	10.32105006	7.758580447
9.204224454	6.895260447	10.10886483	10.23715028	7.466409206
8.852813323	6.890967504	9.679406297	9.35945923	7.537425684
9.236119263	6.660348169	9.028352753	8.135632266	7.267442076
8.575576424	6.69661223	9.444899044	9.540231454	7.224424702
9.167263078	7.735370997	9.524445136	9.238222871	8.084250166
9.096099272	6.265741092	9.567897152	9.446688734	7.175025705
8.417841842	7.254649361	10.12635743	9.627400706	7.560989357
9.153694248	6.173213938	9.598156313	10.87288762	8.03932021
8.497768564	7.683121832	9.023938845	9.24464215	7.62913119
8.60423987	7.133842913	9.408145822	10.60800038	7.31907717
9.108316404	6.442889768	9.739584437	9.581254912	7.712054019
8.531402652	6.598515977	9.085047272	9.616008119	7.338041209
8.974240112	7.372025291	9.013478572	9.335574051	7.862350201
8.590487975	6.506704769	9.463843299	9.834600366	7.652270635
8.535974163	7.017426009	8.88682581	10.40825973	7.946903482
9.0329969	7.315263451	9.259145667	11.30201823	7.622456107
8.5437996	6.411913049	9.131500929	10.37797608	7.759921958
9.753733949	6.417686252	9.708436506	11.83133169	8.413010443
8.296935658	6.795470448	8.902939528	10.73436027	7.010281401
10.79500708	5.767238015	7.979583998	12.08795797	7.676348493
8.89210288	6.87415265	9.840325142	10.3867205	6.874748317
8.85043654	7.212154223	10.13680561	9.685470734	7.745033056
8.832023683	7.176379865	9.727501659	10.32334713	7.384454583
9.94471893	7.452067831	9.330426595	8.733391897	8.336893598
8.915313083	7.160520202	9.323110766	9.563113825	7.195602888
8.262310282	6.392876471	9.065540974	9.41449322	7.574314557
9.279570638	7.292280034	9.525265205	10.14810013	7.489167023
8.709187555	6.106117354	9.647034351	10.41365178	8.09297287
8.013344534	6.897635047	9.792680818	10.6002535	7.318605084
8.349065067	6.674320902	9.746593329	9.011194767	7.36392777
9.28581695	6.914549109	9.442225614	11.11059627	7.224570607
9.281393027	7.19251889	9.181382658	11.62913596	7.5802828
9.669353377	7.83234263	8.037795142	11.00845044	7.940831917
8.310435404	7.086334406	9.02699832	9.570439887	7.802896856
8.167635312	7.127298929	9.851594269	9.358265808	7.349642782
8.484148832	6.943998054	9.143548772	8.24923483	7.54202667
8.068252854	7.335034469	9.569133085	10.18660288	7.363644619

8.222430506	6.716186562	10.03082218	9.190197425	7.340620366
9.762750242	6.720751831	9.163548376	10.67137861	7.856956327
8.753838339	6.243494399	7.514618585	8.878434704	7.51673858
8.2393489	6.487736854	9.046296663	6.94732085	7.857412278
8.64863778	7.363474661	9.806953643	10.41911235	6.848480025
8.760434712	6.958473	10.37984515	9.633857675	7.869014681
9.862573319	7.092966067	9.798973752	10.44795851	7.545294778
8.159366667	6.192552972	9.494897914	10.83438074	7.378790009
8.88232078	7.08191844	9.759308347	9.854689118	7.121308095
8.862252612	7.48436491	9.526153722	10.61342994	7.11118555
8.439791369	6.801709571	9.963307802	9.77982578	7.676705085
8.293096881	6.507668298	9.032265358	9.292550184	7.517252924
8.374052624	6.272047302	9.160449127	8.494219048	7.643691399
11.18449591	6.416367894	8.12389775	12.50307053	7.829493814
8.674058876	6.729230156	9.196075472	9.606914522	7.891176564
8.456375107	7.116214029	9.110327052	8.494085963	7.092474973
9.259019257	6.690567905	8.270167647	9.822494049	7.313229605
8.636849772	6.389923231	8.738615218	10.49705275	7.459897173
8.248358161	7.115611348	10.16462356	10.06610397	7.333296699
8.963058601	6.739033731	9.84651428	9.728638352	7.8229886
8.4818628	7.108739535	10.28586102	8.882653557	7.292483784
8.678924331	6.665248287	9.576096176	9.68050912	7.08542112
10.68058734	7.66369493	9.831421333	9.124051844	6.905152832
8.674627017	7.608887144	9.898930469	9.644782527	6.442245686
8.130116382	7.502421941	9.495920775	10.0368044	6.994506287
8.10627466	7.359107786	9.397938685	10.10279555	6.595875021
9.769180423	7.586961867	8.866561968	10.13556951	8.418601825
8.290059936	7.271866259	9.253300997	10.50828055	6.810821334
8.593534317	7.601164749	9.456709398	9.961239955	7.241582351
8.677822611	7.436230613	9.065314107	11.42937784	6.849062545
8.404419737	8.120127436	9.971746367	9.342898268	6.714687166
8.597376317	7.753277838	9.562581636	12.14645181	7.355455365
7.783500299	6.798592186	9.545506808	7.587536953	7.359493891
8.616031051	6.91343393	9.83991909	9.530427739	7.568570903
9.119625164	7.368350674	8.924746039	9.363076784	7.439080961
8.658787373	7.305670862	9.00725942	10.51706998	7.434181019
8.798588647	7.86187543	8.924666746	11.72574959	7.669595879
8.646756149	6.654399681	9.808122243	10.77678607	7.56147441
8.887401221	7.353186927	9.68621721	9.606746734	7.079775498
9.458329258	6.696013646	9.832191505	9.026309108	7.183464171
8.363857051	6.903143616	8.661322233	9.427588974	6.53629306
8.804776033	6.422077659	9.492617211	10.5391981	7.477397297
8.68362988	6.761330966	10.07599533	8.488456915	7.508456804
9.110276659	7.350608138	8.583300911	10.12553435	7.29062798
9.888455995	6.855616371	9.233116998	11.15990316	7.735807529
8.621818883	7.25246135	9.060061904	10.81644112	7.632638638
9.044124661	6.494176118	9.445170947	9.263201062	7.568463935
8.449174778	6.981925846	9.190100215	7.957587769	7.248172416
8.267648533	6.951116131	9.77562232	9.344176647	7.240566172
7.997585148	6.850586751	8.931608354	10.67811236	7.133838676
8.832650168	6.577820136	10.10731372	10.35559318	7.586613115
7.982882532	6.890184141	9.258540355	10.06420992	7.498689015
8.372153621	6.623433392	10.32403626	11.37114336	7.257551347

8.848833303	7.290252598	10.10398557	11.29091009	7.813980065
8.300765841	7.175133508	8.694640088	9.042537874	7.455427856
8.268041166	6.866750016	9.709953935	10.18227105	6.977881206
8.509998055	7.83996534	9.558558711	9.695915446	7.07545347
8.372872375	7.434603499	9.824708105	8.226677684	7.364753559
9.350304123	6.794795855	9.657918288	10.95997552	7.628728827
9.690362399	6.720607087	9.539005195	11.26039264	7.576894845
8.36709042	6.905852572	8.949310291	10.37749349	7.711955148
8.202530439	6.868196416	9.02947233	9.712134914	7.184890265
8.286533238	6.791126224	9.746873676	9.446653654	7.504151399
8.190318647	6.755378904	9.280964524	6.059382948	7.138364708
8.158358493	6.784334357	8.627678512	9.920197936	7.267500669
9.406151505	7.374959157	9.63922126	9.962281611	6.89660115
8.061597563	6.612136818	9.881738584	10.53695128	7.193768079
8.478710167	6.795483724	9.900906393	10.10707409	7.45889358
8.369654924	7.227746236	9.269961583	9.768628326	7.179417575
8.370863265	6.972242401	10.3677601	10.54222049	7.275527948
8.784943575	7.323890448	9.0802747	9.988601756	7.041569197
8.511038052	7.36646787	9.279641044	9.034973645	6.617124812
8.663662736	6.86493042	9.680189156	10.7893585	7.250626055
8.609110879	7.702696419	9.75088844	11.40540117	6.786189222
9.245566054	7.11673637	8.598759947	9.86239912	7.341584697
9.281629384	7.069054608	9.175971204	9.080133876	7.006753979
8.815874023	7.246164962	10.426405	9.92658542	6.918791204
8.277022554	7.096856898	9.256893323	10.20544772	7.107572196
9.017469177	6.694785539	9.327728813	11.64336584	7.548105116
8.843547775	7.377479514	9.247936356	9.304189017	7.076810484
9.560110231	7.132858115	9.434079987	10.45512114	7.159892772
8.417956707	7.535720058	9.943399473	9.400433467	6.918126247
8.238100552	6.210520218	8.114907201	10.04770408	6.980517201
8.40261345	7.405949881	9.804832696	9.272237615	7.893735722
8.25978945	6.496053408	9.488917589	8.834516428	7.493653295
8.805192618	6.750357251	9.3803819	11.00845044	7.787748601
8.73263288	7.421481409	9.590623379	8.9603888	7.462999048
8.692954792	6.6906215	9.063482434	11.51909736	7.31333066
8.647245404	7.292513904	9.392454307	10.35042764	7.256688031
8.398978311	7.835715133	9.801045024	9.505327673	7.060265153
8.789590742	7.329062197	9.145344091	11.01327185	7.486196477
8.403434422	7.594985507	9.819751014	10.94382132	6.680292455
8.403004018	7.553662353	9.873981719	9.793207998	7.044457616
8.489446408	6.560931528	9.348161198	11.0984257	7.229138551
8.54850496	7.795652721	8.789013417	9.433188553	7.239658046
8.222247209	6.511937988	8.081313616	8.621634998	7.387093788
8.19711014	7.125127947	9.832127797	8.285199681	7.419601015
9.021629434	7.235883938	8.83586053	7.603817578	7.125734317
8.355918611	7.708554992	9.921777371	9.2672483	6.879731484
8.168971006	7.627894534	9.44456664	9.621218356	6.873002937
8.598072359	7.53946154	9.98993209	7.607861817	7.173869526
8.512915349	7.365854106	9.79014842	10.12771839	7.295296028
9.485052531	6.913055613	9.177568995	10.67753431	7.901451275
9.333905694	7.098444174	9.779711064	12.07809994	7.537086676
8.743356002	6.452902007	9.683707378	9.913083989	7.446182534
8.921844625	7.19632086	10.12676396	10.05052694	7.374452447

8.885783605	7.410218821	9.397765	9.825326465	7.181271627
9.575204904	6.40866376	9.136632771	10.20218992	7.914500175
8.120172501	6.975585006	9.380116004	9.453717764	7.602511029
8.718167198	7.131106702	8.75773208	8.800954694	6.91656918
8.59638645	6.814775048	9.156107502	10.93150377	7.439796524
7.844750278	7.174182132	9.786748968	9.576491943	6.811323451
9.460440183	7.001063136	9.548160127	10.95359111	7.344722484
9.148722183	6.62419421	9.655117879	9.87622506	7.109014157
9.077489986	6.946613413	8.258447511	10.45004053	7.282159074
8.108643444	7.322199584	9.295724857	9.353284701	7.721481663
7.894019143	7.348720158	9.732717225	9.454712035	6.780351046
7.985695178	7.215496734	9.477792079	10.34967677	7.132838143
7.983617121	6.931772728	8.435827647	9.338562285	6.852470349
8.20638294	6.708446495	9.817296874	10.54749742	7.404360563
8.937473285	6.605117281	10.00056655	10.98945571	7.868457941
9.396972514	6.159855437	9.206431143	10.53037933	7.607459563
9.266489668	7.083280349	8.590797327	11.17729239	7.555908466
9.131825156	7.418647978	9.472296916	9.573234958	7.235603409
8.98520907	7.131254671	9.383624952	10.43151456	7.50279461
8.942704458	6.476875902	9.959024399	11.15083105	7.627420085
9.272300395	6.378064482	9.178762368	6.09795913	7.161679748
8.506281891	6.129254409	8.263436617	10.58237866	7.257894727
9.324129161	6.698392059	9.365043941	10.4883159	7.493570311
9.451782037	7.405862871	8.583880662	10.56331794	7.166773334
9.111784517	6.239943731	9.009100715	11.35062231	6.913437897
8.682461209	8.023318721	9.744416411	8.483483079	7.742261896
8.961213612	7.653115127	9.461327376	11.26402811	6.932143841
8.421611055	6.843771661	9.701075101	10.30579193	7.243117191
9.408842442	6.556233399	8.46318432	11.31231663	7.51077277
8.748966638	6.875883968	9.372741084	8.810245046	6.963844237
9.109511021	6.282671465	8.6041104	11.45790083	7.731360508
8.375929849	7.529475009	10.10455423	10.23585405	6.951537792
8.096643235	7.332576959	9.206519396	9.562874113	7.439033523
9.481777585	7.283099487	9.849613572	11.10494643	7.462683152
8.457484818	7.104031385	9.720194288	9.949627936	7.09486354
8.278937932	6.994264173	8.13856978	8.069301947	7.658686362
8.344316335	6.825098255	9.764456992	9.829715433	7.129839967
9.232141545	7.403220944	9.666427355	8.931329429	7.32149359
10.04453953	6.82941529	9.265256731	11.79756494	7.578387695
8.800874196	7.539183104	8.300889517	10.79113866	7.0733596
8.215826429	7.639689784	9.077068252	10.1747657	6.611532855
9.337395035	6.346087458	9.70589944	6.454177594	7.895830537
8.431028143	7.777226091	9.491394883	9.267625234	7.459078478
9.009204858	7.432493173	9.528046165	9.396311984	7.460013691
8.520584982	7.304985079	9.59849894	10.79053363	7.607973134
8.715095287	7.1690975	9.403842152	10.72057623	7.040507067
8.072543637	7.106041338	9.533756015	9.85808946	7.146949799
8.306643163	6.531732368	9.42652139	10.16515422	7.550327948
8.78233396	8.470696945	9.877591219	9.123411335	7.912018133
8.927691368	6.640502164	8.86808151	10.93208633	8.125208525
9.124611033	7.195647794	10.14489604	10.33106376	7.170028702
9.207611051	7.301277198	9.638120885	10.91192355	7.492011151
8.900667462	6.605258208	9.178350355	7.063803677	7.466945479

8.911471636	6.837969471	8.922706949	7.208459009	7.165427342
8.336873267	7.830074094	9.453881407	9.988962468	7.126415999
8.529609324	7.285988718	9.637763945	10.81898584	7.173391372
8.963858495	7.422796948	9.751724953	10.42539926	7.121788562
9.708544151	8.045231225	9.251379416	10.8749359	7.101135928
8.938483977	7.295622912	9.49171646	12.03334473	7.073088157
8.463459902	7.045515826	7.931343448	10.58155911	7.000284112
10.50288299	6.691390806	8.982963133	11.20280706	7.312948761
8.629413755	7.700303593	9.638777368	8.623211639	7.128202201
8.058926732	7.503040972	9.90941201	9.318610031	7.005330029
8.736105406	6.467845567	8.261616185	7.715999462	7.480659945
9.632649999	7.330658103	9.870867135	10.77696156	7.180545239
8.786255162	7.17726226	8.949880786	11.51130754	7.478941526
8.978099617	6.445995679	9.299515623	7.619955571	7.86843651
8.186409669	7.046807151	9.688681171	10.29325237	7.1885017
8.394354719	6.707670969	8.796009156	10.12771839	7.928057719
8.674670826	7.31979602	9.583919203	8.786502294	7.371515278
9.176766591	6.37272159	7.829022145	9.469517978	7.587466423
8.988974321	7.132230992	9.75000082	6.958047819	7.117004166
10.15587888	6.820509305	8.644034116	10.06600611	7.461163932
8.985085403	6.726447132	9.144700897	10.14363987	7.034595339
8.918239108	6.461937921	8.554952825	10.05920479	7.110616884
8.992018258	7.176452779	10.23224144	10.89061282	7.892164525
9.147406323	7.343584992	9.419478574	9.913165202	7.529431661
8.640362121	6.977526407	9.663574335	9.416378861	7.43161793
8.171801999	7.169320416	9.051008166	8.292117922	7.633447195
8.924291791	6.995020245	9.665017994	10.93751674	7.324772187
8.622651929	7.097759978	8.980847627	10.28636374	7.113220527
8.884063198	7.008389405	8.844575295	7.913549882	7.019967453
8.791986559	7.553328324	8.913992332	11.27062709	7.080285671
8.992830614	6.943879952	9.448034763	9.904902731	7.77315267
8.528851539	6.460810031	9.813700812	6.666312811	8.065761371
9.484169749	7.001451598	9.514321738	11.10030265	7.324603724
9.184123259	6.461779944	9.427535927	7.767704038	8.063883452
8.595736247	6.512936904	8.780140057	9.746683094	7.05357712
8.833013975	7.405700325	9.129514135	8.713695692	7.89464076
8.702670313	7.637981034	9.848577491	8.583681429	6.960336896
9.123310988	7.218707092	9.293993009	10.0005432	7.530832104
8.313721688	6.97475826	9.951160022	8.504529621	7.175901525
8.599495151	7.18153792	10.14336474	10.72701098	7.841996765
8.503982564	7.571979309	9.404120186	9.837150837	7.266477185
8.559945504	5.756588818	9.700589136	8.539217327	7.514682954
8.827942777	5.926337209	8.788938859	10.48411666	7.392846537
8.605321637	6.499592365	7.770291474	11.65644009	7.282967134
8.759963219	6.817616645	9.103894181	9.340102817	7.473771226
8.706853839	6.957330212	8.901185828	9.6987348	7.410727402
8.294425041	6.44532891	9.073067204	11.54519403	7.908502739
8.108932511	6.724718106	9.623475043	10.94382132	7.578208859
8.854559033	7.047695927	9.717192438	10.41747635	7.923893971
9.760602352	5.523165552	9.589273408	10.76838506	6.678659394
8.732459437	6.161817533	9.102219704	8.814127528	6.989314537
8.676964415	6.287208671	8.186591785	10.18227105	7.837107265
8.655790856	6.184334011	9.663837237	9.759151556	6.492451674

9.620049402	6.416010124	9.814103233	11.09447868	7.41628261
9.255826541	7.538740911	10.04675552	9.784813347	7.480989834
8.94401306	6.479287491	9.87622244	9.2407814	7.176414574
9.211997165	6.386641223	9.409693178	10.31734346	7.429862788
8.414210732	6.776263964	8.294902389	8.269579487	7.562683674
9.866645554	6.465292999	9.166276665	11.30783514	7.533257302
9.474929282	5.96185246	8.59300394	10.56946842	7.244347736
9.099798069	5.986680563	10.65789605	9.381530427	7.042411274
8.93611534	6.79543789	9.537608851	11.21363201	7.874082637
8.999167453	5.921541692	9.280667615	9.428819284	6.856353697
11.18640391	6.184861894	9.253775293	11.36698984	7.038658636
9.077634862	6.389775409	8.727621616	12.14645181	7.900909721
9.091169296	6.562562292	9.879391454	11.37531397	7.706872084
9.160423701	6.333424869	9.253802449	11.00374089	6.541737115
8.938158277	5.194659671	8.090133535	11.79688542	6.855615371
8.769325698	6.318354083	8.843684694	9.385514633	7.030506139
9.063498312	5.846963609	8.659109081	10.2598785	7.167643111
9.927032649	5.443551328	8.361665795	6.726316635	6.118772224
10.08408105	5.826258987	9.527294322	9.686657161	5.649220057
9.003659658	6.511925745	9.190941031	10.2977418	7.47958065
9.318779009	6.394072709	9.544203668	10.99476344	7.383547959
9.446041441	5.879009594	9.747157981	11.60201184	7.096280528
7.980236234	6.603274205	8.78972594	11.34285422	7.565865594
8.776431505	6.582005779	9.471287017	8.826271276	7.546558403
10.74959894	6.41983264	10.23939482	13.0109962	7.13608704
9.288797421	5.680820038	8.996554857	10.07090411	6.467077688
9.4358475	7.147521439	9.574616747	11.17538031	7.323921609
9.008333552	6.643004349	9.949627936	11.43676738	7.365847909
9.735653489	6.223786532	8.787132449	10.73050735	6.41534057
9.114064122	6.155974318	9.421501801	10.31734346	6.231211947
8.632251228	5.766098493	9.083461478	8.265256921	7.145999714
8.649648968	6.545545185	8.823828573	9.901092263	6.905987848
9.102241784	5.438359297	8.552802552	10.21716309	6.73737087
8.959257675	6.040324598	7.845648008	7.74397845	6.98629887
9.751231912	5.908891634	8.76391383	8.463444087	6.50212659
8.603589307	7.148062839	8.679277234	8.773691246	7.118197291
9.548410159	5.837521724	9.162533956	9.496429576	6.737616275
8.931754798	6.055617711	9.601377601	10.52463046	6.838887921
9.519500183	5.860548496	8.888285778	9.181767536	6.90293152
8.883804099	5.926221461	9.537608851	10.7910614	7.372686032
8.826752233	6.10009102	9.310151871	9.534992703	6.773726903
8.591647318	6.25306502	9.266545191	9.776112722	7.038886311
9.105017544	5.786901122	8.754985143	8.882910646	6.838531721
8.645355999	6.097443467	9.038043189	10.77964784	7.53959579
8.854533586	6.349159261	9.646726907	10.56715012	6.838125109
8.858151526	6.046987258	10.05155101	11.41585024	7.240138081
9.001750261	6.756222837	8.931747462	9.666796104	6.952811574
8.810381516	7.080942532	9.230343974	10.37673568	6.925537859
8.604940479	6.419733802	9.595827742	10.1818852	7.344656507
8.719267564	6.129687579	9.210540151	10.99476344	6.534522528
8.784111927	6.821227618	9.876896691	8.956450755	7.856871173
8.950702885	6.595951036	8.842270904	9.768628326	7.289025239
9.593005164	6.14114223	8.737005993	11.7012715	7.390055782

8.674960959	6.610683433	9.471011751	10.56795889	7.701690734
9.670647702	6.471775022	9.131662719	11.89201482	7.4488891
8.814415531	5.936312495	8.364630466	10.08518564	6.995157175
10.79681311	6.573921388	9.031885721	10.6923392	7.305918895
8.796688996	6.03470437	9.835420408	11.69892019	7.395987
8.870588513	6.940495259	8.538236548	10.33551492	7.85227092
8.632509361	6.707260292	9.283964301	10.24156465	6.995968283
9.696223285	6.373595932	9.260037345	11.59535023	7.008343326
8.784869463	6.58410956	9.55514301	9.706466918	7.270601821
8.953938306	6.387763497	9.629244863	10.33097171	7.208473981
9.038742668	6.602188176	10.01937014	12.30022713	7.37243187
8.549037566	6.148133296	9.032419181	10.26447498	7.244788548
9.289897254	6.35451328	8.450065912	10.41105127	6.619587901
8.593064952	6.111590154	9.902760039	10.14517726	7.040689731
8.916845032	6.003296561	9.698085319	10.28636374	7.45266105
8.632009243	6.638200378	8.286103454	9.598602488	7.355513146
8.994318831	6.156788705	9.047228916	8.865663194	7.251885692
8.314176032	5.931040992	8.572880416	9.291604178	7.626330981
8.952388008	6.577332282	9.235921127	11.32397393	7.25731139
9.086141235	6.201503159	9.185049125	11.80164846	7.684429732
9.481962343	5.929130259	8.499712048	9.986307895	7.917219978
8.495836946	6.939694846	8.668989426	10.17879179	7.475487435
9.456778625	7.064528586	9.25590762	10.74577158	7.896305807
8.941061376	7.195859267	9.610232963	11.77884015	8.141625334
9.300112908	6.82601096	8.86808151	10.68579136	6.727564795
9.024194644	5.562657061	8.064763694	8.641837962	7.02559661
8.471369235	6.59954721	9.719573619	9.447002273	7.121708207
8.993462569	6.409539022	8.77757199	9.724270014	7.796877369
10.3063675	6.615618323	9.435102491	10.07318073	6.95084426
8.958346024	6.572654375	9.936236028	10.38852138	7.369305857
8.263705868	6.416190184	9.492617211	10.1376079	7.399018319
8.633466439	6.814026088	9.149202088	9.332427022	7.414822772
9.273334108	6.865489888	9.971625378	11.46894954	7.255375072
8.388993211	6.377080467	9.398306962	11.28489191	7.07425926
8.830828303	6.557185912	9.318521815	10.60781939	7.516918987
8.695795861	6.824891641	9.324613574	10.48452692	7.086887496
8.288265984	6.543715512	9.395859317	10.12884987	7.451577707
8.768619598	6.223094938	8.256963667	11.42021739	7.490919096
8.075862597	6.259550398	9.095070386	8.501706422	7.750065993
8.759497129	6.172494123	10.49773135	10.30883324	7.181002292
10.11383977	5.842589569	9.415981326	11.48206473	7.847060944
8.760644877	6.17427648	8.290129476	10.17252116	7.590625492
9.257320721	6.110473906	8.726514118	9.98644224	7.168600493
9.109252674	6.804244785	9.21572715	11.04213654	8.146383322
8.643752862	6.915395713	9.929365034	9.776652193	7.790217709
7.790309487	6.380938251	8.680002726	9.844320342	7.459051841
8.87528183	6.061665209	9.181767536	10.45294854	7.591848538
8.476657349	6.110555079	8.74336346	7.432522652	7.557323952
9.03366886	6.157096736	9.575706273	10.59674638	7.780094972
8.434511811	6.464926109	9.168564959	8.335375296	7.859628062
8.571051374	6.681657194	8.406276504	8.604839515	7.703745935
8.645603994	6.855581395	9.68226968	9.698284839	7.432228138
9.007318228	6.562958113	9.667578887	10.32730354	7.620004504

8.570144408	6.678319497	9.720674446	9.822450475	7.357796754
8.65385724	7.239997949	8.194554265	10.96809512	7.394328544
8.53652646	6.592177901	9.004431022	9.554525294	7.914896133
8.767381474	7.018627718	9.987442872	9.07287069	8.118378724
8.566674154	7.046127558	9.387839696	8.802399596	7.355954293
8.930355801	6.738488377	8.94172558	6.745296538	7.011981556
9.390951738	6.416113146	9.776142755	11.4466819	7.363598593
8.945422379	6.697220364	8.733359302	9.64108694	7.329109828
9.054666598	7.646446694	8.772698683	10.03197305	7.416206208
8.462311024	6.405308549	9.528608093	10.05338963	8.326037582
9.396809597	6.49063476	9.077384079	10.30825088	8.157724857
7.843741245	6.678927263	10.29503785	11.4704574	7.608529049
8.380943066	6.477265516	9.801821031	10.17941471	7.442566902
8.876714403	6.82548908	7.748668131	9.246989415	7.891353432
9.075409791	6.377007188	9.401460456	9.741103044	7.686590496
10.06464149	6.78809377	9.154470321	10.76408521	8.135160958
8.286359097	7.406216541	9.607455396	9.371247523	7.473870457
9.1341356	6.293635542	9.210812131	9.162637533	7.788192528
7.980980711	6.539780708	8.700589809	6.484509503	7.285735612
8.682071817	7.278896177	9.303721595	9.660239533	7.523323022
7.975779446	6.267192224	8.968062974	8.541326628	7.826477791
8.787600465	6.268973484	9.938283887	11.43052476	7.261584553
7.914045092	6.519367418	7.876470689	11.45790083	8.818120693
8.734092795	6.542424344	9.63865313	11.1702577	9.017337811
8.795412619	6.798423419	9.799154021	10.15027459	7.966598547
8.570590431	6.312960292	9.337002932	10.2927097	7.865639313
8.476854465	5.60713219	8.851206363	9.891054908	7.550844754
8.319675522	6.848933284	9.655161578	8.284230064	7.837732662
8.405617265	7.207488257	9.509068326	9.493915223	7.635895687
8.627298811	6.224663608	9.525948543	9.258968994	7.384152257
8.918327799	7.206627644	8.699854735	9.84292636	7.669649807
8.23601128	6.395582132	9.080365111	10.1014445	7.464902065
8.997493422	6.163407236	8.722451487	9.439420963	7.514491482
8.265000441	6.635412848	9.496429576	9.055969982	7.479003419
10.72598049	5.933202176	9.485183861	12.56120284	8.331392091
10.57446044	6.314838481	9.341466879	10.856573	8.090458854
8.315978472	6.570033445	9.881115801	9.197090044	8.025502492
8.855040605	6.600549566	9.523815298	8.557858703	7.738196469
8.964772301	6.222340861	9.969903361	10.58559649	7.103482672
8.584494981	6.829639259	9.781339542	10.93403427	8.089967324
8.705633813	6.303776579	9.31917645	9.747404765	7.133063014
9.119348961	6.744629709	9.337852167	9.276387121	7.585488013
8.428155339	6.530251383	9.493880027	7.920683539	7.297121629
8.698849837	6.19083689	8.846237726	9.226294845	7.543668993
8.950779373	6.662295889	9.818877058	11.59127263	7.163269719
8.573732617	7.532555719	9.35945923	9.351526138	7.147883058
8.82897188	6.881535671	10.15670762	9.839610887	7.57379834
8.813567696	6.198056288	8.835311771	10.22131371	7.420842671
8.381137748	5.773871437	8.708445554	10.42819144	7.338711792
8.447195072	7.02470943	9.811813393	8.550234306	7.43726411
9.877021916	7.58073542	9.456272866	10.32081176	7.101188421
9.49108684	5.656794623	8.154120983	9.980862547	7.825286385
8.653137077	6.440913042	9.300112375	10.79270684	8.379377341

8.041977801	6.503174656	9.274105048	9.198492521	7.430519881
8.98525367	6.847242675	9.200225838	11.29401764	7.436007694
9.624040259	6.237639102	7.435267757	11.33679909	8.110755749
9.177286589	7.135802072	8.932639492	7.806039905	7.130713367
8.238253068	6.735173601	8.779883579	10.11892241	7.156873534
10.62015768	7.309336732	8.938625607	9.646190311	7.484898633
9.112266437	6.715467582	9.921128713	10.42200144	6.990918528
8.11943313	5.93564461	9.841047272	9.03108954	7.637864275
10.22780974	7.345790316	8.993769656	10.64759239	7.103356199
9.688054332	6.20795321	10.35039663	8.755073622	7.195289066
9.698912393	6.152191516	9.101398062	11.24159356	7.931058785
9.030815685	5.49774608	8.835350543	8.159564311	7.256972689
8.912493037	6.973442381	9.705257656	8.693967212	7.318372838
8.748661987	6.022418648	9.243217305	9.827703238	7.742906765
8.562684156	5.989986426	8.741728917	9.727764781	7.284781967
8.856030072	6.000983713	9.593677503	11.2886851	7.706633483
8.294858485	7.597454864	9.009100715	9.876117692	7.236486151
8.877252787	6.997898635	9.927648257	9.521919142	7.240741119
8.836773464	7.006765069	10.36738909	8.895974219	7.321201092
8.788909569	5.791719322	8.577976463	9.294876597	7.538390174
8.537391668	6.430612179	9.276403332	9.720733559	7.88710147
8.531825427	6.603477417	8.489410784	11.38191098	6.756719692
8.604288961	6.79195865	9.384311035	10.66413429	7.281230664
8.802670666	7.791940531	9.681687701	10.26572854	7.025292534
9.413749901	6.749908243	8.379721395	9.506154964	7.284987562
8.682606259	7.035148322	10.64428604	10.70945604	8.080403126
9.626443206	6.790977798	9.762468467	10.04922887	7.608714948
9.714220093	6.356035011	10.3446575	10.98937339	7.333046801
8.39270377	6.308805334	9.081382048	9.992690787	7.335156826
8.747481439	6.395143179	9.7306	10.4614736	7.346407289
8.520813209	6.288010661	8.359231228	10.90559384	7.92832327
8.726054491	6.609138326	9.122972774	9.912670027	6.897439217
9.145468231	6.46902649	9.486639359	11.54519403	7.89207011
8.380605496	6.924470385	9.98993209	9.704603935	7.073051875
9.203382254	6.276365195	8.803432582	10.33349819	7.127433369
8.581931494	6.324455645	9.018642211	9.362959854	7.270826208
8.955297988	6.007965596	8.888104504	9.971328947	8.109682272
8.327061719	7.515401556	9.334823785	8.698292449	7.264301592
8.77629126	6.648249429	9.107426946	10.16292898	7.244243101
8.611269236	6.925510237	9.491256739	9.321186653	7.646458358
8.747824099	5.695485337	8.838837394	11.36748114	7.597116393
8.78161964	6.764166314	9.577682547	8.48867517	7.524751089
9.239310571	6.09081266	7.539905871	10.97709236	8.560731413
9.691439205	7.276767761	9.240575468	9.394246399	7.719513734
8.70314861	6.487789059	9.608768893	10.86620926	7.001038451
8.32687219	6.253924091	8.569154775	10.14517726	7.805853099
10.1449848	6.96020767	8.61433201	9.835022218	7.276306355
8.305104284	6.346005737	8.511741363	11.04628813	7.478951421
8.424671938	6.826235339	8.370825095	7.792929147	7.780261783
8.918994112	6.693736165	10.03912866	9.695924528	7.542658376
8.716066488	6.56239889	9.279531079	9.7000756	7.670885486
8.44659699	6.912338433	10.04131062	9.485102405	7.474058486
8.340673108	6.598108299	10.34931481	8.03358657	6.887365032

8.367604169	6.981036129	9.012346617	9.760339777	7.202577398
8.939279901	6.950484586	9.795188141	11.56735828	7.898595995
9.165423653	6.513981695	8.883407199	7.616944465	7.748067168
8.852428619	6.732694803	9.434114034	10.63501198	7.687165084
8.792715593	5.900205109	9.208668264	7.525316932	6.97625472
8.667142429	6.24782702	9.357355062	10.13704987	7.885505342
9.866079548	6.954685246	9.114660512	10.24641504	8.623537303
8.665823123	6.220708757	9.483899448	10.64759239	7.661665301
9.250732167	6.215424347	9.050622289	8.079255372	7.098012131
9.009408464	5.732755205	7.540392045	7.815210511	7.173993803
8.324821377	6.386087999	9.279489421	9.520143737	7.142364741
8.583896971	6.96945267	9.969329894	9.86477811	7.409454593
8.444915524	7.265742377	9.318521815	10.15385676	7.887430433
9.03103289	6.564310316	9.613810017	9.522032677	7.551295692
9.53086474	6.289519669	9.163264302	12.32218819	7.962882999
8.892711607	6.275887139	10.03623216	10.31007522	7.718862198
9.616174913	6.318105589	9.207695068	9.005373957	7.496144964
8.305997138	6.042717696	9.875903526	9.810548132	7.369005756
8.974693178	6.216163948	9.598972402	9.424947012	7.976343641
8.427779969	7.011402984	9.438809344	10.6223821	7.874488225
9.23126772	6.469743497	9.238333188	11.90012452	7.810446923
7.906341296	5.914846995	8.619307774	10.75418638	7.652759375
7.916748592	6.551347951	9.53245947	11.83133169	7.671056967
8.181029782	7.419981981	10.12884664	10.58155911	7.941563454
8.294089279	6.9160106	8.471756381	9.912274956	7.318098834
8.477875776	7.055178588	9.650979023	9.519441483	7.32366334
9.1637851	6.888195544	8.723103566	10.89900986	7.944725718
8.388409724	7.013229852	9.334447459	10.35257179	7.229987668
7.834580422	7.022488165	9.062317629	10.01923528	7.681072373
9.125394573	6.514129139	9.2034516	9.913944087	7.3429033
8.620485867	6.002554272	9.212964008	9.977247573	7.369189125
9.02962181	7.434550578	9.41166089	11.21869657	7.959208092
8.679410133	6.865341949	9.493329581	9.675347561	7.585144249
8.144126335	6.707377759	9.160875856	9.331093464	7.917662658
8.454393197	6.773165577	9.812622522	9.85424497	7.020852931
9.785759634	6.2188922	9.254555713	9.66473024	7.711900981
7.928254907	6.381574499	9.367578781	9.046461206	7.339580478
8.354907617	6.97236187	9.241037109	9.757214561	8.323771834
8.974153525	5.842155712	9.293570135	11.41585024	7.846491992
8.829089585	5.998372127	9.477938182	10.72315806	7.500611325
8.809978921	6.480519892	9.298079809	9.672410426	7.238386854
8.17785897	7.536686716	9.387839696	9.540954956	7.599939527
9.482324827	7.058795252	9.937887847	10.91694569	6.890801351
9.260303752	6.945420881	9.220960352	10.29066542	7.454525192
8.226755371	6.643383995	9.902578726	11.01448743	7.364700259
8.552741538	6.351882721	9.096015472	10.04194008	7.364369872
8.626572676	6.241306619	8.202140669	8.966367547	7.823925907
9.493585488	7.079751275	8.96253848	10.65856582	7.68920435
9.263324255	6.224738685	9.506214972	10.76998627	7.853986913
8.49742123	6.811872153	9.191971707	10.32918233	6.90937912
8.559802469	6.225233356	8.357613577	10.38852138	7.850931123
8.397337238	6.080675873	9.608307773	10.33986305	7.300228576
8.741912936	6.011082852	9.20434481	7.819358022	7.967825536

8.285492565	6.365389148	9.697238755	10.3864015	7.978298053
9.106557424	6.487679506	9.116576443	10.49936891	7.688777472
9.090811455	6.691100045	8.555232301	6.298800152	7.324850528
8.385213589	5.377612887	7.197457752	10.54731948	8.966988532
9.373005438	7.525522472	8.612115272	11.45446824	8.567294893
8.798321655	6.446129497	9.825740231	9.564225841	7.343145739
8.709236324	6.579332652	9.447550057	9.48747832	7.592642743
9.139852077	6.768317448	9.421201417	10.67553978	7.271292278
9.366959904	6.794831358	10.03730626	10.26698069	7.807953413
9.424958557	6.03525778	9.120183979	10.72663423	7.589297356
8.803861572	6.61328496	9.795196082	9.741056316	7.594171449
9.239745012	5.414604132	10.191241	11.5276069	7.394550887
8.499448121	6.359916555	10.00229584	9.496429576	7.645992233
8.517691817	6.229527931	9.50431845	10.50269122	7.192763704
8.41556051	6.928546588	8.925841207	9.502015717	7.514957959
8.879136488	5.834972418	8.545680959	8.367909683	7.316574945
8.93379342	6.531810932	9.823247286	11.12345673	7.197855639
8.792733868	6.676051007	9.584580945	11.10030265	7.155072789
8.854975325	6.661654312	9.541631576	7.970670549	7.855755323
8.976269801	6.224493224	9.289423765	8.719889155	8.097894197
8.826810788	6.590576544	9.129778264	8.192037928	7.735846131
8.645354867	6.919428633	9.289779176	10.39438298	7.463471239
9.170235345	6.359964224	8.962376328	9.266045108	7.517777415
8.628528618	7.183127384	9.849613572	9.71115915	7.678632684
8.375673203	6.71690734	9.57530858	10.2508409	7.587894995
8.100427558	6.262682304	9.394729795	8.937484678	7.802548449
8.958532298	6.397695917	8.137395522	9.706965011	7.627410635
8.669706357	6.741037612	9.280725417	9.411979715	7.411561349
8.94322218	6.035356299	9.479572152	10.00431059	7.905265923
10.37956744	6.713510902	9.421385204	10.77447136	7.02187646
8.829199817	6.808208711	9.937887847	9.871096749	7.258975597
8.870667708	6.594282509	9.344232855	10.54684832	7.30110804
8.613464559	6.641291556	9.751136734	9.464974429	7.336842412
8.932043847	6.068988518	8.689571171	10.16007252	7.350643828
8.2190552	6.511048598	9.326578987	7.971954579	7.441537195
10.53856573	6.956641438	8.32467829	11.97073221	7.386419494
9.027590617	6.721870311	9.680089315	10.87688539	6.883576278
8.165087546	7.108441787	9.942668794	8.51845282	7.619617903
9.274929854	6.246056377	8.860155992	7.096233981	7.448229318
9.08110455	6.678444817	9.804663855	9.20064965	7.643804203
8.202256714	7.149246471	9.856235033	10.8685538	7.855096545
8.418843878	6.397367199	9.336664778	7.818308382	7.315426404
10.02524741	7.071980949	9.622174478	11.41585024	7.138713181
8.341314696	5.972098384	9.218819723	11.19868557	7.606657811
8.481730683	7.077055687	9.69822897	9.660243837	7.231818776
8.842102711	6.796975055	8.752342898	9.017182109	7.612792891
9.175355542	6.409373198	9.460334582	10.31770708	7.754677561
8.848740863	6.241873366	9.848313563	9.75752986	7.213035558
8.802485494	6.311012006	10.35305217	10.77792944	7.200282188
9.138250759	6.533335018	8.957720195	9.702629103	7.269058318
9.175825324	7.075018533	8.818357133	10.45877712	8.188446603
8.374678692	6.129577242	9.792698923	11.22872982	7.594535304
9.408781082	6.234619646	9.52214794	9.666456784	6.929977168

8.19277826	6.482244781	9.454045893	10.99615501	7.498523235
8.976678648	6.599070079	9.640980573	10.0095804	7.554439402
8.707382241	6.923723165	9.26875439	11.63787451	8.247647589
8.409459304	6.682124569	9.261318859	10.19942056	7.243404195
8.832446943	6.75134984	9.764175848	10.67973532	7.181569726
7.519881181	6.163728192	8.305964003	8.018322607	7.733560873
7.9501267	7.371647922	9.829753344	10.79861549	7.92964443
8.544326138	6.856587761	9.380167444	9.216395822	7.242714693
9.100413323	6.386227463	9.457932508	9.94783215	7.541474675
8.689993156	6.937712446	8.798684766	8.083993742	7.150743899
8.701384224	6.848412835	8.873040725	9.67724217	8.08660378
9.322460593	6.190651764	9.282865942	10.09882319	7.834708088
8.408437378	6.645365197	8.909675499	11.17729239	7.118830661
7.895120632	6.368341286	9.554768348	10.4868894	7.768505923
9.070047808	6.017338589	9.603893853	8.974586771	7.706947798
8.858656376	6.924269198	9.20236133	9.891068194	7.066364291
8.681066869	6.737542985	9.484253236	11.50583251	7.170832261
9.123870352	7.340060033	9.526473753	9.98140167	7.652022524
8.538424819	6.376263796	9.545765531	8.395808206	7.512897137
8.797928766	6.268358978	9.251646643	8.719961128	7.251561424
9.373074275	6.564010715	9.533940881	10.73019283	7.085526829
8.695348908	6.344481967	10.0364142	8.834788017	7.763386853
8.409049148	6.416932035	8.346996137	9.528084091	7.568774928
8.635743306	6.89307841	9.605230314	9.519464666	7.274379864
9.979291168	6.708710224	9.134525334	9.762242654	7.425249449
8.413394291	6.790776592	9.113783048	9.868027964	7.694160951
9.277924103	5.770866754	9.261135772	10.37021909	7.282545043
8.472464214	7.097165243	9.28870472	9.984261047	7.083472916
9.116637271	6.819872232	8.346100619	11.81593204	7.960250552
8.762081825	6.695360899	9.420030428	10.58538175	7.566722096
8.488308113	6.644667325	10.16019572	9.018651783	7.344184808
8.677537238	7.088980587	9.666868797	11.22131285	7.846747197
8.769709041	6.340506992	9.135515203	9.058717086	7.686117048
8.573805015	6.780183408	9.176659745	9.242295348	7.277065709
8.921547011	6.949663948	9.543345171	8.15435245	7.700576719
8.859670095	6.733501799	8.610988334	9.220150761	7.817095002
8.130917109	6.113920466	8.160357986	11.28207665	7.215131016
10.21206873	6.557866002	9.601377601	11.48047936	7.271593623
8.976266117	6.599988354	9.608338027	9.300307263	7.371585849
8.509068559	7.397265583	9.340102817	7.294813907	7.642704507
9.002410925	7.393448851	9.005537002	10.46715737	7.413713626
8.41120149	6.531993848	9.169199316	8.638817403	7.45775822
9.006196997	6.068949156	9.240143877	10.51278739	8.069486118
9.415835691	6.908645387	9.427594569	10.76048879	7.470225232
9.756143605	6.445715467	9.749816487	11.65673554	7.058146945
8.141380373	7.66492804	9.067522377	10.00546957	7.667763173
9.733784156	6.307595631	9.457701517	11.17265435	7.623483194
9.289947556	6.128929709	8.941963267	10.78710311	7.231530831
9.102442634	5.970839025	8.969407007	7.166198733	8.316619821
8.961340916	7.717812903	9.50023023	10.51278739	8.092074591
8.364364944	6.752477657	9.063266159	9.745040893	7.926963513
8.646465669	7.070352948	9.317255141	10.95734681	7.412919035
7.999086281	6.548384948	9.267348074	7.33036165	8.128831545

8.290988607	6.507236792	9.967734217	7.996939754	8.007891648
7.668985461	7.868626114	8.894289003	11.05543456	7.266394003
8.917793611	7.326359378	8.831593804	7.969794499	7.589478932
8.848610984	6.861669684	9.0608145	9.915705558	7.106957224
9.136277276	6.358937451	9.14431073	8.988420164	7.879062058
8.021373078	6.755396842	9.434565734	9.757012604	8.156319899
8.698854547	7.284843407	9.884189283	9.18416009	7.521858363
8.142418808	6.791881596	9.742214241	10.856573	7.340527303
8.802073516	6.045006969	8.645220921	8.964753703	7.750996603
8.006674992	7.044482017	9.874403887	11.47310062	8.119870678
9.112193471	6.210382256	8.150059106	10.33048236	7.996607959
8.44738225	7.142542387	9.548735327	9.019982785	7.99812417
9.940248365	6.733714926	9.8913096	11.48552756	8.272552791
7.923381421	6.864028839	9.750460302	10.08870688	7.194475708
9.744928394	6.462260258	9.420963376	10.08931137	7.661611137
9.104397273	6.026177443	8.992595802	10.40538979	7.562501494
8.977607421	6.853038981	8.979046593	9.622028041	7.559381105
8.159259416	6.29415507	8.791587698	7.774110833	7.31877971
11.20211249	6.747136882	8.311831609	10.54299057	8.205501136
9.140173909	6.762638842	9.424762187	10.65210923	7.580062789
9.751523063	7.797297739	8.669340348	10.1445475	7.856500554
9.369749695	7.762059121	9.180319241	8.907333735	8.051330411
8.793679131	7.104239182	10.44094002	10.27066423	7.66380232
8.988714712	6.693487625	9.028909212	10.34169733	7.587445512
8.600692203	7.213089699	8.917595631	11.314309	8.206195438
8.230041142	7.644422796	9.468589828	10.55378028	7.346856592
9.313441896	7.156791343	9.565258324	11.31180683	8.102311729
8.524607356	6.241201253	8.388068263	7.664157747	8.040359734
10.34088801	7.408939269	9.149294899	10.95115111	7.8337227
8.003523247	7.080719401	9.68080561	9.304679572	7.831798955
8.220596982	6.944192479	9.442349446	10.60693801	7.476685345
8.258051582	6.430122792	8.668471607	9.434803861	8.069932559
7.895602391	6.964827273	9.135969944	9.581657091	7.725085638
8.550773169	6.989809103	8.903630521	10.56715012	7.676261307
8.696644308	6.502309165	8.638195054	9.87116999	7.676415067
8.436360566	6.586754243	8.600704936	9.300844264	7.505114678
9.185135493	7.104942388	8.794472344	11.35088862	7.515822765
9.097756639	7.549120112	8.512047262	10.19942056	7.915850666
9.251292493	7.303756157	9.416554442	11.47171563	8.249201721
8.208524772	7.073477826	9.456474129	9.57736415	7.487487917
8.527203896	6.787285151	9.548346463	9.319005344	7.138359301
8.811639873	6.888768811	9.664504133	9.991295989	7.58122411
8.350763494	6.734873454	9.262774513	10.42115998	7.043622434
9.484901267	6.909952316	9.241581997	11.31281732	7.716202266
8.64854984	7.112038038	9.64567398	10.20139837	7.627649898
8.446643288	6.506310234	9.062555033	10.0806781	7.417446359
8.196485205	7.118814231	9.341727732	10.28551904	7.409050019
8.734819974	6.877145997	9.548690762	7.80004093	7.588873356
9.811512278	6.950133091	9.2448729	10.60097011	8.103247351
8.771401754	7.400258238	9.615183163	10.17682815	7.659087672
9.024846109	7.376129886	9.691448923	10.74095277	7.407007602
9.764940752	6.388382471	9.003699782	10.59131402	7.523048646
8.686287304	7.162056806	9.119662348	9.50023023	8.069674418

9.328121688	7.148189995	8.805869918	9.694602808	7.500875621
8.923539886	7.304630325	9.292706553	11.16514234	7.279886503
8.670758372	6.099296578	9.114310498	7.055494642	7.522985844
7.881757151	6.604107168	8.976642102	10.20391386	7.734540446
9.121654026	6.755529938	8.794251654	10.03386505	7.536633038
8.895600921	6.612418352	9.72347621	8.196684372	7.752497081
8.632250838	6.970993913	8.968652184	10.1508927	6.950462445
8.985875026	7.149736597	7.925329124	10.85733596	7.978014902
8.836663834	7.007427227	9.30171828	9.670629195	7.537923602
9.32607048	7.171883897	8.963227627	10.8685538	7.419884523
9.662317741	7.055868557	8.798378807	10.56178966	6.948040958
10.01499166	6.478505554	9.184816468	11.50344301	6.893676948
8.960619733	6.939311933	8.853457766	7.647632084	7.007764012
10.14697657	6.397479927	9.550833718	11.08403382	7.798445509
8.778837119	6.348743564	9.617588673	11.15448365	7.096993051
8.20687729	6.877358419	9.48711956	8.521331754	7.284320022
9.382917439	6.42049703	9.496515506	12.05552519	7.393426367
9.357996666	6.51183898	8.427166738	10.46667397	7.998228331
9.011320053	7.029125761	9.771557241	10.79919087	7.120186531
8.419579617	6.41998827	9.113224735	9.246160892	7.581107479
9.014029974	6.880715618	9.140438578	9.90967929	7.508900767
8.133448228	6.891541612	9.714250162	8.600433062	7.255537271
8.610393105	6.100463897	9.009178063	8.060777933	6.870924677
9.085696876	7.720859119	9.861437181	9.232774567	7.530336576
8.270668098	5.995607708	9.279638369	9.563948388	7.164525233
9.569479323	5.569475579	9.290527406	10.7609233	6.406206246
8.934609395	6.314998184	9.782272859	8.0167114	6.803084378
8.731889598	6.405971764	9.383869636	7.858793488	7.389616512
8.865070559	7.199663322	9.597301922	9.621948686	7.395312709
8.326111639	6.733541492	9.053110222	10.44192065	6.795812829
9.074119703	5.882900674	8.664306956	10.99476344	7.235803736
8.93976757	6.768478955	9.921650289	9.786938168	7.198766606
10.52284777	6.48807775	9.675072392	11.14404619	7.246357152
8.897143979	6.913241765	9.476377675	10.18644478	6.881151666
9.082673613	7.183537355	9.759308347	10.58921675	7.089344259
8.804688723	6.735043623	9.342358229	9.039000486	7.20577459
8.553450127	7.376211322	10.16510265	10.12101337	7.130483704
8.596890884	6.332923617	9.443070976	11.3883912	7.843206635
9.122806092	7.595440705	9.732011342	8.946296265	8.037913241
8.906195357	6.193932202	9.145344091	9.376905038	7.151823375
8.768320484	6.93348755	9.176659745	9.831362477	7.103251318
9.498420267	7.266687621	9.225241437	10.20164328	7.35232967
8.725459104	5.718387795	9.813700812	10.03049903	6.914045499
8.607929229	6.660032546	9.615132233	9.527294322	7.287397195
8.644138769	7.751124264	8.547957301	10.64759239	6.851774038
8.679523175	6.309105023	9.330019926	10.12313607	7.056890137
9.145013348	5.629749581	8.521889076	8.417846676	6.548696929
9.257640935	7.442904612	9.765207302	9.57728954	7.132621274
9.326287811	6.959509135	9.791677574	10.08673784	6.813806488
8.522806745	6.37533504	10.8820257	10.56396516	7.397457716
8.357464497	6.729550275	9.271952775	10.24384187	7.1628595
9.153908121	7.091725344	9.762242654	10.42274373	7.546879265
8.320859609	7.590621685	10.01521102	8.470886578	7.038035059

8.200869947	6.067378407	9.562581636	11.0984257	7.136129476
9.266618313	6.473790659	8.643327581	11.18637528	6.909782946
9.341269989	5.897512987	9.184816468	10.45573456	6.689513627
9.404676949	7.428978434	9.848071904	10.65439507	7.574344638
8.398915798	6.2785917	8.354015801	10.66792052	7.324845495
8.761731275	6.55985186	9.386073922	10.62794534	7.194474723
8.622371511	7.220517802	9.586548263	9.762242654	7.596173688
7.935377049	6.741655675	9.785402745	10.92022786	7.636180151
8.855237845	7.336526429	9.67073298	9.77913999	7.26306866
9.194820792	6.413666187	9.818464983	9.088320942	7.605925535
8.93713853	6.621206579	9.498942742	10.75331333	7.046520783
9.567224814	5.688138741	8.072076992	7.593329565	7.478872645
9.159251644	6.011358752	9.626060118	9.409693178	7.623748161
8.471733657	6.669472142	9.790085376	11.05977372	7.533654282
8.626060441	6.586680513	8.837812302	8.409261839	7.512669865
8.546246608	7.078460093	9.181767536	9.327830183	7.268379791
8.629434328	7.65032216	8.756787008	9.3919056	7.988103563
7.91546249	7.497648476	9.57728954	9.972936878	7.483673791
9.262427069	6.964181622	9.913083989	10.84939814	7.544827477
8.61355904	6.670325906	9.704104178	10.70945604	7.718810995
8.633210582	6.748388544	9.932062225	11.58447511	7.490757212
9.211661849	6.575066677	9.362967141	10.14596671	7.4530886
8.66953132	6.876243112	9.616473121	10.7023975	7.419014728
8.826456428	6.633737524	9.958467891	10.25517511	7.384904246
8.870217171	5.964094816	7.974326568	11.17538031	7.818879716
9.703381057	6.715580828	9.017441445	10.00229584	7.474286351
8.600811443	6.690901857	8.856144205	10.26211319	7.700776667
8.466151806	6.833564682	8.963981416	9.68226968	7.568738714
8.588375634	6.736079627	9.524641695	8.8394395	7.677067118
8.79622075	6.682428983	9.491394883	8.898382901	7.353379367
7.939356002	6.727132188	9.67073298	9.833055489	7.64426689
8.114374348	5.95936943	9.578638025	9.701156445	7.707653094
8.702466171	6.600505829	9.447896591	8.050040578	7.729053645
8.610403931	6.595141869	9.54158038	9.244408341	7.814572316
8.732162247	7.294077208	10.41485481	11.65499135	7.800504645
7.598999136	5.940957573	8.748108386	10.24384187	7.475065322
8.98392043	6.212709336	8.98115994	9.409693178	6.872721128
9.478290093	6.259248509	7.803431177	11.98632586	7.692323479
7.655673382	6.983489662	9.738092324	9.332258866	7.477765422
8.762690337	6.514143353	9.641295065	11.15483157	7.795279333
8.23987334	6.319560348	9.304679572	9.341257377	7.394831216
8.919080284	6.465309464	8.717658278	9.01837398	7.764669876
8.330519386	6.378579422	8.392142723	8.517740184	7.593209956
8.436718078	6.493996932	9.523348923	9.330019926	7.751656274
8.81004584	6.885055508	9.404974059	8.033340756	7.837038845
8.881247003	6.532831381	9.128519266	10.66468066	7.823418353
9.09107949	6.752270541	9.162533956	9.119662348	7.426275478
8.692139755	6.173056074	9.215702913	9.928550856	7.18628081
8.830789892	6.673525563	9.129551752	10.94655569	7.637304632
8.226585795	7.047612383	9.330019926	10.73835412	8.067551543
9.394059028	7.561808419	9.434672017	11.20280706	7.802749685
8.327781537	6.140596438	9.757858112	9.223063801	7.457175893
9.009324709	6.239843044	10.00410465	9.679406297	7.789164464

9.324347054	6.983458194	8.559933838	11.8907851	7.305758088
9.288617116	6.526061048	8.926092322	10.06707501	7.56647186
8.671603675	6.254050839	9.20745274	10.8820257	6.697408271
9.031815385	5.651519148	9.432312837	10.8414152	7.262843609
8.516305349	6.855433615	9.824895328	10.39877829	7.795795991
8.559811444	6.94661649	9.894344554	10.02088594	7.214244908
8.196861549	6.739490806	9.267625234	10.16510265	7.732643539
8.06178003	7.170360356	9.208430972	10.37533252	7.468395429
7.817659084	6.67588549	9.394337792	9.48519715	7.216493852
8.55845175	6.614015683	8.873852163	8.712228262	7.216460776
8.508225455	6.017752507	8.408599142	9.930282041	7.96249704
9.781270929	6.456229495	9.963947749	9.998518586	7.226510215
9.520138279	6.730285763	9.595922754	10.8414152	7.532475679
8.550620837	7.530317129	9.294824689	9.368818137	7.49751615
8.454346649	6.773278892	9.29376056	9.388395811	7.43761018
9.613035617	6.840731699	9.445425203	10.48129861	7.839169776
8.260414487	7.553798197	9.702629103	9.745685521	7.508870092
9.438943438	6.776054364	9.631714177	11.50344301	7.291798887
8.717136907	7.229585239	10.02088594	10.45017129	7.813536054
8.22434249	6.818343382	9.702629103	11.6283203	7.187211366
8.358205451	6.104755548	8.889111704	10.14803205	7.670692864
8.299968052	6.102398781	8.916596254	10.53037933	7.737389221
8.429132684	6.726276953	10.12517844	9.341257377	8.003749515
8.293236112	6.947478923	10.09680922	10.0806781	7.353115601
8.558046806	6.987408538	8.575720674	10.64100943	7.692139569
9.301967033	6.335084627	9.876033837	11.65499135	7.163793769
8.867289883	6.33404121	9.373434407	8.423870112	7.224125343
8.685780289	6.81414497	8.704466382	12.04651123	7.17403083
8.405079922	6.889100342	9.117640098	9.729084609	7.415012004
8.205216547	7.173666881	9.504050084	9.335534721	7.086554365
8.671281323	6.003546777	9.866437045	10.76838506	7.444758703
9.123058985	6.28814116	9.881115801	8.769599789	7.776880836
9.544871463	5.903962801	8.363610367	10.5010092	7.6783708
8.648569582	6.554417476	9.587952181	7.408518128	7.114567312
8.383264099	7.218912254	9.871190738	10.66792052	7.181475337
8.378200568	6.773824209	9.299187065	8.612910986	7.352541715
8.852647355	6.490511815	9.223063801	8.308148294	7.908206576
8.755945708	6.853900275	8.659109081	9.887791769	7.113886023
8.339916426	6.914300998	9.058717086	11.79688542	7.191301416
8.661045355	7.259821779	9.616473121	9.877706171	7.017764459
10.004732	6.503740196	8.651534037	11.20806268	7.215780846
8.603349578	6.488260246	8.720729358	9.381530427	7.361642374
8.564012227	6.79209653	9.182798537	10.45857273	7.175978116
8.919731071	6.5277603	9.619248934	10.8297019	7.053240277
8.343027597	6.779938794	8.403968554	9.756288138	7.451344498
9.611144065	6.071697553	9.362967141	11.53439953	7.600693424
8.404654925	6.229000556	9.88613995	9.685134459	7.338112478
8.848351329	6.031793372	9.550833718	6.768126469	7.817280844
8.412823288	6.696330544	9.933807869	9.980146695	7.206287903
8.842130284	6.057000533	9.422677502	11.75251707	7.076155403
8.144919873	6.416940035	9.705520108	10.39606206	7.119695494
7.957366961	6.74809288	8.972949841	10.95115111	8.278989683
8.817435378	6.679251431	9.899449381	9.481376941	7.523516897

8.315252362	7.136063889	9.633105012	9.786938168	7.012442911
9.088617926	6.414142031	9.68080561	7.716491408	7.640392578
8.299853572	6.324977139	9.306837319	10.94655569	7.520632578
8.920086567	5.97895438	8.870415524	9.446688734	7.359259854
8.963210683	6.668506824	10.54530453	10.73121149	7.029960295
10.75754155	6.169968238	8.420543849	10.18018725	7.327879616
8.126041697	5.998474046	8.580730354	9.72319596	7.573146647
8.377033092	6.361984895	9.965748096	9.331176453	7.150439951
8.911856504	6.819467837	9.470025359	9.015610475	7.148256485
8.852800568	6.827899665	10.15670762	11.05031358	7.114607079
8.46044446	6.377160226	8.923493836	7.984236271	7.414734851
8.976017199	5.980188535	9.930282041	9.01837398	7.47242153
8.662252494	6.58119255	9.744106162	9.239081033	7.397243456
8.500311355	6.785413437	9.561257524	8.98115994	6.966176385
10.58023812	6.437719242	9.417913822	13.23086442	7.930644876
9.397366444	6.409854822	9.125519188	9.385006413	7.652055805
10.95824654	6.209000471	9.887791769	11.2528206	7.819691789
9.228847955	7.063625101	8.481158874	10.07487392	7.770271273
9.859581263	5.800990009	9.440591264	9.578638025	7.444265904
8.470982155	7.068450706	9.652445734	10.54530453	7.127767996
9.041447196	6.518097243	10.17593569	10.95115111	7.810591345
8.903047359	6.562750922	9.399090019	10.42539926	7.208405117
8.772613563	6.252218589	9.640004858	11.50344301	7.283844526
8.420234623	6.76735362	9.223063801	9.565258324	7.560582084
9.335339231	6.101342701	10.07090411	11.75251707	7.127403849
8.373501765	5.925282778	9.312456127	8.387535127	7.388230811
8.565708254	6.481781938	8.729173272	9.482621742	7.522572878
8.227022368	6.513159166	8.594527794	9.443070976	7.520023324
9.684944759	6.879602075	9.115718684	11.8907851	7.728411562
8.612915629	6.685001914	9.403785329	11.73174365	7.233005751
8.265495458	6.217107292	7.999512713	10.95964942	6.832284922
8.625367951	5.506992924	9.385006413	8.77765928	7.38753715
8.193000599	6.725273123	9.566644837	10.4614736	6.671901824
9.444830031	6.526000947	9.263287649	11.42937784	8.427816222
8.800205621	6.400054333	9.025868692	10.10682316	7.546668442
8.545550597	5.929580682	9.748659873	10.59244739	7.576656799
8.7434406	6.083689111	9.657905394	10.25517511	7.627291555
9.338605067	6.136713008	8.817248476	10.05338963	7.705373792
8.556617918	5.802034415	9.802585693	9.808889823	7.639765838
8.273196908	6.679263055	9.544203668	9.154340403	7.48562865
8.332023775	6.751546945	8.855288174	10.18850413	7.690583158
9.362009073	5.939640444	9.683707378	8.460770838	7.453828867
8.540435167	7.557578932	10.73121149	10.06514747	7.353739841
8.412926144	6.471972783	9.473790479	10.37021909	7.359274549
8.895466492	6.605518115	9.004626764	10.7609233	7.196733018
8.530614153	6.671819615	9.167556359	9.570582128	7.195453982
8.226425892	6.08409327	9.261135772	9.981969736	7.956251001
8.640983744	6.270371523	10.0572131	9.981969736	7.231945892
8.598164966	6.867598035	9.151322168	10.12313607	7.959622835
9.358295154	6.225187261	8.804947109	9.262217671	7.619632778
8.04202952	6.472283819	8.970294529	9.727652481	7.088688052
8.364013747	5.923773484	8.282992264	9.721720774	8.243900866
9.134572275	6.330514342	9.7807299	8.733871732	7.447695077

9.280473771	5.691383388	9.124575154	9.594480988	7.689223149
8.847791408	6.259701835	9.86477811	10.80622596	7.464995467
8.404633206	7.041528065	9.199190352	10.52167884	7.014374311
8.958419409	6.711056075	9.656502345	9.646726907	7.485370211
8.732779148	5.621973897	10.14380698	9.906216645	7.407448216
8.798905414	6.926620751	9.812163447	10.60509222	7.487978409
8.446998554	6.398068429	10.38547722	10.89900986	7.240896984
8.364549655	6.199266675	9.41552891	7.662924276	7.970975397
8.365600865	7.329930381	8.235071787	11.9171254	7.742408826
8.989781879	6.693289067	9.431167425	11.47310062	7.530116912
8.577413675	6.609558059	9.340102817	10.04770408	7.040901269
9.104174622	6.604599832	9.76368168	11.48047936	7.370637797
8.799308725	6.752174987	9.932062225	10.88641969	6.901293714
8.609097069	6.684500902	9.785402745	10.70945604	7.142995271
8.61029888	5.639732435	7.794197462	11.2528206	8.086835017
8.282258805	6.699547109	9.54821533	8.986533425	7.507774806
8.053155117	7.012589066	9.849713388	10.48706723	7.599133044
10.40782728	6.654039647	8.378436729	10.2083553	8.399568344
8.120203948	7.009379836	9.239081033	9.621948686	7.239970337
8.470404495	7.071255033	8.976642102	10.61164808	7.331617593
8.200112354	7.371833408	9.438209259	10.2738317	7.01988612
9.51850052	6.473228033	8.549355948	10.84530303	7.207691579
8.252260086	7.428485845	9.589273408	9.278556113	7.593652024
9.393037067	6.900795324	8.851206363	10.28566022	8.351586063
9.152836823	7.21248509	9.732011342	9.41552891	7.864946995
8.988629373	7.052638888	9.744106162	10.75717183	7.875594001
9.509789199	7.765449257	9.836234979	9.009178063	7.347076527
