# Supplementary material for: A ferroptosis-associated gene signature for the prediction of prognosis and therapeutic response in luminal-type breast carcinoma
Source: Sci Rep. 2021 Sep 2;11:17610. doi: 10.1038/s41598-021-97102-z (PMC8413464; doi:10.1038/s41598-021-97102-z)
Supplement: Supplementary file 8 — Supplementary Table S4. [file 41598_2021_97102_MOESM8_ESM.pdf]

TableS4 Complete list of 10 candidate gene in the TCGA cohort

	OS.time	OS	CRYAB	PTGS2	PRKCA
TCGA-A8-A07B-01	1308	0	6.2497	-2.3884	-1.0559
TCGA-A8-A08P-01	943	0	4.8258	-2.2447	0.2029
TCGA-A8-A09T-01	579	0	3.3563	-1.685	-0.6643
TCGA-AO-A03O-01	2483	1	3.8591	-2.8262	-1.4305
TCGA-BH-A0DZ-01	495	0	4.8269	-0.2671	0.9038
TCGA-A2-A04V-01	1920	1	4.845	-2.6349	-1.3921
TCGA-AN-A04A-01	90	0	6.1117	-0.013	-0.013
TCGA-A7-A0CH-01	1079	0	3.8729	-2.4659	0.0014
TCGA-A8-A06T-01	1614	0	3.3148	-1.9379	-0.8863
TCGA-A8-A07F-01	577	0	4.1628	-1.4699	0.2154
TCGA-A8-A08C-01	881	0	3.7149	-4.035	-1.2828
TCGA-A8-A08T-01	3409	1	3.8935	-1.9942	-0.1665
TCGA-AN-A0AJ-01	303	0	4.0909	-4.2934	-1.3548
TCGA-AO-A03P-01	2911	1	2.6161	-3.1714	-2.9324
TCGA-A8-A082-01	549	0	3.5547	-4.6082	0.24
TCGA-A8-A08F-01	1004	0	2.876	-2.2447	0.0014
TCGA-A8-A09K-01	912	0	2.1925	-2.3884	-0.4131
TCGA-A8-A06X-01	943	1	3.0217	-2.6349	0.2762
TCGA-A8-A07J-01	365	0	7.463	-1.8314	1.2696
TCGA-A8-A092-01	942	0	4.5693	-2.7274	-0.7108
TCGA-A8-A09C-01	31	0	4.5577	-3.1714	-1.3548
TCGA-A8-A09Q-01	761	0	3.7072	-1.9942	-0.5973
TCGA-A2-A04Y-01	1099	0	4.9612	-0.1665	-1.3921
TCGA-A2-A0CQ-01	2695	0	3.4817	-0.8599	-1.2828
TCGA-A2-A0CU-01	158	1	5.9312	0.8082	0.7748
TCGA-A8-A06Q-01	31	0	3.4504	-4.2934	-2.1779
TCGA-A7-A0CD-01	1165	0	4.2518	-1.8314	-1.0559
TCGA-A7-A0DB-01	1007	0	5.07588931305	-2.17785750019	0.56004368666
TCGA-A8-A06O-01	396	0	6.1016	-1.4305	-0.4325
TCGA-A8-A076-01	1642	0	4.7608	-3.458	0.03
TCGA-A8-A07P-01	334	0	4.0304	-1.7809	-0.5332
TCGA-A8-A086-01	396	0	5.4702	-2.0529	0.1124
TCGA-A8-A07G-01	577	0	5.2544	-2.3147	0.346
TCGA-A8-A07S-01	243	0	3.6916	-3.0469	-0.5543
TCGA-A8-A08S-01	1004	0	3.8157	-2.9324	-1.1172
TCGA-A8-A097-01	365	0	5.7258	-0.1993	0.9038
TCGA-A8-A09V-01	457	0	3.1844	-3.0469	1.2696
TCGA-A8-A0A2-01	579	0	3.1797	-1.9942	0.1903
TCGA-A8-A0AB-01	518	0	4.3688	-2.8262	-0.3566
TCGA-BH-A0BD-01	554	0	6.3574	-1.6394	-0.3022
TCGA-A8-A09B-01	365	0	5.2646	-1.3548	-0.5332
TCGA-A8-A0A4-01	396	0	6.9276	-1.5105	1.9968
TCGA-BH-A0BV-01	1519	0	3.6972	-1.0559	-0.1504
TCGA-A8-A06Y-01	791	0	5.1002	-5.0116	-1.6394
TCGA-A8-A07L-01	975	0	3.8511	-3.816	-0.0877
TCGA-A8-A09A-01	304	0	6.2887	-0.3752	0.8327
TCGA-A8-A09N-01	31	0	4.4536	-1.9379	-0.6873
TCGA-A8-A0A1-01	365	0	6.0631	-0.5973	0.547
TCGA-A7-A0DC-01	906	0	1.8814244331	-1.7975606089	-0.188241688975
TCGA-A8-A06P-01	396	0	5.682	-2.4659	0.2277
TCGA-A8-A079-01	274	0	2.9281	-2.9324	-1.2481
TCGA-A8-A07W-01	304	0	5.166	-2.2447	0.9862

TCGA-AN-A0AK-01	224	0	2.8321	-4.6082	-1.9379
TCGA-A2-A0CY-01	1673	0	1.7009	-0.1031	-2.2447
TCGA-A2-A0EM-01	3094	0	4.8635	-1.5105	-0.1504
TCGA-A2-A0EO-01	2442	0	6.633	-0.5973	0.4967
TCGA-A2-A0ER-01	2263	0	3.034	0.9789	-1.0262
TCGA-A8-A093-01	546	0	5.597	-2.6349	0.1257
TCGA-A8-A09D-01	1522	0	7.9175	-2.8262	0.1903
TCGA-A8-A09R-01	273	0	4.449	-1.8314	0.5763
TCGA-A8-A0A9-01	822	0	6.2887	-2.5479	-0.4325
TCGA-BH-A0E7-01	1363	0	4.4377	-3.0469	-0.3566
TCGA-BH-A0EB-01	745	0	4.4641	-2.3147	-0.394
TCGA-A1-A0SE-01	1321	0	6.4999	-1.4305	0.4657
TCGA-A2-A0SU-01	1662	0	5.5407	-0.4719	-0.2498
TCGA-A2-A0T4-01	624	0	7.2458	0.8246	-0.1345
TCGA-AR-A0TT-01	3316	0	4.0688	-1.9379	0.547
TCGA-AR-A0TV-01	2288	0	6.0068	-1.8836	-1.1488
TCGA-B6-A0RM-01	2373	1	8.9915	-4.2934	-0.3383
TCGA-B6-A0RN-01	8008	0	3.644	-1.4305	-0.8863
TCGA-BH-A0DH-01	1156	0	4.9449	-2.8262	-1.4699
TCGA-BH-A0DQ-01	98	0	7.3243	-0.9132	0.3115
TCGA-AO-A0JJ-01	1887	0	6.5682	2.3077	0.2642
TCGA-AO-A0JM-01	2184	0	6.2852	-0.6873	-0.8863
TCGA-B6-A0RG-01	2082	0	3.7971	-0.8084	-1.2481
TCGA-B6-A0RI-01	7126	0	5.3388	-1.2481	0.03
TCGA-BH-A0B1-01	1148	0	5.1425	0.1124	-0.3022
TCGA-BH-A0B8-01	1569	0	4.9472	0.3685	-1.5105
TCGA-BH-A0BA-01	1132	0	7.1387	0.7999	0.6969
TCGA-BH-A0BJ-01	660	0	5.9741	-0.3383	0.1124
TCGA-A2-A0CT-01	2289	0	3.7656	0.058	-0.8339
TCGA-A2-A0EU-01	1043	0	4.284	1.1316	-0.6873
TCGA-A7-A0D9-01	1139	0	6.4172	-1.4699	1.0079
TCGA-A8-A08O-01	943	0	6.3329	-1.4699	1.5266
TCGA-A8-A0A6-01	640	0	6.0208	-1.2481	-0.1345
TCGA-A8-A0AD-01	1157	0	3.9232	-0.5973	0.7832
TCGA-AO-A03L-01	2442	0	4.9241	-0.5332	-0.0877
TCGA-AO-A0JA-01	655	0	5.4496	-1.5105	0.2762
TCGA-AO-A0JC-01	1547	0	4.0884	0.24	2.0183
TCGA-AO-A0JD-01	2190	0	4.9691	-1.1488	-1.2142
TCGA-AO-A0JF-01	1980	0	7.2569	-0.4521	1.0007
TCGA-AO-A0JI-01	1528	0	3.9874	0.2154	-0.0277
TCGA-BH-A0BM-01	1876	0	8.0068	1.736	-0.2671
TCGA-BH-A0C0-01	1270	0	3.091	-0.7588	-0.8339
TCGA-BH-A0DK-01	423	0	6.3227	0.0158	-0.0877
TCGA-BH-A0DP-01	476	0	6.6836	-0.5543	0.7493
TCGA-BH-A0DS-01	78	0	5.9315	-0.9686	-1.0862
TCGA-BH-A0E1-01	477	0	5.1478	2.05	-0.394
TCGA-BH-A0E2-01	435	0	5.5094	-0.9406	0.5955
TCGA-BH-A0GY-01	923	0	6.1369	0.1903	-1.0559
TCGA-BH-A0GZ-01	328	0	4.6491	-1.0262	-0.9971
TCGA-BH-A0H0-01	461	0	3.6555	-0.3566	-1.7322
TCGA-BH-A0H6-01	747	0	5.5916	-1.1172	-0.4719
TCGA-BH-A0H9-01	1247	0	5.3639	-0.4325	-0.1504
TCGA-BH-A0HB-01	806	0	4.891	-0.8339	-1.3183

TCGA-BH-A0HF-01	727	0	5.8763	0.9268	0.5955
TCGA-A8-A075-01	518	0	5.1248	-2.1779	0.03
TCGA-AO-A0JG-01	798	0	5.7222	0.5069	0.2998
TCGA-AR-A0TQ-01	2991	0	4.1724	-1.9379	0.3907
TCGA-AR-A0TR-01	160	1	1.614	-3.1714	-1.0559
TCGA-B6-A0RL-01	2469	1	1.7575	-4.6082	-4.035
TCGA-B6-A0RV-01	5156	0	6.031	-1.0862	0.4125
TCGA-BH-A0BC-01	974	0	5.8719	-1.8836	-0.4131
TCGA-A1-A0SM-01	242	0	3.3689	-1.3548	0.6699
TCGA-A2-A0SY-01	1347	0	6.1843	0.517	-0.2328
TCGA-A1-A0SH-01	1437	0	5.5132	-0.7108	1.816
TCGA-A1-A0SJ-01	416	0	7.0572	-1.2481	-0.4719
TCGA-A2-A0EN-01	4088	0	6.3153	-0.1665	0.1124
TCGA-A2-A0SV-01	825	1	5.2586	-1.5951	-1.1811
TCGA-A2-A0SW-01	1365	1	4.3611	-3.458	-1.1811
TCGA-A2-A0T5-01	531	0	6.6169	-0.7108	0.2154
TCGA-A2-A0T6-01	575	0	6.2941	2.3451	1.2023
TCGA-AR-A0TW-01	3009	0	2.4753	-1.3921	-0.013
TCGA-B6-A0RO-01	4929	0	5.3377	-1.7322	-0.2498
TCGA-B6-A0RP-01	3126	1	4.0824	0.0014	-0.2845
TCGA-BH-A0HI-01	620	0	4.2196	-0.5125	-0.9132
TCGA-BH-A0HK-01	178	0	6.0826	0.0014	0.2277
TCGA-BH-A0HX-01	829	0	5.2231	-0.4521	0.1124
TCGA-BH-A0HY-01	1545	0	5.0409	-4.035	-0.8863
TCGA-B6-A0I5-01	8556	0	5.4383	-2.5479	-1.5522
TCGA-B6-A0I8-01	749	1	3.6543	-4.2934	-0.6643
TCGA-B6-A0IA-01	8391	0	4.4726	-3.458	-3.0469
TCGA-B6-A0IB-01	3941	1	3.8541	-3.816	-3.0469
TCGA-B6-A0IC-01	1542	1	1.7617	-3.1714	-2.114
TCGA-B6-A0IE-01	1993	1	5.9878	-3.1714	-1.8836
TCGA-B6-A0IG-01	4456	1	5.6778	-1.685	-1.8836
TCGA-BH-A0HO-01	76	0	4.0046	-2.6349	-0.5543
TCGA-BH-A0HQ-01	1121	0	5.9889	-1.0862	0.8961
TCGA-BH-A0HU-01	392	0	3.6725	-4.2934	-2.5479
TCGA-BH-A0HW-01	1561	0	2.0912	-4.2934	-2.5479
TCGA-AN-A0FN-01	218	0	6.5785	2.2391	0.4865
TCGA-AN-A0FS-01	210	0	6.431	1.7954	0.8082
TCGA-AN-A0FT-01	214	0	3.837	-0.8084	-1.5105
TCGA-A2-A0T7-01	631	0	7.2735	0.9568	0.099
TCGA-AR-A0TZ-01	3262	1	3.0602	0.03	0.1776
TCGA-BH-A0HP-01	414	0	6.3627	-1.7322	0.3796
TCGA-A2-A0ET-01	1066	0	3.7028	0.2642	-0.8599
TCGA-A2-A0EV-01	968	0	5.5607	-1.8314	-0.0725
TCGA-A2-A0EX-01	752	0	5.4084	-0.8084	0.3796
TCGA-A2-A0EY-01	1925	0	5.2502	-1.1172	0.8082
TCGA-AN-A0FD-01	196	0	4.1844	-2.8262	-1.1488
TCGA-AN-A0FF-01	172	0	4.2189	-0.4521	-0.5332
TCGA-AN-A0FK-01	213	0	3.8187	-3.1714	-2.5479
TCGA-AO-A0J3-01	651	0	2.0878	-4.2934	-3.1714
TCGA-AO-A0J5-01	792	1	4.7165	-0.7108	0.7321
TCGA-AO-A0J8-01	680	0	4.6502	-3.458	-1.1172
TCGA-AO-A0J9-01	1613	0	9.1302	-2.0529	-0.8599
TCGA-B6-A0IM-01	3873	1	5.5996	-3.1714	-0.5973

TCGA-B6-A0IN-01	2573	1	6.1233	-0.0877	-0.8599
TCGA-B6-A0IO-01	5042	0	3.6543	-3.0469	-2.8262
TCGA-B6-A0IP-01	3926	1	5.7393	-2.8262	-0.9686
TCGA-BH-A0W4-01	759	0	6.8393	-0.2498	0.4447
TCGA-B6-A0WY-01	3461	1	7.1582	-0.9406	0.3796
TCGA-A2-A0YF-01	1535	0	5.4152	-1.6394	-0.3201
TCGA-A2-A0YT-01	723	1	5.3111	0.7321	-0.8084
TCGA-B6-A0WZ-01	6292	0	3.5994	-2.9324	-1.9379
TCGA-AO-A12C-01	2372	0	5.2312	-1.4699	0.6332
TCGA-AO-A12E-01	2142	0	7.6822	1.0573	1.0711
TCGA-AO-A12G-01	1639	0	4.3639	-2.6349	-0.9971
TCGA-AQ-A04H-01	754	0	3.1813	-0.9406	-0.2498
TCGA-B6-A0X7-01	1781	1	5.2649	-2.6349	-0.1345
TCGA-A2-A0CS-01	2348	1	4.835	-3.1714	-0.8339
TCGA-B6-A0IH-01	3418	1	6.1016	-1.0559	1.2875
TCGA-BH-A0BP-01	2296	1	7.9252	0.3796	1.2455
TCGA-BH-A0BQ-01	2255	0	6.2926	-1.7809	0.0854
TCGA-A2-A0YC-01	990	0	5.393	-1.0262	-0.1187
TCGA-A2-A0YD-01	769	0	6.6029	2.1313	1.0503
TCGA-A2-A0YL-01	1474	0	7.5757	1.0503	0.8726
TCGA-AN-A0XO-01	375	0	6.1721	-0.4521	-0.4719
TCGA-A2-A0YI-01	1505	0	4.7016	-0.2498	1.3225
TCGA-AO-A03M-01	1866	0	4.9732	-1.3183	-0.2159
TCGA-AO-A03N-01	2031	0	4.7903	-4.6082	-1.7322
TCGA-AO-A125-01	3456	0	1.7316	-3.816	-2.9324
TCGA-AO-A126-01	3307	0	5.1506	-2.6349	-0.8863
TCGA-BH-A0DX-01	2156	0	6.2696	-0.5973	0.6332
TCGA-BH-A0W7-01	1363	0	5.5626	-1.1488	-0.2498
TCGA-E2-A105-01	1308	0	4.3625	-2.6349	-1.7322
TCGA-E2-A106-01	2541	0	4.8565	-2.1779	-1.685
TCGA-E2-A109-01	1417	0	3.3787	-3.458	-0.5125
TCGA-E2-A10B-01	1141	0	4.637	-2.6349	0.24
TCGA-E2-A10E-01	865	0	5.3889	-0.6643	-0.3022
TCGA-E2-A10F-01	878	0	5.8107	-0.2159	0.3907
TCGA-BH-A0DE-01	2372	0	7.2336	1.4174	0.4233
TCGA-BH-A0EI-01	1926	0	4.1644	-3.6259	0.6239
TCGA-C8-A12N-01	358	0	3.7635	-1.8314	-0.8084
TCGA-C8-A12O-01	385	0	5.6212	-0.8084	1.2815
TCGA-C8-A12X-01	385	0	2.3649	-4.035	-2.4659
TCGA-E2-A15D-01	526	0	5.7225	-1.7809	0.2029
TCGA-E2-A10A-01	1229	0	3.9496	-2.6349	-0.013
TCGA-D8-A146-01	643	0	6.4922	0.2881	1.3956
TCGA-D8-A13Y-01	1728	0	3.4331	-4.035	0.044
TCGA-AN-A0XL-01	163	0	7.9059	2.1862	0.8883
TCGA-AO-A12B-01	2989	0	2.7763	-3.816	-2.114
TCGA-E2-A10C-01	1220	0	3.5572	-4.035	-1.5522
TCGA-AR-A0TY-01	1699	1	3.7028	-2.6349	1.2636
TCGA-A2-A04N-01	4354	0	4.5766	-0.3383	-0.2328
TCGA-AO-A03V-01	1351	0	5.332	-1.5522	-1.1172
TCGA-E2-A15P-01	595	0	5.4273	-0.9686	0.4125
TCGA-E2-A15R-01	1732	0	3.8935	-0.8863	-0.6643
TCGA-BH-A0W3-01	728	0	1.5465	-2.5479	-1.5105
TCGA-C8-A12U-01	385	0	2.1702	-2.2447	-0.7346

TCGA-C8-A132-01	383	0	6.1165	0.4552	1.1897
TCGA-D8-A141-01	626	0	4.9942	0.4016	1.0293
TCGA-E2-A14T-01	2311	0	5.8912	-2.0529	-0.7834
TCGA-A2-A0CV-01	3011	0	6.0849	1.0915	-0.3752
TCGA-A2-A0CW-01	3283	0	3.1955	-4.035	-2.0529
TCGA-B6-A0WS-01	2965	1	4.6871	-1.5951	1.0079
TCGA-B6-A0X0-01	3945	1	4.6376	-2.8262	-1.8314
TCGA-BH-A0B0-01	2477	0	7.4303	-1.5522	-0.4131
TCGA-C8-A12W-01	385	0	4.5638	-1.3183	0.4016
TCGA-C8-A12M-01	358	0	2.8916	-2.8262	1.1641
TCGA-BH-A0EA-01	991	1	7.5319	0.547	0.3115
TCGA-BH-A0E9-01	2489	0	7.4029	1.5266	1.0363
TCGA-BH-A0BR-01	2330	0	5.5759	0.4233	0.537
TCGA-E2-A15T-01	1563	0	3.6657	-1.4305	-0.8863
TCGA-E2-A15S-01	428	0	3.38	-3.3076	-0.8599
TCGA-E2-A154-01	591	0	3.7867	-3.458	-0.5756
TCGA-E2-A14Z-01	563	1	5.6349	-0.7346	-0.1665
TCGA-D8-A145-01	410	0	6.2948	-0.5543	1.6374
TCGA-BH-A0H5-01	1620	0	5.2107	-1.0262	1.2516
TCGA-D8-A140-01	403	0	3.8208	-1.1172	0.2998
TCGA-E2-A15O-01	1545	0	4.6927	0.2029	-1.0262
TCGA-AO-A12H-01	1234	0	2.9281	-2.7274	-1.8314
TCGA-BH-A0C7-01	2767	0	5.0781	0.7058	0.058
TCGA-A1-A0SD-01	437	0	6.8947	0.605	0.3115
TCGA-A2-A0D3-01	1873	0	4.0419	-3.3076	-1.2481
TCGA-A2-A0ES-01	2190	0	8.0526	2.3366	1.105
TCGA-A2-A0EW-01	1884	1	7.5537	2.5707	1.0433
TCGA-A2-A0T3-01	1516	0	5.1384	-0.5543	0.2642
TCGA-AO-A12A-01	3112	0	7.0451	0.4233	-0.2159
TCGA-C8-A130-01	370	0	5.1607	-2.4659	-1.7322
TCGA-E2-A14O-01	1359	0	3.3856	-5.0116	-3.816
TCGA-E2-A15F-01	658	0	5.2365	0.346	0.2029
TCGA-BH-A0BO-01	2197	0	6.9795	4.2064	0.7916
TCGA-BH-A18I-01	1093	0	5.5479	0.9716	0.8883
TCGA-BH-A18J-01	612	1	4.506	-3.458	0.03
TCGA-BH-A18S-01	2009	1	5.131	-1.8836	-1.8836
TCGA-E2-A14Q-01	1163	0	6.816	-0.5543	-0.7834
TCGA-E2-A14S-01	1009	0	3.4331	-2.0529	-0.5332
TCGA-E2-A155-01	640	0	3.66	-0.3566	-1.3921
TCGA-E2-A156-01	726	0	2.7594	-2.4659	-0.9406
TCGA-A7-A13F-01	765	0	4.2457	-2.8262	-0.8084
TCGA-AR-A1AX-01	2629	0	4.8202	1.1833	1.2934
TCGA-BH-A0AU-01	1914	0	5.0926	-1.9942	0.044
TCGA-BH-A0BZ-01	2255	0	5.1551	-2.3884	0.0718
TCGA-BH-A0C3-01	2709	0	6.4353	-0.2328	1.2147
TCGA-BH-A0DD-01	2486	0	2.9186	-2.4659	-0.8599
TCGA-E2-A15J-01	1640	0	2.4386	-3.3076	-1.5522
TCGA-E2-A15K-01	275	0	4.1732	-2.8262	-0.2671
TCGA-BH-A18F-01	1001	0	8.1864	0.7058	0.8408
TCGA-BH-A18H-01	652	0	2.8096	0.679	-0.4921
TCGA-E2-A1BC-01	501	0	6.9428	1.5998	0.4761
TCGA-BH-A18N-01	1148	1	3.668	-2.3147	-0.8339
TCGA-C8-A12Y-01	1476	0	5.7637	-2.4659	-1.8314

TCGA-E2-A153-01	707	0	5.7442	-1.4699	0.03
TCGA-E2-A15E-01	630	0	7.1476	0.03	1.0573
TCGA-BH-A18L-01	811	1	3.6532	-1.3183	-2.0529
TCGA-BH-A18M-01	2207	1	6.7023	0.6699	0.5069
TCGA-BH-A18U-01	1563	1	4.2773	-0.8863	-1.7809
TCGA-BH-A18K-01	2763	1	5.1575	-0.7834	-0.4325
TCGA-BH-A0DT-01	2403	0	6.3457	-0.0877	0.5763
TCGA-BH-A0DO-01	1644	0	6.8759	1.3397	-0.1031
TCGA-BH-A0C1-01	1411	1	4.3639	-0.0277	0.1388
TCGA-E2-A15H-01	393	0	5.0833	-0.7346	-0.2498
TCGA-E2-A15G-01	554	0	3.0859	-3.6259	-1.1811
TCGA-E2-A14W-01	974	0	4.6916	-3.1714	-2.1779
TCGA-E2-A15A-01	710	0	3.6195	-2.7274	-0.1504
TCGA-E2-A15C-01	694	0	5.5979	0.605	0.4447
TCGA-AR-A1AP-01	2856	0	5.2461	-1.1488	-0.0277
TCGA-AR-A1AU-01	2868	0	7.2277	1.0079	0.099
TCGA-AR-A1AS-01	1150	0	3.3549	-2.6349	-0.2328
TCGA-AR-A1AK-01	3159	0	6.0481	3.3192	0.6239
TCGA-BH-A0AZ-01	1919	0	6.4738	-1.2142	1.125
TCGA-BH-A0B5-01	2136	0	5.0449	0.1257	0.3231
TCGA-BH-A0BF-01	1324	1	4.717	-0.8339	-0.3752
TCGA-BH-A0DG-01	2041	0	6.3994	-0.6873	1.3679
TCGA-BH-A0DI-01	912	0	6.9955	-0.3752	1.0433
TCGA-AR-A1AL-01	2971	0	6.7695	2.0218	1.4494
TCGA-AR-A1AN-01	2920	0	4.7318	-1.2828	-0.013
TCGA-AR-A1AV-01	1864	0	4.0488	-1.1488	0.1124
TCGA-BH-A0DV-01	2064	0	7.2284	1.8524	1.3623
TCGA-E2-A1B1-01	2653	0	6.8125	1.884	0.9862
TCGA-E2-A1B4-01	1004	1	5.4938	-3.458	-1.7322
TCGA-AQ-A04L-01	3957	0	4.9279	-2.8262	-1.2828
TCGA-A8-A06U-01	883	1	4.5199	-3.0469	-0.7834
TCGA-BH-A1EO-01	2798	1	5.3744	0.099	1.5366
TCGA-BH-A1ET-01	2520	1	4.4162	1.0293	0.1903
TCGA-BH-A1EU-01	1286	1	6.9294	2.7029	1.6964
TCGA-BH-A1EW-01	1694	1	4.8131	2.2842	0.4233
TCGA-E2-A15I-01	1692	0	5.436	1.7273	0.7832
TCGA-A2-A0CP-01	2813	0	6.6222	-1.1811	0.058
TCGA-A8-A07E-01	608	0	6.0328	-1.2828	1.4388
TCGA-A8-A09I-01	1371	0	3.1955	-1.9379	-1.2481
TCGA-AO-A0J7-01	618	0	3.6725	-2.2447	-1.5951
TCGA-A2-A1FV-01	714	0	5.8679	1.4911	-0.2845
TCGA-A2-A1FW-01	528	0	5.3709	-2.3147	5.5898
TCGA-E2-A15L-01	626	0	5.0964	-2.7274	0.0158
TCGA-E2-A15M-01	336	1	6.4276	1.177	0.7916
TCGA-E2-A1IN-01	675	0	5.0361	-2.1779	0.0158
TCGA-E2-A1L8-01	2240	0	6.574	-2.8262	-1.3921
TCGA-EW-A1J2-01	403	0	6.9658	-0.8084	1.2023
TCGA-A2-A1G4-01	595	0	3.7149	-1.5951	-0.7108
TCGA-AO-A1KP-01	2953	0	4.0515	-0.9971	-1.9942
TCGA-AO-A1KT-01	541	0	5.0964	-0.5756	-0.3752
TCGA-BH-A1EX-01	1508	1	5.0666	0.6608	0.7493
TCGA-BH-A1FD-01	1009	1	7.3592	-2.4659	-1.3183
TCGA-BH-A1FL-01	1673	1	5.2747	0.058	-0.5125

TCGA-C8-A1HE-01	375	0	3.9709	-2.2447	-0.7346
TCGA-C8-A1HO-01	375	0	3.1653	-1.3921	-1.1811
TCGA-D8-A1JD-01	552	0	4.4874	-1.3548	0.4552
TCGA-E2-A1IE-01	2362	0	4.9279	-1.5105	-1.3548
TCGA-AR-A1AW-01	2632	0	2.8361	-2.5479	0.24
TCGA-BH-A0BS-01	2612	0	4.7719	-4.2934	-1.8314
TCGA-BH-A0BT-01	2365	0	5.1627	-1.5522	-1.3548
TCGA-BH-A0H3-01	1928	0	5.9198	0.3685	1.2147
TCGA-BH-A0HA-01	1611	0	5.2529	-1.9942	-0.9686
TCGA-A1-A0SQ-01	554	0	3.4971	-1.5105	-1.0262
TCGA-E2-A1IF-01	1138	0	7.1155	0.5568	-0.1828
TCGA-E2-A1IG-01	2140	0	6.483	-0.8863	-0.9686
TCGA-E2-A1IO-01	1855	0	5.7541	0.434	1.3397
TCGA-E2-A1LA-01	748	0	5.073	-0.7346	-1.8836
TCGA-E9-A1NA-01	1112	0	3.2526	-4.2934	-0.013
TCGA-EW-A1IX-01	1208	0	4.734	2.1734	1.2455
TCGA-EW-A1OZ-01	1229	0	4.3278	-2.3147	-1.3548
TCGA-EW-A1P5-01	703	0	3.6905	-2.5479	-0.9132
TCGA-C8-A1HG-01	345	0	4.1458	-4.6082	-1.3921
TCGA-C8-A1HI-01	343	0	7.1116	-0.8599	-0.8339
TCGA-C8-A1HL-01	317	0	3.6359	-2.114	-0.5973
TCGA-C8-A1HM-01	375	0	2.2299	-2.5479	-1.3548
TCGA-C8-A1HN-01	394	0	2.8799	-1.5951	-1.3921
TCGA-BH-A0H7-01	702	0	4.5268	-3.0469	-0.1993
TCGA-D8-A1JS-01	371	0	4.5608	-5.5735	-4.035
TCGA-E2-A1IH-01	1026	0	5.1635	1.8323	0.2522
TCGA-E2-A1L9-01	598	0	6.2698	-1.1811	0.0014
TCGA-EW-A1J3-01	504	0	5.3466	-0.9971	-1.2481
TCGA-EW-A1J5-01	477	0	5.124	-1.0559	0.0854
TCGA-D8-A1JU-01	447	0	6.5793	-0.0877	1.6045
TCGA-EW-A1IW-01	371	0	6.8188	2.1013	0.2881
TCGA-EW-A1J6-01	875	0	3.4264	-1.3548	-2.2447
TCGA-A1-A0SG-01	434	0	5.5075	-1.2142	-0.1828
TCGA-A1-A0SI-01	635	0	4.9093	0.2522	0.8246
TCGA-A1-A0SN-01	1196	0	4.2173	-0.1345	1.9931
TCGA-E2-A1IJ-01	865	0	5.847	0.4657	0.9115
TCGA-E2-A1IK-01	1800	0	6.4012	-1.4699	-1.3548
TCGA-E2-A1L7-01	1836	0	5.1052	-1.5951	-0.1993
TCGA-A2-A1G0-01	616	0	6.3777	1.46	0.688
TCGA-A7-A13G-01	718	0	3.11321093753	-1.4941126834	-0.156822852955
TCGA-AQ-A1H2-01	475	0	3.0287	-2.8262	-2.1779
TCGA-AQ-A1H3-01	989	0	6.4106	0.4016	0.5069
TCGA-D8-A1X5-01	565	0	3.6983	-0.8863	-0.6643
TCGA-D8-A1XA-01	839	0	4.5154	0.1388	-0.3201
TCGA-D8-A1XB-01	552	0	5.7181	-0.5543	1.105
TCGA-D8-A1XC-01	377	1	3.3336	-2.9324	-1.0559
TCGA-D8-A1XD-01	522	0	5.3002	-0.5756	0.5568
TCGA-D8-A1XF-01	463	0	3.0181	-3.0469	-0.3201
TCGA-D8-A1XG-01	448	0	6.8604	-0.8863	0.099
TCGA-BH-A1FE-01	2273	1	5.6645	1.6964	1.2696
TCGA-D8-A1JE-01	575	0	4.2427	-1.8314	0.4657
TCGA-D8-A1JH-01	426	0	5.3126	0.9568	1.4281
TCGA-E2-A1IL-01	118	0	6.9261	-0.8339	0.058

TCGA-E2-A1IU-01	337	0	5.9454	-1.3183	-0.1187
TCGA-E9-A1N5-01	1120	0	4.6189	0.6608	0.1388
TCGA-E9-A1N6-01	678	1	4.3385	-2.2447	1.884
TCGA-EW-A1OX-01	911	0	1.6187	-4.6082	1.9527
TCGA-EW-A1OY-01	908	0	4.2381	-0.8599	-1.7322
TCGA-EW-A1P0-01	1251	0	3.4291	-1.1811	-0.9971
TCGA-BH-A1FR-01	1642	1	4.3385	0.0014	-0.2498
TCGA-BH-A1EY-01	538	1	4.0436	-0.9971	0.6425
TCGA-D8-A1JP-01	639	0	5.3147	-3.3076	0.6145
TCGA-AO-A1KQ-01	1882	0	3.773	-2.6349	-2.114
TCGA-BH-A1FG-01	3736	1	4.085	-1.9942	-1.9379
TCGA-A7-A0CG-01	1043	0	6.1675	1.6964	1.1316
TCGA-A7-A0CJ-01	931	0	3.5387	-1.7809	-1.7809
TCGA-E2-A1B5-01	984	0	7.1253	-0.0425	1.3283
TCGA-A2-A04R-01	3709	0	5.5607	-0.8599	-1.2142
TCGA-A2-A0YG-01	666	0	4.0269	-1.7322	-0.4521
TCGA-A2-A0YH-01	659	0	3.944	-2.2447	0.2029
TCGA-BH-A1FN-01	2192	1	5.2269	-0.2159	-0.2328
TCGA-D8-A1JB-01	1688	0	6.6844	0.5069	0.8568
TCGA-D8-A1JI-01	577	0	2.1957	-2.3884	-1.0862
TCGA-D8-A1JN-01	620	0	2.043	-3.1714	-2.8262
TCGA-EW-A1PD-01	424	0	6.4862	-0.2328	1.0573
TCGA-EW-A1PE-01	320	0	4.2987	0.7579	-0.6416
TCGA-EW-A1PF-01	439	0	5.6276	1.4547	0.8647
TCGA-A1-A0SF-01	1463	0	6.1258	-2.3884	-0.1993
TCGA-E9-A1R6-01	339	0	4.0082	-3.458	-0.6873
TCGA-E9-A1RA-01	1369	0	5.0339	-2.7274	-0.1665
TCGA-D8-A1J8-01	431	0	3.7783	0.0158	0.058
TCGA-D8-A1J9-01	532	0	4.2906	-2.8262	0.6517
TCGA-D8-A1JC-01	480	0	5.1619	-1.1488	0.346
TCGA-D8-A1JT-01	405	0	4.5448	-1.6394	-2.5479
TCGA-E2-A1L6-01	1648	0	6.2325	-0.1504	0.3231
TCGA-EW-A1IY-01	258	0	5.4246	0.2522	0.5859
TCGA-EW-A1J1-01	575	0	2.0289	-2.5479	-1.2481
TCGA-A2-A1FX-01	1847	0	4.4977	-0.6873	0.1124
TCGA-AO-A1KS-01	350	0	5.4239	0.5271	-0.5125
TCGA-B6-A1KN-01	4233	0	5.8428	-0.3566	-0.6643
TCGA-BH-A1F2-01	959	1	6.4072	3.1162	0.8246
TCGA-BH-A1F5-01	2712	1	3.8808	0.1776	-0.7346
TCGA-BH-A1F8-01	763	1	4.1302	-2.3884	-0.9971
TCGA-AC-A23E-01	698	0	5.2869	-0.8599	1.6093
TCGA-BH-A0W5-01	1288	0	6.0007	-0.9132	1.6093
TCGA-E9-A22B-01	1167	0	6.987	-1.1488	-0.5332
TCGA-E9-A22D-01	1248	0	2.876	-2.5479	-0.8339
TCGA-E9-A22E-01	1269	0	6.1785	-1.2142	0.3573
TCGA-E9-A22H-01	1232	0	2.9765	-2.8262	-0.5973
TCGA-E9-A1QZ-01	755	0	8.6453	0.5568	0.5568
TCGA-B6-A1KI-01	2236	0	5.9864	-2.8262	0.1124
TCGA-D8-A1XM-01	538	0	6.991	-1.0559	0.7407
TCGA-D8-A1XU-01	395	0	4.89	-1.9379	0.8568
TCGA-D8-A1Y0-01	472	0	4.9644	2.0289	0.8327
TCGA-D8-A1Y1-01	302	1	4.7629	-3.0469	0.058
TCGA-E9-A1R3-01	78	0	6.3254	-0.5125	0.7407

TCGA-E9-A1NF-01	1072	1	5.6005	-0.9971	-0.1187
TCGA-E9-A1NH-01	576	0	5.4787	-1.2142	0.0014
TCGA-E9-A1NI-01	300	0	3.8876	-0.9132	-1.5522
TCGA-E9-A1R2-01	1063	0	5.0618	-2.2447	-0.0277
TCGA-E9-A1R4-01	186	0	4.5491	-2.1779	0.24
TCGA-AN-A0XV-01	162	0	5.1611	-0.2498	0.058
TCGA-AN-A0XW-01	170	0	4.8033	-0.7834	0.7832
TCGA-E9-A1N3-01	1059	0	5.6349	-2.7274	-1.1811
TCGA-E9-A1RB-01	976	1	3.8054	-1.7809	-0.8339
TCGA-E9-A1RC-01	1224	0	4.2381	-2.7274	-0.8863
TCGA-E9-A1RD-01	34	0	3.4358	-1.9379	-0.8084
TCGA-E9-A1RE-01	1419	0	4.232	-1.7809	-0.3383
TCGA-E9-A1RF-01	200	0	5.1014	-2.9324	-0.5543
TCGA-E9-A226-01	1048	1	5.5348	-0.9132	0.9862
TCGA-E9-A227-01	975	0	4.7361	-2.2447	0.0158
TCGA-E9-A228-01	1285	0	2.7227	-2.4659	-0.1031
TCGA-E9-A229-01	1148	0	5.4138	-1.6394	0.8961
TCGA-E9-A22A-01	1189	0	5.9898	-2.3147	-0.8599
TCGA-AR-A24W-01	1550	0	7.6118	3.9308	0.8805
TCGA-AR-A24X-01	3004	0	4.5098	0.7233	-0.2671
TCGA-AR-A24Z-01	3001	0	3.4437	-3.3076	-1.1172
TCGA-AR-A250-01	2707	0	4.9769	-1.4305	0.7321
TCGA-AR-A252-01	2838	0	6.7518	0.9716	1.3956
TCGA-AR-A255-01	2161	0	6.2071	0.2762	0.4865
TCGA-AR-A0U2-01	2551	1	2.5634	-3.0469	0.2154
TCGA-AR-A0U3-01	4080	0	3.7783	-3.1714	-0.1345
TCGA-B6-A0WT-01	5739	0	4.3198	-3.458	0.6239
TCGA-B6-A0WV-01	2417	1	4.8182	-3.816	-1.0559
TCGA-B6-A0WW-01	558	1	4.2126	-2.8262	-0.9132
TCGA-B6-A0X4-01	860	1	3.6669	-3.6259	-1.9379
TCGA-B6-A0X5-01	2097	1	2.9488	-3.816	1.8918
TCGA-BH-A209-01	3959	1	4.0375	-2.0529	-0.2671
TCGA-D8-A1X9-01	727	0	4.8279	-2.2447	0.2277
TCGA-D8-A1Y2-01	433	0	5.1301	-1.8836	0.3907
TCGA-D8-A1Y3-01	430	0	3.1892	-5.5735	-2.5479
TCGA-A2-A25B-01	1291	0	4.4693	-0.7346	0.03
TCGA-A2-A25C-01	523	0	4.327	-0.3201	0.4761
TCGA-A2-A25E-01	3204	0	4.1359	1.3956	-0.7108
TCGA-A7-A26E-01	954	0	3.72296647712	-0.904161480432	0.401666624204
TCGA-A7-A26H-01	724	0	4.4536	-0.7108	0.6969
TCGA-A7-A26J-01	627	0	3.67178246842	-0.913244039614	0.231833889897
TCGA-AC-A23C-01	585	0	5.281	-1.7322	0.4233
TCGA-AR-A24H-01	4894	0	2.6464	-0.2159	-0.0877
TCGA-AR-A24K-01	1548	0	2.328	-2.2447	-1.0862
TCGA-AR-A24L-01	2866	1	5.2518	-1.1488	0.5955
TCGA-AR-A24M-01	3660	0	5.899	-1.1488	1.0151
TCGA-AR-A24N-01	3035	0	3.834	-0.0277	-0.394
TCGA-E9-A247-01	1186	0	6.2683	-1.7322	-0.8339
TCGA-E9-A249-01	217	0	3.1145	-5.0116	-2.2447
TCGA-E9-A24A-01	747	0	4.0799	-2.114	0.8726
TCGA-AQ-A0Y5-01	172	1	4.6798	0.2154	1.0503
TCGA-GM-A2DN-01	3091	0	5.8327	-0.1345	0.0158
TCGA-GM-A2DM-01	3226	0	3.5791	-0.2671	-0.1665

TCGA-AR-A24O-01	3607	0	7.1268	1.9264	1.4335
TCGA-AR-A24P-01	84	0	4.5742	0.099	0.6608
TCGA-AR-A24R-01	3430	0	4.5705	-2.4659	-0.7108
TCGA-AR-A24S-01	2976	0	5.9074	0.7233	0.688
TCGA-AR-A24T-01	3202	0	5.6764	-0.2671	0.8408
TCGA-AR-A24V-01	3203	0	5.1023	-0.3022	0.517
TCGA-BH-A201-01	856	0	6.2839	1.7532	0.6699
TCGA-BH-A202-01	795	0	3.1062	-2.114	0.346
TCGA-D8-A1JJ-01	611	0	4.7464	-1.9942	0.517
TCGA-D8-A1X6-01	541	0	0.8647	-1.4305	-0.0725
TCGA-D8-A1X7-01	509	0	4.3299	-2.4659	0.688
TCGA-D8-A1X8-01	783	0	5.2736	1.412	0.6517
TCGA-BH-A1FB-01	3669	1	7.2084	3.5035	2.1894
TCGA-BH-A1FH-01	1034	1	7.0899	2.0535	2.081
TCGA-BH-A1FJ-01	1927	1	3.4437	-3.0469	-1.3921
TCGA-BH-A1FM-01	1388	1	4.0287	-1.3548	0.2642
TCGA-C8-A27A-01	747	0	3.5449	-1.5951	-0.3383
TCGA-E9-A1RI-01	1449	0	5.6118	-0.7588	0.7664
TCGA-B6-A1KC-01	1326	0	2.0776	-1.4305	-1.2828
TCGA-BH-A204-01	2534	1	3.6635	-1.2481	-1.0559
TCGA-AC-A2B8-01	677	0	6.8551	0.1388	1.3051
TCGA-C8-A26Z-01	470	0	3.8601	0.5666	-0.4325
TCGA-C8-A273-01	513	0	5.3848	0.0854	-0.3566
TCGA-C8-A274-01	508	0	3.208	-4.6082	-1.7322
TCGA-D8-A27E-01	530	0	4.5772	-1.1811	-1.1172
TCGA-E9-A1R5-01	92	0	4.9654	-3.816	-0.1828
TCGA-E9-A1R7-01	1467	0	4.3017	-2.6349	1.105
TCGA-AC-A2FO-01	2255	0	6.0531	0.6517	0.9716
TCGA-AR-A2LE-01	5062	0	3.4398	0.03	-0.5332
TCGA-A2-A259-01	1596	0	5.8344	2.5437	0.7058
TCGA-A2-A25D-01	552	0	5.0597	-0.1504	-0.9406
TCGA-BH-A28Q-01	1119	0	3.7762	-1.3183	0.5069
TCGA-C8-A26V-01	616	0	4.6804	-1.8314	-0.9971
TCGA-C8-A26W-01	381	0	4.8141	-1.2828	-0.6416
TCGA-AC-A2FB-01	1234	0	6.4169	2.4544	0.9343
TCGA-AC-A2FF-01	2759	0	6.8895	0.8961	1.4859
TCGA-AC-A2FG-01	1853	0	7.106	0.6969	1.2085
TCGA-D8-A27V-01	381	0	5.8849	-0.3752	0.8327
TCGA-EW-A2FS-01	1604	0	6.0002	0.4967	-0.6416
TCGA-EW-A2FV-01	788	0	5.9468	1.7995	0.4233
TCGA-EW-A2FW-01	672	0	3.7898	-2.5479	-1.9942
TCGA-D8-A1XY-01	503	0	4.5862	-0.394	0.3231
TCGA-D8-A1XZ-01	466	0	3.9673	-2.4659	-1.0262
TCGA-E9-A1N4-01	1000	0	4.1907	0.1257	0.517
TCGA-E9-A1NE-01	1088	0	5.9572	2.9262	0.8726
TCGA-E9-A1NG-01	786	1	6.0696	0.03	1.5216
TCGA-AR-A2LK-01	1649	1	4.5173	-1.8836	-0.5332
TCGA-AR-A2LL-01	2012	0	3.8391	-1.9379	-0.5125
TCGA-AR-A2LM-01	1935	0	5.7814	-0.0425	1.3283
TCGA-E9-A2JS-01	904	1	4.3155	-3.3076	-0.0574
TCGA-GM-A2DK-01	2645	0	7.9045	-1.3183	-1.0262
TCGA-D8-A27G-01	409	0	6.4785	0.4865	0.517
TCGA-D8-A27I-01	439	0	7.512	1.4011	1.8036

TCGA-D8-A27K-01	1461	0	5.2936	-0.6873	1.2455
TCGA-D8-A27L-01	499	0	5.7337	0.7916	1.6045
TCGA-D8-A27N-01	519	0	6.1412	-2.1779	0.4552
TCGA-D8-A27P-01	49	0	6.4308	-0.4325	0.7493
TCGA-D8-A1XL-01	606	0	3.2435	-2.5479	-1.8314
TCGA-D8-A1XO-01	1682	0	4.2472	-0.0725	0.3796
TCGA-D8-A1XR-01	482	0	4.84	-0.2328	-0.3022
TCGA-D8-A1XS-01	496	0	3.8431	-1.7322	0.0158
TCGA-D8-A1XV-01	461	0	3.6646	-3.816	-2.0529
TCGA-A2-A0D4-01	767	0	5.4111	-3.1714	-0.8599
TCGA-A8-A06Z-01	31	0	1.2333	-4.035	-1.5951
TCGA-A8-A085-01	1124	0	3.4048	-3.816	-0.6873
TCGA-A8-A08I-01	365	0	0.9115	-2.7274	-0.0877
TCGA-A8-A091-01	1004	0	4.1891	-2.8262	0.099
TCGA-EW-A1P3-01	1611	0	5.9179	0.9115	-1.1488
TCGA-EW-A1P6-01	562	0	4.5748	-2.114	-0.5125
TCGA-EW-A1PA-01	575	0	5.1165	0.5271	-0.013
TCGA-A2-A1FZ-01	683	0	8.0429	0.3115	0.7664
TCGA-D8-A27R-01	307	0	4.215	-2.114	-0.3383
TCGA-D8-A27T-01	398	0	5.9132	-0.0277	0.2998
TCGA-D8-A27W-01	373	0	4.1433	-1.9942	-0.1187
TCGA-E9-A1R0-01	860	0	8.7543	-2.1779	-0.6193
TCGA-E9-A295-01	375	0	6.0938	-1.1172	-0.1993
TCGA-GI-A2C8-01	225	0	4.0867	-0.7834	0.9862
TCGA-AR-A2LN-01	1161	0	7.7242	3.1844	1.0573
TCGA-AR-A2LO-01	1198	0	6.9077	1.3511	1.6558
TCGA-BH-A2L8-01	612	0	5.2464	-1.2142	0.3346
TCGA-E9-A2JT-01	288	0	6.051	-1.8314	1.7912
TCGA-B6-A2IU-01	5176	0	6.1248	-1.7322	-1.5951
TCGA-GM-A2D9-01	1812	1	4.9151	-2.5479	-1.5105
TCGA-GM-A2DA-01	6593	1	5.1375	1.0007	-0.5756
TCGA-GM-A2DC-01	2535	0	3.9965	-1.2828	-1.1811
TCGA-GM-A2DI-01	2590	0	5.3133	0.7058	1.8444
TCGA-A8-A095-01	1277	0	5.033	-1.2142	0.1519
TCGA-A8-A084-01	458	0	4.4693	-2.5479	-0.3566
TCGA-A2-A3KC-01	1102	0	7.0865	-0.8599	0.7748
TCGA-A2-A3KD-01	1206	0	5.2597	2.0218	0.4233
TCGA-A7-A3J0-01	313	0	3.0688	-2.4659	-0.3566
TCGA-A7-A3IZ-01	322	0	2.8402	-2.7274	-1.0862
TCGA-AC-A23G-01	2248	0	6.8419	1.0293	1.0573
TCGA-A7-A3J1-01	343	0	6.9615	0.5666	0.688
TCGA-AC-A3HN-01	496	0	6.4497	1.1577	0.6332
TCGA-C8-A3M8-01	394	0	5.3284	-1.2828	-0.4719
TCGA-E2-A3DX-01	1325	0	4.6877	-0.3022	1.8036
TCGA-E9-A3HO-01	1158	0	4.2995	-4.035	-2.0529
TCGA-A7-A2KD-01	679	0	4.3024	-1.8836	-0.5125
TCGA-A7-A3IY-01	345	0	5.917	-0.013	-0.0877
TCGA-AC-A3OD-01	451	0	4.17700822615	-1.48600673297	0.125653751904
TCGA-AC-A2BM-01	3022	0	3.3605	-2.4659	-0.5973
TCGA-C8-A3M7-01	1034	1	6.7652	-0.4325	0.2642
TCGA-E9-A3Q9-01	1001	0	5.911	-0.5973	-1.5951
TCGA-EW-A1PC-01	187	0	2.7422	-4.035	-1.2142
TCGA-GM-A3NY-01	1162	0	5.6189	-1.8836	-1.6394

TCGA-GM-A2DL-01	3519	0	6.3073	-1.0559	-0.7588
TCGA-GM-A2DO-01	2596	0	2.043	-3.1714	0.044
TCGA-BH-A0HL-01	72	0	3.136	-3.1714	-1.2142
TCGA-BH-A0HN-01	516	0	2.0535	-2.6349	-2.0529
TCGA-A8-A06R-01	547	0	4.9486	-1.4699	-0.6643
TCGA-A8-A08Z-01	1217	0	4.8949	-0.6643	1.0983
TCGA-A8-A099-01	304	0	2.8014	-2.7274	-1.1172
TCGA-E2-A1BD-01	1133	0	4.889	-3.1714	-1.2142
TCGA-A8-A09M-01	1006	0	1.7954	-5.0116	-1.2828
TCGA-BH-A1ES-01	3462	1	5.8595	-0.8339	0.4447
TCGA-BH-A0B4-01	1191	0	4.1836	-1.1811	-0.6873
TCGA-A8-A08G-01	607	0	3.927	-1.7322	-0.0574
TCGA-A7-A13H-01	899	0	6.63	-0.0877	0.5666
TCGA-E2-A2P6-01	1051	0	4.6537	0.2154	-2.0529
TCGA-A2-A0CO-01	3492	1	6.8276	2.9655	1.1447
TCGA-A7-A3RF-01	408	0	5.1732	-2.8262	-1.7322
TCGA-E2-A14U-01	1318	0	8.3793	1.2455	0.5568
TCGA-E2-A2P5-01	821	1	4.3841	-1.8836	-1.7809
TCGA-A2-A0EP-01	3603	0	7.4634	2.7657	1.6512
TCGA-A7-A425-01	447	0	8.1691	-1.0559	-0.0425
TCGA-AC-A3QP-01	675	0	5.9693	-2.0529	-0.7588
TCGA-A7-A426-01	364	0	7.4772	-1.5105	0.5955
TCGA-LL-A442-01	889	0	3.3966	-2.9324	0.2642
TCGA-AC-A3YJ-01	754	0	2.6114	-3.0469	-0.5543
TCGA-B6-A401-01	2596	0	5.1306	-0.4131	-0.6873
TCGA-B6-A408-01	2072	0	3.9774	-1.685	-0.5332
TCGA-B6-A40B-01	3152	0	5.0215	0.5859	-0.7346
TCGA-B6-A40C-01	2164	0	6.7079	-3.816	-0.8084
TCGA-BH-A42T-01	320	1	4.8783	0.7233	-1.685
TCGA-D8-A3Z5-01	1015	0	7.8553	1.3791	-0.394
TCGA-BH-A42V-01	635	0	7.7295	0.9191	0.5763
TCGA-D8-A3Z6-01	563	0	5.1851	-2.114	-0.3383
TCGA-E9-A3X8-01	926	0	6.5204	-3.458	-1.6394
TCGA-A2-A0CK-01	4159	0	6.9101	-0.1345	-1.2828
TCGA-A2-A0CR-01	3283	0	7.2891	-0.8084	-0.1345
TCGA-AC-A3TM-01	762	0	5.8071	-3.1714	-1.6394
TCGA-AC-A3TN-01	456	0	5.5239	-5.0116	-1.7809
TCGA-AC-A2FE-01	2636	1	6.8572	0.4761	0.3685
TCGA-AC-A2FM-01	792	1	4.7661	-0.3201	-0.6873
TCGA-AC-A2QI-01	588	0	5.2925	-2.114	0.2029
TCGA-AC-A3EH-01	197	1	4.6142	-3.0469	-0.9686
TCGA-AC-A3BB-01	987	0	6.9134	-0.0574	-0.0277
TCGA-AC-A3QQ-01	734	0	3.90556679417	-1.30932433783	-0.940657126057
TCGA-AC-A3W7-01	471	0	6.2413	0.3685	0.5271
TCGA-AC-A3W6-01	602	0	5.599	-0.5543	0.2762
TCGA-AR-A2LJ-01	2632	0	6.6243	-2.3147	-0.7588
TCGA-AR-A1AM-01	2991	0	6.1206	-0.2159	0.2522
TCGA-BH-A0B6-01	2483	0	3.6793	-2.1779	0.2998
TCGA-E2-A107-01	1047	0	4.8968	-2.6349	-1.685
TCGA-EW-A3E8-01	1035	0	7.3359	-1.1172	-0.6193
TCGA-EW-A423-01	533	0	3.7421	-4.035	-2.2447
TCGA-EW-A424-01	715	0	5.8316	-1.7322	-0.9406
TCGA-GM-A3XG-01	1330	0	9.0814	-0.7588	-0.8863

TCGA-GM-A3XN-01	2019	0	8.2283	0.2154	-0.3022
TCGA-LL-A440-01	759	0	7.2397	1.9149	2.1606
TCGA-JL-A3YX-01	352	0	4.8665	0.0014	-1.685
TCGA-GM-A3NW-01	3361	0	5.931	-1.5951	-0.9406
TCGA-D8-A4Z1-01	659	0	7.9442	1.9675	0.9191
TCGA-A2-A4S3-01	666	0	3.2929	-2.8262	-0.4521
TCGA-A2-A4S2-01	643	0	5.8319	-1.6394	-0.6643
TCGA-A7-A4SC-01	446	0	5.8412	0.537	0.1519
TCGA-AQ-A54O-01	1001	0	3.0163	-5.0116	-1.7809
TCGA-GM-A4E0-01	2191	0	8.8471	-0.9132	0.3346
TCGA-A2-A4RW-01	222	0	5.3053	-2.0529	0.537
TCGA-LL-A50Y-01	762	0	4.0091	-4.035	-1.7809
TCGA-A7-A4SB-01	418	0	5.4562	-0.9971	-1.3921
TCGA-E9-A54X-01	727	0	2.9128	-5.5735	-2.114
TCGA-A2-A4S0-01	706	0	3.3177	-4.035	-1.9379
TCGA-LQ-A4E4-01	849	0	6.5372	-1.9379	-1.2142
TCGA-A7-A4SA-01	454	0	5.4702	0.0718	-0.1031
TCGA-MS-A51U-01	681	0	5.9944	-0.1187	1.0222
TCGA-AC-A5XU-01	455	0	4.4777	-3.3076	0.2762
TCGA-AR-A5QN-01	1013	0	6.2752	-0.2845	0.1648
TCGA-E9-A5UP-01	803	0	4.4297	-3.1714	-1.8314
TCGA-E9-A5UO-01	785	0	3.5136	-4.6082	-2.8262
TCGA-AR-A5QP-01	1185	0	7.1574	1.0983	0.3346
TCGA-GM-A5PV-01	412	0	6.9956	-1.1172	-0.8339
TCGA-GM-A5PX-01	551	0	6.9797	-1.8836	-0.7834
TCGA-OL-A5RX-01	878	0	7.0416	0.3907	0.346
TCGA-OL-A5RV-01	1062	0	6.8167	-0.3383	0.0854
TCGA-A7-A56D-01	448	0	5.5043	-4.6082	-2.0529
TCGA-AR-A5QM-01	2231	0	6.2252	2.6805	0.1124
TCGA-E9-A5FK-01	812	0	6.6238	-2.5479	-1.3921
TCGA-BH-A5J0-01	715	0	5.4848	-0.8599	-0.8339
TCGA-LL-A5YM-01	466	0	6.5765	-4.6082	-1.2828
TCGA-LL-A5YN-01	447	0	4.9727	-4.2934	-1.0262
TCGA-OK-A5Q2-01	64	0	7.0099	-0.9132	0.1519
TCGA-OL-A5D8-01	973	0	3.1079	-2.9324	-1.8314
TCGA-OL-A5DA-01	1783	0	5.6142	-2.3884	-0.6193
TCGA-OL-A5RU-01	1219	0	5.496	-0.0725	-0.0277
TCGA-PE-A5DC-01	1430	1	5.061	-0.1504	-1.5105
TCGA-PE-A5DD-01	1953	0	4.9496	-0.8339	-0.9132
TCGA-PE-A5DE-01	2645	0	4.6882	0.8568	-0.1345
TCGA-HN-A2OB-01	1900	1	6.8838	-1.2481	1.4174
TCGA-A7-A5ZW-01	326	0	6.2867	-0.3383	0.9789
TCGA-A7-A5ZX-01	336	0	7.5889	-0.8863	0.3573
TCGA-AC-A5XS-01	588	0	5.3367	-0.4921	-0.6643
TCGA-AC-A62Y-01	530	0	6.1729	0.9935	-1.1172
TCGA-E2-A56Z-01	252	0	3.4398	-2.3884	-2.3884
TCGA-E2-A570-01	931	0	4.5211	-1.5951	0.099
TCGA-LL-A5YL-01	519	0	4.8218	-3.3076	-0.8339
TCGA-OL-A66J-01	1996	0	8.684	0.3907	-0.2328
TCGA-OL-A66K-01	1275	1	4.7945	-1.5951	0.03
TCGA-AC-A62V-01	348	1	4.1474	-5.0116	-2.0529
TCGA-E2-A576-01	1043	0	4.7725	-2.4659	-2.3884
TCGA-E2-A572-01	1208	0	4.6854	-4.035	-3.458

TCGA-E9-A6HE-01	847	0	5.7035	-3.1714	-1.8314
TCGA-LD-A66U-01	646	0	5.5261	-0.1345	-1.0862
TCGA-LL-A6FQ-01	80	0	3.6635	-3.6259	-2.8262
TCGA-LL-A6FP-01	677	0	2.4649	-5.5735	-3.458
TCGA-OL-A66O-01	528	0	3.7094	-3.816	-2.6349
TCGA-OL-A66L-01	1301	0	7.7094	0.0158	-1.5105
TCGA-OL-A66N-01	792	0	5.0427	-3.816	-0.7108
TCGA-A8-A09E-01	1492	0	5.9136	-3.0469	-1.2828
TCGA-EW-A1IZ-01	554	0	4.6821	-0.9132	-1.3183
TCGA-A8-A07Z-01	1371	0	3.8581	-4.6082	-1.4305
TCGA-E9-A1RG-01	647	0	4.6531	-4.035	-2.0529
TCGA-EW-A6SA-01	510	0	2.3366	-4.035	-1.7322
TCGA-EW-A6SC-01	952	0	5.5386	2.9525	-0.4325
TCGA-LL-A740-01	441	0	7.8422	-0.7588	0.547
TCGA-AC-A6IX-01	373	0	7.2028	-0.2328	0.3685
TCGA-D8-A73X-01	767	0	4.4983	-0.6643	-0.0425
TCGA-S3-A6ZF-01	572	0	4.1145	-2.6349	-0.3022
TCGA-S3-A6ZG-01	562	0	3.4517	-2.7274	-0.9132
TCGA-S3-A6ZH-01	641	0	4.2297	-2.7274	-1.3548
TCGA-BH-A6R8-01	293	0	2.108	-4.6082	-2.4659
TCGA-AC-A6NO-01	51	0	4.8136	-2.7274	-1.1811
TCGA-EW-A6S9-01	463	0	4.5565	-1.5105	-0.8599
TCGA-D8-A73U-01	492	0	3.5947	-1.3548	0.1124
TCGA-A7-A6VX-01	317	0	4.546	-2.2447	0.0014
TCGA-LD-A74U-01	402	0	6.9961	-0.8863	1.1706
TCGA-OL-A6VR-01	1220	0	4.8876	-1.5951	-0.8863
TCGA-AC-A6IV-01	568	0	7.7616	-1.3183	1.0079
TCGA-V7-A7HQ-01	2033	0	5.7431	-3.0469	-2.5479
TCGA-BH-A8G0-01	662	0	6.4097	-0.6643	1.105
TCGA-D8-A73W-01	385	1	5.6482	-1.1488	0.0718
TCGA-LD-A7W5-01	216	0	5.6916	-0.4131	0.2154
TCGA-LL-A7SZ-01	594	0	4.4088	-5.5735	-2.7274
TCGA-LD-A7W6-01	404	0	6.5878	-1.0862	-0.394
TCGA-LL-A7T0-01	376	0	4.2358	-3.6259	-1.0862
TCGA-AC-A7VB-01	250	0	3.2707	-3.6259	-1.2142
TCGA-AQ-A7U7-01	584	1	4.4236	0.0014	1.1184
TCGA-BH-A8FY-01	295	1	3.4477	-2.2447	-0.8339
TCGA-XX-A89A-01	488	0	7.8673	-1.6394	0.3796
TCGA-C8-A8HQ-01	380	0	5.4293	-0.8084	0.4552
TCGA-AC-A8OP-01	614	0	5.6436	2.0844	1.8683
TCGA-XX-A899-01	467	0	6.0455	-0.7588	1.0151
TCGA-W8-A86G-01	347	0	7.9403	-0.6873	-0.394
TCGA-3C-AAAU-01	4047	0	3.5224	-2.7274	-0.6416
TCGA-AC-A4ZE-01	890	0	6.8175	-1.6394	-0.9132
TCGA-WT-AB41-01	1611	0	5.5472	-5.5735	-2.1779
TCGA-S3-AA11-01	421	0	5.0014	-1.9379	-1.0262
TCGA-LL-A9Q3-01	532	0	4.796	-4.6082	-2.0529
TCGA-WT-AB44-01	883	0	4.9151	-2.6349	-1.1811
TCGA-3C-AALJ-01	1474	0	5.6413	-5.0116	-0.2498
TCGA-AC-A8OR-01	40	0	6.6265	-2.114	-1.1488
TCGA-S3-AA12-01	574	0	1.9675	-3.6259	-3.1714
TCGA-OL-A6VQ-01	600	0	6.3263	3.6219	1.3567
TCGA-Z7-A8R5-01	3287	0	8.4266	-0.3201	-0.1345

TCGA-S3-AA14-01	529	0	5.2567	-0.1187	-0.0877
TCGA-AC-A8OS-01	70	0	5.8344	0.6239	0.8961
TCGA-3C-AALK-01	1448	0	5.4712	-1.0862	0.1519
TCGA-4H-AAAK-01	348	0	5.7504	-1.685	-0.2671
TCGA-BH-AB28-01	287	0	6.1258	-1.1488	0.0854
TCGA-S3-AA17-01	424	0	2.0912	-2.1779	-0.2498
TCGA-5L-AAT0-01	1477	0	5.8508	-3.1714	0.2522
TCGA-Z7-A8R6-01	3256	0	4.7383	-2.1779	-1.7322
TCGA-5T-A9QA-01	303	0	1.5514	-4.035	0.0854
TCGA-5L-AAT1-01	1471	0	5.1194	-3.1714	0.0158
TCGA-E2-A9RU-01	538	0	3.6771	-3.3076	-1.685
TCGA-UL-AAZ6-01	518	0	2.362	1.3791	-2.0529
TCGA-E9-A54Y-01	725	0	2.0254	-9.9658	-5.0116

AKR1C3	FANCD2	G6PD	ACSL4	ACO1	NQO1
3.7171	2.7357	5.9035	2.971	2.7572	8.4486
3.0568	3.3076	5.9486	3.3521	4.2788	6.6622
1.642	2.7763	4.8954	2.8994	4.4823	5.6381
2.4805	4.6095	6.6864	3.0515	3.3492	6.9814
3.9289	2.3926	5.9225	3.3535	4.0471	6.465
3.2065	3.3032	4.1716	2.2175	2.8701	5.745
2.6738	2.4252	6.4516	3.2435	2.9052	8.2399
2.2723	3.1062	4.8997	2.8341	3.8935	6.5107
2.3135	3.5324	5.1728	2.3019	4.4964	8.3229
2.6828	3.1294	5.7263	3.9847	5.2208	7.0192
2.7572	2.6185	4.9584	2.1213	4.4751	6.1737
3.867	2.8897	4.3758	2.9148	3.3134	6.8747
1.3791	3.9571	5.4667	2.9655	3.5436	6.5003
1.8078	3.0893	4.5626	1.6234	3.186	4.9108
3.8531	4.2289	6.3309	2.8897	3.9728	8.1078
1.4962	4.2495	6.3252	3.6428	5.0875	8.3313
4.4556	2.7292	5.9658	2.5561	2.6348	6.6799
2.2082	3.9965	5.2884	2.5288	3.705	6.1605
5.2028	2.2753	5.9486	4.3951	4.9421	7.4756
2.2051	4.8699	6.0139	2.5683	3.7204	9.494
2.8838	3.6301	4.6089	2.05	3.4958	8.4962
1.7995	3.4726	5.3256	3.3952	4.1053	6.2042
2.7444	2.8442	4.9991	2.4518	3.5851	6.7815
1.7273	2.8219	4.848	1.4756	2.4144	6.1088
3.5621	3.4371	5.7753	2.362	4.5261	8.6667
1.9452	4.5243	6.6286	2.7594	3.7453	7.911
5.2698	3.3464	4.836	2.6395	4.8968	6.2778
2.91667970867	2.30963521684	3.3383645849	2.92299268128	3.65317086522	5.65281014261
2.9674	4.5711	6.0345	2.6441	3.7614	8.0322
2.8562	4.1571	6.1316	3.5436	4.174	6.3724
3.3966	2.8361	5.4243	3.5086	4.6474	5.8657
3.6771	3.5249	5.1182	3.3814	4.2914	6.5294
5.2799	2.8034	5.8657	3.4251	4.1252	8.1228
1.7446	3.5022	5.3151	2.1313	3.0358	5.813
5.436	3.2929	5.5469	2.328	3.5923	8.2572
3.3731	3.9299	5.7383	3.4958	3.4291	7.7381
5.2457	3.866	4.8939	3.1145	3.7656	7.2081
2.2019	2.7679	4.9232	2.8542	3.325	8.088
3.0533	4.3862	5.7994	2.4492	3.9431	7.9085
5.2653	3.6509	5.434	3.4224	5.1744	9.0693
3.3293	2.8422	5.8674	2.296	4.2616	6.7391
4.2556	3.7149	5.8836	2.8341	4.6212	7.9498
4.1044	3.2002	5.7474	3.0978	4.3492	8.751
2.8974	2.1213	4.783	0.9268	4.8238	7.847
1.9377	5.2005	5.5549	2.8522	5.4999	8.2506
5.9948	3.1409	5.961	4.0055	3.4778	8.0597
2.9507	4.2691	5.783	2.753	3.7815	7.0606
4.1359	3.7204	5.9794	4.2033	5.1875	7.8215
1.1216464512	2.3427402101	3.9712381322	2.15573383233	2.96890647192	5.93011354341
3.8818	2.4597	5.3534	3.6578	4.5832	7.8206
4.4977	4.1899	5.4542	1.2455	4.3709	8.3578
1.9786	4.2181	6.6326	4.1069	3.1781	8.4282

4.5136	5.0497	4.8329	1.8484	3.0446	7.2306
1.7744	3.5312	5.3147	1.3397	2.3926	8.1901
3.1876	3.1179	5.4286	3.0706	3.8896	8.3248
4.2434	2.1894	5.4023	3.7507	3.8983	7.3181
3.2541	3.5073	5.447	2.685	5.803	5.9791
3.2405	3.5161	5.322	2.7117	3.9185	5.1111
5.4509	3.5706	5.2107	3.8808	5.322	7.2301
2.667	3.9118	6.3908	3.7432	4.0233	6.613
2.8402	3.1844	5.8679	2.7993	4.0216	8.9272
1.2576	3.3911	5.7605	2.872	4.4476	7.2648
2.5238	2.6828	5.1883	3.1939	3.449	6.1512
3.4034	3.3563	5.6488	3.0757	4.0073	7.6493
4.6691	3.0446	5.4286	2.8034	3.3883	7.2162
5.1883	3.8269	4.7464	3.5461	4.6218	6.8996
2.9148	4.0969	5.3027	3.6066	4.0884	6.3766
0.8726	4.4074	4.5952	2.2753	4.0593	7.4594
2.0742	3.8147	5.5604	1.8119	3.5887	7.6104
4.0225	2.5462	4.7044	2.6043	3.2435	6.9466
5.153	3.2065	5.5339	2.2513	4.1136	7.4071
4.9184	2.8199	6.2132	3.6771	4.4243	8.171
7.0774	3.034	4.8182	3.8611	4.5796	8.061
3.4648	4.1359	5.4337	3.0446	3.1733	8.3183
6.267	3.5803	6.4381	2.694	2.5263	6.3877
4.6142	3.4477	5.9255	3.1924	3.8511	6.1142
6.0837	3.2587	5.5723	3.6018	3.7345	9.1293
5.7738	2.4035	5.2668	2.9319	4.2751	6.3399
4.7756	4.4404	7.1748	3.3856	4.0688	9.6482
5.1835	2.4675	5.1696	3.6782	5.3495	7.6295
6.5612	4.2773	5.2965	3.3717	3.7061	8.1386
6.6624	3.5935	5.8422	3.7971	4.3506	7.6843
5.5214	1.692	5.7594	3.0305	4.748	6.5446
3.9299	3.7539	5.4219	3.5559	4.0824	7.1205
3.4371	2.9224	4.2189	3.6359	3.7259	7.7001
3.0498	3.074	5.6118	4.0506	4.6416	6.2155
2.2872	3.1781	5.2384	3.2885	3.5706	7.6982
3.38	3.6669	6.1167	3.8147	4.5802	6.5361
5.6387	3.9829	5.0545	3.8798	3.5009	6.0747
6.2983	4.4277	6.773	2.9544	3.7335	9.1054
6.7403	2.6232	5.4479	4.5874	5.0968	7.1774
2.6255	3.6928	3.835	3.7836	4.3292	7.6103
4.8131	1.9822	4.1978	2.828	3.9938	7.8853
6.1894	4.1466	7.1454	3.7846	3.8719	8.3799
5.7288	3.5187	5.082	3.7193	3.3703	7.3696
4.4243	2.8916	4.7458	3.5694	4.1384	6.8455
4.4842	2.1798	4.9924	2.8321	3.3842	6.6512
4.4906	3.325	4.9184	2.7572	4.8334	5.7892
5.8721	4.131	5.5107	3.4791	3.29	6.3074
6.1713	3.4661	6.3801	3.9829	3.9318	8.0336
5.2118	3.5274	4.9079	2.6043	4.8729	8.2384
3.5994	4.5367	4.4836	2.1313	2.296	8.8659
2.3843	3.1604	5.1285	3.5815	4.2669	6.08
4.4236	3.0585	6.6871	2.8877	4.9203	10.2133
6.3468	3.3379	5.9381	2.5707	3.6748	8.2628

8.1325	2.7721	6.44	4.0799	4.2312	8.4484
2.0673	4.1178	5.4536	3.2781	3.7582	7.0421
3.4371	2.6783	4.7474	3.2189	4.3772	9.1634
4.0696	4.1891	6.2145	3.0961	3.245	7.0849
0.8883	3.2826	4.6854	1.0779	4.1458	9.9652
5.7244	4.8269	5.5417	-1.5105	4.4673	8.5438
2.8916	3.6393	5.2788	2.8838	4.0162	7.2283
3.668	3.3003	5.5979	3.6393	4.4457	6.9639
3.3966	3.3307	5.5964	3.5694	4.6894	7.3427
3.9412	2.8602	4.9416	3.3563	4.0994	6.635
3.3577	2.3898	6.3322	4.0136	4.8435	7.0736
5.8985	3.4596	6.4891	3.0252	5.0475	10.0935
4.7082	2.5972	5.0683	4.2103	5.133	7.3351
2.676	4.554	5.9505	2.9948	3.9636	9.4723
2.9413	2.5213	6.7578	2.5804	3.7518	8.6029
3.3235	3.2602	4.9031	2.8381	3.5124	6.1018
3.6184	3.186	5.3913	4.7469	5.1301	6.1516
1.6558	2.7357	6.0438	2.5263	5.0242	6.9485
3.162	2.2175	5.5623	2.0569	3.991	8.9263
4.1466	2.7051	4.837	3.2405	3.6725	3.3619
5.109	2.8622	5.4784	2.8055	4.3917	4.9237
6.6284	1.8918	4.9764	2.9186	3.8827	7.8471
6.4023	3.3773	5.8927	3.5337	3.4635	8.1652
3.3979	4.352	5.6744	2.2901	6.213	9.0821
3.0774	2.1345	4.623	1.4547	2.2268	6.2011
2.8641	1.8078	6.1843	2.3451	3.3993	9.016
1.9264	3.5374	4.4977	0.679	3.3379	7.7391
2.7422	2.9562	5.8151	0.2762	4.6259	8.003
4.1376	3.6703	5.6291	0.4447	4.2548	6.0715
4.7997	1.4962	5.1883	0.1648	3.5887	8.2529
2.9071	3.0944	6.1563	1.7185	2.9375	8.4654
4.554	2.6895	5.3402	2.7314	4.4257	6.4043
3.9468	2.753	5.4912	3.0961	4.2434	7.8733
3.2662	3.0568	4.9041	1.334	3.136	8.8212
2.8974	2.6114	5.2292	1.1641	3.8198	5.0781
4.9141	2.2931	4.7198	4.3604	4.352	6.3225
4.8064	2.9562	5.1835	3.2988	4.1343	7.3674
2.7183	2.0183	4.9388	1.7229	3.1195	6.23
3.4143	3.9412	5.3765	3.863	4.3834	8.4077
3.4907	4.748	4.4582	3.2158	3.5766	7.7152
4.4443	2.7095	4.9412	2.9692	3.9299	7.76
1.3679	4.0064	4.996	2.0638	3.2988	8.1094
4.8105	3.1062	6.1873	3.2811	4.6651	6.7873
3.3407	2.7314	5.2747	2.8701	3.8481	7.2607
3.1045	3.2034	6.6163	2.7051	3.9118	8.8677
1.3679	2.7784	4.735	2.5996	2.9148	7.2295
4.996	3.0127	5.4542	3.0568	4.4156	9.079
2.7657	3.2944	5.0379	1.674	4.5742	7.6143
0.1257	3.2602	5.0462	0.5763	3.074	6.7278
4.0144	2.8602	5.5799	3.5098	4.0454	9.4676
4.2995	2.7117	5.1339	2.8582	4.1836	7.1522
6.3975	1.9968	4.1053	2.8602	4.4542	6.5996
3.9901	3.47	6.1879	3.1908	4.5293	5.4169

4.8395	1.4704	4.7209	2.2573	3.6669	5.1891
4.1636	2.9243	5.393	1.0847	3.5022	9.2724
2.8442	3.8054	5.2358	2.0742	3.8925	6.8999
4.427	3.4437	5.6401	3.5349	5.3705	6.4861
3.7961	2.4623	5.9004	3.864	3.4116	8.5637
4.0994	3.7825	4.8095	2.9857	4.4384	7.7439
2.7763	4.435	7.155	3.2647	4.6195	9.53
2.6533	3.3235	5.3122	0.8164	4.0867	6.8618
5.8319	1.6281	4.435	3.2096	3.8391	5.2721
5.6876	3.309	5.8647	3.8218	4.9421	6.8919
2.9929	2.2452	4.9547	2.2391	4.0867	7.6256
3.245	4.5602	5.8306	2.8137	4.3862	9.3421
3.9645	3.0446	4.8187	2.6418	3.6101	7.4592
3.6972	3.5766	5.1716	2.4035	4.6177	7.069
4.0722	3.0323	4.6674	3.5436	3.8531	7.1711
5.9998	2.5338	5.7377	4.001	5.284	7.8083
3.6566	2.6533	5.4002	3.1095	3.9299	6.379
6.1883	3.2826	5.4774	3.208	3.8561	5.8271
5.6218	2.8602	5.306	4.391	4.9041	7.1907
4.7049	3.6703	5.1958	3.795	4.034	7.2449
3.9524	3.1045	5.62	2.7227	4.4027	8.6686
5.1891	1.8078	4.4542	3.7678	4.8253	8.4837
2.6647	2.8936	7.9737	3.483	3.8551	8.6425
2.7249	1.5758	3.9185	1.9264	2.7227	7.9656
1.8484	2.7314	4.8719	0.4761	3.5149	5.615
2.9911	2.4597	4.3931	2.167	3.5669	7.2294
4.3256	4.5699	6.5627	3.8279	4.8929	9.43
3.6451	3.421	5.4588	3.1765	3.6042	7.4099
2.1894	3.9543	6.3322	1.8078	3.5947	5.3879
1.614	2.8096	6.2056	1.1833	3.5161	10.2001
1.4494	4.2714	6.2275	2.4805	2.6161	8.9348
3.7421	3.8054	5.0095	2.7183	4.7247	8.6915
4.2714	3.6916	5.067	2.9966	4.0653	6.4691
3.3421	3.2189	5.5747	3.837	4.7291	6.5589
5.3792	3.1228	4.7512	4.1228	4.2343	5.4896
3.0127	3.4061	6.3982	3.3076	4.691	6.8536
1.8404	2.3337	5.0229	3.3577	4.0918	5.7711
4.1094	4.1053	7.8997	4.0705	4.0454	9.2894
0.4967	1.8363	4.6154	0.7664	2.3564	8.1619
4.3435	2.7993	5.0807	3.0688	4.7231	6.7776
3.6793	4.7624	4.9313	3.0876	4.7693	7.4641
4.447	2.7227	5.2687	3.8964	5.0972	7.6368
1.9859	5.5392	5.2312	2.9637	3.0428	7.549
6.3718	3.2389	5.0811	3.7752	4.8395	7.1123
0.8488	2.96	4.4881	1.0503	3.0585	2.8199
2.8402	2.5011	5.0584	1.6281	1.8484	8.1766
1.9712	3.8064	5.5723	2.2573	3.1813	8.595
2.7594	3.3379	5.489	2.9618	4.3449	7.295
3.603	3.6759	5.366	2.5972	3.6219	6.9828
4.7991	3.3925	5.4777	3.0893	5.1343	8.0271
0.7664	4.3541	5.6144	2.2603	4.1506	3.8983
5.3837	4.0688	5.7937	2.0218	3.866	6.976
2.876	5.1177	6.8041	2.0004	3.0533	8.8467

3.4304	3.4765	5.904	4.0073	4.1027	5.911
3.4156	3.2112	5.4754	4.0091	4.3234	5.4932
5.1583	3.4318	6.7548	2.4701	4.0322	8.2748
3.7116	2.4675	4.585	2.8199	3.8983	7.053
1.674	6.5874	6.1853	1.6964	3.8944	9.3447
3.3938	6.8333	5.7233	3.0961	4.1852	8.0824
2.4544	2.7594	4.8968	0.8082	3.6347	5.6442
5.1522	3.449	5.1651	2.9857	4.5274	8.0277
2.5188	4.5693	6.5297	3.8571	4.0357	8.3842
1.5854	3.668	5.7616	2.7073	2.362	8.8866
5.3927	2.0535	5.5233	3.3563	4.9117	6.9416
4.3213	3.4477	4.8963	3.4237	4.2212	7.7248
4.9084	3.2359	6.9445	3.869	4.7491	8.0684
2.8701	4.018	5.3071	1.9415	3.6725	6.8807
3.4251	5.3002	4.7027	1.7185	3.8769	7.279
0.537	4.1515	5.1656	2.4805	4.0567	7.3194
2.8562	4.3876	5.0879	2.8361	3.5718	5.2784
4.5154	2.8341	5.0295	3.5035	4.2518	7.2102
3.6578	3.1749	5.2672	3.7116	4.4719	5.5211
7.3017	3.0842	4.8719	3.1701	4.0985	6.995
2.5137	4.423	5.3576	2.1114	3.2781	7.3162
1.8404	2.7444	4.1269	1.5165	4.8269	6.4928
5.1514	4.3428	5.6551	3.4517	3.8033	8.7073
4.8007	3.1145	4.7005	3.7345	4.538	7.1538
2.2603	3.1572	5.0215	1.6327	4.1539	5.8058
5.9026	2.8701	4.9402	4.0144	4.2541	7.6979
6.073	3.1475	5.2869	4.079	5.0492	8.4048
2.6895	4.1351	4.851	2.9338	4.4667	7.6977
3.6101	3.055	5.4142	3.335	4.2781	6.6133
4.1724	4.4263	6.0427	2.4063	3.4504	8.1079
5.7088	3.5682	5.7727	0.7493	3.4237	8.4258
3.8289	4.4784	5.7578	3.5461	4.6617	6.9304
4.9203	2.5413	4.9937	5.8953	4.9232	7.0683
2.8799	3.8023	5.0631	4.6759	4.2563	7.4552
3.2707	3.7846	5.5772	2.5413	4.6118	7.0688
3.8054	1.7702	5.295	1.4547	4.5516	6.9141
3.5073	4.0462	5.2939	3.0038	4.2358	7.4882
2.1574	3.6838	4.9084	2.233	4.1119	4.9388
4.6668	4.2855	5.7093	2.4727	5.0781	8.4367
-0.7346	3.2359	5.778	1.7097	4.9379	5.911
4.717	5.0777	6.3025	2.4117	5.5062	6.9382
4.1425	4.026	6.3674	4.3191	4.2165	7.2645
3.7518	3.4945	6.428	3.7259	4.9079	6.6712
2.4492	3.9251	6.489	3.6312	3.4609	7.04
5.7701	3.1179	7.0032	4.245	5.8844	6.8422
2.128	4.981	5.9435	2.4675	3.8391	7.8352
2.9394	3.0774	5.131	1.3956	4.1458	4.6118
2.9128	4.533	5.8861	2.4144	2.7572	7.3116
5.7043	4.1094	5.789	3.6983	5.1384	8.0097
1.8484	2.9243	5.0189	3.7804	3.9983	7.4975
4.9928	2.1925	5.769	4.2788	5.6244	7.6107
1.9264	3.7961	5.8814	2.4332	4.3779	6.0104
6.1625	2.0254	5.0998	1.9034	3.7345	7.7092

2.3366	2.2513	5.3537	3.4739	4.0348	6.5771
4.2111	3.6804	6.8709	3.5754	4.174	8.249
3.5486	3.644	7.238	1.9452	2.6232	8.9496
4.9449	2.6138	5.8956	3.7215	4.5717	7.3475
2.7205	4.9741	5.2103	2.9784	3.6961	8.4007
3.8095	3.4048	5.3737	2.9394	3.9737	8.2689
3.7017	3.0145	5.2668	3.4007	4.3716	6.2382
4.1595	3.1409	4.7253	3.1426	4.2995	7.3823
2.9875	3.4437	5.8074	3.9318	3.8013	7.9981
3.8581	3.8837	6.3623	2.1957	2.8799	8.4319
4.9782	3.7464	5.978	1.6649	4.3385	6.829
3.1112	3.2944	5.2642	1.0847	3.5911	7.2891
3.3759	3.5815	6.6234	3.1278	4.4443	7.3991
3.3293	3.1749	5.2784	3.0723	3.9251	7.5737
4.3484	2.9205	7.7009	2.9875	3.7857	8.1995
4.5602	3.7804	6.0586	3.4648	5.2899	8.3932
3.1588	3.8778	6.064	1.642	4.01	8.7908
5.1441	3.1892	5.4774	3.8095	3.7006	7.5252
4.6606	2.3788	4.9108	3.5791	4.6809	8.7411
3.8167	5.1065	5.1478	3.309	3.6277	7.4034
3.325	3.6612	5.1052	3.8208	3.3549	6.1671
4.6479	2.5338	5.7156	3.1908	4.6753	8.8804
4.2274	1.6785	5.7871	3.7982	4.3206	7.8838
4.7825	3.2885	4.853	4.5318	3.9213	7.0565
4.1285	2.7572	4.9374	3.5337	4.585	6.6889
2.7227	3.4971	6.0416	3.002	5.0683	8.1349
5.7007	1.9638	5.1301	3.7269	4.9265	7.1073
5.2331	3.0671	4.9514	4.5224	4.466	9.5458
2.7868	2.8014	5.1599	1.0573	3.5486	5.9284
1.2696	2.8381	6.1727	2.2813	3.7699	8.0458
5.2165	4.0251	6.7381	3.3032	3.8126	9.1324
4.1954	3.3134	5.3622	4.6944	5.4565	7.8747
4.9846	2.7249	5.2138	3.6861	4.4932	7.5144
5.5367	3.2587	5.4702	4.7071	5.7885	7.6009
5.6949	4.854	6.4544	5.4273	4.2929	8.9605
4.1788	2.5924	5.0475	3.4984	3.7496	7.4806
4.0171	1.6093	5.0947	2.874	3.8944	5.7568
4.6764	3.8729	5.1077	4.0909	4.4842	7.6204
1.5115	4.6955	6.192	3.0568	2.0254	8.8556
1.177	4.0233	5.2502	0.9343	3.2572	7.6677
4.2847	2.6043	4.916	3.0825	2.8799	7.0491
4.2243	3.74	5.5255	2.7636	3.4034	9.0687
4.5098	4.9021	5.3456	3.4304	4.1458	6.8137
5.336	3.9365	5.336	4.7549	5.0989	8.0469
2.3898	3.3828	5.005	3.3407	4.3263	6.5381
4.3206	2.4857	4.9208	2.96	4.3349	7.3197
5.275	3.0602	4.9686	4.1498	4.4128	7.5655
2.004	4.5085	5.9088	1.9073	4.3758	8.8559
2.3019	3.9865	3.8581	1.0983	2.9186	6.1016
2.561	4.445	4.862	3.055	3.9691	8.8088
2.9167	3.8064	6.3913	3.5742	4.1186	8.7422
4.1612	4.5832	7.3783	3.4143	3.7815	8.1196
3.9617	4.1612	5.4545	2.3479	3.9515	6.7149

2.96	3.2811	5.1098	2.2603	3.7475	6.846
1.8918	3.2112	6.0531	2.05	2.5413	8.3583
3.6497	3.5887	7.0238	3.7867	4.4556	8.643
5.569	4.0401	5.61	1.642	3.7072	7.306
3.1409	3.0927	4.7619	4.1636	3.136	6.3482
4.2281	2.6984	5.605	2.4252	4.3313	3.1572
2.5313	2.7951	5.6496	1.9034	3.3148	7.8454
4.2676	2.8402	4.7148	3.716	4.1086	7.0559
5.5961	1.0847	4.5928	3.2405	3.027	7.0652
1.674	2.9319	5.0032	2.2082	4.8319	8.0399
5.468	2.8779	4.5473	2.1766	3.7378	7.6658
3.6849	2.982	5.429	3.2781	3.9213	7.5049
4.5199	2.2482	6.1789	3.9327	4.5429	7.884
2.0076	2.2573	4.0162	2.5087	3.5815	3.9673
2.2842	2.3222	5.2231	3.0654	3.2988	8.4564
3.6172	2.1862	5.7761	3.3675	4.7698	8.5644
3.6759	4.774	6.5452	2.5288	2.8582	6.5676
6.0512	2.6533	7.4168	2.8562	3.8228	10.5359
1.7053	3.9974	6.9006	3.1162	3.5324	7.335
3.831	2.3106	5.497	2.7615	3.9524	8.229
3.4048	4.5349	4.9327	3.0825	2.3704	9.1092
0.9935	5.3653	5.8136	4.0082	2.5585	8.428
3.0995	4.2601	6.29	2.6043	3.9299	8.0612
4.5534	2.5707	6.3872	3.4609	4.4297	4.17
5.3744	2.2051	4.9672	-0.3201	3.2572	5.5317
3.907	3.2973	4.8079	4.0969	4.479	5.4614
3.6463	2.9109	5.5012	3.7324	4.6416	6.9542
4.2995	4.2165	4.7033	2.5487	3.1112	5.8128
4.352	3.8521	5.2173	3.7149	4.1531	6.8411
5.337	2.2783	5.2721	4.433	5.0871	6.7877
4.1692	4.4169	5.1724	3.5742	4.7898	7.8923
3.2466	3.1162	3.222	1.884	3.1797	5.1863
2.7249	3.1028	5.8951	2.9243	4.3896	4.9887
2.8321	4.3463	5.9105	3.8749	4.5922	7.9691
2.2723	3.7877	7.1547	3.3787	4.3002	9.2213
4.3917	2.8955	4.6882	3.8085	3.9175	6.8669
3.0654	2.8641	5.447	1.8119	4.1724	6.0622
2.2482	4.6577	6.0481	3.3163	3.8759	7.4951
4.0841	2.5437	5.3982	3.616	4.1277	6.0191
2.99218391039	1.90342868298	3.65983045303	2.50713841864	2.61941836779	5.35254400237
3.506	2.9488	5.1326	1.7786	4.2312	6.9549
4.7592	3.7313	5.3005	3.4504	4.6479	5.3527
4.5293	2.7336	5.5015	3.4778	4.8635	8.4441
4.5596	3.0774	6.1229	2.8482	4.3292	4.4816
2.9729	3.1179	5.4909	3.5899	4.7453	6.9186
0.8961	2.8482	5.0781	1.2756	3.6759	6.8853
5.9905	3.9156	5.4855	3.1327	3.5994	5.6871
0.3115	4.7426	4.7115	1.5902	1.3679	7.604
2.5462	3.3661	4.9472	3.3393	3.8208	7.4938
3.8075	3.9746	5.8133	4.6514	4.4013	7.2028
3.5645	3.4881	5.2877	3.7762	3.6324	7.6638
5.2169	2.753	5.6862	3.6928	5.3216	6.7748
5.3086	2.3135	5.5404	3.2112	4.9084	7.1052

4.6657	3.2158	5.0492	2.5163	4.3917	6.8319
3.3032	4.1368	6.258	3.5324	4.2009	7.9433
1.9452	4.2609	6.9779	3.7421	5.2457	8.0267
0.9493	1.9415	4.981	0.8488	3.7335	8.5607
1.4808	4.2563	5.3327	1.7575	3.4739	8.2234
3.705	3.1393	5.2653	3.5199	3.3148	8.2972
5.2788	3.0002	5.1639	2.5804	3.7389	5.0148
2.7227	3.3883	5.9798	3.6359	4.7976	6.9027
3.9496	4.0162	5.5123	3.2662	4.162	6.3567
3.1028	4.0251	6.6247	1.9527	2.4883	8.6483
3.0515	2.1734	5.0798	1.7446	4.2181	6.015
4.3786	2.971	5.2028	3.772	5.0704	7.3372
2.6232	3.836	5.9167	2.1147	3.8075	7.2552
4.951	2.6895	4.856	2.7161	3.3897	6.8813
3.8876	4.0497	4.6697	2.6302	4.0136	6.0542
3.7709	4.9594	6.1665	2.8819	3.9534	9.4774
2.5036	3.7017	5.7347	3.6612	1.949	6.3227
2.1862	4.0584	5.7415	3.4437	4.5723	8.5185
4.3807	2.7095	5.7304	3.8187	4.048	8.4041
2.183	2.6579	5.0968	1.9822	3.4687	4.673
1.3511	3.3321	4.7367	1.5216	3.0446	7.6636
4.4437	4.3002	6.6909	3.705	4.2119	8.2642
3.6861	2.9356	5.9951	3.7335	3.4856	7.4356
3.6497	3.4609	7.4227	3.838	4.7198	8.0389
5.0566	2.59	5.4436	2.7551	3.9431	8.2095
2.299	3.863	6.792	1.949	4.4223	7.4705
2.9262	3.2313	5.7027	2.5996	3.9992	7.6917
3.0757	4.3841	5.2762	4.402	4.7939	7.997
4.0436	3.0774	6.5706	3.3435	3.5149	8.1755
4.5577	4.4423	6.3901	4.0198	4.1844	8.254
4.8978	3.3435	5.2608	2.296	4.2766	8.8771
3.5149	3.6883	5.3544	3.6382	4.6708	7.0209
4.6399	3.3076	4.7835	4.5814	4.2884	7.3113
2.2482	3.1045	4.5886	2.4623	3.7815	6.3832
3.6635	4.2389	5.6235	3.3787	3.6242	5.7148
2.8681	3.9543	5.6602	3.5324	3.3703	7.0827
4.0162	5.1136	6.1414	3.1588	2.3843	10.6691
5.4036	3.6578	5.6346	4.2884	4.9946	8.7759
2.3926	2.6138	4.8465	2.9929	4.5608	7.8035
4.7253	3.7269	5.703	2.9984	4.309	9.5481
4.1244	2.6556	5.3067	3.9346	4.3841	7.3759
3.7204	3.4765	5.2862	3.5803	4.1962	7.5087
4.3827	1.683	5.4536	3.1327	3.7259	8.1951
0.7748	3.9965	5.4483	2.9243	3.0411	7.3204
3.6804	2.1313	4.49	3.907	4.0073	7.9478
5.8146	4.0549	5.4111	2.5996	4.7323	7.5413
6.3978	3.7039	4.9828	3.6793	5.1692	7.1704
4.298	2.4985	5.1297	2.5188	4.7661	7.8738
5.022	3.6872	5.8153	4.1359	5.2698	6.8451
6.4635	2.2901	5.7271	3.0995	4.6877	9.3533
3.6669	3.2112	4.9556	4.3869	3.3119	8.0601
1.9675	3.7389	5.6311	2.9413	3.6301	6.8863
3.6939	2.8582	5.5571	3.7877	4.551	7.7576

3.7667	2.1013	6.0503	3.7815	3.3745	6.7203
3.1294	2.9929	5.4637	3.5461	4.1506	5.6442
2.7292	3.5766	5.7488	2.5437	3.5486	7.142
3.5982	3.1971	5.8428	3.3828	4.1612	8.7776
3.9892	4.2639	7.6445	3.6983	4.2304	8.4516
4.2758	3.1572	5.3834	3.1876	4.1466	8.2029
3.6428	3.6849	5.5395	4.0055	4.0127	8.817
7.5817	2.7007	5.3477	1.4494	4.3876	6.4599
5.0545	4.6294	5.8004	3.6006	3.7116	7.7808
3.0995	3.5111	5.9163	2.2901	4.4836	8.8851
2.299	3.5461	5.6639	2.6738	4.7296	5.9709
1.816	3.5584	5.9409	2.8877	2.9618	7.4305
2.8522	2.7805	5.8485	3.0498	3.8228	7.2547
2.8442	2.9984	5.7626	4.4556	3.7204	7.9325
3.4358	2.945	4.8817	2.6828	3.7028	6.6939
1.9859	4.5299	5.1867	2.4198	3.5424	8.0156
3.4143	3.4251	5.5323	3.9012	4.1449	7.9667
3.2811	2.8482	5.0396	3.9792	3.3221	8.0196
5.119	2.6255	5.3259	4.4726	4.5311	8.5311
2.7615	3.483	5.2765	3.4143	4.2064	7.4562
2.6805	4.5186	5.9253	3.1426	3.652	6.8031
2.3926	3.705	5.4992	4.0064	3.8954	7.5975
5.3374	2.9243	5.5472	4.5236	4.9369	7.6074
5.5398	2.9984	5.3758	4.4529	3.9755	7.5766
2.1638	5.8635	6.4583	3.4129	2.9765	6.4785
4.2601	4.81	7.4037	3.0944	3.5535	7.7945
3.4556	3.8983	5.4813	3.309	4.2706	7.8245
3.2389	4.3285	6.9593	2.1766	4.5442	8.2742
3.7603	2.2783	5.2589	2.4518	3.3814	7.1495
3.8033	1.9968	5.3187	0.6699	4.4006	4.9566
1.0915	4.8729	5.8953	0.9191	4.0189	9.1049
2.3788	4.591	4.322	3.1344	2.8137	6.2683
2.8641	3.6066	6.4038	3.8788	4.6725	7.2222
3.1195	3.6939	5.3646	3.5212	3.9099	7.985
1.5266	4.6525	4.6106	0.9419	1.125	7.6731
1.9675	4.431	5.821	3.5035	2.7931	9.1396
4.8694	4.3111	5.3678	3.4868	3.1701	8.2358
3.1523	4.8778	5.3999	2.9857	3.9543	8.1803
5.65100002661	2.72340950508	5.28769673295	2.6586287698	2.83547935225	9.53723365884
4.4169	3.4143	5.3899	3.7302	4.5681	7.5855
1.84569007513	2.68131040413	3.73631447289	3.51738245346	2.48913083362	5.462990842
5.3889	3.7877	5.7225	3.3633	4.7672	8.5817
2.5087	5.5996	4.3126	2.8916	3.5959	8.1511
2.9243	3.8983	5.2072	1.8643	3.637	4.2327
3.9108	3.5827	5.8829	3.8521	4.9141	5.9873
4.5832	2.9893	4.8177	3.8621	4.4784	7.5368
5.1595	4.0627	5.4026	2.9544	3.6928	5.0514
3.2572	4.0118	4.7725	2.0979	2.8402	7.1415
1.3167	3.9746	5.3089	1.7273	3.4061	7.5701
3.8905	3.4264	4.812	3.9746	4.4621	6.0824
3.0092	2.7487	6.5193	3.8054	4.591	6.9703
3.6172	2.8858	4.784	3.027	3.5839	7.0244
7.1784	2.3077	5.2627	2.0878	5.0989	7.3494

5.404	3.4061	5.2335	4.431	4.8248	6.3574
4.4476	3.6646	5.488	3.6849	4.4881	6.4678
2.5288	3.4129	6.9644	3.3307	4.431	7.1301
3.6827	2.8014	5.4578	5.0734	4.0313	7.6838
4.4562	2.971	4.9194	4.4006	4.079	7.6834
5.0444	3.1344	5.4572	4.431	4.2442	7.9275
3.7464	3.1079	5.0427	4.9792	4.9237	8.4036
1.9968	4.2227	5.8425	4.5748	4.4337	9.4715
2.727	3.7699	5.8329	3.6054	4.2541	8.8903
0.8883	3.9393	5.6103	2.4332	4.4964	5.823
3.6289	3.1876	6.101	3.0515	4.6866	8.5499
4.4236	3.2647	5.0255	3.3897	4.166	6.4798
4.2126	3.1129	5.6705	4.8665	3.7593	6.3681
5.2296	1.7786	5.0566	4.641	4.4693	8.6128
4.0419	4.4906	5.322	1.8996	3.4868	8.397
2.233	3.4143	4.9468	4.0601	3.9089	7.9613
0.3796	5.0453	5.6539	3.6635	3.3017	7.6581
6.5734	3.1393	5.2064	3.9543	4.1962	7.4669
1.6045	3.0706	5.009	2.1957	2.4492	6.7812
4.0601	4.0304	4.9327	2.7657	2.8199	5.5805
5.392	2.5087	5.6032	3.9403	4.7367	7.5436
2.7183	3.1045	5.9584	2.4359	3.9384	8.8246
2.8622	3.6417	6.2676	2.727	4.0401	5.3049
3.3521	2.5112	5.2056	1.6466	3.6827	7.8619
5.033	1.9264	5.172	2.1313	4.6531	3.773
3.1813	3.0791	4.6994	2.7336	4.3646	5.6011
2.909	3.5851	4.5224	2.0776	3.7453	8.8154
4.6387	2.2723	5.1918	3.7453	4.6645	7.9849
2.0878	2.4117	3.8228	2.0878	3.9431	7.5426
2.9186	2.8622	5.2032	5.1404	4.6416	7.7119
3.4804	3.0092	4.6047	2.4779	2.3048	7.1761
2.4908	3.0234	5.6702	3.0995	4.0357	6.6943
4.2158	2.4883	7.272	2.5413	5.2521	6.769
3.2205	4.1587	6.6398	2.7826	3.907	7.9783
4.9714	2.8321	5.1567	4.0109	3.5473	7.2211
5.2091	3.0305	5.1627	3.9421	4.6491	6.3887
5.7908	2.6114	4.7672	3.506	4.2017	7.3697
4.2189	2.5996	5.3692	3.2236	4.481	8.6698
4.686	3.0654	4.3463	1.7744	1.5514	8.2491
3.4958	3.4398	5.6557	3.38	3.833	9.3061
2.4144	4.892	5.2445	1.9931	4.8182	6.7236
4.9074	2.0946	5.6789	3.5596	4.2258	7.8685
1.5266	3.7127	5.853	2.9581	4.5844	8.3619
4.7491	3.2127	4.7661	5.9807	4.6294	5.993
4.3876	3.623	5.5167	4.3364	3.7992	7.5898
5.0653	2.6895	5.3413	4.5522	4.4283	6.1767
3.2174	4.6083	5.8286	3.29	4.01	9.001
3.0859	3.958	5.2495	2.8219	4.3534	6.8483
3.8198	3.1507	4.7312	4.2381	4.3639	6.8135
2.8076	4.061	6.937	2.5852	3.4726	6.9773
5.8736	2.2299	4.1128	2.7444	4.0645	7.9644
3.7982	3.3814	5.5292	3.3993	4.2304	7.4623
6.2441	3.2481	6.0255	3.9242	4.859	9.2065

3.29	3.3717	5.1469	3.5523	4.5772	5.5877
3.5486	3.1637	5.5583	4.0488	4.5311	7.7643
4.2235	2.945	6.0834	3.2313	4.0909	7.8752
4.2833	3.0074	5.4142	2.77	4.5047	7.0536
1.2023	2.5388	5.6393	1.8323	1.8201	8.2759
3.3883	3.3938	4.9237	3.5022	4.5766	8.1191
4.5173	4.1883	4.85	2.4649	4.5423	7.7171
2.9784	4.2196	5.6719	3.449	4.197	8.2604
1.8564	2.6371	4.4667	0.8164	3.6804	5.602
5.9189	3.2112	5.9695	1.4494	2.7444	8.7048
0.7146	3.1507	6.2591	1.412	4.14	8.4158
0.8568	4.0841	6.3402	2.3048	3.4635	8.6529
-0.1828	3.9965	6.8992	3.9242	4.8768	8.5317
4.837	2.9857	5.3306	2.8055	3.5022	7.9803
4.2995	3.5887	5.5088	1.9931	2.9071	6.1035
2.4727	3.7794	5.2961	2.1313	3.9552	7.8414
3.6042	4.4108	5.2676	3.4477	4.5466	6.6729
5.4986	3.7561	5.7214	4.1907	5.5286	8.6434
3.2435	3.7464	6.5956	2.362	3.6006	8.9323
4.0295	3.8601	4.9982	3.4984	4.041	5.4363
4.8719	2.96	5.7085	2.77	3.8451	9.6162
5.439	3.2251	5.3906	2.8096	4.5705	6.3965
4.2189	1.8404	5.2939	2.6487	3.8064	6.8319
3.6405	0.8568	6.056	3.5312	3.5436	8.215
5.5407	2.4597	4.9294	4.0427	4.6224	7.0553
4.2488	3.831	5.014	3.4739	4.2056	6.0657
3.5374	3.0602	5.6112	3.2796	4.4257	6.9779
4.4263	3.9765	5.3948	4.2088	4.6006	6.4259
3.4197	2.9413	4.6645	1.692	4.1433	8.0135
3.3264	2.3366	4.2721	2.0569	3.5791	7.4571
3.7072	1.9749	4.3555	2.9071	2.872	6.3129
3.7909	2.036	5.1891	1.2333	2.4332	7.4392
4.245	3.0568	6.0596	4.4667	4.435	6.9506
3.8591	3.9089	4.9594	3.1278	3.944	8.0926
2.7679	4.3463	6.1964	2.5263	2.3843	7.6802
4.5305	3.2359	4.8793	3.6428	4.3807	6.2882
2.9655	2.6895	5.4624	2.4779	4.6657	5.3491
4.5091	2.753	5.5817	2.77	4.2921	5.4206
4.0073	3.8571	4.9486	2.2482	4.9566	3.7486
4.3111	2.0465	4.9663	3.7992	4.3378	5.4175
5.8365	3.6994	5.4676	3.4907	4.4536	8.4472
4.6933	3.2541	5.3625	3.7094	4.3646	6.946
4.445	3.3421	5.298	2.4008	4.0515	8.7256
4.86	2.5852	5.3035	3.8531	4.2181	6.6875
1.7912	3.5742	4.7377	1.2636	1.7489	7.5072
2.9765	4.1636	6.0926	3.4504	3.9242	8.2482
2.5363	2.1734	4.3931	2.2482	2.9186	6.2676
2.28124940751	1.14730605044	3.1125038118	2.90206246218	2.95472889987	5.68512450683
2.0004	3.9992	5.5245	3.3336	3.5887	6.1785
4.6201	3.1588	4.0348	3.6827	3.2496	6.8555
3.8778	1.0983	4.439	2.2019	4.2676	4.6566
5.054	4.7903	6.6913	1.8879	4.7296	8.9351
3.8847	2.2113	4.01	1.5709	3.0568	7.7319

4.3328	2.7868	4.8856	3.2435	3.8729	6.9938
1.7532	4.0304	4.4397	2.8462	2.5338	5.2788
2.6805	3.7204	5.4604	1.7053	4.6165	5.9743
0.2029	2.9692	5.0562	0.4016	4.4621	7.2593
1.5415	3.7389	5.3632	3.5887	3.0498	7.8803
2.5363	3.5212	5.0392	4.3999	4.9298	6.0999
3.2973	2.9281	4.9327	2.5683	5.3277	6.8607
2.8974	3.2557	4.7296	1.5998	3.864	6.7487
3.9356	3.0127	5.5151	2.1925	3.3883	5.717
2.4225	3.773	6.1987	3.0305	3.2127	6.0968
2.9948	2.609	5.1879	2.7007	2.77	7.3724
3.7898	3.6849	6.2728	2.5756	3.8002	7.2767
4.0497	3.4556	5.6548	3.0145	4.2795	8.1944
4.5374	3.8739	4.1994	1.6558	3.5971	5.4407
5.3632	1.7489	4.8274	3.623	4.402	7.3468
3.9819	1.7009	5.6329	1.0222	3.7182	5.1502
3.616	2.2113	5.2196	2.6647	3.9175	7.4175
2.9032	3.2692	5.2377	1.7617	3.3278	6.4848
5.7504	3.4088	5.096	4.0445	4.7192	6.4838
5.4138	4.2041	5.6713	2.2813	4.1547	7.5688
3.7752	1.934	4.6065	2.1702	3.027	5.8156
4.981	2.7401	5.0317	2.8522	3.7507	7.2037
3.0411	2.9765	5.3495	2.5238	4.5994	6.1879
4.9364	2.3508	4.7507	1.0573	4.4751	6.3714
3.1327	2.7784	5.803	2.8199	3.2856	7.988
3.0585	2.93	5.2891	2.6067	3.1572	7.3379
3.1556	2.7401	5.0879	2.7117	3.2158	6.7041
4.1644	3.7539	5.0635	2.3981	2.8301	6.8225
4.0901	3.5923	5.3799	1.5316	3.7571	5.3913
5.2487	2.328	4.8079	2.5188	3.6983	7.8892
4.7597	2.3508	4.9127	2.9338	3.5187	5.076
7.0179	2.0946	5.8674	2.2421	3.3206	7.2285
4.222	1.9264	3.8441	0.8568	2.7487	4.6966
4.6053	2.7993	4.4588	2.0218	3.7291	6.2298
4.8425	2.362	4.3527	1.9111	2.7183	6.1392
4.2274	2.7805	4.5473	0.7058	2.7972	6.6084
3.1011	2.7868	4.7924	0.5271	2.6647	6.3512
4.7756	2.6579	5.3184	3.417	4.6053	7.4254
3.6543	3.1669	5.4935	1.8762	3.1045	6.4681
3.034	2.1862	5.498	3.1669	4.0558	7.6024
2.8402	3.1523	5.6002	2.0254	3.7345	7.7865
5.3999	2.753	5.1981	3.2174	4.7517	6.9148
2.63946342291	2.67266797652	2.66811274813	1.40791798971	1.70199081603	5.43863206443
4.3148	3.1829	5.0917	2.4386	4.1986	7.8738
3.7646	3.0234	4.9141	2.9637	3.6883	6.6468
4.3639	1.9968	4.9552	2.1345	3.9819	7.4094
5.6059	2.1988	5.2631	3.0723	5.0162	8.271
4.554	2.3451	5.5091	3.2034	4.0019	8.7692
3.7992	3.4102	5.0286	1.595	2.5036	7.1234
5.4909	2.2082	5.4864	3.0757	3.958	7.5597
3.38	2.6805	4.7788	1.0642	2.6067	7.105
3.8002	3.6543	4.9991	1.8683	3.3032	6.8017
4.0662	2.0325	5.0313	2.9562	3.1637	6.7493

5.7364	3.0585	5.0418	2.8442	4.079	6.6118
5.4829	2.6067	5.5043	4.4706	4.4135	6.1459
2.8701	3.3911	5.7764	1.5465	4.1236	7.5665
4.0198	3.027	4.7756	1.934	4.2457	6.4143
4.9393	2.8582	5.6212	3.2466	4.0392	6.8415
1.6281	4.8207	6.0547	2.1509	3.8993	6.3288
5.2642	2.8502	6.0367	3.2435	5.2381	8.4447
4.5934	2.9356	5.1494	3.1733	4.773	7.3241
1.4704	2.3019	4.9213	1.0847	3.0533	8.4468
6.383	2.376	4.1236	3.2632	4.4602	6.5309
3.1311	2.7249	5.5186	3.6725	4.6804	8.0495
4.2126	2.9128	4.9093	1.0007	2.7572	5.8306
3.3017	3.4556	4.4496	2.0638	3.5863	5.9693
2.128	2.5263	5.1169	-0.7588	3.7475	6.5244
2.4008	2.9394	6.0339	1.2085	4.4919	5.2832
4.0952	4.435	4.5085	1.8879	3.5374	7.489
5.0644	3.7367	5.904	2.8055	4.1612	8.1447
5.1367	2.4831	5.6326	3.2466	4.2773	8.0079
2.0147	2.7721	4.8905	2.3816	2.3843	5.0036
5.2525	3.6219	5.1044	3.483	4.3632	5.899
3.9645	2.3732	5.5708	0.5859	2.6371	7.8304
2.6019	3.9185	4.9644	1.0293	4.1828	8.3888
4.431	3.2929	4.4061	2.5388	4.0575	8.4479
3.0808	2.1114	4.3737	1.46	3.8739	7.0649
4.588	2.167	3.8541	2.5263	3.5237	6.3628
4.0198	1.9859	5.0867	2.8661	3.5851	6.3225
4.4926	2.4985	6.2348	2.9356	3.5249	7.591
4.0909	1.9302	4.8949	0.537	1.4494	7.7103
4.5868	2.6624	4.9369	3.2174	4.5255	7.3161
4.3299	1.2756	4.9556	1.6234	2.4883	7.4524
3.7072	3.5362	5.5811	2.0776	2.8602	6.0809
3.728	2.5585	5.0614	0.2029	3.7193	7.8766
2.9581	2.7636	4.8334	1.3051	2.727	7.4641
4.6485	4.0287	5.7301	2.6043	3.8759	7.2017
4.7274	2.8936	6.4735	2.4279	3.6148	5.9928
3.2359	3.3619	5.1704	1.8604	3.2511	6.6166
2.8542	2.0912	5.3031	2.2206	4.5491	8.4502
4.287	4.6871	4.6422	1.5661	2.6185	6.3635
4.7809	3.705	4.9654	2.0912	3.7006	7.9968
2.9243	3.0074	5.773	3.2205	4.0645	7.447
3.9883	1.8801	5.1136	3.6148	4.6894	6.7155
4.4509	2.1606	4.8048	3.8054	3.9874	7.2807
4.0773	2.2144	4.6428	3.4102	3.7836	6.5971
4.641	3.2707	4.6877	2.3953	3.5718	8.011
5.6144	3.1813	4.852	3.0181	4.3177	8.7061
2.6043	3.0757	5.1977	1.2147	2.7051	8.0406
2.9243	2.8779	6.0644	2.9013	3.8511	4.0593
3.6089	1.8242	3.9175	1.6649	2.8522	7.6315
6.1158	3.0927	4.853	3.2297	5.0691	5.6203
4.1923	2.6647	5.1863	2.3451	4.0225	7.9681
2.1313	3.6172	5.9198	0.7058	2.4883	8.5271
2.8402	3.1327	4.2936	0.1648	2.6302	6.516
5.4725	1.4494	3.8621	-1.1172	2.3193	7.3335

4.701	3.4048	5.0709	1.4808	4.0375	6.9906
3.2174	2.4779	4.8739	1.4962	3.2127	6.6815
2.7951	1.6558	5.4618	0.3573	2.7205	3.8054
2.6624	2.118	4.8842	-0.9406	3.1409	8.6024
1.766	3.5935	5.0423	0.3346	2.7784	7.9554
5.4935	1.6512	4.7071	1.3901	3.3883	7.4092
3.5923	2.7951	4.7576	1.5709	2.4332	8.0033
4.2929	4.1515	5.5352	1.5366	4.3456	9.8022
3.772	3.3061	4.4628	2.6487	3.0602	7.1514
5.2791	3.1749	6.1465	1.6281	5.1297	7.2119
3.1079	3.5791	6.4795	0.7579	3.3938	7.1605
0.4657	2.9674	6.0374	1.1316	1.9527	7.0622
4.251	3.4304	5.07	3.4713	4.0375	6.8809
5.0184	2.8462	7.8229	3.3336	3.9089	8.8151
5.5958	2.8916	5.5565	3.2722	5.0786	7.2053
3.1908	3.551	5.009	3.034	4.2563	7.5541
2.9765	4.5742	6.278	4.061	3.7877	8.6687
4.49	3.4331	5.4797	2.4649	3.1327	7.8705
2.4465	4.4881	6.2902	1.9073	3.3061	10.8621
3.8798	2.7444	7.0632	1.6374	4.001	8.295
3.7237	4.0037	5.7656	1.8363	3.0995	6.9896
2.3788	3.6148	6.7047	3.2929	3.0568	7.3994
3.2112	2.8137	5.2327	3.4061	3.8501	5.3327
4.773	3.3336	6.3889	2.7679	4.9824	10.5372
4.6201	2.7636	5.1587	3.8491	4.3234	8.012
2.9948	3.208	5.4938	1.2756	3.5584	6.7862
6.565	2.8602	5.2895	3.8461	5.2411	6.3368
2.2452	2.2482	4.8615	-0.1504	3.4156	6.434
4.4516	2.5263	5.2616	3.3563	4.2304	6.9512
3.1701	2.1798	4.7735	3.7443	3.5399	5.8518
3.7678	3.2856	5.7334	2.8321	4.2631	8.1596
0.8805	3.1409	5.2052	0.3346	1.0915	7.6584
4.9658	3.9728	5.1293	2.6464	3.66	7.362
4.0046	5.0229	5.6608	1.6964	2.1702	7.5374
5.7219	4.8939	7.2289	1.3901	3.4687	9.2453
3.7971	3.4224	5.0862	3.9765	3.7961	5.5913
6.3495	3.8876	4.3965	1.0711	3.6657	7.7927
6.5239	3.3661	5.1522	3.7193	5.1289	6.945
2.9581	4.6866	5.7869	3.2707	3.6089	7.553
5.1297	2.9052	4.8645	2.8681	3.9883	7.0878
4.795	3.6532	5.505	4.1796	4.9194	6.9116
4.8365	2.7931	4.7093	2.96	3.2929	7.4158
1.4911	3.5851	6.2182	1.4547	4.1318	6.5111
5.0418	3.6018	4.6236	2.3106	4.5644	7.0307
3.9515	3.0217	4.215	0.7832	2.4597	8.5674
5.3086	3.7291	5.3951	1.9073	4.5349	6.7577
3.6289	2.4306	4.5862	1.3791	3.3535	5.9338
2.7336	2.2299	3.9737	2.1957	2.9562	5.6011
3.867	3.7291	6.3071	2.4571	3.5779	7.691
0.2029	2.6873	5.5358	1.4011	4.1252	5.8724
2.5112	3.6543	5.4848	1.0983	3.0757	6.3315
4.6294	3.074	4.9672	3.6266	4.2884	6.9051
5.1023	1.5902	4.2327	2.8502	3.1749	6.6499

5.943	3.4102	6.6992	2.9948	4.2258	6.8845
4.5491	2.4779	5.0955	3.3521	4.5583	6.8644
3.4451	3.3535	5.8324	2.7292	3.868	7.3784
3.3478	3.3661	5.0627	3.3828	3.8541	6.8035
3.7378	3.9356	5.225	2.6255	3.9709	2.9052
1.4859	3.7171	5.3544	2.971	3.2018	7.3064
3.7193	3.2781	4.5826	2.6019	3.8759	7.0189
2.5996	4.6077	5.6456	1.8363	3.1426	7.2814
8.0837	4.1676	3.9478	0.24	3.6113	8.9582
3.7302	2.8701	5.1404	2.7095	3.716	8.1436
0.9935	3.9737	4.6416	0.7748	2.8582	6.7226
3.1062	4.1433	6.0921	0.6699	2.3135	8.3069
2.0878	3.29	3.7961	-1.685	2.2144	6.9952

CS
6.9046
7.0164
6.3172
8.0394
6.0438
5.7711
6.4474
6.3844
7.0582
7.2507
7.0615
6.3892
6.918
6.3096
6.9998
7.1446
6.913
7.4132
6.7985
7.1516
6.4585
6.9262
5.8151
5.9404
6.7951
6.5735
6.5696
5.1723982637
6.7974
6.9576
6.4492
6.33
6.8615
7.1924
7.0976
5.7621
6.5752
6.4358
7.1421
6.7463
6.6506
6.7149
6.6963
6.8092
7.041
6.7908
7.8425
7.0647
6.07731908262
6.4162
7.0433
7.1811

7.6078
4.4861
6.6103
6.1102
6.7075
6.6676
6.9394
6.8565
6.888
6.6483
6.1277
6.705
6.5072
6.4338
6.3785
6.7968
6.7027
6.3856
6.4271
6.5594
6.2911
6.7548
6.5198
6.9385
6.3649
7.4655
6.6224
6.7479
6.4285
6.7888
6.4859
7.1212
5.9374
6.4941
7.105
7.2837
5.8785
7.3938
6.5308
7.11
5.6577
7.1642
6.1371
6.3528
5.85
6.4435
6.1025
6.6968
5.7301
6.8914
6.1613
6.8641
6.1248

6.5652
7.0213
6.7145
6.9736
7.1015
6.1619
6.4504
6.8167
6.4082
6.3759
6.4927
5.7594
6.4586
6.6577
6.6791
6.5641
6.5823
7.016
6.3347
6.1258
6.5515
6.4762
6.8653
6.7363
5.4611
6.7382
4.9561
6.6713
7.119
5.8475
5.7999
6.6971
6.29
6.5978
6.5003
5.9279
6.2292
5.1811
6.7925
7.3348
6.676
7.1253
6.5059
6.4585
6.5923
6.1534
7.399
7.2202
5.7509
6.8434
6.4291
5.5233
6.6106

4.981
5.9258
6.4676
6.8596
6.6313
6.8994
8.183
6.2733
6.6345
7.1146
6.0433
6.9423
6.4962
6.6558
6.3397
7.3829
6.4702
6.7666
6.8397
6.9039
6.5725
6.8263
7.1993
5.3288
6.0168
6.4375
7.1536
6.2836
6.6363
6.5124
6.6889
7.7591
6.7929
6.8705
6.4767
6.5806
6.2634
7.0065
5.2676
6.7493
6.8786
6.627
7.9597
6.9868
6.7617
6.1831
6.7714
6.0442
6.7774
6.8789
7.176
7.1233
6.3091

6.4204
6.8239
6.1223
6.3228
6.8022
6.7912
6.941
6.6218
6.4943
6.7038
6.9555
6.8939
7.1355
6.864
7.1236
6.9787
5.9723
6.8828
6.2661
6.5245
7.2149
6.2815
6.7162
6.5612
7.1332
6.2774
6.7278
7.1704
6.6889
6.0295
6.0832
7.2581
6.6313
6.7952
6.7043
6.2913
6.5441
6.8041
7.1101
6.8387
6.8753
6.838
7.3333
6.8758
7.2171
7.2168
7.1025
6.7114
7.2949
6.5526
7.0117
6.7533
6.0811

6.0299
6.7174
6.6888
6.773
6.6387
6.5057
6.8035
6.6789
7.5253
7.1871
6.9742
6.7856
6.9927
6.4464
6.8166
6.7561
6.7263
7.2363
6.2619
7.2313
6.4757
6.6785
6.3851
6.8273
7.0661
7.239
6.1741
6.6518
5.7266
7.1649
7.2349
6.3225
6.7645
7.1064
7.6682
6.9145
5.9461
6.8382
7.6167
7.2408
6.6729
7.4919
6.9307
7.3351
6.2288
6.9173
6.4696
6.6227
6.1928
6.4775
6.4976
7.463
6.9684

6.4911
6.9513
7.418
6.6948
6.0803
7.3642
6.0215
6.9198
5.7552
6.102
5.6938
6.2338
7.508
5.5183
7.1662
6.7275
6.5989
6.5806
6.0555
6.569
6.2048
7.4251
6.8856
6.3472
7.009
6.3934
6.7743
6.4842
6.9384
6.357
6.9756
5.0888
6.2602
6.6541
7.5003
5.8402
6.3567
6.6302
7.0137
5.8147832395
7.1195
7.127
6.6808
7.0733
6.8662
6.757
6.7997
6.7948
6.6572
7.2254
7.1267
6.8396
6.9208

6.6081
6.8262
7.0989
6.6893
6.441
6.5758
6.575
6.6799
5.9928
6.4824
5.9816
6.6156
7.0618
5.9928
6.911
7.0884
6.5417
6.745
6.3272
7.1677
6.5572
6.8364
6.3865
6.9146
6.8328
7.1202
7.1303
7.317
7.0713
7.2841
7.1062
6.8892
7.0902
6.7922
6.9721
7.9897
6.9315
6.9342
6.7904
7.6354
7.0792
6.4941
6.1129
6.6294
5.8256
7.1005
6.8659
6.5342
6.7991
7.2024
7.497
6.8035
6.3005

6.3609
6.2771
6.7514
7.1101
7.0641
6.6334
6.3153
6.7097
7.6337
7.0323
6.6834
6.6246
6.1279
7.545
6.2495
6.6581
6.5974
6.5604
6.7564
7.5675
7.0783
7.0106
6.442
7.3032
8.2578
7.2185
6.4608
7.4034
6.7391
6.5225
7.1367
7.0375
6.6365
6.2286
7.2469
7.5616
6.8203
7.0239
5.73687701829
6.5949
5.86058350719
7.0244
6.1973
6.8969
6.8785
6.8405
6.7829
6.7045
6.991
6.5523
6.7578
6.229
6.4525

6.7947
6.8782
7.1611
7.5226
6.6822
6.4345
6.5578
6.6113
7.2964
6.863
6.567
6.8916
6.5275
6.4401
6.8027
7.0179
6.6696
6.7167
6.7129
7.3524
7.2221
6.6872
6.8007
7.5309
7.0399
6.5421
7.0519
6.7114
6.9414
6.7558
6.203
7.0833
7.1495
6.9994
7.0494
6.7382
6.3982
7.2294
5.17
6.3223
6.6421
6.4
7.1881
7.3309
7.0407
6.4696
6.8805
6.3257
6.2838
6.8299
5.6628
6.9537
6.71

7.0436
6.7648
7.0863
7.2049
6.7485
6.8747
6.6042
6.927
6.3018
6.7584
6.5454
6.8016
6.9553
6.9952
5.738
6.332
6.7294
6.9748
6.9016
6.5974
6.9843
6.4901
6.9328
6.0422
6.3145
6.6528
6.4979
6.4693
6.0622
6.0014
4.9421
6.0422
6.5427
7.0796
6.8141
6.542
6.2024
6.5791
6.5526
6.011
6.8429
6.504
6.2704
6.5245
6.1171
6.3702
5.1494
4.86992253787
7.2537
5.8704
5.8076
6.2304
4.889

6.4534
5.5838
6.6174
5.6074
5.4115
6.7916
7.1322
5.8558
6.7878
6.9976
6.2981
7.012
6.4004
5.49
6.0672
6.5534
6.3768
6.7671
6.4365
6.0343
5.726
5.9862
6.3264
5.7602
5.9074
6.2232
6.4305
6.235
6.3383
5.6982
5.9514
6.7829
4.4958
5.9423
5.1359
5.3604
5.6091
6.3395
6.6999
6.0577
6.8014
6.342
4.87458123503
6.6635
6.2413
5.4423
6.1691
6.3408
7.1085
6.2567
5.4019
6.2968
5.8535

5.978
5.8897
6.6716
6.0577
6.6398
6.8674
6.4411
6.1713
5.0321
5.8568
6.9443
5.6517
5.865
5.9725
6.7478
6.5525
6.6276
6.7003
6.2919
6.3556
5.9165
5.391
6.1601
5.6372
5.43
6.7491
6.8556
4.8763
6.7897
5.3335
5.9289
5.0943
5.5463
6.7288
7.5296
6.1605
6.2529
6.3863
6.1494
5.9558
6.3889
6.1148
5.853
5.8812
5.9734
5.7504
5.9421
5.133
6.2967
6.0678
5.6924
5.4867
5.0691

5.6106
5.9727
5.2713
6.132
5.7861
5.2586
5.6253
6.6585
5.9729
7.6271
6.5878
6.8049
6.6051
5.8637
6.9514
6.1737
6.5563
6.8601
6.669
6.48
7.1265
6.7786
5.8495
6.2978
6.1721
6.4887
6.4817
5.6602
6.202
5.9369
6.2875
4.9833
6.3071
6.877
5.9011
6.1237
5.8822
6.8363
6.1845
7.2718
6.7416
5.9596
6.5127
6.8035
5.0431
7.0688
6.205
4.4536
6.0359
6.6007
6.7707
6.4216
5.3519

6.615
6.2401
6.5812
6.2369
6.413
6.832
6.4259
6.7922
6.1281
6.0121
6.1538
6.0431
4.1796
