# Supplementary material for: A ferroptosis-associated gene signature for the prediction of prognosis and therapeutic response in luminal-type breast carcinoma
Source: Sci Rep. 2021 Sep 2;11:17610. doi: 10.1038/s41598-021-97102-z (PMC8413464; doi:10.1038/s41598-021-97102-z)
Supplement: Supplementary file 9 — Supplementary Table S5. [file 41598_2021_97102_MOESM9_ESM.pdf]

TableS5 Clinical-related data with ferroptosis-related riskscore in METABRIC cohort

ID	Age	Menopausal_State	His_Subtype	Histologic_Grade	Tumor_Stage	OS.time	OS
MB-0002	<60	Pre	Ductal/NST	G3	I+II	84.63333333	0
MB-0005	<60	Pre	Ductal/NST	G1/G2	I+II	163.7	1
MB-0006	<60	Pre	Other	G1/G2	I+II	164.9333333	0
MB-0008	¡Ý60	Post	Other	G3	I+II	41.36666667	1
MB-0010	¡Ý60	Post	Ductal/NST	G3	III+IV	7.8	1
MB-0014	<60	Post	Ductal/NST	G1/G2	I+II	164.3333333	0
MB-0028	¡Ý60	Post	Ductal/NST	G3	I+II	36.56666667	1
MB-0036	¡Ý60	Post	Ductal/NST	G1/G2	III+IV	132.0333333	1
MB-0039	¡Ý60	Post	Ductal/NST	G1/G2	I+II	163.5333333	0
MB-0046	¡Ý60	Post	Ductal/NST	G3	I+II	14.13333333	1
MB-0053	¡Ý60	Post	Ductal/NST	G1/G2	I+II	161.0666667	0
MB-0054	¡Ý60	Post	Ductal/NST	G3	I+II	160.3	0
MB-0056	¡Ý60	Post	Other	G1/G2	I+II	62.86666667	0
MB-0060	<60	Pre	Ductal/NST	G3	I+II	140.8666667	0
MB-0064	¡Ý60	Post	Ductal/NST	G1/G2	I+II	108.9333333	0
MB-0066	¡Ý60	Post	Ductal/NST	G1/G2	I+II	157.4333333	0
MB-0068	<60	Post	Other	G1/G2	I+II	103.1333333	0
MB-0083	¡Ý60	Post	Other	G1/G2	I+II	86.06666667	1
MB-0093	<60	Pre	Other	G3	I+II	153.2	0
MB-0095	¡Ý60	Post	Ductal/NST	G1/G2	III+IV	49.76666667	1
MB-0097	¡Ý60	Post	Ductal/NST	G3	I+II	98.7	0
MB-0102	<60	Post	Other	G1/G2	I+II	140.7666667	1
MB-0106	<60	Pre	Ductal/NST	G1/G2	III+IV	85.33333333	0
MB-0107	¡Ý60	Post	Ductal/NST	G3	I+II	158.0333333	0
MB-0111	<60	Post	Ductal/NST	G1/G2	I+II	127.1	0
MB-0112	¡Ý60	Post	Other	G3	III+IV	39.16666667	1
MB-0114	<60	Pre	Ductal/NST	G1/G2	I+II	13.4	0
MB-0117	¡Ý60	Post	Other	G1/G2	I+II	2.4	0
MB-0119	¡Ý60	Post	Ductal/NST	G1/G2	I+II	95.86666667	1
MB-0120	¡Ý60	Post	Ductal/NST	G3	I+II	29.06666667	1
MB-0121	¡Ý60	Post	Ductal/NST	G1/G2	I+II	152.2	0
MB-0122	<60	Post	Ductal/NST	G1/G2	I+II	138.9	0
MB-0123	¡Ý60	Post	Ductal/NST	G3	I+II	114.2333333	1
MB-0124	<60	Pre	Other	G1/G2	III+IV	118.2	0
MB-0125	¡Ý60	Post	Other	G1/G2	I+II	1.266666667	0
MB-0126	¡Ý60	Post	Ductal/NST	G1/G2	I+II	127.6333333	0
MB-0130	<60	Post	Ductal/NST	G1/G2	I+II	153.5666667	0
MB-0131	¡Ý60	Post	Ductal/NST	G3	I+II	66.63333333	1
MB-0133	<60	Post	Other	G1/G2	I+II	151	0
MB-0134	¡Ý60	Post	Ductal/NST	G3	I+II	12.93333333	1
MB-0135	¡Ý60	Post	Ductal/NST	G3	I+II	116.6333333	0
MB-0136	¡Ý60	Post	Ductal/NST	G1/G2	I+II	88.2	0
MB-0138	<60	Post	Ductal/NST	G1/G2	I+II	150.5666667	0
MB-0139	<60	Pre	Other	G1/G2	I+II	109.2	0
MB-0140	¡Ý60	Post	Ductal/NST	G1/G2	I+II	147.9333333	0
MB-0142	<60	Post	Other	G1/G2	I+II	12.4	0
MB-0143	¡Ý60	Post	Ductal/NST	G3	I+II	54.33333333	1
MB-0144	¡Ý60	Post	Ductal/NST	G3	NA	152.0666667	1
MB-0145	¡Ý60	Post	Other	G1/G2	I+II	147.6666667	0
MB-0146	¡Ý60	Post	Ductal/NST	G3	I+II	122.1666667	1
MB-0147	¡Ý60	Post	Other	G1/G2	I+II	51.76666667	1
MB-0151	¡Ý60	Post	Ductal/NST	G3	III+IV	49.23333333	1

MB-0154	¡Ý60	Post	Ductal/NST	G1/G2	I+II	114.7666667	0
MB-0162	<60	Post	Other	G1/G2	I+II	55.76666667	0
MB-0165	<60	Post	Ductal/NST	G3	III+IV	47.63333333	1
MB-0166	<60	Pre	Ductal/NST	G3	I+II	104.4	0
MB-0167	¡Ý60	Post	Ductal/NST	G3	I+II	43.1	1
MB-0170	¡Ý60	Post	Other	G1/G2	I+II	93.36666667	1
MB-0172	<60	Pre	Ductal/NST	G1/G2	I+II	138.1	0
MB-0173	¡Ý60	Post	Ductal/NST	G3	I+II	3.766666667	0
MB-0175	¡Ý60	Post	Other	G1/G2	I+II	72.36666667	0
MB-0176	<60	Pre	Ductal/NST	G3	I+II	113.4333333	0
MB-0177	¡Ý60	Post	Other	G1/G2	I+II	45.6	1
MB-0178	¡Ý60	Post	Ductal/NST	G3	I+II	104.4666667	1
MB-0180	<60	Post	Ductal/NST	G1/G2	I+II	62.33333333	0
MB-0181	¡Ý60	Post	Ductal/NST	G1/G2	I+II	85.96666667	0
MB-0184	¡Ý60	Post	Other	G1/G2	I+II	109.0333333	0
MB-0193	<60	Post	Ductal/NST	G3	NA	19	0
MB-0194	<60	Post	Ductal/NST	G1/G2	I+II	91.6	0
MB-0195	¡Ý60	Post	Ductal/NST	G3	I+II	146	0
MB-0197	<60	Post	Ductal/NST	G3	I+II	70.73333333	0
MB-0198	<60	Pre	Ductal/NST	G3	I+II	144.7666667	0
MB-0199	<60	Post	Other	G3	I+II	144.4	0
MB-0202	<60	Pre	Ductal/NST	G3	I+II	128.3666667	0
MB-0203	¡Ý60	Post	Ductal/NST	G3	I+II	58.76666667	1
MB-0204	¡Ý60	Post	Ductal/NST	G1/G2	I+II	24.3	1
MB-0207	<60	Pre	Other	G1/G2	NA	173.6333333	0
MB-0215	¡Ý60	Post	Other	G1/G2	I+II	122.2	0
MB-0218	<60	Pre	Ductal/NST	G3	I+II	131.0666667	0
MB-0223	<60	Post	Ductal/NST	G3	I+II	81.33333333	1
MB-0224	<60	Pre	Ductal/NST	G3	NA	176.7666667	0
MB-0225	<60	Post	Ductal/NST	G1/G2	I+II	212.2	0
MB-0226	<60	Post	Ductal/NST	G1/G2	I+II	57.63333333	0
MB-0227	<60	Post	Ductal/NST	G1/G2	I+II	111.8666667	0
MB-0229	<60	Pre	Other	G1/G2	I+II	71.5	0
MB-0231	¡Ý60	Post	Other	NA	NA	19.6	0
MB-0232	<60	Pre	Ductal/NST	G1/G2	I+II	207.6333333	0
MB-0233	¡Ý60	Post	Ductal/NST	G1/G2	I+II	72.43333333	1
MB-0234	<60	Post	Other	NA	I+II	94.23333333	1
MB-0235	<60	Post	Ductal/NST	G1/G2	I+II	142.5666667	1
MB-0236	<60	Pre	Ductal/NST	G3	I+II	205.0333333	0
MB-0239	¡Ý60	Post	Other	G1/G2	III+IV	85.56666667	1
MB-0242	<60	Post	Ductal/NST	G1/G2	I+II	199.1	0
MB-0243	<60	Post	Ductal/NST	G1/G2	I+II	149.7666667	0
MB-0245	<60	Post	Other	NA	I+II	164.7	0
MB-0247	¡Ý60	Post	Ductal/NST	G1/G2	I+II	185.6	0
MB-0248	<60	Pre	Ductal/NST	G3	I+II	71	0
MB-0256	<60	Post	Ductal/NST	G1/G2	I+II	200.7	0
MB-0257	<60	Post	Ductal/NST	G1/G2	I+II	91.73333333	1
MB-0258	¡Ý60	Post	Ductal/NST	G3	I+II	86.13333333	0
MB-0260	¡Ý60	Post	Ductal/NST	NA	I+II	169.8333333	1
MB-0261	¡Ý60	Post	Ductal/NST	G1/G2	I+II	90.23333333	1
MB-0263	¡Ý60	Post	Other	NA	I+II	176.0333333	1
MB-0266	¡Ý60	Post	Other	NA	I+II	90.66666667	1
MB-0268	¡Ý60	Post	Ductal/NST	G3	III+IV	28.5	1

MB-0270	<60	Pre	Ductal/NST	G3	I+II	337.0333333	0
MB-0272	<60	Post	Ductal/NST	G3	I+II	122	1
MB-0273	¡Ý60	Post	Ductal/NST	G1/G2	I+II	186.5333333	0
MB-0287	¡Ý60	Post	Ductal/NST	G1/G2	I+II	94.73333333	1
MB-0290	<60	Pre	Ductal/NST	G1/G2	NA	199.5333333	0
MB-0291	<60	Post	Ductal/NST	G3	I+II	39.2	1
MB-0295	<60	Pre	Other	G1/G2	I+II	164.5	0
MB-0301	¡Ý60	Post	Ductal/NST	G3	I+II	122.7	1
MB-0302	¡Ý60	Post	Other	G3	I+II	84.2	1
MB-0304	¡Ý60	Post	Ductal/NST	G3	NA	111.5333333	1
MB-0306	¡Ý60	Post	Ductal/NST	G3	I+II	42.96666667	1
MB-0308	<60	Post	Other	G1/G2	I+II	183.2666667	0
MB-0309	¡Ý60	Post	Ductal/NST	G1/G2	I+II	42.33333333	1
MB-0310	¡Ý60	Post	Ductal/NST	G1/G2	I+II	185.1666667	0
MB-0311	¡Ý60	Post	Ductal/NST	G1/G2	I+II	151.1666667	1
MB-0312	¡Ý60	Post	Other	G1/G2	I+II	169.6	1
MB-0313	<60	Post	Ductal/NST	G3	I+II	184.7666667	0
MB-0315	<60	Post	Ductal/NST	G1/G2	I+II	186.2	0
MB-0317	¡Ý60	Post	Ductal/NST	G3	NA	151.6666667	1
MB-0319	¡Ý60	Post	Other	G1/G2	I+II	40.7	1
MB-0320	<60	Post	Other	G1/G2	I+II	63.73333333	0
MB-0321	<60	Pre	Ductal/NST	G3	I+II	174.6333333	0
MB-0322	¡Ý60	Post	Ductal/NST	G1/G2	I+II	50.53333333	1
MB-0324	<60	Pre	Ductal/NST	G3	I+II	144.9666667	1
MB-0325	¡Ý60	Post	Ductal/NST	G3	I+II	177.5333333	0
MB-0328	¡Ý60	Post	NA	G3	I+II	125.6	1
MB-0336	¡Ý60	Post	Ductal/NST	G3	I+II	170.6333333	1
MB-0339	¡Ý60	Post	Other	G1/G2	I+II	26.73333333	1
MB-0341	¡Ý60	Post	NA	G1/G2	I+II	173.9	1
MB-0345	<60	Pre	Ductal/NST	G1/G2	I+II	72.66666667	0
MB-0348	¡Ý60	Post	Ductal/NST	G1/G2	I+II	122.7	1
MB-0349	¡Ý60	Post	Ductal/NST	G3	I+II	146.0333333	1
MB-0353	¡Ý60	Post	Ductal/NST	G1/G2	I+II	41.83333333	1
MB-0356	<60	Post	Ductal/NST	G1/G2	I+II	213.5	0
MB-0358	<60	Post	Ductal/NST	G3	I+II	32.86666667	0
MB-0359	<60	Pre	Ductal/NST	G1/G2	I+II	21.6	0
MB-0360	¡Ý60	Post	Ductal/NST	G1/G2	I+II	132.5666667	1
MB-0362	<60	Post	Ductal/NST	G1/G2	I+II	47.03333333	1
MB-0363	¡Ý60	Post	Other	G1/G2	III+IV	89.9	1
MB-0364	<60	Pre	Ductal/NST	G1/G2	III+IV	176.6	0
MB-0366	<60	Post	Other	G1/G2	I+II	64.23333333	0
MB-0368	¡Ý60	Post	Ductal/NST	G3	I+II	61.9	1
MB-0369	¡Ý60	Post	Other	G3	I+II	144.3333333	0
MB-0370	¡Ý60	Post	Ductal/NST	G3	I+II	93.5	1
MB-0371	¡Ý60	Post	Ductal/NST	G3	I+II	131.1333333	0
MB-0373	¡Ý60	Post	Ductal/NST	G3	I+II	27	1
MB-0374	<60	Pre	Ductal/NST	G3	I+II	1.433333333	0
MB-0377	¡Ý60	Post	Ductal/NST	G1/G2	I+II	134.3666667	0
MB-0379	<60	Pre	Ductal/NST	G1/G2	I+II	135.6666667	0
MB-0380	¡Ý60	Post	Ductal/NST	G3	I+II	69.33333333	1
MB-0382	<60	Post	Ductal/NST	G1/G2	I+II	136.4666667	0
MB-0383	¡Ý60	Post	Ductal/NST	G3	I+II	85.73333333	1
MB-0384	¡Ý60	Post	Ductal/NST	G3	I+II	61.1	1

MB-0385	¡Ý60	Post	Ductal/NST	G3	I+II	36.43333333	1
MB-0386	¡Ý60	Post	Ductal/NST	G3	III+IV	138.1333333	0
MB-0389	<60	Pre	Ductal/NST	G3	III+IV	96.96666667	0
MB-0394	<60	Post	Other	G1/G2	I+II	64.23333333	0
MB-0397	<60	Post	Other	G1/G2	I+II	57.23333333	0
MB-0398	<60	Post	Ductal/NST	G3	III+IV	43.2	1
MB-0404	¡Ý60	Post	Ductal/NST	G3	I+II	63.5	0
MB-0406	¡Ý60	Post	Ductal/NST	G3	III+IV	96.9	1
MB-0410	<60	Post	Other	G1/G2	I+II	63	0
MB-0411	<60	Post	Other	G1/G2	I+II	139.1666667	0
MB-0412	<60	Post	Ductal/NST	G3	I+II	136.1666667	0
MB-0413	<60	Post	Ductal/NST	G3	I+II	140.0666667	0
MB-0419	¡Ý60	Post	Ductal/NST	G1/G2	I+II	104.3	1
MB-0422	¡Ý60	Post	Ductal/NST	G1/G2	I+II	33.8	1
MB-0425	¡Ý60	Post	Ductal/NST	G1/G2	I+II	24.4	1
MB-0427	¡Ý60	Post	Other	G1/G2	I+II	116.1	0
MB-0428	¡Ý60	Post	Ductal/NST	G3	I+II	55.93333333	0
MB-0429	¡Ý60	Post	Other	G3	I+II	74.46666667	1
MB-0431	¡Ý60	Post	Other	G1/G2	I+II	85.13333333	0
MB-0434	¡Ý60	Post	Ductal/NST	G3	I+II	45.5	1
MB-0437	¡Ý60	Post	Other	G1/G2	I+II	90.56666667	0
MB-0439	<60	Post	Other	G1/G2	I+II	112.1333333	0
MB-0440	<60	Post	Ductal/NST	G3	I+II	100.8333333	0
MB-0444	<60	Pre	Other	G1/G2	III+IV	55.46666667	1
MB-0445	<60	Pre	Ductal/NST	G1/G2	I+II	132.5333333	0
MB-0449	<60	Post	Ductal/NST	G1/G2	I+II	112.5666667	0
MB-0451	¡Ý60	Post	Ductal/NST	G1/G2	I+II	50.06666667	0
MB-0452	<60	Post	Ductal/NST	G3	I+II	62.8	0
MB-0454	¡Ý60	Post	Other	G1/G2	I+II	46.83333333	1
MB-0455	<60	Post	Ductal/NST	G1/G2	I+II	99.33333333	0
MB-0459	¡Ý60	Post	Ductal/NST	G3	III+IV	35.6	1
MB-0463	¡Ý60	Post	Ductal/NST	G1/G2	I+II	120.5666667	1
MB-0465	¡Ý60	Post	Ductal/NST	G3	I+II	28.6	1
MB-0466	<60	Pre	Ductal/NST	G1/G2	I+II	43.86666667	0
MB-0468	¡Ý60	Post	Ductal/NST	G3	I+II	129.6	1
MB-0469	¡Ý60	Post	Ductal/NST	G3	I+II	131.2666667	0
MB-0471	¡Ý60	Post	Other	G1/G2	I+II	107.4666667	0
MB-0472	<60	Post	Other	NA	III+IV	27.4	0
MB-0474	<60	Post	Ductal/NST	G3	III+IV	111.1	1
MB-0475	<60	Post	Ductal/NST	G3	I+II	130.7	0
MB-0480	<60	Post	Ductal/NST	G3	I+II	94.56666667	0
MB-0483	<60	Pre	Ductal/NST	G3	I+II	74.46666667	1
MB-0485	¡Ý60	Post	Other	G3	III+IV	36.76666667	1
MB-0486	<60	Post	Ductal/NST	G3	I+II	89.96666667	0
MB-0490	¡Ý60	Post	Ductal/NST	G1/G2	III+IV	101.9666667	0
MB-0491	<60	Pre	Other	G1/G2	III+IV	125.8	0
MB-0492	¡Ý60	Post	Other	G3	I+II	81.13333333	1
MB-0497	<60	Post	Ductal/NST	G1/G2	I+II	73.7	0
MB-0501	<60	Pre	Ductal/NST	G3	I+II	71.5	0
MB-0503	¡Ý60	Post	Ductal/NST	G1/G2	I+II	101.2333333	0
MB-0504	¡Ý60	Post	Ductal/NST	G3	I+II	130.4666667	0
MB-0505	<60	Post	Ductal/NST	G1/G2	I+II	60.7	0
MB-0507	<60	Post	Ductal/NST	G3	I+II	23.8	0

MB-0511	<60	Post		Ductal/NST	G1/G2	I+II	61.7	0
MB-0512	<60	Post		Ductal/NST	G1/G2	I+II	121.5333333	0
MB-0513	¡Ý60	Post		Ductal/NST	G1/G2	I+II	117.0333333	0
MB-0514	<60	Pre		NA	G1/G2	I+II	13.4	0
MB-0517	<60	Pre		Ductal/NST	G1/G2	I+II	61.9	0
MB-0521	<60	Post		Other	G1/G2	I+II	212.2	0
MB-0526	¡Ý60	Post		Ductal/NST	G3	NA	139.6333333	1
MB-0528	<60	Post		Ductal/NST	G3	I+II	193.9666667	0
MB-0529	¡Ý60	Post		Ductal/NST	G1/G2	III+IV	48.53333333	1
MB-0532	<60	Post		Ductal/NST	G3	I+II	143.1333333	1
MB-0535	<60	Pre		Ductal/NST	G1/G2	I+II	199.1333333	0
MB-0536	¡Ý60	Post		Ductal/NST	G3	I+II	147.3666667	1
MB-0538	¡Ý60	Post		Ductal/NST	G3	I+II	24.1	1
MB-0541	<60	Pre		Ductal/NST	G3	I+II	174.2666667	0
MB-0544	¡Ý60	Post		Ductal/NST	G3	III+IV	79.33333333	1
MB-0550	¡Ý60	Post		Ductal/NST	G1/G2	NA	132.3333333	1
MB-0553	¡Ý60	Post		Ductal/NST	G1/G2	I+II	85.73333333	1
MB-0554	¡Ý60	Post		Ductal/NST	NA	I+II	111.1666667	1
MB-0559	<60	Pre		Ductal/NST	G3	I+II	161.7666667	0
MB-0568	¡Ý60	Post		Ductal/NST	G1/G2	I+II	181.4666667	0
MB-0569	¡Ý60	Post		Ductal/NST	G3	I+II	59.5	1
MB-0570	¡Ý60	Post		Ductal/NST	G3	I+II	272.2	0
MB-0571	¡Ý60	Post		Ductal/NST	G1/G2	I+II	149.8666667	0
MB-0574	¡Ý60	Post		Ductal/NST	G3	I+II	119.8	0
MB-0575	<60	Pre		Ductal/NST	G1/G2	III+IV	128.5666667	0
MB-0576	¡Ý60	Post		Ductal/NST	G3	I+II	31	1
MB-0577	<60	Post		Other	G1/G2	I+II	65.4	0
MB-0579	¡Ý60	Post		Ductal/NST	G1/G2	I+II	92.76666667	1
MB-0580	<60	Pre		Ductal/NST	G3	I+II	79.36666667	1
MB-0583	¡Ý60	Post		Other	G1/G2	I+II	62.63333333	0
MB-0584	<60	Pre		Ductal/NST	G3	I+II	118.2	0
MB-0585	¡Ý60	Post		Ductal/NST	G3	I+II	115.7	1
MB-0586	¡Ý60	Post		Other	G1/G2	III+IV	77.23333333	1
MB-0587	<60	Post		Other	G1/G2	I+II	91.26666667	0
MB-0589	<60	Post		Other	G1/G2	III+IV	125.8	0
MB-0590	¡Ý60	Post		Ductal/NST	G3	I+II	72.46666667	1
MB-0591	¡Ý60	Post		Ductal/NST	G3	I+II	119.4666667	1
MB-0594	¡Ý60	Post		Ductal/NST	G3	III+IV	44.23333333	0
MB-0596	¡Ý60	Post		Other	G1/G2	I+II	107.1	1
MB-0598	¡Ý60	Post		Ductal/NST	G1/G2	I+II	89.36666667	1
MB-0599	<60	Pre		Ductal/NST	G1/G2	I+II	123.2666667	0
MB-0600	¡Ý60	Post		Other	G3	I+II	111	0
MB-0601	<60	Pre		Ductal/NST	G3	I+II	29.66666667	1
MB-0603	¡Ý60	Post		Ductal/NST	G1/G2	I+II	121.9666667	0
MB-0605	<60	Post		Other	G1/G2	I+II	114.6	0
MB-0606	¡Ý60	Post		Other	G1/G2	I+II	27.46666667	1
MB-0607	<60	Post		Ductal/NST	G3	I+II	87.83333333	0
MB-0609	<60	Post		Ductal/NST	G1/G2	I+II	98.7	1
MB-0611	¡Ý60	Post		Ductal/NST	G3	I+II	104.5333333	0
MB-0614	<60	Pre		Ductal/NST	G3	I+II	119.3333333	0
MB-0616	¡Ý60	Post		Ductal/NST	G3	I+II	29.03333333	1
MB-0618	¡Ý60	Post		Ductal/NST	G1/G2	I+II	101.9	0
MB-0621	¡Ý60	Post		Other	G1/G2	I+II	33.56666667	0

MB-0624	¡Ý60	Post	Ductal/NST	G1/G2	I+II	64.03333333	0
MB-0626	¡Ý60	Post	Ductal/NST	G3	I+II	35.53333333	1
MB-0630	<60	Pre	Ductal/NST	G3	I+II	86.8	0
MB-0631	¡Ý60	Post	Ductal/NST	G1/G2	I+II	47.43333333	1
MB-0632	<60	Post	Ductal/NST	G1/G2	I+II	56.5	0
MB-0636	<60	Pre	Ductal/NST	G3	I+II	32.63333333	1
MB-0637	¡Ý60	Post	Ductal/NST	G3	I+II	80.66666667	0
MB-0642	¡Ý60	Post	Ductal/NST	G1/G2	I+II	84.2	1
MB-0646	¡Ý60	Post	Other	G1/G2	I+II	15.16666667	1
MB-0649	¡Ý60	Post	Ductal/NST	G1/G2	I+II	61.43333333	0
MB-0650	¡Ý60	Post	Ductal/NST	G1/G2	I+II	25.53333333	0
MB-0654	¡Ý60	Post	Ductal/NST	G1/G2	I+II	69.4	0
MB-0661	¡Ý60	Post	Ductal/NST	G1/G2	I+II	20	1
MB-0666	¡Ý60	Post	Ductal/NST	G3	I+II	24.33333333	1
MB-0877	¡Ý60	Post	Ductal/NST	G3	I+II	14.43333333	1
MB-0880	¡Ý60	Post	Ductal/NST	G1/G2	I+II	1.233333333	0
MB-0882	¡Ý60	Post	Ductal/NST	G3	I+II	42.36666667	1
MB-0884	<60	Post	Ductal/NST	G3	I+II	93.66666667	1
MB-0891	¡Ý60	Post	Ductal/NST	G1/G2	I+II	149.4	0
MB-0895	¡Ý60	Post	Ductal/NST	G3	I+II	43.1	1
MB-0899	<60	Post	Ductal/NST	G1/G2	I+II	175.8	0
MB-0904	¡Ý60	Post	Ductal/NST	G1/G2	I+II	144.7	1
MB-2513	<60	Post	Ductal/NST	G1/G2	I+II	59.7	1
MB-2536	<60	Post	Other	G1/G2	I+II	47.9	1
MB-2564	<60	Post	Other	G1/G2	I+II	285.4333333	0
MB-2610	<60	Post	Other	G1/G2	I+II	280.7	0
MB-2613	<60	Post	Ductal/NST	G3	I+II	163.4	0
MB-2614	<60	Pre	Other	G1/G2	III+IV	64.93333333	1
MB-2617	¡Ý60	Post	Ductal/NST	G3	I+II	89.1	1
MB-2618	¡Ý60	Post	Ductal/NST	G1/G2	I+II	89.1	1
MB-2624	<60	Post	Other	G1/G2	I+II	128.5333333	0
MB-2626	¡Ý60	Post	Ductal/NST	G3	I+II	193.7	0
MB-2629	¡Ý60	Post	Ductal/NST	G3	I+II	279.1	0
MB-2634	¡Ý60	Post	Ductal/NST	G3	I+II	274.0333333	0
MB-2642	<60	Pre	Ductal/NST	G3	I+II	45.16666667	1
MB-2669	¡Ý60	Post	Ductal/NST	G1/G2	I+II	73.7	1
MB-2686	¡Ý60	Post	Ductal/NST	G3	I+II	177.2666667	0
MB-2705	¡Ý60	Post	Ductal/NST	G1/G2	I+II	163.7333333	0
MB-2708	¡Ý60	Post	Other	G1/G2	I+II	133.2333333	1
MB-2711	<60	Pre	Ductal/NST	G1/G2	I+II	201.4666667	0
MB-2712	<60	Post	Other	G1/G2	I+II	234.4333333	1
MB-2721	<60	Pre	Other	G1/G2	I+II	251.6333333	0
MB-2725	<60	Pre	Ductal/NST	G1/G2	I+II	236.9333333	0
MB-2728	<60	Pre	Ductal/NST	G3	III+IV	51.4	1
MB-2730	¡Ý60	Post	Ductal/NST	G3	III+IV	133.7333333	0
MB-2735	<60	Pre	Ductal/NST	G3	I+II	274.5	0
MB-2745	<60	Pre	Other	G1/G2	I+II	168.9666667	1
MB-2747	¡Ý60	Post	Ductal/NST	G1/G2	I+II	234.3333333	1
MB-2749	¡Ý60	Post	Ductal/NST	G1/G2	I+II	97.76666667	1
MB-2750	<60	Pre	Other	G1/G2	I+II	145.4333333	1
MB-2752	<60	Post	Other	G1/G2	I+II	81.93333333	1
MB-2760	¡Ý60	Post	Ductal/NST	G1/G2	I+II	118.0333333	1
MB-2763	¡Ý60	Post	Other	G1/G2	I+II	275.6	0

MB-2767	<60	Post	Ductal/NST	G3	I+II	271.3333333	0
MB-2769	<60	Post	Other	G1/G2	I+II	160	0
MB-2770	¡Ý60	Post	Other	G1/G2	III+IV	274.3666667	0
MB-2772	<60	Post	Other	G3	I+II	150.7333333	1
MB-2774	¡Ý60	Post	Ductal/NST	G1/G2	III+IV	131.6666667	1
MB-2779	<60	Pre	Ductal/NST	G1/G2	I+II	275.2333333	0
MB-2781	¡Ý60	Post	Ductal/NST	G1/G2	I+II	40.43333333	1
MB-2786	¡Ý60	Post	Ductal/NST	G3	I+II	68.26666667	1
MB-2790	<60	Pre	Other	G1/G2	I+II	116.9333333	0
MB-2791	<60	Pre	Ductal/NST	G1/G2	I+II	271.9333333	0
MB-2793	¡Ý60	Post	Ductal/NST	G1/G2	I+II	267.2333333	0
MB-2797	<60	Post	Other	G1/G2	I+II	252.9666667	0
MB-2801	¡Ý60	Post	Ductal/NST	G1/G2	I+II	271.2666667	0
MB-2803	¡Ý60	Post	Ductal/NST	G1/G2	I+II	85.86666667	1
MB-2814	¡Ý60	Post	Ductal/NST	G1/G2	I+II	231.0333333	1
MB-2819	¡Ý60	Post	Other	G1/G2	I+II	270.5666667	0
MB-2820	<60	Pre	Ductal/NST	G3	I+II	254.6333333	0
MB-2835	<60	Post	Ductal/NST	G3	I+II	55.03333333	1
MB-2838	¡Ý60	Post	Ductal/NST	G1/G2	I+II	270.3	0
MB-2840	¡Ý60	Post	Other	G1/G2	I+II	269	0
MB-2843	¡Ý60	Post	Ductal/NST	G1/G2	I+II	231.5333333	1
MB-2844	¡Ý60	Post	Ductal/NST	G3	I+II	146.7333333	0
MB-2848	¡Ý60	Post	Ductal/NST	G1/G2	I+II	227.7333333	1
MB-2851	<60	Post	Other	G3	I+II	159	1
MB-2853	<60	Pre	Other	G1/G2	I+II	269.6333333	0
MB-2854	¡Ý60	Post	Other	G1/G2	I+II	270.4333333	0
MB-2858	¡Ý60	Post	Other	G3	I+II	141.5666667	1
MB-2863	<60	Post	Other	G1/G2	I+II	258.3333333	0
MB-2867	<60	Pre	Ductal/NST	G1/G2	I+II	269.3333333	0
MB-2901	<60	Pre	Ductal/NST	G1/G2	I+II	218.3	1
MB-2916	<60	Pre	Other	G1/G2	I+II	266.9333333	0
MB-2919	<60	Post	Other	G1/G2	I+II	240.0333333	1
MB-2927	<60	Post	Other	G1/G2	I+II	263.7	1
MB-2931	<60	Pre	Ductal/NST	G1/G2	I+II	228.1	1
MB-2932	¡Ý60	Post	Ductal/NST	G3	I+II	108.4333333	0
MB-2933	<60	Pre	Other	G1/G2	I+II	161.9333333	1
MB-2939	¡Ý60	Post	Ductal/NST	G3	I+II	101.2666667	1
MB-2944	¡Ý60	Post	Other	G3	I+II	27.2	1
MB-2947	<60	Pre	Ductal/NST	G1/G2	I+II	112	0
MB-2951	<60	Pre	Ductal/NST	G1/G2	I+II	263.3666667	0
MB-2952	<60	Post	Other	G1/G2	I+II	189.1	1
MB-2953	<60	Pre	Ductal/NST	G1/G2	I+II	30.13333333	1
MB-2954	¡Ý60	Post	Ductal/NST	G1/G2	I+II	5.5	1
MB-2960	¡Ý60	Post	Ductal/NST	G3	I+II	222.2	1
MB-2966	<60	Post	Ductal/NST	G3	I+II	265	0
MB-2969	¡Ý60	Post	Ductal/NST	G1/G2	I+II	172.9	1
MB-2970	<60	Pre	Other	G1/G2	I+II	252.8666667	1
MB-2971	¡Ý60	Post	Ductal/NST	G1/G2	I+II	264.2333333	0
MB-2977	<60	Pre	Ductal/NST	G1/G2	I+II	110.6	1
MB-2983	<60	Post	Ductal/NST	G1/G2	I+II	180.8333333	1
MB-2984	¡Ý60	Post	Ductal/NST	G3	I+II	261.2	0
MB-2990	<60	Pre	Other	G1/G2	I+II	260	0
MB-2994	<60	Pre	Ductal/NST	G3	I+II	254.5	0

MB-2996	<60	Post	Ductal/NST	G3	I+II	191.2333333	0
MB-2999	¡Ý60	Post	Ductal/NST	G3	I+II	262	0
MB-3002	¡Ý60	Post	Other	G1/G2	I+II	140.5666667	1
MB-3005	<60	Pre	Ductal/NST	G1/G2	I+II	253.0666667	0
MB-3007	<60	Pre	Ductal/NST	G1/G2	I+II	114.9	1
MB-3008	<60	Post	Other	G1/G2	I+II	149.6	0
MB-3016	<60	Post	Ductal/NST	G3	I+II	258.8666667	0
MB-3021	¡Ý60	Post	Other	G3	I+II	217.5666667	1
MB-3026	<60	Pre	Ductal/NST	G3	I+II	261.2	0
MB-3028	¡Ý60	Post	Ductal/NST	G3	I+II	42.56666667	1
MB-3032	<60	Post	Ductal/NST	G1/G2	I+II	250.1333333	0
MB-3033	¡Ý60	Post	Ductal/NST	G1/G2	I+II	70.4	1
MB-3035	<60	Post	Ductal/NST	G1/G2	I+II	260.7333333	0
MB-3037	<60	Post	Other	G1/G2	I+II	98.83333333	0
MB-3049	<60	Post	Other	G1/G2	I+II	259.9666667	0
MB-3050	<60	Post	Ductal/NST	G3	I+II	255.1	1
MB-3060	¡Ý60	Post	Ductal/NST	G3	I+II	44.6	1
MB-3064	<60	Pre	Other	G1/G2	I+II	165.1666667	1
MB-3079	<60	Post	Other	G1/G2	I+II	149.6	0
MB-3083	¡Ý60	Post	Ductal/NST	G3	I+II	81.1	1
MB-3088	¡Ý60	Post	Ductal/NST	G1/G2	I+II	256.5	0
MB-3092	<60	Post	Other	G3	I+II	152.3333333	1
MB-3102	¡Ý60	Post	Ductal/NST	G1/G2	I+II	258.3333333	0
MB-3103	<60	Post	Ductal/NST	G3	I+II	28.73333333	1
MB-3104	<60	Pre	Ductal/NST	G1/G2	I+II	75.36666667	1
MB-3105	<60	Pre	Other	G1/G2	I+II	252.3333333	0
MB-3110	¡Ý60	Post	Other	G1/G2	I+II	100.7333333	0
MB-3121	¡Ý60	Post	Ductal/NST	G1/G2	I+II	131.3333333	1
MB-3167	¡Ý60	Post	Ductal/NST	G1/G2	I+II	136.9333333	0
MB-3171	<60	Post	Ductal/NST	G1/G2	I+II	219.6666667	1
MB-3181	<60	Pre	Ductal/NST	G3	I+II	178.1666667	1
MB-3222	¡Ý60	Post	Other	G1/G2	I+II	23.93333333	1
MB-3228	<60	Post	Other	G1/G2	I+II	252	0
MB-3235	<60	Post	Ductal/NST	G3	I+II	236.1333333	0
MB-3252	<60	Pre	Other	G1/G2	I+II	99.7	0
MB-3253	<60	Post	Ductal/NST	G3	I+II	55.83333333	1
MB-3254	<60	Post	Ductal/NST	G1/G2	I+II	123.9	0
MB-3266	<60	Post	Other	G3	I+II	51.2	1
MB-3272	¡Ý60	Post	Ductal/NST	G3	III+IV	22.66666667	1
MB-3275	<60	Pre	Ductal/NST	G3	I+II	41.53333333	1
MB-3298	<60	Pre	Ductal/NST	G1/G2	I+II	242.5666667	0
MB-3300	¡Ý60	Post	Ductal/NST	G1/G2	I+II	247	0
MB-3301	<60	Pre	Ductal/NST	G1/G2	I+II	248.7666667	0
MB-3303	¡Ý60	Post	Other	G3	I+II	243.9	0
MB-3328	¡Ý60	Post	Ductal/NST	G1/G2	I+II	191.1333333	1
MB-3341	¡Ý60	Post	Ductal/NST	G1/G2	I+II	148.7666667	1
MB-3344	<60	Pre	Ductal/NST	G1/G2	I+II	241.2666667	0
MB-3350	¡Ý60	Post	Other	G1/G2	I+II	227.8	1
MB-3351	<60	Pre	Ductal/NST	G1/G2	I+II	241.8666667	0
MB-3357	<60	Pre	Other	G3	I+II	38.03333333	1
MB-3360	<60	Pre	Ductal/NST	G3	I+II	26.76666667	1
MB-3361	<60	Post	Ductal/NST	G3	I+II	68.2	1
MB-3365	<60	Post	Ductal/NST	G1/G2	I+II	241.6	0

MB-3371	¡Ý60	Post	Ductal/NST	G1/G2	I+II	241.2666667	0
MB-3378	¡Ý60	Post	Other	G1/G2	I+II	87.53333333	1
MB-3379	¡Ý60	Post	Other	G3	I+II	113.8333333	1
MB-3381	<60	Post	Ductal/NST	G3	I+II	236.6333333	0
MB-3382	¡Ý60	Post	Ductal/NST	G3	I+II	136.2333333	0
MB-3388	<60	Post	Ductal/NST	G3	I+II	228.6	1
MB-3389	¡Ý60	Post	Other	G3	I+II	240.7	0
MB-3402	<60	Pre	Ductal/NST	G3	I+II	50.03333333	1
MB-3403	<60	Post	Ductal/NST	G1/G2	I+II	138.3333333	0
MB-3412	<60	Post	Ductal/NST	G3	III+IV	228.8	0
MB-3417	<60	Post	Other	G1/G2	I+II	103.4666667	0
MB-3430	¡Ý60	Post	Ductal/NST	G3	I+II	219.7666667	1
MB-3436	<60	Pre	Ductal/NST	G3	III+IV	50.76666667	1
MB-3437	¡Ý60	Post	Ductal/NST	G3	I+II	108.7666667	0
MB-3439	<60	Pre	Other	G1/G2	I+II	188.7333333	0
MB-3450	<60	Post	Ductal/NST	G1/G2	I+II	240.4666667	0
MB-3462	<60	Pre	Ductal/NST	G1/G2	I+II	234.2333333	0
MB-3487	<60	Pre	Ductal/NST	G3	I+II	237.1333333	0
MB-3490	<60	Pre	Ductal/NST	G1/G2	I+II	102.4	1
MB-3492	¡Ý60	Post	Ductal/NST	G3	I+II	118.1333333	1
MB-3506	<60	Pre	Other	G3	I+II	240.8333333	0
MB-3510	¡Ý60	Post	Other	G1/G2	I+II	59.6	1
MB-3525	¡Ý60	Post	Ductal/NST	G3	III+IV	236.7	0
MB-3545	<60	Pre	Ductal/NST	G3	I+II	232.7333333	1
MB-3548	¡Ý60	Post	Ductal/NST	G1/G2	I+II	234.5333333	1
MB-3600	¡Ý60	Post	Ductal/NST	G1/G2	I+II	214.7	0
MB-3614	¡Ý60	Post	Ductal/NST	G3	I+II	235.4	0
MB-3711	<60	Pre	Ductal/NST	G1/G2	I+II	222.1	0
MB-3748	<60	Post	Ductal/NST	G3	I+II	228	0
MB-3754	<60	Post	Ductal/NST	G3	I+II	92.73333333	1
MB-3781	<60	Pre	Other	G1/G2	I+II	224.5666667	0
MB-3797	¡Ý60	Post	Other	NA	I+II	228.3333333	0
MB-3824	<60	Pre	Ductal/NST	G3	I+II	126.4666667	1
MB-3838	¡Ý60	Post	Ductal/NST	G3	I+II	134.4666667	1
MB-3840	<60	Post	Ductal/NST	G1/G2	I+II	287.2333333	0
MB-3842	<60	Post	Ductal/NST	G3	I+II	226.0666667	0
MB-3850	¡Ý60	Post	Other	G1/G2	I+II	72.3	0
MB-3852	<60	Pre	Ductal/NST	G3	I+II	225.5	0
MB-3854	¡Ý60	Post	Other	G1/G2	I+II	99.23333333	0
MB-3871	¡Ý60	Post	Ductal/NST	G1/G2	I+II	172.8	1
MB-3874	<60	Pre	Ductal/NST	G1/G2	I+II	186.4333333	0
MB-4000	¡Ý60	Post	Ductal/NST	G1/G2	I+II	28.06666667	1
MB-4001	¡Ý60	Post	Ductal/NST	G1/G2	I+II	49.66666667	1
MB-4004	¡Ý60	Post	Ductal/NST	G3	I+II	256.8666667	1
MB-4008	¡Ý60	Post	Ductal/NST	G3	I+II	44.73333333	1
MB-4011	¡Ý60	Post	Ductal/NST	G3	NA	193.1666667	0
MB-4017	¡Ý60	Post	Other	G3	I+II	139.3	1
MB-4018	¡Ý60	Post	Other	G1/G2	NA	105.6666667	1
MB-4033	¡Ý60	Post	Other	G1/G2	III+IV	15.2	1
MB-4091	¡Ý60	Post	Ductal/NST	G3	I+II	281.3666667	1
MB-4120	<60	Post	Ductal/NST	G1/G2	NA	217.1	1
MB-4139	¡Ý60	Post	Ductal/NST	NA	I+II	165.4	1
MB-4140	¡Ý60	Post	Ductal/NST	G3	I+II	114.5	1

MB-4148	<60	Post	Ductal/NST	G1/G2	NA	63.03333333	1
MB-4154	<60	Post	Ductal/NST	G3	I+II	49.56666667	1
MB-4173	¡Ý60	Post	Ductal/NST	G1/G2	I+II	160.3333333	1
MB-4212	<60	Pre	Ductal/NST	G1/G2	NA	330.3666667	0
MB-4213	¡Ý60	Post	Ductal/NST	G1/G2	I+II	84.5	1
MB-4230	¡Ý60	Post	Ductal/NST	G1/G2	I+II	115.6	1
MB-4233	<60	Post	Ductal/NST	G1/G2	I+II	36.36666667	1
MB-4234	¡Ý60	Post	Ductal/NST	G3	I+II	253.5333333	1
MB-4250	¡Ý60	Post	Ductal/NST	G1/G2	III+IV	100.1666667	1
MB-4264	¡Ý60	Post	Ductal/NST	G1/G2	NA	163.2	1
MB-4266	<60	Pre	Ductal/NST	G3	I+II	33.9	1
MB-4274	¡Ý60	Post	Ductal/NST	G1/G2	I+II	59.93333333	1
MB-4282	<60	Pre	Ductal/NST	G3	I+II	34.1	0
MB-4283	<60	Post	Ductal/NST	G1/G2	I+II	174.8333333	0
MB-4292	<60	Post	Ductal/NST	G3	NA	335.6	1
MB-4293	<60	Post	Other	G1/G2	I+II	219.1666667	1
MB-4306	¡Ý60	Post	Other	G1/G2	I+II	234.7	1
MB-4310	¡Ý60	Post	Ductal/NST	G1/G2	NA	265.5666667	1
MB-4313	<60	Post	Other	G3	I+II	300.7	1
MB-4318	¡Ý60	Post	Ductal/NST	G3	I+II	17.13333333	1
MB-4322	¡Ý60	Post	Ductal/NST	G1/G2	I+II	196.4666667	1
MB-4323	¡Ý60	Post	Ductal/NST	NA	NA	126.6333333	1
MB-4328	¡Ý60	Post	Ductal/NST	G1/G2	NA	199.2666667	1
MB-4329	¡Ý60	Post	Ductal/NST	G3	I+II	168.2666667	1
MB-4333	¡Ý60	Post	Ductal/NST	G1/G2	I+II	207.9666667	1
MB-4339	¡Ý60	Post	Ductal/NST	G1/G2	I+II	173.6	1
MB-4342	¡Ý60	Post	Ductal/NST	NA	III+IV	232.7666667	1
MB-4353	<60	Post	Ductal/NST	G3	I+II	128.5333333	1
MB-4357	¡Ý60	Post	Ductal/NST	G3	NA	114.0333333	1
MB-4368	¡Ý60	Post	Ductal/NST	G1/G2	I+II	119.4666667	1
MB-4374	¡Ý60	Post	Ductal/NST	G3	I+II	42.56666667	1
MB-4390	¡Ý60	Post	Ductal/NST	G1/G2	I+II	164.3333333	1
MB-4418	<60	Post	Ductal/NST	G1/G2	I+II	307.6333333	0
MB-4421	¡Ý60	Post	Ductal/NST	G1/G2	I+II	264.7666667	1
MB-4442	<60	Pre	Ductal/NST	G1/G2	I+II	296.8666667	0
MB-4529	¡Ý60	Post	Ductal/NST	G1/G2	I+II	126.4666667	1
MB-4557	<60	Post	Other	G1/G2	I+II	279.8	0
MB-4564	¡Ý60	Post	Ductal/NST	G3	NA	210.4333333	1
MB-4578	¡Ý60	Post	Ductal/NST	G1/G2	I+II	197.3333333	1
MB-4591	¡Ý60	Post	Ductal/NST	G3	I+II	41.16666667	1
MB-4593	<60	Pre	Ductal/NST	G1/G2	I+II	140.2333333	0
MB-4598	¡Ý60	Post	Ductal/NST	G1/G2	I+II	119.3666667	1
MB-4599	¡Ý60	Post	Ductal/NST	G3	I+II	191.1666667	0
MB-4602	<60	Post	Ductal/NST	G3	I+II	122.8	1
MB-4607	<60	Post	Ductal/NST	G1/G2	I+II	121.7333333	0
MB-4616	¡Ý60	Post	Ductal/NST	G1/G2	I+II	90.13333333	1
MB-4618	<60	Pre	Other	G1/G2	I+II	267.4	0
MB-4623	¡Ý60	Post	Other	G1/G2	I+II	292.0333333	1
MB-4627	<60	Post	Other	G3	I+II	186.6333333	1
MB-4630	¡Ý60	Post	Ductal/NST	NA	NA	95.83333333	1
MB-4633	¡Ý60	Post	Other	G3	I+II	318.2	0
MB-4634	¡Ý60	Post	Other	G3	I+II	109.6	1
MB-4639	¡Ý60	Post	Ductal/NST	G3	I+II	119	1

MB-4641	¡Ý60	Post	Ductal/NST	G3	I+II	148.5666667	1
MB-4642	¡Ý60	Post	Ductal/NST	G3	I+II	285.7	1
MB-4644	<60	Post	Ductal/NST	G1/G2	NA	90	1
MB-4648	¡Ý60	Post	Ductal/NST	G3	I+II	168.5333333	1
MB-4649	¡Ý60	Post	Ductal/NST	G1/G2	I+II	16.83333333	1
MB-4651	¡Ý60	Post	Ductal/NST	G1/G2	I+II	30.3	1
MB-4654	¡Ý60	Post	Ductal/NST	G3	I+II	64.3	1
MB-4655	¡Ý60	Post	Ductal/NST	G3	I+II	99	1
MB-4661	¡Ý60	Post	Ductal/NST	G1/G2	I+II	234.2333333	0
MB-4665	<60	Post	Ductal/NST	G3	I+II	260.2	0
MB-4666	<60	Post	Ductal/NST	G3	I+II	111.5666667	1
MB-4669	¡Ý60	Post	Ductal/NST	G3	I+II	119.4666667	1
MB-4670	¡Ý60	Post	Ductal/NST	G1/G2	I+II	67.8	1
MB-4671	¡Ý60	Post	Ductal/NST	G3	I+II	34.7	1
MB-4672	<60	Post	Ductal/NST	G3	NA	220.9333333	1
MB-4673	¡Ý60	Post	Ductal/NST	G1/G2	III+IV	87	1
MB-4674	¡Ý60	Post	Ductal/NST	G1/G2	I+II	81.06666667	1
MB-4675	<60	Post	Ductal/NST	G3	I+II	263.0333333	0
MB-4681	¡Ý60	Post	Ductal/NST	NA	I+II	251.8	1
MB-4682	¡Ý60	Post	Ductal/NST	G3	NA	139.5333333	1
MB-4685	¡Ý60	Post	Ductal/NST	G3	I+II	43.3	1
MB-4686	<60	Post	Ductal/NST	G3	I+II	48.43333333	1
MB-4687	¡Ý60	Post	Ductal/NST	G3	I+II	74.1	1
MB-4688	¡Ý60	Post	Ductal/NST	G1/G2	III+IV	85.3	1
MB-4691	<60	Pre	Ductal/NST	G3	I+II	282.5666667	1
MB-4692	<60	Post	Ductal/NST	G1/G2	NA	107.2666667	1
MB-4698	<60	Post	Other	G3	I+II	170.6666667	1
MB-4701	<60	Pre	Ductal/NST	G3	I+II	127.5333333	0
MB-4702	<60	Post	Ductal/NST	G3	I+II	300.8666667	0
MB-4704	¡Ý60	Post	Other	G3	I+II	64	1
MB-4705	¡Ý60	Post	Ductal/NST	G1/G2	I+II	213.1	1
MB-4706	¡Ý60	Post	Ductal/NST	G3	I+II	55.66666667	1
MB-4708	¡Ý60	Post	Other	G3	I+II	227.9	1
MB-4709	<60	Pre	Ductal/NST	G1/G2	I+II	146.7666667	0
MB-4710	¡Ý60	Post	Ductal/NST	G3	I+II	57.66666667	0
MB-4716	<60	Post	Other	G3	I+II	76.13333333	1
MB-4719	¡Ý60	Post	Ductal/NST	G1/G2	I+II	81.56666667	1
MB-4721	¡Ý60	Post	Other	G1/G2	III+IV	23.73333333	1
MB-4722	¡Ý60	Post	Ductal/NST	NA	I+II	86.1	0
MB-4723	¡Ý60	Post	Ductal/NST	G3	I+II	211.9666667	0
MB-4729	¡Ý60	Post	Ductal/NST	G3	NA	28	1
MB-4730	<60	Post	Other	G3	I+II	131.3	1
MB-4735	<60	Post	Ductal/NST	G1/G2	I+II	297.8	0
MB-4737	<60	Post	Ductal/NST	G3	I+II	122.2	0
MB-4739	<60	Post	Other	G1/G2	I+II	161.6666667	1
MB-4741	<60	Post	Other	G1/G2	I+II	56.5	1
MB-4742	¡Ý60	Post	Other	NA	I+II	83.13333333	1
MB-4744	¡Ý60	Post	Ductal/NST	G3	I+II	153	1
MB-4749	¡Ý60	Post	Other	G3	I+II	51.46666667	1
MB-4750	¡Ý60	Post	Ductal/NST	G1/G2	I+II	37.73333333	1
MB-4752	¡Ý60	Post	Ductal/NST	G1/G2	III+IV	125.2666667	1
MB-4760	<60	Pre	Ductal/NST	NA	NA	124.8	1
MB-4762	¡Ý60	Post	Other	G1/G2	I+II	252.1	0

MB-4764	<60	Post	Other	G3	I+II	245.5	0
MB-4767	¡Ý60	Post	Ductal/NST	G3	I+II	47.13333333	1
MB-4771	<60	Pre	Ductal/NST	G1/G2	I+II	297.2333333	0
MB-4778	¡Ý60	Post	Other	G1/G2	I+II	206.1333333	1
MB-4779	<60	Post	Ductal/NST	G1/G2	I+II	101.5666667	0
MB-4785	¡Ý60	Post	Ductal/NST	G1/G2	I+II	121.6666667	1
MB-4787	¡Ý60	Post	Ductal/NST	G1/G2	I+II	81.06666667	1
MB-4790	<60	Pre	Ductal/NST	G1/G2	I+II	70.46666667	1
MB-4791	¡Ý60	Post	Ductal/NST	G1/G2	I+II	173.8333333	1
MB-4794	¡Ý60	Post	Ductal/NST	NA	I+II	45.5	1
MB-4796	<60	Post	Ductal/NST	G1/G2	I+II	128.2666667	0
MB-4797	¡Ý60	Post	Other	G1/G2	I+II	99.36666667	1
MB-4800	¡Ý60	Post	Ductal/NST	G3	I+II	110.9333333	1
MB-4801	¡Ý60	Post	Ductal/NST	G3	I+II	130.3666667	1
MB-4802	¡Ý60	Post	Ductal/NST	G1/G2	I+II	43.9	1
MB-4805	<60	Pre	Ductal/NST	G3	I+II	129.8	1
MB-4806	<60	Pre	Ductal/NST	G1/G2	I+II	201.7666667	1
MB-4818	¡Ý60	Post	Other	G1/G2	III+IV	76.23333333	1
MB-4820	¡Ý60	Post	Ductal/NST	NA	NA	57.3	1
MB-4822	¡Ý60	Post	Other	G1/G2	I+II	91.63333333	1
MB-4825	¡Ý60	Post	Ductal/NST	G1/G2	I+II	214.4333333	1
MB-4827	¡Ý60	Post	Other	G1/G2	I+II	45.7	1
MB-4828	¡Ý60	Post	Other	G3	III+IV	42.5	1
MB-4829	¡Ý60	Post	Other	NA	I+II	176.2666667	1
MB-4832	<60	Pre	Ductal/NST	G1/G2	I+II	141.7333333	1
MB-4834	<60	Pre	Ductal/NST	G3	NA	36.16666667	1
MB-4838	¡Ý60	Post	Ductal/NST	G1/G2	III+IV	200.7666667	1
MB-4839	¡Ý60	Post	Ductal/NST	NA	NA	299.4	0
MB-4843	<60	Post	Ductal/NST	NA	I+II	265.1333333	0
MB-4845	¡Ý60	Post	Ductal/NST	NA	I+II	25.46666667	1
MB-4846	¡Ý60	Post	Other	G1/G2	I+II	101.6333333	1
MB-4849	¡Ý60	Post	Ductal/NST	G3	I+II	45.33333333	1
MB-4851	¡Ý60	Post	Ductal/NST	G1/G2	I+II	90.26666667	1
MB-4853	¡Ý60	Post	Other	NA	I+II	265.9333333	0
MB-4855	¡Ý60	Post	Ductal/NST	G3	NA	41.46666667	1
MB-4858	¡Ý60	Post	Ductal/NST	G3	NA	129.3333333	1
MB-4860	<60	Pre	Other	G1/G2	I+II	234.6	0
MB-4862	¡Ý60	Post	Ductal/NST	G3	I+II	187.3	0
MB-4866	<60	Pre	Ductal/NST	G1/G2	I+II	224.3	0
MB-4867	<60	Pre	Ductal/NST	G1/G2	I+II	221.9	0
MB-4869	¡Ý60	Post	Ductal/NST	G1/G2	I+II	29.3	1
MB-4870	<60	Post	Ductal/NST	G1/G2	I+II	229.0666667	0
MB-4872	<60	Post	Other	G1/G2	NA	157.1	0
MB-4873	¡Ý60	Post	Ductal/NST	G1/G2	III+IV	117.6666667	1
MB-4878	¡Ý60	Post	Ductal/NST	G1/G2	I+II	22.46666667	1
MB-4882	¡Ý60	Post	Ductal/NST	G1/G2	I+II	266.1	1
MB-4883	<60	Post	Ductal/NST	G1/G2	I+II	268.9	0
MB-4894	<60	Pre	Ductal/NST	G3	I+II	232.4	0
MB-4898	¡Ý60	Post	Ductal/NST	G1/G2	NA	117.9	1
MB-4899	<60	Pre	Ductal/NST	G1/G2	I+II	275.7333333	0
MB-4900	<60	Pre	Ductal/NST	G1/G2	I+II	224.6	0
MB-4906	¡Ý60	Post	Ductal/NST	G1/G2	I+II	233.8666667	1
MB-4908	¡Ý60	Post	Ductal/NST	G3	I+II	47.9	1

MB-4912	¡Ý60	Post	Ductal/NST	G1/G2	I+II	50	1
MB-4925	¡Ý60	Post	Ductal/NST	G1/G2	I+II	102.7666667	1
MB-4930	¡Ý60	Post	Ductal/NST	G3	I+II	58.43333333	0
MB-4933	<60	Post	Ductal/NST	G1/G2	I+II	272.9	0
MB-4934	¡Ý60	Post	Ductal/NST	G1/G2	I+II	70.23333333	0
MB-4937	<60	Pre	Ductal/NST	G3	I+II	77.23333333	1
MB-4941	<60	Post	Ductal/NST	G1/G2	I+II	58.63333333	1
MB-4944	¡Ý60	Post	Ductal/NST	G1/G2	I+II	237.2666667	1
MB-4949	¡Ý60	Post	Ductal/NST	G3	I+II	216.9666667	0
MB-4950	¡Ý60	Post	Ductal/NST	G3	I+II	150.6	1
MB-4956	¡Ý60	Post	Ductal/NST	G3	III+IV	69.3	1
MB-4957	¡Ý60	Post	Other	G1/G2	I+II	197.6666667	1
MB-4959	¡Ý60	Post	Ductal/NST	G1/G2	I+II	117.5666667	1
MB-4961	<60	Post	Ductal/NST	G1/G2	I+II	219.4666667	0
MB-4962	¡Ý60	Post	Ductal/NST	G1/G2	NA	197.8333333	1
MB-4965	¡Ý60	Post	Other	G1/G2	I+II	263.6	1
MB-4966	<60	Post	Other	G1/G2	I+II	49.3	1
MB-4967	¡Ý60	Post	Ductal/NST	G1/G2	I+II	130.7	1
MB-4968	<60	Post	Other	G1/G2	I+II	78.7	0
MB-4969	¡Ý60	Post	Ductal/NST	G1/G2	I+II	198.1	1
MB-4970	¡Ý60	Post	Ductal/NST	G1/G2	NA	88.8	1
MB-4976	¡Ý60	Post	Ductal/NST	G1/G2	NA	224.1	1
MB-4977	¡Ý60	Post	Ductal/NST	G3	NA	216.7333333	1
MB-4981	¡Ý60	Post	Other	G3	NA	180.7666667	1
MB-4986	<60	Pre	Ductal/NST	G1/G2	I+II	79.8	1
MB-4987	¡Ý60	Post	Ductal/NST	G1/G2	I+II	221.7666667	1
MB-4991	¡Ý60	Post	Ductal/NST	G3	I+II	126.8666667	0
MB-4992	¡Ý60	Post	Other	G1/G2	I+II	165.3666667	0
MB-4994	¡Ý60	Post	Ductal/NST	G1/G2	I+II	174.1333333	1
MB-4996	¡Ý60	Post	Ductal/NST	G1/G2	I+II	99.76666667	1
MB-4998	¡Ý60	Post	Other	G3	I+II	65.56666667	1
MB-4999	<60	Pre	Ductal/NST	G1/G2	I+II	187.9333333	0
MB-5001	¡Ý60	Post	Ductal/NST	G1/G2	I+II	81.8	1
MB-5011	¡Ý60	Post	Other	G1/G2	I+II	80.5	1
MB-5013	¡Ý60	Post	Other	G1/G2	I+II	251.2	1
MB-5014	¡Ý60	Post	Ductal/NST	G3	NA	213.3666667	0
MB-5015	<60	Pre	Ductal/NST	G3	I+II	252.3	1
MB-5018	¡Ý60	Post	Ductal/NST	G1/G2	I+II	108.6	1
MB-5020	¡Ý60	Post	Other	G3	I+II	84.83333333	1
MB-5027	<60	Pre	Other	G3	I+II	189.8666667	0
MB-5033	¡Ý60	Post	Other	G1/G2	I+II	52.73333333	1
MB-5035	<60	Post	Other	G3	I+II	213.2	1
MB-5039	¡Ý60	Post	Ductal/NST	G3	III+IV	62.13333333	1
MB-5040	¡Ý60	Post	Ductal/NST	G3	I+II	79.16666667	1
MB-5043	¡Ý60	Post	Other	G3	III+IV	128.3666667	1
MB-5044	<60	Post	Ductal/NST	G1/G2	I+II	139.4333333	0
MB-5045	¡Ý60	Post	Ductal/NST	G3	I+II	168.3	1
MB-5048	<60	Pre	Ductal/NST	G1/G2	I+II	86.36666667	1
MB-5049	¡Ý60	Post	Other	G1/G2	I+II	255.3	0
MB-5050	<60	Post	Other	G1/G2	I+II	184.3333333	0
MB-5053	¡Ý60	Post	Ductal/NST	G1/G2	NA	150.6	1
MB-5059	<60	Post	Other	G1/G2	I+II	257.6333333	0
MB-5060	¡Ý60	Post	Ductal/NST	G1/G2	I+II	150.1	1

MB-5061	¡Ý60	Post	Ductal/NST	G1/G2	I+II	39.43333333	0
MB-5062	¡Ý60	Post	Ductal/NST	G3	I+II	168.2666667	0
MB-5064	¡Ý60	Post	Ductal/NST	G1/G2	I+II	91.5	0
MB-5066	<60	Post	Ductal/NST	G1/G2	I+II	94.7	0
MB-5068	¡Ý60	Post	Ductal/NST	G1/G2	NA	182.5	0
MB-5074	¡Ý60	Post	Other	G3	I+II	221.6	1
MB-5078	<60	Post	Ductal/NST	G1/G2	I+II	87.1	1
MB-5079	¡Ý60	Post	Ductal/NST	G1/G2	I+II	199.9333333	0
MB-5084	<60	Pre	Ductal/NST	G1/G2	I+II	198.1	1
MB-5086	¡Ý60	Post	Ductal/NST	NA	I+II	84.23333333	1
MB-5088	¡Ý60	Post	Ductal/NST	G1/G2	I+II	199.3666667	1
MB-5092	¡Ý60	Post	Ductal/NST	G3	I+II	174.5666667	1
MB-5097	<60	Post	Ductal/NST	G1/G2	I+II	61.6	0
MB-5098	¡Ý60	Post	Ductal/NST	NA	I+II	186.4	1
MB-5101	¡Ý60	Post	Ductal/NST	G1/G2	III+IV	34.33333333	1
MB-5105	¡Ý60	Post	Ductal/NST	G1/G2	I+II	204.4333333	1
MB-5107	¡Ý60	Post	Ductal/NST	G3	I+II	82.63333333	1
MB-5116	¡Ý60	Post	Ductal/NST	G1/G2	I+II	71.76666667	1
MB-5117	<60	Post	Ductal/NST	G1/G2	I+II	209.2666667	0
MB-5118	¡Ý60	Post	Ductal/NST	G1/G2	I+II	211.7333333	0
MB-5119	¡Ý60	Post	Ductal/NST	G1/G2	I+II	59.76666667	0
MB-5121	¡Ý60	Post	Other	G3	NA	114.9	1
MB-5122	¡Ý60	Post	Other	NA	NA	125.7	1
MB-5123	¡Ý60	Post	Ductal/NST	G1/G2	I+II	90.3	1
MB-5124	¡Ý60	Post	Ductal/NST	G1/G2	III+IV	124.2	1
MB-5127	¡Ý60	Post	Other	G3	I+II	191.4666667	1
MB-5130	<60	Post	Ductal/NST	G3	I+II	255	1
MB-5134	<60	Pre	Ductal/NST	G1/G2	I+II	196.5333333	0
MB-5139	¡Ý60	Post	Ductal/NST	G3	I+II	89.96666667	1
MB-5143	¡Ý60	Post	Ductal/NST	G1/G2	I+II	127.9333333	1
MB-5144	<60	Post	Ductal/NST	G1/G2	I+II	213.0333333	0
MB-5147	<60	Post	Other	G3	I+II	20.26666667	0
MB-5152	¡Ý60	Post	Ductal/NST	G1/G2	I+II	115.9333333	1
MB-5160	¡Ý60	Post	Ductal/NST	G1/G2	I+II	87.73333333	1
MB-5163	¡Ý60	Post	Ductal/NST	G3	I+II	212.2	1
MB-5166	¡Ý60	Post	Ductal/NST	G3	III+IV	80.73333333	1
MB-5167	¡Ý60	Post	Ductal/NST	NA	I+II	208.4	0
MB-5169	¡Ý60	Post	Ductal/NST	G3	I+II	51.96666667	1
MB-5179	¡Ý60	Post	Other	G3	I+II	246.6	1
MB-5182	¡Ý60	Post	Other	G3	NA	134.2666667	1
MB-5183	¡Ý60	Post	Ductal/NST	G1/G2	I+II	118.5333333	1
MB-5184	¡Ý60	Post	Other	G1/G2	I+II	89.33333333	1
MB-5185	¡Ý60	Post	Ductal/NST	G3	I+II	77.46666667	1
MB-5186	<60	Pre	Ductal/NST	NA	I+II	203.2666667	0
MB-5189	¡Ý60	Post	Ductal/NST	G3	I+II	111.9666667	1
MB-5191	<60	Post	Other	G3	I+II	142.1666667	1
MB-5193	¡Ý60	Post	Ductal/NST	G1/G2	I+II	54.26666667	1
MB-5195	¡Ý60	Post	Ductal/NST	G3	I+II	196.8666667	1
MB-5196	<60	Post	Ductal/NST	G1/G2	I+II	94.03333333	1
MB-5197	¡Ý60	Post	Ductal/NST	G1/G2	I+II	223.8333333	1
MB-5200	¡Ý60	Post	Ductal/NST	G3	I+II	52.3	1
MB-5201	¡Ý60	Post	Ductal/NST	G1/G2	I+II	96.96666667	1
MB-5204	¡Ý60	Post	Ductal/NST	NA	I+II	191.9333333	1

MB-5206	¡Ý60	Post	Other	G1/G2	I+II	205.7333333	0
MB-5211	¡Ý60	Post	Ductal/NST	G3	I+II	247.8333333	1
MB-5212	¡Ý60	Post	Ductal/NST	G3	I+II	179.1	1
MB-5215	¡Ý60	Post	Ductal/NST	G3	NA	118.7	1
MB-5218	<60	Pre	Ductal/NST	G3	I+II	254.9666667	0
MB-5221	¡Ý60	Post	Ductal/NST	G1/G2	I+II	131.3	1
MB-5224	¡Ý60	Post	Ductal/NST	G3	I+II	99.4	1
MB-5226	¡Ý60	Post	Ductal/NST	G3	I+II	200.6	0
MB-5227	¡Ý60	Post	Ductal/NST	G1/G2	I+II	187.0333333	1
MB-5228	¡Ý60	Post	Ductal/NST	G3	I+II	180.7333333	1
MB-5230	<60	Pre	Other	G1/G2	I+II	176.5	0
MB-5233	¡Ý60	Post	Ductal/NST	G1/G2	I+II	209.0333333	1
MB-5239	¡Ý60	Post	Other	G1/G2	I+II	192.1333333	0
MB-5240	<60	Post	Other	G1/G2	I+II	103.8333333	1
MB-5243	¡Ý60	Post	Ductal/NST	G1/G2	NA	38.43333333	1
MB-5244	¡Ý60	Post	Ductal/NST	G3	I+II	216.8666667	1
MB-5251	¡Ý60	Post	Ductal/NST	NA	I+II	112.4666667	1
MB-5253	¡Ý60	Post	Other	G1/G2	I+II	102.0666667	1
MB-5256	¡Ý60	Post	Ductal/NST	G1/G2	I+II	108.0666667	1
MB-5260	¡Ý60	Post	Other	G3	I+II	202.1	1
MB-5261	¡Ý60	Post	Ductal/NST	G1/G2	I+II	116.4666667	1
MB-5264	<60	Pre	Ductal/NST	G3	I+II	199.3	0
MB-5266	¡Ý60	Post	Ductal/NST	G3	I+II	83.63333333	1
MB-5267	<60	Post	Ductal/NST	G1/G2	I+II	71.06666667	1
MB-5268	<60	Pre	Other	G1/G2	I+II	186.1333333	0
MB-5270	¡Ý60	Post	Other	G3	I+II	187.8333333	1
MB-5271	<60	Post	Other	G1/G2	I+II	167.5	0
MB-5273	¡Ý60	Post	Ductal/NST	G3	I+II	60.86666667	1
MB-5279	¡Ý60	Post	Ductal/NST	G3	I+II	68.76666667	1
MB-5281	<60	Post	Ductal/NST	G3	I+II	79.3	0
MB-5284	¡Ý60	Post	Other	G3	I+II	65.33333333	1
MB-5287	¡Ý60	Post	Ductal/NST	G1/G2	I+II	124	1
MB-5288	¡Ý60	Post	Ductal/NST	G1/G2	I+II	128.4	1
MB-5290	¡Ý60	Post	Ductal/NST	G1/G2	I+II	36.93333333	1
MB-5291	¡Ý60	Post	Ductal/NST	G1/G2	I+II	153.5333333	0
MB-5292	¡Ý60	Post	Ductal/NST	G1/G2	NA	48.43333333	1
MB-5293	¡Ý60	Post	Other	G1/G2	I+II	208.9666667	1
MB-5300	<60	Post	Ductal/NST	G1/G2	I+II	190.1666667	0
MB-5305	¡Ý60	Post	Other	G1/G2	I+II	149.4333333	1
MB-5306	<60	Post	Ductal/NST	G3	I+II	159.7333333	0
MB-5310	¡Ý60	Post	Other	G1/G2	I+II	153.3	1
MB-5317	¡Ý60	Post	Other	G1/G2	I+II	53.63333333	1
MB-5318	¡Ý60	Post	Ductal/NST	G3	I+II	14.16666667	1
MB-5322	¡Ý60	Post	Other	G1/G2	I+II	102.3	1
MB-5324	¡Ý60	Post	Other	G1/G2	I+II	190.1	1
MB-5328	¡Ý60	Post	Ductal/NST	G1/G2	I+II	14.2	1
MB-5329	¡Ý60	Post	Other	G3	III+IV	45.73333333	1
MB-5330	¡Ý60	Post	Other	G1/G2	NA	114.5333333	1
MB-5331	<60	Post	Ductal/NST	G1/G2	I+II	124.1333333	1
MB-5334	¡Ý60	Post	Ductal/NST	G3	I+II	85.4	1
MB-5338	¡Ý60	Post	Ductal/NST	G1/G2	I+II	51.66666667	1
MB-5341	<60	Post	Ductal/NST	G1/G2	I+II	250.8333333	0
MB-5347	<60	Post	Ductal/NST	G3	I+II	211.9	1

MB-5358	¡Ý60	Post	Other	G1/G2	III+IV	28.83333333	1
MB-5360	¡Ý60	Post	Other	G1/G2	III+IV	161.1333333	1
MB-5361	¡Ý60	Post	Ductal/NST	G1/G2	I+II	15.36666667	1
MB-5365	¡Ý60	Post	Ductal/NST	G1/G2	I+II	23.03333333	1
MB-5369	¡Ý60	Post	Other	G1/G2	I+II	39.3	1
MB-5370	¡Ý60	Post	Ductal/NST	G3	I+II	119.3	1
MB-5373	¡Ý60	Post	Ductal/NST	G3	I+II	2.533333333	0
MB-5377	<60	Pre	Ductal/NST	G3	I+II	102	1
MB-5382	¡Ý60	Post	Ductal/NST	G1/G2	I+II	116.2333333	1
MB-5383	¡Ý60	Post	Ductal/NST	G1/G2	NA	113.6666667	1
MB-5384	¡Ý60	Post	NA	G1/G2	NA	71.16666667	1
MB-5386	¡Ý60	Post	Ductal/NST	G1/G2	I+II	192.2	0
MB-5388	¡Ý60	Post	Other	G1/G2	I+II	146.9333333	1
MB-5389	¡Ý60	Post	Ductal/NST	G1/G2	I+II	124.7666667	1
MB-5393	¡Ý60	Post	Other	G1/G2	I+II	154	1
MB-5395	¡Ý60	Post	Ductal/NST	G3	I+II	165.1666667	0
MB-5396	¡Ý60	Post	Ductal/NST	G3	I+II	165.6666667	0
MB-5397	¡Ý60	Post	Ductal/NST	G3	NA	172.8666667	1
MB-5398	¡Ý60	Post	Ductal/NST	G1/G2	I+II	213	0
MB-5399	¡Ý60	Post	Ductal/NST	NA	I+II	106.8	1
MB-5401	¡Ý60	Post	Other	G1/G2	I+II	70.16666667	0
MB-5402	¡Ý60	Post	Ductal/NST	G1/G2	I+II	107.7666667	1
MB-5403	¡Ý60	Post	Ductal/NST	G1/G2	I+II	95.73333333	1
MB-5404	¡Ý60	Post	Ductal/NST	G1/G2	I+II	74.73333333	1
MB-5406	¡Ý60	Post	Other	NA	I+II	97.43333333	1
MB-5407	<60	Pre	Ductal/NST	G3	I+II	205.6	0
MB-5410	¡Ý60	Post	Ductal/NST	G3	I+II	202.7666667	0
MB-5412	<60	Post	Ductal/NST	G1/G2	I+II	220.2333333	0
MB-5414	¡Ý60	Post	Ductal/NST	G3	I+II	140.6	1
MB-5418	¡Ý60	Post	Ductal/NST	G3	I+II	207.1666667	0
MB-5422	<60	Pre	Ductal/NST	G1/G2	I+II	46.06666667	1
MB-5424	¡Ý60	Post	Other	G1/G2	I+II	83.53333333	1
MB-5425	¡Ý60	Post	Ductal/NST	G1/G2	I+II	49.46666667	1
MB-5428	<60	Post	Other	G1/G2	I+II	150.4666667	0
MB-5429	¡Ý60	Post	Ductal/NST	G1/G2	I+II	30.7	1
MB-5432	¡Ý60	Post	Ductal/NST	G1/G2	I+II	98.5	1
MB-5433	¡Ý60	Post	Ductal/NST	G1/G2	I+II	176.3666667	1
MB-5434	¡Ý60	Post	Ductal/NST	G3	III+IV	45.16666667	1
MB-5435	¡Ý60	Post	Ductal/NST	NA	I+II	104	1
MB-5444	<60	Pre	Other	G1/G2	NA	240.4333333	0
MB-5447	¡Ý60	Post	Ductal/NST	NA	III+IV	148.8666667	1
MB-5451	<60	Pre	Other	G1/G2	I+II	189.9	0
MB-5452	¡Ý60	Post	Ductal/NST	G1/G2	I+II	16.16666667	1
MB-5454	<60	Post	Ductal/NST	G3	I+II	50.66666667	1
MB-5455	¡Ý60	Post	Other	G1/G2	I+II	61.8	1
MB-5457	<60	Post	Ductal/NST	G1/G2	I+II	164.5666667	1
MB-5459	¡Ý60	Post	Ductal/NST	G3	I+II	90.8	1
MB-5463	¡Ý60	Post	Other	NA	I+II	58.13333333	1
MB-5464	¡Ý60	Post	Ductal/NST	NA	I+II	116.5333333	1
MB-5471	¡Ý60	Post	Ductal/NST	G3	I+II	185.7666667	0
MB-5472	¡Ý60	Post	Ductal/NST	G1/G2	NA	19.56666667	1
MB-5473	¡Ý60	Post	Ductal/NST	G3	I+II	125.9	1
MB-5475	¡Ý60	Post	Ductal/NST	G1/G2	I+II	2.533333333	1

MB-5477	<60	Post	Other	G1/G2	I+II	110.4666667	0
MB-5478	¡Ý60	Post	Other	G3	III+IV	34.3	1
MB-5481	<60	Post	Ductal/NST	G3	I+II	46.36666667	1
MB-5484	<60	Pre	Other	G1/G2	I+II	238.5	0
MB-5485	¡Ý60	Post	Ductal/NST	G3	I+II	31.8	1
MB-5486	¡Ý60	Post	Ductal/NST	G3	I+II	123.3	1
MB-5489	<60	Post	Ductal/NST	G1/G2	I+II	216.9666667	0
MB-5490	¡Ý60	Post	Ductal/NST	G1/G2	I+II	51.2	1
MB-5491	¡Ý60	Post	Ductal/NST	G3	I+II	65.83333333	1
MB-5492	¡Ý60	Post	Other	G3	NA	23.36666667	1
MB-5493	¡Ý60	Post	Other	G3	NA	57.23333333	1
MB-5495	<60	Pre	Ductal/NST	G1/G2	NA	228.8	0
MB-5497	¡Ý60	Post	Ductal/NST	G1/G2	I+II	237.5	0
MB-5499	¡Ý60	Post	Other	G1/G2	I+II	123.7333333	0
MB-5502	<60	Post	Ductal/NST	G1/G2	I+II	189.7333333	0
MB-5505	¡Ý60	Post	Other	G1/G2	I+II	164.6	1
MB-5510	¡Ý60	Post	Other	G3	NA	103.1	1
MB-5514	¡Ý60	Post	Ductal/NST	G1/G2	I+II	182.9	0
MB-5518	¡Ý60	Post	Ductal/NST	G3	I+II	30.86666667	1
MB-5519	¡Ý60	Post	Ductal/NST	G1/G2	NA	169.2333333	1
MB-5520	<60	Pre	Other	G3	I+II	16.3	1
MB-5521	¡Ý60	Post	Ductal/NST	G3	I+II	124.8	0
MB-5525	¡Ý60	Post	Other	G3	I+II	2	0
MB-5532	¡Ý60	Post	Ductal/NST	G1/G2	I+II	79.86666667	1
MB-5540	¡Ý60	Post	Other	G3	NA	48.43333333	1
MB-5541	¡Ý60	Post	Ductal/NST	G1/G2	I+II	200.1333333	0
MB-5543	¡Ý60	Post	Ductal/NST	G3	I+II	34.43333333	1
MB-5550	¡Ý60	Post	Ductal/NST	G1/G2	I+II	171.3	1
MB-5552	¡Ý60	Post	Ductal/NST	NA	NA	34.7	1
MB-5553	¡Ý60	Post	Ductal/NST	G1/G2	I+II	154.5	1
MB-5554	<60	Post	Other	G1/G2	I+II	229.8333333	0
MB-5556	<60	Post	Ductal/NST	G3	I+II	225.4	0
MB-5562	¡Ý60	Post	Ductal/NST	G1/G2	I+II	102.9666667	1
MB-5563	¡Ý60	Post	Other	G1/G2	I+II	117.6666667	1
MB-5567	¡Ý60	Post	Other	G1/G2	I+II	178.5666667	1
MB-5571	¡Ý60	Post	Ductal/NST	G1/G2	I+II	153.8666667	1
MB-5575	<60	Post	Ductal/NST	G1/G2	I+II	117.6666667	1
MB-5576	¡Ý60	Post	Other	G3	I+II	194	0
MB-5579	¡Ý60	Post	Other	G3	I+II	90.6	1
MB-5580	¡Ý60	Post	Ductal/NST	G1/G2	I+II	71.63333333	1
MB-5582	<60	Pre	Other	G1/G2	I+II	222.3333333	0
MB-5583	<60	Post	Ductal/NST	G1/G2	I+II	111.3666667	0
MB-5589	¡Ý60	Post	Ductal/NST	G1/G2	NA	185.1333333	0
MB-5590	¡Ý60	Post	Ductal/NST	G3	I+II	17.83333333	0
MB-5591	<60	Pre	Ductal/NST	G1/G2	NA	155.3666667	1
MB-5592	¡Ý60	Post	Other	G1/G2	NA	26.33333333	1
MB-5596	<60	Post	Other	G1/G2	NA	167.9333333	1
MB-5597	¡Ý60	Post	Ductal/NST	G1/G2	I+II	32.73333333	1
MB-5599	¡Ý60	Post	Ductal/NST	G1/G2	NA	224.8666667	0
MB-5601	¡Ý60	Post	Ductal/NST	G1/G2	NA	214.8	1
MB-5603	¡Ý60	Post	Ductal/NST	G1/G2	NA	171.6333333	1
MB-5604	¡Ý60	Post	Ductal/NST	G1/G2	I+II	145.6333333	0
MB-5605	<60	Pre	Ductal/NST	G1/G2	I+II	123.7	0

MB-5613	¡Ý60	Post	Ductal/NST	G1/G2	III+IV	212.3666667	1
MB-5614	¡Ý60	Post	Ductal/NST	G3	I+II	116.4333333	1
MB-5617	¡Ý60	Post	Ductal/NST	G1/G2	I+II	128.2	1
MB-5620	¡Ý60	Post	Ductal/NST	G1/G2	NA	42.06666667	1
MB-5622	¡Ý60	Post	Ductal/NST	G3	I+II	182.9333333	0
MB-5623	<60	Pre	Ductal/NST	G1/G2	I+II	137.8	1
MB-5626	¡Ý60	Post	Ductal/NST	G1/G2	I+II	82.96666667	0
MB-5628	¡Ý60	Post	Ductal/NST	G3	NA	42.6	1
MB-5629	¡Ý60	Post	Ductal/NST	G1/G2	NA	103.8	1
MB-5632	¡Ý60	Post	Ductal/NST	G1/G2	I+II	38.16666667	1
MB-5635	<60	Post	Ductal/NST	G1/G2	I+II	21.06666667	1
MB-5636	¡Ý60	Post	Ductal/NST	NA	NA	108.3	1
MB-5638	¡Ý60	Post	Ductal/NST	G1/G2	I+II	195.7	1
MB-5641	¡Ý60	Post	Ductal/NST	G3	I+II	181.8666667	0
MB-5642	¡Ý60	Post	Ductal/NST	G1/G2	III+IV	156.8	1
MB-5645	¡Ý60	Post	Ductal/NST	G1/G2	I+II	22.23333333	1
MB-5646	<60	Post	Other	G3	I+II	167.1	1
MB-5647	¡Ý60	Post	Ductal/NST	G3	I+II	30.8	1
MB-5653	¡Ý60	Post	Ductal/NST	G3	I+II	194.6	0
MB-5654	¡Ý60	Post	Ductal/NST	G1/G2	NA	173.0333333	1
MB-6001	¡Ý60	Post	Other	G1/G2	NA	135.3	1
MB-6006	¡Ý60	Post	Other	G1/G2	I+II	63.56666667	1
MB-6008	¡Ý60	Post	Other	G3	NA	69.1	1
MB-6010	¡Ý60	Post	Ductal/NST	G3	NA	218.2333333	0
MB-6011	<60	Post	Ductal/NST	NA	NA	211.1333333	1
MB-6012	¡Ý60	Post	Other	G1/G2	NA	144.4333333	1
MB-6016	¡Ý60	Post	Ductal/NST	G1/G2	NA	143.5333333	1
MB-6017	¡Ý60	Post	Ductal/NST	G1/G2	NA	70.9	1
MB-6018	¡Ý60	Post	Ductal/NST	G1/G2	NA	143.1666667	1
MB-6021	<60	Post	Other	G1/G2	NA	301.2333333	0
MB-6022	¡Ý60	Post	Ductal/NST	G1/G2	NA	52.06666667	1
MB-6024	¡Ý60	Post	Ductal/NST	G1/G2	NA	252.2666667	1
MB-6026	¡Ý60	Post	Ductal/NST	G1/G2	NA	49.73333333	1
MB-6029	¡Ý60	Post	Other	G3	NA	278.3666667	1
MB-6030	¡Ý60	Post	Ductal/NST	G1/G2	NA	145.3666667	1
MB-6050	<60	Post	Ductal/NST	G1/G2	NA	192.2	0
MB-6051	¡Ý60	Post	Ductal/NST	G1/G2	NA	186.8333333	1
MB-6053	¡Ý60	Post	Ductal/NST	G1/G2	NA	250.8	0
MB-6060	<60	Post	Ductal/NST	G3	NA	38.03333333	1
MB-6065	¡Ý60	Post	Ductal/NST	G1/G2	NA	188.5333333	1
MB-6069	¡Ý60	Post	Ductal/NST	G1/G2	NA	258.1333333	1
MB-6071	¡Ý60	Post	Ductal/NST	G1/G2	NA	57.4	1
MB-6075	¡Ý60	Post	Ductal/NST	G1/G2	NA	182.3333333	1
MB-6079	¡Ý60	Post	Ductal/NST	G1/G2	NA	239.1666667	1
MB-6080	¡Ý60	Post	Other	G3	III+IV	54.1	1
MB-6083	¡Ý60	Post	Ductal/NST	G1/G2	NA	164.0333333	1
MB-6097	¡Ý60	Post	Ductal/NST	G1/G2	NA	159.7	1
MB-6108	<60	Pre	Ductal/NST	G1/G2	I+II	193.9666667	0
MB-6118	<60	Pre	Ductal/NST	G1/G2	I+II	264.6	0
MB-6124	¡Ý60	Post	Ductal/NST	G3	NA	115.6333333	1
MB-6133	¡Ý60	Post	Other	G1/G2	NA	61.8	1
MB-6135	¡Ý60	Post	Ductal/NST	G1/G2	NA	78.6	1
MB-6138	¡Ý60	Post	Other	G3	I+II	25.63333333	1

MB-6141	¡Ý60	Post	Other	G3	NA	78.86666667	1
MB-6145	¡Ý60	Post	Other	G1/G2	NA	34.33333333	1
MB-6149	¡Ý60	Post	Ductal/NST	G1/G2	NA	74.93333333	1
MB-6150	<60	Post	Ductal/NST	G1/G2	NA	77.66666667	1
MB-6154	¡Ý60	Post	Ductal/NST	G1/G2	NA	195.3	0
MB-6163	¡Ý60	Post	Other	G1/G2	NA	88.66666667	1
MB-6164	¡Ý60	Post	Ductal/NST	G1/G2	NA	205.9	0
MB-6167	¡Ý60	Post	Other	G1/G2	NA	122.8	1
MB-6168	¡Ý60	Post	Other	G1/G2	NA	225.7	1
MB-6171	¡Ý60	Post	Ductal/NST	G1/G2	NA	220.3	0
MB-6179	<60	Post	Other	G1/G2	NA	58.46666667	1
MB-6181	¡Ý60	Post	Ductal/NST	G1/G2	NA	54.93333333	1
MB-6183	¡Ý60	Post	Ductal/NST	G1/G2	I+II	79.13333333	1
MB-6185	¡Ý60	Post	Other	G1/G2	NA	197.7333333	1
MB-6189	<60	Post	Other	G1/G2	I+II	240.2	0
MB-6190	¡Ý60	Post	Ductal/NST	G1/G2	NA	24.63333333	1
MB-6192	¡Ý60	Post	Ductal/NST	G1/G2	NA	42.63333333	1
MB-6195	¡Ý60	Post	Other	G1/G2	NA	202.2333333	0
MB-6200	¡Ý60	Post	Ductal/NST	G3	NA	30.06666667	1
MB-6201	¡Ý60	Post	Ductal/NST	G1/G2	I+II	222.7	1
MB-6207	¡Ý60	Post	Ductal/NST	G1/G2	NA	118.3	1
MB-6211	¡Ý60	Post	Ductal/NST	G1/G2	NA	42.3	1
MB-6212	¡Ý60	Post	Other	G1/G2	I+II	224.2333333	0
MB-6214	¡Ý60	Post	Ductal/NST	G1/G2	NA	174.5	1
MB-6217	<60	Pre	Other	G1/G2	NA	211.5333333	1
MB-6218	<60	Pre	Ductal/NST	G3	I+II	147.7666667	1
MB-6225	¡Ý60	Post	Ductal/NST	G1/G2	I+II	117.8666667	0
MB-6232	¡Ý60	Post	Other	G1/G2	I+II	85	1
MB-6233	¡Ý60	Post	Other	G1/G2	I+II	201.1666667	0
MB-6238	¡Ý60	Post	Other	G1/G2	NA	172.9666667	1
MB-6253	¡Ý60	Post	Other	G1/G2	NA	194.3666667	0
MB-6254	¡Ý60	Post	Other	G1/G2	NA	60.9	1
MB-6256	¡Ý60	Post	Ductal/NST	G1/G2	NA	127.8333333	1
MB-6257	<60	Pre	Ductal/NST	G3	NA	91	0
MB-6263	¡Ý60	Post	Ductal/NST	G1/G2	NA	75.86666667	0
MB-6271	¡Ý60	Post	Ductal/NST	G1/G2	NA	157.7333333	0
MB-6273	<60	Pre	Other	G1/G2	NA	189.4333333	0
MB-6281	¡Ý60	Post	Ductal/NST	G1/G2	NA	3.5	1
MB-6283	¡Ý60	Post	Other	G3	NA	92.4	1
MB-6284	¡Ý60	Post	Other	G1/G2	NA	88.33333333	1
MB-6286	¡Ý60	Post	Ductal/NST	G1/G2	NA	49.53333333	1
MB-6287	<60	Post	Ductal/NST	G3	NA	35.2	1
MB-6288	¡Ý60	Post	Ductal/NST	G3	NA	100.3	1
MB-6297	<60	Post	Ductal/NST	G1/G2	NA	170.8666667	0
MB-6300	¡Ý60	Post	Ductal/NST	G1/G2	NA	63.83333333	1
MB-6302	¡Ý60	Post	Ductal/NST	G3	NA	125.8666667	1
MB-6306	¡Ý60	Post	Ductal/NST	G3	NA	64.6	1
MB-6308	¡Ý60	Post	Ductal/NST	G1/G2	NA	151.9333333	1
MB-6312	¡Ý60	Post	Ductal/NST	G3	NA	162.7666667	0
MB-6317	¡Ý60	Post	Ductal/NST	G3	NA	29.23333333	1
MB-6322	¡Ý60	Post	Other	G1/G2	NA	80.36666667	1
MB-6327	¡Ý60	Post	Other	G1/G2	NA	18.26666667	1
MB-6328	¡Ý60	Post	Ductal/NST	G1/G2	NA	126.6666667	0

MB-6329	¡Ý60	Post	Ductal/NST	G3	NA	81.5	1
MB-6344	¡Ý60	Post	Ductal/NST	G1/G2	NA	152.9333333	1
MB-6346	¡Ý60	Post	Other	G1/G2	I+II	281.5	0
MB-7000	¡Ý60	Post	Other	G1/G2	NA	97.4	0
MB-7001	¡Ý60	Post	Ductal/NST	G1/G2	NA	102.7	0
MB-7002	¡Ý60	Post	Ductal/NST	G1/G2	NA	57.93333333	0
MB-7004	¡Ý60	Post	Other	G1/G2	NA	50.46666667	0
MB-7005	¡Ý60	Post	Ductal/NST	G1/G2	NA	45.33333333	0
MB-7006	<60	Post	Ductal/NST	G1/G2	NA	109.9333333	0
MB-7010	¡Ý60	Post	Ductal/NST	G1/G2	NA	74.03333333	0
MB-7011	¡Ý60	Post	Other	G3	NA	70.06666667	1
MB-7013	<60	Pre	Ductal/NST	G1/G2	NA	46.43333333	0
MB-7015	¡Ý60	Post	Ductal/NST	G3	NA	78.16666667	0
MB-7019	¡Ý60	Post	Ductal/NST	G1/G2	NA	80.73333333	1
MB-7022	¡Ý60	Post	Ductal/NST	G1/G2	NA	85.5	0
MB-7024	<60	Post	Ductal/NST	G3	NA	70.26666667	0
MB-7026	<60	Pre	Ductal/NST	G1/G2	NA	111.7	1
MB-7028	¡Ý60	Post	Ductal/NST	G1/G2	NA	89.8	0
MB-7029	<60	Pre	Ductal/NST	G1/G2	NA	76.86666667	0
MB-7032	¡Ý60	Post	NA	G1/G2	NA	71.46666667	0
MB-7034	¡Ý60	Post	Ductal/NST	G1/G2	NA	75.1	0
MB-7037	¡Ý60	Post	Ductal/NST	G1/G2	NA	86.4	0
MB-7040	¡Ý60	Post	Ductal/NST	G1/G2	NA	71.83333333	0
MB-7041	¡Ý60	Post	Other	NA	NA	101.9666667	1
MB-7042	¡Ý60	Post	Ductal/NST	G1/G2	NA	88.5	0
MB-7053	<60	Pre	Ductal/NST	G3	NA	122.1333333	0
MB-7056	¡Ý60	Post	Ductal/NST	G1/G2	NA	117	0
MB-7060	¡Ý60	Post	Other	G1/G2	NA	112.6666667	1
MB-7062	¡Ý60	Post	Ductal/NST	G1/G2	NA	95.86666667	0
MB-7063	¡Ý60	Post	Ductal/NST	G3	NA	86.6	0
MB-7065	<60	Pre	Ductal/NST	G1/G2	NA	107.8666667	0
MB-7072	¡Ý60	Post	Other	G1/G2	NA	52.33333333	1
MB-7075	¡Ý60	Post	Other	G1/G2	NA	111.6333333	0
MB-7076	<60	Post	Ductal/NST	G1/G2	NA	142.6666667	1
MB-7077	<60	Pre	Ductal/NST	G1/G2	NA	101.2666667	0
MB-7080	¡Ý60	Post	Ductal/NST	G1/G2	NA	91.23333333	0
MB-7083	<60	Post	Ductal/NST	G1/G2	NA	105.4666667	0
MB-7086	¡Ý60	Post	Other	G1/G2	NA	123.5333333	0
MB-7091	<60	Post	Ductal/NST	G1/G2	NA	110.2666667	0
MB-7093	¡Ý60	Post	Ductal/NST	G1/G2	NA	125.8333333	1
MB-7094	¡Ý60	Post	Ductal/NST	G3	NA	113.3666667	0
MB-7095	¡Ý60	Post	Ductal/NST	G1/G2	NA	115.6333333	0
MB-7096	<60	Pre	Ductal/NST	G1/G2	NA	124	0
MB-7097	¡Ý60	Post	Ductal/NST	NA	NA	65.16666667	1
MB-7099	¡Ý60	Post	Ductal/NST	G1/G2	NA	163.1666667	1
MB-7100	¡Ý60	Post	Ductal/NST	G3	NA	156.3333333	1
MB-7101	<60	Pre	Other	G1/G2	NA	124.2666667	0
MB-7102	¡Ý60	Post	Other	G1/G2	NA	104.1	1
MB-7106	¡Ý60	Post	Ductal/NST	G1/G2	NA	37.5	1
MB-7107	¡Ý60	Post	Ductal/NST	G3	NA	56.93333333	1
MB-7109	¡Ý60	Post	Ductal/NST	G3	NA	117.5333333	0
MB-7112	<60	Pre	Ductal/NST	G3	NA	64.7	1
MB-7113	¡Ý60	Post	Ductal/NST	G1/G2	NA	136.7	1

MB-7116	¡Ý60	Post	Ductal/NST	G1/G2	NA	33.36666667	1
MB-7118	¡Ý60	Post	Ductal/NST	G1/G2	NA	140.5666667	0
MB-7123	<60	Post	Ductal/NST	G1/G2	NA	137.6666667	0
MB-7124	¡Ý60	Post	Ductal/NST	G1/G2	NA	139.6	0
MB-7128	¡Ý60	Post	NA	G1/G2	NA	60.26666667	1
MB-7130	¡Ý60	Post	Ductal/NST	G3	NA	102.9666667	1
MB-7131	<60	Post	Ductal/NST	G3	NA	134.7333333	0
MB-7132	¡Ý60	Post	Ductal/NST	G1/G2	NA	131.6	0
MB-7133	¡Ý60	Post	Other	G3	NA	23.83333333	1
MB-7137	¡Ý60	Post	Ductal/NST	NA	NA	179.8	0
MB-7138	¡Ý60	Post	Ductal/NST	G3	NA	123	1
MB-7140	<60	Post	Ductal/NST	G3	NA	90.8	1
MB-7141	¡Ý60	Post	Ductal/NST	G1/G2	NA	138.5666667	0
MB-7142	<60	Post	Ductal/NST	G1/G2	NA	175.9	1
MB-7144	<60	Post	Ductal/NST	G3	NA	102.6333333	1
MB-7147	¡Ý60	Post	Other	G3	NA	168.6	1
MB-7149	¡Ý60	Post	Ductal/NST	G3	NA	44.63333333	1
MB-7150	¡Ý60	Post	Ductal/NST	G3	NA	128.9666667	0
MB-7157	<60	Post	Ductal/NST	G1/G2	NA	219.6333333	0
MB-7161	<60	Pre	Ductal/NST	G1/G2	NA	121.5333333	0
MB-7162	<60	Post	Ductal/NST	G1/G2	NA	140.2	0
MB-7163	<60	Pre	Ductal/NST	G1/G2	NA	52.63333333	1
MB-7164	<60	Pre	Ductal/NST	G1/G2	NA	211.4	0
MB-7168	¡Ý60	Post	Ductal/NST	G3	NA	150.5	0
MB-7170	<60	Post	Ductal/NST	G1/G2	NA	155.4	0
MB-7171	¡Ý60	Post	Ductal/NST	G3	NA	160.4	1
MB-7172	¡Ý60	Post	Ductal/NST	G1/G2	NA	149.3	0
MB-7173	¡Ý60	Post	Other	G1/G2	NA	63.53333333	1
MB-7174	¡Ý60	Post	Ductal/NST	G3	NA	140.7666667	0
MB-7176	<60	Post	Ductal/NST	G1/G2	NA	194.4	1
MB-7181	¡Ý60	Post	Ductal/NST	G3	NA	157.5333333	0
MB-7185	¡Ý60	Post	Ductal/NST	G1/G2	NA	125.0333333	1
MB-7186	<60	Pre	Ductal/NST	G1/G2	NA	4.866666667	0
MB-7189	<60	Post	Ductal/NST	G1/G2	NA	143.6	1
MB-7193	¡Ý60	Post	Ductal/NST	G3	NA	87.46666667	1
MB-7194	¡Ý60	Post	Ductal/NST	G1/G2	NA	46.66666667	1
MB-7195	¡Ý60	Post	Other	G1/G2	NA	80.43333333	1
MB-7197	¡Ý60	Post	Ductal/NST	G1/G2	NA	187.8666667	1
MB-7199	¡Ý60	Post	Ductal/NST	G1/G2	NA	76	1
MB-7200	¡Ý60	Post	Ductal/NST	G3	NA	97.6	1
MB-7212	¡Ý60	Post	Other	G3	NA	52.5	1
MB-7216	¡Ý60	Post	Other	G1/G2	NA	207.4666667	1
MB-7217	¡Ý60	Post	Ductal/NST	NA	NA	90.4	1
MB-7218	<60	Post	Other	G1/G2	NA	165.4333333	0
MB-7219	¡Ý60	Post	Ductal/NST	G1/G2	NA	165.2	0
MB-7220	¡Ý60	Post	Ductal/NST	G1/G2	NA	183.2	1
MB-7226	¡Ý60	Post	Ductal/NST	G3	NA	53.36666667	1
MB-7229	¡Ý60	Post	Other	G3	NA	111.6	1
MB-7230	<60	Post	Ductal/NST	G1/G2	NA	182.6	0
MB-7231	¡Ý60	Post	Ductal/NST	NA	NA	199.0333333	1
MB-7232	<60	Post	Ductal/NST	G1/G2	NA	177.6	0
MB-7234	¡Ý60	Post	Ductal/NST	G3	NA	222.2	0
MB-7235	¡Ý60	Post	Other	G1/G2	NA	170.8	0

MB-7236	<60	Post	NA	G1/G2	NA	201.4666667	1
MB-7241	<60	Pre	Ductal/NST	G1/G2	NA	171.1	0
MB-7243	¡Ý60	Post	Ductal/NST	G1/G2	NA	148.8	1
MB-7244	<60	Post	Ductal/NST	G1/G2	NA	184.1666667	0
MB-7249	¡Ý60	Post	Ductal/NST	NA	NA	58.66666667	1
MB-7253	¡Ý60	Post	Ductal/NST	G1/G2	NA	59.96666667	1
MB-7254	¡Ý60	Post	Ductal/NST	G1/G2	NA	192.2	0
MB-7263	¡Ý60	Post	Ductal/NST	G1/G2	NA	164.9666667	1
MB-7266	<60	Post	Ductal/NST	G1/G2	NA	68.16666667	1
MB-7268	<60	Pre	Ductal/NST	G3	NA	227.4666667	0
MB-7276	¡Ý60	Post	Ductal/NST	G1/G2	NA	185.3333333	0
MB-7277	<60	Post	Ductal/NST	G1/G2	NA	187.0333333	0
MB-7278	¡Ý60	Post	Ductal/NST	G1/G2	NA	241.3	1
MB-7280	¡Ý60	Post	Ductal/NST	G1/G2	NA	195.5333333	0
MB-7283	<60	Post	Other	G1/G2	NA	28.86666667	1
MB-7284	¡Ý60	Post	Ductal/NST	G3	NA	203	1
MB-7286	¡Ý60	Post	Ductal/NST	G3	NA	158.6333333	1
MB-7287	¡Ý60	Post	Ductal/NST	G3	NA	96.96666667	1
MB-7288	<60	Post	Ductal/NST	G1/G2	NA	27.06666667	1
MB-7292	<60	Post	Ductal/NST	G1/G2	NA	78.46666667	1
MB-7293	<60	Post	Ductal/NST	G3	NA	199.2333333	0
MB-7294	<60	Post	Ductal/NST	G1/G2	NA	82.73333333	1
MB-7295	<60	Pre	Other	G3	NA	196.8666667	0
MB-7296	<60	Pre	Ductal/NST	G3	NA	44.73333333	1
MB-7297	¡Ý60	Post	Ductal/NST	G3	NA	175.9666667	1
MB-7298	¡Ý60	Post	Ductal/NST	G1/G2	NA	86.23333333	1
MB-7299	¡Ý60	Post	Ductal/NST	G3	NA	201.9	1

risk	riskScore
low	0.215513142
low	-0.324869899
low	0.06871558
high	0.255192433
high	0.337023586
low	-0.602452684
high	0.581361868
low	-0.363697171
low	-0.593550369
low	0.08821305
low	-0.177627983
low	-0.554901661
low	-0.310534508
high	0.347096596
low	-0.123148378
low	0.090771502
low	-0.222285992
low	-0.274394506
low	-0.390219092
high	0.25007311
high	0.256262279
low	0.200499749
low	-0.448539257
high	0.66500429
low	-0.373781709
low	-0.146293455
low	0.187079087
low	-0.233532406
high	0.592688002
high	0.225050785
low	0.01547975
low	-0.203852552
low	0.13898698
low	-0.466170891
low	-0.082399852
low	-0.150189838
low	-0.060117472
low	0.069216837
low	0.134964435
low	-0.200707251
low	0.199213704
low	-0.385029
low	-0.304544935
low	-0.393112092
low	0.2025468
low	0.169529707
high	0.363308324
low	-0.046149928
low	-0.540188761
high	0.627361674
low	0.113299479
low	0.105277967

low	-0.415422994
low	-0.285197398
low	-0.243269236
low	-0.270685127
high	0.38834753
low	-0.441871347
low	-0.228758156
high	0.321023634
low	0.005265334
low	-0.105026307
low	-0.332706636
high	0.544033434
low	-0.176825122
low	-0.27568339
low	-0.321763213
low	-0.164324955
low	-0.590474705
high	0.59791697
high	0.22615967
low	-0.222937458
low	-0.225777111
high	0.263004949
low	0.049737209
low	-0.564606506
low	-0.234273667
low	0.17658808
low	-0.014813735
low	-0.07900191
low	-0.408465406
high	0.479925381
low	-0.090568654
low	-0.614752901
low	-0.002971679
low	-0.297314939
low	0.012921226
low	-0.520070127
low	0.125490629
low	-0.21120537
low	-0.096422132
low	-0.483690208
low	-0.158492022
low	-0.395884804
low	-0.200280878
low	-0.611953271
low	0.171950269
low	-0.424876908
low	0.21827591
low	0.064535869
low	-0.326646394
low	0.112346292
low	-0.311276171
low	-0.349230726
low	0.205644965

high	0.430709918
low	-0.343670899
low	-0.0533409
low	0.010573388
low	-0.192357502
high	0.235902837
low	-0.179686307
low	-0.211055947
low	-0.363732579
high	0.85195699
low	0.115457809
low	-0.26986722
low	-0.258675949
low	-0.37476165
low	0.073170892
low	0.072199672
high	0.555621927
low	-0.082938611
low	-0.185448145
low	0.134463013
low	-0.59251592
high	0.388744919
high	0.366657515
low	0.13608446
low	0.066087024
low	0.158118893
low	0.166654323
low	-0.244235026
low	-0.084625298
low	-0.159409651
low	-0.090932674
high	0.254861525
low	0.049435078
low	-0.156249463
low	0.000406194
low	-0.215301117
high	0.31910616
low	0.160605414
low	0.184245782
low	-0.289997202
low	0.091172527
high	0.643892771
high	0.36593946
high	0.604471021
low	0.207924459
high	0.37227441
low	0.071927722
low	-0.875151655
low	-0.114949857
high	0.638007974
low	-0.224192677
low	-0.259275521
low	0.02726937

high	0.482871936
low	-0.165848298
high	0.247282448
low	-0.295738282
low	-0.342405651
low	0.036066175
high	0.273220739
low	-0.131211918
low	-0.72287825
low	-0.105172897
high	0.435901785
low	-0.085002517
low	0.208113704
low	0.056796246
low	-0.248755636
low	-0.075271576
low	-0.124738533
high	0.587460963
low	-0.154574377
high	0.708268672
low	0.19069471
low	0.215638744
low	0.124327325
low	-0.037832203
low	-0.183721612
low	-0.114785425
low	0.112628204
low	0.04564434
low	-0.072899054
low	0.127789013
low	0.2115233
low	-0.144657683
high	0.382531319
low	-0.191292873
high	0.269377861
high	0.556574454
low	0.059300011
high	0.368537917
high	0.431804637
low	0.012966825
low	0.013301401
high	0.317560513
low	-0.030089639
low	-0.114445149
low	-0.266731169
high	0.363604752
low	0.054398417
low	-0.281138639
low	-0.235687338
low	-0.076255342
low	-0.423378064
low	-0.611482015
low	-0.309689784

low	-0.317894106
low	-0.281013077
low	0.027190875
low	0.206640517
low	0.138005619
low	-0.361774669
high	0.335141157
low	-0.024848199
low	0.042981118
high	0.289232858
low	0.090918611
low	0.090453662
low	0.00492016
low	-0.055508706
low	-0.035506665
low	0.161204751
low	-0.056901415
low	-0.10330695
low	-0.114480729
low	-0.359084073
low	0.060602374
low	0.191106051
low	-0.183187316
high	0.299569649
low	-0.102308588
low	-0.059502602
low	0.018858603
low	-0.12690454
low	0.160439593
low	-0.011243037
low	0.103974519
low	-0.003300328
low	0.038540142
high	0.586756022
low	-0.009099432
high	0.970194307
low	0.015463532
low	0.01586268
low	-0.310135723
low	0.11836678
low	-0.491698542
low	-0.012636574
low	0.070230806
low	0.129678985
low	-0.452851921
low	-0.1285299
low	-0.055768883
low	0.176520912
high	0.419976739
low	-0.000257823
low	-0.072180852
low	-0.155671687
low	-0.494948212

low	-0.880104776
high	0.660268719
high	0.467136927
low	-0.490945294
low	-0.597227214
low	-0.195237591
high	0.395217515
low	0.071166472
low	-0.12935962
low	-0.251636164
low	-0.028111489
low	-0.048975898
low	-0.090167048
high	0.746256019
high	0.56516799
low	-0.493810582
high	0.440051593
high	0.466917325
low	-0.614367681
low	-0.240761747
low	-0.431748994
low	-0.341937321
low	0.082941814
low	-0.848133094
low	-0.418816515
low	-0.505302911
high	0.43653394
low	-0.277730033
low	-0.11820523
low	0.085840798
low	-0.859967334
low	-0.024792439
low	-0.265289935
low	0.152287113
high	0.225118855
low	-0.117680083
high	0.53239619
high	0.23000342
low	-0.32591054
low	-0.290413697
low	-0.374927204
low	-0.014153437
low	-0.653206712
low	0.078250187
high	0.471185551
low	0.119842972
low	0.105773666
low	-0.2956365
low	-0.56214726
low	-0.271067348
low	-0.13878169
low	-0.125959229
low	-0.093005714

low	0.121545078
low	-0.077940324
low	-0.431097933
low	-0.328928741
low	-0.39998237
low	0.075626829
high	0.306672708
low	-0.012341049
low	-0.16559137
low	-0.227851948
low	-0.297350292
low	0.015867251
low	-0.281531889
low	-0.124359068
low	-0.068553078
low	-0.199030375
low	-0.37901268
low	-0.233139348
low	-0.392173461
low	-0.208977681
low	-0.360381102
high	0.295704239
low	-0.398707363
low	-0.649295714
low	-0.252567968
low	0.12509319
low	-0.025372514
low	-0.05348305
low	-0.582305057
high	0.332461411
low	-0.384869001
low	-0.214342379
low	0.171729502
low	-0.277062212
low	0.213671005
low	-0.179046549
low	-0.136744274
low	0.078807484
low	-0.619776714
low	-0.548828539
low	0.002702116
low	-0.059853571
low	0.12731179
low	-0.437063023
low	-0.455908748
low	-0.595166209
low	-0.399562342
low	-0.667622072
low	-0.538607475
high	0.487592056
low	-0.033754354
low	-0.165164579
low	-0.337897574

low	-0.35971546
high	0.34364689
low	-0.205864963
low	-0.286347602
low	0.037975713
low	-0.713936602
low	0.122255626
low	-0.210119154
low	0.156709573
low	-0.054609954
low	-0.717562083
low	-0.386397632
low	-0.273501048
low	-0.213383448
low	-0.069891924
low	0.195500411
high	0.405013445
low	-0.425239796
low	0.05862014
high	0.364263659
low	-0.382381539
high	0.258211504
low	0.101336916
low	0.016599636
low	-0.048460737
low	-0.297373415
low	-0.508338747
low	-0.235940114
high	0.439240243
low	-0.494351562
high	0.4596483
low	-0.450837015
low	-0.340101078
low	0.090944863
low	-0.583522311
low	0.093373327
low	-0.534882727
low	-0.337442478
high	0.41083864
low	-0.06992757
low	-0.635231444
low	-0.061624858
low	-0.222739773
low	0.013484388
low	-0.104932232
low	-0.03393425
low	-0.376660421
low	0.04971365
low	-0.002724454
high	0.735143359
low	0.122980611
low	0.214887781
low	-0.38282273

low	-0.182908354
low	-0.506699861
low	-0.063230255
low	-0.323000562
low	0.119706373
low	0.155891223
high	0.357594761
high	0.333253353
low	-0.314989061
low	-0.517746781
low	0.146271311
low	-0.242251799
high	0.262029789
low	-0.080460966
low	-0.263705115
high	0.461367025
low	-0.53598268
high	0.345166164
low	-0.353902701
low	0.188060264
low	0.084410354
low	-0.094141827
low	0.144514581
low	-0.321529438
low	0.152314431
high	0.268177123
low	-0.086209734
low	-0.134747269
low	-0.436168104
low	-0.053214092
low	-0.063538388
low	-0.151259588
high	0.306585405
low	0.056047075
low	0.102270288
low	-0.052189561
low	-0.707565131
low	0.027614895
low	-0.773418538
low	-0.29006252
low	-0.244167135
low	-0.117530422
high	0.278077451
high	0.55685875
low	0.009869804
low	0.066733915
high	0.738514317
low	-0.054280524
low	-0.015088714
low	-0.198211914
low	0.027850283
high	0.57115698
low	-0.470667505

high	0.250391733
low	-0.184121906
low	-0.180849381
low	-0.236068528
low	-0.221410156
low	0.174426778
high	0.467458675
low	-0.296719531
low	0.049266669
low	-0.092784335
low	0.167857043
high	0.467102025
low	0.161027015
low	-0.381117812
high	0.42158652
low	-0.337300384
low	0.202527074
low	-0.104045633
low	-0.557756673
high	0.320956195
low	0.056864784
low	0.172865504
low	0.119058336
low	-0.208377347
low	-0.039815338
low	-0.048781841
low	-0.141666036
high	0.327025304
low	-0.141634604
low	-0.354464879
low	0.027153564
low	-0.141109645
high	0.3253554
low	0.191739723
low	0.035939295
low	-0.363122816
low	-0.198166925
low	-0.05040839
low	0.009775088
high	0.279094241
low	-0.230536716
low	0.064552586
low	0.131880467
high	0.622550134
low	-0.163738304
low	-0.058743423
low	-0.66434038
low	-0.028948802
low	-0.001213348
high	0.27446574
low	-0.205159945
low	0.08061203
high	0.388995928

low	-0.060261507
high	0.345463039
high	0.321890488
high	0.31748317
high	0.231144562
high	0.456364044
low	-0.026094631
low	0.143771317
low	-0.082289975
low	-0.063769908
low	0.130676719
low	0.059472481
low	-0.01756822
low	-0.083508186
low	0.157161876
low	0.035717697
low	-0.016391625
low	0.167645249
low	-0.019811137
high	0.459037356
high	0.544447024
low	-0.101181211
low	0.104526917
high	0.52869897
low	-0.041596388
low	0.164847306
low	-0.465340414
low	0.179317301
high	0.249821539
low	-0.305244753
low	-0.249150007
low	-0.128679713
low	-0.07127254
low	-0.179028812
low	-0.21482311
low	-0.198493213
low	-0.041965494
high	0.269024028
low	-0.028410719
low	-0.148855055
high	0.53966182
high	0.252157687
low	-0.120594956
high	0.455404336
low	-0.054527583
low	0.054857052
low	0.061880262
low	-0.033029627
high	0.230218571
low	0.081247014
low	-0.037888133
low	-0.256342078
low	-0.07361391

low	-0.287908844
high	0.255428751
low	0.037099658
low	-0.244792639
low	-0.502537884
low	-0.291400844
low	0.168475356
low	-0.025229858
low	0.121031816
high	0.315164681
high	0.640787765
low	0.02277329
low	-0.112217255
high	0.417507888
high	0.378243588
low	0.182892939
low	-0.164370264
low	0.107253173
low	-0.401010267
low	-0.036800503
low	0.087412008
low	-0.14691379
high	0.635708235
high	0.582378437
low	0.025680125
high	0.488806169
high	0.476908599
low	-0.194545045
low	-0.153565461
low	-0.289424133
high	0.382595277
high	0.357200576
high	0.268824582
low	-0.182523454
high	0.651396154
high	0.654755884
low	0.122109683
low	0.028427403
low	-0.136017443
low	0.161778639
low	-0.131431573
low	-0.189137556
low	-0.409667998
low	0.154995314
low	-0.189846315
low	-0.444197804
low	-0.337006339
high	0.302460935
high	0.327750986
low	-0.25869702
low	0.217664786
high	0.366439345
high	0.563754452

low	0.156294693
low	0.059459036
high	1.011935981
low	-0.064599154
low	0.12552098
high	0.505141733
low	-0.414892802
low	0.120854959
low	0.061587425
low	-0.303930077
high	0.419912565
low	0.174612013
low	-0.368797884
low	0.18311833
low	0.165570403
high	0.308417218
low	-0.11776922
low	-0.384848165
low	-0.258331314
high	0.36130064
high	0.53554716
low	-0.130265557
low	-0.032625828
low	-0.586244109
low	0.033048919
low	-0.124848614
high	0.226824286
low	-0.045689405
low	0.212406391
low	-0.114058297
high	0.473914542
low	-0.337861654
high	0.335064666
low	-0.444283346
low	0.049359977
low	0.111357225
high	0.417694208
low	-0.110869378
low	-0.017804464
low	-0.23674279
high	0.457426024
low	-0.077367001
high	0.797864647
high	0.429580921
low	-0.063882259
high	0.355487782
high	0.372814212
high	0.28907425
low	0.075270579
low	-0.125788078
low	-0.037212957
low	-0.370782849
low	-0.405277011

low	-0.146669806
high	0.537465473
low	-0.416603367
low	0.040483766
low	-0.351724398
high	0.27635744
high	0.821443077
low	0.187635917
low	-0.361330271
low	0.191496828
low	-0.1730677
low	-0.578455767
low	0.065626339
low	0.008208571
high	0.582935627
low	0.094112151
high	0.412932227
low	0.076664334
low	0.165443691
low	0.105500571
low	0.214874791
high	0.429313692
low	0.043772229
low	-0.141316438
low	0.066773895
low	0.049295362
high	0.347268303
low	-0.340345761
low	0.060839078
low	-0.056736863
high	0.271554889
low	0.053829048
low	-0.058494082
high	0.256798794
low	-0.325927091
high	0.504549308
low	-0.010788161
high	0.280155823
high	0.337827739
low	0.045398974
low	-0.206278546
low	-0.521882726
low	-0.180642712
low	-0.02551198
low	-0.079822382
low	-0.260799455
high	0.23172034
high	0.249911009
high	0.353232832
low	-0.220087918
high	0.647026516
low	0.066770674
low	-0.086695217

low	0.100585834
high	0.338689746
low	-0.160215975
high	0.898386923
high	0.663558563
low	-0.133434834
low	-0.033394915
low	0.003668236
low	0.111923578
high	0.428139126
low	-0.293224859
high	0.447823402
low	0.048504222
low	-0.339906836
low	0.080667455
low	0.222892278
low	-0.04318291
low	-0.17782894
low	-0.07485393
high	0.282215973
low	0.134317765
low	-0.169089873
low	0.039917547
low	-0.240520213
low	-0.278959957
low	0.156888194
high	0.402144394
low	-0.033637334
high	0.348364744
low	-0.097313616
low	-0.319527537
low	0.06364807
low	-0.028507826
high	0.226821894
low	-0.099295724
high	0.40284025
low	-0.139247645
low	-0.252089579
low	0.030032488
low	0.106091981
low	-0.097282712
low	-0.284939827
low	0.183825401
high	0.232680288
low	-0.295048425
low	-0.049979522
low	0.133053333
low	-0.042135379
low	-0.086518401
low	0.026807991
high	0.568390127
low	0.210647286
low	-0.052539137

low	-0.040337315
low	-0.016458964
high	0.494173093
low	-0.037339521
low	-0.058895801
low	0.081885024
low	-0.012902768
low	-0.112413823
low	0.020933467
low	-0.469526733
low	0.2019676
low	0.14636655
low	0.048215723
low	-0.034612688
low	-0.161569689
low	-0.370377759
low	0.166970332
low	-0.100850782
low	-0.254542555
low	-0.267546637
low	-0.010351965
low	-0.072370381
low	0.06578399
low	-0.170469296
low	0.192577898
low	-0.136742747
high	0.248571739
low	-0.483976167
high	0.700426926
low	0.196761983
low	-0.242287294
low	0.085214066
low	-0.068020615
low	-0.369165436
low	-0.214647334
high	0.283470628
high	0.231013377
low	0.191370931
low	0.056037506
low	-0.562351428
low	0.09188176
low	-0.203247516
high	0.305457816
high	0.283718687
low	-0.040840093
low	-0.113173627
low	-0.45859195
low	0.023566655
low	0.107033966
low	0.064323982
high	0.308596521
low	0.199444082
low	-0.058497471

low	0.105149278
low	-0.166602444
low	-0.231715453
low	0.007335387
high	0.469398453
high	0.382951373
low	-0.293272062
low	-0.12245035
high	0.40081501
low	-0.259878671
high	0.66666942
low	-0.119986663
high	0.468010892
low	-0.258869817
high	0.310203315
high	0.234547907
low	0.189652374
low	-0.307020363
high	1.057056509
high	0.277642148
high	0.333389086
high	0.267964598
low	0.15906804
high	0.264188875
high	0.624533097
low	-0.555752574
low	0.190741599
high	0.307546386
high	0.55589253
low	-0.054188785
low	0.126670866
high	0.326939802
low	0.210985394
high	0.244367676
low	0.188303189
low	0.061475289
high	0.319531283
high	0.340153151
low	0.141496103
low	-0.366652759
low	-0.478234368
low	-0.158181622
low	-0.240140273
high	0.292217174
low	-0.099985632
low	0.176671283
low	-0.392111852
low	-0.21618431
low	0.117589022
low	-0.044935849
low	-0.255069148
high	0.377870883
high	0.295645978

low	0.160122252
low	-0.233776565
low	-0.163823242
low	-0.039222553
high	0.241896718
low	0.016935457
low	-0.134585183
high	0.547291726
low	0.034632055
high	0.2775906
low	0.029214621
high	0.351242033
low	-0.20760001
high	0.372510578
low	0.047322022
low	-0.252203937
high	0.335812159
high	0.493827024
low	-0.247775621
low	0.110847409
low	0.029922984
low	-0.345769143
low	0.042783566
low	-0.045960817
high	0.238489823
low	0.000978308
low	-0.488138308
low	-0.467576193
low	-0.108998631
low	-0.476720686
high	0.370448553
low	-0.384244437
high	0.282673991
low	-0.190325283
low	-0.088934602
low	-0.305343241
low	-0.493124318
low	0.099490646
high	0.343415661
low	-0.14118696
low	-0.243558436
low	0.035890176
low	0.04447553
low	-0.032300661
low	0.043960572
low	-0.133887188
low	-0.085984964
low	-0.330168269
low	-0.406452303
low	-0.21485188
low	-0.054968752
high	0.262909808
low	-0.523545937

low	0.174115791
high	0.324651249
low	0.059816246
low	-0.097755997
high	0.418163182
low	0.105966602
low	0.132121983
low	-0.147143996
low	-0.421797844
low	-0.062624331
low	0.033770001
high	0.343396743
low	0.070479998
low	-0.151373629
low	0.141770596
low	-0.096768487
high	0.399335846
low	-0.432057655
high	0.363652005
low	0.128797261
low	-0.016207866
low	0.061480334
low	-0.283640038
low	-0.221802232
high	0.784701357
low	0.102797433
low	-0.012817998
low	0.007068767
low	-0.186366989
low	-0.231477716
low	-0.148018845
low	0.078099721
low	-0.341823066
low	0.061776796
low	-0.108229205
low	0.170577793
low	-0.42626044
high	0.41398556
low	-0.274284441
low	0.10599124
low	-0.201905087
low	0.120215951
low	-0.054746953
low	0.065237758
low	-0.281945708
high	0.250232028
low	0.052637945
low	-0.111969676
high	0.248085638
high	0.385160225
low	0.098893687
low	0.008678538
low	0.137960839

high	0.550464055
high	0.228148684
low	-0.04517455
low	0.056005884
low	0.010415686
low	-0.239405934
low	-0.029918442
low	-0.153333431
low	-0.394585059
low	-0.196276955
high	0.557895386
low	-0.082316432
high	0.412888838
low	-0.059432019
low	-0.06405623
high	0.510790411
low	-0.260233433
low	0.115792804
low	-0.137531469
low	-0.061549874
high	0.253277948
low	0.179932332
low	-0.217125669
low	-0.295892768
high	0.33505631
low	0.015636464
low	-0.151278674
low	0.138159853
low	-0.077443932
low	-0.422047818
low	0.100119553
low	0.141516622
high	0.512152952
low	-0.57447689
low	-0.381982778
low	-0.178984212
low	-0.008242712
low	-0.102382591
low	-0.298451463
low	-0.592862801
high	0.408251056
low	0.081054006
low	-0.176564184
low	0.036490708
high	0.269726347
high	0.370413796
low	-0.31847226
low	-0.104451755
low	-0.579810014
high	0.332001488
low	0.132749945
high	0.386134051
low	-0.016457206

low	-0.063775909
low	-0.198778754
low	-0.111146848
low	0.177132719
low	-0.336195841
high	0.5539809
low	0.208107577
low	-0.498000114
low	-0.188724305
low	-0.338128479
low	-0.050302249
low	0.012640751
low	-0.250279804
low	-0.199147201
high	0.798964997
high	0.308051487
high	0.459183188
high	0.417512688
high	0.265443168
low	-0.216917199
low	0.025975153
low	-0.257926316
low	-0.002870928
low	-0.131106383
low	-0.171083256
low	-0.116914142
low	-0.047936177
low	0.118939304
high	0.424393132
low	0.037994852
low	0.077640489
low	0.107668694
low	-0.041805404
high	0.469107003
high	0.257178132
high	0.30483842
low	0.027138381
high	0.387464418
low	0.183459295
low	-0.157493426
low	-0.026243629
low	0.006363587
low	-0.62118636
low	-0.167362245
low	-0.091424608
low	-0.115194352
high	0.287690749
low	-0.082640472
high	0.228975443
low	0.116946066
low	-0.137846407
high	0.46686693
low	-0.125666181

low	0.15036789
low	-0.030692445
low	-0.286750685
low	-0.243322402
low	-0.177672429
low	0.052649473
low	-0.210194976
low	0.218169546
high	0.450679543
low	0.146824765
low	-0.235208979
low	0.161226449
low	-0.543334738
low	-0.194065873
high	0.87671786
low	-0.176424952
low	0.009057722
high	0.875901848
low	-0.095299565
high	0.253860743
low	-0.441474331
high	0.231820896
low	-0.205564999
high	0.831510598
low	0.185931564
low	0.11861419
low	-0.176829396
