# Supplementary material for: A ferroptosis-associated gene signature for the prediction of prognosis and therapeutic response in luminal-type breast carcinoma
Source: Sci Rep. 2021 Sep 2;11:17610. doi: 10.1038/s41598-021-97102-z (PMC8413464; doi:10.1038/s41598-021-97102-z)
Supplement: Supplementary file 10 — Supplementary Table S6. [file 41598_2021_97102_MOESM10_ESM.pdf]

TableS6 Clinical-related data with ferroptosis-related riskscore in TCGA cohort

	ID	Age	Menopausal_State	His_Subtype	Histologic_Grade	Tumor_Stage
1	TCGA-3C-AAAU	<60	Pre	Other	NA	NA
2	TCGA-3C-AALJ	¡Ý60	Post	Ductal/NST	NA	I+II
3	TCGA-3C-AALK	<60	Pre	Ductal/NST	NA	I+II
4	TCGA-4H-AAAK	<60	Post	Other	NA	III+IV
5	TCGA-5L-AAT0	<60	Post	Other	NA	I+II
6	TCGA-5L-AAT1	¡Ý60	Post	Other	NA	III+IV
7	TCGA-5T-A9QA	<60	Pre	Other	NA	I+II
8	TCGA-A1-A0SD	<60	Pre	Ductal/NST	NA	I+II
9	TCGA-A1-A0SE	<60	Pre	Other	NA	I+II
10	TCGA-A1-A0SF	<60	Pre	Ductal/NST	NA	I+II
11	TCGA-A1-A0SG	¡Ý60	Post	Other	NA	I+II
12	TCGA-A1-A0SH	<60	Pre	Ductal/NST	NA	I+II
13	TCGA-A1-A0SI	<60	Pre	Ductal/NST	NA	I+II
14	TCGA-A1-A0SJ	<60	Pre	Ductal/NST	NA	III+IV
15	TCGA-A1-A0SM	¡Ý60	Pre	Ductal/NST	NA	NA
16	TCGA-A1-A0SN	<60	Post	Ductal/NST	NA	I+II
17	TCGA-A1-A0SQ	<60	Pre	Ductal/NST	NA	I+II
18	TCGA-A2-A04N	¡Ý60	Post	Ductal/NST	NA	I+II
19	TCGA-A2-A04R	<60	Pre	Ductal/NST	NA	I+II
20	TCGA-A2-A04V	<60	Pre	Ductal/NST	NA	I+II
21	TCGA-A2-A04Y	<60	Post	Ductal/NST	NA	I+II
22	TCGA-A2-A0CK	¡Ý60	Post	Other	NA	III+IV
23	TCGA-A2-A0CO	¡Ý60	Post	Other	NA	I+II
24	TCGA-A2-A0CP	¡Ý60	Post	Ductal/NST	NA	I+II
25	TCGA-A2-A0CQ	¡Ý60	Post	Ductal/NST	NA	I+II
26	TCGA-A2-A0CR	<60	Post	Other	NA	I+II
27	TCGA-A2-A0CS	¡Ý60	Post	Ductal/NST	NA	III+IV
28	TCGA-A2-A0CT	¡Ý60	Post	Ductal/NST	NA	I+II
29	TCGA-A2-A0CU	¡Ý60	Post	Ductal/NST	NA	I+II
30	TCGA-A2-A0CV	<60	Post	Ductal/NST	NA	I+II
31	TCGA-A2-A0CW	¡Ý60	Post	Ductal/NST	NA	I+II
32	TCGA-A2-A0CY	¡Ý60	Post	Ductal/NST	NA	I+II
33	TCGA-A2-A0D3	<60	Post	Ductal/NST	NA	I+II
34	TCGA-A2-A0D4	<60	Pre	Ductal/NST	NA	I+II
35	TCGA-A2-A0EM	¡Ý60	Post	Ductal/NST	NA	I+II
36	TCGA-A2-A0EN	¡Ý60	Post	Other	NA	I+II
37	TCGA-A2-A0EO	<60	Pre	Ductal/NST	NA	I+II
38	TCGA-A2-A0EP	<60	Post	Other	NA	I+II
39	TCGA-A2-A0ER	¡Ý60	Post	Ductal/NST	NA	I+II
40	TCGA-A2-A0ES	<60	Post	Other	NA	I+II
41	TCGA-A2-A0ET	<60	Post	Ductal/NST	NA	III+IV
42	TCGA-A2-A0EU	¡Ý60	Post	Ductal/NST	NA	I+II
43	TCGA-A2-A0EV	¡Ý60	Post	Ductal/NST	NA	I+II
44	TCGA-A2-A0EW	<60	Post	Other	NA	III+IV
45	TCGA-A2-A0EX	<60	Post	Other	NA	I+II
46	TCGA-A2-A0EY	¡Ý60	Post	Ductal/NST	NA	I+II
47	TCGA-A2-A0SU	¡Ý60	Post	Ductal/NST	NA	I+II
48	TCGA-A2-A0SV	¡Ý60	Post	Ductal/NST	NA	III+IV
49	TCGA-A2-A0SW	¡Ý60	Post	Ductal/NST	NA	III+IV
50	TCGA-A2-A0SY	¡Ý60	Post	Other	NA	III+IV
51	TCGA-A2-A0T3	<60	Pre	Ductal/NST	NA	I+II
52	TCGA-A2-A0T4	¡Ý60	Post	Other	NA	I+II

53	TCGA-A2-A0T5	<60	Pre	Ductal/NST	NA	I+II
54	TCGA-A2-A0T6	<60	Pre	Other	NA	I+II
55	TCGA-A2-A0T7	<60	Pre	Ductal/NST	NA	I+II
56	TCGA-A2-A0YC	<60	Post	Ductal/NST	NA	I+II
57	TCGA-A2-A0YD	¡Ý60	Post	Other	NA	I+II
58	TCGA-A2-A0YF	¡Ý60	Post	Ductal/NST	NA	I+II
59	TCGA-A2-A0YG	¡Ý60	Post	Ductal/NST	NA	III+IV
60	TCGA-A2-A0YH	<60	Post	Ductal/NST	NA	III+IV
61	TCGA-A2-A0YI	¡Ý60	Post	Ductal/NST	NA	I+II
62	TCGA-A2-A0YL	<60	Pre	Other	NA	III+IV
63	TCGA-A2-A0YT	<60	Post	Ductal/NST	NA	III+IV
64	TCGA-A2-A1FV	¡Ý60	Post	Other	NA	I+II
65	TCGA-A2-A1FW	¡Ý60	Post	Ductal/NST	NA	III+IV
66	TCGA-A2-A1FX	¡Ý60	Post	Ductal/NST	NA	III+IV
67	TCGA-A2-A1FZ	¡Ý60	Post	Ductal/NST	NA	I+II
68	TCGA-A2-A1G0	<60	Pre	Other	NA	I+II
69	TCGA-A2-A1G4	¡Ý60	Post	Ductal/NST	NA	III+IV
70	TCGA-A2-A259	¡Ý60	Post	Ductal/NST	NA	I+II
71	TCGA-A2-A25B	<60	Pre	Ductal/NST	NA	I+II
72	TCGA-A2-A25C	<60	Pre	Ductal/NST	NA	I+II
73	TCGA-A2-A25D	¡Ý60	Post	Other	NA	III+IV
74	TCGA-A2-A25E	<60	Pre	Ductal/NST	NA	III+IV
75	TCGA-A2-A3KC	<60	Pre	Other	NA	I+II
76	TCGA-A2-A3KD	<60	Pre	Other	NA	III+IV
77	TCGA-A2-A4RW	<60	Pre	Other	NA	III+IV
78	TCGA-A2-A4S0	¡Ý60	Post	Other	NA	I+II
79	TCGA-A2-A4S2	¡Ý60	Post	Other	NA	III+IV
80	TCGA-A2-A4S3	<60	Post	Ductal/NST	NA	I+II
81	TCGA-A7-A0CD	¡Ý60	Pre	Ductal/NST	NA	I+II
82	TCGA-A7-A0CG	¡Ý60	Post	Other	NA	I+II
83	TCGA-A7-A0CH	¡Ý60	Post	Ductal/NST	NA	I+II
84	TCGA-A7-A0CJ	<60	Post	Ductal/NST	NA	I+II
85	TCGA-A7-A0D9	<60	Pre	Ductal/NST	NA	I+II
86	TCGA-A7-A0DB	<60	Post	Ductal/NST	NA	I+II
87	TCGA-A7-A0DC	¡Ý60	Post	Ductal/NST	NA	I+II
88	TCGA-A7-A13F	<60	Pre	Ductal/NST	NA	III+IV
89	TCGA-A7-A13G	¡Ý60	Post	Ductal/NST	NA	I+II
90	TCGA-A7-A13H	¡Ý60	Post	Other	NA	I+II
91	TCGA-A7-A26E	¡Ý60	Post	Other	NA	III+IV
92	TCGA-A7-A26H	¡Ý60	Post	Ductal/NST	NA	I+II
93	TCGA-A7-A26J	<60	Pre	Ductal/NST	NA	I+II
94	TCGA-A7-A2KD	<60	Post	Ductal/NST	NA	III+IV
95	TCGA-A7-A3IY	¡Ý60	Post	Other	NA	I+II
96	TCGA-A7-A3IZ	¡Ý60	Post	Other	NA	I+II
97	TCGA-A7-A3J0	¡Ý60	Post	Other	NA	I+II
98	TCGA-A7-A3J1	¡Ý60	Post	Other	NA	I+II
99	TCGA-A7-A3RF	¡Ý60	Post	Other	NA	I+II
100	TCGA-A7-A425	¡Ý60	Post	Other	NA	III+IV
101	TCGA-A7-A426	<60	Pre	Other	NA	III+IV
102	TCGA-A7-A4SA	<60	Pre	Other	NA	I+II
103	TCGA-A7-A4SB	<60	Post	Other	NA	III+IV
104	TCGA-A7-A4SC	¡Ý60	Post	Other	NA	I+II
105	TCGA-A7-A56D	¡Ý60	Post	Ductal/NST	NA	I+II

106	TCGA-A7-A5ZW	<60	Pre	Ductal/NST	NA	I+II
107	TCGA-A7-A5ZX	<60	Pre	Other	NA	III+IV
108	TCGA-A7-A6VX	¡Ý60	Post	Ductal/NST	NA	I+II
109	TCGA-A8-A06O	¡Ý60	Post	Ductal/NST	NA	I+II
110	TCGA-A8-A06P	¡Ý60	Post	Ductal/NST	NA	III+IV
111	TCGA-A8-A06Q	¡Ý60	Post	Ductal/NST	NA	III+IV
112	TCGA-A8-A06R	¡Ý60	Post	Ductal/NST	NA	I+II
113	TCGA-A8-A06T	¡Ý60	Post	Ductal/NST	NA	III+IV
114	TCGA-A8-A06U	¡Ý60	Post	Ductal/NST	NA	I+II
115	TCGA-A8-A06X	¡Ý60	Post	Ductal/NST	NA	I+II
116	TCGA-A8-A06Y	¡Ý60	Post	Ductal/NST	NA	I+II
117	TCGA-A8-A06Z	¡Ý60	Post	Ductal/NST	NA	I+II
118	TCGA-A8-A075	<60	Pre	Ductal/NST	NA	I+II
119	TCGA-A8-A076	¡Ý60	Post	Ductal/NST	NA	I+II
120	TCGA-A8-A079	¡Ý60	Post	Ductal/NST	NA	III+IV
121	TCGA-A8-A07B	¡Ý60	Post	Ductal/NST	NA	I+II
122	TCGA-A8-A07E	¡Ý60	Post	Ductal/NST	NA	NA
123	TCGA-A8-A07F	¡Ý60	Post	Ductal/NST	NA	I+II
124	TCGA-A8-A07G	¡Ý60	Post	Ductal/NST	NA	I+II
125	TCGA-A8-A07J	<60	Pre	Ductal/NST	NA	I+II
126	TCGA-A8-A07L	<60	Post	Ductal/NST	NA	III+IV
127	TCGA-A8-A07P	¡Ý60	Post	Ductal/NST	NA	I+II
128	TCGA-A8-A07S	¡Ý60	Post	Other	NA	I+II
129	TCGA-A8-A07W	¡Ý60	Post	Ductal/NST	NA	III+IV
130	TCGA-A8-A07Z	¡Ý60	Post	Ductal/NST	NA	I+II
131	TCGA-A8-A082	<60	Post	Ductal/NST	NA	I+II
132	TCGA-A8-A084	¡Ý60	Post	Ductal/NST	NA	I+II
133	TCGA-A8-A085	<60	Pre	Ductal/NST	NA	NA
134	TCGA-A8-A086	<60	Post	Ductal/NST	NA	I+II
135	TCGA-A8-A08C	¡Ý60	Post	Ductal/NST	NA	I+II
136	TCGA-A8-A08F	<60	Post	Ductal/NST	NA	III+IV
137	TCGA-A8-A08G	<60	Pre	Ductal/NST	NA	I+II
138	TCGA-A8-A08I	<60	Post	Ductal/NST	NA	I+II
139	TCGA-A8-A08O	<60	Pre	Ductal/NST	NA	III+IV
140	TCGA-A8-A08P	¡Ý60	Post	Ductal/NST	NA	III+IV
141	TCGA-A8-A08S	¡Ý60	Post	Ductal/NST	NA	I+II
142	TCGA-A8-A08T	¡Ý60	Post	Ductal/NST	NA	III+IV
143	TCGA-A8-A08Z	¡Ý60	Post	Ductal/NST	NA	NA
144	TCGA-A8-A091	¡Ý60	Post	Ductal/NST	NA	I+II
145	TCGA-A8-A092	<60	Pre	Ductal/NST	NA	III+IV
146	TCGA-A8-A093	¡Ý60	Post	Ductal/NST	NA	I+II
147	TCGA-A8-A095	<60	Pre	Ductal/NST	NA	I+II
148	TCGA-A8-A097	¡Ý60	Post	Ductal/NST	NA	I+II
149	TCGA-A8-A099	¡Ý60	Post	Ductal/NST	NA	NA
150	TCGA-A8-A09A	<60	Pre	Ductal/NST	NA	NA
151	TCGA-A8-A09B	<60	Post	Ductal/NST	NA	III+IV
152	TCGA-A8-A09C	¡Ý60	Post	Ductal/NST	NA	NA
153	TCGA-A8-A09D	<60	Post	Ductal/NST	NA	I+II
154	TCGA-A8-A09E	¡Ý60	Post	Ductal/NST	NA	III+IV
155	TCGA-A8-A09I	¡Ý60	Post	Ductal/NST	NA	I+II
156	TCGA-A8-A09K	¡Ý60	Post	Ductal/NST	NA	I+II
157	TCGA-A8-A09M	¡Ý60	Post	Ductal/NST	NA	III+IV
158	TCGA-A8-A09N	<60	Post	Ductal/NST	NA	III+IV

159	TCGA-A8-A09Q	¡Ý60	Post	Ductal/NST	NA	III+IV
160	TCGA-A8-A09R	¡Ý60	Post	Ductal/NST	NA	I+II
161	TCGA-A8-A09T	¡Ý60	Post	Other	NA	NA
162	TCGA-A8-A09V	<60	Pre	Other	NA	I+II
163	TCGA-A8-A0A1	¡Ý60	Post	Other	NA	I+II
164	TCGA-A8-A0A2	¡Ý60	Post	Other	NA	I+II
165	TCGA-A8-A0A4	¡Ý60	Post	Other	NA	I+II
166	TCGA-A8-A0A6	¡Ý60	Post	Other	NA	III+IV
167	TCGA-A8-A0A9	¡Ý60	Post	Ductal/NST	NA	I+II
168	TCGA-A8-A0AB	<60	Post	Other	NA	I+II
169	TCGA-A8-A0AD	¡Ý60	Post	Other	NA	I+II
170	TCGA-AC-A23C	¡Ý60	Post	Ductal/NST	NA	I+II
171	TCGA-AC-A23E	<60	Pre	Ductal/NST	NA	I+II
172	TCGA-AC-A23G	¡Ý60	Post	Other	NA	I+II
173	TCGA-AC-A2B8	¡Ý60	Post	Other	NA	I+II
174	TCGA-AC-A2BM	<60	Pre	Ductal/NST	NA	I+II
175	TCGA-AC-A2FB	¡Ý60	Pre	Other	NA	I+II
176	TCGA-AC-A2FE	¡Ý60	Post	Other	NA	III+IV
177	TCGA-AC-A2FF	<60	Pre	Other	NA	I+II
178	TCGA-AC-A2FG	¡Ý60	Post	Other	NA	I+II
179	TCGA-AC-A2FM	¡Ý60	Post	Other	NA	I+II
180	TCGA-AC-A2FO	¡Ý60	Post	Other	NA	I+II
181	TCGA-AC-A2QI	¡Ý60	Post	Other	NA	III+IV
182	TCGA-AC-A3BB	<60	Pre	Other	NA	III+IV
183	TCGA-AC-A3EH	¡Ý60	Post	Other	NA	III+IV
184	TCGA-AC-A3HN	¡Ý60	Post	Other	NA	I+II
185	TCGA-AC-A3OD	¡Ý60	Post	Other	NA	I+II
186	TCGA-AC-A3QP	¡Ý60	Post	Other	NA	I+II
187	TCGA-AC-A3QQ	<60	Pre	Other	NA	I+II
188	TCGA-AC-A3TM	<60	Pre	Other	NA	III+IV
189	TCGA-AC-A3TN	¡Ý60	Post	Other	NA	I+II
190	TCGA-AC-A3W6	¡Ý60	Post	Other	NA	III+IV
191	TCGA-AC-A3W7	¡Ý60	Post	Other	NA	I+II
192	TCGA-AC-A3YJ	¡Ý60	Post	Other	NA	I+II
193	TCGA-AC-A4ZE	¡Ý60	Post	Other	NA	I+II
194	TCGA-AC-A5XS	¡Ý60	Pre	Other	NA	I+II
195	TCGA-AC-A5XU	¡Ý60	Pre	Ductal/NST	NA	I+II
196	TCGA-AC-A62V	<60	Pre	Ductal/NST	NA	NA
197	TCGA-AC-A62Y	¡Ý60	Post	Other	NA	I+II
198	TCGA-AC-A6IV	<60	Pre	Other	NA	I+II
199	TCGA-AC-A6IX	<60	Pre	Other	NA	III+IV
200	TCGA-AC-A6NO	<60	Pre	Ductal/NST	NA	I+II
201	TCGA-AC-A7VB	<60	Pre	Ductal/NST	NA	I+II
202	TCGA-AC-A8OP	¡Ý60	Post	Ductal/NST	NA	I+II
203	TCGA-AC-A8OR	¡Ý60	Post	Other	NA	I+II
204	TCGA-AC-A8OS	¡Ý60	Post	Other	NA	I+II
205	TCGA-AN-A04A	<60	Pre	Ductal/NST	NA	III+IV
206	TCGA-AN-A0AJ	¡Ý60	Post	Ductal/NST	NA	I+II
207	TCGA-AN-A0AK	¡Ý60	Post	Ductal/NST	NA	I+II
208	TCGA-AN-A0FD	¡Ý60	Post	Ductal/NST	NA	I+II
209	TCGA-AN-A0FF	<60	Pre	Ductal/NST	NA	I+II
210	TCGA-AN-A0FK	¡Ý60	Post	Ductal/NST	NA	III+IV
211	TCGA-AN-A0FN	¡Ý60	Post	Other	NA	I+II

212	TCGA-AN-A0FS	<60	Pre	Other	NA	I+II
213	TCGA-AN-A0FT	¡Ý60	Post	Ductal/NST	NA	I+II
214	TCGA-AN-A0XL	¡Ý60	Post	Ductal/NST	NA	I+II
215	TCGA-AN-A0XO	<60	Post	Ductal/NST	NA	III+IV
216	TCGA-AN-A0XV	¡Ý60	Post	Ductal/NST	NA	III+IV
217	TCGA-AN-A0XW	<60	Pre	Ductal/NST	NA	III+IV
218	TCGA-AO-A03L	<60	Pre	Ductal/NST	NA	III+IV
219	TCGA-AO-A03M	<60	Pre	Ductal/NST	NA	I+II
220	TCGA-AO-A03N	<60	Post	Ductal/NST	NA	I+II
221	TCGA-AO-A03O	¡Ý60	Post	Ductal/NST	NA	I+II
222	TCGA-AO-A03P	<60	Post	Ductal/NST	NA	I+II
223	TCGA-AO-A03V	<60	Pre	Ductal/NST	NA	I+II
224	TCGA-AO-A0J3	¡Ý60	Post	Other	NA	I+II
225	TCGA-AO-A0J5	<60	Pre	Other	NA	III+IV
226	TCGA-AO-A0J7	¡Ý60	Post	Other	NA	I+II
227	TCGA-AO-A0J8	¡Ý60	Post	Other	NA	I+II
228	TCGA-AO-A0J9	¡Ý60	Post	Other	NA	III+IV
229	TCGA-AO-A0JA	<60	Pre	Ductal/NST	NA	III+IV
230	TCGA-AO-A0JC	¡Ý60	Post	Ductal/NST	NA	I+II
231	TCGA-AO-A0JD	<60	Post	Ductal/NST	NA	III+IV
232	TCGA-AO-A0JF	¡Ý60	Post	Ductal/NST	NA	I+II
233	TCGA-AO-A0JG	<60	Post	Other	NA	III+IV
234	TCGA-AO-A0JI	<60	Post	Ductal/NST	NA	I+II
235	TCGA-AO-A0JJ	<60	Post	Other	NA	I+II
236	TCGA-AO-A0JM	<60	Pre	Ductal/NST	NA	I+II
237	TCGA-AO-A125	¡Ý60	Post	Other	NA	I+II
238	TCGA-AO-A126	<60	Pre	Ductal/NST	NA	I+II
239	TCGA-AO-A12A	<60	Pre	Ductal/NST	NA	I+II
240	TCGA-AO-A12B	¡Ý60	Post	Ductal/NST	NA	I+II
241	TCGA-AO-A12C	<60	Pre	Ductal/NST	NA	I+II
242	TCGA-AO-A12E	<60	Pre	Other	NA	I+II
243	TCGA-AO-A12G	¡Ý60	Post	Other	NA	I+II
244	TCGA-AO-A12H	¡Ý60	Pre	Other	NA	I+II
245	TCGA-AO-A1KP	¡Ý60	Post	Ductal/NST	NA	I+II
246	TCGA-AO-A1KQ	¡Ý60	Pre	Ductal/NST	NA	NA
247	TCGA-AO-A1KS	¡Ý60	Post	Other	NA	I+II
248	TCGA-AO-A1KT	¡Ý60	Post	Ductal/NST	NA	I+II
249	TCGA-AQ-A04H	¡Ý60	Post	Ductal/NST	NA	III+IV
250	TCGA-AQ-A04L	<60	Pre	Ductal/NST	NA	I+II
251	TCGA-AQ-A0Y5	¡Ý60	Post	Ductal/NST	NA	III+IV
252	TCGA-AQ-A1H2	¡Ý60	Post	Ductal/NST	NA	III+IV
253	TCGA-AQ-A1H3	<60	Pre	Ductal/NST	NA	III+IV
254	TCGA-AQ-A54O	<60	Pre	Ductal/NST	NA	NA
255	TCGA-AQ-A7U7	<60	Post	Other	NA	III+IV
256	TCGA-AR-A0TQ	<60	Pre	Ductal/NST	NA	III+IV
257	TCGA-AR-A0TR	¡Ý60	Post	Ductal/NST	NA	I+II
258	TCGA-AR-A0TT	<60	Pre	Ductal/NST	NA	III+IV
259	TCGA-AR-A0TV	¡Ý60	Post	Ductal/NST	NA	I+II
260	TCGA-AR-A0TW	<60	Pre	Ductal/NST	NA	III+IV
261	TCGA-AR-A0TY	<60	Post	Ductal/NST	NA	I+II
262	TCGA-AR-A0TZ	<60	Pre	Ductal/NST	NA	NA
263	TCGA-AR-A0U2	<60	Pre	Ductal/NST	NA	III+IV
264	TCGA-AR-A0U3	<60	Post	Ductal/NST	NA	I+II

265	TCGA-AR-A1AK	¡Ý60	Post	Other	NA	I+II
266	TCGA-AR-A1AL	¡Ý60	Post	Other	NA	III+IV
267	TCGA-AR-A1AM	<60	Post	Other	NA	III+IV
268	TCGA-AR-A1AN	<60	Pre	Ductal/NST	NA	I+II
269	TCGA-AR-A1AP	¡Ý60	Post	Ductal/NST	NA	I+II
270	TCGA-AR-A1AS	<60	Post	Ductal/NST	NA	I+II
271	TCGA-AR-A1AU	<60	Pre	Ductal/NST	NA	III+IV
272	TCGA-AR-A1AV	¡Ý60	Pre	Ductal/NST	NA	NA
273	TCGA-AR-A1AW	¡Ý60	Post	Ductal/NST	NA	I+II
274	TCGA-AR-A1AX	¡Ý60	Post	Ductal/NST	NA	I+II
275	TCGA-AR-A24H	¡Ý60	Post	Other	NA	I+II
276	TCGA-AR-A24K	<60	Pre	Ductal/NST	NA	I+II
277	TCGA-AR-A24L	<60	Pre	Ductal/NST	NA	I+II
278	TCGA-AR-A24M	<60	Pre	Other	NA	III+IV
279	TCGA-AR-A24N	<60	Pre	Ductal/NST	NA	I+II
280	TCGA-AR-A24O	<60	Pre	Other	NA	III+IV
281	TCGA-AR-A24P	<60	Post	Ductal/NST	NA	I+II
282	TCGA-AR-A24R	<60	Pre	Ductal/NST	NA	III+IV
283	TCGA-AR-A24S	¡Ý60	Post	Ductal/NST	NA	I+II
284	TCGA-AR-A24T	<60	Pre	Other	NA	III+IV
285	TCGA-AR-A24V	<60	Pre	Ductal/NST	NA	I+II
286	TCGA-AR-A24W	<60	Post	Ductal/NST	NA	I+II
287	TCGA-AR-A24X	<60	Pre	Other	NA	I+II
288	TCGA-AR-A24Z	<60	Post	Ductal/NST	NA	I+II
289	TCGA-AR-A250	<60	Post	Ductal/NST	NA	I+II
290	TCGA-AR-A252	<60	Pre	Ductal/NST	NA	I+II
291	TCGA-AR-A255	¡Ý60	Post	Ductal/NST	NA	I+II
292	TCGA-AR-A2LE	¡Ý60	Post	Other	NA	I+II
293	TCGA-AR-A2LJ	<60	Pre	Other	NA	III+IV
294	TCGA-AR-A2LK	¡Ý60	Post	Other	NA	III+IV
295	TCGA-AR-A2LL	¡Ý60	Post	Other	NA	I+II
296	TCGA-AR-A2LM	<60	Pre	Other	NA	I+II
297	TCGA-AR-A2LN	¡Ý60	Post	Other	NA	I+II
298	TCGA-AR-A2LO	<60	Pre	Other	NA	I+II
299	TCGA-AR-A5QM	¡Ý60	Post	Other	NA	I+II
300	TCGA-AR-A5QN	¡Ý60	Post	Other	NA	III+IV
301	TCGA-AR-A5QP	<60	Post	Other	NA	I+II
302	TCGA-B6-A0I5	<60	Pre	Ductal/NST	NA	I+II
303	TCGA-B6-A0I8	<60	Pre	Ductal/NST	NA	NA
304	TCGA-B6-A0IA	<60	Pre	Other	NA	I+II
305	TCGA-B6-A0IB	¡Ý60	Pre	Ductal/NST	NA	III+IV
306	TCGA-B6-A0IC	¡Ý60	Post	Other	NA	NA
307	TCGA-B6-A0IE	<60	Pre	Other	NA	III+IV
308	TCGA-B6-A0IG	<60	Pre	Ductal/NST	NA	I+II
309	TCGA-B6-A0IH	¡Ý60	Post	Other	NA	III+IV
310	TCGA-B6-A0IM	¡Ý60	Post	Other	NA	I+II
311	TCGA-B6-A0IN	<60	Pre	Other	NA	NA
312	TCGA-B6-A0IO	¡Ý60	Pre	Ductal/NST	NA	I+II
313	TCGA-B6-A0IP	¡Ý60	Post	Other	NA	I+II
314	TCGA-B6-A0RG	<60	Pre	Ductal/NST	NA	I+II
315	TCGA-B6-A0RI	<60	Pre	Ductal/NST	NA	III+IV
316	TCGA-B6-A0RL	¡Ý60	Pre	Ductal/NST	NA	I+II
317	TCGA-B6-A0RM	<60	Pre	Ductal/NST	NA	NA

318	TCGA-B6-A0RN	¡Ý60	Post	Ductal/NST	NA	I+II
319	TCGA-B6-A0RO	¡Ý60	Post	Ductal/NST	NA	III+IV
320	TCGA-B6-A0RP	¡Ý60	Post	Other	NA	NA
321	TCGA-B6-A0RV	<60	Pre	Other	NA	III+IV
322	TCGA-B6-A0WS	<60	Pre	Ductal/NST	NA	NA
323	TCGA-B6-A0WT	¡Ý60	Post	Ductal/NST	NA	I+II
324	TCGA-B6-A0WV	¡Ý60	Post	Ductal/NST	NA	I+II
325	TCGA-B6-A0WW	<60	Pre	Ductal/NST	NA	NA
326	TCGA-B6-A0WY	<60	Pre	Ductal/NST	NA	III+IV
327	TCGA-B6-A0WZ	<60	Pre	Ductal/NST	NA	I+II
328	TCGA-B6-A0X0	<60	Pre	Other	NA	I+II
329	TCGA-B6-A0X4	¡Ý60	Post	Ductal/NST	NA	I+II
330	TCGA-B6-A0X5	¡Ý60	Post	Ductal/NST	NA	I+II
331	TCGA-B6-A0X7	¡Ý60	Post	Other	NA	NA
332	TCGA-B6-A1KC	¡Ý60	Post	Ductal/NST	NA	I+II
333	TCGA-B6-A1KI	¡Ý60	Post	Ductal/NST	NA	I+II
334	TCGA-B6-A1KN	<60	Post	Ductal/NST	NA	III+IV
335	TCGA-B6-A2IU	¡Ý60	Pre	Other	NA	I+II
336	TCGA-B6-A401	<60	Pre	Ductal/NST	NA	I+II
337	TCGA-B6-A408	<60	Pre	Other	NA	III+IV
338	TCGA-B6-A40B	¡Ý60	Post	Other	NA	I+II
339	TCGA-B6-A40C	<60	Pre	Other	NA	I+II
340	TCGA-BH-A0AU	<60	Pre	Ductal/NST	NA	I+II
341	TCGA-BH-A0AZ	<60	Pre	Ductal/NST	NA	III+IV
342	TCGA-BH-A0B0	<60	Pre	Ductal/NST	NA	I+II
343	TCGA-BH-A0B1	¡Ý60	Post	Ductal/NST	NA	I+II
344	TCGA-BH-A0B4	¡Ý60	Pre	Ductal/NST	NA	NA
345	TCGA-BH-A0B5	<60	Pre	Other	NA	III+IV
346	TCGA-BH-A0B6	<60	Pre	Ductal/NST	NA	I+II
347	TCGA-BH-A0B8	¡Ý60	Post	Ductal/NST	NA	I+II
348	TCGA-BH-A0BA	<60	Pre	Other	NA	III+IV
349	TCGA-BH-A0BC	¡Ý60	Post	Ductal/NST	NA	III+IV
350	TCGA-BH-A0BD	<60	Pre	Ductal/NST	NA	I+II
351	TCGA-BH-A0BF	<60	Post	Ductal/NST	NA	I+II
352	TCGA-BH-A0BJ	<60	Pre	Ductal/NST	NA	I+II
353	TCGA-BH-A0BM	<60	Post	Ductal/NST	NA	I+II
354	TCGA-BH-A0BO	<60	Pre	Ductal/NST	NA	I+II
355	TCGA-BH-A0BP	¡Ý60	Post	Ductal/NST	NA	I+II
356	TCGA-BH-A0BQ	<60	Pre	Ductal/NST	NA	I+II
357	TCGA-BH-A0BR	<60	Post	Ductal/NST	NA	I+II
358	TCGA-BH-A0BS	<60	Post	Ductal/NST	NA	III+IV
359	TCGA-BH-A0BT	<60	Pre	Ductal/NST	NA	I+II
360	TCGA-BH-A0BV	¡Ý60	Post	Ductal/NST	NA	I+II
361	TCGA-BH-A0BZ	<60	Post	Ductal/NST	NA	III+IV
362	TCGA-BH-A0C0	¡Ý60	Post	Ductal/NST	NA	I+II
363	TCGA-BH-A0C1	¡Ý60	Post	Other	NA	III+IV
364	TCGA-BH-A0C3	<60	Pre	Other	NA	I+II
365	TCGA-BH-A0C7	<60	Pre	Ductal/NST	NA	I+II
366	TCGA-BH-A0DD	<60	Pre	Ductal/NST	NA	NA
367	TCGA-BH-A0DE	¡Ý60	Post	Ductal/NST	NA	I+II
368	TCGA-BH-A0DG	<60	Pre	Ductal/NST	NA	I+II
369	TCGA-BH-A0DH	¡Ý60	Post	Ductal/NST	NA	I+II
370	TCGA-BH-A0DI	¡Ý60	Post	Ductal/NST	NA	I+II

371	TCGA-BH-A0DK	<60	Pre	Ductal/NST	NA	I+II
372	TCGA-BH-A0DO	¡Ý60	Post	Ductal/NST	NA	I+II
373	TCGA-BH-A0DP	¡Ý60	Post	Other	NA	I+II
374	TCGA-BH-A0DQ	<60	Pre	Ductal/NST	NA	I+II
375	TCGA-BH-A0DS	¡Ý60	Post	Ductal/NST	NA	III+IV
376	TCGA-BH-A0DT	<60	Pre	Ductal/NST	NA	I+II
377	TCGA-BH-A0DV	<60	Pre	Ductal/NST	NA	III+IV
378	TCGA-BH-A0DX	¡Ý60	Post	Ductal/NST	NA	I+II
379	TCGA-BH-A0DZ	<60	Pre	Ductal/NST	NA	I+II
380	TCGA-BH-A0E1	<60	Pre	Ductal/NST	NA	I+II
381	TCGA-BH-A0E2	<60	Pre	Ductal/NST	NA	III+IV
382	TCGA-BH-A0E7	¡Ý60	Post	Ductal/NST	NA	I+II
383	TCGA-BH-A0E9	<60	Post	Other	NA	I+II
384	TCGA-BH-A0EA	¡Ý60	Post	Ductal/NST	NA	I+II
385	TCGA-BH-A0EB	¡Ý60	Post	Ductal/NST	NA	I+II
386	TCGA-BH-A0EI	<60	Pre	Ductal/NST	NA	I+II
387	TCGA-BH-A0GY	¡Ý60	Post	Ductal/NST	NA	I+II
388	TCGA-BH-A0GZ	¡Ý60	Post	Ductal/NST	NA	I+II
389	TCGA-BH-A0H0	¡Ý60	Post	Ductal/NST	NA	I+II
390	TCGA-BH-A0H3	<60	Pre	Ductal/NST	NA	I+II
391	TCGA-BH-A0H5	<60	Post	Ductal/NST	NA	I+II
392	TCGA-BH-A0H6	¡Ý60	Post	Ductal/NST	NA	I+II
393	TCGA-BH-A0H7	¡Ý60	Post	Ductal/NST	NA	III+IV
394	TCGA-BH-A0H9	¡Ý60	Post	Ductal/NST	NA	I+II
395	TCGA-BH-A0HA	<60	Pre	Ductal/NST	NA	I+II
396	TCGA-BH-A0HB	<60	Post	Ductal/NST	NA	I+II
397	TCGA-BH-A0HF	¡Ý60	Post	Ductal/NST	NA	I+II
398	TCGA-BH-A0HI	¡Ý60	Post	Ductal/NST	NA	I+II
399	TCGA-BH-A0HK	¡Ý60	Post	Ductal/NST	NA	I+II
400	TCGA-BH-A0HL	<60	Post	Ductal/NST	NA	I+II
401	TCGA-BH-A0HN	¡Ý60	Post	Ductal/NST	NA	I+II
402	TCGA-BH-A0HO	<60	Pre	Ductal/NST	NA	I+II
403	TCGA-BH-A0HP	¡Ý60	Post	Other	NA	III+IV
404	TCGA-BH-A0HQ	<60	Post	Ductal/NST	NA	I+II
405	TCGA-BH-A0HU	<60	Pre	Ductal/NST	NA	I+II
406	TCGA-BH-A0HW	¡Ý60	Post	Ductal/NST	NA	I+II
407	TCGA-BH-A0HX	<60	Post	Ductal/NST	NA	I+II
408	TCGA-BH-A0HY	¡Ý60	Post	Ductal/NST	NA	I+II
409	TCGA-BH-A0W3	<60	Pre	Ductal/NST	NA	I+II
410	TCGA-BH-A0W4	<60	Pre	Ductal/NST	NA	I+II
411	TCGA-BH-A0W5	¡Ý60	Pre	Ductal/NST	NA	I+II
412	TCGA-BH-A0W7	<60	Pre	Ductal/NST	NA	I+II
413	TCGA-BH-A18F	<60	Pre	Ductal/NST	NA	I+II
414	TCGA-BH-A18H	¡Ý60	Post	Ductal/NST	NA	I+II
415	TCGA-BH-A18I	<60	Post	Ductal/NST	NA	I+II
416	TCGA-BH-A18J	<60	Pre	Ductal/NST	NA	III+IV
417	TCGA-BH-A18K	<60	Pre	Ductal/NST	NA	I+II
418	TCGA-BH-A18L	<60	Pre	Ductal/NST	NA	III+IV
419	TCGA-BH-A18M	<60	Pre	Ductal/NST	NA	III+IV
420	TCGA-BH-A18N	¡Ý60	Pre	Ductal/NST	NA	I+II
421	TCGA-BH-A18S	¡Ý60	Post	Other	NA	I+II
422	TCGA-BH-A18U	¡Ý60	Post	Ductal/NST	NA	III+IV
423	TCGA-BH-A1EO	¡Ý60	Post	Other	NA	I+II

424	TCGA-BH-A1ES	<60	Pre	Ductal/NST	NA	I+II
425	TCGA-BH-A1ET	<60	Pre	Ductal/NST	NA	I+II
426	TCGA-BH-A1EU	¡Ý60	Post	Ductal/NST	NA	I+II
427	TCGA-BH-A1EW	<60	Pre	Ductal/NST	NA	I+II
428	TCGA-BH-A1EX	¡Ý60	Post	Ductal/NST	NA	I+II
429	TCGA-BH-A1EY	¡Ý60	Post	Ductal/NST	NA	I+II
430	TCGA-BH-A1F2	<60	Pre	Ductal/NST	NA	III+IV
431	TCGA-BH-A1F5	¡Ý60	Pre	Ductal/NST	NA	I+II
432	TCGA-BH-A1F8	¡Ý60	Post	Ductal/NST	NA	III+IV
433	TCGA-BH-A1FB	¡Ý60	Post	Ductal/NST	NA	I+II
434	TCGA-BH-A1FD	¡Ý60	Post	Ductal/NST	NA	I+II
435	TCGA-BH-A1FE	<60	Pre	Ductal/NST	NA	I+II
436	TCGA-BH-A1FG	¡Ý60	Post	Ductal/NST	NA	I+II
437	TCGA-BH-A1FH	<60	Pre	Ductal/NST	NA	III+IV
438	TCGA-BH-A1FJ	¡Ý60	Pre	Ductal/NST	NA	III+IV
439	TCGA-BH-A1FL	¡Ý60	Pre	Ductal/NST	NA	I+II
440	TCGA-BH-A1FM	<60	Pre	Ductal/NST	NA	III+IV
441	TCGA-BH-A1FN	<60	Pre	Ductal/NST	NA	I+II
442	TCGA-BH-A1FR	¡Ý60	Pre	Other	NA	III+IV
443	TCGA-BH-A201	¡Ý60	Post	Ductal/NST	NA	I+II
444	TCGA-BH-A202	¡Ý60	Post	Ductal/NST	NA	I+II
445	TCGA-BH-A204	¡Ý60	Post	Ductal/NST	NA	I+II
446	TCGA-BH-A209	¡Ý60	Post	Other	NA	I+II
447	TCGA-BH-A28Q	<60	Pre	Other	NA	I+II
448	TCGA-BH-A2L8	<60	Pre	Other	NA	I+II
449	TCGA-BH-A42T	¡Ý60	Post	Other	NA	I+II
450	TCGA-BH-A42V	<60	Pre	Ductal/NST	NA	I+II
451	TCGA-BH-A5J0	¡Ý60	Post	Ductal/NST	NA	I+II
452	TCGA-BH-A6R8	<60	Pre	Ductal/NST	NA	I+II
453	TCGA-BH-A8FY	¡Ý60	Post	Other	NA	I+II
454	TCGA-BH-A8G0	<60	Pre	Other	NA	I+II
455	TCGA-BH-AB28	<60	Pre	Other	NA	III+IV
456	TCGA-C8-A12M	¡Ý60	Post	Ductal/NST	NA	I+II
457	TCGA-C8-A12N	<60	Post	Ductal/NST	NA	I+II
458	TCGA-C8-A12O	<60	Pre	Ductal/NST	NA	I+II
459	TCGA-C8-A12U	<60	Pre	Ductal/NST	NA	I+II
460	TCGA-C8-A12W	<60	Pre	Ductal/NST	NA	III+IV
461	TCGA-C8-A12X	¡Ý60	Post	Other	NA	I+II
462	TCGA-C8-A12Y	<60	Pre	Ductal/NST	NA	I+II
463	TCGA-C8-A130	<60	Pre	Ductal/NST	NA	III+IV
464	TCGA-C8-A132	<60	Post	Ductal/NST	NA	I+II
465	TCGA-C8-A1HE	<60	Post	Ductal/NST	NA	I+II
466	TCGA-C8-A1HG	<60	Pre	Ductal/NST	NA	I+II
467	TCGA-C8-A1HI	<60	Pre	Ductal/NST	NA	III+IV
468	TCGA-C8-A1HL	<60	Pre	Other	NA	III+IV
469	TCGA-C8-A1HM	¡Ý60	Post	Ductal/NST	NA	I+II
470	TCGA-C8-A1HN	<60	Post	Ductal/NST	NA	I+II
471	TCGA-C8-A1HO	<60	Pre	Ductal/NST	NA	III+IV
472	TCGA-C8-A26V	<60	Pre	Ductal/NST	NA	III+IV
473	TCGA-C8-A26W	<60	Post	Ductal/NST	NA	I+II
474	TCGA-C8-A26Z	<60	Post	Ductal/NST	NA	I+II
475	TCGA-C8-A273	<60	Pre	Ductal/NST	NA	I+II
476	TCGA-C8-A274	¡Ý60	Post	Ductal/NST	NA	I+II

477	TCGA-C8-A27A	<60	Pre	Ductal/NST	NA	I+II
478	TCGA-C8-A3M7	¡Ý60	Post	Other	NA	III+IV
479	TCGA-C8-A3M8	¡Ý60	Post	Other	NA	I+II
480	TCGA-C8-A8HQ	<60	Post	Other	NA	I+II
481	TCGA-D8-A13Y	<60	Post	Ductal/NST	NA	I+II
482	TCGA-D8-A140	¡Ý60	Post	Ductal/NST	NA	I+II
483	TCGA-D8-A141	<60	Pre	Ductal/NST	NA	NA
484	TCGA-D8-A145	¡Ý60	Post	Ductal/NST	NA	I+II
485	TCGA-D8-A146	<60	Post	Ductal/NST	NA	I+II
486	TCGA-D8-A1J8	¡Ý60	Post	Ductal/NST	NA	I+II
487	TCGA-D8-A1J9	<60	Post	Ductal/NST	NA	I+II
488	TCGA-D8-A1JB	<60	Post	Ductal/NST	NA	I+II
489	TCGA-D8-A1JC	<60	Post	Ductal/NST	NA	III+IV
490	TCGA-D8-A1JD	<60	Pre	Ductal/NST	NA	I+II
491	TCGA-D8-A1JE	¡Ý60	Post	Ductal/NST	NA	I+II
492	TCGA-D8-A1JH	<60	Post	Ductal/NST	NA	I+II
493	TCGA-D8-A1JI	<60	Post	Ductal/NST	NA	I+II
494	TCGA-D8-A1JJ	<60	Post	Ductal/NST	NA	I+II
495	TCGA-D8-A1JN	¡Ý60	Post	Other	NA	III+IV
496	TCGA-D8-A1JP	¡Ý60	Post	Ductal/NST	NA	I+II
497	TCGA-D8-A1JS	¡Ý60	Post	Other	NA	I+II
498	TCGA-D8-A1JT	¡Ý60	Post	Other	NA	I+II
499	TCGA-D8-A1JU	<60	Post	Ductal/NST	NA	I+II
500	TCGA-D8-A1X5	¡Ý60	Post	Ductal/NST	NA	III+IV
501	TCGA-D8-A1X6	¡Ý60	Post	Ductal/NST	NA	III+IV
502	TCGA-D8-A1X7	<60	Pre	Other	NA	I+II
503	TCGA-D8-A1X8	¡Ý60	Post	Other	NA	III+IV
504	TCGA-D8-A1X9	¡Ý60	Post	Ductal/NST	NA	I+II
505	TCGA-D8-A1XA	¡Ý60	Post	Ductal/NST	NA	I+II
506	TCGA-D8-A1XB	¡Ý60	Post	Ductal/NST	NA	I+II
507	TCGA-D8-A1XC	¡Ý60	Post	Other	NA	III+IV
508	TCGA-D8-A1XD	<60	Pre	Ductal/NST	NA	III+IV
509	TCGA-D8-A1XF	<60	Pre	Ductal/NST	NA	I+II
510	TCGA-D8-A1XG	¡Ý60	Post	Ductal/NST	NA	III+IV
511	TCGA-D8-A1XL	<60	Pre	Ductal/NST	NA	I+II
512	TCGA-D8-A1XM	<60	Post	Ductal/NST	NA	I+II
513	TCGA-D8-A1XO	<60	Post	Other	NA	I+II
514	TCGA-D8-A1XR	<60	Post	Ductal/NST	NA	I+II
515	TCGA-D8-A1XS	<60	Pre	Other	NA	NA
516	TCGA-D8-A1XU	<60	Post	Ductal/NST	NA	I+II
517	TCGA-D8-A1XV	¡Ý60	Post	Other	NA	I+II
518	TCGA-D8-A1XY	¡Ý60	Post	Ductal/NST	NA	I+II
519	TCGA-D8-A1XZ	¡Ý60	Post	Ductal/NST	NA	III+IV
520	TCGA-D8-A1Y0	¡Ý60	Post	Ductal/NST	NA	III+IV
521	TCGA-D8-A1Y1	¡Ý60	Post	Ductal/NST	NA	III+IV
522	TCGA-D8-A1Y2	¡Ý60	Post	Ductal/NST	NA	I+II
523	TCGA-D8-A1Y3	¡Ý60	Post	Ductal/NST	NA	III+IV
524	TCGA-D8-A27E	¡Ý60	Post	Other	NA	I+II
525	TCGA-D8-A27G	¡Ý60	Post	Other	NA	I+II
526	TCGA-D8-A27I	<60	Post	Other	NA	III+IV
527	TCGA-D8-A27K	<60	Post	Ductal/NST	NA	I+II
528	TCGA-D8-A27L	<60	Post	Ductal/NST	NA	III+IV
529	TCGA-D8-A27N	<60	Pre	Ductal/NST	NA	III+IV

530	TCGA-D8-A27P	¡Ý60	Post	Ductal/NST	NA	I+II
531	TCGA-D8-A27R	<60	Post	Ductal/NST	NA	III+IV
532	TCGA-D8-A27T	<60	Post	Other	NA	III+IV
533	TCGA-D8-A27V	¡Ý60	Post	Other	NA	I+II
534	TCGA-D8-A27W	<60	Post	Other	NA	III+IV
535	TCGA-D8-A3Z5	<60	Post	Other	NA	III+IV
536	TCGA-D8-A3Z6	<60	Post	Other	NA	III+IV
537	TCGA-D8-A4Z1	¡Ý60	Post	Other	NA	I+II
538	TCGA-D8-A73U	¡Ý60	Post	Other	NA	I+II
539	TCGA-D8-A73W	¡Ý60	Post	Other	NA	III+IV
540	TCGA-D8-A73X	<60	Post	Other	NA	I+II
541	TCGA-E2-A105	¡Ý60	Post	Ductal/NST	NA	I+II
542	TCGA-E2-A106	<60	Pre	Ductal/NST	NA	I+II
543	TCGA-E2-A107	<60	Post	Other	NA	III+IV
544	TCGA-E2-A109	¡Ý60	Post	Ductal/NST	NA	I+II
545	TCGA-E2-A10A	<60	Pre	Ductal/NST	NA	I+II
546	TCGA-E2-A10B	¡Ý60	Post	Ductal/NST	NA	I+II
547	TCGA-E2-A10C	<60	Post	Ductal/NST	NA	I+II
548	TCGA-E2-A10E	¡Ý60	Post	Ductal/NST	NA	I+II
549	TCGA-E2-A10F	<60	Pre	Other	NA	I+II
550	TCGA-E2-A14O	¡Ý60	Post	Ductal/NST	NA	III+IV
551	TCGA-E2-A14Q	<60	Pre	Ductal/NST	NA	I+II
552	TCGA-E2-A14S	¡Ý60	Post	Ductal/NST	NA	I+II
553	TCGA-E2-A14T	<60	Post	Ductal/NST	NA	I+II
554	TCGA-E2-A14U	¡Ý60	Post	Other	NA	I+II
555	TCGA-E2-A14W	¡Ý60	Pre	Ductal/NST	NA	NA
556	TCGA-E2-A14Z	¡Ý60	Post	Ductal/NST	NA	I+II
557	TCGA-E2-A153	<60	Pre	Ductal/NST	NA	I+II
558	TCGA-E2-A154	¡Ý60	Post	Ductal/NST	NA	I+II
559	TCGA-E2-A155	<60	Post	Ductal/NST	NA	I+II
560	TCGA-E2-A156	¡Ý60	Post	Ductal/NST	NA	I+II
561	TCGA-E2-A15A	<60	Pre	Ductal/NST	NA	III+IV
562	TCGA-E2-A15C	¡Ý60	Post	Ductal/NST	NA	I+II
563	TCGA-E2-A15D	<60	Pre	Ductal/NST	NA	I+II
564	TCGA-E2-A15E	<60	Pre	Ductal/NST	NA	I+II
565	TCGA-E2-A15F	¡Ý60	Post	Ductal/NST	NA	I+II
566	TCGA-E2-A15G	¡Ý60	Post	Other	NA	I+II
567	TCGA-E2-A15H	<60	Pre	Ductal/NST	NA	I+II
568	TCGA-E2-A15I	<60	Pre	Ductal/NST	NA	I+II
569	TCGA-E2-A15J	<60	Pre	Ductal/NST	NA	I+II
570	TCGA-E2-A15K	<60	Post	Ductal/NST	NA	I+II
571	TCGA-E2-A15L	¡Ý60	Post	Other	NA	I+II
572	TCGA-E2-A15M	¡Ý60	Post	Other	NA	I+II
573	TCGA-E2-A15O	¡Ý60	Post	Ductal/NST	NA	I+II
574	TCGA-E2-A15P	¡Ý60	Post	Ductal/NST	NA	I+II
575	TCGA-E2-A15R	¡Ý60	Post	Ductal/NST	NA	I+II
576	TCGA-E2-A15S	<60	Post	Ductal/NST	NA	I+II
577	TCGA-E2-A15T	¡Ý60	Post	Ductal/NST	NA	I+II
578	TCGA-E2-A1B1	<60	Post	Ductal/NST	NA	I+II
579	TCGA-E2-A1B4	¡Ý60	Post	Ductal/NST	NA	III+IV
580	TCGA-E2-A1B5	<60	Pre	Other	NA	I+II
581	TCGA-E2-A1BC	¡Ý60	Post	Ductal/NST	NA	I+II
582	TCGA-E2-A1BD	<60	Post	Ductal/NST	NA	I+II

583	TCGA-E2-A1IE	¡Ý60	Post	Ductal/NST	NA	I+II
584	TCGA-E2-A1IF	¡Ý60	Post	Ductal/NST	NA	I+II
585	TCGA-E2-A1IG	<60	Pre	Ductal/NST	NA	I+II
586	TCGA-E2-A1IH	¡Ý60	Post	Other	NA	I+II
587	TCGA-E2-A1IJ	<60	Post	Other	NA	I+II
588	TCGA-E2-A1IK	¡Ý60	Post	Ductal/NST	NA	I+II
589	TCGA-E2-A1IL	¡Ý60	Post	Other	NA	I+II
590	TCGA-E2-A1IN	¡Ý60	Post	Ductal/NST	NA	I+II
591	TCGA-E2-A1IO	<60	Pre	Ductal/NST	NA	I+II
592	TCGA-E2-A1IU	¡Ý60	Post	Ductal/NST	NA	I+II
593	TCGA-E2-A1L6	<60	Pre	Ductal/NST	NA	I+II
594	TCGA-E2-A1L7	<60	Pre	Ductal/NST	NA	III+IV
595	TCGA-E2-A1L8	<60	Post	Other	NA	I+II
596	TCGA-E2-A1L9	<60	Pre	Ductal/NST	NA	I+II
597	TCGA-E2-A1LA	<60	Post	Ductal/NST	NA	I+II
598	TCGA-E2-A2P5	¡Ý60	Post	Other	NA	III+IV
599	TCGA-E2-A2P6	¡Ý60	Post	Other	NA	I+II
600	TCGA-E2-A3DX	<60	Pre	Other	NA	III+IV
601	TCGA-E2-A56Z	¡Ý60	Post	Ductal/NST	NA	I+II
602	TCGA-E2-A570	<60	Pre	Ductal/NST	NA	I+II
603	TCGA-E2-A572	¡Ý60	Post	Ductal/NST	NA	III+IV
604	TCGA-E2-A576	¡Ý60	Post	Other	NA	I+II
605	TCGA-E2-A9RU	¡Ý60	Post	Ductal/NST	NA	III+IV
606	TCGA-E9-A1N3	¡Ý60	Post	Other	NA	III+IV
607	TCGA-E9-A1N4	<60	Pre	Other	NA	III+IV
608	TCGA-E9-A1N5	<60	Post	Other	NA	I+II
609	TCGA-E9-A1N6	<60	Post	Ductal/NST	NA	I+II
610	TCGA-E9-A1NA	<60	Post	Other	NA	I+II
611	TCGA-E9-A1NE	<60	Pre	Ductal/NST	NA	I+II
612	TCGA-E9-A1NF	¡Ý60	Post	Other	NA	I+II
613	TCGA-E9-A1NG	¡Ý60	Post	Ductal/NST	NA	I+II
614	TCGA-E9-A1NH	¡Ý60	Post	Ductal/NST	NA	I+II
615	TCGA-E9-A1NI	<60	Post	Other	NA	I+II
616	TCGA-E9-A1QZ	¡Ý60	Post	Ductal/NST	NA	I+II
617	TCGA-E9-A1R0	<60	Post	Ductal/NST	NA	I+II
618	TCGA-E9-A1R2	<60	Post	Ductal/NST	NA	III+IV
619	TCGA-E9-A1R3	¡Ý60	Post	Ductal/NST	NA	III+IV
620	TCGA-E9-A1R4	¡Ý60	Post	Ductal/NST	NA	I+II
621	TCGA-E9-A1R5	¡Ý60	Post	Ductal/NST	NA	I+II
622	TCGA-E9-A1R6	¡Ý60	Post	Ductal/NST	NA	I+II
623	TCGA-E9-A1R7	¡Ý60	Post	Ductal/NST	NA	I+II
624	TCGA-E9-A1RA	<60	Pre	Ductal/NST	NA	I+II
625	TCGA-E9-A1RB	<60	Pre	Ductal/NST	NA	I+II
626	TCGA-E9-A1RC	<60	Post	Other	NA	III+IV
627	TCGA-E9-A1RD	¡Ý60	Post	Ductal/NST	NA	I+II
628	TCGA-E9-A1RE	¡Ý60	Post	Ductal/NST	NA	III+IV
629	TCGA-E9-A1RF	¡Ý60	Post	Ductal/NST	NA	III+IV
630	TCGA-E9-A1RG	¡Ý60	Post	Ductal/NST	NA	III+IV
631	TCGA-E9-A1RI	<60	Pre	Other	NA	III+IV
632	TCGA-E9-A226	<60	Pre	Ductal/NST	NA	III+IV
633	TCGA-E9-A227	<60	Pre	Ductal/NST	NA	I+II
634	TCGA-E9-A228	<60	Post	Ductal/NST	NA	I+II
635	TCGA-E9-A229	<60	Pre	Ductal/NST	NA	I+II

636	TCGA-E9-A22A	¡Ý60	Post	Ductal/NST	NA	I+II
637	TCGA-E9-A22B	¡Ý60	Post	Other	NA	I+II
638	TCGA-E9-A22D	<60	Pre	Ductal/NST	NA	I+II
639	TCGA-E9-A22E	<60	Post	Ductal/NST	NA	III+IV
640	TCGA-E9-A22H	<60	Pre	Ductal/NST	NA	I+II
641	TCGA-E9-A247	<60	Post	Ductal/NST	NA	I+II
642	TCGA-E9-A249	<60	Pre	Ductal/NST	NA	I+II
643	TCGA-E9-A24A	¡Ý60	Post	Ductal/NST	NA	I+II
644	TCGA-E9-A295	¡Ý60	Post	Other	NA	I+II
645	TCGA-E9-A2JS	¡Ý60	Post	Other	NA	I+II
646	TCGA-E9-A2JT	¡Ý60	Post	Other	NA	I+II
647	TCGA-E9-A3HO	<60	Pre	Other	NA	I+II
648	TCGA-E9-A3Q9	¡Ý60	Post	Other	NA	III+IV
649	TCGA-E9-A3X8	<60	Post	Other	NA	I+II
650	TCGA-E9-A54X	¡Ý60	Post	Other	NA	I+II
651	TCGA-E9-A54Y	¡Ý60	Post	Other	NA	III+IV
652	TCGA-E9-A5FK	¡Ý60	Post	Other	NA	III+IV
653	TCGA-E9-A5UO	<60	Pre	Other	NA	I+II
654	TCGA-E9-A5UP	¡Ý60	Post	Other	NA	I+II
655	TCGA-E9-A6HE	<60	Pre	Other	NA	III+IV
656	TCGA-EW-A1IW	¡Ý60	Post	Other	NA	I+II
657	TCGA-EW-A1IX	<60	Pre	Other	NA	I+II
658	TCGA-EW-A1IY	<60	Pre	Ductal/NST	NA	I+II
659	TCGA-EW-A1IZ	<60	Post	Ductal/NST	NA	III+IV
660	TCGA-EW-A1J1	<60	Pre	Ductal/NST	NA	I+II
661	TCGA-EW-A1J2	<60	Pre	Other	NA	NA
662	TCGA-EW-A1J3	¡Ý60	Post	Other	NA	I+II
663	TCGA-EW-A1J5	<60	Post	Other	NA	I+II
664	TCGA-EW-A1J6	¡Ý60	Post	Ductal/NST	NA	I+II
665	TCGA-EW-A1OX	<60	Pre	Other	NA	I+II
666	TCGA-EW-A1OY	¡Ý60	Post	Ductal/NST	NA	I+II
667	TCGA-EW-A1OZ	<60	Post	Ductal/NST	NA	I+II
668	TCGA-EW-A1P0	<60	Post	Other	NA	I+II
669	TCGA-EW-A1P3	<60	Pre	Ductal/NST	NA	I+II
670	TCGA-EW-A1P5	¡Ý60	Post	Ductal/NST	NA	I+II
671	TCGA-EW-A1P6	¡Ý60	Post	Ductal/NST	NA	I+II
672	TCGA-EW-A1PA	<60	Post	Ductal/NST	NA	I+II
673	TCGA-EW-A1PC	¡Ý60	Post	Ductal/NST	NA	I+II
674	TCGA-EW-A1PD	¡Ý60	Pre	Ductal/NST	NA	NA
675	TCGA-EW-A1PE	<60	Post	Ductal/NST	NA	I+II
676	TCGA-EW-A1PF	<60	Post	Ductal/NST	NA	I+II
677	TCGA-EW-A2FS	<60	Pre	Ductal/NST	NA	I+II
678	TCGA-EW-A2FV	<60	Pre	Other	NA	III+IV
679	TCGA-EW-A2FW	<60	Post	Other	NA	I+II
680	TCGA-EW-A3E8	¡Ý60	Pre	Other	NA	I+II
681	TCGA-EW-A423	¡Ý60	Post	Other	NA	I+II
682	TCGA-EW-A424	<60	Pre	Other	NA	III+IV
683	TCGA-EW-A6S9	<60	Pre	Ductal/NST	NA	I+II
684	TCGA-EW-A6SA	<60	Pre	Ductal/NST	NA	NA
685	TCGA-EW-A6SC	¡Ý60	Pre	Other	NA	I+II
686	TCGA-GI-A2C8	¡Ý60	Pre	Ductal/NST	NA	III+IV
687	TCGA-GM-A2D9	¡Ý60	Post	Ductal/NST	NA	I+II
688	TCGA-GM-A2DA	<60	Post	Ductal/NST	NA	I+II

689	TCGA-GM-A2DC	<60	Post	Ductal/NST	NA	I+II
690	TCGA-GM-A2DI	<60	Pre	Ductal/NST	NA	I+II
691	TCGA-GM-A2DK	<60	Post	Ductal/NST	NA	I+II
692	TCGA-GM-A2DL	<60	Pre	Ductal/NST	NA	I+II
693	TCGA-GM-A2DM	<60	Post	Ductal/NST	NA	I+II
694	TCGA-GM-A2DN	<60	Post	Ductal/NST	NA	I+II
695	TCGA-GM-A2DO	<60	Post	Other	NA	I+II
696	TCGA-GM-A3NW	¡Ý60	Post	Other	NA	I+II
697	TCGA-GM-A3NY	¡Ý60	Post	Other	NA	I+II
698	TCGA-GM-A3XG	<60	Pre	Other	NA	III+IV
699	TCGA-GM-A3XN	<60	Post	Other	NA	III+IV
700	TCGA-GM-A4E0	¡Ý60	Post	Other	NA	III+IV
701	TCGA-GM-A5PV	¡Ý60	Post	Other	NA	I+II
702	TCGA-GM-A5PX	¡Ý60	Post	Other	NA	I+II
703	TCGA-HN-A2OB	<60	Pre	Other	NA	I+II
704	TCGA-JL-A3YX	<60	Post	Other	NA	I+II
705	TCGA-LD-A66U	<60	Pre	Other	NA	I+II
706	TCGA-LD-A74U	¡Ý60	Pre	Other	NA	III+IV
707	TCGA-LD-A7W5	<60	Pre	Ductal/NST	NA	III+IV
708	TCGA-LD-A7W6	<60	Post	Other	NA	I+II
709	TCGA-LL-A440	¡Ý60	Post	Other	NA	I+II
710	TCGA-LL-A442	<60	Post	Ductal/NST	NA	I+II
711	TCGA-LL-A50Y	¡Ý60	Post	Other	NA	I+II
712	TCGA-LL-A5YL	¡Ý60	Post	Other	NA	I+II
713	TCGA-LL-A5YM	¡Ý60	Post	Ductal/NST	NA	III+IV
714	TCGA-LL-A5YN	<60	Post	Ductal/NST	NA	I+II
715	TCGA-LL-A6FP	¡Ý60	Post	Other	NA	I+II
716	TCGA-LL-A6FQ	¡Ý60	Post	Ductal/NST	NA	III+IV
717	TCGA-LL-A740	¡Ý60	Post	Ductal/NST	NA	I+II
718	TCGA-LL-A7SZ	<60	Pre	Ductal/NST	NA	I+II
719	TCGA-LL-A7T0	¡Ý60	Post	Ductal/NST	NA	I+II
720	TCGA-LL-A9Q3	¡Ý60	Post	Other	NA	III+IV
721	TCGA-LQ-A4E4	¡Ý60	Post	Other	NA	III+IV
722	TCGA-MS-A51U	<60	Pre	Other	NA	I+II
723	TCGA-OK-A5Q2	<60	Pre	Other	NA	I+II
724	TCGA-OL-A5D8	<60	Pre	Ductal/NST	NA	I+II
725	TCGA-OL-A5DA	¡Ý60	Post	Other	NA	I+II
726	TCGA-OL-A5RU	¡Ý60	Post	Ductal/NST	NA	I+II
727	TCGA-OL-A5RV	<60	Pre	Ductal/NST	NA	III+IV
728	TCGA-OL-A5RX	<60	Pre	Ductal/NST	NA	I+II
729	TCGA-OL-A66J	¡Ý60	Post	Other	NA	I+II
730	TCGA-OL-A66K	¡Ý60	Post	Other	NA	I+II
731	TCGA-OL-A66L	¡Ý60	Post	Ductal/NST	NA	I+II
732	TCGA-OL-A66N	<60	Post	Other	NA	III+IV
733	TCGA-OL-A66O	<60	Pre	Ductal/NST	NA	I+II
734	TCGA-OL-A6VQ	<60	Pre	Other	NA	I+II
735	TCGA-OL-A6VR	<60	Pre	Ductal/NST	NA	I+II
736	TCGA-PE-A5DC	¡Ý60	Post	Other	NA	III+IV
737	TCGA-PE-A5DD	¡Ý60	Post	Other	NA	I+II
738	TCGA-PE-A5DE	<60	Pre	Other	NA	I+II
739	TCGA-S3-A6ZF	¡Ý60	Post	Ductal/NST	NA	I+II
740	TCGA-S3-A6ZG	¡Ý60	Post	Other	NA	I+II
741	TCGA-S3-A6ZH	<60	Pre	Ductal/NST	NA	III+IV

742	TCGA-S3-AA11	¡Ý60	Post	Ductal/NST	NA	I+II
743	TCGA-S3-AA12	¡Ý60	Post	Ductal/NST	NA	III+IV
744	TCGA-S3-AA14	<60	Post	Ductal/NST	NA	I+II
745	TCGA-S3-AA17	¡Ý60	Post	Ductal/NST	NA	I+II
746	TCGA-UL-AAZ6	¡Ý60	Post	Ductal/NST	NA	I+II
747	TCGA-V7-A7HQ	¡Ý60	Post	Ductal/NST	NA	III+IV
748	TCGA-W8-A86G	¡Ý60	Post	Other	NA	I+II
749	TCGA-WT-AB41	<60	Post	Ductal/NST	NA	I+II
750	TCGA-WT-AB44	¡Ý60	Post	Other	NA	I+II
751	TCGA-XX-A899	<60	Post	Other	NA	III+IV
752	TCGA-XX-A89A	¡Ý60	Post	Other	NA	I+II
753	TCGA-Z7-A8R5	¡Ý60	Post	Other	NA	III+IV
754	TCGA-Z7-A8R6	<60	Pre	Other	NA	I+II

OS.time	OS	risk	riskScore
134.9	0	low	0.106158408549611
49.1333333333333	0	low	0.136918730291429
48.2666666666667	0	low	0.011340232125268
11.6	0	low	-0.108671284982425
49.2333333333333	0	low	-0.11095026881126
49.0333333333333	0	low	-0.066749405664056
10.1	0	low	0.115134404910346
14.5666666666667	0	low	-0.265168241133607
44.0333333333333	0	low	0.00317498529507
48.7666666666667	0	low	0.034565429473141
14.4666666666667	0	low	-0.299496507503118
47.9	0	low	-0.369577157689957
21.1666666666667	0	low	0.056150774461327
13.8666666666667	0	low	0.019427753962645
8.06666666666667	0	low	-0.143652871206597
39.8666666666667	0	high	0.299294207457679
18.4666666666667	0	low	-0.059424857300153
145.133333333333	0	low	-0.134956340200852
123.633333333333	0	low	0.063609120420012
64	1	low	-0.125494944440726
36.6333333333333	0	low	-0.145432269226049
138.633333333333	0	low	-0.279793479956713
116.4	1	low	-0.614358444879784
93.7666666666667	0	low	-0.526342849500922
89.8333333333333	0	low	-0.033266260272224
109.433333333333	0	low	-0.498798094069854
78.2666666666667	1	low	0.04173483962615
76.3	0	low	0.167606935036393
5.26666666666667	1	low	0.013662554719774
100.366666666667	0	low	-0.262504510441589
109.433333333333	0	high	0.939089278826964
55.7666666666667	0	low	0.061346604631335
62.4333333333333	0	low	0.086382713839087
25.5666666666667	0	high	0.360001974773363
103.133333333333	0	low	0.088971580426603
136.266666666667	0	low	-0.330865559896501
81.4	0	low	-0.326155399782584
120.1	0	low	-0.487877356323258
75.4333333333333	0	low	-0.193309577290218
73	0	low	-0.394506993159825
35.5333333333333	0	high	0.384279211922754
34.7666666666667	0	low	0.043192213579773
32.2666666666667	0	low	-0.135190763908725
62.8	1	low	-0.264165998466772
25.0666666666667	0	low	-0.152566863902322
64.1666666666667	0	low	0.122319478272732
55.4	0	low	-0.030705052270677
27.5	1	high	0.488859183392814
45.5	1	high	0.287159897438564
44.9	0	low	-0.24868385773536
50.5333333333333	0	low	0.127303117951039
20.8	0	low	-0.208795565500162

17.7	0	low	-0.160115937004223
19.1666666666667	0	low	-0.447513449146809
21.0333333333333	0	low	0.074795396725498
33	0	low	-0.141595465059555
25.6333333333333	0	low	-0.343530025534863
51.1666666666667	0	low	0.080275349420046
22.2	0	high	0.574660380653444
21.9666666666667	0	low	0.181354907631547
50.1666666666667	0	low	-0.346199130685036
49.1333333333333	0	low	-0.105608682340538
24.1	1	high	0.709412696088475
23.8	0	low	-0.08044005026153
17.6	0	low	-0.033541713964713
61.5666666666667	0	low	0.059265430593465
22.7666666666667	0	low	-0.07072437186611
20.5333333333333	0	low	-0.291910384728743
19.8333333333333	0	high	0.37289912810951
53.2	0	low	-0.22494138389235
43.0333333333333	0	high	0.630234996845329
17.4333333333333	0	high	0.232585594000366
18.4	0	low	0.037229348807297
106.8	0	high	0.360148331794162
36.7333333333333	0	low	-0.311885868103006
40.2	0	low	-0.496257472765485
7.4	0	low	-0.04323494131694
23.5333333333333	0	low	0.045681811683964
21.4333333333333	0	low	-0.062162380581328
22.2	0	high	0.304176179852182
38.8333333333333	0	low	-0.148573834032368
34.7666666666667	0	low	-0.354441296334475
35.9666666666667	0	low	-0.116051193004339
31.0333333333333	0	high	0.380670256361272
37.9666666666667	0	low	-0.482055342494045
33.5666666666667	0	low	-0.675338082319038
30.2	0	low	-0.23694235632184
25.5	0	low	0.2144060627341
23.9333333333333	0	low	-0.43139806785734
29.9666666666667	0	low	-0.070936567150815
31.8	0	low	-0.014185157169388
24.1333333333333	0	low	-0.119909618473782
20.9	0	low	-0.339438477443991
22.6333333333333	0	high	0.241535594382074
11.5	0	low	-0.493718383633248
10.7333333333333	0	low	-0.254130292221724
10.4333333333333	0	low	-0.21099446811512
11.4333333333333	0	low	-0.01839485087358
13.6	0	low	-0.187118765689095
14.9	0	low	-0.060186500221838
12.1333333333333	0	low	-0.309215142887707
15.1333333333333	0	low	0.0811278137743
13.9333333333333	0	low	-0.162710999111103
14.8666666666667	0	low	-0.312709920231017
14.9333333333333	0	low	-0.028780577012679

10.8666666666667	0	low	-0.422484859905344
11.2	0	low	-0.460319704311948
10.5666666666667	0	low	0.177889846777387
13.2	0	high	0.338119904624607
13.2	0	low	-0.182464813923581
1.03333333333333	0	high	0.564272718796536
18.2333333333333	0	low	0.030248413289632
53.8	0	high	0.25875426002232
29.4333333333333	1	high	0.5326278748984
31.4333333333333	1	low	0.198554751497274
26.3666666666667	0	low	0.04602711091662
1.03333333333333	0	high	0.374727917572149
17.2666666666667	0	low	0.204284826150588
54.7333333333333	0	low	0.151926836355599
9.13333333333333	0	high	0.391626926266452
43.6	0	high	0.32391910287915
20.2666666666667	0	low	-0.097170224991065
19.2333333333333	0	low	-0.031569275139628
19.2333333333333	0	low	0.019969095395857
12.1666666666667	0	low	-0.304602529739665
32.5	0	high	0.339208126831055
11.1333333333333	0	low	-0.227249115185497
8.1	0	low	0.213508903005906
10.1333333333333	0	high	0.414611498445972
45.7	0	high	0.275754981302498
18.3	0	high	0.358991357428264
15.2666666666667	0	high	0.466616895728952
37.4666666666667	0	high	0.517673288336405
13.2	0	low	-0.162290309287128
29.3666666666667	0	low	0.014672061571122
33.4666666666667	0	high	0.314718170859746
20.2333333333333	0	low	0.203592237362094
12.1666666666667	0	high	0.383962987893136
31.4333333333333	0	low	-0.03250117718753
31.4333333333333	0	low	0.024498275169776
33.4666666666667	0	high	0.326543730340592
113.633333333333	1	low	-0.104852434837307
40.5666666666667	0	low	-0.268219238231074
33.4666666666667	0	low	0.138708123628888
31.4	0	high	0.654863315960611
18.2	0	low	-0.152131817171901
42.5666666666667	0	low	0.178606552749726
12.1666666666667	0	low	-0.100648793996209
10.1333333333333	0	low	-0.00064264186814
10.1333333333333	0	low	0.004442010315477
12.1666666666667	0	low	-0.059101363426453
1.03333333333333	0	high	0.273963803875859
50.7333333333333	0	low	-0.11964015580195
49.7333333333333	0	high	0.419200044917028
45.7	0	high	0.901966953035526
30.4	0	low	0.164974008040442
33.5333333333333	0	low	0.128475489993778
1.03333333333333	0	high	0.456928998438682

25.3666666666667	0	low	0.061223617696415
9.1	0	low	0.080791732373168
19.3	0	low	-0.247551057144386
15.2333333333333	0	low	-0.052000207110834
12.1666666666667	0	low	9.461658132974E-05
19.3	0	low	0.033294317213948
13.2	0	low	-0.117576288264799
21.3333333333333	0	low	-0.206084063749527
27.4	0	high	0.254617931719774
17.2666666666667	0	high	0.381589400580762
38.5666666666667	0	low	-0.278541699112327
19.5	0	low	0.118877321519477
23.2666666666667	0	low	-0.199467455786321
74.9333333333333	0	low	-0.683279380883793
22.5666666666667	0	low	-0.222717835741808
100.733333333333	0	high	0.260334452593885
41.1333333333333	0	low	-0.159306493391359
87.8666666666667	1	low	-0.309454520488396
91.9666666666667	0	low	-0.403779862169817
61.7666666666667	0	low	-0.396803441030132
26.4	1	low	0.052575813150259
75.1666666666667	0	low	-0.291140726796753
19.6	0	low	-0.229205922659636
32.9	0	low	-0.329677294667415
6.56666666666667	1	high	0.223355623193161
16.5333333333333	0	low	-0.252899490702066
15.0333333333333	0	low	-0.773029388820711
22.5	0	low	-0.368210875213517
24.4666666666667	0	low	-0.421844203519898
25.4	0	low	-0.151432144476247
15.2	0	low	-0.025102346001537
20.0666666666667	0	low	-0.226501414372536
15.7	0	low	-0.107728015665639
25.1333333333333	0	low	-0.370629596179608
29.6666666666667	0	low	-0.023477121239522
19.6	0	low	-0.080318479803458
15.1666666666667	0	low	-0.165293565578797
11.6	1	high	0.420306793777598
17.6666666666667	0	low	-0.094711428478076
18.9333333333333	0	low	-0.477327283971325
12.4333333333333	0	low	-0.213741045066866
1.7	0	high	0.388605797997293
8.33333333333333	0	high	0.508180346001345
20.4666666666667	0	low	-0.238774797963283
1.33333333333333	0	low	-0.061979383189487
2.33333333333333	0	low	-0.441355037746577
3	0	low	0.076955978099602
10.1	0	high	0.322419002727768
7.46666666666667	0	high	0.690715423285322
6.53333333333333	0	low	0.066075821109003
5.73333333333333	0	high	0.238610267323552
7.1	0	high	0.340395787142978
7.26666666666667	0	low	-0.592385088140267

7	0	low	-0.321061769228406
7.13333333333333	0	low	-0.337301477337782
5.43333333333333	0	low	-0.293319803177686
12.5	0	low	0.052344085261876
5.4	0	low	-0.004842255210091
5.66666666666667	0	low	0.030385142610927
81.4	0	low	0.147781771691972
62.2	0	high	0.369965235427437
67.7	0	low	-0.147482279180575
82.7666666666667	1	high	0.719868887439675
97.0333333333333	1	low	0.066709211150657
45.0333333333333	0	low	0.167152579997584
21.7	0	high	0.257319036306721
26.4	1	low	0.093813054470151
20.6	0	high	0.511510061745226
22.6666666666667	0	low	-0.039627396524332
53.7666666666667	0	low	-0.575445327283028
21.8333333333333	0	low	0.054561620412594
51.5666666666667	0	low	-0.389992231013331
73	0	high	0.598279701941617
66	0	low	-0.416168893784626
26.6	0	low	-0.05044407511896
50.9333333333333	0	low	0.025852597426828
62.9	0	low	-0.309797039000324
72.8	0	high	0.336255405034282
115.2	0	low	0.041671733824966
110.233333333333	0	low	-0.061050672609277
103.733333333333	0	low	-0.157749646888058
99.6333333333333	0	low	-0.074287886103301
79.0666666666667	0	low	-0.522982881544382
71.4	0	low	-0.221878544149287
54.6333333333333	0	low	-0.130480245202637
41.1333333333333	0	low	-0.134524654748323
98.4333333333333	0	low	0.096328729291999
62.7333333333333	0	high	0.618826722808381
11.6666666666667	0	high	0.40650174489824
18.0333333333333	0	high	0.230538068465897
25.1333333333333	0	high	0.408371081200057
131.9	0	high	0.354804985777216
5.73333333333333	1	low	-0.184237835328533
15.8333333333333	0	low	0.218192385127429
32.9666666666667	0	low	-0.217134719261735
33.3666666666667	0	low	0.006705790184877
19.4666666666667	1	low	-0.371128590119525
99.7	0	high	0.246684536603588
5.33333333333333	1	high	0.474877014137831
110.533333333333	0	low	-0.079161804264164
76.2666666666667	0	high	0.259400740256904
100.3	0	low	-0.032224604596998
56.6333333333333	1	high	0.245090555392517
108.733333333333	1	high	0.303175750470143
85.0333333333333	1	high	0.774427718219758
136	0	high	0.543225343374699

105.3	0	low	-0.036979522570395
99.0333333333333	0	low	-0.257251107993499
99.7	0	low	-0.347790945873518
97.3333333333333	0	low	-0.138091980048668
95.2	0	low	0.201433549438442
38.3333333333333	0	high	0.324311046250483
95.6	0	low	-0.018637163739274
62.1333333333333	0	low	0.150626370839421
87.7333333333333	0	low	-0.15477253934203
87.6333333333333	0	low	-0.02423010479482
163.133333333333	0	high	0.256013799591526
51.6	0	low	0.023519084764431
95.5333333333333	1	low	-0.17245044308291
122	0	low	-0.189108160942131
101.166666666667	0	low	-0.062827727811533
120.233333333333	0	low	-0.3878561692376
2.8	0	low	-0.132703713115814
114.333333333333	0	high	0.246326477977057
99.2	0	low	0.018224533789059
106.733333333333	0	low	-0.16555784982267
106.766666666667	0	low	-0.133270977367826
51.6666666666667	0	low	-0.256762293883046
100.133333333333	0	low	0.181042893021548
100.033333333333	0	high	0.410154734484469
90.2333333333333	0	low	0.108740829799382
94.6	0	low	-0.371211322928496
72.0333333333333	0	low	-0.014922673159074
168.733333333333	0	low	-0.059049996860131
87.7333333333333	0	low	-0.361646349031692
54.9666666666667	1	high	0.434773930293392
67.0666666666667	0	low	-0.000198480361043
64.5	0	low	-0.375056099109741
38.7	0	low	-0.533240704663936
39.9333333333333	0	low	-0.323923007215042
74.3666666666667	0	low	-0.254715759515564
33.7666666666667	0	low	-0.283540515330316
39.5	0	low	-0.181995580263649
285.2	0	low	-0.184822949641008
24.9666666666667	1	high	0.233333804581198
279.7	0	low	0.079217398408329
131.366666666667	1	high	0.329002068609229
51.4	1	high	0.266388512961842
66.4333333333333	1	low	-0.090766282274602
148.533333333333	1	high	0.228261879979695
113.933333333333	1	low	-0.280270028524242
129.1	1	low	-0.093440735836045
85.7666666666667	1	low	-0.733463930936006
168.066666666667	0	high	0.314679409248845
130.866666666667	1	low	0.102591097373375
69.4	0	low	0.193489440600079
237.533333333333	0	low	-0.016871602587184
82.3	1	high	0.582032626300518
79.1	1	low	0.216543156746784

266.933333333333	0	low	-0.062000296225609
164.3	0	low	-0.009142590969566
104.2	1	low	-0.534455683380647
171.866666666667	0	low	-0.061530125201426
98.8333333333333	1	high	0.438750036751554
191.3	0	low	0.047306267796349
80.5666666666667	1	high	0.541534698951716
18.6	1	low	0.042629136350744
115.366666666667	1	low	0.015485752408632
209.733333333333	0	low	0.106010812112446
131.5	1	low	0.064827732302027
28.6666666666667	1	low	-0.206431236785719
69.9	1	high	0.427128972092218
59.3666666666667	1	low	-0.02141209800397
44.2	0	high	0.240481717887943
74.5333333333333	0	low	-0.168012058156246
141.1	0	high	0.785505648449916
172.533333333333	0	low	-0.022556277910731
86.5333333333333	0	low	-0.01614984818743
69.0666666666667	0	low	0.016711878280035
105.066666666667	0	low	-0.079862037638124
72.1333333333333	0	low	0.109963223209227
63.8	0	low	0.069603024858674
63.9666666666667	0	low	-0.299859311852633
82.5666666666667	0	low	-0.01049472057053
38.2666666666667	0	low	0.079192848947351
39.7	0	low	0.029751074192095
71.2	0	high	0.292496861138383
82.7666666666667	0	low	-0.059399938733215
52.3	0	low	-0.012861930573057
37.7333333333333	0	high	0.282851845004793
32.4666666666667	0	low	-0.004931691973201
18.4666666666667	0	low	0.082211963625037
44.1333333333333	1	low	-0.028422740234143
22	0	low	-0.275851776220369
62.5333333333333	0	low	-0.473379864687563
73.2333333333333	0	low	-0.474696691395762
76.5333333333333	1	low	-0.232011489207986
75.1666666666667	0	low	-0.209655902079025
77.6666666666667	0	low	0.054147724494449
87.0666666666667	0	low	-0.113661678073641
78.8333333333333	0	low	0.07766398973619
50.6333333333333	0	low	0.115831703492662
75.1666666666667	0	high	0.235500003773442
42.3333333333333	0	high	0.441411260581369
47.0333333333333	1	high	0.262917714775844
90.3	0	low	-0.230216644719863
92.2333333333333	0	low	0.21437116896722
82.8666666666667	0	high	0.546517046320177
79.0666666666667	0	low	-0.435360136001421
68.0333333333333	0	low	-0.16523819918935
38.5333333333333	0	low	0.076093568935653
30.4	0	low	-0.356155791005616

14.1	0	low	-0.107410603685297
54.8	0	low	-0.15890141854314
15.8666666666667	0	low	-0.323160513615844
3.26666666666667	0	low	-0.085681653838729
2.6	0	low	-0.261182945488121
80.1	0	low	-0.242137512956837
68.8	0	low	-0.628922982021437
71.8666666666667	0	high	0.314489160460463
16.5	0	low	-0.387735676998695
15.9	0	low	-0.338920465637582
14.5	0	low	-0.132053206084176
45.4333333333333	0	low	0.085898156323694
82.9666666666667	0	low	-0.141556641977541
33.0333333333333	1	low	-0.333358516760544
24.8333333333333	0	low	-0.170357852832874
64.2	0	low	-0.047578853299103
30.7666666666667	0	low	0.15056599464142
10.9333333333333	0	low	-0.139015891656546
15.3666666666667	0	high	0.586155705288833
64.2666666666667	0	low	-0.244452588098365
54	0	low	-0.430251229255255
24.9	0	low	-0.226374239978466
23.4	0	low	-0.376750105372974
41.5666666666667	0	high	0.226267608409353
53.7	0	low	-0.365985444866396
26.8666666666667	0	low	0.101453446625646
24.2333333333333	0	low	-0.132561813525125
20.6666666666667	0	low	-0.277235581889215
5.93333333333333	0	low	-0.283620441603389
2.4	0	low	0.043592898520597
17.2	0	low	-0.02342637070858
2.53333333333333	0	low	-0.126764854668418
13.8	0	low	-0.106607984492667
37.3666666666667	0	low	-0.211458446849391
13.0666666666667	0	high	0.445716395481206
52.0333333333333	0	low	0.019665211156207
27.6333333333333	0	low	0.124427245496363
51.5	0	high	0.225273076086222
24.2666666666667	0	high	0.352613120674593
25.3	0	low	-0.23758603495569
42.9333333333333	0	low	-0.186937038492989
45.4333333333333	0	low	-0.004799910557776
33.3666666666667	0	low	0.005753158260002
21.7333333333333	0	low	-0.027798565597087
36.4333333333333	0	low	-0.075506236723542
20.4	1	low	0.04331744443171
92.1	1	low	0.082861197546739
27.0333333333333	1	high	0.627028944087998
73.5666666666667	1	low	-0.215704299865565
38.2666666666667	1	low	0.084885651476147
66.9666666666667	1	low	-0.183107692268572
52.1	1	high	0.470059950824763
93.2666666666667	1	low	-0.34917942439871

115.4	1	low	0.099214420279024
84	1	low	-0.178189901000241
42.8666666666667	1	low	-0.35412179044778
56.4666666666667	1	high	0.407902822147625
50.2666666666667	1	low	0.100765665070768
17.9333333333333	1	low	-0.110622468275302
31.9666666666667	1	low	-0.073051067410628
90.4	1	low	-0.042253028598936
25.4333333333333	1	high	0.521361221855684
122.3	1	low	-0.423169569480619
33.6333333333333	1	high	0.616530649506449
75.7666666666667	1	low	-0.023804882371249
124.533333333333	1	low	-0.226700397658045
34.4666666666667	1	low	-0.485642201871569
64.2333333333333	1	high	0.475194809262696
55.7666666666667	1	low	0.125392718919603
46.2666666666667	1	low	0.123894348950026
73.0666666666667	1	low	0.188427579832242
54.7333333333333	1	low	-0.271716530464257
28.5333333333333	0	low	-0.210334108561422
26.5	0	high	0.293671888755533
84.4666666666667	1	high	0.252527734392052
131.966666666667	1	high	0.251856103944526
37.3	0	low	-0.008112771456451
20.4	0	low	-0.158940206094509
10.6666666666667	1	low	-0.077000462093812
21.1666666666667	0	low	-0.58041850309842
23.8333333333333	0	low	-0.048196096102912
9.76666666666667	0	high	0.387111096042779
9.83333333333333	1	low	-0.002985138299556
22.0666666666667	0	low	-0.390110248215243
9.56666666666667	0	low	-0.397250218893291
11.9333333333333	0	high	0.357848306205333
11.9333333333333	0	low	-0.248445076258573
12.8333333333333	0	high	0.339332733496632
12.8333333333333	0	high	0.583735559106581
12.8333333333333	0	high	0.255561690490449
12.8333333333333	0	low	0.086772106360695
49.2	0	low	-0.096167803906209
12.3333333333333	0	high	0.328429806340627
12.7666666666667	0	low	-0.286464532900913
12.5	0	low	0.031569752587792
11.5	0	high	0.318525751068787
11.4333333333333	0	low	-0.014718089979386
10.5666666666667	0	high	0.426854007519481
12.5	0	high	0.866007564639877
13.1333333333333	0	high	0.43764012263369
12.5	0	high	0.47187438104271
20.5333333333333	0	low	0.026241313833926
12.7	0	high	0.370101182761172
15.6666666666667	0	low	0.178442358136086
17.1	0	low	-0.053528104106717
16.9333333333333	0	high	0.384885520489941

24.9	0	high	0.418917844560881
34.4666666666667	1	low	-0.307172983780474
13.1333333333333	0	low	0.060450734830301
12.6666666666667	0	low	0.104325498117294
57.6	0	high	0.720460936093147
13.4333333333333	0	low	-0.205389646279965
20.8666666666667	0	low	-0.294900919485025
13.6666666666667	0	low	-0.258809889917875
21.4333333333333	0	low	-0.354050337679559
14.3666666666667	0	low	0.212522221979568
17.7333333333333	0	low	0.216978275457844
56.2666666666667	0	low	-0.175820476948229
16	0	high	0.323852368715341
18.4	0	high	0.312020493438817
19.1666666666667	0	low	0.14576548800819
14.2	0	low	-0.395673701707589
19.2333333333333	0	low	-0.006539839215965
20.3666666666667	0	high	0.300509059824746
20.6666666666667	0	high	0.417066504941387
21.3	0	low	-0.169316193459956
12.3666666666667	0	high	0.244434903456803
13.5	0	high	0.400956369402782
14.9	0	low	-0.563264210136132
18.8333333333333	0	low	-0.00439994125057
18.0333333333333	0	low	0.044902592781229
16.9666666666667	0	low	0.016639225415613
26.1	0	low	-0.203638989963417
24.2333333333333	0	low	0.020435560788078
27.9666666666667	0	low	-0.199455761022258
18.4	0	low	-0.198744298036672
12.5666666666667	1	low	0.128259877759675
17.4	0	low	-0.107318823187269
15.4333333333333	0	high	0.582801409538664
14.9333333333333	0	low	-0.013535999015289
20.2	0	high	0.479816592229297
17.9333333333333	0	low	-0.192249608541463
56.0666666666667	0	low	-0.013855152491402
16.0666666666667	0	low	0.03279625806624
16.5333333333333	0	high	0.265281463566076
13.1666666666667	0	low	0.006587194648705
15.3666666666667	0	low	-0.052339621362546
16.7666666666667	0	low	-0.22379958892788
15.5333333333333	0	high	0.366109744141422
15.7333333333333	0	low	0.112431132628331
10.0666666666667	1	low	0.147381012730963
14.4333333333333	0	low	0.003361825636663
14.3333333333333	0	high	0.907068507200183
17.6666666666667	0	low	-0.405831262683845
13.6333333333333	0	low	-0.040263320776991
14.6333333333333	0	low	-0.164914402474165
48.7	0	low	-0.263794615525726
16.6333333333333	0	low	-0.194975062518025
17.3	0	low	0.072934710781807

1.63333333333333	0	low	-0.110364097684118
10.2333333333333	0	high	0.412069107149097
13.2666666666667	0	low	-0.214878916126505
12.7	0	low	-0.016065976214069
12.4333333333333	0	high	0.268350693003829
33.8333333333333	0	low	-0.343792884336399
18.7666666666667	0	low	-0.046760133825002
21.9666666666667	0	low	-0.292965656130003
16.4	0	low	-0.383950384179277
12.8333333333333	1	low	-0.417879231742249
25.5666666666667	0	low	-0.097087271112168
43.6	0	low	0.204125199825346
84.7	0	high	0.460559371363942
34.9	0	high	0.380551888160375
47.2333333333333	0	high	0.587086395913052
40.9666666666667	0	low	0.159436168712215
38.0333333333333	0	high	0.309400666997212
40.6666666666667	0	high	0.293155239630222
28.8333333333333	0	low	-0.035231478334708
29.2666666666667	0	low	-0.165338556633336
45.3	0	high	0.481578113496473
38.7666666666667	0	low	0.079596737125881
33.6333333333333	0	low	-0.075566348297993
77.0333333333333	0	low	0.11464444302256
43.9333333333333	0	low	-0.319045268654799
32.4666666666667	0	high	0.292447033822047
18.7666666666667	1	low	-0.159066492813791
23.5666666666667	0	low	-0.317124137138982
19.7	0	high	0.298268091366564
21.3333333333333	0	high	0.312447267983596
24.2	0	low	0.033967091913389
23.6666666666667	0	low	0.190258332298648
23.1333333333333	0	low	-0.118247475976496
17.5333333333333	0	low	-0.21362515346157
21	0	low	0.051791347085596
21.9333333333333	0	low	0.117694362425456
18.4666666666667	0	low	0.220938576772419
13.1	0	high	0.445031597598724
56.4	0	low	-0.16483379197646
54.6666666666667	0	low	0.006516334946768
9.16666666666667	0	high	0.380096310533732
20.8666666666667	0	low	0.187048879486058
11.2	1	low	-0.012614381860107
51.5	0	high	0.391417922315535
19.8333333333333	0	low	-0.061824084593811
57.7333333333333	0	low	0.031093100767279
14.2666666666667	0	high	0.433803292467637
52.1	0	low	0.219613207718052
88.4333333333333	0	low	-0.113180458841555
33.4666666666667	1	low	-0.13480326084066
32.8	0	low	-0.403421591183654
16.7	0	low	-0.329199111142281
37.7666666666667	0	low	-0.087158612129773

78.7333333333333	0	high	0.225874425783666
37.9333333333333	0	low	-0.313227808399765
71.3333333333333	0	low	-0.047479056137959
34.2	0	low	-0.401228811258738
28.8333333333333	0	low	-0.430173108244953
60	0	low	-0.124440892124256
3.93333333333333	0	low	-0.235715872523766
22.5	0	low	-0.172278176228849
61.8333333333333	0	low	-0.095891816097074
11.2333333333333	0	low	-0.142825675357139
54.9333333333333	0	low	-0.074081371581471
61.2	0	high	0.259823454609797
74.6666666666667	0	low	0.006476030661321
19.9333333333333	0	low	-0.139576105793207
24.9333333333333	0	low	-0.517584300320265
27.3666666666667	1	low	0.183164824744087
35.0333333333333	0	low	-0.227010770297908
44.1666666666667	0	low	-0.395328764245063
8.4	0	high	0.233272603525585
31.0333333333333	0	low	-0.417012333626078
40.2666666666667	0	low	-0.125431488868455
34.7666666666667	0	low	-0.004482844105434
17.9333333333333	0	high	0.241333478278954
35.3	0	low	-0.118952988094123
33.3333333333333	0	low	-0.197245900141495
37.3333333333333	0	low	0.169960472357473
22.6	1	low	0.120031903618314
37.0666666666667	0	high	0.237919545443154
36.2666666666667	0	low	-0.028571238794411
35.7333333333333	1	low	-0.169872267175069
26.2	1	low	-0.462181081084225
19.2	0	low	-0.279139330160187
10	0	high	0.248823282063079
25.1666666666667	0	low	-0.242594531953665
28.6666666666667	0	low	-0.186170006856793
35.4333333333333	0	high	0.229418098531498
2.6	0	low	-0.234913911702719
6.2	0	high	0.393747268650164
3.06666666666667	0	low	-0.210266568409405
11.3	0	high	0.347724552394198
48.9	0	low	0.167960060890426
45.6333333333333	0	low	0.186454697913133
32.5333333333333	1	high	0.507268291125433
40.8	0	high	0.339110349822179
1.13333333333333	0	low	-0.0246677738681
47.3	0	high	0.2396464629292
6.66666666666667	0	low	-0.044538950497422
21.5666666666667	0	high	0.376815045072252
48.3	0	low	-0.167293203821793
34.9333333333333	1	low	0.145376059972109
32.5	0	low	-0.161533693793848
42.8333333333333	0	high	0.321603979828283
38.2666666666667	0	low	-0.027845739982309

39.6333333333333	0	low	0.109921441505467
38.9	0	low	-0.193383156219181
41.6	0	high	0.33490395825029
42.3	0	low	-0.362381482013871
41.0666666666667	0	low	0.189968584788992
39.5333333333333	0	high	0.226119444190132
7.23333333333333	0	high	0.545589994272832
24.9	0	low	-0.259157559386276
12.5	0	low	-0.162605059840819
30.1333333333333	1	high	0.301031868277316
9.6	0	low	-0.273494566294026
38.6	0	high	0.393647681806318
33.3666666666667	0	low	-0.645618524025645
30.8666666666667	0	low	-0.634355905032195
24.2333333333333	0	low	0.034768038995433
24.1666666666667	0	high	0.231160839785127
27.0666666666667	0	low	-0.256705748922765
26.1666666666667	0	low	0.214826484079535
26.7666666666667	0	low	0.131508086736517
28.2333333333333	0	low	-0.078843153982622
12.3666666666667	0	low	0.02602581462688
40.2666666666667	0	low	-0.242899762539593
8.6	0	low	-0.095457875307514
18.4666666666667	0	low	-0.012680087229882
19.1666666666667	0	low	0.076415663384252
13.4333333333333	0	low	-0.283617926764215
16.8	0	low	0.070063165986253
15.9	0	low	0.018557885885307
29.1666666666667	0	low	-0.356717170370176
30.3666666666667	0	low	-0.059603878734585
30.2666666666667	0	high	0.381090360714874
40.9666666666667	0	high	0.417913614932811
41.7	0	low	0.19927811121597
53.7	0	low	-0.109231775004522
23.4333333333333	0	high	0.400278616701601
18.7333333333333	0	low	0.11843007399837
19.1666666666667	0	low	-0.016111126521816
6.23333333333333	0	high	0.399475895681547
14.1333333333333	0	low	0.144453777349985
10.6666666666667	0	low	0.009189193380829
14.6333333333333	0	low	0.037882674474131
53.4666666666667	0	low	-0.072722174639566
26.2666666666667	0	low	0.042844227281933
22.4	0	high	0.271335012193973
34.5	0	low	-0.19364032620834
17.7666666666667	0	low	0.024144659546761
23.8333333333333	0	low	0.046300185131774
15.4333333333333	0	high	0.334623370091238
17	0	high	0.493097268126299
31.7333333333333	0	low	-0.129291162434777
7.5	0	low	-0.345108618798368
60.4	1	low	-0.102104579038895
219.766666666667	1	low	-0.545324701146189

84.5	0	low	-0.009589444753103
86.3333333333333	0	low	-0.311858248653675
88.1666666666667	0	low	-0.338569428161241
117.3	0	low	-0.122743698538082
107.533333333333	0	low	-0.323534688812921
103.033333333333	0	low	-0.1963940929266
86.5333333333333	0	low	-0.127873931478301
112.033333333333	0	low	-0.224676108187327
38.7333333333333	0	low	-0.294983653554877
44.3333333333333	0	low	-0.294814545801352
67.3	0	low	-0.335170900700298
73.0333333333333	0	low	-0.591991937258568
13.7333333333333	0	low	-0.360383192536512
18.3666666666667	0	low	-0.47269010056568
63.3333333333333	1	low	-0.51913574820658
11.7333333333333	0	low	0.178599944978855
21.5333333333333	0	low	-0.172667243978095
13.4	0	low	-0.30264004780725
7.2	0	low	-0.058408908423623
13.4666666666667	0	low	0.017652155881679
25.3	0	low	-0.689832315349199
29.6333333333333	0	low	-0.232971615126601
25.4	0	low	-0.073020282720161
17.3	0	low	-0.314324179501774
15.5333333333333	0	low	-0.178886644587316
14.9	0	low	-0.020297944221523
22.5666666666667	0	high	0.357744723075653
2.66666666666667	0	low	-0.329855367127442
14.7	0	low	-0.045626992068379
19.8	0	high	0.328609729332314
12.5333333333333	0	high	0.606864488440739
17.7333333333333	0	low	-0.056371470952841
28.3	0	low	0.213375663083745
22.7	0	low	-0.195249366361268
2.13333333333333	0	low	0.059923473181703
32.4333333333333	0	high	0.298033797598239
59.4333333333333	0	low	-0.019381803842485
40.6333333333333	0	low	-0.202426526953554
35.4	0	low	-0.001805549944038
29.2666666666667	0	low	-0.301649738994516
66.5333333333333	0	low	-0.486555216932988
42.5	1	low	-0.157637173947252
43.3666666666667	0	low	-0.404510036032577
26.4	0	low	0.022344559044511
17.6	0	high	0.343107255285707
20	0	low	-0.41800903062047
40.6666666666667	0	low	0.053534927079631
47.6666666666667	1	low	0.214552077499859
65.1	0	low	0.05926956995716
88.1666666666667	0	low	-0.171437033503751
19.0666666666667	0	high	0.385100326046446
18.7333333333333	0	high	0.300602562143697
21.3666666666667	0	high	0.748944274263866

14.0333333333333	0	low	0.102888915137119
19.1333333333333	0	high	0.445138651445926
17.6333333333333	0	low	-0.040746335538293
14.1333333333333	0	high	0.258927999805244
17.2666666666667	0	high	0.438445607079057
67.7666666666667	0	low	-0.100686091857631
11.5666666666667	0	low	-0.199272417368151
53.7	0	low	0.100346739966678
29.4333333333333	0	low	-0.560257189594463
15.5666666666667	0	low	-0.201281377876958
16.2666666666667	0	low	-0.22985043135975
109.566666666667	0	low	-0.586973171071045
108.533333333333	0	high	0.46359855745153
