# Supplementary material for: A ferroptosis-associated gene signature for the prediction of prognosis and therapeutic response in luminal-type breast carcinoma
Source: Sci Rep. 2021 Sep 2;11:17610. doi: 10.1038/s41598-021-97102-z (PMC8413464; doi:10.1038/s41598-021-97102-z)
Supplement: Supplementary file 11 — Supplementary Table S7. [file 41598_2021_97102_MOESM11_ESM.pdf]

TableS7 immune infiltration score in the TCGA cohort

	V1	Response	CD4_naive	CD8_naive	Cytotoxic	Exhausted	Tr1	nTreg
1	TCGA-E9-A1NI-01A	NR	0	0.194	0.249	0.097	0.125	0
2	TCGA-A1-A0SP-01A	R	0	0.054	0.334	0.102	0.103	0
3	TCGA-A8-A06X-01A	R	0	0.092	0.37	0.189	0.063	0.096
4	TCGA-E2-A14T-01A	R	0	0.126	0.293	0	0.114	0
5	TCGA-AC-A8OS-01A	R	0.043	0.112	0.434	0.077	0.147	0.038
6	TCGA-A8-A09K-01A	R	0	0.125	0.499	0.136	0.203	0.057
7	TCGA-OL-A5RY-01A	R	0	0.008	0.473	0.09	0.244	0.008
8	TCGA-BH-A0DG-01A	R	0	0.12	0.416	0.104	0.153	0.028
9	TCGA-B6-A0I9-01A	R	0	0.128	0.307	0.067	0.064	0.093
10	TCGA-A2-A0CP-01A	R	0.024	0.114	0.368	0.053	0.063	0
11	TCGA-A8-A07S-01A	NR	0.037	0.128	0.219	0.036	0.133	0.031
12	TCGA-AO-A12H-01A	R	0	0.117	0.307	0.087	0.157	0.004
13	TCGA-AR-A1AU-01A	R	0.008	0.111	0.318	0.047	0.127	0.049
14	TCGA-D8-A1X9-01A	NR	0	0.115	0.343	0.114	0.093	0.13
15	TCGA-A8-A07R-01A	R	0	0.114	0.235	0.074	0	0.007
16	TCGA-AO-A12C-01A	NR	0.026	0.133	0.228	0	0.103	0
17	TCGA-V7-A7HQ-01A	R	0	0.096	0.333	0.052	0.099	0
18	TCGA-A2-A1FZ-01A	R	0	0.08	0.397	0	0.141	0.002
19	TCGA-AR-A251-01A	R	0	0.101	0.339	0.083	0.132	0.126
20	TCGA-EW-A1PF-01A	R	0	0.134	0.365	0.077	0.197	0.048
21	TCGA-BH-A18T-01A	R	0	0.046	0.375	0.104	0.099	0.107
22	TCGA-AR-A24S-01A	R	0	0.074	0.362	0.105	0.223	0.036
23	TCGA-AN-A041-01A	NR	0.015	0.126	0.236	0	0.09	0.021
24	TCGA-E2-A1LL-01A	R	0.022	0	0.153	0.048	0.075	0.081
25	TCGA-E2-A1IJ-01A	R	0	0.108	0.447	0.084	0.176	0.032
26	TCGA-AR-A24O-01A	R	0	0.096	0.391	0.06	0.149	0.06
27	TCGA-XX-A899-01A	R	0.017	0.068	0.484	0.081	0.21	0.012
28	TCGA-D8-A1XK-01A	R	0	0	0.48	0.178	0.083	0.085
29	TCGA-AC-A5XS-01A	R	0	0	0.498	0.123	0.123	0
30	TCGA-BH-A18L-01A	R	0	0.204	0.343	0.057	0.115	0.053
31	TCGA-OL-A66J-01A	R	0	0.118	0.415	0.054	0.101	0
32	TCGA-PE-A5DD-01A	R	0	0.132	0.443	0.09	0.078	0.022
33	TCGA-AN-A04D-01A	R	0	0.154	0.242	0	0.112	0.07
34	TCGA-4H-AAAK-01A	R	0	0.121	0.381	0.027	0.188	0.068
35	TCGA-E9-A5FL-01A	R	0	0.033	0.269	0.083	0.093	0.037
36	TCGA-E9-A24A-01A	R	0	0.098	0.313	0.066	0.089	0.095
37	TCGA-BH-A1FM-01A	NR	0	0.154	0.287	0	0.154	0
38	TCGA-A8-A07C-01A	R	0	0.014	0.463	0.13	0.202	0.123
39	TCGA-AR-A24Q-01A	R	0	0.057	0.426	0.08	0.24	0.038
40	TCGA-AR-A2LE-01A	R	0.012	0.163	0.338	0	0.057	0.032
41	TCGA-AR-A0U4-01A	R	0	0	0.452	0.165	0.152	0.079
42	TCGA-GM-A2DN-01A	R	0	0.017	0.504	0.13	0.23	0.047
43	TCGA-A7-A13D-01A	R	0.021	0.106	0.103	0	0.007	0
44	TCGA-D8-A1JG-01B	R	0	0.081	0.46	0.145	0.258	0.079
45	TCGA-E2-A570-01A	NR	0.018	0.168	0.309	0.104	0.057	0.05
46	TCGA-A2-A04Q-01A	R	0	0	0.562	0.211	0.255	0.057
47	TCGA-A7-A6VV-01A	R	0	0.051	0.362	0.108	0.111	0.048
48	TCGA-E2-A1IG-01A	R	0	0.146	0.285	0	0.093	0
49	TCGA-E9-A1N6-01A	NR	0	0.134	0.342	0.093	0.129	0.058
50	TCGA-E9-A1NC-01A	R	0	0.021	0.482	0.145	0.112	0.037
51	TCGA-A7-A3IZ-01A	R	0.012	0.176	0.232	0.022	0.115	0.017
52	TCGA-AN-A0AK-01A	NR	0	0.052	0.345	0.206	0.073	0.115

53	TCGA-D8-A1JM-01A	R	0	0.003	0.434	0.119	0.084	0.11
54	TCGA-OL-A5S0-01A	R	0	0.088	0.354	0.147	0.088	0
55	TCGA-A8-A06Z-01A	NR	0.057	0.121	0.135	0	0.153	0.022
56	TCGA-AQ-A54O-01A	R	0	0.206	0.326	0.082	0.049	0
57	TCGA-E2-A15S-01A	NR	0.075	0.229	0.275	0	0	0
58	TCGA-EW-A423-01A	R	0	0.125	0.467	0.089	0.05	0.015
59	TCGA-C8-A273-01A	R	0	0.117	0.293	0.086	0.091	0.031
60	TCGA-OL-A5RV-01A	NR	0.05	0.128	0.315	0.058	0.064	0.029
61	TCGA-AC-A2FM-01A	R	0.007	0.172	0.329	0	0.088	0.064
62	TCGA-AO-A0JM-01A	R	0	0.055	0.291	0.113	0.062	0.049
63	TCGA-BH-A0DP-01A	R	0.006	0.097	0.393	0.09	0.058	0
64	TCGA-A2-A25E-01A	R	0	0.158	0.307	0.092	0.148	0.037
65	TCGA-B6-A2IU-01A	R	0	0.18	0.282	0	0.021	0.014
66	TCGA-BH-A18P-01A	R	0	0.123	0.306	0.188	0.079	0.106
67	TCGA-E9-A22B-01A	R	0	0.113	0.338	0.08	0.088	0.001
68	TCGA-A2-A0SW-01A	R	0	0.077	0.519	0.146	0.293	0.016
69	TCGA-E2-A107-01A	R	0	0.177	0.342	0.016	0.067	0
70	TCGA-E2-A1B6-01A	R	0	0.004	0.545	0.137	0.204	0.06
71	TCGA-D8-A1XW-01A	R	0	0.084	0.403	0.102	0.189	0.062
72	TCGA-A7-A0DC-01A	NR	0.121	0.149	0	0	0	0
73	TCGA-EW-A6SD-01A	R	0	0.022	0.46	0.152	0.169	0.115
74	TCGA-AO-A0J3-01A	NR	0	0.201	0.379	0.013	0.079	0
75	TCGA-AN-A03X-01A	R	0	0.095	0.418	0.047	0.109	0
76	TCGA-A8-A099-01A	R	0	0.146	0.252	0.04	0.077	0.129
77	TCGA-E2-A14Z-01A	R	0	0.104	0.505	0.126	0.221	0.044
78	TCGA-AR-A5QP-01A	R	0	0.102	0.339	0.078	0.074	0.044
79	TCGA-AC-A62X-01A	R	0	0.079	0.188	0.067	0.085	0.136
80	TCGA-BH-A42V-01A	R	0	0.066	0.407	0.074	0.106	0.02
81	TCGA-B6-A0RS-01A	R	0	0.064	0.436	0.164	0.129	0.063
82	TCGA-D8-A1JL-01A	R	0	0.043	0.524	0.13	0.163	0.097
83	TCGA-BH-A0DZ-01A	R	0	0.086	0.422	0.153	0.111	0.032
84	TCGA-A8-A06Y-01A	NR	0.04	0.097	0.172	0	0.103	0.022
85	TCGA-B6-A1KC-01B	NR	0.027	0.216	0.238	0.013	0.089	0.028
86	TCGA-C8-A135-01A	R	0	0.097	0.401	0.041	0.172	0.039
87	TCGA-5L-AAT1-01A	R	0	0.05	0.531	0.154	0.146	0.103
88	TCGA-AR-A24R-01A	R	0	0.156	0.384	0.113	0.16	0.055
89	TCGA-S3-AA0Z-01A	R	0	0	0.392	0.16	0.068	0.076
90	TCGA-D8-A1J8-01A	R	0	0.065	0.514	0.169	0.121	0.081
91	TCGA-A2-A0ER-01A	R	0.006	0.12	0.272	0.066	0	0.061
92	TCGA-E9-A1ND-01A	R	0	0.015	0.544	0.178	0.147	0.047
93	TCGA-A2-A1G1-01A	R	0	0.032	0.461	0.147	0.207	0.086
94	TCGA-A2-A0SU-01A	R	0	0.075	0.356	0.063	0.139	0.027
95	TCGA-E2-A1LB-01A	NR	0	0.111	0.269	0	0.218	0.091
96	TCGA-E9-A1RD-01A	R	0	0.164	0.292	0.014	0.111	0.081
97	TCGA-OL-A6VQ-01A	R	0.017	0.097	0.323	0.083	0.141	0
98	TCGA-AQ-A04J-01A	R	0	0.073	0.436	0.045	0.236	0
99	TCGA-AQ-A1H3-01A	R	0	0.088	0.358	0.07	0.138	0.041
100	TCGA-A2-A259-01A	R	0.001	0.103	0.313	0.06	0.22	0.092
101	TCGA-WT-AB44-01A	NR	0	0.105	0.325	0.11	0.034	0.033
102	TCGA-B6-A0IN-01A	R	0	0.012	0.435	0.108	0.187	0.002
103	TCGA-E9-A1R3-01A	NR	0.02	0.102	0.301	0.094	0.219	0.022
104	TCGA-A8-A086-01A	NR	0.035	0.108	0.202	0	0.156	0.01
105	TCGA-AC-A3BB-01A	R	0	0	0.512	0.129	0.172	0.003

106	TCGA-AC-A2QI-01A	R	0	0.074	0.415	0.086	0.087	0.053
107	TCGA-BH-A1F5-01A	NR	0.035	0.119	0.157	0.002	0.001	0.052
108	TCGA-AC-A3HN-01A	R	0	0.077	0.498	0.07	0.172	0.025
109	TCGA-BH-A1FG-01A	NR	0.009	0.139	0.295	0	0.114	0.016
110	TCGA-BH-A0DV-01A	R	0	0.091	0.432	0.038	0.078	0
111	TCGA-UL-AAZ6-01A	NR	0.002	0.179	0.273	0	0.12	0.039
112	TCGA-AR-A255-01A	R	0	0.073	0.422	0.099	0.226	0.098
113	TCGA-LD-A7W6-01A	R	0	0.12	0.337	0.051	0.041	0.037
114	TCGA-AR-A24Z-01A	NR	0	0.203	0.312	0.058	0.097	0.04
115	TCGA-A1-A0SO-01A	R	0	0.072	0.267	0.1	0.116	0.118
116	TCGA-A2-A3XS-01A	R	0.004	0.056	0.415	0.078	0.143	0.136
117	TCGA-D8-A1X6-01A	R	0	0.121	0.291	0.085	0.114	0.049
118	TCGA-BH-A0HF-01A	R	0	0.092	0.367	0.069	0.12	0
119	TCGA-A7-A3J0-01A	NR	0.02	0.093	0.349	0.183	0.095	0.117
120	TCGA-AC-A23C-01A	R	0	0.124	0.395	0.094	0.141	0.016
121	TCGA-LL-A442-01A	NR	0.051	0.082	0.258	0.089	0.118	0.016
122	TCGA-S3-A6ZH-01A	NR	0	0.082	0.337	0.095	0.106	0.065
123	TCGA-AC-A8OR-01A	NR	0.024	0.125	0.336	0	0.253	0.012
124	TCGA-AR-A1AX-01A	R	0	0.021	0.527	0.129	0.334	0.061
125	TCGA-D8-A1XO-01A	R	0	0.11	0.391	0.064	0.118	0.057
126	TCGA-E9-A1R2-01A	R	0	0.115	0.364	0.126	0.113	0.07
127	TCGA-D8-A27M-01A	R	0	0.028	0.517	0.154	0.249	0.023
128	TCGA-AN-A0FY-01A	NR	0.013	0.125	0.269	0.057	0.084	0.054
129	TCGA-BH-A0DX-01A	R	0	0.095	0.361	0.072	0.156	0
130	TCGA-EW-A1IY-01A	R	0	0.053	0.5	0.142	0.194	0.082
131	TCGA-A2-A04N-01A	R	0.003	0.15	0.298	0.035	0.181	0.007
132	TCGA-A2-A0YF-01A	R	0	0.106	0.23	0.003	0.154	0.023
133	TCGA-PE-A5DE-01A	R	0	0	0.569	0.195	0.199	0
134	TCGA-EW-A1J5-01A	R	0	0.07	0.48	0.14	0.215	0.079
135	TCGA-AO-A0JE-01A	R	0	0.077	0.462	0.172	0.251	0.139
136	TCGA-BH-A1FN-01A	NR	0.006	0.128	0.236	0.128	0.071	0.032
137	TCGA-BH-A18V-01A	R	0	0.029	0.426	0.134	0.128	0.08
138	TCGA-BH-A0BF-01A	R	0	0.038	0.559	0.172	0.277	0.04
139	TCGA-B6-A409-01A	R	0.023	0.088	0.355	0.041	0.099	0.02
140	TCGA-A2-A0CL-01A	R	0	0.059	0.529	0.125	0.333	0.026
141	TCGA-A2-A0CO-01A	R	0	0.033	0.505	0.087	0.233	0.024
142	TCGA-AC-A3QP-01A	R	0	0.075	0.404	0.123	0.062	0
143	TCGA-AC-A2QJ-01A	R	0	0.077	0.541	0.148	0.194	0
144	TCGA-B6-A0RM-01A	NR	0.065	0.132	0.244	0	0.087	0.043
145	TCGA-D8-A1JS-01A	R	0.001	0.121	0.219	0.038	0.072	0.065
146	TCGA-A7-A26J-01A	NR	0	0.149	0.259	0.079	0.091	0.027
147	TCGA-A7-A13H-01A	R	0	0.038	0.497	0.081	0.172	0.041
148	TCGA-AO-A126-01A	R	0	0.109	0.363	0.142	0.062	0.072
149	TCGA-BH-A6R8-01A	R	0	0.07	0.403	0.196	0.069	0.046
150	TCGA-A7-A0DB-01C	NR	0.049	0.113	0.218	0	0	0.047
151	TCGA-AC-A3TM-01A	R	0	0.072	0.417	0.101	0.018	0.011
152	TCGA-AR-A0TQ-01A	R	0	0.102	0.462	0.101	0.209	0.036
153	TCGA-GM-A2DH-01A	R	0	0.079	0.504	0.1	0.239	0.053
154	TCGA-B6-A0RI-01A	NR	0	0.185	0.297	0.009	0.137	0.036
155	TCGA-A2-A1G0-01A	R	0	0.057	0.392	0.097	0.171	0.035
156	TCGA-A2-A1FX-01A	R	0	0.107	0.383	0.068	0.152	0.029
157	TCGA-A7-A26G-01A	R	0	0.09	0.386	0.055	0.288	0.09
158	TCGA-E2-A1LI-01A	R	0.009	0.094	0.361	0.12	0.097	0.107

159	TCGA-A8-A08P-01A	R	0	0.037	0.486	0.14	0.173	0.062
160	TCGA-E2-A1IN-01A	NR	0.066	0.157	0.171	0.017	0.136	0.036
161	TCGA-BH-A0HA-01A	R	0	0.11	0.39	0.126	0.138	0.044
162	TCGA-AO-A1KR-01A	R	0	0.045	0.46	0.22	0	0.105
163	TCGA-3C-AALK-01A	R	0	0.164	0.358	0.095	0.105	0.045
164	TCGA-BH-A0BV-01A	R	0	0.128	0.4	0.039	0.109	0.027
165	TCGA-E2-A1IH-01A	R	0	0.051	0.468	0.133	0.2	0.045
166	TCGA-XX-A89A-01A	R	0.04	0.127	0.513	0.038	0.2	0.015
167	TCGA-E9-A1RH-01A	NR	0.003	0.14	0.239	0.16	0	0.068
168	TCGA-A8-A085-01A	NR	0.075	0.158	0	0	0.089	0
169	TCGA-E9-A1N3-01A	NR	0.042	0.114	0.216	0	0.066	0.069
170	TCGA-D8-A13Y-01A	NR	0.051	0.264	0.127	0.135	0.045	0
171	TCGA-AO-A0JB-01A	R	0	0.059	0.308	0.092	0.047	0
172	TCGA-AC-A6IV-01A	R	0	0.076	0.488	0.064	0.165	0.021
173	TCGA-A8-A09M-01A	R	0	0.08	0.477	0.167	0.156	0.028
174	TCGA-BH-A0GY-01A	NR	0	0.134	0.366	0.113	0.182	0.095
175	TCGA-AR-A2LL-01A	NR	0	0.143	0.273	0.037	0.131	0.031
176	TCGA-BH-A208-01A	R	0	0.154	0.376	0.043	0.114	0.032
177	TCGA-EW-A1PE-01A	R	0	0.075	0.485	0.144	0.162	0.042
178	TCGA-AO-A125-01A	NR	0.091	0.172	0	0.019	0.093	0.04
179	TCGA-E2-A1B1-01A	R	0	0.12	0.421	0.105	0.214	0.057
180	TCGA-BH-A0C0-01A	R	0	0.009	0.538	0.207	0.212	0.061
181	TCGA-A7-A26I-01B	R	0.007	0.163	0.33	0.032	0.106	0.062
182	TCGA-AO-A0JJ-01A	R	0	0.104	0.421	0.111	0.201	0.019
183	TCGA-AR-A2LH-01A	R	0	0.04	0.534	0.071	0.184	0.028
184	TCGA-A2-A3XW-01A	R	0	0.078	0.389	0.046	0.036	0.003
185	TCGA-AR-A0TS-01A	R	0.004	0.078	0.453	0.064	0.286	0.031
186	TCGA-BH-A0AV-01A	NR	0.073	0.107	0.284	0.03	0.099	0.014
187	TCGA-A8-A07B-01A	NR	0.004	0.152	0.262	0.116	0.152	0.054
188	TCGA-LL-A73Z-01A	R	0	0.048	0.412	0.102	0.088	0.097
189	TCGA-B6-A402-01A	NR	0.05	0.13	0.305	0.022	0.075	0.012
190	TCGA-AR-A0TT-01A	R	0	0.005	0.51	0.162	0.343	0.069
191	TCGA-EW-A6SB-01A	R	0	0	0.534	0.236	0.264	0.023
192	TCGA-AC-A2FB-01A	R	0	0.028	0.535	0.058	0.291	0.039
193	TCGA-AC-A62V-01A	R	0.003	0.143	0.285	0.028	0.125	0
194	TCGA-D8-A1XQ-01A	R	0	0	0.465	0.135	0.175	0.066
195	TCGA-BH-A0DD-01A	NR	0	0.154	0.36	0.143	0.06	0.071
196	TCGA-Z7-A8R6-01A	NR	0	0.163	0.303	0.085	0.076	0.041
197	TCGA-OL-A5DA-01A	R	0	0.088	0.379	0.112	0.045	0.076
198	TCGA-WT-AB41-01A	R	0	0	0.545	0.133	0.133	0.033
199	TCGA-E2-A1LA-01A	R	0	0	0.515	0.214	0.067	0.068
200	TCGA-AR-A24N-01A	R	0	0.147	0.282	0.085	0.102	0.087
201	TCGA-E9-A3QA-01A	R	0	0	0.553	0.136	0.241	0.06
202	TCGA-E2-A15J-01A	R	0.005	0.058	0.128	0.033	0.09	0.045
203	TCGA-LL-A73Y-01A	R	0.021	0.081	0.423	0.06	0.249	0.069
204	TCGA-A2-A4RY-01A	R	0.076	0.091	0.418	0.061	0.14	0
205	TCGA-D8-A1XU-01A	R	0	0.182	0.278	0.055	0.088	0.096
206	TCGA-AN-A0FJ-01A	R	0	0.022	0.39	0.014	0.087	0.065
207	TCGA-BH-A0DK-01A	R	0	0.083	0.487	0.109	0.215	0.03
208	TCGA-B6-A0IJ-01A	R	0	0	0.481	0.174	0.181	0.064
209	TCGA-LL-A7SZ-01A	R	0	0.007	0.532	0.147	0.189	0.075
210	TCGA-C8-A26Y-01A	NR	0	0.097	0.354	0.135	0.052	0.087
211	TCGA-A8-A06R-01A	R	0	0.118	0.4	0.123	0.18	0.079

212	TCGA-AO-A128-01A	R	0	0	0.554	0.229	0.212	0
213	TCGA-E2-A1BC-01A	R	0.008	0.13	0.258	0.047	0.099	0.011
214	TCGA-B6-A0I6-01A	NR	0.011	0.149	0.288	0.037	0.163	0.022
215	TCGA-BH-A1ET-01A	R	0.019	0.114	0.343	0.087	0.046	0.095
216	TCGA-A2-A04V-01A	R	0.01	0.164	0.243	0.002	0.093	0
217	TCGA-A7-A2KD-01A	R	0.01	0.115	0.399	0.118	0.186	0.128
218	TCGA-A7-A0CE-01A	R	0	0.078	0.317	0.132	0.051	0.094
219	TCGA-D8-A1JE-01A	R	0	0.156	0.397	0.054	0.157	0.052
220	TCGA-B6-A0WX-01A	R	0.055	0.09	0.338	0.068	0.156	0.008
221	TCGA-A7-A4SB-01A	R	0.015	0.138	0.321	0.094	0.062	0
222	TCGA-EW-A1J1-01A	R	0	0.031	0.385	0.137	0.121	0.127
223	TCGA-A2-A0YM-01A	R	0	0.053	0.365	0.11	0.095	0.095
224	TCGA-A8-A0A4-01A	R	0	0.151	0.389	0.052	0.063	0
225	TCGA-A2-A0EM-01A	NR	0.045	0.157	0.286	0.039	0.031	0.033
226	TCGA-AR-A24X-01A	R	0	0.049	0.296	0.071	0.078	0.084
227	TCGA-C8-A132-01A	R	0	0.065	0.485	0.094	0.239	0.054
228	TCGA-AN-A049-01A	R	0	0.129	0.391	0.07	0.16	0.032
229	TCGA-A2-A0SY-01A	R	0	0.09	0.378	0.042	0.175	0.009
230	TCGA-B6-A0RU-01A	R	0	0.146	0.293	0.034	0.065	0
231	TCGA-AR-A0TX-01A	R	0	0.056	0.53	0.144	0.249	0.058
232	TCGA-BH-A0HQ-01A	NR	0.03	0.128	0.248	0.011	0.14	0.012
233	TCGA-E2-A15C-01A	R	0.014	0.132	0.363	0.066	0.165	0.036
234	TCGA-AQ-A54N-01A	R	0	0.023	0.322	0.103	0.09	0.054
235	TCGA-A8-A07I-01A	R	0	0.103	0.377	0.12	0.139	0.109
236	TCGA-B6-A1KI-01A	R	0.003	0.174	0.267	0	0.012	0
237	TCGA-B6-A0RH-01A	R	0	0.135	0.396	0.155	0.107	0.026
238	TCGA-BH-A5IZ-01A	NR	0.011	0.084	0.361	0.063	0.068	0
239	TCGA-AN-A0XU-01A	R	0	0.027	0.491	0.232	0.026	0.061
240	TCGA-LD-A74U-01A	R	0	0.1	0.476	0.069	0.173	0.026
241	TCGA-B6-A401-01A	R	0	0.082	0.453	0.1	0.109	0
242	TCGA-PL-A8LV-01A	R	0	0.075	0.475	0.131	0.165	0.04
243	TCGA-BH-A0BR-01A	R	0	0.091	0.413	0.105	0.2	0.092
244	TCGA-A8-A07G-01A	R	0.033	0.132	0.371	0.072	0.141	0.061
245	TCGA-A2-A0EN-01A	R	0	0.037	0.512	0.099	0.204	0.04
246	TCGA-OK-A5Q2-01A	R	0	0.038	0.474	0.094	0.156	0.106
247	TCGA-A8-A084-01A	NR	0.045	0.093	0.166	0.061	0.028	0.049
248	TCGA-BH-A0DS-01A	R	0	0.041	0.421	0.122	0.12	0.02
249	TCGA-A8-A09I-01A	NR	0	0.077	0.423	0.215	0.064	0.092
250	TCGA-GM-A3XL-01A	R	0	0	0.515	0.167	0.05	0.039
251	TCGA-W8-A86G-01A	NR	0.088	0.184	0.298	0.009	0.051	0.009
252	TCGA-AO-A12F-01A	R	0	0.111	0.316	0.04	0.123	0.046
253	TCGA-BH-A0HK-01A	R	0	0.118	0.339	0.045	0.089	0
254	TCGA-A8-A06Q-01A	NR	0.051	0.108	0.249	0.021	0	0.101
255	TCGA-BH-A42U-01A	R	0.006	0.066	0.466	0.122	0.075	0
256	TCGA-AQ-A04L-01B	NR	0.044	0.148	0.157	0.084	0.148	0.085
257	TCGA-AR-A1AO-01A	R	0	0.016	0.541	0.141	0.201	0
258	TCGA-Z7-A8R5-01A	R	0	0	0.49	0.14	0.026	0
259	TCGA-B6-A0I1-01A	NR	0.07	0.122	0.199	0.025	0.066	0.019
260	TCGA-EW-A1PA-01A	R	0	0.143	0.342	0.094	0.164	0.064
261	TCGA-BH-A0W4-01A	NR	0.02	0.184	0.261	0	0.078	0
262	TCGA-A2-A3XZ-01A	R	0	0.02	0.439	0.148	0.247	0.016
263	TCGA-A7-A26H-01A	R	0	0.092	0.384	0.115	0.191	0.053
264	TCGA-E2-A1B0-01A	R	0	0.077	0.405	0.101	0.169	0.084

265	TCGA-BH-A1EU-01A	R	0	0.07	0.443	0.105	0.258	0.079
266	TCGA-AC-A23H-01A	NR	0.018	0.166	0.233	0.056	0.083	0.028
267	TCGA-EW-A1P4-01A	R	0	0	0.49	0.215	0.025	0.072
268	TCGA-E2-A10E-01A	R	0	0.111	0.325	0.116	0.113	0.083
269	TCGA-EW-A1P0-01A	R	0.002	0.176	0.307	0.045	0.107	0.075
270	TCGA-EW-A6S9-01A	NR	0.018	0.141	0.226	0.037	0.147	0.048
271	TCGA-A2-A25B-01A	R	0	0.095	0.379	0.116	0.168	0.015
272	TCGA-LL-A6FR-01A	NR	0.059	0.061	0.145	0.029	0.128	0
273	TCGA-AR-A5QM-01A	R	0	0.113	0.433	0.101	0.174	0
274	TCGA-EW-A1P5-01A	R	0	0.105	0.354	0.13	0.057	0.037
275	TCGA-E2-A14O-01A	R	0	0.117	0.339	0.06	0.082	0
276	TCGA-AN-A0AR-01A	NR	0.024	0.078	0.274	0.021	0.164	0.037
277	TCGA-E9-A22D-01A	R	0	0.071	0.528	0.208	0.198	0.026
278	TCGA-A2-A0CS-01A	NR	0.056	0.148	0.251	0.047	0.114	0.052
279	TCGA-E2-A1IF-01A	R	0	0.118	0.323	0.074	0.044	0.002
280	TCGA-E2-A1IE-01A	R	0	0.188	0.222	0.006	0.019	0
281	TCGA-BH-A0BW-01A	R	0	0	0.532	0.181	0.15	0.07
282	TCGA-E2-A1AZ-01A	R	0	0.098	0.45	0.097	0.238	0.071
283	TCGA-D8-A27N-01A	R	0	0.063	0.437	0.06	0.302	0.024
284	TCGA-A2-A0CT-01A	NR	0	0.147	0.288	0.034	0.076	0.02
285	TCGA-A8-A09R-01A	R	0	0.075	0.37	0.189	0.051	0.119
286	TCGA-B6-A0RE-01A	R	0	0.039	0.256	0.047	0.082	0.087
287	TCGA-E9-A249-01A	NR	0	0.095	0.363	0.105	0.063	0.098
288	TCGA-E2-A1B5-01A	R	0	0.057	0.459	0.124	0.244	0.059
289	TCGA-A2-A25A-01A	R	0	0.079	0.391	0.047	0.102	0.031
290	TCGA-AO-A03O-01A	R	0	0.113	0.408	0.119	0.121	0.169
291	TCGA-E9-A22A-01A	R	0	0.083	0.491	0.124	0.143	0.049
292	TCGA-BH-A0GZ-01A	R	0	0.136	0.275	0.023	0.052	0
293	TCGA-BH-A1FD-01A	NR	0	0.165	0.155	0.078	0.135	0.102
294	TCGA-A1-A0SH-01A	NR	0.028	0.161	0.316	0.023	0.207	0.024
295	TCGA-E2-A10A-01A	R	0	0.101	0.165	0.011	0.104	0.01
296	TCGA-B6-A0IO-01A	R	0	0.146	0.331	0.073	0.022	0.022
297	TCGA-BH-A1FR-01A	R	0	0.055	0.388	0.05	0.1	0.021
298	TCGA-D8-A143-01A	R	0	0.063	0.39	0.131	0.159	0.1
299	TCGA-AO-A03R-01A	R	0	0.109	0.465	0.102	0.152	0.01
300	TCGA-BH-A0E0-01A	R	0	0.051	0.214	0	0.148	0.113
301	TCGA-A8-A0AD-01A	NR	0.032	0.149	0.237	0	0.149	0.001
302	TCGA-GM-A2DI-01A	R	0	0.037	0.547	0.106	0.324	0.053
303	TCGA-E9-A6HE-01A	R	0	0.069	0.463	0.168	0.123	0
304	TCGA-BH-A0B3-01A	R	0	0.018	0.438	0.162	0.123	0.079
305	TCGA-AR-A0TU-01A	R	0	0.019	0.496	0.192	0.09	0.13
306	TCGA-LQ-A4E4-01A	NR	0.023	0.196	0.332	0.038	0.167	0.03
307	TCGA-PL-A8LY-01A	R	0	0.12	0.421	0.024	0	0.08
308	TCGA-BH-A1FU-01A	R	0	0.114	0.389	0.048	0.235	0.055
309	TCGA-A8-A09D-01A	R	0	0.111	0.377	0.041	0.143	0.04
310	TCGA-B6-A3ZX-01A	R	0	0.036	0.478	0.112	0.336	0.08
311	TCGA-A7-A5ZV-01A	R	0	0.115	0.27	0.116	0.111	0
312	TCGA-BH-A0AY-01A	R	0	0.066	0.472	0.081	0.208	0.017
313	TCGA-A7-A4SD-01A	R	0	0	0.524	0.155	0.234	0.115
314	TCGA-AO-A03L-01A	R	0	0.144	0.343	0.096	0.09	0.025
315	TCGA-A2-A0EX-01A	R	0.016	0.104	0.307	0	0.068	0
316	TCGA-GM-A2DM-01A	R	0	0.007	0.393	0.013	0.221	0.017
317	TCGA-BH-A0DO-01B	NR	0.072	0.148	0.314	0.036	0.034	0

318	TCGA-HN-A2OB-01A	NR	0.063	0.14	0.35	0.091	0.096	0.016
319	TCGA-A2-A0CM-01A	R	0	0.048	0.555	0.124	0.194	0.073
320	TCGA-A2-A4S0-01A	NR	0.074	0.086	0.276	0	0.108	0.056
321	TCGA-BH-A0H9-01A	NR	0.023	0.149	0.295	0.037	0.091	0
322	TCGA-B6-A0IP-01A	R	0.022	0.12	0.374	0.062	0.092	0.061
323	TCGA-C8-A12X-01A	R	0	0.143	0.486	0.07	0.225	0.019
324	TCGA-AN-A0AL-01A	R	0	0.044	0.414	0.123	0.166	0
325	TCGA-LD-A9QF-01A	R	0	0.001	0.565	0.144	0.249	0.075
326	TCGA-E2-A152-01A	NR	0	0.163	0.345	0.072	0.188	0.093
327	TCGA-BH-A1FE-06A	R	0.015	0.091	0.346	0.158	0.095	0
328	TCGA-BH-A18N-01A	R	0.02	0.092	0.078	0	0.136	0.006
329	TCGA-C8-A1HN-01A	NR	0	0.15	0.311	0.057	0.166	0.023
330	TCGA-AN-A0FK-01A	R	0.004	0.153	0.304	0.044	0.107	0.013
331	TCGA-B6-A0IE-01A	NR	0.042	0.204	0.325	0.015	0.094	0.023
332	TCGA-B6-A400-01A	R	0	0.068	0.318	0.107	0.142	0.086
333	TCGA-GM-A2DB-01A	R	0	0.029	0.445	0.13	0.109	0.04
334	TCGA-AC-A2FK-01A	R	0.044	0.049	0.38	0.045	0.126	0.024
335	TCGA-BH-A0HU-01A	NR	0	0.151	0.397	0.087	0.049	0.083
336	TCGA-D8-A140-01A	R	0	0.093	0.438	0.076	0.272	0.03
337	TCGA-A7-A0CD-01A	NR	0.029	0.142	0.265	0.009	0.085	0.022
338	TCGA-D8-A1JF-01A	R	0	0.085	0.373	0.022	0.157	0.073
339	TCGA-A2-A4S1-01A	R	0	0.094	0.48	0.083	0.188	0.009
340	TCGA-5T-A9QA-01A	R	0	0.121	0.276	0.089	0.054	0.009
341	TCGA-AR-A2LK-01A	NR	0	0.169	0.284	0.052	0.142	0
342	TCGA-AR-A1AH-01A	R	0	0.087	0.132	0	0.101	0.08
343	TCGA-AR-A254-01A	R	0	0.057	0.461	0.138	0.167	0.087
344	TCGA-E2-A1IO-01A	R	0.029	0.112	0.418	0.068	0.188	0.05
345	TCGA-S3-A6ZF-01A	R	0	0.126	0.359	0.12	0.154	0.103
346	TCGA-B6-A0IB-01A	R	0	0.181	0.291	0.023	0.069	0.03
347	TCGA-A8-A07J-01A	R	0	0.093	0.402	0.09	0.201	0.023
348	TCGA-AN-A0FV-01A	NR	0.097	0.152	0.042	0.026	0.14	0.038
349	TCGA-AN-A0XP-01A	NR	0.023	0.129	0.201	0.053	0.077	0.034
350	TCGA-A8-A08Z-01A	R	0	0.089	0.369	0.011	0.182	0.025
351	TCGA-E9-A226-01A	NR	0.002	0.121	0.163	0.049	0.064	0.115
352	TCGA-AR-A1AW-01A	R	0	0.016	0.562	0.176	0.345	0.009
353	TCGA-GI-A2C9-01A	R	0	0.061	0.363	0.137	0.202	0.064
354	TCGA-EW-A6SA-01A	NR	0.025	0.23	0.214	0	0.13	0.019
355	TCGA-E9-A1NG-01A	R	0	0.083	0.399	0.03	0.203	0.043
356	TCGA-A8-A07L-01A	NR	0.021	0.169	0.207	0.021	0.166	0.029
357	TCGA-E2-A15L-01A	NR	0.038	0.076	0.012	0	0.14	0.074
358	TCGA-D8-A1JI-01A	R	0	0.212	0.304	0.108	0.071	0.021
359	TCGA-GM-A2DO-01A	R	0	0.025	0.552	0.172	0.246	0.019
360	TCGA-EW-A1P6-01A	NR	0.007	0.149	0.297	0.047	0.123	0.058
361	TCGA-AR-A1AI-01A	R	0	0	0.538	0.172	0.282	0.073
362	TCGA-AR-A0U3-01A	NR	0	0.166	0.276	0.1	0.138	0.094
363	TCGA-A2-A0ES-01A	R	0	0.073	0.389	0.046	0.082	0.068
364	TCGA-A8-A06P-01A	R	0	0.149	0.402	0	0.253	0.025
365	TCGA-S3-AA12-01A	NR	0.011	0.17	0.189	0.002	0.109	0.078
366	TCGA-E9-A3Q9-01A	R	0	0.095	0.405	0.08	0.006	0.034
367	TCGA-BH-A0HX-01A	R	0	0.148	0.314	0.024	0.111	0
368	TCGA-AC-A3W7-01A	NR	0.065	0.157	0.356	0.037	0.074	0.04
369	TCGA-AO-A124-01A	NR	0	0.087	0.31	0.143	0.057	0.118
370	TCGA-AC-A5XU-01A	R	0.014	0.194	0.326	0.055	0.069	0

371	TCGA-LL-A6FQ-01A	R	0	0.148	0.357	0.104	0.115	0
372	TCGA-AQ-A1H2-01A	R	0	0.133	0.327	0.143	0.082	0.073
373	TCGA-BH-A0DQ-01A	R	0.007	0.158	0.354	0.022	0.193	0
374	TCGA-E2-A106-01A	NR	0.053	0.161	0.208	0	0.095	0
375	TCGA-A2-A25F-01A	R	0	0	0.528	0.134	0.333	0
376	TCGA-AN-A0AT-01A	R	0	0.059	0.387	0.145	0.115	0.082
377	TCGA-D8-A27T-01A	R	0	0.093	0.477	0.05	0.171	0.026
378	TCGA-A2-A04X-01A	R	0	0.057	0.52	0.153	0.138	0.035
379	TCGA-A2-A3XT-01A	R	0	0.003	0.441	0.109	0.145	0.008
380	TCGA-AR-A0TY-01A	R	0	0.141	0.271	0.007	0.23	0
381	TCGA-A2-A0YG-01A	R	0	0.155	0.25	0.058	0.13	0.06
382	TCGA-B6-A0WZ-01A	NR	0.013	0.207	0.34	0.028	0.084	0.033
383	TCGA-A7-A5ZX-01A	NR	0.075	0.167	0.302	0.068	0.059	0.047
384	TCGA-AC-A23G-01A	R	0	0.074	0.336	0.053	0.061	0
385	TCGA-GM-A2DA-01A	R	0	0.118	0.532	0.09	0.132	0
386	TCGA-B6-A0X1-01A	R	0	0	0.503	0.2	0	0.082
387	TCGA-AO-A12E-01A	R	0	0.135	0.334	0.046	0.169	0
388	TCGA-AR-A250-01A	NR	0	0.146	0.356	0.117	0.178	0.075
389	TCGA-A8-A06O-01A	R	0	0.153	0.324	0.052	0.151	0.07
390	TCGA-AO-A12G-01A	R	0	0.097	0.308	0.048	0.105	0.019
391	TCGA-A7-A3IY-01A	R	0	0.107	0.436	0.102	0.216	0
392	TCGA-BH-A1EY-01A	R	0	0.078	0.415	0.112	0.221	0.085
393	TCGA-A8-A079-01A	R	0	0.207	0.282	0.1	0.122	0
394	TCGA-AO-A03P-01A	NR	0	0.122	0.211	0.08	0.128	0.026
395	TCGA-E2-A572-01A	R	0	0.134	0.3	0.034	0.048	0
396	TCGA-A2-A0YH-01A	R	0	0.067	0.409	0.124	0.224	0.064
397	TCGA-E2-A15G-01A	R	0.006	0.161	0.275	0	0.066	0
398	TCGA-C8-A12M-01A	R	0	0.106	0.378	0.091	0.157	0.077
399	TCGA-AR-A0TV-01A	R	0	0.113	0.454	0.198	0.125	0.028
400	TCGA-A7-A13G-01B	NR	0.062	0.153	0.115	0	0	0
401	TCGA-A8-A07E-01A	R	0	0.092	0.35	0.03	0.151	0.053
402	TCGA-E9-A1N9-01A	R	0	0.138	0.276	0.056	0.105	0.066
403	TCGA-BH-A0H3-01A	R	0	0.124	0.253	0.044	0.074	0.074
404	TCGA-AR-A1AY-01A	NR	0.066	0.064	0.197	0.029	0	0.036
405	TCGA-A2-A0D4-01A	R	0.02	0.193	0.176	0	0.122	0.022
406	TCGA-E9-A5UO-01A	NR	0.057	0.114	0.043	0.035	0.098	0.025
407	TCGA-GM-A2DL-01A	NR	0	0.105	0.177	0.114	0.104	0.051
408	TCGA-EW-A1PD-01A	R	0.002	0.16	0.29	0.045	0.16	0.001
409	TCGA-A1-A0SI-01A	R	0	0.131	0.39	0.141	0.163	0.041
410	TCGA-A2-A0CV-01A	R	0	0.089	0.321	0.099	0.027	0.023
411	TCGA-E2-A15E-06A	R	0.052	0.094	0.226	0.059	0.115	0.019
412	TCGA-AO-A12B-01A	NR	0.03	0.132	0.16	0	0.045	0.021
413	TCGA-B6-A408-01A	R	0	0.111	0.392	0.054	0.184	0.026
414	TCGA-OL-A5D7-01A	R	0	0.025	0.434	0.164	0.151	0.054
415	TCGA-A2-A0EW-01A	R	0.012	0.095	0.289	0.087	0.089	0
416	TCGA-A2-A0T4-01A	R	0	0.104	0.327	0.151	0.112	0.008
417	TCGA-C8-A131-01A	R	0	0.021	0.304	0.145	0.008	0.025
418	TCGA-BH-A1EN-01A	R	0	0.145	0.288	0.01	0.182	0.034
419	TCGA-D8-A3Z5-01A	R	0.004	0.114	0.322	0.081	0.012	0
420	TCGA-E9-A247-01A	NR	0	0.119	0.143	0.046	0.069	0
421	TCGA-C8-A12Q-01A	NR	0	0.098	0.286	0.079	0.131	0.085
422	TCGA-E2-A1BD-01A	NR	0	0.169	0.268	0.086	0.026	0
423	TCGA-E9-A1RI-01A	R	0	0.11	0.242	0.057	0.13	0.058

424	TCGA-E2-A105-01A	NR	0.039	0.199	0.174	0.061	0.117	0.026
425	TCGA-D8-A1JK-01A	R	0	0	0.419	0.212	0.08	0.037
426	TCGA-BH-A0BJ-01A	R	0	0.096	0.276	0.005	0.109	0
427	TCGA-S3-AA14-01A	R	0.045	0.158	0.217	0.036	0.056	0.032
428	TCGA-C8-A26V-01A	R	0	0.128	0.278	0.228	0.009	0.084
429	TCGA-BH-A1FB-01A	R	0.012	0.09	0.349	0.103	0.248	0.075
430	TCGA-D8-A1JP-01A	R	0	0.081	0.319	0.084	0.126	0.07
431	TCGA-E2-A14X-01A	R	0	0.045	0.333	0.128	0.298	0.009
432	TCGA-BH-A0E2-01A	R	0	0.147	0.274	0.07	0.074	0
433	TCGA-PL-A8LZ-01A	R	0	0.034	0.446	0.132	0.165	0.036
434	TCGA-LL-A740-01A	NR	0	0.022	0.14	0.049	0.188	0.028
435	TCGA-D8-A1X7-01A	R	0.008	0.1	0.142	0.037	0.187	0
436	TCGA-AO-A0JD-01A	NR	0	0.127	0.274	0.137	0.06	0.08
437	TCGA-D8-A1X5-01A	R	0	0.102	0.251	0.14	0.135	0.027
438	TCGA-E9-A22E-01A	R	0	0.129	0.362	0.105	0.111	0.094
439	TCGA-AC-A23E-01A	NR	0.022	0.089	0.202	0.001	0.219	0.038
440	TCGA-AR-A0U2-01A	R	0	0.086	0.379	0.108	0.127	0.101
441	TCGA-A2-A1FV-01A	NR	0.039	0.174	0.073	0	0.063	0.078
442	TCGA-A8-A09B-01A	R	0	0.16	0.27	0.035	0.055	0.027
443	TCGA-AN-A0XO-01A	NR	0.046	0.133	0.072	0	0.117	0.02
444	TCGA-A7-A425-01A	R	0	0.091	0.308	0	0.046	0
445	TCGA-A2-A0CZ-01A	R	0	0.044	0.319	0.088	0.159	0.019
446	TCGA-GM-A3NY-01A	R	0	0.143	0.227	0.11	0.002	0.015
447	TCGA-AR-A0U0-01A	R	0	0	0.451	0.19	0.163	0.03
448	TCGA-AR-A24P-01A	R	0.024	0.162	0.189	0.067	0.081	0.061
449	TCGA-AN-A0G0-01A	R	0	0.033	0.272	0.177	0.077	0.101
450	TCGA-AN-A0AM-01A	R	0	0.042	0.352	0.2	0.01	0.091
451	TCGA-BH-A0E6-01A	R	0	0	0.292	0.183	0.063	0.052
452	TCGA-E9-A248-01A	R	0	0.027	0.458	0.152	0.297	0.033
453	TCGA-B6-A1KN-01A	R	0	0.161	0.235	0.077	0.053	0.065
454	TCGA-LL-A9Q3-01A	R	0	0.058	0.336	0.081	0.082	0.07
455	TCGA-A8-A08B-01A	R	0	0.036	0.364	0.157	0.019	0.038
456	TCGA-A1-A0SG-01A	R	0.022	0.092	0.239	0.05	0.049	0.022
457	TCGA-BH-A0H0-01A	R	0	0.181	0.237	0.058	0.139	0.037
458	TCGA-D8-A1XR-01A	NR	0.022	0.163	0.169	0.011	0.089	0.055
459	TCGA-C8-A12P-01A	R	0	0.144	0.289	0.121	0.081	0.057
460	TCGA-LL-A50Y-01A	R	0	0.061	0.237	0.145	0.006	0.055
461	TCGA-AO-A03N-01B	NR	0	0.171	0.232	0.11	0.07	0
462	TCGA-E2-A1IU-01A	R	0	0.122	0.242	0.114	0.067	0.006
463	TCGA-EW-A1IX-01A	R	0	0.069	0.175	0.086	0.062	0.042
464	TCGA-A2-A0EV-01A	R	0	0.103	0.31	0.092	0.04	0.048
465	TCGA-AC-A3W5-01A	R	0	0.071	0.413	0.108	0.16	0.043
466	TCGA-AR-A2LN-01A	R	0.047	0.075	0.265	0.07	0.095	0.028
467	TCGA-E9-A1QZ-01A	R	0	0.117	0.418	0.119	0.154	0.011
468	TCGA-BH-A8FY-01A	R	0.023	0.145	0.067	0.014	0.055	0
469	TCGA-AC-A6IX-06A	R	0	0.08	0.383	0.12	0.201	0.022
470	TCGA-AR-A1AL-01A	R	0.04	0.105	0.256	0.04	0.133	0.027
471	TCGA-BH-A42T-01A	R	0	0.102	0.304	0.148	0.072	0
472	TCGA-BH-A0B9-01A	R	0	0.009	0.393	0.141	0.148	0.056
473	TCGA-AO-A0J8-01A	R	0	0.095	0.266	0.159	0.072	0.05
474	TCGA-BH-A2L8-01A	R	0	0.09	0.444	0.114	0.168	0.041
475	TCGA-E9-A1N5-01A	R	0	0.15	0.347	0.086	0.085	0.014
476	TCGA-A8-A075-01A	R	0	0.02	0.36	0.208	0.165	0.069

477	TCGA-AN-A0FZ-01A	NR	0.113	0.134	0.074	0	0.032	0
478	TCGA-D8-A1XJ-01A	NR	0.042	0.062	0.076	0.064	0.175	0.057
479	TCGA-E2-A15F-01A	R	0.021	0.108	0.137	0.094	0.06	0.03
480	TCGA-A2-A0EP-01A	R	0	0.006	0.461	0.124	0.244	0.027
481	TCGA-BH-A18K-01A	NR	0.005	0.174	0.207	0.08	0.06	0
482	TCGA-AR-A5QN-01A	R	0.04	0.109	0.246	0.087	0.084	0.003
483	TCGA-E9-A244-01A	R	0	0.053	0.207	0.084	0.072	0.059
484	TCGA-A8-A0A2-01A	R	0	0.109	0.325	0.115	0.087	0.084
485	TCGA-B6-A40C-01A	R	0	0.113	0.315	0.048	0.111	0.035
486	TCGA-AR-A2LQ-01A	R	0.055	0.133	0.253	0.034	0.151	0
487	TCGA-EW-A1J6-01A	R	0	0.052	0.287	0.157	0.102	0.034
488	TCGA-EW-A1IZ-01A	R	0	0.004	0.46	0.24	0.194	0.054
489	TCGA-AC-A2FG-01A	R	0.011	0.12	0.219	0	0.005	0
490	TCGA-A8-A09Z-01A	R	0	0.084	0.385	0.204	0.058	0.097
491	TCGA-EW-A1P1-01A	R	0	0.028	0.37	0.101	0.161	0.03
492	TCGA-A8-A0A9-01A	R	0	0.172	0.273	0.059	0.094	0.036
493	TCGA-BH-A0DT-01A	R	0	0.098	0.284	0.087	0.088	0.047
494	TCGA-A2-A0T2-01A	R	0	0.073	0.28	0.126	0.021	0.075
495	TCGA-A7-A4SA-01A	R	0.028	0.085	0.402	0.096	0.228	0
496	TCGA-D8-A27F-01A	NR	0.074	0.07	0.166	0.047	0.081	0
497	TCGA-D8-A146-01A	R	0	0.086	0.29	0.104	0.136	0.046
498	TCGA-D8-A27E-01A	R	0	0.056	0.233	0.093	0.023	0.107
499	TCGA-C8-A138-01A	R	0	0.113	0.426	0.151	0.101	0.015
500	TCGA-E2-A14V-01A	NR	0.006	0.167	0.2	0.01	0.1	0.085
501	TCGA-AC-A5EH-01A	R	0	0.138	0.319	0.12	0.159	0.041
502	TCGA-A2-A0YL-01A	R	0	0.104	0.348	0.062	0.113	0.033
503	TCGA-E2-A1LK-01A	NR	0.027	0.109	0.144	0.077	0.05	0.076
504	TCGA-D8-A13Z-01A	R	0	0.085	0.292	0.126	0.111	0.075
505	TCGA-D8-A27V-01A	R	0	0.159	0.273	0.029	0	0.037
506	TCGA-EW-A3U0-01A	R	0	0.018	0.469	0.167	0.267	0.039
507	TCGA-A2-A4S2-01A	R	0	0.091	0.439	0.127	0.094	0.046
508	TCGA-D8-A1XV-01A	NR	0.085	0.148	0.023	0	0	0.084
509	TCGA-BH-A1F0-01A	R	0	0	0.434	0.144	0.235	0.028
510	TCGA-AN-A0FT-01A	R	0	0.124	0.337	0.089	0.07	0
511	TCGA-E2-A15K-01A	R	0	0.095	0.416	0.094	0.185	0
512	TCGA-BH-A0DL-01A	R	0	0	0.41	0.183	0.105	0.04
513	TCGA-A2-A0EY-01A	R	0.006	0.081	0.336	0.071	0.168	0.049
514	TCGA-B6-A0IG-01A	NR	0	0.179	0.229	0.105	0.035	0.042
515	TCGA-BH-A0HI-01A	R	0	0.139	0.186	0.1	0.074	0.011
516	TCGA-E2-A576-01A	R	0	0.127	0.298	0.085	0.034	0.013
517	TCGA-BH-A201-01A	R	0	0.152	0.21	0.149	0.054	0
518	TCGA-D8-A1JB-01A	R	0	0.022	0.404	0.145	0.231	0.072
519	TCGA-A1-A0SN-01A	R	0	0.1	0.37	0.053	0.158	0.079
520	TCGA-A8-A08A-01A	R	0	0.068	0.229	0.088	0.022	0.013
521	TCGA-A2-A0YI-01A	R	0	0.053	0.388	0.128	0.209	0
522	TCGA-AR-A1AN-01A	R	0	0.097	0.227	0.037	0.079	0
523	TCGA-AR-A2LM-01A	R	0.03	0.107	0.295	0.034	0.062	0.022
524	TCGA-AR-A5QQ-01A	R	0	0	0.465	0.114	0.18	0.015
525	TCGA-AR-A0TR-01A	NR	0.012	0.08	0.173	0.082	0.072	0.076
526	TCGA-GM-A3XG-01A	NR	0.066	0.142	0.135	0.049	0	0
527	TCGA-BH-A1FC-01A	R	0	0	0.446	0.175	0.243	0.066
528	TCGA-A2-A0D1-01A	R	0	0.142	0.26	0.061	0.036	0.089
529	TCGA-EW-A1P7-01A	R	0	0.017	0.395	0.11	0.295	0

530	TCGA-AR-A1AP-01A	R	0	0.132	0.246	0.112	0.085	0.047
531	TCGA-E2-A150-01A	R	0	0.073	0.271	0.091	0.137	0.141
532	TCGA-EW-A2FW-01A	R	0	0.126	0.235	0.046	0.074	0
533	TCGA-AR-A1AV-01A	NR	0	0.106	0.149	0	0.108	0
534	TCGA-A2-A0T1-01A	R	0	0.037	0.45	0.149	0.231	0.033
535	TCGA-AO-A0JF-01A	R	0	0.099	0.329	0.002	0.157	0
536	TCGA-OL-A6VR-01A	NR	0.072	0.118	0.071	0	0.023	0.01
537	TCGA-A8-A090-01A	R	0	0.078	0.269	0.111	0.044	0.07
538	TCGA-BH-A0BG-01A	R	0	0	0.409	0.122	0.123	0.033
539	TCGA-AO-A1KP-01A	NR	0.023	0.147	0.053	0.051	0	0.079
540	TCGA-LL-A441-01A	R	0	0.062	0.384	0.085	0.265	0.03
541	TCGA-C8-A133-01A	NR	0.035	0.016	0.153	0	0.233	0.058
542	TCGA-BH-A8FZ-01A	R	0	0.028	0.336	0.113	0.229	0.034
543	TCGA-E2-A1II-01A	R	0	0	0.445	0.193	0.207	0.05
544	TCGA-EW-A6SC-01A	R	0	0.092	0.254	0.108	0.134	0
545	TCGA-AN-A0XT-01A	NR	0.028	0.14	0.186	0.027	0.039	0.034
546	TCGA-BH-A0RX-01A	R	0	0	0.456	0.209	0.169	0.044
547	TCGA-A8-A08X-01A	R	0	0.038	0.359	0.12	0.218	0.065
548	TCGA-AO-A03V-01A	R	0.019	0.146	0.196	0.033	0.054	0
549	TCGA-EW-A1PH-01A	R	0	0.091	0.31	0.111	0.022	0.068
550	TCGA-A2-A0ST-01A	R	0	0	0.452	0.168	0.268	0
551	TCGA-BH-A0BC-01A	R	0	0.092	0.29	0.071	0.12	0.03
552	TCGA-E9-A1NH-01A	R	0	0.119	0.26	0.062	0.087	0.031
553	TCGA-B6-A0X5-01A	NR	0.069	0.221	0.098	0.11	0.019	0
554	TCGA-AC-A4ZE-01A	R	0	0.143	0.277	0.054	0.063	0
555	TCGA-A8-A07Z-01A	R	0	0.107	0.324	0.118	0.062	0
556	TCGA-OL-A66P-01A	R	0	0	0.483	0.246	0.176	0
557	TCGA-E2-A1LE-01A	R	0	0.054	0.4	0.112	0.157	0.037
558	TCGA-C8-A8HR-01A	R	0	0.075	0.403	0.134	0.084	0.019
559	TCGA-A8-A09T-01A	R	0	0.125	0.272	0.082	0.242	0.001
560	TCGA-BH-A0HB-01A	R	0	0.08	0.363	0.111	0.125	0.047
561	TCGA-A8-A08G-01A	R	0	0.159	0.255	0.121	0.095	0.073
562	TCGA-A2-A0EQ-01A	R	0	0.013	0.453	0.165	0.231	0.049
563	TCGA-A8-A081-01A	R	0	0.062	0.404	0.169	0.168	0.108
564	TCGA-AO-A1KO-01A	R	0.006	0.078	0.264	0.036	0	0.06
565	TCGA-BH-A0HW-01A	NR	0.088	0.141	0.021	0	0.069	0.075
566	TCGA-OL-A66H-01A	R	0	0.052	0.351	0.148	0.149	0.007
567	TCGA-A2-A0T0-01A	R	0	0.023	0.323	0.127	0.084	0.104
568	TCGA-AN-A04C-01A	R	0	0.078	0.256	0.113	0.077	0.017
569	TCGA-E2-A573-01A	R	0	0	0.447	0.141	0.134	0.02
570	TCGA-AN-A0XS-01A	R	0	0.077	0.348	0.101	0.179	0.05
571	TCGA-D8-A3Z6-01A	R	0.019	0.166	0.204	0.066	0.052	0.039
572	TCGA-EW-A3E8-01B	R	0	0.082	0.362	0.124	0.166	0.014
573	TCGA-C8-A137-01A	NR	0.036	0.144	0.088	0.001	0.131	0.037
574	TCGA-GM-A4E0-01A	R	0	0.071	0.386	0.13	0.006	0
575	TCGA-E2-A1B4-01A	NR	0.053	0.16	0.079	0.024	0.057	0.093
576	TCGA-EW-A2FS-01A	R	0	0.092	0.339	0.101	0.109	0.016
577	TCGA-S3-A6ZG-01A	R	0	0.125	0.263	0.029	0.038	0.052
578	TCGA-GM-A3XN-01A	R	0	0.077	0.353	0.063	0.044	0
579	TCGA-EW-A1P8-01A	R	0	0.014	0.244	0.084	0.115	0.054
580	TCGA-E2-A156-01A	NR	0.065	0.064	0	0	0.153	0.054
581	TCGA-AC-A8OP-01A	R	0.004	0.121	0.264	0.099	0.13	0.058
582	TCGA-AN-A0FS-01A	NR	0.016	0.187	0.201	0.077	0.021	0

583	TCGA-B6-A0IM-01A	NR	0.028	0.197	0.215	0	0.065	0
584	TCGA-E9-A3HO-01A	R	0	0.112	0.315	0.087	0.063	0.061
585	TCGA-D8-A27K-01A	R	0.028	0.121	0.315	0.091	0.164	0.068
586	TCGA-E9-A2JT-01A	R	0	0.03	0.423	0.164	0.327	0.033
587	TCGA-GM-A2DK-01A	R	0	0.094	0.35	0.114	0.115	0.029
588	TCGA-AR-A1AK-01A	R	0	0.08	0.372	0.132	0.158	0.022
589	TCGA-E2-A15R-01A	NR	0.051	0.236	0.044	0	0	0.019
590	TCGA-BH-A18Q-01A	R	0	0.053	0.363	0.129	0.003	0.059
591	TCGA-E2-A153-01A	R	0	0.072	0.259	0.025	0.219	0.007
592	TCGA-BH-A0BT-01A	NR	0	0.068	0.244	0.164	0.015	0.049
593	TCGA-E9-A54Y-01A	NR	0	0.148	0.153	0.049	0.056	0.077
594	TCGA-E2-A14Q-01A	R	0.014	0.124	0.228	0.07	0.041	0.001
595	TCGA-BH-A0HN-01A	NR	0.114	0.149	0.028	0.03	0.07	0.062
596	TCGA-E2-A1IK-01A	R	0	0.056	0.326	0.108	0.116	0.054
597	TCGA-E2-A10C-01A	R	0	0.114	0.33	0.091	0.155	0.032
598	TCGA-A8-A095-01A	R	0	0.168	0.282	0.08	0.124	0.029
599	TCGA-A7-A0DB-01A	NR	0.008	0.144	0.282	0.019	0.103	0
600	TCGA-BH-A18J-01A	R	0.006	0.069	0.022	0.039	0.104	0.001
601	TCGA-BH-A28O-01A	R	0.049	0.131	0.3	0.054	0	0.036
602	TCGA-AC-A3TN-01A	R	0.023	0.151	0.255	0.003	0.022	0
603	TCGA-A7-A13E-01A	NR	0.045	0.112	0.212	0.052	0.105	0.036
604	TCGA-BH-A0W5-01A	R	0	0.058	0.364	0.116	0.317	0.032
605	TCGA-GM-A2DD-01A	R	0	0.031	0.437	0.086	0.186	0.058
606	TCGA-AN-A0XV-01A	R	0	0.12	0.286	0.114	0.124	0.027
607	TCGA-E2-A1IL-01A	R	0	0.105	0.235	0.079	0.059	0.018
608	TCGA-EW-A2FR-01A	R	0	0.108	0.305	0.117	0.108	0.06
609	TCGA-3C-AAAU-01A	NR	0.102	0.119	0.032	0	0.119	0.042
610	TCGA-AR-A24W-01A	R	0.053	0.151	0.182	0.024	0	0
611	TCGA-OL-A5RZ-01A	R	0.017	0.175	0.24	0.019	0.084	0.039
612	TCGA-E9-A1RB-01A	NR	0	0.081	0.139	0.102	0.072	0.055
613	TCGA-AN-A0AJ-01A	R	0	0.086	0.387	0.141	0.149	0.073
614	TCGA-AR-A1AT-01A	R	0	0.032	0.451	0.116	0.182	0.051
615	TCGA-A8-A09Q-01A	R	0	0.046	0.347	0.111	0.223	0.032
616	TCGA-A8-A083-01A	R	0.007	0.155	0.264	0.036	0.036	0.028
617	TCGA-AN-A0XL-01A	R	0	0.148	0.294	0.062	0	0
618	TCGA-E9-A1RC-01A	NR	0.054	0.114	0.014	0	0.063	0
619	TCGA-D8-A27I-01A	R	0	0.058	0.312	0.084	0.169	0.02
620	TCGA-A2-A04Y-01A	R	0	0.097	0.373	0.076	0.128	0.04
621	TCGA-PE-A5DC-01A	R	0.002	0.161	0.277	0.091	0.024	0
622	TCGA-OL-A5D8-01A	R	0	0.145	0.276	0.137	0.069	0.056
623	TCGA-A7-A26E-01A	R	0.017	0.152	0.176	0.016	0	0.027
624	TCGA-AN-A0FL-01A	NR	0.056	0.088	0.256	0.022	0.1	0.01
625	TCGA-A8-A0A1-01A	R	0	0.088	0.391	0.12	0.156	0.065
626	TCGA-AN-A0FN-01A	R	0	0.066	0.383	0.133	0.215	0.038
627	TCGA-D8-A1J9-01A	NR	0	0.14	0.25	0.181	0.202	0.107
628	TCGA-BH-A1EO-01A	R	0	0.101	0.264	0.051	0.248	0.057
629	TCGA-B6-A0I2-01A	R	0	0	0.441	0.233	0.063	0
630	TCGA-E9-A5UP-01A	NR	0.076	0.054	0.254	0.099	0.144	0.019
631	TCGA-A2-A0T5-01A	R	0	0.111	0.154	0.029	0.03	0.027
632	TCGA-A7-A0DA-01A	NR	0.103	0.088	0.203	0.033	0.094	0.089
633	TCGA-B6-A0IC-01A	NR	0.112	0.101	0	0	0.017	0.066
634	TCGA-BH-A5J0-01A	R	0	0.044	0.373	0.102	0.105	0
635	TCGA-A2-A0CY-01A	NR	0.042	0.231	0.174	0.052	0.068	0.003

636	TCGA-E9-A245-01A	R	0.021	0.135	0.147	0.069	0.011	0.071
637	TCGA-GM-A3NW-01A	R	0	0.123	0.257	0.144	0.043	0.02
638	TCGA-A1-A0SB-01A	NR	0.107	0.131	0.062	0.02	0	0.013
639	TCGA-OL-A66N-01A	R	0.014	0.085	0.331	0.163	0.003	0
640	TCGA-A2-A0CQ-01A	R	0	0.151	0.228	0.074	0.047	0.008
641	TCGA-C8-A1HG-01A	R	0	0.109	0.435	0.135	0.171	0.086
642	TCGA-AC-A2BK-01A	NR	0.054	0.16	0.21	0.065	0.133	0.073
643	TCGA-A8-A08J-01A	NR	0	0.148	0.24	0.131	0.079	0.045
644	TCGA-A8-A09V-01A	NR	0.111	0.153	0.027	0	0.068	0.011
645	TCGA-A2-A0T3-01A	NR	0.038	0.179	0.177	0.044	0.025	0.024
646	TCGA-C8-A1HK-01A	NR	0	0.127	0.284	0.13	0.128	0.112
647	TCGA-AN-A04A-01A	R	0	0.122	0.229	0.057	0.022	0
648	TCGA-E9-A1RG-01A	R	0	0.095	0.317	0.144	0.06	0
649	TCGA-JL-A3YW-01A	R	0	0.052	0.411	0.134	0.129	0.061
650	TCGA-E2-A154-01A	NR	0.004	0.186	0.219	0.018	0.166	0.029
651	TCGA-A2-A04U-01A	R	0	0.082	0.18	0.083	0.067	0.068
652	TCGA-BH-A0EE-01A	NR	0	0.069	0.292	0.145	0	0.042
653	TCGA-BH-A0H5-01A	R	0	0.023	0.468	0.166	0.308	0.065
654	TCGA-EW-A1IW-01A	R	0	0.157	0.248	0.085	0.111	0.056
655	TCGA-AO-A0J4-01A	R	0	0.036	0.394	0.083	0.206	0.026
656	TCGA-A7-A3J1-01A	R	0	0.065	0.431	0.117	0.176	0.006
657	TCGA-E9-A243-01A	R	0	0	0.44	0.182	0.269	0.026
658	TCGA-C8-A134-01A	R	0	0.03	0.386	0.141	0.085	0.103
659	TCGA-D8-A1Y1-01A	NR	0.008	0.123	0.291	0.062	0.118	0.012
660	TCGA-AO-A12A-01A	R	0.003	0.087	0.268	0.071	0.046	0.053
661	TCGA-E2-A108-01A	R	0	0.046	0.435	0.181	0.239	0.066
662	TCGA-BH-A0E1-01A	R	0.015	0.155	0.173	0.014	0	0.009
663	TCGA-E2-A14U-01A	R	0.061	0.153	0.216	0.037	0	0
664	TCGA-BH-A0HL-01A	NR	0.091	0.215	0	0	0.09	0.012
665	TCGA-E9-A229-01A	R	0	0.143	0.238	0.095	0.132	0.062
666	TCGA-A8-A08T-01A	NR	0	0.147	0.205	0.092	0.102	0.036
667	TCGA-E9-A1RF-01A	R	0	0.094	0.428	0.14	0.076	0.06
668	TCGA-A2-A04T-01A	R	0	0.063	0.409	0.144	0.129	0.074
669	TCGA-E2-A15P-01A	NR	0.029	0.15	0.213	0.032	0.1	0.08
670	TCGA-C8-A3M8-01A	R	0	0.12	0.233	0	0.063	0.004
671	TCGA-LL-A5YO-01A	R	0	0	0.458	0.18	0.234	0.042
672	TCGA-OL-A66L-01A	R	0	0.034	0.391	0.115	0.014	0
673	TCGA-OL-A66K-01A	NR	0.04	0.162	0.259	0	0.029	0.029
674	TCGA-E2-A1LG-01A	R	0	0.008	0.218	0.111	0	0.013
675	TCGA-E2-A1LS-01A	NR	0.024	0.114	0.122	0	0	0.03
676	TCGA-A7-A426-01A	R	0.017	0.113	0.375	0.09	0	0
677	TCGA-BH-A0B7-01A	R	0.009	0.054	0.392	0.107	0.21	0.007
678	TCGA-AC-A2FO-01A	R	0	0.013	0.37	0.083	0.217	0.055
679	TCGA-D8-A27L-01A	R	0	0.112	0.291	0.063	0.22	0.084
680	TCGA-AC-A2FF-01A	R	0	0.138	0.36	0.073	0.191	0.052
681	TCGA-GM-A2D9-01A	R	0	0.14	0.198	0.089	0.02	0
682	TCGA-A2-A0D3-01A	R	0	0.054	0.299	0.118	0.078	0.125
683	TCGA-A2-A0EO-01A	R	0	0.045	0.309	0.053	0.037	0.014
684	TCGA-A2-A0T7-01A	R	0	0.096	0.273	0.041	0.107	0.029
685	TCGA-OL-A6VO-01A	R	0	0.019	0.219	0.106	0.039	0.092
686	TCGA-BH-A0EI-01A	NR	0.08	0.147	0.041	0	0.162	0.004
687	TCGA-A8-A094-01A	R	0	0.027	0.41	0.182	0.141	0.085
688	TCGA-A8-A092-01A	R	0	0.118	0.194	0.125	0.068	0.086

689	TCGA-AC-A3W6-01A	R	0.003	0.085	0.419	0.044	0.122	0.018
690	TCGA-BH-A0BA-01A	R	0	0.126	0.301	0.068	0.136	0.026
691	TCGA-EW-A1OX-01A	NR	0.032	0.156	0.045	0	0.132	0
692	TCGA-A2-A0CR-01A	R	0	0	0.467	0.169	0.16	0.048
693	TCGA-D8-A1JD-01A	NR	0	0.118	0.281	0.103	0.211	0.127
694	TCGA-C8-A26X-01A	R	0	0.004	0.461	0.184	0.221	0.054
695	TCGA-A1-A0SE-01A	R	0	0.142	0.228	0.018	0.069	0.003
696	TCGA-A7-A4SF-01A	NR	0.009	0.185	0.293	0.173	0.105	0.024
697	TCGA-OL-A66O-01A	R	0	0.187	0.164	0.054	0.051	0
698	TCGA-C8-A26W-01A	R	0	0.093	0.364	0.086	0.12	0.018
699	TCGA-E2-A9RU-01A	NR	0	0.083	0.179	0.126	0.066	0.068
700	TCGA-AN-A0FW-01A	R	0	0.143	0.315	0.06	0.039	0
701	TCGA-D8-A1JC-01A	R	0	0.106	0.347	0.174	0.116	0.089
702	TCGA-C8-A12V-01A	R	0	0	0.451	0.193	0.236	0
703	TCGA-A2-A04P-01A	R	0	0.051	0.279	0.075	0.101	0.07
704	TCGA-BH-A0W3-01A	R	0	0.144	0.085	0	0.093	0.053
705	TCGA-BH-A0B5-01A	R	0	0.14	0.3	0.069	0.081	0.061
706	TCGA-S3-AA10-01A	R	0	0	0.464	0.185	0.24	0.036
707	TCGA-BH-A0B0-01A	R	0	0.14	0.271	0.122	0.147	0.079
708	TCGA-E2-A574-01A	NR	0.059	0.16	0.148	0.078	0	0.037
709	TCGA-AN-A0XN-01A	R	0	0.008	0.31	0.126	0.216	0.028
710	TCGA-AN-A0XW-01A	R	0	0.061	0.436	0.172	0.138	0.064
711	TCGA-C8-A1HF-01A	R	0	0.03	0.436	0.225	0.141	0.061
712	TCGA-AR-A2LO-01A	R	0	0.058	0.42	0.101	0.175	0.02
713	TCGA-D8-A1XA-01A	R	0	0.103	0.258	0.04	0.159	0.045
714	TCGA-BH-A0BL-01A	R	0	0.043	0.394	0.143	0.127	0.036
715	TCGA-EW-A1J2-01A	R	0	0.058	0.332	0.103	0.154	0.017
716	TCGA-BH-A0AZ-01A	R	0	0.128	0.294	0.028	0.07	0.008
717	TCGA-E9-A227-01A	R	0	0.031	0.406	0.096	0.104	0.053
718	TCGA-A2-A0D2-01A	R	0	0.026	0.373	0.116	0.065	0.078
719	TCGA-D8-A1XM-01A	R	0	0.081	0.342	0.091	0.182	0.073
720	TCGA-D8-A1Y3-01A	R	0	0.025	0.31	0.157	0.031	0.066
721	TCGA-C8-A275-01A	R	0	0.047	0.44	0.168	0.208	0.054
722	TCGA-E2-A3DX-01A	R	0	0.033	0.411	0.08	0.209	0.034
723	TCGA-C8-A1HM-01A	R	0	0	0.478	0.24	0.143	0.063
724	TCGA-E2-A158-01A	NR	0.045	0.088	0.232	0.11	0.09	0.074
725	TCGA-AR-A0TP-01A	NR	0.132	0.168	0.051	0	0.121	0.048
726	TCGA-A7-A26F-01B	R	0.019	0.098	0.332	0.045	0.204	0.072
727	TCGA-A2-A1FW-01A	NR	0.063	0.161	0.213	0.066	0.004	0.036
728	TCGA-E2-A15A-06A	R	0	0.109	0.385	0.163	0.191	0.016
729	TCGA-BH-A0C7-01B	R	0.051	0.144	0.184	0.037	0.069	0.002
730	TCGA-AR-A1AJ-01A	R	0	0.005	0.457	0.144	0.262	0
731	TCGA-E9-A1R5-01A	NR	0.048	0.139	0.23	0.042	0.078	0.049
732	TCGA-A8-A09A-01A	R	0	0.084	0.283	0.073	0.101	0.087
733	TCGA-GM-A2DC-01A	NR	0	0.108	0.306	0.081	0.055	0.051
734	TCGA-A2-A3KC-01A	R	0	0.124	0.277	0.063	0.077	0
735	TCGA-AO-A0J9-01A	R	0	0.05	0.388	0.103	0.055	0.027
736	TCGA-E2-A14Y-01A	R	0	0.065	0.256	0.13	0.038	0.03
737	TCGA-D8-A4Z1-01A	R	0.003	0.076	0.388	0.078	0.064	0.038
738	TCGA-3C-AALI-01A	R	0	0.03	0.382	0.148	0.094	0.037
739	TCGA-E2-A159-01A	R	0	0	0.448	0.14	0.223	0.074
740	TCGA-E2-A155-01A	NR	0.038	0.177	0.149	0.023	0	0.06
741	TCGA-A8-A082-01A	NR	0.016	0.139	0.097	0	0.135	0.024

742	TCGA-E2-A14R-01A	R	0	0.056	0.374	0.144	0.123	0.076
743	TCGA-D8-A141-01A	R	0	0.04	0.432	0.151	0.239	0.026
744	TCGA-BH-A204-01A	NR	0.052	0.185	0.064	0	0	0
745	TCGA-E2-A109-01A	NR	0.025	0.195	0.243	0.101	0.119	0.014
746	TCGA-C8-A1HJ-01A	R	0	0.079	0.277	0.149	0.052	0.097
747	TCGA-BH-A0C1-01B	R	0	0.119	0.371	0.101	0.054	0.04
748	TCGA-D8-A1Y2-01A	R	0	0	0.445	0.125	0.186	0.022
749	TCGA-AR-A2LJ-01A	R	0	0.041	0.362	0.095	0.091	0
750	TCGA-AC-A2QH-01B	NR	0.019	0.194	0.209	0.041	0	0.064
751	TCGA-A2-A0YK-01A	R	0	0.063	0.358	0.119	0.262	0.023
752	TCGA-E9-A22H-01A	R	0	0.159	0.315	0.138	0.125	0.08
753	TCGA-E2-A1L8-01A	R	0.007	0.146	0.22	0.096	0.047	0.01
754	TCGA-BH-A0B8-01A	R	0	0.153	0.239	0.006	0.089	0.013
755	TCGA-BH-A18G-01A	R	0	0.04	0.287	0.116	0.01	0.065
756	TCGA-JL-A3YX-01A	R	0	0.097	0.256	0.077	0.091	0.004
757	TCGA-S3-AA17-01A	R	0	0.022	0.466	0.119	0.296	0.029
758	TCGA-E2-A15M-01A	R	0	0.094	0.38	0.107	0.252	0.03
759	TCGA-E2-A56Z-01A	NR	0	0.138	0.247	0.105	0.024	0.041
760	TCGA-A7-A0CH-01A	NR	0.028	0.127	0.187	0.018	0.073	0.035
761	TCGA-AR-A0TZ-01A	R	0	0.175	0.178	0.015	0.11	0.025
762	TCGA-E2-A15I-01A	R	0.007	0.083	0.377	0.093	0.168	0.038
763	TCGA-BH-A0B4-01A	R	0	0.077	0.288	0.08	0.096	0.078
764	TCGA-E2-A15K-06A	NR	0.053	0.168	0.094	0.06	0.074	0.044
765	TCGA-BH-A0WA-01A	R	0	0.089	0.262	0.133	0.026	0.052
766	TCGA-A2-A0ET-01A	R	0	0.13	0.288	0.113	0.007	0.101
767	TCGA-OL-A5RW-01A	R	0	0.015	0.157	0.108	0.056	0
768	TCGA-A7-A13D-01B	R	0	0.134	0.018	0	0	0.094
769	TCGA-AN-A0FD-01A	R	0	0.125	0.331	0.149	0.018	0.027
770	TCGA-BH-A0H7-01A	R	0	0.16	0.263	0.069	0.091	0.006
771	TCGA-LL-A5YL-01A	R	0	0.053	0.344	0.175	0.003	0.031
772	TCGA-A2-A0D0-01A	R	0	0	0.373	0.194	0	0.15
773	TCGA-AO-A129-01A	R	0	0	0.446	0.148	0.127	0.042
774	TCGA-BH-A1EW-01A	R	0	0.104	0.337	0.074	0.162	0.065
775	TCGA-AO-A03M-01B	R	0	0.023	0.419	0.146	0.178	0.036
776	TCGA-B6-A0RP-01A	R	0	0.088	0.34	0.068	0.032	0.018
777	TCGA-E2-A15T-01A	NR	0.053	0.171	0.147	0.05	0.093	0.042
778	TCGA-A2-A4RW-01A	R	0.029	0.136	0.322	0.12	0.086	0.057
779	TCGA-A8-A08O-01A	NR	0.036	0.156	0.187	0	0.118	0.005
780	TCGA-BH-A18I-01A	R	0	0.133	0.33	0.077	0.184	0.069
781	TCGA-BH-A0BM-01A	R	0	0.095	0.253	0.081	0.018	0.019
782	TCGA-E2-A14N-01A	R	0	0.054	0.296	0.125	0.142	0.05
783	TCGA-5L-AAT0-01A	R	0	0.067	0.384	0.081	0.109	0.017
784	TCGA-BH-A6R9-01A	R	0	0.067	0.378	0.174	0.121	0.062
785	TCGA-EW-A1PG-01A	R	0.019	0.095	0.175	0.047	0.031	0.026
786	TCGA-AN-A03Y-01A	R	0.002	0.121	0.309	0.097	0.108	0.076
787	TCGA-B6-A0RT-01A	R	0	0.047	0.448	0.172	0.307	0
788	TCGA-A2-A25D-01A	R	0	0.042	0.436	0.132	0.169	0.053
789	TCGA-A7-A56D-01A	NR	0	0.073	0.355	0.199	0.095	0
790	TCGA-D8-A73W-01A	R	0	0.072	0.325	0.085	0.147	0.06
791	TCGA-AO-A0J5-01A	R	0.009	0.184	0.256	0.032	0.121	0
792	TCGA-A2-A1G6-01A	R	0	0.103	0.308	0.027	0.063	0.004
793	TCGA-AC-A3OD-01B	R	0	0.116	0.407	0.147	0.148	0.049
794	TCGA-A7-A0CJ-01A	NR	0	0.12	0.246	0.147	0.04	0.042

795	TCGA-A8-A07U-01A	R	0	0.005	0.462	0.176	0.252	0.004
796	TCGA-D8-A1XS-01A	R	0	0.105	0.235	0.103	0.091	0.097
797	TCGA-B6-A0I8-01A	NR	0.044	0.145	0.149	0	0	0
798	TCGA-LL-A5YM-01A	R	0	0.09	0.357	0.054	0.015	0
799	TCGA-E2-A15E-01A	NR	0.054	0.146	0.193	0.07	0.025	0.012
800	TCGA-D8-A27G-01A	R	0	0.115	0.377	0.045	0.153	0.114
801	TCGA-BH-A1EX-01A	R	0	0.064	0.319	0.072	0.128	0.125
802	TCGA-A8-A076-01A	NR	0	0.165	0.244	0.062	0.09	0.132
803	TCGA-EW-A1OV-01A	R	0	0.022	0.459	0.115	0.256	0.078
804	TCGA-B6-A0X7-01A	NR	0.045	0.202	0.259	0.004	0.023	0.067
805	TCGA-B6-A0WT-01A	NR	0.003	0.173	0.144	0.029	0.083	0.147
806	TCGA-A2-A4S3-01A	R	0	0.088	0.395	0.156	0.099	0.163
807	TCGA-BH-A0BZ-01A	R	0	0.055	0.473	0.139	0.116	0.082
808	TCGA-OL-A5RU-01A	R	0	0.026	0.441	0.049	0.173	0.164
809	TCGA-D8-A73U-01A	R	0	0	0.51	0.146	0.232	0.051
810	TCGA-A2-A0YT-01A	NR	0.016	0.121	0.362	0.072	0.118	0.035
811	TCGA-A1-A0SK-01A	NR	0.098	0.184	0.228	0.037	0.07	0.084
812	TCGA-E9-A295-01A	R	0	0.203	0.313	0.024	0.01	0.071
813	TCGA-AR-A1AR-01A	R	0	0.039	0.443	0.043	0.179	0.101
814	TCGA-D8-A1XY-01A	NR	0	0.08	0.235	0.038	0.193	0.111
815	TCGA-LL-A8F5-01A	R	0	0	0.398	0.154	0.075	0.082
816	TCGA-E9-A1R6-01A	NR	0.01	0.135	0.206	0.006	0.083	0.137
817	TCGA-BH-A1FL-01A	NR	0.016	0.122	0.237	0.005	0.043	0.124
818	TCGA-E9-A1RE-01A	NR	0.034	0.158	0.208	0.012	0.049	0.085
819	TCGA-D8-A1JN-01A	NR	0.006	0.084	0.298	0.069	0.025	0.14
820	TCGA-D8-A145-01A	R	0.065	0.117	0.325	0.073	0.162	0.053
821	TCGA-B6-A0IK-01A	R	0	0.093	0.302	0.057	0.092	0.11
822	TCGA-C8-A1HE-01A	R	0.03	0.077	0.243	0.021	0.036	0.09
823	TCGA-A8-A0AB-01A	NR	0.054	0.145	0.113	0	0.034	0.124
824	TCGA-EW-A1J3-01A	NR	0	0.144	0.167	0.046	0.003	0.114
825	TCGA-GI-A2C8-01A	R	0.025	0.107	0.337	0.082	0.044	0.09
826	TCGA-A2-A0YJ-01A	NR	0.088	0.053	0.255	0.014	0	0.125
827	TCGA-A8-A0A7-01A	R	0	0	0.494	0.146	0.229	0.083
828	TCGA-A8-A08L-01A	R	0	0.089	0.436	0.137	0.089	0.108
829	TCGA-BH-A0H6-01A	NR	0.061	0.149	0.179	0	0.097	0.076
830	TCGA-D8-A1XL-01A	R	0	0	0.446	0.13	0.05	0.098
831	TCGA-E2-A10B-01A	R	0	0.149	0.293	0.092	0.073	0.09
832	TCGA-E9-A228-01A	NR	0.042	0.167	0.189	0	0.096	0.034
833	TCGA-A2-A3KD-01A	R	0.008	0.101	0.284	0.051	0.114	0.069
834	TCGA-AQ-A7U7-01A	R	0	0.033	0.449	0.118	0.264	0.062
835	TCGA-AO-A1KS-01A	R	0	0.115	0.243	0.031	0.138	0.136
836	TCGA-A7-A0DC-01B	NR	0.079	0.165	0.051	0	0	0.08
837	TCGA-BH-A0EB-01A	R	0	0.091	0.325	0.062	0.096	0.104
838	TCGA-C8-A12W-01A	R	0	0.093	0.372	0.062	0.255	0.093
839	TCGA-AR-A24L-01A	NR	0.018	0.194	0.243	0	0.029	0.085
840	TCGA-EW-A1PB-01A	R	0	0	0.455	0.216	0	0.077
841	TCGA-D8-A1JJ-01A	NR	0	0.143	0.277	0.058	0.18	0.071
842	TCGA-C8-A12Z-01A	NR	0.094	0.072	0.172	0	0.116	0.103
843	TCGA-A8-A09X-01A	R	0	0.061	0.432	0.093	0.192	0.098
844	TCGA-AC-A62Y-01A	R	0	0.123	0.332	0.075	0	0.077
845	TCGA-AC-A3YJ-01A	R	0	0.1	0.303	0.065	0.016	0.074
846	TCGA-E9-A22G-01A	NR	0	0.112	0.254	0.127	0	0.192
847	TCGA-GM-A5PX-01A	R	0.04	0.111	0.323	0.042	0	0.036

848	TCGA-A2-A4RX-01A	R	0.032	0.107	0.308	0.04	0.152	0.058
849	TCGA-B6-A0WW-01A	R	0	0.088	0.23	0.117	0.039	0.143
850	TCGA-A2-A0SV-01A	R	0	0.087	0.288	0.085	0.058	0.086
851	TCGA-AR-A24U-01A	R	0	0.049	0.42	0.043	0.251	0.114
852	TCGA-B6-A0X0-01A	R	0.017	0.113	0.255	0	0.029	0.105
853	TCGA-BH-A0EA-01A	R	0.015	0.08	0.265	0.051	0	0.029
854	TCGA-LL-A440-01A	R	0	0.06	0.306	0.082	0.187	0.045
855	TCGA-A1-A0SQ-01A	R	0	0.118	0.321	0.049	0.139	0.046
856	TCGA-A8-A09N-01A	NR	0	0.178	0.295	0.057	0.043	0.1
857	TCGA-AC-A3QQ-01B	R	0.034	0.16	0.246	0	0	0
858	TCGA-BH-A18V-06A	R	0	0	0.466	0.186	0.149	0.064
859	TCGA-BH-A18H-01A	R	0	0.068	0.442	0.11	0.216	0.131
860	TCGA-E2-A1LH-01A	R	0	0.004	0.479	0.14	0.151	0.058
861	TCGA-C8-A27A-01A	NR	0.047	0.2	0.171	0.061	0.023	0.09
862	TCGA-E9-A1R4-01A	R	0	0.038	0.425	0.076	0.179	0.101
863	TCGA-A2-A3XU-01A	NR	0	0.09	0.12	0	0.008	0.015
864	TCGA-HN-A2NL-01A	R	0.005	0.026	0.384	0.079	0.129	0.129
865	TCGA-BH-A0BO-01A	R	0	0.044	0.219	0.056	0.026	0.059
866	TCGA-D8-A1Y0-01A	R	0	0.136	0.386	0.079	0.178	0.088
867	TCGA-B6-A40B-01A	R	0	0.159	0.158	0.112	0.035	0.088
868	TCGA-PL-A8LX-01A	R	0	0.035	0.272	0.079	0.102	0.083
869	TCGA-B6-A1KF-01A	NR	0.083	0.099	0	0	0.043	0.083
870	TCGA-AO-A0J7-01A	NR	0.01	0.14	0.165	0	0.07	0.1
871	TCGA-EW-A2FV-01A	R	0	0.091	0.365	0.085	0.114	0.083
872	TCGA-AR-A252-01A	R	0	0.039	0.462	0.131	0.251	0.069
873	TCGA-AN-A0XR-01A	NR	0.02	0.187	0.119	0.022	0.062	0.053
874	TCGA-A2-A0YC-01A	R	0	0.181	0.272	0.022	0.087	0.092
875	TCGA-A8-A07P-01A	R	0.004	0.127	0.233	0	0.092	0.048
876	TCGA-E9-A3X8-01A	R	0	0.037	0.453	0.1	0	0.079
877	TCGA-AC-A2B8-01A	R	0	0.082	0.373	0.016	0.113	0.1
878	TCGA-AO-A0JL-01A	R	0	0.128	0.265	0.12	0.017	0.027
879	TCGA-D8-A27W-01A	R	0	0.11	0.404	0.035	0.154	0.089
880	TCGA-B6-A0RO-01A	R	0	0.137	0.214	0.001	0	0.039
881	TCGA-BH-A0DE-01A	R	0	0.117	0.281	0.061	0.027	0.095
882	TCGA-D8-A147-01A	R	0	0.05	0.369	0.074	0.128	0.126
883	TCGA-BH-A202-01A	R	0	0.076	0.348	0.139	0.026	0.161
884	TCGA-BH-A18R-01A	NR	0.045	0.155	0.299	0.108	0.042	0.088
885	TCGA-B6-A0RV-01A	R	0.009	0.098	0.295	0.064	0.123	0.092
886	TCGA-D8-A27P-01A	NR	0.043	0.178	0.225	0.006	0.027	0.07
887	TCGA-BH-A0W7-01A	R	0	0.04	0.435	0.079	0.154	0.086
888	TCGA-AC-A7VB-01A	NR	0.069	0.184	0.145	0	0.077	0.051
889	TCGA-AN-A046-01A	R	0	0.081	0.484	0.101	0.129	0.085
890	TCGA-A2-A0SX-01A	R	0	0.032	0.433	0.087	0.244	0.075
891	TCGA-A8-A06N-01A	NR	0.078	0.138	0.212	0	0.161	0.061
892	TCGA-BH-A8G0-01A	R	0.009	0	0.366	0.092	0.118	0.045
893	TCGA-BH-A0HP-01A	R	0	0.126	0.301	0.059	0	0.109
894	TCGA-BH-A18U-01A	NR	0	0.094	0.257	0.144	0.016	0.17
895	TCGA-AN-A0AS-01A	NR	0	0.077	0.33	0.004	0.199	0.131
896	TCGA-AO-A0JC-01A	R	0	0.066	0.472	0.129	0.269	0.089
897	TCGA-BH-A1F2-01A	R	0.007	0.155	0.207	0	0.033	0.016
898	TCGA-BH-A18M-01A	R	0.014	0.094	0.294	0.037	0.074	0.004
899	TCGA-C8-A12L-01A	NR	0	0.053	0.306	0.167	0.022	0.142
900	TCGA-E9-A1R7-01A	NR	0.086	0.176	0.179	0	0	0.082

901	TCGA-BH-A1FH-01A	R	0.029	0.14	0.302	0.041	0.099	0.05
902	TCGA-AR-A24V-01A	R	0	0.118	0.304	0.038	0.157	0.089
903	TCGA-AC-A6IX-01A	R	0	0.112	0.306	0.093	0.061	0.106
904	TCGA-D8-A1JH-01A	R	0	0.078	0.348	0.025	0.114	0.129
905	TCGA-BH-A1FE-01A	R	0.012	0.121	0.375	0.071	0.2	0.128
906	TCGA-A2-A25C-01A	R	0.001	0.11	0.272	0.007	0.095	0.116
907	TCGA-B6-A0IQ-01A	R	0	0.021	0.322	0.116	0.036	0.125
908	TCGA-AO-A0JI-01A	NR	0.063	0.126	0.252	0	0.102	0.052
909	TCGA-B6-A0IA-01A	NR	0.035	0.092	0.051	0	0	0.053
910	TCGA-E2-A1L7-01A	R	0	0.079	0.437	0.114	0.136	0.093
911	TCGA-BH-A18F-01A	R	0.023	0.134	0.287	0.058	0.118	0.044
912	TCGA-C8-A1HI-01A	R	0	0.12	0.271	0.028	0	0.034
913	TCGA-A2-A0YD-01A	R	0	0.096	0.34	0.078	0.123	0.058
914	TCGA-A8-A06U-01A	R	0	0.108	0.362	0.125	0.056	0.195
915	TCGA-BH-A18S-01A	R	0	0.087	0.219	0.025	0.023	0.077
916	TCGA-A8-A08S-01A	R	0	0.196	0.239	0.08	0.028	0.058
917	TCGA-A8-A08C-01A	NR	0.03	0.169	0.265	0.034	0.032	0.131
918	TCGA-C8-A1HO-01A	R	0	0.167	0.235	0.045	0	0.051
919	TCGA-AO-A0JG-01A	R	0	0.058	0.417	0.075	0.121	0.049
920	TCGA-E9-A1NF-01A	R	0	0.115	0.323	0.085	0.066	0.093
921	TCGA-AO-A0J6-01A	R	0	0	0.432	0.19	0	0.105
922	TCGA-LL-A5YP-01A	R	0	0.066	0.334	0.086	0.093	0.099
923	TCGA-BH-A0DI-01A	R	0	0.098	0.353	0.088	0.092	0.027
924	TCGA-BH-A1FJ-01A	NR	0.078	0.151	0.106	0	0.069	0.088
925	TCGA-UU-A93S-01A	R	0	0.102	0.33	0.131	0	0.131
926	TCGA-A2-A0YE-01A	R	0.019	0.111	0.222	0.137	0.053	0.092
927	TCGA-BH-A209-01A	R	0	0.001	0.463	0.143	0.255	0.074
928	TCGA-OL-A5RX-01A	R	0.047	0.096	0.25	0.091	0.004	0.051
929	TCGA-E2-A15D-01A	R	0	0.082	0.318	0.057	0.021	0.088
930	TCGA-D8-A27H-01A	NR	0.06	0.143	0.231	0.001	0.162	0.039
931	TCGA-BH-A1F6-01A	R	0	0.03	0.435	0.083	0.149	0.114
932	TCGA-A1-A0SD-01A	R	0	0.099	0.242	0.032	0.027	0.078
933	TCGA-A7-A26J-01B	R	0.077	0.07	0.282	0.035	0.129	0.047
934	TCGA-D8-A1XC-01A	NR	0.116	0.074	0.128	0	0.175	0.114
935	TCGA-A2-A04R-01A	R	0.011	0.146	0.144	0	0.046	0.019
936	TCGA-LD-A66U-01A	R	0	0.043	0.409	0.142	0.028	0.043
937	TCGA-E2-A15O-01A	NR	0.049	0.129	0.129	0.016	0.079	0.096
938	TCGA-AR-A24T-01A	R	0	0.135	0.285	0.025	0.071	0.065
939	TCGA-BH-A0HO-01A	R	0	0.11	0.275	0.075	0.153	0.087
940	TCGA-A7-A6VW-01A	R	0	0.013	0.336	0.086	0.023	0.011
941	TCGA-BH-A0HY-01A	NR	0.02	0.127	0.193	0.024	0.033	0.038
942	TCGA-3C-AALJ-01A	R	0.017	0.163	0.401	0.04	0.14	0.089
943	TCGA-BH-AB28-01A	R	0.014	0.104	0.291	0.095	0.119	0.05
944	TCGA-A7-A0CG-01A	R	0.016	0.068	0.387	0.073	0.164	0.096
945	TCGA-A8-A08H-01A	R	0	0.028	0.476	0.068	0.137	0.052
946	TCGA-B6-A0WV-01A	NR	0.048	0.215	0.172	0	0	0.09
947	TCGA-D8-A1XF-01A	NR	0	0.102	0.157	0.023	0.136	0.086
948	TCGA-BH-A0BQ-01A	R	0	0.044	0.338	0.076	0.02	0.033
949	TCGA-EW-A1PC-01B	NR	0	0.183	0.118	0.089	0.022	0.115
950	TCGA-A8-A08I-01A	R	0	0.063	0.413	0.181	0.171	0.072
951	TCGA-B6-A0X4-01A	NR	0.111	0.054	0	0	0.023	0.148
952	TCGA-E9-A2JS-01A	R	0	0.101	0.274	0.093	0.089	0.143
953	TCGA-A8-A096-01A	R	0	0.055	0.445	0.102	0.092	0.144

954	TCGA-B6-A0WY-01A	R	0	0.085	0.257	0.116	0	0.121
955	TCGA-A8-A09E-01A	NR	0	0.138	0.171	0	0.047	0.109
956	TCGA-BH-A1ES-06A	NR	0.057	0.08	0.126	0.001	0	0.061
957	TCGA-EW-A1OW-01/	R	0	0.052	0.262	0.146	0.025	0.117
958	TCGA-D8-A1XD-01A	NR	0.096	0.146	0.098	0	0.105	0.122
959	TCGA-E9-A54X-01A	NR	0.12	0.16	0.009	0	0.058	0.057
960	TCGA-B6-A0RG-01A	NR	0.007	0.181	0.13	0	0.006	0.022
961	TCGA-A8-A097-01A	R	0	0.132	0.337	0.089	0.143	0.114
962	TCGA-A2-A0CX-01A	R	0	0.091	0.358	0.117	0.057	0.088
963	TCGA-E9-A5FK-01A	R	0	0	0.477	0.13	0.115	0.074
964	TCGA-C8-A8HQ-01A	R	0	0.03	0.404	0.155	0.024	0.105
965	TCGA-BH-A0BP-01A	NR	0.058	0.174	0.27	0.053	0.113	0.037
966	TCGA-B6-A0RL-01A	NR	0.09	0.184	0.005	0	0	0.092
967	TCGA-A8-A08F-01A	R	0	0.072	0.393	0.161	0.008	0.113
968	TCGA-A2-A0CK-01A	R	0	0.102	0.351	0.022	0	0.054
969	TCGA-E9-A1N4-01A	R	0	0.07	0.369	0.068	0.112	0.127
970	TCGA-D8-A1XZ-01A	R	0	0.1	0.308	0.047	0.195	0.087
971	TCGA-AR-A1AS-01A	NR	0.027	0.057	0	0	0.13	0.087
972	TCGA-EW-A424-01A	NR	0.022	0.195	0.221	0.021	0.004	0.022
973	TCGA-A7-A3RF-01A	NR	0.114	0.138	0.225	0.066	0.067	0.085
974	TCGA-AR-A24H-01A	R	0	0.144	0.335	0.055	0.121	0.148
975	TCGA-A8-A06T-01A	R	0	0.061	0.312	0.135	0.035	0.11
976	TCGA-C8-A1HL-01A	NR	0	0.125	0.279	0.036	0.147	0.026
977	TCGA-D8-A1X8-01A	R	0	0.112	0.297	0.03	0.062	0.041
978	TCGA-AO-A1KT-01A	R	0	0.114	0.203	0.109	0.031	0.099
979	TCGA-B6-A0RQ-01A	NR	0.067	0.144	0.284	0.001	0	0.056
980	TCGA-AR-A1AQ-01A	R	0	0	0.471	0.142	0.24	0.104
981	TCGA-OL-A5D6-01A	R	0	0.115	0.273	0.03	0.096	0.052
982	TCGA-A8-A091-01A	R	0.024	0.159	0.305	0.031	0.048	0.091
983	TCGA-A7-A4SE-01A	R	0.03	0.071	0.246	0.124	0.024	0.125
984	TCGA-AC-A2FE-01A	R	0	0.015	0.455	0.111	0.17	0.074
985	TCGA-C8-A26Z-01A	NR	0.09	0.175	0.218	0.044	0.024	0.076
986	TCGA-C8-A12Y-01A	R	0	0.112	0.271	0.152	0	0.098
987	TCGA-A7-A13F-01A	NR	0.014	0.129	0.286	0.002	0.115	0.03
988	TCGA-BH-A28Q-01A	R	0.007	0.099	0.241	0.037	0.017	0.053
989	TCGA-GM-A5PV-01A	R	0	0.085	0.305	0.009	0	0.007
990	TCGA-A7-A4SC-01A	R	0.04	0.095	0.37	0.017	0.033	0.105
991	TCGA-A8-A09G-01A	R	0	0.063	0.418	0.093	0.161	0.146
992	TCGA-E2-A15H-01A	NR	0.009	0.089	0.222	0.036	0.062	0.034
993	TCGA-A7-A13E-01B	R	0.07	0.038	0.343	0.012	0.117	0.101
994	TCGA-B6-A0IH-01A	R	0.059	0.083	0.38	0.057	0.102	0.07
995	TCGA-A2-A1G4-01A	R	0	0.137	0.281	0.018	0.147	0.026
996	TCGA-A8-A093-01A	NR	0.051	0.11	0.141	0	0.055	0.076
997	TCGA-C8-A12O-01A	R	0	0.087	0.374	0.088	0.173	0.103
998	TCGA-AO-A1KQ-01A	R	0	0.049	0.441	0.104	0.031	0.091
999	TCGA-AR-A256-01A	NR	0	0.105	0.229	0.046	0.087	0.117
1000	TCGA-OL-A66I-01A	R	0	0.014	0.471	0.112	0.121	0.119
1001	TCGA-C8-A3M7-01A	R	0.038	0.088	0.352	0.016	0.025	0.082
1002	TCGA-A2-A04W-01A	R	0	0.147	0.254	0.074	0.033	0.065
1003	TCGA-OL-A97C-01A	R	0	0.105	0.415	0.068	0.048	0.155
1004	TCGA-AR-A1AM-01A	R	0	0.083	0.33	0.024	0.064	0.079
1005	TCGA-AR-A2LR-01A	R	0.028	0.117	0.33	0.013	0.165	0.048
1006	TCGA-EW-A1OZ-01A	R	0	0.111	0.225	0.073	0.073	0.093

1007	TCGA-A1-A0SF-01A	R	0.009	0.127	0.401	0.058	0.099	0.081
1008	TCGA-A1-A0SJ-01A	R	0.017	0.107	0.235	0	0	0.085
1009	TCGA-D8-A1JU-01A	R	0	0.059	0.386	0.06	0.088	0.111
1010	TCGA-A8-A07F-01A	R	0	0.072	0.346	0.011	0.134	0.136
1011	TCGA-A7-A6VY-01A	R	0	0	0.46	0.159	0.139	0.1
1012	TCGA-A2-A0CU-01A	R	0.017	0.177	0.249	0.063	0.039	0.048
1013	TCGA-E9-A1NE-01A	R	0	0.049	0.471	0.152	0.235	0.067
1014	TCGA-A7-A0D9-01A	NR	0.069	0.157	0.128	0	0.031	0.053
1015	TCGA-C8-A12K-01A	R	0	0	0.474	0.168	0.068	0.105
1016	TCGA-A8-A07O-01A	R	0	0.005	0.295	0.105	0.042	0.094
1017	TCGA-C8-A12U-01A	NR	0	0.077	0.426	0.157	0.031	0.091
1018	TCGA-C8-A8HP-01A	R	0	0	0.48	0.147	0.121	0.117
1019	TCGA-A2-A3XV-01A	R	0	0.077	0.232	0	0.018	0.075
1020	TCGA-BH-A0AW-01A	R	0	0.026	0.455	0.095	0.18	0.118
1021	TCGA-AN-A0FF-01A	R	0	0.095	0.302	0.12	0.029	0.115
1022	TCGA-AR-A24M-01A	NR	0.026	0.16	0.171	0	0.066	0.086
1023	TCGA-E2-A2P6-01A	R	0.023	0.156	0.218	0.078	0.026	0.032
1024	TCGA-C8-A130-01A	R	0	0.118	0.354	0.045	0.018	0.108
1025	TCGA-C8-A274-01A	NR	0.062	0.153	0.226	0.09	0.074	0.144
1026	TCGA-D8-A73X-01A	NR	0	0.119	0.29	0.048	0.064	0.113
1027	TCGA-AO-A0JA-01A	R	0	0.055	0.344	0.056	0.108	0.093
1028	TCGA-LL-A5YN-01A	R	0	0	0.476	0.142	0.159	0.088
1029	TCGA-D8-A1XB-01A	NR	0.014	0.184	0.277	0.001	0.17	0.182
1030	TCGA-LL-A6FP-01A	NR	0.069	0.082	0.037	0	0.038	0.078
1031	TCGA-A7-A26E-01B	R	0.028	0.087	0.087	0	0	0.069
1032	TCGA-A7-A6VX-01A	NR	0.034	0.162	0.247	0.11	0.117	0.13
1033	TCGA-AC-A6IW-01A	R	0	0	0.404	0.108	0.069	0.09
1034	TCGA-BH-A0C3-01A	R	0	0.107	0.447	0.015	0.157	0.056
1035	TCGA-E2-A1L9-01A	NR	0	0.151	0.289	0.067	0.098	0.102
1036	TCGA-BH-A1EV-01A	NR	0.077	0.125	0.135	0	0.079	0.105
1037	TCGA-A2-A3XX-01A	R	0	0.049	0.326	0.104	0.048	0.079
1038	TCGA-E9-A1NA-01A	NR	0	0.174	0.233	0.062	0.054	0.053
1039	TCGA-EW-A1OY-01A	R	0	0.117	0.23	0.087	0.054	0.078
1040	TCGA-BH-A0AU-01A	R	0	0.075	0.29	0.063	0.111	0.127
1041	TCGA-B6-A0WS-01A	R	0	0.088	0.437	0.089	0.211	0.129
1042	TCGA-B6-A0I5-01A	R	0	0.105	0.244	0.033	0	0.01
1043	TCGA-E2-A14P-01A	R	0	0.08	0.316	0.082	0.077	0.13
1044	TCGA-GM-A2DF-01A	R	0	0.028	0.403	0.088	0.19	0.13
1045	TCGA-AQ-A04H-01B	NR	0.058	0.171	0.242	0.066	0.065	0.108
1046	TCGA-AC-A6NO-01A	NR	0.031	0.12	0.23	0.038	0.022	0.054
1047	TCGA-BH-A0E9-01B	NR	0.074	0.114	0.286	0.022	0	0.086
1048	TCGA-BH-A0B1-01A	R	0.017	0.159	0.212	0.083	0.03	0.052
1049	TCGA-AR-A0TW-01A	R	0	0.019	0.412	0.127	0.136	0.149
1050	TCGA-AN-A0FX-01A	R	0.033	0.119	0.262	0.024	0.041	0.051
1051	TCGA-BH-A0BD-01A	R	0	0.074	0.334	0.141	0.082	0.107
1052	TCGA-D8-A1JA-01A	NR	0	0.094	0.143	0.074	0	0.139
1053	TCGA-S3-AA11-01A	NR	0.056	0.153	0.1	0	0.05	0.077
1054	TCGA-AO-A03T-01A	R	0	0.143	0.299	0.138	0	0.068
1055	TCGA-AC-A3YI-01A	R	0.018	0.107	0.333	0.077	0.042	0.075
1056	TCGA-BH-A0B2-01A	R	0	0.067	0.398	0.08	0.132	0.071
1057	TCGA-LL-A7T0-01A	R	0	0.046	0.371	0.07	0.087	0.091
1058	TCGA-BH-A0DH-01A	R	0	0.113	0.289	0.04	0.052	0.085
1059	TCGA-AC-A8OQ-01A	R	0	0.061	0.43	0.093	0.281	0.103

1060	TCGA-C8-A12N-01A	NR	0.022	0.132	0.24	0.082	0.074	0.087
1061	TCGA-AO-A03U-01B	R	0	0	0.458	0.059	0.087	0.057
1062	TCGA-C8-A12T-01A	R	0	0.057	0.324	0.141	0.075	0.165
1063	TCGA-AO-A0J2-01A	R	0	0	0.373	0.173	0	0.12
1064	TCGA-D8-A27R-01A	R	0	0.067	0.361	0.061	0.133	0.109
1065	TCGA-A2-A0EU-01A	NR	0.017	0.115	0.249	0.008	0.03	0.149
1066	TCGA-AO-A12D-01A	R	0	0.021	0.44	0.117	0.217	0.114
1067	TCGA-D8-A1XT-01A	R	0	0.051	0.234	0.082	0.004	0.152
1068	TCGA-A2-A0CW-01A	NR	0.01	0.141	0.289	0.102	0.016	0.13
1069	TCGA-E2-A10F-01A	R	0	0.042	0.368	0.045	0.091	0.101
1070	TCGA-BH-A0E7-01A	NR	0.073	0.098	0.025	0	0	0.06
1071	TCGA-D8-A1XG-01A	NR	0.105	0	0.001	0	0.037	0.101
1072	TCGA-E9-A1N8-01A	NR	0.075	0.091	0.235	0.102	0.03	0.108
1073	TCGA-E2-A2P5-01A	R	0	0.089	0.296	0.069	0.013	0.047
1074	TCGA-C8-A278-01A	R	0	0.049	0.427	0.159	0.233	0.098
1075	TCGA-A8-A08R-01A	R	0	0.043	0.458	0.136	0.154	0.1
1076	TCGA-E2-A15A-01A	R	0	0.102	0.358	0.119	0.053	0.095
1077	TCGA-D8-A1JT-01A	NR	0.04	0.065	0.066	0.04	0	0.166
1078	TCGA-D8-A142-01A	R	0	0.024	0.349	0.086	0.187	0.095
1079	TCGA-A2-A3Y0-01A	R	0	0	0.459	0.163	0.013	0.128
1080	TCGA-AQ-A0Y5-01A	NR	0.068	0.024	0.068	0	0.139	0.14
1081	TCGA-A8-A07W-01A	R	0	0.129	0.296	0.086	0.078	0.172
1082	TCGA-A1-A0SM-01A	NR	0.068	0.075	0.178	0.019	0.046	0.05
1083	TCGA-AC-A3EH-01A	NR	0.036	0.138	0.295	0.027	0.021	0.077
1084	TCGA-MS-A51U-01A	R	0.019	0.118	0.407	0.008	0.108	0.085
1085	TCGA-AR-A24K-01A	NR	0.057	0.12	0.077	0	0	0.126
1086	TCGA-AC-A2BM-01A	R	0.009	0.163	0.345	0	0.148	0.079
1087	TCGA-A2-A3XY-01A	R	0	0.074	0.406	0.05	0.147	0.08
1088	TCGA-E2-A14W-01A	NR	0.044	0.146	0.167	0	0.016	0.07
1089	TCGA-BH-A1F8-01A	R	0	0.139	0.266	0.113	0	0.126
1090	TCGA-E9-A1RA-01A	R	0.025	0.088	0.28	0.041	0.016	0.055
1091	TCGA-A8-A0A6-01A	R	0	0.015	0.459	0.113	0.183	0.131
1092	TCGA-AC-A7VC-01A	R	0	0.15	0.203	0.098	0.024	0.022
1093	TCGA-C8-A27B-01A	R	0	0.005	0.401	0.161	0.03	0.114
1094	TCGA-S3-AA15-01A	R	0	0	0.518	0.138	0.258	0.043
1095	TCGA-BH-A1ES-01A	NR	0.105	0.178	0.034	0	0	0.108
1096	TCGA-E2-A1L6-01A	R	0.034	0.143	0.211	0.057	0	0.04
1097	TCGA-E9-A1R0-01A	R	0	0.083	0.366	0.037	0.069	0.055
1098	TCGA-BH-A0B6-01A	R	0	0	0.467	0.18	0.156	0.066
1099	TCGA-B6-A0RN-01A	NR	0.026	0.113	0.166	0.009	0.025	0.105
1100	TCGA-A8-A09W-01A	R	0	0.159	0.299	0.012	0.132	0.067
1101	TCGA-EW-A1P3-01A	R	0	0.156	0.208	0.079	0	0.032
1102	TCGA-A2-A0T6-01A	R	0	0.05	0.348	0.063	0.191	0.114
1103	TCGA-A7-A5ZW-01A	R	0.018	0.131	0.318	0.023	0	0.179
1104	TCGA-BH-A203-01A	R	0	0.073	0.346	0.113	0.108	0.139
1105	TCGA-BH-A1EU-11A	NR	0.071	0.133	0.331	0	0	0.042
1106	TCGA-BH-A203-11A	R	0.044	0.132	0.301	0	0	0.064
1107	TCGA-E9-A1RH-11A	R	0.001	0.136	0.345	0.022	0	0.078
1108	TCGA-BH-A18J-11A	R	0.02	0.065	0.27	0	0.014	0.06
1109	TCGA-BH-A0E0-11A	R	0	0.127	0.262	0.057	0	0.1
1110	TCGA-BH-A18U-11A	R	0.03	0.108	0.335	0.009	0.041	0.007
1111	TCGA-BH-A1ET-11B	R	0.018	0.102	0.322	0.054	0.019	0.097
1112	TCGA-BH-A18R-11A	R	0.033	0.11	0.278	0.034	0	0.101

1113	TCGA-A7-A0DC-11A	NR	0.032	0.14	0.263	0.014	0.004	0.078
1114	TCGA-BH-A1FM-11B	R	0.031	0.144	0.343	0.005	0	0.067
1115	TCGA-GI-A2C8-11A	R	0	0.066	0.209	0	0	0
1116	TCGA-BH-A1FN-11A	R	0	0.023	0.437	0.108	0.072	0.075
1117	TCGA-AC-A23H-11A	R	0	0.115	0.412	0	0.013	0.017
1118	TCGA-E2-A158-11A	R	0.037	0.093	0.325	0	0	0
1119	TCGA-E9-A1RB-11A	R	0.008	0.042	0.426	0.023	0	0.053
1120	TCGA-BH-A1EW-11B	R	0	0.096	0.334	0.054	0.045	0.127
1121	TCGA-E2-A15I-11A	R	0.014	0.117	0.291	0.027	0	0
1122	TCGA-BH-A0BA-11A	R	0	0.071	0.341	0.067	0.097	0.104
1123	TCGA-BH-A0B3-11B	R	0.05	0.086	0.346	0.039	0.023	0.068
1124	TCGA-BH-A0DO-11A	R	0.049	0.111	0.337	0.022	0	0.072
1125	TCGA-E2-A1LS-11A	R	0.029	0.098	0.246	0	0	0
1126	TCGA-GI-A2C9-11A	R	0	0.076	0.33	0.072	0.078	0.091
1127	TCGA-BH-A0BQ-11A	R	0.025	0.148	0.286	0.053	0	0.034
1128	TCGA-A7-A0CE-11A	R	0.01	0.08	0.31	0.08	0.009	0.087
1129	TCGA-BH-A0C3-11A	R	0.038	0.093	0.354	0	0.029	0.105
1130	TCGA-BH-A0BW-11A	R	0	0.047	0.478	0.094	0.286	0.053
1131	TCGA-E9-A1N6-11A	R	0.029	0.103	0.395	0.017	0.117	0.01
1132	TCGA-BH-A18Q-11A	R	0.061	0.153	0.324	0	0.002	0.043
1133	TCGA-BH-A0DZ-11A	R	0	0.104	0.3	0.075	0	0.088
1134	TCGA-BH-A1EV-11A	R	0.005	0.118	0.326	0.044	0	0.104
1135	TCGA-BH-A0DQ-11A	R	0.04	0.116	0.272	0	0.019	0.069
1136	TCGA-BH-A0BV-11A	NR	0.055	0.15	0.236	0.057	0.026	0.099
1137	TCGA-BH-A0DG-11A	R	0.057	0.083	0.285	0.05	0	0.118
1138	TCGA-BH-A208-11A	NR	0.05	0.104	0.266	0.04	0	0.101
1139	TCGA-BH-A1F0-11B	R	0	0.084	0.317	0.012	0	0.053
1140	TCGA-BH-A18K-11A	R	0.049	0.101	0.242	0.049	0	0.129
1141	TCGA-E9-A1N5-11A	NR	0.037	0.163	0.287	0.043	0.11	0.056
1142	TCGA-E2-A1IG-11A	R	0.029	0.137	0.283	0.082	0	0.031
1143	TCGA-BH-A18M-11A	R	0.015	0.099	0.352	0.028	0.102	0.123
1144	TCGA-E9-A1N9-11A	R	0.021	0.14	0.315	0	0	0.063
1145	TCGA-BH-A0AZ-11A	R	0.051	0.094	0.284	0.014	0	0.007
1146	TCGA-BH-A0DD-11A	R	0	0.097	0.46	0.067	0.047	0
1147	TCGA-BH-A0BJ-11A	R	0.023	0.111	0.257	0.008	0	0.102
1148	TCGA-BH-A0BT-11A	NR	0.075	0.171	0.236	0.032	0	0.107
1149	TCGA-BH-A0BS-11A	R	0.059	0.111	0.281	0	0	0
1150	TCGA-A7-A0D9-11A	R	0	0.09	0.399	0.005	0.028	0
1151	TCGA-BH-A0BM-11A	R	0.021	0.098	0.286	0	0	0.045
1152	TCGA-BH-A0C0-11A	R	0.038	0.099	0.253	0.019	0	0.08
1153	TCGA-BH-A0H7-11A	R	0.017	0.135	0.244	0.018	0	0.037
1154	TCGA-A7-A0DB-11A	R	0	0.054	0.386	0.019	0.039	0
1155	TCGA-BH-A209-11A	R	0	0.052	0.419	0.082	0.097	0.072
1156	TCGA-E2-A15M-11A	R	0	0.058	0.332	0.017	0.064	0.006
1157	TCGA-E2-A1L7-11A	R	0.013	0.057	0.357	0.089	0	0.064
1158	TCGA-BH-A1EN-11A	R	0	0.051	0.433	0.006	0.004	0.071
1159	TCGA-BH-A0DV-11A	R	0.053	0.133	0.279	0	0	0
1160	TCGA-BH-A0DH-11A	R	0	0.132	0.429	0.01	0	0.021
1161	TCGA-BH-A1FD-11B	R	0.019	0.104	0.293	0.032	0.04	0.111
1162	TCGA-BH-A1FJ-11B	R	0.009	0.116	0.327	0.042	0.032	0.075
1163	TCGA-BH-A1FH-11B	R	0	0.095	0.373	0.042	0.033	0.076
1164	TCGA-BH-A0B8-11A	R	0.001	0.097	0.387	0	0.026	0
1165	TCGA-AC-A2FM-11B	R	0	0.09	0.384	0.047	0	0.036

1166	TCGA-BH-A1FC-11A	R	0	0.082	0.456	0.079	0.053	0.059
1167	TCGA-BH-A1F8-11B	R	0	0.123	0.414	0.011	0	0.064
1168	TCGA-BH-A204-11A	R	0.03	0.11	0.266	0	0	0
1169	TCGA-BH-A0H5-11A	NR	0.099	0.133	0.299	0.016	0	0.061
1170	TCGA-A7-A0CH-11A	R	0	0.056	0.402	0.072	0.095	0
1171	TCGA-E9-A1NA-11A	R	0	0.062	0.416	0.048	0.087	0.079
1172	TCGA-E9-A1RC-11A	R	0	0.1	0.466	0.006	0	0
1173	TCGA-BH-A0DK-11A	NR	0.046	0.131	0.196	0.021	0	0.027
1174	TCGA-BH-A0AU-11A	R	0.061	0.082	0.32	0	0	0.061
1175	TCGA-E9-A1RI-11A	R	0	0.111	0.381	0.022	0	0.007
1176	TCGA-E2-A1LH-11A	R	0.058	0.051	0.301	0.028	0	0.044
1177	TCGA-BH-A0B5-11A	NR	0.032	0.084	0.243	0	0	0.037
1178	TCGA-AC-A2FF-11A	R	0	0.07	0.332	0.071	0.117	0.088
1179	TCGA-BH-A0B7-11A	R	0.059	0.12	0.329	0	0	0.011
1180	TCGA-E9-A1NG-11A	R	0	0.116	0.386	0	0.034	0
1181	TCGA-BH-A0DL-11A	R	0.041	0.066	0.381	0.05	0.12	0.073
1182	TCGA-E9-A1R7-11A	R	0	0.086	0.395	0.007	0	0
1183	TCGA-BH-A0H9-11A	R	0	0.09	0.403	0.045	0	0
1184	TCGA-BH-A18S-11A	R	0.017	0.081	0.413	0.036	0.008	0
1185	TCGA-BH-A0BC-11A	R	0.011	0.173	0.323	0	0	0
1186	TCGA-E2-A1LB-11A	NR	0.069	0.129	0.187	0.044	0	0.172
1187	TCGA-BH-A0HK-11A	R	0	0.103	0.312	0.04	0	0.011
1188	TCGA-E2-A1BC-11A	R	0	0.117	0.364	0.001	0	0
1189	TCGA-BH-A1FR-11B	R	0.01	0.061	0.38	0.018	0.111	0.038
1190	TCGA-BH-A1FB-11A	R	0.04	0.082	0.332	0	0.035	0.092
1191	TCGA-BH-A0HA-11A	NR	0.078	0.093	0.374	0	0	0.019
1192	TCGA-BH-A0E1-11A	R	0.018	0.102	0.36	0	0	0
1193	TCGA-AC-A2FB-11A	R	0	0.042	0.418	0	0.063	0.012
1194	TCGA-E2-A153-11A	R	0.041	0.095	0.27	0	0.031	0.012
1195	TCGA-E9-A1RF-11A	R	0	0.124	0.427	0.058	0	0
1196	TCGA-BH-A1F6-11B	R	0	0.087	0.358	0	0.041	0.046
1197	TCGA-BH-A18V-11A	R	0	0.088	0.328	0.002	0.013	0
1198	TCGA-BH-A1FG-11B	R	0.023	0.118	0.255	0.04	0	0.012
1199	TCGA-BH-A18N-11A	R	0.009	0.097	0.353	0	0.1	0.079
1200	TCGA-E2-A15K-11A	R	0	0.047	0.477	0.052	0.019	0
1201	TCGA-BH-A1F2-11A	R	0.031	0.132	0.269	0.029	0.015	0.109
1202	TCGA-E9-A1ND-11A	R	0	0.066	0.488	0.042	0.062	0
1203	TCGA-A7-A13E-11A	R	0	0.066	0.364	0.038	0.096	0.104
1204	TCGA-A7-A13G-11A	R	0.021	0.076	0.261	0	0.044	0.073
1205	TCGA-BH-A1FU-11A	R	0.056	0.101	0.34	0	0.039	0.084
1206	TCGA-BH-A0BZ-11A	R	0	0.071	0.416	0.05	0.059	0.024
1207	TCGA-BH-A18L-11A	R	0	0.12	0.353	0.043	0.093	0.035
1208	TCGA-BH-A0DP-11A	R	0.058	0.121	0.279	0	0	0.05
1209	TCGA-E9-A1RD-11A	R	0	0.11	0.438	0.01	0	0
1210	TCGA-E9-A1NF-11A	R	0	0.062	0.416	0	0.054	0
1211	TCGA-E9-A1N4-11A	R	0.013	0.073	0.314	0.025	0	0.033
1212	TCGA-BH-A0AY-11A	R	0	0.12	0.396	0.01	0	0
1213	TCGA-BH-A18P-11A	NR	0.054	0.15	0.294	0.023	0	0.071
1214	TCGA-BH-A1FE-11B	R	0.023	0.073	0.277	0.059	0	0.119
1215	TCGA-BH-A1EO-11A	R	0	0.072	0.454	0.037	0	0
1216	TCGA-BH-A0DT-11A	NR	0.064	0.141	0.216	0.022	0	0.11
1217	TCGA-A7-A13F-11A	R	0	0.091	0.479	0.042	0.013	0.05

iTreg	Th1	Th2	Th17	Tfh	Central_memory	Effector_memory	NKT	MAIT	DC	B_cell
0.071	0.07	0.251	0.235	0.074	0.135	0.048	0.02	0.097	0.154	0.077
0.256	0.108	0.468	0.079	0.326	0.096	0	0.028	0.274	0.481	0.131
0.228	0.142	0.356	0.156	0.141	0.028	0.072	0	0.234	0.21	0.098
0	0.041	0.408	0.169	0.156	0.087	0	0.09	0.244	0.289	0.101
0.076	0.175	0.311	0.205	0.408	0.164	0.013	0.095	0.259	0.13	0.105
0.164	0.347	0.37	0.077	0.421	0.123	0.066	0	0.113	0.153	0.117
0.183	0.153	0.382	0.062	0.47	0.145	0	0.048	0.274	0.411	0.361
0.202	0.145	0.321	0.139	0.365	0.164	0.011	0.044	0.238	0.26	0.141
0.163	0.169	0.377	0.233	0.229	0.147	0.025	0	0.204	0.308	0.095
0	0.056	0.442	0.232	0.227	0.128	0.004	0.1	0.179	0.142	0.073
0.012	0.033	0.141	0.243	0.007	0.091	0	0.114	0.052	0.136	0.104
0	0.008	0.263	0.283	0.142	0.063	0.003	0.045	0.072	0.114	0.062
0.027	0.054	0.548	0.233	0.172	0.135	0	0.042	0.279	0.174	0.054
0.235	0.165	0.425	0.138	0.129	0.121	0.088	0	0.15	0.254	0.088
0.037	0.133	0.292	0.351	0	0	0	0.145	0.143	0.187	0.211
0	0.076	0.402	0.283	0	0.165	0	0.057	0.168	0.169	0.093
0	0.018	0.334	0.207	0.16	0.036	0.037	0.07	0.145	0.198	0.15
0.007	0.079	0.363	0.219	0.338	0.158	0	0.091	0.263	0.256	0.068
0.229	0.099	0.488	0.318	0.165	0.137	0.003	0.046	0.201	0.209	0.113
0.156	0.094	0.403	0.193	0.318	0.163	0.017	0.025	0.254	0.167	0.123
0.251	0.194	0.577	0.137	0.117	0.01	0.036	0.004	0.181	0.282	0.109
0.199	0.131	0.298	0.153	0.282	0.136	0	0.03	0.234	0.434	0.116
0.06	0.106	0.276	0.247	0.055	0.156	0	0.041	0.242	0.156	0.106
0.152	0.149	0.33	0.172	0.138	0.122	0.011	0.11	0.061	0.225	0.083
0.172	0.166	0.371	0.202	0.45	0.163	0.031	0.023	0.267	0.195	0.066
0.091	0.131	0.32	0.163	0.447	0.167	0	0.1	0.319	0.166	0.094
0.124	0.171	0.323	0.096	0.437	0.198	0	0.028	0.289	0.199	0.228
0.182	0.374	0.441	0.066	0.228	0.047	0.07	0.015	0.19	0.337	0.097
0.1	0.195	0.309	0.126	0.494	0.115	0.03	0.039	0.212	0.276	0.166
0.094	0.121	0.198	0.162	0.151	0.041	0.029	0.021	0.078	0.125	0.084
0	0.094	0.494	0.192	0.283	0.065	0.028	0.098	0.23	0.213	0.045
0.029	0.182	0.355	0.187	0.469	0.152	0.01	0.075	0.31	0.08	0.077
0.022	0.012	0.411	0.225	0.068	0.06	0.005	0.05	0.211	0.138	0.117
0.098	0.14	0.363	0.218	0.334	0.162	0.028	0.071	0.148	0.151	0.074
0.028	0.079	0.467	0.101	0.162	0.039	0.013	0.089	0.183	0.407	0.152
0.137	0.14	0.467	0.231	0.077	0.126	0.057	0	0.239	0.138	0.049
0	0.03	0.348	0.225	0	0.176	0	0.003	0.171	0.205	0.068
0.246	0.213	0.411	0.051	0.38	0.125	0.01	0	0.235	0.274	0.13
0.2	0.18	0.529	0.034	0.386	0.188	0.026	0.011	0.269	0.249	0.124
0.031	0.121	0.326	0.234	0.256	0.143	0.041	0.06	0.259	0.188	0.041
0.267	0.149	0.528	0.055	0.204	0	0.017	0	0.189	0.371	0.161
0.2	0.262	0.428	0.183	0.427	0.154	0.001	0	0.25	0.327	0.119
0	0.103	0.343	0.286	0.089	0.101	0	0.144	0.107	0.139	0.111
0.229	0.277	0.305	0.148	0.291	0.14	0.034	0	0.194	0.313	0.168
0.087	0.118	0.383	0.257	0.122	0.109	0.047	0.05	0.138	0.135	0.078
0.237	0.291	0.32	0.093	0.404	0.081	0	0	0.235	0.392	0.272
0.169	0.196	0.401	0.135	0.233	0.059	0.019	0.104	0.212	0.326	0.143
0	0.062	0.428	0.234	0.171	0.125	0	0.007	0.266	0.16	0.078
0.17	0.033	0.328	0.123	0.055	0.095	0.024	0	0.145	0.239	0.08
0.185	0.311	0.5	0.066	0.358	0.046	0.058	0	0.217	0.259	0.155
0.036	0	0.205	0.258	0.072	0.101	0	0.028	0.226	0.157	0.089
0.16	0.21	0.36	0.056	0.241	0.135	0.084	0	0.123	0.256	0.098

0.144	0.237	0.493	0.109	0.36	0.158	0.085	0	0.253	0.246	0.082
0.093	0.173	0.474	0.068	0.44	0.032	0.002	0.042	0.259	0.331	0.142
0	0.034	0.169	0.27	0	0.099	0.006	0.059	0.057	0.191	0.091
0.025	0.123	0.493	0.131	0.299	0.048	0.06	0.035	0.225	0.202	0.102
0	0.099	0.294	0.236	0.072	0.099	0.008	0	0.256	0.118	0.056
0.047	0.174	0.306	0.181	0.294	0.064	0.078	0.017	0.219	0.176	0.088
0.056	0.13	0.282	0.187	0.171	0.095	0.044	0	0.268	0.185	0.075
0	0.125	0.421	0.281	0.319	0.135	0	0.115	0.194	0.135	0.077
0.119	0.084	0.408	0.194	0.183	0.093	0.013	0.044	0.227	0.078	0.075
0.169	0.176	0.314	0.204	0.247	0.16	0.056	0.082	0.153	0.26	0.046
0.007	0.132	0.365	0.232	0.361	0.167	0	0.053	0.264	0.183	0.078
0.12	0.105	0.351	0.201	0.345	0.153	0	0.014	0.211	0.233	0.12
0.009	0.049	0.337	0.269	0.275	0.173	0.006	0.026	0.188	0.129	0.065
0.16	0.215	0.359	0.24	0.173	0.156	0.068	0.01	0.181	0.159	0.097
0.067	0.006	0.324	0.249	0.208	0.118	0.071	0.008	0.217	0.247	0.053
0.19	0.314	0.271	0.029	0.387	0.19	0.06	0	0.196	0.237	0.185
0	0.069	0.29	0.253	0.313	0.183	0.012	0	0.216	0.206	0.072
0.204	0.281	0.376	0.025	0.415	0.141	0	0	0.232	0.23	0.445
0.198	0.144	0.433	0.235	0.156	0.049	0.015	0.062	0.23	0.18	0.125
0.016	0.099	0.298	0.277	0.075	0.119	0	0.007	0.255	0.026	0.095
0.178	0.247	0.302	0.176	0.238	0.169	0.048	0.013	0.178	0.237	0.153
0.005	0.104	0.329	0.165	0.066	0.11	0.072	0	0.266	0.12	0.073
0	0.062	0.305	0.231	0.255	0.107	0	0.091	0.137	0.149	0.034
0.147	0.169	0.319	0.287	0.197	0.168	0.037	0.002	0.2	0.129	0.075
0.198	0.254	0.275	0.119	0.473	0.146	0	0	0.161	0.3	0.117
0.028	0.167	0.419	0.22	0.447	0.158	0.049	0.059	0.263	0.217	0.1
0.056	0.019	0.195	0.185	0.053	0.125	0.002	0.085	0.117	0.175	0.166
0.07	0.16	0.34	0.192	0.519	0.196	0.025	0.075	0.274	0.252	0.058
0.12	0.175	0.518	0.183	0.385	0.088	0.027	0	0.221	0.293	0.094
0.251	0.302	0.476	0.161	0.315	0.074	0.003	0.059	0.181	0.216	0.118
0.12	0.086	0.396	0.085	0.182	0.085	0.034	0	0.259	0.219	0.082
0	0.088	0.12	0.302	0	0.166	0.003	0.076	0.116	0.099	0.058
0.17	0.076	0.325	0.208	0.019	0.048	0	0	0.21	0.169	0.112
0.146	0.189	0.368	0.108	0.384	0.171	0.013	0.036	0.243	0.164	0.113
0.202	0.316	0.281	0.156	0.377	0.109	0.017	0	0.236	0.209	0.118
0.218	0.241	0.316	0.175	0.298	0.162	0.032	0	0.209	0.219	0.1
0.163	0.149	0.344	0.21	0.405	0	0.037	0.099	0.301	0.222	0.139
0.252	0.324	0.25	0.218	0.364	0.171	0.052	0	0.164	0.293	0.063
0.009	0.113	0.523	0.237	0.236	0.174	0	0	0.156	0.16	0.089
0.261	0.329	0.321	0.188	0.348	0.053	0.013	0.063	0.211	0.277	0.117
0.262	0.27	0.411	0.076	0.253	0.091	0.065	0	0.18	0.349	0.157
0.06	0.155	0.514	0.172	0.409	0.213	0	0.048	0.24	0.24	0.074
0.023	0.151	0.263	0.251	0.112	0.127	0.025	0.029	0.168	0.229	0.098
0.103	0.06	0.253	0.262	0.037	0.097	0.033	0.058	0.193	0.117	0.073
0	0.149	0.45	0.247	0.449	0.186	0	0.068	0.265	0.187	0.064
0.192	0.135	0.479	0.171	0.427	0.157	0	0.024	0.298	0.206	0.347
0.102	0.147	0.366	0.259	0.482	0.224	0	0.038	0.253	0.288	0.089
0.213	0.112	0.466	0.159	0.401	0.171	0	0.04	0.266	0.176	0.132
0.031	0.105	0.339	0.281	0.291	0.079	0.078	0.049	0.122	0.236	0.031
0.11	0.13	0.369	0.135	0.456	0.081	0.011	0.025	0.306	0.398	0.24
0.031	0.086	0.251	0.255	0.12	0.174	0.011	0.047	0.271	0.329	0.047
0.065	0.012	0.389	0.21	0	0.112	0	0.008	0.198	0.15	0.125
0.133	0.168	0.286	0.169	0.525	0.176	0	0.081	0.254	0.246	0.16

0.158	0.143	0.29	0.247	0.357	0.157	0.027	0.046	0.241	0.134	0.114
0.005	0.049	0.411	0.285	0.177	0.144	0.021	0	0.133	0.116	0.091
0.14	0.173	0.313	0.219	0.392	0.093	0	0.082	0.244	0.176	0.13
0.011	0.057	0.49	0.256	0.102	0.11	0.044	0	0.132	0.163	0.081
0	0.063	0.426	0.223	0.385	0.134	0	0.055	0.291	0.271	0.076
0.155	0.025	0.23	0.255	0.054	0.064	0.057	0	0.11	0.31	0.091
0.226	0.188	0.343	0.194	0.326	0.149	0.025	0.021	0.172	0.218	0.104
0.09	0.15	0.43	0.244	0.463	0.189	0.035	0.066	0.261	0.111	0.044
0.083	0.055	0.249	0.136	0	0.131	0.026	0.028	0.154	0.131	0.073
0.108	0.178	0.474	0.074	0.121	0.096	0	0.029	0.227	0.192	0.144
0.156	0.175	0.459	0.178	0.342	0.087	0.019	0.093	0.17	0.211	0.184
0.091	0.121	0.451	0.122	0.03	0.024	0.033	0	0.215	0.107	0.118
0.006	0.126	0.404	0.145	0.334	0.08	0	0.065	0.322	0.356	0.084
0.113	0.25	0.383	0.151	0.199	0.045	0.103	0	0.221	0.087	0.116
0.171	0.187	0.341	0.191	0.27	0.208	0.058	0.033	0.229	0.17	0.103
0	0.031	0.32	0.16	0	0.059	0	0.036	0.148	0.092	0.069
0.048	0.205	0.47	0.19	0.288	0.189	0.068	0.02	0.054	0.262	0.073
0	0	0.202	0.154	0.04	0.083	0	0.01	0.16	0.152	0.076
0.234	0.203	0.285	0.146	0.392	0.185	0	0	0.193	0.085	0.308
0.146	0.096	0.302	0.268	0.185	0.208	0.014	0.007	0.216	0.139	0.08
0.161	0.145	0.365	0.157	0.311	0.188	0.049	0.011	0.258	0.118	0.08
0.226	0.258	0.36	0.116	0.348	0.138	0.028	0.011	0.205	0.229	0.3
0.151	0.115	0.511	0.145	0.146	0.137	0.046	0.011	0.19	0.197	0.082
0.02	0.101	0.35	0.186	0.379	0.156	0	0.099	0.289	0.401	0.059
0.243	0.304	0.351	0.139	0.422	0.192	0.024	0	0.2	0.352	0.119
0.02	0.091	0.372	0.21	0.257	0.176	0.012	0.067	0.163	0.13	0.13
0.019	0.137	0.5	0.285	0.066	0.07	0	0.042	0.219	0.31	0.102
0.152	0.291	0.318	0.119	0.478	0.166	0	0	0.21	0.151	0.238
0.252	0.265	0.237	0.169	0.382	0.165	0.015	0	0.085	0.178	0.126
0.236	0.291	0.326	0.048	0.344	0.114	0.054	0	0.211	0.206	0.106
0.136	0.095	0.272	0.273	0.124	0.058	0.007	0	0.204	0.254	0.104
0.279	0.212	0.382	0.125	0.238	0.074	0.023	0	0.215	0.301	0.142
0.226	0.252	0.305	0.115	0.432	0.149	0	0	0.182	0.254	0.159
0.066	0.082	0.39	0.215	0.241	0.066	0.066	0.055	0.17	0.207	0.132
0.278	0.214	0.207	0.081	0.494	0.158	0	0	0.237	0.107	0.322
0.2	0.154	0.269	0.157	0.497	0.156	0	0.034	0.279	0.167	0.202
0	0.133	0.413	0.156	0.279	0.027	0	0.068	0.18	0.159	0.143
0.001	0.111	0.271	0.107	0.258	0	0	0.054	0.255	0.349	0.126
0.098	0.018	0.447	0.27	0.113	0.171	0	0.099	0.155	0.136	0.058
0	0.083	0.166	0.218	0	0.083	0	0.174	0.063	0.098	0.05
0.194	0.182	0.393	0.259	0.166	0.16	0.034	0	0.106	0.168	0.075
0.2	0.19	0.33	0.211	0.405	0.117	0	0.045	0.226	0.373	0.081
0.097	0.126	0.39	0.21	0.337	0.085	0.06	0.033	0.227	0.13	0.057
0.215	0.225	0.404	0.105	0.186	0.088	0.081	0	0.194	0.188	0.067
0.197	0.054	0.226	0.308	0.21	0.081	0	0.08	0.243	0.115	0.152
0.056	0.171	0.356	0.161	0.396	0.049	0.044	0.058	0.228	0.208	0.116
0.171	0.2	0.33	0.085	0.453	0.175	0	0	0.181	0.271	0.115
0.255	0.27	0.322	0.103	0.436	0.143	0.01	0	0.228	0.324	0.164
0.031	0	0.23	0.262	0.047	0.105	0	0.05	0.109	0.18	0.086
0.041	0.117	0.438	0.202	0.461	0.194	0	0.059	0.269	0.151	0.106
0.127	0.082	0.342	0.214	0.146	0.082	0.019	0	0.243	0.116	0.09
0.287	0.125	0.493	0.094	0.327	0.161	0	0.016	0.197	0.267	0.157
0.199	0.193	0.373	0.089	0.34	0.068	0.063	0.05	0.22	0.203	0.177

0.248	0.31	0.267	0.134	0.353	0.121	0.013	0	0.232	0.318	0.097
0.105	0.087	0.245	0.288	0.029	0.158	0.003	0.058	0.078	0.139	0.081
0.207	0.133	0.435	0.172	0.429	0.157	0.048	0	0.177	0.176	0.097
0.105	0.225	0.393	0.094	0.331	0	0.032	0.045	0.262	0.198	0.154
0.111	0.159	0.365	0.195	0.365	0.161	0.012	0.054	0.256	0.213	0.07
0.136	0.175	0.301	0.188	0.42	0.156	0.036	0.011	0.261	0.158	0.043
0.215	0.283	0.302	0.132	0.498	0.193	0.004	0	0.222	0.326	0.161
0.191	0.171	0.31	0.099	0.397	0.114	0	0.088	0.253	0.244	0.074
0.073	0.158	0.244	0.276	0	0.104	0	0.054	0.13	0.217	0.114
0.049	0	0	0.131	0.003	0.145	0	0.114	0.049	0.121	0.12
0.039	0.059	0.376	0.261	0.01	0.048	0.005	0.02	0.134	0.14	0.093
0	0.109	0.245	0.213	0	0.019	0	0.013	0.256	0.152	0.177
0	0.088	0.421	0.168	0.358	0.05	0	0.083	0.294	0.283	0.111
0.199	0.157	0.382	0.129	0.429	0.13	0	0.06	0.296	0.215	0.073
0.216	0.292	0.333	0.1	0.362	0.083	0.047	0	0.225	0.184	0.124
0.188	0.167	0.396	0.109	0.268	0.15	0.051	0	0.138	0.296	0.09
0.07	0.086	0.395	0.196	0.175	0.1	0.009	0	0.141	0.367	0.104
0.134	0.129	0.442	0.156	0.26	0.051	0.03	0.035	0.303	0.256	0.14
0.227	0.215	0.37	0.172	0.312	0.07	0.014	0	0.207	0.198	0.103
0	0.018	0.247	0.182	0.123	0.157	0	0.105	0.046	0	0.068
0.237	0.205	0.312	0.155	0.461	0.173	0	0	0.244	0.348	0.084
0.234	0.273	0.307	0.112	0.417	0.098	0	0	0.226	0.344	0.174
0.299	0.148	0.207	0.131	0.178	0	0	0.147	0.274	0.096	0.126
0.077	0.121	0.472	0.166	0.473	0.207	0	0	0.299	0.167	0.126
0.205	0.2	0.336	0.148	0.441	0.157	0	0.015	0.243	0.235	0.239
0.088	0.12	0.311	0.379	0.35	0.007	0.004	0.118	0.261	0.312	0.079
0.251	0.148	0.479	0.09	0.368	0.166	0	0	0.211	0.193	0.406
0.083	0.087	0.508	0.157	0.045	0.068	0	0.064	0.133	0.164	0.127
0.166	0.138	0.227	0.243	0.07	0.12	0.034	0.015	0.158	0.146	0.095
0.126	0.189	0.452	0.142	0.371	0.097	0.035	0.064	0.297	0.22	0.135
0	0.026	0.369	0.257	0	0.018	0.014	0.113	0.149	0.159	0.123
0.231	0.267	0.263	0.058	0.387	0.158	0	0	0.216	0.234	0.251
0.155	0.321	0.485	0.041	0.455	0.017	0.032	0.008	0.204	0.33	0.325
0.268	0.164	0.265	0.171	0.564	0.161	0	0	0.186	0.207	0.224
0	0.024	0.29	0.176	0.065	0.086	0	0.106	0.036	0.248	0.094
0.297	0.321	0.492	0.073	0.332	0.058	0.017	0	0.189	0.364	0.154
0.169	0.185	0.343	0.164	0.161	0.131	0.07	0	0.092	0.165	0.064
0.085	0.147	0.34	0.229	0.129	0.054	0.064	0.045	0.237	0.212	0.072
0.096	0.177	0.443	0.226	0.239	0.162	0.081	0.005	0.153	0.082	0.055
0.129	0.192	0.264	0.077	0.497	0.113	0	0	0.256	0.3	0.267
0.111	0.283	0.386	0.125	0.449	0.08	0.018	0	0.263	0.282	0.144
0.16	0.161	0.364	0.28	0.14	0.118	0.036	0	0.224	0.158	0.099
0.227	0.285	0.433	0.058	0.368	0.039	0	0.022	0.237	0.411	0.241
0.048	0.069	0.153	0.276	0.082	0.127	0	0.051	0.048	0.139	0.082
0.182	0.147	0.515	0.131	0.468	0.128	0	0.006	0.252	0.482	0.135
0	0.113	0.459	0.213	0.435	0.16	0	0.086	0.274	0.178	0.145
0.101	0.109	0.438	0.277	0.009	0.071	0.003	0.025	0.242	0.183	0.095
0.168	0.082	0.583	0.184	0.189	0.097	0.03	0.058	0.252	0.227	0.09
0.222	0.18	0.323	0.118	0.47	0.185	0	0	0.276	0.187	0.162
0.212	0.273	0.487	0.125	0.362	0.054	0.034	0	0.23	0.328	0.205
0.154	0.251	0.306	0.099	0.41	0.112	0	0	0.143	0.347	0.281
0.137	0.129	0.327	0.28	0.196	0.099	0.073	0.041	0.177	0.302	0.188
0.198	0.175	0.324	0.073	0.227	0.122	0.059	0	0.246	0.245	0.128

0.234	0.278	0.346	0.172	0.48	0.091	0	0	0.181	0.277	0.2
0.048	0.073	0.391	0.27	0.19	0.203	0	0.091	0.248	0.106	0.074
0.099	0.057	0.415	0.233	0.127	0.056	0.005	0.05	0.224	0.211	0.148
0.092	0.175	0.407	0.243	0.258	0.179	0.05	0	0.215	0.114	0.068
0	0.076	0.516	0.216	0.103	0.127	0.013	0.011	0.328	0.264	0.064
0.164	0.157	0.36	0.134	0.266	0.177	0.021	0.017	0.229	0.169	0.101
0.099	0.126	0.314	0.236	0.122	0.068	0	0.091	0.203	0.239	0.091
0.084	0.1	0.478	0.221	0.032	0.119	0	0	0.196	0.134	0.068
0.118	0.123	0.485	0.113	0.463	0.189	0.024	0.058	0.186	0.218	0.196
0	0.004	0.42	0.232	0.217	0.125	0.019	0.08	0.21	0.05	0.092
0.145	0.2	0.489	0.138	0.29	0.151	0.072	0	0.221	0.154	0.096
0.27	0.151	0.523	0.128	0.207	0.076	0.014	0.007	0.168	0.341	0.112
0	0.144	0.412	0.244	0.192	0.085	0	0.181	0.264	0.13	0.077
0.014	0.003	0.428	0.273	0	0.143	0	0.039	0.118	0.192	0.072
0.103	0.15	0.293	0.248	0.458	0.252	0.064	0.047	0.191	0.077	0.116
0.224	0.202	0.311	0.111	0.437	0.189	0.001	0.025	0.257	0.291	0.118
0.079	0.099	0.416	0.164	0.348	0.113	0.005	0.041	0.207	0.245	0.085
0.098	0.143	0.37	0.177	0.487	0.186	0	0.02	0.264	0.205	0.216
0.008	0.147	0.582	0.173	0.316	0.147	0	0.062	0.258	0.215	0.092
0.221	0.249	0.316	0.172	0.399	0.126	0	0	0.192	0.334	0.154
0	0.077	0.253	0.301	0.006	0.162	0	0.074	0.135	0.252	0.071
0.094	0.116	0.331	0.238	0.427	0.185	0.04	0.014	0.19	0.086	0.147
0.039	0.12	0.515	0.156	0.095	0	0.038	0.022	0.268	0.127	0.17
0.255	0.198	0.307	0.211	0.169	0.113	0.029	0	0.115	0.268	0.115
0	0.016	0.226	0.329	0.029	0.125	0	0.258	0.272	0.04	0.064
0.089	0.112	0.274	0.249	0.024	0.034	0.041	0.027	0.207	0.158	0.136
0	0.006	0.396	0.211	0.047	0.118	0	0.058	0.146	0.274	0.121
0.121	0.371	0.285	0.169	0.236	0.001	0.046	0.05	0.21	0.302	0.123
0.049	0.101	0.325	0.209	0.268	0.095	0	0.078	0.298	0.303	0.071
0.046	0.176	0.284	0.231	0.41	0.155	0.019	0.071	0.223	0.167	0.132
0.13	0.252	0.323	0.106	0.415	0.147	0.049	0.157	0.083	0.22	0.243
0.228	0.118	0.324	0.175	0.281	0.115	0.024	0.001	0.26	0.328	0.076
0.046	0.126	0.399	0.215	0.195	0.147	0.02	0.002	0.279	0.195	0.123
0.18	0.22	0.392	0.149	0.391	0.167	0	0.042	0.273	0.265	0.116
0.096	0.165	0.354	0.178	0.435	0.193	0	0.101	0.241	0.219	0.242
0.051	0.041	0.13	0.27	0	0.185	0.01	0.116	0	0.145	0.072
0.096	0.162	0.331	0.208	0.394	0.136	0.025	0.029	0.268	0.268	0.093
0.238	0.298	0.306	0.131	0.083	0.051	0.104	0	0.145	0.315	0.109
0.205	0.339	0.366	0.188	0.378	0.039	0.039	0	0.216	0.392	0.156
0	0.026	0.337	0.286	0.045	0.09	0.01	0.133	0.187	0.044	0.055
0.017	0.029	0.248	0.175	0.105	0.106	0	0.06	0.262	0.247	0.105
0	0.079	0.31	0.224	0.339	0.181	0	0.071	0.282	0.215	0.077
0.021	0.075	0.35	0.149	0.084	0.116	0	0	0.197	0.222	0.155
0.031	0.206	0.453	0.183	0.54	0.107	0	0.107	0.264	0.224	0.185
0.095	0	0.334	0.231	0.048	0.073	0.06	0.025	0.072	0.15	0.104
0.192	0.206	0.293	0.049	0.488	0.132	0	0	0.269	0.446	0.132
0.076	0.158	0.436	0.197	0.46	0.046	0.012	0.055	0.254	0.275	0.159
0	0.105	0.194	0.302	0.02	0.066	0.011	0.102	0.163	0.129	0.17
0.131	0.148	0.425	0.185	0.274	0.172	0.043	0.023	0.255	0.147	0.13
0	0.063	0.357	0.272	0.137	0.141	0	0.09	0.179	0.219	0.048
0.227	0.277	0.307	0.161	0.409	0.137	0.032	0.019	0.093	0.406	0.338
0.167	0.118	0.435	0.271	0.314	0.16	0.008	0	0.195	0.235	0.057
0.218	0.191	0.42	0.106	0.26	0.089	0.013	0	0.271	0.289	0.103

0.236	0.186	0.309	0.15	0.445	0.199	0	0	0.238	0.316	0.18
0.044	0.118	0.309	0.266	0	0.171	0	0	0.191	0.189	0.077
0.241	0.302	0.378	0.131	0.338	0.041	0.03	0.056	0.236	0.33	0.117
0.091	0.186	0.39	0.232	0.253	0.171	0.051	0.035	0.193	0.163	0.044
0.191	0.18	0.343	0.192	0.414	0.119	0.066	0	0.075	0.203	0.059
0.093	0.112	0.268	0.193	0.123	0.181	0.031	0.035	0.133	0.161	0.129
0.136	0.195	0.342	0.132	0.277	0.189	0.056	0	0.192	0.157	0.084
0.003	0.017	0.136	0.265	0	0.026	0	0.165	0.012	0.201	0.14
0.041	0.145	0.375	0.213	0.361	0.058	0.029	0.086	0.204	0.279	0.103
0.3	0.151	0.435	0.16	0.12	0.022	0.049	0	0.188	0.124	0.061
0	0.097	0.372	0.324	0.043	0.082	0.016	0	0.198	0.195	0.102
0.022	0.052	0.44	0.073	0.032	0.014	0.055	0.038	0.183	0.309	0.148
0.25	0.318	0.395	0.098	0.371	0.124	0.001	0	0.184	0.224	0.165
0.076	0.054	0.215	0.251	0.024	0.131	0	0.144	0.164	0.29	0.055
0	0.092	0.279	0.24	0.417	0.162	0.021	0.092	0.249	0.211	0.084
0	0.005	0.403	0.245	0.102	0.13	0	0.051	0.317	0.078	0.085
0.234	0.271	0.521	0.196	0.286	0.068	0.012	0.079	0.117	0.237	0.125
0.247	0.257	0.358	0.131	0.408	0.155	0	0	0.225	0.208	0.179
0.075	0.148	0.35	0.157	0.291	0.109	0.027	0	0.232	0.248	0.09
0.079	0.04	0.311	0.261	0.082	0.124	0	0.024	0.159	0.222	0.083
0.227	0.299	0.326	0.157	0.177	0.043	0.098	0	0.163	0.128	0.096
0.13	0.051	0.441	0.193	0.121	0.039	0.04	0.092	0.125	0.299	0.137
0.145	0.156	0.425	0.144	0.129	0.107	0.091	0	0.203	0.194	0.105
0.259	0.272	0.285	0.1	0.517	0.192	0	0.028	0.261	0.245	0.285
0.08	0.146	0.388	0.204	0.477	0.164	0	0.063	0.252	0.226	0.098
0.229	0.245	0.306	0.146	0.352	0.084	0.042	0	0.183	0.253	0.1
0.252	0.186	0.341	0.065	0.381	0.17	0.047	0	0.263	0.194	0.074
0	0.119	0.565	0.276	0.219	0.135	0.003	0.007	0.255	0.181	0.082
0.206	0.061	0.308	0.163	0.062	0.097	0.038	0	0.096	0.166	0.093
0.02	0.023	0.403	0.235	0.046	0.142	0	0.087	0.16	0.163	0.081
0.04	0.013	0.368	0.276	0.063	0.157	0	0.012	0.174	0.129	0.091
0.026	0.166	0.521	0.251	0.282	0.12	0.051	0	0.224	0.153	0.032
0.019	0.178	0.465	0.2	0.317	0.124	0.049	0	0.202	0.073	0.127
0.151	0.246	0.378	0.108	0.3	0.09	0.049	0.066	0.296	0.294	0.172
0.08	0.169	0.398	0.07	0.52	0.144	0.002	0	0.247	0.252	0.149
0.211	0.157	0.327	0.279	0.33	0.113	0.005	0.028	0.237	0.54	0.114
0.026	0.018	0.36	0.245	0	0.155	0	0.003	0.157	0.141	0.084
0.232	0.165	0.348	0.156	0.459	0.147	0	0	0.201	0.244	0.203
0	0.135	0.448	0.177	0.319	0.001	0	0.027	0.194	0.204	0.072
0.267	0.244	0.489	0.148	0.172	0	0.026	0	0.193	0.295	0.17
0.192	0.339	0.428	0	0.269	0.03	0.086	0	0.208	0.425	0.104
0	0.059	0.211	0.246	0.044	0.09	0	0.096	0.147	0.136	0.06
0	0.191	0.532	0.231	0.427	0.046	0.029	0.073	0.229	0.176	0.101
0.182	0.157	0.431	0.112	0.426	0.194	0	0.034	0.291	0.219	0.146
0.142	0.124	0.412	0.11	0.239	0.129	0.028	0.061	0.251	0.319	0.08
0.271	0.224	0.457	0.033	0.396	0.138	0	0	0.232	0.169	0.448
0	0.015	0.218	0.283	0.021	0.076	0	0.046	0.193	0.277	0.127
0.035	0.163	0.344	0.156	0.408	0.089	0	0.05	0.246	0.31	0.08
0.277	0.296	0.365	0.181	0.348	0.069	0.031	0	0.101	0.195	0.164
0.081	0.192	0.509	0.131	0.342	0.159	0.042	0.016	0.24	0.127	0.099
0.029	0.132	0.4	0.244	0.429	0.206	0	0.057	0.254	0.167	0.162
0.059	0.066	0.51	0.128	0.129	0.065	0	0	0.203	0.277	0.067
0	0.052	0.338	0.276	0.24	0.158	0	0.072	0.224	0.09	0.117

0.026	0.039	0.488	0.259	0.176	0.155	0	0.077	0.234	0.191	0.061
0.254	0.31	0.387	0.029	0.359	0.103	0.053	0	0.135	0.216	0.145
0	0.017	0.295	0.191	0.052	0.082	0.003	0.024	0.128	0.103	0.064
0.005	0.073	0.398	0.247	0.294	0.194	0.026	0.065	0.119	0.157	0.079
0.066	0.15	0.405	0.252	0.29	0.153	0.016	0.011	0.183	0.11	0.239
0.053	0.076	0.31	0.124	0.106	0.037	0.021	0	0.166	0.146	0.077
0.125	0.168	0.43	0.093	0.429	0.09	0	0.033	0.278	0.218	0.296
0.227	0.174	0.305	0.075	0.444	0.113	0	0	0.208	0.382	0.14
0.189	0.129	0.274	0.162	0.04	0.094	0.018	0	0.22	0.203	0.094
0	0	0.385	0.19	0.047	0.109	0	0.015	0.098	0.182	0
0.104	0.033	0.309	0.099	0.032	0.109	0	0.024	0.218	0.103	0.075
0.143	0.097	0.388	0.078	0.059	0.101	0.055	0	0.23	0.156	0.109
0	0.041	0.29	0.222	0.064	0.097	0.036	0.029	0.229	0.111	0.104
0	0.121	0.356	0.109	0.199	0.152	0.034	0.122	0.196	0.122	0.181
0.246	0.16	0.531	0.079	0.257	0.067	0	0.005	0.233	0.584	0.075
0.069	0.215	0.409	0.078	0.377	0.163	0.052	0	0.235	0.352	0.294
0.044	0.105	0.478	0.244	0.42	0.151	0	0.05	0.305	0.22	0.075
0.076	0.181	0.379	0.162	0.234	0.086	0.109	0	0.209	0.155	0.083
0.222	0.199	0.381	0.157	0.324	0.17	0.05	0	0.165	0.116	0.126
0.035	0.039	0.384	0.274	0.05	0.16	0	0.061	0.206	0.122	0.067
0.198	0.158	0.288	0.146	0.275	0.204	0.037	0.048	0.213	0.229	0.144
0.167	0.092	0.317	0.104	0.17	0.02	0	0.085	0.289	0.16	0.071
0	0.112	0.111	0.16	0.066	0.066	0.045	0.059	0.196	0.177	0.108
0.089	0.104	0.374	0.219	0.036	0.119	0	0	0.206	0.218	0.116
0.09	0.082	0.278	0.097	0.111	0.179	0.028	0.027	0.127	0.131	0.147
0.236	0.208	0.345	0.18	0.306	0.105	0.012	0	0.223	0.333	0.095
0.189	0.133	0.402	0.149	0.449	0.194	0.011	0.068	0.207	0.142	0.073
0.141	0.159	0.394	0.195	0.294	0.152	0.014	0	0.204	0.227	0.167
0	0.053	0.185	0.249	0.099	0.156	0.015	0.109	0.189	0.03	0.11
0.111	0.101	0.299	0.174	0.14	0.082	0	0.059	0.279	0.354	0.102
0.121	0.048	0.092	0.24	0	0.151	0	0.018	0	0.128	0.129
0.006	0.066	0.203	0.291	0	0.128	0.018	0.089	0.162	0.145	0.079
0.15	0.143	0.358	0.196	0.267	0.147	0	0.008	0.224	0.304	0.078
0.184	0.17	0.283	0.188	0.099	0.174	0.037	0.047	0.087	0.137	0.067
0.244	0.251	0.245	0.177	0.46	0.147	0	0	0.177	0.262	0.411
0.107	0.044	0.384	0.226	0.059	0	0.001	0.033	0.178	0.375	0.131
0	0	0.37	0.111	0.099	0.122	0	0.057	0.096	0.003	0.114
0.187	0.077	0.352	0.192	0.123	0.117	0.007	0.093	0.222	0.29	0.067
0.023	0.1	0.442	0.149	0.031	0.109	0.015	0	0.175	0.158	0.105
0.108	0.066	0.162	0.286	0.127	0.16	0	0.058	0.064	0.128	0.088
0	0.11	0.464	0.266	0.093	0.051	0.001	0.002	0.21	0.085	0.06
0.206	0.314	0.373	0.043	0.442	0.146	0	0	0.184	0.202	0.458
0.047	0.133	0.47	0.251	0.133	0.141	0.049	0.038	0.092	0.114	0.144
0.226	0.299	0.518	0.043	0.329	0.121	0	0	0.192	0.364	0.201
0.178	0.202	0.407	0.218	0.122	0.155	0.083	0	0.153	0.206	0.088
0.102	0.124	0.483	0.222	0.428	0.154	0	0.032	0.245	0.316	0.027
0.027	0.035	0.474	0.215	0.023	0.079	0.005	0.08	0.143	0.154	0.102
0.166	0.05	0.321	0.177	0.084	0.176	0.022	0	0.139	0.117	0.061
0.046	0.157	0.293	0.256	0.295	0.094	0.051	0.053	0.266	0.115	0.089
0.038	0.124	0.356	0.176	0.36	0.17	0	0.033	0.293	0.229	0.055
0.012	0.079	0.387	0.273	0.26	0.127	0.042	0.116	0.212	0.132	0.053
0.171	0.113	0.455	0.253	0	0.095	0.056	0.045	0.182	0.212	0.121
0.18	0.156	0.41	0.193	0.134	0.033	0.042	0.038	0.184	0.152	0.099

0	0.068	0.133	0.142	0.175	0.016	0.031	0.056	0.104	0.267	0.09
0	0.184	0.334	0.135	0.031	0.034	0.035	0.05	0.135	0.122	0.058
0.028	0.106	0.355	0.131	0.183	0.133	0.017	0.129	0.209	0.237	0.073
0.036	0.042	0.318	0.189	0.104	0.11	0.013	0.106	0.165	0.068	0.059
0.271	0.26	0.269	0.157	0.517	0.131	0	0	0.212	0.439	0.251
0.193	0.272	0.451	0.068	0.284	0.108	0.083	0	0.186	0.321	0.123
0.179	0.187	0.426	0.202	0.432	0.18	0	0.011	0.179	0.245	0.063
0.173	0.271	0.317	0.121	0.379	0.146	0.048	0	0.217	0.288	0.171
0.135	0.175	0.383	0.183	0.338	0.064	0	0.088	0.244	0.414	0.184
0	0.1	0.248	0.13	0.261	0.216	0.027	0.004	0.229	0.13	0.232
0.164	0.181	0.335	0.186	0.199	0.144	0.03	0.008	0.188	0.168	0.096
0	0.043	0.291	0.242	0.007	0.137	0.006	0.072	0.21	0.105	0.057
0.049	0.048	0.457	0.276	0.031	0.131	0	0.081	0.158	0.199	0.06
0.039	0.086	0.457	0.215	0.334	0.091	0	0.08	0.233	0.354	0.042
0.116	0.282	0.293	0.153	0.533	0.191	0.046	0.024	0.252	0.165	0.099
0.265	0.365	0.323	0.298	0.145	0	0.046	0	0.162	0.14	0.17
0	0.086	0.329	0.232	0.157	0.108	0	0.076	0.236	0.211	0.094
0.229	0.174	0.371	0.056	0.199	0.168	0.07	0	0.196	0.237	0.085
0.148	0.155	0.404	0.193	0.225	0.049	0.014	0.038	0.23	0.23	0.109
0.014	0.116	0.349	0.228	0.31	0.136	0.011	0.05	0.312	0.185	0.116
0.075	0.048	0.529	0.148	0.112	0	0	0	0.322	0.207	0.079
0.224	0.127	0.285	0.155	0.407	0.164	0.007	0.007	0.24	0.321	0.067
0.005	0.117	0.367	0.156	0.075	0.081	0	0	0.201	0.193	0.143
0.064	0.125	0.35	0.135	0.112	0.155	0.072	0	0.219	0.188	0.086
0	0.067	0.271	0.19	0.19	0.041	0.048	0.108	0.054	0.145	0.085
0.284	0.161	0.307	0.149	0.183	0.085	0.011	0	0.267	0.25	0.125
0	0.057	0.456	0.283	0.08	0.138	0	0.01	0.229	0.059	0.067
0.189	0.199	0.48	0.169	0.282	0.176	0.054	0	0.2	0.078	0.082
0.175	0.206	0.405	0.137	0.38	0.099	0.029	0	0.196	0.283	0.042
0.198	0.051	0.071	0.289	0.309	0.051	0	0.264	0.33	0.131	0.118
0.156	0.112	0.322	0.12	0.405	0.101	0.019	0.046	0.322	0.265	0.108
0.164	0.112	0.33	0.144	0.462	0.145	0.044	0	0.277	0.205	0.068
0.116	0.092	0.32	0.211	0.466	0.119	0.035	0.05	0.285	0.23	0.049
0.051	0.002	0.363	0.13	0.106	0.066	0	0.101	0.138	0.29	0.101
0.118	0.004	0.232	0.193	0.093	0.047	0	0.106	0.163	0.136	0.066
0	0.03	0.119	0.151	0.042	0.073	0.011	0.128	0.031	0	0.084
0.074	0.028	0.229	0.143	0.238	0.066	0.036	0.063	0.185	0.429	0.054
0	0	0.205	0.118	0.154	0.068	0	0.094	0.213	0.26	0.078
0.183	0.19	0.239	0.15	0.438	0.073	0.045	0	0.202	0.2	0.077
0.048	0.17	0.34	0.192	0.481	0.063	0.061	0.046	0.268	0.224	0.092
0.05	0.124	0.341	0.186	0.403	0.135	0.024	0.051	0.177	0.158	0.253
0	0.005	0.391	0.232	0.192	0.074	0.074	0	0.219	0.101	0.074
0.248	0.166	0.278	0.133	0.389	0.049	0.036	0	0.246	0.194	0.117
0.23	0.278	0.427	0	0.405	0	0.039	0	0.156	0.281	0.248
0.094	0.064	0.321	0.187	0.559	0.113	0	0.083	0.295	0.213	0.064
0.128	0.13	0.398	0.137	0.474	0.059	0	0.034	0.288	0.315	0.062
0.111	0.095	0.366	0.187	0.407	0.093	0.059	0.052	0.273	0.32	0.057
0.02	0.039	0.246	0.226	0.139	0.032	0	0.009	0.171	0.27	0.061
0	0.052	0.42	0.237	0.279	0.034	0	0.125	0.242	0.119	0.075
0.042	0.102	0.325	0.165	0.097	0.115	0.114	0	0.135	0.233	0.124
0.207	0.079	0.271	0.202	0.27	0.1	0.051	0.003	0.144	0.372	0.095
0	0	0.247	0.246	0.133	0.099	0.058	0.043	0.205	0.172	0.063
0.137	0.12	0.463	0.21	0.268	0.111	0.058	0.018	0.192	0.205	0.052

0.081	0.019	0.186	0.115	0.18	0.022	0.062	0.053	0.053	0.241	0.043
0.18	0.284	0.247	0.127	0.331	0.004	0.064	0.07	0.29	0.396	0.086
0.042	0.094	0.341	0.177	0.454	0.105	0.036	0.061	0.295	0.214	0.063
0.056	0.122	0.278	0.217	0.384	0.095	0.027	0.074	0.261	0.192	0.048
0.147	0.073	0.239	0.2	0.091	0.104	0.063	0	0.212	0.196	0.055
0.248	0.123	0.346	0.12	0.517	0.124	0	0	0.251	0.253	0.174
0.172	0.129	0.276	0.101	0.282	0.036	0.065	0.054	0.274	0.226	0.092
0.159	0.178	0.31	0.133	0.51	0.083	0.03	0	0.272	0.188	0.23
0.103	0.115	0.409	0.115	0.373	0.074	0.087	0	0.257	0.177	0.047
0.243	0.254	0.322	0	0.413	0.035	0.016	0.029	0.296	0.268	0.155
0.129	0.099	0.227	0.124	0.171	0.033	0.046	0.149	0.208	0.451	0.077
0.02	0.058	0.279	0.256	0.198	0.126	0.005	0.005	0.192	0.141	0.12
0.213	0.222	0.305	0.164	0.255	0.022	0.069	0	0.226	0.37	0.089
0.167	0.144	0.353	0.171	0.144	0	0.046	0	0.162	0.224	0.093
0.252	0.141	0.307	0.099	0.326	0.023	0.091	0	0.295	0.268	0.056
0.071	0	0.202	0.213	0.056	0.087	0	0.045	0.073	0.226	0.075
0.238	0.215	0.271	0.08	0.364	0.11	0.08	0	0.304	0.214	0.06
0.032	0	0.209	0.298	0.228	0.058	0	0.101	0.082	0.158	0.03
0.004	0.031	0.374	0.215	0.206	0.082	0.018	0.046	0.192	0.154	0.046
0	0.013	0.251	0.278	0.095	0.094	0	0.049	0.11	0.238	0.062
0	0.063	0.265	0.169	0.394	0	0	0.101	0.32	0.195	0.06
0.103	0.07	0.425	0.14	0.527	0.093	0.04	0.068	0.264	0.314	0.042
0.046	0.08	0.363	0.225	0.439	0.093	0.077	0.061	0.268	0.144	0.054
0.24	0.286	0.207	0.115	0.388	0.022	0.071	0.018	0.21	0.365	0.129
0.121	0.114	0.234	0.262	0.316	0.141	0.007	0.011	0.206	0.194	0.079
0.136	0.171	0.258	0.091	0.103	0	0.025	0.038	0.215	0.305	0.07
0.185	0.276	0.317	0.131	0.251	0	0.092	0	0.294	0.24	0.087
0.128	0.099	0.402	0.031	0.308	0	0.094	0.039	0.284	0.245	0.255
0.236	0.258	0.235	0.183	0.479	0.087	0.009	0	0.16	0.319	0.262
0.19	0.114	0.323	0.231	0.301	0.125	0.048	0	0.261	0.258	0.054
0.193	0.106	0.269	0.164	0.364	0	0.093	0.02	0.266	0.313	0.052
0.121	0.225	0.318	0.175	0.356	0.063	0.067	0	0.227	0.285	0.097
0.083	0.097	0.336	0.188	0.408	0.138	0.014	0.016	0.2	0.166	0.225
0.091	0.127	0.2	0.107	0.284	0.111	0.05	0.01	0.273	0.12	0.062
0	0.008	0.096	0.245	0.064	0.083	0.035	0.089	0.195	0.235	0.02
0.201	0.158	0.386	0.173	0.151	0.002	0.082	0	0.171	0.27	0.106
0.07	0.126	0.269	0.185	0.233	0	0.084	0.138	0.206	0.274	0.14
0	0.008	0.282	0.247	0.078	0.072	0.089	0	0.195	0.171	0.112
0.004	0.139	0.35	0.224	0.31	0.082	0.021	0.028	0.279	0.19	0.13
0.173	0.056	0.318	0.174	0.376	0.058	0.046	0.093	0.277	0.319	0.058
0.219	0.101	0.28	0.172	0.37	0.104	0.046	0	0.219	0.277	0.066
0.201	0.156	0.214	0.137	0.485	0.05	0.02	0.024	0.257	0.291	0.111
0.127	0.041	0.247	0.222	0.513	0.112	0.026	0.072	0.283	0.196	0.101
0.21	0.207	0.234	0.084	0.548	0.152	0.009	0	0.242	0.264	0.197
0	0	0.22	0.19	0.091	0.019	0.033	0.106	0.105	0.138	0.069
0.145	0.154	0.352	0.131	0.501	0.122	0	0	0.281	0.212	0.356
0.039	0.091	0.282	0.266	0.499	0.153	0	0.041	0.29	0.219	0.082
0.066	0.041	0.363	0.096	0.415	0	0.003	0.065	0.206	0.238	0.071
0.233	0.292	0.44	0.06	0.447	0.025	0.049	0	0.213	0.329	0.168
0.152	0.133	0.241	0.224	0.342	0.093	0.061	0.031	0.2	0.255	0.057
0.251	0.243	0.259	0.076	0.431	0.106	0.015	0	0.231	0.319	0.161
0.183	0.134	0.232	0.114	0.424	0.083	0.015	0.05	0.271	0.251	0.072
0.11	0.198	0.316	0.105	0.357	0.079	0.057	0.002	0.196	0.349	0.116

0	0	0.081	0.237	0.135	0.122	0.015	0.074	0.068	0.173	0.096
0.062	0.081	0.1	0.228	0.076	0.036	0.043	0	0.072	0.274	0.094
0.02	0.121	0.398	0.179	0.342	0.108	0.046	0.002	0.231	0.213	0.106
0.244	0.107	0.174	0.062	0.556	0.038	0	0	0.314	0.57	0.256
0.017	0.07	0.326	0.229	0.055	0.025	0.061	0.054	0.202	0.267	0.069
0.004	0.096	0.275	0.245	0.392	0.136	0.016	0.055	0.266	0.188	0.075
0.128	0.076	0.425	0.124	0.324	0.027	0.102	0.027	0.194	0.277	0.124
0.123	0.155	0.252	0.205	0.325	0.086	0.075	0.023	0.137	0.142	0.095
0.156	0.09	0.233	0.194	0.245	0.034	0.071	0.075	0.219	0.156	0.094
0	0.055	0.336	0.208	0.348	0.048	0	0.147	0.292	0.238	0.075
0.133	0.163	0.423	0.066	0.422	0	0.105	0	0.271	0.243	0.148
0.237	0.207	0.28	0	0.5	0.092	0.005	0	0.235	0.196	0.287
0	0.062	0.361	0.238	0.423	0.112	0	0.171	0.309	0.251	0.039
0.176	0.155	0.23	0.125	0.417	0.033	0.046	0	0.193	0.339	0.065
0.108	0.109	0.303	0.114	0.518	0.09	0.002	0.059	0.313	0.381	0.039
0.105	0.06	0.325	0.166	0.231	0.056	0.04	0	0.168	0.27	0.037
0.131	0.102	0.389	0.195	0.462	0.157	0.023	0.046	0.241	0.233	0.079
0.153	0.071	0.45	0.227	0.091	0	0.05	0.087	0.242	0.21	0.108
0.167	0.208	0.259	0.034	0.505	0.106	0	0.047	0.295	0.227	0.087
0	0	0.455	0.14	0.088	0.08	0	0.109	0.127	0.325	0.124
0.104	0.079	0.308	0.193	0.382	0.122	0.039	0.065	0.282	0.236	0.122
0.11	0.1	0.293	0.258	0.162	0	0.058	0	0.233	0.16	0.054
0.156	0.313	0.247	0.126	0.465	0.059	0.057	0	0.25	0.371	0.114
0.105	0.033	0.317	0.235	0.157	0.117	0.045	0	0.225	0.205	0.048
0.124	0.108	0.293	0.171	0.283	0	0.085	0.013	0.236	0.199	0.068
0.069	0.136	0.403	0.196	0.509	0.115	0.014	0.048	0.266	0.248	0.053
0.093	0	0.145	0.259	0.054	0	0	0.037	0.167	0.332	0.122
0.22	0.146	0.342	0.101	0.287	0.069	0.053	0.014	0.248	0.458	0.136
0.084	0.135	0.299	0.232	0.394	0.154	0.034	0.035	0.266	0.19	0.041
0.241	0.209	0.282	0	0.49	0.037	0	0	0.214	0.397	0.283
0.186	0.151	0.339	0.165	0.404	0.077	0.009	0	0.165	0.247	0.055
0.012	0.09	0.355	0.22	0.066	0.051	0.023	0	0.103	0.193	0.072
0.226	0.178	0.265	0.134	0.493	0.058	0	0	0.267	0.372	0.224
0	0.007	0.222	0.194	0.325	0.038	0.029	0	0.222	0.296	0.046
0.182	0.204	0.24	0.128	0.436	0.145	0.042	0.028	0.206	0.296	0.184
0.225	0.256	0.18	0.137	0.386	0	0.045	0.047	0.277	0.496	0.139
0.124	0.114	0.244	0.155	0.436	0.083	0.037	0.085	0.25	0.235	0.105
0.145	0.11	0.311	0.236	0.281	0.099	0.048	0.002	0.168	0.223	0.066
0.028	0.068	0.285	0.239	0.256	0.038	0.011	0.028	0.267	0.193	0.05
0.049	0.16	0.25	0.188	0.33	0	0.098	0.119	0.258	0.209	0.103
0.005	0.118	0.186	0.216	0.231	0.073	0	0.085	0.184	0.315	0.053
0.212	0.22	0.291	0.13	0.547	0.11	0.033	0	0.233	0.234	0.171
0.23	0.181	0.252	0.096	0.449	0.102	0.036	0	0.277	0.267	0.066
0.057	0.103	0.297	0.226	0.268	0.07	0	0.111	0.201	0.241	0.058
0.077	0.149	0.264	0.131	0.432	0.121	0.005	0.045	0.319	0.172	0.095
0	0.046	0.343	0.227	0.309	0.111	0	0.086	0.288	0.241	0.103
0.031	0.099	0.331	0.249	0.414	0.147	0	0.089	0.236	0.217	0.043
0.196	0.215	0.314	0.116	0.446	0	0	0.039	0.276	0.516	0.166
0.074	0.116	0.357	0.202	0.323	0.124	0.099	0.004	0.153	0.141	0.143
0	0.044	0.407	0.252	0.265	0.033	0.04	0.113	0.209	0.173	0.05
0.305	0.271	0.26	0.113	0.421	0.018	0.039	0	0.214	0.23	0.217
0.116	0.014	0.413	0.098	0.104	0.01	0.068	0.019	0.253	0.182	0.07
0.23	0.205	0.429	0	0.508	0.101	0	0	0.273	0.325	0.406

0.166	0.091	0.309	0.19	0.352	0.064	0.053	0.055	0.207	0.319	0.069
0.253	0.095	0.322	0.192	0.26	0.026	0.049	0.06	0.274	0.319	0.13
0	0.05	0.398	0.157	0.232	0.072	0.028	0	0.282	0.187	0.057
0.004	0	0.155	0.239	0.175	0.108	0.017	0.057	0.11	0.248	0.074
0.232	0.202	0.305	0.027	0.439	0.062	0.005	0	0.276	0.416	0.126
0.039	0.076	0.328	0.15	0.444	0.104	0	0.067	0.342	0.323	0.054
0	0.05	0.236	0.224	0.194	0.081	0.023	0.163	0.144	0.163	0.07
0.201	0.081	0.443	0.201	0.276	0.009	0.054	0	0.165	0.296	0.082
0.213	0.243	0.457	0.244	0.432	0.066	0.042	0	0.192	0.202	0.183
0.081	0	0.285	0.251	0.206	0.12	0.017	0	0.179	0.099	0.023
0.228	0.145	0.412	0	0.487	0.093	0	0.019	0.251	0.218	0.453
0.007	0.056	0.282	0.121	0	0	0.07	0.003	0.104	0.216	0.078
0.18	0.11	0.262	0.144	0.51	0.077	0	0	0.298	0.562	0.098
0.276	0.269	0.415	0	0.436	0.019	0.014	0	0.224	0.303	0.174
0.046	0.042	0.424	0.156	0.506	0.074	0.028	0.061	0.253	0.255	0.058
0	0.003	0.228	0.15	0.284	0.079	0.066	0.081	0.297	0.142	0.119
0.228	0.248	0.386	0	0.41	0.019	0.007	0	0.283	0.394	0.203
0.234	0.212	0.232	0.095	0.357	0.048	0.045	0.009	0.254	0.333	0.216
0	0.047	0.37	0.214	0.349	0.108	0.047	0.061	0.333	0.154	0.078
0.211	0.132	0.48	0.19	0.218	0	0.098	0	0.185	0.26	0.099
0.224	0.218	0.337	0.159	0.53	0.032	0	0	0.226	0.517	0.353
0.245	0.114	0.394	0.051	0.385	0.049	0.027	0	0.325	0.33	0.106
0.032	0.077	0.379	0.224	0.218	0.066	0.048	0.039	0.275	0.226	0.072
0	0	0.066	0.166	0.094	0.068	0.061	0.023	0.118	0.164	0.088
0.085	0.137	0.248	0.176	0.392	0.133	0.019	0.093	0.373	0.073	0.013
0.061	0.089	0.436	0.09	0.176	0	0.056	0	0.241	0.282	0.056
0.14	0.27	0.227	0.003	0.521	0	0.016	0	0.267	0.37	0.238
0.141	0.133	0.25	0	0.495	0.112	0.054	0.013	0.316	0.262	0.075
0.109	0.162	0.277	0.073	0.52	0.065	0.024	0.047	0.324	0.262	0.099
0.018	0	0.214	0.185	0	0.05	0	0	0.222	0.171	0.063
0.208	0.145	0.275	0.051	0.455	0.088	0.054	0	0.26	0.446	0.046
0.215	0.138	0.366	0.09	0.137	0	0.069	0	0.231	0.222	0.056
0.23	0.237	0.191	0.129	0.505	0.088	0.017	0	0.241	0.357	0.217
0.244	0.265	0.188	0.104	0.404	0.105	0.037	0	0.198	0.23	0.133
0.071	0.113	0.441	0.286	0.419	0.079	0	0.055	0.296	0.252	0.039
0	0.013	0.097	0.287	0.09	0.06	0.017	0.038	0.141	0.208	0.033
0.069	0.135	0.406	0.173	0.346	0	0.025	0.044	0.279	0.271	0.035
0.196	0.077	0.36	0.134	0.234	0.031	0.066	0	0.282	0.351	0.062
0.054	0.133	0.302	0.118	0.381	0.147	0.036	0.005	0.306	0.288	0.096
0.169	0.301	0.455	0	0.489	0.076	0.063	0	0.206	0.373	0.175
0.183	0.13	0.243	0.081	0.435	0.111	0.015	0.051	0.266	0.203	0.108
0.021	0.043	0.154	0.237	0.123	0.025	0.026	0.075	0.263	0.177	0.066
0.191	0.199	0.224	0.112	0.491	0.159	0.03	0	0.295	0.214	0.178
0.044	0.067	0.206	0.217	0.074	0.112	0.06	0.065	0.174	0.205	0.081
0.047	0.114	0.295	0.071	0.534	0.065	0.009	0.114	0.342	0.22	0.04
0.127	0.017	0.216	0.236	0.09	0.118	0.059	0.07	0.048	0.091	0.027
0.064	0.142	0.409	0.077	0.512	0.124	0.052	0.022	0.334	0.25	0.179
0.136	0.125	0.401	0.181	0.401	0.104	0.053	0.021	0.292	0.191	0.091
0.049	0.148	0.368	0.187	0.534	0.077	0.004	0.097	0.327	0.258	0.061
0.218	0.109	0.361	0.111	0.283	0	0.033	0.093	0.246	0.383	0.121
0.013	0	0.226	0.248	0	0.079	0	0.046	0.032	0.167	0.043
0.042	0.085	0.378	0.164	0.501	0.09	0.037	0.065	0.291	0.178	0.096
0	0	0.124	0.267	0.205	0.107	0	0.133	0.153	0.249	0.044

0.086	0	0.309	0.233	0.107	0.07	0	0.017	0.23	0.243	0.045
0.207	0.101	0.254	0.102	0.433	0.099	0.087	0	0.29	0.189	0.095
0.121	0.128	0.338	0.201	0.445	0.162	0.022	0	0.167	0.175	0.114
0.258	0.206	0.318	0.101	0.497	0.097	0	0	0.22	0.131	0.496
0.072	0.056	0.387	0.095	0.445	0.023	0.012	0.038	0.285	0.433	0.072
0.209	0.179	0.329	0.109	0.502	0.005	0.001	0.013	0.301	0.222	0.079
0.153	0.012	0.045	0.255	0.099	0.04	0	0.061	0.159	0.086	0.068
0.153	0.197	0.445	0.158	0.415	0	0.024	0.016	0.296	0.215	0.1
0.016	0	0.223	0.238	0.143	0.053	0.011	0.044	0.13	0.247	0.063
0.131	0.229	0.411	0.186	0.365	0.119	0.102	0.026	0.097	0.23	0.07
0.058	0	0.002	0.217	0.188	0	0.087	0.016	0.154	0.18	0.197
0.004	0.136	0.409	0.213	0.497	0.124	0.028	0.068	0.286	0.185	0.052
0.011	0.005	0.042	0.158	0.077	0.075	0.048	0.037	0.024	0.151	0.088
0.126	0.153	0.294	0.135	0.52	0.048	0.064	0	0.285	0.159	0.089
0.17	0.197	0.222	0.109	0.449	0.107	0.052	0	0.264	0.194	0.1
0.1	0.041	0.303	0.207	0.268	0.127	0	0.008	0.239	0.239	0.084
0	0.047	0.302	0.217	0.136	0.047	0	0.07	0.264	0.365	0.079
0.069	0.06	0.37	0.225	0.02	0.041	0.055	0.054	0.227	0.21	0.082
0.054	0.113	0.412	0.214	0.494	0.069	0.013	0.139	0.315	0.176	0.04
0	0.083	0.318	0.228	0.259	0.045	0.034	0.082	0.212	0.179	0.031
0.098	0.073	0.251	0.214	0.146	0.027	0.05	0.007	0.189	0.216	0.111
0.265	0.213	0.349	0.074	0.452	0.158	0	0	0.206	0.209	0.333
0.214	0.17	0.25	0.094	0.553	0.071	0.016	0	0.292	0.414	0.145
0.083	0.12	0.335	0.151	0.478	0.169	0.071	0.004	0.273	0.286	0.062
0	0.026	0.422	0.23	0.164	0.09	0	0.087	0.212	0.187	0.014
0.201	0.082	0.311	0.157	0.283	0.025	0.068	0.004	0.252	0.259	0.064
0	0	0.313	0.199	0.163	0.073	0.029	0.031	0.04	0.16	0.126
0	0.068	0.411	0.255	0.323	0.081	0	0.081	0.28	0.186	0.024
0.002	0.019	0.252	0.097	0.239	0.058	0.023	0.054	0.179	0.202	0.073
0.145	0.028	0.353	0.175	0.075	0.046	0.03	0	0.135	0.239	0.102
0.232	0.26	0.238	0.114	0.402	0.083	0.067	0	0.213	0.426	0.087
0.217	0.196	0.267	0.155	0.463	0.076	0	0	0.236	0.305	0.122
0.17	0.145	0.327	0.124	0.421	0.076	0.021	0	0.272	0.336	0.151
0.002	0.033	0.309	0.247	0.133	0.079	0.029	0.031	0.166	0.182	0
0	0.021	0.277	0.212	0.215	0.083	0	0.112	0.267	0.208	0.058
0	0.006	0.167	0.258	0.044	0.129	0.002	0.059	0.057	0.184	0.077
0.075	0.059	0.325	0.144	0.523	0.126	0	0.074	0.329	0.314	0.1
0.17	0.15	0.288	0.12	0.465	0.067	0.067	0	0.212	0.144	0.054
0	0.069	0.293	0.112	0.358	0.074	0.068	0.025	0.302	0.149	0.116
0.096	0.143	0.345	0.055	0.284	0.035	0.048	0	0.338	0.196	0.049
0.04	0.08	0.32	0.271	0.172	0.078	0	0	0.287	0.179	0.06
0	0.013	0.175	0.103	0.117	0.097	0.015	0.104	0.143	0.166	0.089
0.243	0.236	0.208	0.137	0.399	0.071	0.032	0	0.231	0.369	0.062
0.225	0.18	0.267	0.052	0.538	0.119	0.004	0.007	0.282	0.26	0.186
0.233	0.08	0.171	0.176	0	0	0.039	0	0.198	0.257	0.11
0.197	0.074	0.341	0.163	0.308	0.116	0.012	0.006	0.249	0.328	0.048
0.187	0.306	0.378	0.149	0.444	0	0.042	0.056	0.2	0.232	0.147
0.048	0.049	0.117	0.15	0.125	0	0.024	0.034	0.026	0.288	0.058
0.027	0.114	0.378	0.265	0.199	0.074	0.042	0.044	0.197	0.208	0.108
0.068	0	0.294	0.244	0.016	0	0	0.103	0.142	0.152	0.125
0	0	0.138	0.271	0.057	0.061	0	0.042	0	0.165	0.059
0.087	0.208	0.236	0.132	0.472	0.031	0.037	0.033	0.308	0.411	0.082
0	0	0.12	0.179	0.157	0.003	0.006	0.079	0.158	0.165	0.076

0.044	0.083	0.348	0.207	0.158	0.007	0.055	0.033	0.272	0.136	0.056
0.048	0.08	0.381	0.201	0.408	0.116	0.026	0.026	0.217	0.188	0.042
0	0	0.216	0.314	0.098	0.021	0	0.143	0.114	0.053	0.057
0	0.127	0.29	0.205	0.435	0.067	0.009	0.034	0.3	0.246	0.155
0.011	0.044	0.353	0.167	0.248	0.038	0.007	0	0.222	0.184	0.07
0.238	0.232	0.216	0.007	0.428	0.03	0.068	0	0.289	0.34	0.119
0.106	0.109	0.417	0.092	0.054	0.014	0	0.096	0.303	0.209	0.059
0.193	0.103	0.29	0.184	0.021	0.032	0.056	0	0.199	0.29	0.092
0.002	0.016	0.212	0.271	0.039	0.156	0	0.077	0.092	0.131	0.094
0.058	0.087	0.446	0.119	0.229	0.119	0.038	0.039	0.284	0.117	0.058
0.182	0.207	0.33	0.103	0.21	0.105	0.094	0	0.135	0.214	0.087
0	0.112	0.41	0.221	0.24	0.071	0	0	0.374	0.229	0.038
0.098	0.109	0.278	0.153	0.272	0.023	0.039	0	0.221	0.272	0.114
0.224	0.229	0.232	0.092	0.43	0	0.057	0.031	0.279	0.379	0.076
0	0.008	0.205	0.257	0.01	0.067	0.014	0.077	0.091	0.181	0.063
0.046	0.008	0.171	0.129	0.121	0	0.067	0.106	0.274	0.206	0.133
0.127	0.188	0.363	0.127	0.31	0.052	0.13	0.028	0.273	0.241	0.141
0.284	0.22	0.256	0.071	0.495	0.082	0	0	0.207	0.348	0.349
0.187	0.113	0.32	0.204	0.505	0.103	0.003	0.009	0.209	0.259	0.084
0.272	0.169	0.469	0.074	0.47	0.079	0.006	0	0.211	0.236	0.243
0.195	0.121	0.243	0.157	0.531	0.121	0	0.02	0.21	0.162	0.161
0.333	0.246	0.276	0.119	0.469	0.061	0	0	0.228	0.303	0.233
0.266	0.257	0.485	0.18	0.311	0	0.04	0	0.234	0.359	0.106
0.096	0.02	0.222	0.138	0.038	0.129	0.053	0	0.139	0.228	0.091
0.118	0.079	0.403	0.224	0.426	0.102	0.042	0.061	0.266	0.216	0.079
0.228	0.203	0.274	0.015	0.519	0.082	0	0	0.278	0.316	0.144
0	0.038	0.164	0.272	0.197	0.072	0	0.082	0.217	0.181	0.069
0	0.015	0.477	0.226	0.334	0.067	0	0.068	0.24	0.236	0.005
0.075	0	0.186	0.249	0.07	0.071	0.001	0.053	0.021	0.132	0.038
0.233	0.107	0.326	0.108	0.26	0.118	0.071	0.001	0.263	0.199	0.083
0.076	0.083	0.321	0.251	0.179	0.068	0.042	0	0.251	0.19	0.081
0.228	0.238	0.258	0.124	0.364	0.061	0.061	0	0.165	0.36	0.058
0.257	0.221	0.455	0.015	0.427	0.007	0.042	0	0.243	0.421	0.068
0.066	0.063	0.339	0.162	0.193	0.096	0.056	0.052	0.214	0.205	0.071
0	0.024	0.339	0.199	0.201	0.088	0.032	0.058	0.214	0.203	0.095
0.236	0.286	0.232	0	0.439	0.041	0.009	0	0.236	0.391	0.39
0.057	0.178	0.333	0.163	0.546	0	0.009	0.068	0.318	0.331	0.085
0	0.059	0.231	0.217	0.33	0.061	0.068	0.064	0.188	0.182	0.056
0.069	0.189	0.371	0.349	0.344	0.027	0.088	0.022	0.204	0.245	0.143
0.012	0	0.244	0.352	0.202	0.079	0	0.057	0.232	0.251	0.096
0.04	0.155	0.334	0.191	0.444	0.095	0.018	0.104	0.296	0.146	0.078
0.146	0.107	0.268	0.112	0.531	0.136	0.038	0.01	0.316	0.298	0.146
0.2	0.129	0.341	0.175	0.533	0.155	0	0.012	0.271	0.194	0.178
0.226	0.066	0.308	0.152	0.427	0.11	0.009	0.046	0.257	0.256	0.094
0.202	0.104	0.242	0.093	0.482	0.138	0	0.075	0.293	0.299	0.139
0.051	0.045	0.342	0.229	0.338	0.032	0.06	0.035	0.286	0.222	0.05
0.143	0.153	0.257	0.211	0.453	0.149	0.082	0	0.134	0.149	0.113
0.064	0.103	0.448	0.197	0.431	0.135	0	0	0.26	0.282	0.069
0.069	0.118	0.355	0.187	0.423	0.083	0.016	0.058	0.281	0.345	0.05
0.21	0.146	0.328	0.187	0.276	0	0.081	0.043	0.241	0.306	0.091
0.101	0	0.142	0.225	0.044	0.117	0	0.034	0.075	0.179	0.06
0.184	0.281	0.295	0.126	0.353	0.074	0.061	0.002	0.224	0.383	0.106
0.158	0.107	0.313	0.189	0.067	0.029	0.016	0	0.184	0.279	0.086

0.164	0.151	0.239	0.091	0.481	0.085	0.014	0.114	0.293	0.245	0.086
0.106	0.139	0.278	0.183	0.513	0.151	0.03	0.048	0.32	0.145	0.112
0	0.062	0.189	0.171	0.117	0.101	0.02	0.062	0.135	0.121	0.072
0.148	0.207	0.338	0.007	0.546	0.054	0	0	0.246	0.302	0.335
0.26	0.138	0.308	0.114	0.119	0.018	0.046	0	0.204	0.296	0.096
0.192	0.254	0.22	0.098	0.485	0.004	0.018	0	0.279	0.434	0.192
0.007	0.125	0.427	0.247	0.377	0.142	0	0.077	0.258	0.181	0.068
0.175	0.185	0.211	0.148	0.284	0.098	0.078	0	0.152	0.209	0.108
0.039	0.085	0.312	0.133	0.256	0.018	0.089	0.042	0.146	0.102	0.099
0.134	0.137	0.318	0.12	0.496	0.122	0.027	0	0.283	0.188	0.133
0.066	0.022	0.112	0.215	0.107	0.062	0.07	0	0.081	0.323	0.075
0.064	0.089	0.414	0.217	0.265	0.076	0.02	0.075	0.159	0.243	0.074
0.21	0.165	0.324	0.125	0.16	0.032	0.053	0	0.282	0.193	0.074
0.283	0.249	0.394	0.062	0.472	0.034	0	0	0.25	0.459	0.219
0.123	0.114	0.379	0.081	0.542	0.005	0.036	0.088	0.295	0.276	0.233
0.043	0.058	0.113	0.262	0.173	0.072	0	0.031	0.119	0.141	0.094
0.226	0.107	0.248	0.181	0.437	0.097	0.02	0.025	0.233	0.332	0.062
0.265	0.271	0.385	0	0.518	0.024	0	0	0.152	0.329	0.219
0.214	0.095	0.261	0.184	0.466	0.073	0.037	0.05	0.293	0.391	0.068
0.014	0.007	0.093	0.221	0	0	0.089	0.092	0.065	0.23	0.142
0.142	0	0.336	0.163	0.223	0.072	0.054	0	0.246	0.243	0.064
0.263	0.251	0.255	0.069	0.446	0.084	0.041	0	0.26	0.274	0.08
0.221	0.287	0.262	0.026	0.421	0.044	0.081	0	0.242	0.304	0.097
0.22	0.134	0.297	0.079	0.58	0.114	0	0.079	0.265	0.226	0.148
0.116	0.027	0.275	0.245	0.174	0.044	0.049	0	0.172	0.212	0.078
0.24	0.225	0.409	0.104	0.435	0.057	0.016	0.034	0.258	0.398	0.167
0.095	0.093	0.362	0.189	0.414	0.119	0.025	0.044	0.24	0.339	0.026
0.021	0.056	0.459	0.206	0.305	0.077	0.007	0.038	0.319	0.227	0.059
0.114	0.145	0.25	0.149	0.465	0.094	0.069	0.055	0.25	0.466	0.022
0.232	0.174	0.387	0.027	0.403	0.058	0.029	0.031	0.288	0.298	0.124
0.239	0.121	0.349	0.183	0.39	0.098	0	0.016	0.187	0.291	0.083
0.169	0.179	0.253	0.1	0.191	0	0.084	0	0.166	0.269	0.14
0.189	0.266	0.286	0.034	0.426	0.075	0.047	0	0.255	0.399	0.125
0.123	0.103	0.369	0.145	0.523	0.129	0	0.007	0.268	0.237	0.16
0.203	0.282	0.22	0.161	0.448	0.041	0.002	0	0.288	0.293	0.121
0.134	0.076	0.257	0.116	0.201	0.003	0.043	0.067	0.217	0.188	0.106
0.031	0	0.181	0.068	0.021	0.035	0	0.148	0.132	0.293	0.104
0.344	0.091	0.207	0.223	0.342	0.05	0	0.161	0.282	0.272	0.091
0.075	0.134	0.348	0.22	0.254	0.105	0.048	0.043	0.167	0.166	0.049
0.204	0.257	0.155	0.016	0.465	0.133	0.029	0	0.346	0.223	0.255
0.052	0.104	0.241	0.178	0.429	0.132	0.024	0.039	0.373	0.204	0.049
0.263	0.221	0.212	0.192	0.481	0.028	0	0	0.224	0.48	0.229
0.069	0.074	0.263	0.273	0.034	0.104	0.041	0.072	0.105	0.158	0.032
0.119	0.155	0.369	0.17	0.35	0.134	0.026	0.011	0.348	0.256	0.045
0.11	0.069	0.237	0.176	0.263	0.027	0.098	0	0.242	0.192	0.074
0.029	0.13	0.358	0.214	0.455	0.089	0.003	0.069	0.288	0.264	0.095
0.083	0.085	0.332	0.112	0.438	0	0.018	0.081	0.347	0.418	0.056
0.078	0.117	0.18	0.154	0.229	0	0.054	0.015	0.189	0.302	0.083
0.064	0.115	0.374	0.204	0.471	0.07	0.024	0.08	0.299	0.107	0.077
0.107	0.304	0.419	0.082	0.398	0.111	0.06	0	0.303	0.2	0.123
0.238	0.274	0.265	0.115	0.401	0.072	0.043	0	0.249	0.412	0.16
0.056	0	0.331	0.221	0.228	0.122	0.026	0.004	0.123	0.154	0.089
0.015	0	0.208	0.17	0.065	0.039	0	0.015	0.122	0.168	0.105

0.202	0.278	0.215	0.086	0.468	0.039	0.08	0	0.213	0.328	0.145
0.245	0.238	0.198	0.123	0.51	0.112	0.005	0	0.215	0.242	0.142
0.055	0	0.094	0.196	0.043	0.051	0	0.004	0.116	0.203	0.093
0.138	0.185	0.28	0.164	0.269	0.082	0.069	0	0.243	0.176	0.094
0.132	0.219	0.403	0.063	0.306	0.01	0.057	0.111	0.248	0.265	0.122
0.159	0.175	0.271	0.2	0.438	0.162	0	0	0.239	0.195	0.052
0.189	0.147	0.216	0.087	0.471	0.042	0.021	0	0.264	0.349	0.119
0.061	0.127	0.352	0.127	0.499	0	0.009	0.065	0.255	0.428	0.098
0.179	0.099	0.215	0.151	0.126	0	0	0.039	0.188	0.228	0.126
0.233	0.18	0.292	0.13	0.516	0.119	0	0	0.298	0.232	0.264
0.179	0.157	0.264	0.159	0.18	0.035	0.089	0	0.238	0.165	0.089
0.098	0.129	0.352	0.248	0.278	0.097	0.048	0.055	0.234	0.278	0.064
0.045	0.051	0.335	0.231	0.142	0.057	0	0.016	0.197	0.236	0.066
0.112	0.126	0.439	0.289	0.099	0	0.016	0.037	0.226	0.167	0.038
0	0.008	0.234	0.261	0.251	0.059	0.032	0.047	0.241	0.145	0.051
0.27	0.183	0.303	0.093	0.45	0.096	0	0	0.21	0.254	0.238
0.232	0.215	0.298	0.062	0.482	0.145	0.018	0	0.288	0.309	0.125
0.167	0.123	0.333	0.188	0.262	0.042	0.111	0	0.246	0.205	0.028
0.032	0.01	0.261	0.272	0.124	0.127	0.035	0.083	0.129	0.233	0.027
0.028	0.045	0.373	0.216	0.25	0.05	0	0.021	0.153	0.18	0.048
0.118	0.169	0.248	0.194	0.543	0.139	0.039	0	0.206	0.207	0.087
0.126	0.151	0.365	0.076	0.336	0.01	0.057	0.012	0.306	0.268	0.072
0	0	0	0.148	0.131	0.072	0.006	0.129	0.133	0.209	0.079
0.187	0.159	0.507	0.134	0.224	0.035	0.081	0.03	0.254	0.312	0.084
0.156	0.164	0.248	0.098	0.391	0.077	0.1	0	0.166	0.172	0.024
0.054	0.092	0.275	0.356	0.183	0	0.028	0.063	0.119	0.22	0.186
0.285	0.193	0.244	0.245	0.302	0.058	0	0.276	0.315	0.133	0.103
0.13	0.099	0.349	0.121	0.188	0.007	0.092	0	0.277	0.213	0.083
0.042	0.067	0.313	0.229	0.2	0.074	0.034	0	0.219	0.202	0.073
0.064	0.163	0.367	0.191	0.364	0	0.071	0.023	0.231	0.252	0.185
0.096	0.25	0.505	0.085	0.26	0	0.087	0.05	0.234	0.334	0.138
0.168	0.251	0.446	0.037	0.432	0	0.058	0.024	0.269	0.279	0.212
0.204	0.134	0.341	0.194	0.479	0.123	0.003	0	0.248	0.15	0.036
0.246	0.248	0.269	0.04	0.464	0.108	0.045	0	0.197	0.25	0.133
0.071	0.087	0.39	0.224	0.371	0.077	0.027	0.001	0.248	0.145	0.043
0.001	0.065	0.383	0.201	0.177	0.085	0	0	0.217	0.197	0.094
0.134	0.141	0.283	0.146	0.3	0.096	0.062	0.079	0.206	0.135	0.105
0.058	0.009	0.304	0.196	0.126	0.116	0	0.08	0.201	0.17	0.077
0.274	0.137	0.259	0.14	0.463	0.125	0.029	0	0.219	0.323	0.077
0.011	0.094	0.364	0.226	0.44	0.119	0.034	0.061	0.31	0.202	0.076
0.208	0.184	0.374	0.05	0.437	0.055	0.046	0.027	0.237	0.322	0.123
0.1	0.167	0.243	0.153	0.444	0.127	0.046	0.05	0.258	0.224	0.114
0.289	0.156	0.32	0.06	0.248	0	0.025	0.116	0.212	0.396	0.071
0.048	0.161	0.291	0.261	0.264	0.106	0	0.112	0.181	0.25	0.068
0.203	0.169	0.244	0.154	0.369	0.091	0.069	0	0.146	0.159	0.092
0.289	0.243	0.196	0.105	0.499	0.068	0	0	0.195	0.245	0.326
0.215	0.219	0.178	0.093	0.502	0.1	0.019	0	0.232	0.274	0.157
0.053	0.061	0.05	0.167	0.088	0	0.034	0.019	0.129	0.318	0.142
0.109	0.041	0.354	0.225	0.194	0.03	0.004	0.001	0.261	0.202	0.07
0.008	0.05	0.281	0.21	0.179	0.067	0.011	0.121	0.196	0.232	0.072
0.026	0.097	0.459	0.179	0.478	0.059	0.025	0.042	0.285	0.353	0.056
0.3	0.24	0.275	0.096	0.428	0.146	0	0	0.212	0.238	0.177
0.157	0.148	0.219	0.056	0.167	0.058	0.091	0	0.241	0.294	0.053

0.234	0.255	0.285	0.188	0.456	0	0	0.004	0.226	0.341	0.273
0.224	0.131	0.285	0.187	0.198	0.062	0.063	0	0.232	0.214	0.087
0	0	0.026	0.307	0.052	0.042	0	0.098	0.116	0.199	0.092
0	0.093	0.324	0.188	0.308	0	0.046	0.087	0.115	0.299	0.093
0.006	0.054	0.259	0.241	0.15	0.067	0	0.083	0.267	0.188	0.094
0.226	0.176	0.215	0.139	0.448	0.113	0.03	0	0.247	0.237	0.111
0.237	0.133	0.251	0.192	0.351	0.104	0.03	0	0.243	0.265	0.105
0.216	0.115	0.337	0.137	0.061	0.067	0.046	0	0.194	0.321	0.132
0.255	0.271	0.24	0.077	0.461	0.112	0	0	0.205	0.249	0.328
0.043	0.063	0.293	0.254	0.058	0.073	0.008	0.107	0.237	0.182	0.102
0.26	0.083	0.407	0.217	0.005	0.1	0.03	0	0.322	0.163	0.12
0.224	0.202	0.295	0.065	0.342	0.017	0.046	0	0.258	0.303	0.114
0.243	0.284	0.26	0.038	0.405	0.063	0.057	0	0.231	0.264	0.117
0.224	0.179	0.252	0.151	0.481	0.152	0	0.001	0.262	0.177	0.285
0.246	0.265	0.279	0.1	0.42	0.063	0	0	0.276	0.302	0.182
0.016	0.122	0.311	0.197	0.151	0.047	0.057	0	0.178	0.262	0.104
0.105	0.046	0.072	0.088	0.026	0.001	0	0.009	0.159	0.185	0.149
0.121	0.124	0.285	0.263	0.278	0.056	0.063	0.097	0.23	0.244	0.062
0.305	0.169	0.309	0.117	0.405	0.044	0.01	0	0.272	0.35	0.16
0.174	0.095	0.166	0.228	0.043	0.071	0.037	0	0.144	0.24	0.073
0.172	0.289	0.328	0.116	0.261	0	0.076	0	0.284	0.354	0.307
0.189	0.018	0.299	0.122	0.03	0.039	0.033	0.011	0.175	0.129	0.114
0.088	0.098	0.276	0.254	0.268	0.099	0.07	0.066	0.155	0.111	0.174
0.106	0.159	0.463	0.196	0.203	0.127	0.045	0.068	0.232	0.059	0.125
0.167	0.205	0.315	0.254	0.329	0.079	0.12	0.005	0.236	0.119	0.116
0.058	0.111	0.332	0.165	0.381	0.11	0.01	0.055	0.244	0.15	0.166
0.13	0.174	0.334	0.091	0.38	0.11	0.086	0.01	0.304	0.232	0.149
0.059	0.091	0.419	0.253	0.077	0.085	0.01	0.012	0.287	0.181	0.099
0.058	0.024	0.233	0.254	0.055	0.123	0	0	0.144	0.151	0.091
0.137	0.093	0.318	0.238	0.273	0.102	0.064	0	0.291	0.208	0.076
0.136	0.08	0.256	0.212	0.173	0.023	0.007	0	0.33	0.198	0.111
0.136	0	0.174	0.221	0	0	0	0.111	0.21	0.269	0.104
0.249	0.263	0.301	0.043	0.427	0.041	0	0	0.243	0.276	0.316
0.254	0.255	0.253	0.13	0.355	0.089	0.019	0	0.29	0.264	0.149
0.073	0	0.26	0.229	0.055	0.074	0	0.022	0.179	0.191	0.073
0.223	0.329	0.222	0.02	0.456	0.035	0.075	0	0.257	0.266	0.197
0.229	0.13	0.31	0.22	0.264	0.06	0.051	0	0.237	0.184	0.101
0.029	0.038	0.254	0.107	0.001	0.053	0	0	0.209	0.337	0.166
0.055	0.141	0.398	0.239	0.324	0.064	0	0.026	0.304	0.136	0.11
0.287	0.241	0.299	0.055	0.456	0.111	0	0	0.265	0.147	0.259
0.237	0.161	0.349	0.151	0.285	0.123	0.039	0	0.268	0.279	0.094
0.266	0.038	0.156	0.246	0.354	0.024	0	0.171	0.441	0.08	0.118
0.286	0.148	0.274	0.148	0.267	0.044	0.03	0.009	0.325	0.217	0.119
0.175	0.153	0.294	0.097	0.084	0	0.039	0	0.25	0.212	0.189
0.09	0.034	0.447	0.191	0.054	0.092	0.025	0	0.282	0.184	0.115
0.177	0.334	0.339	0.162	0.344	0	0.034	0.018	0.204	0.425	0.192
0.138	0.081	0.35	0.236	0.055	0.028	0.044	0	0.217	0.277	0.106
0.006	0.065	0.191	0.291	0	0.035	0.026	0.019	0.135	0.153	0.145
0.29	0.286	0.254	0	0.372	0.073	0.037	0	0.243	0.273	0.2
0.133	0.075	0.433	0.198	0.232	0	0.032	0.022	0.275	0.251	0.1
0.071	0.1	0.312	0.094	0.109	0	0.05	0	0.329	0.204	0.089
0.28	0.101	0.549	0.043	0.094	0.025	0.057	0	0.269	0.374	0.114
0.047	0.109	0.305	0.207	0.414	0.107	0.017	0.116	0.307	0.207	0.054

0.233	0.153	0.293	0.137	0.439	0.092	0.014	0.028	0.286	0.187	0.24
0.155	0.148	0.186	0.254	0.142	0.028	0.07	0	0.269	0.15	0.097
0.23	0.151	0.239	0.187	0.245	0	0.041	0	0.212	0.296	0.11
0.283	0.16	0.286	0.116	0.449	0.087	0.005	0	0.269	0.38	0.219
0.068	0.054	0.309	0.169	0.201	0.1	0.042	0.034	0.181	0.117	0.091
0.011	0.101	0.413	0.232	0.316	0.113	0	0.072	0.302	0.304	0.084
0.132	0.032	0.35	0.102	0.404	0.034	0	0.067	0.37	0.449	0.096
0	0.078	0.4	0.191	0.189	0.059	0	0	0.316	0.153	0.103
0.142	0.181	0.343	0.152	0.216	0.066	0.104	0	0.253	0.17	0.102
0.05	0.115	0.418	0.24	0.44	0.104	0	0.105	0.233	0.136	0.235
0.287	0.285	0.239	0.173	0.413	0.008	0.001	0.002	0.256	0.266	0.273
0.283	0.234	0.283	0.072	0.436	0.1	0.024	0	0.293	0.214	0.135
0.219	0.266	0.391	0.087	0.411	0	0.034	0	0.246	0.342	0.27
0.146	0.115	0.382	0.12	0	0.066	0.081	0	0.233	0.185	0.168
0.285	0.26	0.4	0.034	0.306	0.052	0.054	0	0.241	0.249	0.14
0	0.049	0.115	0.275	0.071	0	0.077	0.146	0.208	0.18	0.169
0.184	0.236	0.403	0	0.342	0.078	0.031	0	0.345	0.271	0.177
0.14	0.08	0.292	0.344	0.287	0.04	0	0.128	0.277	0.165	0.038
0.258	0.163	0.253	0.064	0.448	0.07	0.019	0	0.264	0.222	0.147
0.067	0.065	0.266	0.293	0.191	0	0.019	0.04	0.223	0.207	0.104
0.09	0.052	0.32	0.132	0	0	0	0	0.265	0.129	0.079
0.009	0	0	0.254	0	0.052	0	0.135	0.089	0.172	0.169
0.066	0.057	0.305	0.2	0.071	0.037	0.08	0.037	0.015	0.169	0.099
0.201	0.157	0.3	0.124	0.461	0.096	0.055	0	0.263	0.315	0.113
0.289	0.228	0.192	0.162	0.475	0.095	0	0	0.348	0.366	0.183
0.081	0.055	0.093	0.21	0.017	0.062	0.044	0.136	0.062	0.217	0.061
0.194	0.111	0.384	0.178	0.147	0.057	0.019	0.013	0.286	0.265	0.09
0.059	0.119	0.413	0.21	0.139	0.07	0	0	0.231	0.263	0.116
0.107	0.245	0.348	0.141	0.378	0	0.056	0.069	0.287	0.202	0.153
0.207	0.126	0.237	0.196	0.43	0.103	0	0.052	0.319	0.213	0.095
0.018	0.033	0.504	0.14	0.094	0	0.006	0.049	0.309	0.217	0.14
0.188	0.163	0.387	0.087	0.276	0.056	0.018	0	0.296	0.215	0.144
0	0.154	0.405	0.235	0.195	0.003	0.009	0.008	0.239	0.166	0.089
0.162	0.113	0.245	0.187	0.413	0.065	0.048	0.063	0.328	0.222	0.085
0.295	0.165	0.47	0.135	0.274	0.049	0.037	0.015	0.2	0.283	0.124
0.279	0.23	0.307	0.122	0.249	0.006	0.067	0	0.277	0.369	0.118
0.21	0.16	0.301	0.144	0.368	0.106	0.024	0	0.209	0.251	0.14
0.19	0.153	0.289	0.144	0.446	0.121	0.055	0	0.319	0.161	0.139
0.017	0.067	0.397	0.261	0.037	0.039	0	0.065	0.248	0.204	0.097
0.306	0.215	0.293	0.077	0.415	0.106	0.02	0	0.236	0.206	0.188
0.073	0.009	0.191	0.174	0	0.058	0.056	0.072	0.163	0.102	0.117
0.258	0.197	0.243	0.114	0.421	0.088	0.031	0	0.179	0.243	0.109
0.281	0.24	0.301	0.111	0.44	0.093	0.007	0	0.218	0.318	0.22
0.055	0.017	0.197	0.283	0.006	0.028	0.005	0.007	0.116	0.136	0.079
0.118	0.12	0.354	0.161	0.45	0.104	0	0.022	0.324	0.324	0.289
0.126	0.162	0.308	0.215	0.356	0.074	0.043	0.033	0.335	0.207	0.108
0.248	0.175	0.242	0.159	0.086	0.007	0.099	0	0.211	0.323	0.143
0.16	0	0.109	0.237	0	0	0.052	0	0.2	0.287	0.117
0.322	0.261	0.271	0.088	0.542	0.072	0	0	0.19	0.317	0.511
0.012	0.038	0.312	0.157	0.183	0.101	0.003	0.038	0.258	0.278	0.089
0.06	0.14	0.431	0.175	0.322	0.101	0.016	0.056	0.311	0.155	0.079
0.191	0.232	0.33	0.156	0.135	0.035	0.074	0	0.249	0.283	0.121
0.065	0.077	0.386	0.181	0.084	0.081	0.041	0.032	0.26	0.08	0.112

0.049	0.082	0.415	0.219	0.365	0.078	0	0.051	0.312	0.159	0.096
0.273	0.044	0.349	0.181	0.009	0.019	0.046	0	0.281	0.168	0.096
0.147	0.151	0.248	0.163	0.427	0.072	0.032	0.09	0.308	0.191	0.115
0.242	0.082	0.35	0.162	0.342	0.083	0.014	0.056	0.285	0.299	0.095
0.268	0.144	0.265	0.087	0.473	0.166	0	0	0.244	0.206	0.196
0.157	0.161	0.325	0.201	0.304	0.132	0.046	0.001	0.325	0.207	0.131
0.183	0.211	0.413	0.069	0.287	0.013	0.092	0.013	0.288	0.326	0.192
0	0.133	0.297	0.194	0.348	0.138	0.01	0	0.285	0	0.162
0	0.011	0.135	0.284	0.063	0.066	0.04	0.035	0.159	0.18	0.095
0.298	0.231	0.214	0.059	0.427	0.021	0.038	0	0.262	0.357	0.14
0.135	0.129	0.288	0.177	0.467	0.135	0	0.037	0.314	0.246	0.133
0	0.131	0.468	0.23	0.148	0.01	0.016	0.009	0.278	0.284	0.132
0.154	0.13	0.281	0.188	0.366	0.092	0.014	0.039	0.268	0.181	0.094
0.24	0.216	0.316	0.109	0.116	0	0.06	0	0.196	0.328	0.114
0.006	0.103	0.372	0.22	0.117	0.058	0.029	0	0.244	0.227	0.081
0.173	0.117	0.172	0.252	0	0	0.023	0.076	0.178	0.239	0.092
0.089	0.059	0.306	0.241	0.006	0.099	0.027	0	0.203	0.139	0.066
0.134	0.153	0.398	0.181	0.222	0.067	0.079	0	0.272	0.146	0.077
0.199	0.205	0.231	0.138	0.498	0.178	0.049	0	0.269	0.218	0.111
0.22	0.128	0.32	0.141	0.187	0.025	0.082	0	0.311	0.248	0.103
0.257	0.31	0.501	0.04	0.237	0	0.068	0.023	0.228	0.227	0.134
0.225	0.115	0.534	0.092	0.26	0	0	0.029	0.245	0.362	0.144
0.093	0.144	0.347	0.126	0.401	0.075	0.007	0.037	0.289	0.355	0.111
0.053	0.037	0.116	0.273	0.051	0.147	0.029	0	0.114	0.167	0.092
0.181	0.123	0.222	0.225	0.069	0	0.066	0	0.208	0.214	0.156
0.144	0.09	0.387	0.115	0.062	0	0.014	0.149	0.179	0.232	0.125
0.261	0.291	0.246	0.131	0.395	0.048	0.014	0	0.246	0.296	0.303
0.056	0.137	0.387	0.237	0.453	0.096	0.024	0.039	0.308	0.151	0.16
0.086	0.174	0.314	0.24	0.377	0.147	0.066	0.064	0.271	0.125	0.093
0.094	0.026	0.467	0.134	0.08	0.014	0	0.083	0.231	0.14	0.146
0.272	0.164	0.36	0.149	0.301	0.004	0.029	0	0.257	0.309	0.169
0.137	0.147	0.426	0.241	0.326	0.091	0.051	0.028	0.261	0.24	0.091
0.352	0.243	0.286	0.239	0.435	0.127	0.011	0	0.185	0.17	0.13
0	0.021	0.038	0.201	0	0.041	0	0.016	0.106	0.111	0.12
0.011	0.062	0.122	0.155	0.159	0.111	0	0.104	0.185	0.157	0.103
0.112	0.239	0.362	0.104	0.481	0.05	0.036	0.044	0.273	0.186	0.142
0.205	0.156	0.233	0.096	0.29	0.155	0.077	0	0.154	0.268	0.094
0.09	0.081	0.415	0.213	0.115	0.086	0	0	0.281	0.245	0.116
0.092	0.083	0.228	0.195	0.047	0.022	0.034	0.014	0.282	0.175	0.083
0	0.038	0.137	0.109	0.14	0	0.022	0.055	0.247	0.302	0.197
0.063	0.082	0.228	0.251	0.095	0.069	0.047	0.094	0.237	0.191	0.123
0.167	0.183	0.262	0	0.432	0.082	0.043	0.013	0.306	0.252	0.125
0.193	0.165	0.288	0.189	0.376	0.107	0.016	0.013	0.281	0.155	0.213
0.198	0.174	0.25	0.172	0.51	0.151	0	0	0.285	0.284	0.168
0.201	0.142	0.306	0.042	0.334	0	0	0.024	0.367	0.367	0.163
0.044	0.074	0.267	0.173	0.063	0.106	0	0.015	0.274	0.104	0.076
0.103	0.11	0.206	0.119	0.024	0.047	0.082	0	0.242	0.178	0.201
0.062	0.132	0.329	0.219	0.488	0.11	0.009	0.047	0.267	0.265	0.114
0.154	0.119	0.167	0.262	0.068	0	0.057	0	0.213	0.221	0.123
0.266	0.271	0.294	0.174	0.383	0	0.008	0	0.232	0.44	0.147
0.135	0.035	0.139	0.172	0.054	0.012	0.066	0.036	0.031	0.044	0.111
0.165	0.116	0.301	0.158	0.031	0	0.073	0	0.176	0.148	0.087
0.273	0.239	0.191	0.134	0.35	0.054	0.048	0	0.29	0.197	0.099

0.155	0.132	0.393	0.125	0.285	0.047	0.05	0.021	0.31	0.198	0.127
0.061	0.092	0.269	0.268	0.184	0.064	0.043	0	0.039	0.202	0.117
0.06	0	0.139	0.346	0.143	0.009	0	0.084	0.282	0.03	0.016
0.212	0.138	0.484	0.07	0.123	0	0.056	0.072	0.248	0.326	0.119
0.118	0.023	0.245	0.159	0.061	0.113	0.017	0.027	0.042	0.136	0.106
0	0.027	0.305	0.19	0.02	0.037	0.057	0	0.093	0.021	0.121
0.074	0.077	0.211	0.154	0.113	0.058	0.031	0.08	0.284	0.207	0.106
0.221	0.085	0.227	0.212	0.087	0.036	0.045	0	0.272	0.205	0.118
0.204	0.172	0.293	0.146	0.265	0.068	0	0.004	0.278	0.296	0.099
0.177	0.27	0.293	0.109	0.477	0.074	0.012	0	0.272	0.264	0.305
0.25	0.195	0.373	0.129	0.307	0.032	0.052	0	0.247	0.198	0.141
0.054	0	0.263	0.162	0.064	0.086	0	0.072	0.217	0.247	0.09
0.078	0.029	0.12	0.269	0.064	0.033	0.003	0	0.106	0.183	0.144
0.223	0.219	0.233	0.156	0.2	0.028	0.036	0	0.251	0.28	0.122
0.073	0.151	0.302	0.195	0.487	0.066	0.048	0.056	0.314	0.189	0.104
0.195	0.107	0.241	0.123	0.351	0.08	0.026	0	0.287	0.181	0.152
0.198	0.165	0.242	0.084	0.284	0.092	0.125	0	0.261	0.163	0.146
0.052	0.019	0	0.188	0	0.1	0	0.122	0	0.212	0.131
0.036	0.009	0.18	0.237	0.139	0.079	0.015	0.096	0.243	0.117	0.109
0.012	0.016	0.09	0.19	0.034	0.017	0.012	0.03	0.19	0.116	0.083
0.297	0.197	0.348	0	0.368	0.015	0.074	0	0.297	0.222	0.11
0.25	0.212	0.253	0.128	0.358	0.077	0.114	0	0.215	0.173	0.113
0.087	0.056	0.286	0.195	0.153	0.053	0.038	0	0.238	0.289	0.149
0.072	0.137	0.338	0.205	0.41	0.098	0	0.039	0.297	0.214	0.095
0.199	0.101	0.341	0.216	0.045	0	0.064	0.009	0.203	0.181	0.138
0	0.02	0.344	0.235	0.072	0	0	0.097	0.232	0.236	0.082
0.283	0.282	0.278	0.038	0.353	0.02	0.029	0	0.264	0.411	0.289
0.073	0.145	0.291	0.201	0.232	0.029	0.047	0.083	0.325	0.292	0.129
0.112	0	0.245	0.26	0.129	0.085	0.005	0.018	0.18	0.144	0.138
0.133	0.098	0.441	0.153	0.243	0.006	0.025	0.03	0.247	0.303	0.112
0.236	0.258	0.298	0.071	0.501	0.101	0	0	0.291	0.285	0.09
0.006	0.105	0.265	0.262	0.141	0.084	0	0	0.164	0.126	0.106
0.169	0.127	0.372	0.088	0.141	0.002	0.075	0.015	0.273	0.181	0.137
0.011	0.018	0.1	0.159	0.03	0.068	0	0.043	0.22	0.318	0.143
0.038	0.145	0.357	0.248	0.333	0.162	0	0	0.272	0.141	0.11
0.001	0.155	0.41	0.17	0.435	0.062	0.023	0.101	0.259	0.254	0.049
0.105	0.175	0.249	0.226	0.449	0.091	0	0.043	0.344	0.138	0.109
0.243	0.226	0.233	0.195	0.377	0.085	0.021	0	0.294	0.222	0.186
0.127	0.119	0.378	0.224	0.116	0.068	0.049	0.011	0.188	0.149	0.116
0.431	0.25	0.306	0.159	0.445	0.147	0	0	0.21	0.237	0.228
0.097	0.118	0.272	0.2	0.387	0.11	0.007	0.052	0.326	0.172	0.117
0.004	0.132	0.401	0.177	0.22	0.106	0.024	0	0.275	0.115	0.191
0.005	0	0.221	0.25	0	0.116	0	0.059	0.109	0.182	0.105
0.238	0.149	0.396	0.132	0.161	0.013	0.048	0	0.261	0.19	0.145
0.186	0.235	0.254	0.051	0.475	0.045	0.054	0	0.308	0.21	0.13
0.225	0.081	0.466	0.161	0.004	0.022	0	0	0.233	0.295	0.157
0.332	0.302	0.381	0	0.442	0.039	0	0	0.291	0.344	0.179
0.068	0.172	0.275	0.215	0.378	0.115	0.035	0.113	0.314	0.148	0.117
0.044	0.072	0.365	0.216	0.221	0	0	0	0.283	0.235	0.112
0.194	0.198	0.393	0.15	0.374	0.043	0	0.042	0.289	0.225	0.042
0.128	0.15	0.414	0.184	0.401	0.075	0.046	0.079	0.285	0.222	0.104
0.038	0.045	0.356	0.071	0.256	0.05	0	0.058	0.282	0.261	0.108
0.185	0.166	0.224	0.095	0.22	0.057	0.038	0.048	0.214	0.321	0.109

0.204	0.154	0.255	0.146	0.452	0.142	0.026	0.02	0.248	0.195	0.105
0.062	0.121	0.41	0.22	0.353	0.148	0	0.026	0.321	0.214	0.108
0.144	0.174	0.288	0.237	0.36	0.023	0.033	0.083	0.303	0.307	0.114
0.258	0.091	0.373	0.106	0.234	0.153	0.04	0	0.193	0.225	0.095
0.278	0.297	0.29	0.083	0.27	0.012	0.038	0	0.215	0.427	0.163
0.017	0.016	0.447	0.178	0.205	0.076	0	0.018	0.265	0.128	0.144
0.288	0.263	0.199	0.102	0.532	0.107	0	0	0.221	0.194	0.208
0.039	0.08	0.383	0.324	0.108	0.071	0.006	0.052	0.235	0.18	0.045
0.304	0.285	0.42	0.008	0.365	0	0.019	0	0.255	0.364	0.177
0.187	0.106	0.417	0.189	0.151	0	0.014	0.005	0.266	0.295	0.137
0.208	0.371	0.198	0	0.307	0	0.143	0	0.254	0.216	0.161
0.228	0.258	0.268	0.086	0.373	0.049	0.062	0.022	0.176	0.368	0.201
0.041	0.152	0.217	0.148	0.256	0	0.098	0.039	0.249	0.238	0.116
0.244	0.265	0.271	0.082	0.397	0.087	0.018	0	0.319	0.355	0.162
0.125	0.147	0.328	0.17	0.392	0.061	0.049	0	0.218	0.167	0.121
0.116	0.009	0.232	0.241	0	0.12	0	0.018	0.156	0.195	0.124
0.027	0.017	0.341	0.199	0.192	0.04	0.043	0.021	0.314	0.186	0.126
0.149	0.17	0.316	0.181	0.268	0.033	0.066	0	0.376	0.153	0.124
0.083	0.065	0.161	0.238	0	0.061	0.013	0	0.157	0.121	0.1
0.122	0.067	0.316	0.245	0.092	0.06	0.038	0	0.234	0.323	0.071
0.189	0.157	0.293	0.152	0.271	0.128	0.018	0.014	0.341	0.243	0.107
0.21	0.289	0.28	0.038	0.435	0.053	0	0	0.162	0.264	0.4
0.241	0.064	0.255	0.213	0.034	0.058	0.013	0.001	0.209	0.161	0.096
0	0.032	0.045	0.281	0.014	0.065	0.064	0.101	0.055	0.114	0.107
0.181	0.074	0.08	0.274	0.35	0	0	0.11	0.332	0.145	0.124
0.179	0.041	0.215	0.239	0.014	0	0.038	0	0.257	0.192	0.111
0.276	0.288	0.394	0.129	0.367	0	0	0.034	0.26	0.373	0.198
0.192	0.186	0.235	0.171	0.399	0.106	0.006	0.055	0.327	0.27	0.129
0.264	0.118	0.343	0.186	0.075	0.019	0.068	0.013	0.206	0.282	0.102
0.04	0.011	0.233	0.257	0	0.044	0.024	0.003	0.135	0.179	0.121
0.166	0.141	0.445	0.106	0.351	0.008	0.007	0.041	0.242	0.203	0.219
0.197	0.107	0.209	0.182	0.084	0.046	0.031	0	0.217	0.291	0.129
0.103	0.11	0.356	0.195	0.165	0.045	0.068	0	0.238	0.145	0.138
0.235	0.138	0.363	0.121	0.365	0.155	0.044	0	0.263	0.209	0.102
0.289	0.197	0.227	0.071	0.407	0.12	0.019	0	0.288	0.245	0.143
0	0.084	0.291	0.207	0.353	0.041	0.039	0.1	0.228	0.248	0.16
0.179	0.241	0.362	0.089	0.469	0.096	0.087	0	0.232	0.277	0.178
0.161	0.114	0.449	0.057	0.462	0	0	0	0.335	0.302	0.271
0.175	0.053	0.348	0.163	0.083	0.098	0.028	0	0.244	0.163	0.11
0.029	0.024	0.3	0.235	0.155	0.103	0.039	0.08	0.216	0.172	0.138
0.116	0.157	0.368	0.221	0.439	0.081	0	0.05	0.279	0.215	0.105
0.054	0.092	0.32	0.251	0.266	0.089	0.005	0	0.257	0.22	0.098
0.221	0.27	0.257	0.095	0.363	0.014	0.083	0	0.217	0.221	0.124
0.054	0.056	0.495	0.088	0.162	0.053	0.012	0.108	0.314	0.207	0.147
0.22	0.167	0.407	0.143	0.367	0.091	0.023	0	0.247	0.209	0.102
0.207	0.134	0.284	0.256	0.095	0.095	0.007	0	0.135	0.26	0.079
0.069	0.046	0.217	0.254	0.126	0.103	0.039	0.069	0.085	0.161	0.078
0.039	0.145	0.434	0.105	0.408	0.036	0.055	0.012	0.359	0.239	0.104
0.073	0.14	0.306	0.219	0.287	0.01	0.02	0.065	0.24	0.166	0.076
0.242	0.176	0.246	0.145	0.416	0.1	0	0	0.312	0.185	0.195
0.188	0.175	0.284	0.067	0.291	0.048	0.082	0.013	0.275	0.248	0.143
0.103	0.21	0.367	0.123	0.207	0.034	0.083	0.073	0.224	0.154	0.139
0.255	0.213	0.258	0	0.415	0.029	0	0.042	0.254	0.216	0.262

0.191	0.078	0.331	0.2	0	0.042	0.021	0.037	0.244	0.218	0.106
0.122	0.116	0.412	0.219	0.242	0	0	0.071	0.301	0.244	0.109
0.231	0.118	0.238	0.123	0.06	0.057	0.077	0	0.234	0.252	0.096
0.167	0.217	0.32	0.146	0.413	0.026	0.079	0	0.229	0.295	0.113
0.184	0.155	0.281	0.095	0.408	0.065	0.05	0	0.304	0.244	0.153
0.167	0.005	0.342	0.146	0.182	0.089	0.029	0	0.258	0.262	0.069
0.276	0.246	0.219	0.11	0.379	0.085	0.032	0	0.288	0.273	0.155
0.249	0.146	0.36	0.138	0.187	0.059	0.104	0	0.23	0.255	0.121
0.2	0.17	0.283	0.095	0.22	0.06	0.092	0	0.304	0.254	0.15
0.167	0.15	0.387	0.184	0.365	0.088	0.033	0	0.29	0.215	0.094
0.075	0.017	0.273	0.234	0	0.077	0	0.047	0.125	0.144	0.1
0.069	0.005	0.121	0.276	0	0.055	0	0.059	0.116	0.193	0.123
0.108	0.081	0.372	0.084	0.029	0.016	0.02	0.076	0.229	0.193	0.151
0.144	0.171	0.333	0.132	0.312	0.084	0.067	0.03	0.321	0.156	0.078
0.27	0.259	0.236	0.023	0.354	0.047	0.05	0	0.252	0.389	0.247
0.271	0.281	0.378	0.115	0.274	0	0.015	0.015	0.241	0.283	0.177
0.179	0.173	0.236	0.085	0.345	0.093	0.05	0	0.322	0.197	0.128
0.175	0.105	0.166	0.152	0.065	0.061	0.08	0	0	0.319	0.087
0.171	0.123	0.331	0.079	0.238	0.05	0.04	0.068	0.254	0.209	0.207
0.216	0.345	0.285	0.098	0.369	0.001	0.058	0	0.276	0.36	0.164
0.12	0.099	0.233	0.259	0.04	0.051	0	0	0.117	0.272	0.117
0.257	0.118	0.31	0.083	0.083	0.041	0.062	0	0.224	0.219	0.124
0.03	0.038	0.263	0.258	0.127	0.116	0	0.001	0.151	0.182	0.124
0.032	0.103	0.307	0.283	0.083	0.073	0.013	0	0.194	0.216	0.081
0.128	0.202	0.39	0.189	0.418	0.118	0.033	0.042	0.268	0.143	0.078
0.137	0.076	0.176	0.261	0.073	0.084	0	0.009	0.122	0.09	0.104
0.215	0.154	0.388	0.102	0.478	0.111	0.022	0	0.267	0.105	0.07
0.266	0.19	0.408	0	0.442	0.039	0	0.046	0.307	0.221	0.193
0.048	0	0.127	0.203	0.084	0.062	0.024	0	0.172	0.145	0.103
0.193	0.131	0.303	0.202	0.064	0.068	0.054	0	0.263	0.21	0.13
0.098	0.144	0.304	0.197	0.3	0.074	0.047	0.028	0.296	0.162	0.174
0.25	0.234	0.252	0.134	0.385	0.099	0.011	0	0.298	0.32	0.152
0.045	0	0.049	0.077	0.113	0	0.005	0.112	0.246	0.285	0.179
0.228	0.288	0.481	0.035	0.284	0.01	0.08	0	0.158	0.414	0.15
0.164	0.204	0.363	0.008	0.44	0.012	0	0	0.288	0.256	0.466
0.108	0.057	0.182	0.18	0.097	0.12	0.021	0.024	0.075	0.141	0.063
0.021	0.12	0.391	0.17	0.278	0.107	0.003	0.101	0.285	0.154	0.121
0.103	0.133	0.265	0.185	0.42	0.144	0.005	0.073	0.321	0.202	0.159
0.291	0.241	0.201	0.095	0.412	0.075	0.006	0	0.303	0.256	0.312
0.084	0.021	0.324	0.26	0.215	0.134	0.037	0	0.191	0.151	0.134
0.077	0.149	0.334	0.128	0.191	0.087	0.002	0	0.279	0.222	0.128
0.018	0.143	0.391	0.225	0.32	0.045	0.032	0.006	0.319	0.15	0.109
0.253	0.099	0.276	0.213	0.522	0.119	0	0.019	0.232	0.204	0.14
0.218	0.145	0.353	0.209	0.344	0.062	0.054	0.059	0.246	0.231	0.059
0.27	0.18	0.25	0.178	0.36	0.08	0.02	0	0.253	0.281	0.121
0.03	0.101	0.282	0.229	0.363	0.088	0	0.028	0.328	0.273	0.112
0.059	0.121	0.418	0.225	0.337	0.083	0	0.066	0.309	0.244	0.11
0.066	0.005	0.267	0.146	0.086	0.052	0	0.1	0.231	0.291	0.137
0.046	0.087	0.39	0.203	0.298	0.071	0	0.129	0.305	0.304	0.092
0.1	0.119	0.396	0.198	0.308	0	0.015	0.073	0.358	0.168	0.109
0	0.122	0.444	0.184	0.377	0.063	0	0.047	0.378	0.219	0.094
0.127	0.097	0.374	0.198	0.413	0.081	0.022	0.06	0.349	0.213	0.092
0.13	0.103	0.403	0.217	0.436	0.095	0.026	0.059	0.354	0.151	0.114

0.082	0.04	0.23	0.203	0.278	0.103	0	0.079	0.345	0.247	0.128
0.044	0.124	0.356	0.204	0.366	0.056	0	0.057	0.363	0.265	0.098
0	0.117	0.269	0.343	0.248	0.013	0	0.105	0.259	0.044	0
0.162	0.157	0.362	0.117	0.501	0.001	0.021	0.002	0.351	0.323	0.174
0.031	0.092	0.385	0.223	0.307	0.026	0	0.034	0.358	0.33	0.133
0	0	0.263	0.109	0.37	0.116	0	0.157	0.247	0.229	0.116
0.083	0.12	0.381	0.152	0.486	0.124	0	0.081	0.342	0.251	0.139
0.137	0.132	0.427	0.198	0.418	0.105	0.024	0	0.319	0.161	0.093
0	0.121	0.358	0.161	0.367	0.084	0	0.11	0.334	0.169	0.153
0.159	0.094	0.39	0.191	0.456	0.086	0	0.028	0.351	0.313	0.147
0.115	0.158	0.384	0.191	0.465	0.118	0.007	0.02	0.367	0.279	0.085
0.076	0.141	0.418	0.233	0.471	0.07	0.027	0.026	0.333	0.194	0.087
0	0.03	0.163	0.177	0.247	0.089	0	0.131	0.137	0.062	0.119
0.144	0.061	0.289	0.183	0.489	0.004	0	0.057	0.343	0.33	0.109
0.034	0.116	0.433	0.185	0.476	0.097	0	0.059	0.326	0.16	0.106
0.137	0.123	0.396	0.175	0.409	0.024	0	0.031	0.319	0.362	0.088
0.117	0.142	0.404	0.219	0.416	0.061	0.03	0.031	0.384	0.227	0.107
0.29	0.15	0.335	0.118	0.525	0.076	0	0	0.242	0.384	0.424
0.019	0.086	0.462	0.152	0.468	0.106	0	0.042	0.349	0.32	0.143
0.022	0.124	0.42	0.222	0.431	0.101	0	0.076	0.362	0.153	0.117
0.094	0.154	0.407	0.167	0.335	0	0.014	0.052	0.382	0.189	0.115
0.136	0.1	0.412	0.181	0.412	0.087	0	0.015	0.336	0.288	0.118
0.057	0.117	0.407	0.203	0.422	0.11	0	0.123	0.354	0.109	0.089
0.082	0	0.287	0.247	0.148	0.05	0	0.048	0.256	0.176	0.09
0.126	0.123	0.348	0.226	0.419	0.09	0	0.058	0.314	0.254	0.094
0.083	0.118	0.457	0.256	0.385	0.039	0.034	0.02	0.311	0.193	0.114
0.049	0.103	0.356	0.188	0.502	0.077	0.014	0.125	0.353	0.184	0.142
0.115	0.112	0.306	0.222	0.338	0.115	0	0.062	0.343	0.118	0.104
0.04	0.096	0.312	0.204	0.209	0.037	0	0.062	0.275	0.303	0.126
0	0.042	0.308	0.156	0.29	0.062	0	0.054	0.344	0.247	0.12
0.146	0.013	0.382	0.175	0.29	0.079	0	0.035	0.345	0.197	0.087
0.063	0.109	0.327	0.206	0.412	0.093	0.004	0.054	0.325	0.145	0.167
0	0.082	0.42	0.25	0.394	0.108	0	0.032	0.36	0.292	0.079
0	0	0.154	0.101	0.201	0.016	0	0.146	0.162	0.345	0.125
0.104	0.093	0.369	0.225	0.325	0.054	0	0.049	0.342	0.22	0.093
0.093	0.094	0.383	0.236	0.181	0.003	0.021	0.039	0.243	0.223	0.072
0	0.159	0.427	0.217	0.453	0.144	0	0.043	0.339	0.214	0.138
0	0	0.215	0.108	0.339	0.105	0	0.117	0.228	0.322	0.119
0.063	0.12	0.462	0.218	0.457	0.076	0	0.053	0.316	0.241	0.08
0.071	0.13	0.459	0.224	0.417	0.075	0.012	0.042	0.321	0.215	0.1
0.023	0.154	0.41	0.21	0.413	0.041	0.002	0.073	0.341	0.266	0.084
0	0	0.175	0.086	0.306	0.064	0	0.094	0.192	0.426	0.105
0.144	0.208	0.354	0.129	0.476	0.093	0.01	0.031	0.317	0.327	0.114
0.015	0.067	0.447	0.163	0.434	0.089	0	0.12	0.347	0.271	0.063
0.094	0.144	0.423	0.141	0.423	0.049	0	0.029	0.333	0.277	0.099
0.105	0.181	0.363	0.112	0.413	0.088	0	0.11	0.368	0.238	0.121
0	0.124	0.449	0.198	0.339	0.081	0	0.057	0.33	0.236	0.085
0.035	0.178	0.428	0.148	0.393	0.008	0	0.053	0.391	0.278	0.08
0.109	0	0.281	0.179	0.321	0.123	0	0.067	0.323	0.207	0.145
0.079	0.049	0.317	0.181	0.34	0.072	0.001	0.048	0.295	0.328	0.137
0.128	0.108	0.373	0.163	0.39	0	0.01	0.041	0.39	0.327	0.091
0	0	0.244	0.117	0.242	0.068	0	0.117	0.156	0.198	0.134
0.052	0.092	0.462	0.175	0.357	0	0.005	0.042	0.344	0.255	0.083

0.098	0.186	0.348	0.134	0.444	0.06	0.026	0.075	0.349	0.232	0.108
0.088	0.095	0.418	0.194	0.226	0	0	0.028	0.352	0.261	0.093
0	0	0.191	0.154	0.23	0.042	0	0.149	0.171	0.287	0.126
0.05	0.123	0.418	0.211	0.376	0.101	0	0.043	0.323	0.214	0.08
0.031	0.05	0.316	0.081	0.431	0.054	0	0.104	0.335	0.347	0.201
0.118	0.086	0.22	0.188	0.355	0.004	0	0.15	0.345	0.237	0.107
0	0.035	0.235	0.109	0.23	0.04	0	0.126	0.18	0.226	0.123
0	0.106	0.284	0.195	0.249	0.073	0	0.043	0.294	0.28	0.085
0.046	0.142	0.403	0.22	0.449	0.079	0.01	0.061	0.333	0.166	0.083
0.028	0.013	0.33	0.125	0.211	0.062	0	0.083	0.217	0.232	0.16
0.032	0.134	0.447	0.207	0.46	0.111	0.011	0.04	0.27	0.273	0.131
0	0.004	0.153	0.128	0.225	0.086	0	0.138	0.091	0.165	0.126
0.14	0.106	0.384	0.125	0.367	0.042	0	0.091	0.372	0.337	0.15
0	0.143	0.399	0.213	0.409	0.067	0	0.158	0.337	0.17	0.086
0	0	0.281	0.138	0.364	0.092	0	0.094	0.276	0.347	0.148
0.155	0.129	0.398	0.13	0.455	0.079	0	0.031	0.359	0.339	0.093
0.037	0.067	0.385	0.121	0.322	0.091	0	0.113	0.268	0.358	0.16
0	0	0.181	0.092	0.285	0.041	0	0.192	0.185	0.187	0.108
0	0.08	0.384	0.119	0.409	0.062	0	0.048	0.368	0.362	0.1
0.012	0.087	0.349	0.18	0.304	0	0	0.051	0.384	0.325	0.119
0.136	0.084	0.34	0.228	0.286	0.035	0	0.077	0.277	0.069	0.103
0.009	0.097	0.347	0.185	0.369	0.001	0	0.059	0.345	0.348	0.105
0	0.017	0.148	0.215	0.262	0.06	0	0.143	0.227	0.137	0.056
0.077	0.087	0.329	0.143	0.44	0.045	0.009	0.031	0.377	0.405	0.106
0.114	0.154	0.417	0.225	0.472	0.07	0.019	0.033	0.355	0.159	0.12
0	0.221	0.46	0.224	0.49	0.101	0	0.022	0.257	0.154	0.101
0	0.162	0.438	0.241	0.494	0.065	0	0.056	0.292	0.156	0.095
0.081	0.087	0.443	0.168	0.486	0.054	0	0.06	0.368	0.322	0.139
0.006	0.094	0.414	0.187	0.423	0.077	0	0.05	0.362	0.35	0.101
0.006	0.039	0.281	0.119	0.27	0.09	0	0.105	0.208	0.372	0.116
0.073	0.052	0.387	0.158	0.284	0.046	0	0.037	0.282	0.397	0.141
0	0.104	0.429	0.183	0.429	0	0	0.145	0.382	0.277	0.122
0	0.139	0.452	0.193	0.311	0.059	0.012	0	0.333	0.257	0.087
0.081	0.031	0.32	0.195	0.349	0.071	0	0.131	0.287	0.216	0.099
0.052	0.146	0.421	0.105	0.449	0	0	0.095	0.339	0.353	0.081
0.088	0.047	0.299	0.216	0.329	0.084	0	0.078	0.323	0.211	0.099
0.021	0.113	0.374	0.081	0.38	0	0	0.081	0.36	0.42	0.192
0.131	0.069	0.32	0.106	0.36	0.063	0	0.089	0.313	0.291	0.13
0.027	0	0.143	0.152	0.228	0.077	0	0.109	0.111	0.212	0.115
0.088	0.102	0.405	0.189	0.441	0.107	0	0.049	0.35	0.172	0.093
0.033	0.062	0.39	0.127	0.387	0.054	0	0.079	0.324	0.299	0.114
0.043	0	0.314	0.163	0.204	0.028	0	0.053	0.272	0.298	0.115
0.036	0.112	0.438	0.215	0.416	0.08	0	0.071	0.329	0.226	0.06
0	0.113	0.385	0.11	0.3	0.056	0	0.067	0.33	0.362	0.14
0	0	0.18	0.085	0.336	0.079	0	0.119	0.121	0.428	0.144
0.03	0.152	0.422	0.201	0.462	0.06	0	0.064	0.302	0.154	0.086
0	0.118	0.441	0.155	0.454	0.099	0	0.074	0.379	0.288	0.112
0.032	0.074	0.263	0.218	0.243	0.078	0	0.053	0.318	0.084	0.107
0.146	0.144	0.445	0.214	0.455	0.123	0.013	0	0.3	0.194	0.124
0.007	0.072	0.287	0.073	0.446	0.05	0	0.091	0.27	0.395	0.131
0.061	0.074	0.303	0.225	0.27	0.075	0	0.052	0.322	0.181	0.115
0.038	0.117	0.302	0.167	0.369	0.046	0	0.087	0.337	0.29	0.112

Monocyte	Macrophage	NK	Neutrophil	Gamma_delta	CD4_T	CD8_T	InfiltrationScore
0.197	0.29	0.077	0.219	0.005	0.107	0.118	0.533
0.262	0.322	0.113	0.16	0.088	0.144	0.207	0.782
0.154	0.301	0.18	0.208	0.093	0.062	0.246	0.627
0.247	0.338	0.165	0.227	0.071	0.105	0.193	0.716
0.128	0.188	0.256	0.148	0.083	0.238	0.22	0.608
0.086	0.174	0.307	0.188	0.15	0.15	0.293	0.631
0.132	0.246	0.248	0.022	0.088	0.209	0.311	0.835
0.149	0.309	0.223	0.135	0.031	0.183	0.184	0.681
0.244	0.227	0.13	0.239	0.049	0.09	0.192	0.657
0.17	0.19	0.227	0.239	0.07	0.16	0.174	0.592
0.228	0.349	0.039	0.26	0.101	0.071	0.099	0.554
0.155	0.298	0.084	0.266	0.087	0.097	0.103	0.507
0.197	0.212	0.186	0.234	0.02	0.138	0.147	0.577
0.204	0.364	0.174	0.181	0.043	0.11	0.178	0.668
0.142	0.275	0.123	0.268	0	0.041	0.078	0.57
0.227	0.245	0.171	0.245	0	0.122	0.068	0.576
0.155	0.298	0.181	0.247	0.075	0.072	0.197	0.644
0.337	0.384	0.155	0.2	0.022	0.137	0.171	0.735
0.135	0.242	0.244	0.21	0.016	0.187	0.12	0.628
0.19	0.272	0.172	0.161	0.039	0.198	0.18	0.63
0.201	0.259	0.136	0.226	0.115	0.046	0.234	0.642
0.252	0.425	0.06	0.142	0.055	0.172	0.143	0.75
0.213	0.23	0.076	0.261	0	0.114	0.086	0.534
0.163	0.123	0.183	0.236	0.091	0.108	0.077	0.516
0.11	0.259	0.27	0.149	0.059	0.199	0.247	0.643
0.144	0.259	0.189	0.162	0.077	0.202	0.221	0.618
0.201	0.281	0.231	0.111	0.058	0.243	0.248	0.749
0.178	0.351	0.154	0.209	0.095	0.088	0.179	0.685
0.053	0.349	0.267	0.161	0.129	0.174	0.291	0.748
0.149	0.245	0.12	0.276	0.1	0.106	0.21	0.565
0.24	0.252	0.322	0.223	0.108	0.088	0.21	0.686
0.14	0.127	0.279	0.168	0.14	0.209	0.291	0.59
0.188	0.092	0.173	0.265	0.07	0.074	0.125	0.504
0.193	0.285	0.1	0.181	0.03	0.156	0.243	0.594
0.248	0.285	0.29	0.193	0.055	0.124	0.11	0.778
0.21	0.415	0.209	0.22	0	0.188	0.101	0.658
0.168	0.229	0.189	0.23	0	0.088	0.07	0.537
0.199	0.248	0.19	0.168	0.094	0.158	0.255	0.698
0.113	0.2	0.256	0.129	0.093	0.197	0.249	0.653
0.166	0.143	0.151	0.207	0.054	0.12	0.252	0.546
0.268	0.432	0.265	0.243	0.073	0.111	0.092	0.836
0.102	0.209	0.316	0.076	0.08	0.186	0.313	0.709
0.136	0.161	0.148	0.297	0	0.093	0.039	0.484
0.232	0.285	0.211	0.152	0.054	0.145	0.207	0.737
0.162	0.177	0.091	0.235	0.002	0.072	0.157	0.477
0.124	0.266	0.38	0.036	0.059	0.177	0.324	0.848
0.234	0.366	0.136	0.156	0.011	0.166	0.148	0.721
0.229	0.302	0.13	0.259	0.019	0.116	0.124	0.601
0.287	0.362	0.089	0.211	0.057	0.07	0.131	0.631
0.165	0.332	0.394	0.099	0.095	0.177	0.279	0.8
0.155	0.224	0.087	0.261	0.114	0.092	0.165	0.529
0.196	0.3	0.15	0.237	0.09	0.061	0.206	0.647

0.053	0.038	0.171	0.242	0.134	0.105	0.243	0.508
0.217	0.305	0.178	0.147	0.038	0.203	0.246	0.761
0.242	0.252	0.166	0.295	0.003	0.091	0.05	0.593
0.153	0.271	0.129	0.169	0.088	0.081	0.289	0.601
0.205	0.092	0.096	0.304	0.055	0.089	0.158	0.481
0.154	0.335	0.238	0.197	0.137	0.143	0.276	0.69
0.188	0.289	0.128	0.243	0.099	0.079	0.164	0.581
0.171	0.086	0.154	0.205	0.038	0.228	0.201	0.541
0.155	0.114	0.165	0.296	0.053	0.105	0.221	0.52
0.229	0.303	0.042	0.175	0.068	0.129	0.16	0.578
0.183	0.279	0.101	0.184	0.055	0.136	0.252	0.601
0.137	0.222	0.078	0.209	0.001	0.169	0.138	0.561
0.136	0.145	0.08	0.257	0.006	0.13	0.206	0.494
0.165	0.183	0.118	0.182	0.059	0.111	0.217	0.53
0.182	0.309	0.112	0.215	0.078	0.106	0.16	0.595
0.155	0.281	0.259	0.075	0.102	0.165	0.313	0.718
0.153	0.116	0.096	0.267	0.066	0.152	0.275	0.575
0.048	0.171	0.32	0.006	0.092	0.251	0.401	0.805
0.144	0.404	0.311	0.168	0	0.15	0.159	0.706
0.224	0.008	0.138	0.305	0.001	0.059	0.053	0.391
0.202	0.301	0.17	0.195	0.044	0.127	0.249	0.703
0.129	0.134	0.191	0.266	0.126	0.07	0.216	0.516
0.19	0.347	0.221	0.241	0.02	0.123	0.13	0.618
0.15	0.209	0.134	0.22	0	0.147	0.199	0.544
0.202	0.327	0.267	0.11	0.072	0.194	0.291	0.778
0.108	0.181	0.225	0.162	0.047	0.192	0.204	0.597
0.083	0.047	0.098	0.335	0.014	0.067	0.13	0.473
0.124	0.136	0.256	0.18	0.095	0.224	0.258	0.641
0.196	0.366	0.24	0.118	0.063	0.127	0.19	0.699
0.169	0.334	0.336	0.12	0.077	0.188	0.204	0.725
0.199	0.361	0.177	0.189	0.104	0.07	0.179	0.635
0.144	0.066	0.062	0.283	0	0.165	0.095	0.418
0.163	0.215	0.016	0.251	0	0.087	0.175	0.511
0.095	0.229	0.219	0.177	0.082	0.157	0.216	0.589
0.085	0.338	0.325	0.084	0.092	0.163	0.373	0.729
0.152	0.236	0.128	0.16	0.085	0.16	0.239	0.6
0.26	0.295	0.185	0.176	0.046	0.161	0.244	0.724
0.091	0.284	0.239	0.149	0.085	0.112	0.2	0.616
0.124	0.24	0.153	0.246	0.012	0.065	0.183	0.542
0.204	0.382	0.285	0.142	0.043	0.131	0.27	0.777
0.225	0.357	0.181	0.13	0.062	0.176	0.169	0.75
0.093	0.12	0.169	0.205	0.025	0.193	0.274	0.589
0.181	0.236	0.046	0.225	0	0.117	0.119	0.538
0.201	0.264	0.202	0.221	0.097	0.105	0.144	0.571
0.146	0.128	0.243	0.174	0.063	0.216	0.189	0.579
0.076	0.176	0.228	0.051	0.07	0.221	0.285	0.685
0.102	0.172	0.153	0.162	0.002	0.196	0.232	0.6
0.181	0.195	0.215	0.189	0.028	0.196	0.2	0.638
0.16	0.264	0.161	0.187	0.01	0.149	0.156	0.579
0.161	0.305	0.236	0.091	0.03	0.166	0.242	0.791
0.217	0.318	0.012	0.187	0	0.168	0.137	0.609
0.203	0.318	0.176	0.276	0	0.091	0.081	0.611
0.156	0.265	0.314	0.08	0.095	0.208	0.334	0.759

0.118	0.291	0.189	0.195	0.06	0.188	0.233	0.629
0.175	0.172	0.082	0.277	0	0.105	0.135	0.497
0.23	0.322	0.275	0.135	0.084	0.199	0.224	0.727
0.208	0.25	0.164	0.252	0.06	0.071	0.21	0.602
0.16	0.254	0.238	0.178	0.074	0.145	0.209	0.659
0.194	0.245	0.167	0.245	0.054	0.07	0.131	0.625
0.152	0.308	0.201	0.161	0.052	0.193	0.171	0.648
0.093	0.137	0.289	0.164	0.117	0.219	0.237	0.557
0.285	0.33	0.085	0.287	0.082	0.088	0.174	0.625
0.193	0.131	0.03	0.22	0.091	0.066	0.191	0.502
0.107	0.18	0.307	0.14	0.043	0.249	0.286	0.716
0.212	0.453	0.132	0.277	0.039	0.105	0.168	0.676
0.283	0.332	0.215	0.18	0.052	0.112	0.211	0.763
0.143	0.188	0.186	0.179	0.087	0.206	0.243	0.58
0.123	0.261	0.195	0.168	0.071	0.163	0.183	0.588
0.229	0.37	0.151	0.315	0.028	0.128	0.006	0.585
0.109	0.181	0.196	0.212	0.094	0.098	0.231	0.586
0.23	0.38	0.041	0.271	0.056	0.108	0.135	0.599
0.037	0.194	0.328	0.1	0.094	0.226	0.319	0.687
0.13	0.295	0.136	0.212	0	0.14	0.183	0.565
0.117	0.149	0.237	0.202	0.108	0.18	0.237	0.568
0.213	0.307	0.288	0.082	0.04	0.193	0.209	0.783
0.242	0.241	0.142	0.212	0.058	0.116	0.166	0.602
0.236	0.396	0.114	0.141	0.051	0.168	0.136	0.71
0.045	0.188	0.278	0.063	0.089	0.185	0.24	0.632
0.214	0.204	0.145	0.192	0	0.159	0.2	0.591
0.128	0.346	0.114	0.218	0	0.114	0.113	0.622
0.083	0.259	0.402	0.047	0.12	0.192	0.382	0.755
0.132	0.325	0.218	0.127	0.106	0.22	0.251	0.678
0.151	0.337	0.239	0.095	0.06	0.155	0.274	0.672
0.261	0.312	0.091	0.22	0.004	0.079	0.113	0.617
0.232	0.368	0.112	0.219	0.03	0.173	0.143	0.727
0.141	0.362	0.34	0.077	0.072	0.19	0.296	0.782
0.175	0.224	0.25	0.247	0.067	0.16	0.242	0.704
0.098	0.276	0.262	0.065	0.092	0.231	0.294	0.712
0.092	0.227	0.305	0.115	0.086	0.256	0.289	0.711
0.179	0.408	0.153	0.204	0.106	0.124	0.246	0.695
0.299	0.445	0.218	0.178	0.077	0.156	0.179	0.839
0.128	0.123	0.172	0.261	0.023	0.129	0.18	0.512
0.156	0.213	0.175	0.312	0.12	0.025	0.108	0.489
0.178	0.316	0.021	0.216	0	0.146	0.17	0.555
0.188	0.362	0.305	0.169	0.041	0.158	0.219	0.798
0.152	0.24	0.175	0.169	0.117	0.133	0.214	0.547
0.158	0.33	0.28	0.185	0.094	0.112	0.236	0.67
0.201	0.169	0.059	0.256	0	0.224	0.1	0.549
0.139	0.279	0.246	0.144	0.169	0.135	0.292	0.671
0.17	0.28	0.164	0.141	0.11	0.182	0.278	0.689
0.089	0.171	0.278	0.083	0.121	0.19	0.321	0.697
0.264	0.369	0.102	0.264	0	0.071	0.125	0.629
0.118	0.208	0.207	0.184	0.092	0.205	0.203	0.594
0.186	0.38	0.117	0.238	0.095	0.152	0.22	0.645
0.163	0.249	0.2	0.105	0.073	0.192	0.199	0.659
0.156	0.173	0.095	0.208	0.083	0.27	0.155	0.618

0.197	0.387	0.253	0.166	0.087	0.155	0.265	0.791
0.191	0.277	0.053	0.228	0	0.095	0.059	0.483
0.115	0.231	0.305	0.14	0.078	0.151	0.293	0.649
0.102	0	0.283	0.173	0.185	0.079	0.326	0.566
0.125	0.186	0.178	0.157	0.07	0.17	0.219	0.567
0.168	0.347	0.168	0.185	0.078	0.153	0.272	0.643
0.109	0.269	0.276	0.052	0.06	0.197	0.287	0.722
0.281	0.326	0.232	0.156	0.033	0.209	0.213	0.746
0.229	0.248	0.033	0.253	0.054	0	0.049	0.492
0.209	0.159	0.049	0.302	0	0.1	0.018	0.464
0.193	0.207	0.199	0.292	0	0.13	0.123	0.593
0.202	0.142	0.207	0.217	0	0.121	0.115	0.574
0.103	0.231	0.156	0.219	0.062	0.143	0.24	0.639
0.203	0.37	0.329	0.157	0.072	0.213	0.255	0.781
0.165	0.318	0.272	0.183	0.114	0.141	0.217	0.691
0.224	0.314	0.158	0.208	0.026	0.159	0.148	0.687
0.254	0.421	0.108	0.217	0.01	0.114	0.192	0.765
0.183	0.242	0.128	0.155	0.036	0.125	0.14	0.589
0.263	0.384	0.28	0.153	0.098	0.172	0.198	0.754
0.185	0.026	0.192	0.311	0	0.147	0.004	0.401
0.155	0.295	0.199	0.082	0.078	0.216	0.197	0.678
0.09	0.339	0.331	0.13	0.073	0.143	0.318	0.805
0.271	0.357	0.071	0.231	0.044	0.249	0.089	0.641
0.075	0.266	0.305	0.13	0.061	0.183	0.273	0.656
0.054	0.266	0.283	0.111	0.088	0.215	0.291	0.729
0.178	0.292	0.254	0.168	0.114	0.183	0.272	0.747
0.125	0.205	0.34	0.072	0.034	0.274	0.299	0.823
0.27	0.3	0.165	0.203	0.036	0.146	0.062	0.618
0.173	0.267	0.058	0.205	0.064	0.073	0.103	0.482
0.151	0.287	0.297	0.111	0.076	0.191	0.266	0.713
0.256	0.206	0.115	0.276	0.025	0.183	0.105	0.612
0.184	0.35	0.262	0.12	0.063	0.181	0.321	0.819
0.158	0.168	0.332	0.105	0.096	0.14	0.345	0.819
0	0.213	0.338	0.098	0.125	0.218	0.299	0.687
0.225	0.328	0.234	0.231	0.069	0.093	0.093	0.665
0.153	0.373	0.293	0.134	0.059	0.15	0.189	0.778
0.195	0.249	0.1	0.223	0.088	0.135	0.242	0.59
0.238	0.269	0.098	0.193	0.071	0.096	0.206	0.595
0.096	0.131	0.26	0.218	0.116	0.143	0.247	0.53
0.035	0.264	0.316	0.096	0.16	0.189	0.384	0.797
0.156	0.312	0.379	0.102	0.106	0.132	0.336	0.793
0.135	0.202	0.193	0.194	0	0.143	0.151	0.548
0.145	0.312	0.343	0.132	0.079	0.168	0.296	0.881
0.234	0.24	0.026	0.272	0.021	0.116	0.051	0.499
0.227	0.106	0.201	0.098	0	0.275	0.222	0.751
0.157	0.12	0.228	0.142	0.046	0.276	0.236	0.637
0.161	0.297	0.22	0.231	0.003	0.126	0.115	0.614
0.229	0.119	0.229	0.191	0.114	0.116	0.304	0.647
0.066	0.252	0.303	0.105	0.064	0.186	0.332	0.686
0.198	0.3	0.247	0.135	0.068	0.118	0.271	0.775
0.124	0.332	0.306	0.059	0.116	0.149	0.346	0.836
0.218	0.235	0.152	0.222	0.051	0.099	0.182	0.687
0.229	0.342	0.148	0.17	0.077	0.148	0.145	0.669

0.09	0.378	0.379	0.124	0.07	0.176	0.29	0.824
0.224	0.118	0.072	0.224	0	0.118	0.142	0.463
0.253	0.24	0.09	0.219	0	0.127	0.106	0.6
0.191	0.216	0.179	0.219	0.037	0.124	0.193	0.561
0.241	0.279	0.149	0.192	0.05	0.131	0.195	0.652
0.108	0.3	0.105	0.176	0.033	0.185	0.249	0.599
0.152	0.184	0.081	0.232	0.142	0.15	0.199	0.571
0.13	0.38	0.211	0.201	0.045	0.098	0.171	0.599
0.087	0.137	0.221	0.11	0.026	0.242	0.299	0.649
0.179	0.064	0.191	0.271	0.043	0.139	0.191	0.507
0.09	0.28	0.255	0.152	0.107	0.129	0.248	0.604
0.182	0.351	0.145	0.216	0.047	0.2	0.128	0.72
0.15	0.164	0.289	0.222	0.069	0.143	0.19	0.587
0.173	0.252	0.125	0.251	0.026	0.077	0.183	0.57
0.137	0.159	0.095	0.192	0.033	0.15	0.214	0.49
0.129	0.146	0.235	0.111	0.114	0.212	0.3	0.664
0.232	0.331	0.167	0.163	0.039	0.168	0.202	0.685
0.108	0.289	0.195	0.132	0.043	0.173	0.24	0.67
0.138	0.117	0.171	0.202	0.048	0.149	0.187	0.547
0.232	0.272	0.31	0.123	0.095	0.152	0.271	0.795
0.342	0.267	0.122	0.245	0	0.099	0.114	0.651
0.116	0.127	0.173	0.208	0.042	0.191	0.223	0.546
0.217	0.234	0.15	0.227	0.074	0.1	0.193	0.61
0.207	0.381	0.144	0.175	0.021	0.153	0.163	0.691
0.231	0.004	0.146	0.271	0.063	0.102	0.148	0.433
0.126	0.271	0.221	0.18	0.114	0.096	0.132	0.568
0.27	0.305	0.092	0.287	0.035	0.104	0.104	0.67
0.205	0.251	0.318	0.16	0.091	0.078	0.193	0.701
0.248	0.349	0.264	0.156	0.082	0.176	0.186	0.754
0.194	0.258	0.232	0.134	0.11	0.219	0.235	0.675
0.143	0.142	0.226	0.078	0.071	0.211	0.281	0.664
0.236	0.368	0.159	0.154	0.034	0.148	0.133	0.689
0.151	0.249	0.177	0.177	0.034	0.121	0.168	0.585
0.176	0.295	0.316	0.117	0.051	0.203	0.296	0.768
0.065	0.096	0.26	0.128	0.082	0.199	0.328	0.661
0.216	0.239	0.078	0.32	0.086	0.082	0.047	0.516
0.201	0.356	0.197	0.144	0.077	0.156	0.221	0.704
0.184	0.386	0.184	0.222	0.073	0.062	0.131	0.685
0.141	0.255	0.26	0.121	0.129	0.102	0.297	0.742
0.241	0.06	0.25	0.258	0.036	0.185	0.138	0.529
0.239	0.218	0.046	0.251	0.023	0.118	0.178	0.603
0.154	0.165	0.157	0.253	0.002	0.118	0.198	0.575
0.171	0.225	0.109	0.28	0	0.036	0.161	0.584
0.03	0.145	0.378	0.085	0.108	0.221	0.281	0.666
0.189	0.285	0	0.251	0.023	0.089	0.076	0.492
0.192	0.462	0.331	0.118	0.084	0.176	0.236	0.9
0.169	0.353	0.464	0.114	0.103	0.183	0.255	0.848
0.153	0.099	0.08	0.269	0.043	0.088	0.163	0.496
0.121	0.127	0.189	0.177	0.082	0.213	0.206	0.563
0.204	0.177	0.123	0.248	0.007	0.127	0.128	0.548
0.065	0.185	0.266	0.102	0.061	0.182	0.278	0.784
0.205	0.336	0.25	0.172	0.029	0.154	0.159	0.674
0.201	0.309	0.173	0.175	0.071	0.141	0.176	0.674

0.094	0.265	0.25	0.077	0.08	0.198	0.282	0.715
0.171	0.231	0.076	0.267	0.026	0.097	0.078	0.511
0.191	0.26	0.308	0.167	0.103	0.11	0.27	0.755
0.141	0.201	0.217	0.187	0.072	0.161	0.197	0.564
0.158	0.264	0.082	0.186	0.094	0.179	0.263	0.6
0.226	0.305	0.05	0.196	0.015	0.124	0.08	0.547
0.161	0.355	0.053	0.213	0.025	0.141	0.189	0.582
0.227	0.32	0.18	0.269	0	0.142	0.091	0.676
0.195	0.329	0.258	0.147	0.109	0.173	0.225	0.735
0.251	0.396	0.311	0.19	0.066	0.123	0.211	0.717
0.23	0.279	0.204	0.208	0.097	0.081	0.234	0.66
0.205	0.211	0.128	0.241	0.089	0.077	0.248	0.674
0.1	0.297	0.436	0.044	0.107	0.167	0.39	0.784
0.209	0.244	0.08	0.191	0.043	0.095	0.179	0.578
0.181	0.17	0.217	0.174	0.038	0.19	0.238	0.631
0.192	0.1	0.181	0.287	0.002	0.067	0.15	0.49
0.191	0.302	0.354	0.203	0.037	0.174	0.161	0.752
0.169	0.244	0.197	0.094	0.081	0.214	0.255	0.672
0.222	0.452	0.153	0.189	0.05	0.11	0.168	0.702
0.153	0.22	0.047	0.295	0	0.055	0.148	0.527
0.256	0.359	0.187	0.195	0.062	0.134	0.21	0.673
0.225	0.23	0.147	0.217	0.021	0.056	0.16	0.633
0.178	0.231	0.121	0.204	0.061	0.131	0.197	0.586
0.016	0.107	0.365	0.01	0.065	0.222	0.407	0.713
0.14	0.239	0.245	0.162	0.098	0.219	0.24	0.675
0.186	0.273	0.156	0.178	0.086	0.131	0.271	0.666
0.109	0.319	0.275	0.164	0.095	0.169	0.228	0.659
0.158	0.138	0.241	0.22	0.019	0.175	0.215	0.607
0.204	0.35	0.124	0.231	0.009	0.106	0.112	0.597
0.217	0.285	0.145	0.22	0	0.096	0.14	0.58
0.315	0.329	0.123	0.24	0	0.144	0.078	0.623
0.228	0.111	0.202	0.204	0.144	0.128	0.232	0.555
0.099	0.291	0.256	0.215	0.079	0.234	0.202	0.644
0.14	0.177	0.177	0.172	0.085	0.113	0.233	0.636
0.078	0.175	0.228	0.114	0.136	0.162	0.318	0.636
0.121	0.126	0.095	0.211	0	0.132	0.203	0.664
0.246	0.396	0.125	0.265	0	0.162	0.059	0.636
0.149	0.243	0.373	0.052	0.109	0.227	0.359	0.796
0.232	0.366	0.199	0.18	0.104	0.111	0.214	0.679
0.176	0.364	0.283	0.151	0.058	0.098	0.24	0.764
0.164	0.308	0.22	0.142	0.141	0.093	0.275	0.745
0.219	0.222	0.113	0.275	0.048	0.137	0.162	0.57
0.052	0.136	0.312	0.18	0.107	0.109	0.272	0.576
0.14	0.142	0.225	0.136	0.039	0.215	0.24	0.629
0.243	0.422	0.138	0.228	0.047	0.139	0.136	0.734
0.067	0.012	0.37	0.02	0.086	0.257	0.419	0.758
0.168	0.364	0.179	0.277	0.019	0.104	0.126	0.697
0.184	0.346	0.167	0.178	0.121	0.13	0.249	0.707
0.207	0.283	0.216	0.107	0.048	0.205	0.29	0.717
0.148	0.26	0.224	0.167	0.051	0.138	0.28	0.62
0.164	0.15	0.188	0.179	0.026	0.179	0.218	0.606
0.278	0.403	0.202	0.257	0.022	0.049	0.153	0.725
0.248	0.07	0.157	0.221	0.019	0.157	0.217	0.549

0.198	0.173	0.207	0.209	0.009	0.154	0.173	0.588
0.179	0.33	0.23	0.164	0.081	0.173	0.263	0.732
0.209	0.213	0.178	0.252	0.064	0.227	0.11	0.584
0.206	0.169	0.119	0.252	0.016	0.098	0.246	0.57
0.09	0.108	0.217	0.18	0.076	0.143	0.225	0.564
0.169	0.334	0.149	0.283	0.161	0.075	0.184	0.609
0.202	0.247	0.225	0.107	0.085	0.183	0.203	0.723
0.192	0.391	0.35	0.079	0.083	0.207	0.339	0.895
0.216	0.348	0.088	0.224	0.009	0.095	0.1	0.588
0.23	0.329	0.37	0.249	0.028	0.157	0.018	0.66
0.176	0.243	0.218	0.29	0	0.201	0.027	0.574
0.2	0.337	0.048	0.255	0.038	0.067	0.18	0.582
0.153	0.206	0.046	0.216	0.074	0.113	0.235	0.509
0.177	0.109	0.214	0.226	0.054	0.102	0.193	0.57
0.217	0.284	0.174	0.151	0.025	0.215	0.153	0.797
0.157	0.146	0.22	0.093	0.117	0.151	0.28	0.728
0.114	0.118	0.293	0.179	0.076	0.227	0.218	0.621
0.177	0.17	0.165	0.202	0.108	0.071	0.261	0.553
0.094	0.223	0.269	0.182	0.096	0.163	0.211	0.595
0.129	0.173	0.182	0.24	0.062	0.093	0.172	0.506
0.118	0.234	0.24	0.176	0.039	0.154	0.181	0.635
0.279	0.419	0.28	0.213	0.051	0.137	0.18	0.748
0.19	0.251	0.149	0.284	0.09	0.015	0.119	0.556
0.239	0.383	0.081	0.236	0	0.12	0.178	0.675
0.235	0.151	0.002	0.253	0.036	0.129	0.189	0.532
0.213	0.4	0.185	0.179	0.046	0.159	0.142	0.734
0.149	0.142	0.263	0.164	0.097	0.196	0.264	0.6
0.148	0.24	0.107	0.141	0.067	0.165	0.317	0.651
0.108	0.024	0.118	0.272	0.1	0.067	0.241	0.418
0.265	0.452	0.152	0.193	0.071	0.126	0.133	0.764
0.243	0.29	0.124	0.231	0	0.111	0	0.54
0.266	0.248	0.136	0.254	0.061	0.114	0.079	0.568
0.289	0.451	0.122	0.205	0	0.151	0.144	0.751
0.236	0.236	0.035	0.231	0	0.061	0.006	0.434
0.059	0.27	0.357	0.041	0.032	0.208	0.311	0.826
0.243	0.318	0.114	0.208	0.058	0.063	0.104	0.669
0.135	0.009	0.141	0.312	0.056	0.124	0.169	0.433
0.254	0.393	0.116	0.153	0	0.121	0.133	0.657
0.229	0.23	0.112	0.272	0	0.101	0.125	0.573
0.296	0.237	0.06	0.259	0	0.109	0.003	0.508
0.138	0.241	0.185	0.25	0.064	0.102	0.185	0.537
0.011	0.062	0.402	0	0.1	0.279	0.373	0.769
0.192	0.21	0.197	0.247	0	0.11	0.188	0.603
0.123	0.311	0.333	0.135	0.049	0.138	0.246	0.797
0.131	0.161	0.111	0.214	0.035	0.165	0.2	0.549
0.152	0.223	0.229	0.172	0.08	0.205	0.177	0.646
0.224	0.329	0.182	0.235	0.003	0.093	0.173	0.642
0.152	0.196	0.079	0.266	0.03	0.122	0.143	0.489
0.105	0.366	0.155	0.215	0.102	0.142	0.195	0.594
0.155	0.235	0.101	0.225	0.052	0.142	0.254	0.601
0.155	0.137	0.251	0.211	0.067	0.158	0.204	0.56
0.198	0.138	0.158	0.211	0.066	0.114	0.19	0.577
0.153	0.181	0.147	0.231	0.09	0.101	0.252	0.566

0.228	0.348	0.151	0.219	0.112	0.081	0.167	0.667
0.145	0.35	0.117	0.287	0.06	0.112	0.16	0.581
0.212	0.294	0.142	0.229	0.04	0.131	0.203	0.654
0.13	0.092	0.065	0.31	0.019	0.121	0.156	0.431
0.139	0.34	0.336	0.077	0.069	0.191	0.274	0.88
0.193	0.213	0.135	0.161	0.113	0.138	0.253	0.661
0.181	0.326	0.33	0.137	0.092	0.181	0.21	0.72
0.125	0.289	0.316	0.109	0.101	0.159	0.299	0.755
0.213	0.396	0.126	0.165	0.061	0.179	0.172	0.795
0.152	0.161	0.01	0.21	0.024	0.131	0.173	0.516
0.148	0.216	0.075	0.252	0.001	0.121	0.168	0.536
0.227	0.139	0.179	0.258	0.028	0.102	0.133	0.516
0.162	0.149	0.17	0.227	0.016	0.153	0.172	0.555
0.25	0.348	0.238	0.163	0.063	0.142	0.197	0.746
0.12	0.182	0.315	0.108	0.053	0.239	0.289	0.652
0.173	0.302	0.319	0.197	0.087	0.071	0.21	0.68
0.189	0.399	0.093	0.24	0.023	0.103	0.165	0.644
0.198	0.354	0.097	0.189	0.023	0.144	0.153	0.627
0.208	0.337	0.163	0.209	0.05	0.105	0.205	0.674
0.14	0.223	0.103	0.207	0.024	0.14	0.207	0.569
0.212	0.48	0.165	0.201	0.056	0.12	0.134	0.687
0.184	0.326	0.198	0.147	0.062	0.188	0.208	0.705
0.221	0.341	0.071	0.234	0.052	0.06	0.223	0.64
0.127	0.205	0.058	0.222	0.032	0.123	0.186	0.514
0.122	0.18	0.266	0.252	0.139	0.076	0.178	0.561
0.166	0.454	0.159	0.21	0.03	0.116	0.207	0.726
0.216	0.186	0.274	0.263	0.016	0.108	0.224	0.601
0.06	0.096	0.255	0.206	0.097	0.14	0.249	0.502
0.159	0.286	0.292	0.188	0.089	0.166	0.242	0.714
0.191	0.174	0.105	0.242	0.07	0.261	0.078	0.52
0.177	0.246	0.198	0.189	0.088	0.139	0.216	0.615
0.145	0.145	0.157	0.21	0.108	0.139	0.28	0.539
0.149	0.214	0.177	0.201	0.027	0.142	0.247	0.563
0.307	0.256	0.106	0.27	0.069	0.077	0.096	0.601
0.162	0.165	0.192	0.296	0.041	0.041	0.06	0.447
0.19	0.117	0.16	0.32	0.062	0.1	0.028	0.399
0.263	0.4	0.044	0.212	0	0.093	0.103	0.639
0.267	0.284	0.09	0.247	0.043	0.084	0.163	0.589
0.157	0.307	0.201	0.128	0.117	0.125	0.249	0.577
0.08	0.258	0.213	0.153	0.106	0.145	0.25	0.566
0.054	0.158	0.096	0.195	0.017	0.157	0.319	0.556
0.142	0.211	0.075	0.29	0.094	0.108	0.185	0.474
0.177	0.355	0.242	0.163	0.083	0.157	0.28	0.674
0.153	0.292	0.267	0.142	0.153	0.12	0.323	0.73
0.162	0.251	0.147	0.182	0.062	0.183	0.226	0.571
0.167	0.321	0.246	0.161	0.127	0.122	0.255	0.659
0.28	0.26	0.167	0.201	0.099	0.083	0.175	0.617
0.21	0.313	0.066	0.273	0.033	0.033	0.159	0.553
0.132	0.045	0.266	0.261	0.122	0.106	0.247	0.5
0.17	0.196	0.162	0.269	0.043	0.135	0.168	0.582
0.289	0.365	0.124	0.213	0.042	0.093	0.177	0.691
0.222	0.198	0.132	0.259	0.095	0.07	0.187	0.521
0.181	0.292	0.198	0.178	0.028	0.133	0.206	0.578

0.232	0.248	0.078	0.302	0.126	0.035	0.199	0.551
0.224	0.379	0.261	0.146	0.097	0.072	0.237	0.72
0.206	0.263	0.189	0.215	0.054	0.105	0.269	0.61
0.2	0.19	0.137	0.216	0.054	0.13	0.152	0.506
0.159	0.266	0.225	0.207	0.103	0.04	0.165	0.525
0.118	0.234	0.316	0.09	0.072	0.175	0.281	0.657
0.16	0.37	0.161	0.238	0.081	0.064	0.22	0.612
0.072	0.144	0.242	0.185	0.051	0.207	0.278	0.618
0.16	0.204	0.225	0.246	0.125	0.063	0.224	0.538
0.149	0.352	0.276	0.126	0.13	0.164	0.272	0.705
0.257	0.355	0.074	0.223	0.072	0.119	0.069	0.65
0.178	0.306	0.051	0.235	0.025	0.105	0.086	0.489
0.172	0.262	0.139	0.212	0.089	0.047	0.214	0.602
0.254	0.448	0.131	0.239	0.016	0.054	0.156	0.639
0.129	0.267	0.245	0.132	0.1	0.058	0.302	0.582
0.29	0.424	0.034	0.265	0	0.044	0.087	0.578
0.165	0.213	0.172	0.187	0.096	0.128	0.226	0.546
0.162	0.114	0.118	0.306	0.001	0.096	0.173	0.463
0.202	0.263	0.23	0.278	0.077	0.062	0.206	0.576
0.229	0.111	0.134	0.293	0	0.058	0.13	0.502
0.341	0.401	0.27	0.254	0.058	0.124	0.244	0.756
0.182	0.259	0.27	0.15	0.087	0.146	0.277	0.656
0.187	0.139	0.212	0.191	0.096	0.119	0.229	0.509
0.144	0.345	0.303	0.153	0.106	0.112	0.287	0.734
0.195	0.289	0.087	0.208	0	0.148	0.135	0.533
0.297	0.287	0.167	0.269	0.15	0.011	0.1	0.602
0.217	0.338	0.211	0.182	0.095	0.05	0.242	0.627
0.152	0.172	0.221	0.181	0.158	0.099	0.313	0.655
0.083	0.279	0.314	0.094	0.107	0.129	0.301	0.712
0.127	0.209	0.11	0.212	0.048	0.142	0.176	0.515
0.161	0.333	0.23	0.202	0.142	0.085	0.221	0.638
0.114	0.253	0.265	0.206	0.11	0.127	0.241	0.634
0.191	0.173	0.108	0.223	0.032	0.164	0.248	0.599
0.142	0.214	0.105	0.254	0.096	0.087	0.178	0.464
0.198	0.248	0.048	0.264	0.053	0.081	0.175	0.508
0.182	0.4	0.174	0.178	0.098	0.066	0.181	0.623
0.193	0.336	0.17	0.211	0.111	0.089	0.245	0.663
0.239	0.142	0.062	0.222	0.085	0.056	0.221	0.489
0.145	0.188	0.161	0.212	0.07	0.086	0.255	0.546
0.2	0.303	0.103	0.224	0.052	0.147	0.156	0.604
0.25	0.356	0.207	0.171	0.114	0.101	0.258	0.674
0.231	0.391	0.233	0.151	0.101	0.148	0.256	0.725
0.152	0.228	0.165	0.206	0.103	0.164	0.218	0.572
0.036	0.213	0.276	0.106	0.102	0.17	0.31	0.629
0.219	0.312	0.189	0.284	0.09	0.111	0.031	0.541
0.013	0.149	0.343	0.118	0.072	0.171	0.317	0.671
0.152	0.184	0.143	0.191	0.057	0.172	0.244	0.555
0.182	0.387	0.09	0.239	0.107	0.082	0.235	0.609
0.2	0.259	0.271	0.117	0.141	0.085	0.351	0.711
0.209	0.369	0.136	0.166	0.092	0.092	0.253	0.614
0.113	0.325	0.279	0.077	0.131	0.115	0.357	0.698
0.204	0.316	0.158	0.167	0.145	0.119	0.267	0.621
0.3	0.361	0.17	0.178	0.062	0.081	0.192	0.699

0.237	0.165	0.02	0.331	0.023	0.083	0.097	0.48
0.22	0.325	0.019	0.287	0.018	0.04	0.089	0.539
0.154	0.218	0.089	0.244	0.04	0.167	0.197	0.555
0.174	0.377	0.307	0.041	0.053	0.178	0.346	0.9
0.323	0.269	0.098	0.229	0.046	0.037	0.22	0.605
0.142	0.236	0.12	0.208	0.063	0.139	0.216	0.529
0.163	0.215	0.144	0.234	0.083	0.194	0.186	0.614
0.122	0.274	0.157	0.226	0.132	0.106	0.221	0.537
0.226	0.371	0.088	0.253	0.076	0.101	0.224	0.605
0.222	0.272	0.138	0.209	0.019	0.131	0.154	0.575
0.105	0.309	0.27	0.162	0.083	0.139	0.253	0.651
0.007	0.224	0.392	0.088	0.159	0.1	0.403	0.678
0.166	0.191	0.227	0.211	0.11	0.135	0.186	0.562
0.203	0.307	0.234	0.173	0.144	0.022	0.287	0.651
0.225	0.338	0.245	0.159	0.125	0.165	0.226	0.711
0.283	0.366	0.142	0.225	0.092	0.068	0.212	0.641
0.121	0.141	0.2	0.178	0.071	0.156	0.267	0.549
0.173	0.275	0.279	0.202	0.124	0.029	0.229	0.601
0.141	0.263	0.29	0.147	0.098	0.173	0.317	0.657
0.27	0.265	0.123	0.278	0.022	0.122	0.081	0.635
0.173	0.279	0.173	0.182	0.061	0.128	0.166	0.583
0.149	0.334	0.091	0.252	0.09	0.11	0.176	0.53
0.067	0.271	0.27	0.081	0.126	0.126	0.351	0.66
0.2	0.152	0.099	0.272	0.056	0.051	0.204	0.492
0.248	0.389	0.092	0.193	0.08	0.031	0.235	0.582
0.128	0.182	0.261	0.168	0.106	0.153	0.232	0.57
0.258	0.29	0.154	0.281	0.026	0.073	0.075	0.634
0.208	0.349	0.204	0.15	0.065	0.13	0.171	0.722
0.142	0.174	0.167	0.231	0.089	0.109	0.178	0.492
0.153	0.359	0.316	0.069	0.106	0.135	0.343	0.822
0.238	0.321	0.342	0.152	0.126	0.181	0.252	0.715
0.172	0.233	0.117	0.274	0.029	0.092	0.062	0.486
0.172	0.453	0.247	0.138	0.056	0.139	0.232	0.79
0.192	0.337	0.118	0.249	0.14	0.085	0.179	0.6
0.126	0.225	0.178	0.111	0.121	0.127	0.339	0.634
0.249	0.389	0.162	0.175	0.07	0.087	0.261	0.783
0.245	0.291	0.121	0.252	0.083	0.121	0.231	0.64
0.181	0.107	0.057	0.264	0.086	0.075	0.215	0.475
0.269	0.298	0.12	0.233	0.041	0.048	0.198	0.563
0.13	0.174	0.164	0.23	0.119	0.042	0.285	0.535
0.254	0.295	0.109	0.22	0.037	0.075	0.091	0.564
0.028	0.205	0.326	0.094	0.124	0.158	0.344	0.624
0.164	0.283	0.16	0.166	0.1	0.15	0.275	0.612
0.189	0.391	0.126	0.244	0.092	0.075	0.18	0.601
0.197	0.374	0.238	0.184	0.091	0.153	0.252	0.666
0.262	0.303	0.029	0.227	0.023	0.111	0.214	0.596
0.141	0.221	0.163	0.21	0.06	0.154	0.229	0.551
0.13	0.362	0.31	0.099	0.108	0.114	0.304	0.8
0.021	0.075	0.163	0.271	0.061	0.157	0.277	0.499
0.142	0.014	0.213	0.234	0.065	0.11	0.197	0.453
0.128	0.28	0.203	0.13	0.083	0.114	0.327	0.651
0.231	0.132	0.171	0.265	0.131	0.075	0.18	0.522
0.114	0.158	0.349	0.032	0.096	0.192	0.337	0.765

0.236	0.377	0.108	0.231	0.021	0.14	0.18	0.664
0.196	0.304	0.176	0.16	0.036	0.14	0.172	0.638
0.23	0.229	0.172	0.27	0.086	0.054	0.223	0.569
0.296	0.315	0.047	0.273	0.022	0.078	0.094	0.57
0.168	0.336	0.304	0.088	0.112	0.153	0.311	0.76
0.213	0.316	0.178	0.205	0.083	0.113	0.235	0.655
0.184	0.091	0.116	0.312	0.059	0.097	0.153	0.474
0.238	0.452	0.226	0.213	0.053	0.081	0.211	0.719
0.163	0.233	0.315	0.12	0.111	0.136	0.303	0.662
0.125	0	0.133	0.283	0.05	0.139	0.135	0.374
0.081	0.044	0.376	0.072	0.083	0.229	0.336	0.723
0.162	0.317	0.19	0.311	0.076	0.039	0.046	0.543
0.154	0.387	0.234	0.176	0.043	0.146	0.212	0.788
0.133	0.324	0.324	0.109	0.131	0.132	0.376	0.75
0.212	0.32	0.131	0.193	0.057	0.147	0.18	0.598
0.151	0.04	0.123	0.266	0.105	0.111	0.201	0.461
0.146	0.327	0.387	0.116	0.118	0.109	0.324	0.802
0.169	0.343	0.144	0.145	0.07	0.129	0.238	0.687
0.142	0.122	0.08	0.24	0.06	0.125	0.26	0.48
0.095	0.184	0.273	0.209	0.168	0.126	0.18	0.57
0.064	0.24	0.37	0.05	0.082	0.139	0.353	0.834
0.2	0.389	0.201	0.165	0.103	0.118	0.261	0.708
0.198	0.312	0.105	0.214	0.057	0.087	0.181	0.558
0.184	0.209	0.052	0.265	0.081	0.04	0.164	0.467
0.125	0.187	0.182	0.236	0.08	0.118	0.231	0.465
0.136	0.31	0.297	0.229	0.155	0.057	0.256	0.649
0.143	0.364	0.405	0.039	0.134	0.126	0.353	0.815
0.205	0.296	0.187	0.163	0.153	0.155	0.31	0.661
0.151	0.3	0.297	0.138	0.133	0.17	0.246	0.664
0.221	0.359	0.086	0.27	0.068	0.028	0.162	0.543
0.152	0.341	0.231	0.184	0.103	0.089	0.26	0.7
0.256	0.365	0.193	0.232	0.072	0.055	0.207	0.634
0.107	0.229	0.3	0.098	0.127	0.11	0.372	0.716
0.091	0.28	0.204	0.192	0.133	0.086	0.297	0.605
0.123	0.08	0.243	0.224	0.008	0.101	0.175	0.495
0.177	0.1	0.079	0.322	0.037	0.088	0.12	0.451
0.211	0.44	0.201	0.211	0.069	0.106	0.197	0.669
0.244	0.311	0.137	0.214	0.079	0.055	0.231	0.642
0.105	0.234	0.063	0.226	0.096	0.118	0.204	0.534
0.134	0.268	0.355	0.115	0.124	0.148	0.281	0.74
0.179	0.288	0.172	0.169	0.107	0.15	0.337	0.642
0.245	0.26	0.19	0.242	0.055	0.085	0.163	0.571
0.11	0.249	0.295	0.098	0.094	0.164	0.317	0.65
0.248	0.164	0.025	0.258	0	0.018	0.155	0.461
0.265	0.357	0.343	0.212	0.128	0.17	0.224	0.732
0.223	0.095	0.082	0.268	0.076	0.097	0.174	0.422
0.104	0.129	0.316	0.153	0.102	0.142	0.224	0.599
0.167	0.276	0.197	0.217	0.108	0.073	0.241	0.581
0.261	0.278	0.266	0.137	0.071	0.183	0.264	0.683
0.252	0.474	0.056	0.191	0.069	0.089	0.232	0.719
0.223	0.266	0.14	0.322	0	0.064	0	0.49
0.147	0.171	0.21	0.217	0.059	0.178	0.196	0.557
0.166	0.203	0.089	0.247	0.022	0.089	0.121	0.483

0.319	0.253	0.105	0.26	0.05	0.065	0.182	0.589
0.091	0.168	0.206	0.205	0.132	0.154	0.332	0.576
0.145	0.21	0.14	0.217	0.049	0.156	0.224	0.552
0	0.057	0.411	0	0.092	0.232	0.386	0.685
0.256	0.326	0.205	0.175	0.094	0.117	0.161	0.698
0.121	0.401	0.25	0.16	0.134	0.166	0.262	0.664
0.185	0.156	0.031	0.362	0.001	0.046	0.109	0.417
0.178	0.204	0.301	0.211	0.112	0.153	0.216	0.631
0.236	0.41	0.123	0.233	0.021	0.059	0.089	0.584
0.116	0.204	0.231	0.181	0.118	0.143	0.239	0.565
0.225	0.242	0.165	0.28	0.1	0.059	0.214	0.625
0.12	0.06	0.149	0.178	0.094	0.157	0.3	0.48
0.209	0.323	0.04	0.316	0.045	0.107	0.061	0.518
0.073	0.298	0.173	0.156	0.167	0.146	0.299	0.557
0.217	0.278	0.144	0.164	0.132	0.124	0.356	0.63
0.265	0.248	0.142	0.205	0.073	0.089	0.257	0.611
0.266	0.416	0.055	0.225	0.009	0.046	0.182	0.653
0.254	0.364	0.084	0.256	0.008	0.031	0.085	0.546
0.151	0.09	0.286	0.186	0.058	0.203	0.215	0.538
0.179	0.16	0.182	0.266	0.099	0.085	0.245	0.531
0.311	0.263	0	0.281	0.053	0.056	0.181	0.567
0.039	0.144	0.31	0.073	0.064	0.199	0.359	0.666
0.158	0.332	0.234	0.072	0.119	0.172	0.331	0.743
0.14	0.115	0.158	0.15	0.09	0.166	0.241	0.527
0.198	0.205	0.177	0.254	0.07	0.099	0.149	0.513
0.275	0.315	0.082	0.182	0.07	0.093	0.188	0.583
0.238	0.212	0.126	0.288	0	0.157	0.112	0.567
0.201	0.117	0.169	0.242	0.053	0.083	0.21	0.493
0.196	0.224	0.055	0.3	0.081	0.053	0.172	0.51
0.319	0.337	0.057	0.23	0.057	0.064	0.145	0.597
0.138	0.323	0.18	0.149	0.101	0.102	0.287	0.676
0.149	0.322	0.263	0.143	0.13	0.119	0.272	0.678
0.182	0.399	0.128	0.142	0.091	0.138	0.248	0.69
0.146	0.186	0.199	0.286	0.045	0.141	0.143	0.513
0.194	0.236	0.174	0.24	0.107	0.065	0.148	0.529
0.232	0.234	0.043	0.327	0	0.063	0.069	0.492
0.124	0.284	0.256	0.187	0.103	0.176	0.237	0.671
0.184	0.376	0.122	0.164	0.091	0.171	0.262	0.59
0.155	0.146	0.187	0.234	0.116	0.09	0.235	0.524
0.174	0.218	0.165	0.242	0.081	0.063	0.228	0.534
0.163	0.313	0.131	0.287	0	0.081	0.174	0.554
0.138	0.17	0.163	0.263	0.121	0.05	0.125	0.465
0.224	0.405	0.195	0.161	0.079	0.11	0.217	0.697
0.121	0.275	0.23	0.062	0.098	0.171	0.321	0.65
0.235	0.386	0.097	0.233	0.025	0.014	0.1	0.573
0.309	0.413	0.125	0.197	0	0.133	0.154	0.682
0.199	0.272	0.355	0.098	0.113	0.091	0.37	0.705
0.264	0.34	0.027	0.352	0.094	0.036	0.096	0.584
0.15	0.171	0.12	0.271	0	0.113	0.204	0.537
0.236	0.269	0.218	0.267	0	0.116	0.109	0.596
0.202	0.207	0.174	0.314	0.012	0.075	0.057	0.501
0.257	0.443	0.216	0.127	0.106	0.144	0.278	0.782
0.18	0.065	0.078	0.331	0.017	0.042	0.194	0.452

0.175	0.225	0.162	0.278	0.065	0.102	0.161	0.517
0.095	0.183	0.167	0.216	0.127	0.108	0.222	0.489
0.257	0.06	0.179	0.312	0.017	0.091	0.119	0.451
0.089	0.191	0.208	0.172	0.106	0.173	0.291	0.609
0.186	0.394	0.108	0.247	0.062	0.1	0.186	0.59
0.146	0.297	0.257	0.132	0.154	0.091	0.358	0.696
0.221	0.109	0.168	0.24	0.053	0	0.183	0.476
0.256	0.415	0.055	0.218	0.062	0.042	0.179	0.619
0.203	0.156	0.144	0.293	0.015	0.081	0.097	0.479
0.155	0.104	0.211	0.23	0.055	0.1	0.245	0.487
0.125	0.226	0.111	0.251	0.09	0.059	0.216	0.516
0.174	0.274	0.114	0.214	0.044	0.088	0.22	0.54
0.21	0.352	0.149	0.197	0.136	0.105	0.28	0.671
0.222	0.422	0.196	0.134	0.061	0.129	0.211	0.707
0.22	0.25	0.073	0.321	0.015	0.042	0.149	0.519
0.211	0.205	0.09	0.265	0.108	0.048	0.206	0.545
0.068	0.124	0.215	0.241	0.117	0.091	0.213	0.533
0.043	0.221	0.396	0.012	0.094	0.169	0.383	0.768
0.126	0.236	0.176	0.19	0.069	0.145	0.202	0.567
0.15	0.215	0.282	0.154	0.057	0.202	0.278	0.703
0.096	0.277	0.31	0.173	0.156	0.197	0.263	0.655
0.186	0.35	0.345	0.1	0.065	0.107	0.291	0.766
0.154	0.256	0.297	0.167	0.048	0.139	0.216	0.677
0.247	0.429	0.082	0.241	0.054	0.061	0.168	0.618
0.136	0.213	0.211	0.171	0.049	0.163	0.266	0.582
0.202	0.38	0.37	0.069	0.102	0.145	0.33	0.782
0.25	0.15	0.128	0.258	0.055	0.064	0.094	0.477
0.116	0.128	0.166	0.279	0.114	0.109	0.197	0.495
0.2	0.049	0.152	0.321	0	0.131	0.079	0.441
0.243	0.395	0.108	0.213	0.015	0.107	0.205	0.621
0.208	0.255	0.07	0.268	0.016	0.029	0.244	0.538
0.217	0.36	0.229	0.159	0.124	0.117	0.228	0.691
0.142	0.314	0.248	0.172	0.098	0.115	0.267	0.698
0.187	0.183	0.114	0.252	0.053	0.14	0.213	0.545
0.223	0.2	0.173	0.274	0.063	0.077	0.16	0.562
0.083	0.313	0.319	0.052	0.112	0.131	0.378	0.822
0.142	0.328	0.373	0.152	0.142	0.123	0.267	0.72
0.176	0.212	0.167	0.265	0.038	0.077	0.226	0.544
0.177	0.132	0.006	0.241	0.117	0.044	0.204	0.476
0.182	0.083	0.039	0.272	0	0.085	0.17	0.471
0.133	0.104	0.35	0.193	0.116	0.195	0.263	0.585
0.158	0.225	0.216	0.117	0.088	0.164	0.306	0.651
0.105	0.225	0.308	0.126	0.112	0.168	0.279	0.633
0.174	0.271	0.138	0.171	0.058	0.156	0.222	0.593
0.148	0.221	0.24	0.139	0.095	0.175	0.246	0.642
0.178	0.263	0.113	0.201	0.086	0.093	0.234	0.542
0.114	0.148	0.072	0.208	0.109	0.116	0.29	0.484
0.172	0.276	0.198	0.177	0.125	0.124	0.233	0.612
0.171	0.301	0.133	0.198	0.034	0.123	0.234	0.622
0.233	0.289	0.07	0.239	0.087	0.117	0.221	0.627
0.192	0.195	0.163	0.278	0.01	0.134	0.097	0.519
0.179	0.369	0.211	0.17	0.105	0.085	0.2	0.681
0.246	0.385	0.086	0.25	0.06	0.044	0.186	0.625

0.159	0.297	0.291	0.132	0.105	0.174	0.308	0.676
0.095	0.127	0.172	0.176	0.08	0.151	0.265	0.497
0.099	0.064	0.141	0.315	0.039	0.085	0.065	0.384
0.01	0.26	0.442	0.041	0.127	0.182	0.428	0.8
0.241	0.418	0.073	0.209	0.019	0.067	0.138	0.615
0.147	0.363	0.294	0.101	0.094	0.105	0.301	0.774
0.154	0.116	0.2	0.226	0.06	0.138	0.249	0.532
0.178	0.231	0.146	0.195	0.118	0.092	0.252	0.564
0.143	0.098	0.183	0.255	0.098	0.044	0.254	0.471
0.127	0.301	0.195	0.177	0.129	0.132	0.277	0.612
0.257	0.405	0.092	0.231	0.091	0.046	0.126	0.621
0.157	0.206	0.258	0.246	0.133	0.061	0.236	0.592
0.15	0.279	0.205	0.208	0.1	0.093	0.206	0.563
0.089	0.359	0.409	0.063	0.083	0.139	0.327	0.826
0.141	0.235	0.163	0.103	0.1	0.122	0.322	0.637
0.19	0.322	0.045	0.299	0.016	0.112	0.135	0.535
0.242	0.309	0.085	0.153	0.093	0.127	0.254	0.625
0.148	0.336	0.321	0.133	0.106	0.129	0.295	0.764
0.097	0.218	0.191	0.128	0.053	0.143	0.264	0.599
0.258	0.025	0.076	0.301	0.019	0.053	0.156	0.496
0.256	0.485	0.116	0.231	0.025	0.095	0.124	0.645
0.135	0.35	0.315	0.108	0.107	0.125	0.27	0.662
0.176	0.338	0.297	0.098	0.146	0.114	0.282	0.683
0.097	0.203	0.277	0.159	0.088	0.162	0.28	0.62
0.217	0.363	0.142	0.258	0.077	0.081	0.187	0.615
0.201	0.311	0.311	0.107	0.082	0.151	0.229	0.749
0.226	0.338	0.188	0.168	0.084	0.161	0.198	0.658
0.207	0.259	0.22	0.194	0.066	0.098	0.207	0.588
0.159	0.247	0.247	0.141	0.205	0.134	0.281	0.679
0.224	0.28	0.238	0.158	0.125	0.12	0.246	0.675
0.197	0.369	0.243	0.166	0.104	0.132	0.224	0.682
0.203	0.481	0.145	0.235	0.133	0.061	0.243	0.71
0.128	0.331	0.273	0.164	0.09	0.091	0.279	0.715
0.155	0.228	0.294	0.131	0.152	0.182	0.316	0.681
0.093	0.328	0.318	0.118	0.129	0.083	0.359	0.685
0.178	0.044	0.063	0.284	0.029	0.117	0.229	0.483
0.188	0.175	0.194	0.303	0	0.078	0.058	0.557
0.295	0.348	0.152	0.212	0.043	0.196	0.092	0.662
0.171	0.254	0.058	0.262	0.089	0.032	0.185	0.471
0.08	0.208	0.289	0.129	0.087	0.145	0.296	0.65
0.142	0.156	0.069	0.215	0.115	0.117	0.226	0.471
0.151	0.393	0.276	0.112	0.078	0.137	0.266	0.817
0.23	0.21	0.072	0.293	0	0.079	0.153	0.49
0.167	0.248	0.199	0.197	0.047	0.117	0.26	0.595
0.188	0.179	0.162	0.248	0.074	0.092	0.233	0.547
0.201	0.286	0.201	0.188	0.036	0.169	0.199	0.64
0.223	0.381	0.309	0.128	0.143	0.096	0.346	0.782
0.179	0.174	0.145	0.299	0.102	0.091	0.114	0.554
0.173	0.153	0.364	0.186	0.117	0.197	0.233	0.596
0.146	0.222	0.296	0.159	0.123	0.186	0.254	0.634
0.159	0.343	0.259	0.111	0.092	0.125	0.316	0.754
0.159	0.138	0.13	0.296	0.016	0.065	0.192	0.488
0.234	0.404	0.137	0.303	0.006	0.103	0.081	0.614

0.136	0.309	0.19	0.131	0.115	0.091	0.256	0.634
0.169	0.329	0.271	0.072	0.128	0.164	0.303	0.677
0.235	0.275	0.054	0.316	0	0.014	0.091	0.512
0.154	0.199	0.038	0.227	0.052	0.112	0.254	0.501
0.14	0.112	0.119	0.189	0.116	0.104	0.211	0.505
0.106	0.178	0.177	0.182	0.121	0.16	0.26	0.524
0.205	0.502	0.22	0.153	0.1	0.151	0.259	0.783
0.194	0.455	0.29	0.147	0.109	0.149	0.286	0.819
0.293	0.156	0.033	0.312	0.089	0.068	0.17	0.553
0.069	0.155	0.302	0.085	0.132	0.178	0.316	0.64
0.187	0.241	0.241	0.208	0.087	0.063	0.233	0.571
0.128	0.178	0.063	0.182	0.08	0.112	0.203	0.483
0.155	0.265	0.13	0.277	0.072	0.096	0.19	0.566
0.198	0.279	0.314	0.178	0.096	0.04	0.165	0.551
0.234	0.181	0.085	0.264	0.087	0.071	0.207	0.495
0.128	0.308	0.327	0.117	0.09	0.193	0.295	0.743
0.162	0.328	0.241	0.087	0.056	0.161	0.277	0.675
0.152	0.127	0.185	0.228	0.083	0.087	0.252	0.505
0.169	0.251	0.044	0.266	0.073	0.057	0.161	0.483
0.199	0.262	0.068	0.275	0.042	0.036	0.213	0.512
0.136	0.122	0.174	0.193	0.139	0.18	0.28	0.551
0.161	0.334	0.227	0.182	0.118	0.096	0.276	0.646
0.163	0.26	0.048	0.3	0.037	0.046	0.14	0.497
0.219	0.236	0.221	0.189	0.084	0.17	0.162	0.638
0.091	0.275	0.106	0.219	0.151	0.097	0.263	0.499
0.209	0.368	0.142	0.219	0.048	0.103	0.195	0.657
0.162	0.085	0.202	0.319	0	0.151	0.087	0.496
0.165	0.234	0.225	0.216	0.137	0.005	0.245	0.554
0.175	0.222	0.115	0.254	0.074	0.083	0.231	0.542
0.145	0.314	0.301	0.16	0.128	0.105	0.269	0.692
0.1	0.226	0.241	0.224	0.144	0.055	0.271	0.635
0.209	0.264	0.334	0.109	0.114	0.133	0.292	0.732
0.247	0.268	0.276	0.234	0.089	0.143	0.211	0.626
0.112	0.323	0.232	0.144	0.146	0.105	0.305	0.641
0.149	0.305	0.297	0.213	0.073	0.118	0.252	0.608
0.171	0.175	0.107	0.282	0	0.068	0.162	0.502
0.154	0.182	0.24	0.198	0.113	0.138	0.279	0.572
0.227	0.178	0.142	0.258	0.005	0.09	0.189	0.532
0.137	0.283	0.149	0.12	0.072	0.156	0.269	0.605
0.125	0.118	0.233	0.186	0.071	0.135	0.215	0.516
0.142	0.219	0.071	0.189	0.11	0.124	0.229	0.568
0.153	0.27	0.26	0.166	0.12	0.146	0.299	0.652
0.272	0.43	0.249	0.138	0.071	0.088	0.215	0.743
0.196	0.209	0.148	0.243	0.007	0.107	0.112	0.533
0.136	0.272	0.124	0.244	0.081	0.129	0.268	0.57
0.078	0.255	0.352	0.04	0.098	0.193	0.385	0.75
0.082	0.341	0.241	0.116	0.098	0.155	0.315	0.672
0.243	0.411	0.176	0.205	0.151	0.018	0.188	0.68
0.241	0.41	0.198	0.231	0.069	0.123	0.197	0.668
0.198	0.287	0.088	0.263	0.065	0.042	0.142	0.529
0.106	0.22	0.285	0.191	0.095	0.158	0.219	0.635
0.079	0.255	0.387	0.104	0.074	0.181	0.259	0.672
0.182	0.312	0.164	0.255	0.118	0.07	0.148	0.592

0.204	0.35	0.319	0.123	0.048	0.119	0.266	0.797
0.196	0.313	0.113	0.213	0.056	0.081	0.205	0.568
0.239	0.211	0.079	0.324	0.072	0	0.141	0.514
0.17	0.325	0.257	0.233	0.154	0.011	0.217	0.642
0.194	0.175	0.093	0.25	0.045	0.104	0.159	0.502
0.219	0.329	0.185	0.234	0.095	0.178	0.262	0.648
0.23	0.434	0.085	0.251	0.029	0.177	0.225	0.654
0.28	0.47	0.069	0.293	0.009	0.075	0.172	0.669
0.139	0.339	0.305	0.142	0.129	0.18	0.396	0.767
0.229	0.194	0.171	0.332	0.063	0.122	0.184	0.56
0.226	0.252	0.183	0.316	0	0.072	0.168	0.553
0.223	0.324	0.185	0.217	0.112	0.162	0.329	0.686
0.24	0.416	0.286	0.197	0.137	0.156	0.334	0.742
0.064	0.242	0.269	0.144	0.115	0.194	0.388	0.65
0.182	0.463	0.426	0.16	0.071	0.19	0.395	0.849
0.221	0.363	0.043	0.339	0.128	0.09	0.21	0.602
0.318	0.089	0	0.368	0.085	0.122	0.236	0.541
0.169	0.184	0.191	0.293	0.089	0.117	0.321	0.584
0.263	0.448	0.187	0.217	0.069	0.167	0.265	0.759
0.223	0.416	0.132	0.31	0.043	0.114	0.153	0.613
0.215	0.298	0.265	0.207	0.084	0.102	0.282	0.749
0.263	0.327	0.158	0.366	0.033	0.087	0.19	0.603
0.185	0.219	0.14	0.304	0.065	0.129	0.247	0.557
0.186	0.165	0.141	0.356	0.016	0.118	0.241	0.514
0.182	0.201	0.109	0.305	0.084	0.089	0.283	0.518
0.248	0.217	0.216	0.288	0.103	0.144	0.239	0.615
0.177	0.244	0.161	0.258	0.112	0.118	0.322	0.614
0.25	0.349	0.26	0.326	0.038	0.13	0.191	0.659
0.254	0.222	0.106	0.391	0	0.087	0.125	0.526
0.261	0.334	0.083	0.278	0.013	0.157	0.163	0.575
0.232	0.478	0.155	0.289	0.046	0.136	0.181	0.657
0.269	0.273	0.111	0.358	0	0.107	0.152	0.607
0.144	0.416	0.389	0.149	0.126	0.142	0.384	0.818
0.132	0.324	0.23	0.244	0.103	0.161	0.276	0.657
0.315	0.321	0.078	0.342	0.008	0.102	0.104	0.563
0.138	0.423	0.264	0.23	0.13	0.145	0.319	0.731
0.187	0.337	0.155	0.278	0.071	0.125	0.267	0.603
0.315	0.403	0.122	0.337	0.021	0.077	0.14	0.7
0.197	0.329	0.149	0.306	0.006	0.142	0.235	0.591
0.106	0.343	0.325	0.149	0.081	0.232	0.367	0.712
0.201	0.29	0.077	0.278	0.038	0.141	0.216	0.582
0.31	0.152	0.101	0.345	0.022	0.226	0.133	0.541
0.178	0.429	0.18	0.233	0.058	0.159	0.277	0.661
0.3	0.45	0.102	0.277	0.096	0.072	0.217	0.672
0.209	0.287	0.205	0.308	0	0.103	0.189	0.591
0.202	0.466	0.393	0.237	0.069	0.087	0.315	0.855
0.325	0.443	0.083	0.293	0	0.066	0.176	0.653
0.258	0.278	0.076	0.371	0.039	0.05	0.125	0.537
0.179	0.439	0.221	0.179	0.11	0.16	0.322	0.728
0.214	0.519	0.269	0.253	0.065	0.132	0.23	0.726
0.251	0.466	0.172	0.328	0.09	0.117	0.234	0.687
0.158	0.169	0.19	0.289	0.105	0.124	0.238	0.611
0.186	0.228	0.185	0.247	0.086	0.126	0.293	0.563

0.184	0.187	0.131	0.193	0.043	0.269	0.26	0.609
0.205	0.294	0.185	0.317	0.053	0.071	0.191	0.557
0.272	0.532	0.033	0.268	0.064	0.129	0.176	0.67
0.204	0.426	0.256	0.169	0.06	0.166	0.237	0.76
0.172	0.107	0.157	0.347	0.096	0.119	0.307	0.523
0.209	0.261	0.271	0.277	0.063	0.132	0.282	0.671
0.305	0.485	0.243	0.242	0.033	0.219	0.183	0.82
0.185	0.357	0.138	0.318	0.056	0.07	0.243	0.578
0.168	0.254	0.172	0.294	0.074	0.072	0.318	0.572
0.217	0.249	0.109	0.266	0.096	0.217	0.236	0.614
0.207	0.403	0.273	0.206	0.066	0.146	0.327	0.775
0.165	0.312	0.313	0.161	0.13	0.161	0.399	0.687
0.191	0.375	0.342	0.134	0.114	0.21	0.338	0.813
0.196	0.239	0.078	0.314	0.037	0.118	0.163	0.539
0.19	0.455	0.273	0.248	0.051	0.166	0.281	0.738
0.266	0.207	0.162	0.341	0	0.02	0.262	0.593
0.185	0.254	0.211	0.226	0.16	0.148	0.339	0.668
0.279	0.402	0.181	0.401	0.082	0.13	0.135	0.639
0.136	0.321	0.28	0.23	0.127	0.21	0.274	0.671
0.25	0.327	0.062	0.309	0	0.087	0.229	0.581
0.26	0.374	0.2	0.366	0.065	0.123	0.094	0.6
0.232	0.104	0.209	0.404	0	0.083	0.075	0.535
0.238	0.278	0.045	0.334	0.09	0.075	0.22	0.539
0.157	0.332	0.171	0.233	0.103	0.165	0.247	0.64
0.086	0.326	0.386	0.119	0.084	0.17	0.354	0.735
0.177	0.264	0.015	0.351	0.095	0.102	0.123	0.483
0.215	0.392	0.224	0.284	0.068	0.131	0.218	0.672
0.196	0.405	0.142	0.315	0.024	0.119	0.22	0.655
0.133	0.287	0.419	0.172	0.149	0.162	0.412	0.716
0.259	0.413	0.173	0.262	0.082	0.167	0.229	0.669
0.243	0.184	0.194	0.323	0.106	0.149	0.179	0.602
0.182	0.391	0.245	0.285	0.059	0.104	0.214	0.657
0.217	0.258	0.091	0.387	0.028	0	0.294	0.555
0.178	0.389	0.179	0.253	0.063	0.171	0.25	0.637
0.21	0.348	0.233	0.225	0.062	0.223	0.272	0.708
0.214	0.403	0.149	0.236	0.086	0.111	0.241	0.68
0.152	0.239	0.045	0.27	0.099	0.099	0.195	0.513
0.101	0.212	0.221	0.266	0.08	0.169	0.292	0.576
0.286	0.253	0.178	0.313	0.041	0.075	0.177	0.584
0.159	0.388	0.288	0.162	0.111	0.173	0.378	0.717
0.204	0.173	0.163	0.377	0.076	0.1	0.181	0.523
0.21	0.377	0.229	0.206	0.121	0.165	0.319	0.686
0.241	0.355	0.246	0.163	0.099	0.201	0.303	0.755
0.181	0.258	0.111	0.39	0.105	0.087	0.168	0.521
0.253	0.411	0.286	0.202	0.02	0.22	0.225	0.815
0.179	0.317	0.162	0.266	0.055	0.135	0.234	0.593
0.25	0.443	0.108	0.282	0.073	0.048	0.18	0.656
0.328	0.478	0.155	0.291	0.071	0.071	0.159	0.696
0	0.146	0.43	0	0.059	0.268	0.435	0.778
0.239	0.358	0.096	0.325	0	0.061	0.172	0.597
0.199	0.332	0.244	0.261	0.081	0.17	0.247	0.623
0.37	0.288	0.133	0.324	0.078	0.055	0.238	0.669
0.225	0.107	0.14	0.38	0.038	0.059	0.239	0.495

0.232	0.277	0.222	0.26	0.076	0.172	0.229	0.608
0.209	0.477	0.189	0.298	0.021	0.105	0.176	0.634
0.19	0.35	0.203	0.256	0.055	0.158	0.253	0.633
0.188	0.383	0.262	0.238	0.071	0.175	0.208	0.682
0.108	0.227	0.264	0.177	0.094	0.193	0.354	0.636
0.169	0.268	0.132	0.296	0.007	0.178	0.222	0.592
0.259	0.156	0.15	0.218	0.146	0.101	0.361	0.651
0.176	0.045	0.054	0.348	0.037	0.177	0.29	0.462
0.269	0.2	0.067	0.377	0.031	0.071	0.151	0.521
0.209	0.341	0.246	0.208	0.135	0.157	0.347	0.74
0.232	0.246	0.105	0.245	0.085	0.166	0.285	0.612
0.145	0.315	0.206	0.302	0.069	0.069	0.236	0.623
0.225	0.402	0.197	0.241	0.092	0.181	0.218	0.642
0.3	0.347	0.247	0.256	0.121	0.05	0.29	0.713
0.18	0.364	0.057	0.331	0.074	0.076	0.222	0.568
0.242	0.343	0.162	0.332	0.089	0.08	0.201	0.624
0.191	0.303	0.075	0.33	0.081	0.115	0.208	0.527
0.159	0.221	0.198	0.328	0.128	0.103	0.229	0.539
0.099	0.184	0.281	0.214	0.135	0.199	0.355	0.613
0.268	0.446	0.162	0.221	0.086	0.094	0.179	0.635
0.216	0.323	0.375	0.256	0.122	0.038	0.315	0.695
0.259	0.421	0.216	0.247	0.05	0.132	0.205	0.733
0.273	0.372	0.169	0.217	0.135	0.156	0.254	0.704
0.192	0.245	0.087	0.326	0.026	0.14	0.131	0.51
0.238	0.315	0.19	0.283	0.109	0.057	0.191	0.607
0.288	0.241	0.078	0.299	0.071	0.064	0.258	0.585
0.151	0.394	0.294	0.149	0.087	0.151	0.34	0.767
0.117	0.187	0.202	0.248	0.033	0.189	0.272	0.564
0.175	0.192	0.196	0.275	0.069	0.178	0.269	0.555
0.257	0.315	0.14	0.308	0	0.199	0.156	0.613
0.216	0.48	0.249	0.253	0.065	0.152	0.215	0.754
0.174	0.289	0.184	0.263	0.063	0.172	0.262	0.618
0.21	0.254	0.113	0.24	0.081	0.286	0.208	0.595
0.249	0.329	0.043	0.395	0.033	0.15	0.071	0.542
0.231	0.196	0.122	0.351	0.038	0.096	0.145	0.517
0.095	0.24	0.385	0.188	0.145	0.218	0.399	0.683
0.245	0.265	0.018	0.309	0.059	0.154	0.191	0.57
0.192	0.4	0.219	0.291	0.04	0.12	0.202	0.659
0.271	0.482	0.073	0.305	0.048	0.094	0.184	0.615
0.277	0.476	0.154	0.329	0.141	0.087	0.192	0.743
0.239	0.241	0.087	0.353	0.054	0.091	0.142	0.541
0.242	0.302	0.184	0.185	0.138	0.198	0.31	0.664
0.134	0.296	0.143	0.254	0.03	0.218	0.254	0.615
0.105	0.263	0.246	0.207	0.083	0.18	0.299	0.646
0.357	0.555	0.347	0.278	0.075	0.161	0.21	0.9
0.194	0.111	0.159	0.362	0.049	0.083	0.194	0.474
0.224	0.214	0.083	0.375	0.093	0.11	0.168	0.573
0.152	0.277	0.14	0.225	0.142	0.182	0.299	0.61
0.199	0.289	0.074	0.34	0.038	0.063	0.177	0.548
0.159	0.413	0.372	0.221	0.07	0.113	0.319	0.806
0.217	0.143	0.212	0.371	0.008	0.154	0.053	0.482
0.299	0.398	0.117	0.326	0.103	0.096	0.191	0.614
0.243	0.465	0.222	0.227	0.071	0.15	0.238	0.679

0.142	0.291	0.171	0.25	0.115	0.163	0.259	0.591
0.209	0.316	0.121	0.346	0.049	0.082	0.199	0.588
0.313	0.215	0.183	0.413	0.02	0.186	0.201	0.575
0.25	0.327	0.158	0.246	0.108	0.16	0.178	0.651
0.25	0.209	0.143	0.35	0.032	0.115	0.111	0.524
0.15	0.147	0.087	0.368	0.043	0.111	0.121	0.416
0.244	0.194	0.157	0.362	0.026	0.043	0.193	0.556
0.229	0.417	0.116	0.284	0.045	0.131	0.195	0.626
0.293	0.391	0.09	0.298	0.058	0.119	0.231	0.67
0.084	0.257	0.411	0.132	0.13	0.199	0.421	0.765
0.272	0.39	0.254	0.217	0.111	0.121	0.249	0.68
0.245	0.368	0.12	0.323	0.023	0.095	0.196	0.622
0.135	0.173	0.098	0.407	0.036	0.018	0.156	0.485
0.242	0.503	0.193	0.268	0.134	0.098	0.214	0.708
0.147	0.26	0.27	0.253	0.13	0.137	0.328	0.623
0.405	0.414	0.156	0.215	0.129	0.141	0.255	0.708
0.186	0.374	0.117	0.285	0.077	0.1	0.266	0.605
0.372	0.442	0.039	0.358	0.023	0.061	0.013	0.601
0.214	0.171	0.215	0.33	0.029	0.143	0.167	0.541
0.226	0.256	0.089	0.345	0.087	0.15	0.13	0.515
0.235	0.379	0.141	0.272	0.084	0.133	0.235	0.637
0.137	0.255	0.149	0.273	0.139	0.137	0.329	0.578
0.221	0.375	0.044	0.299	0.038	0.116	0.213	0.63
0.164	0.398	0.28	0.304	0.034	0.154	0.228	0.678
0.2	0.426	0.098	0.327	0.074	0.101	0.177	0.608
0.179	0.169	0.209	0.333	0.092	0.125	0.204	0.567
0.165	0.391	0.307	0.137	0.093	0.138	0.337	0.803
0.233	0.343	0.062	0.265	0.043	0.13	0.216	0.616
0.188	0.309	0.151	0.37	0.124	0.095	0.278	0.618
0.284	0.322	0.19	0.263	0.037	0.177	0.239	0.698
0.181	0.395	0.353	0.209	0.124	0.208	0.319	0.754
0.24	0.218	0.086	0.355	0.001	0.114	0.176	0.524
0.206	0.334	0.27	0.267	0.114	0.059	0.227	0.621
0.242	0.38	0.084	0.345	0.011	0.084	0.125	0.635
0.195	0.286	0.105	0.314	0.04	0.167	0.224	0.569
0.129	0.284	0.197	0.278	0.167	0.12	0.295	0.593
0.213	0.243	0.236	0.29	0.022	0.226	0.236	0.624
0.113	0.294	0.212	0.233	0.114	0.146	0.286	0.625
0.326	0.266	0.167	0.298	0.014	0.132	0.211	0.614
0.194	0.242	0.207	0.224	0	0.237	0.177	0.644
0.213	0.163	0.262	0.272	0.131	0.183	0.313	0.625
0.178	0.329	0.124	0.343	0.011	0.057	0.211	0.571
0.26	0.275	0.185	0.366	0.015	0.093	0.179	0.607
0.247	0.449	0.165	0.259	0.058	0.122	0.204	0.658
0.194	0.329	0.262	0.195	0.173	0.157	0.397	0.691
0.273	0.372	0.073	0.314	0.046	0.09	0.174	0.646
0.156	0.374	0.339	0.168	0.112	0.178	0.413	0.794
0.241	0.159	0.25	0.296	0.125	0.158	0.25	0.597
0.246	0.317	0.134	0.301	0.011	0.118	0.161	0.599
0.275	0.214	0.296	0.198	0.152	0.143	0.357	0.646
0.273	0.313	0.266	0.26	0.06	0.19	0.256	0.695
0.315	0.382	0.139	0.318	0.039	0.137	0.17	0.675
0.268	0.435	0.046	0.273	0.059	0.146	0.213	0.668

0.2	0.123	0.255	0.237	0.16	0.154	0.38	0.609
0.203	0.179	0.139	0.317	0.034	0.131	0.266	0.575
0.212	0.384	0.228	0.247	0.059	0.118	0.195	0.667
0.227	0.355	0.142	0.302	0.073	0.139	0.249	0.64
0.247	0.386	0.27	0.193	0.09	0.129	0.285	0.775
0.209	0.252	0.191	0.339	0	0.121	0.207	0.587
0.085	0.291	0.333	0.136	0.132	0.196	0.352	0.662
0.255	0.179	0.139	0.354	0	0.104	0.16	0.522
0.217	0.465	0.384	0.228	0.083	0.113	0.327	0.84
0.327	0.36	0.161	0.324	0.113	0.096	0.183	0.695
0.149	0.271	0.265	0.25	0.178	0.085	0.36	0.648
0.271	0.391	0.292	0.191	0.104	0.137	0.309	0.797
0.184	0.325	0.135	0.271	0.136	0.096	0.208	0.58
0.15	0.363	0.322	0.188	0.116	0.149	0.381	0.764
0.211	0.42	0.201	0.266	0.077	0.141	0.249	0.655
0.276	0.364	0.096	0.325	0	0.113	0.143	0.604
0.194	0.344	0.144	0.294	0.001	0.137	0.217	0.606
0.224	0.286	0.175	0.24	0.127	0.123	0.287	0.595
0.195	0.309	0.089	0.376	0	0.076	0.203	0.542
0.241	0.344	0.162	0.307	0.053	0.123	0.187	0.649
0.192	0.383	0.054	0.294	0.014	0.137	0.212	0.599
0.142	0.297	0.37	0.117	0.148	0.186	0.455	0.824
0.245	0.276	0.167	0.302	0	0.086	0.158	0.55
0.21	0.212	0.105	0.407	0.131	0.033	0.165	0.499
0.254	0.291	0.129	0.391	0	0.195	0.109	0.605
0.273	0.389	0.112	0.295	0.039	0.073	0.171	0.597
0.218	0.488	0.252	0.224	0.105	0.159	0.326	0.826
0.268	0.329	0.237	0.245	0.071	0.197	0.233	0.704
0.254	0.474	0.123	0.273	0.036	0.103	0.139	0.646
0.231	0.36	0.16	0.373	0.012	0.116	0.104	0.607
0.224	0.285	0.229	0.246	0.055	0.216	0.277	0.701
0.278	0.519	0.05	0.3	0.025	0.08	0.243	0.698
0.281	0.33	0.1	0.307	0.075	0.104	0.26	0.614
0.199	0.275	0.228	0.26	0.065	0.186	0.291	0.646
0.138	0.256	0.267	0.196	0.092	0.178	0.329	0.646
0.185	0.304	0.09	0.277	0.144	0.126	0.267	0.612
0.191	0.224	0.164	0.255	0.112	0.109	0.265	0.614
0.189	0.379	0.238	0.176	0.082	0.199	0.306	0.76
0.263	0.246	0.104	0.318	0.04	0.116	0.206	0.563
0.202	0.16	0.083	0.337	0.082	0.133	0.275	0.554
0.128	0.191	0.235	0.266	0.049	0.198	0.331	0.616
0.208	0.214	0.108	0.331	0	0.128	0.261	0.579
0.199	0.369	0.182	0.259	0.13	0.087	0.297	0.642
0.232	0.223	0.279	0.294	0.046	0.156	0.199	0.641
0.205	0.379	0.259	0.248	0.098	0.143	0.233	0.656
0.221	0.392	0.033	0.359	0.012	0.047	0.04	0.528
0.23	0.231	0.104	0.342	0.07	0.146	0.14	0.528
0.109	0.163	0.228	0.26	0.123	0.147	0.28	0.565
0.269	0.382	0.229	0.295	0.111	0.158	0.213	0.66
0.138	0.392	0.275	0.208	0.081	0.179	0.312	0.695
0.247	0.349	0.192	0.268	0.135	0.114	0.306	0.689
0.137	0.346	0.213	0.343	0.053	0.045	0.279	0.611
0.251	0.344	0.212	0.187	0.096	0.211	0.317	0.738

0.295	0.383	0.144	0.289	0.04	0.087	0.143	0.614
0.327	0.51	0.455	0.226	0.073	0.073	0.243	0.807
0.253	0.389	0.116	0.288	0.1	0.045	0.205	0.607
0.243	0.286	0.283	0.23	0.153	0.076	0.333	0.686
0.197	0.399	0.14	0.263	0.095	0.158	0.27	0.673
0.2	0.228	0.142	0.32	0.065	0.106	0.306	0.603
0.21	0.371	0.214	0.207	0.061	0.182	0.293	0.703
0.289	0.459	0.191	0.275	0.05	0.13	0.15	0.69
0.201	0.27	0.139	0.326	0.105	0.123	0.222	0.622
0.357	0.478	0.256	0.248	0.038	0.158	0.23	0.751
0.284	0.278	0.146	0.375	0	0.062	0.093	0.547
0.22	0.332	0.146	0.354	0	0.168	0.087	0.598
0.272	0.227	0.117	0.333	0.064	0.091	0.187	0.579
0.183	0.208	0.14	0.276	0.142	0.15	0.318	0.556
0.193	0.415	0.188	0.195	0.078	0.135	0.23	0.736
0.237	0.405	0.338	0.251	0.081	0.189	0.179	0.76
0.247	0.387	0.185	0.284	0.103	0.133	0.251	0.669
0.173	0.405	0.138	0.361	0.049	0.082	0	0.578
0.244	0.386	0.166	0.245	0.07	0.173	0.23	0.687
0.177	0.3	0.246	0.244	0.109	0.126	0.361	0.73
0.294	0.382	0.051	0.36	0	0.108	0.016	0.591
0.273	0.462	0.158	0.3	0.065	0.068	0.188	0.662
0.258	0.393	0.098	0.318	0	0.121	0.091	0.585
0.29	0.297	0.115	0.327	0.089	0.105	0.228	0.612
0.253	0.22	0.328	0.222	0.075	0.216	0.268	0.637
0.222	0.261	0.059	0.351	0	0.066	0.175	0.49
0.151	0.318	0.185	0.246	0.085	0.194	0.309	0.583
0.223	0.352	0.328	0.197	0.048	0.227	0.251	0.735
0.257	0.267	0.072	0.371	0	0.103	0.199	0.56
0.189	0.268	0.151	0.291	0.048	0.049	0.214	0.554
0.212	0.281	0.101	0.254	0.105	0.168	0.297	0.608
0.128	0.327	0.291	0.171	0.1	0.193	0.315	0.7
0.375	0.319	0.084	0.26	0.137	0.143	0.057	0.628
0.277	0.393	0.281	0.285	0.09	0.112	0.242	0.795
0.24	0.348	0.355	0.135	0.092	0.191	0.311	0.849
0.235	0.197	0.076	0.331	0.024	0.113	0.134	0.476
0.3	0.261	0.11	0.298	0.057	0.165	0.198	0.593
0.13	0.222	0.234	0.28	0.124	0.134	0.33	0.624
0.066	0.284	0.413	0.139	0.134	0.16	0.424	0.758
0.18	0.195	0.158	0.34	0	0.11	0.25	0.56
0.215	0.393	0.136	0.288	0.011	0.13	0.255	0.652
0.176	0.269	0.168	0.283	0.038	0.182	0.255	0.587
0.195	0.387	0.166	0.208	0.045	0.199	0.247	0.644
0.178	0.256	0.248	0.226	0.057	0.152	0.304	0.61
0.241	0.349	0.155	0.256	0.084	0.163	0.231	0.663
0.204	0.245	0.243	0.279	0.071	0.153	0.207	0.633
0.182	0.126	0.324	0.299	0.064	0.136	0.208	0.601
0.291	0.309	0.246	0.335	0.06	0.09	0.141	0.679
0.221	0.322	0.312	0.295	0.098	0.149	0.189	0.695
0.13	0.102	0.259	0.301	0.044	0.1	0.214	0.51
0.18	0.269	0.324	0.289	0.044	0.188	0.207	0.654
0.198	0.22	0.333	0.291	0.076	0.178	0.19	0.633
0.184	0.091	0.289	0.268	0.089	0.16	0.239	0.551

0.275	0.25	0.185	0.342	0	0.137	0.114	0.619
0.16	0.179	0.355	0.281	0.051	0.144	0.197	0.62
0.18	0.137	0.27	0.352	0.06	0.113	0.09	0.437
0.147	0.347	0.383	0.198	0.103	0.193	0.247	0.742
0.218	0.25	0.375	0.283	0.082	0.137	0.169	0.7
0.258	0.29	0.264	0.35	0.012	0.155	0.14	0.666
0.224	0.294	0.284	0.35	0.065	0.145	0.241	0.712
0.223	0.21	0.353	0.269	0.082	0.149	0.226	0.622
0.207	0.12	0.184	0.322	0.07	0.094	0.214	0.54
0.174	0.243	0.288	0.239	0.069	0.171	0.268	0.68
0.112	0.239	0.35	0.244	0.065	0.234	0.241	0.658
0.159	0.125	0.331	0.247	0.063	0.174	0.298	0.596
0.212	0.097	0.273	0.39	0.121	0.067	0.128	0.497
0.229	0.309	0.355	0.254	0.061	0.171	0.141	0.7
0.168	0.178	0.195	0.244	0.074	0.208	0.247	0.555
0.224	0.36	0.264	0.252	0.083	0.19	0.208	0.719
0.182	0.128	0.32	0.256	0.041	0.222	0.265	0.63
0.071	0.192	0.4	0.098	0.045	0.25	0.318	0.788
0.144	0.31	0.306	0.245	0.05	0.209	0.218	0.7
0.155	0.114	0.33	0.251	0.054	0.206	0.249	0.581
0.149	0.209	0.303	0.279	0.08	0.111	0.247	0.591
0.184	0.301	0.267	0.266	0.008	0.155	0.191	0.654
0.193	0.119	0.269	0.298	0.092	0.216	0.244	0.567
0.22	0.157	0.233	0.319	0.05	0.13	0.157	0.547
0.167	0.176	0.31	0.259	0.003	0.193	0.199	0.609
0.161	0.135	0.286	0.266	0.043	0.154	0.239	0.572
0.188	0.198	0.288	0.276	0.058	0.147	0.229	0.609
0.166	0.112	0.28	0.305	0.047	0.153	0.174	0.521
0.228	0.312	0.178	0.27	0.008	0.15	0.138	0.629
0.254	0.292	0.284	0.311	0.048	0.075	0.172	0.648
0.231	0.295	0.356	0.306	0.094	0.199	0.204	0.692
0.187	0.124	0.252	0.286	0.053	0.16	0.253	0.581
0.166	0.19	0.203	0.274	0.044	0.206	0.213	0.599
0.329	0.358	0.306	0.337	0.126	0.059	0.145	0.739
0.19	0.192	0.309	0.282	0.032	0.162	0.228	0.618
0.127	0.146	0.277	0.307	0.088	0.139	0.226	0.56
0.102	0.105	0.2	0.266	0.031	0.186	0.301	0.558
0.308	0.379	0.234	0.363	0.085	0.097	0.147	0.727
0.114	0.105	0.269	0.296	0.093	0.151	0.299	0.574
0.14	0.112	0.279	0.292	0.072	0.183	0.285	0.593
0.138	0.135	0.244	0.291	0.04	0.158	0.293	0.594
0.394	0.473	0.247	0.336	0.071	0.095	0.07	0.791
0.203	0.3	0.325	0.226	0.128	0.178	0.228	0.702
0.174	0.261	0.353	0.28	0.085	0.139	0.235	0.655
0.199	0.303	0.316	0.235	0.102	0.204	0.238	0.691
0.247	0.243	0.384	0.285	0.095	0.16	0.205	0.695
0.144	0.079	0.218	0.321	0.039	0.147	0.241	0.543
0.128	0.224	0.449	0.284	0.128	0.115	0.248	0.666
0.193	0.283	0.219	0.306	0.024	0.139	0.178	0.616
0.195	0.3	0.214	0.266	0.004	0.14	0.159	0.642
0.215	0.369	0.419	0.308	0.042	0.129	0.178	0.751
0.248	0.312	0.262	0.347	0.084	0.072	0.153	0.637
0.142	0.354	0.406	0.29	0.103	0.133	0.257	0.709

0.15	0.271	0.438	0.256	0.112	0.171	0.226	0.684
0.237	0.264	0.385	0.282	0.108	0.079	0.212	0.669
0.285	0.255	0.288	0.337	0.061	0.07	0.068	0.633
0.144	0.084	0.261	0.285	0.047	0.185	0.253	0.556
0.209	0.371	0.274	0.274	0.129	0.165	0.244	0.77
0.325	0.395	0.327	0.305	0.067	0.098	0.165	0.723
0.301	0.326	0.331	0.359	0.112	0.046	0.159	0.69
0.148	0.207	0.23	0.334	0.01	0.124	0.152	0.576
0.142	0.092	0.348	0.285	0.097	0.2	0.3	0.596
0.234	0.293	0.223	0.353	0.086	0.045	0.153	0.625
0.078	0.195	0.269	0.26	0.105	0.187	0.284	0.619
0.266	0.254	0.217	0.382	0.071	0.044	0.106	0.575
0.172	0.351	0.29	0.228	0.093	0.204	0.232	0.725
0.166	0.092	0.261	0.269	0.105	0.178	0.207	0.528
0.281	0.383	0.236	0.308	0.02	0.109	0.129	0.716
0.237	0.257	0.352	0.236	0.073	0.222	0.27	0.74
0.282	0.35	0.306	0.312	0.046	0.076	0.159	0.739
0.228	0.22	0.314	0.337	0.154	0.046	0.206	0.608
0.21	0.435	0.367	0.269	0.072	0.126	0.21	0.767
0.196	0.285	0.318	0.297	0.046	0.061	0.205	0.666
0.153	0	0.259	0.337	0	0.148	0.233	0.481
0.207	0.315	0.291	0.293	0.05	0.137	0.177	0.691
0.277	0.192	0.202	0.379	0.08	0.047	0.204	0.551
0.253	0.424	0.291	0.232	0.097	0.193	0.23	0.787
0.125	0.24	0.293	0.243	0.082	0.191	0.251	0.598
0.09	0.014	0.3	0.263	0.06	0.156	0.338	0.522
0.11	0.039	0.265	0.318	0.067	0.12	0.274	0.508
0.205	0.332	0.305	0.266	0.099	0.143	0.254	0.725
0.243	0.343	0.224	0.273	0.004	0.223	0.215	0.727
0.285	0.351	0.174	0.341	0.053	0.062	0.137	0.678
0.257	0.354	0.324	0.297	0.029	0.129	0.106	0.74
0.157	0.296	0.337	0.265	0.075	0.171	0.237	0.688
0.188	0.207	0.25	0.287	0.007	0.102	0.193	0.58
0.213	0.306	0.319	0.293	0.047	0.16	0.181	0.66
0.226	0.336	0.454	0.262	0.149	0.149	0.294	0.795
0.235	0.191	0.288	0.321	0.052	0.15	0.143	0.605
0.272	0.533	0.354	0.254	0.098	0.108	0.226	0.871
0.27	0.369	0.264	0.321	0.024	0.125	0.156	0.711
0.288	0.29	0.239	0.37	0.071	0.061	0.08	0.61
0.182	0.166	0.324	0.274	0.089	0.209	0.248	0.616
0.215	0.362	0.427	0.298	0.068	0.131	0.132	0.73
0.243	0.333	0.284	0.287	0.108	0.109	0.192	0.687
0.143	0.161	0.224	0.283	0.049	0.172	0.246	0.559
0.298	0.342	0.275	0.286	0.095	0.077	0.206	0.733
0.358	0.467	0.218	0.362	0.073	0.099	0.129	0.814
0.164	0.176	0.292	0.263	0.095	0.146	0.286	0.579
0.159	0.161	0.311	0.275	0.057	0.155	0.256	0.634
0.198	0.113	0.252	0.323	0.081	0.083	0.212	0.506
0.137	0.146	0.313	0.249	0.025	0.185	0.268	0.596
0.293	0.477	0.305	0.336	0.076	0.092	0.199	0.822
0.178	0.089	0.205	0.324	0.021	0.152	0.195	0.531
0.203	0.26	0.341	0.293	0.126	0.1	0.188	0.659
