# Supplementary material for: A ferroptosis-associated gene signature for the prediction of prognosis and therapeutic response in luminal-type breast carcinoma
Source: Sci Rep. 2021 Sep 2;11:17610. doi: 10.1038/s41598-021-97102-z (PMC8413464; doi:10.1038/s41598-021-97102-z)
Supplement: Supplementary file 12 — Supplementary Table S8. [file 41598_2021_97102_MOESM12_ESM.pdf]

TableS8 The correlation between risk score and pathway score

	gene	mRNAs	cor	p.value
cor1	riskScore	TumorPurity	0.315705072659519	8.00452883883463E-14
cor2	riskScore	ProliferationScore	0.683130051295377	2.16555359394587E-80
cor3	riskScore	Apoptosis score	0.175483894735102	0.000223558698575
cor4	riskScore	Cell cycle score	0.559759427552205	1.74902036451691E-37
cor5	riskScore	DNA damage response score	0.235286820903646	6.35175316747666E-07
cor6	riskScore	EMT score	-0.270006286876436	9.37594319568351E-09
cor7	riskScore	Hormone_a score	0.191684053927787	5.39806206027592E-05
cor8	riskScore	Hormone_b score	0.027583687777354	0.564788425709768
cor9	riskScore	PI3K/Akt score	-0.119241263024333	0.01251362429265
cor10	riskScore	Ras/MAPK score	-0.32789930820089	1.94545401948275E-12
cor11	riskScore	RTK score	-0.110871214977504	0.020292709539853
cor12	riskScore	TSC/mTOR score	0.177143938523084	0.000194338840829
