# Supplementary material for: A ferroptosis-associated gene signature for the prediction of prognosis and therapeutic response in luminal-type breast carcinoma
Source: Sci Rep. 2021 Sep 2;11:17610. doi: 10.1038/s41598-021-97102-z (PMC8413464; doi:10.1038/s41598-021-97102-z)
Supplement: Supplementary file 15 — Supplementary Table S11. [file 41598_2021_97102_MOESM15_ESM.pdf]

Table S11: Estimated AUC value of commonly used ferroptosis inducer

	drug	auc	p	s	group
1	erastin	0.422826	1.32E-05	***	High risk
2	erastin	0.451664	1.32E-05	***	High risk
3	erastin	0.425867	1.32E-05	***	High risk
4	erastin	0.406067	1.32E-05	***	High risk
5	erastin	0.414931	1.32E-05	***	High risk
6	erastin	0.423308	1.32E-05	***	High risk
7	erastin	0.419738	1.32E-05	***	High risk
8	erastin	0.380552	1.32E-05	***	High risk
9	erastin	0.424647	1.32E-05	***	High risk
10	erastin	0.446905	1.32E-05	***	High risk
11	erastin	0.438775	1.32E-05	***	High risk
12	erastin	0.437646	1.32E-05	***	High risk
13	erastin	0.432948	1.32E-05	***	High risk
14	erastin	0.428263	1.32E-05	***	High risk
15	erastin	0.423752	1.32E-05	***	High risk
16	erastin	0.410177	1.32E-05	***	High risk
17	erastin	0.446187	1.32E-05	***	High risk
18	erastin	0.462996	1.32E-05	***	High risk
19	erastin	0.40074	1.32E-05	***	High risk
20	erastin	0.464462	1.32E-05	***	High risk
21	erastin	0.439769	1.32E-05	***	High risk
22	erastin	0.424103	1.32E-05	***	High risk
23	erastin	0.443375	1.32E-05	***	High risk
24	erastin	0.43656	1.32E-05	***	High risk
25	erastin	0.41782	1.32E-05	***	High risk
26	erastin	0.440252	1.32E-05	***	High risk
27	erastin	0.424573	1.32E-05	***	High risk
28	erastin	0.447964	1.32E-05	***	High risk
29	erastin	0.421448	1.32E-05	***	High risk
30	erastin	0.431127	1.32E-05	***	High risk
31	erastin	0.44389	1.32E-05	***	High risk
32	erastin	0.417457	1.32E-05	***	High risk
33	erastin	0.434769	1.32E-05	***	High risk
34	erastin	0.417776	1.32E-05	***	High risk
35	erastin	0.439755	1.32E-05	***	High risk
36	erastin	0.427645	1.32E-05	***	High risk
37	erastin	0.427186	1.32E-05	***	High risk
38	erastin	0.405368	1.32E-05	***	High risk
39	erastin	0.448875	1.32E-05	***	High risk
40	erastin	0.428564	1.32E-05	***	High risk
41	erastin	0.451861	1.32E-05	***	High risk
42	erastin	0.410911	1.32E-05	***	High risk
43	erastin	0.429275	1.32E-05	***	High risk
44	erastin	0.417514	1.32E-05	***	High risk
45	erastin	0.414849	1.32E-05	***	High risk
46	erastin	0.38549	1.32E-05	***	High risk
47	erastin	0.441779	1.32E-05	***	High risk
48	erastin	0.44316	1.32E-05	***	High risk
49	erastin	0.420755	1.32E-05	***	High risk
50	erastin	0.395946	1.32E-05	***	High risk

51	erastin	0.418678	1.32E-05	***	High risk
52	erastin	0.424739	1.32E-05	***	High risk
53	erastin	0.423506	1.32E-05	***	High risk
54	erastin	0.446026	1.32E-05	***	High risk
55	erastin	0.43939	1.32E-05	***	High risk
56	erastin	0.432384	1.32E-05	***	High risk
57	erastin	0.440057	1.32E-05	***	High risk
58	erastin	0.417461	1.32E-05	***	High risk
59	erastin	0.409138	1.32E-05	***	High risk
60	erastin	0.413692	1.32E-05	***	High risk
61	erastin	0.423032	1.32E-05	***	High risk
62	erastin	0.41945	1.32E-05	***	High risk
63	erastin	0.437118	1.32E-05	***	High risk
64	erastin	0.429073	1.32E-05	***	High risk
65	erastin	0.412834	1.32E-05	***	High risk
66	erastin	0.431707	1.32E-05	***	High risk
67	erastin	0.451536	1.32E-05	***	High risk
68	erastin	0.401219	1.32E-05	***	High risk
69	erastin	0.415064	1.32E-05	***	High risk
70	erastin	0.427692	1.32E-05	***	High risk
71	erastin	0.3814	1.32E-05	***	High risk
72	erastin	0.4164	1.32E-05	***	High risk
73	erastin	0.395207	1.32E-05	***	High risk
74	erastin	0.418158	1.32E-05	***	High risk
75	erastin	0.407772	1.32E-05	***	High risk
76	erastin	0.4225	1.32E-05	***	High risk
77	erastin	0.399149	1.32E-05	***	Low risk
78	erastin	0.413322	1.32E-05	***	Low risk
79	erastin	0.416261	1.32E-05	***	Low risk
80	erastin	0.40891	1.32E-05	***	Low risk
81	erastin	0.414857	1.32E-05	***	Low risk
82	erastin	0.447635	1.32E-05	***	Low risk
83	erastin	0.429979	1.32E-05	***	Low risk
84	erastin	0.416771	1.32E-05	***	Low risk
85	erastin	0.413981	1.32E-05	***	Low risk
86	erastin	0.398449	1.32E-05	***	Low risk
87	erastin	0.409522	1.32E-05	***	Low risk
88	erastin	0.416792	1.32E-05	***	Low risk
89	erastin	0.415941	1.32E-05	***	Low risk
90	erastin	0.390474	1.32E-05	***	Low risk
91	erastin	0.405089	1.32E-05	***	Low risk
92	erastin	0.4165	1.32E-05	***	Low risk
93	erastin	0.406964	1.32E-05	***	Low risk
94	erastin	0.421385	1.32E-05	***	Low risk
95	erastin	0.417202	1.32E-05	***	Low risk
96	erastin	0.405212	1.32E-05	***	Low risk
97	erastin	0.419997	1.32E-05	***	Low risk
98	erastin	0.415795	1.32E-05	***	Low risk
99	erastin	0.421936	1.32E-05	***	Low risk
100	erastin	0.414556	1.32E-05	***	Low risk
101	erastin	0.442021	1.32E-05	***	Low risk

102	erastin	0.42129	1.32E-05	***	Low risk
103	erastin	0.398463	1.32E-05	***	Low risk
104	erastin	0.43793	1.32E-05	***	Low risk
105	erastin	0.396509	1.32E-05	***	Low risk
106	erastin	0.442066	1.32E-05	***	Low risk
107	erastin	0.409035	1.32E-05	***	Low risk
108	erastin	0.421879	1.32E-05	***	Low risk
109	erastin	0.413208	1.32E-05	***	Low risk
110	erastin	0.424228	1.32E-05	***	Low risk
111	erastin	0.399306	1.32E-05	***	Low risk
112	erastin	0.410923	1.32E-05	***	Low risk
113	erastin	0.40604	1.32E-05	***	Low risk
114	erastin	0.435818	1.32E-05	***	Low risk
115	erastin	0.419651	1.32E-05	***	Low risk
116	erastin	0.41985	1.32E-05	***	Low risk
117	erastin	0.397433	1.32E-05	***	Low risk
118	erastin	0.408556	1.32E-05	***	Low risk
119	erastin	0.404637	1.32E-05	***	Low risk
120	erastin	0.406755	1.32E-05	***	Low risk
121	erastin	0.413937	1.32E-05	***	Low risk
122	erastin	0.425873	1.32E-05	***	Low risk
123	erastin	0.420057	1.32E-05	***	Low risk
124	erastin	0.427411	1.32E-05	***	Low risk
125	erastin	0.422392	1.32E-05	***	Low risk
126	erastin	0.448641	1.32E-05	***	Low risk
127	erastin	0.434755	1.32E-05	***	Low risk
128	erastin	0.385932	1.32E-05	***	Low risk
129	erastin	0.400345	1.32E-05	***	Low risk
130	erastin	0.37755	1.32E-05	***	Low risk
131	erastin	0.4306	1.32E-05	***	Low risk
132	erastin	0.428725	1.32E-05	***	Low risk
133	erastin	0.416166	1.32E-05	***	Low risk
134	erastin	0.421415	1.32E-05	***	Low risk
135	erastin	0.411103	1.32E-05	***	Low risk
136	erastin	0.423516	1.32E-05	***	Low risk
137	erastin	0.389112	1.32E-05	***	Low risk
138	erastin	0.401569	1.32E-05	***	Low risk
139	erastin	0.410615	1.32E-05	***	Low risk
140	erastin	0.404405	1.32E-05	***	Low risk
141	erastin	0.434028	1.32E-05	***	Low risk
142	erastin	0.402211	1.32E-05	***	Low risk
143	erastin	0.389079	1.32E-05	***	Low risk
144	erastin	0.405082	1.32E-05	***	Low risk
145	erastin	0.458547	1.32E-05	***	Low risk
146	erastin	0.419562	1.32E-05	***	Low risk
147	erastin	0.408979	1.32E-05	***	Low risk
148	erastin	0.40492	1.32E-05	***	Low risk
149	erastin	0.40836	1.32E-05	***	Low risk
150	erastin	0.417616	1.32E-05	***	Low risk
151	erastin	0.432877	1.32E-05	***	Low risk
152	erastin	0.39365	1.32E-05	***	Low risk

153	1S,3R-RSL-	0.273151	1.42E-10	***	High risk
154	1S,3R-RSL-	0.292643	1.42E-10	***	High risk
155	1S,3R-RSL-	0.266164	1.42E-10	***	High risk
156	1S,3R-RSL-	0.257333	1.42E-10	***	High risk
157	1S,3R-RSL-	0.273085	1.42E-10	***	High risk
158	1S,3R-RSL-	0.284835	1.42E-10	***	High risk
159	1S,3R-RSL-	0.336799	1.42E-10	***	High risk
160	1S,3R-RSL-	0.250157	1.42E-10	***	High risk
161	1S,3R-RSL-	0.296934	1.42E-10	***	High risk
162	1S,3R-RSL-	0.317451	1.42E-10	***	High risk
163	1S,3R-RSL-	0.291938	1.42E-10	***	High risk
164	1S,3R-RSL-	0.305095	1.42E-10	***	High risk
165	1S,3R-RSL-	0.278224	1.42E-10	***	High risk
166	1S,3R-RSL-	0.233493	1.42E-10	***	High risk
167	1S,3R-RSL-	0.247264	1.42E-10	***	High risk
168	1S,3R-RSL-	0.28322	1.42E-10	***	High risk
169	1S,3R-RSL-	0.295144	1.42E-10	***	High risk
170	1S,3R-RSL-	0.358918	1.42E-10	***	High risk
171	1S,3R-RSL-	0.257537	1.42E-10	***	High risk
172	1S,3R-RSL-	0.322736	1.42E-10	***	High risk
173	1S,3R-RSL-	0.279674	1.42E-10	***	High risk
174	1S,3R-RSL-	0.290201	1.42E-10	***	High risk
175	1S,3R-RSL-	0.286234	1.42E-10	***	High risk
176	1S,3R-RSL-	0.297095	1.42E-10	***	High risk
177	1S,3R-RSL-	0.296315	1.42E-10	***	High risk
178	1S,3R-RSL-	0.322076	1.42E-10	***	High risk
179	1S,3R-RSL-	0.250877	1.42E-10	***	High risk
180	1S,3R-RSL-	0.302315	1.42E-10	***	High risk
181	1S,3R-RSL-	0.283131	1.42E-10	***	High risk
182	1S,3R-RSL-	0.288105	1.42E-10	***	High risk
183	1S,3R-RSL-	0.282643	1.42E-10	***	High risk
184	1S,3R-RSL-	0.262325	1.42E-10	***	High risk
185	1S,3R-RSL-	0.243947	1.42E-10	***	High risk
186	1S,3R-RSL-	0.275262	1.42E-10	***	High risk
187	1S,3R-RSL-	0.296034	1.42E-10	***	High risk
188	1S,3R-RSL-	0.222581	1.42E-10	***	High risk
189	1S,3R-RSL-	0.285066	1.42E-10	***	High risk
190	1S,3R-RSL-	0.262305	1.42E-10	***	High risk
191	1S,3R-RSL-	0.299446	1.42E-10	***	High risk
192	1S,3R-RSL-	0.287194	1.42E-10	***	High risk
193	1S,3R-RSL-	0.321468	1.42E-10	***	High risk
194	1S,3R-RSL-	0.274482	1.42E-10	***	High risk
195	1S,3R-RSL-	0.315391	1.42E-10	***	High risk
196	1S,3R-RSL-	0.251093	1.42E-10	***	High risk
197	1S,3R-RSL-	0.308859	1.42E-10	***	High risk
198	1S,3R-RSL-	0.274216	1.42E-10	***	High risk
199	1S,3R-RSL-	0.298839	1.42E-10	***	High risk
200	1S,3R-RSL-	0.268054	1.42E-10	***	High risk
201	1S,3R-RSL-	0.253672	1.42E-10	***	High risk
202	1S,3R-RSL-	0.230966	1.42E-10	***	High risk
203	1S,3R-RSL-	0.2804	1.42E-10	***	High risk

204	1S,3R-RSL-	0.259393	1.42E-10	***	High risk
205	1S,3R-RSL-	0.257158	1.42E-10	***	High risk
206	1S,3R-RSL-	0.337307	1.42E-10	***	High risk
207	1S,3R-RSL-	0.305625	1.42E-10	***	High risk
208	1S,3R-RSL-	0.286198	1.42E-10	***	High risk
209	1S,3R-RSL-	0.26579	1.42E-10	***	High risk
210	1S,3R-RSL-	0.283865	1.42E-10	***	High risk
211	1S,3R-RSL-	0.26703	1.42E-10	***	High risk
212	1S,3R-RSL-	0.306262	1.42E-10	***	High risk
213	1S,3R-RSL-	0.263225	1.42E-10	***	High risk
214	1S,3R-RSL-	0.308085	1.42E-10	***	High risk
215	1S,3R-RSL-	0.321516	1.42E-10	***	High risk
216	1S,3R-RSL-	0.295459	1.42E-10	***	High risk
217	1S,3R-RSL-	0.271715	1.42E-10	***	High risk
218	1S,3R-RSL-	0.293954	1.42E-10	***	High risk
219	1S,3R-RSL-	0.337375	1.42E-10	***	High risk
220	1S,3R-RSL-	0.256796	1.42E-10	***	High risk
221	1S,3R-RSL-	0.250181	1.42E-10	***	High risk
222	1S,3R-RSL-	0.276663	1.42E-10	***	High risk
223	1S,3R-RSL-	0.273162	1.42E-10	***	High risk
224	1S,3R-RSL-	0.283947	1.42E-10	***	High risk
225	1S,3R-RSL-	0.258851	1.42E-10	***	High risk
226	1S,3R-RSL-	0.297319	1.42E-10	***	High risk
227	1S,3R-RSL-	0.30995	1.42E-10	***	High risk
228	1S,3R-RSL-	0.272393	1.42E-10	***	High risk
229	1S,3R-RSL-	0.239458	1.42E-10	***	Low risk
230	1S,3R-RSL-	0.277468	1.42E-10	***	Low risk
231	1S,3R-RSL-	0.252292	1.42E-10	***	Low risk
232	1S,3R-RSL-	0.271672	1.42E-10	***	Low risk
233	1S,3R-RSL-	0.246352	1.42E-10	***	Low risk
234	1S,3R-RSL-	0.283312	1.42E-10	***	Low risk
235	1S,3R-RSL-	0.265425	1.42E-10	***	Low risk
236	1S,3R-RSL-	0.260633	1.42E-10	***	Low risk
237	1S,3R-RSL-	0.268094	1.42E-10	***	Low risk
238	1S,3R-RSL-	0.253653	1.42E-10	***	Low risk
239	1S,3R-RSL-	0.246553	1.42E-10	***	Low risk
240	1S,3R-RSL-	0.262056	1.42E-10	***	Low risk
241	1S,3R-RSL-	0.246431	1.42E-10	***	Low risk
242	1S,3R-RSL-	0.242745	1.42E-10	***	Low risk
243	1S,3R-RSL-	0.22899	1.42E-10	***	Low risk
244	1S,3R-RSL-	0.235263	1.42E-10	***	Low risk
245	1S,3R-RSL-	0.247107	1.42E-10	***	Low risk
246	1S,3R-RSL-	0.270552	1.42E-10	***	Low risk
247	1S,3R-RSL-	0.247129	1.42E-10	***	Low risk
248	1S,3R-RSL-	0.244517	1.42E-10	***	Low risk
249	1S,3R-RSL-	0.271608	1.42E-10	***	Low risk
250	1S,3R-RSL-	0.240256	1.42E-10	***	Low risk
251	1S,3R-RSL-	0.28895	1.42E-10	***	Low risk
252	1S,3R-RSL-	0.259686	1.42E-10	***	Low risk
253	1S,3R-RSL-	0.275266	1.42E-10	***	Low risk
254	1S,3R-RSL-	0.302842	1.42E-10	***	Low risk

255	1S,3R-RSL-	0.252583	1.42E-10	***	Low risk
256	1S,3R-RSL-	0.294642	1.42E-10	***	Low risk
257	1S,3R-RSL-	0.224368	1.42E-10	***	Low risk
258	1S,3R-RSL-	0.298182	1.42E-10	***	Low risk
259	1S,3R-RSL-	0.259081	1.42E-10	***	Low risk
260	1S,3R-RSL-	0.273295	1.42E-10	***	Low risk
261	1S,3R-RSL-	0.246882	1.42E-10	***	Low risk
262	1S,3R-RSL-	0.262192	1.42E-10	***	Low risk
263	1S,3R-RSL-	0.248286	1.42E-10	***	Low risk
264	1S,3R-RSL-	0.2372	1.42E-10	***	Low risk
265	1S,3R-RSL-	0.234752	1.42E-10	***	Low risk
266	1S,3R-RSL-	0.256448	1.42E-10	***	Low risk
267	1S,3R-RSL-	0.276293	1.42E-10	***	Low risk
268	1S,3R-RSL-	0.239693	1.42E-10	***	Low risk
269	1S,3R-RSL-	0.235586	1.42E-10	***	Low risk
270	1S,3R-RSL-	0.231582	1.42E-10	***	Low risk
271	1S,3R-RSL-	0.238231	1.42E-10	***	Low risk
272	1S,3R-RSL-	0.245317	1.42E-10	***	Low risk
273	1S,3R-RSL-	0.261301	1.42E-10	***	Low risk
274	1S,3R-RSL-	0.26722	1.42E-10	***	Low risk
275	1S,3R-RSL-	0.268965	1.42E-10	***	Low risk
276	1S,3R-RSL-	0.27158	1.42E-10	***	Low risk
277	1S,3R-RSL-	0.261252	1.42E-10	***	Low risk
278	1S,3R-RSL-	0.286185	1.42E-10	***	Low risk
279	1S,3R-RSL-	0.270601	1.42E-10	***	Low risk
280	1S,3R-RSL-	0.223229	1.42E-10	***	Low risk
281	1S,3R-RSL-	0.245371	1.42E-10	***	Low risk
282	1S,3R-RSL-	0.241243	1.42E-10	***	Low risk
283	1S,3R-RSL-	0.259093	1.42E-10	***	Low risk
284	1S,3R-RSL-	0.248089	1.42E-10	***	Low risk
285	1S,3R-RSL-	0.253113	1.42E-10	***	Low risk
286	1S,3R-RSL-	0.252107	1.42E-10	***	Low risk
287	1S,3R-RSL-	0.244534	1.42E-10	***	Low risk
288	1S,3R-RSL-	0.256211	1.42E-10	***	Low risk
289	1S,3R-RSL-	0.233449	1.42E-10	***	Low risk
290	1S,3R-RSL-	0.251054	1.42E-10	***	Low risk
291	1S,3R-RSL-	0.288026	1.42E-10	***	Low risk
292	1S,3R-RSL-	0.237002	1.42E-10	***	Low risk
293	1S,3R-RSL-	0.276391	1.42E-10	***	Low risk
294	1S,3R-RSL-	0.231167	1.42E-10	***	Low risk
295	1S,3R-RSL-	0.24412	1.42E-10	***	Low risk
296	1S,3R-RSL-	0.249749	1.42E-10	***	Low risk
297	1S,3R-RSL-	0.302084	1.42E-10	***	Low risk
298	1S,3R-RSL-	0.249651	1.42E-10	***	Low risk
299	1S,3R-RSL-	0.239206	1.42E-10	***	Low risk
300	1S,3R-RSL-	0.259281	1.42E-10	***	Low risk
301	1S,3R-RSL-	0.252204	1.42E-10	***	Low risk
302	1S,3R-RSL-	0.254374	1.42E-10	***	Low risk
303	1S,3R-RSL-	0.257391	1.42E-10	***	Low risk
304	1S,3R-RSL-	0.284628	1.42E-10	***	Low risk
305	ML162	0.33391	1.08E-14	***	High risk

306	ML162	0.33912	1.08E-14	***	High risk
307	ML162	0.302955	1.08E-14	***	High risk
308	ML162	0.321941	1.08E-14	***	High risk
309	ML162	0.333981	1.08E-14	***	High risk
310	ML162	0.350742	1.08E-14	***	High risk
311	ML162	0.37925	1.08E-14	***	High risk
312	ML162	0.325812	1.08E-14	***	High risk
313	ML162	0.349382	1.08E-14	***	High risk
314	ML162	0.351557	1.08E-14	***	High risk
315	ML162	0.358424	1.08E-14	***	High risk
316	ML162	0.360122	1.08E-14	***	High risk
317	ML162	0.342874	1.08E-14	***	High risk
318	ML162	0.278986	1.08E-14	***	High risk
319	ML162	0.297685	1.08E-14	***	High risk
320	ML162	0.341982	1.08E-14	***	High risk
321	ML162	0.321092	1.08E-14	***	High risk
322	ML162	0.369049	1.08E-14	***	High risk
323	ML162	0.324189	1.08E-14	***	High risk
324	ML162	0.355274	1.08E-14	***	High risk
325	ML162	0.339547	1.08E-14	***	High risk
326	ML162	0.34236	1.08E-14	***	High risk
327	ML162	0.33217	1.08E-14	***	High risk
328	ML162	0.367675	1.08E-14	***	High risk
329	ML162	0.338072	1.08E-14	***	High risk
330	ML162	0.393945	1.08E-14	***	High risk
331	ML162	0.318748	1.08E-14	***	High risk
332	ML162	0.360438	1.08E-14	***	High risk
333	ML162	0.331687	1.08E-14	***	High risk
334	ML162	0.33962	1.08E-14	***	High risk
335	ML162	0.329091	1.08E-14	***	High risk
336	ML162	0.306816	1.08E-14	***	High risk
337	ML162	0.30212	1.08E-14	***	High risk
338	ML162	0.331484	1.08E-14	***	High risk
339	ML162	0.352083	1.08E-14	***	High risk
340	ML162	0.295652	1.08E-14	***	High risk
341	ML162	0.320053	1.08E-14	***	High risk
342	ML162	0.318384	1.08E-14	***	High risk
343	ML162	0.343114	1.08E-14	***	High risk
344	ML162	0.328933	1.08E-14	***	High risk
345	ML162	0.371579	1.08E-14	***	High risk
346	ML162	0.302452	1.08E-14	***	High risk
347	ML162	0.35881	1.08E-14	***	High risk
348	ML162	0.308073	1.08E-14	***	High risk
349	ML162	0.36169	1.08E-14	***	High risk
350	ML162	0.345046	1.08E-14	***	High risk
351	ML162	0.338193	1.08E-14	***	High risk
352	ML162	0.323405	1.08E-14	***	High risk
353	ML162	0.329958	1.08E-14	***	High risk
354	ML162	0.28933	1.08E-14	***	High risk
355	ML162	0.353328	1.08E-14	***	High risk
356	ML162	0.311636	1.08E-14	***	High risk

357	ML162	0.326908	1.08E-14	***	High risk
358	ML162	0.373183	1.08E-14	***	High risk
359	ML162	0.374864	1.08E-14	***	High risk
360	ML162	0.339613	1.08E-14	***	High risk
361	ML162	0.315173	1.08E-14	***	High risk
362	ML162	0.335717	1.08E-14	***	High risk
363	ML162	0.321019	1.08E-14	***	High risk
364	ML162	0.360617	1.08E-14	***	High risk
365	ML162	0.317797	1.08E-14	***	High risk
366	ML162	0.349861	1.08E-14	***	High risk
367	ML162	0.392937	1.08E-14	***	High risk
368	ML162	0.343518	1.08E-14	***	High risk
369	ML162	0.325742	1.08E-14	***	High risk
370	ML162	0.348116	1.08E-14	***	High risk
371	ML162	0.377659	1.08E-14	***	High risk
372	ML162	0.324797	1.08E-14	***	High risk
373	ML162	0.297121	1.08E-14	***	High risk
374	ML162	0.318275	1.08E-14	***	High risk
375	ML162	0.336461	1.08E-14	***	High risk
376	ML162	0.343313	1.08E-14	***	High risk
377	ML162	0.314578	1.08E-14	***	High risk
378	ML162	0.358399	1.08E-14	***	High risk
379	ML162	0.361996	1.08E-14	***	High risk
380	ML162	0.345585	1.08E-14	***	High risk
381	ML162	0.306019	1.08E-14	***	Low risk
382	ML162	0.325896	1.08E-14	***	Low risk
383	ML162	0.304342	1.08E-14	***	Low risk
384	ML162	0.312301	1.08E-14	***	Low risk
385	ML162	0.299961	1.08E-14	***	Low risk
386	ML162	0.328724	1.08E-14	***	Low risk
387	ML162	0.294971	1.08E-14	***	Low risk
388	ML162	0.296707	1.08E-14	***	Low risk
389	ML162	0.314053	1.08E-14	***	Low risk
390	ML162	0.305728	1.08E-14	***	Low risk
391	ML162	0.305977	1.08E-14	***	Low risk
392	ML162	0.300237	1.08E-14	***	Low risk
393	ML162	0.29576	1.08E-14	***	Low risk
394	ML162	0.300388	1.08E-14	***	Low risk
395	ML162	0.282239	1.08E-14	***	Low risk
396	ML162	0.276938	1.08E-14	***	Low risk
397	ML162	0.297552	1.08E-14	***	Low risk
398	ML162	0.305123	1.08E-14	***	Low risk
399	ML162	0.30388	1.08E-14	***	Low risk
400	ML162	0.285586	1.08E-14	***	Low risk
401	ML162	0.313155	1.08E-14	***	Low risk
402	ML162	0.284402	1.08E-14	***	Low risk
403	ML162	0.330921	1.08E-14	***	Low risk
404	ML162	0.296705	1.08E-14	***	Low risk
405	ML162	0.305789	1.08E-14	***	Low risk
406	ML162	0.333011	1.08E-14	***	Low risk
407	ML162	0.311303	1.08E-14	***	Low risk

408	ML162	0.342311	1.08E-14	***	Low risk
409	ML162	0.278694	1.08E-14	***	Low risk
410	ML162	0.366989	1.08E-14	***	Low risk
411	ML162	0.318322	1.08E-14	***	Low risk
412	ML162	0.31675	1.08E-14	***	Low risk
413	ML162	0.285995	1.08E-14	***	Low risk
414	ML162	0.286619	1.08E-14	***	Low risk
415	ML162	0.30398	1.08E-14	***	Low risk
416	ML162	0.280392	1.08E-14	***	Low risk
417	ML162	0.297078	1.08E-14	***	Low risk
418	ML162	0.307945	1.08E-14	***	Low risk
419	ML162	0.327166	1.08E-14	***	Low risk
420	ML162	0.292306	1.08E-14	***	Low risk
421	ML162	0.296589	1.08E-14	***	Low risk
422	ML162	0.284452	1.08E-14	***	Low risk
423	ML162	0.296423	1.08E-14	***	Low risk
424	ML162	0.283307	1.08E-14	***	Low risk
425	ML162	0.301716	1.08E-14	***	Low risk
426	ML162	0.319259	1.08E-14	***	Low risk
427	ML162	0.3231	1.08E-14	***	Low risk
428	ML162	0.292148	1.08E-14	***	Low risk
429	ML162	0.307439	1.08E-14	***	Low risk
430	ML162	0.335069	1.08E-14	***	Low risk
431	ML162	0.301851	1.08E-14	***	Low risk
432	ML162	0.28014	1.08E-14	***	Low risk
433	ML162	0.304535	1.08E-14	***	Low risk
434	ML162	0.311353	1.08E-14	***	Low risk
435	ML162	0.315221	1.08E-14	***	Low risk
436	ML162	0.285994	1.08E-14	***	Low risk
437	ML162	0.319453	1.08E-14	***	Low risk
438	ML162	0.305104	1.08E-14	***	Low risk
439	ML162	0.294252	1.08E-14	***	Low risk
440	ML162	0.306168	1.08E-14	***	Low risk
441	ML162	0.308332	1.08E-14	***	Low risk
442	ML162	0.309054	1.08E-14	***	Low risk
443	ML162	0.321265	1.08E-14	***	Low risk
444	ML162	0.305921	1.08E-14	***	Low risk
445	ML162	0.334848	1.08E-14	***	Low risk
446	ML162	0.268108	1.08E-14	***	Low risk
447	ML162	0.311841	1.08E-14	***	Low risk
448	ML162	0.310749	1.08E-14	***	Low risk
449	ML162	0.323823	1.08E-14	***	Low risk
450	ML162	0.283642	1.08E-14	***	Low risk
451	ML162	0.279138	1.08E-14	***	Low risk
452	ML162	0.316286	1.08E-14	***	Low risk
453	ML162	0.295948	1.08E-14	***	Low risk
454	ML162	0.300826	1.08E-14	***	Low risk
455	ML162	0.298581	1.08E-14	***	Low risk
456	ML162	0.327383	1.08E-14	***	Low risk
457	ML210	0.376256	7.64E-15	***	High risk
458	ML210	0.383354	7.64E-15	***	High risk

459	ML210	0.354036	7.64E-15	***	High risk
460	ML210	0.343955	7.64E-15	***	High risk
461	ML210	0.371894	7.64E-15	***	High risk
462	ML210	0.377349	7.64E-15	***	High risk
463	ML210	0.412016	7.64E-15	***	High risk
464	ML210	0.328164	7.64E-15	***	High risk
465	ML210	0.365731	7.64E-15	***	High risk
466	ML210	0.396226	7.64E-15	***	High risk
467	ML210	0.370904	7.64E-15	***	High risk
468	ML210	0.386616	7.64E-15	***	High risk
469	ML210	0.377583	7.64E-15	***	High risk
470	ML210	0.309246	7.64E-15	***	High risk
471	ML210	0.332778	7.64E-15	***	High risk
472	ML210	0.382928	7.64E-15	***	High risk
473	ML210	0.357398	7.64E-15	***	High risk
474	ML210	0.423666	7.64E-15	***	High risk
475	ML210	0.350626	7.64E-15	***	High risk
476	ML210	0.434104	7.64E-15	***	High risk
477	ML210	0.36841	7.64E-15	***	High risk
478	ML210	0.366308	7.64E-15	***	High risk
479	ML210	0.350451	7.64E-15	***	High risk
480	ML210	0.411791	7.64E-15	***	High risk
481	ML210	0.371749	7.64E-15	***	High risk
482	ML210	0.427823	7.64E-15	***	High risk
483	ML210	0.344806	7.64E-15	***	High risk
484	ML210	0.386695	7.64E-15	***	High risk
485	ML210	0.367882	7.64E-15	***	High risk
486	ML210	0.363651	7.64E-15	***	High risk
487	ML210	0.374923	7.64E-15	***	High risk
488	ML210	0.346888	7.64E-15	***	High risk
489	ML210	0.319959	7.64E-15	***	High risk
490	ML210	0.370702	7.64E-15	***	High risk
491	ML210	0.374161	7.64E-15	***	High risk
492	ML210	0.315346	7.64E-15	***	High risk
493	ML210	0.349788	7.64E-15	***	High risk
494	ML210	0.356271	7.64E-15	***	High risk
495	ML210	0.39405	7.64E-15	***	High risk
496	ML210	0.361294	7.64E-15	***	High risk
497	ML210	0.402858	7.64E-15	***	High risk
498	ML210	0.325937	7.64E-15	***	High risk
499	ML210	0.397681	7.64E-15	***	High risk
500	ML210	0.343502	7.64E-15	***	High risk
501	ML210	0.386704	7.64E-15	***	High risk
502	ML210	0.398805	7.64E-15	***	High risk
503	ML210	0.395274	7.64E-15	***	High risk
504	ML210	0.353148	7.64E-15	***	High risk
505	ML210	0.344806	7.64E-15	***	High risk
506	ML210	0.300972	7.64E-15	***	High risk
507	ML210	0.363079	7.64E-15	***	High risk
508	ML210	0.340791	7.64E-15	***	High risk
509	ML210	0.340787	7.64E-15	***	High risk

510	ML210	0.452605	7.64E-15	***	High risk
511	ML210	0.397112	7.64E-15	***	High risk
512	ML210	0.391056	7.64E-15	***	High risk
513	ML210	0.336641	7.64E-15	***	High risk
514	ML210	0.363407	7.64E-15	***	High risk
515	ML210	0.355538	7.64E-15	***	High risk
516	ML210	0.412172	7.64E-15	***	High risk
517	ML210	0.343841	7.64E-15	***	High risk
518	ML210	0.40961	7.64E-15	***	High risk
519	ML210	0.419656	7.64E-15	***	High risk
520	ML210	0.3485	7.64E-15	***	High risk
521	ML210	0.351937	7.64E-15	***	High risk
522	ML210	0.382749	7.64E-15	***	High risk
523	ML210	0.408638	7.64E-15	***	High risk
524	ML210	0.326891	7.64E-15	***	High risk
525	ML210	0.334552	7.64E-15	***	High risk
526	ML210	0.348226	7.64E-15	***	High risk
527	ML210	0.370303	7.64E-15	***	High risk
528	ML210	0.366869	7.64E-15	***	High risk
529	ML210	0.356275	7.64E-15	***	High risk
530	ML210	0.393208	7.64E-15	***	High risk
531	ML210	0.409821	7.64E-15	***	High risk
532	ML210	0.392086	7.64E-15	***	High risk
533	ML210	0.320192	7.64E-15	***	Low risk
534	ML210	0.351769	7.64E-15	***	Low risk
535	ML210	0.31904	7.64E-15	***	Low risk
536	ML210	0.318302	7.64E-15	***	Low risk
537	ML210	0.31241	7.64E-15	***	Low risk
538	ML210	0.353733	7.64E-15	***	Low risk
539	ML210	0.336517	7.64E-15	***	Low risk
540	ML210	0.31331	7.64E-15	***	Low risk
541	ML210	0.334948	7.64E-15	***	Low risk
542	ML210	0.316899	7.64E-15	***	Low risk
543	ML210	0.321144	7.64E-15	***	Low risk
544	ML210	0.347757	7.64E-15	***	Low risk
545	ML210	0.31996	7.64E-15	***	Low risk
546	ML210	0.32338	7.64E-15	***	Low risk
547	ML210	0.29512	7.64E-15	***	Low risk
548	ML210	0.327362	7.64E-15	***	Low risk
549	ML210	0.320777	7.64E-15	***	Low risk
550	ML210	0.32904	7.64E-15	***	Low risk
551	ML210	0.319795	7.64E-15	***	Low risk
552	ML210	0.318281	7.64E-15	***	Low risk
553	ML210	0.312417	7.64E-15	***	Low risk
554	ML210	0.307751	7.64E-15	***	Low risk
555	ML210	0.387478	7.64E-15	***	Low risk
556	ML210	0.342034	7.64E-15	***	Low risk
557	ML210	0.326291	7.64E-15	***	Low risk
558	ML210	0.370344	7.64E-15	***	Low risk
559	ML210	0.323108	7.64E-15	***	Low risk
560	ML210	0.372003	7.64E-15	***	Low risk

561	ML210	0.295166	7.64E-15	***	Low risk
562	ML210	0.397926	7.64E-15	***	Low risk
563	ML210	0.334397	7.64E-15	***	Low risk
564	ML210	0.325107	7.64E-15	***	Low risk
565	ML210	0.316301	7.64E-15	***	Low risk
566	ML210	0.328386	7.64E-15	***	Low risk
567	ML210	0.311587	7.64E-15	***	Low risk
568	ML210	0.309279	7.64E-15	***	Low risk
569	ML210	0.302359	7.64E-15	***	Low risk
570	ML210	0.326823	7.64E-15	***	Low risk
571	ML210	0.352465	7.64E-15	***	Low risk
572	ML210	0.309365	7.64E-15	***	Low risk
573	ML210	0.320879	7.64E-15	***	Low risk
574	ML210	0.306	7.64E-15	***	Low risk
575	ML210	0.315174	7.64E-15	***	Low risk
576	ML210	0.304486	7.64E-15	***	Low risk
577	ML210	0.331881	7.64E-15	***	Low risk
578	ML210	0.334279	7.64E-15	***	Low risk
579	ML210	0.340306	7.64E-15	***	Low risk
580	ML210	0.310824	7.64E-15	***	Low risk
581	ML210	0.324199	7.64E-15	***	Low risk
582	ML210	0.371626	7.64E-15	***	Low risk
583	ML210	0.348768	7.64E-15	***	Low risk
584	ML210	0.293376	7.64E-15	***	Low risk
585	ML210	0.326729	7.64E-15	***	Low risk
586	ML210	0.321744	7.64E-15	***	Low risk
587	ML210	0.33848	7.64E-15	***	Low risk
588	ML210	0.317041	7.64E-15	***	Low risk
589	ML210	0.339465	7.64E-15	***	Low risk
590	ML210	0.318648	7.64E-15	***	Low risk
591	ML210	0.31588	7.64E-15	***	Low risk
592	ML210	0.333207	7.64E-15	***	Low risk
593	ML210	0.329406	7.64E-15	***	Low risk
594	ML210	0.319686	7.64E-15	***	Low risk
595	ML210	0.342582	7.64E-15	***	Low risk
596	ML210	0.318763	7.64E-15	***	Low risk
597	ML210	0.36434	7.64E-15	***	Low risk
598	ML210	0.285483	7.64E-15	***	Low risk
599	ML210	0.321341	7.64E-15	***	Low risk
600	ML210	0.324929	7.64E-15	***	Low risk
601	ML210	0.360564	7.64E-15	***	Low risk
602	ML210	0.314756	7.64E-15	***	Low risk
603	ML210	0.321133	7.64E-15	***	Low risk
604	ML210	0.344446	7.64E-15	***	Low risk
605	ML210	0.312875	7.64E-15	***	Low risk
606	ML210	0.341543	7.64E-15	***	Low risk
607	ML210	0.35781	7.64E-15	***	Low risk
608	ML210	0.37782	7.64E-15	***	Low risk
