# Supplementary material for: A ferroptosis-associated gene signature for the prediction of prognosis and therapeutic response in luminal-type breast carcinoma
Source: Sci Rep. 2021 Sep 2;11:17610. doi: 10.1038/s41598-021-97102-z (PMC8413464; doi:10.1038/s41598-021-97102-z)
Supplement: Supplementary file 13 — Supplementary Table S9. [file 41598_2021_97102_MOESM13_ESM.pdf]

TableS9 gistic scores data of high risk subgroup

	Variant_Classification	Chromosome	Start_Position	End_Position	fdr	G_Score
1	Amp	1	3302046	3309193	0	0.019598
2	Amp	1	3309376	3318992	0	0.015931
3	Amp	1	3319477	3345118	0	0.013517
4	Amp	1	3350785	3490227	0	0.011465
5	Amp	1	3503571	3584494	0	0.016885
6	Amp	1	3586411	3725120	0	0.013785
7	Amp	1	3725129	3797918	0	0.008365
8	Amp	1	3800840	4215231	0	0.005139
9	Amp	1	4220110	5874501	0	0.002898
10	Amp	1	5882687	5996669	0	0.008748
11	Amp	1	5997665	6116631	0	0.010889
12	Amp	1	6117854	6132805	0	0.008748
13	Amp	1	6135369	6216107	0	0.014969
14	Amp	1	6217422	6750728	0	0.02008
15	Amp	1	6756629	7065073	0	0.012651
16	Amp	1	7065353	7469027	0	0.006701
17	Amp	1	7469184	7567847	0	0.010971
18	Amp	1	7570664	7589655	0	0.009219
19	Amp	1	7596443	7713320	0	0.00495
20	Amp	1	7714011	7819070	0	0.002898
21	Amp	1	7819173	8561036	0	0
22	Amp	1	8565390	9159324	0	0.003561
23	Amp	1	9160512	9225959	0	0
24	Amp	1	9226329	10520834	0	0.003611
25	Amp	1	10520966	10608839	0	0.00719
26	Amp	1	10626217	10720289	0	0.008586
27	Amp	1	10720670	11102313	0	0.00719
28	Amp	1	11105344	11121928	0	0.009247
29	Amp	1	11126284	11181886	0	0.005668
30	Amp	1	11182278	11241784	0	0.008421
31	Amp	1	11242008	11255055	0	0.005668
32	Amp	1	11259259	11732615	0	0.010609
33	Amp	1	11737769	12119361	0	0.006998
34	Amp	1	12120067	12494896	0	0.002057
35	Amp	1	12499081	12612531	0	0.004172
36	Amp	1	12615874	14090672	0	0.002057
37	Amp	1	14092712	14224200	0	0.007606
38	Amp	1	14224608	15455589	0	0.002057
39	Amp	1	15455815	15778497	0	0.015654
40	Amp	1	15779765	15889026	0	0.006806
41	Amp	1	15891098	16123041	0	0.002057
42	Amp	1	16123185	16413458	0	0.00582
43	Amp	1	16413540	16470247	0	0.002057
44	Amp	1	16471261	17351414	0	0.009352
45	Amp	1	17353180	17475960	0	0.014517
46	Amp	1	17481614	17590424	0	0.016335
47	Amp	1	17590467	18007487	0	0.01117
48	Amp	1	18009739	18210450	0	0.003875
49	Amp	1	18215562	18272912	0	0.018828
50	Amp	1	18275108	18335089	0	0.022515
51	Amp	1	18335701	18365120	0	0.015322
52	Amp	1	18365190	18446477	0	0.021685

53	Amp	1	18451435	18577325	0	0.005744
54	Amp	1	18577609	18602432	0	0.032141
55	Amp	1	18607587	18719991	0	0.028454
56	Amp	1	18722427	19324911	0	0.011336
57	Amp	1	19332012	19380808	0	0.018394
58	Amp	1	19381365	19764502	0	0.011336
59	Amp	1	19764563	20137141	0	0.015429
60	Amp	1	20140938	20144986	0	0.02263
61	Amp	1	20147121	20177217	0	0.009258
62	Amp	1	20182972	20216935	0	0.019102
63	Amp	1	20218271	20306787	0	0.011901
64	Amp	1	20307336	20844026	0	0.015213
65	Amp	1	20844218	21743411	0	0.018812
66	Amp	1	21744043	21815733	0	0.015499
67	Amp	1	21820584	22109230	0	0.011901
68	Amp	1	22109542	22791680	0	0.004617
69	Amp	1	22796047	22926769	0	0.00991
70	Amp	1	22933134	23145272	0	0.004617
71	Amp	1	23145305	23188539	0	0.002057
72	Amp	1	23191387	23506662	0	0.005436
73	Amp	1	23507778	24226394	0	0.002057
74	Amp	1	24228070	24507028	0	0.007671
75	Amp	1	24507348	24528687	0	0.01372
76	Amp	1	24528703	24846739	0	0.028746
77	Amp	1	24850561	25065138	0	0.007671
78	Amp	1	25065238	25955905	0	0.002057
79	Amp	1	25957343	26089054	0	0.015208
80	Amp	1	26089769	27546490	0	0.002057
81	Amp	1	27560514	27821204	0	0.010712
82	Amp	1	27828388	27890551	0	0.018587
83	Amp	1	27893907	28223937	0	0.023173
84	Amp	1	28226080	28623464	0	0.024632
85	Amp	1	28624350	28728193	0	0.020046
86	Amp	1	28732181	29249093	0	0.018587
87	Amp	1	29252645	29487723	0	0.023908
88	Amp	1	29488197	29544312	0	0.018587
89	Amp	1	29556921	29721559	0	0.020965
90	Amp	1	29730840	29888870	0	0.018587
91	Amp	1	29892192	30379166	0	0.020996
92	Amp	1	30379250	30532907	0	0.032043
93	Amp	1	30533459	30605990	0	0.036773
94	Amp	1	30607414	30640385	0	0.043276
95	Amp	1	30641573	30667100	0	0.038546
96	Amp	1	30789158	30821565	0	0.051246
97	Amp	1	30827002	30899941	0	0.056553
98	Amp	1	30903048	31104540	0	0.05005
99	Amp	1	31110133	31244203	0	0.051711
100	Amp	1	31244722	31441117	0	0.057263
101	Amp	1	31442682	31690116	0	0.069906
102	Amp	1	31693969	31713228	0	0.076931
103	Amp	1	31719102	31742019	0	0.078964
104	Amp	1	31747834	31834567	0	0.071938
105	Amp	1	31835352	31835880	0	0.078285

106	Amp	1	31839122	32132589	0	0.083805
107	Amp	1	32135832	32374651	0	0.081772
108	Amp	1	32378118	32511446	0	0.074559
109	Amp	1	32513610	32513610	0	0.06904
110	Amp	1	32514921	32610108	0	0.062693
111	Amp	1	32613906	32669947	0	0.059047
112	Amp	1	32674618	32675337	0	0.067914
113	Amp	1	32677093	32695277	0	0.078761
114	Amp	1	32697685	32703884	0	0.059047
115	Amp	1	32704812	32728037	0	0.062235
116	Amp	1	32729071	32791044	0	0.079537
117	Amp	1	32797320	32841399	0	0.069375
118	Amp	1	32846572	33040123	0	0.056732
119	Amp	1	33043554	33273639	0	0.063181
120	Amp	1	33275217	33334283	0	0.059993
121	Amp	1	33334621	33347337	0	0.057584
122	Amp	1	33354213	33428857	0	0.046537
123	Amp	1	33432565	33440276	0	0.05219
124	Amp	1	33441187	33464746	0	0.062121
125	Amp	1	33468189	33480031	0	0.046537
126	Amp	1	33480084	33643453	0	0.052567
127	Amp	1	33645497	33677591	0	0.046537
128	Amp	1	33678608	33771921	0	0.055638
129	Amp	1	33774469	33957611	0	0.058615
130	Amp	1	33958792	34169998	0	0.055183
131	Amp	1	34173987	34304572	0	0.059324
132	Amp	1	34304901	34708927	0	0.055183
133	Amp	1	34717923	34823438	0	0.064881
134	Amp	1	34824272	34997105	0	0.055183
135	Amp	1	35001064	35182470	0	0.059569
136	Amp	1	35185044	35185044	0	0.051285
137	Amp	1	35189124	35249883	0	0.042267
138	Amp	1	35250387	35453577	0	0.034391
139	Amp	1	35453860	35543793	0	0.02529
140	Amp	1	35544959	35554361	0	0.035695
141	Amp	1	35555125	35567522	0	0.037757
142	Amp	1	35575795	35651142	0	0.048438
143	Amp	1	35653763	35777293	0	0.062487
144	Amp	1	35788052	35828050	0	0.046682
145	Amp	1	35830510	35851440	0	0.065086
146	Amp	1	35851854	36032086	0	0.049525
147	Amp	1	36033741	36073106	0	0.062033
148	Amp	1	36075934	36122801	0	0.046682
149	Amp	1	36128572	36162550	0	0.064666
150	Amp	1	36162890	36480112	0	0.046682
151	Amp	1	36489632	36732119	0	0.048548
152	Amp	1	36734800	36803098	0	0.053826
153	Amp	1	36807439	36829121	0	0.058952
154	Amp	1	36830441	36836746	0	0.054567
155	Amp	1	36838355	36862539	0	0.052701
156	Amp	1	36863830	37059855	0	0.058827
157	Amp	1	37060075	37182487	0	0.064823
158	Amp	1	37186722	37269650	0	0.072492

159 Amp	1	37277286	37464644	0	0.064823
160 Amp	1	37464693	37873469	0	0.059696
161 Amp	1	37877214	37930275	0	0.056332
162 Amp	1	37938378	37943639	0	0.060946
163 Amp	1	37944918	38003722	0	0.055662
164 Amp	1	38003828	38008975	0	0.059959
165 Amp	1	38016684	38068193	0	0.067625
166 Amp	1	38068888	38143653	0	0.059959
167 Amp	1	38144168	38218841	0	0.068895
168 Amp	1	38219885	38248210	0	0.074574
169 Amp	1	38251196	38377917	0	0.068448
170 Amp	1	38379285	38421978	0	0.074082
171 Amp	1	38429401	38438700	0	0.062531
172 Amp	1	38439228	38563712	0	0.067768
173 Amp	1	38563890	38633346	0	0.072726
174 Amp	1	38635858	38738174	0	0.064072
175 Amp	1	38740200	39004815	0	0.058835
176 Amp	1	39013615	39159089	0	0.062603
177 Amp	1	39164761	39183941	0	0.070265
178 Amp	1	39186518	39624418	0	0.076375
179 Amp	1	39630381	39630381	0	0.070741
180 Amp	1	39631846	39631846	0	0.06868
181 Amp	1	39638789	39703193	0	0.063
182 Amp	1	39704052	39943141	0	0.070539
183 Amp	1	39945932	40133403	0	0.06526
184 Amp	1	40145209	40159005	0	0.057598
185 Amp	1	40159372	40603710	0	0.048685
186 Amp	1	40607012	40932423	0	0.053758
187 Amp	1	40932767	40938093	0	0.057081
188 Amp	1	40939992	41369389	0	0.071561
189 Amp	1	41371844	41572255	0	0.073441
190 Amp	1	41572784	41707102	0	0.071561
191 Amp	1	41708959	41722312	0	0.075123
192 Amp	1	41723785	41815631	0	0.081225
193 Amp	1	41815656	41860136	0	0.073432
194 Amp	1	41861170	41946367	0	0.058952
195 Amp	1	41950845	42000098	0	0.062962
196 Amp	1	42004014	42137320	0	0.070062
197 Amp	1	42138661	42238743	0	0.076959
198 Amp	1	42239376	42280643	0	0.083015
199 Amp	1	42289367	42289367	0	0.075914
200 Amp	1	42294258	42324660	0	0.071904
201 Amp	1	42332094	42563672	0	0.074401
202 Amp	1	42567463	42569579	0	0.071904
203 Amp	1	42576454	42781655	0	0.08468
204 Amp	1	42782969	42821170	0	0.087782
205 Amp	1	42821330	42900832	0	0.081672
206 Amp	1	42901220	42921631	0	0.074955
207 Amp	1	42928018	43057989	0	0.061104
208 Amp	1	43058099	43157973	0	0.070974
209 Amp	1	43162305	43180668	0	0.072944
210 Amp	1	43181492	43199065	0	0.070321
211 Amp	1	43201984	43371507	0	0.060451

212 Amp	1	43374139	43381234	0	0.077338
213 Amp	1	43388835	43458378	0	0.075368
214 Amp	1	43458447	43630826	0	0.064442
215 Amp	1	43634413	43685264	0	0.074452
216 Amp	1	43688808	43704645	0	0.062705
217 Amp	1	43712995	43725544	0	0.052695
218 Amp	1	43728081	43900669	0	0.063781
219 Amp	1	43903232	43903232	0	0.066483
220 Amp	1	43911001	43993713	0	0.059576
221 Amp	1	44004703	44019705	0	0.064199
222 Amp	1	44021417	44075678	0	0.055808
223 Amp	1	44080667	44091691	0	0.061882
224 Amp	1	44100549	44102752	0	0.057259
225 Amp	1	44102806	44170809	0	0.061051
226 Amp	1	44171718	44288916	0	0.058349
227 Amp	1	44291527	44512647	0	0.054557
228 Amp	1	44513183	44643429	0	0.059597
229 Amp	1	44643864	44936625	0	0.061617
230 Amp	1	44944256	45381300	0	0.059597
231 Amp	1	45383259	45492413	0	0.053522
232 Amp	1	45493287	45621241	0	0.048483
233 Amp	1	45630602	45774377	0	0.046612
234 Amp	1	45791218	45800529	0	0.035598
235 Amp	1	45803152	46129164	0	0.0414
236 Amp	1	46130011	46152309	0	0.036327
237 Amp	1	46152962	46768663	0	0.030269
238 Amp	1	46768878	46835307	0	0.036397
239 Amp	1	46837969	47025920	0	0.033421
240 Amp	1	47027226	47038003	0	0.030098
241 Amp	1	47039045	47059549	0	0.044865
242 Amp	1	47067958	47599666	0	0.030098
243 Amp	1	47606857	47638505	0	0.032023
244 Amp	1	47639080	47797335	0	0.037639
245 Amp	1	47798170	47933067	0	0.032023
246 Amp	1	47938217	48064684	0	0.037065
247 Amp	1	48067681	48208019	0	0.038531
248 Amp	1	48208073	48216362	0	0.041273
249 Amp	1	48232680	48332296	0	0.035471
250 Amp	1	48332723	48595486	0	0.037862
251 Amp	1	48599436	48802922	0	0.048408
252 Amp	1	48804608	48805477	0	0.046017
253 Amp	1	48805639	48919794	0	0.055956
254 Amp	1	48920933	48975858	0	0.067466
255 Amp	1	48976351	48986477	0	0.081272
256 Amp	1	48997792	49141329	0	0.073219
257 Amp	1	49141481	49242229	0	0.063279
258 Amp	1	49249600	49396045	0	0.049474
259 Amp	1	49396151	49414232	0	0.046732
260 Amp	1	49416869	49443468	0	0.058739
261 Amp	1	49448451	49903216	0	0.06633
262 Amp	1	49908020	49934370	0	0.080754
263 Amp	1	49942995	50022832	0	0.064437
264 Amp	1	50025526	50117724	0	0.054819

265 Amp	1	50120842	50394249	0	0.044273
266 Amp	1	50403012	50723503	0	0.053559
267 Amp	1	50724201	50749914	0	0.057587
268 Amp	1	50757541	50976326	0	0.059961
269 Amp	1	50980314	51052457	0	0.063003
270 Amp	1	51054309	51073608	0	0.058976
271 Amp	1	51079376	51337803	0	0.055933
272 Amp	1	51338357	51399130	0	0.061801
273 Amp	1	51405941	51716515	0	0.055933
274 Amp	1	51717288	51843523	0	0.057579
275 Amp	1	51843742	51845084	0	0.061345
276 Amp	1	51853322	52130746	0	0.049338
277 Amp	1	52139484	52140488	0	0.067947
278 Amp	1	52149899	52170833	0	0.065574
279 Amp	1	52171266	52879235	0	0.061807
280 Amp	1	52879304	52959036	0	0.069705
281 Amp	1	52960768	53038070	0	0.074604
282 Amp	1	53038086	53117524	0	0.077576
283 Amp	1	53121263	53140408	0	0.084378
284 Amp	1	53142753	53267820	0	0.081406
285 Amp	1	53269062	53273344	0	0.085119
286 Amp	1	53279206	53321038	0	0.087375
287 Amp	1	53321626	53333251	0	0.080573
288 Amp	1	53333626	53521010	0	0.084653
289 Amp	1	53521327	53564183	0	0.08871
290 Amp	1	53564220	53867980	0	0.087052
291 Amp	1	53870720	54023422	0	0.091796
292 Amp	1	54024956	54092858	0	0.085609
293 Amp	1	54093410	54149976	0	0.081552
294 Amp	1	54150265	54311889	0	0.091347
295 Amp	1	54318188	54365331	0	0.081552
296 Amp	1	54371188	54578085	0	0.087867
297 Amp	1	54580310	54659790	0	0.091907
298 Amp	1	54660999	54809137	0	0.089651
299 Amp	1	54809662	54850067	0	0.085571
300 Amp	1	54852887	54975403	0	0.087203
301 Amp	1	54977932	54977960	0	0.079612
302 Amp	1	54979889	55047388	0	0.073297
303 Amp	1	55047496	55193688	0	0.07912
304 Amp	1	55197137	55305589	0	0.086371
305 Amp	1	55308724	55319256	0	0.080547
306 Amp	1	55319334	55331338	0	0.073749
307 Amp	1	55333419	55341828	0	0.076404
308 Amp	1	55342559	55502093	0	0.084278
309 Amp	1	55503299	55576152	0	0.086325
310 Amp	1	55576288	55611406	0	0.098292
311 Amp	1	55612140	55638443	0	0.086325
312 Amp	1	55639556	55785707	0	0.087837
313 Amp	1	55792696	55827469	0	0.098202
314 Amp	1	55829348	55926133	0	0.087837
315 Amp	1	55926306	56326085	0	0.085181
316 Amp	1	56327007	56853429	0	0.093953
317 Amp	1	56853807	56853807	0	0.097582

318	Amp	1	56859716	56902549	0	0.086392
319	Amp	1	56905460	56911403	0	0.095971
320	Amp	1	56911499	57149841	0	0.093924
321	Amp	1	57150002	57198232	0	0.089025
322	Amp	1	57198250	57488744	0	0.095077
323	Amp	1	57489005	58027916	0	0.100215
324	Amp	1	58027999	58226005	0	0.096764
325	Amp	1	58226128	58240737	0	0.087185
326	Amp	1	58241439	58744743	0	0.079446
327	Amp	1	58748753	58820634	0	0.083735
328	Amp	1	58820984	58877296	0	0.080106
329	Amp	1	58877424	58889578	0	0.114029
330	Amp	1	58889833	58941181	0	0.116434
331	Amp	1	58941228	59072661	0	0.104015
332	Amp	1	59072800	59078560	0	0.112358
333	Amp	1	59078763	59169530	0	0.115937
334	Amp	1	59171871	59444939	0	0.121854
335	Amp	1	59445080	59526987	0	0.126586
336	Amp	1	59592607	60366741	0	0.118712
337	Amp	1	60367276	60626589	0	0.112795
338	Amp	1	60628824	60739797	0	0.109216
339	Amp	1	60742067	60791094	0	0.114846
340	Amp	1	60792706	60821180	0	0.110114
341	Amp	1	60826310	61310099	0	0.104484
342	Amp	1	61310469	61393957	0	0.112927
343	Amp	1	61394130	61464390	0	0.104484
344	Amp	1	61464925	61506340	0	0.10905
345	Amp	1	61506572	61595188	0	0.112214
346	Amp	1	61597301	61673182	0	0.107648
347	Amp	1	61676223	61686101	0	0.106003
348	Amp	1	61688821	61968912	0	0.104173
349	Amp	1	61968989	62084385	0	0.099131
350	Amp	1	62086416	62364948	0	0.084894
351	Amp	1	62367882	62463883	0	0.079473
352	Amp	1	62475245	62894766	0	0.095885
353	Amp	1	62898154	63023206	0	0.079473
354	Amp	1	63026109	63031120	0	0.084506
355	Amp	1	63033903	63227962	0	0.079473
356	Amp	1	63228432	63228450	0	0.075184
357	Amp	1	63230017	63411885	0	0.07202
358	Amp	1	63419429	63776262	0	0.074362
359	Amp	1	63783841	64298871	0	0.070594
360	Amp	1	64299387	64345848	0	0.058185
361	Amp	1	64348782	64454280	0	0.054266
362	Amp	1	64456752	64916800	0	0.055682
363	Amp	1	64917109	64995507	0	0.059677
364	Amp	1	64998526	65028772	0	0.067333
365	Amp	1	65029580	65071177	0	0.074623
366	Amp	1	65076043	65665101	0	0.066966
367	Amp	1	65672819	65694222	0	0.072011
368	Amp	1	65704711	65708375	0	0.090613
369	Amp	1	65710324	65715111	0	0.077393
370	Amp	1	65724369	65725392	0	0.082953

371	Amp	1	65727865	65738080	0	0.077571
372	Amp	1	65742278	65761212	0	0.092664
373	Amp	1	65771983	65774146	0	0.090259
374	Amp	1	65774160	65961757	0	0.075166
375	Amp	1	65962260	65964221	0	0.076975
376	Amp	1	65964789	66154436	0	0.082735
377	Amp	1	66154791	66191603	0	0.077175
378	Amp	1	66193972	66432527	0	0.069827
379	Amp	1	66440850	66649547	0	0.066258
380	Amp	1	66649888	66853823	0	0.064449
381	Amp	1	66853842	66936653	0	0.071005
382	Amp	1	66941206	67044915	0	0.064449
383	Amp	1	67045174	67061738	0	0.054751
384	Amp	1	67064745	67279001	0	0.059279
385	Amp	1	67281340	67343760	0	0.061939
386	Amp	1	67344148	68257253	0	0.059279
387	Amp	1	68263901	68406078	0	0.072274
388	Amp	1	68408182	68424181	0	0.059279
389	Amp	1	68431262	68442818	0	0.075101
390	Amp	1	68443186	68585451	0	0.059279
391	Amp	1	68588727	68813364	0	0.043019
392	Amp	1	68814272	69333236	0	0.04769
393	Amp	1	69334402	69529340	0	0.054078
394	Amp	1	69533950	69782701	0	0.049106
395	Amp	1	69788045	69995365	0	0.051768
396	Amp	1	70003888	70105291	0	0.045308
397	Amp	1	70106050	70216867	0	0.049182
398	Amp	1	70218589	70355624	0	0.04652
399	Amp	1	70359871	70433729	0	0.055299
400	Amp	1	70435652	70531469	0	0.04652
401	Amp	1	70533103	70538052	0	0.044888
402	Amp	1	70538067	70636437	0	0.049193
403	Amp	1	70636632	70739693	0	0.047777
404	Amp	1	70740403	70822027	0	0.055308
405	Amp	1	70822847	71228170	0	0.047777
406	Amp	1	71230334	71770280	0	0.050146
407	Amp	1	71771947	71787894	0	0.06053
408	Amp	1	71790759	71902269	0	0.050146
409	Amp	1	71906558	71983620	0	0.055724
410	Amp	1	71984255	71989215	0	0.06028
411	Amp	1	71989353	72007541	0	0.062065
412	Amp	1	72008431	72066507	0	0.05819
413	Amp	1	72066791	72137348	0	0.050828
414	Amp	1	72138995	72252060	0	0.055861
415	Amp	1	72254355	72362629	0	0.074038
416	Amp	1	72362638	72517795	0	0.069483
417	Amp	1	72518186	72791646	0	0.064449
418	Amp	1	72795204	73011960	0	0.067892
419	Amp	1	73015080	73445737	0	0.064449
420	Amp	1	73447116	73449192	0	0.066942
421	Amp	1	73453840	73467430	0	0.048765
422	Amp	1	73467999	73493989	0	0.050488
423	Amp	1	73494550	73599836	0	0.055102

424 Amp	1	73601306	73609364	0	0.062594
425 Amp	1	73610006	73627923	0	0.060225
426 Amp	1	73637681	73705113	0	0.052733
427 Amp	1	73708274	73901465	0	0.048428
428 Amp	1	73905825	74018113	0	0.054083
429 Amp	1	74056596	74094969	0	0.065043
430 Amp	1	74095005	74095005	0	0.059387
431 Amp	1	74106089	74192013	0	0.045935
432 Amp	1	74192645	74711223	0	0.051399
433 Amp	1	74738224	74794644	0	0.057686
434 Amp	1	74794809	74847955	0	0.052222
435 Amp	1	74850804	75090972	0	0.047608
436 Amp	1	75091874	75195959	0	0.052927
437 Amp	1	75196088	75413655	0	0.057544
438 Amp	1	75413908	75617982	0	0.052224
439 Amp	1	75619957	75685113	0	0.057621
440 Amp	1	75685408	75774336	0	0.052224
441 Amp	1	75774375	76137824	0	0.07418
442 Amp	1	76137963	76152308	0	0.067513
443 Amp	1	76154568	76175413	0	0.069107
444 Amp	1	76175502	76197735	0	0.074676
445 Amp	1	76201252	76232427	0	0.079865
446 Amp	1	76232891	76346377	0	0.075663
447 Amp	1	76349203	76361311	0	0.093758
448 Amp	1	76361565	76496275	0	0.075663
449 Amp	1	76496478	77069587	0	0.070093
450 Amp	1	77079058	77325441	0	0.079322
451 Amp	1	77330234	77359975	0	0.0812
452 Amp	1	77360222	77510226	0	0.079607
453 Amp	1	77513302	77557488	0	0.08948
454 Amp	1	77559414	77751992	0	0.073037
455 Amp	1	77760195	77905306	0	0.063809
456 Amp	1	77909082	78096929	0	0.071573
457 Amp	1	78097305	78438018	0	0.054675
458 Amp	1	78439625	78518627	0	0.048425
459 Amp	1	78519512	78709270	0	0.05469
460 Amp	1	78714329	78761341	0	0.049501
461 Amp	1	78761699	78773694	0	0.03882
462 Amp	1	78780916	79961205	0	0.032555
463 Amp	1	79964099	80227278	0	0.041121
464 Amp	1	80227949	80319852	0	0.032555
465 Amp	1	80320184	80345620	0	0.041503
466 Amp	1	80345668	80440857	0	0.035436
467 Amp	1	80440949	80481214	0	0.037743
468 Amp	1	80481631	80542461	0	0.044911
469 Amp	1	80547086	80695652	0	0.037743
470 Amp	1	80704042	80758821	0	0.046289
471 Amp	1	80759687	80835582	0	0.037743
472 Amp	1	80837263	80905147	0	0.045389
473 Amp	1	80905259	80935145	0	0.042507
474 Amp	1	80935192	80990894	0	0.0402
475 Amp	1	80995612	81093230	0	0.032555
476 Amp	1	81238791	81765729	0	0.022122

477 Amp	1	81765896	81996757	0	0.015835
478 Amp	1	82003925	82156806	0	0.022693
479 Amp	1	82157175	83244507	0	0.015835
480 Amp	1	83252575	83514399	0	0.020786
481 Amp	1	83516421	84179035	0	0.015835
482 Amp	1	84179610	85655413	0	0.013956
483 Amp	1	85656102	85761243	0	0.020568
484 Amp	1	85765432	85791236	0	0.011102
485 Amp	1	85794083	86141251	0	0.017826
486 Amp	1	86144995	86367970	0	0.021046
487 Amp	1	86370670	87069570	0	0.017826
488 Amp	1	87075012	87211852	0	0.027936
489 Amp	1	87212996	87421931	0	0.01865
490 Amp	1	87422400	87478424	0	0.022431
491 Amp	1	87482310	87608652	0	0.025533
492 Amp	1	87610556	87653615	0	0.035345
493 Amp	1	87654569	87668879	0	0.022431
494 Amp	1	87670017	87732660	0	0.031957
495 Amp	1	87733436	88069554	0	0.023914
496 Amp	1	88070177	88247823	0	0.015261
497 Amp	1	88249684	88609714	0	0.022204
498 Amp	1	88612760	88671350	0	0.026834
499 Amp	1	88677622	88766326	0	0.022204
500 Amp	1	88766638	88850729	0	0.034497
501 Amp	1	88851889	89533666	0	0.040145
502 Amp	1	89533737	90348801	0	0.034497
503 Amp	1	90353136	90483948	0	0.022204
504 Amp	1	90484405	90704642	0	0.030617
505 Amp	1	90711890	91130781	0	0.051425
506 Amp	1	91133805	91390515	0	0.030617
507 Amp	1	91390554	91410083	0	0.025379
508 Amp	1	91410432	91487946	0	0.026977
509 Amp	1	91490789	91581155	0	0.04356
510 Amp	1	91582453	91907380	0	0.055
511 Amp	1	91912700	92024752	0	0.048057
512 Amp	1	92024939	92069820	0	0.041451
513 Amp	1	92072312	92099684	0	0.062084
514 Amp	1	92103638	92249447	0	0.050643
515 Amp	1	92249844	92462087	0	0.030011
516 Amp	1	92465706	92516861	0	0.041694
517 Amp	1	92517021	92733857	0	0.030011
518 Amp	1	92734327	92824236	0	0.032715
519 Amp	1	92831008	92831008	0	0.038357
520 Amp	1	92837742	92931885	0	0.042741
521 Amp	1	92932714	92950894	0	0.033634
522 Amp	1	92954193	93085870	0	0.048631
523 Amp	1	93089626	93089626	0	0.044247
524 Amp	1	93092574	93571833	0	0.026666
525 Amp	1	93572554	93943352	0	0.028469
526 Amp	1	93944429	94211656	0	0.024541
527 Amp	1	94212365	94310373	0	0.03074
528 Amp	1	94314618	94503203	0	0.028042
529 Amp	1	94512643	94772117	0	0.024541

530 Amp	1	94773102	95027705	0	0.030738
531 Amp	1	95027910	95078468	0	0.034353
532 Amp	1	95079068	95236828	0	0.028526
533 Amp	1	95237738	95268167	0	0.022329
534 Amp	1	95269938	95493983	0	0.019744
535 Amp	1	95495418	96412694	0	0.016128
536 Amp	1	96413257	97457393	0	0.010141
537 Amp	1	97462940	97621744	0	0.021255
538 Amp	1	97623381	97712759	0	0.018551
539 Amp	1	97715051	98822299	0	0.016952
540 Amp	1	98822333	99192804	0	0.012597
541 Amp	1	99193049	100341341	0	0.001483
542 Amp	1	100342807	100773820	0	0.004585
543 Amp	1	100777502	101128001	0	0.007153
544 Amp	1	101134098	101174784	0	0.014503
545 Amp	1	101176050	101475677	0	0.007153
546 Amp	1	101480098	101504739	0	0.004585
547 Amp	1	101507282	102485947	0	0.011402
548 Amp	1	102487529	102500115	0	0.016842
549 Amp	1	102500129	103111782	0	0.021632
550 Amp	1	103112620	103470556	0	0.033073
551 Amp	1	103477949	103524378	0	0.028284
552 Amp	1	103793015	103991879	0	0.04412
553 Amp	1	104003415	104078845	0	0.037304
554 Amp	1	104086397	104096106	0	0.031863
555 Amp	1	104098957	104132654	0	0.034535
556 Amp	1	104135396	104156571	0	0.039145
557 Amp	1	104157967	104210511	0	0.041446
558 Amp	1	104221125	104252882	0	0.045818
559 Amp	1	104256007	104271920	0	0.043146
560 Amp	1	104275078	104322690	0	0.046804
561 Amp	1	104329708	104437458	0	0.052423
562 Amp	1	104441395	104520924	0	0.059524
563 Amp	1	104526276	104526276	0	0.056421
564 Amp	1	104528034	104528034	0	0.052763
565 Amp	1	104529243	104610967	0	0.041644
566 Amp	1	104613041	104637751	0	0.036026
567 Amp	1	104649495	104763135	0	0.050494
568 Amp	1	104768671	104943317	0	0.061543
569 Amp	1	104943867	104965533	0	0.064077
570 Amp	1	104966195	105272304	0	0.059965
571 Amp	1	105272977	105335244	0	0.066127
572 Amp	1	105337134	105365249	0	0.074411
573 Amp	1	105370841	105377833	0	0.06825
574 Amp	1	105382878	105710691	0	0.059965
575 Amp	1	105713331	105998788	0	0.044198
576 Amp	1	106003491	106008235	0	0.036869
577 Amp	1	106008748	106302810	0	0.038289
578 Amp	1	106302909	106707784	0	0.035755
579 Amp	1	106709731	106865436	0	0.0372
580 Amp	1	106869931	106869931	0	0.030099
581 Amp	1	106887857	106947222	0	0.038162
582 Amp	1	106949972	106981109	0	0.038571

583	Amp	1	106986033	107047688	0	0.044574
584	Amp	1	107059990	107151113	0	0.064878
585	Amp	1	107151498	107297631	0	0.069865
586	Amp	1	107297897	107541903	0	0.08015
587	Amp	1	107544628	107610008	0	0.081618
588	Amp	1	107611783	107670456	0	0.08015
589	Amp	1	107671586	107693184	0	0.081759
590	Amp	1	107693582	107743746	0	0.078531
591	Amp	1	107744010	107937600	0	0.082902
592	Amp	1	107946623	108100804	0	0.08806
593	Amp	1	108103935	108330131	0	0.086451
594	Amp	1	108330251	108336315	0	0.081463
595	Amp	1	108345982	108353751	0	0.085148
596	Amp	1	108357483	108532183	0	0.092389
597	Amp	1	108534401	108615891	0	0.088018
598	Amp	1	108621496	108621496	0	0.099374
599	Amp	1	108621775	108767365	0	0.092133
600	Amp	1	108773583	108827892	0	0.080777
601	Amp	1	108829252	108865108	0	0.077092
602	Amp	1	108867754	108930299	0	0.080959
603	Amp	1	108937665	108971725	0	0.077092
604	Amp	1	108972341	108978695	0	0.096547
605	Amp	1	108981372	108985482	0	0.099904
606	Amp	1	109006710	109028555	0	0.103678
607	Amp	1	109067737	109131659	0	0.084223
608	Amp	1	109146692	109339469	0	0.104515
609	Amp	1	109342419	109373700	0	0.102774
610	Amp	1	109376967	109576863	0	0.099509
611	Amp	1	109579745	109718120	0	0.079217
612	Amp	1	109721990	109734781	0	0.075442
613	Amp	1	109735520	109735520	0	0.095271
614	Amp	1	109738403	109751050	0	0.093851
615	Amp	1	109752427	109819099	0	0.101248
616	Amp	1	109820397	109880680	0	0.093685
617	Amp	1	109881907	110246828	0	0.0834
618	Amp	1	110247515	110303740	0	0.093247
619	Amp	1	110303888	110541921	0	0.090865
620	Amp	1	110542987	110650703	0	0.097726
621	Amp	1	110652313	110785692	0	0.094309
622	Amp	1	110786444	110950522	0	0.072626
623	Amp	1	110950819	111121721	0	0.077986
624	Amp	1	111124496	111222365	0	0.072626
625	Amp	1	111224153	111382001	0	0.065229
626	Amp	1	111382127	111633262	0	0.061162
627	Amp	1	111633377	112082946	0	0.050075
628	Amp	1	112083615	112314964	0	0.044087
629	Amp	1	112316472	112370100	0	0.052906
630	Amp	1	112373633	112666413	0	0.073565
631	Amp	1	112667588	112859430	0	0.068767
632	Amp	1	112860550	112863281	0	0.066466
633	Amp	1	112870172	113183125	0	0.056619
634	Amp	1	113184198	113193686	0	0.054977
635	Amp	1	113195051	113225129	0	0.064782

636	Amp	1	113231359	113437276	0	0.054977
637	Amp	1	113442834	113462357	0	0.046158
638	Amp	1	113468376	113579027	0	0.027141
639	Amp	1	113579069	113741510	0	0.029811
640	Amp	1	113744064	113976671	0	0.03139
641	Amp	1	113980031	113988858	0	0.077651
642	Amp	1	113992482	114027181	0	0.082594
643	Amp	1	114033966	114247913	0	0.077651
644	Amp	1	114249796	114380480	0	0.086751
645	Amp	1	114381014	114421592	0	0.093638
646	Amp	1	114428024	114477088	0	0.112414
647	Amp	1	114477874	114531159	0	0.130885
648	Amp	1	114531255	114531255	0	0.123998
649	Amp	1	114535697	114564848	0	0.12777
650	Amp	1	114567154	114646792	0	0.123998
651	Amp	1	114648826	114736201	0	0.118189
652	Amp	1	114742891	114849148	0	0.099718
653	Amp	1	114849179	114857263	0	0.09434
654	Amp	1	114857319	114908280	0	0.106888
655	Amp	1	114916223	114916635	0	0.100821
656	Amp	1	114932146	115064137	0	0.104811
657	Amp	1	115065234	115126840	0	0.13963
658	Amp	1	115127801	115135325	0	0.104811
659	Amp	1	115138450	115271358	0	0.100821
660	Amp	1	115271862	115301435	0	0.139352
661	Amp	1	115304925	115318644	0	0.104292
662	Amp	1	115318683	115324650	0	0.101002
663	Amp	1	115325121	115658443	0	0.133277
664	Amp	1	115660062	115664330	0	0.134851
665	Amp	1	115664748	115681485	0	0.143176
666	Amp	1	115682304	115768577	0	0.130628
667	Amp	1	115770371	115854155	0	0.094882
668	Amp	1	115854407	116030960	0	0.096423
669	Amp	1	116032880	116254756	0	0.077647
670	Amp	1	116257394	116367997	0	0.069322
671	Amp	1	116370856	116527793	0	0.081584
672	Amp	1	116533925	116545157	0	0.085201
673	Amp	1	116547742	116664271	0	0.072939
674	Amp	1	116665778	116726289	0	0.083106
675	Amp	1	116727764	116785353	0	0.078862
676	Amp	1	116790857	116857410	0	0.093154
677	Amp	1	116857593	116874316	0	0.091613
678	Amp	1	116880717	116937130	0	0.077321
679	Amp	1	116939650	116966159	0	0.090673
680	Amp	1	116971426	116971426	0	0.108939
681	Amp	1	116971712	116972493	0	0.114813
682	Amp	1	116973251	117044628	0	0.10146
683	Amp	1	117046316	117058997	0	0.122934
684	Amp	1	117059005	117108377	0	0.103271
685	Amp	1	117108741	117119085	0	0.091137
686	Amp	1	117123969	117140094	0	0.080971
687	Amp	1	117142250	117225114	0	0.059497
688	Amp	1	117225234	117341391	0	0.062125

689	Amp	1	117341433	117407823	0	0.065982
690	Amp	1	117408094	117438133	0	0.061902
691	Amp	1	117438166	117444548	0	0.06678
692	Amp	1	117448224	117510870	0	0.075784
693	Amp	1	117512150	117581356	0	0.06678
694	Amp	1	117584951	117924916	0	0.070597
695	Amp	1	117925016	118003458	0	0.081744
696	Amp	1	118006233	118096032	0	0.070597
697	Amp	1	118096554	118114753	0	0.080534
698	Amp	1	118117777	118172283	0	0.077293
699	Amp	1	118172902	118181399	0	0.072891
700	Amp	1	118196310	118210538	0	0.081571
701	Amp	1	118212766	118256816	0	0.088655
702	Amp	1	118257045	118257304	0	0.0917
703	Amp	1	118258988	118403004	0	0.101463
704	Amp	1	118409524	118450204	0	0.10408
705	Amp	1	118451764	118511164	0	0.1119
706	Amp	1	118511861	118537880	0	0.105498
707	Amp	1	118540342	118568114	0	0.116282
708	Amp	1	118574544	118724998	0	0.105498
709	Amp	1	118727246	118728785	0	0.097478
710	Amp	1	118732964	118745075	0	0.105314
711	Amp	1	118748812	118761801	0	0.113013
712	Amp	1	118764243	118890895	0	0.10325
713	Amp	1	118894153	118896521	0	0.100622
714	Amp	1	118896692	118939793	0	0.103442
715	Amp	1	118942021	119053326	0	0.105354
716	Amp	1	119067312	119093978	0	0.155877
717	Amp	1	119100937	119122506	0	0.157273
718	Amp	1	119123471	119150466	0	0.163672
719	Amp	1	119151764	119160948	0	0.161054
720	Amp	1	119161501	119257095	0	0.162704
721	Amp	1	119258672	119326092	0	0.183279
722	Amp	1	119332482	119364849	0	0.180459
723	Amp	1	119366585	119380185	0	0.185108
724	Amp	1	119383313	119526175	0	0.175729
725	Amp	1	119529459	119556421	0	0.186326
726	Amp	1	119559935	119698152	0	0.17599
727	Amp	1	119702784	119737931	0	0.181249
728	Amp	1	119765652	119801421	0.007199	0.189546
729	Amp	1	119801482	119816579	0.038273	0.193352
730	Amp	1	119817520	119890454	0	0.187752
731	Amp	1	119890730	119906441	0.079463	0.198122
732	Amp	1	119908008	119915295	0.033661	0.192863
733	Amp	1	119916539	119929205	0.012341	0.189818
734	Amp	1	119932796	119979137	0	0.173951
735	Amp	1	119979978	119979978	0	0.16921
736	Amp	1	119981279	119981279	0	0.155383
737	Amp	1	119981332	119981332	0	0.163535
738	Amp	1	119984738	119984738	0	0.183842
739	Amp	1	119984772	119984772	0.223025	0.21328
740	Amp	1	146716041	146716041	0.556748	0.243796
741	Amp	1	149906351	149906351	1.637826	0.322336

742 Amp	1	149907993	149909846	1.453628	0.310422
743 Amp	1	149910462	149913375	1.364151	0.304149
744 Amp	1	149914031	149918981	1.324884	0.301332
745 Amp	1	149927059	149963240	1.295233	0.299054
746 Amp	1	149966299	149966299	1.354645	0.303077
747 Amp	1	149966820	149970206	1.305256	0.300072
748 Amp	1	149970984	149985935	1.364151	0.304309
749 Amp	1	149990380	150028207	1.432145	0.308503
750 Amp	1	150029763	150029941	1.525294	0.314959
751 Amp	1	150030136	150057713	1.463463	0.310774
752 Amp	1	150057994	150086810	1.49455	0.313059
753 Amp	1	150090787	150184413	1.534719	0.315808
754 Amp	1	150187606	150196378	1.394558	0.306244
755 Amp	1	150202007	150213486	2.049537	0.349643
756 Amp	1	150216986	150245502	2.192225	0.358292
757 Amp	1	150246041	150372994	2.260813	0.362298
758 Amp	1	150377292	150377292	2.393797	0.370792
759 Amp	1	150494291	150494291	2.530938	0.378861
760 Amp	1	150494449	150598626	3.243024	0.421138
761 Amp	1	150601435	150611627	3.415716	0.431419
762 Amp	1	150612358	150650220	3.383286	0.429205
763 Amp	1	150655804	150704541	3.243024	0.420711
764 Amp	1	150705243	150765897	2.902384	0.400655
765 Amp	1	150766223	150809251	2.774551	0.393026
766 Amp	1	150809856	150825804	2.692246	0.388833
767 Amp	1	150828700	150840478	2.761965	0.39256
768 Amp	1	150840593	150864342	2.692246	0.388202
769 Amp	1	150873363	150880582	2.483202	0.376237
770 Amp	1	150883915	150883915	2.436112	0.373517
771 Amp	1	150886666	150929866	2.272343	0.363236
772 Amp	1	150936430	150940120	1.667574	0.324812
773 Amp	1	150941402	150941402	1.463463	0.310992
774 Amp	1	150943117	150968759	1.088806	0.284741
775 Amp	1	150970183	151046704	0.702615	0.255994
776 Amp	1	151056453	151115703	0.66072	0.252137
777 Amp	1	151116579	151139451	0.508503	0.23951
778 Amp	1	151143521	151231796	0.613517	0.248646
779 Amp	1	151237523	151252036	0.532449	0.241381
780 Amp	1	151266930	151437510	0.430191	0.232244
781 Amp	1	151438511	151439145	0.532449	0.241446
782 Amp	1	151439159	151486314	0.564815	0.244449
783 Amp	1	151487113	151498967	0.51566	0.240443
784 Amp	1	151500420	151544952	0.430191	0.2323
785 Amp	1	151548255	151613299	0.111991	0.201428
786 Amp	1	151614064	151621444	0.056238	0.19566
787 Amp	1	151623438	151630186	0.073369	0.197238
788 Amp	1	151633929	151633929	0.09318	0.199271
789 Amp	1	151636247	151658036	0.452609	0.234334
790 Amp	1	151659216	151695242	0.09318	0.199271
791 Amp	1	151697079	151748235	0.105051	0.201185
792 Amp	1	151752786	151770799	0.201819	0.211252
793 Amp	1	151772452	151807688	0.230572	0.213669
794 Amp	1	151807701	151851613	0.128931	0.203601

795	Amp	1	151851700	151858962	0.09318	0.199586
796	Amp	1	151859055	151867493	0.117531	0.202394
797	Amp	1	151868548	151905451	0.09318	0.199845
798	Amp	1	151907911	151920867	0.056238	0.195607
799	Amp	1	151921741	151944216	0.230572	0.214218
800	Amp	1	151944275	152019898	0.398217	0.229496
801	Amp	1	152020589	152206229	0.51566	0.240257
802	Amp	1	152210946	152288336	0.548372	0.242961
803	Amp	1	152295561	152423239	0.743132	0.259271
804	Amp	1	152424116	152424116	0.760395	0.261016
805	Amp	1	152425732	152437990	0.548372	0.242759
806	Amp	1	152440790	152666718	0.564815	0.244536
807	Amp	1	152667725	152752479	0.814697	0.26487
808	Amp	1	152753153	152807663	0.685617	0.254349
809	Amp	1	152807801	152828698	0.852608	0.267427
810	Amp	1	152838707	152855958	0.41418	0.230874
811	Amp	1	152861395	152995736	0.523647	0.240628
812	Amp	1	152999142	153049281	0.636206	0.250153
813	Amp	1	153050084	153050084	0.51566	0.240399
814	Amp	1	153055402	153143757	0.444673	0.234255
815	Amp	1	153149533	153180447	0.564815	0.24437
816	Amp	1	153180796	153200676	0.532449	0.241368
817	Amp	1	153201304	153242295	0.652914	0.252031
818	Amp	1	153242538	153248240	0.532449	0.241162
819	Amp	1	153251073	153256299	0.548372	0.243012
820	Amp	1	153257097	153313558	0.508503	0.239202
821	Amp	1	153313742	153314040	0.483654	0.237457
822	Amp	1	153314540	153322177	0.444673	0.233659
823	Amp	1	153329051	153402908	0.355162	0.225693
824	Amp	1	153405027	153412880	0.333141	0.223319
825	Amp	1	153414604	153418315	0.175335	0.208394
826	Amp	1	153422908	153514749	0.117531	0.202531
827	Amp	1	153516020	153587655	0.13565	0.204337
828	Amp	1	153593211	153622728	0.223025	0.213151
829	Amp	1	153625804	153629765	0.149443	0.205772
830	Amp	1	153630385	153682575	0.028398	0.191756
831	Amp	1	153684174	153773946	0.007199	0.189052
832	Amp	1	153775140	153782053	0.068197	0.19707
833	Amp	1	153787628	153828954	0.105051	0.200745
834	Amp	1	153831860	153835315	0.367646	0.2274
835	Amp	1	153840801	153864145	0.325321	0.222654
836	Amp	1	153874357	153932130	0.001198	0.188353
837	Amp	1	153937817	153954576	0.050005	0.194543
838	Amp	1	153956889	153983762	0.02372	0.191267
839	Amp	1	153983915	154020216	0	0.185077
840	Amp	1	154020258	154032528	0	0.180018
841	Amp	1	154037222	154108582	0	0.184378
842	Amp	1	154116932	154141353	0	0.185941
843	Amp	1	154146810	154331500	0	0.175825
844	Amp	1	154331951	154336165	0.195309	0.210344
845	Amp	1	154336204	154346088	0.636206	0.250351
846	Amp	1	154346601	154369831	0.580011	0.245702
847	Amp	1	154372448	154524853	0.230572	0.214341

848	Amp	1	154529010	154579119	0.49169	0.238209
849	Amp	1	154579241	154587538	0.564815	0.244463
850	Amp	1	154588218	154731249	0.613517	0.248167
851	Amp	1	154732585	154810975	1.029845	0.280768
852	Amp	1	154811103	154844226	1.088806	0.284702
853	Amp	1	154845877	154850041	1.2565	0.296205
854	Amp	1	154852794	154881541	1.334042	0.302113
855	Amp	1	154882579	154902617	1.534719	0.31557
856	Amp	1	154907910	154964227	1.424227	0.308439
857	Amp	1	154968467	154972995	1.525294	0.315313
858	Amp	1	154977746	154989230	1.473764	0.31129
859	Amp	1	154989340	154993514	1.442925	0.309571
860	Amp	1	154995890	155012119	2.135909	0.354655
861	Amp	1	155014970	155024487	2.272343	0.363236
862	Amp	1	155025240	155050384	2.203122	0.359302
863	Amp	1	155050389	155051833	2.039783	0.348639
864	Amp	1	155058900	155113887	1.972699	0.344533
865	Amp	1	155123178	155135164	1.324884	0.301257
866	Amp	1	155140628	155242476	1.383826	0.30513
867	Amp	1	155243333	155266833	1.463463	0.311064
868	Amp	1	155269337	155301964	1.542961	0.316668
869	Amp	1	155305169	155313038	1.646768	0.323429
870	Amp	1	155313634	155330989	1.553308	0.317174
871	Amp	1	155333027	155336880	1.534719	0.315596
872	Amp	1	155340004	155353140	1.150167	0.289153
873	Amp	1	155353390	155353390	0.963924	0.275696
874	Amp	1	155356825	155364069	0.452609	0.234449
875	Amp	1	155368997	155380060	0.62858	0.249703
876	Amp	1	155380519	155424706	0.556748	0.243522
877	Amp	1	155429147	155513458	0.476502	0.236761
878	Amp	1	155526176	155529012	0.073369	0.197428
879	Amp	1	155529466	155542642	0.033661	0.192406
880	Amp	1	155546401	155613050	0	0.180433
881	Amp	1	155613394	155659692	0	0.185875
882	Amp	1	155666664	155677371	0	0.180732
883	Amp	1	155680870	155793212	0	0.174824
884	Amp	1	155793241	155893940	0	0.167949
885	Amp	1	155896610	155897591	0	0.166099
886	Amp	1	155898208	155988209	0	0.175391
887	Amp	1	156001901	156039096	0	0.187143
888	Amp	1	156047611	156085218	0	0.148951
889	Amp	1	156085394	156108458	0	0.145597
890	Amp	1	156109343	156144856	0	0.141093
891	Amp	1	156148518	156170495	0	0.153651
892	Amp	1	156171141	156278161	0	0.155312
893	Amp	1	156291463	156305490	0	0.142754
894	Amp	1	156305517	156321591	0	0.132423
895	Amp	1	156323013	156325019	0	0.124405
896	Amp	1	156326249	156328996	0	0.11738
897	Amp	1	156334538	156351363	0	0.130933
898	Amp	1	156358168	156424339	0	0.137195
899	Amp	1	156424689	156472463	0	0.130933
900	Amp	1	156473440	156476088	0	0.12706

901	Amp	1	156477597	156510532	0	0.129203
902	Amp	1	156510597	156511039	0	0.131172
903	Amp	1	156511317	156526396	0	0.12573
904	Amp	1	156528209	156622710	0	0.113848
905	Amp	1	156626307	156645402	0	0.120041
906	Amp	1	156648010	156713005	0	0.133377
907	Amp	1	156717637	156755161	0	0.139982
908	Amp	1	156755209	156899922	0	0.14602
909	Amp	1	156903355	156969023	0	0.126274
910	Amp	1	156971603	156979088	0	0.116982
911	Amp	1	156981387	157001875	0	0.103646
912	Amp	1	157002336	157121157	0	0.10184
913	Amp	1	157127111	157143703	0	0.093472
914	Amp	1	157144379	157157224	0	0.09875
915	Amp	1	157157326	157198281	0	0.09669
916	Amp	1	157198630	157203764	0	0.114376
917	Amp	1	157205856	157364089	0	0.112814
918	Amp	1	157367750	157408737	0	0.126233
919	Amp	1	157408847	157419823	0	0.120955
920	Amp	1	157422647	157471861	0	0.130522
921	Amp	1	157477428	157477428	0	0.136554
922	Amp	1	157482352	157541826	0	0.123134
923	Amp	1	157541949	157550105	0	0.135645
924	Amp	1	157553053	157614304	0	0.143877
925	Amp	1	157614511	157614929	0	0.151517
926	Amp	1	157616377	157645471	0	0.164402
927	Amp	1	157649704	157658565	0	0.167984
928	Amp	1	157658586	157693148	0	0.160343
929	Amp	1	157693770	157704775	0.012341	0.190224
930	Amp	1	157710324	157713937	0.175335	0.20848
931	Amp	1	157718162	157724965	0.061778	0.195969
932	Amp	1	157727418	157765836	0	0.183084
933	Amp	1	157775246	157797603	0	0.186263
934	Amp	1	157798128	157798210	0.086298	0.198982
935	Amp	1	157798562	157828936	0.056238	0.1954
936	Amp	1	157834395	157838648	0.105051	0.200687
937	Amp	1	157839752	157847627	0.230572	0.21412
938	Amp	1	157848884	157867237	0.142507	0.205149
939	Amp	1	157867895	157900302	0.098998	0.199939
940	Amp	1	157901199	157903347	0	0.186506
941	Amp	1	157907587	157944540	0.309523	0.221765
942	Amp	1	157952090	158023519	0	0.186506
943	Amp	1	158025115	158097044	0.073369	0.197295
944	Amp	1	158097057	158098688	0.056238	0.195634
945	Amp	1	158101341	158122095	0	0.182915
946	Amp	1	158124276	158157744	0	0.176281
947	Amp	1	158158548	158197421	0	0.170993
948	Amp	1	158197519	158204655	0	0.169558
949	Amp	1	158205356	158215454	0	0.176602
950	Amp	1	158216246	158244692	0	0.169985
951	Amp	1	158248110	158280527	0	0.184228
952	Amp	1	158281426	158295797	0	0.166674
953	Amp	1	158297916	158326364	0	0.158237

954	Amp	1	158330516	158353037	0	0.187855
955	Amp	1	158354635	158405868	0.02372	0.191528
956	Amp	1	158408747	158416737	0	0.18713
957	Amp	1	158417356	158440172	0.033661	0.192485
958	Amp	1	158441755	158528732	0.007199	0.189356
959	Amp	1	158528982	158533560	0	0.17985
960	Amp	1	158541869	158544421	0	0.171167
961	Amp	1	158544436	158544436	0	0.161599
962	Amp	1	158544724	158545233	0	0.14594
963	Amp	1	158545295	158545295	0	0.180337
964	Amp	1	158580102	158590278	0	0.185371
965	Amp	1	158595832	158627717	0	0.165258
966	Amp	1	158629477	158634116	0	0.172199
967	Amp	1	158634353	158663977	0	0.178239
968	Amp	1	158665271	158665518	0	0.180569
969	Amp	1	158667074	158698408	0	0.174529
970	Amp	1	158700973	158707950	0.007199	0.189124
971	Amp	1	158709833	158711390	0	0.173212
972	Amp	1	158711654	158880783	0	0.175227
973	Amp	1	158882730	158917512	0	0.178498
974	Amp	1	158917527	158973950	0	0.16931
975	Amp	1	158974009	159013653	0	0.164276
976	Amp	1	159014532	159092646	0	0.159625
977	Amp	1	159094778	159106367	0	0.172749
978	Amp	1	159107250	159132064	0	0.174528
979	Amp	1	159133669	159138907	0	0.185473
980	Amp	1	159146891	159178511	0	0.174888
981	Amp	1	159183347	159183690	0	0.144041
982	Amp	1	159184522	159185759	0	0.132446
983	Amp	1	159187158	159192234	0	0.134861
984	Amp	1	159192345	159204333	0	0.128229
985	Amp	1	159206897	159212406	0	0.134247
986	Amp	1	159212646	159216991	0	0.14463
987	Amp	1	159217645	159226975	0	0.142851
988	Amp	1	159227554	159314746	0	0.137876
989	Amp	1	159317991	159393018	0	0.147553
990	Amp	1	159403800	159408804	0	0.153244
991	Amp	1	159414278	159530548	0	0.169133
992	Amp	1	159530761	159601177	0	0.163633
993	Amp	1	159610613	159649566	0	0.165245
994	Amp	1	159650907	159673954	0	0.163276
995	Amp	1	159674341	159741019	0	0.16582
996	Amp	1	159741778	159779398	0	0.174784
997	Amp	1	159779884	159881411	0	0.185336
998	Amp	1	159884105	159888944	0.056238	0.195477
999	Amp	1	159888960	159897745	0.018387	0.190731
1000	Amp	1	159902328	159905665	0.111991	0.201456
1001	Amp	1	159910870	160001606	0.209057	0.211781
1002	Amp	1	160001994	160024923	0.236222	0.214429
1003	Amp	1	160025108	160130885	0.293283	0.22012
1004	Amp	1	160132067	160185135	0.460107	0.23529
1005	Amp	1	160189313	160189366	0.367646	0.227084
1006	Amp	1	160191416	160210027	0.421967	0.231897

1007	Amp	1	160212609	160248343	0.468576	0.236261
1008	Amp	1	160248876	160255473	0.444673	0.233717
1009	Amp	1	160255967	160345144	0.367646	0.227328
1010	Amp	1	160345555	160382306	0.430191	0.232398
1011	Amp	1	160382364	160450533	0.367646	0.226834
1012	Amp	1	160451248	160496566	0.500055	0.238659
1013	Amp	1	160497887	160505380	0.564815	0.244055
1014	Amp	1	160509343	160537294	0.421967	0.231877
1015	Amp	1	160537750	160555831	0.452609	0.234314
1016	Amp	1	160563062	160605709	0.878688	0.269837
1017	Amp	1	160610738	160650361	0.814697	0.264767
1018	Amp	1	160650813	160690667	1.208462	0.293349
1019	Amp	1	160698619	160710145	1.404612	0.306586
1020	Amp	1	160710615	160727927	1.49455	0.313257
1021	Amp	1	160730173	160749785	1.463463	0.311035
1022	Amp	1	160752274	160840003	1.315861	0.300652
1023	Amp	1	160843953	160857437	1.394558	0.306019
1024	Amp	1	160857458	160901404	1.265443	0.297408
1025	Amp	1	160904210	160920736	1.708768	0.327622
1026	Amp	1	160922482	160942110	1.918998	0.340986
1027	Amp	1	160947137	160957580	1.821927	0.334878
1028	Amp	1	160963275	161057702	1.657426	0.324262
1029	Amp	1	161062320	161124298	1.789205	0.332971
1030	Amp	1	161124623	161230697	1.463463	0.310642
1031	Amp	1	161232683	161249765	1.553308	0.317389
1032	Amp	1	161251343	161307385	1.484449	0.312022
1033	Amp	1	161308059	161309388	1.374261	0.304938
1034	Amp	1	161311578	161319748	1.344429	0.30229
1035	Amp	1	161321834	161321834	1.484449	0.312148
1036	Amp	1	161326230	161373395	1.415328	0.30758
1037	Amp	1	161374274	161374707	1.442925	0.309423
1038	Amp	1	161389176	161397337	1.334042	0.302068
1039	Amp	1	161401195	161401195	1.484449	0.311955
1040	Amp	1	161402248	161439440	1.2565	0.296785
1041	Amp	1	161476338	161476341	1.344429	0.30286
1042	Amp	1	161686708	161717471	1.190324	0.29172
1043	Amp	1	161718132	161772228	1.2565	0.296388
1044	Amp	1	161774856	161817342	1.424227	0.308417
1045	Amp	1	161821937	161861106	1.484449	0.312096
1046	Amp	1	161861196	161924688	1.727244	0.328485
1047	Amp	1	161929671	161961377	1.542961	0.316456
1048	Amp	1	161961470	161963258	1.593569	0.320085
1049	Amp	1	161966331	161970464	1.159718	0.28987
1050	Amp	1	161972701	161978337	1.295233	0.299055
1051	Amp	1	161981287	161993585	1.563139	0.317486
1052	Amp	1	161993817	161994000	1.593569	0.320137
1053	Amp	1	161994746	161997370	1.626265	0.321599
1054	Amp	1	161997497	162003620	1.553308	0.317006
1055	Amp	1	162005491	162154155	1.285475	0.298575
1056	Amp	1	162156253	162164667	1.218154	0.293906
1057	Amp	1	162164848	162175663	1.130387	0.287451
1058	Amp	1	162178531	162209376	1.049532	0.282055
1059	Amp	1	162210791	162335119	1.150167	0.289145

1060	Amp	1	162335370	162335370	1.059215	0.282963
1061	Amp	1	162338915	162359781	0.604912	0.24744
1062	Amp	1	162361110	162384716	0.860481	0.268229
1063	Amp	1	162386350	162446855	0.613517	0.248604
1064	Amp	1	162446968	162446968	0.375927	0.228065
1065	Amp	1	162447317	162475536	0.362221	0.226453
1066	Amp	1	162481911	162514125	0.556748	0.243276
1067	Amp	1	162518671	162584349	0.362221	0.226453
1068	Amp	1	162588915	162736725	0	0.175957
1069	Amp	1	162740863	162744763	0.362221	0.226296
1070	Amp	1	162745116	162761443	0.398217	0.23003
1071	Amp	1	162763619	162791178	0.038273	0.193203
1072	Amp	1	162793454	162856541	0	0.179691
1073	Amp	1	162857783	162878965	0	0.173242
1074	Amp	1	162879112	162893350	0	0.1819
1075	Amp	1	162894523	162961590	0.001198	0.188316
1076	Amp	1	162961667	162976869	0.012341	0.18976
1077	Amp	1	162977403	163049350	0.25804	0.216818
1078	Amp	1	163049407	163066418	0.27943	0.218603
1079	Amp	1	163066690	163066690	0.236222	0.214869
1080	Amp	1	163068910	163326503	0.362221	0.226048
1081	Amp	1	163326898	163363639	0.452609	0.234514
1082	Amp	1	163363858	163370719	0.523647	0.240706
1083	Amp	1	163371709	163414403	0.468576	0.236198
1084	Amp	1	163415319	163439461	0.66072	0.252266
1085	Amp	1	163439638	163465881	0.580011	0.245851
1086	Amp	1	163466050	163540952	0.667776	0.253114
1087	Amp	1	163541635	163625805	0.596354	0.246842
1088	Amp	1	163626286	163635639	0.613517	0.248298
1089	Amp	1	163637907	163646961	0.540744	0.242106
1090	Amp	1	163650357	163705697	0.556748	0.24351
1091	Amp	1	163707583	163838889	0.375927	0.227621
1092	Amp	1	163841022	163841163	0.201819	0.21125
1093	Amp	1	163846190	163974784	0.340434	0.224503
1094	Amp	1	163975475	164192512	0.375927	0.22752
1095	Amp	1	164193257	164202551	0.187743	0.209642
1096	Amp	1	164203195	164316734	0.230572	0.214004
1097	Amp	1	164322451	164324163	0.293283	0.220277
1098	Amp	1	164324478	164424730	0.636206	0.250636
1099	Amp	1	164424811	164479165	0.709359	0.256442
1100	Amp	1	164480512	164519220	0.843361	0.267165
1101	Amp	1	164519263	164554769	1.120124	0.286694
1102	Amp	1	164560385	164602134	1.020188	0.279753
1103	Amp	1	164602346	164602346	1.190324	0.291812
1104	Amp	1	164609415	164610871	1.088806	0.284714
1105	Amp	1	164612808	164691467	0.934277	0.273991
1106	Amp	1	164691584	164691584	1.039426	0.281529
1107	Amp	1	164695550	164703078	1.078696	0.283766
1108	Amp	1	164705773	164723108	0.888878	0.270422
1109	Amp	1	164726957	164741683	0.823952	0.265227
1110	Amp	1	164745185	164808192	0.796213	0.263441
1111	Amp	1	164810994	164853962	0.644689	0.251383
1112	Amp	1	164855770	164900024	0.508503	0.239098

1113 Amp	1	164904001	164904486	0.355162	0.225845
1114 Amp	1	164906058	164974783	0.508503	0.239612
1115 Amp	1	164976279	164979095	0.25804	0.216727
1116 Amp	1	164979408	164990099	0.175335	0.208636
1117 Amp	1	164993226	165005570	0.452609	0.234459
1118 Amp	1	165010578	165010578	0.532449	0.241411
1119 Amp	1	165011571	165012155	0.743132	0.259436
1120 Amp	1	165014356	165014760	0.523647	0.240779
1121 Amp	1	165021809	165067152	0.667776	0.252905
1122 Amp	1	165067699	165076297	0.619816	0.248894
1123 Amp	1	165076317	165116661	0.444673	0.234228
1124 Amp	1	165118255	165122048	0.333141	0.223503
1125 Amp	1	165122543	165150174	0.236222	0.214928
1126 Amp	1	165151396	165157711	0.215373	0.212513
1127 Amp	1	165157803	165196440	0.161592	0.206838
1128 Amp	1	165196524	165207334	0.142507	0.205393
1129 Amp	1	165207704	165213118	0.128931	0.203937
1130 Amp	1	165217138	165231329	0.068197	0.196674
1131 Amp	1	165231443	165248886	0.056238	0.19527
1132 Amp	1	165248967	165260658	0.033661	0.192389
1133 Amp	1	165267208	165277120	0	0.166521
1134 Amp	1	165280582	165305654	0.012341	0.190152
1135 Amp	1	165306476	165366171	0	0.166521
1136 Amp	1	165366381	165372659	0	0.163504
1137 Amp	1	165372813	165378642	0	0.162022
1138 Amp	1	165379850	165421437	0	0.17362
1139 Amp	1	165421556	165474126	0	0.185966
1140 Amp	1	165474223	165480102	0	0.176919
1141 Amp	1	165483631	165498628	0	0.167553
1142 Amp	1	165501389	165594703	0	0.171887
1143 Amp	1	165594964	165611875	0	0.170044
1144 Amp	1	165614494	165676283	0	0.168582
1145 Amp	1	165680478	165683078	0	0.17284
1146 Amp	1	165685211	165725681	0	0.176068
1147 Amp	1	165726342	165726489	0	0.157463
1148 Amp	1	165729946	165768230	0	0.170384
1149 Amp	1	165772306	165805478	0.02372	0.191092
1150 Amp	1	165809553	165835186	0	0.183606
1151 Amp	1	165838469	165914066	0	0.162897
1152 Amp	1	165914213	165976471	0	0.158563
1153 Amp	1	165976522	166013314	0	0.14412
1154 Amp	1	166014377	166029606	0	0.137168
1155 Amp	1	166030010	166126867	0	0.141652
1156 Amp	1	166127005	166143398	0	0.155371
1157 Amp	1	166146623	166153341	0	0.141652
1158 Amp	1	166153703	166189529	0	0.125263
1159 Amp	1	166191521	166227575	0	0.135535
1160 Amp	1	166227663	166323085	0	0.138106
1161 Amp	1	166327112	166390411	0	0.128655
1162 Amp	1	166393273	166542230	0	0.118384
1163 Amp	1	166542495	166659023	0	0.129605
1164 Amp	1	166660877	166661797	0	0.160534
1165 Amp	1	166663958	166744182	0	0.149312

1166	Amp	1	166744989	166776034	0	0.144828
1167	Amp	1	166777656	166808854	0	0.156463
1168	Amp	1	166809517	166840941	0	0.139375
1169	Amp	1	166851047	166945021	0	0.143607
1170	Amp	1	166950105	166977814	0	0.153721
1171	Amp	1	166978444	167080453	0	0.159037
1172	Amp	1	167081642	167107297	0	0.185896
1173	Amp	1	167108308	167192336	0	0.183217
1174	Amp	1	167194477	167226691	0.007199	0.189141
1175	Amp	1	167228695	167273666	0	0.176306
1176	Amp	1	167274330	167297608	0	0.164181
1177	Amp	1	167299972	167385297	0	0.1382
1178	Amp	1	167385402	167580737	0	0.132567
1179	Amp	1	167584993	167702205	0	0.13976
1180	Amp	1	167708907	167746599	0	0.165685
1181	Amp	1	167749262	167753081	0	0.137819
1182	Amp	1	167755489	167791494	0	0.139515
1183	Amp	1	167791918	167884165	0	0.123316
1184	Amp	1	167893308	167937334	0	0.136083
1185	Amp	1	167943428	168082028	0	0.130666
1186	Amp	1	168084386	168313481	0	0.12482
1187	Amp	1	168314926	168367323	0	0.129916
1188	Amp	1	168367385	168396691	0	0.123636
1189	Amp	1	168396823	168435043	0	0.127451
1190	Amp	1	168435535	168525818	0	0.145766
1191	Amp	1	168528038	168550967	0	0.177131
1192	Amp	1	168552377	168572842	0	0.171207
1193	Amp	1	168574993	168707008	0	0.187546
1194	Amp	1	168707367	168727418	0	0.18245
1195	Amp	1	168727877	168873663	0	0.166111
1196	Amp	1	168874065	169044697	0	0.173624
1197	Amp	1	169046691	169130513	0	0.166111
1198	Amp	1	169133957	169137992	0	0.170725
1199	Amp	1	169141602	169145229	0	0.157354
1200	Amp	1	169148401	169179122	0	0.139039
1201	Amp	1	169179145	169323260	0	0.146666
1202	Amp	1	169323602	169341991	0	0.139473
1203	Amp	1	169343472	169370592	0	0.129609
1204	Amp	1	169374630	169407411	0	0.152087
1205	Amp	1	169409597	169420050	0	0.154567
1206	Amp	1	169424286	169424650	0	0.161935
1207	Amp	1	169426902	169442321	0	0.165919
1208	Amp	1	169447081	169496048	0	0.14344
1209	Amp	1	169505116	169574805	0	0.139431
1210	Amp	1	169579654	169614057	0	0.147899
1211	Amp	1	169614072	169654356	0	0.143596
1212	Amp	1	169657371	169699307	0	0.141117
1213	Amp	1	169699806	169706643	0	0.145463
1214	Amp	1	169707082	169799440	0	0.155737
1215	Amp	1	169800507	169845907	0	0.145463
1216	Amp	1	169847176	169848495	0	0.148217
1217	Amp	1	169848699	169879189	0	0.135354
1218	Amp	1	169886435	169957371	0	0.127985

1219	Amp	1	169958435	169977536	0	0.143108
1220	Amp	1	169980772	169987309	0	0.137793
1221	Amp	1	169988820	170017511	0	0.133178
1222	Amp	1	170022535	170022535	0	0.156263
1223	Amp	1	170025180	170062906	0	0.167381
1224	Amp	1	170068046	170072811	0	0.173864
1225	Amp	1	170075022	170208342	0	0.15078
1226	Amp	1	170209451	170241672	0	0.166787
1227	Amp	1	170243636	170279645	0	0.153245
1228	Amp	1	170284080	170343533	0	0.135245
1229	Amp	1	170345940	170535061	0	0.117658
1230	Amp	1	170536476	170536476	0	0.114895
1231	Amp	1	170541338	170550196	0	0.154773
1232	Amp	1	170550568	170593942	0	0.161562
1233	Amp	1	170600342	170664755	0	0.140142
1234	Amp	1	170668760	170683293	0	0.161562
1235	Amp	1	170687911	170694334	0	0.121685
1236	Amp	1	170696369	170732312	0	0.117338
1237	Amp	1	170734944	170763006	0	0.108852
1238	Amp	1	170764014	170899468	0	0.11892
1239	Amp	1	170901120	170908265	0	0.107801
1240	Amp	1	170908678	170917449	0	0.101004
1241	Amp	1	170923411	170990167	0	0.10599
1242	Amp	1	170991057	171193596	0	0.145448
1243	Amp	1	171194428	171330864	0	0.143102
1244	Amp	1	171331032	171419131	0	0.141464
1245	Amp	1	171423464	171424843	0	0.105477
1246	Amp	1	171425010	171525775	0	0.102206
1247	Amp	1	171528636	171550926	0	0.113096
1248	Amp	1	171554607	171830850	0	0.115555
1249	Amp	1	171830934	171942335	0	0.117973
1250	Amp	1	171942751	171982577	0	0.109505
1251	Amp	1	171982895	171995076	0	0.116487
1252	Amp	1	171998390	172013949	0	0.132753
1253	Amp	1	172014580	172055388	0	0.130294
1254	Amp	1	172066357	172082050	0	0.123313
1255	Amp	1	172082792	172119541	0	0.128235
1256	Amp	1	172120812	172327448	0	0.134958
1257	Amp	1	172327461	172422727	0	0.129972
1258	Amp	1	172422958	172508677	0	0.12505
1259	Amp	1	172511284	172551825	0	0.138289
1260	Amp	1	172552374	172666284	0	0.135871
1261	Amp	1	172666771	172670442	0	0.122632
1262	Amp	1	172678235	172907223	0	0.109865
1263	Amp	1	172909729	172964238	0	0.114729
1264	Amp	1	172967496	173047397	0	0.116964
1265	Amp	1	173055125	173116071	0	0.10491
1266	Amp	1	173117377	173164518	0	0.11717
1267	Amp	1	173169742	173191793	0	0.124588
1268	Amp	1	173193079	173214962	0	0.106975
1269	Amp	1	173218636	173379295	0	0.099557
1270	Amp	1	173383259	173563824	0	0.096809
1271	Amp	1	173565605	173963666	0	0.092597

1272	Amp	1	173963983	174047636	0	0.105455
1273	Amp	1	174050752	174552582	0	0.092597
1274	Amp	1	174554982	174568213	0	0.109311
1275	Amp	1	174568534	174669806	0	0.11261
1276	Amp	1	174673046	174800072	0	0.106126
1277	Amp	1	174803184	174842513	0	0.102828
1278	Amp	1	174850198	174882485	0	0.116421
1279	Amp	1	174882798	174947026	0	0.114187
1280	Amp	1	174957818	175009729	0	0.119438
1281	Amp	1	175014071	175022666	0	0.122637
1282	Amp	1	175031221	175227197	0	0.109043
1283	Amp	1	175229462	175337253	0	0.100168
1284	Amp	1	175337348	175345245	0	0.116073
1285	Amp	1	175349625	175349747	0	0.128229
1286	Amp	1	175353004	175461672	0	0.12503
1287	Amp	1	175793886	175793886	0	0.109078
1288	Amp	1	175796478	175863770	0	0.103734
1289	Amp	1	175864092	175897185	0	0.105891
1290	Amp	1	175898173	175955899	0	0.116748
1291	Amp	1	175960946	175969436	0	0.10668
1292	Amp	1	175975781	176245434	0	0.103762
1293	Amp	1	176251077	176251077	0	0.110582
1294	Amp	1	176251121	176329992	0	0.120919
1295	Amp	1	176341361	176371568	0	0.155858
1296	Amp	1	176373704	176390643	0	0.149038
1297	Amp	1	176398105	176402829	0	0.15099
1298	Amp	1	176407436	176457758	0	0.153025
1299	Amp	1	176457817	176729250	0	0.154819
1300	Amp	1	176729850	176745487	0	0.164604
1301	Amp	1	176746870	176817542	0	0.169944
1302	Amp	1	176818931	176829364	0	0.164604
1303	Amp	1	176830007	176918433	0	0.165991
1304	Amp	1	176918677	176918677	0	0.170099
1305	Amp	1	176918791	176958790	0	0.187922
1306	Amp	1	176959387	177269256	0.068197	0.197015
1307	Amp	1	177272294	177336261	0.230572	0.213949
1308	Amp	1	177336669	177409253	0.068197	0.197015
1309	Amp	1	177410329	177458324	0.128931	0.203508
1310	Amp	1	177464080	177620817	0.175335	0.208696
1311	Amp	1	177627533	177629866	0.09318	0.199603
1312	Amp	1	177630057	177649485	0.073369	0.197568
1313	Amp	1	177651455	177684754	0	0.162629
1314	Amp	1	177685729	177708144	0	0.147884
1315	Amp	1	177709434	177758657	0	0.133949
1316	Amp	1	177767243	177786692	0	0.117235
1317	Amp	1	177786768	177797481	0	0.115848
1318	Amp	1	177797559	177930427	0	0.11066
1319	Amp	1	177932578	177933347	0	0.106553
1320	Amp	1	177935360	178061466	0	0.104601
1321	Amp	1	178064517	178099489	0	0.102806
1322	Amp	1	178105379	178138764	0	0.132661
1323	Amp	1	178151546	178197424	0	0.149997
1324	Amp	1	178210615	178280124	0	0.120142

1325	Amp	1	178282509	178446265	0	0.113649
1326	Amp	1	178447173	178468034	0	0.118718
1327	Amp	1	178468309	178564697	0	0.108934
1328	Amp	1	178565086	178605082	0	0.1115
1329	Amp	1	178614714	178651961	0	0.123469
1330	Amp	1	178652420	178740831	0	0.1115
1331	Amp	1	178746125	178932518	0	0.136129
1332	Amp	1	178939171	179101106	0	0.133375
1333	Amp	1	179101199	179125528	0	0.128305
1334	Amp	1	179125533	179155234	0	0.13144
1335	Amp	1	179155276	179203821	0	0.12999
1336	Amp	1	179203837	179370199	0	0.127424
1337	Amp	1	179370280	179527644	0	0.135717
1338	Amp	1	179527884	179637319	0	0.138368
1339	Amp	1	179640923	179656366	0	0.178682
1340	Amp	1	179657636	180006980	0	0.181078
1341	Amp	1	180009449	180073398	0	0.185873
1342	Amp	1	180074404	180144888	0	0.187866
1343	Amp	1	180146475	180153677	0.033661	0.192897
1344	Amp	1	180153902	180174660	0.098998	0.200402
1345	Amp	1	180181043	180244400	0.056238	0.19515
1346	Amp	1	180245029	180267329	0.098998	0.200093
1347	Amp	1	180268630	180349068	0.056238	0.19515
1348	Amp	1	180352364	180479646	0.018387	0.190356
1349	Amp	1	180481085	180593408	0.068197	0.196526
1350	Amp	1	180593603	180697969	0.018387	0.190356
1351	Amp	1	180705097	180707511	0	0.178247
1352	Amp	1	180713711	180736071	0	0.181912
1353	Amp	1	180737639	180764211	0	0.176882
1354	Amp	1	180766662	180834410	0	0.144747
1355	Amp	1	180840845	180876180	0	0.104433
1356	Amp	1	180881584	181060575	0	0.135058
1357	Amp	1	181060721	181693708	0	0.139741
1358	Amp	1	181698106	181808658	0	0.109116
1359	Amp	1	181809029	182070149	0	0.11373
1360	Amp	1	182070307	182083141	0	0.11742
1361	Amp	1	182084079	182266857	0	0.112556
1362	Amp	1	182267300	182301668	0	0.117345
1363	Amp	1	182302842	182326589	0	0.112663
1364	Amp	1	182331438	182344007	0	0.102375
1365	Amp	1	182347270	182380334	0	0.112004
1366	Amp	1	182388418	182568464	0	0.107214
1367	Amp	1	182569447	182582202	0	0.113389
1368	Amp	1	182583608	182777096	0	0.107214
1369	Amp	1	182778068	183130425	0	0.111271
1370	Amp	1	183135392	183161651	0	0.122237
1371	Amp	1	183163489	183207295	0	0.126889
1372	Amp	1	183209494	183239951	0	0.122237
1373	Amp	1	183242306	183251802	0	0.128483
1374	Amp	1	183252115	183281817	0	0.122237
1375	Amp	1	183285211	183291919	0	0.127481
1376	Amp	1	183301603	183445443	0	0.122237
1377	Amp	1	183445666	183645720	0	0.128284

1378	Amp	1	183651143	183825129	0	0.138512
1379	Amp	1	183826350	183991920	0	0.139899
1380	Amp	1	183992509	184479276	0	0.147718
1381	Amp	1	184482715	184546958	0	0.143011
1382	Amp	1	184547928	184931609	0	0.131847
1383	Amp	1	184931645	185179528	0	0.13759
1384	Amp	1	185180936	185243495	0	0.143528
1385	Amp	1	185243554	185352240	0	0.141777
1386	Amp	1	185352336	185366400	0	0.144246
1387	Amp	1	185367130	185372246	0	0.14994
1388	Amp	1	185376639	185414715	0	0.160565
1389	Amp	1	185414740	185537746	0	0.1569
1390	Amp	1	185539483	185556911	0	0.13987
1391	Amp	1	185560560	185568312	0	0.143502
1392	Amp	1	185568340	185614736	0	0.140367
1393	Amp	1	185615168	185632881	0	0.13268
1394	Amp	1	185634640	185816007	0	0.14019
1395	Amp	1	185819584	185902341	0	0.144925
1396	Amp	1	185904814	186123478	0	0.141293
1397	Amp	1	186132407	186144965	0	0.123958
1398	Amp	1	186145193	186182361	0	0.113332
1399	Amp	1	186182781	186203311	0	0.115672
1400	Amp	1	186203711	186368095	0	0.117504
1401	Amp	1	186370746	186429051	0	0.115164
1402	Amp	1	186432777	186494866	0	0.11061
1403	Amp	1	186501242	186569699	0	0.105875
1404	Amp	1	186577969	186592132	0	0.118416
1405	Amp	1	186594600	186657277	0	0.105875
1406	Amp	1	186657333	186681031	0	0.110529
1407	Amp	1	186681575	186699960	0	0.111919
1408	Amp	1	186699999	186711949	0	0.113963
1409	Amp	1	186719975	186744701	0	0.122548
1410	Amp	1	186748024	186835872	0	0.124094
1411	Amp	1	186836637	186866897	0	0.125514
1412	Amp	1	186876299	186990013	0	0.118004
1413	Amp	1	186990558	187109539	0	0.135652
1414	Amp	1	187110000	187210190	0	0.143481
1415	Amp	1	187212837	187317002	0	0.14681
1416	Amp	1	187321019	187421088	0	0.154095
1417	Amp	1	187425229	187463047	0	0.14681
1418	Amp	1	187465635	187791350	0	0.13898
1419	Amp	1	187791921	187995392	0	0.140558
1420	Amp	1	187998392	188130443	0	0.13898
1421	Amp	1	188131072	188174883	0	0.142424
1422	Amp	1	188177073	188204655	0	0.136485
1423	Amp	1	188211490	188340648	0	0.13267
1424	Amp	1	188343571	188370361	0	0.139755
1425	Amp	1	188370722	188392379	0	0.136312
1426	Amp	1	188394855	188412909	0	0.163657
1427	Amp	1	188413319	188534494	0.09318	0.19955
1428	Amp	1	188537750	188641371	0	0.172204
1429	Amp	1	188646626	188724867	0	0.178682
1430	Amp	1	188725360	188836521	0	0.180997

1431	Amp	1	188836961	188949261	0	0.150931
1432	Amp	1	188949398	189120301	0	0.146277
1433	Amp	1	189120409	189577974	0	0.1398
1434	Amp	1	189578371	189578398	0	0.144033
1435	Amp	1	189578508	189584937	0	0.136948
1436	Amp	1	189585024	189636553	0	0.141452
1437	Amp	1	189637011	189720888	0	0.148836
1438	Amp	1	189722880	189867155	0	0.1542
1439	Amp	1	189867559	189915202	0	0.141601
1440	Amp	1	189918358	189985845	0	0.153693
1441	Amp	1	189988970	190028609	0	0.14937
1442	Amp	1	190028800	190059039	0	0.152336
1443	Amp	1	190059474	190201589	0	0.169661
1444	Amp	1	190201602	190349634	0	0.165731
1445	Amp	1	190353016	190439044	0	0.157697
1446	Amp	1	190446863	190556648	0	0.164032
1447	Amp	1	190558492	190575229	0	0.150237
1448	Amp	1	190575570	190625890	0	0.155109
1449	Amp	1	190626359	190700728	0	0.148774
1450	Amp	1	190701567	190740362	0	0.153866
1451	Amp	1	190740470	190774327	0	0.174314
1452	Amp	1	190777243	191046442	0	0.161994
1453	Amp	1	191048150	191351518	0	0.171229
1454	Amp	1	191352381	191408534	0	0.173517
1455	Amp	1	191411626	191441375	0	0.181528
1456	Amp	1	191442778	191451530	0	0.173517
1457	Amp	1	191454949	191464057	0	0.168903
1458	Amp	1	191464358	191469520	0	0.173815
1459	Amp	1	191470947	191544098	0	0.180367
1460	Amp	1	191545581	191615526	0	0.185538
1461	Amp	1	191619293	191664863	0.018387	0.190835
1462	Amp	1	191664939	191712666	0	0.185923
1463	Amp	1	191719053	191780043	0.13565	0.20408
1464	Amp	1	191782793	191789400	0.293283	0.220296
1465	Amp	1	191789531	191843584	0.161592	0.207213
1466	Amp	1	191843871	191961161	0.098998	0.199956
1467	Amp	1	191961507	191998812	0.079463	0.198437
1468	Amp	1	191998939	192118189	0.156508	0.206178
1469	Amp	1	192119552	192165257	0.168144	0.208114
1470	Amp	1	192166894	192250285	0.273081	0.218102
1471	Amp	1	192250632	192394707	0.168144	0.208114
1472	Amp	1	192400736	192425120	0	0.185238
1473	Amp	1	192425526	192509959	0	0.167081
1474	Amp	1	192510529	192556067	0.073369	0.197348
1475	Amp	1	192561418	192614783	0	0.184257
1476	Amp	1	192615238	192687315	0	0.179165
1477	Amp	1	192689843	192728282	0	0.173994
1478	Amp	1	192732245	192782288	0	0.163781
1479	Amp	1	192784909	192820614	0	0.162062
1480	Amp	1	192821377	192942978	0	0.159096
1481	Amp	1	192947185	192992254	0	0.165089
1482	Amp	1	192992580	193058704	0	0.163258
1483	Amp	1	193058867	193157311	0	0.157265

1484	Amp	1	193166042	193174718	0	0.140089
1485	Amp	1	193174733	193209517	0	0.156927
1486	Amp	1	193209529	193209584	0	0.159572
1487	Amp	1	193211355	193355781	0	0.161767
1488	Amp	1	193356715	193402980	0	0.163534
1489	Amp	1	193406076	193513752	0	0.166714
1490	Amp	1	193514622	193562180	0	0.169068
1491	Amp	1	193567036	193640634	0	0.182574
1492	Amp	1	193648539	193654986	0	0.184557
1493	Amp	1	193656148	193671871	0	0.186103
1494	Amp	1	193672133	193914702	0.028398	0.192086
1495	Amp	1	193914850	193935341	0.086298	0.198992
1496	Amp	1	193948283	194152510	0	0.183449
1497	Amp	1	194153087	194412005	0.028398	0.191928
1498	Amp	1	194412392	194454381	0	0.183337
1499	Amp	1	194454761	194694365	0	0.16193
1500	Amp	1	194695239	194793123	0	0.16973
1501	Amp	1	194794524	195417767	0	0.154831
1502	Amp	1	195418457	195524583	0	0.159344
1503	Amp	1	195527027	195717802	0	0.152802
1504	Amp	1	195717877	195738360	0	0.147336
1505	Amp	1	195747646	195752611	0	0.153426
1506	Amp	1	195753230	196099187	0	0.157409
1507	Amp	1	196099363	196207809	0	0.151319
1508	Amp	1	196211071	196310276	0	0.142396
1509	Amp	1	196312485	196570709	0	0.148807
1510	Amp	1	196571763	196659115	0	0.146492
1511	Amp	1	196662072	196663226	0	0.167457
1512	Amp	1	196667305	196729142	0	0.165697
1513	Amp	1	196973325	197026072	0	0.144731
1514	Amp	1	197027174	197202936	0	0.147281
1515	Amp	1	197215829	197666004	0	0.153376
1516	Amp	1	197674468	197853639	0	0.175921
1517	Amp	1	197853832	197942960	0	0.187468
1518	Amp	1	197944027	198155004	0	0.18522
1519	Amp	1	198159104	198226853	0	0.16577
1520	Amp	1	198232938	198515213	0	0.169221
1521	Amp	1	198515253	198691595	0	0.166575
1522	Amp	1	198692077	198708674	0	0.171702
1523	Amp	1	198712608	198817824	0	0.17631
1524	Amp	1	198817945	198884741	0	0.187186
1525	Amp	1	198889748	198943422	0.086298	0.198663
1526	Amp	1	198943575	199065111	0	0.183735
1527	Amp	1	199068914	199134796	0	0.175916
1528	Amp	1	199134811	199194965	0	0.16504
1529	Amp	1	199196939	199312881	0	0.166623
1530	Amp	1	199315231	199344276	0	0.165076
1531	Amp	1	199345157	199397510	0	0.163686
1532	Amp	1	199399053	199410480	0	0.183231
1533	Amp	1	199410841	199451371	0.012341	0.19006
1534	Amp	1	199452894	199503499	0	0.181057
1535	Amp	1	199504177	199569935	0	0.170515
1536	Amp	1	199577059	199590377	0.250263	0.215901

1537	Amp	1	199590458	199592774	0.264784	0.217675
1538	Amp	1	199596610	199693582	0.117531	0.202169
1539	Amp	1	199711195	199711195	0.038273	0.193363
1540	Amp	1	199715415	199777994	0.079463	0.198497
1541	Amp	1	199782731	199809515	0.111991	0.201708
1542	Amp	1	199809815	199813353	0.09318	0.199239
1543	Amp	1	199813442	199819734	0	0.171588
1544	Amp	1	199820140	199869374	0	0.170201
1545	Amp	1	199869919	199881824	0	0.168157
1546	Amp	1	199955268	199963391	0	0.163023
1547	Amp	1	199964258	200079264	0	0.161603
1548	Amp	1	200079505	200168650	0	0.182611
1549	Amp	1	200168678	200180367	0	0.187028
1550	Amp	1	200181036	200219465	0	0.185481
1551	Amp	1	200220177	200291315	0	0.167746
1552	Amp	1	200293269	200430578	0	0.165196
1553	Amp	1	200430601	200500941	0	0.162843
1554	Amp	1	200502222	200586497	0	0.158391
1555	Amp	1	200586499	200590135	0	0.159777
1556	Amp	1	200590352	200603896	0	0.15734
1557	Amp	1	200603927	200623419	0	0.165637
1558	Amp	1	200624306	200713358	0	0.160822
1559	Amp	1	200713496	200723262	0	0.159211
1560	Amp	1	200723410	200738233	0	0.163299
1561	Amp	1	200740864	200800165	0	0.156704
1562	Amp	1	200805955	200884305	0	0.166637
1563	Amp	1	200888646	200888646	0	0.161491
1564	Amp	1	200894147	200894147	0	0.163867
1565	Amp	1	200894458	200954404	0	0.165261
1566	Amp	1	200955881	200984367	0	0.162987
1567	Amp	1	200985596	200994697	0	0.183523
1568	Amp	1	200995045	200995154	0	0.185611
1569	Amp	1	200997894	201034478	0	0.187761
1570	Amp	1	201035031	201046839	0	0.177827
1571	Amp	1	201046996	201161686	0	0.181534
1572	Amp	1	201161798	201187056	0	0.187399
1573	Amp	1	201187386	201273691	0.007199	0.189509
1574	Amp	1	201277111	201288936	0	0.184018
1575	Amp	1	201289391	201389551	0	0.187457
1576	Amp	1	201390569	201488086	0.073369	0.19754
1577	Amp	1	201488689	201511532	0.111991	0.201693
1578	Amp	1	201512341	201592547	0.128931	0.203803
1579	Amp	1	201593191	201609893	0.175335	0.208749
1580	Amp	1	201611678	201659449	0.187743	0.210217
1581	Amp	1	201659862	201694973	0.142507	0.20509
1582	Amp	1	201695509	201721350	0.18072	0.209097
1583	Amp	1	201721417	201811267	0.347934	0.225234
1584	Amp	1	201814592	201867633	0.564815	0.244027
1585	Amp	1	201870870	201967037	0.460107	0.235336
1586	Amp	1	201970406	202004437	0.51566	0.240173
1587	Amp	1	202005314	202026266	0.62858	0.249683
1588	Amp	1	202026298	202029403	0.709359	0.256744
1589	Amp	1	202034027	202154898	0.62858	0.249926

1590	Amp	1	202155381	202168612	0.571214	0.24498
1591	Amp	1	202168801	202255392	0.66072	0.252653
1592	Amp	1	202256072	202276389	0.619816	0.248947
1593	Amp	1	202277100	202288038	0.685617	0.254538
1594	Amp	1	202289576	202379786	0.66072	0.252388
1595	Amp	1	202381052	202409128	0.604912	0.24778
1596	Amp	1	202410381	202437580	0.676804	0.253739
1597	Amp	1	202441055	202461912	0.500055	0.238819
1598	Amp	1	202463032	202471533	0.652914	0.252057
1599	Amp	1	202472372	202487271	0.685617	0.254869
1600	Amp	1	202487587	202564752	0.843361	0.266836
1601	Amp	1	202570828	202634532	1.534719	0.316003
1602	Amp	1	202635258	202708050	1.637826	0.322414
1603	Amp	1	202715262	202777092	1.473764	0.311345
1604	Amp	1	202779237	202824872	1.374261	0.304935
1605	Amp	1	202826957	202857410	1.453628	0.310531
1606	Amp	1	202867255	202973458	1.678649	0.325372
1607	Amp	1	202976278	203038739	1.484449	0.312316
1608	Amp	1	203039158	203103549	1.2565	0.296711
1609	Amp	1	203109620	203151522	1.199568	0.292558
1610	Amp	1	203153042	203179794	1.646768	0.323196
1611	Amp	1	203180026	203310238	1.605306	0.320384
1612	Amp	1	203311166	203346232	1.295233	0.299138
1613	Amp	1	203348548	203403848	1.374261	0.304734
1614	Amp	1	203405017	203413241	1.503938	0.313834
1615	Amp	1	203418205	203490814	1.748357	0.329862
1616	Amp	1	203491545	203547632	1.800655	0.333762
1617	Amp	1	203549358	203669100	1.877398	0.338159
1618	Amp	1	203671637	203782843	1.918998	0.341397
1619	Amp	1	203791286	203791528	1.995109	0.34619
1620	Amp	1	203793862	203963808	1.856336	0.337313
1621	Amp	1	203963865	204028798	1.953191	0.343248
1622	Amp	1	204036925	204060965	1.866286	0.337652
1623	Amp	1	204063447	204105404	1.768015	0.331693
1624	Amp	1	204105737	204124361	1.844531	0.336198
1625	Amp	1	204126350	204126350	1.708768	0.327097
1626	Amp	1	204126419	204264648	1.877398	0.338605
1627	Amp	1	204267092	204274604	1.646768	0.32328
1628	Amp	1	204275758	204288891	1.748357	0.330134
1629	Amp	1	204291470	204302423	1.877398	0.338219
1630	Amp	1	204306015	204309100	2.005494	0.346885
1631	Amp	1	204309899	204311126	2.338154	0.367391
1632	Amp	1	204311876	204311947	2.272343	0.362886
1633	Amp	1	204313756	204334494	2.148338	0.355368
1634	Amp	1	204337919	204365912	1.768015	0.331173
1635	Amp	1	204367619	204396101	2.113683	0.353456
1636	Amp	1	204398175	204401286	2.005494	0.346602
1637	Amp	1	204401728	204419193	1.697113	0.326778
1638	Amp	1	204420186	204453403	1.646768	0.323323
1639	Amp	1	204453480	204498548	1.778794	0.332294
1640	Amp	1	204501001	204522707	1.657426	0.323628
1641	Amp	1	204525155	204545827	1.757929	0.331037
1642	Amp	1	204545831	204545895	1.918998	0.340952

1643	Amp	1	204546171	204572147	2.102209	0.352638
1644	Amp	1	204573360	204589698	1.832922	0.335314
1645	Amp	1	204591549	204601523	1.898353	0.339787
1646	Amp	1	204605874	204643484	2.180628	0.357931
1647	Amp	1	204643676	204645608	2.113683	0.353738
1648	Amp	1	204646272	204648155	2.158849	0.356495
1649	Amp	1	204649445	204683495	2.113683	0.353256
1650	Amp	1	204683770	204685806	2.039783	0.34886
1651	Amp	1	204686279	204698686	1.898353	0.339889
1652	Amp	1	204699014	204706513	1.953191	0.343271
1653	Amp	1	204708203	204734352	2.071528	0.350538
1654	Amp	1	204735453	204894842	1.953191	0.343271
1655	Amp	1	204900983	204962478	1.821927	0.334686
1656	Amp	1	204964905	204974774	1.708768	0.327299
1657	Amp	1	204975539	205095499	1.687998	0.325752
1658	Amp	1	205095548	205135673	1.484449	0.312322
1659	Amp	1	205137110	205180839	1.344429	0.302459
1660	Amp	1	205183468	205345846	1.542961	0.316202
1661	Amp	1	205346695	205351273	1.563139	0.31784
1662	Amp	1	205351494	205355766	1.463463	0.310786
1663	Amp	1	205364841	205379003	1.605306	0.320473
1664	Amp	1	205382575	205420464	1.727244	0.32854
1665	Amp	1	205422831	205473757	1.637826	0.322437
1666	Amp	1	205476697	205508311	1.572486	0.318219
1667	Amp	1	205513996	205604935	1.334042	0.302192
1668	Amp	1	205614738	205618985	1.295233	0.299266
1669	Amp	1	205619015	205654955	1.2565	0.296157
1670	Amp	1	205657722	205702002	1.374261	0.304762
1671	Amp	1	205708998	205804251	1.463463	0.311077
1672	Amp	1	205805294	205901294	1.295233	0.298999
1673	Amp	1	205901507	206033148	1.24764	0.295518
1674	Amp	1	206036160	206057416	0.604912	0.247395
1675	Amp	1	206058126	206076064	0.508503	0.239328
1676	Amp	1	206076722	206087555	0.476502	0.236929
1677	Amp	1	206088075	206165498	0.264784	0.217701
1678	Amp	1	206166649	206397447	0.195309	0.21064
1679	Amp	1	206403492	206403492	0.264784	0.217406
1680	Amp	1	206410277	206422085	0.355162	0.225668
1681	Amp	1	206422618	206429946	0.390264	0.228872
1682	Amp	1	206432695	206452351	0.286675	0.219185
1683	Amp	1	206452524	206487811	0.355162	0.225776
1684	Amp	1	206487941	206496625	0.367646	0.227238
1685	Amp	1	206500262	206551331	0.27943	0.218633
1686	Amp	1	206551409	206674030	0.309523	0.221772
1687	Amp	1	206675560	206741989	0.286675	0.219774
1688	Amp	1	206744723	206766559	0.243425	0.215229
1689	Amp	1	206769952	206806804	0.293283	0.219876
1690	Amp	1	206807632	206843303	0.347934	0.224838
1691	Amp	1	206843527	206845966	0.317596	0.222462
1692	Amp	1	206853768	207001692	0.925715	0.272985
1693	Amp	1	207005555	207018728	0.317596	0.222462
1694	Amp	1	207019832	207074489	0.293283	0.220374
1695	Amp	1	207075265	207105106	0.243425	0.215412

1696	Amp	1	207112643	207126914	0.223025	0.213302
1697	Amp	1	207126933	207148249	0.209057	0.211917
1698	Amp	1	207150041	207158496	0.09318	0.199625
1699	Amp	1	207159411	207172397	0.156508	0.206266
1700	Amp	1	207183152	207230912	0.264784	0.217646
1701	Amp	1	207233544	207353248	0.286675	0.219351
1702	Amp	1	207353796	207433747	0.325321	0.222895
1703	Amp	1	207434180	207509802	0.27943	0.218794
1704	Amp	1	207511477	207658012	0.209057	0.212204
1705	Amp	1	207665341	207678266	0.264784	0.217439
1706	Amp	1	207679585	207685486	0.362221	0.226069
1707	Amp	1	207686231	207692767	0.347934	0.224675
1708	Amp	1	207694393	207699795	0.95395	0.275175
1709	Amp	1	207700927	207744154	0.982622	0.277255
1710	Amp	1	207744837	207747443	1.049532	0.281786
1711	Amp	1	207748123	207769292	1.315861	0.300638
1712	Amp	1	207774962	207778376	1.364151	0.303796
1713	Amp	1	207779312	207785976	1.49455	0.312935
1714	Amp	1	207792786	207856895	1.637826	0.32288
1715	Amp	1	207856958	207879303	1.463463	0.310924
1716	Amp	1	207885579	207895819	1.324884	0.300979
1717	Amp	1	207895956	207895956	0.636206	0.25048
1718	Amp	1	207898844	208022736	0.652914	0.252083
1719	Amp	1	208024537	208052012	0.49169	0.237951
1720	Amp	1	208053887	208146376	0.604912	0.247959
1721	Amp	1	208150167	208166393	0.760395	0.261015
1722	Amp	1	208169501	208206515	0.852608	0.267873
1723	Amp	1	208206760	208284828	0.760395	0.260807
1724	Amp	1	208285746	208562592	0.734472	0.258697
1725	Amp	1	208565196	208596993	0.62858	0.249846
1726	Amp	1	208600172	208615481	0.175335	0.208174
1727	Amp	1	208623939	208624825	0	0.177362
1728	Amp	1	208625511	208693109	0	0.182529
1729	Amp	1	208695234	208695234	0	0.175889
1730	Amp	1	208700415	209039430	0	0.171241
1731	Amp	1	209040868	209085668	0.128931	0.203667
1732	Amp	1	209088319	209104427	0.068197	0.196739
1733	Amp	1	209105057	209158125	0.007199	0.189482
1734	Amp	1	209158444	209171627	0	0.183594
1735	Amp	1	209172502	209234420	0.018387	0.190494
1736	Amp	1	209237536	209248964	0	0.17738
1737	Amp	1	209249147	209252031	0	0.173953
1738	Amp	1	209252458	209287578	0	0.163079
1739	Amp	1	209287774	209468180	0	0.159921
1740	Amp	1	209468465	209484551	0.068197	0.196992
1741	Amp	1	209486789	209493821	0	0.186655
1742	Amp	1	209494023	209669344	0.012341	0.190195
1743	Amp	1	209671172	209725194	0	0.184147
1744	Amp	1	209725638	209850752	0.001198	0.188541
1745	Amp	1	209852482	209857956	0.028398	0.192339
1746	Amp	1	209857986	209857986	0.061778	0.196347
1747	Amp	1	209865657	210040127	0.02372	0.191214
1748	Amp	1	210040465	210049929	0.098998	0.200459

1749	Amp	1	210053883	210165529	0.215373	0.212698
1750	Amp	1	210165934	210165934	0.175335	0.208304
1751	Amp	1	210168397	210179296	0.264784	0.217306
1752	Amp	1	210181097	210216072	0	0.181849
1753	Amp	1	210216575	210369340	0.018387	0.190694
1754	Amp	1	210369902	210406179	0	0.181693
1755	Amp	1	210406322	210416651	0	0.178976
1756	Amp	1	210416809	210523462	0	0.170131
1757	Amp	1	210524679	210528055	0	0.172568
1758	Amp	1	210529529	210563164	0	0.166966
1759	Amp	1	210563493	210663733	0	0.163168
1760	Amp	1	210665185	211047189	0	0.150928
1761	Amp	1	211051772	211073399	0	0.149325
1762	Amp	1	211075201	211284479	0	0.146521
1763	Amp	1	211285892	211382133	0	0.148517
1764	Amp	1	211382554	211520670	0	0.141835
1765	Amp	1	211520856	211558613	0	0.156881
1766	Amp	1	211563532	211587389	0	0.147253
1767	Amp	1	211591313	211608615	0	0.157537
1768	Amp	1	211610697	211887976	0	0.15892
1769	Amp	1	211895993	211918681	0	0.164275
1770	Amp	1	211919458	212044660	0	0.150971
1771	Amp	1	212047318	212053220	0	0.158929
1772	Amp	1	212053945	212109545	0	0.170324
1773	Amp	1	212115999	212125503	0	0.164969
1774	Amp	1	212126368	212158996	0	0.160963
1775	Amp	1	212160269	212283747	0	0.158526
1776	Amp	1	212288870	212451407	0	0.171829
1777	Amp	1	212451485	212556992	0	0.168358
1778	Amp	1	212557365	212572822	0	0.164668
1779	Amp	1	212579312	212601835	0	0.153272
1780	Amp	1	212604944	212631623	0	0.154724
1781	Amp	1	212631887	212807785	0	0.161532
1782	Amp	1	212812572	212880831	0	0.153574
1783	Amp	1	212882972	212909162	0	0.159855
1784	Amp	1	212909618	212932836	0	0.153048
1785	Amp	1	212934341	212944583	0	0.141342
1786	Amp	1	212945252	212971509	0	0.143509
1787	Amp	1	212972062	212994420	0	0.148925
1788	Amp	1	212994693	213048873	0	0.147542
1789	Amp	1	213052904	213059370	0	0.157783
1790	Amp	1	213064151	213090488	0	0.15617
1791	Amp	1	213095840	213350084	0	0.150935
1792	Amp	1	213350590	213390475	0	0.152765
1793	Amp	1	213390665	213415856	0	0.154213
1794	Amp	1	213416059	213547514	0	0.142238
1795	Amp	1	213548771	213557594	0	0.143659
1796	Amp	1	213557657	213706504	0	0.142207
1797	Amp	1	213713766	214327847	0	0.14385
1798	Amp	1	214328512	214376042	0	0.146979
1799	Amp	1	214377933	214386868	0	0.149206
1800	Amp	1	214388770	214434292	0	0.143607
1801	Amp	1	214435361	214500102	0	0.167289

1802	Amp	1	214505057	214535187	0	0.133877
1803	Amp	1	214535355	214645701	0	0.141241
1804	Amp	1	214647151	214656573	0	0.143415
1805	Amp	1	214660839	214817412	0	0.137527
1806	Amp	1	214818267	214853173	0	0.139057
1807	Amp	1	214858459	214914312	0	0.128816
1808	Amp	1	214917463	214928799	0	0.130582
1809	Amp	1	214929079	214961527	0	0.132058
1810	Amp	1	214962043	215099217	0	0.144307
1811	Amp	1	215100469	215210896	0	0.152541
1812	Amp	1	215211004	215269270	0	0.154168
1813	Amp	1	215272262	215370653	0	0.165718
1814	Amp	1	215371398	215460531	0.018387	0.190484
1815	Amp	1	215468053	215510224	0	0.163491
1816	Amp	1	215512487	215649163	0	0.180277
1817	Amp	1	215651825	215673212	0	0.163491
1818	Amp	1	215673485	215693896	0.001198	0.188248
1819	Amp	1	215697341	215707343	0.043943	0.194391
1820	Amp	1	215714144	215720217	0	0.169634
1821	Amp	1	215727308	215727836	0	0.132041
1822	Amp	1	215728150	215790032	0	0.13607
1823	Amp	1	215794723	215794736	0	0.129927
1824	Amp	1	215795121	216232430	0	0.124511
1825	Amp	1	216236066	216238616	0	0.167963
1826	Amp	1	216240879	216293326	0	0.172578
1827	Amp	1	216300180	216360458	0	0.18345
1828	Amp	1	216362370	216405826	0.444673	0.23395
1829	Amp	1	216410371	216443561	0	0.18345
1830	Amp	1	216446930	216598369	0	0.1719
1831	Amp	1	216598475	216762639	0	0.177601
1832	Amp	1	216763034	216816619	0.043943	0.194101
1833	Amp	1	216819271	216860707	0.007199	0.189487
1834	Amp	1	216861020	217042901	0	0.183785
1835	Amp	1	217046081	217047102	0.02372	0.191247
1836	Amp	1	217047232	217313055	0	0.18458
1837	Amp	1	217313110	217336866	0	0.173708
1838	Amp	1	217340874	217410764	0	0.171712
1839	Amp	1	217410823	217415326	0	0.169776
1840	Amp	1	217417427	217474639	0	0.151878
1841	Amp	1	217483048	217558206	0	0.142045
1842	Amp	1	217558527	217576260	0	0.139602
1843	Amp	1	217576711	217584209	0	0.15836
1844	Amp	1	217586535	217680434	0	0.165376
1845	Amp	1	217684193	217876661	0	0.137499
1846	Amp	1	217876921	217911157	0	0.130483
1847	Amp	1	217917072	218199332	0	0.132005
1848	Amp	1	218201112	218325370	0	0.133526
1849	Amp	1	218328224	218713955	0	0.152462
1850	Amp	1	218717316	218758894	0	0.148264
1851	Amp	1	218760158	218807481	0	0.15376
1852	Amp	1	218807759	218970801	0	0.15649
1853	Amp	1	218971177	219041122	0	0.155014
1854	Amp	1	219044533	219173274	0	0.151203

1855	Amp	1	219177800	219417424	0	0.136466
1856	Amp	1	219417791	219431111	0	0.129003
1857	Amp	1	219431252	219561656	0	0.127377
1858	Amp	1	219566123	219567111	0	0.12521
1859	Amp	1	219567991	219697934	0	0.123734
1860	Amp	1	219698063	219762527	0	0.11348
1861	Amp	1	219762898	219789507	0	0.111715
1862	Amp	1	219790570	219896797	0	0.110193
1863	Amp	1	219900189	220179774	0	0.104697
1864	Amp	1	220180621	220247233	0	0.111086
1865	Amp	1	220251004	220576296	0	0.116109
1866	Amp	1	220577721	220667466	0	0.113744
1867	Amp	1	220671766	220710045	0	0.112222
1868	Amp	1	220710527	220820169	0	0.114189
1869	Amp	1	220822613	220822613	0	0.108031
1870	Amp	1	220823091	220901438	0	0.103138
1871	Amp	1	220902527	220942053	0	0.105435
1872	Amp	1	220942259	221152194	0	0.103138
1873	Amp	1	221155004	221192507	0	0.104956
1874	Amp	1	221194472	221211526	0	0.123584
1875	Amp	1	221217036	221298948	0	0.139607
1876	Amp	1	221299065	221382307	0	0.145722
1877	Amp	1	221387144	221693815	0	0.139607
1878	Amp	1	221695064	221753484	0	0.154999
1879	Amp	1	221753892	221830566	0	0.139607
1880	Amp	1	221832864	221839608	0	0.159414
1881	Amp	1	221840874	222124900	0	0.181588
1882	Amp	1	222125146	222182214	0	0.180167
1883	Amp	1	222183115	222244614	0.073369	0.19762
1884	Amp	1	222246111	222246183	0.111991	0.201658
1885	Amp	1	222250763	222402985	0.02372	0.191381
1886	Amp	1	222403667	222417621	0	0.172753
1887	Amp	1	222420673	222467949	0	0.179586
1888	Amp	1	222484862	222578061	0.007199	0.189578
1889	Amp	1	222582857	222703545	0.195309	0.210425
1890	Amp	1	222703872	222746553	0.223025	0.213417
1891	Amp	1	222748259	222787411	0.149443	0.206004
1892	Amp	1	222788313	222899136	0.187743	0.2099
1893	Amp	1	222903379	222941801	0.149443	0.206004
1894	Amp	1	222943232	223007081	0.230572	0.214296
1895	Amp	1	223012075	223021873	0.215373	0.212765
1896	Amp	1	223023741	223025800	0.050005	0.194433
1897	Amp	1	223030659	223118172	0.09318	0.199405
1898	Amp	1	223119208	223377942	0.123589	0.202849
1899	Amp	1	223381982	223435162	0.18072	0.20922
1900	Amp	1	223435284	223502727	0	0.16724
1901	Amp	1	223503943	223520224	0	0.165273
1902	Amp	1	223520946	223533651	0	0.16363
1903	Amp	1	223639485	223848844	0	0.168165
1904	Amp	1	223851146	223888412	0	0.171563
1905	Amp	1	223897298	223979655	0	0.150715
1906	Amp	1	223991344	224522024	0	0.159542
1907	Amp	1	224522469	224522469	0	0.161556

1908 Amp	1	224525205	224530039	0	0.151565
1909 Amp	1	224530909	224565663	0	0.156636
1910 Amp	1	224566817	224613811	0	0.141606
1911 Amp	1	224614250	224642839	0	0.145763
1912 Amp	1	224643639	224663278	0	0.13893
1913 Amp	1	224666567	224897169	0	0.164587
1914 Amp	1	224900393	224921013	0	0.151284
1915 Amp	1	224921033	224958475	0	0.147563
1916 Amp	1	224958593	224980283	0	0.158536
1917 Amp	1	224982589	225113983	0	0.148206
1918 Amp	1	225119251	225155273	0	0.145556
1919 Amp	1	225157212	225201788	0	0.142375
1920 Amp	1	225278947	225286027	0	0.150832
1921 Amp	1	225289967	225296796	0	0.148818
1922 Amp	1	225298150	225513248	0	0.139682
1923 Amp	1	225513614	225769146	0	0.14461
1924 Amp	1	225769181	225827940	0	0.136153
1925 Amp	1	225837474	225873366	0	0.137558
1926 Amp	1	225873958	226038016	0	0.140451
1927 Amp	1	226038916	226058143	0	0.137053
1928 Amp	1	226065426	226099341	0	0.145092
1929 Amp	1	226099921	226195751	0	0.151833
1930 Amp	1	226199652	226414183	0	0.155062
1931 Amp	1	226414585	226498462	0	0.150905
1932 Amp	1	226499512	226841864	0	0.142866
1933 Amp	1	226844143	227156820	0	0.136125
1934 Amp	1	227161205	227161205	0	0.14431
1935 Amp	1	227163492	227173569	0	0.154287
1936 Amp	1	227175156	227513611	0	0.130776
1937 Amp	1	227517157	227533532	0	0.136088
1938 Amp	1	227538346	227719679	0	0.133195
1939 Amp	1	227721669	227759733	0	0.131791
1940 Amp	1	227761372	227782216	0	0.115767
1941 Amp	1	227782855	227934431	0	0.113117
1942 Amp	1	227934545	228228900	0	0.107805
1943 Amp	1	228230556	228656037	0	0.115184
1944 Amp	1	228657499	228694598	0	0.133526
1945 Amp	1	228697004	228705524	0	0.139292
1946 Amp	1	228713161	228775202	0	0.126802
1947 Amp	1	228777692	228985874	0	0.12095
1948 Amp	1	228988932	229233738	0	0.135309
1949 Amp	1	229235770	229304443	0	0.152778
1950 Amp	1	229304462	229625395	0	0.148715
1951 Amp	1	229628627	229666577	0	0.135309
1952 Amp	1	229666804	229672745	0	0.128376
1953 Amp	1	229673660	229702329	0	0.138522
1954 Amp	1	229706612	229849609	0	0.131059
1955 Amp	1	229849648	230315666	0	0.132617
1956 Amp	1	230316577	230332905	0	0.151945
1957 Amp	1	230333211	230772966	0	0.132617
1958 Amp	1	230774674	230888907	0	0.129413
1959 Amp	1	230892286	230934927	0	0.132373
1960 Amp	1	230940444	231077692	0	0.147045

1961 Amp	1	231078014	231197179	0	0.145486
1962 Amp	1	231198103	231220555	0	0.149709
1963 Amp	1	231222622	231418041	0	0.127971
1964 Amp	1	231419321	231513533	0	0.115371
1965 Amp	1	231514198	231571153	0	0.1352
1966 Amp	1	231680391	231757942	0	0.115371
1967 Amp	1	231758318	231855385	0	0.121867
1968 Amp	1	231855945	231897973	0	0.127019
1969 Amp	1	231904450	231952486	0	0.139447
1970 Amp	1	231955186	232030548	0	0.135223
1971 Amp	1	232030572	232159803	0	0.141507
1972 Amp	1	232161725	232186015	0	0.134948
1973 Amp	1	232187371	232357367	0	0.129976
1974 Amp	1	232359413	232438037	0	0.151657
1975 Amp	1	232438431	232438766	0	0.154689
1976 Amp	1	232439189	232745162	0	0.133008
1977 Amp	1	232745605	232757002	0	0.124071
1978 Amp	1	232772723	232972877	0	0.110083
1979 Amp	1	232975350	233097811	0	0.128625
1980 Amp	1	233098442	233098442	0	0.149347
1981 Amp	1	233098785	233185766	0	0.144195
1982 Amp	1	233191425	233278440	0	0.125653
1983 Amp	1	233278741	233291874	0	0.136054
1984 Amp	1	233292616	233369599	0	0.124731
1985 Amp	1	233369741	233920740	0	0.130418
1986 Amp	1	233920920	233998229	0	0.127458
1987 Amp	1	233999076	234025053	0	0.130377
1988 Amp	1	234028094	234032097	0	0.136707
1989 Amp	1	234032111	234173185	0	0.173773
1990 Amp	1	234173214	234382205	0	0.167444
1991 Amp	1	234383784	234390272	0	0.172103
1992 Amp	1	234390586	234423679	0.007199	0.189433
1993 Amp	1	234428189	234437809	0.061778	0.195909
1994 Amp	1	234442901	234513089	0	0.177132
1995 Amp	1	234513898	234732186	0	0.187291
1996 Amp	1	234736417	234767459	0	0.177132
1997 Amp	1	234767524	234772540	0.033661	0.192481
1998 Amp	1	234776541	234815027	0	0.186005
1999 Amp	1	234815579	234872571	0.028398	0.192055
2000 Amp	1	234873289	234993113	0.043943	0.193875
2001 Amp	1	234994779	235153395	0.073369	0.197519
2002 Amp	1	235163265	235303249	0.02372	0.191469
2003 Amp	1	235303911	235441656	0.007199	0.189295
2004 Amp	1	235445307	235500574	0.068197	0.196665
2005 Amp	1	235515448	235530117	0	0.181316
2006 Amp	1	235531886	235542322	0	0.173946
2007 Amp	1	235542373	235822642	0	0.172108
2008 Amp	1	235822663	235854741	0	0.166342
2009 Amp	1	235860669	236046597	0	0.17419
2010 Amp	1	236051947	236105686	0	0.175746
2011 Amp	1	236112616	236113488	0	0.17419
2012 Amp	1	236114916	236346041	0	0.179251
2013 Amp	1	236347695	236481216	0	0.16827

2014 Amp	1	236488295	236504677	0.007199	0.189412
2015 Amp	1	236505545	236660649	0	0.171476
2016 Amp	1	236665356	236678349	0	0.162077
2017 Amp	1	236684633	236701720	0	0.157016
2018 Amp	1	236703536	236851800	0	0.106516
2019 Amp	1	236852328	237006457	0	0.108523
2020 Amp	1	237008086	237058950	0	0.120232
2021 Amp	1	237064443	237122834	0	0.112385
2022 Amp	1	237131707	237176691	0	0.120427
2023 Amp	1	237178128	237188054	0	0.117221
2024 Amp	1	237188195	237217888	0	0.109179
2025 Amp	1	237220323	237347614	0	0.111119
2026 Amp	1	237348619	237496290	0	0.1082
2027 Amp	1	237499270	237610040	0	0.109939
2028 Amp	1	237610127	237685013	0	0.111867
2029 Amp	1	237687842	237735135	0	0.100158
2030 Amp	1	237735429	237860118	0	0.103653
2031 Amp	1	237860277	237861041	0	0.101712
2032 Amp	1	237861141	238231855	0	0.114312
2033 Amp	1	238232389	238373208	0	0.118732
2034 Amp	1	238380041	238456715	0	0.121474
2035 Amp	1	238457072	238552011	0	0.123332
2036 Amp	1	238552503	238782297	0	0.119688
2037 Amp	1	238783246	238839392	0	0.115268
2038 Amp	1	238840222	238998098	0	0.113409
2039 Amp	1	238999962	239192855	0	0.124514
2040 Amp	1	239192994	239310255	0	0.122506
2041 Amp	1	239310381	239367618	0	0.125868
2042 Amp	1	239371233	239388378	0	0.123127
2043 Amp	1	239389088	239594389	0	0.115512
2044 Amp	1	239600130	239801172	0	0.11215
2045 Amp	1	239801807	239876798	0	0.115139
2046 Amp	1	239878754	239897028	0	0.152432
2047 Amp	1	239898823	240028354	0	0.104034
2048 Amp	1	240041029	240041029	0	0.11432
2049 Amp	1	240042757	240066481	0	0.112581
2050 Amp	1	240068425	240194337	0	0.124061
2051 Amp	1	240194873	240347101	0	0.128518
2052 Amp	1	240348174	240563927	0	0.124061
2053 Amp	1	240577323	240918042	0	0.112581
2054 Amp	1	240919388	240996417	0	0.121323
2055 Amp	1	240997365	241129221	0	0.119295
2056 Amp	1	241134600	241149036	0	0.121001
2057 Amp	1	241149548	241240544	0	0.110715
2058 Amp	1	241241874	241383712	0	0.109009
2059 Amp	1	241386942	241391340	0	0.126486
2060 Amp	1	241394008	241445103	0	0.12897
2061 Amp	1	241446458	241730636	0	0.136662
2062 Amp	1	241731586	241759937	0	0.12897
2063 Amp	1	241762409	241900849	0	0.127345
2064 Amp	1	241901331	242858507	0	0.130117
2065 Amp	1	242860040	242868583	0	0.132704
2066 Amp	1	242873365	243227523	0	0.128044

2067	Amp	1	243244815	243272562	0	0.137613
2068	Amp	1	243283565	243311039	0	0.140474
2069	Amp	1	243313692	243355985	0	0.130905
2070	Amp	1	243356099	243427402	0	0.128044
2071	Amp	1	243428254	243487556	0	0.134029
2072	Amp	1	243489705	243625029	0	0.148288
2073	Amp	1	243628010	243720827	0	0.153857
2074	Amp	1	243722546	243834278	0	0.157852
2075	Amp	1	243834285	243894192	0	0.153296
2076	Amp	1	243895822	244139405	0	0.168762
2077	Amp	1	244140802	244165415	0	0.188044
2078	Amp	1	244165428	244296459	0.073369	0.197405
2079	Amp	1	244296481	244830840	0	0.18194
2080	Amp	1	244832543	245255129	0	0.167274
2081	Amp	1	245255323	245285584	0	0.169123
2082	Amp	1	245286615	245371767	0	0.170748
2083	Amp	1	245371962	245397642	0	0.175029
2084	Amp	1	245397783	245428027	0	0.180586
2085	Amp	1	245428036	245770997	0	0.177814
2086	Amp	1	245777032	245802249	0.001198	0.188277
2087	Amp	1	245825909	245927516	0.033661	0.192537
2088	Amp	1	245927624	245927624	0	0.185752
2089	Amp	1	245927813	246435044	0	0.181471
2090	Amp	1	246436444	246917020	0	0.186899
2091	Amp	1	246917156	246992523	0.007199	0.189275
2092	Amp	1	246993082	247038420	0	0.183291
2093	Amp	1	247040355	247217308	0	0.179296
2094	Amp	1	247222815	247299399	0	0.175619
2095	Amp	1	247300251	247362664	0	0.173242
2096	Amp	1	247364781	247381465	0.061778	0.196137
2097	Amp	1	247384032	247544430	0	0.173242
2098	Amp	1	247547053	247586102	0	0.1708
2099	Amp	1	247586270	247634167	0	0.166508
2100	Amp	1	247635220	247650984	0	0.163014
2101	Amp	2	480597	548277	0	0.051152
2102	Amp	2	548746	590698	0	0.058483
2103	Amp	2	594741	609520	0	0.055921
2104	Amp	2	614031	678988	0	0.04859
2105	Amp	2	681453	3803791	0	0.052247
2106	Amp	2	3803868	3916943	0	0.04859
2107	Amp	2	3918850	4457078	0	0.043499
2108	Amp	2	4464555	4708232	0	0.04859
2109	Amp	2	4708261	5297300	0	0.043499
2110	Amp	2	5297440	6201037	0	0.039948
2111	Amp	2	6201678	6360554	0	0.043317
2112	Amp	2	6364652	6619124	0	0.039948
2113	Amp	2	6619419	6922415	0	0.044075
2114	Amp	2	6923243	7145213	0	0.047273
2115	Amp	2	7148235	7351086	0	0.044075
2116	Amp	2	7353717	7381395	0	0.047326
2117	Amp	2	7386886	7421063	0	0.050954
2118	Amp	2	7421295	7435967	0	0.047326
2119	Amp	2	7546188	8602523	0	0.044075

2120	Amp	2	8606297	8635363	0	0.056364
2121	Amp	2	8639694	8683423	0	0.044075
2122	Amp	2	8683447	8773275	0	0.039948
2123	Amp	2	8777847	9889955	0	0.041423
2124	Amp	2	9890835	9961540	0	0.046878
2125	Amp	2	9963285	10310985	0	0.041423
2126	Amp	2	10311039	10724990	0	0.035768
2127	Amp	2	10725042	10792622	0	0.04157
2128	Amp	2	10795098	11021418	0	0.043419
2129	Amp	2	11021843	11263893	0	0.037616
2130	Amp	2	11265120	11371589	0	0.048681
2131	Amp	2	11371911	11422085	0	0.054013
2132	Amp	2	11422543	11499537	0	0.052302
2133	Amp	2	11501727	11506593	0	0.050827
2134	Amp	2	11506873	11518411	0	0.045495
2135	Amp	2	11521414	11993540	0	0.03443
2136	Amp	2	11993713	12166999	0	0.032582
2137	Amp	2	12167049	12359539	0	0.034336
2138	Amp	2	12359589	13183096	0	0.032935
2139	Amp	2	13183231	14205667	0	0.037354
2140	Amp	2	14208790	15550271	0	0.032935
2141	Amp	2	15551527	15832019	0	0.044181
2142	Amp	2	15832488	16575350	0	0.049165
2143	Amp	2	16579829	16612844	0	0.059485
2144	Amp	2	16613477	16653947	0	0.064802
2145	Amp	2	16657698	16693066	0	0.059485
2146	Amp	2	16703907	16899772	0	0.054501
2147	Amp	2	16900007	17760107	0	0.059319
2148	Amp	2	17760156	18018882	0	0.048999
2149	Amp	2	18024897	18215066	0	0.037752
2150	Amp	2	18219531	18259798	0	0.049257
2151	Amp	2	18260104	18269347	0	0.037752
2152	Amp	2	18269845	18451458	0	0.032935
2153	Amp	2	18451543	18685334	0	0.041971
2154	Amp	2	18686922	19019149	0	0.032935
2155	Amp	2	19025330	19318934	0	0.036794
2156	Amp	2	19319210	20582165	0	0.032935
2157	Amp	2	20585433	20613897	0	0.028696
2158	Amp	2	20614658	20641348	0	0.023023
2159	Amp	2	20641408	20647958	0	0.028673
2160	Amp	2	20652054	20692246	0	0.008705
2161	Amp	2	20693673	20813062	0	0.012025
2162	Amp	2	20816486	20982585	0	0.018048
2163	Amp	2	20983180	21217025	0	0.012025
2164	Amp	2	21217598	21385482	0	0.008705
2165	Amp	2	21388087	21428528	0	0.021369
2166	Amp	2	21432390	23007801	0	0.008705
2167	Amp	2	23008058	23015132	0	0.01037
2168	Amp	2	23015456	23816459	0	0.008705
2169	Amp	2	23821482	23826583	0	0.011246
2170	Amp	2	23826664	24191133	0	0.009177
2171	Amp	2	24192592	24237554	0	0.012881
2172	Amp	2	24237628	24248292	0	0.010341

2173	Amp	2	24250005	24290048	0	0.012894
2174	Amp	2	24290741	24423344	0	0.010341
2175	Amp	2	24425990	24856901	0	0.011896
2176	Amp	2	24857086	24995150	0	0.016588
2177	Amp	2	25016395	25260014	0	0.015033
2178	Amp	2	25260101	25371494	0	0.010341
2179	Amp	2	25371672	25398031	0	0.018281
2180	Amp	2	25399642	25410499	0	0.010341
2181	Amp	2	25410552	25581141	0	0.011774
2182	Amp	2	25583500	25647128	0	0.017633
2183	Amp	2	25650199	25691487	0	0.020933
2184	Amp	2	25692793	25852560	0	0.015075
2185	Amp	2	25853557	25874672	0	0.01654
2186	Amp	2	25874738	25875185	0	0.013239
2187	Amp	2	25878720	25889184	0	0.029672
2188	Amp	2	25889672	26028492	0	0.013239
2189	Amp	2	26030451	26107439	0	0.032225
2190	Amp	2	26108107	26149411	0	0.034249
2191	Amp	2	26150255	26586603	0	0.038932
2192	Amp	2	26586738	26805706	0	0.029552
2193	Amp	2	26806107	26958586	0	0.035029
2194	Amp	2	26958686	27130639	0	0.033564
2195	Amp	2	27131781	27165436	0	0.028087
2196	Amp	2	27167776	27738702	0	0.029578
2197	Amp	2	27746967	27791452	0	0.04597
2198	Amp	2	27794548	27842350	0	0.051366
2199	Amp	2	27847568	28020090	0	0.056378
2200	Amp	2	28021813	28042146	0	0.063558
2201	Amp	2	28042489	28188088	0	0.058161
2202	Amp	2	28188288	28376898	0	0.064891
2203	Amp	2	28381533	28464176	0	0.086507
2204	Amp	2	28469195	28505387	0	0.078286
2205	Amp	2	28508481	28702687	0	0.073797
2206	Amp	2	28705321	28763484	0	0.088616
2207	Amp	2	29003693	29040119	0	0.075609
2208	Amp	2	29040617	29074443	0	0.104085
2209	Amp	2	29077873	29112977	0	0.079661
2210	Amp	2	29116739	29170396	0	0.053047
2211	Amp	2	29178599	29179182	0	0.079661
2212	Amp	2	29179439	29195082	0	0.07535
2213	Amp	2	29195358	29216390	0	0.070101
2214	Amp	2	29219560	29363881	0	0.045275
2215	Amp	2	29365137	29535600	0	0.028471
2216	Amp	2	29535637	29547897	0	0.031747
2217	Amp	2	29548644	29628524	0	0.03487
2218	Amp	2	29628850	29636939	0	0.040163
2219	Amp	2	29636985	29743347	0	0.036887
2220	Amp	2	29746129	29766585	0	0.033764
2221	Amp	2	29767805	29779927	0	0.030475
2222	Amp	2	29782144	29875378	0	0.025182
2223	Amp	2	29878150	29891288	0	0.019755
2224	Amp	2	29891707	29927958	0	0.025207
2225	Amp	2	29928211	29942429	0	0.033705

2226	Amp	2	29942576	30007250	0	0.021558
2227	Amp	2	30010334	30020181	0	0.041204
2228	Amp	2	30023516	30030833	0	0.048827
2229	Amp	2	30033527	30055962	0	0.05668
2230	Amp	2	30067435	30104592	0	0.076354
2231	Amp	2	30107951	30130106	0	0.080834
2232	Amp	2	30130265	30150500	0	0.063225
2233	Amp	2	30152245	30189557	0	0.057773
2234	Amp	2	30197827	30239439	0	0.050149
2235	Amp	2	30243019	30628580	0	0.067758
2236	Amp	2	30630434	30694568	0	0.072451
2237	Amp	2	30694869	30709521	0	0.064213
2238	Amp	2	30714586	30738780	0	0.05952
2239	Amp	2	30739131	30854733	0	0.065989
2240	Amp	2	30855130	30942479	0	0.070521
2241	Amp	2	30945110	30962156	0	0.066041
2242	Amp	2	30962845	30981732	0	0.081658
2243	Amp	2	30981755	31013498	0	0.070551
2244	Amp	2	31013541	31147845	0	0.075923
2245	Amp	2	31150325	31245510	0	0.079217
2246	Amp	2	31247991	31252893	0	0.073845
2247	Amp	2	31255826	31279935	0	0.10534
2248	Amp	2	31280066	31587991	0	0.091957
2249	Amp	2	31599185	31740396	0	0.087447
2250	Amp	2	31747681	31861384	0	0.091062
2251	Amp	2	31861603	32013038	0	0.105144
2252	Amp	2	32022873	32114814	0	0.093957
2253	Amp	2	32115413	32273359	0	0.102195
2254	Amp	2	32277042	32402929	0	0.119635
2255	Amp	2	33108804	33111125	0	0.081188
2256	Amp	2	33111242	33257519	0	0.077573
2257	Amp	2	33259143	33302232	0	0.082489
2258	Amp	2	33302265	33345827	0	0.077573
2259	Amp	2	33353784	33400021	0	0.089004
2260	Amp	2	33402902	34114828	0	0.077573
2261	Amp	2	34114898	34547473	0	0.08146
2262	Amp	2	34552014	34775379	0	0.077573
2263	Amp	2	34809665	35119977	0	0.068498
2264	Amp	2	35120380	35158327	0	0.065205
2265	Amp	2	35166985	35171695	0	0.083491
2266	Amp	2	35186359	35199285	0	0.077164
2267	Amp	2	35199391	35224240	0	0.079413
2268	Amp	2	35224758	35590727	0	0.077164
2269	Amp	2	35866763	36061037	0	0.043854
2270	Amp	2	36061177	36213111	0	0.049675
2271	Amp	2	36219531	36308200	0	0.043854
2272	Amp	2	36314205	36343996	0	0.025568
2273	Amp	2	36344159	36353141	0	0.030735
2274	Amp	2	36359329	36368128	0	0.026991
2275	Amp	2	36369520	36429129	0	0.035867
2276	Amp	2	36430162	36449484	0	0.038081
2277	Amp	2	36451885	36632605	0	0.041226
2278	Amp	2	36633597	36822420	0	0.036059

2279	Amp	2	36823383	36931658	0	0.03947
2280	Amp	2	36934343	36953064	0	0.030594
2281	Amp	2	36960863	37120944	0	0.02838
2282	Amp	2	37125868	37174806	0	0.038122
2283	Amp	2	37174881	37352968	0	0.042265
2284	Amp	2	37357500	38059802	0	0.037733
2285	Amp	2	38062217	38381825	0	0.046572
2286	Amp	2	38381968	38551960	0	0.04254
2287	Amp	2	38553132	38596829	0	0.051956
2288	Amp	2	38600318	38600318	0	0.042214
2289	Amp	2	38605708	38727277	0	0.032799
2290	Amp	2	38753905	38868465	0	0.039145
2291	Amp	2	38871056	39383014	0	0.04964
2292	Amp	2	39384213	39572893	0	0.05137
2293	Amp	2	39581983	39780656	0	0.057407
2294	Amp	2	39786255	39788169	0	0.065731
2295	Amp	2	39793594	39824076	0	0.059695
2296	Amp	2	39825411	40304799	0	0.05137
2297	Amp	2	40304836	40381231	0	0.066586
2298	Amp	2	40393920	40454450	0	0.075971
2299	Amp	2	40454639	41004302	0	0.071828
2300	Amp	2	41006786	41139834	0	0.073702
2301	Amp	2	41140264	41363208	0	0.077186
2302	Amp	2	41366073	41722760	0	0.047696
2303	Amp	2	41723557	41739174	0	0.030172
2304	Amp	2	41742456	41929911	0	0.028257
2305	Amp	2	41930515	41978364	0	0.042318
2306	Amp	2	41978810	42042147	0	0.028257
2307	Amp	2	42042612	42080481	0	0.034231
2308	Amp	2	42090201	42090609	0	0.028257
2309	Amp	2	42090943	42097916	0	0.022946
2310	Amp	2	42102189	42174007	0	0.030476
2311	Amp	2	42175875	42189363	0	0.035388
2312	Amp	2	42192689	42498733	0	0.027857
2313	Amp	2	42499101	42584266	0	0.026288
2314	Amp	2	42586654	42733805	0	0.040119
2315	Amp	2	42738250	42788781	0	0.030734
2316	Amp	2	42792004	42876474	0	0.016903
2317	Amp	2	42876814	42928803	0	0.011992
2318	Amp	2	42934040	43012286	0	0.005285
2319	Amp	2	43015004	43015168	0	0.020261
2320	Amp	2	43018256	43079686	0	0.046426
2321	Amp	2	43087037	43153454	0	0.031451
2322	Amp	2	43154333	43213775	0	0.055808
2323	Amp	2	43214443	43276430	0	0.005285
2324	Amp	2	43282796	43730995	0	0.03711
2325	Amp	2	43732904	43814621	0	0.029979
2326	Amp	2	43821207	43960767	0	0.043303
2327	Amp	2	43962468	44014642	0	0.018608
2328	Amp	2	44014894	44078322	0	0.02508
2329	Amp	2	44079445	44091513	0	0.029647
2330	Amp	2	44091618	44622652	0	0.031089
2331	Amp	2	44624195	44658861	0	0.036873

2332 Amp	2	44659015	44786399	0	0.032307
2333 Amp	2	44786586	44890840	0	0.036188
2334 Amp	2	44891134	44899240	0	0.038816
2335 Amp	2	44899352	45023845	0	0.034934
2336 Amp	2	45025576	45139861	0	0.032307
2337 Amp	2	45141399	45166207	0	0.034153
2338 Amp	2	45173321	45316700	0	0.039318
2339 Amp	2	45318268	45455132	0	0.050943
2340 Amp	2	45456363	45481609	0	0.043312
2341 Amp	2	45484308	45494550	0	0.031688
2342 Amp	2	45496949	45537515	0	0.026523
2343 Amp	2	45541795	45559496	0	0.020051
2344 Amp	2	45560534	46197982	0	0.023068
2345 Amp	2	46200220	46449501	0	0.026133
2346 Amp	2	46450059	46531173	0	0.024691
2347 Amp	2	46531607	46534980	0	0.041292
2348 Amp	2	46535020	46610866	0	0.058428
2349 Amp	2	46611108	46656600	0	0.074806
2350 Amp	2	46671213	46756102	0	0.058428
2351 Amp	2	46758786	46781436	0	0.060262
2352 Amp	2	46783235	46900895	0	0.06428
2353 Amp	2	46901264	46977357	0	0.072938
2354 Amp	2	46980156	47096155	0	0.064962
2355 Amp	2	47111792	47132527	0	0.071128
2356 Amp	2	47133563	47133882	0	0.068541
2357 Amp	2	47144255	47244521	0	0.078152
2358 Amp	2	47245206	47364508	0	0.074134
2359 Amp	2	47365379	47411770	0	0.067967
2360 Amp	2	47413427	47957060	0	0.066134
2361 Amp	2	47962854	48274724	0	0.057509
2362 Amp	2	48276931	48513161	0	0.053356
2363 Amp	2	48513954	48582734	0	0.047273
2364 Amp	2	48586913	48799789	0	0.049272
2365 Amp	2	48829828	49064234	0	0.057025
2366 Amp	2	49065202	49318679	0	0.059445
2367 Amp	2	49321236	49522272	0	0.051692
2368 Amp	2	49522533	49591185	0	0.054326
2369 Amp	2	49594582	49674248	0	0.041002
2370 Amp	2	49676091	49711876	0	0.034725
2371 Amp	2	49714818	49725484	0	0.039864
2372 Amp	2	49727074	49731791	0	0.068925
2373 Amp	2	49732020	50285921	0	0.039864
2374 Amp	2	50287608	50536003	0	0.034725
2375 Amp	2	50536123	50571371	0	0.039853
2376 Amp	2	50578669	50965967	0	0.072229
2377 Amp	2	51110349	51578812	0	0.062787
2378 Amp	2	51581914	51725344	0	0.054832
2379 Amp	2	51734474	51847314	0	0.068354
2380 Amp	2	51847619	52372830	0	0.072127
2381 Amp	2	52373171	52473331	0	0.059263
2382 Amp	2	52475346	52504828	0	0.064006
2383 Amp	2	52504841	52504841	0	0.058878
2384 Amp	2	52624435	52716652	0	0.065408

2385	Amp	2	52717257	52789677	0	0.070678
2386	Amp	2	52790122	52954310	0	0.065408
2387	Amp	2	52956739	53007520	0	0.058878
2388	Amp	2	53007753	53092063	0	0.067024
2389	Amp	2	53092862	53134376	0	0.073919
2390	Amp	2	53134593	53347048	0	0.091168
2391	Amp	2	53350292	53420651	0	0.099255
2392	Amp	2	53421312	53562166	0	0.091168
2393	Amp	2	53570459	53612388	0	0.077359
2394	Amp	2	53612884	53677322	0	0.062355
2395	Amp	2	53679798	54479883	0	0.058582
2396	Amp	2	54480019	54484388	0	0.061852
2397	Amp	2	54487225	54668882	0	0.067596
2398	Amp	2	54670451	54896364	0	0.061852
2399	Amp	2	54897275	54931906	0	0.068764
2400	Amp	2	54937629	55027029	0	0.07407
2401	Amp	2	55031568	55286656	0	0.067159
2402	Amp	2	55288954	55680328	0	0.061852
2403	Amp	2	55714305	55735945	0	0.058413
2404	Amp	2	55749451	55978689	0	0.053286
2405	Amp	2	55980613	56011222	0	0.057839
2406	Amp	2	56014162	56161078	0	0.067855
2407	Amp	2	56175280	56225284	0	0.063302
2408	Amp	2	56226453	56561293	0	0.046391
2409	Amp	2	56562904	56982612	0	0.042198
2410	Amp	2	56982883	57598893	0	0.039564
2411	Amp	2	57600501	57961173	0	0.045355
2412	Amp	2	57961325	58145843	0	0.05217
2413	Amp	2	58148427	58265210	0	0.055647
2414	Amp	2	58265306	58371584	0	0.06351
2415	Amp	2	58375550	58397352	0	0.055647
2416	Amp	2	58407758	58881805	0	0.048832
2417	Amp	2	58883193	58897834	0	0.053449
2418	Amp	2	58897864	58972268	0	0.049972
2419	Amp	2	58975452	59012999	0	0.045355
2420	Amp	2	59013341	59086985	0	0.047975
2421	Amp	2	59092893	59541035	0	0.042184
2422	Amp	2	59543218	60014897	0	0.039564
2423	Amp	2	60016820	60025335	0	0.048975
2424	Amp	2	60025416	60063225	0	0.052068
2425	Amp	2	60064069	60318768	0	0.042658
2426	Amp	2	60318976	60346785	0	0.040783
2427	Amp	2	60349127	60461777	0	0.02878
2428	Amp	2	60462613	60482124	0	0.034564
2429	Amp	2	60485449	60539927	0	0.042867
2430	Amp	2	60544669	60562176	0	0.041442
2431	Amp	2	60563308	60827022	0	0.044005
2432	Amp	2	60827618	60877311	0	0.039444
2433	Amp	2	60877850	61019118	0	0.033661
2434	Amp	2	61022541	61067987	0	0.03697
2435	Amp	2	61068338	61163656	0	0.031784
2436	Amp	2	61164348	61382376	0	0.040226
2437	Amp	2	61384137	61419952	0	0.048474

2438	Amp	2	61424190	61494855	0	0.052584
2439	Amp	2	61499263	61545288	0	0.058818
2440	Amp	2	61549767	61660465	0	0.05057
2441	Amp	2	61661567	61759781	0	0.0535
2442	Amp	2	61759954	61827142	0	0.056884
2443	Amp	2	61835553	61888008	0	0.061829
2444	Amp	2	61891195	62140440	0	0.057718
2445	Amp	2	62140585	62169136	0	0.054335
2446	Amp	2	62172079	62207644	0	0.060993
2447	Amp	2	62211933	62228810	0	0.056048
2448	Amp	2	62235078	62256130	0	0.04939
2449	Amp	2	62256469	62311911	0	0.040948
2450	Amp	2	62312214	62550724	0	0.034714
2451	Amp	2	62553002	62604933	0	0.046024
2452	Amp	2	62616619	62701012	0	0.034714
2453	Amp	2	62702956	62748384	0	0.041655
2454	Amp	2	62749263	62947186	0	0.034714
2455	Amp	2	62949134	62999789	0	0.031303
2456	Amp	2	63003599	63247165	0	0.040684
2457	Amp	2	63247769	63486128	0	0.031303
2458	Amp	2	63486573	63573154	0	0.021692
2459	Amp	2	63576125	63771965	0	0.023094
2460	Amp	2	63776762	63898207	0	0.0307
2461	Amp	2	63902210	63933876	0	0.023094
2462	Amp	2	63937472	64498846	0	0.021692
2463	Amp	2	64499667	65451299	0	0.018599
2464	Amp	2	65454038	65579893	0	0.015669
2465	Amp	2	65581709	65683042	0	0.021116
2466	Amp	2	65686445	65861838	0	0.017845
2467	Amp	2	65862055	66000267	0	0.012398
2468	Amp	2	66000961	66810155	0	0.015914
2469	Amp	2	66814545	67083385	0	0.020424
2470	Amp	2	67084092	67161371	0	0.015914
2471	Amp	2	67161665	67309310	0	0.019362
2472	Amp	2	67309333	67309570	0	0.02103
2473	Amp	2	67310097	67692877	0	0.017582
2474	Amp	2	67693224	67965401	0	0.022305
2475	Amp	2	67965544	68253839	0	0.017582
2476	Amp	2	68257781	68346569	0	0.020699
2477	Amp	2	68350000	68351226	0	0.033128
2478	Amp	2	68351626	68360851	0	0.03001
2479	Amp	2	68361437	68771874	0	0.017582
2480	Amp	2	68779589	68821472	0	0.025686
2481	Amp	2	68823254	68879607	0	0.017582
2482	Amp	2	68883621	69460092	0	0.027399
2483	Amp	2	69460170	69579736	0	0.021539
2484	Amp	2	69586326	69677649	0	0.025959
2485	Amp	2	69682724	69735837	0	0.034122
2486	Amp	2	69738344	69845332	0	0.037306
2487	Amp	2	69850699	69885448	0	0.029003
2488	Amp	2	69888147	69933715	0	0.02084
2489	Amp	2	69933850	70055959	0	0.017656
2490	Amp	2	70059560	70060392	0	0.023627

2491	Amp	2	70063948	70227380	0	0.027365
2492	Amp	2	70242952	70297010	0	0.024789
2493	Amp	2	70297090	70631117	0	0.021051
2494	Amp	2	70634189	70674444	0	0.026501
2495	Amp	2	70677967	70819404	0	0.021051
2496	Amp	2	70824987	70839147	0	0.028322
2497	Amp	2	70849342	70955583	0	0.043009
2498	Amp	2	70955770	71008607	0	0.052782
2499	Amp	2	71008875	71081682	0	0.045726
2500	Amp	2	71082456	71141435	0	0.040866
2501	Amp	2	71141478	71376362	0	0.045778
2502	Amp	2	71378274	71463105	0	0.038147
2503	Amp	2	71463670	71502003	0	0.028322
2504	Amp	2	71502656	71875573	0	0.029714
2505	Amp	2	71879316	72094865	0	0.03438
2506	Amp	2	72095833	72562188	0	0.029714
2507	Amp	2	72566672	72688159	0	0.023743
2508	Amp	2	72688642	72769307	0	0.022351
2509	Amp	2	72773786	73130477	0	0.030599
2510	Amp	2	73130508	73442334	0	0.03487
2511	Amp	2	73453690	73460088	0	0.052248
2512	Amp	2	73466674	73477961	0	0.063708
2513	Amp	2	73479395	73764609	0	0.04633
2514	Amp	2	73776484	73879703	0	0.049356
2515	Amp	2	73879989	74127352	0	0.037665
2516	Amp	2	74132732	74142720	0	0.035997
2517	Amp	2	74144868	74173277	0	0.047828
2518	Amp	2	74179895	74194036	0	0.059447
2519	Amp	2	74196126	74303786	0	0.061994
2520	Amp	2	74307281	74339058	0	0.066999
2521	Amp	2	74344433	74466857	0	0.071216
2522	Amp	2	74469619	74470227	0	0.059386
2523	Amp	2	74479931	74518953	0	0.064579
2524	Amp	2	74519195	74532065	0	0.062032
2525	Amp	2	74532210	74845410	0	0.06938
2526	Amp	2	74846339	75002104	0	0.064187
2527	Amp	2	75004227	75011234	0	0.05997
2528	Amp	2	75011562	75181892	0	0.062285
2529	Amp	2	75182069	75301611	0	0.068273
2530	Amp	2	75304233	75393110	0	0.079851
2531	Amp	2	75398155	75592270	0	0.077536
2532	Amp	2	75593565	75593565	0	0.071549
2533	Amp	2	75692129	75741725	0	0.05997
2534	Amp	2	75742817	75822655	0	0.064409
2535	Amp	2	75827879	75882285	0	0.061384
2536	Amp	2	75886583	75914291	0	0.068193
2537	Amp	2	75917556	75957690	0	0.075254
2538	Amp	2	75958915	75966004	0	0.07665
2539	Amp	2	75966680	75966680	0	0.069589
2540	Amp	2	75975998	75989803	0	0.06278
2541	Amp	2	75990181	75996175	0	0.068411
2542	Amp	2	75997490	76011990	0	0.071264
2543	Amp	2	76016936	76134441	0	0.077985

2544	Amp	2	76137597	76137597	0	0.071264
2545	Amp	2	76144328	76370126	0	0.064237
2546	Amp	2	76372163	76393657	0	0.065679
2547	Amp	2	76393815	77106275	0	0.058555
2548	Amp	2	77106293	77239256	0	0.062577
2549	Amp	2	77239875	77294195	0	0.07433
2550	Amp	2	77299819	77326244	0	0.084757
2551	Amp	2	77328780	77351684	0	0.07433
2552	Amp	2	77352066	77898588	0	0.070308
2553	Amp	2	77899104	78055275	0	0.064535
2554	Amp	2	78055644	78077316	0	0.057187
2555	Amp	2	78077669	78141013	0	0.068191
2556	Amp	2	78141798	78407977	0	0.063752
2557	Amp	2	78411610	78756907	0	0.052748
2558	Amp	2	78756922	78756922	0	0.04879
2559	Amp	2	79470645	79472285	0	0.041507
2560	Amp	2	79472456	79721933	0	0.046419
2561	Amp	2	79725385	79758404	0	0.052119
2562	Amp	2	79758479	79812483	0	0.059741
2563	Amp	2	79813797	80030021	0	0.052119
2564	Amp	2	80030206	80331168	0	0.046419
2565	Amp	2	80331215	80702243	0	0.040519
2566	Amp	2	80702275	81387105	0	0.035606
2567	Amp	2	81388304	81614871	0	0.043856
2568	Amp	2	81616898	81670680	0	0.071914
2569	Amp	2	81672653	81969821	0	0.063665
2570	Amp	2	82090732	82524686	0	0.069103
2571	Amp	2	82534319	82921044	0	0.091153
2572	Amp	2	82921372	83656424	0	0.059857
2573	Amp	2	83656838	83761345	0	0.061561
2574	Amp	2	83764462	83798217	0	0.082908
2575	Amp	2	83800625	83825750	0	0.061561
2576	Amp	2	83827796	83851036	0	0.050403
2577	Amp	2	83851102	84081985	0	0.058372
2578	Amp	2	84082654	84206772	0	0.06791
2579	Amp	2	84207270	84283967	0	0.076872
2580	Amp	2	84292539	84300084	0	0.091701
2581	Amp	2	84301135	84308063	0	0.097383
2582	Amp	2	84309836	84339538	0	0.092296
2583	Amp	2	84341900	84394026	0	0.082554
2584	Amp	2	84395793	84473375	0	0.087748
2585	Amp	2	84477130	84604141	0	0.078786
2586	Amp	2	84604643	84699360	0	0.087867
2587	Amp	2	84699389	84807495	0	0.09591
2588	Amp	2	84807609	84839056	0	0.097455
2589	Amp	2	84841264	84844851	0	0.092261
2590	Amp	2	84849916	84869490	0	0.078415
2591	Amp	2	84872127	84872127	0	0.075273
2592	Amp	2	84874613	85143707	0	0.069591
2593	Amp	2	85148068	85417470	0	0.073932
2594	Amp	2	85420423	85478511	0	0.064394
2595	Amp	2	85491001	85540743	0	0.060053
2596	Amp	2	85542852	85583869	0	0.072645

2597	Amp	2	85589211	85742397	0	0.076818
2598	Amp	2	85742677	85782550	0	0.071704
2599	Amp	2	85783120	85801481	0	0.06753
2600	Amp	2	85802709	85858022	0	0.070524
2601	Amp	2	85863363	85874776	0	0.059407
2602	Amp	2	85874897	85886440	0	0.063729
2603	Amp	2	85886573	85942866	0	0.053935
2604	Amp	2	85942956	85945218	0	0.050942
2605	Amp	2	85945520	86307794	0	0.046619
2606	Amp	2	86308271	86319023	0	0.04215
2607	Amp	2	86323119	86562473	0	0.038184
2608	Amp	2	86570575	86628877	0	0.047617
2609	Amp	2	86630024	86843936	0	0.049582
2610	Amp	2	86846819	86878530	0	0.048036
2611	Amp	2	86878753	86881088	0	0.038603
2612	Amp	2	87894092	88318119	0	0.031396
2613	Amp	2	88318577	88831554	0	0.036961
2614	Amp	2	94675825	94675975	0	0.078524
2615	Amp	2	94680914	94685288	0	0.085282
2616	Amp	2	94693073	94984458	0	0.089281
2617	Amp	2	94999988	95228730	0	0.099437
2618	Amp	2	95231972	95247542	0	0.08548
2619	Amp	2	95248731	95292627	0	0.079526
2620	Amp	2	95299538	95300673	0	0.083217
2621	Amp	2	95301778	95329356	0	0.073061
2622	Amp	2	95332191	95362574	0	0.06937
2623	Amp	2	95363411	95386725	0	0.070862
2624	Amp	2	95387203	95405756	0	0.066863
2625	Amp	2	95416225	95962873	0	0.061779
2626	Amp	2	95964522	96089334	0	0.067387
2627	Amp	2	96091371	96251870	0	0.072248
2628	Amp	2	96252507	96359572	0	0.081911
2629	Amp	2	96370471	96437496	0	0.072248
2630	Amp	2	96440714	96714824	0	0.067387
2631	Amp	2	96719417	96907892	0	0.07153
2632	Amp	2	96911580	96965848	0	0.07516
2633	Amp	2	96965961	96985483	0	0.071953
2634	Amp	2	96985926	97048036	0	0.081699
2635	Amp	2	97049417	97049417	0	0.092075
2636	Amp	2	97658064	97660859	0	0.090371
2637	Amp	2	97662587	97704399	0	0.098837
2638	Amp	2	97708337	97845583	0	0.109663
2639	Amp	2	97846094	98440299	0	0.113289
2640	Amp	2	98441177	98447290	0	0.105057
2641	Amp	2	98451079	98640725	0	0.101431
2642	Amp	2	98643329	98721187	0	0.082534
2643	Amp	2	98722065	98875307	0	0.074068
2644	Amp	2	98879416	99200766	0	0.063242
2645	Amp	2	99201151	99366542	0	0.059099
2646	Amp	2	99368099	99434070	0	0.061924
2647	Amp	2	99434662	99657583	0	0.057426
2648	Amp	2	99658770	99684501	0	0.053795
2649	Amp	2	99687256	99772398	0	0.05966

2650	Amp	2	99772941	99833748	0	0.053491
2651	Amp	2	99834104	99859793	0	0.055094
2652	Amp	2	99861234	99938713	0	0.050683
2653	Amp	2	99943907	100103107	0	0.044818
2654	Amp	2	100111978	100179786	0	0.033645
2655	Amp	2	100181262	100195508	0	0.025668
2656	Amp	2	100198396	100202017	0	0.040178
2657	Amp	2	100202749	100302550	0	0.038408
2658	Amp	2	100308386	100970993	0	0.040417
2659	Amp	2	100975963	101005691	0	0.06209
2660	Amp	2	101009666	101022426	0	0.040417
2661	Amp	2	101024937	101032965	0	0.037592
2662	Amp	2	101033032	101219433	0	0.062417
2663	Amp	2	101220595	101430786	0	0.073606
2664	Amp	2	101434341	101817571	0	0.065358
2665	Amp	2	101820851	101941052	0	0.071308
2666	Amp	2	101944332	102015602	0	0.090218
2667	Amp	2	102016616	102210783	0	0.098388
2668	Amp	2	102210871	102298194	0	0.090218
2669	Amp	2	102299202	102314488	0	0.039695
2670	Amp	2	102316412	102769766	0	0.020785
2671	Amp	2	102776577	102831183	0	0.032163
2672	Amp	2	102833295	102911853	0	0.034978
2673	Amp	2	102916093	102968480	0	0.036423
2674	Amp	2	102968964	103046380	0	0.034931
2675	Amp	2	103046853	103081917	0	0.038022
2676	Amp	2	103082926	103103044	0	0.047367
2677	Amp	2	103105552	103128747	0	0.049029
2678	Amp	2	103132349	103152633	0	0.039684
2679	Amp	2	103158977	103178014	0	0.028306
2680	Amp	2	103182880	103196092	0	0.03014
2681	Amp	2	103197157	103257202	0	0.028698
2682	Amp	2	103259563	103267379	0	0.036431
2683	Amp	2	103268109	103312014	0	0.034987
2684	Amp	2	103317011	103430802	0	0.027254
2685	Amp	2	103430872	103460942	0	0.023784
2686	Amp	2	103463510	103485515	0	0.02195
2687	Amp	2	103486308	103643878	0	0.019135
2688	Amp	2	103648367	103657096	0	0.016654
2689	Amp	2	103660773	103811849	0	0.01928
2690	Amp	2	103814706	104010023	0	0.016188
2691	Amp	2	104015885	104075696	0	0.060816
2692	Amp	2	104077911	104087280	0	0.037986
2693	Amp	2	104090109	104158332	0	0.016188
2694	Amp	2	104158418	104267576	0	0.02239
2695	Amp	2	104269231	104411633	0	0.016188
2696	Amp	2	104412016	104561085	0	0.021154
2697	Amp	2	104561182	104612194	0	0.027332
2698	Amp	2	104615412	104623631	0	0.041422
2699	Amp	2	104623999	104701308	0	0.027332
2700	Amp	2	104703068	104718589	0	0.025323
2701	Amp	2	104720188	104825941	0	0.019145
2702	Amp	2	104826320	104951411	0	0.021258

2703	Amp	2	104952590	105031875	0	0.016292
2704	Amp	2	105032359	105088864	0	0.022316
2705	Amp	2	105089352	105098729	0	0.01969
2706	Amp	2	105099342	105148319	0	0.018028
2707	Amp	2	105148503	105223578	0	0.023435
2708	Amp	2	105229830	105279713	0	0.031864
2709	Amp	2	105282062	105351727	0	0.023435
2710	Amp	2	105352117	105420283	0	0.015868
2711	Amp	2	105422134	105768041	0	0.012005
2712	Amp	2	105775456	105857018	0	0.020516
2713	Amp	2	105860636	105871955	0	0.016954
2714	Amp	2	105872939	105914604	0	0.019044
2715	Amp	2	105917231	106016762	0	0.016954
2716	Amp	2	106016945	106223865	0	0.021565
2717	Amp	2	106226560	106271414	0	0.019961
2718	Amp	2	106271472	106713965	0	0.025982
2719	Amp	2	106714018	106753764	0	0.023285
2720	Amp	2	106754502	106801206	0	0.025238
2721	Amp	2	106801580	106962134	0	0.019217
2722	Amp	2	106963948	107030309	0	0.023159
2723	Amp	2	107040355	107072269	0	0.034977
2724	Amp	2	107072294	107180278	0	0.023159
2725	Amp	2	107180507	107286141	0	0.021207
2726	Amp	2	107289329	107294807	0	0.065627
2727	Amp	2	107301515	107305982	0	0.057116
2728	Amp	2	107307626	107320301	0	0.012696
2729	Amp	2	107416458	107440248	0	0.016331
2730	Amp	2	107447953	107466883	0	0.060334
2731	Amp	2	107472243	107493218	0	0.02893
2732	Amp	2	107493977	107494021	0	0.024987
2733	Amp	2	107495018	107541069	0	0.055954
2734	Amp	2	107543546	107726251	0	0.062006
2735	Amp	2	107728143	107820730	0	0.065029
2736	Amp	2	107823682	107958370	0	0.115552
2737	Amp	2	107966475	107973183	0	0.113638
2738	Amp	2	107973397	107990738	0	0.115995
2739	Amp	2	107991638	108009503	0	0.088139
2740	Amp	2	108012703	108048505	0	0.091955
2741	Amp	2	108048649	108141177	0	0.119811
2742	Amp	2	108143047	108172186	0	0.116176
2743	Amp	2	108174152	108247515	0	0.114063
2744	Amp	2	108247989	108275272	0	0.118309
2745	Amp	2	108281450	108368033	0	0.0913
2746	Amp	2	108371078	108390953	0	0.085127
2747	Amp	2	108397234	108449463	0	0.0897
2748	Amp	2	108463682	108491315	0	0.116709
2749	Amp	2	108493590	108562048	0	0.113686
2750	Amp	2	108562157	108727501	0	0.104867
2751	Amp	2	108740889	109195670	0	0.114826
2752	Amp	2	109201398	109205292	0	0.087817
2753	Amp	2	109206990	109255246	0	0.054971
2754	Amp	2	109259288	109260171	0	0.082417
2755	Amp	2	109262410	109388057	0	0.086169

2756	Amp	2	109388752	109485171	0	0.090665
2757	Amp	2	109485619	109640822	0	0.086169
2758	Amp	2	109651654	109701928	0	0.083574
2759	Amp	2	109704919	109704919	0	0.07011
2760	Amp	2	110640222	110640222	0	0.026106
2761	Amp	2	110643085	110694239	0	0.020054
2762	Amp	2	110702039	110720389	0	0.030138
2763	Amp	2	110720419	111190770	0	0.032055
2764	Amp	2	111191232	111759305	0	0.036855
2765	Amp	2	111762497	111858909	0	0.026772
2766	Amp	2	111865911	112103038	0	0.030938
2767	Amp	2	112103357	112512047	0	0.03269
2768	Amp	2	112514035	112665158	0	0.036352
2769	Amp	2	112672410	112728123	0	0.032599
2770	Amp	2	112730611	112891641	0	0.038225
2771	Amp	2	112892598	112940559	0	0.041815
2772	Amp	2	112940733	113031739	0	0.045378
2773	Amp	2	113032064	113037675	0	0.040651
2774	Amp	2	113039988	113040107	0	0.036799
2775	Amp	2	113043853	113054199	0	0.040774
2776	Amp	2	113057823	113218966	0	0.083466
2777	Amp	2	113225031	113377446	0	0.086107
2778	Amp	2	113382285	113382838	0	0.08419
2779	Amp	2	113385740	113478959	0	0.080024
2780	Amp	2	113478973	113646549	0	0.067436
2781	Amp	2	113648819	113798588	0	0.064794
2782	Amp	2	113802628	113886830	0	0.053622
2783	Amp	2	113887719	114100221	0	0.060919
2784	Amp	2	114103804	114162622	0	0.071482
2785	Amp	2	114164913	114227715	0	0.060919
2786	Amp	2	114229207	114403133	0	0.031569
2787	Amp	2	114403317	114561104	0	0.040042
2788	Amp	2	114563836	114751887	0	0.031569
2789	Amp	2	114755253	115114060	0	0.040305
2790	Amp	2	115119059	115256501	0	0.083492
2791	Amp	2	115259106	115431028	0	0.074443
2792	Amp	2	115435209	115522515	0	0.124966
2793	Amp	2	115526049	115554585	0	0.08637
2794	Amp	2	115557547	116186647	0	0.074443
2795	Amp	2	116192916	116426114	0	0.062867
2796	Amp	2	116426277	116561337	0	0.064756
2797	Amp	2	116562444	116624545	0	0.05602
2798	Amp	2	116625329	116712422	0	0.051221
2799	Amp	2	116713597	116714158	0	0.059772
2800	Amp	2	116714666	116718438	0	0.064992
2801	Amp	2	117175447	117175447	0	0.01566
2802	Amp	2	117176374	117488744	0	0.020138
2803	Amp	2	117488844	117817639	0	0.01566
2804	Amp	2	117817766	118003946	0	0.01044
2805	Amp	2	118004659	118372546	0	0.013296
2806	Amp	2	118373972	118904050	0	0.004745
2807	Amp	2	118904296	119146393	0	0.006151
2808	Amp	2	119146861	119475228	0	0.00774

2809	Amp	2	119475296	119650824	0	0.012277
2810	Amp	2	119652040	119865969	0	0.013782
2811	Amp	2	119868994	119907961	0	0.011893
2812	Amp	2	119908661	119920046	0	0.007356
2813	Amp	2	119921590	120065821	0	0.016005
2814	Amp	2	120074672	120074672	0	0.024038
2815	Amp	2	120074845	120311535	0	0.022533
2816	Amp	2	120315886	120340621	0	0.024496
2817	Amp	2	120352811	121165866	0	0.016464
2818	Amp	2	121172383	121627423	0	0.014501
2819	Amp	2	121627482	121710252	0	0.018578
2820	Amp	2	121711332	121847140	0	0.020537
2821	Amp	2	121850724	121860405	0	0.016459
2822	Amp	2	121860599	121901461	0	0.019666
2823	Amp	2	121904531	121947227	0	0.016459
2824	Amp	2	121947966	122191013	0	0.01487
2825	Amp	2	122191218	122249169	0	0.013464
2826	Amp	2	122249489	122431997	0	0.004815
2827	Amp	2	122441149	122574606	0	0.01333
2828	Amp	2	122574908	122667919	0	0.017724
2829	Amp	2	122668320	122694313	0	0.01333
2830	Amp	2	122696708	122791717	0	0.018369
2831	Amp	2	122791851	123298448	0	0.01333
2832	Amp	2	123405705	123430190	0	0.010474
2833	Amp	2	123430408	123628060	0	0.01472
2834	Amp	2	123635151	123803054	0	0.012761
2835	Amp	2	123807934	124112435	0	0.014859
2836	Amp	2	124113588	124265167	0	0.012153
2837	Amp	2	124265289	124265289	0	0.003638
2838	Amp	2	125319206	125319206	0	0.011809
2839	Amp	2	125319688	125695805	0	0.013802
2840	Amp	2	125696248	125775475	0	0.005631
2841	Amp	2	125781041	126049097	0	0.01191
2842	Amp	2	126049176	126083437	0	0.010371
2843	Amp	2	126085583	127320669	0	0.008272
2844	Amp	2	127324545	127793796	0	0.010087
2845	Amp	2	127795429	128604427	0	0.008272
2846	Amp	2	128607306	128727333	0	0.022295
2847	Amp	2	128729982	128737917	0	0.025742
2848	Amp	2	128738946	128782256	0	0.027873
2849	Amp	2	128783541	128836270	0	0.029529
2850	Amp	2	128836352	128858570	0	0.038459
2851	Amp	2	128866878	128941836	0	0.024436
2852	Amp	2	128942000	129702523	0	0.020989
2853	Amp	2	129702542	129759838	0	0.027947
2854	Amp	2	129767395	130003736	0	0.043596
2855	Amp	2	130004164	131109439	0	0.04194
2856	Amp	2	131123506	131171632	0	0.026291
2857	Amp	2	131174851	131605965	0	0.020012
2858	Amp	2	131606002	131734413	0	0.013054
2859	Amp	2	131736931	132742935	0	0.010923
2860	Amp	2	132743729	132918641	0	0.014493
2861	Amp	2	132919389	133398502	0	0.010923

2862	Amp	2	133398683	133589971	0	0.022315
2863	Amp	2	133590594	133777578	0	0.0261
2864	Amp	2	133782154	133830069	0	0.017171
2865	Amp	2	133831045	134349416	0	0.005779
2866	Amp	2	134353868	134520843	0	0.016476
2867	Amp	2	134521315	134832996	0	0.01269
2868	Amp	2	134835984	135472566	0	0.001994
2869	Amp	2	135474981	135541811	0	0.004614
2870	Amp	2	135548526	135624851	0	0.007561
2871	Amp	2	135628399	135756892	0	0.004614
2872	Amp	2	135761361	135773901	0	0.010609
2873	Amp	2	135777397	135803987	0	0.028814
2874	Amp	2	135805871	135920063	0	0.016456
2875	Amp	2	135924578	136022784	0	0.031397
2876	Amp	2	136025031	136408905	0	0.060291
2877	Amp	2	136413389	136475608	0	0.054295
2878	Amp	2	136478005	137035965	0	0.010461
2879	Amp	2	137039763	137097445	0	0.018866
2880	Amp	2	137097988	137171381	0	0.010461
2881	Amp	2	137173294	137294697	0	0.011861
2882	Amp	2	137295443	137625180	0	0.010461
2883	Amp	2	138311046	138670325	0	0.004614
2884	Amp	2	138671821	140198103	0	0.006081
2885	Amp	2	140198160	140324642	0	0.011057
2886	Amp	2	140326826	141368820	0	0.006081
2887	Amp	2	141368837	141433732	0	0.017153
2888	Amp	2	141438187	141776198	0	0.027677
2889	Amp	2	141776396	141911973	0	0.017153
2890	Amp	2	141912369	141962812	0	0.021443
2891	Amp	2	141965856	141984096	0	0.01037
2892	Amp	2	141989099	142273019	0	0.012091
2893	Amp	2	142273133	142639881	0	0.006081
2894	Amp	2	142640198	142869528	0	0.004614
2895	Amp	2	142880270	144245209	0	0.009106
2896	Amp	2	144245888	148409906	0	0.004614
2897	Amp	2	148410092	148481412	0	0.012221
2898	Amp	2	148481923	149135892	0	0.004614
2899	Amp	2	149137641	149166507	0	0.011125
2900	Amp	2	149166949	150750928	0	0.004614
2901	Amp	2	150751130	152405017	0	0.006697
2902	Amp	2	152410283	152617280	0	0.05722
2903	Amp	2	152619042	152831588	0	0.074871
2904	Amp	2	152838180	152839919	0	0.041105
2905	Amp	2	152840013	152855153	0	0.05238
2906	Amp	2	152856386	152913239	0	0.035622
2907	Amp	2	152914767	152914820	0	0.037338
2908	Amp	2	152944754	153005728	0	0.023633
2909	Amp	2	153005776	153220341	0	0.014203
2910	Amp	2	153220603	153557943	0	0.012487
2911	Amp	2	153558577	153696916	0	0.014085
2912	Amp	2	153702199	153732970	0	0.008295
2913	Amp	2	153733404	153968457	0	0.009881
2914	Amp	2	153970008	154294971	0	0.015786

2915	Amp	2	154295132	154489435	0	0.028369
2916	Amp	2	154490649	154607197	0	0.032266
2917	Amp	2	154611398	154646191	0	0.055091
2918	Amp	2	154649549	154726291	0	0.068365
2919	Amp	2	154728814	154783560	0	0.070992
2920	Amp	2	154790174	154798180	0	0.065408
2921	Amp	2	154798796	154866767	0	0.06119
2922	Amp	2	154870453	154931629	0	0.062643
2923	Amp	2	154933506	154969723	0	0.064423
2924	Amp	2	154971061	155020028	0	0.049551
2925	Amp	2	155020144	155021803	0	0.047771
2926	Amp	2	155023159	155150265	0	0.038108
2927	Amp	2	155150554	155379213	0	0.036654
2928	Amp	2	155379907	155510424	0	0.013829
2929	Amp	2	155544028	156053200	0	0.033341
2930	Amp	2	156053950	156456620	0	0.031755
2931	Amp	2	156458957	156784915	0	0.038751
2932	Amp	2	156785014	157642671	0	0.035831
2933	Amp	2	157646494	158063770	0	0.033205
2934	Amp	2	158066704	158106201	0	0.045266
2935	Amp	2	158108643	158264209	0	0.037391
2936	Amp	2	158264430	158447627	0	0.047333
2937	Amp	2	158455133	158617703	0	0.055033
2938	Amp	2	158622483	158667786	0	0.062786
2939	Amp	2	158669004	158730744	0	0.055033
2940	Amp	2	158732457	159220681	0	0.03357
2941	Amp	2	159223922	159238249	0	0.039408
2942	Amp	2	159238575	159462586	0	0.029465
2943	Amp	2	159465060	159481122	0	0.027381
2944	Amp	2	159486564	159535137	0	0.03279
2945	Amp	2	159537752	159634128	0	0.023684
2946	Amp	2	159635164	159917056	0	0.026511
2947	Amp	2	159919879	159956315	0	0.029146
2948	Amp	2	159957942	160199331	0	0.036192
2949	Amp	2	160201849	160247353	0	0.047782
2950	Amp	2	160253524	160309723	0	0.036192
2951	Amp	2	160311361	160421101	0	0.033558
2952	Amp	2	160421236	160639215	0	0.026511
2953	Amp	2	160640230	161195270	0	0.019845
2954	Amp	2	161195411	161470840	0	0.026568
2955	Amp	2	161471229	161899868	0	0.019845
2956	Amp	2	161902830	162132606	0	0.070368
2957	Amp	2	162134217	162394242	0	0.07452
2958	Amp	2	162399314	162507276	0	0.070368
2959	Amp	2	162511769	163394484	0	0.062546
2960	Amp	2	163399080	163762315	0	0.019845
2961	Amp	2	163763416	163974248	0	0.02551
2962	Amp	2	163975206	164111477	0	0.019845
2963	Amp	2	164113027	164376227	0	0.014437
2964	Amp	2	164378977	164612240	0	0.016613
2965	Amp	2	164615402	164643381	0	0.022416
2966	Amp	2	164643959	164724375	0	0.032095
2967	Amp	2	164725160	165055160	0	0.026291

2968 Amp	2	165063411	165328730	0	0.035419
2969 Amp	2	165328783	165363421	0	0.028423
2970 Amp	2	165366348	165693618	0	0.030516
2971 Amp	2	165694567	165728162	0	0.038735
2972 Amp	2	165731192	165800732	0	0.046345
2973 Amp	2	165801320	166096889	0	0.052819
2974 Amp	2	166101584	166107229	0	0.045209
2975 Amp	2	166108948	166156642	0	0.038735
2976 Amp	2	166157293	166224469	0	0.048425
2977 Amp	2	166224939	166362159	0	0.056189
2978 Amp	2	166362230	166542068	0	0.058364
2979 Amp	2	166542411	166835319	0	0.059902
2980 Amp	2	166835417	167132476	0	0.041084
2981 Amp	2	167132721	167197578	0	0.044094
2982 Amp	2	167202409	167369181	0	0.058914
2983 Amp	2	167369701	167430594	0	0.044452
2984 Amp	2	167430620	167933567	0	0.049903
2985 Amp	2	167944572	168057163	0	0.047165
2986 Amp	2	168057940	168071283	0	0.041714
2987 Amp	2	168073340	168105567	0	0.078285
2988 Amp	2	168108201	168239790	0	0.082751
2989 Amp	2	168252422	168380113	0	0.04618
2990 Amp	2	168382119	168383696	0	0.042744
2991 Amp	2	168387431	168529998	0	0.044415
2992 Amp	2	168531968	168583338	0	0.042744
2993 Amp	2	168583499	168614112	0	0.044359
2994 Amp	2	168615123	168631257	0	0.039893
2995 Amp	2	168633024	168841869	0	0.046468
2996 Amp	2	168843665	168901302	0	0.055286
2997 Amp	2	168902178	168928445	0	0.057063
2998 Amp	2	168928557	168940550	0	0.048244
2999 Amp	2	168941168	168992121	0	0.064697
3000 Amp	2	168999401	169013869	0	0.072908
3001 Amp	2	169014425	169113843	0	0.064013
3002 Amp	2	169114413	169136918	0	0.0962
3003 Amp	2	169137621	169222293	0	0.07551
3004 Amp	2	169223035	169224086	0	0.073895
3005 Amp	2	169229027	169385819	0	0.067092
3006 Amp	2	169388797	169411962	0	0.058882
3007 Amp	2	169412810	169653919	0	0.056261
3008 Amp	2	169654869	169766958	0	0.060329
3009 Amp	2	169769815	169781728	0	0.068446
3010 Amp	2	169781814	170058274	0	0.061167
3011 Amp	2	170060713	170078935	0	0.071135
3012 Amp	2	170079279	170215002	0	0.062259
3013 Amp	2	170215325	170285353	0	0.077683
3014 Amp	2	170296957	170493160	0	0.052895
3015 Amp	2	170494170	170562722	0	0.063268
3016 Amp	2	170564135	170919019	0	0.113103
3017 Amp	2	170923997	171059571	0	0.10273
3018 Amp	2	171059610	171288171	0	0.109542
3019 Amp	2	171288690	171364264	0	0.10273
3020 Amp	2	171367375	171587025	0	0.097244

3021 Amp	2	171589565	171713685	0	0.106551
3022 Amp	2	171723634	172698121	0	0.097244
3023 Amp	2	172701405	172720058	0	0.100581
3024 Amp	2	172720509	172962298	0	0.101999
3025 Amp	2	172965455	173023528	0	0.110051
3026 Amp	2	173023910	173288627	0	0.101999
3027 Amp	2	173291825	173298092	0	0.097466
3028 Amp	2	173298592	173317934	0	0.092711
3029 Amp	2	173318257	173340492	0	0.042876
3030 Amp	2	173343505	173443880	0	0.035225
3031 Amp	2	173443900	173479628	0	0.04994
3032 Amp	2	173479878	173697423	0	0.037055
3033 Amp	2	173699233	174080229	0	0.034879
3034 Amp	2	174089253	174644523	0	0.038324
3035 Amp	2	174645812	174863685	0	0.031747
3036 Amp	2	174864094	174981169	0	0.041435
3037 Amp	2	174981556	175000068	0	0.091958
3038 Amp	2	175007529	175402596	0	0.041435
3039 Amp	2	175404869	175715442	0	0.044238
3040 Amp	2	175715704	175869971	0	0.048996
3041 Amp	2	175883393	176321948	0	0.046193
3042 Amp	2	176322467	176372524	0	0.048033
3043 Amp	2	176375095	176625845	0	0.043275
3044 Amp	2	176632790	176892005	0	0.096865
3045 Amp	2	176892191	176927009	0	0.095025
3046 Amp	2	176929143	177047475	0	0.105882
3047 Amp	2	177053234	177151832	0	0.095025
3048 Amp	2	177152037	177340401	0	0.08965
3049 Amp	2	177340879	177572618	0	0.09755
3050 Amp	2	177577560	177633287	0	0.099415
3051 Amp	2	177634915	177730455	0	0.09755
3052 Amp	2	177738860	177756982	0	0.084761
3053 Amp	2	177765704	177812229	0	0.090163
3054 Amp	2	177812379	177854651	0	0.117048
3055 Amp	2	177854941	177906229	0	0.140686
3056 Amp	2	177906915	177935324	0	0.142264
3057 Amp	2	177935795	177971606	0	0.14557
3058 Amp	2	177971902	178353718	0	0.143993
3059 Amp	2	178358410	178449632	0	0.153354
3060 Amp	2	178453166	178534432	0	0.105139
3061 Amp	2	178539140	178613882	0	0.095778
3062 Amp	2	178619002	179051445	0	0.079213
3063 Amp	2	179053170	179071445	0	0.064479
3064 Amp	2	179072043	179227106	0	0.079213
3065 Amp	2	179235699	179401137	0	0.090667
3066 Amp	2	179403069	179427622	0	0.079213
3067 Amp	2	179430054	179863333	0	0.076593
3068 Amp	2	179863346	180169709	0	0.075046
3069 Amp	2	180175044	180807771	0	0.069644
3070 Amp	2	180810339	181072956	0	0.019121
3071 Amp	2	181076977	181828565	0	0.025604
3072 Amp	2	181829061	181900773	0	0.019121
3073 Amp	2	181901346	182252461	0	0.015676

3074	Amp	2	182252877	182302654	0	0.012369
3075	Amp	2	182310206	182776508	0	0.018012
3076	Amp	2	182780742	182904561	0	0.020897
3077	Amp	2	182904697	183232792	0	0.023882
3078	Amp	2	183236418	183488765	0	0.020996
3079	Amp	2	183489068	183946943	0	0.023317
3080	Amp	2	183982067	184642502	0	0.020996
3081	Amp	2	184642694	184804428	0	0.022425
3082	Amp	2	184807567	184985579	0	0.062532
3083	Amp	2	184987449	185068835	0	0.076448
3084	Amp	2	185069357	185175384	0	0.085863
3085	Amp	2	185298004	185433729	0	0.08022
3086	Amp	2	186024241	186054703	0	0.041788
3087	Amp	2	186055545	186168499	0	0.054135
3088	Amp	2	186175601	186196600	0	0.063265
3089	Amp	2	186196753	186346340	0	0.058608
3090	Amp	2	186347895	186512754	0	0.044692
3091	Amp	2	186514311	186601702	0	0.050778
3092	Amp	2	186603023	186643815	0	0.044692
3093	Amp	2	186649879	186676615	0	0.055845
3094	Amp	2	186680148	186791129	0	0.065657
3095	Amp	2	186791454	186821233	0	0.071465
3096	Amp	2	186824610	186876151	0	0.075863
3097	Amp	2	186878501	187417603	0	0.063776
3098	Amp	2	187425796	187577364	0	0.061782
3099	Amp	2	187581641	187695661	0	0.074153
3100	Amp	2	187696023	187696023	0	0.068141
3101	Amp	2	187700035	187749969	0	0.051243
3102	Amp	2	187751705	187796202	0	0.040089
3103	Amp	2	187919046	187919046	0	0.046284
3104	Amp	2	187923920	188069582	0	0.057094
3105	Amp	2	188167460	188419634	0	0.067562
3106	Amp	2	188420530	188433125	0	0.071304
3107	Amp	2	188433391	188651969	0	0.075803
3108	Amp	2	188652783	188678787	0	0.071304
3109	Amp	2	188680189	188770864	0	0.066401
3110	Amp	2	188772339	188845871	0	0.055934
3111	Amp	2	188849421	188863170	0	0.063824
3112	Amp	2	188863265	189006122	0	0.069044
3113	Amp	2	189006640	189224871	0	0.062497
3114	Amp	2	189233265	189255054	0	0.08502
3115	Amp	2	189256239	189288716	0	0.075889
3116	Amp	2	189292042	189406002	0	0.084631
3117	Amp	2	189406530	189424930	0	0.068883
3118	Amp	2	189432128	189432128	0	0.05188
3119	Amp	2	189436163	189455326	0	0.102403
3120	Amp	2	189455357	189599850	0	0.106596
3121	Amp	2	189599870	189895607	0	0.098897
3122	Amp	2	189896425	190175848	0	0.093377
3123	Amp	2	190183501	190279695	0	0.103459
3124	Amp	2	190281011	190308058	0	0.111436
3125	Amp	2	190309201	190312741	0	0.104818
3126	Amp	2	190312775	190448710	0	0.101155

3127 Amp	2	190449508	190466625	0	0.086265
3128 Amp	2	190469305	190614883	0	0.089967
3129 Amp	2	190621621	190763419	0	0.100801
3130 Amp	2	190764021	190818845	0	0.110998
3131 Amp	2	190819250	190863318	0	0.101627
3132 Amp	2	190863325	190896718	0	0.116822
3133 Amp	2	190904564	190931621	0	0.105989
3134 Amp	2	190943814	191028008	0	0.093062
3135 Amp	2	191028361	191390607	0	0.091524
3136 Amp	2	191400384	191458957	0	0.079154
3137 Amp	2	191459420	191710489	0	0.081924
3138 Amp	2	191714957	191802034	0	0.072477
3139 Amp	2	191806097	191861140	0	0.079536
3140 Amp	2	191910683	192167186	0	0.072477
3141 Amp	2	192167629	192182273	0	0.057282
3142 Amp	2	192187973	192205107	0	0.054512
3143 Amp	2	192215908	192255862	0	0.044315
3144 Amp	2	192256524	192376045	0	0.045762
3145 Amp	2	192377049	192573538	0	0.051709
3146 Amp	2	192574116	192664598	0	0.069058
3147 Amp	2	192668333	192792142	0	0.080366
3148 Amp	2	192792681	192806343	0	0.069058
3149 Amp	2	192810342	192831145	0	0.063111
3150 Amp	2	192841594	192841741	0	0.066627
3151 Amp	2	193057320	193064261	0	0.049278
3152 Amp	2	193072334	193177758	0	0.059411
3153 Amp	2	193195092	193241878	0	0.081671
3154 Amp	2	193245366	193279426	0	0.076151
3155 Amp	2	193280629	193295771	0	0.078157
3156 Amp	2	193303995	193688837	0	0.099358
3157 Amp	2	193689027	193704998	0	0.097352
3158 Amp	2	193722432	193873399	0	0.10556
3159 Amp	2	193899942	194043206	0	0.093581
3160 Amp	2	194043831	194043831	0	0.122408
3161 Amp	2	194124834	194138261	0	0.132717
3162 Amp	2	194141183	194436006	0	0.129733
3163 Amp	2	194436201	194465873	0	0.123055
3164 Amp	2	194466432	194536981	0	0.128941
3165 Amp	2	194538008	194653834	0	0.131178
3166 Amp	2	194654212	194676733	0	0.125292
3167 Amp	2	194678114	194854741	0	0.123844
3168 Amp	2	195351205	195351205	0	0.114623
3169 Amp	2	195352602	195429828	0	0.11891
3170 Amp	2	195430431	195920188	0	0.121189
3171 Amp	2	195924104	196147605	0	0.110552
3172 Amp	2	196150733	196260096	0	0.116992
3173 Amp	2	196260424	196538851	0	0.153693
3174 Amp	2	196545122	196676572	0	0.146546
3175 Amp	2	196686323	196735570	0	0.148673
3176 Amp	2	196745621	196846312	0	0.15913
3177 Amp	2	196850925	196915035	0	0.171212
3178 Amp	2	196919791	197110892	0	0.187368
3179 Amp	2	197112373	197233034	0.007199	0.189108

3180	Amp	2	197233908	197273293	0	0.184821
3181	Amp	2	197273878	197416173	0.007199	0.189183
3182	Amp	2	197420143	197473123	0.079463	0.198197
3183	Amp	2	197473784	197794442	0.068197	0.196769
3184	Amp	2	197797534	197803741	0	0.177299
3185	Amp	2	197806201	197953902	0	0.127104
3186	Amp	2	197957930	198129983	0	0.076581
3187	Amp	2	198130461	198220937	0	0.074344
3188	Amp	2	198221541	198279830	0	0.069982
3189	Amp	2	198280377	198360500	0	0.078219
3190	Amp	2	198360860	198458959	0	0.069982
3191	Amp	2	198459195	198618626	0	0.028076
3192	Amp	2	198625404	198630754	0	0.021881
3193	Amp	2	198638112	199252299	0	0.027368
3194	Amp	2	199255700	199295094	0	0.037325
3195	Amp	2	199297702	199347814	0	0.087848
3196	Amp	2	199348944	199455295	0	0.07789
3197	Amp	2	199472056	200217022	0	0.027368
3198	Amp	2	200217734	200273320	0	0.032523
3199	Amp	2	200273725	200468731	0	0.036373
3200	Amp	2	200468904	200983919	0	0.032523
3201	Amp	2	200984390	201073113	0	0.03666
3202	Amp	2	201076793	201139552	0	0.031505
3203	Amp	2	201140472	201140472	0	0.026019
3204	Amp	2	201143575	201193514	0	0.03097
3205	Amp	2	201196097	201213771	0	0.022762
3206	Amp	2	201224747	201567012	0	0.017811
3207	Amp	2	201569670	201944892	0	0.015683
3208	Amp	2	201945041	202067474	0	0.021991
3209	Amp	2	202075475	202131991	0	0.015683
3210	Amp	2	202134858	202260668	0	0.024546
3211	Amp	2	202264578	202506427	0	0.03105
3212	Amp	2	202507174	202522980	0	0.033513
3213	Amp	2	202525340	202619832	0	0.039662
3214	Amp	2	202620373	202663256	0	0.037598
3215	Amp	2	202664282	202823788	0	0.031094
3216	Amp	2	202826447	202858276	0	0.024945
3217	Amp	2	202860066	203073802	0	0.027544
3218	Amp	2	203087860	203195364	0	0.0325
3219	Amp	2	203196166	203399207	0	0.027544
3220	Amp	2	203406141	203556561	0	0.025081
3221	Amp	2	203560875	203740620	0	0.030935
3222	Amp	2	203740732	203895341	0	0.025081
3223	Amp	2	203895611	203960563	0	0.022481
3224	Amp	2	203960861	204221399	0	0.016041
3225	Amp	2	204221817	204243952	0	0.024746
3226	Amp	2	204244291	204398255	0	0.032473
3227	Amp	2	204401364	204486086	0	0.016041
3228	Amp	2	204486415	205257470	0	0.033609
3229	Amp	2	205257823	205611980	0	0.045066
3230	Amp	2	205613534	206463871	0	0.027497
3231	Amp	2	206471773	207469600	0	0.03595
3232	Amp	2	207475093	207498032	0	0.037985

3233	Amp	2	207498192	207698403	0	0.03595
3234	Amp	2	207699388	207790949	0	0.045096
3235	Amp	2	207792763	207869173	0	0.036643
3236	Amp	2	207871469	208195839	0	0.04343
3237	Amp	2	208198537	209224503	0	0.033597
3238	Amp	2	209253289	209257753	0	0.027497
3239	Amp	2	209258950	209316372	0	0.035927
3240	Amp	2	209318493	209322874	0	0.024471
3241	Amp	2	209324421	210490771	0	0.016041
3242	Amp	2	210490927	210793127	0	0.019207
3243	Amp	2	210800459	211123543	0	0.021256
3244	Amp	2	211123653	211123653	0	0.018429
3245	Amp	2	211123680	211125464	0	0.030856
3246	Amp	2	211125630	211191191	0	0.033987
3247	Amp	2	211192400	211271986	0	0.029849
3248	Amp	2	211272158	211547856	0	0.032067
3249	Amp	2	211549869	211583853	0	0.03556
3250	Amp	2	211584011	211620370	0	0.038973
3251	Amp	2	211621371	211643409	0	0.03548
3252	Amp	2	211646617	211660314	0	0.0371
3253	Amp	2	211660491	211673690	0	0.042564
3254	Amp	2	211673986	211676872	0	0.051094
3255	Amp	2	211681887	211723902	0	0.05952
3256	Amp	2	211724095	211760096	0	0.057302
3257	Amp	2	211761735	211822786	0	0.065703
3258	Amp	2	211825083	211843285	0	0.057172
3259	Amp	2	211845073	211980330	0	0.060405
3260	Amp	2	211980567	211998710	0	0.054941
3261	Amp	2	212005976	212111215	0	0.051528
3262	Amp	2	212116862	212164943	0	0.043128
3263	Amp	2	212168251	212291272	0	0.041507
3264	Amp	2	212293141	213386939	0	0.038137
3265	Amp	2	213389773	214030951	0	0.029915
3266	Amp	2	214032356	214077923	0	0.080438
3267	Amp	2	214083570	214612637	0	0.086458
3268	Amp	2	214616160	214783270	0	0.091231
3269	Amp	2	214783998	214802636	0	0.085212
3270	Amp	2	214807018	215238510	0	0.034689
3271	Amp	2	215238560	215930368	0	0.040333
3272	Amp	2	215930568	216429101	0	0.038284
3273	Amp	2	216434463	216472146	0	0.040073
3274	Amp	2	216473060	216540480	0	0.057156
3275	Amp	2	216543380	216714302	0	0.055367
3276	Amp	2	216714751	216984786	0	0.059002
3277	Amp	2	216985263	217009693	0	0.055367
3278	Amp	2	217010208	217604599	0	0.060464
3279	Amp	2	217607744	217659109	0	0.055367
3280	Amp	2	217663470	217668796	0	0.038284
3281	Amp	2	217671913	218276470	0	0.041083
3282	Amp	2	218279507	218525884	0	0.044047
3283	Amp	2	218526071	218603104	0	0.041248
3284	Amp	2	218605832	218926813	0	0.045779
3285	Amp	2	218928766	219171103	0	0.048736

3286 Amp	2	219175608	219273686	0	0.052883
3287 Amp	2	219286328	219329763	0	0.067848
3288 Amp	2	219330137	219548412	0	0.060353
3289 Amp	2	219550356	219620649	0	0.045389
3290 Amp	2	219633628	222143903	0	0.041241
3291 Amp	2	222144606	222551951	0	0.048072
3292 Amp	2	222555043	223008019	0	0.045794
3293 Amp	2	223008418	223021845	0	0.052852
3294 Amp	2	223022925	223050132	0	0.06667
3295 Amp	2	223051135	223244182	0	0.068396
3296 Amp	2	223246646	223468421	0	0.073768
3297 Amp	2	223470701	223730325	0	0.075251
3298 Amp	2	223735312	223833038	0	0.073768
3299 Amp	2	223833592	223840885	0	0.075939
3300 Amp	2	223841551	223951210	0	0.062121
3301 Amp	2	223952013	224876786	0	0.057348
3302 Amp	2	224878251	225585873	0	0.054391
3303 Amp	2	225587229	225985003	0	0.052665
3304 Amp	2	225991973	226001245	0	0.038776
3305 Amp	2	226010628	226347708	0	0.0504
3306 Amp	2	226350063	226634043	0	0.038776
3307 Amp	2	226634242	226908750	0	0.041048
3308 Amp	2	226909137	227320988	0	0.035404
3309 Amp	2	227326486	227737902	0	0.030031
3310 Amp	2	227738744	228462454	0	0.033202
3311 Amp	2	228465032	229613864	0	0.03093
3312 Amp	2	229616047	230095627	0	0.027759
3313 Amp	2	230095928	230405779	0	0.033031
3314 Amp	2	230406957	230549015	0	0.027759
3315 Amp	2	230549228	230959966	0	0.029301
3316 Amp	2	230966049	231125386	0	0.032318
3317 Amp	2	231130379	232059390	0	0.029301
3318 Amp	2	232061692	232328858	0	0.032686
3319 Amp	2	232454022	232706323	0	0.04285
3320 Amp	2	232712193	232741375	0	0.049549
3321 Amp	2	232786583	232835821	0	0.046164
3322 Amp	2	232840136	234733691	0	0.039465
3323 Amp	2	234735938	235950274	0	0.043107
3324 Amp	2	235953182	236499735	0	0.039465
3325 Amp	2	236500163	237038645	0	0.042727
3326 Amp	2	237043341	237758405	0	0.039465
3327 Amp	2	237760140	237858342	0	0.041872
3328 Amp	2	237861037	238679676	0	0.031708
3329 Amp	2	238680246	239132679	0	0.033724
3330 Amp	2	239135147	239171804	0	0.035282
3331 Amp	2	239174544	239302072	0	0.033724
3332 Amp	2	239304744	239800034	0	0.031708
3333 Amp	2	239801005	240432585	0	0.034362
3334 Amp	2	240433704	240472786	0	0.039545
3335 Amp	2	240472805	240555775	0	0.034362
3336 Amp	2	240557350	241023430	0	0.044433
3337 Amp	2	241024553	241074746	0	0.042026
3338 Amp	2	241077474	241188357	0	0.04818

3339 Amp	2	241195442	241219993	0	0.049823
3340 Amp	2	241222858	241278212	0	0.04818
3341 Amp	2	241278760	241314748	0	0.042026
3342 Amp	2	241317412	241409643	0	0.047321
3343 Amp	2	241414289	241480882	0	0.050281
3344 Amp	2	241480909	241493275	0	0.047627
3345 Amp	2	241494131	241505921	0	0.049101
3346 Amp	2	241512300	241537572	0	0.047627
3347 Amp	3	2170634	2277811	0	0.18545
3348 Amp	3	2283419	2323933	0.001198	0.188737
3349 Amp	3	2780268	2826366	0.018387	0.190855
3350 Amp	3	2829270	3130439	0	0.181439
3351 Amp	3	3136151	3174217	0.161592	0.207144
3352 Amp	3	3176969	3314360	0.111991	0.201745
3353 Amp	3	3314697	3393381	0.128931	0.203726
3354 Amp	3	3394902	3577820	0.079463	0.198107
3355 Amp	3	3577900	3702255	0.123589	0.203241
3356 Amp	3	3706749	3714901	0.061778	0.195839
3357 Amp	3	3718766	3869461	0	0.187381
3358 Amp	3	3870026	3870739	0.061778	0.196001
3359 Amp	3	4286151	4287971	0.437983	0.233036
3360 Amp	3	4288315	4288315	0.51566	0.239952
3361 Amp	3	4353083	4360965	0.580011	0.245479
3362 Amp	3	4361592	4377385	0.51566	0.240344
3363 Amp	3	4378032	4471540	0.604912	0.247576
3364 Amp	3	4471859	4533811	0.49169	0.23824
3365 Amp	3	4536436	4587175	1.068928	0.28322
3366 Amp	3	4590440	4600228	1.150167	0.289182
3367 Amp	3	4615806	4616657	1.344429	0.302587
3368 Amp	3	4617151	4623238	1.099256	0.285567
3369 Amp	3	4623439	4628838	1.190324	0.291765
3370 Amp	3	4629345	4650752	1.275021	0.298157
3371 Amp	3	4651778	4672573	1.199568	0.292195
3372 Amp	3	4674530	4747558	1.237378	0.295321
3373 Amp	3	4749995	4778768	1.424227	0.308121
3374 Amp	3	4782447	4971135	1.374261	0.304995
3375 Amp	3	4972191	4972473	1.285475	0.298796
3376 Amp	3	4983086	5013455	1.169408	0.290176
3377 Amp	3	5116914	5121732	0.571214	0.245196
3378 Amp	3	5123084	5177255	0.724993	0.257664
3379 Amp	3	5178201	5202865	0.76883	0.261521
3380 Amp	3	5210445	5227952	0.652914	0.25174
3381 Amp	3	5235414	5265239	0.508503	0.239273
3382 Amp	3	5265386	5336724	0.460107	0.235103
3383 Amp	3	5337939	5346678	0.508503	0.239649
3384 Amp	3	5346859	5458146	0.444673	0.234122
3385 Amp	3	6105882	6221160	0.13565	0.204195
3386 Amp	3	6221175	6378582	0.117531	0.202213
3387 Amp	3	6379292	6945008	0.175335	0.208831
3388 Amp	3	6952224	6969847	0.250263	0.215853
3389 Amp	3	6973984	7068407	0.500055	0.23857
3390 Amp	3	7068421	7204554	0.743132	0.25901
3391 Amp	3	7207263	7227669	0.897922	0.2707

3392	Amp	3	7231360	7292222	0.468576	0.235977
3393	Amp	3	7294260	7382525	0.483654	0.237526
3394	Amp	3	7390271	7482416	0.355162	0.225836
3395	Amp	3	7482826	7483333	0.293283	0.220365
3396	Amp	3	7485685	7716420	0	0.176085
3397	Amp	3	7726985	7741629	0.362221	0.226091
3398	Amp	3	7742939	7851873	0.398217	0.22983
3399	Amp	3	7854103	7871721	0.250263	0.216049
3400	Amp	3	7871829	7922146	0.209057	0.211782
3401	Amp	3	7924180	8124614	0.273081	0.218305
3402	Amp	3	8125100	8159541	0.340434	0.224499
3403	Amp	3	8160839	8166718	0	0.177243
3404	Amp	3	8170230	8186943	0.028398	0.192031
3405	Amp	3	8188134	8255818	0	0.174493
3406	Amp	3	8255855	8453112	0	0.168299
3407	Amp	3	8454130	8608946	0	0.187908
3408	Amp	3	8618457	8634128	0.175335	0.208301
3409	Amp	3	8637906	8675655	0	0.186362
3410	Amp	3	8729817	8780118	0	0.172817
3411	Amp	3	8825517	8959988	0	0.170479
3412	Amp	3	8960036	9063797	0	0.179379
3413	Amp	3	9064118	9072263	0	0.176091
3414	Amp	3	9073075	9099539	0	0.169571
3415	Amp	3	9099854	9159260	0	0.172059
3416	Amp	3	9160012	9244881	0	0.176784
3417	Amp	3	9245052	9258146	0	0.175262
3418	Amp	3	9267515	9267515	0	0.180987
3419	Amp	3	9267587	9406401	0	0.179506
3420	Amp	3	9407371	9410030	0	0.186141
3421	Amp	3	9412711	9428501	0.073369	0.197617
3422	Amp	3	9431518	9457764	0.209057	0.212083
3423	Amp	3	9461128	9482763	0.073369	0.197617
3424	Amp	3	9486190	9495054	0.149443	0.205474
3425	Amp	3	9502870	9516121	0.619816	0.248876
3426	Amp	3	9516392	9542686	0.702615	0.256109
3427	Amp	3	9547779	9558120	0.7517	0.260231
3428	Amp	3	9559089	9615403	0.693887	0.255419
3429	Amp	3	9618801	9647877	0.86979	0.268998
3430	Amp	3	9650465	9684962	0.897922	0.270812
3431	Amp	3	9685318	9697551	0.925715	0.272959
3432	Amp	3	9699437	9838945	0.86979	0.269219
3433	Amp	3	9842231	9877366	0.619816	0.249004
3434	Amp	3	9879043	9998989	0.71758	0.256991
3435	Amp	3	9999712	10050335	0.814697	0.264785
3436	Amp	3	10055423	10145384	0.709359	0.256798
3437	Amp	3	10145397	10329623	0.62858	0.249585
3438	Amp	3	10335628	10586019	0.156508	0.206183
3439	Amp	3	10588023	10715477	0.050005	0.194708
3440	Amp	3	10716074	10769045	0.168144	0.207818
3441	Amp	3	10770490	10916018	0.18072	0.209402
3442	Amp	3	10918877	10949660	0.061778	0.196291
3443	Amp	3	10953706	10981986	0.508503	0.239693
3444	Amp	3	10982504	11034911	0.49169	0.23788

3445 Amp	3	11037570	11049203	0.452609	0.234767
3446 Amp	3	11050418	11052147	0.437983	0.233184
3447 Amp	3	11054444	11119669	0.012341	0.189782
3448 Amp	3	11119977	11130954	0.02372	0.191374
3449 Amp	3	11131166	11235962	0.073369	0.197414
3450 Amp	3	11240790	11438024	0	0.183831
3451 Amp	3	11442295	11482196	0	0.187727
3452 Amp	3	11483876	11541557	0.043943	0.194074
3453 Amp	3	11542833	11553456	0	0.187727
3454 Amp	3	11557635	11751071	0.028398	0.191825
3455 Amp	3	11753124	11835242	0.012341	0.189678
3456 Amp	3	11837214	11839342	0.056238	0.195428
3457 Amp	3	11840607	11881918	0.187743	0.209883
3458 Amp	3	11883204	11932796	0.09318	0.199905
3459 Amp	3	11932938	11957667	0.02372	0.191532
3460 Amp	3	11957979	11968190	0.230572	0.214123
3461 Amp	3	11969075	11969075	0.293283	0.220497
3462 Amp	3	11969689	12003230	0.09318	0.19953
3463 Amp	3	12003473	12045097	0	0.17261
3464 Amp	3	12049234	12383294	0.273081	0.218154
3465 Amp	3	12385352	12391121	0.340434	0.224447
3466 Amp	3	12391799	12444387	0.317596	0.222021
3467 Amp	3	12450038	12540093	0.293283	0.220187
3468 Amp	3	12540343	12568438	0.421967	0.232093
3469 Amp	3	12570079	12574680	0.375927	0.227971
3470 Amp	3	12575034	12577584	0.340434	0.22431
3471 Amp	3	12577981	12590205	0.333141	0.223795
3472 Amp	3	12590307	12590307	0.27943	0.218734
3473 Amp	3	12590315	12594756	0.223025	0.213509
3474 Amp	3	12596176	12598315	0.187743	0.209969
3475 Amp	3	12598495	12608077	0.123589	0.203125
3476 Amp	3	12609213	12631840	0.243425	0.215204
3477 Amp	3	12631856	12631872	0.264784	0.217327
3478 Amp	3	12715395	12727195	1.424227	0.308082
3479 Amp	3	12733766	12734217	1.534719	0.315733
3480 Amp	3	12736593	12743076	1.563139	0.317753
3481 Amp	3	12747339	12748253	1.626265	0.322158
3482 Amp	3	12749360	12756627	1.678649	0.324971
3483 Amp	3	12758846	12798416	1.748357	0.329813
3484 Amp	3	12798738	12837237	1.811302	0.334405
3485 Amp	3	12841778	12844107	1.078696	0.283882
3486 Amp	3	12848708	12888427	1.226647	0.294715
3487 Amp	3	12888993	12946525	1.305256	0.299808
3488 Amp	3	12946811	12982043	1.226647	0.294715
3489 Amp	3	13013995	13143993	1.983885	0.345237
3490 Amp	3	13145915	13194327	1.811302	0.334405
3491 Amp	3	13194385	13289246	1.678649	0.325326
3492 Amp	3	13298510	13304750	1.615314	0.321305
3493 Amp	3	13306003	13379686	1.525294	0.314931
3494 Amp	3	13380442	13391658	1.2565	0.296389
3495 Amp	3	13398612	13404998	1.275021	0.298032
3496 Amp	3	13407547	13492082	1.020188	0.279701
3497 Amp	3	13493575	13541076	1.059215	0.282591

3498	Amp	3	13541398	13583399	0.636206	0.25061
3499	Amp	3	13584264	13672211	0.66072	0.252648
3500	Amp	3	13673338	13718250	0.62858	0.249759
3501	Amp	3	13724412	13749978	0.833763	0.265919
3502	Amp	3	13750641	13770082	0.778204	0.26182
3503	Amp	3	13770975	13817302	0.437983	0.233327
3504	Amp	3	13817835	13935131	0.362221	0.226446
3505	Amp	3	13936563	13980168	0.41418	0.231258
3506	Amp	3	13981983	14167222	0.508503	0.23952
3507	Amp	3	14168131	14238773	0.571214	0.244706
3508	Amp	3	14242039	14253659	0.500055	0.238611
3509	Amp	3	14253917	14279948	0.897922	0.270924
3510	Amp	3	14283568	14399791	0.66072	0.252664
3511	Amp	3	14406465	14427259	0.604912	0.247896
3512	Amp	3	14427690	14476235	0.571214	0.244894
3513	Amp	3	14478656	14531089	1.208462	0.293027
3514	Amp	3	14531715	14564480	1.364151	0.303725
3515	Amp	3	14564552	14573249	1.383826	0.305152
3516	Amp	3	14573549	14619953	1.218154	0.293833
3517	Amp	3	14620369	14645805	1.068928	0.283134
3518	Amp	3	14647368	14799046	1.687998	0.325993
3519	Amp	3	14801050	14931813	1.727244	0.328585
3520	Amp	3	14934386	14995515	1.687998	0.325993
3521	Amp	3	14996344	15011439	1.748357	0.330068
3522	Amp	3	15011465	15017449	1.727244	0.328642
3523	Amp	3	15022019	15033853	1.099256	0.285783
3524	Amp	3	15034177	15069942	1.159718	0.289804
3525	Amp	3	15074365	15317139	1.324884	0.300922
3526	Amp	3	15317688	15373881	1.364151	0.304039
3527	Amp	3	15378146	15472790	0.702615	0.255907
3528	Amp	3	15474188	15479170	0.564815	0.244505
3529	Amp	3	15479331	15674955	0.702615	0.255907
3530	Amp	3	15676107	15736825	0.7517	0.260122
3531	Amp	3	15738043	15759233	0.644689	0.251304
3532	Amp	3	15759584	16074779	0.613517	0.248492
3533	Amp	3	16075921	16289264	0.437983	0.233547
3534	Amp	3	16295019	16298228	0.317596	0.222429
3535	Amp	3	16298963	16329168	0.500055	0.238811
3536	Amp	3	16329722	16382308	0.636206	0.25076
3537	Amp	3	16382501	16421864	0.852608	0.26736
3538	Amp	3	16425512	16446795	0.25804	0.217009
3539	Amp	3	16447825	16512462	0.230572	0.213893
3540	Amp	3	16518841	16658827	0.111991	0.201944
3541	Amp	3	16660278	16963917	0.156508	0.206654
3542	Amp	3	16967193	17021735	0	0.162922
3543	Amp	3	17025593	17088160	0	0.16589
3544	Amp	3	17090513	17551244	0	0.165726
3545	Amp	3	17554031	17589043	0	0.162758
3546	Amp	3	17589517	17879652	0	0.167386
3547	Amp	3	17885781	17889947	0	0.173495
3548	Amp	3	17891915	17916143	0	0.16158
3549	Amp	3	17925682	17950918	0	0.183817
3550	Amp	3	17953091	18376873	0	0.16158

3551 Amp	3	18382888	18406637	0	0.157364
3552 Amp	3	18407161	18420590	0	0.152737
3553 Amp	3	18428985	18476670	0	0.147602
3554 Amp	3	18491767	18735722	0	0.141567
3555 Amp	3	18737702	19242135	0	0.139756
3556 Amp	3	19247332	19317407	0	0.133646
3557 Amp	3	19324969	19545259	0	0.129571
3558 Amp	3	19550338	19745251	0	0.134126
3559 Amp	3	19745302	19783667	0	0.143979
3560 Amp	3	19791432	19795046	0	0.134126
3561 Amp	3	19800119	19931274	0	0.129571
3562 Amp	3	19931300	19968554	0	0.140037
3563 Amp	3	19976808	20185705	0	0.146596
3564 Amp	3	20188597	20208305	0	0.150756
3565 Amp	3	20214665	20372738	0	0.146596
3566 Amp	3	20378187	20378235	0	0.13397
3567 Amp	3	20380340	20380340	0	0.136067
3568 Amp	3	20405351	20427466	0	0.130353
3569 Amp	3	20435584	20459017	0	0.13663
3570 Amp	3	20459538	20979534	0	0.130353
3571 Amp	3	20983163	21048951	0	0.116985
3572 Amp	3	21050376	21052254	0	0.114435
3573 Amp	3	21310145	21310880	0	0.103969
3574 Amp	3	21313746	21549390	0	0.101873
3575 Amp	3	21554365	21740479	0	0.113389
3576 Amp	3	21740672	21740672	0	0.128798
3577 Amp	3	22176672	22176672	0	0.124088
3578 Amp	3	22177673	22355166	0	0.112571
3579 Amp	3	22355517	23105349	0	0.110929
3580 Amp	3	23105736	23347943	0	0.106091
3581 Amp	3	23355335	23396286	0	0.090682
3582 Amp	3	23399778	23437661	0	0.086195
3583 Amp	3	23439035	23731593	0	0.075963
3584 Amp	3	23732684	23930279	0	0.069404
3585 Amp	3	23931376	24138277	0	0.074376
3586 Amp	3	24148693	24447374	0	0.09371
3587 Amp	3	24451094	24556277	0	0.117922
3588 Amp	3	24557407	24787597	0	0.12245
3589 Amp	3	24787751	24794963	0	0.128017
3590 Amp	3	24795617	24808981	0	0.117798
3591 Amp	3	24809451	25075091	0	0.113941
3592 Amp	3	25075352	25408159	0	0.125113
3593 Amp	3	25411260	25555025	0	0.091786
3594 Amp	3	25555122	25728377	0	0.105043
3595 Amp	3	25733179	25800717	0	0.119728
3596 Amp	3	25800835	25831163	0	0.106471
3597 Amp	3	25835173	25939090	0	0.091786
3598 Amp	3	26419435	26926438	0	0.121785
3599 Amp	3	27040272	27424212	0	0.115401
3600 Amp	3	27428618	27495308	0	0.121597
3601 Amp	3	27500243	27580675	0	0.109988
3602 Amp	3	27581571	28397976	0	0.085402
3603 Amp	3	28406661	28487437	0	0.080874

3604 Amp	3	28488698	28709986	0	0.082517
3605 Amp	3	28711607	28979842	0	0.087499
3606 Amp	3	28979918	29118858	0	0.093167
3607 Amp	3	29121195	29145957	0	0.087499
3608 Amp	3	29147652	29562618	0	0.085857
3609 Amp	3	29562708	29897308	0	0.091418
3610 Amp	3	29898015	29928025	0	0.085857
3611 Amp	3	29929257	30577121	0	0.094313
3612 Amp	3	30577219	30825470	0	0.099441
3613 Amp	3	30827568	31020009	0	0.103803
3614 Amp	3	31022993	31161680	0	0.095347
3615 Amp	3	31163376	31318238	0	0.090985
3616 Amp	3	31318382	31469596	0	0.092391
3617 Amp	3	31469797	31624633	0	0.087604
3618 Amp	3	31627659	31668475	0	0.082036
3619 Amp	3	31671154	31730448	0	0.08469
3620 Amp	3	31732958	31849538	0	0.082676
3621 Amp	3	31850936	32003870	0	0.088342
3622 Amp	3	32004688	32414297	0	0.098344
3623 Amp	3	32414724	32475660	0	0.094442
3624 Amp	3	32477903	32709738	0	0.091788
3625 Amp	3	32712891	32933224	0	0.081785
3626 Amp	3	32947463	33044406	0	0.083816
3627 Amp	3	33049969	33273815	0	0.09732
3628 Amp	3	33280167	33559210	0	0.083816
3629 Amp	3	33559577	33754006	0	0.078687
3630 Amp	3	33764069	33825243	0	0.076657
3631 Amp	3	33825437	34078292	0	0.078749
3632 Amp	3	34084216	34306314	0	0.072592
3633 Amp	3	34306438	34341476	0	0.066976
3634 Amp	3	34345571	34409812	0	0.059183
3635 Amp	3	34420982	34494603	0	0.0542
3636 Amp	3	34496485	34497066	0	0.048534
3637 Amp	3	34498977	34776603	0	0.050196
3638 Amp	3	34779540	34948322	0	0.055566
3639 Amp	3	34967553	35603196	0	0.050516
3640 Amp	3	35605188	35968353	0	0.044127
3641 Amp	3	35974287	35974969	0	0.042465
3642 Amp	3	35975112	36382165	0	0.046689
3643 Amp	3	36383530	36515233	0	0.049956
3644 Amp	3	36519649	36814837	0	0.047839
3645 Amp	3	36816093	36934640	0	0.052768
3646 Amp	3	36935287	37293752	0	0.047839
3647 Amp	3	37297271	37820344	0	0.044572
3648 Amp	3	37820809	37912665	0	0.045984
3649 Amp	3	37917047	38015555	0	0.04176
3650 Amp	3	38016115	38122883	0	0.049813
3651 Amp	3	38126144	38638052	0	0.04176
3652 Amp	3	38639137	38754614	0	0.040348
3653 Amp	3	38755178	39359617	0	0.034369
3654 Amp	3	39366445	39481512	0	0.041045
3655 Amp	3	39481615	39645582	0	0.052569
3656 Amp	3	39652079	40191121	0	0.041045

3657 Amp	3	40196577	40311501	0	0.035213
3658 Amp	3	40312099	40879766	0	0.038749
3659 Amp	3	40883255	41821701	0	0.032074
3660 Amp	3	41831682	41872696	0	0.037521
3661 Amp	3	42174077	42310575	0	0.035491
3662 Amp	3	42313622	42323013	0	0.037703
3663 Amp	3	42325229	42652888	0	0.035491
3664 Amp	3	42657628	42815487	0	0.026591
3665 Amp	3	42824999	42953665	0	0.02277
3666 Amp	3	42965923	43244549	0	0.018069
3667 Amp	3	43245983	45065335	0	0.028039
3668 Amp	3	45065476	46049017	0	0.018069
3669 Amp	3	46049869	46377198	0	0.020081
3670 Amp	3	46378143	46402842	0	0.027902
3671 Amp	3	46402855	46403303	0	0.02939
3672 Amp	3	46405230	46673894	0	0.021569
3673 Amp	3	46675671	46713488	0	0.028672
3674 Amp	3	46718217	46732421	0	0.021569
3675 Amp	3	46733286	46733286	0	0.022991
3676 Amp	3	46828459	46984645	0	0.026641
3677 Amp	3	46985395	47002048	0	0.03622
3678 Amp	3	47008372	47008372	0	0.026641
3679 Amp	3	47013276	47051914	0	0.024629
3680 Amp	3	47052132	47088954	0	0.020979
3681 Amp	3	47092202	47529076	0	0.017442
3682 Amp	3	47532876	47553491	0	0.029156
3683 Amp	3	47561353	47726865	0	0.023709
3684 Amp	3	47731184	48143738	0	0.011994
3685 Amp	3	48145667	48399494	0	0.010402
3686 Amp	3	48399921	48434274	0	0.018327
3687 Amp	3	48436607	48558857	0	0.010402
3688 Amp	3	48559317	48597999	0	0.020133
3689 Amp	3	48599799	48749786	0	0.010402
3690 Amp	3	48751947	48862117	0	0.008279
3691 Amp	3	48866825	49640249	0	0.002909
3692 Amp	3	49641335	49723146	0	0.007253
3693 Amp	3	49728703	49776022	0	0.002909
3694 Amp	3	49777978	49910004	0	0.005156
3695 Amp	3	49913921	49996003	0	0.002909
3696 Amp	3	49998109	50013818	0	0.016325
3697 Amp	3	50016076	50054806	0	0.002909
3698 Amp	3	50055890	50118830	0	0.005666
3699 Amp	3	50120238	50528778	0	0.00959
3700 Amp	3	50530292	50579991	0	0.019411
3701 Amp	3	50580160	50614320	0	0.015487
3702 Amp	3	50615038	50661106	0	0.017913
3703 Amp	3	50661849	51122764	0	0.008091
3704 Amp	3	51123212	51637161	0	0.005335
3705 Amp	3	51639064	51826801	0	0.008529
3706 Amp	3	51830672	52218224	0	0.014563
3707 Amp	3	52223619	52348735	0	0.006104
3708 Amp	3	52349595	52402408	0	0.013703
3709 Amp	3	52407590	52479011	0	0.006104

3710	Amp	3	52482715	52528215	0	0.014738
3711	Amp	3	52532898	53105126	0	0.006104
3712	Amp	3	53109885	53126116	0	0.00952
3713	Amp	3	53128973	53400742	0	0.008099
3714	Amp	3	53400902	53540025	0	0.006611
3715	Amp	3	53543517	53626003	0	0.003417
3716	Amp	3	53628591	53725860	0	0.012326
3717	Amp	3	53728994	53743949	0	0.02465
3718	Amp	3	53750631	53812714	0	0.012326
3719	Amp	3	53812897	54002578	0	0.003417
3720	Amp	3	54003962	54082357	0	0.005173
3721	Amp	3	54086218	54895473	0	0.015444
3722	Amp	3	54895519	55085249	0	0.018385
3723	Amp	3	55086878	55362544	0	0.015444
3724	Amp	3	55369142	55906610	0	0.020011
3725	Amp	3	55907410	56190377	0	0.025017
3726	Amp	3	56193861	57179696	0	0.015444
3727	Amp	3	57181076	57274145	0	0.021851
3728	Amp	3	57283204	57581879	0	0.027729
3729	Amp	3	57582126	57824152	0	0.029544
3730	Amp	3	57831488	57913639	0	0.036646
3731	Amp	3	57917436	57962639	0	0.049302
3732	Amp	3	57964401	58327708	0	0.042201
3733	Amp	3	58329668	58647598	0	0.029544
3734	Amp	3	58648824	59706442	0	0.027729
3735	Amp	3	59706544	59715181	0	0.025974
3736	Amp	3	59715251	59717095	0	0.020095
3737	Amp	3	59719409	59720118	0	0.003417
3738	Amp	3	61040294	61057116	0	0.008404
3739	Amp	3	61057188	61077966	0	0.014491
3740	Amp	3	61078759	61097926	0	0.008404
3741	Amp	3	61106963	61279789	0	0.003417
3742	Amp	3	61282447	61513159	0	0.007618
3743	Amp	3	61513698	61834182	0	0.012618
3744	Amp	3	61834405	62592766	0	0.007618
3745	Amp	3	62596503	62846555	0	0.022641
3746	Amp	3	62860321	63547690	0	0.007618
3747	Amp	3	63548916	63804357	0	0.018007
3748	Amp	3	63806234	63980934	0	0.024201
3749	Amp	3	63981577	64524712	0	0.027947
3750	Amp	3	64527012	64637165	0	0.032693
3751	Amp	3	64641234	64859500	0	0.029928
3752	Amp	3	64865149	64972440	0	0.024201
3753	Amp	3	64972566	66031114	0	0.030196
3754	Amp	3	66040192	66072436	0	0.032711
3755	Amp	3	66075781	66122534	0	0.038922
3756	Amp	3	66124065	66727236	0	0.022539
3757	Amp	3	66729467	66902126	0	0.02699
3758	Amp	3	66902263	67106068	0	0.02078
3759	Amp	3	67107246	67114679	0	0.026456
3760	Amp	3	67122882	67167921	0	0.023039
3761	Amp	3	67169025	67230992	0	0.025467
3762	Amp	3	67232491	67327811	0	0.023039

3763	Amp	3	67327993	67408138	0	0.018587
3764	Amp	3	67408232	67421840	0	0.02082
3765	Amp	3	67432354	67645312	0	0.030485
3766	Amp	3	67647767	67665718	0	0.02082
3767	Amp	3	67667023	68191016	0	0.018304
3768	Amp	3	68194315	68627808	0	0.027844
3769	Amp	3	68634663	68826817	0	0.018304
3770	Amp	3	68832011	68955689	0	0.016071
3771	Amp	3	68960795	69096481	0	0.019252
3772	Amp	3	69099901	69166430	0	0.033297
3773	Amp	3	69167707	69171933	0	0.030116
3774	Amp	3	69173712	69316114	0	0.016071
3775	Amp	3	69316377	69367067	0	0.030487
3776	Amp	3	69367103	69382893	0	0.024293
3777	Amp	3	69383201	69623864	0	0.028179
3778	Amp	3	69628810	69863444	0	0.022503
3779	Amp	3	69863545	69908649	0	0.027292
3780	Amp	3	69908706	69917782	0	0.029088
3781	Amp	3	69926168	70946629	0	0.022503
3782	Amp	3	70947508	71448264	0	0.008087
3783	Amp	3	71451216	71486187	0	0.013186
3784	Amp	3	71526660	71720992	0	0.021806
3785	Amp	3	71721423	72443164	0	0.026523
3786	Amp	3	72444999	72474576	0	0.0326
3787	Amp	3	72480644	72785442	0	0.026523
3788	Amp	3	72786479	72812071	0	0.027918
3789	Amp	3	72814353	73419376	0	0.026523
3790	Amp	3	73423235	73525562	0	0.013186
3791	Amp	3	73528034	74395697	0	0.008087
3792	Amp	3	74397939	74535454	0	0.050794
3793	Amp	3	74544548	74831014	0	0.008087
3794	Amp	3	74834930	74905409	0	0.051628
3795	Amp	3	74910357	75272102	0	0.05594
3796	Amp	3	75273092	75315279	0	0.061215
3797	Amp	3	75793727	75805867	0	0.109347
3798	Amp	3	75812545	75923014	0	0.065806
3799	Amp	3	75924086	75950365	0	0.067392
3800	Amp	3	75985252	76136808	0	0.095791
3801	Amp	3	76142860	76172899	0	0.079391
3802	Amp	3	76175851	76375824	0	0.047659
3803	Amp	3	76377619	76701954	0	0.049951
3804	Amp	3	76702051	76994799	0	0.047659
3805	Amp	3	76999257	77469231	0	0.019259
3806	Amp	3	77472155	77482775	0	0.025537
3807	Amp	3	77482843	77584457	0	0.028718
3808	Amp	3	77588516	77802326	0	0.032817
3809	Amp	3	77811523	77903590	0	0.021264
3810	Amp	3	77905136	78671506	0	0.019023
3811	Amp	3	78673228	78784872	0	0.032391
3812	Amp	3	78786402	78818051	0	0.036302
3813	Amp	3	78823012	78838265	0	0.022934
3814	Amp	3	78845197	78870836	0	0.037966
3815	Amp	3	78875767	78941385	0	0.022934

3816 Amp	3	78945890	78945890	0	0.032939
3817 Amp	3	78950398	78993126	0	0.02884
3818 Amp	3	78996915	79022917	0	0.024929
3819 Amp	3	79025909	79031101	0	0.038717
3820 Amp	3	79038742	79180742	0	0.048065
3821 Amp	3	79183464	79192798	0	0.034276
3822 Amp	3	79197211	79263687	0	0.044111
3823 Amp	3	79266967	79308961	0	0.038662
3824 Amp	3	79309940	79379673	0	0.040268
3825 Amp	3	79387320	79456014	0	0.058626
3826 Amp	3	79456678	79485994	0	0.060753
3827 Amp	3	79491634	79582278	0	0.058626
3828 Amp	3	79583935	79603504	0	0.048622
3829 Amp	3	79607976	79770960	0	0.093275
3830 Amp	3	79776795	79902472	0	0.105426
3831 Amp	3	79902628	79914041	0	0.098679
3832 Amp	3	79915379	80014572	0	0.086528
3833 Amp	3	80021657	80026323	0	0.068169
3834 Amp	3	80028792	80028792	0	0.023516
3835 Amp	3	80034648	80129811	0	0.035516
3836 Amp	3	80135121	80187393	0	0.040701
3837 Amp	3	80188357	80229937	0	0.03391
3838 Amp	3	80234474	80242280	0	0.021911
3839 Amp	3	80242583	80480477	0	0.018012
3840 Amp	3	80484558	80805241	0	0.022415
3841 Amp	3	80817318	80891141	0	0.018012
3842 Amp	3	80899292	80899292	0	0.042627
3843 Amp	3	80901225	81030910	0	0.055656
3844 Amp	3	81031960	81047851	0	0.062004
3845 Amp	3	81048389	81170906	0	0.073096
3846 Amp	3	81177283	81190718	0	0.074868
3847 Amp	3	81197912	81205647	0	0.079781
3848 Amp	3	81205903	81271794	0	0.068689
3849 Amp	3	81272629	81370953	0	0.066917
3850 Amp	3	81376615	82126410	0	0.031041
3851 Amp	3	82129174	82204029	0	0.036978
3852 Amp	3	82209642	82280403	0	0.044087
3853 Amp	3	82284836	82370171	0	0.046021
3854 Amp	3	82373110	82392598	0	0.064657
3855 Amp	3	82392661	82410940	0	0.07372
3856 Amp	3	82415680	82540323	0	0.067782
3857 Amp	3	82540563	82574355	0	0.05872
3858 Amp	3	82576078	82730607	0	0.051611
3859 Amp	3	82730609	83013078	0	0.046845
3860 Amp	3	83015027	83022564	0	0.028208
3861 Amp	3	83025534	83093156	0	0.033021
3862 Amp	3	83094657	83226603	0	0.034542
3863 Amp	3	83229612	83306188	0	0.039388
3864 Amp	3	83312286	83401380	0	0.044686
3865 Amp	3	83402210	83456888	0	0.046265
3866 Amp	3	83457131	83527229	0	0.040968
3867 Amp	3	83527867	83798762	0	0.046204
3868 Amp	3	83803611	83816644	0	0.040968

3869	Amp	3	83826893	84038496	0	0.048482
3870	Amp	3	84040462	84070742	0	0.053556
3871	Amp	3	84077386	84110633	0	0.046041
3872	Amp	3	84117798	84173793	0	0.050855
3873	Amp	3	84174011	84208881	0	0.056798
3874	Amp	3	84212100	84267664	0	0.06709
3875	Amp	3	84278110	84283104	0	0.072045
3876	Amp	3	84284095	84285864	0	0.115846
3877	Amp	3	84286581	84332993	0	0.125845
3878	Amp	3	84334000	84341436	0	0.171628
3879	Amp	3	84342081	84376543	0	0.155749
3880	Amp	3	84376744	84430510	0	0.168603
3881	Amp	3	84432891	84484749	0	0.16266
3882	Amp	3	84484813	84613493	0	0.145469
3883	Amp	3	84616297	84756198	0	0.165885
3884	Amp	3	84757652	84985142	0	0.163951
3885	Amp	3	84999859	85038597	0.086298	0.19871
3886	Amp	3	85042269	85044478	0.111991	0.201887
3887	Amp	3	85045049	85171909	0.195309	0.210886
3888	Amp	3	85224056	85224056	0	0.177885
3889	Amp	3	85231336	85259896	0.050005	0.194564
3890	Amp	3	85274126	85362129	0.033661	0.192806
3891	Amp	3	85371210	85386890	0.362221	0.226306
3892	Amp	3	85390464	85403814	0.033661	0.192806
3893	Amp	3	85408648	85441637	0	0.187732
3894	Amp	3	85589241	85674119	0	0.144919
3895	Amp	3	85679035	85693056	0	0.165835
3896	Amp	3	85697254	86141794	0	0.144919
3897	Amp	3	86142511	86244905	0	0.150593
3898	Amp	3	86245074	86277837	0	0.144919
3899	Amp	3	86281944	86630389	0	0.160266
3900	Amp	3	86633599	86745910	0	0.164161
3901	Amp	3	86746311	87033664	0	0.170996
3902	Amp	3	87034947	87055749	0	0.128784
3903	Amp	3	87063988	87227912	0	0.119786
3904	Amp	3	87230118	87271872	0	0.112951
3905	Amp	3	87272238	87497852	0	0.118005
3906	Amp	3	87500564	87644056	0	0.11457
3907	Amp	3	87645410	87647475	0	0.1357
3908	Amp	3	87647973	87760453	0	0.091698
3909	Amp	3	87909425	88278658	0	0.070569
3910	Amp	3	88279103	88604493	0	0.06899
3911	Amp	3	88606074	88642066	0	0.074405
3912	Amp	3	88644311	88668511	0	0.075883
3913	Amp	3	88675124	88689966	0	0.08151
3914	Amp	3	88696000	88785103	0	0.084195
3915	Amp	3	88785962	88812855	0	0.08151
3916	Amp	3	88820719	88852302	0	0.082916
3917	Amp	3	88855272	88940168	0	0.129996
3918	Amp	3	88940484	89137973	0	0.139823
3919	Amp	3	89147494	89385089	0	0.183814
3920	Amp	3	89386017	89503594	0	0.185475
3921	Amp	3	89506877	89604134	0.02372	0.19134

3922 Amp	3	89609257	89668713	0.156508	0.206378
3923 Amp	3	89669108	89702789	0.117531	0.202483
3924 Amp	3	89710257	89718941	0.033661	0.192656
3925 Amp	3	89723874	89746363	0.111991	0.201713
3926 Amp	3	89753290	89761630	0.073369	0.197621
3927 Amp	3	89762335	89821796	0.001198	0.188564
3928 Amp	3	89824163	90028462	0.061778	0.196393
3929 Amp	3	90029473	90060888	0.018387	0.190978
3930 Amp	3	90064664	90147967	0	0.185352
3931 Amp	3	90154574	90169207	0	0.183691
3932 Amp	3	90186901	90246881	0	0.168653
3933 Amp	3	90252860	90287340	0	0.124661
3934 Amp	3	90293527	90369509	0	0.095136
3935 Amp	3	90375879	90375879	0	0.091656
3936 Amp	3	90376841	90436486	0	0.046173
3937 Amp	3	90436812	90453712	0	0.061907
3938 Amp	3	91390926	91390926	0	0.063986
3939 Amp	3	91462299	91462299	0	0.062331
3940 Amp	3	94015345	94015345	0	0.100077
3941 Amp	3	94015827	94018736	0	0.114236
3942 Amp	3	94021848	94126870	0	0.112592
3943 Amp	3	94130729	94297719	0	0.103679
3944 Amp	3	94298757	94373616	0	0.077574
3945 Amp	3	94373837	94596235	0	0.081738
3946 Amp	3	94597988	94669289	0	0.084399
3947 Amp	3	94678984	94802716	0	0.105715
3948 Amp	3	94803426	94832206	0	0.114841
3949 Amp	3	94832335	94832335	0	0.110677
3950 Amp	3	94832701	94962580	0	0.147681
3951 Amp	3	94971286	95034571	0	0.179829
3952 Amp	3	95039996	95189751	0	0.141993
3953 Amp	3	95194091	95315478	0	0.132867
3954 Amp	3	95316860	95316860	0	0.145723
3955 Amp	3	95319560	95366547	0	0.124424
3956 Amp	3	95369769	95375577	0	0.129922
3957 Amp	3	95375872	95437174	0	0.136342
3958 Amp	3	95437235	95572851	0	0.133254
3959 Amp	3	95579583	95579583	0	0.128847
3960 Amp	3	95591606	95844270	0	0.122026
3961 Amp	3	95856205	96013014	0	0.132094
3962 Amp	3	96017026	96065874	0	0.122026
3963 Amp	3	96070124	96209242	0	0.10486
3964 Amp	3	96211917	96448863	0	0.102199
3965 Amp	3	96463340	96585044	0	0.082882
3966 Amp	3	96608620	96729141	0	0.089377
3967 Amp	3	96730157	96792183	0	0.09564
3968 Amp	3	96794315	96916429	0	0.075802
3969 Amp	3	96922632	96946032	0	0.070304
3970 Amp	3	96955769	97255742	0	0.090783
3971 Amp	3	97260207	97307516	0	0.066747
3972 Amp	3	97315993	97489188	0	0.068883
3973 Amp	3	97492790	97557947	0	0.048403
3974 Amp	3	97559412	97609765	0	0.052604

3975	Amp	3	97612207	97635165	0	0.097915
3976	Amp	3	97637556	97648466	0	0.052604
3977	Amp	3	97655693	97778805	0	0.055828
3978	Amp	3	97779126	97781475	0	0.061745
3979	Amp	3	97785433	97863751	0	0.066922
3980	Amp	3	97869115	98088155	0	0.061006
3981	Amp	3	98090374	98158504	0	0.059409
3982	Amp	3	98159311	98371156	0	0.055209
3983	Amp	3	98373642	98389526	0	0.050031
3984	Amp	3	98389795	98784932	0	0.060093
3985	Amp	3	98785244	98902275	0	0.050031
3986	Amp	3	98902429	98946369	0	0.089665
3987	Amp	3	98951174	99008104	0	0.121103
3988	Amp	3	99008214	99120070	0	0.114221
3989	Amp	3	99125145	99137095	0	0.141548
3990	Amp	3	99137299	99443869	0	0.159778
3991	Amp	3	99443961	99449669	0	0.163637
3992	Amp	3	99463008	99534829	0	0.173387
3993	Amp	3	99535067	99581558	0	0.159229
3994	Amp	3	99588180	99610560	0	0.140998
3995	Amp	3	99615048	99822289	0	0.135009
3996	Amp	3	99825536	99829352	0	0.132873
3997	Amp	3	99829430	99945860	0	0.138323
3998	Amp	3	99950044	100137257	0	0.110996
3999	Amp	3	100139017	100463974	0	0.105547
4000	Amp	3	100464505	100736295	0	0.126441
4001	Amp	3	100736617	100760598	0	0.12224
4002	Amp	3	100764216	100777836	0	0.126541
4003	Amp	3	100780477	100799806	0	0.12224
4004	Amp	3	100803009	100980085	0	0.127952
4005	Amp	3	100980716	100990152	0	0.12224
4006	Amp	3	100990705	101458682	0	0.115861
4007	Amp	3	101458800	101730889	0	0.094966
4008	Amp	3	101734146	101742396	0	0.10504
4009	Amp	3	101745654	101748946	0	0.129543
4010	Amp	3	101755654	101790527	0	0.141653
4011	Amp	3	101791681	101792049	0	0.154879
4012	Amp	3	101796211	101798248	0	0.140981
4013	Amp	3	101803004	101848500	0	0.148415
4014	Amp	3	101853446	101854208	0	0.151641
4015	Amp	3	101855059	101855059	0	0.169819
4016	Amp	3	101859863	101859863	0	0.163763
4017	Amp	3	101860929	101895545	0.09318	0.199505
4018	Amp	3	101897932	101931615	0.149443	0.205564
4019	Amp	3	101932614	101948003	0.230572	0.213965
4020	Amp	3	101948027	102039869	0.168144	0.207906
4021	Amp	3	102042152	102138897	0.098998	0.200472
4022	Amp	3	102141042	102182251	0.209057	0.2121
4023	Amp	3	102182920	102189891	0.18072	0.208874
4024	Amp	3	102192214	102213137	0.073369	0.197378
4025	Amp	3	102217294	102269754	0	0.1792
4026	Amp	3	102273069	102314816	0	0.16709
4027	Amp	3	102317204	102353364	0	0.168846

4028	Amp	3	102356591	102359675	0	0.158773
4029	Amp	3	102363122	102389570	0	0.153575
4030	Amp	3	102390806	102392805	0	0.146405
4031	Amp	3	102396164	102497109	0	0.109589
4032	Amp	3	102498608	102580761	0	0.093361
4033	Amp	3	102594134	102619444	0	0.082047
4034	Amp	3	102624114	102693802	0	0.075923
4035	Amp	3	102700362	102759793	0	0.081358
4036	Amp	3	102760378	102814910	0	0.072718
4037	Amp	3	102828424	103288998	0	0.067063
4038	Amp	3	103291072	103903074	0	0.060644
4039	Amp	3	103911094	104029674	0	0.0692
4040	Amp	3	104030177	104030218	0	0.074608
4041	Amp	3	104038697	104080113	0	0.06298
4042	Amp	3	104085544	104147346	0	0.108954
4043	Amp	3	104147479	104167867	0	0.1147
4044	Amp	3	104172063	104172638	0	0.109292
4045	Amp	3	104178262	104400718	0	0.119029
4046	Amp	3	104417865	104456442	0	0.113283
4047	Amp	3	104456521	104494117	0	0.111527
4048	Amp	3	104495455	104557983	0	0.061004
4049	Amp	3	104559738	104674125	0	0.06806
4050	Amp	3	104675348	104768079	0	0.061004
4051	Amp	3	104768198	104845488	0	0.051267
4052	Amp	3	104851216	105151833	0	0.071139
4053	Amp	3	105152058	105200646	0	0.085476
4054	Amp	3	105201495	105244944	0	0.092569
4055	Amp	3	105249693	105350093	0	0.081386
4056	Amp	3	105355484	105449254	0	0.066533
4057	Amp	3	105452513	105501746	0	0.07322
4058	Amp	3	105513025	105535947	0	0.074977
4059	Amp	3	105537851	105767718	0	0.078553
4060	Amp	3	105769348	105829719	0	0.084499
4061	Amp	3	105830058	105962569	0	0.080923
4062	Amp	3	105968787	105985319	0	0.087415
4063	Amp	3	105987966	106171448	0	0.074977
4064	Amp	3	106171898	106371226	0	0.068289
4065	Amp	3	106371615	106405286	0	0.094402
4066	Amp	3	106405383	106701183	0	0.096303
4067	Amp	3	106705199	107069303	0	0.104789
4068	Amp	3	107071551	107154075	0	0.119895
4069	Amp	3	107164740	107208017	0	0.111367
4070	Amp	3	107209500	107311241	0	0.122749
4071	Amp	3	107313898	107348038	0	0.118456
4072	Amp	3	107352902	107426785	0	0.12461
4073	Amp	3	107426842	107556784	0	0.118456
4074	Amp	3	107559306	107705243	0	0.122717
4075	Amp	3	107705994	107857896	0	0.115628
4076	Amp	3	107857972	108648113	0	0.10465
4077	Amp	3	108663713	108877854	0	0.081029
4078	Amp	3	109300867	109361219	0	0.100263
4079	Amp	3	109364602	109614761	0	0.081029
4080	Amp	3	109618607	110141323	0	0.076768

4081	Amp	3	110145818	110234977	0	0.1029
4082	Amp	3	110235378	110616207	0	0.08874
4083	Amp	3	110619222	110646179	0	0.099962
4084	Amp	3	110649367	110981339	0	0.110989
4085	Amp	3	110981820	111448795	0	0.123288
4086	Amp	3	111452525	111483290	0	0.127074
4087	Amp	3	111483508	111593458	0	0.123288
4088	Amp	3	111597462	111644399	0	0.103416
4089	Amp	3	111644891	111752370	0	0.126939
4090	Amp	3	111753032	111921527	0	0.121394
4091	Amp	3	111924714	112435856	0	0.128517
4092	Amp	3	112437509	112723364	0	0.11749
4093	Amp	3	112730383	112756044	0	0.127548
4094	Amp	3	112756683	112761025	0	0.11367
4095	Amp	3	112761485	112811940	0	0.118772
4096	Amp	3	112812493	112878859	0	0.130255
4097	Amp	3	112878997	112902884	0	0.125153
4098	Amp	3	112904882	113048315	0	0.135625
4099	Amp	3	113050977	113140382	0	0.14138
4100	Amp	3	113141752	113234811	0	0.143116
4101	Amp	3	113237655	113470521	0	0.153053
4102	Amp	3	113478797	113516530	0	0.156715
4103	Amp	3	113516704	113601077	0	0.165088
4104	Amp	3	113601212	113695189	0	0.154461
4105	Amp	3	113702270	113741327	0	0.161384
4106	Amp	3	113741582	113744639	0	0.168186
4107	Amp	3	113749515	113771316	0	0.156703
4108	Amp	3	113773555	113953562	0	0.130093
4109	Amp	3	113954871	113997416	0	0.138233
4110	Amp	3	113998851	114004309	0	0.129901
4111	Amp	3	114004960	114024793	0	0.119965
4112	Amp	3	114034176	114171721	0	0.113387
4113	Amp	3	114175453	114177676	0	0.110741
4114	Amp	3	114179799	114339055	0	0.116533
4115	Amp	3	114339566	114357512	0	0.121615
4116	Amp	3	114362525	114406204	0	0.103003
4117	Amp	3	114411497	114508685	0	0.097921
4118	Amp	3	114510748	114631340	0	0.096002
4119	Amp	3	114634112	114634293	0	0.101946
4120	Amp	3	114634321	114674913	0	0.097062
4121	Amp	3	114680449	114904171	0	0.123672
4122	Amp	3	114904346	114956799	0	0.097062
4123	Amp	3	114957718	114984193	0	0.098936
4124	Amp	3	114987667	114996027	0	0.092993
4125	Amp	3	114998257	115050364	0	0.120086
4126	Amp	3	115050659	115154061	0	0.116977
4127	Amp	3	115164724	115208109	0	0.124158
4128	Amp	3	115209035	115273805	0	0.138247
4129	Amp	3	115275000	115282102	0	0.131065
4130	Amp	3	115282466	115373125	0	0.125274
4131	Amp	3	115377484	115707923	0	0.13178
4132	Amp	3	115711736	115770999	0	0.122493
4133	Amp	3	115771163	115837812	0	0.132063

4134	Amp	3	115838615	115985587	0	0.122493
4135	Amp	3	115985849	116016969	0	0.120757
4136	Amp	3	116019466	116121598	0	0.118856
4137	Amp	3	116123930	116169322	0	0.121257
4138	Amp	3	116177403	116184124	0	0.097772
4139	Amp	3	116188246	116289731	0	0.095898
4140	Amp	3	116293546	116354657	0	0.104045
4141	Amp	3	116355209	116366410	0	0.118377
4142	Amp	3	116366433	116390066	0	0.098204
4143	Amp	3	116390303	116447779	0	0.069245
4144	Amp	3	116450197	116594557	0	0.07352
4145	Amp	3	116594690	116735112	0	0.084396
4146	Amp	3	116740404	116808908	0	0.068374
4147	Amp	3	116808954	116880307	0	0.074408
4148	Amp	3	116886124	116886124	0	0.069497
4149	Amp	3	116887001	117028050	0	0.063532
4150	Amp	3	117030171	117046440	0	0.067829
4151	Amp	3	117052260	117085211	0	0.079027
4152	Amp	3	117092325	117171685	0	0.074752
4153	Amp	3	117172247	117193891	0	0.072351
4154	Amp	3	117194478	117266524	0	0.061152
4155	Amp	3	117272022	117296984	0	0.067734
4156	Amp	3	117304710	117342137	0	0.065977
4157	Amp	3	117347522	117427460	0	0.059396
4158	Amp	3	117429472	118513819	0	0.054342
4159	Amp	3	118514058	118576243	0	0.041801
4160	Amp	3	118577956	118705593	0	0.045432
4161	Amp	3	118706139	118866159	0	0.041801
4162	Amp	3	118868874	119001132	0	0.046184
4163	Amp	3	119115295	119115295	0	0.052757
4164	Amp	3	119117086	119400601	0	0.048461
4165	Amp	3	119402963	119405515	0	0.044078
4166	Amp	3	119405898	119887404	0	0.05007
4167	Amp	3	119887523	120691173	0	0.037505
4168	Amp	3	120702526	120822757	0	0.041038
4169	Amp	3	120824102	120869303	0	0.044823
4170	Amp	3	120870880	120986900	0	0.05969
4171	Amp	3	120987220	121063822	0	0.055905
4172	Amp	3	121065939	121308326	0	0.041038
4173	Amp	3	121318935	121976849	0	0.037505
4174	Amp	3	121979434	122121166	0	0.042912
4175	Amp	3	122129109	122275011	0	0.037505
4176	Amp	3	122279258	123363551	0	0.028948
4177	Amp	3	123363937	124097811	0	0.03385
4178	Amp	3	124099253	124507750	0	0.028948
4179	Amp	3	124508379	124630152	0	0.033337
4180	Amp	3	124631591	124823684	0	0.042109
4181	Amp	3	124823728	124835124	0	0.048678
4182	Amp	3	124835283	125204141	0	0.042109
4183	Amp	3	125204324	125271851	0	0.037721
4184	Amp	3	125272044	126980540	0	0.031226
4185	Amp	3	126990359	127040945	0	0.043731
4186	Amp	3	127048288	127152287	0	0.031226

4187 Amp	3	127154043	127285129	0	0.055351
4188 Amp	3	127286157	127669993	0	0.046578
4189 Amp	3	127670779	127696204	0	0.053854
4190 Amp	3	127697299	127700275	0	0.046578
4191 Amp	3	127700333	127705367	0	0.031103
4192 Amp	3	127706041	128081698	0	0.046824
4193 Amp	3	128085998	128171906	0	0.027868
4194 Amp	3	128176233	128311163	0	0.034332
4195 Amp	3	128311592	128374591	0	0.042475
4196 Amp	3	128375663	128401031	0	0.044656
4197 Amp	3	128404193	128424891	0	0.050402
4198 Amp	3	128427842	128515865	0	0.044656
4199 Amp	3	128516622	128551050	0	0.042475
4200 Amp	3	128631841	128633674	0	0.027868
4201 Amp	3	128634475	128648733	0	0.025789
4202 Amp	3	128654829	128873499	0	0.023007
4203 Amp	3	128874140	128899715	0	0.035859
4204 Amp	3	128904602	129327076	0	0.038277
4205 Amp	3	129327591	129376321	0	0.035052
4206 Amp	3	129387861	129410310	0	0.036896
4207 Amp	3	129410838	129474067	0	0.039447
4208 Amp	3	129477920	129604922	0	0.044092
4209 Amp	3	129611303	129611757	0	0.05682
4210 Amp	3	129616601	129641771	0	0.052175
4211 Amp	3	129645477	129721919	0	0.049757
4212 Amp	3	129723968	129971349	0	0.036905
4213 Amp	3	129972212	130258530	0	0.024177
4214 Amp	3	130259341	130309874	0	0.025921
4215 Amp	3	130311396	130406506	0	0.024177
4216 Amp	3	130406599	130417390	0	0.018993
4217 Amp	3	130417424	130581490	0	0.024425
4218 Amp	3	130585222	130869631	0	0.027261
4219 Amp	3	130871385	130929954	0	0.043575
4220 Amp	3	130942139	131257495	0	0.027261
4221 Amp	3	131257683	131288384	0	0.030872
4222 Amp	3	131290289	132048818	0	0.040287
4223 Amp	3	132055798	132847923	0	0.034855
4224 Amp	3	132850600	133292827	0	0.030361
4225 Amp	3	133293636	133316329	0	0.041428
4226 Amp	3	133316530	133584828	0	0.030361
4227 Amp	3	133592652	133634749	0	0.025441
4228 Amp	3	133638301	133934095	0	0.034312
4229 Amp	3	133937589	134222842	0	0.043774
4230 Amp	3	134234229	134430114	0	0.050147
4231 Amp	3	134432295	134900882	0	0.043774
4232 Amp	3	134900910	134900910	0	0.048315
4233 Amp	3	134904858	134981456	0	0.050421
4234 Amp	3	134983191	135037918	0	0.053434
4235 Amp	3	135040180	135227598	0	0.040248
4236 Amp	3	135229020	135811944	0	0.037235
4237 Amp	3	135831264	135872262	0	0.035129
4238 Amp	3	135878918	135957570	0	0.044272
4239 Amp	3	135959425	136696544	0	0.049459

4240	Amp	3	136699921	136763422	0	0.052661
4241	Amp	3	136766082	136971526	0	0.05604
4242	Amp	3	136971549	137075187	0	0.066
4243	Amp	3	137077208	137091770	0	0.061458
4244	Amp	3	137099021	137291845	0	0.051499
4245	Amp	3	137293690	137389973	0	0.046353
4246	Amp	3	137398299	137717825	0	0.070212
4247	Amp	3	137723070	137881503	0	0.065025
4248	Amp	3	137883608	138048612	0	0.055882
4249	Amp	3	138129483	138155428	0	0.077961
4250	Amp	3	138160266	138213667	0	0.066922
4251	Amp	3	138215780	138331659	0	0.069632
4252	Amp	3	138334345	138717433	0	0.071021
4253	Amp	3	138720532	138788238	0	0.076346
4254	Amp	3	138791541	138884517	0	0.0924
4255	Amp	3	138886348	139056535	0	0.087076
4256	Amp	3	139057002	139369331	0	0.100955
4257	Amp	3	139371634	139372480	0	0.110897
4258	Amp	3	139374510	139466648	0	0.125578
4259	Amp	3	139469207	139804291	0	0.127234
4260	Amp	3	139805932	140001186	0	0.113913
4261	Amp	3	140001289	140040541	0	0.11612
4262	Amp	3	140041879	140046794	0	0.11341
4263	Amp	3	140047882	140059007	0	0.118402
4264	Amp	3	140061876	140330279	0	0.089843
4265	Amp	3	140332904	140372279	0	0.073788
4266	Amp	3	140373230	140384355	0	0.114382
4267	Amp	3	140386367	140395436	0	0.073788
4268	Amp	3	140395681	140428611	0	0.071581
4269	Amp	3	140428804	140605170	0	0.069924
4270	Amp	3	140609983	140680955	0	0.058884
4271	Amp	3	140682116	140770223	0	0.064087
4272	Amp	3	140770327	140771304	0	0.058884
4273	Amp	3	140773165	140844343	0	0.062686
4274	Amp	3	140845233	140907024	0	0.076982
4275	Amp	3	140912626	141201324	0	0.062686
4276	Amp	3	141214389	141288161	0	0.071955
4277	Amp	3	141288379	141314160	0	0.062686
4278	Amp	3	141320265	141571347	0	0.06804
4279	Amp	3	141574695	141866752	0	0.062686
4280	Amp	3	141870479	141946434	0	0.038826
4281	Amp	3	141947832	141991196	0	0.031734
4282	Amp	3	141992662	142100008	0	0.027932
4283	Amp	3	142375789	142644590	0	0.026856
4284	Amp	3	142645650	143166481	0	0.02294
4285	Amp	3	143166947	143190629	0	0.026375
4286	Amp	3	143191821	143685513	0	0.036897
4287	Amp	3	143688845	143688845	0	0.026375
4288	Amp	3	143689881	143789184	0	0.041117
4289	Amp	3	143792288	143904375	0	0.033194
4290	Amp	3	143905953	144558743	0	0.037592
4291	Amp	3	144575748	144832493	0	0.052942
4292	Amp	3	144833418	144870851	0	0.048544

4293	Amp	3	144871042	144992242	0	0.033194
4294	Amp	3	144993956	145293513	0	0.026375
4295	Amp	3	145294169	145989216	0	0.023172
4296	Amp	3	145990277	146320895	0	0.019737
4297	Amp	3	146324610	146649834	0	0.018348
4298	Amp	3	146650017	146657279	0	0.019757
4299	Amp	3	146657797	147171868	0	0.023523
4300	Amp	3	147306036	147437811	0	0.049946
4301	Amp	3	147750170	147892751	0	0.048398
4302	Amp	3	147978864	147984663	0	0.046335
4303	Amp	3	147984723	148035678	0	0.04921
4304	Amp	3	148035769	148268556	0	0.058658
4305	Amp	3	148269290	148313359	0	0.06295
4306	Amp	3	148320992	148727269	0	0.078577
4307	Amp	3	148730631	149300249	0	0.081973
4308	Amp	3	149301316	149345489	0	0.05555
4309	Amp	3	149347622	149373969	0	0.039923
4310	Amp	3	149374600	149641827	0	0.038514
4311	Amp	3	149645721	149923484	0	0.062277
4312	Amp	3	149924661	150052721	0	0.057985
4313	Amp	3	150053965	150197721	0	0.061969
4314	Amp	3	150203377	151092758	0	0.076514
4315	Amp	3	151093507	151212275	0	0.067065
4316	Amp	3	151213597	151223398	0	0.093825
4317	Amp	3	151225275	151295108	0	0.07928
4318	Amp	3	151295871	151645575	0	0.081676
4319	Amp	3	151645983	151687777	0	0.052952
4320	Amp	3	151687866	151775808	0	0.05595
4321	Amp	3	151776115	151891927	0	0.07241
4322	Amp	3	151900547	151985228	0	0.081959
4323	Amp	3	151988659	152175058	0	0.088008
4324	Amp	3	152176376	152274266	0	0.081959
4325	Amp	3	152276385	152374866	0	0.079084
4326	Amp	3	152375249	152426030	0	0.08832
4327	Amp	3	152428276	152443066	0	0.093345
4328	Amp	3	152446763	152547807	0	0.08411
4329	Amp	3	152548006	152603614	0	0.091335
4330	Amp	3	152606308	152658048	0	0.097557
4331	Amp	3	152663124	152824565	0	0.090135
4332	Amp	3	152825760	152917152	0	0.092568
4333	Amp	3	152918515	152925173	0	0.067772
4334	Amp	3	152927628	152950749	0	0.073996
4335	Amp	3	152953106	153266549	0	0.083965
4336	Amp	3	153269991	153505379	0	0.088468
4337	Amp	3	153505833	153580238	0	0.078499
4338	Amp	3	153581208	153648010	0	0.084553
4339	Amp	3	153648885	153672019	0	0.078499
4340	Amp	3	153672822	153684306	0	0.086193
4341	Amp	3	153684548	153747636	0	0.08169
4342	Amp	3	153748053	153785268	0	0.062019
4343	Amp	3	153786421	153861700	0	0.054325
4344	Amp	3	153861778	153970905	0	0.051892
4345	Amp	3	153995856	154005655	0	0.066558

4346	Amp	3	154007833	154177371	0	0.071421
4347	Amp	3	154180880	154193206	0	0.057695
4348	Amp	3	154197947	154244164	0	0.071299
4349	Amp	3	154247518	154510096	0	0.05496
4350	Amp	3	154510226	154510226	0	0.045551
4351	Amp	3	154730714	154769282	0	0.059668
4352	Amp	3	154769540	154785535	0	0.045551
4353	Amp	3	154789970	154827717	0	0.061842
4354	Amp	3	154827731	154862298	0	0.079709
4355	Amp	3	154869865	155011901	0	0.074847
4356	Amp	3	155012170	155167322	0	0.081641
4357	Amp	3	155172496	155197441	0	0.086558
4358	Amp	3	155197992	155377207	0	0.082574
4359	Amp	3	155377573	155499096	0	0.086125
4360	Amp	3	155499558	155757661	0	0.079331
4361	Amp	3	155757674	155876087	0	0.08678
4362	Amp	3	155876952	155897953	0	0.092358
4363	Amp	3	155899722	155913825	0	0.095046
4364	Amp	3	155915845	155947989	0	0.087596
4365	Amp	3	155953165	155983052	0	0.083601
4366	Amp	3	155983975	156214694	0	0.089476
4367	Amp	3	156217544	156293720	0	0.085926
4368	Amp	3	156297626	156335498	0	0.094199
4369	Amp	3	156337896	156507959	0	0.090433
4370	Amp	3	156508429	156551424	0	0.095213
4371	Amp	3	156551596	156597939	0	0.099589
4372	Amp	3	156600178	156870690	0	0.091315
4373	Amp	3	156873522	156896530	0	0.086058
4374	Amp	3	156898895	156919129	0	0.084214
4375	Amp	3	156920968	156940112	0	0.090229
4376	Amp	3	156941199	156964746	0	0.084651
4377	Amp	3	156965347	157150016	0	0.081964
4378	Amp	3	157150220	157154455	0	0.075949
4379	Amp	3	157163094	157220505	0	0.083654
4380	Amp	3	157221202	157408160	0	0.087602
4381	Amp	3	157409501	157611837	0	0.089648
4382	Amp	3	157615751	157618329	0	0.096941
4383	Amp	3	157618775	157653238	0	0.09216
4384	Amp	3	157654102	157739613	0	0.069965
4385	Amp	3	157746446	157748004	0	0.062673
4386	Amp	3	157752452	157924577	0	0.052615
4387	Amp	3	157931748	157940523	0	0.062969
4388	Amp	3	157942874	158130209	0	0.067422
4389	Amp	3	158131002	158217570	0	0.064586
4390	Amp	3	158227814	158342800	0	0.054233
4391	Amp	3	158347120	158386873	0	0.0609
4392	Amp	3	158389844	158545170	0	0.051148
4393	Amp	3	158547126	158688216	0	0.044482
4394	Amp	3	158690259	158909697	0	0.04865
4395	Amp	3	158909782	159824289	0	0.044482
4396	Amp	3	159829683	160295647	0	0.040028
4397	Amp	3	160304826	160616920	0	0.023738
4398	Amp	3	160621402	160845005	0	0.040422

4399	Amp	3	160848728	160904108	0	0.049369
4400	Amp	3	160909390	161351812	0	0.05628
4401	Amp	3	161354159	161355404	0	0.064283
4402	Amp	3	161363882	161377312	0	0.066703
4403	Amp	3	161377808	161424526	0	0.057756
4404	Amp	3	161432252	161651414	0	0.049753
4405	Amp	3	161651955	161693274	0	0.051235
4406	Amp	3	161834016	161863978	0	0.063621
4407	Amp	3	161866239	161881503	0	0.051235
4408	Amp	3	161881906	161911255	0	0.052748
4409	Amp	3	161912472	161945296	0	0.050328
4410	Amp	3	161947258	162256628	0	0.043417
4411	Amp	3	162259891	162622962	0	0.0385
4412	Amp	3	162624786	162655245	0	0.047372
4413	Amp	3	162656681	163286956	0	0.040926
4414	Amp	3	163293526	163321912	0	0.047437
4415	Amp	3	163331014	163421963	0	0.041561
4416	Amp	3	163438181	163596397	0	0.051482
4417	Amp	3	163606880	163674487	0	0.041561
4418	Amp	3	163675198	163774325	0	0.045614
4419	Amp	3	163779016	163786747	0	0.051218
4420	Amp	3	163788591	163846449	0	0.059706
4421	Amp	3	163846771	163894220	0	0.051218
4422	Amp	3	163901622	163928747	0	0.05751
4423	Amp	3	163931377	164012260	0	0.047449
4424	Amp	3	164015664	164286863	0	0.040825
4425	Amp	3	164304130	164326198	0	0.051004
4426	Amp	3	164327140	164406285	0	0.055814
4427	Amp	3	164409518	164618027	0	0.06175
4428	Amp	3	164620501	164684947	0	0.068302
4429	Amp	3	164694913	164960860	0	0.063493
4430	Amp	3	164962767	164973003	0	0.06198
4431	Amp	3	165172941	165219205	0	0.055933
4432	Amp	3	165219420	165250934	0	0.058338
4433	Amp	3	165257558	165305126	0	0.068225
4434	Amp	3	165312891	165393929	0	0.074277
4435	Amp	3	165394120	165394120	0	0.081093
4436	Amp	3	165412299	165651313	0	0.09301
4437	Amp	3	165654918	165657192	0	0.081093
4438	Amp	3	165660002	165666129	0	0.078667
4439	Amp	3	165670247	165726558	0	0.085103
4440	Amp	3	165726913	165729169	0	0.089244
4441	Amp	3	165730044	165795786	0	0.082808
4442	Amp	3	166162696	166162790	0	0.103423
4443	Amp	3	166165773	166165773	0	0.106047
4444	Amp	3	166165780	166169246	0	0.113381
4445	Amp	3	166175658	166226838	0	0.092766
4446	Amp	3	166232551	166243323	0	0.105379
4447	Amp	3	166243385	166299556	0	0.099327
4448	Amp	3	166300264	166317462	0	0.122333
4449	Amp	3	166319518	166362635	0	0.099327
4450	Amp	3	166364813	166401003	0	0.094018
4451	Amp	3	166403872	166413503	0	0.104261

4452 Amp	3	166413907	166417480	0	0.118663
4453 Amp	3	166422625	166444234	0	0.111848
4454 Amp	3	166454665	166615185	0	0.099235
4455 Amp	3	166623262	166651368	0	0.092682
4456 Amp	3	166659417	166751940	0	0.082439
4457 Amp	3	166752388	167074523	0	0.101524
4458 Amp	3	167082588	167189778	0	0.106433
4459 Amp	3	167189962	167226517	0	0.111949
4460 Amp	3	167226538	167254115	0	0.115671
4461 Amp	3	167254812	167260706	0	0.110762
4462 Amp	3	167264923	167333026	0	0.117457
4463 Amp	3	167333083	167382374	0	0.134318
4464 Amp	3	167383690	167622151	0	0.119915
4465 Amp	3	167625676	167721419	0	0.116193
4466 Amp	3	167723197	167740594	0	0.121085
4467 Amp	3	167752797	167769965	0	0.111198
4468 Amp	3	167772697	167884604	0	0.115552
4469 Amp	3	167884889	168037826	0	0.117029
4470 Amp	3	168040335	168073898	0	0.127103
4471 Amp	3	168078858	168358884	0	0.122748
4472 Amp	3	168359468	168413687	0	0.136002
4473 Amp	3	168418555	168418915	0	0.129307
4474 Amp	3	168421786	168495447	0	0.133892
4475 Amp	3	168497103	168564224	0	0.120638
4476 Amp	3	168567965	168657004	0	0.116054
4477 Amp	3	168662876	168757089	0	0.117875
4478 Amp	3	168760731	168782129	0	0.116054
4479 Amp	3	168782164	168785290	0	0.10598
4480 Amp	3	168785310	168877682	0	0.109599
4481 Amp	3	168882482	168906428	0	0.119659
4482 Amp	3	168908251	168980065	0	0.133624
4483 Amp	3	169003792	169194117	0	0.127679
4484 Amp	3	169196565	169401694	0	0.14084
4485 Amp	3	169401895	169416703	0	0.149857
4486 Amp	3	169417883	169420873	0	0.134086
4487 Amp	3	169421210	169426823	0	0.13693
4488 Amp	3	169428787	169561606	0	0.154804
4489 Amp	3	169561681	169615327	0	0.177577
4490 Amp	3	169615782	169678282	0	0.159703
4491 Amp	3	169678593	169732280	0	0.150032
4492 Amp	3	169739769	169900054	0	0.186547
4493 Amp	3	169900092	169915406	0.001198	0.188869
4494 Amp	3	169915423	169925969	0.168144	0.207883
4495 Amp	3	169926327	170046288	0	0.186075
4496 Amp	3	170048007	170062924	0	0.182554
4497 Amp	3	170063890	170117689	0	0.163906
4498 Amp	3	170118034	170199131	0	0.168301
4499 Amp	3	170200273	170230118	0	0.179097
4500 Amp	3	170240764	170281582	0.117531	0.20202
4501 Amp	3	170281626	170515855	0.175335	0.208234
4502 Amp	3	170524832	170536889	0.13565	0.204294
4503 Amp	3	170538169	170752154	0.18072	0.209367
4504 Amp	3	170755701	170768975	0.086298	0.19857

4505	Amp	3	170770797	170801846	0.128931	0.203858
4506	Amp	3	170804047	170805933	0.317596	0.222253
4507	Amp	3	170808109	170826767	0.09318	0.19933
4508	Amp	3	170833713	170878013	0	0.180935
4509	Amp	3	170889763	170959334	0	0.17542
4510	Amp	3	170959895	170969402	0	0.186461
4511	Amp	3	170980098	171150138	0.033661	0.192821
4512	Amp	3	171152312	171159369	0	0.183291
4513	Amp	3	171160614	171168252	0	0.165056
4514	Amp	3	171170409	171237716	0	0.171472
4515	Amp	3	171242784	171366204	0	0.18258
4516	Amp	3	171368189	171390810	0.079463	0.19812
4517	Amp	3	171391082	171428695	0	0.185501
4518	Amp	3	171428837	171453624	0	0.177704
4519	Amp	3	171455838	171533554	0.028398	0.191823
4520	Amp	3	171534436	171544862	0	0.187462
4521	Amp	3	171544872	171584219	0	0.170397
4522	Amp	3	171584276	171608411	0	0.166927
4523	Amp	3	171610114	171610114	0	0.16874
4524	Amp	3	171612676	171973180	0	0.163816
4525	Amp	3	171973936	172013271	0	0.159539
4526	Amp	3	172014491	172117745	0	0.154251
4527	Amp	3	172117778	172128182	0	0.145412
4528	Amp	3	172129325	172140733	0	0.14777
4529	Amp	3	172142596	172363052	0	0.132895
4530	Amp	3	172363232	172474449	0	0.138479
4531	Amp	3	172475842	172499444	0	0.146227
4532	Amp	3	172501687	172582161	0	0.150333
4533	Amp	3	172582916	172635782	0	0.162285
4534	Amp	3	172638909	172639332	0	0.15789
4535	Amp	3	172639709	172639878	0	0.149635
4536	Amp	3	172641219	172659277	0	0.14072
4537	Amp	3	172660787	172730994	0	0.136614
4538	Amp	3	172731956	172732506	0	0.128866
4539	Amp	3	172733098	172739454	0	0.130531
4540	Amp	3	172743558	172758188	0	0.11821
4541	Amp	3	172762057	172775284	0	0.114827
4542	Amp	3	172779582	172959343	0	0.126792
4543	Amp	3	172963826	172995100	0	0.151979
4544	Amp	3	172995401	173026084	0	0.167091
4545	Amp	3	173029377	173137217	0	0.151979
4546	Amp	3	173138452	173174709	0	0.158445
4547	Amp	3	173176601	173209882	0	0.14648
4548	Amp	3	173215266	173233880	0	0.140895
4549	Amp	3	173239229	173253695	0	0.176806
4550	Amp	3	173256741	173402313	0	0.140895
4551	Amp	3	173403431	173444122	0	0.145906
4552	Amp	3	173444214	173444687	0	0.142288
4553	Amp	3	173445369	173938269	0	0.154889
4554	Amp	3	173938967	173970867	0	0.145039
4555	Amp	3	173971267	174053014	0	0.151233
4556	Amp	3	174054794	174054794	0	0.145039
4557	Amp	3	174055885	174055987	0	0.132438

4558	Amp	3	174061813	174116102	0	0.141716
4559	Amp	3	174118994	174297320	0	0.136704
4560	Amp	3	174300263	174331615	0	0.132564
4561	Amp	3	174333125	174700748	0	0.144163
4562	Amp	3	174701345	174742456	0	0.175669
4563	Amp	3	174748007	174857438	0	0.185036
4564	Amp	3	174861648	174898300	0	0.186935
4565	Amp	3	174898432	174917585	0.325321	0.222846
4566	Amp	3	174919948	174951976	0	0.186935
4567	Amp	3	174958616	174984095	0.068197	0.196663
4568	Amp	3	174985365	175113644	0.142507	0.20505
4569	Amp	3	175113697	175141964	0.362221	0.226052
4570	Amp	3	175142739	175193829	0.444673	0.23363
4571	Amp	3	175198570	175223466	0.619816	0.248703
4572	Amp	3	175226150	175316912	0.51566	0.240316
4573	Amp	3	175317515	175360691	0.243425	0.21513
4574	Amp	3	175360729	175360729	0.347934	0.225328
4575	Amp	3	176041258	176270270	0.111991	0.201482
4576	Amp	3	176274351	176306672	0.215373	0.212292
4577	Amp	3	176307355	176331984	0.111991	0.201482
4578	Amp	3	176333264	176465464	0.398217	0.229825
4579	Amp	3	176465589	176492773	0.273081	0.218225
4580	Amp	3	176493272	176505875	0.142507	0.204978
4581	Amp	3	176507254	176558191	0.398217	0.229477
4582	Amp	3	176558929	176570578	0.468576	0.2363
4583	Amp	3	176578657	176662956	0.644689	0.25103
4584	Amp	3	176667568	176690417	0.587735	0.246587
4585	Amp	3	176690887	176742459	0.317596	0.222088
4586	Amp	3	176746347	176767997	0.430191	0.232443
4587	Amp	3	176770610	176791414	0.317596	0.222088
4588	Amp	3	176794082	176817612	0.362221	0.226446
4589	Amp	3	176818799	176879427	0.468576	0.236192
4590	Amp	3	176880101	176881200	0.355162	0.225994
4591	Amp	3	176881657	176962734	0.286675	0.219171
4592	Amp	3	176965646	176972093	0.340434	0.224163
4593	Amp	3	177177049	177177049	0.028398	0.192108
4594	Amp	3	177180486	177180486	0.061778	0.196022
4595	Amp	3	177180624	177240238	0.02372	0.191664
4596	Amp	3	177245733	177363731	0.001198	0.188446
4597	Amp	3	177367935	177885372	0.033661	0.192634
4598	Amp	3	177885731	177898123	0.001198	0.188446
4599	Amp	3	177899059	178021431	0	0.185433
4600	Amp	3	178024270	178383564	0	0.180442
4601	Amp	3	178383752	178517677	0	0.187053
4602	Amp	3	178520882	178525945	0	0.174295
4603	Amp	3	178526382	178586240	0	0.185865
4604	Amp	3	178588293	178608822	0	0.175011
4605	Amp	3	178609963	178834883	0	0.163441
4606	Amp	3	178836125	178897814	0	0.167515
4607	Amp	3	178900762	178960706	0.056238	0.1955
4608	Amp	3	178961963	179037328	0.038273	0.193688
4609	Amp	3	179037835	179145078	0.018387	0.190347
4610	Amp	3	179146357	179158823	0	0.186434

4611	Amp	3	179158837	179160068	0	0.182455
4612	Amp	3	179160098	179169203	0	0.178147
4613	Amp	3	179169416	179210318	0	0.173255
4614	Amp	3	179210387	179232797	0	0.168261
4615	Amp	3	179233162	179234001	0	0.186366
4616	Amp	3	179234016	179234432	0.012341	0.189957
4617	Amp	3	179234447	179234447	0.043943	0.194177
4618	Amp	3	179235965	179246058	0.086298	0.198924
4619	Amp	3	179246543	179265081	0.123589	0.203322
4620	Amp	3	179267409	179384600	0.355162	0.225739
4621	Amp	3	179384654	179418564	0.27943	0.219128
4622	Amp	3	179418940	179583989	0.098998	0.200512
4623	Amp	3	179586828	179669668	0.500055	0.238779
4624	Amp	3	179670618	179705009	0.264784	0.217182
4625	Amp	3	179705456	179723323	0.390264	0.228947
4626	Amp	3	179725596	179841985	0.613517	0.248702
4627	Amp	3	179843714	179880099	0.390264	0.228947
4628	Amp	3	179880668	179947116	0.362221	0.226625
4629	Amp	3	179947137	179949269	0.41418	0.231308
4630	Amp	3	179949311	179953634	0.444673	0.234247
4631	Amp	3	179953776	179970883	0.405699	0.230174
4632	Amp	3	179979505	180232637	0.452609	0.234343
4633	Amp	3	180235430	180257606	0.564815	0.244089
4634	Amp	3	180262849	180486221	0.333141	0.223654
4635	Amp	3	180504164	180592506	0.476502	0.236705
4636	Amp	3	180594386	180667187	0.333141	0.223654
4637	Amp	3	180668425	180742452	0.309523	0.221672
4638	Amp	3	180746734	180746734	0.444673	0.23416
4639	Amp	3	180746961	180901112	0.709359	0.256577
4640	Amp	3	180903992	181705432	0.362221	0.226501
4641	Amp	3	181709143	181762176	0.175335	0.208792
4642	Amp	3	181764842	181861781	0.09318	0.199725
4643	Amp	3	181861921	182529960	0.175335	0.208792
4644	Amp	3	182535496	182552025	0	0.18409
4645	Amp	3	182554333	182675768	0.012341	0.189953
4646	Amp	3	182676194	182800716	0	0.183442
4647	Amp	3	182801640	182825163	0.073369	0.197521
4648	Amp	3	182825394	182905580	0.156508	0.206524
4649	Amp	3	182915361	182983432	0.033661	0.192445
4650	Amp	3	182984925	183105553	0.117531	0.202578
4651	Amp	3	183107083	183143565	0.161592	0.206949
4652	Amp	3	183144710	183159647	0.056238	0.195132
4653	Amp	3	183159857	183176548	0.007199	0.189528
4654	Amp	3	183180370	183195754	0.123589	0.202846
4655	Amp	3	183198479	183409000	0.007199	0.189528
4656	Amp	3	184179380	184179380	0	0.15828
4657	Amp	3	184180057	184180057	0	0.167772
4658	Amp	3	184183404	184369669	0	0.175886
4659	Amp	3	184370418	184378921	0	0.166393
4660	Amp	3	184382287	184461455	0	0.15828
4661	Amp	3	184461585	184466084	0	0.172564
4662	Amp	3	184466158	184577153	0	0.174936
4663	Amp	3	184580517	184638567	0	0.167144

4664	Amp	3	184638870	184661009	0	0.170802
4665	Amp	3	184661373	185082477	0	0.166695
4666	Amp	3	185083502	185166089	0	0.176357
4667	Amp	3	185166718	185211515	0	0.169032
4668	Amp	3	185211997	185293083	0	0.167556
4669	Amp	3	185297777	185330526	0	0.163276
4670	Amp	3	185330873	185360096	0	0.158905
4671	Amp	3	185361933	185445197	0	0.163116
4672	Amp	3	185452124	185724437	0	0.167119
4673	Amp	3	185724666	185783404	0	0.180935
4674	Amp	3	185793096	185821115	0	0.178562
4675	Amp	3	185827529	185831080	0	0.152964
4676	Amp	3	185832612	186081962	0	0.148754
4677	Amp	3	186083531	186118892	0	0.157951
4678	Amp	3	186122480	186124734	0	0.127124
4679	Amp	3	186129652	186152630	0	0.141408
4680	Amp	3	186156020	186181451	0	0.118892
4681	Amp	3	186182580	186373036	0	0.132547
4682	Amp	3	186378140	186564110	0	0.118892
4683	Amp	3	186572378	186577956	0	0.104608
4684	Amp	3	186578341	186772445	0	0.108961
4685	Amp	3	186773494	186858185	0	0.100964
4686	Amp	3	186859338	186950352	0	0.106587
4687	Amp	3	186950441	186964707	0	0.105104
4688	Amp	3	186964718	187101712	0	0.108502
4689	Amp	3	187102843	187217650	0	0.112041
4690	Amp	3	187219412	187244238	0	0.116389
4691	Amp	3	187245342	187251846	0	0.122748
4692	Amp	3	187252363	187546782	0	0.125029
4693	Amp	3	187552505	187660927	0	0.135042
4694	Amp	3	187666067	187674130	0	0.147136
4695	Amp	3	187675118	187686330	0	0.14935
4696	Amp	3	187687429	187996265	0	0.140557
4697	Amp	3	187998247	188159120	0	0.17042
4698	Amp	3	188159248	188215573	0	0.183997
4699	Amp	3	188217061	188363120	0	0.180695
4700	Amp	3	188364381	188494824	0	0.160056
4701	Amp	3	188497711	188614715	0	0.150086
4702	Amp	3	188614830	188615064	0	0.161712
4703	Amp	3	188619560	188727846	0	0.17171
4704	Amp	3	188731264	188860569	0	0.180146
4705	Amp	3	188863049	188868785	0	0.17171
4706	Amp	3	188869860	188988660	0	0.157344
4707	Amp	3	188989082	189059884	0	0.153948
4708	Amp	3	189068160	189501923	0	0.16342
4709	Amp	3	189505235	189514826	0	0.167896
4710	Amp	3	189514852	189563207	0	0.169933
4711	Amp	3	189564023	189736551	0	0.164748
4712	Amp	3	189737598	189807312	0	0.167089
4713	Amp	3	189807856	189907672	0	0.160744
4714	Amp	3	189908093	190005708	0	0.158403
4715	Amp	3	190006181	190073358	0	0.156367
4716	Amp	3	190073823	190248666	0	0.13737

4717	Amp	3	190249383	190366456	0	0.145689
4718	Amp	3	190371628	190515861	0	0.13737
4719	Amp	3	190515886	190636457	0	0.135269
4720	Amp	3	190637462	190697717	0	0.126367
4721	Amp	3	190701752	190770736	0	0.138021
4722	Amp	3	190775313	190832904	0	0.143741
4723	Amp	3	190838124	190878959	0	0.132086
4724	Amp	3	190879163	191225929	0	0.126367
4725	Amp	3	191235768	191249990	0	0.141053
4726	Amp	3	191251248	191270488	0	0.144228
4727	Amp	3	191272374	191414959	0	0.155511
4728	Amp	3	191421268	191484023	0	0.167063
4729	Amp	3	191484683	191666260	0	0.179174
4730	Amp	3	191677980	191743572	0	0.155781
4731	Amp	3	191746660	191851784	0	0.1694
4732	Amp	3	191851845	191958681	0	0.166913
4733	Amp	3	191961684	192177648	0	0.184228
4734	Amp	3	192302266	192302266	0	0.152227
4735	Amp	3	192302511	192425126	0	0.157304
4736	Amp	3	192428255	192466364	0	0.162582
4737	Amp	3	192467813	192552430	0	0.169229
4738	Amp	3	192552492	192757504	0	0.162582
4739	Amp	3	192762969	192884896	0	0.168285
4740	Amp	3	192884948	193093616	0	0.173695
4741	Amp	3	193094432	193119883	0	0.177686
4742	Amp	3	193120602	193186365	0	0.16241
4743	Amp	3	193186714	193290254	0	0.172509
4744	Amp	3	193298490	193340096	0	0.174164
4745	Amp	3	193340484	193358635	0	0.176209
4746	Amp	3	193358721	193411832	0	0.171732
4747	Amp	3	193412018	193621136	0	0.176713
4748	Amp	3	193624158	193624400	0.111991	0.201558
4749	Amp	3	193625087	193694883	0.142507	0.205158
4750	Amp	3	193695943	193717542	0.156508	0.206558
4751	Amp	3	193719863	193785173	0.215373	0.212644
4752	Amp	3	193785940	193795363	0.168144	0.207567
4753	Amp	3	193797111	193919375	0.142507	0.205005
4754	Amp	3	193919535	193958445	0.038273	0.193452
4755	Amp	3	193958573	194262066	0.061778	0.196227
4756	Amp	3	194262775	194391622	0.012341	0.190141
4757	Amp	3	194394190	194473517	0	0.187366
4758	Amp	3	194476302	194508430	0	0.181955
4759	Amp	3	194508575	194629352	0	0.180556
4760	Amp	3	194639901	194710368	0.073369	0.197702
4761	Amp	3	194713915	194749373	0	0.184636
4762	Amp	3	194751727	194797501	0	0.182591
4763	Amp	3	194799298	194921899	0	0.186963
4764	Amp	3	194926085	195343039	0	0.182883
4765	Amp	3	195343345	195423709	0	0.179011
4766	Amp	3	195428692	195428692	0.02372	0.191299
4767	Amp	3	196017117	196026580	0.050005	0.195036
4768	Amp	3	196026895	196245509	0.117531	0.202218
4769	Amp	3	196246373	196248065	0.079463	0.198482

4770	Amp	3	196252550	196402011	0.02372	0.191299
4771	Amp	3	196405284	196486099	0	0.182288
4772	Amp	3	196489174	196619435	0	0.154462
4773	Amp	3	196620566	197242908	0	0.157403
4774	Amp	3	197243254	197245963	0	0.1517
4775	Amp	3	197247312	197247312	0	0.163947
4776	Amp	3	197247511	197483798	0	0.160671
4777	Amp	3	197484264	197551626	0	0.153682
4778	Amp	3	197551766	197754242	0	0.160162
4779	Amp	3	197755173	197767955	0	0.162423
4780	Amp	3	197768463	197812401	0	0.165488
4781	Amp	4	1059384	1075031	0	0.080171
4782	Amp	4	1075962	1165203	0	0.078095
4783	Amp	4	1167692	1192677	0	0.079788
4784	Amp	4	1197254	1243226	0	0.082364
4785	Amp	4	1250089	1586938	0	0.079788
4786	Amp	4	1587793	1646725	0	0.089161
4787	Amp	4	1646769	1661943	0	0.093866
4788	Amp	4	1661960	1737005	0	0.092173
4789	Amp	4	1750693	1816117	0	0.096712
4790	Amp	4	1816867	1998531	0	0.092173
4791	Amp	4	2005789	2005789	0	0.100886
4792	Amp	4	2006084	2164202	0	0.078876
4793	Amp	4	2167287	2208452	0	0.069503
4794	Amp	4	2210268	2342965	0	0.056085
4795	Amp	4	2349175	2516075	0	0.043683
4796	Amp	4	2519134	3134226	0	0.039605
4797	Amp	4	3135947	3361116	0	0.04119
4798	Amp	4	3361850	3437624	0	0.050602
4799	Amp	4	3452072	3593370	0	0.04119
4800	Amp	4	3622609	3629450	0	0.038114
4801	Amp	4	3631833	3686626	0	0.039844
4802	Amp	4	3686845	3747009	0	0.042208
4803	Amp	4	3748558	4287349	0	0.040071
4804	Amp	4	4288000	5216409	0	0.041535
4805	Amp	4	5218671	5639780	0	0.04716
4806	Amp	4	5641560	5759639	0	0.0598
4807	Amp	4	5760119	5916827	0	0.056447
4808	Amp	4	5920208	6094360	0	0.070978
4809	Amp	4	6096005	6376340	0	0.066325
4810	Amp	4	6376541	6568514	0	0.054965
4811	Amp	4	6571951	6661592	0	0.042325
4812	Amp	4	6662370	6696979	0	0.039962
4813	Amp	4	6699809	6756840	0	0.042442
4814	Amp	4	6759345	6932416	0	0.047561
4815	Amp	4	6934022	6948201	0	0.042442
4816	Amp	4	6952635	7028733	0	0.046899
4817	Amp	4	7029350	7037203	0	0.048441
4818	Amp	4	7037396	7048627	0	0.045961
4819	Amp	4	7048650	7633664	0	0.055372
4820	Amp	4	7635962	7720731	0	0.050916
4821	Amp	4	7720833	7748360	0	0.053275
4822	Amp	4	7748375	7766028	0	0.050916

4823	Amp	4	7766165	7865706	0	0.041504
4824	Amp	4	7870031	7937406	0	0.032708
4825	Amp	4	7941135	8354075	0	0.083231
4826	Amp	4	8354163	8569049	0	0.081824
4827	Amp	4	8571629	8682018	0	0.083646
4828	Amp	4	8684447	8695449	0	0.082104
4829	Amp	4	8697040	9587686	0	0.080187
4830	Amp	4	9594294	9609556	0	0.086737
4831	Amp	4	9652366	9686860	0	0.067472
4832	Amp	4	9686966	9804310	0	0.065833
4833	Amp	4	9806882	9844685	0	0.064282
4834	Amp	4	9879143	9902136	0	0.076996
4835	Amp	4	9907624	10003119	0	0.079047
4836	Amp	4	10003208	10119274	0	0.077462
4837	Amp	4	10119565	10283757	0	0.07564
4838	Amp	4	10285338	10602536	0	0.073588
4839	Amp	4	10602542	10616406	0	0.048641
4840	Amp	4	10616970	10675472	0	0.058533
4841	Amp	4	10679481	11029831	0	0.04685
4842	Amp	4	11030218	11106071	0	0.045387
4843	Amp	4	11106802	11407678	0	0.021602
4844	Amp	4	11414901	12926693	0	0.044816
4845	Amp	4	12936272	13156236	0	0.056529
4846	Amp	4	13157153	13578908	0	0.044816
4847	Amp	4	13580448	13760493	0	0.054046
4848	Amp	4	13763022	14094047	0	0.044816
4849	Amp	4	14094064	14201978	0	0.055704
4850	Amp	4	14204129	14223913	0	0.044816
4851	Amp	4	14231656	14338578	0	0.057385
4852	Amp	4	14339472	14578138	0	0.021602
4853	Amp	4	14579811	14602862	0	0.038041
4854	Amp	4	14605334	14683885	0	0.021602
4855	Amp	4	14686862	14687366	0	0.051302
4856	Amp	4	14693808	14919623	0	0.068704
4857	Amp	4	14924429	15665864	0	0.063554
4858	Amp	4	15666436	15693960	0	0.066912
4859	Amp	4	15695412	15932330	0	0.055273
4860	Amp	4	15933874	16380013	0	0.04951
4861	Amp	4	16380425	16508508	0	0.057072
4862	Amp	4	16512125	16533715	0	0.053713
4863	Amp	4	16533848	17531035	0	0.056447
4864	Amp	4	17531157	17643727	0	0.048885
4865	Amp	4	17647085	17728924	0	0.02622
4866	Amp	4	17741372	17807915	0	0.019185
4867	Amp	4	17808222	18537461	0	0.030396
4868	Amp	4	18537996	18629111	0	0.019185
4869	Amp	4	18633466	18853469	0	0.016451
4870	Amp	4	18853936	19021630	0	0.022103
4871	Amp	4	19024099	19332406	0	0.016451
4872	Amp	4	19337677	19661042	0	0.01931
4873	Amp	4	19665502	19707105	0	0.029301
4874	Amp	4	19711545	19719341	0	0.01931
4875	Amp	4	19723018	20543133	0	0.022994

4876 Amp	4	20544433	20637613	0	0.032622
4877 Amp	4	20638089	20776198	0	0.022994
4878 Amp	4	20778412	20786400	0	0.027128
4879 Amp	4	20786709	21503559	0	0.019641
4880 Amp	4	21505316	21508491	0	0.027072
4881 Amp	4	21508993	22354098	0	0.02352
4882 Amp	4	22355942	22472912	0	0.015703
4883 Amp	4	22474285	22618969	0	0.008272
4884 Amp	4	22619871	23408803	0	0.002859
4885 Amp	4	23408850	23455166	0	0.00972
4886 Amp	4	23460843	23879854	0	0.002859
4887 Amp	4	23882173	23890735	0	0.006233
4888 Amp	4	23891220	24062998	0	0.014141
4889 Amp	4	24064272	24181832	0	0.023399
4890 Amp	4	24183778	24784289	0	0.021408
4891 Amp	4	24788476	25109688	0	0.018034
4892 Amp	4	25113724	25213466	0	0.019617
4893 Amp	4	25213649	25680349	0	0.018034
4894 Amp	4	25682672	25952797	0	0.019524
4895 Amp	4	25952910	26848153	0	0.016665
4896 Amp	4	26848217	27137625	0	0.008757
4897 Amp	4	27138062	27209456	0	0.013087
4898 Amp	4	27210601	27332585	0	0.014716
4899 Amp	4	27336165	27839856	0	0.010386
4900 Amp	4	27849568	28029111	0	0.016006
4901 Amp	4	28033786	28161349	0	0.010386
4902 Amp	4	28163746	28271855	0	0.012989
4903 Amp	4	28276073	29203869	0	0.027662
4904 Amp	4	29211575	29546701	0	0.018767
4905 Amp	4	29548662	29683892	0	0.022332
4906 Amp	4	29691065	30261650	0	0.027865
4907 Amp	4	30272186	30582960	0	0.047416
4908 Amp	4	30591825	30599355	0	0.032743
4909 Amp	4	30600926	31365766	0	0.034217
4910 Amp	4	31366087	31543064	0	0.028684
4911 Amp	4	31547287	32171122	0	0.023886
4912 Amp	4	32172212	32719392	0	0.009133
4913 Amp	4	32722519	32800076	0	0.016593
4914 Amp	4	32800731	32800731	0	0.012499
4915 Amp	4	32802372	33184541	0	0.005039
4916 Amp	4	33187382	33394495	0	0.02594
4917 Amp	4	33394629	33394742	0	0.028424
4918 Amp	4	33396765	33510811	0	0.055618
4919 Amp	4	33510932	33697588	0	0.053134
4920 Amp	4	33716001	34023083	0	0.02594
4921 Amp	4	34026275	34065883	0	0.04263
4922 Amp	4	34067637	34845678	0	0.02594
4923 Amp	4	34845925	35128262	0	0.029269
4924 Amp	4	35128924	35474678	0	0.043395
4925 Amp	4	35475969	35605362	0	0.040066
4926 Amp	4	35613324	36114349	0	0.037804
4927 Amp	4	36114831	36155174	0	0.045445
4928 Amp	4	36162781	36209043	0	0.037966

4929	Amp	4	36213253	36214083	0	0.040088
4930	Amp	4	36216714	36216714	0	0.055219
4931	Amp	4	36218065	36401557	0	0.052595
4932	Amp	4	36403265	36501689	0	0.047711
4933	Amp	4	36503546	36574445	0	0.044146
4934	Amp	4	36575811	36588311	0	0.012838
4935	Amp	4	36592858	36629533	0	0.039974
4936	Amp	4	36629688	36759879	0	0.053681
4937	Amp	4	36762566	36842087	0	0.055132
4938	Amp	4	36842638	37096363	0	0.024658
4939	Amp	4	37096736	37383706	0	0.018119
4940	Amp	4	37384172	37798311	0	0.025806
4941	Amp	4	37798499	37883710	0	0.030218
4942	Amp	4	37887106	37908552	0	0.031747
4943	Amp	4	37913841	37983940	0	0.045073
4944	Amp	4	37985841	38451703	0	0.031419
4945	Amp	4	38468085	38767719	0	0.037487
4946	Amp	4	38774449	39262766	0	0.051403
4947	Amp	4	39264346	39273774	0	0.059669
4948	Amp	4	39276472	39404466	0	0.061534
4949	Amp	4	39407796	39413784	0	0.067237
4950	Amp	4	39415971	39430011	0	0.058971
4951	Amp	4	39431878	39569881	0	0.053268
4952	Amp	4	39571770	39641858	0	0.058215
4953	Amp	4	39643064	39653837	0	0.069703
4954	Amp	4	39658323	39712720	0	0.076012
4955	Amp	4	39716590	39767652	0	0.084189
4956	Amp	4	39769914	39988834	0	0.069703
4957	Amp	4	39993535	40015187	0	0.073743
4958	Amp	4	40022270	40044368	0	0.062256
4959	Amp	4	40044391	40051090	0	0.060727
4960	Amp	4	40051457	40051470	0	0.066195
4961	Amp	4	40178657	40178657	0	0.07801
4962	Amp	4	40178820	40204743	0	0.086656
4963	Amp	4	40212934	40276865	0	0.07484
4964	Amp	4	40277914	40291538	0	0.078331
4965	Amp	4	40291667	40308320	0	0.071243
4966	Amp	4	40311122	40395880	0	0.073149
4967	Amp	4	40395896	40449377	0	0.067682
4968	Amp	4	40450097	40651794	0	0.064191
4969	Amp	4	40654795	40797059	0	0.06015
4970	Amp	4	40800102	40868889	0	0.062149
4971	Amp	4	40869970	40890183	0	0.060698
4972	Amp	4	40890268	40913371	0	0.080915
4973	Amp	4	40918289	41020468	0	0.08275
4974	Amp	4	41026425	41204089	0	0.080885
4975	Amp	4	41207616	41225900	0	0.065934
4976	Amp	4	41228990	41374244	0	0.072222
4977	Amp	4	41377468	41464836	0	0.074502
4978	Amp	4	41467868	41475543	0	0.068214
4979	Amp	4	41480905	41560289	0	0.066215
4980	Amp	4	41561149	41741709	0	0.06095
4981	Amp	4	41753353	41976090	0	0.047624

4982 Amp	4	41977828	42063276	0	0.043376
4983 Amp	4	42064294	42380867	0	0.047509
4984 Amp	4	42381998	42572356	0	0.045603
4985 Amp	4	42588957	42900637	0	0.035935
4986 Amp	4	42900717	43522031	0	0.037789
4987 Amp	4	43526378	43606516	0	0.045062
4988 Amp	4	43608069	43659910	0	0.046944
4989 Amp	4	43668196	43691319	0	0.039672
4990 Amp	4	43696313	43850912	0	0.046966
4991 Amp	4	43853984	43964068	0	0.049575
4992 Amp	4	43964318	44083776	0	0.042281
4993 Amp	4	44086916	44206290	0	0.049384
4994 Amp	4	44210495	44477302	0	0.042281
4995 Amp	4	44478641	44524375	0	0.044096
4996 Amp	4	44530446	44765652	0	0.042281
4997 Amp	4	44767851	45018825	0	0.046583
4998 Amp	4	45018955	45384415	0	0.049407
4999 Amp	4	45384563	45527194	0	0.044459
5000 Amp	4	45528085	45636830	0	0.052718
5001 Amp	4	45644252	45762388	0	0.080014
5002 Amp	4	45763648	45808809	0	0.050864
5003 Amp	4	45816053	45923046	0	0.100913
5004 Amp	4	45927469	45944302	0	0.070295
5005 Amp	4	45944601	46094346	0	0.072391
5006 Amp	4	46107187	46118855	0	0.070004
5007 Amp	4	46122095	46314244	0	0.077058
5008 Amp	4	46314344	46357046	0	0.074962
5009 Amp	4	46361715	46629957	0	0.069512
5010 Amp	4	46630186	46662670	0	0.075847
5011 Amp	4	46666560	46873203	0	0.068222
5012 Amp	4	46874174	46882245	0	0.074108
5013 Amp	4	46884559	46905001	0	0.076874
5014 Amp	4	46905132	46950454	0	0.074051
5015 Amp	4	46952951	47135459	0	0.079811
5016 Amp	4	47135658	47145136	0	0.086923
5017 Amp	4	47148980	47173006	0	0.081036
5018 Amp	4	47178196	47227876	0	0.073983
5019 Amp	4	47229399	47478541	0	0.080493
5020 Amp	4	47478771	47539832	0	0.094661
5021 Amp	4	47540254	47563464	0	0.082459
5022 Amp	4	47565562	47644282	0	0.089338
5023 Amp	4	47647753	47683146	0	0.085035
5024 Amp	4	47686796	47891241	0	0.109198
5025 Amp	4	47893201	47956270	0	0.090228
5026 Amp	4	47961772	47988999	0	0.094593
5027 Amp	4	47990946	48022141	0	0.087715
5028 Amp	4	48022386	48036362	0	0.078016
5029 Amp	4	48036567	48043574	0	0.076133
5030 Amp	4	48043674	48066809	0	0.074011
5031 Amp	4	48067890	48067890	0	0.085392
5032 Amp	4	51832470	51832470	0	0.062017
5033 Amp	4	51832591	51872753	0	0.059251
5034 Amp	4	51873441	51943918	0	0.067816

5035 Amp	4	51951567	51967567	0	0.053949
5036 Amp	4	51967573	52054323	0	0.043185
5037 Amp	4	52055026	52161921	0	0.029237
5038 Amp	4	52162036	52230584	0	0.039174
5039 Amp	4	52231892	52246921	0	0.040994
5040 Amp	4	52248688	52424187	0	0.042718
5041 Amp	4	52425405	52499361	0	0.02759
5042 Amp	4	52510106	52536156	0	0.038623
5043 Amp	4	52544267	52681570	0	0.041779
5044 Amp	4	52689298	52873095	0	0.052098
5045 Amp	4	52873689	52947185	0	0.056123
5046 Amp	4	52948362	53066042	0	0.052098
5047 Amp	4	53066883	53083661	0	0.048942
5048 Amp	4	53086970	53174720	0	0.061049
5049 Amp	4	53185046	53260628	0	0.065975
5050 Amp	4	53265649	53659340	0	0.061049
5051 Amp	4	53659663	53676449	0	0.05073
5052 Amp	4	53678726	53690405	0	0.038713
5053 Amp	4	53693073	53767208	0	0.050128
5054 Amp	4	53769591	54241451	0	0.047848
5055 Amp	4	54249536	54339877	0	0.045887
5056 Amp	4	54346988	54382035	0	0.06125
5057 Amp	4	54382436	54419909	0	0.045887
5058 Amp	4	54421752	54579485	0	0.051428
5059 Amp	4	54579585	54581296	0	0.045887
5060 Amp	4	54581933	54780469	0	0.049994
5061 Amp	4	54782202	55441047	0	0.056383
5062 Amp	4	55445081	55470548	0	0.06732
5063 Amp	4	55474023	55545574	0	0.063212
5064 Amp	4	55547636	55791029	0	0.052276
5065 Amp	4	55797689	55812899	0	0.034902
5066 Amp	4	55814074	55890035	0	0.047947
5067 Amp	4	55892683	56531941	0	0.046222
5068 Amp	4	56534451	56579828	0	0.051395
5069 Amp	4	56582705	57054292	0	0.034546
5070 Amp	4	57056824	57098495	0	0.029373
5071 Amp	4	57098625	57125501	0	0.038792
5072 Amp	4	57129201	57291315	0	0.043239
5073 Amp	4	57295634	57301342	0	0.03382
5074 Amp	4	57304976	57336951	0	0.040265
5075 Amp	4	57338301	57416138	0	0.044184
5076 Amp	4	57416249	57610960	0	0.039737
5077 Amp	4	57611071	57933707	0	0.044425
5078 Amp	4	57933750	58069251	0	0.047608
5079 Amp	4	58073831	58109573	0	0.068626
5080 Amp	4	58110115	58121308	0	0.063938
5081 Amp	4	58124168	58234294	0	0.04292
5082 Amp	4	58234910	58349485	0	0.039737
5083 Amp	4	58352329	58403011	0	0.049292
5084 Amp	4	58403552	58407021	0	0.086018
5085 Amp	4	58407228	58414284	0	0.091597
5086 Amp	4	58415057	58425452	0	0.083946
5087 Amp	4	58429894	58562234	0	0.074391

5088 Amp	4	58562316	58596428	0	0.088037
5089 Amp	4	58605152	58791835	0	0.092458
5090 Amp	4	58794430	58835033	0	0.08854
5091 Amp	4	58835091	58935379	0	0.084118
5092 Amp	4	58936153	58980956	0	0.070473
5093 Amp	4	58983300	59194636	0	0.041397
5094 Amp	4	59194944	60074474	0	0.049867
5095 Amp	4	60077546	60184691	0	0.037844
5096 Amp	4	60185761	60300086	0	0.044967
5097 Amp	4	60300143	60306924	0	0.037844
5098 Amp	4	60307852	60360930	0	0.029373
5099 Amp	4	60363052	60403801	0	0.037444
5100 Amp	4	60404241	60521656	0	0.043667
5101 Amp	4	60528075	60723736	0	0.050461
5102 Amp	4	60727966	60908941	0	0.056025
5103 Amp	4	60909927	60922000	0	0.049803
5104 Amp	4	60928050	60977899	0	0.044239
5105 Amp	4	60978117	61265775	0	0.046943
5106 Amp	4	61266252	61457683	0	0.049532
5107 Amp	4	61464125	61495741	0	0.041461
5108 Amp	4	61499099	61540727	0	0.049209
5109 Amp	4	61541175	61770975	0	0.066441
5110 Amp	4	61771569	62123720	0	0.073345
5111 Amp	4	62129115	62217460	0	0.066441
5112 Amp	4	62217645	62315755	0	0.059646
5113 Amp	4	62315876	62424663	0	0.06212
5114 Amp	4	62432709	62581280	0	0.073223
5115 Amp	4	62581332	62686862	0	0.06145
5116 Amp	4	62690057	62690213	0	0.103876
5117 Amp	4	62692628	62819705	0	0.096127
5118 Amp	4	62828172	62860748	0	0.053702
5119 Amp	4	62863301	63093342	0	0.051867
5120 Amp	4	63093980	63309158	0	0.050393
5121 Amp	4	63310735	63350048	0	0.03929
5122 Amp	4	63351510	63660429	0	0.045418
5123 Amp	4	63665477	63674704	0	0.03929
5124 Amp	4	63679870	63694427	0	0.045874
5125 Amp	4	63702413	63714993	0	0.053561
5126 Amp	4	63715908	63789442	0	0.03929
5127 Amp	4	63789449	64850985	0	0.036586
5128 Amp	4	64853914	64919908	0	0.039338
5129 Amp	4	64931515	65134209	0	0.043702
5130 Amp	4	65134891	65283054	0	0.051136
5131 Amp	4	65284666	65398981	0	0.043702
5132 Amp	4	65399952	65512744	0	0.039338
5133 Amp	4	65514821	65601469	0	0.046689
5134 Amp	4	65608594	65775868	0	0.043936
5135 Amp	4	65775943	65780362	0	0.050243
5136 Amp	4	65780434	65836275	0	0.056805
5137 Amp	4	65844484	65989410	0	0.073333
5138 Amp	4	66002588	66124159	0	0.064698
5139 Amp	4	66126520	66221863	0	0.070821
5140 Amp	4	66225179	66233769	0	0.074115

5141	Amp	4	66234456	66361078	0	0.080736
5142	Amp	4	66363543	66452187	0	0.082294
5143	Amp	4	66457656	66534149	0	0.070528
5144	Amp	4	66534850	66535708	0	0.075887
5145	Amp	4	66541267	66585610	0	0.088644
5146	Amp	4	66585857	66650311	0	0.083479
5147	Amp	4	66651121	66686721	0	0.080185
5148	Amp	4	66695588	66725129	0	0.10454
5149	Amp	4	66725632	66728088	0	0.117205
5150	Amp	4	66729306	66764961	0	0.086288
5151	Amp	4	66765147	67011098	0	0.079667
5152	Amp	4	67012655	67146235	0	0.077193
5153	Amp	4	67150861	67281747	0	0.081587
5154	Amp	4	67283886	67363113	0	0.080029
5155	Amp	4	67363774	67389436	0	0.067364
5156	Amp	4	67394164	67486212	0	0.091885
5157	Amp	4	67487866	67587471	0	0.10545
5158	Amp	4	67587486	67674053	0	0.112202
5159	Amp	4	67674067	67691068	0	0.116381
5160	Amp	4	67691162	67876485	0	0.11255
5161	Amp	4	67881165	67914323	0	0.125214
5162	Amp	4	67914681	67976407	0	0.12671
5163	Amp	4	67977123	68251012	0	0.135231
5164	Amp	4	68255214	68264865	0	0.137109
5165	Amp	4	68269149	68295006	0	0.149973
5166	Amp	4	68295732	68322637	0	0.151806
5167	Amp	4	68323022	68333313	0	0.145054
5168	Amp	4	68335040	68335040	0	0.142919
5169	Amp	4	68338682	68339321	0	0.130425
5170	Amp	4	69452478	69459991	0	0.127932
5171	Amp	4	69460790	69462664	0	0.123753
5172	Amp	4	69464173	69493426	0	0.141283
5173	Amp	4	69500154	69514038	0	0.120923
5174	Amp	4	69516046	69804841	0	0.122956
5175	Amp	4	69808112	69838824	0	0.084927
5176	Amp	4	69839158	69846443	0	0.092736
5177	Amp	4	69853815	69972136	0	0.143259
5178	Amp	4	69979965	69994144	0	0.155971
5179	Amp	4	69995568	70028454	0.038273	0.193619
5180	Amp	4	70030491	70206033	0.073369	0.197801
5181	Amp	4	70207659	70238500	0.038273	0.193619
5182	Amp	4	70240962	70251805	0	0.178559
5183	Amp	4	70274327	70361495	0.038273	0.193332
5184	Amp	4	70385052	70394875	0	0.178559
5185	Amp	4	70395050	70592828	0	0.17597
5186	Amp	4	70594707	70662592	0.001198	0.188765
5187	Amp	4	70665943	70696077	0.161592	0.20716
5188	Amp	4	70701817	70769792	0	0.169512
5189	Amp	4	70770505	70805256	0	0.179866
5190	Amp	4	70811386	70816687	0	0.182424
5191	Amp	4	70821503	70918197	0	0.171919
5192	Amp	4	70919804	70928932	0.09318	0.199261
5193	Amp	4	70930295	71044792	0	0.16514

5194 Amp	4	71049864	71097774	0	0.137799
5195 Amp	4	71098072	71483876	0	0.139833
5196 Amp	4	71487722	71581704	0	0.144676
5197 Amp	4	71581921	71611287	0	0.141063
5198 Amp	4	71612083	71629570	0	0.142708
5199 Amp	4	71636253	71651316	0	0.132354
5200 Amp	4	71656320	71732866	0	0.136288
5201 Amp	4	71735715	71769795	0	0.150825
5202 Amp	4	71770907	71783690	0	0.157243
5203 Amp	4	71784057	71799681	0	0.138848
5204 Amp	4	71800772	71920705	0	0.143684
5205 Amp	4	71926175	71929874	0	0.13975
5206 Amp	4	71931826	72068915	0	0.146626
5207 Amp	4	72068929	72086909	0	0.139075
5208 Amp	4	72087138	72270434	0	0.147525
5209 Amp	4	72281705	72285091	0	0.161007
5210 Amp	4	72285217	72331213	0	0.171591
5211 Amp	4	72331410	72339374	0	0.163782
5212 Amp	4	72344228	72389219	0.018387	0.190856
5213 Amp	4	72390329	72403157	0.09318	0.19927
5214 Amp	4	72407888	72469381	0.073369	0.197236
5215 Amp	4	72472843	72524292	0	0.183754
5216 Amp	4	72532963	72663725	0.13565	0.204201
5217 Amp	4	72664442	72666091	0.073369	0.197814
5218 Amp	4	72708359	72833308	0.286675	0.219161
5219 Amp	4	72833750	72959669	0.333141	0.223362
5220 Amp	4	72966041	73006229	0.652914	0.251814
5221 Amp	4	73009451	73032473	0.71758	0.25703
5222 Amp	4	73032926	73062106	0.852608	0.267323
5223 Amp	4	73065809	73070191	1.305256	0.299884
5224 Amp	4	73077449	73093740	1.190324	0.29147
5225 Amp	4	73094615	73158965	1.394558	0.306126
5226 Amp	4	73162744	73233515	1.24764	0.295542
5227 Amp	4	73241329	73257421	1.179216	0.291268
5228 Amp	4	73261137	73280867	0.667776	0.253086
5229 Amp	4	73291631	73318435	0.548372	0.242597
5230 Amp	4	73326871	73327091	0.604912	0.247756
5231 Amp	4	73327375	73337384	0.66072	0.252472
5232 Amp	4	73337415	73631958	0.613517	0.248271
5233 Amp	4	73632748	73689333	0.685617	0.254695
5234 Amp	4	73689349	73692748	0.587735	0.2463
5235 Amp	4	73692771	73692771	0.685617	0.254328
5236 Amp	4	73693013	73695671	0.532449	0.241437
5237 Amp	4	73696515	73779999	0.685617	0.254339
5238 Amp	4	73780598	73780898	0.724993	0.258093
5239 Amp	4	73781312	73782131	0.786952	0.26305
5240 Amp	4	73782375	73896468	0.709359	0.256626
5241 Amp	4	73896612	73929648	0.676804	0.253894
5242 Amp	4	73930474	73935245	0.375927	0.227811
5243 Amp	4	73940634	73965463	0.76883	0.261396
5244 Amp	4	73965646	73983756	0.852608	0.267366
5245 Amp	4	73985472	74025607	0.991911	0.278073
5246 Amp	4	74035220	74155360	1.120124	0.286759

5247	Amp	4	74157992	74184708	0.843361	0.267196
5248	Amp	4	74196476	74219929	1.000924	0.278745
5249	Amp	4	74222796	74222796	0.823952	0.265842
5250	Amp	4	74222811	74222899	0.430191	0.232837
5251	Amp	4	74222950	74224091	0.340434	0.224101
5252	Amp	4	74224153	74224153	0.944128	0.274348
5253	Amp	4	74224195	74275753	1.078696	0.283908
5254	Amp	4	74276008	74374943	1.130387	0.287404
5255	Amp	4	74377985	74404970	1.295233	0.29891
5256	Amp	4	74407015	74462849	0.823952	0.265795
5257	Amp	4	74463089	74487900	0.685617	0.254288
5258	Amp	4	74490422	74540333	0.906818	0.271498
5259	Amp	4	74542237	74618473	0.685617	0.254288
5260	Amp	4	74618753	74632484	0.888878	0.27034
5261	Amp	4	74634274	74674482	1.130387	0.287543
5262	Amp	4	74674676	74676718	1.099256	0.285665
5263	Amp	4	74677543	74781460	0.66072	0.25241
5264	Amp	4	74782225	74871772	0.548372	0.242678
5265	Amp	4	74871793	74949774	0.62858	0.249398
5266	Amp	4	74951847	74965616	0.709359	0.2565
5267	Amp	4	74967951	75213707	0.860481	0.267998
5268	Amp	4	75214391	75322838	0.786952	0.26248
5269	Amp	4	75323355	75329536	0.676804	0.253553
5270	Amp	4	75329736	75357969	0.62858	0.2498
5271	Amp	4	75359081	75374385	0.564815	0.243972
5272	Amp	4	75374738	75376813	0.49169	0.238003
5273	Amp	4	75377455	75387085	0.523647	0.240782
5274	Amp	4	75389083	75459989	0.564815	0.244112
5275	Amp	4	75462203	75492574	0.613517	0.248418
5276	Amp	4	75492849	75527939	0.66072	0.252442
5277	Amp	4	75530707	75803890	0.476502	0.236559
5278	Amp	4	75811466	75931686	0.286675	0.219637
5279	Amp	4	75931916	75967070	0.398217	0.229736
5280	Amp	4	75967683	75978346	0.340434	0.224519
5281	Amp	4	75981389	75985821	0.41418	0.231252
5282	Amp	4	75989786	76010722	0.476502	0.236364
5283	Amp	4	76012952	76060053	0.390264	0.229414
5284	Amp	4	76060119	76216939	0.230572	0.213956
5285	Amp	4	76218357	76300672	0.123589	0.202801
5286	Amp	4	76304160	76304160	0.476502	0.236426
5287	Amp	4	76309202	76488792	0.564815	0.244223
5288	Amp	4	76490243	76580847	0.437983	0.233471
5289	Amp	4	76583247	76654289	0.51566	0.240133
5290	Amp	4	76656538	76712911	0.390264	0.229215
5291	Amp	4	76715490	76815448	0.168144	0.20816
5292	Amp	4	76817010	76915512	0.043943	0.194361
5293	Amp	4	76915527	76939653	0	0.176782
5294	Amp	4	76940235	76962401	0	0.168433
5295	Amp	4	76969640	76975648	0	0.165425
5296	Amp	4	76978829	76992218	0	0.170586
5297	Amp	4	76995552	76998112	0	0.157205
5298	Amp	4	77000161	77010423	0	0.163357
5299	Amp	4	77011244	77066695	0	0.171277

5300	Amp	4	77067332	77080093	0	0.160515
5301	Amp	4	77082284	77148512	0	0.152575
5302	Amp	4	77149828	77220671	0	0.14827
5303	Amp	4	77224126	77241450	0	0.141513
5304	Amp	4	77242968	77458978	0	0.138622
5305	Amp	4	77463000	77570182	0	0.132232
5306	Amp	4	77573074	77623030	0	0.147238
5307	Amp	4	77623083	77821363	0	0.145405
5308	Amp	4	77824487	77864652	0	0.142477
5309	Amp	4	77866660	77938865	0	0.125859
5310	Amp	4	77944853	77945536	0	0.114636
5311	Amp	4	77947172	77975294	0	0.116298
5312	Amp	4	77983018	78224314	0	0.111305
5313	Amp	4	78226956	78325446	0	0.103508
5314	Amp	4	78326549	78344448	0	0.098857
5315	Amp	4	78344642	78455640	0	0.107629
5316	Amp	4	78455739	78455753	0	0.104913
5317	Amp	4	78456471	78517927	0	0.096121
5318	Amp	4	78522387	78595291	0	0.093733
5319	Amp	4	78598055	78607659	0	0.092071
5320	Amp	4	78607803	78762409	0	0.100745
5321	Amp	4	78762413	78806637	0	0.095629
5322	Amp	4	78809742	78928298	0	0.100599
5323	Amp	4	78929560	78950959	0	0.094205
5324	Amp	4	78954018	79099847	0	0.090548
5325	Amp	4	79100366	79168570	0	0.096748
5326	Amp	4	79178292	79202379	0	0.117244
5327	Amp	4	79203288	79388671	0	0.108754
5328	Amp	4	79393489	79525801	0	0.122695
5329	Amp	4	79531678	79584689	0	0.108754
5330	Amp	4	79588245	79623230	0	0.095717
5331	Amp	4	79624090	79630822	0	0.092511
5332	Amp	4	79631045	79713588	0	0.091015
5333	Amp	4	79713804	79719585	0	0.09402
5334	Amp	4	79736730	79935468	0	0.078449
5335	Amp	4	79975155	80204929	0	0.071668
5336	Amp	4	80208383	80553462	0	0.072412
5337	Amp	4	80565118	80764812	0	0.075523
5338	Amp	4	80765290	81010103	0	0.064754
5339	Amp	4	81018835	81189259	0	0.069947
5340	Amp	4	81192486	81278114	0	0.064754
5341	Amp	4	81281247	81345493	0	0.094295
5342	Amp	4	81345712	81357081	0	0.097969
5343	Amp	4	81357827	81365988	0	0.087262
5344	Amp	4	81367410	81622537	0	0.083588
5345	Amp	4	81629857	81672444	0	0.105094
5346	Amp	4	81680049	81821506	0	0.083588
5347	Amp	4	81821685	81859294	0	0.086867
5348	Amp	4	81859865	81976605	0	0.095567
5349	Amp	4	81977552	82056892	0	0.101605
5350	Amp	4	82057038	82117221	0	0.095567
5351	Amp	4	82118308	82190533	0	0.075071
5352	Amp	4	82190878	82197410	0	0.082112

5353	Amp	4	82202927	82266967	0	0.048547
5354	Amp	4	82267478	82288193	0	0.039586
5355	Amp	4	82301336	82366546	0	0.043349
5356	Amp	4	82368220	82614044	0	0.039586
5357	Amp	4	82614065	82632827	0	0.032805
5358	Amp	4	82633067	82635255	0	0.026625
5359	Amp	4	82637525	82655480	0	0.061748
5360	Amp	4	82655724	82690224	0	0.026625
5361	Amp	4	82691994	82817009	0	0.024016
5362	Amp	4	82818924	82819224	0	0.052055
5363	Amp	4	82821183	82992229	0	0.054364
5364	Amp	4	82993578	83027974	0	0.04492
5365	Amp	4	83030934	83131287	0	0.041809
5366	Amp	4	83138221	83171265	0	0.04776
5367	Amp	4	83172552	83219407	0	0.023214
5368	Amp	4	83221481	83590964	0	0.020905
5369	Amp	4	83591236	83746807	0	0.022727
5370	Amp	4	83747791	83797960	0	0.038589
5371	Amp	4	83800305	83807011	0	0.022727
5372	Amp	4	83812419	83901739	0	0.025455
5373	Amp	4	83906652	83980140	0	0.032748
5374	Amp	4	83980665	83981369	0	0.035004
5375	Amp	4	83983083	84283468	0	0.032276
5376	Amp	4	84291629	84314619	0	0.034868
5377	Amp	4	84320383	84507440	0	0.038251
5378	Amp	4	84507957	84724477	0	0.034868
5379	Amp	4	84726415	84837784	0	0.047454
5380	Amp	4	84844388	85068764	0	0.034868
5381	Amp	4	85074703	85402962	0	0.040132
5382	Amp	4	85410262	85617340	0	0.047096
5383	Amp	4	85621987	85649121	0	0.049418
5384	Amp	4	85650009	85661369	0	0.044154
5385	Amp	4	85661414	85988060	0	0.041833
5386	Amp	4	85989730	86227725	0	0.05442
5387	Amp	4	86229932	86286218	0	0.051579
5388	Amp	4	86286948	87123949	0	0.054494
5389	Amp	4	87124258	87625709	0	0.047632
5390	Amp	4	87631632	87793496	0	0.035045
5391	Amp	4	87793566	87855608	0	0.032129
5392	Amp	4	87855884	88023429	0	0.035407
5393	Amp	4	88023856	88061793	0	0.040506
5394	Amp	4	88065829	88398078	0	0.037228
5395	Amp	4	88398144	88782418	0	0.032129
5396	Amp	4	88782732	88879333	0	0.035208
5397	Amp	4	88879434	88987473	0	0.031085
5398	Amp	4	88989743	89053882	0	0.027418
5399	Amp	4	89055209	89078067	0	0.024339
5400	Amp	4	89079392	89133405	0	0.033535
5401	Amp	4	89135734	89249229	0	0.045315
5402	Amp	4	89253663	89259408	0	0.033535
5403	Amp	4	89265608	89265608	0	0.024339
5404	Amp	4	89266952	89389478	0	0.032675
5405	Amp	4	89389839	89481231	0	0.024339

5406	Amp	4	89483766	89501058	0	0.027765
5407	Amp	4	89502219	89780675	0	0.03409
5408	Amp	4	89782368	89880604	0	0.023759
5409	Amp	4	89883492	89916979	0	0.038889
5410	Amp	4	89923841	89930396	0	0.048002
5411	Amp	4	90033399	90054964	0	0.032871
5412	Amp	4	90055262	90090538	0	0.026547
5413	Amp	4	90094491	90130486	0	0.036405
5414	Amp	4	90136643	90153251	0	0.024699
5415	Amp	4	90159447	90355112	0	0.021725
5416	Amp	4	90361995	90397887	0	0.037407
5417	Amp	4	90401496	90734068	0	0.021725
5418	Amp	4	90741395	90741395	0	0.026873
5419	Amp	4	90741800	90830655	0	0.040548
5420	Amp	4	90839112	90965057	0	0.044345
5421	Amp	4	90977787	91017908	0	0.050209
5422	Amp	4	91017951	91045464	0	0.045061
5423	Amp	4	91054172	91430435	0	0.039197
5424	Amp	4	91434967	91560210	0	0.064131
5425	Amp	4	91560384	91560384	0	0.073807
5426	Amp	4	91560923	91584883	0	0.066924
5427	Amp	4	91591470	91622389	0	0.072222
5428	Amp	4	91622591	91890724	0	0.075802
5429	Amp	4	91892842	91966494	0	0.093147
5430	Amp	4	92021553	92098318	0	0.087848
5431	Amp	4	92105556	92144389	0	0.0796
5432	Amp	4	92150014	92634413	0	0.054666
5433	Amp	4	92634477	92648155	0	0.040991
5434	Amp	4	92652648	92830150	0	0.037411
5435	Amp	4	92911496	94105283	0	0.027735
5436	Amp	4	94105305	94471029	0	0.032403
5437	Amp	4	94471325	94547357	0	0.027735
5438	Amp	4	94554030	94561852	0	0.03144
5439	Amp	4	94565626	95104151	0	0.027642
5440	Amp	4	95107815	95142741	0	0.036802
5441	Amp	4	95143640	95216526	0	0.027642
5442	Amp	4	95219510	95850038	0	0.023938
5443	Amp	4	95850361	97263981	0	0.037384
5444	Amp	4	97267978	97555331	0	0.039864
5445	Amp	4	97560732	97708106	0	0.050106
5446	Amp	4	97709334	97886192	0	0.054916
5447	Amp	4	97890722	98235316	0	0.057863
5448	Amp	4	98237330	98387342	0	0.054916
5449	Amp	4	98391197	98617282	0	0.066444
5450	Amp	4	98618139	98702330	0	0.070832
5451	Amp	4	98707363	98780573	0	0.059304
5452	Amp	4	98782085	98941925	0	0.056824
5453	Amp	4	98944642	99196519	0	0.052436
5454	Amp	4	99199842	99591255	0	0.042194
5455	Amp	4	99597575	99704825	0	0.048046
5456	Amp	4	99705392	100138205	0	0.051106
5457	Amp	4	100139066	100394494	0	0.048046
5458	Amp	4	100397108	100745330	0	0.050114

5459 Amp	4	100747794	100910258	0	0.037263
5460 Amp	4	100911676	101062212	0	0.040496
5461 Amp	4	101062450	101289438	0	0.037263
5462 Amp	4	101291333	101303984	0	0.033089
5463 Amp	4	101315177	101517303	0	0.030815
5464 Amp	4	101521667	101524185	0	0.038033
5465 Amp	4	101524619	101676011	0	0.040551
5466 Amp	4	101679406	101887148	0	0.042096
5467 Amp	4	101901350	101992539	0	0.037287
5468 Amp	4	101993239	102078944	0	0.034769
5469 Amp	4	102080284	102084510	0	0.025484
5470 Amp	4	102085740	102254555	0	0.03063
5471 Amp	4	102257598	102317606	0	0.038523
5472 Amp	4	102318664	102391822	0	0.078613
5473 Amp	4	102392223	102522412	0	0.083087
5474 Amp	4	102524447	102550671	0	0.093519
5475 Amp	4	102556346	102731819	0	0.091974
5476 Amp	4	102735220	102835415	0	0.106953
5477 Amp	4	102842733	102875385	0	0.114744
5478 Amp	4	102877829	103006566	0	0.079153
5479 Amp	4	103006890	103050316	0	0.073169
5480 Amp	4	103051728	103788522	0	0.063469
5481 Amp	4	103790161	103806489	0	0.060044
5482 Amp	4	103812403	103901573	0	0.05557
5483 Amp	4	103903326	104025708	0	0.058241
5484 Amp	4	104031266	104188520	0	0.040638
5485 Amp	4	104188973	104513189	0	0.035491
5486 Amp	4	104533416	104680287	0	0.045156
5487 Amp	4	104686272	104849512	0	0.03374
5488 Amp	4	104850390	105056395	0	0.042817
5489 Amp	4	105060660	105387609	0	0.03374
5490 Amp	4	105389363	105743256	0	0.042303
5491 Amp	4	105747473	105966006	0	0.03374
5492 Amp	4	105968333	106391303	0	0.040473
5493 Amp	4	106393900	106511893	0	0.06531
5494 Amp	4	106515478	106780941	0	0.061995
5495 Amp	4	106781306	106899437	0	0.040473
5496 Amp	4	106904297	107273346	0	0.03374
5497 Amp	4	107273377	107668553	0	0.028317
5498 Amp	4	107669900	107761754	0	0.009097
5499 Amp	4	107768095	107957663	0	0
5500 Amp	4	107963231	107970830	0	0.039345
5501 Amp	4	107973856	108253858	0	0.049416
5502 Amp	4	108254533	108794296	0	0.010072
5503 Amp	4	108795097	108881273	0	0
5504 Amp	4	108886071	109027484	0	0.010101
5505 Amp	4	109028104	109056073	0	0.012859
5506 Amp	4	109057669	109154574	0	0.002759
5507 Amp	4	109157822	109260283	0	0.006851
5508 Amp	4	109262145	109418817	0	0.002759
5509 Amp	4	109419070	109482007	0	0.012141
5510 Amp	4	109485701	109507214	0	0.002759
5511 Amp	4	109510026	109525591	0	0.005798

5512 Amp	4	109529502	109644419	0	0.01601
5513 Amp	4	109651983	109790785	0	0.017549
5514 Amp	4	109797869	109844478	0	0.019576
5515 Amp	4	109844780	109849493	0	0.022168
5516 Amp	4	109850295	109918798	0	0.011957
5517 Amp	4	109922272	110501746	0	0.022573
5518 Amp	4	110507581	110915090	0	0.019981
5519 Amp	4	110916139	111471933	0	0.02699
5520 Amp	4	111473755	113444572	0	0.019981
5521 Amp	4	113445348	113852022	0	0.022526
5522 Amp	4	113859701	114394914	0	0.031168
5523 Amp	4	114394926	114863580	0	0.028623
5524 Amp	4	114863942	115412508	0	0.043205
5525 Amp	4	115417444	115417444	0	0.032589
5526 Amp	4	115417774	115455399	0	0.03849
5527 Amp	4	115462065	115500362	0	0.049782
5528 Amp	4	115500513	115674246	0	0.04114
5529 Amp	4	115679493	115748539	0	0.051689
5530 Amp	4	115875096	115875096	0	0.045788
5531 Amp	4	115881620	116104857	0	0.034497
5532 Amp	4	116107967	116281044	0	0.03093
5533 Amp	4	116286462	116311087	0	0.016347
5534 Amp	4	116311127	116520324	0	0.019373
5535 Amp	4	116523714	116769657	0	0.028145
5536 Amp	4	116770767	116930354	0	0.022749
5537 Amp	4	116966550	117074276	0	0.0122
5538 Amp	4	117074358	117231302	0	0.015339
5539 Amp	4	117238042	117255025	0	0.027468
5540 Amp	4	117256710	117348468	0	0.015339
5541 Amp	4	117352498	117478182	0	0.026629
5542 Amp	4	117478860	117542241	0	0.023491
5543 Amp	4	117548173	117607215	0	0.0122
5544 Amp	4	117610840	117699400	0	0.022279
5545 Amp	4	117699771	117841982	0	0.0122
5546 Amp	4	117843381	117948054	0	0.036945
5547 Amp	4	117954535	118241915	0	0.046673
5548 Amp	4	118242629	118410114	0	0.043914
5549 Amp	4	118410208	118999759	0	0.045435
5550 Amp	4	119005820	119108458	0	0.035707
5551 Amp	4	119108559	119490588	0	0.046483
5552 Amp	4	119492811	119549794	0	0.057198
5553 Amp	4	119554266	119705327	0	0.072563
5554 Amp	4	119706266	119993000	0	0.061787
5555 Amp	4	120002061	120341804	0	0.051072
5556 Amp	4	120348710	120382469	0	0.062652
5557 Amp	4	120384118	120495321	0	0.051072
5558 Amp	4	120496348	120668191	0	0.058982
5559 Amp	4	120668938	120958919	0	0.064346
5560 Amp	4	120960050	121215966	0	0.058982
5561 Amp	4	121216259	121317169	0	0.063617
5562 Amp	4	121320454	121381516	0	0.0689
5563 Amp	4	121385318	121409652	0	0.111293
5564 Amp	4	121478183	121508223	0	0.076198

5565	Amp	4	121518954	121519116	0	0.071563
5566	Amp	4	121528750	121577616	0	0.062626
5567	Amp	4	121577893	121651696	0	0.073607
5568	Amp	4	121653471	121750232	0	0.095922
5569	Amp	4	121751815	122215863	0	0.073607
5570	Amp	4	122219168	122272175	0	0.052959
5571	Amp	4	122272715	122327739	0	0.057765
5572	Amp	4	122339394	122600025	0	0.085397
5573	Amp	4	122602470	123058998	0	0.113868
5574	Amp	4	123061310	123181954	0	0.110842
5575	Amp	4	123183617	123252480	0	0.086097
5576	Amp	4	123254852	124530864	0	0.072371
5577	Amp	4	124534550	124703389	0	0.057246
5578	Amp	4	124703925	124861382	0	0.050708
5579	Amp	4	124864025	125324694	0	0.056538
5580	Amp	4	125328300	125328442	0	0.041793
5581	Amp	4	125331053	125434944	0	0.046531
5582	Amp	4	125435471	125459535	0	0.059556
5583	Amp	4	125460128	125578425	0	0.065393
5584	Amp	4	125583446	125667840	0	0.068043
5585	Amp	4	125675181	125743660	0	0.082102
5586	Amp	4	125746712	125782463	0	0.08634
5587	Amp	4	125782740	126102858	0	0.080503
5588	Amp	4	126102924	126184304	0	0.086977
5589	Amp	4	126184702	126459182	0	0.084327
5590	Amp	4	126462231	126727535	0	0.06829
5591	Amp	4	126733050	127617044	0	0.047764
5592	Amp	4	127620785	128017262	0	0.033705
5593	Amp	4	128020619	128159131	0	0.040566
5594	Amp	4	128160117	128846017	0	0.054047
5595	Amp	4	128849818	129062656	0	0.0488
5596	Amp	4	129066752	129162799	0	0.04381
5597	Amp	4	129163288	129163288	0	0.046649
5598	Amp	4	129172148	129399119	0	0.04361
5599	Amp	4	129399574	129451879	0	0.050791
5600	Amp	4	129452572	129565968	0	0.04361
5601	Amp	4	129567203	129597927	0	0.048273
5602	Amp	4	129598000	129740246	0	0.034792
5603	Amp	4	129765352	130089691	0	0.038366
5604	Amp	4	130091911	130243129	0	0.033703
5605	Amp	4	130243239	130250258	0	0.026777
5606	Amp	4	130250270	130251039	0	0.034735
5607	Amp	4	130333466	130497001	0	0.043721
5608	Amp	4	130498179	131019052	0	0.040882
5609	Amp	4	131440551	131864217	0	0.026824
5610	Amp	4	131866121	131964859	0	0.037301
5611	Amp	4	131974296	132055927	0	0.041768
5612	Amp	4	132059658	133111858	0	0.046382
5613	Amp	4	133113310	133571905	0	0.037396
5614	Amp	4	133573499	133882060	0	0.03293
5615	Amp	4	133882526	134307475	0	0.037634
5616	Amp	4	134308964	134493467	0	0.042805
5617	Amp	4	134496028	134978273	0	0.04912

5618	Amp	4	134979932	134988644	0	0.061395
5619	Amp	4	134992785	135002731	0	0.046304
5620	Amp	4	135004875	135103581	0	0.034029
5621	Amp	4	135103887	135141059	0	0.028859
5622	Amp	4	135161225	135912996	0	0.025284
5623	Amp	4	135914934	135939583	0	0.031904
5624	Amp	4	135943516	136013000	0	0.027312
5625	Amp	4	136013085	136152786	0	0.025589
5626	Amp	4	136159822	136397242	0	0.018969
5627	Amp	4	136398668	136681524	0	0.014264
5628	Amp	4	136684911	136725070	0	0.029343
5629	Amp	4	136725870	136733811	0	0.020211
5630	Amp	4	136737617	136758509	0	0.025066
5631	Amp	4	136761120	136819808	0	0.01912
5632	Amp	4	136823860	136843086	0	0.024286
5633	Amp	4	136850249	136857746	0	0.019431
5634	Amp	4	136857974	136905638	0	0.014264
5635	Amp	4	136906104	137097168	0	0.017927
5636	Amp	4	137098072	137243631	0	0.020972
5637	Amp	4	137243704	137458334	0	0.024226
5638	Amp	4	137458528	138482330	0	0.020972
5639	Amp	4	138482709	138790902	0	0.027059
5640	Amp	4	138791615	138846343	0	0.020972
5641	Amp	4	138849924	139023954	0	0.027583
5642	Amp	4	139024007	139052467	0	0.025107
5643	Amp	4	139053186	139322581	0	0.018496
5644	Amp	4	139322668	139718923	0	0.021139
5645	Amp	4	139720086	139876515	0	0.035032
5646	Amp	4	139881535	139933593	0	0.039997
5647	Amp	4	139933774	140747517	0	0.035032
5648	Amp	4	140748137	140814253	0	0.040066
5649	Amp	4	140814443	140856214	0	0.052095
5650	Amp	4	140856314	140911820	0	0.058437
5651	Amp	4	140912243	141314339	0	0.052095
5652	Amp	4	141314414	141708549	0	0.05615
5653	Amp	4	141711007	141798733	0	0.064366
5654	Amp	4	141802644	141999325	0	0.060311
5655	Amp	4	141999677	142115260	0	0.067641
5656	Amp	4	142119520	142135501	0	0.074187
5657	Amp	4	142142131	142202755	0	0.065971
5658	Amp	4	142203885	142203885	0	0.081261
5659	Amp	4	142211498	142619051	0	0.074716
5660	Amp	4	142620932	142720049	0	0.082251
5661	Amp	4	142721005	142721005	0	0.053067
5662	Amp	4	142723337	143040642	0	0.060696
5663	Amp	4	143042528	143177634	0	0.074726
5664	Amp	4	143189265	143199762	0	0.078054
5665	Amp	4	143206372	143357122	0	0.082032
5666	Amp	4	143358536	143384269	0	0.077208
5667	Amp	4	143386814	143487365	0	0.081798
5668	Amp	4	143491445	143570557	0	0.078135
5669	Amp	4	143571222	143577615	0	0.066621
5670	Amp	4	143577711	143617985	0	0.059525

5671 Amp	4	143621946	143681847	0	0.066292
5672 Amp	4	143690424	143694339	0	0.058662
5673 Amp	4	143703174	143720238	0	0.069663
5674 Amp	4	143721339	143738394	0	0.058662
5675 Amp	4	143738642	143796553	0	0.051896
5676 Amp	4	143796736	143796736	0	0.061602
5677 Amp	4	143797654	143805014	0	0.06987
5678 Amp	4	143805015	143822725	0	0.087684
5679 Amp	4	143822893	143824849	0	0.06987
5680 Amp	4	143834429	143841450	0	0.051896
5681 Amp	4	144126461	144204578	0	0.072477
5682 Amp	4	144206825	144209933	0	0.076752
5683 Amp	4	144213647	144386027	0	0.084992
5684 Amp	4	144405258	144648803	0	0.078147
5685 Amp	4	144650029	144660444	0	0.093987
5686 Amp	4	144662909	144856905	0	0.08685
5687 Amp	4	144857180	145311221	0	0.083523
5688 Amp	4	145311254	145639084	0	0.088051
5689 Amp	4	145639686	145923935	0	0.091752
5690 Amp	4	145929117	146134466	0	0.081451
5691 Amp	4	146134736	146986954	0	0.077176
5692 Amp	4	146992285	147346875	0	0.090687
5693 Amp	4	147346952	147699353	0	0.094774
5694 Amp	4	147704498	147704641	0	0.085232
5695 Amp	4	147706481	147820957	0	0.081144
5696 Amp	4	147821163	148119906	0	0.086357
5697 Amp	4	148121142	148317162	0	0.090835
5698 Amp	4	148339081	148504489	0	0.101797
5699 Amp	4	148510464	149303190	0	0.134426
5700 Amp	4	149304126	149316160	0	0.129947
5701 Amp	4	149321519	149517793	0	0.091353
5702 Amp	4	149518863	149527247	0	0.086141
5703 Amp	4	149532603	149619224	0	0.084457
5704 Amp	4	149619460	149627698	0	0.07837
5705 Amp	4	149631055	149667470	0	0.124551
5706 Amp	4	149668327	149682688	0	0.128468
5707 Amp	4	149689288	149782106	0	0.13391
5708 Amp	4	149787152	150111547	0	0.125551
5709 Amp	4	150118062	150609245	0	0.118127
5710 Amp	4	150609544	150631614	0	0.103561
5711 Amp	4	150682377	150746994	0	0.113596
5712 Amp	4	150752611	150787362	0	0.123723
5713 Amp	4	150790211	150889030	0	0.118281
5714 Amp	4	150896008	150896200	0	0.111636
5715 Amp	4	150896803	150997179	0	0.118226
5716 Amp	4	151103174	151123586	0	0.111636
5717 Amp	4	151144532	151667977	0	0.090358
5718 Amp	4	151668287	151763355	0	0.095704
5719 Amp	4	151764019	151925708	0	0.100603
5720 Amp	4	151930485	151957945	0	0.097216
5721 Amp	4	151959268	151993462	0	0.092317
5722 Amp	4	151995904	152623195	0	0.087178
5723 Amp	4	152623224	152912572	0	0.091895

5724 Amp	4	152916694	152961459	0	0.087178
5725 Amp	4	152961561	153323801	0	0.095148
5726 Amp	4	153327754	153545787	0	0.086752
5727 Amp	4	153546524	153882795	0	0.094123
5728 Amp	4	153884322	153885192	0	0.100399
5729 Amp	4	153885273	153898565	0	0.105518
5730 Amp	4	153901058	154318940	0	0.099242
5731 Amp	4	154319104	154354537	0	0.102929
5732 Amp	4	154356544	154583796	0	0.099242
5733 Amp	4	154584775	154614472	0	0.114741
5734 Amp	4	154623920	154770456	0	0.110824
5735 Amp	4	154770556	155035849	0	0.114312
5736 Amp	4	155036072	155098823	0	0.118691
5737 Amp	4	155103155	155423467	0	0.094953
5738 Amp	4	155425107	155561735	0	0.101025
5739 Amp	4	155564321	155664314	0	0.093654
5740 Amp	4	155672936	155838126	0	0.080565
5741 Amp	4	155841852	155885936	0	0.094473
5742 Amp	4	155887136	155915859	0	0.090094
5743 Amp	4	155917543	155955970	0	0.087528
5744 Amp	4	155959729	156103238	0	0.07362
5745 Amp	4	156110713	156302511	0	0.067547
5746 Amp	4	156302594	156527009	0	0.07171
5747 Amp	4	156527023	156607095	0	0.075284
5748 Amp	4	156607832	156828498	0	0.078381
5749 Amp	4	156830919	156855897	0	0.08241
5750 Amp	4	156856222	157031592	0	0.08013
5751 Amp	4	157032252	157215941	0	0.088883
5752 Amp	4	157216710	157275331	0	0.068101
5753 Amp	4	157276358	157454836	0	0.080043
5754 Amp	4	157457068	157531904	0	0.096838
5755 Amp	4	157532307	157560463	0	0.080043
5756 Amp	4	157563552	157844218	0	0.086661
5757 Amp	4	157846894	158196872	0	0.078469
5758 Amp	4	158198866	158216199	0	0.091515
5759 Amp	4	158216865	158295774	0	0.078469
5760 Amp	4	158304458	158374207	0	0.086895
5761 Amp	4	158376668	158496527	0	0.078469
5762 Amp	4	158497280	159434788	0	0.074981
5763 Amp	4	159440074	159543620	0	0.081306
5764 Amp	4	159545673	160035677	0	0.074981
5765 Amp	4	160038494	161092019	0	0.071805
5766 Amp	4	161174755	161273387	0	0.079927
5767 Amp	4	161273991	161284500	0	0.076354
5768 Amp	4	161298971	161409987	0	0.079041
5769 Amp	4	161411958	161413382	0	0.090623
5770 Amp	4	161427757	161550428	0	0.098944
5771 Amp	4	161555775	161579817	0	0.087363
5772 Amp	4	161584231	161712798	0	0.105348
5773 Amp	4	161713144	161737133	0	0.111276
5774 Amp	4	161750082	161822505	0	0.121921
5775 Amp	4	161822976	161822976	0	0.115994
5776 Amp	4	161824832	161828397	0	0.099683

5777 Amp	4	161828759	161845301	0	0.077818
5778 Amp	4	161845314	161849914	0	0.093301
5779 Amp	4	161852893	161900726	0	0.095299
5780 Amp	4	161907461	161944744	0	0.093301
5781 Amp	4	161945254	162377249	0	0.088397
5782 Amp	4	162378332	162420599	0	0.092688
5783 Amp	4	162421014	162475306	0	0.088397
5784 Amp	4	162487620	162821604	0	0.092692
5785 Amp	4	162821652	163033897	0	0.088397
5786 Amp	4	163034025	163040501	0	0.069121
5787 Amp	4	163040786	163735863	0	0.086945
5788 Amp	4	163740287	164177450	0	0.09632
5789 Amp	4	164185424	164241561	0	0.072292
5790 Amp	4	164247769	164344347	0	0.081056
5791 Amp	4	164345318	164584433	0	0.072292
5792 Amp	4	164989248	165257175	0	0.078974
5793 Amp	4	165260340	165373261	0	0.081891
5794 Amp	4	165375112	166507917	0	0.084503
5795 Amp	4	166507978	166527805	0	0.079438
5796 Amp	4	166528637	166548358	0	0.090092
5797 Amp	4	166549954	167392560	0	0.079438
5798 Amp	4	167392714	167854518	0	0.076521
5799 Amp	4	167856863	168648978	0	0.080389
5800 Amp	4	168649478	169058705	0	0.071014
5801 Amp	4	169064351	169283969	0	0.082132
5802 Amp	4	169287098	169420147	0	0.093762
5803 Amp	4	169420203	169529178	0	0.082132
5804 Amp	4	169530284	170050131	0	0.085889
5805 Amp	4	170051750	170644295	0	0.082132
5806 Amp	4	170649175	170761739	0	0.124363
5807 Amp	4	170765697	170851367	0	0.079087
5808 Amp	4	170853781	170907436	0	0.111185
5809 Amp	4	170909421	170968812	0	0.122179
5810 Amp	4	170969020	170994827	0	0.105618
5811 Amp	4	170997619	171030539	0	0.094624
5812 Amp	4	171033622	171105070	0	0.107389
5813 Amp	4	171110795	171285678	0	0.091096
5814 Amp	4	171292319	171898134	0	0.062111
5815 Amp	4	171909566	172353312	0	0.078625
5816 Amp	4	172355585	172748781	0	0.072876
5817 Amp	4	172758720	173277258	0	0.076797
5818 Amp	4	173278144	173732892	0	0.072876
5819 Amp	4	173734763	173795796	0	0.082187
5820 Amp	4	173798562	174012262	0	0.072876
5821 Amp	4	174012379	174138921	0	0.08125
5822 Amp	4	174139123	175005733	0	0.072876
5823 Amp	4	175005783	175032061	0	0.074596
5824 Amp	4	175033340	176425313	0	0.072876
5825 Amp	4	176426696	177172463	0	0.056362
5826 Amp	4	177172806	177280513	0	0.066412
5827 Amp	4	177282309	177610215	0	0.056362
5828 Amp	4	177610255	177734239	0	0.061053
5829 Amp	4	177734382	177868479	0	0.065004

5830	Amp	4	177868529	177931122	0	0.071921
5831	Amp	4	177936415	178147955	0	0.077961
5832	Amp	4	178149915	178358907	0	0.07327
5833	Amp	4	178359216	178785802	0	0.070986
5834	Amp	4	178786164	179317330	0	0.067036
5835	Amp	4	179321784	179367967	0	0.072381
5836	Amp	4	179406453	179509571	0	0.062346
5837	Amp	4	179512323	179566278	0	0.073028
5838	Amp	4	179569094	179657648	0	0.081229
5839	Amp	4	179657874	180132116	0	0.076595
5840	Amp	4	180132610	180175061	0	0.079302
5841	Amp	4	180175234	180412096	0	0.071101
5842	Amp	4	180412600	181242754	0	0.068394
5843	Amp	4	181244967	181277665	0	0.072249
5844	Amp	4	181277880	181561820	0	0.078212
5845	Amp	4	181565884	181652516	0	0.099373
5846	Amp	4	181652596	181667321	0	0.104108
5847	Amp	4	181667351	181821290	0	0.092955
5848	Amp	4	181830044	181935377	0	0.087296
5849	Amp	4	181938069	182017841	0	0.091907
5850	Amp	4	182018558	182221497	0	0.087172
5851	Amp	4	182221774	182228510	0	0.082561
5852	Amp	4	182228639	182394184	0	0.087216
5853	Amp	4	182397633	182417527	0	0.073049
5854	Amp	4	182420462	182614883	0	0.076897
5855	Amp	4	183286625	183292405	0	0.122949
5856	Amp	4	183293608	183295254	0	0.118429
5857	Amp	4	183295435	183411361	0	0.124633
5858	Amp	4	183412949	183608956	0	0.107957
5859	Amp	4	183610084	183629556	0	0.101752
5860	Amp	4	184172093	184204082	0	0.122899
5861	Amp	4	184204290	184807110	0	0.072377
5862	Amp	4	184807657	184824036	0	0.074133
5863	Amp	4	184827652	185555185	0	0.08488
5864	Amp	4	185563510	185578392	0	0.074133
5865	Amp	4	185582757	185683751	0	0.084183
5866	Amp	4	185684224	185820106	0	0.105292
5867	Amp	4	185820951	186124059	0	0.107069
5868	Amp	4	186172834	186240713	0	0.105018
5869	Amp	4	186240752	186336626	0	0.10117
5870	Amp	4	186336964	186451615	0	0.099414
5871	Amp	4	186453436	187075508	0	0.078305
5872	Amp	4	187076143	187076143	0	0.087965
5873	Amp	4	187079499	187829265	0	0.074621
5874	Amp	4	187829690	187842528	0	0.067279
5875	Amp	5	914118	995155	0	0.11849
5876	Amp	5	996420	1021104	0	0.113835
5877	Amp	5	1287276	1368882	0	0.117978
5878	Amp	5	1370185	1428399	0	0.116263
5879	Amp	5	1428688	1891707	0	0.102505
5880	Amp	5	1892645	1894165	0	0.10555
5881	Amp	5	1897256	2117800	0	0.102006
5882	Amp	5	2118329	2172942	0	0.095454

5883	Amp	5	2176472	2206076	0	0.091311
5884	Amp	5	2207913	2388958	0	0.086505
5885	Amp	5	2389948	2389948	0	0.084859
5886	Amp	5	2390120	2395345	0	0.09285
5887	Amp	5	2397503	2440955	0	0.090693
5888	Amp	5	2445890	2775775	0	0.082702
5889	Amp	5	2778255	2852653	0	0.080953
5890	Amp	5	2855358	2998716	0	0.088252
5891	Amp	5	2999060	3021030	0	0.084549
5892	Amp	5	3024296	3116415	0	0.077249
5893	Amp	5	3117066	3140891	0	0.084785
5894	Amp	5	3142047	3156605	0	0.082838
5895	Amp	5	3157067	3222466	0	0.081133
5896	Amp	5	3222753	3225725	0	0.088943
5897	Amp	5	3225982	3239009	0	0.097748
5898	Amp	5	3239023	3361051	0	0.088943
5899	Amp	5	3361067	3585681	0	0.081133
5900	Amp	5	3588324	3654937	0	0.087097
5901	Amp	5	3655690	3655690	0	0.11035
5902	Amp	5	3671100	4052303	0	0.104385
5903	Amp	5	4058240	4070119	0	0.113516
5904	Amp	5	4070954	4332056	0	0.080896
5905	Amp	5	4335843	4352164	0	0.078491
5906	Amp	5	4352420	4405510	0	0.072661
5907	Amp	5	4405692	4443741	0	0.067611
5908	Amp	5	4445863	4523737	0	0.061093
5909	Amp	5	4523820	4523820	0	0.06539
5910	Amp	5	4528282	4568343	0	0.062777
5911	Amp	5	4568832	4633162	0	0.073405
5912	Amp	5	4633296	4634381	0	0.078787
5913	Amp	5	4638772	4652862	0	0.07449
5914	Amp	5	4654516	4668279	0	0.080271
5915	Amp	5	4668480	4739055	0	0.07449
5916	Amp	5	4741382	5282096	0	0.069108
5917	Amp	5	5282170	5297989	0	0.079212
5918	Amp	5	5298029	5385941	0	0.077151
5919	Amp	5	5390252	6018974	0	0.067047
5920	Amp	5	6023043	6141023	0	0.063665
5921	Amp	5	6141575	6923088	0	0.067118
5922	Amp	5	6923836	7206444	0	0.069029
5923	Amp	5	7208952	7336646	0	0.075452
5924	Amp	5	7336773	7364254	0	0.08485
5925	Amp	5	7367847	7407781	0	0.089564
5926	Amp	5	7409916	7779390	0	0.083141
5927	Amp	5	7779635	7799679	0	0.088734
5928	Amp	5	7804297	8096321	0	0.078257
5929	Amp	5	8098178	8203819	0	0.070604
5930	Amp	5	8206145	8643178	0	0.068859
5931	Amp	5	8645161	10071469	0	0.075307
5932	Amp	5	10072135	10273531	0	0.073465
5933	Amp	5	10275758	10505473	0	0.076568
5934	Amp	5	10505605	10658401	0	0.073465
5935	Amp	5	10661816	11019658	0	0.075078

5936 Amp	5	11026660	11088206	0	0.064736
5937 Amp	5	11089090	11257445	0	0.068207
5938 Amp	5	11263083	11328915	0	0.056446
5939 Amp	5	11329880	11814268	0	0.058282
5940 Amp	5	11816225	12115693	0	0.063768
5941 Amp	5	12115708	12331088	0	0.073268
5942 Amp	5	12333631	12348426	0	0.101545
5943 Amp	5	12352383	12487670	0	0.091373
5944 Amp	5	12488203	12533559	0	0.094171
5945 Amp	5	12534140	12595499	0	0.084671
5946 Amp	5	12595786	12774845	0	0.082263
5947 Amp	5	12775593	13506488	0	0.07767
5948 Amp	5	13507507	13695026	0	0.083959
5949 Amp	5	13695071	13898404	0	0.082346
5950 Amp	5	13898885	13964479	0	0.079549
5951 Amp	5	13972146	14290165	0	0.088495
5952 Amp	5	14290479	14370659	0	0.086698
5953 Amp	5	14370722	14499286	0	0.081104
5954 Amp	5	14502086	14502238	0	0.075296
5955 Amp	5	14503010	14562676	0	0.066351
5956 Amp	5	14570623	14802002	0	0.060054
5957 Amp	5	14802401	14909225	0	0.057748
5958 Amp	5	14911459	15209365	0	0.059598
5959 Amp	5	15209659	15329041	0	0.049273
5960 Amp	5	15329069	15331010	0	0.042831
5961 Amp	5	15331022	15332597	0	0.034085
5962 Amp	5	15332903	15337599	0	0.052307
5963 Amp	5	15337964	15337964	0	0.054454
5964 Amp	5	15338055	15338236	0	0.060777
5965 Amp	5	15338427	15345729	0	0.058171
5966 Amp	5	15353833	16330768	0	0.050302
5967 Amp	5	16332390	16359880	0	0.055359
5968 Amp	5	16360260	16507421	0	0.0666
5969 Amp	5	16508177	16756646	0	0.060576
5970 Amp	5	16759361	16774220	0	0.075114
5971 Amp	5	16777825	16784968	0	0.093265
5972 Amp	5	16785209	16946125	0	0.091416
5973 Amp	5	16947932	16947932	0	0.098474
5974 Amp	5	16949393	17211540	0	0.105157
5975 Amp	5	17213210	17220907	0	0.112633
5976 Amp	5	17228093	17228093	0	0.142908
5977 Amp	5	17228825	17286788	0	0.136224
5978 Amp	5	17287669	17458365	0	0.121686
5979 Amp	5	17462220	17747964	0	0.123246
5980 Amp	5	17756092	18051157	0	0.111714
5981 Amp	5	18053919	18182517	0	0.104656
5982 Amp	5	18184240	18334829	0	0.115224
5983 Amp	5	18334842	18479069	0	0.122543
5984 Amp	5	18480736	18513713	0	0.115224
5985 Amp	5	18514209	18558599	0	0.109982
5986 Amp	5	18558957	18736537	0	0.116917
5987 Amp	5	18740489	18817834	0	0.109982
5988 Amp	5	18818360	18979758	0	0.108406

5989	Amp	5	18981088	19285140	0	0.097236
5990	Amp	5	19312824	19635615	0	0.069049
5991	Amp	5	19636011	20033944	0	0.082465
5992	Amp	5	20034573	20081895	0	0.096149
5993	Amp	5	20088019	20176778	0	0.092903
5994	Amp	5	20182113	20258764	0	0.082335
5995	Amp	5	20263717	20264285	0	0.085143
5996	Amp	5	20274886	20778797	0	0.07146
5997	Amp	5	20778939	21050144	0	0.078153
5998	Amp	5	21052063	21068138	0	0.07146
5999	Amp	5	21069254	21231593	0	0.076117
6000	Amp	5	21232015	21307918	0	0.08085
6001	Amp	5	21308002	21339080	0	0.069988
6002	Amp	5	21341983	21361673	0	0.068134
6003	Amp	5	21391680	21583342	0	0.083053
6004	Amp	5	21586773	21611645	0	0.07832
6005	Amp	5	21612050	21813757	0	0.084575
6006	Amp	5	21819491	21832471	0	0.048796
6007	Amp	5	21834121	21922573	0	0.046648
6008	Amp	5	21926325	21968623	0	0.043358
6009	Amp	5	21973797	21976378	0	0.037184
6010	Amp	5	21990005	22211065	0	0.030929
6011	Amp	5	22222352	22419490	0	0.043053
6012	Amp	5	22424080	23589410	0	0.041396
6013	Amp	5	23591346	23772618	0	0.043709
6014	Amp	5	23773485	23791668	0	0.052661
6015	Amp	5	23797480	23869390	0	0.058949
6016	Amp	5	23869890	23909739	0	0.052661
6017	Amp	5	23918874	23954020	0	0.064683
6018	Amp	5	23960226	23996512	0	0.052661
6019	Amp	5	23996781	24235742	0	0.05992
6020	Amp	5	24235761	24335357	0	0.066301
6021	Amp	5	24337032	24367150	0	0.072408
6022	Amp	5	24370240	24405734	0	0.066027
6023	Amp	5	24407704	24460890	0	0.07397
6024	Amp	5	24462892	24692295	0	0.081442
6025	Amp	5	24692459	24705207	0	0.075335
6026	Amp	5	24707125	24891789	0	0.073506
6027	Amp	5	24892619	25480195	0	0.068849
6028	Amp	5	25485709	25516335	0	0.067044
6029	Amp	5	25516497	25546099	0	0.076789
6030	Amp	5	25551703	25607993	0	0.06953
6031	Amp	5	25615482	25812152	0	0.078363
6032	Amp	5	25824774	25841214	0	0.076051
6033	Amp	5	25844187	25905889	0	0.067098
6034	Amp	5	25906276	26083114	0	0.071653
6035	Amp	5	26084850	26431824	0	0.067098
6036	Amp	5	26431976	26482797	0	0.0648
6037	Amp	5	26487659	26662285	0	0.055055
6038	Amp	5	26663879	26817834	0	0.067991
6039	Amp	5	26818018	26841667	0	0.072019
6040	Amp	5	26846784	26900675	0	0.083163
6041	Amp	5	26900794	26919816	0	0.089987

6042	Amp	5	26921464	26944373	0	0.078844
6043	Amp	5	26945259	27136796	0	0.072019
6044	Amp	5	27143188	27356326	0	0.067991
6045	Amp	5	27356344	27395403	0	0.074379
6046	Amp	5	27395920	27430078	0	0.07748
6047	Amp	5	27433051	27489655	0	0.072143
6048	Amp	5	27490678	27535409	0	0.08021
6049	Amp	5	27536132	27589099	0	0.072143
6050	Amp	5	27589862	27662303	0	0.083165
6051	Amp	5	27663462	27671762	0	0.08557
6052	Amp	5	27672516	27717837	0	0.074548
6053	Amp	5	27726201	27877045	0	0.080028
6054	Amp	5	27877809	27882851	0	0.0757
6055	Amp	5	27882885	28059810	0	0.07022
6056	Amp	5	28066979	28225550	0	0.087471
6057	Amp	5	28225928	28309610	0	0.075347
6058	Amp	5	28309678	28456241	0	0.069954
6059	Amp	5	28457075	28525989	0	0.072547
6060	Amp	5	28531406	28572749	0	0.076202
6061	Amp	5	28575008	28635768	0	0.084617
6062	Amp	5	28729999	28822754	0	0.076202
6063	Amp	5	28823631	29000786	0	0.11847
6064	Amp	5	29000970	29047361	0	0.116327
6065	Amp	5	29050456	29050693	0	0.111717
6066	Amp	5	29052575	29179153	0	0.069449
6067	Amp	5	29183193	29236228	0	0.057093
6068	Amp	5	29238710	29440802	0	0.067194
6069	Amp	5	29441301	29581537	0	0.079753
6070	Amp	5	29582019	29617924	0	0.092297
6071	Amp	5	29640708	29655133	0	0.062299
6072	Amp	5	29655191	29694887	0	0.066751
6073	Amp	5	29697199	29783921	0	0.05665
6074	Amp	5	29785378	29914239	0	0.061993
6075	Amp	5	29914385	30302095	0	0.064014
6076	Amp	5	30302263	30302263	0	0.075985
6077	Amp	5	30311234	30344488	0	0.072144
6078	Amp	5	30350369	30580715	0	0.060173
6079	Amp	5	30584966	30774574	0	0.061631
6080	Amp	5	30781843	30795248	0	0.057976
6081	Amp	5	30799414	30939862	0	0.059707
6082	Amp	5	30941115	31028624	0	0.068448
6083	Amp	5	31040432	31053461	0	0.063996
6084	Amp	5	31053677	31275976	0	0.06573
6085	Amp	5	31276292	31301230	0	0.063137
6086	Amp	5	31306611	31336411	0	0.060733
6087	Amp	5	31337977	31566495	0	0.057942
6088	Amp	5	31568754	31689539	0	0.061034
6089	Amp	5	31690452	31769073	0	0.063673
6090	Amp	5	31770626	31877602	0	0.065738
6091	Amp	5	31879841	32048988	0	0.074833
6092	Amp	5	32049104	32248380	0	0.077477
6093	Amp	5	32249337	32455367	0	0.073055
6094	Amp	5	32458839	32549966	0	0.084161

6095 Amp	5	32550022	32573117	0	0.093375
6096 Amp	5	32575808	32592630	0	0.08227
6097 Amp	5	32592855	32737477	0	0.084546
6098 Amp	5	32738500	32786600	0	0.075332
6099 Amp	5	32787311	32881784	0	0.073267
6100 Amp	5	32882856	33146281	0	0.069423
6101 Amp	5	33147239	33208136	0	0.066656
6102 Amp	5	33208625	33321665	0	0.064017
6103 Amp	5	33326208	33531575	0	0.065804
6104 Amp	5	33534955	33902506	0	0.067776
6105 Amp	5	33904466	33944112	0	0.064685
6106 Amp	5	34754143	35029057	0	0.097056
6107 Amp	5	35029121	35460175	0	0.094852
6108 Amp	5	35463399	35473539	0	0.104062
6109 Amp	5	35474352	35587955	0	0.094852
6110 Amp	5	35591095	35792940	0	0.099356
6111 Amp	5	35794642	36613099	0	0.096664
6112 Amp	5	36616712	36813960	0	0.098379
6113 Amp	5	36816559	36854563	0	0.100438
6114 Amp	5	36855372	36914077	0	0.106174
6115 Amp	5	36915061	36953263	0	0.104154
6116 Amp	5	36958336	37039767	0	0.098573
6117 Amp	5	37043830	37255481	0	0.092837
6118 Amp	5	37259058	37335742	0	0.087664
6119 Amp	5	37336543	37439445	0	0.099278
6120 Amp	5	37651330	37651330	0	0.065449
6121 Amp	5	37667338	37700843	0	0.068172
6122 Amp	5	37703690	37778630	0	0.082176
6123 Amp	5	37778813	37894586	0	0.070536
6124 Amp	5	37896658	38149898	0	0.072222
6125 Amp	5	38150344	38167482	0	0.069859
6126 Amp	5	38169299	38388491	0	0.078314
6127 Amp	5	38389100	38519691	0	0.0974
6128 Amp	5	38522377	38563802	0	0.078314
6129 Amp	5	38567886	38705146	0	0.076527
6130 Amp	5	38706538	38814076	0	0.068072
6131 Amp	5	38817054	38883376	0	0.080095
6132 Amp	5	38883558	39359138	0	0.087002
6133 Amp	5	39359174	39395327	0	0.08074
6134 Amp	5	39395397	39639272	0	0.088969
6135 Amp	5	39639508	39663878	0	0.084465
6136 Amp	5	39664128	39700562	0	0.081363
6137 Amp	5	39701678	39999257	0	0.049496
6138 Amp	5	39999375	39999375	0	0.047765
6139 Amp	5	39999554	40174440	0	0.053257
6140 Amp	5	40174644	40181820	0	0.047765
6141 Amp	5	40183118	40253800	0	0.05268
6142 Amp	5	40255202	40265373	0	0.082541
6143 Amp	5	40269613	40330982	0	0.080854
6144 Amp	5	40331136	40359576	0	0.075938
6145 Amp	5	40365385	40437115	0	0.079344
6146 Amp	5	40437743	40448393	0	0.07416
6147 Amp	5	40452118	40565901	0	0.0443

6148	Amp	5	40567286	40951937	0	0.046379
6149	Amp	5	40952988	40953185	0	0.053321
6150	Amp	5	40953974	41251193	0	0.054822
6151	Amp	5	41253699	41265762	0	0.058525
6152	Amp	5	41266365	41326117	0	0.060413
6153	Amp	5	41327098	41338952	0	0.058304
6154	Amp	5	41339133	42381626	0	0.055659
6155	Amp	5	42381804	42429535	0	0.057719
6156	Amp	5	42431349	42590646	0	0.050777
6157	Amp	5	42599276	42600570	0	0.058917
6158	Amp	5	42606539	42624693	0	0.073919
6159	Amp	5	42626322	42632711	0	0.058917
6160	Amp	5	42635052	42955971	0	0.050777
6161	Amp	5	42956594	43276367	0	0.060342
6162	Amp	5	43278504	43639321	0	0.05191
6163	Amp	5	43641746	43808039	0	0.057484
6164	Amp	5	43809221	43910402	0	0.074187
6165	Amp	5	43910646	43948006	0	0.058926
6166	Amp	5	43949193	43959432	0	0.07606
6167	Amp	5	43959701	44000611	0	0.077583
6168	Amp	5	44002068	44047613	0	0.080639
6169	Amp	5	44054829	44089973	0	0.087299
6170	Amp	5	44090027	44103302	0	0.089425
6171	Amp	5	44104008	44189910	0	0.087902
6172	Amp	5	44194054	44211365	0	0.089518
6173	Amp	5	44216244	44252798	0	0.097809
6174	Amp	5	44253490	44280732	0	0.10834
6175	Amp	5	44283751	44294976	0	0.114429
6176	Amp	5	44299298	44321554	0	0.124706
6177	Amp	5	44323478	44346222	0	0.136761
6178	Amp	5	44365691	44417868	0	0.144027
6179	Amp	5	44422531	44457946	0	0.150418
6180	Amp	5	44462022	44462022	0	0.179864
6181	Amp	5	44462546	44480915	0	0.186913
6182	Amp	5	44487256	44571042	0.028398	0.192088
6183	Amp	5	44585098	44638017	0.09318	0.199339
6184	Amp	5	44641278	44649413	0.02372	0.191047
6185	Amp	5	44652800	44652800	0.098998	0.200428
6186	Amp	5	44653449	44714228	0.038273	0.193379
6187	Amp	5	44715272	44718608	0	0.18454
6188	Amp	5	44719614	44719764	0	0.178149
6189	Amp	5	44722462	44747593	0	0.167872
6190	Amp	5	44759484	44759484	0	0.161082
6191	Amp	5	44761521	44762231	0	0.154422
6192	Amp	5	44765420	44768354	0	0.145045
6193	Amp	5	44770850	44776454	0	0.1156
6194	Amp	5	44797975	44798305	0	0.106218
6195	Amp	5	44800563	44832612	0	0.111699
6196	Amp	5	44836454	44858349	0	0.117742
6197	Amp	5	44860326	44889954	0	0.096341
6198	Amp	5	44890091	45220234	0	0.102309
6199	Amp	5	45223020	45224283	0	0.116459
6200	Amp	5	45228711	45353842	0	0.106894

6201	Amp	5	45354697	45364957	0	0.110078
6202	Amp	5	45377176	45377176	0	0.104258
6203	Amp	5	45378354	45378354	0	0.101202
6204	Amp	5	45383300	45383300	0	0.104124
6205	Amp	5	50828231	50828231	0	0.111679
6206	Amp	5	50829022	50829022	0	0.101148
6207	Amp	5	50837510	50837542	0	0.095505
6208	Amp	5	50837635	50840797	0	0.089463
6209	Amp	5	50843301	50843796	0	0.08749
6210	Amp	5	50847727	50856621	0	0.090045
6211	Amp	5	50856633	50865893	0	0.094958
6212	Amp	5	50866352	50987266	0	0.093457
6213	Amp	5	50991478	51053630	0	0.087489
6214	Amp	5	51054523	51077264	0	0.081254
6215	Amp	5	51080135	51143661	0	0.090081
6216	Amp	5	51145904	51294172	0	0.094258
6217	Amp	5	51297242	51312403	0	0.065493
6218	Amp	5	51315374	51337012	0	0.06917
6219	Amp	5	51339678	51442613	0	0.066615
6220	Amp	5	51442644	51498632	0	0.057788
6221	Amp	5	51499198	51509473	0	0.064515
6222	Amp	5	51521462	51574646	0	0.077486
6223	Amp	5	51578124	51626362	0	0.059989
6224	Amp	5	51626837	51678041	0	0.068489
6225	Amp	5	51680738	51826596	0	0.061762
6226	Amp	5	51826631	51827615	0	0.058578
6227	Amp	5	51829914	51837111	0	0.045606
6228	Amp	5	51837534	51854004	0	0.041429
6229	Amp	5	51858768	52214998	0	0.037752
6230	Amp	5	52223022	52532676	0	0.043779
6231	Amp	5	52542025	52575050	0	0.058996
6232	Amp	5	52575182	52635550	0	0.05326
6233	Amp	5	52637299	52981019	0	0.055962
6234	Amp	5	52983630	53135183	0	0.05326
6235	Amp	5	53135558	53276767	0	0.037752
6236	Amp	5	53276965	53336818	0	0.044708
6237	Amp	5	53337605	53365067	0	0.05431
6238	Amp	5	53365367	53526695	0	0.060485
6239	Amp	5	53535737	53552763	0	0.064062
6240	Amp	5	53556260	53573342	0	0.072502
6241	Amp	5	53575150	53611693	0	0.079455
6242	Amp	5	53612695	53710513	0	0.091713
6243	Amp	5	53713439	53845294	0	0.0822
6244	Amp	5	53845509	53862778	0	0.075247
6245	Amp	5	53866559	53897211	0	0.073168
6246	Amp	5	53902750	53980267	0	0.079583
6247	Amp	5	53980442	54055064	0	0.07491
6248	Amp	5	54071230	54081006	0	0.065308
6249	Amp	5	54086232	54088078	0	0.058893
6250	Amp	5	54090579	54313597	0	0.068874
6251	Amp	5	54315900	54344409	0	0.075619
6252	Amp	5	54352350	54352350	0	0.078503
6253	Amp	5	54352650	54408489	0	0.07359

6254 Amp	5	54411196	54448245	0	0.070014
6255 Amp	5	54451477	54451966	0	0.074092
6256 Amp	5	54452695	54632546	0	0.087654
6257 Amp	5	54634415	54642407	0	0.076468
6258 Amp	5	54642496	54686997	0	0.065203
6259 Amp	5	54689637	54806367	0	0.075955
6260 Amp	5	54807541	54902077	0	0.084379
6261 Amp	5	54902145	54907301	0	0.070899
6262 Amp	5	54907646	54927652	0	0.073628
6263 Amp	5	54928831	55145323	0	0.070899
6264 Amp	5	55147673	55364416	0	0.050546
6265 Amp	5	55366505	55397149	0	0.046468
6266 Amp	5	55397286	55417193	0	0.049977
6267 Amp	5	55419696	55428714	0	0.052317
6268 Amp	5	55431431	55464542	0	0.058228
6269 Amp	5	55467246	55483938	0	0.052747
6270 Amp	5	55492016	55606028	0	0.039168
6271 Amp	5	55608977	55610166	0	0.02934
6272 Amp	5	55615559	55690947	0	0.046536
6273 Amp	5	55698236	55799485	0	0.055491
6274 Amp	5	55801036	55840604	0	0.046536
6275 Amp	5	55841527	55976686	0	0.050709
6276 Amp	5	55979274	56070877	0	0.052359
6277 Amp	5	56071286	56113951	0	0.035163
6278 Amp	5	56114482	56221975	0	0.024772
6279 Amp	5	56227742	56453279	0	0.026342
6280 Amp	5	56453474	56459254	0	0.022169
6281 Amp	5	56466215	56732622	0	0.020599
6282 Amp	5	56732766	56826517	0	0.028814
6283 Amp	5	56826628	56863100	0	0.020599
6284 Amp	5	56865960	56928462	0	0.033967
6285 Amp	5	56928512	56955950	0	0.04442
6286 Amp	5	56956482	56997541	0	0.050767
6287 Amp	5	56998649	57043990	0	0.044219
6288 Amp	5	57044844	57228772	0	0.052952
6289 Amp	5	57230487	57294967	0	0.071704
6290 Amp	5	57296637	57494615	0	0.058336
6291 Amp	5	57495774	57595865	0	0.054431
6292 Amp	5	57596125	57621050	0	0.047923
6293 Amp	5	57621558	57638199	0	0.06065
6294 Amp	5	57639194	57771458	0	0.068087
6295 Amp	5	57778963	57847506	0	0.065203
6296 Amp	5	57850546	57909586	0	0.057009
6297 Amp	5	57910252	57959070	0	0.069308
6298 Amp	5	57960039	57989844	0	0.074601
6299 Amp	5	57990040	58063821	0	0.082794
6300 Amp	5	58064843	58064843	0	0.088878
6301 Amp	5	58071766	58074258	0	0.095592
6302 Amp	5	58074828	58198626	0	0.100887
6303 Amp	5	58202172	58286147	0	0.095592
6304 Amp	5	58294644	58364087	0	0.088878
6305 Amp	5	58365375	58419714	0	0.084888
6306 Amp	5	58422912	58443204	0	0.104524

6307 Amp	5	58447080	58468110	0	0.099231
6308 Amp	5	58469648	58490716	0	0.100634
6309 Amp	5	58490832	58535542	0	0.137477
6310 Amp	5	58543985	58545596	0	0.100634
6311 Amp	5	58546222	58592916	0	0.098764
6312 Amp	5	58594123	58598628	0	0.060566
6313 Amp	5	58599267	58700800	0	0.04093
6314 Amp	5	58702076	58879311	0	0.046566
6315 Amp	5	58880357	58962671	0	0.036443
6316 Amp	5	58964470	58964470	0	0.050105
6317 Amp	5	58965702	58993676	0	0.05538
6318 Amp	5	58995118	58995118	0	0.049296
6319 Amp	5	58995381	59073892	0	0.053683
6320 Amp	5	59074614	59097505	0	0.049296
6321 Amp	5	59098933	59134852	0	0.06737
6322 Amp	5	59138257	59152405	0	0.069099
6323 Amp	5	59152592	59152592	0	0.064083
6324 Amp	5	59153190	59174456	0	0.067859
6325 Amp	5	59180291	59182866	0	0.040734
6326 Amp	5	59182946	59195712	0	0.045259
6327 Amp	5	59201147	59247083	0	0.054792
6328 Amp	5	59247223	59318863	0	0.059953
6329 Amp	5	59319104	59319104	0	0.055428
6330 Amp	5	59319671	59360426	0	0.060399
6331 Amp	5	59360502	59377966	0	0.055238
6332 Amp	5	59378898	59394425	0	0.060005
6333 Amp	5	59400781	59513800	0	0.055238
6334 Amp	5	59514540	59817490	0	0.050267
6335 Amp	5	59818808	59845678	0	0.046515
6336 Amp	5	59848072	59895785	0	0.051653
6337 Amp	5	59897690	59913249	0	0.046515
6338 Amp	5	59914352	59939111	0	0.038015
6339 Amp	5	59944749	59981463	0	0.057613
6340 Amp	5	59981945	59994208	0	0.04808
6341 Amp	5	60005086	60015636	0	0.052277
6342 Amp	5	60016837	60232370	0	0.059437
6343 Amp	5	60232646	60529473	0	0.057721
6344 Amp	5	60529652	60608780	0	0.024462
6345 Amp	5	60612755	60673719	0	0.051231
6346 Amp	5	60677671	60678800	0	0.030373
6347 Amp	5	60682675	60697464	0	0.061108
6348 Amp	5	60697960	60713236	0	0.030373
6349 Amp	5	60714428	60855940	0	0.024741
6350 Amp	5	60870281	61151935	0	0.056559
6351 Amp	5	61153999	61188833	0	0.038229
6352 Amp	5	61190019	61339096	0	0.043308
6353 Amp	5	61340302	61347832	0	0.03911
6354 Amp	5	61351656	61370455	0	0.04779
6355 Amp	5	61377459	61377459	0	0.067036
6356 Amp	5	61378476	61488286	0	0.064468
6357 Amp	5	61489032	61527731	0	0.076506
6358 Amp	5	61531706	61661645	0	0.03786
6359 Amp	5	61663392	61664559	0	0.040515

6360	Amp	5	61664717	61688434	0	0.044673
6361	Amp	5	61695565	61717898	0	0.035994
6362	Amp	5	61717949	61761743	0	0.031836
6363	Amp	5	61762950	61922980	0	0.019799
6364	Amp	5	61924030	62033855	0	0.023386
6365	Amp	5	62041432	62277162	0	0.032062
6366	Amp	5	62316232	62370982	0	0.035751
6367	Amp	5	62377705	62386192	0	0.040761
6368	Amp	5	62386498	62393889	0	0.047139
6369	Amp	5	62395638	62581801	0	0.038462
6370	Amp	5	62591003	62665898	0	0.034875
6371	Amp	5	62666148	62680180	0	0.039107
6372	Amp	5	62681802	62688021	0	0.032729
6373	Amp	5	62692962	62774091	0	0.027719
6374	Amp	5	62774122	62812351	0	0.023487
6375	Amp	5	62812477	63004692	0	0.02773
6376	Amp	5	63006694	63027425	0	0.037005
6377	Amp	5	63030991	63231101	0	0.032762
6378	Amp	5	63239573	63252077	0	0.029073
6379	Amp	5	63256687	63301236	0	0.019799
6380	Amp	5	63885700	63885700	0	0.050258
6381	Amp	5	63886716	63886716	0	0.053771
6382	Amp	5	63887110	63889871	0	0.062757
6383	Amp	5	63893413	64019477	0	0.065184
6384	Amp	5	64023760	64099962	0	0.034724
6385	Amp	5	64113483	64214659	0	0.029246
6386	Amp	5	64226909	64623798	0	0.050402
6387	Amp	5	64625687	64630372	0	0.056015
6388	Amp	5	64630707	65021239	0	0.037383
6389	Amp	5	65021557	65816901	0	0.017044
6390	Amp	5	65819175	65883393	0	0.021587
6391	Amp	5	65883952	66244131	0	0.013317
6392	Amp	5	66244220	67156283	0	0.017825
6393	Amp	5	67159113	68493841	0	0.013282
6394	Amp	5	68495203	68778812	0	0.006935
6395	Amp	5	68785410	68884485	0	0.013426
6396	Amp	5	68888560	69038309	0	0.008919
6397	Amp	5	69038880	69074671	0	0.010737
6398	Amp	5	69081303	69289310	0	0.01354
6399	Amp	5	69291860	69374274	0	0.011722
6400	Amp	5	69375885	71366831	0	0.005231
6401	Amp	5	71367614	71788442	0	0.002427
6402	Amp	5	71792521	71817922	0	0.006451
6403	Amp	5	71818553	71973122	0	0.012815
6404	Amp	5	71975063	72166238	0	0.016375
6405	Amp	5	72166701	72567459	0	0.012815
6406	Amp	5	72571464	72584709	0	0.01759
6407	Amp	5	72591767	74096133	0	0.013567
6408	Amp	5	74096841	74135610	0	0.022413
6409	Amp	5	74136176	74305023	0	0.013567
6410	Amp	5	74310174	74651766	0	0.017683
6411	Amp	5	74651992	74760893	0	0.013567
6412	Amp	5	74764026	75431688	0	0.008791

6413	Amp	5	75436863	75716647	0	0.031099
6414	Amp	5	75720697	75748451	0	0.042534
6415	Amp	5	75750228	76289308	0	0.020226
6416	Amp	5	76295636	76620080	0	0.008791
6417	Amp	5	76620609	77402913	0	0.026391
6418	Amp	5	77403220	77748952	0	0.033318
6419	Amp	5	77749691	78047686	0	0.015718
6420	Amp	5	78048225	78168720	0	0.019454
6421	Amp	5	78203742	78346197	0	0.020881
6422	Amp	5	78351549	78780710	0	0.019454
6423	Amp	5	78782886	78809524	0	0.031445
6424	Amp	5	78821761	79053186	0	0.019454
6425	Amp	5	79053662	79127023	0	0.023553
6426	Amp	5	79128048	79149648	0	0.025194
6427	Amp	5	79196213	79266743	0	0.018267
6428	Amp	5	79275565	79288490	0	0.014167
6429	Amp	5	79291159	79353618	0	0.017331
6430	Amp	5	79355270	79425621	0	0.013596
6431	Amp	5	79427202	79954797	0	0.017504
6432	Amp	5	79955538	80105549	0	0.025397
6433	Amp	5	80106620	81212851	0	0.020593
6434	Amp	5	81214117	82547320	0	0.0127
6435	Amp	5	82547907	82624665	0	0.029052
6436	Amp	5	82625226	83503012	0	0.025144
6437	Amp	5	83507699	83644729	0	0.028982
6438	Amp	5	83645611	85217684	0	0.025144
6439	Amp	5	85219000	86779303	0	0.03195
6440	Amp	5	86779884	87176730	0	0.025144
6441	Amp	5	87181888	87359398	0	0.027096
6442	Amp	5	87361655	87421186	0	0.020732
6443	Amp	5	87421611	87540645	0	0.02706
6444	Amp	5	87545149	87977169	0	0.020732
6445	Amp	5	87980087	88226919	0	0.024633
6446	Amp	5	88229224	88802101	0	0.037839
6447	Amp	5	88803854	89048496	0	0.042985
6448	Amp	5	89052305	89144803	0	0.050087
6449	Amp	5	89150932	89440811	0	0.066058
6450	Amp	5	89444164	89756040	0	0.062158
6451	Amp	5	89756063	89909943	0	0.060205
6452	Amp	5	89970141	90659308	0	0.037132
6453	Amp	5	90660084	91104535	0	0.031986
6454	Amp	5	91107358	91132810	0	0.034663
6455	Amp	5	91133082	91478551	0	0.031986
6456	Amp	5	91483031	92098132	0	0.042275
6457	Amp	5	92100248	92801594	0	0.039848
6458	Amp	5	92801982	92819431	0	0.043674
6459	Amp	5	92822992	92911721	0	0.039848
6460	Amp	5	92912209	93855234	0	0.042529
6461	Amp	5	93857775	93991201	0	0.046672
6462	Amp	5	93993303	94063387	0	0.03032
6463	Amp	5	94065323	94312390	0	0.050212
6464	Amp	5	94312426	95256959	0	0.046069
6465	Amp	5	95269537	95404669	0	0.06355

6466	Amp	5	95406269	95486921	0	0.053261
6467	Amp	5	95497228	95751907	0	0.054822
6468	Amp	5	95752316	95791325	0	0.065047
6469	Amp	5	95791550	95871785	0	0.069272
6470	Amp	5	95872184	96069834	0	0.074711
6471	Amp	5	96071084	96097771	0	0.076903
6472	Amp	5	96100215	96176764	0	0.066679
6473	Amp	5	96176900	96368169	0	0.062454
6474	Amp	5	96368601	96693229	0	0.071565
6475	Amp	5	96694323	96714437	0	0.054085
6476	Amp	5	96717901	96816680	0	0.044973
6477	Amp	5	96819615	96855656	0	0.049238
6478	Amp	5	96863494	97003131	0	0.076851
6479	Amp	5	97003995	97041958	0	0.049238
6480	Amp	5	97042296	97169702	0	0.081408
6481	Amp	5	97171313	97198915	0	0.049238
6482	Amp	5	97203820	97460752	0	0.073279
6483	Amp	5	97462867	97612383	0	0.098488
6484	Amp	5	97614077	97900991	0	0.049238
6485	Amp	5	97905758	97933204	0	0.060681
6486	Amp	5	97933806	98280963	0	0.049238
6487	Amp	5	98282685	98509243	0	0.05851
6488	Amp	5	98510690	98877617	0	0.056949
6489	Amp	5	98880606	99167137	0	0.047677
6490	Amp	5	100226861	100226861	0	0.025103
6491	Amp	5	100227386	100364227	0	0.024262
6492	Amp	5	100367918	101383089	0	0.019997
6493	Amp	5	101423699	102682681	0	0.026209
6494	Amp	5	102700073	102867680	0	0.038142
6495	Amp	5	102874334	103230711	0	0.026209
6496	Amp	5	103230737	104161411	0	0.036834
6497	Amp	5	104168143	104357324	0	0.042461
6498	Amp	5	104357435	105546273	0	0.036834
6499	Amp	5	105549969	105564758	0	0.043266
6500	Amp	5	105565075	105765412	0	0.045295
6501	Amp	5	105776417	106109871	0	0.038863
6502	Amp	5	106114905	106235753	0	0.025656
6503	Amp	5	106236388	106456498	0	0.023627
6504	Amp	5	106458750	107006981	0	0.036118
6505	Amp	5	107011281	107209613	0	0.023627
6506	Amp	5	107219206	107281487	0	0.046568
6507	Amp	5	107282407	107327036	0	0.023627
6508	Amp	5	107327277	107478389	0	0.047001
6509	Amp	5	107478732	107546480	0	0.026231
6510	Amp	5	107551474	107753009	0	0.03688
6511	Amp	5	107754683	107841867	0	0.049784
6512	Amp	5	107841938	107892598	0	0.056821
6513	Amp	5	107893061	107925421	0	0.049784
6514	Amp	5	107927879	107997701	0	0.03688
6515	Amp	5	108000127	108034390	0	0.023627
6516	Amp	5	108035066	108299198	0	0.036663
6517	Amp	5	108305244	108310470	0	0.042043
6518	Amp	5	108317360	108335411	0	0.055028

6519	Amp	5	108335635	109164723	0	0.042043
6520	Amp	5	109164754	109349975	0	0.045948
6521	Amp	5	109356542	111080988	0	0.042043
6522	Amp	5	111119258	111682572	0	0.031419
6523	Amp	5	111684333	112023105	0	0.032894
6524	Amp	5	112025568	112423688	0	0.039001
6525	Amp	5	112425612	112752596	0	0.025965
6526	Amp	5	112752754	112902603	0	0.029583
6527	Amp	5	112903282	112920609	0	0.025965
6528	Amp	5	112924706	113024261	0	0.031864
6529	Amp	5	113027999	113072480	0	0.034315
6530	Amp	5	113080932	113088640	0	0.028936
6531	Amp	5	113089161	113171196	0	0.023037
6532	Amp	5	113171349	113235033	0	0.020586
6533	Amp	5	113236877	113343676	0	0.014479
6534	Amp	5	113351629	113914656	0	0.018771
6535	Amp	5	113915101	114562504	0	0.024951
6536	Amp	5	114645562	114804997	0	0.042722
6537	Amp	5	114812579	114890926	0	0.03143
6538	Amp	5	114895802	114912690	0	0.024951
6539	Amp	5	114915032	115293971	0	0.020659
6540	Amp	5	115300227	115401428	0	0.027681
6541	Amp	5	115402432	115542197	0	0.033859
6542	Amp	5	115542223	116116195	0	0.027681
6543	Amp	5	116116448	117787378	0	0.021501
6544	Amp	5	117788221	118355543	0	0.028713
6545	Amp	5	118355632	119502865	0	0.027238
6546	Amp	5	119503060	120127129	0	0.020025
6547	Amp	5	120137291	120187239	0	0.026372
6548	Amp	5	120187906	120474353	0	0.020025
6549	Amp	5	120478617	121247860	0	0.021945
6550	Amp	5	121248369	121373928	0	0.02741
6551	Amp	5	121375494	121414691	0	0.031104
6552	Amp	5	121416370	122162427	0	0.025638
6553	Amp	5	122162972	122403491	0	0.031924
6554	Amp	5	122405214	123518697	0	0.033917
6555	Amp	5	123524930	123569466	0	0.037499
6556	Amp	5	123573651	123681444	0	0.039669
6557	Amp	5	123681565	123946703	0	0.041066
6558	Amp	5	123950492	124138623	0	0.05078
6559	Amp	5	124144932	124313473	0	0.041066
6560	Amp	5	124316605	124472850	0	0.061503
6561	Amp	5	124473708	124710270	0	0.055869
6562	Amp	5	124710599	125739513	0	0.051966
6563	Amp	5	125743496	125813665	0	0.036086
6564	Amp	5	125819016	126462581	0	0.029801
6565	Amp	5	126464141	126553411	0	0.039693
6566	Amp	5	126554803	126896334	0	0.029801
6567	Amp	5	126900108	127270746	0	0.03545
6568	Amp	5	127270973	127293079	0	0.039998
6569	Amp	5	127293322	127535892	0	0.04456
6570	Amp	5	127538856	127613531	0	0.049429
6571	Amp	5	127614305	127679990	0	0.051404

6572 Amp	5	127684873	127753414	0	0.068056
6573 Amp	5	127755007	127883613	0	0.070269
6574 Amp	5	127886013	127898882	0	0.064621
6575 Amp	5	127899252	127900528	0	0.063012
6576 Amp	5	127902825	127949460	0	0.04636
6577 Amp	5	127953370	128038944	0	0.041811
6578 Amp	5	128041339	128056300	0	0.039598
6579 Amp	5	128062217	128206978	0	0.036338
6580 Amp	5	128207930	128932817	0	0.047319
6581 Amp	5	128933454	129578847	0	0.043156
6582 Amp	5	129588590	129730428	0	0.045172
6583 Amp	5	129734172	129832370	0	0.034191
6584 Amp	5	129836938	130765715	0	0.045585
6585 Amp	5	130770986	130782221	0	0.04361
6586 Amp	5	130782432	130849687	0	0.041444
6587 Amp	5	130851247	130999467	0	0.039525
6588 Amp	5	130999675	131059524	0	0.036704
6589 Amp	5	131062866	131985814	0	0.034688
6590 Amp	5	131986333	132471932	0	0.027665
6591 Amp	5	132474927	132485328	0	0.042855
6592 Amp	5	132485554	132584067	0	0.036642
6593 Amp	5	132588722	132831850	0	0.043639
6594 Amp	5	132836356	132883095	0	0.053845
6595 Amp	5	132886714	132902447	0	0.043639
6596 Amp	5	132909094	133029587	0	0.035066
6597 Amp	5	133029699	133256080	0	0.036909
6598 Amp	5	133259317	133315558	0	0.041647
6599 Amp	5	133316840	133654632	0	0.036909
6600 Amp	5	133656510	133843097	0	0.029913
6601 Amp	5	133845502	134215754	0	0.028069
6602 Amp	5	134216870	134306164	0	0.036398
6603 Amp	5	134306600	134461889	0	0.028069
6604 Amp	5	134468250	134816292	0	0.012879
6605 Amp	5	134816311	134849625	0	0.017353
6606 Amp	5	134850013	135228998	0	0.019405
6607 Amp	5	135230958	135291463	0	0.033453
6608 Amp	5	135295677	135316880	0	0.035632
6609 Amp	5	135317306	135413325	0	0.037941
6610 Amp	5	135419354	135537760	0	0.044999
6611 Amp	5	135539676	135551743	0	0.056066
6612 Amp	5	135557749	135968068	0	0.049276
6613 Amp	5	135973648	136310517	0	0.038209
6614 Amp	5	136314610	136316534	0	0.0359
6615 Amp	5	136316635	136532940	0	0.039835
6616 Amp	5	136536839	136978231	0	0.032778
6617 Amp	5	136983050	137072688	0	0.018729
6618 Amp	5	137073598	137202069	0	0.023946
6619 Amp	5	137202859	137284535	0	0.029278
6620 Amp	5	137284893	137413879	0	0.024061
6621 Amp	5	137415718	137661163	0	0.028586
6622 Amp	5	137663996	137705637	0	0.035182
6623 Amp	5	137708165	138109311	0	0.038201
6624 Amp	5	138112277	138150737	0	0.036149

6625 Amp	5	138152758	138152758	0	0.04082
6626 Amp	5	138154458	138254108	0	0.045442
6627 Amp	5	138257088	138257088	0	0.038846
6628 Amp	5	138258741	138335337	0	0.034175
6629 Amp	5	138342368	138491221	0	0.047731
6630 Amp	5	138491236	138634163	0	0.043206
6631 Amp	5	138641257	138950265	0	0.046091
6632 Amp	5	138959143	139016670	0	0.040904
6633 Amp	5	139017302	139059957	0	0.03643
6634 Amp	5	139070305	139088748	0	0.085971
6635 Amp	5	139095207	139257505	0	0.083085
6636 Amp	5	139259943	139599279	0	0.06616
6637 Amp	5	139600533	139606096	0	0.073716
6638 Amp	5	139606109	139640337	0	0.078333
6639 Amp	5	139642570	139684143	0	0.082074
6640 Amp	5	139685565	139717868	0	0.049459
6641 Amp	5	139724296	139763925	0	0.083287
6642 Amp	5	139766162	139770667	0	0.079546
6643 Amp	5	139779376	139865501	0	0.095258
6644 Amp	5	139870659	139897278	0	0.090641
6645 Amp	5	139898723	139900004	0	0.100837
6646 Amp	5	139900240	140081305	0	0.081969
6647 Amp	5	140081866	140131696	0	0.085268
6648 Amp	5	140135379	140216967	0	0.061391
6649 Amp	5	140222615	140226294	0	0.084887
6650 Amp	5	140230237	140355756	0	0.088855
6651 Amp	5	140356965	140502115	0	0.086422
6652 Amp	5	140504828	140526485	0	0.052831
6653 Amp	5	140526853	140540726	0	0.084688
6654 Amp	5	140542780	140570553	0	0.082509
6655 Amp	5	140590116	140597766	0	0.050652
6656 Amp	5	140603613	140723434	0	0.046716
6657 Amp	5	140724838	141051518	0	0.056061
6658 Amp	5	141054186	141098348	0	0.0602
6659 Amp	5	141098721	141510365	0	0.050855
6660 Amp	5	141517109	141687733	0	0.046716
6661 Amp	5	141687771	141687977	0	0.048589
6662 Amp	5	141688001	141779671	0	0.052701
6663 Amp	5	141781210	141781210	0	0.048733
6664 Amp	5	141781316	141783840	0	0.04686
6665 Amp	5	141787128	142234499	0	0.029257
6666 Amp	5	142237060	142438189	0	0.03674
6667 Amp	5	142438722	142922992	0	0.043823
6668 Amp	5	142924620	143000346	0	0.046086
6669 Amp	5	143002109	143036333	0	0.039003
6670 Amp	5	143036509	143154653	0	0.045049
6671 Amp	5	143155675	143253802	0	0.049788
6672 Amp	5	143256891	143530828	0	0.058052
6673 Amp	5	143537167	143711629	0	0.052006
6674 Amp	5	143711695	143813893	0	0.053389
6675 Amp	5	143817110	143851820	0	0.098107
6676 Amp	5	143858655	143942327	0	0.053389
6677 Amp	5	143944733	144082443	0	0.05613

6678	Amp	5	144083664	144210386	0	0.058024
6679	Amp	5	144211221	144436497	0	0.055282
6680	Amp	5	144437293	145729655	0	0.047018
6681	Amp	5	145730002	145930968	0	0.042279
6682	Amp	5	145932770	146703798	0	0.037657
6683	Amp	5	146704019	146780137	0	0.039747
6684	Amp	5	146782987	146786089	0	0.037485
6685	Amp	5	146788192	146892962	0	0.044894
6686	Amp	5	146893466	146913481	0	0.037485
6687	Amp	5	146913945	146972330	0	0.041988
6688	Amp	5	146979883	147221232	0	0.051568
6689	Amp	5	147226149	147236818	0	0.041988
6690	Amp	5	147236829	147422610	0	0.044341
6691	Amp	5	147423051	147594238	0	0.049572
6692	Amp	5	147599406	147745645	0	0.05846
6693	Amp	5	147749049	147804160	0	0.066839
6694	Amp	5	147804871	147924892	0	0.05846
6695	Amp	5	147930801	148107228	0	0.062248
6696	Amp	5	148107956	148380713	0	0.05846
6697	Amp	5	148383034	148740476	0	0.056107
6698	Amp	5	148740621	149256533	0	0.054724
6699	Amp	5	149258075	149296547	0	0.053014
6700	Amp	5	149300634	149303524	0	0.05647
6701	Amp	5	149303962	149336433	0	0.059481
6702	Amp	5	149337506	149579324	0	0.064688
6703	Amp	5	149585314	149629458	0	0.061231
6704	Amp	5	149630834	149637241	0	0.058221
6705	Amp	5	149640603	149673755	0	0.053014
6706	Amp	5	149676673	149723097	0	0.047783
6707	Amp	5	149723244	149829205	0	0.051987
6708	Amp	5	149829325	149851224	0	0.047783
6709	Amp	5	149851359	149888174	0	0.06159
6710	Amp	5	149895399	150104105	0	0.047783
6711	Amp	5	150104198	150203097	0	0.045889
6712	Amp	5	150203354	150343214	0	0.056856
6713	Amp	5	150344037	150759994	0	0.045889
6714	Amp	5	150760578	151409490	0	0.053997
6715	Amp	5	151409503	151533577	0	0.051906
6716	Amp	5	151534391	151729220	0	0.053413
6717	Amp	5	151729744	151840950	0	0.057165
6718	Amp	5	151842260	152134366	0	0.053413
6719	Amp	5	152140936	152730402	0	0.04593
6720	Amp	5	152731654	152983974	0	0.049115
6721	Amp	5	152985823	153224506	0	0.04593
6722	Amp	5	153225505	154051429	0	0.054381
6723	Amp	5	154054718	154348377	0	0.058761
6724	Amp	5	154348905	154413500	0	0.062048
6725	Amp	5	154417327	154901578	0	0.06697
6726	Amp	5	154902552	155063190	0	0.063683
6727	Amp	5	155065028	155383967	0	0.055232
6728	Amp	5	155385800	155526424	0	0.050852
6729	Amp	5	155532282	155548720	0	0.07352
6730	Amp	5	155549546	155807260	0	0.050852

6731	Amp	5	155811555	156734334	0	0.05867
6732	Amp	5	156735848	156797846	0	0.065717
6733	Amp	5	156798148	156803572	0	0.068786
6734	Amp	5	156812529	156890786	0	0.060968
6735	Amp	5	156892064	156958697	0	0.059357
6736	Amp	5	156963969	156993692	0	0.056288
6737	Amp	5	157000282	157065427	0	0.04924
6738	Amp	5	157066255	157677296	0	0.047734
6739	Amp	5	157678180	157726610	0	0.060284
6740	Amp	5	157727510	158180423	0	0.071308
6741	Amp	5	158187568	158400203	0	0.068468
6742	Amp	5	158401781	158416434	0	0.079077
6743	Amp	5	158418715	158547882	0	0.087065
6744	Amp	5	158552637	158568795	0	0.095003
6745	Amp	5	158575621	158896218	0	0.087065
6746	Amp	5	158896906	158936449	0	0.090635
6747	Amp	5	158936503	158939255	0	0.082647
6748	Amp	5	158948528	159150280	0	0.072038
6749	Amp	5	159152647	159183401	0	0.084684
6750	Amp	5	159183847	159694179	0	0.068468
6751	Amp	5	159696851	159819606	0	0.093747
6752	Amp	5	159821007	159857720	0	0.058758
6753	Amp	5	159857786	160140471	0	0.071168
6754	Amp	5	160146039	160392271	0	0.058758
6755	Amp	5	160392362	160430521	0	0.055966
6756	Amp	5	160430757	161068024	0	0.062217
6757	Amp	5	161068684	161153087	0	0.069605
6758	Amp	5	161156160	161225434	0	0.062217
6759	Amp	5	161226160	162062324	0	0.063673
6760	Amp	5	162071731	162832835	0	0.067027
6761	Amp	5	162835802	163684846	0	0.071299
6762	Amp	5	163685190	163737266	0	0.074399
6763	Amp	5	163738461	163906013	0	0.071045
6764	Amp	5	163908014	164636227	0	0.073274
6765	Amp	5	164637044	164803841	0	0.079683
6766	Amp	5	164806184	164845226	0	0.077454
6767	Amp	5	164845397	164874655	0	0.071045
6768	Amp	5	164887786	164947233	0	0.075762
6769	Amp	5	164953449	165169527	0	0.077557
6770	Amp	5	165170104	165188738	0	0.075762
6771	Amp	5	165194212	165342621	0	0.126285
6772	Amp	5	165343217	166147369	0	0.139569
6773	Amp	5	166148760	166157512	0	0.131161
6774	Amp	5	166160057	166496642	0	0.108622
6775	Amp	5	166500997	166614619	0	0.112323
6776	Amp	5	166615806	166726351	0	0.114103
6777	Amp	5	166726769	166868304	0	0.108348
6778	Amp	5	166871441	166916372	0	0.111851
6779	Amp	5	166918972	166940065	0	0.107134
6780	Amp	5	166940735	167034158	0	0.126071
6781	Amp	5	167038003	167124983	0	0.106495
6782	Amp	5	167129061	167641164	0	0.108413
6783	Amp	5	167643644	167717755	0	0.106495

6784	Amp	5	167718812	167726317	0	0.110512
6785	Amp	5	167727622	167729228	0	0.106495
6786	Amp	5	167730030	167750798	0	0.105039
6787	Amp	5	167751920	167869435	0	0.10676
6788	Amp	5	167874287	167940052	0	0.105039
6789	Amp	5	167943258	168091839	0	0.106661
6790	Amp	5	168091973	169236446	0	0.113467
6791	Amp	5	169240024	169654009	0	0.117862
6792	Amp	5	169655359	169995331	0	0.111833
6793	Amp	5	169998226	170487460	0	0.119525
6794	Amp	5	170487960	170539665	0	0.114734
6795	Amp	5	170547480	170718580	0	0.119268
6796	Amp	5	170719814	170870104	0	0.124541
6797	Amp	5	170878599	171153148	0	0.121038
6798	Amp	5	171156565	171436132	0	0.141131
6799	Amp	5	171436505	171654169	0	0.142656
6800	Amp	5	171654902	171773616	0	0.145956
6801	Amp	5	171773993	172383084	0	0.14151
6802	Amp	5	172385348	172385348	0.028398	0.191925
6803	Amp	5	172393048	172410457	0.086298	0.198786
6804	Amp	5	172412030	172426786	0	0.14978
6805	Amp	5	172427359	172440991	0	0.177476
6806	Amp	5	172441989	172484407	0.007199	0.189156
6807	Amp	5	172488315	172497285	0	0.173475
6808	Amp	5	172501424	172535562	0	0.14837
6809	Amp	5	172541418	172569192	0	0.181988
6810	Amp	5	172569251	172607576	0	0.186105
6811	Amp	5	172608401	172629354	0	0.174573
6812	Amp	5	172632416	172744542	0	0.179653
6813	Amp	5	172746375	172753966	0.02372	0.191665
6814	Amp	5	172762223	172776665	0.223025	0.213094
6815	Amp	5	172777997	172791096	0.168144	0.207821
6816	Amp	5	172791377	172819507	0.340434	0.22458
6817	Amp	5	172819569	172836610	0.540744	0.242129
6818	Amp	5	172841053	172920820	0.038273	0.193534
6819	Amp	5	172921591	172939152	0.362221	0.226102
6820	Amp	5	172939812	172985671	0.375927	0.227707
6821	Amp	5	172998252	173070863	0.056238	0.195139
6822	Amp	5	173071650	173146882	0	0.17759
6823	Amp	5	173148730	173208137	0.175335	0.208394
6824	Amp	5	173208380	173298415	0.25804	0.216559
6825	Amp	5	173305124	173321210	0.286675	0.219433
6826	Amp	5	173321736	173360612	0.362221	0.226342
6827	Amp	5	173361632	173371739	0.460107	0.235281
6828	Amp	5	173372134	173429880	0.693887	0.254893
6829	Amp	5	173430083	173443389	0.778204	0.262177
6830	Amp	5	173443453	173459302	0.604912	0.247495
6831	Amp	5	173464882	173466120	0.702615	0.256044
6832	Amp	5	173467493	173536009	0.644689	0.250849
6833	Amp	5	173537945	173560280	0.209057	0.212215
6834	Amp	5	173560556	173580646	0.149443	0.205825
6835	Amp	5	173580833	173592000	0.09318	0.199561
6836	Amp	5	173592830	173592830	0.195309	0.21034

6837 Amp	5	173593992	173615670	0.111991	0.201401
6838 Amp	5	173617535	173671510	0	0.187443
6839 Amp	5	173674684	173680756	0	0.179947
6840 Amp	5	173686158	173980491	0	0.177073
6841 Amp	5	173982950	173983915	0	0.175548
6842 Amp	5	173985813	174136865	0	0.173943
6843 Amp	5	174139713	174269692	0	0.175496
6844 Amp	5	174271027	174284500	0	0.172265
6845 Amp	5	174284721	174348604	0	0.173828
6846 Amp	5	174349996	174445870	0	0.178525
6847 Amp	5	174447198	174447983	0	0.16413
6848 Amp	5	174448604	174487123	0	0.159596
6849 Amp	5	174493280	174623803	0	0.154899
6850 Amp	5	174624114	174643063	0	0.153336
6851 Amp	5	174643080	174774167	0	0.157187
6852 Amp	5	174779524	175073101	0	0.171022
6853 Amp	5	175077326	175158435	0	0.137424
6854 Amp	5	175161308	175276284	0	0.130563
6855 Amp	5	175278566	175306349	0	0.159592
6856 Amp	5	175306625	175435417	0	0.154381
6857 Amp	5	175435667	175439877	0	0.147961
6858 Amp	5	175441626	175531740	0	0.14263
6859 Amp	5	175536772	175541576	0	0.141076
6860 Amp	5	175542130	175570393	0	0.147136
6861 Amp	5	175570679	175902239	0	0.150204
6862 Amp	5	175905543	175916541	0	0.136621
6863 Amp	5	175919594	175933010	0	0.138609
6864 Amp	5	175935472	176241574	0	0.134757
6865 Amp	5	176245027	176245097	0	0.113252
6866 Amp	5	176246312	176533675	0	0.111264
6867 Amp	5	176533850	176932575	0	0.117748
6868 Amp	5	176936096	177057788	0	0.157212
6869 Amp	5	177061670	177148730	0	0.16472
6870 Amp	5	177167117	177272327	0	0.16855
6871 Amp	5	177273016	177444685	0	0.172988
6872 Amp	5	177445493	177507024	0	0.154114
6873 Amp	5	177510760	177530671	0	0.146607
6874 Amp	5	177534746	177600860	0	0.156567
6875 Amp	5	177602292	177627167	0	0.150297
6876 Amp	5	177631377	178050723	0	0.14723
6877 Amp	5	178054262	178084432	0	0.14987
6878 Amp	5	178087839	178161018	0	0.164051
6879 Amp	5	178170941	178225391	0	0.165894
6880 Amp	5	178275580	178370335	0	0.17076
6881 Amp	5	178370533	178370533	0	0.145731
6882 Amp	5	178371335	178380351	0	0.143091
6883 Amp	5	178381187	179088195	0	0.138225
6884 Amp	5	179089053	179113655	0	0.131741
6885 Amp	5	179117083	179237462	0	0.136019
6886 Amp	5	179237804	179289388	0	0.139767
6887 Amp	5	179293967	179362755	0	0.136988
6888 Amp	5	179363850	179516344	0	0.135145
6889 Amp	5	179520653	179624883	0	0.133646

6890	Amp	5	179625153	179681642	0	0.125911
6891	Amp	5	179683849	179839475	0	0.133951
6892	Amp	5	179840783	180016133	0	0.145087
6893	Amp	5	180016190	180496366	0	0.150499
6894	Amp	5	180497384	180586575	0	0.139831
6895	Amp	5	180588141	180799516	0	0.14602
6896	Amp	5	180799571	180828963	0	0.139831
6897	Amp	5	180829003	180879768	0	0.123072
6898	Amp	5	180895735	180934240	0	0.120676
6899	Amp	6	1011760	1057247	0	0.153043
6900	Amp	6	1057755	1066495	0	0.10277
6901	Amp	6	1067049	1232697	0	0.094308
6902	Amp	6	1236271	1304656	0	0.083505
6903	Amp	6	1305137	1455588	0	0.07918
6904	Amp	6	1459797	1494722	0	0.089174
6905	Amp	6	1495163	1628667	0	0.077953
6906	Amp	6	1629623	1727776	0	0.099574
6907	Amp	6	1729358	1875649	0	0.082231
6908	Amp	6	1877724	1881857	0	0.087194
6909	Amp	6	1882213	2176790	0	0.09119
6910	Amp	6	2179044	2369841	0	0.069569
6911	Amp	6	2375914	2502104	0	0.065802
6912	Amp	6	2502909	2522218	0	0.071396
6913	Amp	6	2523769	2526530	0	0.078698
6914	Amp	6	2528994	2571967	0	0.065802
6915	Amp	6	2575349	2642147	0	0.076951
6916	Amp	6	2642854	2813499	0	0.065802
6917	Amp	6	2814008	2883687	0	0.076194
6918	Amp	6	2889261	2976574	0	0.082099
6919	Amp	6	2978845	3245618	0	0.071707
6920	Amp	6	3251515	3307629	0	0.064611
6921	Amp	6	3308009	3500605	0	0.076122
6922	Amp	6	3502644	3580001	0	0.089293
6923	Amp	6	3587239	3626872	0	0.107249
6924	Amp	6	3627389	3659898	0	0.078588
6925	Amp	6	3663547	3672972	0	0.089446
6926	Amp	6	3683923	3685429	0	0.100138
6927	Amp	6	3685469	3740073	0	0.093387
6928	Amp	6	3745003	3755809	0	0.082695
6929	Amp	6	3755989	3899439	0	0.071185
6930	Amp	6	3899646	3920483	0	0.068287
6931	Amp	6	3921369	4011678	0	0.074261
6932	Amp	6	4012151	4053122	0	0.079548
6933	Amp	6	4053138	4138009	0	0.089405
6934	Amp	6	4138358	4253179	0	0.077288
6935	Amp	6	4498631	4660324	0	0.105988
6936	Amp	6	4664028	4700523	0	0.11859
6937	Amp	6	4700896	4725875	0	0.105988
6938	Amp	6	4725974	4731342	0	0.114549
6939	Amp	6	4735881	4943169	0	0.107239
6940	Amp	6	4946122	4964355	0	0.097101
6941	Amp	6	4964657	5035961	0	0.102657
6942	Amp	6	5036433	5084176	0	0.109416

6943	Amp	6	5097862	5203718	0	0.10386
6944	Amp	6	5206027	5270217	0	0.100189
6945	Amp	6	5272401	5324909	0	0.105656
6946	Amp	6	5392145	5427041	0	0.066098
6947	Amp	6	5430123	5475717	0	0.059339
6948	Amp	6	5478841	5481625	0	0.055806
6949	Amp	6	5482270	5506608	0	0.05988
6950	Amp	6	5506949	5510681	0	0.055806
6951	Amp	6	5512561	5556890	0	0.064316
6952	Amp	6	5557710	5759478	0	0.05211
6953	Amp	6	5762340	5783935	0	0.059878
6954	Amp	6	5788144	5918913	0	0.067279
6955	Amp	6	5919053	6274021	0	0.055963
6956	Amp	6	6274337	6502893	0	0.050883
6957	Amp	6	6504083	6513890	0	0.068483
6958	Amp	6	6514739	6560309	0	0.080035
6959	Amp	6	6560499	6655660	0	0.085433
6960	Amp	6	6655916	6753541	0	0.083394
6961	Amp	6	6753875	6753875	0	0.092254
6962	Amp	6	6754814	6853208	0	0.114885
6963	Amp	6	6854276	6879523	0	0.109487
6964	Amp	6	6882292	7032138	0	0.100627
6965	Amp	6	7033433	7118757	0	0.116544
6966	Amp	6	7122059	7144822	0	0.125421
6967	Amp	6	7147131	7162337	0	0.116544
6968	Amp	6	7165762	7247979	0	0.117978
6969	Amp	6	7248226	7293563	0	0.124842
6970	Amp	6	7299474	7725824	0	0.108926
6971	Amp	6	7728195	7730711	0	0.103354
6972	Amp	6	7738698	7773736	0	0.119959
6973	Amp	6	7775448	7820436	0	0.149737
6974	Amp	6	7823869	7865985	0	0.134106
6975	Amp	6	7866194	7919491	0	0.141715
6976	Amp	6	7921871	8001045	0	0.132151
6977	Amp	6	8001084	8143212	0	0.124878
6978	Amp	6	8146245	8258002	0	0.102247
6979	Amp	6	8258316	8361158	0	0.104515
6980	Amp	6	8361331	8362752	0	0.106675
6981	Amp	6	8364200	8431006	0	0.112045
6982	Amp	6	8435650	8483905	0	0.115862
6983	Amp	6	8484396	8484396	0	0.119709
6984	Amp	6	8484421	8494895	0	0.121165
6985	Amp	6	8495602	8525004	0	0.114301
6986	Amp	6	8530345	8592266	0	0.116321
6987	Amp	6	8592345	8594386	0	0.126194
6988	Amp	6	8598045	8633973	0	0.129964
6989	Amp	6	8635339	8653014	0	0.124595
6990	Amp	6	8658021	8734660	0	0.110529
6991	Amp	6	8736289	9003005	0	0.116521
6992	Amp	6	9003024	9031352	0	0.112702
6993	Amp	6	9031818	9066401	0	0.1141
6994	Amp	6	9068395	9286857	0	0.115982
6995	Amp	6	9288913	9294061	0	0.14103

6996 Amp	6	9296635	9358180	0	0.147462
6997 Amp	6	9358532	9449627	0	0.149555
6998 Amp	6	9452722	9482936	0	0.158349
6999 Amp	6	9484230	9524042	0	0.162382
7000 Amp	6	9524471	9537501	0	0.153589
7001 Amp	6	9538159	9544473	0	0.151496
7002 Amp	6	9548113	9631843	0	0.155526
7003 Amp	6	9641961	9693600	0	0.161315
7004 Amp	6	9694337	9729512	0	0.155778
7005 Amp	6	9735823	9794829	0	0.126734
7006 Amp	6	9795649	9821897	0	0.132527
7007 Amp	6	9821924	9865647	0	0.130645
7008 Amp	6	9868060	9983631	0	0.128377
7009 Amp	6	9986637	10018401	0	0.134925
7010 Amp	6	10018521	10047711	0	0.136633
7011 Amp	6	10048189	10099745	0	0.146213
7012 Amp	6	10105849	10106386	0	0.140745
7013 Amp	6	10108958	10128038	0	0.134953
7014 Amp	6	10128120	10149946	0	0.133518
7015 Amp	6	10150529	10151076	0	0.132121
7016 Amp	6	10151122	10206489	0	0.130664
7017 Amp	6	10206727	10232511	0	0.128644
7018 Amp	6	10235681	10253380	0	0.126936
7019 Amp	6	10254061	10263630	0	0.124762
7020 Amp	6	10266122	10375162	0	0.138124
7021 Amp	6	10386669	10451874	0	0.145901
7022 Amp	6	10456473	10546167	0	0.14316
7023 Amp	6	10742788	10828737	0	0.13939
7024 Amp	6	10829324	10872237	0	0.145527
7025 Amp	6	10874909	10879939	0	0.155678
7026 Amp	6	10880817	10895851	0	0.142317
7027 Amp	6	10897255	10946949	0	0.132166
7028 Amp	6	10953894	10978007	0	0.117216
7029 Amp	6	10979409	11029570	0	0.131545
7030 Amp	6	11030874	11085211	0	0.143265
7031 Amp	6	11089827	11138285	0	0.135438
7032 Amp	6	11145712	11202971	0	0.182652
7033 Amp	6	11203673	11359930	0	0.186061
7034 Amp	6	11364292	11418494	0	0.174341
7035 Amp	6	11424277	11459360	0	0.170524
7036 Amp	6	11460532	11519800	0	0.1747
7037 Amp	6	11523653	11570973	0	0.169736
7038 Amp	6	11571512	11615237	0	0.165561
7039 Amp	6	11615283	11774393	0	0.155728
7040 Amp	6	11774511	11852418	0	0.157463
7041 Amp	6	11852605	11951628	0	0.153466
7042 Amp	6	11951839	11983682	0	0.139024
7043 Amp	6	11983809	11989648	0	0.137231
7044 Amp	6	11991561	12039856	0	0.104431
7045 Amp	6	12044118	12048023	0	0.093787
7046 Amp	6	12050800	12075696	0	0.109493
7047 Amp	6	12078453	12089994	0	0.11784
7048 Amp	6	12098908	12147381	0	0.107966

7049	Amp	6	12149567	12170694	0	0.113004
7050	Amp	6	12173083	12432128	0	0.117812
7051	Amp	6	12435941	12719160	0	0.164079
7052	Amp	6	12719310	12923096	0	0.161919
7053	Amp	6	12924146	12936350	0	0.179504
7054	Amp	6	12936456	12936456	0	0.185216
7055	Amp	6	12942834	12957593	0	0.167631
7056	Amp	6	12962927	13017697	0	0.12844
7057	Amp	6	13021029	13081213	0	0.117954
7058	Amp	6	13084822	13085103	0	0.123706
7059	Amp	6	13085650	13099357	0	0.117569
7060	Amp	6	13099869	13285180	0	0.1341
7061	Amp	6	13286158	13313857	0	0.146562
7062	Amp	6	13314012	13321624	0	0.142473
7063	Amp	6	13326498	13350892	0	0.130011
7064	Amp	6	13358754	13519140	0	0.12426
7065	Amp	6	13521591	13585174	0	0.139104
7066	Amp	6	13589641	13621923	0	0.149147
7067	Amp	6	13625181	13678685	0	0.143254
7068	Amp	6	13688757	13693706	0	0.145743
7069	Amp	6	13694561	13716535	0	0.154945
7070	Amp	6	13719254	13720709	0	0.139239
7071	Amp	6	13722291	13727607	0	0.130507
7072	Amp	6	13732518	13753783	0	0.149564
7073	Amp	6	13754119	13767484	0	0.130507
7074	Amp	6	13773577	13931728	0	0.123487
7075	Amp	6	13937849	13944836	0	0.114833
7076	Amp	6	13944956	13976489	0	0.129155
7077	Amp	6	13978623	14074128	0	0.114833
7078	Amp	6	14078130	14264311	0	0.126791
7079	Amp	6	14265128	14497792	0	0.125402
7080	Amp	6	14499098	14604902	0	0.120593
7081	Amp	6	14605476	14864346	0	0.116563
7082	Amp	6	14864554	14887815	0	0.121059
7083	Amp	6	14888039	14898879	0	0.11691
7084	Amp	6	14899254	14925778	0	0.114421
7085	Amp	6	14932776	14978906	0	0.128799
7086	Amp	6	14979949	15049509	0	0.133373
7087	Amp	6	15050435	15177055	0	0.14276
7088	Amp	6	15178691	15191424	0	0.135724
7089	Amp	6	15197756	15249369	0	0.131149
7090	Amp	6	15249821	15358413	0	0.135081
7091	Amp	6	15536578	15536578	0	0.119692
7092	Amp	6	15537094	15727772	0	0.112084
7093	Amp	6	15730039	15731116	0	0.11385
7094	Amp	6	15734946	15850946	0	0.124751
7095	Amp	6	15851242	15883458	0	0.118319
7096	Amp	6	15884133	15909537	0	0.129319
7097	Amp	6	15912791	15999162	0	0.118319
7098	Amp	6	16002423	16007704	0	0.147733
7099	Amp	6	16007749	16023742	0	0.13581
7100	Amp	6	16023814	16075695	0	0.126423
7101	Amp	6	16076290	16162277	0	0.11268

7102	Amp	6	16164319	16225631	0	0.106504
7103	Amp	6	16244154	16452260	0	0.090833
7104	Amp	6	16452915	16758471	0	0.077128
7105	Amp	6	16758908	16758908	0	0.07852
7106	Amp	6	16771384	16780639	0	0.086682
7107	Amp	6	16784857	16859991	0	0.07852
7108	Amp	6	16862754	16871918	0	0.084389
7109	Amp	6	16872001	16872001	0	0.082622
7110	Amp	6	16875433	16877413	0	0.087407
7111	Amp	6	16881726	16949988	0	0.086015
7112	Amp	6	16958280	16979550	0	0.080874
7113	Amp	6	16984228	17010519	0	0.088431
7114	Amp	6	17011099	17021237	0	0.082645
7115	Amp	6	17021805	17098356	0	0.073673
7116	Amp	6	17108555	17108555	0	0.068888
7117	Amp	6	17109116	17260456	0	0.072638
7118	Amp	6	17260995	17276290	0	0.085127
7119	Amp	6	17284151	17432142	0	0.072638
7120	Amp	6	17441828	17448494	0	0.084223
7121	Amp	6	17451062	17466860	0	0.072638
7122	Amp	6	17468412	17475232	0	0.066878
7123	Amp	6	17476230	17512815	0	0.06452
7124	Amp	6	17515773	17744098	0	0.115043
7125	Amp	6	17744360	17816767	0	0.129894
7126	Amp	6	17816783	17944003	0	0.12394
7127	Amp	6	17947183	18031266	0	0.073417
7128	Amp	6	18032963	18224670	0	0.069668
7129	Amp	6	18224729	18224984	0	0.065091
7130	Amp	6	18225023	18262983	0	0.058655
7131	Amp	6	18263021	18286379	0	0.062955
7132	Amp	6	18292792	18345196	0	0.070012
7133	Amp	6	18347035	18362746	0	0.077209
7134	Amp	6	18364231	18379195	0	0.087807
7135	Amp	6	18379707	18379791	0	0.089547
7136	Amp	6	18381922	18430356	0	0.100493
7137	Amp	6	18439664	18439664	0	0.099072
7138	Amp	6	18442086	18495752	0	0.108698
7139	Amp	6	18496969	18506520	0	0.12006
7140	Amp	6	18506533	18616259	0	0.122479
7141	Amp	6	18618504	18665149	0	0.111881
7142	Amp	6	18672987	18688228	0	0.119775
7143	Amp	6	18688252	18717954	0	0.114063
7144	Amp	6	18721208	18753292	0	0.098972
7145	Amp	6	18756787	19150319	0	0.094524
7146	Amp	6	19154580	19385858	0	0.096586
7147	Amp	6	19385916	19401583	0	0.089029
7148	Amp	6	19412501	19440067	0	0.108556
7149	Amp	6	19441264	19494515	0	0.094368
7150	Amp	6	19500945	19530564	0	0.084742
7151	Amp	6	19535284	19565612	0	0.07338
7152	Amp	6	19569032	19754926	0	0.097751
7153	Amp	6	19755503	19763422	0	0.117973
7154	Amp	6	19773322	19821095	0	0.131021

7155 Amp	6	19823007	20318412	0	0.161323
7156 Amp	6	20321575	20380268	0	0.1108
7157 Amp	6	20390852	20439394	0	0.108738
7158 Amp	6	20442123	20512142	0	0.10733
7159 Amp	6	20516833	20688240	0	0.115778
7160 Amp	6	20688503	20729135	0	0.124834
7161 Amp	6	20729829	20758042	0	0.128618
7162 Amp	6	20758116	20844510	0	0.133932
7163 Amp	6	20851604	20898372	0	0.12042
7164 Amp	6	20901184	20934227	0	0.108868
7165 Amp	6	20943217	20974149	0	0.099812
7166 Amp	6	20978267	20998988	0	0.11109
7167 Amp	6	21004656	21129863	0	0.125429
7168 Amp	6	21131922	21168137	0	0.139912
7169 Amp	6	21172978	21181170	0	0.123612
7170 Amp	6	21182747	21338378	0	0.109129
7171 Amp	6	21338612	21356553	0	0.106288
7172 Amp	6	21356997	21397179	0	0.088078
7173 Amp	6	21398774	21457719	0	0.102519
7174 Amp	6	21458593	21479689	0	0.117109
7175 Amp	6	21483444	21533170	0	0.123844
7176 Amp	6	21540198	21543383	0	0.13061
7177 Amp	6	21544616	21711260	0	0.133513
7178 Amp	6	21711737	21923739	0	0.184036
7179 Amp	6	21927680	22046226	0	0.180252
7180 Amp	6	22046528	22078270	0	0.181815
7181 Amp	6	22078967	22112415	0	0.17508
7182 Amp	6	22112891	22141533	0	0.168314
7183 Amp	6	22145608	22150832	0	0.165411
7184 Amp	6	22155191	22256152	0	0.167112
7185 Amp	6	22261340	22374723	0	0.169126
7186 Amp	6	22375419	22445426	0	0.159396
7187 Amp	6	22445704	22470022	0	0.168845
7188 Amp	6	22470923	22484575	0	0.166371
7189 Amp	6	22486435	22709633	0	0.167853
7190 Amp	6	22709812	22887415	0	0.161419
7191 Amp	6	22888400	22888400	0	0.150473
7192 Amp	6	22896114	22902790	0	0.148054
7193 Amp	6	22903102	22946965	0	0.156801
7194 Amp	6	22948997	22987823	0	0.106279
7195 Amp	6	22987858	23214398	0	0.09679
7196 Amp	6	23219774	23309584	0	0.098509
7197 Amp	6	23312875	23312875	0	0.089762
7198 Amp	6	23313104	23394937	0	0.063991
7199 Amp	6	23395161	23433800	0	0.058623
7200 Amp	6	23433815	23468012	0	0.062782
7201 Amp	6	23468930	23561120	0	0.070086
7202 Amp	6	23561206	23572705	0	0.079446
7203 Amp	6	23575838	23631311	0	0.092359
7204 Amp	6	23631885	23633642	0	0.107193
7205 Amp	6	23634891	23644682	0	0.105179
7206 Amp	6	23647806	23655001	0	0.110122
7207 Amp	6	23655063	23722616	0	0.100762

7208	Amp	6	23724568	23803057	0	0.093458
7209	Amp	6	23809850	23809850	0	0.078625
7210	Amp	6	23821354	23907997	0	0.08608
7211	Amp	6	23909153	23918812	0	0.084598
7212	Amp	6	23921810	23934415	0	0.088505
7213	Amp	6	23935352	23981238	0	0.084598
7214	Amp	6	23981537	24055445	0	0.077143
7215	Amp	6	24059923	24153363	0	0.06423
7216	Amp	6	24155365	24181261	0	0.060071
7217	Amp	6	24182968	24212035	0	0.058405
7218	Amp	6	24268525	24278602	0	0.076639
7219	Amp	6	24278641	24300659	0	0.068297
7220	Amp	6	24304925	24342144	0	0.070021
7221	Amp	6	24344423	24827640	0	0.068319
7222	Amp	6	24828331	24875692	0	0.081385
7223	Amp	6	24880910	25366994	0	0.068319
7224	Amp	6	25368707	25450258	0	0.07249
7225	Amp	6	25735847	25739416	0	0.099133
7226	Amp	6	25739659	25744650	0	0.101244
7227	Amp	6	25747180	25763964	0	0.113442
7228	Amp	6	25764180	25782794	0	0.12075
7229	Amp	6	25784253	25847146	0	0.108552
7230	Amp	6	25847453	25866841	0	0.094283
7231	Amp	6	25868324	25950169	0	0.101505
7232	Amp	6	25950414	26005233	0	0.099766
7233	Amp	6	26008032	26030264	0	0.119961
7234	Amp	6	26031385	26033565	0	0.11785
7235	Amp	6	26033600	26033600	0	0.119918
7236	Amp	6	26072168	26072286	0	0.110534
7237	Amp	6	26076280	26076940	0	0.098715
7238	Amp	6	26080586	26121046	0	0.091493
7239	Amp	6	26121741	26137207	0	0.097367
7240	Amp	6	26138150	26138150	0	0.090059
7241	Amp	6	26139082	26179933	0	0.094895
7242	Amp	6	26183813	26190100	0	0.086519
7243	Amp	6	26197579	26197579	0	0.081576
7244	Amp	6	26197818	26201692	0	0.076569
7245	Amp	6	26202671	26211120	0	0.0745
7246	Amp	6	26211167	26256298	0	0.072781
7247	Amp	6	26256338	26261790	0	0.066907
7248	Amp	6	26264494	26329625	0	0.05954
7249	Amp	6	26334598	26409623	0	0.05069
7250	Amp	6	26411988	26488185	0	0.045853
7251	Amp	6	26495899	26527524	0	0.056482
7252	Amp	6	26527717	26644748	0	0.059536
7253	Amp	6	26644778	26670022	0	0.067855
7254	Amp	6	26936980	26936980	0	0.056482
7255	Amp	6	26937049	26960338	0	0.060742
7256	Amp	6	26960438	26975280	0	0.075524
7257	Amp	6	26983428	27014901	0	0.090622
7258	Amp	6	27014916	27037247	0	0.098656
7259	Amp	6	27037280	27146273	0	0.090983
7260	Amp	6	27146582	27146582	0	0.089259

7261	Amp	6	27200479	27203932	0	0.081225
7262	Amp	6	27241863	27293592	0	0.066128
7263	Amp	6	27295098	27459178	0	0.05485
7264	Amp	6	27461212	27508616	0	0.051894
7265	Amp	6	27509320	27509320	0	0.05856
7266	Amp	6	27737819	27742386	0	0.043777
7267	Amp	6	27742470	28285082	0	0.039518
7268	Amp	6	28290810	28426794	0	0.027513
7269	Amp	6	28435826	28441867	0	0.032579
7270	Amp	6	28447141	29374459	0	0.034096
7271	Amp	6	29374998	29375048	0	0.035859
7272	Amp	6	29384007	29458426	0	0.039884
7273	Amp	6	29459360	29466637	0	0.042487
7274	Amp	6	29467578	29527120	0	0.054932
7275	Amp	6	29527503	29540496	0	0.06618
7276	Amp	6	29541921	29765965	0	0.054932
7277	Amp	6	29787674	29800928	0	0.0593
7278	Amp	6	29836041	30234178	0	0.061586
7279	Amp	6	30234794	30316598	0	0.059822
7280	Amp	6	30317344	30410419	0	0.069181
7281	Amp	6	30410670	30611538	0	0.073341
7282	Amp	6	30617994	30667816	0	0.080768
7283	Amp	6	30690462	30901916	0	0.088722
7284	Amp	6	30901937	30945681	0	0.081296
7285	Amp	6	30946775	30954105	0	0.088927
7286	Amp	6	30954793	31022713	0	0.082711
7287	Amp	6	31025285	31208825	0	0.088668
7288	Amp	6	31210138	31237840	0	0.0843
7289	Amp	6	31238631	31238631	0	0.074942
7290	Amp	6	31238839	31238839	0	0.06754
7291	Amp	6	32899215	32903759	0	0.047069
7292	Amp	6	32912768	33056877	0	0.041111
7293	Amp	6	33061660	33091097	0	0.059193
7294	Amp	6	33092651	33103545	0	0.041111
7295	Amp	6	33104762	33245978	0	0.036045
7296	Amp	6	33250403	33274715	0	0.050418
7297	Amp	6	33282699	33360741	0	0.042786
7298	Amp	6	33361745	33451026	0	0.038943
7299	Amp	6	33458062	33674816	0	0.024571
7300	Amp	6	33674905	34018380	0	0.032915
7301	Amp	6	34022120	34085889	0	0.02889
7302	Amp	6	34086129	34115787	0	0.044297
7303	Amp	6	34118449	34633334	0	0.066783
7304	Amp	6	34634898	34752016	0	0.062671
7305	Amp	6	34919539	35036979	0	0.054327
7306	Amp	6	35037042	35246253	0	0.066957
7307	Amp	6	35248963	35347550	0	0.047815
7308	Amp	6	35353927	35679646	0	0.066552
7309	Amp	6	35690550	36359718	0	0.016434
7310	Amp	6	36360550	36458677	0	0.025513
7311	Amp	6	36459499	36803913	0	0.054224
7312	Amp	6	36806969	36994250	0	0.095089
7313	Amp	6	36995596	37003235	0	0.054224

7314	Amp	6	37004539	37220718	0	0.082833
7315	Amp	6	37225923	37534809	0	0.090894
7316	Amp	6	37535093	37629185	0	0.107735
7317	Amp	6	37629353	37663766	0	0.095959
7318	Amp	6	37665248	37684954	0	0.062183
7319	Amp	6	37685660	37763107	0	0.064761
7320	Amp	6	37771451	37799278	0	0.0567
7321	Amp	6	37799727	37858849	0	0.055183
7322	Amp	6	37865827	37921468	0	0.042594
7323	Amp	6	37921843	38069109	0	0.045284
7324	Amp	6	38069741	38069741	0	0.036205
7325	Amp	6	38073829	38093103	0	0.054517
7326	Amp	6	38100850	38172882	0	0.058476
7327	Amp	6	38173546	38235046	0	0.067484
7328	Amp	6	38237439	38298770	0	0.053937
7329	Amp	6	38304056	38329775	0	0.043449
7330	Amp	6	38329914	38357050	0	0.052196
7331	Amp	6	38358492	38530098	0	0.059806
7332	Amp	6	38533453	38621390	0	0.050798
7333	Amp	6	38621513	38690363	0	0.046838
7334	Amp	6	38690440	38753226	0	0.044149
7335	Amp	6	38754410	38818365	0	0.025105
7336	Amp	6	38824388	38855129	0	0.067432
7337	Amp	6	38856534	38960468	0	0.059823
7338	Amp	6	38964880	38992477	0	0.057245
7339	Amp	6	38992777	39092986	0	0.058721
7340	Amp	6	39099091	39116577	0	0.060325
7341	Amp	6	39117183	39184681	0	0.03789
7342	Amp	6	39190738	39263877	0	0.036286
7343	Amp	6	39266716	39449521	0	0.031986
7344	Amp	6	39450801	39476370	0	0.046688
7345	Amp	6	39476495	39567447	0	0.033957
7346	Amp	6	39574643	39638859	0	0.031986
7347	Amp	6	39639355	39671232	0	0.046392
7348	Amp	6	39676421	39772843	0	0.026499
7349	Amp	6	39776878	40070661	0	0.025023
7350	Amp	6	40071292	40135568	0	0.054478
7351	Amp	6	40139064	40444434	0	0.040073
7352	Amp	6	40447998	40490520	0	0.03432
7353	Amp	6	40492763	40546653	0	0.047545
7354	Amp	6	40550670	40907126	0	0.023843
7355	Amp	6	40908942	41128410	0	0.010617
7356	Amp	6	41132970	41213730	0	0.015012
7357	Amp	6	41216121	41337716	0	0.030329
7358	Amp	6	41339856	41664053	0	0.015012
7359	Amp	6	41673751	41732652	0	0.06006
7360	Amp	6	41741200	41758822	0	0.109543
7361	Amp	6	41760960	41806992	0	0.114175
7362	Amp	6	41808714	41898342	0	0.103745
7363	Amp	6	41902320	41924968	0	0.115756
7364	Amp	6	41925071	41991820	0	0.103745
7365	Amp	6	41994084	42031387	0	0.111145
7366	Amp	6	42033993	42114071	0	0.103745

7367	Amp	6	42116408	42379622	0	0.054262
7368	Amp	6	42380088	42449491	0	0.063513
7369	Amp	6	42450457	42588046	0	0.072301
7370	Amp	6	42596322	42613260	0	0.091079
7371	Amp	6	42614518	42685980	0	0.072301
7372	Amp	6	42691461	42985475	0	0.089657
7373	Amp	6	42986754	43200379	0	0.071754
7374	Amp	6	43200426	43420316	0	0.082553
7375	Amp	6	43420336	43437787	0	0.084843
7376	Amp	6	43437927	43546984	0	0.086805
7377	Amp	6	43549425	43667658	0	0.090459
7378	Amp	6	43669326	43677170	0	0.084867
7379	Amp	6	43678664	43713017	0	0.093675
7380	Amp	6	43715253	43777370	0	0.073609
7381	Amp	6	43778673	43814382	0	0.069954
7382	Amp	6	43814836	43920200	0	0.082234
7383	Amp	6	43922205	44109619	0	0.069954
7384	Amp	6	44110920	44131246	0	0.073728
7385	Amp	6	44137568	44153274	0	0.076533
7386	Amp	6	44156928	44212987	0	0.067282
7387	Amp	6	44215021	44313979	0	0.063112
7388	Amp	6	44314147	44413584	0	0.060822
7389	Amp	6	44415393	44685473	0	0.078764
7390	Amp	6	44686634	44773555	0	0.067702
7391	Amp	6	44777405	44836513	0	0.082124
7392	Amp	6	44840629	44842280	0	0.060999
7393	Amp	6	44842512	44844322	0	0.049852
7394	Amp	6	44844720	44844720	0	0.051806
7395	Amp	6	44844828	44890652	0	0.049001
7396	Amp	6	44892930	45168610	0	0.05411
7397	Amp	6	45171472	45235317	0	0.049861
7398	Amp	6	45238546	45492824	0	0.056333
7399	Amp	6	45497174	45498016	0	0.049861
7400	Amp	6	45499289	45596741	0	0.05914
7401	Amp	6	45602126	45676138	0	0.049861
7402	Amp	6	45681882	45774888	0	0.057584
7403	Amp	6	45774929	46128634	0	0.049861
7404	Amp	6	46129040	46137760	0	0.051761
7405	Amp	6	46138668	46148113	0	0.050097
7406	Amp	6	46148595	46174815	0	0.044987
7407	Amp	6	46175973	46224620	0	0.041213
7408	Amp	6	46232726	46326540	0	0.033282
7409	Amp	6	46336265	46630545	0	0.037143
7410	Amp	6	46631106	46648880	0	0.041789
7411	Amp	6	46650875	46742542	0	0.044449
7412	Amp	6	46743287	46763708	0	0.053569
7413	Amp	6	46765332	47007685	0	0.044449
7414	Amp	6	47010639	47062083	0	0.048743
7415	Amp	6	47062254	47119204	0	0.05209
7416	Amp	6	47119216	47140399	0	0.047444
7417	Amp	6	47142034	47192378	0	0.067128
7418	Amp	6	47194845	47224509	0	0.071776
7419	Amp	6	47224702	47252969	0	0.077702

7420	Amp	6	47253661	47319050	0	0.085765
7421	Amp	6	47320033	47338865	0	0.079839
7422	Amp	6	47339591	47418263	0	0.086426
7423	Amp	6	47419017	47541673	0	0.090118
7424	Amp	6	47542020	47547243	0	0.133736
7425	Amp	6	47547523	47647197	0	0.130044
7426	Amp	6	47647889	47663886	0	0.139725
7427	Amp	6	47666191	47698859	0	0.131662
7428	Amp	6	47700549	47716429	0	0.135837
7429	Amp	6	47719063	47734635	0	0.116152
7430	Amp	6	47734832	47744540	0	0.111977
7431	Amp	6	47744577	47747357	0	0.108116
7432	Amp	6	47752103	47836700	0	0.111845
7433	Amp	6	47836908	47865865	0	0.117675
7434	Amp	6	47877415	47953822	0	0.113946
7435	Amp	6	47954900	47997134	0	0.063741
7436	Amp	6	48000715	48189662	0	0.055486
7437	Amp	6	48196773	48231627	0	0.039666
7438	Amp	6	48232374	48302696	0	0.042399
7439	Amp	6	48311728	48318875	0	0.051944
7440	Amp	6	48318919	48413916	0	0.049448
7441	Amp	6	48425662	48509130	0	0.039902
7442	Amp	6	48510552	48551578	0	0.037418
7443	Amp	6	48557420	48685839	0	0.033124
7444	Amp	6	48687135	48817058	0	0.034974
7445	Amp	6	48818431	48855720	0	0.033124
7446	Amp	6	48856126	48945818	0	0.036126
7447	Amp	6	48949115	48963228	0	0.037862
7448	Amp	6	48979553	49038400	0	0.044548
7449	Amp	6	49039771	49224338	0	0.046242
7450	Amp	6	49224781	49498689	0	0.033678
7451	Amp	6	49498973	49554276	0	0.026993
7452	Amp	6	49554780	49593272	0	0.055816
7453	Amp	6	49597675	49643328	0	0.060195
7454	Amp	6	49643640	49771126	0	0.076076
7455	Amp	6	49774023	49967022	0	0.107567
7456	Amp	6	49967644	49969792	0	0.103188
7457	Amp	6	49970628	49997330	0	0.101452
7458	Amp	6	49999731	50036493	0	0.064424
7459	Amp	6	50039952	50096646	0	0.074885
7460	Amp	6	50099361	50125936	0	0.080577
7461	Amp	6	50134027	50184519	0	0.074885
7462	Amp	6	50192230	50548564	0	0.064424
7463	Amp	6	50549040	50655651	0	0.067831
7464	Amp	6	50660887	50777033	0	0.117646
7465	Amp	6	50778303	50835337	0	0.073536
7466	Amp	6	50836636	50954880	0	0.080181
7467	Amp	6	50957618	51031239	0	0.076774
7468	Amp	6	51039323	51243764	0	0.078982
7469	Amp	6	51245963	51258411	0	0.083292
7470	Amp	6	51262238	51325322	0	0.078982
7471	Amp	6	51325485	51645194	0	0.068638
7472	Amp	6	51647512	51754646	0	0.085425

7473	Amp	6	51754784	51791590	0	0.091142
7474	Amp	6	51791701	51834606	0	0.09335
7475	Amp	6	51836124	51972369	0	0.096214
7476	Amp	6	51972709	52125300	0	0.100031
7477	Amp	6	52125482	52135916	0	0.097822
7478	Amp	6	52137593	52176206	0	0.104564
7479	Amp	6	52180940	52185555	0	0.106064
7480	Amp	6	52185988	52269703	0	0.10984
7481	Amp	6	52269732	52272193	0	0.106023
7482	Amp	6	52273381	52356177	0	0.10316
7483	Amp	6	52357366	52360868	0	0.108117
7484	Amp	6	52372170	52373126	0	0.126033
7485	Amp	6	52376160	52398946	0	0.133926
7486	Amp	6	52401253	52415103	0	0.12821
7487	Amp	6	52417614	52442405	0	0.110294
7488	Amp	6	52446938	52608328	0	0.108395
7489	Amp	6	52610560	52612498	0	0.100501
7490	Amp	6	52612795	52612817	0	0.1103
7491	Amp	6	52616422	52626619	0	0.118077
7492	Amp	6	52630403	52683582	0	0.1103
7493	Amp	6	52683970	52731694	0	0.112697
7494	Amp	6	52731777	52746320	0	0.11851
7495	Amp	6	52799684	52872295	0	0.125542
7496	Amp	6	52874692	52883165	0	0.140286
7497	Amp	6	52886795	52928056	0	0.157245
7498	Amp	6	52928654	52936889	0	0.155037
7499	Amp	6	52937230	52939880	0	0.145446
7500	Amp	6	52939969	52943415	0	0.142776
7501	Amp	6	52945552	52947531	0	0.135744
7502	Amp	6	52948380	52953255	0	0.142707
7503	Amp	6	52953268	53169896	0	0.149284
7504	Amp	6	53170707	53220818	0	0.157115
7505	Amp	6	53222737	53309650	0	0.170351
7506	Amp	6	53311739	53318996	0	0.163894
7507	Amp	6	53319470	53413934	0	0.154096
7508	Amp	6	53416853	53440244	0	0.168873
7509	Amp	6	53445431	53466346	0	0.155637
7510	Amp	6	53466538	53469386	0	0.160866
7511	Amp	6	53469499	53495321	0	0.172553
7512	Amp	6	53495349	53524663	0	0.176496
7513	Amp	6	53525922	53530262	0	0.162207
7514	Amp	6	53537201	53549445	0	0.17649
7515	Amp	6	53550006	53615674	0	0.17059
7516	Amp	6	53618946	53619687	0	0.159219
7517	Amp	6	53622284	53628662	0	0.160674
7518	Amp	6	53628810	53673539	0	0.151821
7519	Amp	6	53674000	53824826	0	0.147082
7520	Amp	6	53828118	53856961	0	0.141689
7521	Amp	6	53857020	53869994	0	0.143973
7522	Amp	6	53872884	53884655	0	0.127324
7523	Amp	6	53884876	53931214	0	0.132911
7524	Amp	6	53932762	53941092	0	0.127683
7525	Amp	6	53944181	54189888	0	0.136768

7526	Amp	6	54191396	54191743	0	0.140648
7527	Amp	6	54192088	54208727	0	0.153731
7528	Amp	6	54209408	54272708	0	0.148499
7529	Amp	6	54273309	54335910	0	0.153723
7530	Amp	6	54336316	54468484	0	0.163248
7531	Amp	6	54472332	54494204	0	0.159305
7532	Amp	6	54497401	54527260	0.050005	0.195037
7533	Amp	6	54535993	54536533	0	0.183724
7534	Amp	6	54537593	54561750	0	0.149123
7535	Amp	6	54563631	54597930	0	0.146121
7536	Amp	6	54598584	54857215	0	0.140896
7537	Amp	6	54860144	55298087	0	0.119106
7538	Amp	6	55301487	55379931	0	0.076121
7539	Amp	6	55381090	55398589	0	0.092384
7540	Amp	6	55399492	55452204	0	0.083488
7541	Amp	6	55452263	55458632	0	0.076912
7542	Amp	6	55458648	55522695	0	0.075457
7543	Amp	6	55525687	55539540	0	0.073174
7544	Amp	6	55541135	55586760	0	0.075352
7545	Amp	6	55591282	55697642	0	0.08431
7546	Amp	6	55699095	55730916	0	0.092361
7547	Amp	6	55733499	55733499	0	0.094455
7548	Amp	6	55742054	55808397	0	0.109624
7549	Amp	6	55809027	55868040	0	0.088524
7550	Amp	6	55871382	55902078	0	0.093584
7551	Amp	6	55903074	55948158	0	0.09851
7552	Amp	6	55952963	55986200	0	0.106537
7553	Amp	6	55987356	56096299	0	0.113573
7554	Amp	6	56105010	56269934	0	0.127098
7555	Amp	6	56269953	56313218	0	0.122006
7556	Amp	6	56314651	56359394	0	0.127352
7557	Amp	6	56359681	56428212	0	0.130846
7558	Amp	6	56430859	56477183	0	0.121888
7559	Amp	6	56479873	56482063	0	0.116543
7560	Amp	6	56484646	56630444	0	0.120948
7561	Amp	6	56633024	56652953	0	0.128265
7562	Amp	6	56656399	56684514	0	0.126321
7563	Amp	6	56687838	56738282	0	0.129846
7564	Amp	6	56739425	56772479	0	0.125441
7565	Amp	6	56776454	56776454	0	0.116228
7566	Amp	6	56779707	56862992	0	0.121439
7567	Amp	6	56864627	56882820	0	0.113412
7568	Amp	6	56884059	56897687	0	0.109171
7569	Amp	6	56899015	56957530	0	0.091301
7570	Amp	6	56957820	57168540	0	0.087776
7571	Amp	6	57173925	57220111	0	0.100416
7572	Amp	6	57224546	57224546	0	0.096922
7573	Amp	6	57225274	57229897	0	0.093815
7574	Amp	6	57233801	57268376	0	0.08651
7575	Amp	6	57274935	57284353	0	0.111866
7576	Amp	6	57287480	57302322	0	0.107471
7577	Amp	6	57304508	57347009	0	0.082116
7578	Amp	6	61527062	61527062	0	0.073465

7579	Amp	6	61530738	61530738	0	0.067449
7580	Amp	6	61596503	61603245	0	0.067428
7581	Amp	6	61607085	61699746	0	0.09079
7582	Amp	6	61699774	61823500	0	0.086802
7583	Amp	6	61823514	61829466	0	0.096924
7584	Amp	6	61829755	61897502	0	0.09523
7585	Amp	6	61901347	61941628	0	0.090427
7586	Amp	6	61946171	62203089	0	0.096275
7587	Amp	6	62208079	62208079	0	0.072913
7588	Amp	6	62211669	62337327	0	0.080755
7589	Amp	6	62344324	62489898	0	0.072913
7590	Amp	6	62500369	62576066	0	0.062791
7591	Amp	6	62582068	62718524	0	0.068323
7592	Amp	6	62722178	62838954	0	0.063691
7593	Amp	6	62872346	62902150	0	0.095159
7594	Amp	6	62902777	62947698	0	0.102943
7595	Amp	6	62955241	62960879	0	0.183387
7596	Amp	6	62966895	62981554	0	0.177698
7597	Amp	6	62984507	63018164	0	0.169913
7598	Amp	6	63018620	63188817	0	0.168116
7599	Amp	6	63189011	63442062	0	0.173602
7600	Amp	6	63444132	63712722	0	0.184942
7601	Amp	6	63713088	63842055	0	0.186723
7602	Amp	6	63845570	63907016	0	0.18119
7603	Amp	6	63907797	63972500	0	0.187072
7604	Amp	6	63974253	63976610	0.038273	0.193128
7605	Amp	6	63976653	64079302	0.098998	0.20004
7606	Amp	6	64105247	64164795	0.043943	0.193984
7607	Amp	6	64169223	64283543	0.128931	0.203514
7608	Amp	6	64284821	64498382	0.056238	0.195274
7609	Amp	6	64499191	64513771	0.111991	0.201805
7610	Amp	6	64518013	64605836	0.201819	0.211023
7611	Amp	6	64605978	64671707	0.168144	0.208001
7612	Amp	6	64673607	64675733	0.264784	0.217588
7613	Amp	6	64676445	64697064	0.355162	0.225728
7614	Amp	6	64699063	64705549	0.250263	0.216141
7615	Amp	6	64708357	64714132	0.390264	0.229214
7616	Amp	6	64716921	64840232	0.476502	0.236485
7617	Amp	6	64840605	64854497	0.556748	0.243349
7618	Amp	6	64856844	64893744	0.468576	0.236078
7619	Amp	6	64893923	64919103	0.523647	0.240865
7620	Amp	6	64921233	65029178	0.430191	0.232801
7621	Amp	6	65030279	65051148	0.286675	0.219728
7622	Amp	6	65054802	65112412	0.230572	0.214266
7623	Amp	6	65213064	65245250	0.355162	0.225354
7624	Amp	6	65245410	65291856	0.273081	0.218441
7625	Amp	6	65302362	65390396	0.128931	0.203842
7626	Amp	6	65390534	65411108	0.201819	0.211373
7627	Amp	6	65411176	65419492	0.264784	0.217137
7628	Amp	6	65419512	65426563	0.340434	0.224049
7629	Amp	6	65437777	65450860	0.398217	0.229775
7630	Amp	6	65456968	65464406	0.223025	0.213027
7631	Amp	6	65469205	65507235	0.161592	0.2073

7632 Amp	6	65510544	65602868	0.105051	0.200769
7633 Amp	6	65603914	65609224	0.123589	0.202733
7634 Amp	6	65609587	65623729	0.079463	0.19796
7635 Amp	6	65623779	65678932	0.149443	0.205455
7636 Amp	6	65679058	65720040	0.195309	0.210636
7637 Amp	6	65723556	65807804	0.532449	0.241624
7638 Amp	6	65808496	65833111	0.25804	0.216767
7639 Amp	6	65839781	65881081	0.309523	0.221712
7640 Amp	6	65881554	65894368	0.230572	0.214217
7641 Amp	6	65894532	65894532	0.273081	0.218307
7642 Amp	6	65897205	65912328	0.340434	0.224542
7643 Amp	6	65918382	65930522	0	0.123497
7644 Amp	6	65935648	65940669	0	0.116581
7645 Amp	6	65941334	65996572	0	0.114851
7646 Amp	6	66002268	66100157	0	0.120208
7647 Amp	6	66100911	66118822	0	0.1247
7648 Amp	6	66121912	66187896	0	0.113211
7649 Amp	6	66188906	66205120	0	0.108719
7650 Amp	6	66210837	66221295	0	0.102955
7651 Amp	6	66221315	66267437	0	0.091868
7652 Amp	6	66267743	66281941	0	0.087081
7653 Amp	6	66283562	66406658	0	0.0812
7654 Amp	6	66407375	66802876	0	0.074964
7655 Amp	6	66809380	66825399	0	0.07682
7656 Amp	6	66831529	67133200	0	0.082014
7657 Amp	6	67137258	67149597	0	0.121804
7658 Amp	6	67150737	67202305	0	0.082014
7659 Amp	6	67203302	67214408	0	0.083418
7660 Amp	6	67220254	67257865	0	0.098273
7661 Amp	6	67258804	67418895	0	0.093092
7662 Amp	6	67419539	67422808	0	0.101319
7663 Amp	6	67449934	67457396	0.117531	0.202365
7664 Amp	6	67457837	67475115	0.043943	0.194138
7665 Amp	6	67476203	68094475	0	0.093092
7666 Amp	6	68095055	68137615	0	0.126588
7667 Amp	6	68139591	68249332	0	0.093092
7668 Amp	6	68251774	68330859	0	0.101772
7669 Amp	6	68332282	68611293	0	0.096578
7670 Amp	6	68611603	68688117	0	0.099546
7671 Amp	6	68694150	68779601	0	0.110939
7672 Amp	6	68780519	68804597	0	0.116788
7673 Amp	6	68804651	68819337	0	0.110939
7674 Amp	6	68824147	68866138	0	0.118121
7675 Amp	6	68868705	68923727	0.286675	0.219167
7676 Amp	6	68931204	68954167	0	0.118121
7677 Amp	6	68965628	68965628	0	0.135086
7678 Amp	6	68965946	68975066	0	0.144646
7679 Amp	6	68990430	69020560	0.580011	0.245692
7680 Amp	6	69021299	69039512	0.382767	0.228727
7681 Amp	6	69041092	69045957	0	0.186369
7682 Amp	6	69047260	69116142	0.056238	0.195206
7683 Amp	6	69116807	69422104	0	0.164102
7684 Amp	6	69422328	69561376	0	0.155265

7685	Amp	6	69561621	69565263	0	0.146585
7686	Amp	6	69578557	69633323	0	0.141099
7687	Amp	6	69646471	69718308	0	0.146628
7688	Amp	6	69719023	69719023	0	0.045582
7689	Amp	6	69719378	69736056	0	0.030727
7690	Amp	6	69739731	69838732	0	0.03409
7691	Amp	6	69838818	69838818	0	0.032126
7692	Amp	6	69843290	70142601	0	0.028763
7693	Amp	6	70147116	70202072	0	0.026907
7694	Amp	6	70202540	70339316	0	0.021378
7695	Amp	6	70348524	70553840	0	0.071901
7696	Amp	6	70557863	70596171	0	0.074538
7697	Amp	6	70597644	70639324	0	0.079986
7698	Amp	6	70640051	71137919	0	0.084403
7699	Amp	6	71137948	71743830	0	0.082103
7700	Amp	6	71745241	72076690	0	0.072573
7701	Amp	6	72079346	72190527	0	0.173619
7702	Amp	6	72195046	72230960	0	0.072573
7703	Amp	6	72235663	72676138	0	0.0819
7704	Amp	6	72677119	72836490	0	0.090877
7705	Amp	6	72841801	73212068	0	0.0819
7706	Amp	6	73215122	73291375	0	0.182946
7707	Amp	6	73291697	73340055	0	0.173619
7708	Amp	6	73344723	73445623	0	0.072573
7709	Amp	6	73446775	74057655	0	0.066752
7710	Amp	6	74059509	74085712	0	0.073774
7711	Amp	6	74085752	74152548	0	0.06883
7712	Amp	6	74208320	74446535	0	0.076943
7713	Amp	6	74452493	74537527	0	0.084353
7714	Amp	6	74541163	74648906	0	0.077331
7715	Amp	6	74653517	74866429	0	0.123042
7716	Amp	6	74867381	74867381	0	0.114929
7717	Amp	6	74880313	75122372	0	0.064406
7718	Amp	6	75124532	75725635	0	0.069378
7719	Amp	6	75725852	75787256	0	0.074196
7720	Amp	6	75796773	75812885	0	0.081974
7721	Amp	6	75814508	75948536	0	0.074564
7722	Amp	6	75951684	75998673	0	0.081069
7723	Amp	6	75999917	76144602	0	0.083774
7724	Amp	6	76145131	76590546	0	0.077921
7725	Amp	6	76592518	76913408	0	0.081811
7726	Amp	6	76913435	76928578	0	0.074878
7727	Amp	6	76931109	76949618	0	0.078331
7728	Amp	6	76950087	77014792	0	0.085446
7729	Amp	6	77015106	77175471	0	0.077669
7730	Amp	6	77183010	77281122	0	0.071588
7731	Amp	6	77283607	77345226	0	0.087868
7732	Amp	6	77349634	77637608	0	0.071588
7733	Amp	6	77640066	77679665	0	0.074238
7734	Amp	6	77681987	77690728	0	0.087162
7735	Amp	6	77691034	77703854	0	0.094354
7736	Amp	6	77709740	77853797	0	0.099021
7737	Amp	6	77860753	77997667	0	0.091829

7738	Amp	6	77998064	78458807	0	0.088956
7739	Amp	6	78466707	78474190	0	0.095575
7740	Amp	6	78474741	78496593	0	0.084716
7741	Amp	6	78496623	78660255	0	0.078097
7742	Amp	6	78660395	78758186	0	0.088393
7743	Amp	6	78762986	79036957	0	0.083657
7744	Amp	6	79037174	79073383	0	0.037946
7745	Amp	6	79075677	79179332	0	0.044205
7746	Amp	6	79183190	79276595	0	0.053536
7747	Amp	6	79277852	79478265	0	0.044205
7748	Amp	6	79479425	79674429	0	0.033909
7749	Amp	6	79678551	79863940	0	0.029242
7750	Amp	6	79864309	79878317	0	0.030711
7751	Amp	6	79883038	80021297	0	0.032626
7752	Amp	6	80024192	80067783	0	0.030711
7753	Amp	6	80069504	80108270	0	0.029242
7754	Amp	6	80112261	80351342	0	0.042168
7755	Amp	6	80352383	80382642	0	0.046804
7756	Amp	6	80382750	80426076	0	0.048448
7757	Amp	6	80427286	80463417	0	0.035523
7758	Amp	6	80463654	80766408	0	0.037133
7759	Amp	6	80771637	80787583	0	0.035489
7760	Amp	6	80791378	80863193	0	0.037588
7761	Amp	6	80866441	80885132	0	0.031329
7762	Amp	6	80886594	80887349	0	0.041242
7763	Amp	6	80890637	80911590	0	0.044273
7764	Amp	6	80922705	80939489	0	0.03436
7765	Amp	6	80939671	81003798	0	0.031329
7766	Amp	6	81012526	81266799	0	0.026511
7767	Amp	6	81268025	81495066	0	0.021874
7768	Amp	6	81495095	81521884	0	0.025473
7769	Amp	6	81524976	81585955	0	0.033019
7770	Amp	6	81586736	81635095	0	0.04089
7771	Amp	6	81635174	81756049	0	0.03928
7772	Amp	6	81764236	81768232	0	0.037181
7773	Amp	6	81778793	81817600	0	0.039236
7774	Amp	6	81820615	81834790	0	0.034264
7775	Amp	6	81835846	81867250	0	0.0377
7776	Amp	6	81878096	81917243	0	0.034264
7777	Amp	6	81924195	81969908	0	0.026393
7778	Amp	6	81971273	82256502	0	0.029898
7779	Amp	6	82262379	82270008	0	0.032491
7780	Amp	6	82273572	82300736	0	0.028892
7781	Amp	6	82300885	82354539	0	0.043024
7782	Amp	6	82359979	82399263	0	0.060754
7783	Amp	6	82402533	82405893	0	0.065254
7784	Amp	6	82408575	82460122	0	0.062605
7785	Amp	6	82462590	82532585	0	0.044875
7786	Amp	6	82533721	82644607	0	0.04137
7787	Amp	6	82651207	82710804	0	0.061371
7788	Amp	6	82711156	82773638	0	0.089299
7789	Amp	6	82780958	82811335	0	0.055378
7790	Amp	6	82816570	83001374	0	0.038777

7791	Amp	6	83002990	83345336	0	0.036721
7792	Amp	6	83351139	83365562	0	0.028815
7793	Amp	6	83368845	83592703	0	0.026178
7794	Amp	6	83593870	83859529	0	0.032923
7795	Amp	6	83861171	83870483	0	0.012047
7796	Amp	6	83875511	84523180	0	0.007546
7797	Amp	6	84523594	85882644	0	0.012301
7798	Amp	6	85883114	86354325	0	0.021965
7799	Amp	6	86354918	86381436	0	0.031474
7800	Amp	6	86382798	87011236	0	0.021965
7801	Amp	6	87011989	87330374	0	0.017429
7802	Amp	6	87330681	87334840	0	0.023515
7803	Amp	6	87339590	87544202	0	0.034405
7804	Amp	6	87544249	87567679	0	0.023515
7805	Amp	6	87571083	87606435	0	0.025095
7806	Amp	6	87608412	87642318	0	0.031413
7807	Amp	6	87647919	87994738	0	0.025095
7808	Amp	6	87995954	88177703	0	0.031748
7809	Amp	6	88183282	88489078	0	0.042371
7810	Amp	6	88493771	88753298	0	0.018856
7811	Amp	6	88754491	89119597	0	0.017276
7812	Amp	6	89120390	89492312	0	0.010623
7813	Amp	6	89494005	89557261	0	0.033196
7814	Amp	6	89557716	89715929	0	0.023737
7815	Amp	6	89718013	89762351	0	0.015638
7816	Amp	6	89763541	89783685	0	0.010623
7817	Amp	6	89787592	90052203	0	0.018868
7818	Amp	6	90059845	90389074	0	0.010623
7819	Amp	6	90393145	90822234	0	0
7820	Amp	6	90827719	90927971	0	0.019031
7821	Amp	6	90930497	91685371	0	0.021042
7822	Amp	6	91687617	91811210	0	0.019031
7823	Amp	6	91812191	91867990	0	0.029672
7824	Amp	6	91869881	91874503	0	0.031726
7825	Amp	6	91883520	92098739	0	0.012695
7826	Amp	6	92102327	92374805	0	0.018732
7827	Amp	6	92375180	92481088	0	0.016679
7828	Amp	6	92494001	92642291	0	0.010642
7829	Amp	6	92647209	93272973	0	0
7830	Amp	6	93275311	93318764	0	0.010328
7831	Amp	6	93319279	93646674	0	0
7832	Amp	6	93648796	93758791	0	0.0016
7833	Amp	6	93759175	93765426	0	0.007009
7834	Amp	6	93768888	95388284	0	0.016954
7835	Amp	6	95388756	95438211	0	0.02171
7836	Amp	6	95443405	96975408	0	0.0163
7837	Amp	6	96976389	97064763	0	0.021837
7838	Amp	6	97065215	97075519	0	0.0163
7839	Amp	6	97079194	97278792	0	0.021572
7840	Amp	6	97280691	97910742	0	0.0163
7841	Amp	6	97913678	98024877	0	0.021208
7842	Amp	6	98025998	98461310	0	0.016452
7843	Amp	6	98464069	98743523	0	0.011544

7844	Amp	6	98745097	99131047	0	0.013486
7845	Amp	6	99134624	99191992	0	0.016746
7846	Amp	6	99194254	99809792	0	0.014804
7847	Amp	6	99810759	99932567	0	0.024296
7848	Amp	6	99942349	100268892	0	0.029497
7849	Amp	6	100270843	100425178	0	0.069211
7850	Amp	6	100425381	100513322	0	0.066397
7851	Amp	6	100515818	100705789	0	0.063137
7852	Amp	6	100782506	100848221	0	0.065549
7853	Amp	6	100852162	100856535	0	0.083236
7854	Amp	6	100856802	100970079	0	0.099119
7855	Amp	6	100971322	101052488	0	0.092843
7856	Amp	6	101062329	101100591	0	0.097429
7857	Amp	6	101111662	101112468	0	0.113958
7858	Amp	6	101118320	101313829	0	0.077058
7859	Amp	6	101320263	101655562	0	0.088861
7860	Amp	6	101658362	101925560	0	0.077909
7861	Amp	6	101930391	101965879	0	0.089235
7862	Amp	6	101965958	102001151	0	0.081927
7863	Amp	6	102001951	102237926	0	0.077909
7864	Amp	6	102238071	102250820	0	0.063561
7865	Amp	6	102274886	102506638	0	0.057984
7866	Amp	6	102510120	102522015	0	0.094252
7867	Amp	6	102523905	102586906	0	0.057984
7868	Amp	6	102610750	102621781	0	0.077014
7869	Amp	6	102631362	102707917	0	0.069092
7870	Amp	6	102708507	102710699	0	0.074459
7871	Amp	6	102713828	102802183	0	0.121571
7872	Amp	6	102802530	102895219	0	0.116204
7873	Amp	6	102906510	102919256	0	0.11382
7874	Amp	6	102923484	103253188	0	0.106597
7875	Amp	6	103255102	103280350	0	0.153171
7876	Amp	6	103322942	103378000	0	0.163519
7877	Amp	6	103381090	103477462	0	0.153171
7878	Amp	6	103510929	103808460	0.033661	0.192754
7879	Amp	6	103809951	104247602	0.018387	0.190343
7880	Amp	6	104248912	104277015	0.079463	0.198382
7881	Amp	6	104277773	104277814	0.187743	0.20992
7882	Amp	6	104282309	104295982	0	0.159468
7883	Amp	6	104297539	104304458	0	0.151429
7884	Amp	6	104304472	104340743	0	0.149829
7885	Amp	6	104348241	104351737	0	0.160577
7886	Amp	6	104352492	104370128	0	0.164526
7887	Amp	6	104380960	104495910	0	0.153778
7888	Amp	6	104496476	104518699	0	0.15604
7889	Amp	6	104526947	104732768	0	0.160484
7890	Amp	6	104747356	104756575	0	0.176746
7891	Amp	6	104757285	104791416	0	0.167953
7892	Amp	6	104798822	104805606	0.230572	0.213664
7893	Amp	6	104808348	104858945	0.317596	0.222342
7894	Amp	6	104869915	105001843	0.41418	0.231411
7895	Amp	6	105006903	105096244	0.833763	0.266052
7896	Amp	6	105097515	105101885	1.315861	0.300312

7897	Amp	6	105102150	105135671	1.237378	0.295112
7898	Amp	6	105137821	105346691	1.453628	0.310248
7899	Amp	6	105350240	105422550	2.082332	0.351142
7900	Amp	6	105426176	105511731	1.453628	0.310248
7901	Amp	6	105512837	105552955	1.424227	0.307836
7902	Amp	6	105554227	105797898	2.082332	0.351587
7903	Amp	6	105798151	105809124	1.832922	0.335882
7904	Amp	6	105809297	105951773	2.061276	0.350008
7905	Amp	6	105953777	106003688	1.424227	0.30842
7906	Amp	6	106003809	106040063	1.305256	0.299955
7907	Amp	6	106043157	106043157	2.071528	0.350478
7908	Amp	6	106043419	106084671	2.170217	0.356888
7909	Amp	6	106086699	106153480	2.458756	0.374649
7910	Amp	6	106154061	106236578	2.625007	0.384693
7911	Amp	6	106236892	106317501	2.703608	0.389334
7912	Amp	6	106318599	106366400	2.727427	0.390743
7913	Amp	6	106367161	106500776	2.404616	0.371582
7914	Amp	6	106505853	106558612	2.22615	0.360499
7915	Amp	6	106559071	106734170	2.294081	0.364801
7916	Amp	6	106736954	106810759	2.994162	0.406389
7917	Amp	6	106814563	106837955	2.948071	0.403735
7918	Amp	6	106841990	106851542	2.260813	0.362147
7919	Amp	6	106854839	106855080	2.948071	0.403849
7920	Amp	6	106988904	107267248	2.878516	0.399263
7921	Amp	6	107268451	107445764	2.98257	0.405591
7922	Amp	6	107452150	107470457	2.796399	0.394483
7923	Amp	6	107474863	107579220	2.028562	0.348304
7924	Amp	6	107579927	107583867	1.963469	0.34386
7925	Amp	6	107584598	107721130	1.898353	0.339558
7926	Amp	6	107725129	107814487	1.324884	0.301364
7927	Amp	6	107816502	107827443	0.702615	0.255653
7928	Amp	6	107828293	108057266	0.852608	0.267282
7929	Amp	6	108072019	108094188	0.86979	0.269212
7930	Amp	6	108099711	108127407	0.95395	0.275057
7931	Amp	6	108128641	108146008	0.906818	0.271757
7932	Amp	6	108148716	108154534	0.963924	0.275615
7933	Amp	6	108159324	108234995	0.86979	0.269287
7934	Amp	6	108238782	108263321	0.852608	0.267878
7935	Amp	6	108266199	108275875	0.833763	0.265949
7936	Amp	6	108276355	108291313	0.963924	0.275864
7937	Amp	6	108298622	108303523	0.897922	0.271222
7938	Amp	6	108304904	108330998	0.852608	0.267364
7939	Amp	6	108334599	108376919	0.25804	0.216841
7940	Amp	6	108378693	108476381	0	0.166319
7941	Amp	6	108476625	108520839	0	0.15469
7942	Amp	6	108524808	108554605	0	0.137541
7943	Amp	6	108557201	108630893	0	0.146323
7944	Amp	6	108631110	108642391	0	0.16075
7945	Amp	6	108646668	108660049	0	0.165486
7946	Amp	6	108660822	108762355	0	0.162376
7947	Amp	6	108762829	108842406	0	0.151931
7948	Amp	6	108845989	108860038	0	0.147195
7949	Amp	6	108861200	108874744	0	0.157692

7950	Amp	6	108875503	108919359	0	0.163567
7951	Amp	6	108925688	109010924	0	0.17406
7952	Amp	6	109013502	109105536	0	0.183604
7953	Amp	6	109105924	109285875	0	0.173112
7954	Amp	6	109286791	109292361	0	0.164434
7955	Amp	6	109292414	109551234	0	0.173112
7956	Amp	6	109557312	109669784	0	0.161309
7957	Amp	6	109671681	110662374	0	0.110786
7958	Amp	6	110666591	110768170	0	0.116884
7959	Amp	6	110776905	110858969	0	0.111009
7960	Amp	6	110877169	110882843	0	0.107519
7961	Amp	6	110885938	110929245	0	0.109021
7962	Amp	6	110966817	111080462	0	0.113215
7963	Amp	6	111082231	111254812	0	0.119772
7964	Amp	6	111258795	111272472	0	0.112078
7965	Amp	6	111273277	111345587	0	0.107885
7966	Amp	6	111356341	111532040	0	0.101327
7967	Amp	6	111538449	111668494	0	0.108564
7968	Amp	6	111672621	111832380	0	0.09862
7969	Amp	6	111832462	111944266	0	0.09347
7970	Amp	6	111945144	112058033	0	0.076192
7971	Amp	6	112061631	112208227	0	0.078083
7972	Amp	6	112215137	112278767	0	0.070846
7973	Amp	6	112278795	112321124	0	0.062064
7974	Amp	6	112326282	112387332	0	0.065554
7975	Amp	6	112392762	112763087	0	0.019783
7976	Amp	6	112768507	112822317	0	0.038514
7977	Amp	6	112823066	113251473	0	0.028002
7978	Amp	6	113254017	113406128	0	0.073745
7979	Amp	6	113408164	113578759	0	0.028002
7980	Amp	6	113578971	114027378	0	0.036749
7981	Amp	6	114029681	114165125	0	0.048692
7982	Amp	6	114167560	114285163	0	0.094191
7983	Amp	6	114288020	114701015	0	0.098667
7984	Amp	6	114701270	115388301	0	0.094191
7985	Amp	6	115404741	115446947	0	0.095666
7986	Amp	6	115454663	115730220	0	0.0787
7987	Amp	6	115730763	116254210	0	0.083564
7988	Amp	6	116255630	116757612	0	0.033429
7989	Amp	6	116757621	116856992	0	0.028564
7990	Amp	6	116863951	116872426	0	0.035794
7991	Amp	6	116872999	116907422	0	0.028564
7992	Amp	6	116907753	117026212	0	0.02709
7993	Amp	6	117028106	117074857	0	0.025588
7994	Amp	6	117079600	117250502	0	0.030873
7995	Amp	6	117251440	117280588	0	0.045319
7996	Amp	6	117280811	117438572	0	0.037846
7997	Amp	6	117438966	117457823	0	0.033693
7998	Amp	6	117459904	117481975	0	0.055343
7999	Amp	6	117486165	117563314	0	0.045126
8000	Amp	6	117571941	117589192	0	0.059373
8001	Amp	6	117596577	117714589	0	0.045126
8002	Amp	6	117719521	117737714	0	0.039841

8003 Amp	6	117740933	117813347	0	0.048526
8004 Amp	6	117822683	117824317	0	0.053745
8005 Amp	6	117824659	117873622	0	0.056492
8006 Amp	6	117876082	118011870	0	0.051273
8007 Amp	6	118012614	118016295	0	0.048526
8008 Amp	6	118017458	118062140	0	0.052053
8009 Amp	6	118066133	118222644	0	0.058247
8010 Amp	6	118226362	118816495	0	0.049562
8011 Amp	6	118816865	118816865	0	0.047993
8012 Amp	6	118817120	118938494	0	0.052678
8013 Amp	6	118938523	118998378	0	0.061871
8014 Amp	6	118998676	119065991	0	0.069585
8015 Amp	6	119067415	119073477	0	0.067628
8016 Amp	6	119075929	119146711	0	0.081493
8017 Amp	6	119148269	119399663	0	0.084124
8018 Amp	6	119405216	119713390	0	0.074931
8019 Amp	6	119713469	119812391	0	0.030413
8020 Amp	6	119812680	119892792	0	0.039674
8021 Amp	6	119896950	120502749	0	0.030413
8022 Amp	6	120506063	120530755	0	0.04194
8023 Amp	6	120533326	120645561	0	0.030413
8024 Amp	6	120646094	121301484	0	0.034029
8025 Amp	6	121303941	121484587	0	0.036431
8026 Amp	6	121488873	121516168	0	0.032415
8027 Amp	6	121517663	121585760	0	0.043634
8028 Amp	6	121588422	121678012	0	0.035836
8029 Amp	6	121686998	121830245	0	0.044808
8030 Amp	6	121830546	122600980	0	0.040885
8031 Amp	6	122601087	122754906	0	0.031914
8032 Amp	6	122754968	122783060	0	0.03737
8033 Amp	6	122783162	122838031	0	0.048096
8034 Amp	6	122847317	122851616	0	0.042639
8035 Amp	6	122860728	123046369	0	0.050042
8036 Amp	6	123050335	123076230	0	0.047946
8037 Amp	6	123080614	123171133	0	0.052464
8038 Amp	6	123171220	123242551	0	0.045062
8039 Amp	6	123248637	123308726	0	0.038067
8040 Amp	6	123314481	123351865	0	0.026124
8041 Amp	6	123359437	123532411	0	0.034237
8042 Amp	6	123533490	123653877	0	0.025987
8043 Amp	6	123654096	123698586	0	0.021302
8044 Amp	6	123700791	123788717	0	0.024867
8045 Amp	6	123804563	123830879	0	0.016754
8046 Amp	6	123832579	123945937	0	0.035626
8047 Amp	6	124033622	124046706	0	0.079993
8048 Amp	6	124046786	124051696	0	0.076428
8049 Amp	6	124054539	124380040	0	0.025905
8050 Amp	6	124382355	124390025	0	0.036987
8051 Amp	6	124398517	124407105	0	0.044391
8052 Amp	6	124410864	124431956	0	0.054201
8053 Amp	6	124434450	124437907	0	0.044391
8054 Amp	6	124437974	124574160	0	0.031675
8055 Amp	6	124574522	124598443	0	0.02427

8056	Amp	6	124599694	124656687	0	0.026015
8057	Amp	6	124662077	125023150	0	0.034727
8058	Amp	6	125023367	125179110	0	0.026015
8059	Amp	6	125181853	125234814	0	0.014933
8060	Amp	6	125247901	125271245	0	0.027478
8061	Amp	6	125271648	125456865	0	0.031757
8062	Amp	6	125457012	126077054	0	0.030013
8063	Amp	6	126081272	126511860	0	0.017468
8064	Amp	6	126522813	126703998	0	0.032827
8065	Amp	6	126707265	126828565	0	0.043396
8066	Amp	6	126833027	126914418	0	0.053537
8067	Amp	6	126920151	126947352	0	0.043396
8068	Amp	6	126953424	126977185	0	0.054329
8069	Amp	6	126977249	127134691	0	0.043396
8070	Amp	6	127134977	127166912	0	0.037323
8071	Amp	6	127169454	127694706	0	0.04264
8072	Amp	6	127695457	127705091	0	0.044157
8073	Amp	6	127710575	127923505	0	0.086002
8074	Amp	6	127923947	128008407	0	0.092466
8075	Amp	6	128009564	128033185	0	0.095331
8076	Amp	6	128034105	128054312	0	0.092466
8077	Amp	6	128057674	128057948	0	0.101678
8078	Amp	6	128067377	128090031	0	0.111341
8079	Amp	6	128090827	128250136	0	0.101678
8080	Amp	6	128256892	128392171	0	0.105583
8081	Amp	6	128394512	128432898	0	0.111464
8082	Amp	6	128433346	128474742	0	0.131531
8083	Amp	6	128477836	128545487	0	0.13804
8084	Amp	6	128552737	128568543	0	0.145605
8085	Amp	6	128570134	128570134	0	0.13804
8086	Amp	6	128580341	128649628	0	0.134135
8087	Amp	6	128649850	128749324	0	0.123175
8088	Amp	6	128751009	128866962	0	0.121658
8089	Amp	6	128872725	128954512	0	0.115776
8090	Amp	6	128960663	129194695	0	0.09571
8091	Amp	6	129195195	129279817	0	0.101005
8092	Amp	6	129281404	129362949	0	0.091793
8093	Amp	6	129371562	129413585	0	0.049948
8094	Amp	6	129415244	129632130	0	0.059939
8095	Amp	6	129632300	129717328	0	0.054645
8096	Amp	6	129721687	129751411	0	0.08555
8097	Amp	6	129751624	129831534	0	0.094019
8098	Amp	6	129831656	129909335	0	0.085939
8099	Amp	6	129909679	129916689	0	0.089866
8100	Amp	6	129916949	130040777	0	0.094296
8101	Amp	6	130049910	130144256	0	0.07147
8102	Amp	6	130144404	130175157	0	0.072995
8103	Amp	6	130177412	130272267	0	0.118706
8104	Amp	6	130272522	130432171	0	0.124123
8105	Amp	6	130434522	130481332	0	0.135877
8106	Amp	6	130482522	130698653	0	0.144247
8107	Amp	6	130700667	130816008	0	0.141145
8108	Amp	6	130819903	130874805	0	0.135728

8109	Amp	6	130875892	131151072	0	0.143681
8110	Amp	6	131152221	131380445	0	0.13369
8111	Amp	6	131381495	131402387	0	0.121936
8112	Amp	6	131432084	131534222	0	0.109553
8113	Amp	6	131545239	131546002	0	0.119576
8114	Amp	6	131547815	131668090	0	0.109572
8115	Amp	6	131670424	131781746	0	0.120954
8116	Amp	6	131782168	132285899	0	0.128635
8117	Amp	6	132288421	132694132	0	0.137503
8118	Amp	6	132695903	132770937	0	0.129821
8119	Amp	6	132774033	132922599	0	0.133791
8120	Amp	6	132922635	132932115	0	0.142058
8121	Amp	6	132933531	132981864	0	0.147705
8122	Amp	6	132985052	133004706	0	0.139438
8123	Amp	6	133011254	133033207	0	0.145461
8124	Amp	6	133033509	133055560	0	0.160941
8125	Amp	6	133057457	133079057	0	0.154917
8126	Amp	6	133086461	133086461	0	0.150947
8127	Amp	6	133086900	133178847	0	0.135467
8128	Amp	6	133188516	133227050	0	0.129821
8129	Amp	6	133233314	133248494	0	0.138546
8130	Amp	6	133249807	133432881	0	0.129821
8131	Amp	6	133434069	133522685	0	0.134762
8132	Amp	6	133523203	133532894	0	0.145071
8133	Amp	6	133534277	133569400	0	0.134762
8134	Amp	6	133574143	133640236	0	0.143169
8135	Amp	6	133641117	133667457	0	0.134762
8136	Amp	6	133667664	133727826	0	0.103792
8137	Amp	6	133727948	133818312	0	0.095421
8138	Amp	6	133821522	134043480	0	0.098808
8139	Amp	6	134043875	134053268	0	0.11105
8140	Amp	6	134055466	134086600	0	0.098808
8141	Amp	6	134092399	134120511	0	0.097283
8142	Amp	6	134123032	134136064	0	0.093896
8143	Amp	6	134137072	134209109	0	0.110035
8144	Amp	6	134214021	134229617	0	0.114391
8145	Amp	6	134234116	134732459	0	0.110035
8146	Amp	6	134732573	134743434	0	0.105094
8147	Amp	6	134745888	134828820	0	0.114396
8148	Amp	6	134830412	134903741	0	0.105094
8149	Amp	6	134910557	135001263	0	0.114377
8150	Amp	6	135004780	135150584	0	0.109361
8151	Amp	6	135161428	135186379	0	0.100464
8152	Amp	6	135192358	135353064	0	0.107904
8153	Amp	6	135353253	135683211	0	0.110389
8154	Amp	6	135685163	135714311	0	0.115405
8155	Amp	6	135729648	135754494	0	0.106121
8156	Amp	6	135755906	135791078	0	0.12672
8157	Amp	6	135792182	135809760	0	0.081009
8158	Amp	6	135809909	135833851	0	0.078524
8159	Amp	6	135838462	136257772	0	0.063197
8160	Amp	6	136262374	136477120	0	0.067567
8161	Amp	6	136482239	136670718	0	0.07291

8162	Amp	6	136671241	136876524	0	0.065201
8163	Amp	6	136883376	136924940	0	0.059858
8164	Amp	6	136925575	137015959	0	0.0675
8165	Amp	6	137024499	137102642	0	0.059858
8166	Amp	6	137108033	137158579	0	0.066356
8167	Amp	6	137163526	137188483	0	0.061039
8168	Amp	6	137189841	137999486	0	0.066156
8169	Amp	6	138006624	138117776	0	0.058716
8170	Amp	6	138117994	138139084	0	0.065224
8171	Amp	6	138140227	138290175	0	0.072928
8172	Amp	6	138290487	138325327	0	0.075534
8173	Amp	6	138325718	138326902	0	0.078755
8174	Amp	6	138329423	138358590	0	0.091735
8175	Amp	6	138359503	138415289	0	0.094642
8176	Amp	6	138415949	138415949	0	0.103319
8177	Amp	6	138419104	138432636	0	0.127207
8178	Amp	6	138433186	138468398	0	0.12209
8179	Amp	6	138468686	138487698	0	0.098202
8180	Amp	6	138488669	138488669	0	0.112804
8181	Amp	6	138490191	138571301	0	0.097629
8182	Amp	6	138573657	138705855	0	0.089435
8183	Amp	6	138710785	138912439	0	0.100222
8184	Amp	6	138913002	138913218	0	0.105041
8185	Amp	6	138915969	139084175	0	0.111238
8186	Amp	6	139089134	139090992	0	0.12035
8187	Amp	6	139097439	139097439	0	0.13733
8188	Amp	6	139103986	139120963	0	0.123743
8189	Amp	6	139129701	139256302	0	0.101437
8190	Amp	6	139261187	139325381	0	0.092324
8191	Amp	6	139326533	139380016	0	0.109508
8192	Amp	6	139385085	139386448	0	0.103
8193	Amp	6	139386568	139465162	0	0.107326
8194	Amp	6	139465733	139702191	0	0.102055
8195	Amp	6	139703208	139975991	0	0.103522
8196	Amp	6	139979247	140068617	0	0.1003
8197	Amp	6	140069278	140154951	0	0.095481
8198	Amp	6	140156872	140236841	0	0.10753
8199	Amp	6	140237849	140260033	0	0.111558
8200	Amp	6	140264586	140318024	0	0.113265
8201	Amp	6	140322714	140647155	0	0.115674
8202	Amp	6	140936436	141745094	0	0.103625
8203	Amp	6	141753178	141862412	0	0.106184
8204	Amp	6	141870390	142198316	0	0.104477
8205	Amp	6	142201755	142295372	0	0.100915
8206	Amp	6	142295439	142416038	0	0.096886
8207	Amp	6	142417865	142417865	0	0.10112
8208	Amp	6	142425554	142444048	0	0.083936
8209	Amp	6	142444268	143477892	0	0.082469
8210	Amp	6	143478256	143529672	0	0.08006
8211	Amp	6	143530157	143747978	0	0.086506
8212	Amp	6	143757509	143762662	0	0.09534
8213	Amp	6	143765257	143771768	0	0.097234
8214	Amp	6	143775372	143877651	0	0.11976

8215 Amp	6	143879083	144025002	0	0.097234
8216 Amp	6	144031760	144034537	0	0.099108
8217 Amp	6	144036800	144301321	0	0.090274
8218 Amp	6	144302217	144396839	0	0.0884
8219 Amp	6	144398320	144439260	0	0.090815
8220 Amp	6	144443327	144788997	0	0.115824
8221 Amp	6	144793598	144866018	0	0.113408
8222 Amp	6	144866215	145264345	0	0.10864
8223 Amp	6	145264645	145565333	0	0.114126
8224 Amp	6	145567120	145603770	0	0.10864
8225 Amp	6	145604459	146076662	0	0.085213
8226 Amp	6	146077161	146154139	0	0.079718
8227 Amp	6	146157739	146162563	0	0.077824
8228 Amp	6	146167031	146427741	0	0.100544
8229 Amp	6	146428918	146598008	0	0.104553
8230 Amp	6	146601190	146763600	0	0.09453
8231 Amp	6	146767438	147024087	0	0.113089
8232 Amp	6	147026431	147084488	0	0.125085
8233 Amp	6	147086990	147185408	0	0.120851
8234 Amp	6	147186932	147218446	0	0.123437
8235 Amp	6	147224057	147278587	0	0.13097
8236 Amp	6	147284846	147401949	0	0.110654
8237 Amp	6	147406069	147577197	0	0.120301
8238 Amp	6	147577422	147623618	0	0.154483
8239 Amp	6	147623693	147668264	0	0.155975
8240 Amp	6	147669019	147721864	0	0.148442
8241 Amp	6	147726442	148002194	0	0.111673
8242 Amp	6	148005864	148007489	0	0.094565
8243 Amp	6	148007634	148154668	0	0.111166
8244 Amp	6	148154930	148327090	0	0.113569
8245 Amp	6	148332058	148417038	0	0.10956
8246 Amp	6	148417637	148443916	0	0.120425
8247 Amp	6	148446700	148522994	0	0.130649
8248 Amp	6	148525474	148574216	0	0.122219
8249 Amp	6	148574430	148657897	0	0.116894
8250 Amp	6	148660770	148682648	0	0.131336
8251 Amp	6	148684147	148687445	0	0.110247
8252 Amp	6	148687626	148707830	0	0.118386
8253 Amp	6	148708823	148727209	0	0.127137
8254 Amp	6	148728274	148736360	0	0.13305
8255 Amp	6	148750828	148863020	0	0.114204
8256 Amp	6	148865625	148891918	0	0.138498
8257 Amp	6	148896699	148900158	0	0.114204
8258 Amp	6	148902749	148972372	0	0.112711
8259 Amp	6	148973440	149040334	0	0.13708
8260 Amp	6	149041483	149139048	0	0.118872
8261 Amp	6	149145045	149336716	0	0.11296
8262 Amp	6	149343658	149343745	0	0.130903
8263 Amp	6	149344709	149577547	0	0.135721
8264 Amp	6	149582111	149710603	0	0.145304
8265 Amp	6	149711896	149783616	0	0.135721
8266 Amp	6	149790418	149826402	0	0.157439
8267 Amp	6	149830852	149877355	0	0.168031

8268	Amp	6	149880584	149912567	0	0.175324
8269	Amp	6	149918194	149982265	0	0.168031
8270	Amp	6	149985832	150373571	0	0.148266
8271	Amp	6	150374324	150400746	0	0.158117
8272	Amp	6	150406123	150416964	0	0.16652
8273	Amp	6	150422228	150486945	0	0.158117
8274	Amp	6	150487056	150511449	0	0.155211
8275	Amp	6	150511750	150515467	0	0.168864
8276	Amp	6	150521141	150521141	0	0.175569
8277	Amp	6	150522541	150587477	0	0.165923
8278	Amp	6	150592392	150668562	0	0.130962
8279	Amp	6	150671174	150689478	0	0.14842
8280	Amp	6	150690854	150719374	0	0.166449
8281	Amp	6	150720035	150964987	0	0.152795
8282	Amp	6	150965761	151084241	0	0.157988
8283	Amp	6	151084255	151289301	0	0.148137
8284	Amp	6	151289889	151408160	0	0.152938
8285	Amp	6	151409437	151420853	0	0.154748
8286	Amp	6	151422449	151445900	0	0.162456
8287	Amp	6	151447000	151455029	0	0.165428
8288	Amp	6	151456015	151458855	0	0.17573
8289	Amp	6	151460905	151464851	0	0.173777
8290	Amp	6	151465042	151473260	0.028398	0.191787
8291	Amp	6	151476786	151486023	0.187743	0.210225
8292	Amp	6	151488465	151492189	0.273081	0.218335
8293	Amp	6	151494959	151509463	0.355162	0.225389
8294	Amp	6	151514103	151551828	0.243425	0.215087
8295	Amp	6	151556555	151576612	0.347934	0.225299
8296	Amp	6	151577371	151577371	0.483654	0.237443
8297	Amp	6	151578401	151593887	0.693887	0.255015
8298	Amp	6	151595533	151612010	0.596354	0.247307
8299	Amp	6	151612040	151615715	0.66072	0.25234
8300	Amp	6	151615877	151643069	0.676804	0.253844
8301	Amp	6	151650105	151690109	0.743132	0.259662
8302	Amp	6	151691604	151737080	0.62858	0.249748
8303	Amp	6	151739277	151744751	0.548372	0.242694
8304	Amp	6	151746734	151749474	0.405699	0.230551
8305	Amp	6	151751627	151756524	0.264784	0.217566
8306	Amp	6	151757576	151766195	0.223025	0.212985
8307	Amp	6	151768889	151769400	0.309523	0.221679
8308	Amp	6	151772771	151774197	0.25804	0.216646
8309	Amp	6	151774325	151780114	0.156508	0.206434
8310	Amp	6	151783416	151820487	0.286675	0.219201
8311	Amp	6	151821167	151834932	0.236222	0.214736
8312	Amp	6	151834952	151835062	0.033661	0.192632
8313	Amp	6	151835136	151858527	0.123589	0.202721
8314	Amp	6	151858705	151865979	0.033661	0.192632
8315	Amp	6	151865993	151883075	0.105051	0.201248
8316	Amp	6	151884176	151888300	0.028398	0.191987
8317	Amp	6	151903601	151910153	0	0.176665
8318	Amp	6	151913175	151949969	0	0.163425
8319	Amp	6	151954580	151987838	0	0.167803
8320	Amp	6	151989259	151998974	0	0.165993

8321	Amp	6	151999013	152084177	0	0.173954
8322	Amp	6	152084830	152098985	0	0.169575
8323	Amp	6	152098998	152099042	0	0.171466
8324	Amp	6	152099995	152111436	0	0.177653
8325	Amp	6	152114407	152135893	0	0.156137
8326	Amp	6	152140246	152147172	0	0.143946
8327	Amp	6	152147308	152187044	0	0.139992
8328	Amp	6	152187062	152264874	0	0.138488
8329	Amp	6	152271616	152273884	0	0.132301
8330	Amp	6	152277895	152715165	0	0.124191
8331	Amp	6	152724778	152823857	0	0.136635
8332	Amp	6	152826788	152859280	0	0.131443
8333	Amp	6	152859378	152936585	0	0.129849
8334	Amp	6	152942658	153001298	0	0.136714
8335	Amp	6	153002031	153089960	0	0.138118
8336	Amp	6	153090120	153147363	0	0.1333
8337	Amp	6	153150857	153177091	0	0.124549
8338	Amp	6	153180556	153284027	0	0.126009
8339	Amp	6	153286187	153287973	0	0.131729
8340	Amp	6	153288959	153356635	0	0.129326
8341	Amp	6	153358848	153554764	0	0.124302
8342	Amp	6	153557639	153671786	0	0.118582
8343	Amp	6	153672562	153712427	0	0.122512
8344	Amp	6	153721068	153838537	0	0.121051
8345	Amp	6	153838694	153917555	0	0.114506
8346	Amp	6	153919961	154087567	0	0.107641
8347	Amp	6	154088859	154115101	0	0.106237
8348	Amp	6	154115579	154135053	0	0.056308
8349	Amp	6	154135571	154169524	0	0.054417
8350	Amp	6	154169890	154186260	0	0.098497
8351	Amp	6	154188467	154201527	0	0.054417
8352	Amp	6	154203091	154428219	0	0.061242
8353	Amp	6	154428231	154444474	0	0.067329
8354	Amp	6	154446674	154451079	0	0.074208
8355	Amp	6	154456801	154467499	0	0.067383
8356	Amp	6	154467618	154920314	0	0.071936
8357	Amp	6	154921927	154925584	0	0.070275
8358	Amp	6	154931418	155061516	0	0.065723
8359	Amp	6	155063441	155088485	0	0.067252
8360	Amp	6	155092903	155681295	0	0.054808
8361	Amp	6	155683529	155755140	0	0.047929
8362	Amp	6	155763290	155901263	0	0.056109
8363	Amp	6	155902339	156352228	0	0.063056
8364	Amp	6	156354361	156515322	0	0.073401
8365	Amp	6	156516434	156731251	0	0.063056
8366	Amp	6	156732369	157101442	0	0.056969
8367	Amp	6	157110544	157139421	0	0.062874
8368	Amp	6	157146023	157194429	0	0.068964
8369	Amp	6	157214426	157498120	0	0.066333
8370	Amp	6	157502450	157781962	0	0.072774
8371	Amp	6	157782418	157825344	0	0.080158
8372	Amp	6	157825615	158007555	0	0.074069
8373	Amp	6	158008276	158050700	0	0.078126

8374	Amp	6	158050928	158257838	0	0.083729
8375	Amp	6	158259301	158292791	0	0.069904
8376	Amp	6	158292982	158438863	0	0.064301
8377	Amp	6	158442440	158496132	0	0.068493
8378	Amp	6	158496606	158710710	0	0.072735
8379	Amp	6	158711433	158715095	0	0.077231
8380	Amp	6	158719697	159109311	0	0.072989
8381	Amp	6	159110211	159122583	0	0.084971
8382	Amp	6	159123400	159142338	0	0.072989
8383	Amp	6	159142516	159171745	0	0.064301
8384	Amp	6	159173132	159182926	0	0.060371
8385	Amp	6	159185841	159205711	0	0.057813
8386	Amp	6	159206020	159539716	0	0.062339
8387	Amp	6	159540016	159743507	0	0.057813
8388	Amp	6	159749022	160096806	0	0.063644
8389	Amp	6	160100199	160191227	0	0.066474
8390	Amp	6	160192675	160240066	0	0.060643
8391	Amp	6	160244811	160570446	0	0.065312
8392	Amp	6	160571049	160594248	0	0.060643
8393	Amp	6	160601499	160605941	0	0.054737
8394	Amp	6	160647076	161187984	0	0.04779
8395	Amp	6	161188115	161237403	0	0.05385
8396	Amp	6	161245622	161283953	0	0.073289
8397	Amp	6	161285910	161307844	0	0.075782
8398	Amp	6	161312784	161423246	0	0.106859
8399	Amp	6	161423807	161434438	0	0.109598
8400	Amp	6	161434753	161459406	0	0.082959
8401	Amp	6	161460090	161515034	0	0.0769
8402	Amp	6	161515153	161515966	0	0.066845
8403	Amp	6	161516075	161819738	0	0.078212
8404	Amp	6	161819754	161835891	0	0.080288
8405	Amp	6	161835989	161911074	0	0.078212
8406	Amp	6	161914157	161941501	0	0.079921
8407	Amp	6	161945234	162062582	0	0.078212
8408	Amp	6	162062922	162062922	0	0.083855
8409	Amp	6	162173909	162703120	0	0.081116
8410	Amp	6	162704934	163438777	0	0.100981
8411	Amp	6	163444396	163975756	0	0.057238
8412	Amp	6	164142925	164881345	0	0.063565
8413	Amp	6	164881802	164970880	0	0.065963
8414	Amp	6	164971212	165096612	0	0.063565
8415	Amp	6	165097097	165284673	0	0.065066
8416	Amp	6	165285462	165308676	0	0.062573
8417	Amp	6	165323080	166092765	0	0.073172
8418	Amp	6	166093621	166195900	0	0.074647
8419	Amp	6	166197350	166291144	0	0.064048
8420	Amp	6	166291256	166302452	0	0.067972
8421	Amp	6	166313078	166441809	0	0.0738
8422	Amp	6	166446237	166506267	0	0.069875
8423	Amp	6	166506625	166949985	0	0.075021
8424	Amp	6	166950460	166978557	0	0.071814
8425	Amp	6	166985794	167042125	0	0.081086
8426	Amp	6	167045257	167862052	0	0.071814

8427	Amp	6	167862339	167922549	0	0.074203
8428	Amp	6	167924209	168461894	0	0.071814
8429	Amp	6	168466737	168496167	0	0.066978
8430	Amp	6	168564608	168609477	0	0.079945
8431	Amp	6	168616528	168708494	0	0.091498
8432	Amp	6	169097598	169102946	0	0.066978
8433	Amp	6	169103115	169217631	0	0.070216
8434	Amp	6	169217761	169508853	0	0.068742
8435	Amp	6	169510216	169674083	0	0.073448
8436	Amp	6	169675930	169695889	0	0.07021
8437	Amp	6	169699302	169719764	0	0.065503
8438	Amp	6	169722607	169763095	0	0.069762
8439	Amp	6	169767293	169767522	0	0.065503
8440	Amp	6	170238867	170484200	0	0.075079
8441	Amp	6	170486852	170555643	0	0.069252
8442	Amp	6	170555865	170572042	0	0.065194
8443	Amp	6	170576704	170596889	0	0.072414
8444	Amp	7	665647	785857	0	0.07148
8445	Amp	7	786149	793348	0	0.073614
8446	Amp	7	796202	823656	0	0.062248
8447	Amp	7	823919	855193	0	0.071405
8448	Amp	7	858016	873118	0	0.080009
8449	Amp	7	879502	896168	0	0.083701
8450	Amp	7	902112	902112	0	0.075097
8451	Amp	7	1204095	1216602	0	0.072169
8452	Amp	7	1221000	1276832	0	0.0815
8453	Amp	7	1278706	1654767	0	0.083316
8454	Amp	7	1656994	1846697	0	0.079624
8455	Amp	7	1846810	1897057	0	0.090627
8456	Amp	7	1909042	1946346	0	0.081295
8457	Amp	7	1948789	1954264	0	0.097628
8458	Amp	7	1963098	1992556	0	0.077469
8459	Amp	7	1995946	2145267	0	0.084501
8460	Amp	7	2147836	2149661	0	0.082749
8461	Amp	7	2152435	2558569	0	0.075717
8462	Amp	7	2570769	2570769	0	0.059384
8463	Amp	7	2571320	3112769	0	0.051062
8464	Amp	7	3112802	3156936	0	0.049548
8465	Amp	7	3157831	3184559	0	0.047414
8466	Amp	7	3185169	3269548	0	0.04602
8467	Amp	7	3273238	4652236	0	0.048031
8468	Amp	7	4655371	4899368	0	0.049642
8469	Amp	7	4899729	5166313	0	0.044771
8470	Amp	7	5169891	5336294	0	0.052835
8471	Amp	7	5336401	5365415	0	0.050926
8472	Amp	7	5366951	5438969	0	0.061252
8473	Amp	7	5442243	5469099	0	0.063722
8474	Amp	7	5469510	5591633	0	0.070497
8475	Amp	7	5597081	5624426	0	0.068027
8476	Amp	7	5625497	5653282	0	0.061252
8477	Amp	7	5656494	5672470	0	0.050926
8478	Amp	7	5676414	5689766	0	0.049246
8479	Amp	7	5691294	5709591	0	0.047236

8480	Amp	7	5999270	5999449	0	0.052655
8481	Amp	7	5999498	5999498	0	0.044592
8482	Amp	7	6002602	6027516	0	0.046106
8483	Amp	7	6027612	6388266	0	0.044684
8484	Amp	7	6390644	6625354	0	0.046706
8485	Amp	7	6626870	6695312	0	0.050749
8486	Amp	7	6698852	6703120	0	0.045329
8487	Amp	7	6703466	6735057	0	0.043538
8488	Amp	7	6736405	7115269	0	0.041286
8489	Amp	7	7119014	7128862	0	0.043036
8490	Amp	7	7129374	7304349	0	0.041015
8491	Amp	7	7304419	7308023	0	0.038631
8492	Amp	7	7308077	7791730	0	0.036561
8493	Amp	7	7791939	7886657	0	0.03495
8494	Amp	7	7890476	8112612	0	0.041594
8495	Amp	7	8113625	8493103	0	0.043596
8496	Amp	7	8493214	8500467	0	0.036952
8497	Amp	7	8500606	8522832	0	0.038424
8498	Amp	7	8522928	8761082	0	0.035218
8499	Amp	7	9192894	9381514	0	0.02966
8500	Amp	7	9383693	9634700	0	0.027658
8501	Amp	7	9640392	9707566	0	0.033192
8502	Amp	7	9996166	10204235	0	0.057778
8503	Amp	7	10206368	10858412	0	0.06253
8504	Amp	7	10861057	11136664	0	0.064366
8505	Amp	7	11136765	11351421	0	0.047101
8506	Amp	7	11353507	11437737	0	0.052852
8507	Amp	7	11437852	11478312	0	0.061862
8508	Amp	7	11480268	11566443	0	0.080908
8509	Amp	7	11570408	11606886	0	0.076156
8510	Amp	7	11607531	11862186	0	0.070405
8511	Amp	7	11862726	12530751	0	0.058392
8512	Amp	7	12530763	13110237	0	0.061354
8513	Amp	7	13118171	13439053	0	0.052866
8514	Amp	7	13439075	13882550	0	0.051031
8515	Amp	7	13885046	14026578	0	0.031985
8516	Amp	7	14041079	14461908	0	0.026451
8517	Amp	7	14464528	14523332	0	0.023263
8518	Amp	7	14525294	14686719	0	0.029083
8519	Amp	7	14687078	14698063	0	0.021802
8520	Amp	7	14698374	14804711	0	0.032582
8521	Amp	7	14806209	15026050	0	0.036311
8522	Amp	7	15030557	15123385	0	0.029352
8523	Amp	7	15127545	15132787	0	0.031818
8524	Amp	7	15132838	15364113	0	0.035201
8525	Amp	7	15381539	15489442	0	0.021802
8526	Amp	7	15497071	15528480	0	0.028253
8527	Amp	7	15528974	15783347	0	0.022433
8528	Amp	7	15786581	15843927	0	0.030431
8529	Amp	7	15843966	15909420	0	0.027482
8530	Amp	7	15910066	16267560	0	0.031991
8531	Amp	7	16269127	16284560	0	0.02554
8532	Amp	7	16284588	16377469	0	0.029806

8533	Amp	7	16377549	16391969	0	0.032788
8534	Amp	7	16394067	16609749	0	0.041467
8535	Amp	7	16609866	16648521	0	0.043856
8536	Amp	7	16649277	16740455	0	0.03454
8537	Amp	7	16740530	17759976	0	0.039399
8538	Amp	7	17761192	17769504	0	0.0483
8539	Amp	7	17769740	17782557	0	0.043791
8540	Amp	7	17782762	17805198	0	0.035112
8541	Amp	7	17805641	18563870	0	0.03213
8542	Amp	7	18566051	18772599	0	0.038054
8543	Amp	7	18772791	18866614	0	0.044391
8544	Amp	7	18866918	18867407	0	0.038054
8545	Amp	7	19748494	19748494	0	0.03213
8546	Amp	7	19749151	19872840	0	0.033621
8547	Amp	7	19878240	20220306	0	0.029221
8548	Amp	7	20221254	20248295	0	0.032647
8549	Amp	7	20261644	20326905	0	0.039717
8550	Amp	7	20326955	20376621	0	0.032647
8551	Amp	7	20380528	20403827	0	0.043982
8552	Amp	7	20403889	20425438	0	0.041593
8553	Amp	7	20427324	20435072	0	0.046247
8554	Amp	7	20435721	20497561	0	0.042822
8555	Amp	7	20497795	20524587	0	0.048738
8556	Amp	7	20528822	20603743	0	0.047266
8557	Amp	7	20604187	20689297	0	0.04135
8558	Amp	7	20696954	20716919	0	0.063679
8559	Amp	7	20726008	20754042	0	0.099264
8560	Amp	7	20755699	20869398	0	0.076935
8561	Amp	7	20871808	20959983	0	0.081129
8562	Amp	7	20960341	20960341	0	0.079639
8563	Amp	7	20960726	21020899	0	0.100831
8564	Amp	7	21024072	21054993	0	0.079639
8565	Amp	7	21055118	21153518	0	0.095707
8566	Amp	7	21155251	21240333	0	0.093225
8567	Amp	7	21244924	21248594	0	0.088723
8568	Amp	7	21248800	21324469	0	0.081785
8569	Amp	7	21324497	21381593	0	0.084328
8570	Amp	7	21381997	21403014	0	0.091812
8571	Amp	7	21405199	21503273	0	0.097228
8572	Amp	7	21506072	21601000	0	0.092257
8573	Amp	7	21601572	21646515	0	0.094131
8574	Amp	7	21646654	21741516	0	0.088716
8575	Amp	7	21744522	21750042	0	0.081231
8576	Amp	7	21750692	21853886	0	0.086388
8577	Amp	7	21854093	21982047	0	0.092236
8578	Amp	7	21982306	22027590	0	0.100322
8579	Amp	7	22033770	22415274	0	0.094473
8580	Amp	7	22417117	22793577	0	0.112322
8581	Amp	7	22793781	23064267	0	0.119108
8582	Amp	7	23065133	23184417	0	0.110247
8583	Amp	7	23190613	23190613	0	0.125157
8584	Amp	7	23190935	23256294	0	0.12
8585	Amp	7	23256742	23695351	0	0.105091

8586	Amp	7	23697004	23706453	0	0.119926
8587	Amp	7	23706539	24091189	0	0.127038
8588	Amp	7	24093051	24147181	0	0.125164
8589	Amp	7	24147194	24168916	0	0.118378
8590	Amp	7	24172378	24172378	0	0.110275
8591	Amp	7	24173077	24264259	0	0.112748
8592	Amp	7	24271213	24272598	0	0.108553
8593	Amp	7	24273406	24608027	0	0.113157
8594	Amp	7	24618459	24635836	0	0.083013
8595	Amp	7	24636534	24840701	0	0.075136
8596	Amp	7	24840992	24876974	0	0.088019
8597	Amp	7	24880315	24978343	0	0.106416
8598	Amp	7	24983449	25024426	0	0.132
8599	Amp	7	25030895	25086222	0	0.136793
8600	Amp	7	25089169	25117262	0	0.136878
8601	Amp	7	25120815	25192500	0	0.134011
8602	Amp	7	25193777	25193777	0	0.131538
8603	Amp	7	25204357	25415761	0	0.101708
8604	Amp	7	25419242	25426310	0	0.09475
8605	Amp	7	25427204	25521085	0	0.105126
8606	Amp	7	25523928	25547963	0	0.115806
8607	Amp	7	25548758	25684730	0	0.103151
8608	Amp	7	25684843	25709198	0	0.122639
8609	Amp	7	25710042	25833490	0	0.120096
8610	Amp	7	25835371	26198480	0	0.123988
8611	Amp	7	26198837	26432141	0	0.108951
8612	Amp	7	26432254	26469001	0	0.118603
8613	Amp	7	26471976	26478493	0	0.12387
8614	Amp	7	26478956	26512202	0	0.109567
8615	Amp	7	26518610	26679458	0	0.1043
8616	Amp	7	26684660	26705467	0	0.099725
8617	Amp	7	26706015	26813985	0	0.10322
8618	Amp	7	26819304	26830726	0	0.100147
8619	Amp	7	26831766	26929851	0	0.096652
8620	Amp	7	26936819	27030978	0	0.089771
8621	Amp	7	27039170	27134223	0	0.083902
8622	Amp	7	27134697	27310589	0	0.088643
8623	Amp	7	27310701	27657330	0	0.084976
8624	Amp	7	27660863	27724894	0	0.111205
8625	Amp	7	27730500	27837308	0	0.103441
8626	Amp	7	27842270	27902696	0	0.097104
8627	Amp	7	27904293	27910336	0	0.1102
8628	Amp	7	27917345	28024245	0	0.101047
8629	Amp	7	28024718	28387240	0	0.097155
8630	Amp	7	28389175	28766252	0	0.105474
8631	Amp	7	28768774	29067262	0	0.102812
8632	Amp	7	29070361	29167159	0	0.107397
8633	Amp	7	29167315	29167315	0	0.090377
8634	Amp	7	29499378	29628162	0	0.072592
8635	Amp	7	29752062	30007691	0	0.076596
8636	Amp	7	30009684	30171093	0	0.083546
8637	Amp	7	30174562	30625425	0	0.078961
8638	Amp	7	30633135	30676420	0	0.085618

8639	Amp	7	30677027	31324199	0	0.078961
8640	Amp	7	31330264	31436659	0	0.110969
8641	Amp	7	31437780	31483062	0	0.115722
8642	Amp	7	31483379	31679125	0	0.130804
8643	Amp	7	31680040	31696878	0	0.136028
8644	Amp	7	31697511	31753026	0	0.138213
8645	Amp	7	31754120	31785753	0	0.110239
8646	Amp	7	31786481	31811423	0	0.108054
8647	Amp	7	31811743	31844507	0	0.100984
8648	Amp	7	31845462	31977800	0	0.109658
8649	Amp	7	31977881	32096309	0	0.114244
8650	Amp	7	32100914	32143032	0	0.130228
8651	Amp	7	32144065	32167564	0	0.12537
8652	Amp	7	32167970	32254032	0	0.131786
8653	Amp	7	32254382	32265253	0	0.097793
8654	Amp	7	32265662	32265779	0	0.093208
8655	Amp	7	32267174	32316787	0	0.088998
8656	Amp	7	32316878	32341945	0	0.097031
8657	Amp	7	32342957	32376295	0	0.091725
8658	Amp	7	32379057	32419822	0	0.081759
8659	Amp	7	32420621	32500373	0	0.077005
8660	Amp	7	32504827	32530324	0	0.066262
8661	Amp	7	32531192	32578280	0	0.073994
8662	Amp	7	32580888	32717282	0	0.066262
8663	Amp	7	32717770	32717770	0	0.076364
8664	Amp	7	32725825	32745957	0	0.08167
8665	Amp	7	32751955	32796488	0	0.086299
8666	Amp	7	32796745	32895057	0	0.076196
8667	Amp	7	32895912	32985576	0	0.108405
8668	Amp	7	32988301	33039402	0	0.111044
8669	Amp	7	33039532	33084393	0	0.113181
8670	Amp	7	33086063	33086063	0	0.085441
8671	Amp	7	33150545	33225765	0	0.091554
8672	Amp	7	33231132	33319743	0	0.086926
8673	Amp	7	33327392	33491798	0	0.084287
8674	Amp	7	33493716	33584478	0	0.087409
8675	Amp	7	33586978	33645379	0	0.096939
8676	Amp	7	33645565	33953371	0	0.09247
8677	Amp	7	33953827	33966369	0	0.09863
8678	Amp	7	33972460	34079761	0	0.09168
8679	Amp	7	34084771	34196216	0	0.073299
8680	Amp	7	34196858	34538377	0	0.079141
8681	Amp	7	34540330	35029184	0	0.081193
8682	Amp	7	35033119	35427211	0	0.092493
8683	Amp	7	35428183	35474289	0	0.056908
8684	Amp	7	35480749	35630083	0	0.045608
8685	Amp	7	35632767	35702418	0	0.039192
8686	Amp	7	35703475	35756767	0	0.042376
8687	Amp	7	35761923	36581144	0	0.048616
8688	Amp	7	36586551	36877279	0	0.059804
8689	Amp	7	36878390	37026863	0	0.064189
8690	Amp	7	37026926	37104461	0	0.053001
8691	Amp	7	37104987	37180265	0	0.064932

8692	Amp	7	37180442	37363179	0	0.062795
8693	Amp	7	37377527	37402077	0	0.050864
8694	Amp	7	37404299	37446874	0	0.046479
8695	Amp	7	37451585	38225779	0	0.034397
8696	Amp	7	38226749	38419189	0	0.031212
8697	Amp	7	38419301	38964504	0	0.033731
8698	Amp	7	38964547	39810548	0	0.037826
8699	Amp	7	39814008	40022228	0	0.041743
8700	Amp	7	40026426	40135967	0	0.039225
8701	Amp	7	40136125	40143079	0	0.049199
8702	Amp	7	40145413	40176295	0	0.047469
8703	Amp	7	40180618	40242475	0	0.037495
8704	Amp	7	40242782	40331576	0	0.04466
8705	Amp	7	40341671	40676810	0	0.037495
8706	Amp	7	40677102	40677627	0	0.0334
8707	Amp	7	40685332	40923607	0	0.031126
8708	Amp	7	40923876	41299906	0	0.035915
8709	Amp	7	41302296	41537910	0	0.033863
8710	Amp	7	41542843	41606670	0	0.029074
8711	Amp	7	41612765	41656547	0	0.060962
8712	Amp	7	41660662	41899140	0	0.065609
8713	Amp	7	41902494	41998585	0	0.067947
8714	Amp	7	42000502	42041503	0	0.0633
8715	Amp	7	42041627	42116659	0	0.081361
8716	Amp	7	42116764	42123943	0	0.064917
8717	Amp	7	42124261	42425227	0	0.042675
8718	Amp	7	42425329	42647310	0	0.044407
8719	Amp	7	42649966	43263085	0	0.046771
8720	Amp	7	43263185	43819887	0	0.044983
8721	Amp	7	43820292	43834159	0	0.042587
8722	Amp	7	43839138	44139144	0	0.046007
8723	Amp	7	44143911	44210375	0	0.077635
8724	Amp	7	44215435	44290290	0	0.086275
8725	Amp	7	44293948	44397806	0	0.077635
8726	Amp	7	44403856	44428965	0	0.046007
8727	Amp	7	44430481	44504414	0	0.065005
8728	Amp	7	44506453	44612198	0	0.046007
8729	Amp	7	44615159	44915164	0	0.049205
8730	Amp	7	44918525	45004213	0	0.051919
8731	Amp	7	45007019	45156865	0	0.061236
8732	Amp	7	45160775	45208436	0	0.062816
8733	Amp	7	45208619	45353748	0	0.059396
8734	Amp	7	45355671	45529392	0	0.063153
8735	Amp	7	45529506	45913974	0	0.061422
8736	Amp	7	45914561	45917517	0	0.059058
8737	Amp	7	45918928	45922075	0	0.061618
8738	Amp	7	45925015	46011030	0	0.057014
8739	Amp	7	46011187	46021093	0	0.059073
8740	Amp	7	46021883	46085193	0	0.0714
8741	Amp	7	46086390	46423975	0	0.059073
8742	Amp	7	46425432	46451586	0	0.061561
8743	Amp	7	46451796	46621773	0	0.059223
8744	Amp	7	46624273	46892366	0	0.055465

8745 Amp	7	46892666	47009205	0	0.052343
8746 Amp	7	47012272	47217734	0	0.053764
8747 Amp	7	47218903	47286056	0	0.056955
8748 Amp	7	47286094	47333972	0	0.060528
8749 Amp	7	47335172	47360241	0	0.064788
8750 Amp	7	47363969	47364066	0	0.063348
8751 Amp	7	47366984	47447564	0	0.066505
8752 Amp	7	47448137	47516051	0	0.060146
8753 Amp	7	47516501	47729999	0	0.063418
8754 Amp	7	47732488	47749740	0	0.060227
8755 Amp	7	47750320	47881040	0	0.058806
8756 Amp	7	47881190	47885835	0	0.057226
8757 Amp	7	47893338	48018184	0	0.062092
8758 Amp	7	48022427	48030897	0	0.057226
8759 Amp	7	48031960	48050357	0	0.054269
8760 Amp	7	48052986	48080103	0	0.051111
8761 Amp	7	48082475	48155182	0	0.056126
8762 Amp	7	48155992	48206078	0	0.061787
8763 Amp	7	48206294	48226108	0	0.056126
8764 Amp	7	48226388	48344988	0	0.051866
8765 Amp	7	48348325	48511227	0	0.046851
8766 Amp	7	48511508	48582540	0	0.050519
8767 Amp	7	48583206	48889621	0	0.039257
8768 Amp	7	48890043	48902043	0	0.035985
8769 Amp	7	48902065	48977175	0	0.032412
8770 Amp	7	48984890	49215581	0	0.028743
8771 Amp	7	49216979	49492109	0	0.032914
8772 Amp	7	49492581	49507334	0	0.038253
8773 Amp	7	49507594	49773257	0	0.046394
8774 Amp	7	49778361	49923210	0	0.041056
8775 Amp	7	49924325	49944041	0	0.036737
8776 Amp	7	49949375	49959921	0	0.040929
8777 Amp	7	49960101	50122239	0	0.050771
8778 Amp	7	50133600	50192169	0	0.055414
8779 Amp	7	50193056	50519319	0	0.06864
8780 Amp	7	50523372	50701837	0	0.077982
8781 Amp	7	50702932	50870509	0	0.08409
8782 Amp	7	50870747	50892846	0	0.077982
8783 Amp	7	50895763	51035209	0	0.073339
8784 Amp	7	51037519	51195187	0	0.069147
8785 Amp	7	51204037	51273048	0	0.070723
8786 Amp	7	51277358	51336839	0	0.072155
8787 Amp	7	51339363	51355194	0	0.067968
8788 Amp	7	51355829	51450066	0	0.07289
8789 Amp	7	51451313	51479219	0	0.079749
8790 Amp	7	51479422	51493506	0	0.074594
8791 Amp	7	51493840	51531012	0	0.078569
8792 Amp	7	51537348	51676047	0	0.082235
8793 Amp	7	51679842	51732242	0	0.07171
8794 Amp	7	51740606	52272403	0	0.076421
8795 Amp	7	52279296	52309413	0	0.066578
8796 Amp	7	52311221	52334263	0	0.061657
8797 Amp	7	52341209	52363996	0	0.06503

8798	Amp	7	52364846	52367431	0	0.070973
8799	Amp	7	52370238	52422691	0	0.086745
8800	Amp	7	52426557	52479844	0	0.091637
8801	Amp	7	52481192	52591481	0	0.093031
8802	Amp	7	52591547	52593955	0	0.08814
8803	Amp	7	52596340	52682077	0	0.097908
8804	Amp	7	52687319	52828822	0	0.105002
8805	Amp	7	52829323	52843955	0	0.110935
8806	Amp	7	52846015	52883579	0	0.105572
8807	Amp	7	52884366	52890186	0	0.109047
8808	Amp	7	52890647	52941605	0	0.105673
8809	Amp	7	52941687	53007007	0	0.110152
8810	Amp	7	53008673	53129456	0	0.100043
8811	Amp	7	53129510	53129510	0	0.091663
8812	Amp	7	53140373	53170796	0	0.095539
8813	Amp	7	53171525	53249136	0	0.104606
8814	Amp	7	53250694	53251092	0	0.101132
8815	Amp	7	53253990	53286783	0	0.102636
8816	Amp	7	53291940	53379288	0	0.097926
8817	Amp	7	53379564	53383781	0	0.104718
8818	Amp	7	53536037	53618704	0	0.091312
8819	Amp	7	53620064	53668157	0	0.08452
8820	Amp	7	53673369	53828834	0	0.078586
8821	Amp	7	53829436	53848157	0	0.085094
8822	Amp	7	53848554	53851786	0	0.102387
8823	Amp	7	53853455	53971548	0	0.105966
8824	Amp	7	53971933	54054565	0	0.104496
8825	Amp	7	54059862	54080613	0	0.102991
8826	Amp	7	54083092	54117015	0	0.09882
8827	Amp	7	54117146	54159607	0	0.076227
8828	Amp	7	54159924	54329793	0	0.074833
8829	Amp	7	54333159	54336246	0	0.079321
8830	Amp	7	54336292	54390281	0	0.081679
8831	Amp	7	54390669	54403491	0	0.084034
8832	Amp	7	54409794	54584774	0	0.078104
8833	Amp	7	54584818	54616406	0	0.07664
8834	Amp	7	54621151	54701717	0	0.066033
8835	Amp	7	54703793	54726261	0	0.062454
8836	Amp	7	54727577	54729010	0	0.058219
8837	Amp	7	54729789	54732728	0	0.073364
8838	Amp	7	54732814	54842970	0	0.07825
8839	Amp	7	54843688	54914815	0	0.096894
8840	Amp	7	54917425	54947654	0	0.100363
8841	Amp	7	54948807	55007093	0	0.095477
8842	Amp	7	55008114	55192842	0	0.109063
8843	Amp	7	55195177	55202271	0	0.101089
8844	Amp	7	55203753	55248536	0	0.095544
8845	Amp	7	55249558	55256626	0	0.104624
8846	Amp	7	55258817	55305743	0	0.102266
8847	Amp	7	55306309	55323118	0	0.093186
8848	Amp	7	55323309	55430912	0	0.079755
8849	Amp	7	55431062	55537736	0	0.083242
8850	Amp	7	55540043	55583659	0	0.081183

8851	Amp	7	55588093	55634014	0	0.093543
8852	Amp	7	55637286	55755888	0	0.085944
8853	Amp	7	55765381	55938298	0	0.088637
8854	Amp	7	55949241	56105935	0	0.076277
8855	Amp	7	56112023	56112023	0	0.079107
8856	Amp	7	56112866	56130034	0	0.081592
8857	Amp	7	56130508	56247091	0	0.083437
8858	Amp	7	56251776	56257539	0	0.064132
8859	Amp	7	56257550	56305580	0	0.072834
8860	Amp	7	56314362	56343876	0	0.060251
8861	Amp	7	56356123	56356123	0	0.05155
8862	Amp	7	56357046	56641562	0	0.050118
8863	Amp	7	57130116	57130954	0	0.05261
8864	Amp	7	57134459	57166220	0	0.063643
8865	Amp	7	57170718	57176334	0	0.066556
8866	Amp	7	57184979	57184979	0	0.068815
8867	Amp	7	57185111	57854817	0	0.071854
8868	Amp	7	57861998	57864241	0	0.068385
8869	Amp	7	57876479	57876479	0	0.078682
8870	Amp	7	63672748	63672748	0	0.070541
8871	Amp	7	63675854	63675854	0	0.104623
8872	Amp	7	63677725	63690237	0	0.168663
8873	Amp	7	63856364	63857929	0	0.173727
8874	Amp	7	63860912	63879429	0	0.171391
8875	Amp	7	63887239	63904796	0	0.173586
8876	Amp	7	63906355	63919204	0	0.160416
8877	Amp	7	63921298	63924054	0	0.16549
8878	Amp	7	63925505	63960261	0	0.162833
8879	Amp	7	63960688	64025321	0	0.171287
8880	Amp	7	64026945	64044797	0	0.166212
8881	Amp	7	64045459	64060367	0	0.178695
8882	Amp	7	64330045	64363059	0	0.141079
8883	Amp	7	64363664	64413771	0	0.15534
8884	Amp	7	64414288	64420499	0	0.141079
8885	Amp	7	64420964	64524347	0	0.144675
8886	Amp	7	64534471	64625850	0	0.146178
8887	Amp	7	64625862	64671727	0	0.142377
8888	Amp	7	64672511	64717253	0	0.138108
8889	Amp	7	64722099	64796104	0	0.136604
8890	Amp	7	64804363	64804567	0	0.132066
8891	Amp	7	64804946	64857763	0	0.143466
8892	Amp	7	64861878	64862184	0	0.153415
8893	Amp	7	64863857	64887244	0	0.156303
8894	Amp	7	64887425	64963274	0	0.144903
8895	Amp	7	64966353	64966353	0	0.138819
8896	Amp	7	64966621	64984586	0	0.146048
8897	Amp	7	64991039	64997366	0	0.136099
8898	Amp	7	64999173	65052476	0	0.130691
8899	Amp	7	65714988	65720303	0	0.111706
8900	Amp	7	65721325	65733190	0	0.100673
8901	Amp	7	65735810	65796311	0	0.108402
8902	Amp	7	65827971	66050328	0	0.123101
8903	Amp	7	66051132	66168580	0	0.118285

8904	Amp	7	66169334	66265811	0	0.122012
8905	Amp	7	66279389	66295072	0	0.128999
8906	Amp	7	66295995	66410562	0	0.146715
8907	Amp	7	66416428	66462626	0	0.140601
8908	Amp	7	66465381	66567655	0	0.114516
8909	Amp	7	66571905	67034713	0	0.093887
8910	Amp	7	67034893	67046046	0	0.090849
8911	Amp	7	67049271	67056110	0	0.09399
8912	Amp	7	67058534	67321387	0	0.087003
8913	Amp	7	67346326	67448782	0	0.088621
8914	Amp	7	67449168	67536780	0	0.091209
8915	Amp	7	67539527	67565256	0	0.102852
8916	Amp	7	67567824	67618282	0	0.100264
8917	Amp	7	67624096	67681876	0	0.096536
8918	Amp	7	67682245	67682596	0	0.109201
8919	Amp	7	67686531	67788243	0	0.097558
8920	Amp	7	67788489	67817736	0	0.084893
8921	Amp	7	67823579	67842968	0	0.090973
8922	Amp	7	67846547	67882884	0	0.093776
8923	Amp	7	67883038	68189093	0	0.090889
8924	Amp	7	68189285	68416032	0	0.092953
8925	Amp	7	68419095	68434659	0	0.088885
8926	Amp	7	68435432	68469423	0	0.094763
8927	Amp	7	68471877	68550028	0	0.092751
8928	Amp	7	68550218	68664591	0	0.095836
8929	Amp	7	68666988	68690582	0	0.100678
8930	Amp	7	68691627	68742798	0	0.10916
8931	Amp	7	68745474	68776775	0	0.11104
8932	Amp	7	68778135	68863611	0	0.108211
8933	Amp	7	68864358	68994747	0	0.113632
8934	Amp	7	69000044	69006829	0	0.110491
8935	Amp	7	69010754	69032661	0	0.105623
8936	Amp	7	69035566	69065727	0	0.107039
8937	Amp	7	69065806	69070385	0	0.103433
8938	Amp	7	69073913	69254263	0	0.106371
8939	Amp	7	69257165	69280172	0	0.108711
8940	Amp	7	69283494	69300521	0	0.110634
8941	Amp	7	69300576	69437913	0	0.119079
8942	Amp	7	69439260	69532048	0	0.125299
8943	Amp	7	69532403	69532403	0	0.12342
8944	Amp	7	69535729	69550284	0	0.129573
8945	Amp	7	69559941	69567751	0	0.138884
8946	Amp	7	69568004	69576243	0	0.13453
8947	Amp	7	69579309	69710667	0	0.114254
8948	Amp	7	69710705	69715612	0	0.116223
8949	Amp	7	69722064	69801093	0	0.125481
8950	Amp	7	69804315	69812389	0	0.11617
8951	Amp	7	69816125	69826736	0	0.111329
8952	Amp	7	69827169	69845690	0	0.11609
8953	Amp	7	69847029	69912035	0	0.121127
8954	Amp	7	69917002	69919576	0	0.125628
8955	Amp	7	69919673	69924444	0	0.117145
8956	Amp	7	69928356	69976722	0	0.112384

8957	Amp	7	69980355	69999521	0	0.110044
8958	Amp	7	69999943	70021785	0	0.105006
8959	Amp	7	70021977	70114300	0	0.114636
8960	Amp	7	70114314	70208434	0	0.105006
8961	Amp	7	70211079	70322807	0	0.095749
8962	Amp	7	70325945	70338494	0	0.092664
8963	Amp	7	70339588	70361608	0	0.094503
8964	Amp	7	70371162	70383306	0	0.098827
8965	Amp	7	70385071	70447453	0	0.091151
8966	Amp	7	70447833	70472415	0	0.10094
8967	Amp	7	70472964	70498627	0	0.097323
8968	Amp	7	70504120	70575462	0	0.107402
8969	Amp	7	70578430	70584070	0	0.102901
8970	Amp	7	70584547	70623127	0	0.106941
8971	Amp	7	70623670	70655958	0	0.112547
8972	Amp	7	70658687	70677482	0	0.118592
8973	Amp	7	70678691	70744830	0	0.114268
8974	Amp	7	70745973	70751084	0	0.122661
8975	Amp	7	70751443	70759323	0	0.117054
8976	Amp	7	70759757	70766084	0	0.111009
8977	Amp	7	70766663	70778494	0	0.116827
8978	Amp	7	70778682	70806671	0	0.106615
8979	Amp	7	70806922	70835745	0	0.111319
8980	Amp	7	70835964	70999992	0	0.105502
8981	Amp	7	71000743	71034208	0	0.100798
8982	Amp	7	71034363	71054178	0	0.098828
8983	Amp	7	71055560	71110579	0	0.098641
8984	Amp	7	71114723	71280541	0	0.088562
8985	Amp	7	71280647	71355685	0	0.08017
8986	Amp	7	71355834	71771869	0	0.072675
8987	Amp	7	71772213	71977511	0	0.070835
8988	Amp	7	71979823	72015296	0	0.07657
8989	Amp	7	72015858	72052811	0	0.079947
8990	Amp	7	72054071	72164074	0	0.071503
8991	Amp	7	72167144	72396017	0	0.068125
8992	Amp	7	72398000	72411961	0	0.072961
8993	Amp	7	72413805	72435625	0	0.078015
8994	Amp	7	72435790	72465472	0	0.073179
8995	Amp	7	72467061	72471705	0	0.071209
8996	Amp	7	72473458	72523231	0	0.07415
8997	Amp	7	72523346	72854426	0	0.069096
8998	Amp	7	72859231	72859231	0	0.076578
8999	Amp	7	72872372	72872372	0	0.079243
9000	Amp	7	72880997	72880997	0	0.084988
9001	Amp	7	72889156	72889156	0	0.086827
9002	Amp	7	72897281	72930043	0	0.104964
9003	Amp	7	72931090	72931090	0	0.102769
9004	Amp	7	72931874	72931874	0	0.116997
9005	Amp	7	73312738	73312738	0	0.11243
9006	Amp	7	73317954	73444153	0	0.113896
9007	Amp	7	73444592	73444592	0	0.116335
9008	Amp	7	73447763	73464468	0	0.10941
9009	Amp	7	73468199	73560832	0	0.112179

9010	Amp	7	73563534	73563568	0	0.113977
9011	Amp	7	73567041	73572764	0	0.106495
9012	Amp	7	73573024	73615549	0	0.107978
9013	Amp	7	73626554	73694594	0	0.102233
9014	Amp	7	73694883	73703271	0	0.097537
9015	Amp	7	73710341	73862465	0	0.094597
9016	Amp	7	73864150	73894020	0	0.104268
9017	Amp	7	73895847	74025992	0	0.10183
9018	Amp	7	74030021	74117319	0	0.099344
9019	Amp	7	74122993	74415437	0	0.089284
9020	Amp	7	74420565	74468045	0	0.091485
9021	Amp	7	74475361	74595956	0	0.10993
9022	Amp	7	74615750	74625440	0	0.122662
9023	Amp	7	74631330	74647486	0	0.125688
9024	Amp	7	74650961	74655499	0	0.114784
9025	Amp	7	74658981	74727168	0	0.117768
9026	Amp	7	74727766	74727766	0	0.105035
9027	Amp	7	74840595	74935977	0	0.102009
9028	Amp	7	74944290	75415352	0	0.099808
9029	Amp	7	75415813	75431737	0	0.101947
9030	Amp	7	75433973	75462627	0	0.105426
9031	Amp	7	75466500	75637381	0	0.092275
9032	Amp	7	75641856	75687963	0	0.100115
9033	Amp	7	75689662	75698034	0	0.097131
9034	Amp	7	75701444	75809462	0	0.100131
9035	Amp	7	75810378	75819949	0	0.111705
9036	Amp	7	75822339	75845979	0	0.109782
9037	Amp	7	75847634	75962111	0	0.107013
9038	Amp	7	75971851	76067288	0	0.139876
9039	Amp	7	76067626	76337858	0	0.13439
9040	Amp	7	76338339	76386905	0	0.137547
9041	Amp	7	76393107	76393107	0	0.132166
9042	Amp	7	76407292	76416723	0	0.130027
9043	Amp	7	76417007	76417007	0	0.13542
9044	Amp	7	76420672	76426530	0	0.123846
9045	Amp	7	77130464	77130464	0	0.088405
9046	Amp	7	77135653	77136126	0	0.092872
9047	Amp	7	77138566	77140991	0	0.096322
9048	Amp	7	77141748	77141748	0	0.094855
9049	Amp	7	77148625	77153003	0	0.093372
9050	Amp	7	77155440	77179907	0	0.091575
9051	Amp	7	77180017	77190972	0	0.094243
9052	Amp	7	77191483	77205085	0	0.100988
9053	Amp	7	77205468	77308656	0	0.094821
9054	Amp	7	77311972	77387184	0	0.098052
9055	Amp	7	77388125	77472215	0	0.103275
9056	Amp	7	77477480	77519547	0	0.114651
9057	Amp	7	77521287	77560284	0	0.110062
9058	Amp	7	77565304	77621488	0	0.098687
9059	Amp	7	77625083	77625083	0	0.093463
9060	Amp	7	77627162	77668611	0	0.114674
9061	Amp	7	77669435	77705235	0	0.110208
9062	Amp	7	77711940	77722388	0	0.106977

9063 Amp	7	77725563	77776581	0	0.143916
9064 Amp	7	77777087	77802670	0	0.145598
9065 Amp	7	77803092	77822829	0	0.150992
9066 Amp	7	77827058	77831901	0	0.147992
9067 Amp	7	77834078	77847535	0	0.104308
9068 Amp	7	77850032	77904161	0	0.097232
9069 Amp	7	77904714	77999089	0	0.099008
9070 Amp	7	78000926	78013644	0	0.100414
9071 Amp	7	78013679	78066920	0	0.102479
9072 Amp	7	78066941	78155325	0	0.105194
9073 Amp	7	78156120	78431337	0	0.103355
9074 Amp	7	78431431	78449933	0	0.105075
9075 Amp	7	78450456	79042197	0	0.103355
9076 Amp	7	79045287	79076205	0	0.098369
9077 Amp	7	79079220	79271169	0	0.100762
9078 Amp	7	79273268	79457001	0	0.090465
9079 Amp	7	79461605	79480326	0	0.095451
9080 Amp	7	79482896	79688502	0	0.090474
9081 Amp	7	79691212	79691477	0	0.099041
9082 Amp	7	79703631	79779409	0	0.110058
9083 Amp	7	79780757	80072377	0	0.101491
9084 Amp	7	80078071	80232749	0	0.109578
9085 Amp	7	80232962	80301834	0	0.119997
9086 Amp	7	80302442	80329491	0	0.109578
9087 Amp	7	80330073	80417785	0	0.101491
9088 Amp	7	80437661	80650876	0	0.106989
9089 Amp	7	80651310	80670335	0	0.101491
9090 Amp	7	80674495	80724280	0	0.11047
9091 Amp	7	80725894	80730705	0	0.099645
9092 Amp	7	80734638	80778485	0	0.083726
9093 Amp	7	80779409	80876995	0	0.089659
9094 Amp	7	80878833	80915215	0	0.086369
9095 Amp	7	80917429	80944814	0	0.068728
9096 Amp	7	80945571	81078453	0	0.073504
9097 Amp	7	81083229	81091983	0	0.079104
9098 Amp	7	81093746	81104662	0	0.074328
9099 Amp	7	81105638	81244143	0	0.080022
9100 Amp	7	81244282	81400332	0	0.084877
9101 Amp	7	81400997	81494971	0	0.079183
9102 Amp	7	81496475	81533645	0	0.077118
9103 Amp	7	81534338	81549466	0	0.074763
9104 Amp	7	81550629	81895865	0	0.069908
9105 Amp	7	81900975	81943306	0	0.080505
9106 Amp	7	81952021	82151440	0	0.060929
9107 Amp	7	82151453	82181386	0	0.066336
9108 Amp	7	82194712	82279409	0	0.054996
9109 Amp	7	82285397	82317988	0	0.064313
9110 Amp	7	82320284	82558498	0	0.054996
9111 Amp	7	82558634	82628259	0	0.061334
9112 Amp	7	82635043	82773057	0	0.059928
9113 Amp	7	82773706	82857151	0	0.065134
9114 Amp	7	82857299	83266565	0	0.059534
9115 Amp	7	83268020	83300936	0	0.054328

9116	Amp	7	83300962	83398525	0	0.04799
9117	Amp	7	83400649	83608907	0	0.069238
9118	Amp	7	83621110	83922541	0	0.063503
9119	Amp	7	83923203	84433054	0	0.061728
9120	Amp	7	84438961	84640830	0	0.066983
9121	Amp	7	84644144	84756071	0	0.053636
9122	Amp	7	84760590	84851363	0	0.057904
9123	Amp	7	84851537	84883327	0	0.053636
9124	Amp	7	84883832	85444314	0	0.04838
9125	Amp	7	85592729	85667950	0	0.060905
9126	Amp	7	85668036	85689548	0	0.066902
9127	Amp	7	85690455	85702430	0	0.054376
9128	Amp	7	85702713	85903582	0	0.057894
9129	Amp	7	85913711	86095687	0	0.062671
9130	Amp	7	86098803	86113344	0	0.068272
9131	Amp	7	86118195	86373909	0	0.079362
9132	Amp	7	86374171	86388517	0	0.105207
9133	Amp	7	86391346	86434253	0	0.094117
9134	Amp	7	86442246	86543912	0	0.105486
9135	Amp	7	86555535	86566061	0	0.094117
9136	Amp	7	86568546	86735411	0	0.100599
9137	Amp	7	86737189	87030252	0	0.112977
9138	Amp	7	87030835	87208752	0	0.116747
9139	Amp	7	87217404	87217871	0	0.112977
9140	Amp	7	87226691	87357655	0	0.114833
9141	Amp	7	87361430	87401587	0	0.102456
9142	Amp	7	87401886	87432218	0	0.095973
9143	Amp	7	87432977	87611076	0	0.086842
9144	Amp	7	87612398	87963431	0	0.081242
9145	Amp	7	87965246	88112508	0	0.083189
9146	Amp	7	88115299	88479908	0	0.073675
9147	Amp	7	88480078	88517017	0	0.078584
9148	Amp	7	90266400	90267056	0	0.085228
9149	Amp	7	90267461	90267461	0	0.090468
9150	Amp	7	90272439	90383708	0	0.09514
9151	Amp	7	90385094	90644288	0	0.093283
9152	Amp	7	90645082	90770738	0	0.084041
9153	Amp	7	90774401	90804120	0	0.0788
9154	Amp	7	90812801	90886718	0	0.076084
9155	Amp	7	90889953	91149324	0	0.079258
9156	Amp	7	91149688	91255109	0	0.076084
9157	Amp	7	91262609	91271294	0	0.069925
9158	Amp	7	91272962	91446795	0	0.065253
9159	Amp	7	91447172	91541134	0	0.067951
9160	Amp	7	91542693	91545172	0	0.065253
9161	Amp	7	91545578	91577351	0	0.060013
9162	Amp	7	91577468	91596584	0	0.066144
9163	Amp	7	91599247	91765720	0	0.071123
9164	Amp	7	91767121	91939161	0	0.074901
9165	Amp	7	91944763	92042830	0	0.071123
9166	Amp	7	92051729	92092267	0	0.076766
9167	Amp	7	92093247	92159939	0	0.082415
9168	Amp	7	92160228	92180445	0	0.086676

9169	Amp	7	92181872	92245372	0	0.081027
9170	Amp	7	92245395	92635096	0	0.076766
9171	Amp	7	92635146	92655788	0	0.071123
9172	Amp	7	92658285	93082238	0	0.064993
9173	Amp	7	93085950	93161516	0	0.066433
9174	Amp	7	93169528	93195566	0	0.06104
9175	Amp	7	93199680	93467919	0	0.064477
9176	Amp	7	93468192	93580443	0	0.069233
9177	Amp	7	93583917	93987342	0	0.067793
9178	Amp	7	93991723	94059721	0	0.056776
9179	Amp	7	94060841	94311102	0	0.069666
9180	Amp	7	94314923	94701173	0	0.056776
9181	Amp	7	94704920	94812333	0	0.063056
9182	Amp	7	94812772	94830152	0	0.08164
9183	Amp	7	94833112	94988226	0	0.054863
9184	Amp	7	94991377	95102998	0	0.073864
9185	Amp	7	95103713	95308864	0	0.076664
9186	Amp	7	95308945	95321160	0	0.070384
9187	Amp	7	95321380	95504578	0	0.067584
9188	Amp	7	95506302	95687559	0	0.069107
9189	Amp	7	95691637	95698098	0	0.087026
9190	Amp	7	95708345	95708752	0	0.103084
9191	Amp	7	95718635	95889016	0	0.097529
9192	Amp	7	95889505	95920788	0	0.095582
9193	Amp	7	95925604	95964226	0	0.084506
9194	Amp	7	95967435	96293575	0	0.091181
9195	Amp	7	96294470	96518274	0	0.084506
9196	Amp	7	96519738	96597937	0	0.07651
9197	Amp	7	96604479	97295857	0	0.060452
9198	Amp	7	97302247	97496081	0	0.067081
9199	Amp	7	97496174	97703294	0	0.062199
9200	Amp	7	97704958	97870806	0	0.059361
9201	Amp	7	97870817	97873828	0	0.056646
9202	Amp	7	97882049	98111901	0	0.05455
9203	Amp	7	98112422	98131459	0	0.056126
9204	Amp	7	98135909	98137771	0	0.062305
9205	Amp	7	98138106	98220555	0	0.068328
9206	Amp	7	98226278	98446061	0	0.070114
9207	Amp	7	98446663	98450771	0	0.071686
9208	Amp	7	98452898	98522511	0	0.083365
9209	Amp	7	98522566	98583166	0	0.079915
9210	Amp	7	98588256	98648166	0	0.083792
9211	Amp	7	98648778	98816033	0	0.072112
9212	Amp	7	99033743	99033743	0	0.094377
9213	Amp	7	99034810	99255788	0	0.088355
9214	Amp	7	99259931	99336267	0	0.096233
9215	Amp	7	99337470	99383671	0	0.094447
9216	Amp	7	99384402	99403404	0	0.086569
9217	Amp	7	99405167	99517366	0	0.088063
9218	Amp	7	99521557	99549342	0	0.092524
9219	Amp	7	99560779	99655171	0	0.090948
9220	Amp	7	99662194	99799569	0	0.084769
9221	Amp	7	99800547	99976753	0	0.086352

9222	Amp	7	99977598	100644740	0	0.088333
9223	Amp	7	100646304	100773961	0	0.075259
9224	Amp	7	100780319	101075858	0	0.07028
9225	Amp	7	101077416	101096524	0	0.067887
9226	Amp	7	101101756	101146230	0	0.082647
9227	Amp	7	101147163	101167811	0	0.067887
9228	Amp	7	101184867	101238835	0	0.057399
9229	Amp	7	101241496	101274027	0	0.060777
9230	Amp	7	101275204	101677518	0	0.058408
9231	Amp	7	101681406	101753343	0	0.054531
9232	Amp	7	101753368	101794462	0	0.056533
9233	Amp	7	101796228	101947435	0	0.054552
9234	Amp	7	101948758	102175051	0	0.058045
9235	Amp	7	102175356	102202256	0	0.064462
9236	Amp	7	102206116	102282046	0	0.078759
9237	Amp	7	102297475	102449027	0	0.064462
9238	Amp	7	102449522	102451288	0	0.062891
9239	Amp	7	102766233	102766233	0	0.056474
9240	Amp	7	102766300	102771192	0	0.0615
9241	Amp	7	102778267	103018573	0	0.059916
9242	Amp	7	103023929	103030486	0	0.062422
9243	Amp	7	103031787	103448095	0	0.067208
9244	Amp	7	103449307	103623914	0	0.06383
9245	Amp	7	103628511	103741035	0	0.065289
9246	Amp	7	103750556	104125272	0	0.062198
9247	Amp	7	104127457	104646445	0	0.070882
9248	Amp	7	104647730	104980743	0	0.065856
9249	Amp	7	104981178	105044725	0	0.067619
9250	Amp	7	105046306	105287900	0	0.073503
9251	Amp	7	105289908	105372232	0	0.067619
9252	Amp	7	105557943	105602470	0	0.073654
9253	Amp	7	105604684	105888857	0	0.09778
9254	Amp	7	105893716	105934741	0	0.050235
9255	Amp	7	105934756	106075058	0	0.048472
9256	Amp	7	106076735	106084062	0	0.050113
9257	Amp	7	106085523	106092000	0	0.048112
9258	Amp	7	106094596	106129847	0	0.052685
9259	Amp	7	106132547	106172079	0	0.04665
9260	Amp	7	106173735	106411926	0	0.052171
9261	Amp	7	106414774	106731730	0	0.04665
9262	Amp	7	106731773	106745313	0	0.02863
9263	Amp	7	106745567	106752973	0	0.023844
9264	Amp	7	106753257	106999453	0	0.015945
9265	Amp	7	107010001	107197841	0	0.020939
9266	Amp	7	107200503	107398694	0	0.030351
9267	Amp	7	107399957	107746365	0	0.01948
9268	Amp	7	107755309	107788715	0	0.027522
9269	Amp	7	107791836	107810766	0	0.042305
9270	Amp	7	107830393	107982640	0	0.032448
9271	Amp	7	107982894	108012720	0	0.027522
9272	Amp	7	108013211	108631035	0	0.045242
9273	Amp	7	108631879	108780238	0	0.052561
9274	Amp	7	108782801	108876078	0	0.046632

9275	Amp	7	108880109	108955511	0	0.01948
9276	Amp	7	108955588	108997544	0	0.039743
9277	Amp	7	109006516	109172070	0	0.01948
9278	Amp	7	109172631	109291813	0	0.021064
9279	Amp	7	109297069	109433197	0	0.030287
9280	Amp	7	109433887	109852584	0	0.049049
9281	Amp	7	109852824	110248407	0	0.047464
9282	Amp	7	110248742	110288625	0	0.04125
9283	Amp	7	110289491	110439384	0	0.022488
9284	Amp	7	110441735	110517573	0	0.029507
9285	Amp	7	110518189	110719773	0	0.038148
9286	Amp	7	110720376	110742009	0	0.033153
9287	Amp	7	110742550	110810973	0	0.024513
9288	Amp	7	110819025	110937936	0	0.015422
9289	Amp	7	110943219	110960537	0	0.019861
9290	Amp	7	110966347	111047104	0	0.030058
9291	Amp	7	111052512	111082068	0	0.019861
9292	Amp	7	111084377	111135042	0	0.029249
9293	Amp	7	111135533	111135533	0	0.03878
9294	Amp	7	111712844	111798747	0	0.046908
9295	Amp	7	111799198	111805993	0	0.049428
9296	Amp	7	111812635	111819631	0	0.04004
9297	Amp	7	111822409	111877421	0	0.044179
9298	Amp	7	111877884	112110551	0	0.034648
9299	Amp	7	112110676	112157876	0	0.027497
9300	Amp	7	112158093	112229119	0	0.023358
9301	Amp	7	112233336	112748340	0	0.020838
9302	Amp	7	112748516	112910256	0	0.01271
9303	Amp	7	112911289	112976529	0	0.015256
9304	Amp	7	112981465	112981465	0	0.02032
9305	Amp	7	112987854	113019921	0	0.021906
9306	Amp	7	113021060	113284909	0	0.027922
9307	Amp	7	113285460	113363728	0	0.02379
9308	Amp	7	113365558	113485179	0	0.027213
9309	Amp	7	113488859	113489127	0	0.022436
9310	Amp	7	113490427	113507462	0	0.027084
9311	Amp	7	113508762	113668653	0	0.02899
9312	Amp	7	113669437	113721140	0	0.024341
9313	Amp	7	113721990	113876268	0	0.019276
9314	Amp	7	113876348	114187548	0	0.024048
9315	Amp	7	114187717	114528797	0	0.022142
9316	Amp	7	114529376	114703547	0	0.027457
9317	Amp	7	114705445	115002874	0	0.031761
9318	Amp	7	115003055	115057251	0	0.036148
9319	Amp	7	115060024	115252923	0	0.031844
9320	Amp	7	115261331	115610537	0	0.025829
9321	Amp	7	115615063	115671816	0	0.027674
9322	Amp	7	115677484	115842404	0	0.022359
9323	Amp	7	115846157	116098784	0	0.018936
9324	Amp	7	116105743	116246231	0	0.014164
9325	Amp	7	116247722	116493023	0	0.023463
9326	Amp	7	116497431	116527541	0	0.043081
9327	Amp	7	116530786	116565647	0	0.03229

9328	Amp	7	116565987	116595325	0	0.036679
9329	Amp	7	116596682	116615718	0	0.02738
9330	Amp	7	116616261	116949584	0	0.032104
9331	Amp	7	116958991	116994994	0	0.037445
9332	Amp	7	116995699	117195310	0	0.033058
9333	Amp	7	117197556	117279924	0	0.041503
9334	Amp	7	117282276	117333081	0	0.033058
9335	Amp	7	117333234	117440430	0	0.03448
9336	Amp	7	117444948	117478159	0	0.04456
9337	Amp	7	117481166	117525067	0	0.04772
9338	Amp	7	117531558	117709173	0	0.049169
9339	Amp	7	117712482	117879771	0	0.064091
9340	Amp	7	117882633	117952264	0	0.054979
9341	Amp	7	117952748	118057808	0	0.067844
9342	Amp	7	118063809	118183061	0	0.05951
9343	Amp	7	118185495	118399158	0	0.026817
9344	Amp	7	118401679	118549995	0	0.036869
9345	Amp	7	118556585	118662094	0	0.026817
9346	Amp	7	118665325	118711943	0	0.038821
9347	Amp	7	118715875	118769765	0	0.026817
9348	Amp	7	118772229	118821934	0	0.033717
9349	Amp	7	118824094	119185056	0	0.032268
9350	Amp	7	119204172	119214537	0	0.025368
9351	Amp	7	119223907	119294830	0	0.020026
9352	Amp	7	119300224	119326879	0	0.025534
9353	Amp	7	119327903	119363631	0	0.036059
9354	Amp	7	119366808	119621964	0	0.025534
9355	Amp	7	119623777	119652721	0	0.029057
9356	Amp	7	119659585	119696539	0	0.036749
9357	Amp	7	119700111	119806867	0	0.035326
9358	Amp	7	119807154	119924905	0	0.036712
9359	Amp	7	119925002	120014345	0	0.03892
9360	Amp	7	120015449	120303999	0	0.036712
9361	Amp	7	120304427	120340075	0	0.034867
9362	Amp	7	120343063	120584271	0	0.040061
9363	Amp	7	120585020	120680775	0	0.034554
9364	Amp	7	120681264	120861431	0	0.033168
9365	Amp	7	120865332	120882429	0	0.07741
9366	Amp	7	120919649	120931073	0	0.085738
9367	Amp	7	120931546	121081225	0	0.10823
9368	Amp	7	121085093	121200899	0	0.063988
9369	Amp	7	121206753	121300939	0	0.056551
9370	Amp	7	121301982	121497767	0	0.064816
9371	Amp	7	121499957	121578818	0	0.075733
9372	Amp	7	121578939	122384293	0	0.0785
9373	Amp	7	122387571	122651666	0	0.067584
9374	Amp	7	122652657	122714305	0	0.06239
9375	Amp	7	122716438	122761492	0	0.072132
9376	Amp	7	122761941	122831607	0	0.043711
9377	Amp	7	122833254	122885275	0	0.053246
9378	Amp	7	122887144	123000951	0	0.058838
9379	Amp	7	123003175	123171273	0	0.053246
9380	Amp	7	123249281	123487516	0	0.039007

9381	Amp	7	123488966	123516025	0	0.041206
9382	Amp	7	123521421	123650404	0	0.04593
9383	Amp	7	123659801	124056452	0	0.066786
9384	Amp	7	124058718	124694158	0	0.060512
9385	Amp	7	124699307	125170345	0	0.083263
9386	Amp	7	125172292	125319006	0	0.089441
9387	Amp	7	125320705	125414231	0	0.076562
9388	Amp	7	125417412	126011886	0	0.081367
9389	Amp	7	126012296	126349374	0	0.100196
9390	Amp	7	126352831	126514182	0	0.105248
9391	Amp	7	126514271	126667243	0	0.100196
9392	Amp	7	126669713	126688026	0	0.102712
9393	Amp	7	126688676	126701821	0	0.099218
9394	Amp	7	126704680	126903825	0	0.093225
9395	Amp	7	126906433	127008312	0	0.048695
9396	Amp	7	127008720	127017491	0	0.04389
9397	Amp	7	127024098	127227393	0	0.089326
9398	Amp	7	127228628	127361603	0	0.090792
9399	Amp	7	127364692	127508099	0	0.054789
9400	Amp	7	127508578	127718433	0	0.061598
9401	Amp	7	127721153	127742211	0	0.09588
9402	Amp	7	127743116	128046138	0	0.045357
9403	Amp	7	128046970	128176093	0	0.05914
9404	Amp	7	128182216	128328398	0	0.055616
9405	Amp	7	128328796	128391244	0	0.068083
9406	Amp	7	128394575	128446458	0	0.080767
9407	Amp	7	128446778	128446778	0	0.088055
9408	Amp	7	128448919	128448919	0	0.092853
9409	Amp	7	128736608	128764375	0	0.070393
9410	Amp	7	128767848	129618558	0	0.076478
9411	Amp	7	129625302	129768857	0	0.0703
9412	Amp	7	129787788	130136342	0	0.085729
9413	Amp	7	130146726	130509220	0	0.08194
9414	Amp	7	130509530	130897965	0	0.088344
9415	Amp	7	130900864	131050787	0	0.090767
9416	Amp	7	131055220	131094732	0	0.103446
9417	Amp	7	131094919	131115246	0	0.101023
9418	Amp	7	131122667	131170142	0	0.110155
9419	Amp	7	131173930	131381670	0	0.094725
9420	Amp	7	131391593	131772384	0	0.098746
9421	Amp	7	131773247	131895556	0	0.09728
9422	Amp	7	131898477	131979256	0	0.117688
9423	Amp	7	131994531	132195099	0	0.092512
9424	Amp	7	132195493	132409846	0	0.088491
9425	Amp	7	132410432	132636663	0	0.094126
9426	Amp	7	132638710	132645154	0	0.108698
9427	Amp	7	132651768	132654902	0	0.091655
9428	Amp	7	132655287	132748001	0	0.077082
9429	Amp	7	132748438	132793778	0	0.088875
9430	Amp	7	132795606	132921242	0	0.077082
9431	Amp	7	132922411	132996402	0	0.081163
9432	Amp	7	132996666	133217043	0	0.088398
9433	Amp	7	133222972	133399457	0	0.095095

9434 Amp	7	133403187	133409014	0	0.088398
9435 Amp	7	133411085	133794285	0	0.081163
9436 Amp	7	133794311	133928362	0	0.086157
9437 Amp	7	133929668	133957137	0	0.081163
9438 Amp	7	133959095	134626948	0	0.083622
9439 Amp	7	134627769	134828597	0	0.081163
9440 Amp	7	134829408	134881320	0	0.090149
9441 Amp	7	134882425	135275623	0	0.081163
9442 Amp	7	135276525	135928047	0	0.084551
9443 Amp	7	135934864	136063356	0	0.081163
9444 Amp	7	136066716	136501312	0	0.08279
9445 Amp	7	136503980	136614083	0	0.091407
9446 Amp	7	136618641	136648654	0	0.08279
9447 Amp	7	136650497	136756493	0	0.085413
9448 Amp	7	136756901	136783187	0	0.090363
9449 Amp	7	136785468	136843589	0	0.088737
9450 Amp	7	136848232	136865379	0	0.100243
9451 Amp	7	136872603	136886404	0	0.088737
9452 Amp	7	136891603	136960274	0	0.090195
9453 Amp	7	136977860	137140493	0	0.088737
9454 Amp	7	137147516	137391258	0	0.09235
9455 Amp	7	137394426	137396743	0	0.088737
9456 Amp	7	137399992	137412340	0	0.094672
9457 Amp	7	137413339	137755832	0	0.090591
9458 Amp	7	137758304	137894151	0	0.094277
9459 Amp	7	137894347	137949526	0	0.100334
9460 Amp	7	137962586	137988619	0	0.143472
9461 Amp	7	137989074	137997928	0	0.137415
9462 Amp	7	137998139	138245366	0	0.148232
9463 Amp	7	138245842	138282453	0	0.137415
9464 Amp	7	138284591	138598491	0	0.12556
9465 Amp	7	138600227	138701802	0	0.094277
9466 Amp	7	138702047	138757599	0	0.09575
9467 Amp	7	138760487	139096583	0	0.100428
9468 Amp	7	139098477	139550380	0	0.087724
9469 Amp	7	139551766	139711617	0	0.091507
9470 Amp	7	139714231	139724223	0	0.099084
9471 Amp	7	139727562	140419829	0	0.117829
9472 Amp	7	140420746	140688393	0	0.114466
9473 Amp	7	140689560	140744235	0	0.112993
9474 Amp	7	140750191	140795837	0	0.111603
9475 Amp	7	140796063	140870033	0	0.113467
9476 Amp	7	140870769	141013460	0	0.119116
9477 Amp	7	141015497	141370524	0	0.120569
9478 Amp	7	141374239	141416168	0	0.133342
9479 Amp	7	141417944	141432099	0	0.148541
9480 Amp	7	141435314	141757176	0	0.120569
9481 Amp	7	141762501	141840620	0	0.124014
9482 Amp	7	141840748	141934571	0	0.118364
9483 Amp	7	141943364	142286632	0	0.114678
9484 Amp	7	142293530	142554630	0	0.126967
9485 Amp	7	142554640	142563940	0	0.177474
9486 Amp	7	142581256	142581256	0	0.165185

9487 Amp	7	142768623	142810692	0	0.132842
9488 Amp	7	142810923	142890021	0	0.139399
9489 Amp	7	142891756	143053146	0	0.133211
9490 Amp	7	143053224	143066190	0	0.138779
9491 Amp	7	143066610	143225569	0	0.134312
9492 Amp	7	143226872	143506678	0	0.116148
9493 Amp	7	143507648	143968472	0	0.126428
9494 Amp	7	143970275	144046911	0	0.116148
9495 Amp	7	144047185	144582035	0	0.126435
9496 Amp	7	144582903	144677822	0	0.128564
9497 Amp	7	144678382	144755851	0	0.130435
9498 Amp	7	144757464	144976790	0	0.128564
9499 Amp	7	144977426	145009987	0	0.122629
9500 Amp	7	145016098	145186231	0	0.120539
9501 Amp	7	145186746	145666299	0	0.116757
9502 Amp	7	145666858	145760544	0	0.120866
9503 Amp	7	145762626	146078010	0	0.110579
9504 Amp	7	146078367	146610733	0	0.114214
9505 Amp	7	146611703	147361899	0	0.110769
9506 Amp	7	147364386	147867432	0	0.120984
9507 Amp	7	147869680	147882949	0	0.119532
9508 Amp	7	147885627	148033930	0	0.121169
9509 Amp	7	148034183	148339285	0	0.115601
9510 Amp	7	148341135	148372263	0	0.144728
9511 Amp	7	148372970	148627252	0	0.132224
9512 Amp	7	148630439	149511617	0	0.115601
9513 Amp	7	149523295	149670224	0	0.105387
9514 Amp	7	149674457	149717333	0	0.128492
9515 Amp	7	149717389	149767991	0	0.139895
9516 Amp	7	149768391	149827576	0	0.149465
9517 Amp	7	149834127	149834127	0	0.139895
9518 Amp	7	150164351	150310262	0	0.11679
9519 Amp	7	150311101	150362513	0	0.115146
9520 Amp	7	150362628	150415352	0	0.118176
9521 Amp	7	150415518	150655799	0	0.115146
9522 Amp	7	150655943	150768253	0	0.118103
9523 Amp	7	150775090	150855785	0	0.103894
9524 Amp	7	150877476	150940746	0	0.113894
9525 Amp	7	150940760	151140442	0	0.11824
9526 Amp	7	151141685	151291086	0	0.113894
9527 Amp	7	151291325	151322625	0	0.103894
9528 Amp	7	151326556	151828380	0	0.118801
9529 Amp	7	151843639	151883949	0	0.115844
9530 Amp	7	151884139	152363083	0	0.102341
9531 Amp	7	152365691	152389446	0	0.084829
9532 Amp	7	152389986	152838729	0	0.065734
9533 Amp	7	152838823	152842423	0	0.067249
9534 Amp	7	152859405	153236156	0	0.070126
9535 Amp	7	154067655	154134791	0	0.109047
9536 Amp	7	154139155	154160726	0	0.114274
9537 Amp	7	154160915	154571637	0	0.128218
9538 Amp	7	154580128	154960796	0	0.145465
9539 Amp	7	154962422	155197793	0	0.149854

9540	Amp	7	155203439	155239289	0	0.151252
9541	Amp	7	155243635	155307797	0	0.158344
9542	Amp	7	155307927	155634255	0	0.162947
9543	Amp	7	155639485	155784581	0	0.172764
9544	Amp	7	155793406	155828461	0	0.162947
9545	Amp	7	155829806	156042227	0	0.158344
9546	Amp	7	156043868	156249490	0	0.153955
9547	Amp	7	156249549	156372242	0	0.161112
9548	Amp	7	156372258	156706390	0	0.153955
9549	Amp	7	156713306	156787045	0	0.136708
9550	Amp	7	156787608	156906076	0	0.153463
9551	Amp	7	156917377	156981581	0	0.163078
9552	Amp	7	156982023	157073705	0	0.153463
9553	Amp	7	157081393	157111145	0	0.163283
9554	Amp	7	157111519	157276525	0	0.153463
9555	Amp	7	157282962	157379163	0	0.144936
9556	Amp	7	157379243	157731109	0	0.147014
9557	Amp	7	157737201	158142921	0	0.136157
9558	Amp	7	158149251	158389337	0	0.13328
9559	Amp	7	158389988	158592540	0	0.128649
9560	Amp	8	667667	1480114	0	0.014518
9561	Amp	8	1483886	1515348	0	0.025035
9562	Amp	8	1518816	1758509	0	0.014518
9563	Amp	8	1762772	1784590	0	0.024216
9564	Amp	8	1822296	2157708	0	0.014518
9565	Amp	8	2157803	2940812	0	0.020656
9566	Amp	8	2941847	3113856	0	0.023048
9567	Amp	8	3115838	3178470	0	0.020656
9568	Amp	8	3184839	3270652	0	0.04972
9569	Amp	8	3271490	3449492	0	0.020656
9570	Amp	8	3449962	3500911	0	0.030623
9571	Amp	8	3502627	3993512	0	0.041624
9572	Amp	8	3997465	4383418	0	0.020656
9573	Amp	8	4383532	4432011	0	0.012841
9574	Amp	8	4559124	4588338	0	0.028179
9575	Amp	8	4588826	4595699	0	0.036794
9576	Amp	8	4595785	4782875	0	0.040886
9577	Amp	8	4927010	5035703	0	0.017247
9578	Amp	8	5035831	5119420	0	0.027483
9579	Amp	8	5124097	5152011	0	0.032903
9580	Amp	8	5155153	5271143	0	0.022667
9581	Amp	8	5275714	5352963	0	0.017247
9582	Amp	8	5354095	5390815	0	0.011282
9583	Amp	8	6348805	6348805	0	0.029174
9584	Amp	8	6350198	6414617	0	0.020558
9585	Amp	8	6418232	6967890	0	0.002666
9586	Amp	8	6968014	6968014	0	0.006548
9587	Amp	8	8398802	8545632	0	0.057071
9588	Amp	8	8565425	8621223	0	0.006548
9589	Amp	8	8623408	8772220	0	0.02572
9590	Amp	8	8772285	8866300	0	0.03227
9591	Amp	8	8868494	8877761	0	0.024346
9592	Amp	8	8877850	8902628	0	0.029706

9593 Amp	8	8904460	8918153	0	0.053075
9594 Amp	8	8920584	8974152	0	0.02094
9595 Amp	8	8977018	8990199	0	0.029526
9596 Amp	8	8991483	9004414	0	0.035227
9597 Amp	8	9017462	9055195	0	0.070895
9598 Amp	8	9059563	9183592	0	0.059827
9599 Amp	8	9325829	9679366	0	0.057161
9600 Amp	8	9683183	9792181	0	0.053279
9601 Amp	8	9813543	10015413	0	0.038926
9602 Amp	8	10017412	10110165	0	0.058969
9603 Amp	8	10115076	10116622	0	0.067606
9604 Amp	8	10518434	10518434	0	0.041863
9605 Amp	8	10519974	10842042	0	0.046061
9606 Amp	8	10843604	11121944	0	0.012835
9607 Amp	8	11127922	11253973	0	0.022159
9608 Amp	8	11254383	12704441	0	0.012835
9609 Amp	8	12704946	12704946	0	0.016757
9610 Amp	8	12709141	12730676	0	0.00812
9611 Amp	8	12730774	12886622	0	0.003922
9612 Amp	8	12900208	12929500	0	0.049491
9613 Amp	8	12939853	13139533	0	0.051873
9614 Amp	8	13143271	13199228	0	0.058359
9615 Amp	8	13201748	13231581	0	0.088618
9616 Amp	8	13232138	13242736	0	0.082132
9617 Amp	8	13247196	13398402	0	0.051873
9618 Amp	8	13398417	13481689	0	0.065338
9619 Amp	8	13482883	13497168	0	0.090301
9620 Amp	8	13497915	13606434	0	0.122253
9621 Amp	8	13796271	13816434	0	0.076836
9622 Amp	8	13816809	13823896	0	0.063694
9623 Amp	8	13833409	13874027	0	0.069979
9624 Amp	8	13878898	13882524	0	0.036264
9625 Amp	8	13884284	13893012	0	0.040423
9626 Amp	8	13893026	13938547	0	0.03746
9627 Amp	8	13938681	14051465	0	0.027502
9628 Amp	8	14051673	14141268	0	0.023405
9629 Amp	8	14144064	14148385	0	0.030794
9630 Amp	8	14150874	14230842	0	0.024913
9631 Amp	8	14231464	14264083	0	0.02908
9632 Amp	8	14274352	14331827	0	0.079603
9633 Amp	8	14334310	14456127	0	0.082019
9634 Amp	8	14459505	14610225	0	0.087333
9635 Amp	8	14610612	14627442	0	0.082019
9636 Amp	8	14629695	15080360	0	0.083564
9637 Amp	8	15080895	15097041	0	0.081148
9638 Amp	8	15101274	15180460	0	0.046242
9639 Amp	8	15181060	15257068	0	0.047941
9640 Amp	8	15259029	15380855	0	0.052748
9641 Amp	8	15381169	15489730	0	0.064601
9642 Amp	8	15493620	15503209	0	0.059057
9643 Amp	8	15512906	15523241	0	0.093964
9644 Amp	8	15528207	15654282	0	0.092265
9645 Amp	8	15655192	15678207	0	0.097602

9646	Amp	8	15678469	15679260	0	0.096057
9647	Amp	8	15680154	15740973	0	0.11335
9648	Amp	8	15741189	15801565	0	0.123401
9649	Amp	8	15802005	15808637	0	0.118064
9650	Amp	8	15809845	15819778	0	0.067541
9651	Amp	8	15819937	15983893	0	0.061232
9652	Amp	8	15984781	16176694	0	0.043938
9653	Amp	8	16177113	16231475	0	0.035378
9654	Amp	8	16235891	16308888	0	0.063838
9655	Amp	8	16309290	16538960	0	0.06633
9656	Amp	8	16539831	16697688	0	0.063838
9657	Amp	8	16703487	16746752	0	0.040592
9658	Amp	8	16747054	16821903	0	0.04748
9659	Amp	8	16828778	17111834	0	0.052898
9660	Amp	8	17115561	17178692	0	0.046009
9661	Amp	8	17178752	17205336	0	0.040592
9662	Amp	8	17205749	17257348	0	0.03221
9663	Amp	8	17258085	17264709	0	0.043654
9664	Amp	8	17272029	17274373	0	0.066196
9665	Amp	8	17275536	17288802	0	0.076923
9666	Amp	8	17295348	17318157	0	0.085763
9667	Amp	8	17318618	17417962	0	0.079172
9668	Amp	8	17489501	17489501	0	0.047059
9669	Amp	8	17499476	17511692	0	0.052553
9670	Amp	8	17512143	17537515	0	0.054356
9671	Amp	8	17538163	17560425	0	0.046637
9672	Amp	8	17564193	17578682	0	0.069046
9673	Amp	8	17580981	17590041	0	0.048141
9674	Amp	8	17592148	17661542	0	0.084757
9675	Amp	8	17662092	17773080	0	0.082954
9676	Amp	8	17773128	17794594	0	0.075518
9677	Amp	8	17799725	17900087	0	0.066677
9678	Amp	8	17900176	17915973	0	0.076948
9679	Amp	8	17916258	17925359	0	0.083766
9680	Amp	8	17925923	17976817	0	0.090466
9681	Amp	8	17978692	18026157	0	0.093341
9682	Amp	8	18029574	18048461	0	0.090466
9683	Amp	8	18051567	18078520	0	0.093591
9684	Amp	8	18079623	18089215	0	0.081899
9685	Amp	8	18089578	18163187	0	0.078773
9686	Amp	8	18167534	18331265	0	0.122477
9687	Amp	8	18331499	18437697	0	0.12771
9688	Amp	8	18440345	18474055	0	0.116983
9689	Amp	8	18475461	18516832	0	0.110284
9690	Amp	8	18517082	18563445	0	0.105734
9691	Amp	8	18564725	18658093	0	0.086742
9692	Amp	8	18660552	18737593	0	0.04142
9693	Amp	8	18738633	18747244	0	0.033931
9694	Amp	8	18748037	18822909	0	0.028698
9695	Amp	8	18823225	18891754	0	0.03206
9696	Amp	8	18892071	18977309	0	0.036998
9697	Amp	8	18978015	19022342	0	0.040227
9698	Amp	8	19022845	19333948	0	0.045219

9699	Amp	8	19368824	19717442	0	0.015843
9700	Amp	8	19717458	19976270	0	0.005201
9701	Amp	8	19981374	20130700	0	0.013464
9702	Amp	8	20131165	20252369	0	0.005201
9703	Amp	8	20257008	20259431	0	0
9704	Amp	8	20262746	20348091	0	0.001679
9705	Amp	8	20353102	20544017	0	0
9706	Amp	8	20545300	20628117	0	0.050523
9707	Amp	8	20632329	20644983	0	0
9708	Amp	8	20647179	20675701	0	0.006462
9709	Amp	8	20679192	20796499	0	0.013765
9710	Amp	8	20797697	20816532	0	0.064288
9711	Amp	8	20819469	20997015	0	0.013765
9712	Amp	8	21002204	21142982	0	0.020315
9713	Amp	8	21143383	21269536	0	0.013765
9714	Amp	8	21270410	21612726	0	0.020548
9715	Amp	8	21613127	21683572	0	0.029857
9716	Amp	8	21686454	21727879	0	0.041774
9717	Amp	8	21731191	21740900	0	0.025682
9718	Amp	8	21745304	21793481	0	0.03384
9719	Amp	8	21804635	21951464	0	0.045858
9720	Amp	8	21952022	22090173	0	0.051733
9721	Amp	8	22092915	22094715	0	0.043652
9722	Amp	8	22097173	22152415	0	0.049983
9723	Amp	8	22162380	22231310	0	0.040272
9724	Amp	8	22237747	22266104	0	0.032115
9725	Amp	8	22271605	22348381	0	0.006331
9726	Amp	8	22525267	22546143	0	0.028117
9727	Amp	8	22552644	22564637	0	0.006331
9728	Amp	8	22574075	23515893	0	0
9729	Amp	8	23517722	23686218	0	0.012497
9730	Amp	8	23688078	23865719	0	0.004621
9731	Amp	8	23868315	24659547	0	0
9732	Amp	8	24665345	24668384	0	0.008849
9733	Amp	8	24675131	24709253	0	0.010541
9734	Amp	8	24713890	25255439	0	0.008849
9735	Amp	8	25257724	26157957	0	0
9736	Amp	8	26160823	26353214	0	0.003053
9737	Amp	8	26354897	26392780	0	0.027556
9738	Amp	8	26402104	26764945	0	0.035957
9739	Amp	8	26765884	26874663	0	0.040681
9740	Amp	8	26884151	26894811	0	0.059408
9741	Amp	8	26899468	26980653	0	0.04707
9742	Amp	8	26981040	27005192	0	0.042346
9743	Amp	8	27005916	27014077	0	0.039293
9744	Amp	8	27016962	27058188	0	0.043114
9745	Amp	8	27064595	27070850	0	0.062808
9746	Amp	8	27072885	27124172	0	0.06937
9747	Amp	8	27124468	27298985	0	0.062808
9748	Amp	8	27299704	27348260	0	0.06716
9749	Amp	8	27349406	27427652	0	0.063339
9750	Amp	8	27430146	27535549	0	0.068662
9751	Amp	8	27547573	27562290	0	0.048968

9752	Amp	8	27565821	27569310	0	0.037256
9753	Amp	8	27571043	27631904	0	0.028855
9754	Amp	8	27638563	27716014	0	0.014983
9755	Amp	8	27716410	28171732	0	0.031615
9756	Amp	8	28173828	28249089	0	0.014983
9757	Amp	8	28249267	28390964	0	0.016768
9758	Amp	8	28398071	28689835	0	0.020063
9759	Amp	8	28690053	28825077	0	0.030225
9760	Amp	8	28830863	28852854	0	0.039534
9761	Amp	8	28855172	29027953	0	0.030225
9762	Amp	8	29027975	29075927	0	0.043912
9763	Amp	8	29076193	29107153	0	0.030225
9764	Amp	8	29107317	29215486	0	0.025242
9765	Amp	8	29219220	29219220	0	0.02734
9766	Amp	8	29221034	29296362	0	0.036061
9767	Amp	8	29296482	29735779	0	0.033963
9768	Amp	8	29737612	29902443	0	0.084486
9769	Amp	8	29905208	30167612	0	0.109889
9770	Amp	8	30173398	30199673	0	0.118479
9771	Amp	8	30200433	30329940	0	0.109889
9772	Amp	8	30332261	30344664	0	0.113521
9773	Amp	8	30344905	30412108	0	0.118954
9774	Amp	8	30413513	30413513	0	0.145877
9775	Amp	8	30414299	30420739	0	0.142244
9776	Amp	8	30421540	30482249	0	0.091721
9777	Amp	8	30482760	30598578	0	0.086289
9778	Amp	8	30602270	30810000	0	0.103219
9779	Amp	8	30814632	30872812	0	0.125536
9780	Amp	8	30873655	30882587	0	0.150656
9781	Amp	8	30883310	30978019	0	0.129091
9782	Amp	8	30978454	31126670	0	0.100133
9783	Amp	8	31132174	31136129	0	0.106341
9784	Amp	8	31136972	31164800	0	0.096934
9785	Amp	8	31168937	31192759	0	0.104029
9786	Amp	8	31194559	31257725	0	0.087099
9787	Amp	8	31257824	31580637	0	0.069583
9788	Amp	8	31586904	31682346	0	0.075962
9789	Amp	8	31682426	31723980	0	0.072447
9790	Amp	8	31724848	31777419	0	0.102296
9791	Amp	8	31778154	31807178	0	0.111971
9792	Amp	8	31813831	32106476	0	0.102296
9793	Amp	8	32109137	32122269	0	0.110177
9794	Amp	8	32124765	32139239	0	0.102296
9795	Amp	8	32139275	32250979	0	0.099603
9796	Amp	8	32251423	32273633	0	0.077287
9797	Amp	8	32274151	32276074	0	0.070908
9798	Amp	8	32276870	32380722	0	0.07528
9799	Amp	8	32384283	32428220	0	0.070792
9800	Amp	8	32433626	32450347	0	0.121315
9801	Amp	8	32451066	32512385	0	0.126637
9802	Amp	8	32513110	32554841	0	0.119542
9803	Amp	8	32558104	32645557	0	0.162808
9804	Amp	8	32647608	32773660	0	0.157899

9805 Amp	8	32775557	32842987	0	0.147676
9806 Amp	8	32843027	32870279	0	0.15729
9807 Amp	8	32876020	33017609	0	0.151968
9808 Amp	8	33019074	33129885	0	0.154013
9809 Amp	8	33133629	33180889	0	0.108399
9810 Amp	8	33181969	33246730	0	0.111876
9811 Amp	8	33252835	33258944	0	0.115944
9812 Amp	8	33259999	33280961	0	0.117794
9813 Amp	8	33281756	33303824	0	0.115944
9814 Amp	8	33304882	33312735	0	0.127142
9815 Amp	8	33313104	33342340	0	0.177664
9816 Amp	8	33344089	33444103	0.038273	0.19329
9817 Amp	8	33445498	33507108	0.012341	0.189814
9818 Amp	8	33507770	33516919	0	0.188029
9819 Amp	8	33518681	33552559	0.236222	0.214621
9820 Amp	8	33552572	33565552	0.325321	0.223015
9821 Amp	8	33570759	33593612	0.934277	0.273538
9822 Amp	8	33597439	33629912	0.852608	0.267425
9823 Amp	8	33637472	33719449	0.963924	0.275633
9824 Amp	8	33726109	33742368	0.644689	0.250881
9825 Amp	8	33743715	33812861	1.049532	0.282001
9826 Amp	8	33815048	33869527	0.897922	0.270803
9827 Amp	8	33875502	33888410	1.199568	0.292552
9828 Amp	8	33890254	33943005	1.275021	0.298069
9829 Amp	8	33945168	33962777	1.646768	0.323439
9830 Amp	8	33971042	33999146	1.199568	0.292319
9831 Amp	8	34002629	34019288	0.532449	0.241796
9832 Amp	8	34021549	34034081	0.604912	0.247941
9833 Amp	8	34034271	34163951	0.860481	0.268102
9834 Amp	8	34164976	34232445	0.685617	0.254764
9835 Amp	8	34239862	34392537	0.437983	0.233016
9836 Amp	8	34394414	34490484	0.460107	0.235529
9837 Amp	8	34495898	34496228	0.571214	0.245011
9838 Amp	8	34496293	34496293	0.273081	0.218419
9839 Amp	8	34521560	34534939	0.508503	0.239326
9840 Amp	8	34537147	34557945	0.540744	0.241909
9841 Amp	8	34558750	34560858	0.430191	0.232426
9842 Amp	8	34560950	34563539	0.571214	0.244633
9843 Amp	8	34572861	34640721	0.636206	0.250117
9844 Amp	8	34640868	34645745	0.760395	0.260955
9845 Amp	8	34651036	34693281	0.709359	0.256786
9846 Amp	8	34694766	34707070	0.596354	0.247264
9847 Amp	8	34707169	34718835	0.724993	0.257716
9848 Amp	8	34723420	34871121	0.66072	0.252198
9849 Amp	8	34872413	34897120	0.734472	0.258907
9850 Amp	8	34899864	34945055	0.814697	0.264984
9851 Amp	8	34997908	34998616	1.130387	0.287809
9852 Amp	8	34999936	35000038	0.982622	0.277357
9853 Amp	8	35002859	35033098	0.367646	0.226834
9854 Amp	8	35036183	35107343	0.195309	0.210913
9855 Amp	8	35107432	35128981	0.111991	0.201693
9856 Amp	8	35131513	35140618	0.073369	0.197653
9857 Amp	8	35250447	35250447	0.49169	0.237979

9858	Amp	8	35253171	35253171	0.62858	0.249558
9859	Amp	8	35257733	35269471	0.7517	0.260311
9860	Amp	8	35270446	35307014	1.190324	0.291664
9861	Amp	8	35307999	35309260	1.000924	0.278897
9862	Amp	8	35315351	35338899	1.737394	0.32942
9863	Amp	8	35346166	35350531	2.447622	0.374047
9864	Amp	8	35351703	35411271	2.22615	0.360355
9865	Amp	8	35412577	35433672	2.483202	0.376205
9866	Amp	8	35440525	35442058	2.580481	0.381784
9867	Amp	8	35446821	35465577	2.625007	0.384641
9868	Amp	8	35465892	35475479	2.866312	0.398877
9869	Amp	8	35480132	35481925	2.039783	0.348354
9870	Amp	8	35486328	35492188	2.327403	0.36633
9871	Amp	8	35497402	35504960	2.238069	0.360751
9872	Amp	8	35509275	35510139	2.614747	0.383803
9873	Amp	8	35516514	35538281	2.30333	0.36497
9874	Amp	8	35538335	35543861	2.71525	0.389831
9875	Amp	8	35546251	35564625	2.294081	0.364461
9876	Amp	8	35566276	35593358	2.591575	0.382376
9877	Amp	8	35596943	35599601	2.436112	0.373347
9878	Amp	8	35606737	35622393	2.727427	0.39025
9879	Amp	8	35624145	35638131	2.866312	0.398657
9880	Amp	8	35638343	35651026	2.424855	0.372879
9881	Amp	8	35653476	35660338	2.737964	0.391088
9882	Amp	8	35661303	35672154	2.98257	0.405711
9883	Amp	8	35674696	35701128	2.683697	0.387502
9884	Amp	8	35701248	35723795	2.866312	0.398829
9885	Amp	8	35729273	35733630	2.170217	0.357065
9886	Amp	8	35735408	35759510	2.393797	0.370839
9887	Amp	8	35763872	35776174	2.436112	0.373154
9888	Amp	8	35781975	35793137	4.551777	0.49063
9889	Amp	8	35796622	35807040	4.355429	0.480494
9890	Amp	8	35808961	35836668	4.116436	0.468765
9891	Amp	8	35850244	35896582	4.540237	0.490407
9892	Amp	8	35897945	35905746	4.793807	0.502546
9893	Amp	8	35907588	35941030	4.581809	0.492018
9894	Amp	8	35941872	35993884	4.809024	0.502967
9895	Amp	8	36003435	36003726	5.222669	0.52251
9896	Amp	8	36005205	36021963	5.739571	0.546253
9897	Amp	8	36024008	36043927	5.982794	0.558544
9898	Amp	8	36045214	36069177	5.769527	0.547596
9899	Amp	8	36070418	36071016	5.925645	0.555151
9900	Amp	8	36075276	36091789	5.739571	0.546758
9901	Amp	8	36096694	36096694	5.622975	0.541348
9902	Amp	8	36098596	36117038	5.967002	0.557291
9903	Amp	8	36122284	36126731	5.84242	0.551147
9904	Amp	8	36133240	36139556	4.751695	0.500624
9905	Amp	8	36145577	36148666	5.459172	0.533667
9906	Amp	8	36152736	36153061	4.957051	0.509924
9907	Amp	8	36153989	36165158	4.694037	0.497633
9908	Amp	8	36168992	36275827	3.701758	0.44711
9909	Amp	8	36276437	36341579	3.801418	0.452681
9910	Amp	8	36346496	36348741	3.906866	0.457727

9911	Amp	8	36351260	36351260	3.587309	0.440598
9912	Amp	8	36352751	36369125	3.688516	0.446232
9913	Amp	8	36371625	36374898	4.090482	0.467551
9914	Amp	8	36378052	36380565	3.855237	0.455297
9915	Amp	8	36381847	36381847	5.295952	0.526141
9916	Amp	8	36582891	36584631	9.19854	0.6939
9917	Amp	8	36586692	36602856	9.931775	0.723206
9918	Amp	8	36603354	36649662	11.237152	0.773426
9919	Amp	8	36652877	36655132	13.28499	0.851409
9920	Amp	8	36655250	36664601	12.953497	0.839209
9921	Amp	8	36666736	36684060	13.0458	0.842525
9922	Amp	8	36686161	36748614	13.231261	0.849294
9923	Amp	8	36752131	36780962	14.103975	0.881465
9924	Amp	8	36784822	36805162	13.231261	0.849294
9925	Amp	8	36810292	36813855	13.436135	0.856895
9926	Amp	8	36814811	36814811	13.6031	0.863099
9927	Amp	8	36818647	36818874	14.329744	0.890179
9928	Amp	8	36820944	36822295	14.633269	0.901
9929	Amp	8	36822662	36828595	14.828326	0.907784
9930	Amp	8	36835282	36880688	15.532141	0.933641
9931	Amp	8	36886499	36896612	15.685307	0.938697
9932	Amp	8	36897710	36897710	15.612603	0.935841
9933	Amp	8	36902754	36904954	16.993677	0.986364
9934	Amp	8	36907381	36907417	17.2159	0.993617
9935	Amp	8	36908132	36923576	17.718565	1.011143
9936	Amp	8	36930713	36940747	17.8364	1.015374
9937	Amp	8	36946887	36946887	19.378804	1.065897
9938	Amp	8	36947564	36971409	19.233054	1.060841
9939	Amp	8	36972443	36975154	20.796398	1.111364
9940	Amp	8	36984444	36991506	21.532386	1.134675
9941	Amp	8	36992428	36992500	21.335552	1.128471
9942	Amp	8	36993473	37014962	22.96925	1.178994
9943	Amp	8	37018779	37019168	21.335552	1.128471
9944	Amp	8	37021337	37023433	21.877376	1.145844
9945	Amp	8	37024526	37026481	21.656796	1.13906
9946	Amp	8	37031043	37033507	23.305271	1.189583
9947	Amp	8	37034255	37034255	23.509629	1.195421
9948	Amp	8	37039519	37039519	25.162568	1.245944
9949	Amp	8	37040734	37061814	25.692774	1.261402
9950	Amp	8	37064080	37066601	25.758442	1.26344
9951	Amp	8	37067019	37068563	26.059034	1.272942
9952	Amp	8	37071854	37071854	27.760903	1.323465
9953	Amp	8	37071920	37076534	28.219176	1.337136
9954	Amp	8	37076828	37081353	28.41792	1.343176
9955	Amp	8	37084024	37107802	28.240813	1.337824
9956	Amp	8	37112248	37114460	28.714825	1.352272
9957	Amp	8	37120107	37143217	28.855201	1.356197
9958	Amp	8	37144676	37148457	27.166977	1.305674
9959	Amp	8	37148544	37176472	27.421534	1.312967
9960	Amp	8	37176854	37181044	27.651122	1.319809
9961	Amp	8	37181115	37186350	27.189678	1.306477
9962	Amp	8	37187485	37189552	27.377383	1.312045
9963	Amp	8	37189638	37189709	27.057841	1.302463

9964	Amp	8	37190115	37217420	27.150152	1.30467
9965	Amp	8	37217712	37217712	27.283162	1.308888
9966	Amp	8	37217930	37230732	27.400122	1.312601
9967	Amp	8	37233595	37267175	27.651122	1.320129
9968	Amp	8	37267620	37274389	27.805541	1.3249
9969	Amp	8	37274549	37292230	28.260508	1.338796
9970	Amp	8	37297969	37324333	28.627386	1.349867
9971	Amp	8	37324611	37338998	28.396066	1.342902
9972	Amp	8	37347983	37350348	27.760903	1.323546
9973	Amp	8	37351522	37379308	27.896225	1.32749
9974	Amp	8	37379415	37385177	28.280169	1.33928
9975	Amp	8	37385473	37385473	29.252659	1.36827
9976	Amp	8	37386066	37389403	29.337765	1.37103
9977	Amp	8	37391868	37417498	30.389263	1.40185
9978	Amp	8	37424260	37448672	31.584784	1.436385
9979	Amp	8	37449659	37457516	31.347536	1.429213
9980	Amp	8	37458054	37482859	31.419857	1.431071
9981	Amp	8	37485390	37518733	31.89834	1.445378
9982	Amp	8	37523454	37530397	32.084162	1.450492
9983	Amp	8	37532467	37550612	33.19646	1.482037
9984	Amp	8	37552935	37552935	33.12558	1.480116
9985	Amp	8	37558060	37592120	34.106466	1.508034
9986	Amp	8	37597282	37603800	34.401282	1.516285
9987	Amp	8	37606088	37619237	33.698575	1.496825
9988	Amp	8	37623213	37657959	33.56167	1.492594
9989	Amp	8	37660465	37703829	33.988075	1.505035
9990	Amp	8	37708884	37708884	34.697768	1.524915
9991	Amp	8	37712117	37735060	35.464251	1.546295
9992	Amp	8	37740892	37740892	34.351743	1.515431
9993	Amp	8	37744700	37758165	33.866545	1.501792
9994	Amp	8	37785605	37809500	32.253817	1.455355
9995	Amp	8	37817347	37834582	31.277057	1.427019
9996	Amp	8	37844412	37846796	30.13489	1.394385
9997	Amp	8	37858784	37859546	29.252659	1.368092
9998	Amp	8	37861376	37862990	28.855201	1.356301
9999	Amp	8	37864226	37867903	28.219176	1.337115
10000	Amp	8	37877519	37901020	29.573024	1.377994
10001	Amp	8	37906387	37931840	29.299351	1.369273
10002	Amp	8	37942994	37971241	29.503846	1.375462
10003	Amp	8	37971436	37979382	29.828619	1.385298
10004	Amp	8	37985152	37999429	30.13489	1.39451
10005	Amp	8	38006937	38043883	29.923156	1.388434
10006	Amp	8	38045379	38084349	30.13489	1.394437
10007	Amp	8	38086028	38086028	29.966919	1.389247
10008	Amp	8	38089568	38089568	29.686518	1.38093
10009	Amp	8	38094625	38107738	29.618421	1.379232
10010	Amp	8	38112562	38112562	29.040508	1.361766
10011	Amp	8	38114424	38131229	27.896225	1.327721
10012	Amp	8	38131787	38135757	27.826464	1.325936
10013	Amp	8	38138657	38138809	27.98291	1.330126
10014	Amp	8	38141137	38141137	26.825119	1.295591
10015	Amp	8	38146907	38150660	26.894587	1.297405
10016	Amp	8	38157974	38162759	26.662828	1.290536

10017 Amp	8	38166565	38171208	27.557582	1.317039
10018 Amp	8	38172190	38186240	27.805541	1.324628
10019 Amp	8	38188441	38214802	27.441822	1.313734
10020 Amp	8	38218299	38220559	26.173087	1.276096
10021 Amp	8	38223900	38223900	25.278212	1.249594
10022 Amp	8	38229806	38237797	23.755589	1.203433
10023 Amp	8	38240352	38248513	23.509629	1.195843
10024 Amp	8	38248606	38254588	23.820212	1.205461
10025 Amp	8	38255288	38266279	24.522607	1.226475
10026 Amp	8	38270728	38272601	24.634721	1.230151
10027 Amp	8	38276275	38282222	25.440402	1.254014
10028 Amp	8	38286684	38293209	27.510951	1.315865
10029 Amp	8	38297876	38309665	27.080967	1.303098
10030 Amp	8	38310131	38313968	26.848413	1.296062
10031 Amp	8	38327805	38349605	25.897609	1.268144
10032 Amp	8	38352395	38352395	26.196038	1.276547
10033 Amp	8	38379076	38409988	25.602213	1.258892
10034 Amp	8	38413087	38413087	24.634721	1.230367
10035 Amp	8	38413105	38413175	23.975802	1.210299
10036 Amp	8	38413201	38422182	23.777851	1.204188
10037 Amp	8	38428191	38433875	23.509629	1.195786
10038 Amp	8	38433902	38433974	23.596551	1.198776
10039 Amp	8	38434429	38455274	24.295284	1.220107
10040 Amp	8	38455399	38489066	23.885844	1.20737
10041 Amp	8	38493684	38493684	23.124223	1.184063
10042 Amp	8	38499435	38521732	22.343118	1.16032
10043 Amp	8	38524519	38531258	22.165137	1.154482
10044 Amp	8	38542325	38553039	21.899238	1.146146
10045 Amp	8	38560919	38560919	21.465588	1.132269
10046 Amp	8	38561633	38571854	21.61392	1.1375
10047 Amp	8	38576848	38592274	21.57179	1.135687
10048 Amp	8	38592613	38592613	21.246626	1.12566
10049 Amp	8	38596499	38611163	20.966664	1.116428
10050 Amp	8	38611785	38624738	20.818036	1.11228
10051 Amp	8	38628627	38629452	21.202464	1.124349
10052 Amp	8	38634282	38634431	22.165137	1.154459
10053 Amp	8	38639315	38672959	22.343118	1.160128
10054 Amp	8	38675826	38693583	21.789975	1.142602
10055 Amp	8	38695180	38722993	21.532386	1.134676
10056 Amp	8	38729500	38740209	20.130476	1.090048
10057 Amp	8	38745099	38763780	21.357124	1.128905
10058 Amp	8	38796095	38796445	20.818036	1.112036
10059 Amp	8	38797549	38843891	19.593966	1.072729
10060 Amp	8	38845587	38852497	19.316796	1.063772
10061 Amp	8	38853098	38855095	19.190972	1.059884
10062 Amp	8	38857769	38861124	20.214809	1.092517
10063 Amp	8	38861208	38876492	19.612583	1.073155
10064 Amp	8	38876610	38883657	19.233054	1.060853
10065 Amp	8	38889656	38903001	18.125364	1.024816
10066 Amp	8	38904745	38906098	17.475385	1.002597
10067 Amp	8	38910266	38923088	17.356477	0.998671
10068 Amp	8	38923522	38925050	16.65678	0.974017
10069 Amp	8	38925109	38973540	16.755306	0.977113

10070	Amp	8	38977750	38977827	15.339812	0.92659
10071	Amp	8	38978129	38981418	15.397291	0.928297
10072	Amp	8	38982666	38995448	16.316047	0.961248
10073	Amp	8	39002819	39050798	15.984625	0.95011
10074	Amp	8	39052989	39103260	16.770066	0.977789
10075	Amp	8	39103960	39136086	16.848673	0.980823
10076	Amp	8	39138570	39151161	16.580308	0.970771
10077	Amp	8	39151639	39170507	16.848673	0.980619
10078	Amp	8	39171249	39198188	16.956055	0.984848
10079	Amp	8	39213087	39239997	15.650561	0.937716
10080	Amp	8	39242218	39242218	15.320079	0.925925
10081	Amp	8	39245508	39267279	14.962133	0.912489
10082	Amp	8	39271012	39298441	15.20645	0.921826
10083	Amp	8	39300121	39307863	14.791074	0.906552
10084	Amp	8	39308402	39316760	15.302159	0.925332
10085	Amp	8	39316780	39349476	15.115662	0.918079
10086	Amp	8	39349835	39349881	14.653486	0.901884
10087	Amp	8	39352527	39352527	13.696984	0.867002
10088	Amp	8	39538338	39538338	12.860528	0.835509
10089	Amp	8	39539496	39539496	12.807917	0.83386
10090	Amp	8	39540213	39540470	12.772159	0.83239
10091	Amp	8	39540503	39558024	12.879705	0.836677
10092	Amp	8	39564532	39587163	13.657948	0.86507
10093	Amp	8	39589804	39603602	12.879705	0.836677
10094	Amp	8	39603975	39607589	12.523772	0.822503
10095	Amp	8	39609917	39609917	12.345058	0.815915
10096	Amp	8	39611300	39620262	12.647575	0.827438
10097	Amp	8	39620329	39621442	12.807917	0.833463
10098	Amp	8	39622847	39746972	12.330001	0.815183
10099	Amp	8	39747685	39767163	11.840328	0.796403
10100	Amp	8	39771138	39771531	10.971689	0.763228
10101	Amp	8	39772600	39780618	10.738968	0.753932
10102	Amp	8	39789593	39789593	10.468401	0.743909
10103	Amp	8	39790133	39800700	10.36543	0.73968
10104	Amp	8	39809042	39858555	10.220127	0.734029
10105	Amp	8	39860134	39865270	10.166941	0.732322
10106	Amp	8	39866561	39866561	9.686	0.713738
10107	Amp	8	39874932	39888699	9.043857	0.688119
10108	Amp	8	39893887	39901299	9.436384	0.704053
10109	Amp	8	39902091	39935849	9.843004	0.719745
10110	Amp	8	39936162	39951833	9.752822	0.716235
10111	Amp	8	39952339	39972684	10.220127	0.733941
10112	Amp	8	39977832	39977832	9.948936	0.724093
10113	Amp	8	39978609	39978609	9.843004	0.719398
10114	Amp	8	39985693	39998204	10.166941	0.732075
10115	Amp	8	39999128	40003606	9.931775	0.722863
10116	Amp	8	40005420	40006960	10.075027	0.728524
10117	Amp	8	40007231	40011489	10.328803	0.737906
10118	Amp	8	40011564	40024129	10.921883	0.76054
10119	Amp	8	40024172	40029109	11.059715	0.766247
10120	Amp	8	40032650	40049184	11.731641	0.792208
10121	Amp	8	40053146	40053922	11.416123	0.78043
10122	Amp	8	40054770	40071636	11.595716	0.787262

10123	Amp	8	40073291	40076405	11.840328	0.796798
10124	Amp	8	40084672	40146419	11.894261	0.798569
10125	Amp	8	40156292	40164340	11.840328	0.796798
10126	Amp	8	40167122	40167122	11.696276	0.79113
10127	Amp	8	40168922	40212272	11.416123	0.7801
10128	Amp	8	40213750	40216666	11.57725	0.786554
10129	Amp	8	40218229	40227158	11.696276	0.790826
10130	Amp	8	40227637	40250042	11.632145	0.788067
10131	Amp	8	40252740	40256725	11.857661	0.797337
10132	Amp	8	40256871	40269575	10.56213	0.746815
10133	Amp	8	40280523	40301597	10.255423	0.735283
10134	Amp	8	40305994	40307146	10.868671	0.758798
10135	Amp	8	40310177	40321012	12.097158	0.806293
10136	Amp	8	40321038	40336937	11.94724	0.801035
10137	Amp	8	40338120	40345720	11.595716	0.786921
10138	Amp	8	40346023	40398797	11.524618	0.784322
10139	Amp	8	40398829	40406894	11.57725	0.786399
10140	Amp	8	40409706	40409706	11.218179	0.772546
10141	Amp	8	40410263	40467543	10.989748	0.763487
10142	Amp	8	40470473	40476309	10.831215	0.757506
10143	Amp	8	40476374	40536721	10.95657	0.762022
10144	Amp	8	40542202	40588309	11.183333	0.771255
10145	Amp	8	40593881	40619804	10.40036	0.740778
10146	Amp	8	40621909	40625302	10.148437	0.731508
10147	Amp	8	40625573	40635481	10.220127	0.73378
10148	Amp	8	40637181	40643649	10.887353	0.759168
10149	Amp	8	40653982	40667514	10.003731	0.725871
10150	Amp	8	40670259	40708790	9.561649	0.708929
10151	Amp	8	40709009	40712078	9.162856	0.692994
10152	Amp	8	40715297	40715297	8.166506	0.652286
10153	Amp	8	40715407	40718395	8.064725	0.648087
10154	Amp	8	40720341	40737471	6.856545	0.597565
10155	Amp	8	40738905	40761411	6.149531	0.565462
10156	Amp	8	40761839	40794728	6.013103	0.559801
10157	Amp	8	40797720	40825876	6.541945	0.583634
10158	Amp	8	40826204	40826204	6.013103	0.559801
10159	Amp	8	40826798	40840844	5.967002	0.557717
10160	Amp	8	40843655	40870856	5.798921	0.549093
10161	Amp	8	40871979	40890148	5.84242	0.551252
10162	Amp	8	40893634	40894724	5.997601	0.558841
10163	Amp	8	40899031	40902730	6.042741	0.561269
10164	Amp	8	40904267	40908672	6.791498	0.594863
10165	Amp	8	40928593	40948284	6.541945	0.583339
10166	Amp	8	40952803	40963642	6.920476	0.600269
10167	Amp	8	40970161	40979074	7.16706	0.610564
10168	Amp	8	40979479	40988955	6.920476	0.600269
10169	Amp	8	40992028	40999810	7.199544	0.611516
10170	Amp	8	41000422	41012844	7.833881	0.638811
10171	Amp	8	41015426	41026027	7.5669	0.627564
10172	Amp	8	41026303	41059585	7.346492	0.618293
10173	Amp	8	41063999	41080132	7.850108	0.639385
10174	Amp	8	41085236	41112872	6.364443	0.575211
10175	Amp	8	41114156	41136009	6.435656	0.578806

10176 Amp	8	41139907	41184352	6.526336	0.582759
10177 Amp	8	41187836	41192170	5.813919	0.550156
10178 Amp	8	41192561	41213641	6.028267	0.560004
10179 Amp	8	41217387	41241887	6.378996	0.576288
10180 Amp	8	41244831	41267147	6.494707	0.581663
10181 Amp	8	41267979	41299720	6.619468	0.586858
10182 Amp	8	41300485	41337579	6.745443	0.592868
10183 Amp	8	41342974	41351777	6.936308	0.60113
10184 Amp	8	41356501	41356501	6.712483	0.591389
10185 Amp	8	41359505	41418711	6.619468	0.586853
10186 Amp	8	41426137	41445217	6.557256	0.584581
10187 Amp	8	41445940	41450438	6.650629	0.588761
10188 Amp	8	41453647	41465246	6.604027	0.586333
10189 Amp	8	41469080	41469080	6.728678	0.592067
10190 Amp	8	41471003	41494556	6.952285	0.601156
10191 Amp	8	41494940	41508189	6.392987	0.576861
10192 Amp	8	41509360	41519880	6.227003	0.569038
10193 Amp	8	41520250	41587486	6.40864	0.577749
10194 Amp	8	41587990	41663492	6.557256	0.584007
10195 Amp	8	41663951	41684721	6.40864	0.577434
10196 Amp	8	41688700	41711406	6.480211	0.58054
10197 Amp	8	41717921	41719010	6.117292	0.56412
10198 Amp	8	41720510	41730668	5.813919	0.549655
10199 Amp	8	41730749	41754276	5.769527	0.54811
10200 Amp	8	41755978	41761059	6.197199	0.567747
10201 Amp	8	41768443	41847769	6.364443	0.575562
10202 Amp	8	41851020	41884040	6.494707	0.581709
10203 Amp	8	41885875	41904328	6.712483	0.590957
10204 Amp	8	41909736	41919201	6.966987	0.60233
10205 Amp	8	41922839	41938525	6.889312	0.598797
10206 Amp	8	41942096	41947731	7.067371	0.606487
10207 Amp	8	41950578	41953330	6.889312	0.598897
10208 Amp	8	41953759	41965127	6.634781	0.587524
10209 Amp	8	41967245	41974516	6.133613	0.565077
10210 Amp	8	41974778	42011425	6.421599	0.578289
10211 Amp	8	42012732	42014532	6.197199	0.568092
10212 Amp	8	42018986	42028811	7.10077	0.607629
10213 Amp	8	42029956	42029956	6.728678	0.592008
10214 Amp	8	42038820	42059934	5.638575	0.541698
10215 Amp	8	42066757	42071634	5.351891	0.528487
10216 Amp	8	42077039	42094390	5.724602	0.546125
10217 Amp	8	42101036	42114330	6.378996	0.576214
10218 Amp	8	42118461	42130871	6.696475	0.590436
10219 Amp	8	42130930	42136633	6.465884	0.579994
10220 Amp	8	42143465	42165441	6.87258	0.597948
10221 Amp	8	42166007	42173806	6.936308	0.601065
10222 Amp	8	42174474	42182310	6.667173	0.589203
10223 Amp	8	42182432	42211185	6.435656	0.578831
10224 Amp	8	42214744	42223646	6.013103	0.559649
10225 Amp	8	42224199	42245703	5.783449	0.548731
10226 Amp	8	42245804	42259530	5.56363	0.538358
10227 Amp	8	42275584	42283425	5.135526	0.518359
10228 Amp	8	42283867	42283867	4.927103	0.509067

10229	Amp	8	42285489	42298312	4.497567	0.48816
10230	Amp	8	42298469	42298499	5.413899	0.531331
10231	Amp	8	42300125	42300125	5.681709	0.543759
10232	Amp	8	42300300	42321627	6.08935	0.562921
10233	Amp	8	42323198	42328629	6.149531	0.565829
10234	Amp	8	42328734	42332060	6.392987	0.576632
10235	Amp	8	42332201	42389733	5.592909	0.539641
10236	Amp	8	42395770	42403138	5.208342	0.521851
10237	Amp	8	42404318	42409358	5.367722	0.529634
10238	Amp	8	42416746	42417516	5.47413	0.534128
10239	Amp	8	42417909	42417909	6.364443	0.575321
10240	Amp	8	42423912	42434987	6.557256	0.584396
10241	Amp	8	42435369	42435434	7.363353	0.618425
10242	Amp	8	42442237	42442237	7.282206	0.615308
10243	Amp	8	42444860	42445892	7.232556	0.613448
10244	Amp	8	42449295	42462293	6.712483	0.590868
10245	Amp	8	42472584	42477270	5.953304	0.556839
10246	Amp	8	42483424	42491712	5.84242	0.551183
10247	Amp	8	42496200	42503920	6.181334	0.567354
10248	Amp	8	42507855	42538785	5.429672	0.532048
10249	Amp	8	42545269	42555582	5.337978	0.528114
10250	Amp	8	42564653	42593842	4.911986	0.508032
10251	Amp	8	42596383	42626737	4.470731	0.486581
10252	Amp	8	42629720	42634935	4.497567	0.488174
10253	Amp	8	42635132	42654300	4.809024	0.50358
10254	Amp	8	42662300	42681912	4.470731	0.486893
10255	Amp	8	42684075	42684075	4.402792	0.483184
10256	Amp	8	42688195	42688195	3.560951	0.439144
10257	Amp	8	42694679	42701270	3.461541	0.434002
10258	Amp	8	42704599	42705022	3.573122	0.440175
10259	Amp	8	42708879	42708879	3.637615	0.443324
10260	Amp	8	42709483	42730190	3.600858	0.441731
10261	Amp	8	42733252	42749648	3.335884	0.426216
10262	Amp	8	42749685	42778100	3.230962	0.42044
10263	Amp	8	42778379	42800292	2.727427	0.390736
10264	Amp	8	42805261	42808491	3.348874	0.427031
10265	Amp	8	42811772	42851633	3.243024	0.42089
10266	Amp	8	42856463	42872309	3.371695	0.428129
10267	Amp	8	42874242	42874242	3.470866	0.434406
10268	Amp	8	42874836	42900086	3.288383	0.423602
10269	Amp	8	42917263	42928572	2.935659	0.402839
10270	Amp	8	42934585	42942814	2.818034	0.396063
10271	Amp	8	42946512	42946512	2.866312	0.398769
10272	Amp	8	42949116	42949116	2.88973	0.400507
10273	Amp	8	42951363	42985297	3.066577	0.410231
10274	Amp	8	42989678	43013908	2.902384	0.400983
10275	Amp	8	43014849	43024369	2.994162	0.406485
10276	Amp	8	43025271	43028711	2.796399	0.394652
10277	Amp	8	43031275	43034923	2.866312	0.399158
10278	Amp	8	43036958	43044471	2.948071	0.403718
10279	Amp	8	43045774	43053246	2.866312	0.398845
10280	Amp	8	43067847	43077335	2.737964	0.391405
10281	Amp	8	43078229	43078229	2.935659	0.403044

10282	Amp	8	43080575	43080575	3.906866	0.457675
10283	Amp	8	46547532	46547532	1.295233	0.299057
10284	Amp	8	46549323	46554650	0.743132	0.259188
10285	Amp	8	46557601	46557601	0.604912	0.2477
10286	Amp	8	46558899	46581638	0.62858	0.249435
10287	Amp	8	46581901	46694262	0.66072	0.25262
10288	Amp	8	46699568	46718557	0.564815	0.244486
10289	Amp	8	46721803	46725770	0.613517	0.248582
10290	Amp	8	46726141	46732868	0.66072	0.252516
10291	Amp	8	46737651	46739194	0.805493	0.264086
10292	Amp	8	46740347	46763172	0.693887	0.255356
10293	Amp	8	46767311	46874450	0.734472	0.258623
10294	Amp	8	46876115	46890357	0.991911	0.278065
10295	Amp	8	46895700	46967391	0.906818	0.271912
10296	Amp	8	46967718	46980274	1.049532	0.281706
10297	Amp	8	46980664	46997177	1.374261	0.304639
10298	Amp	8	46998518	47028268	1.473764	0.311855
10299	Amp	8	47028353	47128124	1.324884	0.301359
10300	Amp	8	47128395	47176999	0.972663	0.276489
10301	Amp	8	47177757	47191163	1.199568	0.292623
10302	Amp	8	47194827	47235513	1.130387	0.287492
10303	Amp	8	47237135	47252758	1.424227	0.308045
10304	Amp	8	47322675	47322675	0.734472	0.258492
10305	Amp	8	47326583	47339482	1.10923	0.28597
10306	Amp	8	47343262	47378124	1.442925	0.309648
10307	Amp	8	47379939	47435134	1.717946	0.328136
10308	Amp	8	47438958	47473014	1.442925	0.309648
10309	Amp	8	47475358	47475595	1.404612	0.306462
10310	Amp	8	47476188	47627000	1.484449	0.312157
10311	Amp	8	47628078	47663565	1.525294	0.314696
10312	Amp	8	47667799	47669267	1.678649	0.325207
10313	Amp	8	47669552	47675436	1.727244	0.329011
10314	Amp	8	47676767	47724528	1.646768	0.323316
10315	Amp	8	47728887	47748626	1.275021	0.297557
10316	Amp	8	47758902	47758902	1.199568	0.292056
10317	Amp	8	47766760	47776495	1.099256	0.285433
10318	Amp	8	47776805	47779345	1.029845	0.280939
10319	Amp	8	47781881	47834051	1.364151	0.304337
10320	Amp	8	47839964	47891194	1.2565	0.296521
10321	Amp	8	47894613	47980162	1.199568	0.292587
10322	Amp	8	47983064	48046516	0.925715	0.273145
10323	Amp	8	48048154	48143205	0.982622	0.277359
10324	Amp	8	48145344	48163631	0.934277	0.273806
10325	Amp	8	48172237	48227725	0.972663	0.276723
10326	Amp	8	48230744	48250004	0.814697	0.265153
10327	Amp	8	48256555	48331711	1.000924	0.278754
10328	Amp	8	48334020	48480283	0.778204	0.262287
10329	Amp	8	48480916	48506423	0.843361	0.26687
10330	Amp	8	48509510	48511700	0.636206	0.250735
10331	Amp	8	48513573	48602307	0.693887	0.255273
10332	Amp	8	48602333	48617626	0.636206	0.250691
10333	Amp	8	48618529	48669079	0.66072	0.252185
10334	Amp	8	48671254	48903528	0.805493	0.264027

10335	Amp	8	48904240	48913047	0.852608	0.267793
10336	Amp	8	48916601	48931818	0.702615	0.255951
10337	Amp	8	48932112	48937227	0.652914	0.251737
10338	Amp	8	48940182	48976311	0.398217	0.230117
10339	Amp	8	48976906	49037858	0.375927	0.228043
10340	Amp	8	49038251	49089387	0.483654	0.237097
10341	Amp	8	49090635	49105961	0.430191	0.232559
10342	Amp	8	49111022	49157809	0.390264	0.229292
10343	Amp	8	49160977	49220916	0.375927	0.227798
10344	Amp	8	49220926	49220926	0.301081	0.220582
10345	Amp	8	49221379	49248638	0.25804	0.216779
10346	Amp	8	49249443	49275832	0.382767	0.228176
10347	Amp	8	49276802	49288121	0.340434	0.22408
10348	Amp	8	49292368	49333274	0.215373	0.212683
10349	Amp	8	49346286	49353739	0.367646	0.226896
10350	Amp	8	49355373	49355373	0.382767	0.228653
10351	Amp	8	49357138	49415749	0.667776	0.252936
10352	Amp	8	49436543	49436543	0.71758	0.25729
10353	Amp	8	49436777	49481495	0.934277	0.273619
10354	Amp	8	49483195	49483195	0.786952	0.263108
10355	Amp	8	49488600	49503168	0.483654	0.237067
10356	Amp	8	49503215	49652912	0.398217	0.229894
10357	Amp	8	49659065	49665360	0.41418	0.231499
10358	Amp	8	49666857	49671917	0.340434	0.224275
10359	Amp	8	49673991	49692048	0.187743	0.210062
10360	Amp	8	49694129	49776363	0.540744	0.241989
10361	Amp	8	49778601	49946976	0.685617	0.25442
10362	Amp	8	50076101	50076101	0.317596	0.222493
10363	Amp	8	50076146	50087456	0.390264	0.229431
10364	Amp	8	50090435	50108067	0.636206	0.250643
10365	Amp	8	50108088	50160727	0.702615	0.255876
10366	Amp	8	50164366	50220505	0.452609	0.234664
10367	Amp	8	50220586	50309669	0.405699	0.230482
10368	Amp	8	50310165	50310165	0.362221	0.226128
10369	Amp	8	50317441	50379356	0.286675	0.21919
10370	Amp	8	50380189	50527315	0.187743	0.209946
10371	Amp	8	50530279	50685284	0.25804	0.216423
10372	Amp	8	50687501	50960332	0.149443	0.205483
10373	Amp	8	50960946	50970847	0.49169	0.237951
10374	Amp	8	50975309	50998664	0.596354	0.24717
10375	Amp	8	50999365	51029302	0.734472	0.258686
10376	Amp	8	51034139	51092306	0.833763	0.265943
10377	Amp	8	51098829	51109480	0.437983	0.233474
10378	Amp	8	51109570	51117470	0.460107	0.235601
10379	Amp	8	51125159	51141766	0.362221	0.226382
10380	Amp	8	51146821	51221062	0.236222	0.214867
10381	Amp	8	51222522	51254442	0.168144	0.208088
10382	Amp	8	51255145	51375998	0.209057	0.211904
10383	Amp	8	51382446	51388411	0.25804	0.216432
10384	Amp	8	51394750	51400083	0.18072	0.209175
10385	Amp	8	51401924	51489888	0.230572	0.214172
10386	Amp	8	51489935	51500998	0.18072	0.209175
10387	Amp	8	51501970	51503151	0.25804	0.216528

10388 Amp	8	51503641	51554153	0.18072	0.208934
10389 Amp	8	51554827	51556795	0.25804	0.216803
10390 Amp	8	51561238	51587113	0.13565	0.204372
10391 Amp	8	51589069	51661209	0.09318	0.199812
10392 Amp	8	51666678	51686192	0.161592	0.2071
10393 Amp	8	51686209	51707590	0.187743	0.209952
10394 Amp	8	51707659	51711840	0	0.185684
10395 Amp	8	51712357	51734818	0	0.184079
10396 Amp	8	51740123	51742782	0.001198	0.188923
10397 Amp	8	51742814	51762067	0	0.184395
10398 Amp	8	51764803	51833290	0.02372	0.191516
10399 Amp	8	51833440	51998735	0.209057	0.211924
10400 Amp	8	52000587	52007563	0.117531	0.202595
10401 Amp	8	52011220	52016671	0.050005	0.194611
10402 Amp	8	52019203	52019332	0.123589	0.202863
10403 Amp	8	52032819	52048593	0.223025	0.213316
10404 Amp	8	52051381	52086375	0.286675	0.219317
10405 Amp	8	52086899	52135418	0.201819	0.211065
10406 Amp	8	52136072	52142793	0.27943	0.218863
10407 Amp	8	52145585	52217863	0.175335	0.20841
10408 Amp	8	52223214	52244654	0	0.188003
10409 Amp	8	52249623	52278815	0.187743	0.209779
10410 Amp	8	52280155	52357798	0.243425	0.215442
10411 Amp	8	52357918	52412635	0.038273	0.193666
10412 Amp	8	52414821	52452089	0.230572	0.214011
10413 Amp	8	52452180	52470477	0.168144	0.208097
10414 Amp	8	52477254	52490804	0.273081	0.21798
10415 Amp	8	52491217	52501591	0.508503	0.239399
10416 Amp	8	52508678	52543360	0.27943	0.219054
10417 Amp	8	52544118	52580313	0.264784	0.217606
10418 Amp	8	52591269	52615948	0.375927	0.228022
10419 Amp	8	52617090	52630505	0.156508	0.206602
10420 Amp	8	52631082	52631082	0.215373	0.212475
10421 Amp	8	52636024	52636024	0.142507	0.205354
10422 Amp	8	52636620	52636620	0.195309	0.210337
10423 Amp	8	52793185	52883728	0.149443	0.205494
10424 Amp	8	52893043	52915408	0.195309	0.210737
10425 Amp	8	52918722	52944147	0.105051	0.200854
10426 Amp	8	52948564	52955221	0.301081	0.221144
10427 Amp	8	52962923	52968571	0.250263	0.216161
10428 Amp	8	52968665	53047187	0.195309	0.210287
10429 Amp	8	53162471	53162471	0.243425	0.215655
10430 Amp	8	53163454	53234646	0.340434	0.224419
10431 Amp	8	53236829	53240563	0.667776	0.253045
10432 Amp	8	53240641	53264853	0.685617	0.254448
10433 Amp	8	53268565	53280535	0.580011	0.245684
10434 Amp	8	53284436	53394636	0.508503	0.239682
10435 Amp	8	53394713	53608575	0.476502	0.236775
10436 Amp	8	53608688	53679257	0.540744	0.241994
10437 Amp	8	53679525	53706850	0.587735	0.246448
10438 Amp	8	53710089	53734518	0.66072	0.252189
10439 Amp	8	53735865	53763987	0.702615	0.256072
10440 Amp	8	53778108	53804729	0.636206	0.250703

10441 Amp	8	53807991	53867614	0.71758	0.257472
10442 Amp	8	53867649	53931053	0.636206	0.250703
10443 Amp	8	53937735	54110632	0.452609	0.234374
10444 Amp	8	54114181	54327931	0.556748	0.243317
10445 Amp	8	54332578	54481251	0.596354	0.247046
10446 Amp	8	54481650	54508666	0.556748	0.243317
10447 Amp	8	54508802	54608821	0.613517	0.248252
10448 Amp	8	54611193	54666792	0.375927	0.227962
10449 Amp	8	54672611	54680583	0.223025	0.213587
10450 Amp	8	54681906	54704783	0.195309	0.210671
10451 Amp	8	54706948	54742151	0.317596	0.222235
10452 Amp	8	54742328	54817952	0.223025	0.213293
10453 Amp	8	54818168	54846379	0.27943	0.218706
10454 Amp	8	54846480	54893879	0.325321	0.223173
10455 Amp	8	54893945	54971545	0.27943	0.219093
10456 Amp	8	54971583	55012148	0.243425	0.215277
10457 Amp	8	55020894	55072805	0.483654	0.237614
10458 Amp	8	55074281	55159522	0.564815	0.244456
10459 Amp	8	55160432	55207323	0.604912	0.24739
10460 Amp	8	55207902	55430902	0.71758	0.25728
10461 Amp	8	55431544	55479130	0.778204	0.262259
10462 Amp	8	55482614	55535121	0.734472	0.258377
10463 Amp	8	55535721	55579076	0.709359	0.256399
10464 Amp	8	55579120	55586546	0.571214	0.245089
10465 Amp	8	55586780	55608959	0.51566	0.240154
10466 Amp	8	55611112	55723501	0.702615	0.255715
10467 Amp	8	55727489	55728664	0.676804	0.253638
10468 Amp	8	55734393	55787632	0.556748	0.243222
10469 Amp	8	55788156	56011499	0.702615	0.255838
10470 Amp	8	56011803	56032424	0.796213	0.263341
10471 Amp	8	56033001	56057014	0.86979	0.269151
10472 Amp	8	56060254	56073619	1.218154	0.293537
10473 Amp	8	56085921	56186628	1.285475	0.298328
10474 Amp	8	56187590	56226804	1.140058	0.287997
10475 Amp	8	56236125	56236962	0.796213	0.263611
10476 Amp	8	56237568	56259673	0.685617	0.254583
10477 Amp	8	56260770	56325123	0.652914	0.251561
10478 Amp	8	56336856	56427246	0.743132	0.259301
10479 Amp	8	56434567	56518815	1.049532	0.281724
10480 Amp	8	56519911	56524867	0.944128	0.274371
10481 Amp	8	56528451	56544419	1.078696	0.284338
10482 Amp	8	56544763	56548916	1.150167	0.289117
10483 Amp	8	56549281	56703440	1.020188	0.279897
10484 Amp	8	56703491	56703497	0.878688	0.26993
10485 Amp	8	56703506	56738256	1.049532	0.281841
10486 Amp	8	56739697	56825669	1.190324	0.291417
10487 Amp	8	56830243	56830828	0.915677	0.272095
10488 Amp	8	56831522	57005385	0.843361	0.266876
10489 Amp	8	57025292	57221284	0.805493	0.263942
10490 Amp	8	57223942	57284684	0.709359	0.256281
10491 Amp	8	57287614	57295979	0.66072	0.252692
10492 Amp	8	57296346	57377551	0.796213	0.263326
10493 Amp	8	57378252	57387478	0.860481	0.268323

10494	Amp	8	57387542	57441651	0.743132	0.259482
10495	Amp	8	57441683	57458599	0.86979	0.268696
10496	Amp	8	57482222	57492262	0.843361	0.266928
10497	Amp	8	57493704	57509380	0.991911	0.277699
10498	Amp	8	57515071	57524366	0.915677	0.272465
10499	Amp	8	57525892	57567212	0.852608	0.267467
10500	Amp	8	57567406	57630020	0.693887	0.254996
10501	Amp	8	57632681	57636411	0.709359	0.256496
10502	Amp	8	57649004	57681558	0.580011	0.245725
10503	Amp	8	57682606	57715172	0.734472	0.258965
10504	Amp	8	57715473	57762718	0.760395	0.260469
10505	Amp	8	57765635	57829592	1.2565	0.296377
10506	Amp	8	57832009	57833948	1.344429	0.302498
10507	Amp	8	57841977	57957299	1.199568	0.292384
10508	Amp	8	57965830	57973892	1.344429	0.302718
10509	Amp	8	57976915	58079972	1.572486	0.318243
10510	Amp	8	58089053	58180829	1.424227	0.307909
10511	Amp	8	58181905	58338851	1.678649	0.325017
10512	Amp	8	58339117	58362766	1.646768	0.323207
10513	Amp	8	58363297	58376487	1.800655	0.333334
10514	Amp	8	58376788	58386587	1.687998	0.325714
10515	Amp	8	58390018	58469473	1.832922	0.33583
10516	Amp	8	58471009	58577074	1.963469	0.344197
10517	Amp	8	58579472	58618061	1.930731	0.341872
10518	Amp	8	58621223	58652537	2.113683	0.35367
10519	Amp	8	58655326	58699194	1.963469	0.343543
10520	Amp	8	58699344	58702853	1.844531	0.336062
10521	Amp	8	58703486	58758209	1.800655	0.333381
10522	Amp	8	58758272	58802865	1.708768	0.327331
10523	Amp	8	58806101	58897226	1.887705	0.338865
10524	Amp	8	58897281	58960675	2.039783	0.348781
10525	Amp	8	58962748	58970768	1.887705	0.338865
10526	Amp	8	58972419	59011242	2.148338	0.355863
10527	Amp	8	59011335	59073233	1.963469	0.344062
10528	Amp	8	59073867	59122964	2.049537	0.349667
10529	Amp	8	59128229	59224382	1.877398	0.338132
10530	Amp	8	59224518	59317134	1.907576	0.340612
10531	Amp	8	59322615	59351857	2.071528	0.350723
10532	Amp	8	59354754	59398941	2.260813	0.362364
10533	Amp	8	59404821	59473116	2.327403	0.3666
10534	Amp	8	59473352	59479666	2.260813	0.362364
10535	Amp	8	59486273	59488256	2.124253	0.354426
10536	Amp	8	59489466	59532058	2.294081	0.364645
10537	Amp	8	59532490	59665270	2.158849	0.356278
10538	Amp	8	59669201	59675202	2.005494	0.346384
10539	Amp	8	59678796	59695802	2.124253	0.353847
10540	Amp	8	59698415	59770557	2.203122	0.358974
10541	Amp	8	59772281	59792319	2.071528	0.350509
10542	Amp	8	59802172	59858921	2.214215	0.35958
10543	Amp	8	59859688	59922314	1.983885	0.3455
10544	Amp	8	59925104	59977744	1.717946	0.328327
10545	Amp	8	59977855	59996295	1.572486	0.318215
10546	Amp	8	60004739	60004739	1.887705	0.339309

10547	Amp	8	60007046	60034423	1.727244	0.328676
10548	Amp	8	60037906	60071654	1.778794	0.332034
10549	Amp	8	60071706	60157886	1.727244	0.328596
10550	Amp	8	60166583	60167709	1.887705	0.339356
10551	Amp	8	60171940	60173833	1.49455	0.313112
10552	Amp	8	60177762	60238033	1.687998	0.326023
10553	Amp	8	60238591	60264392	1.453628	0.310499
10554	Amp	8	60264650	60317832	1.553308	0.317293
10555	Amp	8	60320277	60322489	1.657426	0.324025
10556	Amp	8	60324432	60504841	1.572486	0.318214
10557	Amp	8	60507155	60593354	1.354645	0.303629
10558	Amp	8	60595678	60671473	1.768015	0.331268
10559	Amp	8	60671882	60676395	1.605306	0.320508
10560	Amp	8	60683047	60791524	1.525294	0.314705
10561	Amp	8	60792258	60799784	1.404612	0.306522
10562	Amp	8	60799811	60831494	1.463463	0.310692
10563	Amp	8	60832841	60859354	1.295233	0.29948
10564	Amp	8	60863368	60863368	1.169408	0.29041
10565	Amp	8	60867122	60867122	1.120124	0.286954
10566	Amp	8	60868922	60899205	1.275021	0.297483
10567	Amp	8	60899994	60908469	1.208462	0.293313
10568	Amp	8	60914266	60914266	1.364151	0.304231
10569	Amp	8	60915117	61012674	1.49455	0.312808
10570	Amp	8	61013679	61053560	1.334042	0.302279
10571	Amp	8	61054140	61056569	1.190324	0.291361
10572	Amp	8	61058100	61072108	1.059215	0.282784
10573	Amp	8	61074622	61113970	0.76883	0.261594
10574	Amp	8	61114939	61219056	0.888878	0.270069
10575	Amp	8	61227176	61262141	0.7517	0.260187
10576	Amp	8	61262355	61279089	0.652914	0.251713
10577	Amp	8	61279470	61311782	0.743132	0.259387
10578	Amp	8	61311914	61370692	0.760395	0.260863
10579	Amp	8	61377190	61382670	1.010597	0.279101
10580	Amp	8	61386077	61402539	1.208462	0.293211
10581	Amp	8	61403096	61416673	1.315861	0.300503
10582	Amp	8	61417465	61423895	1.208462	0.292829
10583	Amp	8	61424685	61437843	1.275021	0.297672
10584	Amp	8	61437936	61437936	1.140058	0.288255
10585	Amp	8	61442575	61467029	1.394558	0.305826
10586	Amp	8	61489435	61532403	1.140058	0.288255
10587	Amp	8	61534323	61578903	1.305256	0.299565
10588	Amp	8	61581425	61589561	1.130387	0.287453
10589	Amp	8	61589788	61615013	1.159718	0.28953
10590	Amp	8	61615117	61678441	1.190324	0.291851
10591	Amp	8	61685527	61706648	0.972663	0.276739
10592	Amp	8	61708637	61749398	1.039426	0.28145
10593	Amp	8	61749727	61840655	1.159718	0.289651
10594	Amp	8	61843212	61843212	1.049532	0.282359
10595	Amp	8	61843522	61915136	0.944128	0.274159
10596	Amp	8	61916838	62001050	0.963924	0.275779
10597	Amp	8	62002390	62009096	0.944128	0.274302
10598	Amp	8	62009482	62023111	0.76883	0.261501
10599	Amp	8	62024420	62073175	0.888878	0.2704

10600	Amp	8	62080433	62089060	1.010597	0.279138
10601	Amp	8	62089704	62185007	0.982622	0.277011
10602	Amp	8	62186227	62259414	0.915677	0.272318
10603	Amp	8	62260513	62347885	0.760395	0.261008
10604	Amp	8	62348167	62358126	0.702615	0.256165
10605	Amp	8	62358692	62363939	0.734472	0.258866
10606	Amp	8	62369177	62466205	0.613517	0.248336
10607	Amp	8	62470522	62523917	0.709359	0.256311
10608	Amp	8	62526320	62587839	0.613517	0.248571
10609	Amp	8	62589268	62605016	0.644689	0.25083
10610	Amp	8	62605031	62631653	0.587735	0.245987
10611	Amp	8	62633204	62638062	0.71758	0.257561
10612	Amp	8	62642617	62738284	0.796213	0.263471
10613	Amp	8	62742007	62754815	0.823952	0.265755
10614	Amp	8	62756694	62764683	0.760395	0.260525
10615	Amp	8	62769838	62817900	0.604912	0.247998
10616	Amp	8	62818063	62832808	0.580011	0.24592
10617	Amp	8	62835054	62936124	0.437983	0.233213
10618	Amp	8	62936967	62959496	0.580011	0.245702
10619	Amp	8	62960155	63009843	0.71758	0.257626
10620	Amp	8	63011205	63042601	0.571214	0.245137
10621	Amp	8	63042632	63088140	0.613517	0.248515
10622	Amp	8	63091288	63109351	0.347934	0.225017
10623	Amp	8	63111369	63161506	0.317596	0.222316
10624	Amp	8	63165467	63178125	0.355162	0.225849
10625	Amp	8	63189450	63235749	0.421967	0.231784
10626	Amp	8	63236659	63269899	0.325321	0.223045
10627	Amp	8	63270688	63359867	0.264784	0.217206
10628	Amp	8	63361172	63386364	0.230572	0.21393
10629	Amp	8	63388067	63406652	0.325321	0.222795
10630	Amp	8	63406696	63454475	0.347934	0.225066
10631	Amp	8	63457082	63505194	0.333141	0.223445
10632	Amp	8	63509067	63521571	0.51566	0.240339
10633	Amp	8	63523423	63582719	0.382767	0.228542
10634	Amp	8	63585802	63691660	0.897922	0.271028
10635	Amp	8	63692278	63721969	0.382767	0.228542
10636	Amp	8	63724071	63796150	0.209057	0.211648
10637	Amp	8	63797134	63815041	0.243425	0.215608
10638	Amp	8	63822181	63851234	0.209057	0.212075
10639	Amp	8	63854312	64063745	0.243425	0.215515
10640	Amp	8	64064121	64122818	0.430191	0.232601
10641	Amp	8	64137104	64178291	0.483654	0.237117
10642	Amp	8	64179628	64205950	0.382767	0.228743
10643	Amp	8	64205982	64206465	0.523647	0.24095
10644	Amp	8	64206523	64230986	0.613517	0.248642
10645	Amp	8	64233400	64330191	0.51566	0.23993
10646	Amp	8	64346546	64357166	0.814697	0.264914
10647	Amp	8	64359921	64397797	1.442925	0.309537
10648	Amp	8	64400314	64424023	1.315861	0.300671
10649	Amp	8	64426424	64519471	1.169408	0.290497
10650	Amp	8	64523599	64533872	1.088806	0.284563
10651	Amp	8	64534336	64535880	0.878688	0.269752
10652	Amp	8	64536611	64631876	0.906818	0.271386

10653	Amp	8	64636704	64745702	0.972663	0.27637
10654	Amp	8	64746294	64778501	0.709359	0.256471
10655	Amp	8	64790371	64899600	0.483654	0.23747
10656	Amp	8	64907018	65002555	0.367646	0.227211
10657	Amp	8	65003592	65019194	0.587735	0.246148
10658	Amp	8	65021722	65112688	0.709359	0.256425
10659	Amp	8	65118195	65142844	0.209057	0.211802
10660	Amp	8	65145623	65168337	0.033661	0.192865
10661	Amp	8	65168354	65198555	0.236222	0.214452
10662	Amp	8	65199430	65308366	0.033661	0.192865
10663	Amp	8	65311590	65376652	0.105051	0.200947
10664	Amp	8	65379649	65387585	0.421967	0.23186
10665	Amp	8	65387808	65440255	0.452609	0.23443
10666	Amp	8	65442452	65568088	0.508503	0.239482
10667	Amp	8	65568127	65624668	0.367646	0.227372
10668	Amp	8	65624793	65624793	0.421967	0.232079
10669	Amp	8	65638386	65643952	0.367646	0.22689
10670	Amp	8	65654255	65744582	0.702615	0.256086
10671	Amp	8	65745714	65800149	0.778204	0.262072
10672	Amp	8	65807542	65828737	0.532449	0.241756
10673	Amp	8	65835170	65846568	0.215373	0.21256
10674	Amp	8	65849143	65852362	0.293283	0.220386
10675	Amp	8	65860156	65905500	0.117531	0.202152
10676	Amp	8	65907156	66029099	0.390264	0.228875
10677	Amp	8	66031066	66067009	0.7517	0.259797
10678	Amp	8	66072326	66127154	0.963924	0.275995
10679	Amp	8	66127370	66133702	1.059215	0.282513
10680	Amp	8	66134356	66182366	1.190324	0.291835
10681	Amp	8	66184155	66186276	1.078696	0.284181
10682	Amp	8	66186742	66187393	1.190324	0.291854
10683	Amp	8	66189034	66249831	1.503938	0.313515
10684	Amp	8	66250074	66250074	1.334042	0.302224
10685	Amp	8	66252991	66294667	1.2565	0.296608
10686	Amp	8	66294889	66314987	1.218154	0.294038
10687	Amp	8	66315874	66333613	1.179216	0.291186
10688	Amp	8	66335369	66369653	1.068928	0.28336
10689	Amp	8	66371687	66373599	1.226647	0.29465
10690	Amp	8	66373758	66417897	1.295233	0.298935
10691	Amp	8	66418110	66426482	1.344429	0.302847
10692	Amp	8	66431770	66431960	1.514375	0.31456
10693	Amp	8	66433386	66552163	1.285475	0.298362
10694	Amp	8	66553168	66553353	0.906818	0.27164
10695	Amp	8	66558480	66571265	0.925715	0.273372
10696	Amp	8	66573484	66607830	1.039426	0.281446
10697	Amp	8	66608551	66686034	1.344429	0.302395
10698	Amp	8	66686060	66714719	1.463463	0.310741
10699	Amp	8	66715671	66715904	1.324884	0.301419
10700	Amp	8	66718567	66718567	1.237378	0.294901
10701	Amp	8	66720094	66800620	1.898353	0.339438
10702	Amp	8	66801401	66812593	1.930731	0.341918
10703	Amp	8	66818702	66845580	1.844531	0.336236
10704	Amp	8	66848033	66857147	1.942393	0.342797
10705	Amp	8	66860633	66892368	1.866286	0.337376

10706	Amp	8	66898167	66921900	2.061276	0.35023
10707	Amp	8	66922831	66976569	1.963469	0.343668
10708	Amp	8	66985620	67008123	1.757929	0.330815
10709	Amp	8	67020990	67052156	1.020188	0.280292
10710	Amp	8	67056498	67222888	0.95395	0.27524
10711	Amp	8	67229323	67283868	1.199568	0.292595
10712	Amp	8	67287324	67338193	1.169408	0.290115
10713	Amp	8	67342575	67388913	0.823952	0.265474
10714	Amp	8	67390292	67406728	0.693887	0.255519
10715	Amp	8	67407976	67413837	0.786952	0.262946
10716	Amp	8	67417127	67686964	0.852608	0.267382
10717	Amp	8	67687310	67692631	0.76883	0.261352
10718	Amp	8	67695933	67884836	0.786952	0.262757
10719	Amp	8	67885569	67885569	0.580011	0.245402
10720	Amp	8	67885804	67924329	0.596354	0.247292
10721	Amp	8	67925926	68016218	0.644689	0.250821
10722	Amp	8	68018932	68018932	0.743132	0.259236
10723	Amp	8	68022413	68047834	0.580011	0.245728
10724	Amp	8	68050626	68065258	0.49169	0.238302
10725	Amp	8	68067143	68083965	0.833763	0.26597
10726	Amp	8	68084285	68230652	0.888878	0.270162
10727	Amp	8	68231601	68282163	1.159718	0.289285
10728	Amp	8	68285801	68354077	1.078696	0.284247
10729	Amp	8	68360409	68420482	0.786952	0.26303
10730	Amp	8	68421500	68539568	0.925715	0.272811
10731	Amp	8	68540370	68553075	1.059215	0.282401
10732	Amp	8	68555423	68603383	1.120124	0.286973
10733	Amp	8	68608562	68608562	1.000924	0.278558
10734	Amp	8	68611708	68699394	0.86979	0.268969
10735	Amp	8	68699425	68730363	0.944128	0.274716
10736	Amp	8	68734297	68771623	1.010597	0.279274
10737	Amp	8	68774725	68787521	1.130387	0.287198
10738	Amp	8	68788072	68790463	1.190324	0.291365
10739	Amp	8	68794005	68844129	1.020188	0.280074
10740	Amp	8	68844252	68861227	1.179216	0.291057
10741	Amp	8	68864485	68880984	1.099256	0.285466
10742	Amp	8	68881619	68886847	0.991911	0.278195
10743	Amp	8	68894144	68895065	1.020188	0.280302
10744	Amp	8	68895635	68902626	1.159718	0.289914
10745	Amp	8	68907506	68940263	1.068928	0.28364
10746	Amp	8	68943272	68946645	0.760395	0.260542
10747	Amp	8	68946848	68982786	0.915677	0.272112
10748	Amp	8	68986186	69045516	0.833763	0.266365
10749	Amp	8	69046870	69046870	0.925715	0.272897
10750	Amp	8	69051061	69062272	1.000924	0.278524
10751	Amp	8	69065622	69142530	0.843361	0.266955
10752	Amp	8	69142966	69180602	1.000924	0.27892
10753	Amp	8	69184603	69231736	1.265443	0.297169
10754	Amp	8	69232500	69268016	1.432145	0.308746
10755	Amp	8	69268337	69317426	1.593569	0.319491
10756	Amp	8	69320383	69358397	1.415328	0.307526
10757	Amp	8	69359131	69368825	1.159718	0.289276
10758	Amp	8	69369634	69489144	1.24764	0.295431

10759	Amp	8	69491003	69536695	1.190324	0.291903
10760	Amp	8	69537055	69541898	1.344429	0.302898
10761	Amp	8	69543002	69585373	1.503938	0.313513
10762	Amp	8	69587956	69632755	1.344429	0.302768
10763	Amp	8	69633241	69728084	1.315861	0.300878
10764	Amp	8	69733119	69774431	1.364151	0.304285
10765	Amp	8	69775202	69796887	1.344429	0.302553
10766	Amp	8	69803008	69808822	1.534719	0.315588
10767	Amp	8	69810627	69830062	1.514375	0.314183
10768	Amp	8	69832208	69842440	1.442925	0.309625
10769	Amp	8	69842606	69845336	1.593569	0.320045
10770	Amp	8	69849559	69899559	1.667574	0.324397
10771	Amp	8	69903414	69968856	1.572486	0.318242
10772	Amp	8	69969608	69995354	1.394558	0.306111
10773	Amp	8	69995766	70040438	1.334042	0.30192
10774	Amp	8	70040504	70040504	1.029845	0.280874
10775	Amp	8	70041692	70042441	0.398217	0.230103
10776	Amp	8	70042470	70049279	0.437983	0.233214
10777	Amp	8	70050863	70051137	0.347934	0.225335
10778	Amp	8	70051197	70051197	0.7517	0.259842
10779	Amp	8	70052137	70096607	1.059215	0.283011
10780	Amp	8	70099677	70100696	1.208462	0.293075
10781	Amp	8	70101951	70113884	1.534719	0.315364
10782	Amp	8	70117930	70121234	1.657426	0.324253
10783	Amp	8	70122178	70142104	1.778794	0.332133
10784	Amp	8	70144813	70149663	1.678649	0.325165
10785	Amp	8	70150144	70249042	1.727244	0.328424
10786	Amp	8	70255759	70429338	1.553308	0.316847
10787	Amp	8	70429718	70456379	1.637826	0.322643
10788	Amp	8	70457134	70651997	1.737394	0.329759
10789	Amp	8	70652304	70662624	1.605306	0.320587
10790	Amp	8	70674344	70694961	1.789205	0.332973
10791	Amp	8	70733452	70814822	1.887705	0.338787
10792	Amp	8	70815948	70827129	1.930731	0.341754
10793	Amp	8	70837114	70908880	1.995109	0.345736
10794	Amp	8	70911078	70937035	2.170217	0.356695
10795	Amp	8	70937640	70972413	1.963469	0.343966
10796	Amp	8	70974512	71024325	1.757929	0.330648
10797	Amp	8	71031518	71119210	1.708768	0.327389
10798	Amp	8	71119278	71149861	1.757929	0.330942
10799	Amp	8	71149963	71188820	1.678649	0.325383
10800	Amp	8	71189514	71205625	1.525294	0.314963
10801	Amp	8	71207887	71208247	1.563139	0.317838
10802	Amp	8	71209615	71278558	1.484449	0.312362
10803	Amp	8	71281354	71281354	1.542961	0.316044
10804	Amp	8	71282345	71352676	1.717946	0.328061
10805	Amp	8	71352747	71385184	1.646768	0.322983
10806	Amp	8	71387371	71481429	1.484449	0.311988
10807	Amp	8	71482756	71536401	1.646768	0.32301
10808	Amp	8	71541248	71541814	1.687998	0.325946
10809	Amp	8	71542689	71601506	1.582671	0.318831
10810	Amp	8	71602261	71707583	1.856336	0.336771
10811	Amp	8	71707638	71708952	1.697113	0.326708

10812 Amp	8	71709959	71741043	1.525294	0.314691
10813 Amp	8	71747557	71758473	1.667574	0.324915
10814 Amp	8	71761831	71774808	1.503938	0.313893
10815 Amp	8	71775353	71788741	1.615314	0.32136
10816 Amp	8	71789145	71790877	1.463463	0.311136
10817 Amp	8	71793572	71854540	1.727244	0.328444
10818 Amp	8	71856839	71867404	1.354645	0.303049
10819 Amp	8	71879508	72071053	1.199568	0.292089
10820 Amp	8	72074077	72147314	1.24764	0.295903
10821 Amp	8	72153932	72301608	1.415328	0.307476
10822 Amp	8	72302244	72309181	1.020188	0.279808
10823 Amp	8	72309324	72328002	0.963924	0.275994
10824 Amp	8	72328830	72373091	0.906818	0.271642
10825 Amp	8	72380391	72397080	0.7517	0.260069
10826 Amp	8	72397248	72417855	0.86979	0.268874
10827 Amp	8	72429144	72453729	1.068928	0.283671
10828 Amp	8	72458847	72467215	1.120124	0.286897
10829 Amp	8	72471104	72514792	0.906818	0.271436
10830 Amp	8	72515653	72554177	0.760395	0.260909
10831 Amp	8	72554411	72562314	0.823952	0.265771
10832 Amp	8	72563065	72743227	0.71758	0.256965
10833 Amp	8	72746023	72831516	0.852608	0.267848
10834 Amp	8	72831547	72873629	0.760395	0.260382
10835 Amp	8	72873729	72963649	0.71758	0.257414
10836 Amp	8	72965894	73010064	0.676804	0.25396
10837 Amp	8	73012696	73087424	0.760395	0.260528
10838 Amp	8	73092017	73126419	0.702615	0.255636
10839 Amp	8	73127079	73225401	0.556748	0.243284
10840 Amp	8	73226345	73407401	0.62858	0.249993
10841 Amp	8	73410222	73421730	0.676804	0.253621
10842 Amp	8	73426801	73507044	0.814697	0.265122
10843 Amp	8	73508463	73580231	0.702615	0.255668
10844 Amp	8	73593344	73594764	0.823952	0.265516
10845 Amp	8	73603525	73654517	1.088806	0.285005
10846 Amp	8	73659223	73686691	0.925715	0.273259
10847 Amp	8	73691381	73726944	0.734472	0.258646
10848 Amp	8	73727769	73766406	0.693887	0.254972
10849 Amp	8	73769938	73849005	0.760395	0.26084
10850 Amp	8	73850347	73883775	0.587735	0.246541
10851 Amp	8	73888449	73939841	0.693887	0.255531
10852 Amp	8	73943524	73968139	0.888878	0.270642
10853 Amp	8	73969114	74041807	0.66072	0.25223
10854 Amp	8	74042592	74051425	0.724993	0.257844
10855 Amp	8	74052875	74053108	0.972663	0.276819
10856 Amp	8	74056880	74058014	0.860481	0.268176
10857 Amp	8	74060887	74066251	0.71758	0.257293
10858 Amp	8	74066404	74066404	0.760395	0.260685
10859 Amp	8	74077404	74215475	0.580011	0.245745
10860 Amp	8	74216315	74218039	0.62858	0.249609
10861 Amp	8	74220186	74257702	0.644689	0.251122
10862 Amp	8	74259366	74288679	0.66072	0.252798
10863 Amp	8	74291694	74298136	0.709359	0.256826
10864 Amp	8	74299839	74306490	0.62858	0.249417

10865	Amp	8	74307787	74378255	0.398217	0.229927
10866	Amp	8	74378679	74473494	0.382767	0.228251
10867	Amp	8	74485600	74546939	0.355162	0.225551
10868	Amp	8	74547684	74591151	0.430191	0.232708
10869	Amp	8	74600256	74643737	0.532449	0.241235
10870	Amp	8	74644077	74651367	0.743132	0.259066
10871	Amp	8	74652793	74652793	0.693887	0.255156
10872	Amp	8	74653568	74658692	0.604912	0.248
10873	Amp	8	74659066	74684429	0.66072	0.252164
10874	Amp	8	74684957	74724685	0.760395	0.260564
10875	Amp	8	74724920	74744871	0.709359	0.256882
10876	Amp	8	74751315	74751315	0.814697	0.264713
10877	Amp	8	74760292	74835435	0.523647	0.241117
10878	Amp	8	74837999	74889531	0.468576	0.23616
10879	Amp	8	74889897	74923919	0.405699	0.230409
10880	Amp	8	74923973	74985463	0.49169	0.237818
10881	Amp	8	74986083	75029931	0.444673	0.233907
10882	Amp	8	75034460	75143216	0.362221	0.226076
10883	Amp	8	75143601	75143601	0.25804	0.217086
10884	Amp	8	75146721	75212202	0.309523	0.221808
10885	Amp	8	75212723	75259446	0.355162	0.225665
10886	Amp	8	75264010	75344251	0.476502	0.236606
10887	Amp	8	75358422	75522613	0.317596	0.222211
10888	Amp	8	75527078	75538863	0.483654	0.237648
10889	Amp	8	75549289	75577189	0.580011	0.245619
10890	Amp	8	75587900	75630325	0.676804	0.253712
10891	Amp	8	75630538	75691353	0.71758	0.257183
10892	Amp	8	75693037	75706475	0.523647	0.241036
10893	Amp	8	75707520	75750652	0.644689	0.250892
10894	Amp	8	75750706	75766754	0.437983	0.233294
10895	Amp	8	75767326	75777387	0.580011	0.245807
10896	Amp	8	75781033	75784727	0.540744	0.242226
10897	Amp	8	75788830	75789259	0.604912	0.247525
10898	Amp	8	75795191	75808602	0.644689	0.250824
10899	Amp	8	75812367	75845284	0.523647	0.240969
10900	Amp	8	75846552	75859759	0.540744	0.242436
10901	Amp	8	75864174	75974937	0.613517	0.248662
10902	Amp	8	75977332	75977332	1.199568	0.292257
10903	Amp	8	75986367	76072053	1.226647	0.294351
10904	Amp	8	76073581	76100273	1.159718	0.289629
10905	Amp	8	76100783	76139159	1.354645	0.303388
10906	Amp	8	76139233	76149588	1.265443	0.297163
10907	Amp	8	76150465	76150465	1.364151	0.304064
10908	Amp	8	76152110	76155008	1.514375	0.314019
10909	Amp	8	76155387	76255569	1.315861	0.300259
10910	Amp	8	76262155	76313774	1.169408	0.290305
10911	Amp	8	76318968	76394977	1.768015	0.331242
10912	Amp	8	76395497	76408824	1.453628	0.310347
10913	Amp	8	76411389	76435111	0.833763	0.266184
10914	Amp	8	76436017	76478811	0.934277	0.27369
10915	Amp	8	76479610	76488621	0.878688	0.269826
10916	Amp	8	76489927	76489927	0.906818	0.271646
10917	Amp	8	76494311	76517238	0.878688	0.269793

10918	Amp	8	76520899	76536566	1.140058	0.288189
10919	Amp	8	76551573	76620194	1.324884	0.301445
10920	Amp	8	76620778	76655526	1.432145	0.309047
10921	Amp	8	76658031	76696744	1.534719	0.31586
10922	Amp	8	76709609	76767693	1.424227	0.308258
10923	Amp	8	76768799	76801986	1.190324	0.291967
10924	Amp	8	76805552	76878897	1.315861	0.300484
10925	Amp	8	76879624	76932236	1.295233	0.298971
10926	Amp	8	76932283	76932283	1.199568	0.292157
10927	Amp	8	76932499	76934293	1.140058	0.2883
10928	Amp	8	76939742	76939742	1.354645	0.303548
10929	Amp	8	76945037	76959209	1.800655	0.333403
10930	Amp	8	76965554	76972159	1.615314	0.321035
10931	Amp	8	76972583	76972736	1.534719	0.315473
10932	Amp	8	76975818	76988864	1.983885	0.345282
10933	Amp	8	76993082	77087879	1.856336	0.336765
10934	Amp	8	77094722	77151282	1.995109	0.346019
10935	Amp	8	77155223	77181160	2.091935	0.352303
10936	Amp	8	77181958	77259961	2.039783	0.348396
10937	Amp	8	77260058	77265858	2.071528	0.350986
10938	Amp	8	77267045	77305821	2.238069	0.361089
10939	Amp	8	77306367	77363843	2.383144	0.370014
10940	Amp	8	77371029	77412128	2.635842	0.384791
10941	Amp	8	77413197	77481913	2.383144	0.370014
10942	Amp	8	77486089	77496626	2.614747	0.383546
10943	Amp	8	77543760	77550532	2.383144	0.370014
10944	Amp	8	77551303	77566432	2.625007	0.384055
10945	Amp	8	77566791	77583018	2.383144	0.370155
10946	Amp	8	77583468	77627599	2.238069	0.360902
10947	Amp	8	77629312	77653704	2.349236	0.367979
10948	Amp	8	77660008	77666825	2.625007	0.384124
10949	Amp	8	77671028	77685022	2.784457	0.393807
10950	Amp	8	77693545	77771702	2.507231	0.377662
10951	Amp	8	77772100	77797552	2.315177	0.365974
10952	Amp	8	77797847	77887816	2.750393	0.391617
10953	Amp	8	77890782	77950807	2.922915	0.402126
10954	Amp	8	77952977	77973741	2.830877	0.396499
10955	Amp	8	77977628	77978309	2.483202	0.375831
10956	Amp	8	77980439	77995425	2.192225	0.358525
10957	Amp	8	78000543	78063463	2.283191	0.363684
10958	Amp	8	78067064	78196565	2.447622	0.374064
10959	Amp	8	78199097	78204445	2.238069	0.360808
10960	Amp	8	78206928	78210276	2.393797	0.370415
10961	Amp	8	78216947	78218002	1.887705	0.339001
10962	Amp	8	78221862	78230097	2.249752	0.362041
10963	Amp	8	78232559	78246108	2.091935	0.351937
10964	Amp	8	78246181	78286407	1.942393	0.342254
10965	Amp	8	78292461	78292461	2.124253	0.353942
10966	Amp	8	78292900	78307218	1.963469	0.343562
10967	Amp	8	78310247	78365577	1.995109	0.345741
10968	Amp	8	78366856	78483963	1.354645	0.303564
10969	Amp	8	78484036	78488955	1.453628	0.310377
10970	Amp	8	78493719	78501490	1.344429	0.302745

10971	Amp	8	78506864	78579483	1.463463	0.31064
10972	Amp	8	78582829	78591275	1.344429	0.302745
10973	Amp	8	78595952	78639332	0.66072	0.252337
10974	Amp	8	78640274	78664393	0.596354	0.247178
10975	Amp	8	78669296	78718355	1.049532	0.28221
10976	Amp	8	78722903	78725345	1.169408	0.29013
10977	Amp	8	78738000	78741833	1.150167	0.288663
10978	Amp	8	78741910	78750101	1.029845	0.280742
10979	Amp	8	78751565	78778723	1.000924	0.278922
10980	Amp	8	78782015	78831454	1.140058	0.288192
10981	Amp	8	78835013	78836938	0.667776	0.253159
10982	Amp	8	78840595	78864199	0.556748	0.24389
10983	Amp	8	78943550	79024539	0.41418	0.231101
10984	Amp	8	79025050	79187114	0.476502	0.236854
10985	Amp	8	79192215	79196373	0.906818	0.271477
10986	Amp	8	79201759	79211537	1.029845	0.280931
10987	Amp	8	79211654	79248810	1.199568	0.292496
10988	Amp	8	79253477	79287668	1.059215	0.282379
10989	Amp	8	79287739	79311751	0.925715	0.272925
10990	Amp	8	79312608	79327911	1.068928	0.283731
10991	Amp	8	79328214	79343519	1.190324	0.291521
10992	Amp	8	79343771	79380716	1.503938	0.313334
10993	Amp	8	79393642	79399363	1.605306	0.32053
10994	Amp	8	79404473	79409397	1.646768	0.323506
10995	Amp	8	79414096	79419479	1.727244	0.328537
10996	Amp	8	79422083	79472729	1.877398	0.338586
10997	Amp	8	79474226	79474226	2.082332	0.35128
10998	Amp	8	79476205	79492561	1.918998	0.34103
10999	Amp	8	79494690	79521485	1.637826	0.322474
11000	Amp	8	79527664	79569359	1.757929	0.330583
11001	Amp	8	79573243	79617718	1.898353	0.339975
11002	Amp	8	79620884	79656700	1.789205	0.332538
11003	Amp	8	79656737	79672914	1.697113	0.326784
11004	Amp	8	79673578	79681934	1.605306	0.32049
11005	Amp	8	79682701	79696983	1.907576	0.340608
11006	Amp	8	79698832	79794853	2.091935	0.352247
11007	Amp	8	79797186	79802234	1.907576	0.340608
11008	Amp	8	79802444	79869733	2.028562	0.348091
11009	Amp	8	79873432	79888316	2.158849	0.356487
11010	Amp	8	79897467	79908918	2.878516	0.399192
11011	Amp	8	79909111	79933946	3.243024	0.42107
11012	Amp	8	79942051	79980988	3.207076	0.418891
11013	Amp	8	80009230	80083342	2.774551	0.393189
11014	Amp	8	80083781	80167544	2.914035	0.401672
11015	Amp	8	80168163	80205025	3.312745	0.425257
11016	Amp	8	80207809	80316405	3.26417	0.421905
11017	Amp	8	80316591	80320960	2.948071	0.403509
11018	Amp	8	80321732	80323890	2.855041	0.39821
11019	Amp	8	80325481	80356607	2.914035	0.401634
11020	Amp	8	80361766	80378219	3.403911	0.430171
11021	Amp	8	80380480	80386543	3.288383	0.423456
11022	Amp	8	80386850	80390032	3.217724	0.419183
11023	Amp	8	80391198	80406657	2.914035	0.401384

11024	Amp	8	80409594	80478951	3.790167	0.451638
11025	Amp	8	80491187	80511566	3.394201	0.429825
11026	Amp	8	80516432	80527482	3.637615	0.443598
11027	Amp	8	80530377	80605224	3.394201	0.429825
11028	Amp	8	80607569	80669869	3.171522	0.416399
11029	Amp	8	80670489	80671971	3.26417	0.422009
11030	Amp	8	80672650	80713387	3.207076	0.418987
11031	Amp	8	80714094	80773303	3.041652	0.408996
11032	Amp	8	80773908	80798602	2.878516	0.399619
11033	Amp	8	80804599	80821521	3.041652	0.408841
11034	Amp	8	80824721	80923012	3.006013	0.407214
11035	Amp	8	80923456	80936140	3.089932	0.412008
11036	Amp	8	80941890	80985266	2.922915	0.401959
11037	Amp	8	80985612	80993079	2.784457	0.39385
11038	Amp	8	80995623	81025560	2.102209	0.35304
11039	Amp	8	81027451	81122076	1.856336	0.336748
11040	Amp	8	81122216	81124365	1.637826	0.322269
11041	Amp	8	81124816	81158226	1.49455	0.312877
11042	Amp	8	81162951	81193715	1.424227	0.308083
11043	Amp	8	81194696	81194696	1.169408	0.290059
11044	Amp	8	81194791	81214749	0.693887	0.255436
11045	Amp	8	81214786	81221972	0.66072	0.252713
11046	Amp	8	81227615	81251489	0.796213	0.263147
11047	Amp	8	81255735	81259644	1.078696	0.284311
11048	Amp	8	81262845	81332991	0.915677	0.272571
11049	Amp	8	81333125	81335740	0.982622	0.277515
11050	Amp	8	81337729	81340502	1.708768	0.327702
11051	Amp	8	81342696	81344825	1.432145	0.308958
11052	Amp	8	81348248	81422500	1.130387	0.287793
11053	Amp	8	81425928	81497119	0.564815	0.244013
11054	Amp	8	81499246	81530625	0.596354	0.24715
11055	Amp	8	81532161	81540067	0.564815	0.244013
11056	Amp	8	81542934	81562157	0.483654	0.237606
11057	Amp	8	81569770	81583122	0.195309	0.210232
11058	Amp	8	81586154	81593186	0.038273	0.193156
11059	Amp	8	81594275	81597406	0.007199	0.189304
11060	Amp	8	81611091	81612389	0.201819	0.211527
11061	Amp	8	81614241	81624058	0.105051	0.200731
11062	Amp	8	81624088	81663548	0.056238	0.195122
11063	Amp	8	81669506	81701768	0	0.183608
11064	Amp	8	81702093	81764511	0	0.170914
11065	Amp	8	81768032	81785114	0	0.181404
11066	Amp	8	81788708	81907576	0	0.145631
11067	Amp	8	81907938	81961079	0	0.13514
11068	Amp	8	81961171	81990115	0	0.131159
11069	Amp	8	81992164	82006910	0	0.123962
11070	Amp	8	82008123	82082449	0	0.125915
11071	Amp	8	82083689	82093014	0	0.138704
11072	Amp	8	82097018	82172209	0	0.15119
11073	Amp	8	82175458	82175972	0	0.138401
11074	Amp	8	82186610	82218008	0	0.151504
11075	Amp	8	82218186	82301057	0	0.1501
11076	Amp	8	82326959	82349081	0	0.136998

11077	Amp	8	82354576	82386874	0	0.148283
11078	Amp	8	82386910	82426559	0	0.142268
11079	Amp	8	82426977	82453763	0	0.152378
11080	Amp	8	82457708	82588600	0	0.141093
11081	Amp	8	82592647	82594879	0	0.160663
11082	Amp	8	82596620	82602936	0	0.173452
11083	Amp	8	82602940	82720462	0	0.175803
11084	Amp	8	82723783	82823020	0.056238	0.195757
11085	Amp	8	82863191	82917712	0	0.175803
11086	Amp	8	82918182	82961334	0	0.165315
11087	Amp	8	82963337	83001839	0	0.160365
11088	Amp	8	83004422	83022067	0	0.162708
11089	Amp	8	83023881	83034506	0	0.167947
11090	Amp	8	83035568	83038257	0	0.178632
11091	Amp	8	83043494	83081003	0	0.187863
11092	Amp	8	83086387	83101133	0	0.185513
11093	Amp	8	83101965	83121099	0.033661	0.192889
11094	Amp	8	83123459	83166271	0.018387	0.190698
11095	Amp	8	83175188	83212888	0	0.168408
11096	Amp	8	83215325	83265611	0.27943	0.218832
11097	Amp	8	83270979	83405653	0.149443	0.206042
11098	Amp	8	83411243	83411243	0.483654	0.237473
11099	Amp	8	83413039	83454931	0.273081	0.217903
11100	Amp	8	83455096	83479453	0.309523	0.221245
11101	Amp	8	83479915	83486886	0.13565	0.204383
11102	Amp	8	83491304	83683539	0.09318	0.199439
11103	Amp	8	83686766	83702274	0.250263	0.216168
11104	Amp	8	83703456	83731577	0.168144	0.208155
11105	Amp	8	83732066	83777419	0.073369	0.197446
11106	Amp	8	83778025	83787613	0.123589	0.202674
11107	Amp	8	83793001	83869110	0	0.185945
11108	Amp	8	83871061	83903661	0	0.16854
11109	Amp	8	83904737	84004818	0.001198	0.188651
11110	Amp	8	84007342	84025580	0.111991	0.201315
11111	Amp	8	84026166	84029473	0.028398	0.192083
11112	Amp	8	84034652	84036242	0.149443	0.205616
11113	Amp	8	84042731	84062332	0.033661	0.192951
11114	Amp	8	84066818	84087130	0	0.179783
11115	Amp	8	84089054	84122218	0	0.181706
11116	Amp	8	84125267	84128134	0	0.179363
11117	Amp	8	84136834	84179885	0	0.184348
11118	Amp	8	84187760	84205666	0.073369	0.197517
11119	Amp	8	84206025	84269284	0	0.177406
11120	Amp	8	84270837	84305324	0.086298	0.19882
11121	Amp	8	84308604	84316268	0	0.185288
11122	Amp	8	84318411	84358614	0	0.163873
11123	Amp	8	84359094	84374478	0	0.174651
11124	Amp	8	84377218	84439885	0	0.135894
11125	Amp	8	84439916	84458512	0	0.137606
11126	Amp	8	84461901	84529481	0	0.147946
11127	Amp	8	84532580	84540651	0	0.155947
11128	Amp	8	84541839	84837294	0	0.164
11129	Amp	8	84844800	84868160	0	0.141655

11130	Amp	8	84874809	84891294	0	0.159015
11131	Amp	8	84891462	84984685	0	0.143742
11132	Amp	8	84986909	84989808	0	0.14852
11133	Amp	8	85098420	85098495	0	0.167102
11134	Amp	8	85098512	85127648	0	0.16515
11135	Amp	8	85127671	85127671	0	0.172376
11136	Amp	8	85128081	85128353	0	0.17444
11137	Amp	8	85133155	85202443	0	0.1831
11138	Amp	8	85202500	85209076	0.001198	0.188648
11139	Amp	8	85209234	85243363	0.056238	0.195136
11140	Amp	8	85244478	85341691	0.128931	0.203921
11141	Amp	8	85341753	85353173	0.061778	0.195868
11142	Amp	8	85360083	85380423	0.273081	0.217931
11143	Amp	8	85382047	85405133	0.250263	0.216008
11144	Amp	8	85409564	85493308	0.430191	0.232426
11145	Amp	8	85498814	85499290	0.398217	0.23011
11146	Amp	8	85501953	85545833	0.571214	0.244614
11147	Amp	8	85551400	85556639	0.843361	0.26686
11148	Amp	8	85559512	85561168	0.86979	0.268645
11149	Amp	8	85563819	85601279	0.897922	0.270923
11150	Amp	8	85602329	85603620	0.709359	0.256419
11151	Amp	8	85608821	85608821	0.991911	0.277651
11152	Amp	8	85828731	85828939	0.702615	0.255677
11153	Amp	8	85829000	85913118	0.571214	0.2449
11154	Amp	8	85913639	85920759	0.405699	0.230352
11155	Amp	8	85930874	86005464	0.421967	0.232056
11156	Amp	8	86008108	86103639	0.483654	0.237668
11157	Amp	8	86106063	86125618	0.41418	0.231508
11158	Amp	8	86126234	86168801	0.452609	0.234314
11159	Amp	8	86323850	86355705	0.532449	0.241238
11160	Amp	8	86375531	86394594	0.636206	0.250431
11161	Amp	8	86398725	86490125	0.76883	0.261167
11162	Amp	8	86494776	86494776	0.667776	0.253439
11163	Amp	8	86496727	86564946	0.963924	0.27597
11164	Amp	8	86565625	86567322	0.852608	0.267796
11165	Amp	8	86570265	86578666	1.049532	0.281828
11166	Amp	8	86581023	86627363	0.852608	0.267796
11167	Amp	8	86631513	86635959	1.226647	0.294616
11168	Amp	8	86638132	86647872	0.685617	0.254313
11169	Amp	8	86649285	86671354	0.652914	0.251632
11170	Amp	8	86672446	86684697	0.760395	0.260931
11171	Amp	8	86685051	86704619	0.652914	0.251632
11172	Amp	8	86708081	86708081	0.27943	0.218797
11173	Amp	8	86708122	86723817	0.230572	0.213907
11174	Amp	8	86726840	86755364	0.652914	0.251906
11175	Amp	8	86755632	86773132	0.62858	0.250036
11176	Amp	8	86773769	86841756	0.250263	0.216077
11177	Amp	8	86842187	87038332	0.430191	0.232864
11178	Amp	8	87045432	87046140	0.556748	0.243885
11179	Amp	8	87064660	87190522	0.667776	0.253163
11180	Amp	8	87192511	87231014	0.540744	0.242142
11181	Amp	8	87231509	87243736	0.709359	0.256563
11182	Amp	8	87255034	87255034	0.390264	0.22921

11183	Amp	8	87255177	87263721	0.367646	0.227014
11184	Amp	8	87267014	87459533	0.347934	0.225302
11185	Amp	8	87466475	87539044	0.483654	0.237397
11186	Amp	8	87542235	87544224	0.604912	0.247841
11187	Amp	8	87546222	87608126	0.587735	0.246137
11188	Amp	8	87623714	87644072	0.444673	0.234043
11189	Amp	8	87654800	87815501	0.778204	0.261894
11190	Amp	8	87816794	87817156	0.925715	0.273041
11191	Amp	8	87819402	87833146	0.982622	0.27745
11192	Amp	8	87837806	87903130	1.150167	0.289032
11193	Amp	8	87906103	87925222	1.039426	0.281032
11194	Amp	8	87925343	88001073	1.078696	0.283751
11195	Amp	8	88001741	88019621	0.915677	0.272169
11196	Amp	8	88021694	88067001	1.150167	0.289108
11197	Amp	8	88073054	88073223	1.295233	0.299409
11198	Amp	8	88077198	88118754	1.190324	0.291665
11199	Amp	8	88118811	88129920	1.000924	0.278758
11200	Amp	8	88132613	88160586	0.852608	0.267612
11201	Amp	8	88162171	88199517	1.059215	0.282922
11202	Amp	8	88202919	88263156	0.95395	0.274944
11203	Amp	8	88272026	88289984	0.852608	0.267718
11204	Amp	8	88294374	88352186	0.66072	0.252408
11205	Amp	8	88353977	88403858	0.843361	0.267129
11206	Amp	8	88407268	88448224	0.934277	0.273982
11207	Amp	8	88448528	88450220	1.068928	0.283217
11208	Amp	8	88453117	88504683	1.099256	0.285585
11209	Amp	8	88505067	88505693	1.020188	0.279973
11210	Amp	8	88511074	88547557	1.218154	0.293505
11211	Amp	8	88550063	88723417	1.315861	0.300816
11212	Amp	8	88734139	88785688	1.190324	0.291538
11213	Amp	8	88786995	88949837	1.2565	0.296238
11214	Amp	8	88959126	89009599	1.039426	0.281517
11215	Amp	8	89010187	89068177	1.646768	0.323582
11216	Amp	8	89069922	89085657	1.757929	0.330519
11217	Amp	8	89090010	89121183	1.778794	0.332333
11218	Amp	8	89121354	89132265	1.582671	0.318909
11219	Amp	8	89132402	89302621	1.415328	0.307693
11220	Amp	8	89304941	89366386	1.442925	0.309337
11221	Amp	8	89368984	89415271	1.463463	0.310767
11222	Amp	8	89417476	89417856	1.737394	0.329601
11223	Amp	8	89424859	89488130	1.866286	0.337915
11224	Amp	8	89488608	89491920	1.821927	0.335196
11225	Amp	8	89498333	89508420	1.593569	0.319984
11226	Amp	8	89544455	89638079	1.637826	0.322524
11227	Amp	8	89642021	89795535	1.942393	0.342694
11228	Amp	8	89795916	89859906	2.102209	0.353082
11229	Amp	8	89863111	89893163	1.963469	0.343831
11230	Amp	8	89893370	89983575	2.148338	0.355818
11231	Amp	8	89983743	89996055	2.124253	0.354216
11232	Amp	8	89998958	90008606	1.963469	0.343828
11233	Amp	8	90009054	90009090	1.930731	0.342014
11234	Amp	8	90011468	90078593	2.192225	0.358037
11235	Amp	8	90089111	90116226	2.294081	0.364551

11236	Amp	8	90116409	90142872	2.458756	0.374979
11237	Amp	8	90147185	90163646	2.359227	0.368464
11238	Amp	8	90166059	90336398	2.294081	0.364542
11239	Amp	8	90343071	90345684	1.514375	0.314354
11240	Amp	8	90352031	90360970	1.484449	0.311987
11241	Amp	8	90361946	90381083	1.226647	0.294715
11242	Amp	8	90384924	90421719	1.354645	0.303188
11243	Amp	8	90427323	90483766	1.473764	0.311482
11244	Amp	8	90493464	90599550	1.324884	0.301055
11245	Amp	8	90604457	90811761	1.199568	0.292582
11246	Amp	8	90814479	90830644	1.415328	0.307277
11247	Amp	8	90830974	90926577	1.275021	0.298009
11248	Amp	8	90926689	90952330	1.415328	0.307545
11249	Amp	8	90956299	90992075	1.687998	0.325691
11250	Amp	8	90993857	90997748	1.49455	0.312724
11251	Amp	8	90998249	91014289	1.605306	0.320803
11252	Amp	8	91027661	91036446	2.113683	0.353188
11253	Amp	8	91042088	91092932	1.983885	0.345109
11254	Amp	8	91094256	91106779	1.697113	0.326963
11255	Amp	8	91174785	91176913	1.844531	0.336188
11256	Amp	8	91182010	91259635	1.364151	0.303803
11257	Amp	8	91264226	91312478	1.404612	0.306587
11258	Amp	8	91314931	91324926	1.463463	0.311173
11259	Amp	8	91327022	91407996	1.49455	0.312734
11260	Amp	8	91409717	91499991	1.667574	0.32466
11261	Amp	8	91503029	91640987	1.811302	0.334527
11262	Amp	8	91641512	91672251	1.667574	0.32466
11263	Amp	8	91672617	91826180	1.953191	0.343093
11264	Amp	8	91826221	91826791	1.768015	0.331167
11265	Amp	8	91841277	91901135	1.442925	0.309317
11266	Amp	8	91903573	91944823	1.553308	0.316887
11267	Amp	8	91945859	91993302	1.484449	0.312477
11268	Amp	8	91993340	91993355	1.404612	0.306476
11269	Amp	8	91993448	92007104	1.265443	0.297023
11270	Amp	8	92007199	92007424	0.95395	0.27509
11271	Amp	8	92007733	92007901	0.76883	0.261461
11272	Amp	8	92008360	92008633	0.860481	0.268249
11273	Amp	8	92008669	92013309	1.334042	0.302008
11274	Amp	8	92013322	92013322	1.404612	0.306899
11275	Amp	8	92013435	92054117	1.563139	0.317976
11276	Amp	8	92060698	92116087	1.789205	0.333018
11277	Amp	8	92121715	92211508	1.708768	0.327265
11278	Amp	8	92211822	92211883	1.646768	0.323233
11279	Amp	8	92213810	92226829	1.553308	0.317344
11280	Amp	8	92227405	92449954	1.415328	0.307264
11281	Amp	8	92450638	92554983	1.49455	0.31273
11282	Amp	8	92561009	92567619	1.374261	0.304703
11283	Amp	8	92569439	92583578	1.963469	0.344191
11284	Amp	8	92584934	92652115	2.082332	0.351473
11285	Amp	8	92652391	92656314	2.049537	0.349638
11286	Amp	8	92663414	92851425	1.963469	0.344172
11287	Amp	8	92854313	92885970	2.061276	0.350138
11288	Amp	8	92891776	92969970	1.844531	0.336606

11289	Amp	8	92976899	93080688	1.887705	0.338758
11290	Amp	8	93081044	93095985	1.930731	0.341606
11291	Amp	8	93106021	93127820	2.082332	0.3517
11292	Amp	8	93128097	93207244	2.272343	0.363074
11293	Amp	8	93208753	93229610	2.102209	0.352981
11294	Amp	8	93232834	93248452	1.983885	0.345444
11295	Amp	8	93248759	93289192	2.113683	0.353518
11296	Amp	8	93290590	93340574	2.005494	0.346582
11297	Amp	8	93341957	93365691	2.158849	0.356128
11298	Amp	8	93366023	93444915	2.005494	0.346572
11299	Amp	8	93446755	93456916	1.953191	0.34311
11300	Amp	8	93459503	93459503	2.214215	0.359601
11301	Amp	8	93461363	93461363	2.349236	0.367631
11302	Amp	8	93463615	93504660	2.272343	0.362931
11303	Amp	8	93505769	93538285	2.214215	0.359392
11304	Amp	8	93538636	93603344	2.338154	0.367334
11305	Amp	8	93610327	93621005	1.972699	0.344328
11306	Amp	8	93621060	93676662	2.005494	0.346291
11307	Amp	8	93677970	93680331	2.436112	0.373077
11308	Amp	8	93681409	93681409	2.495444	0.376651
11309	Amp	8	93691945	93716983	2.935659	0.402615
11310	Amp	8	93717510	93753487	3.134208	0.414386
11311	Amp	8	93760091	93772168	2.98257	0.406009
11312	Amp	8	93774320	93789037	2.866312	0.399023
11313	Amp	8	93791597	93798312	2.922915	0.402274
11314	Amp	8	93811492	93830977	3.146436	0.415051
11315	Amp	8	93831453	94011598	3.288383	0.423505
11316	Amp	8	94013588	94184199	3.394201	0.42985
11317	Amp	8	94187074	94199950	3.312745	0.424959
11318	Amp	8	94204038	94244675	2.580481	0.381423
11319	Amp	8	94245837	94306969	2.774551	0.393577
11320	Amp	8	94314440	94326483	2.88973	0.400318
11321	Amp	8	94329645	94342908	2.922915	0.402105
11322	Amp	8	94347564	94398813	2.750393	0.391641
11323	Amp	8	94406560	94447120	2.830877	0.396486
11324	Amp	8	94450371	94504252	2.614747	0.38377
11325	Amp	8	94504280	94544175	2.580481	0.381808
11326	Amp	8	94544215	94558223	2.135909	0.354615
11327	Amp	8	94564122	94564122	2.203122	0.358724
11328	Amp	8	94566121	94633689	2.483202	0.375963
11329	Amp	8	94634189	94682670	2.370685	0.369111
11330	Amp	8	94683902	94709265	2.580481	0.381764
11331	Amp	8	94711048	94714512	2.774551	0.393133
11332	Amp	8	94714832	94714832	2.948071	0.403619
11333	Amp	8	94717275	94745628	2.994162	0.406192
11334	Amp	8	94750239	94832210	3.079122	0.411058
11335	Amp	8	94834059	94905643	3.029636	0.408486
11336	Amp	8	94910482	94915471	2.902384	0.400545
11337	Amp	8	94915474	94918073	2.806723	0.395678
11338	Amp	8	94919530	94951570	2.404616	0.371527
11339	Amp	8	94951661	94952297	2.238069	0.36104
11340	Amp	8	94956862	95026687	2.349236	0.368079
11341	Amp	8	95031601	95045990	2.238069	0.361338

11342	Amp	8	95048487	95050373	2.272343	0.36322
11343	Amp	8	95053570	95103672	2.543219	0.379289
11344	Amp	8	95107274	95112904	2.327403	0.366637
11345	Amp	8	95114802	95156779	2.424855	0.372733
11346	Amp	8	95159877	95214023	2.170217	0.356663
11347	Amp	8	95214112	95295276	2.249752	0.361957
11348	Amp	8	95295322	95350181	2.148338	0.355861
11349	Amp	8	95351015	95381486	2.005494	0.346712
11350	Amp	8	95382015	95435005	2.124253	0.35391
11351	Amp	8	95444562	95500623	1.877398	0.33841
11352	Amp	8	95521102	95548057	1.821927	0.335158
11353	Amp	8	95548131	95560713	1.748357	0.329864
11354	Amp	8	95561611	95565546	1.866286	0.337485
11355	Amp	8	95572330	95687368	2.061276	0.350296
11356	Amp	8	95687660	95694103	2.192225	0.358095
11357	Amp	8	95694394	95699194	2.158849	0.356308
11358	Amp	8	95700562	95852922	2.091935	0.352199
11359	Amp	8	95859001	95921351	2.180628	0.357925
11360	Amp	8	95926313	96063882	2.404616	0.371304
11361	Amp	8	96064299	96069024	2.359227	0.3686
11362	Amp	8	96072613	96092675	2.170217	0.35723
11363	Amp	8	96093167	96219684	2.315177	0.365672
11364	Amp	8	96223367	96270116	2.158849	0.356126
11365	Amp	8	96277985	96277985	2.383144	0.369658
11366	Amp	8	96278734	96280902	2.349236	0.368097
11367	Amp	8	96283817	96338727	2.30333	0.365313
11368	Amp	8	96341332	96416726	2.272343	0.362775
11369	Amp	8	96417965	96499993	2.049537	0.349243
11370	Amp	8	96501007	96559712	2.359227	0.368608
11371	Amp	8	96562354	96567212	2.180628	0.357542
11372	Amp	8	96567547	96572962	2.082332	0.351408
11373	Amp	8	96575273	96609122	2.005494	0.346563
11374	Amp	8	96609748	96631728	2.158849	0.356493
11375	Amp	8	96631976	96650135	2.102209	0.35292
11376	Amp	8	96658516	96672136	2.214215	0.359338
11377	Amp	8	96675947	96678504	1.708768	0.327359
11378	Amp	8	96684493	96721571	1.856336	0.337189
11379	Amp	8	96721787	96725939	2.028562	0.348299
11380	Amp	8	96727285	96858443	2.727427	0.390283
11381	Amp	8	96862540	96867144	2.555572	0.380453
11382	Amp	8	96867957	96902391	2.483202	0.375841
11383	Amp	8	96905824	97038942	2.635842	0.385012
11384	Amp	8	97048337	97138850	2.458756	0.375082
11385	Amp	8	97145393	97214779	2.061276	0.350124
11386	Amp	8	97215737	97369368	2.113683	0.353773
11387	Amp	8	97369483	97386777	2.028562	0.348164
11388	Amp	8	97386870	97388427	2.214215	0.359565
11389	Amp	8	97389010	97444404	2.158849	0.356169
11390	Amp	8	97446607	97537371	2.294081	0.364467
11391	Amp	8	97538541	97551205	2.470601	0.375358
11392	Amp	8	97554387	97566349	2.737964	0.39111
11393	Amp	8	97568452	97577914	2.855041	0.397796
11394	Amp	8	97578107	97636837	2.796399	0.394405

11395	Amp	8	97637863	97655549	2.614747	0.383513
11396	Amp	8	97655998	97659800	2.530938	0.379078
11397	Amp	8	97662828	97802689	2.692246	0.388708
11398	Amp	8	97802742	97881636	2.591575	0.382021
11399	Amp	8	97888386	97922961	2.935659	0.4032
11400	Amp	8	97923923	97979180	3.243024	0.421015
11401	Amp	8	97979322	98067526	3.359177	0.427354
11402	Amp	8	98074658	98103973	3.099988	0.412275
11403	Amp	8	98107137	98110931	2.958746	0.404305
11404	Amp	8	98116520	98146248	3.099988	0.412345
11405	Amp	8	98150033	98180191	2.458756	0.374817
11406	Amp	8	98180369	98193024	2.670502	0.38688
11407	Amp	8	98194646	98199629	2.71525	0.389584
11408	Amp	8	98200746	98240801	3.348874	0.427111
11409	Amp	8	98240864	98251631	3.461541	0.433675
11410	Amp	8	98254043	98310488	3.509701	0.436424
11411	Amp	8	98311293	98365925	3.394201	0.429837
11412	Amp	8	98366503	98375555	2.727427	0.390843
11413	Amp	8	98375793	98450920	2.796399	0.394364
11414	Amp	8	98454978	98478683	2.88973	0.400274
11415	Amp	8	98480647	98480647	3.05489	0.409523
11416	Amp	8	98481810	98627820	3.146436	0.415
11417	Amp	8	98632589	98707735	3.171522	0.416457
11418	Amp	8	98708603	98775373	3.312745	0.424768
11419	Amp	8	98777741	98821934	3.535174	0.4383
11420	Amp	8	98826457	98863830	3.348874	0.426899
11421	Amp	8	98864496	98894399	3.600858	0.441121
11422	Amp	8	98900614	99034841	3.675606	0.445295
11423	Amp	8	99035027	99040273	3.600858	0.441646
11424	Amp	8	99045010	99088804	3.335884	0.426145
11425	Amp	8	99090217	99131829	3.099988	0.412277
11426	Amp	8	99137846	99220722	3.006013	0.406801
11427	Amp	8	99224712	99301259	2.843135	0.397171
11428	Amp	8	99301343	99318962	2.784457	0.393732
11429	Amp	8	99319551	99338333	2.902384	0.400768
11430	Amp	8	99338516	99338516	2.670502	0.387235
11431	Amp	8	99338613	99353455	2.602347	0.382713
11432	Amp	8	99354654	99457762	2.495444	0.376804
11433	Amp	8	99458379	99458379	2.383144	0.369766
11434	Amp	8	99465010	99586679	2.625007	0.384418
11435	Amp	8	99593475	99790750	3.403911	0.430122
11436	Amp	8	99801462	99801462	2.625007	0.384418
11437	Amp	8	99802697	99887565	2.507231	0.377383
11438	Amp	8	99888206	100136293	2.727427	0.390443
11439	Amp	8	100136433	100143262	2.703608	0.388986
11440	Amp	8	100147920	100162582	2.866312	0.398602
11441	Amp	8	100166630	100178443	2.806723	0.395695
11442	Amp	8	100179422	100183272	2.994162	0.406162
11443	Amp	8	100191509	100199750	2.737964	0.39151
11444	Amp	8	100206331	100286682	2.866312	0.399131
11445	Amp	8	100287299	100311897	2.774551	0.393456
11446	Amp	8	100315727	100344106	3.509701	0.436407
11447	Amp	8	100345110	100380723	3.625861	0.442935

11448 Amp	8	100382022	100469964	3.45062	0.43332
11449 Amp	8	100472454	100488427	3.688516	0.446411
11450 Amp	8	100489093	100517722	3.45062	0.43332
11451 Amp	8	100519677	100543033	3.415716	0.431226
11452 Amp	8	100543421	100634162	3.549256	0.439007
11453 Amp	8	100639763	100680650	2.914035	0.401544
11454 Amp	8	100683106	100698310	2.761965	0.392866
11455 Amp	8	100699925	100705461	2.806723	0.395303
11456 Amp	8	100710388	100801402	2.970355	0.405211
11457 Amp	8	100801840	100843048	3.124015	0.413935
11458 Amp	8	100845435	100891352	3.523011	0.436995
11459 Amp	8	100893452	101015197	3.560951	0.439015
11460 Amp	8	101016478	101022461	3.76435	0.450047
11461 Amp	8	101027479	101036325	4.444356	0.485545
11462 Amp	8	101036691	101200227	3.998681	0.462485
11463 Amp	8	101201403	101217135	4.27183	0.476368
11464 Amp	8	101221011	101367875	4.391125	0.48248
11465 Amp	8	101370698	101371568	4.24321	0.474859
11466 Amp	8	101396080	101410502	4.355429	0.480709
11467 Amp	8	101410832	101437951	4.090482	0.467624
11468 Amp	8	101438038	101446151	3.985282	0.461867
11469 Amp	8	101446287	101457507	3.906866	0.457692
11470 Amp	8	101458916	101495697	4.103115	0.46846
11471 Amp	8	101504276	101535467	4.215025	0.473918
11472 Amp	8	101536506	101617168	4.022326	0.46401
11473 Amp	8	101617416	101622269	3.801418	0.452175
11474 Amp	8	101623042	101631742	3.72674	0.448352
11475 Amp	8	101633428	101661255	3.32332	0.425939
11476 Amp	8	101661258	101669532	3.461541	0.433564
11477 Amp	8	101670014	101671103	3.359177	0.427713
11478 Amp	8	101677091	101709160	3.252252	0.421601
11479 Amp	8	101716131	101720299	3.099988	0.412352
11480 Amp	8	101724785	101753948	3.26417	0.422237
11481 Amp	8	101758750	101759154	3.359177	0.427995
11482 Amp	8	101764935	101766935	3.243024	0.42105
11483 Amp	8	101768364	101835288	3.05489	0.410018
11484 Amp	8	101837088	101924665	3.006013	0.407269
11485 Amp	8	101926433	101933076	2.866312	0.399044
11486 Amp	8	101937935	102007598	2.902384	0.40087
11487 Amp	8	102008163	102104702	3.05489	0.409566
11488 Amp	8	102106569	102249996	2.843135	0.39718
11489 Amp	8	102251320	102275649	2.914035	0.401733
11490 Amp	8	102278243	102468526	2.958746	0.404241
11491 Amp	8	102472111	102497397	2.796399	0.394356
11492 Amp	8	102499421	102558965	3.146436	0.415621
11493 Amp	8	102559214	102584574	3.335884	0.426135
11494 Amp	8	102586589	102625325	2.970355	0.40487
11495 Amp	8	102627407	102627407	2.737964	0.391521
11496 Amp	8	102628050	102648136	2.818034	0.396146
11497 Amp	8	102662615	102725155	3.066577	0.410514
11498 Amp	8	102729821	102755416	2.818034	0.396146
11499 Amp	8	102756531	102846260	2.737964	0.391113
11500 Amp	8	102846274	102856127	2.878516	0.399186

11501	Amp	8	102862333	102862696	2.843135	0.397123
11502	Amp	8	102866733	102904164	2.784457	0.393815
11503	Amp	8	102918172	103018796	2.692246	0.388692
11504	Amp	8	103019945	103066088	3.006013	0.406908
11505	Amp	8	103066474	103071423	3.041652	0.409017
11506	Amp	8	103071558	103092909	2.902384	0.400706
11507	Amp	8	103093331	103119973	2.843135	0.39727
11508	Amp	8	103120145	103218223	2.750393	0.392011
11509	Amp	8	103223977	103235185	3.394201	0.429551
11510	Amp	8	103237410	103279068	3.299582	0.424093
11511	Amp	8	103280737	103281437	2.750393	0.392257
11512	Amp	8	103283870	103316790	2.818034	0.396317
11513	Amp	8	103320284	103339442	2.727427	0.390285
11514	Amp	8	103339456	103390315	2.580481	0.381589
11515	Amp	8	103392712	103492789	2.647166	0.385497
11516	Amp	8	103493739	103516224	2.703608	0.389472
11517	Amp	8	103517198	103539854	2.483202	0.376411
11518	Amp	8	103553213	103573565	2.349236	0.368061
11519	Amp	8	103574427	103575851	2.436112	0.373328
11520	Amp	8	103589015	103615195	2.774551	0.393479
11521	Amp	8	103625054	103681932	2.692246	0.388212
11522	Amp	8	103693380	103739884	2.349236	0.368061
11523	Amp	8	103741298	103803144	2.327403	0.366494
11524	Amp	8	103814129	103863832	2.383144	0.370102
11525	Amp	8	103865786	103889016	2.470601	0.375362
11526	Amp	8	103893746	103893996	2.519	0.377914
11527	Amp	8	103898541	103902657	2.71525	0.390109
11528	Amp	8	103903171	103907674	3.112016	0.413296
11529	Amp	8	103910161	103910161	3.335884	0.426357
11530	Amp	8	103912131	103913546	3.243024	0.420665
11531	Amp	8	103917671	103922607	3.48305	0.434985
11532	Amp	8	103931281	103933754	3.32332	0.425657
11533	Amp	8	103934628	103950910	3.573122	0.440214
11534	Amp	8	103957366	104023187	3.348874	0.427154
11535	Amp	8	104023450	104036069	3.099988	0.412467
11536	Amp	8	104039373	104039709	3.066577	0.410195
11537	Amp	8	104051224	104066717	2.796399	0.394392
11538	Amp	8	104067043	104082280	2.935659	0.402622
11539	Amp	8	104089619	104094305	2.683697	0.388065
11540	Amp	8	104101837	104141558	2.635842	0.384766
11541	Amp	8	104142796	104157798	2.249752	0.361578
11542	Amp	8	104163568	104204873	2.192225	0.357969
11543	Amp	8	104205571	104278390	2.039783	0.348668
11544	Amp	8	104279935	104279935	1.972699	0.344759
11545	Amp	8	104280143	104298677	1.953191	0.343163
11546	Amp	8	104299136	104401947	1.757929	0.330913
11547	Amp	8	104407906	104452426	1.821927	0.334666
11548	Amp	8	104453645	104459122	1.930731	0.341474
11549	Amp	8	104463943	104492909	2.148338	0.355661
11550	Amp	8	104493527	104502260	2.113683	0.353509
11551	Amp	8	104508640	104586561	2.283191	0.363855
11552	Amp	8	104592422	104592967	2.135909	0.354684
11553	Amp	8	104593189	104652513	2.061276	0.349814

11554	Amp	8	104656422	104737302	2.203122	0.358636
11555	Amp	8	104739564	104739564	2.124253	0.353891
11556	Amp	8	104751416	104765951	2.016329	0.347403
11557	Amp	8	104767155	104770344	2.180628	0.357922
11558	Amp	8	104772037	104804047	2.567803	0.381189
11559	Amp	8	104807264	104819622	2.016329	0.347403
11560	Amp	8	104821838	104886494	2.238069	0.361074
11561	Amp	8	104886726	104887245	2.082332	0.351631
11562	Amp	8	104889804	104927967	2.170217	0.356578
11563	Amp	8	104931067	104959061	2.082332	0.351631
11564	Amp	8	104966140	105012349	2.124253	0.354271
11565	Amp	8	105016945	105051328	2.148338	0.355827
11566	Amp	8	105055937	105061327	2.124253	0.353818
11567	Amp	8	105064819	105082136	1.844531	0.336482
11568	Amp	8	105086425	105212657	2.061276	0.349729
11569	Amp	8	105218903	105248880	1.918998	0.340907
11570	Amp	8	105251098	105333624	1.953191	0.342897
11571	Amp	8	105336577	105361710	1.821927	0.334806
11572	Amp	8	105364039	105389762	1.757929	0.331053
11573	Amp	8	105392086	105521930	1.605306	0.320707
11574	Amp	8	105525823	105573694	1.637826	0.322418
11575	Amp	8	105573866	105598582	1.534719	0.31561
11576	Amp	8	105598597	105667645	1.646768	0.323034
11577	Amp	8	105672708	105673065	1.484449	0.312323
11578	Amp	8	105673293	105673377	1.553308	0.317041
11579	Amp	8	105677738	105718355	1.415328	0.30752
11580	Amp	8	105718775	105743531	1.582671	0.318857
11581	Amp	8	105744346	105748490	1.737394	0.329422
11582	Amp	8	105755230	105783724	1.708768	0.327596
11583	Amp	8	105785151	105866606	1.768015	0.331222
11584	Amp	8	105866958	105931948	1.800655	0.333255
11585	Amp	8	105932301	105932301	1.907576	0.340099
11586	Amp	8	105945597	106483031	1.983885	0.345089
11587	Amp	8	106486331	106496976	1.697113	0.326558
11588	Amp	8	106497409	106498096	1.877398	0.338425
11589	Amp	8	106499947	106509117	1.963469	0.343969
11590	Amp	8	106511473	106704225	2.091935	0.351808
11591	Amp	8	106704798	106830822	2.22615	0.360561
11592	Amp	8	106833629	106900156	2.393797	0.370305
11593	Amp	8	106905544	106918495	2.113683	0.353736
11594	Amp	8	106919232	106951580	1.942393	0.342399
11595	Amp	8	106954154	107078040	1.866286	0.337409
11596	Amp	8	107082232	107097251	1.463463	0.311094
11597	Amp	8	107099192	107107191	1.678649	0.325257
11598	Amp	8	107107736	107113818	1.844531	0.336413
11599	Amp	8	107113962	107219604	1.637826	0.322742
11600	Amp	8	107223775	107297160	1.473764	0.311586
11601	Amp	8	107302569	107323286	1.354645	0.303281
11602	Amp	8	107326104	107365328	1.285475	0.298728
11603	Amp	8	107372600	107410560	1.534719	0.31561
11604	Amp	8	107411943	107415732	1.667574	0.324458
11605	Amp	8	107417635	107440408	1.415328	0.307576
11606	Amp	8	107441224	107541421	1.907576	0.340263

11607	Amp	8	107547149	107590914	1.514375	0.314109
11608	Amp	8	107596114	107657499	1.383826	0.305356
11609	Amp	8	107659235	107681594	1.265443	0.296967
11610	Amp	8	107688599	107851761	1.394558	0.306358
11611	Amp	8	107853783	107953131	1.305256	0.299826
11612	Amp	8	107958349	107985626	1.424227	0.308042
11613	Amp	8	107987395	108074191	1.285475	0.298298
11614	Amp	8	108077360	108116425	1.2565	0.296587
11615	Amp	8	108130079	108146269	1.463463	0.311029
11616	Amp	8	108147876	108295581	1.525294	0.315229
11617	Amp	8	108296062	108539939	1.563139	0.317767
11618	Amp	8	108541342	108564526	1.687998	0.325691
11619	Amp	8	108567283	108599980	1.563139	0.317475
11620	Amp	8	108600497	108688361	1.534719	0.315366
11621	Amp	8	108688434	108722938	1.49455	0.312727
11622	Amp	8	108729402	108747104	1.285475	0.298284
11623	Amp	8	108750075	108755976	1.415328	0.307257
11624	Amp	8	108758005	108998657	1.553308	0.316922
11625	Amp	8	108998856	109190001	1.415328	0.307531
11626	Amp	8	109193361	109328584	1.315861	0.300255
11627	Amp	8	109328776	109600295	1.24764	0.296055
11628	Amp	8	109600771	109631117	1.542961	0.316225
11629	Amp	8	109631576	109637876	1.605306	0.320611
11630	Amp	8	109638381	109653941	1.582671	0.319055
11631	Amp	8	109661371	109748780	1.563139	0.317427
11632	Amp	8	109756524	109879241	1.626265	0.322147
11633	Amp	8	109880429	109921944	1.708768	0.327137
11634	Amp	8	109922396	109993001	1.404612	0.306967
11635	Amp	8	109993688	110035276	1.582671	0.319189
11636	Amp	8	110035649	110056980	1.525294	0.314804
11637	Amp	8	110060475	110287924	1.563139	0.318033
11638	Amp	8	110291720	110321803	1.667574	0.324734
11639	Amp	8	110327406	110365423	1.898353	0.339517
11640	Amp	8	110365956	110380191	1.963469	0.34417
11641	Amp	8	110382582	110585179	1.898353	0.339451
11642	Amp	8	110585325	110602809	1.866286	0.337666
11643	Amp	8	110606496	110607825	1.930731	0.34186
11644	Amp	8	110608908	110609036	1.963469	0.343689
11645	Amp	8	110609174	110655236	2.028562	0.348136
11646	Amp	8	110655496	110722822	2.071528	0.350644
11647	Amp	8	110727508	110737580	2.761965	0.392683
11648	Amp	8	110739993	110874580	2.519	0.3779
11649	Amp	8	110880435	110986723	2.393797	0.370633
11650	Amp	8	110987812	111013460	2.495444	0.376702
11651	Amp	8	111014586	111024437	2.338154	0.367518
11652	Amp	8	111026005	111069426	2.470601	0.375658
11653	Amp	8	111071591	111158207	2.404616	0.371005
11654	Amp	8	111159881	111264705	2.082332	0.351232
11655	Amp	8	111269132	111269714	2.102209	0.352764
11656	Amp	8	111290954	111326810	2.061276	0.350366
11657	Amp	8	111328156	111331508	1.930731	0.341475
11658	Amp	8	111335233	111357724	1.953191	0.343475
11659	Amp	8	111358287	111394699	2.049537	0.349116

11660	Amp	8	111401567	111403692	2.294081	0.364526
11661	Amp	8	111405959	111417366	2.483202	0.376424
11662	Amp	8	111421099	111438924	2.315177	0.366022
11663	Amp	8	111443026	111443026	2.580481	0.381575
11664	Amp	8	111443029	111443780	2.818034	0.396035
11665	Amp	8	111447471	111447661	2.914035	0.401875
11666	Amp	8	111449015	111544458	2.349236	0.36771
11667	Amp	8	111545201	111630710	2.543219	0.379844
11668	Amp	8	111631048	111656633	2.294081	0.36429
11669	Amp	8	111662934	111715697	2.192225	0.358086
11670	Amp	8	111716404	111732857	2.283191	0.363472
11671	Amp	8	111734410	111741547	2.102209	0.35293
11672	Amp	8	111744762	111872479	2.283191	0.363864
11673	Amp	8	111874949	111903022	2.082332	0.351256
11674	Amp	8	111911110	111939943	2.625007	0.384284
11675	Amp	8	111944942	111946822	2.878516	0.399169
11676	Amp	8	111946838	112048403	3.124015	0.413953
11677	Amp	8	112052505	112090808	3.079122	0.411146
11678	Amp	8	112092914	112128034	2.866312	0.398717
11679	Amp	8	112133999	112195445	3.029636	0.408109
11680	Amp	8	112199585	112475243	2.774551	0.39365
11681	Amp	8	112476782	112513917	2.806723	0.395626
11682	Amp	8	112518702	112573587	2.71525	0.389786
11683	Amp	8	112573999	112582635	3.066577	0.410274
11684	Amp	8	112585720	112601574	2.935659	0.402857
11685	Amp	8	112604916	112697461	2.830877	0.396788
11686	Amp	8	112705487	112741588	2.948071	0.403955
11687	Amp	8	112745980	112803434	3.134208	0.414425
11688	Amp	8	112806417	112881504	2.88973	0.400033
11689	Amp	8	112881596	112881986	3.016502	0.407528
11690	Amp	8	112882102	113053761	3.089932	0.412161
11691	Amp	8	113060744	113073253	3.713958	0.447395
11692	Amp	8	113073808	113115931	2.948071	0.403899
11693	Amp	8	113116339	113149794	3.099988	0.412698
11694	Amp	8	113158627	113206762	3.920712	0.458831
11695	Amp	8	113207213	113286180	4.049098	0.465607
11696	Amp	8	113290532	113300775	3.217724	0.419474
11697	Amp	8	113305443	113315112	3.112016	0.413378
11698	Amp	8	113316106	113327835	3.26417	0.422326
11699	Amp	8	113331997	113362936	3.230962	0.41988
11700	Amp	8	113364814	113380288	3.32332	0.425678
11701	Amp	8	113380825	113387383	3.48305	0.435153
11702	Amp	8	113392587	113476485	3.625861	0.442963
11703	Amp	8	113479545	113480454	3.496514	0.436187
11704	Amp	8	113483927	113560308	3.134208	0.414578
11705	Amp	8	113561752	113561897	3.099988	0.412578
11706	Amp	8	113567382	113591453	3.029636	0.408732
11707	Amp	8	113591938	113591938	3.089932	0.411536
11708	Amp	8	113594850	113685815	3.182419	0.417173
11709	Amp	8	113686047	113712376	3.32332	0.425474
11710	Amp	8	113712427	113735341	3.415716	0.431323
11711	Amp	8	113736780	113745939	3.45062	0.432929
11712	Amp	8	113746199	113790971	3.470866	0.434707

11713	Amp	8	113792991	113851117	3.243024	0.421141
11714	Amp	8	113851597	113865231	3.276597	0.422553
11715	Amp	8	113868412	113944672	3.359177	0.427627
11716	Amp	8	113945148	114006709	3.43725	0.432715
11717	Amp	8	114009512	114041916	3.470866	0.434882
11718	Amp	8	114047119	114089127	3.713958	0.447727
11719	Amp	8	114094469	114150772	3.675606	0.445845
11720	Amp	8	114151333	114151333	2.602347	0.383251
11721	Amp	8	114156278	114156672	2.567803	0.381084
11722	Amp	8	114169343	114218721	3.650307	0.444093
11723	Amp	8	114219260	114292286	3.560951	0.43902
11724	Amp	8	114292762	114300583	3.587309	0.440465
11725	Amp	8	114370289	114421044	3.470866	0.434827
11726	Amp	8	114422482	114441068	3.600858	0.441084
11727	Amp	8	114446734	114465371	4.103115	0.467891
11728	Amp	8	114467709	114475810	4.364676	0.481464
11729	Amp	8	114480261	114487396	4.497567	0.48789
11730	Amp	8	114488582	114529210	4.525461	0.489286
11731	Amp	8	114529491	114543507	4.765369	0.500853
11732	Amp	8	114611050	114719680	4.897525	0.507612
11733	Amp	8	114719701	114719701	4.651516	0.495593
11734	Amp	8	114879397	114896720	5.638575	0.541685
11735	Amp	8	114897691	114978488	5.827612	0.550932
11736	Amp	8	114982154	115054313	5.654252	0.542738
11737	Amp	8	115054886	115120693	5.857214	0.551769
11738	Amp	8	115123816	115130920	5.953304	0.556749
11739	Amp	8	115131645	115172231	5.884474	0.553264
11740	Amp	8	115173480	115305346	6.10345	0.563721
11741	Amp	8	115307295	115322069	5.121758	0.517629
11742	Amp	8	115328927	115369326	6.197199	0.568152
11743	Amp	8	115371906	115391312	6.421599	0.578146
11744	Amp	8	115391630	115397206	6.57161	0.585327
11745	Amp	8	115402196	115410054	6.903728	0.59965
11746	Amp	8	115413632	115439858	6.808231	0.595009
11747	Amp	8	115440112	115456588	6.421599	0.578146
11748	Amp	8	115458243	115495905	6.392987	0.576749
11749	Amp	8	115503555	115504626	5.857214	0.552281
11750	Amp	8	115506132	115529544	5.669031	0.543002
11751	Amp	8	115537535	115540683	5.897742	0.554362
11752	Amp	8	115541190	115549566	6.165023	0.566395
11753	Amp	8	115549597	115585489	6.378996	0.576351
11754	Amp	8	115585563	115621472	6.510151	0.582031
11755	Amp	8	115621500	115681586	6.288489	0.572037
11756	Amp	8	115682885	115703241	6.028267	0.560005
11757	Amp	8	115704696	115747860	5.813919	0.550049
11758	Amp	8	115749434	115759567	5.753822	0.546915
11759	Amp	8	115760235	115771843	5.592909	0.539734
11760	Amp	8	115774582	115803992	5.710355	0.545414
11761	Amp	8	115804214	115891415	5.940125	0.555846
11762	Amp	8	115892093	115902743	6.181334	0.566845
11763	Amp	8	115902828	116008200	6.40864	0.577334
11764	Amp	8	116012792	116020279	6.541945	0.583888
11765	Amp	8	116022699	116029893	6.840128	0.596765

11766	Amp	8	116029947	116074755	6.889312	0.598492
11767	Amp	8	116080071	116136939	6.634781	0.588003
11768	Amp	8	116145466	116145933	6.364443	0.575127
11769	Amp	8	116147930	116184377	6.588	0.585428
11770	Amp	8	116186375	116209770	6.348497	0.574428
11771	Amp	8	116211084	116282169	6.212874	0.568748
11772	Amp	8	116283017	116294872	5.982794	0.558291
11773	Amp	8	116296542	116304741	5.940125	0.556061
11774	Amp	8	116305578	116316060	5.724602	0.545629
11775	Amp	8	116319639	116325426	5.753822	0.547107
11776	Amp	8	116332831	116354150	5.953304	0.556817
11777	Amp	8	116355689	116362810	5.827612	0.550665
11778	Amp	8	116363991	116392078	5.608185	0.540641
11779	Amp	8	116396584	116444899	5.489676	0.53496
11780	Amp	8	116448233	116483160	5.608185	0.540232
11781	Amp	8	116485410	116489565	5.753822	0.546898
11782	Amp	8	116494292	116511660	5.548259	0.537889
11783	Amp	8	116513116	116576184	5.399054	0.530391
11784	Amp	8	116582625	116601358	4.854009	0.50533
11785	Amp	8	116603346	116673763	4.651516	0.49562
11786	Amp	8	116677666	116724156	4.623661	0.494142
11787	Amp	8	116728124	116829816	4.444356	0.485699
11788	Amp	8	116830974	116860729	4.497567	0.488264
11789	Amp	8	116861572	116906911	4.285602	0.476903
11790	Amp	8	116909416	116911350	4.623661	0.494317
11791	Amp	8	116911977	116967932	4.738141	0.500034
11792	Amp	8	116969105	116984568	4.429725	0.485025
11793	Amp	8	116985053	116996155	4.511044	0.489143
11794	Amp	8	116997022	117002340	4.402792	0.483426
11795	Amp	8	117002437	117034059	4.090482	0.467746
11796	Amp	8	117039065	117100147	4.14557	0.470452
11797	Amp	8	117106933	117113327	3.230962	0.419929
11798	Amp	8	117122442	117157696	3.675606	0.445515
11799	Amp	8	117158184	117182349	3.496514	0.436056
11800	Amp	8	117184564	117200135	3.461541	0.433547
11801	Amp	8	117200223	117208857	3.424445	0.431931
11802	Amp	8	117212915	117222803	3.089932	0.412058
11803	Amp	8	117222889	117249436	3.05489	0.40955
11804	Amp	8	117249610	117297124	3.348874	0.426766
11805	Amp	8	117297262	117338819	3.312745	0.424989
11806	Amp	8	117347613	117417866	3.243024	0.420871
11807	Amp	8	117424857	117431286	2.970355	0.404744
11808	Amp	8	117432217	117583747	2.784457	0.393831
11809	Amp	8	117584753	117584753	2.737964	0.390956
11810	Amp	8	117585854	117613649	2.625007	0.384196
11811	Amp	8	117622374	117668350	2.180628	0.357389
11812	Amp	8	117668680	117672792	2.148338	0.35556
11813	Amp	8	117673625	117697141	2.102209	0.352995
11814	Amp	8	117697156	117891470	1.995109	0.345739
11815	Amp	8	117891512	117916589	2.061276	0.349733
11816	Amp	8	117918176	117929457	2.028562	0.348288
11817	Amp	8	117929470	117952109	1.983885	0.345456
11818	Amp	8	117953970	117976303	2.483202	0.376433

11819	Amp	8	117978591	118256302	2.683697	0.387558
11820	Amp	8	118256310	118256310	2.483202	0.376433
11821	Amp	8	118260254	118317230	2.294081	0.364298
11822	Amp	8	118318879	118508580	2.349236	0.36783
11823	Amp	8	118509082	118526826	2.458756	0.374937
11824	Amp	8	118526840	118565232	2.436112	0.373393
11825	Amp	8	118567110	118582758	2.784457	0.394257
11826	Amp	8	118585833	118617919	2.935659	0.403158
11827	Amp	8	118621516	118624719	2.737964	0.391168
11828	Amp	8	118628367	118673124	2.683697	0.387635
11829	Amp	8	118679679	118711658	2.580481	0.381391
11830	Amp	8	118712342	118737014	3.079122	0.41105
11831	Amp	8	118737828	118753330	3.637615	0.443805
11832	Amp	8	118756372	118781573	3.842634	0.454195
11833	Amp	8	118782769	118831380	3.625861	0.442935
11834	Amp	8	118833072	118890221	3.383286	0.429162
11835	Amp	8	118893225	118900132	3.43725	0.432644
11836	Amp	8	118900624	118921073	3.650307	0.444304
11837	Amp	8	118923362	119008810	3.124015	0.413657
11838	Amp	8	119008925	119024503	3.252252	0.421565
11839	Amp	8	119028129	119043335	3.171522	0.416997
11840	Amp	8	119043368	119257271	3.243024	0.420552
11841	Amp	8	119259903	119552835	3.415716	0.430835
11842	Amp	8	119555779	119623058	3.371695	0.428557
11843	Amp	8	119625090	119645885	3.230962	0.419821
11844	Amp	8	119650855	119733435	3.383286	0.429303
11845	Amp	8	119737136	119744930	2.922915	0.402007
11846	Amp	8	119756068	119796196	2.658709	0.386784
11847	Amp	8	119799435	119885261	2.393797	0.370628
11848	Amp	8	119886619	119887008	2.349236	0.367583
11849	Amp	8	119890666	120036144	2.424855	0.372885
11850	Amp	8	120041883	120069966	2.614747	0.383709
11851	Amp	8	120073269	120142300	2.647166	0.385501
11852	Amp	8	120142986	120308443	2.761965	0.392414
11853	Amp	8	120308712	120313431	2.878516	0.39943
11854	Amp	8	120313720	120367966	3.016502	0.407919
11855	Amp	8	120368143	120375924	3.066577	0.41029
11856	Amp	8	120377114	120445923	2.878516	0.399193
11857	Amp	8	120446077	120492298	2.958746	0.404355
11858	Amp	8	120499207	120561934	3.05489	0.409566
11859	Amp	8	120562935	120623903	3.252252	0.421184
11860	Amp	8	120625397	120675905	3.05489	0.409566
11861	Amp	8	120678808	120678808	2.994162	0.406084
11862	Amp	8	120679355	120797463	2.855041	0.398459
11863	Amp	8	120797907	120819412	2.958746	0.404503
11864	Amp	8	120823636	120827376	3.112016	0.413438
11865	Amp	8	120831225	120857699	3.288383	0.423507
11866	Amp	8	120860489	120875870	3.112016	0.413438
11867	Amp	8	120879464	120940167	3.134208	0.41488
11868	Amp	8	120943544	121118416	3.276597	0.423152
11869	Amp	8	121127312	121131980	3.549256	0.438723
11870	Amp	8	121132027	121226930	3.72674	0.448461
11871	Amp	8	121227849	121297511	2.855041	0.397938

11872	Amp	8	121304820	121377411	2.683697	0.387655
11873	Amp	8	121379877	121460964	2.424855	0.372759
11874	Amp	8	121464297	121465499	2.519	0.378086
11875	Amp	8	121470840	121477710	2.359227	0.368349
11876	Amp	8	121480938	121511853	2.530938	0.378819
11877	Amp	8	121513371	121513420	2.436112	0.373609
11878	Amp	8	121521549	121620783	2.683697	0.387912
11879	Amp	8	121624601	121634250	2.470601	0.375371
11880	Amp	8	121646608	121672577	2.658709	0.386464
11881	Amp	8	121673213	121684937	3.348874	0.426969
11882	Amp	8	121690719	121714054	3.415716	0.43111
11883	Amp	8	121716184	121722464	3.299582	0.424343
11884	Amp	8	121724077	121768565	3.48305	0.435392
11885	Amp	8	121769550	121779414	3.461541	0.433726
11886	Amp	8	121779471	121816864	3.288383	0.42363
11887	Amp	8	121817626	121928954	2.436112	0.373589
11888	Amp	8	121932624	122058934	2.349236	0.368177
11889	Amp	8	122059794	122163096	2.447622	0.373898
11890	Amp	8	122163732	122180134	2.830877	0.39646
11891	Amp	8	122183462	122255006	3.207076	0.418708
11892	Amp	8	122255030	122263374	3.05489	0.409773
11893	Amp	8	122263408	122309625	3.089932	0.411972
11894	Amp	8	122312554	122322253	3.243024	0.421014
11895	Amp	8	122322992	122326616	2.98257	0.405442
11896	Amp	8	122327011	122424237	2.796399	0.394972
11897	Amp	8	122424445	122458348	2.625007	0.384399
11898	Amp	8	122459594	122465354	2.71525	0.390173
11899	Amp	8	122467577	122518183	2.902384	0.400746
11900	Amp	8	122519968	122684374	2.796399	0.395025
11901	Amp	8	122684502	122685632	2.970355	0.405047
11902	Amp	8	122691500	122705289	3.252252	0.421563
11903	Amp	8	122706048	122731684	3.496514	0.436201
11904	Amp	8	122732498	122732823	3.312745	0.425152
11905	Amp	8	122735291	122838426	3.348874	0.426803
11906	Amp	8	122840352	122940831	3.312745	0.42489
11907	Amp	8	122945671	122984838	3.461541	0.433932
11908	Amp	8	122988101	123016239	3.288383	0.423506
11909	Amp	8	123016391	123109529	3.348874	0.427094
11910	Amp	8	123110274	123116758	3.288383	0.423636
11911	Amp	8	123116955	123133401	3.394201	0.43005
11912	Amp	8	123136919	123257584	3.461541	0.434104
11913	Amp	8	123258172	123269498	3.424445	0.431905
11914	Amp	8	123270144	123278634	3.613533	0.442275
11915	Amp	8	123282910	123367515	3.650307	0.44407
11916	Amp	8	123377368	123388140	3.32332	0.425377
11917	Amp	8	123392085	123395606	3.403911	0.430379
11918	Amp	8	123403764	123405721	3.146436	0.415402
11919	Amp	8	123410097	123451194	2.935659	0.402863
11920	Amp	8	123451240	123460618	3.066577	0.410706
11921	Amp	8	123460915	123461899	3.207076	0.418946
11922	Amp	8	123462660	123486407	3.560951	0.439032
11923	Amp	8	123488598	123525993	3.299582	0.424511
11924	Amp	8	123531730	123573895	2.970355	0.405007

11925	Amp	8	123574065	123600584	2.796399	0.394985
11926	Amp	8	123603376	123665503	2.774551	0.393334
11927	Amp	8	123665984	123711391	2.703608	0.389193
11928	Amp	8	123711540	123743220	2.774551	0.393623
11929	Amp	8	123745949	123745949	2.614747	0.383499
11930	Amp	8	123746033	123783373	2.635842	0.385312
11931	Amp	8	123796052	123866224	3.207076	0.418759
11932	Amp	8	123866392	123961439	3.171522	0.416946
11933	Amp	8	123963614	123972918	3.600858	0.441559
11934	Amp	8	123996868	124023293	3.509701	0.436557
11935	Amp	8	124025902	124118725	3.394201	0.429856
11936	Amp	8	124119662	124304666	3.549256	0.438657
11937	Amp	8	124310770	124350654	3.243024	0.420534
11938	Amp	8	124351758	124388629	3.535174	0.437656
11939	Amp	8	124394158	124519439	3.461541	0.434101
11940	Amp	8	124521890	124561458	3.801418	0.452383
11941	Amp	8	124563562	124837386	4.540237	0.490528
11942	Amp	8	124838927	124911455	4.215025	0.473406
11943	Amp	8	124913726	124935437	4.257976	0.475452
11944	Amp	8	124935690	124954586	4.076638	0.46665
11945	Amp	8	124956960	125176492	3.86957	0.455846
11946	Amp	8	125181503	125229295	3.777346	0.451309
11947	Amp	8	125232644	125333905	3.613533	0.442361
11948	Amp	8	125334028	125391856	3.424445	0.432103
11949	Amp	8	125394022	125421867	3.230962	0.419994
11950	Amp	8	125422488	125500784	3.394201	0.429894
11951	Amp	8	125503955	125609591	3.461541	0.433854
11952	Amp	8	125609667	125689970	3.560951	0.439667
11953	Amp	8	125692012	125839338	3.701758	0.447133
11954	Amp	8	125843039	125916046	3.933803	0.458954
11955	Amp	8	125916604	125926317	3.893345	0.456879
11956	Amp	8	125930102	125940562	3.790167	0.451963
11957	Amp	8	125946134	125947030	3.688516	0.446189
11958	Amp	8	125949454	125949454	3.777346	0.450867
11959	Amp	8	125950362	125993034	4.103115	0.468248
11960	Amp	8	125995352	126133001	5.150026	0.518715
11961	Amp	8	126134614	126161904	5.077576	0.515373
11962	Amp	8	126161937	126163891	4.854009	0.505346
11963	Amp	8	126163988	126260956	4.738141	0.499533
11964	Amp	8	126263312	126310410	4.927103	0.509013
11965	Amp	8	126313157	126320226	3.920712	0.458545
11966	Amp	8	126320968	126320968	3.675606	0.445231
11967	Amp	8	126321801	126332290	3.587309	0.440553
11968	Amp	8	126338697	126368414	3.470866	0.434442
11969	Amp	8	126368521	126387625	3.880586	0.456631
11970	Amp	8	126387742	126397357	3.998681	0.462747
11971	Amp	8	126398352	126426783	3.625861	0.442995
11972	Amp	8	126427094	126704953	3.663326	0.44463
11973	Amp	8	126715728	126727908	3.777346	0.450698
11974	Amp	8	126731531	126773481	3.86957	0.455807
11975	Amp	8	126773500	126835777	3.688516	0.446013
11976	Amp	8	126837382	126862840	2.994162	0.406232
11977	Amp	8	126863532	126882347	2.692246	0.388851

11978	Amp	8	126883679	126892869	2.507231	0.377626
11979	Amp	8	126895932	126962371	2.902384	0.4011
11980	Amp	8	126967446	126968297	3.157622	0.415905
11981	Amp	8	126968439	126993696	3.496514	0.435867
11982	Amp	8	126997405	127097685	4.327765	0.479491
11983	Amp	8	127102216	127117424	3.880586	0.456287
11984	Amp	8	127120831	127139564	4.376421	0.481943
11985	Amp	8	127140282	127223869	3.880586	0.456287
11986	Amp	8	127226867	127234904	4.022326	0.463941
11987	Amp	8	127235453	127285011	4.063271	0.466182
11988	Amp	8	127291743	127305340	4.809024	0.503484
11989	Amp	8	127321656	127328129	4.30031	0.477947
11990	Amp	8	127328427	127395716	5.337978	0.52825
11991	Amp	8	127395871	127395871	5.696665	0.544216
11992	Amp	8	127396291	127509957	5.857214	0.552127
11993	Amp	8	127510669	127547405	5.56363	0.538029
11994	Amp	8	127549994	127606393	6.10345	0.563567
11995	Amp	8	127607194	127632710	6.288489	0.571607
11996	Amp	8	127633425	127634318	6.421599	0.577839
11997	Amp	8	127637389	127645286	6.494707	0.581588
11998	Amp	8	127649708	127662907	7.664726	0.632056
11999	Amp	8	127663526	127712420	7.424522	0.621419
12000	Amp	8	127714525	127759804	7.523003	0.62554
12001	Amp	8	127765285	127832475	7.330797	0.6175
12002	Amp	8	127836003	127990248	7.37867	0.619124
12003	Amp	8	127990871	128020402	7.067371	0.605956
12004	Amp	8	128021626	128056513	6.775614	0.593682
12005	Amp	8	128056781	128087010	7.151076	0.609986
12006	Amp	8	128090265	128104287	7.553488	0.626632
12007	Amp	8	128104492	128109092	7.647929	0.631158
12008	Amp	8	128112456	128136557	6.920476	0.600315
12009	Amp	8	128136874	128149207	6.823902	0.595664
12010	Amp	8	128151484	128151484	6.728678	0.591915
12011	Amp	8	128151491	128206613	6.889312	0.598537
12012	Amp	8	128206991	128209047	7.083784	0.606732
12013	Amp	8	128211625	128229364	7.151076	0.609445
12014	Amp	8	128233504	128236803	7.033351	0.604919
12015	Amp	8	128241577	128245719	6.712483	0.591203
12016	Amp	8	128245823	128274639	6.683082	0.589579
12017	Amp	8	128275164	128290926	6.604027	0.586649
12018	Amp	8	128297153	128307467	6.40864	0.577672
12019	Amp	8	128308924	128381395	6.288489	0.571603
12020	Amp	8	128383735	128403870	6.450429	0.579235
12021	Amp	8	128406286	128413278	6.40864	0.577203
12022	Amp	8	128413903	128413903	6.557256	0.584341
12023	Amp	8	128414554	128423538	5.47413	0.533874
12024	Amp	8	128424089	128501944	5.534438	0.537148
12025	Amp	8	128503195	128565383	5.669031	0.543194
12026	Amp	8	128568898	128577212	5.753822	0.547425
12027	Amp	8	128577364	128653518	5.798921	0.549246
12028	Amp	8	128666811	128673399	5.592909	0.539346
12029	Amp	8	128674387	128728852	5.669031	0.542784
12030	Amp	8	128729290	128864057	5.710355	0.544874

12031	Amp	8	128867890	128891443	5.548259	0.537548
12032	Amp	8	128900494	128952588	5.337978	0.528068
12033	Amp	8	128952627	128970986	5.267482	0.524794
12034	Amp	8	128974886	128989920	5.31131	0.526444
12035	Amp	8	128990223	129055773	5.519766	0.536137
12036	Amp	8	129055794	129068166	5.710355	0.545276
12037	Amp	8	129076359	129131620	5.519766	0.535971
12038	Amp	8	129132280	129167964	5.31131	0.526278
12039	Amp	8	129171955	129195432	5.077576	0.515455
12040	Amp	8	129197098	129199082	5.295952	0.526018
12041	Amp	8	129209533	129210505	4.986444	0.5117
12042	Amp	8	129213185	129252977	5.193344	0.520951
12043	Amp	8	129256744	129279216	4.971485	0.510948
12044	Amp	8	129279728	129294057	4.444356	0.48541
12045	Amp	8	129298050	129322874	4.651516	0.495749
12046	Amp	8	129329677	129341448	4.594893	0.493035
12047	Amp	8	129345040	129380959	4.470731	0.486413
12048	Amp	8	129385575	129435114	4.416662	0.484051
12049	Amp	8	129437075	129489852	3.815003	0.453092
12050	Amp	8	129492612	129524827	3.637615	0.443722
12051	Amp	8	129526598	129611291	3.470866	0.434202
12052	Amp	8	129611859	129695914	3.523011	0.437207
12053	Amp	8	129696087	129714770	3.45062	0.433109
12054	Amp	8	129715732	129860054	2.602347	0.382806
12055	Amp	8	129860602	129860602	2.703608	0.389165
12056	Amp	8	129864069	129878936	2.658709	0.38616
12057	Amp	8	129879680	129887784	2.602347	0.382821
12058	Amp	8	129906289	129907336	2.404616	0.371075
12059	Amp	8	129909567	129916889	2.902384	0.400789
12060	Amp	8	129921833	129947039	3.041652	0.408866
12061	Amp	8	129951013	129973617	3.32332	0.425622
12062	Amp	8	129978437	129979572	3.509701	0.436923
12063	Amp	8	129980701	130008740	3.403911	0.430243
12064	Amp	8	130012634	130036895	2.88973	0.400136
12065	Amp	8	130037532	130052549	2.948071	0.403348
12066	Amp	8	130053917	130133720	3.276597	0.423071
12067	Amp	8	130135151	130140821	3.146436	0.414994
12068	Amp	8	130143627	130169839	2.806723	0.39533
12069	Amp	8	130171059	130174516	3.016502	0.407522
12070	Amp	8	130175957	130235978	3.089932	0.411894
12071	Amp	8	130244152	130249225	2.958746	0.404552
12072	Amp	8	130249395	130273423	2.843135	0.397305
12073	Amp	8	130274027	130291941	3.146436	0.415329
12074	Amp	8	130292646	130325590	3.252252	0.421347
12075	Amp	8	130327127	130388747	2.948071	0.403686
12076	Amp	8	130390579	130462170	3.099988	0.412813
12077	Amp	8	130465528	130476800	2.922915	0.402474
12078	Amp	8	130477053	130505238	3.099988	0.412758
12079	Amp	8	130505259	130505463	3.394201	0.429661
12080	Amp	8	130505584	130505584	3.182419	0.417239
12081	Amp	8	130509682	130589698	3.029636	0.408112
12082	Amp	8	130589729	130593088	2.88973	0.40038
12083	Amp	8	130602197	130628833	2.692246	0.388188

12084	Amp	8	130628850	130647770	2.935659	0.403186
12085	Amp	8	130654065	130666168	2.591575	0.382013
12086	Amp	8	130667294	130695276	2.338154	0.367142
12087	Amp	8	130696788	130740075	2.393797	0.370866
12088	Amp	8	130740595	130810511	2.338154	0.367044
12089	Amp	8	130811756	130815182	2.458756	0.374776
12090	Amp	8	130816040	130833706	2.507231	0.377373
12091	Amp	8	130848756	130867413	2.436112	0.373413
12092	Amp	8	130872999	130873696	2.530938	0.379026
12093	Amp	8	130875348	130891033	2.359227	0.368743
12094	Amp	8	130894078	130912927	2.393797	0.37099
12095	Amp	8	130913859	130915370	2.102209	0.353069
12096	Amp	8	130916077	131029772	2.071528	0.350822
12097	Amp	8	131030407	131046276	2.530938	0.379214
12098	Amp	8	131049163	131068760	2.404616	0.371371
12099	Amp	8	131070505	131070505	2.283191	0.363625
12100	Amp	8	131070723	131137047	2.370685	0.369302
12101	Amp	8	131137655	131147959	2.283191	0.363689
12102	Amp	8	131148728	131223242	2.071528	0.350887
12103	Amp	8	131225737	131274891	2.016329	0.347163
12104	Amp	8	131276043	131331707	1.800655	0.333769
12105	Amp	8	131331754	131446702	1.963469	0.344031
12106	Amp	8	131450770	131483933	1.995109	0.345771
12107	Amp	8	131484902	131602432	1.811302	0.334066
12108	Amp	8	131605064	131704147	1.687998	0.326212
12109	Amp	8	131704220	131738641	1.717946	0.328265
12110	Amp	8	131746801	131855974	1.637826	0.322222
12111	Amp	8	131856038	132068636	1.778794	0.332351
12112	Amp	8	132068755	132110651	1.811302	0.33432
12113	Amp	8	132110713	132199502	1.657426	0.324191
12114	Amp	8	132200837	132214817	1.778794	0.332045
12115	Amp	8	132218391	132298202	1.866286	0.337754
12116	Amp	8	132298494	132299775	2.005494	0.3467
12117	Amp	8	132300314	132329204	2.113683	0.353709
12118	Amp	8	132331939	132439939	1.972699	0.344763
12119	Amp	8	132440555	132446610	2.294081	0.364711
12120	Amp	8	132449506	132537152	1.972699	0.344763
12121	Amp	8	132538934	132559901	2.170217	0.357197
12122	Amp	8	132560810	132579705	2.249752	0.36144
12123	Amp	8	132583728	132588771	2.349236	0.368061
12124	Amp	8	132592270	132666141	2.315177	0.366092
12125	Amp	8	132673290	132755378	3.171522	0.4166
12126	Amp	8	132755418	132801306	3.335884	0.426552
12127	Amp	8	132803331	132844987	3.230962	0.419931
12128	Amp	8	132847088	132856429	3.194439	0.418142
12129	Amp	8	132860077	132880437	3.299582	0.424431
12130	Amp	8	132883044	132903456	3.394201	0.429803
12131	Amp	8	132905343	132923847	2.543219	0.379296
12132	Amp	8	132928350	132990716	2.71525	0.389849
12133	Amp	8	132990818	133034047	2.658709	0.386636
12134	Amp	8	133037552	133071788	2.495444	0.376685
12135	Amp	8	133071922	133136369	2.447622	0.374088
12136	Amp	8	133141329	133189966	2.249752	0.361654

12137	Amp	8	133193354	133253042	2.082332	0.351101
12138	Amp	8	133253505	133260306	2.214215	0.359609
12139	Amp	8	133260536	133355962	2.447622	0.374044
12140	Amp	8	133356965	133376350	2.555572	0.380164
12141	Amp	8	133376976	133388071	2.404616	0.371656
12142	Amp	8	133388299	133442639	2.359227	0.368275
12143	Amp	8	133443023	133471075	2.530938	0.378998
12144	Amp	8	133471388	133597528	2.415622	0.371989
12145	Amp	8	133598564	133674979	3.124015	0.414177
12146	Amp	8	133675096	133787318	3.243024	0.420467
12147	Amp	8	133788024	133859499	3.124015	0.413982
12148	Amp	8	133863048	133876107	2.958746	0.404102
12149	Amp	8	133881418	133884526	2.249752	0.361913
12150	Amp	8	133886427	133894455	2.393797	0.370498
12151	Amp	8	133895209	134032918	2.436112	0.373522
12152	Amp	8	134033061	134039565	2.249752	0.36186
12153	Amp	8	134040526	134063431	2.203122	0.359284
12154	Amp	8	134064833	134068901	2.359227	0.368624
12155	Amp	8	134068916	134124100	2.519	0.377972
12156	Amp	8	134124414	134142354	2.614747	0.384013
12157	Amp	8	134150435	134154292	2.519	0.377972
12158	Amp	8	134156845	134171265	2.692246	0.388396
12159	Amp	8	134172264	134217918	2.750393	0.391669
12160	Amp	8	134223596	134237829	2.591575	0.382321
12161	Amp	8	134238631	134238631	2.737964	0.391407
12162	Amp	8	134239268	134281461	2.635842	0.385287
12163	Amp	8	134284046	134333870	2.614747	0.383466
12164	Amp	8	134337490	134359536	2.555572	0.380223
12165	Amp	8	134360497	134360497	2.784457	0.393969
12166	Amp	8	134363764	134431548	2.625007	0.384629
12167	Amp	8	134436301	134448472	2.970355	0.405165
12168	Amp	8	134448706	134474011	3.006013	0.406834
12169	Amp	8	134474097	134485523	3.625861	0.443118
12170	Amp	8	134485918	134553025	3.006013	0.406834
12171	Amp	8	134553110	134599135	2.914035	0.401661
12172	Amp	8	134602314	134700048	3.45062	0.432904
12173	Amp	8	134701937	134740461	3.359177	0.427912
12174	Amp	8	134746762	134771504	3.394201	0.429975
12175	Amp	8	134776236	134804143	3.006013	0.406816
12176	Amp	8	134806349	134826906	3.496514	0.435776
12177	Amp	8	134828636	134865287	2.948071	0.403724
12178	Amp	8	134865872	134868539	2.88973	0.400451
12179	Amp	8	134872786	134952728	2.647166	0.385959
12180	Amp	8	134953107	134988235	2.88973	0.400451
12181	Amp	8	134997251	135029137	2.958746	0.404116
12182	Amp	8	135029342	135059140	3.006013	0.407271
12183	Amp	8	135059780	135084170	2.806723	0.395413
12184	Amp	8	135085988	135160519	2.692246	0.388191
12185	Amp	8	135160658	135242328	2.635842	0.385167
12186	Amp	8	135251246	135255827	2.727427	0.390503
12187	Amp	8	135265154	135326624	2.580481	0.381416
12188	Amp	8	135327030	135387846	1.778794	0.331924
12189	Amp	8	135397693	135429830	1.626265	0.321785

12190	Amp	8	135429890	135444207	1.572486	0.31812
12191	Amp	8	135447896	135612722	1.49455	0.312785
12192	Amp	8	135618027	135618027	1.514375	0.314327
12193	Amp	8	135618382	135637434	1.757929	0.330886
12194	Amp	8	135640861	135749535	2.580481	0.381409
12195	Amp	8	135750828	135804557	3.006013	0.407096
12196	Amp	8	135806956	135844970	3.194439	0.418137
12197	Amp	8	135854934	135861070	3.276597	0.422705
12198	Amp	8	135862080	135894901	3.134208	0.414464
12199	Amp	8	135895247	135955898	2.855041	0.397906
12200	Amp	8	135958565	136029549	2.415622	0.372219
12201	Amp	8	136029699	136039144	2.614747	0.383863
12202	Amp	8	136039358	136061790	2.415622	0.372219
12203	Amp	8	136063706	136068267	1.667574	0.32492
12204	Amp	8	136068732	136079352	1.605306	0.320352
12205	Amp	8	136084168	136124673	1.667574	0.324458
12206	Amp	8	136124831	136139066	1.811302	0.334276
12207	Amp	8	136140330	136182390	1.757929	0.331052
12208	Amp	8	136186149	136189423	2.272343	0.362883
12209	Amp	8	136199016	136205963	2.158849	0.356121
12210	Amp	8	136208293	136209155	2.005494	0.34687
12211	Amp	8	136211134	136249431	2.294081	0.364552
12212	Amp	8	136252093	136267575	1.930731	0.341944
12213	Amp	8	136269227	136294487	1.646768	0.322904
12214	Amp	8	136294504	136312616	1.778794	0.332387
12215	Amp	8	136314367	136557804	1.898353	0.339609
12216	Amp	8	136559087	136654485	2.415622	0.371922
12217	Amp	8	136655366	136655366	2.647166	0.38592
12218	Amp	8	136870980	136872020	2.580481	0.381671
12219	Amp	8	136873251	136879828	2.424855	0.372902
12220	Amp	8	136880323	136881227	2.530938	0.378552
12221	Amp	8	136895774	137012050	2.294081	0.364554
12222	Amp	8	137017626	137098004	2.005494	0.346872
12223	Amp	8	137098799	137100805	2.124253	0.35435
12224	Amp	8	137102514	137155136	2.071528	0.350449
12225	Amp	8	137158595	137179675	2.148338	0.35585
12226	Amp	8	137182465	137193695	2.039783	0.348372
12227	Amp	8	137196370	137307265	2.170217	0.356876
12228	Amp	8	137307508	137333866	2.082332	0.351225
12229	Amp	8	137336423	137368533	2.049537	0.349162
12230	Amp	8	137369657	137396383	1.907576	0.340658
12231	Amp	8	137400035	137532033	1.942393	0.342486
12232	Amp	8	137533262	137549923	1.821927	0.334935
12233	Amp	8	137550357	137577096	1.866286	0.337345
12234	Amp	8	137577622	137818794	1.717946	0.327862
12235	Amp	8	137819447	137921697	1.657426	0.323756
12236	Amp	8	137922319	137925818	1.708768	0.327677
12237	Amp	8	137930670	137945138	1.737394	0.329398
12238	Amp	8	137945911	138000302	1.800655	0.333313
12239	Amp	8	138000975	138029523	1.768015	0.331593
12240	Amp	8	138029568	138035432	1.832922	0.335312
12241	Amp	8	138035536	138131668	1.963469	0.344032
12242	Amp	8	138132671	138146785	1.995109	0.345855

12243	Amp	8	138146836	138178450	2.158849	0.356392
12244	Amp	8	138182150	138277263	2.028562	0.347673
12245	Amp	8	138277904	138296309	2.124253	0.354225
12246	Amp	8	138296926	138325235	2.091935	0.35243
12247	Amp	8	138327931	138351085	2.602347	0.382697
12248	Amp	8	138352041	138367801	2.737964	0.391424
12249	Amp	8	138368917	138476057	2.591575	0.382516
12250	Amp	8	138477136	138523388	2.647166	0.386069
12251	Amp	8	138527716	138650508	2.148338	0.355802
12252	Amp	8	138652901	138696538	2.192225	0.358375
12253	Amp	8	138697926	138749763	2.591575	0.38214
12254	Amp	8	138750757	138762757	2.495444	0.377051
12255	Amp	8	138770295	138785298	2.359227	0.368325
12256	Amp	8	138787529	138798683	2.249752	0.361925
12257	Amp	8	138802023	138934581	2.383144	0.370282
12258	Amp	8	138934722	138934722	2.283191	0.363645
12259	Amp	8	138935640	139004186	3.124015	0.414168
12260	Amp	8	139007698	139098174	3.207076	0.418469
12261	Amp	8	139105176	139168464	3.066577	0.410614
12262	Amp	8	139168820	139171019	3.157622	0.416015
12263	Amp	8	139171590	139240104	3.243024	0.420502
12264	Amp	8	139241137	139289510	3.461541	0.433656
12265	Amp	8	139289520	139293730	3.48305	0.435503
12266	Amp	8	139295041	139307091	3.625861	0.442941
12267	Amp	8	139307464	139307840	3.801418	0.452178
12268	Amp	8	139311345	139311345	3.713958	0.447877
12269	Amp	8	139313505	139317306	3.523011	0.437339
12270	Amp	8	139319871	139366630	3.777346	0.450989
12271	Amp	8	139366845	139372133	3.737077	0.448942
12272	Amp	8	139375935	139423172	2.855041	0.398419
12273	Amp	8	139425870	139441609	3.066577	0.410552
12274	Amp	8	139444302	139624213	2.914035	0.401316
12275	Amp	8	139624881	139641795	2.958746	0.404613
12276	Amp	8	139641860	139690970	2.914035	0.401327
12277	Amp	8	139691098	139729308	2.958746	0.404363
12278	Amp	8	139734454	139745211	3.134208	0.414861
12279	Amp	8	139748767	139781616	2.424855	0.3729
12280	Amp	8	139786564	139876862	2.203122	0.359251
12281	Amp	8	139877849	139924600	1.995109	0.346097
12282	Amp	8	139930370	139945228	1.717946	0.327982
12283	Amp	8	139949611	139982141	1.687998	0.32573
12284	Amp	8	139982347	140166985	1.637826	0.322695
12285	Amp	8	140167440	140286814	1.678649	0.325106
12286	Amp	8	140290278	140290576	1.637826	0.322695
12287	Amp	8	140291981	140354093	1.678649	0.325195
12288	Amp	8	140356271	140436214	1.778794	0.331961
12289	Amp	8	140436803	140504085	1.877398	0.338081
12290	Amp	8	140504738	140507372	1.930731	0.341494
12291	Amp	8	140520764	140532136	2.750393	0.391819
12292	Amp	8	140532651	140619100	2.818034	0.395876
12293	Amp	8	140620316	140620316	2.914035	0.401271
12294	Amp	8	141150299	141155241	2.806723	0.395151
12295	Amp	8	141161758	141166181	2.272343	0.362838

12296	Amp	8	141166213	141199444	2.383144	0.369824
12297	Amp	8	141199468	141264555	2.272343	0.363058
12298	Amp	8	141264737	141276663	2.315177	0.365568
12299	Amp	8	141277435	141310438	2.192225	0.358582
12300	Amp	8	141311437	141319311	2.102209	0.352932
12301	Amp	8	141323295	141323326	2.530938	0.37885
12302	Amp	8	141323524	141323933	2.327403	0.366718
12303	Amp	8	142423108	142430065	1.542961	0.316393
12304	Amp	8	142433073	142445166	1.563139	0.317976
12305	Amp	8	142447039	142507081	1.727244	0.328639
12306	Amp	8	142521484	142548838	1.687998	0.326139
12307	Amp	8	142548908	142890532	1.534719	0.315476
12308	Amp	8	142899050	143000623	1.572486	0.318373
12309	Amp	8	143001625	143104371	1.534719	0.315476
12310	Amp	8	143105334	143228447	1.563139	0.317423
12311	Amp	8	143232207	143251666	1.582671	0.3192
12312	Amp	8	143254347	143288616	1.789205	0.332812
12313	Amp	8	143299833	143317866	2.071528	0.350993
12314	Amp	8	143325445	143495933	2.039783	0.348583
12315	Amp	8	143496578	143566339	2.249752	0.361823
12316	Amp	8	143580513	143658611	1.821927	0.334971
12317	Amp	8	143665147	143856219	1.572486	0.318601
12318	Amp	8	143857216	143857216	1.737394	0.329485
12319	Amp	8	143872894	143905656	2.124253	0.353883
12320	Amp	8	143910115	144067190	2.082332	0.351374
12321	Amp	8	144071799	144076662	1.757929	0.33071
12322	Amp	8	144080674	144081683	1.626265	0.32175
12323	Amp	8	144084619	144090784	1.514375	0.31431
12324	Amp	8	144091114	144123152	1.334042	0.301687
12325	Amp	8	144164776	144182542	1.305256	0.299741
12326	Amp	9	789794	923709	0	0.077761
12327	Amp	9	955794	962184	0	0.06718
12328	Amp	9	962232	965006	0	0.060292
12329	Amp	9	1540009	1540009	0	0.057374
12330	Amp	9	1540026	1639619	0	0.055723
12331	Amp	9	1646820	1716095	0	0.066701
12332	Amp	9	1716479	1894807	0	0.065136
12333	Amp	9	1900689	1902868	0	0.076695
12334	Amp	9	1905973	1909586	0	0.065717
12335	Amp	9	1915848	1928054	0	0.074384
12336	Amp	9	1928115	2287088	0	0.065717
12337	Amp	9	2287113	3020299	0	0.067997
12338	Amp	9	3024580	3035385	0	0.080505
12339	Amp	9	3036131	3310999	0	0.085296
12340	Amp	9	3311334	3344994	0	0.089275
12341	Amp	9	3345373	3440251	0	0.083468
12342	Amp	9	3440355	3735468	0	0.085827
12343	Amp	9	3741244	3831932	0	0.081847
12344	Amp	9	3832611	3958783	0	0.05778
12345	Amp	9	3963376	4061476	0	0.08234
12346	Amp	9	4064655	4087982	0	0.070479
12347	Amp	9	4088131	4204021	0	0.05778
12348	Amp	9	4280840	4328194	0	0.06541

12349	Amp	9	4328732	4353554	0	0.05778
12350	Amp	9	4356408	4378014	0	0.064171
12351	Amp	9	4378639	4578846	0	0.056843
12352	Amp	9	4580444	4581370	0	0.055401
12353	Amp	9	4582082	4605023	0	0.04901
12354	Amp	9	4611875	4651982	0	0.047172
12355	Amp	9	4652363	4673641	0	0.045257
12356	Amp	9	4673941	4691073	0	0.070733
12357	Amp	9	4691540	4883257	0	0.067269
12358	Amp	9	4884546	4981269	0	0.058329
12359	Amp	9	4987165	5024452	0	0.062269
12360	Amp	9	5024480	5047342	0	0.058329
12361	Amp	9	5047393	5087087	0	0.077959
12362	Amp	9	5097281	5097281	0	0.093567
12363	Amp	9	5233728	5302095	0	0.121225
12364	Amp	9	5302538	5429108	0	0.130193
12365	Amp	9	5560487	5607230	0	0.09749
12366	Amp	9	5607481	5719212	0	0.092699
12367	Amp	9	5721253	5722792	0	0.117685
12368	Amp	9	5728918	5737923	0	0.119356
12369	Amp	9	5738056	5760616	0	0.124619
12370	Amp	9	5765418	6046655	0	0.099633
12371	Amp	9	6048753	6051203	0	0.094994
12372	Amp	9	6051924	6053089	0	0.097194
12373	Amp	9	6053098	6256586	0	0.100135
12374	Amp	9	7055236	7154923	0	0.124249
12375	Amp	9	7155713	7244787	0	0.091998
12376	Amp	9	7245009	7252973	0	0.100942
12377	Amp	9	7295075	7302941	0	0.060077
12378	Amp	9	7302975	7434628	0	0.051134
12379	Amp	9	7435059	7541473	0	0.048193
12380	Amp	9	7541582	7541969	0	0.051214
12381	Amp	9	7543336	7550870	0	0.066002
12382	Amp	9	7554208	7560870	0	0.051214
12383	Amp	9	7561043	7887951	0	0.065321
12384	Amp	9	7892157	8129544	0	0.051214
12385	Amp	9	8131371	8342969	0	0.080482
12386	Amp	9	8343237	8387554	0	0.08779
12387	Amp	9	8393996	8524854	0	0.080482
12388	Amp	9	8525484	8654867	0	0.103942
12389	Amp	9	8662440	8705964	0	0.086703
12390	Amp	9	8706583	8901758	0	0.083471
12391	Amp	9	9039222	9464090	0	0.057494
12392	Amp	9	9465190	9531452	0	0.064945
12393	Amp	9	9533062	9687679	0	0.068867
12394	Amp	9	9687791	9874215	0	0.061416
12395	Amp	9	9874677	9874677	0	0.057494
12396	Amp	9	9985419	10106336	0	0.053631
12397	Amp	9	10106632	10131974	0	0.056603
12398	Amp	9	10132607	10135016	0	0.064081
12399	Amp	9	10165285	10217683	0	0.089844
12400	Amp	9	10346862	10347222	0	0.09002
12401	Amp	9	10348710	10361350	0	0.058679

12402	Amp	9	10363716	10372205	0	0.053241
12403	Amp	9	10373948	10542631	0	0.045763
12404	Amp	9	10542652	10547558	0	0.047271
12405	Amp	9	10547648	10583273	0	0.045763
12406	Amp	9	10585369	10790911	0	0.04828
12407	Amp	9	10791428	10880972	0	0.054673
12408	Amp	9	10894210	11271267	0	0.036791
12409	Amp	9	11274289	12722400	0	0.033819
12410	Amp	9	12723132	12757059	0	0.039858
12411	Amp	9	12758081	12780671	0	0.033465
12412	Amp	9	12786836	12830448	0	0.043248
12413	Amp	9	12840929	12847725	0	0.050168
12414	Amp	9	12847827	12902978	0	0.044128
12415	Amp	9	12906963	12925922	0	0.079741
12416	Amp	9	12972383	13052571	0	0.044128
12417	Amp	9	13052852	13054471	0	0.037209
12418	Amp	9	13057115	13094442	0	0.039635
12419	Amp	9	13096712	13123919	0	0.037209
12420	Amp	9	13129006	13262790	0	0.05524
12421	Amp	9	13262960	13307716	0	0.074509
12422	Amp	9	13345537	13375145	0	0.082705
12423	Amp	9	13376654	13491505	0	0.072921
12424	Amp	9	13494944	13970432	0	0.035621
12425	Amp	9	13976615	14163478	0	0.032047
12426	Amp	9	14168030	14226493	0	0.019168
12427	Amp	9	14231005	14339237	0	0.014547
12428	Amp	9	14339757	14434740	0	0.017382
12429	Amp	9	14435034	14510368	0	0.014864
12430	Amp	9	14517557	14596594	0	0.012029
12431	Amp	9	14597664	14972085	0	0
12432	Amp	9	15006536	15120494	0	0.012566
12433	Amp	9	15125717	15321544	0	0
12434	Amp	9	15322345	15476526	0	0.013361
12435	Amp	9	15482357	15504931	0	0
12436	Amp	9	15507774	15543810	0	0.008418
12437	Amp	9	15544390	16540448	0	0.01425
12438	Amp	9	16540696	16881561	0	0.020558
12439	Amp	9	16881701	16895899	0	0.030102
12440	Amp	9	16897590	17081685	0	0.027049
12441	Amp	9	17086815	17130362	0	0.020741
12442	Amp	9	17132396	17210516	0	0.024768
12443	Amp	9	17216959	17528477	0	0.020741
12444	Amp	9	17530606	17873009	0	0.023392
12445	Amp	9	17875601	17879266	0	0.028641
12446	Amp	9	17879592	18119272	0	0.023392
12447	Amp	9	18125923	18259825	0	0.028085
12448	Amp	9	18260421	18496848	0	0.034993
12449	Amp	9	18496877	18538879	0	0.028085
12450	Amp	9	18540752	18552495	0	0.02272
12451	Amp	9	18554335	18870443	0	0.026646
12452	Amp	9	18873245	19112911	0	0.041255
12453	Amp	9	19116024	19161854	0	0.044775
12454	Amp	9	19161937	19181927	0	0.040849

12455	Amp	9	19189215	19437429	0	0.02624
12456	Amp	9	19438281	19459293	0	0.036399
12457	Amp	9	19463400	19499282	0	0.04726
12458	Amp	9	19508887	19733647	0	0.056198
12459	Amp	9	19736873	19740318	0	0.052678
12460	Amp	9	19740790	19903873	0	0.041818
12461	Amp	9	19909671	19941807	0	0.057569
12462	Amp	9	19941867	20154690	0	0.052876
12463	Amp	9	20159177	20308261	0	0.050224
12464	Amp	9	20309958	20338431	0	0.040066
12465	Amp	9	20338714	20372800	0	0.050686
12466	Amp	9	20373654	20685892	0	0.056439
12467	Amp	9	20688118	20960863	0	0.050686
12468	Amp	9	20961907	21044022	0	0.072243
12469	Amp	9	21044045	21186156	0	0.059581
12470	Amp	9	21186569	21356932	0	0.057473
12471	Amp	9	21359702	21391365	0	0.061732
12472	Amp	9	21401194	21708884	0	0.057473
12473	Amp	9	21714307	21760397	0	0.047929
12474	Amp	9	21763567	21819478	0	0.037309
12475	Amp	9	21828111	21882135	0	0.046886
12476	Amp	9	21887381	22621296	0	0.009578
12477	Amp	9	22622677	22672075	0	0.015711
12478	Amp	9	22673695	23297362	0	0.027876
12479	Amp	9	23303967	23950905	0	0.077673
12480	Amp	9	23954351	24062633	0	0.075809
12481	Amp	9	24064959	24442010	0	0.069675
12482	Amp	9	24450830	24741498	0	0.072839
12483	Amp	9	24744458	24866801	0	0.074537
12484	Amp	9	24867808	25992540	0	0.072839
12485	Amp	9	25993274	26263020	0	0.04263
12486	Amp	9	26433574	26433574	0	0.079788
12487	Amp	9	26433919	26713337	0	0.08636
12488	Amp	9	26713800	27148500	0	0.090591
12489	Amp	9	27150764	27382087	0	0.084019
12490	Amp	9	27382246	27441539	0	0.077711
12491	Amp	9	27443245	27494817	0	0.08306
12492	Amp	9	27495420	27901737	0	0.041671
12493	Amp	9	27905144	28125258	0	0.023948
12494	Amp	9	28126767	28413463	0	0.041843
12495	Amp	9	28414843	29037965	0	0.080158
12496	Amp	9	29039634	29089612	0	0.085554
12497	Amp	9	29105292	29541945	0	0.080158
12498	Amp	9	29543052	29711283	0	0.08925
12499	Amp	9	29712028	30117409	0	0.071354
12500	Amp	9	30125950	30168475	0	0.087137
12501	Amp	9	30171448	30189670	0	0.088937
12502	Amp	9	30194093	30255387	0	0.100492
12503	Amp	9	30255444	30396361	0	0.10939
12504	Amp	9	30397125	30621118	0	0.100492
12505	Amp	9	30621229	30722890	0	0.102519
12506	Amp	9	30725871	30830159	0	0.104951
12507	Amp	9	30835548	30992627	0	0.108698

12508	Amp	9	30995027	31068072	0	0.106672
12509	Amp	9	31083833	31154556	0	0.111451
12510	Amp	9	31156026	31394338	0	0.107703
12511	Amp	9	31394700	31456575	0	0.105271
12512	Amp	9	31456607	31530660	0	0.113318
12513	Amp	9	31531694	31615045	0	0.130115
12514	Amp	9	31619336	31874036	0	0.108539
12515	Amp	9	31875996	31987048	0	0.135208
12516	Amp	9	31988716	32003876	0	0.120011
12517	Amp	9	32003950	32255127	0	0.121438
12518	Amp	9	32256071	32283646	0	0.109966
12519	Amp	9	32286016	32373515	0	0.099384
12520	Amp	9	32375434	32576685	0	0.051977
12521	Amp	9	32587546	32597017	0	0.06121
12522	Amp	9	32597873	32621417	0	0.066329
12523	Amp	9	32621867	32905901	0	0.075469
12524	Amp	9	32907805	32991331	0	0.069965
12525	Amp	9	32992665	33024068	0	0.0847
12526	Amp	9	33024105	33079765	0	0.075805
12527	Amp	9	33082971	33128431	0	0.080839
12528	Amp	9	33130642	33198440	0	0.100805
12529	Amp	9	33201280	33305033	0	0.107668
12530	Amp	9	33307676	33352748	0	0.100805
12531	Amp	9	33353214	33501635	0	0.108029
12532	Amp	9	33506303	33769475	0	0.100805
12533	Amp	9	33769590	33883806	0	0.112724
12534	Amp	9	33887406	33993282	0	0.108223
12535	Amp	9	34003108	34118062	0	0.096304
12536	Amp	9	34120722	34198060	0	0.109219
12537	Amp	9	34210874	34237482	0	0.112496
12538	Amp	9	34238127	34298012	0	0.100972
12539	Amp	9	34298145	34728033	0	0.109214
12540	Amp	9	34728942	34743063	0	0.116127
12541	Amp	9	34743401	34771537	0	0.126207
12542	Amp	9	34771774	34849058	0	0.115973
12543	Amp	9	34856175	35000923	0	0.125031
12544	Amp	9	35002134	35070180	0	0.129443
12545	Amp	9	35084369	35179447	0	0.111001
12546	Amp	9	35179930	35227687	0	0.115676
12547	Amp	9	35231050	35519362	0	0.123126
12548	Amp	9	35520930	35526073	0	0.114068
12549	Amp	9	35526245	35739490	0	0.117084
12550	Amp	9	35740233	35833451	0	0.129031
12551	Amp	9	35835867	35835867	0	0.139163
12552	Amp	9	35836262	35843006	0	0.143063
12553	Amp	9	35843083	35843083	0	0.149726
12554	Amp	9	35848191	35927179	0	0.145314
12555	Amp	9	35929442	36021359	0	0.150556
12556	Amp	9	36023249	36066388	0	0.159515
12557	Amp	9	36073436	36091260	0	0.17603
12558	Amp	9	36099402	36099625	0	0.170911
12559	Amp	9	36105216	36117681	0	0.177899
12560	Amp	9	36118419	36135427	0	0.164943

12561	Amp	9	36137372	36220137	0	0.174101
12562	Amp	9	36222540	36251861	0	0.157683
12563	Amp	9	36263827	36339769	0	0.167277
12564	Amp	9	36347534	36424443	0	0.159834
12565	Amp	9	36424618	36471215	0	0.145598
12566	Amp	9	36476956	36633901	0	0.151625
12567	Amp	9	36636088	36717505	0	0.143384
12568	Amp	9	36719567	36751650	0	0.14721
12569	Amp	9	36753064	36765048	0	0.135263
12570	Amp	9	36767917	36820607	0	0.14075
12571	Amp	9	36821954	36931904	0	0.131157
12572	Amp	9	36932322	36950304	0	0.120428
12573	Amp	9	36954847	36985146	0	0.127771
12574	Amp	9	36988854	36997899	0	0.123945
12575	Amp	9	36998419	37033162	0	0.114786
12576	Amp	9	37034620	37069839	0	0.112958
12577	Amp	9	37076000	37125914	0	0.102827
12578	Amp	9	37132118	37134120	0	0.092746
12579	Amp	9	37135183	37187002	0	0.098524
12580	Amp	9	37201429	37281827	0	0.138033
12581	Amp	9	37291076	37387221	0	0.135825
12582	Amp	9	37388874	37439354	0	0.12551
12583	Amp	9	37442360	37454877	0	0.13024
12584	Amp	9	37455158	37584996	0	0.142281
12585	Amp	9	37589438	37589885	0	0.100723
12586	Amp	9	37597598	37622780	0	0.105559
12587	Amp	9	37625872	37649203	0	0.12249
12588	Amp	9	37650176	37737062	0	0.119375
12589	Amp	9	37743252	37763827	0	0.113348
12590	Amp	9	37765493	37857282	0	0.103231
12591	Amp	9	37858864	37918948	0	0.0863
12592	Amp	9	37921690	38056963	0	0.088737
12593	Amp	9	38058427	38062430	0	0.094924
12594	Amp	9	38070923	38382343	0	0.087474
12595	Amp	9	38382472	38398829	0	0.086047
12596	Amp	9	38399689	38459826	0	0.088923
12597	Amp	9	38465781	38520414	0	0.099077
12598	Amp	9	38520462	38534725	0	0.09265
12599	Amp	9	38534968	38561296	0	0.1151
12600	Amp	9	38566839	38657641	0	0.104946
12601	Amp	9	38658964	38678743	0	0.112526
12602	Amp	9	38679725	38708589	0	0.104946
12603	Amp	9	38708762	38747691	0	0.101782
12604	Amp	9	38752071	38752071	0	0.091812
12605	Amp	9	68423153	68423153	0	0.028268
12606	Amp	9	68424128	68424128	0	0.02505
12607	Amp	9	68425024	68425024	0	0.033345
12608	Amp	9	68426962	68444455	0	0.030328
12609	Amp	9	68444973	68464477	0	0.027052
12610	Amp	9	68470315	69086258	0	0.037571
12611	Amp	9	69094199	69388669	0	0.049993
12612	Amp	9	69392142	69402044	0	0.054603
12613	Amp	9	69405647	69413658	0	0.057523

12614	Amp	9	69434688	69434688	0	0.053721
12615	Amp	9	69441481	69610011	0	0.050801
12616	Amp	9	69610405	69631124	0	0.042506
12617	Amp	9	69637086	69637353	0	0.030085
12618	Amp	9	69637580	69768236	0	0.039957
12619	Amp	9	69768795	69799236	0	0.030085
12620	Amp	9	69801997	69843118	0	0.040749
12621	Amp	9	69845255	70006874	0	0.030085
12622	Amp	9	70008012	71048720	0	0.019565
12623	Amp	9	71051448	71409229	0	0.030749
12624	Amp	9	71413463	71429946	0	0.040553
12625	Amp	9	71430080	71582509	0	0.030749
12626	Amp	9	71582736	71617727	0	0.019565
12627	Amp	9	71621308	71635909	0	0.022375
12628	Amp	9	71638399	71650619	0	0.028898
12629	Amp	9	71652431	71786207	0	0.022375
12630	Amp	9	71786906	71872329	0	0.032476
12631	Amp	9	71875849	72184347	0	0.029666
12632	Amp	9	72184628	72363190	0	0.034931
12633	Amp	9	72367723	72417522	0	0.044029
12634	Amp	9	72429675	72739414	0	0.034931
12635	Amp	9	72744196	72789683	0	0.029666
12636	Amp	9	72790943	73397513	0	0.019565
12637	Amp	9	73414583	73498167	0	0.033287
12638	Amp	9	73498326	73498326	0	0.023284
12639	Amp	9	73498983	73577946	0	0.039138
12640	Amp	9	73596240	73649307	0	0.009561
12641	Amp	9	73649402	73769423	0	0.048465
12642	Amp	9	73770606	74117647	0	0.043855
12643	Amp	9	74119995	74126827	0	0.058996
12644	Amp	9	74133299	74218795	0	0.043855
12645	Amp	9	74228266	74265398	0	0.004951
12646	Amp	9	74265878	74352841	0	0.011371
12647	Amp	9	74356235	75574851	0	0.004951
12648	Amp	9	75575607	75757991	0	0.018898
12649	Amp	9	75765867	75840888	0	0.004951
12650	Amp	9	75841375	76080410	0	0.013233
12651	Amp	9	76081972	76180663	0	0.017834
12652	Amp	9	76181269	76318958	0	0.009552
12653	Amp	9	76323127	76337513	0	0.01514
12654	Amp	9	76342655	76964330	0	0.009552
12655	Amp	9	76966402	77444584	0	0.004951
12656	Amp	9	77445050	77785984	0	0.012173
12657	Amp	9	77788310	78147507	0	0.018804
12658	Amp	9	78148308	78346818	0	0.022223
12659	Amp	9	78346948	78501967	0	0.025728
12660	Amp	9	78505504	78630246	0	0.019097
12661	Amp	9	78632737	78753820	0	0.050311
12662	Amp	9	78754225	78914291	0	0.044445
12663	Amp	9	78918628	79035465	0	0.041026
12664	Amp	9	79035503	79448364	0	0.047975
12665	Amp	9	79454757	79552554	0	0.054134
12666	Amp	9	79553555	79772350	0	0.047975

12667	Amp	9	79772533	79931367	0	0.054522
12668	Amp	9	79936173	80297925	0	0.022226
12669	Amp	9	80304115	80349208	0	0.034523
12670	Amp	9	80349396	80447984	0	0.027301
12671	Amp	9	80454025	80552297	0	0.015004
12672	Amp	9	80588138	80690300	0	0.027351
12673	Amp	9	80693663	80809748	0	0.022399
12674	Amp	9	80812987	81074363	0	0.010053
12675	Amp	9	81076992	81119565	0	0.018937
12676	Amp	9	81122805	81254415	0	0.010053
12677	Amp	9	81254500	81285590	0	0.015061
12678	Amp	9	81286153	81469861	0	0.020699
12679	Amp	9	81470539	81509836	0	0.027845
12680	Amp	9	81513735	81539770	0	0.022207
12681	Amp	9	81540571	81540609	0	0.027173
12682	Amp	9	81547433	81547895	0	0.040789
12683	Amp	9	81556217	81622027	0	0.034241
12684	Amp	9	81624945	81830499	0	0.029276
12685	Amp	9	81833619	81838873	0	0.01566
12686	Amp	9	81842283	81858559	0	0.017733
12687	Amp	9	81860265	82149886	0	0.01566
12688	Amp	9	82151012	82598626	0	0.010651
12689	Amp	9	82599899	82883567	0	0.017349
12690	Amp	9	82884156	82962249	0	0.026147
12691	Amp	9	82975905	83403561	0	0.017349
12692	Amp	9	83404253	83429623	0	0.06213
12693	Amp	9	83429905	83455711	0	0.017349
12694	Amp	9	83456309	83537079	0	0.032682
12695	Amp	9	83537442	84054748	0	0.017349
12696	Amp	9	84055000	84312332	0	0.020513
12697	Amp	9	84312898	84357171	0	0.027322
12698	Amp	9	84358585	84421258	0	0.034036
12699	Amp	9	84423959	84572357	0	0.027227
12700	Amp	9	84572785	84593504	0	0.017024
12701	Amp	9	84594578	84612114	0	0.027694
12702	Amp	9	84614308	84616831	0	0.033391
12703	Amp	9	84618346	84669347	0	0.02272
12704	Amp	9	84672493	84791253	0	0.016007
12705	Amp	9	84793437	85156927	0	0.012843
12706	Amp	9	85161528	86162820	0	0.007146
12707	Amp	9	86163617	87470526	0	0.014168
12708	Amp	9	87472813	87679659	0	0.017362
12709	Amp	9	87684570	88283112	0	0.014168
12710	Amp	9	88289463	88332241	0	0.029012
12711	Amp	9	88336678	88497358	0	0.014168
12712	Amp	9	88497482	88504897	0	0.017678
12713	Amp	9	88505218	88767599	0	0.028376
12714	Amp	9	88768308	90799332	0	0.033187
12715	Amp	9	90799413	90813684	0	0.073146
12716	Amp	9	90816217	90942644	0	0.08371
12717	Amp	9	90944720	91485216	0	0.08005
12718	Amp	9	91486800	91668570	0	0.034745
12719	Amp	9	91672062	91925645	0	0.024047

12720	Amp	9	91925936	92047269	0	0.035507
12721	Amp	9	92049794	92316840	0	0.025651
12722	Amp	9	92598089	92826179	0	0.067683
12723	Amp	9	92836434	92872684	0	0.02249
12724	Amp	9	92877862	92878080	0	0.015344
12725	Amp	9	92878099	93633519	0	0.024011
12726	Amp	9	93635068	94045115	0	0.019199
12727	Amp	9	94054984	95578038	0	0.010532
12728	Amp	9	95581498	96262126	0	0.007022
12729	Amp	9	96266955	96308884	0	0.013405
12730	Amp	9	96309961	96611431	0	0.007022
12731	Amp	9	96617299	96672405	0	0.045807
12732	Amp	9	96683523	97653558	0	0.007022
12733	Amp	9	97654617	97805852	0	0.008499
12734	Amp	9	97805988	97968027	0	0.014598
12735	Amp	9	97974581	98208025	0	0.017936
12736	Amp	9	98208288	98600579	0	0.011836
12737	Amp	9	98600666	98698808	0	0.008499
12738	Amp	9	98700464	98919703	0	0.022055
12739	Amp	9	98923680	100542170	0	0.008499
12740	Amp	9	100542559	100562112	0	0.016197
12741	Amp	9	100562230	101031609	0	0.008499
12742	Amp	9	101031965	101182114	0	0.009911
12743	Amp	9	101189330	101793034	0	0.008499
12744	Amp	9	101795995	102356339	0	0.012839
12745	Amp	9	102359752	102367712	0	0.035147
12746	Amp	9	102369109	103200948	0	0.012839
12747	Amp	9	103230685	103466709	0	0.018461
12748	Amp	9	103470182	104050851	0	0.020518
12749	Amp	9	104092264	104272405	0	0.014896
12750	Amp	9	104274462	104754092	0	0.012839
12751	Amp	9	104756792	107336926	0	0.010481
12752	Amp	9	107337276	107930948	0	0.012008
12753	Amp	9	107931411	108381380	0	0.010481
12754	Amp	9	108387309	108592638	0	0.014704
12755	Amp	9	108598346	108708250	0	0.022074
12756	Amp	9	108708747	108898228	0	0.014704
12757	Amp	9	108898645	108912480	0	0.021201
12758	Amp	9	108912612	108955783	0	0.014704
12759	Amp	9	108956741	109076983	0	0.019166
12760	Amp	9	109080474	109114543	0	0.012144
12761	Amp	9	109121404	109341927	0	0.007682
12762	Amp	9	109342264	109556662	0	0.010961
12763	Amp	9	109556896	109578951	0	0.017553
12764	Amp	9	109579332	109992785	0	0.021563
12765	Amp	9	109994265	110091849	0	0.024621
12766	Amp	9	110093409	110157008	0	0.034281
12767	Amp	9	110310150	110310327	0	0.027689
12768	Amp	9	110313825	110415555	0	0.031079
12769	Amp	9	110423242	110647450	0	0.037156
12770	Amp	9	110647559	110823360	0	0.048119
12771	Amp	9	110824013	111237237	0	0.037156
12772	Amp	9	111241186	111282653	0	0.047372

12773	Amp	9	111282679	111795858	0	0.045674
12774	Amp	9	111796841	111865875	0	0.041665
12775	Amp	9	111874090	111945114	0	0.064598
12776	Amp	9	111948481	112077728	0	0.070211
12777	Amp	9	112082519	112239394	0	0.064598
12778	Amp	9	112240341	112985113	0	0.073818
12779	Amp	9	112986913	113125832	0	0.08109
12780	Amp	9	113126761	113327518	0	0.074483
12781	Amp	9	113328594	113343031	0	0.080166
12782	Amp	9	113343592	113391819	0	0.08693
12783	Amp	9	113397143	113515108	0	0.081247
12784	Amp	9	113515683	113525021	0	0.074483
12785	Amp	9	113526193	113561474	0	0.063708
12786	Amp	9	113563219	113627173	0	0.075468
12787	Amp	9	113637544	113637544	0	0.09479
12788	Amp	9	113637746	113718817	0	0.096495
12789	Amp	9	113720737	113730219	0	0.103317
12790	Amp	9	113731758	113741686	0	0.091556
12791	Amp	9	113742769	113835269	0	0.099406
12792	Amp	9	113836432	114022007	0	0.094853
12793	Amp	9	114022245	114111033	0	0.098775
12794	Amp	9	114112075	114201781	0	0.091503
12795	Amp	9	114202046	114331901	0	0.088206
12796	Amp	9	114332833	114446998	0	0.084541
12797	Amp	9	114448038	115322421	0	0.052847
12798	Amp	9	115322649	115751202	0	0.056307
12799	Amp	9	115752861	115893314	0	0.064399
12800	Amp	9	115894793	116148250	0	0.061009
12801	Amp	9	116152341	116360301	0	0.074976
12802	Amp	9	116363353	116454146	0	0.070408
12803	Amp	9	116461509	116497662	0	0.076913
12804	Amp	9	116498271	116594784	0	0.073856
12805	Amp	9	116595630	116677942	0	0.079571
12806	Amp	9	116683132	116706256	0	0.092056
12807	Amp	9	116707662	116776544	0	0.079571
12808	Amp	9	116777252	116790602	0	0.084974
12809	Amp	9	116796481	116920494	0	0.079259
12810	Amp	9	116925551	116960428	0	0.065292
12811	Amp	9	116963772	116996835	0	0.0572
12812	Amp	9	116999456	117207243	0	0.050695
12813	Amp	9	117207802	117448218	0	0.062948
12814	Amp	9	117451360	117808117	0	0.082266
12815	Amp	9	117810184	118108680	0	0.070985
12816	Amp	9	118109066	118136403	0	0.068317
12817	Amp	9	118139267	118532682	0	0.052443
12818	Amp	9	118653963	118829532	0	0.042658
12819	Amp	9	118830616	118927132	0	0.03938
12820	Amp	9	118928465	119275899	0	0.041329
12821	Amp	9	119276216	119422024	0	0.04702
12822	Amp	9	119422163	119654921	0	0.041329
12823	Amp	9	119654943	120239480	0	0.042905
12824	Amp	9	120243081	120248068	0	0.046375
12825	Amp	9	120248934	120275798	0	0.058841

12826	Amp	9	120282917	120387213	0	0.085416
12827	Amp	9	120388175	120853245	0	0.070437
12828	Amp	9	120864255	120952705	0	0.058841
12829	Amp	9	120956640	121333429	0	0.075641
12830	Amp	9	121334411	121386639	0	0.074065
12831	Amp	9	121389545	121449551	0	0.072678
12832	Amp	9	121450785	121581314	0	0.069209
12833	Amp	9	121581421	121594079	0	0.064809
12834	Amp	9	121595191	121899229	0	0.067983
12835	Amp	9	121902365	121916332	0	0.078334
12836	Amp	9	121918088	122055770	0	0.067983
12837	Amp	9	122058947	122063308	0	0.079217
12838	Amp	9	122063443	122201121	0	0.089357
12839	Amp	9	122202919	122378417	0	0.093989
12840	Amp	9	122379154	122538517	0	0.092236
12841	Amp	9	122544123	123520101	0	0.089061
12842	Amp	9	123523367	123929248	0	0.114901
12843	Amp	9	123933491	124002355	0	0.118432
12844	Amp	9	124004953	124266672	0	0.108292
12845	Amp	9	124267457	124344864	0	0.114159
12846	Amp	9	124345867	124469682	0	0.108292
12847	Amp	9	124471544	124502213	0	0.120382
12848	Amp	9	124510146	124567145	0	0.108292
12849	Amp	9	124573639	124808848	0	0.094047
12850	Amp	9	124818725	124948118	0	0.106596
12851	Amp	9	124949139	125005925	0	0.125087
12852	Amp	9	125009099	125059499	0	0.130954
12853	Amp	9	125063624	125176207	0	0.141193
12854	Amp	9	125180920	125219962	0	0.12854
12855	Amp	9	125222203	125312132	0	0.13279
12856	Amp	9	125312528	125537326	0	0.135866
12857	Amp	9	125549734	125647117	0	0.13279
12858	Amp	9	125647302	125930216	0	0.126923
12859	Amp	9	125932901	126404438	0	0.120193
12860	Amp	9	126404635	126622646	0	0.128286
12861	Amp	9	126623548	126709618	0	0.132776
12862	Amp	9	126711023	127227714	0	0.128286
12863	Amp	9	127241456	127284029	0	0.140404
12864	Amp	9	127284437	127495373	0	0.137022
12865	Amp	9	127497822	127563119	0	0.126783
12866	Amp	9	127565266	127684214	0	0.122072
12867	Amp	9	127693412	127772785	0	0.107356
12868	Amp	9	127775139	127851031	0	0.105879
12869	Amp	9	127865753	128026508	0	0.09564
12870	Amp	9	128028815	128071066	0	0.101999
12871	Amp	9	128076187	128147050	0	0.085605
12872	Amp	9	128149375	128519987	0	0.079247
12873	Amp	9	128525602	128558639	0	0.091515
12874	Amp	9	128563519	128659184	0	0.079247
12875	Amp	9	128661698	128775576	0	0.064819
12876	Amp	9	128777945	129687250	0	0.053586
12877	Amp	9	129687686	129703433	0	0.057838
12878	Amp	9	129704402	130169790	0	0.055856

12879	Amp	9	130174499	130187585	0	0.059028
12880	Amp	9	130194935	130380480	0	0.040538
12881	Amp	9	130387434	130388363	0	0.049402
12882	Amp	9	130388709	130496996	0	0.044769
12883	Amp	9	130499199	130736306	0	0.041597
12884	Amp	9	130736352	130798561	0	0.044337
12885	Amp	9	130799756	130890485	0	0.041597
12886	Amp	9	130890621	131029050	0	0.046157
12887	Amp	9	131034924	131094684	0	0.059073
12888	Amp	9	131110453	131350185	0	0.054513
12889	Amp	9	131353084	131582991	0	0.058896
12890	Amp	9	131588395	131592367	0	0.054974
12891	Amp	9	131592562	131916533	0	0.050591
12892	Amp	9	131916573	132037227	0	0.056316
12893	Amp	9	132037291	132407549	0	0.053641
12894	Amp	9	132412874	132569709	0	0.050591
12895	Amp	9	132587246	132853826	0	0.0533
12896	Amp	9	132860263	132879747	0	0.063945
12897	Amp	9	132880178	133059798	0	0.061996
12898	Amp	9	133090275	133234843	0	0.051351
12899	Amp	9	133237183	133320784	0	0.060061
12900	Amp	9	133321704	133338901	0	0.056012
12901	Amp	9	133345211	133345211	0	0.051351
12902	Amp	9	133345692	133425375	0	0.054185
12903	Amp	9	133425943	133451244	0	0.060383
12904	Amp	9	133456044	133538411	0	0.073619
12905	Amp	9	133542347	133582688	0	0.081974
12906	Amp	9	133593282	133610214	0	0.070786
12907	Amp	9	133610759	133639458	0	0.05755
12908	Amp	9	133640522	134071515	0	0.062091
12909	Amp	9	134077945	134079343	0	0.065835
12910	Amp	9	134081211	134212949	0	0.059636
12911	Amp	9	134215550	134480000	0	0.069074
12912	Amp	9	134482834	134548982	0	0.075901
12913	Amp	9	134550852	134699359	0	0.080054
12914	Amp	9	134702648	134800936	0	0.073227
12915	Amp	9	134806861	134830798	0	0.078041
12916	Amp	9	134839110	134848359	0	0.068604
12917	Amp	9	134853364	134865479	0	0.066417
12918	Amp	9	134878871	135174246	0	0.07291
12919	Amp	9	135176999	135248141	0	0.066417
12920	Amp	9	135418642	135545928	0	0.109562
12921	Amp	9	135560751	135607058	0	0.093516
12922	Amp	9	135611602	135616985	0	0.075075
12923	Amp	9	135626923	135684056	0	0.07272
12924	Amp	9	135684616	135729601	0	0.084088
12925	Amp	9	135740145	135787340	0	0.090462
12926	Amp	9	135792823	135913852	0	0.081902
12927	Amp	9	135915320	136374886	0	0.084103
12928	Amp	9	136376424	136482147	0	0.086678
12929	Amp	9	136487473	136715446	0	0.080304
12930	Amp	9	136720710	136779743	0	0.085975
12931	Amp	9	136780050	137156786	0	0.090416

12932 Amp	9	137190033	137325416	0	0.085975
12933 Amp	9	137333703	137496163	0	0.095459
12934 Amp	9	137563956	137572613	0	0.085975
12935 Amp	9	137576245	137599242	0	0.080304
12936 Amp	9	137616769	137633679	0	0.078103
12937 Amp	9	137638926	137709106	0	0.075295
12938 Amp	9	137716984	137814427	0	0.077194
12939 Amp	9	137816151	138044505	0	0.068536
12940 Amp	10	366509	397134	0	0.039733
12941 Amp	10	400331	706253	0	0.042681
12942 Amp	10	708888	869606	0	0.040864
12943 Amp	10	870640	891107	0	0.051803
12944 Amp	10	891136	985755	0	0.061635
12945 Amp	10	985900	1652555	0	0.063895
12946 Amp	10	1653099	1776305	0	0.061635
12947 Amp	10	1778660	1881273	0	0.053761
12948 Amp	10	1881306	2331101	0	0.049883
12949 Amp	10	2332696	2616561	0	0.043958
12950 Amp	10	2618354	2645902	0	0.0466
12951 Amp	10	3094525	3096481	0	0.05198
12952 Amp	10	3098269	3212406	0	0.056782
12953 Amp	10	3213602	3269880	0	0.051401
12954 Amp	10	3270953	3687912	0	0.04876
12955 Amp	10	3690783	3732043	0	0.062144
12956 Amp	10	3732587	3745508	0	0.112413
12957 Amp	10	3745769	3753179	0	0.107612
12958 Amp	10	3754377	3829332	0	0.112948
12959 Amp	10	3830684	4472405	0	0.107612
12960 Amp	10	4473445	4497427	0	0.117994
12961 Amp	10	4498719	4614787	0	0.124053
12962 Amp	10	4616390	4981269	0	0.11367
12963 Amp	10	4982202	5013134	0	0.107262
12964 Amp	10	5014375	5049525	0	0.10865
12965 Amp	10	5055280	5109118	0	0.095266
12966 Amp	10	5109625	5146970	0	0.044997
12967 Amp	10	5153642	5184755	0	0.046463
12968 Amp	10	5185419	5204122	0	0.048936
12969 Amp	10	5211563	5470313	0	0.061208
12970 Amp	10	5475688	5564504	0	0.071156
12971 Amp	10	5564686	5678549	0	0.075688
12972 Amp	10	5680348	5724214	0	0.125957
12973 Amp	10	5727490	5759885	0	0.075688
12974 Amp	10	5759901	5780707	0	0.074161
12975 Amp	10	5780803	6010245	0	0.068103
12976 Amp	10	6018470	6116746	0	0.075995
12977 Amp	10	6119019	6308627	0	0.068103
12978 Amp	10	6309083	6376792	0	0.065629
12979 Amp	10	6377317	6454474	0	0.062883
12980 Amp	10	6454587	6956616	0	0.061417
12981 Amp	10	6957915	7056457	0	0.063307
12982 Amp	10	7063546	7156172	0	0.058775
12983 Amp	10	7157092	7177495	0	0.056373
12984 Amp	10	7177663	7350874	0	0.060196

12985	Amp	10	7350902	7468292	0	0.065772
12986	Amp	10	7472621	7558333	0	0.090967
12987	Amp	10	7567305	7862424	0	0.065772
12988	Amp	10	7866460	7866460	0	0.060144
12989	Amp	10	7866869	8068061	0	0.062154
12990	Amp	10	8068975	8114532	0	0.066516
12991	Amp	10	8114594	8184483	0	0.068271
12992	Amp	10	8190019	8326290	0	0.118154
12993	Amp	10	8331133	8407364	0	0.068271
12994	Amp	10	8407492	8407492	0	0.069794
12995	Amp	10	8410099	8444198	0	0.067904
12996	Amp	10	8444481	8459620	0	0.070226
12997	Amp	10	8459858	8493210	0	0.066403
12998	Amp	10	8494246	8496529	0	0.068017
12999	Amp	10	8497042	8531648	0	0.084771
13000	Amp	10	8532111	8628433	0	0.086647
13001	Amp	10	8629772	8629772	0	0.074375
13002	Amp	10	8633442	8685505	0	0.080468
13003	Amp	10	8686456	8701936	0	0.094053
13004	Amp	10	8702058	8725864	0	0.096217
13005	Amp	10	8725982	8760949	0	0.101627
13006	Amp	10	8760957	8866608	0	0.105384
13007	Amp	10	8867961	8918380	0	0.11067
13008	Amp	10	8918443	8936096	0	0.132836
13009	Amp	10	8937098	8937098	0	0.149625
13010	Amp	10	8937497	8978271	0	0.155713
13011	Amp	10	8979301	8981662	0	0.177129
13012	Amp	10	8983075	8985343	0	0.181809
13013	Amp	10	8985840	8996171	0	0.184856
13014	Amp	10	8999871	9041857	0	0.178763
13015	Amp	10	9043340	9051887	0	0.187799
13016	Amp	10	9056421	9066580	0.13565	0.204402
13017	Amp	10	9066595	9095411	0.250263	0.215838
13018	Amp	10	9095583	9098982	0.111991	0.201883
13019	Amp	10	9101913	9102286	0.012341	0.1898
13020	Amp	10	9102582	9149186	0	0.182347
13021	Amp	10	9152371	9161005	0	0.170911
13022	Amp	10	9162202	9175275	0	0.164676
13023	Amp	10	9179033	9198313	0	0.160918
13024	Amp	10	9198767	9222861	0	0.165935
13025	Amp	10	9224331	9224928	0	0.15761
13026	Amp	10	9228111	9259460	0	0.143594
13027	Amp	10	9259724	9276666	0	0.138577
13028	Amp	10	9277094	9305839	0	0.125486
13029	Amp	10	9306510	9311814	0	0.119398
13030	Amp	10	9313321	9327620	0	0.10903
13031	Amp	10	9330184	9346131	0	0.114874
13032	Amp	10	9346283	9384832	0	0.109588
13033	Amp	10	9385258	9475092	0	0.107712
13034	Amp	10	9475175	9518161	0	0.106324
13035	Amp	10	9519142	9825945	0	0.103376
13036	Amp	10	9828717	9852939	0	0.099014
13037	Amp	10	9853587	9868379	0	0.09726

13038	Amp	10	9868472	9868531	0	0.095645
13039	Amp	10	9869005	9913694	0	0.093481
13040	Amp	10	9913934	9933745	0	0.087637
13041	Amp	10	9933780	9938964	0	0.085627
13042	Amp	10	9939247	9953904	0	0.083667
13043	Amp	10	9954791	10142239	0	0.081345
13044	Amp	10	10144619	10246583	0	0.099668
13045	Amp	10	10254156	10284416	0	0.104238
13046	Amp	10	10286666	10292222	0	0.090096
13047	Amp	10	10292609	10330254	0	0.092403
13048	Amp	10	10330319	10388312	0	0.09088
13049	Amp	10	10389572	10544459	0	0.089234
13050	Amp	10	10544670	10586572	0	0.085497
13051	Amp	10	10586721	10638155	0	0.08387
13052	Amp	10	10641051	10845382	0	0.094716
13053	Amp	10	10845494	10855739	0	0.096173
13054	Amp	10	10859230	11007676	0	0.091549
13055	Amp	10	11008014	11223199	0	0.085658
13056	Amp	10	11223393	11516292	0	0.087063
13057	Amp	10	11516824	11538355	0	0.090948
13058	Amp	10	11548983	11548983	0	0.095458
13059	Amp	10	11553255	11600480	0	0.098096
13060	Amp	10	11604983	11632967	0	0.142637
13061	Amp	10	11640356	11647872	0	0.148112
13062	Amp	10	11655593	11702030	0	0.145786
13063	Amp	10	11706059	11758230	0	0.140311
13064	Amp	10	11758519	12216706	0	0.141908
13065	Amp	10	12217020	12281246	0	0.140311
13066	Amp	10	12281465	12819946	0	0.138003
13067	Amp	10	12820337	12834247	0	0.13956
13068	Amp	10	12834792	12836938	0	0.14451
13069	Amp	10	12851545	12965745	0	0.149433
13070	Amp	10	12965881	13116364	0	0.14451
13071	Amp	10	13116412	13135644	0	0.142814
13072	Amp	10	13137444	13150397	0	0.14474
13073	Amp	10	13152398	13390194	0	0.147802
13074	Amp	10	13390745	13495204	0	0.152453
13075	Amp	10	13498537	13514487	0	0.161617
13076	Amp	10	13515945	14121649	0	0.157732
13077	Amp	10	14124990	14148068	0	0.15308
13078	Amp	10	14151684	14182208	0	0.141455
13079	Amp	10	14183270	14190684	0	0.096915
13080	Amp	10	14192353	14263454	0	0.095211
13081	Amp	10	14263545	14336251	0	0.099186
13082	Amp	10	14336253	14481612	0	0.091599
13083	Amp	10	14482698	14531108	0	0.100921
13084	Amp	10	14532785	14556958	0	0.09712
13085	Amp	10	14568102	14579397	0	0.109715
13086	Amp	10	14585847	14598747	0	0.107869
13087	Amp	10	14599360	14640481	0	0.10557
13088	Amp	10	14645774	14694367	0	0.094724
13089	Amp	10	14694680	14797072	0	0.091238
13090	Amp	10	14800948	14900233	0	0.081291

13091	Amp	10	14901290	15067262	0	0.071336
13092	Amp	10	15069619	15096616	0	0.065972
13093	Amp	10	15107981	15197499	0	0.075678
13094	Amp	10	15199323	15244691	0	0.065972
13095	Amp	10	15245494	15264348	0	0.067945
13096	Amp	10	15264521	15296919	0	0.070757
13097	Amp	10	15299220	15571155	0	0.068752
13098	Amp	10	15573303	15768811	0	0.086819
13099	Amp	10	15772218	16222521	0	0.082299
13100	Amp	10	16225248	16416637	0	0.093208
13101	Amp	10	16436393	16734667	0	0.097461
13102	Amp	10	16734806	16870623	0	0.103007
13103	Amp	10	16870819	16886590	0	0.098222
13104	Amp	10	16886617	17441248	0	0.109881
13105	Amp	10	17443588	17495820	0	0.096086
13106	Amp	10	17496890	17682255	0	0.078881
13107	Amp	10	17682435	17686181	0	0.081009
13108	Amp	10	17686307	17735537	0	0.072884
13109	Amp	10	17953706	18017201	0	0.053848
13110	Amp	10	18018088	18172675	0	0.049595
13111	Amp	10	18173373	18191019	0	0.045621
13112	Amp	10	18191298	18192794	0	0.043686
13113	Amp	10	18193762	18210747	0	0.054106
13114	Amp	10	18213820	18257695	0	0.043686
13115	Amp	10	18259830	18299802	0	0.050112
13116	Amp	10	18301290	18376754	0	0.039855
13117	Amp	10	18379994	18456652	0	0.04373
13118	Amp	10	18458157	18458681	0	0.056535
13119	Amp	10	18460416	18471548	0	0.073935
13120	Amp	10	18473148	18596811	0	0.04373
13121	Amp	10	18596923	18647401	0	0.039855
13122	Amp	10	18650329	18655757	0	0.045125
13123	Amp	10	18655986	18800629	0	0.040913
13124	Amp	10	18803713	18890461	0	0.045354
13125	Amp	10	18895125	18900984	0	0.040083
13126	Amp	10	18901023	18933947	0	0.035643
13127	Amp	10	18934183	18945795	0	0.037816
13128	Amp	10	18945817	19081504	0	0.041253
13129	Amp	10	19083842	19134657	0	0.043384
13130	Amp	10	19135679	19173452	0	0.05269
13131	Amp	10	19177386	19211208	0	0.043384
13132	Amp	10	19211223	19289666	0	0.048289
13133	Amp	10	19294739	19382207	0	0.054315
13134	Amp	10	19389357	19404627	0	0.052142
13135	Amp	10	19404803	19536253	0	0.050011
13136	Amp	10	19536443	19676510	0	0.045106
13137	Amp	10	19681959	19787685	0	0.095629
13138	Amp	10	19791246	19987165	0	0.045106
13139	Amp	10	19987233	20105850	0	0.029924
13140	Amp	10	20106757	20178301	0	0.033799
13141	Amp	10	20178500	20267367	0	0.027774
13142	Amp	10	20268166	20478133	0	0.023593
13143	Amp	10	20478227	20489122	0	0.028792

13144	Amp	10	20489512	20907968	0	0.023593
13145	Amp	10	20909451	21030903	0	0.027672
13146	Amp	10	21031644	21459672	0	0.025543
13147	Amp	10	21462682	21525698	0	0.021464
13148	Amp	10	21536480	21605738	0	0.027221
13149	Amp	10	21607804	21657079	0	0.034418
13150	Amp	10	21662059	21709067	0	0.044267
13151	Amp	10	21709637	21813355	0	0.04604
13152	Amp	10	21818240	21939701	0	0.049219
13153	Amp	10	21945664	21970916	0	0.059199
13154	Amp	10	21972492	21981104	0	0.063638
13155	Amp	10	21981316	22015309	0	0.059844
13156	Amp	10	22015650	22154517	0	0.061816
13157	Amp	10	22155041	22226913	0	0.062089
13158	Amp	10	22244615	22342624	0	0.052109
13159	Amp	10	22346588	22648353	0	0.04893
13160	Amp	10	22648831	22760440	0	0.045493
13161	Amp	10	22763343	22785490	0	0.061594
13162	Amp	10	22798369	22829140	0	0.056882
13163	Amp	10	22831374	23047735	0	0.040781
13164	Amp	10	23050117	23207518	0	0.046334
13165	Amp	10	23209219	23257399	0	0.04044
13166	Amp	10	23260198	23580261	0	0.032182
13167	Amp	10	23580604	23941295	0	0.039588
13168	Amp	10	23941311	24083376	0	0.035601
13169	Amp	10	24096443	24201354	0	0.039411
13170	Amp	10	24203963	24216447	0	0.037439
13171	Amp	10	24217679	24298000	0	0.041355
13172	Amp	10	24299314	24610685	0	0.037545
13173	Amp	10	24612171	24978174	0	0.02105
13174	Amp	10	24988643	25023378	0	0.022492
13175	Amp	10	25025674	25211687	0	0.018617
13176	Amp	10	25215074	25215074	0	0.008768
13177	Amp	10	25219108	25267051	0	0.010653
13178	Amp	10	25267105	25326678	0	0.0051
13179	Amp	10	25332029	25840964	0	0.003658
13180	Amp	10	25841483	26072757	0	0.006299
13181	Amp	10	26074506	26585598	0	0.003658
13182	Amp	10	26586137	26870939	0	0.007793
13183	Amp	10	26871061	26926580	0	0.005909
13184	Amp	10	26926718	26974327	0	0.0124
13185	Amp	10	26976868	26998089	0	0.017275
13186	Amp	10	26998836	27030536	0	0.024906
13187	Amp	10	27032043	27101749	0	0.0124
13188	Amp	10	27103593	27423487	0	0.010626
13189	Amp	10	27429412	27526427	0	0.013411
13190	Amp	10	27531281	27791144	0	0.017004
13191	Amp	10	27793604	27937896	0	0.022435
13192	Amp	10	27939936	28128899	0	0.026571
13193	Amp	10	28131891	28246896	0	0.022435
13194	Amp	10	28247207	28354464	0	0.024268
13195	Amp	10	28357689	28446299	0	0.022435
13196	Amp	10	28450248	28579026	0	0.029337

13197	Amp	10	28581226	28640168	0	0.025202
13198	Amp	10	28640789	28674135	0	0.029466
13199	Amp	10	28674854	28764209	0	0.031768
13200	Amp	10	28765241	28985810	0	0.038124
13201	Amp	10	28986099	29179507	0	0.031768
13202	Amp	10	29181507	29273919	0	0.038913
13203	Amp	10	29277054	29977812	0	0.031768
13204	Amp	10	29977992	30100897	0	0.036755
13205	Amp	10	30100907	30105838	0	0.031768
13206	Amp	10	30107001	30220255	0	0.028983
13207	Amp	10	30222087	30255059	0	0.023552
13208	Amp	10	30259082	30288522	0	0.031788
13209	Amp	10	30290282	30387930	0	0.023833
13210	Amp	10	30392213	30554479	0	0.021531
13211	Amp	10	30555966	30603146	0	0.072054
13212	Amp	10	30604949	30656541	0	0.077152
13213	Amp	10	30661115	30781282	0	0.026629
13214	Amp	10	30788467	30857686	0	0.023037
13215	Amp	10	30857778	31063264	0	0.054952
13216	Amp	10	31064132	31070049	0	0.105475
13217	Amp	10	31072009	31135424	0	0.090327
13218	Amp	10	31135515	31149715	0	0.09459
13219	Amp	10	31153081	31248398	0	0.044067
13220	Amp	10	31248661	31459273	0	0.047334
13221	Amp	10	31465987	31468102	0	0.097857
13222	Amp	10	31477616	31959616	0	0.102596
13223	Amp	10	31961352	31983063	0	0.097498
13224	Amp	10	31984499	31991785	0	0.106339
13225	Amp	10	31996240	32301014	0	0.055817
13226	Amp	10	32301648	32578186	0	0.051553
13227	Amp	10	32578836	32691669	0	0.063319
13228	Amp	10	32692433	32692635	0	0.051553
13229	Amp	10	32693309	32868741	0	0.058464
13230	Amp	10	32879254	32946279	0	0.063547
13231	Amp	10	32950263	33151251	0	0.064961
13232	Amp	10	33151852	33164784	0	0.059878
13233	Amp	10	33165787	33187213	0	0.064247
13234	Amp	10	33194169	33295203	0	0.059702
13235	Amp	10	33295866	33355614	0	0.069055
13236	Amp	10	33359331	33400994	0	0.064686
13237	Amp	10	33401469	33413172	0	0.055333
13238	Amp	10	33415620	33435210	0	0.068512
13239	Amp	10	33438309	33562228	0	0.056962
13240	Amp	10	33562249	33704269	0	0.062083
13241	Amp	10	33709645	33756116	0	0.057188
13242	Amp	10	33757835	33808252	0	0.043596
13243	Amp	10	33808690	33886257	0	0.038856
13244	Amp	10	33887318	34199964	0	0.032767
13245	Amp	10	34200976	34241837	0	0.027646
13246	Amp	10	34245282	34246093	0	0.034342
13247	Amp	10	34247486	35157050	0	0.02744
13248	Amp	10	35160751	35761374	0	0.02398
13249	Amp	10	35764061	35772626	0	0.039789

13250	Amp	10	35775304	36124134	0	0.02398
13251	Amp	10	36127396	36740206	0	0.032046
13252	Amp	10	36741148	37053848	0	0.04314
13253	Amp	10	37054229	37095776	0	0.046385
13254	Amp	10	37097849	37107592	0	0.043148
13255	Amp	10	37113848	37123421	0	0.050711
13256	Amp	10	37123566	37127962	0	0.039616
13257	Amp	10	37128043	37270750	0	0.047093
13258	Amp	10	37273915	37273915	0	0.041421
13259	Amp	10	37276059	37326699	0	0.038177
13260	Amp	10	37327375	37359998	0	0.036286
13261	Amp	10	37360256	37398692	0	0.038321
13262	Amp	10	37401728	37492619	0	0.02843
13263	Amp	10	37497327	37517117	0	0.046399
13264	Amp	10	37519189	37564494	0	0.02843
13265	Amp	10	37570681	37668730	0	0.029908
13266	Amp	10	37668745	37976858	0	0.02843
13267	Amp	10	37977423	37994192	0	0.027015
13268	Amp	10	38001645	38297980	0	0.028459
13269	Amp	10	38301077	38656421	0	0.020394
13270	Amp	10	38658070	38770869	0	0.018949
13271	Amp	10	38770921	38770921	0	0.048434
13272	Amp	10	42450960	42450960	0	0.148309
13273	Amp	10	42452522	42452522	0	0.151127
13274	Amp	10	42457632	42457632	0	0.160567
13275	Amp	10	42457683	42457683	0	0.166479
13276	Amp	10	42459603	42472992	0	0.172231
13277	Amp	10	42473252	42505034	0	0.176432
13278	Amp	10	42505362	42661690	0	0.181458
13279	Amp	10	42667646	42869858	0	0.182882
13280	Amp	10	42875902	42902746	0	0.132359
13281	Amp	10	42904353	42923646	0	0.126446
13282	Amp	10	42923818	42965777	0	0.122246
13283	Amp	10	42968596	43007809	0	0.127332
13284	Amp	10	43010596	43062355	0	0.076809
13285	Amp	10	43066597	43196623	0	0.075385
13286	Amp	10	43198648	43199601	0	0.089221
13287	Amp	10	43200036	43256590	0	0.085289
13288	Amp	10	43258084	43309500	0	0.071453
13289	Amp	10	43310988	43715152	0	0.068635
13290	Amp	10	43726843	43772132	0	0.086219
13291	Amp	10	43772162	44005675	0	0.090934
13292	Amp	10	44006110	44071234	0	0.084051
13293	Amp	10	44076729	44264083	0	0.079025
13294	Amp	10	44274500	44394277	0	0.06854
13295	Amp	10	44398416	44405721	0	0.062788
13296	Amp	10	44409770	44434874	0	0.069501
13297	Amp	10	44437874	44541866	0	0.075554
13298	Amp	10	44544531	44544531	0	0.084116
13299	Amp	10	44544822	44582722	0	0.090576
13300	Amp	10	44583495	44608280	0	0.084116
13301	Amp	10	44608967	44662493	0	0.087672
13302	Amp	10	44664977	44670301	0	0.082957

13303	Amp	10	44671282	44878509	0	0.087106
13304	Amp	10	44879049	44896272	0	0.099096
13305	Amp	10	44896900	44920670	0	0.107794
13306	Amp	10	44923885	44934705	0	0.099096
13307	Amp	10	44935014	44946855	0	0.094948
13308	Amp	10	44946866	44957820	0	0.104891
13309	Amp	10	44960515	45035087	0	0.111462
13310	Amp	10	45037990	45163779	0	0.099472
13311	Amp	10	45165952	45239692	0	0.088166
13312	Amp	10	45240001	45349943	0	0.09291
13313	Amp	10	45353397	45373441	0	0.082967
13314	Amp	10	45379542	46041001	0	0.07502
13315	Amp	10	47084490	47198512	0	0.062576
13316	Amp	10	47201063	47231578	0	0.069913
13317	Amp	10	47233219	47256786	0	0.058734
13318	Amp	10	47258326	47304942	0	0.051398
13319	Amp	10	47305627	47327025	0	0.040886
13320	Amp	10	48188464	48261616	0	0.043102
13321	Amp	10	48265803	48532181	0	0.047135
13322	Amp	10	48533090	48594823	0	0.040547
13323	Amp	10	48597323	48686457	0	0.050347
13324	Amp	10	48687496	48731272	0	0.088606
13325	Amp	10	48736204	48841188	0	0.057922
13326	Amp	10	48846529	48949548	0	0.050347
13327	Amp	10	48950927	48971156	0	0.048131
13328	Amp	10	48971821	49042687	0	0.038331
13329	Amp	10	49046455	49434447	0	0.046371
13330	Amp	10	49434540	49467258	0	0.05017
13331	Amp	10	49467878	49524234	0	0.044662
13332	Amp	10	49528473	49863777	0	0.070234
13333	Amp	10	49868126	49868126	0	0.057107
13334	Amp	10	50716389	50731879	0	0.043765
13335	Amp	10	50732087	50910142	0	0.039966
13336	Amp	10	50910179	51177368	0	0.04794
13337	Amp	10	51178106	51288791	0	0.040209
13338	Amp	10	51290084	51800775	0	0.046058
13339	Amp	10	51801138	51888167	0	0.030924
13340	Amp	10	51888254	51895368	0	0.038004
13341	Amp	10	51897888	52073911	0	0.032155
13342	Amp	10	52074092	52656654	0	0.025075
13343	Amp	10	52656765	52690891	0	0.029093
13344	Amp	10	52692359	52782251	0	0.038804
13345	Amp	10	52783017	52857039	0	0.055041
13346	Amp	10	52857298	52926850	0	0.051023
13347	Amp	10	52927071	53106404	0	0.054201
13348	Amp	10	53110950	53137056	0	0.04449
13349	Amp	10	53137263	53419659	0	0.049358
13350	Amp	10	53420768	53437111	0	0.04449
13351	Amp	10	53439470	54101357	0	0.056057
13352	Amp	10	54106148	54159375	0	0.058102
13353	Amp	10	54162222	54171417	0	0.056057
13354	Amp	10	54174915	54368754	0	0.0608
13355	Amp	10	54370707	54618138	0	0.111323

13356	Amp	10	54618213	54685841	0	0.101822
13357	Amp	10	54713613	54725483	0	0.0608
13358	Amp	10	54730249	55148139	0	0.056057
13359	Amp	10	55579314	55579314	0	0.061429
13360	Amp	10	55581565	55587271	0	0.087432
13361	Amp	10	55617034	55633728	0	0.099051
13362	Amp	10	55641281	55641989	0	0.087432
13363	Amp	10	55642547	55653768	0	0.083399
13364	Amp	10	55657201	55666777	0	0.078027
13365	Amp	10	55668142	56176359	0	0.083295
13366	Amp	10	56176534	56501911	0	0.088542
13367	Amp	10	56504664	56515343	0	0.09069
13368	Amp	10	56516784	56522124	0	0.056503
13369	Amp	10	56522407	56568759	0	0.060968
13370	Amp	10	56569449	56581386	0	0.095156
13371	Amp	10	56583464	56786944	0	0.093008
13372	Amp	10	56789822	56792373	0	0.04759
13373	Amp	10	56792440	56903250	0	0.042488
13374	Amp	10	56904387	56972972	0	0.054704
13375	Amp	10	56974102	57231186	0	0.059267
13376	Amp	10	57235534	57292087	0	0.083468
13377	Amp	10	57292649	57348857	0	0.042553
13378	Amp	10	57351058	57733717	0	0.047186
13379	Amp	10	57735732	57863851	0	0.042468
13380	Amp	10	57864397	57883971	0	0.047099
13381	Amp	10	57887645	57961039	0	0.042537
13382	Amp	10	57964350	58003886	0	0.044126
13383	Amp	10	58004843	58080923	0	0.048222
13384	Amp	10	58081733	58104788	0	0.053653
13385	Amp	10	58105202	58117453	0	0.045373
13386	Amp	10	58119659	58174360	0	0.041573
13387	Amp	10	58175346	58239455	0	0.039984
13388	Amp	10	58239562	58350440	0	0.036453
13389	Amp	10	58350575	58479576	0	0.041363
13390	Amp	10	58482705	58663435	0	0.043307
13391	Amp	10	58667796	58896649	0	0.046636
13392	Amp	10	58897741	59292766	0	0.052219
13393	Amp	10	59293588	59388894	0	0.04254
13394	Amp	10	59388992	59663408	0	0.037631
13395	Amp	10	59663910	59677705	0	0.042479
13396	Amp	10	59680366	59764986	0	0.085919
13397	Amp	10	59765333	59903879	0	0.075404
13398	Amp	10	59907508	60088144	0	0.087018
13399	Amp	10	60092348	60092348	0	0.097728
13400	Amp	10	60093839	60138688	0	0.148251
13401	Amp	10	60139575	60139575	0	0.152496
13402	Amp	10	60142035	60142442	0.086298	0.198564
13403	Amp	10	60143412	60170514	0.571214	0.244891
13404	Amp	10	60170641	60185853	0.619816	0.249245
13405	Amp	10	60185885	60197154	0.500055	0.238535
13406	Amp	10	60197410	60244409	0.437983	0.233478
13407	Amp	10	60245443	60245443	0.421967	0.231534
13408	Amp	10	60248423	60311139	0	0.185206

13409	Amp	10	60315881	60352694	0	0.187126
13410	Amp	10	60353653	60483474	0	0.185206
13411	Amp	10	60487572	60511733	0	0.186859
13412	Amp	10	60514172	60529442	0.012341	0.189715
13413	Amp	10	60532054	60832567	0	0.188063
13414	Amp	10	60836304	61200580	0.038273	0.193119
13415	Amp	10	61203650	61439030	0.105051	0.200963
13416	Amp	10	61441808	61500312	0.038273	0.193119
13417	Amp	10	61503536	61664685	0	0.170037
13418	Amp	10	61664701	61787883	0	0.177004
13419	Amp	10	61787960	61808465	0	0.17508
13420	Amp	10	61808501	61950345	0	0.182501
13421	Amp	10	61950531	62073104	0.012341	0.189789
13422	Amp	10	62074163	62090031	0	0.187787
13423	Amp	10	62090524	62292255	0	0.183648
13424	Amp	10	62297701	62390496	0	0.174245
13425	Amp	10	62396572	62762651	0	0.123722
13426	Amp	10	62762738	62877581	0	0.133304
13427	Amp	10	62878229	62918440	0	0.139419
13428	Amp	10	62918776	63262557	0	0.133304
13429	Amp	10	63269914	63435707	0	0.128413
13430	Amp	10	63439434	63468716	0	0.136889
13431	Amp	10	63470433	63530074	0	0.093448
13432	Amp	10	63535865	63541565	0	0.098031
13433	Amp	10	63541729	63591774	0	0.102005
13434	Amp	10	63593913	63674293	0	0.128885
13435	Amp	10	63675049	63675049	0	0.124302
13436	Amp	10	63675408	63809664	0	0.099435
13437	Amp	10	63816262	63866801	0	0.096153
13438	Amp	10	63869406	64038383	0	0.098249
13439	Amp	10	64038572	64149566	0	0.101924
13440	Amp	10	64151533	64909837	0	0.062595
13441	Amp	10	64909892	65237077	0	0.059266
13442	Amp	10	65239960	65309685	0	0.064884
13443	Amp	10	65310176	65477793	0	0.051626
13444	Amp	10	65478197	65672947	0	0.044913
13445	Amp	10	65672982	65952808	0	0.050812
13446	Amp	10	65953676	66005618	0	0.047526
13447	Amp	10	66006713	66016734	0	0.075107
13448	Amp	10	66020440	66132278	0	0.047526
13449	Amp	10	66134663	66254543	0	0.091944
13450	Amp	10	66256876	66789964	0	0.086326
13451	Amp	10	66791662	66900688	0	0.034056
13452	Amp	10	66905627	66943071	0	0.039588
13453	Amp	10	66943472	66946821	0	0.043057
13454	Amp	10	66948805	67307552	0	0.037524
13455	Amp	10	67308089	67487209	0	0.041045
13456	Amp	10	67487411	67492583	0	0.037524
13457	Amp	10	67496325	67505081	0	0.033059
13458	Amp	10	67505151	67576996	0	0.030963
13459	Amp	10	67586608	67676791	0	0.034572
13460	Amp	10	67676915	67734020	0	0.040508
13461	Amp	10	67734277	67756381	0	0.044738

13462	Amp	10	67763257	67763604	0	0.040508
13463	Amp	10	67764801	67851330	0	0.039027
13464	Amp	10	67855426	68018755	0	0.030993
13465	Amp	10	68021439	68222995	0	0.025058
13466	Amp	10	68223692	68441912	0	0.01884
13467	Amp	10	68442786	68477885	0	0.031962
13468	Amp	10	68482708	68510208	0	0.042786
13469	Amp	10	68511687	68850849	0	0.029664
13470	Amp	10	68851487	68972789	0	0.026055
13471	Amp	10	68976977	69098306	0	0.03774
13472	Amp	10	69102154	69188082	0	0.043757
13473	Amp	10	69189951	69203144	0	0.047576
13474	Amp	10	69204621	69439138	0	0.076088
13475	Amp	10	69439238	69550375	0	0.08018
13476	Amp	10	69550995	69603425	0	0.082621
13477	Amp	10	69606809	69625374	0	0.120413
13478	Amp	10	69626268	69905298	0	0.115983
13479	Amp	10	69910197	69979860	0	0.15575
13480	Amp	10	69980064	70020603	0	0.168785
13481	Amp	10	70027240	70049022	0	0.184569
13482	Amp	10	70051381	70090876	0	0.168785
13483	Amp	10	70096206	70182766	0	0.179541
13484	Amp	10	70186337	70292413	0	0.129018
13485	Amp	10	70294609	70327142	0	0.15112
13486	Amp	10	70329933	70333746	0	0.105418
13487	Amp	10	70340928	70384666	0	0.113901
13488	Amp	10	70391654	70399324	0	0.124195
13489	Amp	10	70402490	70445205	0	0.127723
13490	Amp	10	70446834	70874058	0	0.11924
13491	Amp	10	70874210	70892620	0	0.077693
13492	Amp	10	70892882	71110533	0	0.07914
13493	Amp	10	71116049	71144982	0	0.083955
13494	Amp	10	71145569	71240693	0	0.087927
13495	Amp	10	71240929	71250307	0	0.091498
13496	Amp	10	71250724	71289707	0	0.087525
13497	Amp	10	71290374	71455723	0	0.096466
13498	Amp	10	71462134	71490717	0	0.137134
13499	Amp	10	71493566	71509539	0	0.150875
13500	Amp	10	71509579	71691816	0	0.15288
13501	Amp	10	71691840	71767966	0	0.183344
13502	Amp	10	71770398	71882087	0	0.181897
13503	Amp	10	71882197	71937801	0	0.18479
13504	Amp	10	71939387	71968661	0.362221	0.226353
13505	Amp	10	71980855	72047030	0.056238	0.19531
13506	Amp	10	72047151	72073101	0.033661	0.192417
13507	Amp	10	72073597	72107696	0.079463	0.198408
13508	Amp	10	72109905	72109905	0.068197	0.196636
13509	Amp	10	72122059	72247535	0	0.155073
13510	Amp	10	72250755	72267782	0	0.138127
13511	Amp	10	72268339	72399913	0	0.134599
13512	Amp	10	72400018	72474840	0	0.133117
13513	Amp	10	72477583	72503916	0	0.165197
13514	Amp	10	72506442	72836582	0	0.182143

13515	Amp	10	72838807	72933386	0	0.176153
13516	Amp	10	72947058	73028293	0	0.18058
13517	Amp	10	73028985	73041621	0	0.184591
13518	Amp	10	73051082	73141025	0.215373	0.212557
13519	Amp	10	73146546	73212576	0.086298	0.19878
13520	Amp	10	73217387	73298843	0.128931	0.203864
13521	Amp	10	73304725	73360301	0.09318	0.199436
13522	Amp	10	73376184	73430981	0.156508	0.206509
13523	Amp	10	73434458	73601545	0.209057	0.211897
13524	Amp	10	73601752	73880258	0.156508	0.206151
13525	Amp	10	73884428	73918899	0.195309	0.210364
13526	Amp	10	73918957	73919066	0.123589	0.203292
13527	Amp	10	73926475	73990558	0.596354	0.247036
13528	Amp	10	73995773	74046195	0.693887	0.255079
13529	Amp	10	74055870	74080814	0.7517	0.260228
13530	Amp	10	74085191	74088706	0.702615	0.256015
13531	Amp	10	74096922	74108418	0.823952	0.265241
13532	Amp	10	74111132	74117758	0.483654	0.237275
13533	Amp	10	74124399	74208661	0.540744	0.242314
13534	Amp	10	74211237	74211337	1.208462	0.292837
13535	Amp	10	74216391	74245561	1.285475	0.298837
13536	Amp	10	74249407	74321741	1.049532	0.282311
13537	Amp	10	74327566	74333431	1.582671	0.318783
13538	Amp	10	74339285	74369074	1.453628	0.310261
13539	Amp	10	74369770	74389912	1.582671	0.318957
13540	Amp	10	74390631	74399566	1.415328	0.307187
13541	Amp	10	74402087	74433054	1.553308	0.316731
13542	Amp	10	74433339	74492070	1.383826	0.305705
13543	Amp	10	74492119	74535133	1.453628	0.310208
13544	Amp	10	74536622	74568390	1.856336	0.337235
13545	Amp	10	74579363	74623313	1.887705	0.338991
13546	Amp	10	74629727	74647543	2.214215	0.359786
13547	Amp	10	74649311	74661458	2.370685	0.369018
13548	Amp	10	74667299	74669821	2.519	0.378459
13549	Amp	10	74671909	74685304	3.134208	0.414542
13550	Amp	10	74692022	74766246	3.099988	0.412536
13551	Amp	10	74770712	74801968	2.602347	0.382719
13552	Amp	10	74803400	74882900	2.806723	0.395464
13553	Amp	10	74883033	74908959	2.495444	0.377001
13554	Amp	10	74910553	74911091	2.349236	0.367775
13555	Amp	10	74911592	74911592	2.22615	0.360289
13556	Amp	10	74911632	74935765	2.404616	0.371585
13557	Amp	10	74936047	74980229	2.683697	0.388119
13558	Amp	10	74984419	74985163	2.082332	0.351647
13559	Amp	10	74987484	75018455	1.821927	0.335113
13560	Amp	10	75020395	75089200	1.657426	0.323926
13561	Amp	10	75089639	75129081	1.800655	0.333764
13562	Amp	10	75129723	75156944	1.898353	0.339717
13563	Amp	10	75159333	75177817	2.071528	0.350743
13564	Amp	10	75178073	75187279	2.124253	0.353898
13565	Amp	10	75196875	75241575	2.039783	0.348815
13566	Amp	10	75251361	75261450	1.542961	0.316382
13567	Amp	10	75266592	75303343	1.757929	0.330778

13568	Amp	10	75304562	75332289	1.887705	0.339084
13569	Amp	10	75338375	75357847	1.942393	0.342698
13570	Amp	10	75359307	75359307	1.877398	0.3381
13571	Amp	10	75363007	75388705	1.811302	0.334487
13572	Amp	10	75389181	75415176	1.542961	0.316185
13573	Amp	10	75416236	75571232	1.748357	0.330432
13574	Amp	10	75585203	75596018	1.503938	0.313852
13575	Amp	10	75596836	75686022	1.525294	0.315265
13576	Amp	10	75686425	75769754	1.582671	0.319341
13577	Amp	10	75773994	75773994	1.678649	0.325544
13578	Amp	10	75774495	75858606	1.534719	0.316
13579	Amp	10	75859286	75860263	1.484449	0.311989
13580	Amp	10	75861762	75876292	1.415328	0.307174
13581	Amp	10	75878108	75901365	1.344429	0.302542
13582	Amp	10	75901583	76001041	1.553308	0.316954
13583	Amp	10	76001161	76041963	1.503938	0.313428
13584	Amp	10	76042773	76074908	1.821927	0.335099
13585	Amp	10	76074933	76409406	1.708768	0.327635
13586	Amp	10	76410390	76603588	1.748357	0.330133
13587	Amp	10	76608074	76610633	1.768015	0.331564
13588	Amp	10	76614755	76695888	1.605306	0.320268
13589	Amp	10	76698017	76783303	1.553308	0.317365
13590	Amp	10	76787483	76942610	1.099256	0.285143
13591	Amp	10	76942893	77060505	1.029845	0.28064
13592	Amp	10	77061809	77070517	0.982622	0.277256
13593	Amp	10	77072704	77079331	0.888878	0.270307
13594	Amp	10	77079961	77080718	0.963924	0.275836
13595	Amp	10	77081607	77144530	0.991911	0.277837
13596	Amp	10	77144725	77175413	0.915677	0.272308
13597	Amp	10	77178190	77352076	1.208462	0.292948
13598	Amp	10	77353806	77357541	0.823952	0.265499
13599	Amp	10	77357885	77363563	0.906818	0.271922
13600	Amp	10	77364836	77403250	0.702615	0.255627
13601	Amp	10	77403435	77494228	0.7517	0.260118
13602	Amp	10	77494394	77516747	0.852608	0.267723
13603	Amp	10	77517091	77517091	0.897922	0.27133
13604	Amp	10	77524456	77536127	1.059215	0.28286
13605	Amp	10	77539240	77576836	0.906818	0.27182
13606	Amp	10	77577500	77600073	1.059215	0.28288
13607	Amp	10	77600182	77616287	0.95395	0.275275
13608	Amp	10	77617917	77673339	0.86979	0.269259
13609	Amp	10	77675450	77692219	0.743132	0.259343
13610	Amp	10	77693001	77695670	0.71758	0.257341
13611	Amp	10	77698582	77765559	1.169408	0.290025
13612	Amp	10	77766635	77833679	1.218154	0.293942
13613	Amp	10	77836272	77844068	1.354645	0.303262
13614	Amp	10	77848019	78059739	1.424227	0.308156
13615	Amp	10	78062439	78215523	1.354645	0.303035
13616	Amp	10	78217164	78253568	1.374261	0.304749
13617	Amp	10	78257288	78264044	1.000924	0.278576
13618	Amp	10	78264137	78301788	1.068928	0.283173
13619	Amp	10	78303693	78337276	1.039426	0.281459
13620	Amp	10	78337736	78339769	0.982622	0.276968

13621	Amp	10	78340284	78352143	0.86979	0.268931
13622	Amp	10	78352189	78379382	0.963924	0.27569
13623	Amp	10	78380290	78456072	0.897922	0.270797
13624	Amp	10	78457411	78467502	1.415328	0.307356
13625	Amp	10	78467564	78570137	1.432145	0.309053
13626	Amp	10	78572273	78592806	1.415328	0.307356
13627	Amp	10	78594170	78635005	1.334042	0.302207
13628	Amp	10	78637093	78770829	1.218154	0.293411
13629	Amp	10	78772511	78790242	1.24764	0.295436
13630	Amp	10	78790630	78816797	1.199568	0.29228
13631	Amp	10	78823267	78824997	1.039426	0.281183
13632	Amp	10	78825526	78830561	1.453628	0.310469
13633	Amp	10	78830967	78854536	1.424227	0.308445
13634	Amp	10	78855973	79036171	1.374261	0.304369
13635	Amp	10	79037682	79064932	1.503938	0.313876
13636	Amp	10	79065692	79112897	1.374261	0.304369
13637	Amp	10	79115456	79428905	1.424227	0.308056
13638	Amp	10	79430841	79484771	1.404612	0.306625
13639	Amp	10	79487437	79487437	1.344429	0.302937
13640	Amp	10	80248789	80248789	0.897922	0.271123
13641	Amp	10	80252343	80342191	0.991911	0.277915
13642	Amp	10	80343437	80383161	0.963924	0.275497
13643	Amp	10	80389899	80490437	1.453628	0.310236
13644	Amp	10	80493422	80646518	1.534719	0.315467
13645	Amp	10	80646815	80704878	1.2565	0.296677
13646	Amp	10	80708970	80893590	0.991911	0.27767
13647	Amp	10	80895673	80959478	1.078696	0.284372
13648	Amp	10	80961249	80989744	0.991911	0.277613
13649	Amp	10	80996872	81054998	0.548372	0.242874
13650	Amp	10	81055061	81168149	0.483654	0.237643
13651	Amp	10	81176270	81216553	0.613517	0.248257
13652	Amp	10	81216904	81258462	0.724993	0.258291
13653	Amp	10	81259791	81259958	0.613517	0.248257
13654	Amp	10	81260655	81269772	0.709359	0.256815
13655	Amp	10	81272499	81398821	0.796213	0.263387
13656	Amp	10	81408966	81479316	1.068928	0.283166
13657	Amp	10	81488203	81498844	0.571214	0.244775
13658	Amp	10	81499121	81501888	0.66072	0.252544
13659	Amp	10	81505541	81663558	0.676804	0.254111
13660	Amp	10	81663594	81673694	0.66072	0.252705
13661	Amp	10	81678579	81720963	0.644689	0.251138
13662	Amp	10	81721306	81781664	0.702615	0.25594
13663	Amp	10	81782604	81787999	1.315861	0.30052
13664	Amp	10	81790702	81790702	0.743132	0.259591
13665	Amp	10	81795272	81815029	0.571214	0.245109
13666	Amp	10	81816322	81925343	0.532449	0.241459
13667	Amp	10	81926046	81926693	0.444673	0.234181
13668	Amp	10	81926802	81997443	0.333141	0.223568
13669	Amp	10	81998100	81998530	0.362221	0.226568
13670	Amp	10	82003259	82017377	0.012341	0.189736
13671	Amp	10	82022165	82071763	0.293283	0.220325
13672	Amp	10	82072548	82111611	0.243425	0.215267
13673	Amp	10	82112409	82121629	0.390264	0.229361

13674	Amp	10	82124522	82155520	0.500055	0.238487
13675	Amp	10	82156668	82181036	0.421967	0.231975
13676	Amp	10	82181758	82217313	0.390264	0.22932
13677	Amp	10	82220939	82234368	0.301081	0.220845
13678	Amp	10	82238866	82423719	0.362221	0.226141
13679	Amp	10	82423815	82457667	0.301081	0.221159
13680	Amp	10	82503700	82527412	0.156508	0.206189
13681	Amp	10	82528792	82725085	0.043943	0.193888
13682	Amp	10	82728666	82837740	0.128931	0.203868
13683	Amp	10	82842338	82857555	0.243425	0.215582
13684	Amp	10	82858316	82858840	0.128931	0.203868
13685	Amp	10	82858853	82938523	0.056238	0.19517
13686	Amp	10	82942497	82946897	0	0.169763
13687	Amp	10	82946931	83024113	0	0.159784
13688	Amp	10	83028923	83029451	0	0.149818
13689	Amp	10	83030345	83075558	0	0.158918
13690	Amp	10	83077482	83115949	0	0.167573
13691	Amp	10	83120178	83293178	0	0.157647
13692	Amp	10	83298054	83388317	0	0.148992
13693	Amp	10	83394612	83649610	0	0.113255
13694	Amp	10	83649653	84047457	0	0.103417
13695	Amp	10	84047474	84079002	0	0.107906
13696	Amp	10	84079805	84337944	0	0.103417
13697	Amp	10	84339642	84341822	0	0.09599
13698	Amp	10	84342427	84979003	0	0.099197
13699	Amp	10	84983744	85030561	0	0.118258
13700	Amp	10	85036056	85038005	0	0.123977
13701	Amp	10	85038795	85140768	0	0.1422
13702	Amp	10	85143098	85325536	0	0.136482
13703	Amp	10	85330261	85581490	0	0.123244
13704	Amp	10	85583730	85672774	0	0.126
13705	Amp	10	85676414	85865551	0	0.108732
13706	Amp	10	85866257	86334865	0	0.111957
13707	Amp	10	86336290	86352168	0	0.093734
13708	Amp	10	86354781	86577343	0	0.043211
13709	Amp	10	86584222	86696122	0	0.061998
13710	Amp	10	86698487	86861273	0	0.055497
13711	Amp	10	86861274	86884982	0	0.052272
13712	Amp	10	86885424	86929645	0	0.057739
13713	Amp	10	86932998	87105532	0	0.055168
13714	Amp	10	87108914	87108914	0	0.0497
13715	Amp	10	87116095	87116095	0	0.052828
13716	Amp	10	87406076	87545362	0	0.040542
13717	Amp	10	87546925	87773321	0	0.049782
13718	Amp	10	87781131	87786069	0	0.046654
13719	Amp	10	87789495	87828714	0	0.037414
13720	Amp	10	87838937	87870165	0	0.018353
13721	Amp	10	87871755	87975840	0	0.013839
13722	Amp	10	87976151	88614881	0	0.017591
13723	Amp	10	88617220	88706786	0	0.023727
13724	Amp	10	88707310	88837871	0	0.032116
13725	Amp	10	88838987	88975149	0	0.023727
13726	Amp	10	88976642	89006115	0	0.027386

13727 Amp	10	89007883	89672646	0	0.023727
13728 Amp	10	89677406	89754823	0	0.025776
13729 Amp	10	89754990	89857564	0	0.035612
13730 Amp	10	89857626	90717501	0	0.030445
13731 Amp	10	90721817	90838701	0	0.034082
13732 Amp	10	90838866	91092996	0	0.043193
13733 Amp	10	91099615	91245082	0	0.059197
13734 Amp	10	91246215	91408779	0	0.06553
13735 Amp	10	91414741	91435138	0	0.032109
13736 Amp	10	91438249	91981257	0	0.025776
13737 Amp	10	91984291	92694399	0	0.024363
13738 Amp	10	92697907	93150939	0	0.02212
13739 Amp	10	93151315	93176571	0	0.020071
13740 Amp	10	93176764	94395541	0	0.022482
13741 Amp	10	94420618	94420618	0	0.028405
13742 Amp	10	94420720	94460755	0	0.041413
13743 Amp	10	94467637	95238701	0	0.028405
13744 Amp	10	95240103	95757054	0	0.022269
13745 Amp	10	95761712	96204505	0	0.019858
13746 Amp	10	96204662	96378883	0	0.022637
13747 Amp	10	96379079	96634172	0	0.024722
13748 Amp	10	96635237	96860848	0	0.019858
13749 Amp	10	96862746	97122668	0	0.030293
13750 Amp	10	97123199	97315869	0	0.035492
13751 Amp	10	97318820	97372326	0	0.025057
13752 Amp	10	97378082	97491794	0	0.030649
13753 Amp	10	97496800	97574571	0	0.041236
13754 Amp	10	97574890	97867438	0	0.030649
13755 Amp	10	97873146	97916646	0	0.035628
13756 Amp	10	97917337	98109204	0	0.030649
13757 Amp	10	98111665	98226250	0	0.025451
13758 Amp	10	98227020	98552287	0	0.031388
13759 Amp	10	98553285	99093734	0	0.033782
13760 Amp	10	99094140	99327719	0	0.036778
13761 Amp	10	99333306	100033847	0	0.043042
13762 Amp	10	100034770	100085677	0	0.048174
13763 Amp	10	100098264	100136294	0	0.045178
13764 Amp	10	100138177	100180333	0	0.050242
13765 Amp	10	100180682	100401639	0	0.056808
13766 Amp	10	100402085	100478884	0	0.045413
13767 Amp	10	100486339	100510565	0	0.052585
13768 Amp	10	100512924	100539458	0	0.04752
13769 Amp	10	100539482	100686237	0	0.0496
13770 Amp	10	100692042	100710785	0	0.043035
13771 Amp	10	100711999	100841818	0	0.035862
13772 Amp	10	100842369	100968862	0	0.029939
13773 Amp	10	100975021	101374876	0	0.03648
13774 Amp	10	101376187	101644012	0	0.034085
13775 Amp	10	101644844	101910316	0	0.035659
13776 Amp	10	101912770	102101589	0	0.034085
13777 Amp	10	102102662	102276598	0	0.0388
13778 Amp	10	102276692	102539892	0	0.053176
13779 Amp	10	102541755	102595725	0	0.045733

13780	Amp	10	102598481	102732187	0	0.050975
13781	Amp	10	102737415	103099866	0	0.045038
13782	Amp	10	103107393	103155553	0	0.051684
13783	Amp	10	103155833	103385185	0	0.049603
13784	Amp	10	103388931	103755398	0	0.054249
13785	Amp	10	103756892	104001486	0	0.055981
13786	Amp	10	104003175	104175677	0	0.058182
13787	Amp	10	104179912	104450106	0	0.042515
13788	Amp	10	104451011	104463063	0	0.040314
13789	Amp	10	104467950	104729787	0	0.035072
13790	Amp	10	104731378	104829507	0	0.028426
13791	Amp	10	104833187	104844472	0	0.04505
13792	Amp	10	104844948	105046900	0	0.039753
13793	Amp	10	105049519	105243401	0	0.046564
13794	Amp	10	105243748	105453261	0	0.03309
13795	Amp	10	105457039	105482415	0	0.042163
13796	Amp	10	105484776	105497764	0	0.030116
13797	Amp	10	105501526	105631629	0	0.036644
13798	Amp	10	105632231	105848951	0	0.027571
13799	Amp	10	105852299	105930609	0	0.016767
13800	Amp	10	105930762	105943292	0	0.010238
13801	Amp	10	105943484	106020332	0	0.015928
13802	Amp	10	106023290	106054289	0	0.021958
13803	Amp	10	106054925	106171218	0	0.017313
13804	Amp	10	106178217	106363979	0	0.00569
13805	Amp	10	106368543	106371517	0	0
13806	Amp	10	106372694	106495612	0	0.002596
13807	Amp	10	106496664	106514981	0	0.007258
13808	Amp	10	106516017	106572821	0	0.015929
13809	Amp	10	106574215	106682769	0	0.007258
13810	Amp	10	106682792	106689873	0	0.002596
13811	Amp	10	106690424	107301060	0	0
13812	Amp	10	107301701	107338691	0	0.001466
13813	Amp	10	107339114	108224553	0	0.003144
13814	Amp	10	108224761	111941829	0	0.001679
13815	Amp	10	111945859	111960206	0	0.005475
13816	Amp	10	111963838	112768664	0	0.001679
13817	Amp	10	112769054	112973123	0	0.004509
13818	Amp	10	112973147	113472309	0	0.010635
13819	Amp	10	113472976	113744625	0	0.007805
13820	Amp	10	113746860	114240111	0	0.001679
13821	Amp	10	114242317	114471861	0	0.010965
13822	Amp	10	114472402	114483739	0	0.01751
13823	Amp	10	114484870	114693513	0	0.021325
13824	Amp	10	114695296	114710156	0	0.027777
13825	Amp	10	114710852	114807214	0	0.047027
13826	Amp	10	114807335	114847908	0	0.040575
13827	Amp	10	114848627	114885705	0	0.042185
13828	Amp	10	114894815	114902037	0	0.027881
13829	Amp	10	114902791	114945225	0	0.029576
13830	Amp	10	114948125	115186766	0	0.069168
13831	Amp	10	115187397	115187466	0	0.070989
13832	Amp	10	115190232	115198508	0	0.050533

13833	Amp	10	115208588	115218882	0	0.043988
13834	Amp	10	115220036	115234751	0	0.045455
13835	Amp	10	115234926	115439170	0	0.048635
13836	Amp	10	115443712	115600376	0	0.034597
13837	Amp	10	115600794	115681664	0	0.069092
13838	Amp	10	115681726	115787106	0	0.065911
13839	Amp	10	115787298	116052369	0	0.064444
13840	Amp	10	116052670	116120990	0	0.040045
13841	Amp	10	116126105	116159032	0	0.02995
13842	Amp	10	116159320	116326089	0	0.028341
13843	Amp	10	116327708	116342574	0	0.031545
13844	Amp	10	116342669	116355517	0	0.066793
13845	Amp	10	116356443	116524400	0	0.064971
13846	Amp	10	116527026	116540143	0	0.061767
13847	Amp	10	116542009	116645189	0	0.060072
13848	Amp	10	116649017	116803034	0	0.01478
13849	Amp	10	116803489	117063439	0	0.019332
13850	Amp	10	117070154	117497986	0	0.022293
13851	Amp	10	117501470	117526067	0	0.017741
13852	Amp	10	117538312	117982728	0	0.01478
13853	Amp	10	117988142	118573814	0	0.024341
13854	Amp	10	118576995	119173812	0	0.01869
13855	Amp	10	119175289	119254295	0	0.017012
13856	Amp	10	119263103	119463613	0	0.013101
13857	Amp	10	119465964	119696417	0	0.018181
13858	Amp	10	119707870	120298581	0	0.058803
13859	Amp	10	120300487	120309652	0	0.061184
13860	Amp	10	120311038	120366760	0	0.076012
13861	Amp	10	120368732	120395612	0	0.082185
13862	Amp	10	120397274	120686941	0	0.079804
13863	Amp	10	120689729	120823662	0	0.114155
13864	Amp	10	120824934	120880754	0	0.121328
13865	Amp	10	120880880	120931186	0	0.127783
13866	Amp	10	120939334	121001578	0	0.148694
13867	Amp	10	121030305	121037583	0	0.184126
13868	Amp	10	121038054	121046066	0	0.17767
13869	Amp	10	121050180	121052848	0	0.185367
13870	Amp	10	121055155	121078040	0.13565	0.204713
13871	Amp	10	121078067	121098449	0.201819	0.211137
13872	Amp	10	121101781	121217052	0.028398	0.191791
13873	Amp	10	121217291	121235627	0	0.185367
13874	Amp	10	121235798	121318482	0	0.178195
13875	Amp	10	121321538	121425789	0	0.140804
13876	Amp	10	121426042	121458957	0	0.136147
13877	Amp	10	121462396	121633096	0	0.165958
13878	Amp	10	121639381	121709163	0	0.136147
13879	Amp	10	121715198	121736140	0	0.1161
13880	Amp	10	121738707	121811210	0	0.06584
13881	Amp	10	121818336	121913691	0	0.040185
13882	Amp	10	121913897	122097514	0	0.043444
13883	Amp	10	122103401	122161589	0	0.040185
13884	Amp	10	122166795	122188706	0	0.046342
13885	Amp	10	122189564	122216514	0	0.04983

13886	Amp	10	122218449	122297259	0	0.043674
13887	Amp	10	122300723	122423575	0	0.040185
13888	Amp	10	122430605	122797707	0	0.067181
13889	Amp	10	122805167	122874713	0	0.040185
13890	Amp	10	122921307	123165436	0	0.055366
13891	Amp	10	123165692	123205403	0	0.051861
13892	Amp	10	123205763	123608849	0	0.055284
13893	Amp	10	123609090	123808420	0	0.050776
13894	Amp	10	123815834	123890288	0	0.0538
13895	Amp	10	123890734	123943507	0	0.067349
13896	Amp	10	123951019	123951883	0	0.054451
13897	Amp	10	123952366	124321058	0	0.10471
13898	Amp	10	124323830	124343157	0	0.116048
13899	Amp	10	124347115	124441107	0	0.065788
13900	Amp	10	124443960	124470107	0	0.056136
13901	Amp	10	124474639	124707119	0	0.037058
13902	Amp	10	124707783	124866961	0	0.087317
13903	Amp	10	124881753	124942542	0	0.123215
13904	Amp	10	124946003	125008758	0	0.111877
13905	Amp	10	125011435	125107179	0	0.114531
13906	Amp	10	125107660	125135342	0	0.103092
13907	Amp	10	125135499	125214499	0	0.099669
13908	Amp	10	125216941	125302694	0	0.101326
13909	Amp	10	125315673	125361580	0	0.051067
13910	Amp	10	125364697	125403172	0	0.04941
13911	Amp	10	125404278	125434779	0	0.062984
13912	Amp	10	125438758	125488080	0	0.056812
13913	Amp	10	125491278	125620304	0	0.059809
13914	Amp	10	125620602	125661917	0	0.072298
13915	Amp	10	125663457	125770911	0	0.102931
13916	Amp	10	125771095	125864450	0	0.099934
13917	Amp	10	125866547	125943916	0	0.069301
13918	Amp	10	125944166	126160211	0	0.084173
13919	Amp	10	126161487	126301858	0	0.134432
13920	Amp	10	126307902	126417297	0	0.129858
13921	Amp	10	126417320	126427134	0	0.134432
13922	Amp	10	126429196	126504416	0	0.11956
13923	Amp	10	126506123	126736477	0	0.069301
13924	Amp	10	126739120	127012556	0	0.062157
13925	Amp	10	127014626	127082912	0	0.112416
13926	Amp	10	127091927	127098673	0	0.103314
13927	Amp	10	127102428	127384738	0	0.105553
13928	Amp	10	127384997	127418069	0	0.077028
13929	Amp	10	127419304	127496303	0	0.071276
13930	Amp	10	127498455	127560653	0	0.069037
13931	Amp	10	127561066	127596270	0	0.078686
13932	Amp	10	127597489	127663883	0	0.088203
13933	Amp	10	127666944	127894654	0	0.037944
13934	Amp	10	127899313	128171907	0	0.02637
13935	Amp	10	128175039	128232326	0	0.038195
13936	Amp	10	128235069	128550319	0	0.02637
13937	Amp	10	128552840	128658662	0	0.031167
13938	Amp	10	128658680	128980188	0	0.037161

13939 Amp	10	128986089	129505126	0	0.031167
13940 Amp	10	129505642	129510308	0	0.081426
13941 Amp	10	129513799	129785274	0	0.073835
13942 Amp	10	129785289	129979159	0	0.079029
13943 Amp	10	129984024	130297903	0	0.028769
13944 Amp	10	130302437	130380941	0	0.031141
13945 Amp	10	130380993	130383003	0	0.025947
13946 Amp	10	131110205	131235586	0	0.031017
13947 Amp	10	131236421	132277871	0	0.032785
13948 Amp	10	132302850	132703729	0	0.04841
13949 Amp	10	132708461	132747703	0	0.051449
13950 Amp	10	133038543	133132423	0	0.046651
13951 Amp	10	133143045	133286029	0	0.043613
13952 Amp	10	133298340	133312629	0	0.050532
13953 Amp	10	133339792	133370501	0	0.048763
13954 Amp	10	133370917	133376854	0	0.050724
13955 Amp	10	133376925	133411599	0	0.053226
13956 Amp	11	456120	502217	0	0.036062
13957 Amp	11	515931	630331	0	0.041206
13958 Amp	11	641563	692415	0	0.036062
13959 Amp	11	692445	692908	0	0.034012
13960 Amp	11	709861	723658	0	0.025302
13961 Amp	11	724088	755712	0	0.035481
13962 Amp	11	756176	772982	0	0.037638
13963 Amp	11	774078	906708	0	0.04111
13964 Amp	11	909492	909492	0	0.046599
13965 Amp	11	911992	963980	0	0.043126
13966 Amp	11	964271	964271	0	0.04097
13967 Amp	11	969080	1117867	0	0.03079
13968 Amp	11	1122714	1147952	0	0.03235
13969 Amp	11	1156810	1157084	0	0.03079
13970 Amp	11	1209581	1311005	0	0.042508
13971 Amp	11	1324332	1348770	0	0.03079
13972 Amp	11	1359534	1662334	0	0.025302
13973 Amp	11	1878795	1922972	0	0.013706
13974 Amp	11	1932597	2709955	0	0.029356
13975 Amp	11	2710157	2743719	0	0.032347
13976 Amp	11	2744664	2786292	0	0.029356
13977 Amp	11	2795471	2836833	0	0.02549
13978 Amp	11	2840483	2871329	0	0.030682
13979 Amp	11	2872708	3070014	0	0.02549
13980 Amp	11	3082502	3170643	0	0.032976
13981 Amp	11	3172698	3212175	0	0.034565
13982 Amp	11	3228599	3657448	0	0.032976
13983 Amp	11	3658428	3685383	0	0.038609
13984 Amp	11	3686903	3951853	0	0.036778
13985 Amp	11	3952027	4077821	0	0.035008
13986 Amp	11	4078022	4470078	0	0.027523
13987 Amp	11	4470602	5287666	0	0.021889
13988 Amp	11	5287702	5626161	0	0.029092
13989 Amp	11	5630105	5924672	0	0.02688
13990 Amp	11	5926696	6948281	0	0.01123
13991 Amp	11	6948445	7011072	0	0.004028

13992 Amp	11	7011341	7017968	0	0.028867
13993 Amp	11	7024427	7098662	0	0.004028
13994 Amp	11	7101915	7262018	0	0.012866
13995 Amp	11	7269563	7553143	0	0.01938
13996 Amp	11	7562783	7574566	0	0.029044
13997 Amp	11	7576213	7580841	0	0.062523
13998 Amp	11	7582792	7592118	0	0.029044
13999 Amp	11	7597810	7624489	0	0.039246
14000 Amp	11	7625179	7702136	0	0.068099
14001 Amp	11	7703907	7705779	0	0.057897
14002 Amp	11	7706060	7714487	0	0.047285
14003 Amp	11	7725350	7754070	0	0.064616
14004 Amp	11	7755407	7788131	0	0.067774
14005 Amp	11	7820250	7830362	0	0.045506
14006 Amp	11	7831643	7846416	0	0.042348
14007 Amp	11	7847983	7924268	0	0.048037
14008 Amp	11	7924271	8328994	0	0.042348
14009 Amp	11	8329087	8479124	0	0.044651
14010 Amp	11	8481400	8532868	0	0.042348
14011 Amp	11	8533002	8603549	0	0.051363
14012 Amp	11	8606509	8625123	0	0.070109
14013 Amp	11	8629585	8887309	0	0.060445
14014 Amp	11	8887821	9067304	0	0.066667
14015 Amp	11	9069836	9258915	0	0.057652
14016 Amp	11	9274337	9384476	0	0.060259
14017 Amp	11	9385764	9386879	0	0.062204
14018 Amp	11	9394496	9760941	0	0.05017
14019 Amp	11	9761623	9768541	0	0.04503
14020 Amp	11	9771159	9898144	0	0.038489
14021 Amp	11	9898911	10008450	0	0.036544
14022 Amp	11	10009324	10068339	0	0.049428
14023 Amp	11	10069593	10138505	0	0.047857
14024 Amp	11	10144749	10192908	0	0.039659
14025 Amp	11	10197597	10281213	0	0.033437
14026 Amp	11	10281630	10457073	0	0.03083
14027 Amp	11	10459758	10556402	0	0.033605
14028 Amp	11	10558948	10580026	0	0.0475
14029 Amp	11	10584817	10623052	0	0.049047
14030 Amp	11	10624975	10709206	0	0.042177
14031 Amp	11	10711013	10876200	0	0.028282
14032 Amp	11	10881947	10894156	0	0.042353
14033 Amp	11	10895136	10984348	0	0.028282
14034 Amp	11	10985776	11055023	0	0.023596
14035 Amp	11	11061166	11107728	0	0.03988
14036 Amp	11	11107756	11108141	0	0.035959
14037 Amp	11	11115168	11129564	0	0.047575
14038 Amp	11	11135176	11139701	0	0.057189
14039 Amp	11	11142002	11154436	0	0.040905
14040 Amp	11	11157412	11241071	0	0.02929
14041 Amp	11	11247351	11268617	0	0.019675
14042 Amp	11	11271471	11386102	0	0.023314
14043 Amp	11	11390633	11396393	0	0.033006
14044 Amp	11	11397847	11456463	0	0.023314

14045 Amp	11	11458319	11476863	0	0.033247
14046 Amp	11	11477436	11890889	0	0.023314
14047 Amp	11	11893772	11983501	0	0.031115
14048 Amp	11	11985530	11988272	0	0.023314
14049 Amp	11	11988583	11996840	0	0.019675
14050 Amp	11	12007535	12029105	0	0.027532
14051 Amp	11	12030150	12252848	0	0.019675
14052 Amp	11	12255375	12342948	0	0.010837
14053 Amp	11	12343548	12577832	0	0.008061
14054 Amp	11	12580387	12621589	0	0.011933
14055 Amp	11	12624033	12643129	0	0.008061
14056 Amp	11	12643889	12913543	0	0.014049
14057 Amp	11	12914191	13047393	0	0.023078
14058 Amp	11	13047428	13085199	0	0.02449
14059 Amp	11	13097761	13103340	0	0.023078
14060 Amp	11	13106268	13109012	0	0.02656
14061 Amp	11	13109082	13190330	0	0.03404
14062 Amp	11	13190646	13197388	0	0.037404
14063 Amp	11	13197936	13203777	0	0.053032
14064 Amp	11	13204403	13234149	0	0.059376
14065 Amp	11	13234424	13349241	0	0.051896
14066 Amp	11	13351042	13425330	0	0.050349
14067 Amp	11	13436591	13567670	0	0.058599
14068 Amp	11	13576869	13597257	0	0.068267
14069 Amp	11	13598625	13647732	0	0.087265
14070 Amp	11	13652941	13675990	0	0.099682
14071 Amp	11	13682108	13931038	0	0.071016
14072 Amp	11	13931857	14070077	0	0.055388
14073 Amp	11	14071429	14276088	0	0.066518
14074 Amp	11	14286015	14737534	0	0.05992
14075 Amp	11	14742553	14805300	0	0.064515
14076 Amp	11	14806994	15076521	0	0.067699
14077 Amp	11	15079724	15111964	0	0.078944
14078 Amp	11	15114454	15136461	0	0.067699
14079 Amp	11	15137775	15275085	0	0.05992
14080 Amp	11	15278296	15608165	0	0.066327
14081 Amp	11	15609930	15873878	0	0.05992
14082 Amp	11	15874121	16003920	0	0.0541
14083 Amp	11	16004628	16160924	0	0.056155
14084 Amp	11	16162835	16298168	0	0.083134
14085 Amp	11	16302903	16336360	0	0.093094
14086 Amp	11	16337133	16340036	0	0.08415
14087 Amp	11	16340892	16429550	0	0.088266
14088 Amp	11	16432546	16498429	0	0.098136
14089 Amp	11	16508905	16616183	0	0.114089
14090 Amp	11	16616495	16637801	0	0.112035
14091 Amp	11	16642477	16725041	0	0.138561
14092 Amp	11	16732791	16849679	0	0.140195
14093 Amp	11	16852134	16871798	0	0.144503
14094 Amp	11	16873294	16936120	0	0.158404
14095 Amp	11	16937487	17245785	0	0.152038
14096 Amp	11	17247964	17904437	0	0.179315
14097 Amp	11	17904978	17978648	0	0.152038

14098	Amp	11	17986829	18086998	0	0.15989
14099	Amp	11	18091180	18350959	0	0.138138
14100	Amp	11	18351258	18470330	0	0.148759
14101	Amp	11	18473422	18660642	0	0.155856
14102	Amp	11	18665369	18867319	0	0.164951
14103	Amp	11	18871790	18904545	0	0.152696
14104	Amp	11	18957582	19056853	0	0.135418
14105	Amp	11	19056921	19085196	0	0.140969
14106	Amp	11	19085683	19134007	0	0.130183
14107	Amp	11	19134546	19181618	0	0.153183
14108	Amp	11	19183389	19187503	0	0.162399
14109	Amp	11	19187588	19402732	0.007199	0.189236
14110	Amp	11	19403356	19498419	0.043943	0.193854
14111	Amp	11	19499043	19591783	0	0.176437
14112	Amp	11	19593101	19631974	0.033661	0.193052
14113	Amp	11	19632673	19702987	0.079463	0.198487
14114	Amp	11	19709924	19719185	0.123589	0.202688
14115	Amp	11	19722727	19805383	0.27943	0.21899
14116	Amp	11	19805436	19911104	0.236222	0.214789
14117	Amp	11	19911473	19919832	0	0.178302
14118	Amp	11	19925922	19946883	0	0.168883
14119	Amp	11	19950559	19963955	0	0.147495
14120	Amp	11	19965209	20102753	0	0.152855
14121	Amp	11	20108755	20279663	0	0.143271
14122	Amp	11	20283510	20294647	0	0.136083
14123	Amp	11	20294669	20357730	0	0.130722
14124	Amp	11	20359791	20403305	0	0.125971
14125	Amp	11	20403782	20529206	0	0.130178
14126	Amp	11	20531414	20767774	0	0.132691
14127	Amp	11	20768965	20877204	0	0.121631
14128	Amp	11	20877617	20880229	0	0.119118
14129	Amp	11	20881926	21064544	0	0.112088
14130	Amp	11	21065894	21084826	0	0.108606
14131	Amp	11	21088922	21106015	0	0.102261
14132	Amp	11	21107723	21145586	0	0.096273
14133	Amp	11	21148310	21160866	0	0.103425
14134	Amp	11	21162447	21252622	0	0.110541
14135	Amp	11	21253621	21261706	0	0.097894
14136	Amp	11	21261981	21342569	0	0.095936
14137	Amp	11	21343028	21439942	0	0.106408
14138	Amp	11	21497985	21613736	0	0.102182
14139	Amp	11	21615150	21754732	0	0.107881
14140	Amp	11	21755122	21878194	0	0.100562
14141	Amp	11	21886935	21935123	0	0.094864
14142	Amp	11	21946729	21967784	0	0.087747
14143	Amp	11	21970981	22119625	0	0.094218
14144	Amp	11	22134532	22516195	0	0.112331
14145	Amp	11	22517043	22589709	0	0.131114
14146	Amp	11	22589889	22834307	0	0.117172
14147	Amp	11	22841980	22972050	0	0.100871
14148	Amp	11	22978288	23039805	0	0.119418
14149	Amp	11	23039964	23062771	0	0.133297
14150	Amp	11	23063355	23079628	0	0.11475

14151	Amp	11	23080096	23114912	0	0.103362
14152	Amp	11	23115064	23164923	0	0.115082
14153	Amp	11	23167075	23176091	0	0.123264
14154	Amp	11	23178774	23219707	0	0.139612
14155	Amp	11	23221908	23225371	0	0.125401
14156	Amp	11	23225617	23331836	0	0.121285
14157	Amp	11	23335695	23481660	0	0.101125
14158	Amp	11	23487162	23551175	0	0.112285
14159	Amp	11	23560489	23833062	0	0.104102
14160	Amp	11	23836120	23909395	0	0.099794
14161	Amp	11	23910502	23927746	0	0.090009
14162	Amp	11	23927831	23945923	0	0.076833
14163	Amp	11	23949108	23996925	0	0.083868
14164	Amp	11	23997976	24070223	0	0.090335
14165	Amp	11	24076777	24176449	0	0.099516
14166	Amp	11	24243232	24358265	0	0.090335
14167	Amp	11	24361109	24411236	0	0.0833
14168	Amp	11	24420560	24420628	0	0.087614
14169	Amp	11	24433630	24674283	0	0.08425
14170	Amp	11	24678466	24702766	0	0.105403
14171	Amp	11	24703342	25112297	0	0.101088
14172	Amp	11	25274907	25275147	0	0.070225
14173	Amp	11	25275362	25714470	0	0.094721
14174	Amp	11	25716041	25826277	0	0.118147
14175	Amp	11	25829688	25847977	0	0.123202
14176	Amp	11	25860253	25936256	0	0.132758
14177	Amp	11	25939895	25940552	0	0.109333
14178	Amp	11	25941461	25942284	0	0.113599
14179	Amp	11	25946493	26091328	0	0.117829
14180	Amp	11	26094019	26120039	0	0.110316
14181	Amp	11	26120181	26150654	0	0.10605
14182	Amp	11	26151307	26246016	0	0.094097
14183	Amp	11	26248037	26389383	0	0.089867
14184	Amp	11	26391551	26663606	0	0.101058
14185	Amp	11	26663850	26699095	0	0.105646
14186	Amp	11	26702364	26960656	0	0.110498
14187	Amp	11	26971148	26983394	0	0.100026
14188	Amp	11	26983686	27010717	0	0.093809
14189	Amp	11	27013061	27018127	0	0.087295
14190	Amp	11	27020108	27064972	0	0.093815
14191	Amp	11	27077665	27209359	0	0.103871
14192	Amp	11	27210695	27219530	0	0.092679
14193	Amp	11	27226269	27318053	0	0.082624
14194	Amp	11	27318441	27448863	0	0.077772
14195	Amp	11	27453632	27590441	0	0.088907
14196	Amp	11	27592171	27602064	0	0.083429
14197	Amp	11	27602177	27849529	0	0.071689
14198	Amp	11	27849877	27849877	0	0.064698
14199	Amp	11	27857305	27869986	0	0.053563
14200	Amp	11	27871959	28058312	0	0.072322
14201	Amp	11	28071020	28156856	0	0.066366
14202	Amp	11	28157176	28650803	0	0.071371
14203	Amp	11	28654312	28679591	0	0.073816

14204	Amp	11	28688578	28702003	0	0.067552
14205	Amp	11	28702019	28833660	0	0.072302
14206	Amp	11	28834033	28848993	0	0.067552
14207	Amp	11	28850753	29023963	0	0.073757
14208	Amp	11	29024359	29153861	0	0.077317
14209	Amp	11	29157507	29228310	0	0.071112
14210	Amp	11	29229133	29368769	0	0.075542
14211	Amp	11	29369642	29432112	0	0.08318
14212	Amp	11	29437671	29629079	0	0.109346
14213	Amp	11	29629752	29687951	0	0.104915
14214	Amp	11	29688261	29744514	0	0.10669
14215	Amp	11	29745835	29861927	0	0.109993
14216	Amp	11	29865644	29977433	0	0.117933
14217	Amp	11	29982010	30041284	0	0.109993
14218	Amp	11	30045582	30122819	0	0.115562
14219	Amp	11	30123016	30141002	0	0.117005
14220	Amp	11	30141094	30141094	0	0.109367
14221	Amp	11	30141379	30208497	0	0.112531
14222	Amp	11	30211068	30234320	0	0.121257
14223	Amp	11	30237172	30254781	0	0.129971
14224	Amp	11	30273824	30340692	0	0.112531
14225	Amp	11	30342352	30362353	0	0.109227
14226	Amp	11	30365480	30406715	0	0.110822
14227	Amp	11	30410735	30512075	0	0.079088
14228	Amp	11	30512241	30665463	0	0.086467
14229	Amp	11	30667556	30831235	0	0.096229
14230	Amp	11	30831964	30912134	0	0.097911
14231	Amp	11	30916343	30917536	0	0.107873
14232	Amp	11	30917913	30985004	0	0.103654
14233	Amp	11	30985143	31141106	0	0.096274
14234	Amp	11	31141323	31191020	0	0.086312
14235	Amp	11	31194040	31260355	0	0.081205
14236	Amp	11	31263165	31333905	0	0.077096
14237	Amp	11	31338769	31706059	0	0.087437
14238	Amp	11	31710221	31742845	0	0.094706
14239	Amp	11	31750239	31793141	0	0.104589
14240	Amp	11	31801110	31829323	0	0.094706
14241	Amp	11	31831237	31845608	0	0.137218
14242	Amp	11	31846153	31953865	0	0.135522
14243	Amp	11	31955356	32007897	0	0.09301
14244	Amp	11	32011622	32045002	0	0.131271
14245	Amp	11	32045235	32095054	0	0.134088
14246	Amp	11	32097517	32111902	0	0.095827
14247	Amp	11	32112187	32215821	0	0.13627
14248	Amp	11	32217994	32362840	0	0.095827
14249	Amp	11	32364434	32383809	0	0.088558
14250	Amp	11	32385957	32491213	0	0.138833
14251	Amp	11	32496524	32565933	0	0.142029
14252	Amp	11	32567644	32617646	0	0.131688
14253	Amp	11	32618299	32746709	0	0.121927
14254	Amp	11	32750995	32751932	0	0.127625
14255	Amp	11	32752443	32761075	0	0.156899
14256	Amp	11	32773889	33082367	0.161592	0.20711

14257 Amp	11	33093390	33246908	0.098998	0.20059
14258 Amp	11	33251794	33280928	0.050005	0.194891
14259 Amp	11	33463183	33463228	0.086298	0.198628
14260 Amp	11	33463561	33579035	0.056238	0.195432
14261 Amp	11	33581300	33607487	0.161592	0.207251
14262 Amp	11	33607674	33622968	0.18072	0.209403
14263 Amp	11	33624275	33714885	0.128931	0.203408
14264 Amp	11	33716490	33753992	0	0.161927
14265 Amp	11	33754248	33839098	0	0.156104
14266 Amp	11	33842413	33975036	0	0.12683
14267 Amp	11	33979996	34112750	0	0.136057
14268 Amp	11	34113151	34161753	0	0.14477
14269 Amp	11	34164960	34354770	0	0.137723
14270 Amp	11	34355335	34456329	0	0.139303
14271 Amp	11	34456526	34467705	0	0.14127
14272 Amp	11	34467796	34532302	0	0.147657
14273 Amp	11	34533727	34552748	0	0.14127
14274 Amp	11	34552910	34617953	0	0.145548
14275 Amp	11	34767796	34920766	0	0.142492
14276 Amp	11	34921029	34930108	0	0.136761
14277 Amp	11	34930180	34945595	0	0.131056
14278 Amp	11	34947565	35052620	0	0.122262
14279 Amp	11	35055532	35277061	0	0.156918
14280 Amp	11	35285261	35336889	0	0.166236
14281 Amp	11	35339511	35357402	0	0.161958
14282 Amp	11	35357729	35361512	0	0.160413
14283 Amp	11	35361668	35370847	0	0.158481
14284 Amp	11	35371208	35379664	0	0.1517
14285 Amp	11	35384476	35407437	0	0.135231
14286 Amp	11	35416316	35458187	0	0.138012
14287 Amp	11	35458371	35489359	0	0.128694
14288 Amp	11	35489546	35565276	0	0.127114
14289 Amp	11	35565697	35682229	0	0.125147
14290 Amp	11	35687534	35755202	0	0.11326
14291 Amp	11	35759142	35849457	0	0.118765
14292 Amp	11	35849666	35909139	0	0.129508
14293 Amp	11	35912925	35936955	0	0.137158
14294 Amp	11	35939773	36002251	0	0.129508
14295 Amp	11	36002918	36033163	0	0.136321
14296 Amp	11	36033435	36104883	0	0.130022
14297 Amp	11	36105028	36110910	0	0.133354
14298 Amp	11	36113120	36126655	0	0.124545
14299 Amp	11	36127119	36318123	0	0.122611
14300 Amp	11	36320085	36454127	0	0.113785
14301 Amp	11	36454822	36564841	0	0.106868
14302 Amp	11	36565467	36593803	0	0.113293
14303 Amp	11	36596695	36623657	0	0.120691
14304 Amp	11	36623759	36634922	0	0.114267
14305 Amp	11	36636805	36795557	0	0.125844
14306 Amp	11	36795606	36856238	0	0.133166
14307 Amp	11	36859296	36889512	0	0.12084
14308 Amp	11	36889696	36942293	0	0.126115
14309 Amp	11	36942730	36942730	0	0.119933

14310	Amp	11	37635347	37646484	0	0.112535
14311	Amp	11	37646530	37654443	0	0.105721
14312	Amp	11	37655954	37673560	0	0.074495
14313	Amp	11	37676021	37688462	0	0.072136
14314	Amp	11	37690282	37696104	0	0.078636
14315	Amp	11	37698826	37733866	0	0.064886
14316	Amp	11	37821027	37821027	0	0.055659
14317	Amp	11	37821229	37956414	0	0.057631
14318	Amp	11	37961011	38002559	0	0.04798
14319	Amp	11	38003434	38018126	0	0.050749
14320	Amp	11	38019093	38266909	0	0.069196
14321	Amp	11	38270922	38539546	0	0.066032
14322	Amp	11	38540346	38613890	0	0.077436
14323	Amp	11	38616911	38635023	0	0.058159
14324	Amp	11	38639933	38783823	0	0.05166
14325	Amp	11	38792758	38848318	0	0.054359
14326	Amp	11	38857870	38874264	0	0.066929
14327	Amp	11	38876216	38894025	0	0.054359
14328	Amp	11	38894448	39083172	0	0.05159
14329	Amp	11	39097990	39306975	0	0.055186
14330	Amp	11	39307161	39532991	0	0.061751
14331	Amp	11	39535580	39579891	0	0.070607
14332	Amp	11	39584318	39592370	0	0.061751
14333	Amp	11	39593371	39685849	0	0.056695
14334	Amp	11	39692379	39713132	0	0.060257
14335	Amp	11	39713464	39725381	0	0.053693
14336	Amp	11	39729687	39854855	0	0.061766
14337	Amp	11	39858254	39887542	0	0.078665
14338	Amp	11	39896586	39978791	0	0.084862
14339	Amp	11	39980807	40086903	0	0.067962
14340	Amp	11	40088768	40415485	0	0.056631
14341	Amp	11	40415660	40450010	0	0.055189
14342	Amp	11	40450724	40630500	0	0.062013
14343	Amp	11	40631997	40810148	0	0.055189
14344	Amp	11	40811432	40813847	0	0.049914
14345	Amp	11	40815699	40966624	0	0.036089
14346	Amp	11	40970112	41147883	0	0.025516
14347	Amp	11	41150975	41396432	0	0.02192
14348	Amp	11	41396517	41435657	0	0.023309
14349	Amp	11	41436586	41443120	0	0.036189
14350	Amp	11	41445150	41506957	0	0.031237
14351	Amp	11	41507719	41599519	0	0.022103
14352	Amp	11	41600261	41674201	0	0.025805
14353	Amp	11	41674643	41697372	0	0.060448
14354	Amp	11	41701064	41801495	0	0.055148
14355	Amp	11	41801896	41954959	0	0.051402
14356	Amp	11	41966355	42131808	0	0.056169
14357	Amp	11	42131963	42146627	0	0.061709
14358	Amp	11	42146841	42201456	0	0.063962
14359	Amp	11	42204456	42284666	0	0.054304
14360	Amp	11	42285170	42354656	0	0.061577
14361	Amp	11	42357376	42364818	0	0.041767
14362	Amp	11	42372817	42392012	0	0.037

14363	Amp	11	42397743	42579721	0	0.029852
14364	Amp	11	42580980	42699306	0	0.024313
14365	Amp	11	42699495	42737452	0	0.03052
14366	Amp	11	42737500	42838904	0	0.024324
14367	Amp	11	42842887	42955811	0	0.020621
14368	Amp	11	42955889	43089178	0	0.033015
14369	Amp	11	43093376	43275282	0	0.038834
14370	Amp	11	43276713	43372149	0	0.040264
14371	Amp	11	43376713	43450287	0	0.033162
14372	Amp	11	43453433	43499730	0	0.030909
14373	Amp	11	43507036	43529866	0	0.034282
14374	Amp	11	43529990	43590804	0	0.037444
14375	Amp	11	43593905	43622074	0	0.040902
14376	Amp	11	43626314	43688415	0	0.085218
14377	Amp	11	43690704	43749056	0	0.081845
14378	Amp	11	43761574	43771041	0	0.044301
14379	Amp	11	43774107	43782880	0	0.032751
14380	Amp	11	43783955	43807209	0	0.027459
14381	Amp	11	43807992	43871273	0	0.024001
14382	Amp	11	43881767	43883380	0	0.074524
14383	Amp	11	43885146	43898144	0	0.084079
14384	Amp	11	43898699	43945037	0	0.078492
14385	Amp	11	43946518	43946802	0	0.084232
14386	Amp	11	43947530	44048039	0	0.087761
14387	Amp	11	44049720	44202286	0	0.078206
14388	Amp	11	44208226	44276125	0	0.075044
14389	Amp	11	44276840	44341524	0	0.069304
14390	Amp	11	44342050	44424590	0	0.074065
14391	Amp	11	44426241	44539525	0	0.07982
14392	Amp	11	44540674	44608533	0	0.084162
14393	Amp	11	44610664	44683556	0	0.078408
14394	Amp	11	44685306	44929996	0	0.083995
14395	Amp	11	44930004	45063974	0	0.082565
14396	Amp	11	45065260	45197443	0	0.086729
14397	Amp	11	45199449	45221961	0	0.036206
14398	Amp	11	45221992	45304987	0	0.045261
14399	Amp	11	45305297	45378354	0	0.039442
14400	Amp	11	45379862	45520673	0	0.03414
14401	Amp	11	45520792	45549694	0	0.032542
14402	Amp	11	45551893	45603517	0	0.028199
14403	Amp	11	45603593	45633794	0	0.034626
14404	Amp	11	45640485	45644664	0	0.030462
14405	Amp	11	45645316	45693334	0	0.02849
14406	Amp	11	45697322	45767190	0	0.031278
14407	Amp	11	45770333	45972451	0	0.021099
14408	Amp	11	45973494	45996563	0	0.01757
14409	Amp	11	45996588	46241547	0	0.020698
14410	Amp	11	46247838	46248890	0	0.022436
14411	Amp	11	46249295	46397248	0	0.027321
14412	Amp	11	46398233	46702053	0	0.030811
14413	Amp	11	46707279	46707515	0	0.028022
14414	Amp	11	46710112	46815996	0	0.024895
14415	Amp	11	46816341	46834595	0	0.030275

14416	Amp	11	46837874	46899179	0	0.033938
14417	Amp	11	46902685	47048032	0	0.028558
14418	Amp	11	47048874	47157336	0	0.032939
14419	Amp	11	47159194	47410532	0	0.035809
14420	Amp	11	47413435	47421342	0	0.032319
14421	Amp	11	47427521	47555015	0	0.029448
14422	Amp	11	47557734	47631492	0	0.039614
14423	Amp	11	47633416	47714762	0	0.043061
14424	Amp	11	47715536	47886253	0	0.041632
14425	Amp	11	47889271	47938379	0	0.057942
14426	Amp	11	47940946	47956268	0	0.061273
14427	Amp	11	47958998	47988179	0	0.070182
14428	Amp	11	47989817	48057845	0	0.072382
14429	Amp	11	48061600	48106637	0	0.068935
14430	Amp	11	48106717	48309141	0	0.062065
14431	Amp	11	48317491	48341564	0	0.063891
14432	Amp	11	48343895	48343895	0	0.059006
14433	Amp	11	48351028	48352939	0	0.04884
14434	Amp	11	48953076	49002025	0	0.04106
14435	Amp	11	49007426	49012211	0	0.042767
14436	Amp	11	49012307	49040162	0	0.044742
14437	Amp	11	49040603	49088271	0	0.047164
14438	Amp	11	49089915	49119778	0	0.052557
14439	Amp	11	49123089	49135294	0	0.071013
14440	Amp	11	49139969	49197613	0	0.047164
14441	Amp	11	49198049	49235545	0	0.069789
14442	Amp	11	49237217	49264384	0	0.064426
14443	Amp	11	49266230	49364580	0	0.041801
14444	Amp	11	49366946	49437667	0	0.039379
14445	Amp	11	49440815	49554515	0	0.042098
14446	Amp	11	49559138	49598348	0	0.052101
14447	Amp	11	49602012	49672641	0	0.042098
14448	Amp	11	49678015	49748543	0	0.048191
14449	Amp	11	49748716	49768267	0	0.042098
14450	Amp	11	49768442	49839758	0	0.045461
14451	Amp	11	49840798	49990649	0	0.058667
14452	Amp	11	49992133	50092125	0	0.055303
14453	Amp	11	50096159	50096159	0	0.024158
14454	Amp	11	50101778	50101778	0	0.066909
14455	Amp	11	56244856	56244856	0	0.065083
14456	Amp	11	56247303	56250069	0.508503	0.239628
14457	Amp	11	56253349	56269101	0.548372	0.243145
14458	Amp	11	56270130	56275224	0.476502	0.23676
14459	Amp	11	56275504	56313348	0.619816	0.248932
14460	Amp	11	56313957	56354581	0.814697	0.265181
14461	Amp	11	56355373	56378213	1.099256	0.285655
14462	Amp	11	56378372	56412910	1.208462	0.29272
14463	Amp	11	56415530	56434217	1.078696	0.284232
14464	Amp	11	56436845	56453930	1.039426	0.281415
14465	Amp	11	56460972	56474684	0.41418	0.231366
14466	Amp	11	56474772	56489388	0.340434	0.2243
14467	Amp	11	56493287	56515501	0.934277	0.273848
14468	Amp	11	56516383	56543671	0.888878	0.270248

14469	Amp	11	56546400	56546606	0.934277	0.273877
14470	Amp	11	56547032	56610396	0.906818	0.271481
14471	Amp	11	56613029	56671589	0.860481	0.26815
14472	Amp	11	56674349	56696577	0.702615	0.256107
14473	Amp	11	56697842	56701604	0.786952	0.262917
14474	Amp	11	56703718	56719545	0.613517	0.248264
14475	Amp	11	56720600	56739069	0.66072	0.252779
14476	Amp	11	56739666	56770217	0.523647	0.240606
14477	Amp	11	56771711	56827526	0.652914	0.252136
14478	Amp	11	56828426	56840206	0.76883	0.261238
14479	Amp	11	56840224	56840224	1.010597	0.279186
14480	Amp	11	56840293	56908347	1.130387	0.287474
14481	Amp	11	56909196	56909876	1.364151	0.304215
14482	Amp	11	56910970	56979383	1.394558	0.305912
14483	Amp	11	56981089	57004408	1.099256	0.285439
14484	Amp	11	57004874	57018150	1.463463	0.310552
14485	Amp	11	57021907	57031387	0.934277	0.273753
14486	Amp	11	57033495	57056539	1.10923	0.28648
14487	Amp	11	57060104	57061764	1.088806	0.284462
14488	Amp	11	57068624	57112365	0.452609	0.234914
14489	Amp	11	57113380	57150937	0.437983	0.233356
14490	Amp	11	57151360	57182744	0.540744	0.24201
14491	Amp	11	57182790	57193673	0.421967	0.231602
14492	Amp	11	57200532	57201981	0.27943	0.218874
14493	Amp	11	57203880	57223123	0	0.17876
14494	Amp	11	57229571	57278271	0	0.181994
14495	Amp	11	57279421	57375007	0	0.185239
14496	Amp	11	57375519	57419019	0	0.170348
14497	Amp	11	57421413	57494645	0	0.180928
14498	Amp	11	57495520	57528391	0.02372	0.19166
14499	Amp	11	57529505	57559630	0	0.179027
14500	Amp	11	57561240	57561240	0	0.183123
14501	Amp	11	57561929	57661063	0	0.179878
14502	Amp	11	57663999	57674915	0	0.171223
14503	Amp	11	57676024	57708542	0	0.162121
14504	Amp	11	57709869	57816074	0.195309	0.210299
14505	Amp	11	57816196	57946192	0.273081	0.21811
14506	Amp	11	57955474	57966913	0.068197	0.196797
14507	Amp	11	57971735	57973056	0.293283	0.220497
14508	Amp	11	57983068	58019567	0.476502	0.236804
14509	Amp	11	58023881	58095448	0.375927	0.228054
14510	Amp	11	58095944	58112338	0.201819	0.211201
14511	Amp	11	58112920	58190262	0.250263	0.216169
14512	Amp	11	58196711	58251650	0	0.167991
14513	Amp	11	58251770	58295712	0	0.159702
14514	Amp	11	58298393	58336664	0	0.165088
14515	Amp	11	58349059	58438648	0	0.174885
14516	Amp	11	58443102	58580023	0	0.15656
14517	Amp	11	58580292	58580872	0	0.146763
14518	Amp	11	58586212	58599815	0	0.141389
14519	Amp	11	58603410	58650488	0	0.13776
14520	Amp	11	58653538	58696146	0	0.150584
14521	Amp	11	58704909	58712202	0.105051	0.20092

14522	Amp	11	58715827	58743234	0.18072	0.20932
14523	Amp	11	58745643	58786269	0.724993	0.258146
14524	Amp	11	58790890	58795173	0.878688	0.26955
14525	Amp	11	58798903	58913897	0.27943	0.219027
14526	Amp	11	58917661	58951577	0.362221	0.226405
14527	Amp	11	58952433	58957329	0.444673	0.233708
14528	Amp	11	58957614	58963487	0.362221	0.22633
14529	Amp	11	58963547	58994290	0.444673	0.234139
14530	Amp	11	58994609	59038470	0.508503	0.239091
14531	Amp	11	59039768	59101750	0.405699	0.23018
14532	Amp	11	59111709	59114762	0.325321	0.222877
14533	Amp	11	59115109	59123819	0.243425	0.215068
14534	Amp	11	59124191	59148565	0.27943	0.219061
14535	Amp	11	59153196	59229677	0.230572	0.213676
14536	Amp	11	59229969	59242332	0.301081	0.220808
14537	Amp	11	59252946	59255195	0.325321	0.223223
14538	Amp	11	59255322	59284952	0.398217	0.229598
14539	Amp	11	59285296	59342713	0.437983	0.233229
14540	Amp	11	59344263	59362121	0	0.182892
14541	Amp	11	59364820	59379602	0.437983	0.233229
14542	Amp	11	59388932	59403530	0.355162	0.225418
14543	Amp	11	59408469	59437790	0.823952	0.265832
14544	Amp	11	59447736	59457748	0.243425	0.215495
14545	Amp	11	59459166	59475522	0.209057	0.211865
14546	Amp	11	59476233	59497886	0.264784	0.217777
14547	Amp	11	59506197	59510969	0.243425	0.215283
14548	Amp	11	59513484	59538674	0.273081	0.218436
14549	Amp	11	59547844	59578204	0	0.178022
14550	Amp	11	59580907	59581220	0	0.176315
14551	Amp	11	59581621	59614798	0	0.173162
14552	Amp	11	59614833	59719061	0	0.183784
14553	Amp	11	59722190	59725021	0	0.179404
14554	Amp	11	59726914	59789955	0	0.184398
14555	Amp	11	59790394	59839153	0.02372	0.191435
14556	Amp	11	59845671	59845671	0.187743	0.209774
14557	Amp	11	59847918	59852733	0.309523	0.221801
14558	Amp	11	59853202	59858538	0.347934	0.224873
14559	Amp	11	59861832	59867716	0.27943	0.218962
14560	Amp	11	59867876	59932322	0.301081	0.220845
14561	Amp	11	59932467	60058112	0.230572	0.213809
14562	Amp	11	60058642	60092158	0.652914	0.252069
14563	Amp	11	60095780	60115007	0.460107	0.235252
14564	Amp	11	60116074	60123201	0.355162	0.225803
14565	Amp	11	60123615	60370606	0.71758	0.257118
14566	Amp	11	60374386	60409659	1.24764	0.296082
14567	Amp	11	60410812	60444824	1.364151	0.304238
14568	Amp	11	60447593	60511474	1.295233	0.299287
14569	Amp	11	60511507	60516450	1.534719	0.31559
14570	Amp	11	60522622	60563653	0.972663	0.276626
14571	Amp	11	60564290	60606374	0.7517	0.260323
14572	Amp	11	60609842	60617023	0.51566	0.240102
14573	Amp	11	60617067	60617908	0.340434	0.224571
14574	Amp	11	60625272	60627737	0.51566	0.240381

14575 Amp	11	60627863	60722181	0.444673	0.234149
14576 Amp	11	60727289	60742226	0.805493	0.264348
14577 Amp	11	60744291	60744291	0.786952	0.262464
14578 Amp	11	60747256	60792332	0.430191	0.232265
14579 Amp	11	60792379	60792379	0.398217	0.229814
14580 Amp	11	60797331	60816821	0.724993	0.257642
14581 Amp	11	60817807	60824859	0.76883	0.261472
14582 Amp	11	60827532	60882537	0.963924	0.275806
14583 Amp	11	60888482	60888482	0.76883	0.261472
14584 Amp	11	60892106	60944605	0.734472	0.258399
14585 Amp	11	60945605	60969285	0.778204	0.262442
14586 Amp	11	60970385	61081485	0.564815	0.244102
14587 Amp	11	61086625	61117244	1.099256	0.285338
14588 Amp	11	61120529	61130533	1.029845	0.28037
14589 Amp	11	61131504	61165678	0.66072	0.252542
14590 Amp	11	61168506	61197989	0.564815	0.244307
14591 Amp	11	61198004	61198004	0.66072	0.252788
14592 Amp	11	61252993	61275018	1.039426	0.281037
14593 Amp	11	61279404	61318915	0.51566	0.2398
14594 Amp	11	61321007	61330321	0.209057	0.211935
14595 Amp	11	61331857	61390432	0.382767	0.228641
14596 Amp	11	61397808	61403030	0.982622	0.27727
14597 Amp	11	61412256	61447076	1.199568	0.292159
14598 Amp	11	61447877	61455641	1.130387	0.287424
14599 Amp	11	61457893	61458926	1.039426	0.281445
14600 Amp	11	61459029	61488580	0.906818	0.27191
14601 Amp	11	61491914	61495260	0.644689	0.251362
14602 Amp	11	61503723	61521350	0.523647	0.240521
14603 Amp	11	61521634	61636580	0.613517	0.248093
14604 Amp	11	61640977	61648472	0.128931	0.203937
14605 Amp	11	61651521	61660771	0.161592	0.207333
14606 Amp	11	61663516	61663516	0	0.172703
14607 Amp	11	61671062	61781986	0	0.164538
14608 Amp	11	61785617	61890385	0	0.175666
14609 Amp	11	61901041	61919953	0	0.180499
14610 Amp	11	61920202	61947644	0	0.183152
14611 Amp	11	61949461	62078623	0	0.166411
14612 Amp	11	62085431	62104399	0	0.170348
14613 Amp	11	62105185	62157233	0	0.187313
14614 Amp	11	62161826	62237447	0.056238	0.195475
14615 Amp	11	62237473	62301893	0	0.184961
14616 Amp	11	62312570	62314632	0.09318	0.199878
14617 Amp	11	62316058	62316531	0.556748	0.24347
14618 Amp	11	62317350	62321517	0.685617	0.254645
14619 Amp	11	62389946	62479683	0.050005	0.194437
14620 Amp	11	62482188	62482188	0.571214	0.24496
14621 Amp	11	62482765	62495406	0.540744	0.242306
14622 Amp	11	62503078	62547074	0.667776	0.253354
14623 Amp	11	62551058	62560566	0.571214	0.244873
14624 Amp	11	62561801	62595607	0.51566	0.239878
14625 Amp	11	62596022	62620378	0.209057	0.212078
14626 Amp	11	62627409	62637854	0.367646	0.22688
14627 Amp	11	62651415	62681104	0	0.176357

14628	Amp	11	62688853	62702009	0	0.186107
14629	Amp	11	62703247	62730490	0	0.169142
14630	Amp	11	62738173	62746827	0	0.176964
14631	Amp	11	62759612	62764728	0	0.147188
14632	Amp	11	62767274	62780771	0	0.158429
14633	Amp	11	62791235	62806406	0	0.143627
14634	Amp	11	62810056	62824275	0	0.135805
14635	Amp	11	62824968	62847813	0	0.131975
14636	Amp	11	62849839	62920080	0	0.1208
14637	Amp	11	62922198	62947319	0	0.16609
14638	Amp	11	62949825	63192820	0	0.115567
14639	Amp	11	63214889	63214889	0	0.12113
14640	Amp	11	63217225	63269800	0	0.109889
14641	Amp	11	63272590	63290373	0	0.136373
14642	Amp	11	63290661	63293303	0	0.138055
14643	Amp	11	63301137	63415239	0	0.121666
14644	Amp	11	63415384	63473456	0	0.11426
14645	Amp	11	63476922	63533547	0	0.118729
14646	Amp	11	63539617	63557729	0	0.163245
14647	Amp	11	63564880	63567171	0	0.155083
14648	Amp	11	63575166	63612378	0	0.163063
14649	Amp	11	63612454	63693638	0	0.158594
14650	Amp	11	63698855	63751031	0	0.168224
14651	Amp	11	63751575	63770037	0	0.160437
14652	Amp	11	63776812	63819867	0	0.154875
14653	Amp	11	63824389	63925331	0	0.160469
14654	Amp	11	63928715	64163853	0	0.17859
14655	Amp	11	64168158	64196595	0.033661	0.192597
14656	Amp	11	64197133	64205924	0.061778	0.196403
14657	Amp	11	64216132	64240002	0	0.150205
14658	Amp	11	64242772	64242772	0	0.136518
14659	Amp	11	64258382	64326957	0	0.130923
14660	Amp	11	64333962	64364313	0	0.100864
14661	Amp	11	64367826	64395830	0	0.151387
14662	Amp	11	64398787	64412995	0	0.156567
14663	Amp	11	64429552	64509144	0	0.153356
14664	Amp	11	64511165	64806290	0	0.148176
14665	Amp	11	64817487	64817543	0	0.152202
14666	Amp	11	64825254	64825436	0	0.148396
14667	Amp	11	64828200	64828200	0	0.161018
14668	Amp	11	65902628	65902628	0	0.162995
14669	Amp	11	65903039	65906669	0	0.188021
14670	Amp	11	65906682	65906682	0.079463	0.198288
14671	Amp	11	65906792	65943236	0.018387	0.190307
14672	Amp	11	65943281	65983923	0.09318	0.199626
14673	Amp	11	65985114	66003187	0.437983	0.233394
14674	Amp	11	66008121	66009114	0.580011	0.245678
14675	Amp	11	66010787	66013945	1.120124	0.286967
14676	Amp	11	66018482	66068230	1.010597	0.278988
14677	Amp	11	66073601	66073601	1.179216	0.29129
14678	Amp	11	66074331	66081805	1.930731	0.341813
14679	Amp	11	66087670	66097833	2.555572	0.380128
14680	Amp	11	66100180	66117988	2.404616	0.371552

14681	Amp	11	66118101	66119205	2.359227	0.368269
14682	Amp	11	66119635	66139024	2.158849	0.355966
14683	Amp	11	66142658	66210885	2.238069	0.360696
14684	Amp	11	66213641	66230101	2.135909	0.355027
14685	Amp	11	66230427	66233476	2.458756	0.375024
14686	Amp	11	66235633	66264698	2.750393	0.391779
14687	Amp	11	66270638	66270638	2.878516	0.39918
14688	Amp	11	66285772	66285772	3.359177	0.427343
14689	Amp	11	66286060	66324239	3.403911	0.430296
14690	Amp	11	66324343	66324343	3.72674	0.448133
14691	Amp	11	66328681	66333189	4.444356	0.48551
14692	Amp	11	66339254	66341981	4.551777	0.490823
14693	Amp	11	66343195	66349330	5.445472	0.532877
14694	Amp	11	66351205	66351318	6.08935	0.56314
14695	Amp	11	66351564	66361468	5.897742	0.553821
14696	Amp	11	66361739	66403140	6.058244	0.561557
14697	Amp	11	66412397	66433764	7.199544	0.61208
14698	Amp	11	66436824	66436824	7.664726	0.631824
14699	Amp	11	66439241	66451833	7.833881	0.638911
14700	Amp	11	66453906	66475158	8.064725	0.648246
14701	Amp	11	66476983	66509105	8.556974	0.668
14702	Amp	11	66513049	66529338	8.704996	0.674351
14703	Amp	11	66535198	66536990	9.061723	0.688913
14704	Amp	11	66540908	66540908	9.265068	0.697217
14705	Amp	11	66558891	66565728	9.825427	0.719086
14706	Amp	11	66566367	66582642	9.948936	0.723488
14707	Amp	11	66584269	66584269	10.201495	0.733663
14708	Amp	11	66595411	66622215	9.650316	0.711794
14709	Amp	11	66623859	66653736	9.87821	0.721362
14710	Amp	11	66655287	66659167	9.63277	0.711187
14711	Amp	11	66660040	66660040	9.859793	0.72006
14712	Amp	11	66662919	66669099	10.129941	0.730424
14713	Amp	11	66670089	66676097	10.417812	0.741835
14714	Amp	11	66684491	66713992	11.076964	0.767018
14715	Amp	11	66720062	66762182	11.983124	0.802098
14716	Amp	11	66767166	66767166	12.167312	0.80883
14717	Amp	11	66775640	66803124	12.55982	0.824295
14718	Amp	11	66805802	66808218	13.094926	0.844368
14719	Amp	11	66811300	66831470	13.587928	0.862302
14720	Amp	11	66835990	66835990	13.153847	0.846837
14721	Amp	11	66841050	66873601	12.628241	0.827093
14722	Amp	11	66876485	66876485	13.990439	0.877616
14723	Amp	11	66877783	66882589	13.513811	0.859779
14724	Amp	11	66889132	66899346	14.81056	0.907002
14725	Amp	11	66905608	66909895	13.417924	0.856479
14726	Amp	11	66910363	66910537	12.680413	0.829032
14727	Amp	11	66912116	66990942	12.134117	0.807896
14728	Amp	11	66993461	66993992	11.632145	0.788532
14729	Amp	11	66999239	67025858	11.712793	0.792053
14730	Amp	11	67036566	67037277	12.185197	0.8099
14731	Amp	11	67046835	67060923	11.928463	0.799857
14732	Amp	11	67064607	67082396	12.185197	0.80994
14733	Amp	11	67084617	67084617	12.455926	0.81988

14734 Amp	11	67091644	67093470	13.080267	0.843925
14735 Amp	11	67100374	67100374	13.474556	0.858109
14736 Amp	11	67103077	67112637	14.276024	0.887878
14737 Amp	11	67117271	67126829	14.516909	0.896929
14738 Amp	11	67127283	67156104	15.302159	0.925207
14739 Amp	11	67161007	67174570	15.359376	0.927138
14740 Amp	11	67176401	67180162	14.902815	0.910727
14741 Amp	11	67182158	67190484	15.910177	0.94693
14742 Amp	11	67192236	67199191	16.848673	0.980676
14743 Amp	11	67200105	67200105	17.196379	0.993206
14744 Amp	11	67207765	67213347	18.64417	1.041676
14745 Amp	11	67219296	67221477	18.957283	1.052416
14746 Amp	11	67223010	67226807	18.828995	1.048068
14747 Amp	11	67230774	67232206	19.171581	1.058925
14748 Amp	11	67235918	67235918	17.778878	1.012845
14749 Amp	11	67236827	67236827	18.375365	1.032849
14750 Amp	11	67248979	67249435	19.128428	1.057939
14751 Amp	11	67251953	67251953	18.62318	1.041215
14752 Amp	11	67253191	67253960	18.807658	1.047236
14753 Amp	11	67257903	67273727	19.171581	1.059192
14754 Amp	11	67274739	67274739	19.000192	1.053325
14755 Amp	11	67286673	67292735	17.475385	1.002802
14756 Amp	11	67293901	67324281	17.054065	0.988173
14757 Amp	11	67326389	67331320	17.317675	0.99726
14758 Amp	11	67337301	67346734	17.556752	1.005175
14759 Amp	11	67348402	67358493	17.759187	1.01235
14760 Amp	11	67361399	67386141	17.89516	1.016827
14761 Amp	11	67386997	67386997	18.204677	1.027285
14762 Amp	11	67390233	67402340	18.64417	1.042188
14763 Amp	11	67403095	67413540	18.456472	1.035869
14764 Amp	11	67422921	67472846	19.400441	1.066696
14765 Amp	11	67473110	67489030	19.632814	1.073873
14766 Amp	11	67490085	67490085	19.82634	1.080477
14767 Amp	11	67490111	67501654	20.151803	1.090768
14768 Amp	11	67508998	67526837	20.534209	1.103261
14769 Amp	11	67529387	67529387	20.68587	1.107781
14770 Amp	11	68003122	68003122	19.572281	1.072013
14771 Amp	11	68003149	68003149	18.375365	1.033052
14772 Amp	11	68007631	68032264	17.317675	0.997127
14773 Amp	11	68032282	68051786	17.412935	1.000748
14774 Amp	11	68052144	68053512	17.778878	1.013191
14775 Amp	11	68053569	68073888	17.617137	1.007676
14776 Amp	11	68075354	68096955	16.203162	0.957153
14777 Amp	11	68098983	68126000	15.965304	0.948997
14778 Amp	11	68133959	68149620	15.632845	0.937057
14779 Amp	11	68150265	68183041	15.283117	0.924613
14780 Amp	11	68185159	68192140	15.743591	0.940671
14781 Amp	11	68192360	68215814	15.927057	0.947932
14782 Amp	11	68216822	68216822	16.142788	0.955322
14783 Amp	11	68221697	68221697	15.743591	0.940784
14784 Amp	11	68223596	68245676	16.003748	0.950342
14785 Amp	11	68245975	68254538	16.062424	0.952349
14786 Amp	11	68255732	68270417	15.812503	0.943522

14787	Amp	11	68270432	68277104	15.927057	0.947413
14788	Amp	11	68281331	68284257	16.302613	0.960607
14789	Amp	11	68288005	68288005	16.865574	0.981315
14790	Amp	11	68288172	68289302	16.003748	0.950422
14791	Amp	11	68289837	68289874	16.062424	0.952488
14792	Amp	11	68292635	68326825	16.755306	0.977049
14793	Amp	11	68328273	68369505	16.65678	0.973917
14794	Amp	11	68376815	68376815	16.240018	0.958919
14795	Amp	11	68380922	68385254	16.451457	0.966129
14796	Amp	11	68399426	68416451	15.650561	0.937137
14797	Amp	11	68432454	68440465	16.487757	0.967643
14798	Amp	11	68444152	68455977	17.915554	1.018166
14799	Amp	11	68456574	68489918	17.475385	1.002797
14800	Amp	11	68493759	68493759	17.2159	0.993923
14801	Amp	11	68498305	68499317	18.104201	1.024316
14802	Amp	11	68514307	68529965	18.560988	1.039246
14803	Amp	11	68530487	68530487	18.333504	1.03139
14804	Amp	11	68536161	68536161	19.233054	1.061052
14805	Amp	11	68539210	68540882	20.279519	1.094441
14806	Amp	11	68541372	68541640	20.554413	1.103971
14807	Amp	11	68543020	68545969	20.108824	1.089041
14808	Amp	11	68547096	68548196	20.1733	1.091014
14809	Amp	11	68554019	68555541	21.12129	1.12162
14810	Amp	11	68558570	68559207	21.443814	1.132203
14811	Amp	11	68563983	68564247	23.146739	1.184496
14812	Amp	11	68564345	68574805	22.563712	1.167206
14813	Amp	11	68576499	68578809	24.204047	1.216767
14814	Amp	11	68583446	68585778	24.385388	1.222885
14815	Amp	11	68589744	68592792	26.081962	1.273407
14816	Amp	11	68599490	68599490	26.731348	1.292962
14817	Amp	11	68599539	68600235	26.429831	1.283771
14818	Amp	11	68610134	68612528	26.685451	1.291335
14819	Amp	11	68617866	68621934	25.003247	1.240812
14820	Amp	11	68637532	68637532	26.593495	1.288479
14821	Amp	11	68640536	68647767	26.081962	1.273638
14822	Amp	11	68652187	68673286	27.783453	1.32416
14823	Amp	11	68676629	68676629	28.41792	1.343435
14824	Amp	11	68678413	68680823	28.604764	1.348961
14825	Amp	11	68694616	68694616	29.594899	1.378182
14826	Amp	11	68701287	68701287	29.088175	1.36362
14827	Amp	11	68704011	68704576	28.737699	1.353227
14828	Amp	11	68707000	68719040	29.135861	1.364485
14829	Amp	11	68721832	68721832	29.252659	1.368161
14830	Amp	11	68721918	68727713	29.923156	1.388018
14831	Amp	11	68731558	68745998	30.40879	1.402617
14832	Amp	11	68748153	68756486	30.767965	1.412784
14833	Amp	11	68760430	68772848	30.274004	1.398295
14834	Amp	11	68780102	68782836	32.012464	1.448818
14835	Amp	11	68787127	68787127	31.009347	1.419598
14836	Amp	11	68788912	68818518	32.588221	1.464822
14837	Amp	11	68819703	68820358	31.776494	1.441894
14838	Amp	11	68823832	68825298	33.56167	1.492417
14839	Amp	11	68829636	68829636	34.893168	1.530178

14840 Amp	11	68831066	68838202	34.574252	1.521338
14841 Amp	11	68838360	68840119	35.704042	1.552809
14842 Amp	11	68840542	68845459	35.97934	1.560876
14843 Amp	11	68855964	68860067	36.761406	1.581938
14844 Amp	11	68860153	68860153	37.115616	1.591425
14845 Amp	11	68862814	68884734	37.317882	1.596843
14846 Amp	11	68885097	68887306	37.668228	1.606822
14847 Amp	11	68893329	68894936	38.610291	1.632111
14848 Amp	11	68896036	68908532	38.485659	1.628863
14849 Amp	11	68911023	68914185	38.737414	1.635598
14850 Amp	11	68914525	68914525	39.352381	1.651966
14851 Amp	11	68914934	68928429	39.633289	1.659733
14852 Amp	11	68929237	68948399	39.759159	1.663162
14853 Amp	11	68949498	68953903	39.480892	1.655095
14854 Amp	11	68956801	68956801	41.363012	1.705618
14855 Amp	11	68958518	68969593	41.937432	1.720752
14856 Amp	11	68976193	68976193	42.299212	1.730377
14857 Amp	11	68977376	68978214	42.037974	1.72312
14858 Amp	11	68978430	68978430	42.403732	1.732673
14859 Amp	11	68981734	68984439	43.982412	1.774239
14860 Amp	11	68985943	68987575	45.746875	1.82023
14861 Amp	11	68996968	68996968	46.549324	1.840538
14862 Amp	11	68998066	69008843	47.083961	1.854782
14863 Amp	11	69012165	69025499	48.540748	1.891781
14864 Amp	11	69031123	69033149	48.95739	1.902792
14865 Amp	11	69037338	69037338	48.631864	1.894441
14866 Amp	11	69048055	69048055	50.636431	1.944964
14867 Amp	11	69052961	69053408	51.532982	1.967718
14868 Amp	11	69060158	69065262	51.862739	1.976561
14869 Amp	11	69075023	69075023	53.855281	2.025767
14870 Amp	11	69075127	69079537	54.971535	2.053429
14871 Amp	11	69079791	69085565	56.852471	2.098883
14872 Amp	11	69088015	69090681	57.415784	2.112605
14873 Amp	11	69093249	69095162	59.465765	2.163128
14874 Amp	11	69095856	69096135	59.918869	2.174152
14875 Amp	11	69096881	69109119	60.287918	2.182669
14876 Amp	11	69109694	69121714	60.764069	2.19495
14877 Amp	11	69122922	69124816	61.295011	2.20757
14878 Amp	11	69125340	69129401	62.100115	2.227209
14879 Amp	11	69129807	69150315	64.154456	2.27572
14880 Amp	11	69153373	69156525	65.540493	2.308752
14881 Amp	11	69159054	69165431	65.831174	2.315756
14882 Amp	11	69165497	69165764	66.3835	2.328466
14883 Amp	11	69167494	69180225	68.207808	2.371986
14884 Amp	11	69180237	69189642	68.719874	2.384567
14885 Amp	11	69191192	69192923	69.457031	2.4017
14886 Amp	11	69196009	69200189	71.586195	2.450936
14887 Amp	11	69205892	69205892	71.849467	2.456798
14888 Amp	11	69206090	69229315	71.301252	2.444088
14889 Amp	11	69230205	69235483	72.600536	2.474375
14890 Amp	11	69236833	69241110	73.041713	2.484722
14891 Amp	11	69242000	69257843	73.432572	2.493657
14892 Amp	11	69257999	69257999	73.959063	2.505754

14893	Amp	11	69258086	69259237	73.688419	2.499888
14894	Amp	11	69272662	69280718	74.420884	2.517353
14895	Amp	11	69281441	69301278	74.656621	2.522895
14896	Amp	11	69305402	69305749	75.002667	2.531449
14897	Amp	11	69306180	69328516	75.572887	2.545014
14898	Amp	11	69329579	69333346	75.888213	2.55234
14899	Amp	11	69335955	69371512	75.975162	2.554411
14900	Amp	11	69372432	69372432	76.555118	2.568192
14901	Amp	11	69372653	69372653	75.976035	2.55561
14902	Amp	11	69380172	69383940	77.397486	2.587448
14903	Amp	11	69385925	69415653	79.286057	2.631894
14904	Amp	11	69415686	69429259	79.628319	2.639631
14905	Amp	11	69429308	69429308	80.287519	2.655285
14906	Amp	11	69431765	69431765	80.533842	2.660507
14907	Amp	11	69436673	69436673	80.822928	2.668373
14908	Amp	11	69440030	69440908	81.161792	2.67535
14909	Amp	11	69442131	69449854	81.493722	2.684686
14910	Amp	11	69452739	69455897	81.599301	2.687478
14911	Amp	11	69460294	69461097	81.257766	2.678414
14912	Amp	11	69462310	69462338	81.404951	2.682561
14913	Amp	11	69463318	69463318	84.204757	2.746179
14914	Amp	11	69466459	69467061	85.626699	2.778045
14915	Amp	11	69472725	69479371	86.980073	2.808221
14916	Amp	11	69480882	69480882	87.27994	2.815405
14917	Amp	11	69489745	69506105	89.118477	2.858076
14918	Amp	11	69508111	69524849	90.435696	2.902901
14919	Amp	11	69528300	69537143	90.193699	2.891847
14920	Amp	11	69546044	69555054	89.753096	2.875409
14921	Amp	11	69557015	69557015	89.657608	2.872163
14922	Amp	11	69558044	69562593	89.421034	2.866745
14923	Amp	11	69562960	69584415	89.006269	2.853814
14924	Amp	11	69584881	69585449	89.305616	2.864058
14925	Amp	11	69590840	69590840	89.183823	2.861266
14926	Amp	11	69591344	69598492	89.71495	2.87318
14927	Amp	11	69616738	69619535	89.753096	2.875275
14928	Amp	11	69619812	69619812	90.193699	2.887785
14929	Amp	11	69620462	69620859	90.364985	2.900167
14930	Amp	11	69625586	69650389	90.193699	2.886954
14931	Amp	11	69659758	69659758	90.042967	2.883968
14932	Amp	11	69661094	69661094	89.86003	2.879878
14933	Amp	11	69661481	69677679	88.637828	2.844358
14934	Amp	11	69678503	69688161	87.466965	2.819083
14935	Amp	11	69689162	69689162	86.997792	2.80884
14936	Amp	11	69689509	69699730	86.274662	2.792528
14937	Amp	11	69707465	69708300	85.933158	2.784644
14938	Amp	11	69708781	69708781	85.651944	2.778455
14939	Amp	11	69715985	69715985	85.251063	2.769731
14940	Amp	11	69716657	69718811	84.567155	2.754948
14941	Amp	11	69720554	69724133	84.233227	2.747082
14942	Amp	11	69724944	69738395	81.979545	2.696559
14943	Amp	11	69738836	69741702	82.316527	2.70455
14944	Amp	11	69744833	69746737	82.64603	2.711969
14945	Amp	11	69749625	69749625	82.256864	2.703126

14946	Amp	11	69753669	69754650	80.594154	2.661578
14947	Amp	11	69757916	69782674	81.161792	2.675481
14948	Amp	11	69783019	69802381	81.257766	2.678233
14949	Amp	11	69803076	69808838	80.744084	2.665643
14950	Amp	11	69808873	69832611	80.822928	2.667583
14951	Amp	11	69832982	69836687	80.744084	2.665643
14952	Amp	11	69843442	69854915	78.557991	2.614937
14953	Amp	11	69854948	69881218	77.788559	2.597127
14954	Amp	11	69882400	69884020	78.610551	2.616402
14955	Amp	11	69884534	69885212	78.163793	2.606283
14956	Amp	11	69954050	69954050	78.251641	2.608488
14957	Amp	11	69959977	69984330	77.481794	2.589751
14958	Amp	11	69991152	69991545	76.859796	2.57559
14959	Amp	11	69991645	69991817	77.246729	2.584306
14960	Amp	11	69992280	69992893	76.885442	2.575753
14961	Amp	11	69993211	70000098	77.527314	2.591326
14962	Amp	11	70002038	70008612	75.492216	2.542677
14963	Amp	11	70013220	70022890	75.267332	2.537653
14964	Amp	11	70023573	70037764	75.870015	2.551825
14965	Amp	11	70041956	70055168	75.38088	2.540451
14966	Amp	11	70056961	70071323	74.888978	2.528612
14967	Amp	11	70071927	70071927	75.002667	2.531598
14968	Amp	11	70074028	70074028	75.432377	2.541876
14969	Amp	11	70074911	70089341	75.060477	2.532433
14970	Amp	11	70091924	70149983	74.420884	2.51686
14971	Amp	11	70150099	70181807	74.200503	2.511237
14972	Amp	11	70182038	70190001	73.432572	2.493454
14973	Amp	11	70190611	70207441	73.018611	2.483858
14974	Amp	11	70210571	70210571	72.657677	2.47534
14975	Amp	11	70225613	70227742	71.586195	2.451057
14976	Amp	11	70229260	70230481	71.152919	2.440778
14977	Amp	11	70242901	70242901	70.525432	2.426179
14978	Amp	11	70243747	70243747	70.078346	2.416013
14979	Amp	11	70245530	70250039	69.162495	2.3947
14980	Amp	11	70250479	70255966	68.42756	2.377331
14981	Amp	11	70260564	70275513	67.945482	2.365915
14982	Amp	11	70280279	70311538	67.603958	2.357683
14983	Amp	11	70313181	70334487	68.407667	2.376707
14984	Amp	11	70335370	70343411	68.662662	2.38268
14985	Amp	11	70344127	70346616	67.889665	2.364649
14986	Amp	11	70346954	70366943	67.462113	2.354123
14987	Amp	11	70367349	70375611	66.439793	2.330138
14988	Amp	11	70377725	70385201	67.142035	2.346791
14989	Amp	11	70385410	70390687	65.831174	2.315867
14990	Amp	11	70394310	70395998	63.923282	2.270438
14991	Amp	11	70402628	70403081	63.314621	2.256064
14992	Amp	11	70405706	70409269	61.956896	2.223849
14993	Amp	11	70412126	70431107	61.160875	2.20425
14994	Amp	11	70431560	70453844	60.232778	2.181948
14995	Amp	11	70455226	70455226	59.69221	2.168665
14996	Amp	11	70455528	70455528	59.521282	2.164782
14997	Amp	11	70461610	70462065	59.806052	2.171358
14998	Amp	11	70465032	70474991	60.709982	2.193641

14999	Amp	11	70480702	70491578	60.482595	2.187823
15000	Amp	11	70501941	70503251	58.95652	2.150872
15001	Amp	11	70507636	70514354	57.808298	2.122409
15002	Amp	11	70516102	70518051	58.983423	2.151484
15003	Amp	11	70518404	70524002	58.730777	2.145109
15004	Amp	11	70524083	70546349	58.340417	2.135313
15005	Amp	11	70547213	70553754	58.004288	2.127423
15006	Amp	11	70555049	70555049	57.695429	2.120052
15007	Amp	11	70557032	70557032	55.642347	2.06953
15008	Amp	11	70557106	70557280	55.054522	2.055131
15009	Amp	11	70562750	70564025	52.995277	2.004608
15010	Amp	11	70574714	70581179	51.289702	1.962045
15011	Amp	11	70583495	70599056	50.800527	1.949529
15012	Amp	11	70604583	70604719	50.286541	1.93669
15013	Amp	11	70605607	70614759	50.367715	1.938699
15014	Amp	11	70617712	70652205	48.590494	1.892887
15015	Amp	11	70655522	70655522	49.472299	1.915887
15016	Amp	11	70655781	70666765	49.717313	1.921705
15017	Amp	11	70671399	70671399	47.729022	1.871182
15018	Amp	11	70671822	70676903	46.844457	1.848182
15019	Amp	11	70677888	70694346	45.057887	1.802728
15020	Amp	11	70696689	70728820	44.74848	1.794081
15021	Amp	11	70729255	70733462	44.19223	1.779953
15022	Amp	11	70737755	70749600	42.455805	1.734161
15023	Amp	11	70750254	70750254	40.998621	1.696032
15024	Amp	11	70759623	70759623	37.241952	1.594987
15025	Amp	11	70760305	70763702	36.737219	1.581478
15026	Amp	11	70765864	70776238	34.91784	1.531238
15027	Amp	11	70780953	70793744	35.610624	1.550544
15028	Amp	11	70796715	70802375	35.265904	1.540427
15029	Amp	11	70803425	70803657	35.778828	1.554851
15030	Amp	11	70806899	70806899	37.115616	1.59128
15031	Amp	11	70817197	70820148	35.681556	1.552414
15032	Amp	11	70825755	70837455	34.747423	1.526409
15033	Amp	11	70838600	70849532	33.603439	1.494256
15034	Amp	11	70849761	70851929	33.538555	1.491919
15035	Amp	11	70855163	70876988	33.149978	1.481159
15036	Amp	11	70880532	70880532	32.416235	1.460128
15037	Amp	11	70884341	70906199	32.299441	1.456508
15038	Amp	11	70906910	70908815	32.012464	1.448731
15039	Amp	11	70912479	70915852	32.084162	1.450384
15040	Amp	11	70916707	70927782	31.754483	1.440816
15041	Amp	11	70930202	70931618	30.839975	1.415081
15042	Amp	11	70931844	70936136	30.226475	1.397126
15043	Amp	11	70937447	70938094	29.828619	1.385125
15044	Amp	11	70940536	70948969	30.274004	1.398602
15045	Amp	11	70949160	71096315	29.480339	1.374809
15046	Amp	11	71096727	71096727	30.154207	1.394655
15047	Amp	11	71097018	71102882	30.5992	1.407764
15048	Amp	11	71109713	71109713	29.594899	1.378706
15049	Amp	11	71110013	71113917	29.966919	1.389667
15050	Amp	11	71117422	71121768	28.581394	1.348238
15051	Amp	11	71125421	71135358	28.487956	1.345568

15052	Amp	11	71135915	71136635	28.325336	1.340838
15053	Amp	11	71139338	71148608	28.808907	1.355413
15054	Amp	11	71160763	71171342	27.715579	1.321964
15055	Amp	11	71172147	71175957	28.668066	1.351022
15056	Amp	11	71176744	71177991	28.897355	1.358046
15057	Amp	11	71179998	71183209	29.664925	1.380537
15058	Amp	11	71184267	71185386	30.40879	1.402631
15059	Amp	11	71185457	71196641	29.71033	1.381883
15060	Amp	11	71197049	71226168	29.299351	1.369726
15061	Amp	11	71231578	71233341	28.148773	1.335149
15062	Amp	11	71233432	71233432	27.826464	1.32587
15063	Amp	11	71235268	71235268	28.148773	1.335027
15064	Amp	11	71236042	71237563	27.715579	1.322211
15065	Amp	11	71239775	71239775	28.301768	1.339866
15066	Amp	11	71245281	71255951	26.616743	1.289343
15067	Amp	11	71258636	71266545	25.117698	1.244819
15068	Amp	11	71270001	71274752	24.54421	1.227516
15069	Amp	11	71274891	71275850	24.181702	1.216058
15070	Amp	11	71277364	71278947	22.585145	1.167477
15071	Amp	11	71279408	71291364	22.122239	1.152903
15072	Amp	11	71302169	71312044	19.675402	1.075335
15073	Amp	11	71312062	71314285	19.252125	1.061771
15074	Amp	11	71316946	71332205	18.49898	1.036765
15075	Amp	11	71333698	71347538	17.956929	1.019316
15076	Amp	11	71348743	71352473	17.915554	1.017663
15077	Amp	11	71352709	71360056	17.856649	1.016097
15078	Amp	11	71360463	71389168	16.993677	0.986328
15079	Amp	11	71389198	71398870	17.317675	0.997019
15080	Amp	11	71399101	71399936	16.755306	0.97753
15081	Amp	11	71402258	71416865	16.41095	0.965127
15082	Amp	11	71416925	71423021	16.103101	0.953826
15083	Amp	11	71423920	71423920	15.984625	0.949834
15084	Amp	11	71429110	71429440	17.317675	0.996999
15085	Amp	11	71429532	71437258	17.256623	0.995143
15086	Amp	11	71438810	71471313	17.797592	1.013416
15087	Amp	11	71479586	71479847	17.576426	1.005913
15088	Amp	11	71480492	71494329	17.176603	0.992191
15089	Amp	11	71494362	71494362	16.528835	0.969281
15090	Amp	11	71496159	71499024	15.339812	0.92676
15091	Amp	11	71499359	71525087	15.302159	0.925362
15092	Amp	11	71525717	71538404	15.18674	0.920661
15093	Amp	11	71542601	71542601	13.990439	0.877641
15094	Amp	11	71544576	71556497	12.718133	0.830346
15095	Amp	11	71557473	71560198	11.524618	0.784525
15096	Amp	11	71560406	71560406	11.416123	0.779825
15097	Amp	11	71560804	71573179	11.291459	0.775498
15098	Amp	11	71574175	71580444	12.612387	0.826021
15099	Amp	11	71582841	71582841	12.275031	0.813221
15100	Amp	11	71588129	71592950	11.469971	0.781925
15101	Amp	11	71615907	71641093	11.451245	0.781611
15102	Amp	11	71645745	71649248	9.111233	0.690636
15103	Amp	11	71649989	71649989	8.82287	0.679044
15104	Amp	11	71652965	71659754	7.952174	0.643453

15105	Amp	11	71713405	71713405	7.134252	0.609331
15106	Amp	11	71714949	71719310	6.920476	0.600207
15107	Amp	11	71719657	71723459	6.450429	0.579738
15108	Amp	11	71729525	71735202	6.392987	0.576487
15109	Amp	11	71736632	71753030	6.348497	0.574545
15110	Amp	11	71802409	71802409	5.429672	0.532012
15111	Amp	11	71802551	71802551	5.337978	0.52811
15112	Amp	11	71812272	71816665	4.971485	0.51085
15113	Amp	11	71859610	71878244	4.444356	0.485334
15114	Amp	11	71880515	71880515	4.391125	0.482947
15115	Amp	11	71889740	71900042	3.790167	0.451494
15116	Amp	11	71903690	71903690	3.041652	0.409413
15117	Amp	11	71908877	71943975	3.112016	0.413318
15118	Amp	11	71943991	71991166	2.784457	0.394292
15119	Amp	11	71991533	71998540	2.016329	0.347126
15120	Amp	11	72008357	72019669	1.285475	0.298169
15121	Amp	11	72028377	72028377	1.190324	0.291578
15122	Amp	11	72031193	72037100	0.587735	0.246354
15123	Amp	11	72040559	72046201	0.843361	0.267049
15124	Amp	11	72047606	72058906	0.888878	0.270475
15125	Amp	11	72059556	72060134	1.473764	0.31131
15126	Amp	11	72066505	72073854	1.49455	0.312842
15127	Amp	11	72079117	72085780	2.061276	0.35008
15128	Amp	11	72087933	72096671	2.039783	0.348549
15129	Amp	11	72101365	72118565	1.473764	0.31131
15130	Amp	11	72119455	72134495	1.553308	0.316827
15131	Amp	11	72183005	72183005	1.415328	0.307333
15132	Amp	11	72189920	72205834	2.016329	0.346961
15133	Amp	11	72206725	72248149	1.953191	0.343395
15134	Amp	11	72251257	72279374	1.995109	0.345888
15135	Amp	11	72280065	72320436	2.393797	0.370645
15136	Amp	11	72327811	72348661	2.98257	0.405708
15137	Amp	11	72357853	72371028	3.171522	0.416951
15138	Amp	11	72376974	72376974	3.383286	0.429269
15139	Amp	11	72379492	72419907	3.549256	0.43848
15140	Amp	11	72420831	72436831	3.252252	0.42167
15141	Amp	11	72443192	72444840	4.416662	0.483725
15142	Amp	11	72447531	72448220	3.790167	0.451792
15143	Amp	11	72454083	72456391	4.778542	0.501889
15144	Amp	11	72465532	72466601	3.971602	0.461053
15145	Amp	11	72468972	72487383	3.933803	0.45908
15146	Amp	11	72492399	72514910	3.75156	0.449886
15147	Amp	11	72516805	72537322	3.920712	0.458846
15148	Amp	11	72539451	72540347	4.185786	0.47236
15149	Amp	11	72541327	72548123	4.009908	0.4634
15150	Amp	11	72552018	72552866	3.75156	0.449886
15151	Amp	11	72556445	72576306	4.009908	0.463614
15152	Amp	11	72577681	72582998	3.855237	0.455067
15153	Amp	11	72594629	72594629	3.971602	0.461546
15154	Amp	11	72597527	72612106	3.587309	0.440888
15155	Amp	11	72616161	72637459	3.252252	0.421593
15156	Amp	11	72640243	72653567	3.587309	0.440596
15157	Amp	11	72654148	72667293	3.701758	0.4472

15158	Amp	11	72668848	72668848	4.416662	0.483995
15159	Amp	11	72672727	72681075	4.30031	0.477516
15160	Amp	11	72683401	72731501	3.958753	0.460443
15161	Amp	11	72733702	72764980	4.090482	0.467671
15162	Amp	11	72765453	72825202	4.27183	0.476673
15163	Amp	11	72831222	72860230	3.72674	0.448153
15164	Amp	11	72872475	72895484	3.893345	0.457217
15165	Amp	11	72896192	72921868	3.933803	0.459301
15166	Amp	11	72929555	72942246	3.75156	0.449532
15167	Amp	11	72947047	72955574	4.035317	0.464488
15168	Amp	11	72960411	72964422	4.24321	0.475307
15169	Amp	11	72965273	72975822	4.364676	0.481054
15170	Amp	11	72978048	73005853	4.313672	0.478567
15171	Amp	11	73009064	73009064	4.131279	0.469503
15172	Amp	11	73011396	73033785	3.906866	0.458107
15173	Amp	11	73037339	73040788	4.24321	0.474868
15174	Amp	11	73044961	73067237	4.131279	0.469513
15175	Amp	11	73067417	73129464	4.090482	0.467693
15176	Amp	11	73146269	73146269	4.911986	0.508225
15177	Amp	11	73150843	73173337	5.077576	0.515846
15178	Amp	11	73174212	73204734	5.16411	0.519546
15179	Amp	11	73205309	73227375	5.489676	0.53465
15180	Amp	11	73232205	73247653	5.84242	0.551049
15181	Amp	11	73255409	73265201	6.212874	0.568337
15182	Amp	11	73267993	73269349	6.08935	0.562821
15183	Amp	11	73269444	73273825	5.997601	0.55887
15184	Amp	11	73277421	73285036	6.435656	0.578939
15185	Amp	11	73286876	73287642	6.288489	0.571874
15186	Amp	11	73288145	73290675	6.936308	0.600567
15187	Amp	11	73294076	73310039	7.631244	0.630136
15188	Amp	11	73316804	73333306	8.181109	0.652748
15189	Amp	11	73337059	73404269	7.314626	0.616279
15190	Amp	11	73409108	73424969	8.332564	0.659181
15191	Amp	11	73437538	73458928	8.080264	0.648968
15192	Amp	11	73473325	73524376	8.233051	0.655119
15193	Amp	11	73534113	73544483	8.283481	0.6568
15194	Amp	11	73547597	73567991	7.183547	0.610809
15195	Amp	11	73572945	73584783	7.392813	0.62028
15196	Amp	11	73590613	73598927	7.183547	0.610809
15197	Amp	11	73603532	73633138	7.282206	0.615429
15198	Amp	11	73633442	73652165	7.199544	0.611559
15199	Amp	11	73653926	73681128	7.952174	0.643091
15200	Amp	11	73681588	73706627	7.714123	0.634087
15201	Amp	11	73706643	73735157	7.440283	0.622089
15202	Amp	11	73737554	73764777	7.816717	0.637722
15203	Amp	11	73766000	73766000	7.614335	0.629663
15204	Amp	11	73781428	73809850	6.936308	0.600725
15205	Amp	11	73810101	73810101	7.314626	0.616273
15206	Amp	11	73820603	73832452	6.494707	0.58147
15207	Amp	11	73841485	73892406	7.664726	0.631993
15208	Amp	11	73895353	73947169	7.631244	0.630312
15209	Amp	11	73947492	74002546	7.664726	0.631852
15210	Amp	11	74005232	74009120	7.363353	0.618724

15211	Amp	11	74011576	74048574	7.583482	0.62798
15212	Amp	11	74049462	74070669	8.002024	0.645169
15213	Amp	11	74070957	74089256	7.765225	0.636033
15214	Amp	11	74089730	74116062	7.363353	0.618843
15215	Amp	11	74119370	74152063	8.215812	0.65408
15216	Amp	11	74154679	74343378	8.658307	0.672076
15217	Amp	11	74343459	74430739	8.806401	0.678428
15218	Amp	11	74435480	74455275	7.583482	0.628294
15219	Amp	11	74456041	74480933	7.440283	0.621942
15220	Amp	11	74481257	74500360	7.583482	0.628445
15221	Amp	11	74501338	74545007	7.37867	0.61919
15222	Amp	11	74545389	74655469	7.282206	0.614898
15223	Amp	11	74655710	74690012	7.614335	0.62944
15224	Amp	11	74690426	74694303	7.714123	0.63382
15225	Amp	11	74695857	74703874	8.181109	0.653342
15226	Amp	11	74707597	74793655	7.473408	0.623142
15227	Amp	11	74796064	74818371	7.538447	0.626517
15228	Amp	11	74819329	74819974	8.131547	0.651089
15229	Amp	11	74822407	74828526	8.316825	0.658691
15230	Amp	11	74830743	74833619	8.7383	0.675641
15231	Amp	11	74834030	74884235	8.87384	0.680865
15232	Amp	11	74893700	74893873	9.63277	0.711189
15233	Amp	11	74894127	74920657	9.333718	0.699869
15234	Amp	11	74935198	74967043	8.858746	0.680347
15235	Amp	11	74968999	74978327	9.297696	0.698047
15236	Amp	11	74978479	74982628	9.4207	0.703004
15237	Amp	11	74991039	74998194	9.507136	0.706474
15238	Amp	11	75001679	75003524	9.368074	0.70125
15239	Amp	11	75012082	75022293	8.888709	0.681704
15240	Amp	11	75026872	75033457	8.992196	0.685729
15241	Amp	11	75033550	75039731	9.265068	0.697253
15242	Amp	11	75040348	75080716	9.471331	0.705331
15243	Amp	11	75082128	75097950	9.686	0.71339
15244	Amp	11	75100212	75100976	9.843004	0.719734
15245	Amp	11	75103220	75109113	9.80717	0.718194
15246	Amp	11	75114778	75154225	10.705434	0.752718
15247	Amp	11	75154671	75190556	10.417812	0.74174
15248	Amp	11	75192631	75217832	9.351643	0.700122
15249	Amp	11	75222593	75279610	8.591044	0.669797
15250	Amp	11	75280652	75336854	9.146337	0.691946
15251	Amp	11	75339863	75339863	9.229684	0.695477
15252	Amp	11	75340223	75343132	9.146337	0.692347
15253	Amp	11	75346373	75348464	9.333718	0.699494
15254	Amp	11	75355359	75368056	9.19854	0.694012
15255	Amp	11	75369638	75370214	9.009204	0.686865
15256	Amp	11	75374470	75393630	8.922319	0.683334
15257	Amp	11	75396292	75403282	8.772488	0.676989
15258	Amp	11	75405443	75407114	8.233051	0.654841
15259	Amp	11	75416164	75424402	9.19854	0.693905
15260	Amp	11	75427333	75441986	9.281415	0.697679
15261	Amp	11	75445062	75467204	10.452292	0.742903
15262	Amp	11	75473292	75485661	10.34731	0.738877
15263	Amp	11	75489256	75490046	11.416123	0.780091

15264	Amp	11	75490173	75498353	11.451245	0.781512
15265	Amp	11	75501329	75516402	10.166941	0.731996
15266	Amp	11	75523440	75523590	8.939239	0.683686
15267	Amp	11	75528519	75534540	8.790054	0.677667
15268	Amp	11	75537146	75586839	8.858746	0.680296
15269	Amp	11	75587215	75619305	8.7383	0.675915
15270	Amp	11	75622933	75666473	9.009204	0.68646
15271	Amp	11	75675808	75724134	8.672968	0.672755
15272	Amp	11	75724380	75726585	9.896323	0.722
15273	Amp	11	75727879	75745522	10.183792	0.732779
15274	Amp	11	75748307	75772578	10.971689	0.76313
15275	Amp	11	75773805	75789059	11.750046	0.793454
15276	Amp	11	75799139	75799139	12.001211	0.803033
15277	Amp	11	75800140	75824477	11.696276	0.79124
15278	Amp	11	75829723	75848804	11.50723	0.78328
15279	Amp	11	75854213	75854227	11.731641	0.792352
15280	Amp	11	75862533	75869112	12.647575	0.82738
15281	Amp	11	75869510	75869510	12.991623	0.840316
15282	Amp	11	75876425	75883147	13.007244	0.841444
15283	Amp	11	75883675	75894897	12.55982	0.824405
15284	Amp	11	75900402	75912385	12.345058	0.815501
15285	Amp	11	75914805	75925148	12.114948	0.806905
15286	Amp	11	75929182	75944196	11.147569	0.769622
15287	Amp	11	75944394	76005591	10.689829	0.752072
15288	Amp	11	76007569	76007569	10.723194	0.753486
15289	Amp	11	76018848	76031374	10.452292	0.742941
15290	Amp	11	76035590	76035590	10.757147	0.754895
15291	Amp	11	76036506	76058377	11.613928	0.787702
15292	Amp	11	76062540	76064344	11.272465	0.77472
15293	Amp	11	76067785	76073876	11.632145	0.788391
15294	Amp	11	76085775	76112073	10.653252	0.750438
15295	Amp	11	76121828	76125569	10.868671	0.758837
15296	Amp	11	76126247	76126247	10.616849	0.749018
15297	Amp	11	76130309	76141404	11.909413	0.799541
15298	Amp	11	76142886	76142886	11.678994	0.790468
15299	Amp	11	76144284	76181697	11.50723	0.783801
15300	Amp	11	76185673	76188877	11.416123	0.77987
15301	Amp	11	76189483	76203043	10.40036	0.741247
15302	Amp	11	76204095	76265470	11.254828	0.773643
15303	Amp	11	76266720	76269481	11.488703	0.782814
15304	Amp	11	76275110	76275110	11.396997	0.779344
15305	Amp	11	76281546	76310875	11.112261	0.768564
15306	Amp	11	76314787	76321035	11.237152	0.773338
15307	Amp	11	76323198	76325226	11.876832	0.797814
15308	Amp	11	76327126	76327126	12.097158	0.805892
15309	Amp	11	76329419	76342483	12.362697	0.816615
15310	Amp	11	76342863	76342863	12.523772	0.822755
15311	Amp	11	76345412	76371247	12.735133	0.831139
15312	Amp	11	76382975	76388483	11.894261	0.798743
15313	Amp	11	76388762	76388825	11.731641	0.792602
15314	Amp	11	76389121	76417249	11.524618	0.784435
15315	Amp	11	76419295	76465995	12.541299	0.823508
15316	Amp	11	76467680	76469287	12.647575	0.827785

15317	Amp	11	76479322	76479877	11.218179	0.772488
15318	Amp	11	76480593	76485829	11.129193	0.769398
15319	Amp	11	76490135	76494322	11.928463	0.800007
15320	Amp	11	76498720	76527938	12.097158	0.80626
15321	Amp	11	76529077	76529077	12.058994	0.804553
15322	Amp	11	76530094	76531327	12.507948	0.821899
15323	Amp	11	76532857	76541603	12.330001	0.815296
15324	Amp	11	76542217	76548264	12.097158	0.80609
15325	Amp	11	76551872	76572037	11.291459	0.775481
15326	Amp	11	76572995	76611208	11.488703	0.782549
15327	Amp	11	76611506	76638514	11.378673	0.778775
15328	Amp	11	76639395	76696218	11.025256	0.765105
15329	Amp	11	76699924	76716059	11.166314	0.770355
15330	Amp	11	76716235	76733223	11.007149	0.764204
15331	Amp	11	76733615	76733615	11.237152	0.773195
15332	Amp	11	76736415	76749230	11.059715	0.766502
15333	Amp	11	76751735	76779672	11.166314	0.77053
15334	Amp	11	76785553	76830634	9.931775	0.723097
15335	Amp	11	76831165	76832632	10.505863	0.744881
15336	Amp	11	76837146	76842687	11.539522	0.7849
15337	Amp	11	76842946	76859016	12.860528	0.835423
15338	Amp	11	76862486	76869666	14.048553	0.879703
15339	Amp	11	76870551	76914754	15.150849	0.919589
15340	Amp	11	76917943	76949894	15.743591	0.94085
15341	Amp	11	76956128	76959978	15.417214	0.928895
15342	Amp	11	76960599	76995385	15.650561	0.93733
15343	Amp	11	77001571	77001614	14.259155	0.887472
15344	Amp	11	77012740	77017691	14.366019	0.89139
15345	Amp	11	77020006	77022803	13.173641	0.84711
15346	Amp	11	77024719	77024719	12.915337	0.838055
15347	Amp	11	77028687	77052706	12.772159	0.832024
15348	Amp	11	77056750	77062463	12.827194	0.834146
15349	Amp	11	77063401	77070014	13.064561	0.843316
15350	Amp	11	77081385	77081424	13.52858	0.860466
15351	Amp	11	77083706	77083706	13.858672	0.873005
15352	Amp	11	77087919	77088346	14.276024	0.88776
15353	Amp	11	77088638	77116946	14.463105	0.894549
15354	Amp	11	77120349	77126506	13.696984	0.866481
15355	Amp	11	77136867	77154518	14.922956	0.911668
15356	Amp	11	77157383	77164799	14.791074	0.906418
15357	Amp	11	77166240	77206175	15.022625	0.91503
15358	Amp	11	77214030	77215490	15.359376	0.927081
15359	Amp	11	77216980	77222248	15.302159	0.924959
15360	Amp	11	77223937	77257328	15.743591	0.940835
15361	Amp	11	77257452	77257873	15.66735	0.938269
15362	Amp	11	77265967	77281619	15.894232	0.946422
15363	Amp	11	77286883	77307369	16.003748	0.950665
15364	Amp	11	77310835	77310835	15.910177	0.946978
15365	Amp	11	77312465	77325918	16.808776	0.979557
15366	Amp	11	77326354	77326354	16.735182	0.976259
15367	Amp	11	77334211	77334211	14.423095	0.893157
15368	Amp	11	77334792	77382533	14.259155	0.88752
15369	Amp	11	77384758	77416371	15.24597	0.922782

15370	Amp	11	77420178	77420930	15.760688	0.941857
15371	Amp	11	77428002	77432569	15.612603	0.935785
15372	Amp	11	77460846	77481776	15.685307	0.939083
15373	Amp	11	77482787	77552158	16.355051	0.963091
15374	Amp	11	77553002	77553834	15.781148	0.942256
15375	Amp	11	77560702	77565463	16.042896	0.951554
15376	Amp	11	77573003	77589003	16.828307	0.979888
15377	Amp	11	77598663	77623894	16.203162	0.957454
15378	Amp	11	77624191	77628786	15.965304	0.949301
15379	Amp	11	77630083	77630083	16.162536	0.955991
15380	Amp	11	77633247	77646656	16.316047	0.961628
15381	Amp	11	77649980	77714261	15.894232	0.946644
15382	Amp	11	77716337	77745405	15.723975	0.940509
15383	Amp	11	77747489	77764022	16.162536	0.955962
15384	Amp	11	77767690	77804238	16.302613	0.960567
15385	Amp	11	77805102	77847087	16.618944	0.972457
15386	Amp	11	77850882	77864229	16.142788	0.954979
15387	Amp	11	77866800	77902154	16.302613	0.960925
15388	Amp	11	77904567	77915698	17.412935	1.0005
15389	Amp	11	77915917	77917245	17.778878	1.013076
15390	Amp	11	77929801	77931156	15.812503	0.943478
15391	Amp	11	77939083	77939083	15.377461	0.927602
15392	Amp	11	77945822	78016333	15.795475	0.942863
15393	Amp	11	78016877	78023729	16.103101	0.954067
15394	Amp	11	78024098	78028818	15.397291	0.928278
15395	Amp	11	78033359	78110036	14.734881	0.904684
15396	Amp	11	78113818	78128304	13.359728	0.854399
15397	Amp	11	78137903	78217064	13.89726	0.874014
15398	Amp	11	78219453	78237049	14.576253	0.898924
15399	Amp	11	78248585	78284396	14.81056	0.907437
15400	Amp	11	78285212	78414200	13.789953	0.870113
15401	Amp	11	78423016	78450642	13.173641	0.847107
15402	Amp	11	78451938	78477288	12.879705	0.836556
15403	Amp	11	78479294	78485720	12.753451	0.831733
15404	Amp	11	78491099	78504284	12.523772	0.822561
15405	Amp	11	78508944	78540439	13.30367	0.851952
15406	Amp	11	78543440	78548542	12.807917	0.833523
15407	Amp	11	78551486	78557012	12.879705	0.83639
15408	Amp	11	78557479	78568222	12.381595	0.817519
15409	Amp	11	78571374	78572758	12.436905	0.819353
15410	Amp	11	78573688	78589267	11.129193	0.76883
15411	Amp	11	78592867	78650124	10.794696	0.755765
15412	Amp	11	78650570	78655923	10.34731	0.738626
15413	Amp	11	78656203	78741172	11.307418	0.775951
15414	Amp	11	78741791	78766598	11.183333	0.771354
15415	Amp	11	78768631	78779606	11.112261	0.768488
15416	Amp	11	78781767	78828608	11.291459	0.77497
15417	Amp	11	78829638	78846720	11.059715	0.766359
15418	Amp	11	78851504	78893047	10.36543	0.739383
15419	Amp	11	78895894	78902224	9.561649	0.708412
15420	Amp	11	78911728	78922656	9.436384	0.704135
15421	Amp	11	78923224	79008164	9.702856	0.71441
15422	Amp	11	79008867	79034457	9.843004	0.719949

15423 Amp	11	79039174	79066349	9.702856	0.71441
15424 Amp	11	79066474	79067704	9.859793	0.720043
15425 Amp	11	79072538	79136262	9.4207	0.703275
15426 Amp	11	79144995	79146393	9.543403	0.708278
15427 Amp	11	79146638	79157391	8.299661	0.657755
15428 Amp	11	79157534	79189924	8.591044	0.669177
15429 Amp	11	79199013	79261449	8.400894	0.661742
15430 Amp	11	79266128	79276156	8.299661	0.657824
15431 Amp	11	79292881	79325161	8.050507	0.647549
15432 Amp	11	79325404	79435532	8.166506	0.652544
15433 Amp	11	79436996	79479813	8.332564	0.659324
15434 Amp	11	79481667	79481667	8.622652	0.670668
15435 Amp	11	79481756	79502551	8.316825	0.658778
15436 Amp	11	79502678	79511270	7.330797	0.617293
15437 Amp	11	79511549	79539124	7.952174	0.643313
15438 Amp	11	79544166	79546632	8.720964	0.674919
15439 Amp	11	79549268	79601315	8.87384	0.681329
15440 Amp	11	79603064	79611441	8.790054	0.678031
15441 Amp	11	79614413	79649860	8.68932	0.673629
15442 Amp	11	79651510	79774093	7.583482	0.628405
15443 Amp	11	79774764	79818392	8.080264	0.64891
15444 Amp	11	79818730	79828442	8.181109	0.653274
15445 Amp	11	79837413	79947756	7.553488	0.627084
15446 Amp	11	79949648	80018272	6.683082	0.589759
15447 Amp	11	80020591	80062417	6.760799	0.593466
15448 Amp	11	80066202	80075100	7.10077	0.607613
15449 Amp	11	80077445	80130934	5.953304	0.557103
15450 Amp	11	80132088	80160516	5.884474	0.553397
15451 Amp	11	80162066	80173722	5.783449	0.548375
15452 Amp	11	80174062	80195261	5.56363	0.538217
15453 Amp	11	80195458	80197445	5.252642	0.523672
15454 Amp	11	80201362	80208566	5.351891	0.528705
15455 Amp	11	80219602	80236076	5.489676	0.535184
15456 Amp	11	80236996	80244506	4.581809	0.492305
15457 Amp	11	80245107	80255788	4.416662	0.484138
15458 Amp	11	80274240	80300675	4.076638	0.466987
15459 Amp	11	80304366	80344009	4.14557	0.470196
15460 Amp	11	80345621	80358462	4.063271	0.466213
15461 Amp	11	80358616	80364327	4.185786	0.472501
15462 Amp	11	80365978	80373221	3.26417	0.421978
15463 Amp	11	80375408	80422537	2.98257	0.40569
15464 Amp	11	80425267	80453727	3.079122	0.411182
15465 Amp	11	80455087	80470611	3.549256	0.438536
15466 Amp	11	80471130	80490235	3.637615	0.443286
15467 Amp	11	80491832	80491832	3.72674	0.448544
15468 Amp	11	80497905	80584596	4.009908	0.463335
15469 Amp	11	80588608	80638613	3.842634	0.45472
15470 Amp	11	80639231	80639231	3.933803	0.459354
15471 Amp	11	80640674	80651264	3.217724	0.419401
15472 Amp	11	80652064	80656566	3.171522	0.416772
15473 Amp	11	80661197	80661337	2.470601	0.375373
15474 Amp	11	80665771	80686561	2.830877	0.396672
15475 Amp	11	80688043	80694197	2.737964	0.391414

15476	Amp	11	80696646	80698969	2.383144	0.370115
15477	Amp	11	80706018	80712360	3.05489	0.410068
15478	Amp	11	80714428	80778084	3.75156	0.449948
15479	Amp	11	80778572	80778572	4.355429	0.480483
15480	Amp	11	80779275	80788420	4.14557	0.470215
15481	Amp	11	80788518	80804102	3.86957	0.455518
15482	Amp	11	80815492	80830912	3.737077	0.449038
15483	Amp	11	80832563	80866628	4.30031	0.478016
15484	Amp	11	80868965	80882640	3.906866	0.457764
15485	Amp	11	80885696	80924057	3.815003	0.453015
15486	Amp	11	80926821	80934943	3.880586	0.456697
15487	Amp	11	80935302	80955800	3.801418	0.452137
15488	Amp	11	80966053	81009000	3.32332	0.425871
15489	Amp	11	81011184	81021778	3.45062	0.433223
15490	Amp	11	81022628	81036559	3.299582	0.424492
15491	Amp	11	81037162	81056787	3.182419	0.417139
15492	Amp	11	81060398	81082645	3.112016	0.413457
15493	Amp	11	81086281	81130273	4.27183	0.476698
15494	Amp	11	81131078	81134297	4.651516	0.495741
15495	Amp	11	81134548	81183187	4.765369	0.501112
15496	Amp	11	81186574	81217766	3.920712	0.458871
15497	Amp	11	81221699	81223113	4.063271	0.465862
15498	Amp	11	81226161	81240536	3.842634	0.454519
15499	Amp	11	81240824	81271219	3.933803	0.459075
15500	Amp	11	81307644	81313274	3.041652	0.409196
15501	Amp	11	81313557	81344563	2.878516	0.399802
15502	Amp	11	81345033	81378441	2.958746	0.404486
15503	Amp	11	81382456	81392512	3.288383	0.423597
15504	Amp	11	81393196	81415202	2.878516	0.399555
15505	Amp	11	81418555	81436746	2.784457	0.394184
15506	Amp	11	81440859	81455969	2.192225	0.358215
15507	Amp	11	81457031	81493894	2.567803	0.380763
15508	Amp	11	81494033	81500839	2.404616	0.371262
15509	Amp	11	81504890	81505804	2.447622	0.374088
15510	Amp	11	81515838	81525332	2.082332	0.35154
15511	Amp	11	81528412	81588423	2.203122	0.359208
15512	Amp	11	81589605	81635395	2.359227	0.368747
15513	Amp	11	81637194	81638144	2.447622	0.373829
15514	Amp	11	81642674	81729535	2.555572	0.380006
15515	Amp	11	81730360	81763407	2.415622	0.372339
15516	Amp	11	81767467	81776480	2.338154	0.367336
15517	Amp	11	81777987	81837716	2.102209	0.353072
15518	Amp	11	81864971	81864971	2.71525	0.389748
15519	Amp	11	81865927	81887752	2.158849	0.35643
15520	Amp	11	81890250	81900729	2.338154	0.367151
15521	Amp	11	81905181	81913687	2.914035	0.401302
15522	Amp	11	81916661	81925034	2.750393	0.391762
15523	Amp	11	81928037	81974087	2.180628	0.357611
15524	Amp	11	81974268	81979565	2.102209	0.352927
15525	Amp	11	81984747	82043772	2.180628	0.35763
15526	Amp	11	82044676	82073775	2.082332	0.351127
15527	Amp	11	82075925	82142180	1.737394	0.329176
15528	Amp	11	82146845	82190992	1.832922	0.335297

15529 Amp	11	82191271	82199284	1.963469	0.344195
15530 Amp	11	82208307	82246155	2.124253	0.354441
15531 Amp	11	82246923	82273186	1.963469	0.344195
15532 Amp	11	82273917	82311989	1.898353	0.339492
15533 Amp	11	82314176	82342495	1.757929	0.330898
15534 Amp	11	82365345	82373962	1.394558	0.305729
15535 Amp	11	82376305	82401014	1.503938	0.313398
15536 Amp	11	82414382	82447693	1.582671	0.319358
15537 Amp	11	82448109	82463927	0.888878	0.270113
15538 Amp	11	82466504	82473130	0.580011	0.245311
15539 Amp	11	82476372	82516510	0.362221	0.226314
15540 Amp	11	82521298	82524136	0.483654	0.237508
15541 Amp	11	82648805	82658085	0.734472	0.258843
15542 Amp	11	82658565	82679475	0.76883	0.261127
15543 Amp	11	82679749	82710144	0.724993	0.258272
15544 Amp	11	82711859	82729826	1.130387	0.287703
15545 Amp	11	82734568	82744328	0.483654	0.237577
15546 Amp	11	82746904	82841884	0.421967	0.231617
15547 Amp	11	82842035	82862746	0.452609	0.234905
15548 Amp	11	82863830	82982027	0.580011	0.245533
15549 Amp	11	82984917	83007859	0.613517	0.248389
15550 Amp	11	83008728	83017572	0.587735	0.246105
15551 Amp	11	83019480	83148065	0.375927	0.228073
15552 Amp	11	83149335	83177118	0.430191	0.232875
15553 Amp	11	83179960	83249442	0.508503	0.239616
15554 Amp	11	83250016	83260227	0.286675	0.219408
15555 Amp	11	83260580	83322144	0.508503	0.239616
15556 Amp	11	83330891	83346471	0.382767	0.228529
15557 Amp	11	83346740	83369540	0.398217	0.230002
15558 Amp	11	83373229	83418585	0.508503	0.239647
15559 Amp	11	83429270	83482944	0.693887	0.255147
15560 Amp	11	83483637	83488818	0.508503	0.239647
15561 Amp	11	83488973	83557894	0.587735	0.246192
15562 Amp	11	83558003	83733595	0.532449	0.241653
15563 Amp	11	83738817	83738817	0.460107	0.235108
15564 Amp	11	83739699	83743728	0.421967	0.23182
15565 Amp	11	83745988	83763041	0.033661	0.192384
15566 Amp	11	83767314	83815181	0	0.165456
15567 Amp	11	83816968	83855518	0	0.154827
15568 Amp	11	83868236	83917770	0	0.151619
15569 Amp	11	83918868	83938681	0	0.130284
15570 Amp	11	83939160	84012424	0	0.137371
15571 Amp	11	84014416	84055995	0	0.126722
15572 Amp	11	84056033	84134536	0	0.133332
15573 Amp	11	84135450	84136567	0	0.128006
15574 Amp	11	84136924	84148355	0	0.135496
15575 Amp	11	84153641	84261660	0	0.115606
15576 Amp	11	84267999	84301990	0	0.110078
15577 Amp	11	84305581	84358092	0	0.127684
15578 Amp	11	84472844	84514722	0	0.159464
15579 Amp	11	84522817	84726002	0	0.120403
15580 Amp	11	84726514	84874132	0	0.115528
15581 Amp	11	84878367	85054827	0	0.091921

15582	Amp	11	85080090	85119129	0	0.085797
15583	Amp	11	85122104	85135420	0	0.102212
15584	Amp	11	85135903	85147988	0	0.100798
15585	Amp	11	85153519	85207054	0	0.122037
15586	Amp	11	85208600	85216478	0	0.100798
15587	Amp	11	85218335	85239835	0	0.140431
15588	Amp	11	85246112	85246968	0	0.100798
15589	Amp	11	85251998	85269777	0	0.105779
15590	Amp	11	85276998	85281206	0	0.138639
15591	Amp	11	85281595	85287707	0	0.135024
15592	Amp	11	85293522	85379226	0	0.165917
15593	Amp	11	85383557	85466188	0.250263	0.216333
15594	Amp	11	85472602	85494969	0	0.185439
15595	Amp	11	85495311	85536078	0.273081	0.217995
15596	Amp	11	85538546	85653967	0.41418	0.231158
15597	Amp	11	85662927	85684675	0.086298	0.198602
15598	Amp	11	85685093	85718031	0.18072	0.209202
15599	Amp	11	85762205	85762205	0.175335	0.208803
15600	Amp	11	85764397	85798763	0.333141	0.223913
15601	Amp	11	85799259	85861916	0.286675	0.219357
15602	Amp	11	85862209	85869159	0.236222	0.21439
15603	Amp	11	85870235	85997076	0.13565	0.204247
15604	Amp	11	85998086	86008242	0.636206	0.250346
15605	Amp	11	86020533	86046663	0.580011	0.245365
15606	Amp	11	86054113	86148441	0.050005	0.194949
15607	Amp	11	86148612	86154109	0	0.18757
15608	Amp	11	86154149	86164947	0.038273	0.193306
15609	Amp	11	86165180	86172261	0	0.183105
15610	Amp	11	86172521	86283925	0	0.186735
15611	Amp	11	86288038	86292003	0.068197	0.196541
15612	Amp	11	86292300	86466877	0	0.18097
15613	Amp	11	86470228	86669294	0	0.174255
15614	Amp	11	86674251	86771998	0	0.167388
15615	Amp	11	86779538	86872505	0	0.174592
15616	Amp	11	86875638	86924146	0.209057	0.212205
15617	Amp	11	86924313	87015908	0.142507	0.204837
15618	Amp	11	87015984	87029531	0.209057	0.211677
15619	Amp	11	87029608	87044511	0.223025	0.213197
15620	Amp	11	87053883	87060207	0	0.182953
15621	Amp	11	87060446	87069002	0.098998	0.20003
15622	Amp	11	87069407	87128018	0.117531	0.202444
15623	Amp	11	87128055	87173991	0.098998	0.20003
15624	Amp	11	87174039	87219661	0.123589	0.202724
15625	Amp	11	87220475	87225259	0.098998	0.20003
15626	Amp	11	87235913	87235982	0.111991	0.201828
15627	Amp	11	87236575	87236646	0.375927	0.228087
15628	Amp	11	87251292	87282034	0.500055	0.238513
15629	Amp	11	87282174	87311148	0.437983	0.233482
15630	Amp	11	87312042	87350800	0.468576	0.235684
15631	Amp	11	87353672	87456703	0.18072	0.209424
15632	Amp	11	87460065	87527420	0.161592	0.207223
15633	Amp	11	87527794	87565383	0.201819	0.210981
15634	Amp	11	87565704	87595834	0.13565	0.204141

15635	Amp	11	87600176	87613747	0.236222	0.214867
15636	Amp	11	87613854	87652717	0.13565	0.204141
15637	Amp	11	87660289	87802482	0.236222	0.214376
15638	Amp	11	87803287	87851358	0.309523	0.221664
15639	Amp	11	87855382	87911041	0.128931	0.20337
15640	Amp	11	87911151	87954636	0.175335	0.208401
15641	Amp	11	87957913	87970164	0.142507	0.204772
15642	Amp	11	88007638	88095015	0.18072	0.208938
15643	Amp	11	88095413	88130250	0.123589	0.203202
15644	Amp	11	88132185	88184110	0.18072	0.209123
15645	Amp	11	88188817	88357684	0.123589	0.203202
15646	Amp	11	88366789	88393170	0.209057	0.212086
15647	Amp	11	88403928	88505800	0.123589	0.203202
15648	Amp	11	88511156	88595793	0.033661	0.192775
15649	Amp	11	88598606	88786390	0	0.186479
15650	Amp	11	88788180	88833926	0.02372	0.191124
15651	Amp	11	88833939	88970682	0.007199	0.189355
15652	Amp	11	88971653	88999361	0	0.18581
15653	Amp	11	89001816	89014994	0.056238	0.195132
15654	Amp	11	89015142	89341778	0	0.164909
15655	Amp	11	89342113	89378026	0	0.156275
15656	Amp	11	89384122	89677295	0	0.146953
15657	Amp	11	89681629	89695811	0	0.122842
15658	Amp	11	90098873	90098873	0	0.180966
15659	Amp	11	90098900	90098900	0	0.173402
15660	Amp	11	90099675	90099675	0	0.167883
15661	Amp	11	90100568	90173983	0	0.163238
15662	Amp	11	90174799	90215344	0	0.157234
15663	Amp	11	90215929	90629197	0	0.151868
15664	Amp	11	90630408	90641138	0.09318	0.199739
15665	Amp	11	90642337	90898819	0	0.151868
15666	Amp	11	90901264	90934463	0	0.164373
15667	Amp	11	90936343	90938911	0	0.11385
15668	Amp	11	90939565	90946288	0	0.14262
15669	Amp	11	90948992	90952532	0	0.130115
15670	Amp	11	90952822	90991877	0	0.120059
15671	Amp	11	90995602	91104965	0	0.111616
15672	Amp	11	91112484	91173351	0	0.081087
15673	Amp	11	91179165	91224153	0	0.083884
15674	Amp	11	91226338	91233709	0	0.094529
15675	Amp	11	91234406	91281436	0	0.086928
15676	Amp	11	91284845	91638820	0	0.126012
15677	Amp	11	91641274	91716542	0	0.128069
15678	Amp	11	91720868	91901679	0	0.125273
15679	Amp	11	91902640	92214583	0	0.123752
15680	Amp	11	92216618	92349610	0	0.13284
15681	Amp	11	92350514	92492096	0	0.122195
15682	Amp	11	92492480	92648610	0	0.113107
15683	Amp	11	92650551	92678609	0	0.150133
15684	Amp	11	92682533	93020721	0	0.147307
15685	Amp	11	93023385	93023385	0	0.141281
15686	Amp	11	93027097	93040806	0	0.146869
15687	Amp	11	93048042	93051762	0	0.154045

15688	Amp	11	93491739	93819689	0	0.146869
15689	Amp	11	93820477	94068507	0	0.154205
15690	Amp	11	94076452	94164747	0	0.155917
15691	Amp	11	94174968	94215712	0	0.144418
15692	Amp	11	94219369	94231286	0	0.151484
15693	Amp	11	94242774	94242774	0	0.144418
15694	Amp	11	94242993	94242993	0	0.152633
15695	Amp	11	94246478	94444256	0	0.176743
15696	Amp	11	94446949	94590746	0	0.137659
15697	Amp	11	94592797	94671149	0	0.114867
15698	Amp	11	94671274	94692583	0	0.102597
15699	Amp	11	94697835	94708853	0	0.135122
15700	Amp	11	94709613	94720737	0	0.185645
15701	Amp	11	94721728	94722753	0.168144	0.207483
15702	Amp	11	94725002	94729990	0	0.15696
15703	Amp	11	94730091	94832854	0.168144	0.207483
15704	Amp	11	94833739	94887879	0.149443	0.205771
15705	Amp	11	94893008	95034387	0	0.167527
15706	Amp	11	95041147	95115579	0	0.118822
15707	Amp	11	95117557	95363651	0	0.117004
15708	Amp	11	95365082	95538701	0	0.13813
15709	Amp	11	95542646	95669521	0	0.127555
15710	Amp	11	95671435	95719328	0	0.101505
15711	Amp	11	95728060	95741600	0	0.068979
15712	Amp	11	95741898	96051745	0	0.063392
15713	Amp	11	96052081	96097859	0	0.030603
15714	Amp	11	96103420	96292545	0	0.081126
15715	Amp	11	96294469	96334993	0	0.030603
15716	Amp	11	96335011	96597138	0	0.050497
15717	Amp	11	96597679	97139216	0	0.052267
15718	Amp	11	97139288	97173570	0	0.034129
15719	Amp	11	97175879	97220672	0	0.023579
15720	Amp	11	97222507	97524083	0	0.033227
15721	Amp	11	97526506	97537343	0	0.038606
15722	Amp	11	97542144	97692890	0	0.043932
15723	Amp	11	97693109	97849776	0	0.040119
15724	Amp	11	97851738	98011810	0	0.030471
15725	Amp	11	98017740	98091369	0	0.032083
15726	Amp	11	98096034	98216893	0	0.041411
15727	Amp	11	98217451	98222054	0	0.043331
15728	Amp	11	98223945	98234731	0	0.038005
15729	Amp	11	98243288	98669417	0	0.036085
15730	Amp	11	98672488	98708515	0	0.043136
15731	Amp	11	98712141	99094839	0	0.032318
15732	Amp	11	99095665	99100424	0	0.022989
15733	Amp	11	99102404	99122731	0	0.073512
15734	Amp	11	99126292	99151465	0	0.068888
15735	Amp	11	99151503	99188519	0	0.079134
15736	Amp	11	99191870	99258693	0	0.068888
15737	Amp	11	99260123	99320398	0	0.073512
15738	Amp	11	99331024	99365956	0	0.085232
15739	Amp	11	99366126	99451351	0	0.073512
15740	Amp	11	99458153	99458438	0	0.083444

15741	Amp	11	99459680	99550292	0	0.063156
15742	Amp	11	99550309	99595702	0	0.078313
15743	Amp	11	99595848	99640757	0	0.063156
15744	Amp	11	99641525	99694692	0	0.064608
15745	Amp	11	99703816	99812791	0	0.071253
15746	Amp	11	99816373	99842487	0	0.064608
15747	Amp	11	99842683	99863204	0	0.057557
15748	Amp	11	99864031	99920000	0	0.056105
15749	Amp	11	99921604	100018977	0	0.039739
15750	Amp	11	100019371	100051980	0	0.03436
15751	Amp	11	100055621	100209864	0	0.035923
15752	Amp	11	100210581	100245837	0	0.037574
15753	Amp	11	100245984	100254667	0	0.027642
15754	Amp	11	100263991	100357998	0	0.034964
15755	Amp	11	100360267	100499165	0	0.033313
15756	Amp	11	100502521	100506822	0	0.043619
15757	Amp	11	100508451	100604278	0	0.047187
15758	Amp	11	100606430	100628156	0	0.036881
15759	Amp	11	100628228	100873847	0	0.058726
15760	Amp	11	100878298	100940098	0	0.051404
15761	Amp	11	100940198	100943907	0	0.056126
15762	Amp	11	100946924	100979853	0	0.065795
15763	Amp	11	100985714	101032263	0	0.069116
15764	Amp	11	101037939	101042509	0	0.06669
15765	Amp	11	101042684	101067058	0	0.055129
15766	Amp	11	101067118	101071705	0	0.051561
15767	Amp	11	101076055	101093177	0	0.044318
15768	Amp	11	101093225	101116417	0	0.041093
15769	Amp	11	101118580	101476362	0	0.039529
15770	Amp	11	101479689	101598120	0	0.025661
15771	Amp	11	101599814	101626093	0	0.032888
15772	Amp	11	101626406	101634398	0	0.031391
15773	Amp	11	101640270	101783420	0	0.036645
15774	Amp	11	101787678	101841907	0	0.029417
15775	Amp	11	101843429	102116371	0	0.01752
15776	Amp	11	102392217	102395912	0	0.020584
15777	Amp	11	102399478	102425341	0	0.030135
15778	Amp	11	102427271	102525516	0	0.065434
15779	Amp	11	102526668	102540359	0	0.068941
15780	Amp	11	102543212	102551260	0	0.084354
15781	Amp	11	102552043	102554677	0	0.073203
15782	Amp	11	102554760	102883125	0	0.080342
15783	Amp	11	102883907	102973691	0	0.073203
15784	Amp	11	102974124	103084362	0	0.064887
15785	Amp	11	103093410	103352633	0	0.072788
15786	Amp	11	103352667	103366097	0	0.074595
15787	Amp	11	103378508	103383829	0	0.055675
15788	Amp	11	103385693	103428322	0	0.047774
15789	Amp	11	103429439	103448231	0	0.045967
15790	Amp	11	103593639	103854096	0	0.012267
15791	Amp	11	103854304	103948605	0	0.005091
15792	Amp	11	103948828	104988540	0	0.012058
15793	Amp	11	104990672	105001074	0	0.041216

15794	Amp	11	105001376	105255641	0	0.029799
15795	Amp	11	105256150	105911212	0	0.038989
15796	Amp	11	105912707	105944043	0	0.045374
15797	Amp	11	105946899	106100186	0	0.018442
15798	Amp	11	106100747	106323380	0	0.012058
15799	Amp	11	106327000	106499792	0	0.016202
15800	Amp	11	106500856	107098901	0	0.01783
15801	Amp	11	107101316	107570212	0	0.026216
15802	Amp	11	107573980	108849499	0	0.013686
15803	Amp	11	108853573	109070522	0	0.012058
15804	Amp	11	109071024	110343846	0	0.008737
15805	Amp	11	110344436	110511188	0	0.006967
15806	Amp	11	110511442	111587855	0	0.014481
15807	Amp	11	111590972	111657322	0	0.006967
15808	Amp	11	111661277	111781612	0	0.014374
15809	Amp	11	111784909	112141952	0	0.007406
15810	Amp	11	112145791	112506825	0	0.016944
15811	Amp	11	112508436	112546351	0	0.009538
15812	Amp	11	112552118	113515321	0	0
15813	Amp	11	113517819	113543328	0	0.001854
15814	Amp	11	113547756	114357916	0	0
15815	Amp	11	114359539	115097686	0	0.001946
15816	Amp	11	115109271	115124678	0	0.010568
15817	Amp	11	115125688	116630967	0	0.001946
15818	Amp	11	116631063	116684545	0	0
15819	Amp	11	116685001	116691280	0	0.009484
15820	Amp	11	116701489	116762259	0	0.038074
15821	Amp	11	116763841	116939923	0	0.017997
15822	Amp	11	116941606	117287214	0	0
15823	Amp	11	117292927	117502340	0	0.008138
15824	Amp	11	117502717	117544925	0	0.0158
15825	Amp	11	117549475	117737898	0	0.008138
15826	Amp	11	117739672	117761200	0	0.019811
15827	Amp	11	117764730	117792194	0	0.034683
15828	Amp	11	117798904	117845738	0	0.019811
15829	Amp	11	117847636	117883809	0	0.029832
15830	Amp	11	117884826	118075671	0	0.039576
15831	Amp	11	118075976	118109255	0	0.045296
15832	Amp	11	118115173	118182320	0	0.072995
15833	Amp	11	118182441	118195389	0	0.068836
15834	Amp	11	118199380	118264734	0	0.060068
15835	Amp	11	118265030	118319892	0	0.078686
15836	Amp	11	118336938	118432011	0	0.051618
15837	Amp	11	118435617	118485066	0	0.070162
15838	Amp	11	118485090	118485325	0	0.067924
15839	Amp	11	118487003	118563706	0	0.072952
15840	Amp	11	118564650	118667478	0	0.066692
15841	Amp	11	118667654	118798199	0	0.05891
15842	Amp	11	118801805	119007103	0	0.027056
15843	Amp	11	119008858	119072889	0	0.022028
15844	Amp	11	119076206	119092106	0	0.012283
15845	Amp	11	119099504	119261831	0	0.022535
15846	Amp	11	119267603	119283268	0	0.012283

15847	Amp	11	119287193	119487112	0	0
15848	Amp	11	119487710	119689739	0	0.001994
15849	Amp	11	119690070	120058736	0	0.006285
15850	Amp	11	120058923	120268835	0	0.001994
15851	Amp	11	120269528	120328019	0	0.00778
15852	Amp	11	120334263	121297469	0	0.001994
15853	Amp	11	121313102	121347807	0	0
15854	Amp	11	121349015	121386385	0	0.006977
15855	Amp	11	121388312	121401897	0	0
15856	Amp	11	121404597	121439855	0	0.007455
15857	Amp	11	121446449	121908759	0	0
15858	Amp	11	121908822	122057711	0	0.00941
15859	Amp	11	122059999	122704229	0	0
15860	Amp	11	122705899	123126116	0	0.007318
15861	Amp	11	123130376	123137759	0	0.057841
15862	Amp	11	123142528	123340266	0	0.050523
15863	Amp	11	123343410	123381490	0	0.058034
15864	Amp	11	123382094	123427380	0	0.073936
15865	Amp	11	123433942	123467184	0	0.058034
15866	Amp	11	123470909	123519947	0	0.082686
15867	Amp	11	123520261	123567270	0	0.100986
15868	Amp	11	123567410	123642505	0	0.093475
15869	Amp	11	123643425	123658271	0	0.051401
15870	Amp	11	123659762	123670945	0	0.093163
15871	Amp	11	123671312	123712908	0	0.051401
15872	Amp	11	123717071	123732237	0	0.058972
15873	Amp	11	123735229	124039624	0	0.062213
15874	Amp	11	124046070	124121108	0	0.01169
15875	Amp	11	124123131	125892996	0	0.008449
15876	Amp	11	125893851	125910021	0	0.025951
15877	Amp	11	125913212	126366419	0	0.017502
15878	Amp	11	126367586	126380525	0	0.022266
15879	Amp	11	126388167	126517348	0	0.017502
15880	Amp	11	126517595	126714102	0	0
15881	Amp	11	126716259	126883036	0	0.010424
15882	Amp	11	126885579	126965606	0	0.013824
15883	Amp	11	126965725	127161871	0	0.0034
15884	Amp	11	127163983	127287003	0	0
15885	Amp	11	127290200	127421252	0	0.004467
15886	Amp	11	127423154	127664538	0	0.011714
15887	Amp	11	127664554	127748420	0	0.043111
15888	Amp	11	127751153	127845590	0	0.044523
15889	Amp	11	127848273	128206547	0	0.040056
15890	Amp	11	128206701	128534550	0	0.027478
15891	Amp	11	128538329	128865771	0	0.026066
15892	Amp	11	128869468	128903655	0	0.047017
15893	Amp	11	128908420	128910802	0	0.055167
15894	Amp	11	128911444	128935919	0	0.060254
15895	Amp	11	128937028	129009303	0	0.055167
15896	Amp	11	129014413	129069587	0	0.068137
15897	Amp	11	129074948	129093632	0	0.072391
15898	Amp	11	129094530	129257934	0	0.076456
15899	Amp	11	129262422	129391269	0	0.063327

15900 Amp	11	129402160	129554363	0	0.059073
15901 Amp	11	129554491	129650311	0	0.071393
15902 Amp	11	129652073	129870253	0	0.063243
15903 Amp	11	129876793	130074556	0	0.072255
15904 Amp	11	130077207	130086205	0	0.076375
15905 Amp	11	130086743	130438984	0	0.086325
15906 Amp	11	130440033	130537625	0	0.065558
15907 Amp	11	130540519	130563838	0	0.095636
15908 Amp	11	130571702	130571702	0	0.091517
15909 Amp	11	130759533	130791319	0	0.042619
15910 Amp	11	130794396	130915310	0	0.035808
15911 Amp	11	130921347	130966328	0	0.031454
15912 Amp	11	130971084	130971084	0	0.011311
15913 Amp	11	130974820	131352364	0	0.017935
15914 Amp	11	131354553	131696699	0	0.035188
15915 Amp	11	131698074	132097164	0	0.017935
15916 Amp	11	132097764	132413595	0	0.037912
15917 Amp	11	132413813	132857642	0	0.031288
15918 Amp	11	132858891	132925743	0	0.041267
15919 Amp	11	132930674	132952941	0	0.031288
15920 Amp	11	132953650	133122224	0	0.035594
15921 Amp	11	133393701	133926781	0	0.052734
15922 Amp	11	134117835	134272740	0	0.06769
15923 Amp	12	780472	865284	0	0.148431
15924 Amp	12	866110	891685	0	0.152048
15925 Amp	12	891909	1034147	0	0.145111
15926 Amp	12	1038668	1095711	0	0.136242
15927 Amp	12	1096536	1112660	0	0.126521
15928 Amp	12	1117070	1159910	0	0.121749
15929 Amp	12	1168297	1197489	0	0.129001
15930 Amp	12	1752621	1752814	0	0.136576
15931 Amp	12	1752992	1757633	0	0.141842
15932 Amp	12	1758235	1763521	0	0.136046
15933 Amp	12	1766046	1815039	0	0.150101
15934 Amp	12	1819540	1877084	0	0.151516
15935 Amp	12	1878079	1949499	0	0.153294
15936 Amp	12	1949529	2000292	0	0.148028
15937 Amp	12	2011392	2060101	0	0.110026
15938 Amp	12	2062425	2208478	0	0.113124
15939 Amp	12	2210040	2246640	0	0.116404
15940 Amp	12	2247269	2302834	0	0.108619
15941 Amp	12	2305509	2329644	0	0.118314
15942 Amp	12	2331298	2815379	0	0.101187
15943 Amp	12	2820078	2936823	0	0.091491
15944 Amp	12	2986968	2990794	0	0.083875
15945 Amp	12	2991767	3086778	0	0.089089
15946 Amp	12	3089592	3107303	0	0.079801
15947 Amp	12	3110694	3289301	0	0.072978
15948 Amp	12	3289924	3319593	0	0.082444
15949 Amp	12	3321202	3377110	0	0.072978
15950 Amp	12	3377689	3387520	0	0.06941
15951 Amp	12	3391672	3431731	0	0.080441
15952 Amp	12	3433287	3538365	0	0.06941

15953 Amp	12	3545285	3563563	0	0.08478
15954 Amp	12	3566397	3717477	0	0.109154
15955 Amp	12	3717920	3771745	0	0.121181
15956 Amp	12	3775736	3804026	0	0.125639
15957 Amp	12	3804766	3902486	0	0.123806
15958 Amp	12	3903084	3996305	0	0.108436
15959 Amp	12	3996744	4151739	0	0.10702
15960 Amp	12	4151805	4294223	0	0.102562
15961 Amp	12	4294377	4431126	0	0.097348
15962 Amp	12	4431476	4562324	0	0.098898
15963 Amp	12	4562499	4688001	0	0.08687
15964 Amp	12	4694990	4717206	0	0.097915
15965 Amp	12	4719637	4742214	0	0.080255
15966 Amp	12	4743346	5625153	0	0.06784
15967 Amp	12	5628495	5795769	0	0.058324
15968 Amp	12	5796973	5808990	0	0.062116
15969 Amp	12	5813779	5954808	0	0.065698
15970 Amp	12	5955904	5971124	0	0.081261
15971 Amp	12	5975025	6042707	0	0.090166
15972 Amp	12	6043134	6110436	0	0.083198
15973 Amp	12	6117381	6342424	0	0.091219
15974 Amp	12	6345932	6381018	0	0.089591
15975 Amp	12	6381443	6387997	0	0.086009
15976 Amp	12	6388185	6643624	0	0.08113
15977 Amp	12	6645025	6691963	0	0.091313
15978 Amp	12	6702591	6736879	0	0.087521
15979 Amp	12	6737366	6876328	0	0.097187
15980 Amp	12	6878319	6957905	0	0.086566
15981 Amp	12	6965929	7010959	0	0.076384
15982 Amp	12	7013092	7215718	0	0.066718
15983 Amp	12	7222528	7222989	0	0.051155
15984 Amp	12	7223650	7259755	0	0.047458
15985 Amp	12	7262050	7274392	0	0.04568
15986 Amp	12	7274714	7280867	0	0.095129
15987 Amp	12	7285080	7336757	0	0.087575
15988 Amp	12	7344666	7480055	0	0.096487
15989 Amp	12	7485258	7502541	0	0.047038
15990 Amp	12	7502682	7566435	0	0.034748
15991 Amp	12	7570749	7583252	0	0.050987
15992 Amp	12	7584500	7638198	0	0.048111
15993 Amp	12	7642316	7647052	0	0.040611
15994 Amp	12	7651050	7801455	0	0.024373
15995 Amp	12	7805181	7993566	0	0.026561
15996 Amp	12	7994093	8098141	0	0.042086
15997 Amp	12	8100930	8157111	0	0.024373
15998 Amp	12	8443757	8596147	0	0.012326
15999 Amp	12	8597572	8639985	0	0.022542
16000 Amp	12	8647970	8685203	0	0.031209
16001 Amp	12	8685427	8938072	0	0.036906
16002 Amp	12	8938263	8996707	0	0.044732
16003 Amp	12	9000550	9184428	0	0.031209
16004 Amp	12	9187150	9764510	0	0.026459
16005 Amp	12	9764815	9939974	0	0.017792

16006 Amp	12	9942044	10305422	0	0.031737
16007 Amp	12	10305588	10344125	0	0.023057
16008 Amp	12	10350985	10375714	0	0.030528
16009 Amp	12	10375734	10446882	0	0.023057
16010 Amp	12	10447769	10571258	0	0.017792
16011 Amp	12	10578116	10629369	0	0.023011
16012 Amp	12	10632074	10836935	0	0.017792
16013 Amp	12	10837524	11000226	0	0.024659
16014 Amp	12	11200674	11200674	0	0.017792
16015 Amp	12	11203172	12073806	0	0.007576
16016 Amp	12	12074161	13212879	0	0
16017 Amp	12	13217302	13463601	0	0.003127
16018 Amp	12	13463967	13654132	0	0.009086
16019 Amp	12	13657469	13845684	0	0.00596
16020 Amp	12	13847579	14720678	0	0.014641
16021 Amp	12	14724010	14735831	0	0.019253
16022 Amp	12	14736917	14861359	0	0.027741
16023 Amp	12	14868147	14963668	0	0.043072
16024 Amp	12	14963966	15083313	0	0.055389
16025 Amp	12	15083365	15115865	0	0.059148
16026 Amp	12	15116297	15116297	0	0.057221
16027 Amp	12	15117047	15127522	0	0.064416
16028 Amp	12	15127981	15152066	0	0.06824
16029 Amp	12	15153072	15240278	0	0.059753
16030 Amp	12	15242614	15252935	0	0.066854
16031 Amp	12	15254251	15256610	0	0.055835
16032 Amp	12	15265811	15322188	0	0.051222
16033 Amp	12	15329947	15386222	0	0.044121
16034 Amp	12	15386946	15585592	0	0.040362
16035 Amp	12	15586386	15758462	0	0.052296
16036 Amp	12	15759194	15810000	0	0.046337
16037 Amp	12	15814670	16965055	0	0.034403
16038 Amp	12	16984418	17348378	0	0.040235
16039 Amp	12	17348527	17366369	0	0.044059
16040 Amp	12	17366713	17568160	0	0.031742
16041 Amp	12	17576239	18014977	0	0.039235
16042 Amp	12	18021269	18162387	0	0.04439
16043 Amp	12	18163015	18188890	0	0.058096
16044 Amp	12	18191232	18229736	0	0.077401
16045 Amp	12	18229773	18229867	0	0.063997
16046 Amp	12	18235011	18235011	0	0.04578
16047 Amp	12	18235012	18235637	0	0.032075
16048 Amp	12	18235732	18235793	0	0.042325
16049 Amp	12	18235993	18235993	0	0.057618
16050 Amp	12	18247102	18347263	0	0.069853
16051 Amp	12	18347345	18573364	0	0.072475
16052 Amp	12	18573771	18805700	0	0.07418
16053 Amp	12	18806409	18963951	0	0.081392
16054 Amp	12	18967989	19160520	0	0.07418
16055 Amp	12	19160855	19590675	0	0.06865
16056 Amp	12	19590721	19647169	0	0.063495
16057 Amp	12	19647685	19683200	0	0.048202
16058 Amp	12	19683692	19791283	0	0.059281

16059	Amp	12	19791411	19860537	0	0.048202
16060	Amp	12	19864506	19865288	0	0.042781
16061	Amp	12	19866036	19980253	0	0.058448
16062	Amp	12	19981359	20094429	0	0.042781
16063	Amp	12	20094714	20154334	0	0.057837
16064	Amp	12	20154797	20154797	0	0.062819
16065	Amp	12	20164267	20188524	0	0.064348
16066	Amp	12	20191363	20340562	0	0.047762
16067	Amp	12	20340848	20476944	0	0.035921
16068	Amp	12	20482692	20508599	0	0.041033
16069	Amp	12	20512940	20585906	0	0.035921
16070	Amp	12	20590097	20604634	0	0.062519
16071	Amp	12	20608460	20855049	0	0.035921
16072	Amp	12	20884733	20992687	0	0.048519
16073	Amp	12	20992865	21188322	0	0.056947
16074	Amp	12	21205014	21218228	0	0.051965
16075	Amp	12	21219410	21257155	0	0.037956
16076	Amp	12	21262713	21302525	0	0.043308
16077	Amp	12	21305866	21313030	0	0.04977
16078	Amp	12	21313183	21427274	0	0.06404
16079	Amp	12	21429570	21640341	0	0.058689
16080	Amp	12	21641040	21735359	0	0.047535
16081	Amp	12	21740315	22023092	0	0.06037
16082	Amp	12	22030052	22084538	0	0.058966
16083	Amp	12	22084563	22142832	0	0.052504
16084	Amp	12	22156477	22362959	0	0.055057
16085	Amp	12	22363689	22703086	0	0.051023
16086	Amp	12	22703127	22847699	0	0.049443
16087	Amp	12	22848032	22952336	0	0.051288
16088	Amp	12	22953191	23070178	0	0.060868
16089	Amp	12	23087460	23122395	0	0.08425
16090	Amp	12	23123365	23323486	0	0.07467
16091	Amp	12	23332192	23358313	0	0.083701
16092	Amp	12	23363209	23451435	0	0.090888
16093	Amp	12	23453202	23650287	0	0.080638
16094	Amp	12	23652289	23688680	0	0.071998
16095	Amp	12	23688877	23738680	0	0.07555
16096	Amp	12	23743678	24035312	0	0.068235
16097	Amp	12	24037072	24055163	0	0.073141
16098	Amp	12	24055375	24055544	0	0.069589
16099	Amp	12	24055811	24096387	0	0.074582
16100	Amp	12	24098647	24284172	0	0.078715
16101	Amp	12	24287085	24295020	0	0.06789
16102	Amp	12	24304282	24365110	0	0.059254
16103	Amp	12	24366129	24368921	0	0.068594
16104	Amp	12	24372326	24576422	0	0.064462
16105	Amp	12	24576747	24649654	0	0.072841
16106	Amp	12	24651495	24690644	0	0.064462
16107	Amp	12	24693588	25169730	0	0.055121
16108	Amp	12	25172979	25524106	0	0.066983
16109	Amp	12	25530562	25555886	0	0.06265
16110	Amp	12	25556598	25647802	0	0.06968
16111	Amp	12	25647826	25664945	0	0.08969

16112 Amp	12	25665928	25713592	0	0.091342
16113 Amp	12	25715924	25761847	0	0.100332
16114 Amp	12	25764474	25811415	0	0.093302
16115 Amp	12	25814535	25848718	0	0.090056
16116 Amp	12	25855763	26027665	0	0.117437
16117 Amp	12	26028116	26511692	0	0.113706
16118 Amp	12	26512043	26711598	0	0.119039
16119 Amp	12	26712347	26841458	0	0.117048
16120 Amp	12	26841720	27353522	0	0.113763
16121 Amp	12	27355663	27556982	0	0.107384
16122 Amp	12	27558982	27601534	0	0.102727
16123 Amp	12	27610742	27706071	0	0.089453
16124 Amp	12	27707031	27728356	0	0.092176
16125 Amp	12	27733701	27967532	0	0.107825
16126 Amp	12	27974006	28094665	0	0.112466
16127 Amp	12	28098702	28112651	0	0.117442
16128 Amp	12	28112669	28228259	0	0.127052
16129 Amp	12	28228580	28262779	0	0.135273
16130 Amp	12	28267437	28322188	0	0.130298
16131 Amp	12	28326183	28461248	0	0.122076
16132 Amp	12	28461296	28542577	0	0.115006
16133 Amp	12	28544333	28613183	0	0.117057
16134 Amp	12	28614687	28615685	0	0.130256
16135 Amp	12	28615938	29001377	0	0.128205
16136 Amp	12	29003840	29006580	0	0.131459
16137 Amp	12	29015050	29043311	0	0.168649
16138 Amp	12	29044814	29127293	0	0.177181
16139 Amp	12	29127694	29157228	0	0.168649
16140 Amp	12	29157369	29225523	0	0.175755
16141 Amp	12	29225576	29272356	0	0.171313
16142 Amp	12	29273098	29277752	0	0.17561
16143 Amp	12	29279548	29312425	0	0.178266
16144 Amp	12	29318914	29385336	0	0.161498
16145 Amp	12	29390589	29422119	0	0.153545
16146 Amp	12	29422309	29633204	0	0.160702
16147 Amp	12	29633588	29707776	0	0.146471
16148 Amp	12	29710473	29726104	0	0.142173
16149 Amp	12	29743995	29788140	0	0.136401
16150 Amp	12	29789364	29826890	0	0.139718
16151 Amp	12	29826992	29942209	0	0.146784
16152 Amp	12	29946459	29951542	0	0.148975
16153 Amp	12	29954626	29977940	0	0.133799
16154 Amp	12	29978310	29990199	0	0.126732
16155 Amp	12	29990853	30184554	0	0.132448
16156 Amp	12	30189001	30217892	0	0.137506
16157 Amp	12	30218784	30229220	0	0.140937
16158 Amp	12	30229783	30365604	0	0.138154
16159 Amp	12	30365643	30464369	0	0.135498
16160 Amp	12	30474963	30689533	0	0.133307
16161 Amp	12	30692031	30692031	0	0.135316
16162 Amp	12	30693626	30716240	0	0.144199
16163 Amp	12	30716601	30780350	0	0.156045
16164 Amp	12	30780676	30865521	0	0.149713

16165	Amp	12	30866604	30899209	0	0.146282
16166	Amp	12	30899549	30946406	0	0.150219
16167	Amp	12	30947015	31012208	0	0.164676
16168	Amp	12	31016794	31018378	0	0.155792
16169	Amp	12	31020049	31080036	0	0.159314
16170	Amp	12	31084222	31442895	0	0.156761
16171	Amp	12	31458579	31619019	0	0.151703
16172	Amp	12	31621671	31724355	0	0.133982
16173	Amp	12	31726000	31737550	0	0.122137
16174	Amp	12	31764869	31846713	0	0.093502
16175	Amp	12	31915248	31979968	0	0.08998
16176	Amp	12	31980592	32360868	0	0.093663
16177	Amp	12	32360954	32411326	0	0.08998
16178	Amp	12	32413873	32505703	0	0.10108
16179	Amp	12	32506343	32628487	0	0.106615
16180	Amp	12	32628896	32711593	0	0.101974
16181	Amp	12	32718926	32718926	0	0.087855
16182	Amp	12	32722039	32722472	0	0.090077
16183	Amp	12	32722516	32741983	0	0.092189
16184	Amp	12	32744010	32765721	0	0.098082
16185	Amp	12	32766639	32800835	0	0.095455
16186	Amp	12	32801813	32811663	0	0.084355
16187	Amp	12	32814033	32831396	0	0.07882
16188	Amp	12	32831854	33027559	0	0.08616
16189	Amp	12	33028292	33129292	0	0.091455
16190	Amp	12	33129371	33329714	0	0.098792
16191	Amp	12	33338266	33342383	0	0.091455
16192	Amp	12	33342598	33357482	0	0.089343
16193	Amp	12	33358796	33358982	0	0.086026
16194	Amp	12	33361111	33365705	0	0.084181
16195	Amp	12	33365925	33366590	0	0.092952
16196	Amp	12	33368172	33368172	0	0.084396
16197	Amp	12	33368216	33368216	0	0.077239
16198	Amp	12	38827290	38827290	0	0.05978
16199	Amp	12	38829764	38834281	0	0.050696
16200	Amp	12	38834977	38871673	0	0.043356
16201	Amp	12	38879636	38882783	0	0.044804
16202	Amp	12	38883944	39083525	0	0.042563
16203	Amp	12	39094246	39232108	0	0.047979
16204	Amp	12	39233995	39278600	0	0.035685
16205	Amp	12	39284909	39292307	0	0.038725
16206	Amp	12	39295176	39346232	0	0.035633
16207	Amp	12	39354337	39354358	0	0.048033
16208	Amp	12	39356728	39363601	0	0.050469
16209	Amp	12	39364756	39671088	0	0.047428
16210	Amp	12	39672074	39773079	0	0.050927
16211	Amp	12	39774201	39852184	0	0.053874
16212	Amp	12	39856079	39880800	0	0.041474
16213	Amp	12	39881504	39907722	0	0.056661
16214	Amp	12	39909703	39987417	0	0.058866
16215	Amp	12	39990100	40113403	0	0.062293
16216	Amp	12	40114292	40150880	0	0.049235
16217	Amp	12	40153577	40169104	0	0.059068

16218	Amp	12	40173123	40227283	0	0.049235
16219	Amp	12	40232039	40350028	0	0.052476
16220	Amp	12	40356467	40522928	0	0.075704
16221	Amp	12	40527686	40612884	0	0.072604
16222	Amp	12	40613085	40620389	0	0.070475
16223	Amp	12	40623065	40655620	0	0.06827
16224	Amp	12	40659931	41060336	0	0.079314
16225	Amp	12	41061091	41076896	0	0.085952
16226	Amp	12	41078959	41151090	0	0.098244
16227	Amp	12	41151393	41198938	0	0.095809
16228	Amp	12	41202194	41220939	0	0.073107
16229	Amp	12	41221686	41324736	0	0.059951
16230	Amp	12	41325411	41333456	0	0.06594
16231	Amp	12	41335584	41358061	0	0.059302
16232	Amp	12	41359013	41429881	0	0.055875
16233	Amp	12	41429970	41436571	0	0.066603
16234	Amp	12	41436969	41970681	0	0.061279
16235	Amp	12	41971283	42134982	0	0.067182
16236	Amp	12	42135059	42147127	0	0.069311
16237	Amp	12	42155270	42158004	0	0.080133
16238	Amp	12	42158725	42194357	0	0.084312
16239	Amp	12	42194762	42239501	0	0.087187
16240	Amp	12	42241529	42485324	0	0.090369
16241	Amp	12	42488351	42770327	0	0.081185
16242	Amp	12	42770808	42815499	0	0.082901
16243	Amp	12	42818897	42818897	0	0.088154
16244	Amp	12	43344680	43345446	0	0.09298
16245	Amp	12	43347737	43414831	0	0.108283
16246	Amp	12	43417849	43440062	0	0.101718
16247	Amp	12	43441800	43507530	0	0.13665
16248	Amp	12	43508398	43532329	0	0.130661
16249	Amp	12	43627099	43851414	0	0.134784
16250	Amp	12	43851442	43883222	0	0.129958
16251	Amp	12	43884855	44005216	0	0.082485
16252	Amp	12	44005428	44038693	0	0.079303
16253	Amp	12	44041395	44116566	0	0.084493
16254	Amp	12	44116806	44171567	0	0.094725
16255	Amp	12	44172272	44209127	0	0.089535
16256	Amp	12	44209977	44228698	0	0.095661
16257	Amp	12	44229236	44234885	0	0.102377
16258	Amp	12	44237667	44312244	0	0.096251
16259	Amp	12	44317772	44415272	0	0.10006
16260	Amp	12	44418688	44563580	0	0.095937
16261	Amp	12	44563835	44606427	0	0.098935
16262	Amp	12	44606445	44613821	0	0.105533
16263	Amp	12	44614763	44630834	0	0.114357
16264	Amp	12	44633225	44696078	0	0.102534
16265	Amp	12	44698075	44717125	0	0.100819
16266	Amp	12	44721061	44840142	0	0.104717
16267	Amp	12	44841206	44938546	0	0.081548
16268	Amp	12	44942404	44959405	0	0.091332
16269	Amp	12	44962886	45125772	0	0.101273
16270	Amp	12	45130728	45261834	0	0.12532

16271 Amp	12	45263184	45360067	0	0.112851
16272 Amp	12	45370396	45532471	0	0.106135
16273 Amp	12	45532485	45846405	0	0.107677
16274 Amp	12	45848874	45915562	0	0.087661
16275 Amp	12	45916055	45935816	0	0.092703
16276 Amp	12	45938714	45945250	0	0.098933
16277 Amp	12	45949259	45954519	0	0.14686
16278 Amp	12	45955005	46184865	0	0.155803
16279 Amp	12	46185605	46372112	0	0.153042
16280 Amp	12	46374736	46490315	0	0.140201
16281 Amp	12	46493362	46590611	0	0.159463
16282 Amp	12	46595038	46655379	0	0.140201
16283 Amp	12	46655452	46898627	0	0.119006
16284 Amp	12	46900449	46931848	0	0.106861
16285 Amp	12	46932801	47027575	0	0.108438
16286 Amp	12	47027853	47031882	0	0.106792
16287 Amp	12	47032278	47132947	0	0.127319
16288 Amp	12	47133816	47189599	0	0.129762
16289 Amp	12	47190267	47550395	0	0.127319
16290 Amp	12	47664492	47720595	0	0.111361
16291 Amp	12	47723582	47875627	0	0.11558
16292 Amp	12	47875867	47937523	0	0.122789
16293 Amp	12	47938727	48017195	0	0.11558
16294 Amp	12	48017319	48030633	0	0.124533
16295 Amp	12	48030714	48043903	0	0.127007
16296 Amp	12	48044552	48069730	0	0.136727
16297 Amp	12	48072258	48185882	0	0.131103
16298 Amp	12	48186727	48259625	0	0.121384
16299 Amp	12	48264750	48268887	0	0.112431
16300 Amp	12	48269870	48306499	0	0.127329
16301 Amp	12	48308019	48380377	0	0.124075
16302 Amp	12	48380678	48391302	0	0.121852
16303 Amp	12	48391706	48418413	0	0.091965
16304 Amp	12	48419202	48454857	0	0.074593
16305 Amp	12	48459283	48678258	0	0.077168
16306 Amp	12	48678288	48773477	0	0.06591
16307 Amp	12	48785775	48855901	0	0.060017
16308 Amp	12	48857472	48898889	0	0.056377
16309 Amp	12	48903586	48905507	0	0.069783
16310 Amp	12	48908922	48920584	0	0.075311
16311 Amp	12	48922834	48975364	0	0.083667
16312 Amp	12	48991174	49026295	0	0.122273
16313 Amp	12	49028576	49120277	0	0.113917
16314 Amp	12	49126022	49127889	0	0.109698
16315 Amp	12	49135790	49257201	0	0.115133
16316 Amp	12	49258728	49291300	0	0.124404
16317 Amp	12	49292869	49340514	0	0.121929
16318 Amp	12	49341557	49396484	0	0.083323
16319 Amp	12	49397689	49477181	0	0.077795
16320 Amp	12	49478393	49493344	0	0.075018
16321 Amp	12	49501305	49540782	0	0.061785
16322 Amp	12	49551898	49654257	0	0.053776
16323 Amp	12	49655753	49695590	0	0.056395

16324	Amp	12	49696760	49858091	0	0.044824
16325	Amp	12	49858127	49978243	0	0.049882
16326	Amp	12	49981865	50047968	0	0.056696
16327	Amp	12	50059612	50059612	0	0.052809
16328	Amp	12	50059810	50222885	0	0.047793
16329	Amp	12	50225993	50328358	0	0.045175
16330	Amp	12	50332182	50473336	0	0.049082
16331	Amp	12	50474929	50630646	0	0.054813
16332	Amp	12	50632840	50639351	0	0.065519
16333	Amp	12	50643142	50787916	0	0.06109
16334	Amp	12	50788629	50879201	0	0.059115
16335	Amp	12	50880439	51062695	0	0.061471
16336	Amp	12	51064719	51270557	0	0.05574
16337	Amp	12	51274402	51516800	0	0.053384
16338	Amp	12	51517025	51593524	0	0.056735
16339	Amp	12	51593826	51662583	0	0.046029
16340	Amp	12	51665064	51702652	0	0.042122
16341	Amp	12	51705369	51714302	0	0.0397
16342	Amp	12	51714914	51782451	0	0.041731
16343	Amp	12	51783856	52096077	0	0.043401
16344	Amp	12	52100838	52165054	0	0.041452
16345	Amp	12	52165380	52252300	0	0.043466
16346	Amp	12	52252788	52282133	0	0.045551
16347	Amp	12	52282619	52527495	0	0.061851
16348	Amp	12	52530197	52676904	0	0.068261
16349	Amp	12	52677598	52745505	0	0.063492
16350	Amp	12	52748291	52761561	0	0.075495
16351	Amp	12	52763742	52767045	0	0.063492
16352	Amp	12	52768744	52808596	0	0.056623
16353	Amp	12	52810163	52852120	0	0.054608
16354	Amp	12	52856648	52885175	0	0.052967
16355	Amp	12	52885195	52892993	0	0.050883
16356	Amp	12	52896619	52963839	0	0.097787
16357	Amp	12	52964087	53017314	0	0.049213
16358	Amp	12	53017933	53485857	0	0.044156
16359	Amp	12	53485953	54114977	0	0.047249
16360	Amp	12	54118217	54192534	0	0.044156
16361	Amp	12	54195499	54236067	0	0.053492
16362	Amp	12	54236733	54470258	0	0.041278
16363	Amp	12	54473400	54537130	0	0.046076
16364	Amp	12	54538619	54558904	0	0.055938
16365	Amp	12	54559765	54597165	0	0.058027
16366	Amp	12	54601420	54609873	0	0.067965
16367	Amp	12	54610147	54679795	0	0.07842
16368	Amp	12	54680652	54783148	0	0.067965
16369	Amp	12	54795066	54899682	0	0.077479
16370	Amp	12	54900003	55303832	0	0.067965
16371	Amp	12	55303961	55651075	0	0.076003
16372	Amp	12	55653923	55663241	0	0.073915
16373	Amp	12	55669871	55838392	0	0.065031
16374	Amp	12	55842534	55949499	0	0.066737
16375	Amp	12	55952263	56071905	0	0.071531
16376	Amp	12	56073803	56268256	0	0.082428

16377	Amp	12	56272763	56289214	0	0.077635
16378	Amp	12	56296064	56471554	0	0.07263
16379	Amp	12	56471897	56866408	0	0.070923
16380	Amp	12	56872529	56929937	0	0.090603
16381	Amp	12	56930072	57123223	0	0.093877
16382	Amp	12	57123561	57167668	0	0.085827
16383	Amp	12	57168496	57219437	0	0.095824
16384	Amp	12	57223164	57262922	0	0.085827
16385	Amp	12	57263779	57355765	0	0.080822
16386	Amp	12	57359416	57402325	0	0.123306
16387	Amp	12	57406898	57428693	0	0.101737
16388	Amp	12	57429409	57477357	0	0.125287
16389	Amp	12	57477384	57543491	0	0.140885
16390	Amp	12	57543582	57543733	0	0.172924
16391	Amp	12	57543750	57575888	0	0.182399
16392	Amp	12	57579142	57592941	0.347934	0.22472
16393	Amp	12	57593426	57593426	0.398217	0.22989
16394	Amp	12	57595260	57707548	1.020188	0.280246
16395	Amp	12	57711099	57721488	1.226647	0.294052
16396	Amp	12	57722795	57779215	1.534719	0.31545
16397	Amp	12	57779683	57809594	0.814697	0.265094
16398	Amp	12	57810526	57862368	0.556748	0.243696
16399	Amp	12	57863079	57926520	0.243425	0.215542
16400	Amp	12	57927735	57956848	0.156508	0.206154
16401	Amp	12	57963224	57971991	0.13565	0.204577
16402	Amp	12	57976150	57976150	0.587735	0.246131
16403	Amp	12	57980542	58037743	0.556748	0.243309
16404	Amp	12	58041222	58043205	0.685617	0.254557
16405	Amp	12	58044045	58049492	0.724993	0.25774
16406	Amp	12	58076366	58084815	0.805493	0.264288
16407	Amp	12	58085369	58091984	0.906818	0.271584
16408	Amp	12	58092238	58167261	1.010597	0.279597
16409	Amp	12	58173312	58217090	0.49169	0.238044
16410	Amp	12	58223810	58291815	0.243425	0.215077
16411	Amp	12	58297240	58431552	0.195309	0.210229
16412	Amp	12	58432459	58439747	0.073369	0.197256
16413	Amp	12	58442848	58572887	0	0.156472
16414	Amp	12	58575453	58658760	0	0.12956
16415	Amp	12	58662635	58670335	0	0.121429
16416	Amp	12	58670821	58780747	0	0.10978
16417	Amp	12	58784913	58797947	0	0.121437
16418	Amp	12	58801308	58822389	0	0.130667
16419	Amp	12	58822475	58822475	0	0.116035
16420	Amp	12	58828192	58828192	0	0.099211
16421	Amp	12	58828198	58828198	0	0.075545
16422	Amp	12	58828282	58828282	0	0.069092
16423	Amp	12	58828295	58828895	0	0.057435
16424	Amp	12	58828938	58828938	0	0.111881
16425	Amp	12	58828985	58833794	0	0.122018
16426	Amp	12	58833809	58838601	0	0.129712
16427	Amp	12	58838671	58840007	0	0.134661
16428	Amp	12	58840286	58840290	0	0.138832
16429	Amp	12	58840301	58924706	0	0.131387

16430	Amp	12	58932883	58998952	0	0.179014
16431	Amp	12	59004263	59140351	0	0.176321
16432	Amp	12	59142684	59208466	0	0.128695
16433	Amp	12	59209005	59243201	0	0.132252
16434	Amp	12	59247232	59363781	0	0.125948
16435	Amp	12	59364123	59391262	0	0.133869
16436	Amp	12	59391352	59391352	0	0.13059
16437	Amp	12	59396056	59455896	0	0.122669
16438	Amp	12	59456376	59507087	0	0.113439
16439	Amp	12	59512149	59561403	0	0.110257
16440	Amp	12	59562559	59568982	0	0.124409
16441	Amp	12	59571355	59579272	0	0.136054
16442	Amp	12	59579539	59604366	0	0.1305
16443	Amp	12	59606072	59664313	0	0.142819
16444	Amp	12	59669146	59720369	0	0.127942
16445	Amp	12	59723238	59774103	0	0.124894
16446	Amp	12	59776118	59812246	0	0.140499
16447	Amp	12	59812671	59819373	0	0.13687
16448	Amp	12	59820999	60003484	0	0.140647
16449	Amp	12	60005910	60069063	0	0.143353
16450	Amp	12	60074171	60137726	0	0.137675
16451	Amp	12	60148143	60155162	0	0.120177
16452	Amp	12	60155390	60251336	0	0.127915
16453	Amp	12	60252303	60256064	0	0.151237
16454	Amp	12	60257075	60320973	0	0.14853
16455	Amp	12	60325899	60499255	0	0.153005
16456	Amp	12	60500142	60504503	0	0.131409
16457	Amp	12	60508871	60509628	0	0.127034
16458	Amp	12	60510656	60697494	0	0.116054
16459	Amp	12	60698951	60712139	0	0.120752
16460	Amp	12	60714302	60874990	0	0.124192
16461	Amp	12	60877843	60907092	0	0.140612
16462	Amp	12	60914738	60926558	0	0.132874
16463	Amp	12	60929107	60989446	0	0.146438
16464	Amp	12	60995284	61016160	0	0.128293
16465	Amp	12	61016936	61122496	0	0.134805
16466	Amp	12	61126396	61166203	0	0.143762
16467	Amp	12	61201531	61291597	0	0.146774
16468	Amp	12	61291661	61312496	0	0.136046
16469	Amp	12	61313772	61524879	0	0.142253
16470	Amp	12	61531900	61550604	0	0.13924
16471	Amp	12	61552071	61583211	0	0.128814
16472	Amp	12	61583668	61597541	0	0.122607
16473	Amp	12	61598561	61677340	0	0.127101
16474	Amp	12	61681561	61706319	0	0.164152
16475	Amp	12	61709708	61710655	0	0.152508
16476	Amp	12	61713683	61713683	0	0.136053
16477	Amp	12	61719809	61804490	0	0.143823
16478	Amp	12	61808789	61867284	0	0.136053
16479	Amp	12	61885690	61940578	0	0.185736
16480	Amp	12	61940741	62002153	0.018387	0.190486
16481	Amp	12	62002187	62016658	0.09318	0.19927
16482	Amp	12	62024308	62024841	0.264784	0.217665

16483	Amp	12	62025709	62029532	0.215373	0.212915
16484	Amp	12	62032159	62108935	0.050005	0.194519
16485	Amp	12	62115547	62132551	0.236222	0.214724
16486	Amp	12	62132762	62155963	0.325321	0.223082
16487	Amp	12	62157002	62216815	0.13565	0.20468
16488	Amp	12	62217079	62223453	0.333141	0.223475
16489	Amp	12	62223849	62223849	0.564815	0.243919
16490	Amp	12	62228204	62356999	0.676804	0.253649
16491	Amp	12	62362853	62382785	0.833763	0.266223
16492	Amp	12	62383166	62388687	1.49455	0.313
16493	Amp	12	62388798	62388798	1.525294	0.315233
16494	Amp	12	62390187	62399356	1.615314	0.32124
16495	Amp	12	62405212	62451814	1.525294	0.314729
16496	Amp	12	62452892	62478969	1.150167	0.289049
16497	Amp	12	62482059	62545777	1.237378	0.295377
16498	Amp	12	62549046	62549046	1.821927	0.335025
16499	Amp	12	62551437	62551437	1.099256	0.285342
16500	Amp	12	62553015	62553015	1.404612	0.307061
16501	Amp	12	62553770	62557506	1.2565	0.296455
16502	Amp	12	62557914	62594747	1.503938	0.313676
16503	Amp	12	62605374	62630844	0.944128	0.274683
16504	Amp	12	62632071	62647032	0.548372	0.24291
16505	Amp	12	62653884	62661992	0.476502	0.236903
16506	Amp	12	62662059	62662168	0.421967	0.231836
16507	Amp	12	62664031	62682310	0.652914	0.251848
16508	Amp	12	62685061	62692811	0.724993	0.257764
16509	Amp	12	62695076	62720774	0.243425	0.2153
16510	Amp	12	62723527	62745408	0.571214	0.244821
16511	Amp	12	62747820	62781813	0.201819	0.210987
16512	Amp	12	62784250	62811452	0.580011	0.24559
16513	Amp	12	62813835	62861608	0.76883	0.261576
16514	Amp	12	62865478	62900677	0.944128	0.274414
16515	Amp	12	62907063	62918948	1.169408	0.290364
16516	Amp	12	62920724	62955157	1.275021	0.297803
16517	Amp	12	62958806	62973082	1.453628	0.309968
16518	Amp	12	62981486	63134929	1.615314	0.320984
16519	Amp	12	63135035	63208816	1.748357	0.330232
16520	Amp	12	63209208	63210287	1.605306	0.320576
16521	Amp	12	63210712	63210712	1.646768	0.323167
16522	Amp	12	63211083	63251055	1.687998	0.325862
16523	Amp	12	63251561	63255879	1.593569	0.319726
16524	Amp	12	63260257	63332811	1.563139	0.317994
16525	Amp	12	63338195	63352301	1.856336	0.337264
16526	Amp	12	63355123	63399305	1.424227	0.308462
16527	Amp	12	63400043	63488485	1.394558	0.305767
16528	Amp	12	63496347	63501542	1.424227	0.308253
16529	Amp	12	63508022	63525708	1.463463	0.310656
16530	Amp	12	63526209	63526209	1.553308	0.316761
16531	Amp	12	63751525	63849269	1.667574	0.324375
16532	Amp	12	63851492	63880900	1.800655	0.333315
16533	Amp	12	63883555	63901662	1.697113	0.326835
16534	Amp	12	63904987	63921377	1.757929	0.330473
16535	Amp	12	63928353	64007418	1.542961	0.3161

16536	Amp	12	64007458	64007458	1.484449	0.312463
16537	Amp	12	64007632	64022678	1.525294	0.315204
16538	Amp	12	64024038	64090510	1.49455	0.312756
16539	Amp	12	64094349	64099614	0.833763	0.265987
16540	Amp	12	64102253	64125425	0.972663	0.276795
16541	Amp	12	64127122	64231642	0.888878	0.27066
16542	Amp	12	64235817	64244095	1.059215	0.282402
16543	Amp	12	64244667	64255941	0.852608	0.267357
16544	Amp	12	64258762	64258762	0.652914	0.25167
16545	Amp	12	64262291	64262291	0.693887	0.254958
16546	Amp	12	64263363	64279021	0.760395	0.260453
16547	Amp	12	64282057	64351533	0.564815	0.244005
16548	Amp	12	64351646	64363733	0.652914	0.252031
16549	Amp	12	64363765	64372421	0.636206	0.250322
16550	Amp	12	64374403	64381205	0.972663	0.276562
16551	Amp	12	64385236	64396999	0.805493	0.264043
16552	Amp	12	64400217	64436786	0.724993	0.258126
16553	Amp	12	64438989	64450861	1.059215	0.282584
16554	Amp	12	64467289	64518211	0.897922	0.270892
16555	Amp	12	64524972	64530692	0.778204	0.261983
16556	Amp	12	64533532	64567904	0.66072	0.252735
16557	Amp	12	64576316	64598656	0.878688	0.269681
16558	Amp	12	64606062	64619482	0.944128	0.274474
16559	Amp	12	64621634	64624412	0.852608	0.267549
16560	Amp	12	64630604	64630924	0.71758	0.257474
16561	Amp	12	64634091	64659455	0.580011	0.245733
16562	Amp	12	64660453	64663497	0.897922	0.270791
16563	Amp	12	64666584	64685608	0.778204	0.262166
16564	Amp	12	64685631	64708285	0.833763	0.26616
16565	Amp	12	64709388	64767879	1.010597	0.279
16566	Amp	12	64768663	64833857	1.068928	0.283171
16567	Amp	12	64835453	64840981	1.10923	0.28645
16568	Amp	12	64843086	64865180	0.7517	0.26021
16569	Amp	12	64871907	64876130	0.66072	0.252333
16570	Amp	12	64877984	64878439	0.7517	0.259929
16571	Amp	12	64881988	64885776	0.796213	0.263706
16572	Amp	12	64886565	64936549	0.693887	0.255425
16573	Amp	12	64936986	64990782	0.814697	0.26452
16574	Amp	12	64990926	65059263	0.76883	0.261241
16575	Amp	12	65060768	65069173	1.24764	0.295656
16576	Amp	12	65069235	65162399	1.344429	0.302485
16577	Amp	12	65171221	65197246	1.315861	0.300252
16578	Amp	12	65201331	65201331	1.404612	0.306729
16579	Amp	12	65201845	65215276	1.563139	0.31741
16580	Amp	12	65216478	65242542	1.737394	0.329179
16581	Amp	12	65250492	65314078	1.563139	0.31741
16582	Amp	12	65317955	65317955	2.180628	0.357638
16583	Amp	12	65323181	65355252	2.203122	0.359086
16584	Amp	12	65355311	65383365	2.113683	0.35338
16585	Amp	12	65386165	65386165	1.918998	0.341252
16586	Amp	12	65386642	65424763	2.028562	0.34814
16587	Amp	12	65430444	65445627	2.260813	0.362356
16588	Amp	12	65446178	65461696	2.349236	0.367571

16589	Amp	12	65462201	65611512	2.415622	0.372078
16590	Amp	12	65611864	65630579	2.349236	0.367571
16591	Amp	12	65632055	65663916	2.415622	0.37202
16592	Amp	12	65664765	65664890	2.249752	0.361473
16593	Amp	12	65666177	65697060	2.170217	0.357024
16594	Amp	12	65697103	65713604	2.249752	0.361827
16595	Amp	12	65715183	65726528	2.30333	0.365431
16596	Amp	12	65734081	65734867	2.249752	0.361827
16597	Amp	12	65749877	65807597	1.887705	0.339232
16598	Amp	12	65810293	65823614	1.667574	0.324721
16599	Amp	12	65830681	65862615	2.091935	0.351972
16600	Amp	12	65862926	65873530	2.158849	0.356107
16601	Amp	12	65877243	65878952	2.238069	0.361103
16602	Amp	12	65884904	66001055	2.415622	0.371783
16603	Amp	12	66010844	66093940	1.972699	0.344532
16604	Amp	12	66099271	66119331	2.249752	0.362009
16605	Amp	12	66122019	66161943	2.393797	0.370797
16606	Amp	12	66164037	66164630	2.796399	0.394529
16607	Amp	12	66166887	66181379	2.71525	0.389736
16608	Amp	12	66181477	66448355	2.614747	0.383498
16609	Amp	12	66448646	66540601	2.647166	0.385433
16610	Amp	12	66541300	66614487	2.555572	0.380437
16611	Amp	12	66617790	66728743	2.948071	0.403846
16612	Amp	12	66729503	66748477	3.146436	0.415496
16613	Amp	12	66748755	66813658	4.035317	0.465084
16614	Amp	12	66818604	66905284	4.158928	0.471212
16615	Amp	12	66905393	67039370	4.364676	0.481429
16616	Amp	12	67039619	67039619	4.525461	0.48937
16617	Amp	12	67044814	67065951	4.355429	0.480582
16618	Amp	12	67073155	67086529	4.608746	0.493868
16619	Amp	12	67089107	67107371	4.724007	0.499242
16620	Amp	12	67108653	67166303	5.31131	0.526833
16621	Amp	12	67170372	67187867	4.724007	0.499242
16622	Amp	12	67189856	67232353	5.324711	0.527589
16623	Amp	12	67234269	67264729	5.459172	0.533519
16624	Amp	12	67266436	67266436	5.654252	0.542076
16625	Amp	12	67271319	67359786	5.696665	0.544147
16626	Amp	12	67360893	67398121	5.56363	0.538218
16627	Amp	12	67398873	67445382	5.870027	0.553008
16628	Amp	12	67447616	67498224	5.295952	0.526051
16629	Amp	12	67503464	67515786	5.813919	0.550111
16630	Amp	12	67515890	67564920	6.042741	0.560619
16631	Amp	12	67565257	67592965	5.953304	0.556682
16632	Amp	12	67595538	67604927	5.534438	0.536899
16633	Amp	12	67605131	67636716	5.622975	0.540835
16634	Amp	12	67640140	67694220	5.267482	0.524315
16635	Amp	12	67699102	67765358	5.696665	0.544617
16636	Amp	12	67774215	67787615	5.739571	0.546222
16637	Amp	12	67793453	67810912	6.08935	0.56303
16638	Amp	12	67811133	67814715	6.165023	0.566221
16639	Amp	12	67821109	67841632	5.953304	0.556492
16640	Amp	12	67851079	67851079	5.383269	0.530347
16641	Amp	12	67852653	67942048	5.267482	0.52422

16642	Amp	12	67944414	68071929	5.897742	0.553868
16643	Amp	12	68071977	68074931	5.753822	0.54691
16644	Amp	12	68075499	68087631	5.31131	0.526609
16645	Amp	12	68091795	68155173	5.769527	0.547576
16646	Amp	12	68155469	68166865	6.197199	0.568147
16647	Amp	12	68170584	68170584	6.435656	0.578787
16648	Amp	12	68173108	68173108	6.745443	0.592378
16649	Amp	12	68173909	68192247	7.016776	0.60445
16650	Amp	12	68192953	68210136	7.16706	0.610527
16651	Amp	12	68214120	68260516	6.683082	0.58956
16652	Amp	12	68260612	68260625	6.619468	0.586969
16653	Amp	12	68262867	68268143	6.696475	0.590579
16654	Amp	12	68268681	68280182	6.604027	0.586408
16655	Amp	12	68281061	68283125	6.650629	0.588194
16656	Amp	12	68283389	68342323	6.952285	0.601393
16657	Amp	12	68342436	68360947	7.363353	0.618989
16658	Amp	12	68361119	68386307	7.523003	0.625404
16659	Amp	12	68388225	68393002	8.064725	0.648203
16660	Amp	12	68394541	68439430	8.249161	0.655485
16661	Amp	12	68443343	68453937	8.469083	0.664731
16662	Amp	12	68460568	68478916	8.87384	0.681434
16663	Amp	12	68483457	68486149	9.009204	0.68644
16664	Amp	12	68487269	68628660	9.19854	0.694045
16665	Amp	12	68628940	68706170	9.095386	0.690434
16666	Amp	12	68708289	68784240	9.265068	0.697298
16667	Amp	12	68789498	68811169	9.061723	0.68874
16668	Amp	12	68811393	68817137	9.247887	0.696081
16669	Amp	12	68817264	68834820	9.385128	0.701762
16670	Amp	12	68834838	68874798	9.597162	0.710319
16671	Amp	12	68876682	68940591	10.905321	0.76053
16672	Amp	12	68943030	68973552	11.025256	0.765033
16673	Amp	12	68973611	68973611	11.291459	0.775105
16674	Amp	12	68976658	69067550	11.095564	0.767501
16675	Amp	12	69070521	69075701	11.341542	0.777071
16676	Amp	12	69075850	69154695	11.806876	0.794943
16677	Amp	12	69154742	69197044	11.678994	0.790609
16678	Amp	12	69203865	69224082	11.876832	0.798151
16679	Amp	12	69225217	69259202	12.149668	0.807978
16680	Amp	12	69259366	69303586	12.313728	0.814333
16681	Amp	12	69311076	69313386	12.097158	0.806392
16682	Amp	12	69313534	69391968	12.222572	0.811034
16683	Amp	12	69392690	69408229	12.078285	0.805791
16684	Amp	12	69409147	69415251	11.964103	0.801288
16685	Amp	12	69417589	69437855	11.666631	0.78968
16686	Amp	12	69438287	69518146	11.806876	0.794957
16687	Amp	12	69518327	69552343	11.678994	0.790315
16688	Amp	12	69552574	69586361	11.876832	0.79773
16689	Amp	12	69589735	69630746	10.5993	0.748368
16690	Amp	12	69634273	69643110	9.752822	0.716448
16691	Amp	12	69643879	69645873	9.077474	0.689491
16692	Amp	12	69646835	69677113	8.906445	0.682475
16693	Amp	12	69677162	69679456	8.720964	0.675302
16694	Amp	12	69682921	69684203	8.539219	0.667613

16695	Amp	12	69684795	69715509	8.34915	0.659805
16696	Amp	12	69716927	69800655	7.952174	0.643446
16697	Amp	12	69811545	69854689	7.5669	0.627782
16698	Amp	12	69865718	69875497	8.34915	0.659985
16699	Amp	12	69880205	69881067	8.504106	0.666029
16700	Amp	12	69883526	69909443	8.906445	0.682789
16701	Amp	12	69909739	69909739	9.212677	0.694663
16702	Amp	12	69910730	69960969	8.790054	0.677904
16703	Amp	12	69963355	69965788	9.281415	0.697702
16704	Amp	12	69966066	69974055	9.212677	0.695103
16705	Amp	12	69977273	69988357	8.26608	0.656361
16706	Amp	12	69989940	69993703	8.790054	0.677909
16707	Amp	12	69993913	70026704	8.704996	0.674572
16708	Amp	12	70026744	70026744	8.181109	0.653023
16709	Amp	12	70027448	70037255	8.720964	0.675199
16710	Amp	12	70043895	70050428	8.992196	0.686056
16711	Amp	12	70051070	70074994	10.166941	0.73188
16712	Amp	12	70078559	70081108	10.236812	0.73465
16713	Amp	12	70081269	70108670	10.671482	0.751409
16714	Amp	12	70108711	70166785	10.383055	0.740551
16715	Amp	12	70170257	70216822	10.022	0.726702
16716	Amp	12	70227036	70234636	8.87384	0.680878
16717	Amp	12	70239141	70298039	10.057544	0.72797
16718	Amp	12	70299358	70325565	9.650316	0.712354
16719	Amp	12	70325679	70359064	9.825427	0.719208
16720	Amp	12	70359188	70428153	9.717504	0.715065
16721	Amp	12	70433417	70449646	9.436384	0.70405
16722	Amp	12	70454794	70490840	9.247887	0.696076
16723	Amp	12	70491125	70512579	9.180995	0.69387
16724	Amp	12	70517732	70546903	8.114435	0.649994
16725	Amp	12	70546916	70572004	7.900934	0.641106
16726	Amp	12	70574116	70621351	7.664726	0.631536
16727	Amp	12	70621419	70643635	7.392813	0.620243
16728	Amp	12	70644384	70670568	7.50594	0.624563
16729	Amp	12	70672692	70762330	7.408763	0.621006
16730	Amp	12	70764626	70824962	7.232556	0.612897
16731	Amp	12	70829826	70863301	6.808231	0.595548
16732	Amp	12	70871481	70907496	6.745443	0.592356
16733	Amp	12	70909278	70931239	6.683082	0.589586
16734	Amp	12	70932233	70945520	6.57161	0.585266
16735	Amp	12	70946305	71047199	6.274109	0.571363
16736	Amp	12	71052096	71058749	5.56363	0.537957
16737	Amp	12	71060268	71065496	5.997601	0.558857
16738	Amp	12	71066084	71097725	5.925645	0.555614
16739	Amp	12	71105261	71116475	6.133613	0.564799
16740	Amp	12	71127747	71183589	5.925645	0.555614
16741	Amp	12	71184485	71184485	5.870027	0.552417
16742	Amp	12	71191963	71251059	5.445472	0.532619
16743	Amp	12	71253448	71280849	5.739571	0.546522
16744	Amp	12	71281222	71281794	5.001595	0.512322
16745	Amp	12	71282275	71285888	4.868506	0.506278
16746	Amp	12	71289950	71297375	5.150026	0.51931
16747	Amp	12	71297829	71300730	4.971485	0.510999

16748	Amp	12	71305074	71321951	5.107515	0.517278
16749	Amp	12	71328749	71341747	4.27183	0.476702
16750	Amp	12	71472182	71504578	4.957051	0.510448
16751	Amp	12	71505933	71507413	4.27183	0.476702
16752	Amp	12	71511010	71533024	4.483097	0.487516
16753	Amp	12	71533435	71543214	4.364676	0.481237
16754	Amp	12	71546373	71546373	4.24321	0.474823
16755	Amp	12	71546505	71568307	4.327765	0.478908
16756	Amp	12	71568686	71628524	4.364676	0.481031
16757	Amp	12	71630600	71636719	4.581809	0.492443
16758	Amp	12	71639485	71654579	4.809024	0.503206
16759	Amp	12	71654762	71695114	4.927103	0.508495
16760	Amp	12	71700434	71853928	4.765369	0.500899
16761	Amp	12	71854962	71889499	4.608746	0.493617
16762	Amp	12	71894982	71920626	4.911986	0.507935
16763	Amp	12	71922386	71926290	4.623661	0.49465
16764	Amp	12	71926651	71944865	4.724007	0.499138
16765	Amp	12	71946293	71946293	4.402792	0.483641
16766	Amp	12	71949477	72022214	4.090482	0.467664
16767	Amp	12	72027721	72063078	4.009908	0.463177
16768	Amp	12	72071277	72090801	3.880586	0.456849
16769	Amp	12	72098265	72268558	4.355429	0.480541
16770	Amp	12	72269539	72279043	3.880586	0.456849
16771	Amp	12	72279384	72281897	3.688516	0.446087
16772	Amp	12	72285214	72331215	3.371695	0.42829
16773	Amp	12	72331507	72460518	3.312745	0.425179
16774	Amp	12	72466718	72581567	3.523011	0.436955
16775	Amp	12	72585289	72618793	2.658709	0.386432
16776	Amp	12	72619695	72621137	2.530938	0.379113
16777	Amp	12	72621882	72630177	2.238069	0.360692
16778	Amp	12	72631128	72670984	2.580481	0.381549
16779	Amp	12	72707335	72710268	1.768015	0.331329
16780	Amp	12	72714742	72722009	1.963469	0.343656
16781	Amp	12	72729816	72733949	2.338154	0.367447
16782	Amp	12	72734025	72788027	2.30333	0.365324
16783	Amp	12	72793021	72806145	2.507231	0.377582
16784	Amp	12	72806577	72837599	2.737964	0.39108
16785	Amp	12	72838560	72986661	3.509701	0.436846
16786	Amp	12	72991414	72992356	2.737964	0.39108
16787	Amp	12	72992372	72995909	2.670502	0.387144
16788	Amp	12	72995930	73016529	2.591575	0.382585
16789	Amp	12	73016794	73022481	2.148338	0.355739
16790	Amp	12	73023040	73173669	2.272343	0.362765
16791	Amp	12	73179465	73202312	1.687998	0.325933
16792	Amp	12	73203483	73212216	1.821927	0.334879
16793	Amp	12	73219376	73261583	1.615314	0.321381
16794	Amp	12	73269017	73358048	2.415622	0.371904
16795	Amp	12	73359065	73408095	2.483202	0.376463
16796	Amp	12	73408809	73431892	2.283191	0.363707
16797	Amp	12	73433613	73504587	2.349236	0.367832
16798	Amp	12	73514920	73542933	2.543219	0.379424
16799	Amp	12	73544125	73553402	2.614747	0.383491
16800	Amp	12	73553611	73553611	2.283191	0.363865

16801	Amp	12	73553983	73554565	2.530938	0.379029
16802	Amp	12	73559994	73594001	2.635842	0.385001
16803	Amp	12	73597646	73605286	2.530938	0.379029
16804	Amp	12	73611559	73611559	2.727427	0.390875
16805	Amp	12	73613694	73645728	2.470601	0.375215
16806	Amp	12	73649891	73665028	3.112016	0.413133
16807	Amp	12	73692041	73717100	3.573122	0.440088
16808	Amp	12	73718599	73719338	3.424445	0.431969
16809	Amp	12	73723130	73758622	3.920712	0.458867
16810	Amp	12	73761044	73781253	4.525461	0.489646
16811	Amp	12	73781974	73804579	3.998681	0.462748
16812	Amp	12	73804904	73856430	3.842634	0.454771
16813	Amp	12	73860075	73877858	3.650307	0.444124
16814	Amp	12	73880233	73893898	3.383286	0.429354
16815	Amp	12	73896118	73897690	3.348874	0.426868
16816	Amp	12	73900539	73900539	3.252252	0.421579
16817	Amp	12	73901715	73906191	3.182419	0.417453
16818	Amp	12	73913012	74143453	2.591575	0.382047
16819	Amp	12	74143734	74152752	2.458756	0.375021
16820	Amp	12	74156739	74157002	2.028562	0.348066
16821	Amp	12	74161433	74207690	1.324884	0.300975
16822	Amp	12	74213383	74252622	0.860481	0.268573
16823	Amp	12	74259829	74301644	0.644689	0.251002
16824	Amp	12	74301900	74351713	0.587735	0.245995
16825	Amp	12	74356868	74419407	0.500055	0.238654
16826	Amp	12	74424318	74433292	0.41418	0.231112
16827	Amp	12	74436122	74465857	0.293283	0.220298
16828	Amp	12	74466443	74472933	0.340434	0.224641
16829	Amp	12	74473912	74584902	0.580011	0.245551
16830	Amp	12	74584916	74613069	0.702615	0.255854
16831	Amp	12	74617757	74628806	0.652914	0.252128
16832	Amp	12	74629879	74657670	0.556748	0.243734
16833	Amp	12	74658736	74658736	0.86979	0.268839
16834	Amp	12	74662876	74685589	0.273081	0.218317
16835	Amp	12	74686043	74744640	0.340434	0.224633
16836	Amp	12	74745362	74815359	0.195309	0.210818
16837	Amp	12	74817571	74893435	0.230572	0.214217
16838	Amp	12	74897033	74924299	0.355162	0.225628
16839	Amp	12	74924489	74937544	0.532449	0.241661
16840	Amp	12	74939001	75032234	0.405699	0.230251
16841	Amp	12	75033183	75064657	0.897922	0.270945
16842	Amp	12	75067783	75069604	1.029845	0.280842
16843	Amp	12	75070746	75074382	0.888878	0.270539
16844	Amp	12	75074630	75088048	1.020188	0.279713
16845	Amp	12	75091095	75137472	1.159718	0.289417
16846	Amp	12	75140510	75223981	1.010597	0.27952
16847	Amp	12	75224298	75248380	0.963924	0.276121
16848	Amp	12	75252157	75329256	0.7517	0.260087
16849	Amp	12	75338662	75402941	0.580011	0.245534
16850	Amp	12	75410597	75426992	0.09318	0.199399
16851	Amp	12	75445290	75486226	0.49169	0.238341
16852	Amp	12	75486635	75486635	0.619816	0.249071
16853	Amp	12	75486917	75500334	0.375927	0.227806

16854	Amp	12	75502785	75509411	0	0.178444
16855	Amp	12	75510020	75529011	0.09318	0.1997
16856	Amp	12	75531372	75543976	0.175335	0.208594
16857	Amp	12	75545892	75677216	0.223025	0.213603
16858	Amp	12	75683866	75698967	0.001198	0.188899
16859	Amp	12	75701726	75895212	0	0.17817
16860	Amp	12	75896906	76023555	0.382767	0.228692
16861	Amp	12	76027869	76147924	0	0.17817
16862	Amp	12	76148617	76287306	0	0.187904
16863	Amp	12	76287950	76291578	0.018387	0.190939
16864	Amp	12	76297997	76635306	0	0.174959
16865	Amp	12	76639459	76639459	0.02372	0.191234
16866	Amp	12	76641181	76730526	0.09318	0.199486
16867	Amp	12	76730978	76772357	0	0.183211
16868	Amp	12	76775611	76789239	0.215373	0.212667
16869	Amp	12	76792643	76841155	0.301081	0.22113
16870	Amp	12	76845496	76867554	0.195309	0.210323
16871	Amp	12	76869704	76884151	0.149443	0.205521
16872	Amp	12	76886347	76901227	0.068197	0.196626
16873	Amp	12	76905396	76923080	0.156508	0.206234
16874	Amp	12	76923468	76986197	0.068197	0.1965
16875	Amp	12	76991117	76991399	0.596354	0.247023
16876	Amp	12	76992760	77187927	0.564815	0.243987
16877	Amp	12	77192450	77405636	0.468576	0.235735
16878	Amp	12	77406429	77410069	0.540744	0.24251
16879	Amp	12	77411389	77423577	0.398217	0.229663
16880	Amp	12	77430546	77441666	0.437983	0.233348
16881	Amp	12	77446162	77544835	0.398217	0.229663
16882	Amp	12	77545109	77590052	0.476502	0.236939
16883	Amp	12	77591958	77612103	0.963924	0.275774
16884	Amp	12	77615139	77685110	0.476502	0.236939
16885	Amp	12	77687167	77732734	0.293283	0.22033
16886	Amp	12	77735107	77737800	0	0.169807
16887	Amp	12	77745611	77836171	0	0.119284
16888	Amp	12	77840313	77973210	0	0.121508
16889	Amp	12	77973467	78104273	0	0.125081
16890	Amp	12	78106551	78181116	0	0.128802
16891	Amp	12	78181763	78325040	0	0.13275
16892	Amp	12	78326780	78331373	0	0.129028
16893	Amp	12	78338233	78367811	0	0.125081
16894	Amp	12	78368355	78416427	0	0.133396
16895	Amp	12	78421317	78427581	0	0.125081
16896	Amp	12	78433021	78569248	0	0.121508
16897	Amp	12	78611420	78637678	0	0.114233
16898	Amp	12	78640635	78866313	0	0.121501
16899	Amp	12	78867119	79062675	0	0.148818
16900	Amp	12	79066292	79131524	0	0.17842
16901	Amp	12	79133135	79173201	0.086298	0.198736
16902	Amp	12	79173285	79203668	0.123589	0.203084
16903	Amp	12	79206351	79211856	0.050005	0.194614
16904	Amp	12	79218729	79270948	0	0.174298
16905	Amp	12	79287062	79299603	0	0.156447
16906	Amp	12	79303507	79315071	0	0.160866

16907	Amp	12	79320107	79394125	0	0.156517
16908	Amp	12	79399532	79399532	0	0.13935
16909	Amp	12	79400184	79417506	0	0.140833
16910	Amp	12	79418210	79494707	0	0.138609
16911	Amp	12	79499354	79655109	0	0.144776
16912	Amp	12	79655450	79684759	0	0.153094
16913	Amp	12	79687174	79759913	0	0.177796
16914	Amp	12	79765255	79773581	0	0.173453
16915	Amp	12	79774707	79824537	0	0.184293
16916	Amp	12	79826902	79980474	0	0.176854
16917	Amp	12	79981284	80037863	0	0.182828
16918	Amp	12	80041399	80098022	0	0.178409
16919	Amp	12	80098289	80173133	0	0.170091
16920	Amp	12	80175537	80340690	0	0.167954
16921	Amp	12	80347211	80437071	0	0.133271
16922	Amp	12	80440879	80530745	0	0.139625
16923	Amp	12	80535332	80560306	0	0.172178
16924	Amp	12	80564018	80823364	0	0.163914
16925	Amp	12	80827457	80889808	0	0.176579
16926	Amp	12	80889967	81070863	0	0.185093
16927	Amp	12	81073594	81202712	0.018387	0.190538
16928	Amp	12	81206370	81360356	0.073369	0.197321
16929	Amp	12	81362064	81442825	0.061778	0.195837
16930	Amp	12	81444780	81447201	0.142507	0.205269
16931	Amp	12	81450714	81482506	0.340434	0.224005
16932	Amp	12	81482950	81535193	0.309523	0.221907
16933	Amp	12	81537599	81545118	0.123589	0.203171
16934	Amp	12	81545224	81560069	0.050005	0.194945
16935	Amp	12	81563195	81563195	0.215373	0.212736
16936	Amp	12	81565556	81613360	0.123589	0.203305
16937	Amp	12	81614280	81700079	0.050005	0.19459
16938	Amp	12	81708347	81802535	0	0.157454
16939	Amp	12	81805341	81805590	0	0.165963
16940	Amp	12	81805963	81845365	0	0.160519
16941	Amp	12	81856341	82005878	0	0.168255
16942	Amp	12	82006402	82011375	0	0.16148
16943	Amp	12	82012727	82012727	0	0.153744
16944	Amp	12	82015701	82038574	0	0.163069
16945	Amp	12	82039253	82176246	0	0.15456
16946	Amp	12	82176732	82234704	0	0.158539
16947	Amp	12	82237456	82357704	0	0.166249
16948	Amp	12	82357719	82357719	0	0.158539
16949	Amp	12	82371044	82411199	0	0.140747
16950	Amp	12	82412259	82436135	0	0.147584
16951	Amp	12	82436243	82529096	0	0.151956
16952	Amp	12	82531920	82540630	0	0.146947
16953	Amp	12	82540797	82676913	0	0.151414
16954	Amp	12	82679338	82745055	0	0.147435
16955	Amp	12	82750285	82871667	0	0.141031
16956	Amp	12	82871683	82898724	0	0.136728
16957	Amp	12	82913792	82964640	0	0.187251
16958	Amp	12	82968532	82968987	0	0.136728
16959	Amp	12	82975100	83033198	0	0.148147

16960	Amp	12	83038398	83039843	0.086298	0.19867
16961	Amp	12	83044334	83172564	0.156508	0.206405
16962	Amp	12	83173362	83222618	0.09318	0.199567
16963	Amp	12	83227132	83272075	0	0.18529
16964	Amp	12	83275322	83282462	0.086298	0.198931
16965	Amp	12	83288528	83330472	0.286675	0.219659
16966	Amp	12	83335050	83576619	0	0.169136
16967	Amp	12	83579174	83579174	0	0.176667
16968	Amp	12	83584237	83768973	0	0.165248
16969	Amp	12	83769130	84192996	0.02372	0.191149
16970	Amp	12	84195375	84359876	0.061778	0.196221
16971	Amp	12	84361272	84401453	0.001198	0.18869
16972	Amp	12	84406545	84511519	0	0.144362
16973	Amp	12	84515973	84553411	0	0.173351
16974	Amp	12	84557470	84576320	0	0.184281
16975	Amp	12	84579939	84652935	0.068197	0.196652
16976	Amp	12	84653849	84869522	0	0.186062
16977	Amp	12	84872705	84887483	0	0.16016
16978	Amp	12	84888783	84979100	0	0.177857
16979	Amp	12	84986915	85012809	0.028398	0.192302
16980	Amp	12	85018345	85455631	0.018387	0.19092
16981	Amp	12	85456790	85510143	0	0.186453
16982	Amp	12	85512314	85553173	0	0.173742
16983	Amp	12	85554727	85565230	0	0.185898
16984	Amp	12	85574180	85689237	0.27943	0.218724
16985	Amp	12	85690168	85895963	0.333141	0.223748
16986	Amp	12	85897950	85972786	0.390264	0.229083
16987	Amp	12	85975573	86124108	0.476502	0.236692
16988	Amp	12	86128464	86234468	0.62858	0.249625
16989	Amp	12	86239588	86364377	0.375927	0.227595
16990	Amp	12	86364863	86429131	0.390264	0.229254
16991	Amp	12	86433399	86458877	0.250263	0.216321
16992	Amp	12	86459863	86499895	0.111991	0.201877
16993	Amp	12	86501065	86611527	0.333141	0.223907
16994	Amp	12	86616525	86660833	0.367646	0.226794
16995	Amp	12	86667255	86710541	0.636206	0.250122
16996	Amp	12	86714516	86810575	0.367646	0.226794
16997	Amp	12	86815598	87000345	0.347934	0.225136
16998	Amp	12	87002253	87221737	0.382767	0.228211
16999	Amp	12	87231599	87308438	0.347934	0.225323
17000	Amp	12	87322931	87396005	0.382767	0.228665
17001	Amp	12	87397767	87415051	0.347934	0.225323
17002	Amp	12	87415569	87613560	0.325321	0.223206
17003	Amp	12	87614565	87679802	0	0.172683
17004	Amp	12	87680104	87856071	0	0.154809
17005	Amp	12	87863784	87891760	0	0.156482
17006	Amp	12	87896008	87901018	0	0.172384
17007	Amp	12	87901885	87949539	0.007199	0.189116
17008	Amp	12	87950700	87969640	0	0.173213
17009	Amp	12	87975919	88065888	0.333141	0.223736
17010	Amp	12	88068531	88171070	0.421967	0.231962
17011	Amp	12	88171782	88236823	0.532449	0.241805
17012	Amp	12	88245570	88288162	0.437983	0.233579

17013 Amp	12	88289971	88342658	0.405699	0.230504
17014 Amp	12	88342703	88371406	0.483654	0.237042
17015 Amp	12	88373589	88463976	0.508503	0.239635
17016 Amp	12	88472055	88496894	0.743132	0.259137
17017 Amp	12	88503279	88690430	0.587735	0.246209
17018 Amp	12	88691461	88739424	0.652914	0.251526
17019 Amp	12	88740342	88750015	0.709359	0.25686
17020 Amp	12	88753024	89188541	0.652914	0.251543
17021 Amp	12	89191452	89502576	0.778204	0.262205
17022 Amp	12	89504876	89602991	0.250263	0.215932
17023 Amp	12	89612978	90030640	0.098998	0.200098
17024 Amp	12	90036958	90077153	0.111991	0.201598
17025 Amp	12	90077187	90100419	0	0.18586
17026 Amp	12	90103244	90107071	0.111991	0.201598
17027 Amp	12	90111662	90156203	0.168144	0.207859
17028 Amp	12	90160121	90297530	0.156508	0.206186
17029 Amp	12	90297902	90343251	0.050005	0.194935
17030 Amp	12	90350724	90666404	0.068197	0.196771
17031 Amp	12	90666683	90821905	0.038273	0.193317
17032 Amp	12	90826139	90826139	0.007199	0.189067
17033 Amp	12	90826647	90976648	0	0.182522
17034 Amp	12	90987547	90990596	0.086298	0.199191
17035 Amp	12	91016819	91016819	0.187743	0.209685
17036 Amp	12	91020136	91112772	0.7517	0.260207
17037 Amp	12	91112942	91140473	0.823952	0.265435
17038 Amp	12	91144327	91479776	0.906818	0.271969
17039 Amp	12	91486985	91550327	0.760395	0.260687
17040 Amp	12	91550872	91780804	0.743132	0.259187
17041 Amp	12	91781399	91827679	0.852608	0.267638
17042 Amp	12	91828429	91835098	0.833763	0.266073
17043 Amp	12	91841055	92234606	0.702615	0.25558
17044 Amp	12	92235962	92254055	0.613517	0.248084
17045 Amp	12	92256901	92311428	0.556748	0.243739
17046 Amp	12	92320147	92562227	0.540744	0.241953
17047 Amp	12	92562374	92617631	0.508503	0.239361
17048 Amp	12	92617935	92726154	0.41418	0.23091
17049 Amp	12	92726953	92727118	0.49169	0.238292
17050 Amp	12	92727219	92795281	0.382767	0.228511
17051 Amp	12	92810173	92833928	0.18072	0.209275
17052 Amp	12	92834432	92836413	0.111991	0.201772
17053 Amp	12	92836500	92972207	0.215373	0.212302
17054 Amp	12	92981260	93043791	0.236222	0.214828
17055 Amp	12	93044395	93196500	0.195309	0.210449
17056 Amp	12	93196668	93470101	0.156508	0.206273
17057 Amp	12	93470135	93470237	0.007199	0.189057
17058 Amp	12	93470514	93489958	0	0.179353
17059 Amp	12	93491726	93506021	0.001198	0.188722
17060 Amp	12	93506229	93506229	0.098998	0.200499
17061 Amp	12	93508669	93520266	0.325321	0.222741
17062 Amp	12	93522624	93524457	0.02372	0.191454
17063 Amp	12	93530500	93636703	0	0.181362
17064 Amp	12	93638105	93706751	0	0.169585
17065 Amp	12	93707155	93983140	0	0.182954

17066	Amp	12	93989653	93989653	0	0.174655
17067	Amp	12	93989686	94138989	0	0.167329
17068	Amp	12	94141943	94195444	0	0.170546
17069	Amp	12	94198860	94287292	0.043943	0.194237
17070	Amp	12	94290027	94306043	0	0.173586
17071	Amp	12	94309133	94398921	0	0.168517
17072	Amp	12	94401470	94500821	0	0.178299
17073	Amp	12	94504824	94512365	0	0.175092
17074	Amp	12	94517404	94518641	0	0.165311
17075	Amp	12	94524113	95068458	0	0.146474
17076	Amp	12	95070035	95102831	0	0.140833
17077	Amp	12	95103477	95212255	0	0.187359
17078	Amp	12	95212619	95307016	0	0.140833
17079	Amp	12	95310266	95354221	0	0.150887
17080	Amp	12	95358227	95372432	0	0.145553
17081	Amp	12	95373470	95382038	0	0.135499
17082	Amp	12	95382482	95385572	0	0.144203
17083	Amp	12	95386818	95413911	0	0.141677
17084	Amp	12	95414085	95520750	0	0.148446
17085	Amp	12	95522445	95762291	0	0.143028
17086	Amp	12	95762890	95847679	0	0.16389
17087	Amp	12	95861926	95895366	0	0.172222
17088	Amp	12	95929868	96066189	0	0.181256
17089	Amp	12	96069399	96085436	0	0.178216
17090	Amp	12	96090392	96120903	0	0.188147
17091	Amp	12	96121299	96442252	0	0.16847
17092	Amp	12	96453027	96563783	0	0.187338
17093	Amp	12	96565662	96583438	0.007199	0.189281
17094	Amp	12	96586016	96608434	0.033661	0.192764
17095	Amp	12	96618445	96638079	0	0.182833
17096	Amp	12	96642211	96714238	0	0.184361
17097	Amp	12	96714446	96963112	0	0.180879
17098	Amp	12	96965676	96988768	0	0.186035
17099	Amp	12	96995181	97284533	0	0.181035
17100	Amp	12	97286727	97418946	0	0.130513
17101	Amp	12	97422364	97487655	0	0.116644
17102	Amp	12	97490011	97973664	0	0.110578
17103	Amp	12	97976996	97995011	0	0.109131
17104	Amp	12	97995563	98011942	0	0.103974
17105	Amp	12	98021632	98068399	0	0.087003
17106	Amp	12	98070498	98089404	0	0.088833
17107	Amp	12	98090888	98183211	0	0.087003
17108	Amp	12	98185191	98199946	0	0.083646
17109	Amp	12	98207879	98215508	0	0.082118
17110	Amp	12	98221392	98400383	0	0.077046
17111	Amp	12	98402179	98629753	0	0.063102
17112	Amp	12	98630539	98754463	0	0.068569
17113	Amp	12	98754664	98776151	0	0.058402
17114	Amp	12	98776494	98796142	0	0.060348
17115	Amp	12	98796594	99124465	0	0.054882
17116	Amp	12	99125626	99157918	0	0.075398
17117	Amp	12	99168790	99219513	0	0.09452
17118	Amp	12	99227341	99268434	0	0.075398

17119 Amp	12	99272423	99295810	0	0.073452
17120 Amp	12	99297670	99444312	0	0.075359
17121 Amp	12	99445209	99478596	0	0.079417
17122 Amp	12	99479238	99714135	0	0.06826
17123 Amp	12	99720655	99734100	0	0.064202
17124 Amp	12	99740688	99767798	0	0.051149
17125 Amp	12	99773157	99788154	0	0.049242
17126 Amp	12	99791447	99849776	0	0.059438
17127 Amp	12	99850455	100141745	0	0.062722
17128 Amp	12	100148505	100248361	0	0.037076
17129 Amp	12	100249032	100491329	0	0.033791
17130 Amp	12	100493689	100625501	0	0.042613
17131 Amp	12	100629324	100635962	0	0.032418
17132 Amp	12	100643180	101394367	0	0.036475
17133 Amp	12	101403512	101581372	0	0.032418
17134 Amp	12	101581519	101761583	0	0.037152
17135 Amp	12	101763673	102175643	0	0.032418
17136 Amp	12	102177966	102241743	0	0.044979
17137 Amp	12	102260133	102600688	0	0.032418
17138 Amp	12	102600689	102843690	0	0.029201
17139 Amp	12	102843847	103054493	0	0.032083
17140 Amp	12	103057444	103102128	0	0.059748
17141 Amp	12	103110133	103255056	0	0.06477
17142 Amp	12	103262648	103348883	0	0.037105
17143 Amp	12	103353400	103419928	0	0.028282
17144 Amp	12	103420422	104169609	0	0.030651
17145 Amp	12	104178367	104179726	0	0.052467
17146 Amp	12	104179752	104182857	0	0.057656
17147 Amp	12	104183716	104211450	0	0.065667
17148 Amp	12	104217880	104355679	0	0.072828
17149 Amp	12	104355715	104381867	0	0.065667
17150 Amp	12	104384376	104545227	0	0.060478
17151 Amp	12	104547304	104549957	0	0.062939
17152 Amp	12	104550519	104551093	0	0.082673
17153 Amp	12	104578700	104582096	0	0.080212
17154 Amp	12	104584894	104586532	0	0.060478
17155 Amp	12	104586614	104777741	0	0.062062
17156 Amp	12	104778142	104821319	0	0.059291
17157 Amp	12	104821402	104910774	0	0.062753
17158 Amp	12	104913219	105448505	0	0.059291
17159 Amp	12	105449532	105642267	0	0.064357
17160 Amp	12	105646637	105785929	0	0.056346
17161 Amp	12	105786470	105811720	0	0.059929
17162 Amp	12	105812740	105830530	0	0.058345
17163 Amp	12	105832303	106068358	0	0.067197
17164 Amp	12	106072049	106811263	0	0.071394
17165 Amp	12	106811635	106842619	0	0.076494
17166 Amp	12	106846169	106896055	0	0.071473
17167 Amp	12	106902063	106920473	0	0.066372
17168 Amp	12	106922508	106925883	0	0.078381
17169 Amp	12	106931870	107012972	0	0.092573
17170 Amp	12	107017177	107019079	0	0.074993
17171 Amp	12	107027904	107111862	0	0.066142

17172 Amp	12	107113950	107184256	0	0.080887
17173 Amp	12	107184277	107294341	0	0.078518
17174 Amp	12	107296372	107337090	0	0.080461
17175 Amp	12	107339644	107392856	0	0.08446
17176 Amp	12	107393106	107789168	0	0.079394
17177 Amp	12	107793600	107968731	0	0.075395
17178 Amp	12	107969669	108004491	0	0.073453
17179 Amp	12	108092446	108346821	0	0.075878
17180 Amp	12	108352283	108668183	0	0.073453
17181 Amp	12	108670633	108702242	0	0.08269
17182 Amp	12	108711110	108895203	0	0.093384
17183 Amp	12	108896578	109275610	0	0.062981
17184 Amp	12	109277431	109283862	0	0.054781
17185 Amp	12	109284664	109324643	0	0.049313
17186 Amp	12	109326080	109459888	0	0.055136
17187 Amp	12	109465162	109536599	0	0.045899
17188 Amp	12	109539581	109651227	0	0.049495
17189 Amp	12	109652086	109700724	0	0.046613
17190 Amp	12	109719176	109934405	0	0.035564
17191 Amp	12	109934893	109960050	0	0.037787
17192 Amp	12	109962676	110084710	0	0.041863
17193 Amp	12	110087629	110265716	0	0.037787
17194 Amp	12	110266682	110407571	0	0.047781
17195 Amp	12	110411212	110675513	0	0.038413
17196 Amp	12	110679296	111040642	0	0.028418
17197 Amp	12	111041900	111152688	0	0.032396
17198 Amp	12	111154576	111356739	0	0.076573
17199 Amp	12	111363603	111457127	0	0.040254
17200 Amp	12	111460535	111489097	0	0.043281
17201 Amp	12	111491366	111506225	0	0.039303
17202 Amp	12	111508749	111533429	0	0.049951
17203 Amp	12	111534350	111660732	0	0.046355
17204 Amp	12	111661673	111883947	0	0.044132
17205 Amp	12	111885173	111887343	0	0.033484
17206 Amp	12	111889253	111901804	0	0.035978
17207 Amp	12	111903019	112160517	0	0.032951
17208 Amp	12	112162505	112367457	0	0.048206
17209 Amp	12	112369266	112457195	0	0.032951
17210 Amp	12	112457228	112907215	0	0.029142
17211 Amp	12	112907362	113091146	0	0.031578
17212 Amp	12	113091392	113347249	0	0.023719
17213 Amp	12	113347670	113386054	0	0.019522
17214 Amp	12	113392268	113735753	0	0.020974
17215 Amp	12	113737040	113768271	0	0.029136
17216 Amp	12	113772855	114140806	0	0.020974
17217 Amp	12	114140826	114657253	0	0.024016
17218 Amp	12	114660445	114663716	0	0.033447
17219 Amp	12	114673462	114693817	0	0.040496
17220 Amp	12	114694011	114750278	0	0.044252
17221 Amp	12	114752409	114772568	0	0.060429
17222 Amp	12	114780717	114824978	0	0.074228
17223 Amp	12	114916356	115114129	0	0.034822
17224 Amp	12	115114632	115135288	0	0.027772

17225	Amp	12	115137467	115157094	0	0.039785
17226	Amp	12	115158742	115276724	0	0.027772
17227	Amp	12	115276770	115349775	0	0.025337
17228	Amp	12	115351395	115370004	0	0.021754
17229	Amp	12	115375508	115977849	0	0.05833
17230	Amp	12	115979433	116110681	0	0.06563
17231	Amp	12	116110744	116152975	0	0.068986
17232	Amp	12	116165809	116239228	0	0.067535
17233	Amp	12	116243489	116653926	0	0.064178
17234	Amp	12	116655881	116776463	0	0.069881
17235	Amp	12	116776812	117082995	0	0.07671
17236	Amp	12	117091177	117104460	0	0.027132
17237	Amp	12	117113963	117129871	0	0.038855
17238	Amp	12	117133724	117194172	0	0.045923
17239	Amp	12	117210428	117263909	0	0.038855
17240	Amp	12	117266889	117266889	0	0.04465
17241	Amp	12	117267969	117385464	0	0.047199
17242	Amp	12	117386337	117451964	0	0.053583
17243	Amp	12	117454942	117464518	0	0.056131
17244	Amp	12	117469886	117624560	0	0.047787
17245	Amp	12	117629614	117791244	0	0.053928
17246	Amp	12	117795309	118015543	0	0.064629
17247	Amp	12	118018285	118069924	0	0.07355
17248	Amp	12	118070991	118145921	0	0.062848
17249	Amp	12	118146312	118167355	0	0.053928
17250	Amp	12	118167766	118170475	0	0.051378
17251	Amp	12	118172048	118208625	0	0.048669
17252	Amp	12	118209496	118442040	0	0.042528
17253	Amp	12	118446306	118848333	0	0.044721
17254	Amp	12	118850355	118890982	0	0.046832
17255	Amp	12	118894162	118897833	0	0.063593
17256	Amp	12	118899154	119135288	0	0.078575
17257	Amp	12	119138559	119186432	0	0.095767
17258	Amp	12	119186530	119346332	0	0.078575
17259	Amp	12	119347502	119589753	0	0.071095
17260	Amp	12	119596233	119625020	0	0.059073
17261	Amp	12	119637796	120076653	0	0.046832
17262	Amp	12	120077539	120091955	0	0.053841
17263	Amp	12	120094778	120173813	0	0.05173
17264	Amp	12	120178189	120514819	0	0.048902
17265	Amp	12	120518108	120649512	0	0.041893
17266	Amp	12	120651648	120845490	0	0.039398
17267	Amp	12	120846181	120948690	0	0.032569
17268	Amp	12	120951159	120966253	0	0.038998
17269	Amp	12	120967323	120999999	0	0.047466
17270	Amp	12	121004261	121141221	0	0.049747
17271	Amp	12	121149024	121160849	0	0.052247
17272	Amp	12	121162377	121294838	0	0.092955
17273	Amp	12	121299285	121299779	0	0.086527
17274	Amp	12	121303970	121456103	0	0.045819
17275	Amp	12	121460241	121524500	0	0.039952
17276	Amp	12	121528927	121627894	0	0.067304
17277	Amp	12	121633605	121944323	0	0.031484

17278	Amp	12	121946438	122191029	0	0.033903
17279	Amp	12	122194060	122535203	0	0.031622
17280	Amp	12	122537745	122810163	0	0.037352
17281	Amp	12	122811548	122942192	0	0.031622
17282	Amp	12	122944638	122970879	0	0.03953
17283	Amp	12	122994180	123203227	0	0.031622
17284	Amp	12	123204974	123309374	0	0.053757
17285	Amp	12	123309475	123571569	0	0.051782
17286	Amp	12	123572622	123612981	0	0.068365
17287	Amp	12	123613219	123613301	0	0.076205
17288	Amp	12	123614065	123625822	0	0.085458
17289	Amp	12	123722551	123736951	0	0.04674
17290	Amp	12	123737554	123786382	0	0.048134
17291	Amp	12	123786392	123969369	0	0.049737
17292	Amp	12	123975620	124048054	0	0.053856
17293	Amp	12	124048141	124069313	0	0.056369
17294	Amp	12	124073862	124087415	0	0.052251
17295	Amp	12	124090970	124199195	0	0.078884
17296	Amp	12	124199662	124203567	0	0.080777
17297	Amp	12	124203609	124298275	0	0.082342
17298	Amp	12	124298664	124310604	0	0.089761
17299	Amp	12	124313867	124318485	0	0.091803
17300	Amp	12	124330466	124370357	0	0.098344
17301	Amp	12	124370730	124371451	0	0.096302
17302	Amp	12	124379419	124396339	0	0.089761
17303	Amp	12	124403634	124514019	0	0.099988
17304	Amp	12	124515515	124525901	0	0.101384
17305	Amp	12	124526161	124553750	0	0.125189
17306	Amp	12	124559245	124560594	0	0.101384
17307	Amp	12	124561490	124604916	0	0.095414
17308	Amp	12	124607797	124610407	0	0.120121
17309	Amp	12	124611797	124670894	0	0.124624
17310	Amp	12	124670970	124677570	0	0.130858
17311	Amp	12	124679860	124681500	0	0.124624
17312	Amp	12	124682392	124710880	0	0.099917
17313	Amp	12	124711829	124719679	0	0.08969
17314	Amp	12	124723570	124763839	0	0.088125
17315	Amp	12	124765886	124838694	0	0.086729
17316	Amp	12	124839079	124848409	0	0.084836
17317	Amp	12	124848518	124978264	0	0.080333
17318	Amp	12	124982441	125026997	0	0.089863
17319	Amp	12	125028256	125123876	0	0.080333
17320	Amp	12	125126334	125187853	0	0.086542
17321	Amp	12	125188990	125208482	0	0.085093
17322	Amp	12	125211198	125283177	0	0.078884
17323	Amp	12	125283868	125377296	0	0.076371
17324	Amp	12	125378388	125568952	0	0.079468
17325	Amp	12	125579857	126387289	0	0.071628
17326	Amp	12	126390995	126790204	0	0.070234
17327	Amp	12	126790363	126823105	0	0.06691
17328	Amp	12	126832949	126841082	0	0.075136
17329	Amp	12	126844567	126881366	0	0.06691
17330	Amp	12	126886378	126891612	0	0.077017

17331	Amp	12	126893516	126909155	0	0.075414
17332	Amp	12	126916942	127017346	0	0.065306
17333	Amp	12	127017353	127650906	0	0.061284
17334	Amp	12	127652810	127702330	0	0.067211
17335	Amp	12	127705740	127705740	0	0.071598
17336	Amp	12	127707250	128551931	0	0.068501
17337	Amp	12	128558003	128717308	0	0.07351
17338	Amp	12	128717692	129155607	0	0.076618
17339	Amp	12	129157531	129295310	0	0.078961
17340	Amp	12	129295962	129312264	0	0.080576
17341	Amp	12	129318981	130751970	0	0.06964
17342	Amp	12	130755362	130784251	0	0.065253
17343	Amp	12	130786873	130821733	0	0.06731
17344	Amp	12	130829072	130984447	0	0.072009
17345	Amp	12	130985017	131054314	0	0.07628
17346	Amp	12	131055026	131104426	0	0.072009
17347	Amp	12	131110018	131167119	0	0.06731
17348	Amp	12	131172672	131532954	0	0.064966
17349	Amp	12	131538583	131806941	0	0.066404
17350	Amp	12	131807802	132049578	0	0.064789
17351	Amp	12	132060098	132126981	0	0.066764
17352	Amp	12	132127961	132449408	0	0.063008
17353	Amp	12	132449560	132605822	0	0.072811
17354	Amp	13	18874255	18886418	0	0.091164
17355	Amp	13	18887344	19199239	0	0.095757
17356	Amp	13	19234783	19326137	0	0.113661
17357	Amp	13	19330322	19430792	0	0.094848
17358	Amp	13	19530371	19561562	0	0.083052
17359	Amp	13	19952621	19952621	0	0.077273
17360	Amp	13	19953271	19989089	0	0.070771
17361	Amp	13	19992103	20010502	0	0.082111
17362	Amp	13	20017277	20034598	0	0.053851
17363	Amp	13	20035109	20553525	0	0.051313
17364	Amp	13	20575580	20743892	0	0.046242
17365	Amp	13	20747770	20782771	0	0.034902
17366	Amp	13	20788975	20953532	0	0.056384
17367	Amp	13	20953623	20967367	0	0.060477
17368	Amp	13	20967792	21213217	0	0.068133
17369	Amp	13	21221051	21353356	0	0.088985
17370	Amp	13	21357047	21504463	0	0.077273
17371	Amp	13	21508925	21550937	0	0.079878
17372	Amp	13	21551186	21929025	0	0.091088
17373	Amp	13	21931328	22012652	0	0.098091
17374	Amp	13	22012752	22124366	0	0.104705
17375	Amp	13	22129391	22183216	0	0.11687
17376	Amp	13	22183363	22410389	0	0.110256
17377	Amp	13	22411114	22518745	0	0.104036
17378	Amp	13	22540513	22663433	0	0.081118
17379	Amp	13	22667527	23111360	0	0.068953
17380	Amp	13	23111455	23342597	0	0.066357
17381	Amp	13	23343001	23445207	0	0.070979
17382	Amp	13	23447557	23474969	0	0.064983
17383	Amp	13	23478904	23493088	0	0.07164

17384	Amp	13	23493399	23510670	0	0.069035
17385	Amp	13	23510906	24080908	0	0.062945
17386	Amp	13	24082012	24337542	0	0.070189
17387	Amp	13	24341939	25311147	0	0.063533
17388	Amp	13	25314770	25518438	0	0.084332
17389	Amp	13	25519719	25586564	0	0.11316
17390	Amp	13	25590883	25767394	0	0.084332
17391	Amp	13	25769812	25794813	0	0.088606
17392	Amp	13	25799939	25809288	0	0.073312
17393	Amp	13	25809424	25810442	0	0.081497
17394	Amp	13	25817337	25871499	0	0.091587
17395	Amp	13	25874411	25932677	0	0.114157
17396	Amp	13	25933674	25936077	0	0.109438
17397	Amp	13	25942204	26040433	0	0.125591
17398	Amp	13	26044222	26120305	0	0.13603
17399	Amp	13	26125552	26142828	0	0.182466
17400	Amp	13	26144866	26453602	0	0.175264
17401	Amp	13	26454103	26564307	0	0.181188
17402	Amp	13	26564335	26572274	0	0.182946
17403	Amp	13	26579471	26686204	0	0.169524
17404	Amp	13	26689591	26727218	0	0.163825
17405	Amp	13	26729091	26763594	0	0.156822
17406	Amp	13	26768684	26973060	0	0.151615
17407	Amp	13	26973204	26994965	0	0.156299
17408	Amp	13	26996607	27118826	0	0.150375
17409	Amp	13	27123915	27131560	0	0.187207
17410	Amp	13	27132019	27389754	0	0.185449
17411	Amp	13	27391407	27475369	0	0.152434
17412	Amp	13	27475891	27800149	0	0.154649
17413	Amp	13	27801099	27844106	0	0.114831
17414	Amp	13	27851779	27865736	0	0.154649
17415	Amp	13	27869506	27971131	0	0.118405
17416	Amp	13	27972629	28054137	0	0.12131
17417	Amp	13	28054154	28097673	0	0.119096
17418	Amp	13	28097717	28184039	0	0.122747
17419	Amp	13	28184456	28319347	0	0.149396
17420	Amp	13	28319505	28422823	0	0.150883
17421	Amp	13	28423263	28463653	0	0.154509
17422	Amp	13	28471715	28489675	0	0.134158
17423	Amp	13	28490202	28492334	0	0.135799
17424	Amp	13	28493034	28612630	0	0.144248
17425	Amp	13	28613074	28663048	0	0.157922
17426	Amp	13	28663072	28742425	0	0.162917
17427	Amp	13	28745574	28778355	0	0.136268
17428	Amp	13	28780590	28794998	0	0.155599
17429	Amp	13	28795180	28805219	0	0.159032
17430	Amp	13	28810277	28837833	0	0.139701
17431	Amp	13	28838581	28843116	0	0.132457
17432	Amp	13	28843754	28864836	0	0.136483
17433	Amp	13	28865890	28872305	0	0.141543
17434	Amp	13	28873214	28889564	0	0.131484
17435	Amp	13	28892961	28932038	0	0.139135
17436	Amp	13	28932139	28960488	0	0.13414

17437	Amp	13	28963642	29003321	0	0.14612
17438	Amp	13	29003859	29008983	0	0.13414
17439	Amp	13	29010375	29030209	0	0.142002
17440	Amp	13	29034768	29310286	0	0.13414
17441	Amp	13	29311240	29411346	0	0.141029
17442	Amp	13	29413288	29429388	0	0.139144
17443	Amp	13	29429998	29547752	0	0.146812
17444	Amp	13	29548246	29598321	0	0.160579
17445	Amp	13	29598358	29599130	0	0.155556
17446	Amp	13	29599341	29691361	0	0.143417
17447	Amp	13	29694597	29729904	0	0.153333
17448	Amp	13	29730428	29777129	0	0.164325
17449	Amp	13	29779208	29832947	0	0.153333
17450	Amp	13	29833261	29860330	0	0.16449
17451	Amp	13	29862412	29952894	0	0.170942
17452	Amp	13	29955037	30050362	0.098998	0.199928
17453	Amp	13	30061238	30072928	0	0.187349
17454	Amp	13	30074963	30203468	0.105051	0.201066
17455	Amp	13	30204644	30222511	0.382767	0.228146
17456	Amp	13	30223154	30242092	0.317596	0.222017
17457	Amp	13	30245138	30467458	0.362221	0.226329
17458	Amp	13	30468186	30468828	0.293283	0.219876
17459	Amp	13	30473112	30502189	0.149443	0.205798
17460	Amp	13	30502396	30508587	0.325321	0.222651
17461	Amp	13	30512203	30634462	0.444673	0.233687
17462	Amp	13	30634944	30707993	0.823952	0.26545
17463	Amp	13	30708202	30711395	0.693887	0.255556
17464	Amp	13	30711418	30719443	0.564815	0.24452
17465	Amp	13	30719643	30749205	0.548372	0.242728
17466	Amp	13	30752124	30816354	0.596354	0.24676
17467	Amp	13	30817852	30835428	0.444673	0.233759
17468	Amp	13	30835941	30842919	0.390264	0.229448
17469	Amp	13	30846035	30852633	0.340434	0.224494
17470	Amp	13	30855484	30880129	0	0.174033
17471	Amp	13	30880607	30903593	0	0.170001
17472	Amp	13	30909151	31028777	0	0.15786
17473	Amp	13	31030980	31040044	0	0.14711
17474	Amp	13	31041260	31120106	0	0.15786
17475	Amp	13	31120463	31151480	0	0.159784
17476	Amp	13	31315918	31317024	0	0.138367
17477	Amp	13	31318549	31331805	0	0.142296
17478	Amp	13	31332004	31381444	0	0.115317
17479	Amp	13	31384690	31509321	0	0.110532
17480	Amp	13	31510265	31619896	0	0.099376
17481	Amp	13	31627026	31723722	0	0.094316
17482	Amp	13	31728330	31748034	0	0.105749
17483	Amp	13	31750689	31752465	0	0.11056
17484	Amp	13	31754191	31843940	0	0.10663
17485	Amp	13	31847962	31970936	0	0.114183
17486	Amp	13	31971635	32126407	0	0.11875
17487	Amp	13	32133526	32137515	0	0.130013
17488	Amp	13	32137566	32142351	0	0.140896
17489	Amp	13	32147478	32158400	0	0.125845

17490 Amp	13	32159001	32199422	0	0.114962
17491 Amp	13	32293372	32296306	0	0.105486
17492 Amp	13	32299016	32366904	0	0.100919
17493 Amp	13	32367178	32379392	0	0.084902
17494 Amp	13	32385103	32387065	0	0.069195
17495 Amp	13	32391593	32446976	0	0.078987
17496 Amp	13	32448850	32636717	0	0.071555
17497 Amp	13	32644896	32647065	0	0.081107
17498 Amp	13	32649662	32669938	0	0.064377
17499 Amp	13	32672679	32802746	0	0.068421
17500 Amp	13	32803480	32907655	0	0.073835
17501 Amp	13	32908244	33201160	0	0.08334
17502 Amp	13	33201882	33294031	0	0.079313
17503 Amp	13	33296968	33346108	0	0.084181
17504 Amp	13	33348962	33369548	0	0.090412
17505 Amp	13	33379454	33425850	0	0.073589
17506 Amp	13	33428056	33453628	0	0.077812
17507 Amp	13	33458790	33734662	0	0.067928
17508 Amp	13	33735110	33746084	0	0.080117
17509 Amp	13	33748208	33788408	0	0.067928
17510 Amp	13	33788632	34015041	0	0.075661
17511 Amp	13	34020320	34047783	0	0.086164
17512 Amp	13	34048636	34293111	0	0.083464
17513 Amp	13	34293153	34334906	0	0.081767
17514 Amp	13	34340603	34342587	0	0.105271
17515 Amp	13	34344735	34382991	0	0.099392
17516 Amp	13	34391274	34410710	0	0.086965
17517 Amp	13	34411031	34426191	0	0.10238
17518 Amp	13	34427644	34442631	0	0.091303
17519 Amp	13	34443149	34551268	0	0.075888
17520 Amp	13	34553309	34660298	0	0.072829
17521 Amp	13	34661032	35171668	0	0.075671
17522 Amp	13	35174618	35204385	0	0.10053
17523 Amp	13	35220015	35289199	0	0.123885
17524 Amp	13	35299411	35305649	0	0.129521
17525 Amp	13	35312231	35383968	0	0.124108
17526 Amp	13	35385475	35616405	0	0.140366
17527 Amp	13	35618677	35689171	0	0.151275
17528 Amp	13	35697001	35709183	0	0.127206
17529 Amp	13	35709266	36007876	0	0.132266
17530 Amp	13	36007924	36068145	0	0.127548
17531 Amp	13	36069854	36204635	0	0.109709
17532 Amp	13	36206095	36316046	0	0.106466
17533 Amp	13	36319648	36352509	0	0.095963
17534 Amp	13	36353910	36369640	0	0.090903
17535 Amp	13	36370595	36380510	0	0.084457
17536 Amp	13	36386546	36609435	0	0.075842
17537 Amp	13	36609761	36646706	0	0.078476
17538 Amp	13	36647184	36658332	0	0.097
17539 Amp	13	36658656	36847297	0	0.075842
17540 Amp	13	36848450	37225197	0	0.072938
17541 Amp	13	37227904	37295393	0	0.07878
17542 Amp	13	37296023	37627483	0	0.072938

17543	Amp	13	37629086	37882743	0	0.082396
17544	Amp	13	37894074	37933845	0	0.088668
17545	Amp	13	37934013	37946864	0	0.044115
17546	Amp	13	37947900	37968015	0	0.030441
17547	Amp	13	37968546	38104547	0	0.024169
17548	Amp	13	38105864	38258190	0	0.037034
17549	Amp	13	38258489	38357159	0	0.027575
17550	Amp	13	38362251	38377376	0	0.031204
17551	Amp	13	38379072	38407835	0	0.049677
17552	Amp	13	38411687	38544945	0	0.031204
17553	Amp	13	38546025	38560579	0	0.042733
17554	Amp	13	38561892	38752118	0	0.041109
17555	Amp	13	38752884	39152779	0	0.03748
17556	Amp	13	39160963	39759365	0	0.03151
17557	Amp	13	39759630	39855267	0	0.033013
17558	Amp	13	39856577	39972993	0	0.055255
17559	Amp	13	39980785	40395301	0	0.021484
17560	Amp	13	40396002	40405882	0	0.019981
17561	Amp	13	40406215	41198044	0	0.021545
17562	Amp	13	41204754	41529593	0	0.015909
17563	Amp	13	41530162	41894752	0	0.003044
17564	Amp	13	41899985	42608851	0	0.015914
17565	Amp	13	42610366	42629160	0	0.034862
17566	Amp	13	42655690	44044695	0	0.015914
17567	Amp	13	44045757	44275776	0	0.033895
17568	Amp	13	44275801	45679058	0	0.015914
17569	Amp	13	45679167	46261264	0	0.014351
17570	Amp	13	46261825	49105491	0	0.016735
17571	Amp	13	49107583	49920448	0	0.023185
17572	Amp	13	49923945	49923945	0	0.020801
17573	Amp	13	51050074	51220419	0	0.071324
17574	Amp	13	51227300	51848075	0	0.079996
17575	Amp	13	51848342	52214956	0	0.083713
17576	Amp	13	52374407	52483236	0	0.039449
17577	Amp	13	52599178	52690909	0	0.060747
17578	Amp	13	52691283	52768919	0	0.062911
17579	Amp	13	52770062	52819038	0	0.048157
17580	Amp	13	52823933	52851706	0	0.043211
17581	Amp	13	52855516	53026154	0	0.03988
17582	Amp	13	53027078	53847180	0	0.027491
17583	Amp	13	53850458	54471030	0	0.02601
17584	Amp	13	54472076	54521625	0	0.01956
17585	Amp	13	54525981	54561613	0	0.026674
17586	Amp	13	54563454	55318432	0	0.011641
17587	Amp	13	55324375	56343384	0	0.017892
17588	Amp	13	56344750	56596925	0	0.022939
17589	Amp	13	56608508	57036364	0	0.016687
17590	Amp	13	57036675	57086315	0	0.009573
17591	Amp	13	57476563	57476563	0	0.011878
17592	Amp	13	57477107	57479530	0	0.006832
17593	Amp	13	57483107	61880468	0	0.013992
17594	Amp	13	61880690	62199365	0	0.015964
17595	Amp	13	62199428	63532576	0	0.013992

17596	Amp	13	63532685	63680723	0	0.006832
17597	Amp	13	63681260	64825923	0	0.012156
17598	Amp	13	64898325	65238083	0	0.019921
17599	Amp	13	65247656	65266426	0	0.045578
17600	Amp	13	65272834	65291330	0	0.062081
17601	Amp	13	65292398	65523958	0	0.045578
17602	Amp	13	65526006	66299225	0	0.029789
17603	Amp	13	66309598	66675328	0	0.012156
17604	Amp	13	66675585	67137411	0	0.006832
17605	Amp	13	67140492	67461239	0	0.009048
17606	Amp	13	67462810	67884650	0	0.016648
17607	Amp	13	67885451	67953535	0	0.014432
17608	Amp	13	67955324	68429317	0	0.020958
17609	Amp	13	68429661	68590759	0	0.030547
17610	Amp	13	68591356	69162528	0	0.022947
17611	Amp	13	69164526	69572751	0	0.013358
17612	Amp	13	69576290	69598848	0	0.024063
17613	Amp	13	69601311	69619956	0	0.013358
17614	Amp	13	69620491	69646025	0	0.028717
17615	Amp	13	69647811	69773340	0	0.013358
17616	Amp	13	69790192	69851967	0	0.034579
17617	Amp	13	69857351	69858582	0	0.036457
17618	Amp	13	69860538	69880928	0	0.018171
17619	Amp	13	69891806	69945790	0	0.015237
17620	Amp	13	69946148	69956768	0	0.013358
17621	Amp	13	69963716	69978228	0	0.015013
17622	Amp	13	69978336	70020548	0	0.021016
17623	Amp	13	70020664	70282349	0	0.01936
17624	Amp	13	70285709	70417633	0	0.037152
17625	Amp	13	70422821	70657752	0	0.03115
17626	Amp	13	70664298	71024684	0	0.020374
17627	Amp	13	71027935	71242440	0	0.013358
17628	Amp	13	71245306	71268059	0	0.016071
17629	Amp	13	71268299	71432883	0	0.013358
17630	Amp	13	71435799	71536609	0	0.0297
17631	Amp	13	71539105	71543096	0	0.02705
17632	Amp	13	71550048	71648388	0	0.035778
17633	Amp	13	71651911	71701273	0	0.02705
17634	Amp	13	71701898	71793315	0	0.035949
17635	Amp	13	71796066	72005051	0	0.022257
17636	Amp	13	72008190	72127569	0	0.028424
17637	Amp	13	72131158	72317872	0	0.06786
17638	Amp	13	72329817	72356473	0	0.028424
17639	Amp	13	72356914	72600601	0	0.021897
17640	Amp	13	72604052	72682538	0	0.041802
17641	Amp	13	72685383	73017110	0	0.021897
17642	Amp	13	73021002	73025890	0	0.012998
17643	Amp	13	73028290	74894997	0	0.008471
17644	Amp	13	74897322	74921579	0	0.01762
17645	Amp	13	74923263	74949771	0	0.008471
17646	Amp	13	74950887	76359244	0	0.017513
17647	Amp	13	76359484	76488613	0	0.02431
17648	Amp	13	76492311	76795900	0	0.015269

17649	Amp	13	76796274	77310505	0	0.019721
17650	Amp	13	77311098	77378516	0	0.027161
17651	Amp	13	77381251	77813678	0	0.020364
17652	Amp	13	77813828	78335803	0	0.02481
17653	Amp	13	78335881	78457835	0	0.01737
17654	Amp	13	78458083	78477034	0	0.03185
17655	Amp	13	78477762	78483319	0	0.046745
17656	Amp	13	78485161	78524474	0	0.048498
17657	Amp	13	78525279	78607446	0	0.044051
17658	Amp	13	78614284	78644984	0	0.014676
17659	Amp	13	78647324	79222606	0	0.021826
17660	Amp	13	79225699	80170855	0	0.014676
17661	Amp	13	80175037	80221631	0	0.010224
17662	Amp	13	80221822	80950577	0	0.012064
17663	Amp	13	80950976	82147880	0	0.015976
17664	Amp	13	82148504	82545128	0	0.012064
17665	Amp	13	82548920	82633553	0	0.019756
17666	Amp	13	82640829	82748133	0	0.024006
17667	Amp	13	82750482	82840711	0	0.025571
17668	Amp	13	82841175	84440576	0	0.024006
17669	Amp	13	84441109	84543764	0	0.028822
17670	Amp	13	84546046	84575796	0	0.024006
17671	Amp	13	84576455	84870593	0	0.022253
17672	Amp	13	84872387	85373615	0	0.029858
17673	Amp	13	85374486	85633006	0	0.028018
17674	Amp	13	85633094	85788633	0	0.032174
17675	Amp	13	85788859	85848162	0	0.028018
17676	Amp	13	85851269	86996139	0	0.030001
17677	Amp	13	86999484	87220254	0	0.028018
17678	Amp	13	87224140	89250772	0	0.042141
17679	Amp	13	89251084	89970961	0	0.053481
17680	Amp	13	89975255	89983186	0	0.07932
17681	Amp	13	89983858	90000958	0	0.057533
17682	Amp	13	90001705	90545265	0	0.042141
17683	Amp	13	90548490	90890370	0	0.049458
17684	Amp	13	90894504	91355604	0	0.035335
17685	Amp	13	91358403	91577530	0	0.044288
17686	Amp	13	91580936	91920063	0	0.056776
17687	Amp	13	91922107	92042328	0	0.053871
17688	Amp	13	92043909	92482392	0	0.062342
17689	Amp	13	92483944	92505080	0	0.087757
17690	Amp	13	92506600	92852278	0	0.062342
17691	Amp	13	92858656	93290719	0	0.060037
17692	Amp	13	93296873	93858812	0	0.078067
17693	Amp	13	93861537	94368701	0	0.08279
17694	Amp	13	94370030	94494050	0	0.089002
17695	Amp	13	94494519	94852462	0	0.093797
17696	Amp	13	94853008	94941181	0	0.098593
17697	Amp	13	94943122	95027878	0	0.103766
17698	Amp	13	95028031	95044286	0	0.09897
17699	Amp	13	95051276	95752879	0	0.105014
17700	Amp	13	95753871	96120813	0	0.100219
17701	Amp	13	96121939	96190326	0	0.095495

17702	Amp	13	96191299	96418213	0	0.090322
17703	Amp	13	96418857	96483885	0	0.096985
17704	Amp	13	96489298	96863201	0	0.090322
17705	Amp	13	96863529	97024970	0	0.095495
17706	Amp	13	97026648	97066608	0	0.10024
17707	Amp	13	97066836	97281114	0	0.115054
17708	Amp	13	97281919	97338925	0	0.10885
17709	Amp	13	97343211	97352519	0	0.104106
17710	Amp	13	97354761	97383438	0	0.110888
17711	Amp	13	97383740	97405911	0	0.115747
17712	Amp	13	97412808	98047566	0	0.100354
17713	Amp	13	98047963	98064461	0	0.103175
17714	Amp	13	98065388	98075438	0	0.104564
17715	Amp	13	98080595	98271831	0	0.101744
17716	Amp	13	98276686	98284817	0	0.082813
17717	Amp	13	98285277	98458142	0	0.077954
17718	Amp	13	98459083	98640313	0	0.087778
17719	Amp	13	98640488	98757845	0	0.069377
17720	Amp	13	98761449	98984416	0	0.077876
17721	Amp	13	98984733	99204017	0	0.069526
17722	Amp	13	99211296	99465539	0	0.061027
17723	Amp	13	99465658	99593884	0	0.062958
17724	Amp	13	99597513	99677192	0	0.046675
17725	Amp	13	99679882	99814461	0	0.054116
17726	Amp	13	99815251	99840444	0	0.046675
17727	Amp	13	99840906	99909199	0	0.056118
17728	Amp	13	99918181	100236801	0	0.050074
17729	Amp	13	100240967	100708976	0	0.040631
17730	Amp	13	100709518	100769115	0	0.047215
17731	Amp	13	100771096	100816553	0	0.041049
17732	Amp	13	100821443	100890309	0	0.056727
17733	Amp	13	100899980	100977483	0	0.050143
17734	Amp	13	100977548	101304027	0	0.048754
17735	Amp	13	101305920	101336693	0	0.055422
17736	Amp	13	101342459	102203141	0	0.048754
17737	Amp	13	102204156	103302018	0	0.050229
17738	Amp	13	103307336	103793810	0	0.034551
17739	Amp	13	103794222	103926625	0	0.044017
17740	Amp	13	103927585	103942628	0	0.048843
17741	Amp	13	103945189	104192632	0	0.039376
17742	Amp	13	104198444	104459410	0	0.045589
17743	Amp	13	104462542	104510708	0	0.039376
17744	Amp	13	104511872	104655135	0	0.044486
17745	Amp	13	104656949	105128361	0	0.048606
17746	Amp	13	105128737	105843549	0	0.051416
17747	Amp	13	105845638	106080464	0	0.055524
17748	Amp	13	106081326	106224046	0	0.052715
17749	Amp	13	106227206	106392890	0	0.062671
17750	Amp	13	106396254	106487442	0	0.058562
17751	Amp	13	106489001	107200567	0	0.048606
17752	Amp	13	107201238	107518023	0	0.051993
17753	Amp	13	107518089	107829337	0	0.046883
17754	Amp	13	107831129	108035394	0	0.054702

17755 Amp	13	108036357	108174806	0	0.062434
17756 Amp	13	108180936	108226133	0	0.054615
17757 Amp	13	108227793	108440156	0	0.046883
17758 Amp	13	108440704	108498691	0	0.050017
17759 Amp	13	108498881	108891925	0	0.056858
17760 Amp	13	108895901	108973515	0	0.063268
17761 Amp	13	108975238	109112798	0	0.073154
17762 Amp	13	109113254	109214793	0	0.081171
17763 Amp	13	109261543	109317741	0	0.090792
17764 Amp	13	109322722	109767506	0	0.081171
17765 Amp	13	109769104	110067348	0	0.087382
17766 Amp	13	110068878	110332630	0	0.089059
17767 Amp	13	110332875	110450884	0	0.079173
17768 Amp	13	110450909	110671373	0	0.075786
17769 Amp	13	110675796	110701763	0	0.068469
17770 Amp	13	110703207	110759098	0	0.062058
17771 Amp	13	110760538	110858262	0	0.063641
17772 Amp	13	110862400	110878704	0	0.06822
17773 Amp	13	110880991	111180055	0	0.063641
17774 Amp	13	111181684	111551547	0	0.067662
17775 Amp	13	111553808	111667195	0	0.060821
17776 Amp	13	111667749	111668142	0	0.0568
17777 Amp	13	111851441	112058535	0	0.063077
17778 Amp	13	112064341	112121661	0	0.0568
17779 Amp	13	112124887	112158231	0	0.058266
17780 Amp	13	112159518	112462317	0	0.061713
17781 Amp	13	112462496	112825234	0	0.067403
17782 Amp	13	112826599	112862607	0	0.063956
17783 Amp	13	113081740	113664174	0	0.07035
17784 Amp	13	113877048	114136775	0	0.083283
17785 Amp	13	114137065	114137065	0	0.080149
17786 Amp	13	114149650	114226675	0	0.067216
17787 Amp	14	20033191	20070235	0.079463	0.197892
17788 Amp	14	20070536	20293351	0	0.154745
17789 Amp	14	20293457	20308930	0	0.152893
17790 Amp	14	20313799	20321736	0	0.164518
17791 Amp	14	20322213	20403486	0	0.174434
17792 Amp	14	20403513	20551029	0	0.178285
17793 Amp	14	20555289	20607475	0	0.182358
17794 Amp	14	20609676	20675788	0.007199	0.189357
17795 Amp	14	20675883	20787565	0	0.182358
17796 Amp	14	20787967	20797478	0	0.177177
17797 Amp	14	20798547	21006080	0	0.168095
17798 Amp	14	21006947	21201170	0	0.156469
17799 Amp	14	21201442	21284260	0	0.152619
17800 Amp	14	21284364	21352295	0	0.155619
17801 Amp	14	21358741	21359914	0	0.153641
17802 Amp	14	21360830	21380012	0	0.155954
17803 Amp	14	21380640	21410148	0	0.154353
17804 Amp	14	21414490	21414490	0	0.148596
17805 Amp	14	21419614	21436963	0	0.144755
17806 Amp	14	21440988	21634218	0	0.150421
17807 Amp	14	21634589	21634589	0	0.145912

17808 Amp	14	21634977	21634977	0	0.153134
17809 Amp	14	21635439	21635439	0	0.146538
17810 Amp	14	21635545	21635545	0	0.175323
17811 Amp	14	22664303	22664303	0	0.170581
17812 Amp	14	22664866	22796272	0	0.174455
17813 Amp	14	22796827	22801891	0	0.179751
17814 Amp	14	22802062	22806422	0	0.172529
17815 Amp	14	22808213	22945992	0	0.169487
17816 Amp	14	22950472	22975462	0	0.1804
17817 Amp	14	22989947	22989947	0	0.18628
17818 Amp	14	22996260	23017877	0.079463	0.198452
17819 Amp	14	23020579	23047301	0.056238	0.195452
17820 Amp	14	23050766	23051430	0.105051	0.201031
17821 Amp	14	23055920	23055920	0.156508	0.206766
17822 Amp	14	23063776	23132940	0.068197	0.19685
17823 Amp	14	23136216	23229467	0.086298	0.199236
17824 Amp	14	23229808	23267069	0.117531	0.202467
17825 Amp	14	23268293	23429262	0.068197	0.196732
17826 Amp	14	23432821	23501042	0	0.18456
17827 Amp	14	23503490	23631262	0	0.17841
17828 Amp	14	23632223	23634924	0	0.180347
17829 Amp	14	23639178	23642153	0.02372	0.191532
17830 Amp	14	23642522	23678085	0.086298	0.198848
17831 Amp	14	23680250	23710558	0.061778	0.195848
17832 Amp	14	23714995	23715513	0.02372	0.191379
17833 Amp	14	23717366	23719172	0.007199	0.189442
17834 Amp	14	23719375	23843784	0	0.182126
17835 Amp	14	23845836	23906228	0	0.176247
17836 Amp	14	23907120	23926567	0	0.183513
17837 Amp	14	23932083	23938512	0	0.179007
17838 Amp	14	23938707	23945088	0	0.170916
17839 Amp	14	23945103	24024956	0	0.165337
17840 Amp	14	24025034	24025034	0	0.159636
17841 Amp	14	24025440	24031436	0	0.143427
17842 Amp	14	24031630	24031792	0	0.160534
17843 Amp	14	24041000	24049930	0	0.171516
17844 Amp	14	24050053	24061086	0	0.177542
17845 Amp	14	24065832	24076775	0.02372	0.191393
17846 Amp	14	24078812	24192971	0	0.186861
17847 Amp	14	24199204	24203282	0.061778	0.196087
17848 Amp	14	24204769	24307138	0.018387	0.19034
17849 Amp	14	24317276	24337594	0	0.176489
17850 Amp	14	24340589	24366267	0	0.163092
17851 Amp	14	24369415	24376460	0	0.170966
17852 Amp	14	24379921	24394800	0	0.180978
17853 Amp	14	24395023	24439525	0	0.182961
17854 Amp	14	24440156	24648957	0	0.176937
17855 Amp	14	24650267	24658974	0	0.158797
17856 Amp	14	24668046	24746273	0	0.173081
17857 Amp	14	24750179	24867830	0	0.174579
17858 Amp	14	24868670	24878316	0	0.158039
17859 Amp	14	24878709	24891977	0	0.152479
17860 Amp	14	24893473	24909067	0	0.16917

17861	Amp	14	24909700	25010524	0	0.175332
17862	Amp	14	25011063	25043837	0	0.16917
17863	Amp	14	25048766	25090584	0	0.17356
17864	Amp	14	25090617	25115180	0.243425	0.215202
17865	Amp	14	25117444	25253122	0.347934	0.22529
17866	Amp	14	25253257	25266878	0.540744	0.242384
17867	Amp	14	25271669	25272155	0.430191	0.232296
17868	Amp	14	25274118	25288539	0.483654	0.237301
17869	Amp	14	25288567	25301190	0.532449	0.241403
17870	Amp	14	25306435	25312539	0.476502	0.237014
17871	Amp	14	25312986	25314249	0.523647	0.240738
17872	Amp	14	25314567	25327513	0.619816	0.24891
17873	Amp	14	25330683	25358100	0.367646	0.226884
17874	Amp	14	25358933	25376371	0.444673	0.234229
17875	Amp	14	25377831	25465038	0.760395	0.260657
17876	Amp	14	25466379	25470068	0.702615	0.255652
17877	Amp	14	25470421	25552075	0.86979	0.268828
17878	Amp	14	25552368	25561879	0.906818	0.27146
17879	Amp	14	25562970	25567227	0.963924	0.275926
17880	Amp	14	25570646	25633075	1.120124	0.286731
17881	Amp	14	25634041	25805741	0.963924	0.275926
17882	Amp	14	25806074	25839845	0.991911	0.277945
17883	Amp	14	25841023	26009736	0.944128	0.274714
17884	Amp	14	26010076	26072714	1.000924	0.278667
17885	Amp	14	26077882	26077882	1.120124	0.287144
17886	Amp	14	26087428	26148230	1.218154	0.293936
17887	Amp	14	26152756	26167982	1.078696	0.283828
17888	Amp	14	26168632	26216955	0.991911	0.27803
17889	Amp	14	26219484	26234661	0.796213	0.263197
17890	Amp	14	26234954	26287624	0.685617	0.254627
17891	Amp	14	26290000	26294421	0.833763	0.266202
17892	Amp	14	26300516	26300517	0.778204	0.261831
17893	Amp	14	26300519	26300519	0.860481	0.268117
17894	Amp	14	26300708	26303393	0.991911	0.277856
17895	Amp	14	26304876	26372539	0.7517	0.259875
17896	Amp	14	26372624	26402752	0.833763	0.266205
17897	Amp	14	26403225	26533866	1.000924	0.278248
17898	Amp	14	26536425	26580613	1.088806	0.285043
17899	Amp	14	26583088	26617627	1.000924	0.278917
17900	Amp	14	26618337	26626472	1.099256	0.285559
17901	Amp	14	26627536	26663456	0.888878	0.270183
17902	Amp	14	26666431	26692813	0.963924	0.275642
17903	Amp	14	26693069	26718472	1.088806	0.284681
17904	Amp	14	26724507	26797775	1.059215	0.282611
17905	Amp	14	26801027	26801027	0.963924	0.275816
17906	Amp	14	26802118	26955258	1.130387	0.287647
17907	Amp	14	26955797	27208300	1.265443	0.29713
17908	Amp	14	27210588	27242444	0.613517	0.248115
17909	Amp	14	27243026	27433220	0.548372	0.242656
17910	Amp	14	27434613	27449803	0.460107	0.235538
17911	Amp	14	27453620	27485979	0.508503	0.239313
17912	Amp	14	27486050	27784225	0.382767	0.228324
17913	Amp	14	27787293	27789615	0.286675	0.219285

17914	Amp	14	27790300	27974142	0.347934	0.224714
17915	Amp	14	27974291	28067673	0.444673	0.23361
17916	Amp	14	28070085	28072301	0.51566	0.240018
17917	Amp	14	28073236	28083520	0.580011	0.245562
17918	Amp	14	28086520	28094473	0.7517	0.259766
17919	Amp	14	28096493	28117505	0.580011	0.245562
17920	Amp	14	28128223	28128223	0.51566	0.240133
17921	Amp	14	28128703	28129746	0.667776	0.252998
17922	Amp	14	28131309	28227832	0.709359	0.256828
17923	Amp	14	28231813	28312320	0.685617	0.254739
17924	Amp	14	28319683	28333220	0.619816	0.249195
17925	Amp	14	28339219	28369256	0.709359	0.256944
17926	Amp	14	28372303	28475523	0.62858	0.249607
17927	Amp	14	28477025	28491299	0.564815	0.244454
17928	Amp	14	28492204	28515829	0.548372	0.242572
17929	Amp	14	28520026	28530634	0.452609	0.234822
17930	Amp	14	28532052	28555430	0.405699	0.230356
17931	Amp	14	28556113	28620187	0.430191	0.232409
17932	Amp	14	28620527	28696222	0.355162	0.226002
17933	Amp	14	28698495	28709459	0.483654	0.237688
17934	Amp	14	28709730	28763608	0.444673	0.233914
17935	Amp	14	28764665	28764665	0.51566	0.24009
17936	Amp	14	28780143	28838809	0.693887	0.255147
17937	Amp	14	28839172	29006031	0.500055	0.238555
17938	Amp	14	29008093	29023287	0.604912	0.247875
17939	Amp	14	29030549	29052047	0.66072	0.252457
17940	Amp	14	29052062	29160432	0.548372	0.243137
17941	Amp	14	29168230	29218979	0.468576	0.235918
17942	Amp	14	29218997	29229849	0.398217	0.229493
17943	Amp	14	29239365	29253192	0.62858	0.249991
17944	Amp	14	29254408	29285807	0.693887	0.255197
17945	Amp	14	29289972	29466982	0.452609	0.234699
17946	Amp	14	29472097	29493640	0.325321	0.223012
17947	Amp	14	29495243	29800386	0.925715	0.273273
17948	Amp	14	29800824	29897406	0.972663	0.276785
17949	Amp	14	29901724	29948629	1.099256	0.285253
17950	Amp	14	29953222	30015844	1.150167	0.289002
17951	Amp	14	30019507	30322957	1.218154	0.293675
17952	Amp	14	30326126	30357683	1.130387	0.287499
17953	Amp	14	30361531	30373236	1.265443	0.2974
17954	Amp	14	30373414	30441390	1.068928	0.283528
17955	Amp	14	30441733	30454165	1.159718	0.289563
17956	Amp	14	30454493	30467211	1.2565	0.296451
17957	Amp	14	30482525	30577840	1.354645	0.303168
17958	Amp	14	30578758	30583988	1.484449	0.312471
17959	Amp	14	30585402	30682956	1.534719	0.315583
17960	Amp	14	30692211	30754366	1.563139	0.317585
17961	Amp	14	30755267	30805738	1.424227	0.308282
17962	Amp	14	30810476	30846924	1.637826	0.322775
17963	Amp	14	30847448	30887607	1.856336	0.336647
17964	Amp	14	30887861	30972225	1.737394	0.329759
17965	Amp	14	30974990	30995898	1.687998	0.325929
17966	Amp	14	30996035	30999056	1.582671	0.319212

17967	Amp	14	31004327	31153535	1.708768	0.327092
17968	Amp	14	31157171	31257017	1.615314	0.321038
17969	Amp	14	31261139	31282833	1.789205	0.332713
17970	Amp	14	31283695	31307981	1.866286	0.33745
17971	Amp	14	31311891	31340280	1.687998	0.325775
17972	Amp	14	31340550	31397244	1.757929	0.330755
17973	Amp	14	31404166	31404166	1.708768	0.327243
17974	Amp	14	31413002	31459450	1.424227	0.308296
17975	Amp	14	31467146	31469803	1.140058	0.288418
17976	Amp	14	31472717	31477407	1.324884	0.301023
17977	Amp	14	31479751	31484875	1.24764	0.296044
17978	Amp	14	31491296	31500294	1.068928	0.283438
17979	Amp	14	31503083	31503083	1.130387	0.287798
17980	Amp	14	31503444	31648323	1.295233	0.299446
17981	Amp	14	31648690	31720596	1.265443	0.297444
17982	Amp	14	31722493	31792039	1.344429	0.302554
17983	Amp	14	31800093	31833698	1.442925	0.309375
17984	Amp	14	31834160	31884359	1.542961	0.316565
17985	Amp	14	31884813	31891389	1.778794	0.332269
17986	Amp	14	31892405	31912768	1.953191	0.343089
17987	Amp	14	31915343	31936948	1.856336	0.336758
17988	Amp	14	31939597	32039810	1.748357	0.329937
17989	Amp	14	32040337	32041584	1.768015	0.331367
17990	Amp	14	32041699	32074903	1.605306	0.320548
17991	Amp	14	32083812	32130123	1.717946	0.327953
17992	Amp	14	32134422	32134422	1.657426	0.324262
17993	Amp	14	32134741	32244086	1.717946	0.328002
17994	Amp	14	32251137	32278872	1.748357	0.330015
17995	Amp	14	32283390	32327673	1.657426	0.324032
17996	Amp	14	32330823	32444017	1.678649	0.325556
17997	Amp	14	32446019	32466262	1.020188	0.280189
17998	Amp	14	32468635	32489773	1.088806	0.284846
17999	Amp	14	32489799	32519354	1.159718	0.289448
18000	Amp	14	32524655	32531138	1.179216	0.291015
18001	Amp	14	32532480	32550626	1.208462	0.292691
18002	Amp	14	32553790	32643613	1.265443	0.29691
18003	Amp	14	32645240	32657860	1.525294	0.314811
18004	Amp	14	32658820	32691370	1.453628	0.31021
18005	Amp	14	32691922	32694576	1.354645	0.303417
18006	Amp	14	32700239	32719642	1.404612	0.307012
18007	Amp	14	32722019	32731525	1.324884	0.301346
18008	Amp	14	32733438	32748624	1.2565	0.29669
18009	Amp	14	32756780	32777294	1.049532	0.282197
18010	Amp	14	32780005	32982972	1.218154	0.293632
18011	Amp	14	32983475	32996582	1.150167	0.288895
18012	Amp	14	32998585	33028938	1.364151	0.303929
18013	Amp	14	33031369	33057039	1.424227	0.307967
18014	Amp	14	33057268	33079046	1.354645	0.303608
18015	Amp	14	33080508	33128675	1.473764	0.311516
18016	Amp	14	33129662	33154991	1.354645	0.303608
18017	Amp	14	33155634	33161599	1.484449	0.312246
18018	Amp	14	33163264	33173932	1.534719	0.315704
18019	Amp	14	33174196	33190636	2.238069	0.360857

18020 Amp	14	33194278	33429290	2.327403	0.366348
18021 Amp	14	33430668	33448462	2.806723	0.395585
18022 Amp	14	33448949	33451502	2.591575	0.382301
18023 Amp	14	33451936	33468075	2.727427	0.390596
18024 Amp	14	33468479	33505295	2.796399	0.394477
18025 Amp	14	33506980	33569073	3.194439	0.418203
18026 Amp	14	33577805	33577805	3.05489	0.409908
18027 Amp	14	33581078	33596315	2.994162	0.40645
18028 Amp	14	33606003	33620821	3.496514	0.435618
18029 Amp	14	33624171	33652941	3.217724	0.419111
18030 Amp	14	33653265	33783009	3.079122	0.411192
18031 Amp	14	33783120	33792701	2.958746	0.404002
18032 Amp	14	33796335	33807972	2.818034	0.396105
18033 Amp	14	33810096	33820790	2.796399	0.394581
18034 Amp	14	33821450	33821596	2.830877	0.396659
18035 Amp	14	33827453	33827453	2.602347	0.382899
18036 Amp	14	33834308	33855190	2.458756	0.374431
18037 Amp	14	33857576	33954981	2.614747	0.383651
18038 Amp	14	33957002	33967751	3.112016	0.41323
18039 Amp	14	33978464	34007261	2.784457	0.394158
18040 Amp	14	34007774	34160397	2.806723	0.395637
18041 Amp	14	34160434	34163121	2.878516	0.399602
18042 Amp	14	34165357	34183952	3.124015	0.414104
18043 Amp	14	34184090	34189902	2.703608	0.389096
18044 Amp	14	34197734	34203698	2.806723	0.395157
18045 Amp	14	34209209	34217872	2.774551	0.393589
18046 Amp	14	34217942	34252003	2.703608	0.3894
18047 Amp	14	34252416	34274825	2.914035	0.401386
18048 Amp	14	34278300	34286555	2.774551	0.39333
18049 Amp	14	34286706	34380917	2.625007	0.38411
18050 Amp	14	34380935	34450143	2.591575	0.382434
18051 Amp	14	34452646	34470555	2.567803	0.381003
18052 Amp	14	34475196	34497730	2.483202	0.376398
18053 Amp	14	34500200	34500227	2.383144	0.369757
18054 Amp	14	34502615	34502615	2.170217	0.357221
18055 Amp	14	34502916	34502916	1.563139	0.31798
18056 Amp	14	34505580	34513258	1.534719	0.315694
18057 Amp	14	34521237	34521938	1.442925	0.309633
18058 Amp	14	34521995	34522419	0.317596	0.222126
18059 Amp	14	34527119	34569707	0.175335	0.20853
18060 Amp	14	34569888	34579436	2.071528	0.35091
18061 Amp	14	34579478	34579478	2.658709	0.386311
18062 Amp	14	34589610	34589610	2.878516	0.399484
18063 Amp	14	34591792	34598336	3.182419	0.417561
18064 Amp	14	34600290	34728222	3.312745	0.424692
18065 Amp	14	34731664	34800638	3.016502	0.408009
18066 Amp	14	34801596	34809441	2.902384	0.400878
18067 Amp	14	34813037	34906876	3.182419	0.417561
18068 Amp	14	34908595	34996430	3.112016	0.413216
18069 Amp	14	34996578	35221772	3.089932	0.411537
18070 Amp	14	35224963	35276436	3.146436	0.415041
18071 Amp	14	35278830	35326338	3.171522	0.416439
18072 Amp	14	35326789	35380264	2.994162	0.406075

18073 Amp	14	35382013	35401264	3.029636	0.408227
18074 Amp	14	35401490	35435613	3.124015	0.414249
18075 Amp	14	35442901	35448061	3.006013	0.406824
18076 Amp	14	35449490	35472362	2.774551	0.393459
18077 Amp	14	35473652	35547356	3.05489	0.409858
18078 Amp	14	35551400	35574515	2.994162	0.406308
18079 Amp	14	35578323	35597679	2.71525	0.389909
18080 Amp	14	35597783	35706295	2.683697	0.388099
18081 Amp	14	35709823	35729481	2.625007	0.384507
18082 Amp	14	35729576	35745061	2.555572	0.380502
18083 Amp	14	35745289	35785693	2.22615	0.360454
18084 Amp	14	35787698	35837903	1.811302	0.334256
18085 Amp	14	35841899	35862129	1.930731	0.341505
18086 Amp	14	35863337	36009032	2.039783	0.348368
18087 Amp	14	36009390	36016237	1.995109	0.346194
18088 Amp	14	36017872	36019197	1.930731	0.342121
18089 Amp	14	36019547	36022870	1.832922	0.335523
18090 Amp	14	36025233	36091193	2.249752	0.36172
18091 Amp	14	36091338	36163195	2.148338	0.355633
18092 Amp	14	36163349	36175124	2.203122	0.358865
18093 Amp	14	36175595	36347162	2.170217	0.356787
18094 Amp	14	36347898	36392151	2.393797	0.370606
18095 Amp	14	36393117	36402532	2.061276	0.349913
18096 Amp	14	36404222	36452726	2.113683	0.353431
18097 Amp	14	36453216	36497882	2.249752	0.361445
18098 Amp	14	36498489	36577052	2.283191	0.364044
18099 Amp	14	36577190	36587207	2.327403	0.366642
18100 Amp	14	36588264	36645691	2.113683	0.353551
18101 Amp	14	36645776	36673349	2.170217	0.35722
18102 Amp	14	36676467	36708211	1.983885	0.345155
18103 Amp	14	36714409	36793042	2.192225	0.358478
18104 Amp	14	36793198	36888072	2.260813	0.362691
18105 Amp	14	36888207	36900653	2.294081	0.364698
18106 Amp	14	36904216	36949816	2.260813	0.362099
18107 Amp	14	36950146	36954361	2.180628	0.357425
18108 Amp	14	36954422	37050845	2.039783	0.34881
18109 Amp	14	37053611	37072848	1.582671	0.319228
18110 Amp	14	37076474	37106620	1.553308	0.317003
18111 Amp	14	37114054	37174312	1.687998	0.3257
18112 Amp	14	37179175	37205410	1.877398	0.338675
18113 Amp	14	37206715	37232860	1.930731	0.342034
18114 Amp	14	37235490	37274741	1.844531	0.336559
18115 Amp	14	37275088	37282368	1.930731	0.342055
18116 Amp	14	37285368	37316355	1.995109	0.345624
18117 Amp	14	37316372	37323742	2.082332	0.35141
18118 Amp	14	37327168	37394902	2.359227	0.368851
18119 Amp	14	37397905	37416118	2.470601	0.375763
18120 Amp	14	37417505	37448191	2.591575	0.382577
18121 Amp	14	37459560	37459560	2.830877	0.396483
18122 Amp	14	37463100	37500069	2.958746	0.403993
18123 Amp	14	37501361	37576057	3.146436	0.415608
18124 Amp	14	37577553	37577553	3.029636	0.408697
18125 Amp	14	37579902	37604999	3.230962	0.41979

18126	Amp	14	37607943	37610883	3.112016	0.412976
18127	Amp	14	37617010	37650645	3.016502	0.40748
18128	Amp	14	37673871	37684929	2.98257	0.405472
18129	Amp	14	37687564	37694989	2.922915	0.402114
18130	Amp	14	37695659	37707041	2.796399	0.394604
18131	Amp	14	37709574	37721177	2.703608	0.388997
18132	Amp	14	37723308	37734241	2.727427	0.390721
18133	Amp	14	37734515	37749337	2.602347	0.383333
18134	Amp	14	37751117	37751117	2.635842	0.384735
18135	Amp	14	37754497	37754497	2.272343	0.363105
18136	Amp	14	37766666	37766666	1.887705	0.339382
18137	Amp	14	37774010	37827292	1.717946	0.327766
18138	Amp	14	37839669	37873471	1.572486	0.318378
18139	Amp	14	37873507	37926064	1.514375	0.313994
18140	Amp	14	37928178	37949968	1.344429	0.302364
18141	Amp	14	37957632	37977887	1.10923	0.286212
18142	Amp	14	37978056	38032826	1.226647	0.294428
18143	Amp	14	38037501	38056251	1.088806	0.28471
18144	Amp	14	38063696	38133479	1.237378	0.295232
18145	Amp	14	38133954	38190203	1.637826	0.322353
18146	Amp	14	38196708	38208447	1.708768	0.327602
18147	Amp	14	38214542	38216671	1.344429	0.302904
18148	Amp	14	38218068	38231402	1.49455	0.312734
18149	Amp	14	38233156	38282433	1.453628	0.310136
18150	Amp	14	38283052	38283052	1.218154	0.293474
18151	Amp	14	38288226	38376836	1.049532	0.281765
18152	Amp	14	38377444	38392296	1.068928	0.283263
18153	Amp	14	38393781	38462150	0.580011	0.245435
18154	Amp	14	38462163	38469783	0.175335	0.208657
18155	Amp	14	38469797	38475342	0.301081	0.221064
18156	Amp	14	38475617	38481423	0.223025	0.213079
18157	Amp	14	38481810	38481810	0.161592	0.207357
18158	Amp	14	38585376	38650769	0.02372	0.191668
18159	Amp	14	38655651	38716649	0	0.178345
18160	Amp	14	38718605	38719175	0	0.179811
18161	Amp	14	38722150	38723707	0.033661	0.193036
18162	Amp	14	38727488	38771504	0.086298	0.199116
18163	Amp	14	38777060	38785992	0.175335	0.208251
18164	Amp	14	38786377	38793852	0.079463	0.197863
18165	Amp	14	38799352	38806969	0.27943	0.219043
18166	Amp	14	38807174	38889615	0.223025	0.213037
18167	Amp	14	38890130	38918212	0.317596	0.222042
18168	Amp	14	38918678	38941994	0.215373	0.212907
18169	Amp	14	38943987	38995531	0.460107	0.235229
18170	Amp	14	38995859	39068630	0.123589	0.20331
18171	Amp	14	39070737	39115780	0	0.185647
18172	Amp	14	39117340	39149293	0	0.184148
18173	Amp	14	39149482	39175167	0.02372	0.191647
18174	Amp	14	39178202	39212603	0	0.186399
18175	Amp	14	39215761	39260403	0.156508	0.206117
18176	Amp	14	39264156	39347577	0	0.1878
18177	Amp	14	39347607	39362819	0.038273	0.193233
18178	Amp	14	39365437	39495174	0	0.182866

18179	Amp	14	39497435	39615328	0.001198	0.188857
18180	Amp	14	39618944	39687728	0.123589	0.203102
18181	Amp	14	39694271	39709549	0.273081	0.218256
18182	Amp	14	39709676	39728171	0.128931	0.20401
18183	Amp	14	39739771	39915370	0	0.174537
18184	Amp	14	39916433	39943309	0	0.168323
18185	Amp	14	39943520	40198605	0	0.164804
18186	Amp	14	40201995	40201995	0	0.169477
18187	Amp	14	40202237	40343381	0	0.171769
18188	Amp	14	40344044	40376359	0	0.166413
18189	Amp	14	40383354	40470194	0	0.157473
18190	Amp	14	40471904	40471904	0	0.160927
18191	Amp	14	40472868	40579893	0	0.164288
18192	Amp	14	40580290	40600018	0	0.15229
18193	Amp	14	40600789	40735583	0	0.157411
18194	Amp	14	40735599	40775455	0.117531	0.202142
18195	Amp	14	40775877	40819577	0.018387	0.190707
18196	Amp	14	40820043	40863787	0	0.187347
18197	Amp	14	40864165	40873306	0	0.185838
18198	Amp	14	40877459	40912460	0	0.180633
18199	Amp	14	40913140	41230772	0	0.178645
18200	Amp	14	41233378	41233651	0.007199	0.189002
18201	Amp	14	41234571	41320267	0.105051	0.200823
18202	Amp	14	41321114	41331709	0.018387	0.190466
18203	Amp	14	41334029	41341627	0.050005	0.194953
18204	Amp	14	41341785	41341872	0.111991	0.201984
18205	Amp	14	41344761	41397386	0.079463	0.19853
18206	Amp	14	41398047	41426931	0	0.184907
18207	Amp	14	41428887	41477464	0	0.182669
18208	Amp	14	41480226	41484499	0	0.176302
18209	Amp	14	41484559	41524794	0	0.171815
18210	Amp	14	41525660	41579577	0	0.181992
18211	Amp	14	41580905	41597261	0	0.171815
18212	Amp	14	41597631	41672540	0	0.17543
18213	Amp	14	41676612	41788731	0	0.169997
18214	Amp	14	41788796	41868892	0	0.166382
18215	Amp	14	41868908	41875619	0	0.171432
18216	Amp	14	41875724	41899881	0	0.173669
18217	Amp	14	41902727	41906267	0	0.186403
18218	Amp	14	41918277	41968314	0	0.180413
18219	Amp	14	41971348	41971816	0	0.173008
18220	Amp	14	41973854	42034141	0	0.178363
18221	Amp	14	42045473	42059759	0	0.165629
18222	Amp	14	42061634	42202542	0	0.160579
18223	Amp	14	42203968	42380790	0	0.165473
18224	Amp	14	42392289	42396555	0	0.156764
18225	Amp	14	42399065	42430871	0	0.150176
18226	Amp	14	42431223	42489042	0	0.156573
18227	Amp	14	42491448	42895899	0	0.162654
18228	Amp	14	42898209	43047451	0	0.1572
18229	Amp	14	43048651	43100455	0	0.158637
18230	Amp	14	43101031	43290044	0	0.160509
18231	Amp	14	43290647	43350726	0	0.172995

18232	Amp	14	43779976	43853084	0	0.123143
18233	Amp	14	43854861	43859381	0	0.116547
18234	Amp	14	44031914	44142165	0	0.13309
18235	Amp	14	44142504	44171407	0	0.127734
18236	Amp	14	44171567	44184884	0	0.147658
18237	Amp	14	44184986	44279793	0	0.135173
18238	Amp	14	44280947	44295742	0	0.115248
18239	Amp	14	44301734	44602297	0	0.113811
18240	Amp	14	44602609	44819945	0	0.112088
18241	Amp	14	44822788	44885409	0	0.110216
18242	Amp	14	44891998	44908578	0	0.114215
18243	Amp	14	44909437	44955031	0	0.123107
18244	Amp	14	44957124	45190693	0	0.114215
18245	Amp	14	45197321	45300525	0	0.108299
18246	Amp	14	45300952	45339990	0	0.105187
18247	Amp	14	45593519	45648402	0	0.111489
18248	Amp	14	45648976	45680126	0	0.105187
18249	Amp	14	45761842	45849252	0	0.10717
18250	Amp	14	45858317	45984559	0	0.103171
18251	Amp	14	45986364	46153741	0	0.104796
18252	Amp	14	46154521	46404573	0	0.107351
18253	Amp	14	46405258	46486000	0	0.115314
18254	Amp	14	46487241	46514989	0	0.107351
18255	Amp	14	46518500	46624678	0	0.104796
18256	Amp	14	46626085	46645063	0	0.102812
18257	Amp	14	46645159	47032125	0	0.108204
18258	Amp	14	47032520	47078541	0	0.111445
18259	Amp	14	47078697	47160455	0	0.115842
18260	Amp	14	47162695	47264091	0	0.122024
18261	Amp	14	47271841	47690492	0	0.112601
18262	Amp	14	47691443	47740259	0	0.105571
18263	Amp	14	47742941	47830921	0	0.116561
18264	Amp	14	47831083	47856664	0	0.111169
18265	Amp	14	47858473	47979126	0	0.100179
18266	Amp	14	47982703	47992529	0	0.104781
18267	Amp	14	47996568	48008471	0	0.113198
18268	Amp	14	48010800	48029561	0	0.109539
18269	Amp	14	48047980	48103429	0	0.104781
18270	Amp	14	48104273	48116508	0	0.109506
18271	Amp	14	48119817	48218648	0	0.114495
18272	Amp	14	48219544	48244307	0	0.109892
18273	Amp	14	48249504	48263682	0	0.119669
18274	Amp	14	48264531	48264531	0	0.134561
18275	Amp	14	48397525	48424582	0	0.129837
18276	Amp	14	48429351	48435928	0	0.121305
18277	Amp	14	48444296	48444296	0	0.109956
18278	Amp	14	48447780	48461294	0	0.116979
18279	Amp	14	48468026	48470722	0	0.119763
18280	Amp	14	48477578	48493662	0	0.11274
18281	Amp	14	48495717	48516062	0	0.109956
18282	Amp	14	48518445	48528763	0	0.119948
18283	Amp	14	48536062	48595595	0	0.124739
18284	Amp	14	48598554	48616704	0	0.109764

18285	Amp	14	48617425	48626565	0	0.117343
18286	Amp	14	48629869	48773211	0	0.112552
18287	Amp	14	48773505	48773505	0	0.119609
18288	Amp	14	48777352	48780866	0	0.126868
18289	Amp	14	48781036	48807270	0	0.132302
18290	Amp	14	48807595	48842767	0	0.133994
18291	Amp	14	48842798	48935161	0	0.141196
18292	Amp	14	48941603	48961956	0	0.144935
18293	Amp	14	48962220	49056366	0	0.149996
18294	Amp	14	49056546	49143575	0	0.144752
18295	Amp	14	49143746	49167193	0	0.153887
18296	Amp	14	49170036	49305537	0	0.167966
18297	Amp	14	49305744	49305744	0	0.17301
18298	Amp	14	49305814	49335245	0	0.182324
18299	Amp	14	49335548	49540289	0	0.17319
18300	Amp	14	49544480	49576562	0	0.17919
18301	Amp	14	49578285	49581354	0	0.183913
18302	Amp	14	49581729	49590512	0	0.177056
18303	Amp	14	49592951	49645931	0	0.162976
18304	Amp	14	49645994	49716856	0	0.160182
18305	Amp	14	49719753	49861352	0	0.164994
18306	Amp	14	49871956	49988369	0	0.160182
18307	Amp	14	49992891	50114371	0	0.148083
18308	Amp	14	50131842	50202113	0	0.14336
18309	Amp	14	50204247	50275531	0	0.136257
18310	Amp	14	50280619	50292391	0	0.146463
18311	Amp	14	50303443	50487859	0	0.137148
18312	Amp	14	50489748	50502239	0	0.132066
18313	Amp	14	50502492	50519378	0	0.137766
18314	Amp	14	50520811	50611818	0	0.14404
18315	Amp	14	50616876	50737995	0	0.153112
18316	Amp	14	50740346	50828040	0	0.147411
18317	Amp	14	50830010	50839679	0	0.132066
18318	Amp	14	50840012	51199674	0	0.12186
18319	Amp	14	51200786	51224807	0	0.127761
18320	Amp	14	51224810	51466922	0	0.12056
18321	Amp	14	51475280	51515697	0	0.116821
18322	Amp	14	51515970	51606459	0	0.114808
18323	Amp	14	51606583	51678387	0	0.121777
18324	Amp	14	51679458	51768503	0	0.112
18325	Amp	14	51768693	51858190	0	0.116807
18326	Amp	14	51858679	51978945	0	0.140807
18327	Amp	14	51979401	52086973	0	0.133228
18328	Amp	14	52089093	52220228	0	0.145098
18329	Amp	14	52225273	52320728	0	0.156241
18330	Amp	14	52322041	52410084	0	0.145098
18331	Amp	14	52410228	52428656	0	0.129664
18332	Amp	14	52430901	52560734	0	0.141291
18333	Amp	14	52562567	52658089	0	0.134322
18334	Amp	14	52658332	52692356	0	0.139353
18335	Amp	14	52692734	52692734	0	0.160729
18336	Amp	14	52698519	52841244	0	0.168122
18337	Amp	14	52846631	52861657	0	0.157413

18338	Amp	14	52864961	52978828	0	0.153195
18339	Amp	14	52982454	53081113	0	0.170838
18340	Amp	14	53082819	53110435	0	0.163446
18341	Amp	14	53131428	53310649	0	0.145802
18342	Amp	14	53311914	53425717	0	0.140771
18343	Amp	14	53439266	53475418	0	0.147034
18344	Amp	14	53475667	53553117	0	0.151611
18345	Amp	14	53555103	53606762	0	0.145611
18346	Amp	14	53614775	53685470	0	0.15031
18347	Amp	14	53688982	53706821	0	0.138683
18348	Amp	14	53707578	53989403	0	0.149657
18349	Amp	14	53992486	54021898	0	0.155856
18350	Amp	14	54034462	54146147	0	0.147291
18351	Amp	14	54151676	54183413	0	0.153645
18352	Amp	14	54183537	54233277	0	0.147383
18353	Amp	14	54247056	54274562	0	0.117311
18354	Amp	14	54274743	54284094	0	0.112611
18355	Amp	14	54284356	54344008	0	0.118556
18356	Amp	14	54346865	54395745	0	0.119996
18357	Amp	14	54398245	54434301	0	0.118556
18358	Amp	14	54437124	54674574	0	0.115057
18359	Amp	14	54679508	54761392	0	0.112998
18360	Amp	14	54761991	54807012	0	0.116297
18361	Amp	14	54809974	54869961	0	0.123477
18362	Amp	14	54870033	54924863	0	0.119291
18363	Amp	14	54925664	54967240	0	0.111179
18364	Amp	14	54968074	55282388	0	0.106666
18365	Amp	14	55284930	55446222	0	0.103492
18366	Amp	14	55448675	55464589	0	0.10527
18367	Amp	14	55464829	55477647	0	0.107976
18368	Amp	14	55481432	55510221	0	0.105198
18369	Amp	14	55510806	55543552	0	0.10342
18370	Amp	14	55548439	56050061	0	0.096834
18371	Amp	14	56050343	56116064	0	0.10316
18372	Amp	14	56120954	56474756	0	0.110556
18373	Amp	14	56476757	56651350	0	0.10316
18374	Amp	14	56651721	57412161	0	0.090162
18375	Amp	14	57412353	57539186	0	0.095553
18376	Amp	14	57545677	57638536	0	0.105142
18377	Amp	14	57639002	58010501	0	0.118159
18378	Amp	14	58010606	58227231	0	0.133041
18379	Amp	14	58236774	58271010	0	0.127589
18380	Amp	14	58276223	58440701	0	0.137122
18381	Amp	14	58441291	58522080	0	0.127589
18382	Amp	14	58522143	58549962	0	0.131115
18383	Amp	14	58553594	58566757	0	0.143262
18384	Amp	14	58570481	58577635	0	0.132288
18385	Amp	14	58580257	58626147	0	0.128989
18386	Amp	14	58627246	58665455	0	0.130465
18387	Amp	14	58668679	58723713	0	0.141724
18388	Amp	14	58724195	58806450	0	0.138198
18389	Amp	14	58811122	58854448	0	0.125819
18390	Amp	14	58857779	58878529	0	0.114559

18391	Amp	14	58878812	59082062	0	0.108688
18392	Amp	14	59084496	59166959	0	0.105588
18393	Amp	14	59175134	59521589	0	0.116907
18394	Amp	14	59522976	59575234	0	0.104761
18395	Amp	14	59576221	59817856	0	0.107465
18396	Amp	14	59820625	59882302	0	0.078889
18397	Amp	14	59884200	60199149	0	0.081878
18398	Amp	14	60201806	60573255	0	0.077121
18399	Amp	14	60576175	60606157	0	0.070794
18400	Amp	14	60608989	61091867	0	0.067805
18401	Amp	14	61091995	61407377	0	0.055268
18402	Amp	14	61407579	61416769	0	0.067237
18403	Amp	14	61418722	61523343	0	0.06161
18404	Amp	14	61523487	61566532	0	0.067003
18405	Amp	14	61569365	61975737	0	0.06161
18406	Amp	14	61976615	62391173	0	0.05029
18407	Amp	14	62395140	62450664	0	0.047787
18408	Amp	14	62456013	62496391	0	0.049509
18409	Amp	14	62497428	62598854	0	0.047787
18410	Amp	14	62603429	62657077	0	0.033838
18411	Amp	14	62660441	62667513	0	0.027638
18412	Amp	14	62668679	62683697	0	0.02945
18413	Amp	14	62688686	62960829	0	0.042975
18414	Amp	14	62963545	63300126	0	0.045927
18415	Amp	14	63300268	63624083	0	0.050606
18416	Amp	14	63625662	63652787	0	0.054853
18417	Amp	14	63655257	63678226	0	0.052275
18418	Amp	14	63678680	63809518	0	0.045676
18419	Amp	14	63809801	63915632	0	0.047124
18420	Amp	14	63922800	64087083	0	0.045313
18421	Amp	14	64090467	64321788	0	0.040633
18422	Amp	14	64324142	64409915	0	0.045413
18423	Amp	14	64411162	64426064	0	0.029686
18424	Amp	14	64429887	64697966	0	0.023605
18425	Amp	14	64707868	64711923	0	0.018236
18426	Amp	14	64714429	65096277	0	0.029806
18427	Amp	14	65103551	65187290	0	0.02556
18428	Amp	14	65191204	66091230	0	0.01399
18429	Amp	14	66091365	66136560	0	0.016461
18430	Amp	14	66140837	66208461	0	0.01399
18431	Amp	14	66212453	67221135	0	0.011037
18432	Amp	14	67221571	67322510	0	0.016914
18433	Amp	14	67322840	67513630	0	0.02431
18434	Amp	14	67516499	67615081	0	0.014721
18435	Amp	14	67618336	67687388	0	0.023181
18436	Amp	14	67698770	67715892	0	0.018069
18437	Amp	14	67718697	67762329	0	0.014721
18438	Amp	14	67767822	67776645	0	0.020075
18439	Amp	14	67778905	67933159	0	0.012679
18440	Amp	14	67933302	68100851	0	0.01819
18441	Amp	14	68102472	68143350	0	0.012837
18442	Amp	14	68147447	68180914	0	0.022408
18443	Amp	14	68180948	68199970	0	0.02748

18444 Amp	14	68202328	68358739	0	0.021968
18445 Amp	14	68365358	68392064	0	0.016091
18446 Amp	14	68397594	68413705	0	0.040047
18447 Amp	14	68417884	68579159	0	0.016091
18448 Amp	14	68582887	68938383	0	0.01102
18449 Amp	14	68938540	68989555	0	0.018307
18450 Amp	14	68994396	69005442	0	0.027351
18451 Amp	14	69008861	69021905	0	0.019708
18452 Amp	14	69022402	69144709	0	0.02764
18453 Amp	14	69162128	69375560	0	0.018307
18454 Amp	14	69375943	69410866	0	0.020737
18455 Amp	14	69411695	69630651	0	0.018307
18456 Amp	14	69631286	69763018	0	0.008736
18457 Amp	14	69766060	69926025	0	0.016594
18458 Amp	14	69926189	70336923	0	0.021128
18459 Amp	14	70338531	70653417	0	0.025132
18460 Amp	14	70656843	70665518	0	0.038139
18461 Amp	14	70666042	70711786	0	0.034135
18462 Amp	14	70717454	70717454	0	0.031394
18463 Amp	14	70717935	70742491	0	0.023535
18464 Amp	14	70745441	71543028	0	0.010528
18465 Amp	14	71548782	71691797	0	0.022369
18466 Amp	14	71698029	71868186	0	0.010528
18467 Amp	14	71868386	71896671	0	0.015107
18468 Amp	14	71897925	71959274	0	0.010528
18469 Amp	14	71960039	72081918	0	0.015754
18470 Amp	14	72084702	72162104	0	0.042197
18471 Amp	14	72168157	72857892	0	0.010528
18472 Amp	14	72858657	73523917	0	0.014977
18473 Amp	14	73599605	73599605	0	0.007053
18474 Amp	14	73599951	73676484	0	0.005605
18475 Amp	14	73678578	73711939	0	0.002901
18476 Amp	14	73724417	73801775	0	0.008292
18477 Amp	14	73812613	74185741	0	0.002901
18478 Amp	14	74190733	74485664	0	0.008738
18479 Amp	14	74487363	74507037	0	0.032058
18480 Amp	14	74513258	74587998	0	0.019546
18481 Amp	14	74588523	74812336	0	0.008738
18482 Amp	14	74816459	74937114	0	0.011371
18483 Amp	14	74939686	74969749	0	0.026627
18484 Amp	14	74969763	75005799	0	0.02079
18485 Amp	14	75007769	75296471	0	0.027934
18486 Amp	14	75299030	75645217	0	0.039903
18487 Amp	14	75645389	75880248	0	0.037271
18488 Amp	14	75880553	75892948	0	0.047953
18489 Amp	14	75894725	76095892	0	0.051565
18490 Amp	14	76095907	76465231	0	0.039596
18491 Amp	14	76469482	76888033	0	0.045835
18492 Amp	14	76888259	76912344	0	0.050161
18493 Amp	14	76912559	77009751	0	0.056765
18494 Amp	14	77011321	77483616	0	0.059402
18495 Amp	14	77483780	77664226	0	0.046165
18496 Amp	14	77664401	77721661	0	0.043265

18497 Amp	14	77731282	78325661	0	0.034233
18498 Amp	14	78326763	78644808	0	0.038959
18499 Amp	14	78649865	78810847	0	0.034233
18500 Amp	14	78811467	79034136	0	0.031596
18501 Amp	14	79035389	79040758	0	0.034187
18502 Amp	14	79041134	79171166	0	0.032654
18503 Amp	14	79176925	80316934	0	0.031011
18504 Amp	14	80319636	80390332	0	0.041026
18505 Amp	14	80394225	80590075	0	0.031011
18506 Amp	14	80601294	80728928	0	0.008092
18507 Amp	14	80731845	80843955	0	0.01947
18508 Amp	14	80847092	80927779	0	0.039483
18509 Amp	14	80931567	81181534	0	0.028105
18510 Amp	14	81186631	81205762	0	0.038063
18511 Amp	14	81207214	81589001	0	0.028105
18512 Amp	14	81600029	81609246	0	0.008092
18513 Amp	14	81612885	81785358	0	0.021515
18514 Amp	14	81790398	81832684	0	0.029936
18515 Amp	14	81832829	81847474	0	0.032546
18516 Amp	14	81853282	81868202	0	0.024124
18517 Amp	14	81868499	81917748	0	0.033793
18518 Amp	14	81917948	82001341	0	0.0257
18519 Amp	14	82001849	82326597	0	0.031766
18520 Amp	14	82330018	82855711	0	0.018344
18521 Amp	14	82859882	83085980	0	0.015753
18522 Amp	14	83087193	83112423	0	0.028725
18523 Amp	14	83117497	83268923	0	0.015753
18524 Amp	14	83276142	83462688	0	0.008111
18525 Amp	14	83463814	83762910	0	0.010908
18526 Amp	14	83765150	83805641	0	0.008111
18527 Amp	14	83806111	83838982	0	0.005501
18528 Amp	14	83840737	83872282	0	0.016508
18529 Amp	14	83873627	84304671	0	0.005501
18530 Amp	14	84305260	84767447	0	0.008779
18531 Amp	14	84767497	85126810	0	0.005501
18532 Amp	14	85127350	85228469	0	0.01355
18533 Amp	14	85232215	85303391	0	0.005501
18534 Amp	14	85306592	85360712	0	0.0162
18535 Amp	14	85364931	85437288	0	0.005501
18536 Amp	14	85439574	85485257	0	0.016511
18537 Amp	14	85487574	85729063	0	0.005501
18538 Amp	14	85735362	85779770	0	0.02191
18539 Amp	14	85780946	85853271	0	0.012113
18540 Amp	14	85855362	85962912	0	0.023155
18541 Amp	14	85963694	85985567	0	0.012113
18542 Amp	14	86023990	86272425	0	0.027428
18543 Amp	14	86277145	86285567	0	0.037911
18544 Amp	14	86288725	86462891	0	0.058298
18545 Amp	14	86463172	86562813	0	0.041203
18546 Amp	14	86566474	86722810	0	0.025888
18547 Amp	14	86726626	86970386	0	0.042887
18548 Amp	14	86979578	87099142	0	0.049357
18549 Amp	14	87100564	87374213	0	0.032358

18550	Amp	14	87379420	87513919	0	0.040359
18551	Amp	14	87517894	87517894	0	0.052415
18552	Amp	14	87519627	87568479	0	0.045945
18553	Amp	14	87570079	87739184	0	0.03389
18554	Amp	14	87743119	87801119	0	0.044701
18555	Amp	14	87802393	87811236	0	0.046182
18556	Amp	14	87811667	88063974	0	0.058832
18557	Amp	14	88064085	88678811	0	0.050831
18558	Amp	14	88686922	88695459	0	0.061419
18559	Amp	14	88696199	89167231	0	0.076271
18560	Amp	14	89170142	89531247	0	0.078003
18561	Amp	14	89532751	89679212	0	0.057616
18562	Amp	14	89681867	89730663	0	0.044966
18563	Amp	14	89736420	89764036	0	0.04811
18564	Amp	14	89764468	89803755	0	0.049521
18565	Amp	14	89804740	89889695	0	0.051397
18566	Amp	14	89897859	90206663	0	0.036545
18567	Amp	14	90212846	90507254	0	0.039464
18568	Amp	14	90507406	90759852	0	0.043053
18569	Amp	14	90761357	90937186	0	0.032464
18570	Amp	14	90941015	91249930	0	0.02932
18571	Amp	14	91260421	91267674	0	0.039309
18572	Amp	14	91270829	91330777	0	0.042235
18573	Amp	14	91331744	91509281	0	0.040359
18574	Amp	14	91515024	91990291	0	0.03677
18575	Amp	14	91998921	92137614	0	0.055173
18576	Amp	14	92143068	92865961	0	0.059192
18577	Amp	14	92867388	92888499	0	0.055173
18578	Amp	14	92891766	93023221	0	0.05827
18579	Amp	14	93027339	93079932	0	0.048281
18580	Amp	14	93087286	93175270	0	0.06258
18581	Amp	14	93178240	93203198	0	0.067624
18582	Amp	14	93216923	93643378	0	0.049221
18583	Amp	14	93661108	93908756	0	0.059819
18584	Amp	14	93908850	94223870	0	0.065551
18585	Amp	14	94227484	94335013	0	0.070125
18586	Amp	14	94335031	94422330	0	0.059527
18587	Amp	14	94424564	94641732	0	0.063967
18588	Amp	14	94642050	94934802	0	0.065746
18589	Amp	14	94936989	95056303	0	0.076711
18590	Amp	14	95056490	95251072	0	0.089942
18591	Amp	14	95251311	95362142	0	0.076711
18592	Amp	14	95364159	95399555	0	0.082329
18593	Amp	14	95403309	95536571	0	0.059424
18594	Amp	14	95538009	95556270	0	0.065451
18595	Amp	14	95558252	95589213	0	0.068711
18596	Amp	14	95591315	95627829	0	0.065451
18597	Amp	14	95628257	95753229	0	0.07074
18598	Amp	14	95753744	95796415	0	0.065451
18599	Amp	14	95796973	95869030	0	0.071048
18600	Amp	14	95877933	95911911	0	0.065429
18601	Amp	14	95912045	97050979	0	0.059832
18602	Amp	14	97055215	97107770	0	0.078938

18603 Amp	14	97112228	97367644	0	0.068127
18604 Amp	14	97369307	97398592	0	0.062395
18605 Amp	14	97400093	97462447	0	0.068458
18606 Amp	14	97463089	97502823	0	0.062395
18607 Amp	14	97508187	97568016	0	0.068409
18608 Amp	14	97570573	97663665	0	0.086746
18609 Amp	14	97680464	97694866	0	0.084967
18610 Amp	14	97695727	97913766	0	0.06663
18611 Amp	14	97913993	97973469	0	0.059215
18612 Amp	14	97974955	98314637	0	0.060864
18613 Amp	14	98319583	98756665	0	0.059215
18614 Amp	14	98756699	98783478	0	0.061294
18615 Amp	14	98974537	99102302	0	0.072376
18616 Amp	14	99115837	99427757	0	0.093155
18617 Amp	14	99428996	99509841	0	0.077715
18618 Amp	14	99512900	99608330	0	0.085184
18619 Amp	14	99608654	99640025	0	0.077715
18620 Amp	14	99647919	99663043	0	0.090257
18621 Amp	14	99667755	99723175	0	0.107472
18622 Amp	14	99729380	99969622	0	0.09373
18623 Amp	14	99974616	100176037	0	0.073226
18624 Amp	14	100184415	100244427	0	0.08668
18625 Amp	14	100250104	100257240	0	0.104883
18626 Amp	14	100261473	100300327	0	0.096926
18627 Amp	14	100308456	100451035	0	0.083473
18628 Amp	14	100451189	100480950	0	0.089948
18629 Amp	14	100481876	100487118	0	0.10288
18630 Amp	14	100489859	100619014	0	0.096853
18631 Amp	14	100627816	100673689	0	0.090378
18632 Amp	14	100673903	100692482	0	0.099345
18633 Amp	14	100693079	100798900	0	0.090378
18634 Amp	14	100804988	100813573	0	0.09796
18635 Amp	14	100813740	100823502	0	0.087051
18636 Amp	14	100827129	100847649	0	0.089831
18637 Amp	14	100853087	100883961	0	0.082249
18638 Amp	14	100885679	101237338	0	0.076235
18639 Amp	14	101240429	101244666	0	0.086744
18640 Amp	14	101247073	101410840	0	0.084721
18641 Amp	14	101415120	101512951	0	0.08984
18642 Amp	14	101513198	101549944	0	0.098995
18643 Amp	14	101555070	101571053	0	0.08984
18644 Amp	14	101571290	101649521	0	0.087059
18645 Amp	14	101654159	101687183	0	0.081941
18646 Amp	14	101753923	102143339	0	0.084466
18647 Amp	14	102155120	102158296	0	0.093481
18648 Amp	14	102166760	102197669	0	0.101721
18649 Amp	14	102204240	102208524	0	0.092706
18650 Amp	14	102211670	102261513	0	0.100553
18651 Amp	14	102278072	102719619	0	0.151076
18652 Amp	14	102722447	102819758	0	0.156152
18653 Amp	14	102823404	102855089	0	0.176432
18654 Amp	14	102859578	102871761	0	0.156152
18655 Amp	14	102873574	103069047	0	0.1589

18656	Amp	14	103075221	103145318	0	0.163001
18657	Amp	14	103145941	103200526	0	0.160252
18658	Amp	14	103201550	103398376	0	0.163934
18659	Amp	14	103402929	103402929	0	0.160252
18660	Amp	14	103403291	103437180	0	0.157726
18661	Amp	14	103437724	103456053	0	0.140703
18662	Amp	14	103462482	103562120	0	0.147843
18663	Amp	14	103566760	103575235	0	0.134851
18664	Amp	14	103580785	103580785	0	0.153091
18665	Amp	14	103581365	103595407	0	0.145951
18666	Amp	14	103595424	103692980	0	0.130606
18667	Amp	14	103697541	103721415	0	0.138291
18668	Amp	14	103725717	103865220	0	0.130606
18669	Amp	14	103868767	103893234	0	0.142312
18670	Amp	14	103893488	103893488	0	0.139416
18671	Amp	14	103895506	103936510	0	0.12771
18672	Amp	14	103937549	104075017	0	0.130002
18673	Amp	14	104076931	104214691	0	0.141652
18674	Amp	14	104215796	104241839	0	0.1333
18675	Amp	14	104242453	104568156	0	0.103126
18676	Amp	14	104570413	104570413	0	0.113072
18677	Amp	14	104570691	104737896	0	0.105716
18678	Amp	14	104740797	104760000	0	0.09577
18679	Amp	14	104760912	105015651	0	0.100312
18680	Amp	14	105019048	105137078	0	0.09577
18681	Amp	14	105167207	105205043	0	0.099339
18682	Amp	14	105219567	105354932	0	0.106988
18683	Amp	14	105360751	105468051	0	0.112579
18684	Amp	14	105468704	105521701	0	0.106988
18685	Amp	15	23437561	23573909	0	0.099114
18686	Amp	15	23581140	23590855	0	0.093056
18687	Amp	15	23599578	23639661	0	0.081466
18688	Amp	15	23639982	23696796	0	0.093032
18689	Amp	15	23698600	23765135	0	0.081466
18690	Amp	15	23766299	23846579	0	0.084725
18691	Amp	15	23849097	23996195	0	0.088317
18692	Amp	15	23997070	24551555	0	0.086301
18693	Amp	15	24556575	24574674	0	0.08271
18694	Amp	15	24576698	24632632	0	0.092223
18695	Amp	15	24634185	24660633	0	0.086106
18696	Amp	15	24663420	25095666	0	0.076593
18697	Amp	15	25101218	25171567	0	0.082651
18698	Amp	15	25171977	25249354	0	0.086639
18699	Amp	15	25249955	25350975	0	0.082651
18700	Amp	15	25357861	26058163	0	0.084197
18701	Amp	15	26061244	26517586	0	0.033674
18702	Amp	15	26519283	26533320	0	0.084197
18703	Amp	15	26537218	26663597	0	0.033674
18704	Amp	15	26664268	26671953	0	0.043551
18705	Amp	15	26674859	26693100	0	0.033674
18706	Amp	15	26704940	26817359	0	0.044561
18707	Amp	15	26817883	27439909	0	0.038439
18708	Amp	15	27440501	27527746	0	0.043742

18709	Amp	15	27528232	27543967	0	0.065976
18710	Amp	15	27544553	27838020	0	0.043742
18711	Amp	15	27838061	28247049	0	0.038439
18712	Amp	15	28250353	28315670	0	0.027553
18713	Amp	15	28315870	30839824	0	0.02414
18714	Amp	15	30842699	30848742	0	0.029563
18715	Amp	15	30848884	30886294	0	0.026304
18716	Amp	15	30887331	31059872	0	0.036416
18717	Amp	15	31100696	31361441	0	0.05691
18718	Amp	15	31363005	31617732	0	0.053608
18719	Amp	15	31621699	31663787	0	0.048115
18720	Amp	15	31664417	31664862	0	0.053739
18721	Amp	15	32636613	32735053	0	0.057075
18722	Amp	15	32747097	33080283	0	0.042073
18723	Amp	15	33080367	33313100	0	0.036449
18724	Amp	15	33315903	33654342	0	0.043032
18725	Amp	15	33660889	34301584	0	0.036449
18726	Amp	15	34301660	34313127	0	0.040764
18727	Amp	15	34315256	34359780	0	0.04677
18728	Amp	15	34360482	34361296	0	0.040335
18729	Amp	15	34361534	34916423	0	0.050752
18730	Amp	15	34922943	35465168	0	0.040335
18731	Amp	15	35471243	35484027	0	0.042955
18732	Amp	15	35484375	35538586	0	0.040335
18733	Amp	15	35541038	35874199	0	0.030223
18734	Amp	15	35875371	36026944	0	0.031641
18735	Amp	15	36027010	36151157	0	0.030223
18736	Amp	15	36151222	36309672	0	0.038365
18737	Amp	15	36312642	36327832	0	0.041653
18738	Amp	15	36329657	36362486	0	0.04324
18739	Amp	15	36362861	36481730	0	0.035098
18740	Amp	15	36483861	36502670	0	0.049323
18741	Amp	15	36505628	36589159	0	0.035098
18742	Amp	15	36600343	36612744	0	0.029091
18743	Amp	15	36616222	36754336	0	0.025803
18744	Amp	15	36761719	36817737	0	0.012643
18745	Amp	15	36821811	36828746	0	0.006449
18746	Amp	15	36829597	36854434	0	0.012349
18747	Amp	15	36854617	36871699	0	0.019663
18748	Amp	15	36873048	36981966	0	0.012349
18749	Amp	15	36985986	36994988	0	0.006449
18750	Amp	15	36995319	37397740	0	0.004862
18751	Amp	15	37398133	37527688	0	0.007321
18752	Amp	15	37527790	37860117	0	0.003985
18753	Amp	15	37863141	38977019	0	0.001527
18754	Amp	15	38977639	39129882	0	0.00691
18755	Amp	15	39133920	39348093	0	0.056849
18756	Amp	15	39351494	39377072	0	0.00691
18757	Amp	15	39378649	39578257	0	0.001527
18758	Amp	15	39578476	39585560	0	0.009393
18759	Amp	15	39587780	39637600	0	0.022186
18760	Amp	15	39637846	39690726	0	0.01432
18761	Amp	15	39693077	40032280	0	0.001527

18762	Amp	15	40032355	40717556	0	0
18763	Amp	15	40719540	41200172	0	0.006044
18764	Amp	15	41204608	41861039	0	0
18765	Amp	15	41863936	41887226	0	0.00815
18766	Amp	15	41892061	42928377	0	0
18767	Amp	15	42930807	42979898	0	0.007677
18768	Amp	15	42982345	43250504	0	0.010836
18769	Amp	15	43252621	44097672	0	0.007677
18770	Amp	15	44097699	44273331	0	0.010772
18771	Amp	15	44279409	44628329	0	0.016586
18772	Amp	15	44634881	44808654	0	0.033817
18773	Amp	15	45003915	45220009	0	0.053202
18774	Amp	15	45221952	45390193	0	0.046967
18775	Amp	15	45391597	45438902	0	0.045525
18776	Amp	15	45439467	45564394	0	0.048091
18777	Amp	15	45570254	45666106	0	0.045525
18778	Amp	15	45668699	46121019	0	0.072402
18779	Amp	15	46124857	46188127	0	0.061764
18780	Amp	15	46195694	46693607	0	0.067204
18781	Amp	15	46694313	46930583	0	0.05247
18782	Amp	15	46935102	47165005	0	0.047029
18783	Amp	15	47171630	47353964	0	0.051717
18784	Amp	15	47355320	47362251	0	0.057741
18785	Amp	15	47364365	47665985	0	0.053052
18786	Amp	15	47678351	47826764	0	0.036866
18787	Amp	15	47827054	47871681	0	0.045506
18788	Amp	15	47875115	47890556	0	0.047857
18789	Amp	15	47891325	48240495	0	0.051699
18790	Amp	15	48242473	48329375	0	0.061223
18791	Amp	15	48335720	48414374	0	0.074615
18792	Amp	15	48419860	48681700	0	0.065091
18793	Amp	15	48691337	48774673	0	0.083408
18794	Amp	15	48774793	48834166	0	0.087356
18795	Amp	15	48834659	49086890	0	0.089638
18796	Amp	15	49098787	49351787	0	0.08569
18797	Amp	15	49360327	49698012	0	0.135961
18798	Amp	15	49705099	49744991	0	0.129938
18799	Amp	15	49747493	49865986	0	0.131625
18800	Amp	15	49866498	50021073	0	0.139098
18801	Amp	15	50024702	50036118	0	0.148024
18802	Amp	15	50038828	50099491	0	0.139098
18803	Amp	15	50100242	50419925	0	0.142829
18804	Amp	15	50421958	50491580	0	0.092558
18805	Amp	15	50492857	50608500	0	0.088716
18806	Amp	15	50610664	50684604	0	0.075177
18807	Amp	15	50687223	50698969	0	0.084367
18808	Amp	15	50704027	50754098	0	0.079693
18809	Amp	15	50780118	51064531	0	0.103857
18810	Amp	15	51071722	51090444	0	0.130929
18811	Amp	15	51091997	51491784	0	0.103857
18812	Amp	15	51493729	51579912	0	0.120819
18813	Amp	15	51580976	51634253	0	0.127634
18814	Amp	15	51648932	52364592	0	0.125352

18815	Amp	15	52366161	52391872	0	0.128186
18816	Amp	15	52394173	52519492	0	0.136006
18817	Amp	15	52520765	52523749	0	0.128186
18818	Amp	15	52524145	52733153	0	0.131769
18819	Amp	15	52737897	52759053	0	0.12848
18820	Amp	15	52759974	52779852	0	0.087525
18821	Amp	15	52780476	53068744	0	0.08469
18822	Amp	15	53072656	53390241	0	0.034167
18823	Amp	15	53390840	53490573	0	0.027352
18824	Amp	15	53490604	53618222	0	0.022866
18825	Amp	15	53622488	54500230	0	0.013234
18826	Amp	15	54500317	54862350	0	0.019684
18827	Amp	15	54862877	54937766	0	0.013234
18828	Amp	15	54938020	55009172	0	0.017596
18829	Amp	15	55009194	55151703	0	0.021238
18830	Amp	15	55157074	55635458	0	0.014931
18831	Amp	15	55635500	55925390	0	0.018538
18832	Amp	15	55926415	56156022	0	0.013616
18833	Amp	15	56156105	56234965	0	0.01001
18834	Amp	15	56235079	56244646	0	0.006368
18835	Amp	15	56248111	56867100	0	0
18836	Amp	15	56872374	56889014	0	0.005167
18837	Amp	15	56889950	56946859	0	0
18838	Amp	15	56949729	57155488	0	0.011162
18839	Amp	15	57163521	57820512	0	0.018534
18840	Amp	15	57821133	59300567	0	0.007372
18841	Amp	15	59302464	59459562	0	0.011012
18842	Amp	15	59461274	59696395	0	0.015725
18843	Amp	15	59696507	59739685	0	0.012086
18844	Amp	15	59742118	60233458	0	0.019301
18845	Amp	15	60234955	60448893	0	0.014588
18846	Amp	15	60454386	60536359	0	0.020765
18847	Amp	15	60541625	60660924	0	0.013549
18848	Amp	15	60661825	60806346	0	0.017581
18849	Amp	15	60806445	60850051	0	0.013549
18850	Amp	15	60851092	61109002	0	0.007372
18851	Amp	15	61111018	61336013	0	0.019002
18852	Amp	15	61339111	61735116	0	0.023776
18853	Amp	15	61739917	63183047	0	0.019002
18854	Amp	15	63186077	63335634	0	0.026856
18855	Amp	15	63340136	63445500	0	0.031386
18856	Amp	15	63445726	63816128	0	0.026856
18857	Amp	15	63817399	63888684	0	0.023852
18858	Amp	15	63890192	64438853	0	0.030227
18859	Amp	15	64443906	64678599	0	0.022373
18860	Amp	15	64680389	64794854	0	0.023859
18861	Amp	15	64797980	64868787	0	0.036991
18862	Amp	15	64874147	65372492	0	0.023859
18863	Amp	15	65374321	65506159	0	0.025748
18864	Amp	15	65507549	65536003	0	0.030734
18865	Amp	15	65536298	65734325	0	0.022108
18866	Amp	15	65740154	66030817	0	0.017122
18867	Amp	15	66032664	66235675	0	0.019511

18868 Amp	15	66238394	66349394	0	0.025032
18869 Amp	15	66351449	66811437	0	0.019511
18870 Amp	15	66832579	66839336	0	0.023265
18871 Amp	15	66843527	67114907	0	0.027228
18872 Amp	15	67115302	67786495	0	0.023265
18873 Amp	15	67786750	67898430	0	0.028117
18874 Amp	15	67903828	68301459	0	0.019853
18875 Amp	15	68301668	68815685	0	0.016099
18876 Amp	15	68816017	69199488	0	0.019656
18877 Amp	15	69200959	69683131	0	0.024027
18878 Amp	15	69685118	69862547	0	0.032569
18879 Amp	15	69863107	69996682	0	0.026784
18880 Amp	15	69998421	70284729	0	0.022412
18881 Amp	15	70286443	70312347	0	0.028811
18882 Amp	15	70313182	70378349	0	0.0325
18883 Amp	15	70378841	70396583	0	0.029743
18884 Amp	15	70396707	70448502	0	0.037259
18885 Amp	15	70454252	70483944	0	0.049434
18886 Amp	15	70487252	70496874	0	0.044582
18887 Amp	15	70497458	70885869	0	0.040893
18888 Amp	15	70888143	70888143	0	0.049167
18889 Amp	15	70889658	70937621	0	0.052647
18890 Amp	15	70943566	71017254	0	0.055154
18891 Amp	15	71017289	71091818	0	0.04688
18892 Amp	15	71093343	71130251	0	0.044373
18893 Amp	15	71131670	71227614	0	0.047484
18894 Amp	15	71227939	71317183	0	0.054317
18895 Amp	15	71317756	71426508	0	0.060376
18896 Amp	15	71426667	71460803	0	0.076101
18897 Amp	15	71463255	71516424	0	0.07299
18898 Amp	15	71521212	71576155	0	0.058746
18899 Amp	15	71577045	71648321	0	0.066067
18900 Amp	15	71649002	72000474	0	0.059234
18901 Amp	15	72002511	72270595	0	0.062816
18902 Amp	15	72278352	72278352	0	0.066248
18903 Amp	15	72283394	72293238	0	0.070684
18904 Amp	15	72301613	72336623	0	0.077415
18905 Amp	15	72337380	72500479	0	0.071699
18906 Amp	15	72507973	72533855	0	0.083495
18907 Amp	15	72534851	72602289	0	0.095175
18908 Amp	15	72610271	72732527	0	0.090739
18909 Amp	15	72734320	72962691	0	0.065318
18910 Amp	15	72965384	73205707	0	0.053521
18911 Amp	15	73205859	73255812	0	0.049956
18912 Amp	15	73257753	73264603	0	0.05439
18913 Amp	15	73265095	73379176	0	0.057318
18914 Amp	15	73381361	73411823	0	0.05542
18915 Amp	15	73418519	73430159	0	0.060619
18916 Amp	15	73430364	73649923	0	0.065279
18917 Amp	15	73650001	73711418	0	0.071949
18918 Amp	15	73711595	73825171	0	0.067289
18919 Amp	15	73832202	73855637	0	0.064361
18920 Amp	15	73857246	73916815	0	0.057691

18921	Amp	15	73921337	73998682	0	0.05096
18922	Amp	15	74001937	74011809	0	0.048537
18923	Amp	15	74011919	74031793	0	0.045956
18924	Amp	15	74031947	74035142	0	0.052549
18925	Amp	15	74035700	74063307	0	0.049773
18926	Amp	15	74102654	74551688	0	0.053469
18927	Amp	15	74552503	74665351	0	0.064785
18928	Amp	15	74667540	74817633	0	0.053469
18929	Amp	15	74818117	74904658	0	0.060643
18930	Amp	15	74909018	74944435	0	0.069855
18931	Amp	15	74944837	74963650	0	0.074929
18932	Amp	15	74970294	74991262	0	0.065898
18933	Amp	15	74994160	75073842	0	0.060825
18934	Amp	15	75077277	75933379	0	0.051612
18935	Amp	15	75936106	76204636	0	0.057675
18936	Amp	15	76210954	76687806	0	0.051612
18937	Amp	15	76697003	76787896	0	0.053992
18938	Amp	15	76792643	77064219	0	0.051612
18939	Amp	15	77081221	77150282	0	0.057179
18940	Amp	15	77151362	77453420	0	0.051612
18941	Amp	15	77455527	77634194	0	0.055507
18942	Amp	15	77636037	78317917	0	0.058631
18943	Amp	15	78319135	78355820	0	0.072381
18944	Amp	15	78414432	78662640	0	0.058631
18945	Amp	15	78664916	79327776	0	0.063094
18946	Amp	15	79329460	79733541	0	0.05997
18947	Amp	15	79734553	80096110	0	0.072356
18948	Amp	15	80097465	80211757	0	0.067893
18949	Amp	15	80216412	80304578	0	0.077809
18950	Amp	15	80307893	80319183	0	0.084339
18951	Amp	15	80319287	80469088	0	0.089177
18952	Amp	15	80470097	80563501	0	0.091308
18953	Amp	15	80563940	80646505	0	0.106072
18954	Amp	15	80650796	80748446	0	0.101235
18955	Amp	15	80749700	80859468	0	0.112949
18956	Amp	15	80859738	80920503	0	0.121897
18957	Amp	15	80921423	81051292	0	0.130041
18958	Amp	15	81052776	81062869	0	0.118327
18959	Amp	15	81065406	81221671	0	0.110183
18960	Amp	15	81223778	81242901	0	0.101563
18961	Amp	15	81251128	81390166	0	0.099465
18962	Amp	15	81392894	81592465	0	0.097076
18963	Amp	15	81595841	81611267	0	0.103367
18964	Amp	15	81611363	81807024	0	0.109634
18965	Amp	15	81809042	81866890	0	0.109191
18966	Amp	15	81867639	81916958	0	0.100011
18967	Amp	15	81917596	81930181	0	0.083201
18968	Amp	15	81930256	82018008	0	0.084896
18969	Amp	15	82018083	82120955	0	0.091473
18970	Amp	15	82123016	82196339	0	0.095333
18971	Amp	15	82203118	82236036	0	0.12005
18972	Amp	15	82238094	82609230	0	0.123073
18973	Amp	15	82610181	82695482	0	0.125054

18974	Amp	15	82696989	82726588	0	0.118478
18975	Amp	15	82726863	82855505	0	0.116496
18976	Amp	15	82855773	83000399	0	0.112254
18977	Amp	15	83000790	83204044	0	0.109594
18978	Amp	15	83259039	83457730	0	0.098526
18979	Amp	15	83462309	83736713	0	0.094665
18980	Amp	15	83736822	84105659	0	0.097187
18981	Amp	15	84106899	84624945	0	0.101421
18982	Amp	15	84625027	84915675	0	0.099289
18983	Amp	15	84960904	84960944	0	0.10877
18984	Amp	15	84964418	84984397	0	0.104537
18985	Amp	15	84985171	85022372	0	0.100641
18986	Amp	15	85022853	85181957	0	0.098119
18987	Amp	15	85182000	85236349	0	0.090247
18988	Amp	15	85572645	85749240	0	0.083957
18989	Amp	15	85749866	85913307	0	0.086735
18990	Amp	15	85919621	86577624	0	0.115285
18991	Amp	15	86580204	86924031	0	0.10656
18992	Amp	15	86928406	87117849	0	0.146001
18993	Amp	15	87119603	87124120	0	0.148379
18994	Amp	15	87125017	87183077	0	0.146001
18995	Amp	15	87190377	87202292	0	0.168404
18996	Amp	15	87202887	87262313	0	0.171125
18997	Amp	15	87264927	87630461	0	0.175978
18998	Amp	15	87634099	87665155	0	0.178233
18999	Amp	15	87665580	88069926	0	0.138792
19000	Amp	15	88072861	88227143	0	0.145759
19001	Amp	15	88228757	88260612	0	0.151425
19002	Amp	15	88265317	88348266	0	0.145759
19003	Amp	15	88355913	88355913	0	0.149076
19004	Amp	15	88356255	88388889	0	0.150664
19005	Amp	15	88395280	88407456	0	0.154225
19006	Amp	15	88412636	88430657	0	0.156317
19007	Amp	15	88431396	88628660	0	0.151464
19008	Amp	15	88629804	88724971	0	0.158882
19009	Amp	15	88725474	88776740	0	0.151464
19010	Amp	15	88777720	88838325	0	0.147289
19011	Amp	15	88839617	88890676	0	0.140322
19012	Amp	15	88891187	88936139	0	0.143245
19013	Amp	15	88936644	89087474	0	0.14136
19014	Amp	15	89103899	89103899	0	0.144026
19015	Amp	15	89108023	89132642	0	0.147526
19016	Amp	15	89137276	89165907	0	0.14486
19017	Amp	15	89171846	89219975	0	0.162867
19018	Amp	15	89220372	89264214	0	0.164597
19019	Amp	15	89264915	89331382	0	0.16861
19020	Amp	15	89337393	89341193	0	0.147679
19021	Amp	15	89341352	89353823	0	0.143666
19022	Amp	15	89354111	89434923	0	0.152818
19023	Amp	15	89440423	89470242	0.028398	0.192259
19024	Amp	15	89472248	89489087	0.056238	0.195136
19025	Amp	15	89491928	89505641	0.123589	0.202962
19026	Amp	15	89509824	89555523	0.056238	0.195136

19027	Amp	15	89556869	89668913	0	0.185985
19028	Amp	15	89675243	89729944	0.028398	0.192118
19029	Amp	15	89730456	89761038	0	0.185603
19030	Amp	15	89761200	89915265	0.061778	0.196194
19031	Amp	15	89916945	90022763	0.050005	0.194464
19032	Amp	15	90025728	90030505	0.117531	0.20238
19033	Amp	15	90034423	90150299	0.156508	0.206558
19034	Amp	15	90155154	90181066	0.117531	0.20238
19035	Amp	15	90184008	90193450	0.161592	0.207442
19036	Amp	15	90194573	90261474	0.187743	0.210038
19037	Amp	15	90261581	90261581	0.128931	0.203581
19038	Amp	15	90266401	90273107	0.079463	0.198519
19039	Amp	15	90277489	90277766	0	0.159078
19040	Amp	15	90283732	90404326	0	0.154894
19041	Amp	15	90410416	90834099	0	0.152803
19042	Amp	15	90835419	90981967	0	0.138914
19043	Amp	15	90982088	91120132	0	0.140863
19044	Amp	15	91121097	91174007	0	0.137985
19045	Amp	15	91174098	91217405	0	0.140106
19046	Amp	15	91218770	91242707	0	0.13679
19047	Amp	15	91242728	91747194	0	0.141867
19048	Amp	15	91751712	91752633	0	0.155563
19049	Amp	15	91753575	91901564	0	0.162562
19050	Amp	15	91902352	92079502	0	0.166508
19051	Amp	15	92079718	92258908	0	0.170507
19052	Amp	15	92261205	92261205	0	0.158493
19053	Amp	15	92261369	92261851	0	0.120555
19054	Amp	15	92261879	92261893	0	0.159852
19055	Amp	15	92267541	92395890	0	0.171081
19056	Amp	15	92397034	92545937	0.201819	0.211032
19057	Amp	15	92548495	92732579	0.286675	0.219245
19058	Amp	15	92737466	92750645	0.317596	0.222368
19059	Amp	15	92754769	93003244	0.273081	0.218369
19060	Amp	15	93008116	93058468	0.243425	0.215246
19061	Amp	15	93060379	93069695	0.201819	0.211299
19062	Amp	15	93075075	93108090	0.230572	0.213976
19063	Amp	15	93466223	93735122	0.105051	0.201249
19064	Amp	15	93735920	93848522	0.236222	0.214559
19065	Amp	15	93848843	93980168	0.201819	0.211345
19066	Amp	15	93983943	94115013	0	0.171394
19067	Amp	15	94115124	94213600	0	0.174678
19068	Amp	15	94217416	94331715	0	0.182571
19069	Amp	15	94334152	94369307	0	0.174678
19070	Amp	15	94474335	94566317	0	0.171394
19071	Amp	15	94571174	94622146	0.195309	0.210292
19072	Amp	15	94622626	94982199	0.123589	0.202924
19073	Amp	15	94984218	95196895	0.050005	0.194781
19074	Amp	15	95196958	95351382	0	0.187783
19075	Amp	15	95354379	95480826	0.086298	0.199086
19076	Amp	15	95482082	95545223	0	0.187783
19077	Amp	15	95546348	95633712	0.043943	0.194082
19078	Amp	15	95635794	95836491	0.111991	0.20139
19079	Amp	15	95836694	95841515	0.18072	0.209313

19080	Amp	15	95842513	95866581	0.215373	0.212839
19081	Amp	15	95872889	96089782	0.142507	0.204916
19082	Amp	15	96094214	96298162	0.230572	0.213871
19083	Amp	15	96299040	96299040	0.142507	0.204916
19084	Amp	15	96301872	96526271	0.111991	0.20139
19085	Amp	15	96527623	96788564	0.161592	0.206883
19086	Amp	15	96789239	96957080	0.09318	0.199575
19087	Amp	15	96957690	96979182	0.043943	0.194082
19088	Amp	15	96982750	97050759	0	0.187783
19089	Amp	15	97057421	97112219	0.117531	0.202069
19090	Amp	15	97128636	97395919	0.161592	0.207359
19091	Amp	15	97398941	97622367	0.273081	0.218416
19092	Amp	15	97629769	97989242	0.355162	0.225398
19093	Amp	15	97989311	98228730	0.41418	0.231173
19094	Amp	15	98232313	98576923	0.25804	0.216886
19095	Amp	15	98578346	98588759	0.421967	0.231984
19096	Amp	15	98589061	98608172	0.25804	0.216886
19097	Amp	15	98611432	98654662	0.367646	0.227037
19098	Amp	15	98655234	98661796	0.301081	0.220924
19099	Amp	15	98662924	98690570	0.483654	0.237398
19100	Amp	15	98693273	98898207	0.437983	0.23336
19101	Amp	15	98899910	98959298	0.564815	0.243923
19102	Amp	15	98960516	99069944	0.49169	0.238148
19103	Amp	15	99070096	99227875	0.375927	0.227585
19104	Amp	15	99228375	99333231	0.201819	0.211111
19105	Amp	15	99333609	99496533	0.25804	0.216586
19106	Amp	15	99496746	99734542	0.340434	0.224293
19107	Amp	15	99737090	99843865	0.117531	0.202332
19108	Amp	15	99962160	99968164	0	0.183915
19109	Amp	15	100204464	100236456	0	0.156942
19110	Amp	15	100237359	100239562	0.001198	0.188278
19111	Amp	15	100243039	100408652	0.043943	0.193871
19112	Amp	15	100415538	100440789	0.073369	0.19721
19113	Amp	15	100447727	100541687	0.156508	0.20616
19114	Amp	15	100541858	100589412	0.086298	0.199178
19115	Amp	15	100590222	100677761	0.061778	0.195839
19116	Amp	15	100682097	100686289	0.195309	0.210754
19117	Amp	15	100689191	100705308	0	0.179418
19118	Amp	15	100709794	100727844	0.007199	0.189353
19119	Amp	15	100731600	100861495	0	0.179418
19120	Amp	15	100862571	100924710	0.028398	0.191798
19121	Amp	15	100927355	100954766	0.056238	0.195583
19122	Amp	15	100964982	101264707	0	0.145885
19123	Amp	15	101267169	101344124	0	0.180923
19124	Amp	16	603333	930784	0	0.127438
19125	Amp	16	938889	957763	0	0.130133
19126	Amp	16	965209	978503	0	0.120815
19127	Amp	16	992594	1042871	0	0.118216
19128	Amp	16	1059435	1084458	0	0.142288
19129	Amp	16	1084683	1129031	0	0.140384
19130	Amp	16	1136465	1472669	0	0.139001
19131	Amp	16	1505082	1509837	0	0.136569
19132	Amp	16	1510885	1571827	0	0.131456

19133 Amp	16	1572303	1672384	0	0.124389
19134 Amp	16	1675764	1734141	0	0.121694
19135 Amp	16	1740326	1750949	0	0.129501
19136 Amp	16	1762971	1762971	0	0.136237
19137 Amp	16	1767430	1802206	0	0.134494
19138 Amp	16	1802774	1822458	0	0.122929
19139 Amp	16	1822904	1855873	0	0.124416
19140 Amp	16	1862020	2013825	0	0.133719
19141 Amp	16	2020930	2051748	0	0.142057
19142 Amp	16	2066979	2066979	0	0.143499
19143 Amp	16	2069067	2220112	0	0.135657
19144 Amp	16	2257942	2270508	0	0.156013
19145 Amp	16	2273393	2303205	0	0.154377
19146 Amp	16	2304456	2357258	0	0.146039
19147 Amp	16	2357744	2360151	0	0.151564
19148 Amp	16	2361182	2438692	0	0.142468
19149 Amp	16	2446587	2477074	0	0.147801
19150 Amp	16	2481867	2544037	0	0.141064
19151 Amp	16	2697856	2703179	0	0.135539
19152 Amp	16	2703845	2709498	0	0.134052
19153 Amp	16	2730959	2744306	0	0.127325
19154 Amp	16	2744825	2758491	0	0.136041
19155 Amp	16	2767179	2861319	0	0.133751
19156 Amp	16	2861774	2897013	0	0.128419
19157 Amp	16	2913260	2913260	0	0.084277
19158 Amp	16	2913527	2948628	0	0.075561
19159 Amp	16	2949217	3052175	0	0.071955
19160 Amp	16	3052290	3131995	0	0.079506
19161 Amp	16	3133359	3213937	0	0.071955
19162 Amp	16	3220459	3364495	0	0.065581
19163 Amp	16	3368467	3382216	0	0.064194
19164 Amp	16	3382269	3421059	0	0.058429
19165 Amp	16	3424516	3731312	0	0.054228
19166 Amp	16	3736184	3918980	0	0.100109
19167 Amp	16	3919174	4021657	0	0.085404
19168 Amp	16	4023303	4218934	0	0.079218
19169 Amp	16	4219814	4285128	0	0.083773
19170 Amp	16	4295916	4383670	0	0.10216
19171 Amp	16	4389130	4458250	0	0.083773
19172 Amp	16	4461202	4559218	0	0.100101
19173 Amp	16	4560759	4656377	0	0.095546
19174 Amp	16	4663440	4701185	0	0.098053
19175 Amp	16	4703077	4724353	0	0.099644
19176 Amp	16	4726856	4815632	0	0.068467
19177 Amp	16	4819092	4819657	0	0.070196
19178 Amp	16	4820398	4827957	0	0.071791
19179 Amp	16	4828401	4845037	0	0.067473
19180 Amp	16	4845318	4899945	0	0.069133
19181 Amp	16	4902193	4971658	0	0.070518
19182 Amp	16	4971982	5021543	0	0.068391
19183 Amp	16	5023510	5042090	0	0.069823
19184 Amp	16	5042117	5083089	0	0.066845
19185 Amp	16	5091044	5223193	0	0.072171

19186	Amp	16	5223715	5460605	0	0.069362
19187	Amp	16	5469599	5477410	0	0.082321
19188	Amp	16	5478533	5482609	0	0.069362
19189	Amp	16	5482878	5527588	0	0.070745
19190	Amp	16	5530645	5844781	0	0.067228
19191	Amp	16	5846625	5972749	0	0.062422
19192	Amp	16	5972767	6047062	0	0.066861
19193	Amp	16	6047384	6200849	0	0.060315
19194	Amp	16	6200970	6229305	0	0.064614
19195	Amp	16	6375599	6381921	0	0.074882
19196	Amp	16	6381933	6481393	0	0.070583
19197	Amp	16	6481428	6536271	0	0.072086
19198	Amp	16	6536366	6582887	0	0.059497
19199	Amp	16	6583259	6583259	0	0.049229
19200	Amp	16	7081613	7082742	0	0.038526
19201	Amp	16	7316368	7676755	0	0.051295
19202	Amp	16	7677419	7726361	0	0.057877
19203	Amp	16	7726442	7997956	0	0.052551
19204	Amp	16	7998007	8175748	0	0.056148
19205	Amp	16	8176773	8294510	0	0.065094
19206	Amp	16	8294782	8357158	0	0.053889
19207	Amp	16	8357458	8442547	0	0.058108
19208	Amp	16	8442813	8452242	0	0.049162
19209	Amp	16	8452631	8839164	0	0.058866
19210	Amp	16	9132117	9214224	0	0.066826
19211	Amp	16	9218262	9218925	0	0.079483
19212	Amp	16	9223735	9260899	0	0.071523
19213	Amp	16	9263337	9361059	0	0.067304
19214	Amp	16	9362696	9377411	0	0.074761
19215	Amp	16	9379269	9595617	0	0.097107
19216	Amp	16	9599468	9706723	0	0.113091
19217	Amp	16	9707034	9708982	0	0.116233
19218	Amp	16	9709612	9713759	0	0.112908
19219	Amp	16	9713930	9961220	0	0.111404
19220	Amp	16	9963734	10027336	0	0.107807
19221	Amp	16	10027404	10030338	0	0.112328
19222	Amp	16	10031619	10077254	0	0.092977
19223	Amp	16	10079218	10115056	0	0.104817
19224	Amp	16	10115066	10373899	0	0.103434
19225	Amp	16	10375679	10469341	0	0.102048
19226	Amp	16	10470444	10634580	0	0.100504
19227	Amp	16	10634838	10685963	0	0.099072
19228	Amp	16	10687422	11047464	0	0.097476
19229	Amp	16	11048131	11124749	0	0.09902
19230	Amp	16	11125184	11125184	0	0.088942
19231	Amp	16	11126126	11189845	0	0.090389
19232	Amp	16	11191332	11282172	0	0.08873
19233	Amp	16	11289662	11310106	0	0.084209
19234	Amp	16	11310973	11382238	0	0.094988
19235	Amp	16	11383389	11446129	0	0.093396
19236	Amp	16	11446856	11483511	0	0.096303
19237	Amp	16	11547324	11573421	0	0.091762
19238	Amp	16	11578272	11598802	0	0.101623

19239	Amp	16	11599380	11600551	0	0.095233
19240	Amp	16	11605864	11638388	0	0.084454
19241	Amp	16	11640400	11699242	0	0.038852
19242	Amp	16	11699374	11819526	0	0.03709
19243	Amp	16	11822053	11836480	0	0.025647
19244	Amp	16	11838092	11868489	0	0.015785
19245	Amp	16	11868601	11874698	0	0.014338
19246	Amp	16	11880058	11941719	0	0.025136
19247	Amp	16	11945489	12148206	0	0.029179
19248	Amp	16	12151604	12249420	0	0.023745
19249	Amp	16	12249990	12272877	0	0.019732
19250	Amp	16	12272963	12284361	0	0.021656
19251	Amp	16	12289472	12303862	0	0.018749
19252	Amp	16	12304830	12973809	0	0.013355
19253	Amp	16	12974147	13022719	0	0.024266
19254	Amp	16	13025302	13031979	0	0.013355
19255	Amp	16	13045901	13144065	0	0.062977
19256	Amp	16	13144557	13325264	0	0.068868
19257	Amp	16	13326270	13336514	0	0.076303
19258	Amp	16	13338337	13367773	0	0.083712
19259	Amp	16	13368064	13431154	0	0.086122
19260	Amp	16	13431202	13523861	0	0.087949
19261	Amp	16	13528783	13586003	0	0.038328
19262	Amp	16	13593510	13656429	0	0.044787
19263	Amp	16	13659492	13689126	0	0.037352
19264	Amp	16	13689533	13718975	0	0.041473
19265	Amp	16	13719045	13779008	0	0.037352
19266	Amp	16	13780128	13796637	0	0.031461
19267	Amp	16	13800173	13849260	0	0.037232
19268	Amp	16	13851431	13880628	0	0.035404
19269	Amp	16	13882234	13993319	0	0.029633
19270	Amp	16	13994695	14105733	0	0.033194
19271	Amp	16	14107412	14210318	0	0.040062
19272	Amp	16	14214212	14531477	0	0.050987
19273	Amp	16	14532037	14652868	0	0.047427
19274	Amp	16	14653245	14657199	0	0.052588
19275	Amp	16	18797068	18797068	0	0.060443
19276	Amp	16	18797081	18819616	0	0.055125
19277	Amp	16	18820597	19139808	0	0.052715
19278	Amp	16	19143200	19143599	0	0.06207
19279	Amp	16	19144694	19241441	0	0.060342
19280	Amp	16	19244503	19387382	0	0.050987
19281	Amp	16	19398857	19611459	0	0.059465
19282	Amp	16	19614016	19700147	0	0.068157
19283	Amp	16	19702560	19782970	0	0.059679
19284	Amp	16	19802798	19876784	0	0.034804
19285	Amp	16	19883165	20280538	0	0.038211
19286	Amp	16	20282208	20302511	0	0.042026
19287	Amp	16	20303360	20436358	0	0.051014
19288	Amp	16	20438340	20465821	0	0.06233
19289	Amp	16	20562099	21171602	0	0.053638
19290	Amp	16	21174670	21280370	0	0.044651
19291	Amp	16	21281281	21283416	0	0.063732

19292 Amp	16	21290789	21338882	0	0.060325
19293 Amp	16	21340717	21340717	0	0.063974
19294 Amp	16	21348993	21348993	0	0.048865
19295 Amp	16	22773239	22903627	0	0.057349
19296 Amp	16	22903825	22944093	0	0.055798
19297 Amp	16	22944604	22978086	0	0.047314
19298 Amp	16	22985211	23154989	0	0.058714
19299 Amp	16	23157293	23313914	0	0.067112
19300 Amp	16	23315403	23327612	0	0.056187
19301 Amp	16	23327940	23481033	0	0.047788
19302 Amp	16	23484451	23793141	0	0.054526
19303 Amp	16	23795109	23894414	0	0.050711
19304 Amp	16	23896540	24141565	0	0.062148
19305 Amp	16	24145625	24168351	0	0.058498
19306 Amp	16	24168477	24237722	0	0.059892
19307 Amp	16	24239284	24360479	0	0.084099
19308 Amp	16	24361836	24524176	0	0.094259
19309 Amp	16	24526341	24526657	0	0.105341
19310 Amp	16	24528192	24710493	0	0.112637
19311 Amp	16	24718089	24845454	0	0.101238
19312 Amp	16	24846801	24914580	0	0.093941
19313 Amp	16	24915138	25251274	0	0.073711
19314 Amp	16	25255202	25433388	0	0.069734
19315 Amp	16	25434267	25689625	0	0.059574
19316 Amp	16	25689719	25713600	0	0.074303
19317 Amp	16	25713657	25713657	0	0.069142
19318 Amp	16	25713978	25764359	0	0.054413
19319 Amp	16	25768352	25823968	0	0.059671
19320 Amp	16	25825198	25839741	0	0.061645
19321 Amp	16	25839870	26448600	0	0.056386
19322 Amp	16	26450900	26638150	0	0.049648
19323 Amp	16	26642454	27126074	0	0.051768
19324 Amp	16	27127156	27132575	0	0.067215
19325 Amp	16	27133778	27271963	0	0.065291
19326 Amp	16	27278379	27305080	0	0.054985
19327 Amp	16	27308542	27407538	0	0.064155
19328 Amp	16	27408845	27535628	0	0.066747
19329 Amp	16	27536539	27555501	0	0.097313
19330 Amp	16	27557731	27932984	0	0.100414
19331 Amp	16	27933839	27935952	0	0.111777
19332 Amp	16	27936133	27984073	0	0.110383
19333 Amp	16	27987544	28126645	0	0.111995
19334 Amp	16	28128021	28264921	0	0.117658
19335 Amp	16	28265693	28330451	0	0.115065
19336 Amp	16	28373156	28373156	0	0.093104
19337 Amp	16	28473919	28517277	0	0.070036
19338 Amp	16	28517302	28538187	0	0.078449
19339 Amp	16	28542752	28620700	0	0.098578
19340 Amp	16	29078721	29114764	0	0.074158
19341 Amp	16	29122775	29283930	0	0.080388
19342 Amp	16	29286385	29300932	0	0.074158
19343 Amp	16	29410386	29415091	0	0.078719
19344 Amp	16	29417218	29417224	0	0.086207

19345	Amp	16	30295647	30295647	0	0.105612
19346	Amp	16	30308694	30308694	0	0.136774
19347	Amp	16	30308986	30339440	0	0.141604
19348	Amp	16	30358860	30535185	0	0.149882
19349	Amp	16	30536574	30542838	0	0.142394
19350	Amp	16	30543422	30563634	0	0.149168
19351	Amp	16	30570617	30595830	0	0.155592
19352	Amp	16	30597723	30903305	0	0.159549
19353	Amp	16	30903402	31028472	0	0.14601
19354	Amp	16	31030610	31063604	0	0.139587
19355	Amp	16	31064387	31136894	0	0.132812
19356	Amp	16	31138801	31357495	0	0.139159
19357	Amp	16	31358244	31440758	0	0.129857
19358	Amp	16	31446836	31461282	0	0.129399
19359	Amp	16	31467598	31507932	0	0.132626
19360	Amp	16	31509880	31542860	0	0.131014
19361	Amp	16	31542972	31608242	0	0.134041
19362	Amp	16	31609722	31649106	0	0.129211
19363	Amp	16	31649725	31768797	0	0.120933
19364	Amp	16	31769692	31779169	0	0.117706
19365	Amp	16	31779242	31812037	0	0.106799
19366	Amp	16	31812529	31817777	0	0.102842
19367	Amp	16	31825824	31832698	0	0.09452
19368	Amp	16	31833730	31841585	0	0.099943
19369	Amp	16	46470502	46470502	0	0.144088
19370	Amp	16	46473753	46473753	0	0.14876
19371	Amp	16	46474548	46482583	0	0.143613
19372	Amp	16	46489505	46516500	0	0.149608
19373	Amp	16	46581419	46695757	0	0.119082
19374	Amp	16	46696167	46756606	0	0.129181
19375	Amp	16	46757306	46808873	0	0.131002
19376	Amp	16	46815946	46826622	0	0.140624
19377	Amp	16	46829898	46848146	0	0.13228
19378	Amp	16	46848757	46969373	0	0.116759
19379	Amp	16	46970159	47070938	0	0.105817
19380	Amp	16	47074762	47074762	0	0.084687
19381	Amp	16	47080410	47092804	0	0.098886
19382	Amp	16	47096681	47107303	0	0.094213
19383	Amp	16	47113274	47126371	0	0.109854
19384	Amp	16	47129314	47167168	0	0.113499
19385	Amp	16	47169113	47173056	0	0.097858
19386	Amp	16	47173636	47177321	0	0.101182
19387	Amp	16	47189154	47203574	0	0.114319
19388	Amp	16	47204265	47216294	0	0.132235
19389	Amp	16	47224495	47227601	0	0.153453
19390	Amp	16	47234295	47322243	0	0.147458
19391	Amp	16	47328198	47397081	0	0.136068
19392	Amp	16	47399623	47552436	0	0.137647
19393	Amp	16	47558462	47574761	0	0.128024
19394	Amp	16	47576413	47600661	0	0.138924
19395	Amp	16	47603219	47619210	0	0.14311
19396	Amp	16	47619703	47687719	0	0.14755
19397	Amp	16	47688243	47751045	0	0.166957

19398	Amp	16	47751118	47794137	0	0.171258
19399	Amp	16	47794466	47815447	0	0.168371
19400	Amp	16	47819377	47829521	0	0.175184
19401	Amp	16	47833379	47889097	0	0.182646
19402	Amp	16	47891761	47895247	0	0.171835
19403	Amp	16	47898428	47925263	0	0.165023
19404	Amp	16	47935795	47946645	0	0.160721
19405	Amp	16	47948375	47974465	0	0.167084
19406	Amp	16	47976361	47999559	0	0.174019
19407	Amp	16	48003638	48027227	0	0.167656
19408	Amp	16	48029071	48091944	0	0.151136
19409	Amp	16	48104348	48105185	0	0.159156
19410	Amp	16	48106009	48122960	0	0.152221
19411	Amp	16	48126231	48180992	0	0.16469
19412	Amp	16	48182311	48206852	0	0.118059
19413	Amp	16	48208448	48299443	0	0.111758
19414	Amp	16	48307297	48318710	0	0.1187
19415	Amp	16	48322394	48408440	0	0.12323
19416	Amp	16	48410460	48596023	0	0.115793
19417	Amp	16	48597977	48780587	0	0.111263
19418	Amp	16	48785289	48791593	0	0.121318
19419	Amp	16	48795158	48946801	0	0.10353
19420	Amp	16	48948087	48954132	0	0.093051
19421	Amp	16	48954583	49022646	0	0.087146
19422	Amp	16	49023761	49150634	0	0.054365
19423	Amp	16	49151789	49287422	0	0.052543
19424	Amp	16	49288773	49304419	0	0.058846
19425	Amp	16	49304589	49370009	0	0.061212
19426	Amp	16	49370607	49420551	0	0.057566
19427	Amp	16	49421321	49585031	0	0.062479
19428	Amp	16	49585798	49645550	0	0.052424
19429	Amp	16	49648000	49739268	0	0.032451
19430	Amp	16	49740844	49947754	0	0.015164
19431	Amp	16	49948103	50028700	0	0.012171
19432	Amp	16	50030955	50151398	0	0.022058
19433	Amp	16	50151652	50466468	0	0.027192
19434	Amp	16	50468933	50708151	0	0.051536
19435	Amp	16	50710784	50798821	0	0.059349
19436	Amp	16	50807884	50852092	0	0.061896
19437	Amp	16	50855269	51040182	0	0.071724
19438	Amp	16	51041550	51121706	0	0.085994
19439	Amp	16	51123072	51163730	0	0.076167
19440	Amp	16	51164165	51528038	0	0.089103
19441	Amp	16	51530466	51563307	0	0.083969
19442	Amp	16	51563458	51659100	0	0.087719
19443	Amp	16	51659125	51671426	0	0.093755
19444	Amp	16	51672762	51709709	0	0.085092
19445	Amp	16	51711933	51814471	0	0.093833
19446	Amp	16	51814883	51823545	0	0.059602
19447	Amp	16	51826557	51977193	0	0.048495
19448	Amp	16	51979319	51981098	0	0.034225
19449	Amp	16	51981975	52084541	0	0.037059
19450	Amp	16	52086456	52134742	0	0.047222

19451 Amp	16	52137299	52153112	0	0.054501
19452 Amp	16	52154053	52177526	0	0.044338
19453 Amp	16	52178212	52493715	0	0.038302
19454 Amp	16	52495619	52528918	0	0.054274
19455 Amp	16	52532542	52538920	0	0.066523
19456 Amp	16	52539304	52582090	0	0.063689
19457 Amp	16	52584180	52659366	0	0.069877
19458 Amp	16	52668101	52691096	0	0.066567
19459 Amp	16	52691253	52831764	0	0.060379
19460 Amp	16	52836242	52859931	0	0.052565
19461 Amp	16	52860604	52920735	0	0.061096
19462 Amp	16	52929240	52990967	0	0.083755
19463 Amp	16	52991763	53118759	0	0.104625
19464 Amp	16	53119438	53147414	0	0.102273
19465 Amp	16	53167051	53183673	0	0.116124
19466 Amp	16	53185648	53199675	0	0.103875
19467 Amp	16	53201169	53272888	0	0.101328
19468 Amp	16	53277011	53329284	0	0.098954
19469 Amp	16	53338696	53489453	0	0.082983
19470 Amp	16	53489515	53489702	0	0.080198
19471 Amp	16	53490396	53516986	0	0.0841
19472 Amp	16	53517069	53630685	0	0.087276
19473 Amp	16	53636475	53667452	0	0.097926
19474 Amp	16	53667601	53691489	0	0.094024
19475 Amp	16	53691744	53742630	0	0.099756
19476 Amp	16	53743108	53765066	0	0.10851
19477 Amp	16	53765680	53802572	0	0.099756
19478 Amp	16	53809621	53955066	0	0.113897
19479 Amp	16	53957443	53961849	0	0.12031
19480 Amp	16	53964775	54040964	0	0.151409
19481 Amp	16	54048458	54049255	0	0.144996
19482 Amp	16	54052556	54071911	0	0.117352
19483 Amp	16	54072059	54082253	0	0.129389
19484 Amp	16	54083010	54141733	0	0.135681
19485 Amp	16	54143089	54197283	0	0.129389
19486 Amp	16	54201218	54201528	0	0.117352
19487 Amp	16	54204827	54241629	0	0.125318
19488 Amp	16	54242274	54258063	0	0.113897
19489 Amp	16	54273420	54321711	0	0.064635
19490 Amp	16	54326910	54520877	0	0.068633
19491 Amp	16	54521308	54587424	0	0.070067
19492 Amp	16	54588281	54708793	0	0.059417
19493 Amp	16	54709485	54816804	0	0.045276
19494 Amp	16	54817129	54955736	0	0.050297
19495 Amp	16	54957821	55060086	0	0.058264
19496 Amp	16	55063549	55089541	0	0.054266
19497 Amp	16	55090612	55125012	0	0.061404
19498 Amp	16	55125631	55132023	0	0.053437
19499 Amp	16	55135233	55211887	0	0.059568
19500 Amp	16	55212120	55245421	0	0.05243
19501 Amp	16	55246479	55287185	0	0.045268
19502 Amp	16	55290182	55409075	0	0.031416
19503 Amp	16	55410526	55425708	0	0.022084

19504	Amp	16	55427847	55470915	0	0.023708
19505	Amp	16	55471416	55504019	0	0.010772
19506	Amp	16	55507896	55539409	0	0.012511
19507	Amp	16	55539698	55542249	0	0.011077
19508	Amp	16	55545524	55695924	0	0.009337
19509	Amp	16	55699576	55748372	0	0.029761
19510	Amp	16	55839497	55868994	0	0.028137
19511	Amp	16	55869092	55991821	0	0.029547
19512	Amp	16	55992062	56290965	0	0.009123
19513	Amp	16	56293443	56308147	0	0.013566
19514	Amp	16	56312359	56328418	0	0.011984
19515	Amp	16	56330118	56971389	0	0.02082
19516	Amp	16	56973600	57031084	0	0.066172
19517	Amp	16	57031209	57051452	0	0.067637
19518	Amp	16	57051635	57624060	0	0.066172
19519	Amp	16	57624601	57662252	0	0.028355
19520	Amp	16	57665019	57928158	0	0.016377
19521	Amp	16	57933288	58092574	0	0.010246
19522	Amp	16	58095614	58599145	0	0.008836
19523	Amp	16	58609753	58908269	0	0
19524	Amp	16	58927676	58968237	0	0.001872
19525	Amp	16	58968734	59369150	0	0.003268
19526	Amp	16	59372607	60294446	0	0.012778
19527	Amp	16	60294898	60476001	0	0.021839
19528	Amp	16	60476727	60924798	0	0.012778
19529	Amp	16	60926834	61270221	0	0.031498
19530	Amp	16	61271800	61321281	0	0.044234
19531	Amp	16	61321908	61457515	0	0.031498
19532	Amp	16	61457799	61476165	0	0.069757
19533	Amp	16	61482525	61618245	0	0.085709
19534	Amp	16	61619347	62256067	0	0.093353
19535	Amp	16	62261966	62356892	0	0.087756
19536	Amp	16	62360240	62478271	0	0.080112
19537	Amp	16	62481226	62730426	0	0.04745
19538	Amp	16	62733710	62800746	0	0.054815
19539	Amp	16	62800999	62990529	0	0.057469
19540	Amp	16	62992479	63077400	0	0.065981
19541	Amp	16	63078706	63320049	0	0.05647
19542	Amp	16	63328357	64138899	0	0.021799
19543	Amp	16	64144939	64196588	0	0.013287
19544	Amp	16	64197372	64559371	0	0.015107
19545	Amp	16	64559546	65407788	0	0.021251
19546	Amp	16	65410390	66105912	0	0.019854
19547	Amp	16	66106656	66228991	0	0.024734
19548	Amp	16	66229190	66235233	0	0.026182
19549	Amp	16	66235992	66386102	0	0.024311
19550	Amp	16	66386982	66537518	0	0.021657
19551	Amp	16	66537659	66938470	0	0.019605
19552	Amp	16	66939065	67301066	0	0.012239
19553	Amp	16	67301116	67471141	0	0.015135
19554	Amp	16	67472190	67517216	0	0.017715
19555	Amp	16	67523174	67593732	0	0.015895
19556	Amp	16	67594576	67681364	0	0.012999

19557	Amp	16	67690670	67745641	0	0.019234
19558	Amp	16	67749203	67780962	0	0.027424
19559	Amp	16	67787419	67942008	0	0.039504
19560	Amp	16	67960016	68180237	0	0.060384
19561	Amp	16	68182828	68338420	0	0.071031
19562	Amp	16	68342667	68378596	0	0.0838
19563	Amp	16	68379957	68534976	0	0.076846
19564	Amp	16	68535742	68557327	0	0.083679
19565	Amp	16	68561763	68725318	0	0.07091
19566	Amp	16	68730591	68832915	0	0.060263
19567	Amp	16	68833936	69086967	0	0.046337
19568	Amp	16	69087418	69525793	0	0.049379
19569	Amp	16	69528282	69583610	0	0.054746
19570	Amp	16	69584837	69795972	0	0.050477
19571	Amp	16	69799009	69857024	0	0.047435
19572	Amp	16	69857419	70212430	0	0.054079
19573	Amp	16	70212436	70302944	0	0.038134
19574	Amp	16	70316785	70584387	0	0.043741
19575	Amp	16	70588712	70674807	0	0.045949
19576	Amp	16	70677068	70706336	0	0.051116
19577	Amp	16	70707529	70722163	0	0.045949
19578	Amp	16	70723652	70732621	0	0.047768
19579	Amp	16	70738284	70808194	0	0.050466
19580	Amp	16	71168894	71217892	0	0.055659
19581	Amp	16	71218254	71374645	0	0.064894
19582	Amp	16	71375037	71422239	0	0.063445
19583	Amp	16	71429699	71451760	0	0.069348
19584	Amp	16	71454868	71464015	0	0.063741
19585	Amp	16	71464761	71550430	0	0.061533
19586	Amp	16	71553296	71628823	0	0.055299
19587	Amp	16	71629510	71736240	0	0.0526
19588	Amp	16	71744464	71770170	0	0.046698
19589	Amp	16	71781527	71894162	0	0.026903
19590	Amp	16	71894947	71894947	0	0.032462
19591	Amp	16	71909399	72440352	0	0.038274
19592	Amp	16	72445364	72482053	0	0.041255
19593	Amp	16	72498277	72580421	0	0.052462
19594	Amp	16	72637616	72637616	0	0.041255
19595	Amp	16	72642117	72671337	0	0.038274
19596	Amp	16	72673902	73000789	0	0.035863
19597	Amp	16	73012261	74006119	0	0.023802
19598	Amp	16	74010636	74121949	0	0.030466
19599	Amp	16	74125594	74197334	0	0.035395
19600	Amp	16	74197932	74291845	0	0.028731
19601	Amp	16	74437522	74608495	0	0.031964
19602	Amp	16	74611744	74826658	0	0.028731
19603	Amp	16	74826991	75065668	0	0.031723
19604	Amp	16	75071982	75615377	0	0.034609
19605	Amp	16	75615834	75773564	0	0.031723
19606	Amp	16	75774771	75978732	0	0.03318
19607	Amp	16	75978816	76343988	0	0.028251
19608	Amp	16	76353290	76585067	0	0.043095
19609	Amp	16	76585125	76640067	0	0.055487

19610	Amp	16	76667906	76748211	0	0.037184
19611	Amp	16	76784266	77043774	0	0.022341
19612	Amp	16	77046440	77142627	0	0.019349
19613	Amp	16	77143231	77157956	0	0.02881
19614	Amp	16	77160222	77160222	0	0.032425
19615	Amp	16	77193772	77210940	0	0.022965
19616	Amp	16	77211143	77358844	0	0.021507
19617	Amp	16	77359983	77635946	0	0.017892
19618	Amp	16	77636260	77645363	0	0.01208
19619	Amp	16	77646209	78408201	0	0.020128
19620	Amp	16	78409935	79189115	0	0.01208
19621	Amp	16	79189177	79224440	0	0.019105
19622	Amp	16	79247649	80628984	0	0.01208
19623	Amp	16	80629149	81081220	0	0.015454
19624	Amp	16	81081668	81257026	0	0.01208
19625	Amp	16	81259125	81301670	0	0.026264
19626	Amp	16	81302152	81707858	0	0.02797
19627	Amp	16	81710612	82049689	0	0.026264
19628	Amp	16	82052416	82126060	0	0.017224
19629	Amp	16	82126274	82240602	0	0.01208
19630	Amp	16	82241074	83437190	0	0.037807
19631	Amp	16	83440866	84008053	0	0.01208
19632	Amp	16	84008711	84136867	0	0.018229
19633	Amp	16	84137154	84954505	0	0.020162
19634	Amp	16	84954615	85032144	0	0.02828
19635	Amp	16	85036998	85108461	0	0.029754
19636	Amp	16	85108560	85125137	0	0.032051
19637	Amp	16	85127025	85494946	0	0.034473
19638	Amp	16	85498174	85622752	0	0.022393
19639	Amp	16	85629573	85630337	0	0.010392
19640	Amp	16	85638325	86002114	0	0.008918
19641	Amp	16	86002334	86075629	0	0.006985
19642	Amp	16	86086301	86115902	0	0.020859
19643	Amp	16	86119821	86204565	0	0.016171
19644	Amp	16	86210911	86232426	0	0.002297
19645	Amp	16	86270638	86663402	0	0.015993
19646	Amp	16	86663615	87083257	0	0.013697
19647	Amp	16	87086203	87100730	0	0
19648	Amp	16	87102347	87108560	0	0.012564
19649	Amp	16	87109331	87320765	0	0.014142
19650	Amp	16	87340606	87621890	0	0.001578
19651	Amp	16	87623680	87635801	0	0.009743
19652	Amp	16	87636436	87680240	0	0.001578
19653	Amp	16	87683911	87715835	0	0.004449
19654	Amp	16	87716028	87736452	0	0.001578
19655	Amp	16	87740187	87816375	0	0.004054
19656	Amp	16	87816457	87819369	0	0.00643
19657	Amp	16	87825570	87850911	0	0.011671
19658	Amp	16	87850941	88048457	0	0.00643
19659	Amp	16	88059855	88070485	0	0.02098
19660	Amp	16	88170123	88322821	0	0.014983
19661	Amp	16	88323652	88335725	0	0.020136
19662	Amp	16	88343034	88459876	0	0.023415

19663 Amp	16	89234014	89238494	0	0.018262
19664 Amp	16	89271105	89317317	0	0.015786
19665 Amp	17	1074619	1191979	0	0.017953
19666 Amp	17	1192024	1192024	0	0.015813
19667 Amp	17	1262192	1319840	0	0.021176
19668 Amp	17	1319960	1322646	0	0.015813
19669 Amp	17	1324207	1367133	0	0.017557
19670 Amp	17	1368632	1378465	0	0.011669
19671 Amp	17	1378570	1473375	0	0.01625
19672 Amp	17	1473750	1601870	0	0.011669
19673 Amp	17	1603997	1636246	0	0.01858
19674 Amp	17	1642620	1804724	0	0.016761
19675 Amp	17	1810639	2048318	0	0.028442
19676 Amp	17	2048785	2287966	0	0.016761
19677 Amp	17	2288406	2297140	0	0.009849
19678 Amp	17	2298305	2298305	0	0.019112
19679 Amp	17	2299731	2502462	0	0.023166
19680 Amp	17	2504649	2805995	0	0.019112
19681 Amp	17	2819772	2995998	0	0.014538
19682 Amp	17	3095770	3409769	0	0.025695
19683 Amp	17	3410060	3490150	0	0.03194
19684 Amp	17	3490668	3544620	0	0.030196
19685 Amp	17	3546069	3580491	0	0.023951
19686 Amp	17	3581074	3776484	0	0.026281
19687 Amp	17	3777393	4084581	0	0.035894
19688 Amp	17	4090931	4101027	0	0.026281
19689 Amp	17	4104300	4131162	0	0.028542
19690 Amp	17	4131335	4290949	0	0.034898
19691 Amp	17	4293249	4583273	0	0.032568
19692 Amp	17	4585589	4904836	0	0.030307
19693 Amp	17	4906204	4939421	0	0.035159
19694 Amp	17	4942574	5033123	0	0.032782
19695 Amp	17	5034608	5271496	0	0.030307
19696 Amp	17	5275463	5338772	0	0.038883
19697 Amp	17	5346703	5884262	0	0.030307
19698 Amp	17	5892433	5990153	0	0.01915
19699 Amp	17	5992323	5997709	0	0.031177
19700 Amp	17	5999971	6345981	0	0.027646
19701 Amp	17	6346919	6386299	0	0.015619
19702 Amp	17	6386425	6616632	0	0.017783
19703 Amp	17	6620906	7088835	0	0.002164
19704 Amp	17	7099082	7819047	0	0
19705 Amp	17	7830661	7839781	0	0.011895
19706 Amp	17	7841337	7966447	0	0.013948
19707 Amp	17	7983417	9476246	0	0.011895
19708 Amp	17	9476417	9657629	0	0.041647
19709 Amp	17	9659229	9708011	0	0.047239
19710 Amp	17	9713388	9992394	0	0.040554
19711 Amp	17	9995393	10256832	0	0.034961
19712 Amp	17	10258419	10511479	0	0.036671
19713 Amp	17	10514122	10543271	0	0.024776
19714 Amp	17	10545860	10884989	0	0.039049
19715 Amp	17	10885794	11182856	0	0.024776

19716 Amp	17	11184577	11419494	0	0.031013
19717 Amp	17	11422887	11462414	0	0.024776
19718 Amp	17	11462970	11952092	0	0.031736
19719 Amp	17	11952902	12128752	0	0.04134
19720 Amp	17	12131989	12246373	0	0.031736
19721 Amp	17	12247410	12272700	0	0.03349
19722 Amp	17	12279648	12313374	0	0.041335
19723 Amp	17	12315162	12890625	0	0.03349
19724 Amp	17	12890681	12911608	0	0.041116
19725 Amp	17	12911812	12929662	0	0.03349
19726 Amp	17	12930923	12955832	0	0.038951
19727 Amp	17	12956337	13060711	0	0.020692
19728 Amp	17	13061036	13490674	0	0.015231
19729 Amp	17	13497318	13540932	0	0.027471
19730 Amp	17	13541239	13561814	0	0.042928
19731 Amp	17	13563577	13745215	0	0.010424
19732 Amp	17	13746010	13888069	0	0.020488
19733 Amp	17	13888284	13975996	0	0.028894
19734 Amp	17	13977100	14085485	0	0.02714
19735 Amp	17	14085827	14231324	0	0.025093
19736 Amp	17	15654216	15685870	0	0.028797
19737 Amp	17	15778279	16042632	0	0.027087
19738 Amp	17	16046756	16192940	0	0.052773
19739 Amp	17	16193939	16257529	0	0.029078
19740 Amp	17	16259883	16342901	0	0.033917
19741 Amp	17	16348466	16376258	0	0.060102
19742 Amp	17	16390256	16408798	0	0.066229
19743 Amp	17	16412605	16619425	0	0.078637
19744 Amp	17	16620083	16708434	0	0.07251
19745 Amp	17	16708823	16937892	0	0.078154
19746 Amp	17	16945436	17174281	0	0.065746
19747 Amp	17	17177261	17182095	0	0.049198
19748 Amp	17	17187187	17270217	0	0.099721
19749 Amp	17	17271536	17311806	0	0.120145
19750 Amp	17	17312209	17367804	0	0.138091
19751 Amp	17	17371084	17570120	0	0.144068
19752 Amp	17	17572846	17575038	0	0.132846
19753 Amp	17	17576379	17625356	0	0.126869
19754 Amp	17	17631020	17648209	0	0.137364
19755 Amp	17	17651249	17680034	0	0.119183
19756 Amp	17	17691396	17691396	0	0.120846
19757 Amp	17	17696369	17746147	0	0.070323
19758 Amp	17	17747688	17786776	0	0.088504
19759 Amp	17	17801230	17859431	0	0.037981
19760 Amp	17	17862746	17979551	0	0.070107
19761 Amp	17	17985235	18092593	0	0.064464
19762 Amp	17	18095128	18097007	0	0.053968
19763 Amp	17	18097424	18130141	0	0.059095
19764 Amp	17	18131997	18193916	0	0.026969
19765 Amp	17	18194881	18386413	0	0.025306
19766 Amp	17	18390113	18390113	0	0.023871
19767 Amp	17	18647546	18659407	0	0.074393
19768 Amp	17	18666219	18783417	0	0.06767

19769	Amp	17	18784994	18880623	0	0.075083
19770	Amp	17	18885025	18957450	0	0.06767
19771	Amp	17	18957997	18957997	0	0.073926
19772	Amp	17	19246050	19312967	0	0.148526
19773	Amp	17	19316353	19316353	0	0.098004
19774	Amp	17	19316446	19357907	0	0.123349
19775	Amp	17	19361419	19390414	0	0.118511
19776	Amp	17	19393216	19503078	0	0.104978
19777	Amp	17	19512202	19783133	0	0.107923
19778	Amp	17	19786035	19817838	0	0.101996
19779	Amp	17	19821365	19961118	0	0.106864
19780	Amp	17	19961474	19971349	0	0.128537
19781	Amp	17	19971624	19991889	0	0.167086
19782	Amp	17	19994311	20021651	0	0.128537
19783	Amp	17	20023187	20151401	0	0.144298
19784	Amp	17	20153908	20165258	0	0.138896
19785	Amp	17	20165415	20171849	0	0.124682
19786	Amp	17	20349166	20387400	0	0.132494
19787	Amp	17	20389632	20389632	0	0.129586
19788	Amp	17	20403746	20426761	0	0.122842
19789	Amp	17	20426903	20426903	0	0.113256
19790	Amp	17	20530272	20530272	0	0.127866
19791	Amp	17	20530556	20530556	0	0.134746
19792	Amp	17	20554187	20699136	0	0.127944
19793	Amp	17	20701796	20727234	0	0.132072
19794	Amp	17	20727403	20776621	0	0.130112
19795	Amp	17	20784411	20882601	0	0.123232
19796	Amp	17	20883692	20900697	0	0.127721
19797	Amp	17	20902471	21071786	0	0.1719
19798	Amp	17	21075303	21144114	0	0.176956
19799	Amp	17	21145945	21168918	0	0.181625
19800	Amp	17	21170840	21201314	0.033661	0.192821
19801	Amp	17	21205838	21347106	0	0.181625
19802	Amp	17	21353196	21394819	0	0.176956
19803	Amp	17	21395786	21395786	0	0.178445
19804	Amp	17	21396582	21399695	0.038273	0.193707
19805	Amp	17	21774057	21774057	0	0.14682
19806	Amp	17	26943491	26943491	0.018387	0.190542
19807	Amp	17	26943774	26943774	0.111991	0.201938
19808	Amp	17	26947350	26949301	0.375927	0.227581
19809	Amp	17	26955079	26975284	0.540744	0.242425
19810	Amp	17	26982437	26989417	1.068928	0.283395
19811	Amp	17	26989437	26989437	1.000924	0.27834
19812	Amp	17	26990128	27015487	0.944128	0.274636
19813	Amp	17	27016182	27036401	1.039426	0.28159
19814	Amp	17	27036628	27128903	0.982622	0.27707
19815	Amp	17	27135382	27142475	0.888878	0.270526
19816	Amp	17	27142588	27168751	0.934277	0.274064
19817	Amp	17	27173332	27173480	1.150167	0.28884
19818	Amp	17	27174138	27174138	1.237378	0.295278
19819	Amp	17	27199009	27201129	1.140058	0.288324
19820	Amp	17	27208042	27230524	0.878688	0.269771
19821	Amp	17	27230970	27233265	0.944128	0.2746

19822	Amp	17	27241372	27329865	1.24764	0.295508
19823	Amp	17	27330984	27399084	1.169408	0.290498
19824	Amp	17	27409092	27410913	1.394558	0.306366
19825	Amp	17	27417133	27430853	1.453628	0.309926
19826	Amp	17	27431631	27441519	1.2565	0.296564
19827	Amp	17	27444873	27541982	1.285475	0.298422
19828	Amp	17	27542498	27587284	1.637826	0.322888
19829	Amp	17	27593982	27593982	1.582671	0.318858
19830	Amp	17	27596144	27613792	1.657426	0.323598
19831	Amp	17	27616680	27648235	1.49455	0.312856
19832	Amp	17	27652360	27706234	2.272343	0.363379
19833	Amp	17	27709503	27754003	1.49455	0.312856
19834	Amp	17	27758082	27779649	1.637826	0.322588
19835	Amp	17	27782004	27815136	1.930731	0.341552
19836	Amp	17	27821258	27848356	2.148338	0.355293
19837	Amp	17	27848846	27910683	1.942393	0.342292
19838	Amp	17	27911950	27962122	1.995109	0.34622
19839	Amp	17	27963542	28039825	2.039783	0.348809
19840	Amp	17	28045917	28052523	2.016329	0.346951
19841	Amp	17	28057419	28060577	1.800655	0.333209
19842	Amp	17	28067389	28069188	2.383144	0.369679
19843	Amp	17	28071104	28195769	2.424855	0.372864
19844	Amp	17	28198946	28205828	2.602347	0.38273
19845	Amp	17	28209829	28239010	2.750393	0.391667
19846	Amp	17	28243171	28254026	2.591575	0.382566
19847	Amp	17	28255935	28287993	2.727427	0.390757
19848	Amp	17	28293068	28303834	3.041652	0.409014
19849	Amp	17	28308353	28359422	3.288383	0.423821
19850	Amp	17	28367462	28428533	3.207076	0.418908
19851	Amp	17	28432554	28448412	3.348874	0.427073
19852	Amp	17	28448723	28464370	3.157622	0.415666
19853	Amp	17	28464723	28483228	3.099988	0.412665
19854	Amp	17	28483790	28525513	3.45062	0.43316
19855	Amp	17	28536015	28572484	3.600858	0.441524
19856	Amp	17	28578731	28624958	4.103115	0.468157
19857	Amp	17	28625418	28649928	4.049098	0.465135
19858	Amp	17	28651537	28665349	3.893345	0.456883
19859	Amp	17	28666144	28674762	4.327765	0.479515
19860	Amp	17	28681774	28725326	4.171444	0.471418
19861	Amp	17	28728422	28728422	4.724007	0.499017
19862	Amp	17	28737334	28805609	4.882637	0.50681
19863	Amp	17	28805724	28809674	5.062558	0.514608
19864	Amp	17	28817590	28817590	4.470731	0.487009
19865	Amp	17	28821599	28827705	4.076638	0.466514
19866	Amp	17	28836344	28888684	4.34102	0.479608
19867	Amp	17	28890134	28890688	4.30031	0.478119
19868	Amp	17	28908259	28919135	3.946237	0.459861
19869	Amp	17	28919255	28940490	3.971602	0.461573
19870	Amp	17	28944821	28959644	3.523011	0.436999
19871	Amp	17	28961315	28991843	3.688516	0.446188
19872	Amp	17	28992152	29006266	3.625861	0.44265
19873	Amp	17	29012187	29027337	3.509701	0.436878
19874	Amp	17	29031958	29031958	3.946237	0.460138

19875	Amp	17	29032074	29036975	4.376421	0.482254
19876	Amp	17	29042364	29042597	3.946237	0.459621
19877	Amp	17	29049573	29058792	4.483097	0.48717
19878	Amp	17	29059429	29061939	3.777346	0.450779
19879	Amp	17	29070956	29130675	2.98257	0.405759
19880	Amp	17	29140898	29146710	2.703608	0.389243
19881	Amp	17	29147221	29248698	2.683697	0.387531
19882	Amp	17	29250768	29276068	2.703608	0.389103
19883	Amp	17	29276298	29289644	2.614747	0.383374
19884	Amp	17	29297507	29305577	2.88973	0.399984
19885	Amp	17	29305918	29311338	2.958746	0.40459
19886	Amp	17	29316452	29353857	2.855041	0.398188
19887	Amp	17	29357114	29398174	2.830877	0.396616
19888	Amp	17	29398841	29495113	2.935659	0.403139
19889	Amp	17	29495783	29504782	3.029636	0.408325
19890	Amp	17	29510449	29550320	2.958746	0.404584
19891	Amp	17	29554445	29597161	2.71525	0.389994
19892	Amp	17	29604994	29606214	2.647166	0.385553
19893	Amp	17	29626585	29631205	2.602347	0.382965
19894	Amp	17	29632885	29688372	2.327403	0.366552
19895	Amp	17	29689639	29699473	2.22615	0.360387
19896	Amp	17	29702842	29721992	1.605306	0.32023
19897	Amp	17	29723985	29759404	1.542961	0.316343
19898	Amp	17	29763006	29763297	1.717946	0.328278
19899	Amp	17	29764477	29777288	1.657426	0.324182
19900	Amp	17	29782147	29787172	1.404612	0.306641
19901	Amp	17	29789275	29848086	1.534719	0.315897
19902	Amp	17	29853818	29855667	1.424227	0.307949
19903	Amp	17	29858131	29901674	1.49455	0.312883
19904	Amp	17	29905933	29909627	1.374261	0.304558
19905	Amp	17	29915266	29953330	1.324884	0.301372
19906	Amp	17	29954115	29965623	1.432145	0.308971
19907	Amp	17	29966930	29969309	1.49455	0.312626
19908	Amp	17	29970359	30021298	1.553308	0.3168
19909	Amp	17	30022051	30071959	1.503938	0.313538
19910	Amp	17	30072053	30096031	1.626265	0.321628
19911	Amp	17	30098597	30111430	1.542961	0.316441
19912	Amp	17	30111845	30123649	1.473764	0.311835
19913	Amp	17	30130736	30134181	1.120124	0.286956
19914	Amp	17	30138084	30199761	1.324884	0.30123
19915	Amp	17	30200476	30227068	1.2565	0.2964
19916	Amp	17	30228407	30259113	1.315861	0.300895
19917	Amp	17	30263114	30320734	1.374261	0.304535
19918	Amp	17	30335824	30355435	1.190324	0.291588
19919	Amp	17	30358931	30364082	1.130387	0.287413
19920	Amp	17	30366294	30371756	1.275021	0.298119
19921	Amp	17	30372070	30437404	1.2565	0.296657
19922	Amp	17	30437968	30459795	1.120124	0.28695
19923	Amp	17	30460711	30601980	0.982622	0.277356
19924	Amp	17	30604756	30606411	1.120124	0.287032
19925	Amp	17	30606938	30609191	1.068928	0.283391
19926	Amp	17	30609785	30610646	0.897922	0.270858
19927	Amp	17	30625011	30625268	0.76883	0.261183

19928	Amp	17	30848226	30848226	1.285475	0.298549
19929	Amp	17	30860091	30891673	0.982622	0.277451
19930	Amp	17	30896988	30896988	0.382767	0.228669
19931	Amp	17	30897169	30928011	0.273081	0.217963
19932	Amp	17	30928978	30932934	0.215373	0.212501
19933	Amp	17	30938281	30950096	0.123589	0.202826
19934	Amp	17	30950471	30956050	0.061778	0.196388
19935	Amp	17	30956585	30956706	0.086298	0.198836
19936	Amp	17	30959567	31000110	0.02372	0.191585
19937	Amp	17	31006498	31030515	0.156508	0.206302
19938	Amp	17	31032986	31113536	0.223025	0.213132
19939	Amp	17	31125921	31175862	0.587735	0.246637
19940	Amp	17	31181757	31192037	0.156508	0.206302
19941	Amp	17	31198927	31209115	0.243425	0.215124
19942	Amp	17	31210763	31237813	0.001198	0.188811
19943	Amp	17	31245266	31255046	0.038273	0.193695
19944	Amp	17	31257776	31270475	0	0.184873
19945	Amp	17	31274290	31301474	0	0.18078
19946	Amp	17	31301531	31440177	0	0.164848
19947	Amp	17	31440351	31474187	0	0.159965
19948	Amp	17	31475895	31483735	0	0.167609
19949	Amp	17	31486671	31491956	0	0.165161
19950	Amp	17	31496993	31567713	0	0.150444
19951	Amp	17	31576317	31583817	0.068197	0.196866
19952	Amp	17	31584786	31642048	0.007199	0.189222
19953	Amp	17	31642435	31646303	0	0.171437
19954	Amp	17	31649631	31649631	0.038273	0.193074
19955	Amp	17	31650290	31665332	0.123589	0.203313
19956	Amp	17	31669416	31687177	0	0.181676
19957	Amp	17	31687694	31736755	0	0.187707
19958	Amp	17	31737081	31787844	0.043943	0.19413
19959	Amp	17	31787955	31838646	0	0.178855
19960	Amp	17	31845561	31892590	0	0.172431
19961	Amp	17	31894983	31988128	0	0.152907
19962	Amp	17	31988459	32005593	0	0.147974
19963	Amp	17	32010432	32085782	0	0.150002
19964	Amp	17	32086638	32117728	0	0.156112
19965	Amp	17	32119729	32148412	0	0.147224
19966	Amp	17	32153161	32176982	0	0.140701
19967	Amp	17	32182216	32215587	0	0.185953
19968	Amp	17	32217220	32228977	0	0.145604
19969	Amp	17	32229263	32268994	0	0.155563
19970	Amp	17	32269483	32279282	0	0.158083
19971	Amp	17	32285794	32334231	0.079463	0.198321
19972	Amp	17	32335768	32335768	0.390264	0.229294
19973	Amp	17	32335813	32369841	0.293283	0.219896
19974	Amp	17	32372373	32384578	0.596354	0.246948
19975	Amp	17	32392764	32468005	1.140058	0.288236
19976	Amp	17	32471586	32495957	0.991911	0.277605
19977	Amp	17	32495989	32495989	1.039426	0.281519
19978	Amp	17	32496364	32626596	0.51566	0.240232
19979	Amp	17	32626826	32713264	0.636206	0.250691
19980	Amp	17	32715394	32761392	0.66072	0.25272

19981	Amp	17	32764743	32772414	0.860481	0.26861
19982	Amp	17	32779179	32825326	0.814697	0.264613
19983	Amp	17	32833022	32833022	0.843361	0.267146
19984	Amp	17	32833349	32834086	0.860481	0.268565
19985	Amp	17	32849022	32901611	0.888878	0.270217
19986	Amp	17	32907109	32916008	0.613517	0.248607
19987	Amp	17	32918314	32963468	0.540744	0.242497
19988	Amp	17	32963496	33016562	0.685617	0.254716
19989	Amp	17	33017251	33019409	0.556748	0.243838
19990	Amp	17	33020515	33020529	0.500055	0.238825
19991	Amp	17	33024103	33034989	0.906818	0.271416
19992	Amp	17	33037988	33046815	0.41418	0.231178
19993	Amp	17	33048336	33127866	0	0.184755
19994	Amp	17	33128256	33192211	0	0.174629
19995	Amp	17	33194002	33295499	0.068197	0.197031
19996	Amp	17	33299129	33321487	0.028398	0.191893
19997	Amp	17	33322350	33392804	0	0.183962
19998	Amp	17	33396999	33464791	0.452609	0.234458
19999	Amp	17	33465951	33489024	0.508503	0.239415
20000	Amp	17	33491344	33496090	0.7517	0.259766
20001	Amp	17	33498098	33502005	1.120124	0.286571
20002	Amp	17	33506834	33510234	1.697113	0.326809
20003	Amp	17	33511184	33521666	1.424227	0.308025
20004	Amp	17	33524856	33612592	0.71758	0.257502
20005	Amp	17	33612937	33631275	0.702615	0.256083
20006	Amp	17	34611015	34616624	0.02372	0.191327
20007	Amp	17	34617146	34617146	0.142507	0.205233
20008	Amp	17	34620083	34645453	0.230572	0.213898
20009	Amp	17	34645710	34673247	0.25804	0.216923
20010	Amp	17	34677070	34677670	0.187743	0.210031
20011	Amp	17	34678596	34737585	0.156508	0.206712
20012	Amp	17	34738246	34740567	0.286675	0.219781
20013	Amp	17	34745271	34746867	0.333141	0.223658
20014	Amp	17	34751091	34754833	0.437983	0.233102
20015	Amp	17	34755458	34788200	0.468576	0.235697
20016	Amp	17	34789063	34807582	0.437983	0.233164
20017	Amp	17	34811033	34811033	0.556748	0.243374
20018	Amp	17	34812489	34838034	0.636206	0.250192
20019	Amp	17	34843009	34862273	0.09318	0.199669
20020	Amp	17	34862318	34883670	0.038273	0.193336
20021	Amp	17	34885348	34914734	0.301081	0.220765
20022	Amp	17	34916018	34977374	0.27943	0.219113
20023	Amp	17	34982682	34985560	0.301081	0.220928
20024	Amp	17	34986292	35007263	0.25804	0.217003
20025	Amp	17	35011428	35020493	0.236222	0.214975
20026	Amp	17	35026152	35042498	0.532449	0.241301
20027	Amp	17	35047412	35065257	0.685617	0.254234
20028	Amp	17	35068961	35080319	0.878688	0.269734
20029	Amp	17	35080705	35080705	0.76883	0.261641
20030	Amp	17	35081101	35089986	0.823952	0.265759
20031	Amp	17	35095139	35103090	0.556748	0.243757
20032	Amp	17	35104542	35104542	0.734472	0.258937
20033	Amp	17	35106318	35143830	0.62858	0.249554

20034 Amp	17	35146648	35151934	0.685617	0.254582
20035 Amp	17	35155270	35171922	1.383826	0.305105
20036 Amp	17	35172849	35174117	1.169408	0.289926
20037 Amp	17	35177790	35188145	0.786952	0.26312
20038 Amp	17	35197079	35248101	0.724993	0.258092
20039 Amp	17	35248815	35322896	0.437983	0.233432
20040 Amp	17	35322962	35349487	0.564815	0.243955
20041 Amp	17	35442187	35442187	0.333141	0.223617
20042 Amp	17	35442818	35456095	0.347934	0.225304
20043 Amp	17	35461551	35502939	0.286675	0.219499
20044 Amp	17	35503612	35510232	0.18072	0.208976
20045 Amp	17	35516898	35551205	0.142507	0.2051
20046 Amp	17	35553637	35572587	0.317596	0.222102
20047 Amp	17	35582705	35672498	0.685617	0.254331
20048 Amp	17	35676861	35676861	0.62858	0.25007
20049 Amp	17	35687188	35695509	0.532449	0.241406
20050 Amp	17	35697522	35729651	0.644689	0.251269
20051 Amp	17	35733380	35756446	0.805493	0.264461
20052 Amp	17	35760685	35779671	0.644689	0.250987
20053 Amp	17	35781076	35840194	0.76883	0.261317
20054 Amp	17	35841724	35877803	1.010597	0.279469
20055 Amp	17	35883693	35918805	0.786952	0.262467
20056 Amp	17	35918838	35967043	0.62858	0.249619
20057 Amp	17	35968936	36065936	0.564815	0.244315
20058 Amp	17	36066931	36067159	0.676804	0.254118
20059 Amp	17	36567187	36586260	1.208462	0.293325
20060 Amp	17	36586276	36589910	1.088806	0.284808
20061 Amp	17	36590030	36630669	0.95395	0.275005
20062 Amp	17	36631986	36642371	0.982622	0.277263
20063 Amp	17	36644841	36646278	1.068928	0.28335
20064 Amp	17	36647789	36654519	0.95395	0.2751
20065 Amp	17	36660668	36686511	1.404612	0.306891
20066 Amp	17	36691915	36700577	1.953191	0.342877
20067 Amp	17	36700860	36703544	1.898353	0.339497
20068 Amp	17	36706619	36706680	1.354645	0.303511
20069 Amp	17	36709448	36714926	0.878688	0.269631
20070 Amp	17	36715139	36769629	0.925715	0.27324
20071 Amp	17	36772001	36781105	0.833763	0.266272
20072 Amp	17	36790071	36811048	0.71758	0.257415
20073 Amp	17	36811934	36819516	0.398217	0.230046
20074 Amp	17	36819951	36833005	0.355162	0.225371
20075 Amp	17	36836184	36843288	0.444673	0.23418
20076 Amp	17	36850290	36891145	0.333141	0.22385
20077 Amp	17	36891653	36959931	0.236222	0.215012
20078 Amp	17	36963546	36963546	0.340434	0.22427
20079 Amp	17	36964872	36973537	0.243425	0.215462
20080 Amp	17	36980968	36995874	0.175335	0.208725
20081 Amp	17	36999363	37010499	0.128931	0.203498
20082 Amp	17	37012497	37087893	0	0.184093
20083 Amp	17	37091701	37091701	0.061778	0.196008
20084 Amp	17	37093598	37101128	0	0.169682
20085 Amp	17	37104485	37139167	0	0.151891
20086 Amp	17	37146222	37218189	0	0.177172

20087	Amp	17	37218278	37227337	0	0.172046
20088	Amp	17	37232010	37252988	0	0.176067
20089	Amp	17	37253511	37253511	0	0.185027
20090	Amp	17	37253880	37276563	0.098998	0.200173
20091	Amp	17	37276797	37277130	0.636206	0.250696
20092	Amp	17	37279273	37310220	0.596354	0.246675
20093	Amp	17	37310867	37312387	0.636206	0.250729
20094	Amp	17	37317803	37326084	1.324884	0.301252
20095	Amp	17	37327486	37361579	1.404612	0.306963
20096	Amp	17	37363057	37413714	0.596354	0.24677
20097	Amp	17	37425380	37427296	1.265443	0.297097
20098	Amp	17	37435174	37475765	1.697113	0.326595
20099	Amp	17	37476412	37478481	1.789205	0.332719
20100	Amp	17	37478715	37488196	1.907576	0.340164
20101	Amp	17	37488553	37497091	2.071528	0.350902
20102	Amp	17	37506490	37508035	2.483202	0.376314
20103	Amp	17	37512249	37533494	1.727244	0.328588
20104	Amp	17	37534768	37551941	1.383826	0.30562
20105	Amp	17	37552976	37570871	1.553308	0.317112
20106	Amp	17	37572485	37661709	1.887705	0.338853
20107	Amp	17	37661922	37683523	2.214215	0.359542
20108	Amp	17	37684274	37707951	2.039783	0.348575
20109	Amp	17	37708110	37743574	2.843135	0.397452
20110	Amp	17	37743881	37749832	3.72674	0.447975
20111	Amp	17	37752278	37763774	3.335884	0.426419
20112	Amp	17	38254377	38287144	1.768015	0.331559
20113	Amp	17	38291398	38312348	1.887705	0.338733
20114	Amp	17	38314737	38366870	2.192225	0.35841
20115	Amp	17	38368371	38385960	2.238069	0.361012
20116	Amp	17	38390561	38390561	2.635842	0.384737
20117	Amp	17	38391595	38413030	2.761965	0.392959
20118	Amp	17	38413207	38432884	2.818034	0.396191
20119	Amp	17	38433920	38443161	2.914035	0.401523
20120	Amp	17	38444036	38449123	3.079122	0.411522
20121	Amp	17	38449246	38462688	3.112016	0.41312
20122	Amp	17	38464057	38490098	3.348874	0.426738
20123	Amp	17	38491342	38492212	3.252252	0.421257
20124	Amp	17	38492262	38535957	3.415716	0.431261
20125	Amp	17	38536412	38554277	3.971602	0.461337
20126	Amp	17	38554762	38616242	4.063271	0.465801
20127	Amp	17	38620583	38627112	3.998681	0.462569
20128	Amp	17	38628386	38654096	4.24321	0.474931
20129	Amp	17	38657093	38664792	4.540237	0.490062
20130	Amp	17	38667935	38668462	4.34102	0.4802
20131	Amp	17	38679862	38681055	3.920712	0.458408
20132	Amp	17	38689573	38693724	3.828482	0.453944
20133	Amp	17	38697138	38752808	4.355429	0.48085
20134	Amp	17	38755743	38756085	5.367722	0.529441
20135	Amp	17	38756275	38816555	5.739571	0.546635
20136	Amp	17	38818968	38829805	5.953304	0.557072
20137	Amp	17	38829813	38843564	6.028267	0.560057
20138	Amp	17	38844502	38865150	5.654252	0.542324
20139	Amp	17	38881465	38890618	6.683082	0.589862

20140	Amp	17	38898205	38898824	8.469083	0.664761
20141	Amp	17	38899562	38903311	8.556974	0.668092
20142	Amp	17	38903881	38925685	8.672968	0.672653
20143	Amp	17	38925828	38929943	8.858746	0.680252
20144	Amp	17	38936035	38962432	9.913717	0.722399
20145	Amp	17	38966861	38976304	10.273329	0.736112
20146	Amp	17	38991105	39018882	11.432737	0.780599
20147	Amp	17	39029593	39126781	11.650236	0.788779
20148	Amp	17	39130316	39146941	12.953497	0.839302
20149	Amp	17	39149157	39186594	13.513811	0.859774
20150	Amp	17	39193016	39208740	14.883159	0.910297
20151	Amp	17	39214442	39230646	15.339812	0.926205
20152	Amp	17	39235670	39243087	15.685307	0.938955
20153	Amp	17	39244798	39244798	15.965304	0.94885
20154	Amp	17	39253612	39277235	17.196379	0.993249
20155	Amp	17	39280713	39287369	18.416949	1.034475
20156	Amp	17	39293136	39331052	17.856649	1.01543
20157	Amp	17	39331833	39345112	18.14144	1.025114
20158	Amp	17	39346548	39484477	18.75026	1.045315
20159	Amp	17	39487527	39487527	17.235549	0.994792
20160	Amp	17	39495286	39509301	16.334865	0.96214
20161	Amp	17	39510553	39517450	17.759187	1.012663
20162	Amp	17	39526404	39569245	18.125364	1.024406
20163	Amp	17	39570170	39610186	19.359407	1.065414
20164	Amp	17	39623268	39683156	20.926471	1.115644
20165	Amp	17	39689951	39714286	21.142999	1.122547
20166	Amp	17	39719656	39719962	20.512807	1.102059
20167	Amp	17	39719993	39720645	20.236485	1.09353
20168	Amp	17	39720681	39743435	19.979676	1.085312
20169	Amp	17	39747958	39877180	20.47762	1.101033
20170	Amp	17	39884459	39891218	20.642489	1.106266
20171	Amp	17	39896312	39913748	20.1733	1.091452
20172	Amp	17	39921348	39929538	18.871839	1.04965
20173	Amp	17	39933291	39941897	17.515119	1.003863
20174	Amp	17	39942552	39961648	17.2159	0.993746
20175	Amp	17	39965864	39974426	17.356477	0.998251
20176	Amp	17	39979157	39979157	16.885462	0.981762
20177	Amp	17	39984674	39999677	16.316047	0.961378
20178	Amp	17	40010626	40020205	16.97368	0.985445
20179	Amp	17	40020606	40036380	17.617137	1.007382
20180	Amp	17	40041052	40041052	17.718565	1.0107
20181	Amp	17	40044698	40068878	17.597458	1.006936
20182	Amp	17	40070779	40087611	16.735182	0.976928
20183	Amp	17	40091769	40153585	16.302613	0.960974
20184	Amp	17	40160019	40173757	16.65678	0.973999
20185	Amp	17	40176350	40185351	16.569826	0.97037
20186	Amp	17	40199581	40200563	15.795475	0.942721
20187	Amp	17	40212548	40255387	12.991623	0.840561
20188	Amp	17	40256520	40256520	11.666631	0.790038
20189	Amp	17	40260127	40267006	11.539522	0.784805
20190	Amp	17	40267227	40291317	10.905321	0.760246
20191	Amp	17	40300899	40300899	10.40036	0.740777
20192	Amp	17	40310095	40321019	9.896323	0.721665

20193	Amp	17	40324405	40324848	7.833881	0.638339
20194	Amp	17	40325043	40325043	6.903728	0.599489
20195	Amp	17	40329292	40334276	6.541945	0.583867
20196	Amp	17	40336289	40336376	4.483097	0.487351
20197	Amp	17	40349352	40349509	4.941808	0.509716
20198	Amp	17	40349804	40349894	5.383269	0.529769
20199	Amp	17	40371518	40373855	5.03214	0.513802
20200	Amp	17	40380731	40380731	5.077576	0.515445
20201	Amp	17	40387144	40408095	5.107515	0.516851
20202	Amp	17	40409738	40438559	4.751695	0.500641
20203	Amp	17	40440173	40454413	3.76435	0.450314
20204	Amp	17	40457926	40473508	4.009908	0.463356
20205	Amp	17	40475061	40480767	3.828482	0.453461
20206	Amp	17	40481999	40484392	3.933803	0.459353
20207	Amp	17	40484492	40514076	3.958753	0.460832
20208	Amp	17	40514261	40578262	4.14557	0.470426
20209	Amp	17	40579450	40607624	4.185786	0.472113
20210	Amp	17	40616114	40616408	4.594893	0.493276
20211	Amp	17	40622836	40629466	4.809024	0.503109
20212	Amp	17	40629493	40642947	5.062558	0.51472
20213	Amp	17	40643391	40643391	4.778542	0.501678
20214	Amp	17	40649855	40672771	4.355429	0.480515
20215	Amp	17	40689379	40695864	3.946237	0.45976
20216	Amp	17	40696111	40696111	4.200241	0.472881
20217	Amp	17	40696227	40724279	3.958753	0.460877
20218	Amp	17	40724967	40733973	3.777346	0.451044
20219	Amp	17	40736084	40771762	3.828482	0.453977
20220	Amp	17	40779560	40806666	3.43725	0.432195
20221	Amp	17	40807528	40812999	3.523011	0.437581
20222	Amp	17	40815483	40826819	3.424445	0.431689
20223	Amp	17	40827299	40838349	2.914035	0.401837
20224	Amp	17	40854292	40873923	2.415622	0.372016
20225	Amp	17	40877944	40892522	1.918998	0.341321
20226	Amp	17	40895690	40912480	1.737394	0.329162
20227	Amp	17	40915430	40915982	1.789205	0.332791
20228	Amp	17	40922280	40931731	2.135909	0.355095
20229	Amp	17	40934881	40954668	1.514375	0.314129
20230	Amp	17	40956152	40985421	1.394558	0.305991
20231	Amp	17	40986495	41001850	1.159718	0.289331
20232	Amp	17	41002125	41120530	0.991911	0.277849
20233	Amp	17	41122536	41141463	1.150167	0.289193
20234	Amp	17	41145460	41223419	1.059215	0.282943
20235	Amp	17	41224170	41224178	1.150167	0.28917
20236	Amp	17	41284704	41305121	1.305256	0.29984
20237	Amp	17	41307306	41307306	1.394558	0.305827
20238	Amp	17	41314088	41370168	0.693887	0.255304
20239	Amp	17	41370308	41409616	0.760395	0.261026
20240	Amp	17	41414554	41459973	0.51566	0.240126
20241	Amp	17	41461050	41474954	0.012341	0.189838
20242	Amp	17	41475141	41496870	0	0.188151
20243	Amp	17	41499055	41501085	0	0.175401
20244	Amp	17	41510342	41510342	0	0.186692
20245	Amp	17	41540812	41545095	0.018387	0.190486

20246	Amp	17	41549612	41550474	0.156508	0.206409
20247	Amp	17	41551041	41556332	0.068197	0.197135
20248	Amp	17	41557666	41564587	0.038273	0.193341
20249	Amp	17	41564629	41599830	0.056238	0.195744
20250	Amp	17	41600750	41632423	0.043943	0.194099
20251	Amp	17	41632460	41632460	0.033661	0.19262
20252	Amp	17	41632949	41661019	0	0.175353
20253	Amp	17	41663863	41686506	0	0.17371
20254	Amp	17	41686993	41700905	0	0.157787
20255	Amp	17	41701419	41714095	0	0.167546
20256	Amp	17	41714121	41727839	0	0.165223
20257	Amp	17	41729239	41729239	0	0.159236
20258	Amp	17	41738729	41752860	0	0.125981
20259	Amp	17	41767318	41800335	0	0.116222
20260	Amp	17	41802092	41812449	0	0.129184
20261	Amp	17	41814649	41815232	0	0.125575
20262	Amp	17	41815756	41837496	0	0.135633
20263	Amp	17	41841719	41872315	0	0.124963
20264	Amp	17	41884561	41932666	0	0.084793
20265	Amp	17	41939842	42007296	0	0.070059
20266	Amp	17	42009934	42014982	0	0.063202
20267	Amp	17	42018481	42034110	0	0.058945
20268	Amp	17	42034941	42044296	0	0.048887
20269	Amp	17	42047517	42100949	0	0.057629
20270	Amp	17	42101111	42149825	0	0.048887
20271	Amp	17	42152197	42314231	0	0.055011
20272	Amp	17	42317564	42322195	0	0.067026
20273	Amp	17	42328363	42604836	0	0.071117
20274	Amp	17	42605595	42656393	0	0.059102
20275	Amp	17	42658755	42695849	0	0.053698
20276	Amp	17	42700572	43026222	0	0.074397
20277	Amp	17	43026747	43045257	0	0.069498
20278	Amp	17	43050793	43096059	0	0.059903
20279	Amp	17	43096288	43099914	0	0.056625
20280	Amp	17	43105068	43155084	0	0.069062
20281	Amp	17	43155380	43168187	0	0.063676
20282	Amp	17	43184131	43189750	0	0.052332
20283	Amp	17	43428081	43428081	0	0.049929
20284	Amp	17	43437078	43437078	0	0.042939
20285	Amp	17	43444725	43444725	0	0.065903
20286	Amp	17	43444797	43446818	0	0.076253
20287	Amp	17	43447423	43447595	0	0.081296
20288	Amp	17	43456462	43534982	0	0.077205
20289	Amp	17	43535004	43536239	0	0.084685
20290	Amp	17	43541309	43561612	0	0.086152
20291	Amp	17	43565935	43650099	0	0.073924
20292	Amp	17	43658708	43675569	0	0.063574
20293	Amp	17	43677088	43702266	0	0.114097
20294	Amp	17	43702751	43776302	0	0.119675
20295	Amp	17	43782692	43849916	0	0.108939
20296	Amp	17	43852129	44106903	0	0.058416
20297	Amp	17	44114525	44279877	0	0.045132
20298	Amp	17	44280215	44323112	0	0.041243

20299	Amp	17	44340128	44424324	0	0.086129
20300	Amp	17	44426718	44426718	0	0.053743
20301	Amp	17	44428776	44626081	0	0.057576
20302	Amp	17	44629399	44643548	0	0.053448
20303	Amp	17	44643569	44645140	0	0.056203
20304	Amp	17	44647283	44703780	0	0.05116
20305	Amp	17	44706392	44765467	0	0.038134
20306	Amp	17	44766276	44808791	0	0.043061
20307	Amp	17	44814641	45000579	0	0.034789
20308	Amp	17	45010734	45098554	0	0.039644
20309	Amp	17	45105660	45179669	0	0.033943
20310	Amp	17	45184750	45212511	0	0.029088
20311	Amp	17	45212842	45365868	0	0.033303
20312	Amp	17	45370145	45395141	0	0.040199
20313	Amp	17	45403165	45436394	0	0.04259
20314	Amp	17	45461373	45461373	0	0.047722
20315	Amp	17	46733528	46733528	0.243425	0.215529
20316	Amp	17	46733732	46740644	0.223025	0.213138
20317	Amp	17	46741267	46764265	0.27943	0.218565
20318	Amp	17	46764722	46897053	0.317596	0.222555
20319	Amp	17	46899753	46988901	0	0.172032
20320	Amp	17	46989235	46993168	0	0.166605
20321	Amp	17	46993322	47069958	0	0.165056
20322	Amp	17	47074183	47147856	0	0.139108
20323	Amp	17	47150089	47151194	0	0.147901
20324	Amp	17	47163702	47225203	0	0.137772
20325	Amp	17	47225320	47266411	0	0.143918
20326	Amp	17	47270526	47429814	0	0.138671
20327	Amp	17	47440021	47441718	0	0.131778
20328	Amp	17	47448848	47450036	0	0.127789
20329	Amp	17	47459479	47459815	0	0.13618
20330	Amp	17	47467907	47550228	0	0.131144
20331	Amp	17	47557019	47617256	0	0.138203
20332	Amp	17	47617293	47625700	0	0.145902
20333	Amp	17	47628076	47634096	0	0.151258
20334	Amp	17	47639038	47640747	0.105051	0.201059
20335	Amp	17	47643325	47645905	0.061778	0.195927
20336	Amp	17	47655408	47658609	0.105051	0.201175
20337	Amp	17	47660371	47708034	0.223025	0.213287
20338	Amp	17	47714101	47753576	0.796213	0.26381
20339	Amp	17	47753582	47788858	0.724993	0.257664
20340	Amp	17	47795563	47831817	0.972663	0.276691
20341	Amp	17	47839359	47855975	1.159718	0.289753
20342	Amp	17	47856784	47887410	0.897922	0.270726
20343	Amp	17	47888600	47892239	1.120124	0.286683
20344	Amp	17	47894244	47894244	1.305256	0.300171
20345	Amp	17	47899199	47936284	1.364151	0.304187
20346	Amp	17	47936815	47968271	1.24764	0.295627
20347	Amp	17	47972167	47973589	1.484449	0.312259
20348	Amp	17	47977670	47992546	1.24764	0.295627
20349	Amp	17	47992614	48021669	1.432145	0.309132
20350	Amp	17	48022188	48022188	1.697113	0.32652
20351	Amp	17	48027053	48052957	2.349236	0.367713

20352	Amp	17	48061847	48095765	2.507231	0.377765
20353	Amp	17	48110203	48231196	2.948071	0.403864
20354	Amp	17	48240759	48261315	3.207076	0.418703
20355	Amp	17	48262165	48340929	3.312745	0.424757
20356	Amp	17	48341037	48388506	3.383286	0.428881
20357	Amp	17	48395039	48423311	3.855237	0.454875
20358	Amp	17	48423542	48508365	3.880586	0.456633
20359	Amp	17	48508528	48534681	3.713958	0.44758
20360	Amp	17	48538882	48546738	3.523011	0.437528
20361	Amp	17	48561246	48566799	3.650307	0.444482
20362	Amp	17	48589942	48630445	3.790167	0.452044
20363	Amp	17	48639345	48707038	4.076638	0.466803
20364	Amp	17	48712985	48714711	4.215025	0.473581
20365	Amp	17	48727378	48727378	4.076638	0.466684
20366	Amp	17	48727705	48736169	3.998681	0.46256
20367	Amp	17	48750515	48755936	3.946237	0.459787
20368	Amp	17	48756878	48764631	4.049098	0.465116
20369	Amp	17	48767740	48840842	4.455933	0.485753
20370	Amp	17	48840899	48848497	4.708951	0.498478
20371	Amp	17	48849429	48860874	4.483097	0.487729
20372	Amp	17	48867346	48870856	5.445472	0.532641
20373	Amp	17	48871227	48871602	5.592909	0.539469
20374	Amp	17	48877431	48936765	5.654252	0.542113
20375	Amp	17	48938131	48952063	5.16411	0.519877
20376	Amp	17	48958522	48969629	5.608185	0.540184
20377	Amp	17	48969752	48986224	5.724602	0.545732
20378	Amp	17	48987713	49040685	5.910477	0.554805
20379	Amp	17	49041773	49061659	5.827612	0.55059
20380	Amp	17	49063637	49084985	6.117292	0.564212
20381	Amp	17	49087661	49087661	6.227003	0.569105
20382	Amp	17	49087874	49106533	6.450429	0.579472
20383	Amp	17	49107188	49136891	7.456767	0.62289
20384	Amp	17	49137045	49157145	7.199544	0.611926
20385	Amp	17	49161515	49164242	7.033351	0.604868
20386	Amp	17	49165173	49165991	8.233051	0.654812
20387	Amp	17	49167966	49201267	7.884054	0.640435
20388	Amp	17	49201281	49203290	7.50594	0.625175
20389	Amp	17	49205696	49231971	7.392813	0.620282
20390	Amp	17	49232462	49246934	7.553488	0.626718
20391	Amp	17	49247040	49249588	7.799797	0.637475
20392	Amp	17	49253219	49278738	8.233051	0.655325
20393	Amp	17	49294560	49294560	8.840788	0.680039
20394	Amp	17	49295311	49325114	9.061723	0.688787
20395	Amp	17	49325354	49330730	9.111233	0.690662
20396	Amp	17	49331545	49342487	9.281415	0.697835
20397	Amp	17	49342793	49400273	9.351643	0.700268
20398	Amp	17	49403291	49405568	9.63277	0.711233
20399	Amp	17	49405910	49432418	9.66807	0.712724
20400	Amp	17	49436512	49436544	8.131547	0.651037
20401	Amp	17	49446966	49463859	9.128876	0.691633
20402	Amp	17	49466791	49469749	9.247887	0.69634
20403	Amp	17	49473765	49489224	9.111233	0.691091
20404	Amp	17	49492609	49511453	8.957274	0.684391

20405 Amp	17	49514102	49514440	8.486462	0.665097
20406 Amp	17	49520934	49560008	7.489335	0.624502
20407 Amp	17	49577731	49593136	7.69681	0.633285
20408 Amp	17	49595595	49598321	7.456767	0.622695
20409 Amp	17	49601545	49625645	7.967172	0.644315
20410 Amp	17	49627903	49633477	7.553488	0.626866
20411 Amp	17	49633917	49640826	7.134252	0.609016
20412 Amp	17	49645149	49714142	7.363353	0.618753
20413 Amp	17	49718308	49752189	7.489335	0.624102
20414 Amp	17	49752228	49843153	6.333704	0.574301
20415 Amp	17	49845301	49901622	6.227003	0.568952
20416 Amp	17	49903449	49915587	6.421599	0.578307
20417 Amp	17	49915928	49934462	6.212874	0.568589
20418 Amp	17	49937427	49953292	6.042741	0.5607
20419 Amp	17	49953675	49961943	5.84242	0.551346
20420 Amp	17	49968074	49979692	6.378996	0.575781
20421 Amp	17	49979781	50021947	6.465884	0.579883
20422 Amp	17	50023148	50023148	6.604027	0.586295
20423 Amp	17	50026771	50058553	6.760799	0.593554
20424 Amp	17	50061643	50082859	7.330797	0.617107
20425 Amp	17	50083518	50179793	6.435656	0.579066
20426 Amp	17	50179887	50187356	6.57161	0.584708
20427 Amp	17	50187729	50208408	6.823902	0.59632
20428 Amp	17	50211368	50221605	6.775614	0.593886
20429 Amp	17	50228607	50248951	5.681709	0.54393
20430 Amp	17	50251052	50284646	6.058244	0.561876
20431 Amp	17	50287058	50288821	5.827612	0.550516
20432 Amp	17	50293961	50355817	6.378996	0.576159
20433 Amp	17	50362819	50370662	4.927103	0.508845
20434 Amp	17	50375970	50440713	5.940125	0.556022
20435 Amp	17	50440906	50440906	5.997601	0.558573
20436 Amp	17	50441769	50457171	6.823902	0.595769
20437 Amp	17	50459761	50461674	6.465884	0.580121
20438 Amp	17	50462251	50513619	6.557256	0.584556
20439 Amp	17	50514559	50565233	6.40864	0.577778
20440 Amp	17	50566354	50608939	6.840128	0.596357
20441 Amp	17	50614618	50618396	6.557256	0.584466
20442 Amp	17	50625264	50654270	6.856545	0.597269
20443 Amp	17	50656384	50690307	5.783449	0.548317
20444 Amp	17	50691125	50703520	6.526336	0.582951
20445 Amp	17	50704659	50710203	5.967002	0.557505
20446 Amp	17	50715869	50732954	6.133613	0.564991
20447 Amp	17	50734766	50753041	5.884474	0.553379
20448 Amp	17	50753188	50827703	6.952285	0.601625
20449 Amp	17	50829712	50829712	6.856545	0.597359
20450 Amp	17	50832347	50832347	6.936308	0.601117
20451 Amp	17	50838691	50858639	6.745443	0.592582
20452 Amp	17	50859698	50913996	6.465884	0.580187
20453 Amp	17	50925198	50939163	5.870027	0.552498
20454 Amp	17	50946399	50955808	4.608746	0.493497
20455 Amp	17	50961360	50974490	4.566654	0.491622
20456 Amp	17	50974812	50994809	4.679943	0.496769
20457 Amp	17	50996170	51088284	4.623661	0.494406

20458	Amp	17	51094053	51094053	4.651516	0.495934
20459	Amp	17	51096695	51132389	5.047749	0.514385
20460	Amp	17	51133251	51142270	6.028267	0.560255
20461	Amp	17	51142283	51160201	6.378996	0.576163
20462	Amp	17	51160326	51188568	6.421599	0.577929
20463	Amp	17	51193998	51204217	6.450429	0.579427
20464	Amp	17	51205855	51205855	6.40864	0.577554
20465	Amp	17	51206479	51207382	7.067371	0.606031
20466	Amp	17	51210293	51228102	6.57161	0.58523
20467	Amp	17	51229525	51229965	6.435656	0.578818
20468	Amp	17	51233881	51242201	6.378996	0.576174
20469	Amp	17	51479558	51479558	4.103115	0.468207
20470	Amp	17	51487144	51530264	4.009908	0.463252
20471	Amp	17	51532123	51572839	4.035317	0.465043
20472	Amp	17	51572937	51586637	4.103115	0.468487
20473	Amp	17	51587600	51606790	4.14557	0.47019
20474	Amp	17	51606904	51612231	4.215025	0.47378
20475	Amp	17	51616076	51638862	4.257976	0.476053
20476	Amp	17	51639065	51700476	4.355429	0.480665
20477	Amp	17	51702890	51768903	4.402792	0.483231
20478	Amp	17	51771410	51771410	4.14557	0.47011
20479	Amp	17	51771848	51837306	4.009908	0.46356
20480	Amp	17	51838595	51838595	4.215025	0.473577
20481	Amp	17	51841076	51867694	4.076638	0.466624
20482	Amp	17	51868478	51880122	3.985282	0.461738
20483	Amp	17	51882135	51899753	3.461541	0.433566
20484	Amp	17	51902005	51949882	3.415716	0.431
20485	Amp	17	51950634	51953065	3.348874	0.427243
20486	Amp	17	51955407	51958144	3.112016	0.413105
20487	Amp	17	51959583	52014566	2.283191	0.363596
20488	Amp	17	52014831	52036157	2.866312	0.398981
20489	Amp	17	52052439	52243812	2.683697	0.387943
20490	Amp	17	52246854	52266541	2.249752	0.361856
20491	Amp	17	52269896	52280818	2.404616	0.371024
20492	Amp	17	52281813	52430232	2.338154	0.367191
20493	Amp	17	52436416	52472932	1.657426	0.32426
20494	Amp	17	52474088	52485503	1.856336	0.336962
20495	Amp	17	52488167	52520185	2.393797	0.37042
20496	Amp	17	52520787	52526380	2.458756	0.37477
20497	Amp	17	52536293	52536518	2.495444	0.376662
20498	Amp	17	52593672	52660639	1.953191	0.343204
20499	Amp	17	52661248	52663641	2.203122	0.359105
20500	Amp	17	52665609	52689963	2.727427	0.390868
20501	Amp	17	52690812	52698398	3.05489	0.409628
20502	Amp	17	52699004	52706945	3.359177	0.427817
20503	Amp	17	52708931	52797561	3.26417	0.422448
20504	Amp	17	52799218	52861306	3.089932	0.411616
20505	Amp	17	52863043	52868536	3.171522	0.416661
20506	Amp	17	52870155	52872660	3.029636	0.408612
20507	Amp	17	53239515	53239515	2.591575	0.382442
20508	Amp	17	53240500	53300769	3.124015	0.414227
20509	Amp	17	53302897	53302897	2.935659	0.403205
20510	Amp	17	53306827	53307726	3.182419	0.417433

20511	Amp	17	53309848	53325646	3.230962	0.42001
20512	Amp	17	53329356	53336643	2.148338	0.355259
20513	Amp	17	53341701	53380000	2.102209	0.352682
20514	Amp	17	53380668	53434932	2.806723	0.395679
20515	Amp	17	53437087	53461103	2.774551	0.393534
20516	Amp	17	53461675	53468697	2.750393	0.391642
20517	Amp	17	53471415	53521438	2.71525	0.389765
20518	Amp	17	53529008	53571062	2.519	0.378109
20519	Amp	17	53573664	53593220	2.635842	0.385256
20520	Amp	17	53595451	53603175	1.995109	0.345987
20521	Amp	17	53609609	53614261	1.778794	0.331963
20522	Amp	17	53614394	53614723	1.473764	0.311298
20523	Amp	17	53619928	53695749	1.383826	0.305656
20524	Amp	17	53696564	53714831	1.572486	0.318173
20525	Amp	17	53715501	53763036	1.383826	0.305656
20526	Amp	17	53765761	53866390	1.514375	0.314353
20527	Amp	17	53868605	53941179	1.605306	0.320586
20528	Amp	17	53942983	54066989	1.525294	0.315028
20529	Amp	17	54092172	54092406	1.463463	0.310823
20530	Amp	17	54096040	54150824	1.324884	0.301093
20531	Amp	17	54154624	54207798	1.789205	0.333122
20532	Amp	17	54209130	54235204	1.821927	0.335258
20533	Amp	17	54236353	54278149	2.635842	0.385216
20534	Amp	17	54282738	54291480	3.006013	0.407404
20535	Amp	17	54293925	54353737	3.171522	0.416979
20536	Amp	17	54370002	54424472	3.461541	0.433768
20537	Amp	17	54425476	54494971	3.171522	0.416979
20538	Amp	17	54499481	54529698	2.994162	0.406302
20539	Amp	17	54532674	54558115	2.796399	0.394694
20540	Amp	17	54558266	54617994	3.006013	0.407102
20541	Amp	17	54618877	54655632	3.182419	0.417168
20542	Amp	17	54662101	54771723	2.914035	0.401905
20543	Amp	17	54773494	54801658	3.134208	0.414839
20544	Amp	17	54802088	54842139	3.230962	0.420129
20545	Amp	17	54849465	54849465	3.006013	0.407195
20546	Amp	17	54851893	54857268	3.089932	0.412164
20547	Amp	17	54859474	54904492	3.383286	0.429236
20548	Amp	17	54904603	54930915	3.45062	0.433174
20549	Amp	17	54931512	54947762	3.637615	0.443234
20550	Amp	17	54949336	54967964	3.415716	0.431402
20551	Amp	17	54968144	54984261	3.76435	0.45012
20552	Amp	17	54984819	55002752	4.171444	0.471759
20553	Amp	17	55007429	55037889	4.444356	0.485518
20554	Amp	17	55038258	55038258	4.540237	0.4903
20555	Amp	17	55049235	55073620	4.986444	0.511319
20556	Amp	17	55075600	55110135	4.355429	0.480425
20557	Amp	17	55116420	55137925	3.549256	0.438329
20558	Amp	17	55139097	55163193	3.688516	0.44603
20559	Amp	17	55163656	55177984	3.560951	0.439452
20560	Amp	17	55178111	55187831	3.75156	0.449578
20561	Amp	17	55193459	55312191	4.355429	0.480472
20562	Amp	17	55315183	55315551	3.461541	0.434182
20563	Amp	17	55317531	55332252	3.371695	0.428325

20564 Amp	17	55335233	55338489	3.828482	0.453584
20565 Amp	17	55338737	55352776	3.998681	0.462719
20566 Amp	17	55352867	55356925	3.855237	0.455437
20567 Amp	17	55357472	55375588	3.946237	0.459953
20568 Amp	17	55375742	55411501	3.815003	0.453259
20569 Amp	17	55411640	55420661	3.933803	0.459322
20570 Amp	17	55420910	55437578	3.855237	0.455348
20571 Amp	17	55437956	55465230	4.063271	0.466094
20572 Amp	17	55474400	55524829	4.511044	0.488668
20573 Amp	17	55527946	55544131	4.665596	0.496363
20574 Amp	17	55548781	55556191	5.383269	0.530232
20575 Amp	17	55560209	55572170	4.708951	0.498171
20576 Amp	17	55572211	55572399	4.665596	0.49619
20577 Amp	17	55572844	55575744	4.927103	0.508554
20578 Amp	17	55577075	55623287	4.724007	0.498849
20579 Amp	17	55623406	55650250	4.27183	0.476661
20580 Amp	17	55650422	55666361	4.131279	0.469273
20581 Amp	17	55666448	55699134	3.906866	0.458145
20582 Amp	17	55699145	55703955	3.855237	0.45539
20583 Amp	17	55704106	55744139	3.946237	0.459914
20584 Amp	17	55751949	55862300	4.594893	0.493171
20585 Amp	17	55865076	55874595	4.402792	0.483487
20586 Amp	17	55880626	55906567	3.958753	0.460382
20587 Amp	17	55906770	55920223	3.72674	0.448018
20588 Amp	17	55920515	55929809	3.828482	0.454
20589 Amp	17	55931131	55996470	3.946237	0.459676
20590 Amp	17	56002207	56016143	4.116436	0.469077
20591 Amp	17	56017322	56078110	3.985282	0.46233
20592 Amp	17	56078503	56080528	4.24321	0.474907
20593 Amp	17	56080546	56080851	4.049098	0.465369
20594 Amp	17	56095906	56100460	4.228481	0.474501
20595 Amp	17	56100621	56111407	4.327765	0.479026
20596 Amp	17	56113025	56126113	4.158928	0.47086
20597 Amp	17	56127048	56145871	4.30031	0.47793
20598 Amp	17	56147091	56152581	4.171444	0.471896
20599 Amp	17	56152594	56167778	4.402792	0.482983
20600 Amp	17	56176957	56176957	4.511044	0.489013
20601 Amp	17	56178747	56180363	4.30031	0.477927
20602 Amp	17	56180879	56182293	4.215025	0.47341
20603 Amp	17	56182730	56201269	4.391125	0.482353
20604 Amp	17	56206548	56209267	4.665596	0.496531
20605 Amp	17	56210317	56211405	4.483097	0.487589
20606 Amp	17	56216347	56233192	5.121758	0.517381
20607 Amp	17	56233578	56234893	4.809024	0.503202
20608 Amp	17	56240170	56270471	5.121758	0.517384
20609 Amp	17	56270548	56270548	5.324711	0.527407
20610 Amp	17	56272645	56320658	5.724602	0.545612
20611 Amp	17	56321012	56345155	5.884474	0.553672
20612 Amp	17	56349309	56366078	6.227003	0.56916
20613 Amp	17	56366348	56367030	6.08935	0.563129
20614 Amp	17	56369600	56405869	6.274109	0.571007
20615 Amp	17	56406199	56417934	6.435656	0.578933
20616 Amp	17	56420249	56420249	6.634781	0.587856

20617	Amp	17	56422136	56438788	6.696475	0.59069
20618	Amp	17	56442612	56470647	6.450429	0.579771
20619	Amp	17	56470929	56481936	7.199544	0.611642
20620	Amp	17	56485980	56522209	6.667173	0.589176
20621	Amp	17	56524730	56528260	7.282206	0.615189
20622	Amp	17	56528541	56535084	7.248573	0.613691
20623	Amp	17	56537023	56551671	8.050507	0.64723
20624	Amp	17	56555140	56575049	8.002024	0.645539
20625	Amp	17	56587065	56602621	8.148967	0.651538
20626	Amp	17	56603264	56603264	7.984434	0.644468
20627	Amp	17	56604632	56609063	7.134252	0.608998
20628	Amp	17	56615238	56623425	6.541945	0.583944
20629	Amp	17	56624222	56625086	6.588	0.585534
20630	Amp	17	56626000	56691237	6.421599	0.578074
20631	Amp	17	56691638	56713333	5.857214	0.552062
20632	Amp	17	56713557	56728410	6.227003	0.569329
20633	Amp	17	56729222	56732081	6.378996	0.576048
20634	Amp	17	56737481	56755987	5.925645	0.55525
20635	Amp	17	56756680	56772135	5.884474	0.553639
20636	Amp	17	56772805	56783801	5.798921	0.549107
20637	Amp	17	56784553	56792112	5.925645	0.555353
20638	Amp	17	56794394	56797742	5.739571	0.546209
20639	Amp	17	56798538	56803211	6.24213	0.570004
20640	Amp	17	56806274	56807226	6.197199	0.567731
20641	Amp	17	56807693	56819126	6.319279	0.573292
20642	Amp	17	56821256	56825371	6.450429	0.579717
20643	Amp	17	56828776	56841479	5.459172	0.533387
20644	Amp	17	56841647	56850435	6.197199	0.567765
20645	Amp	17	56850468	56862354	6.333704	0.573991
20646	Amp	17	56862376	56877361	6.588	0.585669
20647	Amp	17	56884823	56887190	5.669031	0.542893
20648	Amp	17	56888571	56895040	5.489676	0.534594
20649	Amp	17	56896721	56907833	5.31131	0.526668
20650	Amp	17	56910523	56931853	5.280955	0.524964
20651	Amp	17	56939571	56939571	5.489676	0.534822
20652	Amp	17	56940024	56943587	5.135526	0.518374
20653	Amp	17	56948758	56948784	5.193344	0.521151
20654	Amp	17	56953782	56967916	7.117295	0.608677
20655	Amp	17	56968293	56969583	7.248573	0.614189
20656	Amp	17	56975809	56981042	6.212874	0.568481
20657	Amp	17	56986521	57002968	6.378996	0.576184
20658	Amp	17	57003057	57030438	6.348497	0.574717
20659	Amp	17	57031373	57055472	6.058244	0.561955
20660	Amp	17	57059111	57059329	7.067371	0.606497
20661	Amp	17	57064232	57092120	7.647929	0.630791
20662	Amp	17	57092995	57092995	7.730271	0.634224
20663	Amp	17	57093435	57096744	7.850108	0.639014
20664	Amp	17	57100840	57100840	7.151076	0.60971
20665	Amp	17	57101967	57106637	7.523003	0.62579
20666	Amp	17	57107408	57108185	8.215812	0.654273
20667	Amp	17	57110686	57118198	7.917239	0.642196
20668	Amp	17	57120441	57125954	7.248573	0.613714
20669	Amp	17	57127846	57155129	7.833881	0.638342

20670	Amp	17	57155743	57222876	8.002024	0.645376
20671	Amp	17	57223480	57251852	8.283481	0.657067
20672	Amp	17	57258794	57268113	8.064725	0.648484
20673	Amp	17	57269377	57269377	8.504106	0.666109
20674	Amp	17	57270272	57291439	8.574393	0.668861
20675	Amp	17	57293322	57293322	8.806401	0.678569
20676	Amp	17	57297245	57297245	9.212677	0.694944
20677	Amp	17	57298228	57309579	9.368074	0.700823
20678	Amp	17	57311095	57349337	9.212677	0.694872
20679	Amp	17	57349504	57362906	8.992196	0.686322
20680	Amp	17	57369003	57370082	8.772488	0.677361
20681	Amp	17	57373857	57448852	8.591044	0.669658
20682	Amp	17	57451144	57478638	7.799797	0.637193
20683	Amp	17	57479740	57540949	8.316825	0.658215
20684	Amp	17	57541336	57541336	8.002024	0.645254
20685	Amp	17	57542868	57544236	8.451341	0.664125
20686	Amp	17	57556107	57600595	8.416339	0.662358
20687	Amp	17	57601214	57603256	8.233051	0.655324
20688	Amp	17	57608651	57651402	8.858746	0.680507
20689	Amp	17	57653989	57662051	9.009204	0.686621
20690	Amp	17	57665695	57672171	9.265068	0.696938
20691	Amp	17	57675456	57675456	9.333718	0.699537
20692	Amp	17	57675898	57676950	9.128876	0.691605
20693	Amp	17	57685888	57690562	9.095386	0.689813
20694	Amp	17	57696487	57732157	9.281415	0.697731
20695	Amp	17	57736187	57736187	8.858746	0.680319
20696	Amp	17	57739760	57755232	8.704996	0.67444
20697	Amp	17	57757138	57758262	8.050507	0.647809
20698	Amp	17	57760549	57761315	7.917239	0.641747
20699	Amp	17	57761347	57763406	8.131547	0.651166
20700	Amp	17	57765103	57766884	7.867314	0.640223
20701	Amp	17	57767828	57770652	7.714123	0.633998
20702	Amp	17	57776791	57778689	8.064725	0.647957
20703	Amp	17	57778948	57788969	7.199544	0.611559
20704	Amp	17	57792917	57800580	6.728678	0.591981
20705	Amp	17	57801119	57810785	6.480211	0.580791
20706	Amp	17	57812265	57812265	6.212874	0.568616
20707	Amp	17	57817156	57833195	6.465884	0.580285
20708	Amp	17	57834636	57840558	5.813919	0.549919
20709	Amp	17	57842069	57842482	5.967002	0.557822
20710	Amp	17	57844591	57848719	5.925645	0.555223
20711	Amp	17	57851849	57917599	5.884474	0.553391
20712	Amp	17	57920798	57944516	6.258466	0.570405
20713	Amp	17	57947252	57976599	5.997601	0.558736
20714	Amp	17	57978610	58020827	6.258466	0.570644
20715	Amp	17	58023109	58039847	5.783449	0.548806
20716	Amp	17	58044810	58058433	5.827612	0.55094
20717	Amp	17	58059698	58107956	5.884474	0.553087
20718	Amp	17	58109877	58150377	6.013103	0.559357
20719	Amp	17	58150642	58201283	5.813919	0.550225
20720	Amp	17	58205402	58211860	5.753822	0.547369
20721	Amp	17	58213195	58226969	6.028267	0.560517
20722	Amp	17	58235367	58240884	6.258466	0.570475

20723	Amp	17	58242797	58244892	6.421599	0.578299
20724	Amp	17	58245984	58248654	6.526336	0.583276
20725	Amp	17	58255788	58272562	6.364443	0.575452
20726	Amp	17	58277664	58283476	6.791498	0.59464
20727	Amp	17	58284957	58286152	7.16706	0.610321
20728	Amp	17	58290942	58297122	8.365343	0.660844
20729	Amp	17	58297673	58298996	8.858746	0.68061
20730	Amp	17	58299513	58329552	8.704996	0.674174
20731	Amp	17	58330818	58351813	8.591044	0.669649
20732	Amp	17	58354092	58354188	8.68932	0.673762
20733	Amp	17	58360147	58360571	8.957274	0.684521
20734	Amp	17	58363181	58373462	9.180995	0.693316
20735	Amp	17	58373926	58408454	8.974763	0.685399
20736	Amp	17	58408852	58437025	8.68932	0.67385
20737	Amp	17	58437097	58445172	8.640562	0.671823
20738	Amp	17	58451268	58465088	8.591044	0.669689
20739	Amp	17	58472159	58475337	8.486462	0.66509
20740	Amp	17	58482587	58484581	8.332564	0.659382
20741	Amp	17	58490329	58492580	8.840788	0.679979
20742	Amp	17	58494589	58502133	8.469083	0.664482
20743	Amp	17	58506813	58525591	8.574393	0.668586
20744	Amp	17	58529997	58534510	8.469083	0.664473
20745	Amp	17	58535626	58550586	8.591044	0.669696
20746	Amp	17	58551010	58600366	8.400894	0.661794
20747	Amp	17	58608674	58621466	8.233051	0.655402
20748	Amp	17	58622046	58662045	7.867314	0.640115
20749	Amp	17	58662634	58668244	7.78239	0.636723
20750	Amp	17	58669769	58696921	8.922319	0.682979
20751	Amp	17	58697584	58704822	8.68932	0.673844
20752	Amp	17	58705269	58711925	8.486462	0.665329
20753	Amp	17	58712843	58730995	8.658307	0.672231
20754	Amp	17	58736508	58738582	8.7383	0.675571
20755	Amp	17	58741734	58748395	8.87384	0.681502
20756	Amp	17	58748818	58857504	8.755614	0.676165
20757	Amp	17	58859540	58861197	8.957274	0.68454
20758	Amp	17	58866655	58887071	9.077474	0.689737
20759	Amp	17	58889863	58889863	10.111383	0.729765
20760	Amp	17	58890487	58897025	9.948936	0.724029
20761	Amp	17	58901272	58907336	9.48898	0.705732
20762	Amp	17	58918962	58939108	9.4207	0.703215
20763	Amp	17	58943405	58972297	8.858746	0.680684
20764	Amp	17	58976060	58976060	9.095386	0.690243
20765	Amp	17	59093350	59093786	10.5993	0.748663
20766	Amp	17	59100301	59111170	11.307418	0.776055
20767	Amp	17	59112118	59124923	10.905321	0.760374
20768	Amp	17	59132708	59164577	12.827194	0.834295
20769	Amp	17	59167465	59167465	12.55982	0.824241
20770	Amp	17	59171552	59210890	12.666108	0.828218
20771	Amp	17	59211264	59229899	12.436905	0.81944
20772	Amp	17	59230940	59246685	12.934185	0.838798
20773	Amp	17	59247176	59258795	12.827194	0.834177
20774	Amp	17	59261312	59269345	13.340239	0.853573
20775	Amp	17	59290376	59290376	13.11458	0.845198

20776	Amp	17	59290754	59326563	12.991623	0.840723
20777	Amp	17	59326591	59344874	13.30367	0.852547
20778	Amp	17	59346940	59347284	14.536938	0.897811
20779	Amp	17	59348072	59354405	14.161599	0.883836
20780	Amp	17	59360425	59362048	15.130646	0.918701
20781	Amp	17	59362206	59376085	15.320079	0.925444
20782	Amp	17	59377164	59463835	15.478508	0.931376
20783	Amp	17	59464266	59527593	15.115662	0.91802
20784	Amp	17	59527953	59539052	15.417214	0.929358
20785	Amp	17	59539832	59587994	15.264758	0.923426
20786	Amp	17	59595384	59621659	16.618944	0.972704
20787	Amp	17	59621957	59631637	17.073694	0.988647
20788	Amp	17	59632497	59640376	17.556752	1.005296
20789	Amp	17	59640778	59662714	17.698739	1.010555
20790	Amp	17	59664894	59675362	18.375365	1.033007
20791	Amp	17	59675417	59689614	17.778878	1.013227
20792	Amp	17	59689781	59689781	17.370974	0.999435
20793	Amp	17	59690289	59732570	16.956055	0.985172
20794	Amp	17	59736130	59742449	16.580308	0.970781
20795	Amp	17	59747149	59755584	16.993677	0.986094
20796	Amp	17	59760458	59760458	18.416949	1.034149
20797	Amp	17	59761582	59767679	18.560988	1.039246
20798	Amp	17	59781770	59788118	19.086077	1.056229
20799	Amp	17	59792385	59799109	19.190972	1.059728
20800	Amp	17	59802327	59814594	18.354873	1.032123
20801	Amp	17	59820498	59838007	18.62318	1.041414
20802	Amp	17	59840502	59845081	18.580761	1.039944
20803	Amp	17	59847814	59853285	19.021733	1.054335
20804	Amp	17	59853360	59861138	18.49898	1.037239
20805	Amp	17	59862408	59890877	18.416949	1.03419
20806	Amp	17	59892264	59892474	16.956055	0.985176
20807	Amp	17	59899297	59929832	16.755306	0.976995
20808	Amp	17	59934474	59961532	16.370143	0.963575
20809	Amp	17	59972128	59972690	15.264758	0.923736
20810	Amp	17	59974510	59977596	14.311382	0.88903
20811	Amp	17	59998150	59998150	14.902815	0.910886
20812	Amp	17	59998235	60003122	15.24597	0.923215
20813	Amp	17	60036209	60076846	16.580308	0.971341
20814	Amp	17	60077618	60103802	16.937173	0.983904
20815	Amp	17	60105722	60111524	16.65678	0.974108
20816	Amp	17	60127486	60129815	16.618944	0.972587
20817	Amp	17	60130677	60151243	17.073694	0.989048
20818	Amp	17	60153559	60156662	16.97368	0.985385
20819	Amp	17	60163681	60176880	16.618944	0.972728
20820	Amp	17	60181043	60219133	16.451457	0.966494
20821	Amp	17	60224140	60240953	16.54971	0.97
20822	Amp	17	60245298	60263583	16.355051	0.963151
20823	Amp	17	60263774	60295459	16.885462	0.981906
20824	Amp	17	60298020	60312662	16.694254	0.975466
20825	Amp	17	60317687	60324931	15.910177	0.947362
20826	Amp	17	60327323	60400888	16.470478	0.96716
20827	Amp	17	60404137	60419777	15.910177	0.947362
20828	Amp	17	60421102	60430199	16.355051	0.963005

20829	Amp	17	60430268	60563025	16.569826	0.970212
20830	Amp	17	60563564	60623156	16.221184	0.958323
20831	Amp	17	60623831	60651064	16.569826	0.970469
20832	Amp	17	60659066	60683322	16.042896	0.95192
20833	Amp	17	60686487	60722440	15.927057	0.948077
20834	Amp	17	60725498	60730462	16.103101	0.95366
20835	Amp	17	60733967	60748309	16.865574	0.981225
20836	Amp	17	60750927	60752006	16.082558	0.953159
20837	Amp	17	60752810	60793324	15.632845	0.936699
20838	Amp	17	60798211	60805228	16.142788	0.955249
20839	Amp	17	60808500	60915465	15.781148	0.942225
20840	Amp	17	60921651	60921651	16.122032	0.954866
20841	Amp	17	60922010	60945034	16.302613	0.96097
20842	Amp	17	60945411	61002835	17.356477	0.998345
20843	Amp	17	61012184	61024554	16.808776	0.979615
20844	Amp	17	61029363	61058718	17.276926	0.996075
20845	Amp	17	61068171	61074446	16.865574	0.981489
20846	Amp	17	61074869	61083321	15.478508	0.931473
20847	Amp	17	61084285	61123277	15.080746	0.916567
20848	Amp	17	61126705	61146727	15.377461	0.927647
20849	Amp	17	61149166	61150524	15.115662	0.91849
20850	Amp	17	61151905	61165081	15.041026	0.915247
20851	Amp	17	61165488	61213563	14.86676	0.909266
20852	Amp	17	61218555	61278585	14.068098	0.880233
20853	Amp	17	61278801	61315624	13.858672	0.872984
20854	Amp	17	61316404	61318447	14.010452	0.878203
20855	Amp	17	61318925	61319088	14.24056	0.886581
20856	Amp	17	61330597	61346059	14.405997	0.892984
20857	Amp	17	61346454	61347899	14.576253	0.898766
20858	Amp	17	61351452	61364852	15.061207	0.91632
20859	Amp	17	61365684	61365684	14.81056	0.906896
20860	Amp	17	61367005	61395042	14.86676	0.909437
20861	Amp	17	61404012	61424555	15.150849	0.919322
20862	Amp	17	61424958	61434211	15.095714	0.917353
20863	Amp	17	61439111	61451007	14.922956	0.911571
20864	Amp	17	61459249	61459249	14.612981	0.90049
20865	Amp	17	61461302	61484379	14.405997	0.892775
20866	Amp	17	61485275	61490398	14.276024	0.887843
20867	Amp	17	61490430	61500547	14.068098	0.880636
20868	Amp	17	61502583	61550516	14.2928	0.888379
20869	Amp	17	61555000	61568037	14.405997	0.892963
20870	Amp	17	61568141	61568141	14.595077	0.899675
20871	Amp	17	61568224	61601713	14.24056	0.886288
20872	Amp	17	61603974	61613192	14.443321	0.893884
20873	Amp	17	61622225	61667120	14.349793	0.890923
20874	Amp	17	61667650	61667650	14.536938	0.89791
20875	Amp	17	61674874	61689598	13.247277	0.850282
20876	Amp	17	61690263	61709013	13.321924	0.852621
20877	Amp	17	61710576	61756355	12.991623	0.840363
20878	Amp	17	61759184	61790078	12.680413	0.829026
20879	Amp	17	61791950	61820468	12.915337	0.837492
20880	Amp	17	61820636	61835013	13.064561	0.843277
20881	Amp	17	61835667	61835797	12.330001	0.815046

20882	Amp	17	61848230	61882404	12.612387	0.82615
20883	Amp	17	61892224	61898011	13.13436	0.845786
20884	Amp	17	61906501	61908513	12.827194	0.834682
20885	Amp	17	61909920	61938073	12.507948	0.821966
20886	Amp	17	61940527	61982685	12.827194	0.83439
20887	Amp	17	61993172	62004761	12.991623	0.840302
20888	Amp	17	62009094	62070547	12.55982	0.823995
20889	Amp	17	62074435	62096731	12.313728	0.814309
20890	Amp	17	62101958	62119835	13.436135	0.85701
20891	Amp	17	62120580	62133360	13.359728	0.854024
20892	Amp	17	62142842	62143088	12.418144	0.818608
20893	Amp	17	62143514	62188842	12.313728	0.81463
20894	Amp	17	62190081	62245492	12.149668	0.808073
20895	Amp	17	62247491	62286763	12.455926	0.820012
20896	Amp	17	62289615	62296315	12.647575	0.827637
20897	Amp	17	62300456	62304175	12.718133	0.829967
20898	Amp	17	62304415	62336047	12.843784	0.834993
20899	Amp	17	62338740	62340162	13.080267	0.844077
20900	Amp	17	62340177	62375289	13.821655	0.871562
20901	Amp	17	62378561	62391018	13.30367	0.85231
20902	Amp	17	62400384	62430026	12.612387	0.826029
20903	Amp	17	62435834	62446311	11.632145	0.788425
20904	Amp	17	62446641	62518129	12.79065	0.833133
20905	Amp	17	62522436	62530043	13.28499	0.851703
20906	Amp	17	62533450	62535721	13.231261	0.849364
20907	Amp	17	62536174	62554847	13.455219	0.857661
20908	Amp	17	62556470	62556470	15.437405	0.930167
20909	Amp	17	62558090	62558090	15.852778	0.94505
20910	Amp	17	62559465	62607564	15.910177	0.947338
20911	Amp	17	62613603	62643703	15.492166	0.931943
20912	Amp	17	62654656	62665114	15.080746	0.916695
20913	Amp	17	62668913	62676543	13.772971	0.869476
20914	Amp	17	62678862	62683813	13.455219	0.857896
20915	Amp	17	62685852	62711871	13.772971	0.869479
20916	Amp	17	62712005	62745782	14.595077	0.899688
20917	Amp	17	62754882	62773719	14.010452	0.878298
20918	Amp	17	62780156	62821817	14.259155	0.887582
20919	Amp	17	62822234	62850242	13.789953	0.869836
20920	Amp	17	62850731	62850993	14.048553	0.879464
20921	Amp	17	62861823	62866244	14.311382	0.889473
20922	Amp	17	62869899	62870616	14.443321	0.893938
20923	Amp	17	62871403	62901582	14.536938	0.897848
20924	Amp	17	62905345	62927849	15.359376	0.92714
20925	Amp	17	62929844	62929844	14.2928	0.888457
20926	Amp	17	62935796	62951565	13.933402	0.875699
20927	Amp	17	62953884	62969903	14.068098	0.880547
20928	Amp	17	62979274	62979274	14.220401	0.886211
20929	Amp	17	63014897	63029010	14.751183	0.904856
20930	Amp	17	63039767	63062101	15.478508	0.931364
20931	Amp	17	63062442	63121157	16.885462	0.981887
20932	Amp	17	63121455	63122345	17.113337	0.990126
20933	Amp	17	63129300	63142954	17.392085	1.000267
20934	Amp	17	63144381	63262315	16.921597	0.983815

20935 Amp	17	63263953	63271424	16.674143	0.974531
20936 Amp	17	63275644	63275644	15.437405	0.929822
20937 Amp	17	63287981	63334177	15.66735	0.938195
20938 Amp	17	63337539	63402727	14.276024	0.887672
20939 Amp	17	63403653	63403653	14.385783	0.892092
20940 Amp	17	63404057	63413883	14.122401	0.88272
20941 Amp	17	63417857	63479193	14.349793	0.890657
20942 Amp	17	63480815	63484395	13.789953	0.870109
20943 Amp	17	63485810	63487831	13.455219	0.857684
20944 Amp	17	63488670	63488974	13.30367	0.852392
20945 Amp	17	63490292	63496183	12.991623	0.840453
20946 Amp	17	63498784	63534062	12.55982	0.824068
20947 Amp	17	63536995	63537240	12.330001	0.815294
20948 Amp	17	63540141	63555561	12.735133	0.83123
20949 Amp	17	63560060	63616912	12.345058	0.81556
20950 Amp	17	63624488	63625126	12.436905	0.819579
20951 Amp	17	63625629	63632013	12.574862	0.824523
20952 Amp	17	63636915	63665776	12.666108	0.828365
20953 Amp	17	63666210	63666210	12.475004	0.82036
20954 Amp	17	63671236	63671956	12.418144	0.81886
20955 Amp	17	63672794	63679513	12.647575	0.827513
20956 Amp	17	63685575	63731243	12.507948	0.822359
20957 Amp	17	63734168	63768392	13.513811	0.859479
20958 Amp	17	63772941	63772941	13.657948	0.865321
20959 Amp	17	63774083	63795093	13.417924	0.856591
20960 Amp	17	63795947	63814307	13.513811	0.86007
20961 Amp	17	63817621	63832807	13.89726	0.874178
20962 Amp	17	63833263	63860642	14.029362	0.878994
20963 Amp	17	63865580	63959920	13.6031	0.863058
20964 Amp	17	63960810	63968195	13.474556	0.858172
20965 Amp	17	63969381	63989826	13.231261	0.84946
20966 Amp	17	63989907	63992793	13.378853	0.855201
20967 Amp	17	63995277	64018579	13.007244	0.841093
20968 Amp	17	64029381	64054376	13.266071	0.851111
20969 Amp	17	64054585	64056128	13.6031	0.863041
20970 Amp	17	64056377	64073949	13.513811	0.859563
20971 Amp	17	64075322	64088597	13.247277	0.850047
20972 Amp	17	64091885	64092134	13.39854	0.855497
20973 Amp	17	64094026	64101139	12.753451	0.831901
20974 Amp	17	64102936	64122786	12.843784	0.834738
20975 Amp	17	64123105	64226560	13.080267	0.84411
20976 Amp	17	64226762	64233866	12.860528	0.835737
20977 Amp	17	64233879	64237971	12.753451	0.831721
20978 Amp	17	64238478	64326982	13.587928	0.862409
20979 Amp	17	64327030	64329884	13.696984	0.866595
20980 Amp	17	64332226	64332226	13.474556	0.858217
20981 Amp	17	64333211	64464879	12.475004	0.8205
20982 Amp	17	64468704	64503583	12.718133	0.830269
20983 Amp	17	64504635	64510804	13.494087	0.859132
20984 Amp	17	64511513	64532547	13.696984	0.866402
20985 Amp	17	64535373	64542568	13.789953	0.87023
20986 Amp	17	64544572	64544682	13.933402	0.875608
20987 Amp	17	64546746	64556214	13.789953	0.870172

20988	Amp	17	64556593	64602542	13.840197	0.872274
20989	Amp	17	64603016	64612193	13.657948	0.865004
20990	Amp	17	64615101	64627915	13.513811	0.86006
20991	Amp	17	64636266	64661066	13.0458	0.842813
20992	Amp	17	64663439	64666389	12.735133	0.831158
20993	Amp	17	64667139	64697678	12.418144	0.818332
20994	Amp	17	64698620	64709869	12.507948	0.821863
20995	Amp	17	64713862	64720662	11.183333	0.77134
20996	Amp	17	64725497	64761044	11.041758	0.7656
20997	Amp	17	64765218	64802468	10.616849	0.748938
20998	Amp	17	64802530	64808025	9.98547	0.72528
20999	Amp	17	64816408	64816408	9.717504	0.71471
21000	Amp	17	64843932	64867438	8.858746	0.680756
21001	Amp	17	64929599	64936097	8.772488	0.676857
21002	Amp	17	64936226	64951737	8.82287	0.678807
21003	Amp	17	64952455	64952455	9.009204	0.686809
21004	Amp	17	64952808	64962687	9.368074	0.701368
21005	Amp	17	64968312	64968312	9.525066	0.707024
21006	Amp	17	64971989	65018886	9.061723	0.688506
21007	Amp	17	65019891	65023923	8.806401	0.678737
21008	Amp	17	65029025	65055775	8.68932	0.67381
21009	Amp	17	65060911	65061533	9.453092	0.704553
21010	Amp	17	65064355	65074741	9.507136	0.706292
21011	Amp	17	65077238	65083792	9.686	0.713282
21012	Amp	17	65083805	65087820	9.4207	0.703045
21013	Amp	17	65088877	65088877	9.453092	0.70452
21014	Amp	17	65094815	65099227	9.368074	0.700989
21015	Amp	17	65102530	65115975	9.63277	0.711694
21016	Amp	17	65120554	65162079	10.039611	0.726991
21017	Amp	17	65164577	65206786	10.95657	0.762072
21018	Amp	17	65206902	65209770	10.738968	0.75407
21019	Amp	17	65211747	65274472	10.689829	0.751968
21020	Amp	17	65276310	65291615	10.29192	0.736906
21021	Amp	17	65292530	65337586	10.723194	0.753494
21022	Amp	17	65339191	65344691	9.98547	0.725211
21023	Amp	17	65345346	65354917	10.383055	0.740325
21024	Amp	17	65355322	65360770	11.059715	0.76638
21025	Amp	17	65366999	65378333	11.166314	0.770324
21026	Amp	17	65378603	65383788	10.757147	0.755028
21027	Amp	17	65383898	65383898	10.812328	0.756514
21028	Amp	17	65383957	65472261	10.940241	0.761701
21029	Amp	17	65472709	65473306	11.183333	0.77095
21030	Amp	17	65476910	65490062	11.112261	0.76862
21031	Amp	17	65490217	65528964	11.341542	0.777273
21032	Amp	17	65541780	65598637	11.94724	0.800525
21033	Amp	17	65598911	65626487	12.204051	0.810186
21034	Amp	17	65627571	65635820	12.362697	0.81653
21035	Amp	17	65637141	65664112	12.612387	0.825957
21036	Amp	17	65674713	65693554	12.475004	0.820579
21037	Amp	17	65693604	65724408	12.718133	0.830021
21038	Amp	17	65726689	65736127	12.55982	0.824318
21039	Amp	17	65736855	65754744	12.680413	0.82881
21040	Amp	17	65755039	65755039	12.541299	0.823226

21041	Amp	17	65755525	65792224	12.79065	0.832671
21042	Amp	17	65792296	65803227	12.666108	0.828179
21043	Amp	17	65803947	65913131	13.064561	0.843644
21044	Amp	17	65914577	65923193	13.28499	0.851474
21045	Amp	17	65928420	65947398	13.173641	0.847104
21046	Amp	17	65949558	65957752	13.28499	0.851395
21047	Amp	17	65958822	65966482	12.507948	0.822236
21048	Amp	17	65966697	65971853	12.475004	0.820497
21049	Amp	17	65975101	66013886	12.507948	0.822147
21050	Amp	17	66018457	66019450	12.255767	0.812263
21051	Amp	17	66021213	66057554	12.167312	0.808963
21052	Amp	17	66057680	66094364	11.983124	0.801973
21053	Amp	17	66096575	66097452	12.239687	0.811474
21054	Amp	17	66101654	66109683	12.275031	0.813125
21055	Amp	17	66113571	66113571	12.167312	0.808834
21056	Amp	17	66114136	66151880	11.840328	0.796882
21057	Amp	17	66153863	66201141	11.323402	0.77695
21058	Amp	17	66204123	66208623	11.095564	0.767449
21059	Amp	17	66208946	66233675	11.199579	0.771716
21060	Amp	17	66235413	66244619	10.971689	0.763063
21061	Amp	17	66244651	66245183	10.738968	0.754082
21062	Amp	17	66245322	66312420	10.435377	0.742081
21063	Amp	17	66312594	66402698	10.723194	0.753424
21064	Amp	17	66402957	66493013	10.95657	0.762144
21065	Amp	17	66493389	66499061	10.738968	0.754335
21066	Amp	17	66499129	66514292	11.041758	0.765408
21067	Amp	17	66514424	66522315	11.469971	0.782135
21068	Amp	17	66522389	66552855	11.524618	0.784504
21069	Amp	17	66565600	66571209	11.237152	0.773431
21070	Amp	17	66572509	66593405	11.696276	0.791404
21071	Amp	17	66595357	66665772	12.239687	0.81196
21072	Amp	17	66666885	66685672	13.064561	0.843634
21073	Amp	17	66687253	66694140	13.513811	0.859763
21074	Amp	17	66694645	66730423	13.772971	0.869236
21075	Amp	17	66734768	66736847	13.913757	0.874784
21076	Amp	17	66737444	66748195	13.54844	0.861296
21077	Amp	17	66749572	66788085	13.789953	0.86978
21078	Amp	17	66788327	66813717	14.161599	0.883579
21079	Amp	17	66814213	66834611	14.311382	0.889463
21080	Amp	17	66835539	66870338	13.933402	0.875664
21081	Amp	17	66872299	66874731	14.366019	0.89132
21082	Amp	17	66874952	66954915	14.463105	0.894727
21083	Amp	17	66955163	66959616	14.576253	0.898676
21084	Amp	17	66959905	66980172	15.115662	0.918407
21085	Amp	17	66983368	67030770	15.080746	0.916756
21086	Amp	17	67032564	67055437	15.16796	0.920077
21087	Amp	17	67056188	67077445	14.142245	0.883266
21088	Amp	17	67077841	67085903	14.083932	0.880897
21089	Amp	17	67086048	67130818	14.848289	0.908319
21090	Amp	17	67133818	67140727	14.349793	0.890859
21091	Amp	17	67142587	67200861	13.6031	0.863438
21092	Amp	17	67202603	67202603	12.879705	0.836284
21093	Amp	17	67203419	67223844	12.843784	0.834809

21094 Amp	17	67229937	67241275	13.007244	0.841372
21095 Amp	17	67242635	67283990	12.972471	0.839885
21096 Amp	17	67286976	67302959	13.080267	0.844333
21097 Amp	17	67311714	67332924	12.666108	0.828082
21098 Amp	17	67337046	67413825	12.134117	0.807527
21099 Amp	17	67414184	67469039	12.49015	0.821349
21100 Amp	17	67479822	67480498	13.080267	0.84391
21101 Amp	17	67484993	67517967	13.715145	0.867452
21102 Amp	17	67520779	67535543	13.39854	0.855379
21103 Amp	17	67540499	67542386	13.587928	0.862451
21104 Amp	17	67552424	67562045	13.321924	0.853012
21105 Amp	17	67562455	67582757	13.417924	0.856073
21106 Amp	17	67583227	67597717	13.618548	0.864004
21107 Amp	17	67598322	67598322	13.359728	0.854445
21108 Amp	17	67603380	67637981	12.523772	0.822887
21109 Amp	17	67638528	67650947	12.039993	0.804074
21110 Amp	17	67651847	67654092	12.718133	0.830129
21111 Amp	17	67659301	67687065	12.896494	0.836826
21112 Amp	17	67688395	67742216	12.666108	0.828091
21113 Amp	17	67742710	67784771	12.574862	0.82503
21114 Amp	17	67786035	67795077	12.418144	0.818332
21115 Amp	17	67795479	67798370	12.313728	0.814491
21116 Amp	17	67798390	67861922	12.55982	0.824411
21117 Amp	17	67865583	67915279	11.894261	0.798357
21118 Amp	17	67921748	67956612	12.381595	0.817079
21119 Amp	17	67962578	67970542	12.593011	0.825344
21120 Amp	17	67970904	67975785	12.167312	0.808692
21121 Amp	17	67976503	67983025	12.058994	0.804742
21122 Amp	17	67983168	67998511	11.416123	0.780227
21123 Amp	17	68000822	68000822	10.634439	0.749619
21124 Amp	17	68019847	68026544	9.770577	0.7168
21125 Amp	17	68029767	68029767	9.48898	0.705901
21126 Amp	17	68041227	68062805	9.650316	0.712212
21127 Amp	17	68066200	68066200	9.913717	0.722739
21128 Amp	17	68178765	68187451	10.723194	0.753334
21129 Amp	17	68187783	68189132	10.580546	0.747492
21130 Amp	17	68189163	68227684	10.40036	0.740807
21131 Amp	17	68227876	68227876	10.52455	0.74577
21132 Amp	17	68228476	68249300	10.887353	0.759736
21133 Amp	17	68255019	68255019	11.539522	0.785228
21134 Amp	17	68255688	68278275	11.613928	0.787872
21135 Amp	17	68280916	68280916	12.49015	0.821153
21136 Amp	17	68281830	68297843	12.666108	0.828036
21137 Amp	17	68300310	68341731	12.381595	0.81751
21138 Amp	17	68344557	68357870	11.696276	0.791305
21139 Amp	17	68360440	68372311	11.768863	0.794094
21140 Amp	17	68375132	68389257	11.894261	0.798623
21141 Amp	17	68390500	68404360	12.134117	0.807483
21142 Amp	17	68423088	68424298	11.147569	0.770119
21143 Amp	17	68427870	68443464	10.616849	0.749099
21144 Amp	17	68444373	68451868	9.351643	0.700249
21145 Amp	17	68452349	68459197	9.146337	0.692012
21146 Amp	17	68460522	68469002	8.888709	0.682092

21147	Amp	17	68473049	68479678	9.043857	0.687709
21148	Amp	17	68480286	68539318	9.19854	0.694539
21149	Amp	17	68541735	68603904	9.095386	0.69001
21150	Amp	17	68605630	68632456	8.283481	0.656903
21151	Amp	17	68633024	68633024	8.064725	0.648414
21152	Amp	17	68633040	68700110	7.553488	0.627082
21153	Amp	17	68700570	68708868	7.523003	0.62569
21154	Amp	17	68717640	68766174	8.064725	0.647984
21155	Amp	17	68770254	68837813	8.017325	0.645953
21156	Amp	17	68839691	68855303	7.833881	0.638848
21157	Amp	17	68861037	68865707	7.489335	0.624429
21158	Amp	17	68866070	68876661	7.183547	0.610957
21159	Amp	17	68879819	68885488	6.856545	0.597167
21160	Amp	17	68888155	68889385	6.650629	0.588682
21161	Amp	17	68890848	68932547	6.465884	0.579961
21162	Amp	17	68936862	68944483	6.258466	0.570712
21163	Amp	17	68947071	68957938	6.073269	0.562473
21164	Amp	17	68960817	69006918	6.212874	0.568753
21165	Amp	17	69007098	69012195	6.073269	0.562442
21166	Amp	17	69012904	69035741	6.149531	0.565513
21167	Amp	17	69042759	69055254	5.592909	0.539679
21168	Amp	17	69055591	69064735	5.503852	0.535575
21169	Amp	17	69065666	69114709	5.222669	0.522743
21170	Amp	17	69122476	69135746	5.56363	0.537982
21171	Amp	17	69142737	69189286	5.267482	0.524506
21172	Amp	17	69190305	69191800	5.047749	0.51413
21173	Amp	17	69192404	69199672	5.413899	0.531744
21174	Amp	17	69200211	69233783	5.548259	0.537294
21175	Amp	17	69239564	69239564	5.84242	0.551368
21176	Amp	17	69243566	69245341	6.302827	0.572887
21177	Amp	17	69248298	69260097	6.619468	0.586885
21178	Amp	17	69261959	69277261	6.08935	0.562963
21179	Amp	17	69277471	69277471	5.897742	0.554149
21180	Amp	17	69281166	69299135	5.696665	0.544432
21181	Amp	17	69299918	69340204	5.857214	0.552014
21182	Amp	17	69344509	69352023	5.696665	0.544758
21183	Amp	17	69352351	69355424	5.622975	0.540753
21184	Amp	17	69363554	69367936	5.769527	0.548034
21185	Amp	17	69369786	69407352	5.910477	0.555098
21186	Amp	17	69409867	69412080	6.10345	0.563758
21187	Amp	17	69416490	69416975	5.967002	0.557415
21188	Amp	17	69418739	69446727	6.149531	0.565981
21189	Amp	17	69446789	69476771	5.982794	0.558398
21190	Amp	17	69477738	69477738	6.24213	0.569941
21191	Amp	17	69480329	69480672	6.274109	0.571465
21192	Amp	17	69485772	69487514	6.634781	0.587483
21193	Amp	17	69488937	69494089	6.435656	0.578918
21194	Amp	17	69505997	69525358	6.288489	0.571929
21195	Amp	17	69525400	69586522	6.333704	0.574005
21196	Amp	17	69587410	69607222	6.212874	0.568457
21197	Amp	17	69608527	69608527	6.557256	0.58441
21198	Amp	17	69608891	69620541	6.712483	0.591127
21199	Amp	17	69620859	69628414	6.619468	0.587

21200	Amp	17	69629000	69733768	6.480211	0.580913
21201	Amp	17	69734510	69801482	6.319279	0.573293
21202	Amp	17	69802289	69807038	6.480211	0.580592
21203	Amp	17	69809018	69851101	6.333704	0.574311
21204	Amp	17	69862292	69879063	5.47413	0.534466
21205	Amp	17	69881203	69915426	6.181334	0.566874
21206	Amp	17	69916275	69935286	5.982794	0.55839
21207	Amp	17	69935445	69945939	6.073269	0.562245
21208	Amp	17	69963203	69977271	5.897742	0.554201
21209	Amp	17	69982192	69995274	5.208342	0.521793
21210	Amp	17	69997177	70008857	5.337978	0.527636
21211	Amp	17	70012549	70024859	6.149531	0.565441
21212	Amp	17	70025008	70028630	6.364443	0.575621
21213	Amp	17	70029880	70040594	6.450429	0.579303
21214	Amp	17	70040628	70071117	6.319279	0.57346
21215	Amp	17	70071740	70082745	6.058244	0.561917
21216	Amp	17	70091768	70109486	5.252642	0.524112
21217	Amp	17	70109759	70162690	5.445472	0.532977
21218	Amp	17	70162769	70162769	5.252642	0.524118
21219	Amp	17	70163488	70195243	5.179405	0.520263
21220	Amp	17	70197902	70211518	5.399054	0.530496
21221	Amp	17	70214655	70222267	5.534438	0.537189
21222	Amp	17	70225876	70225876	5.413899	0.531638
21223	Amp	17	70226094	70311708	5.222669	0.522433
21224	Amp	17	70314023	70433561	4.416662	0.484061
21225	Amp	17	70436455	70451045	4.364676	0.481224
21226	Amp	17	70452588	70466104	4.035317	0.464672
21227	Amp	17	70467338	70492750	3.72674	0.448165
21228	Amp	17	70493008	70526104	3.675606	0.445592
21229	Amp	17	70526994	70562549	3.613533	0.44191
21230	Amp	17	70566930	70572288	3.777346	0.451273
21231	Amp	17	70573184	70639700	3.625861	0.442932
21232	Amp	17	70644204	70666354	3.348874	0.42726
21233	Amp	17	70667082	70693958	3.194439	0.417896
21234	Amp	17	70696713	70710903	3.348874	0.427014
21235	Amp	17	70712483	70715909	3.496514	0.436247
21236	Amp	17	70720897	70773047	3.587309	0.440564
21237	Amp	17	70773107	70773783	3.637615	0.443217
21238	Amp	17	70777473	70796050	3.470866	0.434658
21239	Amp	17	70796133	70812533	3.587309	0.440961
21240	Amp	17	70812829	70841662	3.998681	0.46303
21241	Amp	17	70842433	70890192	3.920712	0.458713
21242	Amp	17	70896957	70896957	3.815003	0.453152
21243	Amp	17	70897275	70931578	3.72674	0.448563
21244	Amp	17	70931812	71085298	3.86957	0.455909
21245	Amp	17	71086645	71113489	4.313672	0.478461
21246	Amp	17	71114146	71114957	4.185786	0.47247
21247	Amp	17	71117031	71117031	4.131279	0.469817
21248	Amp	17	71120089	71170537	4.429725	0.484545
21249	Amp	17	71173664	71184862	3.985282	0.461993
21250	Amp	17	71190740	71195226	3.496514	0.435938
21251	Amp	17	71197146	71396906	3.089932	0.411645
21252	Amp	17	71402088	71403045	2.922915	0.402093

21253	Amp	17	71403364	71432363	3.146436	0.415478
21254	Amp	17	71433002	71501522	2.994162	0.406246
21255	Amp	17	71505033	71525589	3.335884	0.426441
21256	Amp	17	71534517	71644433	3.415716	0.431262
21257	Amp	17	71648376	71661204	3.713958	0.447811
21258	Amp	17	71661429	71755005	3.76435	0.450113
21259	Amp	17	71756699	71823517	3.461541	0.434095
21260	Amp	17	71826146	71830158	3.496514	0.43613
21261	Amp	17	71831614	71841635	3.613533	0.442063
21262	Amp	17	71842045	71933414	3.637615	0.443567
21263	Amp	17	71935333	71949406	3.348874	0.427018
21264	Amp	17	71952194	71960353	3.45062	0.433389
21265	Amp	17	71962763	72026379	3.48305	0.435333
21266	Amp	17	72027686	72090456	3.777346	0.451023
21267	Amp	17	72094012	72097342	3.713958	0.447712
21268	Amp	17	72101466	72101466	3.587309	0.44043
21269	Amp	17	72102020	72102861	3.701758	0.447009
21270	Amp	17	72103300	72246356	3.75156	0.449574
21271	Amp	17	72246787	72303491	3.893345	0.457514
21272	Amp	17	72303520	72316657	3.933803	0.459009
21273	Amp	17	72319151	72358948	3.998681	0.462606
21274	Amp	17	72359246	72364187	3.76435	0.450671
21275	Amp	17	72366438	72370135	3.815003	0.452936
21276	Amp	17	72375062	72383056	3.76435	0.450634
21277	Amp	17	72383375	72470272	3.424445	0.431811
21278	Amp	17	72472517	72481911	3.613533	0.442324
21279	Amp	17	72485513	72505001	3.171522	0.416426
21280	Amp	17	72506018	72540994	3.029636	0.408167
21281	Amp	17	72541767	72560304	2.994162	0.406223
21282	Amp	17	72561207	72568281	3.26417	0.421884
21283	Amp	17	72572369	72613727	3.842634	0.454522
21284	Amp	17	72616827	72654313	3.713958	0.447473
21285	Amp	17	72654873	72664763	3.688516	0.445949
21286	Amp	17	72665934	72696601	3.946237	0.46014
21287	Amp	17	72701900	72711222	4.103115	0.468074
21288	Amp	17	72711233	72729694	4.185786	0.472243
21289	Amp	17	72729982	72773952	4.30031	0.47813
21290	Amp	17	72774808	72787595	4.158928	0.471252
21291	Amp	17	72787618	72789302	4.30031	0.477584
21292	Amp	17	72791023	72791023	4.402792	0.482989
21293	Amp	17	72792267	72857301	4.444356	0.4852
21294	Amp	17	72857544	72863175	4.636424	0.495063
21295	Amp	17	72868047	72870657	5.696665	0.544681
21296	Amp	17	72871584	72875733	5.534438	0.536741
21297	Amp	17	72875798	72931546	5.445472	0.532545
21298	Amp	17	72936611	72996427	5.121758	0.517825
21299	Amp	17	72996932	73030072	5.179405	0.520249
21300	Amp	17	73030708	73054470	4.971485	0.510758
21301	Amp	17	73058174	73062575	4.402792	0.483099
21302	Amp	17	73065750	73092361	4.738141	0.499604
21303	Amp	17	73094130	73122775	5.150026	0.519337
21304	Amp	17	73124447	73161126	4.838865	0.504668
21305	Amp	17	73163167	73169179	5.001595	0.512378

21306	Amp	17	73173244	73178007	5.208342	0.521968
21307	Amp	17	73180345	73184288	5.337978	0.527781
21308	Amp	17	73187917	73221231	5.399054	0.530435
21309	Amp	17	73227828	73243459	5.459172	0.533233
21310	Amp	17	73247150	73322104	5.669031	0.543409
21311	Amp	17	73324526	73333045	5.047749	0.514085
21312	Amp	17	73335409	73357297	5.208342	0.522032
21313	Amp	17	73361721	73366381	4.566654	0.491574
21314	Amp	17	73373143	73412442	4.957051	0.509783
21315	Amp	17	73416047	73424366	6.013103	0.559282
21316	Amp	17	73424609	73432766	6.042741	0.560923
21317	Amp	17	73433103	73433637	6.212874	0.568843
21318	Amp	17	73435028	73450084	6.40864	0.577294
21319	Amp	17	73450098	73472207	6.557256	0.584024
21320	Amp	17	73474912	73518434	5.459172	0.533501
21321	Amp	17	73518488	73565774	5.813919	0.549832
21322	Amp	17	73570593	73576239	6.302827	0.57291
21323	Amp	17	73576894	73590256	5.940125	0.556405
21324	Amp	17	73591585	73670759	5.681709	0.543955
21325	Amp	17	73671042	73679420	5.897742	0.554084
21326	Amp	17	73679511	73685288	5.84242	0.551661
21327	Amp	17	73691604	73691854	5.710355	0.545098
21328	Amp	17	73692206	73697851	5.669031	0.542886
21329	Amp	17	73701909	73730783	5.135526	0.51804
21330	Amp	17	73737501	73745262	5.062558	0.515243
21331	Amp	17	73755406	73769196	5.03214	0.513852
21332	Amp	17	73769275	73783724	4.868506	0.505933
21333	Amp	17	73784268	73790495	4.809024	0.502998
21334	Amp	17	73794897	73796730	4.738141	0.500006
21335	Amp	17	73798372	73817945	3.855237	0.455195
21336	Amp	17	73820787	73820787	4.076638	0.466677
21337	Amp	17	73829384	73829384	4.022326	0.464384
21338	Amp	17	74695492	74695492	3.359177	0.427517
21339	Amp	17	74695789	74706388	2.750393	0.392066
21340	Amp	17	74710490	74725013	2.866312	0.399091
21341	Amp	17	74725599	74731034	2.830877	0.396437
21342	Amp	17	74732122	74740709	2.796399	0.394795
21343	Amp	17	74746524	74769076	2.750393	0.391696
21344	Amp	17	74769592	74774192	2.806723	0.395042
21345	Amp	17	74776210	74894376	2.658709	0.386592
21346	Amp	17	74897033	74897033	2.970355	0.40506
21347	Amp	17	74897639	74909037	3.252252	0.42141
21348	Amp	17	74909375	74909560	2.935659	0.402941
21349	Amp	17	74909975	74985708	2.750393	0.392253
21350	Amp	17	74999282	75004738	3.496514	0.435742
21351	Amp	17	75009435	75020526	3.613533	0.442023
21352	Amp	17	75021866	75036245	3.26417	0.421895
21353	Amp	17	75041973	75106406	3.403911	0.430307
21354	Amp	17	75114084	75127320	3.230962	0.420247
21355	Amp	17	75141754	75141754	3.675606	0.445587
21356	Amp	17	75143862	75248953	3.613533	0.442009
21357	Amp	17	75256972	75256972	2.948071	0.403403
21358	Amp	17	75260266	75286555	2.843135	0.397133

21359	Amp	17	75292850	75314882	2.914035	0.401365
21360	Amp	17	75317704	75323706	2.483202	0.376026
21361	Amp	17	75329035	75341034	2.683697	0.387627
21362	Amp	17	75342629	75378864	2.658709	0.386241
21363	Amp	17	75379878	75432295	2.614747	0.38378
21364	Amp	17	75433634	75454128	2.88973	0.400435
21365	Amp	17	75454144	75470162	2.958746	0.404234
21366	Amp	17	75475238	75571454	3.45062	0.433107
21367	Amp	17	75572844	75585895	3.403911	0.43044
21368	Amp	17	75586707	75587851	3.523011	0.437346
21369	Amp	17	75590479	75626909	3.600858	0.441103
21370	Amp	17	75641916	75651788	3.371695	0.428593
21371	Amp	17	75654606	75797966	3.207076	0.418475
21372	Amp	17	75804098	75806953	3.312745	0.424669
21373	Amp	17	75815541	75833738	3.946237	0.459746
21374	Amp	17	75834108	75841090	3.312745	0.424669
21375	Amp	17	75846985	75846985	3.76435	0.450356
21376	Amp	17	75848329	75886726	3.828482	0.454085
21377	Amp	17	75903403	75903403	3.157622	0.415652
21378	Amp	17	75904067	75982472	3.079122	0.411487
21379	Amp	17	75984357	75986113	3.182419	0.417651
21380	Amp	17	75995916	75999246	2.914035	0.401377
21381	Amp	17	76000087	76024659	2.958746	0.404566
21382	Amp	17	76025223	76032299	2.902384	0.401195
21383	Amp	17	76033132	76053345	3.243024	0.42103
21384	Amp	17	76057534	76060860	3.276597	0.422733
21385	Amp	17	76061208	76156285	3.348874	0.427318
21386	Amp	17	76157565	76166916	3.230962	0.420407
21387	Amp	17	76167275	76182277	2.948071	0.403752
21388	Amp	17	76200640	76362462	2.878516	0.3998
21389	Amp	17	76372007	76438735	3.182419	0.417386
21390	Amp	17	76438793	76461367	4.090482	0.467313
21391	Amp	17	76466526	76470935	4.215025	0.473497
21392	Amp	17	76479963	76503459	3.288383	0.423571
21393	Amp	17	76504250	76513298	3.182419	0.417376
21394	Amp	17	76520461	76633855	3.383286	0.428981
21395	Amp	17	76634554	76655610	3.276597	0.422817
21396	Amp	17	76656192	76705635	3.424445	0.431503
21397	Amp	17	76705685	76739260	3.171522	0.416551
21398	Amp	17	76739295	76746723	3.041652	0.409066
21399	Amp	17	76747684	76755550	3.066577	0.410794
21400	Amp	17	76763737	76859736	3.32332	0.425888
21401	Amp	17	76867813	76867904	3.182419	0.417489
21402	Amp	17	76874881	76876823	3.335884	0.426363
21403	Amp	17	76880812	76913017	3.383286	0.428855
21404	Amp	17	76914310	76975872	2.970355	0.404956
21405	Amp	17	76979096	76979096	2.914035	0.40161
21406	Amp	17	76982798	76982798	2.855041	0.398113
21407	Amp	17	76985181	76992758	3.217724	0.419368
21408	Amp	17	76994917	77174591	3.72674	0.448091
21409	Amp	17	77176951	77179175	3.906866	0.45764
21410	Amp	17	77181129	77219404	3.933803	0.459585
21411	Amp	17	77221097	77251197	4.257976	0.475457

21412 Amp	17	77251581	77256513	3.933803	0.459585
21413 Amp	17	77263535	77287459	4.941808	0.509344
21414 Amp	17	77292989	77315254	4.391125	0.482757
21415 Amp	17	77318404	77375740	3.45062	0.432997
21416 Amp	17	77375892	77416252	3.415716	0.431269
21417 Amp	17	77424154	77449922	4.200241	0.473006
21418 Amp	17	77452076	77452076	4.022326	0.46432
21419 Amp	17	77567552	77581221	4.103115	0.467907
21420 Amp	17	77581295	77604896	4.30031	0.477857
21421 Amp	17	77605290	77644738	4.497567	0.488194
21422 Amp	17	77657739	77672830	5.077576	0.51575
21423 Amp	17	77672834	77674817	5.324711	0.527268
21424 Amp	17	77679362	77742853	5.208342	0.521864
21425 Amp	17	77745398	77785320	5.739571	0.546343
21426 Amp	17	77785437	77855384	5.324711	0.527519
21427 Amp	17	77857196	77994242	5.280955	0.525353
21428 Amp	17	78002155	78081752	5.56363	0.538495
21429 Amp	17	78081813	78090939	5.696665	0.544642
21430 Amp	17	78091097	78136611	5.56363	0.53845
21431 Amp	17	78151739	78155085	6.10345	0.563663
21432 Amp	17	78155305	78233930	6.319279	0.573538
21433 Amp	17	78234607	78241793	5.503852	0.535345
21434 Amp	17	78250213	78253601	5.681709	0.543692
21435 Amp	17	78257556	78272425	4.941808	0.509711
21436 Amp	17	78274006	78388706	5.252642	0.523775
21437 Amp	17	78390193	78412343	5.077576	0.515428
21438 Amp	17	78456702	78456702	4.971485	0.511042
21439 Amp	17	78459996	78508455	5.047749	0.514018
21440 Amp	17	78509342	78511630	4.594893	0.493192
21441 Amp	17	78511884	78539927	4.778542	0.50185
21442 Amp	17	78540130	78561861	4.897525	0.507619
21443 Amp	17	78562691	78563081	4.566654	0.491675
21444 Amp	17	78732558	78779591	5.092203	0.516303
21445 Amp	17	78782838	78788116	4.200241	0.473192
21446 Amp	17	78791019	78801415	4.049098	0.465618
21447 Amp	17	78807719	78821381	4.636424	0.495047
21448 Amp	17	78826086	78829640	3.815003	0.45331
21449 Amp	17	78830473	78872709	3.72674	0.448124
21450 Amp	17	78873593	78911363	3.777346	0.451348
21451 Amp	17	78915410	78998287	3.72674	0.448371
21452 Amp	17	78999123	79003355	3.893345	0.456984
21453 Amp	17	79006681	79073327	3.790167	0.451527
21454 Amp	17	79073976	79085068	4.313672	0.478213
21455 Amp	17	79088192	79119130	4.34102	0.480037
21456 Amp	17	79119305	79147378	4.171444	0.471424
21457 Amp	17	79154698	79162921	4.200241	0.473071
21458 Amp	17	79238441	79240493	4.158928	0.471247
21459 Amp	17	79241401	79241401	4.103115	0.468024
21460 Amp	17	79245328	79245328	3.43725	0.432801
21461 Amp	17	79245485	79254965	3.112016	0.413466
21462 Amp	17	79255108	79261041	3.675606	0.445371
21463 Amp	17	79261053	79261263	3.842634	0.454178
21464 Amp	17	79262073	79262073	3.880586	0.456734

21465	Amp	17	79266984	79291116	4.090482	0.467572
21466	Amp	17	79298895	79315075	4.313672	0.478352
21467	Amp	17	79315188	79365496	4.355429	0.480367
21468	Amp	17	79365779	79365779	4.200241	0.473192
21469	Amp	17	79406159	79408012	4.376421	0.481942
21470	Amp	17	79410326	79410326	4.497567	0.488401
21471	Amp	17	79411690	79415537	4.809024	0.503364
21472	Amp	17	79417772	79438402	4.986444	0.511577
21473	Amp	17	79440229	79470107	5.121758	0.517755
21474	Amp	17	79471697	79549025	6.08935	0.562948
21475	Amp	17	79656995	79656995	5.31131	0.526641
21476	Amp	17	79667705	79680848	6.696475	0.590204
21477	Amp	17	79683660	79732460	6.936308	0.601076
21478	Amp	17	79734466	79749785	7.614335	0.629955
21479	Amp	17	79754807	79792183	7.900934	0.64126
21480	Amp	17	79796051	79818238	8.574393	0.669078
21481	Amp	17	79818387	79826944	8.34915	0.659932
21482	Amp	17	79827637	79827667	8.080264	0.648628
21483	Amp	17	79827705	79846779	7.10077	0.607555
21484	Amp	17	79847864	79860398	5.982794	0.557955
21485	Amp	17	79863417	79868380	6.24213	0.569686
21486	Amp	17	79872870	79925135	6.028267	0.560276
21487	Amp	17	79935639	79935639	5.534438	0.536658
21488	Amp	17	79936215	79950088	5.608185	0.540026
21489	Amp	17	79950106	79965008	5.534438	0.536658
21490	Amp	17	79970506	80033814	4.751695	0.500331
21491	Amp	17	80045981	80087554	5.135526	0.518518
21492	Amp	17	80088079	80103199	5.222669	0.522552
21493	Amp	17	80103915	80116282	5.16411	0.52006
21494	Amp	17	80116524	80124691	4.854009	0.50515
21495	Amp	17	80124721	80129214	4.470731	0.487027
21496	Amp	17	80129554	80132197	4.376421	0.481947
21497	Amp	17	80135102	80156511	4.257976	0.475769
21498	Amp	17	80161337	80210594	3.998681	0.463022
21499	Amp	17	80211728	80227984	3.933803	0.459222
21500	Amp	17	80232700	80237933	4.8233	0.503659
21501	Amp	17	80238407	80245008	5.208342	0.521769
21502	Amp	17	80352467	80361246	4.708951	0.498484
21503	Amp	17	80361572	80371874	4.651516	0.495839
21504	Amp	17	80374832	80374832	3.828482	0.453865
21505	Amp	17	80375211	80393633	3.933803	0.459239
21506	Amp	17	80394251	80503330	3.625861	0.443107
21507	Amp	17	80506091	80506457	3.790167	0.451426
21508	Amp	17	80509760	80550292	3.842634	0.454402
21509	Amp	17	80550330	80553289	3.112016	0.413202
21510	Amp	17	80553674	80622528	2.970355	0.405295
21511	Amp	17	80641561	80641561	3.041652	0.408815
21512	Amp	17	80641736	80641736	2.948071	0.403441
21513	Amp	17	80697103	80747371	2.670502	0.387118
21514	Amp	17	80750185	80768654	2.530938	0.378714
21515	Amp	17	80769032	80880339	2.495444	0.37708
21516	Amp	17	80880423	80882434	2.737964	0.39099
21517	Amp	17	80885310	80899214	2.830877	0.396918

21518	Amp	17	80900130	80906957	2.091935	0.352278
21519	Amp	17	80908200	80916329	2.049537	0.349303
21520	Amp	17	80917720	80978587	2.082332	0.351414
21521	Amp	17	80989081	81176854	2.424855	0.372495
21522	Amp	17	81177709	81204932	2.294081	0.364491
21523	Amp	17	81207825	81291901	2.272343	0.363027
21524	Amp	17	81292757	81310502	2.192225	0.358455
21525	Amp	17	81320655	81321846	2.294081	0.36471
21526	Amp	17	81326387	81402211	2.260813	0.362155
21527	Amp	17	81415451	81421889	2.158849	0.356481
21528	Amp	17	81457300	81470706	2.203122	0.358738
21529	Amp	17	81485136	81508566	2.294081	0.364592
21530	Amp	17	81510690	81664386	2.338154	0.367182
21531	Amp	17	81666247	81718026	2.866312	0.398678
21532	Amp	17	81726106	81985599	3.276597	0.422708
21533	Amp	17	82018602	82035188	3.496514	0.436155
21534	Amp	17	82041501	82073964	3.383286	0.429252
21535	Amp	17	82100360	82109109	3.288383	0.423373
21536	Amp	17	82110558	82112457	3.424445	0.432017
21537	Amp	17	82113173	82121899	2.670502	0.387149
21538	Amp	17	82125680	82125680	2.855041	0.398023
21539	Amp	17	82256888	82256888	2.692246	0.388421
21540	Amp	17	82257710	82261192	2.774551	0.393588
21541	Amp	17	82261464	82382648	2.855041	0.39831
21542	Amp	17	82399121	82399121	2.750393	0.392055
21543	Amp	17	82403173	82403173	2.914035	0.401896
21544	Amp	17	82403670	82544467	2.948071	0.403369
21545	Amp	17	82546088	82576759	2.796399	0.394724
21546	Amp	17	82576823	82600814	2.737964	0.390994
21547	Amp	17	82601291	82611607	2.806723	0.395332
21548	Amp	17	82613184	82681363	2.683697	0.387624
21549	Amp	17	82681460	82688308	2.507231	0.377782
21550	Amp	17	82690977	82695842	2.727427	0.390527
21551	Amp	17	82695857	82700570	3.089932	0.411756
21552	Amp	17	82702812	82726217	2.658709	0.386312
21553	Amp	17	82726459	82745569	2.750393	0.391876
21554	Amp	17	82754905	82768384	2.774551	0.393613
21555	Amp	17	82782265	82816050	2.914035	0.401639
21556	Amp	17	82818167	82827769	2.843135	0.397407
21557	Amp	17	82828539	82946072	2.727427	0.390425
21558	Amp	17	82947423	82949377	2.647166	0.385604
21559	Amp	17	82949683	82959812	2.543219	0.379693
21560	Amp	18	326691	352057	0	0.023626
21561	Amp	18	352074	367725	0	0.021892
21562	Amp	18	891647	1038276	0	0.037995
21563	Amp	18	1039385	1082022	0	0.023618
21564	Amp	18	1082334	1327311	0	0.032203
21565	Amp	18	1328355	1358035	0	0.028632
21566	Amp	18	1359324	1438094	0	0.048392
21567	Amp	18	1438392	1482909	0	0.028632
21568	Amp	18	1483440	1584081	0	0.025207
21569	Amp	18	1585585	1595868	0	0.016621
21570	Amp	18	1600556	1600556	0	0.009176

21571	Amp	18	1600944	2108164	0	0.013249
21572	Amp	18	2108766	2162726	0	0.011524
21573	Amp	18	2163157	2211348	0	0.012954
21574	Amp	18	2211483	2330829	0	0.023692
21575	Amp	18	2336090	2383813	0	0.028978
21576	Amp	18	2399867	2478269	0	0.035039
21577	Amp	18	2480669	2806505	0	0.042012
21578	Amp	18	2809882	2872653	0	0.048509
21579	Amp	18	2872681	2922902	0	0.04066
21580	Amp	18	2923083	3015146	0	0.032485
21581	Amp	18	3015921	3368238	0	0.028178
21582	Amp	18	3377844	3485359	0	0.021205
21583	Amp	18	3490128	3540089	0	0.029642
21584	Amp	18	3548694	3725189	0	0.030377
21585	Amp	18	3725553	3824077	0	0.033752
21586	Amp	18	3824165	3825114	0	0.032322
21587	Amp	18	3825148	3830266	0	0.026756
21588	Amp	18	3830606	3991340	0	0.031545
21589	Amp	18	3991395	4106357	0	0.024095
21590	Amp	18	4110103	4211925	0	0.026245
21591	Amp	18	4217165	4350156	0	0.024095
21592	Amp	18	4354518	4603011	0	0.016812
21593	Amp	18	4608852	4631619	0	0.023698
21594	Amp	18	4632976	4661208	0	0.030742
21595	Amp	18	4664609	4686502	0	0.028854
21596	Amp	18	4688684	4849251	0	0.022793
21597	Amp	18	4853491	5013859	0	0.018004
21598	Amp	18	5014085	5039501	0	0.02287
21599	Amp	18	5042020	5121535	0	0.030415
21600	Amp	18	5121580	5211033	0	0.023529
21601	Amp	18	5214513	5751942	0	0.019455
21602	Amp	18	5755337	5922928	0	0.027632
21603	Amp	18	5923281	5958555	0	0.032295
21604	Amp	18	5958576	5959259	0	0.024118
21605	Amp	18	6602285	6653591	0	0.011707
21606	Amp	18	6657258	6691692	0	0.020094
21607	Amp	18	6692101	6936252	0	0.011707
21608	Amp	18	6936457	7427518	0	0.019063
21609	Amp	18	7431279	7456673	0	0.023262
21610	Amp	18	7460381	7555390	0	0.016218
21611	Amp	18	7556210	7774935	0	0.012019
21612	Amp	18	7777035	7902816	0	0.007355
21613	Amp	18	7905636	8070506	0	0.015677
21614	Amp	18	8077300	8331684	0	0.019892
21615	Amp	18	8332402	8519369	0	0.01157
21616	Amp	18	8524029	8629722	0	0.02637
21617	Amp	18	8633357	8800393	0	0.01157
21618	Amp	18	8800549	8964796	0	0.004215
21619	Amp	18	8968374	8995264	0	0.009651
21620	Amp	18	8998803	9191422	0	0.004215
21621	Amp	18	9192310	10115536	0	0.011968
21622	Amp	18	10119544	10371748	0	0.004215
21623	Amp	18	10373768	10402882	0	0.008619

21624	Amp	18	10403296	10403296	0	0.004404
21625	Amp	18	10404206	10504977	0	0.011622
21626	Amp	18	10509963	10611391	0	0.019089
21627	Amp	18	10611417	10691111	0	0.025111
21628	Amp	18	10695159	10752184	0	0.038248
21629	Amp	18	10752239	10811096	0	0.054026
21630	Amp	18	10811337	10826236	0	0.059265
21631	Amp	18	10830999	10974188	0	0.051798
21632	Amp	18	10974450	11030517	0	0.056363
21633	Amp	18	11032029	11054338	0	0.063193
21634	Amp	18	11063528	11070642	0	0.068207
21635	Amp	18	11071006	11165198	0	0.061377
21636	Amp	18	11170341	11211135	0	0.065301
21637	Amp	18	11213284	11271978	0	0.072402
21638	Amp	18	11272255	11279346	0	0.068479
21639	Amp	18	11281734	11297067	0	0.051144
21640	Amp	18	11307113	11312515	0	0.056735
21641	Amp	18	11312774	11318730	0	0.060679
21642	Amp	18	11318883	11466336	0	0.055088
21643	Amp	18	11468329	11502297	0	0.043508
21644	Amp	18	11503485	11561887	0	0.056834
21645	Amp	18	11565966	11594936	0	0.06958
21646	Amp	18	11598991	11607752	0	0.078494
21647	Amp	18	11648760	11673157	0	0.072473
21648	Amp	18	11678947	11749613	0	0.083591
21649	Amp	18	11753181	11893318	0	0.091501
21650	Amp	18	11893392	12077566	0	0.081697
21651	Amp	18	12078843	12189118	0	0.073787
21652	Amp	18	12192232	12493713	0	0.069383
21653	Amp	18	12499292	12537715	0	0.072036
21654	Amp	18	12542711	12549851	0	0.073831
21655	Amp	18	12558135	12630449	0	0.067226
21656	Amp	18	12639896	12714218	0	0.060124
21657	Amp	18	12728128	12741060	0	0.051209
21658	Amp	18	12741371	12752250	0	0.057293
21659	Amp	18	12761429	12779948	0	0.087852
21660	Amp	18	12782762	12853821	0	0.094208
21661	Amp	18	12857272	12859072	0	0.086057
21662	Amp	18	12863143	12882062	0	0.089354
21663	Amp	18	12889218	12928542	0	0.106341
21664	Amp	18	12933252	12960487	0	0.090047
21665	Amp	18	12963182	12998356	0	0.097426
21666	Amp	18	13002643	13015247	0	0.085203
21667	Amp	18	13016479	13091464	0	0.081259
21668	Amp	18	13093198	13097534	0	0.085495
21669	Amp	18	13109258	13207551	0	0.088334
21670	Amp	18	13211223	13314873	0	0.095501
21671	Amp	18	13316254	13340015	0	0.097005
21672	Amp	18	13340113	13432524	0	0.091991
21673	Amp	18	13436441	13669419	0	0.099911
21674	Amp	18	13671936	13689282	0	0.082924
21675	Amp	18	13697294	13703458	0	0.075706
21676	Amp	18	13704829	14060447	0	0.06854

21677	Amp	18	14062285	14407180	0	0.057864
21678	Amp	18	14461445	14481714	0	0.050404
21679	Amp	18	14482921	14632450	0	0.056565
21680	Amp	18	14641845	14642857	0	0.043238
21681	Amp	18	14648458	14648458	0	0.036433
21682	Amp	18	14653029	14660483	0	0.032197
21683	Amp	18	14661644	14661644	0	0.024277
21684	Amp	18	21109102	21109102	0	0.048492
21685	Amp	18	21109786	21114061	0	0.063064
21686	Amp	18	21117668	21150874	0	0.06041
21687	Amp	18	21151436	21161899	0	0.055291
21688	Amp	18	21164577	21335966	0	0.040719
21689	Amp	18	21336430	21395941	0	0.03788
21690	Amp	18	21398621	21424183	0	0.036376
21691	Amp	18	21427218	21478581	0	0.050922
21692	Amp	18	21479664	21574444	0	0.04587
21693	Amp	18	21576384	21869405	0	0.031324
21694	Amp	18	21875043	21948482	0	0.033239
21695	Amp	18	21955170	22443672	0	0.023556
21696	Amp	18	22450154	22473405	0	0.039609
21697	Amp	18	22488786	22642887	0	0.023556
21698	Amp	18	22643022	22674022	0	0.030336
21699	Amp	18	22674073	22684025	0	0.037264
21700	Amp	18	22684180	22726604	0	0.031979
21701	Amp	18	22726801	22800381	0	0.039239
21702	Amp	18	22802390	22983169	0	0.054277
21703	Amp	18	22986150	23078281	0	0.059328
21704	Amp	18	23082318	23100328	0	0.061736
21705	Amp	18	23101243	23120835	0	0.052982
21706	Amp	18	23124039	23126329	0	0.037944
21707	Amp	18	23129159	23138761	0	0.032893
21708	Amp	18	23138854	23308991	0	0.036022
21709	Amp	18	23310754	23380509	0	0.027179
21710	Amp	18	23384084	23625602	0	0.021018
21711	Amp	18	23628944	23666778	0	0.027653
21712	Amp	18	23670108	23910070	0	0.030908
21713	Amp	18	23911647	23915519	0	0.027653
21714	Amp	18	23917300	24076781	0	0.025245
21715	Amp	18	24077410	24185111	0	0.039518
21716	Amp	18	24186147	24343671	0	0.025245
21717	Amp	18	24344694	24362608	0	0.034601
21718	Amp	18	24363017	24390125	0	0.025245
21719	Amp	18	24392993	24425948	0	0.04008
21720	Amp	18	24430260	24445184	0	0.025245
21721	Amp	18	24446957	24808621	0	0.022115
21722	Amp	18	24809950	24938114	0	0.039374
21723	Amp	18	24940035	25035938	0	0.034685
21724	Amp	18	25036426	25131527	0	0.038381
21725	Amp	18	25133597	25459513	0	0.04276
21726	Amp	18	25460632	25601821	0	0.025502
21727	Amp	18	25603551	25619187	0	0.04282
21728	Amp	18	25621355	25641741	0	0.059238
21729	Amp	18	25643715	25697689	0	0.061612

21730	Amp	18	25698219	25802817	0	0.059238
21731	Amp	18	25803020	25896484	0	0.065475
21732	Amp	18	25900144	25952647	0	0.074634
21733	Amp	18	25954801	25977719	0	0.115568
21734	Amp	18	25980457	26070118	0	0.137206
21735	Amp	18	26085719	26284388	0	0.115568
21736	Amp	18	26293742	26330217	0	0.168056
21737	Amp	18	26330691	26576461	0	0.161819
21738	Amp	18	26578560	26703856	0	0.166956
21739	Amp	18	26706198	26737873	0	0.116864
21740	Amp	18	26739390	26830973	0	0.118747
21741	Amp	18	26831201	26836092	0	0.149218
21742	Amp	18	26839174	27583744	0	0.169072
21743	Amp	18	27583826	27706503	0	0.16516
21744	Amp	18	27706541	27771839	0	0.170317
21745	Amp	18	27772684	27949291	0	0.168435
21746	Amp	18	27952020	27956217	0	0.164055
21747	Amp	18	27965642	27965642	0	0.119162
21748	Amp	18	27967096	27991502	0	0.115465
21749	Amp	18	27998279	28061773	0	0.110034
21750	Amp	18	28063433	28114945	0	0.126017
21751	Amp	18	28117548	28334868	0	0.110034
21752	Amp	18	28335176	28450494	0	0.118174
21753	Amp	18	28455117	28616887	0	0.107384
21754	Amp	18	28621763	28666293	0	0.082856
21755	Amp	18	28666923	28837976	0	0.088837
21756	Amp	18	28838120	28977127	0	0.093119
21757	Amp	18	28980883	29548115	0	0.087961
21758	Amp	18	29550314	29683653	0	0.079821
21759	Amp	18	29685057	29798689	0	0.070727
21760	Amp	18	29799424	29957054	0	0.087223
21761	Amp	18	29960583	30325355	0	0.070727
21762	Amp	18	30327990	30427388	0	0.075252
21763	Amp	18	30427389	30496733	0	0.081277
21764	Amp	18	30506568	30508848	0	0.115293
21765	Amp	18	30511758	30554356	0	0.108658
21766	Amp	18	30555268	30800954	0	0.118564
21767	Amp	18	30801779	30902790	0	0.077242
21768	Amp	18	30903877	30966418	0	0.07126
21769	Amp	18	30966851	31155716	0	0.073872
21770	Amp	18	31160751	31176416	0	0.087621
21771	Amp	18	31177325	31303544	0	0.081596
21772	Amp	18	31303658	31347544	0	0.086396
21773	Amp	18	31347917	31384671	0	0.083784
21774	Amp	18	31384848	31472670	0	0.068413
21775	Amp	18	31473041	31506085	0	0.05542
21776	Amp	18	31506217	31544160	0	0.072948
21777	Amp	18	31551285	31662329	0	0.074957
21778	Amp	18	31663900	32002027	0	0.07317
21779	Amp	18	32003793	32058699	0	0.080525
21780	Amp	18	32069992	32099712	0	0.089916
21781	Amp	18	32099959	32213249	0	0.080525
21782	Amp	18	32213432	32392986	0	0.075725

21783	Amp	18	32393768	32447649	0	0.078299
21784	Amp	18	32448125	32488036	0	0.07323
21785	Amp	18	32491069	32525445	0	0.083455
21786	Amp	18	32526407	32546414	0	0.07323
21787	Amp	18	32546503	32706525	0	0.060724
21788	Amp	18	32710922	32774234	0	0.065778
21789	Amp	18	32774336	33026657	0	0.067176
21790	Amp	18	33034222	33063779	0	0.02276
21791	Amp	18	33064190	33181211	0	0.017705
21792	Amp	18	33182332	33187388	0	0.022441
21793	Amp	18	33189507	33275907	0	0.029145
21794	Amp	18	33276014	33283871	0	0.022441
21795	Amp	18	33284005	33308177	0	0.017705
21796	Amp	18	33310869	33327137	0	0.023462
21797	Amp	18	33333991	33519352	0	0.02056
21798	Amp	18	33526526	33974217	0	0.024962
21799	Amp	18	33977149	34806161	0	0.013156
21800	Amp	18	34806628	34867736	0	0.028091
21801	Amp	18	34872884	35035692	0	0.032919
21802	Amp	18	35037353	35059795	0	0.031521
21803	Amp	18	35060841	35422195	0	0.016586
21804	Amp	18	35425701	35770275	0	0.00923
21805	Amp	18	35770953	35971248	0	0.004827
21806	Amp	18	35977503	36115585	0	0.036192
21807	Amp	18	36118973	36134730	0	0.044013
21808	Amp	18	36138252	36351007	0	0.007822
21809	Amp	18	36357113	36831555	0	0
21810	Amp	18	36831639	37148426	0	0.003648
21811	Amp	18	37152949	37967718	0	0
21812	Amp	18	37968726	39323659	0	0.001655
21813	Amp	18	39327678	39845699	0	0.007511
21814	Amp	18	39845935	40877745	0	0.00912
21815	Amp	18	40878034	41024876	0	0.006366
21816	Amp	18	41031090	41273938	0	0.004757
21817	Amp	18	41274333	41658252	0	0.009672
21818	Amp	18	41660531	41770277	0	0.001655
21819	Amp	18	41770328	42133952	0	0.01669
21820	Amp	18	42138648	43582645	0	0.029093
21821	Amp	18	43594980	44343414	0	0.034212
21822	Amp	18	44344072	44461011	0	0.039842
21823	Amp	18	44461098	44572502	0	0.034212
21824	Amp	18	44576635	44774210	0	0.041186
21825	Amp	18	44774343	44801513	0	0.039532
21826	Amp	18	44801819	44936341	0	0.027128
21827	Amp	18	44939497	45105797	0	0.032645
21828	Amp	18	45106357	45192606	0	0.027541
21829	Amp	18	45195256	45485606	0	0.025671
21830	Amp	18	45488110	45715129	0	0.033348
21831	Amp	18	45715183	45807168	0	0.037203
21832	Amp	18	45813629	46317343	0	0.029526
21833	Amp	18	46319799	46804550	0	0.037127
21834	Amp	18	46807778	47147723	0	0.022092
21835	Amp	18	47149450	47309562	0	0.016973

21836	Amp	18	47309919	47348029	0	0.033134
21837	Amp	18	47354392	47436490	0	0.016973
21838	Amp	18	47438809	47519334	0	0.018725
21839	Amp	18	47520119	47649031	0	0.013208
21840	Amp	18	47651137	47857956	0	0.011456
21841	Amp	18	47858030	47905410	0	0.027417
21842	Amp	18	47906459	47908026	0	0.015431
21843	Amp	18	47911449	48036727	0	0.026849
21844	Amp	18	48038591	48085096	0	0.030077
21845	Amp	18	48086054	48090557	0	0.007083
21846	Amp	18	48091638	48151376	0	0.01036
21847	Amp	18	48156261	48275769	0	0.007083
21848	Amp	18	48277870	48369200	0	0.015857
21849	Amp	18	48371168	48372228	0	0.014332
21850	Amp	18	48372876	48835070	0	0.035393
21851	Amp	18	48835373	48938250	0	0.040313
21852	Amp	18	48955771	49013586	0	0.056741
21853	Amp	18	49019851	49130417	0	0.051822
21854	Amp	18	49135395	49135395	0	0.056802
21855	Amp	18	49141510	49325745	0	0.040374
21856	Amp	18	49326085	49626164	0	0.037146
21857	Amp	18	49628842	49642949	0	0.054882
21858	Amp	18	49643314	49702882	0	0.072297
21859	Amp	18	49708720	49875629	0	0.05456
21860	Amp	18	49875815	50010452	0	0.016084
21861	Amp	18	50016539	50069592	0	0.008835
21862	Amp	18	50074425	50351950	0	0.016229
21863	Amp	18	50356385	50369088	0	0.043468
21864	Amp	18	50371616	51511741	0	0.036074
21865	Amp	18	51512008	51522082	0	0.037767
21866	Amp	18	51526320	51595097	0	0.045224
21867	Amp	18	51595652	52224949	0	0.040244
21868	Amp	18	52225362	52793233	0	0.047381
21869	Amp	18	52793850	52835193	0	0.051609
21870	Amp	18	52837197	52893123	0	0.04493
21871	Amp	18	52893186	52898127	0	0.049666
21872	Amp	18	52898652	52925027	0	0.042529
21873	Amp	18	52927448	52991406	0	0.059079
21874	Amp	18	52996203	54321319	0	0.039975
21875	Amp	18	54321906	54420350	0	0.045284
21876	Amp	18	54420495	54438168	0	0.066079
21877	Amp	18	54445023	54724343	0	0.045284
21878	Amp	18	54726513	54944905	0	0.039975
21879	Amp	18	54949333	55325865	0	0.032787
21880	Amp	18	55326244	55396528	0	0.039718
21881	Amp	18	55398111	55631009	0	0.032787
21882	Amp	18	55633183	55660432	0	0.038835
21883	Amp	18	55662373	55691468	0	0.047155
21884	Amp	18	55699355	56047635	0	0.038835
21885	Amp	18	56048834	56474560	0	0.046728
21886	Amp	18	56476711	56795357	0	0.038835
21887	Amp	18	56797229	56959544	0	0.021536
21888	Amp	18	56959952	57032468	0	0.019843

21889	Amp	18	57034536	57222283	0	0.044506
21890	Amp	18	57222577	57792096	0	0.049693
21891	Amp	18	57794631	57848301	0	0.055181
21892	Amp	18	57851872	57899309	0	0.050553
21893	Amp	18	57900427	57907876	0	0.030518
21894	Amp	18	57909746	57937887	0	0.038629
21895	Amp	18	57940626	57942075	0	0.042474
21896	Amp	18	57945371	57951409	0	0.050134
21897	Amp	18	57952646	58008928	0	0.05194
21898	Amp	18	58013225	58038482	0	0.069401
21899	Amp	18	58040147	58169265	0	0.05194
21900	Amp	18	58171726	58299003	0	0.060371
21901	Amp	18	58300853	58336932	0	0.052711
21902	Amp	18	58337393	58444395	0	0.050905
21903	Amp	18	58449494	58474803	0	0.071389
21904	Amp	18	58475080	58500114	0	0.077926
21905	Amp	18	58503397	58549733	0	0.080756
21906	Amp	18	58552358	58612315	0	0.075569
21907	Amp	18	58617819	58658037	0	0.089016
21908	Amp	18	58658933	58718592	0	0.09815
21909	Amp	18	58762812	58909286	0	0.107444
21910	Amp	18	58909903	59252169	0	0.12182
21911	Amp	18	59253326	59606755	0	0.110328
21912	Amp	18	59607281	59718725	0	0.114945
21913	Amp	18	59719071	59801789	0	0.109457
21914	Amp	18	59805760	59851878	0	0.114192
21915	Amp	18	59854230	59934231	0	0.099816
21916	Amp	18	59938005	60019941	0	0.098254
21917	Amp	18	60020619	60120808	0	0.101448
21918	Amp	18	60121618	60202919	0	0.105062
21919	Amp	18	60202946	61195470	0	0.100445
21920	Amp	18	61198254	61587166	0	0.109998
21921	Amp	18	61589211	61646864	0	0.115705
21922	Amp	18	61652049	61699539	0	0.122095
21923	Amp	18	61700438	61809897	0	0.118901
21924	Amp	18	61842053	61876197	0	0.069037
21925	Amp	18	61876274	62068760	0	0.073953
21926	Amp	18	62070081	62167136	0	0.078513
21927	Amp	18	62168775	62309712	0	0.082873
21928	Amp	18	62310157	62553545	0	0.099444
21929	Amp	18	62553756	62560197	0	0.096615
21930	Amp	18	62565540	62649177	0	0.147138
21931	Amp	18	62661036	62706594	0	0.130567
21932	Amp	18	62707350	62874231	0	0.113505
21933	Amp	18	62878780	63038955	0	0.116567
21934	Amp	18	63039970	63085922	0	0.118306
21935	Amp	18	63091086	63110032	0	0.115244
21936	Amp	18	63110573	63147682	0	0.110884
21937	Amp	18	63151728	63326282	0	0.120689
21938	Amp	18	63326412	63438413	0	0.11895
21939	Amp	18	63440957	63546085	0	0.110979
21940	Amp	18	63551914	63605655	0	0.128631
21941	Amp	18	63608960	63671944	0	0.110979

21942	Amp	18	63672438	63887188	0	0.117874
21943	Amp	18	63887380	63923851	0	0.132324
21944	Amp	18	63928275	64028109	0	0.117874
21945	Amp	18	64031057	64108773	0	0.120986
21946	Amp	18	64117720	64210925	0	0.117874
21947	Amp	18	64214388	64624169	0	0.129536
21948	Amp	18	64625377	64646271	0	0.124621
21949	Amp	18	64646570	64740902	0	0.075174
21950	Amp	18	64751208	65471686	0	0.070614
21951	Amp	18	65480316	65532281	0	0.065879
21952	Amp	18	65532329	65532809	0	0.058819
21953	Amp	18	66065592	66735841	0	0.045681
21954	Amp	18	66743604	66964097	0	0.064737
21955	Amp	18	66969375	67010720	0	0.058348
21956	Amp	18	67014755	67149023	0	0.063717
21957	Amp	18	67152832	67211915	0	0.058348
21958	Amp	18	67212818	67453768	0	0.070851
21959	Amp	18	67461686	67619757	0	0.076114
21960	Amp	18	67620212	67695974	0	0.062965
21961	Amp	18	67697799	67790664	0	0.057702
21962	Amp	18	67795608	67865952	0	0.062514
21963	Amp	18	67869215	67938472	0	0.056607
21964	Amp	18	68118231	68171504	0	0.051795
21965	Amp	18	68177478	68346818	0	0.034688
21966	Amp	18	68563349	68739510	0	0.043724
21967	Amp	18	68741190	68846995	0	0.048475
21968	Amp	18	68848636	68939976	0	0.061386
21969	Amp	18	68940341	68984729	0	0.048475
21970	Amp	18	68986237	69070193	0	0.038572
21971	Amp	18	69070359	69071897	0	0.034727
21972	Amp	18	69100687	69717427	0	0.040613
21973	Amp	18	69721718	69726111	0	0.047321
21974	Amp	18	69726462	69733311	0	0.042569
21975	Amp	18	69734893	69751659	0	0.047313
21976	Amp	18	69755995	69884770	0	0.040605
21977	Amp	18	69887248	69965679	0	0.084199
21978	Amp	18	69967327	70186712	0	0.080169
21979	Amp	18	70191234	70247534	0	0.085335
21980	Amp	18	70248064	70303309	0	0.086967
21981	Amp	18	70305843	70379644	0	0.094825
21982	Amp	18	70462786	70568966	0	0.101413
21983	Amp	18	70571499	70631862	0	0.096248
21984	Amp	18	70635227	70849465	0	0.059378
21985	Amp	18	70850038	70909122	0	0.054634
21986	Amp	18	70918549	71200022	0	0.091504
21987	Amp	18	71203777	71218138	0	0.081699
21988	Amp	18	71218990	71350482	0	0.094438
21989	Amp	18	71352086	71382253	0	0.095873
21990	Amp	18	73699619	74022039	0	0.038363
21991	Amp	18	74022997	74030583	0	0.033173
21992	Amp	18	74036122	74223917	0	0.037983
21993	Amp	18	74224215	74344918	0	0.033173
21994	Amp	18	74346540	74582245	0	0.048711

21995 Amp	18	74586502	74599818	0	0.055562
21996 Amp	18	74599934	74631582	0	0.040025
21997 Amp	18	74632267	74672902	0	0.043842
21998 Amp	18	74675179	74716892	0	0.05244
21999 Amp	18	74723770	74741850	0	0.043842
22000 Amp	18	74745477	75138633	0	0.040685
22001 Amp	18	75153610	75178019	0	0.044516
22002 Amp	18	75182595	75264894	0	0.040685
22003 Amp	18	75268227	75442093	0	0.035274
22004 Amp	18	75443125	75733006	0	0.031579
22005 Amp	18	75733027	76065735	0	0.027762
22006 Amp	18	76065917	76249175	0	0.030451
22007 Amp	18	76249545	76312673	0	0.0358
22008 Amp	18	76323157	76515915	0	0.027941
22009 Amp	18	76521109	76972061	0	0.035021
22010 Amp	18	76972255	76989373	0	0.052234
22011 Amp	18	76989686	77023590	0	0.057255
22012 Amp	18	77024629	77031590	0	0.095435
22013 Amp	18	77032201	77234506	0	0.078222
22014 Amp	18	77234704	77285306	0	0.060238
22015 Amp	18	77295805	78105792	0	0.055217
22016 Amp	18	78106407	78274414	0	0.057122
22017 Amp	18	78274527	78307494	0	0.072862
22018 Amp	18	78313538	78365152	0	0.057122
22019 Amp	18	78365807	78920709	0	0.051773
22020 Amp	18	78921269	78996673	0	0.05543
22021 Amp	18	78998599	79057231	0	0.070173
22022 Amp	18	79057246	79127893	0	0.05543
22023 Amp	18	79131372	79349796	0	0.051773
22024 Amp	19	283868	335055	0	0.065282
22025 Amp	19	335323	634032	0	0.040001
22026 Amp	19	634095	715430	0	0.060449
22027 Amp	19	750173	756844	0	0.047146
22028 Amp	19	761789	807592	0	0.045218
22029 Amp	19	826420	827975	0	0.035167
22030 Amp	19	865374	865374	0	0.014719
22031 Amp	19	939673	939777	0	0.02387
22032 Amp	19	967584	988136	0	0.042654
22033 Amp	19	988246	1135486	0	0.033503
22034 Amp	19	1156398	1210939	0	0.029518
22035 Amp	19	1228278	1439043	0	0.010734
22036 Amp	19	1441984	1525886	0	0.013425
22037 Amp	19	1530034	1613848	0	0.019551
22038 Amp	19	1626156	1626156	0	0.02123
22039 Amp	19	1636590	1939741	0	0.018538
22040 Amp	19	1943800	2027583	0	0.029913
22041 Amp	19	2036591	2048599	0	0.028235
22042 Amp	19	2053995	2166396	0	0.016859
22043 Amp	19	2166995	2284285	0	0.02744
22044 Amp	19	2299390	2354789	0	0.021568
22045 Amp	19	2376903	2403566	0	0.018076
22046 Amp	19	2405118	2473199	0	0.019767
22047 Amp	19	2473583	2499529	0	0.015058

22048	Amp	19	2503630	2536863	0	0.025795
22049	Amp	19	2538168	2538168	0	0.027654
22050	Amp	19	2538670	2582156	0	0.031118
22051	Amp	19	2582552	2592252	0	0.034825
22052	Amp	19	2646942	2663851	0	0.024088
22053	Amp	19	2668613	2748357	0	0.028842
22054	Amp	19	2754550	2832569	0	0.037563
22055	Amp	19	2833433	2836770	0	0.050714
22056	Amp	19	2836847	2852522	0	0.055112
22057	Amp	19	2853568	2888916	0	0.041961
22058	Amp	19	2891099	3210140	0	0.050081
22059	Amp	19	3214116	3214116	0	0.053799
22060	Amp	19	3227104	3321712	0	0.080471
22061	Amp	19	3327945	3364414	0	0.075937
22062	Amp	19	3365641	3405428	0	0.101105
22063	Amp	19	3411648	3467020	0	0.108563
22064	Amp	19	3481580	3499309	0	0.083395
22065	Amp	19	3501286	3501286	0	0.079677
22066	Amp	19	3501756	3503008	0	0.076107
22067	Amp	19	3509794	3599136	0	0.07171
22068	Amp	19	3605541	3705112	0	0.07464
22069	Amp	19	3705662	3750692	0	0.072782
22070	Amp	19	3750871	3864709	0	0.065324
22071	Amp	19	3877751	3886676	0	0.053965
22072	Amp	19	3895678	3942434	0	0.075184
22073	Amp	19	3942973	3987802	0	0.069058
22074	Amp	19	3993130	4063069	0	0.070977
22075	Amp	19	4073730	4106786	0	0.07727
22076	Amp	19	4113314	4299133	0	0.075579
22077	Amp	19	4300624	4406163	0	0.070825
22078	Amp	19	4673369	4673369	0	0.062003
22079	Amp	19	4673502	4809242	0	0.075406
22080	Amp	19	4821587	4853291	0	0.09107
22081	Amp	19	4853488	4866427	0	0.087196
22082	Amp	19	4870918	4895650	0	0.096041
22083	Amp	19	4908081	5016608	0	0.107387
22084	Amp	19	5025379	5098580	0	0.112351
22085	Amp	19	5105397	5155297	0	0.128246
22086	Amp	19	5155614	5170329	0	0.121162
22087	Amp	19	5171097	5189956	0	0.108298
22088	Amp	19	5193985	5375077	0	0.106071
22089	Amp	19	5376374	5377740	0	0.094425
22090	Amp	19	5377807	5433855	0	0.083254
22091	Amp	19	5434638	5566221	0	0.085421
22092	Amp	19	5570172	5581553	0	0.069525
22093	Amp	19	5582016	5633227	0	0.089868
22094	Amp	19	5633867	5638043	0	0.086858
22095	Amp	19	5641443	5831096	0	0.078738
22096	Amp	19	5870270	5882858	0	0.08413
22097	Amp	19	5884662	5885354	0	0.085681
22098	Amp	19	5893602	5893602	0	0.065338
22099	Amp	19	5914614	6017302	0	0.090682
22100	Amp	19	6017900	6299337	0	0.088516

22101	Amp	19	6305990	6382140	0	0.075251
22102	Amp	19	6382901	6399426	0	0.065013
22103	Amp	19	6399907	6499394	0	0.05161
22104	Amp	19	6499625	6691620	0	0.048324
22105	Amp	19	6699731	6720290	0	0.037882
22106	Amp	19	6722011	6811634	0	0.041452
22107	Amp	19	6818803	6832090	0	0.051702
22108	Amp	19	6833766	6854435	0	0.079328
22109	Amp	19	6869670	6885312	0	0.067528
22110	Amp	19	7112112	7187617	0	0.186853
22111	Amp	19	7188968	7229666	0	0.18159
22112	Amp	19	7230427	7333344	0	0.18581
22113	Amp	19	7339450	7453398	0	0.173657
22114	Amp	19	7455331	7455331	0	0.185721
22115	Amp	19	7462544	7510032	0	0.150314
22116	Amp	19	7510522	7546066	0	0.168077
22117	Amp	19	7549791	7559505	0	0.153174
22118	Amp	19	7588332	7592618	0	0.125547
22119	Amp	19	7597408	7602987	0	0.113483
22120	Amp	19	7616626	7709539	0	0.116836
22121	Amp	19	7716549	7786139	0	0.111443
22122	Amp	19	7787451	7826806	0	0.06092
22123	Amp	19	7833709	8109008	0	0.057568
22124	Amp	19	8109324	8144700	0	0.076753
22125	Amp	19	8150736	8222975	0	0.079498
22126	Amp	19	8223251	8250759	0	0.064546
22127	Amp	19	8251426	8314410	0	0.0618
22128	Amp	19	8315317	8394076	0	0.087919
22129	Amp	19	8395650	8433976	0	0.063557
22130	Amp	19	8440476	8512984	0	0.060651
22131	Amp	19	8515718	8520034	0	0.074142
22132	Amp	19	8522898	8662168	0	0.070572
22133	Amp	19	8662244	8668521	0	0.072811
22134	Amp	19	8668610	8720922	0	0.079395
22135	Amp	19	8723736	8768031	0	0.082583
22136	Amp	19	8795410	8854027	0	0.084677
22137	Amp	19	8854042	8903030	0	0.061246
22138	Amp	19	8907777	8924180	0	0.059152
22139	Amp	19	8924638	8924638	0	0.065503
22140	Amp	19	8930143	8950555	0	0.075914
22141	Amp	19	8953002	9022722	0	0.074157
22142	Amp	19	9024139	9068723	0	0.071227
22143	Amp	19	9073930	9107275	0	0.060616
22144	Amp	19	9107378	9213029	0	0.070007
22145	Amp	19	9217032	9253614	0	0.080469
22146	Amp	19	9255816	9270302	0	0.064739
22147	Amp	19	9271573	9443514	0	0.07047
22148	Amp	19	9452604	9527369	0	0.063061
22149	Amp	19	9528829	9539095	0	0.054843
22150	Amp	19	9552462	9574461	0	0.071438
22151	Amp	19	9575143	9586287	0	0.054843
22152	Amp	19	9600910	9681224	0	0.04438
22153	Amp	19	9682675	9781435	0	0.055783

22154 Amp	19	9785093	9809665	0	0.04438
22155 Amp	19	9813324	9830545	0	0.037458
22156 Amp	19	9836241	9921069	0	0.04419
22157 Amp	19	9923070	9942635	0	0.035138
22158 Amp	19	9945333	9948843	0	0.041289
22159 Amp	19	9950627	10005400	0	0.051258
22160 Amp	19	10014130	10046222	0	0.054239
22161 Amp	19	10046528	10055699	0	0.06144
22162 Amp	19	10064792	10102310	0	0.080483
22163 Amp	19	10103147	10301842	0	0.075317
22164 Amp	19	10303362	10364976	0	0.079563
22165 Amp	19	10371675	10371675	0	0.06052
22166 Amp	19	10373876	10387105	0	0.072974
22167 Amp	19	10390969	10422127	0	0.06052
22168 Amp	19	10428607	10461930	0	0.054369
22169 Amp	19	10464035	10507986	0	0.047167
22170 Amp	19	10509520	10531054	0	0.052845
22171 Amp	19	10534819	10545766	0	0.049923
22172 Amp	19	10546128	10598160	0	0.055267
22173 Amp	19	10606674	10631494	0	0.060863
22174 Amp	19	10632616	10754166	0	0.048139
22175 Amp	19	10754927	10904261	0	0.041406
22176 Amp	19	10904326	10954733	0	0.036062
22177 Amp	19	10963057	11043453	0	0.030331
22178 Amp	19	11047358	11079146	0	0.03931
22179 Amp	19	11084147	11168579	0	0.047083
22180 Amp	19	11174863	11372549	0	0.056578
22181 Amp	19	11374206	11415407	0	0.072009
22182 Amp	19	11415721	11467869	0	0.056578
22183 Amp	19	11469634	11679697	0	0.060813
22184 Amp	19	11680921	11850377	0	0.051834
22185 Amp	19	11890245	11959189	0	0.045483
22186 Amp	19	11959770	11987887	0	0.049932
22187 Amp	19	11993954	12015789	0	0.067043
22188 Amp	19	12016389	12037347	0	0.090292
22189 Amp	19	12041177	12152809	0	0.067043
22190 Amp	19	12160284	12162014	0	0.064062
22191 Amp	19	12170246	12388078	0	0.046951
22192 Amp	19	12389106	12519514	0	0.041355
22193 Amp	19	12529097	12557003	0	0.049387
22194 Amp	19	12557409	12579493	0	0.047835
22195 Amp	19	12593981	12735346	0	0.039322
22196 Amp	19	12735709	12798558	0	0.046173
22197 Amp	19	12803471	12831266	0	0.061196
22198 Amp	19	12831936	12842597	0	0.067458
22199 Amp	19	12843050	12857022	0	0.07225
22200 Amp	19	12857822	12909071	0	0.06372
22201 Amp	19	12913884	12942516	0	0.067439
22202 Amp	19	12948155	13020577	0	0.063193
22203 Amp	19	13033158	13090993	0	0.073904
22204 Amp	19	13107504	13181876	0	0.075512
22205 Amp	19	13182293	13266195	0	0.07072
22206 Amp	19	13272091	13284912	0	0.079713

22207	Amp	19	13285861	13343433	0	0.088209
22208	Amp	19	13346981	13387506	0	0.077499
22209	Amp	19	13388161	13415237	0	0.082702
22210	Amp	19	13421690	13434562	0	0.099501
22211	Amp	19	13441859	13465843	0	0.111821
22212	Amp	19	13470555	13497157	0	0.127836
22213	Amp	19	13497780	13503193	0	0.139337
22214	Amp	19	13511519	13533905	0	0.122538
22215	Amp	19	13535043	13554372	0	0.1174
22216	Amp	19	13554721	13684509	0	0.105899
22217	Amp	19	13685596	13724180	0	0.099607
22218	Amp	19	13725819	13772285	0	0.089862
22219	Amp	19	13777533	13807167	0	0.08087
22220	Amp	19	13809393	13809393	0	0.08989
22221	Amp	19	13814596	13969689	0	0.079549
22222	Amp	19	13972591	14051622	0	0.074346
22223	Amp	19	14055856	14124713	0	0.085075
22224	Amp	19	14124828	14273924	0	0.092318
22225	Amp	19	14274123	14275249	0	0.087868
22226	Amp	19	14276073	14398750	0	0.091169
22227	Amp	19	14402969	14447009	0	0.093272
22228	Amp	19	14454236	14583587	0	0.083927
22229	Amp	19	14584541	14588557	0	0.067912
22230	Amp	19	14590257	14680211	0	0.078102
22231	Amp	19	14680457	14688689	0	0.08897
22232	Amp	19	14691292	14732896	0	0.09307
22233	Amp	19	14735488	14825425	0	0.089351
22234	Amp	19	14827899	14830165	0	0.07916
22235	Amp	19	14832726	14836843	0	0.083497
22236	Amp	19	14837257	14844217	0	0.09475
22237	Amp	19	14845796	14867472	0	0.08547
22238	Amp	19	14870555	14873323	0	0.076449
22239	Amp	19	14874993	14997117	0	0.092039
22240	Amp	19	14997851	15095560	0	0.113003
22241	Amp	19	15095850	15114945	0	0.115817
22242	Amp	19	15115261	15266792	0	0.120244
22243	Amp	19	15269438	15301077	0	0.11743
22244	Amp	19	15302409	15446489	0	0.113094
22245	Amp	19	15453905	15565939	0	0.102226
22246	Amp	19	15590267	15630615	0	0.094453
22247	Amp	19	15635661	15728649	0	0.090353
22248	Amp	19	15732156	15772388	0	0.074763
22249	Amp	19	15773403	15926549	0	0.082413
22250	Amp	19	15927490	15957807	0	0.091819
22251	Amp	19	15958351	15989057	0	0.100362
22252	Amp	19	15990122	16010770	0	0.10213
22253	Amp	19	16012807	16176409	0	0.100362
22254	Amp	19	16179552	16203681	0	0.150805
22255	Amp	19	16210090	16535269	0	0.103007
22256	Amp	19	16537421	16721785	0	0.089521
22257	Amp	19	16731854	16758152	0	0.095222
22258	Amp	19	16758655	16787008	0	0.097696
22259	Amp	19	16787484	16799893	0	0.096226

22260	Amp	19	16800862	16909878	0	0.101406
22261	Amp	19	16912776	16930362	0	0.09596
22262	Amp	19	16934264	16959930	0	0.108485
22263	Amp	19	16975963	17088157	0	0.149644
22264	Amp	19	17088616	17089348	0	0.145217
22265	Amp	19	17094472	17135402	0	0.136918
22266	Amp	19	17138171	17146992	0	0.132781
22267	Amp	19	17147665	17164240	0	0.120294
22268	Amp	19	17165636	17180933	0	0.11782
22269	Amp	19	17184214	17187806	0	0.107357
22270	Amp	19	17191071	17202905	0	0.11207
22271	Amp	19	17207350	17319295	0	0.116736
22272	Amp	19	17326040	17326040	0	0.119522
22273	Amp	19	17345413	17406560	0	0.114857
22274	Amp	19	17415701	17450645	0	0.118001
22275	Amp	19	17452160	17460329	0	0.110352
22276	Amp	19	17462767	17537362	0	0.105639
22277	Amp	19	17544314	17618787	0	0.108776
22278	Amp	19	17621903	17685187	0	0.096252
22279	Amp	19	17689683	17705690	0	0.109063
22280	Amp	19	17719125	17729362	0	0.112193
22281	Amp	19	17730160	17754289	0	0.119788
22282	Amp	19	17763934	17871001	0	0.112699
22283	Amp	19	17878597	17882018	0	0.118667
22284	Amp	19	17887255	17916070	0	0.123998
22285	Amp	19	17919638	17991910	0	0.130089
22286	Amp	19	17992819	18004679	0	0.123998
22287	Amp	19	18013156	18069426	0	0.132051
22288	Amp	19	18070213	18095286	0	0.122897
22289	Amp	19	18096053	18096053	0	0.114845
22290	Amp	19	18100050	18163767	0	0.12019
22291	Amp	19	18174473	18184261	0	0.111695
22292	Amp	19	18184580	18202705	0	0.12699
22293	Amp	19	18211331	18212023	0	0.136399
22294	Amp	19	18213607	18265005	0	0.133255
22295	Amp	19	18270802	18282872	0	0.166052
22296	Amp	19	18283486	18322119	0	0.176276
22297	Amp	19	18324609	18364920	0	0.160981
22298	Amp	19	18365058	18413576	0	0.145577
22299	Amp	19	18414891	18496770	0	0.138376
22300	Amp	19	18497042	18519020	0	0.128966
22301	Amp	19	18526680	18625692	0	0.139408
22302	Amp	19	18634853	18690365	0	0.143772
22303	Amp	19	18690989	18690989	0	0.135188
22304	Amp	19	18696613	18705229	0	0.129857
22305	Amp	19	18711128	18736798	0	0.135508
22306	Amp	19	18737022	18779166	0	0.132863
22307	Amp	19	18791783	18846409	0	0.128499
22308	Amp	19	18848287	18911529	0	0.132434
22309	Amp	19	18912444	18921871	0	0.117313
22310	Amp	19	18935077	18993228	0	0.108817
22311	Amp	19	18993837	18994964	0	0.119949
22312	Amp	19	18997367	19001657	0	0.115718

22313	Amp	19	19003260	19043757	0	0.121054
22314	Amp	19	19047309	19122015	0	0.123945
22315	Amp	19	19124121	19124121	0	0.121967
22316	Amp	19	19126265	19181596	0	0.115998
22317	Amp	19	19182194	19235571	0	0.110663
22318	Amp	19	19242301	19288010	0	0.116299
22319	Amp	19	19294143	19582490	0	0.118309
22320	Amp	19	19584521	19603223	0	0.131824
22321	Amp	19	19603627	19676473	0	0.118309
22322	Amp	19	19677002	19764062	0	0.122322
22323	Amp	19	19765106	19768005	0	0.134042
22324	Amp	19	19773382	19834971	0	0.137027
22325	Amp	19	19835282	19835282	0	0.137951
22326	Amp	19	19836130	19836130	0	0.149244
22327	Amp	19	21064698	21064698	0	0.118195
22328	Amp	19	21065501	21077361	0	0.124094
22329	Amp	19	21080712	21126830	0	0.12917
22330	Amp	19	21135992	21219960	0	0.124994
22331	Amp	19	21223217	21241658	0	0.136515
22332	Amp	19	21245434	21245434	0	0.132502
22333	Amp	19	21245668	21249373	0	0.136175
22334	Amp	19	21254023	21258726	0	0.146219
22335	Amp	19	21267711	21339415	0	0.149618
22336	Amp	19	21341600	21369050	0	0.147462
22337	Amp	19	21371748	21478228	0	0.150545
22338	Amp	19	21478235	21534501	0	0.145469
22339	Amp	19	21538872	21538872	0	0.14207
22340	Amp	19	21539503	21615751	0	0.147365
22341	Amp	19	21619732	21620182	0	0.153279
22342	Amp	19	21620894	21687768	0	0.150294
22343	Amp	19	21700824	21700824	0	0.180849
22344	Amp	19	21703134	21728314	0.033661	0.192673
22345	Amp	19	21730165	21773146	0	0.175706
22346	Amp	19	21781455	21827337	0	0.181384
22347	Amp	19	21827700	21836794	0	0.16956
22348	Amp	19	21840844	22009436	0	0.182198
22349	Amp	19	22015805	22019364	0.001198	0.188353
22350	Amp	19	22020218	22059252	0	0.180486
22351	Amp	19	22059602	22320695	0	0.171136
22352	Amp	19	22324903	22334082	0	0.176516
22353	Amp	19	22334444	22452651	0	0.163479
22354	Amp	19	22456547	22636041	0	0.159735
22355	Amp	19	22636487	22805326	0	0.148368
22356	Amp	19	22808784	22827117	0	0.152798
22357	Amp	19	22828117	22851625	0	0.142312
22358	Amp	19	22852218	22889617	0	0.13992
22359	Amp	19	22891907	22968988	0	0.117232
22360	Amp	19	22970162	22983764	0	0.128277
22361	Amp	19	22984083	23049683	0	0.155313
22362	Amp	19	23049973	23096201	0	0.153273
22363	Amp	19	23101548	23101569	0	0.126238
22364	Amp	19	23102216	23133736	0	0.133426
22365	Amp	19	23144414	23256825	0	0.165843

22366	Amp	19	23257372	23406543	0	0.181352
22367	Amp	19	23408850	23483072	0	0.184801
22368	Amp	19	23490019	23491088	0	0.186598
22369	Amp	19	23491562	23500446	0	0.181218
22370	Amp	19	23500887	23547628	0	0.178284
22371	Amp	19	23549223	23549223	0	0.171976
22372	Amp	19	23549397	23582690	0	0.168041
22373	Amp	19	23593586	23664120	0	0.163114
22374	Amp	19	23675047	23848522	0	0.16732
22375	Amp	19	23849040	23870190	0	0.154033
22376	Amp	19	23872082	23872082	0	0.152143
22377	Amp	19	27749470	27749586	0	0.160898
22378	Amp	19	27749601	27749601	0	0.149504
22379	Amp	19	27752285	27752285	0.061778	0.196203
22380	Amp	19	27757509	27757927	0.086298	0.199216
22381	Amp	19	27758174	27839130	0.117531	0.202438
22382	Amp	19	27839631	27920622	0.09318	0.199425
22383	Amp	19	27921063	27959019	0.117531	0.202495
22384	Amp	19	27960122	27969641	0.215373	0.212299
22385	Amp	19	27971593	27990506	0.117531	0.202495
22386	Amp	19	27991753	28001049	0.230572	0.21397
22387	Amp	19	28001431	28002554	0.117531	0.202495
22388	Amp	19	28003851	28010616	0.175335	0.208379
22389	Amp	19	28011708	28062677	0.273081	0.217869
22390	Amp	19	28065290	28137890	0.293283	0.220011
22391	Amp	19	28139156	28199590	0.195309	0.210521
22392	Amp	19	28200013	28202728	0.050005	0.194835
22393	Amp	19	28206472	28293330	0.086298	0.198645
22394	Amp	19	28296349	28316162	0.033661	0.192761
22395	Amp	19	28317294	28499427	0.056238	0.195191
22396	Amp	19	28500385	28754078	0.028398	0.192121
22397	Amp	19	28756376	28859544	0	0.17468
22398	Amp	19	28859690	28871159	0	0.182564
22399	Amp	19	28875594	28936633	0	0.172435
22400	Amp	19	28941692	29211975	0	0.16709
22401	Amp	19	29212012	29315442	0	0.171299
22402	Amp	19	29316399	29340634	0	0.175359
22403	Amp	19	29341261	29643774	0.02372	0.191045
22404	Amp	19	29646128	29646128	0.250263	0.216264
22405	Amp	19	29646206	29684642	0.195309	0.210258
22406	Amp	19	29685090	29774393	0.619816	0.249292
22407	Amp	19	29774925	29824363	0.676804	0.254169
22408	Amp	19	29824828	29836830	0.317596	0.222479
22409	Amp	19	29840336	29938581	0	0.184087
22410	Amp	19	29942076	30343596	0	0.17921
22411	Amp	19	30345941	30386244	0	0.180763
22412	Amp	19	30389196	30398505	0.325321	0.222892
22413	Amp	19	30400192	30400387	0.347934	0.224689
22414	Amp	19	30402174	30440291	0.421967	0.232189
22415	Amp	19	30440346	30462143	0.382767	0.22838
22416	Amp	19	30472471	30478056	0	0.173648
22417	Amp	19	30480153	30486670	0.09318	0.199922
22418	Amp	19	30487332	30510892	0.079463	0.198125

22419	Amp	19	30514592	30586518	0.09318	0.199654
22420	Amp	19	30587312	30612959	0.325321	0.222969
22421	Amp	19	30619326	30622663	0.02372	0.191211
22422	Amp	19	30624174	30624174	0.012341	0.189682
22423	Amp	19	30624298	30661391	0.187743	0.209698
22424	Amp	19	30663928	30677952	0.564815	0.24403
22425	Amp	19	30681500	30707114	0.613517	0.248451
22426	Amp	19	30716277	30728435	0.667776	0.252904
22427	Amp	19	30736771	30767422	0.460107	0.235168
22428	Amp	19	30770894	30782515	0	0.185579
22429	Amp	19	30785425	30793899	0.056238	0.19544
22430	Amp	19	30797211	30797633	0.007199	0.189399
22431	Amp	19	30799692	30858851	0.02372	0.191116
22432	Amp	19	30859017	30874556	0.056238	0.19539
22433	Amp	19	30875525	30900368	0.068197	0.197001
22434	Amp	19	30905089	31029235	0.033661	0.192727
22435	Amp	19	31032189	31042951	0.012341	0.189893
22436	Amp	19	31047756	31096919	0.043943	0.194259
22437	Amp	19	31106213	31121383	0	0.184397
22438	Amp	19	31123804	31166634	0	0.188017
22439	Amp	19	31168123	31177753	0	0.17639
22440	Amp	19	31177817	31232452	0	0.172024
22441	Amp	19	31236163	31402138	0	0.173459
22442	Amp	19	31407689	31458730	0	0.177457
22443	Amp	19	31462724	31796210	0	0.159918
22444	Amp	19	31796662	31889196	0	0.140669
22445	Amp	19	31890165	31892758	0	0.155104
22446	Amp	19	31896321	31940463	0	0.169521
22447	Amp	19	31942105	32157261	0	0.185043
22448	Amp	19	32159595	32184501	0.033661	0.19286
22449	Amp	19	32185509	32260951	0.02372	0.191307
22450	Amp	19	32263234	32325166	0	0.176871
22451	Amp	19	32327206	32337232	0	0.186484
22452	Amp	19	32338087	32373715	0	0.18791
22453	Amp	19	32375342	32519400	0	0.182623
22454	Amp	19	32519982	32634577	0	0.18048
22455	Amp	19	32636116	32645984	0	0.182304
22456	Amp	19	32647161	32653624	0	0.174459
22457	Amp	19	32655565	32771678	0	0.159679
22458	Amp	19	32775254	32814434	0	0.157855
22459	Amp	19	32814621	33060155	0	0.147032
22460	Amp	19	33068787	33135736	0	0.137832
22461	Amp	19	33137624	33137624	0	0.147794
22462	Amp	19	33137848	33189876	0	0.152966
22463	Amp	19	33190481	33304320	0	0.159348
22464	Amp	19	33304335	33379418	0	0.152966
22465	Amp	19	33379581	33550563	0	0.157066
22466	Amp	19	33553213	33597355	0	0.152966
22467	Amp	19	33599971	33660791	0	0.170152
22468	Amp	19	33664792	33671336	0	0.119629
22469	Amp	19	33671834	33801016	0	0.110885
22470	Amp	19	33803962	33803962	0	0.119408
22471	Amp	19	33805600	33839678	0	0.111591

22472	Amp	19	33840453	33888583	0	0.117615
22473	Amp	19	33888831	33935063	0	0.120912
22474	Amp	19	33935617	33952127	0	0.128622
22475	Amp	19	33955020	34082669	0	0.126976
22476	Amp	19	34084148	34163064	0	0.111578
22477	Amp	19	34164154	34179820	0	0.120124
22478	Amp	19	34184872	34192537	0	0.122843
22479	Amp	19	34193695	34471156	0	0.119106
22480	Amp	19	34474355	34494561	0	0.11138
22481	Amp	19	34495444	34584550	0	0.108661
22482	Amp	19	34593388	34647875	0	0.094114
22483	Amp	19	34648402	34713099	0	0.086859
22484	Amp	19	34713181	34814122	0	0.096699
22485	Amp	19	34816159	34844064	0	0.105053
22486	Amp	19	34844573	34919894	0	0.096699
22487	Amp	19	34921344	35189404	0	0.103135
22488	Amp	19	35190264	35221189	0	0.110718
22489	Amp	19	35221548	35226167	0	0.107194
22490	Amp	19	35226532	35283653	0	0.104764
22491	Amp	19	35288519	35294260	0	0.098328
22492	Amp	19	35294356	35465488	0	0.09646
22493	Amp	19	35465872	35571553	0	0.091224
22494	Amp	19	35576717	35713788	0	0.068717
22495	Amp	19	35725155	35780287	0	0.084757
22496	Amp	19	35802365	35945544	0	0.083147
22497	Amp	19	35946909	36025830	0	0.08779
22498	Amp	19	36027424	36051707	0	0.091355
22499	Amp	19	36056544	36112840	0	0.099944
22500	Amp	19	36115091	36146712	0	0.091355
22501	Amp	19	36164505	36207756	0	0.09348
22502	Amp	19	36208132	36224069	0	0.098129
22503	Amp	19	36226209	36258756	0	0.099656
22504	Amp	19	36260813	36260813	0	0.09472
22505	Amp	19	36261319	36262089	0	0.120321
22506	Amp	19	36265950	36265950	0	0.123092
22507	Amp	19	36356789	36362538	0	0.085522
22508	Amp	19	36362590	36380828	0	0.076452
22509	Amp	19	36387551	36494984	0	0.053959
22510	Amp	19	36497320	36497320	0	0.051834
22511	Amp	19	36509261	36510004	0	0.043288
22512	Amp	19	36514729	36585128	0	0.054958
22513	Amp	19	36590247	36595743	0	0.071043
22514	Amp	19	36597480	36823672	0	0.059373
22515	Amp	19	36827990	36893875	0	0.095142
22516	Amp	19	36898897	36944968	0	0.053455
22517	Amp	19	36945174	36970948	0	0.050685
22518	Amp	19	36974175	37014600	0	0.052374
22519	Amp	19	37016131	37329618	0	0.059819
22520	Amp	19	37332909	37550854	0	0.056597
22521	Amp	19	37554129	37588396	0	0.064924
22522	Amp	19	37588490	37641202	0	0.070637
22523	Amp	19	37644145	37650065	0	0.064254
22524	Amp	19	37650091	37663801	0	0.072188

22525	Amp	19	37676912	37900832	0	0.093028
22526	Amp	19	37902572	38139678	0	0.084019
22527	Amp	19	38144179	38333941	0	0.090401
22528	Amp	19	38337667	38359790	0	0.078571
22529	Amp	19	38363459	38405744	0	0.072188
22530	Amp	19	38412459	38552560	0	0.077134
22531	Amp	19	38555821	38672547	0	0.075607
22532	Amp	19	38676649	38761598	0	0.073248
22533	Amp	19	38767337	38788941	0	0.0246
22534	Amp	19	38793127	39034196	0	0.016272
22535	Amp	19	39091645	39095008	0	0.023232
22536	Amp	19	39097627	39117513	0	0.026184
22537	Amp	19	39128908	39144632	0	0.019224
22538	Amp	19	39146421	39149475	0	0.02811
22539	Amp	19	39150506	39462491	0	0.025159
22540	Amp	19	39464303	39535783	0	0.032249
22541	Amp	19	39538243	39725584	0	0.082772
22542	Amp	19	39726141	39874001	0	0.095888
22543	Amp	19	39907346	39983746	0	0.087018
22544	Amp	19	39986062	40183334	0	0.09629
22545	Amp	19	40183689	40214461	0	0.07138
22546	Amp	19	40214544	40246423	0	0.055755
22547	Amp	19	40249130	40283871	0	0.067715
22548	Amp	19	40294167	40298396	0	0.055755
22549	Amp	19	40303944	40314635	0	0.080665
22550	Amp	19	40315044	40400309	0	0.030142
22551	Amp	19	40400794	40476468	0	0.025561
22552	Amp	19	40481169	40509015	0	0.033736
22553	Amp	19	40510665	40713344	0	0.032047
22554	Amp	19	40720510	40757440	0	0.08257
22555	Amp	19	40760168	40785214	0	0.032047
22556	Amp	19	40787216	40803620	0	0.06596
22557	Amp	19	40804666	40888431	0	0.060247
22558	Amp	19	40889145	40940044	0	0.079379
22559	Amp	19	40940664	41038859	0	0.060247
22560	Amp	19	41039039	41111988	0	0.052072
22561	Amp	19	41121275	41672828	0	0.01816
22562	Amp	19	41673535	41737643	0	0.021916
22563	Amp	19	41741403	41862174	0	0.043543
22564	Amp	19	41863295	41886253	0	0.047794
22565	Amp	19	41889215	41965182	0	0.063196
22566	Amp	19	41980184	42044996	0	0.049134
22567	Amp	19	42045157	42065261	0	0.058452
22568	Amp	19	42068892	42140854	0	0.054202
22569	Amp	19	42141822	42205731	0	0.046636
22570	Amp	19	42209683	42218870	0	0.022348
22571	Amp	19	42219845	42380706	0	0.026802
22572	Amp	19	42383304	42418355	0	0.017529
22573	Amp	19	42435212	42535098	0	0.013075
22574	Amp	19	42538670	42548751	0	0.026934
22575	Amp	19	42550436	42638714	0	0.015445
22576	Amp	19	42641265	42709707	0	0.011688
22577	Amp	19	42711176	42712819	0	0.015388

22578	Amp	19	43458545	43458545	0	0.026107
22579	Amp	19	43459491	43510305	0	0.014418
22580	Amp	19	43514023	43667873	0	0.021213
22581	Amp	19	43674285	43786274	0	0.017513
22582	Amp	19	43786683	44531020	0	0.019036
22583	Amp	19	44538275	44615856	0	0.04874
22584	Amp	19	44623056	44623056	0	0.058136
22585	Amp	19	44624135	44624135	0	0.060373
22586	Amp	19	44626079	44711537	0	0.05885
22587	Amp	19	44711970	45099162	0	0.048131
22588	Amp	19	45099523	45192480	0	0.058983
22589	Amp	19	45194653	45322368	0	0.061519
22590	Amp	19	45322440	45400216	0	0.078043
22591	Amp	19	45407710	45409148	0	0.071248
22592	Amp	19	45412709	45725512	0	0.068712
22593	Amp	19	45729065	45901004	0	0.07487
22594	Amp	19	45903347	46010823	0	0.065473
22595	Amp	19	46011539	46013326	0	0.115996
22596	Amp	19	46017995	46190660	0	0.086293
22597	Amp	19	46191937	46268118	0	0.09669
22598	Amp	19	46274250	46503974	0	0.046167
22599	Amp	19	46504327	46683196	0	0.039372
22600	Amp	19	46684238	46836196	0	0.044222
22601	Amp	19	46837385	46840714	0	0.039372
22602	Amp	19	46841269	47026992	0	0.037136
22603	Amp	19	47029776	47382025	0	0.039302
22604	Amp	19	47387398	47443257	0	0.075108
22605	Amp	19	47444999	47474620	0	0.079998
22606	Amp	19	47476896	47582599	0	0.087214
22607	Amp	19	47583390	47591383	0	0.089385
22608	Amp	19	47594000	47823958	0	0.05358
22609	Amp	19	47831641	47881800	0	0.047422
22610	Amp	19	47883100	47961014	0	0.049605
22611	Amp	19	47965532	47965928	0	0.059032
22612	Amp	19	47966522	48046691	0	0.062266
22613	Amp	19	48046729	48153367	0	0.068936
22614	Amp	19	48155267	48187084	0	0.07171
22615	Amp	19	48192529	48369800	0	0.074148
22616	Amp	19	48371455	48438000	0	0.077042
22617	Amp	19	48438019	48520353	0	0.074268
22618	Amp	19	48520414	48538662	0	0.067053
22619	Amp	19	48542842	48572658	0	0.070545
22620	Amp	19	48580236	48594903	0	0.070846
22621	Amp	19	48607051	48641533	0	0.081178
22622	Amp	19	48643210	48679378	0	0.079012
22623	Amp	19	48710920	48851678	0	0.112696
22624	Amp	19	48853060	49060867	0	0.102364
22625	Amp	19	49067856	49145794	0	0.108664
22626	Amp	19	49154077	49160967	0	0.112346
22627	Amp	19	49161163	49195756	0	0.135617
22628	Amp	19	49207230	49483107	0	0.141113
22629	Amp	19	49485199	49527197	0	0.148064
22630	Amp	19	49533187	49588745	0	0.164041

22631	Amp	19	49602198	49629082	0	0.170689
22632	Amp	19	49631033	49719657	0	0.147418
22633	Amp	19	49720562	49728871	0	0.144184
22634	Amp	19	49731091	49867421	0	0.10383
22635	Amp	19	49871239	49874886	0	0.098334
22636	Amp	19	50090736	50157268	0	0.082356
22637	Amp	19	50158191	50352678	0	0.075405
22638	Amp	19	50352692	50394760	0	0.083137
22639	Amp	19	50403911	50403911	0	0.123167
22640	Amp	19	50404235	50491079	0	0.12656
22641	Amp	19	50491167	50526345	0	0.124389
22642	Amp	19	50530624	50544642	0	0.135238
22643	Amp	19	50555831	50634403	0	0.127506
22644	Amp	19	50635641	50657831	0	0.1357
22645	Amp	19	50671507	50676640	0	0.142099
22646	Amp	19	50676698	50683314	0	0.135452
22647	Amp	19	50683338	50977726	0	0.14114
22648	Amp	19	50981938	51062940	0.02372	0.191663
22649	Amp	19	51062974	51062974	0.056238	0.195566
22650	Amp	19	51063011	51148092	0.243425	0.215424
22651	Amp	19	51148842	51164567	0.317596	0.222281
22652	Amp	19	51164843	51297087	0.421967	0.231634
22653	Amp	19	51298362	51306646	0.375927	0.227951
22654	Amp	19	51306710	51345715	0.340434	0.224048
22655	Amp	19	51349581	51435140	0.175335	0.208267
22656	Amp	19	51435334	51483420	0.230572	0.21391
22657	Amp	19	51483835	51498231	0.25804	0.216989
22658	Amp	19	51498271	51555166	0.340434	0.224269
22659	Amp	19	51555401	51610411	0.375927	0.227662
22660	Amp	19	51610569	51624018	0.500055	0.23899
22661	Amp	19	51654184	51654184	0.604912	0.247395
22662	Amp	19	51654299	51687306	0.468576	0.236067
22663	Amp	19	51692221	51716766	0.390264	0.228786
22664	Amp	19	51722272	51754420	0.293283	0.220382
22665	Amp	19	51756318	51756318	0.333141	0.223704
22666	Amp	19	51756598	51766834	0.421967	0.232103
22667	Amp	19	51767765	51767765	0.571214	0.244736
22668	Amp	19	52118391	52125358	0.043943	0.194422
22669	Amp	19	52127660	52144059	0.079463	0.198314
22670	Amp	19	52149866	52220842	0	0.187984
22671	Amp	19	52221573	52248799	0	0.18655
22672	Amp	19	52249419	52331957	0.056238	0.195442
22673	Amp	19	52332211	52352780	0.073369	0.197615
22674	Amp	19	52355034	52360998	0.056238	0.195442
22675	Amp	19	52364461	52366281	0.13565	0.204616
22676	Amp	19	52366799	52470970	0	0.188088
22677	Amp	19	52471092	52534137	0	0.184765
22678	Amp	19	52537854	52555923	0.098998	0.200075
22679	Amp	19	52559278	52559278	0.079463	0.19787
22680	Amp	19	52560883	52568835	0.195309	0.210868
22681	Amp	19	52576480	52576480	0.043943	0.193978
22682	Amp	19	52580768	52699649	0	0.171703
22683	Amp	19	52702132	52702132	0	0.184757

22684	Amp	19	52982662	53004046	0	0.175583
22685	Amp	19	53005178	53005259	0	0.182384
22686	Amp	19	53006207	53092614	0	0.172749
22687	Amp	19	53092916	53093918	0	0.175794
22688	Amp	19	53096269	53316310	0.001198	0.188713
22689	Amp	19	53319790	53343525	0	0.185293
22690	Amp	19	53344575	53380212	0.007199	0.18901
22691	Amp	19	53380956	53391809	0	0.185965
22692	Amp	19	53392626	53404859	0.086298	0.19888
22693	Amp	19	53514004	53534635	0	0.179022
22694	Amp	19	53534886	53847513	0	0.183836
22695	Amp	19	53847931	53871273	0.111991	0.201431
22696	Amp	19	53872572	53885122	0.068197	0.196777
22697	Amp	19	53887334	53917705	0.175335	0.208223
22698	Amp	19	53917832	53925944	0.195309	0.210429
22699	Amp	19	53926196	54003737	0.13565	0.20415
22700	Amp	19	54007463	54087875	0.111991	0.201945
22701	Amp	19	54088052	54194014	0.25804	0.216614
22702	Amp	19	54213181	54384137	0.309523	0.221459
22703	Amp	19	54389714	54470018	0.25804	0.216645
22704	Amp	19	54470034	54478884	0.309523	0.221688
22705	Amp	19	54481677	54515214	0.421967	0.232058
22706	Amp	19	54518264	54535837	0.27943	0.219139
22707	Amp	19	54537051	54631479	0.325321	0.223049
22708	Amp	19	54639945	54714912	0.223025	0.213224
22709	Amp	19	54714974	54715476	0.317596	0.222583
22710	Amp	19	54715554	54904326	0.209057	0.212213
22711	Amp	19	54904949	54904949	0.079463	0.198009
22712	Amp	19	54905255	54933920	0.111991	0.201865
22713	Amp	19	54935930	55036566	0.142507	0.204981
22714	Amp	19	55037257	55043521	0.156508	0.206555
22715	Amp	19	55044212	55233139	0.209057	0.212264
22716	Amp	19	55242819	55268530	0.243425	0.215143
22717	Amp	19	55277077	55322092	0.355162	0.225684
22718	Amp	19	55332824	55338620	0.301081	0.220641
22719	Amp	19	55349789	55462601	0.398217	0.229803
22720	Amp	19	55472020	55508418	0.556748	0.243624
22721	Amp	19	55511245	55682391	0.613517	0.248326
22722	Amp	19	55682641	55795036	0.540744	0.242358
22723	Amp	19	55796481	55819962	0.038273	0.193223
22724	Amp	19	55820038	55840801	0.079463	0.198292
22725	Amp	19	55846466	56119859	0.038273	0.193223
22726	Amp	19	56119948	56130366	0.086298	0.198852
22727	Amp	19	56141223	56173153	0.117531	0.202535
22728	Amp	19	56175024	56195145	0.250263	0.215901
22729	Amp	19	56229658	56251411	0.079463	0.198305
22730	Amp	19	56256494	56287935	0.068197	0.196731
22731	Amp	19	56288833	56364258	0.012341	0.189881
22732	Amp	19	56367276	56465342	0	0.183805
22733	Amp	19	56467156	56490768	0	0.174643
22734	Amp	19	56492828	56496966	0	0.167672
22735	Amp	19	56497005	56572222	0	0.169713
22736	Amp	19	56572755	56818669	0.117531	0.202569

22737	Amp	19	56820322	56927370	0.098998	0.200528
22738	Amp	19	56932871	56933754	0	0.18054
22739	Amp	19	56933960	57114049	0	0.185131
22740	Amp	19	57116825	57225759	0	0.177418
22741	Amp	19	57226667	57305805	0	0.186005
22742	Amp	19	57310521	57312423	0	0.176712
22743	Amp	19	57312827	57332552	0	0.166172
22744	Amp	19	57336158	57345354	0.142507	0.205191
22745	Amp	19	57349717	57349862	0	0.166172
22746	Amp	19	57350368	57350368	0	0.170037
22747	Amp	19	57351137	57408056	0	0.174526
22748	Amp	19	57408576	57419672	0	0.170661
22749	Amp	19	57424919	57458825	0	0.156578
22750	Amp	19	57459443	57563049	0	0.1693
22751	Amp	19	57568438	57734463	0	0.161928
22752	Amp	19	57734808	57778641	0	0.183151
22753	Amp	19	57783618	57862267	0	0.169786
22754	Amp	19	57884476	57914670	0	0.177087
22755	Amp	19	57919256	57946563	0	0.181178
22756	Amp	19	57950175	57989309	0	0.177087
22757	Amp	19	58011102	58062191	0	0.174208
22758	Amp	19	58063446	58086038	0	0.165822
22759	Amp	19	58089854	58169321	0.001198	0.188655
22760	Amp	19	58171974	58177063	0	0.184973
22761	Amp	19	58191718	58222013	0.007199	0.189599
22762	Amp	19	58222964	58264725	0	0.149572
22763	Amp	19	58265102	58273366	0	0.145082
22764	Amp	19	58273737	58349320	0	0.117053
22765	Amp	19	58353716	58370362	0	0.122524
22766	Amp	20	472817	512599	0	0.039991
22767	Amp	20	512668	1215468	0	0.037471
22768	Amp	20	1223558	1345043	0	0.048653
22769	Amp	20	1359746	1396677	0	0.038691
22770	Amp	20	1400134	1908831	0	0.042101
22771	Amp	20	1913689	2170752	0	0.038691
22772	Amp	20	2173566	2367555	0	0.032914
22773	Amp	20	2367609	2849268	0	0.030367
22774	Amp	20	2849681	3040624	0	0.036134
22775	Amp	20	3041696	3656993	0	0.037522
22776	Amp	20	3657463	3661684	0	0.041779
22777	Amp	20	3661999	3737202	0	0.045518
22778	Amp	20	3740809	3748858	0	0.053279
22779	Amp	20	3752840	3797508	0	0.062515
22780	Amp	20	3800281	3822799	0	0.058258
22781	Amp	20	3823017	3823017	0	0.050498
22782	Amp	20	4123473	4178460	0	0.039315
22783	Amp	20	4178632	4205433	0	0.037927
22784	Amp	20	4206868	4209877	0	0.033258
22785	Amp	20	4210411	4784663	0	0.029519
22786	Amp	20	4791356	4911113	0	0.038305
22787	Amp	20	4912632	4963668	0	0.039937
22788	Amp	20	4964649	5013614	0	0.046742
22789	Amp	20	5016318	5059124	0	0.04934

22790	Amp	20	5059406	5163671	0	0.050914
22791	Amp	20	5172576	5193665	0	0.044109
22792	Amp	20	5193954	5234067	0	0.046334
22793	Amp	20	5235438	5399991	0	0.050738
22794	Amp	20	5400781	5417495	0	0.055344
22795	Amp	20	5417599	5756393	0	0.046108
22796	Amp	20	5762636	5979702	0	0.047687
22797	Amp	20	5980495	6002180	0	0.045461
22798	Amp	20	6003881	6101411	0	0.041473
22799	Amp	20	6103497	6110232	0	0.037069
22800	Amp	20	6112183	6185988	0	0.031301
22801	Amp	20	6188725	6325935	0	0.022515
22802	Amp	20	6331321	6608452	0	0.028094
22803	Amp	20	6609517	6756289	0	0.035261
22804	Amp	20	6756555	6791419	0	0.066539
22805	Amp	20	6791616	6866716	0	0.06496
22806	Amp	20	6867785	6889544	0	0.062363
22807	Amp	20	6891516	6937182	0	0.076702
22808	Amp	20	6937815	6942090	0	0.083295
22809	Amp	20	6942163	6955493	0	0.081663
22810	Amp	20	7136070	7136070	0	0.085326
22811	Amp	20	7137726	7179348	0	0.090017
22812	Amp	20	7181432	7516965	0	0.068511
22813	Amp	20	7517125	7629617	0	0.06382
22814	Amp	20	7630609	8044359	0	0.057226
22815	Amp	20	8052730	8081707	0	0.078695
22816	Amp	20	8092952	8103757	0	0.065372
22817	Amp	20	8103825	8104743	0	0.061709
22818	Amp	20	8605879	8605879	0	0.057102
22819	Amp	20	8606650	8735687	0	0.069709
22820	Amp	20	8737609	8869752	0	0.065855
22821	Amp	20	8869916	8944951	0	0.057989
22822	Amp	20	8945555	9311529	0	0.062226
22823	Amp	20	9313992	9417785	0	0.057989
22824	Amp	20	9418775	9569901	0	0.061066
22825	Amp	20	9570909	9627929	0	0.057989
22826	Amp	20	9630611	9643504	0	0.065076
22827	Amp	20	9644979	9694498	0	0.071302
22828	Amp	20	9695163	9721185	0	0.065076
22829	Amp	20	9721514	9765697	0	0.068893
22830	Amp	20	9771760	9868206	0	0.065076
22831	Amp	20	9869601	9942982	0	0.051038
22832	Amp	20	9945561	10465893	0	0.043951
22833	Amp	20	10469958	10515696	0	0.056511
22834	Amp	20	10518309	10621775	0	0.073109
22835	Amp	20	10621918	10654408	0	0.066395
22836	Amp	20	10656409	10665125	0	0.09723
22837	Amp	20	10667910	10670187	0	0.108073
22838	Amp	20	10673873	10806264	0	0.110706
22839	Amp	20	10809552	10881715	0	0.116955
22840	Amp	20	10881944	10892095	0	0.114323
22841	Amp	20	10896640	10943348	0	0.164846
22842	Amp	20	10944996	11094812	0	0.114323

22843	Amp	20	11095523	11106906	0	0.108073
22844	Amp	20	11110810	11147105	0	0.109461
22845	Amp	20	11154259	11157399	0	0.103883
22846	Amp	20	11157789	11171902	0	0.106851
22847	Amp	20	11173335	11198682	0	0.132912
22848	Amp	20	11199110	11200220	0	0.131524
22849	Amp	20	11209065	11312932	0	0.105463
22850	Amp	20	11313445	11323563	0	0.098842
22851	Amp	20	11324543	11366655	0	0.145622
22852	Amp	20	11370685	11475375	0	0.098842
22853	Amp	20	11482193	11492869	0	0.095874
22854	Amp	20	11493242	11500629	0	0.081402
22855	Amp	20	11502137	11502137	0	0.091569
22856	Amp	20	11512448	11588600	0	0.060733
22857	Amp	20	11592576	11623868	0	0.070936
22858	Amp	20	11625977	11647582	0	0.121042
22859	Amp	20	11650555	11694546	0	0.070936
22860	Amp	20	11701517	11745652	0	0.079064
22861	Amp	20	11748940	11758425	0	0.068861
22862	Amp	20	11760625	11861347	0	0.073509
22863	Amp	20	11867748	12014705	0	0.068861
22864	Amp	20	12018497	12018644	0	0.081532
22865	Amp	20	12020186	12042003	0	0.10685
22866	Amp	20	12044776	12355201	0	0.082528
22867	Amp	20	12356771	12403996	0	0.073287
22868	Amp	20	12404147	12475471	0	0.066753
22869	Amp	20	12481554	12622857	0	0.074896
22870	Amp	20	12630485	12660639	0	0.064053
22871	Amp	20	12660688	12660736	0	0.05591
22872	Amp	20	12668419	12676741	0	0.067372
22873	Amp	20	12677640	12704784	0	0.05591
22874	Amp	20	12707098	12740889	0	0.07502
22875	Amp	20	12741600	12765316	0	0.077141
22876	Amp	20	12770159	12780732	0	0.080173
22877	Amp	20	12782492	12818127	0	0.08602
22878	Amp	20	12911119	12912540	0	0.129251
22879	Amp	20	12913062	12921778	0	0.135204
22880	Amp	20	12924540	13065301	0	0.116095
22881	Amp	20	13069173	13072023	0	0.110141
22882	Amp	20	13072370	13137100	0	0.107109
22883	Amp	20	13138254	13175247	0	0.111371
22884	Amp	20	13182536	13204725	0	0.133456
22885	Amp	20	13205555	13213298	0	0.147707
22886	Amp	20	13215031	13235610	0	0.143446
22887	Amp	20	13237119	13276823	0	0.11413
22888	Amp	20	13276877	13444097	0	0.120145
22889	Amp	20	13451050	13517900	0	0.083549
22890	Amp	20	13521934	13581220	0	0.076876
22891	Amp	20	13585749	13650338	0	0.087458
22892	Amp	20	13650368	13716116	0	0.092555
22893	Amp	20	13719179	13784777	0	0.090434
22894	Amp	20	13785661	13797766	0	0.085337
22895	Amp	20	13804574	13874181	0	0.116246

22896	Amp	20	13877128	13937537	0	0.120777
22897	Amp	20	13938690	13984479	0	0.12574
22898	Amp	20	13993903	14027057	0	0.130389
22899	Amp	20	14040812	14062870	0	0.108304
22900	Amp	20	14065095	14109865	0	0.105885
22901	Amp	20	14121063	14186199	0	0.095303
22902	Amp	20	14186297	14322229	0	0.083171
22903	Amp	20	14323868	14372700	0	0.086837
22904	Amp	20	14372740	14386224	0	0.082187
22905	Amp	20	14391118	14427444	0	0.078521
22906	Amp	20	14434582	14436713	0	0.07399
22907	Amp	20	14473274	14710476	0	0.05518
22908	Amp	20	14711021	14711021	0	0.047392
22909	Amp	20	15182862	15240191	0	0.057637
22910	Amp	20	15242129	15294759	0	0.074622
22911	Amp	20	15295448	15341445	0	0.08582
22912	Amp	20	15341595	15419339	0	0.074622
22913	Amp	20	15419557	15640717	0	0.072067
22914	Amp	20	15641928	15836127	0	0.062048
22915	Amp	20	15839140	15917385	0	0.051804
22916	Amp	20	15918007	15993563	0	0.058398
22917	Amp	20	15993828	16022954	0	0.064134
22918	Amp	20	16023638	16185882	0	0.073267
22919	Amp	20	16187214	16265006	0	0.064134
22920	Amp	20	16265091	16303923	0	0.068749
22921	Amp	20	16305985	16507438	0	0.073062
22922	Amp	20	16509623	16509623	0	0.080372
22923	Amp	20	16513511	16583677	0	0.058493
22924	Amp	20	16583715	16654903	0	0.053879
22925	Amp	20	16661502	16722606	0	0.07433
22926	Amp	20	16725239	16811042	0	0.078751
22927	Amp	20	16811555	17024908	0	0.074437
22928	Amp	20	17028057	17158077	0	0.081976
22929	Amp	20	17158880	17341580	0	0.086785
22930	Amp	20	17341740	17383559	0	0.085211
22931	Amp	20	17387518	17406995	0	0.080402
22932	Amp	20	17409120	17484568	0	0.087039
22933	Amp	20	17485267	17606864	0	0.080402
22934	Amp	20	17608975	17616247	0	0.082374
22935	Amp	20	17617382	17636886	0	0.096111
22936	Amp	20	17640556	18101890	0	0.082374
22937	Amp	20	18102081	18666154	0	0.074835
22938	Amp	20	18668088	18671911	0	0.072863
22939	Amp	20	18672371	18683234	0	0.068442
22940	Amp	20	18685249	18867534	0	0.07517
22941	Amp	20	18867684	19250488	0	0.068442
22942	Amp	20	19250562	19334816	0	0.061132
22943	Amp	20	19336725	19365117	0	0.066708
22944	Amp	20	19365540	19427815	0	0.071741
22945	Amp	20	19428686	19508308	0	0.0748
22946	Amp	20	19509340	19522395	0	0.071741
22947	Amp	20	19523827	19760014	0	0.066708
22948	Amp	20	19770161	19844573	0	0.06866

22949	Amp	20	19844874	19880373	0	0.072258
22950	Amp	20	19886683	20043846	0	0.075179
22951	Amp	20	20045489	20058415	0	0.073226
22952	Amp	20	20058825	20072476	0	0.076171
22953	Amp	20	20075438	20133149	0	0.073226
22954	Amp	20	20133615	20213032	0	0.076699
22955	Amp	20	20213590	20352774	0	0.056248
22956	Amp	20	20360466	20708313	0	0.049654
22957	Amp	20	20726349	20913748	0	0.046181
22958	Amp	20	20916046	21046739	0	0.041177
22959	Amp	20	21047374	21097432	0	0.049333
22960	Amp	20	21097487	21097896	0	0.041177
22961	Amp	20	21110856	21298226	0	0.046547
22962	Amp	20	21302458	21448777	0	0.062493
22963	Amp	20	21449178	21527856	0	0.056918
22964	Amp	20	21531894	21628101	0	0.027834
22965	Amp	20	21630345	21723554	0	0.022465
22966	Amp	20	21724553	21725895	0	0.027857
22967	Amp	20	21733714	21792655	0	0.048586
22968	Amp	20	21793700	21982240	0	0.043193
22969	Amp	20	21987312	22061780	0	0.045141
22970	Amp	20	22064379	22161861	0	0.053726
22971	Amp	20	22162012	22263375	0	0.040225
22972	Amp	20	22268229	22292165	0	0.045535
22973	Amp	20	22295661	22316077	0	0.043588
22974	Amp	20	22316409	22330906	0	0.037069
22975	Amp	20	22331983	22354273	0	0.031759
22976	Amp	20	22354291	22480226	0	0.044354
22977	Amp	20	22481532	22616241	0	0.049641
22978	Amp	20	22616381	22759713	0	0.053822
22979	Amp	20	22762661	22983953	0	0.058306
22980	Amp	20	22984120	23348445	0	0.059903
22981	Amp	20	23352310	23642243	0	0.040081
22982	Amp	20	23643567	23697275	0	0.045149
22983	Amp	20	23698253	23722537	0	0.055419
22984	Amp	20	23723651	23951201	0	0.039473
22985	Amp	20	23952548	24098856	0	0.043998
22986	Amp	20	24099038	24179913	0	0.039473
22987	Amp	20	24182140	24196855	0	0.029202
22988	Amp	20	24198438	24215058	0	0.03067
22989	Amp	20	24215185	24280106	0	0.026187
22990	Amp	20	24282014	24350769	0	0.030714
22991	Amp	20	24352095	24372558	0	0.022129
22992	Amp	20	24373169	24423749	0	0.019412
22993	Amp	20	24424346	24464914	0	0.022367
22994	Amp	20	24466743	24692391	0	0.01708
22995	Amp	20	24693061	24890443	0	0.014125
22996	Amp	20	24890749	25197169	0	0.016974
22997	Amp	20	25197284	25272964	0	0.014125
22998	Amp	20	25274052	25319324	0	0.02266
22999	Amp	20	25320386	25533631	0	0.014125
23000	Amp	20	25534541	25550029	0	0.009943
23001	Amp	20	25552858	25576790	0	0.02345

23002 Amp	20	25577662	25578410	0	0.01382
23003 Amp	20	25580918	25613235	0	0.020435
23004 Amp	20	25613776	25692712	0	0.018838
23005 Amp	20	25693414	25764087	0	0.029491
23006 Amp	20	25774116	26009805	0	0.031757
23007 Amp	20	26010660	26010660	0	0.029491
23008 Amp	20	26091946	26096020	0	0.03846
23009 Amp	20	26098835	26098835	0	0.047365
23010 Amp	20	26105478	26172890	0	0.052532
23011 Amp	20	26175590	26198217	0	0.05509
23012 Amp	20	26204548	26215250	0	0.059272
23013 Amp	20	26215627	26217263	0	0.072037
23014 Amp	20	26217295	26217295	0	0.075751
23015 Amp	20	31247668	31247668	0	0.059731
23016 Amp	20	31256075	31263032	0	0.060591
23017 Amp	20	31263351	31318384	0	0.059124
23018 Amp	20	31318915	31350024	0	0.075382
23019 Amp	20	31351605	31446807	0	0.059124
23020 Amp	20	31449137	31514078	0	0.056703
23021 Amp	20	31517899	31526460	0	0.054727
23022 Amp	20	31529632	31584314	0	0.068363
23023 Amp	20	31589229	31615226	0	0.072696
23024 Amp	20	31615679	31631139	0	0.070191
23025 Amp	20	31633619	31759373	0	0.074096
23026 Amp	20	31764527	31764527	0	0.087444
23027 Amp	20	31768788	31798820	0	0.091091
23028 Amp	20	31803091	31826117	0	0.087773
23029 Amp	20	31828693	31831371	0	0.083591
23030 Amp	20	31834775	32020243	0	0.079685
23031 Amp	20	32023396	32127894	0	0.084306
23032 Amp	20	32129176	32146824	0	0.087932
23033 Amp	20	32147484	32227858	0	0.091605
23034 Amp	20	32232773	32273726	0	0.095158
23035 Amp	20	32276476	32360478	0	0.090537
23036 Amp	20	32360511	32487844	0	0.086911
23037 Amp	20	32487858	32728100	0	0.082574
23038 Amp	20	32728417	33000553	0	0.076989
23039 Amp	20	33001967	33072737	0	0.081854
23040 Amp	20	33073194	33350240	0	0.0783
23041 Amp	20	33351826	33399514	0	0.073436
23042 Amp	20	33400310	33479756	0	0.087193
23043 Amp	20	33499195	33500567	0	0.091641
23044 Amp	20	33501177	33501435	0	0.077884
23045 Amp	20	33506307	33622195	0	0.081532
23046 Amp	20	33624737	33645165	0	0.077084
23047 Amp	20	33648210	33710868	0	0.08403
23048 Amp	20	33726061	33786508	0	0.093421
23049 Amp	20	33789224	33833936	0	0.106542
23050 Amp	20	33837597	33846879	0	0.143339
23051 Amp	20	33848917	33990697	0	0.139691
23052 Amp	20	33996961	34016047	0	0.147292
23053 Amp	20	34016433	34221443	0	0.150583
23054 Amp	20	34222360	34232531	0	0.144918

23055	Amp	20	34244169	34400372	0	0.157269
23056	Amp	20	34401668	34410848	0.168144	0.207792
23057	Amp	20	34412678	34421099	0.13565	0.204501
23058	Amp	20	34421838	34458221	0.105051	0.200828
23059	Amp	20	34467575	34545380	0.175335	0.208276
23060	Amp	20	34563828	34566799	0.556748	0.243545
23061	Amp	20	34566893	34685599	0.644689	0.251013
23062	Amp	20	34687382	34712903	0.685617	0.254542
23063	Amp	20	34716999	34752477	0.596354	0.246941
23064	Amp	20	34752510	34912124	0.644689	0.25092
23065	Amp	20	34913386	34923580	0.613517	0.248361
23066	Amp	20	34925930	35038098	1.140058	0.288061
23067	Amp	20	35038413	35171451	1.190324	0.291888
23068	Amp	20	35171469	35252136	1.140058	0.287909
23069	Amp	20	35255776	35279445	1.020188	0.279787
23070	Amp	20	35279894	35355872	0.963924	0.276157
23071	Amp	20	35362003	35362003	1.159718	0.28953
23072	Amp	20	35363146	35384631	1.199568	0.292465
23073	Amp	20	35389918	35491411	1.315861	0.300911
23074	Amp	20	35493817	35505339	1.190324	0.29152
23075	Amp	20	35505860	35522859	1.334042	0.302067
23076	Amp	20	35524364	35575804	1.24764	0.295849
23077	Amp	20	35582633	35672836	1.315861	0.300266
23078	Amp	20	35674089	35751278	1.473764	0.311399
23079	Amp	20	35798377	35834820	1.432145	0.308961
23080	Amp	20	35840156	35867735	1.49455	0.312759
23081	Amp	20	35871099	35896323	1.605306	0.320153
23082	Amp	20	35897523	35918225	1.687998	0.326185
23083	Amp	20	35918782	35947469	1.757929	0.331049
23084	Amp	20	35947869	35968044	1.898353	0.339674
23085	Amp	20	35969697	36078206	1.800655	0.333356
23086	Amp	20	36081991	36128725	2.039783	0.34899
23087	Amp	20	36133633	36169578	2.102209	0.352993
23088	Amp	20	36170390	36194824	1.983885	0.345525
23089	Amp	20	36204403	36276177	1.717946	0.328102
23090	Amp	20	36297685	36300679	1.667574	0.324304
23091	Amp	20	36300873	36304686	1.534719	0.315859
23092	Amp	20	36323899	36323899	1.484449	0.312005
23093	Amp	20	36335898	36439615	1.748357	0.330032
23094	Amp	20	36440382	36685133	1.717946	0.327883
23095	Amp	20	36688688	36726605	1.534719	0.315348
23096	Amp	20	36727217	36776800	1.344429	0.302441
23097	Amp	20	36782500	36839310	1.000924	0.278262
23098	Amp	20	36842313	36868185	0.805493	0.264461
23099	Amp	20	36871001	36871001	0.636206	0.250652
23100	Amp	20	36871655	36887811	0.587735	0.24632
23101	Amp	20	36891543	36891543	0.540744	0.242493
23102	Amp	20	36896408	36896408	0.18072	0.209111
23103	Amp	20	36896959	36896959	0.13565	0.204694
23104	Amp	20	36898585	36901371	0.117531	0.202023
23105	Amp	20	36906647	36943662	0.028398	0.191697
23106	Amp	20	36944613	36945492	0.168144	0.207694
23107	Amp	20	36945676	36970994	0.243425	0.215205

23108	Amp	20	36972730	36972730	0.187743	0.209897
23109	Amp	20	36975321	36978502	0.123589	0.202951
23110	Amp	20	36984415	36998124	0.007199	0.189578
23111	Amp	20	37003756	37003756	0.421967	0.231549
23112	Amp	20	37003824	37010545	0.375927	0.227546
23113	Amp	20	37017537	37023427	0	0.185576
23114	Amp	20	37025639	37056247	0.068197	0.196927
23115	Amp	20	37068156	37068156	0	0.183002
23116	Amp	20	37068159	37072082	0	0.180067
23117	Amp	20	37072144	37078624	0	0.175417
23118	Amp	20	37083754	37090176	0	0.138695
23119	Amp	20	37090367	37093618	0	0.142829
23120	Amp	20	37093952	37094427	0	0.14439
23121	Amp	20	37097999	37119070	0	0.164991
23122	Amp	20	37120013	37120013	0	0.167707
23123	Amp	20	37120491	37129861	0	0.173161
23124	Amp	20	37133091	37133091	0	0.178514
23125	Amp	20	37134211	37134211	0	0.182652
23126	Amp	20	37144677	37147626	0.309523	0.221824
23127	Amp	20	37148663	37152321	0.362221	0.226163
23128	Amp	20	37155091	37170697	0.437983	0.233343
23129	Amp	20	37171650	37174432	0.49169	0.237956
23130	Amp	20	37176354	37195742	0.76883	0.261403
23131	Amp	20	37198011	37198280	0.195309	0.21088
23132	Amp	20	37198506	37202862	0.223025	0.213429
23133	Amp	20	37205215	37209895	0.571214	0.245196
23134	Amp	20	37209969	37251614	0.636206	0.250186
23135	Amp	20	37253053	37319430	0.405699	0.23082
23136	Amp	20	37321843	37329858	0.362221	0.226687
23137	Amp	20	37335674	37376436	0.437983	0.233472
23138	Amp	20	37376872	37388937	0.51566	0.239775
23139	Amp	20	37390709	37449070	0.532449	0.241216
23140	Amp	20	37449677	37459168	0.452609	0.234431
23141	Amp	20	37461549	37596422	0.62858	0.249919
23142	Amp	20	37596571	37661734	0.508503	0.239667
23143	Amp	20	37669140	37697413	0.375927	0.227415
23144	Amp	20	37697445	37698267	0.49169	0.237942
23145	Amp	20	37699980	37745772	0.452609	0.234295
23146	Amp	20	37746669	37839237	0.27943	0.218791
23147	Amp	20	37839355	37887201	0.347934	0.224884
23148	Amp	20	37887522	37914039	0.250263	0.21637
23149	Amp	20	37916777	37986191	0.201819	0.211212
23150	Amp	20	37986780	38057165	0.111991	0.201857
23151	Amp	20	38057295	38058493	0.286675	0.219715
23152	Amp	20	38059597	38109369	0.556748	0.243813
23153	Amp	20	38109958	38250447	0.540744	0.242372
23154	Amp	20	38259220	38329435	0.991911	0.277658
23155	Amp	20	38331343	38405310	0.925715	0.272794
23156	Amp	20	38406571	38412977	0.897922	0.271233
23157	Amp	20	38418813	38512353	0.86979	0.268645
23158	Amp	20	38513160	38515681	0.760395	0.260769
23159	Amp	20	38516053	38539826	0.796213	0.263558
23160	Amp	20	38542533	38555784	0.724993	0.258103

23161	Amp	20	38557366	38590870	0.982622	0.277127
23162	Amp	20	38590911	38646963	1.010597	0.279047
23163	Amp	20	38649464	38703838	0.644689	0.251229
23164	Amp	20	38704143	39001373	0.619816	0.249059
23165	Amp	20	39001458	39030774	0.636206	0.250529
23166	Amp	20	39035740	39061842	1.324884	0.301052
23167	Amp	20	39062219	39073756	1.344429	0.302538
23168	Amp	20	39075169	39081792	1.364151	0.304048
23169	Amp	20	39082156	39174142	1.394558	0.305854
23170	Amp	20	39192353	39195630	1.130387	0.287744
23171	Amp	20	39198536	39280311	1.534719	0.315831
23172	Amp	20	39280672	39285264	1.442925	0.309281
23173	Amp	20	39285535	39304531	1.503938	0.313453
23174	Amp	20	39305066	39365475	1.2565	0.296278
23175	Amp	20	39365539	39387873	1.190324	0.291665
23176	Amp	20	39390864	39390864	1.130387	0.28727
23177	Amp	20	39390993	39413403	0.963924	0.275542
23178	Amp	20	39417485	39535351	0.982622	0.277033
23179	Amp	20	39538874	39554115	1.275021	0.298072
23180	Amp	20	39555184	39573955	1.324884	0.300924
23181	Amp	20	39575706	39669688	1.169408	0.290518
23182	Amp	20	39671490	39682727	1.140058	0.288185
23183	Amp	20	39683574	39771332	1.354645	0.303155
23184	Amp	20	39774707	39788137	1.315861	0.300304
23185	Amp	20	39793314	39840579	1.364151	0.303861
23186	Amp	20	39841881	39885590	1.2565	0.296369
23187	Amp	20	39887993	39970437	1.208462	0.292811
23188	Amp	20	39973967	40161619	0.897922	0.271322
23189	Amp	20	40161768	40239965	1.059215	0.282758
23190	Amp	20	40243080	40330760	1.237378	0.295102
23191	Amp	20	40332868	40356618	0.843361	0.267089
23192	Amp	20	40357346	40357346	0.460107	0.234975
23193	Amp	20	40358946	40416364	0.325321	0.222631
23194	Amp	20	40418676	40464070	0.390264	0.228924
23195	Amp	20	40464255	40485764	0.061778	0.195891
23196	Amp	20	40485949	40557685	0.390264	0.228924
23197	Amp	20	40561354	40618716	0	0.178401
23198	Amp	20	40625275	40679498	0	0.187897
23199	Amp	20	40686030	40706165	0.243425	0.21504
23200	Amp	20	40709288	40746144	0	0.178401
23201	Amp	20	40746954	40758785	0.390264	0.228924
23202	Amp	20	40759500	40777935	0.375927	0.227414
23203	Amp	20	40778003	40800213	0.347934	0.224699
23204	Amp	20	40800472	40806719	0.273081	0.218405
23205	Amp	20	40806830	40811614	0.340434	0.224277
23206	Amp	20	40814721	40825921	0.86979	0.269193
23207	Amp	20	40827228	40848312	0.523647	0.240957
23208	Amp	20	40850566	40990067	0.13565	0.204676
23209	Amp	20	40996570	41058480	0.175335	0.208842
23210	Amp	20	41064630	41091185	0.548372	0.243037
23211	Amp	20	41092190	41153562	0.483654	0.237181
23212	Amp	20	41155908	41169523	0.230572	0.213719
23213	Amp	20	41171888	41173409	0.293283	0.219861

23214 Amp	20	41232176	41256874	0.25804	0.216489
23215 Amp	20	41261794	41282745	0.068197	0.197121
23216 Amp	20	41283285	41313260	0	0.152184
23217 Amp	20	41313309	41319547	0	0.140629
23218 Amp	20	41322165	41398036	0	0.138321
23219 Amp	20	41398079	41501560	0	0.151946
23220 Amp	20	41501960	41580237	0	0.146361
23221 Amp	20	41586242	41586242	0	0.136082
23222 Amp	20	41596137	41629315	0	0.124971
23223 Amp	20	41632434	41649844	0	0.130799
23224 Amp	20	41649956	41653129	0	0.141109
23225 Amp	20	41654037	41661666	0	0.129672
23226 Amp	20	41662167	41694596	0	0.127158
23227 Amp	20	41695605	41723118	0	0.136403
23228 Amp	20	41725159	41726601	0	0.147151
23229 Amp	20	41728262	41731388	0	0.12272
23230 Amp	20	41732145	41732656	0	0.125891
23231 Amp	20	41732893	41732966	0	0.136852
23232 Amp	20	41733178	41810785	0	0.170857
23233 Amp	20	41813604	41881133	0	0.174081
23234 Amp	20	41881265	41891423	0	0.169915
23235 Amp	20	41896125	41974375	0	0.157206
23236 Amp	20	41974729	42049180	0	0.152951
23237 Amp	20	42049790	42061214	0	0.147977
23238 Amp	20	42062131	42083485	0	0.184839
23239 Amp	20	42085013	42088439	0.012341	0.190128
23240 Amp	20	42088989	42093335	0.043943	0.194258
23241 Amp	20	42095000	42119500	0.098998	0.199959
23242 Amp	20	42123341	42125654	0.223025	0.213041
23243 Amp	20	42125777	42164094	0.187743	0.209817
23244 Amp	20	42166171	42216507	0.215373	0.212723
23245 Amp	20	42216640	42254320	0.286675	0.219397
23246 Amp	20	42255989	42277858	0	0.181064
23247 Amp	20	42278834	42352645	0	0.183144
23248 Amp	20	42352745	42356325	0	0.186406
23249 Amp	20	42357376	42383652	0.050005	0.195031
23250 Amp	20	42388928	42396142	0.111991	0.20149
23251 Amp	20	42398477	42450170	0.149443	0.205527
23252 Amp	20	42453155	42480486	0.111991	0.20149
23253 Amp	20	42481062	42485700	0.201819	0.210934
23254 Amp	20	42487651	42503252	0.111991	0.20149
23255 Amp	20	42510127	42671051	0.02372	0.191438
23256 Amp	20	42672686	42674203	0	0.163505
23257 Amp	20	42674353	42708814	0	0.153892
23258 Amp	20	42712220	42777911	0	0.15157
23259 Amp	20	42781261	42784729	0	0.156765
23260 Amp	20	42785437	42794740	0	0.163267
23261 Amp	20	42796598	42865968	0	0.157932
23262 Amp	20	42866086	42872338	0	0.160166
23263 Amp	20	42874044	42921675	0	0.167482
23264 Amp	20	42921854	42966536	0	0.172031
23265 Amp	20	42968931	42970355	0	0.177762
23266 Amp	20	42980252	42980252	0	0.16468

23267 Amp	20	42980548	43019078	0	0.169934
23268 Amp	20	43019260	43046262	0	0.167854
23269 Amp	20	43046280	43120691	0	0.162459
23270 Amp	20	43121445	43136850	0	0.164024
23271 Amp	20	43136865	43174295	0	0.162076
23272 Amp	20	43177093	43185524	0	0.160606
23273 Amp	20	43194481	43199269	0.201819	0.211129
23274 Amp	20	43199518	43225036	0.187743	0.209683
23275 Amp	20	43225149	43230963	0.175335	0.208197
23276 Amp	20	43231245	43278645	0.156508	0.206705
23277 Amp	20	43280704	43296454	0.195309	0.210336
23278 Amp	20	43297219	43343808	0.156508	0.206206
23279 Amp	20	43343821	43368301	0.168144	0.208145
23280 Amp	20	43372778	43373810	0.195309	0.210452
23281 Amp	20	43374068	43383995	0.375927	0.227959
23282 Amp	20	43384041	43422958	0.604912	0.247937
23283 Amp	20	43423245	43467963	0.667776	0.253269
23284 Amp	20	43468584	43474374	0.230572	0.213863
23285 Amp	20	43475023	43517387	0	0.16334
23286 Amp	20	43517629	43573161	0	0.173503
23287 Amp	20	43574148	43598372	0	0.169691
23288 Amp	20	43602055	43648004	0	0.175636
23289 Amp	20	43648076	43663321	0	0.163818
23290 Amp	20	43664941	43798226	0	0.158486
23291 Amp	20	43800193	43812761	0	0.15996
23292 Amp	20	43822149	43832006	0	0.136325
23293 Amp	20	43834538	43865143	0	0.157437
23294 Amp	20	43866324	43888983	0	0.136325
23295 Amp	20	43892048	43958136	0	0.141054
23296 Amp	20	43959316	44003043	0	0.14406
23297 Amp	20	44003061	44114814	0	0.154578
23298 Amp	20	44117437	44128617	0	0.150947
23299 Amp	20	44130752	44131434	0	0.161029
23300 Amp	20	44136775	44147704	0	0.148797
23301 Amp	20	44149663	44154872	0	0.160334
23302 Amp	20	44154905	44184810	0	0.150252
23303 Amp	20	44185162	44334156	0	0.140367
23304 Amp	20	44334807	44509328	0	0.133908
23305 Amp	20	44512534	44517232	0	0.151412
23306 Amp	20	44524579	44636257	0.111991	0.201935
23307 Amp	20	44638624	44642655	0.098998	0.199996
23308 Amp	20	44647146	44708313	0	0.149473
23309 Amp	20	44708514	44770166	0	0.147584
23310 Amp	20	44771621	44791946	0	0.145611
23311 Amp	20	44792087	45027808	0	0.158763
23312 Amp	20	45032454	45148865	0	0.136686
23313 Amp	20	45149214	45200027	0	0.144709
23314 Amp	20	45200722	45235504	0	0.148384
23315 Amp	20	45238747	45256220	0	0.143654
23316 Amp	20	45257396	45269499	0	0.13766
23317 Amp	20	45286303	45389526	0	0.148269
23318 Amp	20	45392311	45467204	0	0.151684
23319 Amp	20	45472044	45753445	0	0.169134

23320	Amp	20	45754535	45908039	0	0.16553
23321	Amp	20	45916874	45947863	0	0.158524
23322	Amp	20	45950542	45950542	0	0.136911
23323	Amp	20	45951440	45972654	0	0.145911
23324	Amp	20	45988151	45990010	0	0.151021
23325	Amp	20	45999282	46073185	0	0.168715
23326	Amp	20	46073447	46093372	0	0.163514
23327	Amp	20	46093681	46176159	0.230572	0.213943
23328	Amp	20	46184665	46201544	0	0.163514
23329	Amp	20	46204028	46279888	0	0.163015
23330	Amp	20	46285993	46298238	0	0.173706
23331	Amp	20	46304172	46369063	0	0.177749
23332	Amp	20	46371319	46395555	0.382767	0.228272
23333	Amp	20	46395641	46427048	0.347934	0.224856
23334	Amp	20	46433767	46452860	0.230572	0.214165
23335	Amp	20	46453266	46459863	0	0.163642
23336	Amp	20	46461041	46469035	0	0.159968
23337	Amp	20	46473627	46504404	0	0.172582
23338	Amp	20	46507805	46543922	0	0.187806
23339	Amp	20	46545217	46570589	0.073369	0.197795
23340	Amp	20	46571406	46574067	0.111991	0.201974
23341	Amp	20	46574132	46668832	0.156508	0.206314
23342	Amp	20	46671457	46757138	0.301081	0.220809
23343	Amp	20	46761555	46956583	0.209057	0.211809
23344	Amp	20	46957152	46964571	0.460107	0.235517
23345	Amp	20	46971613	46999445	0.405699	0.230407
23346	Amp	20	47001597	47102472	1.029845	0.28093
23347	Amp	20	47102570	47115518	0.972663	0.276591
23348	Amp	20	47115716	47137458	0.95395	0.275026
23349	Amp	20	47137580	47148329	0.934277	0.273552
23350	Amp	20	47162110	47278749	1.010597	0.279061
23351	Amp	20	47285041	47293706	0.382767	0.228539
23352	Amp	20	47301632	47325548	0.51566	0.240144
23353	Amp	20	47327640	47366836	0.390264	0.229004
23354	Amp	20	47369653	47378190	0.273081	0.217877
23355	Amp	20	47385120	47432355	0.405699	0.23041
23356	Amp	20	47432542	47439289	0.362221	0.226231
23357	Amp	20	47439355	47515821	0.236222	0.214625
23358	Amp	20	47516380	47590479	0.468576	0.235933
23359	Amp	20	47598305	47608081	0.209057	0.212225
23360	Amp	20	47611121	47627855	0.786952	0.262748
23361	Amp	20	47633373	47659841	1.029845	0.280394
23362	Amp	20	47662738	47718507	0.95395	0.274885
23363	Amp	20	47722590	47767261	1.150167	0.288638
23364	Amp	20	47773657	47790502	1.887705	0.339161
23365	Amp	20	47792860	47810018	1.687998	0.326008
23366	Amp	20	47810059	47856336	1.514375	0.314394
23367	Amp	20	47857430	47884704	1.542961	0.316355
23368	Amp	20	47884769	47907116	1.364151	0.303822
23369	Amp	20	47909337	47917654	0.667776	0.253299
23370	Amp	20	47919236	47947062	0.702615	0.255841
23371	Amp	20	47947970	48049094	0.540744	0.242089
23372	Amp	20	48050349	48066509	0.587735	0.246378

23373	Amp	20	48067016	48079834	0.564815	0.244417
23374	Amp	20	48080201	48114507	0.676804	0.253538
23375	Amp	20	48117540	48118555	1.099256	0.285737
23376	Amp	20	48123622	48206705	1.150167	0.289112
23377	Amp	20	48208679	48251571	0.982622	0.277289
23378	Amp	20	48251954	48261540	0.944128	0.274746
23379	Amp	20	48262499	48264375	1.120124	0.286534
23380	Amp	20	48265834	48379098	1.159718	0.28924
23381	Amp	20	48381773	48449135	1.24764	0.2959
23382	Amp	20	48454783	48489075	1.265443	0.297409
23383	Amp	20	48489519	48489519	1.334042	0.301899
23384	Amp	20	48489530	48519062	1.432145	0.308628
23385	Amp	20	48519356	48572693	1.582671	0.319417
23386	Amp	20	48574384	48595462	1.768015	0.331654
23387	Amp	20	48600074	48671830	1.856336	0.336919
23388	Amp	20	48673117	48711317	1.768015	0.331654
23389	Amp	20	48712604	48712604	1.844531	0.336145
23390	Amp	20	48714925	48726191	1.404612	0.306804
23391	Amp	20	48726277	48726522	1.190324	0.29158
23392	Amp	20	48726850	48791771	1.265443	0.297262
23393	Amp	20	48791877	48804164	1.442925	0.309609
23394	Amp	20	48804527	48840477	1.364151	0.303927
23395	Amp	20	48844158	48844355	1.525294	0.315023
23396	Amp	20	48847175	48879119	1.503938	0.313606
23397	Amp	20	48879178	48888059	1.473764	0.3113
23398	Amp	20	48889488	48925852	1.295233	0.298953
23399	Amp	20	48930540	48940127	1.169408	0.290276
23400	Amp	20	48943957	48957515	1.237378	0.295062
23401	Amp	20	48958137	48998372	1.334042	0.30211
23402	Amp	20	49000633	49020484	1.275021	0.297619
23403	Amp	20	49023474	49041106	1.208462	0.292832
23404	Amp	20	49043365	49054581	1.315861	0.30079
23405	Amp	20	49054893	49060834	1.364151	0.304145
23406	Amp	20	49062233	49082988	1.049532	0.281898
23407	Amp	20	49084227	49103674	1.190324	0.291645
23408	Amp	20	49103849	49184998	1.039426	0.281656
23409	Amp	20	49186437	49239695	0.41418	0.231134
23410	Amp	20	49239772	49241381	0.309523	0.221386
23411	Amp	20	49242385	49292084	0.27943	0.219105
23412	Amp	20	49294394	49336930	0.355162	0.225709
23413	Amp	20	49338594	49353727	0.444673	0.234051
23414	Amp	20	49356603	49359651	0.709359	0.256299
23415	Amp	20	49363104	49392908	0.805493	0.264374
23416	Amp	20	49394665	49427654	0.897922	0.270828
23417	Amp	20	49432334	49479298	1.099256	0.285555
23418	Amp	20	49480944	49500145	0.897922	0.270828
23419	Amp	20	49500361	49504240	0.843361	0.267157
23420	Amp	20	49509483	49529692	1.265443	0.297217
23421	Amp	20	49622075	49622075	1.415328	0.307507
23422	Amp	20	49623107	49649406	1.866286	0.337455
23423	Amp	20	49649755	49657680	1.737394	0.329379
23424	Amp	20	49663013	49681321	1.953191	0.34347
23425	Amp	20	49738173	49738173	3.079122	0.411313

23426 Amp	20	49742021	49845455	3.252252	0.421239
23427 Amp	20	49848258	49859162	3.006013	0.407149
23428 Amp	20	49863023	49866684	3.194439	0.418268
23429 Amp	20	49867086	49879826	3.394201	0.429954
23430 Amp	20	49881119	49904753	3.496514	0.436059
23431 Amp	20	49908290	49934112	2.914035	0.40171
23432 Amp	20	49936974	49936974	2.994162	0.406043
23433 Amp	20	49937166	49948419	2.806723	0.395183
23434 Amp	20	49949335	49955136	3.05489	0.409693
23435 Amp	20	49999680	49999680	4.116436	0.469216
23436 Amp	20	50001086	50007103	3.815003	0.452901
23437 Amp	20	50012420	50102403	3.461541	0.433927
23438 Amp	20	50102468	50115885	3.76435	0.450242
23439 Amp	20	50116773	50208920	3.415716	0.431195
23440 Amp	20	50210630	50241747	3.383286	0.428996
23441 Amp	20	50243701	50313216	3.217724	0.419591
23442 Amp	20	50313829	50377732	3.276597	0.422575
23443 Amp	20	50379182	50380840	3.600858	0.441622
23444 Amp	20	50383850	50441492	3.470866	0.4343
23445 Amp	20	50443691	50530318	3.415716	0.431316
23446 Amp	20	50530767	50545704	3.312745	0.425211
23447 Amp	20	50545958	50550174	3.288383	0.423671
23448 Amp	20	50555207	50558213	2.507231	0.377456
23449 Amp	20	50558229	50568538	2.635842	0.38494
23450 Amp	20	50569112	50569112	2.692246	0.388792
23451 Amp	20	50571723	50571723	2.866312	0.399084
23452 Amp	20	50571762	50574931	2.902384	0.400551
23453 Amp	20	50580393	50607032	3.041652	0.408913
23454 Amp	20	50611428	50617584	2.970355	0.405061
23455 Amp	20	50621309	50645357	3.079122	0.411003
23456 Amp	20	50652059	50804934	1.963469	0.343913
23457 Amp	20	50807606	50828909	2.135909	0.354562
23458 Amp	20	50830566	50840732	1.953191	0.342875
23459 Amp	20	50843836	50849178	1.525294	0.314821
23460 Amp	20	50856205	50879584	0.805493	0.264298
23461 Amp	20	50879688	50879688	0.734472	0.258814
23462 Amp	20	50880231	50896966	0.613517	0.248522
23463 Amp	20	50900458	50909163	1.295233	0.299045
23464 Amp	20	50909450	50923881	1.394558	0.306401
23465 Amp	20	50924048	50924048	1.514375	0.314075
23466 Amp	20	50924586	50934749	1.534719	0.315973
23467 Amp	20	50935560	50956703	1.748357	0.329879
23468 Amp	20	50957437	50969716	2.005494	0.34628
23469 Amp	20	50971849	50974027	1.963469	0.343569
23470 Amp	20	50974750	50984521	1.708768	0.327168
23471 Amp	20	50984942	51006045	1.844531	0.336043
23472 Amp	20	51006224	51009480	2.192225	0.358542
23473 Amp	20	51011742	51013381	2.170217	0.357033
23474 Amp	20	51017593	51027085	2.124253	0.354326
23475 Amp	20	51031065	51036345	1.983885	0.345452
23476 Amp	20	51036724	51045048	1.877398	0.338037
23477 Amp	20	51051070	51066740	2.327403	0.366456
23478 Amp	20	51068878	51068878	2.102209	0.352549

23479	Amp	20	51069424	51092037	2.180628	0.357651
23480	Amp	20	51092837	51108211	2.049537	0.349258
23481	Amp	20	51109316	51120465	1.748357	0.329781
23482	Amp	20	51121928	51122131	2.102209	0.352788
23483	Amp	20	51122171	51139689	2.192225	0.358404
23484	Amp	20	51140869	51140869	2.005494	0.346863
23485	Amp	20	51143362	51176644	1.953191	0.34336
23486	Amp	20	51179246	51217029	1.778794	0.332163
23487	Amp	20	51218611	51268832	1.898353	0.339467
23488	Amp	20	51269770	51281567	1.657426	0.323993
23489	Amp	20	51282542	51303370	1.553308	0.316883
23490	Amp	20	51305688	51311883	1.415328	0.3076
23491	Amp	20	51313156	51334994	1.49455	0.313268
23492	Amp	20	51344680	51358974	1.768015	0.331332
23493	Amp	20	51362368	51371520	1.907576	0.340439
23494	Amp	20	51372398	51383347	1.383826	0.305389
23495	Amp	20	51384294	51395916	1.305256	0.299773
23496	Amp	20	51396011	51411065	1.2565	0.29644
23497	Amp	20	51411195	51419806	1.315861	0.300758
23498	Amp	20	51425683	51425683	1.605306	0.320234
23499	Amp	20	51429660	51447067	1.811302	0.334196
23500	Amp	20	51449796	51454587	1.667574	0.3248
23501	Amp	20	51458429	51460204	2.005494	0.346792
23502	Amp	20	51468230	51471439	1.877398	0.338513
23503	Amp	20	51471726	51502908	2.071528	0.350471
23504	Amp	20	51503795	51525673	2.383144	0.370156
23505	Amp	20	51527437	51539420	2.249752	0.361396
23506	Amp	20	51547601	51556401	2.830877	0.396503
23507	Amp	20	51557519	51581487	3.424445	0.431863
23508	Amp	20	51581808	51616170	3.790167	0.451466
23509	Amp	20	51617892	51698542	3.252252	0.421273
23510	Amp	20	51706078	51775438	3.146436	0.415356
23511	Amp	20	51775452	51798120	3.207076	0.418868
23512	Amp	20	51802856	51806135	2.830877	0.396585
23513	Amp	20	51832686	51887598	2.530938	0.378585
23514	Amp	20	51891138	51894327	2.591575	0.382653
23515	Amp	20	51895640	51903082	2.447622	0.374008
23516	Amp	20	51904697	51934711	2.530938	0.378574
23517	Amp	20	51946940	51951581	2.591575	0.382048
23518	Amp	20	51955629	52005195	2.519	0.378537
23519	Amp	20	52006649	52088464	2.383144	0.369907
23520	Amp	20	52088582	52094863	2.359227	0.36844
23521	Amp	20	52096100	52096100	2.327403	0.366609
23522	Amp	20	52098274	52109853	2.294081	0.364376
23523	Amp	20	52112459	52118826	2.370685	0.369541
23524	Amp	20	52120996	52145709	2.30333	0.364875
23525	Amp	20	52149563	52168639	1.727244	0.328996
23526	Amp	20	52168644	52187221	1.442925	0.309311
23527	Amp	20	52195554	52195554	1.415328	0.307251
23528	Amp	20	52196969	52199515	1.463463	0.311124
23529	Amp	20	52201408	52204436	1.49455	0.312882
23530	Amp	20	52205786	52211819	1.442925	0.30962
23531	Amp	20	52220647	52227895	1.525294	0.315025

23532	Amp	20	52230305	52230410	1.404612	0.306606
23533	Amp	20	52235635	52237348	1.918998	0.340839
23534	Amp	20	52239085	52257981	1.534719	0.315995
23535	Amp	20	52261585	52268444	2.049537	0.349386
23536	Amp	20	52268873	52277319	2.458756	0.374496
23537	Amp	20	52278796	52300383	2.567803	0.381204
23538	Amp	20	52303502	52315423	2.670502	0.387032
23539	Amp	20	52315553	52318935	2.447622	0.374343
23540	Amp	20	52322708	52358175	3.171522	0.416577
23541	Amp	20	52360825	52389145	2.774551	0.393057
23542	Amp	20	52392723	52392975	3.112016	0.413455
23543	Amp	20	52392990	52408465	3.029636	0.408289
23544	Amp	20	52410941	52430078	3.348874	0.427003
23545	Amp	20	52430282	52430282	3.43725	0.43222
23546	Amp	20	52438254	52459229	3.48305	0.435498
23547	Amp	20	52459734	52478927	3.276597	0.423097
23548	Amp	20	52480342	52482675	3.348874	0.426977
23549	Amp	20	52483224	52601930	3.371695	0.428472
23550	Amp	20	52602461	52624065	3.299582	0.423907
23551	Amp	20	52625747	52676818	3.230962	0.420388
23552	Amp	20	52677103	52686534	3.066577	0.410462
23553	Amp	20	52692805	52696021	3.394201	0.429999
23554	Amp	20	52697243	52702582	3.688516	0.446271
23555	Amp	20	52729148	52744518	3.371695	0.428512
23556	Amp	20	52744745	52746469	3.637615	0.443608
23557	Amp	20	52747442	52776919	4.158928	0.471209
23558	Amp	20	52779053	52786983	4.679943	0.497399
23559	Amp	20	52787455	52842961	4.854009	0.505446
23560	Amp	20	52843566	52909511	5.047749	0.514075
23561	Amp	20	52909801	52909801	5.280955	0.52497
23562	Amp	20	52913853	52932721	5.193344	0.520903
23563	Amp	20	52934856	52937534	4.838865	0.504842
23564	Amp	20	52938156	52938156	4.540237	0.489971
23565	Amp	20	52940622	52953967	4.665596	0.496661
23566	Amp	20	52957680	52962433	4.971485	0.510711
23567	Amp	20	52962962	52973995	4.868506	0.505776
23568	Amp	20	52974738	52979977	5.179405	0.520647
23569	Amp	20	52982503	52996894	4.838865	0.504375
23570	Amp	20	53005383	53024832	4.497567	0.487812
23571	Amp	20	53026590	53037986	4.540237	0.490254
23572	Amp	20	53041464	53041464	4.327765	0.478884
23573	Amp	20	53042634	53108626	4.022326	0.463788
23574	Amp	20	53110370	53115084	4.391125	0.482345
23575	Amp	20	53117584	53117962	4.651516	0.495577
23576	Amp	20	53121481	53147226	5.179405	0.520235
23577	Amp	20	53153065	53166147	5.337978	0.528097
23578	Amp	20	53167661	53169551	5.696665	0.544124
23579	Amp	20	53170211	53171178	5.654252	0.542568
23580	Amp	20	53171305	53172063	5.31131	0.526541
23581	Amp	20	53175482	53245509	5.669031	0.543192
23582	Amp	20	53246899	53251480	5.638575	0.541433
23583	Amp	20	53252587	53276718	5.997601	0.559155
23584	Amp	20	53280576	53280576	6.10345	0.563839

23585 Amp	20	53291517	53303166	5.710355	0.545241
23586 Amp	20	53303599	53312303	6.604027	0.586197
23587 Amp	20	53315842	53329936	6.212874	0.568475
23588 Amp	20	53333816	53342792	6.133613	0.565197
23589 Amp	20	53343426	53398872	5.897742	0.553741
23590 Amp	20	53402873	53408464	5.696665	0.544186
23591 Amp	20	53410420	53427809	6.058244	0.561681
23592 Amp	20	53433789	53433789	6.117292	0.564511
23593 Amp	20	53434226	53434904	6.013103	0.559294
23594 Amp	20	53439069	53439410	6.073269	0.562092
23595 Amp	20	53441149	53510548	6.288489	0.57161
23596 Amp	20	53513125	53513125	6.903728	0.599629
23597 Amp	20	53513902	53520661	7.952174	0.643462
23598 Amp	20	53525664	53540388	9.297696	0.698026
23599 Amp	20	53545858	53546628	10.468401	0.743373
23600 Amp	20	53547098	53547194	10.831215	0.757372
23601 Amp	20	53548676	53738792	11.666631	0.789695
23602 Amp	20	53743012	53747068	10.34731	0.739172
23603 Amp	20	53748221	53811681	10.075027	0.728789
23604 Amp	20	53814340	53889922	10.452292	0.74275
23605 Amp	20	53891365	53891365	10.111383	0.72997
23606 Amp	20	53898126	53936241	9.686	0.713228
23607 Amp	20	53937819	53940155	9.788989	0.717364
23608 Amp	20	53942148	53951542	9.896323	0.721497
23609 Amp	20	53953690	53958303	9.948936	0.723714
23610 Amp	20	53958507	53967272	9.436384	0.703776
23611 Amp	20	53967367	53987347	9.368074	0.700978
23612 Amp	20	53987877	54008043	9.061723	0.688721
23613 Amp	20	54012265	54028228	8.504106	0.666103
23614 Amp	20	54050346	54061069	7.867314	0.639826
23615 Amp	20	54061557	54122985	8.050507	0.647284
23616 Amp	20	54124819	54200372	7.538447	0.626227
23617 Amp	20	54200852	54202971	7.265098	0.614506
23618 Amp	20	54204148	54210499	7.538447	0.626206
23619 Amp	20	54210729	54249296	7.714123	0.633938
23620 Amp	20	54251351	54261209	7.523003	0.62582
23621 Amp	20	54261239	54275376	7.314626	0.616463
23622 Amp	20	54276705	54339799	6.966987	0.602259
23623 Amp	20	54343315	54343525	7.346492	0.618089
23624 Amp	20	54343850	54368619	7.183547	0.61138
23625 Amp	20	54377895	54380888	7.392813	0.620046
23626 Amp	20	54381888	54441894	6.760799	0.593066
23627 Amp	20	54442231	54443344	6.840128	0.596857
23628 Amp	20	54448614	54458447	5.940125	0.555901
23629 Amp	20	54459698	54498646	5.982794	0.558483
23630 Amp	20	54506136	54514051	5.489676	0.534854
23631 Amp	20	54514507	54548733	5.897742	0.554056
23632 Amp	20	54549073	54575372	5.367722	0.529431
23633 Amp	20	54576175	54627659	4.957051	0.510229
23634 Amp	20	54630160	54650572	5.489676	0.534832
23635 Amp	20	54651465	54672085	5.870027	0.55287
23636 Amp	20	54675549	54676668	6.08935	0.562834
23637 Amp	20	54680465	54689937	5.710355	0.545187

23638	Amp	20	54691925	54698688	5.413899	0.531712
23639	Amp	20	54700477	54739821	4.364676	0.481283
23640	Amp	20	54743554	54751619	4.30031	0.477492
23641	Amp	20	54757016	54797778	4.063271	0.466483
23642	Amp	20	54798000	54816560	4.215025	0.473773
23643	Amp	20	54820478	54877451	3.971602	0.461179
23644	Amp	20	54877489	54897841	4.022326	0.463752
23645	Amp	20	54908976	54909293	3.777346	0.451274
23646	Amp	20	54911778	54921672	3.893345	0.456912
23647	Amp	20	54942654	54994747	3.790167	0.451576
23648	Amp	20	54995601	55023269	3.675606	0.445749
23649	Amp	20	55025848	55051924	3.348874	0.426767
23650	Amp	20	55059827	55077492	3.26417	0.421854
23651	Amp	20	55078383	55093267	3.32332	0.42583
23652	Amp	20	55093626	55098899	3.403911	0.430242
23653	Amp	20	55104286	55118734	2.88973	0.400234
23654	Amp	20	55120490	55129442	3.157622	0.416186
23655	Amp	20	55130423	55138184	3.079122	0.411502
23656	Amp	20	55141064	55145990	2.635842	0.385373
23657	Amp	20	55146169	55174500	2.796399	0.394693
23658	Amp	20	55174719	55199963	2.614747	0.383849
23659	Amp	20	55200099	55284748	2.30333	0.364946
23660	Amp	20	55285679	55320035	2.683697	0.387936
23661	Amp	20	55320743	55322871	3.171522	0.416914
23662	Amp	20	55323018	55330410	3.079122	0.411509
23663	Amp	20	55333515	55398865	2.98257	0.405829
23664	Amp	20	55412978	55438182	2.495444	0.376662
23665	Amp	20	55445659	55469393	2.061276	0.350335
23666	Amp	20	55469504	55478628	2.039783	0.34884
23667	Amp	20	55478773	55492803	2.180628	0.357511
23668	Amp	20	55493641	55497046	2.124253	0.354011
23669	Amp	20	55499842	55563648	2.294081	0.364242
23670	Amp	20	55564305	55585507	1.800655	0.333377
23671	Amp	20	55589786	55606804	2.470601	0.375263
23672	Amp	20	55607106	55621283	1.877398	0.33815
23673	Amp	20	55623046	55709462	2.203122	0.359127
23674	Amp	20	55714731	55715737	2.647166	0.385817
23675	Amp	20	55716346	55719303	2.483202	0.376239
23676	Amp	20	55727340	55728230	2.148338	0.355261
23677	Amp	20	55729234	55753639	1.995109	0.346154
23678	Amp	20	55755759	55781918	2.170217	0.356991
23679	Amp	20	55784586	55784586	2.294081	0.36451
23680	Amp	20	55784804	55789773	2.283191	0.364086
23681	Amp	20	55789981	55806510	2.359227	0.368261
23682	Amp	20	55810593	55813369	2.994162	0.40649
23683	Amp	20	55816265	55835879	2.507231	0.377512
23684	Amp	20	55843792	55845571	1.697113	0.326989
23685	Amp	20	55846073	55859068	1.856336	0.337281
23686	Amp	20	55869237	55956463	2.005494	0.346915
23687	Amp	20	55957006	56027228	2.214215	0.359445
23688	Amp	20	56027696	56126907	2.507231	0.37723
23689	Amp	20	56127356	56151575	2.543219	0.379702
23690	Amp	20	56158572	56162992	2.148338	0.355692

23691	Amp	20	56166079	56171497	2.806723	0.395711
23692	Amp	20	56171971	56206760	2.98257	0.40584
23693	Amp	20	56209922	56224660	3.288383	0.423299
23694	Amp	20	56225590	56242106	2.658709	0.386332
23695	Amp	20	56242766	56250370	3.26417	0.421875
23696	Amp	20	56251582	56264799	2.98257	0.405663
23697	Amp	20	56265650	56270449	2.843135	0.397367
23698	Amp	20	56273921	56304152	2.866312	0.398964
23699	Amp	20	56304852	56313111	2.737964	0.391525
23700	Amp	20	56313929	56324911	3.335884	0.42606
23701	Amp	20	56326941	56327734	2.935659	0.40311
23702	Amp	20	56328410	56329670	2.393797	0.370736
23703	Amp	20	56329768	56357446	2.238069	0.361278
23704	Amp	20	56358048	56371124	2.567803	0.381258
23705	Amp	20	56373185	56383044	3.403911	0.430184
23706	Amp	20	56384985	56384985	3.029636	0.408578
23707	Amp	20	56388247	56401897	3.32332	0.425631
23708	Amp	20	56408717	56444783	3.613533	0.441995
23709	Amp	20	56449349	56459548	3.573122	0.440204
23710	Amp	20	56460938	56465112	3.600858	0.441739
23711	Amp	20	56465609	56491577	3.288383	0.423878
23712	Amp	20	56492079	56492079	3.32332	0.425284
23713	Amp	20	56492115	56509176	3.394201	0.429726
23714	Amp	20	56512409	56516601	4.022326	0.46434
23715	Amp	20	56521396	56541941	3.946237	0.460136
23716	Amp	20	56558355	56558355	3.637615	0.443771
23717	Amp	20	56560330	56560330	4.416662	0.484297
23718	Amp	20	56563359	56573752	4.455933	0.485864
23719	Amp	20	56574229	56582080	4.483097	0.487472
23720	Amp	20	56584542	56596638	4.30031	0.477928
23721	Amp	20	56598490	56611158	4.391125	0.482354
23722	Amp	20	56615053	56615053	4.30031	0.477928
23723	Amp	20	56615410	56646968	3.359177	0.427685
23724	Amp	20	56647905	56662442	3.613533	0.442268
23725	Amp	20	56666547	56667414	3.45062	0.433266
23726	Amp	20	56673474	56719483	3.72674	0.448321
23727	Amp	20	56781498	56802539	3.920712	0.45884
23728	Amp	20	56803125	56827779	4.131279	0.469723
23729	Amp	20	56854768	56858185	4.285602	0.477415
23730	Amp	20	56858456	56922490	4.444356	0.485046
23731	Amp	20	56924954	56944268	4.623661	0.494204
23732	Amp	20	56944289	56960009	4.497567	0.487801
23733	Amp	20	56960026	56971521	4.355429	0.480533
23734	Amp	20	56972093	56976391	4.497567	0.488198
23735	Amp	20	56976426	56989636	4.809024	0.503411
23736	Amp	20	56991257	57029753	5.001595	0.512413
23737	Amp	20	57029793	57036756	5.252642	0.524066
23738	Amp	20	57038156	57038156	5.16411	0.519624
23739	Amp	20	57038942	57052158	4.882637	0.506417
23740	Amp	20	57061636	57115657	4.751695	0.50064
23741	Amp	20	57120021	57120782	4.665596	0.496663
23742	Amp	20	57121595	57141327	4.402792	0.483254
23743	Amp	20	57143082	57177948	4.444356	0.485048

23744	Amp	20	57178026	57181853	4.793807	0.502303
23745	Amp	20	57189680	57210562	4.765369	0.500869
23746	Amp	20	57216402	57223515	4.009908	0.463257
23747	Amp	20	57225240	57231671	3.985282	0.461851
23748	Amp	20	57232491	57277413	3.933803	0.459458
23749	Amp	20	57282855	57307267	4.355429	0.480625
23750	Amp	20	57307719	57361826	4.327765	0.479059
23751	Amp	20	57362310	57400022	4.511044	0.489007
23752	Amp	20	57403942	57421679	5.337978	0.528206
23753	Amp	20	57421772	57437618	5.608185	0.540335
23754	Amp	20	57440299	57440299	5.383269	0.529926
23755	Amp	20	57441456	57461232	5.121758	0.517735
23756	Amp	20	57462639	57473765	5.608185	0.540305
23757	Amp	20	57479473	57487747	5.910477	0.554888
23758	Amp	20	57490787	57498582	5.576912	0.538783
23759	Amp	20	57501075	57514360	5.519766	0.536242
23760	Amp	20	57519300	57529936	6.133613	0.565
23761	Amp	20	57532163	57547669	5.724602	0.546149
23762	Amp	20	57548163	57558205	5.884474	0.553416
23763	Amp	20	57562751	57562751	5.489676	0.534699
23764	Amp	20	57562778	57568069	4.868506	0.505941
23765	Amp	20	57568113	57578843	4.551777	0.490864
23766	Amp	20	57578964	57586679	4.30031	0.477763
23767	Amp	20	57589592	57600534	5.324711	0.527089
23768	Amp	20	57600696	57600696	5.503852	0.535611
23769	Amp	20	57604819	57628541	4.765369	0.500997
23770	Amp	20	57630443	57650391	4.897525	0.507279
23771	Amp	20	57650563	57680342	5.001595	0.511873
23772	Amp	20	57684073	57722465	5.179405	0.520097
23773	Amp	20	57722559	57724100	5.399054	0.530625
23774	Amp	20	57724596	57727961	5.534438	0.536956
23775	Amp	20	57728592	57785486	5.940125	0.556129
23776	Amp	20	57786511	57787496	6.274109	0.57152
23777	Amp	20	57794957	57801271	5.324711	0.526966
23778	Amp	20	57804838	57809468	5.813919	0.550057
23779	Amp	20	57810479	57867460	5.324711	0.526966
23780	Amp	20	57868332	57888607	5.534438	0.53666
23781	Amp	20	57890601	57897078	5.769527	0.547626
23782	Amp	20	57898770	57912333	5.870027	0.552633
23783	Amp	20	57912752	57945554	5.769527	0.547794
23784	Amp	20	57945599	57973335	5.870027	0.553014
23785	Amp	20	57975718	58015178	5.84242	0.551406
23786	Amp	20	58016667	58092489	5.31131	0.526425
23787	Amp	20	58093032	58148814	5.107515	0.517274
23788	Amp	20	58150004	58196113	4.868506	0.505692
23789	Amp	20	58200075	58213032	4.651516	0.496019
23790	Amp	20	58213073	58237681	4.551777	0.491012
23791	Amp	20	58238293	58249402	4.376421	0.48175
23792	Amp	20	58251612	58426637	4.470731	0.486588
23793	Amp	20	58428350	58470360	4.694037	0.498072
23794	Amp	20	58470611	58481874	4.854009	0.505625
23795	Amp	20	58490397	58569956	4.679943	0.49697
23796	Amp	20	58570145	58574730	4.765369	0.501275

23797	Amp	20	58574910	58589024	4.868506	0.506105
23798	Amp	20	58589475	58685234	4.623661	0.494621
23799	Amp	20	58685864	58704865	4.540237	0.490316
23800	Amp	20	58709977	58719097	4.566654	0.491841
23801	Amp	20	58720032	58743738	4.215025	0.473631
23802	Amp	20	58743774	58756836	3.893345	0.457364
23803	Amp	20	58757318	58757318	3.985282	0.462196
23804	Amp	20	58760793	58761247	4.927103	0.508418
23805	Amp	20	58762972	58766850	5.798921	0.549183
23806	Amp	20	58766873	58776648	5.681709	0.543943
23807	Amp	20	58777109	58777109	5.459172	0.533712
23808	Amp	20	58778527	58778527	5.236731	0.522999
23809	Amp	20	58779351	58782339	5.107515	0.517197
23810	Amp	20	58786824	58858287	4.158928	0.470975
23811	Amp	20	58862062	58900339	5.107515	0.51702
23812	Amp	20	58902687	58934254	4.364676	0.481015
23813	Amp	20	58938052	59009835	5.062558	0.514598
23814	Amp	20	59012333	59036339	4.941808	0.509765
23815	Amp	20	59038947	59059332	5.324711	0.52754
23816	Amp	20	59060631	59077546	5.193344	0.52118
23817	Amp	20	59078361	59080987	5.092203	0.51635
23818	Amp	20	59082222	59091822	4.868506	0.505777
23819	Amp	20	59092564	59288966	4.581809	0.492485
23820	Amp	20	59290821	59316934	4.793807	0.502289
23821	Amp	20	59317619	59336740	4.679943	0.49707
23822	Amp	20	59336837	59402380	4.391125	0.482449
23823	Amp	20	59408822	59429936	4.34102	0.479999
23824	Amp	20	59430928	59494525	3.958753	0.460886
23825	Amp	20	59495489	59573058	3.701758	0.446815
23826	Amp	20	59580341	59640727	3.371695	0.428281
23827	Amp	20	59645553	59679313	3.217724	0.419421
23828	Amp	20	59679885	59748968	3.43725	0.432322
23829	Amp	20	59749143	59776574	3.549256	0.438968
23830	Amp	20	59777735	59782884	3.650307	0.44431
23831	Amp	20	59784185	59817148	3.86957	0.455775
23832	Amp	20	59817947	59845181	3.146436	0.415009
23833	Amp	20	59848006	59857564	3.252252	0.421731
23834	Amp	20	59859232	59862601	3.312745	0.424894
23835	Amp	20	59878803	59901012	3.394201	0.42944
23836	Amp	20	59905838	59907417	3.32332	0.425399
23837	Amp	20	59912717	59931696	3.029636	0.408593
23838	Amp	20	59936107	59943710	3.32332	0.42578
23839	Amp	20	59947198	59948854	3.573122	0.440014
23840	Amp	20	59949474	59974532	3.288383	0.423559
23841	Amp	20	59977070	59986935	3.509701	0.436272
23842	Amp	20	59990466	60018184	3.625861	0.442615
23843	Amp	20	60018435	60046878	3.958753	0.460903
23844	Amp	20	60049357	60099970	3.790167	0.451996
23845	Amp	20	60101461	60161551	3.079122	0.411208
23846	Amp	20	60167379	60174874	3.461541	0.433939
23847	Amp	20	60175309	60227854	3.790167	0.451996
23848	Amp	20	60228190	60237982	3.650307	0.444444
23849	Amp	20	60239370	60283384	3.403911	0.430209

23850	Amp	20	60289982	60310619	3.230962	0.419955
23851	Amp	20	60310633	60363111	2.703608	0.38942
23852	Amp	20	60363653	60378241	2.415622	0.372165
23853	Amp	20	60378667	60489368	2.283191	0.363465
23854	Amp	20	60489786	60515850	2.30333	0.364958
23855	Amp	20	60523338	60526857	2.994162	0.406547
23856	Amp	20	60532296	60594159	3.089932	0.411759
23857	Amp	20	60595301	60613671	2.830877	0.396813
23858	Amp	20	60614880	60696010	3.207076	0.419042
23859	Amp	20	60696532	60710904	3.112016	0.413366
23860	Amp	20	60711612	60758364	3.029636	0.408154
23861	Amp	20	60761032	60776816	3.194439	0.418099
23862	Amp	20	60777120	60778074	3.312745	0.424801
23863	Amp	20	60780659	60785768	2.71525	0.389984
23864	Amp	20	60789206	60840114	2.415622	0.371695
23865	Amp	20	60843124	60892575	3.112016	0.413479
23866	Amp	20	60892771	60896370	3.394201	0.430053
23867	Amp	20	60899371	60910241	3.26417	0.422425
23868	Amp	20	60910572	60915234	2.958746	0.40465
23869	Amp	20	60915639	60918444	2.647166	0.385749
23870	Amp	20	60918503	60925166	2.750393	0.392071
23871	Amp	20	60927832	60928562	2.180628	0.357914
23872	Amp	20	60928861	60949614	2.370685	0.369298
23873	Amp	20	60950132	60973708	2.124253	0.353968
23874	Amp	20	60974064	60974794	1.942393	0.342584
23875	Amp	20	60975108	60989035	1.678649	0.325198
23876	Amp	20	61032969	61045023	2.703608	0.389047
23877	Amp	20	61048793	61051920	2.591575	0.382346
23878	Amp	20	61052622	61077834	2.902384	0.400542
23879	Amp	20	61078297	61081706	3.006013	0.406961
23880	Amp	20	61085348	61122464	3.637615	0.443599
23881	Amp	20	61122632	61154970	3.523011	0.43718
23882	Amp	20	61158176	61206711	3.713958	0.447652
23883	Amp	20	61209353	61210070	2.843135	0.397223
23884	Amp	20	61210361	61216486	2.866312	0.398674
23885	Amp	20	61217600	61261070	2.383144	0.369887
23886	Amp	20	61274641	61274641	2.214215	0.359415
23887	Amp	20	61278882	61295317	2.30333	0.364823
23888	Amp	20	61295888	61319399	2.806723	0.39547
23889	Amp	20	61320231	61369822	2.902384	0.400682
23890	Amp	20	61370842	61380012	2.784457	0.393725
23891	Amp	20	61382133	61403923	2.948071	0.403493
23892	Amp	20	61406011	61424972	2.727427	0.390804
23893	Amp	20	61434025	61439901	2.580481	0.381904
23894	Amp	20	61440237	61444881	2.519	0.378434
23895	Amp	20	61510606	61510606	3.461541	0.433778
23896	Amp	20	61511162	61511162	3.777346	0.451321
23897	Amp	20	61512800	61548444	4.228481	0.474102
23898	Amp	20	61550971	61551101	4.257976	0.475745
23899	Amp	20	61551302	61572088	4.470731	0.48657
23900	Amp	20	61572804	61584921	4.257976	0.475745
23901	Amp	20	61589644	61589644	4.497567	0.487823
23902	Amp	20	61592662	61655233	4.665596	0.496438

23903	Amp	20	61655686	61707906	4.751695	0.500501
23904	Amp	20	61713053	61715436	4.285602	0.476811
23905	Amp	20	61715477	61732375	4.483097	0.487234
23906	Amp	20	61733789	61733789	4.364676	0.481248
23907	Amp	20	61733835	61763816	3.946237	0.460283
23908	Amp	20	61763977	61783213	3.998681	0.462587
23909	Amp	20	61789636	61806439	4.049098	0.46566
23910	Amp	20	61811589	61825779	4.103115	0.468452
23911	Amp	20	61829284	61829284	3.737077	0.448822
23912	Amp	20	61834745	61847144	3.335884	0.426401
23913	Amp	20	61847174	61859736	3.05489	0.410074
23914	Amp	20	61860426	61867884	3.359177	0.427989
23915	Amp	20	61868276	61883012	3.470866	0.434675
23916	Amp	20	61886923	61915858	4.313672	0.478409
23917	Amp	20	61917580	61936132	4.391125	0.482608
23918	Amp	20	61940219	62009304	3.76435	0.450474
23919	Amp	20	62011419	62041108	3.006013	0.406741
23920	Amp	20	62041977	62061571	2.692246	0.388545
23921	Amp	20	62061673	62115992	2.625007	0.384482
23922	Amp	20	62116015	62124283	2.692246	0.388302
23923	Amp	20	62125182	62136161	3.312745	0.425257
23924	Amp	20	62137038	62153711	2.922915	0.402347
23925	Amp	20	62161807	62170056	3.099988	0.412788
23926	Amp	20	62175120	62188341	2.922915	0.402347
23927	Amp	20	62193129	62223256	3.066577	0.410201
23928	Amp	20	62231695	62246779	2.922915	0.402347
23929	Amp	20	62250539	62258814	2.878516	0.399491
23930	Amp	20	62259273	62263060	2.970355	0.405083
23931	Amp	20	62263656	62275054	3.146436	0.414986
23932	Amp	20	62280406	62296652	3.207076	0.418581
23933	Amp	20	62297639	62316232	3.288383	0.423226
23934	Amp	20	62331766	62338071	2.948071	0.40368
23935	Amp	20	62340293	62340293	2.737964	0.391234
23936	Amp	20	62344904	62345851	2.866312	0.398626
23937	Amp	20	62345988	62405352	2.914035	0.401676
23938	Amp	20	62422274	62426487	2.806723	0.395066
23939	Amp	20	62431058	62479776	3.182419	0.417512
23940	Amp	20	62479805	62479907	3.157622	0.415869
23941	Amp	20	62483744	62546280	3.394201	0.429444
23942	Amp	20	62547734	62547989	3.470866	0.434431
23943	Amp	20	62549702	62611524	3.359177	0.427744
23944	Amp	20	62632769	62633163	3.146436	0.415064
23945	Amp	20	62636772	62658391	3.312745	0.42519
23946	Amp	20	62663245	62741749	3.600858	0.441281
23947	Amp	20	62741794	62751641	3.523011	0.437393
23948	Amp	20	62753168	62814690	3.650307	0.443872
23949	Amp	20	62816407	62876389	3.560951	0.439228
23950	Amp	20	62883022	63027448	3.45062	0.433354
23951	Amp	20	63035453	63070677	3.600858	0.44117
23952	Amp	20	63074600	63075398	3.403911	0.430388
23953	Amp	20	63077356	63080185	3.613533	0.442311
23954	Amp	20	63141749	63148134	3.75156	0.449923
23955	Amp	20	63148543	63148632	3.523011	0.43722

23956 Amp	20	63155226	63212292	3.600858	0.441714
23957 Amp	20	63222837	63269158	3.893345	0.456977
23958 Amp	20	63278747	63335424	3.650307	0.444149
23959 Amp	20	63335685	63345036	3.535174	0.438181
23960 Amp	20	63357759	63398358	3.371695	0.428054
23961 Amp	20	63400682	63401318	4.228481	0.474099
23962 Amp	20	63403750	63468033	4.257976	0.475845
23963 Amp	20	63474178	63526293	4.470731	0.48677
23964 Amp	20	63526406	63531914	4.581809	0.49216
23965 Amp	20	63539265	63556063	5.252642	0.523657
23966 Amp	20	63556289	63556493	5.548259	0.537345
23967 Amp	20	63556719	63588502	5.31131	0.52642
23968 Amp	21	13974127	14222050	0	0.047923
23969 Amp	21	14222144	14407485	0	0.049792
23970 Amp	21	14409873	14696137	0	0.053958
23971 Amp	21	14700815	14716931	0	0.08117
23972 Amp	21	14719054	15060614	0	0.062708
23973 Amp	21	15062902	15100864	0	0.074919
23974 Amp	21	15101436	15141022	0	0.078583
23975 Amp	21	15149296	15378105	0	0.088095
23976 Amp	21	15378770	15419055	0	0.086226
23977 Amp	21	15422260	15782437	0	0.087668
23978 Amp	21	15782767	15788391	0	0.083503
23979 Amp	21	15789003	15911483	0	0.091153
23980 Amp	21	15924059	16127920	0	0.085771
23981 Amp	21	16135498	16361926	0	0.09917
23982 Amp	21	16362365	16409783	0	0.1055
23983 Amp	21	16418755	16449779	0	0.076789
23984 Amp	21	16449916	16606602	0	0.075347
23985 Amp	21	16606699	16674974	0	0.089092
23986 Amp	21	16675274	16697960	0	0.075347
23987 Amp	21	16701138	16710688	0	0.064565
23988 Amp	21	16710837	16843735	0	0.047601
23989 Amp	21	16844862	16891705	0	0.035602
23990 Amp	21	16895214	17485565	0	0.025272
23991 Amp	21	17485590	17700740	0	0.01743
23992 Amp	21	17700964	17766681	0	0.018848
23993 Amp	21	17768106	18060106	0	0.017259
23994 Amp	21	18061123	18421572	0	0.018904
23995 Amp	21	18422688	21418616	0	0.022239
23996 Amp	21	21418807	21422852	0	0.020822
23997 Amp	21	21426535	21441302	0	0.03874
23998 Amp	21	21446493	21449116	0	0.0402
23999 Amp	21	21449323	21637211	0	0.043544
24000 Amp	21	21651410	21957383	0	0.046174
24001 Amp	21	21959749	22206936	0	0.036509
24002 Amp	21	22214829	22358849	0	0.033879
24003 Amp	21	22359384	22379557	0	0.032234
24004 Amp	21	22576499	22644664	0	0.023269
24005 Amp	21	22673071	23304957	0	0.019925
24006 Amp	21	23305334	23543064	0	0.024655
24007 Amp	21	23548743	23690318	0	0.030356
24008 Amp	21	23690559	23734300	0	0.037438

24009	Amp	21	23828538	23836756	0	0.025626
24010	Amp	21	23836840	23933114	0	0.019925
24011	Amp	21	23933295	23995526	0	0.010413
24012	Amp	21	23996439	25002925	0	0.001461
24013	Amp	21	25004750	25188473	0	0
24014	Amp	21	25192750	25220971	0	0.001479
24015	Amp	21	25222056	25280482	0	0
24016	Amp	21	25282634	25297799	0	0.005333
24017	Amp	21	25302149	25647424	0	0
24018	Amp	21	25648738	25805996	0	0.005842
24019	Amp	21	25879390	25892272	0	0.014832
24020	Amp	21	25894163	25927322	0	0.031744
24021	Amp	21	25929026	26176012	0	0.019532
24022	Amp	21	26177119	26286180	0	0.016912
24023	Amp	21	26286586	27769893	0	0
24024	Amp	21	27770775	27842943	0	0.001934
24025	Amp	21	27843039	28519227	0	0
24026	Amp	21	28522929	28568305	0	0.008329
24027	Amp	21	28571861	28585401	0	0.020537
24028	Amp	21	28586571	28665556	0	0.008329
24029	Amp	21	28666142	29148391	0	0
24030	Amp	21	29148586	29513805	0	0.001994
24031	Amp	21	29516557	29947779	0	0
24032	Amp	21	29948391	30346807	0	0.009294
24033	Amp	21	30348682	30572911	0	0.013524
24034	Amp	21	30573704	30638389	0	0.043821
24035	Amp	21	30638739	30706741	0	0.03959
24036	Amp	21	30707396	30707396	0	0.022184
24037	Amp	21	30879355	31025402	0	0.031345
24038	Amp	21	31025583	31046770	0	0.06793
24039	Amp	21	31049439	31106006	0	0.058636
24040	Amp	21	31111760	31183363	0	0.083203
24041	Amp	21	31183479	31620260	0	0.069465
24042	Amp	21	31626585	31738124	0	0.070931
24043	Amp	21	31742367	31791299	0	0.042794
24044	Amp	21	31795042	31912023	0	0.035194
24045	Amp	21	31912037	31977629	0	0.050906
24046	Amp	21	31981052	32002488	0	0.059629
24047	Amp	21	32009187	32039601	0	0.050906
24048	Amp	21	32042863	32098850	0	0.055616
24049	Amp	21	32099019	32133687	0	0.053703
24050	Amp	21	32135492	32144322	0	0.043199
24051	Amp	21	32145542	32186606	0	0.045281
24052	Amp	21	32188154	32211032	0	0.052229
24053	Amp	21	32211506	32328157	0	0.050763
24054	Amp	21	32331727	32349624	0	0.066511
24055	Amp	21	32350724	32427943	0	0.075042
24056	Amp	21	32430198	32636222	0	0.079562
24057	Amp	21	32636259	32660306	0	0.090541
24058	Amp	21	32663495	32762223	0	0.071767
24059	Amp	21	32766659	32770912	0	0.07895
24060	Amp	21	32772697	32856308	0	0.063451
24061	Amp	21	32858923	33102447	0	0.061369

24062 Amp	21	33112544	33133961	0	0.064352
24063 Amp	21	33134000	33165395	0	0.060825
24064 Amp	21	33166506	33208728	0	0.068491
24065 Amp	21	33210880	33227587	0	0.071924
24066 Amp	21	33230944	33320706	0	0.068941
24067 Amp	21	33329071	33360774	0	0.079421
24068 Amp	21	33361336	33420603	0	0.075764
24069 Amp	21	33425808	33430243	0	0.079684
24070 Amp	21	33434808	33463622	0	0.08949
24071 Amp	21	33464158	33484000	0	0.102425
24072 Amp	21	33499733	33511491	0	0.08949
24073 Amp	21	33512954	33537914	0	0.095439
24074 Amp	21	33542841	33553344	0	0.092006
24075 Amp	21	33558748	33582476	0	0.098287
24076 Amp	21	33582823	33594460	0	0.094368
24077 Amp	21	33599151	33647807	0	0.101576
24078 Amp	21	33693310	33836673	0	0.09177
24079 Amp	21	33837105	33980754	0	0.085489
24080 Amp	21	33981573	34015961	0	0.103189
24081 Amp	21	34016335	34113938	0	0.085489
24082 Amp	21	34116452	34125660	0	0.075009
24083 Amp	21	34126118	34216838	0	0.07828
24084 Amp	21	34218042	34294923	0	0.085977
24085 Amp	21	34298687	34311204	0	0.078769
24086 Amp	21	34312670	34534792	0	0.084746
24087 Amp	21	34534878	34568534	0	0.078769
24088 Amp	21	34570746	34573449	0	0.09062
24089 Amp	21	34578400	34606773	0	0.084671
24090 Amp	21	34606830	34616483	0	0.102265
24091 Amp	21	34617810	34640603	0	0.086517
24092 Amp	21	34642609	34644923	0	0.068923
24093 Amp	21	34646696	34752817	0	0.08649
24094 Amp	21	34752936	34920203	0	0.06793
24095 Amp	21	34922137	35162989	0	0.075564
24096 Amp	21	35164158	35214947	0	0.085923
24097 Amp	21	35216262	35234289	0	0.074072
24098 Amp	21	35235450	35245773	0	0.085358
24099 Amp	21	35249206	35294436	0	0.074072
24100 Amp	21	35297149	35403301	0	0.063713
24101 Amp	21	35403424	35865049	0	0.069457
24102 Amp	21	35871831	35920135	0	0.073459
24103 Amp	21	35920757	36062147	0	0.08397
24104 Amp	21	36064338	36472015	0	0.070328
24105 Amp	21	36472664	36488749	0	0.055042
24106 Amp	21	36489260	36513491	0	0.075982
24107 Amp	21	36514475	36554210	0	0.107479
24108 Amp	21	36560880	36560880	0	0.124313
24109 Amp	21	36563590	36721165	0	0.092816
24110 Amp	21	36724815	36725971	0	0.104909
24111 Amp	21	36728505	36849033	0	0.110137
24112 Amp	21	36853826	36985997	0	0.125722
24113 Amp	21	36986332	37012765	0	0.119983
24114 Amp	21	37013458	37029682	0	0.134696

24115	Amp	21	37031534	37123813	0	0.180468
24116	Amp	21	37124542	37132773	0	0.187797
24117	Amp	21	37137322	37188655	0	0.176674
24118	Amp	21	37189077	37310900	0	0.179309
24119	Amp	21	37319970	37362508	0.043943	0.193887
24120	Amp	21	37372591	37414694	0	0.186739
24121	Amp	21	37415115	37431526	0.033661	0.192514
24122	Amp	21	37434170	37436560	0	0.165396
24123	Amp	21	37436925	37487876	0	0.150818
24124	Amp	21	37495245	37502517	0	0.139844
24125	Amp	21	37505545	37625372	0	0.105196
24126	Amp	21	37628210	37647381	0	0.101924
24127	Amp	21	37648633	37689497	0	0.142353
24128	Amp	21	37690466	37742817	0	0.11819
24129	Amp	21	37742921	37819437	0	0.111205
24130	Amp	21	37819835	37861757	0	0.10857
24131	Amp	21	37862042	37942765	0	0.11933
24132	Amp	21	37946151	37989243	0	0.106884
24133	Amp	21	37989848	38009793	0	0.126952
24134	Amp	21	38010056	38034890	0	0.106884
24135	Amp	21	38036673	38213974	0	0.081197
24136	Amp	21	38217052	38535804	0	0.091005
24137	Amp	21	38535856	38566173	0	0.102035
24138	Amp	21	38566488	38584905	0	0.117095
24139	Amp	21	38587655	38712480	0	0.104665
24140	Amp	21	38718046	38732027	0	0.094001
24141	Amp	21	38783137	38810253	0	0.087994
24142	Amp	21	38815826	38818612	0	0.084106
24143	Amp	21	38818682	38915314	0	0.091973
24144	Amp	21	38919263	38922431	0	0.103507
24145	Amp	21	38923252	38935301	0	0.116631
24146	Amp	21	38936477	39028082	0	0.140461
24147	Amp	21	39028196	39070913	0	0.152295
24148	Amp	21	39071690	39134268	0	0.139483
24149	Amp	21	39134798	39159380	0	0.125808
24150	Amp	21	39159764	39170487	0	0.150299
24151	Amp	21	39176161	39206620	0	0.141138
24152	Amp	21	39207758	39276326	0	0.167392
24153	Amp	21	39290918	39291255	0	0.159594
24154	Amp	21	39291694	39346235	0	0.166629
24155	Amp	21	39346895	39486369	0	0.168117
24156	Amp	21	39486469	39538085	0	0.169734
24157	Amp	21	39538792	39554012	0	0.171142
24158	Amp	21	39554726	39554791	0	0.161576
24159	Amp	21	39632879	39652751	0	0.121818
24160	Amp	21	39653630	39727192	0	0.110787
24161	Amp	21	39727715	40174232	0	0.086785
24162	Amp	21	40175028	40207522	0	0.078433
24163	Amp	21	40208566	40227253	0	0.086296
24164	Amp	21	40239166	40340249	0	0.110936
24165	Amp	21	40348810	40368151	0	0.116517
24166	Amp	21	40371957	40423834	0	0.113066
24167	Amp	21	40424915	40482755	0	0.107485

24168	Amp	21	40483213	40635525	0	0.114143
24169	Amp	21	40644088	40664294	0	0.108734
24170	Amp	21	40666248	40723822	0	0.116406
24171	Amp	21	40728141	40959282	0	0.128484
24172	Amp	21	40961982	41015349	0	0.130941
24173	Amp	21	41021761	41062576	0	0.096135
24174	Amp	21	41067836	41123518	0	0.093678
24175	Amp	21	41124424	41124424	0	0.069038
24176	Amp	21	41125415	41233236	0	0.06755
24177	Amp	21	41234597	41356506	0	0.087587
24178	Amp	21	41356533	41469760	0	0.08597
24179	Amp	21	41469836	41499448	0	0.078298
24180	Amp	21	41499476	41696398	0	0.087666
24181	Amp	21	41697446	41783802	0	0.092316
24182	Amp	21	41788438	41793068	0	0.094048
24183	Amp	21	41794049	42002416	0	0.095463
24184	Amp	21	42007383	42058026	0	0.106981
24185	Amp	21	42065420	42072948	0	0.126368
24186	Amp	21	42078871	42113938	0	0.106981
24187	Amp	21	42114572	42128129	0	0.108364
24188	Amp	21	42128279	42268556	0	0.101694
24189	Amp	21	42270947	42290638	0	0.067151
24190	Amp	21	42290799	42298373	0	0.063731
24191	Amp	21	42302599	42516385	0	0.071695
24192	Amp	21	42517137	42536789	0	0.063731
24193	Amp	21	42537167	42545485	0	0.096155
24194	Amp	21	42546537	42578554	0	0.080619
24195	Amp	21	42579308	42618903	0	0.079237
24196	Amp	21	42619487	42824169	0	0.062348
24197	Amp	21	42827914	42866080	0	0.060316
24198	Amp	21	42868938	43034042	0	0.055395
24199	Amp	21	43034905	43034905	0	0.062373
24200	Amp	21	43572694	43618504	0	0.094163
24201	Amp	21	43621644	43640452	0	0.116797
24202	Amp	21	43645080	43681639	0	0.114857
24203	Amp	21	43682554	43700356	0	0.130756
24204	Amp	21	43703900	43711854	0	0.12304
24205	Amp	21	43716612	43742486	0	0.1265
24206	Amp	21	43744973	43766788	0	0.130358
24207	Amp	21	43767242	43826445	0	0.127154
24208	Amp	21	43826541	43853805	0	0.123693
24209	Amp	21	43855383	43888657	0	0.119775
24210	Amp	21	43889659	43977960	0	0.124977
24211	Amp	21	43986214	44095664	0	0.132094
24212	Amp	21	44253766	44254692	0	0.14358
24213	Amp	21	44259207	44265930	0	0.11886
24214	Amp	21	44269191	44335477	0	0.115704
24215	Amp	21	44339726	44357867	0	0.135625
24216	Amp	21	44448356	44448469	0	0.132442
24217	Amp	21	44452104	44528465	0	0.121412
24218	Amp	21	44533286	44721051	0	0.104674
24219	Amp	21	44721103	44755363	0	0.096434
24220	Amp	21	44756124	45214724	0	0.108102

24221 Amp	21	45218478	45227190	0	0.113124
24222 Amp	21	45227667	45227667	0	0.121773
24223 Amp	21	45670561	45674233	0	0.127331
24224 Amp	21	45675076	45684532	0	0.132383
24225 Amp	21	45690088	45851171	0	0.164365
24226 Amp	21	45854495	45903608	0	0.155716
24227 Amp	21	45903828	45918093	0	0.157134
24228 Amp	21	45920391	45964261	0	0.166524
24229 Amp	21	45979081	46022662	0	0.174716
24230 Amp	21	46030942	46076997	0	0.177091
24231 Amp	21	46083397	46104695	0	0.185645
24232 Amp	21	46106805	46182047	0	0.180623
24233 Amp	21	46188666	46214481	0	0.188054
24234 Amp	21	46220078	46262057	0.012341	0.190297
24235 Amp	22	16934932	16952107	0	0.097454
24236 Amp	22	16952820	17055291	0	0.103072
24237 Amp	22	17055501	17163374	0	0.109098
24238 Amp	22	17168860	17236923	0	0.063527
24239 Amp	22	17237779	17632626	0	0.0575
24240 Amp	22	17633315	17780180	0	0.052992
24241 Amp	22	17782984	18016541	0	0.065709
24242 Amp	22	18022317	18037816	0	0.07997
24243 Amp	22	18037888	18048218	0	0.08357
24244 Amp	22	18054379	18094572	0	0.090542
24245 Amp	22	18098866	18100728	0	0.104932
24246 Amp	22	18101648	18124603	0	0.106382
24247 Amp	22	18125120	18128031	0	0.108982
24248 Amp	22	18128862	18130970	0	0.102009
24249 Amp	22	18131162	18131162	0	0.105394
24250 Amp	22	21741482	21741482	0	0.098266
24251 Amp	22	21742513	21747226	0	0.102997
24252 Amp	22	21749134	21751293	0	0.0941
24253 Amp	22	21752246	21938623	0	0.0905
24254 Amp	22	21939017	21949041	0	0.085769
24255 Amp	22	23474170	23474241	0	0.090723
24256 Amp	22	23475053	23479094	0	0.086612
24257 Amp	22	23483227	23641308	0	0.083227
24258 Amp	22	23646701	23754809	0	0.092044
24259 Amp	22	23754828	24020015	0	0.108919
24260 Amp	22	24020100	24154414	0	0.115579
24261 Amp	22	24157567	24173545	0	0.120565
24262 Amp	22	24173852	24177482	0	0.123849
24263 Amp	22	24186073	24186771	0	0.132038
24264 Amp	22	24195941	24209217	0	0.127053
24265 Amp	22	24212956	24339545	0	0.108685
24266 Amp	22	24489781	24514161	0	0.113395
24267 Amp	22	24514419	24514419	0	0.108685
24268 Amp	22	24515474	24845481	0	0.103582
24269 Amp	22	24846625	24875077	0	0.105584
24270 Amp	22	24877579	24912539	0	0.107032
24271 Amp	22	24915337	25140990	0	0.108499
24272 Amp	22	25142234	25612136	0	0.095481
24273 Amp	22	25620770	25828129	0	0.080913

24274 Amp	22	25832573	25946563	0	0.079465
24275 Amp	22	25947095	26190904	0	0.071458
24276 Amp	22	26192779	26363781	0	0.075055
24277 Amp	22	26364702	26689935	0	0.073054
24278 Amp	22	26690072	26770076	0	0.069457
24279 Amp	22	26775613	26795979	0	0.080458
24280 Amp	22	26797881	26896905	0	0.081903
24281 Amp	22	26898580	26922339	0	0.073792
24282 Amp	22	26923959	26944833	0	0.065407
24283 Amp	22	26945154	27035899	0	0.061694
24284 Amp	22	27038636	27134460	0	0.052739
24285 Amp	22	27136964	27142363	0	0.062462
24286 Amp	22	27143241	27169569	0	0.069398
24287 Amp	22	27169926	27789152	0	0.067863
24288 Amp	22	27789464	27905755	0	0.066419
24289 Amp	22	27906126	28045431	0	0.074302
24290 Amp	22	28045483	28166477	0	0.071702
24291 Amp	22	28170737	28290996	0	0.063819
24292 Amp	22	28298222	28424883	0	0.070552
24293 Amp	22	28425215	28451987	0	0.076882
24294 Amp	22	28463079	28517103	0	0.065113
24295 Amp	22	28518835	28519550	0	0.061506
24296 Amp	22	28523100	28558834	0	0.06799
24297 Amp	22	28560672	28561510	0	0.06986
24298 Amp	22	28568186	28573526	0	0.063376
24299 Amp	22	28584475	28747364	0	0.073214
24300 Amp	22	28749423	28749551	0	0.081917
24301 Amp	22	28749700	28874003	0	0.090989
24302 Amp	22	28875421	28937684	0	0.08339
24303 Amp	22	28938612	29002389	0	0.086948
24304 Amp	22	29002471	29115632	0	0.092083
24305 Amp	22	29116823	29153393	0	0.100174
24306 Amp	22	29156034	29158195	0	0.108917
24307 Amp	22	29166271	29205215	0	0.105562
24308 Amp	22	29207807	29207807	0	0.097472
24309 Amp	22	29210775	29409818	0	0.096005
24310 Amp	22	29413741	29437094	0	0.092448
24311 Amp	22	29438777	29564183	0	0.083745
24312 Amp	22	29564346	29717971	0	0.069606
24313 Amp	22	29732405	29863353	0	0.080248
24314 Amp	22	29865230	30082428	0	0.069606
24315 Amp	22	30083144	30234026	0	0.060863
24316 Amp	22	30234154	30308234	0	0.051493
24317 Amp	22	30315454	30367041	0	0.058783
24318 Amp	22	30369908	30383778	0	0.070517
24319 Amp	22	30383968	30389432	0	0.058783
24320 Amp	22	30390706	30424562	0	0.067284
24321 Amp	22	30425618	30469350	0	0.069183
24322 Amp	22	30469352	30473349	0	0.067284
24323 Amp	22	30473443	30534188	0	0.058783
24324 Amp	22	30547039	30562717	0	0.073818
24325 Amp	22	30563257	30617562	0	0.085145
24326 Amp	22	30617909	30619481	0	0.078815

24327	Amp	22	30627906	30641445	0	0.06378
24328	Amp	22	30644152	30648299	0	0.072609
24329	Amp	22	30653083	30653083	0	0.065319
24330	Amp	22	30653946	30667432	0	0.063449
24331	Amp	22	30672827	30686808	0	0.06957
24332	Amp	22	30695947	31069198	0	0.063449
24333	Amp	22	31074065	31084083	0	0.066046
24334	Amp	22	31089360	31119618	0	0.07337
24335	Amp	22	31124352	31156716	0	0.063449
24336	Amp	22	31156749	31190959	0	0.070631
24337	Amp	22	31192342	31205322	0	0.084898
24338	Amp	22	31207815	31260279	0	0.063449
24339	Amp	22	31263115	31264047	0	0.070116
24340	Amp	22	31268510	31349288	0	0.079602
24341	Amp	22	31351895	31367883	0	0.072935
24342	Amp	22	31368045	31389295	0	0.066115
24343	Amp	22	31393025	31544860	0	0.063449
24344	Amp	22	31545926	31832379	0	0.068783
24345	Amp	22	31834199	32033883	0	0.070546
24346	Amp	22	32035660	32086619	0	0.061718
24347	Amp	22	32087363	32108908	0	0.063814
24348	Amp	22	32113966	32144559	0	0.065762
24349	Amp	22	32158998	32989945	0	0.041233
24350	Amp	22	32995613	33014061	0	0.037948
24351	Amp	22	33018179	33135420	0	0.021486
24352	Amp	22	33136267	33374636	0	0.049596
24353	Amp	22	33376679	33462197	0	0.057046
24354	Amp	22	33464800	33551395	0	0.050766
24355	Amp	22	33554551	33554551	0	0.052313
24356	Amp	22	33560094	33715992	0	0.058247
24357	Amp	22	33723880	34020610	0	0.069194
24358	Amp	22	34022031	34135601	0	0.072912
24359	Amp	22	34136417	34284608	0	0.076477
24360	Amp	22	34284944	34404130	0	0.068558
24361	Amp	22	34405307	34462627	0	0.063367
24362	Amp	22	34464186	34525254	0	0.05242
24363	Amp	22	34526459	34533604	0	0.037347
24364	Amp	22	34534530	34535892	0	0.044334
24365	Amp	22	34536307	34621401	0	0.071117
24366	Amp	22	34623159	34848920	0	0.057478
24367	Amp	22	34849466	35013890	0	0.063878
24368	Amp	22	35014476	35100070	0	0.047914
24369	Amp	22	35101734	35114998	0	0.023847
24370	Amp	22	35118897	35164897	0	0.017913
24371	Amp	22	35170790	35413611	0	0.019405
24372	Amp	22	35419616	35436769	0	0.021954
24373	Amp	22	35440186	35717380	0	0.019405
24374	Amp	22	35720033	35720033	0	0.017858
24375	Amp	22	35721807	35951577	0	0.016365
24376	Amp	22	35951598	36002233	0	0.014269
24377	Amp	22	36002483	36362781	0	0.012321
24378	Amp	22	36362804	36452480	0	0.015508
24379	Amp	22	36460761	36471417	0	0.01927

24380	Amp	22	36471769	36599289	0	0.016082
24381	Amp	22	36601092	36603621	0	0.010748
24382	Amp	22	36606017	36677702	0	0.015972
24383	Amp	22	36678142	36720413	0	0.010748
24384	Amp	22	36721010	37039591	0	0.003761
24385	Amp	22	37042611	37422307	0	0.006936
24386	Amp	22	37424039	37493033	0	0.011039
24387	Amp	22	37493623	37811061	0	0.006936
24388	Amp	22	37822630	37875789	0	0.016247
24389	Amp	22	37876922	38093277	0	0.006936
24390	Amp	22	38094049	38180308	0	0.010515
24391	Amp	22	38180581	38229003	0	0.013065
24392	Amp	22	38229027	38820662	0	0.009486
24393	Amp	22	38820687	39172990	0	0.006312
24394	Amp	22	39180863	39205268	0	0.01109
24395	Amp	22	39228047	39382162	0	0.006312
24396	Amp	22	39382322	39790730	0	0.003761
24397	Amp	22	39792370	39912696	0	0.012912
24398	Amp	22	39979144	40175534	0	0.003761
24399	Amp	22	40175869	40208961	0	0.018183
24400	Amp	22	40209185	40749600	0	0.028104
24401	Amp	22	40770888	40779259	0	0.013682
24402	Amp	22	40779381	40809133	0	0.01888
24403	Amp	22	40814036	40847718	0	0.020791
24404	Amp	22	40850503	41223828	0	0.01888
24405	Amp	22	41236179	41552579	0	0.021631
24406	Amp	22	41553255	41616075	0	0.027358
24407	Amp	22	41616282	41839348	0	0.017437
24408	Amp	22	41839798	42093925	0	0.011709
24409	Amp	22	42094329	42144203	0	0.016532
24410	Amp	22	42149230	42302568	0	0.018353
24411	Amp	22	42302794	42335534	0	0.033746
24412	Amp	22	42336829	42380124	0	0.018353
24413	Amp	22	42380270	42648759	0	0.034609
24414	Amp	22	42650367	42757704	0	0.040365
24415	Amp	22	42762383	42762383	0	0.044581
24416	Amp	22	42768404	42851409	0	0.038824
24417	Amp	22	42853194	42860792	0	0.035662
24418	Amp	22	42863947	42893467	0	0.047249
24419	Amp	22	42896772	42939484	0	0.05298
24420	Amp	22	42939965	42956392	0	0.048568
24421	Amp	22	42958328	43054344	0	0.038883
24422	Amp	22	43055320	43124154	0	0.037062
24423	Amp	22	43124453	43135123	0	0.032846
24424	Amp	22	43137354	43148817	0	0.035829
24425	Amp	22	43148926	43604831	0	0.050312
24426	Amp	22	43613502	44149665	0	0.035829
24427	Amp	22	44156192	44270333	0	0.032846
24428	Amp	22	44272270	44288159	0	0.04385
24429	Amp	22	44293292	44455210	0	0.032846
24430	Amp	22	44455471	44595209	0	0.038395
24431	Amp	22	44600319	44617353	0	0.0423
24432	Amp	22	44621827	44794870	0	0.038395

24433	Amp	22	44799666	44865012	0	0.031824
24434	Amp	22	44879238	45224620	0	0.036721
24435	Amp	22	45226470	45337318	0	0.038628
24436	Amp	22	45337966	45339888	0	0.032898
24437	Amp	22	45340166	45564390	0	0.034799
24438	Amp	22	45564672	45870451	0	0.038892
24439	Amp	22	45874650	45874650	0	0.041143
24440	Amp	22	45875727	46029658	0	0.042875
24441	Amp	22	46029766	46087068	0	0.041143
24442	Amp	22	46091336	46093321	0	0.04567
24443	Amp	22	46098802	46135882	0	0.043419
24444	Amp	22	46136901	46247877	0	0.038892
24445	Amp	22	46254411	46256406	0	0.027305
24446	Amp	22	46256475	46310463	0	0.031005
24447	Amp	22	46315536	46407877	0	0.042176
24448	Amp	22	46411025	46419908	0	0.045015
24449	Amp	22	46424387	46464166	0	0.054561
24450	Amp	22	46467277	46490139	0	0.051722
24451	Amp	22	46494815	46706628	0	0.048022
24452	Amp	22	46708409	46761098	0	0.050414
24453	Amp	22	46769688	46842529	0	0.053226
24454	Amp	22	46843076	46962850	0	0.049133
24455	Amp	22	46970359	47200118	0	0.077966
24456	Amp	22	47200209	47296886	0	0.065956
24457	Amp	22	47301288	47390565	0	0.067847
24458	Amp	22	47390860	47408042	0	0.069626
24459	Amp	22	47408493	47437863	0	0.067847
24460	Amp	22	47438355	47490694	0	0.081392
24461	Amp	22	47491015	48595928	0	0.051024
24462	Amp	22	48598207	48675956	0	0.047839
24463	Amp	22	48685333	48848072	0	0.051311
24464	Amp	22	48885900	48935200	0	0.057356
24465	Del	1	3302046	3302046	6.64879	0.227771
24466	Del	1	3302359	3451567	6.370507	0.223038
24467	Del	1	3451579	3486887	5.865505	0.213572
24468	Del	1	3487663	3623098	5.793339	0.212389
24469	Del	1	3623660	3632268	5.392808	0.204698
24470	Del	1	3634034	3659269	5.383834	0.204106
24471	Del	1	3659365	3680479	5.300956	0.202331
24472	Del	1	3680520	3698012	5.35617	0.203515
24473	Del	1	3698042	3749303	5.369172	0.204106
24474	Del	1	3749737	3780042	5.386944	0.204698
24475	Del	1	3780075	3797469	5.383773	0.204106
24476	Del	1	3797918	3854839	6.246029	0.220671
24477	Del	1	3855795	3888691	6.347202	0.223038
24478	Del	1	3889073	3939532	5.398307	0.204698
24479	Del	1	3939577	3955322	5.380503	0.204106
24480	Del	1	3957975	4648927	5.769259	0.211797
24481	Del	1	4652424	5559299	5.503316	0.206473
24482	Del	1	5563560	5862018	5.414027	0.204698
24483	Del	1	5862221	5862761	4.921578	0.19464
24484	Del	1	5862774	5951845	4.721039	0.191091
24485	Del	1	5954503	6019642	4.847366	0.193457

24486	Del	1	6020227	6028304	4.885119	0.194049
24487	Del	1	6028611	6094386	4.899356	0.19464
24488	Del	1	6094681	6176837	4.944788	0.195232
24489	Del	1	6183275	6263905	5.001579	0.196415
24490	Del	1	6264330	6362615	4.890682	0.19464
24491	Del	1	6362699	6394399	4.873186	0.194049
24492	Del	1	6394534	6431233	5.007035	0.196415
24493	Del	1	6438103	6457443	4.98342	0.195824
24494	Del	1	6459544	6507189	5.013394	0.196415
24495	Del	1	6508890	6553130	4.968925	0.195824
24496	Del	1	6555047	6590790	4.998787	0.196415
24497	Del	1	6591033	6605539	4.988807	0.195824
24498	Del	1	6610335	6636818	5.013394	0.196415
24499	Del	1	6638914	6702875	4.788891	0.192866
24500	Del	1	6703077	6783774	4.921578	0.19464
24501	Del	1	6783868	6873021	4.786144	0.192274
24502	Del	1	6877711	6892266	4.754606	0.191682
24503	Del	1	6895273	7769373	3.655979	0.169793
24504	Del	1	7769713	7781489	4.759391	0.192274
24505	Del	1	7786423	7845814	4.693993	0.190499
24506	Del	1	7845948	7854594	4.776126	0.192274
24507	Del	1	7854775	7986078	5.375258	0.204106
24508	Del	1	7993075	8208118	5.389679	0.204698
24509	Del	1	8208655	8315853	4.767602	0.192274
24510	Del	1	8316537	8363450	4.738558	0.191682
24511	Del	1	8365437	8413753	4.722313	0.191091
24512	Del	1	8420102	8435345	4.765513	0.192274
24513	Del	1	8435459	8486854	4.28802	0.182808
24514	Del	1	8487820	8495974	4.785239	0.192274
24515	Del	1	8503397	8859335	4.28802	0.182808
24516	Del	1	8859635	8945210	4.242037	0.181625
24517	Del	1	8945261	9027949	4.219886	0.181033
24518	Del	1	9030291	9038363	5.212208	0.199965
24519	Del	1	9040939	9074131	5.220774	0.200556
24520	Del	1	9078408	9146408	5.386944	0.204698
24521	Del	1	9147248	9147248	5.376845	0.204106
24522	Del	1	9151464	9152724	5.414027	0.204698
24523	Del	1	9153118	9180199	5.376845	0.204106
24524	Del	1	9181688	9247978	5.392808	0.204698
24525	Del	1	9255788	9372103	5.563988	0.207656
24526	Del	1	9372331	9524383	5.636817	0.208839
24527	Del	1	9528050	9586562	5.608782	0.208247
24528	Del	1	9587064	9606629	5.616057	0.208839
24529	Del	1	9609028	9616944	5.605615	0.208247
24530	Del	1	9617009	9729792	5.962016	0.215347
24531	Del	1	9734247	9842347	5.922557	0.214755
24532	Del	1	9842842	9914107	5.553427	0.207656
24533	Del	1	9914746	9952956	5.495194	0.206473
24534	Del	1	9957375	9965785	5.458934	0.205881
24535	Del	1	9967328	9967482	5.516258	0.206473
24536	Del	1	9969855	9987812	5.458934	0.205881
24537	Del	1	9989119	10030667	5.492809	0.206473
24538	Del	1	10031603	10224178	5.352222	0.203515

24539	Del	1	10225186	10228739	5.516258	0.206473
24540	Del	1	10229443	10298108	5.352222	0.203515
24541	Del	1	10298533	10305205	5.516258	0.206473
24542	Del	1	10305228	10321714	5.352222	0.203515
24543	Del	1	10321730	10385876	5.257976	0.201148
24544	Del	1	10387735	10445075	5.315592	0.202331
24545	Del	1	10446134	10449254	5.296161	0.20174
24546	Del	1	10449646	10472924	5.313824	0.202331
24547	Del	1	10477033	10633983	5.0812	0.19819
24548	Del	1	10634068	10798689	5.128892	0.198782
24549	Del	1	10809488	11010551	5.257976	0.201148
24550	Del	1	11010638	11068018	4.29299	0.182808
24551	Del	1	11068597	11097442	4.271409	0.182217
24552	Del	1	11098048	11105344	4.302648	0.182808
24553	Del	1	11106118	11263560	4.236867	0.181625
24554	Del	1	11265464	11291667	4.412533	0.185175
24555	Del	1	11293314	11300136	4.39884	0.184583
24556	Del	1	11300900	11538613	4.675509	0.190499
24557	Del	1	11539717	11694530	4.719182	0.191091
24558	Del	1	11697946	11721916	4.684222	0.190499
24559	Del	1	11722711	11756035	4.713128	0.191091
24560	Del	1	11761718	11774397	4.68705	0.190499
24561	Del	1	11774997	11783110	4.721039	0.191091
24562	Del	1	11783141	11783141	4.68705	0.190499
24563	Del	1	11783840	11809839	4.707318	0.191091
24564	Del	1	11813694	11838976	4.654022	0.189907
24565	Del	1	11839776	11932127	4.719182	0.191091
24566	Del	1	11932953	11977700	4.693993	0.190499
24567	Del	1	11982204	12011376	4.669902	0.189907
24568	Del	1	12013007	12057844	4.722313	0.191091
24569	Del	1	12059512	12144226	4.601406	0.188724
24570	Del	1	12145468	12170574	4.724333	0.191091
24571	Del	1	12171597	12190922	4.636496	0.189316
24572	Del	1	12190990	12196889	4.724333	0.191091
24573	Del	1	12197646	12209650	4.636496	0.189316
24574	Del	1	12209703	12504511	4.957632	0.195824
24575	Del	1	12505205	12508967	5.296161	0.20174
24576	Del	1	12513690	12513690	4.957632	0.195824
24577	Del	1	12514795	12577132	5.250281	0.201148
24578	Del	1	12578936	12579140	5.296161	0.20174
24579	Del	1	12588404	12601635	5.250281	0.201148
24580	Del	1	12603299	12623415	5.21558	0.200556
24581	Del	1	12624831	12739779	5.25585	0.201148
24582	Del	1	12739885	12764432	6.428891	0.224221
24583	Del	1	12765316	13471994	6.479818	0.224813
24584	Del	1	13473667	13580155	5.232425	0.200556
24585	Del	1	13582647	13618073	5.212208	0.199965
24586	Del	1	13619382	13704218	5.927872	0.214755
24587	Del	1	13704299	13744331	6.068977	0.217122
24588	Del	1	13745350	13789833	6.156699	0.218896
24589	Del	1	13790733	13828560	5.934535	0.214755
24590	Del	1	13830156	15071744	6.709062	0.234278
24591	Del	1	15073450	15105161	6.673787	0.233095

24592	Del	1	15108761	15155558	7.686395	0.254393
24593	Del	1	15157969	15157969	7.56241	0.252618
24594	Del	1	15158643	15173606	7.806164	0.256168
24595	Del	1	15173836	15243216	7.56241	0.252618
24596	Del	1	15245637	15407805	7.378325	0.24966
24597	Del	1	15407891	15494905	6.750404	0.237236
24598	Del	1	15495090	15569033	6.750404	0.236053
24599	Del	1	15569475	15592181	6.750404	0.236645
24600	Del	1	15592244	15659445	6.815951	0.240194
24601	Del	1	15659575	15761329	6.86791	0.241378
24602	Del	1	15761501	15761501	6.843452	0.240786
24603	Del	1	15761822	15806512	6.86791	0.241378
24604	Del	1	15806855	15850269	6.815951	0.239603
24605	Del	1	15854897	15868075	6.758324	0.237828
24606	Del	1	15870264	15907072	6.815951	0.239011
24607	Del	1	15907564	15933318	6.758324	0.237828
24608	Del	1	15940652	15976487	6.815951	0.239011
24609	Del	1	15977607	16123251	6.815951	0.239603
24610	Del	1	16123711	16193529	6.815951	0.239011
24611	Del	1	16193697	16237453	6.73617	0.235462
24612	Del	1	16268499	16352669	6.673787	0.233095
24613	Del	1	16353185	16459312	6.730989	0.23487
24614	Del	1	16459673	17016429	6.73617	0.235462
24615	Del	1	17018588	17065857	6.705692	0.234278
24616	Del	1	17066485	17122610	6.673787	0.232504
24617	Del	1	17129208	17245727	6.682451	0.233687
24618	Del	1	17247493	17265184	6.713264	0.234278
24619	Del	1	17266821	17307027	6.750404	0.235462
24620	Del	1	17307077	17364420	6.673787	0.233095
24621	Del	1	17365232	17534910	6.708213	0.234278
24622	Del	1	17536907	17577423	6.64879	0.230729
24623	Del	1	17578695	17611502	6.668694	0.231912
24624	Del	1	17613939	17753317	6.673787	0.233095
24625	Del	1	17753364	17827617	6.673787	0.232504
24626	Del	1	17830369	18105007	6.736971	0.235462
24627	Del	1	18105530	18378977	6.330095	0.222446
24628	Del	1	18379006	18625618	6.736971	0.235462
24629	Del	1	18629415	18738880	6.004609	0.215938
24630	Del	1	18739292	18751212	5.960271	0.215347
24631	Del	1	18751331	18878683	6.162731	0.218896
24632	Del	1	18882051	18886145	6.19434	0.219488
24633	Del	1	18886497	18895348	6.156699	0.218896
24634	Del	1	18896973	18899793	6.19434	0.219488
24635	Del	1	18903769	18903769	6.147987	0.218896
24636	Del	1	18905539	18958070	6.119582	0.218305
24637	Del	1	18958420	19199091	6.682451	0.233687
24638	Del	1	19199869	19230985	6.815951	0.239011
24639	Del	1	19231219	19243289	6.775063	0.23842
24640	Del	1	19243410	19247253	6.758324	0.237828
24641	Del	1	19250337	19296937	6.815951	0.239011
24642	Del	1	19300512	19314095	6.180406	0.219488
24643	Del	1	19314182	19334376	6.162731	0.218896
24644	Del	1	19336589	19372367	5.798331	0.212389

24645	Del	1	19372852	19490109	5.888995	0.214164
24646	Del	1	19490149	19599867	6.190993	0.219488
24647	Del	1	19601059	19605393	6.126513	0.218305
24648	Del	1	19606106	19609154	6.19434	0.219488
24649	Del	1	19612955	19629880	6.126513	0.218305
24650	Del	1	19632235	19637980	6.07478	0.217122
24651	Del	1	19638481	19682387	6.170997	0.219488
24652	Del	1	19682539	19813847	5.910755	0.214164
24653	Del	1	19813896	19878465	5.688595	0.210022
24654	Del	1	19878764	19914097	5.660441	0.209431
24655	Del	1	19915012	20069094	5.68581	0.210022
24656	Del	1	20069323	20093655	5.207474	0.199965
24657	Del	1	20094038	20154347	5.242677	0.201148
24658	Del	1	20156557	20182972	5.226828	0.200556
24659	Del	1	20183947	20289228	5.265165	0.201148
24660	Del	1	20289275	20431547	5.149217	0.199373
24661	Del	1	20431699	20585792	5.265165	0.201148
24662	Del	1	20587732	20619193	5.210151	0.199965
24663	Del	1	20625633	20635058	5.265165	0.201148
24664	Del	1	20636122	20662264	5.223793	0.200556
24665	Del	1	20664946	20715062	5.164604	0.199373
24666	Del	1	20717297	20788340	5.238113	0.200556
24667	Del	1	20794600	20940761	5.323864	0.202923
24668	Del	1	20940964	21168749	5.413746	0.204698
24669	Del	1	21169167	21188277	5.265213	0.201148
24670	Del	1	21189326	21281995	5.611876	0.208247
24671	Del	1	21287656	21314008	5.68581	0.210022
24672	Del	1	21314072	21349164	5.516881	0.206473
24673	Del	1	21349351	21503843	5.652932	0.209431
24674	Del	1	21508618	21670789	5.555356	0.207656
24675	Del	1	21671058	21722171	5.891524	0.214164
24676	Del	1	21722224	21783535	5.984473	0.215938
24677	Del	1	21783579	21837671	6.068977	0.217122
24678	Del	1	21838233	21938800	5.891524	0.214164
24679	Del	1	21939621	22116490	6.07478	0.217122
24680	Del	1	22117267	22146835	6.040404	0.21653
24681	Del	1	22146917	22449346	6.07478	0.217122
24682	Del	1	22450841	22533154	5.919171	0.214755
24683	Del	1	22534111	22590178	6.053611	0.217122
24684	Del	1	22591319	22606021	6.033902	0.21653
24685	Del	1	22608151	22650729	6.07478	0.217122
24686	Del	1	22651390	22705401	6.046492	0.21653
24687	Del	1	22707372	22717730	5.584624	0.208247
24688	Del	1	22719450	22721570	6.07478	0.217122
24689	Del	1	22722786	22860422	5.584624	0.208247
24690	Del	1	22862566	22865846	5.760551	0.211205
24691	Del	1	22866069	22925771	5.584624	0.208247
24692	Del	1	22926464	23016239	5.735071	0.211205
24693	Del	1	23019076	23036684	5.678878	0.210022
24694	Del	1	23044086	23049200	5.760551	0.211205
24695	Del	1	23049345	23051177	5.678878	0.210022
24696	Del	1	23055122	23059673	5.760551	0.211205
24697	Del	1	23059827	23084270	5.678878	0.210022

24698	Del	1	23084901	23188539	5.633241	0.208839
24699	Del	1	23191387	23194847	5.315592	0.202331
24700	Del	1	23196132	23302147	5.654594	0.209431
24701	Del	1	23304450	23348813	5.616057	0.208839
24702	Del	1	23349390	23376625	5.643663	0.209431
24703	Del	1	23380160	23486582	5.5577	0.207656
24704	Del	1	23486742	23519471	5.616275	0.208839
24705	Del	1	23521550	23833167	5.653094	0.209431
24706	Del	1	23833883	23870467	5.636859	0.208839
24707	Del	1	23871355	23914555	5.35617	0.203515
24708	Del	1	23920830	24055744	5.383773	0.204106
24709	Del	1	24055994	24107727	5.322408	0.202923
24710	Del	1	24118051	24148762	5.348141	0.203515
24711	Del	1	24149880	24191620	5.345928	0.202923
24712	Del	1	24191716	24314845	5.36388	0.203515
24713	Del	1	24315475	24368661	5.337913	0.202923
24714	Del	1	24368675	24412352	5.315217	0.202331
24715	Del	1	24412572	24455650	5.32435	0.202923
24716	Del	1	24456486	24468086	5.300591	0.202331
24717	Del	1	24468604	24477614	5.296161	0.20174
24718	Del	1	24483579	24507028	5.379917	0.204106
24719	Del	1	24507348	24538086	5.35617	0.203515
24720	Del	1	24538984	24575585	4.237572	0.181625
24721	Del	1	24577584	24610439	4.202013	0.180442
24722	Del	1	24610712	24741027	4.237572	0.181625
24723	Del	1	24742750	24846546	3.712401	0.170976
24724	Del	1	24846739	24934895	3.801787	0.172751
24725	Del	1	24935139	24965969	3.762074	0.171567
24726	Del	1	24966461	25027455	3.822318	0.172751
24727	Del	1	25030228	25613529	4.03372	0.176892
24728	Del	1	25615754	25791902	3.794115	0.172159
24729	Del	1	25792419	25828293	3.712085	0.170976
24730	Del	1	25828595	25850237	4.28991	0.182808
24731	Del	1	25855944	25957790	4.322139	0.1834
24732	Del	1	25957876	25999781	4.278278	0.182217
24733	Del	1	26001180	26044311	4.327445	0.1834
24734	Del	1	26049086	26071473	4.748917	0.191682
24735	Del	1	26072739	26181880	4.765513	0.192274
24736	Del	1	26184800	26212399	5.207474	0.199965
24737	Del	1	26212565	26233560	5.238458	0.200556
24738	Del	1	26235540	26262561	5.174199	0.199373
24739	Del	1	26268640	26321890	5.218312	0.200556
24740	Del	1	26322043	26358480	5.165917	0.199373
24741	Del	1	26358699	26373796	5.210151	0.199965
24742	Del	1	26374140	26429718	5.21277	0.200556
24743	Del	1	26429892	26457095	5.347213	0.202923
24744	Del	1	26460238	26477518	5.381568	0.204106
24745	Del	1	26477915	26546754	5.503316	0.206473
24746	Del	1	26549798	26555954	5.546417	0.207064
24747	Del	1	26556437	26576203	5.50447	0.206473
24748	Del	1	26576390	26699310	6.673787	0.232504
24749	Del	1	26705860	26793484	6.658444	0.23132
24750	Del	1	26801444	26823515	5.544423	0.207064

24751	Del	1	26824331	26861325	5.496733	0.206473
24752	Del	1	26861590	27097451	5.546417	0.207064
24753	Del	1	27098414	27229890	5.49213	0.206473
24754	Del	1	27229966	27308758	5.33065	0.202923
24755	Del	1	27308826	27401456	5.381568	0.204106
24756	Del	1	27401536	27609806	5.337913	0.202923
24757	Del	1	27610945	27723023	4.998787	0.196415
24758	Del	1	27725146	27829006	4.968925	0.195824
24759	Del	1	27829963	27889927	4.530899	0.187541
24760	Del	1	27890551	27948473	4.509625	0.186949
24761	Del	1	27958304	27968199	4.542514	0.187541
24762	Del	1	27969698	27972835	4.426894	0.185175
24763	Del	1	27975171	28098329	4.433599	0.185766
24764	Del	1	28098399	28194968	4.522682	0.186949
24765	Del	1	28200153	28252464	4.52807	0.187541
24766	Del	1	28255562	28285526	4.17023	0.17985
24767	Del	1	28286371	28338452	4.189926	0.180442
24768	Del	1	28340155	28403427	4.481544	0.186358
24769	Del	1	28408901	28600374	4.530899	0.187541
24770	Del	1	28604957	28639469	4.513555	0.186949
24771	Del	1	28640324	28733592	4.551092	0.187541
24772	Del	1	28734075	28808169	4.342017	0.183991
24773	Del	1	28808333	28873816	3.874577	0.173934
24774	Del	1	28882444	28898023	3.141164	0.159144
24775	Del	1	28912086	29067841	3.203822	0.160327
24776	Del	1	29070435	29116074	3.141164	0.159144
24777	Del	1	29116468	29145923	3.335647	0.162693
24778	Del	1	29146182	29185815	3.297559	0.162102
24779	Del	1	29186335	29235048	3.52298	0.166835
24780	Del	1	29235558	29327324	3.441165	0.16506
24781	Del	1	29329144	30006149	3.537362	0.167426
24782	Del	1	30006923	30666733	3.464348	0.165651
24783	Del	1	30667100	30867774	2.845836	0.152044
24784	Del	1	30868159	30930496	2.581658	0.14672
24785	Del	1	30930879	30935209	2.167455	0.137254
24786	Del	1	30935262	30938231	2.258915	0.139029
24787	Del	1	30940072	30945463	2.167455	0.137254
24788	Del	1	30947040	30957585	2.258915	0.139029
24789	Del	1	30958329	30968074	2.167455	0.137254
24790	Del	1	30968135	30978297	2.258915	0.139029
24791	Del	1	30982831	31175087	2.167455	0.137254
24792	Del	1	31177972	31240837	2.124259	0.136071
24793	Del	1	31241005	31301656	2.165861	0.137254
24794	Del	1	31301681	31360859	2.127713	0.136071
24795	Del	1	31364785	31493478	2.174603	0.137254
24796	Del	1	31495473	31516983	1.614076	0.122464
24797	Del	1	31517921	31572874	1.411539	0.116548
24798	Del	1	31576149	31651771	1.219473	0.110631
24799	Del	1	31652178	31706216	1.188087	0.11004
24800	Del	1	31706245	31787204	1.204691	0.110631
24801	Del	1	31787704	32045061	1.015508	0.104124
24802	Del	1	32045455	32068504	1.009212	0.103532
24803	Del	1	32070198	32103130	1.011799	0.104124

24804	Del	1	32104116	32175043	1.005451	0.103532
24805	Del	1	32175403	32538409	1.01777	0.104124
24806	Del	1	32538854	32606756	1.008233	0.103532
24807	Del	1	32607934	33102692	1.013499	0.104124
24808	Del	1	33104527	33123391	1.009212	0.103532
24809	Del	1	33123715	33177187	1.009456	0.104124
24810	Del	1	33177449	33252417	1.008233	0.103532
24811	Del	1	33252430	33301105	0.86264	0.098799
24812	Del	1	33301627	33364336	0.884068	0.099391
24813	Del	1	33365879	33380399	0.859838	0.098799
24814	Del	1	33380639	33510969	1.457277	0.118322
24815	Del	1	33512471	33854804	0.711395	0.094066
24816	Del	1	33857886	33865716	0.84514	0.098208
24817	Del	1	33865789	33866173	0.711395	0.094066
24818	Del	1	33866561	33886351	0.837292	0.098208
24819	Del	1	33889126	34156185	0.711395	0.094066
24820	Del	1	34156748	34668116	1.059448	0.105899
24821	Del	1	34668244	34849240	0.992065	0.10294
24822	Del	1	34850136	34862511	0.775839	0.095841
24823	Del	1	34862644	34931551	0.96332	0.102349
24824	Del	1	34932374	35059419	0.992065	0.10294
24825	Del	1	35059744	35073824	0.975924	0.102349
24826	Del	1	35078938	35261188	0.982064	0.10294
24827	Del	1	35262199	35422090	0.93296	0.101166
24828	Del	1	35422546	35561504	1.05252	0.105307
24829	Del	1	35561853	35632406	0.897255	0.099982
24830	Del	1	35633513	35641909	0.88623	0.099391
24831	Del	1	35643104	35771188	0.903449	0.099982
24832	Del	1	35777293	35929514	1.562908	0.12128
24833	Del	1	35929726	36075934	1.52412	0.120097
24834	Del	1	36081054	36216890	1.006646	0.103532
24835	Del	1	36217080	36300311	0.97497	0.102349
24836	Del	1	36300537	36327204	1.005913	0.103532
24837	Del	1	36327505	36389991	0.98722	0.10294
24838	Del	1	36390093	36412571	1.008741	0.103532
24839	Del	1	36417599	36425761	0.990814	0.10294
24840	Del	1	36426693	36464792	0.997249	0.103532
24841	Del	1	36467023	36489632	0.991571	0.10294
24842	Del	1	36490148	37158944	1.005644	0.103532
24843	Del	1	37159461	37379639	0.454939	0.084009
24844	Del	1	37383192	37714104	0.252764	0.075135
24845	Del	1	37714613	37757133	0.246242	0.074543
24846	Del	1	37758989	37852558	0.25536	0.075135
24847	Del	1	37854376	37949643	0.366128	0.080459
24848	Del	1	37954598	37990820	0.371123	0.081051
24849	Del	1	37999351	38886411	0.2098	0.072768
24850	Del	1	38889679	38943463	0.198245	0.072177
24851	Del	1	38944344	39077065	0.2098	0.072768
24852	Del	1	39078476	39406225	0.138103	0.068627
24853	Del	1	39406792	39420305	0.210298	0.072768
24854	Del	1	39421268	39487757	0.138103	0.068627
24855	Del	1	39488273	39838328	0.206334	0.072768
24856	Del	1	39838975	39884361	0.202822	0.072177

24857	Del	1	39888660	40038107	0.211083	0.072768
24858	Del	1	40038796	40068581	0.202822	0.072177
24859	Del	1	40069170	40159372	0.20359	0.072768
24860	Del	1	40161246	40241322	0.19877	0.072177
24861	Del	1	40244941	40620254	0.20989	0.072768
24862	Del	1	40620552	40688735	0.202822	0.072177
24863	Del	1	40688878	40692486	0.162215	0.06981
24864	Del	1	40693557	40734483	0.143298	0.069219
24865	Del	1	40734716	40780187	0.153856	0.06981
24866	Del	1	40780629	40841771	0.147426	0.069219
24867	Del	1	40843046	41046439	0.162563	0.06981
24868	Del	1	41051967	41165158	0.135239	0.068627
24869	Del	1	41169284	41246381	0.121631	0.068035
24870	Del	1	41246988	41505541	0.157762	0.06981
24871	Del	1	41505556	42036809	0.050976	0.063302
24872	Del	1	42037903	42172835	0.105108	0.066261
24873	Del	1	42174637	42340520	0.080687	0.065077
24874	Del	1	42342523	42451774	0.092131	0.065669
24875	Del	1	42454655	42532730	0.10539	0.066261
24876	Del	1	42534116	42655150	0.080982	0.065077
24877	Del	1	42656131	42706102	0.099702	0.066261
24878	Del	1	42712398	42915394	0.282216	0.076318
24879	Del	1	42921631	42955853	0.159471	0.06981
24880	Del	1	42956259	43162305	0.165613	0.070402
24881	Del	1	43162778	43174057	0.018119	0.060344
24882	Del	1	43174873	43212385	0.008911	0.059753
24883	Del	1	43212652	43255323	0.000516	0.059161
24884	Del	1	43256039	43370704	0.018569	0.060344
24885	Del	1	43371507	43390494	0.173468	0.070402
24886	Del	1	43390962	43444575	0.153935	0.06981
24887	Del	1	43447515	43455015	0.173853	0.070402
24888	Del	1	43455713	43623961	0.217653	0.07336
24889	Del	1	43624195	43693832	0.23434	0.073952
24890	Del	1	43695142	43728081	0.246416	0.074543
24891	Del	1	43728628	43833169	0.191059	0.071585
24892	Del	1	43837594	43839092	0.247213	0.074543
24893	Del	1	43839964	43931754	0.191059	0.071585
24894	Del	1	43932531	44247017	0.24707	0.074543
24895	Del	1	44247565	44247565	0.208784	0.072768
24896	Del	1	44251149	44255119	0.247213	0.074543
24897	Del	1	44258607	44358237	0.208784	0.072768
24898	Del	1	44362473	44423318	0.73695	0.094658
24899	Del	1	44423493	44650765	0.744158	0.09525
24900	Del	1	44650931	44678654	0.876776	0.099391
24901	Del	1	44679178	44720421	0.595537	0.089925
24902	Del	1	44721613	44722242	0.620811	0.090517
24903	Del	1	44729408	44729408	0.595537	0.089925
24904	Del	1	44730915	44768812	0.620811	0.090517
24905	Del	1	44770304	44816032	0.185757	0.070993
24906	Del	1	44823546	44844138	0.173538	0.070402
24907	Del	1	44847195	44870639	0.153204	0.069219
24908	Del	1	44871422	44991606	0.157386	0.06981
24909	Del	1	44991974	45018113	0.183991	0.070993

24910	Del	1	45023602	45211591	0.150139	0.069219
24911	Del	1	45212033	45344419	0.185757	0.070993
24912	Del	1	45348269	45492413	0.154899	0.06981
24913	Del	1	45493287	45619440	0.093718	0.065669
24914	Del	1	45619684	45687413	0.03832	0.062119
24915	Del	1	45706176	45755945	0	0.054428
24916	Del	1	45757414	45856106	0.024976	0.061528
24917	Del	1	45856171	46183220	0.032221	0.062119
24918	Del	1	46184392	46319697	0.083415	0.065077
24919	Del	1	46321609	46549401	0.45949	0.084601
24920	Del	1	46557088	46603005	0.66414	0.092291
24921	Del	1	46604173	46632376	0.687404	0.092883
24922	Del	1	46632586	46668649	0.667119	0.092291
24923	Del	1	46670340	46692363	0.686574	0.092883
24924	Del	1	46692814	46722478	0.657787	0.092291
24925	Del	1	46722780	46955890	0.673615	0.092883
24926	Del	1	46955948	47057396	0.654021	0.0917
24927	Del	1	47059549	47132976	0.213014	0.07336
24928	Del	1	47136464	47247989	0.081603	0.065077
24929	Del	1	47249998	47332905	0.071386	0.064486
24930	Del	1	47333133	47378864	0.036233	0.062119
24931	Del	1	47379261	47409955	0.029542	0.061528
24932	Del	1	47414606	47418557	0.039521	0.062119
24933	Del	1	47418910	47429331	0.029542	0.061528
24934	Del	1	47430543	47441525	0.039159	0.062119
24935	Del	1	47441932	48100702	0.279635	0.076318
24936	Del	1	48101445	48182571	0.157851	0.06981
24937	Del	1	48183818	48293602	0.181123	0.070993
24938	Del	1	48294959	48473303	0.154899	0.06981
24939	Del	1	48473971	48724360	0.232316	0.073952
24940	Del	1	48724562	48779578	0.367767	0.080459
24941	Del	1	48782364	49372335	0.232316	0.073952
24942	Del	1	49373930	49533594	0.301686	0.077501
24943	Del	1	49536318	50025526	0.232316	0.073952
24944	Del	1	50026796	50099248	0.275462	0.076318
24945	Del	1	50099760	50104993	0.291459	0.07691
24946	Del	1	50105207	50181354	0.304625	0.077501
24947	Del	1	50182360	50204421	0.292537	0.07691
24948	Del	1	50207659	50438110	0.541677	0.087559
24949	Del	1	50440694	50956567	0.505726	0.086375
24950	Del	1	50961890	51079376	0.539758	0.087559
24951	Del	1	51100599	51149351	0.523085	0.086967
24952	Del	1	51150684	51331737	0.528985	0.087559
24953	Del	1	51331971	51348391	0.526376	0.086967
24954	Del	1	51348602	51422712	0.366696	0.080459
24955	Del	1	51424273	51606648	0.346	0.079868
24956	Del	1	51613295	51727284	0.179639	0.070993
24957	Del	1	51727729	51789811	0.192793	0.072177
24958	Del	1	51789976	51815812	0.18782	0.071585
24959	Del	1	51831387	51836907	0.202822	0.072177
24960	Del	1	51837333	51897127	0.18782	0.071585
24961	Del	1	51898624	51991165	0.185757	0.070993
24962	Del	1	51992581	52065261	0.193021	0.072177

24963	Del	1	52067942	52139484	0	0.05502
24964	Del	1	52140488	52345054	0	0.053837
24965	Del	1	52345909	52420963	0	0.05502
24966	Del	1	52422281	52554702	0	0.054428
24967	Del	1	52554929	52723418	0	0.05502
24968	Del	1	52724144	52834789	0	0.053837
24969	Del	1	52838206	52996666	0	0.044371
24970	Del	1	52997944	53011528	0	0.043188
24971	Del	1	53014871	53052141	0	0.043779
24972	Del	1	53052270	53085621	0	0.044371
24973	Del	1	53086208	53103820	0	0.043779
24974	Del	1	53104987	53188157	0	0.044371
24975	Del	1	53188870	53243687	0	0.042004
24976	Del	1	53246063	53331111	0	0.041413
24977	Del	1	53331229	53468174	0	0.03668
24978	Del	1	53468418	53764309	0	0.038455
24979	Del	1	53765040	53915733	0	0.03668
24980	Del	1	53916312	53941831	0	0.041413
24981	Del	1	53944364	54171107	0	0.045554
24982	Del	1	54171494	54225615	0	0.03668
24983	Del	1	54226155	54230674	0	0.034905
24984	Del	1	54231070	54239709	0	0.03668
24985	Del	1	54241373	54407436	0	0.034905
24986	Del	1	54408515	54604905	0	0.036088
24987	Del	1	54606491	54641346	0	0.03668
24988	Del	1	54641382	54705851	0	0.036088
24989	Del	1	54713332	54808979	0	0.03668
24990	Del	1	54809137	54842717	0	0.036088
24991	Del	1	54842737	55067069	0	0.03668
24992	Del	1	55068954	55212826	0	0.035497
24993	Del	1	55212843	56491652	0	0.03668
24994	Del	1	56492536	56587751	0	0.034313
24995	Del	1	56595907	56656417	0	0.037863
24996	Del	1	56656445	56719406	0	0.037272
24997	Del	1	56719687	56819763	0	0.03668
24998	Del	1	56819766	56919157	0	0.037272
24999	Del	1	56919641	56994921	0	0.037863
25000	Del	1	56997653	57858271	0	0.026031
25001	Del	1	57860522	57863922	0	0.037863
25002	Del	1	57870386	58250986	0	0.026031
25003	Del	1	58251738	58549158	0	0.042596
25004	Del	1	58553848	58648766	0	0.043188
25005	Del	1	58648946	58703041	0	0.042596
25006	Del	1	58705121	58900380	0	0.047921
25007	Del	1	58900580	59088335	0	0.048512
25008	Del	1	59088743	59293611	0	0.049104
25009	Del	1	59294082	59513890	0	0.046146
25010	Del	1	59514486	59732566	0	0.047329
25011	Del	1	59732758	59733919	0	0.049104
25012	Del	1	59734492	59766290	0	0.047329
25013	Del	1	59766986	59988495	0	0.049104
25014	Del	1	59990125	60077662	0	0.048512
25015	Del	1	60077688	60939045	0	0.054428

25016	Del	1	60939627	61062438	0	0.049104
25017	Del	1	61066272	61248632	0	0.044963
25018	Del	1	61248825	61254976	0	0.049104
25019	Del	1	61255725	61464390	0	0.044963
25020	Del	1	61464925	61737338	0	0.048512
25021	Del	1	61740065	62073441	0	0.043779
25022	Del	1	62073807	62080187	0	0.048512
25023	Del	1	62081091	62164690	0	0.043779
25024	Del	1	62167811	62213530	0	0.048512
25025	Del	1	62213619	62434457	0.220775	0.07336
25026	Del	1	62435572	62456989	0	0.057386
25027	Del	1	62463883	62583922	0	0.055612
25028	Del	1	62583991	62592109	0	0.056795
25029	Del	1	62596132	62606771	0	0.057386
25030	Del	1	62607294	62865643	0	0.056795
25031	Del	1	62866380	63311502	0.028531	0.061528
25032	Del	1	63313675	63364808	0.060057	0.063302
25033	Del	1	63365203	63441209	0.043245	0.062711
25034	Del	1	63441538	63522694	0	0.053837
25035	Del	1	63522813	63587897	0	0.054428
25036	Del	1	63589899	63622576	0	0.053837
25037	Del	1	63623100	63771733	0	0.054428
25038	Del	1	63773053	64197836	0	0.053837
25039	Del	1	64202883	64468493	0	0.052653
25040	Del	1	64469299	64577915	0	0.052062
25041	Del	1	64578172	64584998	0	0.054428
25042	Del	1	64592685	64693455	0	0.052062
25043	Del	1	64695438	64827415	0	0.053837
25044	Del	1	64831333	64969380	0	0.055612
25045	Del	1	64969600	65142009	0	0.054428
25046	Del	1	65142418	65261719	0	0.053837
25047	Del	1	65263711	65304411	0	0.053245
25048	Del	1	65308735	65386381	0	0.053837
25049	Del	1	65387966	65491255	0	0.054428
25050	Del	1	65491413	65788286	0	0.053245
25051	Del	1	65788474	65911963	0	0.047329
25052	Del	1	65912823	65992103	0	0.048512
25053	Del	1	65992121	66041942	0	0.049104
25054	Del	1	66041959	66051367	0	0.054428
25055	Del	1	66052075	66331928	0	0.049104
25056	Del	1	66331986	66353220	0	0.053837
25057	Del	1	66353812	66532012	0	0.054428
25058	Del	1	66532050	66624416	0	0.050879
25059	Del	1	66625158	66630021	0	0.054428
25060	Del	1	66636161	66746204	0	0.050879
25061	Del	1	66747456	66795383	0	0.054428
25062	Del	1	66795463	66804252	0	0.052062
25063	Del	1	66804425	66920521	0	0.05147
25064	Del	1	66921838	66949978	0	0.052062
25065	Del	1	66951872	67136456	0	0.05147
25066	Del	1	67136526	67261236	0	0.057978
25067	Del	1	67261605	67398620	0.155279	0.06981
25068	Del	1	67399098	67698815	0.180181	0.070993

25069	Del	1	67698999	67834930	0.141544	0.069219
25070	Del	1	67834989	68041855	0.135103	0.068627
25071	Del	1	68042882	68052876	0.245798	0.074543
25072	Del	1	68055767	68098070	0.135103	0.068627
25073	Del	1	68098124	68121226	0.192262	0.072177
25074	Del	1	68125051	68180000	0.208407	0.072768
25075	Del	1	68180206	68185169	0.247213	0.074543
25076	Del	1	68185328	68234281	0.208407	0.072768
25077	Del	1	68234486	68450440	0.237075	0.073952
25078	Del	1	68455753	68497714	0.238945	0.074543
25079	Del	1	68499756	69561133	0.23434	0.073952
25080	Del	1	69562123	69759657	0.031092	0.062119
25081	Del	1	69760149	69912221	0.071807	0.064486
25082	Del	1	69917798	69920681	0.141544	0.068627
25083	Del	1	69922520	70127565	0.071807	0.064486
25084	Del	1	70128845	70202611	0.130519	0.068035
25085	Del	1	70205421	70702959	0.139154	0.068627
25086	Del	1	70705808	70833939	0	0.059161
25087	Del	1	70850149	70948151	0	0.057978
25088	Del	1	70948625	71044736	0.000508	0.059161
25089	Del	1	71046477	71082050	0.00709	0.059753
25090	Del	1	71087677	71402279	0	0.05857
25091	Del	1	71402375	71791622	0	0.054428
25092	Del	1	71791982	71846449	0.044557	0.062711
25093	Del	1	71852062	72353207	0	0.054428
25094	Del	1	72362629	74017936	0.00167	0.059161
25095	Del	1	74018113	74193474	0	0.057978
25096	Del	1	74194782	74566199	0.369843	0.081051
25097	Del	1	74566796	74572544	0.236937	0.073952
25098	Del	1	74573816	74630194	0.253765	0.075135
25099	Del	1	74630211	74676521	0.236937	0.073952
25100	Del	1	74677038	75125252	0.264621	0.075726
25101	Del	1	75125647	75198687	0.165613	0.070402
25102	Del	1	75199277	75723672	0.089852	0.065669
25103	Del	1	75723760	75783805	0.026449	0.061528
25104	Del	1	75784043	75796229	0	0.053837
25105	Del	1	75796310	75915269	0.000193	0.059161
25106	Del	1	75916195	76019448	0.017367	0.060344
25107	Del	1	76021199	76577308	0.149247	0.069219
25108	Del	1	76577699	76632401	0.135676	0.068627
25109	Del	1	76632798	77055314	0.153575	0.069219
25110	Del	1	77058203	77060797	0.185757	0.070993
25111	Del	1	77064491	77068688	0.153575	0.069219
25112	Del	1	77069007	77280560	0.1611	0.06981
25113	Del	1	77281563	77559866	0	0.049695
25114	Del	1	77559999	77693674	0	0.053245
25115	Del	1	77694209	77772085	0	0.047921
25116	Del	1	77774432	77877169	0	0.047329
25117	Del	1	77877751	78040688	0	0.047921
25118	Del	1	78044697	78225954	0	0.047329
25119	Del	1	78226329	78370085	0	0.046737
25120	Del	1	78370628	78547360	0	0.047329
25121	Del	1	78547592	79089920	0.033548	0.062119

25122	Del	1	79098506	80637116	0.258515	0.075726
25123	Del	1	80637229	80646981	0.293018	0.07691
25124	Del	1	80647681	81558720	0.393344	0.082234
25125	Del	1	81563820	81800220	0.937081	0.101166
25126	Del	1	81800330	81992942	0.990478	0.10294
25127	Del	1	81994892	83572897	1.193113	0.11004
25128	Del	1	83574078	83798613	1.006536	0.103532
25129	Del	1	83800591	84143675	1.161253	0.108857
25130	Del	1	84144200	84406294	1.186947	0.11004
25131	Del	1	84406965	84417213	1.210948	0.110631
25132	Del	1	84417267	84507751	1.266476	0.112406
25133	Del	1	84508466	84578297	1.258453	0.111815
25134	Del	1	84584723	84641904	1.259855	0.112406
25135	Del	1	84643411	84810603	1.243903	0.111815
25136	Del	1	84812296	84998055	1.212411	0.110631
25137	Del	1	84999343	85060873	1.25805	0.111815
25138	Del	1	85061090	85131779	1.222283	0.111223
25139	Del	1	85132944	85156774	1.275412	0.112406
25140	Del	1	85157551	85206658	1.25805	0.111815
25141	Del	1	85207066	85313710	1.26973	0.112406
25142	Del	1	85314199	85404657	1.222283	0.111223
25143	Del	1	85405329	85467266	1.166296	0.109448
25144	Del	1	85467806	85571868	1.093509	0.107082
25145	Del	1	85574424	85649218	1.468614	0.118322
25146	Del	1	85649327	85720696	1.441586	0.117731
25147	Del	1	85721071	86156602	1.46216	0.118322
25148	Del	1	86169562	86352321	1.904838	0.130155
25149	Del	1	86352656	86361854	1.931389	0.130746
25150	Del	1	86361950	86467250	1.907804	0.130155
25151	Del	1	86468110	86500648	1.742429	0.126013
25152	Del	1	86501033	86581447	1.765733	0.126605
25153	Del	1	86583072	86621580	1.742429	0.126013
25154	Del	1	86624971	86656284	1.783135	0.127197
25155	Del	1	86657031	86674919	1.742429	0.126013
25156	Del	1	86674961	86709846	1.793146	0.127197
25157	Del	1	86710116	86918436	1.765733	0.126605
25158	Del	1	86921436	87111776	1.678937	0.124239
25159	Del	1	87114907	87138562	1.78915	0.127197
25160	Del	1	87139185	87253005	1.745123	0.126013
25161	Del	1	87253392	87348985	1.766895	0.126605
25162	Del	1	87349948	88537206	1.773456	0.127197
25163	Del	1	88537339	88682820	1.539524	0.120689
25164	Del	1	88683110	88850729	1.57598	0.121872
25165	Del	1	88851889	88933748	1.446938	0.117731
25166	Del	1	88934107	88978323	1.419749	0.117139
25167	Del	1	88979421	89129857	1.46417	0.118322
25168	Del	1	89130115	89176173	1.445878	0.117731
25169	Del	1	89178766	89425414	1.458324	0.118322
25170	Del	1	89425859	89555359	1.84157	0.128971
25171	Del	1	89558197	89597909	1.874598	0.129563
25172	Del	1	89598054	89635767	2.041871	0.133704
25173	Del	1	89636069	89819066	1.959815	0.131929
25174	Del	1	89819631	89939631	1.819187	0.12838

25175	Del	1	89940735	90780785	1.90562	0.130155
25176	Del	1	90781970	90837991	1.84306	0.128971
25177	Del	1	90841722	91406817	1.828022	0.12838
25178	Del	1	91406999	91679170	1.927709	0.130746
25179	Del	1	91679376	91907273	1.636734	0.123055
25180	Del	1	91907380	92018258	1.715207	0.125422
25181	Del	1	92022293	92065545	1.748019	0.126013
25182	Del	1	92069820	92077698	1.769885	0.126605
25183	Del	1	92079135	92149876	1.72127	0.125422
25184	Del	1	92151153	92190635	1.75881	0.126605
25185	Del	1	92191542	92241760	2.08151	0.134888
25186	Del	1	92244482	92299393	2.540432	0.145537
25187	Del	1	92305897	92391230	2.494466	0.144945
25188	Del	1	92394751	92506271	2.550205	0.146128
25189	Del	1	92508218	92824236	2.517346	0.145537
25190	Del	1	92831008	92842116	2.363675	0.141395
25191	Del	1	92845320	92963530	2.250284	0.139029
25192	Del	1	92964617	93141277	2.293643	0.140212
25193	Del	1	93141383	93184780	2.318893	0.140804
25194	Del	1	93185943	93287725	2.267901	0.13962
25195	Del	1	93288178	93341662	2.295144	0.140212
25196	Del	1	93343134	93440684	2.317003	0.140804
25197	Del	1	93443728	93554740	2.259991	0.139029
25198	Del	1	93557168	93590333	2.298044	0.140212
25199	Del	1	93590722	93590818	2.262661	0.13962
25200	Del	1	93591698	93601078	2.324035	0.140804
25201	Del	1	93601162	93615850	2.262661	0.13962
25202	Del	1	93617360	93683396	2.196409	0.137846
25203	Del	1	93683772	93845234	2.045842	0.134296
25204	Del	1	93846461	93991964	2.338031	0.140804
25205	Del	1	93991999	94121945	2.123372	0.136071
25206	Del	1	94123198	94519383	2.261324	0.13962
25207	Del	1	94519411	94546229	2.313234	0.140212
25208	Del	1	94546355	94743397	2.571807	0.14672
25209	Del	1	94745729	94746049	2.845836	0.152044
25210	Del	1	94746261	94819075	2.571807	0.14672
25211	Del	1	94819222	94895801	2.72249	0.149678
25212	Del	1	94896237	94928796	2.645611	0.147903
25213	Del	1	94928979	94976986	2.624721	0.147311
25214	Del	1	94977059	95073520	2.568499	0.146128
25215	Del	1	95074149	95110604	2.910821	0.153819
25216	Del	1	95111741	95197940	2.938542	0.154411
25217	Del	1	95198342	95247891	2.867983	0.152636
25218	Del	1	95248084	95321823	2.938848	0.154411
25219	Del	1	95322132	95516270	2.975263	0.155002
25220	Del	1	95517738	96253486	3.335639	0.162693
25221	Del	1	96253902	96375248	2.573819	0.14672
25222	Del	1	96378345	97076923	2.808453	0.151453
25223	Del	1	97077241	97086822	2.477515	0.144353
25224	Del	1	97094035	97332414	2.975553	0.155002
25225	Del	1	97332922	97717486	2.477515	0.144353
25226	Del	1	97719258	97789337	3.054231	0.156777
25227	Del	1	97789788	97801193	3.243819	0.160918

25228	Del	1	97802804	97927699	3.054231	0.156777
25229	Del	1	97929972	98036854	3.195991	0.160327
25230	Del	1	98037817	98208141	3.246151	0.160918
25231	Del	1	98209172	98888756	3.172815	0.159735
25232	Del	1	98890112	99149917	2.885699	0.153228
25233	Del	1	99152529	99309640	3.00542	0.156186
25234	Del	1	99310148	99635636	3.054231	0.157369
25235	Del	1	99644393	99695251	3.282044	0.162102
25236	Del	1	99695548	99704546	3.066786	0.157369
25237	Del	1	99705490	99849298	3.259416	0.16151
25238	Del	1	99849413	99924211	3.227045	0.160918
25239	Del	1	99924243	100027241	3.524733	0.166835
25240	Del	1	100027311	100179307	3.760745	0.171567
25241	Del	1	100179332	100249980	3.736693	0.170976
25242	Del	1	100250611	100267141	3.791947	0.172159
25243	Del	1	100269222	100280400	3.758433	0.171567
25244	Del	1	100280610	100284108	3.794115	0.172159
25245	Del	1	100284732	100343443	3.758433	0.171567
25246	Del	1	100344138	100500127	3.596704	0.168609
25247	Del	1	100500920	100522119	3.570361	0.168018
25248	Del	1	100522616	100625007	3.785334	0.172159
25249	Del	1	100625041	100898223	3.749794	0.171567
25250	Del	1	100905366	100980951	3.709571	0.170384
25251	Del	1	100982033	101026734	3.749794	0.171567
25252	Del	1	101027915	101087911	3.719956	0.170976
25253	Del	1	101088642	101321293	4.049593	0.177484
25254	Del	1	101323280	101797858	3.975687	0.175709
25255	Del	1	101798353	101847292	3.757199	0.171567
25256	Del	1	101850296	101870021	3.464333	0.165651
25257	Del	1	101870350	101895066	3.829948	0.173342
25258	Del	1	101895394	102870917	3.464333	0.165651
25259	Del	1	102873555	103403395	3.092397	0.15796
25260	Del	1	103412238	103524255	3.070076	0.157369
25261	Del	1	103524378	104070434	3.133494	0.158552
25262	Del	1	104071000	105585913	2.462364	0.143762
25263	Del	1	105589666	105602040	2.409561	0.142578
25264	Del	1	105602073	105620227	2.419141	0.14317
25265	Del	1	105620240	107136112	2.678296	0.148495
25266	Del	1	107136860	107564876	2.222592	0.138437
25267	Del	1	107565938	107689008	2.083476	0.134888
25268	Del	1	107693184	107893960	2.017457	0.133113
25269	Del	1	107896947	107897462	2.232974	0.138437
25270	Del	1	107897607	107963109	2.017457	0.133113
25271	Del	1	107963121	108131286	2.392461	0.142578
25272	Del	1	108133825	108357561	2.031684	0.133704
25273	Del	1	108358141	108556501	1.769885	0.126605
25274	Del	1	108559142	108641961	1.702599	0.12483
25275	Del	1	108644076	108712891	1.761472	0.126605
25276	Del	1	108712919	108742932	1.744918	0.126013
25277	Del	1	108743203	108814834	1.710141	0.125422
25278	Del	1	108815562	108854286	1.634489	0.123055
25279	Del	1	108854362	108872702	1.681367	0.124239
25280	Del	1	108875420	108927664	1.643786	0.123647

25281	Del	1	108928038	108967060	1.671802	0.124239
25282	Del	1	108968731	109067737	1.643786	0.123647
25283	Del	1	109069836	109129593	1.633481	0.123055
25284	Del	1	109131659	109184907	1.578616	0.121872
25285	Del	1	109185210	109206848	1.608581	0.122464
25286	Del	1	109207235	109244767	1.636545	0.123055
25287	Del	1	109244871	109310269	1.46417	0.118322
25288	Del	1	109311865	109395480	1.420836	0.117139
25289	Del	1	109395703	109426671	1.44921	0.117731
25290	Del	1	109429412	109553275	1.461733	0.118322
25291	Del	1	109553331	109595914	1.450059	0.117731
25292	Del	1	109595978	109908568	1.470767	0.118322
25293	Del	1	109909068	109983008	1.233197	0.111223
25294	Del	1	109983974	109995242	1.215978	0.110631
25295	Del	1	109996304	110079191	1.226949	0.111223
25296	Del	1	110080931	110110258	1.219859	0.110631
25297	Del	1	110111876	110147955	1.237159	0.111223
25298	Del	1	110148002	110203842	1.200368	0.11004
25299	Del	1	110206182	110283073	1.23952	0.111223
25300	Del	1	110283910	110334463	1.189589	0.11004
25301	Del	1	110337964	110352364	1.234165	0.111223
25302	Del	1	110355952	110391656	1.390784	0.115956
25303	Del	1	110392367	110590817	1.408257	0.116548
25304	Del	1	110591550	110602146	1.376436	0.115956
25305	Del	1	110602333	110609527	1.410586	0.116548
25306	Del	1	110609642	110633365	1.376436	0.115956
25307	Del	1	110633655	110788274	1.396727	0.116548
25308	Del	1	110869657	110902257	1.190558	0.11004
25309	Del	1	110903233	110965195	1.217138	0.110631
25310	Del	1	110967655	111185107	1.391554	0.115956
25311	Del	1	111185663	111224153	1.396727	0.116548
25312	Del	1	111226615	111243606	1.384087	0.115956
25313	Del	1	111243629	111297256	1.398011	0.116548
25314	Del	1	111301836	111320684	1.373379	0.115956
25315	Del	1	111320829	111383659	1.401886	0.116548
25316	Del	1	111384187	111413584	1.122007	0.107673
25317	Del	1	111414040	111429346	1.103637	0.107082
25318	Del	1	111432313	111482002	1.119341	0.107673
25319	Del	1	111482359	111495823	1.104817	0.107082
25320	Del	1	111499042	111507328	1.120844	0.107673
25321	Del	1	111531439	111565047	1.07765	0.10649
25322	Del	1	111565553	111619013	1.120034	0.107673
25323	Del	1	111619254	111717059	1.081795	0.10649
25324	Del	1	111717145	111772200	1.119341	0.107673
25325	Del	1	111772828	111852810	1.052062	0.105307
25326	Del	1	111852970	111859428	1.23952	0.111223
25327	Del	1	111859558	111905486	1.052062	0.105307
25328	Del	1	111905671	111911573	1.23952	0.111223
25329	Del	1	111912501	111985854	1.052062	0.105307
25330	Del	1	111988111	112395518	1.233197	0.111223
25331	Del	1	112395875	112465599	0.817138	0.097616
25332	Del	1	112466001	112507240	0.637595	0.091108
25333	Del	1	112507301	112521846	0.661027	0.092291

25334	Del	1	112527620	112669207	0.639857	0.0917
25335	Del	1	112670914	112730227	0.66605	0.092291
25336	Del	1	112730661	112852540	0.766216	0.095841
25337	Del	1	112858766	112917117	0.992911	0.103532
25338	Del	1	112922836	112925910	1.020447	0.104124
25339	Del	1	112929902	112955315	0.992911	0.103532
25340	Del	1	112955388	113008291	1.018768	0.104124
25341	Del	1	113008600	113073642	0.990814	0.10294
25342	Del	1	113074012	113193686	0.906879	0.100574
25343	Del	1	113195051	113385160	0.753026	0.09525
25344	Del	1	113388169	113687348	0.645408	0.0917
25345	Del	1	113687730	113761186	0.762853	0.095841
25346	Del	1	113762492	113811087	0.752895	0.09525
25347	Del	1	113811220	113872746	0.762853	0.095841
25348	Del	1	113872792	113874720	0.759821	0.09525
25349	Del	1	113874815	113887734	0.776999	0.095841
25350	Del	1	113888106	113893860	0.759821	0.09525
25351	Del	1	113893879	113982930	0.772058	0.095841
25352	Del	1	113982952	114386761	0.782532	0.096433
25353	Del	1	114389525	114513584	0.499012	0.085784
25354	Del	1	114513756	114594150	0.52267	0.086967
25355	Del	1	114597892	114761069	0.504835	0.086375
25356	Del	1	114761372	114783903	0.52267	0.086967
25357	Del	1	114789606	115029491	0.913022	0.100574
25358	Del	1	115029744	115045809	0.654907	0.0917
25359	Del	1	115046618	115281087	0.613642	0.090517
25360	Del	1	115281695	115770371	0.46413	0.084601
25361	Del	1	115770642	115925967	0.497921	0.085784
25362	Del	1	115926770	115977603	0.477617	0.085192
25363	Del	1	115978436	116071909	0.455798	0.084601
25364	Del	1	116072176	116370856	0.489763	0.085784
25365	Del	1	116371662	116381170	0.341408	0.079276
25366	Del	1	116382329	116423207	0.345249	0.079868
25367	Del	1	116423513	116478865	0.332033	0.079276
25368	Del	1	116481869	116575340	0.346	0.079868
25369	Del	1	116577445	116693694	0.333333	0.079276
25370	Del	1	116693787	116898362	0.265501	0.075726
25371	Del	1	116900746	116998432	0.130356	0.068035
25372	Del	1	117001303	117036635	0.096142	0.066261
25373	Del	1	117040676	117058997	0.090652	0.065669
25374	Del	1	117059005	117103012	0.098676	0.066261
25375	Del	1	117103122	117143370	0.264017	0.075726
25376	Del	1	117143531	117211597	0.246242	0.074543
25377	Del	1	117213178	117364076	0.25614	0.075726
25378	Del	1	117364282	117526312	0.237357	0.073952
25379	Del	1	117526815	117609619	0.262652	0.075726
25380	Del	1	117609728	117928198	0.25527	0.075135
25381	Del	1	117929246	117964606	0.266746	0.075726
25382	Del	1	117965230	118880290	0.21253	0.07336
25383	Del	1	118881404	118991186	0.084405	0.065669
25384	Del	1	118991200	119028642	0.114479	0.066852
25385	Del	1	119029434	119043103	0.090705	0.065669
25386	Del	1	119044668	119067312	0.113619	0.066852

25387	Del	1	119067560	119140949	0.090705	0.065669
25388	Del	1	119142221	119186741	0	0.05857
25389	Del	1	119187779	119234361	0	0.057978
25390	Del	1	119234757	119367866	0	0.057386
25391	Del	1	119367882	119556421	0	0.053245
25392	Del	1	119559935	119648979	0	0.049104
25393	Del	1	119650141	119898203	0	0.053245
25394	Del	1	119899280	146716041	0	0.059161
25395	Del	1	149906351	149906351	0	0.021298
25396	Del	1	149907993	149918981	0	0.017748
25397	Del	1	149927059	150615125	0	0.015382
25398	Del	1	150619563	151359451	0	0.011832
25399	Del	1	151359541	151538792	0	0.010649
25400	Del	1	151542649	154274778	0	0.009466
25401	Del	1	154282794	154697101	0	0.004141
25402	Del	1	154697190	154920147	0	0.00355
25403	Del	1	154922740	154937631	0	0.001183
25404	Del	1	154938100	154938781	0	0.000592
25405	Del	1	154940147	155182632	0	0.001183
25406	Del	1	155184471	155184471	0	0.000592
25407	Del	1	155184594	155617962	0	0.001183
25408	Del	1	155621583	155748593	0	0.006508
25409	Del	1	155749293	156243466	0	0.010649
25410	Del	1	156244321	156344836	0	0.013607
25411	Del	1	156348599	156574026	0	0.010649
25412	Del	1	156575928	156940696	0	0.015382
25413	Del	1	156941411	157091445	0	0.01479
25414	Del	1	157091758	157989825	0	0.011832
25415	Del	1	157993139	158101341	0	0.011241
25416	Del	1	158102916	158698252	0	0.011832
25417	Del	1	158698408	160957147	0	0.005325
25418	Del	1	160957196	161148820	0	0.013607
25419	Del	1	161153394	161285543	0	0.005325
25420	Del	1	161285714	161757028	0	0.013607
25421	Del	1	161763537	161966331	0	0.015974
25422	Del	1	161966893	162068066	0	0.017157
25423	Del	1	162068488	162156253	0	0.011241
25424	Del	1	162156654	162158656	0	0.013607
25425	Del	1	162159032	162342037	0	0.011241
25426	Del	1	162342537	162342963	0	0.013607
25427	Del	1	162343330	162361618	0	0.011241
25428	Del	1	162362731	162630329	0	0.013607
25429	Del	1	162631124	162781031	0	0.012424
25430	Del	1	162781172	163138213	0	0.013607
25431	Del	1	163138238	163158254	0	0.013015
25432	Del	1	163160640	163213215	0	0.013607
25433	Del	1	163215449	163294650	0	0.013015
25434	Del	1	163295539	163424789	0	0.013607
25435	Del	1	163425021	164768312	0	0.020706
25436	Del	1	164768351	164774942	0	0.023073
25437	Del	1	164778331	165201483	0	0.020706
25438	Del	1	165201535	165357763	0	0.015974
25439	Del	1	165357779	165476293	0	0.017157

25440	Del	1	165476459	165542893	0	0.016565
25441	Del	1	165543591	165564743	0	0.017157
25442	Del	1	165564893	165585338	0	0.016565
25443	Del	1	165586985	165818794	0	0.017157
25444	Del	1	165819084	165904382	0	0.016565
25445	Del	1	165907468	165908041	0	0.017157
25446	Del	1	165908338	166153341	0	0.016565
25447	Del	1	166153703	166155573	0	0.017157
25448	Del	1	166156002	166602395	0	0.016565
25449	Del	1	166603893	167215157	0	0.011832
25450	Del	1	167218382	167324681	0	0.011241
25451	Del	1	167326601	167430266	0	0.011832
25452	Del	1	167431661	167518643	0	0.011241
25453	Del	1	167521077	167557236	0	0.020706
25454	Del	1	167557271	167643460	0	0.026031
25455	Del	1	167643775	167913161	0	0.025439
25456	Del	1	167916174	167937334	0	0.026031
25457	Del	1	167943428	167991997	0	0.025439
25458	Del	1	167993307	167999787	0	0.026031
25459	Del	1	168000216	168077152	0	0.025439
25460	Del	1	168077534	168314926	0	0.026031
25461	Del	1	168315644	168694036	0	0.027806
25462	Del	1	168694160	168729214	0	0.027214
25463	Del	1	168729642	168902304	0	0.027806
25464	Del	1	168903763	169092320	0	0.026031
25465	Del	1	169092543	169133957	0	0.027806
25466	Del	1	169134085	169511438	0	0.026031
25467	Del	1	169511938	169585992	0	0.025439
25468	Del	1	169588720	169679367	0	0.034313
25469	Del	1	169679985	169806563	0	0.026031
25470	Del	1	169817618	169852603	0	0.025439
25471	Del	1	169852928	169913566	0	0.026031
25472	Del	1	169913659	170075022	0	0.024848
25473	Del	1	170075230	171005156	0	0.026031
25474	Del	1	171010287	171068019	0	0.025439
25475	Del	1	171069074	171483435	0	0.026031
25476	Del	1	171484063	171594722	0	0.025439
25477	Del	1	171595591	171837664	0	0.026031
25478	Del	1	171838919	171864066	0	0.024256
25479	Del	1	171864106	171866177	0	0.026031
25480	Del	1	171866353	172142359	0	0.024256
25481	Del	1	172146014	172420058	0	0.02189
25482	Del	1	172420218	173182017	0	0.020115
25483	Del	1	173182393	173230047	0	0.01479
25484	Del	1	173233224	173416334	0	0.013607
25485	Del	1	173416528	173464446	0	0.01479
25486	Del	1	173467367	173474687	0	0.013607
25487	Del	1	173475676	173495207	0	0.01479
25488	Del	1	173496994	173602889	0	0.018932
25489	Del	1	173606753	174157604	0	0.019523
25490	Del	1	174158380	174796308	0	0.024256
25491	Del	1	174799389	174867388	0	0.027214
25492	Del	1	174869587	174958330	0	0.028397

25493	Del	1	174960326	174997294	0	0.027214
25494	Del	1	174998741	175060796	0	0.023664
25495	Del	1	175061984	175148090	0	0.023073
25496	Del	1	175148443	175320263	0	0.023664
25497	Del	1	175321430	175793886	0	0.022481
25498	Del	1	175796478	175935673	0	0.023664
25499	Del	1	175942575	175965971	0	0.022481
25500	Del	1	175966605	175969436	0	0.023664
25501	Del	1	175975781	176461129	0	0.022481
25502	Del	1	176462831	176700495	0	0.013607
25503	Del	1	176701241	177028127	0	0.013015
25504	Del	1	177029171	177030816	0	0.01479
25505	Del	1	177031067	177165573	0	0.013015
25506	Del	1	177168704	177286791	0	0.014199
25507	Del	1	177287357	178096916	0	0.01479
25508	Del	1	178097064	178337663	0	0.013607
25509	Del	1	178339776	178481169	0	0.014199
25510	Del	1	178483494	178717674	0	0.01479
25511	Del	1	178722231	178839035	0	0.014199
25512	Del	1	178848563	178873591	0	0.01479
25513	Del	1	178874546	178922870	0	0.014199
25514	Del	1	178923702	179097207	0	0.01479
25515	Del	1	179097936	179151067	0	0.014199
25516	Del	1	179151608	179233074	0	0.013607
25517	Del	1	179234795	179370127	0	0.01479
25518	Del	1	179370199	179549324	0	0.014199
25519	Del	1	179549950	179724343	0	0.01479
25520	Del	1	179727651	179727651	0	0.014199
25521	Del	1	179728851	179745112	0	0.01479
25522	Del	1	179745360	179816520	0	0.014199
25523	Del	1	179816602	179951101	0	0.01479
25524	Del	1	179954321	180119626	0	0.014199
25525	Del	1	180120555	180200814	0	0.019523
25526	Del	1	180201226	180283287	0	0.020115
25527	Del	1	180284113	180433533	0	0.019523
25528	Del	1	180433734	180438506	0	0.020115
25529	Del	1	180441773	180504497	0	0.019523
25530	Del	1	180504995	180631463	0	0.020115
25531	Del	1	180631518	180840845	0	0.019523
25532	Del	1	180841202	180891554	0	0.018932
25533	Del	1	180892763	181480787	0	0.020115
25534	Del	1	181483121	181808560	0	0.017748
25535	Del	1	181808602	182202886	0	0.020115
25536	Del	1	182203320	182314436	0	0.014199
25537	Del	1	182317246	182443565	0	0.01479
25538	Del	1	182444497	182573176	0	0.014199
25539	Del	1	182573218	182613672	0	0.007691
25540	Del	1	182613885	182785410	0	0.004733
25541	Del	1	182785980	182835063	0	0.001183
25542	Del	1	182835324	182888000	0	0.000592
25543	Del	1	182888873	182954587	0	0.001183
25544	Del	1	182954683	183184560	0	0.007099
25545	Del	1	183184660	183245291	0	0.000592

25546	Del	1	183245748	183478195	0	0.001183
25547	Del	1	183485040	183552761	0	0.000592
25548	Del	1	183555158	184385100	0	0.001183
25549	Del	1	184385272	184629503	0	0.000592
25550	Del	1	184630380	185117225	0	0.001183
25551	Del	1	185117859	185294255	0	0.000592
25552	Del	1	185294496	186314690	0	0.001183
25553	Del	1	186318139	186373372	0	0.000592
25554	Del	1	186373593	190802305	0	0.001183
25555	Del	1	190804435	193114832	0	0.008874
25556	Del	1	193114915	193135537	0	0.007691
25557	Del	1	193135674	193138076	0	0.008874
25558	Del	1	193138091	193175517	0	0.007691
25559	Del	1	193178606	193191928	0	0.008874
25560	Del	1	193192075	193255965	0	0.007691
25561	Del	1	193264546	196218838	0	0.008874
25562	Del	1	196219175	196579878	0	0.007099
25563	Del	1	196582088	196582603	0	0.007691
25564	Del	1	196584453	196610909	0	0.007099
25565	Del	1	196611004	196973325	0	0.007691
25566	Del	1	196973491	197014101	0	0.000592
25567	Del	1	197018951	197083978	0	0.001183
25568	Del	1	197084011	197147260	0	0.000592
25569	Del	1	197148156	197202936	0	0.001183
25570	Del	1	197215829	197479363	0	0.000592
25571	Del	1	197480010	197910132	0	0.001183
25572	Del	1	197910610	197914282	0	0.000592
25573	Del	1	197914694	198991074	0	0.001183
25574	Del	1	198992439	199085537	0	0.000592
25575	Del	1	199125422	200339692	0	0.005325
25576	Del	1	200342215	200376802	0	0.000592
25577	Del	1	200379496	200884305	0	0.001183
25578	Del	1	200888646	200894147	0	0.000592
25579	Del	1	200894458	201131620	0	0.001183
25580	Del	1	201133504	201229830	0	0.000592
25581	Del	1	201230521	201358308	0	0.001183
25582	Del	1	201358967	201376224	0	0.000592
25583	Del	1	201378123	201475011	0	0.001183
25584	Del	1	201475570	201508884	0	0.000592
25585	Del	1	201510680	201950546	0	0.001183
25586	Del	1	201954677	201971096	0	0.000592
25587	Del	1	201972821	202192516	0	0.001183
25588	Del	1	202192585	202202211	0	0.000592
25589	Del	1	202202897	202876260	0	0.001183
25590	Del	1	202876470	202890472	0	0.000592
25591	Del	1	202890939	202939306	0	0.001183
25592	Del	1	202939408	202958899	0	0.000592
25593	Del	1	202960458	203168353	0	0.001183
25594	Del	1	203169648	203176173	0	0.000592
25595	Del	1	203176865	203621856	0	0.001183
25596	Del	1	203625674	203728840	0	0.000592
25597	Del	1	203729251	203737012	0	0.001183
25598	Del	1	203741462	203747619	0	0.000592

25599	Del	1	203748252	203812288	0	0.001183
25600	Del	1	203815660	203856548	0	0.000592
25601	Del	1	203857127	204028394	0	0.001183
25602	Del	1	204028798	204139365	0	0.000592
25603	Del	1	204140482	204145368	0	0.001183
25604	Del	1	204146420	204153598	0	0.000592
25605	Del	1	204153984	204199728	0	0.001183
25606	Del	1	204201529	204214647	0	0.000592
25607	Del	1	204216056	204503151	0	0.001183
25608	Del	1	204509302	204560590	0	0.000592
25609	Del	1	204560680	205685686	0	0.001183
25610	Del	1	205687455	206028050	0	0.004733
25611	Del	1	206030931	206126681	0	0.008874
25612	Del	1	206134718	206468719	0	0.017157
25613	Del	1	206469582	208021660	0	0.004733
25614	Del	1	208022171	208246621	0	0.004141
25615	Del	1	208246775	209426131	0	0.01479
25616	Del	1	209426913	209735178	0	0.013607
25617	Del	1	209735531	209742873	0	0.013015
25618	Del	1	209743444	209935128	0	0.013607
25619	Del	1	209935941	210164346	0	0.013015
25620	Del	1	210164381	210316066	0	0.02189
25621	Del	1	210317611	211134774	0	0.018932
25622	Del	1	211135861	211494211	0	0.02189
25623	Del	1	211494515	211639668	0	0.027214
25624	Del	1	211639687	211938963	0	0.02189
25625	Del	1	211939187	212035149	0	0.021298
25626	Del	1	212035452	212225026	0	0.02189
25627	Del	1	212225109	212278707	0	0.030172
25628	Del	1	212283747	212556469	0	0.030764
25629	Del	1	212556992	212604944	0	0.035497
25630	Del	1	212605165	212721276	0	0.036088
25631	Del	1	212721289	212788823	0	0.035497
25632	Del	1	212791393	213048274	0	0.036088
25633	Del	1	213048873	213818514	0	0.034905
25634	Del	1	213818571	213922550	0	0.034313
25635	Del	1	213922601	213926758	0	0.036088
25636	Del	1	213926840	213981376	0	0.034313
25637	Del	1	213981483	214278721	0	0.036088
25638	Del	1	214280414	214341018	0	0.035497
25639	Del	1	214345295	214553500	0	0.028397
25640	Del	1	214554040	214665694	0	0.030764
25641	Del	1	214665832	215077884	0	0.037272
25642	Del	1	215078297	215242022	0	0.038455
25643	Del	1	215242040	215623331	0	0.039638
25644	Del	1	215623679	216071707	0	0.037863
25645	Del	1	216072132	216087790	0	0.045554
25646	Del	1	216090314	216172806	0	0.037863
25647	Del	1	216173236	216501223	0	0.043188
25648	Del	1	216502245	216726497	0	0.042596
25649	Del	1	216728984	216805460	0	0.042004
25650	Del	1	216806526	216941970	0	0.039638
25651	Del	1	216942285	217090063	0	0.039046

25652	Del	1	217090849	217425968	0	0.038455
25653	Del	1	217425988	217607037	0	0.037863
25654	Del	1	217607130	217631613	0	0.039046
25655	Del	1	217631793	217778927	0	0.037272
25656	Del	1	217780397	217788051	0	0.039046
25657	Del	1	217791674	217870121	0	0.037272
25658	Del	1	217872128	218041621	0	0.039046
25659	Del	1	218041691	218339293	0	0.031947
25660	Del	1	218339869	218348939	0	0.02189
25661	Del	1	218350962	218438795	0	0.021298
25662	Del	1	218440997	218446304	0	0.02189
25663	Del	1	218446680	219080176	0	0.017748
25664	Del	1	219080364	219172951	0	0.021298
25665	Del	1	219173274	219431828	0	0.02189
25666	Del	1	219435097	219561413	0	0.020706
25667	Del	1	219561618	219968436	0	0.030764
25668	Del	1	219968509	220047913	0	0.030172
25669	Del	1	220048660	220152669	0	0.030764
25670	Del	1	220153384	220191858	0	0.030172
25671	Del	1	220194445	220201853	0	0.030764
25672	Del	1	220202582	220264447	0	0.030172
25673	Del	1	220264565	220269027	0	0.030764
25674	Del	1	220269222	220277030	0	0.030172
25675	Del	1	220285134	220582981	0	0.030764
25676	Del	1	220583041	220666360	0	0.030172
25677	Del	1	220667422	220705910	0	0.03668
25678	Del	1	220706275	221325340	0	0.042004
25679	Del	1	221325712	222814101	0	0.027214
25680	Del	1	222814251	223006387	0	0.026031
25681	Del	1	223006472	223220544	0	0.027214
25682	Del	1	223220676	223388460	0	0.026031
25683	Del	1	223390731	223698457	0	0.017748
25684	Del	1	223699082	223776703	0	0.017157
25685	Del	1	223777329	224435576	0	0.017748
25686	Del	1	224437846	224613811	0	0.015382
25687	Del	1	224614250	224743583	0	0.016565
25688	Del	1	224743877	224967950	0	0.017748
25689	Del	1	224969036	225025785	0	0.017157
25690	Del	1	225025825	225400227	0	0.015974
25691	Del	1	225400674	225433715	0	0.017748
25692	Del	1	225434562	225657612	0	0.023664
25693	Del	1	225658509	225873306	0	0.024256
25694	Del	1	225873366	226629998	0	0.017748
25695	Del	1	226631372	226655438	0	0.017157
25696	Del	1	226655511	226676326	0	0.017748
25697	Del	1	226676824	226739636	0	0.017157
25698	Del	1	226741627	226991261	0	0.026623
25699	Del	1	226991336	227563411	0	0.024256
25700	Del	1	227563510	227679757	0	0.017157
25701	Del	1	227682006	228219917	0	0.017748
25702	Del	1	228219930	228309769	0	0.017157
25703	Del	1	228312480	230054128	0	0.017748
25704	Del	1	230055271	230065181	0	0.015974

25705	Del	1	230065396	230092739	0	0.017748
25706	Del	1	230092758	230285757	0	0.015974
25707	Del	1	230285976	230427014	0	0.017157
25708	Del	1	230427709	231476775	0	0.017748
25709	Del	1	231481234	231827591	0	0.047329
25710	Del	1	231831101	232397269	0	0.044371
25711	Del	1	232397775	232516163	0	0.020706
25712	Del	1	232517091	232979306	0	0.02189
25713	Del	1	232980704	233293223	0	0.019523
25714	Del	1	233293246	233322655	0	0.02189
25715	Del	1	233323202	233623892	0	0.021298
25716	Del	1	233624118	233625559	0	0.02189
25717	Del	1	233627155	233673125	0	0.021298
25718	Del	1	233673255	234325338	0	0.026623
25719	Del	1	234325437	234390586	0	0.032539
25720	Del	1	234391122	234480445	0	0.021298
25721	Del	1	234480741	235163265	0	0.02189
25722	Del	1	235163380	235185924	0	0.021298
25723	Del	1	235187636	235191240	0	0.02189
25724	Del	1	235192821	235325869	0	0.021298
25725	Del	1	235325920	235515448	0	0.02189
25726	Del	1	235515585	235547272	0	0.020706
25727	Del	1	235547476	235654850	0	0.021298
25728	Del	1	235656289	235658515	0	0.020706
25729	Del	1	235658550	235822663	0	0.029581
25730	Del	1	235825266	235970251	0	0.030172
25731	Del	1	235973732	236488295	0	0.029581
25732	Del	1	236489063	236556734	0	0.030172
25733	Del	1	236556839	236785336	0	0.029581
25734	Del	1	236786909	236908536	0	0.028989
25735	Del	1	236910092	237005074	0	0.030172
25736	Del	1	237005949	237861041	0	0.037863
25737	Del	1	237861141	237933586	0	0.034905
25738	Del	1	237933642	238486457	0	0.035497
25739	Del	1	238489372	239703073	0	0.043779
25740	Del	1	239704885	239719146	0	0.047329
25741	Del	1	239719299	239894035	0	0.043779
25742	Del	1	239897028	239900411	0	0.047329
25743	Del	1	239900521	239911006	0	0.043779
25744	Del	1	239915248	240013300	0	0.047329
25745	Del	1	240013333	240475484	0	0.055612
25746	Del	1	240475749	240770595	0.165613	0.070402
25747	Del	1	240771983	241496059	0.057666	0.063302
25748	Del	1	241496402	241521531	0	0.053245
25749	Del	1	241522201	241592623	0.030624	0.061528
25750	Del	1	241593059	241628655	0.035867	0.062119
25751	Del	1	241628732	241636158	0.048332	0.062711
25752	Del	1	241636236	241646728	0.035867	0.062119
25753	Del	1	241651974	241890353	0.023863	0.060936
25754	Del	1	241890369	242083169	0.030769	0.061528
25755	Del	1	242083541	242456375	0	0.056203
25756	Del	1	242456523	242880979	0	0.05502
25757	Del	1	242881005	243263904	0	0.049695

25758	Del	1	243264005	243342539	0	0.046737
25759	Del	1	243345215	243346642	0	0.049695
25760	Del	1	243347760	243487414	0	0.046737
25761	Del	1	243487556	243914072	0	0.050879
25762	Del	1	243915992	244053612	0	0.048512
25763	Del	1	244054271	244061883	0	0.043188
25764	Del	1	244061944	244390205	0	0.046737
25765	Del	1	244390355	244963357	0	0.047329
25766	Del	1	244964887	244973029	0	0.03668
25767	Del	1	244976994	245099803	0	0.036088
25768	Del	1	245100730	245152296	0	0.035497
25769	Del	1	245152685	245704619	0	0.034905
25770	Del	1	245705006	247008955	0	0.04023
25771	Del	1	247014654	247033237	0	0.039046
25772	Del	1	247033634	247502879	0	0.04023
25773	Del	1	247503221	247523152	0	0.039638
25774	Del	1	247524498	247534862	0	0.04023
25775	Del	1	247535364	247547053	0	0.039638
25776	Del	1	247547322	247650984	0	0.04023
25777	Del	2	480597	557322	0	0.027806
25778	Del	2	557590	678386	0	0.025439
25779	Del	2	678988	2894834	0	0.035497
25780	Del	2	2895004	3128258	0	0.023664
25781	Del	2	3129126	3380425	0	0.025439
25782	Del	2	3381717	3483995	0	0.024848
25783	Del	2	3485983	3644908	0	0.025439
25784	Del	2	3646149	3705948	0	0.028397
25785	Del	2	3706242	3848775	0	0.026623
25786	Del	2	3848808	5633362	0	0.03313
25787	Del	2	5633737	5688982	0	0.032539
25788	Del	2	5689247	5931625	0	0.03313
25789	Del	2	5932355	5972384	0	0.032539
25790	Del	2	5973876	5976733	0	0.03313
25791	Del	2	5976811	5979138	0	0.032539
25792	Del	2	5981174	5988434	0	0.03313
25793	Del	2	5988497	6726781	0	0.047329
25794	Del	2	6728394	6920753	0	0.038455
25795	Del	2	6921485	7046190	0	0.03668
25796	Del	2	7046195	8015429	0	0.038455
25797	Del	2	8016121	8330972	0	0.035497
25798	Del	2	8330993	8727726	0	0.038455
25799	Del	2	8728558	8849054	0	0.037863
25800	Del	2	8849316	9007245	0	0.035497
25801	Del	2	9008649	9400720	0	0.05147
25802	Del	2	9401252	9414936	0	0.054428
25803	Del	2	9415857	9492268	0.01199	0.060344
25804	Del	2	9492542	9829559	0.006559	0.059753
25805	Del	2	9835428	9935860	0.000193	0.059161
25806	Del	2	9936739	10019955	0.006559	0.059753
25807	Del	2	10020702	10133117	0.018569	0.060344
25808	Del	2	10135619	10191787	0.061713	0.063894
25809	Del	2	10191793	10194885	0.075677	0.064486
25810	Del	2	10201231	10298495	0.061713	0.063894

25811	Del	2	10300861	10328371	0.044618	0.062711
25812	Del	2	10328700	10365807	0.050976	0.063302
25813	Del	2	10366970	10433072	0.060437	0.063894
25814	Del	2	10433446	10456147	0.074185	0.064486
25815	Del	2	10457794	10694314	0.191059	0.071585
25816	Del	2	10700560	10814066	0.332647	0.079276
25817	Del	2	10816908	10877523	0.323603	0.078684
25818	Del	2	10877875	11180542	0.179304	0.070993
25819	Del	2	11180751	11392789	0.154605	0.06981
25820	Del	2	11393585	11466904	0.081603	0.065077
25821	Del	2	11467272	11537323	0.693552	0.093475
25822	Del	2	11537389	11598405	0.726089	0.094658
25823	Del	2	11600051	11656911	0.693552	0.093475
25824	Del	2	11657823	11675500	0.570811	0.088742
25825	Del	2	11675597	11722888	0.505232	0.086375
25826	Del	2	11723758	11763313	0.549087	0.08815
25827	Del	2	11763714	11770001	0.57425	0.088742
25828	Del	2	11770376	11784368	0.549087	0.08815
25829	Del	2	11785209	11835246	0.53937	0.087559
25830	Del	2	11836693	12007050	0.10539	0.066261
25831	Del	2	12007063	12085547	0.080736	0.065077
25832	Del	2	12085804	12131720	0.0669	0.063894
25833	Del	2	12133896	12197878	0	0.056795
25834	Del	2	12198042	12200051	0.10539	0.066261
25835	Del	2	12201105	12580057	0	0.056795
25836	Del	2	12580185	12955905	0.068346	0.064486
25837	Del	2	12957300	14205667	0.0669	0.063894
25838	Del	2	14208790	14401269	0	0.057978
25839	Del	2	14401333	15166372	0.030373	0.061528
25840	Del	2	15166586	15563314	0	0.057386
25841	Del	2	15564820	16046332	0.026449	0.061528
25842	Del	2	16048851	16086271	0.002017	0.059753
25843	Del	2	16086758	17510402	0.378428	0.081051
25844	Del	2	17510527	17533735	0.25527	0.075135
25845	Del	2	17538522	17656474	0.220202	0.07336
25846	Del	2	17656537	17752008	0.326392	0.078684
25847	Del	2	17753095	17790693	0.339902	0.079276
25848	Del	2	17790748	17877126	0.354776	0.079868
25849	Del	2	17877595	17933516	0.337748	0.079276
25850	Del	2	17933600	18985650	0.587353	0.089333
25851	Del	2	18985663	19029193	0.333333	0.079276
25852	Del	2	19029665	19862282	0.354776	0.079868
25853	Del	2	19862797	19902887	0.251068	0.075135
25854	Del	2	19904811	19978325	0.246068	0.074543
25855	Del	2	19979459	20244947	0.254464	0.075135
25856	Del	2	20246002	20329209	0.241479	0.074543
25857	Del	2	20330339	20351814	0.236682	0.073952
25858	Del	2	20352232	20596185	0.25536	0.075135
25859	Del	2	20597108	20662826	0.481843	0.085192
25860	Del	2	20663648	20682296	0.448618	0.084009
25861	Del	2	20682653	20829120	0.396415	0.082234
25862	Del	2	20835321	23381741	0.454127	0.084009
25863	Del	2	23381873	23710486	0.226179	0.073952

25864	Del	2	23710579	23933677	0.291459	0.07691
25865	Del	2	23938061	24086694	0.322799	0.078684
25866	Del	2	24086830	24114644	0.308119	0.077501
25867	Del	2	24114724	24128139	0.32603	0.078684
25868	Del	2	24128766	24169473	0.320796	0.078093
25869	Del	2	24172590	24201010	0.324584	0.078684
25870	Del	2	24202751	24216179	0.317373	0.078093
25871	Del	2	24219326	24363254	0.32105	0.078684
25872	Del	2	24363280	24770803	0.534079	0.087559
25873	Del	2	24770964	24814757	0.604091	0.089925
25874	Del	2	24816007	24923001	0.708405	0.094066
25875	Del	2	24925225	24973397	0.751164	0.09525
25876	Del	2	24973621	25040071	0.738814	0.094658
25877	Del	2	25048320	25158519	0.189847	0.071585
25878	Del	2	25159148	25255584	0.2098	0.072768
25879	Del	2	25255618	25279669	0.191167	0.071585
25880	Del	2	25280074	25325681	0.201999	0.072177
25881	Del	2	25326033	25335036	0.211554	0.072768
25882	Del	2	25338675	25344360	0.201999	0.072177
25883	Del	2	25347291	25655497	0.357272	0.080459
25884	Del	2	25655528	25728149	0.352571	0.079868
25885	Del	2	25731145	25905021	0.289391	0.07691
25886	Del	2	25906461	26032394	0	0.057386
25887	Del	2	26032827	26139646	0	0.047921
25888	Del	2	26141573	26349200	0	0.048512
25889	Del	2	26350584	26398887	0	0.044963
25890	Del	2	26401029	26459778	0	0.044371
25891	Del	2	26463858	26481436	0	0.043188
25892	Del	2	26481539	26559753	0	0.042004
25893	Del	2	26560970	26690457	0	0.043188
25894	Del	2	26691496	26806107	0	0.04023
25895	Del	2	26813076	26954478	0	0.039638
25896	Del	2	26954677	27780307	0	0.04023
25897	Del	2	27781205	27885523	0	0.039638
25898	Del	2	27885538	27893656	0	0.04023
25899	Del	2	27900027	27994373	0	0.03668
25900	Del	2	27994460	27997243	0	0.04023
25901	Del	2	27997351	28148327	0	0.03668
25902	Del	2	28148425	28207664	0	0.04023
25903	Del	2	28208151	28306067	0	0.03668
25904	Del	2	28306475	28312194	0	0.04023
25905	Del	2	28312835	28339048	0	0.03668
25906	Del	2	28339836	28496030	0	0.037863
25907	Del	2	28496061	28561843	0	0.036088
25908	Del	2	28564537	28644319	0	0.037272
25909	Del	2	28644540	29003693	0	0.037863
25910	Del	2	29004515	29052406	0	0.037272
25911	Del	2	29052746	29095234	0	0.037863
25912	Del	2	29096313	29111565	0	0.037272
25913	Del	2	29112977	29178599	0	0.037863
25914	Del	2	29178603	29192502	0	0.037272
25915	Del	2	29192559	29469094	0	0.028989
25916	Del	2	29469356	29475517	0	0.03313

25917	Del	2	29475893	29922561	0	0.028989
25918	Del	2	29922925	30163838	0	0.03668
25919	Del	2	30166389	30446272	0	0.042004
25920	Del	2	30446499	30644707	0	0.04023
25921	Del	2	30645313	30901337	0	0.040821
25922	Del	2	30903633	31142176	0	0.039638
25923	Del	2	31142237	31232361	0	0.041413
25924	Del	2	31232882	31331881	0	0.042004
25925	Del	2	31334120	31416979	0	0.041413
25926	Del	2	31417589	31582803	0	0.042004
25927	Del	2	31582871	32003928	0.018569	0.060344
25928	Del	2	32008768	32055630	0.030373	0.061528
25929	Del	2	32057465	32222859	0.023136	0.060936
25930	Del	2	32222936	32306873	0.027338	0.061528
25931	Del	2	32314167	33108804	0.166488	0.070402
25932	Del	2	33108895	33134223	0.007636	0.059753
25933	Del	2	33134390	33400021	0.017318	0.060344
25934	Del	2	33402902	33435897	0.068346	0.063894
25935	Del	2	33436081	33472687	0.030769	0.061528
25936	Del	2	33474300	33580247	0.039521	0.062119
25937	Del	2	33582490	33704389	0.020604	0.060936
25938	Del	2	33706008	33723925	0	0.047921
25939	Del	2	33726033	33729977	0	0.055612
25940	Del	2	33730302	34677130	0	0.047921
25941	Del	2	34677386	35472413	0	0.016565
25942	Del	2	35473883	36551744	0	0.039638
25943	Del	2	36551939	36598605	0	0.032539
25944	Del	2	36598789	36834637	0	0.046146
25945	Del	2	36837445	37083078	0	0.036088
25946	Del	2	37083709	37249185	0	0.03668
25947	Del	2	37252436	37319677	0	0.034313
25948	Del	2	37329123	38045199	0	0.056795
25949	Del	2	38045289	38067071	0	0.056203
25950	Del	2	38067294	38125032	0	0.056795
25951	Del	2	38125191	38290264	0	0.056203
25952	Del	2	38290373	38381825	0	0.034905
25953	Del	2	38381968	38516577	0	0.043188
25954	Del	2	38516739	38659249	0	0.043779
25955	Del	2	38660827	38721006	0	0.043188
25956	Del	2	38727277	38775480	0	0.043779
25957	Del	2	38775495	38794790	0	0.034905
25958	Del	2	38795751	38873618	0	0.034313
25959	Del	2	38874067	38978847	0	0.034905
25960	Del	2	38979949	39129030	0	0.034313
25961	Del	2	39129591	39246635	0	0.034905
25962	Del	2	39247538	39602145	0	0.033722
25963	Del	2	39604090	39720581	0	0.034313
25964	Del	2	39720741	39901737	0	0.034905
25965	Del	2	39902025	40255577	0	0.029581
25966	Del	2	40256233	40430509	0	0.028989
25967	Del	2	40430864	40512518	0	0.028397
25968	Del	2	40515144	42161122	0	0.031947
25969	Del	2	42163124	42332807	0	0.034313

25970	Del	2	42333248	42498037	0	0.035497
25971	Del	2	42498733	42559563	0	0.033722
25972	Del	2	42562085	42714927	0	0.034905
25973	Del	2	42715037	42757567	0	0.034313
25974	Del	2	42757605	42764681	0	0.035497
25975	Del	2	42766295	42795828	0	0.038455
25976	Del	2	42795864	43040821	0	0.044963
25977	Del	2	43043756	43230133	0.09402	0.065669
25978	Del	2	43230434	43634338	0.03832	0.062119
25979	Del	2	43634412	43667871	0	0.052062
25980	Del	2	43674474	43686030	0	0.053837
25981	Del	2	43686081	43767995	0	0.052062
25982	Del	2	43768352	43839724	0	0.053837
25983	Del	2	43840680	43882368	0	0.044963
25984	Del	2	43885513	44166470	0	0.043779
25985	Del	2	44167431	44362830	0	0.029581
25986	Del	2	44362927	44773531	0	0.026623
25987	Del	2	44774376	45166207	0	0.031355
25988	Del	2	45173321	45255361	0	0.028989
25989	Del	2	45255690	45648887	0	0.042596
25990	Del	2	45649311	46190557	0	0.037863
25991	Del	2	46190669	46387063	0	0.043188
25992	Del	2	46387407	46544779	0	0.044371
25993	Del	2	46547030	46781436	0	0.039046
25994	Del	2	46783235	46827703	0	0.057386
25995	Del	2	46827842	46901264	0	0.039046
25996	Del	2	46901564	46948940	0	0.023664
25997	Del	2	46949147	47079852	0	0.022481
25998	Del	2	47082778	47191743	0	0.023664
25999	Del	2	47191934	47332582	0	0.023073
26000	Del	2	47334391	47335898	0	0.023664
26001	Del	2	47340190	47346929	0	0.023073
26002	Del	2	47348476	47517673	0	0.023664
26003	Del	2	47518386	47805173	0	0.007099
26004	Del	2	47806349	48312691	0	0.021298
26005	Del	2	48313466	48679702	0	0.007099
26006	Del	2	48681602	48683794	0	0.006508
26007	Del	2	48683873	48758887	0	0.007099
26008	Del	2	48761881	48778409	0	0.006508
26009	Del	2	48779037	49158360	0	0.028397
26010	Del	2	49159070	50636135	0	0.035497
26011	Del	2	50636524	51110349	0	0.037272
26012	Del	2	51110458	52701167	0	0.020115
26013	Del	2	52701874	53669630	0	0.017748
26014	Del	2	53669776	53765722	0	0.017157
26015	Del	2	53766350	53775404	0	0.017748
26016	Del	2	53775876	53784242	0	0.017157
26017	Del	2	53784672	53821579	0	0.017748
26018	Del	2	53823074	53845218	0	0.017157
26019	Del	2	53847638	53855893	0	0.017748
26020	Del	2	53863335	53968616	0	0.021298
26021	Del	2	53969520	54475237	0	0.02189
26022	Del	2	54475339	54528645	0	0.020706

26023	Del	2	54528949	54529922	0	0.02189
26024	Del	2	54532554	54538015	0	0.020706
26025	Del	2	54538037	54663989	0	0.021298
26026	Del	2	54664066	54718036	0	0.020706
26027	Del	2	54718637	54971932	0	0.020115
26028	Del	2	54972112	55051496	0	0.02189
26029	Del	2	55054299	55138175	0	0.021298
26030	Del	2	55138186	55286656	0	0.02189
26031	Del	2	55288954	55422313	0	0.021298
26032	Del	2	55423962	55925345	0	0.02189
26033	Del	2	55939695	55981024	0	0.033722
26034	Del	2	55981312	55989769	0	0.034313
26035	Del	2	55989811	55998399	0	0.033722
26036	Del	2	55998453	56002249	0	0.034313
26037	Del	2	56007964	56052232	0	0.033722
26038	Del	2	56054468	56186210	0	0.034313
26039	Del	2	56195313	57907295	0	0.032539
26040	Del	2	57907469	58042489	0	0.007691
26041	Del	2	58045025	58158157	0	0.008283
26042	Del	2	58158218	58519028	0	0.013015
26043	Del	2	58520566	59064498	0	0.006508
26044	Del	2	59065127	61194166	0	0.008283
26045	Del	2	61197527	61416643	0	0.007691
26046	Del	2	61416928	61419952	0	0.008283
26047	Del	2	61424190	61472121	0	0.007691
26048	Del	2	61474316	61887299	0	0.008283
26049	Del	2	61888008	62186853	0	0.011832
26050	Del	2	62193980	62671621	0	0.008283
26051	Del	2	62673504	63036727	0	0.007691
26052	Del	2	63037612	63121057	0	0.008283
26053	Del	2	63121570	63438471	0	0.007691
26054	Del	2	63438989	63586324	0	0.007099
26055	Del	2	63588072	63889455	0	0.008283
26056	Del	2	63889731	64026607	0	0.025439
26057	Del	2	64029496	65226986	0	0.026031
26058	Del	2	65227076	65367389	0	0.008283
26059	Del	2	65368337	65439992	0	0.007691
26060	Del	2	65440138	66425823	0	0.008283
26061	Del	2	66431773	66569873	0	0.012424
26062	Del	2	66572404	66698242	0	0.013015
26063	Del	2	66699334	66704029	0	0.008283
26064	Del	2	66705729	67083385	0	0.016565
26065	Del	2	67084092	67122309	0	0.010649
26066	Del	2	67122579	67245413	0	0.011832
26067	Del	2	67245498	67290927	0	0.010649
26068	Del	2	67291105	68731129	0	0.011832
26069	Del	2	68732339	68770543	0	0.011241
26070	Del	2	68771874	69009383	0	0.011832
26071	Del	2	69010451	69103152	0	0.011241
26072	Del	2	69103337	69103884	0	0.011832
26073	Del	2	69106568	69172983	0	0.011241
26074	Del	2	69173752	69253380	0	0.010649
26075	Del	2	69254179	69395240	0	0.020706

26076	Del	2	69396004	69450644	0	0.011832
26077	Del	2	69451433	69519558	0	0.011241
26078	Del	2	69519710	69521455	0	0.011832
26079	Del	2	69522162	69735837	0	0.011241
26080	Del	2	69738344	70128495	0	0.011832
26081	Del	2	70130164	70447020	0	0.015382
26082	Del	2	70447101	70463002	0	0.01479
26083	Del	2	70466233	70484475	0	0.015382
26084	Del	2	70484570	70769661	0	0.01479
26085	Del	2	70769679	71452620	0	0.015382
26086	Del	2	71453502	71687308	0	0.014199
26087	Del	2	71688527	72174757	0	0.01834
26088	Del	2	72175681	72836281	0	0.015974
26089	Del	2	72836597	73062758	0	0.01834
26090	Del	2	73066077	73066163	0	0.017748
26091	Del	2	73069557	73294738	0	0.01834
26092	Del	2	73294754	73611957	0	0.026623
26093	Del	2	73612928	73983315	0	0.027806
26094	Del	2	73987129	74109659	0	0.027214
26095	Del	2	74109912	74215589	0	0.027806
26096	Del	2	74215924	74344433	0	0.027214
26097	Del	2	74344827	74831886	0	0.027806
26098	Del	2	74832312	75044041	0	0.01834
26099	Del	2	75044669	75086964	0	0.017157
26100	Del	2	75089180	75092575	0	0.01834
26101	Del	2	75093230	75202571	0	0.017157
26102	Del	2	75207119	75523541	0	0.017748
26103	Del	2	75523811	75542756	0	0.01834
26104	Del	2	75544778	75570202	0	0.017748
26105	Del	2	75570314	75711125	0	0.01834
26106	Del	2	75714801	77505540	0	0.032539
26107	Del	2	77506509	77737781	0	0.037863
26108	Del	2	77738395	77905631	0	0.025439
26109	Del	2	77907855	77918589	0	0.028397
26110	Del	2	77919296	77952701	0	0.025439
26111	Del	2	77953604	77961271	0	0.028397
26112	Del	2	77961315	78086913	0	0.025439
26113	Del	2	78087148	78130545	0	0.028397
26114	Del	2	78131528	78541255	0	0.025439
26115	Del	2	78543785	78569320	0	0.01834
26116	Del	2	78572255	79489018	0	0.027806
26117	Del	2	79492817	80297159	0	0.023073
26118	Del	2	80297731	80306296	0	0.028397
26119	Del	2	80307752	80649329	0	0.025439
26120	Del	2	80649348	81468228	0	0.028397
26121	Del	2	81468621	84283898	0	0.041413
26122	Del	2	84283967	84416896	0	0.033722
26123	Del	2	84417929	84505510	0	0.030172
26124	Del	2	84506190	84819794	0	0.027806
26125	Del	2	84821043	85130210	0	0.030172
26126	Del	2	85131433	85206976	0	0.028989
26127	Del	2	85211107	85219901	0	0.030172
26128	Del	2	85219970	85310473	0	0.028989

26129	Del	2	85314505	86055837	0	0.030172
26130	Del	2	86057163	86339578	0	0.037272
26131	Del	2	86339958	86503059	0	0.037863
26132	Del	2	86503319	86570575	0	0.030172
26133	Del	2	86582723	86599630	0	0.028989
26134	Del	2	86601159	86624376	0	0.030172
26135	Del	2	86625358	86705034	0	0.028989
26136	Del	2	86707622	86846819	0	0.030172
26137	Del	2	86859047	88066681	0	0.039046
26138	Del	2	88067427	88079246	0	0.038455
26139	Del	2	88080088	88083617	0	0.039046
26140	Del	2	88083731	88116921	0	0.038455
26141	Del	2	88117361	88831240	0	0.039046
26142	Del	2	88831554	94754549	0	0.033722
26143	Del	2	94754662	95186653	0	0.031355
26144	Del	2	95191049	95292627	0	0.035497
26145	Del	2	95299538	95343844	0	0.034905
26146	Del	2	95345136	95416225	0	0.035497
26147	Del	2	95832876	96497235	0	0.028989
26148	Del	2	96497432	96899403	0	0.024848
26149	Del	2	96899553	96913658	0	0.024256
26150	Del	2	96915029	96925933	0	0.024848
26151	Del	2	96928405	96990145	0	0.024256
26152	Del	2	96990226	97665171	0	0.031947
26153	Del	2	97669772	97754995	0	0.02189
26154	Del	2	97755818	97997899	0	0.020706
26155	Del	2	97999767	98313572	0	0.027806
26156	Del	2	98315266	98440299	0	0.030172
26157	Del	2	98441177	98591601	0	0.029581
26158	Del	2	98591746	98666196	0	0.030172
26159	Del	2	98668499	98740361	0	0.029581
26160	Del	2	98740686	98788520	0	0.030172
26161	Del	2	98793568	98996628	0	0.029581
26162	Del	2	98997081	99129397	0	0.027214
26163	Del	2	99129995	99401297	0	0.027806
26164	Del	2	99403095	99492539	0	0.027214
26165	Del	2	99492789	100142995	0	0.030764
26166	Del	2	100143120	100817852	0	0.03668
26167	Del	2	100818352	100969726	0	0.034905
26168	Del	2	100970993	100977992	0	0.03668
26169	Del	2	100978396	100997860	0	0.034905
26170	Del	2	100998451	101020547	0	0.03668
26171	Del	2	101020649	101147737	0	0.035497
26172	Del	2	101148798	101393825	0	0.03668
26173	Del	2	101394535	101694823	0	0.036088
26174	Del	2	101695428	101899496	0	0.031355
26175	Del	2	101900299	102057979	0	0.032539
26176	Del	2	102059668	102102237	0	0.031355
26177	Del	2	102102715	102152913	0	0.031947
26178	Del	2	102153467	102472767	0	0.032539
26179	Del	2	102473219	102711183	0	0.031947
26180	Del	2	102714520	102738080	0	0.032539
26181	Del	2	102740433	102820981	0	0.031947

26182	Del	2	102822046	102984592	0	0.032539
26183	Del	2	102989122	104433783	0	0.039046
26184	Del	2	104434275	104487640	0	0.038455
26185	Del	2	104488223	104510840	0	0.039046
26186	Del	2	104512266	104514064	0	0.038455
26187	Del	2	104515874	104933690	0	0.039046
26188	Del	2	104934941	105039499	0	0.038455
26189	Del	2	105040174	105104770	0	0.036088
26190	Del	2	105112494	105238147	0	0.042004
26191	Del	2	105241362	105247082	0	0.036088
26192	Del	2	105248369	105332058	0	0.035497
26193	Del	2	105332415	105404484	0	0.036088
26194	Del	2	105404502	105439214	0	0.035497
26195	Del	2	105439227	105743241	0	0.036088
26196	Del	2	105743894	105845366	0	0.034905
26197	Del	2	105851050	105895267	0	0.035497
26198	Del	2	105895390	106139241	0	0.039046
26199	Del	2	106139345	106198442	0	0.037863
26200	Del	2	106203937	106801206	0	0.043779
26201	Del	2	106801580	106812636	0	0.039046
26202	Del	2	106812979	106891246	0	0.039638
26203	Del	2	106892275	107752192	0	0.048512
26204	Del	2	107752238	107819855	0	0.035497
26205	Del	2	107820730	108058237	0	0.036088
26206	Del	2	108060610	108219046	0	0.045554
26207	Del	2	108221308	108311312	0	0.046146
26208	Del	2	108312393	108531227	0	0.055612
26209	Del	2	108533322	108617624	0	0.056203
26210	Del	2	108617749	108791289	0	0.056795
26211	Del	2	108797633	108893323	0	0.056203
26212	Del	2	108893696	108989442	0	0.055612
26213	Del	2	108991769	109130883	0	0.056795
26214	Del	2	109131789	109139914	0	0.05147
26215	Del	2	109139945	109141986	0	0.056795
26216	Del	2	109142768	109312135	0	0.05147
26217	Del	2	109312845	109314235	0	0.056795
26218	Del	2	109314401	109505194	0	0.05147
26219	Del	2	109505300	109619969	0	0.056795
26220	Del	2	109621519	110726968	0.114479	0.066852
26221	Del	2	110730576	111096115	0.053064	0.063302
26222	Del	2	111097716	111150071	0.136089	0.068627
26223	Del	2	111150089	111170796	0.129575	0.068035
26224	Del	2	111171915	111773105	0.135654	0.068627
26225	Del	2	111779890	111886554	0.130128	0.068035
26226	Del	2	111891895	112051391	0.21265	0.07336
26227	Del	2	112051642	112106833	0.129272	0.068035
26228	Del	2	112109181	112476739	0.136497	0.068627
26229	Del	2	112480953	112536764	0.129575	0.068035
26230	Del	2	112537631	112910511	0.141544	0.068627
26231	Del	2	112911022	113020743	0.118025	0.067444
26232	Del	2	113021522	113053370	0.113319	0.066852
26233	Del	2	113053480	113209498	0.116667	0.067444
26234	Del	2	113214969	113232560	0.112657	0.066852

26235	Del	2	113232795	113267269	0.12071	0.067444
26236	Del	2	113267777	113280772	0.112657	0.066852
26237	Del	2	113283164	113701610	0.119548	0.067444
26238	Del	2	113703584	113714683	0.113055	0.066852
26239	Del	2	113720734	113723132	0.120258	0.067444
26240	Del	2	113725697	113735683	0.113055	0.066852
26241	Del	2	113741786	113827678	0.118025	0.067444
26242	Del	2	113827728	113963821	0.114364	0.066852
26243	Del	2	113964411	114438504	0.119895	0.067444
26244	Del	2	114441309	114461398	0	0.057978
26245	Del	2	114461481	114827788	0	0.05857
26246	Del	2	114828127	114833667	0.119895	0.067444
26247	Del	2	114833781	115061445	0	0.05857
26248	Del	2	115063604	115141010	0.059113	0.063302
26249	Del	2	115141325	115163970	0.118025	0.067444
26250	Del	2	115164388	115919444	0.071807	0.064486
26251	Del	2	115920252	117910053	0.168611	0.070402
26252	Del	2	117914471	118014296	0.147705	0.069219
26253	Del	2	118014470	118941673	0.168611	0.070402
26254	Del	2	118942023	119006377	0.161173	0.06981
26255	Del	2	119006886	119219679	0.619887	0.090517
26256	Del	2	119219755	119241848	0.584337	0.089333
26257	Del	2	119243990	119250136	0.615541	0.090517
26258	Del	2	119250409	119365632	0.584337	0.089333
26259	Del	2	119365701	119438689	0.620811	0.090517
26260	Del	2	119439005	119525583	0.569856	0.088742
26261	Del	2	119525597	119656891	0.584971	0.089333
26262	Del	2	119659211	119758125	0.619094	0.090517
26263	Del	2	119758330	119989684	0.569334	0.088742
26264	Del	2	119990187	120111726	0.593938	0.089925
26265	Del	2	120112313	120179195	0.571787	0.088742
26266	Del	2	120180456	120251357	0.617294	0.090517
26267	Del	2	120251789	120300836	0.602781	0.089925
26268	Del	2	120301348	120796389	0.620811	0.090517
26269	Del	2	120796728	121215793	0.510753	0.086375
26270	Del	2	121216151	121336599	0.153935	0.06981
26271	Del	2	121336900	121522967	0.120892	0.068035
26272	Del	2	121528842	121531972	0.173853	0.070402
26273	Del	2	121534832	121641520	0.120892	0.068035
26274	Del	2	121648480	121652545	0.172477	0.070402
26275	Del	2	121652592	121724566	0.156418	0.06981
26276	Del	2	121725752	121770016	0.17003	0.070402
26277	Del	2	121770504	125319206	1.122205	0.107673
26278	Del	2	125319688	126697294	1.311192	0.11359
26279	Del	2	126699892	127180402	1.345478	0.114773
26280	Del	2	127180649	127344105	1.329543	0.114181
26281	Del	2	127344373	127476600	1.347894	0.114773
26282	Del	2	127477572	127534704	1.315416	0.114181
26283	Del	2	127534924	127550592	1.301677	0.11359
26284	Del	2	127552056	127571359	1.345405	0.114773
26285	Del	2	127571807	127624019	1.301677	0.11359
26286	Del	2	127625082	127705363	1.345405	0.114773
26287	Del	2	127707157	127732591	1.297269	0.11359

26288	Del	2	127732739	127811269	1.317683	0.114181
26289	Del	2	127811626	127939698	1.345405	0.114773
26290	Del	2	127940155	128086238	1.298435	0.11359
26291	Del	2	128086346	128199211	1.291233	0.112998
26292	Del	2	128206451	128858206	1.317683	0.114181
26293	Del	2	128858570	129240488	0.879901	0.099391
26294	Del	2	129241079	129920977	0.667814	0.092291
26295	Del	2	129922090	130726392	0.600789	0.089925
26296	Del	2	130728626	130753000	0.655895	0.0917
26297	Del	2	130753460	130783105	0.604091	0.089925
26298	Del	2	130783445	131045081	0.783872	0.096433
26299	Del	2	131045582	131095286	0.833825	0.098208
26300	Del	2	131102824	131153308	0.8337	0.097616
26301	Del	2	131156275	131802808	0.847067	0.098208
26302	Del	2	131814020	132603497	1.125324	0.107673
26303	Del	2	132604568	132642769	1.084313	0.10649
26304	Del	2	132642836	132673983	1.100058	0.107082
26305	Del	2	132674409	132914888	0.686208	0.092883
26306	Del	2	132915163	132931566	1.11824	0.107673
26307	Del	2	132931700	133262972	0.686208	0.092883
26308	Del	2	133263399	133284815	1.122007	0.107673
26309	Del	2	133285643	133568952	0.686208	0.092883
26310	Del	2	133571124	134132093	1.816786	0.127788
26311	Del	2	134132656	134455248	1.850385	0.128971
26312	Del	2	134455685	134720476	1.589757	0.121872
26313	Del	2	134725790	134854777	1.753594	0.126605
26314	Del	2	134854852	134860335	1.816786	0.127788
26315	Del	2	134861375	134866438	1.753594	0.126605
26316	Del	2	134866493	134963967	1.784118	0.127197
26317	Del	2	134964195	134979792	1.769885	0.126605
26318	Del	2	134980338	135050858	1.783135	0.127197
26319	Del	2	135051768	135193452	1.744673	0.126013
26320	Del	2	135195918	135527207	1.651663	0.123647
26321	Del	2	135527722	135580304	1.704954	0.12483
26322	Del	2	135581272	135663120	1.720849	0.125422
26323	Del	2	135663338	135669452	1.816786	0.127788
26324	Del	2	135669711	135731408	1.720849	0.125422
26325	Del	2	135731922	135788274	1.7917	0.127197
26326	Del	2	135789065	135817964	1.768667	0.126605
26327	Del	2	135818317	135824643	1.812499	0.127788
26328	Del	2	135833176	135835685	1.768667	0.126605
26329	Del	2	135838029	135901925	1.779954	0.127197
26330	Del	2	135902537	135979036	1.769885	0.126605
26331	Del	2	135984793	136764474	1.800496	0.127788
26332	Del	2	136764665	137621453	1.222283	0.111223
26333	Del	2	137625180	138311046	1.837801	0.12838
26334	Del	2	138312265	138667110	2.044474	0.133704
26335	Del	2	138668967	138782141	1.956296	0.131929
26336	Del	2	138782542	140223495	2.089881	0.134888
26337	Del	2	140227228	140577168	1.941839	0.131338
26338	Del	2	140583057	140587301	3.028126	0.156186
26339	Del	2	140587511	142134303	1.941839	0.131338
26340	Del	2	142136707	142989727	1.972473	0.131929

26341	Del	2	142990054	143128868	1.931389	0.131338
26342	Del	2	143129014	143769321	1.799316	0.127788
26343	Del	2	143771495	143942708	2.071247	0.134888
26344	Del	2	143943139	144212051	2.357349	0.141395
26345	Del	2	144212263	144237999	2.338031	0.140804
26346	Del	2	144238451	144383974	2.300325	0.140212
26347	Del	2	144384305	144510085	1.957854	0.131929
26348	Del	2	144511130	144528098	2.088483	0.134888
26349	Del	2	144528950	144664829	2.026006	0.133704
26350	Del	2	144665128	145079540	1.793932	0.127197
26351	Del	2	145082811	146594155	2.703697	0.149086
26352	Del	2	146597710	148024896	2.876825	0.153228
26353	Del	2	148025209	148632332	2.72249	0.149678
26354	Del	2	148642920	148871324	2.440127	0.14317
26355	Del	2	148874102	148946388	2.413225	0.142578
26356	Del	2	148947155	149036562	2.472174	0.144353
26357	Del	2	149037071	149077122	1.578979	0.121872
26358	Del	2	149077408	149109687	1.624065	0.123055
26359	Del	2	149116490	149326652	1.667118	0.124239
26360	Del	2	149327214	149475238	1.657172	0.123647
26361	Del	2	149475526	149584701	1.760334	0.126605
26362	Del	2	149584747	150467234	2.237424	0.139029
26363	Del	2	150467886	150548717	1.375162	0.115956
26364	Del	2	150552032	151245374	0.807409	0.097024
26365	Del	2	151246540	151261266	0.752071	0.09525
26366	Del	2	151261965	151333442	1.066175	0.105899
26367	Del	2	151336845	151354257	0.738814	0.094658
26368	Del	2	151354289	151405390	0.753026	0.09525
26369	Del	2	151408317	151480910	0.698924	0.093475
26370	Del	2	151480932	151737281	1.009212	0.104124
26371	Del	2	151737424	151831172	1.14842	0.108857
26372	Del	2	151832318	151975454	1.077528	0.10649
26373	Del	2	151975967	152110936	1.006646	0.103532
26374	Del	2	152111464	152176276	0.747152	0.09525
26375	Del	2	152176704	152643826	1.006646	0.103532
26376	Del	2	152647710	152703130	1.060485	0.105899
26377	Del	2	152712745	153473704	1.070466	0.10649
26378	Del	2	153475876	153870918	0.499012	0.085784
26379	Del	2	153871060	153938890	0.212741	0.07336
26380	Del	2	153939640	154431314	0.236253	0.073952
26381	Del	2	154434575	154457782	0.35809	0.080459
26382	Del	2	154457892	156016166	0.521606	0.086967
26383	Del	2	156016427	156319922	0.335367	0.079276
26384	Del	2	156322694	156426731	0.210581	0.072768
26385	Del	2	156432464	156587159	0.177614	0.070993
26386	Del	2	156589954	157313536	0.32105	0.078093
26387	Del	2	157315341	157367074	0.340587	0.079276
26388	Del	2	157376630	157521373	0.153678	0.069219
26389	Del	2	157521520	157732487	0.137091	0.068627
26390	Del	2	157735953	157877096	0.119895	0.067444
26391	Del	2	157879405	158100335	0.299968	0.077501
26392	Del	2	158100565	158157246	0.329609	0.078684
26393	Del	2	158158653	158236352	0.31876	0.078093

26394	Del	2	158238314	158337354	0.25527	0.075135
26395	Del	2	158337510	158462165	0.313699	0.078093
26396	Del	2	158462714	158683681	0.277257	0.076318
26397	Del	2	158685892	158736323	0.201418	0.072177
26398	Del	2	158736439	158960801	0.234436	0.073952
26399	Del	2	158964335	159185572	0.162563	0.06981
26400	Del	2	159186731	159188002	0.202822	0.072177
26401	Del	2	159189035	159232282	0.162563	0.06981
26402	Del	2	159232712	159318768	0.187696	0.071585
26403	Del	2	159318799	159613224	0.139876	0.068627
26404	Del	2	159613490	159769671	0.202822	0.072177
26405	Del	2	159770111	159798696	0.191167	0.071585
26406	Del	2	159798762	159802228	0.156418	0.06981
26407	Del	2	159802287	159936904	0.172477	0.070402
26408	Del	2	159937929	160064289	0.161173	0.06981
26409	Del	2	160068730	160201849	0.181079	0.070993
26410	Del	2	160202279	160275251	0.199251	0.072177
26411	Del	2	160275585	160403174	0.157197	0.06981
26412	Del	2	160403301	160408588	0.202822	0.072177
26413	Del	2	160411984	160498483	0.157197	0.06981
26414	Del	2	160500252	161220168	0.268882	0.075726
26415	Del	2	161220183	161241806	0.281731	0.076318
26416	Del	2	161244126	161249226	0.295252	0.07691
26417	Del	2	161252058	161304794	0.281731	0.076318
26418	Del	2	161305526	161413285	0.252799	0.075135
26419	Del	2	161413545	161621918	0.293018	0.07691
26420	Del	2	161623767	161988897	0.230243	0.073952
26421	Del	2	161989929	162075900	0.308119	0.077501
26422	Del	2	162076392	162142342	0.313933	0.078093
26423	Del	2	162142431	162154825	0.32566	0.078684
26424	Del	2	162156220	162316406	0.313933	0.078093
26425	Del	2	162316690	162367495	0.32105	0.078684
26426	Del	2	162369724	162421140	0.225268	0.07336
26427	Del	2	162422132	162738104	0.23897	0.074543
26428	Del	2	162740149	162808318	0.32105	0.078684
26429	Del	2	162815208	163604437	0.23897	0.074543
26430	Del	2	163604490	163742792	0	0.057978
26431	Del	2	163743945	164571315	0.378881	0.081051
26432	Del	2	164572081	164679332	0.362708	0.080459
26433	Del	2	164680159	164683333	0.193553	0.072177
26434	Del	2	164684102	164689804	0.237357	0.073952
26435	Del	2	164694691	164822708	0.193553	0.072177
26436	Del	2	164825069	164896904	0.237357	0.073952
26437	Del	2	164896959	164955918	0.224927	0.07336
26438	Del	2	164956209	165205551	0.339175	0.079276
26439	Del	2	165205597	165287278	0.743461	0.09525
26440	Del	2	165293790	165465726	0.775537	0.095841
26441	Del	2	165469352	165569222	0.286179	0.07691
26442	Del	2	165569365	165690284	0.32105	0.078684
26443	Del	2	165693618	165795583	0.34088	0.079276
26444	Del	2	165795854	165876017	0.347407	0.079868
26445	Del	2	165877228	165932631	0.333951	0.079276
26446	Del	2	165932814	165954843	0.351525	0.079868

26447	Del	2	165955255	166038642	0.559806	0.08815
26448	Del	2	166039309	166075792	0.541925	0.087559
26449	Del	2	166077706	166152241	0.50461	0.086375
26450	Del	2	166156642	166192594	0.487158	0.085784
26451	Del	2	166193643	166377784	0.508884	0.086375
26452	Del	2	166377839	166886596	0.554537	0.08815
26453	Del	2	166886784	167121310	0.240585	0.074543
26454	Del	2	167122785	167141077	0.32105	0.078684
26455	Del	2	167141468	167184652	0.240585	0.074543
26456	Del	2	167186473	167812832	0.31266	0.078093
26457	Del	2	167813955	167816733	0.296786	0.077501
26458	Del	2	167818252	167873472	0.32105	0.078684
26459	Del	2	167874558	167953163	0.296786	0.077501
26460	Del	2	167953522	168451539	0.225724	0.073952
26461	Del	2	168453223	168578106	0.208333	0.072768
26462	Del	2	168580830	168583118	0.32105	0.078093
26463	Del	2	168583338	168767751	0.208333	0.072768
26464	Del	2	168769797	168791460	0.242853	0.074543
26465	Del	2	168793774	168802324	0.210581	0.072768
26466	Del	2	168802434	168865012	0.218173	0.07336
26467	Del	2	168865434	168892095	0.234798	0.073952
26468	Del	2	168892333	168917669	0.242046	0.074543
26469	Del	2	168918312	169032232	0.219835	0.07336
26470	Del	2	169032562	169123752	0.24324	0.074543
26471	Del	2	169124354	169366574	0.171642	0.070402
26472	Del	2	169367020	169580391	0.242046	0.074543
26473	Del	2	169583555	169683304	0.236337	0.073952
26474	Del	2	169692233	169708738	0.246242	0.074543
26475	Del	2	169709383	169796819	0.232561	0.073952
26476	Del	2	169797366	169823513	0.009595	0.059753
26477	Del	2	169823939	170172742	0.148531	0.069219
26478	Del	2	170175538	170332961	0.119895	0.067444
26479	Del	2	170333179	170351695	0.162215	0.06981
26480	Del	2	170351732	170655797	0.119895	0.067444
26481	Del	2	170656842	170711218	0.141685	0.069219
26482	Del	2	170711332	170719912	0.162563	0.06981
26483	Del	2	170720375	170764312	0.141685	0.069219
26484	Del	2	170764875	170983572	0.162215	0.06981
26485	Del	2	170988537	171067080	0.062618	0.063894
26486	Del	2	171068718	171169379	0.046943	0.062711
26487	Del	2	171169999	171232830	0.030903	0.062119
26488	Del	2	171233632	171431827	0.059113	0.063302
26489	Del	2	171433623	171486739	0.068155	0.063894
26490	Del	2	171488477	171684868	0.070466	0.064486
26491	Del	2	171685099	171751282	0.066485	0.063894
26492	Del	2	171752016	171898533	0.2098	0.072768
26493	Del	2	171904336	172079922	0.224438	0.07336
26494	Del	2	172081799	172110488	0.23657	0.073952
26495	Del	2	172111273	172507476	0.614671	0.090517
26496	Del	2	172508023	172600468	0.658161	0.092291
26497	Del	2	172607436	172625657	0.649733	0.0917
26498	Del	2	172628350	172736748	0.666911	0.092291
26499	Del	2	172737656	172818448	0.471409	0.085192

26500	Del	2	172820337	172859206	0.507966	0.086375
26501	Del	2	172859821	172924645	0.528285	0.087559
26502	Del	2	172927897	173055731	0.565685	0.088742
26503	Del	2	173056666	173227221	0.604292	0.089925
26504	Del	2	173227591	173269801	0.576489	0.089333
26505	Del	2	173270062	173286249	0.457257	0.084601
26506	Del	2	173288627	173903441	0.663963	0.092291
26507	Del	2	173903875	173968223	0.620811	0.090517
26508	Del	2	173968239	174069356	0.636022	0.091108
26509	Del	2	174070047	174323080	0.561297	0.088742
26510	Del	2	174323587	174430873	0.184619	0.070993
26511	Del	2	174431070	174557164	0.171717	0.070402
26512	Del	2	174557761	174558250	0.018569	0.060344
26513	Del	2	174558335	174635101	0.004642	0.059753
26514	Del	2	174638567	174683592	0	0.059161
26515	Del	2	174685950	174797725	0.018569	0.060344
26516	Del	2	174798521	174847961	0.008911	0.059753
26517	Del	2	174850066	175070153	0	0.057978
26518	Del	2	175070170	175113912	0	0.044963
26519	Del	2	175114035	175186905	0	0.045554
26520	Del	2	175188811	176265265	0	0.057386
26521	Del	2	176266875	176338630	0	0.047921
26522	Del	2	176340923	177266968	0	0.048512
26523	Del	2	177268260	177547224	0	0.053837
26524	Del	2	177548037	177613674	0	0.05502
26525	Del	2	177613852	177623553	0	0.048512
26526	Del	2	177623967	177672948	0	0.046146
26527	Del	2	177673295	177727678	0	0.048512
26528	Del	2	177730455	177923761	0	0.046146
26529	Del	2	177930455	178078062	0	0.044371
26530	Del	2	178078984	178109632	0	0.043779
26531	Del	2	178111787	178188741	0	0.035497
26532	Del	2	178188930	178318006	0	0.033722
26533	Del	2	178320149	178403905	0	0.034905
26534	Del	2	178404658	178511911	0	0.035497
26535	Del	2	178512553	178621496	0	0.037863
26536	Del	2	178624009	178743465	0	0.036088
26537	Del	2	178743480	178824141	0	0.037863
26538	Del	2	178828985	179051445	0	0.028397
26539	Del	2	179053170	179565342	0	0.028989
26540	Del	2	179565604	179587131	0	0.028397
26541	Del	2	179588004	179746258	0	0.027214
26542	Del	2	179746630	179854642	0	0.026031
26543	Del	2	179857259	180009994	0	0.029581
26544	Del	2	180011975	180917561	0.09402	0.066261
26545	Del	2	180919915	181119583	0.12071	0.068035
26546	Del	2	181122826	181302075	0	0.059161
26547	Del	2	181305318	181305679	0.039521	0.062119
26548	Del	2	181309525	181400477	0	0.059161
26549	Del	2	181403045	181476820	0.034432	0.062119
26550	Del	2	181480309	181675173	0.023347	0.060936
26551	Del	2	181675956	181690622	0	0.049695
26552	Del	2	181690825	181952825	0.1945	0.072177

26553	Del	2	181953573	181979817	0.160076	0.06981
26554	Del	2	181983824	182117848	0.164177	0.070402
26555	Del	2	182128473	182135136	0.161637	0.06981
26556	Del	2	182137079	182709778	0.113223	0.066852
26557	Del	2	182710307	183043792	0	0.057386
26558	Del	2	183047128	183116592	0	0.057978
26559	Del	2	183116720	184378902	0	0.057386
26560	Del	2	184378913	184564802	0	0.053245
26561	Del	2	184565455	185164267	0	0.045554
26562	Del	2	185164861	186032339	0	0.043188
26563	Del	2	186032596	186588626	0	0.036088
26564	Del	2	186588660	186764768	0	0.035497
26565	Del	2	186765023	187335590	0	0.036088
26566	Del	2	187338784	187555197	0	0.035497
26567	Del	2	187555511	188289715	0	0.049104
26568	Del	2	188293662	188293662	0	0.040821
26569	Del	2	188294102	188300991	0	0.042004
26570	Del	2	188301527	188598224	0	0.040821
26571	Del	2	188598443	188969558	0	0.049695
26572	Del	2	188975600	188978636	0	0.050287
26573	Del	2	188979274	188993217	0	0.049695
26574	Del	2	188993267	188995946	0	0.050287
26575	Del	2	188997270	189030397	0	0.049695
26576	Del	2	189030722	189128174	0	0.049104
26577	Del	2	189128566	189136403	0	0.050287
26578	Del	2	189138435	189180902	0	0.049104
26579	Del	2	189183535	189657584	0	0.050287
26580	Del	2	189661158	189807520	0	0.037863
26581	Del	2	189807959	189878322	0	0.037272
26582	Del	2	189880012	190065230	0	0.037863
26583	Del	2	190065254	190207661	0	0.055612
26584	Del	2	190208179	190321772	0	0.037272
26585	Del	2	190324962	190404443	0	0.037863
26586	Del	2	190404894	190502554	0	0.037272
26587	Del	2	190503140	190877022	0	0.037863
26588	Del	2	190877079	191014691	0	0.028989
26589	Del	2	191015207	191033306	0	0.032539
26590	Del	2	191033599	191241916	0	0.031355
26591	Del	2	191244388	191425436	0	0.030764
26592	Del	2	191425589	191830381	0	0.039638
26593	Del	2	191832142	191948727	0	0.032539
26594	Del	2	191948824	192171513	0	0.024256
26595	Del	2	192172908	192748503	0	0.026031
26596	Del	2	192748907	192777812	0	0.022481
26597	Del	2	192780644	194423990	0	0.039046
26598	Del	2	194426840	194837122	0	0.039638
26599	Del	2	194837153	195740850	0	0.054428
26600	Del	2	195744519	196074831	0	0.052062
26601	Del	2	196075542	196196428	0	0.05502
26602	Del	2	196197110	196259468	0	0.052653
26603	Del	2	196259882	196264292	0	0.05502
26604	Del	2	196276224	196362829	0	0.052653
26605	Del	2	196364142	196626122	0	0.049695

26606	Del	2	196629303	196696112	0	0.041413
26607	Del	2	196699615	196713978	0	0.042004
26608	Del	2	196717290	196761490	0	0.041413
26609	Del	2	196761510	196983798	0	0.03313
26610	Del	2	196983843	197096924	0	0.030172
26611	Del	2	197098404	197192793	0	0.031355
26612	Del	2	197196863	197200600	0	0.03313
26613	Del	2	197200839	197249216	0	0.031355
26614	Del	2	197249643	197566356	0	0.03313
26615	Del	2	197569055	197677977	0	0.032539
26616	Del	2	197678432	197789530	0	0.03313
26617	Del	2	197790283	198152121	0.060057	0.063302
26618	Del	2	198154368	199267874	0.106041	0.066852
26619	Del	2	199268467	199386002	0.054282	0.063302
26620	Del	2	199391874	199759911	0.076844	0.065077
26621	Del	2	199760177	199856450	0.068811	0.064486
26622	Del	2	199860003	200302138	0.080839	0.065077
26623	Del	2	200302792	200302792	0	0.049695
26624	Del	2	200308704	200483206	0	0.050287
26625	Del	2	200485706	200585011	0	0.052653
26626	Del	2	200585062	200694043	0	0.05147
26627	Del	2	200695545	200711412	0	0.050879
26628	Del	2	200711561	200735936	0	0.052653
26629	Del	2	200739314	200778761	0	0.050879
26630	Del	2	200779943	200973550	0	0.052653
26631	Del	2	200977937	201069527	0	0.052062
26632	Del	2	201070440	201091741	0	0.052653
26633	Del	2	201096103	201175421	0	0.053837
26634	Del	2	201180072	201230530	0.035548	0.062119
26635	Del	2	201232457	201255044	0.030624	0.061528
26636	Del	2	201257778	201289123	0.035927	0.062119
26637	Del	2	201289197	201439604	0.030373	0.061528
26638	Del	2	201441663	201488120	0.036233	0.062119
26639	Del	2	201488144	201534155	0.020376	0.060936
26640	Del	2	201534269	201619087	0.025799	0.061528
26641	Del	2	201619849	201644388	0.03832	0.062119
26642	Del	2	201645091	201758285	0.026638	0.061528
26643	Del	2	201758342	201805822	0.035927	0.062119
26644	Del	2	201806075	201896097	0.026638	0.061528
26645	Del	2	201896858	202067181	0.039023	0.062119
26646	Del	2	202067474	202086390	0.016564	0.060344
26647	Del	2	202086989	202110537	0.029542	0.061528
26648	Del	2	202111145	202117826	0.038637	0.062119
26649	Del	2	202119696	202201696	0.016564	0.060344
26650	Del	2	202203146	202370685	0.055136	0.063302
26651	Del	2	202372276	202571521	0.035927	0.062119
26652	Del	2	202576659	202810496	0.055527	0.063302
26653	Del	2	202813490	202813490	0.024976	0.060936
26654	Del	2	202814777	202830542	0.030373	0.061528
26655	Del	2	202834036	202869642	0.024976	0.060936
26656	Del	2	202869657	203011494	0.030373	0.061528
26657	Del	2	203013360	203234217	0.008183	0.059753
26658	Del	2	203236134	203309313	0	0.053837

26659	Del	2	203311995	203364069	0	0.043188
26660	Del	2	203365038	203433781	0	0.043779
26661	Del	2	203439151	203536595	0	0.043188
26662	Del	2	203545957	203964079	0	0.043779
26663	Del	2	203967922	205621303	0	0.044963
26664	Del	2	205621440	205745150	0	0.050287
26665	Del	2	205745264	205991568	0	0.049695
26666	Del	2	205992219	206086818	0	0.042004
26667	Del	2	206087228	206438904	0	0.042596
26668	Del	2	206443085	206638600	0	0.041413
26669	Del	2	206638944	206644482	0	0.034313
26670	Del	2	206648972	206735479	0	0.05857
26671	Del	2	206736967	207134297	0	0.034313
26672	Del	2	207134446	207161233	0	0.033722
26673	Del	2	207161535	207185225	0	0.034313
26674	Del	2	207185425	207255323	0	0.033722
26675	Del	2	207255484	207525689	0	0.034313
26676	Del	2	207528593	207772143	0	0.021298
26677	Del	2	207774449	208033368	0	0.028397
26678	Del	2	208033820	208252574	0	0.029581
26679	Del	2	208252593	208311632	0	0.032539
26680	Del	2	208312017	208358295	0	0.031947
26681	Del	2	208358333	208538460	0	0.037272
26682	Del	2	208541616	209422038	0	0.030764
26683	Del	2	209422112	209575807	0	0.030172
26684	Del	2	209575948	209995002	0	0.031355
26685	Del	2	209995832	210064591	0	0.032539
26686	Del	2	210073118	210173058	0	0.031947
26687	Del	2	210173631	210320959	0	0.032539
26688	Del	2	210321111	210428157	0	0.031947
26689	Del	2	210430295	210475931	0	0.032539
26690	Del	2	210477414	210534795	0	0.036088
26691	Del	2	210534923	210588549	0	0.03668
26692	Del	2	210590907	210612294	0	0.037272
26693	Del	2	210614914	210620153	0	0.037863
26694	Del	2	210621644	211373816	0	0.037272
26695	Del	2	211374257	212422064	0	0.030764
26696	Del	2	212425811	212432139	0	0.032539
26697	Del	2	212433879	212540552	0	0.030764
26698	Del	2	212547702	212924550	0	0.042004
26699	Del	2	212926169	212927088	0	0.032539
26700	Del	2	212927903	213156189	0	0.040821
26701	Del	2	213156601	213319030	0	0.042596
26702	Del	2	213319045	214410885	0	0.041413
26703	Del	2	214411155	214506832	0	0.040821
26704	Del	2	214507272	214536939	0	0.042596
26705	Del	2	214538202	214576181	0	0.040821
26706	Del	2	214579366	214811493	0	0.042004
26707	Del	2	214812033	214965697	0	0.041413
26708	Del	2	214967019	215035130	0	0.031947
26709	Del	2	215035363	215138510	0	0.031355
26710	Del	2	215142292	215359542	0	0.055612
26711	Del	2	215360357	215411652	0	0.05502

26712	Del	2	215412367	215607975	0	0.056203
26713	Del	2	215611315	215716219	0	0.053245
26714	Del	2	215716387	215721173	0	0.056203
26715	Del	2	215721764	215845109	0	0.053245
26716	Del	2	215848625	216036775	0.073572	0.064486
26717	Del	2	216036811	216106126	0	0.032539
26718	Del	2	216106814	216207504	0	0.031947
26719	Del	2	216207871	216257041	0	0.032539
26720	Del	2	216257084	216483403	0	0.031947
26721	Del	2	216484042	216872854	0	0.032539
26722	Del	2	216881200	217344758	0	0.042004
26723	Del	2	217344905	217757254	0	0.037863
26724	Del	2	217757569	217920752	0	0.052062
26725	Del	2	217920877	217945023	0	0.05147
26726	Del	2	217946146	218031768	0	0.053245
26727	Del	2	218032085	218092000	0	0.050287
26728	Del	2	218093591	218298063	0	0.05147
26729	Del	2	218300371	218320822	0	0.050879
26730	Del	2	218321391	218450893	0	0.05147
26731	Del	2	218452445	218565648	0	0.050879
26732	Del	2	218566812	218596627	0	0.05147
26733	Del	2	218597761	218712373	0.188082	0.071585
26734	Del	2	218713250	218758722	0.185259	0.070993
26735	Del	2	218759447	218957733	0.185757	0.071585
26736	Del	2	218957876	219068121	0.018569	0.060344
26737	Del	2	219069055	219159205	0.060057	0.063894
26738	Del	2	219159483	219325472	0.074577	0.064486
26739	Del	2	219325819	219433077	0.047914	0.062711
26740	Del	2	219433333	219457751	0.039283	0.062119
26741	Del	2	219459106	219466980	0.048969	0.062711
26742	Del	2	219470099	219493864	0.039283	0.062119
26743	Del	2	219503543	221415986	0.030769	0.061528
26744	Del	2	221416086	221577754	0.021746	0.060936
26745	Del	2	221578066	222288892	0.038019	0.062119
26746	Del	2	222293320	222362075	0.059256	0.063302
26747	Del	2	222363336	222562791	0.266158	0.075726
26748	Del	2	222562963	222945862	0.296868	0.077501
26749	Del	2	222946019	223750241	0.320026	0.078093
26750	Del	2	223750531	223840885	0.29284	0.07691
26751	Del	2	223841551	223947235	0.301326	0.077501
26752	Del	2	223949469	223974898	0.31876	0.078093
26753	Del	2	223974979	224468118	0.29284	0.07691
26754	Del	2	224469295	224588648	0.281515	0.076318
26755	Del	2	224589067	225006956	0.926503	0.101166
26756	Del	2	225007030	225012230	1.009212	0.103532
26757	Del	2	225012316	225394679	0.926503	0.101166
26758	Del	2	225399169	225657036	0.336985	0.079276
26759	Del	2	225657743	226725064	0.515796	0.086967
26760	Del	2	226725226	226801363	0.498396	0.085784
26761	Del	2	226803428	226999405	0.616003	0.090517
26762	Del	2	227001838	227160293	0.788526	0.096433
26763	Del	2	227162718	227219583	1.187706	0.11004
26764	Del	2	227220801	227320828	1.224786	0.111223

26765	Del	2	227320988	227361058	1.269369	0.112406
26766	Del	2	227361369	227389556	1.284729	0.112998
26767	Del	2	227394141	227475235	1.524981	0.120097
26768	Del	2	227475679	227566149	1.470767	0.118322
26769	Del	2	227566163	227802740	1.499473	0.119506
26770	Del	2	227806707	227870120	0.901364	0.099982
26771	Del	2	227871016	227926810	0.850291	0.098799
26772	Del	2	227927765	229022287	1.067947	0.105899
26773	Del	2	229022843	229233195	0.754319	0.09525
26774	Del	2	229233252	229273682	0.728098	0.094658
26775	Del	2	229274613	229710778	1.55736	0.12128
26776	Del	2	229712080	230031484	1.885541	0.129563
26777	Del	2	230033262	230069466	1.850869	0.128971
26778	Del	2	230071038	230173687	1.929924	0.130746
26779	Del	2	230174194	230220457	1.932867	0.131338
26780	Del	2	230221462	230225063	1.931389	0.130746
26781	Del	2	230225300	230239202	1.987297	0.132521
26782	Del	2	230240555	230414686	1.931389	0.131338
26783	Del	2	230415077	230472831	1.830529	0.12838
26784	Del	2	230472944	230509746	1.799316	0.127788
26785	Del	2	230510985	230520071	1.791818	0.127197
26786	Del	2	230520254	230545805	1.770274	0.126605
26787	Del	2	230547280	230704120	1.852661	0.128971
26788	Del	2	230707953	230824029	1.768667	0.126605
26789	Del	2	230825305	230976811	1.851341	0.128971
26790	Del	2	230983547	231050130	1.140442	0.108265
26791	Del	2	231050320	231175408	1.157519	0.108857
26792	Del	2	231175905	231346600	1.226406	0.111223
26793	Del	2	231350413	231394665	1.219186	0.110631
26794	Del	2	231394808	231402543	1.03853	0.104715
26795	Del	2	231406573	231503112	1.500464	0.119506
26796	Del	2	231504690	231702264	1.258453	0.111815
26797	Del	2	231704107	231732038	1.161801	0.108857
26798	Del	2	231732379	231770004	1.135813	0.108265
26799	Del	2	231774466	231890886	1.162746	0.108857
26800	Del	2	231891818	231930544	1.022647	0.104124
26801	Del	2	231931043	232319832	1.468091	0.118322
26802	Del	2	232328858	232548110	1.684031	0.124239
26803	Del	2	232548124	232584154	1.853662	0.128971
26804	Del	2	232585900	232695202	2.660736	0.148495
26805	Del	2	232695785	232740182	2.457172	0.143762
26806	Del	2	232740875	232740875	2.414665	0.142578
26807	Del	2	232741375	232786583	2.494466	0.144353
26808	Del	2	232789671	232862361	2.414665	0.142578
26809	Del	2	232862772	232878443	2.48919	0.144353
26810	Del	2	232879311	232931765	2.431097	0.14317
26811	Del	2	232932495	233011265	2.332773	0.140804
26812	Del	2	233012511	233035379	2.494466	0.144353
26813	Del	2	233036489	233249710	3.16756	0.159735
26814	Del	2	233250193	233272333	2.61116	0.147311
26815	Del	2	233274261	233277843	2.678296	0.148495
26816	Del	2	233280713	233305954	2.61116	0.147311
26817	Del	2	233305995	233349455	2.436657	0.14317

26818	Del	2	233349894	233473058	3.436277	0.16506
26819	Del	2	233473171	233612283	2.750452	0.150269
26820	Del	2	233612490	233671494	2.08728	0.134888
26821	Del	2	233671659	233678168	2.100574	0.135479
26822	Del	2	233680544	233776881	2.116462	0.136071
26823	Del	2	233777511	233834102	1.924794	0.130746
26824	Del	2	233834375	233855851	1.963701	0.131929
26825	Del	2	233855972	233870804	1.990471	0.132521
26826	Del	2	233872871	234020915	2.09732	0.135479
26827	Del	2	234021042	234490498	2.169408	0.137254
26828	Del	2	234492085	234680232	1.990471	0.132521
26829	Del	2	234682614	234718056	1.963701	0.131929
26830	Del	2	234718443	235493098	2.141476	0.136662
26831	Del	2	235493414	235499217	1.702523	0.12483
26832	Del	2	235499319	235508014	1.995056	0.132521
26833	Del	2	235508316	235857072	1.702523	0.12483
26834	Del	2	235857688	236133046	1.512663	0.120097
26835	Del	2	236134495	236193288	1.995056	0.132521
26836	Del	2	236193456	236320175	1.91999	0.130746
26837	Del	2	236321341	236511727	1.859861	0.128971
26838	Del	2	236512467	236582696	1.954446	0.131929
26839	Del	2	236583294	237101505	2.036436	0.133704
26840	Del	2	237101631	237365500	2.089845	0.134888
26841	Del	2	237366185	237482043	2.148648	0.136662
26842	Del	2	237482500	237506474	2.107777	0.135479
26843	Del	2	237506476	237511498	2.185794	0.137254
26844	Del	2	237512235	237557713	2.107777	0.135479
26845	Del	2	237557757	237566382	2.185794	0.137254
26846	Del	2	237569505	237591750	2.366163	0.141987
26847	Del	2	237592530	237797094	2.808453	0.151453
26848	Del	2	237797485	237858157	1.903266	0.130155
26849	Del	2	237858342	237919292	1.721695	0.125422
26850	Del	2	237920792	238042966	1.914256	0.130746
26851	Del	2	238043765	238059419	1.853864	0.128971
26852	Del	2	238059946	238099426	1.931389	0.131338
26853	Del	2	238099543	238170901	1.96445	0.131929
26854	Del	2	238173799	238203962	1.942187	0.131338
26855	Del	2	238204462	238247942	1.966094	0.131929
26856	Del	2	238250626	238311638	1.945905	0.131338
26857	Del	2	238314644	238420242	1.914945	0.130746
26858	Del	2	238421868	238555669	1.608581	0.122464
26859	Del	2	238562828	239190740	1.637875	0.123055
26860	Del	2	239192441	239196235	1.82937	0.12838
26861	Del	2	239196336	239304744	1.630005	0.123055
26862	Del	2	239304786	239305705	1.837801	0.12838
26863	Del	2	239310443	239345264	1.630005	0.123055
26864	Del	2	239345975	239352670	1.837801	0.12838
26865	Del	2	239353343	239396271	1.630005	0.123055
26866	Del	2	239397060	239411399	1.837801	0.12838
26867	Del	2	239415788	239891926	1.898336	0.130155
26868	Del	2	239892262	239938929	1.744673	0.126013
26869	Del	2	239939023	239945633	1.908675	0.130155
26870	Del	2	239946392	239954858	1.744673	0.126013

26871	Del	2	239956804	239971998	1.816249	0.127788
26872	Del	2	239972117	240026268	1.837801	0.12838
26873	Del	2	240028499	240141237	1.908675	0.130155
26874	Del	2	240141375	240476630	2.139468	0.136662
26875	Del	2	240477920	240555775	2.056869	0.134296
26876	Del	2	240557350	240776486	2.158186	0.136662
26877	Del	2	240788528	240822728	2.13417	0.136071
26878	Del	2	240823162	240950485	2.156771	0.136662
26879	Del	2	240952382	240970090	1.99203	0.132521
26880	Del	2	240970510	241072219	2.795721	0.151453
26881	Del	2	241072273	241093125	2.853286	0.152636
26882	Del	2	241093294	241105676	2.88632	0.153228
26883	Del	2	241106216	241144360	2.843496	0.152044
26884	Del	2	241146319	241168671	2.876969	0.153228
26885	Del	2	241168820	241182305	2.845162	0.152044
26886	Del	2	241188357	241196527	2.889591	0.153228
26887	Del	2	241198650	241278212	2.867863	0.152636
26888	Del	2	241278760	241314580	2.809835	0.151453
26889	Del	2	241314748	241350071	2.867457	0.152636
26890	Del	2	241351844	241467701	3.930584	0.175117
26891	Del	2	241468063	241495311	3.875506	0.173934
26892	Del	2	241498021	241537572	3.036074	0.156777
26893	Del	3	2170634	3058965	0	0.044371
26894	Del	3	3059103	3798027	0	0.032539
26895	Del	3	3798106	3852039	0	0.031947
26896	Del	3	3858107	4359501	0	0.039638
26897	Del	3	4360965	4467396	0	0.03313
26898	Del	3	4469418	4491276	0	0.034313
26899	Del	3	4491474	4849723	0	0.048512
26900	Del	3	4849814	4983086	0	0.057978
26901	Del	3	4983485	5117356	0	0.039638
26902	Del	3	5121732	5190804	0	0.031947
26903	Del	3	5191666	6631900	0	0.028397
26904	Del	3	6632210	6667588	0	0.023664
26905	Del	3	6667985	6855158	0	0.024256
26906	Del	3	6858598	6886245	0	0.022481
26907	Del	3	6886961	6894157	0	0.024256
26908	Del	3	6894366	7519418	0	0.022481
26909	Del	3	7519462	7535850	0	0.024256
26910	Del	3	7536490	7741629	0	0.022481
26911	Del	3	7742939	8018706	0	0.023664
26912	Del	3	8020705	8492807	0	0.025439
26913	Del	3	8499622	8569250	0	0.026623
26914	Del	3	8569384	8655686	0	0.027214
26915	Del	3	8658440	8877115	0	0.03313
26916	Del	3	8877162	8973251	0	0.032539
26917	Del	3	8973258	9249713	0	0.030172
26918	Del	3	9249757	9371727	0	0.034905
26919	Del	3	9393556	9494469	0	0.045554
26920	Del	3	9495054	9644463	0	0.031947
26921	Del	3	9647877	9727785	0	0.017748
26922	Del	3	9730156	9794129	0	0.023664
26923	Del	3	9796602	10127208	0	0.017748

26924	Del	3	10127734	10322954	0	0.020115
26925	Del	3	10323109	10327840	0	0.018932
26926	Del	3	10329623	10335628	0	0.020115
26927	Del	3	10335966	10394095	0	0.018932
26928	Del	3	10394256	10395185	0	0.020115
26929	Del	3	10395555	10507799	0	0.018932
26930	Del	3	10508163	10835065	0	0.020115
26931	Del	3	10835097	10944467	0	0.018932
26932	Del	3	10949339	11004141	0	0.019523
26933	Del	3	11004210	11020926	0	0.020115
26934	Del	3	11021077	11042563	0	0.019523
26935	Del	3	11044681	11221965	0	0.025439
26936	Del	3	11223547	11267672	0	0.026031
26937	Del	3	11269060	11559886	0	0.024256
26938	Del	3	11561735	11605480	0	0.01834
26939	Del	3	11605511	11644915	0	0.017748
26940	Del	3	11645134	11720790	0	0.017157
26941	Del	3	11721527	12003230	0	0.01834
26942	Del	3	12003473	12150894	0	0.007691
26943	Del	3	12150940	12159406	0	0.008283
26944	Del	3	12165880	12347814	0	0.007691
26945	Del	3	12351251	12434887	0	0.008283
26946	Del	3	12435556	12584112	0	0.017157
26947	Del	3	12584248	12715395	0	0.015974
26948	Del	3	12715447	13013995	0	0.017157
26949	Del	3	13014076	13475203	0	0.016565
26950	Del	3	13476688	13883943	0	0.008283
26951	Del	3	13883981	14353855	0	0.01479
26952	Del	3	14359965	14448186	0	0.024848
26953	Del	3	14453947	14491793	0	0.024256
26954	Del	3	14496232	14674380	0	0.018932
26955	Del	3	14674864	14774246	0	0.01834
26956	Del	3	14774745	14818064	0	0.018932
26957	Del	3	14818196	14940055	0	0.01834
26958	Del	3	14941510	15249742	0	0.008874
26959	Del	3	15253857	15343152	0	0.018932
26960	Del	3	15346437	15429210	0	0.019523
26961	Del	3	15430760	15802269	0	0.008874
26962	Del	3	15803204	15865490	0	0.008283
26963	Del	3	15866198	16337874	0	0.008874
26964	Del	3	16339383	16518841	0	0.008283
26965	Del	3	16521499	16882947	0	0.008874
26966	Del	3	16883152	16932213	0	0.008283
26967	Del	3	16932270	16934154	0	0.008874
26968	Del	3	16938263	17154444	0	0.008283
26969	Del	3	17156045	17706903	0	0.007691
26970	Del	3	17707659	17741414	0	0.007099
26971	Del	3	17741892	17961005	0	0.008874
26972	Del	3	17962287	18269920	0	0.007691
26973	Del	3	18272567	19143628	0	0.008874
26974	Del	3	19146029	19309219	0	0.007691
26975	Del	3	19313993	19316839	0	0.008874
26976	Del	3	19317407	19536210	0	0.007691

26977	Del	3	19545259	20034931	0	0.008874
26978	Del	3	20035922	20133405	0	0.008283
26979	Del	3	20135316	20139029	0	0.008874
26980	Del	3	20142444	20156634	0	0.008283
26981	Del	3	20156834	21416726	0	0.008874
26982	Del	3	21416748	21528342	0	0.007691
26983	Del	3	21541164	21582139	0	0.008874
26984	Del	3	21583448	21740479	0	0.007691
26985	Del	3	21740672	23198566	0	0.008874
26986	Del	3	23199180	23593143	0	0.011241
26987	Del	3	23593635	23927735	0	0.013015
26988	Del	3	23930279	24114870	0	0.01479
26989	Del	3	24116648	24150793	0	0.012424
26990	Del	3	24151093	24190281	0	0.01479
26991	Del	3	24190941	24492284	0	0.012424
26992	Del	3	24493999	24529029	0	0.01479
26993	Del	3	24529088	25599818	0	0.015974
26994	Del	3	25603772	27108619	0	0.013015
26995	Del	3	27108989	27218210	0	0.010649
26996	Del	3	27218792	27370689	0	0.010057
26997	Del	3	27371123	27489828	0	0.010649
26998	Del	3	27490050	28330923	0	0.017157
26999	Del	3	28331663	28571630	0	0.025439
27000	Del	3	28574515	28759878	0	0.024848
27001	Del	3	28760382	29280435	0	0.026031
27002	Del	3	29280576	30603086	0	0.030764
27003	Del	3	30603934	30725554	0	0.024848
27004	Del	3	30725622	30895433	0	0.024256
27005	Del	3	30895953	31657860	0	0.032539
27006	Del	3	31658756	31700434	0	0.029581
27007	Del	3	31702358	31724815	0	0.032539
27008	Del	3	31725232	31978408	0	0.029581
27009	Del	3	31978688	32104696	0	0.032539
27010	Del	3	32106225	32173324	0	0.024256
27011	Del	3	32173407	32385520	0	0.031947
27012	Del	3	32389928	32477903	0	0.027214
27013	Del	3	32479698	32996088	0	0.020115
27014	Del	3	32996229	33089578	0	0.019523
27015	Del	3	33090095	33273612	0	0.020115
27016	Del	3	33273815	33383538	0	0.019523
27017	Del	3	33386305	33493919	0	0.020115
27018	Del	3	33496192	33721804	0	0.019523
27019	Del	3	33721969	33874018	0	0.020115
27020	Del	3	33874975	35394459	0	0.024848
27021	Del	3	35396697	35678587	0	0.031355
27022	Del	3	35678639	35689483	0	0.031947
27023	Del	3	35689716	35743804	0	0.031355
27024	Del	3	35744116	35745039	0	0.031947
27025	Del	3	35745871	35905882	0	0.031355
27026	Del	3	35907150	36548364	0	0.032539
27027	Del	3	36549711	36826416	0	0.033722
27028	Del	3	36826616	36945441	0	0.032539
27029	Del	3	36947151	37058075	0	0.033722

27030	Del	3	37059015	37367153	0	0.03313
27031	Del	3	37367938	37450647	0	0.033722
27032	Del	3	37451015	37752911	0	0.030172
27033	Del	3	37753573	37968258	0	0.03313
27034	Del	3	37969217	37969887	0	0.033722
27035	Del	3	37971174	37984896	0	0.03313
27036	Del	3	37985161	38037842	0	0.033722
27037	Del	3	38038610	38120450	0	0.03313
27038	Del	3	38122577	38340569	0	0.041413
27039	Del	3	38341263	38418571	0	0.040821
27040	Del	3	38419250	38529798	0	0.050287
27041	Del	3	38530514	38650328	0	0.055612
27042	Del	3	38652906	38953737	0.032221	0.062119
27043	Del	3	38953977	39097199	0.419509	0.082826
27044	Del	3	39097349	39110978	0.058385	0.063302
27045	Del	3	39111015	39133513	0.049726	0.062711
27046	Del	3	39134085	39138608	0.060057	0.063302
27047	Del	3	39140306	39140306	0.049726	0.062711
27048	Del	3	39140886	39466707	0.060057	0.063302
27049	Del	3	39467477	39499236	0.030493	0.061528
27050	Del	3	39499395	39503804	0.060057	0.063302
27051	Del	3	39503816	39805581	0.030493	0.061528
27052	Del	3	39805698	40098845	0	0.057978
27053	Del	3	40099386	40308457	0.025431	0.061528
27054	Del	3	40308718	40315102	0.059411	0.063302
27055	Del	3	40317147	40452574	0.172163	0.070402
27056	Del	3	40452783	41197615	0.18246	0.070993
27057	Del	3	41197952	41244752	0.143521	0.069219
27058	Del	3	41246202	41348648	0.08755	0.065669
27059	Del	3	41350002	42174077	0.114479	0.067444
27060	Del	3	42176371	42204249	0.068346	0.064486
27061	Del	3	42204507	42227348	0.0669	0.063894
27062	Del	3	42228361	42488451	0.073638	0.064486
27063	Del	3	42489180	42523240	0.068155	0.063894
27064	Del	3	42524650	42532985	0.075677	0.064486
27065	Del	3	42534799	42539026	0.068346	0.063894
27066	Del	3	42540188	42601389	0.071859	0.064486
27067	Del	3	42602443	42608273	0.068346	0.063894
27068	Del	3	42611817	42706378	0.068346	0.064486
27069	Del	3	42707542	42781245	0.064186	0.063894
27070	Del	3	42781378	42805365	0	0.057978
27071	Del	3	42806488	42855307	0.093768	0.065669
27072	Del	3	42863657	42972924	0.10539	0.066261
27073	Del	3	42975703	43057772	0.084421	0.065669
27074	Del	3	43058320	43106511	0.094529	0.066261
27075	Del	3	43109086	43345731	0.241119	0.074543
27076	Del	3	43346442	43362999	0.25536	0.075135
27077	Del	3	43365795	43687645	0.208784	0.072768
27078	Del	3	43688252	43723674	0.16706	0.070402
27079	Del	3	43723948	44233784	0.380097	0.081051
27080	Del	3	44233834	44337227	0.35986	0.080459
27081	Del	3	44338016	44360378	0.25527	0.075135
27082	Del	3	44360571	44410249	0.244476	0.074543

27083	Del	3	44410402	44765735	0.25536	0.075135
27084	Del	3	44766291	44854555	0.242853	0.074543
27085	Del	3	44859950	44874111	0.25536	0.075135
27086	Del	3	44874500	44974088	0.244781	0.074543
27087	Del	3	44975034	45039709	0.344533	0.079868
27088	Del	3	45040185	45108535	0.451625	0.084009
27089	Del	3	45108629	45147206	0.469569	0.084601
27090	Del	3	45147459	45387422	0.483919	0.085192
27091	Del	3	45388191	45472433	0.422781	0.082826
27092	Del	3	45476211	45513873	0.472976	0.085192
27093	Del	3	45514461	45549342	0.422781	0.082826
27094	Del	3	45549900	45676726	0.440277	0.084009
27095	Del	3	45676957	45689783	0.687404	0.092883
27096	Del	3	45689959	45754937	0.652422	0.0917
27097	Del	3	45755165	45800294	0.457122	0.084601
27098	Del	3	45801947	45843868	0.479474	0.085192
27099	Del	3	45845077	45885631	0.447439	0.084009
27100	Del	3	45886249	45904117	0.476611	0.085192
27101	Del	3	45904733	45917163	0.447439	0.084009
27102	Del	3	45917502	45939181	0.438812	0.083417
27103	Del	3	45942436	45948625	0.482049	0.085192
27104	Del	3	45948725	46020348	0.438812	0.083417
27105	Del	3	46020595	46377198	0.366761	0.080459
27106	Del	3	46378143	46403303	0.339	0.079276
27107	Del	3	46405230	46434857	0.367793	0.080459
27108	Del	3	46435728	46486506	0.352415	0.079868
27109	Del	3	46486945	46507698	0.366696	0.080459
27110	Del	3	46515345	46552596	0.349603	0.079868
27111	Del	3	46553570	46561166	0.366696	0.080459
27112	Del	3	46565765	46565765	0.349603	0.079868
27113	Del	3	46571732	46627199	0.365983	0.080459
27114	Del	3	46627902	46828459	0.487617	0.085784
27115	Del	3	46831077	46918203	0.453354	0.084009
27116	Del	3	46921389	46967210	0.424678	0.083417
27117	Del	3	46969820	47014363	0.44489	0.084009
27118	Del	3	47016378	47161344	0.419572	0.082826
27119	Del	3	47162075	47276640	0.432105	0.083417
27120	Del	3	47280597	47347309	0.648832	0.0917
27121	Del	3	47347457	47403633	0.699044	0.093475
27122	Del	3	47403889	47515734	0.872425	0.099391
27123	Del	3	47519373	47585292	1.050196	0.105307
27124	Del	3	47585627	47783227	1.002884	0.103532
27125	Del	3	47783288	47818580	1.050196	0.105307
27126	Del	3	47820670	47848297	1.037284	0.104715
27127	Del	3	47849546	47852431	1.054345	0.105307
27128	Del	3	47855877	47971281	0.984132	0.10294
27129	Del	3	47971307	48090087	1.02176	0.104124
27130	Del	3	48092208	48192136	1.039431	0.104715
27131	Del	3	48194592	48506373	1.054345	0.105307
27132	Del	3	48506668	48547024	1.25805	0.111815
27133	Del	3	48549264	48550487	1.275412	0.112406
27134	Del	3	48552953	48559317	1.25805	0.111815
27135	Del	3	48560120	48742301	1.27404	0.112406

27136	Del	3	48742909	48837870	1.251801	0.111815
27137	Del	3	48845331	48852083	1.275412	0.112406
27138	Del	3	48855201	48921736	1.485602	0.119506
27139	Del	3	48923598	48982641	1.470767	0.118914
27140	Del	3	48983675	49031521	1.497424	0.119506
27141	Del	3	49033066	49092250	1.481983	0.118914
27142	Del	3	49095712	49198763	1.499963	0.119506
27143	Del	3	49200889	49254450	1.476432	0.118914
27144	Del	3	49268108	49279859	1.500464	0.119506
27145	Del	3	49279905	49310624	1.470767	0.118914
27146	Del	3	49311089	49430749	1.489103	0.119506
27147	Del	3	49438064	49532767	1.831434	0.12838
27148	Del	3	49533449	49555986	1.855575	0.128971
27149	Del	3	49556687	49665768	1.781088	0.127197
27150	Del	3	49668647	49708767	1.855575	0.128971
27151	Del	3	49714152	49728703	1.943087	0.131338
27152	Del	3	49729803	49790059	1.926407	0.130746
27153	Del	3	49792220	49857648	1.85389	0.128971
27154	Del	3	49860945	49901980	1.629424	0.123055
27155	Del	3	49907305	49933191	1.699044	0.12483
27156	Del	3	49935383	50079701	1.62238	0.123055
27157	Del	3	50081054	50120238	1.694663	0.12483
27158	Del	3	50120462	50158985	1.684031	0.124239
27159	Del	3	50159578	50167434	1.611371	0.122464
27160	Del	3	50169642	50175537	1.637875	0.123055
27161	Del	3	50179660	50189901	1.611371	0.122464
27162	Del	3	50191088	50278688	1.589957	0.121872
27163	Del	3	50280451	50368578	1.329543	0.114181
27164	Del	3	50369913	50552184	1.263733	0.112406
27165	Del	3	50555832	50587719	1.062859	0.105899
27166	Del	3	50589226	50614187	1.329543	0.114181
27167	Del	3	50614320	50649521	1.305594	0.11359
27168	Del	3	50651608	50681914	1.330386	0.114181
27169	Del	3	50682171	51382990	1.173132	0.109448
27170	Del	3	51384193	51388722	1.500887	0.119506
27171	Del	3	51394410	51500705	1.523224	0.120097
27172	Del	3	51502259	51669048	1.866798	0.129563
27173	Del	3	51669593	51704756	1.912296	0.130746
27174	Del	3	51706524	52073893	1.721695	0.125422
27175	Del	3	52074043	52155842	1.696327	0.12483
27176	Del	3	52160953	52300577	2.002988	0.133113
27177	Del	3	52315396	52397741	1.972473	0.131929
27178	Del	3	52398121	52457431	2.011379	0.133113
27179	Del	3	52457456	52490558	1.992054	0.132521
27180	Del	3	52492820	52544992	2.009616	0.133113
27181	Del	3	52545164	52692360	1.927709	0.130746
27182	Del	3	52693241	52704149	1.816786	0.127788
27183	Del	3	52709808	52771205	1.987618	0.132521
27184	Del	3	52774224	52809597	1.99826	0.133113
27185	Del	3	52809855	52836116	1.931389	0.131338
27186	Del	3	52837900	52839001	1.903108	0.130155
27187	Del	3	52839968	52846979	1.947079	0.131338
27188	Del	3	52847220	52896136	1.903108	0.130155

27189	Del	3	52898992	53050310	2.06636	0.134296
27190	Del	3	53051264	53279275	2.348837	0.141395
27191	Del	3	53283210	53490596	2.332318	0.140804
27192	Del	3	53493183	53812714	1.972473	0.131929
27193	Del	3	53812897	53860220	2.13417	0.136071
27194	Del	3	53863764	53891990	2.156771	0.136662
27195	Del	3	53892620	54627126	2.328494	0.140804
27196	Del	3	54630299	54642752	2.644761	0.147903
27197	Del	3	54643164	54873770	2.328494	0.140804
27198	Del	3	54874237	54903516	3.452729	0.165651
27199	Del	3	54907182	54917905	2.328494	0.140804
27200	Del	3	54918056	54929359	3.523626	0.166835
27201	Del	3	54931653	54968039	3.452729	0.165651
27202	Del	3	54968627	55076124	2.328494	0.140804
27203	Del	3	55081454	55506937	3.535266	0.167426
27204	Del	3	55508149	55655076	2.560519	0.146128
27205	Del	3	55655355	55660033	3.557413	0.167426
27206	Del	3	55662174	55849204	2.560519	0.146128
27207	Del	3	55849242	55853132	3.709908	0.170384
27208	Del	3	55853167	56471023	2.560519	0.146128
27209	Del	3	56471059	56618885	3.452729	0.165651
27210	Del	3	56620113	56685275	3.671908	0.169793
27211	Del	3	56694926	56778131	4.180629	0.180442
27212	Del	3	56779081	56804357	4.127003	0.179258
27213	Del	3	56804642	56918872	3.923984	0.175117
27214	Del	3	56919146	56933054	3.684536	0.170384
27215	Del	3	56933267	56964411	4.203784	0.181033
27216	Del	3	56964467	57059656	3.684536	0.170384
27217	Del	3	57060134	57076212	3.724482	0.170976
27218	Del	3	57085272	57088275	3.684536	0.170384
27219	Del	3	57088334	57189552	3.587701	0.168018
27220	Del	3	57190878	57218035	3.442357	0.16506
27221	Del	3	57219135	57267261	3.405879	0.164468
27222	Del	3	57267767	57283204	3.441082	0.16506
27223	Del	3	57283680	57294839	3.419833	0.164468
27224	Del	3	57297199	57483760	3.230506	0.160918
27225	Del	3	57485937	57545594	3.391967	0.164468
27226	Del	3	57546297	57563630	3.441082	0.16506
27227	Del	3	57567470	57570671	3.362067	0.163877
27228	Del	3	57571305	57600968	3.421844	0.16506
27229	Del	3	57601006	57752585	3.362067	0.163877
27230	Del	3	57755327	57887858	2.94626	0.154411
27231	Del	3	57889658	58001183	3.048324	0.156777
27232	Del	3	58005193	58162006	2.851144	0.152636
27233	Del	3	58162375	58170985	3.070377	0.157369
27234	Del	3	58171354	58173170	2.851144	0.152636
27235	Del	3	58176047	58211116	3.042775	0.156777
27236	Del	3	58211146	58296401	3.39231	0.164468
27237	Del	3	58296946	58321632	3.454796	0.165651
27238	Del	3	58321917	58422520	3.877906	0.173934
27239	Del	3	58423399	58487018	3.999712	0.1763
27240	Del	3	58487692	58504281	3.463466	0.165651
27241	Del	3	58512963	58539540	3.443922	0.16506

27242	Del	3	58539799	58578630	3.45082	0.165651
27243	Del	3	58578710	58587807	3.431028	0.16506
27244	Del	3	58589383	58604544	3.365091	0.163877
27245	Del	3	58604687	58738169	3.412607	0.164468
27246	Del	3	58739584	58813290	3.227266	0.160918
27247	Del	3	58814158	59020289	3.32389	0.162693
27248	Del	3	59022043	59058933	3.227266	0.160918
27249	Del	3	59059438	61040294	3.357732	0.163285
27250	Del	3	61040679	61251724	2.977281	0.155594
27251	Del	3	61252544	62295111	4.797777	0.192866
27252	Del	3	62295683	62317312	3.840181	0.173342
27253	Del	3	62318645	62396454	3.912521	0.174526
27254	Del	3	62398038	63098495	3.144434	0.159144
27255	Del	3	63100395	63276689	2.985322	0.155594
27256	Del	3	63277838	63423389	2.499742	0.144945
27257	Del	3	63423489	63550473	2.802987	0.151453
27258	Del	3	63550975	63617196	2.759494	0.150269
27259	Del	3	63617364	63833403	2.978439	0.155594
27260	Del	3	63833847	63866560	3.003298	0.156186
27261	Del	3	63869328	63896942	2.88508	0.153228
27262	Del	3	63897513	63962890	2.915446	0.153819
27263	Del	3	63965722	64003593	2.978439	0.155594
27264	Del	3	64003620	64063827	3.015029	0.156186
27265	Del	3	64064996	64076097	2.970933	0.155002
27266	Del	3	64076835	64087429	3.006715	0.156186
27267	Del	3	64092333	64102202	2.970933	0.155002
27268	Del	3	64103333	64106437	3.021564	0.156186
27269	Del	3	64107138	64185680	2.774126	0.150861
27270	Del	3	64187352	64206305	2.980432	0.155594
27271	Del	3	64206361	64512300	2.774126	0.150861
27272	Del	3	64515028	64561220	2.745495	0.150269
27273	Del	3	64561239	64591775	3.003298	0.156186
27274	Del	3	64594493	64683416	2.745495	0.150269
27275	Del	3	64684729	65351439	2.996446	0.155594
27276	Del	3	65351496	65890443	2.655394	0.148495
27277	Del	3	65891416	65925645	3.534816	0.167426
27278	Del	3	65928101	66040192	2.658113	0.148495
27279	Del	3	66057454	66506898	3.200069	0.160327
27280	Del	3	66507612	66740723	3.408244	0.164468
27281	Del	3	66740798	67355385	3.570265	0.168018
27282	Del	3	67358884	67371700	3.163725	0.159735
27283	Del	3	67373788	67647923	3.196571	0.160327
27284	Del	3	67648391	67748128	3.566031	0.168018
27285	Del	3	67748202	67983012	3.412607	0.164468
27286	Del	3	67986194	68499086	3.205999	0.160327
27287	Del	3	68499120	68506516	3.682436	0.170384
27288	Del	3	68506644	68547056	3.211041	0.160327
27289	Del	3	68547479	68934077	3.341116	0.163285
27290	Del	3	68935625	69015408	3.635776	0.169201
27291	Del	3	69015822	69044930	3.657555	0.169793
27292	Del	3	69046770	69055601	3.709908	0.170384
27293	Del	3	69055896	69168606	3.670429	0.169793
27294	Del	3	69168637	69386891	3.337247	0.163285

27295	Del	3	69390580	69762404	4.03372	0.176892
27296	Del	3	69762420	69863642	4.089453	0.178667
27297	Del	3	69864288	69975209	4.330299	0.1834
27298	Del	3	69986160	70031714	3.709908	0.170384
27299	Del	3	70031739	71248083	4.201918	0.180442
27300	Del	3	71249047	71286916	4.114419	0.179258
27301	Del	3	71287006	71310460	4.59915	0.188724
27302	Del	3	71313368	71677392	4.114419	0.179258
27303	Del	3	71677865	71729586	3.616505	0.168609
27304	Del	3	71729771	71752561	3.583421	0.168018
27305	Del	3	71752598	72034494	3.709908	0.170384
27306	Del	3	72034778	72102844	3.076964	0.157369
27307	Del	3	72103512	72178567	3.125808	0.158552
27308	Del	3	72178717	72448111	3.738148	0.170976
27309	Del	3	72452843	72665009	3.853885	0.173342
27310	Del	3	72667285	72882257	4.021236	0.176892
27311	Del	3	72886328	72975458	3.992932	0.1763
27312	Del	3	72975807	73016801	4.104852	0.178667
27313	Del	3	73017346	73057129	4.03562	0.177484
27314	Del	3	73057546	73065921	4.139649	0.179258
27315	Del	3	73066211	73379622	4.03562	0.177484
27316	Del	3	73380420	73434235	3.731792	0.170976
27317	Del	3	73435152	73475345	3.645262	0.169201
27318	Del	3	73475923	73519590	3.517745	0.166835
27319	Del	3	73520569	73570410	3.780314	0.172159
27320	Del	3	73570611	73612220	3.427757	0.16506
27321	Del	3	73618766	74260340	3.853885	0.173342
27322	Del	3	74260493	74521899	3.356871	0.163285
27323	Del	3	74522252	77087944	3.794115	0.172159
27324	Del	3	77094425	78595726	3.813355	0.172751
27325	Del	3	78596332	79022438	3.315528	0.162693
27326	Del	3	79022917	79408993	3.018515	0.156186
27327	Del	3	79411525	79435389	3.363792	0.163877
27328	Del	3	79440615	80985612	3.018515	0.156186
27329	Del	3	80992462	81488713	2.198376	0.137846
27330	Del	3	81489477	84633693	1.451807	0.117731
27331	Del	3	84634677	84954801	0.648344	0.0917
27332	Del	3	84956870	86475908	0.435682	0.083417
27333	Del	3	86478241	86993244	0.232316	0.073952
27334	Del	3	86994173	87157679	0.383514	0.081642
27335	Del	3	87159160	87759935	0.407785	0.082234
27336	Del	3	87760453	87979914	0.381212	0.081642
27337	Del	3	87981539	88061879	0.404964	0.082234
27338	Del	3	88064329	88153460	0.378881	0.081051
27339	Del	3	88154033	88164996	0.236178	0.073952
27340	Del	3	88165601	89402228	0.268026	0.075726
27341	Del	3	89403907	91390926	0.253751	0.075135
27342	Del	3	91462299	94059033	0.113619	0.066852
27343	Del	3	94059214	94920924	0	0.027806
27344	Del	3	94924915	96360329	0	0.01479
27345	Del	3	96361596	97010403	0	0.015382
27346	Del	3	97011102	97433810	0	0.012424
27347	Del	3	97433921	97655693	0	0.015382

27348	Del	3	97658366	97755786	0	0.012424
27349	Del	3	97759046	97818568	0	0.015974
27350	Del	3	97818957	97938419	0	0.015382
27351	Del	3	97941262	98349120	0	0.015974
27352	Del	3	98350621	98902275	0	0.011832
27353	Del	3	98902429	99796745	0	0.019523
27354	Del	3	99799481	99825536	0	0.020706
27355	Del	3	99825557	99829352	0	0.018932
27356	Del	3	99829430	99879910	0	0.020706
27357	Del	3	99881456	99962503	0	0.019523
27358	Del	3	99962600	99965647	0	0.020706
27359	Del	3	99966842	100580485	0	0.019523
27360	Del	3	100585258	100752503	0	0.028397
27361	Del	3	100753760	100994283	0	0.026623
27362	Del	3	100994291	101511498	0	0.027806
27363	Del	3	101514215	101826252	0	0.028397
27364	Del	3	101826475	101940101	0	0.031947
27365	Del	3	101940282	102162332	0	0.031355
27366	Del	3	102162929	102482694	0	0.022481
27367	Del	3	102483807	105363816	0	0.039046
27368	Del	3	105364688	105553617	0	0.029581
27369	Del	3	105557629	105577469	0	0.028989
27370	Del	3	105577905	107102597	0	0.035497
27371	Del	3	107108055	107521726	0	0.02189
27372	Del	3	107522323	107811457	0	0.020115
27373	Del	3	107819893	108364100	0	0.02189
27374	Del	3	108379115	108530139	0	0.025439
27375	Del	3	108533322	108877655	0	0.026623
27376	Del	3	108877854	111040435	0	0.02189
27377	Del	3	111044511	111607875	0	0.01834
27378	Del	3	111608212	111666136	0	0.017748
27379	Del	3	111670368	111678441	0	0.01834
27380	Del	3	111680362	111848369	0	0.017157
27381	Del	3	111848391	111848681	0	0.016565
27382	Del	3	111849047	111977471	0	0.017748
27383	Del	3	111978046	112137874	0	0.01834
27384	Del	3	112140887	112294315	0	0.017748
27385	Del	3	112295873	112365842	0	0.01834
27386	Del	3	112366386	112815091	0	0.024256
27387	Del	3	112815222	112848305	0	0.01834
27388	Del	3	112848942	113251775	0	0.027214
27389	Del	3	113251860	113283303	0	0.026623
27390	Del	3	113284036	113361102	0	0.028989
27391	Del	3	113361325	113447752	0	0.029581
27392	Del	3	113449278	113529801	0	0.027214
27393	Del	3	113529901	113593723	0	0.026623
27394	Del	3	113593735	113595163	0	0.027214
27395	Del	3	113597536	113629964	0	0.026623
27396	Del	3	113630604	113944921	0	0.027214
27397	Del	3	113945825	114179799	0	0.031947
27398	Del	3	114180969	114199443	0	0.031355
27399	Del	3	114200162	114309302	0	0.031947
27400	Del	3	114309786	114740872	0	0.036088

27401	Del	3	114741046	114744324	0	0.037272
27402	Del	3	114744911	114872986	0	0.036088
27403	Del	3	114873023	114879490	0	0.037272
27404	Del	3	114879682	115096983	0	0.036088
27405	Del	3	115097786	115104887	0	0.037272
27406	Del	3	115107867	115151133	0	0.036088
27407	Del	3	115154061	115799744	0	0.041413
27408	Del	3	115802203	116447531	0	0.039638
27409	Del	3	116447543	116849760	0.000193	0.059161
27410	Del	3	116849989	116918120	0	0.048512
27411	Del	3	116920914	118504392	0	0.034905
27412	Del	3	118504829	118641471	0	0.034313
27413	Del	3	118641543	118647911	0	0.03668
27414	Del	3	118648097	118899751	0	0.034313
27415	Del	3	118900369	118938552	0	0.027806
27416	Del	3	118938635	118948266	0	0.028397
27417	Del	3	118948558	119143545	0	0.027806
27418	Del	3	119145151	119209587	0	0.028397
27419	Del	3	119211539	119224947	0	0.033722
27420	Del	3	119225063	119297314	0	0.053245
27421	Del	3	119297516	119426665	0	0.052062
27422	Del	3	119427401	119699898	0	0.033722
27423	Del	3	119701607	119780660	0	0.03313
27424	Del	3	119780761	119817871	0	0.033722
27425	Del	3	119817970	120096532	0	0.040821
27426	Del	3	120098139	120244302	0	0.041413
27427	Del	3	120249014	120389157	0	0.042004
27428	Del	3	120390582	120390582	0	0.041413
27429	Del	3	120395281	120395790	0	0.042004
27430	Del	3	120398513	120452441	0	0.041413
27431	Del	3	120452873	120836658	0	0.042004
27432	Del	3	120836805	121435590	0	0.053837
27433	Del	3	121437584	121546039	0	0.048512
27434	Del	3	121546329	121887607	0	0.049104
27435	Del	3	121887891	121944212	0	0.055612
27436	Del	3	121944932	122052276	0	0.056203
27437	Del	3	122052430	122075082	0	0.055612
27438	Del	3	122075780	122178270	0	0.056203
27439	Del	3	122178831	122288033	0	0.05502
27440	Del	3	122290248	122445395	0.027338	0.061528
27441	Del	3	122448833	122515377	0.02202	0.060936
27442	Del	3	122515768	122580995	0.025691	0.061528
27443	Del	3	122583826	122610935	0.017993	0.060344
27444	Del	3	122612347	122882304	0.024976	0.060936
27445	Del	3	122882423	122900445	0.030373	0.061528
27446	Del	3	122902265	122976441	0.018569	0.060936
27447	Del	3	122979245	122993387	0.015478	0.060344
27448	Del	3	122995394	123030943	0.008911	0.059753
27449	Del	3	123031570	123160185	0.013477	0.060344
27450	Del	3	123160293	123198217	0.030769	0.061528
27451	Del	3	123199350	123281099	0.018569	0.060936
27452	Del	3	123281608	123456760	0.010637	0.059753
27453	Del	3	123460544	123579003	0.016072	0.060344

27454	Del	3	123581582	123611085	0.024976	0.060936
27455	Del	3	123612000	123622031	0.030373	0.061528
27456	Del	3	123624610	123633429	0.024976	0.060936
27457	Del	3	123636966	123685956	0	0.05857
27458	Del	3	123688272	123694433	0.030769	0.061528
27459	Del	3	123694895	123889564	0	0.05857
27460	Del	3	123894859	123963214	0.024769	0.060936
27461	Del	3	123963406	124092363	0.027529	0.061528
27462	Del	3	124092858	124131782	0.023863	0.060936
27463	Del	3	124132417	124134008	0.030769	0.061528
27464	Del	3	124136446	124165316	0.023863	0.060936
27465	Del	3	124170602	124369162	0	0.055612
27466	Del	3	124373610	124374950	0.030769	0.061528
27467	Del	3	124382488	124525632	0	0.055612
27468	Del	3	124526074	124581101	0	0.053245
27469	Del	3	124581121	124679246	0.000981	0.059161
27470	Del	3	124681843	124726917	0	0.05857
27471	Del	3	124727057	124752312	0.027338	0.061528
27472	Del	3	124753885	124893546	0.157386	0.06981
27473	Del	3	124900021	124960448	0.185873	0.071585
27474	Del	3	124965479	125081238	0.167146	0.070402
27475	Del	3	125081651	125150509	0.143215	0.069219
27476	Del	3	125151084	125152841	0.191385	0.071585
27477	Del	3	125152898	125213876	0.143215	0.069219
27478	Del	3	125215656	125378238	0.161173	0.06981
27479	Del	3	125380261	125786811	0.181684	0.070993
27480	Del	3	125789127	125887674	0	0.047921
27481	Del	3	125888071	126005047	0	0.053245
27482	Del	3	126005226	126102710	0	0.052653
27483	Del	3	126103160	126178695	0	0.050287
27484	Del	3	126178732	126210325	0	0.050879
27485	Del	3	126210339	126477496	0	0.05147
27486	Del	3	126478942	126515067	0	0.050879
27487	Del	3	126522952	126609258	0	0.05147
27488	Del	3	126610116	126655504	0	0.050879
27489	Del	3	126656936	126699238	0	0.05147
27490	Del	3	126699661	126964307	0	0.050287
27491	Del	3	126964323	127321072	0.027529	0.061528
27492	Del	3	127321881	127394278	0.019708	0.060936
27493	Del	3	127394326	127571777	0.025992	0.061528
27494	Del	3	127572032	127922525	0	0.05147
27495	Del	3	127922980	127988372	0	0.050879
27496	Del	3	127990190	128071933	0	0.059161
27497	Del	3	128072545	128074595	0	0.05857
27498	Del	3	128075440	128126107	0.001228	0.059161
27499	Del	3	128128360	128155093	0	0.05857
27500	Del	3	128156069	128357879	0	0.049104
27501	Del	3	128360176	128362757	0	0.052062
27502	Del	3	128364489	128408800	0	0.049104
27503	Del	3	128409240	128720939	0	0.052062
27504	Del	3	128721175	128815147	0	0.05147
27505	Del	3	128817092	128967202	0	0.052062
27506	Del	3	128969791	129440996	0	0.044371

27507	Del	3	129443438	129520836	0	0.043779
27508	Del	3	129521044	129697805	0	0.044371
27509	Del	3	129699625	129904089	0	0.043188
27510	Del	3	129904396	130212605	0	0.04023
27511	Del	3	130212780	130342333	0	0.039638
27512	Del	3	130343017	130484883	0	0.037863
27513	Del	3	130485516	130677670	0	0.039638
27514	Del	3	130677833	130848946	0	0.037272
27515	Del	3	130849062	131009313	0	0.03668
27516	Del	3	131010159	131030318	0	0.037272
27517	Del	3	131030630	131177261	0	0.03668
27518	Del	3	131177577	131185552	0	0.036088
27519	Del	3	131186088	131324005	0	0.034905
27520	Del	3	131324973	131529597	0	0.037272
27521	Del	3	131533437	132316711	0	0.032539
27522	Del	3	132317241	132413855	0	0.027214
27523	Del	3	132413947	132541559	0	0.026623
27524	Del	3	132541643	132723685	0	0.027214
27525	Del	3	132724817	133037233	0	0.026031
27526	Del	3	133037338	133123724	0	0.024256
27527	Del	3	133124494	133238449	0	0.025439
27528	Del	3	133245612	133260088	0	0.027214
27529	Del	3	133260256	133398214	0	0.025439
27530	Del	3	133399187	133741954	0	0.027214
27531	Del	3	133744351	133779897	0	0.026623
27532	Del	3	133779945	133822857	0	0.027214
27533	Del	3	133822930	133897125	0	0.026623
27534	Del	3	133897357	133931064	0	0.027214
27535	Del	3	133933478	134034124	0	0.026623
27536	Del	3	134035253	134792849	0	0.027214
27537	Del	3	134793204	135260753	0	0.023073
27538	Del	3	135261954	136144380	0	0.026623
27539	Del	3	136148792	136336011	0	0.027214
27540	Del	3	136336140	136811913	0	0.025439
27541	Del	3	136817830	139444698	0	0.023073
27542	Del	3	139445151	139451122	0	0.02189
27543	Del	3	139451262	139478119	0	0.023073
27544	Del	3	139480420	139515241	0	0.02189
27545	Del	3	139516722	139540644	0	0.023073
27546	Del	3	139540945	139558350	0	0.02189
27547	Del	3	139559606	139926668	0	0.022481
27548	Del	3	139933567	141320265	0	0.017748
27549	Del	3	141320660	141455579	0	0.017157
27550	Del	3	141456114	141616279	0	0.022481
27551	Del	3	141622677	141721469	0	0.032539
27552	Del	3	141721575	142002671	0	0.03313
27553	Del	3	142003585	142098901	0	0.032539
27554	Del	3	142100008	142449523	0	0.03313
27555	Del	3	142450330	142653314	0	0.032539
27556	Del	3	142655922	142926036	0	0.03313
27557	Del	3	142926213	143210555	0	0.023073
27558	Del	3	143212052	143850855	0	0.021298
27559	Del	3	143851197	143993106	0	0.02189

27560	Del	3	143996998	146252672	0	0.025439
27561	Del	3	146253151	146546244	0	0.026031
27562	Del	3	146549395	146658754	0	0.034905
27563	Del	3	146660742	147382011	0	0.03313
27564	Del	3	147384096	149508095	0	0.020115
27565	Del	3	149509387	149655515	0	0.017748
27566	Del	3	149655593	149663754	0	0.01834
27567	Del	3	149664409	149703595	0	0.017157
27568	Del	3	149707105	149802988	0	0.01834
27569	Del	3	149806117	149963047	0	0.017748
27570	Del	3	149963907	150973325	0	0.01834
27571	Del	3	150973611	151081071	0	0.017748
27572	Del	3	151081827	151193618	0	0.015974
27573	Del	3	151196402	151204118	0	0.01834
27574	Del	3	151204851	151210404	0	0.015974
27575	Del	3	151210466	151278646	0	0.01834
27576	Del	3	151280369	151291910	0	0.015974
27577	Del	3	151292384	151317209	0	0.01834
27578	Del	3	151317356	151323046	0	0.015974
27579	Del	3	151323725	151329707	0	0.01834
27580	Del	3	151330040	151336164	0	0.015974
27581	Del	3	151336327	151385337	0	0.01834
27582	Del	3	151386265	151433432	0	0.015974
27583	Del	3	151433509	151748451	0	0.01834
27584	Del	3	151750901	151767737	0	0.023073
27585	Del	3	151770118	151790048	0	0.023664
27586	Del	3	151791550	151791550	0	0.023073
27587	Del	3	151792254	151837466	0	0.023664
27588	Del	3	151837943	151872882	0	0.023073
27589	Del	3	151873255	151889693	0	0.023664
27590	Del	3	151891474	151940164	0	0.023073
27591	Del	3	151940265	152261128	0	0.026031
27592	Del	3	152261831	152274266	0	0.023664
27593	Del	3	152276385	152336420	0	0.022481
27594	Del	3	152336433	152343179	0	0.023664
27595	Del	3	152343196	152468428	0	0.022481
27596	Del	3	152468919	153159043	0	0.023664
27597	Del	3	153161650	153505231	0	0.01834
27598	Del	3	153505379	154123579	0	0.020706
27599	Del	3	154126394	154273580	0	0.015382
27600	Del	3	154273970	154429751	0	0.013015
27601	Del	3	154430961	155234255	0	0.020706
27602	Del	3	155240295	155295616	0	0.013015
27603	Del	3	155296404	155707238	0	0.017157
27604	Del	3	155707397	155938506	0	0.01834
27605	Del	3	155938998	156542177	0	0.030764
27606	Del	3	156542292	156672126	0	0.028397
27607	Del	3	156672158	156738271	0	0.023073
27608	Del	3	156746262	156806431	0	0.022481
27609	Del	3	156807773	156812381	0	0.023073
27610	Del	3	156817615	156817615	0	0.022481
27611	Del	3	156824848	156928550	0	0.027214
27612	Del	3	156930027	157051181	0	0.026623

27613	Del	3	157051878	157256784	0	0.027806
27614	Del	3	157257034	157429779	0	0.021298
27615	Del	3	157430120	157445846	0	0.023073
27616	Del	3	157448586	157455766	0	0.021298
27617	Del	3	157455855	157601976	0	0.023073
27618	Del	3	157603007	158097428	0	0.042596
27619	Del	3	158101795	158545033	0	0.043188
27620	Del	3	158545170	158799155	0	0.038455
27621	Del	3	158799559	159067999	0	0.030764
27622	Del	3	159068046	159264304	0	0.030172
27623	Del	3	159264435	159266967	0	0.029581
27624	Del	3	159269277	159280540	0	0.025439
27625	Del	3	159280851	159284103	0	0.027806
27626	Del	3	159285869	159759806	0	0.025439
27627	Del	3	159760209	159768661	0	0.027806
27628	Del	3	159770703	159839266	0	0.025439
27629	Del	3	159839557	159898975	0	0.027806
27630	Del	3	159899661	160016913	0	0.030764
27631	Del	3	160018981	160033011	0	0.03313
27632	Del	3	160033824	160211319	0	0.030764
27633	Del	3	160211602	160255849	0	0.03313
27634	Del	3	160256501	160398039	0	0.032539
27635	Del	3	160399254	160493014	0	0.03313
27636	Del	3	160494252	160570749	0	0.030172
27637	Del	3	160575508	160752876	0	0.040821
27638	Del	3	160754665	160840594	0	0.038455
27639	Del	3	160841671	160870497	0	0.039046
27640	Del	3	160870515	161082024	0	0.039638
27641	Del	3	161082196	161217802	0	0.033722
27642	Del	3	161218576	161343292	0	0.022481
27643	Del	3	161344543	161452432	0	0.017748
27644	Del	3	161452628	163168598	0	0.023073
27645	Del	3	163169139	163310843	0	0.020115
27646	Del	3	163310868	164332629	0	0.024256
27647	Del	3	164336098	164832357	0	0.019523
27648	Del	3	164832759	165205552	0	0.024256
27649	Del	3	165206394	165771762	0	0.023073
27650	Del	3	165772619	166869636	0	0.020115
27651	Del	3	166870271	167441082	0	0.014199
27652	Del	3	167441189	167476785	0	0.010649
27653	Del	3	167477835	167661445	0	0.010057
27654	Del	3	167664797	167732781	0	0.01479
27655	Del	3	167732863	168007935	0	0.01834
27656	Del	3	168008262	168242379	0	0.017748
27657	Del	3	168245918	168899622	0	0.015974
27658	Del	3	168900301	168923146	0	0.010057
27659	Del	3	168925880	169147953	0	0.010649
27660	Del	3	169151324	169763620	0	0.008283
27661	Del	3	169764470	170437223	0	0.002958
27662	Del	3	170440340	170464494	0	0.008283
27663	Del	3	170466029	170668981	0	0.010057
27664	Del	3	170669381	170755701	0	0.009466
27665	Del	3	170755997	171055823	0	0.010649

27666	Del	3	171056419	171102936	0	0.007099
27667	Del	3	171105835	171108895	0	0.008874
27668	Del	3	171109776	171339531	0	0.007099
27669	Del	3	171340032	171466931	0	0.008283
27670	Del	3	171468761	172035314	0	0.008874
27671	Del	3	172037278	172403057	0	0.001183
27672	Del	3	172403148	172524198	0	0.002958
27673	Del	3	172527218	172717370	0	0.013015
27674	Del	3	172718252	172885597	0	0.013607
27675	Del	3	172888954	173394464	0	0.012424
27676	Del	3	173394715	174284810	0	0.011241
27677	Del	3	174284873	176514654	0	0.017157
27678	Del	3	176515098	177440969	0	0.014199
27679	Del	3	177441470	177755446	0	0.012424
27680	Del	3	177755551	178523362	0	0.014199
27681	Del	3	178523699	178866569	0	0.013015
27682	Del	3	178870830	179149798	0	0.014199
27683	Del	3	179150042	179234447	0	0.013015
27684	Del	3	179235965	179633866	0	0.014199
27685	Del	3	179634988	179897642	0	0.021298
27686	Del	3	179897741	179923641	0	0.022481
27687	Del	3	179924155	179979505	0	0.021298
27688	Del	3	179980055	180037440	0	0.020706
27689	Del	3	180045682	180706536	0	0.022481
27690	Del	3	180707221	180872357	0	0.02189
27691	Del	3	180873347	181054687	0	0.022481
27692	Del	3	181054781	181172894	0	0.019523
27693	Del	3	181173031	181224937	0	0.020115
27694	Del	3	181225348	181239123	0	0.022481
27695	Del	3	181239219	181406702	0	0.020115
27696	Del	3	181415683	181450267	0	0.022481
27697	Del	3	181450371	181746609	0	0.020115
27698	Del	3	181747108	182014139	0	0.026623
27699	Del	3	182014389	182784557	0	0.027214
27700	Del	3	182790993	182932906	0	0.016565
27701	Del	3	182934229	182993816	0	0.017157
27702	Del	3	182994612	183175169	0	0.022481
27703	Del	3	183176548	183252136	0	0.021298
27704	Del	3	183252406	183274357	0	0.022481
27705	Del	3	183274360	184179380	0	0.021298
27706	Del	3	184180057	184184700	0	0.016565
27707	Del	3	184189144	184775454	0	0.008283
27708	Del	3	184776195	185056964	0	0.013015
27709	Del	3	185058852	185157808	0	0.013607
27710	Del	3	185162327	185188924	0	0.008283
27711	Del	3	185189672	185293083	0	0.016565
27712	Del	3	185297777	185362510	0	0.015974
27713	Del	3	185362618	185490123	0	0.016565
27714	Del	3	185494827	185639743	0	0.011241
27715	Del	3	185640264	185708705	0	0.010649
27716	Del	3	185709092	185731189	0	0.011241
27717	Del	3	185731625	185764830	0	0.010649
27718	Del	3	185766145	185768302	0	0.011241

27719	Del	3	185768692	185827529	0	0.010649
27720	Del	3	185828028	185986556	0	0.011241
27721	Del	3	185987102	186145386	0	0.017748
27722	Del	3	186146789	186453910	0	0.017157
27723	Del	3	186454425	186498626	0	0.011241
27724	Del	3	186501422	186655385	0	0.016565
27725	Del	3	186655797	186745387	0	0.02189
27726	Del	3	186745554	186859338	0	0.027214
27727	Del	3	186860325	187018451	0	0.030172
27728	Del	3	187018586	187079116	0	0.030764
27729	Del	3	187080640	187196704	0	0.031355
27730	Del	3	187196815	187214084	0	0.026031
27731	Del	3	187214139	187286977	0	0.025439
27732	Del	3	187287967	187976596	0	0.026031
27733	Del	3	187976979	188208104	0	0.033722
27734	Del	3	188212725	188685438	0	0.034313
27735	Del	3	188688008	188689191	0	0.035497
27736	Del	3	188689225	188939508	0	0.034313
27737	Del	3	188939579	189169525	0	0.020706
27738	Del	3	189169905	189237525	0	0.020115
27739	Del	3	189238508	189241406	0	0.020706
27740	Del	3	189241640	189589344	0	0.020115
27741	Del	3	189590432	189628691	0	0.018932
27742	Del	3	189628733	189786715	0	0.019523
27743	Del	3	189787541	189821331	0	0.020115
27744	Del	3	189821531	189831634	0	0.020706
27745	Del	3	189832412	189897342	0	0.020115
27746	Del	3	189899178	190122562	0	0.024848
27747	Del	3	190126890	190511361	0	0.017748
27748	Del	3	190513552	190636457	0	0.017157
27749	Del	3	190637462	190657735	0	0.016565
27750	Del	3	190658954	191263383	0	0.017748
27751	Del	3	191265995	191423569	0	0.026623
27752	Del	3	191424417	191460681	0	0.025439
27753	Del	3	191460963	191461995	0	0.026623
27754	Del	3	191470699	192138030	0	0.025439
27755	Del	3	192139016	192728274	0	0.017157
27756	Del	3	192729751	193232633	0	0.024848
27757	Del	3	193232968	193275216	0	0.024256
27758	Del	3	193275837	193305466	0	0.023664
27759	Del	3	193305637	193315148	0	0.024256
27760	Del	3	193315594	193396854	0	0.023664
27761	Del	3	193398605	193550164	0	0.023073
27762	Del	3	193552321	193589595	0	0.024256
27763	Del	3	193589623	193616804	0	0.023664
27764	Del	3	193617057	193629256	0	0.024256
27765	Del	3	193629844	193697999	0	0.023664
27766	Del	3	193699390	194139106	0	0.024256
27767	Del	3	194142759	194373270	0	0.025439
27768	Del	3	194373381	194481974	0	0.039046
27769	Del	3	194483635	194705013	0	0.025439
27770	Del	3	194705308	195064886	0	0.024848
27771	Del	3	195068083	195086455	0	0.024256

27772	Del	3	195094273	195097088	0	0.025439
27773	Del	3	195097358	195130485	0	0.024256
27774	Del	3	195130621	195134910	0	0.025439
27775	Del	3	195134946	195146400	0	0.024256
27776	Del	3	195147131	195152900	0	0.025439
27777	Del	3	195153226	195273899	0	0.024256
27778	Del	3	195274025	195423709	0	0.024848
27779	Del	3	195428692	196017117	0	0.025439
27780	Del	3	196019900	196234705	0	0.029581
27781	Del	3	196237039	196289275	0	0.03313
27782	Del	3	196289371	196638894	0	0.029581
27783	Del	3	196639048	196640529	0	0.036088
27784	Del	3	196641630	197242908	0	0.044371
27785	Del	3	197243254	197289929	0	0.043779
27786	Del	3	197290309	197495827	0	0.044371
27787	Del	3	197501290	197785297	0	0.036088
27788	Del	3	197789678	197812401	0	0.031947
27789	Del	4	1059384	1505243	0	0.026031
27790	Del	4	1510104	1714966	0	0.030764
27791	Del	4	1718619	2077126	0	0.026031
27792	Del	4	2079294	2226456	0	0.025439
27793	Del	4	2227708	2368407	0	0.026031
27794	Del	4	2368614	2413703	0	0.025439
27795	Del	4	2414271	2622376	0	0.026031
27796	Del	4	2622481	2732448	0	0.025439
27797	Del	4	2736363	2846456	0	0.026031
27798	Del	4	2847441	2929137	0	0.025439
27799	Del	4	2929828	2966507	0	0.026031
27800	Del	4	2968586	3042708	0	0.025439
27801	Del	4	3042814	3071644	0	0.026031
27802	Del	4	3072592	3241138	0	0.03313
27803	Del	4	3241193	3313149	0	0.034313
27804	Del	4	3313239	3436916	0	0.033722
27805	Del	4	3437624	3534275	0	0.034313
27806	Del	4	3534385	3593370	0	0.037272
27807	Del	4	3622609	3771172	0	0.042004
27808	Del	4	3772224	4184322	0	0.043188
27809	Del	4	4187229	4291513	0	0.042004
27810	Del	4	4291827	4324175	0	0.041413
27811	Del	4	4324736	4421849	0	0.042004
27812	Del	4	4422195	4472108	0	0.040821
27813	Del	4	4474189	4482200	0	0.042004
27814	Del	4	4482763	4544915	0	0.040821
27815	Del	4	4545196	4711295	0	0.04023
27816	Del	4	4711581	4912785	0	0.042004
27817	Del	4	4916244	4989354	0	0.041413
27818	Del	4	4989653	5043964	0	0.042004
27819	Del	4	5044612	5501939	0	0.037272
27820	Del	4	5502333	5559631	0	0.042004
27821	Del	4	5561280	5709607	0	0.04023
27822	Del	4	5709641	5758314	0	0.041413
27823	Del	4	5758815	5815967	0	0.040821
27824	Del	4	5817427	5893698	0	0.041413

27825	Del	4	5894071	6191432	0	0.042004
27826	Del	4	6191517	6311249	0	0.057978
27827	Del	4	6311705	6382756	0	0.057386
27828	Del	4	6382947	6421825	0	0.056795
27829	Del	4	6422300	6474078	0	0.056203
27830	Del	4	6474271	6565434	0	0.05502
27831	Del	4	6565912	6635193	0.155767	0.06981
27832	Del	4	6635844	6769764	0	0.05857
27833	Del	4	6772120	6905325	0	0.043188
27834	Del	4	6905638	6948201	0	0.042596
27835	Del	4	6952635	7028733	0	0.043188
27836	Del	4	7029350	7116761	0	0.048512
27837	Del	4	7119184	7750759	0.199449	0.072177
27838	Del	4	7751690	7780325	0.031092	0.062119
27839	Del	4	7780707	7937003	0.008183	0.059753
27840	Del	4	7937406	7961362	0.039521	0.062119
27841	Del	4	7963555	7999543	0	0.05502
27842	Del	4	8000664	8005921	0	0.056203
27843	Del	4	8006608	8077225	0	0.05502
27844	Del	4	8077342	8198161	0	0.054428
27845	Del	4	8198755	8309990	0	0.04023
27846	Del	4	8312093	8420046	0	0.044963
27847	Del	4	8423104	8579225	0	0.052653
27848	Del	4	8579386	8704195	0	0.026623
27849	Del	4	8704538	9825177	0	0.022481
27850	Del	4	9825246	10022679	0	0.021298
27851	Del	4	10023469	10048051	0	0.020706
27852	Del	4	10048642	10460350	0	0.022481
27853	Del	4	10460482	11365388	0	0.042596
27854	Del	4	11365549	11430764	0	0.022481
27855	Del	4	11431074	13365191	0	0.038455
27856	Del	4	13365766	13488182	0	0.032539
27857	Del	4	13488312	13652366	0	0.03313
27858	Del	4	13652637	13932481	0	0.030172
27859	Del	4	13932906	14468649	0	0.03313
27860	Del	4	14468703	14888686	0	0.028989
27861	Del	4	14888928	14990786	0	0.032539
27862	Del	4	14999323	15073233	0	0.03313
27863	Del	4	15073706	15337172	0	0.029581
27864	Del	4	15338318	15425644	0	0.032539
27865	Del	4	15426060	15484953	0	0.03313
27866	Del	4	15485174	15601446	0	0.031947
27867	Del	4	15601997	15769302	0	0.03313
27868	Del	4	15774558	15853557	0	0.032539
27869	Del	4	15853671	15966890	0	0.03313
27870	Del	4	15966951	16076758	0	0.031947
27871	Del	4	16078047	16087564	0	0.031355
27872	Del	4	16089627	16497575	0	0.03313
27873	Del	4	16498210	16741639	0	0.024848
27874	Del	4	16742461	17168115	0	0.026623
27875	Del	4	17169086	17628569	0	0.024256
27876	Del	4	17640962	17789471	0	0.023664
27877	Del	4	17789831	17814266	0	0.024256

27878	Del	4	17817262	17845545	0	0.015382
27879	Del	4	17845658	17880210	0	0.01479
27880	Del	4	17880253	18023745	0	0.015382
27881	Del	4	18023861	20390005	0	0.016565
27882	Del	4	20390298	20397807	0	0.01834
27883	Del	4	20398607	20526056	0	0.016565
27884	Del	4	20526138	20531391	0	0.01834
27885	Del	4	20532176	20621313	0	0.016565
27886	Del	4	20621644	20753617	0	0.017157
27887	Del	4	20754384	21305844	0	0.010057
27888	Del	4	21306158	21454370	0	0.008874
27889	Del	4	21461691	21465774	0	0.013015
27890	Del	4	21468162	21544914	0	0.008874
27891	Del	4	21547653	21699850	0	0.008283
27892	Del	4	21705962	21831471	0	0.007099
27893	Del	4	21832048	21854318	0	0.008283
27894	Del	4	21856917	21950156	0	0.007099
27895	Del	4	21950830	22384491	0	0.008283
27896	Del	4	22385993	22713441	0	0.007691
27897	Del	4	22713804	22824368	0	0.008283
27898	Del	4	22829153	23890735	0	0.013015
27899	Del	4	23891220	24803696	0	0.018932
27900	Del	4	24804530	24921482	0	0.01834
27901	Del	4	24922542	24982130	0	0.017748
27902	Del	4	24984315	25280326	0	0.018932
27903	Del	4	25280788	25529341	0	0.027214
27904	Del	4	25529828	25746639	0	0.024256
27905	Del	4	25746940	25864825	0	0.023664
27906	Del	4	25865496	26314052	0	0.024256
27907	Del	4	26318228	26435259	0	0.009466
27908	Del	4	26437686	26579832	0	0.024256
27909	Del	4	26581605	27285515	0	0.009466
27910	Del	4	27285722	30733344	0	0.013015
27911	Del	4	30735102	31148103	0	0.011241
27912	Del	4	31148519	31995521	0	0.013015
27913	Del	4	31995660	32003107	0	0.012424
27914	Del	4	32004737	32022962	0	0.013015
27915	Del	4	32028439	32155486	0	0.012424
27916	Del	4	32156847	32356023	0	0.017157
27917	Del	4	32359581	36064721	0	0.037863
27918	Del	4	36064848	36249220	0	0.03668
27919	Del	4	36249233	36274293	0	0.023073
27920	Del	4	36275251	36345870	0	0.022481
27921	Del	4	36345983	37246815	0	0.023073
27922	Del	4	37247002	37449102	0	0.021298
27923	Del	4	37449635	37582513	0	0.02189
27924	Del	4	37582543	37602948	0	0.023073
27925	Del	4	37603316	37689584	0	0.022481
27926	Del	4	37689708	37883710	0	0.023073
27927	Del	4	37887106	38106131	0	0.021298
27928	Del	4	38106190	38418196	0	0.023073
27929	Del	4	38418222	38524305	0	0.022481
27930	Del	4	38525214	38795256	0	0.023073

27931	Del	4	38796195	38870788	0	0.018932
27932	Del	4	38871675	38949491	0	0.01834
27933	Del	4	38950409	39180276	0	0.018932
27934	Del	4	39181827	39285329	0	0.01834
27935	Del	4	39286037	39786790	0	0.018932
27936	Del	4	39787228	39894918	0	0.035497
27937	Del	4	39897915	40038909	0	0.036088
27938	Del	4	40041046	40355309	0	0.023664
27939	Del	4	40355513	40686719	0	0.043188
27940	Del	4	40687746	40811277	0	0.026623
27941	Del	4	40813251	40862050	0	0.02189
27942	Del	4	40862104	41218271	0	0.018932
27943	Del	4	41218799	41345385	0	0.02189
27944	Del	4	41347183	41530086	0	0.019523
27945	Del	4	41531183	41611914	0	0.020706
27946	Del	4	41612471	41700887	0	0.021298
27947	Del	4	41700911	41992032	0	0.02189
27948	Del	4	41992628	42087535	0	0.021298
27949	Del	4	42094357	42294301	0	0.02189
27950	Del	4	42298978	42391511	0	0.017748
27951	Del	4	42393803	42404571	0	0.01834
27952	Del	4	42404679	42659549	0	0.031355
27953	Del	4	42660266	43321532	0	0.03313
27954	Del	4	43322138	44165751	0	0.037272
27955	Del	4	44168648	44477302	0	0.022481
27956	Del	4	44478641	44728820	0	0.023073
27957	Del	4	44732829	46210877	0	0.03668
27958	Del	4	46211510	46909362	0	0.017748
27959	Del	4	46915550	47028679	0	0.01834
27960	Del	4	47029476	47427112	0	0.011832
27961	Del	4	47427790	47482628	0	0.013607
27962	Del	4	47483094	47593834	0	0.013015
27963	Del	4	47594153	47636251	0	0.012424
27964	Del	4	47636667	47649147	0	0.013015
27965	Del	4	47649987	47661837	0	0.013607
27966	Del	4	47662105	47827124	0	0.013015
27967	Del	4	47831278	51838342	0	0.013607
27968	Del	4	51840547	52872349	0	0.007691
27969	Del	4	52872357	53369196	0	0.006508
27970	Del	4	53369526	53460566	0	0.007691
27971	Del	4	53462101	53499140	0	0.007099
27972	Del	4	53499666	53524782	0	0.007691
27973	Del	4	53524984	53590202	0	0.007099
27974	Del	4	53590219	53734391	0	0.007691
27975	Del	4	53734456	54227522	0	0.014199
27976	Del	4	54227808	54299800	0	0.013015
27977	Del	4	54300700	54607300	0	0.014199
27978	Del	4	54609242	55070601	0	0.033722
27979	Del	4	55078133	55431280	0	0.01834
27980	Del	4	55431842	55547636	0	0.017748
27981	Del	4	55547664	56163669	0	0.01834
27982	Del	4	56166803	56329803	0	0.017157
27983	Del	4	56331527	56823693	0	0.01834

27984	Del	4	56824108	56982116	0	0.036088
27985	Del	4	56983085	57031698	0	0.01834
27986	Del	4	57032064	57105703	0	0.017748
27987	Del	4	57105753	57466128	0	0.01834
27988	Del	4	57466591	58553007	0	0.027214
27989	Del	4	58553108	60917743	0	0.027806
27990	Del	4	60922000	61191890	0	0.024256
27991	Del	4	61194396	62053734	0	0.019523
27992	Del	4	62057573	62162060	0	0.024256
27993	Del	4	62166771	64416669	0	0.028397
27994	Del	4	64422881	65319272	0	0.03313
27995	Del	4	65319480	65662904	0	0.026623
27996	Del	4	65669116	65700083	0	0.029581
27997	Del	4	65701338	67415722	0	0.031947
27998	Del	4	67416143	67471280	0	0.026031
27999	Del	4	67472086	67557571	0	0.017157
28000	Del	4	67558394	67884996	0	0.010649
28001	Del	4	67885506	68305063	0	0.01479
28002	Del	4	68305147	69922992	0	0.008874
28003	Del	4	69927553	70251485	0	0.004733
28004	Del	4	70251805	71183614	0	0.013015
28005	Del	4	71184325	71326423	0	0.011832
28006	Del	4	71334071	71581029	0	0.013015
28007	Del	4	71581704	72028694	0	0.017748
28008	Del	4	72030740	72571594	0	0.014199
28009	Del	4	72571744	73070191	0	0.015382
28010	Del	4	73077449	73261137	0	0.01479
28011	Del	4	73265584	73736009	0	0.015382
28012	Del	4	73740487	74303864	0	0.009466
28013	Del	4	74304205	74457928	0	0.010057
28014	Del	4	74462391	74931578	0	0.016565
28015	Del	4	74932626	74953954	0	0.015974
28016	Del	4	74954088	74970639	0	0.016565
28017	Del	4	74972638	75051866	0	0.015974
28018	Del	4	75051915	75674255	0	0.024848
28019	Del	4	75674598	75902072	0	0.033722
28020	Del	4	75905030	76009761	0	0.036088
28021	Del	4	76010722	76016341	0	0.034905
28022	Del	4	76018741	76024369	0	0.036088
28023	Del	4	76030397	76031924	0	0.034905
28024	Del	4	76033099	76037181	0	0.036088
28025	Del	4	76038471	76074177	0	0.034905
28026	Del	4	76074310	76156466	0	0.036088
28027	Del	4	76156732	76208119	0	0.035497
28028	Del	4	76208415	76311590	0	0.044963
28029	Del	4	76313605	76429665	0	0.039046
28030	Del	4	76432269	76571501	0	0.030172
28031	Del	4	76572649	76579794	0	0.03313
28032	Del	4	76580204	76786145	0	0.030172
28033	Del	4	76786155	76899479	0	0.03313
28034	Del	4	76900178	77040334	0	0.046146
28035	Del	4	77045320	77172582	0	0.047329
28036	Del	4	77174097	77821363	0	0.052653

28037	Del	4	77824487	78054122	0.019343	0.060936
28038	Del	4	78055419	78451471	0	0.048512
28039	Del	4	78452100	78544765	0	0.046737
28040	Del	4	78549214	78769092	0	0.052653
28041	Del	4	78771865	78817444	0	0.052062
28042	Del	4	78818095	78826050	0	0.052653
28043	Del	4	78826655	78904020	0	0.052062
28044	Del	4	78907896	78970410	0	0.052653
28045	Del	4	78971657	79311761	0	0.049104
28046	Del	4	79313679	79484702	0	0.052653
28047	Del	4	79488077	79576928	0	0.052062
28048	Del	4	79578293	79863841	0	0.052653
28049	Del	4	79867628	79975257	0.20327	0.072768
28050	Del	4	79975588	80180334	0.213167	0.07336
28051	Del	4	80181905	80331565	0.092751	0.065669
28052	Del	4	80334596	80967733	0.010918	0.060344
28053	Del	4	80968220	81083087	0.08527	0.065669
28054	Del	4	81084771	81161584	0.080839	0.065077
28055	Del	4	81162619	81163911	0.068346	0.064486
28056	Del	4	81164043	81195150	0.088286	0.065669
28057	Del	4	81195523	81221397	0.068346	0.064486
28058	Del	4	81221934	82481915	0.173538	0.070402
28059	Del	4	82482064	82566418	0.157251	0.06981
28060	Del	4	82569245	82627966	0.17003	0.070402
28061	Del	4	82627998	82655724	0.114479	0.067444
28062	Del	4	82655820	82750960	0.127723	0.068035
28063	Del	4	82751010	82759399	0.173853	0.070402
28064	Del	4	82761047	82806210	0.127723	0.068035
28065	Del	4	82809265	82889421	0.154443	0.06981
28066	Del	4	82890422	82913432	0.172477	0.070402
28067	Del	4	82914852	83014290	0.324584	0.078684
28068	Del	4	83017171	83114816	0.335265	0.079276
28069	Del	4	83114885	83289563	0.519992	0.086967
28070	Del	4	83292417	83451106	0.506786	0.086375
28071	Del	4	83451845	83530874	0.52148	0.086967
28072	Del	4	83533036	83964139	0.324584	0.078684
28073	Del	4	83966130	84305515	0.030493	0.061528
28074	Del	4	84306627	84579048	0.105108	0.066261
28075	Del	4	84579434	84667297	0.090652	0.065669
28076	Del	4	84667625	84962553	0.054996	0.063302
28077	Del	4	84964893	85471784	0.098676	0.066261
28078	Del	4	85473865	85716836	0	0.059161
28079	Del	4	85717504	85722550	0.010637	0.059753
28080	Del	4	85722589	86012049	0	0.059161
28081	Del	4	86012198	86117611	0	0.055612
28082	Del	4	86119666	86221339	0	0.05857
28083	Del	4	86222282	86374085	0	0.055612
28084	Del	4	86375867	86458500	0	0.05502
28085	Del	4	86460806	86583818	0.010637	0.059753
28086	Del	4	86590418	86816260	0	0.057978
28087	Del	4	86816705	86936439	0.005546	0.059753
28088	Del	4	86940182	87000671	0	0.057386
28089	Del	4	87006382	87142591	0	0.057978

28090	Del	4	87144821	87225819	0.000204	0.059161
28091	Del	4	87229398	87303325	0.439408	0.083417
28092	Del	4	87303766	87472628	0.008183	0.059753
28093	Del	4	87473161	87532285	0	0.059161
28094	Del	4	87532759	87820686	0.00891	0.059753
28095	Del	4	87821166	87851943	0	0.059161
28096	Del	4	87852202	88005469	0.008183	0.059753
28097	Del	4	88006555	88080033	0	0.05857
28098	Del	4	88080741	88160289	0	0.059161
28099	Del	4	88165451	88231505	0	0.05857
28100	Del	4	88231566	88282941	0.006672	0.059753
28101	Del	4	88283205	88443344	0.068346	0.063894
28102	Del	4	88454800	88585395	0.068346	0.064486
28103	Del	4	88586224	88660900	0.064924	0.063894
28104	Del	4	88663273	88687074	0.054752	0.063302
28105	Del	4	88693419	88698405	0.075677	0.064486
28106	Del	4	88699921	88707849	0.054752	0.063302
28107	Del	4	88707915	88738990	0.071606	0.064486
28108	Del	4	88739422	88827731	0.059256	0.063302
28109	Del	4	88828480	89106269	0.021665	0.060936
28110	Del	4	89106700	89116902	0	0.057386
28111	Del	4	89121281	89723357	0.06297	0.063894
28112	Del	4	89723502	89835270	0	0.056203
28113	Del	4	89835399	89883492	0	0.057386
28114	Del	4	89886965	90033399	0	0.056795
28115	Del	4	90036504	91604044	0.046253	0.062711
28116	Del	4	91604883	92300180	0.059713	0.063302
28117	Del	4	92300992	93828767	0	0.057978
28118	Del	4	93828907	94114600	0	0.05502
28119	Del	4	94116052	94251566	0	0.054428
28120	Del	4	94251761	94458024	0	0.05502
28121	Del	4	94458671	94588612	0	0.053837
28122	Del	4	94588732	94668326	0	0.052653
28123	Del	4	94670825	94752838	0.017054	0.060344
28124	Del	4	94754467	94993247	0.158066	0.06981
28125	Del	4	94996129	95048017	0.210177	0.072768
28126	Del	4	95048906	95838297	0.227278	0.073952
28127	Del	4	95838993	95841839	0.191385	0.071585
28128	Del	4	95842826	97553230	0.202189	0.072177
28129	Del	4	97555331	98257197	0.128286	0.068035
28130	Del	4	98257449	98464148	0.092751	0.065669
28131	Del	4	98466676	98661839	0.080136	0.065077
28132	Del	4	98662488	98934090	0.113619	0.066852
28133	Del	4	98934235	98997510	0.191385	0.071585
28134	Del	4	98997742	99064585	0.18246	0.070993
28135	Del	4	99064872	99090677	0.188082	0.071585
28136	Del	4	99092657	99121615	0.157014	0.06981
28137	Del	4	99122354	99155152	0.190784	0.071585
28138	Del	4	99155730	99157733	0.157014	0.06981
28139	Del	4	99158100	99164856	0.191385	0.071585
28140	Del	4	99168003	99202029	0.157014	0.06981
28141	Del	4	99202619	99220044	0.190784	0.071585
28142	Del	4	99222940	99276138	0.157014	0.06981

28143	Del	4	99276202	99292187	0.189847	0.071585
28144	Del	4	99292276	99301933	0.157014	0.06981
28145	Del	4	99303683	99565021	0.188082	0.071585
28146	Del	4	99565161	99870301	0.18314	0.070993
28147	Del	4	99870648	99952742	0.191059	0.071585
28148	Del	4	99953103	100037983	0.179951	0.070993
28149	Del	4	100038214	100397108	0.191059	0.071585
28150	Del	4	100397225	100520638	0.161867	0.06981
28151	Del	4	100521275	100675366	0.189847	0.071585
28152	Del	4	100676043	101787962	0.258557	0.075726
28153	Del	4	101788173	101810943	0.030769	0.061528
28154	Del	4	101813573	102077524	0.034805	0.062119
28155	Del	4	102077710	102261331	0.056294	0.063302
28156	Del	4	102261459	102325223	0.061261	0.063894
28157	Del	4	102326829	102417532	0.060057	0.063302
28158	Del	4	102418518	102503074	0.004389	0.059753
28159	Del	4	102511817	102774743	0	0.059161
28160	Del	4	102775653	102846182	0	0.055612
28161	Del	4	102847107	102857314	0	0.05502
28162	Del	4	102867054	102895956	0	0.055612
28163	Del	4	102911530	103019522	0	0.05502
28164	Del	4	103023233	103100031	0	0.055612
28165	Del	4	103100095	103202515	0.034001	0.062119
28166	Del	4	103203943	103588600	0.048332	0.062711
28167	Del	4	103589405	103720707	0.030373	0.061528
28168	Del	4	103720763	104496433	0.117228	0.067444
28169	Del	4	104496694	104701649	0.080445	0.065077
28170	Del	4	104702092	105243823	0.138927	0.068627
28171	Del	4	105244013	105279964	0.12785	0.068035
28172	Del	4	105283736	105353970	0.120571	0.067444
28173	Del	4	105355420	105560516	0.132644	0.068627
28174	Del	4	105561558	105571911	0.156974	0.06981
28175	Del	4	105574874	105635886	0.13479	0.068627
28176	Del	4	105636272	105681909	0.12206	0.068035
28177	Del	4	105681941	105710080	0.161173	0.06981
28178	Del	4	105710947	105812281	0.135239	0.068627
28179	Del	4	105812602	105828575	0.161173	0.06981
28180	Del	4	105829223	105848177	0.135239	0.068627
28181	Del	4	105849414	105974417	0.149042	0.069219
28182	Del	4	105975256	106029576	0.158718	0.06981
28183	Del	4	106033673	106313012	0.17003	0.070402
28184	Del	4	106313749	106315711	0.211554	0.072768
28185	Del	4	106316493	106918781	0.21253	0.07336
28186	Del	4	106918841	107037845	0.191167	0.071585
28187	Del	4	107038187	107724191	0.21253	0.07336
28188	Del	4	107729221	107877715	0.411538	0.082826
28189	Del	4	107885188	107915970	0.436775	0.083417
28190	Del	4	107916653	107928052	0.42004	0.082826
28191	Del	4	107930839	107987355	0.432942	0.083417
28192	Del	4	107989436	108047065	0.229064	0.073952
28193	Del	4	108047346	108163414	0.210298	0.072768
28194	Del	4	108163678	108536751	0.247213	0.074543
28195	Del	4	108537568	108554876	0	0.056203

28196	Del	4	108555364	108560702	0	0.056795
28197	Del	4	108561639	108617096	0	0.056203
28198	Del	4	108617265	108772770	0	0.056795
28199	Del	4	108773327	109304633	0.008219	0.059753
28200	Del	4	109305250	109482007	0.018569	0.060936
28201	Del	4	109485701	109489513	0.030769	0.061528
28202	Del	4	109491081	109556247	0.018569	0.060936
28203	Del	4	109558999	109686339	0	0.053245
28204	Del	4	109686810	109733484	0	0.054428
28205	Del	4	109739342	109804592	0	0.053837
28206	Del	4	109805166	109844780	0	0.054428
28207	Del	4	109848098	109911150	0.053072	0.063302
28208	Del	4	109911265	110043971	0.030624	0.061528
28209	Del	4	110044315	110200685	0	0.052062
28210	Del	4	110203627	110564861	0	0.053837
28211	Del	4	110565006	110860763	0	0.054428
28212	Del	4	110861222	112294708	0	0.059161
28213	Del	4	112295414	112442972	0	0.057386
28214	Del	4	112443069	112532301	0.00167	0.059161
28215	Del	4	112538249	112621316	0	0.05857
28216	Del	4	112623526	112813278	0.000634	0.059161
28217	Del	4	112817084	113046338	0	0.052062
28218	Del	4	113046786	113105376	0	0.054428
28219	Del	4	113105524	113107846	0.00167	0.059161
28220	Del	4	113108190	113150036	0	0.054428
28221	Del	4	113150971	113154532	0.00167	0.059161
28222	Del	4	113154556	113384076	0	0.054428
28223	Del	4	113386705	113750971	0	0.055612
28224	Del	4	113754303	113896834	0.000634	0.059161
28225	Del	4	113897843	113979801	0	0.057978
28226	Del	4	113982975	114651317	0.086591	0.065669
28227	Del	4	114654823	114656964	0.10539	0.066261
28228	Del	4	114662309	114821442	0.088842	0.065669
28229	Del	4	114824129	116296577	0.043649	0.062711
28230	Del	4	116296972	116305227	0	0.046146
28231	Del	4	116305634	117086397	0	0.049104
28232	Del	4	117090393	118028723	0.074001	0.064486
28233	Del	4	118028996	118258716	0	0.039046
28234	Del	4	118261249	118282588	0	0.040821
28235	Del	4	118287229	118355674	0	0.04023
28236	Del	4	118409574	118720786	0	0.040821
28237	Del	4	118720855	118796558	0	0.04023
28238	Del	4	118849901	118887074	0	0.05857
28239	Del	4	118887423	119040950	0.004389	0.059753
28240	Del	4	119041432	119134644	0	0.059161
28241	Del	4	119135270	119191174	0	0.049695
28242	Del	4	119191317	119203702	0	0.046737
28243	Del	4	119204205	119297953	0	0.057978
28244	Del	4	119301728	119394491	0	0.046737
28245	Del	4	119396355	119450118	0	0.042596
28246	Del	4	119453484	119502490	0	0.045554
28247	Del	4	119502780	119797703	0	0.03668
28248	Del	4	119798662	119965425	0	0.036088

28249	Del	4	119968344	120068698	0	0.042596
28250	Del	4	120068926	120924959	0	0.043188
28251	Del	4	120928873	121172183	0	0.045554
28252	Del	4	121173090	121327497	0	0.044963
28253	Del	4	121327633	121655900	0	0.044371
28254	Del	4	121656357	122167710	0	0.035497
28255	Del	4	122167735	122364394	0	0.034905
28256	Del	4	122366505	122456718	0	0.035497
28257	Del	4	122470309	122621244	0	0.038455
28258	Del	4	122624987	122689551	0	0.037863
28259	Del	4	122689919	122825728	0	0.038455
28260	Del	4	122825787	122890317	0	0.037863
28261	Del	4	122892624	122918872	0	0.038455
28262	Del	4	122918909	123320723	0	0.048512
28263	Del	4	123321313	123649253	0	0.052062
28264	Del	4	123649352	123772666	0	0.049104
28265	Del	4	123773839	123868257	0	0.050879
28266	Del	4	123868390	123930571	0	0.049104
28267	Del	4	123930745	124558604	0	0.05147
28268	Del	4	124560430	125304561	0	0.052062
28269	Del	4	125313572	125503534	0	0.042596
28270	Del	4	125505588	125508116	0	0.041413
28271	Del	4	125509175	127970650	0	0.047329
28272	Del	4	127977878	128040352	0	0.046737
28273	Del	4	128043832	128124162	0	0.047329
28274	Del	4	128128216	128212319	0	0.046737
28275	Del	4	128212785	128426630	0	0.047329
28276	Del	4	128427604	128519848	0	0.046737
28277	Del	4	128520596	129723090	0.2281	0.073952
28278	Del	4	129723404	129774009	0.035096	0.062119
28279	Del	4	129774627	131794311	0.074577	0.064486
28280	Del	4	131797990	132683410	0.079671	0.065077
28281	Del	4	132683872	133148512	0.126899	0.068035
28282	Del	4	133148642	137146964	0.141323	0.068627
28283	Del	4	137154526	137227350	0.075677	0.064486
28284	Del	4	137228783	137532988	0.114364	0.066852
28285	Del	4	137537491	138087258	0.254464	0.075135
28286	Del	4	138087983	138178347	0.371818	0.081051
28287	Del	4	138179218	138302213	0.367207	0.080459
28288	Del	4	138304353	138819697	0.236332	0.073952
28289	Del	4	138819781	139048316	0	0.05147
28290	Del	4	139049166	139247847	0.429033	0.083417
28291	Del	4	139263612	139302839	0.39272	0.081642
28292	Del	4	139304286	139394675	0.181382	0.070993
28293	Del	4	139396727	139492921	0.189847	0.071585
28294	Del	4	139494556	139527949	0.175939	0.070993
28295	Del	4	139528670	139660953	0.18696	0.071585
28296	Del	4	139665547	139704619	0	0.05502
28297	Del	4	139705221	139742467	0	0.054428
28298	Del	4	139743403	140251786	0	0.047921
28299	Del	4	140255844	140341925	0	0.045554
28300	Del	4	140343413	140439743	0	0.046146
28301	Del	4	140441397	140499684	0	0.045554

28302	Del	4	140500092	140619761	0	0.046146
28303	Del	4	140620410	140640073	0	0.044371
28304	Del	4	140640199	140644612	0	0.046146
28305	Del	4	140645899	140865321	0	0.044371
28306	Del	4	140865391	141137620	0	0.043188
28307	Del	4	141142532	141630301	0	0.045554
28308	Del	4	141635823	142022393	0	0.023073
28309	Del	4	142022927	142027678	0	0.013607
28310	Del	4	142028244	142848806	0	0.014199
28311	Del	4	142850172	143327942	0	0.01834
28312	Del	4	143334685	143334685	0	0.017748
28313	Del	4	143343197	143343894	0	0.01834
28314	Del	4	143344260	143475521	0	0.017748
28315	Del	4	143478068	143575127	0	0.01834
28316	Del	4	143575324	143703174	0	0.017748
28317	Del	4	143705857	144648540	0	0.021298
28318	Del	4	144648803	144983475	0	0.020706
28319	Del	4	144992689	145745278	0	0.019523
28320	Del	4	145755053	145943446	0	0.01834
28321	Del	4	145944315	146191308	0	0.019523
28322	Del	4	146191343	146404773	0	0.025439
28323	Del	4	146406555	146417035	0	0.027214
28324	Del	4	146417638	146437969	0	0.025439
28325	Del	4	146440938	146631185	0	0.027214
28326	Del	4	146632651	146701728	0	0.019523
28327	Del	4	146704702	147344036	0	0.018932
28328	Del	4	147344305	147480038	0	0.015974
28329	Del	4	147480246	147546013	0	0.015382
28330	Del	4	147548390	147688098	0	0.015974
28331	Del	4	147691337	148076251	0	0.023073
28332	Del	4	148076401	148448079	0	0.022481
28333	Del	4	148448455	150076820	0	0.024848
28334	Del	4	150077184	150262702	0	0.01834
28335	Del	4	150262719	150572269	0	0.016565
28336	Del	4	150578465	150587561	0	0.019523
28337	Del	4	150588687	151103174	0	0.016565
28338	Del	4	151104687	151402950	0	0.015974
28339	Del	4	151407666	151763355	0	0.015382
28340	Del	4	151764019	152107279	0	0.015974
28341	Del	4	152107314	152382573	0	0.030172
28342	Del	4	152385057	152486860	0	0.029581
28343	Del	4	152489238	152490691	0	0.030172
28344	Del	4	152512299	152529675	0	0.029581
28345	Del	4	152529841	152782628	0	0.030172
28346	Del	4	152785768	152912572	0	0.029581
28347	Del	4	152916694	153151701	0	0.030172
28348	Del	4	153152920	153198781	0	0.028989
28349	Del	4	153199617	153249880	0	0.029581
28350	Del	4	153256204	153278494	0	0.030172
28351	Del	4	153279168	153304436	0	0.028989
28352	Del	4	153304779	153311185	0	0.030172
28353	Del	4	153311370	153341147	0	0.028989
28354	Del	4	153342924	153470738	0	0.030172

28355	Del	4	153470964	153637796	0	0.028989
28356	Del	4	153637812	153706503	0	0.030172
28357	Del	4	153707232	153761411	0	0.029581
28358	Del	4	153764139	154234166	0	0.030172
28359	Del	4	154234251	154492878	0	0.028989
28360	Del	4	154494188	154614331	0	0.030172
28361	Del	4	154614472	154829217	0	0.03313
28362	Del	4	154830365	155662610	0	0.036088
28363	Del	4	155664314	155742338	0	0.029581
28364	Del	4	155743591	155868459	0	0.030172
28365	Del	4	155869264	155955970	0	0.031947
28366	Del	4	155959729	156973222	0	0.033722
28367	Del	4	156974528	157370374	0	0.034905
28368	Del	4	157371890	158521012	0	0.035497
28369	Del	4	158521187	158653811	0	0.034905
28370	Del	4	158656954	158763718	0	0.035497
28371	Del	4	158764591	158908601	0	0.034905
28372	Del	4	158911454	159036028	0	0.034313
28373	Del	4	159041890	159265180	0	0.035497
28374	Del	4	159267302	159360910	0	0.034905
28375	Del	4	159361536	162018735	0	0.036088
28376	Del	4	162019116	162055225	0	0.042004
28377	Del	4	162057999	162165337	0	0.036088
28378	Del	4	162165466	163104376	0	0.04023
28379	Del	4	163106515	163313012	0	0.047329
28380	Del	4	163314841	163520683	0	0.043188
28381	Del	4	163521112	163614467	0	0.042004
28382	Del	4	163614904	164195577	0	0.036088
28383	Del	4	164195961	164201580	0	0.043188
28384	Del	4	164201659	164385702	0	0.036088
28385	Del	4	164386782	165375112	0	0.044371
28386	Del	4	165378427	165383316	0	0.043188
28387	Del	4	165383502	165387517	0	0.044371
28388	Del	4	165387936	165684211	0	0.043188
28389	Del	4	165684444	165724484	0	0.040821
28390	Del	4	165725012	165743466	0	0.041413
28391	Del	4	165743739	165872049	0	0.040821
28392	Del	4	165872291	166031900	0	0.03668
28393	Del	4	166032219	166731635	0	0.036088
28394	Del	4	166731855	167236704	0	0.034313
28395	Del	4	167237817	168213390	0	0.044963
28396	Del	4	168213432	168341032	0	0.043779
28397	Del	4	168354867	168388827	0	0.043188
28398	Del	4	168388867	168493278	0	0.043779
28399	Del	4	168494992	168624019	0	0.039046
28400	Del	4	168628495	168826742	0	0.040821
28401	Del	4	168829848	168931567	0	0.043779
28402	Del	4	168941048	169091503	0	0.044371
28403	Del	4	169092759	169614807	0	0.043188
28404	Del	4	169615536	169724892	0	0.046737
28405	Del	4	169728483	169912994	0	0.044371
28406	Del	4	169915093	169977243	0	0.043779
28407	Del	4	169978203	170100520	0	0.044371

28408	Del	4	170105841	170741956	0	0.053837
28409	Del	4	170742097	171186954	0	0.039046
28410	Del	4	171189186	171810430	0	0.056203
28411	Del	4	171812159	173041855	0	0.057386
28412	Del	4	173042094	173141130	0.130854	0.068035
28413	Del	4	173143216	173167897	0.133393	0.068627
28414	Del	4	173167929	173267011	0.154605	0.06981
28415	Del	4	173267063	173269071	0.185757	0.070993
28416	Del	4	173269073	173324118	0.154605	0.06981
28417	Del	4	173326484	173379866	0.185757	0.070993
28418	Del	4	173379887	173514476	0.254464	0.075135
28419	Del	4	173514776	173587054	0.502646	0.086375
28420	Del	4	173587486	174089322	0.498396	0.085784
28421	Del	4	174091537	174226819	0.213167	0.07336
28422	Del	4	174233156	174274453	0.24707	0.074543
28423	Del	4	174282826	174634571	0.252764	0.075135
28424	Del	4	174641615	174827244	0.200038	0.072177
28425	Del	4	174827864	174913246	0.319248	0.078093
28426	Del	4	174913641	174980622	0.309388	0.077501
28427	Del	4	174980696	175789794	0.686326	0.092883
28428	Del	4	175790040	175813451	0.751164	0.09525
28429	Del	4	175815320	176058018	0.669585	0.092291
28430	Del	4	176063211	176170995	0.639222	0.0917
28431	Del	4	176182453	176211146	0.677002	0.092883
28432	Del	4	176212537	176270634	0.649601	0.0917
28433	Del	4	176271909	176333213	0.681991	0.092883
28434	Del	4	176333421	176797225	0.698924	0.093475
28435	Del	4	176798359	177363461	0.981203	0.10294
28436	Del	4	177365524	177443824	1.263763	0.112406
28437	Del	4	177447580	177992662	2.160629	0.136662
28438	Del	4	177992834	182138243	4.162714	0.17985
28439	Del	4	182138389	182241846	1.005913	0.103532
28440	Del	4	182241904	182613999	0.883184	0.099391
28441	Del	4	182614883	183314788	1.237525	0.111223
28442	Del	4	183314971	183512578	1.258453	0.111815
28443	Del	4	183512904	184336541	1.44003	0.117731
28444	Del	4	184340382	184385856	1.258453	0.111815
28445	Del	4	184385891	184475842	1.168014	0.109448
28446	Del	4	184478744	184753159	1.254226	0.111815
28447	Del	4	184753979	184797573	1.225296	0.111223
28448	Del	4	184797678	184800216	1.258453	0.111815
28449	Del	4	184800270	184805849	1.225296	0.111223
28450	Del	4	184807110	184824036	1.220867	0.110631
28451	Del	4	184827652	185400293	1.258453	0.111815
28452	Del	4	185400570	185433569	1.23952	0.111223
28453	Del	4	185438837	185500412	1.191916	0.11004
28454	Del	4	185500570	185584039	1.167163	0.109448
28455	Del	4	185585011	185657351	1.153269	0.108857
28456	Del	4	185657375	185776340	1.05277	0.105307
28457	Del	4	185777877	185812426	1.03207	0.104715
28458	Del	4	185813326	185958460	0.948972	0.101757
28459	Del	4	185958590	186193766	1.193113	0.11004
28460	Del	4	186193893	186220703	1.175474	0.109448

28461	Del	4	186223046	186292335	0.920759	0.100574
28462	Del	4	186295234	186412637	0.827734	0.097616
28463	Del	4	186413734	186462736	0.896817	0.099982
28464	Del	4	186462751	186583882	0.936194	0.101166
28465	Del	4	186587359	186726070	0.845034	0.098208
28466	Del	4	186731106	187507327	1.01864	0.104124
28467	Del	4	187507586	187673124	1.051702	0.105307
28468	Del	4	187673475	187842528	1.125324	0.107673
28469	Del	5	914118	986438	0	0.018932
28470	Del	5	986593	2757845	0	0.017748
28471	Del	5	2759136	3416258	0	0.02189
28472	Del	5	3417121	3494139	0	0.017157
28473	Del	5	3494574	3505546	0	0.017748
28474	Del	5	3507218	3538259	0	0.017157
28475	Del	5	3538648	3612112	0	0.017748
28476	Del	5	3612334	5033540	0	0.030172
28477	Del	5	5033713	5143690	0	0.024848
28478	Del	5	5146919	5320905	0	0.022481
28479	Del	5	5328277	6304093	0	0.030764
28480	Del	5	6309822	7392820	0	0.024848
28481	Del	5	7394437	7828420	0	0.02189
28482	Del	5	7828454	8330897	0	0.024848
28483	Del	5	8331041	8456597	0	0.017157
28484	Del	5	8456618	9026666	0	0.017748
28485	Del	5	9030939	9050708	0	0.014199
28486	Del	5	9053716	9054972	0	0.017748
28487	Del	5	9057244	9510024	0	0.014199
28488	Del	5	9510965	9519387	0	0.017748
28489	Del	5	9519483	9540742	0	0.014199
28490	Del	5	9542919	9639299	0	0.017748
28491	Del	5	9639895	10221902	0	0.015974
28492	Del	5	10222067	10446002	0	0.012424
28493	Del	5	10446012	10523889	0	0.007099
28494	Del	5	10524391	10658401	0	0.012424
28495	Del	5	10661816	10968760	0	0.013015
28496	Del	5	10970481	11589405	0	0.004733
28497	Del	5	11589426	11905214	0	0.00355
28498	Del	5	11906200	13689765	0	0.007099
28499	Del	5	13689807	14138362	0	0.005916
28500	Del	5	14138753	14512660	0	0.005325
28501	Del	5	14512710	14700155	0	0.007099
28502	Del	5	14705556	14712518	0	0.006508
28503	Del	5	14712600	14716917	0	0.007099
28504	Del	5	14717927	14821408	0	0.006508
28505	Del	5	14821930	14826440	0	0.007099
28506	Del	5	14827165	14875575	0	0.006508
28507	Del	5	14875759	16510960	0	0.007099
28508	Del	5	16512133	16614133	0	0.006508
28509	Del	5	16614589	16659249	0	0.007099
28510	Del	5	16660036	16937866	0	0.006508
28511	Del	5	16938460	19469300	0	0.007099
28512	Del	5	19471539	19992635	0	0.005916
28513	Del	5	19992802	20576293	0	0.004733

28514	Del	5	20582675	21591392	0	0.011241
28515	Del	5	21592829	22222352	0	0.021298
28516	Del	5	22225303	23496520	0	0.01834
28517	Del	5	23497300	23529389	0	0.008283
28518	Del	5	23529953	24646717	0	0.014199
28519	Del	5	24646745	26879530	0	0.01479
28520	Del	5	26879935	27038880	0	0.014199
28521	Del	5	27044180	28282444	0	0.01479
28522	Del	5	28283377	28288879	0	0.014199
28523	Del	5	28289465	29396937	0	0.01834
28524	Del	5	29397764	31192603	0	0.019523
28525	Del	5	31192947	31329224	0	0.010649
28526	Del	5	31329426	31535247	0	0.011832
28527	Del	5	31537442	31559182	0	0.011241
28528	Del	5	31559261	32221100	0	0.039638
28529	Del	5	32226558	32706099	0	0.011241
28530	Del	5	32706392	32792663	0	0.010649
28531	Del	5	32793068	33522036	0	0.011241
28532	Del	5	33522097	33935838	0	0.009466
28533	Del	5	33935971	33942580	0	0.006508
28534	Del	5	33943487	34834247	0	0.014199
28535	Del	5	34837640	35118654	0	0.007691
28536	Del	5	35120749	35230806	0	0.007099
28537	Del	5	35231885	35850873	0	0.017748
28538	Del	5	35852627	36302076	0	0.007691
28539	Del	5	36308066	36688435	0	0.015974
28540	Del	5	36689079	36878795	0	0.016565
28541	Del	5	36880589	37245488	0	0.015974
28542	Del	5	37246385	37376194	0	0.016565
28543	Del	5	37376848	38255829	0	0.009466
28544	Del	5	38255953	38280615	0	0.008283
28545	Del	5	38280698	38291874	0	0.009466
28546	Del	5	38292290	38399059	0	0.008283
28547	Del	5	38399620	38693016	0	0.009466
28548	Del	5	38693207	38707961	0	0.008874
28549	Del	5	38708008	38720903	0	0.009466
28550	Del	5	38721537	38842308	0	0.008874
28551	Del	5	38843096	39104729	0	0.009466
28552	Del	5	39104816	39219953	0	0.001183
28553	Del	5	39220711	39270761	0	0.000592
28554	Del	5	39271545	40055155	0	0.001183
28555	Del	5	40055267	40992902	0	0.002366
28556	Del	5	40993530	41727113	0	0.001183
28557	Del	5	41730005	41862698	0	0.000592
28558	Del	5	41869256	41872411	0	0.001183
28559	Del	5	41873422	42176020	0	0.007691
28560	Del	5	42178480	42547153	0	0.015382
28561	Del	5	42547922	42750474	0	0.015974
28562	Del	5	42755176	44817207	0	0.001183
28563	Del	5	44817255	45378354	0	0.034905
28564	Del	5	45383300	50970623	0	0.043779
28565	Del	5	50970696	51395750	0	0.054428
28566	Del	5	51396561	52802921	0.338518	0.079276

28567	Del	5	52802961	52954054	0.267984	0.075726
28568	Del	5	52955254	53094943	0.301686	0.077501
28569	Del	5	53095400	53472858	0.339175	0.079276
28570	Del	5	53473802	53556260	0.282791	0.076318
28571	Del	5	53559260	54311064	0.250329	0.075135
28572	Del	5	54311310	54418429	0.313699	0.078093
28573	Del	5	54418489	54524437	0.342947	0.079276
28574	Del	5	54524708	54704459	0.329609	0.078684
28575	Del	5	54705403	54747670	0.309388	0.077501
28576	Del	5	54748126	55112788	0.334691	0.079276
28577	Del	5	55112883	55159611	0.32105	0.078684
28578	Del	5	55159878	55168658	0.34021	0.079276
28579	Del	5	55168851	55168851	0.32105	0.078684
28580	Del	5	55170517	55309918	0.342947	0.079276
28581	Del	5	55313594	55507225	0.310716	0.078093
28582	Del	5	55507672	55511248	0.342947	0.079276
28583	Del	5	55511292	55515346	0.320026	0.078093
28584	Del	5	55517640	55610166	0.341247	0.079276
28585	Del	5	55615559	55725113	0.328115	0.078684
28586	Del	5	55735116	55763868	0.320026	0.078093
28587	Del	5	55763922	55989706	0.329191	0.078684
28588	Del	5	55990363	56096718	0.341801	0.079276
28589	Del	5	56098029	56233521	0.298125	0.077501
28590	Del	5	56235356	56506856	0.5653	0.088742
28591	Del	5	56509561	56607812	0.499755	0.086375
28592	Del	5	56608254	56900019	0.532153	0.087559
28593	Del	5	56902116	56929053	0.570872	0.088742
28594	Del	5	56932502	57167707	0.559745	0.08815
28595	Del	5	57172972	57210503	0.373314	0.081051
28596	Del	5	57211302	57563058	0.380433	0.081642
28597	Del	5	57568740	57618434	0.380097	0.081051
28598	Del	5	57619375	58577984	0.381473	0.081642
28599	Del	5	58578682	58859528	0.293623	0.07691
28600	Del	5	58859826	59001385	0.378749	0.081051
28601	Del	5	59001753	59040441	0.361151	0.080459
28602	Del	5	59041415	59318863	0.26571	0.075726
28603	Del	5	59319104	59357069	0.251945	0.075135
28604	Del	5	59360426	59895272	0.234436	0.073952
28605	Del	5	59895785	60485120	0.155349	0.06981
28606	Del	5	60485875	60491631	0.354776	0.079868
28607	Del	5	60492159	60588490	0.338929	0.079276
28608	Del	5	60595477	60848119	0.329609	0.078684
28609	Del	5	60849493	60901252	0.338373	0.079276
28610	Del	5	60901888	60981243	0.343819	0.079868
28611	Del	5	60986148	61151935	0.31484	0.078093
28612	Del	5	61153999	61163213	0.354776	0.079868
28613	Del	5	61163889	61232300	0.329244	0.078684
28614	Del	5	61232345	61546457	0.526448	0.087559
28615	Del	5	61547879	61660800	0.558946	0.08815
28616	Del	5	61661645	61698837	0.578891	0.089333
28617	Del	5	61699001	61711631	0.558946	0.08815
28618	Del	5	61713041	61731847	0.53787	0.087559
28619	Del	5	61732404	62276868	0.603158	0.089925

28620	Del	5	62277162	62387298	0.57425	0.088742
28621	Del	5	62388133	62410219	0.600176	0.089925
28622	Del	5	62410274	63952173	0.522873	0.086967
28623	Del	5	63958278	64226909	0.000769	0.059161
28624	Del	5	64230166	64503151	0	0.057978
28625	Del	5	64503892	64573570	0	0.057386
28626	Del	5	64574782	64614008	0	0.05857
28627	Del	5	64615148	64727280	0	0.059161
28628	Del	5	64727306	64763968	0	0.05857
28629	Del	5	64767131	64812872	0	0.059161
28630	Del	5	64813287	64847604	0	0.05857
28631	Del	5	64849549	65019562	0	0.057386
28632	Del	5	65019867	65489381	0.034592	0.062119
28633	Del	5	65489849	65589155	0.086563	0.065669
28634	Del	5	65589468	65624050	0.08388	0.065077
28635	Del	5	65624453	65669090	0.092601	0.065669
28636	Del	5	65673065	65727736	0.082617	0.065077
28637	Del	5	65729883	65941384	0.068533	0.064486
28638	Del	5	65943109	65946272	0.093718	0.065669
28639	Del	5	65952106	66082554	0.068533	0.064486
28640	Del	5	66083393	66185520	0.087946	0.065669
28641	Del	5	66188374	66593483	0.151654	0.069219
28642	Del	5	66595714	66828113	0.082903	0.065077
28643	Del	5	66828398	66955345	0.108661	0.066852
28644	Del	5	66955645	66997899	0.145035	0.069219
28645	Del	5	67000340	67004762	0.202822	0.072177
28646	Del	5	67006362	67170451	0.159666	0.06981
28647	Del	5	67172486	67202655	0.193935	0.072177
28648	Del	5	67202886	68203845	0.609478	0.090517
28649	Del	5	68213973	68283748	0.355985	0.080459
28650	Del	5	68287140	68966626	0.386207	0.081642
28651	Del	5	68966702	69030301	0.375076	0.081051
28652	Del	5	69030573	69105138	0.390828	0.081642
28653	Del	5	69107628	69133809	0.379528	0.081051
28654	Del	5	69135452	69444194	0.390828	0.081642
28655	Del	5	69444660	71364924	1.054345	0.105307
28656	Del	5	71365381	71370707	1.020447	0.104124
28657	Del	5	71372365	71388937	1.050988	0.105307
28658	Del	5	71389549	71454770	1.020447	0.104124
28659	Del	5	71454824	71570661	1.007406	0.103532
28660	Del	5	71571485	71660684	1.022647	0.104124
28661	Del	5	71660711	72102222	1.054345	0.105307
28662	Del	5	72103902	72168441	1.011215	0.104124
28663	Del	5	72169344	72169907	1.054345	0.105307
28664	Del	5	72175886	72211976	1.03465	0.104715
28665	Del	5	72212936	72315682	1.014654	0.104124
28666	Del	5	72317086	72439420	1.037875	0.104715
28667	Del	5	72439477	72508874	1.009434	0.104124
28668	Del	5	72509323	72572397	1.052352	0.105307
28669	Del	5	72582767	72814713	1.020447	0.104124
28670	Del	5	72815188	72842655	0.921598	0.100574
28671	Del	5	72845580	72945485	0.931926	0.101166
28672	Del	5	72955633	73093228	0.89674	0.099982

28673	Del	5	73093791	73133004	0.951036	0.101757
28674	Del	5	73133622	73150662	0.934998	0.101166
28675	Del	5	73150938	73439180	0.958567	0.101757
28676	Del	5	73442534	73623753	0.407785	0.082234
28677	Del	5	73623872	73810575	0.289889	0.07691
28678	Del	5	73811665	74304200	0.354776	0.079868
28679	Del	5	74305023	74366530	0.25536	0.075135
28680	Del	5	74368424	74538127	0.190637	0.071585
28681	Del	5	74538422	74642627	0.253256	0.075135
28682	Del	5	74643440	74681699	0.174771	0.070993
28683	Del	5	74682681	74779604	0.191059	0.071585
28684	Del	5	74780924	74863103	0.178142	0.070993
28685	Del	5	74863312	75054710	0.191385	0.071585
28686	Del	5	75055711	75241452	0.277426	0.076318
28687	Del	5	75244249	75514662	0.84211	0.098208
28688	Del	5	75515269	75599865	0.576594	0.089333
28689	Del	5	75600361	75671574	0.88743	0.099982
28690	Del	5	75673590	76081642	0.910756	0.100574
28691	Del	5	76081726	76334363	0.770155	0.095841
28692	Del	5	76335161	76355961	0.742227	0.09525
28693	Del	5	76361986	76708503	0.776796	0.095841
28694	Del	5	76711389	76848949	0.105108	0.066261
28695	Del	5	76849040	76924456	0.092131	0.065669
28696	Del	5	76924470	77088037	0.102813	0.066261
28697	Del	5	77093677	77148782	0.091773	0.065669
28698	Del	5	77148929	77429400	0.403863	0.082234
28699	Del	5	77429757	77625889	0.472033	0.085192
28700	Del	5	77627669	77688558	0.103724	0.066261
28701	Del	5	77688724	77882157	0.08527	0.065669
28702	Del	5	77884487	77956282	0.081603	0.065077
28703	Del	5	77956834	77963325	0.10539	0.066261
28704	Del	5	77964670	78297100	0.350033	0.079868
28705	Del	5	78297971	78364798	0.423612	0.082826
28706	Del	5	78366551	78484628	0.376387	0.081051
28707	Del	5	78484761	78654622	0.398592	0.082234
28708	Del	5	78654714	78808919	0.607916	0.090517
28709	Del	5	78809524	78987740	0.625754	0.091108
28710	Del	5	78988293	79019544	0.681886	0.092883
28711	Del	5	79020141	79068692	0.69654	0.093475
28712	Del	5	79068984	79227709	0.716007	0.094066
28713	Del	5	79229650	79372727	0.686991	0.092883
28714	Del	5	79372940	79520878	0.439408	0.083417
28715	Del	5	79521156	79692018	0.470196	0.085192
28716	Del	5	79692919	79933823	0.296226	0.077501
28717	Del	5	79934711	79986975	0.004047	0.059753
28718	Del	5	79990051	80031546	0.511876	0.086967
28719	Del	5	80033436	80065442	0.530076	0.087559
28720	Del	5	80065509	80106893	0.54906	0.08815
28721	Del	5	80111068	80294988	0.490951	0.085784
28722	Del	5	80295994	80442771	0.141544	0.068627
28723	Del	5	80443387	80484424	0.128925	0.068035
28724	Del	5	80485452	80656165	0.13422	0.068627
28725	Del	5	80656215	80945499	0.006408	0.059753

28726	Del	5	80947454	80955560	0	0.053245
28727	Del	5	80955858	81231658	0	0.059161
28728	Del	5	81233937	81304174	0	0.053245
28729	Del	5	81310812	81316560	0	0.043779
28730	Del	5	81316810	81413371	0.029099	0.061528
28731	Del	5	81417277	81757626	0	0.044371
28732	Del	5	81758368	82255853	0	0.044963
28733	Del	5	82256578	83078098	0	0.046737
28734	Del	5	83079964	83311193	0	0.044963
28735	Del	5	83313671	83356502	0	0.044371
28736	Del	5	83360003	83531108	0	0.045554
28737	Del	5	83531243	83564529	0	0.046737
28738	Del	5	83564561	83582700	0	0.045554
28739	Del	5	83584452	83934708	0	0.046146
28740	Del	5	83935934	84385219	0	0.043779
28741	Del	5	84385395	87117031	0	0.053245
28742	Del	5	87118144	87212183	0	0.052062
28743	Del	5	87212484	87239696	0	0.053245
28744	Del	5	87240591	87249685	0	0.052062
28745	Del	5	87250148	87306550	0	0.052653
28746	Del	5	87306828	87324715	0	0.053245
28747	Del	5	87324752	87391130	0	0.052653
28748	Del	5	87392196	87416884	0	0.053245
28749	Del	5	87420028	88270844	0	0.057978
28750	Del	5	88281712	88436705	0	0.057386
28751	Del	5	88438311	88535916	0	0.053245
28752	Del	5	88536141	88662179	0	0.052062
28753	Del	5	88662890	88680872	0	0.053245
28754	Del	5	88684401	88692916	0	0.052062
28755	Del	5	88703061	88825371	0	0.052653
28756	Del	5	88826375	88887834	0	0.052062
28757	Del	5	88888186	88974032	0	0.052653
28758	Del	5	88974162	89032455	0	0.052062
28759	Del	5	89032749	89468398	0	0.048512
28760	Del	5	89468576	90157716	0	0.053245
28761	Del	5	90158010	90291033	0	0.052062
28762	Del	5	90291434	90556220	0	0.053245
28763	Del	5	90557525	91167454	0	0.046737
28764	Del	5	91170479	93407075	0	0.053245
28765	Del	5	93407319	93587868	0	0.049104
28766	Del	5	93588957	93598041	0	0.041413
28767	Del	5	93599217	93729977	0	0.042004
28768	Del	5	93737148	93745326	0	0.044963
28769	Del	5	93755934	94115611	0	0.042004
28770	Del	5	94120872	94621232	0	0.043779
28771	Del	5	94621281	94696417	0	0.040821
28772	Del	5	94700301	95082044	0	0.043188
28773	Del	5	95082254	95286079	0	0.041413
28774	Del	5	95291786	95554420	0	0.050879
28775	Del	5	95554928	95730592	0	0.05147
28776	Del	5	95730788	95797787	0	0.050879
28777	Del	5	95797882	95883557	0	0.05147
28778	Del	5	95883633	95959489	0	0.050879

28779	Del	5	95960057	96656569	0	0.054428
28780	Del	5	96657158	96693229	0	0.050287
28781	Del	5	96694323	96816057	0	0.050879
28782	Del	5	96816680	96889548	0	0.05147
28783	Del	5	96890070	96926795	0	0.050879
28784	Del	5	96927886	97142870	0	0.052653
28785	Del	5	97145771	97504546	0	0.053245
28786	Del	5	97504572	97672878	0	0.052062
28787	Del	5	97673408	98847646	0	0.053245
28788	Del	5	98854826	98924533	0	0.052653
28789	Del	5	98925401	99152826	0	0.053245
28790	Del	5	99167137	100799346	0	0.049695
28791	Del	5	100800848	100814875	0	0.043779
28792	Del	5	100816037	100822455	0	0.044371
28793	Del	5	100832744	100882181	0	0.043779
28794	Del	5	100884961	102232325	0	0.044371
28795	Del	5	102233523	102500239	0	0.042004
28796	Del	5	102501088	102617863	0	0.042596
28797	Del	5	102618721	102751568	0	0.042004
28798	Del	5	102753475	102858802	0	0.039046
28799	Del	5	102865303	103032551	0	0.039638
28800	Del	5	103033235	103125569	0	0.040821
28801	Del	5	103126069	103126069	0	0.04023
28802	Del	5	103128260	105168236	0	0.040821
28803	Del	5	105171909	107373725	0	0.042004
28804	Del	5	107374099	107675858	0	0.038455
28805	Del	5	107676011	108726929	0	0.047921
28806	Del	5	108727429	108744738	0	0.03313
28807	Del	5	108746265	108851788	0	0.032539
28808	Del	5	108851861	108917767	0	0.030764
28809	Del	5	108919705	109201671	0	0.031947
28810	Del	5	109204479	109412293	0	0.03313
28811	Del	5	109412815	109881727	0	0.03668
28812	Del	5	109881967	110402581	0	0.03313
28813	Del	5	110405809	110511643	0	0.029581
28814	Del	5	110511971	110514461	0	0.031355
28815	Del	5	110514940	110737158	0	0.029581
28816	Del	5	110737662	111130631	0	0.026031
28817	Del	5	111131388	111493380	0	0.029581
28818	Del	5	111494511	111514490	0	0.031355
28819	Del	5	111515121	111726850	0	0.029581
28820	Del	5	111728599	111760729	0	0.031355
28821	Del	5	111760855	111909316	0	0.029581
28822	Del	5	111909695	112017530	0	0.030764
28823	Del	5	112017968	112164035	0	0.031355
28824	Del	5	112166032	112211249	0	0.028397
28825	Del	5	112213315	112258900	0	0.031355
28826	Del	5	112258913	112412938	0	0.028397
28827	Del	5	112413302	112423384	0	0.031355
28828	Del	5	112423688	112703395	0	0.032539
28829	Del	5	112703928	112855469	0	0.030172
28830	Del	5	112855945	113016382	0	0.032539
28831	Del	5	113016720	113296904	0	0.050879

28832	Del	5	113301563	113509911	0	0.049104
28833	Del	5	113513070	114359481	0	0.04023
28834	Del	5	114360138	114427113	0	0.034905
28835	Del	5	114428695	114446398	0	0.035497
28836	Del	5	114446793	114774501	0	0.037863
28837	Del	5	114781310	115207315	0	0.039638
28838	Del	5	115207624	115518139	0	0.036088
28839	Del	5	115518988	115887289	0	0.028989
28840	Del	5	115887338	116293826	0	0.032539
28841	Del	5	116294084	116443044	0	0.034313
28842	Del	5	116443156	116443766	0	0.032539
28843	Del	5	116446192	116474101	0	0.034313
28844	Del	5	116474447	116576868	0	0.032539
28845	Del	5	116582643	117414154	0	0.034313
28846	Del	5	117414595	117727655	0	0.03313
28847	Del	5	117729785	118267594	0	0.028397
28848	Del	5	118267690	118344265	0	0.032539
28849	Del	5	118345839	118563768	0	0.031355
28850	Del	5	118564213	118832295	0	0.032539
28851	Del	5	118834440	118963521	0	0.031947
28852	Del	5	118963552	118979493	0	0.032539
28853	Del	5	118980049	118986347	0	0.031947
28854	Del	5	118987426	119071007	0	0.032539
28855	Del	5	119074338	119154042	0	0.031355
28856	Del	5	119154616	119157400	0	0.032539
28857	Del	5	119160449	119332053	0	0.031355
28858	Del	5	119332355	119347872	0	0.031947
28859	Del	5	119352420	119450127	0	0.032539
28860	Del	5	119451887	119543573	0	0.028989
28861	Del	5	119544119	119636273	0	0.029581
28862	Del	5	119636360	120530092	0	0.034313
28863	Del	5	120530869	120687722	0	0.034905
28864	Del	5	120688335	121328784	0	0.039638
28865	Del	5	121329002	121957954	0	0.043188
28866	Del	5	121960296	122305696	0	0.03668
28867	Del	5	122309995	122388888	0	0.035497
28868	Del	5	122390428	122432976	0	0.036088
28869	Del	5	122435865	122625666	0	0.03668
28870	Del	5	122628565	122735454	0	0.027806
28871	Del	5	122736245	122836211	0	0.028397
28872	Del	5	122838336	123010994	0	0.04023
28873	Del	5	123015798	123086998	0	0.041413
28874	Del	5	123087042	123188136	0	0.04023
28875	Del	5	123190972	123426174	0	0.040821
28876	Del	5	123426535	124049963	0	0.044371
28877	Del	5	124052194	124636285	0	0.041413
28878	Del	5	124636587	124745616	0	0.030764
28879	Del	5	124751580	125369955	0	0.050879
28880	Del	5	125370722	126178017	0	0.054428
28881	Del	5	126178956	126357380	0	0.05147
28882	Del	5	126357661	126418953	0	0.050287
28883	Del	5	126421666	126536551	0	0.050879
28884	Del	5	126539517	126596882	0	0.042596

28885	Del	5	126597192	126779141	0	0.043188
28886	Del	5	126779500	126846566	0	0.042596
28887	Del	5	126848421	127032286	0	0.044963
28888	Del	5	127033562	127078242	0	0.046146
28889	Del	5	127078637	127463537	0	0.053245
28890	Del	5	127463754	127703281	0.225625	0.073952
28891	Del	5	127703386	128016712	0.176824	0.070993
28892	Del	5	128019775	128087232	0	0.05502
28893	Del	5	128089658	128191286	0	0.049695
28894	Del	5	128191954	128545730	0	0.05147
28895	Del	5	128546775	129037330	0	0.055612
28896	Del	5	129038833	129395888	0	0.056203
28897	Del	5	129396979	129399464	0	0.046146
28898	Del	5	129399477	129465635	0	0.053245
28899	Del	5	129466822	129747591	0	0.05147
28900	Del	5	129747913	129767328	0	0.047329
28901	Del	5	129771444	130187198	0	0.048512
28902	Del	5	130187257	131259054	0	0.056795
28903	Del	5	131261293	131417384	0	0.056203
28904	Del	5	131423704	131459874	0	0.05502
28905	Del	5	131463233	131804191	0	0.055612
28906	Del	5	131804894	131946371	0	0.056203
28907	Del	5	131946753	132059045	0.030903	0.062119
28908	Del	5	132059422	132179376	0.030624	0.061528
28909	Del	5	132179720	132193064	0	0.056795
28910	Del	5	132194718	132229824	0	0.043188
28911	Del	5	132230967	132289476	0	0.056203
28912	Del	5	132291271	132358384	0	0.055612
28913	Del	5	132360396	132409098	0	0.056203
28914	Del	5	132409193	132479692	0	0.055612
28915	Del	5	132481211	132547271	0	0.056203
28916	Del	5	132552651	132629487	0	0.055612
28917	Del	5	132629612	133041391	0	0.056203
28918	Del	5	133045075	133193759	0	0.043188
28919	Del	5	133194683	133424634	0	0.037863
28920	Del	5	133425007	133428025	0	0.043188
28921	Del	5	133428226	133616829	0	0.037863
28922	Del	5	133617832	134435538	0	0.043188
28923	Del	5	134435790	134521950	0	0.044371
28924	Del	5	134522017	134583917	0	0.043779
28925	Del	5	134585313	134752824	0	0.044371
28926	Del	5	134754725	134832111	0	0.038455
28927	Del	5	134832117	134902373	0	0.039046
28928	Del	5	134911165	134921788	0	0.038455
28929	Del	5	134922493	134930000	0	0.039046
28930	Del	5	134930722	134963080	0	0.038455
28931	Del	5	134966657	135025330	0	0.039046
28932	Del	5	135025907	135036154	0	0.030764
28933	Del	5	135036710	135036821	0	0.027806
28934	Del	5	135036995	135042082	0	0.030764
28935	Del	5	135043621	135235145	0	0.027806
28936	Del	5	135235460	135248898	0	0.029581
28937	Del	5	135250521	135329392	0	0.027806

28938	Del	5	135330110	135443700	0	0.030764
28939	Del	5	135443727	135537760	0	0.024256
28940	Del	5	135539676	136210182	0	0.025439
28941	Del	5	136211868	136220555	0	0.006508
28942	Del	5	136225533	136316252	0	0.007099
28943	Del	5	136316534	136970104	0	0.006508
28944	Del	5	136972538	137125166	0	0.005916
28945	Del	5	137125945	137131015	0	0.008283
28946	Del	5	137133047	137503106	0	0.005916
28947	Del	5	137504275	138349886	0	0.007099
28948	Del	5	138359286	138937082	0	0.005916
28949	Del	5	138944727	139202790	0	0.005325
28950	Del	5	139202916	139401281	0	0.005916
28951	Del	5	139403120	139845232	0	0.007691
28952	Del	5	139846337	140043791	0	0.007099
28953	Del	5	140046119	141098085	0	0.007691
28954	Del	5	141098348	141105446	0	0.016565
28955	Del	5	141109017	141229069	0	0.023664
28956	Del	5	141229157	141353542	0	0.018932
28957	Del	5	141353931	141353931	0	0.01834
28958	Del	5	141353973	141620495	0	0.018932
28959	Del	5	141620604	142326902	0	0.011241
28960	Del	5	142327073	142464523	0	0.010649
28961	Del	5	142465092	142644651	0	0.011241
28962	Del	5	142644691	142698298	0	0.010649
28963	Del	5	142698504	142764193	0	0.011241
28964	Del	5	142766991	142857921	0	0.010649
28965	Del	5	142858554	142869137	0	0.011241
28966	Del	5	142872692	143189752	0	0.010649
28967	Del	5	143191454	143194661	0	0.013015
28968	Del	5	143194865	143229960	0	0.010649
28969	Del	5	143230077	143406518	0	0.013015
28970	Del	5	143409146	143451919	0	0.012424
28971	Del	5	143454530	143810837	0	0.013015
28972	Del	5	143810937	144170825	0	0.011241
28973	Del	5	144174704	144477760	0	0.010057
28974	Del	5	144478005	145934393	0	0.011241
28975	Del	5	145934606	146064428	0	0.010649
28976	Del	5	146065161	146588304	0	0.011241
28977	Del	5	146588635	146879536	0	0.010057
28978	Del	5	146879874	146913481	0	0.008874
28979	Del	5	146913945	146922270	0	0.011241
28980	Del	5	146923912	147083276	0	0.008874
28981	Del	5	147084946	147387907	0	0.010649
28982	Del	5	147388254	147455972	0	0.011241
28983	Del	5	147462449	147518425	0	0.010649
28984	Del	5	147519779	147662157	0	0.011241
28985	Del	5	147662404	147785590	0	0.010649
28986	Del	5	147787916	148448868	0	0.011241
28987	Del	5	148449878	148655311	0	0.010649
28988	Del	5	148658742	149140466	0	0.011241
28989	Del	5	149141106	149261473	0	0.010649
28990	Del	5	149261530	149736489	0	0.011241

28991	Del	5	149738137	149852650	0	0.010649
28992	Del	5	149853547	150051660	0	0.011241
28993	Del	5	150051696	150491584	0	0.012424
28994	Del	5	150491922	150558394	0	0.020706
28995	Del	5	150558641	150947414	0	0.021298
28996	Del	5	150949136	151065397	0	0.026031
28997	Del	5	151066755	151087768	0	0.025439
28998	Del	5	151087840	151142063	0	0.026031
28999	Del	5	151143059	151158068	0	0.025439
29000	Del	5	151159908	151431172	0	0.026031
29001	Del	5	151436204	151498994	0	0.024848
29002	Del	5	151501586	151521501	0	0.024256
29003	Del	5	151521700	151523814	0	0.024848
29004	Del	5	151523831	151658810	0	0.024256
29005	Del	5	151660972	151821926	0	0.023073
29006	Del	5	151821944	152270509	0	0.022481
29007	Del	5	152271549	152617659	0	0.024848
29008	Del	5	152618131	153487751	0	0.023664
29009	Del	5	153487910	153818118	0	0.023073
29010	Del	5	153818136	154060995	0	0.026031
29011	Del	5	154065183	154343499	0	0.037272
29012	Del	5	154344797	154347322	0	0.039638
29013	Del	5	154348377	154386189	0	0.037272
29014	Del	5	154388377	154449155	0	0.039046
29015	Del	5	154449395	154480452	0	0.026031
29016	Del	5	154480714	155021061	0	0.030764
29017	Del	5	155022415	156847623	0	0.035497
29018	Del	5	156848567	157177324	0	0.034905
29019	Del	5	157178210	157257481	0	0.034313
29020	Del	5	157258194	157339700	0	0.033722
29021	Del	5	157340178	157346777	0	0.034905
29022	Del	5	157347044	157360285	0	0.033722
29023	Del	5	157362043	157456150	0	0.034313
29024	Del	5	157456580	157476115	0	0.034905
29025	Del	5	157477242	157576643	0	0.033722
29026	Del	5	157577179	157681487	0	0.034905
29027	Del	5	157681665	157782192	0	0.037863
29028	Del	5	157782564	158316773	0	0.037272
29029	Del	5	158320350	158694682	0	0.035497
29030	Del	5	158695189	159096159	0	0.025439
29031	Del	5	159097509	159211634	0	0.028397
29032	Del	5	159212872	159313842	0	0.032539
29033	Del	5	159314018	159772360	0	0.028397
29034	Del	5	159776216	159872086	0	0.027214
29035	Del	5	159873203	160250736	0	0.028397
29036	Del	5	160250912	160339947	0	0.027806
29037	Del	5	160340471	160559793	0	0.028397
29038	Del	5	160559921	160853333	0	0.026031
29039	Del	5	160853690	160940445	0	0.028397
29040	Del	5	160940969	161549748	0	0.033722
29041	Del	5	161550215	161702845	0	0.036088
29042	Del	5	161708826	162061665	0	0.044371
29043	Del	5	162062324	162155771	0	0.043779

29044	Del	5	162155943	163521302	0	0.046146
29045	Del	5	163521386	164468638	0	0.056795
29046	Del	5	164468859	166904556	0	0.056203
29047	Del	5	166904860	167280708	0	0.047921
29048	Del	5	167284601	168282835	0	0.042004
29049	Del	5	168285790	168472520	0	0.03668
29050	Del	5	168473467	168658715	0	0.038455
29051	Del	5	168659269	168703123	0	0.031355
29052	Del	5	168704067	168785451	0	0.038455
29053	Del	5	168785946	169009352	0	0.031355
29054	Del	5	169009614	169039198	0	0.038455
29055	Del	5	169039773	169261630	0	0.031355
29056	Del	5	169261646	169263905	0	0.03313
29057	Del	5	169265419	169302390	0	0.031355
29058	Del	5	169303263	169610805	0	0.03313
29059	Del	5	169613862	170104795	0	0.042004
29060	Del	5	170105715	170188387	0	0.037863
29061	Del	5	170189362	170352212	0	0.03313
29062	Del	5	170352947	170372671	0	0.029581
29063	Del	5	170373675	170390281	0	0.03313
29064	Del	5	170391565	170423657	0	0.032539
29065	Del	5	170423852	170501651	0	0.029581
29066	Del	5	170501770	170636801	0	0.030764
29067	Del	5	170637709	170682606	0	0.03313
29068	Del	5	170685382	170739590	0	0.030764
29069	Del	5	170740401	170859544	0	0.03313
29070	Del	5	170859754	171300704	0	0.030764
29071	Del	5	171302755	172208249	0	0.03313
29072	Del	5	172208264	172266669	0	0.032539
29073	Del	5	172276353	172323112	0	0.03313
29074	Del	5	172323215	172333316	0	0.031355
29075	Del	5	172333424	172456504	0	0.031947
29076	Del	5	172459258	172833414	0	0.03313
29077	Del	5	172833477	172949175	0	0.031947
29078	Del	5	172952273	173702619	0	0.03313
29079	Del	5	173703278	173746813	0	0.032539
29080	Del	5	173747075	173886504	0	0.03313
29081	Del	5	173887361	173961752	0	0.032539
29082	Del	5	173962453	174335706	0	0.03313
29083	Del	5	174335879	174527560	0	0.030764
29084	Del	5	174527566	174914884	0	0.03313
29085	Del	5	174916846	175000050	0	0.032539
29086	Del	5	175000100	175793425	0	0.037863
29087	Del	5	175794985	175867230	0	0.037272
29088	Del	5	175870374	175885498	0	0.037863
29089	Del	5	175885707	176346182	0	0.046146
29090	Del	5	176347862	176442456	0	0.038455
29091	Del	5	176443777	176510393	0	0.037863
29092	Del	5	176510546	176538658	0	0.038455
29093	Del	5	176539316	176904815	0	0.03313
29094	Del	5	176904831	177121567	0	0.028397
29095	Del	5	177123300	177299634	0	0.027806
29096	Del	5	177299982	178275580	0	0.028397

29097	Del	5	178277026	178595028	0	0.026031
29098	Del	5	178595625	179084406	0	0.028397
29099	Del	5	179084845	179346216	0	0.031355
29100	Del	5	179347399	179954399	0	0.03313
29101	Del	5	179954941	180013908	0	0.032539
29102	Del	5	180014584	180016133	0	0.03313
29103	Del	5	180016190	180243341	0	0.032539
29104	Del	5	180243528	180491722	0	0.03313
29105	Del	5	180492029	180578634	0	0.029581
29106	Del	5	180586575	180862451	0	0.030172
29107	Del	5	180864372	180934240	0	0.03668
29108	Del	6	1011760	1011760	1.275412	0.112406
29109	Del	6	1014046	1101474	1.181741	0.109448
29110	Del	6	1101658	1597922	1.588567	0.121872
29111	Del	6	1597992	1621169	1.23952	0.111223
29112	Del	6	1622611	2176400	0.923841	0.101166
29113	Del	6	2176790	2244487	0.887083	0.099982
29114	Del	6	2244594	2413979	0.960563	0.101757
29115	Del	6	2414195	2662444	1.02195	0.104124
29116	Del	6	2662919	2754560	0.971316	0.102349
29117	Del	6	2755024	2786151	1.020447	0.104124
29118	Del	6	2786329	2947549	1.02909	0.104715
29119	Del	6	2947622	2974153	1.022647	0.104124
29120	Del	6	2974511	2997363	1.037684	0.104715
29121	Del	6	2997531	3016967	1.013657	0.104124
29122	Del	6	3017247	3030981	1.034253	0.104715
29123	Del	6	3031759	3151083	1.169179	0.109448
29124	Del	6	3151097	3269937	1.201892	0.11004
29125	Del	6	3270440	3321363	1.084313	0.10649
29126	Del	6	3321642	3460068	1.119341	0.107673
29127	Del	6	3462651	3755612	1.179049	0.109448
29128	Del	6	3755809	3853672	1.201892	0.11004
29129	Del	6	3853805	4065198	1.275857	0.112998
29130	Del	6	4065519	4080115	1.305318	0.11359
29131	Del	6	4080512	4114942	1.252856	0.111815
29132	Del	6	4115130	4160780	1.289234	0.112998
29133	Del	6	4160930	4608019	1.305318	0.11359
29134	Del	6	4608395	4703145	0.655895	0.0917
29135	Del	6	4705958	4772296	0.400715	0.082234
29136	Del	6	4772852	4827036	0.429866	0.083417
29137	Del	6	4828086	4990439	0.449675	0.084009
29138	Del	6	4994010	5067090	0.416527	0.082826
29139	Del	6	5068291	5106665	0.431075	0.083417
29140	Del	6	5106803	5134340	0.45314	0.084009
29141	Del	6	5135863	5180023	0.470637	0.085192
29142	Del	6	5183355	5258997	0.501961	0.086375
29143	Del	6	5260152	5664155	1.226406	0.111223
29144	Del	6	5664855	5696104	1.497027	0.119506
29145	Del	6	5696523	5995785	1.226406	0.111223
29146	Del	6	5997282	6138508	1.161801	0.108857
29147	Del	6	6139863	6168717	1.11824	0.107673
29148	Del	6	6168985	6170216	1.313923	0.11359
29149	Del	6	6170812	6341740	1.11824	0.107673

29150	Del	6	6342522	6623873	0.953567	0.101757
29151	Del	6	6626030	6655660	0.732885	0.094658
29152	Del	6	6655916	7275708	1.159127	0.108857
29153	Del	6	7276094	7317461	0.960563	0.101757
29154	Del	6	7325041	7377362	0.791211	0.096433
29155	Del	6	7377879	7539016	0.805596	0.097024
29156	Del	6	7539721	7586790	0.774765	0.095841
29157	Del	6	7586961	7725749	0.805596	0.097024
29158	Del	6	7725824	7878333	0.70222	0.093475
29159	Del	6	7879897	7911469	0.786174	0.096433
29160	Del	6	7913313	8066053	0.687266	0.092883
29161	Del	6	8066181	8071329	0.520667	0.086967
29162	Del	6	8071439	8077762	0.556661	0.08815
29163	Del	6	8078545	8430960	0.561297	0.088742
29164	Del	6	8431006	8713095	0.894205	0.099982
29165	Del	6	8715253	10389159	0.852356	0.098799
29166	Del	6	10392612	10456877	0.18246	0.070993
29167	Del	6	10459365	10761879	0.303654	0.077501
29168	Del	6	10768211	10839030	0.291459	0.07691
29169	Del	6	10840510	10889578	0.301686	0.077501
29170	Del	6	10892887	10924759	0.276765	0.076318
29171	Del	6	10931484	10954307	0.303654	0.077501
29172	Del	6	10954367	11042806	0.276765	0.076318
29173	Del	6	11043506	11085211	0.298593	0.077501
29174	Del	6	11089827	11099174	0.293018	0.07691
29175	Del	6	11101685	11112246	0.305648	0.077501
29176	Del	6	11118194	11181528	0.293018	0.07691
29177	Del	6	11181846	11191549	0.116393	0.067444
29178	Del	6	11198581	11234611	0.12071	0.068035
29179	Del	6	11235132	11382481	0.073638	0.064486
29180	Del	6	11383224	11712029	0.118436	0.067444
29181	Del	6	11712755	11988931	0.013229	0.060344
29182	Del	6	11989648	12010063	0	0.037863
29183	Del	6	12011057	12166266	0	0.03668
29184	Del	6	12166689	12706881	0	0.037863
29185	Del	6	12710055	12954175	0	0.028397
29186	Del	6	12956497	13005833	0	0.030764
29187	Del	6	13011567	13288416	0	0.031355
29188	Del	6	13288437	13296788	0	0.024848
29189	Del	6	13296890	13300136	0	0.024256
29190	Del	6	13302699	13329234	0	0.024848
29191	Del	6	13330865	13408266	0	0.034313
29192	Del	6	13408440	13467871	0	0.033722
29193	Del	6	13469147	13619397	0	0.034905
29194	Del	6	13619700	13713680	0	0.04023
29195	Del	6	13716360	13984242	0	0.040821
29196	Del	6	13984507	14138627	0	0.057978
29197	Del	6	14139472	15240478	0.119757	0.067444
29198	Del	6	15241150	15536578	0	0.044371
29199	Del	6	15537094	15668430	0	0.039638
29200	Del	6	15668468	16224833	0.135654	0.068627
29201	Del	6	16225631	16297960	0	0.05502
29202	Del	6	16298117	16771384	0	0.048512

29203	Del	6	16771468	17301455	0	0.054428
29204	Del	6	17305169	17594857	0.0196	0.060936
29205	Del	6	17594998	17613197	0	0.034313
29206	Del	6	17617055	17703262	0	0.033722
29207	Del	6	17704320	17711230	0	0.034313
29208	Del	6	17711532	17949122	0	0.042596
29209	Del	6	17949256	18159941	0	0.044371
29210	Del	6	18161586	18224240	0	0.043779
29211	Del	6	18224299	18381922	0	0.043188
29212	Del	6	18384156	18571240	0	0.019523
29213	Del	6	18571294	18573439	0	0.009466
29214	Del	6	18575904	19449737	0	0.024848
29215	Del	6	19449786	19461323	0	0.015974
29216	Del	6	19461589	19829598	0	0.025439
29217	Del	6	19831587	20095989	0	0.015974
29218	Del	6	20098533	20213448	0	0.015382
29219	Del	6	20213887	20527279	0	0.024848
29220	Del	6	20529966	21483509	0	0.020706
29221	Del	6	21483776	21654733	0	0.01479
29222	Del	6	21661506	22126772	0	0.011832
29223	Del	6	22133165	22149335	0	0.01479
29224	Del	6	22150004	22195104	0	0.011832
29225	Del	6	22195488	22303237	0	0.01479
29226	Del	6	22305262	22642905	0	0.026623
29227	Del	6	22643220	22722740	0	0.026031
29228	Del	6	22723219	24171054	0	0.033722
29229	Del	6	24171151	24353208	0	0.03313
29230	Del	6	24355621	24361325	0	0.033722
29231	Del	6	24365628	24383371	0	0.032539
29232	Del	6	24383738	24427178	0	0.033722
29233	Del	6	24427891	24490283	0	0.03313
29234	Del	6	24491174	24537375	0	0.033722
29235	Del	6	24537637	24641100	0	0.050879
29236	Del	6	24641415	24801860	0	0.052062
29237	Del	6	24804229	24836311	0	0.050879
29238	Del	6	24838408	24880910	0	0.05147
29239	Del	6	24881270	24938049	0	0.050879
29240	Del	6	24942278	25080620	0	0.049695
29241	Del	6	25080721	25107542	0	0.042596
29242	Del	6	25107712	25244611	0	0.043188
29243	Del	6	25244876	25262394	0	0.04023
29244	Del	6	25262513	25751493	0	0.044963
29245	Del	6	25753412	25841490	0	0.028989
29246	Del	6	25843698	26030264	0	0.021298
29247	Del	6	26031385	26495899	0	0.023073
29248	Del	6	26496425	26578719	0	0.021298
29249	Del	6	26581646	26664304	0	0.04023
29250	Del	6	26670022	27128855	0	0.021298
29251	Del	6	27129074	27446482	0	0.01479
29252	Del	6	27449180	27816532	0	0.012424
29253	Del	6	27816650	28277187	0	0.029581
29254	Del	6	28280817	29296878	0	0.036088
29255	Del	6	29296963	29478912	0	0.019523

29256	Del	6	29479078	30216437	0	0.012424
29257	Del	6	30216927	31062345	0	0.019523
29258	Del	6	31062447	31112655	0	0.026031
29259	Del	6	31112694	31145275	0	0.034905
29260	Del	6	31145329	31204878	0	0.028989
29261	Del	6	31208262	32985934	0	0.046146
29262	Del	6	32988199	33056877	0	0.056203
29263	Del	6	33061660	33111989	0	0.048512
29264	Del	6	33112035	33274715	0	0.045554
29265	Del	6	33282699	33397863	0	0.038455
29266	Del	6	33399495	33451011	0	0.044963
29267	Del	6	33451026	33619666	0	0.045554
29268	Del	6	33619949	33627682	0	0.044963
29269	Del	6	33628431	33637190	0	0.045554
29270	Del	6	33642368	33697243	0	0.044963
29271	Del	6	33697748	34018314	0	0.039046
29272	Del	6	34018380	34114798	0	0.038455
29273	Del	6	34115539	34156636	0	0.037863
29274	Del	6	34157504	34255625	0	0.039046
29275	Del	6	34258242	34416011	0	0.043779
29276	Del	6	34417075	34464075	0	0.044371
29277	Del	6	34464481	34514418	0	0.043779
29278	Del	6	34514522	34558549	0	0.044371
29279	Del	6	34558578	34692794	0	0.050879
29280	Del	6	34693295	34697941	0	0.05147
29281	Del	6	34699848	34919539	0	0.057978
29282	Del	6	34922451	35093738	0	0.056203
29283	Del	6	35095923	35329629	0	0.057978
29284	Del	6	35336869	35435008	0	0.050879
29285	Del	6	35435957	35520920	0	0.05147
29286	Del	6	35534839	35662236	0	0.050879
29287	Del	6	35663090	35666118	0	0.05147
29288	Del	6	35666349	35690550	0	0.050879
29289	Del	6	35690564	35721797	0	0.050287
29290	Del	6	35721942	35829554	0	0.05147
29291	Del	6	35830026	36026129	0	0.050879
29292	Del	6	36026295	36112319	0	0.044963
29293	Del	6	36112740	36241846	0	0.045554
29294	Del	6	36242005	36309033	0	0.044963
29295	Del	6	36309036	36337951	0	0.045554
29296	Del	6	36342381	36428042	0	0.053837
29297	Del	6	36431788	36759400	0	0.054428
29298	Del	6	36761727	36787891	0	0.053245
29299	Del	6	36789406	36852789	0	0.053837
29300	Del	6	36854098	36877076	0	0.047329
29301	Del	6	36877840	36929052	0	0.046737
29302	Del	6	36932217	36987173	0	0.047329
29303	Del	6	36987272	37162506	0	0.050287
29304	Del	6	37163600	37268672	0	0.041413
29305	Del	6	37272393	37333341	0	0.040821
29306	Del	6	37334299	37557712	0	0.041413
29307	Del	6	37560201	37808296	0	0.046146
29308	Del	6	37817243	38159333	0	0.043779

29309	Del	6	38159710	38600237	0	0.045554
29310	Del	6	38602327	38641138	0	0.044963
29311	Del	6	38648491	39036948	0	0.050287
29312	Del	6	39047693	39092800	0	0.049695
29313	Del	6	39092986	39183819	0	0.050287
29314	Del	6	39184681	39233395	0	0.049695
29315	Del	6	39233515	39322877	0	0.050287
29316	Del	6	39323058	39791111	0	0.052062
29317	Del	6	39791744	39887336	0	0.046737
29318	Del	6	39888696	39897579	0	0.047921
29319	Del	6	39898347	39902802	0	0.046737
29320	Del	6	39903534	40342679	0	0.047921
29321	Del	6	40343847	40381627	0	0.043188
29322	Del	6	40382660	40588382	0	0.046146
29323	Del	6	40589376	41025779	0	0.047921
29324	Del	6	41026075	41550275	0	0.049104
29325	Del	6	41551032	41595419	0	0.048512
29326	Del	6	41598075	41602100	0	0.049104
29327	Del	6	41602264	41602612	0	0.048512
29328	Del	6	41602774	41682167	0.117228	0.067444
29329	Del	6	41682998	41730396	0.019708	0.060936
29330	Del	6	41732652	41790845	0.030373	0.061528
29331	Del	6	41793076	41897385	0.022792	0.060936
29332	Del	6	41898342	41945921	0.030373	0.061528
29333	Del	6	41947405	42042947	0.014117	0.060344
29334	Del	6	42046402	42222708	0.026449	0.061528
29335	Del	6	42222818	42559026	0	0.059161
29336	Del	6	42562979	42621351	0	0.052062
29337	Del	6	42623978	42693666	0	0.050879
29338	Del	6	42694818	42740890	0	0.052062
29339	Del	6	42746422	42775710	0.018569	0.060344
29340	Del	6	42779511	42791425	0.030493	0.061528
29341	Del	6	42792302	42808847	0.018569	0.060344
29342	Del	6	42818126	42913199	0.026565	0.061528
29343	Del	6	42914758	43072019	0	0.05857
29344	Del	6	43072225	43162435	0.017993	0.060344
29345	Del	6	43165054	43182564	0.023875	0.060936
29346	Del	6	43184186	43223185	0.018569	0.060344
29347	Del	6	43224713	43530935	0.024792	0.060936
29348	Del	6	43534562	43572464	0.018119	0.060344
29349	Del	6	43573189	43651484	0.018569	0.060936
29350	Del	6	43651952	43788315	0	0.05147
29351	Del	6	43789278	43940887	0	0.056795
29352	Del	6	43942427	44009304	0	0.057386
29353	Del	6	44009798	44110920	0	0.056795
29354	Del	6	44111479	44378269	0	0.039638
29355	Del	6	44378337	44430511	0	0.043779
29356	Del	6	44434093	44437516	0	0.044371
29357	Del	6	44437531	44453503	0	0.043779
29358	Del	6	44453715	44552367	0	0.044371
29359	Del	6	44552714	45194585	0	0.049104
29360	Del	6	45196618	45201285	0	0.050879
29361	Del	6	45203585	45325813	0	0.049104

29362	Del	6	45326909	45376134	0	0.050879
29363	Del	6	45381760	45413847	0	0.049104
29364	Del	6	45414724	45553274	0	0.049695
29365	Del	6	45553549	45937932	0	0.050287
29366	Del	6	45939624	46109269	0	0.049695
29367	Del	6	46109939	46152366	0	0.053837
29368	Del	6	46152666	46219981	0	0.044371
29369	Del	6	46220017	46367432	0	0.043779
29370	Del	6	46367581	46487862	0	0.042004
29371	Del	6	46490099	46538178	0	0.044371
29372	Del	6	46538743	46650875	0	0.05857
29373	Del	6	46652515	46690119	0.004389	0.059753
29374	Del	6	46690471	46848736	0	0.044371
29375	Del	6	46851999	46901026	0	0.043188
29376	Del	6	46902158	46914137	0	0.044371
29377	Del	6	46914656	46924475	0	0.043188
29378	Del	6	46924503	46956730	0	0.042596
29379	Del	6	46956767	47017272	0	0.043779
29380	Del	6	47019852	47231139	0	0.044371
29381	Del	6	47231408	47629376	0	0.037863
29382	Del	6	47629419	47694849	0	0.038455
29383	Del	6	47694974	47865865	0	0.044963
29384	Del	6	47877415	48069715	0	0.043779
29385	Del	6	48069750	49686468	0.056762	0.063302
29386	Del	6	49687936	49882638	0	0.057386
29387	Del	6	49884824	49951029	0.268026	0.075726
29388	Del	6	49952065	50007551	0.585072	0.089333
29389	Del	6	50007621	50039952	0.091514	0.065669
29390	Del	6	50043333	50711699	0	0.047329
29391	Del	6	50712569	50849923	0	0.043779
29392	Del	6	50851499	52138966	0	0.052062
29393	Del	6	52139565	52415103	0	0.035497
29394	Del	6	52417614	52495991	0	0.034905
29395	Del	6	52497237	52572227	0	0.039046
29396	Del	6	52576172	53151405	0	0.039638
29397	Del	6	53151508	53292064	0	0.044371
29398	Del	6	53292918	53330234	0	0.044963
29399	Del	6	53330903	53337657	0	0.045554
29400	Del	6	53338779	53351168	0	0.044963
29401	Del	6	53353804	53793413	0	0.045554
29402	Del	6	53793627	53925702	0	0.044371
29403	Del	6	53926266	53987139	0	0.044963
29404	Del	6	53987279	54009638	0	0.045554
29405	Del	6	54011410	54109301	0	0.053245
29406	Del	6	54109324	54307959	0	0.053837
29407	Del	6	54308161	54391033	0	0.044371
29408	Del	6	54391416	54945150	0	0.054428
29409	Del	6	54946412	55283087	0.000634	0.059161
29410	Del	6	55284602	55433537	0.139597	0.068627
29411	Del	6	55434161	55511369	0.018569	0.060936
29412	Del	6	55511532	55750528	0.035073	0.062119
29413	Del	6	55752476	55876083	0	0.056795
29414	Del	6	55876597	56094199	0	0.057978

29415	Del	6	56095222	56249857	0	0.056203
29416	Del	6	56250023	56456681	0	0.053837
29417	Del	6	56457022	56607814	0	0.052062
29418	Del	6	56610087	56644550	0	0.053837
29419	Del	6	56645344	56845183	0	0.050879
29420	Del	6	56846478	56854756	0	0.050287
29421	Del	6	56857437	56957530	0	0.049695
29422	Del	6	56957820	57013355	0	0.044963
29423	Del	6	57014364	57028417	0	0.044371
29424	Del	6	57029975	57310243	0	0.044963
29425	Del	6	57314641	57314641	0	0.038455
29426	Del	6	57315049	61527062	0.027338	0.061528
29427	Del	6	61530738	61596503	0.010637	0.059753
29428	Del	6	61596563	62337327	0.142103	0.069219
29429	Del	6	62344324	63564609	0.573218	0.088742
29430	Del	6	63565664	63692994	0.245798	0.074543
29431	Del	6	63693293	63713088	0.236642	0.073952
29432	Del	6	63717576	65778949	1.258453	0.111815
29433	Del	6	65779055	65792829	0.724792	0.094066
29434	Del	6	65793418	68226499	2.782554	0.150861
29435	Del	6	68226906	68329969	2.267901	0.13962
29436	Del	6	68330158	68624851	2.363675	0.141395
29437	Del	6	68635591	69663454	2.522762	0.145537
29438	Del	6	69663471	69798227	1.665569	0.124239
29439	Del	6	69798271	70215584	2.208106	0.137846
29440	Del	6	70215885	70286887	2.067854	0.134296
29441	Del	6	70286979	70303288	2.040773	0.133704
29442	Del	6	70306977	70408051	2.150174	0.136662
29443	Del	6	70408315	70564116	2.083476	0.134888
29444	Del	6	70564415	70667242	2.143088	0.136662
29445	Del	6	70667580	70859426	2.021927	0.133704
29446	Del	6	70859570	71110207	2.54672	0.146128
29447	Del	6	71110282	71406270	3.004935	0.156186
29448	Del	6	71406416	71884180	2.997994	0.155594
29449	Del	6	71885619	72364621	2.766458	0.150861
29450	Del	6	72364818	72615609	2.798398	0.151453
29451	Del	6	72615796	72627949	2.204515	0.137846
29452	Del	6	72628542	72680985	2.802638	0.151453
29453	Del	6	72681369	72964923	2.204515	0.137846
29454	Del	6	72966618	72969051	2.845836	0.152044
29455	Del	6	72969065	73132056	2.204515	0.137846
29456	Del	6	73132903	73143845	2.825506	0.152044
29457	Del	6	73146946	73199070	2.204515	0.137846
29458	Del	6	73204057	73265681	2.844331	0.152044
29459	Del	6	73271580	73315824	2.806254	0.151453
29460	Del	6	73316055	73457683	2.845836	0.152044
29461	Del	6	73461821	73505256	2.802638	0.151453
29462	Del	6	73507555	73590785	2.840807	0.152044
29463	Del	6	73593269	73654470	2.762197	0.150269
29464	Del	6	73654906	73699816	2.844331	0.152044
29465	Del	6	73700172	73830871	2.642362	0.147903
29466	Del	6	73831638	74692747	2.828988	0.152044
29467	Del	6	74693007	75207449	3.334235	0.162693

29468	Del	6	75207834	75251371	3.502415	0.166243
29469	Del	6	75252092	75283141	3.466709	0.165651
29470	Del	6	75283852	75293827	3.492835	0.166243
29471	Del	6	75294301	75307510	3.276029	0.16151
29472	Del	6	75307898	75378622	3.295544	0.162102
29473	Del	6	75381146	75426552	3.421844	0.16506
29474	Del	6	75427234	75429879	3.504194	0.166243
29475	Del	6	75430924	75680361	3.421844	0.16506
29476	Del	6	75687173	75719448	3.395094	0.164468
29477	Del	6	75724109	75920557	4.221262	0.181033
29478	Del	6	75925657	76072971	3.774623	0.172159
29479	Del	6	76073214	77648832	4.078209	0.178075
29480	Del	6	77649155	78863190	3.817274	0.172751
29481	Del	6	78863205	78904046	3.353941	0.163285
29482	Del	6	78904104	79079295	3.487424	0.166243
29483	Del	6	79083030	79225925	3.570265	0.168018
29484	Del	6	79226282	79313928	3.606388	0.168609
29485	Del	6	79316994	79651263	3.946117	0.175117
29486	Del	6	79651359	79704306	3.869452	0.173934
29487	Del	6	79704485	79912704	3.938436	0.175117
29488	Del	6	79914243	80105085	3.900815	0.174526
29489	Del	6	80106579	81745355	3.773726	0.172159
29490	Del	6	81745477	81806860	3.212979	0.160327
29491	Del	6	81807251	81814826	2.867983	0.152636
29492	Del	6	81816082	82359979	3.408242	0.164468
29493	Del	6	82360006	82370823	2.210664	0.137846
29494	Del	6	82379101	83060458	2.889591	0.153228
29495	Del	6	83063514	83163165	2.983271	0.155594
29496	Del	6	83163667	83188450	3.042775	0.156777
29497	Del	6	83188699	83209402	3.070377	0.157369
29498	Del	6	83209465	83510686	2.876969	0.153228
29499	Del	6	83511051	83541689	2.494466	0.144945
29500	Del	6	83552790	83700999	2.363675	0.141395
29501	Del	6	83703390	83859205	2.494466	0.144945
29502	Del	6	83859529	83961085	2.419777	0.14317
29503	Del	6	83961822	84119903	2.741392	0.150269
29504	Del	6	84120274	84228589	2.40753	0.142578
29505	Del	6	84235760	84475071	2.449262	0.143762
29506	Del	6	84477230	84769682	2.924061	0.154411
29507	Del	6	84776481	85436078	3.092397	0.15796
29508	Del	6	85445938	85595729	3.053962	0.156777
29509	Del	6	85595984	86933850	3.080609	0.15796
29510	Del	6	86934734	87018111	2.510045	0.144945
29511	Del	6	87018444	87097462	2.585401	0.14672
29512	Del	6	87098530	87264305	2.915446	0.153819
29513	Del	6	87264649	87366247	2.993657	0.155594
29514	Del	6	87367148	87405304	3.028126	0.156186
29515	Del	6	87405523	87467579	2.967251	0.155002
29516	Del	6	87467721	87516923	2.98948	0.155594
29517	Del	6	87517836	87590944	2.955253	0.155002
29518	Del	6	87591572	87667512	2.940182	0.154411
29519	Del	6	87667524	87782808	2.983271	0.155594
29520	Del	6	87782932	88044081	2.858058	0.152636

29521	Del	6	88044535	88520761	2.747497	0.150269
29522	Del	6	88520777	88607951	2.73613	0.149678
29523	Del	6	88609891	88966429	2.475422	0.144353
29524	Del	6	88967615	89119597	2.540432	0.145537
29525	Del	6	89120390	89175868	2.833322	0.152044
29526	Del	6	89176711	89252778	2.778472	0.150861
29527	Del	6	89253500	89315393	2.760211	0.150269
29528	Del	6	89315598	89361963	2.817835	0.151453
29529	Del	6	89364308	89430408	2.784023	0.150861
29530	Del	6	89430688	89557261	2.61847	0.147311
29531	Del	6	89557716	89627097	2.750452	0.150269
29532	Del	6	89628158	89639765	2.845836	0.152044
29533	Del	6	89646190	89691415	2.796603	0.151453
29534	Del	6	89691658	89812421	2.654673	0.148495
29535	Del	6	89814033	89877006	2.821918	0.151453
29536	Del	6	89877684	89896612	2.845836	0.152044
29537	Del	6	89897394	90299531	2.924061	0.154411
29538	Del	6	90301250	90511076	3.388083	0.163877
29539	Del	6	90511140	90588440	3.084787	0.15796
29540	Del	6	90590227	91601192	3.586764	0.168018
29541	Del	6	91601461	91696183	3.521686	0.166835
29542	Del	6	91696374	93704030	4.566837	0.188133
29543	Del	6	93706304	95555221	3.471631	0.165651
29544	Del	6	95557976	95574561	2.845836	0.152044
29545	Del	6	95575060	95582508	2.867983	0.152636
29546	Del	6	95583151	95611604	2.836728	0.152044
29547	Del	6	95617125	96217251	3.012405	0.156186
29548	Del	6	96220079	96560772	3.250594	0.16151
29549	Del	6	96560802	96621962	3.23828	0.160918
29550	Del	6	96622224	96839564	3.259463	0.16151
29551	Del	6	96840561	96893601	3.293137	0.162102
29552	Del	6	96893839	96919065	3.508373	0.166835
29553	Del	6	96924218	97006706	3.538727	0.167426
29554	Del	6	97008840	97139742	3.601136	0.168609
29555	Del	6	97140834	97304072	3.629384	0.169201
29556	Del	6	97304948	97711860	3.388034	0.163877
29557	Del	6	97712229	98872092	3.647518	0.169201
29558	Del	6	98873439	98894642	3.544105	0.167426
29559	Del	6	98895004	98954756	3.584972	0.168018
29560	Del	6	98955851	99271050	4.495697	0.186358
29561	Del	6	99272535	99350801	3.506534	0.166835
29562	Del	6	99350959	99423011	4.046736	0.177484
29563	Del	6	99423134	99432504	4.058907	0.178075
29564	Del	6	99432682	99516319	3.95759	0.175709
29565	Del	6	99516558	99541166	4.056255	0.178075
29566	Del	6	99541268	99570964	4.03372	0.177484
29567	Del	6	99571075	99914149	4.078209	0.178075
29568	Del	6	99917848	100387181	4.008014	0.1763
29569	Del	6	100387431	100502979	3.976194	0.175709
29570	Del	6	100507229	101396639	3.772724	0.172159
29571	Del	6	101397136	102072287	2.502729	0.144945
29572	Del	6	102073719	104932148	4.187099	0.180442
29573	Del	6	104934540	104951838	4.139649	0.179258

29574	Del	6	104952227	105084968	4.018977	0.176892
29575	Del	6	105085511	105154813	4.05517	0.178075
29576	Del	6	105156081	105276493	4.096228	0.178667
29577	Del	6	105276766	105407038	3.935401	0.175117
29578	Del	6	105416982	106097881	4.093828	0.178667
29579	Del	6	106098159	106110609	4.123257	0.179258
29580	Del	6	106111727	106317501	4.368859	0.184583
29581	Del	6	106318599	106326688	4.354479	0.183991
29582	Del	6	106327919	106509631	4.545371	0.187541
29583	Del	6	106509848	106569067	4.424278	0.185175
29584	Del	6	106570503	106628169	4.431894	0.185766
29585	Del	6	106628772	106714722	4.507348	0.186949
29586	Del	6	106714773	106783477	4.393203	0.184583
29587	Del	6	106783695	106785572	4.551092	0.187541
29588	Del	6	106786059	106791532	4.393203	0.184583
29589	Del	6	106793180	107024919	4.551092	0.187541
29590	Del	6	107025169	107063692	4.518298	0.186949
29591	Del	6	107065111	107121454	4.470286	0.186358
29592	Del	6	107121767	107486676	4.812605	0.192866
29593	Del	6	107486998	107661721	4.268924	0.182217
29594	Del	6	107661761	107824419	4.537261	0.187541
29595	Del	6	107824597	108037124	4.551092	0.188133
29596	Del	6	108037365	108208374	4.221262	0.181033
29597	Del	6	108209809	108263321	4.202013	0.180442
29598	Del	6	108266199	108526172	5.972961	0.215347
29599	Del	6	108526918	108748767	6.815951	0.239011
29600	Del	6	108751046	108775507	6.199867	0.22008
29601	Del	6	108778515	108982285	6.370791	0.223038
29602	Del	6	108984017	109010548	6.203108	0.22008
29603	Del	6	109010924	109093484	6.097249	0.217713
29604	Del	6	109094022	109120393	6.205784	0.22008
29605	Del	6	109123671	109235664	6.23652	0.220671
29606	Del	6	109236339	109273182	6.177635	0.219488
29607	Del	6	109273584	109365907	6.210031	0.22008
29608	Del	6	109366375	109392096	5.379	0.204106
29609	Del	6	109392130	109442511	5.333293	0.202923
29610	Del	6	109443767	109468314	4.518832	0.186949
29611	Del	6	109468395	109479359	4.488117	0.186358
29612	Del	6	109480929	109485060	4.524043	0.186949
29613	Del	6	109490057	109627633	5.073676	0.197598
29614	Del	6	109628440	109696837	5.254774	0.201148
29615	Del	6	109701082	109747936	5.136004	0.198782
29616	Del	6	109750032	109759583	5.367597	0.203515
29617	Del	6	109760415	109826722	5.136004	0.198782
29618	Del	6	109827621	109980742	5.364553	0.203515
29619	Del	6	109980962	110181628	5.378585	0.204106
29620	Del	6	110182989	110242160	5.235416	0.200556
29621	Del	6	110245381	110387423	5.087567	0.19819
29622	Del	6	110391946	110420994	4.742516	0.191682
29623	Del	6	110424584	110477582	4.691523	0.190499
29624	Del	6	110477998	110802862	5.032291	0.197007
29625	Del	6	110811048	110858415	5.120398	0.198782
29626	Del	6	110858969	110898297	5.174199	0.199373

29627	Del	6	110912656	110978713	5.205378	0.199965
29628	Del	6	110980937	111029497	5.175773	0.199373
29629	Del	6	111031405	111082231	5.265165	0.201148
29630	Del	6	111086441	111227227	5.074711	0.197598
29631	Del	6	111227610	111294848	5.250691	0.201148
29632	Del	6	111296360	111474902	5.044265	0.197007
29633	Del	6	111481590	111552621	5.212208	0.200556
29634	Del	6	111553772	111567409	5.336518	0.202923
29635	Del	6	111573787	111656270	5.25585	0.201148
29636	Del	6	111656711	112051723	4.742516	0.191682
29637	Del	6	112053010	112089798	4.553331	0.188133
29638	Del	6	112089979	112107092	4.540954	0.187541
29639	Del	6	112107443	112255701	4.330632	0.1834
29640	Del	6	112257505	112306838	3.818303	0.172751
29641	Del	6	112306941	113620909	3.935817	0.175117
29642	Del	6	113620985	113652154	3.898847	0.174526
29643	Del	6	113652194	113898114	3.938436	0.175117
29644	Del	6	113898467	113974101	3.902786	0.174526
29645	Del	6	113974320	115632660	3.464333	0.165651
29646	Del	6	115632874	115940844	1.760334	0.126605
29647	Del	6	115941378	116037295	1.684655	0.124239
29648	Del	6	116038615	116040033	1.768667	0.126605
29649	Del	6	116040569	116116048	1.684655	0.124239
29650	Del	6	116118844	116132304	1.767157	0.126605
29651	Del	6	116132898	116246067	1.67666	0.124239
29652	Del	6	116247610	116252777	1.407165	0.116548
29653	Del	6	116254009	116272134	1.492774	0.119506
29654	Del	6	116274746	116452237	1.504335	0.120097
29655	Del	6	116452532	116487724	1.23952	0.111223
29656	Del	6	116493205	116508160	1.212857	0.110631
29657	Del	6	116511480	116635078	1.230825	0.111223
29658	Del	6	116635326	116678964	1.07765	0.10649
29659	Del	6	116680799	116750220	1.590151	0.121872
29660	Del	6	116751758	116872426	1.058251	0.105899
29661	Del	6	116872999	116933341	0.57425	0.089333
29662	Del	6	116935518	117286657	0.604707	0.089925
29663	Del	6	117287155	117428359	0.536439	0.087559
29664	Del	6	117428689	117549214	0.561138	0.088742
29665	Del	6	117558532	117603194	0.584879	0.089333
29666	Del	6	117610425	117711661	0.604091	0.089925
29667	Del	6	117714589	118085639	0.680214	0.092883
29668	Del	6	118085696	118099282	0.859632	0.098799
29669	Del	6	118101650	118816495	0.680214	0.092883
29670	Del	6	118816865	118824820	0.580559	0.089333
29671	Del	6	118825212	118909333	0.596649	0.089925
29672	Del	6	118909350	118909350	0.580559	0.089333
29673	Del	6	118909603	118953492	0.593938	0.089925
29674	Del	6	118954424	119067696	0.582254	0.089333
29675	Del	6	119067783	119070016	0.637595	0.091108
29676	Del	6	119070041	119079309	0.582254	0.089333
29677	Del	6	119081767	119176011	0.555512	0.08815
29678	Del	6	119176358	119352783	0.523876	0.086967
29679	Del	6	119353338	119547665	0.582273	0.089333

29680	Del	6	119547731	120012365	0.510753	0.086375
29681	Del	6	120012394	121068259	0.191385	0.071585
29682	Del	6	121075722	121335044	0.164916	0.070402
29683	Del	6	121335549	122397228	0.210298	0.072768
29684	Del	6	122397384	122474579	0.055527	0.063302
29685	Del	6	122476539	122606905	0.018924	0.060936
29686	Del	6	122606981	122652282	0.034913	0.062119
29687	Del	6	122652287	122775414	0.044487	0.062711
29688	Del	6	122777134	122993555	0.060057	0.063302
29689	Del	6	122994363	123214645	0.042296	0.062711
29690	Del	6	123215440	123347540	0.002998	0.059753
29691	Del	6	123347661	123380249	0.021	0.060936
29692	Del	6	123380793	123478344	0.058762	0.063302
29693	Del	6	123480522	123526884	0.026473	0.061528
29694	Del	6	123527577	123784052	0.034593	0.062119
29695	Del	6	123788717	124964263	0	0.052062
29696	Del	6	124966415	125151632	0	0.050879
29697	Del	6	125151667	125203233	0	0.041413
29698	Del	6	125203572	125264431	0	0.042004
29699	Del	6	125264587	125774925	0	0.042596
29700	Del	6	125777085	125797668	0	0.041413
29701	Del	6	125797748	125820914	0	0.042596
29702	Del	6	125821727	125914281	0	0.041413
29703	Del	6	125918516	126348993	0	0.042596
29704	Del	6	126349449	126481676	0	0.042004
29705	Del	6	126481821	127116431	0	0.042596
29706	Del	6	127116472	127199912	0	0.042004
29707	Del	6	127202143	127459702	0	0.044963
29708	Del	6	127459909	127519665	0	0.044371
29709	Del	6	127519762	127704493	0	0.044963
29710	Del	6	127705091	127863049	0	0.041413
29711	Del	6	127863593	127964392	0	0.040821
29712	Del	6	127968366	127999767	0	0.037863
29713	Del	6	127999909	128026303	0	0.038455
29714	Del	6	128027062	128087305	0	0.042596
29715	Del	6	128090031	128880535	0	0.038455
29716	Del	6	128882518	129576206	0	0.030764
29717	Del	6	129576289	129710348	0	0.026031
29718	Del	6	129711077	130017475	0	0.027214
29719	Del	6	130018435	130223364	0	0.026623
29720	Del	6	130223591	130371399	0	0.025439
29721	Del	6	130371653	130841125	0	0.015382
29722	Del	6	130843758	131001286	0	0.01479
29723	Del	6	131002715	131066884	0	0.014199
29724	Del	6	131072276	131243352	0	0.01479
29725	Del	6	131248698	131787689	0	0.015382
29726	Del	6	131790226	131798226	0	0.004733
29727	Del	6	131802350	133239632	0	0.015382
29728	Del	6	133240173	133532234	0	0.013607
29729	Del	6	133532894	133811100	0	0.013015
29730	Del	6	133818312	133854270	0	0.015382
29731	Del	6	133854907	133878041	0	0.013015
29732	Del	6	133889105	134178042	0	0.015382

29733	Del	6	134179787	134324995	0	0.01479
29734	Del	6	134327276	134544468	0	0.015382
29735	Del	6	134544687	135179752	0	0.020706
29736	Del	6	135180217	135222669	0	0.020115
29737	Del	6	135222789	135282771	0	0.020706
29738	Del	6	135283183	135383608	0	0.019523
29739	Del	6	135387183	135499928	0	0.020115
29740	Del	6	135500252	135691643	0	0.019523
29741	Del	6	135694363	136230223	0	0.027806
29742	Del	6	136230796	136337120	0	0.020706
29743	Del	6	136337264	136467438	0	0.020115
29744	Del	6	136470018	136626402	0	0.019523
29745	Del	6	136626688	136648476	0	0.020706
29746	Del	6	136648715	136738789	0	0.019523
29747	Del	6	136738812	137499652	0	0.020706
29748	Del	6	137502811	138158105	0	0.025439
29749	Del	6	138160454	138213496	0	0.019523
29750	Del	6	138213649	138222483	0	0.020706
29751	Del	6	138222970	138350022	0	0.019523
29752	Del	6	138355595	138415289	0	0.020706
29753	Del	6	138415949	138500571	0	0.025439
29754	Del	6	138501492	138573657	0	0.024848
29755	Del	6	138573715	138801103	0	0.031355
29756	Del	6	138801204	138992158	0	0.030764
29757	Del	6	138995176	139767424	0	0.031355
29758	Del	6	139770594	139852653	0	0.030764
29759	Del	6	139854298	139861208	0	0.031355
29760	Del	6	139862478	140094038	0	0.034905
29761	Del	6	140094686	142045397	0	0.035497
29762	Del	6	142048204	142147616	0	0.015382
29763	Del	6	142149571	142224143	0	0.010057
29764	Del	6	142227434	142640330	0	0.026623
29765	Del	6	142640572	142963675	0	0.043188
29766	Del	6	142964393	143058214	0	0.026031
29767	Del	6	143058660	143058660	0	0.024848
29768	Del	6	143059965	143191537	0	0.026031
29769	Del	6	143191981	143341413	0	0.024848
29770	Del	6	143341426	143493078	0	0.026623
29771	Del	6	143493133	143511989	0	0.026031
29772	Del	6	143512246	143604237	0	0.026623
29773	Del	6	143604283	143672527	0	0.024848
29774	Del	6	143673062	143831364	0	0.025439
29775	Del	6	143832141	143859316	0	0.026623
29776	Del	6	143860175	143943937	0	0.028397
29777	Del	6	143944473	144009919	0	0.022481
29778	Del	6	144010354	144066561	0	0.02189
29779	Del	6	144066729	144191956	0	0.022481
29780	Del	6	144194089	145615067	0	0.033722
29781	Del	6	145616050	145796433	0	0.023664
29782	Del	6	145797847	145884468	0	0.024256
29783	Del	6	145884481	146027163	0	0.034905
29784	Del	6	146027194	146438129	0	0.032539
29785	Del	6	146443346	146601190	0	0.035497

29786	Del	6	146601408	146795724	0	0.033722
29787	Del	6	146799180	146805810	0	0.035497
29788	Del	6	146816925	146841113	0	0.033722
29789	Del	6	146841306	147205673	0	0.032539
29790	Del	6	147206849	147390578	0	0.028397
29791	Del	6	147390758	148340745	0	0.03313
29792	Del	6	148341560	148735327	0	0.027214
29793	Del	6	148736360	148953410	0	0.018932
29794	Del	6	148953995	148965064	0	0.021298
29795	Del	6	148965462	149019525	0	0.018932
29796	Del	6	149026368	149034165	0	0.021298
29797	Del	6	149034317	149077752	0	0.018932
29798	Del	6	149078472	149231167	0	0.021298
29799	Del	6	149231451	149315830	0	0.020115
29800	Del	6	149315970	149390847	0	0.020706
29801	Del	6	149390916	149404154	0	0.021298
29802	Del	6	149404660	149412617	0	0.020706
29803	Del	6	149414294	150139670	0	0.021298
29804	Del	6	150141984	150250711	0	0.020706
29805	Del	6	150251072	150599090	0	0.021298
29806	Del	6	150599261	150632760	0	0.020706
29807	Del	6	150632806	150667767	0	0.021298
29808	Del	6	150668562	150682621	0	0.019523
29809	Del	6	150682704	150720216	0	0.020115
29810	Del	6	150720249	150844548	0	0.020706
29811	Del	6	150846208	150900306	0	0.021298
29812	Del	6	150901128	151103362	0	0.019523
29813	Del	6	151104031	151238574	0	0.021298
29814	Del	6	151238717	151324226	0	0.020706
29815	Del	6	151324434	151392675	0	0.021298
29816	Del	6	151395044	151488465	0	0.042004
29817	Del	6	151492189	151622842	0	0.020706
29818	Del	6	151623016	151801264	0	0.055612
29819	Del	6	151801912	152131793	0	0.056795
29820	Del	6	152133351	152696105	0	0.031947
29821	Del	6	152696763	152762275	0	0.028397
29822	Del	6	152762291	153002031	0	0.037272
29823	Del	6	153008373	153418419	0	0.046737
29824	Del	6	153418585	153420941	0	0.038455
29825	Del	6	153421778	154032236	0	0.056203
29826	Del	6	154033183	154088859	0	0.056795
29827	Del	6	154088979	154093311	0	0.057386
29828	Del	6	154093689	154132442	0	0.056795
29829	Del	6	154133280	154148427	0	0.054428
29830	Del	6	154151192	154332120	0	0.05502
29831	Del	6	154333844	154356878	0	0.054428
29832	Del	6	154359292	154513540	0	0.056203
29833	Del	6	154514048	154836332	0	0.056795
29834	Del	6	154838086	155087283	0	0.057386
29835	Del	6	155088485	155211555	0	0.055612
29836	Del	6	155215472	155258614	0	0.056795
29837	Del	6	155262305	155278608	0	0.057386
29838	Del	6	155280102	155394443	0	0.056795

29839	Del	6	155395338	155457511	0	0.056203
29840	Del	6	155458860	156782487	0	0.057386
29841	Del	6	156793880	157194429	0	0.055612
29842	Del	6	157214426	157354967	0.017054	0.060344
29843	Del	6	157355538	157513830	0	0.05857
29844	Del	6	157514124	157531128	0.018569	0.060344
29845	Del	6	157535098	157674251	0	0.05857
29846	Del	6	157675266	158001045	0.217127	0.07336
29847	Del	6	158001048	158003807	0.25536	0.075135
29848	Del	6	158007555	158013993	0.218738	0.07336
29849	Del	6	158015754	158102110	0.229711	0.073952
29850	Del	6	158105549	158175267	0.244781	0.074543
29851	Del	6	158176152	158305184	0.014865	0.060344
29852	Del	6	158310579	158514035	0	0.057386
29853	Del	6	158514086	158605724	0.004389	0.059753
29854	Del	6	158608310	158612020	0.018569	0.060344
29855	Del	6	158612814	158629508	0.004389	0.059753
29856	Del	6	158634250	158647682	0.017993	0.060344
29857	Del	6	158647736	158656293	0.00167	0.059161
29858	Del	6	158662595	158762965	0.003254	0.059753
29859	Del	6	158763475	158766054	0.018569	0.060344
29860	Del	6	158769768	158815311	0.010107	0.059753
29861	Del	6	158816693	159171745	0.018569	0.060344
29862	Del	6	159173132	159272152	0	0.05857
29863	Del	6	159272292	159396474	0.01761	0.060344
29864	Del	6	159401108	159756975	0.208784	0.072768
29865	Del	6	159759800	159759800	0.19197	0.072177
29866	Del	6	159760070	159905658	0.20989	0.072768
29867	Del	6	159906293	159964760	0.018569	0.060344
29868	Del	6	159968207	160001753	0	0.05857
29869	Del	6	160001813	160007675	0.018482	0.060344
29870	Del	6	160010348	160092611	0	0.05857
29871	Del	6	160092697	160096449	0.018569	0.060344
29872	Del	6	160096646	160112608	0	0.05857
29873	Del	6	160112987	160160342	0.011115	0.060344
29874	Del	6	160160366	160344375	0.030827	0.062119
29875	Del	6	160345738	160453031	0	0.05857
29876	Del	6	160455062	160518195	0.010637	0.060344
29877	Del	6	160518464	160669538	0.146346	0.069219
29878	Del	6	160670561	160712014	0.182525	0.070993
29879	Del	6	160715453	160988440	0.164986	0.070402
29880	Del	6	160990011	161129476	0	0.041413
29881	Del	6	161129528	161156097	0	0.040821
29882	Del	6	161157162	161165808	0	0.042596
29883	Del	6	161166075	161274911	0	0.040821
29884	Del	6	161277226	162731531	0	0.047329
29885	Del	6	162732176	163038150	0	0.03668
29886	Del	6	163040157	163054393	0	0.042596
29887	Del	6	163054833	163180212	0	0.03668
29888	Del	6	163180316	163196592	0	0.042596
29889	Del	6	163198169	163309499	0	0.03668
29890	Del	6	163309640	163420120	0	0.042596
29891	Del	6	163420868	163579087	0	0.041413

29892	Del	6	163580460	163674479	0	0.042596
29893	Del	6	163675702	165323080	0	0.053837
29894	Del	6	165323097	165901500	0.082481	0.065077
29895	Del	6	165902119	165983119	0	0.053245
29896	Del	6	165985722	166170013	0	0.053837
29897	Del	6	166170248	166407763	0	0.05857
29898	Del	6	166408749	166459522	0	0.056795
29899	Del	6	166460335	166467838	0	0.05857
29900	Del	6	166468157	166508553	0	0.056795
29901	Del	6	166508605	166513676	0	0.05857
29902	Del	6	166514010	166544695	0	0.056795
29903	Del	6	166544900	166629757	0	0.05502
29904	Del	6	166630591	166864488	0	0.05147
29905	Del	6	166864783	166929745	0	0.05857
29906	Del	6	166932686	166958278	0	0.057978
29907	Del	6	166960059	167001456	0	0.05857
29908	Del	6	167002117	167045257	0	0.057978
29909	Del	6	167048639	167139546	0	0.05857
29910	Del	6	167141072	167862052	0.342947	0.079276
29911	Del	6	167862339	167930914	0.325913	0.078684
29912	Del	6	167931668	168457458	0.342947	0.079276
29913	Del	6	168461894	168684009	0.315563	0.078093
29914	Del	6	168685087	169239806	0.342947	0.079276
29915	Del	6	169239904	169255805	0.327493	0.078684
29916	Del	6	169255984	169700557	0.897542	0.099982
29917	Del	6	169701185	170251061	1.03853	0.104715
29918	Del	6	170252698	170294466	0.423612	0.082826
29919	Del	6	170294680	170328362	0.377003	0.081051
29920	Del	6	170330685	170334073	0.423612	0.082826
29921	Del	6	170334810	170407993	0.3794	0.081051
29922	Del	6	170408127	170555643	0.555132	0.08815
29923	Del	6	170555865	170596889	0.411423	0.082826
29924	Del	7	665647	730173	1.837801	0.12838
29925	Del	7	742958	788915	1.812168	0.127788
29926	Del	7	793348	845067	2.01316	0.133113
29927	Del	7	845106	879502	1.97881	0.132521
29928	Del	7	880030	1419586	1.518037	0.120097
29929	Del	7	1419643	1706713	1.408257	0.116548
29930	Del	7	1707665	1731977	1.391554	0.115956
29931	Del	7	1732077	1809491	1.407165	0.116548
29932	Del	7	1809777	1836445	1.368977	0.115364
29933	Del	7	1837266	1846697	1.411539	0.116548
29934	Del	7	1846810	1941911	1.368977	0.115364
29935	Del	7	1945404	2304677	1.226406	0.111223
29936	Del	7	2305304	2558338	0.938264	0.101166
29937	Del	7	2558569	2629599	0.913604	0.100574
29938	Del	7	2629953	2674723	0.894051	0.099982
29939	Del	7	2681714	2768404	1.151068	0.108857
29940	Del	7	2768464	2819427	1.142875	0.108265
29941	Del	7	2820182	2903815	1.125324	0.107673
29942	Del	7	2904896	3044847	0.717095	0.094066
29943	Del	7	3046574	3297806	0.832043	0.097616
29944	Del	7	3299057	4127704	0.558417	0.08815

29945	Del	7	4127810	4269062	0.721432	0.094066
29946	Del	7	4269145	4773752	0.813114	0.097024
29947	Del	7	4774611	4810828	0.839239	0.098208
29948	Del	7	4814999	4852982	0.819252	0.097616
29949	Del	7	4856591	4863365	0.84413	0.098208
29950	Del	7	4864876	4934497	0.819252	0.097616
29951	Del	7	4934872	5183185	0.84211	0.098208
29952	Del	7	5183952	5211809	0.833825	0.097616
29953	Del	7	5212643	5304687	0.835641	0.098208
29954	Del	7	5316859	5429171	0.815166	0.097024
29955	Del	7	5433063	5616204	0.836537	0.098208
29956	Del	7	5616670	5658238	0.801428	0.097024
29957	Del	7	5658297	5682707	0.839239	0.098208
29958	Del	7	5688047	5709363	0.801428	0.097024
29959	Del	7	5709591	6025563	0.847067	0.098208
29960	Del	7	6026173	6029688	0.827278	0.097616
29961	Del	7	6030297	6037274	0.84211	0.098208
29962	Del	7	6037665	6161627	0.827278	0.097616
29963	Del	7	6168738	6274279	0.812277	0.097024
29964	Del	7	6274409	6370690	0.839239	0.098208
29965	Del	7	6372700	6448111	0.832805	0.097616
29966	Del	7	6450768	6496420	0.834605	0.098208
29967	Del	7	6496486	6552202	0.832805	0.097616
29968	Del	7	6552755	6667179	0.840098	0.098208
29969	Del	7	6680710	7180603	0.699044	0.093475
29970	Del	7	7181394	7246814	0.636022	0.091108
29971	Del	7	7254984	7357985	0.642599	0.0917
29972	Del	7	7358212	7537190	0.580064	0.089333
29973	Del	7	7537544	7572218	0.662982	0.092291
29974	Del	7	7572615	7634974	0.654142	0.0917
29975	Del	7	7635198	7718928	0.625575	0.091108
29976	Del	7	7719570	7879267	0.509573	0.086375
29977	Del	7	7881007	7971273	0.620811	0.090517
29978	Del	7	7974560	8089314	0.588046	0.089333
29979	Del	7	8090098	8138095	0.609478	0.090517
29980	Del	7	8139207	8492889	0.637595	0.091108
29981	Del	7	8493103	8756025	0.534346	0.087559
29982	Del	7	8756748	10930532	1.03853	0.104715
29983	Del	7	10930807	10969581	0.537518	0.087559
29984	Del	7	10973313	11108170	0.500362	0.086375
29985	Del	7	11108758	11368624	0.472588	0.085192
29986	Del	7	11370251	12210267	0.446865	0.084009
29987	Del	7	12210776	12237539	0.330764	0.079276
29988	Del	7	12238523	12404864	0.620811	0.090517
29989	Del	7	12407007	12570053	0.667119	0.092291
29990	Del	7	12570362	12653767	0.642599	0.0917
29991	Del	7	12654425	13885046	0.687404	0.092883
29992	Del	7	13891105	13994450	0.254464	0.075135
29993	Del	7	13995416	14175001	0.420522	0.082826
29994	Del	7	14175512	15196703	0.437154	0.083417
29995	Del	7	15200269	15609236	0.185757	0.071585
29996	Del	7	15609333	15659902	0.029799	0.061528
29997	Del	7	15667820	15684128	0.044557	0.062711

29998	Del	7	15684532	15686748	0.029799	0.061528
29999	Del	7	15687360	16087084	0.046375	0.062711
30000	Del	7	16087186	16205589	0	0.059161
30001	Del	7	16207996	16270748	0.03148	0.062119
30002	Del	7	16270881	16422338	0	0.059161
30003	Del	7	16422362	16525508	0.048332	0.062711
30004	Del	7	16525715	16647917	0.034432	0.062119
30005	Del	7	16648521	16784669	0	0.053245
30006	Del	7	16785004	16885894	0	0.053837
30007	Del	7	16890902	17759717	0.010637	0.059753
30008	Del	7	17759976	18022819	0.097806	0.066261
30009	Del	7	18022944	18084225	0.000769	0.059161
30010	Del	7	18085874	18125357	0	0.052653
30011	Del	7	18126157	18128694	0.00167	0.059161
30012	Del	7	18129389	18288945	0	0.052653
30013	Del	7	18289454	18484674	0	0.05502
30014	Del	7	18492003	18561726	0	0.059161
30015	Del	7	18561742	18668888	0	0.057386
30016	Del	7	18674687	18867273	0	0.053245
30017	Del	7	18867407	19748494	0.00167	0.059161
30018	Del	7	19749151	19913829	0	0.05857
30019	Del	7	19918763	20131723	0	0.056203
30020	Del	7	20133039	20139730	0	0.057978
30021	Del	7	20139921	20154254	0.000769	0.059161
30022	Del	7	20154356	20217202	0	0.057978
30023	Del	7	20217322	20311779	0.000981	0.059161
30024	Del	7	20312107	20604187	0.180872	0.070993
30025	Del	7	20604717	20646520	0	0.049104
30026	Del	7	20646788	20670414	0	0.050879
30027	Del	7	20671025	20775305	0	0.049695
30028	Del	7	20775952	20832584	0	0.040821
30029	Del	7	20833665	21024072	0	0.039046
30030	Del	7	21024968	21434992	0	0.040821
30031	Del	7	21440360	21468262	0	0.04023
30032	Del	7	21470127	21472234	0	0.040821
30033	Del	7	21472264	21538034	0	0.04023
30034	Del	7	21540083	21872586	0	0.03668
30035	Del	7	21872858	22116378	0	0.040821
30036	Del	7	22116894	22359535	0	0.028397
30037	Del	7	22360350	22502148	0	0.030172
30038	Del	7	22505963	22941019	0	0.030764
30039	Del	7	22941058	23014329	0	0.030172
30040	Del	7	23014386	23106333	0	0.030764
30041	Del	7	23109119	23175436	0	0.030172
30042	Del	7	23176069	23309630	0	0.030764
30043	Del	7	23309821	23484926	0	0.029581
30044	Del	7	23487680	23706453	0	0.023073
30045	Del	7	23706539	24282738	0	0.022481
30046	Del	7	24283190	24793532	0	0.014199
30047	Del	7	24795238	24892727	0	0.013607
30048	Del	7	24892747	24983449	0	0.013015
30049	Del	7	24983481	25945319	0	0.014199
30050	Del	7	25945722	25952357	0	0.007691

30051	Del	7	25953735	26400513	0	0.015382
30052	Del	7	26400761	26498717	0	0.01479
30053	Del	7	26498792	26663945	0	0.015382
30054	Del	7	26664020	27086746	0	0.01479
30055	Del	7	27087612	27182984	0	0.012424
30056	Del	7	27183944	27248705	0	0.009466
30057	Del	7	27252301	27736275	0	0.021298
30058	Del	7	27736611	27830544	0	0.009466
30059	Del	7	27832917	28183574	0	0.008283
30060	Del	7	28189423	28298480	0	0.007691
30061	Del	7	28299238	28412048	0	0.005325
30062	Del	7	28412483	28680992	0	0.005916
30063	Del	7	28682322	28825912	0	0.007099
30064	Del	7	28829202	29013663	0	0.007691
30065	Del	7	29015583	29119096	0	0.007099
30066	Del	7	29119677	29133093	0	0.007691
30067	Del	7	29133432	29140977	0	0.007099
30068	Del	7	29143851	29804354	0	0.019523
30069	Del	7	29804704	29918070	0	0.013015
30070	Del	7	29920049	29991658	0	0.013607
30071	Del	7	29992068	30027691	0	0.020115
30072	Del	7	30027732	30495594	0	0.022481
30073	Del	7	30495912	30648312	0	0.020115
30074	Del	7	30648841	30759066	0	0.019523
30075	Del	7	30759902	30893280	0	0.018932
30076	Del	7	30895044	31330264	0	0.019523
30077	Del	7	31335295	31341016	0	0.015382
30078	Del	7	31341280	31552070	0	0.024848
30079	Del	7	31552734	31654762	0	0.025439
30080	Del	7	31655209	31658880	0	0.024848
30081	Del	7	31659768	31750680	0	0.026031
30082	Del	7	31750866	32300304	0	0.023664
30083	Del	7	32300487	32454823	0	0.022481
30084	Del	7	32454860	32496308	0	0.019523
30085	Del	7	32496688	32717770	0	0.018932
30086	Del	7	32725825	32866234	0	0.019523
30087	Del	7	32868113	33066980	0	0.008874
30088	Del	7	33067936	33903204	0	0.021298
30089	Del	7	33903679	34345579	0	0.018932
30090	Del	7	34346288	34760809	0	0.01834
30091	Del	7	34761651	34834426	0	0.017157
30092	Del	7	34837949	34878728	0	0.016565
30093	Del	7	34878903	35802378	0	0.017748
30094	Del	7	35808346	35907169	0	0.017157
30095	Del	7	35907313	36151967	0	0.017748
30096	Del	7	36152090	36301612	0	0.016565
30097	Del	7	36302150	36508777	0	0.017748
30098	Del	7	36510194	36595874	0	0.016565
30099	Del	7	36596843	36603656	0	0.017748
30100	Del	7	36604909	36727078	0	0.016565
30101	Del	7	36728195	37451585	0	0.019523
30102	Del	7	37453457	37845649	0	0.023664
30103	Del	7	37846533	38177110	0	0.015974

30104	Del	7	38178000	38634990	0	0.01479
30105	Del	7	38635039	38720248	0	0.016565
30106	Del	7	38720437	38971442	0	0.015382
30107	Del	7	38975631	38978805	0	0.008874
30108	Del	7	38979803	39014536	0	0.010649
30109	Del	7	39016988	39391989	0	0.008874
30110	Del	7	39396495	39409031	0	0.010649
30111	Del	7	39409412	39464806	0	0.008874
30112	Del	7	39465460	39620177	0	0.028397
30113	Del	7	39622669	39710077	0	0.027806
30114	Del	7	39711559	39942961	0	0.028397
30115	Del	7	39947980	40100873	0	0.026031
30116	Del	7	40101030	40135967	0	0.026623
30117	Del	7	40136125	40867003	0	0.022481
30118	Del	7	40867098	41675847	0	0.026623
30119	Del	7	41677285	41779879	0	0.008874
30120	Del	7	41780312	42660576	0	0.009466
30121	Del	7	42661302	43250941	0	0.008874
30122	Del	7	43252724	43507201	0	0.007691
30123	Del	7	43508149	43524543	0	0.008874
30124	Del	7	43524617	43565505	0	0.007691
30125	Del	7	43568834	43925401	0	0.008874
30126	Del	7	43925475	44331815	0	0.010057
30127	Del	7	44332588	44491650	0	0.01834
30128	Del	7	44493099	45076637	0	0.018932
30129	Del	7	45077818	45665418	0	0.017748
30130	Del	7	45669805	45721752	0	0.017157
30131	Del	7	45722239	47271185	0	0.017748
30132	Del	7	47272241	47586799	0	0.015974
30133	Del	7	47586816	47769489	0	0.017748
30134	Del	7	47770671	47949556	0	0.022481
30135	Del	7	47953436	48030615	0	0.023664
30136	Del	7	48030897	48158439	0	0.027806
30137	Del	7	48159502	48648120	0	0.024256
30138	Del	7	48648995	48929814	0	0.032539
30139	Del	7	48930198	49923210	0	0.03313
30140	Del	7	49924325	50301181	0	0.033722
30141	Del	7	50302666	50376209	0	0.030172
30142	Del	7	50380400	50405553	0	0.029581
30143	Del	7	50406053	50456510	0	0.030172
30144	Del	7	50458202	50587672	0	0.030764
30145	Del	7	50589324	50732437	0	0.030172
30146	Del	7	50733633	51015843	0	0.029581
30147	Del	7	51016125	51064179	0	0.01834
30148	Del	7	51068499	53034566	0	0.018932
30149	Del	7	53035006	53653431	0	0.013015
30150	Del	7	53653719	54197894	0	0.012424
30151	Del	7	54198159	54349896	0	0.010649
30152	Del	7	54350565	54574291	0	0.011832
30153	Del	7	54575238	55017274	0	0.028989
30154	Del	7	55017506	55177671	0	0.024256
30155	Del	7	55177814	55189235	0	0.027214
30156	Del	7	55189720	55210271	0	0.024256

30157	Del	7	55212022	55364530	0	0.027214
30158	Del	7	55364599	55435616	0	0.026623
30159	Del	7	55436422	55554031	0	0.027214
30160	Del	7	55557791	55574105	0	0.026623
30161	Del	7	55576194	55792547	0	0.034313
30162	Del	7	55792772	55867189	0	0.020115
30163	Del	7	55868115	56122391	0	0.02189
30164	Del	7	56122700	56542963	0	0.027214
30165	Del	7	56544468	57164131	0	0.032539
30166	Del	7	57165389	57184979	0	0.024848
30167	Del	7	57185111	57854817	0	0.022481
30168	Del	7	57861998	64033122	0	0.025439
30169	Del	7	64035211	64436418	0	0.027806
30170	Del	7	64437168	64571153	0	0.031947
30171	Del	7	64573929	64781884	0	0.026623
30172	Del	7	64783751	65011052	0	0.023073
30173	Del	7	65013030	65773545	0	0.032539
30174	Del	7	65776453	66068022	0	0.03668
30175	Del	7	66072009	66197963	0	0.023664
30176	Del	7	66198463	66360996	0	0.023073
30177	Del	7	66362795	66401533	0	0.023664
30178	Del	7	66402028	66686734	0	0.028989
30179	Del	7	66687804	66724872	0	0.028397
30180	Del	7	66725714	66979302	0	0.028989
30181	Del	7	66979463	66990186	0	0.024848
30182	Del	7	66994521	67148046	0	0.043779
30183	Del	7	67151617	68017123	0	0.044371
30184	Del	7	68020240	68063261	0	0.030172
30185	Del	7	68063408	71130639	0	0.052062
30186	Del	7	71131346	71302122	0	0.024848
30187	Del	7	71302334	71317390	0	0.028989
30188	Del	7	71318777	71714331	0	0.024848
30189	Del	7	71720115	72523231	0	0.036088
30190	Del	7	72523346	73312738	0	0.023664
30191	Del	7	73317954	73360297	0	0.017748
30192	Del	7	73361556	74615750	0	0.018932
30193	Del	7	74616516	75530800	0	0.024256
30194	Del	7	75533626	75745339	0	0.023073
30195	Del	7	75746794	75809462	0	0.024256
30196	Del	7	75810378	75891361	0	0.018932
30197	Del	7	75891866	75995911	0	0.035497
30198	Del	7	75997677	76362917	0	0.018932
30199	Del	7	76362980	76386905	0	0.028989
30200	Del	7	76393107	76424968	0	0.034313
30201	Del	7	76426530	77307636	0	0.03668
30202	Del	7	77308656	77410166	0	0.026623
30203	Del	7	77410251	77521287	0	0.027214
30204	Del	7	77522948	77640518	0	0.026623
30205	Del	7	77643054	77786518	0	0.027214
30206	Del	7	77793240	77799672	0	0.020115
30207	Del	7	77802670	77838295	0	0.019523
30208	Del	7	77838691	77926955	0	0.020115
30209	Del	7	77927697	77961807	0	0.019523

30210	Del	7	77962318	79006886	0	0.025439
30211	Del	7	79007127	79014313	0	0.03313
30212	Del	7	79014952	79441151	0	0.025439
30213	Del	7	79451726	79474220	0	0.03313
30214	Del	7	79474327	80220125	0	0.033722
30215	Del	7	80220168	80596831	0	0.034313
30216	Del	7	80596858	80625476	0	0.033722
30217	Del	7	80625554	80737984	0	0.034313
30218	Del	7	80741118	80923635	0	0.032539
30219	Del	7	80924091	81632517	0	0.034313
30220	Del	7	81632581	81742286	0	0.033722
30221	Del	7	81742303	81942832	0	0.034313
30222	Del	7	81943306	82001649	0	0.030764
30223	Del	7	82007813	82031297	0	0.034313
30224	Del	7	82033959	82042943	0	0.030764
30225	Del	7	82044456	82553123	0	0.031355
30226	Del	7	82554167	82816702	0	0.049104
30227	Del	7	82816824	83359527	0	0.049695
30228	Del	7	83361442	83649991	0	0.037272
30229	Del	7	83652092	84528864	0.020455	0.060936
30230	Del	7	84529068	84994358	0	0.04023
30231	Del	7	84995116	85125600	0	0.039638
30232	Del	7	85126104	86643483	0	0.04023
30233	Del	7	86643848	86868493	0	0.032539
30234	Del	7	86869524	86968364	0	0.033722
30235	Del	7	86968786	87060457	0	0.03313
30236	Del	7	87060579	87361430	0	0.034313
30237	Del	7	87361438	87499642	0	0.033722
30238	Del	7	87501081	87624397	0	0.03313
30239	Del	7	87625332	87833200	0	0.033722
30240	Del	7	87833519	87932477	0	0.034313
30241	Del	7	87933357	88184094	0	0.048512
30242	Del	7	88184492	88197642	0	0.047921
30243	Del	7	88201991	90331818	0	0.050287
30244	Del	7	90332562	90593737	0	0.041413
30245	Del	7	90593986	90706956	0	0.038455
30246	Del	7	90707320	91210808	0	0.039638
30247	Del	7	91213056	91935392	0	0.041413
30248	Del	7	91939161	92110499	0	0.04023
30249	Del	7	92110939	92250509	0	0.041413
30250	Del	7	92256745	92401751	0	0.04023
30251	Del	7	92402634	92536548	0	0.041413
30252	Del	7	92542959	92600848	0	0.034313
30253	Del	7	92601702	92832424	0	0.03313
30254	Del	7	92834562	92918650	0	0.035497
30255	Del	7	92919070	93158646	0	0.03668
30256	Del	7	93159577	93280121	0	0.042004
30257	Del	7	93281562	93421518	0	0.041413
30258	Del	7	93423277	93712700	0	0.040821
30259	Del	7	93714957	94581493	0	0.03668
30260	Del	7	94581810	94649834	0	0.036088
30261	Del	7	94650032	94672962	0	0.03668
30262	Del	7	94673822	95302400	0	0.052653

30263	Del	7	95302663	95325307	0	0.034905
30264	Del	7	95325384	95600536	0	0.035497
30265	Del	7	95600949	96114162	0	0.040821
30266	Del	7	96114606	96219030	0	0.042596
30267	Del	7	96219058	96221140	0	0.044371
30268	Del	7	96222991	96325176	0	0.042596
30269	Del	7	96326244	96504219	0	0.044371
30270	Del	7	96504803	96964830	0	0.048512
30271	Del	7	96965543	97991379	0	0.044371
30272	Del	7	97992624	98209828	0	0.044963
30273	Del	7	98210165	98282929	0	0.045554
30274	Del	7	98290859	98405981	0	0.050287
30275	Del	7	98408027	98637526	0	0.050879
30276	Del	7	98637579	99148458	0.008739	0.059753
30277	Del	7	99149664	99266549	0.018482	0.060344
30278	Del	7	99268600	99412043	0	0.041413
30279	Del	7	99414413	99870119	0	0.054428
30280	Del	7	99870544	100534427	0	0.041413
30281	Del	7	100535967	101060385	0	0.03668
30282	Del	7	101061030	101252199	0	0.043779
30283	Del	7	101254531	101498586	0	0.046146
30284	Del	7	101498690	101558984	0	0.045554
30285	Del	7	101561276	101811336	0	0.046146
30286	Del	7	101814008	102274376	0	0.042596
30287	Del	7	102282046	102383740	0	0.046146
30288	Del	7	102383801	102434929	0	0.045554
30289	Del	7	102435977	102816490	0	0.040821
30290	Del	7	102817310	102899663	0	0.03313
30291	Del	7	102906769	102945650	0	0.033722
30292	Del	7	102945855	102969174	0	0.03313
30293	Del	7	102972097	102979635	0	0.033722
30294	Del	7	102985070	103073638	0	0.03313
30295	Del	7	103074191	103346033	0	0.04023
30296	Del	7	103347145	103369965	0	0.033722
30297	Del	7	103372286	103442055	0	0.03313
30298	Del	7	103442754	103456630	0	0.038455
30299	Del	7	103463817	104122502	0	0.043188
30300	Del	7	104125272	104162867	0	0.037863
30301	Del	7	104162885	104209492	0	0.038455
30302	Del	7	104209595	104912189	0.014398	0.060344
30303	Del	7	104913336	104981178	0	0.038455
30304	Del	7	104981515	105019466	0	0.035497
30305	Del	7	105036075	105115859	0	0.034905
30306	Del	7	105116135	105302804	0	0.034313
30307	Del	7	105304739	105371619	0	0.033722
30308	Del	7	105372232	105602470	0	0.035497
30309	Del	7	105604684	105680446	0	0.034905
30310	Del	7	105680918	105759204	0	0.03313
30311	Del	7	105759792	105961069	0	0.034313
30312	Del	7	105962475	106084062	0	0.027806
30313	Del	7	106085523	106909420	0	0.023073
30314	Del	7	106909457	107166544	0	0.030172
30315	Del	7	107167133	107202934	0	0.028989

30316	Del	7	107206590	107466504	0	0.027214
30317	Del	7	107468889	107478755	0	0.028989
30318	Del	7	107484208	107553297	0	0.027214
30319	Del	7	107562057	107922002	0	0.028989
30320	Del	7	107924003	108004148	0	0.028397
30321	Del	7	108004512	108465391	0	0.034905
30322	Del	7	108465439	108527242	0	0.028397
30323	Del	7	108527370	108528818	0	0.027806
30324	Del	7	108532146	108883486	0	0.028397
30325	Del	7	108883946	109962778	0	0.020115
30326	Del	7	109963460	111085615	0	0.026623
30327	Del	7	111088339	111125633	0	0.029581
30328	Del	7	111126694	111718956	0	0.026623
30329	Del	7	111726066	112217003	0	0.017157
30330	Del	7	112222693	114407933	0	0.011241
30331	Del	7	114410433	114914219	0	0.013015
30332	Del	7	114917169	116665719	0	0.011241
30333	Del	7	116665807	116798457	0	0.010057
30334	Del	7	116798600	116960104	0	0.011241
30335	Del	7	116965995	117018392	0	0.010057
30336	Del	7	117019301	117022371	0	0.011241
30337	Del	7	117022761	117070251	0	0.010057
30338	Del	7	117071302	117093197	0	0.011241
30339	Del	7	117095046	117111011	0	0.010057
30340	Del	7	117111880	117145918	0	0.011241
30341	Del	7	117147122	117177378	0	0.010057
30342	Del	7	117179402	117210716	0	0.011241
30343	Del	7	117210926	117237919	0	0.010057
30344	Del	7	117238229	117477709	0	0.011241
30345	Del	7	117478159	117874786	0	0.010649
30346	Del	7	117879771	118242862	0	0.011241
30347	Del	7	118244807	119785793	0	0.026031
30348	Del	7	119786128	120269173	0	0.026623
30349	Del	7	120269853	120750616	0	0.009466
30350	Del	7	120752875	120986820	0	0.011241
30351	Del	7	120987068	121298056	0	0.010057
30352	Del	7	121300939	121400534	0	0.011241
30353	Del	7	121403460	122062223	0	0.023073
30354	Del	7	122065452	122298600	0	0.024256
30355	Del	7	122301287	122316164	0	0.011241
30356	Del	7	122321042	122695298	0	0.008874
30357	Del	7	122696635	122703303	0	0.011241
30358	Del	7	122707103	122887144	0	0.008874
30359	Del	7	122889325	122997214	0	0.011241
30360	Del	7	122997358	123536309	0	0.015382
30361	Del	7	123538693	123819593	0	0.011241
30362	Del	7	123820212	124932751	0	0.023664
30363	Del	7	124935630	125146585	0	0.023073
30364	Del	7	125147155	125379367	0	0.028989
30365	Del	7	125383211	127243932	0	0.042004
30366	Del	7	127245925	127368986	0	0.041413
30367	Del	7	127369103	127617444	0	0.033722
30368	Del	7	127618154	128205371	0	0.045554

30369	Del	7	128206960	128950214	0	0.038455
30370	Del	7	128953732	129047886	0	0.037863
30371	Del	7	129047930	129222990	0	0.038455
30372	Del	7	129223977	129366836	0	0.037272
30373	Del	7	129367979	129608661	0	0.038455
30374	Del	7	129608797	129758182	0	0.037863
30375	Del	7	129758846	129828683	0	0.038455
30376	Del	7	129830327	129959434	0	0.037863
30377	Del	7	129959818	130205624	0	0.038455
30378	Del	7	130206538	130938498	0	0.028397
30379	Del	7	130939025	131111262	0	0.027214
30380	Del	7	131111487	131303707	0	0.025439
30381	Del	7	131308132	131331696	0	0.028397
30382	Del	7	131332681	131498282	0	0.027806
30383	Del	7	131498459	131905467	0	0.028397
30384	Del	7	131908808	131979256	0	0.026031
30385	Del	7	131994531	132258240	0	0.025439
30386	Del	7	132258244	132264617	0	0.029581
30387	Del	7	132265454	132382711	0	0.025439
30388	Del	7	132383074	132577820	0	0.027806
30389	Del	7	132578836	132644165	0	0.026031
30390	Del	7	132645154	132731701	0	0.026623
30391	Del	7	132732067	132784413	0	0.027214
30392	Del	7	132784570	132972920	0	0.025439
30393	Del	7	132973324	133029979	0	0.026623
30394	Del	7	133031077	133036865	0	0.027214
30395	Del	7	133037242	133089143	0	0.026623
30396	Del	7	133089190	133109370	0	0.027214
30397	Del	7	133111730	133488834	0	0.028397
30398	Del	7	133489499	133795911	0	0.024256
30399	Del	7	133798154	133827512	0	0.029581
30400	Del	7	133829370	134044418	0	0.024256
30401	Del	7	134044457	134276536	0	0.028989
30402	Del	7	134277232	134779071	0	0.029581
30403	Del	7	134779304	134890504	0	0.027806
30404	Del	7	134891299	134986056	0	0.029581
30405	Del	7	134986076	135146296	0	0.028989
30406	Del	7	135146481	135358636	0	0.029581
30407	Del	7	135360134	135514763	0	0.028989
30408	Del	7	135518160	135979189	0	0.029581
30409	Del	7	135981304	136895009	0	0.031947
30410	Del	7	136898004	137164639	0	0.036088
30411	Del	7	137168267	137387605	0	0.047921
30412	Del	7	137387618	137849638	0	0.029581
30413	Del	7	137850285	137948709	0	0.032539
30414	Del	7	137949526	137962586	0	0.03313
30415	Del	7	137964501	138119441	0	0.032539
30416	Del	7	138120750	138455833	0	0.03313
30417	Del	7	138457458	138777310	0	0.032539
30418	Del	7	138778900	138796852	0	0.031947
30419	Del	7	138797112	138806130	0	0.03313
30420	Del	7	138806648	138983042	0	0.03668
30421	Del	7	138984621	139041637	0	0.037863

30422	Del	7	139041930	139058059	0	0.037272
30423	Del	7	139058795	139308430	0	0.037863
30424	Del	7	139308732	139558137	0	0.042004
30425	Del	7	139561546	139780403	0	0.04023
30426	Del	7	139781106	139824572	0	0.039638
30427	Del	7	139828863	140022825	0	0.04023
30428	Del	7	140023600	140083508	0	0.042004
30429	Del	7	140083642	140172895	0	0.041413
30430	Del	7	140174589	140448988	0	0.042004
30431	Del	7	140451848	140514238	0	0.037863
30432	Del	7	140514279	140649146	0	0.031947
30433	Del	7	140649462	140708278	0	0.032539
30434	Del	7	140712676	140934507	0	0.035497
30435	Del	7	140934655	141061892	0	0.038455
30436	Del	7	141070777	141485633	0	0.033722
30437	Del	7	141485691	141660973	0	0.037863
30438	Del	7	141663416	141843298	0	0.034905
30439	Del	7	141847047	141994609	0	0.038455
30440	Del	7	141994761	142034594	0	0.037863
30441	Del	7	142038226	143406124	0	0.038455
30442	Del	7	143406287	143506678	0	0.043188
30443	Del	7	143507648	144449686	0	0.043779
30444	Del	7	144450529	144837489	0	0.039638
30445	Del	7	144837740	147079870	0	0.057386
30446	Del	7	147080512	147098765	0.111907	0.066852
30447	Del	7	147100544	147377097	0	0.057386
30448	Del	7	147377842	147378224	0.162563	0.06981
30449	Del	7	147383904	147668398	0	0.057386
30450	Del	7	147671644	147673505	0.161867	0.06981
30451	Del	7	147673608	148421157	0	0.057386
30452	Del	7	148422096	148695251	0.299921	0.077501
30453	Del	7	148695367	148793821	0.272777	0.076318
30454	Del	7	148802964	148891183	0.2872	0.07691
30455	Del	7	148892777	149137195	0.309388	0.077501
30456	Del	7	149137358	149234222	0.289911	0.07691
30457	Del	7	149234333	149297502	0.305648	0.077501
30458	Del	7	149297522	149543191	0.429083	0.083417
30459	Del	7	149544265	149626354	0.407785	0.082234
30460	Del	7	149630807	149834127	0.432942	0.083417
30461	Del	7	150164351	150240296	0.309388	0.077501
30462	Del	7	150243739	150321832	0.234436	0.073952
30463	Del	7	150322677	150449093	0.352395	0.079868
30464	Del	7	150449343	150479726	0.342947	0.079276
30465	Del	7	150479845	150714660	0.352395	0.079868
30466	Del	7	150715229	150733623	0.507058	0.086375
30467	Del	7	150734215	151124920	0.520667	0.086967
30468	Del	7	151125239	151146768	0.507626	0.086375
30469	Del	7	151151174	151340035	0.37487	0.081051
30470	Del	7	151341094	151379368	0.368363	0.080459
30471	Del	7	151379488	151445892	0.369837	0.081051
30472	Del	7	151453760	151521858	0.191167	0.071585
30473	Del	7	151523069	151635255	0.490171	0.085784
30474	Del	7	151639460	151739266	0.454969	0.084009

30475	Del	7	151741778	151815091	0.426236	0.083417
30476	Del	7	151817251	151869358	0.404637	0.082234
30477	Del	7	151874582	151953155	0.526376	0.086967
30478	Del	7	151955054	152019364	0.502646	0.086375
30479	Del	7	152019477	152123748	0.862304	0.098799
30480	Del	7	152128470	152438509	1.793753	0.127197
30481	Del	7	152438990	152644074	1.468091	0.118322
30482	Del	7	152646144	153235178	1.450059	0.117731
30483	Del	7	153236156	154067655	1.162746	0.108857
30484	Del	7	154070879	154589765	0.84211	0.098208
30485	Del	7	154590073	154923442	0.670602	0.092291
30486	Del	7	154923663	154951922	0.109074	0.066852
30487	Del	7	154956511	154999751	0.097034	0.066261
30488	Del	7	155000336	155646471	0.114364	0.066852
30489	Del	7	155646568	155782578	0.081603	0.065077
30490	Del	7	155783945	156468908	0.114479	0.066852
30491	Del	7	156471868	156535160	0.083415	0.065077
30492	Del	7	156535190	156543756	0.114479	0.066852
30493	Del	7	156544054	156603846	0.083415	0.065077
30494	Del	7	156611368	156701941	0.114479	0.066852
30495	Del	7	156703537	156894844	0.080216	0.065077
30496	Del	7	156896065	157016740	0.203083	0.072768
30497	Del	7	157016941	157335574	0.270814	0.076318
30498	Del	7	157336075	157417527	0.172163	0.070402
30499	Del	7	157420609	157501614	0.179304	0.070993
30500	Del	7	157502244	158543782	0.550789	0.08815
30501	Del	7	158544249	158590915	0.53941	0.087559
30502	Del	7	158592426	158592540	0.439408	0.083417
30503	Del	8	667667	667667	3.335647	0.162693
30504	Del	8	667885	727596	3.253767	0.16151
30505	Del	8	733275	741344	3.333745	0.162693
30506	Del	8	743312	1295899	3.0745	0.157369
30507	Del	8	1295964	1302889	2.903555	0.153819
30508	Del	8	1303601	1758390	4.271409	0.182217
30509	Del	8	1758509	1765808	3.386172	0.163877
30510	Del	8	1768450	1781051	3.353941	0.163285
30511	Del	8	1784590	1822296	3.388083	0.163877
30512	Del	8	1822408	1977905	4.59071	0.188724
30513	Del	8	1978290	2038435	4.554851	0.188133
30514	Del	8	2043999	2074299	3.976194	0.175709
30515	Del	8	2075446	2076680	4.109324	0.178667
30516	Del	8	2077588	2157449	3.976194	0.175709
30517	Del	8	2157691	2926896	5.367597	0.203515
30518	Del	8	2935217	6398811	5.24822	0.201148
30519	Del	8	6399012	6410719	2.962706	0.155002
30520	Del	8	6411921	6448226	2.888977	0.153228
30521	Del	8	6448433	6493731	2.510252	0.144945
30522	Del	8	6494581	6563756	2.833322	0.152044
30523	Del	8	6565104	6615439	2.510252	0.144945
30524	Del	8	6615597	6617812	2.829705	0.152044
30525	Del	8	6619105	6622616	2.975263	0.155002
30526	Del	8	6623465	6705149	2.829705	0.152044
30527	Del	8	6705482	6743308	2.876825	0.153228

30528	Del	8	6743971	6746232	2.975263	0.155002
30529	Del	8	6747678	6761525	2.876825	0.153228
30530	Del	8	6761753	6868594	2.967251	0.155002
30531	Del	8	6870245	6879513	2.947407	0.154411
30532	Del	8	6879599	6967890	2.967251	0.155002
30533	Del	8	6968014	8693908	2.093289	0.134888
30534	Del	8	8697355	8704309	0.201999	0.072177
30535	Del	8	8704498	8895785	0.564757	0.088742
30536	Del	8	8902628	9152396	0.639353	0.0917
30537	Del	8	9152532	9552985	1.069378	0.105899
30538	Del	8	9553180	9740301	0.979572	0.10294
30539	Del	8	9740784	9743407	1.069378	0.105899
30540	Del	8	9743803	9782890	0.979572	0.10294
30541	Del	8	9783524	9905631	1.066086	0.105899
30542	Del	8	9910900	10095274	1.505711	0.120097
30543	Del	8	10095397	10523618	1.54721	0.120689
30544	Del	8	10523857	10554606	1.257172	0.111815
30545	Del	8	10556077	10671020	1.269011	0.112406
30546	Del	8	10672452	10701238	1.250682	0.111815
30547	Del	8	10701909	10763630	1.266476	0.112406
30548	Del	8	10764450	10825164	1.206469	0.110631
30549	Del	8	10825267	10825506	1.275412	0.112406
30550	Del	8	10828065	10838801	1.206469	0.110631
30551	Del	8	10839005	10888480	1.264535	0.112406
30552	Del	8	10891871	11034449	1.201476	0.11004
30553	Del	8	11035020	11035856	1.411539	0.116548
30554	Del	8	11036638	11059013	1.201476	0.11004
30555	Del	8	11061972	11069165	1.411539	0.116548
30556	Del	8	11073210	11111462	1.201476	0.11004
30557	Del	8	11111565	11205373	1.342525	0.114773
30558	Del	8	11205494	11324639	1.369925	0.115364
30559	Del	8	11324674	11368068	1.410586	0.116548
30560	Del	8	11368105	11420810	1.839072	0.128971
30561	Del	8	11421091	11490666	1.901671	0.130155
30562	Del	8	11493212	11527332	1.36776	0.115364
30563	Del	8	11528546	11672308	1.411539	0.116548
30564	Del	8	11673075	11695635	1.336082	0.114773
30565	Del	8	11695846	11707578	1.411539	0.116548
30566	Del	8	11712290	11754806	1.351637	0.115364
30567	Del	8	11755189	11764784	1.411539	0.116548
30568	Del	8	11765727	11791560	2.29709	0.140212
30569	Del	8	11791683	12716562	1.569963	0.12128
30570	Del	8	12718352	12727927	0.225146	0.07336
30571	Del	8	12728221	12786988	0.2098	0.072768
30572	Del	8	12787348	12945324	0.213437	0.07336
30573	Del	8	12945622	13011808	0.191167	0.071585
30574	Del	8	13012089	13136073	0.2098	0.072768
30575	Del	8	13136119	13278056	0.169128	0.070402
30576	Del	8	13292365	13302109	0.094136	0.066261
30577	Del	8	13304035	13518052	0.141649	0.069219
30578	Del	8	13518263	14083720	0.225146	0.07336
30579	Del	8	14084259	14850575	0.12206	0.068035
30580	Del	8	14852522	14855034	0.225146	0.07336

30581	Del	8	14855705	15537587	0.12206	0.068035
30582	Del	8	15537635	15766743	0.100084	0.066261
30583	Del	8	15766916	16194000	0.352415	0.079868
30584	Del	8	16194236	17025131	0.507058	0.086375
30585	Del	8	17025643	17152789	0.490171	0.085784
30586	Del	8	17153295	17225457	0.476982	0.085192
30587	Del	8	17226387	17242888	0.504204	0.086375
30588	Del	8	17243684	17299750	0.616829	0.090517
30589	Del	8	17300168	17417200	0.541677	0.087559
30590	Del	8	17417962	17538286	0.573727	0.088742
30591	Del	8	17538715	17570767	0.610052	0.090517
30592	Del	8	17573444	17642610	0.58421	0.089333
30593	Del	8	17642714	17676853	0.605535	0.090517
30594	Del	8	17677066	17679039	0.58421	0.089333
30595	Del	8	17681258	17683328	0.637595	0.091108
30596	Del	8	17683424	17697754	0.58421	0.089333
30597	Del	8	17697869	17722326	0.560587	0.088742
30598	Del	8	17723646	17723646	0.559806	0.08815
30599	Del	8	17724628	17803029	0.506786	0.086375
30600	Del	8	17804524	17920430	0.883184	0.099391
30601	Del	8	17922742	18054396	0.83632	0.098208
30602	Del	8	18054987	18085477	0.673853	0.092883
30603	Del	8	18085541	18163187	0.481843	0.085192
30604	Del	8	18167534	18209183	0.459941	0.084601
30605	Del	8	18209886	18526510	0.477979	0.085192
30606	Del	8	18527181	18810973	0.113571	0.066852
30607	Del	8	18811318	19182223	0.064433	0.063894
30608	Del	8	19182256	19255126	0.038019	0.062119
30609	Del	8	19255358	19396561	0.046943	0.062711
30610	Del	8	19396748	19717442	0.114479	0.067444
30611	Del	8	19717458	19855989	0.192203	0.072177
30612	Del	8	19859476	20139976	0.468891	0.084601
30613	Del	8	20140029	20184238	0.439691	0.084009
30614	Del	8	20186944	20293468	0.475018	0.085192
30615	Del	8	20293641	21691506	0.733677	0.094658
30616	Del	8	21691735	21792685	0.711421	0.094066
30617	Del	8	21793481	21917912	0.740744	0.094658
30618	Del	8	21918510	22007029	0.689157	0.092883
30619	Del	8	22009422	22168102	0.737807	0.094658
30620	Del	8	22168131	22211505	0.724615	0.094066
30621	Del	8	22218820	22271605	0.735238	0.094658
30622	Del	8	22272709	22346699	1.275412	0.112998
30623	Del	8	22348381	22618913	1.313923	0.11359
30624	Del	8	22620072	22675417	2.483339	0.144353
30625	Del	8	22675875	22712849	2.540432	0.145537
30626	Del	8	22713095	22875870	1.571186	0.121872
30627	Del	8	22876663	22888135	1.757269	0.126605
30628	Del	8	22890402	22928637	1.571186	0.121872
30629	Del	8	22928868	22993561	1.742263	0.126013
30630	Del	8	22994656	23026232	1.751231	0.126605
30631	Del	8	23026539	23067397	1.728056	0.126013
30632	Del	8	23068002	23127039	1.760334	0.126605
30633	Del	8	23130480	23182356	1.748019	0.126013

30634	Del	8	23191432	23227351	1.571186	0.121872
30635	Del	8	23232743	23296618	0.99118	0.10294
30636	Del	8	23297087	23335169	0.913907	0.100574
30637	Del	8	23335796	23366949	0.97598	0.102349
30638	Del	8	23368232	23407133	0.913907	0.100574
30639	Del	8	23408376	23525895	1.140396	0.108265
30640	Del	8	23526123	23576638	0.915967	0.100574
30641	Del	8	23577000	23839979	0.95974	0.101757
30642	Del	8	23840649	23855937	0.350516	0.079868
30643	Del	8	23858399	24361184	0.545676	0.08815
30644	Del	8	24363793	24383191	0.452861	0.084009
30645	Del	8	24383852	24406511	0.503647	0.086375
30646	Del	8	24410560	24436707	0.452861	0.084009
30647	Del	8	24439900	24509685	0.48409	0.085192
30648	Del	8	24509824	24549460	0.452861	0.084009
30649	Del	8	24549686	24959631	0.482049	0.085192
30650	Del	8	24959651	25271823	0.914986	0.100574
30651	Del	8	25272432	25343664	0.816737	0.097024
30652	Del	8	25344871	25348022	0.960563	0.101757
30653	Del	8	25348147	25410460	0.816737	0.097024
30654	Del	8	25414848	25427494	0.953116	0.101757
30655	Del	8	25427539	25840942	0.931926	0.101166
30656	Del	8	25840976	26286453	0.657116	0.092291
30657	Del	8	26289158	26373116	0.576594	0.089333
30658	Del	8	26373819	26500086	0.593161	0.089925
30659	Del	8	26502850	26502850	0.407041	0.082234
30660	Del	8	26505511	26571952	0.553274	0.08815
30661	Del	8	26573950	26748107	0.586667	0.089333
30662	Del	8	26748143	26767134	0.565047	0.088742
30663	Del	8	26767176	26868051	0.576047	0.089333
30664	Del	8	26868392	27222131	1.054345	0.105307
30665	Del	8	27227343	27312318	1.03465	0.104715
30666	Del	8	27313839	27459463	0.962515	0.102349
30667	Del	8	27459526	27489854	0.763105	0.095841
30668	Del	8	27490277	27547573	0.419509	0.082826
30669	Del	8	27547587	27627435	0.431437	0.083417
30670	Del	8	27631904	27679399	0.418793	0.082826
30671	Del	8	27679552	27732707	0.439408	0.083417
30672	Del	8	27733058	27767684	0.413731	0.082826
30673	Del	8	27769763	27806448	0.431437	0.083417
30674	Del	8	27809063	27869198	0.423784	0.082826
30675	Del	8	27869308	27885442	0.371411	0.081051
30676	Del	8	27885884	27886847	0.439408	0.083417
30677	Del	8	27891017	27995842	0.371411	0.081051
30678	Del	8	27998814	28191576	0.395083	0.082234
30679	Del	8	28198451	28336329	0.42076	0.082826
30680	Del	8	28338008	28346022	0.43366	0.083417
30681	Del	8	28346747	28425066	0.422781	0.082826
30682	Del	8	28425408	28487374	0.404637	0.082234
30683	Del	8	28491109	28504811	0.411538	0.082826
30684	Del	8	28504942	28505275	0.439408	0.083417
30685	Del	8	28507448	28521582	0.411538	0.082826
30686	Del	8	28530298	28530729	0.439408	0.083417

30687	Del	8	28532402	28576514	0.411538	0.082826
30688	Del	8	28577124	28704261	1.03853	0.104715
30689	Del	8	28705424	28763652	1.011621	0.104124
30690	Del	8	28763741	28892829	0.986212	0.10294
30691	Del	8	28894268	29059530	0.975924	0.102349
30692	Del	8	29065531	29264343	0.951036	0.101757
30693	Del	8	29268907	29752526	1.031298	0.104715
30694	Del	8	29752771	29803234	1.015074	0.104124
30695	Del	8	29804597	29919177	1.034253	0.104715
30696	Del	8	29921059	30054317	0.674238	0.092883
30697	Del	8	30056354	30111513	0.114479	0.067444
30698	Del	8	30111685	30130219	0.113619	0.066852
30699	Del	8	30130487	30376780	0.11934	0.067444
30700	Del	8	30378011	30386488	0.030769	0.061528
30701	Del	8	30387835	30570662	0.009967	0.059753
30702	Del	8	30578386	30638069	0.019977	0.060936
30703	Del	8	30638539	30645969	0.030624	0.061528
30704	Del	8	30646275	30731321	0.019977	0.060936
30705	Del	8	30736124	30991899	0.029542	0.061528
30706	Del	8	30992224	31037636	0	0.052653
30707	Del	8	31038214	31174238	0	0.050879
30708	Del	8	31176479	32022202	0	0.056203
30709	Del	8	32024311	32139624	0.159666	0.06981
30710	Del	8	32142581	32440664	0	0.056203
30711	Del	8	32440742	32443279	0.058762	0.063302
30712	Del	8	32444576	32746301	0	0.056203
30713	Del	8	32747847	33370644	0	0.044371
30714	Del	8	33370681	33475577	0	0.043188
30715	Del	8	33476956	33602186	0	0.044371
30716	Del	8	33603041	34871121	0	0.057978
30717	Del	8	34872413	36780867	0.104111	0.066261
30718	Del	8	36780962	37403361	0	0.057386
30719	Del	8	37405691	37494765	0	0.034905
30720	Del	8	37496263	37785605	0	0.035497
30721	Del	8	37787828	37830415	0	0.041413
30722	Del	8	37834582	37901020	0	0.050879
30723	Del	8	37906387	38161334	0	0.057386
30724	Del	8	38162759	38179909	0	0.050287
30725	Del	8	38186240	38213690	0	0.042596
30726	Del	8	38214802	38240352	0	0.050287
30727	Del	8	38245112	38260263	0	0.049695
30728	Del	8	38260858	38272060	0	0.050287
30729	Del	8	38272601	38313003	0	0.049695
30730	Del	8	38313968	38409988	0	0.050287
30731	Del	8	38413087	38484098	0	0.049695
30732	Del	8	38484236	38529873	0	0.050287
30733	Del	8	38530494	38611163	0	0.057386
30734	Del	8	38611785	38853098	0.062191	0.063894
30735	Del	8	38855095	38969274	0.415748	0.082826
30736	Del	8	38973540	39002819	0.432151	0.083417
30737	Del	8	39004680	39111781	0.405947	0.082234
30738	Del	8	39113207	39286601	0.381977	0.081642
30739	Del	8	39290321	39349881	0.423784	0.082826

30740	Del	8	39352527	39578385	0.439408	0.083417
30741	Del	8	39578770	39741789	0.769478	0.095841
30742	Del	8	39741859	39839269	0.665244	0.092291
30743	Del	8	39839693	39927802	0.701728	0.093475
30744	Del	8	39929581	40146271	0.88743	0.099982
30745	Del	8	40146419	40529270	0.075677	0.064486
30746	Del	8	40530272	41259376	0	0.057978
30747	Del	8	41261523	41267147	0	0.057386
30748	Del	8	41267979	41271191	0	0.057978
30749	Del	8	41273651	41312027	0	0.057386
30750	Del	8	41312357	41578963	0.197631	0.072177
30751	Del	8	41579503	41643747	0.191059	0.071585
30752	Del	8	41643919	41649718	0.141544	0.068627
30753	Del	8	41651718	41799123	0.69629	0.093475
30754	Del	8	41799771	41925651	0.645084	0.0917
30755	Del	8	41928973	42046570	0.231452	0.073952
30756	Del	8	42049600	42174474	0.248065	0.075135
30757	Del	8	42174508	42249587	0.244476	0.074543
30758	Del	8	42252667	42258874	0.083042	0.065077
30759	Del	8	42259530	42332633	0.32105	0.078684
30760	Del	8	42336024	42406639	0.383266	0.081642
30761	Del	8	42409358	42503473	0.373924	0.081051
30762	Del	8	42503920	42545269	0.36541	0.080459
30763	Del	8	42547773	42553667	0	0.056795
30764	Del	8	42554304	42737160	0.195456	0.072177
30765	Del	8	42738753	42835391	0.313933	0.078093
30766	Del	8	42835571	42895355	0	0.05147
30767	Del	8	42895643	43031275	0	0.05502
30768	Del	8	43031340	46835670	0.264621	0.075726
30769	Del	8	46838916	47184373	0	0.028397
30770	Del	8	47187876	47194827	0	0.022481
30771	Del	8	47195788	47736128	0	0.030172
30772	Del	8	47742150	47767137	0	0.022481
30773	Del	8	47772179	47962251	0	0.02189
30774	Del	8	47962344	48544972	0	0.015974
30775	Del	8	48545745	48617626	0	0.011241
30776	Del	8	48618529	48624044	0	0.011832
30777	Del	8	48624127	48699407	0	0.011241
30778	Del	8	48699942	48741559	0	0.011832
30779	Del	8	48755458	49053880	0	0.037272
30780	Del	8	49054101	49076425	0	0.034905
30781	Del	8	49078854	49906094	0	0.043188
30782	Del	8	49906241	50712627	0	0.036088
30783	Del	8	50716071	51317542	0	0.035497
30784	Del	8	51319457	51815925	0	0.014199
30785	Del	8	51816922	51900379	0	0.013607
30786	Del	8	51902208	52191215	0	0.01479
30787	Del	8	52191856	52199826	0	0.016565
30788	Del	8	52201536	52410993	0	0.01479
30789	Del	8	52411226	53710840	0	0.016565
30790	Del	8	53713585	54632288	0	0.004733
30791	Del	8	54632912	55446148	0	0.011241
30792	Del	8	55447674	55455796	0	0.013607

30793	Del	8	55455896	55735493	0	0.011241
30794	Del	8	55736867	56563776	0	0.004733
30795	Del	8	56564207	57273299	0	0.013607
30796	Del	8	57277715	57743209	0	0.004733
30797	Del	8	57743287	57750321	0	0.001183
30798	Del	8	57750430	57991859	0	0.008874
30799	Del	8	57992306	58250694	0	0.008283
30800	Del	8	58253780	58437763	0	0.001183
30801	Del	8	58440610	58451712	0	0.000592
30802	Del	8	58452871	60181702	0	0.001183
30803	Del	8	60184840	60205344	0	0.000592
30804	Del	8	60205572	61282801	0	0.001183
30805	Del	8	61282988	61499648	0	0.000592
30806	Del	8	61500249	63755594	0	0.001183
30807	Del	8	63765419	64367965	0	0.000592
30808	Del	8	64372605	64389881	0	0.001183
30809	Del	8	64390557	64585372	0	0.017157
30810	Del	8	64587039	65159797	0	0.016565
30811	Del	8	65160468	65562771	0	0.009466
30812	Del	8	65563147	65712947	0	0.017157
30813	Del	8	65714571	66117156	0	0.009466
30814	Del	8	66125229	66199949	0	0.008283
30815	Del	8	66200733	66553353	0	0.021298
30816	Del	8	66558480	66682805	0	0.008283
30817	Del	8	66683332	66702829	0	0.007691
30818	Del	8	66705262	67491930	0	0.008283
30819	Del	8	67493520	67746948	0	0.007099
30820	Del	8	67747058	68106046	0	0.007691
30821	Del	8	68106130	68237813	0	0.007099
30822	Del	8	68237948	68331970	0	0.008283
30823	Del	8	68352579	68536274	0	0.007691
30824	Del	8	68539568	68821683	0	0.006508
30825	Del	8	68822795	69107920	0	0.007691
30826	Del	8	69108291	69462857	0	0.008283
30827	Del	8	69464292	69591684	0	0.007691
30828	Del	8	69592195	69668361	0	0.008283
30829	Del	8	69668547	69838380	0	0.007691
30830	Del	8	69839044	70107226	0	0.008283
30831	Del	8	70107427	70741852	0	0.001183
30832	Del	8	70742000	71367078	0	0.007691
30833	Del	8	71369570	71847538	0	0.008283
30834	Del	8	71848814	72015867	0	0.007691
30835	Del	8	72015880	72201073	0	0.01479
30836	Del	8	72201289	72252073	0	0.006508
30837	Del	8	72252763	72729263	0	0.013015
30838	Del	8	72729546	72754034	0	0.015382
30839	Del	8	72754111	72938404	0	0.013015
30840	Del	8	72941326	73446336	0	0.015382
30841	Del	8	73448606	73543308	0	0.014199
30842	Del	8	73543699	73748526	0	0.01479
30843	Del	8	73753023	74231518	0	0.015382
30844	Del	8	74232289	74322033	0	0.01479
30845	Del	8	74327743	74585736	0	0.015382

30846	Del	8	74591151	74703918	0	0.01479
30847	Del	8	74705442	74705854	0	0.015382
30848	Del	8	74706299	74760292	0	0.01479
30849	Del	8	74760422	74855179	0	0.015382
30850	Del	8	74855541	75284738	0	0.020706
30851	Del	8	75290011	75538093	0	0.026031
30852	Del	8	75538863	76404068	0	0.027214
30853	Del	8	76404945	76684912	0	0.005916
30854	Del	8	76687964	76867411	0	0.005325
30855	Del	8	76868067	77477303	0	0.005916
30856	Del	8	77477985	78722903	0	0.01479
30857	Del	8	78723421	78725345	0	0.005916
30858	Del	8	78738000	81532161	0	0.001183
30859	Del	8	81534191	82908958	0	0.008283
30860	Del	8	82908976	82963337	0	0.005916
30861	Del	8	82967580	84179126	0	0.009466
30862	Del	8	84179885	84696897	0	0.007099
30863	Del	8	84703464	85106138	0	0.008874
30864	Del	8	85106325	86570265	0	0.005916
30865	Del	8	86572389	86862720	0	0.005325
30866	Del	8	86863886	87869203	0	0.004733
30867	Del	8	87870156	89760982	0	0.001183
30868	Del	8	89761018	89792413	0	0.000592
30869	Del	8	89792773	89930204	0	0.001183
30870	Del	8	89932511	90052938	0	0.000592
30871	Del	8	90055374	90388226	0	0.001183
30872	Del	8	90391967	90786635	0	0.008283
30873	Del	8	90788848	91099102	0	0.001183
30874	Del	8	91101034	91198986	0	0.000592
30875	Del	8	91205311	92063784	0	0.001183
30876	Del	8	92064325	92106434	0	0.000592
30877	Del	8	92107718	92656314	0	0.001183
30878	Del	8	92663414	92881802	0	0.008874
30879	Del	8	92882104	94121753	0	0.001183
30880	Del	8	94126945	94217707	0	0.000592
30881	Del	8	94218748	94368728	0	0.001183
30882	Del	8	94368867	94426700	0	0.000592
30883	Del	8	94427682	94440016	0	0.001183
30884	Del	8	94441620	94508181	0	0.000592
30885	Del	8	94510810	94717275	0	0.001183
30886	Del	8	94717550	94797531	0	0.000592
30887	Del	8	94799653	94922993	0	0.001183
30888	Del	8	94923286	94951570	0	0.000592
30889	Del	8	94951661	95075245	0	0.001183
30890	Del	8	95077192	95242232	0	0.010649
30891	Del	8	95243728	95811077	0	0.000592
30892	Del	8	95811483	97636837	0	0.001183
30893	Del	8	97637863	97743428	0	0.000592
30894	Del	8	97747062	97862465	0	0.001183
30895	Del	8	97865309	98035821	0	0.000592
30896	Del	8	98036511	98120394	0	0.001183
30897	Del	8	98120427	98160966	0	0.000592
30898	Del	8	98162267	98952775	0	0.001183

30899	Del	8	98953374	99215296	0	0.005916
30900	Del	8	99215476	99531567	0	0.004733
30901	Del	8	99532223	99537251	0	0.006508
30902	Del	8	99537529	99879054	0	0.004733
30903	Del	8	99886207	99958160	0	0.001183
30904	Del	8	99960834	100023470	0	0.000592
30905	Del	8	100023799	100028097	0	0.001183
30906	Del	8	100028903	100106909	0	0.000592
30907	Del	8	100110169	100154227	0	0.001183
30908	Del	8	100155467	100243180	0	0.000592
30909	Del	8	100244109	100502874	0	0.001183
30910	Del	8	100509245	100650467	0	0.000592
30911	Del	8	100650480	101081159	0	0.006508
30912	Del	8	101081398	101367323	0	0.009466
30913	Del	8	101367875	101370698	0	0.002958
30914	Del	8	101371568	101670014	0	0.008874
30915	Del	8	101670305	101791689	0	0.009466
30916	Del	8	101791958	101980771	0	0.007691
30917	Del	8	101983142	101997619	0	0.009466
30918	Del	8	102007598	102119941	0	0.007691
30919	Del	8	102122027	102200781	0	0.009466
30920	Del	8	102200959	103444665	0	0.002958
30921	Del	8	103446644	103818742	0	0.008283
30922	Del	8	103819073	104359048	0	0.009466
30923	Del	8	104360756	104482719	0	0.022481
30924	Del	8	104483757	104592422	0	0.009466
30925	Del	8	104592967	105805176	0	0.01479
30926	Del	8	105805990	106483031	0	0.013607
30927	Del	8	106486331	106655230	0	0.002366
30928	Del	8	106655565	107336884	0	0.002958
30929	Del	8	107339004	108085299	0	0.002366
30930	Del	8	108085448	108599980	0	0.002958
30931	Del	8	108600497	108775627	0	0.002366
30932	Del	8	108778427	109358002	0	0.002958
30933	Del	8	109362002	109531292	0	0.002366
30934	Del	8	109532608	109977927	0	0.002958
30935	Del	8	109978185	111097179	0	0.011241
30936	Del	8	111098602	112222327	0	0.002958
30937	Del	8	112222462	112640136	0	0.001183
30938	Del	8	112643367	112644745	0	0.002958
30939	Del	8	112645334	113438132	0	0.001183
30940	Del	8	113438289	115669892	0	0.016565
30941	Del	8	115670852	116643332	0	0.037863
30942	Del	8	116643877	116649936	0	0.016565
30943	Del	8	116650572	116655312	0	0.017157
30944	Del	8	116655523	116756661	0	0.016565
30945	Del	8	116760110	116947248	0	0.017157
30946	Del	8	116947975	117134319	0	0.015974
30947	Del	8	117134960	117796327	0	0.017157
30948	Del	8	117798597	118187836	0	0.015382
30949	Del	8	118188449	118377615	0	0.014199
30950	Del	8	118377736	118617415	0	0.015382
30951	Del	8	118617919	118728292	0	0.016565

30952	Del	8	118728362	118953514	0	0.017157
30953	Del	8	118953670	119059700	0	0.015974
30954	Del	8	119059848	119204591	0	0.017157
30955	Del	8	119204718	119245793	0	0.008874
30956	Del	8	119245976	119415408	0	0.010057
30957	Del	8	119415616	119593040	0	0.008874
30958	Del	8	119596743	119676406	0	0.008283
30959	Del	8	119677458	119869935	0	0.008874
30960	Del	8	119870902	120375828	0	0.008283
30961	Del	8	120375924	120530454	0	0.008874
30962	Del	8	120530624	120759296	0	0.007691
30963	Del	8	120759721	120777121	0	0.008874
30964	Del	8	120777150	120814194	0	0.007691
30965	Del	8	120814290	121646608	0	0.008874
30966	Del	8	121647252	122568687	0	0.017157
30967	Del	8	122568829	122775675	0	0.01834
30968	Del	8	122776175	122975635	0	0.008283
30969	Del	8	122975731	123849836	0	0.008874
30970	Del	8	123850263	123972195	0	0.007691
30971	Del	8	123972918	124041391	0	0.008874
30972	Del	8	124041497	124042485	0	0.007691
30973	Del	8	124045487	124549760	0	0.008874
30974	Del	8	124549788	124819098	0	0.016565
30975	Del	8	124819574	125095313	0	0.008874
30976	Del	8	125096143	125372853	0	0.008283
30977	Del	8	125375726	127243616	0	0.008874
30978	Del	8	127243866	127399212	0	0.008283
30979	Del	8	127399497	127402253	0	0.008874
30980	Del	8	127403016	127411572	0	0.008283
30981	Del	8	127412547	127419449	0	0.008874
30982	Del	8	127422486	127441520	0	0.008283
30983	Del	8	127442177	127797361	0	0.008874
30984	Del	8	127801545	127935770	0	0.007691
30985	Del	8	127935979	127950518	0	0.008874
30986	Del	8	127951352	127958768	0	0.007691
30987	Del	8	127960475	127961419	0	0.008874
30988	Del	8	127961698	128002287	0	0.007691
30989	Del	8	128002451	128010647	0	0.008874
30990	Del	8	128013941	128047566	0	0.007691
30991	Del	8	128048558	128049654	0	0.008874
30992	Del	8	128050775	128148911	0	0.007691
30993	Del	8	128149207	128151484	0	0.002958
30994	Del	8	128151491	128557753	0	0.010057
30995	Del	8	128557818	129214032	0	0.010649
30996	Del	8	129214049	129347846	0	0.008874
30997	Del	8	129348072	129481434	0	0.007691
30998	Del	8	129481669	129484983	0	0.008874
30999	Del	8	129485335	129580301	0	0.007691
31000	Del	8	129580405	129685134	0	0.007099
31001	Del	8	129685763	130052039	0	0.008874
31002	Del	8	130052053	130079359	0	0.007099
31003	Del	8	130080329	130084983	0	0.008874
31004	Del	8	130087736	130291941	0	0.007099

31005	Del	8	130292646	130297586	0	0.008874
31006	Del	8	130297608	130446505	0	0.007099
31007	Del	8	130447012	131043624	0	0.007691
31008	Del	8	131044861	132119974	0	0.008874
31009	Del	8	132120833	132484405	0	0.007099
31010	Del	8	132484830	132865182	0	0.008874
31011	Del	8	132865693	133033449	0	0.007099
31012	Del	8	133034047	133060898	0	0.008874
31013	Del	8	133061412	133103493	0	0.008283
31014	Del	8	133105786	133135230	0	0.007099
31015	Del	8	133135813	133451920	0	0.008874
31016	Del	8	133453031	133577197	0	0.008283
31017	Del	8	133577631	134594372	0	0.008874
31018	Del	8	134595580	134602314	0	0.010057
31019	Del	8	134602729	134713556	0	0.008874
31020	Del	8	134713980	134886792	0	0.017748
31021	Del	8	134887955	135456007	0	0.021298
31022	Del	8	135456291	135650988	0	0.020115
31023	Del	8	135652405	138128863	0	0.027214
31024	Del	8	138128894	138497636	0	0.026623
31025	Del	8	138500748	139598338	0	0.032539
31026	Del	8	139599511	139707920	0	0.024848
31027	Del	8	139708169	140459598	0	0.032539
31028	Del	8	140465124	140528747	0	0.038455
31029	Del	8	140528897	140619100	0	0.037863
31030	Del	8	140620316	142454818	0	0.038455
31031	Del	8	142455615	143228432	0	0.029581
31032	Del	8	143228447	143300022	0	0.031355
31033	Del	8	143302447	143563196	0	0.03313
31034	Del	8	143563290	143679240	0	0.036088
31035	Del	8	143680108	144182542	0	0.03313
31036	Del	9	789794	789794	0.672701	0.092291
31037	Del	9	789932	2010942	0.726781	0.094658
31038	Del	9	2011588	2155085	0.240109	0.074543
31039	Del	9	2155145	2533850	0.311594	0.078093
31040	Del	9	2534202	2615337	0.286735	0.07691
31041	Del	9	2615553	2658311	0.311594	0.078093
31042	Del	9	2658587	2801321	0.32566	0.078684
31043	Del	9	2801706	2847114	0.315889	0.078093
31044	Del	9	2847195	3216078	0.32105	0.078684
31045	Del	9	3217054	3246491	0.251356	0.075135
31046	Del	9	3246924	3490407	0.281731	0.076318
31047	Del	9	3501511	3529411	0.251356	0.075135
31048	Del	9	3530635	3824004	0.329748	0.078684
31049	Del	9	3824038	3898271	0.300058	0.077501
31050	Del	9	3898509	3901649	0.329445	0.078684
31051	Del	9	3902075	4300881	0.300058	0.077501
31052	Del	9	4301029	4590890	0.52139	0.086967
31053	Del	9	4591655	4658662	0.653195	0.0917
31054	Del	9	4661853	4665668	0.705173	0.093475
31055	Del	9	4668310	4668310	0.653195	0.0917
31056	Del	9	4669084	4680354	0.703449	0.093475
31057	Del	9	4680407	4709004	0.683065	0.092883

31058	Del	9	4709050	4791680	0.699044	0.093475
31059	Del	9	4792098	4836899	0.643791	0.0917
31060	Del	9	4837260	4848630	0.6821	0.092883
31061	Del	9	4848875	4851328	0.706856	0.093475
31062	Del	9	4851440	4978643	0.6821	0.092883
31063	Del	9	4981269	5040386	0.585409	0.089333
31064	Del	9	5040486	5087087	0.65483	0.0917
31065	Del	9	5097281	5341193	0.706856	0.093475
31066	Del	9	5341593	5423103	0.833825	0.098208
31067	Del	9	5429108	5621588	0.88623	0.099391
31068	Del	9	5625673	5774159	0.656876	0.092291
31069	Del	9	5774225	5775859	0.654021	0.0917
31070	Del	9	5777197	5833337	0.681269	0.092883
31071	Del	9	5833923	5916465	0.698303	0.093475
31072	Del	9	5918096	6000551	0.663995	0.092291
31073	Del	9	6003680	6017252	0.706856	0.093475
31074	Del	9	6017418	6235950	0.910756	0.100574
31075	Del	9	6236350	7055236	0.924215	0.101166
31076	Del	9	7055253	7125060	0.825417	0.097616
31077	Del	9	7126151	7175657	0.753683	0.09525
31078	Del	9	7175774	7800297	0.84514	0.098208
31079	Del	9	7802660	10617850	4.513555	0.186949
31080	Del	9	10619052	12688441	0.210298	0.072768
31081	Del	9	12689377	12710342	0	0.053245
31082	Del	9	12710663	12773679	0	0.052062
31083	Del	9	12773724	12823505	0	0.053245
31084	Del	9	12823587	13101490	0	0.055612
31085	Del	9	13101687	13402850	0	0.053837
31086	Del	9	13405073	13432829	0	0.042596
31087	Del	9	13435676	13947667	0	0.050287
31088	Del	9	13954437	14185137	0.02806	0.061528
31089	Del	9	14185247	14319510	0	0.05857
31090	Del	9	14321080	14399901	0	0.057386
31091	Del	9	14401107	14717051	0.062699	0.063894
31092	Del	9	14718856	14731526	0	0.05857
31093	Del	9	14734447	14782624	0	0.057978
31094	Del	9	14784667	14911067	0	0.055612
31095	Del	9	14911084	15031400	0	0.05857
31096	Del	9	15032079	15252038	0.289911	0.07691
31097	Del	9	15253036	15307465	0.276873	0.076318
31098	Del	9	15308121	15462176	0.343819	0.079868
31099	Del	9	15462394	15469735	0.307905	0.077501
31100	Del	9	15470574	15552371	0.309388	0.078093
31101	Del	9	15552401	15973714	0	0.057386
31102	Del	9	15974374	16279110	0	0.057978
31103	Del	9	16279446	17130362	0.129272	0.068035
31104	Del	9	17132396	17168869	0	0.037863
31105	Del	9	17169426	17307120	0	0.03668
31106	Del	9	17307571	17505200	0	0.034905
31107	Del	9	17505230	17797858	0	0.03668
31108	Del	9	17798206	18926999	0	0.041413
31109	Del	9	18927179	19034787	0	0.031355
31110	Del	9	19035853	19046192	0	0.030764

31111	Del	9	19047174	19052722	0	0.032539
31112	Del	9	19052925	19059771	0	0.031947
31113	Del	9	19060939	19064606	0	0.032539
31114	Del	9	19064919	19104664	0	0.031947
31115	Del	9	19106589	19226021	0	0.032539
31116	Del	9	19228387	19376410	0	0.031947
31117	Del	9	19377241	19497272	0	0.026031
31118	Del	9	19499282	19794692	0	0.022481
31119	Del	9	19796863	20407936	0	0.034905
31120	Del	9	20408622	20415620	0	0.037272
31121	Del	9	20416454	20500277	0	0.034905
31122	Del	9	20500723	20502451	0	0.037272
31123	Del	9	20503768	20625229	0	0.034905
31124	Del	9	20625876	20678603	0	0.054428
31125	Del	9	20679615	20684779	0.000981	0.059161
31126	Del	9	20685098	20714015	0	0.054428
31127	Del	9	20715185	20717944	0.00167	0.059161
31128	Del	9	20718241	20996452	0	0.054428
31129	Del	9	20996710	21033558	0	0.05857
31130	Del	9	21034282	21078376	0.00167	0.059161
31131	Del	9	21078876	21171268	0.328351	0.078684
31132	Del	9	21171591	21450475	0.812862	0.097024
31133	Del	9	21453346	21473891	0.732197	0.094658
31134	Del	9	21474576	21487254	0.816737	0.097024
31135	Del	9	21487999	21511924	0.732197	0.094658
31136	Del	9	21512064	21513615	0.816737	0.097024
31137	Del	9	21514365	21562758	0.732197	0.094658
31138	Del	9	21563283	21868909	2.880002	0.153228
31139	Del	9	21869080	21996997	4.17023	0.17985
31140	Del	9	21997724	21999616	3.028126	0.156186
31141	Del	9	22000771	22009961	3.13829	0.159144
31142	Del	9	22011393	22081851	3.028126	0.156186
31143	Del	9	22082341	22432667	2.983271	0.155594
31144	Del	9	22446298	22455519	2.93987	0.154411
31145	Del	9	22455893	23495185	3.002774	0.156186
31146	Del	9	23496119	23672467	0.5608	0.088742
31147	Del	9	23672516	23827182	0.575877	0.089333
31148	Del	9	23827413	25654530	0.797969	0.096433
31149	Del	9	25656013	26030588	0.619094	0.090517
31150	Del	9	26062844	26834141	0.288271	0.07691
31151	Del	9	26834234	26900239	0	0.057978
31152	Del	9	26901070	26946084	0	0.057386
31153	Del	9	26946251	26958441	0	0.057978
31154	Del	9	26962561	26988952	0	0.057386
31155	Del	9	26991528	27009102	0	0.057978
31156	Del	9	27009694	27103416	0	0.057386
31157	Del	9	27108802	27230154	0	0.055612
31158	Del	9	27230511	27280234	0	0.057386
31159	Del	9	27281060	27315625	0	0.057978
31160	Del	9	27322372	27545962	0	0.054428
31161	Del	9	27546464	27944441	0	0.053245
31162	Del	9	27947910	30384254	0	0.05147
31163	Del	9	30385985	32382548	0	0.032539

31164	Del	9	32384287	32453789	0	0.026623
31165	Del	9	32454290	32531273	0	0.026031
31166	Del	9	32531750	33003899	0	0.026623
31167	Del	9	33003989	33036930	0	0.033722
31168	Del	9	33036983	33109373	0	0.038455
31169	Del	9	33110135	33164683	0	0.037863
31170	Del	9	33166236	33284188	0	0.038455
31171	Del	9	33286158	33374937	0	0.037863
31172	Del	9	33377168	33492001	0	0.038455
31173	Del	9	33492017	33743582	0	0.040821
31174	Del	9	33743685	33816224	0	0.033722
31175	Del	9	33816478	33921935	0	0.03313
31176	Del	9	33921979	33934823	0	0.027214
31177	Del	9	33935738	33951021	0	0.026623
31178	Del	9	33952155	33956090	0	0.027214
31179	Del	9	33956634	34050169	0	0.026623
31180	Del	9	34050309	34365274	0	0.027214
31181	Del	9	34365359	35101927	0	0.025439
31182	Del	9	35103725	35153424	0	0.034313
31183	Del	9	35158656	35405546	0	0.03313
31184	Del	9	35407434	35487247	0	0.028397
31185	Del	9	35488216	35599925	0	0.020706
31186	Del	9	35599969	35789613	0	0.018932
31187	Del	9	35790556	36411462	0	0.007691
31188	Del	9	36412614	36823801	0	0.017157
31189	Del	9	36824089	36863453	0	0.024256
31190	Del	9	36863523	36865028	0	0.026623
31191	Del	9	36867498	36893283	0	0.024256
31192	Del	9	36893309	36894636	0	0.026623
31193	Del	9	36894840	37034620	0	0.024256
31194	Del	9	37035184	37091780	0	0.026623
31195	Del	9	37092156	37358610	0	0.044963
31196	Del	9	37359578	37515714	0	0.047329
31197	Del	9	37516503	37578426	0	0.046737
31198	Del	9	37580800	37650176	0	0.047329
31199	Del	9	37650206	37746904	0	0.040821
31200	Del	9	37749216	37914391	0	0.042004
31201	Del	9	37915009	38070923	0	0.04023
31202	Del	9	38071508	38626312	0	0.042004
31203	Del	9	38626355	68532736	0.083415	0.065077
31204	Del	9	68534313	68548804	0	0.01834
31205	Del	9	68552474	68952323	0	0.026623
31206	Del	9	68952456	68980912	0	0.027806
31207	Del	9	68981049	69009184	0	0.026623
31208	Del	9	69009618	69034636	0	0.029581
31209	Del	9	69035469	69118687	0	0.028989
31210	Del	9	69118752	69166392	0	0.028397
31211	Del	9	69167576	69293268	0	0.028989
31212	Del	9	69296051	69318241	0	0.027806
31213	Del	9	69320661	69413646	0	0.027214
31214	Del	9	69413658	69675622	0	0.026031
31215	Del	9	69675917	69860637	0	0.027806
31216	Del	9	69861022	69907785	0	0.027214

31217	Del	9	69908998	70150407	0	0.044371
31218	Del	9	70151498	70178093	0	0.046737
31219	Del	9	70178944	70215734	0	0.044371
31220	Del	9	70216008	70262516	0	0.046737
31221	Del	9	70262642	70355144	0	0.046146
31222	Del	9	70358838	70534186	0	0.046737
31223	Del	9	70534981	70781963	0	0.042596
31224	Del	9	70782174	70808208	0	0.045554
31225	Del	9	70809437	70811937	0	0.046737
31226	Del	9	70811998	70873648	0	0.045554
31227	Del	9	70874126	71682138	0	0.04023
31228	Del	9	71682329	71773025	0	0.012424
31229	Del	9	71773210	72111870	0	0.015382
31230	Del	9	72112980	72148510	0	0.01479
31231	Del	9	72149254	72299260	0	0.015382
31232	Del	9	72301744	72367723	0	0.013015
31233	Del	9	72372344	72839369	0	0.027806
31234	Del	9	72844400	74368163	0	0.030172
31235	Del	9	74368195	74493699	0	0.028397
31236	Del	9	74494694	74688516	0	0.033722
31237	Del	9	74688837	74944800	0	0.034313
31238	Del	9	74946441	75030238	0	0.020115
31239	Del	9	75032382	75889813	0	0.028397
31240	Del	9	75889966	76195971	0	0.024256
31241	Del	9	76196461	76363214	0	0.022481
31242	Del	9	76363587	76609388	0	0.027214
31243	Del	9	76611300	76695515	0	0.026623
31244	Del	9	76695739	76760394	0	0.024256
31245	Del	9	76760455	76787962	0	0.027214
31246	Del	9	76789162	76906820	0	0.024256
31247	Del	9	76907117	77178497	0	0.027214
31248	Del	9	77179035	77418326	0	0.026031
31249	Del	9	77422947	77445796	0	0.025439
31250	Del	9	77445932	77527130	0	0.026623
31251	Del	9	77527490	77715802	0	0.025439
31252	Del	9	77715820	78034294	0	0.019523
31253	Del	9	78036355	79134439	0	0.023664
31254	Del	9	79135194	79569675	0	0.017748
31255	Del	9	79570439	79822280	0	0.017157
31256	Del	9	79822667	81582588	0	0.016565
31257	Del	9	81582934	81680221	0	0.010057
31258	Del	9	81682355	83267408	0	0.010649
31259	Del	9	83267888	83332008	0	0.010057
31260	Del	9	83332104	83470768	0	0.009466
31261	Del	9	83475054	83540636	0	0.008874
31262	Del	9	83541281	83713710	0	0.010649
31263	Del	9	83713743	83832925	0	0.027806
31264	Del	9	83836473	83931014	0	0.024848
31265	Del	9	83931206	84097923	0	0.025439
31266	Del	9	84098139	84341697	0	0.027806
31267	Del	9	84342365	84369414	0	0.027214
31268	Del	9	84370055	84819341	0	0.029581
31269	Del	9	84819545	84884675	0	0.028989

31270	Del	9	84891874	85545323	0	0.027806
31271	Del	9	85545849	85742580	0	0.01479
31272	Del	9	85742648	85926218	0	0.017748
31273	Del	9	85928167	86015305	0	0.012424
31274	Del	9	86016066	86357914	0	0.018932
31275	Del	9	86360614	86954075	0	0.024848
31276	Del	9	86954849	87005076	0	0.024256
31277	Del	9	87005766	87143665	0	0.036088
31278	Del	9	87143775	87494850	0	0.029581
31279	Del	9	87495052	87721839	0	0.016565
31280	Del	9	87723590	87891902	0	0.012424
31281	Del	9	87892102	89007262	0	0.01834
31282	Del	9	89008590	89304803	0	0.017157
31283	Del	9	89304953	89360512	0	0.012424
31284	Del	9	89364243	89482394	0	0.011832
31285	Del	9	89483329	89637175	0	0.012424
31286	Del	9	89637444	89723639	0	0.011832
31287	Del	9	89723694	90042203	0	0.012424
31288	Del	9	90043507	90460907	0	0.026623
31289	Del	9	90461572	90587681	0	0.011832
31290	Del	9	90588094	90643546	0	0.012424
31291	Del	9	90643615	91213189	0	0.018932
31292	Del	9	91213652	91364091	0	0.01834
31293	Del	9	91364217	91713130	0	0.018932
31294	Del	9	91717605	91951086	0	0.017157
31295	Del	9	91951262	93177934	0	0.018932
31296	Del	9	93181871	93316588	0	0.01834
31297	Del	9	93318019	93536179	0	0.018932
31298	Del	9	93536690	93588935	0	0.01834
31299	Del	9	93594297	93595408	0	0.018932
31300	Del	9	93595457	93679866	0	0.01834
31301	Del	9	93679942	94642312	0	0.018932
31302	Del	9	94642980	94755447	0	0.026623
31303	Del	9	94757886	95061842	0	0.027214
31304	Del	9	95062559	95066189	0	0.029581
31305	Del	9	95069745	95080197	0	0.027214
31306	Del	9	95081366	95098441	0	0.029581
31307	Del	9	95098938	95319219	0	0.028397
31308	Del	9	95321535	95463295	0	0.036088
31309	Del	9	95463572	95470047	0	0.037863
31310	Del	9	95470300	95509637	0	0.036088
31311	Del	9	95511655	95519048	0	0.035497
31312	Del	9	95521893	95867168	0	0.039638
31313	Del	9	95867894	95877506	0	0.04023
31314	Del	9	95877962	95970574	0	0.039638
31315	Del	9	95970902	96018324	0	0.039046
31316	Del	9	96018897	96234252	0	0.04023
31317	Del	9	96234445	96319230	0	0.039638
31318	Del	9	96319858	96384243	0	0.04023
31319	Del	9	96385085	96568988	0	0.038455
31320	Del	9	96569092	96621620	0	0.037863
31321	Del	9	96623374	97026628	0	0.038455
31322	Del	9	97027490	97242846	0	0.036088

31323	Del	9	97243608	97363011	0	0.035497
31324	Del	9	97363369	97367425	0	0.036088
31325	Del	9	97367906	97377760	0	0.035497
31326	Del	9	97378747	97505760	0	0.036088
31327	Del	9	97506363	97597273	0	0.035497
31328	Del	9	97598450	97674394	0	0.036088
31329	Del	9	97674776	98120159	0	0.033722
31330	Del	9	98120599	98196077	0	0.03313
31331	Del	9	98197844	98215175	0	0.033722
31332	Del	9	98216090	98227446	0	0.03313
31333	Del	9	98228264	98229944	0	0.033722
31334	Del	9	98232188	98284819	0	0.03313
31335	Del	9	98285474	98711441	0	0.028989
31336	Del	9	98713123	98857121	0	0.033722
31337	Del	9	98857728	99102426	0	0.039046
31338	Del	9	99102829	99377245	0	0.033722
31339	Del	9	99379711	99817185	0	0.054428
31340	Del	9	99818091	99907275	0	0.056203
31341	Del	9	99907595	99974621	0	0.055612
31342	Del	9	99977060	100102710	0	0.05502
31343	Del	9	100109986	100298104	0	0.054428
31344	Del	9	100301871	100354919	0	0.055612
31345	Del	9	100358429	100454241	0	0.056203
31346	Del	9	100455249	100470187	0	0.05502
31347	Del	9	100470495	100575890	0	0.055612
31348	Del	9	100577731	100589649	0	0.056203
31349	Del	9	100589822	101176725	0.102977	0.066261
31350	Del	9	101182114	101326082	0.140971	0.068627
31351	Del	9	101326538	101386963	0.297272	0.077501
31352	Del	9	101388092	101567341	0.129575	0.068035
31353	Del	9	101567671	101585188	0	0.043779
31354	Del	9	101589425	101596410	0	0.045554
31355	Del	9	101596753	101741169	0	0.043779
31356	Del	9	101741180	102657717	0	0.044371
31357	Del	9	102657933	103137761	0	0.045554
31358	Del	9	103138441	103997531	0	0.043779
31359	Del	9	103997558	104098753	0	0.03668
31360	Del	9	104100766	104142810	0	0.036088
31361	Del	9	104142996	104503739	0	0.043779
31362	Del	9	104503886	104553879	0	0.039046
31363	Del	9	104554359	104778645	0	0.032539
31364	Del	9	104779499	105241567	0	0.050879
31365	Del	9	105243333	105397483	0	0.04023
31366	Del	9	105398583	105435585	0	0.039638
31367	Del	9	105436908	105474127	0	0.041413
31368	Del	9	105478842	105509621	0	0.040821
31369	Del	9	105512650	105529942	0	0.041413
31370	Del	9	105530761	105643063	0	0.040821
31371	Del	9	105644793	105690517	0	0.041413
31372	Del	9	105690608	105777318	0	0.029581
31373	Del	9	105784499	106615349	0	0.030172
31374	Del	9	106615461	106856214	0	0.029581
31375	Del	9	106856362	107107790	0	0.028989

31376	Del	9	107112759	107417824	0	0.047921
31377	Del	9	107418455	107467328	0	0.047329
31378	Del	9	107468889	108852724	0	0.047921
31379	Del	9	108853374	108863633	0	0.039046
31380	Del	9	108863810	108925304	0	0.050287
31381	Del	9	108928001	108940994	0	0.05147
31382	Del	9	108943484	109013944	0	0.050879
31383	Del	9	109016639	109043809	0	0.050287
31384	Del	9	109043995	109047250	0	0.05147
31385	Del	9	109057042	109121404	0	0.050287
31386	Del	9	109124939	109171155	0	0.05147
31387	Del	9	109171433	109235229	0	0.049695
31388	Del	9	109238461	109325393	0	0.050287
31389	Del	9	109326964	109430058	0	0.05147
31390	Del	9	109430741	109452889	0	0.050879
31391	Del	9	109453595	109500090	0	0.050287
31392	Del	9	109500362	109639325	0	0.05147
31393	Del	9	109639394	109954955	0	0.050879
31394	Del	9	109955225	110047483	0	0.038455
31395	Del	9	110047708	110123462	0	0.041413
31396	Del	9	110124660	110364016	0	0.043188
31397	Del	9	110365197	110580726	0	0.04023
31398	Del	9	110581211	110872900	0	0.042004
31399	Del	9	110873002	111040153	0	0.040821
31400	Del	9	111040339	111360163	0	0.043188
31401	Del	9	111360483	111523093	0	0.042004
31402	Del	9	111524145	111654445	0	0.039046
31403	Del	9	111654522	111660557	0	0.038455
31404	Del	9	111661153	111677230	0	0.039046
31405	Del	9	111677376	111795721	0	0.038455
31406	Del	9	111795858	112035904	0	0.039046
31407	Del	9	112036339	112176651	0	0.037863
31408	Del	9	112176792	112483569	0	0.038455
31409	Del	9	112483595	112614181	0	0.037863
31410	Del	9	112616014	112831449	0	0.039046
31411	Del	9	112831590	112875003	0	0.038455
31412	Del	9	112878901	112891896	0	0.039046
31413	Del	9	112891966	113165577	0	0.049104
31414	Del	9	113165620	113219571	0	0.048512
31415	Del	9	113219998	113432893	0	0.049104
31416	Del	9	113437511	113462172	0	0.039046
31417	Del	9	113462257	113873210	0	0.04023
31418	Del	9	113873963	114053840	0	0.037863
31419	Del	9	114055532	114151716	0	0.04023
31420	Del	9	114153583	114204286	0	0.038455
31421	Del	9	114209147	114211368	0	0.04023
31422	Del	9	114211475	114315893	0	0.038455
31423	Del	9	114316006	114335437	0	0.04023
31424	Del	9	114335475	114395054	0	0.039638
31425	Del	9	114401726	114505916	0	0.039046
31426	Del	9	114507877	114609567	0	0.04023
31427	Del	9	114609903	114663036	0	0.039638
31428	Del	9	114663715	114930711	0	0.025439

31429	Del	9	114930893	115120108	0	0.028397
31430	Del	9	115120647	115141696	0	0.025439
31431	Del	9	115141796	115410006	0	0.023664
31432	Del	9	115411719	115744582	0	0.039046
31433	Del	9	115744712	116148250	0	0.046146
31434	Del	9	116152341	116424941	0	0.034905
31435	Del	9	116425180	117647381	0	0.026623
31436	Del	9	117647403	117657261	0	0.012424
31437	Del	9	117657311	119163384	0	0.021298
31438	Del	9	119163787	119934357	0	0.007099
31439	Del	9	119934722	120245820	0	0.001183
31440	Del	9	120246039	120582628	0	0.015974
31441	Del	9	120585797	120715451	0	0.016565
31442	Del	9	120716180	120949066	0	0.017157
31443	Del	9	120949883	120997158	0	0.016565
31444	Del	9	120998149	121051074	0	0.017157
31445	Del	9	121053287	121084342	0	0.016565
31446	Del	9	121087894	121202552	0	0.001183
31447	Del	9	121202623	121501403	0	0.011832
31448	Del	9	121502137	121732994	0	0.020706
31449	Del	9	121733560	121818256	0	0.02189
31450	Del	9	121821554	122119736	0	0.020115
31451	Del	9	122120006	122939152	0	0.011832
31452	Del	9	122940016	123031312	0	0.011241
31453	Del	9	123032393	123037715	0	0.011832
31454	Del	9	123038226	123105071	0	0.011241
31455	Del	9	123105908	124472034	0	0.011832
31456	Del	9	124481221	124510146	0	0.001183
31457	Del	9	124514122	124654523	0	0.011832
31458	Del	9	124654621	124699396	0	0.012424
31459	Del	9	124701198	124773638	0	0.011832
31460	Del	9	124773713	124847016	0	0.012424
31461	Del	9	124847597	124934308	0	0.001183
31462	Del	9	124938825	125144468	0	0.018932
31463	Del	9	125146027	125235024	0	0.020115
31464	Del	9	125245860	127583108	0	0.001183
31465	Del	9	127584516	127735841	0	0.004141
31466	Del	9	127736336	128332589	0	0.006508
31467	Del	9	128345153	128663485	0	0.01479
31468	Del	9	128671543	129028808	0	0.015974
31469	Del	9	129028866	129149851	0	0.031355
31470	Del	9	129152237	129477420	0	0.03668
31471	Del	9	129478080	129513732	0	0.031355
31472	Del	9	129514724	129644733	0	0.049104
31473	Del	9	129646385	129736742	0	0.050287
31474	Del	9	129736790	129833792	0	0.047329
31475	Del	9	129834501	129883712	0	0.050287
31476	Del	9	129886820	130045027	0	0.046737
31477	Del	9	130045182	130139124	0	0.047329
31478	Del	9	130139228	130168171	0	0.047921
31479	Del	9	130169790	130194935	0	0.047329
31480	Del	9	130197039	130264361	0	0.047921
31481	Del	9	130264837	130512845	0	0.029581

31482	Del	9	130513148	130580942	0	0.030172
31483	Del	9	130581012	130625439	0	0.029581
31484	Del	9	130625631	130627146	0	0.030172
31485	Del	9	130628720	130641831	0	0.029581
31486	Del	9	130643069	130706544	0	0.030172
31487	Del	9	130709517	130833054	0	0.046737
31488	Del	9	130833546	130890251	0	0.049104
31489	Del	9	130890269	130947709	0	0.050287
31490	Del	9	130948086	131093159	0.092751	0.065669
31491	Del	9	131094323	131127532	0.113852	0.066852
31492	Del	9	131127589	131251571	0.09192	0.065669
31493	Del	9	131255157	131309285	0	0.045554
31494	Del	9	131309686	131483812	0	0.047921
31495	Del	9	131485232	131490853	0	0.048512
31496	Del	9	131490887	131498887	0	0.047921
31497	Del	9	131499214	131534220	0	0.048512
31498	Del	9	131534405	131710351	0	0.052653
31499	Del	9	131710905	132071735	0	0.052062
31500	Del	9	132072465	132160215	0	0.053245
31501	Del	9	132160756	132243197	0	0.052653
31502	Del	9	132257331	132371907	0	0.052062
31503	Del	9	132375086	132407324	0	0.038455
31504	Del	9	132407549	132569591	0	0.037272
31505	Del	9	132569709	132593780	0	0.038455
31506	Del	9	132594789	132657856	0	0.037863
31507	Del	9	132657927	132724673	0	0.038455
31508	Del	9	132724799	132874686	0	0.037272
31509	Del	9	132875115	132890177	0	0.038455
31510	Del	9	132891317	132946903	0.176097	0.070993
31511	Del	9	132949657	132975587	0.058782	0.063302
31512	Del	9	132977950	133660184	0.066619	0.063894
31513	Del	9	133663599	133761583	0.05305	0.063302
31514	Del	9	133761880	133997407	0.021477	0.060936
31515	Del	9	133999813	134316751	0.0669	0.063894
31516	Del	9	134324562	134374858	0.059411	0.063302
31517	Del	9	134374977	134632220	0.068346	0.063894
31518	Del	9	134634207	134646211	0.025774	0.061528
31519	Del	9	134646982	134658852	0.068155	0.063894
31520	Del	9	134660552	134847165	0.025774	0.061528
31521	Del	9	134848359	134853364	0	0.057978
31522	Del	9	134854778	134878871	0	0.057386
31523	Del	9	134879093	135098476	0	0.057978
31524	Del	9	135099632	135134168	0	0.057386
31525	Del	9	135134481	135418642	0	0.057978
31526	Del	9	135472087	135472543	0	0.029581
31527	Del	9	135473649	135528583	0	0.036088
31528	Del	9	135528821	135660388	0	0.045554
31529	Del	9	135660680	135793326	0	0.028989
31530	Del	9	135794725	135930924	0	0.029581
31531	Del	9	135931192	136400830	0	0.023664
31532	Del	9	136401057	137443692	0	0.014199
31533	Del	9	137443787	137599242	0	0.024848
31534	Del	9	137616769	137836309	0	0.041413

31535	Del	9	137839737	137909533	0	0.039046
31536	Del	9	137911306	137942466	0	0.038455
31537	Del	9	137942855	137948328	0	0.039046
31538	Del	9	137951706	138037308	0	0.038455
31539	Del	9	138037754	138044505	0	0.039046
31540	Del	10	366509	366509	0	0.028397
31541	Del	10	369300	803925	0	0.027214
31542	Del	10	804039	929277	0	0.025439
31543	Del	10	930021	1270173	0	0.026031
31544	Del	10	1270227	1521311	0	0.01834
31545	Del	10	1523069	1558906	0	0.021298
31546	Del	10	1559140	1759857	0	0.01834
31547	Del	10	1761331	2075304	0	0.043779
31548	Del	10	2075343	3137471	0	0.021298
31549	Del	10	3137650	3310236	0	0.013607
31550	Del	10	3310687	3753179	0	0.011241
31551	Del	10	3754377	5495606	0	0.013607
31552	Del	10	5497663	6393195	0	0.007099
31553	Del	10	6393878	6578149	0	0.006508
31554	Del	10	6578192	7156172	0	0.007099
31555	Del	10	7157092	7183815	0	0.002958
31556	Del	10	7185592	7187349	0	0.00355
31557	Del	10	7187477	7416421	0	0.002958
31558	Del	10	7419276	7817193	0	0.00355
31559	Del	10	7817575	8015675	0	0.002958
31560	Del	10	8016438	9296013	0	0.00355
31561	Del	10	9296875	10461288	0	0.010649
31562	Del	10	10461715	11332988	0	0.021298
31563	Del	10	11337709	11532915	0	0.010649
31564	Del	10	11535667	11561065	0	0.010057
31565	Del	10	11563767	12158906	0	0.010649
31566	Del	10	12159304	12193559	0	0.016565
31567	Del	10	12193712	12344298	0	0.025439
31568	Del	10	12344352	12574863	0	0.023073
31569	Del	10	12575766	12580810	0	0.025439
31570	Del	10	12582825	12649577	0	0.023073
31571	Del	10	12650155	12653317	0	0.025439
31572	Del	10	12653388	12720881	0	0.023073
31573	Del	10	12723565	12726791	0	0.025439
31574	Del	10	12726980	12835584	0	0.023073
31575	Del	10	12835675	13097245	0	0.023664
31576	Del	10	13098065	13158057	0	0.025439
31577	Del	10	13159212	13643649	0	0.016565
31578	Del	10	13643675	13710507	0	0.015974
31579	Del	10	13714584	14008771	0	0.013607
31580	Del	10	14009961	14071283	0	0.011241
31581	Del	10	14072184	14089153	0	0.016565
31582	Del	10	14089543	14382430	0	0.01479
31583	Del	10	14383186	14575881	0	0.010649
31584	Del	10	14576114	14606638	0	0.010057
31585	Del	10	14609980	14775068	0	0.009466
31586	Del	10	14775083	14905467	0	0.012424
31587	Del	10	14909662	15207776	0	0.010649

31588	Del	10	15208314	15377960	0	0.010057
31589	Del	10	15382174	15776093	0	0.020115
31590	Del	10	15777170	16586548	0	0.010649
31591	Del	10	16586885	16819401	0	0.009466
31592	Del	10	16819865	17134826	0	0.013015
31593	Del	10	17135350	17316029	0	0.010649
31594	Del	10	17319044	17386712	0	0.010057
31595	Del	10	17386911	17408504	0	0.010649
31596	Del	10	17409271	17586798	0	0.010057
31597	Del	10	17587943	18138501	0	0.007099
31598	Del	10	18138617	18260186	0	0.005325
31599	Del	10	18260219	18396478	0	0.005916
31600	Del	10	18396962	18536235	0	0.006508
31601	Del	10	18536733	19028895	0	0.007099
31602	Del	10	19029351	19708645	0	0.004733
31603	Del	10	19710276	19730533	0	0.007099
31604	Del	10	19730699	20291128	0	0.004733
31605	Del	10	20291196	20573498	0	0.01834
31606	Del	10	20577781	21121312	0	0.026031
31607	Del	10	21125264	21150272	0	0.029581
31608	Del	10	21150304	21171566	0	0.026031
31609	Del	10	21171971	21492709	0	0.029581
31610	Del	10	21493913	21561611	0	0.022481
31611	Del	10	21563240	21756168	0	0.02189
31612	Del	10	21756310	22006818	0	0.011832
31613	Del	10	22006889	22331762	0	0.012424
31614	Del	10	22341837	22531253	0	0.017748
31615	Del	10	22531724	22926526	0	0.011241
31616	Del	10	22926644	23038764	0	0.006508
31617	Del	10	23039409	23691794	0	0.014199
31618	Del	10	23692719	24206637	0	0.009466
31619	Del	10	24206657	24274847	0	0.012424
31620	Del	10	24275210	24276252	0	0.014199
31621	Del	10	24276418	24443638	0	0.012424
31622	Del	10	24447169	24464461	0	0.013607
31623	Del	10	24465622	24546917	0	0.014199
31624	Del	10	24547566	24726000	0	0.013607
31625	Del	10	24734899	24955006	0	0.01479
31626	Del	10	24955990	25157316	0	0.015382
31627	Del	10	25157685	25177994	0	0.025439
31628	Del	10	25182274	25649638	0	0.022481
31629	Del	10	25651259	25936305	0	0.015382
31630	Del	10	25936607	26218977	0	0.014199
31631	Del	10	26222975	26435276	0	0.009466
31632	Del	10	26436426	26568132	0	0.008874
31633	Del	10	26569931	27101749	0	0.009466
31634	Del	10	27103593	27420482	0	0.013607
31635	Del	10	27423487	27542322	0	0.015382
31636	Del	10	27543412	27735261	0	0.047329
31637	Del	10	27735803	27811562	0	0.047921
31638	Del	10	27812051	28042128	0	0.046146
31639	Del	10	28042484	28072392	0	0.039046
31640	Del	10	28072901	28078875	0	0.042004

31641	Del	10	28079274	28285633	0	0.039046
31642	Del	10	28285707	28290054	0	0.042004
31643	Del	10	28290119	28431893	0	0.039046
31644	Del	10	28431929	28497866	0	0.020706
31645	Del	10	28498865	28530477	0	0.032539
31646	Del	10	28531380	28674135	0	0.044963
31647	Del	10	28674854	28738773	0	0.033722
31648	Del	10	28738794	28786746	0	0.03313
31649	Del	10	28787128	28796622	0	0.033722
31650	Del	10	28798407	28810955	0	0.03313
31651	Del	10	28811125	29469770	0	0.033722
31652	Del	10	29469890	29489138	0	0.03313
31653	Del	10	29490270	29541499	0	0.031947
31654	Del	10	29542095	29546087	0	0.033722
31655	Del	10	29547235	29601338	0	0.031947
31656	Del	10	29601507	29602575	0	0.033722
31657	Del	10	29603074	29635115	0	0.031947
31658	Del	10	29635543	29744011	0	0.030764
31659	Del	10	29744694	30632251	0	0.033722
31660	Del	10	30632358	30721921	0	0.055612
31661	Del	10	30722623	30724925	0	0.033722
31662	Del	10	30725106	31000689	0	0.052653
31663	Del	10	31000992	31187230	0	0.052062
31664	Del	10	31187558	31263129	0	0.03313
31665	Del	10	31263150	31321516	0	0.033722
31666	Del	10	31321789	31803449	0	0.032539
31667	Del	10	31804456	31930248	0	0.027806
31668	Del	10	31942473	32259555	0	0.028397
31669	Del	10	32266383	32339079	0	0.027806
31670	Del	10	32341591	32438684	0	0.028397
31671	Del	10	32443051	32895730	0	0.040821
31672	Del	10	32895748	32959239	0	0.026623
31673	Del	10	32961538	33195901	0	0.028989
31674	Del	10	33196508	33341992	0	0.029581
31675	Del	10	33343038	34108135	0	0.038455
31676	Del	10	34108722	34309033	0	0.032539
31677	Del	10	34309490	34797709	0	0.034313
31678	Del	10	34797727	35218236	0	0.038455
31679	Del	10	35218285	35599709	0	0.03668
31680	Del	10	35600129	35609834	0	0.030764
31681	Del	10	35611971	37123421	0	0.03313
31682	Del	10	37123566	37233794	0	0.021298
31683	Del	10	37234370	37352620	0	0.023073
31684	Del	10	37352718	37775998	0	0.023664
31685	Del	10	37776088	37792052	0	0.023073
31686	Del	10	37793138	37798073	0	0.023664
31687	Del	10	37798474	37825079	0	0.023073
31688	Del	10	37828592	38002590	0	0.023664
31689	Del	10	38009592	38702069	0	0.016565
31690	Del	10	38702193	42473822	0	0.039638
31691	Del	10	42474909	42569267	0	0.03313
31692	Del	10	42570966	42579810	0	0.032539
31693	Del	10	42579840	42692267	0	0.03313

31694	Del	10	42693931	42868221	0	0.039046
31695	Del	10	42869858	42997817	0	0.03313
31696	Del	10	42998449	43131924	0	0.044963
31697	Del	10	43132006	43618185	0	0.046737
31698	Del	10	43618659	43774215	0	0.052653
31699	Del	10	43774895	43857226	0	0.046737
31700	Del	10	43857635	43909420	0	0.046146
31701	Del	10	43911982	43914615	0	0.046737
31702	Del	10	43915172	43986177	0	0.046146
31703	Del	10	43987009	44700826	0	0.05147
31704	Del	10	44700866	44910629	0	0.050287
31705	Del	10	44910696	44937697	0	0.05147
31706	Del	10	44939364	44957169	0	0.050287
31707	Del	10	44957820	45094335	0	0.05147
31708	Del	10	45099254	45149320	0	0.050879
31709	Del	10	45153929	45297432	0	0.05147
31710	Del	10	45299713	45373074	0	0.049104
31711	Del	10	45373441	45442434	0	0.048512
31712	Del	10	45442544	45453901	0	0.049104
31713	Del	10	45455012	45537734	0	0.048512
31714	Del	10	45537879	45602247	0	0.047921
31715	Del	10	45605479	46031829	0	0.049104
31716	Del	10	46032384	47283186	0	0.041413
31717	Del	10	47283243	48188464	0	0.03668
31718	Del	10	48189189	48251803	0	0.036088
31719	Del	10	48252111	48274918	0	0.035497
31720	Del	10	48277116	48401070	0	0.04023
31721	Del	10	48401135	48440789	0	0.040821
31722	Del	10	48441974	48493679	0	0.049104
31723	Del	10	48498158	48607248	0	0.047329
31724	Del	10	48608144	48681814	0	0.046737
31725	Del	10	48684153	48908528	0	0.044963
31726	Del	10	48909063	48916732	0	0.049695
31727	Del	10	48918818	48985007	0	0.044963
31728	Del	10	48985477	49012366	0	0.049695
31729	Del	10	49012567	49100605	0	0.048512
31730	Del	10	49101860	49445557	0	0.049695
31731	Del	10	49454181	49514923	0	0.049104
31732	Del	10	49514954	49613145	0	0.049695
31733	Del	10	49613197	49665545	0	0.049104
31734	Del	10	49665982	50716389	0	0.049695
31735	Del	10	50719256	50798511	0	0.052062
31736	Del	10	50799038	50980049	0	0.05147
31737	Del	10	50981494	51056164	0	0.043188
31738	Del	10	51061008	51068637	0	0.052062
31739	Del	10	51070875	51071244	0	0.043188
31740	Del	10	51071264	51297832	0	0.043779
31741	Del	10	51299550	51299839	0	0.052062
31742	Del	10	51300012	51694311	0	0.043779
31743	Del	10	51694338	51701506	0	0.052062
31744	Del	10	51701815	52289599	0	0.043779
31745	Del	10	52289627	52471740	0	0.047921
31746	Del	10	52472346	52774554	0.00167	0.059161

31747	Del	10	52774701	53807771	0.024976	0.060936
31748	Del	10	53808394	55586019	0.025691	0.061528
31749	Del	10	55587271	56350086	0	0.043188
31750	Del	10	56350645	58512227	0	0.041413
31751	Del	10	58512368	58710943	0	0.038455
31752	Del	10	58712170	58717554	0	0.041413
31753	Del	10	58719195	58836340	0	0.038455
31754	Del	10	58839689	59169705	0	0.052653
31755	Del	10	59170158	59179302	0	0.041413
31756	Del	10	59179903	59254406	0	0.040821
31757	Del	10	59255186	59365812	0	0.04023
31758	Del	10	59366216	59711206	0	0.040821
31759	Del	10	59711936	59753973	0	0.041413
31760	Del	10	59754244	59955556	0	0.046146
31761	Del	10	59955830	60023710	0	0.043188
31762	Del	10	60026215	60142035	0	0.042004
31763	Del	10	60142120	60390445	0	0.038455
31764	Del	10	60390504	60572967	0	0.037272
31765	Del	10	60573447	60775059	0	0.036088
31766	Del	10	60777553	60866696	0	0.035497
31767	Del	10	60867116	60945271	0	0.034905
31768	Del	10	60948761	61002890	0	0.034313
31769	Del	10	61006934	61656802	0	0.035497
31770	Del	10	61662012	61900851	0	0.034905
31771	Del	10	61900930	62191672	0	0.033722
31772	Del	10	62192181	62409689	0	0.027214
31773	Del	10	62409973	62489381	0	0.026623
31774	Del	10	62491552	62517779	0	0.024848
31775	Del	10	62518251	62672087	0	0.026031
31776	Del	10	62680105	63269914	0	0.027214
31777	Del	10	63271809	63466928	0	0.026031
31778	Del	10	63468716	63524843	0	0.025439
31779	Del	10	63525329	63625760	0	0.021298
31780	Del	10	63627596	65500863	0	0.027214
31781	Del	10	65500959	65767154	0	0.025439
31782	Del	10	65767242	67788537	0	0.05147
31783	Del	10	67791383	67919310	0	0.035497
31784	Del	10	67924773	68008228	0	0.034905
31785	Del	10	68008571	68011466	0	0.035497
31786	Del	10	68013381	68065590	0	0.034905
31787	Del	10	68066222	68100685	0	0.035497
31788	Del	10	68104514	68215276	0	0.034905
31789	Del	10	68215557	68531025	0	0.035497
31790	Del	10	68531085	68720599	0	0.039638
31791	Del	10	68721111	69269709	0	0.030764
31792	Del	10	69278961	69312412	0	0.029581
31793	Del	10	69313380	69400171	0	0.030172
31794	Del	10	69400353	69800611	0	0.030764
31795	Del	10	69801200	69959753	0	0.028989
31796	Del	10	69962677	70110358	0	0.030172
31797	Del	10	70110598	70298433	0	0.030764
31798	Del	10	70298778	70384666	0	0.030172
31799	Del	10	70391654	70474235	0	0.030764

31800	Del	10	70476473	70571441	0	0.030172
31801	Del	10	70571784	71210448	0	0.030764
31802	Del	10	71211981	71215705	0	0.030172
31803	Del	10	71216306	71219739	0	0.030764
31804	Del	10	71221345	71303533	0	0.030172
31805	Del	10	71303681	71365606	0	0.030764
31806	Del	10	71366274	71709202	0	0.03313
31807	Del	10	71710362	71745879	0	0.03668
31808	Del	10	71746372	71746372	0	0.032539
31809	Del	10	71746757	71776432	0	0.03668
31810	Del	10	71777857	71787374	0	0.032539
31811	Del	10	71788943	72056021	0	0.03668
31812	Del	10	72058623	72092998	0	0.030764
31813	Del	10	72098500	72211079	0	0.030172
31814	Del	10	72211567	72365291	0	0.030764
31815	Del	10	72367135	72525113	0	0.026623
31816	Del	10	72525541	72691727	0	0.026031
31817	Del	10	72691769	72719289	0	0.011241
31818	Del	10	72719510	72721421	0	0.011832
31819	Del	10	72722447	72888066	0	0.011241
31820	Del	10	72888126	72955950	0	0.011832
31821	Del	10	72956086	73098178	0	0.030764
31822	Del	10	73107092	73360301	0	0.021298
31823	Del	10	73376184	73434458	0	0.011832
31824	Del	10	73435596	74135318	0	0.006508
31825	Del	10	74138166	74500962	0	0.021298
31826	Del	10	74502050	74535133	0	0.023664
31827	Del	10	74536622	74821820	0	0.021298
31828	Del	10	74822269	74911592	0	0.01479
31829	Del	10	74911632	74940648	0	0.015382
31830	Del	10	74940844	75035964	0	0.01479
31831	Del	10	75039021	75416236	0	0.006508
31832	Del	10	75424236	75551464	0	0.002958
31833	Del	10	75552342	75557828	0	0.006508
31834	Del	10	75558049	75646536	0	0.002958
31835	Del	10	75647196	75709594	0	0.00355
31836	Del	10	75710466	75744214	0	0.006508
31837	Del	10	75744529	76557477	0	0.00355
31838	Del	10	76558814	77351011	0	0.028397
31839	Del	10	77351655	77789347	0	0.026031
31840	Del	10	77789484	77923386	0	0.014199
31841	Del	10	77924337	77970166	0	0.01479
31842	Del	10	77974439	78929276	0	0.008283
31843	Del	10	78929352	79379126	0	0.001183
31844	Del	10	79380066	79447919	0	0.000592
31845	Del	10	79449208	80271441	0	0.008874
31846	Del	10	80271522	80293285	0	0.000592
31847	Del	10	80295340	80452785	0	0.001183
31848	Del	10	80453253	80523782	0	0.000592
31849	Del	10	80524756	80535642	0	0.001183
31850	Del	10	80537777	80646815	0	0.004733
31851	Del	10	80648571	82228886	0	0.01834
31852	Del	10	82229005	82233648	0	0.019523

31853	Del	10	82233949	82991373	0	0.01834
31854	Del	10	82991836	83677827	0	0.026623
31855	Del	10	83677845	84225752	0	0.029581
31856	Del	10	84225927	84272076	0	0.034905
31857	Del	10	84276606	84378071	0	0.023073
31858	Del	10	84379290	84420164	0	0.022481
31859	Del	10	84423637	84518735	0	0.023073
31860	Del	10	84518839	85426663	0	0.034313
31861	Del	10	85431270	85439514	0	0.023073
31862	Del	10	85440148	85607609	0	0.034905
31863	Del	10	85607638	86259192	0	0.028397
31864	Del	10	86259637	86267367	0	0.035497
31865	Del	10	86268612	86372338	0	0.028397
31866	Del	10	86374500	86525367	0	0.034905
31867	Del	10	86525463	86739198	0	0.035497
31868	Del	10	86739927	86929645	0	0.03668
31869	Del	10	86932998	87088376	0	0.046737
31870	Del	10	87092094	87503230	0.009595	0.059753
31871	Del	10	87503615	87653300	0	0.053245
31872	Del	10	87658973	87748576	0	0.052062
31873	Del	10	87750472	87817454	0	0.052653
31874	Del	10	87817989	87857400	0.485064	0.085784
31875	Del	10	87857644	87857644	0.191167	0.071585
31876	Del	10	87861304	88271932	2.192336	0.137846
31877	Del	10	88273666	88589062	0	0.03313
31878	Del	10	88590153	89424947	0	0.026031
31879	Del	10	89425835	89450314	0	0.025439
31880	Del	10	89450905	89468309	0	0.026031
31881	Del	10	89468748	89592539	0	0.025439
31882	Del	10	89592688	89592893	0	0.026031
31883	Del	10	89592910	89646273	0	0.025439
31884	Del	10	89647170	89701363	0	0.039638
31885	Del	10	89701396	89829229	0	0.039046
31886	Del	10	89829291	89841912	0	0.026031
31887	Del	10	89842455	90740497	0	0.037863
31888	Del	10	90740762	90862439	0	0.027214
31889	Del	10	90863076	91062346	0	0.027806
31890	Del	10	91062859	91154665	0	0.039638
31891	Del	10	91156127	91219187	0	0.042004
31892	Del	10	91219608	91284956	0	0.042596
31893	Del	10	91285076	91406074	0	0.046146
31894	Del	10	91408779	91465291	0	0.048512
31895	Del	10	91467386	91517744	0	0.047921
31896	Del	10	91518247	91613530	0	0.046146
31897	Del	10	91618873	91922596	0	0.043188
31898	Del	10	91923365	92042750	0	0.042596
31899	Del	10	92042798	92283528	0	0.042004
31900	Del	10	92290231	92824289	0	0.043188
31901	Del	10	92826670	93054543	0	0.041413
31902	Del	10	93058315	93302111	0	0.043188
31903	Del	10	93304719	93483915	0	0.042004
31904	Del	10	93484051	93609889	0	0.044371
31905	Del	10	93612452	93665357	0	0.043779

31906	Del	10	93666668	93754675	0	0.044371
31907	Del	10	93756433	93801258	0	0.043779
31908	Del	10	93801640	93963700	0	0.044371
31909	Del	10	93965928	94334993	0	0.056795
31910	Del	10	94335177	94392606	0	0.049104
31911	Del	10	94395541	94537651	0	0.048512
31912	Del	10	94538666	94935687	0	0.049104
31913	Del	10	94936985	94989424	0	0.041413
31914	Del	10	94989663	95188573	0	0.053245
31915	Del	10	95190092	95235432	0	0.041413
31916	Del	10	95238063	95311671	0	0.040821
31917	Del	10	95311760	95446862	0	0.04023
31918	Del	10	95448921	95563778	0	0.038455
31919	Del	10	95563875	95655676	0	0.040821
31920	Del	10	95656150	95709142	0	0.041413
31921	Del	10	95710122	95754722	0	0.04023
31922	Del	10	95754860	95878051	0	0.040821
31923	Del	10	95888386	95907163	0	0.039046
31924	Del	10	95907825	95952362	0	0.041413
31925	Del	10	95953802	95998512	0	0.039046
31926	Del	10	95999377	96091355	0	0.041413
31927	Del	10	96091675	96188977	0.018569	0.060936
31928	Del	10	96190730	96302028	0.126787	0.068035
31929	Del	10	96304051	96338859	0	0.040821
31930	Del	10	96340723	96362839	0	0.041413
31931	Del	10	96363489	96512542	0	0.039638
31932	Del	10	96515114	96588967	0	0.040821
31933	Del	10	96589831	96721550	0	0.04023
31934	Del	10	96722665	96837645	0	0.041413
31935	Del	10	96842394	96966754	0	0.040821
31936	Del	10	96967674	96992028	0	0.041413
31937	Del	10	96992827	97102123	0	0.039046
31938	Del	10	97102556	97103804	0	0.041413
31939	Del	10	97104555	97152442	0	0.039046
31940	Del	10	97152478	97216977	0	0.043188
31941	Del	10	97219044	97294295	0	0.043779
31942	Del	10	97296446	97424077	0	0.044371
31943	Del	10	97424407	97507882	0	0.03668
31944	Del	10	97507916	97569377	0	0.036088
31945	Del	10	97571875	97873146	0	0.03668
31946	Del	10	97873995	98033029	0	0.035497
31947	Del	10	98033255	98245149	0	0.036088
31948	Del	10	98245525	98456023	0	0.03668
31949	Del	10	98457024	98921642	0	0.036088
31950	Del	10	98923097	98926381	0	0.042596
31951	Del	10	98930295	99394539	0	0.036088
31952	Del	10	99394683	99778591	0	0.03668
31953	Del	10	99782122	99852563	0	0.036088
31954	Del	10	99856400	99916503	0	0.03668
31955	Del	10	99921928	99925734	0	0.036088
31956	Del	10	99926041	99959911	0	0.03668
31957	Del	10	99966449	100010973	0	0.035497
31958	Del	10	100011199	100083191	0	0.03668

31959	Del	10	100083704	100728148	0	0.037863
31960	Del	10	100732802	100833218	0	0.037272
31961	Del	10	100833593	101348576	0	0.037863
31962	Del	10	101351730	101558546	0	0.03668
31963	Del	10	101559228	101580422	0	0.037863
31964	Del	10	101580586	101610578	0	0.041413
31965	Del	10	101613216	101695560	0	0.040821
31966	Del	10	101698284	101853648	0	0.049104
31967	Del	10	101862091	102059548	0	0.047921
31968	Del	10	102062263	102165233	0	0.049104
31969	Del	10	102168272	102248994	0.030769	0.061528
31970	Del	10	102253494	102385101	0.018569	0.060344
31971	Del	10	102389478	102441624	0.030373	0.061528
31972	Del	10	102442972	102502929	0.25536	0.075135
31973	Del	10	102506100	102739534	0.233983	0.073952
31974	Del	10	102741740	102773156	0.391768	0.081642
31975	Del	10	102774534	102818313	0.406908	0.082234
31976	Del	10	102818774	102837723	0.420903	0.082826
31977	Del	10	102837961	102902701	0.626286	0.091108
31978	Del	10	102902730	102927857	0.652422	0.0917
31979	Del	10	102930195	103084254	0.587353	0.089333
31980	Del	10	103085146	103243715	0.614384	0.090517
31981	Del	10	103244113	103517584	0.605535	0.089925
31982	Del	10	103522635	103588493	0.575877	0.089333
31983	Del	10	103590354	103744857	1.099887	0.107082
31984	Del	10	103745196	103756892	1.255393	0.111815
31985	Del	10	103759242	103858641	1.099887	0.107082
31986	Del	10	103859796	103961830	1.415208	0.117139
31987	Del	10	103966580	104044193	1.400056	0.116548
31988	Del	10	104047725	104048363	1.451632	0.117731
31989	Del	10	104050642	104086193	1.398769	0.116548
31990	Del	10	104086316	104129576	1.450378	0.117731
31991	Del	10	104129958	104218008	1.35399	0.115364
31992	Del	10	104218500	104219613	1.451632	0.117731
31993	Del	10	104219880	104233543	1.35399	0.115364
31994	Del	10	104233772	104271437	1.43388	0.117731
31995	Del	10	104272452	104300451	1.415208	0.117139
31996	Del	10	104301509	104356848	1.446025	0.117731
31997	Del	10	104360608	104455984	1.361091	0.115364
31998	Del	10	104456593	104637859	1.446025	0.117731
31999	Del	10	104640168	104663529	1.021312	0.104124
32000	Del	10	104663940	104666344	1.44921	0.117731
32001	Del	10	104668893	105665232	1.021312	0.104124
32002	Del	10	105673185	105820720	0.484572	0.085784
32003	Del	10	105824980	106189151	0.672328	0.092291
32004	Del	10	106189571	107165485	0.994651	0.103532
32005	Del	10	107165758	109864383	1.586025	0.121872
32006	Del	10	109864670	110287520	1.444405	0.117731
32007	Del	10	110287747	110493072	1.479706	0.118914
32008	Del	10	110493904	110563429	1.382651	0.115956
32009	Del	10	110564257	110640067	1.355445	0.115364
32010	Del	10	110640077	110840928	1.212491	0.110631
32011	Del	10	110842026	110871099	1.390784	0.115956

32012	Del	10	110873200	110897573	1.366009	0.115364
32013	Del	10	110897686	110924076	1.390784	0.115956
32014	Del	10	110924757	110934272	1.33374	0.114773
32015	Del	10	110934993	110939087	1.390784	0.115956
32016	Del	10	110940124	110977120	1.33374	0.114773
32017	Del	10	110988003	110991077	1.390784	0.115956
32018	Del	10	110993963	111018200	1.33374	0.114773
32019	Del	10	111018369	112148302	1.389686	0.115956
32020	Del	10	112148522	112298727	1.068315	0.105899
32021	Del	10	112298805	112300395	1.08465	0.10649
32022	Del	10	112301767	112310140	1.069378	0.105899
32023	Del	10	112310391	112357080	1.046376	0.105307
32024	Del	10	112358659	112427329	1.054478	0.105899
32025	Del	10	112428029	112451452	1.075148	0.10649
32026	Del	10	112451666	112633145	0.876447	0.099391
32027	Del	10	112633222	112635035	1.08465	0.10649
32028	Del	10	112635052	112821434	0.876447	0.099391
32029	Del	10	112822880	112830765	1.082036	0.10649
32030	Del	10	112832964	113044625	1.219405	0.110631
32031	Del	10	113045257	113046101	1.347894	0.114773
32032	Del	10	113047110	113172490	1.219405	0.110631
32033	Del	10	113173852	113552088	1.292887	0.112998
32034	Del	10	113552127	113590838	1.296171	0.11359
32035	Del	10	113591346	113664721	1.271471	0.112406
32036	Del	10	113664806	113750908	1.313923	0.11359
32037	Del	10	113751133	113762826	1.060485	0.105899
32038	Del	10	113764603	113779813	1.07765	0.10649
32039	Del	10	113780893	113783566	1.060485	0.105899
32040	Del	10	113785666	113856226	1.076157	0.10649
32041	Del	10	113856765	113915266	1.067273	0.105899
32042	Del	10	113915933	114094347	1.08465	0.10649
32043	Del	10	114098532	114155093	1.059686	0.105899
32044	Del	10	114159271	114179177	1.077528	0.10649
32045	Del	10	114190062	114233640	1.060485	0.105899
32046	Del	10	114233667	114302751	1.587011	0.121872
32047	Del	10	114309215	114430839	1.551465	0.12128
32048	Del	10	114430924	114527327	1.524981	0.120689
32049	Del	10	114528274	114658690	1.408137	0.116548
32050	Del	10	114659562	114685009	1.37698	0.115956
32051	Del	10	114686679	114763851	1.316372	0.114181
32052	Del	10	114764181	114817716	1.466906	0.118322
32053	Del	10	114819971	114865400	1.446938	0.117731
32054	Del	10	114865606	114902037	1.421925	0.117139
32055	Del	10	114902791	114990798	1.445878	0.117731
32056	Del	10	114991288	115085522	0.922462	0.100574
32057	Del	10	115089975	115171738	0.884595	0.099391
32058	Del	10	115175296	115949093	0.532081	0.087559
32059	Del	10	115949292	116275819	0.705531	0.093475
32060	Del	10	116276312	116381923	0.832805	0.097616
32061	Del	10	116390547	116478905	1.105073	0.107082
32062	Del	10	116486780	116568549	1.13694	0.108265
32063	Del	10	116568731	116610001	1.481983	0.118914
32064	Del	10	116610575	116670645	1.488085	0.119506

32065	Del	10	116671258	116744114	1.448619	0.117731
32066	Del	10	116744796	116840344	1.356609	0.115364
32067	Del	10	116840572	116884159	1.477873	0.118914
32068	Del	10	116884878	117011775	1.440555	0.117731
32069	Del	10	117014507	117123857	1.382303	0.115956
32070	Del	10	117127383	117195881	1.502082	0.119506
32071	Del	10	117196053	117237777	1.396727	0.116548
32072	Del	10	117238377	117282828	1.356204	0.115364
32073	Del	10	117285315	117378148	1.345405	0.114773
32074	Del	10	117381650	117526067	1.369925	0.115364
32075	Del	10	117538312	117999437	1.410586	0.116548
32076	Del	10	118004247	118100864	1.124133	0.107673
32077	Del	10	118100930	118215315	1.061455	0.105899
32078	Del	10	118216498	118343295	1.124133	0.107673
32079	Del	10	118343311	118677707	1.140442	0.108265
32080	Del	10	118680377	118756380	1.104098	0.107082
32081	Del	10	118756742	119041320	1.181741	0.109448
32082	Del	10	119042541	119056010	1.162409	0.108857
32083	Del	10	119056545	119062113	1.181741	0.109448
32084	Del	10	119067278	119138120	1.162409	0.108857
32085	Del	10	119138968	119185169	1.168108	0.109448
32086	Del	10	119188966	119455836	1.931389	0.130746
32087	Del	10	119457549	119571878	2.113068	0.136071
32088	Del	10	119573312	119648559	1.939184	0.131338
32089	Del	10	119649612	119707870	1.924923	0.130746
32090	Del	10	119709117	119799038	1.86231	0.129563
32091	Del	10	119801114	119816534	1.837801	0.12838
32092	Del	10	119818409	119832031	1.89575	0.130155
32093	Del	10	119832470	119945945	1.873141	0.129563
32094	Del	10	119946183	120338987	1.908675	0.130155
32095	Del	10	120346567	120356836	1.411539	0.116548
32096	Del	10	120360062	120590025	1.811203	0.127788
32097	Del	10	120590484	120760530	1.966094	0.131929
32098	Del	10	120760642	120843419	1.111846	0.107673
32099	Del	10	120849292	121475547	1.154193	0.108857
32100	Del	10	121476933	121532433	0.880977	0.099391
32101	Del	10	121532519	121595212	0.819364	0.097616
32102	Del	10	121595801	121606833	0.813191	0.097024
32103	Del	10	121607645	121924040	1.252856	0.111815
32104	Del	10	121925066	121982617	1.351637	0.115364
32105	Del	10	121986932	122112126	1.162746	0.108857
32106	Del	10	122112284	122161269	1.249235	0.111815
32107	Del	10	122161589	122255424	1.292029	0.112998
32108	Del	10	122255849	122347969	1.298435	0.11359
32109	Del	10	122350405	122414812	1.333589	0.114773
32110	Del	10	122415958	122420497	1.369925	0.115364
32111	Del	10	122423575	122432914	1.348857	0.114773
32112	Del	10	122432958	122464294	1.361865	0.115364
32113	Del	10	122464428	122515087	1.315388	0.114181
32114	Del	10	122515627	122655223	1.344396	0.114773
32115	Del	10	122657975	122754266	1.36728	0.115364
32116	Del	10	122756052	122797707	1.313923	0.11359
32117	Del	10	122805167	122977325	1.363287	0.115364

32118	Del	10	122977686	123009383	1.252213	0.111815
32119	Del	10	123014221	123058554	1.223046	0.111223
32120	Del	10	123062196	123664202	1.258453	0.111815
32121	Del	10	123664790	123742788	1.166273	0.109448
32122	Del	10	123744663	124003726	1.097448	0.107082
32123	Del	10	124007414	124094256	0.937348	0.101166
32124	Del	10	124094812	124461089	1.249235	0.111815
32125	Del	10	124461579	124499760	1.23362	0.111223
32126	Del	10	124501663	124614367	1.14144	0.108265
32127	Del	10	124616434	124695330	1.174892	0.109448
32128	Del	10	124696147	124716083	1.245337	0.111815
32129	Del	10	124719155	124756732	1.174892	0.109448
32130	Del	10	124757376	124938318	1.011351	0.104124
32131	Del	10	124941988	125029048	1.000193	0.103532
32132	Del	10	125029580	125029886	0.898476	0.099982
32133	Del	10	125032104	125033966	1.022647	0.104124
32134	Del	10	125034081	125162753	0.898476	0.099982
32135	Del	10	125163766	125578654	1.237159	0.111223
32136	Del	10	125579562	125653363	1.15068	0.108857
32137	Del	10	125655503	125702448	1.219859	0.110631
32138	Del	10	125702816	125721713	1.225485	0.111223
32139	Del	10	125721748	125741542	1.216962	0.110631
32140	Del	10	125743908	125752870	1.238233	0.111223
32141	Del	10	125756205	125756205	1.216962	0.110631
32142	Del	10	125757308	125777505	1.235133	0.111223
32143	Del	10	125783732	125795974	1.216962	0.110631
32144	Del	10	125796685	125853926	1.225485	0.111223
32145	Del	10	125854033	125893688	1.215978	0.110631
32146	Del	10	125893703	125969507	1.163165	0.108857
32147	Del	10	125969535	125973865	1.23952	0.111223
32148	Del	10	125974366	126013242	1.163165	0.108857
32149	Del	10	126014541	126041165	1.090816	0.107082
32150	Del	10	126041185	126391026	1.11715	0.107673
32151	Del	10	126391241	126420880	1.214004	0.110631
32152	Del	10	126421043	126902568	1.404254	0.116548
32153	Del	10	126903452	127134489	1.283261	0.112998
32154	Del	10	127134869	127198387	1.345803	0.114773
32155	Del	10	127198631	127457572	1.283261	0.112998
32156	Del	10	127458071	127553448	1.385266	0.115956
32157	Del	10	127553547	127741639	1.972473	0.131929
32158	Del	10	127742789	127903030	2.261308	0.13962
32159	Del	10	127903681	127981054	2.093289	0.135479
32160	Del	10	127981061	127997457	2.159483	0.136662
32161	Del	10	127997982	128040015	2.176461	0.137254
32162	Del	10	128040341	128055881	2.215338	0.138437
32163	Del	10	128056105	128063528	2.267901	0.13962
32164	Del	10	128065164	128086671	2.215338	0.138437
32165	Del	10	128087206	128284512	2.258369	0.139029
32166	Del	10	128285210	128317845	1.783135	0.127197
32167	Del	10	128318252	129768924	1.924094	0.130746
32168	Del	10	129769080	129970432	2.021927	0.133704
32169	Del	10	129975997	130131667	2.443185	0.143762
32170	Del	10	130134679	130183069	2.391861	0.142578

32171	Del	10	130183138	131338451	2.909467	0.153819
32172	Del	10	131342778	131914143	2.338031	0.140804
32173	Del	10	131914230	131964282	2.29687	0.140212
32174	Del	10	131965391	132063998	2.320564	0.140804
32175	Del	10	132064372	132183081	2.222446	0.138437
32176	Del	10	132183527	132208366	2.331579	0.140804
32177	Del	10	132210126	132256426	2.303471	0.140212
32178	Del	10	132258344	132309515	2.276652	0.13962
32179	Del	10	132311422	132317483	2.259991	0.139029
32180	Del	10	132322783	132385563	2.334858	0.140804
32181	Del	10	132385605	132535811	2.831504	0.152044
32182	Del	10	132536505	132678400	2.675722	0.148495
32183	Del	10	132678718	132747307	2.794687	0.150861
32184	Del	10	132747703	133097553	2.845836	0.152044
32185	Del	10	133097573	133097573	2.817835	0.151453
32186	Del	10	133097985	133279086	2.823389	0.152044
32187	Del	10	133279882	133307142	2.821918	0.151453
32188	Del	10	133307187	133411599	3.495102	0.166243
32189	Del	11	456120	456120	4.03372	0.176892
32190	Del	11	458784	484204	4.007239	0.1763
32191	Del	11	491334	549959	4.025237	0.176892
32192	Del	11	564127	620599	4.252785	0.181625
32193	Del	11	628753	641563	4.139649	0.179258
32194	Del	11	641991	709861	4.096228	0.178667
32195	Del	11	718436	733813	3.150625	0.159144
32196	Del	11	745581	863088	2.637024	0.147903
32197	Del	11	863309	920108	2.840522	0.152044
32198	Del	11	922466	1019194	2.782722	0.150861
32199	Del	11	1021248	1156810	2.358552	0.141395
32200	Del	11	1157084	1264749	2.210664	0.137846
32201	Del	11	1264946	1307664	2.388852	0.141987
32202	Del	11	1307682	1562919	2.414665	0.142578
32203	Del	11	1569075	1594136	2.70707	0.149086
32204	Del	11	1597130	1599137	2.736539	0.149678
32205	Del	11	1599300	1599300	2.70707	0.149086
32206	Del	11	1599554	1660062	2.73456	0.149678
32207	Del	11	1662334	1883471	2.414665	0.142578
32208	Del	11	1883627	1887776	2.387611	0.141987
32209	Del	11	1896642	1962212	2.403512	0.142578
32210	Del	11	2043393	2327519	2.210664	0.137846
32211	Del	11	2327548	2371788	2.171187	0.137254
32212	Del	11	2374337	2439877	2.204508	0.137846
32213	Del	11	2440975	2454345	2.013612	0.133113
32214	Del	11	2457965	2603316	2.022839	0.133704
32215	Del	11	2603683	2702374	2.188696	0.137846
32216	Del	11	2702860	2851644	2.022839	0.133704
32217	Del	11	2851681	2871282	1.931389	0.131338
32218	Del	11	2871329	2872708	1.568504	0.12128
32219	Del	11	2874570	2937015	2.159708	0.136662
32220	Del	11	2937047	2959939	3.168306	0.159735
32221	Del	11	2959968	2965470	3.246151	0.160918
32222	Del	11	2965546	3000006	3.168306	0.159735
32223	Del	11	3000312	3166816	1.864004	0.129563

32224	Del	11	3168052	3356788	1.931389	0.130746
32225	Del	11	3356833	3377432	1.237159	0.111223
32226	Del	11	3378510	3661342	1.249949	0.111815
32227	Del	11	3664188	3675705	1.420631	0.117139
32228	Del	11	3676858	3701657	1.352554	0.115364
32229	Del	11	3709637	3798037	1.371516	0.115956
32230	Del	11	3805057	3843036	1.089626	0.107082
32231	Del	11	3844556	3856283	1.411539	0.116548
32232	Del	11	3859885	4094311	1.263763	0.112406
32233	Del	11	4094732	4108629	0.969068	0.102349
32234	Del	11	4109060	4642729	0.982064	0.10294
32235	Del	11	4643427	4703544	0.974534	0.102349
32236	Del	11	4704437	4987935	0.986845	0.10294
32237	Del	11	4987955	5054651	0.523782	0.086967
32238	Del	11	5058289	5337094	0.46722	0.084601
32239	Del	11	5340042	5351460	0.187366	0.071585
32240	Del	11	5351488	5353125	0.25536	0.075135
32241	Del	11	5355626	5386052	0.187366	0.071585
32242	Del	11	5386067	5390514	0.25536	0.075135
32243	Del	11	5390808	5421716	0.187366	0.071585
32244	Del	11	5421755	5423563	0.254901	0.075135
32245	Del	11	5423656	5439984	0.187366	0.071585
32246	Del	11	5440067	5441915	0.25527	0.075135
32247	Del	11	5442195	5445100	0.187366	0.071585
32248	Del	11	5445546	5466366	0.25536	0.075135
32249	Del	11	5467510	5488459	0.187366	0.071585
32250	Del	11	5488518	5489990	0.25527	0.075135
32251	Del	11	5490059	5505934	0.187366	0.071585
32252	Del	11	5506947	5688227	0.25536	0.075135
32253	Del	11	5688494	5734452	0.207463	0.072768
32254	Del	11	5734980	6388588	0.12071	0.067444
32255	Del	11	6388755	6521451	0	0.057978
32256	Del	11	6522530	6574883	0	0.056795
32257	Del	11	6576090	6764770	0	0.057978
32258	Del	11	6764822	7024427	0	0.040821
32259	Del	11	7024442	7074801	0	0.04023
32260	Del	11	7075581	7247000	0	0.040821
32261	Del	11	7247961	7437172	0	0.037863
32262	Del	11	7437395	7467110	0	0.04023
32263	Del	11	7467435	7470248	0	0.037863
32264	Del	11	7478928	7511997	0	0.040821
32265	Del	11	7518447	7591051	0	0.039046
32266	Del	11	7592118	7654038	0	0.04023
32267	Del	11	7654348	8020143	0	0.040821
32268	Del	11	8020262	8079597	0	0.052653
32269	Del	11	8079626	8106676	0	0.053245
32270	Del	11	8107886	8388076	0	0.052653
32271	Del	11	8389074	8594546	0	0.05147
32272	Del	11	8600995	8692568	0	0.053245
32273	Del	11	8692778	8768078	0	0.052062
32274	Del	11	8768745	8811149	0	0.052653
32275	Del	11	8811322	8811686	0	0.052062
32276	Del	11	8813943	8897357	0	0.050879

32277	Del	11	8897906	9006547	0	0.053245
32278	Del	11	9008153	9084373	0.014397	0.060344
32279	Del	11	9085049	9096351	0.024976	0.060936
32280	Del	11	9096960	9258915	0.068155	0.063894
32281	Del	11	9274337	9379143	0.083819	0.065077
32282	Del	11	9384476	9423555	0.073223	0.064486
32283	Del	11	9424487	9433852	0.083415	0.065077
32284	Del	11	9440446	9450159	0.073223	0.064486
32285	Del	11	9452223	9463717	0.083819	0.065077
32286	Del	11	9467194	9529126	0.074001	0.064486
32287	Del	11	9530958	9660312	0.082003	0.065077
32288	Del	11	9662916	9751293	0.0669	0.063894
32289	Del	11	9751970	9760941	0.083415	0.065077
32290	Del	11	9761623	9816035	0.069246	0.064486
32291	Del	11	9816578	9833720	0	0.059161
32292	Del	11	9834072	9929554	0.031419	0.062119
32293	Del	11	9929710	10300078	0	0.059161
32294	Del	11	10301931	10309235	0	0.050879
32295	Del	11	10309273	10431405	0	0.049104
32296	Del	11	10436533	10507818	0	0.050287
32297	Del	11	10508152	10508152	0	0.042596
32298	Del	11	10510897	10610613	0	0.049104
32299	Del	11	10610705	10653050	0	0.048512
32300	Del	11	10653113	10694174	0	0.047921
32301	Del	11	10694757	10881947	0	0.049104
32302	Del	11	10882223	11346894	0	0.05502
32303	Del	11	11346980	11353382	0.039159	0.062119
32304	Del	11	11353439	11653008	0	0.05502
32305	Del	11	11655617	11782418	0	0.054428
32306	Del	11	11782634	11836804	0	0.047921
32307	Del	11	11840829	12015358	0	0.039638
32308	Del	11	12015519	12106794	0	0.04023
32309	Del	11	12108967	12160913	0	0.039046
32310	Del	11	12161489	12164197	0	0.04023
32311	Del	11	12164539	12208935	0	0.039046
32312	Del	11	12209125	12264582	0	0.038455
32313	Del	11	12265112	12376087	0	0.039638
32314	Del	11	12377064	12536815	0	0.038455
32315	Del	11	12537446	12978609	0	0.047921
32316	Del	11	12979476	13273018	0	0.044963
32317	Del	11	13275750	13388251	0	0.04023
32318	Del	11	13388296	13666281	0	0.036088
32319	Del	11	13666868	13736284	0	0.027214
32320	Del	11	13736374	13956090	0	0.04023
32321	Del	11	13956191	14275141	0	0.037272
32322	Del	11	14276088	14520996	0	0.034313
32323	Del	11	14521031	14873110	0	0.033722
32324	Del	11	14873833	15111652	0	0.034313
32325	Del	11	15111964	15148218	0	0.03313
32326	Del	11	15148385	15248673	0	0.033722
32327	Del	11	15248747	15964026	0	0.034313
32328	Del	11	15964211	15967804	0	0.020115
32329	Del	11	15968079	15972437	0	0.022481

32330	Del	11	15973276	16409651	0	0.020115
32331	Del	11	16409879	16480280	0	0.019523
32332	Del	11	16484543	16776221	0	0.027214
32333	Del	11	16776849	17015325	0	0.024848
32334	Del	11	17017093	17085616	0	0.027214
32335	Del	11	17085644	17208842	0	0.026623
32336	Del	11	17208914	17391413	0	0.027214
32337	Del	11	17392788	17649308	0	0.026623
32338	Del	11	17650565	17787473	0	0.027214
32339	Del	11	17787796	18014550	0	0.025439
32340	Del	11	18016571	18138904	0	0.027214
32341	Del	11	18139218	18205166	0	0.037863
32342	Del	11	18205195	18727272	0	0.027214
32343	Del	11	18727361	18794455	0	0.026623
32344	Del	11	18794698	19053917	0	0.050879
32345	Del	11	19054080	19222866	0	0.03313
32346	Del	11	19222948	19350069	0	0.027214
32347	Del	11	19350360	20154059	0	0.019523
32348	Del	11	20154084	20384261	0	0.017748
32349	Del	11	20386541	20509445	0	0.017157
32350	Del	11	20510106	20663538	0	0.017748
32351	Del	11	20665185	22189941	0	0.012424
32352	Del	11	22190395	22321575	0	0.001183
32353	Del	11	22321664	22493861	0	0.000592
32354	Del	11	22495974	23419574	0	0.001183
32355	Del	11	23420283	26187880	0	0.027806
32356	Del	11	26188724	26331686	0	0.020706
32357	Del	11	26331890	26467060	0	0.021298
32358	Del	11	26468962	26556503	0	0.022481
32359	Del	11	26557077	26573092	0	0.024256
32360	Del	11	26574480	26663409	0	0.022481
32361	Del	11	26663579	26724192	0	0.023664
32362	Del	11	26724621	27040791	0	0.024256
32363	Del	11	27040941	27128715	0	0.023664
32364	Del	11	27129976	27330510	0	0.023073
32365	Del	11	27338313	27501107	0	0.013015
32366	Del	11	27504577	27615467	0	0.021298
32367	Del	11	27617387	27635242	0	0.022481
32368	Del	11	27635308	27648561	0	0.021298
32369	Del	11	27651574	27725635	0	0.022481
32370	Del	11	27726124	28111659	0	0.034905
32371	Del	11	28111873	28335213	0	0.033722
32372	Del	11	28335680	29977301	0	0.034905
32373	Del	11	29977433	30230078	0	0.009466
32374	Del	11	30230113	30587729	0	0.001183
32375	Del	11	30593864	31256293	0	0.009466
32376	Del	11	31260355	31380616	0	0.001183
32377	Del	11	31382270	31429310	0	0.000592
32378	Del	11	31430644	31829323	0	0.001183
32379	Del	11	31831237	31887101	0	0.000592
32380	Del	11	31887179	32043103	0	0.001183
32381	Del	11	32043405	32142536	0	0.007099
32382	Del	11	32142631	32431379	0	0.001183

32383	Del	11	32432076	32433981	0	0.000592
32384	Del	11	32434307	32825627	0	0.001183
32385	Del	11	32825937	32982442	0	0.000592
32386	Del	11	32983255	33871162	0	0.001183
32387	Del	11	33872467	33892881	0	0.000592
32388	Del	11	33893375	34148367	0	0.001183
32389	Del	11	34150561	34517457	0	0.000592
32390	Del	11	34519102	34880522	0	0.001183
32391	Del	11	34880974	34915254	0	0.000592
32392	Del	11	34915529	35656270	0	0.001183
32393	Del	11	35659406	35812883	0	0.000592
32394	Del	11	35812964	36583992	0	0.001183
32395	Del	11	36586360	37938228	0	0.019523
32396	Del	11	37938265	38659250	0	0.009466
32397	Del	11	38659597	40295956	0	0.05147
32398	Del	11	40297051	41713251	0	0.041413
32399	Del	11	41713766	41843060	0	0.025439
32400	Del	11	41843113	43357129	0	0.026031
32401	Del	11	43357956	43495477	0	0.025439
32402	Del	11	43495572	43677406	0	0.026031
32403	Del	11	43677868	43857990	0	0.024848
32404	Del	11	43858810	44093828	0	0.026031
32405	Del	11	44095501	44322125	0	0.025439
32406	Del	11	44322306	44763736	0	0.026031
32407	Del	11	44764194	44931175	0	0.024256
32408	Del	11	44931379	45091415	0	0.026031
32409	Del	11	45093871	45236841	0	0.025439
32410	Del	11	45237415	45885279	0	0.026031
32411	Del	11	45888300	45925913	0	0.011241
32412	Del	11	45926765	46123424	0	0.015974
32413	Del	11	46123950	46386919	0	0.016565
32414	Del	11	46387761	46444294	0	0.015974
32415	Del	11	46448822	46452614	0	0.016565
32416	Del	11	46453756	46600842	0	0.015974
32417	Del	11	46601416	47265445	0	0.007099
32418	Del	11	47268883	47449829	0	0.014199
32419	Del	11	47454205	47655657	0	0.018932
32420	Del	11	47657564	47973747	0	0.015974
32421	Del	11	47974014	48094680	0	0.010649
32422	Del	11	48096265	48097441	0	0.011241
32423	Del	11	48097922	48173669	0	0.010649
32424	Del	11	48175807	48327685	0	0.011241
32425	Del	11	48327878	49020714	0	0.020706
32426	Del	11	49021136	49549679	0	0.019523
32427	Del	11	49554515	49816812	0	0.018932
32428	Del	11	49818589	49955815	0	0.029581
32429	Del	11	49958561	50042616	0	0.034313
32430	Del	11	50051837	56244856	0	0.037272
32431	Del	11	56247303	56253349	0.354776	0.079868
32432	Del	11	56256870	56317516	0.423612	0.082826
32433	Del	11	56317996	56363982	0.309388	0.077501
32434	Del	11	56365157	56840766	0.498564	0.085784
32435	Del	11	56840917	56879464	0.309388	0.078093

32436	Del	11	56883473	57033925	0.342392	0.079276
32437	Del	11	57037282	57192309	0.236869	0.073952
32438	Del	11	57193299	57289556	0.341247	0.079276
32439	Del	11	57289600	57543214	0.158718	0.06981
32440	Del	11	57544451	57615919	0.347989	0.079868
32441	Del	11	57617925	57759156	0.511526	0.086375
32442	Del	11	57760085	57760085	0.483919	0.085192
32443	Del	11	57760763	58019373	0.499012	0.085784
32444	Del	11	58019567	58028537	0.301326	0.077501
32445	Del	11	58028821	58033587	0.367793	0.080459
32446	Del	11	58033687	58116775	0.301326	0.077501
32447	Del	11	58117457	58119810	0.367793	0.080459
32448	Del	11	58121380	58182557	0.301326	0.077501
32449	Del	11	58183182	58352539	0.368363	0.080459
32450	Del	11	58356327	58361695	0.246242	0.074543
32451	Del	11	58362444	58525562	0.436775	0.083417
32452	Del	11	58526175	58569147	0.422781	0.082826
32453	Del	11	58571880	58705326	0.426751	0.083417
32454	Del	11	58706801	58926331	0.240585	0.074543
32455	Del	11	58927578	58939318	0.237075	0.073952
32456	Del	11	58940392	58957329	0.244181	0.074543
32457	Del	11	58957614	59087546	0.217653	0.07336
32458	Del	11	59087573	59138545	0.242046	0.074543
32459	Del	11	59138640	59213076	0.352635	0.079868
32460	Del	11	59216030	59569573	0.454127	0.084009
32461	Del	11	59573682	59591103	0.342947	0.079276
32462	Del	11	59592368	59598112	0.354776	0.079868
32463	Del	11	59602112	59617629	0.342947	0.079276
32464	Del	11	59626360	59751937	0.347407	0.079868
32465	Del	11	59752963	59803581	0.329244	0.078684
32466	Del	11	59804122	59940771	0.353268	0.079868
32467	Del	11	59940784	60022835	0.34088	0.079276
32468	Del	11	60026215	60205735	0.353268	0.079868
32469	Del	11	60206350	60487021	0.247213	0.074543
32470	Del	11	60489820	60609842	0.171642	0.070402
32471	Del	11	60611950	60689426	0.160982	0.06981
32472	Del	11	60694023	60971176	0.171318	0.070402
32473	Del	11	60971360	61024009	0.158718	0.06981
32474	Del	11	61025039	61129817	0.208015	0.072768
32475	Del	11	61130032	61197989	0.200358	0.072177
32476	Del	11	61198004	61301886	0.173853	0.070402
32477	Del	11	61305719	61491914	0	0.053245
32478	Del	11	61492279	61519028	0	0.054428
32479	Del	11	61519220	61577600	0	0.053837
32480	Del	11	61585305	61749438	0	0.054428
32481	Del	11	61750765	61823108	0.080736	0.065077
32482	Del	11	61823823	61823823	0.075677	0.064486
32483	Del	11	61824060	62123420	0.075798	0.065077
32484	Del	11	62123878	62154221	0	0.053837
32485	Del	11	62157233	62432897	0	0.054428
32486	Del	11	62432930	62511159	0	0.053837
32487	Del	11	62514147	62547074	0	0.054428
32488	Del	11	62551058	62608598	0	0.056795

32489	Del	11	62611641	62651415	0.114364	0.066852
32490	Del	11	62654715	62688853	0.059713	0.063302
32491	Del	11	62689937	62740067	0.090705	0.065669
32492	Del	11	62740938	62850708	0.173853	0.070402
32493	Del	11	62851734	62922198	0.074577	0.064486
32494	Del	11	62922258	63079689	0.191059	0.071585
32495	Del	11	63079880	63108552	0.068262	0.063894
32496	Del	11	63113457	63145203	0.073638	0.064486
32497	Del	11	63145313	63288078	0.21253	0.07336
32498	Del	11	63289206	63566746	0.075677	0.065077
32499	Del	11	63567171	63615453	0.075231	0.064486
32500	Del	11	63628912	63675900	0	0.057978
32501	Del	11	63680658	63811719	0	0.057386
32502	Del	11	63812510	63991669	0	0.047921
32503	Del	11	63991682	64168158	0.114479	0.067444
32504	Del	11	64169789	64322402	0.139876	0.068627
32505	Del	11	64322963	64644627	0.32105	0.078093
32506	Del	11	64647178	64724272	0.298705	0.077501
32507	Del	11	64724646	65916513	0.319394	0.078093
32508	Del	11	65916800	66073601	0	0.050879
32509	Del	11	66074331	66248302	0	0.049695
32510	Del	11	66249476	66310131	0	0.050879
32511	Del	11	66313740	66345103	0	0.045554
32512	Del	11	66346497	66416452	0	0.052653
32513	Del	11	66417883	66582642	0	0.042004
32514	Del	11	66584269	66841050	0	0.035497
32515	Del	11	66841520	66925960	0	0.04023
32516	Del	11	66926393	66935626	0	0.040821
32517	Del	11	66946563	66964195	0	0.04023
32518	Del	11	66965350	66985824	0	0.040821
32519	Del	11	66987138	67112637	0	0.049104
32520	Del	11	67117271	67264497	0	0.041413
32521	Del	11	67265037	67425631	0	0.027806
32522	Del	11	67426709	67469694	0	0.031947
32523	Del	11	67471651	67490111	0	0.036088
32524	Del	11	67490352	67526002	0	0.043188
32525	Del	11	67526727	68007631	0	0.052653
32526	Del	11	68007658	68051786	0	0.034905
32527	Del	11	68052144	68123822	0	0.046146
32528	Del	11	68123972	68163594	0	0.050287
32529	Del	11	68164294	68260636	0	0.050879
32530	Del	11	68260785	68276168	0	0.043188
32531	Del	11	68277104	68455977	0	0.047329
32532	Del	11	68456574	68680729	0	0.054428
32533	Del	11	68680823	69143386	0	0.015974
32534	Del	11	69144220	69479371	0	0.007099
32535	Del	11	69480882	69493807	0	0.002958
32536	Del	11	69500287	69822369	0	0.007691
32537	Del	11	69822897	70076466	0	0.017157
32538	Del	11	70077193	70189972	0	0.016565
32539	Del	11	70190001	70265426	0	0.017157
32540	Del	11	70265755	70437962	0	0.012424
32541	Del	11	70449792	71225302	0	0.032539

32542	Del	11	71226168	71450911	0	0.046737
32543	Del	11	71451658	71470807	0	0.046146
32544	Del	11	71471313	71479586	0	0.046737
32545	Del	11	71479847	71501961	0	0.046146
32546	Del	11	71510528	71542601	0	0.046737
32547	Del	11	71544576	71859610	0	0.05857
32548	Del	11	71862236	71929953	0.248065	0.075135
32549	Del	11	71930047	71995791	0.237962	0.074543
32550	Del	11	71998540	72081025	0.502646	0.086375
32551	Del	11	72083500	72134352	0.362794	0.080459
32552	Del	11	72134495	72286451	0.329748	0.078684
32553	Del	11	72287281	72567601	0.229216	0.073952
32554	Del	11	72568506	72568506	0.191167	0.071585
32555	Del	11	72572340	72681075	0.337248	0.079276
32556	Del	11	72683401	72722875	0.250585	0.075135
32557	Del	11	72723910	72753480	0.246242	0.074543
32558	Del	11	72754507	72831222	0.251945	0.075135
32559	Del	11	72831903	73215965	0.379508	0.081051
32560	Del	11	73216074	73262482	0.329609	0.078684
32561	Del	11	73262549	73300061	0.247558	0.075135
32562	Del	11	73303810	73369610	0.245798	0.074543
32563	Del	11	73372419	73398590	0.254531	0.075135
32564	Del	11	73401478	73598839	0.218738	0.07336
32565	Del	11	73598927	73668030	0.250329	0.075135
32566	Del	11	73675343	73865703	0.238945	0.074543
32567	Del	11	73866752	73870863	0.25536	0.075135
32568	Del	11	73875484	73947169	0.374536	0.081051
32569	Del	11	73947492	74011720	0.254464	0.075135
32570	Del	11	74012577	74167643	0.217127	0.07336
32571	Del	11	74168257	74305188	0.245117	0.074543
32572	Del	11	74306274	74327229	0.25527	0.075135
32573	Del	11	74329450	74387688	0.241119	0.074543
32574	Del	11	74387738	74500360	0.25536	0.075135
32575	Del	11	74501338	74646319	0.435768	0.083417
32576	Del	11	74646801	74724476	0.681991	0.092883
32577	Del	11	74724816	74732833	0.671844	0.092291
32578	Del	11	74733519	74837785	0.795442	0.096433
32579	Del	11	74838562	74951505	0.808755	0.097024
32580	Del	11	74953946	75149741	0.443193	0.084009
32581	Del	11	75150500	75207903	0.427603	0.083417
32582	Del	11	75208960	75258203	0.454939	0.084009
32583	Del	11	75258811	75334758	0.413976	0.082826
32584	Del	11	75334856	75336854	0.454939	0.084009
32585	Del	11	75339863	75393630	0.413976	0.082826
32586	Del	11	75396292	75432825	0.25536	0.075135
32587	Del	11	75433039	75528519	0.23443	0.073952
32588	Del	11	75528858	75586813	0.254531	0.075135
32589	Del	11	75586839	75598563	0.236394	0.073952
32590	Del	11	75601815	75675808	0.241119	0.074543
32591	Del	11	75677742	75804379	0.251945	0.075135
32592	Del	11	75806041	76181697	0.261996	0.075726
32593	Del	11	76185673	76437698	0	0.053837
32594	Del	11	76442824	76656706	0	0.053245

32595	Del	11	76657283	76676922	0	0.046737
32596	Del	11	76677272	76721962	0	0.046146
32597	Del	11	76722647	76858539	0	0.046737
32598	Del	11	76859016	77028687	0	0.045554
32599	Del	11	77030205	77126453	0	0.046737
32600	Del	11	77126506	77214030	0	0.046146
32601	Del	11	77215490	77317797	0	0.050879
32602	Del	11	77320361	77474833	0	0.046146
32603	Del	11	77475278	77665202	0	0.046737
32604	Del	11	77665422	77814221	0	0.045554
32605	Del	11	77815603	77872430	0	0.046737
32606	Del	11	77875588	78013628	0	0.046146
32607	Del	11	78015218	78047512	0	0.038455
32608	Del	11	78048753	78062669	0	0.037863
32609	Del	11	78062880	78216990	0	0.038455
32610	Del	11	78217064	78346922	0	0.037863
32611	Del	11	78347675	78414200	0	0.03668
32612	Del	11	78423016	78428616	0	0.038455
32613	Del	11	78435047	78528877	0	0.043188
32614	Del	11	78529219	78560568	0	0.044371
32615	Del	11	78560772	78575987	0	0.043188
32616	Del	11	78576013	80748769	0.269955	0.076318
32617	Del	11	80750987	80765032	0.059256	0.063302
32618	Del	11	80765371	82725750	1.727368	0.126013
32619	Del	11	82729826	82818962	1.054345	0.105307
32620	Del	11	82819079	82888732	1.006536	0.103532
32621	Del	11	82894093	82970226	1.039431	0.104715
32622	Del	11	82972557	83035189	0.47067	0.085192
32623	Del	11	83035323	83041223	0.457257	0.084601
32624	Del	11	83041420	83041756	0.499012	0.085784
32625	Del	11	83043190	83066388	0.457257	0.084601
32626	Del	11	83067178	83217527	0.498396	0.085784
32627	Del	11	83217776	83254842	0.476982	0.085192
32628	Del	11	83257122	83287453	0.488316	0.085784
32629	Del	11	83290156	84923638	0.522873	0.086967
32630	Del	11	84925442	85639778	0.369837	0.081051
32631	Del	11	85645173	85689951	0.245798	0.074543
32632	Del	11	85692458	85762205	0.231838	0.073952
32633	Del	11	85764397	85812382	0.21479	0.07336
32634	Del	11	85812645	85930588	0.25536	0.075135
32635	Del	11	85930912	86239219	0.438268	0.083417
32636	Del	11	86239276	86296539	0.359473	0.080459
32637	Del	11	86297221	86374102	0.163529	0.070402
32638	Del	11	86374192	86425021	0.158718	0.06981
32639	Del	11	86425265	86787565	0.192843	0.072177
32640	Del	11	86788251	86811265	0.081603	0.065077
32641	Del	11	86811887	86854586	0.0562	0.063302
32642	Del	11	86856687	86858549	0.083418	0.065077
32643	Del	11	86861878	86939358	0.0562	0.063302
32644	Del	11	86942957	86957404	0.082481	0.065077
32645	Del	11	86957420	87003754	0.075628	0.064486
32646	Del	11	87004814	87717480	0.088732	0.065669
32647	Del	11	87718216	87723886	0.083418	0.065077

32648	Del	11	87723956	88174574	0.228324	0.073952
32649	Del	11	88176025	88291112	0.25536	0.075135
32650	Del	11	88292321	88319421	0.243456	0.074543
32651	Del	11	88320028	88343687	0.249689	0.075135
32652	Del	11	88347065	89175295	0.266531	0.075726
32653	Del	11	89175737	89297333	0.176097	0.070993
32654	Del	11	89298412	89487054	0.319447	0.078093
32655	Del	11	89488071	89492216	0.368363	0.080459
32656	Del	11	89492920	89492920	0.319394	0.078093
32657	Del	11	89495429	89650686	0.291097	0.07691
32658	Del	11	89658921	90117678	0.195784	0.072177
32659	Del	11	90124386	90192826	0.056983	0.063302
32660	Del	11	90193504	90225147	0.13479	0.068627
32661	Del	11	90225603	90915513	1.020447	0.104124
32662	Del	11	90918928	92896612	1.03207	0.104715
32663	Del	11	92897080	92983098	1.369925	0.115364
32664	Del	11	92984154	93721358	1.433241	0.117731
32665	Del	11	93721461	93725684	1.484839	0.118914
32666	Del	11	93725917	93729441	1.446025	0.117731
32667	Del	11	93729879	93763719	1.483345	0.118914
32668	Del	11	93765667	94012898	1.701289	0.12483
32669	Del	11	94020456	94115385	1.651037	0.123647
32670	Del	11	94116935	94304985	1.699044	0.12483
32671	Del	11	94305630	94414608	1.484839	0.118914
32672	Del	11	94415438	94466106	1.178664	0.109448
32673	Del	11	94466121	94466838	1.23952	0.111223
32674	Del	11	94468258	94493786	1.178664	0.109448
32675	Del	11	94493863	94502208	1.237159	0.111223
32676	Del	11	94505108	94533951	1.40297	0.116548
32677	Del	11	94534010	94550193	1.423034	0.117139
32678	Del	11	94554006	94564016	1.252771	0.111815
32679	Del	11	94567104	94621476	1.374905	0.115956
32680	Del	11	94621739	94764322	1.266305	0.112406
32681	Del	11	94765694	94877228	1.164359	0.109448
32682	Del	11	94877283	95085249	1.226406	0.111223
32683	Del	11	95086283	95132943	1.208057	0.110631
32684	Del	11	95137450	95161772	1.228216	0.111223
32685	Del	11	95161799	95229263	1.188087	0.11004
32686	Del	11	95229335	95767747	1.217854	0.110631
32687	Del	11	95768698	95792953	1.169179	0.109448
32688	Del	11	95802056	95833190	1.16162	0.108857
32689	Del	11	95834224	95974049	1.128339	0.108265
32690	Del	11	95974748	96335709	0.751763	0.09525
32691	Del	11	96335724	96442367	0.777832	0.095841
32692	Del	11	96443277	96506108	0.737807	0.094658
32693	Del	11	96506277	96550823	0.776717	0.095841
32694	Del	11	96553652	100310399	1.740653	0.126013
32695	Del	11	100312594	100682227	1.72127	0.125422
32696	Del	11	100683568	100685402	1.409421	0.116548
32697	Del	11	100686135	100988708	1.612241	0.122464
32698	Del	11	100990763	101024607	1.927153	0.130746
32699	Del	11	101024724	101125539	1.345405	0.114773
32700	Del	11	101125547	101448166	2.100661	0.135479

32701	Del	11	101449353	101519141	1.988157	0.132521
32702	Del	11	101519462	101523810	2.135166	0.136071
32703	Del	11	101525555	101585119	1.988157	0.132521
32704	Del	11	101585708	101918807	2.117889	0.136071
32705	Del	11	101918962	102001332	2.061314	0.134296
32706	Del	11	102002721	102079510	2.114299	0.136071
32707	Del	11	102082575	102086491	2.109367	0.135479
32708	Del	11	102087217	102392542	2.129749	0.136071
32709	Del	11	102395912	102446880	2.099013	0.135479
32710	Del	11	102447125	102466739	2.127149	0.136071
32711	Del	11	102467046	102530902	2.093776	0.135479
32712	Del	11	102533399	102628609	2.299747	0.140212
32713	Del	11	102628628	102708042	2.325008	0.140804
32714	Del	11	102708650	102780856	2.35696	0.141395
32715	Del	11	102784326	102785692	2.271532	0.13962
32716	Del	11	102789366	102798914	2.351293	0.141395
32717	Del	11	102800116	102831127	2.271532	0.13962
32718	Del	11	102832042	103045638	2.355032	0.141395
32719	Del	11	103047129	103093410	2.321866	0.140804
32720	Del	11	103093538	103593639	2.595731	0.14672
32721	Del	11	103593797	103904248	4.200393	0.180442
32722	Del	11	103906289	104035324	4.108073	0.178667
32723	Del	11	104035887	104043230	4.168351	0.17985
32724	Del	11	104043539	104166130	4.108073	0.178667
32725	Del	11	104166286	104557759	4.638003	0.189316
32726	Del	11	104558000	104957553	4.57527	0.188133
32727	Del	11	104957579	104988540	4.534855	0.187541
32728	Del	11	104990672	105023444	4.513555	0.186949
32729	Del	11	105023782	105061029	4.553331	0.188133
32730	Del	11	105061261	105069049	4.549385	0.187541
32731	Del	11	105069183	105145284	4.573422	0.188133
32732	Del	11	105147196	105917636	7.045818	0.244336
32733	Del	11	105919761	106003866	6.929787	0.242561
32734	Del	11	106004582	106027000	6.673787	0.232504
32735	Del	11	106027540	106080079	6.673787	0.233095
32736	Del	11	106080333	106248340	6.673787	0.232504
32737	Del	11	106248644	106672501	6.013441	0.21653
32738	Del	11	106672739	107021746	5.434857	0.205289
32739	Del	11	107023795	107460607	5.784763	0.211797
32740	Del	11	107460608	107570212	5.896021	0.214164
32741	Del	11	107573980	107597955	6.040404	0.21653
32742	Del	11	107599032	107667463	5.914805	0.214164
32743	Del	11	107668203	107776206	6.022817	0.21653
32744	Del	11	107777591	107862784	5.930829	0.214755
32745	Del	11	107865982	108081855	6.043213	0.21653
32746	Del	11	108083284	108150316	5.996621	0.215938
32747	Del	11	108150975	108225787	6.058999	0.217122
32748	Del	11	108226487	108382525	5.975527	0.215347
32749	Del	11	108382719	108467613	6.058999	0.217122
32750	Del	11	108469878	108538252	6.13698	0.218305
32751	Del	11	108539057	108553769	6.089427	0.217713
32752	Del	11	108554469	108595163	6.014784	0.21653
32753	Del	11	108595702	108944509	6.168347	0.219488

32754	Del	11	108944713	110088912	6.815951	0.239011
32755	Del	11	110091706	110173953	6.743884	0.235462
32756	Del	11	110174416	110193472	6.731591	0.23487
32757	Del	11	110195548	110226231	6.750404	0.236053
32758	Del	11	110229363	110299215	6.750404	0.237236
32759	Del	11	110303305	110465458	6.769025	0.237828
32760	Del	11	110465865	111255215	6.815951	0.239011
32761	Del	11	111255833	111287464	6.268028	0.221263
32762	Del	11	111289495	111350847	6.31664	0.222446
32763	Del	11	111351988	111382659	6.270995	0.221263
32764	Del	11	111386477	111464639	6.338818	0.222446
32765	Del	11	111465115	111501446	6.268028	0.221263
32766	Del	11	111506355	111515664	6.338818	0.222446
32767	Del	11	111515776	111537579	6.275522	0.221855
32768	Del	11	111537712	111595875	6.666806	0.23132
32769	Del	11	111595883	111717825	6.64879	0.228954
32770	Del	11	111721263	111781599	6.658444	0.23132
32771	Del	11	111781612	111872071	6.64879	0.230729
32772	Del	11	111872930	111928794	6.673787	0.232504
32773	Del	11	111928829	112025600	6.668694	0.231912
32774	Del	11	112029195	112060921	6.663865	0.23132
32775	Del	11	112062710	112141907	6.673787	0.232504
32776	Del	11	112141952	112163854	6.668694	0.231912
32777	Del	11	112164002	112172802	6.673787	0.232504
32778	Del	11	112173272	112220576	6.64879	0.230729
32779	Del	11	112220671	112244480	6.673787	0.232504
32780	Del	11	112247218	112256978	6.668694	0.231912
32781	Del	11	112259060	112269609	6.673787	0.232504
32782	Del	11	112269913	112365148	6.64879	0.230137
32783	Del	11	112365665	112482431	6.64879	0.230729
32784	Del	11	112483392	112491739	6.673787	0.232504
32785	Del	11	112503355	112544129	6.64879	0.230729
32786	Del	11	112544860	112964422	5.851048	0.213572
32787	Del	11	112965771	113265358	5.26967	0.20174
32788	Del	11	113268528	113268624	5.250691	0.201148
32789	Del	11	113269314	113276761	5.876274	0.213572
32790	Del	11	113276909	113278920	5.250691	0.201148
32791	Del	11	113279328	113367342	5.765271	0.211797
32792	Del	11	113367501	113408042	5.847438	0.21298
32793	Del	11	113409398	113447983	5.727066	0.210614
32794	Del	11	113449333	113451784	5.883313	0.213572
32795	Del	11	113452234	113483765	5.727066	0.210614
32796	Del	11	113487093	113732320	5.847438	0.21298
32797	Del	11	113732763	113775155	5.813279	0.212389
32798	Del	11	113775192	113782530	5.883313	0.213572
32799	Del	11	113783758	113876970	5.892578	0.214164
32800	Del	11	113877582	113946564	5.994421	0.215938
32801	Del	11	113946857	113972755	6.106735	0.217713
32802	Del	11	113977396	114057385	6.108969	0.218305
32803	Del	11	114058139	114253526	5.611338	0.208247
32804	Del	11	114254538	114359539	6.033902	0.21653
32805	Del	11	114360533	114380537	6.004449	0.215938
32806	Del	11	114380581	114405034	6.056923	0.217122

32807	Del	11	114408664	114521388	5.934419	0.214755
32808	Del	11	114521576	114560992	5.654594	0.209431
32809	Del	11	114561348	114677472	5.6747	0.210022
32810	Del	11	114677624	114708527	5.24822	0.201148
32811	Del	11	114708855	115327977	5.362775	0.203515
32812	Del	11	115329060	115341198	5.891524	0.214164
32813	Del	11	115345955	115395647	5.362775	0.203515
32814	Del	11	115396226	115397817	5.910796	0.214164
32815	Del	11	115398443	116634959	5.362775	0.203515
32816	Del	11	116636845	116784996	5.322408	0.202923
32817	Del	11	116786845	116857220	5.364553	0.203515
32818	Del	11	116857409	117129581	4.986343	0.195824
32819	Del	11	117143105	117174151	4.724333	0.191091
32820	Del	11	117177451	117204969	4.725303	0.191682
32821	Del	11	117205280	117233432	4.721039	0.191091
32822	Del	11	117234843	117282704	4.688404	0.190499
32823	Del	11	117283763	117303702	4.748917	0.191682
32824	Del	11	117304442	117320787	4.722834	0.191091
32825	Del	11	117320874	117414054	4.593144	0.188724
32826	Del	11	117414078	117816129	5.309145	0.202331
32827	Del	11	117816405	117832217	4.302648	0.182808
32828	Del	11	117832791	117832791	4.218206	0.181033
32829	Del	11	117834717	117880178	4.236059	0.181625
32830	Del	11	117880305	117929554	4.264953	0.182217
32831	Del	11	117929586	118009424	4.29299	0.182808
32832	Del	11	118012493	118076493	4.150643	0.17985
32833	Del	11	118076718	118121090	4.180629	0.180442
32834	Del	11	118121850	118146650	4.288404	0.182808
32835	Del	11	118146730	118214810	4.26918	0.182217
32836	Del	11	118215652	118224041	4.237983	0.181625
32837	Del	11	118224206	118252773	4.273201	0.182217
32838	Del	11	118252931	118317517	4.297584	0.182808
32839	Del	11	118318187	118359549	4.722313	0.191091
32840	Del	11	118359577	118432011	4.691523	0.190499
32841	Del	11	118435617	118533651	3.054231	0.156777
32842	Del	11	118534500	118570582	2.941519	0.154411
32843	Del	11	118570940	118643001	2.917466	0.153819
32844	Del	11	118643304	118646298	2.947156	0.154411
32845	Del	11	118646867	118657714	2.90078	0.153819
32846	Del	11	118657756	118746338	2.929478	0.154411
32847	Del	11	118747211	118794866	2.904859	0.153819
32848	Del	11	118795858	118966403	2.927304	0.154411
32849	Del	11	118969153	119043282	3.351567	0.163285
32850	Del	11	119043496	119081661	3.335647	0.162693
32851	Del	11	119082492	119197084	3.337247	0.163285
32852	Del	11	119200445	119331233	4.231402	0.181625
32853	Del	11	119332649	119347631	3.912521	0.174526
32854	Del	11	119347906	119382547	4.323878	0.1834
32855	Del	11	119383276	119415629	4.176338	0.180442
32856	Del	11	119416211	119429795	4.357969	0.183991
32857	Del	11	119429885	119505146	4.176338	0.180442
32858	Del	11	119505936	119645390	4.65515	0.189907
32859	Del	11	119645470	119722279	4.677078	0.190499

32860	Del	11	119726062	119740223	4.74069	0.191682
32861	Del	11	119750435	120139741	5.037894	0.197007
32862	Del	11	120141775	120234330	5.057189	0.197598
32863	Del	11	120235377	120239002	4.917969	0.19464
32864	Del	11	120249528	120268778	5.050817	0.197598
32865	Del	11	120268835	120322802	4.9225	0.19464
32866	Del	11	120327384	120335454	4.59915	0.188724
32867	Del	11	120335953	120368478	4.399818	0.185175
32868	Del	11	120379333	120503179	4.45932	0.185766
32869	Del	11	120506099	120559798	4.03372	0.177484
32870	Del	11	120564315	120811312	4.09811	0.178667
32871	Del	11	120811340	120820723	4.593922	0.188724
32872	Del	11	120821830	120864988	4.09811	0.178667
32873	Del	11	120866208	120895966	4.558818	0.188133
32874	Del	11	120898268	120936988	4.09811	0.178667
32875	Del	11	120937715	120959342	4.558818	0.188133
32876	Del	11	120959810	120989716	4.09811	0.178667
32877	Del	11	120990105	121102187	4.798749	0.192866
32878	Del	11	121102528	121191277	4.694275	0.190499
32879	Del	11	121191777	121449455	5.30234	0.202331
32880	Del	11	121451045	121635262	4.901984	0.19464
32881	Del	11	121638699	122088686	4.925094	0.195232
32882	Del	11	122088868	122368137	5.071085	0.197598
32883	Del	11	122368295	122426975	4.998787	0.196415
32884	Del	11	122427084	122815229	5.369688	0.204106
32885	Del	11	122815261	122862104	5.761409	0.211205
32886	Del	11	122862164	122882339	5.793695	0.212389
32887	Del	11	122882472	122910848	5.752473	0.211205
32888	Del	11	122911102	122963293	5.650251	0.209431
32889	Del	11	122963328	123069324	5.812725	0.212389
32890	Del	11	123070972	123195788	5.535297	0.207064
32891	Del	11	123201116	123503879	5.785559	0.211797
32892	Del	11	123506261	123524912	5.531765	0.207064
32893	Del	11	123525005	123558954	5.567215	0.207656
32894	Del	11	123559282	123626194	5.645301	0.209431
32895	Del	11	123627168	123658271	5.735938	0.211205
32896	Del	11	123659762	123731208	5.783219	0.211797
32897	Del	11	123731225	123744002	5.744951	0.211205
32898	Del	11	123744595	124114041	5.785559	0.211797
32899	Del	11	124114193	124124997	5.264409	0.201148
32900	Del	11	124125020	124147847	5.235416	0.200556
32901	Del	11	124148554	124266093	5.296161	0.20174
32902	Del	11	124266327	124320834	6.268028	0.221263
32903	Del	11	124321889	124530792	6.64879	0.230137
32904	Del	11	124535415	124637123	6.270995	0.221263
32905	Del	11	124637810	124671720	6.205784	0.22008
32906	Del	11	124673419	124695492	6.249189	0.220671
32907	Del	11	124696295	124776644	6.270995	0.221263
32908	Del	11	124784030	124836179	6.212421	0.22008
32909	Del	11	124838802	124884715	6.256434	0.221263
32910	Del	11	124885475	124897394	6.246479	0.220671
32911	Del	11	124900915	124920942	6.270995	0.221263
32912	Del	11	124923820	124950629	6.243321	0.220671

32913	Del	11	124952273	125041949	6.172685	0.219488
32914	Del	11	125041972	125089018	6.210031	0.22008
32915	Del	11	125094276	125114086	6.246029	0.220671
32916	Del	11	125116008	125166871	6.270995	0.221263
32917	Del	11	125168558	125434113	5.677386	0.210022
32918	Del	11	125435334	125483631	6.205784	0.22008
32919	Del	11	125487880	125499568	6.269487	0.221263
32920	Del	11	125499655	125657810	6.668694	0.231912
32921	Del	11	125657894	125668218	6.653695	0.23132
32922	Del	11	125669307	125877722	6.673787	0.232504
32923	Del	11	125879422	125892953	6.668694	0.231912
32924	Del	11	125892996	125905376	6.673787	0.232504
32925	Del	11	125906160	125955329	6.666806	0.23132
32926	Del	11	125955813	126066214	6.635027	0.227771
32927	Del	11	126066941	126217436	6.668694	0.231912
32928	Del	11	126221926	126257171	6.662063	0.23132
32929	Del	11	126262821	126297322	6.673787	0.232504
32930	Del	11	126297579	126334266	6.653695	0.23132
32931	Del	11	126340967	126361425	6.673787	0.232504
32932	Del	11	126366419	126400479	6.64879	0.230729
32933	Del	11	126402165	126423344	6.673787	0.232504
32934	Del	11	126423390	126427774	5.636817	0.208839
32935	Del	11	126428447	126651192	5.649608	0.209431
32936	Del	11	126651275	126682664	5.775268	0.211797
32937	Del	11	126682746	127001596	5.649608	0.209431
32938	Del	11	127001744	127066627	4.722779	0.191091
32939	Del	11	127066661	127106114	4.654022	0.189907
32940	Del	11	127112185	128456568	5.274403	0.20174
32941	Del	11	128458143	128521100	2.854938	0.152636
32942	Del	11	128521254	128534223	3.246151	0.160918
32943	Del	11	128534550	128590309	2.803524	0.151453
32944	Del	11	128593902	128700064	3.241489	0.160918
32945	Del	11	128701185	128813345	3.015029	0.156186
32946	Del	11	128814019	128842796	3.239315	0.160918
32947	Del	11	128842810	128899043	3.19807	0.160327
32948	Del	11	128899313	128908420	3.230639	0.160918
32949	Del	11	128909893	128919244	3.193917	0.160327
32950	Del	11	128919365	128959853	3.227045	0.160918
32951	Del	11	128962881	129026335	3.183317	0.159735
32952	Del	11	129026353	129195901	3.024477	0.156186
32953	Del	11	129195985	129609303	3.944443	0.175117
32954	Del	11	129609713	129796743	3.135446	0.159144
32955	Del	11	129801156	129860200	3.098089	0.15796
32956	Del	11	129860667	129950899	3.126907	0.158552
32957	Del	11	129950923	129992412	3.05581	0.157369
32958	Del	11	129996758	130062825	3.160242	0.159144
32959	Del	11	130063336	130145062	3.064495	0.157369
32960	Del	11	130145107	130221150	3.090344	0.15796
32961	Del	11	130226629	130315483	2.445761	0.143762
32962	Del	11	130320726	130445681	2.845836	0.152636
32963	Del	11	130446785	130476746	2.841962	0.152044
32964	Del	11	130477295	130842638	2.867983	0.152636
32965	Del	11	130843357	130862844	2.843557	0.152044

32966	Del	11	130862915	130871520	2.866301	0.152636
32967	Del	11	130875296	131369729	2.796842	0.151453
32968	Del	11	131369909	131531997	2.439227	0.14317
32969	Del	11	131533434	131542823	2.847734	0.152636
32970	Del	11	131543525	131658575	2.439227	0.14317
32971	Del	11	131658620	131665529	2.867457	0.152636
32972	Del	11	131666230	131908677	2.439227	0.14317
32973	Del	11	131910046	132338534	3.092058	0.15796
32974	Del	11	132338907	132945318	4.433599	0.185766
32975	Del	11	132945473	133533195	4.228214	0.181033
32976	Del	11	133536417	133813077	4.271409	0.182217
32977	Del	11	133813743	133848007	4.297584	0.182808
32978	Del	11	133848044	133902940	4.753671	0.191682
32979	Del	11	133902943	133926643	5.657267	0.209431
32980	Del	11	133926781	134152128	5.723866	0.210614
32981	Del	11	134152192	134221155	5.692576	0.210022
32982	Del	11	134223267	134245687	5.738637	0.211205
32983	Del	11	134247121	134272740	5.781788	0.211797
32984	Del	12	780472	780472	0	0.046737
32985	Del	12	780736	909713	0	0.046146
32986	Del	12	910108	953765	0	0.046737
32987	Del	12	953944	992098	0	0.046146
32988	Del	12	992724	1023927	0	0.044963
32989	Del	12	1025098	1752621	0	0.045554
32990	Del	12	1752814	1791569	0	0.033722
32991	Del	12	1792319	1819540	0	0.02189
32992	Del	12	1819904	1838027	0	0.022481
32993	Del	12	1877084	1922947	0	0.02189
32994	Del	12	1923303	2054596	0	0.022481
32995	Del	12	2055728	2218778	0	0.02189
32996	Del	12	2219754	2226630	0	0.026623
32997	Del	12	2226775	2266470	0	0.02189
32998	Del	12	2266652	2289058	0	0.026623
32999	Del	12	2289514	2668239	0	0.02189
33000	Del	12	2668360	3044213	0	0.026623
33001	Del	12	3044999	3381322	0	0.031355
33002	Del	12	3381341	3483301	0	0.025439
33003	Del	12	3483326	3493076	0	0.026623
33004	Del	12	3496747	3759114	0	0.026031
33005	Del	12	3759143	4265207	0	0.026623
33006	Del	12	4265574	4315842	0	0.029581
33007	Del	12	4315956	4807387	0	0.026623
33008	Del	12	4807646	4852570	0	0.026031
33009	Del	12	4854632	5429343	0	0.026623
33010	Del	12	5429968	5490480	0	0.026031
33011	Del	12	5492763	5561284	0	0.026623
33012	Del	12	5561762	5947628	0	0.023073
33013	Del	12	5948811	6127993	0	0.025439
33014	Del	12	6129313	6238728	0	0.026623
33015	Del	12	6241250	6474221	0	0.028397
33016	Del	12	6474237	6534150	0	0.034905
33017	Del	12	6534163	6745745	0	0.028397
33018	Del	12	6747467	6967073	0	0.020706

33019	Del	12	6968214	7058267	0	0.03313
33020	Del	12	7059466	7504756	0	0.031355
33021	Del	12	7504831	8676984	0	0.041413
33022	Del	12	8678022	8818886	0	0.040821
33023	Del	12	8822219	8843098	0	0.031947
33024	Del	12	8843160	9053433	0	0.032539
33025	Del	12	9054117	10067286	0	0.031355
33026	Del	12	10067467	10362109	0	0.034905
33027	Del	12	10362778	10616609	0	0.03313
33028	Del	12	10618440	10696776	0	0.032539
33029	Del	12	10697984	10811574	0	0.029581
33030	Del	12	10813216	10832831	0	0.039046
33031	Del	12	10833396	10934535	0	0.046146
33032	Del	12	10934845	10961814	0	0.046737
33033	Del	12	10962425	11425478	0	0.047329
33034	Del	12	11428840	11643005	0	0.052653
[truncated: 345,716 more chars]
