# Supplementary material for: A ferroptosis-associated gene signature for the prediction of prognosis and therapeutic response in luminal-type breast carcinoma
Source: Sci Rep. 2021 Sep 2;11:17610. doi: 10.1038/s41598-021-97102-z (PMC8413464; doi:10.1038/s41598-021-97102-z)
Supplement: Supplementary file 14 — Supplementary Table S10. [file 41598_2021_97102_MOESM14_ESM.pdf]

TableS10 gistic scores data of low risk subgroup

	Variant_Classification	Chromosome	Start_Position	End_Position	fdr	G_Score
1	Amp	1	3302046	3309193	0	0.008779
2	Amp	1	3309649	3345118	0	0.007464
3	Amp	1	3350785	3854784	0	0.006833
4	Amp	1	3854839	3885264	0	0.008784
5	Amp	1	3885647	4009627	0	0.007413
6	Amp	1	4013004	4018674	0	0.008091
7	Amp	1	4018716	4364085	0	0.00614
8	Amp	1	4365703	4652424	0	0.009927
9	Amp	1	4657280	5034643	0	0.008402
10	Amp	1	5037294	5042084	0	0.00761
11	Amp	1	5119746	5710205	0	0.003823
12	Amp	1	5712931	5742609	0	0.00529
13	Amp	1	5743560	6300087	0	0.003823
14	Amp	1	6304311	6335675	0	0.00725
15	Amp	1	6339720	6548126	0	0.004105
16	Amp	1	6551233	6676330	0	0.005157
17	Amp	1	6676739	6768567	0	0.008977
18	Amp	1	6768948	6976742	0	0.011105
19	Amp	1	6978351	6987540	0	0.007286
20	Amp	1	6990595	7040405	0	0.006234
21	Amp	1	7040695	7303250	0	0.004679
22	Amp	1	7303593	7539272	0	0.007659
23	Amp	1	7539353	7843170	0	0.009135
24	Amp	1	7845814	8039643	0	0.006155
25	Amp	1	8040080	8104440	0	0.005477
26	Amp	1	8105340	8223057	0	0.006094
27	Amp	1	8224597	8379997	0	0.005477
28	Amp	1	8380230	8554218	0	0.003348
29	Amp	1	8559375	8630844	0	0.001872
30	Amp	1	8658852	8800155	0	0.003867
31	Amp	1	8803019	9078959	0	0.006058
32	Amp	1	9080019	9137402	0	0.005514
33	Amp	1	9140655	9419510	0	0.00352
34	Amp	1	9423147	9929146	0	0.002192
35	Amp	1	9933962	10228739	0	0
36	Amp	1	10229443	10285267	0	0.001927
37	Amp	1	10285328	10748530	0	0.004324
38	Amp	1	10763717	10886195	0	0
39	Amp	1	10890669	10935422	0	0.001475
40	Amp	1	10939437	10970785	0	0
41	Amp	1	10972283	11386853	0	0.004347
42	Amp	1	11389688	11474485	0	0.004995
43	Amp	1	11483287	11769360	0	0.004347
44	Amp	1	11769634	11819195	0	0.006709
45	Amp	1	11819210	12087665	0	0.009718
46	Amp	1	12093131	12132249	0	0.007356
47	Amp	1	12134394	12163425	0	0.008006
48	Amp	1	12165294	12335732	0	0.007356
49	Amp	1	12340039	12485724	0	0.004347
50	Amp	1	12486639	12765430	0	0.006491
51	Amp	1	12765866	12774437	0	0.009216
52	Amp	1	13468357	13475857	0	0.004869

53	Amp	1	13475969	14736130	0	0.006096
54	Amp	1	14737886	14823391	0	0.005624
55	Amp	1	14826259	15236684	0	0.009142
56	Amp	1	15237219	15252949	0	0.006889
57	Amp	1	15253283	15770261	0	0.004746
58	Amp	1	15772497	15956281	0	0.001228
59	Amp	1	15960576	16108232	0	0.004153
60	Amp	1	16110276	16192971	0	0.006774
61	Amp	1	16193529	16328359	0	0.004153
62	Amp	1	16328513	17003654	0	0.001228
63	Amp	1	17010382	17236303	0	0.002711
64	Amp	1	17238352	17308916	0	0.005068
65	Amp	1	17309517	17351414	0	0.003584
66	Amp	1	17353180	17422711	0	0.005568
67	Amp	1	17423008	17437538	0	0.003584
68	Amp	1	17440184	17510199	0	0.002357
69	Amp	1	17511109	17955601	0	0.003519
70	Amp	1	17958313	18009739	0	0.004592
71	Amp	1	18010409	18013041	0	0.00343
72	Amp	1	18013254	18026804	0	0.002357
73	Amp	1	18027107	18040192	0	0.003926
74	Amp	1	18042940	18363113	0	0.002357
75	Amp	1	18364119	18371262	0	0.006141
76	Amp	1	18373518	18860632	0	0.002357
77	Amp	1	18860749	19082156	0	0.003471
78	Amp	1	19085006	19175207	0	0.007373
79	Amp	1	19186019	19311815	0	0.009325
80	Amp	1	19314095	19354064	0	0.005423
81	Amp	1	19362686	19509072	0	0.003471
82	Amp	1	19512645	19517189	0	0.004825
83	Amp	1	19524537	19605393	0	0.007226
84	Amp	1	19606106	19847365	0	0.008853
85	Amp	1	19848619	20144986	0	0.007499
86	Amp	1	20147121	20252075	0	0.005142
87	Amp	1	20255925	20584798	0	0.003515
88	Amp	1	20584911	20712487	0	0.005591
89	Amp	1	20713658	20799917	0	0.008082
90	Amp	1	20801444	21358952	0	0.005591
91	Amp	1	21361264	21610416	0	0.012953
92	Amp	1	21610824	21627289	0	0.015258
93	Amp	1	21629815	21684282	0	0.018852
94	Amp	1	21685618	21712494	0	0.017044
95	Amp	1	21715388	21956126	0	0.015929
96	Amp	1	21961373	22292849	0	0.016865
97	Amp	1	22302695	22343478	0	0.014143
98	Amp	1	22345985	22513347	0	0.011838
99	Amp	1	22516935	22659910	0	0.007362
100	Amp	1	22660245	22675602	0	0.008079
101	Amp	1	22675966	22688154	0	0.000718
102	Amp	1	22689915	22755454	0	0
103	Amp	1	22755626	22957443	0	0.002458
104	Amp	1	22959794	23241646	0	0
105	Amp	1	23244756	23330212	0	0.004275

106	Amp	1	23333862	23334629	0	0.002403
107	Amp	1	23335566	23367528	0	0.001204
108	Amp	1	23372022	23757290	0	0
109	Amp	1	23763907	24078925	0	0.001625
110	Amp	1	24080157	24219708	0	0.007429
111	Amp	1	24226054	24357626	0	0.010225
112	Amp	1	24359705	24390399	0	0.01283
113	Amp	1	24402139	24477614	0	0.015745
114	Amp	1	24483579	24492891	0	0.024858
115	Amp	1	24495534	24619164	0	0.022253
116	Amp	1	24620695	24641799	0	0.034947
117	Amp	1	24644578	24727732	0	0.037084
118	Amp	1	24732886	24769293	0	0.033002
119	Amp	1	24771301	24808610	0	0.034908
120	Amp	1	24809796	24860251	0	0.032771
121	Amp	1	24864144	24913549	0	0.020077
122	Amp	1	24924549	25111459	0	0.017162
123	Amp	1	25115233	25118529	0	0.015537
124	Amp	1	25139359	25396759	0	0.006424
125	Amp	1	25407761	25447591	0	0.003767
126	Amp	1	25562931	25665036	0	0.012364
127	Amp	1	25669445	25792419	0	0.016388
128	Amp	1	25802876	25806536	0	0.017978
129	Amp	1	25808435	25929240	0	0.019301
130	Amp	1	25932053	26010018	0	0.015277
131	Amp	1	26016621	26024891	0	0.013687
132	Amp	1	26029149	26087048	0	0.012364
133	Amp	1	26089054	26223723	0	0.010642
134	Amp	1	26233560	26324641	0	0.012693
135	Amp	1	26325696	26363446	0	0.014962
136	Amp	1	26365559	26439753	0	0.012911
137	Amp	1	26442084	26546754	0	0.010642
138	Amp	1	26549798	26728338	0	0.008597
139	Amp	1	26730873	26762777	0	0
140	Amp	1	26763075	26894366	0	0.003619
141	Amp	1	26896264	27014714	0	0.00633
142	Amp	1	27020298	27022452	0	0.008777
143	Amp	1	27072223	27194374	0	0.006066
144	Amp	1	27195013	28035717	0	0.003619
145	Amp	1	28036116	28045064	0	0.007156
146	Amp	1	28045835	28186296	0	0.003538
147	Amp	1	28188781	28665596	0	0
148	Amp	1	28681156	28723213	0	0.001711
149	Amp	1	28728193	29253314	0	0.008228
150	Amp	1	29254639	29379511	0	0.001711
151	Amp	1	29382391	29392963	0	0.002546
152	Amp	1	29400160	29710292	0	0.003032
153	Amp	1	29711579	29717700	0	0.00506
154	Amp	1	29717809	29732145	0	0.004574
155	Amp	1	29733944	29742865	0	0.005504
156	Amp	1	29745789	29827758	0	0.004574
157	Amp	1	29828461	29914856	0	0.005023
158	Amp	1	29920040	30059743	0	0.016998

159	Amp	1	30059821	30177377	0	0.012883
160	Amp	1	30181334	30193293	0	0.013987
161	Amp	1	30193910	30205430	0	0.007621
162	Amp	1	30219475	30234444	0	0.009776
163	Amp	1	30238008	30277832	0	0.007621
164	Amp	1	30281677	30444352	0	0.018427
165	Amp	1	30445319	30538779	0	0.007621
166	Amp	1	30540928	30542027	0	0.007172
167	Amp	1	30542880	30551862	0	0.005144
168	Amp	1	30552120	30570884	0	0.006975
169	Amp	1	30572648	30667100	0	0.005144
170	Amp	1	30789158	30793010	0	0.008249
171	Amp	1	30794326	30899941	0	0.008829
172	Amp	1	30903048	30938231	0	0.024765
173	Amp	1	30940072	30945463	0	0.026296
174	Amp	1	30947040	31032755	0	0.020739
175	Amp	1	31033157	31084473	0	0.017634
176	Amp	1	31085938	31087522	0	0.018313
177	Amp	1	31091134	31110497	0	0.022634
178	Amp	1	31120401	31122022	0	0.026753
179	Amp	1	31126961	31141173	0	0.027849
180	Amp	1	31143757	31152499	0	0.029153
181	Amp	1	31152542	31170201	0	0.0309
182	Amp	1	31175087	31214812	0	0.0285
183	Amp	1	31216669	31278344	0	0.024381
184	Amp	1	31283052	31405396	0	0.031837
185	Amp	1	31407875	31428340	0	0.034186
186	Amp	1	31428805	31435322	0	0.035946
187	Amp	1	31436225	31449930	0	0.038433
188	Amp	1	31454089	31547339	0	0.030976
189	Amp	1	31550174	31583793	0	0.030141
190	Amp	1	31588493	31628031	0	0.028648
191	Amp	1	31628660	31628660	0	0.037236
192	Amp	1	31632609	31658996	0	0.031742
193	Amp	1	31663315	31670797	0	0.033584
194	Amp	1	31673332	31713228	0	0.029982
195	Amp	1	31719102	31742019	0	0.034135
196	Amp	1	31747834	31790485	0	0.035861
197	Amp	1	31800372	31849376	0	0.041149
198	Amp	1	31849950	31920158	0	0.039388
199	Amp	1	31923891	31924039	0	0.03704
200	Amp	1	31926442	31927457	0	0.035197
201	Amp	1	31929917	32049367	0	0.036317
202	Amp	1	32051319	32079583	0	0.021785
203	Amp	1	32079728	32212247	0	0.020059
204	Amp	1	32219725	32474851	0	0.009785
205	Amp	1	32475013	32610108	0	0.012354
206	Amp	1	32613906	32632867	0	0.009785
207	Amp	1	32638492	32650286	0	0.012389
208	Amp	1	32653992	32695277	0	0.018406
209	Amp	1	32697685	32869438	0	0.01592
210	Amp	1	32877136	32899144	0	0.016657
211	Amp	1	32905234	32977463	0	0.015537

212	Amp	1	32978534	32995529	0	0.013433
213	Amp	1	33000959	33084354	0	0.010828
214	Amp	1	33086177	33150007	0	0.004811
215	Amp	1	33152163	33507551	0	0.011839
216	Amp	1	33510969	33576286	0	0.014127
217	Amp	1	33576681	33628076	0	0.014588
218	Amp	1	33631656	33684951	0	0.01717
219	Amp	1	33685570	33728134	0	0.019978
220	Amp	1	33729135	33731623	0	0.01717
221	Amp	1	33732604	33779885	0	0.019309
222	Amp	1	33780255	33965124	0	0.022401
223	Amp	1	33965956	34011474	0	0.019309
224	Amp	1	34011903	34039410	0	0.022331
225	Amp	1	34039514	34080413	0	0.019284
226	Amp	1	34085826	34250086	0	0.015303
227	Amp	1	34250173	34291095	0	0.012721
228	Amp	1	34294621	34296488	0	0.010145
229	Amp	1	34304503	34360124	0	0.008005
230	Amp	1	34362718	34372772	0	0.007269
231	Amp	1	34373900	34374247	0	0.008493
232	Amp	1	34374383	34558372	0	0.009061
233	Amp	1	34559003	34565969	0	0.008493
234	Amp	1	34572695	34701039	0	0.008032
235	Amp	1	34705404	34777292	0	0.005744
236	Amp	1	34779167	34825328	0	0.007861
237	Amp	1	34830840	34841842	0	0.005744
238	Amp	1	34842078	34875700	0	0.007916
239	Amp	1	34875800	34887735	0	0.006691
240	Amp	1	34889221	34958969	0	0.010997
241	Amp	1	34961420	35019469	0	0.012604
242	Amp	1	35023494	35089593	0	0.008299
243	Amp	1	35091641	35095837	0	0.010508
244	Amp	1	35096558	35240219	0	0.010062
245	Amp	1	35241405	35348967	0	0.013947
246	Amp	1	35349333	35426783	0	0.016896
247	Amp	1	35427315	35433730	0	0.018201
248	Amp	1	35435621	35453577	0	0.016594
249	Amp	1	35453860	35549410	0	0.014521
250	Amp	1	35549889	35567522	0	0.013842
251	Amp	1	35575795	35591609	0	0.012029
252	Amp	1	35602801	35663464	0	0.015861
253	Amp	1	35677856	35679130	0	0.012029
254	Amp	1	35684053	35695796	0	0.009858
255	Amp	1	35697192	35731266	0	0.013023
256	Amp	1	35734789	35881785	0	0.010075
257	Amp	1	35892284	35892358	0	0.007866
258	Amp	1	35895885	36085885	0	0.01459
259	Amp	1	36086294	36187939	0	0.016545
260	Amp	1	36189036	36204302	0	0.018887
261	Amp	1	36212053	36212053	0	0.021372
262	Amp	1	36213009	36253068	0	0.022108
263	Amp	1	36256597	36257187	0	0.020803
264	Amp	1	36257616	36288063	0	0.021938

265	Amp	1	36288429	36317391	0	0.018461
266	Amp	1	36321787	36355326	0	0.021684
267	Amp	1	36360577	36443771	0	0.018461
268	Amp	1	36443784	36464387	0	0.019211
269	Amp	1	36464792	36546564	0	0.021812
270	Amp	1	36553039	36594959	0	0.016828
271	Amp	1	36598896	36652427	0	0.014227
272	Amp	1	36654095	36695453	0	0.016642
273	Amp	1	36703812	36849794	0	0.014227
274	Amp	1	36851332	36990619	0	0.012272
275	Amp	1	37003475	37028266	0	0.009787
276	Amp	1	37037523	37059402	0	0.009052
277	Amp	1	37059855	37301413	0	0.013141
278	Amp	1	37305022	37455082	0	0.01143
279	Amp	1	37455442	37697088	0	0.009971
280	Amp	1	37697264	37714613	0	0.009156
281	Amp	1	37723169	37786091	0	0.010964
282	Amp	1	37787820	37828964	0	0.013723
283	Amp	1	37834568	37834568	0	0.009634
284	Amp	1	37837043	37837043	0	0.015762
285	Amp	1	37837353	37999351	0	0.013954
286	Amp	1	38003519	38176853	0	0.015032
287	Amp	1	38179108	38421576	0	0.014518
288	Amp	1	38421679	38469233	0	0.021419
289	Amp	1	38470109	38540935	0	0.023132
290	Amp	1	38544290	38545176	0	0.025077
291	Amp	1	38546246	38738174	0	0.031189
292	Amp	1	38740200	38812362	0	0.033533
293	Amp	1	38813652	38837053	0	0.035277
294	Amp	1	38844389	38844389	0	0.030511
295	Amp	1	38845578	38930211	0	0.029761
296	Amp	1	38930398	39000954	0	0.032863
297	Amp	1	39001020	39027648	0	0.03115
298	Amp	1	39029738	39186518	0	0.03278
299	Amp	1	39192650	39250120	0	0.03515
300	Amp	1	39252365	39362277	0	0.033406
301	Amp	1	39372593	39421323	0	0.036054
302	Amp	1	39422275	39474363	0	0.031951
303	Amp	1	39474551	39550169	0	0.0282
304	Amp	1	39557105	39557105	0	0.032074
305	Amp	1	39559408	39715831	0	0.03412
306	Amp	1	39717357	39841748	0	0.037901
307	Amp	1	39842345	39846274	0	0.035855
308	Amp	1	39848177	39852298	0	0.032074
309	Amp	1	39863020	39878017	0	0.03511
310	Amp	1	39880329	39905075	0	0.050316
311	Amp	1	39912235	39915619	0	0.051949
312	Amp	1	39915781	39985973	0	0.058997
313	Amp	1	39989368	39990071	0	0.055686
314	Amp	1	39993064	40078733	0	0.05359
315	Amp	1	40080953	40166658	0	0.045765
316	Amp	1	40170907	40185117	0	0.044253
317	Amp	1	40186963	40192261	0	0.042623

318	Amp	1	40197207	40244941	0	0.04508
319	Amp	1	40245369	40261444	0	0.047176
320	Amp	1	40263996	40282247	0	0.042749
321	Amp	1	40284410	40306301	0	0.050598
322	Amp	1	40306322	40315366	0	0.049493
323	Amp	1	40329956	40372964	0	0.041041
324	Amp	1	40406684	40406684	0	0.046485
325	Amp	1	40406951	40473424	0	0.044028
326	Amp	1	40474220	40583765	0	0.045794
327	Amp	1	40584821	40672628	0	0.046575
328	Amp	1	40673375	40683331	0	0.047315
329	Amp	1	40688337	40774847	0	0.04971
330	Amp	1	40780187	40853380	0	0.045968
331	Amp	1	40856524	40921184	0	0.048328
332	Amp	1	40921199	40974453	0	0.049866
333	Amp	1	40976444	40981493	0	0.04683
334	Amp	1	40981666	41060351	0	0.046091
335	Amp	1	41060423	41149686	0	0.055392
336	Amp	1	41149924	41171636	0	0.053032
337	Amp	1	41173831	41260030	0	0.054198
338	Amp	1	41263627	41298572	0	0.058149
339	Amp	1	41300425	41355898	0	0.063775
340	Amp	1	41356107	41374525	0.040093	0.065636
341	Amp	1	41374678	41384214	0.089561	0.06777
342	Amp	1	41384510	41400090	0	0.063038
343	Amp	1	41409489	41409489	0	0.061871
344	Amp	1	41412940	41413409	0	0.06001
345	Amp	1	41414912	41429826	0	0.050025
346	Amp	1	41433910	41459379	0	0.051777
347	Amp	1	41460577	41469814	0	0.049643
348	Amp	1	41470767	41525756	0	0.045074
349	Amp	1	41526741	41646340	0	0.049278
350	Amp	1	41648386	41658480	0	0.05001
351	Amp	1	41660008	41672357	0	0.061
352	Amp	1	41673505	41680624	0	0.063121
353	Amp	1	41686813	41742931	0.015369	0.064612
354	Amp	1	41743418	41746337	0.100827	0.068229
355	Amp	1	41747405	41747543	0.112543	0.068781
356	Amp	1	41754953	41928636	0.040093	0.065582
357	Amp	1	41933851	42000057	0.08435	0.067634
358	Amp	1	42000098	42052638	0.1237	0.069075
359	Amp	1	42054202	42077295	0.342649	0.07786
360	Amp	1	42077787	42217600	0.1237	0.069075
361	Amp	1	42217613	42230051	0	0.06112
362	Amp	1	42238452	42260811	0	0.058684
363	Amp	1	42280304	42348972	0.044794	0.065832
364	Amp	1	42349503	42444287	0	0.062395
365	Amp	1	42449380	42454655	0.024438	0.064952
366	Amp	1	42462922	42462922	0.065715	0.066876
367	Amp	1	42463059	42491312	0	0.062844
368	Amp	1	42491666	42495988	0.015369	0.064695
369	Amp	1	42496550	42496550	0	0.059535
370	Amp	1	42497099	42680237	0	0.058982

371	Amp	1	42686905	42689391	0	0.057058
372	Amp	1	42692839	42749604	0	0.058954
373	Amp	1	42762015	42884932	0	0.055412
374	Amp	1	42885165	42885165	0	0.053561
375	Amp	1	42886039	42892307	0	0.054964
376	Amp	1	42893480	42982700	0	0.057863
377	Amp	1	42983238	43011286	0	0.057398
378	Amp	1	43012125	43060354	0	0.054718
379	Amp	1	43061510	43077946	0	0.050913
380	Amp	1	43080068	43170047	0	0.04928
381	Amp	1	43170457	43299010	0	0.041237
382	Amp	1	43308766	43407703	0	0.04314
383	Amp	1	43408245	43438213	0	0.044543
384	Amp	1	43443594	43452783	0	0.051928
385	Amp	1	43455015	43495046	0	0.040938
386	Amp	1	43503250	43513166	0	0.04274
387	Amp	1	43513608	43559471	0	0.046959
388	Amp	1	43560418	43560740	0	0.045556
389	Amp	1	43563682	43667357	0	0.040854
390	Amp	1	43674404	43704645	0	0.03347
391	Amp	1	43712995	43811143	0	0.02638
392	Amp	1	43811876	43897130	0	0.029411
393	Amp	1	43897471	43903232	0	0.030928
394	Amp	1	43911001	44004703	0	0.027897
395	Amp	1	44005072	44032289	0	0.029367
396	Amp	1	44032697	44069988	0	0.02681
397	Amp	1	44070549	44234176	0	0.028935
398	Amp	1	44237006	44272393	0	0.027418
399	Amp	1	44274033	44403934	0	0.025948
400	Amp	1	44412837	44519510	0	0.026954
401	Amp	1	44522288	44533942	0	0.022735
402	Amp	1	44534282	44597423	0	0.021728
403	Amp	1	44599087	44605524	0	0.023233
404	Amp	1	44613548	44616668	0	0.016459
405	Amp	1	44620716	44620716	0	0.014954
406	Amp	1	44622645	44669091	0	0.012829
407	Amp	1	44670462	44754558	0	0.009312
408	Amp	1	44754871	45220531	0	0.010759
409	Amp	1	45221081	45348386	0	0.012557
410	Amp	1	45348431	45439674	0	0.010759
411	Amp	1	45442591	45729224	0	0.014758
412	Amp	1	45730058	45771128	0	0.018154
413	Amp	1	45771414	45772399	0	0.014758
414	Amp	1	45774377	45791218	0	0.01795
415	Amp	1	45796457	46206325	0	0.018704
416	Amp	1	46209488	46230405	0	0.015512
417	Amp	1	46236446	46384885	0	0.014758
418	Amp	1	46393062	46673431	0	0.010759
419	Amp	1	46673908	46691589	0	0.009312
420	Amp	1	46691634	46710592	0	0.011467
421	Amp	1	46710934	46772958	0	0.009312
422	Amp	1	46785370	46823698	0	0.011056
423	Amp	1	46823728	46872530	0	0.014022

424	Amp	1	46878103	46919911	0	0.016223
425	Amp	1	46926570	46955948	0	0.018318
426	Amp	1	46965215	46977720	0	0.019296
427	Amp	1	46981839	47039045	0	0.017201
428	Amp	1	47041486	47059549	0	0.021686
429	Amp	1	47067958	47089593	0	0.017201
430	Amp	1	47094941	47107741	0	0.013503
431	Amp	1	47108149	47295297	0	0.015081
432	Amp	1	47297535	47418910	0	0.018309
433	Amp	1	47426086	47467422	0	0.016731
434	Amp	1	47467691	47599485	0	0.015753
435	Amp	1	47599666	47723959	0	0.019968
436	Amp	1	47725933	47925857	0	0.017209
437	Amp	1	47925996	47979240	0	0.017941
438	Amp	1	47979536	47979536	0	0.016117
439	Amp	1	47979750	48293602	0	0.01866
440	Amp	1	48294959	48371665	0	0.017983
441	Amp	1	48395235	48457210	0	0.017255
442	Amp	1	48465313	48728699	0	0.015055
443	Amp	1	48731415	48877350	0	0.013652
444	Amp	1	48880394	48949172	0	0.016029
445	Amp	1	48949530	49448451	0	0.020255
446	Amp	1	49533594	49655065	0	0.024574
447	Amp	1	49659290	49798053	0	0.020359
448	Amp	1	49798101	49842260	0	0.021124
449	Amp	1	49842884	49925468	0	0.019383
450	Amp	1	49927823	50012278	0	0.022533
451	Amp	1	50012686	50017342	0	0.023947
452	Amp	1	50022174	50030375	0	0.017343
453	Amp	1	50030600	50125820	0	0.0148
454	Amp	1	50125893	50414094	0	0.012549
455	Amp	1	50432288	50472176	0	0.011136
456	Amp	1	50474090	50485971	0	0.010371
457	Amp	1	50487948	50592383	0	0.018809
458	Amp	1	50601278	50757541	0	0.012295
459	Amp	1	50767700	50767700	0	0.014363
460	Amp	1	50772510	50915188	0	0.013631
461	Amp	1	50918024	51029129	0	0.011887
462	Amp	1	51037672	51067956	0	0.014994
463	Amp	1	51073136	51179163	0	0.019946
464	Amp	1	51186552	51438943	0	0.014994
465	Amp	1	51448914	51551897	0	0.017338
466	Amp	1	51557529	51613295	0	0.014994
467	Amp	1	51618789	51725763	0	0.017258
468	Amp	1	51727284	51791212	0	0.019353
469	Amp	1	51798210	51981407	0	0.021008
470	Amp	1	51981596	52005681	0	0.018744
471	Amp	1	52008801	52373308	0	0.017089
472	Amp	1	52374826	52409115	0	0.018471
473	Amp	1	52411092	52462776	0	0.016376
474	Amp	1	52462910	52502019	0	0.018944
475	Amp	1	52506967	52723418	0	0.020157
476	Amp	1	52724144	52759691	0	0.018907

477	Amp	1	52759725	52787297	0	0.016339
478	Amp	1	52791085	52853708	0	0.018787
479	Amp	1	52854602	52879304	0	0.030563
480	Amp	1	52887790	52928334	0	0.018787
481	Amp	1	52932888	53015446	0	0.017573
482	Amp	1	53022198	53022198	0	0.0197
483	Amp	1	53022778	53024068	0	0.018317
484	Amp	1	53026932	53038070	0	0.019942
485	Amp	1	53038086	53066295	0	0.017495
486	Amp	1	53066863	53086208	0	0.015369
487	Amp	1	53088633	53179726	0	0.019461
488	Amp	1	53180868	53201394	0	0.022306
489	Amp	1	53203545	53203545	0	0.023941
490	Amp	1	53210776	53279206	0	0.019848
491	Amp	1	53280923	53322607	0	0.022952
492	Amp	1	53323202	53333626	0	0.025102
493	Amp	1	53336243	53353309	0	0.041038
494	Amp	1	53353354	53386182	0	0.038194
495	Amp	1	53386482	53508909	0	0.035091
496	Amp	1	53516209	53564459	0	0.033023
497	Amp	1	53564486	53600645	0	0.036049
498	Amp	1	53600926	53705539	0	0.03684
499	Amp	1	53706982	53719716	0	0.039209
500	Amp	1	53720329	53737434	0	0.038418
501	Amp	1	53743162	53756898	0	0.040886
502	Amp	1	53757059	53823748	0	0.038735
503	Amp	1	53824946	53891409	0	0.036267
504	Amp	1	53896816	54000129	0	0.033898
505	Amp	1	54005041	54068228	0	0.037868
506	Amp	1	54070021	54084389	0	0.038966
507	Amp	1	54091368	54114339	0	0.041452
508	Amp	1	54114518	54127363	0	0.04684
509	Amp	1	54128590	54140726	0	0.042855
510	Amp	1	54140849	54152762	0	0.041758
511	Amp	1	54153560	54211455	0	0.039871
512	Amp	1	54212113	54351381	0	0.041189
513	Amp	1	54352389	54417490	0	0.043005
514	Amp	1	54419157	54453786	0	0.041603
515	Amp	1	54462820	54604905	0	0.042102
516	Amp	1	54606491	54609389	0	0.038095
517	Amp	1	54619468	54705851	0	0.03647
518	Amp	1	54713332	54716627	0	0.036331
519	Amp	1	54719876	54957108	0	0.034515
520	Amp	1	54957255	54983945	0	0.02702
521	Amp	1	54985651	54992142	0	0.025255
522	Amp	1	54994518	55025942	0	0.029514
523	Amp	1	55030071	55038977	0	0.032757
524	Amp	1	55042283	55074160	0	0.032258
525	Amp	1	55076008	55080453	0	0.034777
526	Amp	1	55081222	55088337	0	0.036728
527	Amp	1	55094642	55111343	0	0.034381
528	Amp	1	55111489	55367890	0	0.031862
529	Amp	1	55368449	55436141	0	0.042208

530	Amp	1	55437153	55457366	0	0.040516
531	Amp	1	55457741	55532732	0	0.042513
532	Amp	1	55532762	55538222	0	0.041241
533	Amp	1	55542933	55621031	0	0.037998
534	Amp	1	55626502	55739245	0	0.04036
535	Amp	1	55739689	55749404	0	0.038424
536	Amp	1	55752409	56071324	0	0.036427
537	Amp	1	56077524	56182242	0	0.028017
538	Amp	1	56186021	56327007	0	0.032281
539	Amp	1	56329747	56492536	0	0.033592
540	Amp	1	56499329	56566795	0	0.032274
541	Amp	1	56567146	56575272	0	0.033553
542	Amp	1	56575586	56667321	0	0.035762
543	Amp	1	56667848	56915462	0	0.0334
544	Amp	1	56919157	56972743	0	0.032583
545	Amp	1	56974569	57122009	0	0.041106
546	Amp	1	57123709	57213581	0	0.035738
547	Amp	1	57220786	57267705	0	0.038592
548	Amp	1	57273396	57356639	0	0.035738
549	Amp	1	57357195	57493371	0	0.037403
550	Amp	1	57495882	57550124	0	0.038618
551	Amp	1	57556529	57939452	0	0.037336
552	Amp	1	57939591	58001242	0	0.038664
553	Amp	1	58004016	58012690	0	0.039389
554	Amp	1	58013465	58013661	0	0.037724
555	Amp	1	58016249	58069336	0	0.03659
556	Amp	1	58069468	58080656	0	0.038401
557	Amp	1	58083422	58124744	0	0.040331
558	Amp	1	58126206	58200404	0	0.038521
559	Amp	1	58200501	58226681	0	0.039723
560	Amp	1	58227370	58354245	0	0.042107
561	Amp	1	58354727	58404053	0	0.033584
562	Amp	1	58406760	58439071	0	0.032255
563	Amp	1	58442473	58460524	0	0.03345
564	Amp	1	58463407	58477823	0	0.032235
565	Amp	1	58482093	58571240	0	0.03151
566	Amp	1	58571437	58581834	0	0.032172
567	Amp	1	58582215	58699370	0	0.030697
568	Amp	1	58703041	58774316	0	0.030035
569	Amp	1	58774876	58811511	0	0.036796
570	Amp	1	58817900	58821019	0	0.035042
571	Amp	1	58823068	58836154	0	0.039772
572	Amp	1	58837504	58868733	0	0.040534
573	Amp	1	58872595	58939323	0	0.043617
574	Amp	1	58940617	58968488	0	0.046
575	Amp	1	58969244	58978700	0	0.043278
576	Amp	1	58979495	58995607	0	0.04471
577	Amp	1	58999025	59108029	0	0.043278
578	Amp	1	59111464	59130554	0	0.037812
579	Amp	1	59130577	59135747	0	0.039968
580	Amp	1	59136273	59169530	0	0.036708
581	Amp	1	59171871	59233818	0	0.035945
582	Amp	1	59239013	59245607	0	0.034859

583 Amp	1	59246848	59291377	0	0.0306
584 Amp	1	59293611	59307560	0	0.035789
585 Amp	1	59307712	59357816	0	0.034368
586 Amp	1	59362877	59391136	0	0.043064
587 Amp	1	59394584	59451472	0	0.041692
588 Amp	1	59453403	59474992	0	0.047175
589 Amp	1	59477133	59507940	0	0.046012
590 Amp	1	59509698	59660079	0	0.040529
591 Amp	1	59662450	59665295	0	0.036658
592 Amp	1	59674010	59734492	0	0.032214
593 Amp	1	59735007	59753339	0	0.037974
594 Amp	1	59754162	59762630	0	0.044479
595 Amp	1	59766290	59867097	0	0.036287
596 Amp	1	59869134	59892423	0	0.045385
597 Amp	1	59893310	59951169	0	0.046862
598 Amp	1	59951264	59955564	0	0.048245
599 Amp	1	59956090	59982192	0	0.043866
600 Amp	1	59982414	60017388	0	0.042389
601 Amp	1	60028371	60037578	0	0.038316
602 Amp	1	60039295	60111392	0	0.035049
603 Amp	1	60117571	60124103	0	0.037755
604 Amp	1	60124988	60147671	0	0.038585
605 Amp	1	60155316	60268667	0	0.044251
606 Amp	1	60273346	60383906	0	0.037962
607 Amp	1	60387048	60403690	0	0.040814
608 Amp	1	60408800	60527470	0	0.037962
609 Amp	1	60528005	60562063	0	0.035257
610 Amp	1	60564697	60715233	0	0.037858
611 Amp	1	60716177	60716177	0	0.039961
612 Amp	1	60719251	60719728	0	0.043066
613 Amp	1	60722868	60790628	0	0.048979
614 Amp	1	60790881	60832727	0	0.048075
615 Amp	1	60833022	60879273	0	0.048512
616 Amp	1	60879337	60928029	0	0.051349
617 Amp	1	60928930	60936213	0	0.050912
618 Amp	1	60937664	60939627	0	0.057875
619 Amp	1	60941680	61020994	0	0.058419
620 Amp	1	61023169	61028264	0	0.059969
621 Amp	1	61028316	61036874	0	0.057735
622 Amp	1	61037526	61044947	0	0.058965
623 Amp	1	61052767	61181484	0	0.05586
624 Amp	1	61181690	61280111	0	0.055258
625 Amp	1	61286927	61343414	0	0.051367
626 Amp	1	61347101	61391491	0	0.047935
627 Amp	1	61392740	61471079	0	0.049636
628 Amp	1	61471092	61478477	0	0.047935
629 Amp	1	61480599	61488497	0	0.056408
630 Amp	1	61489154	61619058	0	0.059218
631 Amp	1	61620002	61655631	0	0.056408
632 Amp	1	61660047	61723297	0	0.055864
633 Amp	1	61723869	61809254	0	0.057552
634 Amp	1	61810248	61864702	0	0.058851
635 Amp	1	61868247	61931808	0.001323	0.063985

636	Amp	1	61932574	61938324	0.060686	0.066654
637	Amp	1	61942126	61949251	0.1237	0.06914
638	Amp	1	61949717	61989112	0.079652	0.067401
639	Amp	1	62000561	62000561	0.050473	0.066102
640	Amp	1	62002981	62011562	0	0.062995
641	Amp	1	62012246	62027199	0	0.060428
642	Amp	1	62029828	62047622	0	0.057942
643	Amp	1	62051011	62056889	0	0.061007
644	Amp	1	62057402	62057402	0.010261	0.064419
645	Amp	1	62057551	62124931	0.029031	0.065323
646	Amp	1	62128846	62131671	0.138391	0.069784
647	Amp	1	62133118	62144614	0.181932	0.071645
648	Amp	1	62146924	62154859	0	0.055708
649	Amp	1	62159179	62180510	0	0.05304
650	Amp	1	62181086	62198029	0	0.051296
651	Amp	1	62202849	62215309	0	0.049252
652	Amp	1	62226599	62313972	0	0.051192
653	Amp	1	62316832	62348809	0	0.049331
654	Amp	1	62352546	62412281	0	0.047392
655	Amp	1	62412747	62463883	0	0.04363
656	Amp	1	62475245	62640045	0	0.044311
657	Amp	1	62641096	62691193	0	0.040727
658	Amp	1	62694022	62721951	0	0.042196
659	Amp	1	62722415	62814882	0	0.043731
660	Amp	1	62817647	62833307	0	0.046777
661	Amp	1	62847648	62924111	0	0.039955
662	Amp	1	62926040	62989742	0	0.038072
663	Amp	1	62989983	63103187	0	0.039832
664	Amp	1	63105681	63200379	0	0.037098
665	Amp	1	63203083	63228450	0	0.035182
666	Amp	1	63230017	63262878	0	0.04181
667	Amp	1	63277029	63296376	0	0.035182
668	Amp	1	63301926	63301963	0	0.039765
669	Amp	1	63302422	63311350	0	0.042677
670	Amp	1	63311502	63313675	0	0.039729
671	Amp	1	63327484	63391764	0	0.041438
672	Amp	1	63393093	63443177	0	0.039729
673	Amp	1	63447222	63524073	0	0.038094
674	Amp	1	63524525	63667643	0	0.031707
675	Amp	1	63671276	63683182	0	0.030803
676	Amp	1	63684304	63699819	0	0.032206
677	Amp	1	63699874	63755972	0	0.032743
678	Amp	1	63756046	63769306	0	0.032206
679	Amp	1	63769639	63776262	0	0.034106
680	Amp	1	63783841	63790347	0	0.033425
681	Amp	1	63794075	63876855	0	0.031524
682	Amp	1	63878023	63890887	0	0.032127
683	Amp	1	63893653	63931720	0	0.031524
684	Amp	1	63935860	64137253	0	0.028612
685	Amp	1	64141390	64254648	0	0.02721
686	Amp	1	64258032	64271273	0	0.052662
687	Amp	1	64279307	64279314	0	0.053401
688	Amp	1	64279924	64311559	0	0.027948

689 Amp	1	64312587	64330680	0	0.032206
690 Amp	1	64338058	64485524	0	0.034143
691 Amp	1	64495393	64561325	0	0.035924
692 Amp	1	64561525	64616915	0	0.035185
693 Amp	1	64617704	64637952	0	0.036282
694 Amp	1	64638514	64659430	0	0.041782
695 Amp	1	64661519	64792854	0	0.040001
696 Amp	1	64798014	64850544	0	0.036967
697 Amp	1	64850558	64869774	0	0.038472
698 Amp	1	64872615	64924820	0	0.036967
699 Amp	1	64930732	65056419	0	0.021031
700 Amp	1	65056445	65300299	0	0.027774
701 Amp	1	65303286	65665101	0	0.021031
702 Amp	1	65672819	65736831	0	0.019521
703 Amp	1	65738080	65799589	0	0.020376
704 Amp	1	65799670	65807607	0	0.017628
705 Amp	1	65808473	65845182	0	0.020325
706 Amp	1	65846077	65917332	0	0.017628
707 Amp	1	65919747	65956204	0	0.018212
708 Amp	1	65961757	66008439	0	0.01934
709 Amp	1	66009271	66011677	0	0.021039
710 Amp	1	66012555	66145285	0	0.019327
711 Amp	1	66146724	66369132	0	0.018747
712 Amp	1	66369575	66470613	0	0.017893
713 Amp	1	66470858	66557660	0	0.015796
714 Amp	1	66557939	66790061	0	0.014097
715 Amp	1	66790357	66882985	0	0.011033
716 Amp	1	66883588	66921838	0	0.011715
717 Amp	1	66925889	67190212	0	0.013642
718 Amp	1	67190271	67429213	0	0.011715
719 Amp	1	67438134	67573659	0	0.012693
720 Amp	1	67576089	67850739	0	0.019172
721 Amp	1	67851121	67940092	0	0.020074
722 Amp	1	67945992	67957395	0	0.02177
723 Amp	1	67959032	68007641	0	0.022947
724 Amp	1	68011295	68075269	0	0.022045
725 Amp	1	68082547	68084538	0	0.015567
726 Amp	1	68087889	68105537	0	0.014589
727 Amp	1	68106523	68170064	0	0.022931
728 Amp	1	68173702	68256176	0	0.028046
729 Amp	1	68256297	68276569	0	0.027364
730 Amp	1	68281649	68283110	0	0.028444
731 Amp	1	68284332	68286694	0	0.023328
732 Amp	1	68294152	68340917	0	0.023984
733 Amp	1	68350912	68379753	0	0.018017
734 Amp	1	68379928	68499756	0	0.017436
735 Amp	1	68504159	68514625	0	0.016259
736 Amp	1	68515055	68603148	0	0.015444
737 Amp	1	68603356	68639974	0	0.014364
738 Amp	1	68643768	69075182	0	0.015844
739 Amp	1	69078125	69259975	0	0.0171
740 Amp	1	69261156	69296984	0	0.015621
741 Amp	1	69300650	69626636	0	0.017466

742	Amp	1	69630213	69691379	0	0.018711
743	Amp	1	69691383	70337111	0	0.017455
744	Amp	1	70341222	70948625	0	0.015609
745	Amp	1	70993290	71354048	0	0.01405
746	Amp	1	71355684	71879365	0	0.019648
747	Amp	1	71882544	71882544	0	0.024776
748	Amp	1	71884305	72043643	0	0.026002
749	Amp	1	72049851	72050849	0	0.03569
750	Amp	1	72054511	72111831	0	0.034463
751	Amp	1	72117692	72245386	0	0.024776
752	Amp	1	72245505	72398059	0	0.026148
753	Amp	1	72398799	72539622	0	0.024776
754	Amp	1	72542279	72542484	0	0.027096
755	Amp	1	72631417	72631417	0	0.036964
756	Amp	1	72631497	72643389	0	0.040139
757	Amp	1	72643449	72656579	0	0.037818
758	Amp	1	72661819	72690912	0	0.040956
759	Amp	1	72692743	72699820	0	0.032184
760	Amp	1	72702390	73101370	0	0.03014
761	Amp	1	73105640	73245645	0	0.031853
762	Amp	1	73250091	73650996	0	0.027258
763	Amp	1	73654171	73719524	0	0.021985
764	Amp	1	73724043	73761619	0	0.017188
765	Amp	1	73761854	73938278	0	0.017842
766	Amp	1	73938573	74883067	0	0.017188
767	Amp	1	74883325	74884054	0	0.018551
768	Amp	1	74886515	75098864	0	0.019137
769	Amp	1	75102013	75102013	0	0.017773
770	Amp	1	75102718	75189369	0	0.019189
771	Amp	1	75189963	75362755	0	0.019854
772	Amp	1	75368769	75400130	0	0.01847
773	Amp	1	75400977	75427199	0	0.020354
774	Amp	1	75429272	75532129	0	0.018658
775	Amp	1	75533010	75771334	0	0.017242
776	Amp	1	75771620	75865988	0	0.018817
777	Amp	1	75873354	76087598	0	0.016577
778	Amp	1	76091265	76152308	0	0.022246
779	Amp	1	76154568	76388163	0	0.021001
780	Amp	1	76393816	76399666	0	0.023588
781	Amp	1	76401611	76444546	0	0.025643
782	Amp	1	76446102	76612646	0	0.023588
783	Amp	1	76612910	77064491	0	0.021001
784	Amp	1	77065538	77174481	0	0.015332
785	Amp	1	77175129	77243000	0	0.014747
786	Amp	1	77245144	77333260	0	0.016215
787	Amp	1	77337245	77443300	0	0.014747
788	Amp	1	77446829	77568079	0	0.015513
789	Amp	1	77571872	77572715	0	0.018281
790	Amp	1	77576580	77709830	0	0.019756
791	Amp	1	77711189	77792266	0	0.021095
792	Amp	1	77794957	77823634	0	0.023158
793	Amp	1	77824722	78145185	0	0.025403
794	Amp	1	78157045	78587591	0	0.024064

795 Amp	1	78588390	78609328	0	0.034999
796 Amp	1	78613481	78623278	0	0.030325
797 Amp	1	78624924	78704611	0	0.029559
798 Amp	1	78709270	78805583	0	0.030126
799 Amp	1	78810379	78838065	0	0.030581
800 Amp	1	78845087	78854115	0	0.029106
801 Amp	1	79089920	79261549	0	0.038603
802 Amp	1	79276508	79948066	0	0.022844
803 Amp	1	79950023	80706081	0	0.019655
804 Amp	1	80707343	80911623	0	0.016517
805 Amp	1	80911843	81054324	0	0.015951
806 Amp	1	81054948	81369596	0	0.013183
807 Amp	1	81372563	81444495	0	0.011299
808 Amp	1	81444792	81591226	0	0.01306
809 Amp	1	81594841	81608719	0	0.014004
810 Amp	1	81612624	82211523	0	0.01306
811 Amp	1	82211906	82307423	0	0.011524
812 Amp	1	82314265	82886070	0	0.012059
813 Amp	1	82887087	82905733	0	0.010298
814 Amp	1	82906260	83429214	0	0.011226
815 Amp	1	83438185	83810806	0	0.018667
816 Amp	1	83813808	83844180	0	0.021256
817 Amp	1	83844510	83857732	0	0.025582
818 Amp	1	83858018	83864341	0	0.022919
819 Amp	1	83864808	83909975	0	0.021256
820 Amp	1	83913209	84019915	0	0.013815
821 Amp	1	84024773	84080491	0	0.01584
822 Amp	1	84084215	84096595	0	0.015305
823 Amp	1	84103331	84172715	0	0.014187
824 Amp	1	84174010	84226850	0	0.017094
825 Amp	1	84239798	84417213	0	0.014187
826 Amp	1	84417267	84610414	0	0.015154
827 Amp	1	84613346	84629316	0	0.013875
828 Amp	1	84630999	84755154	0	0.01185
829 Amp	1	84758575	84803171	0	0.013241
830 Amp	1	84809867	84933802	0	0.011606
831 Amp	1	84934431	85166913	0	0.012156
832 Amp	1	85169730	85412453	0	0.014769
833 Amp	1	85425774	85743698	0	0.012156
834 Amp	1	85758239	85804978	0	0.010765
835 Amp	1	85809602	85949044	0	0.013743
836 Amp	1	85950001	86000156	0	0.014961
837 Amp	1	86003611	86029875	0	0.01441
838 Amp	1	86030066	86077764	0	0.013442
839 Amp	1	86083815	86094378	0	0.012224
840 Amp	1	86095431	87000958	0	0.009635
841 Amp	1	87005564	87502742	0	0.006657
842 Amp	1	87508127	88244365	0	0.008447
843 Amp	1	88247823	88422690	0	0.016465
844 Amp	1	88423242	88527108	0	0.019126
845 Amp	1	88530222	88538133	0	0.019637
846 Amp	1	88539302	89154109	0	0.019126
847 Amp	1	89160087	89278330	0	0.02061

848	Amp	1	89281244	89374781	0	0.019683
849	Amp	1	89376219	89819631	0	0.018199
850	Amp	1	89835044	89849849	0	0.023221
851	Amp	1	89849934	89862703	0	0.021432
852	Amp	1	89868621	89987275	0	0.02346
853	Amp	1	89990538	90601949	0	0.021432
854	Amp	1	90603288	90751078	0	0.018771
855	Amp	1	90752106	91321022	0	0.019908
856	Amp	1	91327260	91333702	0	0.011464
857	Amp	1	91333712	91337198	0	0.014998
858	Amp	1	91337226	91377806	0	0.019712
859	Amp	1	91379423	91407103	0	0.01475
860	Amp	1	91410083	91453883	0	0.016864
861	Amp	1	91453960	91471682	0	0.017332
862	Amp	1	91472138	91483783	0	0.015218
863	Amp	1	91487748	91608690	0	0.01475
864	Amp	1	91609205	91679376	0	0.017437
865	Amp	1	91745053	92028440	0	0.01475
866	Amp	1	92029055	92065545	0	0.022873
867	Amp	1	92069820	92159260	0	0.02594
868	Amp	1	92161113	92170137	0	0.018057
869	Amp	1	92176097	92476909	0	0.033
870	Amp	1	92479712	93002478	0	0.018057
871	Amp	1	93002505	93019705	0	0.01499
872	Amp	1	93021792	93092574	0	0.015597
873	Amp	1	93094568	93128614	0	0.018701
874	Amp	1	93136637	93165502	0	0.015597
875	Amp	1	93174578	93198911	0	0.020733
876	Amp	1	93199387	93347087	0	0.026515
877	Amp	1	93348442	93361490	0	0.027293
878	Amp	1	93372853	93387160	0	0.028724
879	Amp	1	93397975	93431460	0	0.034656
880	Amp	1	93432067	93495632	0	0.036038
881	Amp	1	93499473	93838683	0	0.037366
882	Amp	1	93838789	93975459	0	0.039073
883	Amp	1	93977183	94133728	0	0.028269
884	Amp	1	94140148	94160112	0	0.025168
885	Amp	1	94163574	94586920	0	0.024031
886	Amp	1	94589663	94661129	0	0.022561
887	Amp	1	94661145	94764974	0	0.021179
888	Amp	1	94765813	94910808	0	0.022712
889	Amp	1	94911873	95065443	0	0.024383
890	Amp	1	95065817	95214568	0	0.026686
891	Amp	1	95215344	95392320	0	0.025015
892	Amp	1	95394089	95395007	0	0.032585
893	Amp	1	95396166	95464562	0	0.033241
894	Amp	1	95474211	95612790	0	0.034055
895	Amp	1	95616207	95659379	0	0.028919
896	Amp	1	95660850	95696727	0	0.020534
897	Amp	1	95699010	95833984	0	0.019928
898	Amp	1	95834383	95931946	0	0.016092
899	Amp	1	95932192	96156183	0	0.014385
900	Amp	1	96156720	96345190	0	0.017179

901 Amp	1	96345465	96579390	0	0.023232
902 Amp	1	96580534	96653271	0	0.023811
903 Amp	1	96654211	96694171	0	0.026736
904 Amp	1	96694997	96698004	0	0.026157
905 Amp	1	96705149	96964310	0	0.020371
906 Amp	1	96967364	97001030	0	0.019593
907 Amp	1	97004996	97185793	0	0.018036
908 Amp	1	97186364	97734567	0	0.019447
909 Amp	1	97735163	97874736	0	0.019884
910 Amp	1	97884105	97916926	0	0.021318
911 Amp	1	97927699	97963456	0	0.020881
912 Amp	1	97965628	97977953	0	0.020225
913 Amp	1	97981026	98014572	0	0.02254
914 Amp	1	98016564	98020076	0	0.021106
915 Amp	1	98022207	98062896	0	0.022303
916 Amp	1	98063822	98183904	0	0.023226
917 Amp	1	98186695	98224658	0	0.025593
918 Amp	1	98227662	98430345	0	0.023402
919 Amp	1	98432333	98448287	0	0.024781
920 Amp	1	98449925	98496758	0	0.023276
921 Amp	1	98498942	98636043	0	0.020909
922 Amp	1	98638624	98783583	0	0.02248
923 Amp	1	98789865	98860578	0	0.018968
924 Amp	1	98860788	99016602	0	0.02154
925 Amp	1	99017164	99120960	0	0.022449
926 Amp	1	99123347	99181958	0	0.019877
927 Amp	1	99188299	99226471	0	0.023041
928 Amp	1	99227603	99351242	0	0.019877
929 Amp	1	99353117	99379014	0	0.022827
930 Amp	1	99382221	99464865	0	0.024172
931 Amp	1	99466972	99515889	0	0.022762
932 Amp	1	99516232	99523197	0	0.024601
933 Amp	1	99526816	99786661	0	0.023256
934 Amp	1	99793338	99802852	0	0.021417
935 Amp	1	99806469	99931612	0	0.024176
936 Amp	1	99936000	99941694	0	0.021225
937 Amp	1	99942312	100257351	0	0.021779
938 Amp	1	100258092	100274916	0	0.021225
939 Amp	1	100278704	100330564	0	0.022868
940 Amp	1	100332480	100398275	0	0.017799
941 Amp	1	100402069	100422451	0	0.014426
942 Amp	1	100424720	100428514	0	0.015493
943 Amp	1	100430304	100467010	0	0.012734
944 Amp	1	100471456	100510102	0	0.011092
945 Amp	1	100513237	100531509	0	0.012987
946 Amp	1	100531539	100569605	0	0.016071
947 Amp	1	100571549	100597665	0	0.014176
948 Amp	1	100606010	100819454	0	0.013109
949 Amp	1	100826712	101040570	0	0.010025
950 Amp	1	101047896	101055849	0	0.008455
951 Amp	1	101059192	101082672	0	0.014591
952 Amp	1	101083801	101219482	0	0.016239
953 Amp	1	101224933	101265818	0	0.013877

954	Amp	1	101265853	101279521	0	0.016864
955	Amp	1	101280478	101330407	0	0.013877
956	Amp	1	101332533	101396547	0	0.017113
957	Amp	1	101397338	101406099	0	0.020168
958	Amp	1	101408163	101520332	0	0.024928
959	Amp	1	101521764	101645157	0	0.027204
960	Amp	1	101647296	101750652	0	0.024928
961	Amp	1	101751584	101817983	0	0.02285
962	Amp	1	101819272	101823806	0	0.026393
963	Amp	1	101824797	101844887	0	0.020405
964	Amp	1	101845946	101863632	0	0.016862
965	Amp	1	101864595	101939909	0	0.019418
966	Amp	1	101942301	102151497	0	0.021537
967	Amp	1	102154133	102190128	0	0.023858
968	Amp	1	102393290	102393290	0	0.024473
969	Amp	1	102396750	102467860	0	0.025724
970	Amp	1	102474134	102685320	0	0.024473
971	Amp	1	102686302	102710519	0	0.022715
972	Amp	1	102716165	102839231	0	0.017541
973	Amp	1	102851916	102928545	0	0.02429
974	Amp	1	102934758	103054082	0	0.025023
975	Amp	1	103054273	103122162	0	0.02658
976	Amp	1	103122951	103131925	0	0.024259
977	Amp	1	103134999	103441218	0	0.022846
978	Amp	1	103444883	103524255	0	0.024416
979	Amp	1	103524378	103524378	0	0.022584
980	Amp	1	103793015	103964061	0	0.019434
981	Amp	1	103977129	104153553	0	0.024495
982	Amp	1	104156571	104157967	0	0.02318
983	Amp	1	104158275	104396829	0	0.018119
984	Amp	1	104398193	104447715	0	0.020191
985	Amp	1	104449841	104512552	0	0.01862
986	Amp	1	104520924	104637751	0	0.019919
987	Amp	1	104649495	104656995	0	0.018928
988	Amp	1	104667757	104692669	0	0.016637
989	Amp	1	104702933	105303994	0	0.013684
990	Amp	1	105313521	105412815	0	0.012385
991	Amp	1	105412845	106083834	0	0.013998
992	Amp	1	106087328	106104431	0	0.016949
993	Amp	1	106104486	106162417	0	0.013998
994	Amp	1	106171723	106218066	0	0.022318
995	Amp	1	106218079	106228332	0	0.023969
996	Amp	1	106228581	106293716	0	0.027472
997	Amp	1	106295084	106373864	0	0.02582
998	Amp	1	106374111	106391079	0	0.027857
999	Amp	1	106391157	106395116	0	0.030205
1000	Amp	1	106398959	106426723	0	0.033062
1001	Amp	1	106427428	106498154	0	0.030714
1002	Amp	1	106499917	106563038	0	0.04109
1003	Amp	1	106563643	106704192	0	0.043564
1004	Amp	1	106707784	106802513	0	0.04109
1005	Amp	1	106802542	106972182	0	0.039018
1006	Amp	1	106972857	107030186	0	0.042404

1007	Amp	1	107030897	107079498	0	0.043932
1008	Amp	1	107081853	107207977	0	0.044772
1009	Amp	1	107208604	107236395	0	0.043245
1010	Amp	1	107243039	107331193	0	0.039859
1011	Amp	1	107332154	107471724	0	0.039018
1012	Amp	1	107475930	107508989	0	0.041192
1013	Amp	1	107509452	107533721	0	0.041924
1014	Amp	1	107534480	107546964	0	0.044269
1015	Amp	1	107547122	107597014	0	0.041924
1016	Amp	1	107597353	107618344	0	0.043334
1017	Amp	1	107618975	107629681	0	0.045574
1018	Amp	1	107630997	107695849	0	0.043401
1019	Amp	1	107697302	107713447	0	0.046422
1020	Amp	1	107720441	107776767	0	0.043401
1021	Amp	1	107777345	107785756	0	0.040215
1022	Amp	1	107786293	107859481	0	0.042607
1023	Amp	1	107859987	107872692	0	0.044818
1024	Amp	1	107872717	107875930	0	0.044085
1025	Amp	1	107876735	107912933	0	0.047674
1026	Amp	1	107918099	107920222	0	0.044085
1027	Amp	1	107920797	107947953	0	0.047287
1028	Amp	1	107949619	107968879	0	0.045877
1029	Amp	1	107972725	108130783	0	0.047416
1030	Amp	1	108131152	108171920	0	0.041823
1031	Amp	1	108176950	108330898	0	0.044151
1032	Amp	1	108330960	108471686	0	0.04194
1033	Amp	1	108479495	108479495	0	0.043428
1034	Amp	1	108479650	108481648	0	0.045878
1035	Amp	1	108486251	108509733	0	0.046391
1036	Amp	1	108514114	108541678	0	0.038072
1037	Amp	1	108544068	108564040	0	0.038574
1038	Amp	1	108565773	108597621	0	0.041317
1039	Amp	1	108597978	108634800	0	0.038989
1040	Amp	1	108635364	108652273	0	0.038413
1041	Amp	1	108658962	108665524	0	0.039373
1042	Amp	1	108673894	108808411	0	0.041585
1043	Amp	1	108814834	108851521	0	0.039972
1044	Amp	1	108854286	108868671	0	0.04218
1045	Amp	1	108872601	108872702	0	0.041678
1046	Amp	1	108875420	108914228	0	0.04363
1047	Amp	1	108916674	108941228	0	0.042091
1048	Amp	1	108961034	108966266	0	0.039883
1049	Amp	1	108966416	108967060	0.181932	0.071655
1050	Amp	1	108968731	108985239	0	0.039883
1051	Amp	1	108985482	109131659	0	0.038626
1052	Amp	1	109146692	109277504	0	0.040004
1053	Amp	1	109277968	109489811	0	0.041462
1054	Amp	1	109500895	109553331	0	0.043442
1055	Amp	1	109553771	109714002	0	0.04431
1056	Amp	1	109718120	109866873	0	0.040882
1057	Amp	1	109869470	109903595	0	0.039394
1058	Amp	1	109908341	109936935	0	0.042065
1059	Amp	1	109938856	109983974	0	0.040607

1060	Amp	1	109988250	109993581	0	0.030231
1061	Amp	1	109994869	110009477	0	0.028854
1062	Amp	1	110009646	110206683	0	0.027986
1063	Amp	1	110217174	110399703	0	0.029236
1064	Amp	1	110401565	110435241	0	0.030569
1065	Amp	1	110443411	110465843	0	0.029318
1066	Amp	1	110468753	110480968	0	0.029789
1067	Amp	1	110484902	110503002	0	0.029318
1068	Amp	1	110503706	110504403	0	0.027078
1069	Amp	1	110509377	110599033	0	0.028705
1070	Amp	1	110600189	110619055	0	0.029552
1071	Amp	1	110619391	110664331	0	0.027078
1072	Amp	1	110665098	110683099	0	0.028272
1073	Amp	1	110683584	110780620	0	0.027759
1074	Amp	1	110782105	110788274	0	0.025547
1075	Amp	1	110869657	111060588	0	0.029039
1076	Amp	1	111062018	111107005	0	0.029637
1077	Amp	1	111107794	111194776	0	0.026967
1078	Amp	1	111195556	111224153	0	0.0409
1079	Amp	1	111226615	111249642	0	0.038863
1080	Amp	1	111249795	111285127	0	0.037669
1081	Amp	1	111297256	111321002	0	0.040343
1082	Amp	1	111321320	111332906	0	0.034824
1083	Amp	1	111342307	111351887	0	0.032151
1084	Amp	1	111352248	111382001	0	0.03291
1085	Amp	1	111382127	111927471	0	0.032151
1086	Amp	1	111932927	111933401	0	0.030818
1087	Amp	1	111933489	111935744	0	0.032612
1088	Amp	1	111935822	111951752	0	0.03551
1089	Amp	1	111952223	111968310	0	0.032612
1090	Amp	1	111968346	111995855	0	0.035495
1091	Amp	1	111996121	112022617	0	0.032612
1092	Amp	1	112025050	112025854	0	0.029755
1093	Amp	1	112026382	112163591	0	0.030334
1094	Amp	1	112163596	112363995	0	0.032474
1095	Amp	1	112368702	112753195	0	0.030494
1096	Amp	1	112754875	112942794	0	0.031123
1097	Amp	1	112944259	112958947	0	0.029623
1098	Amp	1	112961996	113026874	0	0.031168
1099	Amp	1	113027938	113061907	0	0.029374
1100	Amp	1	113061928	113207042	0	0.030595
1101	Amp	1	113208087	113231359	0	0.030015
1102	Amp	1	113231492	113243181	0	0.028337
1103	Amp	1	113243301	113284683	0	0.027708
1104	Amp	1	113289591	113318902	0	0.026487
1105	Amp	1	113319881	113319881	0	0.024942
1106	Amp	1	113325701	113488979	0	0.024344
1107	Amp	1	113494003	113569141	0	0.027615
1108	Amp	1	113570197	113625158	0	0.024344
1109	Amp	1	113630788	113631057	0	0.0249
1110	Amp	1	113632670	113729465	0	0.026336
1111	Amp	1	113729952	113729952	0	0.027343
1112	Amp	1	113738095	113762492	0	0.029412

1113	Amp	1	113766234	113803712	0	0.029879
1114	Amp	1	113809084	114101270	0	0.033102
1115	Amp	1	114103867	114128347	0	0.035597
1116	Amp	1	114129003	114148759	0	0.036602
1117	Amp	1	114151650	114170529	0	0.035596
1118	Amp	1	114170737	114213300	0	0.03459
1119	Amp	1	114216468	114339465	0	0.043329
1120	Amp	1	114342617	114357230	0	0.039546
1121	Amp	1	114358056	114464344	0	0.040202
1122	Amp	1	114467106	114525030	0	0.043232
1123	Amp	1	114527285	114672456	0	0.040736
1124	Amp	1	114674339	114744225	0	0.044712
1125	Amp	1	114744314	114789878	0	0.031241
1126	Amp	1	114793492	114857263	0	0.032401
1127	Amp	1	114857319	114934218	0	0.033339
1128	Amp	1	114938697	114954541	0	0.032683
1129	Amp	1	114956105	115012691	0	0.03016
1130	Amp	1	115021450	115038110	0	0.029653
1131	Amp	1	115038729	115095423	0	0.030843
1132	Amp	1	115095793	115138882	0	0.029683
1133	Amp	1	115140120	115167593	0	0.021032
1134	Amp	1	115171117	115215593	0	0.018963
1135	Amp	1	115216378	115258328	0	0.022234
1136	Amp	1	115259165	115403117	0	0.02293
1137	Amp	1	115408162	115443483	0	0.023906
1138	Amp	1	115447442	115491713	0	0.027697
1139	Amp	1	115492232	115494171	0	0.024426
1140	Amp	1	115495773	115544106	0	0.02813
1141	Amp	1	115547733	115617890	0	0.029738
1142	Amp	1	115622077	115649438	0	0.025482
1143	Amp	1	115650138	115665212	0	0.024705
1144	Amp	1	115668447	115672544	0	0.022254
1145	Amp	1	115675042	115703278	0	0.03466
1146	Amp	1	115709145	115742032	0	0.035253
1147	Amp	1	115745955	115878948	0	0.032612
1148	Amp	1	115879537	115904041	0	0.030201
1149	Amp	1	115906026	116200139	0	0.031062
1150	Amp	1	116206560	116208610	0	0.033472
1151	Amp	1	116209313	116302009	0	0.033986
1152	Amp	1	116302530	116362780	0	0.03477
1153	Amp	1	116363729	116386233	0	0.03236
1154	Amp	1	116386713	116465901	0	0.031528
1155	Amp	1	116469777	116509197	0	0.040165
1156	Amp	1	116511534	116525225	0	0.039382
1157	Amp	1	116527793	116548108	0	0.041532
1158	Amp	1	116551600	116708941	0	0.042153
1159	Amp	1	116709685	116790857	0	0.043861
1160	Amp	1	116795104	116802566	0	0.035224
1161	Amp	1	116808457	116841677	0	0.037267
1162	Amp	1	116842883	116881008	0	0.037862
1163	Amp	1	116883820	116951845	0	0.040613
1164	Amp	1	116953482	117112236	0	0.043644
1165	Amp	1	117113466	117113466	0	0.048292

1166	Amp	1	117115233	117172404	0	0.047431
1167	Amp	1	117172419	117184745	0	0.04852
1168	Amp	1	117185870	117336444	0	0.049883
1169	Amp	1	117340625	117370397	0	0.047132
1170	Amp	1	117371020	117533917	0	0.056964
1171	Amp	1	117540280	117711015	0	0.05492
1172	Amp	1	117711587	117776682	0	0.055685
1173	Amp	1	117784228	117857732	0	0.052654
1174	Amp	1	117864022	117938653	0	0.050946
1175	Amp	1	117964606	118011424	0	0.037627
1176	Amp	1	118012883	118041182	0	0.03503
1177	Amp	1	118043611	118096554	0	0.047281
1178	Amp	1	118098195	118108431	0	0.049816
1179	Amp	1	118114351	118125670	0	0.0477
1180	Amp	1	118129161	118154456	0	0.044543
1181	Amp	1	118155027	118212766	0	0.048191
1182	Amp	1	118239162	118323210	0	0.050379
1183	Amp	1	118323364	118340673	0	0.049785
1184	Amp	1	118340808	118355103	0	0.050785
1185	Amp	1	118355440	118484811	0	0.051341
1186	Amp	1	118486443	118502932	0	0.058909
1187	Amp	1	118505558	118578708	0	0.051896
1188	Amp	1	118588329	118728785	0	0.051341
1189	Amp	1	118732964	118774144	0	0.06277
1190	Amp	1	118774165	118820108	0.050473	0.066043
1191	Amp	1	118822299	118827196	0	0.05792
1192	Amp	1	118837324	118901864	0	0.054273
1193	Amp	1	118903378	118936205	0	0.052058
1194	Amp	1	118936711	118970259	0	0.052937
1195	Amp	1	118970290	119028642	0	0.051914
1196	Amp	1	119035302	119039825	0	0.053548
1197	Amp	1	119040719	119081540	0	0.058224
1198	Amp	1	119082344	119105323	0	0.060654
1199	Amp	1	119115201	119189724	0.024438	0.064936
1200	Amp	1	119191969	119282854	0	0.062357
1201	Amp	1	119282867	119403528	0.001323	0.064038
1202	Amp	1	119403575	119413100	0.050473	0.066154
1203	Amp	1	119416666	119475138	0.019169	0.064792
1204	Amp	1	119475331	119491226	0.040093	0.065723
1205	Amp	1	119494326	119682349	0.065715	0.066842
1206	Amp	1	119683689	119801421	0.015369	0.064653
1207	Amp	1	119801482	119853088	0.001323	0.063891
1208	Amp	1	119855157	119864614	0	0.062012
1209	Amp	1	119867080	119884026	0	0.06248
1210	Amp	1	119890454	119904099	0.060686	0.066542
1211	Amp	1	119906441	119952136	0.107017	0.06843
1212	Amp	1	119952779	119955627	0.094967	0.067969
1213	Amp	1	119955930	119965901	0.170723	0.071181
1214	Amp	1	119967232	119978092	0.14896	0.070249
1215	Amp	1	119979030	119979137	0.210594	0.072728
1216	Amp	1	119979978	119979978	0.246818	0.074218
1217	Amp	1	119981279	119981279	0.112543	0.068777
1218	Amp	1	119981332	119981332	0	0.059708

1219	Amp	1	119984738	119984738	0	0.062899
1220	Amp	1	119984772	119984772	0.386426	0.079444
1221	Amp	1	146716041	146716041	1.252022	0.103168
1222	Amp	1	149906351	149906351	2.614791	0.131217
1223	Amp	1	149909846	149909846	2.812598	0.134896
1224	Amp	1	149910462	149910462	2.929372	0.137087
1225	Amp	1	149913375	149914031	2.882945	0.136218
1226	Amp	1	149918981	149918981	3.009724	0.138738
1227	Amp	1	149927059	149928056	3.852343	0.15382
1228	Amp	1	149936215	149966299	4.223463	0.160137
1229	Amp	1	149966820	149966820	4.090086	0.158077
1230	Amp	1	149966973	149972350	4.3738	0.162479
1231	Amp	1	149975408	149991322	4.47072	0.163943
1232	Amp	1	149993982	150002511	4.838749	0.169761
1233	Amp	1	150004041	150057994	4.69701	0.167611
1234	Amp	1	150058787	150071479	4.3738	0.162588
1235	Amp	1	150077351	150086641	4.499013	0.164564
1236	Amp	1	150086810	150131878	4.5989	0.166053
1237	Amp	1	150147495	150147495	4.640474	0.166715
1238	Amp	1	150148513	150176837	4.768128	0.168532
1239	Amp	1	150177309	150315150	4.683031	0.167201
1240	Amp	1	150315443	150520671	4.264196	0.160834
1241	Amp	1	150522687	150533787	4.209693	0.16002
1242	Amp	1	150544285	150593647	4.924414	0.171005
1243	Amp	1	150597511	150645155	5.02373	0.172564
1244	Amp	1	150646156	150700888	4.91152	0.170772
1245	Amp	1	150703805	150704541	4.882872	0.170304
1246	Amp	1	150705243	150765897	4.69701	0.167616
1247	Amp	1	150766223	150838819	4.116393	0.158422
1248	Amp	1	150838949	150883915	4.541384	0.165222
1249	Amp	1	150886666	150886666	4.47072	0.163994
1250	Amp	1	150889547	150891045	4.429656	0.163437
1251	Amp	1	150893765	150979001	4.116393	0.158439
1252	Amp	1	150979329	151002131	4.090086	0.157984
1253	Amp	1	151006598	151070549	3.874391	0.154297
1254	Amp	1	151070735	151070735	3.839479	0.153708
1255	Amp	1	151073507	151099783	3.788687	0.152668
1256	Amp	1	151115703	151115703	3.471122	0.147164
1257	Amp	1	151116579	151198313	3.103562	0.140455
1258	Amp	1	151201646	151222856	2.278531	0.124817
1259	Amp	1	151226395	151288420	2.213223	0.12352
1260	Amp	1	151293382	151358828	2.158817	0.122312
1261	Amp	1	151359311	151359541	1.994915	0.119005
1262	Amp	1	151436260	151437026	1.96212	0.118521
1263	Amp	1	151437367	151498967	2.158817	0.122275
1264	Amp	1	151500420	151501628	2.256542	0.124291
1265	Amp	1	151504717	151504963	2.278531	0.124792
1266	Amp	1	151506001	151506001	2.300463	0.125265
1267	Amp	1	151507898	151529261	2.345513	0.126034
1268	Amp	1	151536508	151557459	2.26699	0.124472
1269	Amp	1	151563440	151563440	1.512549	0.10905
1270	Amp	1	151564677	151568809	1.445847	0.10755
1271	Amp	1	151569239	151578125	1.531891	0.109644

1272	Amp	1	151578197	151630186	1.416817	0.106982
1273	Amp	1	151633929	151686595	1.472629	0.108332
1274	Amp	1	151686890	151689637	1.378521	0.106141
1275	Amp	1	151690251	151759607	1.261401	0.103498
1276	Amp	1	151760224	151768525	1.290368	0.104016
1277	Amp	1	151770667	151770667	1.348715	0.105476
1278	Amp	1	151770799	151770799	1.378521	0.10598
1279	Amp	1	151772452	151782846	1.280185	0.104007
1280	Amp	1	151789185	151845522	0.891602	0.094559
1281	Amp	1	151847822	151901561	0.995635	0.097245
1282	Amp	1	151904006	151904006	1.178324	0.101501
1283	Amp	1	151904502	151938778	1.243254	0.103122
1284	Amp	1	151942422	151998169	1.270711	0.103621
1285	Amp	1	151999021	152033246	1.197658	0.102
1286	Amp	1	152033856	152053260	0.977077	0.096665
1287	Amp	1	152056085	152175092	0.995635	0.097168
1288	Amp	1	152176337	152183308	1.023539	0.097826
1289	Amp	1	152185315	152252565	1.290368	0.104118
1290	Amp	1	152255234	152258902	1.149583	0.100906
1291	Amp	1	152263498	152288336	0.891602	0.094614
1292	Amp	1	152295561	152295561	1.041205	0.098318
1293	Amp	1	152298587	152356893	1.121584	0.10028
1294	Amp	1	152360241	152372861	1.168655	0.101393
1295	Amp	1	152375887	152381528	1.102589	0.099834
1296	Amp	1	152382758	152382758	1.131695	0.100326
1297	Amp	1	152393104	152437590	0.857296	0.093628
1298	Amp	1	152437990	152440790	1.112463	0.099927
1299	Amp	1	152440885	152459649	1.149583	0.100798
1300	Amp	1	152461715	152469813	0.915698	0.095167
1301	Amp	1	152469836	152493458	0.941704	0.095774
1302	Amp	1	152494491	152535661	0.891602	0.094659
1303	Amp	1	152536086	152640996	0.840289	0.093198
1304	Amp	1	152641155	152667725	0.873678	0.094095
1305	Amp	1	152668473	152668473	0.840289	0.093198
1306	Amp	1	152668698	152726279	0.823771	0.092725
1307	Amp	1	152728542	152752479	0.848692	0.093436
1308	Amp	1	152753153	152766750	0.873678	0.094109
1309	Amp	1	152767131	152825329	0.751221	0.090799
1310	Amp	1	152828698	152828698	0.882843	0.094413
1311	Amp	1	152838707	152864958	1.093834	0.099527
1312	Amp	1	152867032	152882330	1.159209	0.101179
1313	Amp	1	152883608	152883608	0.950886	0.096064
1314	Amp	1	152883826	152900077	0.807647	0.092451
1315	Amp	1	152901969	152930644	0.959607	0.096254
1316	Amp	1	152933231	152933231	0.857296	0.093697
1317	Amp	1	152934101	152972410	0.831947	0.092987
1318	Amp	1	152972461	152992003	0.807647	0.092319
1319	Amp	1	152992224	153018584	0.758088	0.091033
1320	Amp	1	153028502	153080915	0.742723	0.090549
1321	Amp	1	153083902	153127918	0.697374	0.089436
1322	Amp	1	153128018	153128018	0.494792	0.083119
1323	Amp	1	153128035	153131706	0.576183	0.085755
1324	Amp	1	153131776	153133389	0.774648	0.091469

1325	Amp	1	153133819	153160508	0.72817	0.090218
1326	Amp	1	153161611	153206707	0.751221	0.090873
1327	Amp	1	153207447	153207621	0.72817	0.090265
1328	Amp	1	153209815	153251073	0.667587	0.088388
1329	Amp	1	153251646	153256299	0.720659	0.089979
1330	Amp	1	153257097	153259007	0.644502	0.087842
1331	Amp	1	153272606	153274022	0.563096	0.085156
1332	Amp	1	153275182	153282541	0.583752	0.085968
1333	Amp	1	153282939	153314040	0.563096	0.085313
1334	Amp	1	153314540	153321922	0.515676	0.083766
1335	Amp	1	153321996	153322177	0.576183	0.08566
1336	Amp	1	153329051	153342229	0.681869	0.088924
1337	Amp	1	153342346	153399125	0.667587	0.088406
1338	Amp	1	153399371	153402908	0.644502	0.087901
1339	Amp	1	153405027	153459085	0.667587	0.088445
1340	Amp	1	153459146	153490450	0.563096	0.085182
1341	Amp	1	153490561	153490561	0.543344	0.084679
1342	Amp	1	153493819	153573458	0.463534	0.081892
1343	Amp	1	153584088	153693134	0.47716	0.082428
1344	Amp	1	153699567	153796506	0.529451	0.084208
1345	Amp	1	153800463	153874357	0.515676	0.083672
1346	Amp	1	153875833	153887765	0.689884	0.089086
1347	Amp	1	153890003	153928787	0.609133	0.086777
1348	Amp	1	153932130	153947928	0.774648	0.091618
1349	Amp	1	153948693	154019235	0.720659	0.089925
1350	Amp	1	154019265	154086400	0.79909	0.092167
1351	Amp	1	154088004	154190635	0.873678	0.094171
1352	Amp	1	154195150	154224650	0.995635	0.097264
1353	Amp	1	154224756	154266807	1.131695	0.10049
1354	Amp	1	154274778	154335486	0.781972	0.091653
1355	Amp	1	154336165	154386703	1.075952	0.099217
1356	Amp	1	154387350	154417346	0.807647	0.092381
1357	Amp	1	154424769	154424784	1.378521	0.106072
1358	Amp	1	154425354	154446403	1.290368	0.104068
1359	Amp	1	154449905	154563051	1.463774	0.107993
1360	Amp	1	154563091	154566641	1.563118	0.110238
1361	Amp	1	154572360	154650092	1.645511	0.111901
1362	Amp	1	154651697	154689862	2.435195	0.127837
1363	Amp	1	154693941	154745413	2.468223	0.128507
1364	Amp	1	154745432	154847591	2.52589	0.129445
1365	Amp	1	154849733	154860921	2.837591	0.135452
1366	Amp	1	154870871	154881541	2.871582	0.135995
1367	Amp	1	154882579	154951763	2.776997	0.134332
1368	Amp	1	154952953	154952953	2.637742	0.13162
1369	Amp	1	154964227	154977746	2.837591	0.135306
1370	Amp	1	154982928	154989340	2.882945	0.136284
1371	Amp	1	154992265	154993514	2.661431	0.132028
1372	Amp	1	154995890	154999851	2.514205	0.129252
1373	Amp	1	155011992	155044688	2.860424	0.135834
1374	Amp	1	155045450	155077709	2.837591	0.135291
1375	Amp	1	155090669	155094807	2.235168	0.123868
1376	Amp	1	155106045	155147675	2.379392	0.12661
1377	Amp	1	155151091	155159197	2.502083	0.129089

1378	Amp	1	155163749	155168172	2.213223	0.123375
1379	Amp	1	155173638	155176826	2.311329	0.125295
1380	Amp	1	155182632	155262283	1.984015	0.118969
1381	Amp	1	155264807	155269959	1.940776	0.117992
1382	Amp	1	155270312	155315261	1.748606	0.114067
1383	Amp	1	155317731	155333027	1.583409	0.110529
1384	Amp	1	155333790	155335328	1.624655	0.111585
1385	Amp	1	155336546	155348517	1.197658	0.101975
1386	Amp	1	155349209	155396615	1.168655	0.101247
1387	Amp	1	155398680	155405570	0.563096	0.085311
1388	Amp	1	155408810	155542642	0.644502	0.087753
1389	Amp	1	155546401	155641923	0.583752	0.085833
1390	Amp	1	155643858	155646487	0.550755	0.084895
1391	Amp	1	155649237	155649237	0.373599	0.078888
1392	Amp	1	155655811	155666664	0.515676	0.083622
1393	Amp	1	155667097	155761313	0.431198	0.080986
1394	Amp	1	155763607	155778679	0.4115	0.080317
1395	Amp	1	155779616	155813579	0.362016	0.078423
1396	Amp	1	155817414	155900727	0.373599	0.078942
1397	Amp	1	155903523	155923806	0.329205	0.077351
1398	Amp	1	155926088	155967566	0.287738	0.075988
1399	Amp	1	155973008	156009242	0.417873	0.080442
1400	Amp	1	156011698	156017008	0.362016	0.078591
1401	Amp	1	156022462	156031555	0.246818	0.074137
1402	Amp	1	156032988	156039096	0.233782	0.073593
1403	Amp	1	156047611	156161826	0.170723	0.071114
1404	Amp	1	156165450	156180434	0.079652	0.067402
1405	Amp	1	156184308	156321235	0.215608	0.072952
1406	Amp	1	156321574	156386688	0.133193	0.069597
1407	Amp	1	156397646	156429151	0.170723	0.07122
1408	Amp	1	156441358	156450669	0.056211	0.066429
1409	Amp	1	156451132	156453066	0	0.062626
1410	Amp	1	156455196	156461549	0	0.063614
1411	Amp	1	156462367	156478149	0.094967	0.068107
1412	Amp	1	156489907	156513264	0.069848	0.067031
1413	Amp	1	156521187	156593291	0.050473	0.066043
1414	Amp	1	156594021	156612355	0.010261	0.064419
1415	Amp	1	156612728	156737983	0.056211	0.066265
1416	Amp	1	156741003	156770863	0	0.060715
1417	Amp	1	156780361	156782789	0	0.062563
1418	Amp	1	156788135	156810923	0.056211	0.066437
1419	Amp	1	156815564	156820205	0.08435	0.067587
1420	Amp	1	156824556	156831013	0.107017	0.068472
1421	Amp	1	156831449	156860426	0.118001	0.068921
1422	Amp	1	156862987	156999474	0.069848	0.067072
1423	Amp	1	157000104	157041533	0.056211	0.06634
1424	Amp	1	157041803	157069645	0.015369	0.064529
1425	Amp	1	157069670	157088632	0.107017	0.068607
1426	Amp	1	157090101	157226910	0.089561	0.06793
1427	Amp	1	157228147	157247031	0.056211	0.066274
1428	Amp	1	157247284	157471861	0	0.062196
1429	Amp	1	157477428	157486927	0	0.060707
1430	Amp	1	157487136	157487136	0	0.062337

1431	Amp	1	157491916	157571164	0.001323	0.064008
1432	Amp	1	157571300	157785419	0	0.06275
1433	Amp	1	157789921	157847627	0	0.060533
1434	Amp	1	157848884	157989825	0	0.060014
1435	Amp	1	157993139	158069635	0	0.058343
1436	Amp	1	158070977	158083176	0	0.063605
1437	Amp	1	158084709	158115775	0.079652	0.067355
1438	Amp	1	158120861	158200394	0.065715	0.066855
1439	Amp	1	158201288	158274869	0.029031	0.065225
1440	Amp	1	158276808	158380337	0	0.059964
1441	Amp	1	158382692	158401983	0	0.063527
1442	Amp	1	158405816	158418644	0	0.058793
1443	Amp	1	158422773	158497936	0	0.056931
1444	Amp	1	158498370	158518070	0	0.056482
1445	Amp	1	158528732	158541869	0	0.05982
1446	Amp	1	158542020	158544217	0.001323	0.064004
1447	Amp	1	158544304	158544407	0	0.059511
1448	Amp	1	158544421	158544421	0	0.050367
1449	Amp	1	158544436	158544436	0	0.049217
1450	Amp	1	158544724	158545154	0	0.043585
1451	Amp	1	158545168	158545295	0	0.040248
1452	Amp	1	158580102	158650857	0	0.049231
1453	Amp	1	158651233	158794775	0	0.051295
1454	Amp	1	158794822	158795142	0	0.053222
1455	Amp	1	158799016	158866716	0	0.049313
1456	Amp	1	158871470	158951561	0	0.051103
1457	Amp	1	158951884	158986622	0	0.052833
1458	Amp	1	158997602	158997602	0	0.05345
1459	Amp	1	159000282	159068194	0	0.050947
1460	Amp	1	159070235	159224476	0	0.053926
1461	Amp	1	159224622	159248028	0	0.051951
1462	Amp	1	159251489	159275184	0	0.054763
1463	Amp	1	159276545	159279025	0	0.06305
1464	Amp	1	159286876	159334014	0.069848	0.067078
1465	Amp	1	159334337	159406113	0.1237	0.069063
1466	Amp	1	159406165	159584728	0.14896	0.070251
1467	Amp	1	159588468	159598058	0.204831	0.072499
1468	Amp	1	159598132	159629024	0.17597	0.071319
1469	Amp	1	159632634	159641030	0.848692	0.093431
1470	Amp	1	159644196	159654647	1.032525	0.098075
1471	Amp	1	159654967	159655385	0.857296	0.093738
1472	Amp	1	159655509	159677005	0.181932	0.071626
1473	Amp	1	159677134	159689641	0.282828	0.075585
1474	Amp	1	159692839	159703306	0.529451	0.084086
1475	Amp	1	159703819	159709846	0.622604	0.087219
1476	Amp	1	159710195	159752266	0.583752	0.085862
1477	Amp	1	159752445	159825688	0.667587	0.088568
1478	Amp	1	159832179	159847698	0.450702	0.081489
1479	Amp	1	159850236	159884105	0.424557	0.080756
1480	Amp	1	159885969	159905665	0.482379	0.082562
1481	Amp	1	159910870	159950929	0.322897	0.077135
1482	Amp	1	159967032	160034011	0.251218	0.074429
1483	Amp	1	160035807	160092169	0.264858	0.074935

1484	Amp	1	160094628	160149919	0.251218	0.074429
1485	Amp	1	160150359	160193082	0.210594	0.072623
1486	Amp	1	160193847	160228410	0.241616	0.074012
1487	Amp	1	160229017	160255473	0.204831	0.07236
1488	Amp	1	160255967	160270643	0.287738	0.075916
1489	Amp	1	160273935	160300053	0.316877	0.076991
1490	Amp	1	160303902	160359846	0.386426	0.07933
1491	Amp	1	160360133	160379910	0.271552	0.075284
1492	Amp	1	160379962	160402319	0.329205	0.077383
1493	Amp	1	160402556	160413260	0.379885	0.079139
1494	Amp	1	160413894	160423211	0.4115	0.08013
1495	Amp	1	160428562	160509343	0.386426	0.079325
1496	Amp	1	160517745	160578473	0.482379	0.082723
1497	Amp	1	160578937	160652624	0.616549	0.086895
1498	Amp	1	160656246	160690484	0.569771	0.085506
1499	Amp	1	160690509	160690667	0.500841	0.083167
1500	Amp	1	160698619	160705312	0.924746	0.095533
1501	Amp	1	160707748	160711725	0.563096	0.08525
1502	Amp	1	160716704	160781315	0.500841	0.083167
1503	Amp	1	160789428	160789880	0.704849	0.089472
1504	Amp	1	160795410	160796352	0.915698	0.095121
1505	Amp	1	160800725	160833928	1.058188	0.098645
1506	Amp	1	160833945	160917506	1.032525	0.098027
1507	Amp	1	160917791	161234748	1.23519	0.102717
1508	Amp	1	161238513	161287888	1.329472	0.105053
1509	Amp	1	161292622	161347992	0.950886	0.095984
1510	Amp	1	161348959	161397337	0.86541	0.093884
1511	Amp	1	161401195	161439440	1.041205	0.098247
1512	Amp	1	161476338	161476341	0.630438	0.087348
1513	Amp	1	161686708	161686708	0.899693	0.094731
1514	Amp	1	161688868	161700551	0.735486	0.090368
1515	Amp	1	161702205	161711762	0.882843	0.094384
1516	Amp	1	161712651	161719600	0.636464	0.087564
1517	Amp	1	161720430	161725067	0.508568	0.083549
1518	Amp	1	161730889	161732594	0.463534	0.081889
1519	Amp	1	161732920	161741462	0.508568	0.083422
1520	Amp	1	161743431	161811410	0.576183	0.085614
1521	Amp	1	161816359	161861196	0.529451	0.084081
1522	Amp	1	161864381	161864656	0.543344	0.084559
1523	Amp	1	161864805	161911507	0.622604	0.08712
1524	Amp	1	161921766	161943757	0.689884	0.089153
1525	Amp	1	161945597	161966998	0.557122	0.084981
1526	Amp	1	161967392	161967392	0.487495	0.08279
1527	Amp	1	161967834	162038289	0.4115	0.080228
1528	Amp	1	162038511	162045950	0.500841	0.083318
1529	Amp	1	162046084	162092592	0.569771	0.085523
1530	Amp	1	162092778	162146250	0.583752	0.085985
1531	Amp	1	162148165	162151430	0.660254	0.088249
1532	Amp	1	162152029	162159627	0.720659	0.090095
1533	Amp	1	162159756	162170478	0.660254	0.088366
1534	Amp	1	162170546	162182887	0.720659	0.090019
1535	Amp	1	162183319	162199376	0.602643	0.08652
1536	Amp	1	162199478	162203587	0.536238	0.084315

1537	Amp	1	162203605	162241744	0.550755	0.084831
1538	Amp	1	162242826	162303677	0.457501	0.081698
1539	Amp	1	162306642	162326048	0.399144	0.079833
1540	Amp	1	162328022	162340523	0.311747	0.076743
1541	Amp	1	162340795	162386680	0.4115	0.080149
1542	Amp	1	162387993	162399384	0.379885	0.079073
1543	Amp	1	162399620	162407466	0.246818	0.074061
1544	Amp	1	162407526	162521519	0.187449	0.071797
1545	Amp	1	162526962	162766573	0.19964	0.072265
1546	Amp	1	162766587	162800857	0.153838	0.070379
1547	Amp	1	162803724	162803724	0.164935	0.07092
1548	Amp	1	162811997	162884873	0.089561	0.067761
1549	Amp	1	162888845	163114900	0.143787	0.070036
1550	Amp	1	163115720	163164150	0.010261	0.064386
1551	Amp	1	163165249	163295539	0	0.062112
1552	Amp	1	163306262	163312192	0	0.063495
1553	Amp	1	163312568	163324694	0.015369	0.064663
1554	Amp	1	163324738	163336446	0.005328	0.064148
1555	Amp	1	163336777	163380030	0.040093	0.065667
1556	Amp	1	163382346	163413346	0.094967	0.068041
1557	Amp	1	163413602	163414403	0.040093	0.065667
1558	Amp	1	163415319	163471496	0.015369	0.064499
1559	Amp	1	163475434	163604938	0.079652	0.067353
1560	Amp	1	163605734	163625473	0.128324	0.069413
1561	Amp	1	163625805	163646468	0.222041	0.073166
1562	Amp	1	163646961	163662937	0.271552	0.075191
1563	Amp	1	163664732	163695228	0.4115	0.080157
1564	Amp	1	163696755	163700550	0.529451	0.08412
1565	Amp	1	163701670	163773211	0.463534	0.08206
1566	Amp	1	163773527	163782196	0.508568	0.083414
1567	Amp	1	163833292	163846190	0.697374	0.089294
1568	Amp	1	163846406	163859605	0.536238	0.084328
1569	Amp	1	163860112	163890599	0.424557	0.080576
1570	Amp	1	163891515	163943238	0.563096	0.085282
1571	Amp	1	163943286	163999344	0.522881	0.084022
1572	Amp	1	164003662	164109110	0.494792	0.082963
1573	Amp	1	164109340	164202551	0.431198	0.080899
1574	Amp	1	164203195	164369190	0.450702	0.081586
1575	Amp	1	164369495	164479165	0.47716	0.082322
1576	Amp	1	164480512	164562863	0.576183	0.085609
1577	Amp	1	164563088	164602346	0.494792	0.083101
1578	Amp	1	164609415	164746339	0.47716	0.082365
1579	Amp	1	164750903	164815216	0.379885	0.079087
1580	Amp	1	164816593	164827125	0.457501	0.081771
1581	Amp	1	164829854	164847719	0.636464	0.087648
1582	Amp	1	164848050	164933991	0.609133	0.086747
1583	Amp	1	164936106	164944457	0.622604	0.087193
1584	Amp	1	164944966	165010578	0.47716	0.082487
1585	Amp	1	165011571	165037498	0.30524	0.07661
1586	Amp	1	165043686	165054437	0.241616	0.073925
1587	Amp	1	165054524	165093570	0.210594	0.072737
1588	Amp	1	165094663	165210937	0.362016	0.078538
1589	Amp	1	165211583	165223243	0.210594	0.072737

1590	Amp	1	165227163	165248886	0.138391	0.069883
1591	Amp	1	165248967	165314403	0.128324	0.069414
1592	Amp	1	165314445	165349399	0.118001	0.068851
1593	Amp	1	165357763	165386259	0.034113	0.065564
1594	Amp	1	165389566	165424850	0	0.057788
1595	Amp	1	165424908	165472489	0	0.056175
1596	Amp	1	165474126	165476459	0	0.055692
1597	Amp	1	165477168	165555480	0	0.058771
1598	Amp	1	165556268	165676283	0	0.058325
1599	Amp	1	165680478	165701282	0	0.060525
1600	Amp	1	165708436	165715998	0	0.057446
1601	Amp	1	165716465	165777498	0	0.056969
1602	Amp	1	165777831	165865907	0	0.055738
1603	Amp	1	165868741	165886965	0	0.057058
1604	Amp	1	165887073	165917901	0	0.059971
1605	Amp	1	165918160	165936636	0	0.058181
1606	Amp	1	165939121	165953100	0	0.062475
1607	Amp	1	165955480	165967349	0	0.058181
1608	Amp	1	165969923	166074011	0	0.058863
1609	Amp	1	166076187	166106813	0	0.058177
1610	Amp	1	166109650	166237236	0	0.055977
1611	Amp	1	166252574	166423066	0	0.057401
1612	Amp	1	166423913	166432965	0	0.060463
1613	Amp	1	166433837	166491280	0	0.05978
1614	Amp	1	166493012	166493503	0.138391	0.069782
1615	Amp	1	166503626	166522201	0.089561	0.067944
1616	Amp	1	166530590	166538349	0.034113	0.065531
1617	Amp	1	166542230	166567447	0	0.057942
1618	Amp	1	166568602	166601993	0	0.0593
1619	Amp	1	166602395	166633612	0	0.061239
1620	Amp	1	166633786	166692954	0	0.061699
1621	Amp	1	166693198	166798728	0	0.05976
1622	Amp	1	166798936	166837945	0	0.058535
1623	Amp	1	166838646	166939648	0	0.059754
1624	Amp	1	166940908	166968635	0.029031	0.065261
1625	Amp	1	166969908	166970858	0.001323	0.064042
1626	Amp	1	166977119	167001643	0	0.058535
1627	Amp	1	167006262	167018761	0	0.062418
1628	Amp	1	167019041	167046752	0.010261	0.064391
1629	Amp	1	167047086	167055371	0.001323	0.063932
1630	Amp	1	167059022	167069699	0	0.061907
1631	Amp	1	167069735	167074474	0	0.058024
1632	Amp	1	167075290	167264344	0.024438	0.065033
1633	Amp	1	167264929	167286806	0.044794	0.065971
1634	Amp	1	167290099	167364080	0.001323	0.063937
1635	Amp	1	167364874	167442147	0.040093	0.065679
1636	Amp	1	167443582	167467645	0.060686	0.066597
1637	Amp	1	167476370	167485677	0	0.06152
1638	Amp	1	167485732	167515903	0	0.059779
1639	Amp	1	167516398	167609842	0	0.058861
1640	Amp	1	167610766	167643062	0	0.062704
1641	Amp	1	167643460	167652633	0.107017	0.068412
1642	Amp	1	167652663	167726064	0.118001	0.068927

1643	Amp	1	167726262	167805599	0.08435	0.067666
1644	Amp	1	167808822	167832356	0.128324	0.069481
1645	Amp	1	167834018	167856543	0.241616	0.07398
1646	Amp	1	167860768	167878372	0.128324	0.069481
1647	Amp	1	167879396	167999787	0.107017	0.068587
1648	Amp	1	168000216	168041982	0.143787	0.070137
1649	Amp	1	168044612	168164534	0.238246	0.073713
1650	Amp	1	168168342	168212716	0.379885	0.079239
1651	Amp	1	168212867	168254026	0.47716	0.082416
1652	Amp	1	168254683	168268292	0.404718	0.080119
1653	Amp	1	168268938	168286535	0.322897	0.077205
1654	Amp	1	168287267	168367385	0.431198	0.08085
1655	Amp	1	168369504	168369504	0.34892	0.078013
1656	Amp	1	168369887	168393560	0.264858	0.074977
1657	Amp	1	168396460	168612187	0.133193	0.069681
1658	Amp	1	168612881	168626283	0.259438	0.074824
1659	Amp	1	168626605	168694160	0.335313	0.077701
1660	Amp	1	168696108	168705822	0.355319	0.07836
1661	Amp	1	168706369	168758326	0.404718	0.080027
1662	Amp	1	168759234	168774357	0.583752	0.085787
1663	Amp	1	168774536	168856794	0.404718	0.080027
1664	Amp	1	168858048	168866506	0.379885	0.079115
1665	Amp	1	168868156	168984086	0.1237	0.069078
1666	Amp	1	168984407	169007204	0.181932	0.071512
1667	Amp	1	169012926	169050984	0.164935	0.070854
1668	Amp	1	169051274	169067410	0.107017	0.068419
1669	Amp	1	169069175	169092320	0.204831	0.072489
1670	Amp	1	169092543	169115853	0.107017	0.068419
1671	Amp	1	169115881	169163534	0.170723	0.071041
1672	Amp	1	169164203	169230187	0.107017	0.068419
1673	Amp	1	169234328	169246873	0.133193	0.069649
1674	Amp	1	169247699	169299454	0.159312	0.070751
1675	Amp	1	169299505	169380527	0.138391	0.069713
1676	Amp	1	169387110	169402310	0.164935	0.070868
1677	Amp	1	169407087	169549990	0.222041	0.073077
1678	Amp	1	169550656	169600047	0.368062	0.078674
1679	Amp	1	169600207	169603709	0.404718	0.080052
1680	Amp	1	169607679	169609348	0.193543	0.071898
1681	Amp	1	169611479	169612357	0.112543	0.068765
1682	Amp	1	169613959	169640827	0.153838	0.070541
1683	Amp	1	169643195	169645462	0.215608	0.072929
1684	Amp	1	169646782	169845036	0.241616	0.074004
1685	Amp	1	169845054	169998107	0.215608	0.072929
1686	Amp	1	170000186	170115044	0.187449	0.071827
1687	Amp	1	170115361	170120080	0.204831	0.072341
1688	Amp	1	170123850	170127275	0.227787	0.073377
1689	Amp	1	170127928	170274741	0.282828	0.075635
1690	Amp	1	170275090	170300051	0.264858	0.07512
1691	Amp	1	170301927	170311826	0.311747	0.076849
1692	Amp	1	170311996	170314016	0.329205	0.077341
1693	Amp	1	170314735	170352336	0.362016	0.078503
1694	Amp	1	170352588	170486547	0.399144	0.079758
1695	Amp	1	170486923	170562245	0.329205	0.0775

1696	Amp	1	170566495	170702265	0.299433	0.076245
1697	Amp	1	170705164	170709703	0.316877	0.077052
1698	Amp	1	170715028	170731617	0.355319	0.078376
1699	Amp	1	170732312	170890018	0.373599	0.079008
1700	Amp	1	170890046	170916222	0.316877	0.077035
1701	Amp	1	170916422	171038518	0.287738	0.075873
1702	Amp	1	171038996	171205761	0.311747	0.076742
1703	Amp	1	171206104	171321991	0.19964	0.072289
1704	Amp	1	171322647	171394293	0.17597	0.07142
1705	Amp	1	171396053	171416234	0.282828	0.075769
1706	Amp	1	171416911	171462672	0.17597	0.07142
1707	Amp	1	171462927	171465297	0.193543	0.071988
1708	Amp	1	171465502	171486525	0.210594	0.072652
1709	Amp	1	171487107	171539000	0.159312	0.070696
1710	Amp	1	171541900	171547323	0.143787	0.070031
1711	Amp	1	171549304	171567285	0.215608	0.072819
1712	Amp	1	171570149	171623413	0.079652	0.067515
1713	Amp	1	171623729	171650397	0.069848	0.067023
1714	Amp	1	171661386	171697384	0.107017	0.068423
1715	Amp	1	171697692	171786349	0.159312	0.070655
1716	Amp	1	171786457	171805969	0.107017	0.068446
1717	Amp	1	171807582	171825768	0.056211	0.066371
1718	Amp	1	171826101	171858383	0.044794	0.065803
1719	Amp	1	171858930	171886179	0.019169	0.064767
1720	Amp	1	171888936	171982895	0.204831	0.072451
1721	Amp	1	171985131	172014580	0.316877	0.076961
1722	Amp	1	172015151	172017818	0.259438	0.074859
1723	Amp	1	172020948	172021331	0.094967	0.068171
1724	Amp	1	172026578	172035351	0	0.063662
1725	Amp	1	172035965	172055388	0.024438	0.065038
1726	Amp	1	172066357	172124794	0.010261	0.064406
1727	Amp	1	172124990	172202137	0.040093	0.065759
1728	Amp	1	172204489	172353657	0.069848	0.066897
1729	Amp	1	172354952	172366441	0.1237	0.069137
1730	Amp	1	172367694	172590390	0.159312	0.070668
1731	Amp	1	172591373	172646255	0.1237	0.069137
1732	Amp	1	172646342	172664835	0.153838	0.070564
1733	Amp	1	172666284	172704182	0.17597	0.071424
1734	Amp	1	172708034	172716735	0.287738	0.075964
1735	Amp	1	172718418	172740114	0.17597	0.071424
1736	Amp	1	172742283	172760464	0.227787	0.073387
1737	Amp	1	172761074	172796720	0.276783	0.075364
1738	Amp	1	172804927	172831665	0.368062	0.078738
1739	Amp	1	172835343	172893961	0.311747	0.076775
1740	Amp	1	172904853	172923812	0.222041	0.073143
1741	Amp	1	172924674	172953785	0.143787	0.069967
1742	Amp	1	172958601	173039847	0.193543	0.072018
1743	Amp	1	173040692	173139256	0.14896	0.070231
1744	Amp	1	173141814	173195672	0.050473	0.066089
1745	Amp	1	173197500	173385062	0.015369	0.064689
1746	Amp	1	173386954	173399532	0.044794	0.065832
1747	Amp	1	173401264	173485297	0.164935	0.070795
1748	Amp	1	173495207	173519606	0.255918	0.074549

1749	Amp	1	173519619	173602478	0.282828	0.07576
1750	Amp	1	173602889	173625042	0.251218	0.074384
1751	Amp	1	173631165	173813720	0.282828	0.075756
1752	Amp	1	173818553	173820391	0.293269	0.076213
1753	Amp	1	173821160	173862963	0.34892	0.078126
1754	Amp	1	173863668	173886481	0.47716	0.082312
1755	Amp	1	173887018	173922963	0.437198	0.081101
1756	Amp	1	173923522	173984699	0.515676	0.083747
1757	Amp	1	173988491	174060209	0.457501	0.081834
1758	Amp	1	174061622	174067134	0.355319	0.078194
1759	Amp	1	174071456	174157455	0.463534	0.082074
1760	Amp	1	174157604	174197402	0.557122	0.084904
1761	Amp	1	174200703	174301068	0.515676	0.083725
1762	Amp	1	174302481	174321084	0.47716	0.082324
1763	Amp	1	174323134	174325413	0.595248	0.086268
1764	Amp	1	174325607	174509998	0.609133	0.086814
1765	Amp	1	174511292	174637805	0.72817	0.090283
1766	Amp	1	174645494	174657083	0.595248	0.086282
1767	Amp	1	174664167	174742253	0.636464	0.087712
1768	Amp	1	174743384	174826008	0.681869	0.089009
1769	Amp	1	174826061	174880627	0.735486	0.090352
1770	Amp	1	174882485	174893906	0.790614	0.091949
1771	Amp	1	174897475	175009729	0.86541	0.093991
1772	Amp	1	175014071	175031221	0.899693	0.094814
1773	Amp	1	175033554	175051170	0.977077	0.09666
1774	Amp	1	175051406	175058848	0.995635	0.097112
1775	Amp	1	175059972	175060411	1.023539	0.097828
1776	Amp	1	175060796	175088986	0.950886	0.095982
1777	Amp	1	175089326	175123704	1.058188	0.09878
1778	Amp	1	175132858	175136642	1.041205	0.098239
1779	Amp	1	175140748	175236035	1.075952	0.099227
1780	Amp	1	175240742	175303532	1.023539	0.097798
1781	Amp	1	175303557	175360867	1.075952	0.099127
1782	Amp	1	175361054	175422605	0.86541	0.093875
1783	Amp	1	175429575	175458019	0.840289	0.093159
1784	Amp	1	175459821	175460288	0.751221	0.090817
1785	Amp	1	175461075	175461075	0.720659	0.09001
1786	Amp	1	175461672	175796653	0.857296	0.093701
1787	Amp	1	175800018	175825153	0.807647	0.092358
1788	Amp	1	175825331	175832596	0.781972	0.091812
1789	Amp	1	175833393	175901001	0.609133	0.086746
1790	Amp	1	175902145	175966605	0.583752	0.085922
1791	Amp	1	175969436	176046981	0.386426	0.079269
1792	Amp	1	176046996	176076520	0.494792	0.083161
1793	Amp	1	176079807	176098233	0.463534	0.082023
1794	Amp	1	176101395	176184323	0.362016	0.078555
1795	Amp	1	176184455	176241539	0.379885	0.07923
1796	Amp	1	176245434	176316411	0.276783	0.075544
1797	Amp	1	176320689	176365536	0.251218	0.074386
1798	Amp	1	176365719	176388319	0.241616	0.073929
1799	Amp	1	176390643	176402829	0.210594	0.072606
1800	Amp	1	176407436	176433120	0.246818	0.074185
1801	Amp	1	176433664	176462831	0.159312	0.070737

1802	Amp	1	176465473	176469540	0.17597	0.07129
1803	Amp	1	176469979	176497473	0.187449	0.071812
1804	Amp	1	176501633	176597181	0.153838	0.070515
1805	Amp	1	176597214	176645148	0.181932	0.071505
1806	Amp	1	176650134	176676480	0.210594	0.072686
1807	Amp	1	176677196	176809474	0.251218	0.074332
1808	Amp	1	176812307	176830007	0.373599	0.079023
1809	Amp	1	176837251	177022690	0.399144	0.07975
1810	Amp	1	177023279	177160901	0.362016	0.078569
1811	Amp	1	177161213	177179023	0.457501	0.081846
1812	Amp	1	177183180	177198483	0.362016	0.078569
1813	Amp	1	177198747	177289357	0.34892	0.078127
1814	Amp	1	177296205	177372805	0.463534	0.082029
1815	Amp	1	177375767	177457636	0.34892	0.078127
1816	Amp	1	177457869	177589544	0.30524	0.076591
1817	Amp	1	177592303	177642183	0.482379	0.082544
1818	Amp	1	177644788	177768254	0.536238	0.084316
1819	Amp	1	177768529	177946677	0.589605	0.086123
1820	Amp	1	177948344	177949151	0.576183	0.085601
1821	Amp	1	177956704	177966068	0.470509	0.082194
1822	Amp	1	177966140	177966140	0.457501	0.08175
1823	Amp	1	177968306	177999481	0.437198	0.081197
1824	Amp	1	177999924	178030632	0.463534	0.081896
1825	Amp	1	178030933	178097064	0.450702	0.081443
1826	Amp	1	178099044	178173003	0.494792	0.083069
1827	Amp	1	178173061	178225389	0.457501	0.081662
1828	Amp	1	178226123	178275600	0.373599	0.078864
1829	Amp	1	178277987	178307749	0.246818	0.074172
1830	Amp	1	178321672	178370370	0.210594	0.072547
1831	Amp	1	178373684	178438683	0.329205	0.077466
1832	Amp	1	178445938	178463010	0.204831	0.072381
1833	Amp	1	178465059	178475772	0.30524	0.07658
1834	Amp	1	178476680	178591024	0.181932	0.07147
1835	Amp	1	178592776	178734846	0.060686	0.066551
1836	Amp	1	178735054	178746125	0.100827	0.068201
1837	Amp	1	178756094	178756094	0.153838	0.070374
1838	Amp	1	178761953	178789212	0.069848	0.066944
1839	Amp	1	178789230	179164599	0.001323	0.063989
1840	Amp	1	179164628	179174661	0	0.063263
1841	Amp	1	179180155	179283457	0.128324	0.069369
1842	Amp	1	179284332	179402260	0.08435	0.067718
1843	Amp	1	179403407	179670065	0.069848	0.066949
1844	Amp	1	179672700	179748696	0.040093	0.065704
1845	Amp	1	179749120	179931509	0.056211	0.066237
1846	Amp	1	179931843	180018679	0.034113	0.065538
1847	Amp	1	180018767	180047080	0.073902	0.067297
1848	Amp	1	180047451	180058113	0.118001	0.068892
1849	Amp	1	180065205	180083659	0.079652	0.067402
1850	Amp	1	180090850	180187532	0.094967	0.068118
1851	Amp	1	180187565	180245029	0.19964	0.072188
1852	Amp	1	180246133	180310767	0.299433	0.076354
1853	Amp	1	180311843	180370763	0.19964	0.072188
1854	Amp	1	180370828	180385127	0.238246	0.07369

1855	Amp	1	180385430	180385430	0.193543	0.071931
1856	Amp	1	180391765	180400633	0.128324	0.069384
1857	Amp	1	180403643	180420057	0.233782	0.073413
1858	Amp	1	180421908	180444407	0.241616	0.073963
1859	Amp	1	180456073	180456073	0.404718	0.079972
1860	Amp	1	180457145	180457145	0.543344	0.084674
1861	Amp	1	180462803	180499955	0.609133	0.086707
1862	Amp	1	180501690	180526534	0.522881	0.083858
1863	Amp	1	180534437	180549122	0.342649	0.077849
1864	Amp	1	180552234	180645596	0.282828	0.075676
1865	Amp	1	180647576	180674749	0.241616	0.074031
1866	Amp	1	180675702	180723679	0.193543	0.071998
1867	Amp	1	180723846	180977656	0.153838	0.070496
1868	Amp	1	180980492	181003505	0.060686	0.066569
1869	Amp	1	181003821	181007637	0.044794	0.065894
1870	Amp	1	181007755	181049280	0.112543	0.068678
1871	Amp	1	181051045	181070775	0.044794	0.065894
1872	Amp	1	181073225	181095233	0.079652	0.067336
1873	Amp	1	181095813	181168596	0.14896	0.070224
1874	Amp	1	181168684	181192707	0.204831	0.072369
1875	Amp	1	181197543	181449389	0.128324	0.069481
1876	Amp	1	181451900	181460870	0.238246	0.073681
1877	Amp	1	181468422	181600755	0.128324	0.069481
1878	Amp	1	181605028	181608185	0.181932	0.071645
1879	Amp	1	181608497	181656566	0.227787	0.073238
1880	Amp	1	181657172	181800418	0.181932	0.071645
1881	Amp	1	181801401	181872914	0.153838	0.070424
1882	Amp	1	181875086	181954433	0.19964	0.07214
1883	Amp	1	181956295	181984574	0.143787	0.069995
1884	Amp	1	181985583	182000768	0.153838	0.070448
1885	Amp	1	182001314	182012245	0.112543	0.068732
1886	Amp	1	182013255	182118842	0.153838	0.070484
1887	Amp	1	182120000	182331438	0.255918	0.074691
1888	Amp	1	182333172	182406106	0.241616	0.073975
1889	Amp	1	182407094	182442493	0.299433	0.076236
1890	Amp	1	182442872	182500607	0.241616	0.073975
1891	Amp	1	182503303	182557704	0.276783	0.075548
1892	Amp	1	182558274	182620196	0.238246	0.073796
1893	Amp	1	182623201	182670397	0.368062	0.078723
1894	Amp	1	182671503	182765453	0.355319	0.07819
1895	Amp	1	182767920	182822649	0.576183	0.085582
1896	Amp	1	182822762	182835324	0.355319	0.07819
1897	Amp	1	182840976	182877607	0.143787	0.069954
1898	Amp	1	182886563	182905678	0.181932	0.071491
1899	Amp	1	182907516	182950467	0.251218	0.07429
1900	Amp	1	182954587	183189008	0.282828	0.075644
1901	Amp	1	183189839	183260243	0.368062	0.078689
1902	Amp	1	183265106	183268177	0.392611	0.079597
1903	Amp	1	183271294	183308836	0.450702	0.081493
1904	Amp	1	183313440	183340114	0.508568	0.083395
1905	Amp	1	183340683	183444030	0.450702	0.081434
1906	Amp	1	183444048	183485747	0.431198	0.080879
1907	Amp	1	183491131	183491131	0.470509	0.082158

1908	Amp	1	183491979	183762567	0.602643	0.08652
1909	Amp	1	183762984	183835475	0.417873	0.080414
1910	Amp	1	183837140	183868762	0.276783	0.075488
1911	Amp	1	183869871	183919724	0.287738	0.075968
1912	Amp	1	183920418	184004377	0.238246	0.073803
1913	Amp	1	184007775	184038024	0.34892	0.077954
1914	Amp	1	184043572	184093663	0.329205	0.077475
1915	Amp	1	184098823	184099197	0.392611	0.079561
1916	Amp	1	184101360	184116285	0.227787	0.073303
1917	Amp	1	184116899	184195951	0.17597	0.071236
1918	Amp	1	184199379	184425123	0.316877	0.07695
1919	Amp	1	184425822	184593415	0.368062	0.078631
1920	Amp	1	184593758	184655059	0.417873	0.080509
1921	Amp	1	184658379	184729797	0.399144	0.079865
1922	Amp	1	184738407	184740448	0.133193	0.069559
1923	Amp	1	184746073	184748591	0.089561	0.067878
1924	Amp	1	184751581	184936129	0.181932	0.071468
1925	Amp	1	184937728	184959033	0.089561	0.067878
1926	Amp	1	184960470	185209154	0.193543	0.072021
1927	Amp	1	185212352	185218711	0.355319	0.078182
1928	Amp	1	185221515	185244009	0.368062	0.078783
1929	Amp	1	185248022	185311039	0.487495	0.082739
1930	Amp	1	185320236	185331277	0.293269	0.076133
1931	Amp	1	185333367	185353357	0.444474	0.081288
1932	Amp	1	185353412	185444650	0.457501	0.08185
1933	Amp	1	185452895	185496837	0.4115	0.080304
1934	Amp	1	185498143	185507583	0.529451	0.084122
1935	Amp	1	185517348	185520547	0.457501	0.081706
1936	Amp	1	185520620	185560560	0.392611	0.079596
1937	Amp	1	185560585	185650460	0.329205	0.077509
1938	Amp	1	185661979	185664628	0.287738	0.075973
1939	Amp	1	185671086	185677426	0.222041	0.073174
1940	Amp	1	185680701	185772037	0.246818	0.074103
1941	Amp	1	185773715	185795183	0.210594	0.07253
1942	Amp	1	185795687	185807555	0.241616	0.073962
1943	Amp	1	185810907	186007923	0.255918	0.074618
1944	Amp	1	186008738	186179382	0.238246	0.07371
1945	Amp	1	186179414	186199149	0.222041	0.073109
1946	Amp	1	186202254	186297486	0.238246	0.073699
1947	Amp	1	186301689	186321654	0.255918	0.074574
1948	Amp	1	186325773	186393508	0.264858	0.07502
1949	Amp	1	186393987	186502169	0.251218	0.074429
1950	Amp	1	186506777	186522908	0.227787	0.073274
1951	Amp	1	186526209	186563439	0.17597	0.071372
1952	Amp	1	186563874	186626970	0.133193	0.069565
1953	Amp	1	186629010	186647778	0.044794	0.065916
1954	Amp	1	186648585	186773815	0.069848	0.06702
1955	Amp	1	186779571	186805761	0.044794	0.065963
1956	Amp	1	186808580	186858008	0.015369	0.064531
1957	Amp	1	186863290	186967030	0	0.061451
1958	Amp	1	186967078	186973994	0	0.06206
1959	Amp	1	186976597	187185171	0	0.058492
1960	Amp	1	187186451	187234882	0	0.055212

1961	Amp	1	187236541	187276141	0	0.057242
1962	Amp	1	187278654	187289221	0	0.056403
1963	Amp	1	187294725	187336241	0	0.053566
1964	Amp	1	187339106	187365248	0	0.056027
1965	Amp	1	187366710	187370743	0	0.052585
1966	Amp	1	187372275	187408877	0	0.056661
1967	Amp	1	187411591	187421088	0	0.057208
1968	Amp	1	187425229	187450843	0	0.053132
1969	Amp	1	187457697	187487566	0	0.05176
1970	Amp	1	187488040	187539704	0	0.050656
1971	Amp	1	187540262	187615567	0	0.049573
1972	Amp	1	187618200	187618547	0	0.054754
1973	Amp	1	187620412	187638601	0	0.052724
1974	Amp	1	187642100	187654839	0	0.055974
1975	Amp	1	187654933	187682531	0	0.052724
1976	Amp	1	187687043	187721405	0	0.055598
1977	Amp	1	187721842	187818129	0	0.051157
1978	Amp	1	187825821	187853859	0	0.050418
1979	Amp	1	187870546	187883709	0	0.051894
1980	Amp	1	187884722	187893525	0	0.050048
1981	Amp	1	187897338	187982615	0	0.055843
1982	Amp	1	187986168	188025291	0	0.055103
1983	Amp	1	188027894	188118001	0	0.049308
1984	Amp	1	188118961	188150986	0	0.051199
1985	Amp	1	188151783	188165938	0	0.052168
1986	Amp	1	188173876	188178429	0	0.048969
1987	Amp	1	188181210	188212545	0	0.050786
1988	Amp	1	188213590	188223362	0	0.047912
1989	Amp	1	188227636	188327577	0	0.048551
1990	Amp	1	188329154	188397151	0	0.050237
1991	Amp	1	188401948	188403731	0	0.047266
1992	Amp	1	188406549	188414043	0	0.063051
1993	Amp	1	188418319	188555171	0.056211	0.066384
1994	Amp	1	188556165	188596872	0.015369	0.064698
1995	Amp	1	188599673	188622609	0	0.061365
1996	Amp	1	188626495	188626495	0.019169	0.064784
1997	Amp	1	188629036	188670882	0.044794	0.065843
1998	Amp	1	188673775	188750007	0.073902	0.067308
1999	Amp	1	188750389	188751900	0.005328	0.064179
2000	Amp	1	188758640	188782249	0.118001	0.068982
2001	Amp	1	188782477	188838716	0.193543	0.071888
2002	Amp	1	188844382	188874531	0.164935	0.07083
2003	Amp	1	188875379	188901236	0.181932	0.071551
2004	Amp	1	188901319	188920275	0.143787	0.070086
2005	Amp	1	188920662	188951767	0.010261	0.064477
2006	Amp	1	188953362	189015846	0.029031	0.065332
2007	Amp	1	189015936	189041209	0.094967	0.067968
2008	Amp	1	189041527	189063349	0.073902	0.067113
2009	Amp	1	189065103	189115234	0.107017	0.068506
2010	Amp	1	189120286	189162715	0.118001	0.068961
2011	Amp	1	189167111	189173963	0.210594	0.07254
2012	Amp	1	189177925	189186324	0.1237	0.069187
2013	Amp	1	189190130	189190942	0.204831	0.0724

2014	Amp	1	189193276	189287661	0.005328	0.064061
2015	Amp	1	189289682	189289682	0	0.063608
2016	Amp	1	189577840	189622012	0.029031	0.065349
2017	Amp	1	189622627	189647647	0.079652	0.06742
2018	Amp	1	189648210	189738542	0.089561	0.067933
2019	Amp	1	189740290	189745259	0.019169	0.06472
2020	Amp	1	189749745	189750106	0.034113	0.065426
2021	Amp	1	189752676	189768259	0.1237	0.069136
2022	Amp	1	189769135	189778301	0.034113	0.065426
2023	Amp	1	189782483	189871223	0.118001	0.068909
2024	Amp	1	189871388	189890589	0.143787	0.070015
2025	Amp	1	189897041	190051072	0.30524	0.076456
2026	Amp	1	190051258	190145433	0.282828	0.075593
2027	Amp	1	190147724	190192022	0.373599	0.078883
2028	Amp	1	190193057	190309133	0.404718	0.080114
2029	Amp	1	190310772	190415169	0.355319	0.078223
2030	Amp	1	190415508	190415508	0.322897	0.077117
2031	Amp	1	190420093	190476968	0.259438	0.074721
2032	Amp	1	190480221	190516188	0.187449	0.07181
2033	Amp	1	190516219	190530345	0.170723	0.071103
2034	Amp	1	190530830	190530830	0.015369	0.064662
2035	Amp	1	190535401	190585831	0	0.063768
2036	Amp	1	190588796	190599315	0.015369	0.064545
2037	Amp	1	190599874	190678694	0	0.063768
2038	Amp	1	190684481	190774327	0.128324	0.069295
2039	Amp	1	190777243	190848699	0.015369	0.064492
2040	Amp	1	190848975	190851264	0.029031	0.065193
2041	Amp	1	190851375	191001526	0.015369	0.06468
2042	Amp	1	191002526	191072602	0.060686	0.066634
2043	Amp	1	191078211	191118103	0.112543	0.068808
2044	Amp	1	191119991	191149747	0.164935	0.070877
2045	Amp	1	191150262	191411626	0.14896	0.070156
2046	Amp	1	191413381	191511018	0.164935	0.070846
2047	Amp	1	191512828	191563902	0.118001	0.068893
2048	Amp	1	191568765	191726069	0.107017	0.068439
2049	Amp	1	191726116	191780043	0.094967	0.067993
2050	Amp	1	191782793	191822761	0.107017	0.06847
2051	Amp	1	191822781	191825299	0	0.062944
2052	Amp	1	191839485	191958940	0.079652	0.067463
2053	Amp	1	191961161	191978644	0.118001	0.068969
2054	Amp	1	191979749	192000853	0.164935	0.070857
2055	Amp	1	192001466	192026685	0.094967	0.06799
2056	Amp	1	192029121	192102980	0.044794	0.065919
2057	Amp	1	192105392	192242406	0.094967	0.067968
2058	Amp	1	192242712	192401769	0.050473	0.06608
2059	Amp	1	192405390	192409892	0	0.06277
2060	Amp	1	192415667	192447070	0.050473	0.066051
2061	Amp	1	192449678	192478814	0.170723	0.071025
2062	Amp	1	192480803	192511133	0.133193	0.069633
2063	Amp	1	192517983	192526044	0.238246	0.073627
2064	Amp	1	192530281	192550123	0.276783	0.075561
2065	Amp	1	192553262	192565172	0.233782	0.073492
2066	Amp	1	192568378	192568734	0.255918	0.074688

2067	Amp	1	192568859	192617557	0.271552	0.075284
2068	Amp	1	192617726	192646932	0.238246	0.073778
2069	Amp	1	192647065	192660013	0.311747	0.076695
2070	Amp	1	192662028	192682753	0.210594	0.072701
2071	Amp	1	192686706	192695463	0.138391	0.069896
2072	Amp	1	192698054	192699663	0.193543	0.072078
2073	Amp	1	192700915	192784909	0.1237	0.06916
2074	Amp	1	192785884	192785884	0.019169	0.064715
2075	Amp	1	192791407	192862166	0	0.062099
2076	Amp	1	192863346	192901661	0.015369	0.064666
2077	Amp	1	192925414	192925414	0	0.062942
2078	Amp	1	192927304	192928246	0	0.059363
2079	Amp	1	192928879	192990627	0	0.056796
2080	Amp	1	192992254	193028120	0	0.055919
2081	Amp	1	193033449	193095659	0	0.061969
2082	Amp	1	193096480	193175517	0.069848	0.067067
2083	Amp	1	193178606	193182180	0.222041	0.073077
2084	Amp	1	193182722	193264546	0.187449	0.071859
2085	Amp	1	193268671	193640634	0.069848	0.066995
2086	Amp	1	193648539	193766389	0.133193	0.069502
2087	Amp	1	193768328	193794823	0.299433	0.076416
2088	Amp	1	193795431	193809717	0.437198	0.081053
2089	Amp	1	193819007	193876563	0.246818	0.074139
2090	Amp	1	193883425	193912179	0.543344	0.084534
2091	Amp	1	193912194	193928014	0.6737	0.08865
2092	Amp	1	193929647	193986523	0.543344	0.084534
2093	Amp	1	193987354	194114683	0.557122	0.085052
2094	Amp	1	194114767	194141080	0.494792	0.082986
2095	Amp	1	194141809	194193617	0.457501	0.081736
2096	Amp	1	194194374	194196381	0.335313	0.077733
2097	Amp	1	194200660	194204667	0.311747	0.076769
2098	Amp	1	194205014	194220072	0.056211	0.066373
2099	Amp	1	194224389	194243354	0	0.060323
2100	Amp	1	194245625	194245625	0	0.058847
2101	Amp	1	194256252	194294703	0	0.05837
2102	Amp	1	194296089	194329616	0	0.060387
2103	Amp	1	194329764	194433409	0	0.056507
2104	Amp	1	194435347	194454761	0	0.058615
2105	Amp	1	194454817	194454817	0	0.059673
2106	Amp	1	194495592	194495592	0.089561	0.067825
2107	Amp	1	194498192	194518804	0.133193	0.069616
2108	Amp	1	194522000	194548636	0.17597	0.071368
2109	Amp	1	194549268	194566260	0.215608	0.072853
2110	Amp	1	194567955	194659639	0.255918	0.074664
2111	Amp	1	194669322	194696048	0.241616	0.073963
2112	Amp	1	194696303	194733145	0.282828	0.075668
2113	Amp	1	194733321	194745027	0.241616	0.073857
2114	Amp	1	194748711	194755861	0.282828	0.075581
2115	Amp	1	194758501	194911254	0.241616	0.073829
2116	Amp	1	194911968	195040200	0.079652	0.067361
2117	Amp	1	195041083	195043680	0.164935	0.070972
2118	Amp	1	195043750	195073186	0.107017	0.068465
2119	Amp	1	195077011	195080943	0.019169	0.064859

2120	Amp	1	195083472	195167781	0.210594	0.072552
2121	Amp	1	195168109	195250032	0.17597	0.071356
2122	Amp	1	195250254	195250405	0.08435	0.067745
2123	Amp	1	195250500	195595397	0.040093	0.065637
2124	Amp	1	195600016	195621521	0.056211	0.066248
2125	Amp	1	195627646	195641742	0.065715	0.066765
2126	Amp	1	195642162	195690566	0.010261	0.064359
2127	Amp	1	195693125	195726758	0	0.063285
2128	Amp	1	195727623	195792076	0.010261	0.064428
2129	Amp	1	195792176	195802332	0.040093	0.065689
2130	Amp	1	195805164	195832231	0.015369	0.064546
2131	Amp	1	195833891	195838363	0	0.063729
2132	Amp	1	195838524	195851760	0.08435	0.067734
2133	Amp	1	195906930	195907162	0.143787	0.070128
2134	Amp	1	195907438	195922485	0.215608	0.072748
2135	Amp	1	195922628	195946353	0.143787	0.070042
2136	Amp	1	195946450	195949820	0.210594	0.072685
2137	Amp	1	195958262	196060606	0.311747	0.076706
2138	Amp	1	196061138	196104662	0.246818	0.074086
2139	Amp	1	196104928	196126269	0.143787	0.070081
2140	Amp	1	196129145	196131960	0.060686	0.066553
2141	Amp	1	196132290	196134829	0.005328	0.064239
2142	Amp	1	196135852	196163723	0	0.062753
2143	Amp	1	196167513	196196156	0.015369	0.064498
2144	Amp	1	196196667	196204925	0	0.062544
2145	Amp	1	196206589	196232581	0.019169	0.064887
2146	Amp	1	196234764	196261869	0.010261	0.064338
2147	Amp	1	196267893	196386394	0	0.062664
2148	Amp	1	196387260	196433683	0.015369	0.064514
2149	Amp	1	196435678	196463242	0	0.062014
2150	Amp	1	196466817	196619833	0.034113	0.065423
2151	Amp	1	196624996	196627527	0	0.057851
2152	Amp	1	196627608	196654830	0	0.059082
2153	Amp	1	196659065	196716846	0	0.056807
2154	Amp	1	196716854	196729142	0	0.054514
2155	Amp	1	196973325	196995755	0	0.055077
2156	Amp	1	196996904	197026072	0	0.061317
2157	Amp	1	197027174	197048593	0	0.059439
2158	Amp	1	197049016	197066454	0	0.060846
2159	Amp	1	197067169	197252000	0	0.060284
2160	Amp	1	197259404	197350615	0.005328	0.064143
2161	Amp	1	197351568	197368538	0.015369	0.064613
2162	Amp	1	197369095	197408930	0.024438	0.065116
2163	Amp	1	197411929	197411929	0.065715	0.066754
2164	Amp	1	197412575	197419110	0.015369	0.06451
2165	Amp	1	197420906	197581891	0.056211	0.066362
2166	Amp	1	197584722	197585329	0	0.062503
2167	Amp	1	197586730	197644121	0.001323	0.063991
2168	Amp	1	197644544	197666004	0.050473	0.066145
2169	Amp	1	197674468	197785837	0.100827	0.068315
2170	Amp	1	197786089	197920141	0.193543	0.071956
2171	Amp	1	197922672	198088842	0.311747	0.076749
2172	Amp	1	198089525	198105634	0.424557	0.080587

2173	Amp	1	198107475	198146432	0.311747	0.076749
2174	Amp	1	198149689	198155004	0.222041	0.073107
2175	Amp	1	198159104	198220288	0.251218	0.074308
2176	Amp	1	198220335	198293361	0.227787	0.073379
2177	Amp	1	198293960	198333605	0.073902	0.067139
2178	Amp	1	198333791	198384814	0.170723	0.07106
2179	Amp	1	198387041	198448649	0.19964	0.072169
2180	Amp	1	198449365	198456622	0.159312	0.070761
2181	Amp	1	198457478	198611125	0.241616	0.07394
2182	Amp	1	198611744	198698592	0.210594	0.072739
2183	Amp	1	198701871	198759907	0.233782	0.073581
2184	Amp	1	198761372	198782411	0.271552	0.075181
2185	Amp	1	198782778	198862478	0.299433	0.076249
2186	Amp	1	198865446	198925600	0.246818	0.074095
2187	Amp	1	198926482	198979470	0.238246	0.073613
2188	Amp	1	198984482	198992439	0.138391	0.069782
2189	Amp	1	198999634	199055444	0.444474	0.081361
2190	Amp	1	199057638	199058451	0.494792	0.083094
2191	Amp	1	199065111	199134811	0.437198	0.081111
2192	Amp	1	199134866	199138468	0.550755	0.084814
2193	Amp	1	199145262	199149585	0.522881	0.08387
2194	Amp	1	199149616	199229830	0.589605	0.086204
2195	Amp	1	199234706	199242229	0.630438	0.08732
2196	Amp	1	199244066	199295493	0.595248	0.086253
2197	Amp	1	199296619	199302405	0.758088	0.091036
2198	Amp	1	199303429	199324265	0.781972	0.09169
2199	Amp	1	199330900	199358986	0.857296	0.093662
2200	Amp	1	199359366	199461256	0.790614	0.092023
2201	Amp	1	199463326	199517915	0.293269	0.076087
2202	Amp	1	199524498	199596882	0.34892	0.078146
2203	Amp	1	199609538	199626239	0.392611	0.079633
2204	Amp	1	199626639	199653960	0.450702	0.081639
2205	Amp	1	199654405	199666386	0.470509	0.082121
2206	Amp	1	199667752	199667752	0.482379	0.082703
2207	Amp	1	199669410	199670408	0.529451	0.084083
2208	Amp	1	199671014	199731024	0.583752	0.085831
2209	Amp	1	199740258	199753720	0.563096	0.085222
2210	Amp	1	199762642	199825822	0.515676	0.083734
2211	Amp	1	199828508	199860487	0.529451	0.08419
2212	Amp	1	199860664	199981355	0.508568	0.083534
2213	Amp	1	199981422	199999655	0.529451	0.084145
2214	Amp	1	200000275	200013822	0.595248	0.086215
2215	Amp	1	200013905	200067662	0.630438	0.087401
2216	Amp	1	200069798	200087110	0.589605	0.086188
2217	Amp	1	200091928	200110652	0.906385	0.094926
2218	Amp	1	200110831	200117682	0.995635	0.097168
2219	Amp	1	200129162	200138268	0.906385	0.095097
2220	Amp	1	200143542	200154834	0.815228	0.092651
2221	Amp	1	200158414	200165217	0.781972	0.09183
2222	Amp	1	200166210	200223150	0.915698	0.095241
2223	Amp	1	200224737	200309270	1.093834	0.099609
2224	Amp	1	200310992	200328034	1.261401	0.103504
2225	Amp	1	200328165	200366156	1.398396	0.106556

2226	Amp	1	200367054	200367794	1.531891	0.109571
2227	Amp	1	200367818	200414772	1.717671	0.113416
2228	Amp	1	200418964	200462515	2.202226	0.123273
2229	Amp	1	200463000	200488758	2.311329	0.125399
2230	Amp	1	200489055	200491435	2.060912	0.120315
2231	Amp	1	200493317	200500542	2.027585	0.119765
2232	Amp	1	200500941	200513783	1.994915	0.119204
2233	Amp	1	200513978	200555421	2.256542	0.124298
2234	Amp	1	200555555	200555555	1.994915	0.119204
2235	Amp	1	200558247	200581819	1.865019	0.116428
2236	Amp	1	200581830	200598153	1.887378	0.116881
2237	Amp	1	200598273	200623419	1.908279	0.117323
2238	Amp	1	200624306	200634574	1.833307	0.115764
2239	Amp	1	200638338	200713695	1.717671	0.113522
2240	Amp	1	200720587	200740864	1.61378	0.111282
2241	Amp	1	200762428	200762428	1.833307	0.115803
2242	Amp	1	200769143	200805955	1.919241	0.117486
2243	Amp	1	200811577	200815678	1.780186	0.114763
2244	Amp	1	200874640	200894147	1.706315	0.113276
2245	Amp	1	200894458	200899752	1.823153	0.115714
2246	Amp	1	200902088	200932301	1.876242	0.116723
2247	Amp	1	200932434	200937345	1.759227	0.114285
2248	Amp	1	200941404	200949283	1.812192	0.115473
2249	Amp	1	200951185	201000615	1.790459	0.114926
2250	Amp	1	201000814	201011570	1.655056	0.112079
2251	Amp	1	201011593	201051723	1.68612	0.112736
2252	Amp	1	201052097	201085226	1.823153	0.115671
2253	Amp	1	201091737	201160199	1.984015	0.118943
2254	Amp	1	201161686	201229830	1.823153	0.115671
2255	Amp	1	201230521	201286071	1.759227	0.114412
2256	Amp	1	201286142	201324310	1.790459	0.114903
2257	Amp	1	201324842	201390569	2.016585	0.119557
2258	Amp	1	201390691	201392869	2.137445	0.121966
2259	Amp	1	201395306	201416370	1.96212	0.118395
2260	Amp	1	201416869	201417428	2.005947	0.11942
2261	Amp	1	201420557	201433920	1.531891	0.109582
2262	Amp	1	201438654	201469988	1.593878	0.110913
2263	Amp	1	201475011	201475011	1.706315	0.11318
2264	Amp	1	201475570	201491124	1.542638	0.10973
2265	Amp	1	201491429	201568206	1.472629	0.108172
2266	Amp	1	201568357	201587242	1.426786	0.107148
2267	Amp	1	201590976	201593525	1.645511	0.112034
2268	Amp	1	201593647	201669082	1.542638	0.109767
2269	Amp	1	201674609	201688581	1.472629	0.108187
2270	Amp	1	201688922	201689321	1.696256	0.112955
2271	Amp	1	201694973	201700579	1.865019	0.116527
2272	Amp	1	201701463	201710199	1.801583	0.115197
2273	Amp	1	201712280	201730267	1.780186	0.114759
2274	Amp	1	201730926	201745627	1.876242	0.116689
2275	Amp	1	201748593	201770802	1.769915	0.114551
2276	Amp	1	201771566	201857212	1.843664	0.116083
2277	Amp	1	201858250	201867633	1.748606	0.114153
2278	Amp	1	201870870	201877998	1.823153	0.115534

2279	Amp	1	201878660	201908738	1.908279	0.117323
2280	Amp	1	201909348	201943780	1.812192	0.11547
2281	Amp	1	201945136	201965661	1.865019	0.116459
2282	Amp	1	201966543	202003761	1.972776	0.118642
2283	Amp	1	202003827	202023038	2.127614	0.121752
2284	Amp	1	202025075	202043607	2.192012	0.123033
2285	Amp	1	202049090	202065182	2.290039	0.125065
2286	Amp	1	202067879	202105425	2.390849	0.126982
2287	Amp	1	202105640	202121513	2.479057	0.128678
2288	Amp	1	202123452	202135968	2.649655	0.131905
2289	Amp	1	202137669	202154898	2.490318	0.128795
2290	Amp	1	202155381	202192585	2.457251	0.128288
2291	Amp	1	202196417	202235085	2.626052	0.131393
2292	Amp	1	202236195	202240605	2.548433	0.130013
2293	Amp	1	202240839	202275394	2.637742	0.131603
2294	Amp	1	202276389	202363431	2.696333	0.132735
2295	Amp	1	202374905	202407067	2.812598	0.135056
2296	Amp	1	202409128	202514621	3.138463	0.140994
2297	Amp	1	202517393	202606492	3.275143	0.143612
2298	Amp	1	202608577	202691087	3.127699	0.140883
2299	Amp	1	202693228	202707297	3.057106	0.139503
2300	Amp	1	202708050	202870075	3.172599	0.141693
2301	Amp	1	202872590	202890472	3.195626	0.142182
2302	Amp	1	202890939	203110336	3.230405	0.142709
2303	Amp	1	203111997	203194239	3.09333	0.140117
2304	Amp	1	203196253	203281365	2.975629	0.137926
2305	Amp	1	203286365	203286690	3.240397	0.1429
2306	Amp	1	203290119	203389048	3.529607	0.148108
2307	Amp	1	203392045	203438495	3.690482	0.150964
2308	Amp	1	203440922	203469200	3.529607	0.148108
2309	Amp	1	203469844	203505281	3.852343	0.153876
2310	Amp	1	203506457	203516679	3.566107	0.148757
2311	Amp	1	203518691	203679508	3.701758	0.151345
2312	Amp	1	203681367	203765857	3.9978	0.156379
2313	Amp	1	203765965	203770089	4.062894	0.15757
2314	Amp	1	203770735	203782213	3.946233	0.155599
2315	Amp	1	203782843	203800988	3.701758	0.151223
2316	Amp	1	203812288	203827987	4.388129	0.162813
2317	Amp	1	203828599	203832923	4.457399	0.163738
2318	Amp	1	203839569	203849615	3.862695	0.154168
2319	Amp	1	203856548	203881584	3.749777	0.152043
2320	Amp	1	203882146	203917154	4.050096	0.15742
2321	Amp	1	203917678	203959284	4.304307	0.161486
2322	Amp	1	203959967	204008420	4.388129	0.162708
2323	Amp	1	204014863	204042909	3.690482	0.151118
2324	Amp	1	204046536	204100830	3.862695	0.154103
2325	Amp	1	204101096	204106020	3.887411	0.154604
2326	Amp	1	204109668	204118644	3.985868	0.156272
2327	Amp	1	204118865	204141532	4.196711	0.159735
2328	Amp	1	204145368	204149232	4.416011	0.163212
2329	Amp	1	204153598	204191898	4.050096	0.157312
2330	Amp	1	204191969	204196055	3.985868	0.15616
2331	Amp	1	204199728	204206600	4.062894	0.15756

2332	Amp	1	204207826	204264648	3.749777	0.152183
2333	Amp	1	204267092	204292867	3.399017	0.145817
2334	Amp	1	204300255	204318948	3.6657	0.150687
2335	Amp	1	204319399	204343302	3.701758	0.151254
2336	Amp	1	204345091	204384824	3.628913	0.149914
2337	Amp	1	204386116	204389868	3.749777	0.152047
2338	Amp	1	204389944	204390205	3.713605	0.151559
2339	Amp	1	204390258	204480306	3.554363	0.148615
2340	Amp	1	204483000	204498548	3.516868	0.147958
2341	Amp	1	204501001	204503151	3.422847	0.146262
2342	Amp	1	204509302	204519677	3.323812	0.144472
2343	Amp	1	204519774	204543632	3.29895	0.143981
2344	Amp	1	204543704	204544372	3.195626	0.142044
2345	Amp	1	204544437	204545241	2.89426	0.136397
2346	Amp	1	204545377	204545818	2.661431	0.132124
2347	Amp	1	204545827	204545831	2.812598	0.134916
2348	Amp	1	204545875	204545881	3.033246	0.139177
2349	Amp	1	204545895	204545895	3.195626	0.142086
2350	Amp	1	204546171	204549714	3.335819	0.144714
2351	Amp	1	204550059	204550059	3.516868	0.147919
2352	Amp	1	204551414	204561796	3.5415	0.148385
2353	Amp	1	204569214	204569214	3.29895	0.144
2354	Amp	1	204570657	204641862	3.230405	0.142779
2355	Amp	1	204643390	204657924	3.446786	0.14673
2356	Amp	1	204660608	204696235	3.6657	0.150481
2357	Amp	1	204696604	204745091	3.411175	0.145997
2358	Amp	1	204745437	204745437	3.09333	0.140218
2359	Amp	1	204748070	204790022	3.033246	0.139027
2360	Amp	1	204795499	204822598	2.871582	0.136093
2361	Amp	1	204822873	204833425	2.696333	0.132866
2362	Amp	1	204835790	204868236	2.837591	0.135459
2363	Amp	1	204872832	204943715	2.755154	0.13379
2364	Amp	1	204945448	204967812	2.673338	0.132348
2365	Amp	1	204969312	204974157	2.788392	0.134624
2366	Amp	1	204974774	205059787	2.765626	0.134058
2367	Amp	1	205060536	205075211	2.637742	0.131715
2368	Amp	1	205076074	205116047	2.848911	0.135684
2369	Amp	1	205116621	205125323	2.800837	0.13468
2370	Amp	1	205125761	205128140	3.160681	0.141477
2371	Amp	1	205130831	205250959	2.614791	0.131331
2372	Amp	1	205252287	205252531	2.649655	0.131846
2373	Amp	1	205253759	205258721	3.505046	0.147783
2374	Amp	1	205258769	205276909	3.422847	0.146268
2375	Amp	1	205277135	205277135	3.335819	0.144736
2376	Amp	1	205280867	205375780	3.311026	0.144214
2377	Amp	1	205377688	205541996	2.457251	0.128278
2378	Amp	1	205543150	205626625	3.09333	0.140138
2379	Amp	1	205629982	205629982	2.457251	0.128278
2380	Amp	1	205630152	205668749	2.537294	0.129828
2381	Amp	1	205672620	205770588	2.743935	0.133545
2382	Amp	1	205772688	205778675	2.537294	0.129849
2383	Amp	1	205782261	205816357	2.345513	0.126132
2384	Amp	1	205816804	205947395	2.22459	0.123571

2385	Amp	1	205949558	206043160	2.256542	0.124206
2386	Amp	1	206047243	206097334	2.22459	0.123593
2387	Amp	1	206100617	206403492	2.300463	0.125083
2388	Amp	1	206410277	206421718	3.127699	0.140893
2389	Amp	1	206422085	206429946	3.172599	0.141613
2390	Amp	1	206432695	206463793	3.207115	0.142371
2391	Amp	1	206464459	206513930	3.264473	0.143409
2392	Amp	1	206514025	206560716	3.127699	0.14074
2393	Amp	1	206561058	206589271	3.361849	0.145206
2394	Amp	1	206591922	206694649	3.578505	0.149016
2395	Amp	1	206694900	206770888	3.422847	0.146188
2396	Amp	1	206771701	206858585	3.690482	0.151129
2397	Amp	1	206859406	206887488	3.762283	0.152376
2398	Amp	1	206887802	206912849	3.874391	0.154194
2399	Amp	1	206913624	206914236	3.654159	0.150301
2400	Amp	1	206918124	206944883	3.8021	0.152871
2401	Amp	1	206950415	206968531	3.701758	0.151276
2402	Amp	1	206969038	206969038	3.726055	0.151724
2403	Amp	1	206969762	206976502	3.615896	0.149673
2404	Amp	1	206980414	206989088	3.103562	0.140317
2405	Amp	1	206989616	207022992	3.286256	0.143952
2406	Amp	1	207024321	207043350	3.361849	0.145062
2407	Amp	1	207049016	207058192	3.516868	0.14789
2408	Amp	1	207061524	207063987	3.483236	0.147388
2409	Amp	1	207066402	207080142	3.446786	0.146629
2410	Amp	1	207082274	207105106	3.387253	0.145672
2411	Amp	1	207112643	207131555	3.349067	0.145037
2412	Amp	1	207131724	207183755	3.435586	0.146552
2413	Amp	1	207184177	207195565	3.387253	0.145571
2414	Amp	1	207198051	207198051	3.172599	0.141602
2415	Amp	1	207201802	207239747	3.045223	0.139287
2416	Amp	1	207240370	207353248	2.940615	0.137255
2417	Amp	1	207353796	207373551	2.707624	0.133077
2418	Amp	1	207373703	207385002	2.673338	0.132394
2419	Amp	1	207385428	207400042	3.057106	0.139427
2420	Amp	1	207400303	207414019	2.975629	0.137936
2421	Amp	1	207414656	207497924	2.594754	0.130904
2422	Amp	1	207500049	207518704	2.413643	0.127269
2423	Amp	1	207518735	207575522	2.278531	0.124796
2424	Amp	1	207577330	207651643	2.425182	0.127529
2425	Amp	1	207652702	207681771	2.290039	0.125033
2426	Amp	1	207684237	207695618	2.245479	0.123996
2427	Amp	1	207697679	207699795	2.626052	0.131521
2428	Amp	1	207700927	207720606	2.661431	0.132056
2429	Amp	1	207720688	207765604	2.684894	0.132547
2430	Amp	1	207766193	207837289	2.743935	0.133546
2431	Amp	1	207837362	207839031	2.33403	0.125917
2432	Amp	1	207840275	207841577	2.368227	0.126386
2433	Amp	1	207844107	207866240	2.300463	0.125255
2434	Amp	1	207866270	207895956	2.245479	0.124016
2435	Amp	1	207898844	207905076	2.345513	0.125946
2436	Amp	1	207907627	207916673	2.049917	0.120203
2437	Amp	1	207917424	207983622	2.560422	0.130204

2438	Amp	1	207983950	208004944	2.594754	0.130734
2439	Amp	1	208013074	208073344	2.446878	0.128
2440	Amp	1	208073987	208074309	2.379392	0.126754
2441	Amp	1	208075969	208120094	2.356694	0.126306
2442	Amp	1	208120168	208121132	2.33403	0.125779
2443	Amp	1	208121959	208151652	2.235168	0.123849
2444	Amp	1	208151899	208264476	2.127614	0.121686
2445	Amp	1	208284575	208287588	1.748606	0.114195
2446	Amp	1	208287603	208291605	1.168655	0.1012
2447	Amp	1	208295995	208328613	0.959607	0.096303
2448	Amp	1	208332390	208363742	0.933745	0.095717
2449	Amp	1	208367586	208387918	0.959607	0.0962
2450	Amp	1	208388008	208451517	1.093834	0.09948
2451	Amp	1	208452386	208519888	1.066493	0.098863
2452	Amp	1	208521707	208577471	1.131695	0.100438
2453	Amp	1	208580127	208613838	1.112463	0.099936
2454	Amp	1	208613942	208692439	1.084294	0.099431
2455	Amp	1	208693109	208780268	1.243254	0.102972
2456	Amp	1	208782159	208793342	1.61378	0.111365
2457	Amp	1	208794997	208890700	1.542638	0.10979
2458	Amp	1	208891071	208893100	1.445847	0.107581
2459	Amp	1	208900562	208935148	1.329472	0.105011
2460	Amp	1	208938001	208940758	1.243254	0.102962
2461	Amp	1	208940877	208968497	1.388256	0.106237
2462	Amp	1	208972105	208991140	1.252022	0.103194
2463	Amp	1	208993222	208993222	1.23519	0.102722
2464	Amp	1	208998226	209035866	1.252022	0.103194
2465	Amp	1	209036109	209038623	0.765474	0.09135
2466	Amp	1	209039430	209064125	0.899693	0.09478
2467	Amp	1	209065666	209137079	1.197658	0.101924
2468	Amp	1	209141225	209150793	1.121584	0.1002
2469	Amp	1	209151257	209312056	1.23519	0.102853
2470	Amp	1	209312681	209368265	1.456037	0.107841
2471	Amp	1	209370918	209417405	1.140417	0.100698
2472	Amp	1	209420095	209436696	1.215958	0.102456
2473	Amp	1	209438699	209440907	1.348715	0.10551
2474	Amp	1	209445372	209446862	1.215958	0.102366
2475	Amp	1	209448236	209461978	1.299876	0.104429
2476	Amp	1	209462210	209514837	1.426786	0.107214
2477	Amp	1	209515137	209515377	1.456037	0.107736
2478	Amp	1	209516440	209558138	1.188121	0.101825
2479	Amp	1	209558197	209561663	0.977077	0.096837
2480	Amp	1	209562492	209583366	1.084294	0.099386
2481	Amp	1	209585172	209598002	1.023539	0.097841
2482	Amp	1	209601489	209613911	1.369395	0.105774
2483	Amp	1	209615342	209647193	1.531891	0.109505
2484	Amp	1	209647284	209648112	1.456037	0.10777
2485	Amp	1	209648223	209654864	1.378521	0.105975
2486	Amp	1	209654894	209699005	1.290368	0.104079
2487	Amp	1	209705040	209733330	1.197658	0.102023
2488	Amp	1	209734403	209852482	0.735486	0.090355
2489	Amp	1	209853251	209925033	0.807647	0.092434
2490	Amp	1	209925362	209934483	0.873678	0.094202

2491	Amp	1	209934884	209961203	0.857296	0.093664
2492	Amp	1	209963874	209989131	0.774648	0.091601
2493	Amp	1	209992583	210028331	0.681869	0.088816
2494	Amp	1	210030113	210074471	0.595248	0.086267
2495	Amp	1	210076141	210112773	0.494792	0.082987
2496	Amp	1	210116737	210122296	0.602643	0.086549
2497	Amp	1	210126859	210127146	0.494792	0.082987
2498	Amp	1	210128698	210138993	0.463534	0.081988
2499	Amp	1	210140864	210211669	0.487495	0.082861
2500	Amp	1	210211860	210231416	0.557122	0.084989
2501	Amp	1	210236482	210313401	0.386426	0.079292
2502	Amp	1	210313550	210360181	0.335313	0.077524
2503	Amp	1	210362934	210362934	0.386426	0.07932
2504	Amp	1	210364281	210424823	0.616549	0.086885
2505	Amp	1	210424830	210469726	0.522881	0.083831
2506	Amp	1	210473874	210476543	0.563096	0.085151
2507	Amp	1	210476708	210503847	1.159209	0.101087
2508	Amp	1	210505741	210558981	1.140417	0.100596
2509	Amp	1	210562747	210587100	0.857296	0.093638
2510	Amp	1	210590384	210622720	0.335313	0.077702
2511	Amp	1	210623495	210623495	0.34892	0.078138
2512	Amp	1	210626707	210707969	0.251218	0.074276
2513	Amp	1	210710206	210743772	0.276783	0.075479
2514	Amp	1	210745272	210810088	0.457501	0.081834
2515	Amp	1	210813667	210818448	0.276783	0.075479
2516	Amp	1	210819729	210824107	0.264858	0.074949
2517	Amp	1	210827381	210918793	0.204831	0.072467
2518	Amp	1	210921770	210949223	0.595248	0.086308
2519	Amp	1	210951283	210955274	0.543344	0.084563
2520	Amp	1	210958292	211055135	0.424557	0.080642
2521	Amp	1	211058856	211116610	0.386426	0.079322
2522	Amp	1	211121537	211199037	0.529451	0.084085
2523	Amp	1	211199196	211311628	0.515676	0.083648
2524	Amp	1	211315845	211354139	0.404718	0.079946
2525	Amp	1	211354623	211420245	0.536238	0.084315
2526	Amp	1	211422152	211475729	0.522881	0.083843
2527	Amp	1	211475772	211479724	0.543344	0.084492
2528	Amp	1	211482896	211527841	0.515676	0.083789
2529	Amp	1	211530385	211570380	0.494792	0.08307
2530	Amp	1	211572847	211674693	0.368062	0.078701
2531	Amp	1	211677441	211687217	0.019169	0.06486
2532	Amp	1	211689362	211872391	0	0.063296
2533	Amp	1	211873105	211917386	0.029031	0.065325
2534	Amp	1	211917480	211919458	0.222041	0.07301
2535	Amp	1	211921656	211938032	0.311747	0.076741
2536	Amp	1	211938902	211968327	0.322897	0.077178
2537	Amp	1	211972057	212008111	0.287738	0.075948
2538	Amp	1	212010180	212024502	0.276783	0.075475
2539	Amp	1	212027223	212027524	0.210594	0.072558
2540	Amp	1	212035149	212086273	0.019169	0.064873
2541	Amp	1	212087050	212109545	0.241616	0.073942
2542	Amp	1	212115999	212125503	0.19964	0.072274
2543	Amp	1	212126368	212162957	0.282828	0.075705

2544	Amp	1	212166227	212170981	0.259438	0.074832
2545	Amp	1	212171147	212256661	0.276783	0.075518
2546	Amp	1	212257414	212283747	0.159312	0.070779
2547	Amp	1	212288870	212398629	0.238246	0.073658
2548	Amp	1	212400287	212460735	0.181932	0.071629
2549	Amp	1	212461216	212604944	0.210594	0.072685
2550	Amp	1	212605165	212678556	0.276783	0.075411
2551	Amp	1	212678846	212721236	0.311747	0.07681
2552	Amp	1	212721276	212744201	0.241616	0.073931
2553	Amp	1	212744638	212752087	0.282828	0.075689
2554	Amp	1	212752379	212867587	0.404718	0.079966
2555	Amp	1	212867683	213000914	0.355319	0.078208
2556	Amp	1	213003378	213136410	0.329205	0.077348
2557	Amp	1	213138715	213181175	0.282828	0.075571
2558	Amp	1	213181365	213307354	0.251218	0.074463
2559	Amp	1	213316289	213342418	0.299433	0.076248
2560	Amp	1	213347319	213374934	0.276783	0.075358
2561	Amp	1	213375446	213482431	0.233782	0.073601
2562	Amp	1	213482521	213544149	0.210594	0.072545
2563	Amp	1	213544474	213706271	0.170723	0.071154
2564	Amp	1	213706504	213816149	0.1237	0.069267
2565	Amp	1	213817641	213874946	0.112543	0.068812
2566	Amp	1	213875547	213911494	0.19964	0.07216
2567	Amp	1	213912496	213920336	0.153838	0.070493
2568	Amp	1	213922525	213941523	0.143787	0.070056
2569	Amp	1	213951475	213963044	0.065715	0.066707
2570	Amp	1	213963882	213966004	0.089561	0.06785
2571	Amp	1	213967650	213999524	0.233782	0.073564
2572	Amp	1	214001542	214015210	0.050473	0.066064
2573	Amp	1	214016440	214082355	0.210594	0.07267
2574	Amp	1	214084887	214118784	0.299433	0.076299
2575	Amp	1	214120301	214140127	0.210594	0.07267
2576	Amp	1	214140848	214321644	0.251218	0.07427
2577	Amp	1	214321796	214375678	0.259438	0.074777
2578	Amp	1	214376028	214404555	0.316877	0.077014
2579	Amp	1	214406134	214513751	0.255918	0.074667
2580	Amp	1	214514267	214573564	0.342649	0.077884
2581	Amp	1	214573914	214583499	0.193543	0.07208
2582	Amp	1	214586976	214592594	0.282828	0.075667
2583	Amp	1	214601179	214743776	0.299433	0.076416
2584	Amp	1	214744939	214831274	0.373599	0.078876
2585	Amp	1	214831804	214836089	0.322897	0.077276
2586	Amp	1	214836633	214838128	0.404718	0.079937
2587	Amp	1	214840044	214883995	0.311747	0.076729
2588	Amp	1	214884314	215053656	0.246818	0.074067
2589	Amp	1	215053698	215193905	0.233782	0.073553
2590	Amp	1	215194437	215256096	0.287738	0.075939
2591	Amp	1	215258569	215313942	0.335313	0.077535
2592	Amp	1	215317231	215398168	0.259438	0.074809
2593	Amp	1	215401824	215426278	0.040093	0.06574
2594	Amp	1	215427410	215550517	0.005328	0.064144
2595	Amp	1	215551389	215608633	0.056211	0.066322
2596	Amp	1	215608676	215660217	0.100827	0.068272

2597	Amp	1	215665748	215682652	0.153838	0.070477
2598	Amp	1	215692667	215739525	0.100827	0.068184
2599	Amp	1	215741304	215769398	0.024438	0.064967
2600	Amp	1	215770138	215820951	0	0.063017
2601	Amp	1	215821166	215927605	0	0.061172
2602	Amp	1	215928168	215934668	0	0.057599
2603	Amp	1	215938906	215978740	0	0.061148
2604	Amp	1	215978959	215986593	0	0.058943
2605	Amp	1	215987291	216021432	0	0.059679
2606	Amp	1	216021606	216031768	0	0.061747
2607	Amp	1	216031939	216067182	0.029031	0.065292
2608	Amp	1	216070469	216462065	0.056211	0.066336
2609	Amp	1	216462469	216484506	0.069848	0.066935
2610	Amp	1	216493016	216495696	0.079652	0.06738
2611	Amp	1	216499708	216587765	0.264858	0.074922
2612	Amp	1	216587995	216769439	0.299433	0.076283
2613	Amp	1	216769587	216776802	0.264858	0.075002
2614	Amp	1	216776958	216923878	0.255918	0.074558
2615	Amp	1	216924694	216933583	0.470509	0.082087
2616	Amp	1	216935116	216977471	0.482379	0.082549
2617	Amp	1	216978673	216981784	0.316877	0.076945
2618	Amp	1	217042123	217203323	0.392611	0.079628
2619	Amp	1	217203568	217308353	0.335313	0.07767
2620	Amp	1	217313055	217350543	0.379885	0.079056
2621	Amp	1	217353682	217417720	0.437198	0.081195
2622	Amp	1	217417845	217483795	0.379885	0.079056
2623	Amp	1	217485285	217485285	0.437198	0.081109
2624	Amp	1	217486758	217600065	0.487495	0.082731
2625	Amp	1	217601287	217661868	0.543344	0.084671
2626	Amp	1	217662005	217843565	0.47716	0.082406
2627	Amp	1	217845614	217865636	0.431198	0.080784
2628	Amp	1	217866343	217877169	0.482379	0.082627
2629	Amp	1	217881345	217968787	0.583752	0.085792
2630	Amp	1	217968932	217968932	0.482379	0.082627
2631	Amp	1	217969079	218025053	0.508568	0.083453
2632	Amp	1	218025273	218039407	0.482379	0.082627
2633	Amp	1	218039937	218070355	0.529451	0.084175
2634	Amp	1	218070802	218188740	0.47716	0.082332
2635	Amp	1	218192548	218223681	0.463534	0.081896
2636	Amp	1	218227700	218265913	0.450702	0.081434
2637	Amp	1	218271714	218278183	0.550755	0.084812
2638	Amp	1	218282636	218337512	0.500841	0.083264
2639	Amp	1	218338199	218437092	0.563096	0.085129
2640	Amp	1	218437209	218533430	0.630438	0.087314
2641	Amp	1	218534336	218658915	0.563096	0.085129
2642	Amp	1	218659872	218693025	0.602643	0.086476
2643	Amp	1	218699129	218767602	0.711903	0.089882
2644	Amp	1	218770271	218999438	0.602643	0.086476
2645	Amp	1	219003293	219018437	0.823771	0.09282
2646	Amp	1	219018628	219044533	0.765474	0.091321
2647	Amp	1	219046189	219084491	0.831947	0.093088
2648	Amp	1	219085296	219091880	0.959607	0.096328
2649	Amp	1	219092038	219121585	0.86541	0.093989

2650	Amp	1	219123348	219141474	1.131695	0.100341
2651	Amp	1	219147602	219222271	1.290368	0.104211
2652	Amp	1	219226258	219269554	1.445847	0.107499
2653	Amp	1	219281520	219396761	1.552619	0.109906
2654	Amp	1	219399214	219538150	1.463774	0.10804
2655	Amp	1	219540388	219561413	1.502257	0.108951
2656	Amp	1	219561618	219575246	1.407997	0.106729
2657	Amp	1	219575476	219588217	1.348715	0.105382
2658	Amp	1	219592974	219648136	1.299876	0.104242
2659	Amp	1	219648167	219751216	1.416817	0.10699
2660	Amp	1	219751451	219777765	1.456037	0.107851
2661	Amp	1	219778611	219854517	1.243254	0.103132
2662	Amp	1	219858976	219910817	1.456037	0.107794
2663	Amp	1	219910887	219964547	1.512549	0.109204
2664	Amp	1	219967372	220022190	1.398396	0.106522
2665	Amp	1	220023296	220054179	1.416817	0.107016
2666	Amp	1	220060049	220060049	1.445847	0.107606
2667	Amp	1	220060054	220139297	1.348715	0.105504
2668	Amp	1	220140053	220160411	1.463774	0.107985
2669	Amp	1	220163026	220207477	0.882843	0.094271
2670	Amp	1	220208465	220285134	1.058188	0.098782
2671	Amp	1	220285986	220303967	0.882843	0.094271
2672	Amp	1	220306265	220334018	0.959607	0.096335
2673	Amp	1	220334087	220370355	0.857296	0.093766
2674	Amp	1	220372797	220439920	0.470509	0.082123
2675	Amp	1	220440171	220480403	0.424557	0.080713
2676	Amp	1	220482011	220683729	0.368062	0.078649
2677	Amp	1	220689960	220808016	0.241616	0.073986
2678	Amp	1	220808916	220859525	0.210594	0.072587
2679	Amp	1	220866392	220926544	0.222041	0.073139
2680	Amp	1	220928254	220983282	0.204831	0.07249
2681	Amp	1	220983793	221133329	0.251218	0.074324
2682	Amp	1	221133442	221157173	0.293269	0.076116
2683	Amp	1	221158370	221217036	0.316877	0.076916
2684	Amp	1	221218444	221288630	0.34892	0.078149
2685	Amp	1	221290697	221353253	0.299433	0.076315
2686	Amp	1	221353750	221497931	0.271552	0.07522
2687	Amp	1	221498077	221636210	0.241616	0.073987
2688	Amp	1	221636384	222403667	0.255918	0.074508
2689	Amp	1	222404929	222467949	0.215608	0.072943
2690	Amp	1	222484862	222544356	0	0.063555
2691	Amp	1	222547946	222641100	0.029031	0.065193
2692	Amp	1	222641466	222735660	0.015369	0.064686
2693	Amp	1	222736232	222748259	0.089561	0.067891
2694	Amp	1	222748826	222931983	0.159312	0.070783
2695	Amp	1	222934646	222998264	0.259438	0.074839
2696	Amp	1	222998314	223043718	0.251218	0.074287
2697	Amp	1	223116005	223156423	0.112543	0.068647
2698	Amp	1	223156942	223345454	0.015369	0.064592
2699	Amp	1	223348279	223474195	0	0.062574
2700	Amp	1	223476469	223502727	0	0.062053
2701	Amp	1	223503943	223639485	0	0.060261
2702	Amp	1	223640201	223646744	0	0.058655

2703	Amp	1	223648960	223718719	0	0.057854
2704	Amp	1	223719596	223777329	0.029031	0.065291
2705	Amp	1	223777551	223876269	0.079652	0.067515
2706	Amp	1	223876411	223897298	0.107017	0.068446
2707	Amp	1	223899161	224047932	0.010261	0.06438
2708	Amp	1	224048462	224238355	0.073902	0.067185
2709	Amp	1	224245320	224328244	0.431198	0.080927
2710	Amp	1	224332055	224365633	0.233782	0.07349
2711	Amp	1	224371965	224398588	0.329205	0.077429
2712	Amp	1	224403210	224429683	0.255918	0.074624
2713	Amp	1	224435576	224508926	0.204831	0.072401
2714	Amp	1	224514997	224542841	0.293269	0.076192
2715	Amp	1	224542992	224556991	0.316877	0.076997
2716	Amp	1	224558875	224613811	0	0.063255
2717	Amp	1	224614250	224634726	0.029031	0.06525
2718	Amp	1	224634780	224647670	0.050473	0.06612
2719	Amp	1	224648812	224672307	0	0.062329
2720	Amp	1	224672580	224795655	0	0.063031
2721	Amp	1	224796665	224803832	0	0.06216
2722	Amp	1	224805664	224814207	0	0.063128
2723	Amp	1	224815538	224905223	0	0.062217
2724	Amp	1	224910977	224938890	0	0.06125
2725	Amp	1	224940013	225055240	0	0.063414
2726	Amp	1	225055947	225109095	0.060686	0.066547
2727	Amp	1	225113983	225131487	0.193543	0.071882
2728	Amp	1	225132619	225132619	0.233782	0.073543
2729	Amp	1	225132806	225155273	0.153838	0.07041
2730	Amp	1	225157212	225283698	0.010261	0.064306
2731	Amp	1	225283759	225308695	0	0.061205
2732	Amp	1	225308997	225334948	0	0.062576
2733	Amp	1	225338890	225374683	0	0.063341
2734	Amp	1	225382173	225401160	0	0.061108
2735	Amp	1	225408628	225449904	0	0.063742
2736	Amp	1	225461457	225623121	0.040093	0.065656
2737	Amp	1	225625209	225709682	0.073902	0.067138
2738	Amp	1	225711110	225731061	0.08435	0.067689
2739	Amp	1	225733602	225739257	0.118001	0.068938
2740	Amp	1	225744086	225792892	0.233782	0.073521
2741	Amp	1	225801413	225881227	0.299433	0.076241
2742	Amp	1	225883370	226156594	0.187449	0.071659
2743	Amp	1	226156675	226300240	0.227787	0.073353
2744	Amp	1	226303527	226430498	0.118001	0.069018
2745	Amp	1	226430681	226844143	0.056211	0.066297
2746	Amp	1	226845840	226894882	0.034113	0.065532
2747	Amp	1	226896959	227072029	0.138391	0.069731
2748	Amp	1	227075534	227130998	0.246818	0.074104
2749	Amp	1	227134374	227400855	0.138391	0.069731
2750	Amp	1	227406946	227562151	0.112543	0.068639
2751	Amp	1	227563411	227616411	0.164935	0.07092
2752	Amp	1	227617044	227622566	0.210594	0.072599
2753	Amp	1	227623479	227623479	0.264858	0.075001
2754	Amp	1	227623813	227744981	0.30524	0.076507
2755	Amp	1	227745543	227758863	0.362016	0.078484

2756	Amp	1	227759334	227835500	0.457501	0.081813
2757	Amp	1	227835653	227901917	0.550755	0.084705
2758	Amp	1	227907915	227922906	0.697374	0.089372
2759	Amp	1	227923033	227930028	0.651644	0.088072
2760	Amp	1	227931233	227939526	0.689884	0.089046
2761	Amp	1	227948831	228014491	0.576183	0.085717
2762	Amp	1	228027677	228109044	0.437198	0.08105
2763	Amp	1	228123322	228185869	0.368062	0.078648
2764	Amp	1	228186551	228228900	0.316877	0.076955
2765	Amp	1	228230556	228245896	0.373599	0.079029
2766	Amp	1	228246901	228247099	0.316877	0.077052
2767	Amp	1	228247328	228295123	0.276783	0.075372
2768	Amp	1	228295668	228295668	0.227787	0.073298
2769	Amp	1	228296929	228309482	0.193543	0.071927
2770	Amp	1	228309769	228309769	0.133193	0.069646
2771	Amp	1	228312480	228657499	0.187449	0.071868
2772	Amp	1	228665291	228667018	0.164935	0.070895
2773	Amp	1	228671781	228679881	0.17597	0.071407
2774	Amp	1	228687141	228775202	0.060686	0.066602
2775	Amp	1	228777692	228904759	0.010261	0.06438
2776	Amp	1	228904842	228918017	0	0.062211
2777	Amp	1	228918756	228980562	0.029031	0.065313
2778	Amp	1	228982609	228999712	0	0.062771
2779	Amp	1	229000012	229004646	0	0.060575
2780	Amp	1	229004705	229007732	0	0.058783
2781	Amp	1	229009174	229183962	0	0.060816
2782	Amp	1	229184321	229237586	0	0.060114
2783	Amp	1	229243212	229262645	0	0.062787
2784	Amp	1	229265205	229329099	0	0.061401
2785	Amp	1	229332283	229449329	0.034113	0.06545
2786	Amp	1	229453783	229478715	0.073902	0.067174
2787	Amp	1	229479243	229493621	0.065715	0.066672
2788	Amp	1	229494815	229550188	0.14896	0.070327
2789	Amp	1	229553491	229556548	0.056211	0.066277
2790	Amp	1	229557097	229567533	0	0.062622
2791	Amp	1	229573646	229782428	0	0.060589
2792	Amp	1	229782564	229794996	0	0.062962
2793	Amp	1	229795161	229977087	0.015369	0.064612
2794	Amp	1	229977275	230055271	0	0.06224
2795	Amp	1	230065181	230099063	0.024438	0.064993
2796	Amp	1	230099285	230099285	0.040093	0.065601
2797	Amp	1	230101356	230177691	0	0.062848
2798	Amp	1	230186228	230219363	0	0.063599
2799	Amp	1	230219502	230279547	0.050473	0.06619
2800	Amp	1	230281548	230292756	0.034113	0.065439
2801	Amp	1	230292878	230372744	0.065715	0.066717
2802	Amp	1	230373142	230414448	0.005328	0.064127
2803	Amp	1	230417011	230711777	0	0.061454
2804	Amp	1	230711810	230711810	0	0.05973
2805	Amp	1	230712043	230769977	0	0.058451
2806	Amp	1	230770828	230923573	0	0.058983
2807	Amp	1	230924038	230946708	0	0.058375
2808	Amp	1	230949491	231001326	0	0.059288

2809	Amp	1	231006692	231179244	0	0.06217
2810	Amp	1	231180158	231188698	0	0.059288
2811	Amp	1	231191920	231403649	0	0.059949
2812	Amp	1	231406910	231697972	0	0.062445
2813	Amp	1	231757426	231761212	0.015369	0.064701
2814	Amp	1	231827591	231876203	0.159312	0.070741
2815	Amp	1	231879294	231904450	0.14896	0.07019
2816	Amp	1	231906398	231989414	0.089561	0.067934
2817	Amp	1	231989635	231996687	0.019169	0.064798
2818	Amp	1	232000932	232046641	0.029031	0.065339
2819	Amp	1	232049685	232117889	0	0.061894
2820	Amp	1	232126268	232365712	0	0.063733
2821	Amp	1	232371026	232472395	0	0.06282
2822	Amp	1	232472597	232572450	0	0.062159
2823	Amp	1	232572654	232591405	0	0.059056
2824	Amp	1	232596873	232605331	0	0.061739
2825	Amp	1	232606280	232812597	0	0.063045
2826	Amp	1	232813158	232865904	0.010261	0.064334
2827	Amp	1	232866526	233027832	0	0.062827
2828	Amp	1	233028167	233081664	0.044794	0.065843
2829	Amp	1	233081763	233125443	0	0.062951
2830	Amp	1	233135897	233347836	0	0.061112
2831	Amp	1	233347985	233353308	0	0.063102
2832	Amp	1	233353359	233402520	0.005328	0.064214
2833	Amp	1	233403389	233415786	0.255918	0.074526
2834	Amp	1	233416519	233474653	0.264858	0.075042
2835	Amp	1	233478227	233572564	0.316877	0.076935
2836	Amp	1	233582550	233635873	0.299433	0.076418
2837	Amp	1	233639534	233700213	0.329205	0.077315
2838	Amp	1	233703433	233718129	0.287738	0.075858
2839	Amp	1	233718996	233748154	0.316877	0.077044
2840	Amp	1	233749752	233780959	0.342649	0.077862
2841	Amp	1	233781287	233784719	0.431198	0.080812
2842	Amp	1	233785067	233920920	0.470509	0.082184
2843	Amp	1	233921477	233986840	0.444474	0.081288
2844	Amp	1	233989256	234106073	0.487495	0.08279
2845	Amp	1	234114111	234159121	0.444474	0.081288
2846	Amp	1	234160998	234312664	0.457501	0.08173
2847	Amp	1	234312784	234376725	0.494792	0.083106
2848	Amp	1	234376757	234480741	0.569771	0.085344
2849	Amp	1	234481216	234493781	0.630438	0.087456
2850	Amp	1	234499713	234507714	0.616549	0.086946
2851	Amp	1	234508470	234513898	0.651644	0.087964
2852	Amp	1	234516109	234541805	0.765474	0.091279
2853	Amp	1	234541991	234580389	0.848692	0.093487
2854	Amp	1	234580966	234722314	0.899693	0.094752
2855	Amp	1	234723963	234746395	0.98589	0.097051
2856	Amp	1	234747435	234780167	0.899693	0.094843
2857	Amp	1	234794424	234854757	0.968522	0.096529
2858	Amp	1	234869375	234869763	0.882843	0.094291
2859	Amp	1	234871309	234914278	1.066493	0.098922
2860	Amp	1	234919629	234928026	0.959607	0.09623
2861	Amp	1	234928065	234983894	0.86541	0.093932

2862	Amp	1	234987631	235085097	0.689884	0.089112
2863	Amp	1	235100678	235100678	0.589605	0.086096
2864	Amp	1	235101118	235101118	0.630438	0.087357
2865	Amp	1	235103144	235153395	0.536238	0.084405
2866	Amp	1	235163265	235177695	0.404718	0.080083
2867	Amp	1	235177796	235192821	0.282828	0.07573
2868	Amp	1	235196467	235220799	0.322897	0.077222
2869	Amp	1	235221523	235303249	0.271552	0.075143
2870	Amp	1	235303911	235361938	0.322897	0.077209
2871	Amp	1	235364332	235418790	0.233782	0.073453
2872	Amp	1	235419634	235489268	0.276783	0.075498
2873	Amp	1	235491121	235531886	0.335313	0.077618
2874	Amp	1	235538014	235552361	0.311747	0.076757
2875	Amp	1	235555399	235767503	0.255918	0.074638
2876	Amp	1	235767544	235928641	0.30524	0.07655
2877	Amp	1	235929546	236015742	0.246818	0.074054
2878	Amp	1	236016661	236021488	0.17597	0.071411
2879	Amp	1	236021631	236128593	0.1237	0.069258
2880	Amp	1	236129215	236129368	0.079652	0.067346
2881	Amp	1	236132779	236186069	0.029031	0.065301
2882	Amp	1	236186082	236321543	0.069848	0.066992
2883	Amp	1	236321650	236411447	0.029031	0.065301
2884	Amp	1	236413723	236427848	0.079652	0.067455
2885	Amp	1	236429931	236431237	0.204831	0.072427
2886	Amp	1	236431986	236442992	0.14896	0.070274
2887	Amp	1	236445018	236445666	0.159312	0.070726
2888	Amp	1	236446915	236481216	0.133193	0.069696
2889	Amp	1	236488295	236507760	0.227787	0.073326
2890	Amp	1	236510395	236518450	0.100827	0.068353
2891	Amp	1	236522753	236548228	0.143787	0.070111
2892	Amp	1	236548286	236614846	0.133193	0.06958
2893	Amp	1	236615109	236730336	0.107017	0.068548
2894	Amp	1	236730417	236730749	0.233782	0.073494
2895	Amp	1	236734076	236737237	0.362016	0.078424
2896	Amp	1	236738518	236805056	0.107017	0.068548
2897	Amp	1	236805196	236833860	0.227787	0.073207
2898	Amp	1	236835514	237015871	0.255918	0.074665
2899	Amp	1	237019124	237300299	0.271552	0.07528
2900	Amp	1	237306112	237375512	0.138391	0.069869
2901	Amp	1	237376367	237610040	0.128324	0.069426
2902	Amp	1	237610127	237617287	0.17597	0.071301
2903	Amp	1	237617727	237657410	0.138391	0.069843
2904	Amp	1	237660930	237783704	0.238246	0.073701
2905	Amp	1	237784021	237801743	0.311747	0.076791
2906	Amp	1	237803055	237845898	0.342649	0.07774
2907	Amp	1	237849470	237850207	0.368062	0.07867
2908	Amp	1	237850244	237898063	0.4115	0.080133
2909	Amp	1	237903052	237910951	0.322897	0.077183
2910	Amp	1	237911409	237988145	0.246818	0.074093
2911	Amp	1	237990047	238029775	0.210594	0.07263
2912	Amp	1	238046466	238065541	0.112543	0.068773
2913	Amp	1	238070033	238095607	0.089561	0.067887
2914	Amp	1	238097730	238468240	0.069848	0.066957

2915	Amp	1	238469174	238472645	0.14896	0.070181
2916	Amp	1	238475272	238571913	0.164935	0.07094
2917	Amp	1	238578264	238692794	0.118001	0.068916
2918	Amp	1	238699149	238765419	0.138391	0.069781
2919	Amp	1	238765475	238816488	0.17597	0.071431
2920	Amp	1	238819469	238893806	0.311747	0.076685
2921	Amp	1	238893941	238989801	0.342649	0.0778
2922	Amp	1	238996311	239004946	0.379885	0.07925
2923	Amp	1	239005802	239092087	0.424557	0.080597
2924	Amp	1	239095100	239101104	0.437198	0.081132
2925	Amp	1	239104595	239144132	0.424557	0.080597
2926	Amp	1	239144630	239363432	0.392611	0.079482
2927	Amp	1	239366303	239441308	0.368062	0.078754
2928	Amp	1	239442019	239608137	0.322897	0.077104
2929	Amp	1	239608518	239612045	0.299433	0.076239
2930	Amp	1	239612517	239663688	0.282828	0.075623
2931	Amp	1	239663911	240068425	0.238246	0.073664
2932	Amp	1	240069117	240103728	0.204831	0.072317
2933	Amp	1	240107213	240196819	0.222041	0.072971
2934	Amp	1	240203040	240205090	0.34892	0.07815
2935	Amp	1	240209596	240227405	0.222041	0.072971
2936	Amp	1	240227537	240286526	0.255918	0.074616
2937	Amp	1	240286573	240444109	0.293269	0.076007
2938	Amp	1	240444264	240535469	0.255918	0.074616
2939	Amp	1	240536381	240641437	0.227787	0.07324
2940	Amp	1	240644181	240647067	0.17597	0.071365
2941	Amp	1	240652898	240722905	0.233782	0.073396
2942	Amp	1	240723058	240776749	0.193543	0.071946
2943	Amp	1	240779644	240854559	0.259438	0.074738
2944	Amp	1	240855196	240959861	0.271552	0.075193
2945	Amp	1	240961189	241057003	0.329205	0.077338
2946	Amp	1	241058555	241183587	0.404718	0.080092
2947	Amp	1	241186465	241345102	0.457501	0.081731
2948	Amp	1	241346495	241406027	0.500841	0.083344
2949	Amp	1	241407411	241425304	0.529451	0.084187
2950	Amp	1	241432793	241443522	0.609133	0.086674
2951	Amp	1	241444337	241523886	0.529451	0.084148
2952	Amp	1	241526580	241562382	0.482379	0.082535
2953	Amp	1	241566754	241605347	0.417873	0.08039
2954	Amp	1	241608858	241628732	0.470509	0.082141
2955	Amp	1	241629109	241651974	0.508568	0.083536
2956	Amp	1	241694837	241846514	0.399144	0.079779
2957	Amp	1	241846770	241847325	0.689884	0.089101
2958	Amp	1	241848232	241849304	0.660254	0.088194
2959	Amp	1	241849737	241893326	0.355319	0.078384
2960	Amp	1	241894238	242166903	0.316877	0.076902
2961	Amp	1	242167781	242239376	0.386426	0.079295
2962	Amp	1	242239700	242292896	0.335313	0.077656
2963	Amp	1	242296055	242322412	0.299433	0.076284
2964	Amp	1	242323300	242437304	0.34892	0.078061
2965	Amp	1	242437355	242498738	0.379885	0.079143
2966	Amp	1	242499206	242511882	0.444474	0.081358
2967	Amp	1	242514276	242561805	0.543344	0.084655

2968	Amp	1	242561955	242672867	0.595248	0.086225
2969	Amp	1	242675497	242735345	0.482379	0.082638
2970	Amp	1	242735799	242751945	0.536238	0.084415
2971	Amp	1	242752063	242810590	0.557122	0.085085
2972	Amp	1	242811442	242814831	0.576183	0.085557
2973	Amp	1	242816604	242828684	0.660254	0.088286
2974	Amp	1	242829562	242873653	0.758088	0.091127
2975	Amp	1	242874036	242878965	0.720659	0.089908
2976	Amp	1	242880979	242881005	0.667587	0.088437
2977	Amp	1	243192159	243225884	0.790614	0.092005
2978	Amp	1	243227523	243334403	0.742723	0.090745
2979	Amp	1	243335010	243351625	0.720659	0.090091
2980	Amp	1	243352351	243451126	0.667587	0.088499
2981	Amp	1	243451213	243487556	0.583752	0.08596
2982	Amp	1	243489705	243680540	0.630438	0.087368
2983	Amp	1	243684422	243719701	0.660254	0.088366
2984	Amp	1	243719986	243729670	0.697374	0.089325
2985	Amp	1	243730937	243747490	0.790614	0.092042
2986	Amp	1	243747904	243832338	0.704849	0.089503
2987	Amp	1	243834245	243846113	0.609133	0.086662
2988	Amp	1	243846976	243915992	0.630438	0.087385
2989	Amp	1	243918762	243942376	0.609133	0.086662
2990	Amp	1	243946356	244043308	0.6737	0.088774
2991	Amp	1	244053612	244152616	0.616549	0.08686
2992	Amp	1	244153021	244242744	0.595248	0.086389
2993	Amp	1	244243210	244308789	0.644502	0.087892
2994	Amp	1	244311968	244327067	0.602643	0.086483
2995	Amp	1	244327408	244331854	0.569771	0.085485
2996	Amp	1	244333884	244361625	0.667587	0.088504
2997	Amp	1	244361780	244458948	0.720659	0.090033
2998	Amp	1	244462758	244463308	0.924746	0.095441
2999	Amp	1	244463841	244472567	0.941704	0.095883
3000	Amp	1	244473659	244709126	0.882843	0.09438
3001	Amp	1	244716022	244771250	0.681869	0.088972
3002	Amp	1	244775196	244785311	0.630438	0.087443
3003	Amp	1	244788707	244825073	0.636464	0.087662
3004	Amp	1	244825104	244856466	0.602643	0.086638
3005	Amp	1	244858082	244936576	0.529451	0.084105
3006	Amp	1	244936916	244979229	0.487495	0.082927
3007	Amp	1	244982650	245101325	0.444474	0.081234
3008	Amp	1	245102091	245203771	0.470509	0.08217
3009	Amp	1	245203896	245204232	0.404718	0.080058
3010	Amp	1	245205578	245248051	0.470509	0.082173
3011	Amp	1	245248542	245371962	0.482379	0.082693
3012	Amp	1	245372560	245383096	0.543344	0.084492
3013	Amp	1	245386660	245395362	0.529451	0.08405
3014	Amp	1	245395526	245397642	0.543344	0.084591
3015	Amp	1	245398146	245445233	0.616549	0.086931
3016	Amp	1	245445517	245489367	0.557122	0.085063
3017	Amp	1	245490690	245570726	0.543344	0.084522
3018	Amp	1	245574470	245621719	0.494792	0.083013
3019	Amp	1	245623445	245735811	0.543344	0.084673
3020	Amp	1	245736399	245873942	0.482379	0.082567

3021	Amp	1	245873990	245930755	0.602643	0.08649
3022	Amp	1	245941074	245973251	0.697374	0.089331
3023	Amp	1	245973464	245989437	0.681869	0.088811
3024	Amp	1	245995189	246022678	0.758088	0.091006
3025	Amp	1	246026700	246026700	0.660254	0.088164
3026	Amp	1	246435044	246436444	0.651644	0.088151
3027	Amp	1	246880731	246891261	0.774648	0.091526
3028	Amp	1	246892125	246928662	0.667587	0.088562
3029	Amp	1	246936216	246993082	0.735486	0.090501
3030	Amp	1	246995621	247121237	0.807647	0.092418
3031	Amp	1	247122204	247133582	0.758088	0.091073
3032	Amp	1	247172433	247184887	0.681869	0.088879
3033	Amp	1	247192162	247459638	0.616549	0.086961
3034	Amp	1	247460305	247547053	0.720659	0.089915
3035	Amp	1	247547322	247626617	0.774648	0.091478
3036	Amp	1	247629386	247629386	0.823771	0.092724
3037	Amp	1	247629900	247650984	0.891602	0.094564
3038	Amp	2	480597	2317843	0	0.002995
3039	Amp	2	2318368	2369395	0	0.004338
3040	Amp	2	2370607	2592170	0	0.009126
3041	Amp	2	2593241	2640571	0	0.0136
3042	Amp	2	2640794	2718523	0	0.009126
3043	Amp	2	2719383	2732591	0	0.010816
3044	Amp	2	2734532	2860614	0	0.0134
3045	Amp	2	2862136	2912784	0	0.010816
3046	Amp	2	2913183	2933798	0	0.012077
3047	Amp	2	2934819	2942559	0	0.010816
3048	Amp	2	2944687	2970791	0	0.01128
3049	Amp	2	2977054	3103263	0	0.006492
3050	Amp	2	3104170	3135789	0	0.006027
3051	Amp	2	3138728	3322646	0	0.006547
3052	Amp	2	3329633	3664458	0	0.005204
3053	Amp	2	3666068	3903576	0	0.004685
3054	Amp	2	3906092	4335707	0	0.006401
3055	Amp	2	4341569	4457078	0	0.007694
3056	Amp	2	4464555	4641119	0	0.005978
3057	Amp	2	4650539	5415953	0	0.004685
3058	Amp	2	5417692	5522548	0	0.002995
3059	Amp	2	5528214	5604871	0	0.004245
3060	Amp	2	5604925	5604925	0	0.005182
3061	Amp	2	5612100	6034728	0	0.003933
3062	Amp	2	6035038	6176766	0	0.00495
3063	Amp	2	6177048	6618825	0	0.003933
3064	Amp	2	6619124	6702850	0	0.006257
3065	Amp	2	6703906	7010963	0	0.003933
3066	Amp	2	7011413	7109447	0	0.005588
3067	Amp	2	7111856	7122700	0	0.005148
3068	Amp	2	7122825	7431839	0	0.003492
3069	Amp	2	7432299	7931788	0	0.002905
3070	Amp	2	7932434	8153834	0	0.00439
3071	Amp	2	8154648	9325196	0	0.003453
3072	Amp	2	9325558	9810242	0	0.004008
3073	Amp	2	9813820	9835428	0	0.007926

3074	Amp	2	9850884	9935860	0	0.004008
3075	Amp	2	9936739	10700801	0	0.003453
3076	Amp	2	10704796	10789027	0	0.003895
3077	Amp	2	10791956	11014686	0	0.003453
3078	Amp	2	11014865	11108318	0	0.005274
3079	Amp	2	11110748	11215068	0	0.00379
3080	Amp	2	11224496	11225874	0	0.001968
3081	Amp	2	11230422	11488349	0	0.003518
3082	Amp	2	11491654	11572580	0	0.006089
3083	Amp	2	11578732	11591557	0	0.009939
3084	Amp	2	11591648	11607911	0	0.006089
3085	Amp	2	11608280	11608459	0	0.004539
3086	Amp	2	11612306	11715683	0	0.001968
3087	Amp	2	11716983	12164599	0	0.00082
3088	Amp	2	12164627	13793804	0	0.001687
3089	Amp	2	13794259	13900538	0	0.00082
3090	Amp	2	13900892	14032314	0	0.003543
3091	Amp	2	14036394	14146803	0	0.00082
3092	Amp	2	14146919	14771339	0	0.001909
3093	Amp	2	14772027	15863592	0	0.00241
3094	Amp	2	15864752	15891005	0	0.007306
3095	Amp	2	15893304	15922926	0	0.003098
3096	Amp	2	15927936	16188419	0	0.00241
3097	Amp	2	16189564	16209586	0	0.009436
3098	Amp	2	16211646	16229184	0	0.00241
3099	Amp	2	16229724	16304882	0	0.004306
3100	Amp	2	16311335	16454507	0	0.00241
3101	Amp	2	16454803	16516636	0	0.00418
3102	Amp	2	16518255	16570508	0	0.00241
3103	Amp	2	16571731	16686348	0	0.004311
3104	Amp	2	16692989	16842485	0	0.00241
3105	Amp	2	16845507	16856441	0	0.0033
3106	Amp	2	16856542	16926720	0	0.00241
3107	Amp	2	16932498	16940361	0	0.006562
3108	Amp	2	16940636	17282983	0	0.00241
3109	Amp	2	17283199	17804760	0	0.001909
3110	Amp	2	17805901	17875901	0	0.002564
3111	Amp	2	17876699	17876699	0	0.003822
3112	Amp	2	17876945	17942145	0	0.00467
3113	Amp	2	17943081	18027831	0	0.004014
3114	Amp	2	18032731	18204636	0	0.001909
3115	Amp	2	18209733	18222235	0	0.004461
3116	Amp	2	18224532	18411292	0	0.001909
3117	Amp	2	18413718	18457344	0	0.008079
3118	Amp	2	18467421	18501817	0	0.001909
3119	Amp	2	18504178	18519443	0	0.003933
3120	Amp	2	18520361	18532607	0	0.001909
3121	Amp	2	18533281	18609068	0	0.003641
3122	Amp	2	18613226	18724109	0	0.001909
3123	Amp	2	18727715	18780532	0	0.003609
3124	Amp	2	18780694	18815041	0	0.001909
3125	Amp	2	18816420	18855367	0	0.003637
3126	Amp	2	18856490	19131098	0	0.001909

3127 Amp	2	19131894	19305423	0	0.002374
3128 Amp	2	19316259	19332596	0	0.008036
3129 Amp	2	19345119	19427746	0	0.002374
3130 Amp	2	19427791	19938103	0	0.001286
3131 Amp	2	19942520	19975141	0	0.004195
3132 Amp	2	19978054	20060787	0	0.006227
3133 Amp	2	20070266	20164164	0	0.004195
3134 Amp	2	20168758	20205516	0	0.010311
3135 Amp	2	20209561	20221247	0	0.004195
3136 Amp	2	20227399	20291871	0	0.005641
3137 Amp	2	20294904	20439727	0	0.002732
3138 Amp	2	20441773	20490489	0	0.001286
3139 Amp	2	20492859	20570168	0	0.003336
3140 Amp	2	20573159	20588293	0	0.001286
3141 Amp	2	20592054	20631864	0	0.006
3142 Amp	2	20634405	20662826	0	0.001286
3143 Amp	2	20663648	20684826	0	0.002186
3144 Amp	2	20685561	20709742	0	0.008057
3145 Amp	2	20717346	20764875	0	0.003427
3146 Amp	2	20766028	20782884	0	0.001286
3147 Amp	2	20786748	20890041	0	0.00082
3148 Amp	2	20893195	20916772	0	0.007071
3149 Amp	2	20919223	20943370	0	0.001724
3150 Amp	2	20945252	20987836	0	0.007312
3151 Amp	2	20994618	21234755	0	0.00082
3152 Amp	2	21236100	21305524	0	0.002972
3153 Amp	2	21306364	21412710	0	0.00082
3154 Amp	2	21415198	21441429	0	0.006551
3155 Amp	2	21442920	21588945	0	0.00082
3156 Amp	2	21589393	21664271	0	0.002956
3157 Amp	2	21666499	21766570	0	0.009154
3158 Amp	2	21768485	21832610	0	0.002956
3159 Amp	2	21833614	21856898	0	0.009229
3160 Amp	2	21862761	21926306	0	0.002956
3161 Amp	2	21929648	21931746	0	0.00469
3162 Amp	2	21936043	22350042	0	0.006173
3163 Amp	2	22352218	22362900	0	0.011538
3164 Amp	2	22365653	22474456	0	0.004439
3165 Amp	2	22479119	22555891	0	0.006688
3166 Amp	2	22556467	22613768	0	0.004439
3167 Amp	2	22620577	22631620	0	0.00817
3168 Amp	2	22632883	22662983	0	0.004439
3169 Amp	2	22663373	22690202	0	0.002303
3170 Amp	2	22697714	22809699	0	0.003943
3171 Amp	2	22810653	22990257	0	0.002303
3172 Amp	2	22990274	23011926	0	0.004181
3173 Amp	2	23015132	23146679	0	0.002303
3174 Amp	2	23156506	23179189	0	0.00372
3175 Amp	2	23184018	23419080	0	0.002303
3176 Amp	2	23419702	23483089	0	0.002826
3177 Amp	2	23483258	23498275	0	0.004335
3178 Amp	2	23501518	23579758	0	0.002826
3179 Amp	2	23583216	23655321	0	0.005209

3180	Amp	2	23659228	23714205	0	0.002826
3181	Amp	2	23717176	23743172	0	0.004376
3182	Amp	2	23745732	23775644	0	0.002826
3183	Amp	2	23796501	23803810	0	0.004527
3184	Amp	2	23811308	23944069	0	0.006017
3185	Amp	2	23947344	23967762	0	0.006712
3186	Amp	2	23975616	24203382	0	0.005222
3187	Amp	2	24205130	24290741	0	0.003521
3188	Amp	2	24292127	24372459	0	0.005139
3189	Amp	2	24407230	24591228	0	0.003521
3190	Amp	2	24591670	24686557	0	0.005583
3191	Amp	2	24688211	24724678	0	0.003521
3192	Amp	2	24724939	24782553	0	0.005595
3193	Amp	2	24789591	24799729	0	0.003521
3194	Amp	2	24808085	24977750	0	0.002826
3195	Amp	2	24986001	25066959	0	0.005337
3196	Amp	2	25070069	25320688	0	0.007182
3197	Amp	2	25323794	25418177	0	0.005337
3198	Amp	2	25419425	25487673	0	0.011401
3199	Amp	2	25492047	25692793	0	0.005337
3200	Amp	2	25700492	25906461	0	0.010809
3201	Amp	2	25927720	25931981	0	0.011534
3202	Amp	2	25934408	26071009	0	0.006061
3203	Amp	2	26079006	26133977	0	0.01279
3204	Amp	2	26134879	26182625	0	0.007083
3205	Amp	2	26187148	26234182	0	0.006061
3206	Amp	2	26243608	26306475	0	0.00785
3207	Amp	2	26309086	26544463	0	0.006061
3208	Amp	2	26546566	26560970	0	0.008848
3209	Amp	2	26561018	26601378	0	0.011107
3210	Amp	2	26614451	26614527	0	0.00832
3211	Amp	2	26616667	26766007	0	0.006061
3212	Amp	2	26766293	26860621	0	0.008915
3213	Amp	2	26861800	27051113	0	0.006061
3214	Amp	2	27056125	27076730	0	0.008381
3215	Amp	2	27080059	27096743	0	0.009831
3216	Amp	2	27099019	27161711	0	0.008845
3217	Amp	2	27164396	27267560	0	0.007511
3218	Amp	2	27270754	27379289	0	0.006988
3219	Amp	2	27387628	27406129	0	0.008795
3220	Amp	2	27408324	27469167	0	0.007345
3221	Amp	2	27481099	27538133	0	0.005538
3222	Amp	2	27561561	27617672	0	0.006089
3223	Amp	2	27620399	27653799	0	0.00755
3224	Amp	2	27653871	27717222	0	0.005538
3225	Amp	2	27717898	27784960	0	0.012338
3226	Amp	2	27790185	27975899	0	0.010793
3227	Amp	2	27976597	28026626	0	0.006616
3228	Amp	2	28027228	28052715	0	0.01184
3229	Amp	2	28056055	28083253	0	0.005538
3230	Amp	2	28096872	28285557	0	0.007558
3231	Amp	2	28287394	28344838	0	0.005538
3232	Amp	2	28346778	28372375	0	0.007559

3233	Amp	2	28374757	28413873	0	0.006739
3234	Amp	2	28417866	28432451	0	0.004718
3235	Amp	2	28432921	28763484	0	0.005649
3236	Amp	2	29003693	29146756	0	0.007673
3237	Amp	2	29148968	29807009	0	0.005649
3238	Amp	2	29807393	29922925	0	0.003138
3239	Amp	2	29923758	29955982	0	0.004923
3240	Amp	2	29956444	31352556	0	0.006699
3241	Amp	2	31353768	33229118	0	0.004923
3242	Amp	2	33229769	35280628	0	0.004198
3243	Amp	2	35280925	35318296	0	0.007643
3244	Amp	2	35341960	35590727	0	0.004198
3245	Amp	2	35866763	36131425	0	0.006395
3246	Amp	2	36132191	38395097	0	0.004198
3247	Amp	2	38398197	38769501	0	0.005395
3248	Amp	2	38775480	38799007	0	0.006692
3249	Amp	2	38799542	39178429	0	0.005496
3250	Amp	2	39182713	40997783	0	0.004198
3251	Amp	2	40999101	42243682	0	0.003268
3252	Amp	2	42250509	42484084	0	0.005581
3253	Amp	2	42491347	42507904	0	0.008467
3254	Amp	2	42513493	42625332	0	0.005581
3255	Amp	2	42629561	42796027	0	0.010307
3256	Amp	2	42796343	42978295	0	0.014022
3257	Amp	2	42979841	43140311	0	0.009419
3258	Amp	2	43144221	43541113	0	0.007105
3259	Amp	2	43552507	43618190	0	0.003268
3260	Amp	2	43618218	43786865	0	0.004917
3261	Amp	2	43789746	43854052	0	0.003268
3262	Amp	2	43872294	43911367	0	0.006216
3263	Amp	2	43922053	44008165	0	0.007837
3264	Amp	2	44010867	44046571	0	0.006216
3265	Amp	2	44076908	44186940	0	0.009204
3266	Amp	2	44189825	44193013	0	0.006216
3267	Amp	2	44199698	45347052	0	0.003268
3268	Amp	2	45347971	45690425	0	0.004948
3269	Amp	2	45697146	45791084	0	0.00637
3270	Amp	2	45791745	45882121	0	0.007438
3271	Amp	2	45882696	45936061	0	0.006016
3272	Amp	2	45939857	46329206	0	0.004335
3273	Amp	2	46329494	46415393	0	0.005292
3274	Amp	2	46415848	46817079	0	0.004335
3275	Amp	2	46824412	47595274	0	0.007996
3276	Amp	2	47596900	47763810	0	0.010213
3277	Amp	2	47769457	47814672	0	0.015135
3278	Amp	2	47815090	48166973	0	0.010213
3279	Amp	2	48168347	48313466	0	0.011321
3280	Amp	2	48315499	48417419	0	0.016459
3281	Amp	2	48419287	48545063	0	0.015391
3282	Amp	2	48548270	48618567	0	0.014283
3283	Amp	2	48618711	48770307	0	0.012738
3284	Amp	2	48771920	48794882	0	0.016862
3285	Amp	2	48799621	48846435	0	0.018547

3286	Amp	2	48848788	48875263	0	0.017064
3287	Amp	2	48876005	48879729	0	0.020297
3288	Amp	2	48882964	48961204	0	0.022831
3289	Amp	2	48962782	48975446	0	0.018707
3290	Amp	2	48977688	49232340	0	0.014488
3291	Amp	2	49237768	49248693	0	0.010895
3292	Amp	2	49251298	49349797	0	0.01208
3293	Amp	2	49350528	49417662	0	0.012676
3294	Amp	2	49417766	49437491	0	0.011491
3295	Amp	2	49442241	49636033	0	0.008258
3296	Amp	2	49644448	49741050	0	0.011082
3297	Amp	2	49744101	49983550	0	0.011735
3298	Amp	2	49986540	50299476	0	0.011082
3299	Amp	2	50303282	50477960	0	0.011781
3300	Amp	2	50480639	50636524	0	0.013746
3301	Amp	2	50965967	51433260	0	0.020052
3302	Amp	2	51437142	51437306	0	0.021415
3303	Amp	2	51437365	51462823	0	0.020716
3304	Amp	2	51463177	51544926	0	0.018227
3305	Amp	2	51546255	51621069	0	0.016864
3306	Amp	2	51623879	51792503	0	0.019509
3307	Amp	2	51794858	51918162	0	0.013048
3308	Amp	2	51922877	52131130	0	0.013872
3309	Amp	2	52139478	52173018	0	0.013048
3310	Amp	2	52358148	52373171	0	0.011082
3311	Amp	2	52430806	52712603	0	0.012355
3312	Amp	2	52716652	52737534	0	0.011082
3313	Amp	2	52737701	52780333	0	0.012499
3314	Amp	2	52784623	52803682	0	0.011082
3315	Amp	2	52803811	52821622	0	0.012337
3316	Amp	2	52824057	52942491	0	0.014521
3317	Amp	2	52950752	52996478	0	0.012737
3318	Amp	2	53003516	53099999	0	0.014601
3319	Amp	2	53101271	53239746	0	0.012417
3320	Amp	2	53240223	53266159	0	0.011045
3321	Amp	2	53269553	53388306	0	0.010201
3322	Amp	2	53390122	53432929	0	0.008945
3323	Amp	2	53435784	53504768	0	0.009947
3324	Amp	2	53507519	53730913	0	0.012082
3325	Amp	2	53730999	53826255	0	0.013365
3326	Amp	2	53826339	54476208	0	0.011231
3327	Amp	2	54479034	54537442	0	0.010634
3328	Amp	2	54538015	54605125	0	0.006973
3329	Amp	2	54606050	54670451	0	0.008557
3330	Amp	2	54677326	55494437	0	0.012248
3331	Amp	2	55499992	55562063	0	0.013435
3332	Amp	2	55565884	55838547	0	0.012248
3333	Amp	2	55841898	55993136	0	0.017567
3334	Amp	2	55998041	56367880	0	0.010665
3335	Amp	2	56371174	56541153	0	0.012555
3336	Amp	2	56541212	57260812	0	0.010665
3337	Amp	2	57261165	58040263	0	0.012264
3338	Amp	2	58042489	58316762	0	0.013413

3339	Amp	2	58322203	58492254	0	0.011814
3340	Amp	2	58492258	58838948	0	0.010665
3341	Amp	2	58839935	59015034	0	0.017139
3342	Amp	2	59015301	59532530	0	0.010665
3343	Amp	2	59533859	59711581	0	0.009663
3344	Amp	2	59715896	59854852	0	0.011754
3345	Amp	2	59862547	60196097	0	0.009663
3346	Amp	2	60197802	60283337	0	0.011829
3347	Amp	2	60286254	60417953	0	0.014609
3348	Amp	2	60423358	60477349	0	0.015844
3349	Amp	2	60477798	60529781	0	0.013678
3350	Amp	2	60533502	60601404	0	0.010898
3351	Amp	2	60607590	60682078	0	0.01283
3352	Amp	2	60690233	60734447	0	0.013674
3353	Amp	2	60735182	60757399	0	0.016363
3354	Amp	2	60759782	60957578	0	0.01283
3355	Amp	2	60958588	61011835	0	0.011595
3356	Amp	2	61017528	61033192	0	0.013233
3357	Amp	2	61034999	61221358	0	0.016238
3358	Amp	2	61221641	61249323	0	0.013233
3359	Amp	2	61253682	61331268	0	0.014702
3360	Amp	2	61335422	61412719	0	0.016193
3361	Amp	2	61412971	61582325	0	0.018898
3362	Amp	2	61584635	61675466	0	0.01804
3363	Amp	2	61680117	61819248	0	0.021174
3364	Amp	2	61820016	61936737	0	0.023001
3365	Amp	2	61937753	62186853	0	0.021174
3366	Amp	2	62193980	62275882	0	0.019683
3367	Amp	2	62279348	62333886	0	0.016978
3368	Amp	2	62335175	62467315	0	0.015508
3369	Amp	2	62467558	62545957	0	0.014434
3370	Amp	2	62546814	62649111	0	0.019014
3371	Amp	2	62656733	62770846	0	0.017497
3372	Amp	2	62773185	62906283	0	0.022553
3373	Amp	2	62909092	62947186	0	0.017497
3374	Amp	2	62949134	62980061	0	0.011279
3375	Amp	2	62983200	63083094	0	0.013861
3376	Amp	2	63083143	63365095	0	0.011279
3377	Amp	2	63365311	63519208	0	0.014001
3378	Amp	2	63521318	63790684	0	0.011279
3379	Amp	2	63792103	63864262	0	0.012387
3380	Amp	2	63864399	64049731	0	0.014346
3381	Amp	2	64052472	64463493	0	0.012387
3382	Amp	2	64465879	65207698	0	0.014668
3383	Amp	2	65207746	66221132	0	0.012387
3384	Amp	2	66221423	66489460	0	0.012859
3385	Amp	2	66490396	66522516	0	0.013362
3386	Amp	2	66525814	66638747	0	0.014661
3387	Amp	2	66639635	66704029	0	0.017872
3388	Amp	2	66705729	66750499	0	0.016573
3389	Amp	2	66758715	67402864	0	0.013362
3390	Amp	2	67403344	67538760	0	0.014568
3391	Amp	2	67541380	67996393	0	0.014065

3392	Amp	2	67997400	68005355	0	0.013592
3393	Amp	2	68006188	68013406	0	0.016888
3394	Amp	2	68019427	68069799	0	0.019302
3395	Amp	2	68073295	68132443	0	0.016006
3396	Amp	2	68139101	68404128	0	0.013592
3397	Amp	2	68406763	69138666	0	0.012387
3398	Amp	2	69143844	69540041	0	0.013878
3399	Amp	2	69541987	69548401	0	0.014444
3400	Amp	2	69549438	69723926	0	0.015731
3401	Amp	2	69726760	69767513	0	0.016707
3402	Amp	2	69767876	70106427	0	0.018957
3403	Amp	2	70108841	70174633	0	0.022641
3404	Amp	2	70179991	70613052	0	0.018957
3405	Amp	2	70613114	70876533	0	0.017981
3406	Amp	2	70881650	71290000	0	0.016694
3407	Amp	2	71291528	71570247	0	0.017706
3408	Amp	2	71570510	71958820	0	0.018997
3409	Amp	2	71959231	72910896	0	0.021194
3410	Amp	2	72922666	73015133	0	0.022651
3411	Amp	2	73018333	73114871	0	0.021543
3412	Amp	2	73117458	73288068	0	0.020087
3413	Amp	2	73294738	73434358	0	0.022283
3414	Amp	2	73439517	73747642	0	0.020087
3415	Amp	2	73748301	73763153	0	0.019311
3416	Amp	2	73764609	73855759	0	0.01782
3417	Amp	2	73856031	74115410	0	0.020204
3418	Amp	2	74115674	74183657	0	0.021847
3419	Amp	2	74186561	74223655	0	0.019983
3420	Amp	2	74224025	74329038	0	0.017733
3421	Amp	2	74332875	74576600	0	0.019205
3422	Amp	2	74578692	74758142	0	0.017733
3423	Amp	2	74760593	74864291	0	0.02005
3424	Amp	2	74864903	74918024	0	0.021004
3425	Amp	2	74925373	75013442	0	0.024995
3426	Amp	2	75015161	75140570	0	0.021004
3427	Amp	2	75140632	75145856	0	0.022937
3428	Amp	2	75145950	75304233	0	0.020621
3429	Amp	2	75304617	75426933	0	0.022004
3430	Amp	2	75427499	75695974	0	0.024017
3431	Amp	2	75696287	75714801	0	0.022083
3432	Amp	2	75715237	75813038	0	0.02007
3433	Amp	2	75814217	75858864	0	0.020621
3434	Amp	2	75859266	76827780	0	0.018977
3435	Amp	2	76829743	76842552	0	0.034913
3436	Amp	2	76844663	77051852	0	0.018977
3437	Amp	2	77055351	78046831	0	0.016593
3438	Amp	2	78047418	78057226	0	0.018873
3439	Amp	2	78064540	78172569	0	0.016593
3440	Amp	2	78173124	78177235	0	0.014395
3441	Amp	2	78256561	78442910	0	0.02267
3442	Amp	2	78446823	78548512	0	0.014395
3443	Amp	2	78548921	79470645	0	0.013384
3444	Amp	2	79470856	79626346	0	0.014506

3445	Amp	2	79626966	79901817	0	0.015663
3446	Amp	2	79907386	80347709	0	0.012839
3447	Amp	2	80347727	80450339	0	0.013411
3448	Amp	2	80452826	80552658	0	0.015803
3449	Amp	2	80554953	80785697	0	0.013411
3450	Amp	2	80788566	81380326	0	0.012254
3451	Amp	2	81386731	81501757	0	0.008563
3452	Amp	2	81508803	81509552	0	0.005896
3453	Amp	2	81512739	83718083	0	0.007564
3454	Amp	2	83719496	83798217	0	0.00654
3455	Amp	2	83800625	83955547	0	0.009981
3456	Amp	2	83965323	84300084	0	0.010427
3457	Amp	2	84301135	84330539	0	0.010949
3458	Amp	2	84332057	84363521	0	0.009139
3459	Amp	2	84368399	84369090	0	0.01095
3460	Amp	2	84375199	84388348	0	0.010503
3461	Amp	2	84389374	84432369	0	0.009981
3462	Amp	2	84434646	84446231	0	0.008171
3463	Amp	2	84454262	84505457	0	0.00654
3464	Amp	2	84505510	84788414	0	0.007936
3465	Amp	2	84797607	84940940	0	0.009115
3466	Amp	2	84943347	85179753	0	0.010682
3467	Amp	2	85181812	85181812	0	0.011155
3468	Amp	2	85183397	85191550	0	0.012958
3469	Amp	2	85192683	85281702	0	0.012407
3470	Amp	2	85282895	85318929	0	0.011934
3471	Amp	2	85319011	85420466	0	0.013351
3472	Amp	2	85427176	85440245	0	0.011549
3473	Amp	2	85441850	85456448	0	0.010426
3474	Amp	2	85457087	85622505	0	0.011341
3475	Amp	2	85622962	85948635	0	0.009924
3476	Amp	2	85951845	86055837	0	0.00783
3477	Amp	2	86057163	86510770	0	0.006263
3478	Amp	2	86523496	88098180	0	0.0076
3479	Amp	2	88098398	88707239	0	0.009771
3480	Amp	2	88707769	88749629	0	0.0076
3481	Amp	2	88751605	88807650	0	0.009621
3482	Amp	2	88807675	88831554	0	0.0076
3483	Amp	2	94675825	94675825	0	0.009753
3484	Amp	2	94675975	94680914	0	0.008357
3485	Amp	2	94681256	94719149	0	0.011387
3486	Amp	2	94721248	94821485	0	0.012531
3487	Amp	2	94833664	94999988	0	0.013799
3488	Amp	2	95001122	95098776	0	0.012183
3489	Amp	2	95107895	96233071	0	0.010915
3490	Amp	2	96233878	96494559	0	0.011759
3491	Amp	2	96497235	96531417	0	0.013423
3492	Amp	2	96533063	96888010	0	0.015783
3493	Amp	2	96889002	96993641	0	0.014939
3494	Amp	2	96994606	97049417	0	0.012579
3495	Amp	2	97658064	98211404	0	0.011546
3496	Amp	2	98211739	98281884	0	0.008956
3497	Amp	2	98282122	98606453	0	0.011082

3498	Amp	2	98607071	98681513	0	0.010451
3499	Amp	2	98682493	98759563	0	0.013044
3500	Amp	2	98760935	98796170	0	0.0124
3501	Amp	2	98797892	98834202	0	0.014178
3502	Amp	2	98837787	98923421	0	0.011585
3503	Amp	2	98947965	99080344	0	0.009458
3504	Amp	2	99082594	99327538	0	0.009995
3505	Amp	2	99328217	99603955	0	0.011104
3506	Amp	2	99604859	99606328	0	0.013275
3507	Amp	2	99607232	99663920	0	0.01432
3508	Amp	2	99666164	99742470	0	0.017019
3509	Amp	2	99744585	99819072	0	0.019554
3510	Amp	2	99823117	99886044	0	0.017019
3511	Amp	2	99886222	99930918	0	0.01432
3512	Amp	2	99931297	99985136	0	0.01559
3513	Amp	2	99986307	100133523	0	0.014109
3514	Amp	2	100136487	100145011	0	0.013065
3515	Amp	2	100146544	100194563	0	0.014132
3516	Amp	2	100195184	100206213	0	0.01356
3517	Amp	2	100208806	100289366	0	0.020329
3518	Amp	2	100290342	100373965	0	0.018551
3519	Amp	2	100374393	100450532	0	0.016087
3520	Amp	2	100451356	100490891	0	0.013963
3521	Amp	2	100492303	100767477	0	0.011783
3522	Amp	2	100769558	100916051	0	0.010512
3523	Amp	2	100916368	101017726	0	0.008341
3524	Amp	2	101020547	101321264	0	0.007197
3525	Amp	2	101322504	101464670	0	0.008705
3526	Amp	2	101471817	101805818	0	0.00947
3527	Amp	2	101812023	101900299	0	0.008705
3528	Amp	2	101902567	102246761	0	0.007197
3529	Amp	2	102246776	102370901	0	0.009116
3530	Amp	2	102371025	102403459	0	0.011479
3531	Amp	2	102403570	102579706	0	0.012367
3532	Amp	2	102581487	102660816	0	0.011479
3533	Amp	2	102664958	102738080	0	0.012561
3534	Amp	2	102740433	102934516	0	0.011479
3535	Amp	2	102935921	103052928	0	0.009578
3536	Amp	2	103057976	103147784	0	0.007197
3537	Amp	2	103151154	103164929	0	0.00979
3538	Amp	2	103168037	103178014	0	0.010235
3539	Amp	2	103182880	103326941	0	0.009167
3540	Amp	2	103327303	103395359	0	0.010258
3541	Amp	2	103395558	103559835	0	0.012115
3542	Amp	2	103560566	103578591	0	0.011024
3543	Amp	2	103579981	103787528	0	0.012456
3544	Amp	2	103792993	103962075	0	0.010149
3545	Amp	2	103962465	104191969	0	0.010719
3546	Amp	2	104192570	104403381	0	0.013095
3547	Amp	2	104403444	104465466	0	0.012525
3548	Amp	2	104467606	104541724	0	0.014096
3549	Amp	2	104542180	104937361	0	0.012525
3550	Amp	2	104941437	104942621	0	0.01208

3551	Amp	2	104942982	105032359	0	0.008135
3552	Amp	2	105032716	105202250	0	0.010025
3553	Amp	2	105206132	105218560	0	0.012759
3554	Amp	2	105220479	105365121	0	0.013423
3555	Amp	2	105365511	105406398	0	0.015176
3556	Amp	2	105406535	105547067	0	0.012442
3557	Amp	2	105548331	105592361	0	0.010689
3558	Amp	2	105597732	105704751	0	0.013391
3559	Amp	2	105706255	105722179	0	0.010689
3560	Amp	2	105722197	105727685	0	0.010025
3561	Amp	2	105729488	106077621	0	0.011215
3562	Amp	2	106079233	106805278	0	0.006547
3563	Amp	2	106807258	106839183	0	0.008135
3564	Amp	2	106839911	107173630	0	0.011089
3565	Amp	2	107173933	107203956	0	0.012505
3566	Amp	2	107208473	107264402	0	0.009757
3567	Amp	2	107268227	107416458	0	0.008454
3568	Amp	2	107418201	107435059	0	0.005392
3569	Amp	2	107439985	107722086	0	0.00396
3570	Amp	2	107726251	107804172	0	0.006347
3571	Amp	2	107808171	107820730	0	0.007108
3572	Amp	2	107903920	107990738	0	0.008625
3573	Amp	2	107991638	108007210	0	0.006687
3574	Amp	2	108009503	108023697	0	0.00517
3575	Amp	2	108026911	108084854	0	0.006426
3576	Amp	2	108087090	108204119	0	0.008453
3577	Amp	2	108207058	108213309	0	0.007196
3578	Amp	2	108213777	108449463	0	0.009688
3579	Amp	2	108463682	108594731	0	0.006734
3580	Amp	2	108596325	108652625	0	0.004242
3581	Amp	2	108659671	108678593	0	0.007845
3582	Amp	2	108681634	108713983	0	0.005818
3583	Amp	2	108715184	108791289	0	0.002215
3584	Amp	2	108797633	108877580	0	0.003071
3585	Amp	2	108880580	109230288	0	0.004056
3586	Amp	2	109235745	109416270	0	0.004702
3587	Amp	2	109417798	109498460	0	0.007228
3588	Amp	2	109499099	109675103	0	0.006582
3589	Amp	2	109675309	110640222	0	0.009222
3590	Amp	2	110643085	110649733	0	0.006582
3591	Amp	2	110652094	110946846	0	0.004056
3592	Amp	2	110947850	111644640	0	0.00231
3593	Amp	2	111645026	111668125	0	0.002766
3594	Amp	2	111669951	111911258	0	0.001877
3595	Amp	2	111911993	111966288	0	0.00266
3596	Amp	2	111968319	112466144	0	0.003958
3597	Amp	2	112472141	112519322	0	0.004496
3598	Amp	2	112520283	112621942	0	0.005207
3599	Amp	2	112622537	112742657	0	0.004669
3600	Amp	2	112743105	112832813	0	0.007351
3601	Amp	2	112832890	112908921	0	0.008324
3602	Amp	2	112910176	113054199	0	0.00669
3603	Amp	2	113057823	113478959	0	0.007735

3604	Amp	2	113478973	113701357	0	0.004931
3605	Amp	2	113701610	114115438	0	0.005405
3606	Amp	2	114116976	114392652	0	0.006913
3607	Amp	2	114395847	114645637	0	0.00594
3608	Amp	2	114645655	114645670	0	0.007268
3609	Amp	2	114646501	114860359	0	0.006412
3610	Amp	2	114862001	115084301	0	0.009772
3611	Amp	2	115085849	115256501	0	0.006412
3612	Amp	2	115259106	115771679	0	0.005591
3613	Amp	2	115771711	115772852	0	0.004448
3614	Amp	2	115919444	116057385	0	0.00315
3615	Amp	2	116065615	116372461	0	0.004625
3616	Amp	2	116377687	116634757	0	0.005726
3617	Amp	2	116640175	116704717	0	0.00719
3618	Amp	2	116705680	117176374	0	0.005726
3619	Amp	2	117179943	117336505	0	0.006347
3620	Amp	2	117336947	117508035	0	0.005726
3621	Amp	2	117508236	117557514	0	0.006224
3622	Amp	2	117557615	117869311	0	0.007887
3623	Amp	2	117870838	117983722	0	0.007413
3624	Amp	2	117986929	118338942	0	0.00575
3625	Amp	2	118340605	118365710	0	0.002948
3626	Amp	2	118366066	119584762	0	0.002165
3627	Amp	2	119585121	120203445	0	0.003712
3628	Amp	2	120208138	120214420	0	0.00424
3629	Amp	2	120217920	120291302	0	0.004858
3630	Amp	2	120291590	120529529	0	0.003311
3631	Amp	2	120529836	120552563	0	0.006833
3632	Amp	2	120590648	121293654	0	0.003311
3633	Amp	2	121296969	121300672	0	0.002693
3634	Amp	2	121303109	121346428	0	0.002196
3635	Amp	2	121354011	121641520	0	0.003444
3636	Amp	2	121648480	121770660	0	0.002342
3637	Amp	2	121776343	121919953	0	0.00363
3638	Amp	2	121922894	122195574	0	0.002383
3639	Amp	2	122198954	122268574	0	0.001855
3640	Amp	2	122269759	123056330	0	0.002355
3641	Amp	2	123058750	123298390	0	0.005839
3642	Amp	2	123298448	123442112	0	0.00975
3643	Amp	2	123449151	123484634	0	0.005839
3644	Amp	2	123602994	123972142	0	0.002355
3645	Amp	2	123978032	124241707	0	0.001067
3646	Amp	2	124241810	125371899	0	0.0005
3647	Amp	2	125374489	125435935	0	0.001114
3648	Amp	2	125695805	126046786	0	0.002492
3649	Amp	2	126049097	126583715	0	0.001879
3650	Amp	2	126586371	127209734	0	0.001378
3651	Amp	2	127214162	127324656	0	0.004446
3652	Amp	2	127327077	128192643	0	0.001378
3653	Amp	2	128199211	129018854	0	0
3654	Amp	2	129023641	129072580	0	0.000528
3655	Amp	2	129075674	129182355	0	0.002652
3656	Amp	2	129183074	129590353	0	0.000528

3657 Amp	2	129596666	129872567	0	0.001805
3658 Amp	2	129876618	130001889	0	0.004558
3659 Amp	2	130003736	131014424	0	0.001805
3660 Amp	2	131016613	131583751	0	0.002325
3661 Amp	2	131585766	131895850	0	0.004747
3662 Amp	2	131897833	131901780	0	0.004227
3663 Amp	2	131905616	132845092	0	0.003699
3664 Amp	2	132845109	132894516	0	0.004939
3665 Amp	2	132900555	133142502	0	0.00748
3666 Amp	2	133142556	133515901	0	0.004939
3667 Amp	2	133516493	133605970	0	0.003699
3668 Amp	2	133606185	133648510	0	0.00496
3669 Amp	2	133656033	134575727	0	0.007337
3670 Amp	2	134578196	134578196	0	0.008542
3671 Amp	2	134581070	135114344	0	0.007282
3672 Amp	2	135119992	135205141	0	0.006077
3673 Amp	2	135207536	135938468	0	0.003699
3674 Amp	2	135942758	136097898	0	0.00469
3675 Amp	2	136101202	136222470	0	0.003699
3676 Amp	2	136224845	136295153	0	0.004151
3677 Amp	2	136297834	136390977	0	0.001729
3678 Amp	2	136391133	136470083	0	0.002532
3679 Amp	2	136474554	136474557	0	0.004541
3680 Amp	2	136474836	136615271	0	0.00409
3681 Amp	2	136619331	136620024	0	0.003273
3682 Amp	2	136623379	136682836	0	0.00208
3683 Amp	2	136691496	136935145	0	0.002775
3684 Amp	2	136939650	137292917	0	0.003957
3685 Amp	2	137293631	137605381	0	0.003262
3686 Amp	2	137610524	138460427	0	0.004353
3687 Amp	2	138461680	138474518	0	0.005929
3688 Amp	2	138475913	138863574	0	0.010672
3689 Amp	2	138868169	138898046	0	0.012192
3690 Amp	2	138900971	139768856	0	0.013559
3691 Amp	2	139771976	139836288	0	0.017223
3692 Amp	2	139836310	140108694	0	0.018101
3693 Amp	2	140113283	140218724	0	0.020892
3694 Amp	2	140223495	140285574	0	0.018101
3695 Amp	2	140301661	140317830	0	0.015014
3696 Amp	2	140317910	140331528	0	0.011351
3697 Amp	2	140332778	140362298	0	0.00983
3698 Amp	2	140364842	140690743	0	0.008255
3699 Amp	2	140691618	140921471	0	0.0117
3700 Amp	2	140922114	141327726	0	0.010043
3701 Amp	2	141327831	141364748	0	0.025917
3702 Amp	2	141365356	141371954	0	0.010043
3703 Amp	2	141377824	141424542	0	0.006598
3704 Amp	2	141427880	141633447	0	0.00523
3705 Amp	2	141636823	141637578	0	0.007948
3706 Amp	2	141639769	141718655	0	0.008467
3707 Amp	2	141720163	141879321	0	0.007664
3708 Amp	2	141882063	141891292	0	0.009585
3709 Amp	2	141892766	141903319	0	0.012654

3710	Amp	2	141903384	141929233	0	0.014428
3711	Amp	2	141929733	141947860	0	0.016385
3712	Amp	2	141950028	142014138	0	0.014465
3713	Amp	2	142023119	142087553	0	0.018872
3714	Amp	2	142087831	142102109	0	0.020849
3715	Amp	2	142105261	142105591	0	0.022599
3716	Amp	2	142109048	142198018	0	0.02208
3717	Amp	2	142202210	142236759	0	0.024589
3718	Amp	2	142236813	142244191	0	0.022632
3719	Amp	2	142244284	142325206	0	0.021665
3720	Amp	2	142325484	142333098	0	0.019156
3721	Amp	2	142336830	142338304	0	0.017179
3722	Amp	2	142339416	142501372	0	0.015997
3723	Amp	2	142506088	142533493	0	0.014247
3724	Amp	2	142539391	142668845	0	0.00984
3725	Amp	2	142670706	142909118	0	0.008749
3726	Amp	2	142909425	143019929	0	0.009786
3727	Amp	2	143020311	143050966	0	0.010878
3728	Amp	2	143052358	143219110	0	0.007809
3729	Amp	2	143219471	143219471	0	0.008615
3730	Amp	2	143223400	143370986	0	0.010438
3731	Amp	2	143372720	143374134	0	0.009632
3732	Amp	2	143375793	143424804	0	0.006914
3733	Amp	2	143425244	143497505	0	0.005091
3734	Amp	2	143497828	144282225	0	0.004283
3735	Amp	2	144284969	144952230	0	0.005482
3736	Amp	2	144958306	145994544	0	0.004446
3737	Amp	2	146007582	147400324	0	0.003246
3738	Amp	2	147400846	147990409	0	0.003711
3739	Amp	2	147997256	148082096	0	0.004943
3740	Amp	2	148084810	148137444	0	0.006123
3741	Amp	2	148139791	148807478	0	0.007
3742	Amp	2	148810555	148999086	0	0.006123
3743	Amp	2	149003226	149362708	0	0.009607
3744	Amp	2	149365177	149533763	0	0.010652
3745	Amp	2	149536496	149714101	0	0.011624
3746	Amp	2	149714852	150108072	0	0.010444
3747	Amp	2	150108554	150234481	0	0.011528
3748	Amp	2	150238764	150552920	0	0.010556
3749	Amp	2	150556126	151739010	0	0.012081
3750	Amp	2	151739328	151946966	0	0.010556
3751	Amp	2	151947661	152031373	0	0.009511
3752	Amp	2	152035682	152125313	0	0.011921
3753	Amp	2	152125559	152231482	0	0.013088
3754	Amp	2	152234123	152613477	0	0.010678
3755	Amp	2	152615488	152619229	0	0.010213
3756	Amp	2	152619783	152671177	0	0.013253
3757	Amp	2	152671320	152680566	0	0.01418
3758	Amp	2	152683667	152831588	0	0.011141
3759	Amp	2	152838180	152889434	0	0.012363
3760	Amp	2	152892081	152901689	0	0.013465
3761	Amp	2	152903601	153268939	0	0.012243
3762	Amp	2	153277240	154088178	0	0.011141

3763	Amp	2	154095281	154277858	0	0.013183
3764	Amp	2	154278255	154322819	0	0.015425
3765	Amp	2	154324576	154434575	0	0.012256
3766	Amp	2	154439857	154453575	0	0.019377
3767	Amp	2	154455080	154488633	0	0.012256
3768	Amp	2	154489435	154516878	0	0.019548
3769	Amp	2	154518400	154548082	0	0.012256
3770	Amp	2	154550113	154556817	0	0.014979
3771	Amp	2	154559969	154591037	0	0.017565
3772	Amp	2	154594279	154613616	0	0.023207
3773	Amp	2	154615272	154813691	0	0.014979
3774	Amp	2	154813825	155360582	0	0.012256
3775	Amp	2	155364365	155820083	0	0.011164
3776	Amp	2	155822022	156015658	0	0.012621
3777	Amp	2	156015934	156103817	0	0.013304
3778	Amp	2	156105056	156105056	0	0.014677
3779	Amp	2	156106771	156298197	0	0.015594
3780	Amp	2	156299909	156395583	0	0.018802
3781	Amp	2	156403826	156445381	0	0.018119
3782	Amp	2	156446439	156575859	0	0.014911
3783	Amp	2	156576964	156601040	0	0.016608
3784	Amp	2	156603044	156675886	0	0.017686
3785	Amp	2	156676037	156686223	0	0.019289
3786	Amp	2	156701490	156863896	0	0.017246
3787	Amp	2	156866019	156907306	0	0.015643
3788	Amp	2	156911511	156911511	0	0.013947
3789	Amp	2	156920111	157020656	0	0.012489
3790	Amp	2	157024757	157631419	0	0.011411
3791	Amp	2	157631798	157767889	0	0.012991
3792	Amp	2	157768149	157944020	0	0.013471
3793	Amp	2	157947273	158132648	0	0.015916
3794	Amp	2	158134607	158276696	0	0.014998
3795	Amp	2	158277340	160137592	0	0.012553
3796	Amp	2	160143832	161097074	0	0.010974
3797	Amp	2	161099765	161219008	0	0.010494
3798	Amp	2	161219985	161328018	0	0.012108
3799	Amp	2	161331823	161994503	0	0.010494
3800	Amp	2	161994637	162029379	0	0.012693
3801	Amp	2	162029611	162029611	0	0.010494
3802	Amp	2	162033707	162148053	0	0.009326
3803	Amp	2	162148648	162175396	0	0.010261
3804	Amp	2	162175700	162192510	0	0.011202
3805	Amp	2	162194629	162355987	0	0.010261
3806	Amp	2	162357213	162934014	0	0.009326
3807	Amp	2	162938403	162959484	0	0.013079
3808	Amp	2	162961708	163028673	0	0.009326
3809	Amp	2	163032290	163078141	0	0.012108
3810	Amp	2	163081479	163120676	0	0.009326
3811	Amp	2	163124855	163316560	0	0.010815
3812	Amp	2	163316647	163482352	0	0.009326
3813	Amp	2	163483387	163523148	0	0.012721
3814	Amp	2	163528334	163689256	0	0.009326
3815	Amp	2	163695802	163770135	0	0.010415

3816	Amp	2	163770646	163962768	0	0.009184
3817	Amp	2	163965626	164016621	0	0.009788
3818	Amp	2	164018815	164209848	0	0.009184
3819	Amp	2	164217519	164257392	0	0.013672
3820	Amp	2	164266955	164304673	0	0.010013
3821	Amp	2	164306695	164415210	0	0.013371
3822	Amp	2	164415914	164428637	0	0.015876
3823	Amp	2	164429261	164544687	0	0.012518
3824	Amp	2	164545724	164635940	0	0.009184
3825	Amp	2	164638156	164971819	0	0.012059
3826	Amp	2	164971881	165001071	0	0.013658
3827	Amp	2	165001537	165010980	0	0.018086
3828	Amp	2	165014203	165082849	0	0.021622
3829	Amp	2	165083294	165108255	0	0.017343
3830	Amp	2	165109768	165598568	0	0.013658
3831	Amp	2	165598669	165894341	0	0.014431
3832	Amp	2	165897642	165904205	0	0.011556
3833	Amp	2	165910843	166056929	0	0.015886
3834	Amp	2	166056965	166064399	0	0.018613
3835	Amp	2	166065491	166122185	0	0.014284
3836	Amp	2	166124218	166140524	0	0.017369
3837	Amp	2	166141017	166156642	0	0.014284
3838	Amp	2	166157293	166198435	0	0.018302
3839	Amp	2	166198543	166215635	0	0.021528
3840	Amp	2	166218512	166266292	0	0.014284
3841	Amp	2	166271337	166282470	0	0.018588
3842	Amp	2	166282825	166377839	0	0.014284
3843	Amp	2	166377922	166421528	0	0.011556
3844	Amp	2	166425242	166435519	0	0.020338
3845	Amp	2	166437827	166475859	0	0.013834
3846	Amp	2	166476562	166496774	0	0.011556
3847	Amp	2	166499195	166651417	0	0.02065
3848	Amp	2	166653114	166664612	0	0.014853
3849	Amp	2	166668835	166797358	0	0.011556
3850	Amp	2	166799359	166924359	0	0.012792
3851	Amp	2	166924934	166959781	0	0.012018
3852	Amp	2	166960895	167139783	0	0.010783
3853	Amp	2	167140670	167538170	0	0.012442
3854	Amp	2	167539506	167566245	0	0.025881
3855	Amp	2	167566635	167683297	0	0.0273
3856	Amp	2	167683415	167696615	0	0.030573
3857	Amp	2	167696831	167740239	0	0.031082
3858	Amp	2	167740277	167746894	0	0.03266
3859	Amp	2	167753441	167849933	0	0.019221
3860	Amp	2	167851510	167932171	0	0.021718
3861	Amp	2	167933567	168061280	0	0.020298
3862	Amp	2	168061899	168124759	0	0.021738
3863	Amp	2	168125408	168227647	0	0.022841
3864	Amp	2	168227974	168416105	0	0.021738
3865	Amp	2	168420977	168484533	0	0.019242
3866	Amp	2	168490550	168727189	0	0.020149
3867	Amp	2	168727593	168826568	0	0.025905
3868	Amp	2	168832237	168893331	0	0.027026

3869	Amp	2	168896441	168934928	0	0.031478
3870	Amp	2	168935911	168937921	0	0.029819
3871	Amp	2	168940550	168990902	0	0.022485
3872	Amp	2	168991034	169045709	0	0.021578
3873	Amp	2	169053793	169162928	0	0.02238
3874	Amp	2	169166150	169194419	0	0.019108
3875	Amp	2	169194657	169312699	0	0.018306
3876	Amp	2	169316654	169333271	0	0.020815
3877	Amp	2	169337301	169394363	0	0.022092
3878	Amp	2	169405656	169530168	0	0.020972
3879	Amp	2	169530773	170255264	0	0.018462
3880	Amp	2	170260307	170380020	0	0.017022
3881	Amp	2	170385617	170402435	0	0.019439
3882	Amp	2	170402454	170442153	0	0.017022
3883	Amp	2	170442903	170508949	0	0.017768
3884	Amp	2	170509379	170593651	0	0.017022
3885	Amp	2	170593843	171222610	0	0.015635
3886	Amp	2	171227789	171395939	0	0.014036
3887	Amp	2	171397246	172526739	0	0.009584
3888	Amp	2	172530495	172658402	0	0.010674
3889	Amp	2	172658726	173720111	0	0.009584
3890	Amp	2	173721052	173829038	0	0.008307
3891	Amp	2	173829121	174065969	0	0.011631
3892	Amp	2	174067024	174111527	0	0.009326
3893	Amp	2	174115611	174269218	0	0.008237
3894	Amp	2	174274565	174495812	0	0.009532
3895	Amp	2	174496092	174533659	0	0.008237
3896	Amp	2	174534877	175855297	0	0.007218
3897	Amp	2	175855789	176138487	0	0.008566
3898	Amp	2	176139143	176697578	0	0.01005
3899	Amp	2	176702518	176755892	0	0.012257
3900	Amp	2	176756460	176768955	0	0.01098
3901	Amp	2	176778093	176822077	0	0.012273
3902	Amp	2	176822806	176877717	0	0.015396
3903	Amp	2	176877801	177074564	0	0.013912
3904	Amp	2	177077727	177129617	0	0.015375
3905	Amp	2	177134176	177231532	0	0.013167
3906	Amp	2	177232938	177356136	0	0.010044
3907	Amp	2	177361281	177570422	0	0.008582
3908	Amp	2	177572618	177590036	0	0.010251
3909	Amp	2	177598186	178475854	0	0.008582
3910	Amp	2	178475983	179006445	0	0.012946
3911	Amp	2	179006648	179075246	0	0.013714
3912	Amp	2	179081696	179224241	0	0.015441
3913	Amp	2	179224877	179643783	0	0.013714
3914	Amp	2	179644630	179818962	0	0.012946
3915	Amp	2	179824009	180139023	0	0.012494
3916	Amp	2	180141744	180281546	0	0.014547
3917	Amp	2	180284912	180603283	0	0.012494
3918	Amp	2	180604955	180613824	0	0.017015
3919	Amp	2	180615959	180709974	0	0.012494
3920	Amp	2	180709988	180732503	0	0.014571
3921	Amp	2	180732834	180760345	0	0.013204

3922	Amp	2	180763022	180858001	0	0.012494
3923	Amp	2	180858372	180944195	0	0.014182
3924	Amp	2	180945548	181102667	0	0.012494
3925	Amp	2	181104982	181319143	0	0.010168
3926	Amp	2	181322108	182059031	0	0.012282
3927	Amp	2	182059561	182293053	0	0.010934
3928	Amp	2	182293210	182816904	0	0.012494
3929	Amp	2	182817150	182860697	0	0.013938
3930	Amp	2	182860927	183014194	0	0.014532
3931	Amp	2	183016841	183139713	0	0.013089
3932	Amp	2	183140878	183271899	0	0.014245
3933	Amp	2	183272363	183534803	0	0.016546
3934	Amp	2	183540055	183576860	0	0.014432
3935	Amp	2	183583473	183598164	0	0.024975
3936	Amp	2	183598930	183685527	0	0.017833
3937	Amp	2	183686003	186047860	0	0.014432
3938	Amp	2	186053280	186164045	0	0.012132
3939	Amp	2	186168093	186288222	0	0.014582
3940	Amp	2	186294632	186294632	0	0.012132
3941	Amp	2	186296063	187123121	0	0.011537
3942	Amp	2	187123504	188247672	0	0.012982
3943	Amp	2	188274349	188400660	0	0.011537
3944	Amp	2	188400899	188640742	0	0.021126
3945	Amp	2	188641271	188724249	0	0.015477
3946	Amp	2	188729762	188730301	0	0.018875
3947	Amp	2	188732596	189233265	0	0.014936
3948	Amp	2	189235757	189309775	0	0.01608
3949	Amp	2	189310612	189328040	0	0.01856
3950	Amp	2	189328141	189401032	0	0.020004
3951	Amp	2	189405096	189635233	0	0.033952
3952	Amp	2	189636091	189665692	0	0.028639
3953	Amp	2	189671200	189928686	0	0.026601
3954	Amp	2	189933844	190170755	0	0.024551
3955	Amp	2	190174644	190297744	0	0.016522
3956	Amp	2	190300113	190418248	0	0.018004
3957	Amp	2	190421538	190479037	0	0.022302
3958	Amp	2	190479406	190930820	0	0.022801
3959	Amp	2	190931621	191270271	0	0.026142
3960	Amp	2	191272466	191490233	0	0.027817
3961	Amp	2	191491336	191681345	0	0.024476
3962	Amp	2	191685334	191926439	0	0.022801
3963	Amp	2	191926479	192205107	0	0.021657
3964	Amp	2	192215908	192264879	0	0.0192
3965	Amp	2	192277451	192447684	0	0.021671
3966	Amp	2	192447770	192738370	0	0.024227
3967	Amp	2	192742424	193490855	0	0.021671
3968	Amp	2	193496094	194546480	0	0.017373
3969	Amp	2	194559207	195725578	0	0.018643
3970	Amp	2	195728211	195818210	0	0.02007
3971	Amp	2	195818458	195936485	0	0.018109
3972	Amp	2	195936772	196015145	0	0.014988
3973	Amp	2	196015743	196035758	0	0.018101
3974	Amp	2	196042499	196399753	0	0.014988

3975 Amp	2	196400113	196602079	0	0.016997
3976 Amp	2	196604831	196748429	0	0.020005
3977 Amp	2	196752382	197495293	0	0.017996
3978 Amp	2	197497294	197497294	0	0.018571
3979 Amp	2	197498898	198478763	0	0.020727
3980 Amp	2	198479835	198934969	0	0.022162
3981 Amp	2	198937920	199423995	0	0.020727
3982 Amp	2	199425482	199428514	0	0.018957
3983 Amp	2	199429201	199615494	0	0.020412
3984 Amp	2	199617302	199660666	0	0.021531
3985 Amp	2	199664857	199690914	0	0.028346
3986 Amp	2	199694000	199749059	0	0.021531
3987 Amp	2	199749437	199911034	0	0.020412
3988 Amp	2	199913877	200898760	0	0.018957
3989 Amp	2	200900911	201114405	0	0.018504
3990 Amp	2	201119683	201209067	0	0.015496
3991 Amp	2	201209375	202628978	0	0.013935
3992 Amp	2	202632184	202664282	0	0.011178
3993 Amp	2	202810496	203013360	0	0.012958
3994 Amp	2	203016111	203087860	0	0.014866
3995 Amp	2	203088287	203113300	0	0.01404
3996 Amp	2	203118062	203174343	0	0.013086
3997 Amp	2	203175483	203292290	0	0.014194
3998 Amp	2	203294586	203480716	0	0.015929
3999 Amp	2	203485119	203527154	0	0.014194
4000 Amp	2	203527515	203527515	0	0.01585
4001 Amp	2	203529107	203575259	0	0.013943
4002 Amp	2	203575358	203608592	0	0.015599
4003 Amp	2	203614145	203748389	0	0.016054
4004 Amp	2	203750448	204000548	0	0.014398
4005 Amp	2	204001231	204260883	0	0.023544
4006 Amp	2	204262755	204486415	0	0.022388
4007 Amp	2	204486992	204506116	0	0.02357
4008 Amp	2	204506493	204626037	0	0.019766
4009 Amp	2	204627545	204841478	0	0.018584
4010 Amp	2	204844623	204965268	0	0.017475
4011 Amp	2	204968619	205014248	0	0.025315
4012 Amp	2	205014778	205191190	0	0.02909
4013 Amp	2	205192667	205257470	0	0.031782
4014 Amp	2	205257823	205366817	0	0.023942
4015 Amp	2	205367520	205372245	0	0.016134
4016 Amp	2	205375305	205409238	0	0.017507
4017 Amp	2	205413257	205569145	0	0.033443
4018 Amp	2	205569892	205642179	0	0.034376
4019 Amp	2	205643663	205680697	0	0.034943
4020 Amp	2	205680741	205819623	0	0.034376
4021 Amp	2	205820532	205821254	0	0.033002
4022 Amp	2	205824734	206005765	0	0.031346
4023 Amp	2	206006060	206542081	0	0.029011
4024 Amp	2	206542616	206772329	0	0.030922
4025 Amp	2	206784626	206904160	0	0.02841
4026 Amp	2	206906589	207089874	0	0.030758
4027 Amp	2	207101600	207615781	0	0.037039

4028	Amp	2	207620133	207669222	0	0.04322
4029	Amp	2	207670741	207747339	0	0.040707
4030	Amp	2	207752224	207817474	0	0.042808
4031	Amp	2	207819617	208204579	0	0.040707
4032	Amp	2	208210822	208269379	0	0.04535
4033	Amp	2	208281676	208494783	0	0.031927
4034	Amp	2	208502936	208518798	0	0.032578
4035	Amp	2	208526805	208640682	0	0.020858
4036	Amp	2	208643286	208967874	0	0.022474
4037	Amp	2	208969257	209067398	0	0.023336
4038	Amp	2	209069125	209203010	0	0.0224
4039	Amp	2	209203649	209293393	0	0.021064
4040	Amp	2	209296500	209457216	0	0.021988
4041	Amp	2	209457383	209564786	0	0.023228
4042	Amp	2	209564875	209699883	0	0.022304
4043	Amp	2	209705573	209764910	0	0.026362
4044	Amp	2	209766899	209829996	0	0.022304
4045	Amp	2	209835799	209863443	0	0.016023
4046	Amp	2	209864793	210015115	0	0.017024
4047	Amp	2	210015917	210490182	0	0.015796
4048	Amp	2	210490771	210534923	0	0.012309
4049	Amp	2	210557802	210577319	0	0.014051
4050	Amp	2	210577991	210662319	0	0.012309
4051	Amp	2	210663230	210688623	0	0.012966
4052	Amp	2	210689850	210718145	0	0.014226
4053	Amp	2	210724824	210769706	0	0.012966
4054	Amp	2	210770178	210810983	0	0.013842
4055	Amp	2	210813505	211502064	0	0.017692
4056	Amp	2	211503463	211531788	0	0.019136
4057	Amp	2	211535784	211640939	0	0.018479
4058	Amp	2	211643409	211657957	0	0.014629
4059	Amp	2	211660248	211992641	0	0.021218
4060	Amp	2	211992700	212062115	0	0.024034
4061	Amp	2	212062278	212100500	0	0.025573
4062	Amp	2	212100551	212104788	0	0.027299
4063	Amp	2	212105894	212126112	0	0.022093
4064	Amp	2	212126143	212152882	0	0.021217
4065	Amp	2	212157419	212273526	0	0.019679
4066	Amp	2	212274385	212341751	0	0.016862
4067	Amp	2	212341764	212425811	0	0.015136
4068	Amp	2	212432139	212500540	0	0.01884
4069	Amp	2	212523983	212524948	0	0.015136
4070	Amp	2	212525040	212540552	0	0.016269
4071	Amp	2	212547702	212588187	0	0.028733
4072	Amp	2	212588207	212653650	0	0.031633
4073	Amp	2	212655505	212749733	0	0.037161
4074	Amp	2	212750511	212790312	0	0.039366
4075	Amp	2	212791463	212848704	0	0.037922
4076	Amp	2	212850918	212858436	0	0.038972
4077	Amp	2	212858576	212959321	0	0.040848
4078	Amp	2	212961427	213167432	0	0.039798
4079	Amp	2	213168089	213205375	0	0.038416
4080	Amp	2	213211702	213268257	0	0.034719

4081	Amp	2	213270840	213323671	0	0.031818
4082	Amp	2	213327531	213341353	0	0.022422
4083	Amp	2	213344255	213522940	0	0.020217
4084	Amp	2	213524283	213615865	0	0.022959
4085	Amp	2	213616957	213695509	0	0.021048
4086	Amp	2	213695936	213720298	0	0.018306
4087	Amp	2	213722172	213736019	0	0.018887
4088	Amp	2	213742238	213747429	0	0.018306
4089	Amp	2	213748147	213884456	0	0.019562
4090	Amp	2	213885269	213905198	0	0.024693
4091	Amp	2	213905817	214030951	0	0.023437
4092	Amp	2	214032356	214212656	0	0.02219
4093	Amp	2	214212794	214229542	0	0.01706
4094	Amp	2	214229786	214447655	0	0.018547
4095	Amp	2	214449630	214475611	0	0.019657
4096	Amp	2	214486070	214496516	0	0.023585
4097	Amp	2	214496956	214554198	0	0.019657
4098	Amp	2	214555516	214594581	0	0.018547
4099	Amp	2	214595800	214610586	0	0.01706
4100	Amp	2	214610660	214756033	0	0.011532
4101	Amp	2	214758666	214812033	0	0.010531
4102	Amp	2	214814962	215011647	0	0.015152
4103	Amp	2	215028012	215077616	0	0.012804
4104	Amp	2	215084407	215093026	0	0.014384
4105	Amp	2	215096178	215291429	0	0.017055
4106	Amp	2	215291712	215331322	0	0.019856
4107	Amp	2	215331927	215352916	0	0.021281
4108	Amp	2	215353168	215492934	0	0.01848
4109	Amp	2	215496570	215521774	0	0.020011
4110	Amp	2	215523181	215576834	0	0.017855
4111	Amp	2	215576937	215675511	0	0.019159
4112	Amp	2	215675612	215743762	0	0.017628
4113	Amp	2	215748485	215868058	0	0.013007
4114	Amp	2	215869347	215974208	0	0.014683
4115	Amp	2	215974718	216000010	0	0.012522
4116	Amp	2	216001716	216007487	0	0.014767
4117	Amp	2	216009850	216011613	0	0.013464
4118	Amp	2	216011804	216676274	0	0.014733
4119	Amp	2	216677461	216841448	0	0.013057
4120	Amp	2	216848176	216964022	0	0.014896
4121	Amp	2	216965941	218071118	0	0.013317
4122	Amp	2	218072496	218157506	0	0.011477
4123	Amp	2	218158279	218220062	0	0.013058
4124	Amp	2	218220316	218281666	0	0.012538
4125	Amp	2	218284518	218302394	0	0.013623
4126	Amp	2	218305168	218448650	0	0.012354
4127	Amp	2	218450893	218795623	0	0.011268
4128	Amp	2	218798344	218816976	0	0.009687
4129	Amp	2	218820761	219147854	0	0.010366
4130	Amp	2	219148727	219381496	0	0.012026
4131	Amp	2	219391711	219433015	0	0.009467
4132	Amp	2	219433077	219466416	0	0.010151
4133	Amp	2	219466980	219686097	0	0.011523

4134	Amp	2	219687075	219744421	0	0.010151
4135	Amp	2	219744596	219828260	0	0.012958
4136	Amp	2	219828579	220095940	0	0.010151
4137	Amp	2	220097176	220274946	0	0.011851
4138	Amp	2	220275526	220544897	0	0.010191
4139	Amp	2	220545931	220592552	0	0.00954
4140	Amp	2	220598480	220752596	0	0.007839
4141	Amp	2	220754008	220851193	0	0.006383
4142	Amp	2	220853568	221287232	0	0.008724
4143	Amp	2	221287251	221444124	0	0.010023
4144	Amp	2	221446346	221589192	0	0.011907
4145	Amp	2	221591415	221720491	0	0.009229
4146	Amp	2	221721721	221791892	0	0.012222
4147	Amp	2	221793986	221856517	0	0.014973
4148	Amp	2	221869122	221879907	0	0.01198
4149	Amp	2	221881763	221963967	0	0.013414
4150	Amp	2	221967490	222005626	0	0.016959
4151	Amp	2	222016720	222146314	0	0.014619
4152	Amp	2	222146836	222749243	0	0.013379
4153	Amp	2	222751377	222812446	0	0.017503
4154	Amp	2	222812945	222959819	0	0.018769
4155	Amp	2	222964788	223108731	0	0.017343
4156	Amp	2	223109713	223605838	0	0.016077
4157	Amp	2	223607611	223737930	0	0.016998
4158	Amp	2	223738907	223750531	0	0.012874
4159	Amp	2	223755950	223825469	0	0.01603
4160	Amp	2	223833038	224041657	0	0.017212
4161	Amp	2	224049507	224219856	0	0.018916
4162	Amp	2	224220047	224307861	0	0.017212
4163	Amp	2	224311950	224421209	0	0.014056
4164	Amp	2	224427243	224455485	0	0.022841
4165	Amp	2	224458111	224536360	0	0.014926
4166	Amp	2	224540378	224554541	0	0.012419
4167	Amp	2	224555079	224583704	0	0.013459
4168	Amp	2	224588648	224664014	0	0.011669
4169	Amp	2	224665865	224823802	0	0.010235
4170	Amp	2	224830382	225088018	0	0.00799
4171	Amp	2	225089575	225110897	0	0.010543
4172	Amp	2	225113847	225272917	0	0.01125
4173	Amp	2	225275603	225767430	0	0.012727
4174	Amp	2	225774591	225788255	0	0.010844
4175	Amp	2	225794322	225825937	0	0.013538
4176	Amp	2	225831020	225831250	0	0.023268
4177	Amp	2	225832712	225835494	0	0.02465
4178	Amp	2	225839662	225855655	0	0.021956
4179	Amp	2	225863182	226001140	0	0.012226
4180	Amp	2	226001245	226034531	0	0.010844
4181	Amp	2	226034996	226076016	0	0.013131
4182	Amp	2	226077689	226105134	0	0.010578
4183	Amp	2	226113888	226123532	0	0.012586
4184	Amp	2	226123546	226203474	0	0.015143
4185	Amp	2	226207167	226426664	0	0.013136
4186	Amp	2	226429049	226709505	0	0.010848

4187	Amp	2	226709684	226789683	0	0.009808
4188	Amp	2	226790674	226820086	0	0.009101
4189	Amp	2	226824983	226846434	0	0.007623
4190	Amp	2	226853640	226869650	0	0.013984
4191	Amp	2	226873004	226976269	0	0.008245
4192	Amp	2	226978215	227010241	0	0.007566
4193	Amp	2	227010323	227181351	0	0.00917
4194	Amp	2	227182404	227343060	0	0.012524
4195	Amp	2	227343490	227361683	0	0.008548
4196	Amp	2	227365993	227679662	0	0.010091
4197	Amp	2	227679687	227756966	0	0.011586
4198	Amp	2	227760141	228222433	0	0.010043
4199	Amp	2	228228573	228228646	0	0.007485
4200	Amp	2	228235251	229712080	0	0.005882
4201	Amp	2	230031484	230033262	0	0.006957
4202	Amp	2	230035049	230309705	0	0.005462
4203	Amp	2	230311348	230400636	0	0.008031
4204	Amp	2	230401563	231350413	0	0.005462
4205	Amp	2	231351383	231433157	0	0.005909
4206	Amp	2	231436959	231504690	0	0.007388
4207	Amp	2	231597793	232470431	0	0.004833
4208	Amp	2	232472772	232552842	0	0.003992
4209	Amp	2	232553351	232555566	0	0.006799
4210	Amp	2	232555711	232741375	0	0.008937
4211	Amp	2	232786583	232895809	0	0.006799
4212	Amp	2	232898354	233376468	0	0.009541
4213	Amp	2	233376904	234695669	0	0.006799
4214	Amp	2	234695767	235523486	0	0.008591
4215	Amp	2	235527875	235539025	0	0.012262
4216	Amp	2	235545899	235988412	0	0.008591
4217	Amp	2	235988801	236355715	0	0.010418
4218	Amp	2	236356642	237537160	0	0.008626
4219	Amp	2	237540069	237898981	0	0.010231
4220	Amp	2	237901625	237939315	0	0.008626
4221	Amp	2	237940602	238030359	0	0.006799
4222	Amp	2	238030391	238053435	0	0.008298
4223	Amp	2	238053980	238090320	0	0.009506
4224	Amp	2	238091450	238146591	0	0.010303
4225	Amp	2	238147187	238167294	0	0.009095
4226	Amp	2	238170901	238322877	0	0.007596
4227	Amp	2	238323597	238842563	0	0.006799
4228	Amp	2	239108788	239474096	0	0.012048
4229	Amp	2	239480057	240473182	0	0.006799
4230	Amp	2	240476630	240671904	0	0.008451
4231	Amp	2	240776486	241328341	0	0.006799
4232	Amp	2	241330180	241517549	0	0.008678
4233	Amp	2	241517979	241518661	0	0.009434
4234	Amp	2	241523854	241537572	0	0.007556
4235	Amp	3	2170634	2232532	0	0.036286
4236	Amp	3	2233271	2236267	0	0.037133
4237	Amp	3	2236385	2780268	0	0.037805
4238	Amp	3	2780466	2833163	0	0.033771
4239	Amp	3	2836375	2875681	0	0.034773

4240	Amp	3	2875753	2879556	0	0.03748
4241	Amp	3	2879777	2937787	0	0.034677
4242	Amp	3	2938024	2994168	0	0.03861
4243	Amp	3	2995332	3075275	0	0.03422
4244	Amp	3	3126491	3155378	0	0.038295
4245	Amp	3	3155610	3265860	0	0.037623
4246	Amp	3	3267202	3296065	0	0.038211
4247	Amp	3	3297800	3321542	0	0.040235
4248	Amp	3	3324928	3570914	0	0.036159
4249	Amp	3	3572598	3618204	0	0.033452
4250	Amp	3	3618990	4353083	0	0.032864
4251	Amp	3	4354435	4354507	0	0.029179
4252	Amp	3	4357533	4378345	0	0.035094
4253	Amp	3	4378465	4410355	0	0.039638
4254	Amp	3	4414436	4487640	0	0.043283
4255	Amp	3	4490742	4491474	0	0.044622
4256	Amp	3	4493796	4587175	0	0.046062
4257	Amp	3	4590440	4628838	0	0.047722
4258	Amp	3	4629345	4683871	0	0.04847
4259	Amp	3	4685710	4785477	0	0.054867
4260	Amp	3	4788048	4825826	0	0.04847
4261	Amp	3	4829357	4838987	0	0.046809
4262	Amp	3	4841016	4911515	0	0.049388
4263	Amp	3	4911783	4972191	0	0.044845
4264	Amp	3	4972473	4989142	0	0.046715
4265	Amp	3	4989152	5013455	0	0.045375
4266	Amp	3	5116914	5116914	0	0.03946
4267	Amp	3	5117356	5238602	0	0.033198
4268	Amp	3	5240849	5248828	0	0.03388
4269	Amp	3	5249344	5264337	0	0.035617
4270	Amp	3	5265239	5275362	0	0.033038
4271	Amp	3	5277833	5408284	0	0.032356
4272	Amp	3	5408784	5457037	0	0.02987
4273	Amp	3	5457906	6299812	0	0.028431
4274	Amp	3	6300414	6312083	0	0.026693
4275	Amp	3	6315274	6814858	0	0.024669
4276	Amp	3	6819766	6906731	0	0.027333
4277	Amp	3	6908502	6981106	0	0.024959
4278	Amp	3	6981281	6994278	0	0.023957
4279	Amp	3	6994569	7035837	0	0.024738
4280	Amp	3	7035945	7100620	0	0.023957
4281	Amp	3	7100859	7114015	0	0.025233
4282	Amp	3	7114819	7137879	0	0.02588
4283	Amp	3	7137995	7257106	0	0.027151
4284	Amp	3	7257644	7309686	0	0.027713
4285	Amp	3	7315201	7468028	0	0.027151
4286	Amp	3	7468246	7681492	0	0.030383
4287	Amp	3	7683181	7910563	0	0.02846
4288	Amp	3	7910699	7946618	0	0.030203
4289	Amp	3	7947169	8067249	0	0.030642
4290	Amp	3	8070226	8124614	0	0.032581
4291	Amp	3	8125100	8307000	0	0.03131
4292	Amp	3	8309941	8320370	0	0.029371

4293	Amp	3	8396891	8402761	0	0.03533
4294	Amp	3	8404043	8467791	0	0.032098
4295	Amp	3	8467811	8591906	0	0.036138
4296	Amp	3	8595770	8632991	0	0.032098
4297	Amp	3	8634092	8670818	0	0.030313
4298	Amp	3	8673573	8772326	0	0.030766
4299	Amp	3	8774151	8774658	0	0.029134
4300	Amp	3	8774943	8838536	0	0.028648
4301	Amp	3	8839161	8848337	0	0.030518
4302	Amp	3	8851862	8856218	0	0.031553
4303	Amp	3	8858710	8900212	0	0.034954
4304	Amp	3	8903418	8989046	0	0.036294
4305	Amp	3	8991213	9078320	0	0.034954
4306	Amp	3	9078743	9105865	0	0.034514
4307	Amp	3	9107415	9203225	0	0.028471
4308	Amp	3	9203845	9271069	0	0.030441
4309	Amp	3	9274101	9318037	0	0.029419
4310	Amp	3	9331862	9339100	0	0.031346
4311	Amp	3	9339438	9371727	0	0.03311
4312	Amp	3	9393556	9467706	0	0.032097
4313	Amp	3	9472896	9585343	0	0.031549
4314	Amp	3	9585846	9636871	0	0.029263
4315	Amp	3	9644027	9772942	0	0.028228
4316	Amp	3	9778908	9805358	0	0.030885
4317	Amp	3	9811753	9847951	0	0.028228
4318	Amp	3	9854980	9958223	0	0.030204
4319	Amp	3	9960088	10084628	0	0.028228
4320	Amp	3	10084698	10100312	0	0.026464
4321	Amp	3	10100676	10266508	0	0.029874
4322	Amp	3	10266642	10411592	0	0.028926
4323	Amp	3	10413209	10416279	0	0.033402
4324	Amp	3	10417749	10524402	0	0.033881
4325	Amp	3	10526926	10599136	0	0.03659
4326	Amp	3	10601207	10770490	0	0.03318
4327	Amp	3	10775385	10788565	0	0.03047
4328	Amp	3	10792279	10995023	0	0.028231
4329	Amp	3	10995939	11230962	0	0.025994
4330	Amp	3	11231473	11957667	0	0.025515
4331	Amp	3	11957979	11977201	0	0.026083
4332	Amp	3	11979929	12306522	0	0.03185
4333	Amp	3	12308425	12380857	0	0.029911
4334	Amp	3	12383200	12574081	0	0.024144
4335	Amp	3	12574365	12938797	0	0.025481
4336	Amp	3	12942699	12976988	0	0.023553
4337	Amp	3	12977485	13181443	0	0.024067
4338	Amp	3	13182071	13202953	0	0.028141
4339	Amp	3	13202983	13524453	0	0.024067
4340	Amp	3	13525428	13602859	0	0.027639
4341	Amp	3	13605642	13677557	0	0.024067
4342	Amp	3	13677949	13687377	0	0.027389
4343	Amp	3	13687594	13764638	0	0.025344
4344	Amp	3	13766284	13928400	0	0.027177
4345	Amp	3	13935131	14213016	0	0.027683

4346	Amp	3	14216478	14289676	0	0.032842
4347	Amp	3	14301388	14453947	0	0.039354
4348	Amp	3	14454566	14511486	0	0.045339
4349	Amp	3	14513708	14541416	0	0.044263
4350	Amp	3	14541432	14558188	0	0.048727
4351	Amp	3	14559567	14727584	0	0.050137
4352	Amp	3	14727833	14797507	0	0.048727
4353	Amp	3	14799046	14949919	0	0.057246
4354	Amp	3	14951705	14977636	0	0.053631
4355	Amp	3	14978842	15011465	0	0.051183
4356	Amp	3	15014153	15036386	0	0.046278
4357	Amp	3	15038479	15200384	0	0.045764
4358	Amp	3	15200581	15261540	0	0.044761
4359	Amp	3	15263234	15343152	0	0.047209
4360	Amp	3	15346437	15534107	0	0.046703
4361	Amp	3	15534428	15611964	0	0.043762
4362	Amp	3	15615125	15665459	0	0.035113
4363	Amp	3	15665636	15753405	0	0.034649
4364	Amp	3	15755173	15793429	0	0.035641
4365	Amp	3	15796277	16129290	0	0.030482
4366	Amp	3	16131217	16220530	0	0.032234
4367	Amp	3	16222003	16458543	0	0.031745
4368	Amp	3	16462589	16721549	0	0.030482
4369	Amp	3	16721856	16729640	0	0.03117
4370	Amp	3	16731429	17045891	0	0.027339
4371	Amp	3	17046342	17090513	0	0.029504
4372	Amp	3	17091906	17109246	0	0.028512
4373	Amp	3	17113783	17177670	0	0.025357
4374	Amp	3	17180309	17400031	0	0.022035
4375	Amp	3	17407481	17721589	0	0.02539
4376	Amp	3	17723879	17773191	0	0.027128
4377	Amp	3	17791079	18049191	0	0.029156
4378	Amp	3	18051588	18128542	0	0.025801
4379	Amp	3	18130670	18382888	0	0.023636
4380	Amp	3	18383233	18471967	0	0.021899
4381	Amp	3	18475128	18633926	0	0.019871
4382	Amp	3	18634207	19332936	0	0.021641
4383	Amp	3	19336764	19355630	0	0.022445
4384	Amp	3	19358421	19413178	0	0.021641
4385	Amp	3	19413467	19520440	0	0.013806
4386	Amp	3	19526517	19558204	0	0.016773
4387	Amp	3	19561013	19561013	0	0.013806
4388	Amp	3	19562937	19676577	0	0.016233
4389	Amp	3	19680765	19702228	0	0.01315
4390	Amp	3	19706852	20043208	0	0.016041
4391	Amp	3	20043295	20554527	0	0.019421
4392	Amp	3	20557486	20881081	0	0.016995
4393	Amp	3	20881457	20964306	0	0.01789
4394	Amp	3	20966143	21026374	0	0.017202
4395	Amp	3	21027432	21034849	0	0.017862
4396	Amp	3	21035883	21395225	0	0.018614
4397	Amp	3	21396420	22177673	0	0.019439
4398	Amp	3	22185876	22213986	0	0.02241

4399	Amp	3	22214651	22278318	0	0.020577
4400	Amp	3	22283706	22842431	0	0.019917
4401	Amp	3	22843248	23109006	0	0.02198
4402	Amp	3	23110178	23259455	0	0.025671
4403	Amp	3	23259722	23379207	0	0.02198
4404	Amp	3	23384468	24114870	0	0.019917
4405	Amp	3	24116648	24185179	0	0.021456
4406	Amp	3	24187084	24438352	0	0.019917
4407	Amp	3	24438440	24805759	0	0.020735
4408	Amp	3	24807331	24832434	0	0.024306
4409	Amp	3	24832803	24945203	0	0.026153
4410	Amp	3	24945776	25152911	0	0.025335
4411	Amp	3	25153433	25166673	0	0.026631
4412	Amp	3	25166755	25273535	0	0.028619
4413	Amp	3	25274712	25340330	0	0.030423
4414	Amp	3	25340698	25413457	0	0.029127
4415	Amp	3	25419105	25560188	0	0.025555
4416	Amp	3	25560203	25607612	0	0.023752
4417	Amp	3	25607693	25723061	0	0.029318
4418	Amp	3	25723786	25938994	0	0.023752
4419	Amp	3	25939090	25939090	0	0.021764
4420	Amp	3	26419435	26439801	0	0.017047
4421	Amp	3	26440080	26474736	0	0.018896
4422	Amp	3	26477528	26530389	0	0.017047
4423	Amp	3	26535549	26535592	0	0.020676
4424	Amp	3	26574458	26737846	0	0.03657
4425	Amp	3	26741745	26921767	0	0.035863
4426	Amp	3	26926438	26926438	0	0.037965
4427	Amp	3	27040272	27061232	0	0.034335
4428	Amp	3	27061975	27101230	0	0.035988
4429	Amp	3	27105165	27255213	0	0.035093
4430	Amp	3	27255331	27279249	0	0.030976
4431	Amp	3	27288844	27336739	0	0.033903
4432	Amp	3	27338216	27420969	0	0.030976
4433	Amp	3	27421732	27610053	0	0.021215
4434	Amp	3	27610219	27652742	0	0.019562
4435	Amp	3	27653958	27868314	0	0.017461
4436	Amp	3	27872782	27940161	0	0.01939
4437	Amp	3	27940701	28095814	0	0.018268
4438	Amp	3	28098831	28138527	0	0.021163
4439	Amp	3	28138758	28270153	0	0.023331
4440	Amp	3	28272591	28491158	0	0.021401
4441	Amp	3	28493078	28903754	0	0.022131
4442	Amp	3	28906099	29364097	0	0.021306
4443	Amp	3	29369031	29723359	0	0.019459
4444	Amp	3	29727726	29727726	0	0.01994
4445	Amp	3	29728413	30134529	0	0.02038
4446	Amp	3	30140884	30666940	0	0.019459
4447	Amp	3	30668019	30793326	0	0.018707
4448	Amp	3	30793607	30917590	0	0.015816
4449	Amp	3	30921548	31253630	0	0.013648
4450	Amp	3	31253764	31636497	0	0.010754
4451	Amp	3	31640421	31693717	0	0.013019

4452	Amp	3	31695431	31813264	0	0.014035
4453	Amp	3	31814142	32366753	0	0.013019
4454	Amp	3	32368092	32395924	0	0.014534
4455	Amp	3	32400985	32441048	0	0.013684
4456	Amp	3	32441323	32896899	0	0.0146
4457	Amp	3	32900449	33012867	0	0.016232
4458	Amp	3	33016103	33064914	0	0.0146
4459	Amp	3	33066102	33285656	0	0.015082
4460	Amp	3	33289612	33367045	0	0.0146
4461	Amp	3	33371016	33371848	0	0.016768
4462	Amp	3	33374463	33446464	0	0.017235
4463	Amp	3	33448081	33493393	0	0.015067
4464	Amp	3	33493661	33550036	0	0.018325
4465	Amp	3	33559090	33765437	0	0.015067
4466	Amp	3	33769090	33858440	0	0.014338
4467	Amp	3	33861678	33971327	0	0.017015
4468	Amp	3	33975392	34121755	0	0.014338
4469	Amp	3	34122982	34165482	0	0.014846
4470	Amp	3	34165534	34399153	0	0.014338
4471	Amp	3	34402141	34544220	0	0.013491
4472	Amp	3	34546376	34546376	0	0.014319
4473	Amp	3	34546691	34799752	0	0.013852
4474	Amp	3	34804532	34829887	0	0.014484
4475	Amp	3	34832666	34909853	0	0.013852
4476	Amp	3	34917056	34996974	0	0.013024
4477	Amp	3	34997830	35044017	0	0.010759
4478	Amp	3	35047813	35151488	0	0.01223
4479	Amp	3	35152484	35203635	0	0.015168
4480	Amp	3	35209322	35404226	0	0.013696
4481	Amp	3	35410510	35905882	0	0.01303
4482	Amp	3	35907150	36269641	0	0.011722
4483	Amp	3	36271456	36515233	0	0.014891
4484	Amp	3	36519649	36551979	0	0.011954
4485	Amp	3	36553711	36580822	0	0.010658
4486	Amp	3	36581058	36756572	0	0.019359
4487	Amp	3	36759868	37011003	0	0.010658
4488	Amp	3	37015194	37107956	0	0.008784
4489	Amp	3	37112877	37159974	0	0.011915
4490	Amp	3	37163317	37198971	0	0.008784
4491	Amp	3	37198986	37267557	0	0.011362
4492	Amp	3	37272404	37368290	0	0.008784
4493	Amp	3	37373007	37393192	0	0.011905
4494	Amp	3	37395813	37753573	0	0.008784
4495	Amp	3	37753791	38531547	0	0.005968
4496	Amp	3	38534260	39010769	0	0.005173
4497	Amp	3	39013014	39129520	0	0.00954
4498	Amp	3	39131777	39224630	0	0.00693
4499	Amp	3	39224716	39272260	0	0.006014
4500	Amp	3	39273083	39439846	0	0.004257
4501	Amp	3	39442407	39536767	0	0.00766
4502	Amp	3	39542374	39653102	0	0.004257
4503	Amp	3	39662697	39709264	0	0.006529
4504	Amp	3	39732056	39852200	0	0.004257

4505 Amp	3	39856646	40049176	0	0.005778
4506 Amp	3	40052571	40218690	0	0.002808
4507 Amp	3	40220494	40574342	0	0.002063
4508 Amp	3	40577431	40669698	0	0.005359
4509 Amp	3	40671089	40783938	0	0.004548
4510 Amp	3	40784304	40852907	0	0.001251
4511 Amp	3	40854107	41006098	0	0.00071
4512 Amp	3	41011276	41044490	0	0.002967
4513 Amp	3	41052084	41348648	0	0.00071
4514 Amp	3	41350002	41872696	0	0.001262
4515 Amp	3	42174077	42174077	0	0
4516 Amp	3	42176371	42784568	0	0.001872
4517 Amp	3	42785457	43365795	0	0
4518 Amp	3	43366474	43407327	0	0.00287
4519 Amp	3	43408503	44488677	0	0
4520 Amp	3	44489204	45093824	0	0.000813
4521 Amp	3	45097609	46127394	0	0.00165
4522 Amp	3	46127774	46194302	0	0.004655
4523 Amp	3	46197275	47803981	0	0.00165
4524 Amp	3	47804056	47961818	0	0.000836
4525 Amp	3	47967770	48241004	0	0.001946
4526 Amp	3	48244725	48295117	0	0.003314
4527 Amp	3	48298209	48822492	0	0.001367
4528 Amp	3	48830888	48951260	0	0.004391
4529 Amp	3	48959992	48992063	0	0.005849
4530 Amp	3	48992967	48992967	0	0.007521
4531 Amp	3	48997084	49017259	0	0.010461
4532 Amp	3	49017727	49171394	0	0.007521
4533 Amp	3	49171432	49422974	0	0.005265
4534 Amp	3	49428845	49471896	0	0.003594
4535 Amp	3	49477304	49679603	0	0.002227
4536 Amp	3	49690326	49865632	0	0.003554
4537 Amp	3	49867922	49983710	0	0.002227
4538 Amp	3	49990690	50045200	0	0.00402
4539 Amp	3	50045481	50131977	0	0.002561
4540 Amp	3	50140578	50251477	0	0.004878
4541 Amp	3	50254185	50331164	0	0.002561
4542 Amp	3	50331842	50487723	0	0.004143
4543 Amp	3	50490261	50589226	0	0.004599
4544 Amp	3	50591114	50614187	0	0.007514
4545 Amp	3	50614320	50619021	0	0.004599
4546 Amp	3	50621081	51036766	0	0.004143
4547 Amp	3	51039396	51910831	0	0.002349
4548 Amp	3	51915371	52846979	0	0.000768
4549 Amp	3	52847220	52963855	0	0.001448
4550 Amp	3	52967279	53067764	0	0.000768
4551 Amp	3	53067810	53314524	0	0.002311
4552 Amp	3	53317860	53352591	0	0.003412
4553 Amp	3	53362094	53415751	0	0.005793
4554 Amp	3	53416306	53435288	0	0.008319
4555 Amp	3	53436836	53451763	0	0.009922
4556 Amp	3	53460796	53484593	0	0.007396
4557 Amp	3	53484956	53609823	0	0.006295

4558	Amp	3	53611535	53628943	0	0.007507
4559	Amp	3	53629163	53724676	0	0.005904
4560	Amp	3	53724886	54322692	0	0.004692
4561	Amp	3	54322726	54974999	0	0.002311
4562	Amp	3	54976280	55105450	0	0.00477
4563	Amp	3	55106186	55844146	0	0.008224
4564	Amp	3	55846169	55860282	0	0.006466
4565	Amp	3	55860475	55919417	0	0.00827
4566	Amp	3	55921186	56021892	0	0.00581
4567	Amp	3	56033392	57241117	0	0.004268
4568	Amp	3	57246227	57605501	0	0.004997
4569	Amp	3	57606121	58406684	0	0.001498
4570	Amp	3	58408367	59330284	0	0.00284
4571	Amp	3	59330605	59714996	0	0.002071
4572	Amp	3	59715181	59715251	0	0.000729
4573	Amp	3	59715704	59720118	0	0
4574	Amp	3	61040294	61045041	0	0.001148
4575	Amp	3	61049540	61057116	0	0.004092
4576	Amp	3	61057188	61130220	0	0.00613
4577	Amp	3	61130336	61156034	0	0.003185
4578	Amp	3	61156548	61173815	0	0.006382
4579	Amp	3	61173843	61191114	0	0.004345
4580	Amp	3	61191606	61203846	0	0.001148
4581	Amp	3	61204234	62128573	0	0.000449
4582	Amp	3	62128754	62644790	0	0.000904
4583	Amp	3	62646865	62647662	0	0.002671
4584	Amp	3	62648792	62648858	0	0.002216
4585	Amp	3	62649218	62876175	0	0.003643
4586	Amp	3	62877306	63777318	0	0.000449
4587	Amp	3	63783193	63834817	0	0.003708
4588	Amp	3	63840948	63897513	0	0.005187
4589	Amp	3	63900985	64050340	0	0.001928
4590	Amp	3	64053140	64969331	0	0.000449
4591	Amp	3	64969511	64970619	0	0.048256
4592	Amp	3	64970847	65126463	0	0.000449
4593	Amp	3	65126517	65357345	0	0.000988
4594	Amp	3	65360911	65382052	0	0.004176
4595	Amp	3	65382355	65524721	0	0.000988
4596	Amp	3	65525233	65595083	0	0.003803
4597	Amp	3	65599863	65611523	0	0.004559
4598	Amp	3	65612305	65631088	0	0.001744
4599	Amp	3	65633596	65706332	0	0.000988
4600	Amp	3	65710705	66059007	0	0.000449
4601	Amp	3	66062744	66115101	0	0.002664
4602	Amp	3	66117448	66133310	0	0.003846
4603	Amp	3	66135372	66136414	0	0.005117
4604	Amp	3	66140268	66140268	0	0.006905
4605	Amp	3	66140874	66512352	0	0.00469
4606	Amp	3	66515318	66592931	0	0.002902
4607	Amp	3	66594208	66619605	0	0.005405
4608	Amp	3	66624413	66639405	0	0.002902
4609	Amp	3	66642519	66849213	0	0.001631
4610	Amp	3	66849343	66913394	0	0.003795

4611	Amp	3	66933255	66974700	0	0.006154
4612	Amp	3	66988770	66994168	0	0.0079
4613	Amp	3	66999779	67135617	0	0.009308
4614	Amp	3	67136342	67645312	0	0.004785
4615	Amp	3	67647767	68000535	0	0.001631
4616	Amp	3	68003393	68360472	0	0.002647
4617	Amp	3	68362413	68474005	0	0.004378
4618	Amp	3	68479276	68876391	0	0.006028
4619	Amp	3	68876413	68896070	0	0.005579
4620	Amp	3	68896249	68940421	0	0.007655
4621	Amp	3	68944509	69017028	0	0.010021
4622	Amp	3	69024602	69162875	0	0.007944
4623	Amp	3	69163442	69176724	0	0.010491
4624	Amp	3	69176760	69259897	0	0.00711
4625	Amp	3	69261431	69556399	0	0.00546
4626	Amp	3	69559685	69606292	0	0.007183
4627	Amp	3	69611193	69614957	0	0.008663
4628	Amp	3	69616151	69693771	0	0.00694
4629	Amp	3	69696274	70127978	0	0.00546
4630	Amp	3	70130512	70364508	0	0.004278
4631	Amp	3	70364725	70561769	0	0.006525
4632	Amp	3	70565027	70590312	0	0.004795
4633	Amp	3	70590608	70980257	0	0.002547
4634	Amp	3	70984380	71004777	0	0.003165
4635	Amp	3	71007350	71020996	0	0.000618
4636	Amp	3	71021344	71076453	0	0.001803
4637	Amp	3	71077262	71219916	0	0.002981
4638	Amp	3	71230709	71316123	0	0.001803
4639	Amp	3	71316220	71456193	0	0.000618
4640	Amp	3	71457769	71551938	0	0.002525
4641	Amp	3	71563065	71727896	0	0.000618
4642	Amp	3	71729586	71913396	0	0.002093
4643	Amp	3	71915806	72240274	0	0.000618
4644	Amp	3	72244701	72255056	0	0.001623
4645	Amp	3	72256395	73584176	0	0.000618
4646	Amp	3	73586869	73655074	0	0.001076
4647	Amp	3	73655256	73679035	0	0.004358
4648	Amp	3	73679973	73864005	0	0.001076
4649	Amp	3	73865365	73947310	0	0.000618
4650	Amp	3	73950562	74128690	0	0.001229
4651	Amp	3	74128882	74980405	0	0.000618
4652	Amp	3	74989339	75117567	0	0.005338
4653	Amp	3	75129078	76030389	0	0.000618
4654	Amp	3	76033336	76350248	0	0
4655	Amp	3	76375601	76484014	0	0.007614
4656	Amp	3	76491422	76520889	0	0.010663
4657	Amp	3	76529025	76537820	0	0.011591
4658	Amp	3	76538440	76616714	0	0.008542
4659	Amp	3	76618168	76694333	0	0.010235
4660	Amp	3	76697140	76728172	0	0.013717
4661	Amp	3	76733646	76821882	0	0.010235
4662	Amp	3	76824001	76870081	0	0.008542
4663	Amp	3	76870249	77058385	0	0.006505

4664	Amp	3	77061171	77546983	0	0.002212
4665	Amp	3	77547206	77783353	0	0.004018
4666	Amp	3	77792275	78016918	0	0.0055
4667	Amp	3	78019596	78029204	0	0.006817
4668	Amp	3	78038299	78131873	0	0.005533
4669	Amp	3	78133747	78564252	0	0.003727
4670	Amp	3	78564337	78941385	0	0.002245
4671	Amp	3	78945890	79197211	0	0.005263
4672	Amp	3	79204357	79387320	0	0.006708
4673	Amp	3	79391504	79405400	0	0.009363
4674	Amp	3	79408993	80026323	0	0.007918
4675	Amp	3	80028792	80976727	0	0.002245
4676	Amp	3	80977796	81018266	0	0.003361
4677	Amp	3	81027141	81097639	0	0.003914
4678	Amp	3	81103465	81182289	0	0.002799
4679	Amp	3	81183472	81489477	0	0.002245
4680	Amp	3	81490962	81650515	0	0.006804
4681	Amp	3	81654467	81745591	0	0.002245
4682	Amp	3	81753267	81826048	0	0.002934
4683	Amp	3	81830391	81928724	0	0.004684
4684	Amp	3	81931406	82690115	0	0.002934
4685	Amp	3	82691312	82860198	0	0.008781
4686	Amp	3	82862860	82891020	0	0.024527
4687	Amp	3	82895274	82945829	0	0.016771
4688	Amp	3	82951710	83015027	0	0.008781
4689	Amp	3	83018916	83372609	0	0.002934
4690	Amp	3	83375162	83659811	0	0.005512
4691	Amp	3	83668281	83700636	0	0.006379
4692	Amp	3	83700993	83712804	0	0.00738
4693	Amp	3	83719826	83730786	0	0.004802
4694	Amp	3	83736837	83783169	0	0.005989
4695	Amp	3	83784412	83852523	0	0.005301
4696	Amp	3	83853536	84027363	0	0.004113
4697	Amp	3	84037233	84173793	0	0.007301
4698	Amp	3	84174011	84349416	0	0.012902
4699	Amp	3	84353966	84712492	0	0.016611
4700	Amp	3	84713870	84740644	0	0.01725
4701	Amp	3	84742700	84884699	0	0.014062
4702	Amp	3	84892552	84902538	0	0.004753
4703	Amp	3	84904775	84946448	0	0.007111
4704	Amp	3	84946777	85024074	0	0.005244
4705	Amp	3	85032639	85255975	0	0.007083
4706	Amp	3	85258811	85338160	0	0.005244
4707	Amp	3	85340405	85433274	0	0.002885
4708	Amp	3	85433445	85629593	0	0.002245
4709	Amp	3	85633738	85640193	0	0.007243
4710	Amp	3	85640993	85679551	0	0.002245
4711	Amp	3	85681264	85927331	0	0.003252
4712	Amp	3	85928112	86101309	0	0.002245
4713	Amp	3	86104453	86485777	0	0.003173
4714	Amp	3	86486138	86640681	0	0.004751
4715	Amp	3	86672451	86738239	0	0.007449
4716	Amp	3	86740999	87310357	0	0.004943

4717	Amp	3	87310911	87340635	0	0.005951
4718	Amp	3	87347555	87591817	0	0.004943
4719	Amp	3	87594861	87760453	0	0.007565
4720	Amp	3	87909425	88022347	0	0.009947
4721	Amp	3	88024235	88064329	0	0.008602
4722	Amp	3	88068753	88132047	0	0.01064
4723	Amp	3	88133918	88201894	0	0.011681
4724	Amp	3	88204037	88370423	0	0.012849
4725	Amp	3	88376521	88419384	0	0.014302
4726	Amp	3	88432847	88639896	0	0.013261
4727	Amp	3	88640840	88640840	0	0.012458
4728	Amp	3	88642066	88881115	0	0.010421
4729	Amp	3	88882327	88920207	0	0.011457
4730	Amp	3	88923479	89080854	0	0.012738
4731	Amp	3	89081192	89126420	0	0.011285
4732	Amp	3	89137139	89137973	0	0.012626
4733	Amp	3	89147494	89176603	0	0.013276
4734	Amp	3	89180262	89219116	0	0.012626
4735	Amp	3	89219494	89220069	0	0.015625
4736	Amp	3	89220259	89221115	0	0.014589
4737	Amp	3	89221163	89408775	0	0.01159
4738	Amp	3	89409462	89783189	0	0.012547
4739	Amp	3	89792404	89803680	0	0.016049
4740	Amp	3	89805967	89824699	0	0.016782
4741	Amp	3	89826440	90230647	0	0.01328
4742	Amp	3	90233924	90315110	0	0.010898
4743	Amp	3	90315775	90401331	0	0.009941
4744	Amp	3	90406012	90453712	0	0.009391
4745	Amp	3	91390926	91390926	0	0.00677
4746	Amp	3	94015345	94015827	0	0.024571
4747	Amp	3	94017382	94095501	0	0.026032
4748	Amp	3	94098136	94099622	0	0.026588
4749	Amp	3	94099916	94114731	0	0.022448
4750	Amp	3	94115355	94126870	0	0.020732
4751	Amp	3	94130729	94220340	0	0.016619
4752	Amp	3	94226916	94297719	0	0.014001
4753	Amp	3	94298757	94302882	0	0.016491
4754	Amp	3	94305586	94311825	0	0.017614
4755	Amp	3	94312103	94346785	0	0.015124
4756	Amp	3	94352637	94516612	0	0.015867
4757	Amp	3	94524869	94580668	0	0.016711
4758	Amp	3	94596235	94715970	0	0.017663
4759	Amp	3	94726136	94775474	0	0.022145
4760	Amp	3	94781632	94835230	0	0.024971
4761	Amp	3	94845090	94855854	0	0.030179
4762	Amp	3	94863754	94916546	0	0.038148
4763	Amp	3	94920924	94962069	0	0.033667
4764	Amp	3	94962580	94962580	0	0.036978
4765	Amp	3	94971286	95107481	0	0.036133
4766	Amp	3	95107852	95396849	0	0.0381
4767	Amp	3	95396893	95439907	0	0.035832
4768	Amp	3	95457526	95464012	0	0.039817
4769	Amp	3	95464160	95561645	0	0.039261

4770	Amp	3	95564925	95715954	0	0.036435
4771	Amp	3	95717807	95734408	0	0.03983
4772	Amp	3	95735624	95836277	0	0.036519
4773	Amp	3	95838241	95838510	0	0.038021
4774	Amp	3	95843876	96059716	0	0.034626
4775	Amp	3	96064635	96064635	0	0.038047
4776	Amp	3	96065127	96092538	0	0.038585
4777	Amp	3	96097299	96155836	0	0.0346
4778	Amp	3	96163416	96233282	0	0.03118
4779	Amp	3	96243487	96430421	0	0.030641
4780	Amp	3	96438648	96497159	0	0.029139
4781	Amp	3	96504143	96504143	0	0.028405
4782	Amp	3	96509134	96628071	0	0.023197
4783	Amp	3	96628127	96628127	0	0.032047
4784	Amp	3	96631049	96815753	0	0.032736
4785	Amp	3	96819709	96969895	0	0.033769
4786	Amp	3	96972348	96976977	0	0.03308
4787	Amp	3	96977693	97062453	0	0.025227
4788	Amp	3	97065582	97111079	0	0.02817
4789	Amp	3	97111301	97231877	0	0.025227
4790	Amp	3	97232748	97327783	0	0.028513
4791	Amp	3	97328739	97328739	0	0.029604
4792	Amp	3	97332842	97371130	0	0.028841
4793	Amp	3	97371969	97489188	0	0.025555
4794	Amp	3	97492790	97589188	0	0.027866
4795	Amp	3	97592231	97655693	0	0.025899
4796	Amp	3	97658366	97714091	0	0.025076
4797	Amp	3	97724206	97724206	0	0.025864
4798	Amp	3	97726189	97761153	0	0.035248
4799	Amp	3	97765808	97790017	0	0.034461
4800	Amp	3	97792711	97804875	0	0.033509
4801	Amp	3	97807480	97857291	0	0.029499
4802	Amp	3	97860139	97905109	0	0.033774
4803	Amp	3	97911582	97916487	0	0.03635
4804	Amp	3	97917905	97941262	0	0.039141
4805	Amp	3	97954120	97977902	0	0.033591
4806	Amp	3	97978684	98009931	0	0.039787
4807	Amp	3	98013923	98099555	0	0.051175
4808	Amp	3	98099979	98099979	0	0.048385
4809	Amp	3	98104785	98141048	0	0.036997
4810	Amp	3	98144444	98251224	0	0.030801
4811	Amp	3	98252873	98343211	0	0.035961
4812	Amp	3	98343783	98366425	0	0.037489
4813	Amp	3	98371156	98401636	0	0.03289
4814	Amp	3	98411994	98482014	0	0.030217
4815	Amp	3	98493107	98526940	0	0.029294
4816	Amp	3	98530821	98737034	0	0.031394
4817	Amp	3	98741628	98840495	0	0.03207
4818	Amp	3	98843925	99021555	0	0.032911
4819	Amp	3	99021685	99101142	0	0.034901
4820	Amp	3	99109027	99170144	0	0.03583
4821	Amp	3	99234917	99279374	0	0.029133
4822	Amp	3	99288854	99389395	0	0.024956

4823	Amp	3	99392291	99483698	0	0.024115
4824	Amp	3	99499665	99764929	0	0.026482
4825	Amp	3	99767717	99829430	0	0.028452
4826	Amp	3	99839587	99976300	0	0.026482
4827	Amp	3	99976975	100064450	0	0.027916
4828	Amp	3	100064778	100076807	0	0.029049
4829	Amp	3	100077082	100219028	0	0.030568
4830	Amp	3	100219293	100565163	0	0.032487
4831	Amp	3	100571707	100855484	0	0.030119
4832	Amp	3	100856909	100896706	0	0.031852
4833	Amp	3	100898171	100955654	0	0.028599
4834	Amp	3	100957278	101136092	0	0.027467
4835	Amp	3	101138817	101140568	0	0.029851
4836	Amp	3	101143050	101202341	0	0.027933
4837	Amp	3	101202559	101225232	0	0.034758
4838	Amp	3	101227569	101229192	0	0.027933
4839	Amp	3	101233515	101525053	0	0.025833
4840	Amp	3	101539806	101559226	0	0.030653
4841	Amp	3	101559326	101588771	0	0.031374
4842	Amp	3	101599140	101630829	0	0.029566
4843	Amp	3	101640484	101652447	0	0.027827
4844	Amp	3	101653078	101668554	0	0.028431
4845	Amp	3	101669771	101755654	0	0.027114
4846	Amp	3	101762699	101791681	0	0.028834
4847	Amp	3	101791790	101853446	0	0.03046
4848	Amp	3	101854208	101854208	0	0.033649
4849	Amp	3	101855059	101859863	0	0.036203
4850	Amp	3	101860929	101932614	0	0.043812
4851	Amp	3	101934372	101940282	0	0.045138
4852	Amp	3	101942309	102217294	0	0.04677
4853	Amp	3	102219533	102254903	0	0.045991
4854	Amp	3	102259656	102280088	0	0.044365
4855	Amp	3	102280281	102291027	0	0.040972
4856	Amp	3	102293442	102298581	0	0.039198
4857	Amp	3	102299090	102325651	0	0.036814
4858	Amp	3	102327038	102347707	0	0.035488
4859	Amp	3	102350469	102353364	0	0.031272
4860	Amp	3	102356591	102454330	0	0.038818
4861	Amp	3	102457281	102474337	0	0.035737
4862	Amp	3	102474831	102574050	0	0.039753
4863	Amp	3	102574307	102598668	0	0.039149
4864	Amp	3	102601132	102688134	0	0.031603
4865	Amp	3	102689507	102692907	0	0.029883
4866	Amp	3	102693252	102973250	0	0.029161
4867	Amp	3	102975749	103086404	0	0.031334
4868	Amp	3	103092660	103475026	0	0.030607
4869	Amp	3	103487801	103507795	0	0.029266
4870	Amp	3	103510876	103653873	0	0.025172
4871	Amp	3	103654064	103693631	0	0.022999
4872	Amp	3	103693753	103750341	0	0.02362
4873	Amp	3	103750789	103773743	0	0.031066
4874	Amp	3	103794755	103874428	0	0.033401
4875	Amp	3	103875744	103935142	0	0.031066

4876	Amp	3	103938276	104042822	0	0.031919
4877	Amp	3	104052803	104142320	0	0.033747
4878	Amp	3	104142983	104297714	0	0.032818
4879	Amp	3	104298897	104312739	0	0.033432
4880	Amp	3	104324972	104334047	0	0.048514
4881	Amp	3	104338884	104374570	0	0.045567
4882	Amp	3	104375056	104418509	0	0.04717
4883	Amp	3	104418555	104481178	0	0.045567
4884	Amp	3	104486929	104529132	0	0.036236
4885	Amp	3	104529191	104551178	0	0.037018
4886	Amp	3	104555396	104593251	0	0.029799
4887	Amp	3	104593576	104631049	0	0.02797
4888	Amp	3	104633616	104697717	0	0.035582
4889	Amp	3	104697747	104805925	0	0.039208
4890	Amp	3	104807283	104874531	0	0.033338
4891	Amp	3	104876946	104902429	0	0.036703
4892	Amp	3	104902571	104914505	0	0.03365
4893	Amp	3	104914519	105004968	0	0.036751
4894	Amp	3	105005007	105213947	0	0.042634
4895	Amp	3	105214266	105344372	0	0.039268
4896	Amp	3	105344543	105416939	0	0.038487
4897	Amp	3	105422582	105443414	0	0.031028
4898	Amp	3	105447409	105493831	0	0.020479
4899	Amp	3	105496759	105756973	0	0.026028
4900	Amp	3	105757454	105826150	0	0.028641
4901	Amp	3	105829719	105898899	0	0.023091
4902	Amp	3	105900169	106114940	0	0.026465
4903	Amp	3	106117418	106131556	0	0.034037
4904	Amp	3	106139483	106146212	0	0.030664
4905	Amp	3	106152073	106181121	0	0.034363
4906	Amp	3	106183629	106218018	0	0.030664
4907	Amp	3	106232202	106262572	0	0.032089
4908	Amp	3	106263091	106354644	0	0.03264
4909	Amp	3	106358044	106371615	0	0.03356
4910	Amp	3	106374883	106411476	0	0.025299
4911	Amp	3	106413309	106441656	0	0.022687
4912	Amp	3	106445722	106530837	0	0.024798
4913	Amp	3	106532283	106776847	0	0.023372
4914	Amp	3	106778380	106858886	0	0.022822
4915	Amp	3	106865992	107178296	0	0.021902
4916	Amp	3	107182684	107250050	0	0.030339
4917	Amp	3	107252594	107487649	0	0.026666
4918	Amp	3	107487955	107492922	0	0.032975
4919	Amp	3	107496356	107561021	0	0.045945
4920	Amp	3	107563602	107564860	0	0.04112
4921	Amp	3	107573714	107587732	0	0.036452
4922	Amp	3	107588205	107702803	0	0.035105
4923	Amp	3	107705243	107766172	0	0.033885
4924	Amp	3	107768368	107843692	0	0.032241
4925	Amp	3	107850908	107949104	0	0.033995
4926	Amp	3	107949574	107958354	0	0.031797
4927	Amp	3	107959919	107995784	0	0.028402
4928	Amp	3	108001114	108025245	0	0.031537

4929	Amp	3	108025453	108033573	0	0.034865
4930	Amp	3	108037441	108041953	0	0.044266
4931	Amp	3	108045604	108045950	0	0.028364
4932	Amp	3	108045978	108220937	0	0.02523
4933	Amp	3	108222661	108401390	0	0.029469
4934	Amp	3	108401403	108428599	0	0.027358
4935	Amp	3	108431267	108462279	0	0.02403
4936	Amp	3	108463543	108558023	0	0.022422
4937	Amp	3	108559230	108601590	0	0.021389
4938	Amp	3	108607155	108634429	0	0.032746
4939	Amp	3	108641466	108648113	0	0.030471
4940	Amp	3	108663713	108731731	0	0.028156
4941	Amp	3	108732313	108805211	0	0.029013
4942	Amp	3	108806653	108852014	0	0.030162
4943	Amp	3	108852695	108856102	0	0.027531
4944	Amp	3	108859152	108877854	0	0.028608
4945	Amp	3	109300867	109462051	0	0.032207
4946	Amp	3	109464448	109500465	0	0.039657
4947	Amp	3	109507766	109572100	0	0.032207
4948	Amp	3	109581115	109675787	0	0.027251
4949	Amp	3	109687663	110025791	0	0.02894
4950	Amp	3	110026823	110074104	0	0.02238
4951	Amp	3	110076618	110124324	0	0.028394
4952	Amp	3	110126643	110147833	0	0.0242
4953	Amp	3	110150994	110158916	0	0.020692
4954	Amp	3	110163647	110200692	0	0.026127
4955	Amp	3	110201442	110356916	0	0.028614
4956	Amp	3	110356968	110391038	0	0.029927
4957	Amp	3	110391126	110577736	0	0.03282
4958	Amp	3	110577884	110588735	0	0.027385
4959	Amp	3	110588749	110764230	0	0.028415
4960	Amp	3	110771036	110968529	0	0.031109
4961	Amp	3	110969362	111117491	0	0.027019
4962	Amp	3	111119888	111196057	0	0.023289
4963	Amp	3	111199078	111317534	0	0.026666
4964	Amp	3	111320930	111386062	0	0.027239
4965	Amp	3	111391006	111448795	0	0.03379
4966	Amp	3	111452525	111496618	0	0.027239
4967	Amp	3	111507223	111518769	0	0.043141
4968	Amp	3	111518933	111533808	0	0.042568
4969	Amp	3	111536614	111773021	0	0.046112
4970	Amp	3	111774892	111891065	0	0.042568
4971	Amp	3	111891141	111904375	0	0.04573
4972	Amp	3	111906723	111911168	0	0.055138
4973	Amp	3	111911940	112003245	0	0.04573
4974	Amp	3	112003649	112046987	0	0.0447
4975	Amp	3	112047274	112071363	0	0.045982
4976	Amp	3	112074948	112258185	0	0.044281
4977	Amp	3	112259453	112416492	0	0.046791
4978	Amp	3	112416852	112583603	0	0.046079
4979	Amp	3	112584384	112637923	0	0.039909
4980	Amp	3	112646026	112735279	0	0.046329
4981	Amp	3	112739073	112855898	0	0.039169

4982	Amp	3	112856233	112958565	0	0.040788
4983	Amp	3	112958682	113002847	0	0.038548
4984	Amp	3	113003479	113058598	0	0.035346
4985	Amp	3	113065000	113195909	0	0.037721
4986	Amp	3	113196720	113239477	0	0.042599
4987	Amp	3	113240049	113297622	0	0.043754
4988	Amp	3	113298470	113372680	0	0.044593
4989	Amp	3	113376316	113496985	0	0.043311
4990	Amp	3	113499359	113521461	0	0.044928
4991	Amp	3	113529801	113597536	0	0.042552
4992	Amp	3	113598016	113615905	0	0.041475
4993	Amp	3	113616160	114066971	0	0.037795
4994	Amp	3	114067643	114096733	0	0.040075
4995	Amp	3	114098139	114098139	0	0.040533
4996	Amp	3	114098399	114254161	0	0.042905
4997	Amp	3	114254227	114309786	0	0.041912
4998	Amp	3	114315251	114339055	0	0.040757
4999	Amp	3	114339566	114395875	0	0.038477
5000	Amp	3	114399459	114583252	0	0.036104
5001	Amp	3	114585306	114861087	0	0.034488
5002	Amp	3	114864658	114915287	0	0.033649
5003	Amp	3	114921349	115013374	0	0.032318
5004	Amp	3	115014638	115164724	0	0.034479
5005	Amp	3	115164800	115262412	0	0.032784
5006	Amp	3	115262484	115436667	0	0.033892
5007	Amp	3	115436801	115438875	0	0.034558
5008	Amp	3	115439429	115614397	0	0.021399
5009	Amp	3	115616185	115677404	0	0.020942
5010	Amp	3	115684846	115817653	0	0.018781
5011	Amp	3	115818216	115886297	0	0.023259
5012	Amp	3	115886459	115947381	0	0.022152
5013	Amp	3	115948044	115960582	0	0.023287
5014	Amp	3	115962459	116050452	0	0.026978
5015	Amp	3	116051243	116169322	0	0.022496
5016	Amp	3	116177403	116249368	0	0.024761
5017	Amp	3	116249734	116336046	0	0.021071
5018	Amp	3	116337383	116408550	0	0.020214
5019	Amp	3	116408726	116415810	0	0.028217
5020	Amp	3	116416150	116432135	0	0.031828
5021	Amp	3	116433009	116597006	0	0.023825
5022	Amp	3	116597243	116635621	0	0.021559
5023	Amp	3	116637163	116685651	0	0.01347
5024	Amp	3	116687352	116762567	0	0.018343
5025	Amp	3	116764118	116914094	0	0.02138
5026	Amp	3	116917156	117687613	0	0.020681
5027	Amp	3	117688440	117805966	0	0.022718
5028	Amp	3	117807223	117946798	0	0.018629
5029	Amp	3	117950562	118285821	0	0.017963
5030	Amp	3	118286501	118342343	0	0.033864
5031	Amp	3	118347252	118392193	0	0.030044
5032	Amp	3	118395843	118446534	0	0.031434
5033	Amp	3	118446587	118456332	0	0.032568
5034	Amp	3	118456896	118542560	0	0.031277

5035	Amp	3	118542735	118565612	0	0.032803
5036	Amp	3	118566835	118613613	0	0.027673
5037	Amp	3	118616322	118773902	0	0.023656
5038	Amp	3	118777036	118796399	0	0.02213
5039	Amp	3	118799578	118855688	0	0.02074
5040	Amp	3	118860657	118878161	0	0.021997
5041	Amp	3	118879349	118899751	0	0.02074
5042	Amp	3	118900369	118982902	0	0.02214
5043	Amp	3	118986944	119117086	0	0.020026
5044	Amp	3	119118411	119239356	0	0.018626
5045	Amp	3	119261341	119262417	0	0.022745
5046	Amp	3	119262919	119278221	0	0.023809
5047	Amp	3	119285467	119394973	0	0.01969
5048	Amp	3	119396876	119411513	0	0.020921
5049	Amp	3	119419828	119455011	0	0.023757
5050	Amp	3	119459109	119666293	0	0.022526
5051	Amp	3	119671997	119735645	0	0.024703
5052	Amp	3	119742179	119892861	0	0.029783
5053	Amp	3	119915500	119919067	0	0.018731
5054	Amp	3	119919390	120020115	0	0.020504
5055	Amp	3	120025154	120049399	0	0.018719
5056	Amp	3	120055086	120299367	0	0.021897
5057	Amp	3	120301642	120431508	0	0.020124
5058	Amp	3	120433043	120552583	0	0.016947
5059	Amp	3	120553180	120702526	0	0.019124
5060	Amp	3	120702961	120820188	0	0.018362
5061	Amp	3	120822757	120983361	0	0.016184
5062	Amp	3	120986900	121048101	0	0.017203
5063	Amp	3	121048321	121135521	0	0.016184
5064	Amp	3	121137169	121361167	0	0.017114
5065	Amp	3	121365240	121406696	0	0.019019
5066	Amp	3	121408413	121449195	0	0.0239
5067	Amp	3	121449497	121804681	0	0.021995
5068	Amp	3	121805510	121838404	0	0.025626
5069	Amp	3	121838686	121874065	0	0.021995
5070	Amp	3	121874922	121943503	0	0.021065
5071	Amp	3	121944212	121975565	0	0.024379
5072	Amp	3	121976849	122410890	0	0.021065
5073	Amp	3	122416373	122743154	0	0.021919
5074	Amp	3	122744279	122923447	0	0.02377
5075	Amp	3	122923568	123003325	0	0.024465
5076	Amp	3	123008481	123103838	0	0.0256
5077	Amp	3	123104210	123438630	0	0.024465
5078	Amp	3	123440567	123538258	0	0.027127
5079	Amp	3	123538499	123556551	0	0.024465
5080	Amp	3	123559893	123696667	0	0.025837
5081	Amp	3	123700066	123772005	0	0.020956
5082	Amp	3	123778809	123941544	0	0.02142
5083	Amp	3	123942109	123997005	0	0.020566
5084	Amp	3	123998530	124056053	0	0.019871
5085	Amp	3	124061189	124189286	0	0.023364
5086	Amp	3	124189436	124201433	0	0.017569
5087	Amp	3	124203417	124203417	0	0.016401

5088	Amp	3	124206448	124212567	0	0.015337
5089	Amp	3	124214943	124393280	0	0.014762
5090	Amp	3	124396471	124574981	0	0.014299
5091	Amp	3	124576602	124607954	0	0.011811
5092	Amp	3	124608400	124744325	0	0.013884
5093	Amp	3	124745135	124764242	0	0.014987
5094	Amp	3	124767432	124784302	0	0.016135
5095	Amp	3	124791364	125199910	0	0.015001
5096	Amp	3	125200035	125252912	0	0.013898
5097	Amp	3	125255057	125523564	0	0.011063
5098	Amp	3	125524898	125637250	0	0.01303
5099	Amp	3	125641293	125705628	0	0.011658
5100	Amp	3	125750578	126041797	0	0.012163
5101	Amp	3	126047943	126251034	0	0.011658
5102	Amp	3	126254815	126827492	0	0.014427
5103	Amp	3	126830300	126892291	0	0.013279
5104	Amp	3	126948598	127258704	0	0.015911
5105	Amp	3	127261032	127327433	0	0.017728
5106	Amp	3	127327945	127365745	0	0.019524
5107	Amp	3	127367218	127701497	0	0.024918
5108	Amp	3	127701510	127777762	0	0.02448
5109	Amp	3	127780709	127885559	0	0.026879
5110	Amp	3	127885618	128036836	0	0.029863
5111	Amp	3	128047215	128130982	0	0.026879
5112	Amp	3	128134572	128708021	0	0.024247
5113	Amp	3	128710092	128720210	0	0.023388
5114	Amp	3	128720939	128741952	0	0.026386
5115	Amp	3	128744091	128764662	0	0.023388
5116	Amp	3	128768400	128878983	0	0.026043
5117	Amp	3	128884307	128913548	0	0.033212
5118	Amp	3	128913611	128987291	0	0.029774
5119	Amp	3	128987663	128991257	0	0.026043
5120	Amp	3	128995453	129264421	0	0.023388
5121	Amp	3	129264616	129327076	0	0.024724
5122	Amp	3	129327591	130391707	0	0.020627
5123	Amp	3	130395239	130899153	0	0.019911
5124	Amp	3	130902928	131150217	0	0.022308
5125	Amp	3	131155250	131211810	0	0.019911
5126	Amp	3	131213148	131319679	0	0.023284
5127	Amp	3	131322361	131529597	0	0.019911
5128	Amp	3	131533437	131682967	0	0.024887
5129	Amp	3	131683905	132030329	0	0.016154
5130	Amp	3	132030350	132037702	0	0.016775
5131	Amp	3	132038728	132056472	0	0.017566
5132	Amp	3	132059475	132214767	0	0.022497
5133	Amp	3	132217514	132563608	0	0.017566
5134	Amp	3	132564871	132628732	0	0.020021
5135	Amp	3	132636841	132718843	0	0.021095
5136	Amp	3	132721175	132803692	0	0.022257
5137	Amp	3	132813165	133400782	0	0.021183
5138	Amp	3	133405410	133410428	0	0.019829
5139	Amp	3	133418820	133445989	0	0.021755
5140	Amp	3	133453530	133550675	0	0.020653

5141	Amp	3	133557638	133599851	0	0.023531
5142	Amp	3	133601983	133656493	0	0.031349
5143	Amp	3	133656801	133786459	0	0.028471
5144	Amp	3	133787084	134060863	0	0.02793
5145	Amp	3	134061457	134108462	0	0.028376
5146	Amp	3	134108752	134180657	0	0.02793
5147	Amp	3	134185296	134597536	0	0.030105
5148	Amp	3	134597811	134643655	0	0.028477
5149	Amp	3	134645943	134807393	0	0.031072
5150	Amp	3	134809081	134881283	0	0.03488
5151	Amp	3	134883172	135076868	0	0.032285
5152	Amp	3	135077443	135081663	0	0.034231
5153	Amp	3	135082820	135090535	0	0.036556
5154	Amp	3	135095618	135342469	0	0.037157
5155	Amp	3	135342572	135582911	0	0.029496
5156	Amp	3	135671490	135675831	0	0.040767
5157	Amp	3	135676274	135696696	0	0.038442
5158	Amp	3	135698714	135749505	0	0.040187
5159	Amp	3	135754229	135804704	0	0.042612
5160	Amp	3	135806245	135896532	0	0.034646
5161	Amp	3	135900104	135906759	0	0.032221
5162	Amp	3	135912540	135924813	0	0.030476
5163	Amp	3	135927198	135927885	0	0.02862
5164	Amp	3	135941035	136121836	0	0.024812
5165	Amp	3	136123961	136132235	0	0.024211
5166	Amp	3	136133548	136332127	0	0.02359
5167	Amp	3	136335980	136618886	0	0.018701
5168	Amp	3	136621295	136639845	0	0.022452
5169	Amp	3	136642892	137046314	0	0.018701
5170	Amp	3	137049168	137100736	0	0.020618
5171	Amp	3	137112469	137542675	0	0.017849
5172	Amp	3	137543199	137759622	0	0.022758
5173	Amp	3	137762500	137803767	0	0.024946
5174	Amp	3	137805515	137817958	0	0.02315
5175	Amp	3	137818187	137833267	0	0.025486
5176	Amp	3	137836044	137860666	0	0.021381
5177	Amp	3	137861386	137915651	0	0.023533
5178	Amp	3	137916238	137948792	0	0.020792
5179	Amp	3	137951919	138692794	0	0.018455
5180	Amp	3	138694299	138769433	0	0.019367
5181	Amp	3	138774616	138822570	0	0.020427
5182	Amp	3	138825636	138957161	0	0.022724
5183	Amp	3	138961449	139064272	0	0.021812
5184	Amp	3	139078165	139222147	0	0.02229
5185	Amp	3	139224201	139231939	0	0.019986
5186	Amp	3	139234817	139331689	0	0.017833
5187	Amp	3	139331798	139386569	0	0.016773
5188	Amp	3	139392804	139588318	0	0.019288
5189	Amp	3	139590036	139861889	0	0.019983
5190	Amp	3	139862265	140155235	0	0.021976
5191	Amp	3	140161495	140333418	0	0.019679
5192	Amp	3	140333859	140588729	0	0.018985
5193	Amp	3	140588857	140617490	0	0.017209

5194	Amp	3	140618805	140955350	0	0.016731
5195	Amp	3	140955644	141060448	0	0.019471
5196	Amp	3	141066992	141288161	0	0.016731
5197	Amp	3	141288379	141345254	0	0.015992
5198	Amp	3	141347800	141401254	0	0.01837
5199	Amp	3	141404583	141819247	0	0.015992
5200	Amp	3	141822263	141958418	0	0.018721
5201	Amp	3	141967908	141979586	0	0.017385
5202	Amp	3	141982461	142100008	0	0.020241
5203	Amp	3	142375789	142701858	0	0.014656
5204	Amp	3	142705704	142897562	0	0.016906
5205	Amp	3	142897867	143045736	0	0.01855
5206	Amp	3	143046124	143147790	0	0.023993
5207	Amp	3	143148094	143212052	0	0.022737
5208	Amp	3	143469691	143874533	0	0.019292
5209	Amp	3	143878084	143920555	0	0.016779
5210	Amp	3	143920813	144263630	0	0.019447
5211	Amp	3	144263719	144333936	0	0.021384
5212	Amp	3	144334727	144366177	0	0.020708
5213	Amp	3	144366454	144738468	0	0.018041
5214	Amp	3	144748087	144756647	0	0.024798
5215	Amp	3	144759276	144759276	0	0.018041
5216	Amp	3	144759771	144998667	0	0.015427
5217	Amp	3	144999590	145243516	0	0.01823
5218	Amp	3	145244387	145286211	0	0.01877
5219	Amp	3	145292958	145303506	0	0.021056
5220	Amp	3	145303807	145415349	0	0.023813
5221	Amp	3	145415956	145467482	0	0.021056
5222	Amp	3	145471570	145497179	0	0.021783
5223	Amp	3	145497249	145540896	0	0.021056
5224	Amp	3	145545823	145637646	0	0.023651
5225	Amp	3	145637849	145800481	0	0.021056
5226	Amp	3	145800767	145821476	0	0.018185
5227	Amp	3	145824654	145869779	0	0.021001
5228	Amp	3	145874656	145887957	0	0.018964
5229	Amp	3	145896244	146018285	0	0.02079
5230	Amp	3	146019014	146090584	0	0.022114
5231	Amp	3	146091098	146142961	0	0.024766
5232	Amp	3	146145951	146300331	0	0.027664
5233	Amp	3	146305419	146466400	0	0.024766
5234	Amp	3	146473111	146501765	0	0.021668
5235	Amp	3	146507883	146655651	0	0.016494
5236	Amp	3	146657279	146660742	0	0.018512
5237	Amp	3	147074820	147074820	0	0.026909
5238	Amp	3	147086979	147130466	0	0.035757
5239	Amp	3	147130822	147171868	0	0.027359
5240	Amp	3	147306036	147782657	0	0.028694
5241	Amp	3	147789438	148063898	0	0.029215
5242	Amp	3	148065879	148078841	0	0.035678
5243	Amp	3	148080711	148150339	0	0.029672
5244	Amp	3	148159565	148179295	0	0.028535
5245	Amp	3	148186579	148310019	0	0.027995
5246	Amp	3	148310633	148462353	0	0.029871

5247	Amp	3	148466822	148791507	0	0.032404
5248	Amp	3	148791777	148874657	0	0.031947
5249	Amp	3	148877838	149078768	0	0.031067
5250	Amp	3	149082914	149519280	0	0.040244
5251	Amp	3	149521664	149595596	0	0.042253
5252	Amp	3	149597816	149717430	0	0.040244
5253	Amp	3	149718625	149754417	0	0.02952
5254	Amp	3	149755659	149847054	0	0.03237
5255	Amp	3	149850961	149899261	0	0.02952
5256	Amp	3	149900519	149944120	0	0.032625
5257	Amp	3	149949952	150023613	0	0.039496
5258	Amp	3	150023846	150110351	0	0.037408
5259	Amp	3	150110827	150127343	0	0.030753
5260	Amp	3	150129367	150169677	0	0.032495
5261	Amp	3	150170001	150245215	0	0.029921
5262	Amp	3	150246522	150629722	0	0.034246
5263	Amp	3	150633481	150647997	0	0.04483
5264	Amp	3	150648028	150686587	0	0.04691
5265	Amp	3	150687183	150699806	0	0.045099
5266	Amp	3	150701796	150778304	0	0.050452
5267	Amp	3	150781808	150872381	0	0.04302
5268	Amp	3	150873967	150891857	0	0.042499
5269	Amp	3	150891907	150955687	0	0.040531
5270	Amp	3	150958100	151065355	0	0.042711
5271	Amp	3	151071607	151320092	0	0.040444
5272	Amp	3	151322077	151365279	0	0.042818
5273	Amp	3	151366624	151422984	0	0.039713
5274	Amp	3	151423466	151438689	0	0.035018
5275	Amp	3	151442793	151455523	0	0.036667
5276	Amp	3	151455956	151891474	0	0.040517
5277	Amp	3	151891927	152057113	0	0.0426
5278	Amp	3	152057210	152311397	0	0.043498
5279	Amp	3	152314706	152357327	0	0.032914
5280	Amp	3	152359070	152468428	0	0.029503
5281	Amp	3	152468919	152488626	0	0.028605
5282	Amp	3	152488729	152616952	0	0.030631
5283	Amp	3	152617000	153043985	0	0.028605
5284	Amp	3	153044673	153094615	0	0.030647
5285	Amp	3	153096683	153736060	0	0.033043
5286	Amp	3	153738327	153760634	0	0.026042
5287	Amp	3	153763908	153879828	0	0.026545
5288	Amp	3	153880020	153927232	0	0.029317
5289	Amp	3	153927406	153970905	0	0.027276
5290	Amp	3	153995856	154150405	0	0.025586
5291	Amp	3	154152463	154181165	0	0.025083
5292	Amp	3	154181357	154201070	0	0.023266
5293	Amp	3	154207998	154272960	0	0.025427
5294	Amp	3	154273311	154281737	0	0.027248
5295	Amp	3	154288552	154380756	0	0.025183
5296	Amp	3	154380769	154415310	0	0.026453
5297	Amp	3	154415508	154730714	0	0.02437
5298	Amp	3	154731376	155211358	0	0.022297
5299	Amp	3	155214659	155386516	0	0.023153

5300	Amp	3	155389781	155655694	0	0.023712
5301	Amp	3	155663964	155733486	0	0.024594
5302	Amp	3	155733873	155807000	0	0.026692
5303	Amp	3	155808009	155878236	0	0.02581
5304	Amp	3	155879998	156051137	0	0.026768
5305	Amp	3	156051591	156421879	0	0.027792
5306	Amp	3	156422960	156441251	0	0.031088
5307	Amp	3	156442595	156677883	0	0.027792
5308	Amp	3	156681391	156782192	0	0.026768
5309	Amp	3	156782272	156788877	0	0.028032
5310	Amp	3	156790921	156896530	0	0.03176
5311	Amp	3	156898895	156994959	0	0.032669
5312	Amp	3	156995355	157303185	0	0.035086
5313	Amp	3	157304799	157397838	0	0.037223
5314	Amp	3	157399881	157520521	0	0.031769
5315	Amp	3	157524112	157572899	0	0.029671
5316	Amp	3	157573982	157623618	0	0.027677
5317	Amp	3	157629324	157868007	0	0.028161
5318	Amp	3	157869058	158533473	0	0.02656
5319	Amp	3	158534485	158731234	0	0.027374
5320	Amp	3	158733834	158854456	0	0.028417
5321	Amp	3	158854488	159009981	0	0.029076
5322	Amp	3	159010469	159187049	0	0.026703
5323	Amp	3	159187070	159446274	0	0.02589
5324	Amp	3	159450302	159471182	0	0.027282
5325	Amp	3	159472091	159705593	0	0.026623
5326	Amp	3	159709907	159760209	0	0.031741
5327	Amp	3	159766439	160342236	0	0.034042
5328	Amp	3	160342785	160441827	0	0.028924
5329	Amp	3	160453506	161041150	0	0.032829
5330	Amp	3	161042358	161191345	0	0.037624
5331	Amp	3	161200531	161224379	0	0.039775
5332	Amp	3	161224999	161238963	0	0.037656
5333	Amp	3	161240332	161377312	0	0.035505
5334	Amp	3	161377808	161641642	0	0.0316
5335	Amp	3	161645537	161654001	0	0.032866
5336	Amp	3	161659227	161664589	0	0.030191
5337	Amp	3	161671005	161693274	0	0.032201
5338	Amp	3	161834016	161866239	0	0.035656
5339	Amp	3	161877221	161990164	0	0.037929
5340	Amp	3	161990311	162053594	0	0.041252
5341	Amp	3	162054225	162113421	0	0.039115
5342	Amp	3	162113454	162135359	0	0.042521
5343	Amp	3	162140427	162146723	0	0.04747
5344	Amp	3	162148105	162148453	0	0.042798
5345	Amp	3	162239158	162391595	0	0.03602
5346	Amp	3	162392607	162552895	0	0.040803
5347	Amp	3	162554871	162718452	0	0.042093
5348	Amp	3	162718608	162760118	0	0.044998
5349	Amp	3	162761828	162762809	0	0.047537
5350	Amp	3	163008827	163008827	0	0.04772
5351	Amp	3	163008889	163013504	0	0.045349
5352	Amp	3	163020549	163054842	0	0.043339

5353	Amp	3	163059522	163119307	0	0.047675
5354	Amp	3	163121330	163264459	0	0.050687
5355	Amp	3	163264685	163534031	0	0.049397
5356	Amp	3	163534890	163549976	0	0.051776
5357	Amp	3	163557031	163866166	0	0.054047
5358	Amp	3	163866257	163965410	0	0.054855
5359	Amp	3	163966494	164126526	0	0.053162
5360	Amp	3	164131529	164157293	0	0.056227
5361	Amp	3	164166372	164344821	0	0.053139
5362	Amp	3	164349262	164382333	0	0.055041
5363	Amp	3	164405579	164475312	0	0.051976
5364	Amp	3	164475616	164557060	0	0.054742
5365	Amp	3	164557273	164570606	0	0.056171
5366	Amp	3	164577821	164681437	0	0.056806
5367	Amp	3	164684198	164720107	0	0.054903
5368	Amp	3	164730328	164868668	0	0.057161
5369	Amp	3	164874189	164880301	0	0.058309
5370	Amp	3	164882802	165219420	0	0.055931
5371	Amp	3	165232042	165293498	0	0.053696
5372	Amp	3	165296991	165315403	0	0.051438
5373	Amp	3	165370734	165422040	0	0.055659
5374	Amp	3	165422159	165541222	0	0.053557
5375	Amp	3	165579837	165591080	0	0.056792
5376	Amp	3	165599896	165604056	0	0.058618
5377	Amp	3	165608677	165640331	0	0.055384
5378	Amp	3	165641626	165726558	0	0.053557
5379	Amp	3	165726913	165791453	0	0.055481
5380	Amp	3	165791562	165795786	0	0.054334
5381	Amp	3	166162696	166165773	0.181932	0.071515
5382	Amp	3	166165780	166177317	0.133193	0.069591
5383	Amp	3	166181411	166192262	0.069848	0.067012
5384	Amp	3	166197739	166364813	0.050473	0.066073
5385	Amp	3	166380227	166438612	0	0.050547
5386	Amp	3	166440919	166455455	0	0.049213
5387	Amp	3	166460712	166644596	0	0.052393
5388	Amp	3	166644631	166846076	0	0.054691
5389	Amp	3	166851377	167001994	0	0.055782
5390	Amp	3	167005992	167109353	0	0.057721
5391	Amp	3	167112044	167112044	0	0.056649
5392	Amp	3	167112711	167193405	0	0.055558
5393	Amp	3	167194067	167362825	0	0.058058
5394	Amp	3	167367507	167500755	0	0.055826
5395	Amp	3	167507006	167557299	0	0.057136
5396	Amp	3	167561248	167565271	0	0.058473
5397	Amp	3	167566012	167566012	0	0.056174
5398	Amp	3	167570263	167795042	0	0.056698
5399	Amp	3	167795616	167840849	0	0.056213
5400	Amp	3	167841006	167867986	0	0.057954
5401	Amp	3	167869897	167936115	0	0.056869
5402	Amp	3	167950827	168281594	0	0.05556
5403	Amp	3	168281657	168307554	0	0.056317
5404	Amp	3	168308584	168418915	0	0.055191
5405	Amp	3	168421786	168589163	0	0.060238

5406	Amp	3	168590660	168607796	0	0.061195
5407	Amp	3	168609501	168671647	0	0.062863
5408	Amp	3	168672407	168704351	0	0.060238
5409	Amp	3	168709888	168756920	0.17597	0.071361
5410	Amp	3	168757089	168808713	0.1237	0.069259
5411	Amp	3	168812844	168877682	0	0.063336
5412	Amp	3	168882482	168908289	0.065715	0.066759
5413	Amp	3	168910408	169058142	0.128324	0.069319
5414	Amp	3	169058538	169100772	0.193543	0.071881
5415	Amp	3	169103286	169147953	0.342649	0.077803
5416	Amp	3	169151324	169249535	0.616549	0.086972
5417	Amp	3	169250470	169306720	0.735486	0.090356
5418	Amp	3	169309139	169337913	0.463534	0.082026
5419	Amp	3	169340221	169466635	0.500841	0.083307
5420	Amp	3	169467611	169476645	0.522881	0.084013
5421	Amp	3	169477561	169500898	0.583752	0.085979
5422	Amp	3	169501069	169549528	0.609133	0.08679
5423	Amp	3	169552231	169630215	0.630438	0.087369
5424	Amp	3	169631156	169666509	0.550755	0.084807
5425	Amp	3	169666945	169712438	0.463534	0.082002
5426	Amp	3	169719000	169731674	0.529451	0.084076
5427	Amp	3	169732280	169739769	0.569771	0.08534
5428	Amp	3	169740411	169740411	0.463534	0.081957
5429	Amp	3	169740559	169820824	0.515676	0.083666
5430	Amp	3	169825896	169830668	0.589605	0.086002
5431	Amp	3	169831062	169831062	0.681869	0.088962
5432	Amp	3	169832971	169925969	0.823771	0.092819
5433	Amp	3	169926327	169949301	0.79909	0.092114
5434	Amp	3	169953427	170112080	0.899693	0.094743
5435	Amp	3	170113396	170176087	0.79909	0.092114
5436	Amp	3	170176555	170207430	1.00505	0.097486
5437	Amp	3	170207876	170214955	1.058188	0.098749
5438	Amp	3	170215046	170221102	0.968522	0.096426
5439	Amp	3	170223577	170229089	1.348715	0.105442
5440	Amp	3	170229800	170254293	1.436692	0.107273
5441	Amp	3	170260864	170310209	0.781972	0.091765
5442	Amp	3	170310397	170310397	0.823771	0.09288
5443	Amp	3	170316873	170448303	0.882843	0.094363
5444	Amp	3	170448486	170506368	0.765474	0.091345
5445	Amp	3	170507315	170525050	0.616549	0.087006
5446	Amp	3	170525431	170575926	0.487495	0.082888
5447	Amp	3	170577965	170584102	0.4115	0.080328
5448	Amp	3	170587420	170592656	0.379885	0.079063
5449	Amp	3	170593235	170599795	0.463534	0.082014
5450	Amp	3	170602166	170622631	0.529451	0.0842
5451	Amp	3	170626919	170660620	0.595248	0.086241
5452	Amp	3	170660662	170809511	0.550755	0.084757
5453	Amp	3	170810879	170867245	0.529451	0.084234
5454	Amp	3	170867619	170939981	0.386426	0.079429
5455	Amp	3	170940729	171042288	0.329205	0.077354
5456	Amp	3	171043251	171102936	0.457501	0.081774
5457	Amp	3	171105835	171152446	0.417873	0.080394
5458	Amp	3	171153189	171175852	0.470509	0.08209

5459	Amp	3	171180221	171188803	1.032525	0.097937
5460	Amp	3	171189637	171198788	0.906385	0.094902
5461	Amp	3	171201597	171290213	0.444474	0.081427
5462	Amp	3	171298039	171425429	0.515676	0.083749
5463	Amp	3	171427922	171428150	0.444474	0.081405
5464	Amp	3	171428596	171508551	0.424557	0.080594
5465	Amp	3	171508888	171585861	0.644502	0.087873
5466	Amp	3	171589803	171610114	0.6737	0.088805
5467	Amp	3	171612676	171622551	0.576183	0.085674
5468	Amp	3	171972471	172050857	0.444474	0.081249
5469	Amp	3	172058108	172069383	0.457501	0.081767
5470	Amp	3	172069981	172074923	0.373599	0.078912
5471	Amp	3	172076305	172197295	0.487495	0.082902
5472	Amp	3	172197424	172260473	0.399144	0.079768
5473	Amp	3	172260662	172279749	0.373599	0.078912
5474	Amp	3	172280453	172320789	0.689884	0.089191
5475	Amp	3	172321379	172398845	0.660254	0.08829
5476	Amp	3	172403057	172404731	0.630438	0.087358
5477	Amp	3	172405681	172425550	0.450702	0.081614
5478	Amp	3	172425588	172550068	0.494792	0.083159
5479	Amp	3	172552325	172582916	0.576183	0.085637
5480	Amp	3	172586316	172633784	0.450702	0.081546
5481	Amp	3	172635718	172687361	0.34892	0.077989
5482	Amp	3	172687907	172727097	0.276783	0.075512
5483	Amp	3	172727305	172730994	0.311747	0.076779
5484	Amp	3	172731956	172762057	0.238246	0.073819
5485	Amp	3	172768083	172768083	0.069848	0.067096
5486	Amp	3	172772412	172855299	0.138391	0.069881
5487	Amp	3	172855519	172919371	0.187449	0.071707
5488	Amp	3	172920290	172920290	0.138391	0.069881
5489	Amp	3	172928380	173014539	0.187449	0.071871
5490	Amp	3	173014696	173037156	0.271552	0.075229
5491	Amp	3	173038981	173039792	0.431198	0.080795
5492	Amp	3	173044075	173144951	0.329205	0.077437
5493	Amp	3	173153700	173174709	0.255918	0.074652
5494	Amp	3	173176601	173209882	0.264858	0.075128
5495	Amp	3	173215266	173269777	0.222041	0.073143
5496	Amp	3	173278812	173318976	0.246818	0.074173
5497	Amp	3	173321402	173324164	0.19964	0.072183
5498	Amp	3	173324708	173350645	0.287738	0.075906
5499	Amp	3	173357923	173444122	0.19964	0.072183
5500	Amp	3	173444214	173444687	0.241616	0.073963
5501	Amp	3	173445369	173445369	0.316877	0.077077
5502	Amp	3	173912890	173914127	0.271552	0.07525
5503	Amp	3	173915081	174007619	0.264858	0.075057
5504	Amp	3	174008548	174069419	0.316877	0.077017
5505	Amp	3	174070160	174082626	0.373599	0.078981
5506	Amp	3	174083900	174134625	0.362016	0.078463
5507	Amp	3	174135019	174155654	0.463534	0.082067
5508	Amp	3	174160311	174160497	0.602643	0.086568
5509	Amp	3	174161282	174222637	0.529451	0.084242
5510	Amp	3	174226989	174267677	0.550755	0.084745
5511	Amp	3	174269647	174333248	0.529451	0.084242

5512 Amp	3	174334939	174369215	0.609133	0.08683
5513 Amp	3	174374551	174389450	0.660254	0.08822
5514 Amp	3	174390752	174418637	0.557122	0.085107
5515 Amp	3	174418827	174432635	0.500841	0.083327
5516 Amp	3	174444045	174446076	0.463534	0.082048
5517 Amp	3	174456704	174500660	0.386426	0.07946
5518 Amp	3	174503683	174569276	0.299433	0.076327
5519 Amp	3	174569624	174572052	0.251218	0.074368
5520 Amp	3	174576127	174636079	0.138391	0.069866
5521 Amp	3	174637368	174643344	0.417873	0.080512
5522 Amp	3	174644208	174657536	0.589605	0.086076
5523 Amp	3	174658242	174664571	0.515676	0.083754
5524 Amp	3	174664866	174669395	0.399144	0.079736
5525 Amp	3	174669514	174678226	0.616549	0.086878
5526 Amp	3	174678295	174917585	0.589605	0.08607
5527 Amp	3	174919948	174959481	0.681869	0.088944
5528 Amp	3	174961770	175111897	0.758088	0.09102
5529 Amp	3	175112560	175144572	0.417873	0.080522
5530 Amp	3	175147232	175296284	0.667587	0.088437
5531 Amp	3	175296333	175296417	0.774648	0.091431
5532 Amp	3	175297677	175317515	0.470509	0.082228
5533 Amp	3	175317925	175358750	0.522881	0.08395
5534 Amp	3	175360537	175360729	0.392611	0.079529
5535 Amp	3	176041258	176240071	0.681869	0.088901
5536 Amp	3	176240085	176244362	0.616549	0.086972
5537 Amp	3	176245258	176319095	0.550755	0.084772
5538 Amp	3	176323072	176439484	0.494792	0.083049
5539 Amp	3	176439525	176531395	0.569771	0.085405
5540 Amp	3	176541396	176777007	0.342649	0.077867
5541 Amp	3	176777337	176788948	0.636464	0.087622
5542 Amp	3	176789296	176879427	0.557122	0.085027
5543 Amp	3	176880101	176909706	0.595248	0.086328
5544 Amp	3	176913185	176940359	0.774648	0.091423
5545 Amp	3	176943520	176970480	0.840289	0.093367
5546 Amp	3	176970709	176971726	0.924746	0.095509
5547 Amp	3	176972093	176972093	0.848692	0.093518
5548 Amp	3	177177049	177177049	1.843664	0.116147
5549 Amp	3	177180486	177295915	1.388256	0.106392
5550 Amp	3	177300682	177360474	1.388256	0.106354
5551 Amp	3	177361679	177414638	1.502257	0.108887
5552 Amp	3	177415494	177499664	1.696256	0.113107
5553 Amp	3	177500101	177565315	1.604336	0.111003
5554 Amp	3	177567399	177587977	1.624655	0.111537
5555 Amp	3	177591101	177627409	1.812192	0.115419
5556 Amp	3	177629008	177671321	1.604336	0.111003
5557 Amp	3	177673204	177734977	1.823153	0.115688
5558 Amp	3	177740003	177744070	1.727286	0.113743
5559 Amp	3	177744253	177751431	1.645511	0.111925
5560 Amp	3	177751767	177777137	1.522708	0.109392
5561 Amp	3	177778126	177806395	1.014678	0.097548
5562 Amp	3	177813283	177859374	1.093834	0.099646
5563 Amp	3	177863617	177891033	1.583409	0.11056
5564 Amp	3	177898123	177918905	1.482378	0.108513

5565	Amp	3	177925733	178082649	1.299876	0.104293
5566	Amp	3	178083981	178162588	1.112463	0.099919
5567	Amp	3	178166476	178239226	1.398396	0.10657
5568	Amp	3	178241865	178247663	1.445847	0.107493
5569	Amp	3	178248128	178252862	1.149583	0.100841
5570	Amp	3	178252906	178299685	1.058188	0.098688
5571	Amp	3	178304497	178318831	1.280185	0.103834
5572	Amp	3	178320510	178328373	1.33853	0.105161
5573	Amp	3	178337974	178398096	1.131695	0.10053
5574	Amp	3	178439726	178480264	0.98589	0.096873
5575	Amp	3	178482049	178482049	1.112463	0.100056
5576	Amp	3	178491791	178502391	1.309159	0.104652
5577	Amp	3	178504358	178526382	1.502257	0.108793
5578	Amp	3	178536156	178570095	1.801583	0.11518
5579	Amp	3	178573842	178668302	1.531891	0.109523
5580	Amp	3	178668350	178683097	1.790459	0.115056
5581	Amp	3	178683287	178764573	1.645511	0.111933
5582	Amp	3	178766871	178786576	1.604336	0.11102
5583	Amp	3	178787263	178811789	1.675319	0.112646
5584	Amp	3	178812068	178832303	1.645511	0.111878
5585	Amp	3	178834883	178848800	1.780186	0.11486
5586	Amp	3	178851025	178893025	1.876242	0.116748
5587	Amp	3	178896944	178897814	1.940776	0.118022
5588	Amp	3	178900762	179066745	1.717671	0.113426
5589	Amp	3	179067741	179122123	1.748606	0.114
5590	Amp	3	179124200	179124440	1.675319	0.112539
5591	Amp	3	179136185	179146357	1.61378	0.111212
5592	Amp	3	179149798	179158587	1.96212	0.11843
5593	Amp	3	179158602	179158823	1.908279	0.117428
5594	Amp	3	179158837	179159507	1.812192	0.115502
5595	Amp	3	179159575	179159717	1.769915	0.114465
5596	Amp	3	179159759	179160118	1.717671	0.113539
5597	Amp	3	179160161	179160372	1.675319	0.112509
5598	Amp	3	179165384	179165882	1.531891	0.109569
5599	Amp	3	179165920	179210303	1.436692	0.107315
5600	Amp	3	179210318	179234243	1.348715	0.105321
5601	Amp	3	179234266	179234432	1.445847	0.107552
5602	Amp	3	179234447	179234447	1.645511	0.111974
5603	Amp	3	179235965	179243711	1.790459	0.115018
5604	Amp	3	179245109	179246058	1.854421	0.116387
5605	Amp	3	179246543	179255237	1.994915	0.119037
5606	Amp	3	179262147	179264846	2.071483	0.120629
5607	Amp	3	179264932	179271046	2.127614	0.121701
5608	Amp	3	179273008	179278838	2.18114	0.12281
5609	Amp	3	179288420	179333011	1.940776	0.117938
5610	Amp	3	179333678	179359905	1.984015	0.118961
5611	Amp	3	179364043	179398321	1.96212	0.118388
5612	Amp	3	179407642	179598799	1.369395	0.105865
5613	Amp	3	179603416	179693549	1.319393	0.104842
5614	Amp	3	179697681	179705009	1.280185	0.103919
5615	Amp	3	179705456	179810798	1.215958	0.102327
5616	Amp	3	179815913	179818008	1.00505	0.097417
5617	Amp	3	179822389	179924155	1.261401	0.103481

5618	Amp	3	179924279	179927171	1.280185	0.103933
5619	Amp	3	179929577	179935409	1.159209	0.101018
5620	Amp	3	179936214	180202309	1.23519	0.102762
5621	Amp	3	180207261	180351171	1.675319	0.112592
5622	Amp	3	180357720	180514768	2.005947	0.119233
5623	Amp	3	180517095	180524570	1.675319	0.112592
5624	Amp	3	180525125	180548280	1.531891	0.109444
5625	Amp	3	180548377	180586489	1.416817	0.107005
5626	Amp	3	180589840	180713084	1.502257	0.108943
5627	Amp	3	180714225	180738703	1.482378	0.108491
5628	Amp	3	180740404	180792658	1.270711	0.103641
5629	Amp	3	180805966	180866783	1.388256	0.106234
5630	Amp	3	180866958	180881348	1.492063	0.108743
5631	Amp	3	180884019	180896328	1.445847	0.107621
5632	Amp	3	180899679	181108003	1.655056	0.112117
5633	Amp	3	181121010	181142075	1.270711	0.103601
5634	Amp	3	181142378	181157586	1.159209	0.101092
5635	Amp	3	181161968	181173031	1.140417	0.100574
5636	Amp	3	181175789	181197719	1.197658	0.102031
5637	Amp	3	181201052	181329453	1.309159	0.104616
5638	Amp	3	181330225	181330225	1.456037	0.107745
5639	Amp	3	181330950	181338555	1.252022	0.103221
5640	Amp	3	181339034	181437198	1.456037	0.107727
5641	Amp	3	181439992	181463010	1.309159	0.104598
5642	Amp	3	181466807	181499316	1.225419	0.102508
5643	Amp	3	181499458	181517622	1.168655	0.101303
5644	Amp	3	181517951	181536263	1.093834	0.099476
5645	Amp	3	181537205	181560708	1.058188	0.098719
5646	Amp	3	181561247	181605239	1.075952	0.09918
5647	Amp	3	181605653	181653007	1.168655	0.10127
5648	Amp	3	181654384	181874886	1.261401	0.103384
5649	Amp	3	181874932	181896519	1.168655	0.101287
5650	Amp	3	181899056	181910133	1.215958	0.10235
5651	Amp	3	181910676	181932312	0.977077	0.096661
5652	Amp	3	181938083	181955934	1.215958	0.102485
5653	Amp	3	181958653	181960121	1.112463	0.099893
5654	Amp	3	181961573	181968412	0.681869	0.088815
5655	Amp	3	181968753	182162552	0.774648	0.091509
5656	Amp	3	182165298	182213139	0.681869	0.088815
5657	Amp	3	182214100	182302568	0.47716	0.082399
5658	Amp	3	182306588	182327802	0.373599	0.078906
5659	Amp	3	182328250	182333119	0.311747	0.07672
5660	Amp	3	182339106	182381631	0.238246	0.07366
5661	Amp	3	182393936	182403844	0.210594	0.072563
5662	Amp	3	182406313	182422074	0.241616	0.074007
5663	Amp	3	182433118	182652125	0.293269	0.076082
5664	Amp	3	182652688	182699321	0.30524	0.076608
5665	Amp	3	182700205	182730905	0.379885	0.079174
5666	Amp	3	182730988	182736327	0.399144	0.079868
5667	Amp	3	182738837	182753544	0.368062	0.078804
5668	Amp	3	182758826	182773627	0.508568	0.083419
5669	Amp	3	182779895	182790993	0.543344	0.084508
5670	Amp	3	182796848	182843921	0.399144	0.079893

5671	Amp	3	182844975	183012385	0.583752	0.085774
5672	Amp	3	183012715	183055526	0.595248	0.086237
5673	Amp	3	183057389	183110356	0.6737	0.088718
5674	Amp	3	183111756	183170246	0.595248	0.086387
5675	Amp	3	183172232	183175169	0.704849	0.089591
5676	Amp	3	183176548	183322849	0.840289	0.093239
5677	Amp	3	183329697	183348135	0.781972	0.091815
5678	Amp	3	183357318	183373773	0.660254	0.088166
5679	Amp	3	183375635	183407876	0.72817	0.090182
5680	Amp	3	183409000	183409000	0.636464	0.087616
5681	Amp	3	184179380	184183601	0.112543	0.068632
5682	Amp	3	184184700	184189144	0.089561	0.067939
5683	Amp	3	184189244	184265365	0.079652	0.067478
5684	Amp	3	184276748	184376936	0.128324	0.069379
5685	Amp	3	184378632	184390100	0.094967	0.06811
5686	Amp	3	184461455	184480655	0.4115	0.080244
5687	Amp	3	184480687	184484624	0.379885	0.079155
5688	Amp	3	184485243	184529837	0.431198	0.080781
5689	Amp	3	184531697	184609076	0.508568	0.083385
5690	Amp	3	184613454	184620030	0.609133	0.086708
5691	Amp	3	184624757	184733455	0.630438	0.087418
5692	Amp	3	184737247	184757177	0.424557	0.080765
5693	Amp	3	184757552	184761079	0.404718	0.080055
5694	Amp	3	184761828	184938242	0.255918	0.074575
5695	Amp	3	184939927	184960606	0.282828	0.075618
5696	Amp	3	184961919	184988254	0.19964	0.072295
5697	Amp	3	184991589	185071941	0.159312	0.070586
5698	Amp	3	185072945	185183233	0.143787	0.069954
5699	Amp	3	185184616	185199329	0.118001	0.06891
5700	Amp	3	185199880	185202792	0.024438	0.064935
5701	Amp	3	185205817	185222648	0.1237	0.069113
5702	Amp	3	185225298	185251030	0.210594	0.072636
5703	Amp	3	185253393	185280587	0.233782	0.073511
5704	Amp	3	185283171	185283171	0.271552	0.075313
5705	Amp	3	185283295	185319618	0.187449	0.07179
5706	Amp	3	185321380	185595659	0.133193	0.069517
5707	Amp	3	185598828	185703859	0.14896	0.070269
5708	Amp	3	185708363	185798666	0.463534	0.082026
5709	Amp	3	185800182	185827529	0.404718	0.079912
5710	Amp	3	185828028	185980858	0.450702	0.081553
5711	Amp	3	185986556	186003442	0.602643	0.086527
5712	Amp	3	186004048	186091016	0.589605	0.086001
5713	Amp	3	186093673	186104153	0.417873	0.080417
5714	Amp	3	186104189	186112102	0.450702	0.081597
5715	Amp	3	186112378	186188087	0.463534	0.082064
5716	Amp	3	186188206	186198933	0.602643	0.086468
5717	Amp	3	186202814	186279751	0.536238	0.084452
5718	Amp	3	186279912	186286737	0.630438	0.087329
5719	Amp	3	186288037	186501775	0.47716	0.082355
5720	Amp	3	186504052	186592512	0.170723	0.071076
5721	Amp	3	186592954	186599574	0.19964	0.072231
5722	Amp	3	186600563	186665435	0.222041	0.073065
5723	Amp	3	186665455	186666398	0.259438	0.074894

5724	Amp	3	186668350	186668636	0.238246	0.073739
5725	Amp	3	186718815	186888071	0.373599	0.078916
5726	Amp	3	186888496	187025265	0.34892	0.078082
5727	Amp	3	187030094	187326080	0.271552	0.075204
5728	Amp	3	187332683	187371932	0.362016	0.07842
5729	Amp	3	187376772	187378466	0.227787	0.073243
5730	Amp	3	187379875	187503034	0.17597	0.071414
5731	Amp	3	187503921	187522765	0.259438	0.074866
5732	Amp	3	187524740	187591086	0.143787	0.070009
5733	Amp	3	187592146	187601484	0.222041	0.073065
5734	Amp	3	187601901	187780562	0.210594	0.072598
5735	Amp	3	187786281	187952229	0.233782	0.073395
5736	Amp	3	187952892	188140460	0.246818	0.074218
5737	Amp	3	188141839	188164955	0.329205	0.077324
5738	Amp	3	188167204	188414540	0.246818	0.074218
5739	Amp	3	188414708	188553062	0.271552	0.075273
5740	Amp	3	188563287	188657502	0.246818	0.074218
5741	Amp	3	188657959	188896140	0.233782	0.073395
5742	Amp	3	188897417	188965811	0.210594	0.072598
5743	Amp	3	188969015	189059884	0.238246	0.073723
5744	Amp	3	189068160	189077000	0.255918	0.074553
5745	Amp	3	189081619	189135328	0.316877	0.076989
5746	Amp	3	189137854	189228944	0.255918	0.074553
5747	Amp	3	189234735	189331071	0.181932	0.071498
5748	Amp	3	189332056	189388992	0.238246	0.073759
5749	Amp	3	189392877	189486606	0.276783	0.075515
5750	Amp	3	189487243	189505913	0.251218	0.074389
5751	Amp	3	189506581	189639209	0.233782	0.073559
5752	Amp	3	189642114	189676701	0.17597	0.071298
5753	Amp	3	189680316	189752672	0.204831	0.07248
5754	Amp	3	189753850	189852567	0.259438	0.074853
5755	Amp	3	189854879	189895022	0.204831	0.07248
5756	Amp	3	189895140	189929978	0.227787	0.073229
5757	Amp	3	189931824	190281963	0.181932	0.071473
5758	Amp	3	190285815	190292780	0.159312	0.070722
5759	Amp	3	190293086	190510559	0.238246	0.073709
5760	Amp	3	190511361	190518969	0.362016	0.078479
5761	Amp	3	190519170	190521359	0.238246	0.073709
5762	Amp	3	190521954	190557977	0.17597	0.071321
5763	Amp	3	190560676	190684936	0.14896	0.070138
5764	Amp	3	190689366	190716019	0.107017	0.06856
5765	Amp	3	190719608	190759962	0.431198	0.08092
5766	Amp	3	190762506	190788707	0.470509	0.082218
5767	Amp	3	190792051	190796188	0.19964	0.072124
5768	Amp	3	190802064	190841154	0.138391	0.069858
5769	Amp	3	190841798	190870915	0.14896	0.070338
5770	Amp	3	190870954	191052634	0.1237	0.069067
5771	Amp	3	191052713	191276178	0.060686	0.066545
5772	Amp	3	191276354	191389204	0.1237	0.06922
5773	Amp	3	191390160	191605490	0.065715	0.066812
5774	Amp	3	191606121	191637360	0.100827	0.068265
5775	Amp	3	191637447	191666131	0.153838	0.07041
5776	Amp	3	191666205	191722006	0.138391	0.069831

5777	Amp	3	191722403	191800053	0.100827	0.068378
5778	Amp	3	191851784	191875865	0.210594	0.072567
5779	Amp	3	191878772	191878772	0.153838	0.070421
5780	Amp	3	191878954	191878954	0.079652	0.067336
5781	Amp	3	191910119	191961684	0.065715	0.066856
5782	Amp	3	191963519	192011418	0.143787	0.069958
5783	Amp	3	192014275	192120964	0.19964	0.072112
5784	Amp	3	192122325	192325749	0.210594	0.072666
5785	Amp	3	192326060	192415914	0.143787	0.069945
5786	Amp	3	192416330	192423049	0.522881	0.083859
5787	Amp	3	192425126	192566201	0.589605	0.086083
5788	Amp	3	192567448	192634025	0.522881	0.083859
5789	Amp	3	192635953	192657611	0.576183	0.085764
5790	Amp	3	192660578	192682016	0.138391	0.069827
5791	Amp	3	192682181	192778565	0.14896	0.070342
5792	Amp	3	192778622	192973315	0.170723	0.071006
5793	Amp	3	192979359	192987352	0.215608	0.072756
5794	Amp	3	192988922	193135057	0.227787	0.073334
5795	Amp	3	193148816	193247545	0.311747	0.076867
5796	Amp	3	193254322	193545226	0.293269	0.076119
5797	Amp	3	193546918	193765007	0.238246	0.073704
5798	Amp	3	193769799	193845059	0.246818	0.074186
5799	Amp	3	193846642	193969263	0.311747	0.076667
5800	Amp	3	193970681	194006398	0.246818	0.074134
5801	Amp	3	194010404	194086432	0.215608	0.072746
5802	Amp	3	194087092	194092803	0.271552	0.07521
5803	Amp	3	194093059	194248772	0.259438	0.074728
5804	Amp	3	194249639	194769475	0.238246	0.073635
5805	Amp	3	194770853	194781675	0.227787	0.073181
5806	Amp	3	194788350	194822241	0.271552	0.075237
5807	Amp	3	194830213	194830213	0.444474	0.081379
5808	Amp	3	194830339	194849405	0.431198	0.080825
5809	Amp	3	194851261	194875921	0.322897	0.077292
5810	Amp	3	194876172	195193813	0.170723	0.07115
5811	Amp	3	195195215	195195402	0.19964	0.072299
5812	Amp	3	195196104	195264165	0.227787	0.073377
5813	Amp	3	195264537	195268144	0.241616	0.073876
5814	Amp	3	195273899	195428692	0.215608	0.072949
5815	Amp	3	196017117	196017117	0.386426	0.079373
5816	Amp	3	196019900	196026895	0.487495	0.082818
5817	Amp	3	196034367	196195556	0.457501	0.081668
5818	Amp	3	196197301	196219664	0.500841	0.083289
5819	Amp	3	196230006	196282228	0.595248	0.086285
5820	Amp	3	196285609	196330745	0.636464	0.087636
5821	Amp	3	196337577	196347257	0.444474	0.081211
5822	Amp	3	196347332	196348339	0.536238	0.084334
5823	Amp	3	196354098	196365802	0.576183	0.085565
5824	Amp	3	196365866	196422796	0.482379	0.082569
5825	Amp	3	196425258	196437963	0.569771	0.085365
5826	Amp	3	196441059	196471488	0.790614	0.092009
5827	Amp	3	196473866	196506924	0.644502	0.087911
5828	Amp	3	196515390	196524756	0.79909	0.092247
5829	Amp	3	196537842	196591150	0.751221	0.090896

5830	Amp	3	196591333	196629554	0.667587	0.088446
5831	Amp	3	196631608	196640529	0.765474	0.091284
5832	Amp	3	196641630	196770477	0.660254	0.088161
5833	Amp	3	197242908	197247447	1.058188	0.098704
5834	Amp	3	197247511	197272543	1.075952	0.099161
5835	Amp	3	197275809	197278817	0.899693	0.094883
5836	Amp	3	197279769	197483798	0.857296	0.093653
5837	Amp	3	197484264	197516065	0.968522	0.096588
5838	Amp	3	197527360	197551766	0.891602	0.094457
5839	Amp	3	197552126	197561209	0.742723	0.090606
5840	Amp	3	197607658	197611842	0.651644	0.088061
5841	Amp	3	197685959	197754242	0.444474	0.081369
5842	Amp	3	197755173	197799169	0.482379	0.08257
5843	Amp	3	197799330	197812401	0.437198	0.081053
5844	Amp	4	1059384	1068450	0	0.0203
5845	Amp	4	1072166	1075962	0	0.019792
5846	Amp	4	1078119	1136187	0	0.021966
5847	Amp	4	1138836	1167692	0	0.032135
5848	Amp	4	1168234	1198455	0	0.033626
5849	Amp	4	1201936	1292817	0	0.033095
5850	Amp	4	1293178	1303781	0	0.033676
5851	Amp	4	1306692	1505243	0	0.042802
5852	Amp	4	1510104	1517775	0	0.035272
5853	Amp	4	1535956	1587793	0	0.03378
5854	Amp	4	1617307	1708788	0	0.036772
5855	Amp	4	1710686	1731823	0	0.03378
5856	Amp	4	1732512	1736525	0	0.03615
5857	Amp	4	1736540	1737005	0	0.038718
5858	Amp	4	1750693	1750693	0	0.043859
5859	Amp	4	1750804	1777074	0	0.04107
5860	Amp	4	1790291	1905354	0	0.045445
5861	Amp	4	1909996	1969958	0	0.043075
5862	Amp	4	1970120	2033971	0	0.043794
5863	Amp	4	2052592	2080282	0	0.041009
5864	Amp	4	2084131	2106191	0	0.038441
5865	Amp	4	2108935	2124047	0	0.021609
5866	Amp	4	2124852	2124852	0	0.02089
5867	Amp	4	2131576	2283983	0	0.018173
5868	Amp	4	2287539	2887944	0	0.020217
5869	Amp	4	2889218	2957446	0	0.018216
5870	Amp	4	2962365	3007405	0	0.021382
5871	Amp	4	3008882	3017327	0	0.016241
5872	Amp	4	3022996	3082542	0	0.013075
5873	Amp	4	3082626	3120495	0	0.014959
5874	Amp	4	3129846	3186538	0	0.011031
5875	Amp	4	3189753	3580911	0	0.01345
5876	Amp	4	3586020	3713923	0	0.017765
5877	Amp	4	3715854	3869335	0	0.01345
5878	Amp	4	3873169	3873169	0	0.011031
5879	Amp	4	4182970	4187229	0	0.012384
5880	Amp	4	4188845	4313525	0	0.012892
5881	Amp	4	4313661	4373516	0	0.015309
5882	Amp	4	4374361	4427058	0	0.01576

5883	Amp	4	4428802	4598751	0	0.015205
5884	Amp	4	4601178	4628934	0	0.014738
5885	Amp	4	4629508	4807087	0	0.015181
5886	Amp	4	4811595	4819086	0	0.01589
5887	Amp	4	4820366	5255885	0	0.015181
5888	Amp	4	5256034	6000759	0	0.014734
5889	Amp	4	6010357	6055039	0	0.015458
5890	Amp	4	6056475	6067148	0	0.014734
5891	Amp	4	6067887	6441318	0	0.018005
5892	Amp	4	6444862	6606938	0	0.01947
5893	Amp	4	6606993	6622845	0	0.019027
5894	Amp	4	6628013	6769764	0	0.019564
5895	Amp	4	6772120	6866997	0	0.0181
5896	Amp	4	6869347	7019380	0	0.018869
5897	Amp	4	7019443	7135609	0	0.015598
5898	Amp	4	7135794	7207858	0	0.016277
5899	Amp	4	7208862	7332697	0	0.016992
5900	Amp	4	7333853	7470684	0	0.016277
5901	Amp	4	7471478	8261771	0	0.015508
5902	Amp	4	8263789	8263789	0	0.013091
5903	Amp	4	8268593	8319982	0	0.015479
5904	Amp	4	8320267	8337617	0	0.019336
5905	Amp	4	8347820	8471323	0	0.015479
5906	Amp	4	8475658	8605860	0	0.018218
5907	Amp	4	8607468	8664936	0	0.017767
5908	Amp	4	8665160	8704195	0	0.015028
5909	Amp	4	8704538	9587686	0	0.011972
5910	Amp	4	9594294	9882433	0	0.009584
5911	Amp	4	9882830	9891739	0	0.009047
5912	Amp	4	9894053	9964481	0	0.010164
5913	Amp	4	9964756	9985399	0	0.007311
5914	Amp	4	9985700	10159324	0	0.006194
5915	Amp	4	10160473	10277933	0	0.005613
5916	Amp	4	10279065	10303839	0	0.005155
5917	Amp	4	10307741	10348420	0	0.006227
5918	Amp	4	10349324	10491990	0	0.005779
5919	Amp	4	10493448	10593889	0	0.008189
5920	Amp	4	10594393	10595945	0	0.007743
5921	Amp	4	10596391	10659442	0	0.005334
5922	Amp	4	10659939	11081337	0	0.004654
5923	Amp	4	11082635	11243168	0	0.007468
5924	Amp	4	11245529	12327288	0	0.008117
5925	Amp	4	12329132	12359751	0	0.007468
5926	Amp	4	12362833	12392445	0	0.005755
5927	Amp	4	12394792	12496216	0	0.006839
5928	Amp	4	12496258	12553819	0	0.006322
5929	Amp	4	12555535	12555535	0	0.008515
5930	Amp	4	12555734	12575805	0	0.011199
5931	Amp	4	12576437	12576626	0	0.009176
5932	Amp	4	12576694	12637970	0	0.008093
5933	Amp	4	12655112	12804256	0	0.005899
5934	Amp	4	12808538	12967314	0	0.007463
5935	Amp	4	12967828	13425431	0	0.005899

5936	Amp	4	13427945	13489034	0	0.007573
5937	Amp	4	13489050	13550285	0	0.008149
5938	Amp	4	13552289	13610960	0	0.011464
5939	Amp	4	13620060	13641432	0	0.019666
5940	Amp	4	13641603	13753867	0	0.018594
5941	Amp	4	13755302	13830138	0	0.022115
5942	Amp	4	13831482	13834421	0	0.02154
5943	Amp	4	13837203	14156577	0	0.009817
5944	Amp	4	14158747	14184452	0	0.012891
5945	Amp	4	14190509	14190509	0	0.009817
5946	Amp	4	14190600	14280507	0	0.01154
5947	Amp	4	14280822	14286244	0	0.010879
5948	Amp	4	14286861	14312318	0	0.011845
5949	Amp	4	14314951	14398530	0	0.014342
5950	Amp	4	14398752	14405774	0	0.012668
5951	Amp	4	14407002	14494691	0	0.010171
5952	Amp	4	14499303	14561729	0	0.009205
5953	Amp	4	14562762	14751206	0	0.009657
5954	Amp	4	14761905	14811067	0	0.015417
5955	Amp	4	14811311	15360112	0	0.012643
5956	Amp	4	15366797	15425644	0	0.009657
5957	Amp	4	15426060	15431571	0	0.006342
5958	Amp	4	15431759	15737767	0	0.008157
5959	Amp	4	15737875	15930594	0	0.005343
5960	Amp	4	15932146	16054237	0	0.003528
5961	Amp	4	16054940	16152383	0	0.006118
5962	Amp	4	16153373	16457476	0	0.003528
5963	Amp	4	16462829	16505713	0	0.001805
5964	Amp	4	16506200	16596174	0	0.004291
5965	Amp	4	16596774	16799733	0	0.001805
5966	Amp	4	16804629	16834510	0	0.008868
5967	Amp	4	16836333	17455776	0	0.010571
5968	Amp	4	17456466	17617090	0	0.011433
5969	Amp	4	17619188	17764134	0	0.019989
5970	Amp	4	17767844	17772933	0	0.022732
5971	Amp	4	17773425	18004158	0	0.029137
5972	Amp	4	18008336	18082496	0	0.025667
5973	Amp	4	18083250	18168299	0	0.024805
5974	Amp	4	18172970	18325331	0	0.022062
5975	Amp	4	18328041	18348571	0	0.020118
5976	Amp	4	18350428	18396866	0	0.018469
5977	Amp	4	18397322	18743571	0	0.003508
5978	Amp	4	18745583	18774739	0	0.006074
5979	Amp	4	18775009	18779379	0	0.005621
5980	Amp	4	18779514	18860026	0	0.006147
5981	Amp	4	18862050	18880569	0	0.00834
5982	Amp	4	18882050	19122609	0	0.006147
5983	Amp	4	19125286	20115268	0	0.005621
5984	Amp	4	20115793	20171706	0	0.007622
5985	Amp	4	20171768	20290616	0	0.008976
5986	Amp	4	20292911	20364957	0	0.011221
5987	Amp	4	20366268	20842557	0	0.012334
5988	Amp	4	20848138	21123838	0	0.005621

5989 Amp	4	21128291	21171823	0	0.012434
5990 Amp	4	21171986	21171986	0	0.01477
5991 Amp	4	21175019	21178475	0	0.031793
5992 Amp	4	21178598	21178660	0	0.01477
5993 Amp	4	21179217	21323498	0	0.012434
5994 Amp	4	21330930	21424811	0	0.01484
5995 Amp	4	21425759	21550849	0	0.012274
5996 Amp	4	21551397	21560897	0	0.019351
5997 Amp	4	21561992	21601355	0	0.012274
5998 Amp	4	21602138	21738688	0	0.009868
5999 Amp	4	21738747	21786252	0	0.008515
6000 Amp	4	21791188	21864817	0	0.016782
6001 Amp	4	21865060	21882883	0	0.019425
6002 Amp	4	21889623	21932419	0	0.012612
6003 Amp	4	21932603	22043756	0	0.013162
6004 Amp	4	22072923	22340909	0	0.025532
6005 Amp	4	22342534	22464872	0	0.013162
6006 Amp	4	22468995	22584909	0	0.025707
6007 Amp	4	22588547	23204118	0	0.00997
6008 Amp	4	23204947	23206952	0	0.014162
6009 Amp	4	23208531	23225309	0	0.015743
6010 Amp	4	23230577	23294388	0	0.014162
6011 Amp	4	23294401	23460843	0	0.00997
6012 Amp	4	23460944	24009985	0	0.009433
6013 Amp	4	24010484	24331628	0	0.001702
6014 Amp	4	24331662	24352925	0	0.005066
6015 Amp	4	24354935	24432236	0	0.006535
6016 Amp	4	24433821	24505612	0	0.005066
6017 Amp	4	24509058	24591228	0	0.006531
6018 Amp	4	24591872	24827177	0	0.005066
6019 Amp	4	24827805	25597835	0	0.011851
6020 Amp	4	25599629	25845929	0	0.005066
6021 Amp	4	25847757	26639917	0	0.001702
6022 Amp	4	26642122	27017726	0	0.004284
6023 Amp	4	27018442	27130560	0	0.003469
6024 Amp	4	27133786	27435547	0	0.001702
6025 Amp	4	27438690	27578032	0	0.003529
6026 Amp	4	27579220	27917151	0	0.004096
6027 Amp	4	27919239	28987125	0	0.002393
6028 Amp	4	28990147	29035641	0	0.008229
6029 Amp	4	29039234	29066869	0	0.002393
6030 Amp	4	29067104	29166495	0	0.00427
6031 Amp	4	29170408	29313742	0	0.006537
6032 Amp	4	29313785	29316627	0	0.00427
6033 Amp	4	29316643	29330361	0	0.006346
6034 Amp	4	29331164	29528188	0	0.00427
6035 Amp	4	29530980	29615379	0	0.005984
6036 Amp	4	29615901	29630685	0	0.005418
6037 Amp	4	29634484	29725847	0	0.003704
6038 Amp	4	29729126	29765011	0	0.007368
6039 Amp	4	29765392	29936756	0	0.003704
6040 Amp	4	29942188	29988540	0	0.004846
6041 Amp	4	29990358	30157797	0	0.006098

6042	Amp	4	30166070	30308357	0	0.004221
6043	Amp	4	30309065	30848223	0	0.00748
6044	Amp	4	30855531	30904107	0	0.004221
6045	Amp	4	30904795	30948339	0	0.008065
6046	Amp	4	30951925	31366907	0	0.004221
6047	Amp	4	31369920	31444873	0	0.002898
6048	Amp	4	31445866	31926237	0	0.004704
6049	Amp	4	31929054	32172212	0	0.008251
6050	Amp	4	32179660	32456886	0	0.004704
6051	Amp	4	32456905	32730077	0	0.005156
6052	Amp	4	32734363	33365488	0	0.00335
6053	Amp	4	33365527	34312191	0	0.002898
6054	Amp	4	34312829	34344306	0	0.003758
6055	Amp	4	34346365	34350741	0	0.00721
6056	Amp	4	34351926	34411244	0	0.007902
6057	Amp	4	34411294	34538976	0	0.00445
6058	Amp	4	34544076	34566087	0	0.003758
6059	Amp	4	34566608	34578545	0	0.004592
6060	Amp	4	34582139	34672378	0	0.003758
6061	Amp	4	34699387	34699387	0	0.005138
6062	Amp	4	34699616	34763561	0	0.008201
6063	Amp	4	34763973	34843994	0	0.005138
6064	Amp	4	34844007	35313191	0	0.007526
6065	Amp	4	35319116	35369593	0	0.005138
6066	Amp	4	35413916	35919191	0	0.003758
6067	Amp	4	35924110	35929660	0	0.011843
6068	Amp	4	35929937	36086565	0	0.003758
6069	Amp	4	36090011	36174409	0	0.004262
6070	Amp	4	36175295	36222879	0	0.003758
6071	Amp	4	36227539	36490373	0	0.007436
6072	Amp	4	36493362	36639757	0	0.008254
6073	Amp	4	36642346	36696391	0	0.012673
6074	Amp	4	36696644	36748959	0	0.013384
6075	Amp	4	36754727	36886079	0	0.029121
6076	Amp	4	36890745	36909023	0	0.02967
6077	Amp	4	36942276	36942276	0	0.014706
6078	Amp	4	36947638	37072747	0	0.010287
6079	Amp	4	37074155	37148111	0	0.01108
6080	Amp	4	37149318	37209027	0	0.011683
6081	Amp	4	37210368	37602948	0	0.010287
6082	Amp	4	37603316	37883710	0	0.011225
6083	Amp	4	37887106	38060909	0	0.01517
6084	Amp	4	38063977	38143848	0	0.014232
6085	Amp	4	38147206	38227299	0	0.014786
6086	Amp	4	38229636	38326286	0	0.014012
6087	Amp	4	38326832	38417342	0	0.021003
6088	Amp	4	38417356	38959181	0	0.021863
6089	Amp	4	38959638	39118999	0	0.024685
6090	Amp	4	39122041	39173255	0	0.025181
6091	Amp	4	39177428	39254058	0	0.024471
6092	Amp	4	39261536	39273370	0	0.022948
6093	Amp	4	39273774	39299952	0	0.030798
6094	Amp	4	39300083	39523522	0	0.029938

6095 Amp	4	39530224	39701485	0	0.031014
6096 Amp	4	39701593	39820301	0	0.031785
6097 Amp	4	39820693	39835508	0	0.03332
6098 Amp	4	39842300	39995084	0	0.034278
6099 Amp	4	40002832	40335637	0	0.034973
6100 Amp	4	40337557	40356857	0	0.037565
6101 Amp	4	40362188	40375483	0	0.034973
6102 Amp	4	40375674	40801962	0	0.033721
6103 Amp	4	40802617	40928882	0	0.03519
6104 Amp	4	40929695	40939908	0	0.037502
6105 Amp	4	40940436	40974679	0	0.035849
6106 Amp	4	40975517	41040805	0	0.035079
6107 Amp	4	41042921	41069504	0	0.036884
6108 Amp	4	41070276	41102330	0	0.034572
6109 Amp	4	41104097	41287603	0	0.032767
6110 Amp	4	41288534	41467868	0	0.034263
6111 Amp	4	41468610	41485671	0	0.032767
6112 Amp	4	41486211	41499912	0	0.032213
6113 Amp	4	41503231	41503231	0	0.033211
6114 Amp	4	41510228	41702622	0	0.036632
6115 Amp	4	41705678	41713765	0	0.032739
6116 Amp	4	41721140	41724817	0	0.033664
6117 Amp	4	41727211	41880947	0	0.030975
6118 Amp	4	41886056	41961728	0	0.029506
6119 Amp	4	41967034	42146685	0	0.030742
6120 Amp	4	42147298	42301153	0	0.030108
6121 Amp	4	42310183	42384821	0	0.031957
6122 Amp	4	42386234	42452943	0	0.032572
6123 Amp	4	42453566	42737954	0	0.030724
6124 Amp	4	42738647	42749315	0	0.030227
6125 Amp	4	42749808	43321532	0	0.029612
6126 Amp	4	43322138	43479975	0	0.028375
6127 Amp	4	43483181	43730159	0	0.027826
6128 Amp	4	43730840	44079200	0	0.02645
6129 Amp	4	44082840	44091625	0	0.02863
6130 Amp	4	44091659	44245367	0	0.030671
6131 Amp	4	44259459	44271708	0	0.028491
6132 Amp	4	44272086	44289984	0	0.029512
6133 Amp	4	44290280	44538516	0	0.027471
6134 Amp	4	44541791	44555094	0	0.02645
6135 Amp	4	44559128	44751261	0	0.025947
6136 Amp	4	44753972	44761893	0	0.027171
6137 Amp	4	44765652	44864880	0	0.02873
6138 Amp	4	44865499	44919548	0	0.030403
6139 Amp	4	44919743	45162296	0	0.029261
6140 Amp	4	45163115	45339323	0	0.027273
6141 Amp	4	45340252	45344775	0	0.029207
6142 Amp	4	45345139	45490737	0	0.027644
6143 Amp	4	45494834	45618957	0	0.024412
6144 Amp	4	45621122	45965216	0	0.025834
6145 Amp	4	45967600	46005583	0	0.022005
6146 Amp	4	46006470	46332477	0	0.032557
6147 Amp	4	46336433	46339089	0	0.036867

6148	Amp	4	46340993	46361715	0	0.031167
6149	Amp	4	46364050	46389064	0	0.028146
6150	Amp	4	46389556	46420385	0	0.023836
6151	Amp	4	46424230	46547642	0	0.022611
6152	Amp	4	46548305	46553025	0	0.021188
6153	Amp	4	46553362	46872771	0	0.024139
6154	Amp	4	46873203	47178196	0	0.022308
6155	Amp	4	47178395	47805259	0	0.022777
6156	Amp	4	47812372	48026135	0	0.018832
6157	Amp	4	48026425	48066809	0	0.018285
6158	Amp	4	48067890	48067890	0	0.020414
6159	Amp	4	51832470	51832470	0	0.018419
6160	Amp	4	51832591	51833610	0	0.015486
6161	Amp	4	51834605	51867903	0	0.016276
6162	Amp	4	51870649	51880805	0	0.016943
6163	Amp	4	51884244	51983276	0	0.016064
6164	Amp	4	51991917	52070850	0	0.015426
6165	Amp	4	52071815	52200108	0	0.016276
6166	Amp	4	52201177	52201982	0	0.017261
6167	Amp	4	52205615	52392781	0	0.018536
6168	Amp	4	52392915	52551106	0	0.017385
6169	Amp	4	52551416	52698293	0	0.016718
6170	Amp	4	52705511	53082898	0	0.017341
6171	Amp	4	53083661	53369526	0	0.016055
6172	Amp	4	53371265	53473311	0	0.017769
6173	Amp	4	53476491	53493513	0	0.020151
6174	Amp	4	53495473	53653740	0	0.017769
6175	Amp	4	53656301	53664069	0	0.016979
6176	Amp	4	53664578	53876388	0	0.017951
6177	Amp	4	53891428	53938219	0	0.020883
6178	Amp	4	53940040	53945091	0	0.016904
6179	Amp	4	53955183	53962073	0	0.019932
6180	Amp	4	53962263	53974944	0	0.018067
6181	Amp	4	53980057	54040137	0	0.017218
6182	Amp	4	54054609	54165328	0	0.014189
6183	Amp	4	54165472	54184729	0	0.013567
6184	Amp	4	54185247	54238483	0	0.012801
6185	Amp	4	54239081	54249593	0	0.01141
6186	Amp	4	54254390	54291938	0	0.012545
6187	Amp	4	54291972	54292229	0	0.01871
6188	Amp	4	54293000	54293015	0	0.050486
6189	Amp	4	54293109	54293308	0	0.05139
6190	Amp	4	54293345	54466769	0	0.013449
6191	Amp	4	54469825	54552872	0	0.012187
6192	Amp	4	54558488	54709340	0	0.014129
6193	Amp	4	54716074	54993081	0	0.01586
6194	Amp	4	54997389	55020113	0	0.019069
6195	Amp	4	55021682	55048766	0	0.017338
6196	Amp	4	55054038	55109552	0	0.020317
6197	Amp	4	55110001	55115972	0	0.017108
6198	Amp	4	55116585	55117003	0	0.015166
6199	Amp	4	55117100	55172075	0	0.016917
6200	Amp	4	55173131	55191616	0	0.020127

6201 Amp	4	55196359	55310622	0	0.016917
6202 Amp	4	55312058	55329459	0	0.019079
6203 Amp	4	55337032	55468800	0	0.017328
6204 Amp	4	55470548	55718522	0	0.018626
6205 Amp	4	55719745	56129436	0	0.019975
6206 Amp	4	56131184	56140816	0	0.020418
6207 Amp	4	56144162	56153402	0	0.021459
6208 Amp	4	56153977	56300193	0	0.019562
6209 Amp	4	56300630	56323332	0	0.017849
6210 Amp	4	56323863	56341704	0	0.016808
6211 Amp	4	56345204	56388402	0	0.014769
6212 Amp	4	56390035	56570766	0	0.016819
6213 Amp	4	56571261	56691793	0	0.017818
6214 Amp	4	56692839	56845135	0	0.016723
6215 Amp	4	56847673	56867438	0	0.018688
6216 Amp	4	56871097	56939140	0	0.016723
6217 Amp	4	56939483	56959884	0	0.01577
6218 Amp	4	56963549	56964047	0	0.01824
6219 Amp	4	56966541	57183027	0	0.017241
6220 Amp	4	57236387	57352049	0	0.019997
6221 Amp	4	57354561	57409451	0	0.017241
6222 Amp	4	57410526	57617274	0	0.012702
6223 Amp	4	57619997	57723592	0	0.013284
6224 Amp	4	57723726	57991918	0	0.012702
6225 Amp	4	57991995	58042487	0	0.011746
6226 Amp	4	58042969	58117086	0	0.012683
6227 Amp	4	58118142	58127741	0	0.01638
6228 Amp	4	58127927	58141142	0	0.012683
6229 Amp	4	58145638	58287880	0	0.013129
6230 Amp	4	58289376	58352661	0	0.01266
6231 Amp	4	58353065	58403552	0	0.012214
6232 Amp	4	58407021	58471960	0	0.011329
6233 Amp	4	58474224	58482563	0	0.011966
6234 Amp	4	58486682	58649289	0	0.014366
6235 Amp	4	58649517	58698299	0	0.012579
6236 Amp	4	58698943	58707223	0	0.014742
6237 Amp	4	58712880	59205300	0	0.019248
6238 Amp	4	59212268	59262021	0	0.017778
6239 Amp	4	59266464	59307236	0	0.026958
6240 Amp	4	59315432	59439480	0	0.021421
6241 Amp	4	59440178	59716620	0	0.020783
6242 Amp	4	59717910	59828226	0	0.018673
6243 Amp	4	59828731	59996349	0	0.021233
6244 Amp	4	59997736	60093686	0	0.020297
6245 Amp	4	60093839	60162791	0	0.01842
6246 Amp	4	60162815	60309713	0	0.019651
6247 Amp	4	60312642	60413791	0	0.016892
6248 Amp	4	60420454	60495001	0	0.021315
6249 Amp	4	60496365	60695510	0	0.016892
6250 Amp	4	60701699	60746466	0	0.018628
6251 Amp	4	60746515	61274459	0	0.019146
6252 Amp	4	61278427	61477155	0	0.022695
6253 Amp	4	61478721	61515683	0	0.023998

6254 Amp	4	61516303	61525370	0	0.027043
6255 Amp	4	61526136	61541880	0	0.029425
6256 Amp	4	61543437	61543437	0	0.027924
6257 Amp	4	61547349	61686469	0	0.025542
6258 Amp	4	61688413	61853609	0	0.02107
6259 Amp	4	61855849	61901579	0	0.022214
6260 Amp	4	61905998	61978558	0	0.026721
6261 Amp	4	61978690	61997693	0	0.024984
6262 Amp	4	62001253	62044311	0	0.022449
6263 Amp	4	62048220	62424663	0	0.021305
6264 Amp	4	62432709	62581489	0	0.023743
6265 Amp	4	62590346	62652084	0	0.026868
6266 Amp	4	62655438	62694586	0	0.02736
6267 Amp	4	62697110	62975101	0	0.026868
6268 Amp	4	62976723	63025443	0	0.029207
6269 Amp	4	63031218	63307902	0	0.02635
6270 Amp	4	63308587	63363576	0	0.021843
6271 Amp	4	63364439	63471987	0	0.018295
6272 Amp	4	63480742	63501987	0	0.020153
6273 Amp	4	63502103	63502103	0	0.019376
6274 Amp	4	63503254	63583213	0	0.0175
6275 Amp	4	63585966	63774466	0	0.015642
6276 Amp	4	63781852	63958511	0	0.017175
6277 Amp	4	63966301	63966301	0	0.018606
6278 Amp	4	63966912	64280162	0	0.015481
6279 Amp	4	64280982	64647670	0	0.013043
6280 Amp	4	64647685	64672798	0	0.014782
6281 Amp	4	64680088	64730347	0	0.01743
6282 Amp	4	64730801	64739204	0	0.019323
6283 Amp	4	64742541	65012948	0	0.017893
6284 Amp	4	65012978	65094708	0	0.019886
6285 Amp	4	65094958	65319480	0	0.021926
6286 Amp	4	65322577	65475745	0	0.02247
6287 Amp	4	65476352	65500385	0	0.0245
6288 Amp	4	65500893	65546474	0	0.025914
6289 Amp	4	65546744	66076336	0	0.02402
6290 Amp	4	66077405	66110906	0	0.024709
6291 Amp	4	66115178	66115178	0	0.032277
6292 Amp	4	66115882	66134955	0	0.032887
6293 Amp	4	66134995	66440340	0	0.034644
6294 Amp	4	66447723	66585857	0	0.035184
6295 Amp	4	66596896	66646653	0	0.034574
6296 Amp	4	66649432	66770729	0	0.038676
6297 Amp	4	66773314	66821174	0	0.034574
6298 Amp	4	66829999	66914817	0	0.033342
6299 Amp	4	66922345	67095014	0	0.035031
6300 Amp	4	67095038	67226876	0	0.033342
6301 Amp	4	67228079	67516667	0	0.031585
6302 Amp	4	67519365	67564632	0	0.035448
6303 Amp	4	67567329	67567329	0	0.02788
6304 Amp	4	67567765	67581482	0	0.029718
6305 Amp	4	67587155	67920505	0	0.037509
6306 Amp	4	67924901	67975879	0	0.03979

6307	Amp	4	67976407	67977123	0	0.041407
6308	Amp	4	68119088	68120853	0	0.041691
6309	Amp	4	68120922	68149710	0	0.043009
6310	Amp	4	68152092	68177646	0	0.040728
6311	Amp	4	68177815	68206144	0	0.036184
6312	Amp	4	68209816	68272588	0	0.039338
6313	Amp	4	68272668	68335040	0	0.040068
6314	Amp	4	68338682	68339321	0	0.039018
6315	Amp	4	69452478	69452478	0	0.041865
6316	Amp	4	69453762	69566288	0	0.045652
6317	Amp	4	69567179	69595911	0	0.047113
6318	Amp	4	69596324	69690989	0	0.045652
6319	Amp	4	69691794	69788302	0	0.043682
6320	Amp	4	69792497	70014394	0	0.042089
6321	Amp	4	70014983	70083263	0	0.040251
6322	Amp	4	70085177	70251805	0	0.040764
6323	Amp	4	70274327	70274327	0	0.039779
6324	Amp	4	70275029	70288397	0	0.039266
6325	Amp	4	70288893	70324797	0	0.038012
6326	Amp	4	70327862	70356095	0	0.038637
6327	Amp	4	70359986	70361495	0	0.038012
6328	Amp	4	70385052	70410150	0	0.037155
6329	Amp	4	70412574	70451734	0	0.037775
6330	Amp	4	70451852	70454078	0	0.0387
6331	Amp	4	70454679	70611968	0	0.035602
6332	Amp	4	70614654	70726337	0	0.03719
6333	Amp	4	70726760	70766265	0	0.040065
6334	Amp	4	70767439	70866958	0	0.041086
6335	Amp	4	70873837	70916530	0	0.03913
6336	Amp	4	70917805	70981039	0	0.040836
6337	Amp	4	70995218	71009569	0	0.044681
6338	Amp	4	71011340	71097774	0	0.043093
6339	Amp	4	71098072	71213842	0	0.044816
6340	Amp	4	71214203	71309683	0	0.046564
6341	Amp	4	71313640	71418252	0	0.047594
6342	Amp	4	71425410	71505193	0	0.050352
6343	Amp	4	71514600	71621529	0	0.049427
6344	Amp	4	71622320	71676476	0	0.048398
6345	Amp	4	71689906	71773107	0	0.049249
6346	Amp	4	71773285	71817660	0	0.04649
6347	Amp	4	71820911	71842289	0	0.04547
6348	Amp	4	71842391	71858037	0	0.044832
6349	Amp	4	71858657	71988739	0	0.047647
6350	Amp	4	71991829	72007836	0	0.046797
6351	Amp	4	72016079	72063100	0	0.062733
6352	Amp	4	72068822	72087138	0	0.050957
6353	Amp	4	72095172	72160467	0	0.052023
6354	Amp	4	72171217	72175591	0	0.062909
6355	Amp	4	72178993	72183418	0.094967	0.068058
6356	Amp	4	72183961	72260477	0.1237	0.069154
6357	Amp	4	72261955	72263674	0.181932	0.071598
6358	Amp	4	72265440	72281705	0.164935	0.070978
6359	Amp	4	72285072	72344391	0.215608	0.072852

6360	Amp	4	72355273	72395769	0.143787	0.070037
6361	Amp	4	72401852	72409000	0.044794	0.065877
6362	Amp	4	72410585	72427472	0.005328	0.064153
6363	Amp	4	72430244	72445405	0	0.062969
6364	Amp	4	72450300	72455190	0.322897	0.077283
6365	Amp	4	72461314	72485662	0.424557	0.08058
6366	Amp	4	72485676	72489893	0.450702	0.081431
6367	Amp	4	72489986	72524292	0.379885	0.079141
6368	Amp	4	72532963	72613534	0.34892	0.078133
6369	Amp	4	72615935	72727920	0.299433	0.076259
6370	Amp	4	72736120	72834454	0.362016	0.078497
6371	Amp	4	72835239	72852636	1.243254	0.103138
6372	Amp	4	72852641	72852641	1.378521	0.105968
6373	Amp	4	72856687	72856687	0.651644	0.088128
6374	Amp	4	72856975	72885162	0.379885	0.079089
6375	Amp	4	72885209	72885209	0.271552	0.075302
6376	Amp	4	72885346	72892933	0.287738	0.07589
6377	Amp	4	72896926	72959669	0.373599	0.07885
6378	Amp	4	72966041	73027520	0.4115	0.08014
6379	Amp	4	73032473	73197959	0.529451	0.084121
6380	Amp	4	73212735	73214973	0.437198	0.08116
6381	Amp	4	73217055	73261137	0.487495	0.082754
6382	Amp	4	73265584	73296174	0.550755	0.084779
6383	Amp	4	73297277	73434893	0.704849	0.089509
6384	Amp	4	73435163	73762400	0.630438	0.087484
6385	Amp	4	73762738	73928229	0.576183	0.085735
6386	Amp	4	73929648	73990078	0.463534	0.082014
6387	Amp	4	73990141	74001561	0.536238	0.084266
6388	Amp	4	74009218	74120959	0.373599	0.078831
6389	Amp	4	74123365	74184708	0.14896	0.07022
6390	Amp	4	74196476	74206025	0.241616	0.07399
6391	Amp	4	74207983	74219929	0.187449	0.071738
6392	Amp	4	74222796	74222796	0.065715	0.066692
6393	Amp	4	74222811	74222811	0	0.060261
6394	Amp	4	74222899	74224091	0	0.058777
6395	Amp	4	74224153	74224153	0.005328	0.064114
6396	Amp	4	74224195	74224195	0.112543	0.068756
6397	Amp	4	74224306	74251146	0.193543	0.071916
6398	Amp	4	74254755	74293161	0.386426	0.079354
6399	Amp	4	74295935	74368769	0.30524	0.076436
6400	Amp	4	74369972	74394615	0.316877	0.076972
6401	Amp	4	74395690	74416087	0.329205	0.077418
6402	Amp	4	74416658	74468225	0.299433	0.076322
6403	Amp	4	74468346	74483740	0.355319	0.078322
6404	Amp	4	74487729	74552387	0.222041	0.072968
6405	Amp	4	74555455	74619109	0.276783	0.075414
6406	Amp	4	74620135	74664203	0.233782	0.073414
6407	Amp	4	74669028	74709788	0.386426	0.079273
6408	Amp	4	74714899	74763498	0.373599	0.078827
6409	Amp	4	74763693	74785912	0.355319	0.078281
6410	Amp	4	74791070	74798763	0.431198	0.080945
6411	Amp	4	74799805	74843082	0.463534	0.081902
6412	Amp	4	74843911	74922577	0.386426	0.079377

6413	Amp	4	74927253	74960174	0.30524	0.076548
6414	Amp	4	74963154	74987624	0.204831	0.072353
6415	Amp	4	74988359	74995248	0.233782	0.073397
6416	Amp	4	74997296	75098408	0.040093	0.06559
6417	Amp	4	75105397	75238606	0.010261	0.064434
6418	Amp	4	75238844	75256588	0	0.062623
6419	Amp	4	75258049	75281372	0	0.06113
6420	Amp	4	75284228	75296217	0.164935	0.070936
6421	Amp	4	75299475	75350307	0.204831	0.072343
6422	Amp	4	75350923	75416232	0.276783	0.075367
6423	Amp	4	75422035	75462203	0.329205	0.077315
6424	Amp	4	75473179	75555738	0.470509	0.08227
6425	Amp	4	75556365	75569396	0.508568	0.083504
6426	Amp	4	75571607	75599233	0.667587	0.08848
6427	Amp	4	75601125	75668811	0.576183	0.085596
6428	Amp	4	75674255	75702644	0.508568	0.083504
6429	Amp	4	75705879	75803890	0.589605	0.086183
6430	Amp	4	75811466	75811466	0.557122	0.084974
6431	Amp	4	75816637	75915672	0.595248	0.086381
6432	Amp	4	75915945	75973325	0.482379	0.082523
6433	Amp	4	75974353	76068743	0.329205	0.07738
6434	Amp	4	76068955	76083307	0.276783	0.07535
6435	Amp	4	76083975	76103201	0.293269	0.076117
6436	Amp	4	76103983	76135352	0.34892	0.078152
6437	Amp	4	76135711	76219580	0.368062	0.078713
6438	Amp	4	76221699	76325682	0.437198	0.081044
6439	Amp	4	76325743	76337520	0.536238	0.084404
6440	Amp	4	76338003	76379668	0.882843	0.094335
6441	Amp	4	76380961	76380961	0.735486	0.090501
6442	Amp	4	76386512	76410575	0.924746	0.095371
6443	Amp	4	76412847	76462047	0.815228	0.092673
6444	Amp	4	76462067	76476432	0.735486	0.09047
6445	Amp	4	76477468	76544879	0.815228	0.09266
6446	Amp	4	76546447	76550715	0.742723	0.090706
6447	Amp	4	76551399	76574418	0.711903	0.089855
6448	Amp	4	76579794	76583247	0.6737	0.08862
6449	Amp	4	76586710	76606350	0.500841	0.083319
6450	Amp	4	76611726	76637624	0.293269	0.076109
6451	Amp	4	76641543	76646345	0.311747	0.07675
6452	Amp	4	76647460	76757046	0.259438	0.074803
6453	Amp	4	76757575	76871039	0.241616	0.073876
6454	Amp	4	76871045	76882856	0.311747	0.076662
6455	Amp	4	76884498	76912095	0.251218	0.074331
6456	Amp	4	76912942	76937914	0.1237	0.069192
6457	Amp	4	76938432	77025061	0.238246	0.073637
6458	Amp	4	77025664	77047041	0.259438	0.074796
6459	Amp	4	77047087	77091640	0.204831	0.072476
6460	Amp	4	77095917	77156989	0.094967	0.068145
6461	Amp	4	77159810	77245743	0	0.061529
6462	Amp	4	77246450	77294377	0	0.060887
6463	Amp	4	77295863	77344546	0	0.055905
6464	Amp	4	77345416	77391832	0	0.055138
6465	Amp	4	77395379	77454136	0	0.05086

6466	Amp	4	77454847	77539671	0	0.049794
6467	Amp	4	77543544	77550381	0	0.04767
6468	Amp	4	77551591	77611535	0	0.049705
6469	Amp	4	77613424	77921929	0	0.051951
6470	Amp	4	77924928	77924928	0	0.049705
6471	Amp	4	77927088	77938865	0	0.051626
6472	Amp	4	77944853	77944853	0	0.059429
6473	Amp	4	77945536	77956824	0	0.055822
6474	Amp	4	77958307	77961341	0	0.054778
6475	Amp	4	77962763	78037520	0	0.059501
6476	Amp	4	78040262	78119723	0	0.054546
6477	Amp	4	78119737	78139161	0	0.049198
6478	Amp	4	78143481	78215104	0	0.047158
6479	Amp	4	78216411	78217229	0	0.042451
6480	Amp	4	78222767	78266873	0	0.043971
6481	Amp	4	78267577	78393482	0	0.042365
6482	Amp	4	78404496	78514376	0	0.047069
6483	Amp	4	78515773	78639804	0	0.046297
6484	Amp	4	78640264	78667948	0	0.044404
6485	Amp	4	78674585	78682766	0	0.047293
6486	Amp	4	78682855	78701254	0	0.044894
6487	Amp	4	78701978	78957077	0	0.044165
6488	Amp	4	78958618	79045181	0	0.046183
6489	Amp	4	79045283	79081513	0	0.050052
6490	Amp	4	79083035	79129955	0	0.044922
6491	Amp	4	79134205	79396618	0	0.04191
6492	Amp	4	79396823	79449757	0	0.039865
6493	Amp	4	79450152	79576928	0	0.045407
6494	Amp	4	79578293	79605860	0	0.052469
6495	Amp	4	79607864	79628156	0	0.045407
6496	Amp	4	79629792	79719071	0	0.051367
6497	Amp	4	79719143	79811847	0	0.045407
6498	Amp	4	79816699	79917559	0	0.040836
6499	Amp	4	79920738	79930322	0	0.04373
6500	Amp	4	79933096	79935468	0	0.042302
6501	Amp	4	79975155	79992651	0	0.03949
6502	Amp	4	79994925	80042254	0	0.04185
6503	Amp	4	80044716	80174360	0	0.047194
6504	Amp	4	80175654	80199768	0	0.045154
6505	Amp	4	80204929	80209407	0	0.046694
6506	Amp	4	80209560	80241877	0	0.045634
6507	Amp	4	80242935	80292962	0	0.044094
6508	Amp	4	80293421	80355106	0	0.035741
6509	Amp	4	80356070	80614239	0	0.034678
6510	Amp	4	80617209	80666945	0	0.03724
6511	Amp	4	80668188	80786940	0	0.036727
6512	Amp	4	80791295	80823417	0	0.038323
6513	Amp	4	80825593	80837073	0	0.03658
6514	Amp	4	80841755	80870119	0	0.035394
6515	Amp	4	80870618	80897920	0	0.03471
6516	Amp	4	80898780	80955375	0	0.037277
6517	Amp	4	80956347	80963606	0	0.038573
6518	Amp	4	80967733	80997467	0	0.036977

6519	Amp	4	80997730	81006613	0	0.032497
6520	Amp	4	81007739	81102736	0	0.029469
6521	Amp	4	81106256	81335010	0	0.028173
6522	Amp	4	81337222	81396851	0	0.025611
6523	Amp	4	81397370	81622537	0	0.027215
6524	Amp	4	81629857	81847731	0	0.024325
6525	Amp	4	81852466	81983381	0	0.021253
6526	Amp	4	81984225	82050411	0	0.023734
6527	Amp	4	82052089	82330760	0	0.021253
6528	Amp	4	82341483	82343607	0	0.027331
6529	Amp	4	82356608	82419429	0	0.025727
6530	Amp	4	82421593	82438316	0	0.024877
6531	Amp	4	82440583	82635255	0	0.025728
6532	Amp	4	82637525	82720673	0	0.024877
6533	Amp	4	82723539	82730558	0	0.030418
6534	Amp	4	82732057	82809265	0	0.024877
6535	Amp	4	82811191	83131287	0	0.025532
6536	Amp	4	83138221	83179278	0	0.028947
6537	Amp	4	83179439	83212347	0	0.027021
6538	Amp	4	83213083	83518487	0	0.029903
6539	Amp	4	83518667	83633276	0	0.031914
6540	Amp	4	83634994	83676627	0	0.029903
6541	Amp	4	83679918	83859175	0	0.027625
6542	Amp	4	83859315	84062385	0	0.029172
6543	Amp	4	84062860	84392527	0	0.030948
6544	Amp	4	84402201	84442703	0	0.029172
6545	Amp	4	84443140	84526030	0	0.036397
6546	Amp	4	84526104	84605956	0	0.034499
6547	Amp	4	84606913	84969731	0	0.037329
6548	Amp	4	84973482	85139691	0	0.033715
6549	Amp	4	85142877	85170717	0	0.03084
6550	Amp	4	85176068	85236260	0	0.032135
6551	Amp	4	85245257	85320125	0	0.027815
6552	Amp	4	85321178	85366116	0	0.03262
6553	Amp	4	85370439	85412575	0	0.035576
6554	Amp	4	85415875	85437696	0	0.030771
6555	Amp	4	85438289	85519605	0	0.028726
6556	Amp	4	85519662	85573832	0	0.02577
6557	Amp	4	85575450	85584789	0	0.023582
6558	Amp	4	85586052	85589972	0	0.028769
6559	Amp	4	85590060	85883503	0	0.023582
6560	Amp	4	85883757	86000596	0	0.021119
6561	Amp	4	86003972	86045486	0	0.019571
6562	Amp	4	86048246	86069538	0	0.021971
6563	Amp	4	86070328	86132000	0	0.021317
6564	Amp	4	86133823	86205956	0	0.018917
6565	Amp	4	86206024	86267062	0	0.021227
6566	Amp	4	86268770	86341626	0	0.018917
6567	Amp	4	86342098	86550793	0	0.020283
6568	Amp	4	86556268	86615463	0	0.022042
6569	Amp	4	86617313	86679046	0	0.024832
6570	Amp	4	86679224	86805872	0	0.023073
6571	Amp	4	86811754	87218593	0	0.025416

6572	Amp	4	87225819	87763868	0	0.023073
6573	Amp	4	87765704	87779307	0.040093	0.065785
6574	Amp	4	87783516	87883129	0	0.023073
6575	Amp	4	87886094	87897741	0	0.021033
6576	Amp	4	87900338	88191958	0	0.024708
6577	Amp	4	88198799	88286740	0	0.022699
6578	Amp	4	88289437	88629336	0	0.021033
6579	Amp	4	88629611	88766380	0	0.019738
6580	Amp	4	88774334	88782418	0	0.011634
6581	Amp	4	88782732	89145403	0	0.008844
6582	Amp	4	89150546	89178931	0	0.01146
6583	Amp	4	89185912	89185963	0	0.008844
6584	Amp	4	89186741	89381510	0	0.010405
6585	Amp	4	89382796	89748769	0	0.009039
6586	Amp	4	89750394	89792108	0	0.00949
6587	Amp	4	89792179	89930396	0	0.009039
6588	Amp	4	90033399	90033399	0	0.017472
6589	Amp	4	90036504	90287457	0	0.018809
6590	Amp	4	90292546	90343499	0	0.020196
6591	Amp	4	90347035	90383849	0	0.0236
6592	Amp	4	90390139	90675567	0	0.020196
6593	Amp	4	90688146	91231845	0	0.022301
6594	Amp	4	91238267	91262950	0	0.024527
6595	Amp	4	91267480	91336440	0	0.026766
6596	Amp	4	91338334	91647296	0	0.024527
6597	Amp	4	91651348	91710801	0	0.025697
6598	Amp	4	91711443	91730826	0	0.024527
6599	Amp	4	91736736	91918393	0	0.025299
6600	Amp	4	91918757	92327796	0	0.026996
6601	Amp	4	92328810	92960214	0	0.025889
6602	Amp	4	93013415	93053028	0	0.02888
6603	Amp	4	93055042	93060458	0	0.028108
6604	Amp	4	93060670	93266504	0	0.025117
6605	Amp	4	93267622	93607515	0	0.017185
6606	Amp	4	93620090	93713151	0	0.02058
6607	Amp	4	93713271	94208195	0	0.022251
6608	Amp	4	94222515	94587604	0	0.019244
6609	Amp	4	94588612	94632220	0	0.027161
6610	Amp	4	94636200	94813876	0	0.028954
6611	Amp	4	94816692	94873988	0	0.030672
6612	Amp	4	94876298	94949115	0	0.03346
6613	Amp	4	94955127	95021107	0	0.034482
6614	Amp	4	95023432	95036926	0	0.035742
6615	Amp	4	95044646	95119817	0	0.025041
6616	Amp	4	95120276	95193174	0	0.024451
6617	Amp	4	95195627	95273552	0	0.022733
6618	Amp	4	95273654	95358222	0	0.019338
6619	Amp	4	95358573	95386064	0	0.01655
6620	Amp	4	95386746	95454980	0	0.01781
6621	Amp	4	95455763	95628817	0	0.01655
6622	Amp	4	95630518	95725690	0	0.017216
6623	Amp	4	95736973	96013474	0	0.01655
6624	Amp	4	96017620	96107628	0	0.020346

6625 Amp	4	96108137	96124814	0	0.017451
6626 Amp	4	96130804	96158266	0	0.020443
6627 Amp	4	96163524	96171022	0	0.01605
6628 Amp	4	96171799	97242821	0	0.013057
6629 Amp	4	97243636	97308855	0	0.010832
6630 Amp	4	97309205	97463138	0	0.009856
6631 Amp	4	97468447	97860593	0	0.015072
6632 Amp	4	97868391	98457879	0	0.009856
6633 Amp	4	98459997	98589812	0	0.012238
6634 Amp	4	98591017	98645671	0	0.010446
6635 Amp	4	98649839	98745091	0	0.011909
6636 Amp	4	98745641	98851984	0	0.011351
6637 Amp	4	98853227	98893003	0	0.013894
6638 Amp	4	98893217	99095580	0	0.011351
6639 Amp	4	99121111	99401629	0	0.008969
6640 Amp	4	99402713	99444543	0	0.011217
6641 Amp	4	99447629	99486110	0	0.008969
6642 Amp	4	99486167	99646091	0	0.009908
6643 Amp	4	99646303	99830742	0	0.008969
6644 Amp	4	99833508	100446997	0	0.011856
6645 Amp	4	100450960	100781617	0	0.008969
6646 Amp	4	100782870	100935454	0	0.01102
6647 Amp	4	100937237	100975519	0	0.008857
6648 Amp	4	100978305	101190334	0	0.007393
6649 Amp	4	101191924	101731137	0	0.009823
6650 Amp	4	101731857	102270630	0	0.007393
6651 Amp	4	102279190	102370849	0	0.009511
6652 Amp	4	102371460	102463388	0	0.011128
6653 Amp	4	102466188	102503074	0	0.009511
6654 Amp	4	102511817	102582667	0	0.011791
6655 Amp	4	102585217	103099624	0	0.009511
6656 Amp	4	103099789	103617064	0	0.010375
6657 Amp	4	103621077	103722022	0	0.011645
6658 Amp	4	103725075	103762701	0	0.014026
6659 Amp	4	103770095	103816743	0	0.013162
6660 Amp	4	103841700	103876748	0	0.013687
6661 Amp	4	103877830	103901573	0	0.011306
6662 Amp	4	103903326	104100463	0	0.012581
6663 Amp	4	104103457	104290201	0	0.010463
6664 Amp	4	104290467	104290467	0	0.012422
6665 Amp	4	104291391	104345499	0	0.010861
6666 Amp	4	104350516	104825245	0	0.006358
6667 Amp	4	104826410	105086367	0	0.005832
6668 Amp	4	105087310	105131307	0	0.004673
6669 Amp	4	105134146	105208958	0	0.008315
6670 Amp	4	105209868	105252727	0	0.011383
6671 Amp	4	105256232	105478030	0	0.00774
6672 Amp	4	105480932	105567677	0	0.004673
6673 Amp	4	105569491	105616711	0	0.00672
6674 Amp	4	105621611	105782376	0	0.004673
6675 Amp	4	105788935	106047313	0	0.006339
6676 Amp	4	106047362	107278256	0	0.004673
6677 Amp	4	107278270	107285712	0	0.007304

6678	Amp	4	107285750	107869215	0	0.004673
6679	Amp	4	107871579	108101650	0	0.008574
6680	Amp	4	108104645	108327206	0	0.004673
6681	Amp	4	108327535	109183023	0	0.005714
6682	Amp	4	109184893	110878261	0	0.003663
6683	Amp	4	110882480	110957449	0	0.005847
6684	Amp	4	110966681	110989302	0	0.006311
6685	Amp	4	110989488	110998660	0	0.005847
6686	Amp	4	111004422	111028287	0	0.006404
6687	Amp	4	111028540	111185986	0	0.005847
6688	Amp	4	111186817	112263248	0	0.003663
6689	Amp	4	112269595	112319373	0	0.005064
6690	Amp	4	112326279	112561562	0	0.003663
6691	Amp	4	112561733	112739669	0	0.005772
6692	Amp	4	112743148	113001595	0	0.008431
6693	Amp	4	113003702	113260385	0	0.003663
6694	Amp	4	113266738	113357121	0	0.006536
6695	Amp	4	113363990	113445461	0	0.010374
6696	Amp	4	113448379	113740012	0	0.006536
6697	Amp	4	113740273	113758138	0	0.007875
6698	Amp	4	113758561	114047012	0	0.005002
6699	Amp	4	114049608	115371004	0	0.003663
6700	Amp	4	115374337	115446264	0	0.006612
6701	Amp	4	115446487	115719097	0	0.007476
6702	Amp	4	115719403	115898087	0	0.009823
6703	Amp	4	115899667	116050449	0	0.006875
6704	Amp	4	116057884	116155406	0	0.008523
6705	Amp	4	116159224	116199764	0	0.010704
6706	Amp	4	116200548	116251469	0	0.012007
6707	Amp	4	116252185	116683014	0	0.011144
6708	Amp	4	116692197	116981258	0	0.009496
6709	Amp	4	117004426	117270960	0	0.007462
6710	Amp	4	117276055	117650334	0	0.010774
6711	Amp	4	117662944	117794157	0	0.01158
6712	Amp	4	117794503	117889958	0	0.008267
6713	Amp	4	117890163	118588341	0	0.006086
6714	Amp	4	118589867	118905499	0	0.004782
6715	Amp	4	118906001	118918240	0	0.005947
6716	Amp	4	118923114	119111185	0	0.006398
6717	Amp	4	119116032	119284861	0	0.007321
6718	Amp	4	119287771	119459233	0	0.009129
6719	Amp	4	119460368	119838014	0	0.008206
6720	Amp	4	119838226	120027213	0	0.007755
6721	Amp	4	120028467	120060483	0	0.009947
6722	Amp	4	120062104	120238929	0	0.007755
6723	Amp	4	120239381	120626306	0	0.006583
6724	Amp	4	120631671	120848486	0	0.007567
6725	Amp	4	120849201	121068094	0	0.011574
6726	Amp	4	121074543	121387087	0	0.007567
6727	Amp	4	121387304	121501974	0	0.017012
6728	Amp	4	121503338	121578510	0	0.018455
6729	Amp	4	121584153	121626759	0	0.020227
6730	Amp	4	121629644	121858819	0	0.018455

6731	Amp	4	121863804	122164552	0	0.013272
6732	Amp	4	122167710	122404814	0	0.015322
6733	Amp	4	122405687	122714270	0	0.016427
6734	Amp	4	122721216	122878105	0	0.014984
6735	Amp	4	122885669	122961448	0	0.01763
6736	Amp	4	122966040	122978264	0	0.013881
6737	Amp	4	122980057	122980057	0	0.019404
6738	Amp	4	122989760	123108454	0	0.018299
6739	Amp	4	123112177	123293695	0	0.017494
6740	Amp	4	123297991	123298647	0	0.016329
6741	Amp	4	123299521	123431305	0	0.012636
6742	Amp	4	123433129	124198272	0	0.014518
6743	Amp	4	124204749	124623571	0	0.017175
6744	Amp	4	124626776	124926594	0	0.014518
6745	Amp	4	124927064	125169013	0	0.010586
6746	Amp	4	125169348	125313572	0	0.01272
6747	Amp	4	125320716	125553298	0	0.016258
6748	Amp	4	125553864	125730953	0	0.015217
6749	Amp	4	125736126	125798691	0	0.010956
6750	Amp	4	125801938	125834586	0	0.012703
6751	Amp	4	125836472	125856944	0	0.013541
6752	Amp	4	125870870	126037884	0	0.011793
6753	Amp	4	126042458	126143031	0	0.0155
6754	Amp	4	126153255	126212137	0	0.011793
6755	Amp	4	126218200	126940124	0	0.009659
6756	Amp	4	126942170	127061132	0	0.010334
6757	Amp	4	127070483	127217395	0	0.008003
6758	Amp	4	127217968	127349199	0	0.008536
6759	Amp	4	127378175	127480168	0	0.010508
6760	Amp	4	127487227	127607462	0	0.01205
6761	Amp	4	127614715	128379069	0	0.014981
6762	Amp	4	128380202	128392498	0	0.017285
6763	Amp	4	128393020	129207657	0	0.014981
6764	Amp	4	129208120	129209759	0	0.013996
6765	Amp	4	129215297	129235464	0	0.016346
6766	Amp	4	129239551	129338590	0	0.015814
6767	Amp	4	129339724	129534860	0	0.015139
6768	Amp	4	129535005	129853674	0	0.017265
6769	Amp	4	129860557	129884580	0	0.021854
6770	Amp	4	129885442	129933780	0	0.017265
6771	Amp	4	129933871	130246311	0	0.018141
6772	Amp	4	130249780	130250350	0.034113	0.065432
6773	Amp	4	130250672	130251039	0	0.018141
6774	Amp	4	130333466	130347990	0	0.013512
6775	Amp	4	130348122	131012610	0	0.014144
6776	Amp	4	131013913	131560611	0	0.012264
6777	Amp	4	131560676	131587727	0	0.009333
6778	Amp	4	131591556	131604552	0	0.011947
6779	Amp	4	131604639	131604696	0	0.011071
6780	Amp	4	131606114	131705361	0	0.00863
6781	Amp	4	131767338	132180528	0	0.006015
6782	Amp	4	132183374	132185493	0	0.008792
6783	Amp	4	132185620	132742512	0	0.008005

6784	Amp	4	132745682	132925948	0	0.009169
6785	Amp	4	132926380	133619199	0	0.008005
6786	Amp	4	133619676	134271522	0	0.005227
6787	Amp	4	134271658	134290051	0	0.00711
6788	Amp	4	134290872	134446538	0	0.010172
6789	Amp	4	134450878	134662979	0	0.008289
6790	Amp	4	134663374	134941729	0	0.009405
6791	Amp	4	134941749	135128410	0	0.010763
6792	Amp	4	135128451	135486338	0	0.011928
6793	Amp	4	135493894	135777648	0	0.010571
6794	Amp	4	135783173	135912996	0	0.009325
6795	Amp	4	135914934	136112783	0	0.00816
6796	Amp	4	136113271	136372051	0	0.005098
6797	Amp	4	136379681	136388774	0	0.006476
6798	Amp	4	136390801	136419937	0	0.007775
6799	Amp	4	136420093	136504900	0	0.009859
6800	Amp	4	136507130	136514255	0	0.010483
6801	Amp	4	136520392	136920976	0	0.012339
6802	Amp	4	136924257	136954637	0	0.014237
6803	Amp	4	136955672	137698165	0	0.012938
6804	Amp	4	137698756	138056826	0	0.01235
6805	Amp	4	138059020	138179636	0	0.010494
6806	Amp	4	138182954	138188598	0	0.009378
6807	Amp	4	138189079	138294580	0	0.011356
6808	Amp	4	138298092	138918647	0	0.009378
6809	Amp	4	138919708	139000723	0	0.012291
6810	Amp	4	139013963	139074422	0	0.009378
6811	Amp	4	139074903	139074968	0	0.00748
6812	Amp	4	139075015	139077296	0	0.00891
6813	Amp	4	139077327	139077327	0	0.010626
6814	Amp	4	139084831	139135901	0	0.009196
6815	Amp	4	139136150	139163033	0	0.0112
6816	Amp	4	139164353	139243904	0	0.014165
6817	Amp	4	139247705	139547921	0	0.012449
6818	Amp	4	139549058	139850306	0	0.013469
6819	Amp	4	139854197	140610450	0	0.015774
6820	Amp	4	140611966	140627986	0	0.014118
6821	Amp	4	140628239	140635636	0	0.015229
6822	Amp	4	140635915	140828308	0	0.017929
6823	Amp	4	140833491	140859194	0	0.021294
6824	Amp	4	140861917	140998883	0	0.014118
6825	Amp	4	140998940	141175270	0	0.01613
6826	Amp	4	141177105	141388588	0	0.014118
6827	Amp	4	141393526	141760842	0	0.015564
6828	Amp	4	141764998	142017907	0	0.016047
6829	Amp	4	142022332	142358680	0	0.017141
6830	Amp	4	142360871	142391837	0	0.020005
6831	Amp	4	142400467	142400756	0	0.021322
6832	Amp	4	142404678	142549126	0	0.024694
6833	Amp	4	142551543	142598753	0	0.024211
6834	Amp	4	142599233	142961079	0	0.022894
6835	Amp	4	142961392	143087991	0	0.025533
6836	Amp	4	143088089	143213777	0	0.026063

6837	Amp	4	143216148	143436353	0	0.023714
6838	Amp	4	143437613	143481311	0	0.022619
6839	Amp	4	143482614	143650483	0	0.02477
6840	Amp	4	143652986	143694339	0	0.025674
6841	Amp	4	143703174	143720238	0	0.030121
6842	Amp	4	143721339	143796736	0	0.025674
6843	Amp	4	143797654	143805016	0	0.029944
6844	Amp	4	143815907	143824849	0	0.031355
6845	Amp	4	143834429	143841450	0	0.027085
6846	Amp	4	144126461	144209933	0	0.019637
6847	Amp	4	144213647	144995761	0	0.018191
6848	Amp	4	145000645	145735643	0	0.019195
6849	Amp	4	145735883	145958251	0	0.021221
6850	Amp	4	145958652	146115674	0	0.023168
6851	Amp	4	146116070	146659760	0	0.022164
6852	Amp	4	146661789	147465770	0	0.021261
6853	Amp	4	147475770	147921356	0	0.019234
6854	Amp	4	147923242	148018539	0	0.025205
6855	Amp	4	148023986	148231090	0	0.019234
6856	Amp	4	148233763	148455181	0	0.021834
6857	Amp	4	148456598	148658892	0	0.019234
6858	Amp	4	148661459	149123387	0	0.014522
6859	Amp	4	149127857	149274811	0	0.016048
6860	Amp	4	149275209	149863030	0	0.018243
6861	Amp	4	149863195	150326847	0	0.019039
6862	Amp	4	150328425	150556465	0	0.017513
6863	Amp	4	150556564	150708987	0	0.016826
6864	Amp	4	150712994	150840648	0	0.016135
6865	Amp	4	150843741	151123586	0	0.01791
6866	Amp	4	151144532	151162561	0	0.021642
6867	Amp	4	151168121	151193082	0	0.01791
6868	Amp	4	151193511	151683684	0	0.016135
6869	Amp	4	151684297	152161542	0	0.01534
6870	Amp	4	152165324	152213876	0	0.016843
6871	Amp	4	152215928	152742612	0	0.01534
6872	Amp	4	152747176	152787147	0	0.017487
6873	Amp	4	152795365	152968975	0	0.01534
6874	Amp	4	152969302	153031812	0	0.017195
6875	Amp	4	153034788	153470738	0	0.01534
6876	Amp	4	153470964	153727754	0	0.016713
6877	Amp	4	153730876	153743354	0	0.01534
6878	Amp	4	153743869	153778198	0	0.016364
6879	Amp	4	153778417	154698510	0	0.01534
6880	Amp	4	154706223	154921454	0	0.016463
6881	Amp	4	154924800	155207621	0	0.01823
6882	Amp	4	155210742	155350547	0	0.016463
6883	Amp	4	155355496	155382403	0	0.019675
6884	Amp	4	155383270	155650955	0	0.016463
6885	Amp	4	155657262	155687357	0	0.019455
6886	Amp	4	155687403	155845166	0	0.017508
6887	Amp	4	155847076	156150653	0	0.016385
6888	Amp	4	156155260	156781100	0	0.017865
6889	Amp	4	156784399	157069518	0	0.016385

6890	Amp	4	157069760	157215941	0	0.018111
6891	Amp	4	157216710	157477211	0	0.015119
6892	Amp	4	157479078	158019028	0	0.013393
6893	Amp	4	158021345	158306801	0	0.012863
6894	Amp	4	158311931	158368949	0	0.014586
6895	Amp	4	158369907	158553629	0	0.017418
6896	Amp	4	158563237	158745669	0	0.015196
6897	Amp	4	158753635	158956221	0	0.016277
6898	Amp	4	158956712	159006558	0	0.015196
6899	Amp	4	159007755	159036028	0	0.012863
6900	Amp	4	159041890	159064640	0	0.016086
6901	Amp	4	159065355	159661134	0	0.012863
6902	Amp	4	159667491	160605607	0	0.028686
6903	Amp	4	160606412	160654265	0	0.029167
6904	Amp	4	160655996	160798619	0	0.031936
6905	Amp	4	160802135	160834754	0	0.033094
6906	Amp	4	160835040	161088291	0	0.030325
6907	Amp	4	161091185	161091519	0	0.014502
6908	Amp	4	161092019	161500884	0	0.017209
6909	Amp	4	161501015	161559759	0	0.016728
6910	Amp	4	161561443	162120922	0	0.014021
6911	Amp	4	162126147	162664475	0	0.015075
6912	Amp	4	162668073	164372664	0	0.014021
6913	Amp	4	164376017	164439365	0	0.009521
6914	Amp	4	164439955	165005356	0	0.012724
6915	Amp	4	165005413	165087782	0	0.014089
6916	Amp	4	165088523	165180217	0	0.016692
6917	Amp	4	165182800	165576513	0	0.014089
6918	Amp	4	165580244	166177471	0	0.01544
6919	Amp	4	166178189	166586738	0	0.014089
6920	Amp	4	166588324	166806240	0	0.015371
6921	Amp	4	166807871	167662108	0	0.014089
6922	Amp	4	167662377	167682938	0	0.015018
6923	Amp	4	167689450	168159908	0	0.013653
6924	Amp	4	168162316	168175229	0	0.017444
6925	Amp	4	168175246	168284283	0	0.016515
6926	Amp	4	168284886	168505111	0	0.017313
6927	Amp	4	168505804	168540536	0	0.017874
6928	Amp	4	168542505	168823416	0	0.018575
6929	Amp	4	168826422	168921009	0	0.020148
6930	Amp	4	168921891	169014730	0	0.021172
6931	Amp	4	169017084	169115978	0	0.020471
6932	Amp	4	169118404	169771224	0	0.022767
6933	Amp	4	169773518	169836461	0	0.024764
6934	Amp	4	169840847	169884754	0	0.023966
6935	Amp	4	169885864	170080508	0	0.023405
6936	Amp	4	170100520	170144096	0	0.024604
6937	Amp	4	170146926	170186724	0	0.025642
6938	Amp	4	170188243	170745334	0	0.021851
6939	Amp	4	170745810	171398788	0	0.022923
6940	Amp	4	171401902	171706560	0	0.021129
6941	Amp	4	171709072	171769950	0	0.023095
6942	Amp	4	171775122	171972120	0	0.023558

6943	Amp	4	171973963	172174940	0	0.024838
6944	Amp	4	172175032	172700025	0	0.023558
6945	Amp	4	172704304	172783055	0	0.024932
6946	Amp	4	172783651	172799283	0	0.02598
6947	Amp	4	172799766	172875881	0	0.025402
6948	Amp	4	172878318	172976201	0	0.027649
6949	Amp	4	172977094	172977094	0	0.029422
6950	Amp	4	172979899	173098618	0	0.031222
6951	Amp	4	173099272	173193044	0	0.029448
6952	Amp	4	173196733	173324118	0	0.026275
6953	Amp	4	173326484	173326484	0	0.025227
6954	Amp	4	173335524	173613455	0	0.023971
6955	Amp	4	173617854	173654927	0	0.024661
6956	Amp	4	173655497	173698622	0	0.022414
6957	Amp	4	173701691	176252866	0	0.021724
6958	Amp	4	176258582	176375682	0	0.024866
6959	Amp	4	176375762	177080602	0	0.026443
6960	Amp	4	177082979	177722888	0	0.024612
6961	Amp	4	177723475	177869135	0	0.025796
6962	Amp	4	177869562	177974753	0	0.026632
6963	Amp	4	177975045	178081160	0	0.025054
6964	Amp	4	178081556	178196864	0	0.024016
6965	Amp	4	178197069	178358907	0	0.02318
6966	Amp	4	178359216	178934739	0	0.024287
6967	Amp	4	178939045	178961108	0	0.026858
6968	Amp	4	178962274	179126392	0	0.028648
6969	Amp	4	179127178	179128199	0	0.029269
6970	Amp	4	179132080	179198069	0	0.025801
6971	Amp	4	179198122	179305041	0	0.027975
6972	Amp	4	179309273	179359828	0	0.026868
6973	Amp	4	179360206	179577757	0	0.024297
6974	Amp	4	179579444	179652211	0	0.02526
6975	Amp	4	179654551	179855725	0	0.024297
6976	Amp	4	179855852	179976379	0	0.023005
6977	Amp	4	179978629	180299374	0	0.023774
6978	Amp	4	180301536	180318981	0	0.022892
6979	Amp	4	180324116	180338660	0	0.02182
6980	Amp	4	180339257	180604429	0	0.019648
6981	Amp	4	181157671	181238603	0	0.026442
6982	Amp	4	181242754	181441875	0	0.023643
6983	Amp	4	181442228	181459291	0	0.024392
6984	Amp	4	181462361	181872867	0	0.025997
6985	Amp	4	181873511	181951296	0	0.02446
6986	Amp	4	181951332	182037801	0	0.026207
6987	Amp	4	182038146	182251048	0	0.02446
6988	Amp	4	182251873	182308068	0	0.029253
6989	Amp	4	182310355	183404974	0	0.023657
6990	Amp	4	183405416	183421596	0	0.022947
6991	Amp	4	183423387	184823666	0	0.023681
6992	Amp	4	184824036	184824036	0	0.025072
6993	Amp	4	184827652	184827652	0	0.024338
6994	Amp	4	185378502	185378502	0	0.026282
6995	Amp	4	185378604	185446588	0	0.028519

6996 Amp	4	185446831	185524298	0	0.026023
6997 Amp	4	185524324	185663555	0	0.024079
6998 Amp	4	185663765	186099782	0	0.024601
6999 Amp	4	186100161	186236164	0	0.024079
7000 Amp	4	186236304	186657755	0	0.022474
7001 Amp	4	186660322	186756778	0	0.025936
7002 Amp	4	186757630	186872725	0	0.028267
7003 Amp	4	186975086	187135672	0	0.025936
7004 Amp	4	187138365	187234339	0	0.027358
7005 Amp	4	187234390	187309562	0	0.025936
7006 Amp	4	187313758	187363659	0	0.029425
7007 Amp	4	187373826	187477880	0	0.025936
7008 Amp	4	187479183	187842528	0	0.022474
7009 Amp	5	914118	943212	0	0.05449
7010 Amp	5	944147	1020239	0	0.051562
7011 Amp	5	1021104	1397614	0	0.050673
7012 Amp	5	1397892	1891707	0	0.049166
7013 Amp	5	1892645	2149096	0	0.050004
7014 Amp	5	2149270	2158439	0	0.049166
7015 Amp	5	2159025	2410218	0	0.048703
7016 Amp	5	2412744	2417911	0	0.049639
7017 Amp	5	2417974	2446015	0	0.05137
7018 Amp	5	2447938	2492806	0	0.050435
7019 Amp	5	2493166	2623628	0	0.049589
7020 Amp	5	2626438	2922000	0	0.049133
7021 Amp	5	2924510	3018134	0	0.048632
7022 Amp	5	3021030	3125381	0	0.046458
7023 Amp	5	3125529	3419009	0	0.047591
7024 Amp	5	3422886	3496522	0	0.050005
7025 Amp	5	3496616	3641907	0	0.047591
7026 Amp	5	3642737	3783788	0	0.05434
7027 Amp	5	3788170	3849062	0	0.053887
7028 Amp	5	3850051	3907957	0	0.049403
7029 Amp	5	3910417	4332056	0	0.042654
7030 Amp	5	4335843	4427062	0	0.04221
7031 Amp	5	4428308	4491160	0	0.044114
7032 Amp	5	4491479	4496033	0	0.04221
7033 Amp	5	4508794	4801006	0	0.030483
7034 Amp	5	4802646	4861167	0	0.02927
7035 Amp	5	4861319	5047624	0	0.027628
7036 Amp	5	5048822	5076922	0	0.02561
7037 Amp	5	5077020	5175095	0	0.026858
7038 Amp	5	5181326	5181326	0	0.031543
7039 Amp	5	5181716	5184139	0	0.028328
7040 Amp	5	5185254	5304917	0	0.02889
7041 Amp	5	5307800	5344075	0	0.024205
7042 Amp	5	5348285	5469553	0	0.023643
7043 Amp	5	5471918	5639017	0	0.022395
7044 Amp	5	5641233	5675265	0	0.024814
7045 Amp	5	5675788	5785913	0	0.022395
7046 Amp	5	5785923	5838771	0	0.019795
7047 Amp	5	5839609	5880300	0	0.017019
7048 Amp	5	5880434	5991369	0	0.017944

7049	Amp	5	5997496	6168426	0	0.014046
7050	Amp	5	6172855	6304093	0	0.013505
7051	Amp	5	6309822	6745762	0	0.015392
7052	Amp	5	6745893	6754554	0	0.016824
7053	Amp	5	6758858	6790157	0	0.017978
7054	Amp	5	6796485	6850483	0	0.015392
7055	Amp	5	6850502	6874651	0	0.014466
7056	Amp	5	6881302	7021848	0	0.013514
7057	Amp	5	7023183	7168823	0	0.01458
7058	Amp	5	7168841	7681094	0	0.016239
7059	Amp	5	7682796	7853028	0	0.016739
7060	Amp	5	7853696	7923503	0	0.017708
7061	Amp	5	7923570	8025904	0	0.016552
7062	Amp	5	8028230	8289086	0	0.018684
7063	Amp	5	8291007	8599016	0	0.017275
7064	Amp	5	8599113	8677057	0	0.018558
7065	Amp	5	8677492	9215219	0	0.019974
7066	Amp	5	9218832	9606097	0	0.018907
7067	Amp	5	9606551	9870475	0	0.019427
7068	Amp	5	9870556	9870556	0	0.018521
7069	Amp	5	9875923	9889366	0	0.016634
7070	Amp	5	9889860	10309651	0	0.016114
7071	Amp	5	10309686	10358741	0	0.016729
7072	Amp	5	10362228	10466630	0	0.020412
7073	Amp	5	10466975	10550861	0	0.026653
7074	Amp	5	10552475	10588894	0	0.026143
7075	Amp	5	10589390	11215020	0	0.026601
7076	Amp	5	11218376	11256003	0	0.022919
7077	Amp	5	11256359	11977237	0	0.021087
7078	Amp	5	11979593	11993489	0	0.022885
7079	Amp	5	11996046	12068011	0	0.02044
7080	Amp	5	12068765	12118077	0	0.019471
7081	Amp	5	12118198	12181165	0	0.018501
7082	Amp	5	12181436	12198861	0	0.015675
7083	Amp	5	12201183	12203499	0	0.020323
7084	Amp	5	12203514	12315625	0	0.015675
7085	Amp	5	12318412	12364529	0	0.014392
7086	Amp	5	12369795	12440112	0	0.012594
7087	Amp	5	12448417	12774845	0	0.014887
7088	Amp	5	12775593	13169467	0	0.015896
7089	Amp	5	13173552	13201360	0	0.015173
7090	Amp	5	13202941	13308065	0	0.021111
7091	Amp	5	13309505	13658969	0	0.020653
7092	Amp	5	13659197	13668610	0	0.021377
7093	Amp	5	13669297	13757107	0	0.021831
7094	Amp	5	13758825	13769678	0	0.037768
7095	Amp	5	13769865	13798710	0	0.039492
7096	Amp	5	13799272	14419443	0	0.042803
7097	Amp	5	14422204	14474569	0	0.04051
7098	Amp	5	14478492	14491860	0	0.03126
7099	Amp	5	14491967	14558883	0	0.029825
7100	Amp	5	14561153	14904527	0	0.028452
7101	Amp	5	14904865	14911588	0	0.028924

7102	Amp	5	14911790	14922321	0	0.02847
7103	Amp	5	14925948	15020088	0	0.027747
7104	Amp	5	15020258	15074024	0	0.027276
7105	Amp	5	15075345	15339178	0	0.012062
7106	Amp	5	15345729	15361869	0	0.010103
7107	Amp	5	16048599	16071608	0	0.008107
7108	Amp	5	16075454	16144583	0	0.007098
7109	Amp	5	16145209	16168985	0	0.008898
7110	Amp	5	16171856	16458862	0	0.010433
7111	Amp	5	16460048	16558293	0	0.008634
7112	Amp	5	16559472	16719592	0	0.010102
7113	Amp	5	16720366	16743843	0	0.008566
7114	Amp	5	16749214	18031231	0	0.007098
7115	Amp	5	18034594	18105574	0	0.008461
7116	Amp	5	18109355	18349488	0	0.006737
7117	Amp	5	18350002	18416402	0	0.008731
7118	Amp	5	18416428	18416428	0	0.006013
7119	Amp	5	18431216	18715021	0	0.015513
7120	Amp	5	18716639	18756970	0	0.014769
7121	Amp	5	18757837	20034573	0	0.016431
7122	Amp	5	20036348	20130020	0	0.015816
7123	Amp	5	20136661	20176778	0	0.019289
7124	Amp	5	20182113	20198128	0	0.02234
7125	Amp	5	20205572	20568729	0	0.019289
7126	Amp	5	20569820	20612561	0	0.016063
7127	Amp	5	20613479	20883776	0	0.014432
7128	Amp	5	20886618	20975120	0	0.016235
7129	Amp	5	20975217	21080286	0	0.017625
7130	Amp	5	21083896	21093485	0	0.014152
7131	Amp	5	21093804	21312104	0	0.012349
7132	Amp	5	21312119	21321035	0	0.015689
7133	Amp	5	21321123	21321123	0	0.012349
7134	Amp	5	21323000	22208699	0	0.010687
7135	Amp	5	22210630	22316917	0	0.01244
7136	Amp	5	22318181	22478953	0	0.013551
7137	Amp	5	22482363	22498040	0	0.01244
7138	Amp	5	22499395	22697848	0	0.013623
7139	Amp	5	22699098	22910271	0	0.01187
7140	Amp	5	22910949	22964681	0	0.01309
7141	Amp	5	22966760	22999941	0	0.01187
7142	Amp	5	23000750	23137851	0	0.012564
7143	Amp	5	23138010	23270410	0	0.01385
7144	Amp	5	23272709	23279385	0	0.01246
7145	Amp	5	23288013	23291338	0	0.011766
7146	Amp	5	23291626	23343769	0	0.012737
7147	Amp	5	23344083	23349187	0	0.012208
7148	Amp	5	23350461	24082741	0	0.012979
7149	Amp	5	24083322	24438358	0	0.014922
7150	Amp	5	24451776	24507117	0	0.015553
7151	Amp	5	24507299	24535260	0	0.01361
7152	Amp	5	24538456	24579985	0	0.019337
7153	Amp	5	24580647	24744075	0	0.01361
7154	Amp	5	24751440	25077135	0	0.012979

7155	Amp	5	25077162	25385351	0	0.015529
7156	Amp	5	25386413	25649108	0	0.016388
7157	Amp	5	25649357	25657662	0	0.018397
7158	Amp	5	25657981	25701887	0	0.017034
7159	Amp	5	25702151	25720193	0	0.018578
7160	Amp	5	25720326	25758604	0	0.019225
7161	Amp	5	25762637	26017369	0	0.019727
7162	Amp	5	26017555	26020006	0	0.020434
7163	Amp	5	26020317	26261061	0	0.021669
7164	Amp	5	26266154	26296040	0	0.022533
7165	Amp	5	26298165	26602771	0	0.021669
7166	Amp	5	26602788	27052792	0	0.020434
7167	Amp	5	27058229	27291051	0	0.022876
7168	Amp	5	27291390	27410058	0	0.020434
7169	Amp	5	27410447	27462171	0	0.02292
7170	Amp	5	27463129	27463569	0	0.022273
7171	Amp	5	27474131	27645651	0	0.02359
7172	Amp	5	27648270	27683001	0	0.024869
7173	Amp	5	27690969	27708910	0	0.0286
7174	Amp	5	27716402	27773081	0	0.024869
7175	Amp	5	27773632	27843495	0	0.02321
7176	Amp	5	27844705	28097263	0	0.021931
7177	Amp	5	28099505	28244265	0	0.023419
7178	Amp	5	28247448	28426542	0	0.024859
7179	Amp	5	28433408	28989861	0	0.023542
7180	Amp	5	28990810	29054426	0	0.024358
7181	Amp	5	29054513	29068245	0	0.025099
7182	Amp	5	29069670	29328383	0	0.027933
7183	Amp	5	29330107	29380199	0	0.019109
7184	Amp	5	29384376	29459988	0	0.01806
7185	Amp	5	29460921	29505761	0	0.016773
7186	Amp	5	29506259	29508122	0	0.015229
7187	Amp	5	29521678	29655133	0	0.018815
7188	Amp	5	29655191	29852732	0	0.015229
7189	Amp	5	29852745	29861043	0	0.014489
7190	Amp	5	29866314	30011089	0	0.016503
7191	Amp	5	30018645	30028568	0	0.019012
7192	Amp	5	30030904	30055655	0	0.018509
7193	Amp	5	30057388	30063474	0	0.017021
7194	Amp	5	30066047	30114921	0	0.020102
7195	Amp	5	30121299	30218005	0	0.021477
7196	Amp	5	30219016	30336950	0	0.018968
7197	Amp	5	30341964	30663078	0	0.021182
7198	Amp	5	30667103	31000178	0	0.022859
7199	Amp	5	31001549	31566495	0	0.021483
7200	Amp	5	31568754	31574977	0	0.022947
7201	Amp	5	31575415	31740869	0	0.024456
7202	Amp	5	31743772	31827318	0	0.022947
7203	Amp	5	31833709	31937007	0	0.024601
7204	Amp	5	31940329	32218214	0	0.027809
7205	Amp	5	32221100	32246941	0	0.028262
7206	Amp	5	32248078	32428605	0	0.026799
7207	Amp	5	32431922	32431922	0	0.025122

7208	Amp	5	32433576	32450381	0	0.026645
7209	Amp	5	32450416	32593680	0	0.027461
7210	Amp	5	32594019	32691619	0	0.029161
7211	Amp	5	32692132	32692132	0	0.030635
7212	Amp	5	32699387	32706099	0	0.033499
7213	Amp	5	32706392	32814922	0	0.03091
7214	Amp	5	32817409	32892023	0	0.029891
7215	Amp	5	32892939	32935267	0	0.029438
7216	Amp	5	32935627	33089872	0	0.030407
7217	Amp	5	33093023	33406004	0	0.027592
7218	Amp	5	33416902	33482444	0	0.030614
7219	Amp	5	33486835	33510013	0	0.029756
7220	Amp	5	33520494	33535786	0	0.032173
7221	Amp	5	33537581	33542807	0	0.040101
7222	Amp	5	33546066	33596771	0	0.032173
7223	Amp	5	33597351	33606926	0	0.035574
7224	Amp	5	33610424	33767747	0	0.032675
7225	Amp	5	33768096	33922975	0	0.029756
7226	Amp	5	33923167	33944112	0	0.030336
7227	Amp	5	34754143	34760358	0	0.033073
7228	Amp	5	34760487	34769506	0	0.034342
7229	Amp	5	34770064	35289426	0	0.032642
7230	Amp	5	35292602	35491083	0	0.031154
7231	Amp	5	35503785	35560969	0	0.035702
7232	Amp	5	35562049	35565259	0	0.031154
7233	Amp	5	35567134	35640911	0	0.034397
7234	Amp	5	35641480	35880285	0	0.032611
7235	Amp	5	35883074	35971503	0	0.029369
7236	Amp	5	35979113	36019337	0	0.030861
7237	Amp	5	36023168	36037015	0	0.033742
7238	Amp	5	36044177	36093285	0	0.0379
7239	Amp	5	36101335	36123241	0	0.03732
7240	Amp	5	36134291	36143290	0	0.033162
7241	Amp	5	36149462	36211824	0	0.034412
7242	Amp	5	36212115	36271178	0	0.035093
7243	Amp	5	36271526	36404072	0	0.036011
7244	Amp	5	36405360	36433215	0	0.036637
7245	Amp	5	36434024	36476023	0	0.036011
7246	Amp	5	36477646	36808864	0	0.035329
7247	Amp	5	36809644	37025769	0	0.038237
7248	Amp	5	37027071	37188260	0	0.039767
7249	Amp	5	37196333	37216655	0	0.038498
7250	Amp	5	37218356	37255481	0	0.037248
7251	Amp	5	37259058	37281370	0	0.035719
7252	Amp	5	37282562	37285474	0	0.034469
7253	Amp	5	37286515	37300876	0	0.041159
7254	Amp	5	37304725	37336543	0	0.031261
7255	Amp	5	37339348	37416936	0	0.042471
7256	Amp	5	37423016	37439445	0	0.033102
7257	Amp	5	37651330	37832418	0	0.038407
7258	Amp	5	37832696	37853246	0	0.034494
7259	Amp	5	37854636	38005002	0	0.037696
7260	Amp	5	38006453	38272572	0	0.036881

7261	Amp	5	38272714	38280698	0	0.037356
7262	Amp	5	38282725	38282725	0	0.028134
7263	Amp	5	38286946	38305842	0	0.028595
7264	Amp	5	38306802	38308353	0	0.026586
7265	Amp	5	38308758	38353586	0	0.035466
7266	Amp	5	38360136	38366428	0	0.027543
7267	Amp	5	38367793	38574958	0	0.032934
7268	Amp	5	38577652	38649136	0	0.034334
7269	Amp	5	38657456	38875334	0	0.035276
7270	Amp	5	38875629	38921686	0	0.031614
7271	Amp	5	38922121	39152178	0	0.030843
7272	Amp	5	39155826	39210033	0	0.033924
7273	Amp	5	39212002	39228918	0	0.027614
7274	Amp	5	39230702	39232395	0	0.052464
7275	Amp	5	39233224	39242418	0	0.05529
7276	Amp	5	39248238	39263612	0	0.03044
7277	Amp	5	39267515	39286852	0	0.031204
7278	Amp	5	39287921	39314645	0	0.033858
7279	Amp	5	39315347	39316320	0	0.040069
7280	Amp	5	39317238	39398840	0	0.038669
7281	Amp	5	39404165	39405544	0	0.041171
7282	Amp	5	39405634	39492163	0	0.042045
7283	Amp	5	39495673	39526149	0	0.033008
7284	Amp	5	39530778	39620353	0	0.038825
7285	Amp	5	39622738	39648462	0	0.042293
7286	Amp	5	39651200	39667441	0	0.039792
7287	Amp	5	39667974	39682430	0	0.030506
7288	Amp	5	39691505	39692063	0	0.031175
7289	Amp	5	39692230	39733749	0	0.033232
7290	Amp	5	39736821	39776157	0	0.032757
7291	Amp	5	39776433	39869684	0	0.029875
7292	Amp	5	39875933	39979070	0	0.027818
7293	Amp	5	39979358	40014189	0	0.031479
7294	Amp	5	40014257	40252209	0	0.027818
7295	Amp	5	40253800	40375443	0	0.024698
7296	Amp	5	40375805	40540283	0	0.023261
7297	Amp	5	40547717	40624459	0	0.026108
7298	Amp	5	40625663	40740847	0	0.024594
7299	Amp	5	40745400	40749602	0	0.023261
7300	Amp	5	40750600	40858479	0	0.024195
7301	Amp	5	40860911	40883889	0	0.027519
7302	Amp	5	40888617	40951937	0	0.024195
7303	Amp	5	40952988	41012044	0	0.02483
7304	Amp	5	41012755	41034499	0	0.031247
7305	Amp	5	41035120	41061182	0	0.02483
7306	Amp	5	41061769	41218838	0	0.028209
7307	Amp	5	41219677	41349751	0	0.02483
7308	Amp	5	41351679	41470068	0	0.024195
7309	Amp	5	41490577	41625464	0	0.025532
7310	Amp	5	41625562	41673777	0	0.02404
7311	Amp	5	41674555	41702239	0	0.026458
7312	Amp	5	41703559	42137045	0	0.02404
7313	Amp	5	42137365	42152899	0	0.023106

7314	Amp	5	42153730	42246402	0	0.026216
7315	Amp	5	42249132	42254682	0	0.023106
7316	Amp	5	42263046	42276367	0	0.026337
7317	Amp	5	42277189	42306279	0	0.028837
7318	Amp	5	42309842	42309842	0	0.034491
7319	Amp	5	42310219	42359930	0	0.035396
7320	Amp	5	42365538	42365538	0	0.039746
7321	Amp	5	42369121	42395928	0	0.034092
7322	Amp	5	42396980	42412167	0	0.04561
7323	Amp	5	42416403	42470483	0	0.034092
7324	Amp	5	42470628	42564646	0	0.04172
7325	Amp	5	42569483	42619214	0	0.040815
7326	Amp	5	42621654	42651122	0	0.033748
7327	Amp	5	42654901	42690532	0	0.044247
7328	Amp	5	42692437	42731473	0	0.034284
7329	Amp	5	42741494	42767344	0	0.040496
7330	Amp	5	42774968	42800622	0	0.042577
7331	Amp	5	42804013	42804013	0	0.041659
7332	Amp	5	42805642	42898151	0	0.039819
7333	Amp	5	42900811	42944576	0	0.034911
7334	Amp	5	42946180	42977816	0	0.040996
7335	Amp	5	42982709	42992335	0	0.038915
7336	Amp	5	42994255	43232535	0	0.03283
7337	Amp	5	43232782	43429102	0	0.03353
7338	Amp	5	43429862	43517047	0	0.03047
7339	Amp	5	43523135	43609170	0	0.032534
7340	Amp	5	43613125	43678250	0	0.037354
7341	Amp	5	43682051	43707298	0	0.032534
7342	Amp	5	43710537	43728995	0	0.038598
7343	Amp	5	43731590	43739620	0	0.037897
7344	Amp	5	43740974	43820622	0	0.034095
7345	Amp	5	43821751	43910402	0	0.042491
7346	Amp	5	43910646	43949751	0	0.043634
7347	Amp	5	43958657	43975773	0	0.049768
7348	Amp	5	43976195	44037396	0	0.046407
7349	Amp	5	44047613	44072744	0	0.041532
7350	Amp	5	44072780	44132606	0	0.037316
7351	Amp	5	44147500	44156168	0	0.046135
7352	Amp	5	44157109	44185413	0	0.035683
7353	Amp	5	44185528	44196322	0	0.037849
7354	Amp	5	44198823	44210869	0	0.048228
7355	Amp	5	44211365	44248088	0	0.046061
7356	Amp	5	44248424	44272494	0	0.036799
7357	Amp	5	44272930	44301567	0	0.037972
7358	Amp	5	44305413	44305647	0	0.036527
7359	Amp	5	44308796	44346222	0	0.035411
7360	Amp	5	44365691	44365691	0	0.034468
7361	Amp	5	44369903	44372958	0	0.036087
7362	Amp	5	44375312	44375312	0	0.033874
7363	Amp	5	44376319	44377001	0	0.035906
7364	Amp	5	44381596	44381959	0	0.037155
7365	Amp	5	44382459	44395458	0	0.040015
7366	Amp	5	44395913	44414706	0	0.043391

7367	Amp	5	44416631	44417868	0	0.04431
7368	Amp	5	44422531	44430722	0	0.046374
7369	Amp	5	44434884	44444998	0	0.058353
7370	Amp	5	44454328	44462022	0	0.060066
7371	Amp	5	44462546	44462546	0	0.063743
7372	Amp	5	44467817	44480811	0.069848	0.066977
7373	Amp	5	44480915	44530020	0.118001	0.068965
7374	Amp	5	44539851	44612045	0.153838	0.070526
7375	Amp	5	44620113	44638017	0.1237	0.069266
7376	Amp	5	44641278	44676417	0.050473	0.066061
7377	Amp	5	44677071	44688124	0.19964	0.072234
7378	Amp	5	44695188	44728595	0.227787	0.073258
7379	Amp	5	44730361	44747593	0.204831	0.072339
7380	Amp	5	44759484	44759484	0.034113	0.065484
7381	Amp	5	44761521	44768354	0	0.06342
7382	Amp	5	44770850	44770850	0	0.062181
7383	Amp	5	44773456	44776454	0	0.053092
7384	Amp	5	44797975	44838900	0	0.035442
7385	Amp	5	44844323	44860326	0	0.034193
7386	Amp	5	44860979	45009304	0	0.030817
7387	Amp	5	45017755	45353165	0	0.031311
7388	Amp	5	45353842	45358981	0	0.031933
7389	Amp	5	45360996	45378354	0	0.030558
7390	Amp	5	45383300	45383300	0	0.023248
7391	Amp	5	50828231	50828231	0	0.023992
7392	Amp	5	50829022	50829022	0	0.022969
7393	Amp	5	50837510	51141961	0	0.017382
7394	Amp	5	51143661	51218693	0	0.01926
7395	Amp	5	51219502	51288949	0	0.018052
7396	Amp	5	51288970	51578422	0	0.017431
7397	Amp	5	51580699	51687670	0	0.019445
7398	Amp	5	51688181	51782718	0	0.017567
7399	Amp	5	51785460	51994647	0	0.018142
7400	Amp	5	51997292	52081425	0	0.017567
7401	Amp	5	52085094	52142210	0	0.01942
7402	Amp	5	52143852	52149866	0	0.017767
7403	Amp	5	52151640	52178412	0	0.019502
7404	Amp	5	52178901	52203212	0	0.017649
7405	Amp	5	52203672	52225813	0	0.015635
7406	Amp	5	52229863	52345178	0	0.017479
7407	Amp	5	52345872	52654158	0	0.015635
7408	Amp	5	52667742	52724462	0	0.016826
7409	Amp	5	52726879	52741327	0	0.015091
7410	Amp	5	52741687	52870578	0	0.016186
7411	Amp	5	52875173	52908460	0	0.020252
7412	Amp	5	52917554	53333813	0	0.016186
7413	Amp	5	53334604	53345156	0	0.019714
7414	Amp	5	53345766	53353540	0	0.016186
7415	Amp	5	53355648	53517183	0	0.022055
7416	Amp	5	53519702	53538184	0	0.016186
7417	Amp	5	53552552	53693590	0	0.015091
7418	Amp	5	53699877	53848329	0	0.016886
7419	Amp	5	53857827	54000105	0	0.015091

7420	Amp	5	54000313	54220885	0	0.016917
7421	Amp	5	54221215	54237815	0	0.030845
7422	Amp	5	54240463	54540554	0	0.016917
7423	Amp	5	54542279	54610983	0	0.017353
7424	Amp	5	54614141	54835487	0	0.021023
7425	Amp	5	54840527	54891253	0	0.017353
7426	Amp	5	54893971	55014913	0	0.015527
7427	Amp	5	55020275	55505433	0	0.014336
7428	Amp	5	55506542	55530580	0	0.015016
7429	Amp	5	55531751	55578381	0	0.019452
7430	Amp	5	55584215	56012021	0	0.015016
7431	Amp	5	56023543	56075208	0	0.021382
7432	Amp	5	56085188	56156678	0	0.023446
7433	Amp	5	56159818	56192212	0	0.01708
7434	Amp	5	56192589	56268923	0	0.0164
7435	Amp	5	56269269	56276695	0	0.019875
7436	Amp	5	56278063	56379258	0	0.006739
7437	Amp	5	56382074	56556574	0	0.016613
7438	Amp	5	56558051	56820663	0	0.006739
7439	Amp	5	56822780	56840808	0	0.008512
7440	Amp	5	56841723	56867403	0	0.008075
7441	Amp	5	56869946	56875049	0	0.0046
7442	Amp	5	56883385	57045669	0	0.002828
7443	Amp	5	57088959	57138807	0	0.013286
7444	Amp	5	57147155	57172972	0	0.000764
7445	Amp	5	57183082	57480984	0	0.003763
7446	Amp	5	57488201	57521696	0	0.001552
7447	Amp	5	57521910	57523692	0	0.000789
7448	Amp	5	57526504	57703197	0	0
7449	Amp	5	57704683	57738949	0	0.002893
7450	Amp	5	57742156	57883950	0	0.005581
7451	Amp	5	57884185	57884342	0	0.017064
7452	Amp	5	57885424	57953758	0	0.019156
7453	Amp	5	57955898	57972823	0	0.004985
7454	Amp	5	57980984	58398893	0	0.002893
7455	Amp	5	58401075	58402467	0	0.00639
7456	Amp	5	58404652	58436920	0	0.008257
7457	Amp	5	58437016	58455616	0	0.011756
7458	Amp	5	58458213	58501019	0	0.008257
7459	Amp	5	58502987	58502987	0	0.00476
7460	Amp	5	58503008	58572274	0	0.002893
7461	Amp	5	58577984	58748556	0	0
7462	Amp	5	58756263	58815640	0	0.001938
7463	Amp	5	58819279	59008903	0	0.003594
7464	Amp	5	59016641	59032734	0	0.001938
7465	Amp	5	59034442	59811995	0	0.007945
7466	Amp	5	59812362	59873509	0	0.009114
7467	Amp	5	59877085	59981945	0	0.011721
7468	Amp	5	59982912	60805953	0	0.013546
7469	Amp	5	60812443	61810830	0	0.014747
7470	Amp	5	61810846	61864173	0	0.016244
7471	Amp	5	61866272	62066525	0	0.017645
7472	Amp	5	62069015	62384504	0	0.015038

7473 Amp	5	62384621	62387298	0	0.013869
7474 Amp	5	62388133	62518686	0	0.011931
7475 Amp	5	62519385	62542812	0	0.009685
7476 Amp	5	62547076	62609385	0	0.012127
7477 Amp	5	62612079	62655942	0	0.010302
7478 Amp	5	62661567	62927613	0	0.008901
7479 Amp	5	62929379	64066005	0	0.006951
7480 Amp	5	64066485	64075680	0	0.010138
7481 Amp	5	64086158	64116861	0	0.009195
7482 Amp	5	64120961	64158355	0	0.012349
7483 Amp	5	64169053	64171739	0	0.013052
7484 Amp	5	64173593	64279193	0	0.016234
7485 Amp	5	64282955	64509676	0	0.014104
7486 Amp	5	64511251	64559386	0	0.025987
7487 Amp	5	64560283	64572489	0	0.014104
7488 Amp	5	64573570	64653905	0	0.011925
7489 Amp	5	64664360	64729555	0	0.010165
7490 Amp	5	64731208	64797954	0	0.009162
7491 Amp	5	64798206	64823198	0	0.007873
7492 Amp	5	64828285	64909549	0	0.00856
7493 Amp	5	64957384	65181590	0	0.022584
7494 Amp	5	65182945	65249126	0	0.024536
7495 Amp	5	65249806	65278652	0	0.032168
7496 Amp	5	65278858	65611625	0	0.026012
7497 Amp	5	65612058	65727736	0	0.02409
7498 Amp	5	65729883	65797826	0	0.023403
7499 Amp	5	65798018	65811874	0	0.026184
7500 Amp	5	65812388	65821355	0	0.027491
7501 Amp	5	65823404	65871970	0	0.0266
7502 Amp	5	65875387	65932423	0	0.021922
7503 Amp	5	65941384	66472141	0	0.019141
7504 Amp	5	66472179	66578837	0	0.019652
7505 Amp	5	66584819	66706960	0	0.006518
7506 Amp	5	66709192	66942537	0	0.00696
7507 Amp	5	66943394	67025047	0	0.00942
7508 Amp	5	67026224	67107275	0	0.011495
7509 Amp	5	67113627	67141294	0	0.013781
7510 Amp	5	67146557	67167226	0	0.01504
7511 Amp	5	67170451	67235117	0	0.017194
7512 Amp	5	67236142	67242188	0	0.01504
7513 Amp	5	67242572	67262083	0	0.014598
7514 Amp	5	67262430	67290190	0	0.011265
7515 Amp	5	67290512	67468149	0	0.008978
7516 Amp	5	67470392	68662275	0	0.006518
7517 Amp	5	68664098	68733644	0	0.007076
7518 Amp	5	68734451	68824300	0	0.009398
7519 Amp	5	68824574	69054300	0	0.007076
7520 Amp	5	69064543	69089254	0	0.019825
7521 Amp	5	69091975	69213744	0	0.022855
7522 Amp	5	69213943	69264043	0	0.022298
7523 Amp	5	69267602	69417660	0	0.02385
7524 Amp	5	69420227	69437871	0	0.015036
7525 Amp	5	69438029	69444194	0	0.012006

7526	Amp	5	69444660	69448492	0	0.014229
7527	Amp	5	71364924	71367629	0	0.023551
7528	Amp	5	71370707	71677216	0	0.021999
7529	Amp	5	71677464	71680676	0	0.031237
7530	Amp	5	71681233	72550894	0	0.021999
7531	Amp	5	72551183	72785740	0	0.022743
7532	Amp	5	72785943	72877320	0	0.021739
7533	Amp	5	72884656	72999777	0	0.023349
7534	Amp	5	73001621	73028523	0	0.025359
7535	Amp	5	73031710	73054157	0	0.023349
7536	Amp	5	73054725	73335750	0	0.021739
7537	Amp	5	73335948	73725420	0	0.022593
7538	Amp	5	73726294	73958715	0	0.021739
7539	Amp	5	73958930	74207372	0	0.020963
7540	Amp	5	74209753	74852403	0	0.02001
7541	Amp	5	74852426	75137338	0	0.019265
7542	Amp	5	75142914	77000585	0	0.007288
7543	Amp	5	77004971	77048926	0	0.01953
7544	Amp	5	77051638	77111846	0	0.018249
7545	Amp	5	77116086	77262476	0	0.006008
7546	Amp	5	77265419	77339253	0	0.008823
7547	Amp	5	77342388	77530695	0	0.010331
7548	Amp	5	77530969	77771990	0	0.009485
7549	Amp	5	77774938	77777982	0	0.007977
7550	Amp	5	77780810	77821916	0	0.008825
7551	Amp	5	77821935	77893527	0	0.007977
7552	Amp	5	77896273	80560057	0	0.006008
7553	Amp	5	80561790	80583963	0	0.009196
7554	Amp	5	80584622	80683746	0	0.006008
7555	Amp	5	80688895	80933279	0	0.010568
7556	Amp	5	80936878	80967511	0	0.013338
7557	Amp	5	80971951	81132453	0	0.010568
7558	Amp	5	81140765	81980422	0	0.016349
7559	Amp	5	81991157	82291747	0	0.010568
7560	Amp	5	82297398	82307763	0	0.006581
7561	Amp	5	82309534	82347694	0	0.010147
7562	Amp	5	82351761	82745448	0	0.007588
7563	Amp	5	82748286	82789103	0	0.009785
7564	Amp	5	82791892	82825096	0	0.012154
7565	Amp	5	82827539	83185968	0	0.009785
7566	Amp	5	83196869	83611656	0	0.007588
7567	Amp	5	83611758	83930714	0	0.006008
7568	Amp	5	83932171	84215665	0	0.011993
7569	Amp	5	84216311	84402926	0	0.006008
7570	Amp	5	84408792	84430780	0	0.009288
7571	Amp	5	84432591	85126646	0	0.006008
7572	Amp	5	85132363	86377603	0	0.006529
7573	Amp	5	86377688	86451899	0	0.010627
7574	Amp	5	86455493	87003253	0	0.006529
7575	Amp	5	87003556	87169922	0	0.008477
7576	Amp	5	87171766	87176730	0	0.006529
7577	Amp	5	87181888	87225218	0	0.010111
7578	Amp	5	87228971	87405286	0	0.006529

7579	Amp	5	87416884	87630118	0	0.009163
7580	Amp	5	87630439	88502238	0	0.008641
7581	Amp	5	88504904	88569983	0	0.006008
7582	Amp	5	88572511	88642739	0	0.002943
7583	Amp	5	88642967	89909943	0	0.005482
7584	Amp	5	89970141	90275434	0	0.008252
7585	Amp	5	90275914	90518300	0	0.010599
7586	Amp	5	90518369	90704137	0	0.008252
7587	Amp	5	90706779	90785975	0	0.005713
7588	Amp	5	90786579	91223704	0	0.002943
7589	Amp	5	91223812	91314220	0	0.003627
7590	Amp	5	91328710	91416020	0	0.005672
7591	Amp	5	91420756	91556198	0	0.006331
7592	Amp	5	91556751	92182380	0	0.004285
7593	Amp	5	92186136	93294584	0	0.003627
7594	Amp	5	93295310	93529566	0	0.006388
7595	Amp	5	93530652	93729977	0	0.008887
7596	Amp	5	93737148	93947303	0	0.006388
7597	Amp	5	93950896	94431900	0	0.012075
7598	Amp	5	94433700	95118749	0	0.006388
7599	Amp	5	95119027	95147427	0	0.005704
7600	Amp	5	95148095	95747762	0	0.007085
7601	Amp	5	95748427	95763121	0	0.005704
7602	Amp	5	95763264	95836627	0	0.00638
7603	Amp	5	95837532	95849768	0	0.007536
7604	Amp	5	95856149	96251465	0	0.009669
7605	Amp	5	96255613	96308255	0	0.008513
7606	Amp	5	96309979	96825407	0	0.00638
7607	Amp	5	96829297	96830323	0	0.007899
7608	Amp	5	96830338	96863585	0	0.010416
7609	Amp	5	96866282	97004912	0	0.007899
7610	Amp	5	97008980	97046489	0	0.00638
7611	Amp	5	97046711	97384122	0	0.003619
7612	Amp	5	97388021	97465490	0	0.005794
7613	Amp	5	97476805	97534737	0	0.007814
7614	Amp	5	97537987	97611826	0	0.01125
7615	Amp	5	97612383	97624424	0	0.011943
7616	Amp	5	97627351	97707887	0	0.011267
7617	Amp	5	97898076	98279403	0	0.007832
7618	Amp	5	98279545	98296818	0	0.011283
7619	Amp	5	98297365	98485748	0	0.010589
7620	Amp	5	98488918	98595136	0	0.011064
7621	Amp	5	98605165	98974244	0	0.007138
7622	Amp	5	98974334	99033208	0	0.009102
7623	Amp	5	99033350	99141104	0	0.007138
7624	Amp	5	99143476	99152826	0	0.009749
7625	Amp	5	99167137	99167137	0	0.007729
7626	Amp	5	100226861	101084638	0	0.005554
7627	Amp	5	101085081	101177623	0	0.00953
7628	Amp	5	101185900	101300279	0	0.007582
7629	Amp	5	101300392	101664170	0	0.00827
7630	Amp	5	101666267	101964286	0	0.007582
7631	Amp	5	101964917	102255873	0	0.004292

7632	Amp	5	102257038	102357625	0	0.005402
7633	Amp	5	102361313	102758794	0	0.004292
7634	Amp	5	102759848	102817710	0	0.005233
7635	Amp	5	102821244	103329295	0	0.004292
7636	Amp	5	103332591	103372974	0	0.007226
7637	Amp	5	103374111	103426338	0	0.004292
7638	Amp	5	103428347	103498284	0	0.006586
7639	Amp	5	103498638	103948266	0	0.004292
7640	Amp	5	103956968	103981745	0	0.007752
7641	Amp	5	103985120	104161065	0	0.004292
7642	Amp	5	104161411	104557081	0	0.004928
7643	Amp	5	104557759	104633867	0	0.003579
7644	Amp	5	104635290	104684009	0	0.006123
7645	Amp	5	104686162	104832320	0	0.003579
7646	Amp	5	104833134	104885864	0	0.004015
7647	Amp	5	104889676	104967050	0	0.003579
7648	Amp	5	104971560	104991377	0	0.004105
7649	Amp	5	104991543	105886279	0	0.003468
7650	Amp	5	105886667	106250437	0	0.002943
7651	Amp	5	106252526	106324977	0	0.004604
7652	Amp	5	106326053	106661980	0	0.002943
7653	Amp	5	106662274	107177812	0	0.003674
7654	Amp	5	107177931	107219927	0	0.004352
7655	Amp	5	107220545	107291902	0	0.005837
7656	Amp	5	107292643	107435831	0	0.004352
7657	Amp	5	107440417	107702633	0	0.003674
7658	Amp	5	107702650	107801780	0	0.004432
7659	Amp	5	107803280	108397914	0	0.003674
7660	Amp	5	108401952	108427420	0	0.008869
7661	Amp	5	108431910	110152357	0	0.003674
7662	Amp	5	110156793	110263914	0	0.006986
7663	Amp	5	110266968	110603028	0	0.003674
7664	Amp	5	110609158	111149876	0	0.004241
7665	Amp	5	111153679	111763459	0	0.003674
7666	Amp	5	111765330	111828873	0	0.005654
7667	Amp	5	111830758	112235945	0	0.003674
7668	Amp	5	112236269	112429172	0	0.005596
7669	Amp	5	112431648	113107681	0	0.003674
7670	Amp	5	113114667	113381983	0	0.004139
7671	Amp	5	113383607	115388603	0	0.003674
7672	Amp	5	115388618	116177785	0	0.005111
7673	Amp	5	116179137	116186308	0	0.005799
7674	Amp	5	116187172	116198730	0	0.004362
7675	Amp	5	116198829	116240535	0	0.003631
7676	Amp	5	116243080	116649483	0	0.004118
7677	Amp	5	116650017	116754206	0	0.006969
7678	Amp	5	116755540	116806393	0	0.009813
7679	Amp	5	116806924	116865158	0	0.009125
7680	Amp	5	116866886	117345447	0	0.006274
7681	Amp	5	117345544	117452746	0	0.006746
7682	Amp	5	117453196	117485496	0	0.00788
7683	Amp	5	117485867	118119334	0	0.006746
7684	Amp	5	118119557	118161463	0	0.007954

7685	Amp	5	118161858	118196062	0	0.010127
7686	Amp	5	118196216	118267690	0	0.007954
7687	Amp	5	118267882	118273741	0	0.009416
7688	Amp	5	118276542	118276542	0	0.008208
7689	Amp	5	118276713	118328568	0	0.007721
7690	Amp	5	118331191	118381825	0	0.007249
7691	Amp	5	118382866	118427780	0	0.007975
7692	Amp	5	118427927	118958011	0	0.010062
7693	Amp	5	118961046	119036213	0	0.018541
7694	Amp	5	119040152	119160449	0	0.010062
7695	Amp	5	119164771	119252308	0	0.016677
7696	Amp	5	119258567	119991068	0	0.010062
7697	Amp	5	119992475	120289123	0	0.005798
7698	Amp	5	120289249	120500342	0	0.004336
7699	Amp	5	120502589	120565105	0	0.005787
7700	Amp	5	120565549	120896472	0	0.007206
7701	Amp	5	120902021	120957397	0	0.008882
7702	Amp	5	120957890	121457048	0	0.008215
7703	Amp	5	121457149	121575376	0	0.009567
7704	Amp	5	121575615	121593665	0	0.007891
7705	Amp	5	121596484	121960296	0	0.006539
7706	Amp	5	121964625	122398521	0	0.007863
7707	Amp	5	122398565	122540677	0	0.009357
7708	Amp	5	122545512	122753036	0	0.01152
7709	Amp	5	122754598	123822311	0	0.010026
7710	Amp	5	123823581	124154825	0	0.00835
7711	Amp	5	124162681	124332579	0	0.012535
7712	Amp	5	124335582	124569514	0	0.00835
7713	Amp	5	124572470	124617374	0	0.010326
7714	Amp	5	124617531	125132828	0	0.01199
7715	Amp	5	125136470	125315718	0	0.010014
7716	Amp	5	125315767	125499891	0	0.006539
7717	Amp	5	125502171	125582941	0	0.009397
7718	Amp	5	125583621	126010453	0	0.006539
7719	Amp	5	126011137	126087985	0	0.00928
7720	Amp	5	126091703	126129039	0	0.009733
7721	Amp	5	126130966	126344047	0	0.009007
7722	Amp	5	126346377	126353747	0	0.013844
7723	Amp	5	126353770	126636932	0	0.014869
7724	Amp	5	126637015	126637138	0	0.014416
7725	Amp	5	126643545	126720039	0	0.011674
7726	Amp	5	126720653	126830499	0	0.013084
7727	Amp	5	126832903	126878797	0	0.013774
7728	Amp	5	126889234	126910608	0	0.010831
7729	Amp	5	126913029	126931659	0	0.009421
7730	Amp	5	126933417	127152240	0	0.013811
7731	Amp	5	127152382	127196239	0	0.008973
7732	Amp	5	127204323	127366200	0	0.004583
7733	Amp	5	127368388	127535892	0	0.003165
7734	Amp	5	127538856	127614411	0	0.004268
7735	Amp	5	127615185	127733677	0	0.005993
7736	Amp	5	127740924	127814701	0	0.004268
7737	Amp	5	127816543	127836624	0	0.005694

7738	Amp	5	127841902	127856013	0	0.004243
7739	Amp	5	127856545	127883613	0	0.003141
7740	Amp	5	127886013	127943081	0	0.005425
7741	Amp	5	127943423	127983284	0	0.003998
7742	Amp	5	127985900	128350686	0	0.005918
7743	Amp	5	128357818	128880134	0	0.005228
7744	Amp	5	128881355	129259214	0	0.00749
7745	Amp	5	129259302	129612523	0	0.008978
7746	Amp	5	129620725	129720837	0	0.00749
7747	Amp	5	129723335	130040499	0	0.010569
7748	Amp	5	130041137	130152771	0	0.012243
7749	Amp	5	130154352	130330700	0	0.01292
7750	Amp	5	130331351	130331351	0	0.011246
7751	Amp	5	130337513	130414090	0	0.014063
7752	Amp	5	130416217	130457488	0	0.011802
7753	Amp	5	130460664	130782432	0	0.008985
7754	Amp	5	130789406	130934865	0	0.011233
7755	Amp	5	130936811	131116793	0	0.008985
7756	Amp	5	131120930	131136900	0	0.00647
7757	Amp	5	131140193	131417384	0	0.013744
7758	Amp	5	131423704	132245158	0	0.013067
7759	Amp	5	132260091	132264799	0	0.013935
7760	Amp	5	132266820	132318321	0	0.01523
7761	Amp	5	132320429	132397705	0	0.018092
7762	Amp	5	132404159	132437502	0	0.015013
7763	Amp	5	132441851	132444463	0	0.010683
7764	Amp	5	132444952	132595931	0	0.007821
7765	Amp	5	132598600	132775961	0	0.011025
7766	Amp	5	132781399	132872424	0	0.010157
7767	Amp	5	132872476	132896568	0	0.011254
7768	Amp	5	132896623	132936664	0	0.008309
7769	Amp	5	132942382	133200298	0	0.005106
7770	Amp	5	133201163	133399530	0	0.005556
7771	Amp	5	133400093	133575109	0	0.006363
7772	Amp	5	133577560	134001920	0	0.005556
7773	Amp	5	134014976	134055825	0	0.006748
7774	Amp	5	134055924	134128966	0	0.008304
7775	Amp	5	134132120	134149344	0	0.007111
7776	Amp	5	134150767	134287009	0	0.006661
7777	Amp	5	134289731	134507529	0	0.005106
7778	Amp	5	134508231	134825305	0	0.003811
7779	Amp	5	134826456	134887903	0	0.002121
7780	Amp	5	134888460	134933096	0	0.003996
7781	Amp	5	134933929	134998216	0	0.004804
7782	Amp	5	135004340	135532414	0	0.002929
7783	Amp	5	135532649	135715727	0	0.002121
7784	Amp	5	135719225	135995864	0	0.003831
7785	Amp	5	135996211	137016298	0	0.004567
7786	Amp	5	137016708	137028583	0	0.005128
7787	Amp	5	137030379	137034799	0	0.004567
7788	Amp	5	137035692	137373920	0	0.005391
7789	Amp	5	137374121	137578303	0	0.004655
7790	Amp	5	137578637	138189731	0	0.002945

7791	Amp	5	138192228	138240621	0	0.003472
7792	Amp	5	138250641	138267612	0	0.004979
7793	Amp	5	138271549	138272977	0	0.005918
7794	Amp	5	138273246	138287642	0	0.005094
7795	Amp	5	138288063	138288063	0	0.008909
7796	Amp	5	138291363	138915516	0	0.009904
7797	Amp	5	138921964	139602739	0	0.011425
7798	Amp	5	139605267	139605267	0	0.012026
7799	Amp	5	139606096	139679004	0	0.010504
7800	Amp	5	139679619	139985761	0	0.013293
7801	Amp	5	139986359	140115734	0	0.01189
7802	Amp	5	140117717	140222615	0	0.009102
7803	Amp	5	140222809	140492354	0	0.008501
7804	Amp	5	140493059	140513290	0	0.007506
7805	Amp	5	140521268	140701838	0	0.010674
7806	Amp	5	140703190	140717390	0	0.016148
7807	Amp	5	140723434	140730366	0	0.010674
7808	Amp	5	140730462	140810787	0	0.009649
7809	Amp	5	140814419	140953996	0	0.008707
7810	Amp	5	140957597	141017184	0	0.015182
7811	Amp	5	141019685	141024623	0	0.013712
7812	Amp	5	141037995	141239171	0	0.011511
7813	Amp	5	141239381	141273689	0	0.009838
7814	Amp	5	141278798	141444562	0	0.01151
7815	Amp	5	141447667	141628560	0	0.009311
7816	Amp	5	141639180	141865656	0	0.012154
7817	Amp	5	141866249	141932717	0	0.013128
7818	Amp	5	141936116	142596092	0	0.010528
7819	Amp	5	142598083	142971111	0	0.00902
7820	Amp	5	142973562	142973562	0	0.010503
7821	Amp	5	142975906	143030748	0	0.011688
7822	Amp	5	143031489	143295037	0	0.013873
7823	Amp	5	143295513	143389282	0	0.012688
7824	Amp	5	143389856	143618116	0	0.014337
7825	Amp	5	143621897	143629519	0	0.015888
7826	Amp	5	143630147	143683894	0	0.013703
7827	Amp	5	143685615	143750958	0	0.015724
7828	Amp	5	143751144	143753102	0	0.014242
7829	Amp	5	143758775	144305867	0	0.012691
7830	Amp	5	144311010	144456552	0	0.009562
7831	Amp	5	144463554	145130776	0	0.009051
7832	Amp	5	145130953	145351173	0	0.007826
7833	Amp	5	145365558	146045286	0	0.008701
7834	Amp	5	146046020	147603295	0	0.007826
7835	Amp	5	147603381	147688684	0	0.006729
7836	Amp	5	147692272	147707196	0	0.008673
7837	Amp	5	147708938	147731468	0	0.007724
7838	Amp	5	147734012	147957601	0	0.00578
7839	Amp	5	147957711	148273948	0	0.007056
7840	Amp	5	148290011	148353583	0	0.00578
7841	Amp	5	148356488	148402436	0	0.006896
7842	Amp	5	148404707	149535253	0	0.007397
7843	Amp	5	149538008	150366149	0	0.008858

7844	Amp	5	150368268	150583535	0	0.011435
7845	Amp	5	150584752	150792250	0	0.013675
7846	Amp	5	150843825	150886484	0	0.011098
7847	Amp	5	150895090	150952092	0	0.008858
7848	Amp	5	150954028	151891364	0	0.006697
7849	Amp	5	151895721	151999749	0	0.008876
7850	Amp	5	152000346	152339854	0	0.006697
7851	Amp	5	152346229	152734153	0	0.011247
7852	Amp	5	152748907	152774389	0	0.012572
7853	Amp	5	152777210	152882165	0	0.015357
7854	Amp	5	152898460	154204803	0	0.010807
7855	Amp	5	154204927	154270702	0	0.013664
7856	Amp	5	154271899	154376568	0	0.010807
7857	Amp	5	154376737	155106411	0	0.011257
7858	Amp	5	155107746	155145717	0	0.013808
7859	Amp	5	155146787	155523055	0	0.011257
7860	Amp	5	155526424	155548720	0	0.01408
7861	Amp	5	155549546	155648356	0	0.008473
7862	Amp	5	155650393	155806699	0	0.016231
7863	Amp	5	155807260	155853416	0	0.011771
7864	Amp	5	155856925	156181642	0	0.012349
7865	Amp	5	156188790	156535293	0	0.011771
7866	Amp	5	156541163	156667681	0	0.008473
7867	Amp	5	156671617	156695914	0	0.021151
7868	Amp	5	156696984	156847623	0	0.008473
7869	Amp	5	156848567	158429946	0	0.007916
7870	Amp	5	158432611	158676558	0	0.011231
7871	Amp	5	158680086	158725440	0	0.007916
7872	Amp	5	158727679	159047249	0	0.011077
7873	Amp	5	159048184	159056274	0	0.010808
7874	Amp	5	159056556	159232699	0	0.007916
7875	Amp	5	159240623	159521954	0	0.010968
7876	Amp	5	159521986	159597758	0	0.013347
7877	Amp	5	159598384	159985817	0	0.010968
7878	Amp	5	159991483	160205744	0	0.011902
7879	Amp	5	160206477	160242365	0	0.012825
7880	Amp	5	160242820	160498190	0	0.011891
7881	Amp	5	160498807	160642733	0	0.015144
7882	Amp	5	160646847	160913448	0	0.015681
7883	Amp	5	160916108	160925977	0	0.034535
7884	Amp	5	160926224	161156651	0	0.015681
7885	Amp	5	161156760	161888904	0	0.012427
7886	Amp	5	161965920	162111603	0	0.015588
7887	Amp	5	162113283	162145612	0	0.01652
7888	Amp	5	162148647	163224608	0	0.015588
7889	Amp	5	163226678	163314292	0	0.015051
7890	Amp	5	163314386	163728799	0	0.021091
7891	Amp	5	163729189	163734801	0	0.023459
7892	Amp	5	163737266	163792003	0	0.017419
7893	Amp	5	163794203	164190113	0	0.014258
7894	Amp	5	164192292	164306682	0	0.017523
7895	Amp	5	164310186	164367169	0	0.018175
7896	Amp	5	164369307	165009777	0	0.018817

7897	Amp	5	165014018	165064650	0	0.01645
7898	Amp	5	165067411	165868700	0	0.015808
7899	Amp	5	165871775	166285400	0	0.027947
7900	Amp	5	166286271	166293601	0	0.015808
7901	Amp	5	166294159	167413220	0	0.017932
7902	Amp	5	167416251	167700687	0	0.023637
7903	Amp	5	167701025	168277311	0	0.021513
7904	Amp	5	168281761	168829235	0	0.022511
7905	Amp	5	168829530	170281687	0	0.016805
7906	Amp	5	170323154	170380100	0	0.019665
7907	Amp	5	170380481	171615238	0	0.016805
7908	Amp	5	171619741	171841776	0	0.01354
7909	Amp	5	171842017	171877622	0	0.014326
7910	Amp	5	171879510	171908979	0	0.018826
7911	Amp	5	171909543	171909543	0	0.019826
7912	Amp	5	172204765	172204765	0	0.022813
7913	Amp	5	172208249	172250336	0	0.024876
7914	Amp	5	172250723	172291695	0	0.021889
7915	Amp	5	172292504	172356576	0	0.02523
7916	Amp	5	172357610	172463926	0	0.026081
7917	Amp	5	172469542	172496707	0	0.027738
7918	Amp	5	172497285	172645362	0	0.024397
7919	Amp	5	172645840	172739256	0	0.027796
7920	Amp	5	172742387	172791096	0	0.034352
7921	Amp	5	172791377	172868343	0	0.035633
7922	Amp	5	172868651	173197048	0	0.033516
7923	Amp	5	173197479	173197714	0	0.038908
7924	Amp	5	173204048	173323837	0	0.03956
7925	Amp	5	173331073	173342498	0	0.037497
7926	Amp	5	173342946	173449734	0	0.033452
7927	Amp	5	173449848	173484966	0	0.025083
7928	Amp	5	173486129	173543279	0	0.022571
7929	Amp	5	173548516	173578489	0	0.025358
7930	Amp	5	173580588	173724424	0	0.024706
7931	Amp	5	173727151	173732423	0	0.026771
7932	Amp	5	173733657	173754500	0	0.027528
7933	Amp	5	173755099	173808036	0	0.026771
7934	Amp	5	173814292	173827557	0	0.024706
7935	Amp	5	173827818	173967163	0	0.025369
7936	Amp	5	173967404	174122412	0	0.026875
7937	Amp	5	174128464	174165031	0	0.025369
7938	Amp	5	174166402	174187751	0	0.024706
7939	Amp	5	174192941	174204791	0	0.025225
7940	Amp	5	174205114	174352299	0	0.027171
7941	Amp	5	174353011	174363592	0	0.025225
7942	Amp	5	174366334	174583900	0	0.026945
7943	Amp	5	174584497	174606820	0	0.025225
7944	Amp	5	174607045	174804853	0	0.022439
7945	Amp	5	174806822	174954577	0	0.021653
7946	Amp	5	174955546	175452063	0	0.021134
7947	Amp	5	175453642	175468399	0	0.022726
7948	Amp	5	175469372	175564954	0	0.030606
7949	Amp	5	175568027	175615522	0	0.029013

7950	Amp	5	175615755	175632109	0	0.031172
7951	Amp	5	175632710	175666418	0	0.033399
7952	Amp	5	175675326	175701414	0	0.03124
7953	Amp	5	175702960	175824575	0	0.031693
7954	Amp	5	175830891	175847802	0	0.034303
7955	Amp	5	175848482	175866152	0	0.036025
7956	Amp	5	175867230	176241574	0	0.035174
7957	Amp	5	176245027	176443777	0	0.037328
7958	Amp	5	176451038	176534603	0	0.039996
7959	Amp	5	176538658	176539316	0	0.037328
7960	Amp	5	176548393	177049105	0	0.040576
7961	Amp	5	177049197	177194856	0	0.042321
7962	Amp	5	177201883	177318172	0	0.040576
7963	Amp	5	177321155	177381194	0	0.037825
7964	Amp	5	177381437	177535247	0	0.036103
7965	Amp	5	177538645	177559445	0	0.038749
7966	Amp	5	177565673	177680789	0	0.036595
7967	Amp	5	177982825	177998062	0	0.032094
7968	Amp	5	178002093	178087839	0	0.024214
7969	Amp	5	178103571	178132142	0	0.026048
7970	Amp	5	178133251	178180895	0	0.028432
7971	Amp	5	178181181	178197073	0	0.029161
7972	Amp	5	178206115	178210446	0	0.027328
7973	Amp	5	178211191	178275580	0	0.024944
7974	Amp	5	178277026	178293424	0	0.0259
7975	Amp	5	178297919	178401361	0	0.024944
7976	Amp	5	178403813	178424689	0	0.026379
7977	Amp	5	178424769	178430348	0	0.025925
7978	Amp	5	178430478	178510862	0	0.024919
7979	Amp	5	178510886	179040396	0	0.023484
7980	Amp	5	179043960	179124095	0	0.020874
7981	Amp	5	179126018	179293967	0	0.021486
7982	Amp	5	179302587	179378611	0	0.028932
7983	Amp	5	179378629	179469650	0	0.028321
7984	Amp	5	179469859	179498842	0	0.029435
7985	Amp	5	179513195	179593796	0	0.021989
7986	Amp	5	179599063	179599063	0	0.02401
7987	Amp	5	179599122	179611876	0	0.026957
7988	Amp	5	179611973	179625184	0	0.02596
7989	Amp	5	179625556	179726796	0	0.023013
7990	Amp	5	179734694	179792191	0	0.019877
7991	Amp	5	179804359	179936040	0	0.020731
7992	Amp	5	179937627	179996167	0	0.022933
7993	Amp	5	180000400	180188580	0	0.020731
7994	Amp	5	180213365	180304014	0	0.02533
7995	Amp	5	180305080	180652970	0	0.022034
7996	Amp	5	180653093	180653187	0	0.022543
7997	Amp	5	180799516	180895735	0	0.024069
7998	Amp	5	180895785	180933469	0	0.033542
7999	Amp	6	1011760	1028445	0	0.029939
8000	Amp	6	1033968	1210392	0	0.033985
8001	Amp	6	1213225	1250644	0	0.030945
8002	Amp	6	1253752	1272001	0	0.029325

8003	Amp	6	1274200	1366438	0	0.0269
8004	Amp	6	1371901	1513148	0	0.023426
8005	Amp	6	1513280	1520637	0	0.024617
8006	Amp	6	1558405	1583678	0	0.031964
8007	Amp	6	1583774	1636101	0	0.031486
8008	Amp	6	1636916	1732536	0	0.033529
8009	Amp	6	1741157	1790004	0	0.026182
8010	Amp	6	1794478	1795315	0	0.023553
8011	Amp	6	1796370	1832702	0	0.024002
8012	Amp	6	1832807	1866371	0	0.02281
8013	Amp	6	1866545	1964413	0	0.024812
8014	Amp	6	1965072	1993051	0	0.040748
8015	Amp	6	2007453	2027509	0	0.050321
8016	Amp	6	2030483	2064776	0	0.043656
8017	Amp	6	2070899	2124943	0	0.040748
8018	Amp	6	2129245	2232574	0	0.038705
8019	Amp	6	2235276	2241911	0	0.045133
8020	Amp	6	2244422	2252192	0	0.047031
8021	Amp	6	2253604	2376437	0	0.040603
8022	Amp	6	2378096	2431531	0	0.038354
8023	Amp	6	2432173	2442947	0	0.03983
8024	Amp	6	2447368	2462150	0	0.038354
8025	Amp	6	2462573	2532240	0	0.036222
8026	Amp	6	2535442	2590691	0	0.039855
8027	Amp	6	2591353	2601935	0	0.036222
8028	Amp	6	2608761	2679050	0	0.039884
8029	Amp	6	2679790	2682532	0	0.02608
8030	Amp	6	2682590	2707415	0	0.029098
8031	Amp	6	2710769	2717365	0	0.025436
8032	Amp	6	2717547	2842733	0	0.022418
8033	Amp	6	2843192	2918313	0	0.021316
8034	Amp	6	2920533	2960835	0	0.037252
8035	Amp	6	2961135	2997531	0	0.036162
8036	Amp	6	3002162	3017247	0	0.020226
8037	Amp	6	3020455	3130194	0	0.021272
8038	Amp	6	3132309	3233410	0	0.019374
8039	Amp	6	3240890	3319637	0	0.020446
8040	Amp	6	3319917	3331166	0	0.021033
8041	Amp	6	3331468	3395613	0	0.019961
8042	Amp	6	3428595	3441300	0	0.035897
8043	Amp	6	3442370	3475945	0	0.037078
8044	Amp	6	3476042	3486812	0	0.055682
8045	Amp	6	3487325	3493790	0	0.042599
8046	Amp	6	3494965	3580001	0	0.036492
8047	Amp	6	3587239	3614731	0	0.039762
8048	Amp	6	3615586	3643553	0	0.039313
8049	Amp	6	3644148	3666133	0	0.040593
8050	Amp	6	3671909	3671909	0	0.042721
8051	Amp	6	3672972	3717479	0	0.0453
8052	Amp	6	3718010	3748872	0	0.049225
8053	Amp	6	3755612	3767164	0	0.051283
8054	Amp	6	3769861	3887825	0	0.047358
8055	Amp	6	3891338	3912041	0	0.050245

8056	Amp	6	3914061	3958029	0	0.046975
8057	Amp	6	3960139	4107727	0	0.049937
8058	Amp	6	4108628	4160930	0	0.05147
8059	Amp	6	4162110	4235838	0	0.048508
8060	Amp	6	4237039	4253179	0	0.045621
8061	Amp	6	4498631	4528404	0	0.051044
8062	Amp	6	4530266	4679048	0	0.048464
8063	Amp	6	4679534	4754508	0	0.050099
8064	Amp	6	4755943	4849675	0	0.044677
8065	Amp	6	4851970	4864347	0	0.046896
8066	Amp	6	4864727	4876568	0	0.05021
8067	Amp	6	4881381	4932963	0	0.048575
8068	Amp	6	4934445	5031372	0	0.046884
8069	Amp	6	5035296	5043545	0	0.032638
8070	Amp	6	5044535	5150620	0	0.034072
8071	Amp	6	5151013	5210614	0	0.030759
8072	Amp	6	5214750	5260152	0	0.02863
8073	Amp	6	5270217	5294139	0	0.02918
8074	Amp	6	5298129	5470936	0	0.028265
8075	Amp	6	5472695	5475717	0	0.031499
8076	Amp	6	5478841	5641895	0	0.033245
8077	Amp	6	5641922	5657424	0	0.035845
8078	Amp	6	5658488	5730453	0	0.035405
8079	Amp	6	5732564	5814636	0	0.039674
8080	Amp	6	5817550	5833830	0	0.037074
8081	Amp	6	5834857	5919053	0	0.034107
8082	Amp	6	5919217	6021645	0	0.032361
8083	Amp	6	6040055	6162173	0	0.034219
8084	Amp	6	6163704	6226801	0	0.034822
8085	Amp	6	6227626	6253313	0	0.030553
8086	Amp	6	6254470	6319481	0	0.037334
8087	Amp	6	6321151	6322745	0	0.030553
8088	Amp	6	6326625	6398269	0	0.031938
8089	Amp	6	6398290	6574742	0	0.032689
8090	Amp	6	6577532	6648151	0	0.03047
8091	Amp	6	6650159	6682175	0	0.027236
8092	Amp	6	6688453	6711447	0	0.031228
8093	Amp	6	6716400	6790632	0	0.03414
8094	Amp	6	6793469	6818994	0	0.041249
8095	Amp	6	6819637	6829927	0	0.040646
8096	Amp	6	6832104	6887140	0	0.036653
8097	Amp	6	6887398	6955214	0	0.034285
8098	Amp	6	6955865	7062859	0	0.031373
8099	Amp	6	7063038	7138973	0	0.032678
8100	Amp	6	7142842	7156668	0	0.030096
8101	Amp	6	7156807	7156807	0	0.030616
8102	Amp	6	7162337	7171174	0	0.032715
8103	Amp	6	7175834	7222580	0	0.035891
8104	Amp	6	7225302	7247979	0	0.039172
8105	Amp	6	7248226	7251646	0	0.040497
8106	Amp	6	7252437	7475473	0	0.043601
8107	Amp	6	7476353	7501131	0	0.045868
8108	Amp	6	7502428	7503552	0	0.049105

8109	Amp	6	7504065	7555016	0	0.050746
8110	Amp	6	7555621	7641259	0	0.047509
8111	Amp	6	7642276	7685210	0	0.045868
8112	Amp	6	7685262	7840072	0	0.046993
8113	Amp	6	7840568	7845701	0	0.048416
8114	Amp	6	7847178	7953756	0	0.046149
8115	Amp	6	7955342	7977003	0	0.049267
8116	Amp	6	7977173	8058784	0	0.049745
8117	Amp	6	8059088	8066181	0	0.051282
8118	Amp	6	8067341	8067521	0	0.048177
8119	Amp	6	8070374	8083745	0	0.048735
8120	Amp	6	8087149	8091604	0	0.048177
8121	Amp	6	8094296	8146245	0	0.051547
8122	Amp	6	8147239	8150371	0	0.048429
8123	Amp	6	8153002	8155950	0	0.050331
8124	Amp	6	8158860	8237170	0	0.051723
8125	Amp	6	8240094	8272886	0	0.049821
8126	Amp	6	8286769	8296221	0	0.048397
8127	Amp	6	8304895	8313690	0	0.051471
8128	Amp	6	8317106	8334515	0	0.053617
8129	Amp	6	8334727	8369820	0	0.050542
8130	Amp	6	8374560	8388961	0	0.048081
8131	Amp	6	8389686	8431006	0	0.046689
8132	Amp	6	8435650	8450202	0	0.04459
8133	Amp	6	8453174	8513195	0	0.045852
8134	Amp	6	8516949	8592345	0	0.043707
8135	Amp	6	8593937	8683526	0	0.042444
8136	Amp	6	8696439	8883061	0	0.045178
8137	Amp	6	8883838	8953361	0	0.046216
8138	Amp	6	8961401	8990028	0	0.042846
8139	Amp	6	8994308	9003024	0	0.046126
8140	Amp	6	9005377	9011376	0	0.049682
8141	Amp	6	9015017	9019250	0	0.057487
8142	Amp	6	9030081	9034968	0	0.05998
8143	Amp	6	9034999	9042981	0	0.060993
8144	Amp	6	9045696	9053832	0	0.053187
8145	Amp	6	9054337	9080252	0	0.051343
8146	Amp	6	9080769	9265515	0	0.051809
8147	Amp	6	9267568	9268080	0	0.050771
8148	Amp	6	9272209	9337336	0	0.049897
8149	Amp	6	9337534	9358532	0	0.049234
8150	Amp	6	9363716	9528811	0	0.053019
8151	Amp	6	9530983	9648986	0	0.055875
8152	Amp	6	9653950	9660021	0	0.057726
8153	Amp	6	9660913	9661803	0	0.060241
8154	Amp	6	9661840	9682486	0	0.05855
8155	Amp	6	9682686	9683358	0	0.054766
8156	Amp	6	9688160	9746020	0	0.056402
8157	Amp	6	9746142	9751826	0	0.058804
8158	Amp	6	9754724	9835996	0	0.062047
8159	Amp	6	9837640	9893255	0	0.059532
8160	Amp	6	9894897	9900935	0	0.059065
8161	Amp	6	9902835	9961900	0	0.060727

8162 Amp	6	9965180	9965180	0	0.058876
8163 Amp	6	9965820	10008724	0	0.062908
8164 Amp	6	10010377	10249677	0.079652	0.067528
8165 Amp	6	10250904	10359229	0.001323	0.063972
8166 Amp	6	10360622	10368475	0.073902	0.067296
8167 Amp	6	10369442	10374956	0.050473	0.066172
8168 Amp	6	10375162	10424235	0	0.06214
8169 Amp	6	10431027	10434987	0.100827	0.068297
8170 Amp	6	10437642	10437642	0.024438	0.064973
8171 Amp	6	10438064	10441241	0	0.06268
8172 Amp	6	10444393	10451874	0	0.057633
8173 Amp	6	10456473	10534810	0.015369	0.064526
8174 Amp	6	10535430	10542877	0	0.063221
8175 Amp	6	10546167	10546167	0	0.058601
8176 Amp	6	10742788	10742788	0	0.062644
8177 Amp	6	10744833	10801155	0.040093	0.065672
8178 Amp	6	10804974	10858037	0.073902	0.067114
8179 Amp	6	10858069	10893446	0.034113	0.065498
8180 Amp	6	10893490	10905680	0	0.062697
8181 Amp	6	10905813	10990040	0	0.061458
8182 Amp	6	10991702	11017248	0	0.063646
8183 Amp	6	11018579	11022651	0.019169	0.064807
8184 Amp	6	11026766	11110423	0.1237	0.069228
8185 Amp	6	11111036	11232516	0.069848	0.067041
8186 Amp	6	11234611	11247408	0	0.062619
8187 Amp	6	11251622	11377924	0.015369	0.064611
8188 Amp	6	11380158	11476182	0.118001	0.068885
8189 Amp	6	11476385	11485238	0.050473	0.06615
8190 Amp	6	11486466	11503331	0.069848	0.06706
8191 Amp	6	11504253	11606311	0.14896	0.070336
8192 Amp	6	11606797	11617954	0.100827	0.068344
8193 Amp	6	11618533	11653324	0.079652	0.067434
8194 Amp	6	11653862	11689674	0.133193	0.069535
8195 Amp	6	11691831	11712029	0.238246	0.073714
8196 Amp	6	11712755	11730039	0.210594	0.072553
8197 Amp	6	11730379	11797767	0.143787	0.070059
8198 Amp	6	11798181	11798776	0.08435	0.0676
8199 Amp	6	11799191	11807441	0.010261	0.064324
8200 Amp	6	11807831	11842929	0	0.063341
8201 Amp	6	11846607	12138646	0	0.060498
8202 Amp	6	12145232	12196371	0	0.057322
8203 Amp	6	12196677	12239186	0	0.056669
8204 Amp	6	12242164	12250689	0.079652	0.067362
8205 Amp	6	12250955	12257253	0.128324	0.069474
8206 Amp	6	12258061	12326487	0.094967	0.068032
8207 Amp	6	12328963	12349505	0.17597	0.071226
8208 Amp	6	12350179	12359070	0.238246	0.073718
8209 Amp	6	12359485	12405596	0.264858	0.075066
8210 Amp	6	12405674	12427431	0.299433	0.076314
8211 Amp	6	12427903	12446631	0.424557	0.080768
8212 Amp	6	12455961	12472024	0.494792	0.083137
8213 Amp	6	12473475	12484915	0.622604	0.087107
8214 Amp	6	12491235	12530604	0.424557	0.080768

8215	Amp	6	12533067	12570772	0.30524	0.076462
8216	Amp	6	12574939	12596400	0.463534	0.081946
8217	Amp	6	12597646	12628733	0.536238	0.084288
8218	Amp	6	12632636	12634053	0.404718	0.079922
8219	Amp	6	12638322	12670423	0.311747	0.076728
8220	Amp	6	12671110	12675959	0.386426	0.079426
8221	Amp	6	12680791	12680791	0.457501	0.081661
8222	Amp	6	12691907	12703620	0.522881	0.083898
8223	Amp	6	12704410	12718894	0.487495	0.08278
8224	Amp	6	12719160	12747130	0.417873	0.080437
8225	Amp	6	12751304	12767331	0.293269	0.076164
8226	Amp	6	12770591	12770591	0.431198	0.080965
8227	Amp	6	12770611	12772677	0.342649	0.077782
8228	Amp	6	12772692	12781392	0.264858	0.075084
8229	Amp	6	12784074	12784074	0.14896	0.070282
8230	Amp	6	12785223	12800965	0.112543	0.068621
8231	Amp	6	12806962	12842053	0.060686	0.066619
8232	Amp	6	12844543	12854011	0.181932	0.07155
8233	Amp	6	12854211	12881832	0.08435	0.067713
8234	Amp	6	12886144	12918916	0.107017	0.068461
8235	Amp	6	12922457	12967413	0.133193	0.069525
8236	Amp	6	12967999	12986480	0.193543	0.072023
8237	Amp	6	12988357	13066143	0.470509	0.082127
8238	Amp	6	13066285	13117151	0.543344	0.08458
8239	Amp	6	13117245	13132476	0.529451	0.084102
8240	Amp	6	13135108	13197349	0.482379	0.082717
8241	Amp	6	13201731	13220183	0.6737	0.088799
8242	Amp	6	13220487	13305718	0.831947	0.092996
8243	Amp	6	13307304	13314012	1.112463	0.099994
8244	Amp	6	13316017	13350014	1.188121	0.101801
8245	Amp	6	13350892	13374655	1.329472	0.105005
8246	Amp	6	13375215	13388222	1.398396	0.10648
8247	Amp	6	13390038	13427114	1.542638	0.109849
8248	Amp	6	13432418	13523602	1.482378	0.108377
8249	Amp	6	13529476	13533840	1.280185	0.103923
8250	Amp	6	13537687	13571568	1.309159	0.104478
8251	Amp	6	13573598	13619352	0.6737	0.088797
8252	Amp	6	13619397	13643059	0.576183	0.085732
8253	Amp	6	13646709	13657953	0.720659	0.089982
8254	Amp	6	13661672	13716491	0.667587	0.088507
8255	Amp	6	13716535	13724202	0.589605	0.086015
8256	Amp	6	13724665	13779248	0.660254	0.08823
8257	Amp	6	13779690	13838627	0.704849	0.089627
8258	Amp	6	13838878	13840616	0.630438	0.08739
8259	Amp	6	13842027	13877461	0.609133	0.086779
8260	Amp	6	13886645	13986686	0.667587	0.088383
8261	Amp	6	13988492	14005856	0.704849	0.089464
8262	Amp	6	14008356	14026705	0.487495	0.082788
8263	Amp	6	14029941	14033654	0.424557	0.080726
8264	Amp	6	14036105	14041361	0.30524	0.07653
8265	Amp	6	14044589	14044939	0.368062	0.07881
8266	Amp	6	14046259	14046895	0.386426	0.079374
8267	Amp	6	14049041	14130802	0.322897	0.07714

8268	Amp	6	14131636	14142079	0.373599	0.078901
8269	Amp	6	14142258	14197037	0.311747	0.076783
8270	Amp	6	14197388	14201403	0.329205	0.077353
8271	Amp	6	14202484	14210188	0.299433	0.076289
8272	Amp	6	14212742	14341570	0.373599	0.079014
8273	Amp	6	14345931	14429117	0.424557	0.080647
8274	Amp	6	14431882	14521737	0.386426	0.079299
8275	Amp	6	14524357	14576465	0.293269	0.076095
8276	Amp	6	14576623	14576623	0.153838	0.070486
8277	Amp	6	14583810	14635056	0.107017	0.06849
8278	Amp	6	14636189	14702436	0.056211	0.066257
8279	Amp	6	14702665	14781832	0.005328	0.064156
8280	Amp	6	14787497	14806402	0.069848	0.066904
8281	Amp	6	14808687	14856126	0.056211	0.06634
8282	Amp	6	14863099	14899254	0	0.063593
8283	Amp	6	14901184	14923473	0.005328	0.064178
8284	Amp	6	14925778	14953306	0.143787	0.06997
8285	Amp	6	14954209	15031620	0.153838	0.070465
8286	Amp	6	15035186	15178691	0.238246	0.073647
8287	Amp	6	15188177	15237645	0.255918	0.074667
8288	Amp	6	15240478	15339843	0.193543	0.071942
8289	Amp	6	15340240	15550859	0.379885	0.079146
8290	Amp	6	15552467	15570324	0.227787	0.073354
8291	Amp	6	15577012	15718525	0.138391	0.069824
8292	Amp	6	15720209	15926418	0.065715	0.066783
8293	Amp	6	15926602	15986846	0.1237	0.069127
8294	Amp	6	15994595	16047503	0.187449	0.071706
8295	Amp	6	16048455	16083427	0.08435	0.067543
8296	Amp	6	16085358	16209736	0.233782	0.073486
8297	Amp	6	16219300	16258282	0.276783	0.075484
8298	Amp	6	16261368	16341046	0.251218	0.074328
8299	Amp	6	16345984	16351312	0.282828	0.075602
8300	Amp	6	16353817	16401566	0.112543	0.06871
8301	Amp	6	16402466	16405479	0.065715	0.066712
8302	Amp	6	16407114	16441012	0	0.06353
8303	Amp	6	16441473	16538340	0.065715	0.066723
8304	Amp	6	16549121	16607179	0.024438	0.064956
8305	Amp	6	16608143	16623567	0.056211	0.066347
8306	Amp	6	16626950	16687314	0.019169	0.064743
8307	Amp	6	16691293	16741887	0.1237	0.069125
8308	Amp	6	16742370	16750670	0.112543	0.06863
8309	Amp	6	16751402	16808406	0.050473	0.066051
8310	Amp	6	16809464	16892416	0.153838	0.070412
8311	Amp	6	16898889	16900182	0	0.062837
8312	Amp	6	16900309	16911264	0.044794	0.066008
8313	Amp	6	16914789	16946808	0.001323	0.064004
8314	Amp	6	16949101	17006612	0.079652	0.067385
8315	Amp	6	17007258	17007258	0.034113	0.06545
8316	Amp	6	17010519	17019195	0.094967	0.068063
8317	Amp	6	17019474	17019795	0.128324	0.06935
8318	Amp	6	17020846	17039132	0.19964	0.072154
8319	Amp	6	17043969	17139452	0.128324	0.06935
8320	Amp	6	17147207	17316695	0.233782	0.073609

8321	Amp	6	17320689	17448494	0.159312	0.070591
8322	Amp	6	17451062	17486660	0.246818	0.074222
8323	Amp	6	17490869	17491511	0.264858	0.075019
8324	Amp	6	17493459	17504271	0.276783	0.075507
8325	Amp	6	17504654	17512815	0.19964	0.072126
8326	Amp	6	17515773	17519824	0.089561	0.067868
8327	Amp	6	17520401	17557843	0.138391	0.069872
8328	Amp	6	17561063	17582276	0.222041	0.073074
8329	Amp	6	17586964	17627359	0.379885	0.079247
8330	Amp	6	17627660	17627660	0.316877	0.076967
8331	Amp	6	17629385	17666132	0.543344	0.084604
8332	Amp	6	17669470	17682600	0.316877	0.076967
8333	Amp	6	17685017	17746282	0.515676	0.083652
8334	Amp	6	17816767	17818980	0.494792	0.083163
8335	Amp	6	17829938	17841420	0.630438	0.087429
8336	Amp	6	17841840	17847790	0.529451	0.084227
8337	Amp	6	17849806	17932010	0.34892	0.078054
8338	Amp	6	17935097	18011809	0.399144	0.079842
8339	Amp	6	18022608	18037956	0.34892	0.078054
8340	Amp	6	18039165	18114889	0.404718	0.080086
8341	Amp	6	18115466	18173287	0.30524	0.076456
8342	Amp	6	18173781	18207957	0.293269	0.076009
8343	Amp	6	18209532	18224515	0.355319	0.07834
8344	Amp	6	18224653	18225070	0.342649	0.07777
8345	Amp	6	18226470	18234304	0	0.05779
8346	Amp	6	18237430	18238337	0.010261	0.064425
8347	Amp	6	18238671	18238671	0.1237	0.069154
8348	Amp	6	18238736	18253188	0.241616	0.073989
8349	Amp	6	18253311	18258386	0.287738	0.075889
8350	Amp	6	18258394	18262983	0.30524	0.0765
8351	Amp	6	18263021	18286379	0.322897	0.077095
8352	Amp	6	18292792	18293160	0.487495	0.082744
8353	Amp	6	18293601	18403496	0.417873	0.080413
8354	Amp	6	18403821	18410390	0.463534	0.081954
8355	Amp	6	18410839	18411283	0.636464	0.087584
8356	Amp	6	18414929	18588575	0.616549	0.086863
8357	Amp	6	18588683	18625051	0.47716	0.08234
8358	Amp	6	18629566	18921943	0.522881	0.084007
8359	Amp	6	18923416	18954940	0.589605	0.086007
8360	Amp	6	18959054	19027163	0.569771	0.085367
8361	Amp	6	19028557	19038361	0.697374	0.089334
8362	Amp	6	19038395	19106396	0.781972	0.091806
8363	Amp	6	19106440	19261531	0.950886	0.096002
8364	Amp	6	19261864	19262506	0.848692	0.093531
8365	Amp	6	19263127	19276371	0.697374	0.089334
8366	Amp	6	19281969	19381372	0.79909	0.092279
8367	Amp	6	19381820	19422774	0.704849	0.089666
8368	Amp	6	19423136	19437352	0.576183	0.085698
8369	Amp	6	19438666	19471953	0.667587	0.088584
8370	Amp	6	19487630	19505674	0.950886	0.096051
8371	Amp	6	19511819	19556009	0.667587	0.088584
8372	Amp	6	19558362	19559933	0.616549	0.087043
8373	Amp	6	19564116	19603338	0.758088	0.091117

8374	Amp	6	19609886	19746940	0.735486	0.090427
8375	Amp	6	19747817	19763422	0.589605	0.086049
8376	Amp	6	19773322	19823007	0.30524	0.076452
8377	Amp	6	19824740	19858237	0.316877	0.076991
8378	Amp	6	19858795	20005319	0.392611	0.079578
8379	Amp	6	20009547	20041721	0.259438	0.074833
8380	Amp	6	20044332	20146708	0.181932	0.071448
8381	Amp	6	20147310	20204722	0.193543	0.071908
8382	Amp	6	20205679	20237791	0.010261	0.064359
8383	Amp	6	20238176	20369234	0	0.062093
8384	Amp	6	20369377	20373897	0	0.063157
8385	Amp	6	20376310	20398820	0.112543	0.068722
8386	Amp	6	20401678	20414785	0.065715	0.066721
8387	Amp	6	20416341	20416341	0.118001	0.068918
8388	Amp	6	20424128	20535099	0.19964	0.072294
8389	Amp	6	20535465	20599397	0.100827	0.068336
8390	Amp	6	20599657	20645991	0.024438	0.064959
8391	Amp	6	20647429	20688503	0.034113	0.065421
8392	Amp	6	20690832	20710078	0.065715	0.066766
8393	Amp	6	20710689	20753334	0.001323	0.063926
8394	Amp	6	20753888	20764670	0.044794	0.065845
8395	Amp	6	20765538	20802868	0.133193	0.06951
8396	Amp	6	20803316	20903104	0.089561	0.067901
8397	Amp	6	20905000	21046087	0.079652	0.067438
8398	Amp	6	21055257	21094959	0.153838	0.070383
8399	Amp	6	21103226	21164897	0.065715	0.066719
8400	Amp	6	21166357	21172978	0.159312	0.070756
8401	Amp	6	21179677	21278265	0	0.062761
8402	Amp	6	21278973	21312324	0	0.061697
8403	Amp	6	21312358	21331676	0.010261	0.064351
8404	Amp	6	21334023	21364110	0.024438	0.064979
8405	Amp	6	21368447	21382666	0.050473	0.066023
8406	Amp	6	21384382	21407316	0.034113	0.065395
8407	Amp	6	21410630	21490960	0	0.063149
8408	Amp	6	21501456	21557362	0.073902	0.067108
8409	Amp	6	21557772	21601814	0	0.063684
8410	Amp	6	21602858	21652510	0.005328	0.064165
8411	Amp	6	21653446	21690778	0	0.060207
8412	Amp	6	21691383	21708632	0	0.058855
8413	Amp	6	21710665	21746794	0.089561	0.067866
8414	Amp	6	21751851	21780915	0.159312	0.070678
8415	Amp	6	21783646	21806789	0.271552	0.075136
8416	Amp	6	21808249	21907862	0.287738	0.075887
8417	Amp	6	21910987	21961679	0.4115	0.080151
8418	Amp	6	21962090	21967094	0.311747	0.076841
8419	Amp	6	21967467	21974520	0.329205	0.077401
8420	Amp	6	21977190	22000073	0.399144	0.079776
8421	Amp	6	22011514	22043580	0.47716	0.08251
8422	Amp	6	22044433	22070998	0.215608	0.072748
8423	Amp	6	22071075	22097146	0.19964	0.072136
8424	Amp	6	22099846	22166956	0.170723	0.071037
8425	Amp	6	22172387	22198515	0.159312	0.070577
8426	Amp	6	22200155	22248546	0.040093	0.065754

8427	Amp	6	22249430	22340349	0	0.060213
8428	Amp	6	22340775	22374723	0	0.058294
8429	Amp	6	22375419	22393246	0	0.060593
8430	Amp	6	22397998	22397999	0	0.058294
8431	Amp	6	22398779	22443257	0	0.059999
8432	Amp	6	22445316	22486435	0	0.063434
8433	Amp	6	22486645	22505788	0.069848	0.066909
8434	Amp	6	22506344	22524642	0.005328	0.064194
8435	Amp	6	22526929	22530914	0	0.062527
8436	Amp	6	22535453	22636343	0	0.056881
8437	Amp	6	22636506	22647575	0	0.055489
8438	Amp	6	22649929	22709812	0	0.053114
8439	Amp	6	22711819	22792633	0	0.05141
8440	Amp	6	22792650	22811046	0	0.053147
8441	Amp	6	22813130	22852950	0	0.05095
8442	Amp	6	22859206	22859344	0	0.059057
8443	Amp	6	22860493	22909104	0	0.056323
8444	Amp	6	22909651	22975190	0	0.054979
8445	Amp	6	22981616	22981616	0	0.060476
8446	Amp	6	22987823	23008647	0	0.062048
8447	Amp	6	23009086	23068198	0.024438	0.065004
8448	Amp	6	23073927	23119274	0	0.058888
8449	Amp	6	23119785	23169786	0	0.062084
8450	Amp	6	23170758	23202546	0	0.061529
8451	Amp	6	23202707	23209994	0	0.058094
8452	Amp	6	23210005	23240413	0	0.060238
8453	Amp	6	23242598	23258921	0	0.063658
8454	Amp	6	23263447	23324045	0	0.062718
8455	Amp	6	23325357	23372267	0	0.061552
8456	Amp	6	23374161	23485148	0	0.059231
8457	Amp	6	23486894	23524192	0	0.05724
8458	Amp	6	23526825	23543143	0	0.05382
8459	Amp	6	23543698	23548198	0	0.054501
8460	Amp	6	23549413	23560392	0	0.053962
8461	Amp	6	23560587	23594959	0	0.054404
8462	Amp	6	23597541	23597662	0	0.057596
8463	Amp	6	23599119	23672102	0	0.056964
8464	Amp	6	23675851	23724568	0	0.061316
8465	Amp	6	23724836	23792012	0	0.060875
8466	Amp	6	23793135	23905687	0	0.062715
8467	Amp	6	23907997	24063555	0	0.061626
8468	Amp	6	24066798	24101619	0	0.058434
8469	Amp	6	24102495	24204195	0	0.061576
8470	Amp	6	24209658	24211776	0	0.058764
8471	Amp	6	24212035	24285278	0	0.05633
8472	Amp	6	24285343	24341864	0	0.057882
8473	Amp	6	24342143	24380643	0	0.05707
8474	Amp	6	24383371	24409678	0	0.062834
8475	Amp	6	24412900	24460479	0	0.060246
8476	Amp	6	24461199	24475072	0	0.062217
8477	Amp	6	24479617	24506826	0	0.057865
8478	Amp	6	24507023	24512609	0	0.060259
8479	Amp	6	24513408	24533204	0	0.063639

8480	Amp	6	24533277	24552530	0	0.062599
8481	Amp	6	24553153	24609681	0	0.059218
8482	Amp	6	24610881	24624340	0	0.055673
8483	Amp	6	24624629	24671255	0	0.057582
8484	Amp	6	24673772	24764694	0	0.056751
8485	Amp	6	24766505	24824689	0	0.055673
8486	Amp	6	24827427	24827640	0	0.054993
8487	Amp	6	24828331	24828331	0	0.056446
8488	Amp	6	24832485	24947577	0	0.059092
8489	Amp	6	24950971	24961023	0	0.058557
8490	Amp	6	24961995	25060118	0	0.060251
8491	Amp	6	25063711	25173658	0	0.060733
8492	Amp	6	25173842	25212854	0	0.059039
8493	Amp	6	25213466	25261279	0	0.056645
8494	Amp	6	25262394	25294171	0	0.055192
8495	Amp	6	25297784	25409849	0	0.056366
8496	Amp	6	25410726	25449798	0	0.053224
8497	Amp	6	25449876	25449876	0	0.050902
8498	Amp	6	25449925	25735847	0	0.053047
8499	Amp	6	25737704	25742301	0	0.056144
8500	Amp	6	25742471	25763964	0	0.053288
8501	Amp	6	25764180	25787589	0	0.054298
8502	Amp	6	25794809	25809570	0	0.062823
8503	Amp	6	25809681	25812979	0.193543	0.071879
8504	Amp	6	25815633	25843698	0.282828	0.075698
8505	Amp	6	25847146	25866841	0.210594	0.072601
8506	Amp	6	25868324	25869143	0	0.063544
8507	Amp	6	25869620	25869620	0	0.05502
8508	Amp	6	25870314	25870585	0	0.055877
8509	Amp	6	25870816	25944924	0	0.052942
8510	Amp	6	25944983	25944983	0	0.049985
8511	Amp	6	25950154	25969808	0	0.049127
8512	Amp	6	25977074	26000657	0	0.054492
8513	Amp	6	26001046	26018984	0	0.058232
8514	Amp	6	26027192	26072168	0	0.060263
8515	Amp	6	26072217	26144563	0	0.043549
8516	Amp	6	26153056	26161384	0	0.045069
8517	Amp	6	26162279	26167834	0	0.043517
8518	Amp	6	26168015	26211387	0	0.040547
8519	Amp	6	26215518	26238693	0	0.036729
8520	Amp	6	26240421	26261790	0	0.036247
8521	Amp	6	26264494	26355530	0	0.038535
8522	Amp	6	26355940	26471347	0	0.040829
8523	Amp	6	26471368	26631506	0	0.039308
8524	Amp	6	26640944	26664304	0	0.042046
8525	Amp	6	26670022	26670022	0	0.039308
8526	Amp	6	26936980	26995155	0	0.024731
8527	Amp	6	27005687	27009790	0	0.028122
8528	Amp	6	27009939	27019162	0	0.034069
8529	Amp	6	27019229	27129074	0	0.036556
8530	Amp	6	27137053	27203932	0	0.031059
8531	Amp	6	27241863	27269437	0	0.027669
8532	Amp	6	27273177	27276425	0	0.02936

8533	Amp	6	27278013	27293545	0	0.032141
8534	Amp	6	27293592	27295098	0	0.033693
8535	Amp	6	27377994	27411006	0	0.035939
8536	Amp	6	27411630	27458893	0	0.037377
8537	Amp	6	27459178	27509320	0	0.038606
8538	Amp	6	27737819	27742386	0	0.03429
8539	Amp	6	27742470	27765286	0	0.029514
8540	Amp	6	27766322	27784576	0	0.03034
8541	Amp	6	27784749	27866728	0	0.032684
8542	Amp	6	27870667	27874275	0	0.035959
8543	Amp	6	27885174	27904981	0	0.038006
8544	Amp	6	27906149	27911135	0	0.036986
8545	Amp	6	27912262	27912262	0	0.035757
8546	Amp	6	27914501	27914501	0	0.033511
8547	Amp	6	27922371	27935794	0	0.03188
8548	Amp	6	27935820	27938499	0	0.029099
8549	Amp	6	27941027	28065396	0	0.026755
8550	Amp	6	28065618	28065745	0	0.025928
8551	Amp	6	28071528	28111963	0	0.023957
8552	Amp	6	28119039	28120553	0	0.02191
8553	Amp	6	28121235	28135913	0	0.021274
8554	Amp	6	28142182	28199145	0	0.019583
8555	Amp	6	28199257	28208244	0	0.018667
8556	Amp	6	28219590	28414485	0	0.015393
8557	Amp	6	28415812	28455380	0	0.016898
8558	Amp	6	28456946	28872144	0	0.017926
8559	Amp	6	28883860	29055437	0	0.019083
8560	Amp	6	29063365	29228360	0	0.017926
8561	Amp	6	29237690	29292654	0	0.018385
8562	Amp	6	29292964	29349190	0	0.017356
8563	Amp	6	29355089	29459360	0	0.016898
8564	Amp	6	29466165	29639699	0	0.017522
8565	Amp	6	29643212	29765965	0	0.021526
8566	Amp	6	29787674	29800928	0	0.023943
8567	Amp	6	29836041	30067680	0	0.021799
8568	Amp	6	30068694	30254243	0	0.020361
8569	Amp	6	30261161	30352153	0	0.017944
8570	Amp	6	30353280	30395359	0	0.019341
8571	Amp	6	30395574	30407535	0	0.024005
8572	Amp	6	30408710	30499918	0	0.025784
8573	Amp	6	30507638	30574075	0	0.028601
8574	Amp	6	30593947	30611538	0	0.027203
8575	Amp	6	30617994	30758064	0	0.030091
8576	Amp	6	30758971	30766478	0	0.031409
8577	Amp	6	30770669	30889765	0	0.027405
8578	Amp	6	30890211	30945681	0	0.025547
8579	Amp	6	30946775	31004694	0	0.026518
8580	Amp	6	31004812	31037949	0	0.028493
8581	Amp	6	31038315	31176123	0	0.025676
8582	Amp	6	31178143	31199721	0	0.027726
8583	Amp	6	31204878	31238839	0	0.023063
8584	Amp	6	32899215	32899215	0	0.029184
8585	Amp	6	32901268	32903759	0	0.027376

8586 Amp	6	32912768	32913750	0	0.029606
8587 Amp	6	32932241	32932241	0	0.026777
8588 Amp	6	32934157	32955131	0	0.026108
8589 Amp	6	32965445	32991993	0	0.023388
8590 Amp	6	32997190	33008877	0	0.026175
8591 Amp	6	33008982	33017518	0	0.026769
8592 Amp	6	33020918	33206065	0	0.02612
8593 Amp	6	33208047	33250403	0	0.027743
8594 Amp	6	33254386	33334889	0	0.021199
8595 Amp	6	33339017	33475720	0	0.019694
8596 Amp	6	33477070	33578410	0	0.020378
8597 Amp	6	33585593	33619298	0	0.016646
8598 Amp	6	33619545	33622694	0	0.014417
8599 Amp	6	33627622	33817087	0	0.016025
8600 Amp	6	33819729	33892524	0	0.013239
8601 Amp	6	33894856	33894856	0	0.01627
8602 Amp	6	33898282	33925375	0	0.018709
8603 Amp	6	33933038	33962394	0	0.01627
8604 Amp	6	33962411	33977829	0	0.018527
8605 Amp	6	33978144	34101409	0	0.020419
8606 Amp	6	34103746	34157777	0	0.018796
8607 Amp	6	34179117	34229638	0	0.024472
8608 Amp	6	34231241	34274296	0	0.022215
8609 Amp	6	34275736	34418858	0	0.024167
8610 Amp	6	34424954	34433504	0	0.026422
8611 Amp	6	34433601	34540177	0	0.02455
8612 Amp	6	34542011	34587932	0	0.027738
8613 Amp	6	34588204	34588204	0	0.029227
8614 Amp	6	34590343	34634898	0	0.025547
8615 Amp	6	34637054	34709326	0	0.021302
8616 Amp	6	34714830	34749511	0	0.019678
8617 Amp	6	34919539	34985133	0	0.021593
8618 Amp	6	34992869	35026749	0	0.019338
8619 Amp	6	35026909	35103879	0	0.020884
8620 Amp	6	35110830	35146765	0	0.027938
8621 Amp	6	35149586	35150219	0	0.020316
8622 Amp	6	35152597	35163155	0	0.019723
8623 Amp	6	35163822	35313542	0	0.022799
8624 Amp	6	35313559	35378164	0	0.019723
8625 Amp	6	35380213	35408510	0	0.018752
8626 Amp	6	35411440	35611598	0	0.016247
8627 Amp	6	35628168	35654872	0	0.014356
8628 Amp	6	35662236	35774923	0	0.008547
8629 Amp	6	35783853	35810942	0	0.014099
8630 Amp	6	35814390	35852068	0	0.01261
8631 Amp	6	35852130	36107394	0	0.010387
8632 Amp	6	36108133	36189415	0	0.012803
8633 Amp	6	36189877	36271276	0	0.010387
8634 Amp	6	36274256	36299154	0	0.012703
8635 Amp	6	36304263	36581207	0	0.010387
8636 Amp	6	36588239	36623837	0	0.012872
8637 Amp	6	36625858	36908107	0	0.013469
8638 Amp	6	36908566	37065153	0	0.010437

8639	Amp	6	37066076	37225923	0	0.007953
8640	Amp	6	37228068	37347736	0	0.009728
8641	Amp	6	37348202	37671411	0	0.013078
8642	Amp	6	37671515	37744564	0	0.011103
8643	Amp	6	37749349	37817243	0	0.011582
8644	Amp	6	37823455	37841036	0	0.020157
8645	Amp	6	37845507	37953829	0	0.016807
8646	Amp	6	37957130	37977218	0	0.023945
8647	Amp	6	37979871	37982585	0	0.021895
8648	Amp	6	37984018	37993807	0	0.021297
8649	Amp	6	37995532	38018691	0	0.025103
8650	Amp	6	38020088	38057199	0	0.021297
8651	Amp	6	38063684	38069109	0	0.025658
8652	Amp	6	38069741	38123254	0	0.022714
8653	Amp	6	38132561	38174984	0	0.020939
8654	Amp	6	38175410	38187165	0	0.02139
8655	Amp	6	38187858	38238890	0	0.022583
8656	Amp	6	38241833	38261823	0	0.015446
8657	Amp	6	38262453	38287769	0	0.014253
8658	Amp	6	38287876	38288268	0	0.016136
8659	Amp	6	38289829	38304056	0	0.019278
8660	Amp	6	38304875	38307700	0	0.018827
8661	Amp	6	38319928	38353753	0	0.025736
8662	Amp	6	38354257	38433925	0	0.028793
8663	Amp	6	38434845	38443254	0	0.032358
8664	Amp	6	38443467	38448079	0	0.029302
8665	Amp	6	38455595	38535958	0	0.025736
8666	Amp	6	38537927	38574012	0	0.021906
8667	Amp	6	38574861	38695118	0	0.018827
8668	Amp	6	38695668	38865002	0	0.015684
8669	Amp	6	38868985	38892303	0	0.017763
8670	Amp	6	38892317	38992477	0	0.021938
8671	Amp	6	38992777	39062134	0	0.018608
8672	Amp	6	39062625	39099091	0	0.008801
8673	Amp	6	39101136	39285785	0	0.011807
8674	Amp	6	39286463	39316017	0	0.008801
8675	Amp	6	39317537	39323833	0	0.013771
8676	Amp	6	39326122	39357302	0	0.011915
8677	Amp	6	39363732	39399474	0	0.008801
8678	Amp	6	39401792	39426382	0	0.009259
8679	Amp	6	39427366	39481113	0	0.008801
8680	Amp	6	39483293	39649093	0	0.006722
8681	Amp	6	39656058	39676421	0	0.009807
8682	Amp	6	39679554	39729293	0	0.006722
8683	Amp	6	39731623	39887336	0	0.002362
8684	Amp	6	39888696	39979255	0	0.002857
8685	Amp	6	39985764	39994461	0	0.000974
8686	Amp	6	39995298	40124592	0	0.003106
8687	Amp	6	40127573	40223383	0	0.006848
8688	Amp	6	40223540	40314768	0	0.003106
8689	Amp	6	40314943	40452901	0	0.002611
8690	Amp	6	40453598	40460354	0	0.003219
8691	Amp	6	40463265	40475034	0	0.005141

8692	Amp	6	40476195	40706044	0	0.003219
8693	Amp	6	40709039	40731479	0	0.004391
8694	Amp	6	40733547	40795637	0	0.004831
8695	Amp	6	40795727	40824752	0	0.003658
8696	Amp	6	40825162	41132970	0	0.003219
8697	Amp	6	41136914	41157402	0	0.009816
8698	Amp	6	41160234	41424826	0	0.003219
8699	Amp	6	41431376	41493194	0	0.006121
8700	Amp	6	41493657	41506144	0	0.005514
8701	Amp	6	41508446	41623516	0	0.007513
8702	Amp	6	41630134	41641482	0	0.004611
8703	Amp	6	41644846	41645350	0	0.006832
8704	Amp	6	41645628	41724061	0	0.007301
8705	Amp	6	41724957	41818683	0	0.006832
8706	Amp	6	41818942	41930358	0	0.004611
8707	Amp	6	41930826	41939412	0	0.006875
8708	Amp	6	41939533	42067928	0	0.007381
8709	Amp	6	42068768	42098498	0	0.005116
8710	Amp	6	42098675	42191518	0	0.003117
8711	Amp	6	42194265	42765375	0	0.002637
8712	Amp	6	42766353	43258517	0	0.005091
8713	Amp	6	43266578	43363247	0	0.006852
8714	Amp	6	43367612	43495960	0	0.015575
8715	Amp	6	43497094	43512457	0	0.017096
8716	Amp	6	43518172	43522292	0	0.020079
8717	Amp	6	43523071	43605277	0	0.018317
8718	Amp	6	43609032	43637382	0	0.023895
8719	Amp	6	43639063	43689592	0	0.027084
8720	Amp	6	43690000	43712725	0	0.027741
8721	Amp	6	43713017	44336639	0	0.028209
8722	Amp	6	44338154	44420920	0	0.028764
8723	Amp	6	44421450	44439769	0	0.029951
8724	Amp	6	44444875	44444875	0	0.031963
8725	Amp	6	44449251	44486280	0	0.02898
8726	Amp	6	44487005	44508559	0	0.027792
8727	Amp	6	44508815	44650161	0	0.029122
8728	Amp	6	44651278	44692065	0	0.030207
8729	Amp	6	44701803	44812964	0	0.028292
8730	Amp	6	44815162	44988885	0	0.027713
8731	Amp	6	44989202	45020588	0	0.03099
8732	Amp	6	45021782	45116335	0	0.029661
8733	Amp	6	45119992	45344942	0	0.027048
8734	Amp	6	45347481	45360528	0	0.024595
8735	Amp	6	45361210	45412311	0	0.021465
8736	Amp	6	45413847	45430420	0	0.023376
8737	Amp	6	45430794	45617131	0	0.025514
8738	Amp	6	45619076	45833014	0	0.028589
8739	Amp	6	45836253	45846387	0	0.033639
8740	Amp	6	45852033	45929454	0	0.031501
8741	Amp	6	45930265	45957459	0	0.032618
8742	Amp	6	45958767	45980842	0	0.029542
8743	Amp	6	46109269	46109939	0	0.028143
8744	Amp	6	46114237	46267162	0	0.029503

8745	Amp	6	46267266	46383436	0	0.032079
8746	Amp	6	46383699	46399037	0	0.030719
8747	Amp	6	46408084	46574878	0	0.02707
8748	Amp	6	46577310	46606378	0	0.015689
8749	Amp	6	46610331	46650875	0	0.015221
8750	Amp	6	46652515	46822387	0	0.012943
8751	Amp	6	46827547	46972096	0	0.024072
8752	Amp	6	46972202	47027614	0	0.023066
8753	Amp	6	47028482	47119216	0	0.014341
8754	Amp	6	47119471	47177636	0	0.014936
8755	Amp	6	47177829	47239345	0	0.015481
8756	Amp	6	47239365	47269964	0	0.016382
8757	Amp	6	47275030	47364833	0	0.02008
8758	Amp	6	47373047	47485642	0	0.016382
8759	Amp	6	47485719	47635375	0	0.021009
8760	Amp	6	47642609	47761186	0	0.023188
8761	Amp	6	47761314	47953822	0	0.021009
8762	Amp	6	47954900	48334915	0	0.025253
8763	Amp	6	48336469	48472914	0	0.025888
8764	Amp	6	48473485	48587454	0	0.024987
8765	Amp	6	48591132	48617906	0	0.025715
8766	Amp	6	48625354	48633521	0	0.024987
8767	Amp	6	48678504	48753754	0	0.026452
8768	Amp	6	48754240	48807612	0	0.030415
8769	Amp	6	48811979	48859648	0	0.032227
8770	Amp	6	48862825	48871524	0	0.032872
8771	Amp	6	48873542	48881377	0	0.021597
8772	Amp	6	48881770	48889960	0	0.019785
8773	Amp	6	48891611	48915194	0	0.019141
8774	Amp	6	48925235	49102273	0	0.02083
8775	Amp	6	49108768	49113912	0	0.02439
8776	Amp	6	49114669	49414176	0	0.027017
8777	Amp	6	49416145	49430490	0	0.029522
8778	Amp	6	49432271	49547812	0	0.028977
8779	Amp	6	49548360	49868365	0	0.025181
8780	Amp	6	49876956	50291443	0	0.023169
8781	Amp	6	50292708	50423809	0	0.021704
8782	Amp	6	50424762	50477245	0	0.019714
8783	Amp	6	50483299	50483802	0	0.022036
8784	Amp	6	50489930	50497391	0	0.018476
8785	Amp	6	50500086	50549040	0	0.026991
8786	Amp	6	50550076	50565240	0	0.027979
8787	Amp	6	50566772	50660887	0	0.027343
8788	Amp	6	50661100	50681831	0	0.028749
8789	Amp	6	50682426	50690901	0	0.023576
8790	Amp	6	50695596	50760415	0	0.024128
8791	Amp	6	50768150	50783596	0	0.020786
8792	Amp	6	50784122	50803261	0	0.022276
8793	Amp	6	50807005	51021085	0	0.03083
8794	Amp	6	51023192	51120809	0	0.036182
8795	Amp	6	51230728	51266601	0	0.058557
8796	Amp	6	51267516	51343859	0	0.05591
8797	Amp	6	51350430	51367475	0	0.052264

8798	Amp	6	51370918	51420607	0	0.044651
8799	Amp	6	51420862	51431850	0	0.042733
8800	Amp	6	51432245	51453336	0	0.041043
8801	Amp	6	51453768	51465673	0	0.048855
8802	Amp	6	51467364	51483289	0	0.03447
8803	Amp	6	51484456	51545009	0	0.036303
8804	Amp	6	51545525	51595356	0	0.028491
8805	Amp	6	51602814	51627747	0	0.026995
8806	Amp	6	51627807	51725280	0	0.028167
8807	Amp	6	51736138	51888633	0	0.0288
8808	Amp	6	51891634	51967589	0	0.026967
8809	Amp	6	51971300	52224761	0	0.032891
8810	Amp	6	52226788	52235651	0	0.034454
8811	Amp	6	52235942	52259746	0	0.035151
8812	Amp	6	52260284	52357366	0	0.034588
8813	Amp	6	52359460	52387875	0	0.036865
8814	Amp	6	52391214	52392720	0	0.039474
8815	Amp	6	52393748	52453527	0	0.041355
8816	Amp	6	52455756	52457702	0	0.043197
8817	Amp	6	52459346	52459607	0	0.042025
8818	Amp	6	52464712	52486040	0	0.04818
8819	Amp	6	52486805	52490930	0	0.052505
8820	Amp	6	52493901	52499974	0	0.053192
8821	Amp	6	52500691	52616767	0	0.050622
8822	Amp	6	52617359	52642487	0	0.052092
8823	Amp	6	52642749	52649091	0	0.054619
8824	Amp	6	52650280	52801660	0	0.052297
8825	Amp	6	52805291	52821528	0	0.053725
8826	Amp	6	52822132	52822132	0	0.051197
8827	Amp	6	52827326	52880263	0	0.0505
8828	Amp	6	52880424	52914409	0	0.047059
8829	Amp	6	52916762	52933573	0	0.049016
8830	Amp	6	52933618	52963326	0	0.04658
8831	Amp	6	52963415	53083388	0	0.047697
8832	Amp	6	53083857	53112132	0	0.046026
8833	Amp	6	53115811	53124158	0	0.05056
8834	Amp	6	53124224	53225751	0	0.051894
8835	Amp	6	53228517	53253984	0	0.049389
8836	Amp	6	53258320	53275250	0	0.055114
8837	Amp	6	53275898	53345557	0	0.057121
8838	Amp	6	53346027	53429755	0	0.056032
8839	Amp	6	53430405	53491004	0	0.05334
8840	Amp	6	53492684	53495947	0	0.056223
8841	Amp	6	53496478	53568782	0	0.054217
8842	Amp	6	53569720	53594321	0	0.054906
8843	Amp	6	53596331	53682017	0	0.052311
8844	Amp	6	53689948	53750763	0	0.055424
8845	Amp	6	53752932	53822917	0	0.053928
8846	Amp	6	53823686	53913448	0	0.048207
8847	Amp	6	53917398	53935997	0	0.043673
8848	Amp	6	53937509	53951415	0	0.04619
8849	Amp	6	53952880	53970179	0	0.051776
8850	Amp	6	53973964	53977899	0	0.048647

8851	Amp	6	53979470	54039622	0	0.04613
8852	Amp	6	54043152	54043152	0	0.047953
8853	Amp	6	54043590	54060135	0	0.047033
8854	Amp	6	54060151	54112832	0	0.047513
8855	Amp	6	54120101	54136756	0	0.041179
8856	Amp	6	54136887	54136887	0	0.03709
8857	Amp	6	54138546	54296813	0	0.035754
8858	Amp	6	54299281	54320459	0	0.038966
8859	Amp	6	54320891	54426023	0	0.03833
8860	Amp	6	54434672	54460149	0	0.034005
8861	Amp	6	54468484	54519754	0	0.030792
8862	Amp	6	54521444	54521444	0	0.028039
8863	Amp	6	54524075	54557275	0	0.029041
8864	Amp	6	54558281	54593758	0	0.026157
8865	Amp	6	54597930	54598584	0	0.026645
8866	Amp	6	54602835	54617833	0	0.027941
8867	Amp	6	54619366	54665663	0	0.028849
8868	Amp	6	54667163	54684422	0	0.035193
8869	Amp	6	54693583	54713240	0	0.034206
8870	Amp	6	54713280	54759003	0	0.037779
8871	Amp	6	54766383	54777930	0	0.035195
8872	Amp	6	54783094	54935691	0	0.039652
8873	Amp	6	54936076	54947102	0	0.043478
8874	Amp	6	54952675	54994909	0	0.039905
8875	Amp	6	54999958	54999958	0	0.030596
8876	Amp	6	55000254	55077597	0	0.029067
8877	Amp	6	55079858	55079858	0	0.0308
8878	Amp	6	55080679	55104637	0	0.025231
8879	Amp	6	55105203	55142580	0	0.02629
8880	Amp	6	55144627	55153388	0	0.026746
8881	Amp	6	55153662	55169703	0	0.027752
8882	Amp	6	55173497	55204196	0	0.038979
8883	Amp	6	55209476	55209476	0	0.037246
8884	Amp	6	55211662	55275320	0	0.040718
8885	Amp	6	55276386	55287857	0	0.043589
8886	Amp	6	55289218	55377179	0	0.044845
8887	Amp	6	55377468	55390939	0	0.044213
8888	Amp	6	55391030	55459132	0	0.042956
8889	Amp	6	55462700	55468617	0	0.044366
8890	Amp	6	55468791	55497136	0	0.041495
8891	Amp	6	55500683	55532168	0	0.040489
8892	Amp	6	55539284	55570243	0	0.030581
8893	Amp	6	55574853	55597403	0	0.034605
8894	Amp	6	55597489	55603664	0	0.033286
8895	Amp	6	55608279	55633277	0	0.034799
8896	Amp	6	55633696	55673557	0	0.033286
8897	Amp	6	55673861	55690574	0	0.034833
8898	Amp	6	55693268	55797913	0	0.033423
8899	Amp	6	55798419	55801216	0	0.0314
8900	Amp	6	55808294	55882919	0	0.027929
8901	Amp	6	55883955	55900142	0	0.028643
8902	Amp	6	55902078	55912558	0	0.032951
8903	Amp	6	55916475	55997401	0	0.029978

8904	Amp	6	56001293	56091471	0	0.028643
8905	Amp	6	56094199	56194596	0	0.032297
8906	Amp	6	56198599	56295010	0	0.035069
8907	Amp	6	56297875	56309415	0	0.038015
8908	Amp	6	56309734	56319870	0	0.037187
8909	Amp	6	56321645	56457022	0	0.037952
8910	Amp	6	56460844	56461967	0	0.040622
8911	Amp	6	56462101	56464867	0	0.037851
8912	Amp	6	56467858	56506900	0	0.036361
8913	Amp	6	56509121	56509820	0	0.037653
8914	Amp	6	56512649	56594678	0	0.037197
8915	Amp	6	56600875	56618003	0	0.05158
8916	Amp	6	56619832	56672549	0	0.052852
8917	Amp	6	56672921	56912755	0	0.054796
8918	Amp	6	56919031	56932884	0	0.051141
8919	Amp	6	56948388	56958627	0	0.054841
8920	Amp	6	56960828	56974365	0	0.047016
8921	Amp	6	56980657	56985284	0	0.043316
8922	Amp	6	56988354	57035193	0	0.046104
8923	Amp	6	57036246	57128330	0	0.043158
8924	Amp	6	57128992	57200709	0	0.041334
8925	Amp	6	57208219	57229219	0	0.041774
8926	Amp	6	57229897	57252826	0	0.04122
8927	Amp	6	57253043	57276595	0	0.040779
8928	Amp	6	57277368	57341687	0	0.045976
8929	Amp	6	57347009	57347009	0	0.044134
8930	Amp	6	61527062	61527062	0.050473	0.066092
8931	Amp	6	61530738	61530738	0	0.063093
8932	Amp	6	61596503	61596503	0.287738	0.075882
8933	Amp	6	61596563	61624695	0.271552	0.075195
8934	Amp	6	61625183	61625183	0.251218	0.074459
8935	Amp	6	61625407	61633719	0.386426	0.079335
8936	Amp	6	61634159	61659801	0.368062	0.07862
8937	Amp	6	61679520	61699746	0.255918	0.074643
8938	Amp	6	61699774	61699774	0.335313	0.077525
8939	Amp	6	61823500	61823500	0.404718	0.080066
8940	Amp	6	61823514	61845242	0.431198	0.080906
8941	Amp	6	61847101	61867135	0.444474	0.08141
8942	Amp	6	61871118	61890013	0.529451	0.08412
8943	Amp	6	61890750	61901790	0.379885	0.079245
8944	Amp	6	61909886	61920958	0.073902	0.067192
8945	Amp	6	61925382	61951771	0.164935	0.070994
8946	Amp	6	61953890	61970734	0.14896	0.070317
8947	Amp	6	61972739	62044130	0.210594	0.072599
8948	Amp	6	62044775	62065970	0.19964	0.072112
8949	Amp	6	62066042	62115040	0.276783	0.075544
8950	Amp	6	62119439	62130381	0.246818	0.074248
8951	Amp	6	62137529	62269585	0.164935	0.070816
8952	Amp	6	62272192	62285229	0.215608	0.07295
8953	Amp	6	62285478	62285478	0.246818	0.074117
8954	Amp	6	62285566	62344324	0.1237	0.069096
8955	Amp	6	62345031	62345031	0.060686	0.066555
8956	Amp	6	62345258	62390090	0.040093	0.065715

8957	Amp	6	62391172	62401906	0	0.063581
8958	Amp	6	62406586	62436201	0.005328	0.064177
8959	Amp	6	62436700	62535309	0.044794	0.065894
8960	Amp	6	62539558	62573676	0.034113	0.06539
8961	Amp	6	62574592	62575461	0.050473	0.066196
8962	Amp	6	62576066	62599770	0.010261	0.064479
8963	Amp	6	62605280	62608480	0	0.062347
8964	Amp	6	62611963	62627977	0.024438	0.065026
8965	Amp	6	62628313	62684105	0.118001	0.068998
8966	Amp	6	62685255	62767102	0.029031	0.065234
8967	Amp	6	62770737	62808629	0	0.062109
8968	Amp	6	62811699	62838954	0	0.059664
8969	Amp	6	62872346	62876877	0	0.063081
8970	Amp	6	62888548	62921906	0.010261	0.064441
8971	Amp	6	62922566	63000624	0.08435	0.067685
8972	Amp	6	63000931	63019295	0.107017	0.068561
8973	Amp	6	63022447	63022784	0.133193	0.0695
8974	Amp	6	63024941	63044706	0.112543	0.068624
8975	Amp	6	63045495	63074629	0.138391	0.069863
8976	Amp	6	63080202	63131122	0.17597	0.071369
8977	Amp	6	63131929	63161343	0.215608	0.072928
8978	Amp	6	63165349	63242450	0.431198	0.080976
8979	Amp	6	63246073	63277079	0.386426	0.07947
8980	Amp	6	63280189	63283523	0.299433	0.076226
8981	Amp	6	63284915	63348992	0.255918	0.074655
8982	Amp	6	63352480	63363724	0.222041	0.073096
8983	Amp	6	63368221	63374411	0.187449	0.071858
8984	Amp	6	63374801	63416492	0.299433	0.076313
8985	Amp	6	63422855	63463112	0.34892	0.078034
8986	Amp	6	63469247	63540620	0.404718	0.080062
8987	Amp	6	63544998	63605276	0.589605	0.086094
8988	Amp	6	63605305	63622948	0.630438	0.087451
8989	Amp	6	63626218	63722523	0.550755	0.084741
8990	Amp	6	63722961	63819142	0.494792	0.08302
8991	Amp	6	63824054	63847037	0.583752	0.085841
8992	Amp	6	63848107	63948198	1.131695	0.100347
8993	Amp	6	63948759	63949509	0.651644	0.088069
8994	Amp	6	63954851	63992209	0.589605	0.086041
8995	Amp	6	63992741	64003645	0.543344	0.084602
8996	Amp	6	64011913	64021263	0.47716	0.082374
8997	Amp	6	64021831	64044347	0.515676	0.08376
8998	Amp	6	64049702	64130256	0.431198	0.080939
8999	Amp	6	64136647	64164795	0.368062	0.078685
9000	Amp	6	64169223	64175445	0.379885	0.079161
9001	Amp	6	64175965	64190656	0.34892	0.077994
9002	Amp	6	64191455	64310578	0.311747	0.076727
9003	Amp	6	64322029	64409255	0	0.060795
9004	Amp	6	64409679	64409679	0	0.062107
9005	Amp	6	64412548	64424223	0	0.060749
9006	Amp	6	64424278	64454879	0	0.059586
9007	Amp	6	64457222	64483927	0	0.061893
9008	Amp	6	64483940	64486558	0	0.061087
9009	Amp	6	64489352	64558681	0	0.060611

9010	Amp	6	64559615	64714113	0	0.062212
9011	Amp	6	64714132	64773422	0	0.061652
9012	Amp	6	64774549	64778133	0	0.06005
9013	Amp	6	64783410	64809019	0	0.061942
9014	Amp	6	64815002	64840963	0.034113	0.065559
9015	Amp	6	64847192	64848670	0	0.061942
9016	Amp	6	64848788	64904868	0	0.060472
9017	Amp	6	64904886	64921233	0	0.062136
9018	Amp	6	64930766	64937688	0.005328	0.064158
9019	Amp	6	64938212	64947631	0.112543	0.068731
9020	Amp	6	64949318	64977414	0.079652	0.06742
9021	Amp	6	64980413	65033972	0.164935	0.070918
9022	Amp	6	65037800	65039756	0.204831	0.072468
9023	Amp	6	65049683	65057727	0.138391	0.06978
9024	Amp	6	65061143	65067457	0.100827	0.06823
9025	Amp	6	65068828	65075622	0.079652	0.067364
9026	Amp	6	65106606	65112100	0.065715	0.0668
9027	Amp	6	65112412	65112412	0.019169	0.064908
9028	Amp	6	65213064	65220792	0	0.05889
9029	Amp	6	65227322	65227481	0	0.059571
9030	Amp	6	65231005	65285086	0.001323	0.063869
9031	Amp	6	65287172	65291856	0	0.061848
9032	Amp	6	65302362	65324815	0	0.063013
9033	Amp	6	65334666	65389388	0	0.061848
9034	Amp	6	65390396	65403785	0	0.063135
9035	Amp	6	65404018	65417358	0.005328	0.064058
9036	Amp	6	65417371	65426563	0	0.063598
9037	Amp	6	65437777	65516233	0.056211	0.066233
9038	Amp	6	65519351	65526247	0	0.053729
9039	Amp	6	65527219	65550084	0.133193	0.069665
9040	Amp	6	65556072	65566563	0.112543	0.068742
9041	Amp	6	65567523	65567523	0.040093	0.065753
9042	Amp	6	65568350	65568350	0.060686	0.066595
9043	Amp	6	65570944	65604257	0	0.062297
9044	Amp	6	65609224	65638729	0	0.061788
9045	Amp	6	65642708	65661440	0.029031	0.065337
9046	Amp	6	65670018	65678932	0.060686	0.066659
9047	Amp	6	65679058	65679198	0.019169	0.06492
9048	Amp	6	65703930	65703930	0.424557	0.080774
9049	Amp	6	65706759	65707491	0.392611	0.079486
9050	Amp	6	65708043	65846040	0.287738	0.075937
9051	Amp	6	65854913	65942486	0.246818	0.074177
9052	Amp	6	65943726	65950539	0.112543	0.068618
9053	Amp	6	65951304	65989654	0.133193	0.069674
9054	Amp	6	65989689	66034430	0.19964	0.072251
9055	Amp	6	66037813	66042187	0.282828	0.07576
9056	Amp	6	66043742	66060052	0.238246	0.073684
9057	Amp	6	66060161	66128877	0.14896	0.070334
9058	Amp	6	66138080	66139151	0.233782	0.073597
9059	Amp	6	66140043	66155714	0.282828	0.075645
9060	Amp	6	66156016	66156016	0.335313	0.077704
9061	Amp	6	66165740	66191130	0.362016	0.078553
9062	Amp	6	66194514	66194514	0.529451	0.084127

9063	Amp	6	66195850	66205120	0.602643	0.086535
9064	Amp	6	66210837	66217529	0.751221	0.090934
9065	Amp	6	66221195	66273094	0.373599	0.078833
9066	Amp	6	66276504	66283625	0.34892	0.077984
9067	Amp	6	66403730	66513403	0.238246	0.073821
9068	Amp	6	66514709	66514709	0.251218	0.074292
9069	Amp	6	66517452	66599041	0.287738	0.075964
9070	Amp	6	66606737	66712197	0.238246	0.073791
9071	Amp	6	66715178	66721145	0.227787	0.07332
9072	Amp	6	66723572	66777921	0.368062	0.078777
9073	Amp	6	66784197	66809449	0.437198	0.081082
9074	Amp	6	66809791	66832891	0.563096	0.085282
9075	Amp	6	66834711	66854173	0.494792	0.082977
9076	Amp	6	66856355	66869945	0.470509	0.082262
9077	Amp	6	66874457	66884661	0.437198	0.081205
9078	Amp	6	66886356	66901872	0.457501	0.081786
9079	Amp	6	66902034	66995037	0.379885	0.079151
9080	Amp	6	66995723	67065601	0.282828	0.075642
9081	Amp	6	67068373	67068634	0.30524	0.076497
9082	Amp	6	67075771	67121775	0.4115	0.080297
9083	Amp	6	67122094	67126784	0.386426	0.079443
9084	Amp	6	67127000	67150737	0.431198	0.080889
9085	Amp	6	67155471	67221044	0.316877	0.076957
9086	Amp	6	67221132	67230339	0.259438	0.074779
9087	Amp	6	67235236	67301679	0	0.058843
9088	Amp	6	67306604	67356591	0	0.062099
9089	Amp	6	67361748	67362892	0.034113	0.065367
9090	Amp	6	67375097	67464657	0	0.059883
9091	Amp	6	67467585	67495053	0	0.056615
9092	Amp	6	67495070	67548149	0	0.055655
9093	Amp	6	67551268	67585690	0	0.056491
9094	Amp	6	67586455	67594347	0	0.056952
9095	Amp	6	67596990	67815618	0	0.062128
9096	Amp	6	67815700	67821077	0	0.057397
9097	Amp	6	67821727	67835409	0	0.057949
9098	Amp	6	67839014	67948894	0	0.059518
9099	Amp	6	67954505	67988778	0	0.058683
9100	Amp	6	67990740	68039606	0	0.054981
9101	Amp	6	68040748	68109977	0	0.050784
9102	Amp	6	68110582	68116378	0	0.054307
9103	Amp	6	68117346	68118457	0	0.053226
9104	Amp	6	68120844	68126946	0	0.055525
9105	Amp	6	68127798	68148903	0	0.052002
9106	Amp	6	68152001	68185242	0	0.054442
9107	Amp	6	68186748	68207968	0	0.052002
9108	Amp	6	68210408	68257668	0	0.054188
9109	Amp	6	68257987	68303616	0	0.052002
9110	Amp	6	68304234	68353071	0	0.054917
9111	Amp	6	68361223	68375470	0	0.053531
9112	Amp	6	68379251	68450368	0	0.054084
9113	Amp	6	68466884	68504798	0	0.056718
9114	Amp	6	68505751	68505751	0	0.059673
9115	Amp	6	68513342	68558014	0	0.061268

9116	Amp	6	68561548	68588768	0.322897	0.077204
9117	Amp	6	68590456	68590456	0.282828	0.075696
9118	Amp	6	68593914	68644632	0.470509	0.082207
9119	Amp	6	68645557	68667501	0.457501	0.08174
9120	Amp	6	68669284	68673917	0.164935	0.070951
9121	Amp	6	68674504	68695479	0.215608	0.072876
9122	Amp	6	68700162	68704730	0.187449	0.07179
9123	Amp	6	68706387	68722727	0	0.063016
9124	Amp	6	68723885	68854161	0	0.061422
9125	Amp	6	68854754	68922335	0.005328	0.064058
9126	Amp	6	68922652	69059688	0	0.063506
9127	Amp	6	69062043	69166178	0.019169	0.064918
9128	Amp	6	69169290	69178156	0	0.061151
9129	Amp	6	69184202	69206878	0	0.059226
9130	Amp	6	69212676	69329888	0	0.052607
9131	Amp	6	69330257	69397475	0	0.050548
9132	Amp	6	69400059	69461909	0	0.048997
9133	Amp	6	69476612	69491373	0	0.033061
9134	Amp	6	69522312	69533129	0	0.026061
9135	Amp	6	69561376	69719378	0	0.029101
9136	Amp	6	69721559	69749389	0	0.027689
9137	Amp	6	69754539	69760761	0	0.025055
9138	Amp	6	69763489	69786123	0	0.028313
9139	Amp	6	69791621	69883911	0	0.025397
9140	Amp	6	69884522	70004679	0	0.02214
9141	Amp	6	70006660	70018310	0	0.024932
9142	Amp	6	70018780	70110591	0	0.024444
9143	Amp	6	70110870	70176892	0	0.027127
9144	Amp	6	70178784	70194351	0	0.02449
9145	Amp	6	70194470	70199369	0	0.02788
9146	Amp	6	70199631	70327797	0	0.02484
9147	Amp	6	70328066	70397307	0	0.025891
9148	Amp	6	70400766	70446318	0	0.02484
9149	Amp	6	70446523	70488642	0	0.021451
9150	Amp	6	70489517	70520576	0	0.024489
9151	Amp	6	70520998	70538700	0	0.025654
9152	Amp	6	70553840	70568533	0	0.02371
9153	Amp	6	70577251	70599508	0	0.026883
9154	Amp	6	70601086	70724624	0	0.029296
9155	Amp	6	70729808	70743966	0	0.031238
9156	Amp	6	70744545	70782309	0	0.0282
9157	Amp	6	70788205	70794155	0	0.032808
9158	Amp	6	70795323	70814248	0	0.031643
9159	Amp	6	70822403	70864461	0	0.047579
9160	Amp	6	70868855	70885448	0	0.056285
9161	Amp	6	70886325	70886325	0	0.047579
9162	Amp	6	71110207	71111518	0	0.039894
9163	Amp	6	71111816	71125815	0	0.037212
9164	Amp	6	71125862	71137746	0	0.03404
9165	Amp	6	71137919	71160477	0	0.03521
9166	Amp	6	71163131	71205611	0	0.03404
9167	Amp	6	71205740	71257706	0	0.035195
9168	Amp	6	71258042	71385752	0	0.036364

9169	Amp	6	71387157	71415350	0	0.03921
9170	Amp	6	71417350	71544988	0	0.036364
9171	Amp	6	71565028	71808941	0	0.034422
9172	Amp	6	71810697	71823356	0	0.03801
9173	Amp	6	71834939	71869064	0	0.034422
9174	Amp	6	71869282	72054361	0	0.03387
9175	Amp	6	72056220	72073460	0	0.031571
9176	Amp	6	72076690	72166170	0	0.033927
9177	Amp	6	72168958	72193085	0	0.034444
9178	Amp	6	72195046	72240544	0	0.031652
9179	Amp	6	72242156	72334871	0	0.030974
9180	Amp	6	72335206	72518705	0	0.031614
9181	Amp	6	72521720	72527495	0	0.036517
9182	Amp	6	72529265	72555590	0	0.038002
9183	Amp	6	72576304	72647091	0	0.022066
9184	Amp	6	72647857	72681442	0	0.019711
9185	Amp	6	72682595	72786547	0	0.020419
9186	Amp	6	72787573	72787573	0	0.019711
9187	Amp	6	72789894	72834777	0	0.022312
9188	Amp	6	72835012	72886710	0	0.019711
9189	Amp	6	72888582	72900900	0	0.018542
9190	Amp	6	72900910	73096377	0	0.019395
9191	Amp	6	73096686	73128693	0	0.020673
9192	Amp	6	73129202	73138535	0	0.022112
9193	Amp	6	73138686	73171600	0	0.021259
9194	Amp	6	73172276	73186626	0	0.020104
9195	Amp	6	73190405	73241715	0	0.018822
9196	Amp	6	73242264	73424476	0	0.017544
9197	Amp	6	73427188	73670509	0	0.013923
9198	Amp	6	73671116	73876472	0	0.017254
9199	Amp	6	73876718	73915232	0	0.015025
9200	Amp	6	73915306	74026424	0	0.017896
9201	Amp	6	74027021	74221858	0	0.019975
9202	Amp	6	74227475	74277217	0	0.017896
9203	Amp	6	74277630	74437777	0	0.013079
9204	Amp	6	74439426	74456226	0	0.01164
9205	Amp	6	74456234	74653517	0	0.012559
9206	Amp	6	74653579	74654319	0	0.015861
9207	Amp	6	74655132	74675334	0	0.019137
9208	Amp	6	74675387	74703654	0	0.021347
9209	Amp	6	74703681	74708263	0	0.018071
9210	Amp	6	74713183	74757249	0	0.019962
9211	Amp	6	74760037	74766314	0	0.018071
9212	Amp	6	74774828	74861642	0	0.0152
9213	Amp	6	74865955	74867381	0	0.017365
9214	Amp	6	74880313	74880501	0	0.014062
9215	Amp	6	74880710	74915535	0	0.013143
9216	Amp	6	74915887	75049982	0	0.015953
9217	Amp	6	75057132	75066980	0	0.019561
9218	Amp	6	75067268	75095067	0	0.015953
9219	Amp	6	75097848	75122372	0	0.013143
9220	Amp	6	75124532	75168469	0	0.01532
9221	Amp	6	75168709	75184767	0	0.013143

9222	Amp	6	75187303	75517397	0	0.010979
9223	Amp	6	75520361	75840121	0	0.012202
9224	Amp	6	75843059	75876820	0	0.01358
9225	Amp	6	75878388	75909532	0	0.014447
9226	Amp	6	75917039	75970727	0	0.017737
9227	Amp	6	75971861	76182957	0	0.014447
9228	Amp	6	76183927	76448435	0	0.01587
9229	Amp	6	76452054	76555022	0	0.015003
9230	Amp	6	76563103	76599200	0	0.017135
9231	Amp	6	76618975	76627766	0	0.019422
9232	Amp	6	76642347	76660336	0	0.017922
9233	Amp	6	76667089	76852229	0	0.023757
9234	Amp	6	76852956	76875593	0	0.021625
9235	Amp	6	76879514	76956084	0	0.025472
9236	Amp	6	76965155	77082368	0	0.024049
9237	Amp	6	77093112	77528187	0	0.020203
9238	Amp	6	77536799	77754705	0	0.024839
9239	Amp	6	77759738	78122001	0	0.02346
9240	Amp	6	78122840	78128942	0	0.027566
9241	Amp	6	78345474	78366606	0	0.031298
9242	Amp	6	78366615	78438281	0	0.035242
9243	Amp	6	78443367	78505821	0	0.040847
9244	Amp	6	78516652	78539320	0	0.04134
9245	Amp	6	78541947	78640995	0	0.040847
9246	Amp	6	78643000	78643000	0	0.03856
9247	Amp	6	78643288	78701206	0	0.039007
9248	Amp	6	78704977	78716607	0	0.054944
9249	Amp	6	78719746	78770151	0	0.039007
9250	Amp	6	78772738	78897340	0	0.043463
9251	Amp	6	78898020	78963702	0	0.053146
9252	Amp	6	78965250	79035570	0	0.050936
9253	Amp	6	79036359	79127700	0	0.04648
9254	Amp	6	79129634	79270210	0	0.044014
9255	Amp	6	79270696	79279206	0	0.035239
9256	Amp	6	79282688	79319091	0	0.026659
9257	Amp	6	79319455	79319455	0	0.02887
9258	Amp	6	79325779	79400604	0	0.030921
9259	Amp	6	79471226	79471226	0	0.028711
9260	Amp	6	79471639	79695138	0	0.02548
9261	Amp	6	79699277	79704768	0	0.021748
9262	Amp	6	79705711	79705711	0	0.013309
9263	Amp	6	79709318	79781011	0	0.011513
9264	Amp	6	79781078	79842717	0	0.010221
9265	Amp	6	79860950	80011315	0	0.005505
9266	Amp	6	80016692	80069504	0	0.007925
9267	Amp	6	80071119	80174716	0	0.008639
9268	Amp	6	80178962	80440448	0	0.006219
9269	Amp	6	80441374	80501169	0	0.011096
9270	Amp	6	80501502	80799671	0	0.004896
9271	Amp	6	80800062	80994423	0	0.00793
9272	Amp	6	80995627	81121498	0	0.004896
9273	Amp	6	81121602	81214235	0	0.00615
9274	Amp	6	81216243	81691395	0	0.004896

9275	Amp	6	81694338	81816321	0	0.008081
9276	Amp	6	81817600	81933483	0	0.008544
9277	Amp	6	81936385	82446773	0	0.009748
9278	Amp	6	82447260	82618769	0	0.0113
9279	Amp	6	82619439	82859924	0	0.009748
9280	Amp	6	82861801	82942394	0	0.011561
9281	Amp	6	82943278	82994814	0	0.009748
9282	Amp	6	82995015	83098201	0	0.011315
9283	Amp	6	83098474	83202082	0	0.009748
9284	Amp	6	83206350	83254076	0	0.010566
9285	Amp	6	83256838	83272632	0	0.009852
9286	Amp	6	83283469	83351446	0	0.010974
9287	Amp	6	83352862	83412163	0	0.00993
9288	Amp	6	83413631	83644298	0	0.008807
9289	Amp	6	83646523	83917596	0	0.009658
9290	Amp	6	83923415	84345173	0	0.00884
9291	Amp	6	84349208	84619819	0	0.00799
9292	Amp	6	84622162	84784623	0	0.005707
9293	Amp	6	84784635	84903781	0	0.003567
9294	Amp	6	84904555	85648218	0	0.002306
9295	Amp	6	85650562	85757821	0	0.014424
9296	Amp	6	85864821	86067234	0	0.004264
9297	Amp	6	86069366	86094608	0	0.002306
9298	Amp	6	86098979	86830734	0	0.015647
9299	Amp	6	86835363	86992617	0	0.004582
9300	Amp	6	87006819	87170391	0	0.002306
9301	Amp	6	87170972	87642318	0	0.001103
9302	Amp	6	87647919	87709934	0	0.002832
9303	Amp	6	87710388	87919404	0	0.00459
9304	Amp	6	87922811	88048576	0	0.002832
9305	Amp	6	88054258	88100211	0	0.006122
9306	Amp	6	88104870	88360764	0	0.008131
9307	Amp	6	88364469	88405514	0	0.009087
9308	Amp	6	88405739	88516935	0	0.007077
9309	Amp	6	88517429	88597603	0	0.015712
9310	Amp	6	88602432	88691504	0	0.008625
9311	Amp	6	88701303	88722794	0	0.006895
9312	Amp	6	88731310	88924172	0	0.003071
9313	Amp	6	88924424	89018502	0	0.018108
9314	Amp	6	89020616	89205415	0	0.003071
9315	Amp	6	89206922	89584369	0	0.004343
9316	Amp	6	89584845	89655619	0	0.010474
9317	Amp	6	89658745	89678429	0	0.024718
9318	Amp	6	89685297	89715929	0	0.025886
9319	Amp	6	89718013	89843222	0	0.011642
9320	Amp	6	89847134	89961395	0	0.010629
9321	Amp	6	89964327	90021445	0	0.009461
9322	Amp	6	90021726	90118692	0	0.006694
9323	Amp	6	90126127	90184181	0	0.008968
9324	Amp	6	90185088	90240672	0	0.01063
9325	Amp	6	90244254	90426659	0	0.018573
9326	Amp	6	90427432	90590227	0	0.016911
9327	Amp	6	90591645	90647202	0	0.018156

9328	Amp	6	90650590	90692315	0	0.021054
9329	Amp	6	90693047	90909043	0	0.019878
9330	Amp	6	90909887	90955290	0	0.020914
9331	Amp	6	90957762	90977552	0	0.021511
9332	Amp	6	90981728	91086870	0	0.020914
9333	Amp	6	91087768	91162883	0	0.019878
9334	Amp	6	91163151	91202498	0	0.01878
9335	Amp	6	91204623	91423081	0	0.022721
9336	Amp	6	91426328	91460256	0	0.025239
9337	Amp	6	91460292	91473530	0	0.024776
9338	Amp	6	91477012	91554304	0	0.020835
9339	Amp	6	91554768	91596640	0	0.022854
9340	Amp	6	91597226	91601192	0	0.020807
9341	Amp	6	91601461	91734702	0	0.017276
9342	Amp	6	91734786	91791066	0	0.015559
9343	Amp	6	91797124	91907397	0	0.012518
9344	Amp	6	91910962	91985208	0	0.014767
9345	Amp	6	91989840	92042990	0	0.018397
9346	Amp	6	92044005	92096935	0	0.020916
9347	Amp	6	92098739	92121755	0	0.025914
9348	Amp	6	92124014	92125044	0	0.028795
9349	Amp	6	92125414	92133145	0	0.023797
9350	Amp	6	92139559	92153916	0	0.020916
9351	Amp	6	92154331	92176735	0	0.018667
9352	Amp	6	92181700	92227885	0	0.015037
9353	Amp	6	92231388	92330377	0	0.012138
9354	Amp	6	92334967	92370150	0	0.021368
9355	Amp	6	92372955	92462653	0	0.018245
9356	Amp	6	92462922	92480980	0	0.021234
9357	Amp	6	92481088	92621711	0	0.023898
9358	Amp	6	92624553	92806985	0	0.027397
9359	Amp	6	92807414	92811558	0	0.028796
9360	Amp	6	92811754	92961920	0	0.023734
9361	Amp	6	92961956	92991838	0	0.022307
9362	Amp	6	92992423	93014304	0	0.018808
9363	Amp	6	93016109	93047350	0	0.022313
9364	Amp	6	93048455	93070015	0	0.024526
9365	Amp	6	93070925	93079048	0	0.022313
9366	Amp	6	93080847	93096322	0	0.020574
9367	Amp	6	93097102	93161911	0	0.01707
9368	Amp	6	93162350	93452086	0	0.017737
9369	Amp	6	93452246	93579966	0	0.017098
9370	Amp	6	93580180	93580180	0	0.018309
9371	Amp	6	93582124	93656613	0	0.01965
9372	Amp	6	93658754	93768888	0	0.022318
9373	Amp	6	94556105	94556105	0	0.025207
9374	Amp	6	94563946	94563946	0	0.016504
9375	Amp	6	94564604	94588794	0	0.018509
9376	Amp	6	94594509	94617621	0	0.011643
9377	Amp	6	94620977	95386776	0	0.012931
9378	Amp	6	95388284	95444034	0	0.014243
9379	Amp	6	95445284	95544553	0	0.013575
9380	Amp	6	95547782	95554313	0	0.012263

9381	Amp	6	95555221	95583151	0	0.010258
9382	Amp	6	95584227	95604854	0	0.015146
9383	Amp	6	95607059	95724994	0	0.012789
9384	Amp	6	95728188	95767895	0	0.014215
9385	Amp	6	95770448	95782890	0	0.019384
9386	Amp	6	95787128	95806591	0	0.021006
9387	Amp	6	95808798	95875854	0	0.024686
9388	Amp	6	95879420	95903305	0	0.020511
9389	Amp	6	95904723	96014292	0	0.024458
9390	Amp	6	96014836	96030804	0	0.022836
9391	Amp	6	96031145	96057929	0	0.018161
9392	Amp	6	96062367	96204047	0	0.016417
9393	Amp	6	96204521	96264302	0	0.019292
9394	Amp	6	96270562	96315322	0	0.016417
9395	Amp	6	96319189	96345302	0	0.019811
9396	Amp	6	96347814	96347814	0	0.016417
9397	Amp	6	96349873	96520649	0	0.018086
9398	Amp	6	96521352	96663814	0	0.026382
9399	Amp	6	96664496	96714812	0	0.031812
9400	Amp	6	96717560	96725929	0	0.033486
9401	Amp	6	96726033	96754050	0	0.031539
9402	Amp	6	96754868	96760583	0	0.026649
9403	Amp	6	96761359	96864655	0	0.027839
9404	Amp	6	96864683	96899863	0	0.035786
9405	Amp	6	96903304	96912427	0	0.027157
9406	Amp	6	96915413	96918884	0	0.025483
9407	Amp	6	96919065	97006107	0	0.028751
9408	Amp	6	97006706	97140834	0	0.03296
9409	Amp	6	97146876	97278792	0	0.029971
9410	Amp	6	97280691	97375113	0	0.024598
9411	Amp	6	97387384	97403016	0	0.029894
9412	Amp	6	97403781	97567476	0	0.028704
9413	Amp	6	97568064	97827222	0	0.023409
9414	Amp	6	97828658	97956027	0	0.020797
9415	Amp	6	97956123	97958928	0	0.029078
9416	Amp	6	97960594	98079596	0	0.029998
9417	Amp	6	98082680	98117974	0	0.030627
9418	Amp	6	98118465	98423162	0	0.029363
9419	Amp	6	98423304	98423304	0	0.030796
9420	Amp	6	98424645	98645195	0	0.029877
9421	Amp	6	98649911	98765859	0	0.038364
9422	Amp	6	98766857	98908859	0	0.028485
9423	Amp	6	98909954	99104289	0	0.024865
9424	Amp	6	99106851	99154295	0	0.027947
9425	Amp	6	99162100	99182162	0	0.026214
9426	Amp	6	99184010	99226105	0	0.024865
9427	Amp	6	99227479	99251042	0	0.027793
9428	Amp	6	99254694	99288757	0	0.030863
9429	Amp	6	99291146	99335734	0	0.022334
9430	Amp	6	99339452	99388206	0	0.021557
9431	Amp	6	99397050	99496466	0	0.018488
9432	Amp	6	99498741	99532646	0	0.026449
9433	Amp	6	99541166	99987003	0	0.028763

9434	Amp	6	99988939	99999914	0	0.039073
9435	Amp	6	100007593	100089587	0	0.026792
9436	Amp	6	100090418	100113028	0	0.026021
9437	Amp	6	100119438	100179616	0	0.02981
9438	Amp	6	100184805	100493600	0	0.03119
9439	Amp	6	100494341	100577308	0	0.027401
9440	Amp	6	100588246	100686201	0	0.03029
9441	Amp	6	100686407	100686407	0	0.029661
9442	Amp	6	100690714	100705789	0	0.038407
9443	Amp	6	100782506	100782506	0	0.035518
9444	Amp	6	100782984	101033873	0	0.029325
9445	Amp	6	101045849	101289669	0	0.029795
9446	Amp	6	101295610	101341710	0	0.027482
9447	Amp	6	101346135	101513145	0	0.019172
9448	Amp	6	101513295	101527754	0	0.015905
9449	Amp	6	101528415	101547591	0	0.019625
9450	Amp	6	101550755	101563137	0	0.015905
9451	Amp	6	101566703	101590007	0	0.028944
9452	Amp	6	101595078	101849315	0	0.033435
9453	Amp	6	101855053	101860967	0	0.035675
9454	Amp	6	101861196	101919521	0	0.033435
9455	Amp	6	101924727	101936908	0	0.036957
9456	Amp	6	101936962	102068385	0	0.038206
9457	Amp	6	102069224	102094324	0	0.040091
9458	Amp	6	102097766	102117543	0	0.039621
9459	Amp	6	102161223	102161474	0	0.032614
9460	Amp	6	102169001	102194459	0	0.029092
9461	Amp	6	102197568	102318836	0	0.027843
9462	Amp	6	102320021	102357443	0	0.034851
9463	Amp	6	102364406	102385980	0	0.040341
9464	Amp	6	102387726	102453782	0	0.03872
9465	Amp	6	102461230	102506638	0	0.03323
9466	Amp	6	102510120	102523905	0	0.040306
9467	Amp	6	102526253	102537776	0	0.043606
9468	Amp	6	102538328	102621781	0	0.040997
9469	Amp	6	102631362	102675062	0	0.044378
9470	Amp	6	102679383	102701142	0	0.0452
9471	Amp	6	102701156	102727320	0	0.043119
9472	Amp	6	102727819	102856913	0	0.041477
9473	Amp	6	102858829	102953307	0	0.042744
9474	Amp	6	102953900	102958485	0	0.048234
9475	Amp	6	102958615	102982998	0	0.049684
9476	Amp	6	102983117	103027359	0	0.052138
9477	Amp	6	103033031	103055804	0	0.046647
9478	Amp	6	103059384	103180606	0	0.043703
9479	Amp	6	103180999	103195583	0	0.042436
9480	Amp	6	103198419	103210902	0	0.04005
9481	Amp	6	103212416	103271975	0	0.036749
9482	Amp	6	103272064	103331241	0	0.041595
9483	Amp	6	103334243	103362717	0	0.039939
9484	Amp	6	103368455	103368982	0	0.037783
9485	Amp	6	103375917	103377649	0	0.038541
9486	Amp	6	103377651	103377653	0	0.04014

9487	Amp	6	103378000	103399720	0	0.042757
9488	Amp	6	103399927	103399927	0	0.04014
9489	Amp	6	103404592	103439206	0	0.043686
9490	Amp	6	103441130	103790784	0	0.037189
9491	Amp	6	103791451	104034253	0	0.03574
9492	Amp	6	104038786	104053082	0	0.037263
9493	Amp	6	104053203	104061215	0	0.039139
9494	Amp	6	104092079	104108410	0	0.039627
9495	Amp	6	104108830	104444713	0	0.042894
9496	Amp	6	104495548	104500459	0	0.045625
9497	Amp	6	104503465	104506623	0	0.042358
9498	Amp	6	104508680	104518684	0	0.039627
9499	Amp	6	104518699	104544520	0	0.042807
9500	Amp	6	104544809	104593160	0	0.044462
9501	Amp	6	104597383	104638250	0	0.043974
9502	Amp	6	104638280	104678848	0	0.052309
9503	Amp	6	104681794	104700566	0	0.056071
9504	Amp	6	104700606	104747356	0	0.05886
9505	Amp	6	104748844	104821253	0	0.056316
9506	Amp	6	104827089	104900703	0	0.060028
9507	Amp	6	104901718	104903119	0	0.062371
9508	Amp	6	104911080	104916642	0.118001	0.068868
9509	Amp	6	104917384	105286977	0.060686	0.066472
9510	Amp	6	105290386	105311835	0.005328	0.064128
9511	Amp	6	105312188	105554859	0.143787	0.06992
9512	Amp	6	105560697	105643375	0.227787	0.073302
9513	Amp	6	105647238	105701043	0.271552	0.075142
9514	Amp	6	105701712	105809297	0.187449	0.07176
9515	Amp	6	105811776	105954321	0.164935	0.071001
9516	Amp	6	105954433	106084571	0.128324	0.06947
9517	Amp	6	106084671	106094552	0.164935	0.071001
9518	Amp	6	106096070	106175055	0.1237	0.069162
9519	Amp	6	106180035	106232752	0.164935	0.070905
9520	Amp	6	106233668	106254359	0.264858	0.075114
9521	Amp	6	106255179	106314079	0.322897	0.077112
9522	Amp	6	106315847	106321723	0.215608	0.072904
9523	Amp	6	106324161	106369462	0.264858	0.07498
9524	Amp	6	106371490	106450546	0.227787	0.073237
9525	Amp	6	106456026	106507257	0.34892	0.078144
9526	Amp	6	106507727	106509848	0.287738	0.075903
9527	Amp	6	106519212	106588784	0.164935	0.070996
9528	Amp	6	106590799	106616981	0.583752	0.085782
9529	Amp	6	106617363	106718680	0.857296	0.093761
9530	Amp	6	106721592	106736954	1.270711	0.103782
9531	Amp	6	106739185	106750421	1.358811	0.105674
9532	Amp	6	106754319	106800910	2.127614	0.12161
9533	Amp	6	106801426	106817434	2.060912	0.120303
9534	Amp	6	106821684	106854839	2.116108	0.121557
9535	Amp	6	106854909	106855080	2.235168	0.123802
9536	Amp	6	106988904	106992777	2.060912	0.120314
9537	Amp	6	106997347	107014103	1.95165	0.118315
9538	Amp	6	107017708	107033147	1.215958	0.102379
9539	Amp	6	107034693	107077565	1.159209	0.101125

9540	Amp	6	107078623	107175504	1.049301	0.098457
9541	Amp	6	107178271	107249277	0.86541	0.094013
9542	Amp	6	107251934	107267248	0.840289	0.09326
9543	Amp	6	107268451	107277432	0.711903	0.08989
9544	Amp	6	107292605	107410354	0.644502	0.087802
9545	Amp	6	107415883	107531079	0.807647	0.092478
9546	Amp	6	107531096	107574177	0.72817	0.090233
9547	Amp	6	107575970	107620203	0.644502	0.087908
9548	Amp	6	107623363	107697655	0.563096	0.085127
9549	Amp	6	107698115	107705667	0.34892	0.078016
9550	Amp	6	107707505	107770399	0.089561	0.067804
9551	Amp	6	107771524	107801164	0	0.06373
9552	Amp	6	107805872	107810457	0	0.06086
9553	Amp	6	107810645	107863373	0.005328	0.064219
9554	Amp	6	107863954	107955200	0	0.06086
9555	Amp	6	107969403	107979708	0	0.058783
9556	Amp	6	107985302	108146008	0	0.05275
9557	Amp	6	108148716	108359940	0	0.053818
9558	Amp	6	108361413	108476381	0	0.037882
9559	Amp	6	108476625	108507702	0	0.033733
9560	Amp	6	108508201	108535246	0	0.03555
9561	Amp	6	108536280	108650949	0	0.036297
9562	Amp	6	108651816	108651816	0	0.034479
9563	Amp	6	108653543	109153691	0	0.033733
9564	Amp	6	109155232	109170692	0	0.032678
9565	Amp	6	109171197	109405689	0	0.048614
9566	Amp	6	109405874	109485060	0	0.046748
9567	Amp	6	109490057	109516989	0	0.043568
9568	Amp	6	109517003	109558038	0	0.04808
9569	Amp	6	109565840	109573709	0	0.039024
9570	Amp	6	109585677	109633315	0	0.05496
9571	Amp	6	109633742	109667233	0	0.06083
9572	Amp	6	109667532	109772068	0	0.058681
9573	Amp	6	109775400	109924709	0	0.059829
9574	Amp	6	109925385	109942616	0	0.058681
9575	Amp	6	109944616	110009741	0	0.051384
9576	Amp	6	110011492	110151855	0	0.043595
9577	Amp	6	110155227	110155227	0	0.038633
9578	Amp	6	110164242	110242160	0	0.041137
9579	Amp	6	110245381	110268966	0	0.0516
9580	Amp	6	110272416	110280396	0	0.035663
9581	Amp	6	110284966	110392380	0	0.032914
9582	Amp	6	110393381	110427438	0	0.037816
9583	Amp	6	110427508	110500712	0	0.032914
9584	Amp	6	110504649	110722651	0	0.029795
9585	Amp	6	110723581	110802862	0	0.031067
9586	Amp	6	110811048	110998980	0	0.034036
9587	Amp	6	110999636	111165098	0	0.034877
9588	Amp	6	111166487	111203025	0	0.036152
9589	Amp	6	111206694	111398532	0	0.031639
9590	Amp	6	111401824	111610776	0	0.030364
9591	Amp	6	111614178	111786668	0	0.03245
9592	Amp	6	111793588	111889830	0	0.036321

9593	Amp	6	111890183	111911148	0	0.037973
9594	Amp	6	111911166	111928427	0	0.035699
9595	Amp	6	111928572	111945747	0	0.033614
9596	Amp	6	111945946	112085021	0	0.031737
9597	Amp	6	112089798	112140266	0	0.03924
9598	Amp	6	112140771	112409422	0	0.041877
9599	Amp	6	112409492	112472076	0	0.044787
9600	Amp	6	112472368	112504770	0	0.039696
9601	Amp	6	112507538	112560816	0	0.037192
9602	Amp	6	112561740	112566535	0	0.029504
9603	Amp	6	112573645	112603658	0	0.031884
9604	Amp	6	112607858	112608342	0	0.03297
9605	Amp	6	112610754	112718160	0	0.033916
9606	Amp	6	112718710	112786524	0	0.030565
9607	Amp	6	112792407	112895210	0	0.041885
9608	Amp	6	112895413	112962212	0	0.045471
9609	Amp	6	112964486	113017424	0	0.044386
9610	Amp	6	113017901	113244626	0	0.034511
9611	Amp	6	113244649	113334404	0	0.036262
9612	Amp	6	113334776	113364279	0	0.03499
9613	Amp	6	113367735	113376002	0	0.032145
9614	Amp	6	113377390	113470461	0	0.034465
9615	Amp	6	113471086	113803286	0	0.037803
9616	Amp	6	113803387	113843946	0	0.039544
9617	Amp	6	113851756	113987854	0	0.024145
9618	Amp	6	113989649	114042421	0	0.026848
9619	Amp	6	114046647	114057806	0	0.029992
9620	Amp	6	114062421	114082300	0	0.027023
9621	Amp	6	114084745	114102592	0	0.023879
9622	Amp	6	114104943	114135763	0	0.018856
9623	Amp	6	114136220	114424056	0	0.021859
9624	Amp	6	114430107	114461675	0	0.024262
9625	Amp	6	114464444	114486772	0	0.022521
9626	Amp	6	114487033	114527878	0	0.023273
9627	Amp	6	114529648	114547260	0	0.02087
9628	Amp	6	114547717	114623676	0	0.022499
9629	Amp	6	114624830	114632538	0	0.02087
9630	Amp	6	114642607	114715153	0	0.025718
9631	Amp	6	114742457	114756465	0	0.028981
9632	Amp	6	114757125	114955141	0	0.025978
9633	Amp	6	114955345	115221135	0	0.024455
9634	Amp	6	115248187	115411891	0	0.025991
9635	Amp	6	115412062	115522013	0	0.029517
9636	Amp	6	115525451	115603773	0	0.025991
9637	Amp	6	115603868	115610999	0	0.027925
9638	Amp	6	115612865	115730763	0	0.029284
9639	Amp	6	115731187	115786787	0	0.027888
9640	Amp	6	115789971	116168387	0	0.022502
9641	Amp	6	116183078	116286413	0	0.026204
9642	Amp	6	116287842	116452532	0	0.032571
9643	Amp	6	116465854	116465854	0	0.045366
9644	Amp	6	116465999	116482833	0	0.039819
9645	Amp	6	116483230	116506926	0	0.036117

9646	Amp	6	116507459	116516859	0	0.038553
9647	Amp	6	116521986	116582372	0	0.053416
9648	Amp	6	116582781	116603963	0	0.047127
9649	Amp	6	116610095	116770102	0	0.032264
9650	Amp	6	116770520	116975861	0	0.034169
9651	Amp	6	116976350	117020529	0	0.030913
9652	Amp	6	117021176	117029814	0	0.028599
9653	Amp	6	117030191	117079876	0	0.030664
9654	Amp	6	117084671	117309040	0	0.028599
9655	Amp	6	117309533	117318457	0	0.027613
9656	Amp	6	117318575	117486165	0	0.030778
9657	Amp	6	117486585	117506804	0	0.028995
9658	Amp	6	117511116	117615267	0	0.031141
9659	Amp	6	117617560	117784053	0	0.027976
9660	Amp	6	117784557	117950622	0	0.029537
9661	Amp	6	117953613	118007835	0	0.039549
9662	Amp	6	118008036	118055116	0	0.042084
9663	Amp	6	118055648	118197591	0	0.045864
9664	Amp	6	118200315	118201576	0	0.055551
9665	Amp	6	118206725	118227095	0	0.045539
9666	Amp	6	118227934	118227934	0	0.04418
9667	Amp	6	118816495	118816495	0	0.0427
9668	Amp	6	118816865	118817120	0	0.044024
9669	Amp	6	118821899	118825212	0	0.043271
9670	Amp	6	118895717	118953492	0	0.037691
9671	Amp	6	118954424	118977273	0	0.039726
9672	Amp	6	118978361	118992468	0	0.049411
9673	Amp	6	118993012	118994173	0	0.050109
9674	Amp	6	118994861	119018169	0	0.051035
9675	Amp	6	119030828	119040092	0	0.035099
9676	Amp	6	119042530	119043191	0	0.029345
9677	Amp	6	119044861	119067415	0	0.02681
9678	Amp	6	119067696	119116816	0	0.026111
9679	Amp	6	119117653	119119548	0	0.023376
9680	Amp	6	119121015	119121015	0	0.039312
9681	Amp	6	119122271	119129306	0	0.03987
9682	Amp	6	119132173	119161543	0	0.041479
9683	Amp	6	119162684	119176358	0	0.042049
9684	Amp	6	119178085	119204435	0	0.041124
9685	Amp	6	119205050	119241834	0	0.041767
9686	Amp	6	119244735	119312460	0	0.041124
9687	Amp	6	119313824	119343907	0	0.042984
9688	Amp	6	119348056	119514789	0	0.044769
9689	Amp	6	119519527	119622238	0	0.028833
9690	Amp	6	119630624	119700464	0	0.027048
9691	Amp	6	119710931	119754328	0	0.027822
9692	Amp	6	119760369	119810633	0	0.043758
9693	Amp	6	119812391	119864639	0	0.042993
9694	Amp	6	119874254	120317636	0	0.037487
9695	Amp	6	120323092	120343738	0	0.031401
9696	Amp	6	120344004	120416620	0	0.034576
9697	Amp	6	120420313	120438462	0	0.041093
9698	Amp	6	120446871	120519213	0	0.048366

9699	Amp	6	120526579	120547097	0	0.054451
9700	Amp	6	120547368	120606876	0	0.055563
9701	Amp	6	120614375	120637336	0	0.049879
9702	Amp	6	120645477	120652149	0	0.033943
9703	Amp	6	120706657	120740091	0	0.020152
9704	Amp	6	120747037	120830453	0	0.01904
9705	Amp	6	120830962	120886567	0	0.01718
9706	Amp	6	120887199	121019909	0	0.018097
9707	Amp	6	121021237	121032144	0	0.016562
9708	Amp	6	121034977	121192531	0	0.019089
9709	Amp	6	121197655	121365510	0	0.01748
9710	Amp	6	121374170	121426819	0	0.018461
9711	Amp	6	121427071	121839724	0	0.018904
9712	Amp	6	121841999	122013466	0	0.017573
9713	Amp	6	122016894	122016894	0	0.016004
9714	Amp	6	122017370	122051512	0	0.015024
9715	Amp	6	122056367	122148826	0	0.024479
9716	Amp	6	122150555	122331866	0	0.030622
9717	Amp	6	122337078	122382060	0	0.025161
9718	Amp	6	122382365	122452235	0	0.029
9719	Amp	6	122454715	122476905	0	0.030082
9720	Amp	6	122479837	122481213	0	0.032567
9721	Amp	6	122482967	122537422	0	0.035963
9722	Amp	6	122544744	122615904	0	0.028956
9723	Amp	6	122616224	122671575	0	0.0304
9724	Amp	6	122673045	122817248	0	0.020945
9725	Amp	6	122818136	123055824	0	0.020375
9726	Amp	6	123060407	123169553	0	0.019931
9727	Amp	6	123169615	123194247	0	0.018487
9728	Amp	6	123197849	123702530	0	0.015959
9729	Amp	6	123703804	123768330	0	0.012785
9730	Amp	6	123777291	123902216	0	0.01393
9731	Amp	6	123902230	123907908	0	0.015556
9732	Amp	6	123908290	124039378	0	0.018507
9733	Amp	6	124040619	124109946	0	0.019001
9734	Amp	6	124150388	124261099	0	0.016625
9735	Amp	6	124268157	124310310	0	0.020098
9736	Amp	6	124310671	124321110	0	0.016625
9737	Amp	6	124323684	124566520	0	0.013674
9738	Amp	6	124568859	124599694	0	0.012049
9739	Amp	6	124599994	124719210	0	0.015247
9740	Amp	6	124720347	124795099	0	0.015807
9741	Amp	6	124795168	124895083	0	0.01943
9742	Amp	6	124899104	124942806	0	0.020623
9743	Amp	6	124942878	125031031	0	0.022212
9744	Amp	6	125031101	125102643	0	0.018588
9745	Amp	6	125105241	125311137	0	0.019317
9746	Amp	6	125311885	125315307	0	0.024026
9747	Amp	6	125322331	125371244	0	0.023297
9748	Amp	6	125372547	125577239	0	0.021262
9749	Amp	6	125582595	125687136	0	0.023682
9750	Amp	6	125687477	125687477	0	0.021262
9751	Amp	6	125690938	125800121	0	0.022441

9752	Amp	6	125802124	125802890	0	0.02288
9753	Amp	6	125804291	125830991	0	0.027302
9754	Amp	6	125834060	125854039	0	0.02796
9755	Amp	6	125856298	125856298	0	0.0274
9756	Amp	6	125868540	125924498	0	0.02941
9757	Amp	6	125927450	125979887	0	0.026571
9758	Amp	6	125981055	125985626	0	0.02573
9759	Amp	6	125987407	125989580	0	0.02455
9760	Amp	6	125990618	126042975	0	0.03763
9761	Amp	6	126044904	126085660	0	0.033662
9762	Amp	6	126088618	126173488	0	0.03763
9763	Amp	6	126173721	126278020	0	0.03923
9764	Amp	6	126278372	126285460	0	0.03736
9765	Amp	6	126285666	126314131	0	0.036702
9766	Amp	6	126315131	126407912	0	0.039652
9767	Amp	6	126416158	126472755	0	0.036702
9768	Amp	6	126473347	126477637	0	0.035557
9769	Amp	6	126481676	126537209	0	0.037807
9770	Amp	6	126545168	126558314	0	0.024728
9771	Amp	6	126564523	126670699	0	0.022477
9772	Amp	6	126681488	126744612	0	0.024072
9773	Amp	6	126745079	126775382	0	0.022483
9774	Amp	6	126780723	126813987	0	0.021566
9775	Amp	6	126815077	126845309	0	0.02321
9776	Amp	6	126846251	126928718	0	0.026992
9777	Amp	6	126931854	127019311	0	0.025392
9778	Amp	6	127023778	127062939	0	0.021345
9779	Amp	6	127066942	127075003	0	0.022916
9780	Amp	6	127076095	127091570	0	0.021272
9781	Amp	6	127091583	127125465	0	0.019701
9782	Amp	6	127130520	127143609	0	0.021135
9783	Amp	6	127148635	127192534	0	0.025129
9784	Amp	6	127195066	127266073	0	0.026688
9785	Amp	6	127272315	127300603	0	0.025129
9786	Amp	6	127302279	127329221	0	0.021931
9787	Amp	6	127330068	127363401	0	0.023149
9788	Amp	6	127363736	127454497	0	0.024418
9789	Amp	6	127459702	127465308	0	0.022984
9790	Amp	6	127469999	127710575	0	0.032013
9791	Amp	6	127717064	127720389	0	0.034109
9792	Amp	6	127722906	127739751	0	0.03284
9793	Amp	6	127740468	128006615	0	0.031647
9794	Amp	6	128008407	128067579	0	0.033875
9795	Amp	6	128067845	128135292	0	0.031779
9796	Amp	6	128138119	128149462	0	0.029551
9797	Amp	6	128150570	128188393	0	0.031097
9798	Amp	6	128197150	128315258	0	0.032906
9799	Amp	6	128328262	128462545	0	0.034151
9800	Amp	6	128468093	128496949	0	0.035649
9801	Amp	6	128501846	128517163	0	0.040824
9802	Amp	6	128519594	128570134	0	0.042892
9803	Amp	6	128580341	128671473	0	0.041393
9804	Amp	6	128673894	128880272	0	0.037399

9805	Amp	6	128880535	128915522	0	0.040938
9806	Amp	6	128915809	129106277	0	0.037399
9807	Amp	6	129111090	129282913	0	0.035331
9808	Amp	6	129283208	129371562	0	0.034252
9809	Amp	6	129378323	129476086	0	0.036455
9810	Amp	6	129476939	129615263	0	0.03521
9811	Amp	6	129623082	129737065	0	0.033401
9812	Amp	6	129739091	129763057	0	0.036082
9813	Amp	6	129763726	129845881	0	0.03844
9814	Amp	6	129850522	129890190	0	0.036082
9815	Amp	6	129890640	130242810	0	0.037894
9816	Amp	6	130246573	130436016	0	0.039306
9817	Amp	6	130440783	130536254	0	0.037894
9818	Amp	6	130538304	130605268	0	0.039137
9819	Amp	6	130610433	130641134	0	0.037894
9820	Amp	6	130641387	130866534	0	0.039194
9821	Amp	6	130867172	131077951	0	0.043154
9822	Amp	6	131079939	131080043	0	0.043647
9823	Amp	6	131088156	131155388	0	0.037137
9824	Amp	6	131162638	131162638	0	0.03857
9825	Amp	6	131162699	131513354	0	0.043108
9826	Amp	6	131513725	131611826	0	0.041784
9827	Amp	6	131619344	131721294	0	0.044926
9828	Amp	6	131723056	131848789	0	0.046957
9829	Amp	6	131848864	131853789	0	0.045381
9830	Amp	6	131857986	131912481	0	0.043948
9831	Amp	6	131912654	131958700	0	0.042844
9832	Amp	6	131958709	131975727	0	0.044816
9833	Amp	6	131976369	131990025	0	0.042572
9834	Amp	6	131994856	131996433	0	0.033183
9835	Amp	6	131997146	132021605	0	0.03269
9836	Amp	6	132021820	132049966	0	0.034589
9837	Amp	6	132055434	132126111	0	0.032994
9838	Amp	6	132126383	132214698	0	0.041583
9839	Amp	6	132217993	132224242	0	0.036391
9840	Amp	6	132227365	132310714	0	0.034492
9841	Amp	6	132312462	132319178	0	0.038381
9842	Amp	6	132322715	132366109	0	0.041616
9843	Amp	6	132366391	132443543	0	0.040316
9844	Amp	6	132452845	132453072	0	0.041465
9845	Amp	6	132454363	132461792	0	0.042967
9846	Amp	6	132461949	132478405	0	0.039732
9847	Amp	6	132478579	132566968	0	0.040644
9848	Amp	6	132574618	132855658	0	0.039697
9849	Amp	6	132858938	133079057	0	0.038225
9850	Amp	6	133086461	133088498	0	0.030014
9851	Amp	6	133092160	133138619	0	0.028512
9852	Amp	6	133138640	133280124	0	0.024731
9853	Amp	6	133280257	133404638	0	0.029153
9854	Amp	6	133404690	133532234	0	0.028241
9855	Amp	6	133532894	133691560	0	0.030097
9856	Amp	6	133692134	133811100	0	0.028285
9857	Amp	6	133818312	133821789	0	0.02956

9858	Amp	6	133828836	133845109	0	0.043523
9859	Amp	6	133846243	133854907	0	0.03743
9860	Amp	6	133857359	133877876	0	0.023467
9861	Amp	6	133878041	133926744	0	0.037699
9862	Amp	6	133929555	133964942	0	0.024488
9863	Amp	6	133966003	134035444	0	0.022325
9864	Amp	6	134036385	134154959	0	0.035536
9865	Amp	6	134160407	134188116	0	0.036983
9866	Amp	6	134188691	134209109	0	0.036544
9867	Amp	6	134214021	134256676	0	0.022312
9868	Amp	6	134257256	134450188	0	0.021818
9869	Amp	6	134450312	134496598	0	0.020669
9870	Amp	6	134496927	134513400	0	0.03148
9871	Amp	6	134520005	134666844	0	0.020669
9872	Amp	6	134667196	134955558	0	0.021816
9873	Amp	6	134959124	135014291	0	0.022311
9874	Amp	6	135021116	135105435	0	0.020108
9875	Amp	6	135105904	135193312	0	0.021188
9876	Amp	6	135193420	135196676	0	0.025378
9877	Amp	6	135198362	135200070	0	0.027999
9878	Amp	6	135200106	135203236	0	0.035068
9879	Amp	6	135203250	135292049	0	0.034158
9880	Amp	6	135293525	135300034	0	0.031537
9881	Amp	6	135302446	135317672	0	0.024468
9882	Amp	6	135318845	135546993	0	0.023389
9883	Amp	6	135554378	135566970	0	0.02741
9884	Amp	6	135567022	135858599	0	0.023221
9885	Amp	6	135859363	135913896	0	0.025361
9886	Amp	6	135918041	136105736	0	0.029461
9887	Amp	6	136109504	136132736	0	0.028966
9888	Amp	6	136132840	136298732	0	0.029562
9889	Amp	6	136299714	136351965	0	0.024628
9890	Amp	6	136356715	136446831	0	0.02244
9891	Amp	6	136447316	136482692	0	0.02806
9892	Amp	6	136492410	136517194	0	0.025714
9893	Amp	6	136523361	136618956	0	0.026947
9894	Amp	6	136621349	136738812	0	0.031693
9895	Amp	6	136864792	136876524	0	0.028419
9896	Amp	6	136883376	137102642	0	0.029539
9897	Amp	6	137108033	137140533	0	0.034149
9898	Amp	6	137141741	137158221	0	0.032723
9899	Amp	6	137158579	137178470	0	0.031074
9900	Amp	6	137180398	137197210	0	0.028953
9901	Amp	6	137200086	137323701	0	0.032155
9902	Amp	6	137327555	137347398	0	0.028953
9903	Amp	6	137349847	137439171	0	0.027933
9904	Amp	6	137439330	137529671	0	0.026487
9905	Amp	6	137530705	137568592	0	0.028843
9906	Amp	6	137570360	137629740	0	0.030597
9907	Amp	6	137631997	137859785	0	0.028843
9908	Amp	6	137860809	137938994	0	0.030881
9909	Amp	6	137944211	137984153	0	0.034469
9910	Amp	6	137989731	138021607	0	0.038722

9911	Amp	6	138025824	138187272	0	0.034469
9912	Amp	6	138188108	138624199	0	0.037745
9913	Amp	6	138628345	138686600	0	0.039405
9914	Amp	6	138686830	138725534	0	0.040703
9915	Amp	6	138727425	138801103	0	0.04142
9916	Amp	6	138801204	138817079	0	0.038145
9917	Amp	6	138819371	138960626	0	0.041551
9918	Amp	6	138964959	139089134	0	0.038145
9919	Amp	6	139090262	139256302	0	0.036847
9920	Amp	6	139261187	139341905	0	0.034717
9921	Amp	6	139342748	139369934	0	0.033148
9922	Amp	6	139370563	139458105	0	0.036642
9923	Amp	6	139458208	139519556	0	0.039801
9924	Amp	6	139521462	139606863	0	0.036642
9925	Amp	6	139607240	139657283	0	0.037427
9926	Amp	6	139657669	139846705	0	0.037895
9927	Amp	6	139846814	139880041	0	0.034401
9928	Amp	6	139881086	140215309	0	0.040938
9929	Amp	6	140216344	140301815	0	0.039791
9930	Amp	6	140305244	140372897	0	0.042015
9931	Amp	6	140373200	140947331	0	0.031106
9932	Amp	6	140948305	140954243	0	0.032363
9933	Amp	6	140957649	141093175	0	0.030006
9934	Amp	6	141093180	141273136	0	0.031362
9935	Amp	6	141275551	141449056	0	0.030644
9936	Amp	6	141451096	141519587	0	0.02842
9937	Amp	6	141522284	141704619	0	0.034617
9938	Amp	6	141704731	141780896	0	0.036392
9939	Amp	6	141781104	141782916	0	0.03712
9940	Amp	6	141783654	141821317	0	0.038555
9941	Amp	6	141821538	141841389	0	0.037322
9942	Amp	6	141843366	141962061	0	0.033362
9943	Amp	6	141997778	142023685	0	0.032635
9944	Amp	6	142024285	142028941	0	0.036522
9945	Amp	6	142038051	142135246	0	0.039328
9946	Amp	6	142143611	142162527	0	0.036522
9947	Amp	6	142172653	142279426	0	0.034747
9948	Amp	6	142279698	142280047	0	0.036012
9949	Amp	6	142280509	142299268	0	0.037583
9950	Amp	6	142300810	142387539	0	0.038619
9951	Amp	6	142388046	142480146	0	0.040212
9952	Amp	6	142483353	142514848	0	0.025379
9953	Amp	6	142518361	142626390	0	0.026302
9954	Amp	6	142626822	142658300	0	0.027966
9955	Amp	6	142662516	142700821	0	0.031344
9956	Amp	6	142701109	142865900	0	0.027966
9957	Amp	6	142867502	142951678	0	0.029598
9958	Amp	6	142958038	143159663	0	0.026373
9959	Amp	6	143163063	143218669	0	0.030808
9960	Amp	6	143227144	143290881	0	0.032912
9961	Amp	6	143291469	143331783	0	0.031249
9962	Amp	6	143332585	143361636	0	0.03389
9963	Amp	6	143366384	143371580	0	0.022102

9964	Amp	6	143373083	143414625	0	0.024482
9965	Amp	6	143423127	143459207	0	0.022102
9966	Amp	6	143477311	143502751	0	0.026915
9967	Amp	6	143502774	143503594	0	0.02172
9968	Amp	6	143503944	143565031	0	0.022517
9969	Amp	6	143570259	143642145	0	0.034789
9970	Amp	6	143642333	143729668	0	0.026486
9971	Amp	6	143730198	143860175	0	0.028766
9972	Amp	6	143869306	143893598	0	0.027501
9973	Amp	6	143894419	143894419	0	0.026354
9974	Amp	6	143895741	143927858	0	0.024766
9975	Amp	6	143936101	144025002	0	0.022086
9976	Amp	6	144031760	144085190	0	0.021449
9977	Amp	6	144085427	144264247	0	0.018471
9978	Amp	6	144265026	144283784	0	0.018956
9979	Amp	6	144293730	144384405	0	0.021678
9980	Amp	6	144388113	144466408	0	0.018956
9981	Amp	6	144469373	144485555	0	0.022885
9982	Amp	6	144490947	144509566	0	0.018114
9983	Amp	6	144511698	144667786	0	0.015834
9984	Amp	6	144674380	144699020	0	0.017697
9985	Amp	6	144703734	144719635	0	0.018978
9986	Amp	6	144719747	144721178	0	0.018054
9987	Amp	6	144722519	144736359	0	0.031112
9988	Amp	6	144738828	144866383	0	0.029979
9989	Amp	6	144867071	144880069	0	0.033394
9990	Amp	6	144883181	144959017	0	0.029979
9991	Amp	6	144961848	145155677	0	0.028544
9992	Amp	6	145156823	145165617	0	0.026817
9993	Amp	6	145170599	145181794	0	0.024456
9994	Amp	6	145182660	145302769	0	0.0266
9995	Amp	6	145306099	145357498	0	0.025382
9996	Amp	6	145359428	145539130	0	0.027402
9997	Amp	6	145539248	145563882	0	0.028003
9998	Amp	6	145564145	145615067	0	0.031961
9999	Amp	6	145616050	145875830	0	0.029809
10000	Amp	6	145880994	146104728	0	0.033797
10001	Amp	6	146110450	146273747	0	0.033204
10002	Amp	6	146274621	146579480	0	0.035007
10003	Amp	6	146580981	146771339	0	0.033204
10004	Amp	6	146776039	147514799	0	0.034244
10005	Amp	6	147515082	147921378	0	0.030257
10006	Amp	6	147922126	148144231	0	0.034366
10007	Amp	6	148150377	148420312	0	0.033326
10008	Amp	6	148422386	149011998	0	0.032069
10009	Amp	6	149012041	149039519	0	0.033723
10010	Amp	6	149039742	149420078	0	0.035125
10011	Amp	6	149420871	149571429	0	0.03666
10012	Amp	6	149571702	149791373	0	0.037337
10013	Amp	6	149803147	149819870	0	0.038539
10014	Amp	6	149825080	149848809	0	0.040784
10015	Amp	6	149849308	149962442	0	0.041595
10016	Amp	6	149963521	150073681	0	0.03935

10017	Amp	6	150076359	150086975	0	0.041318
10018	Amp	6	150087810	150305155	0	0.042109
10019	Amp	6	150308683	150390028	0	0.043513
10020	Amp	6	150391525	150538530	0	0.041978
10021	Amp	6	150539898	150659634	0	0.04332
10022	Amp	6	150659890	150757332	0	0.046498
10023	Amp	6	150758758	150804885	0	0.050538
10024	Amp	6	150805414	150834205	0	0.050986
10025	Amp	6	150834242	150844548	0	0.049019
10026	Amp	6	150846208	150977873	0	0.051403
10027	Amp	6	150978271	150982361	0	0.052768
10028	Amp	6	150982518	150985318	0	0.054843
10029	Amp	6	150985780	151018533	0	0.057456
10030	Amp	6	151019163	151042143	0	0.055072
10031	Amp	6	151048612	151074558	0	0.057258
10032	Amp	6	151074582	151214625	0	0.055072
10033	Amp	6	151215015	151228173	0	0.056353
10034	Amp	6	151231137	151295156	0	0.055905
10035	Amp	6	151296050	151304887	0	0.056346
10036	Amp	6	151307145	151383559	0	0.059592
10037	Amp	6	151384296	151400699	0.005328	0.064143
10038	Amp	6	151407337	151420853	0.056211	0.06624
10039	Amp	6	151422449	151423148	0.094967	0.068063
10040	Amp	6	151436907	151437170	0.056211	0.066374
10041	Amp	6	151439591	151444221	0.107017	0.068523
10042	Amp	6	151444376	151445687	0.08435	0.067712
10043	Amp	6	151445900	151495135	0.112543	0.06878
10044	Amp	6	151499742	151522261	0.005328	0.064229
10045	Amp	6	151524429	151524429	0.019169	0.064869
10046	Amp	6	151525959	151545602	0.079652	0.067325
10047	Amp	6	151551365	151556555	0.1237	0.069163
10048	Amp	6	151556959	151556959	0.14896	0.070284
10049	Amp	6	151560836	151587107	0.233782	0.073408
10050	Amp	6	151592789	151593816	0.316877	0.076938
10051	Amp	6	151593887	151600264	0.342649	0.077877
10052	Amp	6	151604242	151604732	0.271552	0.075156
10053	Amp	6	151610947	151630162	0.470509	0.082158
10054	Amp	6	151630438	151643069	0.557122	0.085076
10055	Amp	6	151650105	151654638	0.508568	0.083514
10056	Amp	6	151656087	151663398	0.424557	0.080596
10057	Amp	6	151664868	151664868	0.34892	0.077983
10058	Amp	6	151666626	151668165	0.368062	0.078778
10059	Amp	6	151674783	151691899	0.4115	0.080146
10060	Amp	6	151694413	151707279	0.222041	0.073144
10061	Amp	6	151708077	151721355	0.133193	0.069614
10062	Amp	6	151721367	151739277	0.060686	0.06649
10063	Amp	6	151742538	151790611	0.128324	0.069356
10064	Amp	6	151792057	151877531	0.159312	0.070664
10065	Amp	6	151878224	151903601	0.222041	0.073127
10066	Amp	6	151905787	151910153	0.271552	0.075301
10067	Amp	6	151913175	151944305	0.215608	0.072845
10068	Amp	6	151944387	151956566	0.159312	0.070771
10069	Amp	6	151957750	152027766	0.100827	0.068308

10070	Amp	6	152037044	152040145	0.181932	0.071502
10071	Amp	6	152047788	152099995	0.133193	0.069663
10072	Amp	6	152101320	152104447	0.056211	0.066276
10073	Amp	6	152107569	152111436	0.005328	0.064127
10074	Amp	6	152114407	152115023	0	0.061261
10075	Amp	6	152115997	152128055	0	0.061843
10076	Amp	6	152131793	152146308	0	0.061166
10077	Amp	6	152146686	152155817	0	0.057626
10078	Amp	6	152156732	152156772	0	0.056448
10079	Amp	6	152157128	152249905	0	0.05538
10080	Amp	6	152250791	152318975	0	0.053822
10081	Amp	6	152319113	152343052	0	0.054405
10082	Amp	6	152347170	152473042	0	0.05361
10083	Amp	6	152475930	152481162	0	0.051956
10084	Amp	6	152481348	152567339	0	0.050554
10085	Amp	6	152571463	152580072	0	0.04736
10086	Amp	6	152581674	152674602	0	0.04672
10087	Amp	6	152674899	152762349	0	0.044546
10088	Amp	6	152763337	152807731	0	0.045536
10089	Amp	6	152811653	152975637	0	0.046384
10090	Amp	6	152976015	153128077	0	0.045801
10091	Amp	6	153132852	153344696	0	0.042515
10092	Amp	6	153345158	153405822	0	0.040123
10093	Amp	6	153406897	153425469	0	0.044192
10094	Amp	6	153426011	153476490	0	0.041014
10095	Amp	6	153479452	153493757	0	0.036945
10096	Amp	6	153496131	153540315	0	0.038277
10097	Amp	6	153541809	153543752	0	0.036945
10098	Amp	6	153544286	153588494	0	0.036183
10099	Amp	6	153588714	153646642	0	0.034815
10100	Amp	6	153647552	153659995	0	0.036157
10101	Amp	6	153661215	153691454	0	0.03669
10102	Amp	6	153695801	153846436	0	0.0357
10103	Amp	6	153847586	153892838	0	0.03367
10104	Amp	6	153892854	154098175	0	0.032822
10105	Amp	6	154098777	154252011	0	0.033553
10106	Amp	6	154254824	154269605	0	0.03489
10107	Amp	6	154272022	154420345	0	0.03085
10108	Amp	6	154420399	154460458	0	0.033864
10109	Amp	6	154460578	154669930	0	0.031845
10110	Amp	6	154673928	155004297	0	0.02883
10111	Amp	6	155004310	155066706	0	0.027488
10112	Amp	6	155071435	155107088	0	0.026697
10113	Amp	6	155131614	155176164	0	0.028665
10114	Amp	6	155181079	155233217	0	0.026697
10115	Amp	6	155234245	155335373	0	0.025966
10116	Amp	6	155340637	155506227	0	0.023428
10117	Amp	6	155507327	155619846	0	0.026805
10118	Amp	6	155621680	155621680	0	0.025463
10119	Amp	6	155622658	155655401	0	0.022086
10120	Amp	6	155658547	156009274	0	0.025239
10121	Amp	6	156009446	156122993	0	0.025835
10122	Amp	6	156128615	156135726	0	0.029071

10123	Amp	6	156136574	156165329	0	0.032547
10124	Amp	6	156218061	156219283	0	0.029311
10125	Amp	6	156219949	156360092	0	0.028351
10126	Amp	6	156368537	156489495	0	0.026948
10127	Amp	6	156494740	156950707	0	0.029047
10128	Amp	6	156950932	157111526	0	0.02988
10129	Amp	6	157121933	157139421	0	0.031472
10130	Amp	6	157146023	157146023	0	0.02988
10131	Amp	6	157161088	157432627	0	0.032135
10132	Amp	6	157433774	157664371	0	0.03341
10133	Amp	6	157665163	157868185	0	0.035688
10134	Amp	6	157868291	157877537	0	0.036158
10135	Amp	6	157880263	157929252	0	0.032683
10136	Amp	6	157937125	158253144	0	0.035915
10137	Amp	6	158253592	158680895	0	0.034641
10138	Amp	6	158681499	158725899	0	0.040187
10139	Amp	6	158734966	159085471	0	0.040864
10140	Amp	6	159085745	159097649	0	0.040187
10141	Amp	6	159107487	159156412	0	0.036954
10142	Amp	6	159159527	159220804	0	0.038948
10143	Amp	6	159221931	159327670	0	0.037882
10144	Amp	6	159328275	159429145	0	0.035889
10145	Amp	6	159436116	160647514	0	0.031389
10146	Amp	6	160647530	160731262	0	0.030856
10147	Amp	6	160731787	160788985	0	0.030295
10148	Amp	6	160793754	160919932	0	0.033668
10149	Amp	6	160921658	161141235	0	0.034199
10150	Amp	6	161150119	161189878	0	0.031173
10151	Amp	6	161193720	161665477	0	0.035878
10152	Amp	6	161665763	161947086	0	0.034602
10153	Amp	6	161948837	161954246	0	0.029411
10154	Amp	6	161955069	162062922	0	0.030721
10155	Amp	6	162173909	162838228	0	0.032001
10156	Amp	6	162838409	162979117	0	0.032555
10157	Amp	6	162987309	163060615	0	0.032001
10158	Amp	6	163062046	163615628	0	0.030721
10159	Amp	6	163615690	163943132	0	0.031879
10160	Amp	6	163945684	164142925	0	0.032416
10161	Amp	6	164144122	164816537	0	0.031879
10162	Amp	6	164816881	164945614	0	0.030721
10163	Amp	6	164954484	164964508	0	0.04223
10164	Amp	6	164970305	165054306	0	0.030721
10165	Amp	6	165059275	165559137	0	0.033604
10166	Amp	6	165559169	165642115	0	0.035195
10167	Amp	6	165643668	167147219	0	0.033604
10168	Amp	6	167147930	167147930	0	0.029796
10169	Amp	6	167862052	167931668	0	0.026914
10170	Amp	6	168457458	169580804	0	0.029737
10171	Amp	6	169581714	169598090	0	0.030253
10172	Amp	6	169606879	169634835	0	0.031437
10173	Amp	6	169635420	169762441	0	0.033929
10174	Amp	6	169763095	170436724	0	0.035993
10175	Amp	6	170436993	170594831	0	0.035476

10176	Amp	7	665647	709285	0	0.03893
10177	Amp	7	716986	763705	0	0.036896
10178	Amp	7	782886	879502	0	0.039216
10179	Amp	7	880030	1213138	0	0.036946
10180	Amp	7	1215257	1370914	0	0.040106
10181	Amp	7	1372323	1377045	0	0.042096
10182	Amp	7	1383419	1500999	0	0.043735
10183	Amp	7	1505238	1512819	0	0.040575
10184	Amp	7	1521818	1617331	0	0.04411
10185	Amp	7	1618869	1718481	0	0.045792
10186	Amp	7	1721528	1741977	0	0.04411
10187	Amp	7	1757846	1779503	0	0.044612
10188	Amp	7	1781642	1809777	0	0.042822
10189	Amp	7	1818309	1832933	0	0.042227
10190	Amp	7	1836445	1941911	0	0.038691
10191	Amp	7	1945404	2210259	0	0.035551
10192	Amp	7	2304677	2629953	0	0.033969
10193	Amp	7	2633338	2712108	0	0.036702
10194	Amp	7	2714033	2775001	0	0.035063
10195	Amp	7	2775100	2907594	0	0.036811
10196	Amp	7	2911712	2972777	0	0.037261
10197	Amp	7	2984996	3039970	0	0.040335
10198	Amp	7	3040259	3254383	0	0.039833
10199	Amp	7	3256169	3288683	0	0.042285
10200	Amp	7	3289876	3320254	0	0.040295
10201	Amp	7	3320781	3482101	0	0.037562
10202	Amp	7	3482171	3562779	0	0.035111
10203	Amp	7	3567008	3598928	0	0.031874
10204	Amp	7	3618708	4015718	0	0.033859
10205	Amp	7	4016801	4030091	0	0.034321
10206	Amp	7	4030244	4050651	0	0.032336
10207	Amp	7	4054625	4190039	0	0.034432
10208	Amp	7	4193590	4268477	0	0.034939
10209	Amp	7	4268573	4283136	0	0.033275
10210	Amp	7	4292155	4293643	0	0.037296
10211	Amp	7	4296845	4307846	0	0.040703
10212	Amp	7	4310055	4311864	0	0.036228
10213	Amp	7	4315439	4425336	0	0.040118
10214	Amp	7	4428619	4428619	0	0.041218
10215	Amp	7	4437319	4439758	0	0.043722
10216	Amp	7	4520589	4520589	0	0.039832
10217	Amp	7	4526963	4535949	0	0.041894
10218	Amp	7	4536283	4536283	0	0.041444
10219	Amp	7	4539593	4542388	0	0.037575
10220	Amp	7	4543637	4646888	0	0.039764
10221	Amp	7	4647542	4693443	0	0.042501
10222	Amp	7	4695508	4703760	0	0.040312
10223	Amp	7	4703812	4828808	0	0.037992
10224	Amp	7	4837381	4860131	0	0.035488
10225	Amp	7	4860407	4885329	0	0.038542
10226	Amp	7	4885381	4906974	0	0.036899
10227	Amp	7	4907246	5044110	0	0.036388
10228	Amp	7	5050416	5133363	0	0.037986

10229	Amp	7	5135074	5373525	0	0.036885
10230	Amp	7	5374971	5437775	0	0.03979
10231	Amp	7	5438291	5446451	0	0.041854
10232	Amp	7	5460362	5529477	0	0.038949
10233	Amp	7	5538858	5597081	0	0.034376
10234	Amp	7	5598591	5616670	0	0.036513
10235	Amp	7	5621055	5709363	0	0.033776
10236	Amp	7	5709591	5709591	0	0.036542
10237	Amp	7	5999270	6002602	0	0.033116
10238	Amp	7	6004292	6038745	0	0.031122
10239	Amp	7	6040654	6081068	0	0.028872
10240	Amp	7	6083054	6116165	0	0.026245
10241	Amp	7	6116242	6139300	0	0.028885
10242	Amp	7	6140476	6152264	0	0.029687
10243	Amp	7	6159441	6250904	0	0.032321
10244	Amp	7	6259468	6405604	0	0.031866
10245	Amp	7	6407410	6514591	0	0.033709
10246	Amp	7	6514784	6579666	0	0.033124
10247	Amp	7	6582236	6701776	0	0.03049
10248	Amp	7	6703120	6736846	0	0.029598
10249	Amp	7	6737427	6739639	0	0.026958
10250	Amp	7	6758397	7115109	0	0.023376
10251	Amp	7	7115182	7161004	0	0.021314
10252	Amp	7	7161250	7270392	0	0.020752
10253	Amp	7	7270635	7502201	0	0.020301
10254	Amp	7	7511477	7511643	0	0.018131
10255	Amp	7	7511657	7878654	0	0.015994
10256	Amp	7	7879267	7930535	0	0.017116
10257	Amp	7	7930929	7945757	0	0.020429
10258	Amp	7	7947025	7965937	0	0.019627
10259	Amp	7	7966150	8138059	0	0.016314
10260	Amp	7	8138095	8138095	0	0.015192
10261	Amp	7	8139207	8500467	0	0.01388
10262	Amp	7	8500606	8517189	0	0.014339
10263	Amp	7	8517855	8578850	0	0.016105
10264	Amp	7	8579226	8656041	0	0.016777
10265	Amp	7	8656154	8757691	0	0.01723
10266	Amp	7	8758068	8761082	0	0.015712
10267	Amp	7	9192894	9223227	0	0.018696
10268	Amp	7	9225516	9267904	0	0.019237
10269	Amp	7	9267923	9398528	0	0.018391
10270	Amp	7	9410439	9491449	0	0.018919
10271	Amp	7	9506647	9556285	0	0.019129
10272	Amp	7	9556672	9599714	0	0.018391
10273	Amp	7	9601163	10010977	0	0.015372
10274	Amp	7	10081314	10231596	0	0.016337
10275	Amp	7	10231685	10414284	0	0.015462
10276	Amp	7	10416231	10495939	0	0.014789
10277	Amp	7	10582054	10691645	0	0.027923
10278	Amp	7	10694427	10701744	0	0.037824
10279	Amp	7	10705171	10735749	0	0.031678
10280	Amp	7	10735786	10836267	0	0.025864
10281	Amp	7	10836566	10959866	0	0.023411

10282	Amp	7	10960264	11310289	0	0.021777
10283	Amp	7	11312647	12163753	0	0.022644
10284	Amp	7	12164444	12273615	0	0.026248
10285	Amp	7	12275550	12377587	0	0.022644
10286	Amp	7	12384403	12477851	0	0.028518
10287	Amp	7	12478004	12513441	0	0.031122
10288	Amp	7	12513603	12705705	0	0.028518
10289	Amp	7	12709002	12710011	0	0.032588
10290	Amp	7	12714412	12721459	0	0.03612
10291	Amp	7	12723343	12801755	0	0.040347
10292	Amp	7	12803095	12885808	0	0.034473
10293	Amp	7	12887496	12907418	0	0.030403
10294	Amp	7	12911059	12947749	0	0.032461
10295	Amp	7	12949225	12950490	0	0.030403
10296	Amp	7	12952537	12995635	0	0.032232
10297	Amp	7	13003687	13091453	0	0.036202
10298	Amp	7	13091589	13160368	0	0.037587
10299	Amp	7	13171582	13278994	0	0.035759
10300	Amp	7	13279871	13309509	0	0.033992
10301	Amp	7	13312942	13354998	0	0.053323
10302	Amp	7	13359070	13434369	0	0.033992
10303	Amp	7	13439053	13465171	0	0.035762
10304	Amp	7	13477329	13515440	0	0.031792
10305	Amp	7	13519356	13596332	0	0.030925
10306	Amp	7	13599094	13717543	0	0.032435
10307	Amp	7	13717843	13837082	0	0.032903
10308	Amp	7	13870143	13898785	0	0.036556
10309	Amp	7	13898839	13929496	0	0.035937
10310	Amp	7	13929521	13929521	0	0.051846
10311	Amp	7	13929641	13929717	0.065715	0.066799
10312	Amp	7	13929828	13937945	0.444474	0.08124
10313	Amp	7	13939184	13958322	0	0.045644
10314	Amp	7	13958336	13958336	0	0.043874
10315	Amp	7	13958367	13973945	0	0.04449
10316	Amp	7	13974004	14026578	0	0.034783
10317	Amp	7	14041079	14056636	0	0.036806
10318	Amp	7	14059617	14097353	0	0.032578
10319	Amp	7	14104181	14143682	0	0.031613
10320	Amp	7	14145989	14158487	0	0.032691
10321	Amp	7	14165891	14240604	0	0.031181
10322	Amp	7	14240923	14363159	0	0.029158
10323	Amp	7	14368738	14447901	0	0.025626
10324	Amp	7	14448190	14467563	0	0.023403
10325	Amp	7	14468253	14579205	0	0.021505
10326	Amp	7	14579401	14583465	0	0.023229
10327	Amp	7	14584279	14635496	0	0.025194
10328	Amp	7	14635868	14691198	0	0.026664
10329	Amp	7	14693870	14745524	0	0.025279
10330	Amp	7	14745627	14868784	0	0.02653
10331	Amp	7	14869167	14886756	0	0.023546
10332	Amp	7	14887918	14911734	0	0.022468
10333	Amp	7	14913951	14950335	0	0.020998
10334	Amp	7	14953462	14969904	0	0.019747

10335 Amp	7	14970188	14970188	0	0.022915
10336 Amp	7	15006368	15171470	0	0.032877
10337 Amp	7	15173752	15294187	0	0.035264
10338 Amp	7	15294345	15546699	0	0.032877
10339 Amp	7	15546766	15633332	0	0.035984
10340 Amp	7	15633480	15674200	0	0.032887
10341 Amp	7	15674309	15969990	0	0.033957
10342 Amp	7	15975010	16250432	0	0.035565
10343 Amp	7	16250846	16525715	0	0.033732
10344 Amp	7	16528801	16536626	0	0.028577
10345 Amp	7	16536842	16536842	0	0.029088
10346 Amp	7	16537353	16649277	0	0.030182
10347 Amp	7	16653200	16706091	0	0.031761
10348 Amp	7	16707924	16836650	0	0.033456
10349 Amp	7	16840602	16946144	0	0.031761
10350 Amp	7	16946466	17016596	0	0.03125
10351 Amp	7	17017095	17759717	0	0.030791
10352 Amp	7	17759976	17782762	0	0.032038
10353 Amp	7	17784000	17821098	0	0.030459
10354 Amp	7	17826185	17873309	0	0.033842
10355 Amp	7	17876402	17910371	0	0.030459
10356 Amp	7	17922069	18106027	0	0.029877
10357 Amp	7	18107283	18276682	0	0.031905
10358 Amp	7	18277741	18444803	0	0.034295
10359 Amp	7	18444957	18449113	0	0.031905
10360 Amp	7	18451180	18484660	0	0.030297
10361 Amp	7	18484674	18543983	0	0.031405
10362 Amp	7	18544583	18588415	0	0.030952
10363 Amp	7	18591204	18754294	0	0.032902
10364 Amp	7	18759207	18867273	0	0.030952
10365 Amp	7	18867407	18867407	0	0.028925
10366 Amp	7	19748494	19867821	0	0.036417
10367 Amp	7	19868198	20014205	0	0.039071
10368 Amp	7	20015130	20108092	0	0.040634
10369 Amp	7	20113178	20207582	0	0.043521
10370 Amp	7	20210462	20337608	0	0.041049
10371 Amp	7	20340246	20604187	0	0.038162
10372 Amp	7	20604717	20746404	0	0.03404
10373 Amp	7	20746691	20761667	0	0.033101
10374 Amp	7	20763634	20764045	0	0.034649
10375 Amp	7	20767922	20833665	0	0.036787
10376 Amp	7	20836394	21011423	0	0.035239
10377 Amp	7	21011518	21069638	0	0.036684
10378 Amp	7	21075491	21112080	0	0.03786
10379 Amp	7	21120838	21155965	0	0.044709
10380 Amp	7	21157471	21194145	0	0.049688
10381 Amp	7	21201161	21279794	0	0.047724
10382 Amp	7	21282820	21293724	0	0.04507
10383 Amp	7	21298457	21385798	0	0.038221
10384 Amp	7	21385890	21405199	0	0.039314
10385 Amp	7	21405698	21518236	0	0.040575
10386 Amp	7	21523706	21537699	0	0.038437
10387 Amp	7	21538034	21591865	0	0.039294

10388	Amp	7	21592489	21601000	0	0.038615
10389	Amp	7	21601572	21646654	0	0.041646
10390	Amp	7	21649963	21687771	0	0.040327
10391	Amp	7	21688409	21820698	0	0.03947
10392	Amp	7	21821188	21872858	0	0.038209
10393	Amp	7	21951914	22067116	0	0.040613
10394	Amp	7	22071756	22112350	0	0.041292
10395	Amp	7	22113259	22196227	0	0.042621
10396	Amp	7	22197860	22228820	0	0.038962
10397	Amp	7	22228943	22236093	0	0.035472
10398	Amp	7	22237217	22271421	0	0.034856
10399	Amp	7	22272032	22301246	0	0.035583
10400	Amp	7	22302364	22369151	0	0.033771
10401	Amp	7	22373122	22407131	0	0.034323
10402	Amp	7	22407989	22431149	0	0.033597
10403	Amp	7	22431812	22448724	0	0.034754
10404	Amp	7	22453337	22526981	0	0.034075
10405	Amp	7	22527792	23314794	0	0.03238
10406	Amp	7	23315622	23316347	0	0.031223
10407	Amp	7	23317973	23343516	0	0.033804
10408	Amp	7	23344020	23369750	0	0.034249
10409	Amp	7	23374400	23386658	0	0.03069
10410	Amp	7	23393180	23396962	0	0.033109
10411	Amp	7	23398355	23539963	0	0.031633
10412	Amp	7	23543294	23555522	0	0.028465
10413	Amp	7	23558973	23674926	0	0.025616
10414	Amp	7	23676297	23706539	0	0.023198
10415	Amp	7	23714842	23766649	0	0.021225
10416	Amp	7	23772677	23778957	0	0.017376
10417	Amp	7	23784394	23784465	0	0.020444
10418	Amp	7	23784857	23819728	0	0.021453
10419	Amp	7	23837530	23895815	0	0.028425
10420	Amp	7	23896492	24036814	0	0.024491
10421	Amp	7	24036866	24076737	0	0.021909
10422	Amp	7	24081171	24200546	0	0.023725
10423	Amp	7	24200768	24212487	0	0.021909
10424	Amp	7	24213352	24226485	0	0.037671
10425	Amp	7	24227739	24248508	0	0.021909
10426	Amp	7	24250223	24276938	0	0.018842
10427	Amp	7	24277087	24381847	0	0.018397
10428	Amp	7	24382222	24465957	0	0.024019
10429	Amp	7	24471592	24541510	0	0.025555
10430	Amp	7	24542681	24542681	0	0.027208
10431	Amp	7	24543155	24571166	0	0.029152
10432	Amp	7	24572464	24659710	0	0.026826
10433	Amp	7	24660093	24674510	0	0.029727
10434	Amp	7	24674981	24793532	0	0.028718
10435	Amp	7	24795238	24930929	0	0.030798
10436	Amp	7	24931870	24943836	0	0.034027
10437	Amp	7	24944217	24944270	0	0.032259
10438	Amp	7	24947651	24947651	0	0.032839
10439	Amp	7	24948871	25032739	0	0.027994
10440	Amp	7	25035496	25072253	0	0.028824

10441 Amp	7	25073635	25101940	0	0.028244
10442 Amp	7	25102577	25124428	0	0.029287
10443 Amp	7	25129190	25152075	0	0.030299
10444 Amp	7	25152592	25255090	0	0.028218
10445 Amp	7	25258136	25268355	0	0.032711
10446 Amp	7	25268650	25329996	0	0.033605
10447 Amp	7	25330416	25412170	0	0.035495
10448 Amp	7	25412406	25413992	0	0.03237
10449 Amp	7	25414562	25459924	0	0.031477
10450 Amp	7	25463484	25484458	0	0.028426
10451 Amp	7	25484807	25500449	0	0.026535
10452 Amp	7	25501706	25553849	0	0.020561
10453 Amp	7	25554157	25561494	0	0.021751
10454 Amp	7	25561508	25587079	0	0.025265
10455 Amp	7	25589469	25637739	0	0.026846
10456 Amp	7	25640178	25766335	0	0.025882
10457 Amp	7	25770232	25791808	0	0.02735
10458 Amp	7	25792857	25814583	0	0.029639
10459 Amp	7	25816496	25865979	0	0.030538
10460 Amp	7	25875219	25937509	0	0.028249
10461 Amp	7	25945319	26072083	0	0.030505
10462 Amp	7	26072262	26178142	0	0.03717
10463 Amp	7	26178414	26179939	0	0.03634
10464 Amp	7	26182832	26248442	0	0.032763
10465 Amp	7	26254637	26275807	0	0.028504
10466 Amp	7	26280903	26304297	0	0.029724
10467 Amp	7	26320974	26320974	0	0.027468
10468 Amp	7	26321634	26547003	0	0.029918
10469 Amp	7	26547115	26593882	0	0.02921
10470 Amp	7	26595206	26753627	0	0.029932
10471 Amp	7	26753896	26791224	0	0.02644
10472 Amp	7	26794468	27134223	0	0.019696
10473 Amp	7	27134697	27171310	0	0.020708
10474 Amp	7	27172046	27205922	0	0.022427
10475 Amp	7	27207660	27347780	0	0.023112
10476 Amp	7	27349579	27350101	0	0.021393
10477 Amp	7	27350824	27425132	0	0.03034
10478 Amp	7	27428731	27480615	0	0.029297
10479 Amp	7	27480707	27826418	0	0.023936
10480 Amp	7	27827744	27890748	0	0.028358
10481 Amp	7	27891347	27940929	0	0.025907
10482 Amp	7	27944026	27955104	0	0.018926
10483 Amp	7	27955139	28134872	0	0.021509
10484 Amp	7	28137682	28216621	0	0.028341
10485 Amp	7	28219126	28238639	0	0.026374
10486 Amp	7	28238753	28280232	0	0.028591
10487 Amp	7	28280343	28401361	0	0.027055
10488 Amp	7	28401789	28473384	0	0.020223
10489 Amp	7	28477404	28603699	0	0.026716
10490 Amp	7	28609126	28609444	0	0.025044
10491 Amp	7	28615084	28680992	0	0.028763
10492 Amp	7	28682322	28752542	0	0.024845
10493 Amp	7	28753154	28760610	0	0.025389

10494	Amp	7	28760993	28832331	0	0.018896
10495	Amp	7	28835171	28886148	0	0.028699
10496	Amp	7	28897253	28918826	0	0.032808
10497	Amp	7	28921467	29060106	0	0.035379
10498	Amp	7	29060336	29134444	0	0.036283
10499	Amp	7	29138876	29624937	0	0.020502
10500	Amp	7	29624952	29628162	0	0.021035
10501	Amp	7	29752062	29987456	0	0.023471
10502	Amp	7	29988252	30166211	0	0.022401
10503	Amp	7	30167115	30182398	0	0.023501
10504	Amp	7	30183818	30302271	0	0.035202
10505	Amp	7	30302643	30539264	0	0.037477
10506	Amp	7	30546604	30552010	0	0.036944
10507	Amp	7	30553240	30564175	0	0.036044
10508	Amp	7	30574892	30679433	0	0.031935
10509	Amp	7	30681381	30753612	0	0.032731
10510	Amp	7	30756914	30799015	0	0.035781
10511	Amp	7	30799224	30807188	0	0.032572
10512	Amp	7	30808321	30979732	0	0.029521
10513	Amp	7	30980282	30997513	0	0.028957
10514	Amp	7	31003319	31081886	0	0.026261
10515	Amp	7	31082056	31139860	0	0.025465
10516	Amp	7	31144874	31178196	0	0.03567
10517	Amp	7	31183666	31199728	0	0.025465
10518	Amp	7	31199864	31426832	0	0.024924
10519	Amp	7	31430079	31750680	0	0.019833
10520	Amp	7	31750866	31754120	0	0.019289
10521	Amp	7	31754185	31804526	0	0.019836
10522	Amp	7	31810263	31812371	0	0.020441
10523	Amp	7	31812874	31939675	0	0.023362
10524	Amp	7	31943585	31954212	0	0.028244
10525	Amp	7	31954513	32006962	0	0.023362
10526	Amp	7	32008369	32092374	0	0.020441
10527	Amp	7	32092558	32133560	0	0.023366
10528	Amp	7	32135952	32177791	0	0.032797
10529	Amp	7	32179123	32249336	0	0.026186
10530	Amp	7	32250015	32256179	0	0.028502
10531	Amp	7	32257142	32267174	0	0.035007
10532	Amp	7	32267712	32287695	0	0.02148
10533	Amp	7	32290724	32346595	0	0.02771
10534	Amp	7	32354930	32364809	0	0.034847
10535	Amp	7	32364976	32445998	0	0.046617
10536	Amp	7	32447368	32656166	0	0.04607
10537	Amp	7	32662014	32717770	0	0.05922
10538	Amp	7	32725825	32733748	0	0.063387
10539	Amp	7	32741845	32849828	0	0.055227
10540	Amp	7	32851070	32852489	0	0.056536
10541	Amp	7	32855543	32895912	0.204831	0.072472
10542	Amp	7	32897783	32975949	0	0.056536
10543	Amp	7	32977043	32978076	0	0.041999
10544	Amp	7	32985576	33179444	0	0.040992
10545	Amp	7	33181254	33198257	0	0.056928
10546	Amp	7	33202862	33222578	0	0.043128

10547 Amp	7	33225559	33252925	0	0.040992
10548 Amp	7	33253245	33329765	0	0.043496
10549 Amp	7	33335497	33385934	0	0.044707
10550 Amp	7	33390396	33488286	0	0.039895
10551 Amp	7	33491798	33560206	0	0.029779
10552 Amp	7	33560291	33751790	0	0.028469
10553 Amp	7	33752555	33807297	0	0.030946
10554 Amp	7	33810773	33898776	0	0.028631
10555 Amp	7	33899024	33910829	0	0.026154
10556 Amp	7	33914555	34050211	0	0.04209
10557 Amp	7	34050460	34073351	0	0.040869
10558 Amp	7	34074218	34088134	0	0.042143
10559 Amp	7	34088872	34295345	0	0.039638
10560 Amp	7	34296445	34442248	0	0.041006
10561 Amp	7	34443721	34465832	0	0.043092
10562 Amp	7	34469126	34559473	0	0.044632
10563 Amp	7	34564786	34626626	0	0.041707
10564 Amp	7	34626922	34965611	0	0.040167
10565 Amp	7	34969381	35024700	0	0.043218
10566 Amp	7	35029184	35111991	0	0.042118
10567 Amp	7	35118043	35226478	0	0.048245
10568 Amp	7	35227365	35289860	0	0.04014
10569 Amp	7	35290168	35463990	0	0.039534
10570 Amp	7	35464063	35552498	0	0.037098
10571 Amp	7	35553483	35704861	0	0.035334
10572 Amp	7	35705786	35891153	0	0.037098
10573 Amp	7	35891205	35901856	0	0.039275
10574 Amp	7	35906411	35913183	0	0.044483
10575 Amp	7	35913262	35969958	0	0.045698
10576 Amp	7	35975281	35975699	0	0.04049
10577 Amp	7	35977115	36002612	0	0.037812
10578 Amp	7	36002712	36110363	0	0.038621
10579 Amp	7	36110904	36139130	0	0.039101
10580 Amp	7	36139594	36146441	0	0.03425
10581 Amp	7	36149110	36188858	0	0.03377
10582 Amp	7	36189798	36239615	0	0.034979
10583 Amp	7	36242099	36303553	0	0.029908
10584 Amp	7	36317052	36363420	0	0.029413
10585 Amp	7	36364746	36463186	0	0.028199
10586 Amp	7	36464079	36506018	0	0.02699
10587 Amp	7	36506410	36779219	0	0.029333
10588 Amp	7	36779697	36875580	0	0.02824
10589 Amp	7	36877279	36949397	0	0.017374
10590 Amp	7	36951844	37228150	0	0.028045
10591 Amp	7	37233148	37308459	0	0.02677
10592 Amp	7	37308618	37350372	0	0.02596
10593 Amp	7	37350941	37351662	0	0.026843
10594 Amp	7	37363179	37415662	0	0.029629
10595 Amp	7	37415678	37453457	0	0.028747
10596 Amp	7	37456245	37514044	0	0.030328
10597 Amp	7	37515195	37675206	0	0.033603
10598 Amp	7	37675887	37713103	0	0.034592
10599 Amp	7	37714789	37732354	0	0.037803

10600	Amp	7	37738537	37766076	0	0.034528
10601	Amp	7	37771423	37772775	0	0.032351
10602	Amp	7	37776229	37776229	0	0.03077
10603	Amp	7	37777553	37831182	0	0.031873
10604	Amp	7	37832737	37842112	0	0.024775
10605	Amp	7	37845519	37861586	0	0.028284
10606	Amp	7	37861751	37890198	0	0.032391
10607	Amp	7	37891879	37896928	0	0.029558
10608	Amp	7	37897431	37933225	0	0.028882
10609	Amp	7	37933412	37974314	0	0.026176
10610	Amp	7	37974469	38026397	0	0.015204
10611	Amp	7	38027975	38419189	0	0.020793
10612	Amp	7	38419301	38461598	0	0.0182
10613	Amp	7	38462602	38556029	0	0.02146
10614	Amp	7	38558417	38565266	0	0.023717
10615	Amp	7	38569059	38576829	0	0.020457
10616	Amp	7	38577422	38606329	0	0.036275
10617	Amp	7	38607150	38637692	0	0.031707
10618	Amp	7	38638582	38750304	0	0.035863
10619	Amp	7	38750952	38755167	0	0.041871
10620	Amp	7	38755326	38755326	0	0.043238
10621	Amp	7	38760950	38770392	0	0.051335
10622	Amp	7	38770456	38814172	0	0.049078
10623	Amp	7	38814312	38861777	0	0.048561
10624	Amp	7	38863135	38884791	0	0.051195
10625	Amp	7	38888236	38891203	0	0.046828
10626	Amp	7	38897683	38957841	0	0.048962
10627	Amp	7	38959249	38987176	0	0.047595
10628	Amp	7	38987294	39113531	0	0.043824
10629	Amp	7	39114141	39211860	0	0.047318
10630	Amp	7	39224145	39347402	0	0.044265
10631	Amp	7	39349348	39449136	0	0.05597
10632	Amp	7	39451267	39500541	0	0.059298
10633	Amp	7	39514696	39541441	0	0.051177
10634	Amp	7	39543223	39579239	0	0.05309
10635	Amp	7	39580396	39612763	0	0.050456
10636	Amp	7	39613034	39616419	0	0.055124
10637	Amp	7	39620177	39657936	0	0.06075
10638	Amp	7	39660427	39782478	0	0.061472
10639	Amp	7	39784133	39815968	0	0.056804
10640	Amp	7	39816755	39888347	0	0.051178
10641	Amp	7	39890919	39897024	0	0.054586
10642	Amp	7	39897202	39917230	0	0.052453
10643	Amp	7	39920071	39927107	0	0.059064
10644	Amp	7	39932217	40022118	0.029031	0.065276
10645	Amp	7	40022228	40145413	0	0.059594
10646	Amp	7	40146383	40149082	0	0.060997
10647	Amp	7	40151519	40176295	0	0.056741
10648	Amp	7	40180618	40259279	0	0.055888
10649	Amp	7	40262291	40300457	0	0.05764
10650	Amp	7	40304329	40329283	0	0.045935
10651	Amp	7	40329304	40623817	0	0.043719
10652	Amp	7	40627088	40791871	0	0.042315

10653	Amp	7	40796692	40818260	0	0.041593
10654	Amp	7	40823164	40999702	0	0.038822
10655	Amp	7	41000910	41024849	0	0.04174
10656	Amp	7	41025944	41154823	0	0.038822
10657	Amp	7	41159894	41176282	0	0.035381
10658	Amp	7	41176459	41245367	0	0.0387
10659	Amp	7	41248234	41276943	0	0.03242
10660	Amp	7	41279662	41293894	0	0.036811
10661	Amp	7	41294012	41483237	0	0.038174
10662	Amp	7	41485994	41549021	0	0.040864
10663	Amp	7	41549778	41735378	0	0.045935
10664	Amp	7	41735680	41772846	0	0.043504
10665	Amp	7	41772885	41792541	0	0.042141
10666	Amp	7	41795637	41796424	0	0.037658
10667	Amp	7	41796577	41857262	0	0.039543
10668	Amp	7	41857570	41879029	0	0.044632
10669	Amp	7	41879878	41886554	0	0.04759
10670	Amp	7	41891123	41891123	0	0.052361
10671	Amp	7	41898217	41935319	0	0.057205
10672	Amp	7	41935522	41949297	0	0.052115
10673	Amp	7	41955781	41963667	0	0.059373
10674	Amp	7	41968830	41972520	0	0.057145
10675	Amp	7	41975840	41998238	0	0.052754
10676	Amp	7	41998585	42044838	0	0.057473
10677	Amp	7	42048285	42061764	0	0.05166
10678	Amp	7	42061894	42074327	0	0.053208
10679	Amp	7	42074796	42093630	0	0.049827
10680	Amp	7	42096620	42221941	0	0.050411
10681	Amp	7	42227003	42227003	0	0.052606
10682	Amp	7	42227737	42337240	0	0.052022
10683	Amp	7	42339049	42388382	0	0.050475
10684	Amp	7	42388861	42420063	0	0.055601
10685	Amp	7	42420783	43250941	0	0.050864
10686	Amp	7	43252724	43345748	0	0.036977
10687	Amp	7	43349338	43459073	0	0.035064
10688	Amp	7	43459115	43578498	0	0.032106
10689	Amp	7	43580584	43667013	0	0.045992
10690	Amp	7	43670989	43767670	0	0.047421
10691	Amp	7	43769595	43810913	0	0.051905
10692	Amp	7	43819887	43861188	0.050473	0.066058
10693	Amp	7	43865174	43868616	0	0.054595
10694	Amp	7	43871083	43931554	0	0.053166
10695	Amp	7	43932391	43943578	0	0.048039
10696	Amp	7	43944635	43954727	0	0.033886
10697	Amp	7	43954744	44032262	0	0.030625
10698	Amp	7	44041495	44041495	0	0.0367
10699	Amp	7	44042643	44043853	0	0.039933
10700	Amp	7	44046467	44060065	0	0.051359
10701	Amp	7	44061176	44078934	0	0.05182
10702	Amp	7	44080443	44083822	0.079652	0.067511
10703	Amp	7	44092741	44137813	0.112543	0.06882
10704	Amp	7	44139144	44184440	0.010261	0.064337
10705	Amp	7	44187114	44473535	0	0.059282

10706	Amp	7	44474360	44509319	0	0.061184
10707	Amp	7	44512211	44570891	0	0.060587
10708	Amp	7	44572630	44589597	0	0.05976
10709	Amp	7	44597588	44654298	0	0.060685
10710	Amp	7	44654987	44773897	0	0.062343
10711	Amp	7	44774573	44775735	0	0.047843
10712	Amp	7	44776251	44782138	0	0.049881
10713	Amp	7	44787918	44822971	0	0.038484
10714	Amp	7	44825348	44951034	0	0.049039
10715	Amp	7	44954299	45016035	0	0.048114
10716	Amp	7	45017071	45019535	0	0.047653
10717	Amp	7	45021654	45024281	0	0.049235
10718	Amp	7	45025887	45072344	0	0.047197
10719	Amp	7	45073028	45073028	0	0.04795
10720	Amp	7	45076637	45243516	0	0.056935
10721	Amp	7	45243570	45339861	0	0.052775
10722	Amp	7	45352744	45390343	0	0.051193
10723	Amp	7	45393601	45415071	0.073902	0.067129
10724	Amp	7	45419405	45435116	0	0.060906
10725	Amp	7	45437247	45544286	0	0.050351
10726	Amp	7	45548396	45584850	0	0.046884
10727	Amp	7	45586339	45665418	0	0.043858
10728	Amp	7	45669805	45760564	0	0.045631
10729	Amp	7	45763230	45780234	0	0.044165
10730	Amp	7	45789996	45792467	0	0.046976
10731	Amp	7	45822404	45851321	0	0.045203
10732	Amp	7	45860658	45876964	0	0.042392
10733	Amp	7	45880750	45892627	0	0.046881
10734	Amp	7	45892698	45910847	0	0.042392
10735	Amp	7	45913798	45954657	0	0.046881
10736	Amp	7	45954810	45970501	0	0.05033
10737	Amp	7	45978250	45980463	0	0.045841
10738	Amp	7	45981516	45995583	0	0.049018
10739	Amp	7	45997886	46194418	0	0.042392
10740	Amp	7	46194485	46368772	0	0.045692
10741	Amp	7	46368982	46457691	0	0.043791
10742	Amp	7	46459427	46547653	0	0.040491
10743	Amp	7	46550509	46826379	0	0.029515
10744	Amp	7	46826814	46886471	0	0.030041
10745	Amp	7	46889731	46889731	0	0.031546
10746	Amp	7	46892366	46892885	0	0.035415
10747	Amp	7	46895041	46932607	0	0.034889
10748	Amp	7	46935586	46973197	0	0.037766
10749	Amp	7	46973499	47040724	0	0.036261
10750	Amp	7	47041316	47106085	0	0.03698
10751	Amp	7	47106226	47106380	0	0.030754
10752	Amp	7	47115017	47183968	0	0.036416
10753	Amp	7	47186601	47270593	0	0.030857
10754	Amp	7	47271185	47360241	0	0.027601
10755	Amp	7	47363969	47396900	0	0.029039
10756	Amp	7	47396961	47410744	0	0.032932
10757	Amp	7	47411034	47411034	0	0.032214
10758	Amp	7	47412320	47523707	0	0.026552

10759	Amp	7	47523901	47550680	0	0.025114
10760	Amp	7	47551151	47875372	0	0.021221
10761	Amp	7	47876053	48221196	0	0.019703
10762	Amp	7	48225409	48283068	0	0.018343
10763	Amp	7	48283433	48331810	0	0.021247
10764	Amp	7	48332233	48403973	0	0.019973
10765	Amp	7	48404191	48474016	0	0.015013
10766	Amp	7	48474307	48510037	0	0.020451
10767	Amp	7	48510906	48521499	0	0.021869
10768	Amp	7	48527238	48572733	0	0.029134
10769	Amp	7	48575544	48623583	0	0.038056
10770	Amp	7	48624484	48660778	0	0.030111
10771	Amp	7	48661071	49142188	0	0.028694
10772	Amp	7	49149362	49427422	0	0.023935
10773	Amp	7	49429033	49474827	0	0.02596
10774	Amp	7	49479534	49517209	0	0.017038
10775	Amp	7	49520259	49623140	0	0.019662
10776	Amp	7	49623348	49644977	0	0.021379
10777	Amp	7	49647917	49837482	0	0.018755
10778	Amp	7	49837844	49846258	0	0.017038
10779	Amp	7	49849837	50120593	0	0.017626
10780	Amp	7	50122239	50202553	0	0.018863
10781	Amp	7	50203892	50281878	0	0.016838
10782	Amp	7	50282294	50663476	0	0.017551
10783	Amp	7	50666460	50911521	0	0.01879
10784	Amp	7	50912611	51105278	0	0.021089
10785	Amp	7	51106704	51418071	0	0.01879
10786	Amp	7	51421481	51687967	0	0.017551
10787	Amp	7	51688367	51695205	0	0.018032
10788	Amp	7	51695930	51880031	0	0.018602
10789	Amp	7	51880412	51940883	0	0.017889
10790	Amp	7	51942612	52005261	0	0.014986
10791	Amp	7	52005374	52019343	0	0.013233
10792	Amp	7	52027921	52279517	0	0.017535
10793	Amp	7	52279594	52326387	0	0.016946
10794	Amp	7	52326990	52465329	0	0.019022
10795	Amp	7	52466719	52858537	0	0.016946
10796	Amp	7	52858655	52909127	0	0.019986
10797	Amp	7	52910140	53018545	0	0.016946
10798	Amp	7	53022421	53041359	0	0.02155
10799	Amp	7	53043833	53115587	0	0.016946
10800	Amp	7	53116049	53169871	0	0.013538
10801	Amp	7	53170796	53226348	0	0.016524
10802	Amp	7	53227265	53536037	0	0.01776
10803	Amp	7	53536776	53781682	0	0.018463
10804	Amp	7	53782820	53968057	0	0.017982
10805	Amp	7	53968647	54236476	0	0.018928
10806	Amp	7	54236490	54329793	0	0.021937
10807	Amp	7	54333159	54727577	0	0.022492
10808	Amp	7	54728670	55131406	0	0.021922
10809	Amp	7	55131489	55199064	0	0.020686
10810	Amp	7	55199467	55256626	0	0.019523
10811	Amp	7	55258817	55375006	0	0.016515

10812	Amp	7	55379240	55665013	0	0.017646
10813	Amp	7	55666112	55755888	0	0.017068
10814	Amp	7	55765381	55965448	0	0.015937
10815	Amp	7	55965464	56443141	0	0.01499
10816	Amp	7	56444131	56531430	0	0.016893
10817	Amp	7	56535439	57135709	0	0.01499
10818	Amp	7	57138060	57176334	0	0.014263
10819	Amp	7	57184979	57185111	0	0.015565
10820	Amp	7	57854817	57876479	0	0.013738
10821	Amp	7	63672748	63677725	0	0.015222
10822	Amp	7	63690237	63856364	0	0.017825
10823	Amp	7	63857880	63896590	0	0.019328
10824	Amp	7	63903052	63921298	0	0.018773
10825	Amp	7	63924054	64044797	0	0.018069
10826	Amp	7	64045459	64060367	0	0.020175
10827	Amp	7	64330045	64476011	0	0.023615
10828	Amp	7	64477208	64731896	0	0.02151
10829	Amp	7	64733284	64892547	0	0.022389
10830	Amp	7	64893313	65041135	0	0.02151
10831	Amp	7	65046526	65050285	0	0.02388
10832	Amp	7	65052476	65052476	0	0.025706
10833	Amp	7	65714988	65727888	0	0.037831
10834	Amp	7	65728909	65827971	0	0.034692
10835	Amp	7	65830040	65854350	0	0.035702
10836	Amp	7	65861729	65891976	0	0.038802
10837	Amp	7	65896711	65902717	0	0.036432
10838	Amp	7	65908527	65942683	0	0.035196
10839	Amp	7	66005756	66063677	0	0.042176
10840	Amp	7	66066060	66093235	0	0.044463
10841	Amp	7	66102096	66244790	0	0.033745
10842	Amp	7	66245001	66583623	0	0.034223
10843	Amp	7	66587683	66673298	0	0.033213
10844	Amp	7	66680876	66681718	0	0.032692
10845	Amp	7	66686734	66746023	0	0.030865
10846	Amp	7	66754357	66781611	0	0.039136
10847	Amp	7	66783098	66922292	0	0.037057
10848	Amp	7	66926911	67017101	0	0.032556
10849	Amp	7	67021914	67070504	0	0.030987
10850	Amp	7	67072636	67072636	0	0.032756
10851	Amp	7	67075488	67293090	0	0.031066
10852	Amp	7	67307705	67460540	0	0.029763
10853	Amp	7	67460738	67487755	0	0.030288
10854	Amp	7	67487873	67552891	0	0.032024
10855	Amp	7	67553034	67579815	0	0.032869
10856	Amp	7	67583271	67605680	0	0.028476
10857	Amp	7	67607546	67698449	0	0.030669
10858	Amp	7	67698508	68280415	0	0.032826
10859	Amp	7	68280603	68330561	0	0.036465
10860	Amp	7	68331020	68390837	0	0.035941
10861	Amp	7	68391408	68419198	0	0.036532
10862	Amp	7	68419300	68586004	0	0.032893
10863	Amp	7	68586021	68634291	0	0.032331
10864	Amp	7	68636293	68659962	0	0.033047

10865	Amp	7	68661759	68967369	0	0.031843
10866	Amp	7	68968611	69131322	0	0.033949
10867	Amp	7	69135839	69208428	0	0.036169
10868	Amp	7	69216776	69243524	0	0.034063
10869	Amp	7	69248936	69313207	0	0.03534
10870	Amp	7	69313319	69340324	0	0.03452
10871	Amp	7	69342609	69362006	0	0.036172
10872	Amp	7	69365429	69379000	0	0.033952
10873	Amp	7	69381278	69396167	0	0.032183
10874	Amp	7	69403475	69403475	0	0.034439
10875	Amp	7	69403716	69407592	0	0.035771
10876	Amp	7	69417532	69419408	0	0.038414
10877	Amp	7	69420189	69493763	0	0.0406
10878	Amp	7	69498382	69504220	0	0.043958
10879	Amp	7	69505706	69575736	0	0.042681
10880	Amp	7	69576243	69583351	0	0.040038
10881	Amp	7	69589974	69710705	0	0.041952
10882	Amp	7	69712858	69785590	0	0.0403
10883	Amp	7	69787043	69787043	0	0.043354
10884	Amp	7	69791489	69797347	0	0.048502
10885	Amp	7	69800703	69827914	0	0.047786
10886	Amp	7	69831661	69860557	0	0.044845
10887	Amp	7	69862830	69945181	0	0.043512
10888	Amp	7	69947797	69964591	0	0.041355
10889	Amp	7	69964921	70163449	0	0.043025
10890	Amp	7	70167766	70214695	0	0.043581
10891	Amp	7	70219377	70432827	0	0.044073
10892	Amp	7	70432840	70497702	0	0.046192
10893	Amp	7	70498627	70498627	0	0.044934
10894	Amp	7	70504120	70504414	0	0.047797
10895	Amp	7	70505868	70520637	0	0.04834
10896	Amp	7	70522961	70527139	0	0.050582
10897	Amp	7	70529644	70625548	0	0.050026
10898	Amp	7	70625866	70628133	0	0.053349
10899	Amp	7	70630474	70666837	0	0.055737
10900	Amp	7	70666891	70670375	0	0.054519
10901	Amp	7	70670461	70671779	0	0.052132
10902	Amp	7	70673359	70701776	0	0.055445
10903	Amp	7	70702050	70724326	0	0.056065
10904	Amp	7	70740467	70759757	0	0.062042
10905	Amp	7	70763963	70779318	0	0.062494
10906	Amp	7	70781926	70797150	0.005328	0.064089
10907	Amp	7	70806671	70827343	0	0.05974
10908	Amp	7	70829310	70909429	0	0.060921
10909	Amp	7	70911241	70939160	0	0.058802
10910	Amp	7	70940093	70941021	0	0.062325
10911	Amp	7	70943280	70987174	0	0.059002
10912	Amp	7	70987415	71106761	0	0.056384
10913	Amp	7	71107250	71497732	0	0.054647
10914	Amp	7	71500889	71594978	0	0.052045
10915	Amp	7	71595645	71695709	0	0.048431
10916	Amp	7	71697062	71697062	0	0.047075
10917	Amp	7	71702254	71733897	0	0.046383

10918	Amp	7	71734145	71752151	0	0.047427
10919	Amp	7	71754096	71764555	0	0.04659
10920	Amp	7	71765954	71766716	0	0.044676
10921	Amp	7	71766939	71921629	0	0.045356
10922	Amp	7	71929241	71948343	0	0.049281
10923	Amp	7	71948399	72040912	0	0.050782
10924	Amp	7	72046037	72060168	0	0.043954
10925	Amp	7	72060211	72183011	0	0.044805
10926	Amp	7	72183614	72194193	0	0.047547
10927	Amp	7	72228448	72366500	0	0.043591
10928	Amp	7	72367144	72413805	0	0.042971
10929	Amp	7	72415237	72448559	0	0.044522
10930	Amp	7	72449167	72454756	0	0.043871
10931	Amp	7	72457559	72470224	0	0.04553
10932	Amp	7	72471304	72476454	0	0.04209
10933	Amp	7	72477253	72853922	0	0.040909
10934	Amp	7	72854426	72872372	0	0.03925
10935	Amp	7	72880997	72880997	0	0.044029
10936	Amp	7	72889156	72889156	0	0.039496
10937	Amp	7	72897281	72911579	0	0.040968
10938	Amp	7	72912917	72931090	0	0.039468
10939	Amp	7	72931874	72931874	0	0.037001
10940	Amp	7	73312738	73312738	0	0.050797
10941	Amp	7	73317954	73325738	0	0.048055
10942	Amp	7	73338359	73341521	0	0.051547
10943	Amp	7	73342335	73351571	0	0.050558
10944	Amp	7	73355394	73420826	0	0.055539
10945	Amp	7	73439787	73474169	0	0.056264
10946	Amp	7	73475535	73538697	0	0.050861
10947	Amp	7	73548101	73569970	0	0.052242
10948	Amp	7	73571446	73811362	0	0.052995
10949	Amp	7	73813311	73914994	0	0.044856
10950	Amp	7	73919095	73944754	0	0.050097
10951	Amp	7	73944846	74092342	0	0.052219
10952	Amp	7	74095174	74191026	0	0.051465
10953	Amp	7	74194985	74288238	0	0.054253
10954	Amp	7	74292303	74323737	0	0.051859
10955	Amp	7	74326601	74478286	0	0.056153
10956	Amp	7	74482925	74482955	0	0.043225
10957	Amp	7	74483258	74499898	0	0.041434
10958	Amp	7	74500058	74503925	0	0.053412
10959	Amp	7	74506964	74545016	0	0.051842
10960	Amp	7	74549434	74549434	0	0.055189
10961	Amp	7	74549850	74571656	0	0.050485
10962	Amp	7	74573803	74616516	0	0.051783
10963	Amp	7	74617136	74635973	0	0.051103
10964	Amp	7	74645101	74701068	0	0.0486
10965	Amp	7	74701388	74721470	0	0.045812
10966	Amp	7	74722165	74727766	0	0.047077
10967	Amp	7	74840595	74870728	0	0.033314
10968	Amp	7	74920689	74944290	0	0.031002
10969	Amp	7	74951861	74953065	0	0.029622
10970	Amp	7	74972480	74972480	0	0.025156

10971 Amp	7	75052263	75067998	0	0.023321
10972 Amp	7	75115775	75428935	0	0.021371
10973 Amp	7	75429099	75453095	0	0.025204
10974 Amp	7	75455754	75546626	0	0.021371
10975 Amp	7	75547002	75654065	0	0.020326
10976 Amp	7	75654096	75693231	0	0.019061
10977 Amp	7	75694264	75752294	0	0.019511
10978 Amp	7	75753821	75790967	0	0.021221
10979 Amp	7	75795224	75826923	0	0.022265
10980 Amp	7	75827997	75887098	0	0.019392
10981 Amp	7	75887968	75961490	0	0.018348
10982 Amp	7	75962111	76026671	0	0.021881
10983 Amp	7	76027788	76119594	0	0.013367
10984 Amp	7	76130770	76424968	0	0.015636
10985 Amp	7	76426530	76426530	0	0.013367
10986 Amp	7	77130464	77135653	0	0.012165
10987 Amp	7	77136126	77437940	0	0.013234
10988 Amp	7	77440878	77453579	0	0.01042
10989 Amp	7	77453902	77472215	0	0.02166
10990 Amp	7	77477480	77627396	0	0.023301
10991 Amp	7	77630518	77725973	0	0.012061
10992 Amp	7	77728096	77743589	0	0.014556
10993 Amp	7	77744721	77915759	0	0.012915
10994 Amp	7	77915787	77985146	0	0.0153
10995 Amp	7	77993234	78008438	0	0.017478
10996 Amp	7	78008759	78028211	0	0.019199
10997 Amp	7	78028543	78092514	0	0.016815
10998 Amp	7	78095071	78196814	0	0.018084
10999 Amp	7	78196932	78204659	0	0.019521
11000 Amp	7	78205467	78284154	0	0.018084
11001 Amp	7	78284601	78532836	0	0.020535
11002 Amp	7	78541271	78552051	0	0.02218
11003 Amp	7	78559644	78613763	0	0.019729
11004 Amp	7	78613824	78759945	0	0.023694
11005 Amp	7	78761222	78851850	0	0.030536
11006 Amp	7	78855655	78866185	0	0.02657
11007 Amp	7	78866521	78933111	0	0.027338
11008 Amp	7	78940833	78943418	0	0.020497
11009 Amp	7	78943714	78949344	0	0.023591
11010 Amp	7	78949389	78954258	0	0.022823
11011 Amp	7	78958376	78962688	0	0.025741
11012 Amp	7	78964751	78964751	0	0.031065
11013 Amp	7	78965719	78985746	0	0.027971
11014 Amp	7	78986104	78998691	0	0.022647
11015 Amp	7	79001873	79051410	0	0.019729
11016 Amp	7	79053481	79090881	0	0.018459
11017 Amp	7	79095060	79127223	0	0.016815
11018 Amp	7	79129775	79159758	0	0.015093
11019 Amp	7	79160386	79273718	0	0.012915
11020 Amp	7	79279670	79296806	0	0.014498
11021 Amp	7	79299038	79309114	0	0.016469
11022 Amp	7	79311699	79318147	0	0.01782
11023 Amp	7	79321459	79370432	0	0.015325

11024	Amp	7	79370434	79621532	0	0.013773
11025	Amp	7	79623495	79703631	0	0.014646
11026	Amp	7	79704070	80012969	0	0.013295
11027	Amp	7	80014642	80181211	0	0.014744
11028	Amp	7	80182155	80236962	0	0.013871
11029	Amp	7	80239541	80348332	0	0.014514
11030	Amp	7	80349317	80520815	0	0.012931
11031	Amp	7	80525147	80570018	0	0.027726
11032	Amp	7	80570434	80682478	0	0.03007
11033	Amp	7	80687758	80766721	0	0.027726
11034	Amp	7	80770195	80837560	0	0.025363
11035	Amp	7	80842219	80885787	0	0.029993
11036	Amp	7	80890700	80966261	0	0.025363
11037	Amp	7	80972846	80987668	0	0.030097
11038	Amp	7	80988707	81358366	0	0.025363
11039	Amp	7	81367271	81533645	0	0.026422
11040	Amp	7	81534338	81549466	0	0.024973
11041	Amp	7	81550629	81773348	0	0.02626
11042	Amp	7	81774578	81779836	0	0.025617
11043	Amp	7	81779913	81792440	0	0.022271
11044	Amp	7	81793046	81984649	0	0.024371
11045	Amp	7	81985813	82033959	0	0.026646
11046	Amp	7	82034112	82201510	0	0.027783
11047	Amp	7	82203949	82279077	0	0.026496
11048	Amp	7	82279409	82279409	0	0.028141
11049	Amp	7	82285397	82553123	0	0.025866
11050	Amp	7	82554167	82583491	0	0.027428
11051	Amp	7	82584277	82600645	0	0.025866
11052	Amp	7	82601669	82626890	0	0.017687
11053	Amp	7	82627800	82719165	0	0.019566
11054	Amp	7	82724992	83106302	0	0.020938
11055	Amp	7	83106342	83580025	0	0.022321
11056	Amp	7	83583948	83608907	0	0.020442
11057	Amp	7	83621110	83655275	0	0.019992
11058	Amp	7	83656595	83857325	0	0.018609
11059	Amp	7	83860523	83866235	0	0.019797
11060	Amp	7	83870422	84405547	0	0.018609
11061	Amp	7	84409267	84445710	0	0.022111
11062	Amp	7	84446121	84515115	0	0.0247
11063	Amp	7	84522713	84662652	0	0.025603
11064	Amp	7	84663236	84669400	0	0.023959
11065	Amp	7	84670154	84736258	0	0.02137
11066	Amp	7	84737558	84841891	0	0.021963
11067	Amp	7	84841973	84883832	0	0.019889
11068	Amp	7	84890696	85054608	0	0.021444
11069	Amp	7	85059542	85174960	0	0.02476
11070	Amp	7	85176712	85212315	0	0.023205
11071	Amp	7	85212489	85708726	0	0.022068
11072	Amp	7	85709557	86053604	0	0.019914
11073	Amp	7	86057602	86190851	0	0.023945
11074	Amp	7	86196473	86485986	0	0.024742
11075	Amp	7	86486221	86568546	0	0.023674
11076	Amp	7	86572765	86598125	0	0.020358

11077	Amp	7	86600010	86687209	0	0.02164
11078	Amp	7	86687663	86757363	0	0.023459
11079	Amp	7	86758358	87332046	0	0.028444
11080	Amp	7	87334113	87348578	0	0.029351
11081	Amp	7	87350026	87736589	0	0.028069
11082	Amp	7	87749767	87881472	0	0.027272
11083	Amp	7	87883582	88004212	0	0.031115
11084	Amp	7	88010584	88187203	0	0.034959
11085	Amp	7	88191552	88230641	0	0.03314
11086	Amp	7	88231309	88235085	0	0.032233
11087	Amp	7	88238419	88255095	0	0.033061
11088	Amp	7	88258230	88363451	0	0.035326
11089	Amp	7	88367905	88498427	0	0.031482
11090	Amp	7	88503107	88509034	0	0.030654
11091	Amp	7	88511864	88517017	0	0.031546
11092	Amp	7	90266400	90424817	0	0.025134
11093	Amp	7	90425430	90684222	0	0.027113
11094	Amp	7	90688742	90795902	0	0.024987
11095	Amp	7	90799750	90845938	0	0.023008
11096	Amp	7	90846183	91824970	0	0.01928
11097	Amp	7	91833191	91935392	0	0.021789
11098	Amp	7	91939161	92522644	0	0.01928
11099	Amp	7	92525795	92666196	0	0.018221
11100	Amp	7	92666661	92870751	0	0.019511
11101	Amp	7	92870847	92871241	0	0.030123
11102	Amp	7	92871531	92874428	0	0.029453
11103	Amp	7	92874444	93230220	0	0.018221
11104	Amp	7	93230333	93720539	0	0.01733
11105	Amp	7	93720650	94036640	0	0.018934
11106	Amp	7	94037066	94356712	0	0.01733
11107	Amp	7	94363370	94638954	0	0.017833
11108	Amp	7	94642122	94982093	0	0.01693
11109	Amp	7	94988226	95352688	0	0.020012
11110	Amp	7	95353865	95779435	0	0.01693
11111	Amp	7	95781867	96193150	0	0.016426
11112	Amp	7	96195423	96326244	0	0.019089
11113	Amp	7	96326727	97251304	0	0.014597
11114	Amp	7	97251535	97339016	0	0.013226
11115	Amp	7	97339224	97846981	0	0.010564
11116	Amp	7	97856901	98015093	0	0.009971
11117	Amp	7	98015139	98122456	0	0.010623
11118	Amp	7	98122820	98300368	0	0.009971
11119	Amp	7	98306359	98816033	0	0.012067
11120	Amp	7	99033743	99177115	0	0.018249
11121	Amp	7	99177203	99515882	0	0.014658
11122	Amp	7	99517366	100052296	0	0.013961
11123	Amp	7	100062261	100111122	0	0.014773
11124	Amp	7	100115339	100217624	0	0.016979
11125	Amp	7	100229612	100255128	0	0.015895
11126	Amp	7	100272870	100447886	0	0.013688
11127	Amp	7	100471279	100647715	0	0.027411
11128	Amp	7	100657847	100723515	0	0.028099
11129	Amp	7	100758170	100783390	0	0.028593

11130	Amp	7	100790683	100817460	0	0.030287
11131	Amp	7	100820211	101083860	0	0.026175
11132	Amp	7	101093388	101190660	0	0.019682
11133	Amp	7	101200312	101289150	0	0.014996
11134	Amp	7	101294727	101311575	0	0.017396
11135	Amp	7	101311641	101518256	0	0.018786
11136	Amp	7	101518850	101595691	0	0.014497
11137	Amp	7	101596646	101810360	0	0.012687
11138	Amp	7	101811336	102038521	0	0.011297
11139	Amp	7	102039221	102128266	0	0.010804
11140	Amp	7	102158380	102207865	0	0.005384
11141	Amp	7	102211855	102326935	0	0.002984
11142	Amp	7	102327247	103364713	0	0.002295
11143	Amp	7	103369965	103542388	0	0.003238
11144	Amp	7	103542586	103633781	0	0.004433
11145	Amp	7	103634120	103832540	0	0.003238
11146	Amp	7	103838622	103876610	0	0.00369
11147	Amp	7	103877286	103978851	0	0.004837
11148	Amp	7	103979066	104022350	0	0.00369
11149	Amp	7	104033156	104398165	0	0.002207
11150	Amp	7	104402424	104428116	0	0.001754
11151	Amp	7	104431485	104581547	0	0.003071
11152	Amp	7	104595300	104724348	0	0.002128
11153	Amp	7	104726317	105019466	0	0.003164
11154	Amp	7	105036075	105372232	0	0.007182
11155	Amp	7	105557943	105575523	0	0.00966
11156	Amp	7	105576247	105637754	0	0.010241
11157	Amp	7	105640067	105666674	0	0.007763
11158	Amp	7	105674124	105731239	0	0.012199
11159	Amp	7	105735169	106105534	0	0.023517
11160	Amp	7	106105879	106559080	0	0.022482
11161	Amp	7	106564630	106653943	0	0.023975
11162	Amp	7	106659297	106715821	0	0.022482
11163	Amp	7	106716200	107202934	0	0.018463
11164	Amp	7	107206590	107276993	0	0.02098
11165	Amp	7	107280403	107374153	0	0.018463
11166	Amp	7	107381747	108348274	0	0.022952
11167	Amp	7	108351123	108441521	0	0.030242
11168	Amp	7	108441931	108489234	0	0.033441
11169	Amp	7	108489338	108566242	0	0.03263
11170	Amp	7	108566621	108618349	0	0.035097
11171	Amp	7	108623722	108653142	0	0.030609
11172	Amp	7	108653427	108739693	0	0.028141
11173	Amp	7	108739887	109329976	0	0.030774
11174	Amp	7	109354667	109412302	0	0.028432
11175	Amp	7	109419663	109491863	0	0.023998
11176	Amp	7	109492997	109663555	0	0.025934
11177	Amp	7	109664613	109716682	0	0.020045
11178	Amp	7	109726965	109740030	0	0.025285
11179	Amp	7	109744385	109839119	0	0.019014
11180	Amp	7	109843683	109958852	0	0.017077
11181	Amp	7	109959328	110013161	0	0.015403
11182	Amp	7	110014156	110051141	0	0.01277

11183	Amp	7	110056346	110198321	0	0.006736
11184	Amp	7	110207927	110404966	0	0.010674
11185	Amp	7	110411205	110594258	0	0.006736
11186	Amp	7	110595552	110804955	0	0.008036
11187	Amp	7	110808252	110835335	0	0.006766
11188	Amp	7	110836045	111010974	0	0.003197
11189	Amp	7	111011057	111135533	0	0.004445
11190	Amp	7	111712844	111827267	0	0.006567
11191	Amp	7	111832984	111845271	0	0.005267
11192	Amp	7	111846472	111979925	0	0.007219
11193	Amp	7	111982427	112332106	0	0.010566
11194	Amp	7	112686150	112755734	0	0.001897
11195	Amp	7	112756565	113042460	0	0.006369
11196	Amp	7	113046949	113409295	0	0.001897
11197	Amp	7	113415583	113505196	0	0.017004
11198	Amp	7	113507462	113820227	0	0.001897
11199	Amp	7	113821409	113852330	0	0.002848
11200	Amp	7	113865767	114107834	0	0.001897
11201	Amp	7	114111936	114344875	0	0.017511
11202	Amp	7	114346501	114424268	0	0.018021
11203	Amp	7	114430786	114727634	0	0.017511
11204	Amp	7	114731971	114952212	0	0.001897
11205	Amp	7	114955003	115155946	0	0.005558
11206	Amp	7	115158429	115177160	0	0.008715
11207	Amp	7	115178378	115279701	0	0.010949
11208	Amp	7	115288574	115373230	0	0.019635
11209	Amp	7	115386686	115564288	0	0.007792
11210	Amp	7	115566436	115752809	0	0.008869
11211	Amp	7	115754464	115997393	0	0.012342
11212	Amp	7	116004479	116154342	0	0.014565
11213	Amp	7	116161878	116164839	0	0.011092
11214	Amp	7	116168975	116449106	0	0.010015
11215	Amp	7	116450297	116450297	0	0.012691
11216	Amp	7	116460131	116575072	0	0.010468
11217	Amp	7	116576918	116779479	0	0.01206
11218	Amp	7	116783613	116793068	0	0.013812
11219	Amp	7	116794105	116798600	0	0.014625
11220	Amp	7	116801480	116945619	0	0.013812
11221	Amp	7	116945670	116977478	0	0.01206
11222	Amp	7	116979720	117080828	0	0.013821
11223	Amp	7	117084243	117227241	0	0.014974
11224	Amp	7	117237919	117249475	0	0.013658
11225	Amp	7	117249664	117312729	0	0.010982
11226	Amp	7	117313381	117386946	0	0.01349
11227	Amp	7	117390644	117491593	0	0.014833
11228	Amp	7	117492865	118094186	0	0.011459
11229	Amp	7	118094263	118191690	0	0.014231
11230	Amp	7	118194670	118204221	0	0.017927
11231	Amp	7	118204329	118401679	0	0.016774
11232	Amp	7	118402618	118617752	0	0.015013
11233	Amp	7	118617962	118702806	0	0.017611
11234	Amp	7	118705349	118832544	0	0.011142
11235	Amp	7	118839540	118902794	0	0.009407

11236	Amp	7	118904007	118982038	0	0.007815
11237	Amp	7	118986127	119144699	0	0.006952
11238	Amp	7	119144988	119156047	0	0.012298
11239	Amp	7	119161700	119338652	0	0.006952
11240	Amp	7	119341837	119393904	0	0.004158
11241	Amp	7	119394115	119442162	0	0.006122
11242	Amp	7	119444871	119465713	0	0.007174
11243	Amp	7	119468394	119600711	0	0.00521
11244	Amp	7	119605020	119691676	0	0.004158
11245	Amp	7	119691967	119700111	0	0.008037
11246	Amp	7	119752257	120112092	0	0.004158
11247	Amp	7	120114624	120843124	0	0.009189
11248	Amp	7	120850675	121314438	0	0.007846
11249	Amp	7	121324169	121336832	0	0.013062
11250	Amp	7	121336983	121484438	0	0.007846
11251	Amp	7	121486002	121704665	0	0.023782
11252	Amp	7	121717213	121909931	0	0.007846
11253	Amp	7	121910325	122677224	0	0.008858
11254	Amp	7	122683682	122753678	0	0.017882
11255	Amp	7	122754095	123149325	0	0.008858
11256	Amp	7	123152216	123162770	0	0.012764
11257	Amp	7	123164299	123171273	0	0.013524
11258	Amp	7	123249281	123249646	0	0.009618
11259	Amp	7	123250459	123402136	0	0.015662
11260	Amp	7	123403046	123489245	0	0.020046
11261	Amp	7	123509695	123570489	0	0.015125
11262	Amp	7	123570628	123845435	0	0.014365
11263	Amp	7	123847756	124591104	0	0.019026
11264	Amp	7	124592948	125123412	0	0.0203
11265	Amp	7	125127241	125213857	0	0.021485
11266	Amp	7	125220972	125339638	0	0.022018
11267	Amp	7	125344483	125975513	0	0.021485
11268	Amp	7	125975926	126052745	0	0.020473
11269	Amp	7	126057577	126072799	0	0.02778
11270	Amp	7	126073409	126089614	0	0.020473
11271	Amp	7	126089937	126208614	0	0.019198
11272	Amp	7	126210581	126238562	0	0.020383
11273	Amp	7	126241638	126515944	0	0.021689
11274	Amp	7	126517302	126793273	0	0.022921
11275	Amp	7	126793483	126963144	0	0.023692
11276	Amp	7	126963208	127618397	0	0.022921
11277	Amp	7	127618870	127618870	0	0.021736
11278	Amp	7	127626828	127791286	0	0.025615
11279	Amp	7	127798179	127982290	0	0.031103
11280	Amp	7	127984304	127984304	0	0.041059
11281	Amp	7	127985197	128444091	0	0.03557
11282	Amp	7	128446458	128446778	0	0.037246
11283	Amp	7	128448919	128448919	0	0.037826
11284	Amp	7	128736608	128797443	0	0.034736
11285	Amp	7	128799496	128855786	0	0.03704
11286	Amp	7	128862737	128863888	0	0.03823
11287	Amp	7	128865151	129432183	0	0.037651
11288	Amp	7	129440592	129600243	0	0.039905

11289	Amp	7	129601091	129654138	0	0.036617
11290	Amp	7	129657447	129789824	0	0.034362
11291	Amp	7	129790171	129872253	0	0.036642
11292	Amp	7	129878747	130036946	0	0.034621
11293	Amp	7	130044167	130046768	0	0.044155
11294	Amp	7	130054898	130098544	0	0.051287
11295	Amp	7	130100458	130253183	0	0.049007
11296	Amp	7	130258819	130505748	0	0.047317
11297	Amp	7	130505834	130701670	0	0.044005
11298	Amp	7	130707948	130736111	0	0.034471
11299	Amp	7	130744163	130782816	0	0.039178
11300	Amp	7	130786856	130939025	0	0.040191
11301	Amp	7	130942255	131289527	0	0.034753
11302	Amp	7	131289934	131314416	0	0.03624
11303	Amp	7	131321144	131467241	0	0.036823
11304	Amp	7	131467559	131471590	0	0.031191
11305	Amp	7	131475189	131513025	0	0.03174
11306	Amp	7	131513769	131525324	0	0.032206
11307	Amp	7	131525843	131655908	0	0.031043
11308	Amp	7	131661514	131680684	0	0.032273
11309	Amp	7	131684951	131900477	0	0.031083
11310	Amp	7	131900592	131973427	0	0.029853
11311	Amp	7	131973950	132305122	0	0.029304
11312	Amp	7	132305269	132581344	0	0.028291
11313	Amp	7	132591091	132610082	0	0.02326
11314	Amp	7	132614079	132616841	0	0.021954
11315	Amp	7	132617107	132682471	0	0.024081
11316	Amp	7	132682821	132921242	0	0.023615
11317	Amp	7	132922411	133020770	0	0.025677
11318	Amp	7	133023775	133647957	0	0.023615
11319	Amp	7	133649233	134039690	0	0.021488
11320	Amp	7	134040294	134185091	0	0.022609
11321	Amp	7	134185891	134303406	0	0.023844
11322	Amp	7	134303414	134590877	0	0.024977
11323	Amp	7	134596233	134672068	0	0.025693
11324	Amp	7	134678200	134702665	0	0.026279
11325	Amp	7	134703331	134740279	0	0.025157
11326	Amp	7	134740419	134920148	0	0.024441
11327	Amp	7	134924546	135031679	0	0.027846
11328	Amp	7	135032905	135048323	0	0.028877
11329	Amp	7	135052953	135223482	0	0.031916
11330	Amp	7	135225686	135264934	0	0.034066
11331	Amp	7	135268476	135302405	0	0.037772
11332	Amp	7	135302990	135313839	0	0.037187
11333	Amp	7	135318183	135390655	0	0.036156
11334	Amp	7	135394639	135677462	0	0.038335
11335	Amp	7	135677921	135773362	0	0.03493
11336	Amp	7	135773813	135791607	0	0.032781
11337	Amp	7	135791823	136189325	0	0.030602
11338	Amp	7	136194121	136211730	0	0.024352
11339	Amp	7	136212001	136366846	0	0.024825
11340	Amp	7	136367386	136408710	0	0.026166
11341	Amp	7	136420314	136450936	0	0.023971

11342	Amp	7	136455846	136725027	0	0.026444
11343	Amp	7	136725850	136935405	0	0.023971
11344	Amp	7	136935753	136960274	0	0.02263
11345	Amp	7	136977860	137230661	0	0.021142
11346	Amp	7	137230802	137256053	0	0.022093
11347	Amp	7	137263550	138267887	0	0.021142
11348	Amp	7	138269853	138399768	0	0.018908
11349	Amp	7	138401621	138711760	0	0.020137
11350	Amp	7	138712445	138766865	0	0.020989
11351	Amp	7	138767140	139160384	0	0.02313
11352	Amp	7	139181882	139426423	0	0.025667
11353	Amp	7	139426495	139427474	0	0.026762
11354	Amp	7	139430057	139821735	0	0.024225
11355	Amp	7	139823426	139838723	0	0.032878
11356	Amp	7	139840056	139944091	0	0.032026
11357	Amp	7	139947850	140091309	0	0.045192
11358	Amp	7	140092225	140092225	0	0.053824
11359	Amp	7	140095264	140308159	0	0.053143
11360	Amp	7	140308668	140524515	0	0.050747
11361	Amp	7	140525950	140661172	0	0.04429
11362	Amp	7	140668108	140910633	0	0.052275
11363	Amp	7	140913992	140922663	0	0.052787
11364	Amp	7	140922696	140989208	0	0.05329
11365	Amp	7	140996758	141116751	0	0.046446
11366	Amp	7	141118977	141131130	0	0.047691
11367	Amp	7	141131997	141310679	0	0.049092
11368	Amp	7	141312140	141358193	0	0.048579
11369	Amp	7	141359623	141458782	0	0.047178
11370	Amp	7	141460519	141492510	0	0.05143
11371	Amp	7	141496064	141637182	0	0.055245
11372	Amp	7	141641909	141873058	0	0.04125
11373	Amp	7	141877220	141904069	0	0.045234
11374	Amp	7	141907048	141927349	0	0.043989
11375	Amp	7	141928139	142032935	0	0.04543
11376	Amp	7	142034594	142293530	0	0.043222
11377	Amp	7	142358543	142554630	0	0.047097
11378	Amp	7	142554640	142554640	0	0.049743
11379	Amp	7	142563940	142563940	0	0.048302
11380	Amp	7	142581256	142768623	0	0.044426
11381	Amp	7	142810364	143112170	0	0.037528
11382	Amp	7	143112640	143221535	0	0.035753
11383	Amp	7	143225088	143434863	0	0.029362
11384	Amp	7	143440750	143506489	0	0.025139
11385	Amp	7	143506678	143506678	0	0.026011
11386	Amp	7	143507648	143507648	0	0.030693
11387	Amp	7	143923163	144142333	0	0.035444
11388	Amp	7	144147555	144407069	0	0.034572
11389	Amp	7	144408848	144896300	0	0.02989
11390	Amp	7	144900126	144983116	0	0.03132
11391	Amp	7	144987277	145066130	0	0.037172
11392	Amp	7	145066449	145192046	0	0.03132
11393	Amp	7	145192193	145280855	0	0.02666
11394	Amp	7	145282933	145523059	0	0.029023

11395	Amp	7	145523475	145601893	0	0.024272
11396	Amp	7	145605854	145615664	0	0.027767
11397	Amp	7	145619415	145670877	0	0.031272
11398	Amp	7	145671284	145744124	0	0.027767
11399	Amp	7	145749601	145755348	0	0.026337
11400	Amp	7	145757122	145859715	0	0.027073
11401	Amp	7	145860491	146064626	0	0.023578
11402	Amp	7	146067737	146086874	0	0.037904
11403	Amp	7	146087575	146095755	0	0.022842
11404	Amp	7	146097238	146244280	0	0.025064
11405	Amp	7	146250538	146260377	0	0.019557
11406	Amp	7	146261704	146595837	0	0.020815
11407	Amp	7	146596005	146700247	0	0.021341
11408	Amp	7	146700755	146876530	0	0.026691
11409	Amp	7	146876614	146919801	0	0.027486
11410	Amp	7	146921637	147039267	0	0.022136
11411	Amp	7	147040975	147041940	0	0.026825
11412	Amp	7	147042214	147090261	0	0.027561
11413	Amp	7	147090274	147177746	0	0.028748
11414	Amp	7	147181839	147684690	0	0.022285
11415	Amp	7	147686696	147732275	0	0.021098
11416	Amp	7	147738995	147856201	0	0.016474
11417	Amp	7	147857166	147885730	0	0.017899
11418	Amp	7	147886886	148091858	0	0.016805
11419	Amp	7	148092998	148230187	0	0.016279
11420	Amp	7	148230709	148252140	0	0.017225
11421	Amp	7	148253116	148282331	0	0.018266
11422	Amp	7	148285580	148357730	0	0.021698
11423	Amp	7	148360510	148510989	0	0.028112
11424	Amp	7	148516499	148593144	0	0.025971
11425	Amp	7	148601802	148783535	0	0.029069
11426	Amp	7	148790237	148790303	0	0.033435
11427	Amp	7	148790773	148900992	0	0.038544
11428	Amp	7	148901181	149004554	0	0.03916
11429	Amp	7	149007542	149012415	0	0.034051
11430	Amp	7	149012718	149455357	0	0.029685
11431	Amp	7	149461344	149738097	0	0.023271
11432	Amp	7	149740738	150305397	0	0.026982
11433	Amp	7	150305745	150315722	0	0.026533
11434	Amp	7	150315870	150489784	0	0.025002
11435	Amp	7	150492383	150548723	0	0.029154
11436	Amp	7	150548900	150634880	0	0.025002
11437	Amp	7	150640304	150741711	0	0.026005
11438	Amp	7	150741861	150820517	0	0.027519
11439	Amp	7	150823655	151026178	0	0.029603
11440	Amp	7	151026913	151054979	0	0.025892
11441	Amp	7	151055521	151231459	0	0.021563
11442	Amp	7	151244540	151281397	0	0.01948
11443	Amp	7	151281457	151319789	0	0.022914
11444	Amp	7	151321159	151365459	0	0.01948
11445	Amp	7	151366978	151491932	0	0.021171
11446	Amp	7	151498227	151624237	0	0.020167
11447	Amp	7	151627724	151690111	0	0.020703

11448	Amp	7	151690878	151739266	0	0.020167
11449	Amp	7	151741778	151755125	0	0.020791
11450	Amp	7	151755542	151766426	0	0.019276
11451	Amp	7	151766715	151804803	0	0.018481
11452	Amp	7	151805216	151806724	0	0.020429
11453	Amp	7	151809069	152076431	0	0.019806
11454	Amp	7	152078971	152133234	0	0.018115
11455	Amp	7	152141363	152217116	0	0.019387
11456	Amp	7	152219465	152259381	0	0.018885
11457	Amp	7	152268649	152393597	0	0.021397
11458	Amp	7	152394992	152414796	0	0.020124
11459	Amp	7	152418752	152622648	0	0.025234
11460	Amp	7	152624395	152755108	0	0.023287
11461	Amp	7	152755649	153040171	0	0.023851
11462	Amp	7	153040616	153236156	0	0.02281
11463	Amp	7	154067655	154067655	0	0.026788
11464	Amp	7	154070879	154070879	0	0.024276
11465	Amp	7	154070951	154071285	0	0.026977
11466	Amp	7	154104301	154712742	0	0.028431
11467	Amp	7	154715590	155235954	0	0.032787
11468	Amp	7	155236821	155390561	0	0.033562
11469	Amp	7	155391137	155394553	0	0.035627
11470	Amp	7	155395284	155537025	0	0.034852
11471	Amp	7	155543868	155667329	0	0.036547
11472	Amp	7	155667605	155799597	0	0.038895
11473	Amp	7	155814378	155829806	0	0.04067
11474	Amp	7	155833641	155837387	0	0.042205
11475	Amp	7	155838178	155865383	0	0.039856
11476	Amp	7	155867982	155916659	0	0.038321
11477	Amp	7	155917433	155927159	0	0.0319
11478	Amp	7	155930157	155930157	0	0.032814
11479	Amp	7	155931052	156002677	0	0.035067
11480	Amp	7	156004992	156021039	0	0.036994
11481	Amp	7	156021424	156111952	0	0.035067
11482	Amp	7	156113255	156200234	0	0.033292
11483	Amp	7	156202374	156218923	0	0.032378
11484	Amp	7	156219536	156392054	0	0.036735
11485	Amp	7	156395207	156649509	0	0.034331
11486	Amp	7	156652015	156652015	0	0.040633
11487	Amp	7	156658120	156704839	0	0.043439
11488	Amp	7	156706068	156778252	0	0.046435
11489	Amp	7	156780673	156835212	0	0.044182
11490	Amp	7	156836334	156872089	0	0.043683
11491	Amp	7	156881431	156938273	0	0.046142
11492	Amp	7	156938606	157048856	0	0.044203
11493	Amp	7	157049458	157138352	0	0.044714
11494	Amp	7	157141577	157257115	0	0.046981
11495	Amp	7	157268780	157288706	0	0.051584
11496	Amp	7	157289062	157299090	0	0.050101
11497	Amp	7	157304033	157374224	0	0.053624
11498	Amp	7	157376414	157391260	0	0.049022
11499	Amp	7	157404628	157404628	0	0.056529
11500	Amp	7	157405245	157433106	0	0.057409

11501	Amp	7	157434045	157437577	0	0.04908
11502	Amp	7	157438309	157503371	0	0.045557
11503	Amp	7	157505313	157558146	0	0.046033
11504	Amp	7	157559061	157712968	0	0.046618
11505	Amp	7	157714920	157828377	0	0.044415
11506	Amp	7	157845270	157907922	0	0.03739
11507	Amp	7	157910117	158034877	0	0.03651
11508	Amp	7	158034895	158091272	0	0.036979
11509	Amp	7	158092911	158205385	0	0.038187
11510	Amp	7	158210962	158236594	0	0.040127
11511	Amp	7	158243769	158244763	0	0.037132
11512	Amp	7	158248430	158363663	0	0.041116
11513	Amp	7	158363872	158388417	0	0.044257
11514	Amp	7	158389337	158422955	0	0.044922
11515	Amp	7	158424071	158486438	0	0.042875
11516	Amp	7	158486720	158499076	0	0.043841
11517	Amp	7	158499481	158568506	0	0.041626
11518	Amp	7	158569085	158592540	0	0.039686
11519	Amp	8	667667	800278	0	0.01321
11520	Amp	8	800572	943453	0	0.011063
11521	Amp	8	980235	980559	0	0.011601
11522	Amp	8	1258082	1290301	0	0.019399
11523	Amp	8	1291392	1296743	0	0.024761
11524	Amp	8	1298536	1303819	0	0.019399
11525	Amp	8	1309780	1367454	0	0.014619
11526	Amp	8	1367720	1368533	0	0.011474
11527	Amp	8	1370137	1491869	0	0.016637
11528	Amp	8	1492224	1501321	0	0.020344
11529	Amp	8	1511066	1667528	0	0.023056
11530	Amp	8	1669225	1710427	0	0.026172
11531	Amp	8	1710530	1843519	0	0.023298
11532	Amp	8	1844694	1960999	0	0.02392
11533	Amp	8	1961403	1970186	0	0.02648
11534	Amp	8	1971482	1971482	0	0.027107
11535	Amp	8	1972160	2009004	0	0.026485
11536	Amp	8	2009163	2026336	0	0.024582
11537	Amp	8	2029164	2077853	0	0.025275
11538	Amp	8	2079552	2083739	0	0.022715
11539	Amp	8	2084292	2084312	0	0.022277
11540	Amp	8	2088664	2102806	0	0.016393
11541	Amp	8	2106222	2157903	0	0.015765
11542	Amp	8	2161451	2166022	0	0.015072
11543	Amp	8	2849664	2861590	0	0.01486
11544	Amp	8	2866784	2926896	0	0.020702
11545	Amp	8	2935217	2995735	0	0.014243
11546	Amp	8	2997472	3098762	0	0.016872
11547	Amp	8	3102350	3123356	0	0.014243
11548	Amp	8	3123454	3136602	0	0.015209
11549	Amp	8	3138628	3270599	0	0.018875
11550	Amp	8	3270652	3284320	0	0.013712
11551	Amp	8	3285425	3376950	0	0.014802
11552	Amp	8	3377541	3475605	0	0.016443
11553	Amp	8	3475950	3579612	0	0.015352

11554	Amp	8	3585212	3662143	0	0.020006
11555	Amp	8	3662172	3662172	0	0.02243
11556	Amp	8	3663079	3690728	0	0.016588
11557	Amp	8	3797835	3972342	0	0.012523
11558	Amp	8	3972558	4016999	0	0.015789
11559	Amp	8	4017881	4036371	0	0.01946
11560	Amp	8	4036773	4042388	0	0.018494
11561	Amp	8	4064299	4101657	0	0.014822
11562	Amp	8	4101902	4108403	0	0.010169
11563	Amp	8	4108477	4123120	0	0.009459
11564	Amp	8	4220531	4259912	0	0.010479
11565	Amp	8	4351875	4569017	0	0.009459
11566	Amp	8	4574857	4588826	0	0.003237
11567	Amp	8	4590246	4947883	0	0.003704
11568	Amp	8	4949589	5038416	0	0.003237
11569	Amp	8	5038695	5073234	0	0.005381
11570	Amp	8	5073475	5295250	0	0.003237
11571	Amp	8	5299841	5358118	0	0.004158
11572	Amp	8	5358843	8398802	0	0.003237
11573	Amp	8	8399076	9183592	0	0.007563
11574	Amp	8	9325829	9334913	0	0.016049
11575	Amp	8	9335991	9722738	0	0.009921
11576	Amp	8	9736225	9753161	0	0.01883
11577	Amp	8	9756293	9792181	0	0.022935
11578	Amp	8	9813543	9884808	0	0.032188
11579	Amp	8	9885015	9905631	0	0.025774
11580	Amp	8	9910900	10537985	0	0.032188
11581	Amp	8	10539926	10679388	0	0.038104
11582	Amp	8	10685296	10745764	0	0.03164
11583	Amp	8	10747940	10860500	0	0.034939
11584	Amp	8	10860551	10910238	0	0.038493
11585	Amp	8	10915248	10926259	0	0.032577
11586	Amp	8	10929033	10941677	0	0.018999
11587	Amp	8	10941947	10951500	0	0.022853
11588	Amp	8	10957350	11044421	0	0.016523
11589	Amp	8	11050311	11130043	0	0.016
11590	Amp	8	11133588	11219244	0	0.012146
11591	Amp	8	11220318	11245173	0	0.015892
11592	Amp	8	11245748	11283955	0	0.012146
11593	Amp	8	11285763	11333025	0	0.013937
11594	Amp	8	11335742	11425726	0	0.012146
11595	Amp	8	11427516	11437066	0	0.016497
11596	Amp	8	11437293	11639949	0	0.013835
11597	Amp	8	11640640	11786406	0	0.012777
11598	Amp	8	11787180	11789003	0	0.013706
11599	Amp	8	11791081	11801118	0	0.015369
11600	Amp	8	12704441	12704946	0	0.018613
11601	Amp	8	12709141	12730774	0	0.017684
11602	Amp	8	12732610	12957086	0	0.016021
11603	Amp	8	12986932	13015248	0	0.017028
11604	Amp	8	13015905	13188703	0	0.016901
11605	Amp	8	13189153	13189502	0	0.017372
11606	Amp	8	13190507	13250540	0	0.014129

11607	Amp	8	13251388	13577412	0	0.012777
11608	Amp	8	13577537	13606434	0	0.012146
11609	Amp	8	13796271	13874027	0	0.013398
11610	Amp	8	13878898	13882524	0	0.017021
11611	Amp	8	13884284	13946915	0	0.020264
11612	Amp	8	13958557	13974889	0	0.012895
11613	Amp	8	13975298	13984283	0	0.010801
11614	Amp	8	13987725	14062732	0	0.009272
11615	Amp	8	14071229	14089559	0	0.006035
11616	Amp	8	14090006	14100687	0	0.013438
11617	Amp	8	14105265	14264083	0	0.006035
11618	Amp	8	14274352	14455880	0	0.002791
11619	Amp	8	14455886	14669472	0	0
11620	Amp	8	14679264	14701154	0	0.006368
11621	Amp	8	14703285	14815804	0	0.00908
11622	Amp	8	14816696	14817437	0	0.006368
11623	Amp	8	14820560	15014522	0	0
11624	Amp	8	15015406	15132718	0	0.004287
11625	Amp	8	15134759	15450924	0	0
11626	Amp	8	15452309	15594012	0	0.001499
11627	Amp	8	15594136	15741576	0	0.005053
11628	Amp	8	15743278	16166714	0	0.004452
11629	Amp	8	16166875	16551958	0	0.005314
11630	Amp	8	16552119	16777206	0	0.003554
11631	Amp	8	16781966	17015837	0	0
11632	Amp	8	17018982	17205826	0	0.001939
11633	Amp	8	17208425	17621502	0	0.005423
11634	Amp	8	17621984	17623417	0	0.008701
11635	Amp	8	17624497	17658475	0	0.010301
11636	Amp	8	17661193	17693553	0	0.025897
11637	Amp	8	17695985	17707609	0	0.023958
11638	Amp	8	17712329	17721247	0	0.025427
11639	Amp	8	17721281	17794594	0	0.024328
11640	Amp	8	17799725	17823788	0	0.022728
11641	Amp	8	17823893	17869485	0	0.021258
11642	Amp	8	17869789	17898304	0	0.023015
11643	Amp	8	17899130	17972047	0	0.023965
11644	Amp	8	17972171	18034876	0	0.023015
11645	Amp	8	18040676	18208232	0	0.011509
11646	Amp	8	18208617	18221770	0	0.013077
11647	Amp	8	18224845	18449651	0	0.022171
11648	Amp	8	18451300	18490830	0	0.013077
11649	Amp	8	18491392	18502033	0	0.013973
11650	Amp	8	18504032	18953289	0	0.012405
11651	Amp	8	18956308	19069292	0	0.011509
11652	Amp	8	19072870	19176512	0	0.021697
11653	Amp	8	19178397	19583557	0	0.011509
11654	Amp	8	19587330	19670065	0	0.013506
11655	Amp	8	19672816	19893800	0	0.011749
11656	Amp	8	19894696	20035536	0	0.009753
11657	Amp	8	20039826	20039826	0	0.011796
11658	Amp	8	20041505	20049938	0	0.015372
11659	Amp	8	20053264	20380607	0	0.009753

11660	Amp	8	20381064	20387903	0	0.025689
11661	Amp	8	20388446	20401438	0	0.009753
11662	Amp	8	20403160	20458090	0	0.014756
11663	Amp	8	20461079	20501107	0	0.016637
11664	Amp	8	20506140	20535695	0	0.021541
11665	Amp	8	20540533	20618155	0	0.016637
11666	Amp	8	20621259	20672533	0	0.012166
11667	Amp	8	20675246	20767580	0	0.005281
11668	Amp	8	20771871	20925295	0	0.0076
11669	Amp	8	20927978	21002580	0	0.005281
11670	Amp	8	21006617	21094360	0	0.009298
11671	Amp	8	21100389	21925518	0	0.005281
11672	Amp	8	21928290	22104035	0	0.002003
11673	Amp	8	22111788	22211505	0	0.005813
11674	Amp	8	22218820	22348381	0	0.007779
11675	Amp	8	22526630	22668467	0	0.013759
11676	Amp	8	22675417	22699255	0	0.017112
11677	Amp	8	22705218	22715221	0	0.013759
11678	Amp	8	22715755	22726426	0	0.021311
11679	Amp	8	22732106	22858621	0	0.013759
11680	Amp	8	22868583	22886381	0	0.014474
11681	Amp	8	22888135	22917799	0	0.012508
11682	Amp	8	22917906	22922162	0	0.011793
11683	Amp	8	22925495	23010756	0	0.012548
11684	Amp	8	23016020	23058846	0	0.011793
11685	Amp	8	23059125	23081742	0	0.00979
11686	Amp	8	23086994	23139266	0	0.006434
11687	Amp	8	23141894	23201532	0	0.009404
11688	Amp	8	23202743	23479897	0	0.00598
11689	Amp	8	23480166	23505888	0	0.009947
11690	Amp	8	23508371	23695484	0	0.00598
11691	Amp	8	23699719	24192112	0	0.010725
11692	Amp	8	24194158	24252394	0	0.012199
11693	Amp	8	24265726	24282582	0	0.013857
11694	Amp	8	24282863	24554168	0	0.01564
11695	Amp	8	24556419	24606637	0	0.024947
11696	Amp	8	24609845	24666053	0	0.014167
11697	Amp	8	24666959	24857708	0	0.012508
11698	Amp	8	24879374	25044923	0	0.010725
11699	Amp	8	25047691	25082727	0	0.012522
11700	Amp	8	25089453	25313058	0	0.015729
11701	Amp	8	25317194	25423105	0	0.010984
11702	Amp	8	25423150	25501042	0	0.013287
11703	Amp	8	25506112	25511117	0	0.009187
11704	Amp	8	25520312	25739144	0	0.011946
11705	Amp	8	25740354	25801968	0	0.010281
11706	Amp	8	25802104	25952216	0	0.008937
11707	Amp	8	25956540	26149778	0	0.007395
11708	Amp	8	26150378	26174724	0	0.010231
11709	Amp	8	26175499	26217330	0	0.007395
11710	Amp	8	26218055	26246519	0	0.008893
11711	Amp	8	26247722	26373819	0	0.00945
11712	Amp	8	26392780	26392780	0	0.019701

11713	Amp	8	26402104	26553634	0	0.02025
11714	Amp	8	26554276	26641135	0	0.01823
11715	Amp	8	26643835	26736534	0	0.017681
11716	Amp	8	26742563	26748107	0	0.028623
11717	Amp	8	26748143	26809029	0	0.027731
11718	Amp	8	26809528	26827088	0	0.036643
11719	Amp	8	26828349	26859155	0	0.038308
11720	Amp	8	26861548	26901903	0	0.041843
11721	Amp	8	26902532	27143204	0	0.038168
11722	Amp	8	27143658	27184003	0	0.03293
11723	Amp	8	27184570	27285081	0	0.035034
11724	Amp	8	27285657	27291272	0	0.037133
11725	Amp	8	27292664	27336472	0	0.038479
11726	Amp	8	27341367	27362793	0	0.037133
11727	Amp	8	27366916	27483498	0	0.035327
11728	Amp	8	27486843	27500988	0	0.0391
11729	Amp	8	27506825	27579326	0	0.036997
11730	Amp	8	27579534	27798913	0	0.038022
11731	Amp	8	27806175	27842817	0	0.031379
11732	Amp	8	27845005	27849018	0	0.030822
11733	Amp	8	27852583	27929707	0	0.028723
11734	Amp	8	27929792	28095510	0	0.027191
11735	Amp	8	28101009	28116403	0	0.026166
11736	Amp	8	28125469	28146587	0	0.027657
11737	Amp	8	28147849	28176472	0	0.030959
11738	Amp	8	28179117	28184593	0	0.032141
11739	Amp	8	28184670	28233549	0	0.030207
11740	Amp	8	28239872	28250157	0	0.022206
11741	Amp	8	28252897	28253999	0	0.021023
11742	Amp	8	28257566	28346747	0	0.015043
11743	Amp	8	28347366	28356608	0	0.018399
11744	Amp	8	28358061	28373113	0	0.01939
11745	Amp	8	28384194	28390194	0	0.021668
11746	Amp	8	28390964	28390964	0	0.028927
11747	Amp	8	28398071	28419821	0	0.025571
11748	Amp	8	28424296	28476644	0	0.018822
11749	Amp	8	28479527	28496789	0	0.029241
11750	Amp	8	28499099	28530298	0	0.018822
11751	Amp	8	28530729	28866154	0	0.015153
11752	Amp	8	28869928	28903677	0	0.014162
11753	Amp	8	28907701	29047498	0	0.014632
11754	Amp	8	29050620	29120091	0	0.017977
11755	Amp	8	29122432	29251470	0	0.021879
11756	Amp	8	29253016	29264343	0	0.026598
11757	Amp	8	29268907	29323081	0	0.042456
11758	Amp	8	29326345	29327042	0	0.043383
11759	Amp	8	29331055	29363373	0	0.053095
11760	Amp	8	29364217	29448964	0	0.054937
11761	Amp	8	29453801	29625446	0	0.056343
11762	Amp	8	29625972	29641389	0	0.052855
11763	Amp	8	29644227	29709485	0	0.053406
11764	Amp	8	29711001	29717963	0	0.061659
11765	Amp	8	29718860	29787145	0	0.060544

11766	Amp	8	29789252	29795509	0.050473	0.066013
11767	Amp	8	29803234	29866624	0	0.050155
11768	Amp	8	29872739	29969182	0.024438	0.065129
11769	Amp	8	29969957	29991150	0	0.059189
11770	Amp	8	29991230	30122979	0	0.057881
11771	Amp	8	30127865	30136078	0	0.057371
11772	Amp	8	30142830	30157473	0	0.056444
11773	Amp	8	30158242	30193697	0	0.060191
11774	Amp	8	30193812	30292609	0	0.062491
11775	Amp	8	30301534	30318725	0.187449	0.071788
11776	Amp	8	30324552	30352758	0	0.063013
11777	Amp	8	30355018	30376780	0.210594	0.072706
11778	Amp	8	30378011	30413513	0.287738	0.07583
11779	Amp	8	30414299	30487075	0.444474	0.081234
11780	Amp	8	30492344	30492344	0.056211	0.066259
11781	Amp	8	30492763	30496936	0.143787	0.070012
11782	Amp	8	30499552	30510913	0.246818	0.074219
11783	Amp	8	30518375	30545300	0.515676	0.083726
11784	Amp	8	30545418	30568388	0.697374	0.089412
11785	Amp	8	30570014	30579491	0.79909	0.092143
11786	Amp	8	30579514	30689159	0.848692	0.093545
11787	Amp	8	30692802	30704503	0.899693	0.094825
11788	Amp	8	30707333	30774422	0.774648	0.09148
11789	Amp	8	30782578	30837052	0.72817	0.090199
11790	Amp	8	30846866	30861081	0.563096	0.085232
11791	Amp	8	30861518	30869395	0.644502	0.087811
11792	Amp	8	30869424	30934083	0.563096	0.085232
11793	Amp	8	30934619	31050312	0.735486	0.090404
11794	Amp	8	31055051	31111532	0.667587	0.088562
11795	Amp	8	31112462	31116638	0.379885	0.079055
11796	Amp	8	31118539	31141912	0.299433	0.076325
11797	Amp	8	31142287	31147795	0.379885	0.079154
11798	Amp	8	31151099	31156389	0.431198	0.080906
11799	Amp	8	31161169	31199115	0.500841	0.083294
11800	Amp	8	31202051	31230743	0.293269	0.07619
11801	Amp	8	31231029	31265825	0.079652	0.067394
11802	Amp	8	31266636	31395461	0.170723	0.071007
11803	Amp	8	31397328	31436645	0.233782	0.07355
11804	Amp	8	31438571	31440854	0.089561	0.067941
11805	Amp	8	31449619	31469300	0.450702	0.081489
11806	Amp	8	31469500	31548798	0.609133	0.08669
11807	Amp	8	31549085	31549832	0.576183	0.085637
11808	Amp	8	31550778	31590117	0.660254	0.088338
11809	Amp	8	31590399	31627711	0.72817	0.090134
11810	Amp	8	31629042	31631394	0.882843	0.09436
11811	Amp	8	31633105	31647501	0.906385	0.094973
11812	Amp	8	31649255	31677075	1.252022	0.103213
11813	Amp	8	31682346	31751316	1.994915	0.119066
11814	Amp	8	31755240	31761708	2.425182	0.127613
11815	Amp	8	31764509	31764509	2.502083	0.129001
11816	Amp	8	31770907	31776037	2.158817	0.122306
11817	Amp	8	31777419	31782732	2.457251	0.128223
11818	Amp	8	31787403	31792702	2.158817	0.122306

11819	Amp	8	31793153	31799346	2.083076	0.120918
11820	Amp	8	31799820	31802356	1.95165	0.118217
11821	Amp	8	31806471	31864786	1.215958	0.102281
11822	Amp	8	31867132	31885597	1.270711	0.103728
11823	Amp	8	31885917	31890487	1.159209	0.101042
11824	Amp	8	31891570	31916048	0.891602	0.094569
11825	Amp	8	31920501	31949147	0.774648	0.091576
11826	Amp	8	31949408	31998669	0.72817	0.090171
11827	Amp	8	32000914	32022202	0.660254	0.088375
11828	Amp	8	32024311	32024311	0.720659	0.090097
11829	Amp	8	32030978	32035589	0.583752	0.085871
11830	Amp	8	32041333	32080659	0.72817	0.090241
11831	Amp	8	32083111	32125551	0.636464	0.087639
11832	Amp	8	32128510	32206981	0.720659	0.090033
11833	Amp	8	32209202	32251423	0.47716	0.082403
11834	Amp	8	32260218	32287466	0.457501	0.081791
11835	Amp	8	32287793	32311921	0.424557	0.080747
11836	Amp	8	32312024	32314510	0.487495	0.082758
11837	Amp	8	32315545	32324432	0.550755	0.084899
11838	Amp	8	32329564	32334416	0.355319	0.07824
11839	Amp	8	32334881	32343568	0.386426	0.079267
11840	Amp	8	32346619	32356801	0.322897	0.077256
11841	Amp	8	32357017	32401569	0.569771	0.085383
11842	Amp	8	32402366	32408661	0.4115	0.080181
11843	Amp	8	32413410	32428220	0.47716	0.082415
11844	Amp	8	32433626	32434715	0.4115	0.080181
11845	Amp	8	32438171	32444900	0.379885	0.079155
11846	Amp	8	32445797	32447435	0.424557	0.08065
11847	Amp	8	32449468	32475816	0.583752	0.085905
11848	Amp	8	32482120	32482187	0.667587	0.088397
11849	Amp	8	32482767	32483529	0.543344	0.084623
11850	Amp	8	32487276	32489629	0.437198	0.081011
11851	Amp	8	32489714	32502704	0.215608	0.072884
11852	Amp	8	32504135	32533748	0.241616	0.073849
11853	Amp	8	32536630	32540415	0.159312	0.070724
11854	Amp	8	32541249	32571417	0.143787	0.069952
11855	Amp	8	32571946	32571946	0.238246	0.073738
11856	Amp	8	32574851	32597120	0.259438	0.074905
11857	Amp	8	32598664	32631288	0.287738	0.075886
11858	Amp	8	32633389	32665107	0.392611	0.079627
11859	Amp	8	32665264	32698752	0.4115	0.080311
11860	Amp	8	32700313	32705845	0.311747	0.076684
11861	Amp	8	32708602	32727339	0.264858	0.074962
11862	Amp	8	32729556	32741031	0.210594	0.072568
11863	Amp	8	32746301	32748214	0.299433	0.076332
11864	Amp	8	32751903	32755206	0.386426	0.079381
11865	Amp	8	32762949	32811333	0.444474	0.081278
11866	Amp	8	32811754	32846701	0.399144	0.079822
11867	Amp	8	32853279	32882369	0.563096	0.085318
11868	Amp	8	32882984	32932871	0.508568	0.083512
11869	Amp	8	32938756	32958987	0.444474	0.081372
11870	Amp	8	32959667	32963895	0.335313	0.077607
11871	Amp	8	32974190	32974190	0.529451	0.084221

11872	Amp	8	32976632	32988832	0.751221	0.090793
11873	Amp	8	32996988	33087271	0.823771	0.092867
11874	Amp	8	33088506	33089933	0.977077	0.096774
11875	Amp	8	33090555	33129153	0.831947	0.092989
11876	Amp	8	33129558	33141467	0.742723	0.090674
11877	Amp	8	33145472	33164203	0.774648	0.091547
11878	Amp	8	33170801	33170856	0.977077	0.096793
11879	Amp	8	33173070	33193295	0.941704	0.09592
11880	Amp	8	33194315	33231201	0.995635	0.097104
11881	Amp	8	33231640	33261618	0.758088	0.091164
11882	Amp	8	33266887	33268280	0.583752	0.085899
11883	Amp	8	33276147	33294402	0.293269	0.076012
11884	Amp	8	33295417	33312735	0.362016	0.078554
11885	Amp	8	33313104	33351877	0.404718	0.080027
11886	Amp	8	33353033	33354583	0.424557	0.080707
11887	Amp	8	33355194	33355194	0.470509	0.08226
11888	Amp	8	33366048	33366894	0.589605	0.086129
11889	Amp	8	33370644	33392278	0.644502	0.087832
11890	Amp	8	33392637	33399284	0.751221	0.090785
11891	Amp	8	33401395	33455811	0.86541	0.093847
11892	Amp	8	33457137	33477219	0.751221	0.090891
11893	Amp	8	33477656	33481464	0.576183	0.085645
11894	Amp	8	33488540	33495145	1.014678	0.097654
11895	Amp	8	33497771	33511564	0.882843	0.09431
11896	Amp	8	33516919	33523167	1.023539	0.097911
11897	Amp	8	33524644	33524644	0.873678	0.094165
11898	Amp	8	33530227	33530227	0.79909	0.09209
11899	Amp	8	33535477	33536086	0.689884	0.089138
11900	Amp	8	33540191	33607521	0.79909	0.092239
11901	Amp	8	33611827	33621901	0.651644	0.087954
11902	Amp	8	33621978	33638024	0.569771	0.085412
11903	Amp	8	33640133	33640133	0.735486	0.090378
11904	Amp	8	33674608	33674608	0.977077	0.096687
11905	Amp	8	33674635	33682516	0.857296	0.093625
11906	Amp	8	33685008	33685008	1.131695	0.100382
11907	Amp	8	33686791	33686791	0.977077	0.096781
11908	Amp	8	33689168	33697814	0.959607	0.096312
11909	Amp	8	33698156	33708090	0.899693	0.094839
11910	Amp	8	33709957	33746564	1.014678	0.097542
11911	Amp	8	33748582	33821563	1.041205	0.098189
11912	Amp	8	33822265	33822265	1.058188	0.098688
11913	Amp	8	33827554	33857522	0.968522	0.096411
11914	Amp	8	33863123	33891299	1.665486	0.112312
11915	Amp	8	33902975	33902975	1.706315	0.113273
11916	Amp	8	33904066	33928827	1.68612	0.112778
11917	Amp	8	33932729	33945168	1.759227	0.114291
11918	Amp	8	33945184	33955781	1.583409	0.110704
11919	Amp	8	33959910	33959910	2.537294	0.12981
11920	Amp	8	33962777	34007485	2.245479	0.123996
11921	Amp	8	34008191	34047739	2.083076	0.120786
11922	Amp	8	34060133	34087746	2.127614	0.121798
11923	Amp	8	34100836	34117052	2.22459	0.12366
11924	Amp	8	34121984	34143638	2.379392	0.126789

11925	Amp	8	34144503	34144503	2.457251	0.128263
11926	Amp	8	34145724	34164976	2.33403	0.125833
11927	Amp	8	34165215	34208487	1.854421	0.116372
11928	Amp	8	34213354	34226136	1.737623	0.11388
11929	Amp	8	34226885	34239862	1.812192	0.115352
11930	Amp	8	34242577	34262232	1.665486	0.112287
11931	Amp	8	34262638	34288085	2.22459	0.123593
11932	Amp	8	34289832	34329061	2.300463	0.125189
11933	Amp	8	34329675	34341327	2.755154	0.133898
11934	Amp	8	34346582	34378393	3.654159	0.150276
11935	Amp	8	34379474	34381999	3.554363	0.148723
11936	Amp	8	34387295	34439632	3.933438	0.155365
11937	Amp	8	34444881	34444881	4.513276	0.164751
11938	Amp	8	34453268	34453268	4.570393	0.165658
11939	Amp	8	34462222	34462536	5.449463	0.17879
11940	Amp	8	34464888	34473702	5.375101	0.177768
11941	Amp	8	34473739	34496228	5.404308	0.178208
11942	Amp	8	34496293	34496293	5.702358	0.182562
11943	Amp	8	34521560	34521560	4.924414	0.170894
11944	Amp	8	34522465	34527773	5.227494	0.175524
11945	Amp	8	34531309	34537147	5.463735	0.179113
11946	Amp	8	34547920	34574584	6.167547	0.18933
11947	Amp	8	34584165	34600747	5.081731	0.173394
11948	Amp	8	34601293	34630373	4.626369	0.166421
11949	Amp	8	34630406	34637091	4.570393	0.165514
11950	Amp	8	34637335	34640868	3.826187	0.153504
11951	Amp	8	34645654	34674233	3.6657	0.150532
11952	Amp	8	34675396	34675396	3.749777	0.152066
11953	Amp	8	34679845	34684367	3.5415	0.148491
11954	Amp	8	34686801	34711008	3.654159	0.150478
11955	Amp	8	34711188	34718835	3.446786	0.146634
11956	Amp	8	34723420	34735701	3.654159	0.150364
11957	Amp	8	34747006	34748598	3.726055	0.151725
11958	Amp	8	34749861	34761549	3.067933	0.139633
11959	Amp	8	34762638	34772383	3.349067	0.144869
11960	Amp	8	34776615	34781228	3.603523	0.149574
11961	Amp	8	34786465	34801719	3.762283	0.152431
11962	Amp	8	34801871	34807589	3.505046	0.147798
11963	Amp	8	34809962	34836902	3.566107	0.148859
11964	Amp	8	34838436	34839338	3.361849	0.145129
11965	Amp	8	34842176	34889378	3.399017	0.14575
11966	Amp	8	34889743	34889743	3.446786	0.146625
11967	Amp	8	34896015	34903058	3.160681	0.14139
11968	Amp	8	34904223	34904223	3.554363	0.148608
11969	Amp	8	34909586	34909586	3.471122	0.147011
11970	Amp	8	34915026	34917880	3.195626	0.142188
11971	Amp	8	34919767	34933142	3.628913	0.149995
11972	Amp	8	34933415	34945045	3.690482	0.150991
11973	Amp	8	34945055	34945055	3.251638	0.143184
11974	Amp	8	34997908	34997908	3.264473	0.143369
11975	Amp	8	34998616	35004106	3.218362	0.142494
11976	Amp	8	35008512	35009117	3.590086	0.149356
11977	Amp	8	35011683	35048930	3.6657	0.150482

11978	Amp	8	35051245	35071941	3.690482	0.151002
11979	Amp	8	35077387	35077387	4.331178	0.161791
11980	Amp	8	35077860	35082784	4.196711	0.159804
11981	Amp	8	35082972	35091040	4.264196	0.160732
11982	Amp	8	35091667	35113573	4.090086	0.157875
11983	Amp	8	35113853	35128981	4.183261	0.15947
11984	Amp	8	35131513	35131513	4.290674	0.16121
11985	Amp	8	35140073	35140073	4.388129	0.162696
11986	Amp	8	35140618	35140618	4.457399	0.16374
11987	Amp	8	35250447	35279792	5.067861	0.173145
11988	Amp	8	35281956	35299385	5.02373	0.172523
11989	Amp	8	35303274	35365952	4.838749	0.169599
11990	Amp	8	35366455	35368261	5.566851	0.180649
11991	Amp	8	35371812	35399927	6.002088	0.186779
11992	Amp	8	35400441	35400441	6.198231	0.189606
11993	Amp	8	35400536	35411271	6.291248	0.190985
11994	Amp	8	35412577	35412577	6.626113	0.195802
11995	Amp	8	35417389	35418312	5.551585	0.180292
11996	Amp	8	35419211	35430885	5.271084	0.176244
11997	Amp	8	35433672	35433672	5.522349	0.179838
11998	Amp	8	35440525	35440525	5.169562	0.174654
11999	Amp	8	35440714	35442058	5.008935	0.172346
12000	Amp	8	35446821	35453675	5.39004	0.178017
12001	Amp	8	35459519	35486738	6.092521	0.188261
12002	Amp	8	35487140	35487140	6.259932	0.190528
12003	Amp	8	35489864	35490220	6.671073	0.196321
12004	Amp	8	35492188	35504960	6.763246	0.197805
12005	Amp	8	35509275	35520068	6.716522	0.1971
12006	Amp	8	35538281	35543861	6.825391	0.198643
12007	Amp	8	35546251	35557212	7.993387	0.214579
12008	Amp	8	35557481	35586503	8.169654	0.217009
12009	Amp	8	35589090	35593358	8.266115	0.21823
12010	Amp	8	35596943	35599601	8.18543	0.217218
12011	Amp	8	35606737	35618964	9.289139	0.231879
12012	Amp	8	35619878	35643344	9.138353	0.22982
12013	Amp	8	35645787	35649516	9.425321	0.233633
12014	Amp	8	35650878	35653476	9.559998	0.235178
12015	Amp	8	35655039	35658263	9.627477	0.236175
12016	Amp	8	35660338	35660338	9.899054	0.239564
12017	Amp	8	35661303	35675430	10.260441	0.244068
12018	Amp	8	35676447	35677123	10.32955	0.245004
12019	Amp	8	35690747	35714787	10.893171	0.252136
12020	Amp	8	35715270	35715270	11.15007	0.255561
12021	Amp	8	35716150	35716802	11.324528	0.257551
12022	Amp	8	35721573	35729273	12.671608	0.274014
12023	Amp	8	35729403	35740014	12.781778	0.275193
12024	Amp	8	35741739	35759510	12.456062	0.271365
12025	Amp	8	35763872	35854694	12.637869	0.273539
12026	Amp	8	35858960	35870816	12.855613	0.276157
12027	Amp	8	35872067	35898146	13.165358	0.279769
12028	Amp	8	35899217	35899230	14.503631	0.295705
12029	Amp	8	35899919	35926798	14.430436	0.294725
12030	Amp	8	35927764	35927764	14.650436	0.297423

12031	Amp	8	35940128	35941030	14.779959	0.29883
12032	Amp	8	35941872	35941872	15.06204	0.302094
12033	Amp	8	35945225	35948728	15.385452	0.305868
12034	Amp	8	35953296	35969027	14.300473	0.29326
12035	Amp	8	35969706	35988219	14.113089	0.291153
12036	Amp	8	35993884	36021963	14.912742	0.300353
12037	Amp	8	36024008	36045214	14.725554	0.298224
12038	Amp	8	36053695	36060376	14.48507	0.295355
12039	Amp	8	36061110	36070418	14.650436	0.297413
12040	Amp	8	36070649	36077672	14.447899	0.295043
12041	Amp	8	36078307	36081952	13.764867	0.286884
12042	Amp	8	36085104	36085983	13.618338	0.285273
12043	Amp	8	36086957	36089581	13.729199	0.28649
12044	Amp	8	36091789	36096694	13.093327	0.278986
12045	Amp	8	36098596	36109058	13.363175	0.282071
12046	Amp	8	36109077	36114323	13.454936	0.283315
12047	Amp	8	36114723	36117038	13.398977	0.282596
12048	Amp	8	36122284	36146138	13.345022	0.281893
12049	Amp	8	36148555	36148666	13.436139	0.282944
12050	Amp	8	36152736	36175913	13.345022	0.282008
12051	Amp	8	36178190	36181897	13.074762	0.278681
12052	Amp	8	36183266	36185184	12.83688	0.275964
12053	Amp	8	36187792	36195055	13.001761	0.277868
12054	Amp	8	36201715	36201715	13.184	0.280028
12055	Amp	8	36201800	36230458	13.328506	0.281697
12056	Amp	8	36234398	36242814	13.038387	0.278339
12057	Amp	8	36243258	36243258	12.81801	0.275641
12058	Amp	8	36245324	36266149	12.671608	0.273983
12059	Amp	8	36266816	36267988	14.022374	0.289919
12060	Amp	8	36271604	36276437	14.300473	0.293322
12061	Amp	8	36280618	36280618	13.691717	0.286109
12062	Amp	8	36284306	36284772	13.436139	0.283065
12063	Amp	8	36289956	36290090	13.636576	0.285378
12064	Amp	8	36290147	36294338	13.729199	0.286488
12065	Amp	8	36296875	36301253	15.55852	0.307879
12066	Amp	8	36301467	36301872	16.397251	0.317399
12067	Amp	8	36305667	36307372	17.276105	0.327213
12068	Amp	8	36308274	36308274	18.008387	0.335187
12069	Amp	8	36309466	36310925	17.651349	0.331263
12070	Amp	8	36312362	36312362	17.870548	0.333543
12071	Amp	8	36317239	36322794	18.128001	0.336568
12072	Amp	8	36323557	36327022	19.044055	0.346537
12073	Amp	8	36327845	36355024	18.428857	0.339756
12074	Amp	8	36356245	36365355	18.56575	0.341152
12075	Amp	8	36369125	36369125	19.206111	0.348155
12076	Amp	8	36371625	36374026	19.024158	0.34617
12077	Amp	8	36374898	36374898	18.824364	0.344121
12078	Amp	8	36378052	36378052	18.624086	0.341841
12079	Amp	8	36380565	36380565	19.389328	0.350229
12080	Amp	8	36381847	36381847	20.399182	0.360905
12081	Amp	8	36582891	36584631	19.225845	0.348411
12082	Amp	8	36586692	36586692	19.59374	0.352285
12083	Amp	8	36588891	36589902	19.47108	0.351061

12084	Amp	8	36590739	36598811	19.328184	0.349527
12085	Amp	8	36602856	36603354	19.024158	0.346225
12086	Amp	8	36606756	36633719	19.852569	0.355158
12087	Amp	8	36640718	36652877	20.764613	0.364862
12088	Amp	8	36653769	36655250	20.482298	0.36177
12089	Amp	8	36660324	36660512	20.646444	0.36355
12090	Amp	8	36661835	36691715	20.073356	0.357532
12091	Amp	8	36693309	36693309	20.154058	0.358457
12092	Amp	8	36695997	36719665	21.685739	0.374393
12093	Amp	8	36722580	36741414	22.782563	0.385803
12094	Amp	8	36743142	36764070	22.591993	0.383734
12095	Amp	8	36765786	36765786	23.825878	0.396349
12096	Amp	8	36765912	36770459	24.042414	0.398504
12097	Amp	8	36770783	36772711	23.847929	0.396682
12098	Amp	8	36773978	36776274	23.998523	0.398208
12099	Amp	8	36780733	36780962	25.092641	0.408956
12100	Amp	8	36784822	36784988	23.934958	0.397547
12101	Amp	8	36792784	36792784	24.740659	0.405614
12102	Amp	8	36796582	36800310	23.891039	0.397008
12103	Amp	8	36801315	36801315	24.501008	0.403188
12104	Amp	8	36803439	36803511	25.328665	0.41132
12105	Amp	8	36804145	36810292	25.524992	0.41327
12106	Amp	8	36811881	36813855	25.765319	0.415713
12107	Amp	8	36814811	36824292	26.112011	0.419274
12108	Amp	8	36825065	36827925	26.556521	0.423532
12109	Amp	8	36828595	36828595	25.744542	0.415415
12110	Amp	8	36835282	36835282	27.025719	0.428023
12111	Amp	8	36835297	36847532	27.245994	0.430258
12112	Amp	8	36847925	36847925	25.872665	0.416702
12113	Amp	8	36852985	36861101	24.284149	0.401034
12114	Amp	8	36861166	36861166	24.521428	0.403286
12115	Amp	8	36863501	36864090	25.221441	0.410291
12116	Amp	8	36872091	36896612	27.025719	0.428092
12117	Amp	8	36897710	36904954	27.538273	0.433028
12118	Amp	8	36907381	36918146	28.423806	0.441744
12119	Amp	8	36920498	36920602	29.459956	0.451708
12120	Amp	8	36920801	36920801	29.636198	0.453477
12121	Amp	8	36921251	36921324	29.681769	0.453923
12122	Amp	8	36922691	36960017	31.29255	0.469449
12123	Amp	8	36962535	36962535	32.209205	0.477981
12124	Amp	8	36966140	36966140	32.364292	0.479577
12125	Amp	8	36967448	36967448	32.829371	0.483984
12126	Amp	8	36967737	36971409	32.619783	0.482041
12127	Amp	8	36972443	36975154	33.226208	0.487502
12128	Amp	8	36984444	36985205	34.802142	0.501871
12129	Amp	8	36988591	36994383	34.348468	0.497773
12130	Amp	8	36994667	36996864	33.798632	0.492892
12131	Amp	8	37000965	37008821	33.70342	0.491917
12132	Amp	8	37009331	37018779	34.418799	0.498496
12133	Amp	8	37019168	37031270	36.048696	0.513202
12134	Amp	8	37033507	37033507	36.360223	0.515931
12135	Amp	8	37034255	37048151	37.603196	0.527088
12136	Amp	8	37049950	37061814	36.921909	0.521092

12137	Amp	8	37064080	37073895	37.313135	0.524545
12138	Amp	8	37075228	37075228	38.158843	0.532044
12139	Amp	8	37076534	37077086	38.085226	0.53147
12140	Amp	8	37080799	37085372	38.892898	0.538722
12141	Amp	8	37087264	37089682	39.286188	0.541987
12142	Amp	8	37090015	37091099	39.554286	0.544499
12143	Amp	8	37093283	37113068	39.138496	0.540808
12144	Amp	8	37113837	37113837	38.843328	0.538145
12145	Amp	8	37114460	37124069	39.409721	0.543127
12146	Amp	8	37124129	37128401	39.978485	0.548267
12147	Amp	8	37134724	37147051	41.520567	0.561552
12148	Amp	8	37148347	37148641	42.18487	0.567519
12149	Amp	8	37149213	37169926	42.992596	0.574436
12150	Amp	8	37176472	37176472	42.662508	0.571703
12151	Amp	8	37176854	37178309	43.703271	0.580567
12152	Amp	8	37178959	37189709	44.103948	0.583964
12153	Amp	8	37190115	37190338	44.935957	0.59117
12154	Amp	8	37190660	37198650	45.27031	0.594
12155	Amp	8	37200656	37230582	47.132612	0.609912
12156	Amp	8	37230732	37230732	48.526752	0.621881
12157	Amp	8	37233595	37233595	50.610767	0.639173
12158	Amp	8	37236176	37237418	50.899372	0.641439
12159	Amp	8	37239603	37258827	52.621013	0.655932
12160	Amp	8	37259919	37267904	53.329284	0.661793
12161	Amp	8	37268624	37270301	53.460079	0.66287
12162	Amp	8	37270317	37273772	53.276195	0.661273
12163	Amp	8	37274389	37278626	51.338365	0.645337
12164	Amp	8	37280553	37321932	51.05734	0.642879
12165	Amp	8	37321976	37328650	50.768776	0.640474
12166	Amp	8	37328723	37347983	51.076291	0.642979
12167	Amp	8	37350301	37350348	51.72599	0.64853
12168	Amp	8	37351522	37353450	52.092224	0.651619
12169	Amp	8	37353817	37353940	52.436609	0.654299
12170	Amp	8	37354066	37368206	52.750114	0.656946
12171	Amp	8	37370213	37379784	54.675637	0.672882
12172	Amp	8	37380547	37383010	54.859851	0.674283
12173	Amp	8	37383559	37388429	54.597654	0.672111
12174	Amp	8	37388598	37389403	54.885959	0.674618
12175	Amp	8	37391868	37395954	54.172234	0.668758
12176	Amp	8	37397096	37398527	55.660696	0.680769
12177	Amp	8	37400838	37407031	55.95567	0.683221
12178	Amp	8	37410956	37433396	57.575037	0.696189
12179	Amp	8	37440347	37440347	57.708815	0.697385
12180	Amp	8	37448672	37448672	57.924427	0.699195
12181	Amp	8	37449659	37449659	58.248502	0.701652
12182	Amp	8	37455569	37455569	58.901215	0.706985
12183	Amp	8	37457516	37466642	60.075652	0.716279
12184	Amp	8	37469101	37472744	60.212044	0.717306
12185	Amp	8	37475770	37478927	61.945132	0.730984
12186	Amp	8	37480246	37482631	62.34718	0.734264
12187	Amp	8	37482859	37493896	62.210831	0.733237
12188	Amp	8	37494765	37496263	62.930457	0.738719
12189	Amp	8	37496959	37550402	63.151757	0.740569

12190	Amp	8	37550612	37550612	63.221953	0.741318
12191	Amp	8	37552935	37552935	64.022401	0.747581
12192	Amp	8	37558060	37568487	64.190355	0.748883
12193	Amp	8	37569366	37569366	63.882587	0.746445
12194	Amp	8	37571366	37574512	64.217232	0.74903
12195	Amp	8	37575125	37581304	63.687998	0.744973
12196	Amp	8	37592120	37592120	64.52499	0.751408
12197	Amp	8	37597282	37597541	66.151578	0.764154
12198	Amp	8	37602006	37611531	65.438973	0.758603
12199	Amp	8	37612018	37612403	65.65252	0.760221
12200	Amp	8	37612480	37615624	65.818933	0.761623
12201	Amp	8	37618138	37619237	66.011531	0.763047
12202	Amp	8	37623213	37629587	65.575861	0.759733
12203	Amp	8	37629742	37629742	66.627906	0.76785
12204	Amp	8	37631990	37639787	66.853734	0.769707
12205	Amp	8	37646963	37649835	67.069486	0.771366
12206	Amp	8	37651148	37657959	66.601771	0.767665
12207	Amp	8	37660465	37690259	67.018575	0.770882
12208	Amp	8	37693357	37693888	68.650129	0.783582
12209	Amp	8	37703829	37716447	66.936592	0.770409
12210	Amp	8	37719244	37719244	66.655795	0.768215
12211	Amp	8	37722277	37724023	65.575861	0.759571
12212	Amp	8	37724786	37725475	65.4116	0.758401
12213	Amp	8	37732928	37744700	65.600307	0.759881
12214	Amp	8	37747026	37747765	66.936592	0.770271
12215	Amp	8	37752837	37752837	66.320423	0.76555
12216	Amp	8	37752988	37758165	65.600307	0.759948
12217	Amp	8	37785605	37785605	65.186526	0.756583
12218	Amp	8	37787828	37793959	64.749509	0.753126
12219	Amp	8	37799047	37824475	65.111392	0.755992
12220	Amp	8	37829659	37830415	61.753297	0.729554
12221	Amp	8	37834582	37834582	61.230909	0.725432
12222	Amp	8	37844412	37846796	61.561238	0.728007
12223	Amp	8	37858784	37861376	61.039571	0.723866
12224	Amp	8	37862990	37884102	59.530442	0.711894
12225	Amp	8	37886003	37886441	60.129855	0.716664
12226	Amp	8	37897222	37901020	59.093004	0.708517
12227	Amp	8	37906387	37906387	57.274893	0.693811
12228	Amp	8	37911375	37921369	56.814499	0.690233
12229	Amp	8	37924060	37931364	55.58062	0.680093
12230	Amp	8	37931840	37979382	55.450079	0.679049
12231	Amp	8	37985152	37994649	54.331533	0.669958
12232	Amp	8	37998001	38006937	53.885452	0.666363
12233	Amp	8	38008001	38015824	53.962701	0.666964
12234	Amp	8	38019365	38020823	54.094081	0.668137
12235	Amp	8	38022839	38025793	53.223117	0.660936
12236	Amp	8	38034774	38034774	53.406678	0.662442
12237	Amp	8	38040266	38054112	52.462789	0.654653
12238	Amp	8	38063458	38066746	54.198287	0.668929
12239	Amp	8	38073432	38073432	53.699542	0.664775
12240	Amp	8	38082834	38086028	55.155084	0.676646
12241	Amp	8	38089568	38089568	53.672954	0.664664
12242	Amp	8	38094625	38107738	52.957354	0.658621

12243	Amp	8	38112562	38112562	51.464832	0.646214
12244	Amp	8	38114424	38135757	51.805106	0.649232
12245	Amp	8	38138657	38147457	51.542021	0.647072
12246	Amp	8	38147775	38161334	51.362106	0.645546
12247	Amp	8	38162759	38162759	51.438495	0.646177
12248	Amp	8	38166565	38171208	52.092224	0.651621
12249	Amp	8	38172190	38173827	53.143244	0.660238
12250	Amp	8	38179909	38186240	52.670403	0.656359
12251	Amp	8	38188441	38199827	51.881587	0.649759
12252	Amp	8	38200941	38204168	51.234115	0.644426
12253	Amp	8	38213690	38213690	51.673526	0.648102
12254	Amp	8	38214802	38214802	51.362106	0.645441
12255	Amp	8	38218299	38223900	50.743014	0.640266
12256	Amp	8	38229806	38245581	49.644638	0.631152
12257	Amp	8	38248363	38249061	49.099547	0.626636
12258	Amp	8	38252697	38255288	48.787048	0.624016
12259	Amp	8	38256159	38256159	48.890926	0.624774
12260	Amp	8	38258196	38258196	49.356236	0.628782
12261	Amp	8	38259191	38260858	49.748684	0.6319
12262	Amp	8	38266279	38266279	49.879905	0.633021
12263	Amp	8	38270728	38272601	50.245407	0.63606
12264	Amp	8	38276275	38276637	48.526752	0.621691
12265	Amp	8	38282145	38282145	48.787048	0.624003
12266	Amp	8	38282222	38293209	48.916489	0.625037
12267	Amp	8	38297876	38307554	48.476184	0.6213
12268	Amp	8	38309665	38310131	49.356236	0.628657
12269	Amp	8	38310849	38310849	48.551566	0.621969
12270	Amp	8	38313003	38313968	47.656352	0.614417
12271	Amp	8	38327805	38330883	47.132612	0.609829
12272	Amp	8	38334285	38334941	47.528532	0.613212
12273	Amp	8	38339886	38346560	49.408182	0.629148
12274	Amp	8	38347134	38380416	49.27894	0.628087
12275	Amp	8	38386347	38387233	48.476184	0.621445
12276	Amp	8	38389588	38396540	48.112258	0.618384
12277	Amp	8	38403297	38409988	48.761263	0.623671
12278	Amp	8	38413087	38413295	48.267715	0.619607
12279	Amp	8	38416868	38422182	47.964035	0.61709
12280	Amp	8	38428191	38428782	47.708129	0.614903
12281	Amp	8	38428834	38433805	46.669752	0.605891
12282	Amp	8	38433828	38433974	46.087238	0.600941
12283	Amp	8	38434429	38455274	47.178485	0.610281
12284	Amp	8	38455399	38484098	46.745804	0.606526
12285	Amp	8	38484236	38520827	47.280531	0.611149
12286	Amp	8	38521732	38568494	47.429953	0.612394
12287	Amp	8	38571139	38578289	47.107637	0.609529
12288	Amp	8	38582535	38624738	46.187387	0.60179
12289	Amp	8	38628627	38629452	46.873881	0.607742
12290	Amp	8	38634282	38634431	45.781157	0.598439
12291	Amp	8	38639315	38639315	45.985904	0.600168
12292	Amp	8	38640132	38640132	45.012804	0.591773
12293	Amp	8	38666364	38672959	44.612767	0.588239
12294	Amp	8	38675826	38703513	44.688712	0.5889
12295	Amp	8	38705327	38712420	45.395499	0.595051

12296	Amp	8	38716247	38717059	43.525112	0.579115
12297	Amp	8	38717970	38729500	43.728093	0.580663
12298	Amp	8	38729525	38729525	43.499755	0.578728
12299	Amp	8	38730540	38730540	43.626762	0.579856
12300	Amp	8	38740209	38745240	43.168561	0.575895
12301	Amp	8	38746378	38746378	42.840387	0.573223
12302	Amp	8	38750664	38761314	42.638752	0.571443
12303	Amp	8	38763462	38763462	40.825634	0.555507
12304	Amp	8	38763780	38796095	40.428506	0.552033
12305	Amp	8	38796445	38796445	40.552865	0.553203
12306	Amp	8	38797549	38801585	40.478375	0.552541
12307	Amp	8	38808170	38845587	41.372396	0.560252
12308	Amp	8	38848468	38883657	41.888663	0.564863
12309	Amp	8	38889656	38910497	41.76769	0.563786
12310	Amp	8	38914644	38914644	42.336344	0.568811
12311	Amp	8	38918967	38930059	42.009654	0.566051
12312	Amp	8	38937043	38959840	41.841095	0.564392
12313	Amp	8	38959894	38969274	41.960273	0.565608
12314	Amp	8	38973540	38978372	41.841095	0.564487
12315	Amp	8	38981418	39004680	41.744349	0.563636
12316	Amp	8	39008699	39008699	41.520567	0.561646
12317	Amp	8	39013106	39013106	41.444978	0.560888
12318	Amp	8	39019330	39019330	41.744349	0.563631
12319	Amp	8	39024798	39050798	40.752056	0.554857
12320	Amp	8	39052989	39060330	41.568451	0.562012
12321	Amp	8	39061888	39092006	40.9996	0.556987
12322	Amp	8	39092465	39103960	40.875616	0.556042
12323	Amp	8	39111781	39123218	40.678332	0.554301
12324	Amp	8	39125091	39125476	39.504688	0.544029
12325	Amp	8	39131419	39156935	39.066099	0.540218
12326	Amp	8	39158330	39171249	38.327083	0.533585
12327	Amp	8	39171434	39214572	38.255172	0.532877
12328	Amp	8	39219613	39220822	37.409975	0.525378
12329	Amp	8	39221650	39239997	37.745652	0.528393
12330	Amp	8	39242218	39242218	37.018668	0.521952
12331	Amp	8	39245508	39245508	36.50578	0.517278
12332	Amp	8	39245944	39280312	36.338929	0.515827
12333	Amp	8	39283593	39292849	34.657736	0.500598
12334	Amp	8	39295608	39307863	34.157533	0.495987
12335	Amp	8	39308402	39308402	33.846472	0.493245
12336	Amp	8	39310341	39319634	35.590513	0.509181
12337	Amp	8	39319812	39337396	35.277215	0.506314
12338	Amp	8	39338374	39343307	35.111443	0.504808
12339	Amp	8	39345929	39352527	34.969783	0.5035
12340	Amp	8	39538338	39538338	34.945679	0.503199
12341	Amp	8	39539496	39539496	34.969783	0.503407
12342	Amp	8	39540213	39548195	34.826079	0.502205
12343	Amp	8	39550827	39552475	35.421326	0.507656
12344	Amp	8	39556324	39558024	36.57899	0.518083
12345	Amp	8	39564532	39592543	35.759294	0.510566
12346	Amp	8	39594011	39594013	36.000151	0.512877
12347	Amp	8	39600811	39603975	36.409031	0.516403
12348	Amp	8	39605844	39615460	36.12019	0.51393

12349	Amp	8	39615604	39615604	35.111443	0.504656
12350	Amp	8	39617691	39636756	34.06293	0.495226
12351	Amp	8	39636983	39636983	33.13262	0.486741
12352	Amp	8	39637155	39644678	33.607711	0.49112
12353	Amp	8	39645397	39663225	33.204331	0.487349
12354	Amp	8	39663900	39682804	32.273296	0.478718
12355	Amp	8	39683025	39688769	32.619783	0.481959
12356	Amp	8	39698189	39719324	32.189017	0.477794
12357	Amp	8	39719784	39726649	32.29486	0.478848
12358	Amp	8	39728050	39736289	31.360049	0.470028
12359	Amp	8	39740226	39741859	31.176554	0.468284
12360	Amp	8	39744000	39744381	30.723588	0.464019
12361	Amp	8	39746972	39776374	30.233001	0.459098
12362	Amp	8	39778802	39780618	30.025733	0.457227
12363	Amp	8	39789593	39789593	28.647613	0.443883
12364	Amp	8	39790133	39790326	28.132847	0.438975
12365	Amp	8	39794403	39794403	26.756945	0.425512
12366	Amp	8	39796464	39800700	26.556521	0.423536
12367	Amp	8	39809042	39829198	27.734305	0.435045
12368	Amp	8	39835068	39840858	27.289486	0.43079
12369	Amp	8	39840889	39840889	27.180986	0.429618
12370	Amp	8	39845930	39845930	27.289486	0.430793
12371	Amp	8	39853971	39853971	25.678	0.414857
12372	Amp	8	39854855	39854855	25.481443	0.412905
12373	Amp	8	39858555	39866561	26.489533	0.422984
12374	Amp	8	39874932	39874932	26.155333	0.419661
12375	Amp	8	39875775	39894051	25.393293	0.412043
12376	Amp	8	39894105	39896768	25.328665	0.411406
12377	Amp	8	39898946	39902821	25.634956	0.414415
12378	Amp	8	39911824	39911856	25.699725	0.415099
12379	Amp	8	39912074	39918235	25.502901	0.413206
12380	Amp	8	39918326	39936619	25.656358	0.414578
12381	Amp	8	39937921	39948305	25.591369	0.413894
12382	Amp	8	39949762	39969026	24.501008	0.403189
12383	Amp	8	39972684	39978609	25.114202	0.409214
12384	Amp	8	39985693	39987746	25.243712	0.410512
12385	Amp	8	39989715	39989715	25.200555	0.409994
12386	Amp	8	39990442	40003547	25.243712	0.410604
12387	Amp	8	40003606	40007231	25.634956	0.414457
12388	Amp	8	40008390	40012406	25.806057	0.416107
12389	Amp	8	40014819	40015785	25.678	0.414808
12390	Amp	8	40015875	40023846	25.765319	0.415806
12391	Amp	8	40024129	40032650	25.415285	0.41228
12392	Amp	8	40033256	40043200	25.135919	0.409367
12393	Amp	8	40043832	40044278	25.350724	0.41161
12394	Amp	8	40047246	40049184	26.089651	0.419045
12395	Amp	8	40053146	40063676	25.938335	0.41754
12396	Amp	8	40066499	40073291	25.200555	0.410169
12397	Amp	8	40076121	40086992	24.87391	0.406867
12398	Amp	8	40089622	40097733	25.307231	0.411143
12399	Amp	8	40098293	40100136	25.028351	0.408412
12400	Amp	8	40101874	40107694	25.980631	0.417851
12401	Amp	8	40111181	40132052	25.894578	0.416935

12402	Amp	8	40132211	40132211	25.744542	0.415487
12403	Amp	8	40133453	40135170	26.067295	0.418696
12404	Amp	8	40136178	40163520	25.157792	0.40966
12405	Amp	8	40164340	40168922	25.092641	0.409004
12406	Amp	8	40175414	40188842	25.243712	0.410456
12407	Amp	8	40189031	40202643	25.049527	0.408629
12408	Amp	8	40202705	40202705	25.243712	0.410478
12409	Amp	8	40203291	40216666	26.846754	0.426414
12410	Amp	8	40218229	40246060	27.245994	0.43021
12411	Amp	8	40246642	40262966	27.581587	0.433578
12412	Amp	8	40265315	40284865	28.001632	0.437742
12413	Amp	8	40288165	40293076	28.220415	0.43973
12414	Amp	8	40293298	40301597	29.681769	0.45392
12415	Amp	8	40305994	40324459	29.526184	0.452468
12416	Amp	8	40324478	40324478	30.365538	0.460343
12417	Amp	8	40336937	40347025	30.857233	0.465272
12418	Amp	8	40350572	40354760	30.365538	0.460343
12419	Amp	8	40358374	40381056	29.888511	0.455907
12420	Amp	8	40381805	40385727	29.956679	0.456437
12421	Amp	8	40386435	40396346	30.502639	0.461712
12422	Amp	8	40396901	40398940	31.10781	0.467677
12423	Amp	8	40399793	40400783	31.269176	0.469041
12424	Amp	8	40401178	40406894	31.130801	0.467897
12425	Amp	8	40409706	40425688	30.746529	0.464032
12426	Amp	8	40425790	40442940	30.87949	0.465498
12427	Amp	8	40445071	40462888	30.836598	0.465003
12428	Amp	8	40464166	40471491	30.94943	0.466199
12429	Amp	8	40471516	40480050	30.456814	0.461366
12430	Amp	8	40480151	40499287	30.635708	0.463061
12431	Amp	8	40502376	40506488	30.433907	0.461095
12432	Amp	8	40510774	40510774	30.274095	0.459534
12433	Amp	8	40511286	40514652	30.433907	0.461087
12434	Amp	8	40515990	40517724	30.163303	0.458601
12435	Amp	8	40517890	40529270	30.567717	0.462315
12436	Amp	8	40530272	40536721	30.635708	0.463033
12437	Amp	8	40542202	40542202	28.984614	0.447097
12438	Amp	8	40543166	40543583	28.557994	0.443096
12439	Amp	8	40547379	40554148	26.177122	0.419828
12440	Amp	8	40554268	40555257	26.489533	0.422918
12441	Amp	8	40559608	40563332	26.264814	0.42075
12442	Amp	8	40563478	40579255	26.668562	0.424591
12443	Amp	8	40583080	40597808	27.158943	0.42933
12444	Amp	8	40597897	40619804	26.646349	0.424473
12445	Amp	8	40621909	40624699	26.601595	0.423863
12446	Amp	8	40625173	40628697	26.668562	0.424699
12447	Amp	8	40630281	40635481	26.001327	0.41806
12448	Amp	8	40637181	40654417	25.481443	0.412926
12449	Amp	8	40654609	40657569	24.851864	0.406652
12450	Amp	8	40661024	40671700	24.371167	0.401913
12451	Amp	8	40672583	40672583	23.934958	0.397563
12452	Amp	8	40676390	40682436	23.457147	0.392659
12453	Amp	8	40685467	40687051	23.111043	0.389183
12454	Amp	8	40688604	40689598	23.028019	0.388286

12455	Amp	8	40690256	40700160	22.967626	0.38763
12456	Amp	8	40704656	40734789	22.824016	0.386145
12457	Amp	8	40735091	40738905	22.88355	0.386789
12458	Amp	8	40753635	40783041	22.591993	0.383768
12459	Amp	8	40785887	40810337	21.034527	0.367831
12460	Amp	8	40815729	40822041	20.482298	0.361867
12461	Amp	8	40825053	40831309	19.87244	0.355456
12462	Amp	8	40835512	40835531	19.633539	0.352664
12463	Amp	8	40838885	40844524	20.785556	0.365016
12464	Amp	8	40845848	40850837	19.792143	0.35441
12465	Amp	8	40853831	40853831	19.83239	0.354971
12466	Amp	8	40856153	40861180	19.87244	0.355422
12467	Amp	8	40863108	40878678	19.732254	0.353925
12468	Amp	8	40882173	40890148	19.389328	0.35009
12469	Amp	8	40893634	40893634	19.024158	0.346297
12470	Amp	8	40893957	40894185	17.812399	0.333008
12471	Amp	8	40894377	40894724	17.928201	0.334259
12472	Amp	8	40899031	40904267	18.428857	0.339759
12473	Amp	8	40904465	40904465	18.56575	0.341267
12474	Amp	8	40904744	40929126	18.824364	0.344122
12475	Amp	8	40930500	40954449	18.506432	0.340594
12476	Amp	8	40957623	40971924	18.068998	0.335741
12477	Amp	8	40972236	40972236	17.552268	0.33024
12478	Amp	8	40975338	40988955	17.373987	0.328175
12479	Amp	8	40992028	40992028	16.475247	0.318212
12480	Amp	8	40995038	41022888	17.910589	0.334148
12481	Amp	8	41022982	41049040	18.248294	0.337747
12482	Amp	8	41055407	41080132	19.712397	0.353683
12483	Amp	8	41085236	41136009	19.61439	0.352633
12484	Amp	8	41139907	41180308	19.693709	0.353484
12485	Amp	8	41184230	41192561	18.248294	0.337767
12486	Amp	8	41196737	41196748	18.624086	0.341843
12487	Amp	8	41200481	41206558	18.923342	0.345177
12488	Amp	8	41208469	41217387	18.428857	0.339656
12489	Amp	8	41218233	41232537	19.004359	0.345907
12490	Amp	8	41234270	41234943	18.546429	0.340922
12491	Amp	8	41236842	41244831	18.049178	0.335657
12492	Amp	8	41244848	41267147	17.910589	0.334103
12493	Amp	8	41267979	41271071	18.227868	0.337585
12494	Amp	8	41271191	41271191	17.651349	0.331335
12495	Amp	8	41273651	41299243	17.711998	0.331949
12496	Amp	8	41299720	41316111	17.571902	0.330287
12497	Amp	8	41316731	41324129	17.472375	0.329233
12498	Amp	8	41329354	41333611	16.551166	0.318997
12499	Amp	8	41334326	41336125	16.80163	0.321845
12500	Amp	8	41337579	41343519	16.475247	0.318246
12501	Amp	8	41343562	41347306	16.204394	0.315109
12502	Amp	8	41349262	41351777	16.282141	0.315941
12503	Amp	8	41356501	41372279	16.475247	0.318266
12504	Amp	8	41375846	41387296	17.196473	0.326155
12505	Amp	8	41391430	41440280	16.841708	0.322442
12506	Amp	8	41441076	41445217	17.137101	0.325573
12507	Amp	8	41445940	41449231	17.078925	0.324855

12508	Amp	8	41450438	41461832	16.165422	0.314776
12509	Amp	8	41462082	41491814	16.107433	0.313992
12510	Amp	8	41494556	41494940	16.704765	0.320909
12511	Amp	8	41504215	41505020	17.255967	0.326815
12512	Amp	8	41505049	41505049	17.196473	0.326263
12513	Amp	8	41506760	41509360	17.77219	0.332533
12514	Amp	8	41513426	41514483	19.206111	0.348118
12515	Amp	8	41514563	41516822	19.47108	0.350996
12516	Amp	8	41517188	41519880	19.206111	0.348148
12517	Amp	8	41520250	41533614	19.348627	0.349712
12518	Amp	8	41534069	41534069	19.12459	0.347401
12519	Amp	8	41539048	41555499	19.024158	0.34615
12520	Amp	8	41555935	41556972	19.225845	0.348475
12521	Amp	8	41560497	41560497	19.348627	0.349713
12522	Amp	8	41560616	41568954	18.644239	0.342069
12523	Amp	8	41569167	41596856	18.865086	0.344591
12524	Amp	8	41597485	41598753	18.923342	0.345067
12525	Amp	8	41607352	41622990	20.216253	0.359146
12526	Amp	8	41623577	41651740	19.993383	0.356707
12527	Amp	8	41652631	41652631	20.357182	0.360505
12528	Amp	8	41659062	41691676	18.865086	0.344569
12529	Amp	8	41693269	41698436	19.348627	0.349663
12530	Amp	8	41701380	41701380	19.59374	0.352334
12531	Amp	8	41706167	41721719	19.652987	0.352938
12532	Amp	8	41721880	41727920	19.91297	0.355888
12533	Amp	8	41728266	41728266	20.011995	0.356918
12534	Amp	8	41730668	41733686	19.67284	0.353121
12535	Amp	8	41733756	41750903	19.004359	0.346014
12536	Amp	8	41754195	41769436	18.428857	0.33977
12537	Amp	8	41771132	41831310	19.91297	0.355706
12538	Amp	8	41831807	41835563	19.652987	0.353033
12539	Amp	8	41837343	41847769	18.18712	0.337097
12540	Amp	8	41851020	41878591	18.865086	0.344542
12541	Amp	8	41879122	41883035	18.664489	0.342274
12542	Amp	8	41884040	41893676	18.467261	0.340139
12543	Amp	8	41893781	41893781	18.428857	0.339663
12544	Amp	8	41894190	41894190	18.56575	0.341343
12545	Amp	8	41898654	41909736	18.664489	0.342333
12546	Amp	8	41910618	41913774	18.307307	0.338416
12547	Amp	8	41914872	41942991	17.552268	0.330255
12548	Amp	8	41947731	41957382	17.333668	0.327816
12549	Amp	8	41957496	41971383	17.176717	0.32612
12550	Amp	8	41974516	41987704	18.088995	0.335979
12551	Amp	8	41988316	41994187	17.928201	0.334242
12552	Amp	8	41996342	41996342	18.267757	0.338052
12553	Amp	8	42006066	42018986	18.327886	0.338556
12554	Amp	8	42019597	42042099	18.428857	0.339771
12555	Amp	8	42046029	42049600	19.287215	0.349113
12556	Amp	8	42059934	42059934	19.104535	0.347125
12557	Amp	8	42066757	42080720	18.943031	0.345441
12558	Amp	8	42081489	42081489	18.664489	0.342232
12559	Amp	8	42086979	42103269	17.910589	0.334053
12560	Amp	8	42112130	42114330	16.475247	0.318154

12561	Amp	8	42118461	42118461	17.910589	0.33409
12562	Amp	8	42118816	42130871	17.611387	0.330896
12563	Amp	8	42130930	42134058	17.235634	0.32662
12564	Amp	8	42136633	42149300	15.652786	0.308835
12565	Amp	8	42150668	42150668	15.538779	0.307672
12566	Amp	8	42158597	42165441	15.461712	0.306641
12567	Amp	8	42166007	42182310	15.081766	0.302477
12568	Amp	8	42182432	42182432	15.156258	0.303329
12569	Amp	8	42188571	42193070	14.412004	0.294525
12570	Amp	8	42193620	42193620	14.355711	0.293971
12571	Amp	8	42193636	42207133	13.874487	0.288333
12572	Amp	8	42207199	42209110	13.746952	0.286693
12573	Amp	8	42211185	42219665	13.600394	0.285004
12574	Amp	8	42222450	42223382	13.417296	0.282842
12575	Amp	8	42223646	42223646	12.927625	0.2769
12576	Amp	8	42224199	42236300	12.347637	0.270048
12577	Amp	8	42241842	42245804	12.184583	0.268123
12578	Amp	8	42249587	42249587	12.546325	0.272389
12579	Amp	8	42252667	42259530	11.840411	0.26394
12580	Amp	8	42275584	42283425	10.791286	0.250912
12581	Amp	8	42283867	42292359	10.14039	0.242669
12582	Amp	8	42292474	42292474	10.087806	0.242109
12583	Amp	8	42295973	42298312	10.651729	0.249166
12584	Amp	8	42298469	42298499	12.021369	0.266173
12585	Amp	8	42300125	42300125	12.310616	0.269476
12586	Amp	8	42300300	42303917	12.671608	0.27389
12587	Amp	8	42304007	42305069	12.800156	0.275435
12588	Amp	8	42305996	42328629	13.276743	0.281026
12589	Amp	8	42328734	42329061	13.398977	0.282647
12590	Amp	8	42329144	42329307	13.618338	0.285235
12591	Amp	8	42329477	42329883	13.546866	0.284245
12592	Amp	8	42329951	42366637	13.600394	0.285043
12593	Amp	8	42366934	42389733	13.673012	0.28582
12594	Amp	8	42395770	42403138	13.258073	0.280899
12595	Amp	8	42404318	42406593	13.454936	0.283224
12596	Amp	8	42406639	42417909	13.381152	0.282334
12597	Amp	8	42423912	42423912	13.111032	0.279064
12598	Amp	8	42426876	42426876	13.258073	0.280912
12599	Amp	8	42427818	42427818	13.220925	0.280466
12600	Amp	8	42429109	42445892	13.381152	0.282417
12601	Amp	8	42449295	42450043	13.129509	0.279331
12602	Amp	8	42458082	42472935	13.220925	0.280332
12603	Amp	8	42476822	42491712	13.510816	0.283871
12604	Amp	8	42496200	42496739	13.746952	0.28679
12605	Amp	8	42498851	42514154	13.691717	0.286146
12606	Amp	8	42515173	42515173	13.345022	0.281881
12607	Amp	8	42516255	42519736	13.600394	0.285066
12608	Amp	8	42523605	42548897	12.27458	0.269208
12609	Amp	8	42549679	42549679	12.763765	0.275045
12610	Amp	8	42550634	42555582	12.927625	0.276966
12611	Amp	8	42564653	42574794	12.546325	0.272437
12612	Amp	8	42580719	42598117	12.800156	0.275337
12613	Amp	8	42599936	42613787	13.947841	0.28923

12614	Amp	8	42618862	42634699	13.473193	0.283501
12615	Amp	8	42634935	42639720	13.146748	0.279467
12616	Amp	8	42643052	42649655	13.001761	0.277922
12617	Amp	8	42649810	42676916	12.781778	0.275148
12618	Amp	8	42681912	42684075	12.057562	0.266488
12619	Amp	8	42688195	42709483	11.930548	0.26496
12620	Amp	8	42727456	42729781	11.857518	0.264138
12621	Amp	8	42729795	42736848	11.787092	0.263306
12622	Amp	8	42737160	42749685	12.219704	0.26854
12623	Amp	8	42751216	42753220	12.401399	0.270565
12624	Amp	8	42753420	42784169	12.347637	0.27004
12625	Amp	8	42787902	42799087	12.401399	0.270747
12626	Amp	8	42799673	42835571	12.347637	0.269971
12627	Amp	8	42846171	42846171	12.094071	0.266896
12628	Amp	8	42846545	42858852	11.857518	0.264167
12629	Amp	8	42861647	42864660	11.662686	0.261787
12630	Amp	8	42868840	42878034	11.716436	0.262352
12631	Amp	8	42890734	42890734	10.756361	0.250405
12632	Amp	8	42891907	42895643	10.600629	0.248454
12633	Amp	8	42900086	42928572	10.791286	0.250865
12634	Amp	8	42934585	42938451	10.756361	0.250424
12635	Amp	8	42942814	42951363	10.808758	0.251032
12636	Amp	8	42955041	42966535	11.698383	0.262296
12637	Amp	8	42967941	42980944	11.360465	0.257981
12638	Amp	8	42984989	42984989	10.465282	0.246717
12639	Amp	8	42985297	43042692	10.414382	0.246076
12640	Amp	8	43043328	43053246	10.294658	0.244674
12641	Amp	8	43067847	43067847	8.6219	0.22304
12642	Amp	8	43068274	43077335	7.92856	0.213781
12643	Amp	8	43078229	43078229	8.02414	0.215057
12644	Amp	8	43080575	43080575	9.69597	0.237097
12645	Amp	8	46547532	46547532	2.071483	0.12069
12646	Amp	8	46549323	46549323	1.696256	0.112926
12647	Amp	8	46552240	46552443	1.159209	0.101021
12648	Amp	8	46553821	46554650	1.102589	0.099741
12649	Amp	8	46557601	46575802	1.075952	0.099137
12650	Amp	8	46581638	46589459	1.463774	0.107945
12651	Amp	8	46598238	46635727	1.075952	0.099137
12652	Amp	8	46637164	46677402	1.102589	0.099718
12653	Amp	8	46678327	46688826	0.915698	0.095315
12654	Amp	8	46694262	46714791	1.075952	0.099039
12655	Amp	8	46718557	46835670	1.112463	0.100096
12656	Amp	8	46838916	46848090	0.915698	0.095191
12657	Amp	8	46859601	46929159	1.023539	0.097811
12658	Amp	8	46935135	47094994	1.093834	0.099592
12659	Amp	8	47109618	47198984	1.049301	0.098535
12660	Amp	8	47205294	47248264	0.831947	0.093142
12661	Amp	8	47250832	47252758	0.735486	0.090522
12662	Amp	8	47322675	47348311	0.941704	0.095915
12663	Amp	8	47368914	47368914	0.790614	0.091977
12664	Amp	8	47372362	47491424	0.735486	0.090458
12665	Amp	8	47492008	47506343	0.437198	0.081019
12666	Amp	8	47507060	47661411	0.463534	0.082062

12667	Amp	8	47661893	47833329	0.557122	0.08502
12668	Amp	8	47834051	47839964	0.609133	0.086733
12669	Amp	8	47840742	47921407	0.720659	0.09007
12670	Amp	8	47924237	47968865	0.667587	0.088534
12671	Amp	8	47970339	48030257	0.636464	0.087682
12672	Amp	8	48030333	48161528	0.616549	0.086881
12673	Amp	8	48162353	48187472	0.470509	0.082253
12674	Amp	8	48187682	48385861	0.482379	0.082603
12675	Amp	8	48390885	48513927	0.630438	0.087349
12676	Amp	8	48514949	48646040	0.482379	0.082603
12677	Amp	8	48648597	48763877	0.470509	0.082119
12678	Amp	8	48767382	48779146	0.276783	0.075546
12679	Amp	8	48779636	48866594	0.251218	0.074287
12680	Amp	8	48869463	48880599	0.287738	0.075786
12681	Amp	8	48884132	48940182	0.17597	0.071372
12682	Amp	8	48943561	49140224	0.153838	0.070559
12683	Amp	8	49141553	49213063	0.112543	0.068778
12684	Amp	8	49213091	49257841	0.204831	0.072466
12685	Amp	8	49259037	49305734	0.227787	0.073218
12686	Amp	8	49309928	49332656	0.138391	0.06988
12687	Amp	8	49333274	49389826	0.069848	0.066922
12688	Amp	8	49393717	49400411	0.065715	0.066776
12689	Amp	8	49403155	49442723	0.187449	0.071707
12690	Amp	8	49445234	49466426	0.065715	0.066776
12691	Amp	8	49466691	49630380	0	0.063608
12692	Amp	8	49632606	49701785	0.010261	0.064301
12693	Amp	8	49705060	49762344	0	0.061215
12694	Amp	8	49762767	49831146	0.040093	0.065749
12695	Amp	8	49831609	49849110	0.100827	0.068205
12696	Amp	8	49851295	49889310	0.029031	0.065304
12697	Amp	8	49893542	49918041	0.050473	0.066212
12698	Amp	8	49920942	49946976	0.14896	0.070221
12699	Amp	8	50076101	50076146	0.118001	0.068984
12700	Amp	8	50076174	50076174	0.164935	0.070881
12701	Amp	8	50077704	50148347	0.181932	0.071653
12702	Amp	8	50159182	50202521	0.065715	0.066768
12703	Amp	8	50205193	50339417	0.024438	0.064963
12704	Amp	8	50339483	50546257	0	0.061275
12705	Amp	8	50546808	50578361	0.010261	0.064453
12706	Amp	8	50581147	50582952	0.060686	0.066448
12707	Amp	8	50591789	50610371	0	0.061808
12708	Amp	8	50611715	50757175	0	0.06117
12709	Amp	8	50758222	50931194	0	0.058714
12710	Amp	8	50931420	50947129	0	0.062649
12711	Amp	8	50949590	51100235	0	0.058714
12712	Amp	8	51109480	51116013	0	0.060009
12713	Amp	8	51116749	51188523	0	0.060653
12714	Amp	8	51188884	51191464	0	0.05961
12715	Amp	8	51191977	51250757	0	0.061992
12716	Amp	8	51250954	51268245	0	0.063556
12717	Amp	8	51268573	51269284	0.001323	0.06405
12718	Amp	8	51275842	51295264	0.050473	0.066181
12719	Amp	8	51295813	51301379	0.118001	0.068861

12720	Amp	8	51302153	51317542	0.100827	0.068367
12721	Amp	8	51319457	51342019	0.143787	0.069987
12722	Amp	8	51342097	51417355	0.100827	0.068367
12723	Amp	8	51418458	51418751	0.14896	0.070224
12724	Amp	8	51419121	51485019	0.034113	0.065413
12725	Amp	8	51485425	51538196	0.019169	0.064769
12726	Amp	8	51545629	51719878	0	0.062912
12727	Amp	8	51720348	51816922	0	0.061223
12728	Amp	8	51817856	51846585	0	0.060471
12729	Amp	8	51848550	51878042	0	0.055831
12730	Amp	8	51880306	51906723	0	0.060554
12731	Amp	8	51907515	51994321	0	0.061227
12732	Amp	8	51998735	52004909	0	0.059663
12733	Amp	8	52007563	52071778	0	0.058755
12734	Amp	8	52072832	52239575	0	0.054032
12735	Amp	8	52240482	52240482	0	0.053261
12736	Amp	8	52241840	52371124	0	0.052588
12737	Amp	8	52371460	52393404	0	0.053166
12738	Amp	8	52399515	52559272	0	0.05699
12739	Amp	8	52560130	52636620	0	0.056328
12740	Amp	8	52793185	52856807	0	0.057538
12741	Amp	8	52857860	52879370	0	0.054376
12742	Amp	8	52883728	52896396	0	0.055912
12743	Amp	8	52896542	52897877	0	0.056584
12744	Amp	8	52900306	53042655	0	0.055085
12745	Amp	8	53047084	53168522	0	0.049758
12746	Amp	8	53169501	53229035	0	0.048156
12747	Amp	8	53230988	53239276	0	0.050063
12748	Amp	8	53239422	53337123	0	0.04892
12749	Amp	8	53337226	53427417	0	0.04985
12750	Amp	8	53431540	53447153	0	0.048924
12751	Amp	8	53452365	53562652	0	0.047713
12752	Amp	8	53562798	53630297	0	0.045807
12753	Amp	8	53631976	53644561	0	0.049859
12754	Amp	8	53644628	54025271	0	0.048928
12755	Amp	8	54026168	54033023	0	0.049447
12756	Amp	8	54047375	54053575	0	0.051567
12757	Amp	8	54067219	54122452	0	0.0555
12758	Amp	8	54124764	54168629	0	0.051567
12759	Amp	8	54172222	54211977	0	0.050896
12760	Amp	8	54216000	54541380	0	0.0489
12761	Amp	8	54541748	54687736	0	0.049925
12762	Amp	8	54690979	54723624	0	0.0489
12763	Amp	8	54730966	54778898	0	0.044882
12764	Amp	8	54782090	54867111	0	0.044364
12765	Amp	8	54869274	54897705	0	0.043069
12766	Amp	8	54897913	54911625	0	0.044799
12767	Amp	8	54912365	54996087	0	0.049652
12768	Amp	8	54996211	55124507	0	0.050374
12769	Amp	8	55125662	55201958	0	0.055188
12770	Amp	8	55204303	55222699	0	0.058547
12771	Amp	8	55223167	55270547	0	0.055188
12772	Amp	8	55272758	55318499	0	0.045521

12773	Amp	8	55322229	55502915	0	0.048243
12774	Amp	8	55507250	55513995	0	0.051611
12775	Amp	8	55514121	55710429	0	0.054151
12776	Amp	8	55714893	55748461	0	0.053429
12777	Amp	8	55748505	55968842	0	0.051699
12778	Amp	8	55969469	56004594	0	0.04833
12779	Amp	8	56010913	56046341	0	0.047603
12780	Amp	8	56049085	56085921	0	0.046171
12781	Amp	8	56087767	56096007	0	0.047265
12782	Amp	8	56097357	56121465	0	0.048063
12783	Amp	8	56122740	56259673	0	0.047453
12784	Amp	8	56260770	56610009	0	0.046359
12785	Amp	8	56620504	56738256	0	0.05614
12786	Amp	8	56739697	56794347	0	0.046359
12787	Amp	8	56795455	56891114	0	0.045429
12788	Amp	8	56893904	56909039	0	0.048673
12789	Amp	8	56909681	57334704	0	0.045429
12790	Amp	8	57335422	57348085	0	0.049975
12791	Amp	8	57348258	57351452	0	0.054736
12792	Amp	8	57353690	57363136	0	0.055404
12793	Amp	8	57364442	57442880	0	0.052372
12794	Amp	8	57445766	57445766	0	0.050858
12795	Amp	8	57457382	57492262	0	0.052685
12796	Amp	8	57493704	57542142	0	0.056961
12797	Amp	8	57546606	57621707	0	0.0522
12798	Amp	8	57621757	57649004	0	0.053994
12799	Amp	8	57649027	57790273	0	0.049718
12800	Amp	8	57790683	57791630	0	0.051637
12801	Amp	8	57793018	57846291	0	0.05295
12802	Amp	8	57846655	57905142	0	0.052282
12803	Amp	8	57905943	57957299	0	0.055118
12804	Amp	8	57965830	57992306	0	0.058209
12805	Amp	8	57999150	58056852	0	0.062787
12806	Amp	8	58057209	58057384	0.094967	0.068145
12807	Amp	8	58064342	58218341	0.040093	0.065606
12808	Amp	8	58219819	58257536	0.073902	0.067214
12809	Amp	8	58258405	58300501	0.029031	0.065296
12810	Amp	8	58301185	58330005	0	0.063729
12811	Amp	8	58337342	58345048	0	0.057779
12812	Amp	8	58346599	58481175	0	0.054688
12813	Amp	8	58482343	58533895	0	0.05389
12814	Amp	8	58534370	58586126	0	0.055817
12815	Amp	8	58586830	58594333	0	0.054503
12816	Amp	8	58599197	58641704	0	0.056281
12817	Amp	8	58642966	58898318	0	0.054354
12818	Amp	8	58898667	58988994	0	0.04991
12819	Amp	8	58989418	59011335	0	0.050785
12820	Amp	8	59015705	59090254	0	0.055025
12821	Amp	8	59093925	59128229	0	0.057428
12822	Amp	8	59128787	59185984	0	0.052851
12823	Amp	8	59186420	59214151	0	0.054669
12824	Amp	8	59216099	59326335	0	0.061267
12825	Amp	8	59326720	59521770	0	0.058049

12826	Amp	8	59523743	59544712	0	0.062537
12827	Amp	8	59545479	59891841	0.001323	0.06398
12828	Amp	8	59901021	59901306	0.143787	0.070035
12829	Amp	8	59903314	59934894	0.450702	0.081516
12830	Amp	8	59935454	60264392	0.399144	0.079721
12831	Amp	8	60264650	60273378	0.47716	0.082386
12832	Amp	8	60275994	60465460	0.437198	0.08115
12833	Amp	8	60466057	60533591	0.362016	0.078486
12834	Amp	8	60542828	60685130	0.651644	0.088045
12835	Amp	8	60685250	60733993	0.616549	0.086899
12836	Amp	8	60734396	60940476	0.697374	0.089398
12837	Amp	8	60941051	61067144	0.569771	0.085456
12838	Amp	8	61067314	61090192	0.522881	0.083981
12839	Amp	8	61090341	61101067	0.563096	0.08517
12840	Amp	8	61103924	61106026	0.508568	0.083392
12841	Amp	8	61110322	61196644	0.557122	0.084962
12842	Amp	8	61201629	61249275	0.417873	0.080474
12843	Amp	8	61255870	61266906	0.681869	0.089018
12844	Amp	8	61266943	61314095	0.720659	0.090044
12845	Amp	8	61317954	61327505	0.857296	0.093602
12846	Amp	8	61328491	61399123	0.751221	0.090812
12847	Amp	8	61402372	61404102	0.667587	0.088409
12848	Amp	8	61407565	61422059	0.482379	0.082655
12849	Amp	8	61423895	61559293	0.222041	0.073096
12850	Amp	8	61562016	61562086	0.282828	0.075681
12851	Amp	8	61562538	61563971	0.47716	0.082434
12852	Amp	8	61564688	61699195	0.494792	0.083106
12853	Amp	8	61703718	61749933	0.444474	0.081288
12854	Amp	8	61755222	61876699	0.342649	0.077807
12855	Amp	8	61879738	61905488	0.362016	0.078454
12856	Amp	8	61908301	61908500	0.711903	0.08968
12857	Amp	8	61914234	61976573	0.781972	0.091657
12858	Amp	8	61982950	62149983	0.704849	0.089663
12859	Amp	8	62150022	62287908	0.557122	0.08491
12860	Amp	8	62288410	62292311	0.487495	0.082933
12861	Amp	8	62292867	62358126	0.563096	0.085254
12862	Amp	8	62358692	62482412	0.602643	0.086504
12863	Amp	8	62483073	62490184	1.159209	0.101165
12864	Amp	8	62490335	62543031	1.215958	0.102432
12865	Amp	8	62543592	62549793	1.33853	0.105187
12866	Amp	8	62553438	62554640	1.531891	0.109553
12867	Amp	8	62562580	62576551	1.624655	0.11151
12868	Amp	8	62576598	62584377	1.552619	0.10994
12869	Amp	8	62587529	62611515	1.032525	0.097955
12870	Amp	8	62617054	62683741	0.915698	0.095279
12871	Amp	8	62689259	62746808	1.00505	0.097435
12872	Amp	8	62747051	62747051	0.899693	0.09485
12873	Amp	8	62747076	62750746	0.98589	0.097054
12874	Amp	8	62750927	62796890	0.941704	0.095804
12875	Amp	8	62797333	62835054	0.977077	0.096843
12876	Amp	8	62836076	62845089	0.933745	0.095627
12877	Amp	8	62845511	62887631	0.536238	0.084401
12878	Amp	8	62890266	62890266	0.4115	0.08024

12879	Amp	8	62895841	62909924	0.494792	0.082957
12880	Amp	8	62912527	62915756	0.536238	0.08435
12881	Amp	8	62918735	62997214	0.482379	0.082664
12882	Amp	8	63004197	63061339	0.630438	0.087459
12883	Amp	8	63067749	63068586	0.482379	0.082664
12884	Amp	8	63068680	63073298	0.299433	0.076221
12885	Amp	8	63078512	63113185	0.563096	0.08519
12886	Amp	8	63117200	63150899	0.431198	0.08095
12887	Amp	8	63156382	63193976	0.355319	0.078233
12888	Amp	8	63194108	63194108	0.47716	0.082456
12889	Amp	8	63194555	63236659	0.500841	0.083227
12890	Amp	8	63246157	63251576	0.450702	0.081642
12891	Amp	8	63257780	63382215	0.689884	0.089222
12892	Amp	8	63382245	63388067	0.583752	0.085842
12893	Amp	8	63389902	63417648	0.287738	0.075941
12894	Amp	8	63418235	63549074	0.417873	0.080495
12895	Amp	8	63549648	63585802	0.667587	0.088552
12896	Amp	8	63587046	63587046	0.924746	0.09552
12897	Amp	8	63593784	63593784	1.023539	0.097887
12898	Amp	8	63600227	63718795	1.206641	0.102085
12899	Amp	8	63720230	63730056	1.084294	0.099377
12900	Amp	8	63738428	63926136	1.407997	0.106645
12901	Amp	8	63926481	63995144	1.665486	0.112369
12902	Amp	8	64003665	64026184	1.790459	0.114955
12903	Amp	8	64029653	64066602	1.940776	0.117999
12904	Amp	8	64067539	64082932	1.727286	0.113775
12905	Amp	8	64085329	64155857	1.665486	0.11243
12906	Amp	8	64159802	64175444	1.463774	0.108054
12907	Amp	8	64175869	64233400	1.398396	0.106427
12908	Amp	8	64237244	64252791	1.552619	0.109965
12909	Amp	8	64253088	64267405	1.759227	0.114421
12910	Amp	8	64310834	64415837	1.929887	0.117835
12911	Amp	8	64420360	64489527	2.147988	0.122074
12912	Amp	8	64493726	64498688	1.929887	0.117835
12913	Amp	8	64506549	64531763	1.865019	0.116547
12914	Amp	8	64533872	64631498	1.887378	0.11702
12915	Amp	8	64631568	64655681	1.727286	0.113628
12916	Amp	8	64655772	64655772	1.780186	0.11466
12917	Amp	8	64665910	64693982	1.398396	0.106569
12918	Amp	8	64694359	64707431	1.482378	0.108378
12919	Amp	8	64711977	64723066	1.552619	0.110025
12920	Amp	8	64724050	64756213	1.369395	0.105827
12921	Amp	8	64757132	64811249	2.116108	0.121542
12922	Amp	8	64815151	64847974	1.984015	0.118956
12923	Amp	8	64849042	64874794	1.665486	0.112317
12924	Amp	8	64875744	64881306	1.512549	0.109053
12925	Amp	8	64882588	64983972	1.737623	0.113934
12926	Amp	8	64987056	65033529	1.634701	0.111733
12927	Amp	8	65034328	65045885	1.717671	0.113426
12928	Amp	8	65047834	65058820	1.812192	0.115375
12929	Amp	8	65060916	65097060	1.61378	0.111297
12930	Amp	8	65098485	65098485	1.645511	0.111979
12931	Amp	8	65099132	65138535	1.696256	0.112998

12932	Amp	8	65139952	65266974	1.929887	0.117777
12933	Amp	8	65269590	65288038	1.759227	0.114342
12934	Amp	8	65290136	65290237	1.887378	0.116851
12935	Amp	8	65295678	65332464	1.737623	0.113963
12936	Amp	8	65337785	65379649	2.202226	0.123259
12937	Amp	8	65385722	65513697	1.833307	0.115879
12938	Amp	8	65518555	65592869	1.748606	0.114187
12939	Amp	8	65594685	65607428	1.309159	0.104653
12940	Amp	8	65612564	65656273	1.833307	0.115741
12941	Amp	8	65659902	65745714	1.748606	0.11404
12942	Amp	8	65759540	65824319	1.95165	0.118287
12943	Amp	8	65827875	65882254	2.049917	0.120108
12944	Amp	8	65882528	65942025	1.854421	0.11631
12945	Amp	8	65946224	66023053	1.897704	0.117236
12946	Amp	8	66023759	66056499	2.005947	0.119319
12947	Amp	8	66059966	66088135	1.887378	0.116939
12948	Amp	8	66088287	66189034	1.972776	0.118648
12949	Amp	8	66190466	66215853	2.147988	0.122165
12950	Amp	8	66221205	66237261	2.18114	0.122848
12951	Amp	8	66237499	66274903	1.994915	0.11908
12952	Amp	8	66276046	66392843	1.780186	0.114833
12953	Amp	8	66397025	66418110	1.727286	0.113644
12954	Amp	8	66424926	66426482	2.071483	0.120555
12955	Amp	8	66431770	66433937	2.235168	0.123869
12956	Amp	8	66434495	66448490	2.33403	0.125744
12957	Amp	8	66448527	66477405	2.245479	0.124022
12958	Amp	8	66478789	66498216	2.26699	0.124488
12959	Amp	8	66509655	66571265	2.479057	0.1287
12960	Amp	8	66573484	66574662	2.649655	0.131864
12961	Amp	8	66574680	66682805	2.743935	0.133724
12962	Amp	8	66683332	66691715	2.661431	0.132078
12963	Amp	8	66693178	66705262	2.548433	0.129994
12964	Amp	8	66714312	66801401	2.626052	0.131545
12965	Amp	8	66806492	66812593	2.719563	0.133268
12966	Amp	8	66818702	66837496	2.560422	0.130104
12967	Amp	8	66837820	66870149	2.33403	0.125892
12968	Amp	8	66870306	66933164	2.311329	0.125426
12969	Amp	8	66935563	67049792	2.52589	0.129534
12970	Amp	8	67051829	67264050	2.425182	0.12753
12971	Amp	8	67273847	67283868	2.213223	0.123422
12972	Amp	8	67287324	67337504	2.311329	0.125426
12973	Amp	8	67337848	67427613	2.18114	0.122794
12974	Amp	8	67434155	67434458	2.22459	0.123755
12975	Amp	8	67438935	67614974	2.192012	0.123073
12976	Amp	8	67615962	67682811	2.582422	0.130577
12977	Amp	8	67686964	67745240	2.614791	0.131271
12978	Amp	8	67745892	67775600	2.311329	0.125396
12979	Amp	8	67777617	67849418	1.624655	0.111438
12980	Amp	8	67849962	67994525	1.178324	0.101598
12981	Amp	8	67997525	68055145	1.206641	0.102087
12982	Amp	8	68056175	68162639	1.252022	0.103299
12983	Amp	8	68165114	68167801	1.290368	0.104018
12984	Amp	8	68172074	68207164	1.215958	0.102466

12985	Amp	8	68208657	68260383	1.407997	0.106735
12986	Amp	8	68261099	68479910	1.887378	0.116899
12987	Amp	8	68497207	68512290	1.542638	0.109711
12988	Amp	8	68512735	68519476	1.280185	0.103919
12989	Amp	8	68523761	68527114	1.388256	0.106247
12990	Amp	8	68530251	68540468	1.348715	0.105529
12991	Amp	8	68541179	68563547	0.924746	0.095385
12992	Amp	8	68565771	68591855	0.950886	0.096102
12993	Amp	8	68591891	68600961	1.112463	0.099952
12994	Amp	8	68601351	68612178	1.084294	0.099258
12995	Amp	8	68613545	68637963	0.906385	0.094989
12996	Amp	8	68643876	68685455	1.309159	0.104654
12997	Amp	8	68688787	68721672	1.112463	0.100016
12998	Amp	8	68728548	68734731	1.472629	0.1082
12999	Amp	8	68737441	68775380	1.436692	0.107483
13000	Amp	8	68776831	68779661	1.270711	0.103633
13001	Amp	8	68780143	68816397	1.378521	0.105974
13002	Amp	8	68817268	68822795	1.482378	0.108555
13003	Amp	8	68823481	68824373	2.060912	0.120389
13004	Amp	8	68826988	68848248	1.759227	0.114273
13005	Amp	8	68855679	68866265	1.737623	0.113784
13006	Amp	8	68868749	68943272	2.049917	0.120139
13007	Amp	8	68943499	68955638	2.071483	0.12064
13008	Amp	8	68956234	68995976	1.95165	0.118312
13009	Amp	8	68996860	69016502	1.593878	0.110923
13010	Amp	8	69017180	69028580	1.388256	0.106323
13011	Amp	8	69029273	69042594	1.542638	0.109714
13012	Amp	8	69043091	69150849	1.563118	0.110302
13013	Amp	8	69151220	69164905	1.456037	0.107721
13014	Amp	8	69165109	69209296	1.801583	0.115109
13015	Amp	8	69212333	69278883	1.512549	0.109113
13016	Amp	8	69286427	69296082	1.329472	0.104914
13017	Amp	8	69299337	69388417	1.61378	0.111186
13018	Amp	8	69388970	69414310	1.984015	0.118783
13019	Amp	8	69425100	69468910	2.192012	0.123107
13020	Amp	8	69468950	69492252	1.801583	0.115149
13021	Amp	8	69492712	69515900	1.102589	0.099713
13022	Amp	8	69518996	69543712	1.348715	0.105514
13023	Amp	8	69544683	69552678	1.512549	0.109006
13024	Amp	8	69553610	69617574	1.252022	0.103206
13025	Amp	8	69622839	69693838	1.445847	0.107613
13026	Amp	8	69693940	69771981	1.398396	0.106587
13027	Amp	8	69772159	69790973	1.178324	0.101606
13028	Amp	8	69796887	69898906	1.299876	0.104333
13029	Amp	8	69899029	69974094	1.023539	0.09772
13030	Amp	8	69978430	70015523	1.112463	0.099896
13031	Amp	8	70015572	70040504	1.225419	0.102603
13032	Amp	8	70041692	70042037	0.482379	0.082693
13033	Amp	8	70042119	70042119	0.444474	0.081299
13034	Amp	8	70042441	70042441	0.47716	0.08244
13035	Amp	8	70042470	70042599	0.529451	0.084234
13036	Amp	8	70048926	70049279	0.595248	0.086294
13037	Amp	8	70050863	70051137	0.543344	0.0845

13038	Amp	8	70051197	70051197	1.112463	0.099904
13039	Amp	8	70052137	70096607	1.243254	0.103016
13040	Amp	8	70099677	70123253	1.329472	0.104959
13041	Amp	8	70125165	70227014	1.512549	0.10902
13042	Amp	8	70227341	70307801	1.329472	0.104959
13043	Amp	8	70307957	70319258	1.243254	0.102949
13044	Amp	8	70320098	70327128	1.456037	0.107811
13045	Amp	8	70327289	70408666	1.634701	0.111743
13046	Amp	8	70411903	70502699	1.801583	0.115168
13047	Amp	8	70507805	70523829	1.502257	0.108839
13048	Amp	8	70524375	70524375	1.261401	0.103367
13049	Amp	8	70527085	70533244	1.188121	0.101825
13050	Amp	8	70533293	70587437	1.215958	0.102276
13051	Amp	8	70589107	70610778	1.472629	0.108299
13052	Amp	8	70613473	70648692	1.188121	0.101665
13053	Amp	8	70651997	70748413	1.717671	0.113439
13054	Amp	8	70750357	70761490	1.482378	0.108483
13055	Amp	8	70763958	70826069	1.759227	0.114297
13056	Amp	8	70827129	70844973	1.041205	0.098361
13057	Amp	8	70852757	70869614	0.840289	0.093308
13058	Amp	8	70872018	70923104	0.86541	0.093962
13059	Amp	8	70927960	70945603	1.00505	0.097427
13060	Amp	8	70948204	71011332	0.848692	0.093495
13061	Amp	8	71011856	71057330	0.720659	0.09003
13062	Amp	8	71059236	71119278	0.968522	0.096608
13063	Amp	8	71126122	71168831	0.660254	0.088278
13064	Amp	8	71169838	71490590	0.557122	0.0851
13065	Amp	8	71494285	71601506	0.47716	0.082463
13066	Amp	8	71602261	71624464	0.543344	0.084508
13067	Amp	8	71627115	71656040	0.515676	0.083617
13068	Amp	8	71656208	71810991	0.569771	0.085417
13069	Amp	8	71813957	71833714	0.342649	0.077757
13070	Amp	8	71834056	71949585	0.494792	0.082974
13071	Amp	8	71952005	72068569	0.563096	0.085165
13072	Amp	8	72070582	72194681	0.697374	0.089327
13073	Amp	8	72195303	72246645	0.823771	0.092912
13074	Amp	8	72248631	72320053	0.79909	0.092229
13075	Amp	8	72327312	72332122	0.616549	0.086968
13076	Amp	8	72332218	72417855	0.576183	0.085719
13077	Amp	8	72429144	72448679	0.950886	0.096114
13078	Amp	8	72451135	72526865	0.742723	0.090732
13079	Amp	8	72533818	72543535	0.831947	0.093084
13080	Amp	8	72548076	72565866	0.616549	0.087016
13081	Amp	8	72566950	72632494	0.689884	0.089236
13082	Amp	8	72633119	72639561	0.735486	0.090475
13083	Amp	8	72651791	72662680	0.622604	0.087172
13084	Amp	8	72665268	72778339	0.790614	0.091958
13085	Amp	8	72779869	72867650	0.751221	0.090817
13086	Amp	8	72871517	72930097	0.823771	0.09284
13087	Amp	8	72932080	72948493	0.906385	0.09506
13088	Amp	8	72949844	72950800	0.98589	0.096855
13089	Amp	8	72951510	72997344	1.329472	0.105083
13090	Amp	8	72998906	73049521	1.463774	0.108034

13091	Amp	8	73051631	73054990	1.329472	0.105083
13092	Amp	8	73062335	73066906	1.456037	0.107916
13093	Amp	8	73067431	73115244	1.634701	0.111692
13094	Amp	8	73115633	73239374	1.604336	0.111038
13095	Amp	8	73241972	73259997	1.23519	0.10281
13096	Amp	8	73261080	73322791	1.290368	0.104045
13097	Amp	8	73326519	73540656	1.197658	0.101854
13098	Amp	8	73541365	73726944	1.140417	0.100704
13099	Amp	8	73727769	73833947	0.995635	0.09725
13100	Amp	8	73833963	73852175	0.950886	0.096185
13101	Amp	8	73852655	73871286	0.891602	0.094627
13102	Amp	8	73872347	73960371	0.807647	0.092407
13103	Amp	8	73963000	73966461	0.86541	0.093885
13104	Amp	8	73966852	74066404	0.933745	0.095586
13105	Amp	8	74077404	74090172	1.058188	0.098633
13106	Amp	8	74090803	74268873	0.933745	0.095586
13107	Amp	8	74274835	74460890	0.831947	0.092959
13108	Amp	8	74461074	74486140	0.950886	0.096119
13109	Amp	8	74488261	74500922	0.765474	0.091333
13110	Amp	8	74501264	74501473	0.711903	0.089855
13111	Amp	8	74503330	74512893	0.765474	0.091354
13112	Amp	8	74522581	74547684	0.790614	0.091906
13113	Amp	8	74551284	74555609	0.906385	0.094999
13114	Amp	8	74555848	74581474	0.941704	0.095769
13115	Amp	8	74585277	74591151	1.084294	0.099438
13116	Amp	8	74600256	74775207	1.188121	0.101657
13117	Amp	8	74776823	74806057	1.084294	0.099304
13118	Amp	8	74807073	74835435	1.131695	0.100484
13119	Amp	8	74837999	74838185	1.00505	0.097324
13120	Amp	8	74844644	74845606	0.950886	0.096025
13121	Amp	8	74849132	74862909	1.058188	0.098626
13122	Amp	8	74863819	74907116	0.924746	0.095533
13123	Amp	8	74910101	74968385	0.807647	0.092414
13124	Amp	8	74968404	74976907	0.72817	0.090195
13125	Amp	8	74979469	75029931	0.781972	0.091839
13126	Amp	8	75034460	75309161	0.689884	0.08903
13127	Amp	8	75310987	75376724	0.924746	0.095363
13128	Amp	8	75379430	75384554	0.781972	0.091695
13129	Amp	8	75385626	75459106	0.569771	0.085362
13130	Amp	8	75463046	75479314	0.602643	0.086565
13131	Amp	8	75479349	75558199	0.616549	0.087047
13132	Amp	8	75561823	75595099	0.557122	0.085113
13133	Amp	8	75599323	75617602	0.482379	0.082633
13134	Amp	8	75629100	75634198	0.355319	0.078367
13135	Amp	8	75645419	75735291	0.392611	0.079486
13136	Amp	8	75738637	75750706	0.368062	0.078716
13137	Amp	8	75752305	75969256	0.487495	0.082887
13138	Amp	8	75971200	76003091	0.660254	0.088266
13139	Amp	8	76004976	76025424	0.711903	0.089682
13140	Amp	8	76025719	76203472	0.536238	0.084303
13141	Amp	8	76203812	76233346	0.6737	0.088656
13142	Amp	8	76233443	76299646	0.536238	0.084303
13143	Amp	8	76299834	76342718	0.550755	0.084873

13144	Amp	8	76343201	76436017	0.536238	0.084392
13145	Amp	8	76441243	76551573	0.4115	0.080221
13146	Amp	8	76553677	76564882	0.293269	0.076163
13147	Amp	8	76572007	76719534	0.482379	0.082659
13148	Amp	8	76720595	76787979	0.536238	0.084283
13149	Amp	8	76788058	77025942	0.482379	0.082659
13150	Amp	8	77027133	77103202	0.392611	0.079482
13151	Amp	8	77103950	77115770	0.311747	0.076818
13152	Amp	8	77116196	77134244	0	0.063546
13153	Amp	8	77141920	77150464	0.015369	0.064687
13154	Amp	8	77150475	77167382	0.005328	0.064236
13155	Amp	8	77168099	77293349	0.015369	0.064682
13156	Amp	8	77293384	77294593	0.181932	0.071539
13157	Amp	8	77294811	77316972	0.193543	0.071989
13158	Amp	8	77317825	77338978	0.299433	0.076413
13159	Amp	8	77344926	77413877	0.193543	0.071989
13160	Amp	8	77415666	77612325	0.233782	0.073507
13161	Amp	8	77619135	77637995	0	0.060154
13162	Amp	8	77638795	77671093	0.073902	0.067142
13163	Amp	8	77671682	77772100	0	0.060154
13164	Amp	8	77773806	77912313	0	0.059386
13165	Amp	8	77914382	78007093	0	0.060336
13166	Amp	8	78007588	78027642	0	0.057553
13167	Amp	8	78033179	78101098	0	0.056434
13168	Amp	8	78104121	78292900	0	0.057603
13169	Amp	8	78295183	78341711	0	0.056461
13170	Amp	8	78345327	78349124	0	0.058942
13171	Amp	8	78358598	78451607	0	0.057763
13172	Amp	8	78452037	78469814	0	0.059087
13173	Amp	8	78472714	78497716	0	0.059887
13174	Amp	8	78498043	78664393	0	0.061505
13175	Amp	8	78669296	78750071	0	0.061059
13176	Amp	8	78750101	78772959	0.010261	0.064356
13177	Amp	8	78774001	79052580	0.060686	0.066498
13178	Amp	8	79056598	79094866	0.079652	0.067505
13179	Amp	8	79095892	79132038	0.044794	0.065888
13180	Amp	8	79134339	79192215	0.335313	0.077624
13181	Amp	8	79192926	79247276	0.271552	0.075143
13182	Amp	8	79248810	79306611	0.222041	0.073001
13183	Amp	8	79306680	79328214	0.138391	0.069704
13184	Amp	8	79328331	79380716	0.17597	0.071233
13185	Amp	8	79393642	79393642	0.450702	0.081622
13186	Amp	8	79393695	79455823	0.404718	0.080092
13187	Amp	8	79456652	79521485	0.470509	0.082218
13188	Amp	8	79527664	79533533	0.529451	0.084048
13189	Amp	8	79541005	79562834	0.463534	0.081922
13190	Amp	8	79564541	79585693	0.515676	0.083758
13191	Amp	8	79587519	79587519	0.536238	0.08431
13192	Amp	8	79589807	79682701	1.049301	0.098422
13193	Amp	8	79683804	79698832	1.112463	0.099973
13194	Amp	8	79702417	79707644	1.178324	0.101598
13195	Amp	8	79708776	79708776	1.280185	0.103909
13196	Amp	8	79722781	79726911	1.00505	0.097425

13197	Amp	8	79726970	79731204	1.058188	0.098729
13198	Amp	8	79732638	79752713	1.112463	0.099941
13199	Amp	8	79755813	79766443	1.398396	0.106485
13200	Amp	8	79769970	79794853	1.502257	0.10881
13201	Amp	8	79797186	79817570	1.416817	0.106981
13202	Amp	8	79817676	79901082	1.436692	0.107468
13203	Amp	8	79901752	80027415	1.252022	0.103307
13204	Amp	8	80032023	80062650	1.887378	0.116992
13205	Amp	8	80063035	80068224	1.929887	0.117701
13206	Amp	8	80068427	80134380	2.094104	0.120986
13207	Amp	8	80135195	80205025	2.213223	0.123424
13208	Amp	8	80207809	80224795	2.435195	0.127694
13209	Amp	8	80225277	80228109	2.368227	0.12639
13210	Amp	8	80230780	80315483	2.52589	0.129562
13211	Amp	8	80316405	80426907	2.605213	0.131002
13212	Amp	8	80432336	80520693	2.435195	0.127831
13213	Amp	8	80523707	80576982	2.356694	0.12628
13214	Amp	8	80583689	80616967	2.696333	0.132701
13215	Amp	8	80622203	80636104	2.413643	0.127418
13216	Amp	8	80637411	80708828	2.290039	0.124981
13217	Amp	8	80711233	80749369	1.593878	0.110869
13218	Amp	8	80754305	80930501	1.696256	0.113077
13219	Amp	8	80931799	80961693	1.645511	0.111865
13220	Amp	8	80983633	80992296	1.041205	0.09818
13221	Amp	8	80993079	81038504	1.102589	0.099698
13222	Amp	8	81038510	81099215	1.00505	0.097387
13223	Amp	8	81101258	81139995	1.041205	0.098273
13224	Amp	8	81140261	81195862	1.014678	0.097565
13225	Amp	8	81199536	81279612	1.140417	0.100747
13226	Amp	8	81282491	81312343	1.225419	0.102589
13227	Amp	8	81313172	81321675	1.280185	0.103818
13228	Amp	8	81332716	81338606	1.140417	0.100753
13229	Amp	8	81340502	81406207	1.032525	0.097932
13230	Amp	8	81415739	81447793	1.00505	0.097381
13231	Amp	8	81448380	81463778	0.924746	0.095538
13232	Amp	8	81464464	81465409	3.067933	0.139838
13233	Amp	8	81465871	81465900	0.924746	0.095538
13234	Amp	8	81467412	81539657	0.882843	0.0944
13235	Amp	8	81540067	81628524	0.933745	0.095701
13236	Amp	8	81629517	81785114	0.815228	0.092519
13237	Amp	8	81788708	81832821	0.470509	0.082131
13238	Amp	8	81836356	81959218	0.494792	0.083038
13239	Amp	8	81961079	82075765	0.515676	0.083666
13240	Amp	8	82081487	82107225	0.487495	0.082759
13241	Amp	8	82107504	82149184	0.386426	0.079473
13242	Amp	8	82149561	82172209	0.424557	0.080628
13243	Amp	8	82175458	82175972	0.287738	0.075848
13244	Amp	8	82186610	82248216	0.181932	0.071578
13245	Amp	8	82249380	82272551	0.112543	0.068806
13246	Amp	8	82283548	82329405	0.187449	0.071802
13247	Amp	8	82332300	82332980	0.251218	0.074385
13248	Amp	8	82336353	82336353	0.215608	0.072867
13249	Amp	8	82338754	82340000	0.335313	0.077684

13250	Amp	8	82349081	82386787	0.417873	0.080479
13251	Amp	8	82386874	82398044	0.282828	0.075662
13252	Amp	8	82401617	82410861	0.193543	0.072071
13253	Amp	8	82410912	82432660	0.128324	0.069275
13254	Amp	8	82438534	82439203	0.204831	0.072366
13255	Amp	8	82439712	82439712	0.255918	0.074484
13256	Amp	8	82443849	82457708	0.227787	0.073289
13257	Amp	8	82464253	82485982	0.14896	0.070198
13258	Amp	8	82510574	82510574	0.227787	0.073263
13259	Amp	8	82510974	82539856	0.159312	0.07068
13260	Amp	8	82540746	82639471	0.204831	0.072312
13261	Amp	8	82643185	82644880	0.251218	0.074446
13262	Amp	8	82647786	82670688	0.238246	0.073646
13263	Amp	8	82672789	82730768	0.159312	0.070581
13264	Amp	8	82730896	82754874	0.118001	0.068949
13265	Amp	8	82758293	82760468	0.204831	0.07249
13266	Amp	8	82763610	82789069	0.259438	0.074848
13267	Amp	8	82789155	82902552	0.271552	0.075329
13268	Amp	8	82906481	82914853	0.227787	0.073211
13269	Amp	8	82914869	83009427	0.204831	0.072414
13270	Amp	8	83017445	83092730	0.335313	0.077648
13271	Amp	8	83094683	83166271	0.246818	0.074107
13272	Amp	8	83175188	83215325	0.322897	0.077298
13273	Amp	8	83215341	83243042	0.246818	0.074107
13274	Amp	8	83243757	83307437	0.431198	0.08082
13275	Amp	8	83308214	83418251	0.392611	0.079518
13276	Amp	8	83418675	83673595	0.424557	0.080598
13277	Amp	8	83675578	83702274	0.536238	0.084347
13278	Amp	8	83703456	83794811	0.583752	0.085776
13279	Amp	8	83800032	83890552	0.660254	0.088375
13280	Amp	8	83893103	83897266	0.616549	0.086946
13281	Amp	8	83899992	83936656	0.342649	0.077862
13282	Amp	8	83939440	84103960	0.399144	0.079718
13283	Amp	8	84113572	84137103	0.299433	0.076223
13284	Amp	8	84137583	84509016	0.259438	0.074898
13285	Amp	8	84511265	84536756	0.589605	0.086199
13286	Amp	8	84539009	84567806	0.4115	0.080284
13287	Amp	8	84570703	84837294	0.311747	0.076699
13288	Amp	8	84844800	84894668	0.210594	0.07253
13289	Amp	8	84895140	84950737	0.276783	0.075364
13290	Amp	8	84951389	85127648	0.233782	0.073487
13291	Amp	8	85127671	85146616	0	0.063521
13292	Amp	8	85148158	85148158	0.019169	0.064719
13293	Amp	8	85148859	85178859	0.159312	0.070646
13294	Amp	8	85187084	85304144	0.14896	0.070164
13295	Amp	8	85305036	85312877	0.164935	0.07092
13296	Amp	8	85315930	85389872	0.210594	0.072675
13297	Amp	8	85398353	85465905	0.079652	0.067524
13298	Amp	8	85468176	85601279	0.255918	0.074596
13299	Amp	8	85602329	85602329	0.193543	0.071997
13300	Amp	8	85603620	85608821	0.044794	0.065824
13301	Amp	8	85828731	85828939	0.368062	0.078731
13302	Amp	8	85829000	85888163	0.392611	0.07966

13303	Amp	8	85888207	85913118	0.470509	0.082248
13304	Amp	8	85913639	85915575	0.550755	0.084867
13305	Amp	8	85916656	85919826	0.609133	0.086741
13306	Amp	8	85920759	85930874	0.697374	0.089264
13307	Amp	8	85934594	85935400	0.616549	0.086947
13308	Amp	8	85937498	86063589	0.720659	0.08996
13309	Amp	8	86063921	86070590	0.616549	0.086885
13310	Amp	8	86071374	86168801	0.522881	0.083872
13311	Amp	8	86323850	86453263	0.742723	0.090589
13312	Amp	8	86453415	86454867	0.651644	0.088066
13313	Amp	8	86457386	86542166	0.72817	0.090271
13314	Amp	8	86542422	86550102	0.781972	0.091739
13315	Amp	8	86553987	86565625	0.899693	0.09484
13316	Amp	8	86567322	86631641	0.644502	0.087769
13317	Amp	8	86631762	86727216	0.576183	0.085564
13318	Amp	8	86728636	86766602	0.515676	0.08369
13319	Amp	8	86768315	86772128	0.609133	0.086746
13320	Amp	8	86772731	87008440	0.583752	0.08597
13321	Amp	8	87010558	87034965	0.557122	0.084963
13322	Amp	8	87035525	87219742	0.450702	0.081549
13323	Amp	8	87231014	87236677	0.431198	0.08099
13324	Amp	8	87239463	87357981	0.399144	0.079792
13325	Amp	8	87360626	87392194	0.417873	0.080474
13326	Amp	8	87392811	87429758	0.17597	0.07128
13327	Amp	8	87433749	87586432	0.246818	0.074259
13328	Amp	8	87588365	87658430	0.373599	0.078824
13329	Amp	8	87661083	87757237	0.500841	0.083227
13330	Amp	8	87757389	87794943	0.522881	0.083967
13331	Amp	8	87799526	87809647	0.742723	0.090635
13332	Amp	8	87811558	87925343	0.807647	0.092476
13333	Amp	8	87930526	88025657	0.6737	0.088762
13334	Amp	8	88026391	88055546	0.576183	0.085576
13335	Amp	8	88061111	88061111	0.322897	0.077122
13336	Amp	8	88063164	88166169	0.246818	0.074144
13337	Amp	8	88168964	88244547	0.282828	0.07571
13338	Amp	8	88244776	88289984	0.246818	0.074242
13339	Amp	8	88294374	88294374	0.362016	0.07855
13340	Amp	8	88297295	88309038	0.379885	0.079134
13341	Amp	8	88309660	88317817	0.259438	0.074827
13342	Amp	8	88318202	88402914	0.30524	0.076472
13343	Amp	8	88402973	88600980	0.282828	0.07562
13344	Amp	8	88618638	88618638	0.379885	0.07912
13345	Amp	8	88619203	88807990	0.322897	0.077279
13346	Amp	8	88818277	88821394	0.30524	0.076539
13347	Amp	8	88822584	89046301	0.368062	0.078819
13348	Amp	8	89046789	89128912	0.322897	0.077174
13349	Amp	8	89131502	89190755	0.392611	0.079521
13350	Amp	8	89192691	89196886	0.550755	0.084786
13351	Amp	8	89202149	89263713	0.529451	0.084201
13352	Amp	8	89264339	89305578	0.569771	0.085554
13353	Amp	8	89306505	89314835	0.758088	0.090986
13354	Amp	8	89315019	89315129	0.941704	0.095969
13355	Amp	8	89315511	89340019	0.735486	0.090537

13356	Amp	8	89340605	89348844	0.636464	0.087719
13357	Amp	8	89350410	89379257	0.487495	0.082736
13358	Amp	8	89382316	89393166	0.4115	0.080289
13359	Amp	8	89401018	89483519	0.959607	0.096225
13360	Amp	8	89488130	89498333	0.899693	0.094873
13361	Amp	8	89499986	89508420	1.058188	0.098693
13362	Amp	8	89544455	89614130	1.168655	0.101203
13363	Amp	8	89614964	89638079	1.23519	0.102815
13364	Amp	8	89642021	89647622	1.388256	0.10634
13365	Amp	8	89650216	89678313	1.188121	0.101774
13366	Amp	8	89686412	89742114	1.929887	0.11771
13367	Amp	8	89743834	89814895	2.256542	0.124265
13368	Amp	8	89816829	89857181	2.169666	0.122621
13369	Amp	8	89858006	89882782	2.311329	0.125488
13370	Amp	8	89885310	90012459	2.345513	0.126044
13371	Amp	8	90016131	90016930	2.425182	0.127482
13372	Amp	8	90019158	90069834	2.290039	0.124928
13373	Amp	8	90069867	90127347	2.368227	0.126551
13374	Amp	8	90130144	90138418	2.871582	0.136063
13375	Amp	8	90138498	90196579	2.964305	0.137827
13376	Amp	8	90196756	90315472	3.207115	0.142302
13377	Amp	8	90315573	90387445	3.230405	0.142826
13378	Amp	8	90388226	90388226	3.08058	0.139954
13379	Amp	8	90391967	90407566	2.9061	0.136799
13380	Amp	8	90407738	90428126	2.871582	0.136134
13381	Amp	8	90431033	90553251	3.172599	0.141719
13382	Amp	8	90553752	90593870	2.871582	0.136134
13383	Amp	8	90593972	90598538	2.719563	0.133303
13384	Amp	8	90599550	90607912	2.345513	0.126051
13385	Amp	8	90611672	90619890	2.26699	0.124613
13386	Amp	8	90621206	90673411	2.18114	0.12285
13387	Amp	8	90675342	90702089	2.278531	0.124734
13388	Amp	8	90703819	90863203	2.322689	0.125705
13389	Amp	8	90867323	90890883	2.614791	0.131156
13390	Amp	8	90902780	90964091	2.322689	0.125705
13391	Amp	8	90965779	91014289	2.060912	0.120489
13392	Amp	8	91027661	91060570	2.202226	0.12329
13393	Amp	8	91061400	91094256	2.235168	0.123979
13394	Amp	8	91097409	91106779	2.060912	0.120479
13395	Amp	8	91174785	91271463	2.379392	0.126778
13396	Amp	8	91272483	91322697	2.435195	0.127741
13397	Amp	8	91323174	91327022	2.094104	0.121073
13398	Amp	8	91329135	91338456	2.22459	0.123586
13399	Amp	8	91347742	91348978	1.994915	0.119111
13400	Amp	8	91349939	91443140	1.854421	0.11631
13401	Amp	8	91444923	91479082	1.801583	0.115214
13402	Amp	8	91482637	91489630	1.972776	0.118661
13403	Amp	8	91499426	91639565	1.865019	0.116521
13404	Amp	8	91640987	91640987	1.843664	0.116004
13405	Amp	8	91641512	91685716	1.940776	0.118033
13406	Amp	8	91697963	91815592	2.016585	0.119596
13407	Amp	8	91825387	91826791	2.425182	0.127581
13408	Amp	8	91841277	91868448	2.837591	0.135454

13409	Amp	8	91868820	91991063	2.755154	0.133841
13410	Amp	8	91992474	92003478	2.614791	0.131316
13411	Amp	8	92003494	92007424	2.049917	0.120108
13412	Amp	8	92007733	92007901	1.876242	0.116661
13413	Amp	8	92008590	92008633	1.940776	0.118121
13414	Amp	8	92008669	92009032	2.514205	0.129328
13415	Amp	8	92012262	92034470	2.649655	0.131882
13416	Amp	8	92034888	92046810	2.514205	0.129328
13417	Amp	8	92047332	92092559	2.605213	0.131087
13418	Amp	8	92092577	92167636	2.848911	0.135654
13419	Amp	8	92168019	92202622	2.614791	0.131327
13420	Amp	8	92206016	92214557	3.138463	0.140928
13421	Amp	8	92216412	92281233	2.290039	0.124992
13422	Amp	8	92281724	92360384	2.169666	0.122669
13423	Amp	8	92360525	92381330	2.413643	0.127377
13424	Amp	8	92388013	92412097	1.908279	0.117328
13425	Amp	8	92412135	92453754	2.127614	0.121812
13426	Amp	8	92454560	92458612	2.18114	0.122831
13427	Amp	8	92462558	92462558	1.940776	0.118124
13428	Amp	8	92462909	92477649	1.823153	0.115611
13429	Amp	8	92483634	92487589	1.552619	0.109948
13430	Amp	8	92488506	92634843	1.68612	0.112892
13431	Amp	8	92640342	92656314	1.812192	0.115414
13432	Amp	8	92663414	92704865	1.727286	0.113655
13433	Amp	8	92712703	92718013	2.235168	0.123838
13434	Amp	8	92722618	92726848	2.435195	0.12783
13435	Amp	8	92737397	92775534	2.278531	0.124735
13436	Amp	8	92776212	92807648	2.345513	0.12614
13437	Amp	8	92809828	92866098	2.537294	0.129752
13438	Amp	8	92868937	92891859	2.765626	0.134055
13439	Amp	8	92893679	92917582	2.626052	0.131545
13440	Amp	8	92921313	92963507	2.952409	0.137587
13441	Amp	8	92969749	93013593	2.755154	0.133785
13442	Amp	8	93015516	93029188	2.882945	0.13635
13443	Amp	8	93031314	93047916	3.150497	0.141349
13444	Amp	8	93048111	93056198	2.882945	0.13635
13445	Amp	8	93068603	93128097	2.940615	0.137338
13446	Amp	8	93130978	93157150	2.755154	0.13397
13447	Amp	8	93157314	93248452	2.860424	0.135803
13448	Amp	8	93248759	93290616	3.057106	0.139503
13449	Amp	8	93291867	93335782	2.788392	0.134508
13450	Amp	8	93336802	93456916	2.594754	0.130897
13451	Amp	8	93459503	93467882	2.696333	0.132831
13452	Amp	8	93469003	93472314	2.649655	0.13186
13453	Amp	8	93476700	93533746	2.137445	0.121929
13454	Amp	8	93535578	93615905	2.446878	0.128085
13455	Amp	8	93618394	93680299	2.614791	0.131242
13456	Amp	8	93680331	93697628	2.673338	0.132323
13457	Amp	8	93708407	93716818	3.033246	0.139006
13458	Amp	8	93716883	93726072	2.696333	0.132849
13459	Amp	8	93732123	93774725	2.765626	0.134041
13460	Amp	8	93782878	93825034	2.235168	0.123858
13461	Amp	8	93825582	93930991	2.413643	0.127272

13462	Amp	8	93931387	93962609	2.368227	0.126567
13463	Amp	8	93962949	94053878	2.245479	0.124046
13464	Amp	8	94056315	94061696	2.311329	0.125444
13465	Amp	8	94062206	94090725	2.26699	0.124473
13466	Amp	8	94094323	94150606	2.300463	0.1251
13467	Amp	8	94154093	94166149	2.788392	0.134432
13468	Amp	8	94167885	94171489	2.860424	0.135827
13469	Amp	8	94172339	94214465	2.929372	0.137107
13470	Amp	8	94216931	94228081	2.490318	0.128867
13471	Amp	8	94230465	94244675	2.707624	0.133055
13472	Amp	8	94245837	94250178	2.755154	0.13378
13473	Amp	8	94252032	94270329	2.468223	0.1285
13474	Amp	8	94270531	94279876	2.929372	0.137216
13475	Amp	8	94280083	94282899	2.975629	0.13792
13476	Amp	8	94287660	94291228	2.514205	0.129204
13477	Amp	8	94292370	94305938	2.637742	0.131708
13478	Amp	8	94306969	94318347	2.571513	0.130312
13479	Amp	8	94324604	94544159	3.057106	0.139511
13480	Amp	8	94544175	94544215	2.776997	0.134269
13481	Amp	8	94544231	94544322	2.26699	0.124563
13482	Amp	8	94544360	94552305	2.169666	0.12247
13483	Amp	8	94555060	94558223	1.790459	0.114917
13484	Amp	8	94564122	94564122	2.446878	0.127946
13485	Amp	8	94566121	94566121	2.882945	0.136211
13486	Amp	8	94567558	94708963	2.9061	0.136698
13487	Amp	8	94709265	94858885	2.743935	0.133597
13488	Amp	8	94859901	94910482	3.349067	0.14487
13489	Amp	8	94910524	94935974	3.422847	0.146272
13490	Amp	8	94936412	95006454	3.483236	0.147393
13491	Amp	8	95009253	95055888	3.566107	0.148886
13492	Amp	8	95056516	95075245	3.505046	0.147742
13493	Amp	8	95077192	95077967	3.021176	0.138783
13494	Amp	8	95084799	95159877	2.882945	0.136247
13495	Amp	8	95160262	95174372	2.812598	0.135056
13496	Amp	8	95176547	95208986	2.765626	0.134108
13497	Amp	8	95212871	95234227	2.158817	0.122314
13498	Amp	8	95234645	95250216	2.256542	0.124403
13499	Amp	8	95250404	95255608	2.390849	0.126978
13500	Amp	8	95255792	95263848	2.322689	0.125576
13501	Amp	8	95264064	95264064	2.245479	0.124082
13502	Amp	8	95266884	95295276	2.368227	0.126463
13503	Amp	8	95295322	95305682	2.22459	0.123706
13504	Amp	8	95307956	95368270	1.908279	0.117265
13505	Amp	8	95380152	95432774	1.843664	0.116144
13506	Amp	8	95434448	95451458	1.812192	0.11544
13507	Amp	8	95452158	95458489	1.696256	0.112936
13508	Amp	8	95460297	95466535	1.593878	0.110933
13509	Amp	8	95471223	95471684	1.482378	0.108358
13510	Amp	8	95472044	95560563	1.378521	0.106078
13511	Amp	8	95560713	95660096	1.463774	0.108084
13512	Amp	8	95660176	95660176	1.33853	0.105226
13513	Amp	8	95662541	95700562	0.977077	0.09674
13514	Amp	8	95700950	95859635	0.848692	0.093499

13515	Amp	8	95862467	96012335	0.751221	0.090934
13516	Amp	8	96019538	96025231	0.697374	0.08931
13517	Amp	8	96026143	96093167	0.72817	0.090135
13518	Amp	8	96093630	96219684	0.711903	0.089824
13519	Amp	8	96223367	96270116	0.583752	0.085773
13520	Amp	8	96277985	96308420	0.622604	0.087176
13521	Amp	8	96311615	96315451	0.569771	0.085434
13522	Amp	8	96318463	96342312	0.457501	0.081772
13523	Amp	8	96343438	96377546	0.444474	0.081285
13524	Amp	8	96378802	96572962	0.508568	0.083476
13525	Amp	8	96575273	96628618	0.4115	0.080153
13526	Amp	8	96631063	96641069	0.34892	0.078146
13527	Amp	8	96643398	96738556	0.368062	0.07879
13528	Amp	8	96746924	96853852	0.444474	0.081375
13529	Amp	8	96856545	96989773	0.589605	0.086045
13530	Amp	8	97003577	97133434	0.47716	0.082386
13531	Amp	8	97134042	97138850	0.557122	0.085066
13532	Amp	8	97145393	97168614	0.437198	0.081082
13533	Amp	8	97174186	97174186	0.404718	0.080071
13534	Amp	8	97174418	97178401	0.329205	0.077486
13535	Amp	8	97179687	97323848	0.667587	0.088591
13536	Amp	8	97323873	97323873	0.72817	0.09031
13537	Amp	8	97324318	97416460	0.751221	0.090805
13538	Amp	8	97417801	97515447	1.407997	0.10673
13539	Amp	8	97515934	97545704	1.329472	0.10501
13540	Amp	8	97551205	97554387	0.689884	0.089085
13541	Amp	8	97555354	97683803	1.041205	0.098362
13542	Amp	8	97684175	97691847	0.950886	0.096171
13543	Amp	8	97696647	97703605	1.041205	0.098231
13544	Amp	8	97708906	97799961	1.058188	0.09876
13545	Amp	8	97800335	97838489	1.032525	0.097936
13546	Amp	8	97838519	97838519	1.168655	0.10137
13547	Amp	8	97839099	97873318	1.502257	0.108818
13548	Amp	8	97881636	97941784	1.573014	0.110434
13549	Amp	8	97944940	98091506	1.717671	0.113373
13550	Amp	8	98093498	98126260	1.854421	0.116226
13551	Amp	8	98127382	98127382	1.897704	0.117213
13552	Amp	8	98127980	98145541	1.737623	0.113879
13553	Amp	8	98146069	98172835	1.168655	0.101276
13554	Amp	8	98177617	98186702	1.049301	0.098424
13555	Amp	8	98186838	98269980	1.131695	0.100375
13556	Amp	8	98274148	98274148	1.358811	0.10562
13557	Amp	8	98276653	98287889	1.940776	0.118019
13558	Amp	8	98291914	98408547	2.457251	0.128141
13559	Amp	8	98409375	98444288	2.673338	0.132313
13560	Amp	8	98445686	98450819	2.707624	0.133028
13561	Amp	8	98450920	98558400	2.755154	0.133908
13562	Amp	8	98561329	98627820	2.673338	0.132342
13563	Amp	8	98632589	98812597	2.800837	0.134771
13564	Amp	8	98814375	98832845	2.89426	0.136458
13565	Amp	8	98842460	98868435	2.812598	0.13506
13566	Amp	8	98879411	98910665	2.929372	0.137179
13567	Amp	8	98910930	98979557	3.08058	0.139977

13568	Amp	8	98984119	99030685	2.964305	0.137858
13569	Amp	8	99034464	99040273	2.837591	0.135428
13570	Amp	8	99045010	99055707	2.684894	0.13263
13571	Amp	8	99061218	99079757	3.286256	0.143879
13572	Amp	8	99081660	99188231	3.195626	0.142168
13573	Amp	8	99190254	99197603	3.08058	0.13985
13574	Amp	8	99205434	99208675	2.537294	0.129728
13575	Amp	8	99208945	99226949	2.614791	0.13126
13576	Amp	8	99229345	99229345	2.548433	0.129857
13577	Amp	8	99231185	99282455	2.661431	0.1321
13578	Amp	8	99282617	99337249	2.871582	0.1361
13579	Amp	8	99338333	99434872	3.021176	0.13876
13580	Amp	8	99436261	99516417	2.707624	0.133023
13581	Amp	8	99519602	99575875	2.402099	0.127069
13582	Amp	8	99579995	99586292	2.278531	0.124809
13583	Amp	8	99586493	99623687	2.235168	0.123929
13584	Amp	8	99626261	99628693	2.356694	0.126312
13585	Amp	8	99629802	99637995	2.837591	0.135294
13586	Amp	8	99641036	99661680	3.195626	0.14205
13587	Amp	8	99664738	99746752	2.788392	0.134512
13588	Amp	8	99746807	99765660	2.964305	0.137844
13589	Amp	8	99774657	99807548	3.103562	0.140357
13590	Amp	8	99807661	99823863	2.997552	0.138441
13591	Amp	8	99824942	99831798	3.09333	0.140146
13592	Amp	8	99835587	99868580	2.964305	0.1378
13593	Amp	8	99868676	99900993	3.09333	0.140244
13594	Amp	8	99904209	99905602	2.825055	0.135125
13595	Amp	8	99910145	99911172	3.033246	0.139155
13596	Amp	8	99912043	99985486	3.207115	0.142272
13597	Amp	8	99985859	100002129	3.103562	0.140393
13598	Amp	8	100002437	100046385	3.251638	0.143135
13599	Amp	8	100054653	100060186	3.033246	0.139104
13600	Amp	8	100061763	100061763	2.9061	0.13666
13601	Amp	8	100070975	100071534	3.033246	0.138996
13602	Amp	8	100072828	100086936	2.986184	0.138282
13603	Amp	8	100087539	100110169	2.626052	0.131526
13604	Amp	8	100110183	100148989	2.502083	0.128999
13605	Amp	8	100154227	100263934	2.89426	0.136456
13606	Amp	8	100265833	100271436	3.033246	0.139147
13607	Amp	8	100276921	100393075	3.264473	0.143357
13608	Amp	8	100395813	100408526	3.033246	0.139147
13609	Amp	8	100411147	100494429	3.446786	0.14669
13610	Amp	8	100497234	100550021	3.29895	0.144029
13611	Amp	8	100553063	100553063	3.762283	0.152283
13612	Amp	8	100553398	100575629	3.6657	0.150578
13613	Amp	8	100580942	100609385	3.387253	0.145559
13614	Amp	8	100613380	100613380	3.349067	0.144916
13615	Amp	8	100613605	100645007	3.207115	0.142329
13616	Amp	8	100650467	100658170	2.765626	0.134074
13617	Amp	8	100658425	100710388	2.860424	0.135884
13618	Amp	8	100712170	100717975	3.183474	0.141858
13619	Amp	8	100719914	100801840	2.975629	0.137898
13620	Amp	8	100801914	100904936	3.103562	0.140289

13621	Amp	8	100907093	100958844	2.776997	0.134315
13622	Amp	8	100959264	100976594	2.952409	0.137527
13623	Amp	8	100976951	101050430	2.89426	0.136541
13624	Amp	8	101050910	101088301	2.929372	0.137162
13625	Amp	8	101088711	101145156	3.127699	0.140827
13626	Amp	8	101145254	101177584	2.997552	0.138491
13627	Amp	8	101177842	101334181	3.195626	0.142074
13628	Amp	8	101335491	101406246	3.103562	0.140448
13629	Amp	8	101406923	101424019	2.940615	0.137332
13630	Amp	8	101432131	101492283	3.172599	0.141712
13631	Amp	8	101492313	101524177	2.684894	0.132646
13632	Amp	8	101528714	101543073	2.800837	0.134753
13633	Amp	8	101548792	101643612	2.975629	0.137934
13634	Amp	8	101643783	101661258	2.800837	0.134753
13635	Amp	8	101663059	101709160	3.021176	0.138852
13636	Amp	8	101716131	101719924	2.788392	0.134473
13637	Amp	8	101720299	101772404	2.917729	0.136874
13638	Amp	8	101772658	101802073	2.765626	0.134183
13639	Amp	8	101806767	101889165	2.871582	0.135962
13640	Amp	8	101890637	101997619	3.172599	0.141721
13641	Amp	8	102007598	102104702	2.837591	0.13545
13642	Amp	8	102106569	102251320	2.975629	0.138007
13643	Amp	8	102251834	102306199	3.138463	0.141028
13644	Amp	8	102323683	102343036	3.411175	0.146091
13645	Amp	8	102351126	102355371	2.837591	0.135416
13646	Amp	8	102356999	102356999	2.940615	0.137288
13647	Amp	8	102358584	102366900	3.009724	0.138614
13648	Amp	8	102370580	102381364	2.743935	0.133552
13649	Amp	8	102382374	102382374	2.848911	0.135505
13650	Amp	8	102387905	102402007	2.882945	0.136216
13651	Amp	8	102410188	102429945	2.848911	0.135527
13652	Amp	8	102436241	102475661	2.743935	0.1337
13653	Amp	8	102480128	102513747	2.940615	0.137331
13654	Amp	8	102516080	102553802	3.103562	0.140439
13655	Amp	8	102553998	102558965	2.848911	0.135539
13656	Amp	8	102559214	102648136	2.446878	0.127983
13657	Amp	8	102662615	102685038	2.594754	0.130722
13658	Amp	8	102685384	102735660	2.755154	0.133776
13659	Amp	8	102740400	102836380	2.940615	0.137445
13660	Amp	8	102846260	102896411	3.033246	0.139168
13661	Amp	8	102899216	102899216	2.776997	0.134381
13662	Amp	8	102900112	102918172	2.684894	0.132661
13663	Amp	8	102918215	103018572	2.390849	0.126868
13664	Amp	8	103018796	103030134	2.776997	0.13438
13665	Amp	8	103032281	103073637	2.952409	0.137654
13666	Amp	8	103073729	103083042	3.195626	0.142066
13667	Amp	8	103085168	103090626	3.08058	0.14001
13668	Amp	8	103090682	103156100	2.848911	0.135598
13669	Amp	8	103156620	103168273	2.776997	0.134239
13670	Amp	8	103170262	103208212	2.871582	0.136023
13671	Amp	8	103209577	103237611	2.776997	0.134239
13672	Amp	8	103237626	103250966	2.940615	0.1373
13673	Amp	8	103259647	103269392	2.997552	0.138387

13674	Amp	8	103270201	103273303	2.537294	0.129641
13675	Amp	8	103273428	103345279	3.387253	0.145577
13676	Amp	8	103345719	103345719	3.311026	0.144251
13677	Amp	8	103347493	103413599	3.275143	0.143646
13678	Amp	8	103413817	103446644	3.138463	0.141044
13679	Amp	8	103447544	103465727	3.009724	0.13862
13680	Amp	8	103466989	103467378	2.917729	0.136896
13681	Amp	8	103467810	103533910	2.89426	0.136367
13682	Amp	8	103539571	103553213	3.230405	0.142748
13683	Amp	8	103559264	103596608	2.964305	0.137768
13684	Amp	8	103597041	103615195	2.882945	0.136149
13685	Amp	8	103625098	103717856	2.402099	0.127099
13686	Amp	8	103719163	103720282	2.300463	0.125189
13687	Amp	8	103721135	103796188	2.571513	0.130473
13688	Amp	8	103797593	103838250	2.743935	0.133721
13689	Amp	8	103845559	103874261	2.9061	0.136589
13690	Amp	8	103878596	103893996	2.871582	0.136066
13691	Amp	8	103898541	103912131	3.160681	0.141467
13692	Amp	8	103912268	103937883	3.138463	0.140973
13693	Amp	8	103944477	104021466	2.637742	0.131686
13694	Amp	8	104022008	104022008	2.743935	0.133726
13695	Amp	8	104023187	104030961	2.514205	0.129345
13696	Amp	8	104036067	104051224	2.605213	0.130969
13697	Amp	8	104055043	104157798	2.661431	0.132081
13698	Amp	8	104163568	104218394	2.73206	0.133456
13699	Amp	8	104227578	104236382	2.560422	0.130208
13700	Amp	8	104237773	104298677	2.594754	0.130791
13701	Amp	8	104299136	104321075	2.825055	0.135179
13702	Amp	8	104325515	104329961	2.379392	0.126666
13703	Amp	8	104330417	104389919	2.322689	0.125551
13704	Amp	8	104393991	104446796	2.278531	0.124811
13705	Amp	8	104447565	104487601	2.964305	0.137879
13706	Amp	8	104489871	104554494	3.446786	0.146596
13707	Amp	8	104559666	104571193	4.156262	0.15905
13708	Amp	8	104572235	104594483	4.47072	0.164001
13709	Amp	8	104603039	104603958	3.516868	0.148065
13710	Amp	8	104604033	104676493	3.374864	0.145275
13711	Amp	8	104676943	104679169	2.696333	0.132821
13712	Amp	8	104679448	104683952	2.788392	0.134513
13713	Amp	8	104685784	104701012	2.929372	0.137086
13714	Amp	8	104701122	104708910	3.033246	0.138977
13715	Amp	8	104710732	104782552	3.898065	0.154784
13716	Amp	8	104785068	104812654	3.701758	0.15125
13717	Amp	8	104812673	104814013	3.505046	0.147699
13718	Amp	8	104814204	104825104	3.446786	0.146587
13719	Amp	8	104830335	104895233	3.839479	0.153677
13720	Amp	8	104897519	104919153	3.738665	0.151946
13721	Amp	8	104920303	104925258	3.713605	0.151363
13722	Amp	8	104926550	104933261	3.8021	0.152883
13723	Amp	8	104933842	104984128	3.678399	0.150843
13724	Amp	8	104985062	104988462	3.640671	0.150132
13725	Amp	8	104990361	105093040	3.826187	0.153382
13726	Amp	8	105093354	105155653	4.809994	0.169318

13727	Amp	8	105156672	105156672	4.897301	0.170597
13728	Amp	8	105156710	105193661	4.795971	0.169082
13729	Amp	8	105195091	105243878	4.668674	0.167191
13730	Amp	8	105246118	105364039	4.513276	0.164759
13731	Amp	8	105364107	105384888	4.304307	0.16151
13732	Amp	8	105385466	105431448	4.416011	0.163175
13733	Amp	8	105434134	105470428	4.739548	0.168142
13734	Amp	8	105477790	105480446	3.749777	0.152206
13735	Amp	8	105481445	105500842	3.483236	0.147239
13736	Amp	8	105501073	105532925	3.387253	0.145612
13737	Amp	8	105532962	105577714	3.554363	0.148539
13738	Amp	8	105582491	105597748	3.615896	0.149725
13739	Amp	8	105598431	105768416	4.31834	0.16166
13740	Amp	8	105777792	105834313	4.264196	0.160807
13741	Amp	8	105839971	105861454	4.359416	0.162421
13742	Amp	8	105866331	105866958	3.946233	0.155538
13743	Amp	8	105868335	105945957	4.037384	0.157165
13744	Amp	8	105946859	105962741	4.142958	0.158923
13745	Amp	8	105962834	105971462	4.290674	0.161206
13746	Amp	8	106483031	106487006	4.050096	0.157365
13747	Amp	8	106490727	106490727	3.8021	0.152988
13748	Amp	8	106491617	106523079	3.908795	0.154969
13749	Amp	8	106524976	106534261	4.250544	0.160645
13750	Amp	8	106536949	106585726	3.908795	0.154969
13751	Amp	8	106594371	106640083	3.985868	0.156273
13752	Amp	8	106640841	106669201	4.183261	0.159446
13753	Amp	8	106671369	106754996	4.129725	0.158616
13754	Amp	8	106761391	106768275	3.349067	0.144981
13755	Amp	8	106773260	106811575	3.29895	0.144106
13756	Amp	8	106813247	106821788	3.5415	0.148361
13757	Amp	8	106823316	106866672	3.446786	0.146747
13758	Amp	8	106868636	106900156	3.103562	0.140361
13759	Amp	8	106905544	107006082	2.929372	0.137188
13760	Amp	8	107011133	107034678	3.009724	0.138671
13761	Amp	8	107034778	107039616	3.138463	0.140967
13762	Amp	8	107041431	107068254	3.311026	0.14422
13763	Amp	8	107068391	107196680	3.554363	0.148597
13764	Amp	8	107199025	107274150	3.446786	0.14675
13765	Amp	8	107274863	107275524	3.529607	0.148195
13766	Amp	8	107276121	107290654	3.492824	0.14763
13767	Amp	8	107293327	107327301	4.223463	0.160206
13768	Amp	8	107327383	107347818	4.129725	0.158627
13769	Amp	8	107348117	107362955	4.499013	0.164418
13770	Amp	8	107365328	107446654	4.5989	0.166075
13771	Amp	8	107446879	107449261	4.640474	0.166591
13772	Amp	8	107452215	107490460	4.304307	0.161345
13773	Amp	8	107492924	107532726	3.749777	0.152108
13774	Amp	8	107536172	107560018	3.775276	0.152588
13775	Amp	8	107566295	107657499	3.628913	0.150031
13776	Amp	8	107659235	107672224	2.776997	0.134251
13777	Amp	8	107672487	107672644	2.719563	0.133233
13778	Amp	8	107673389	107704967	2.743935	0.13369
13779	Amp	8	107711331	107747103	2.719563	0.13321

13780	Amp	8	107748983	107779969	2.684894	0.13246
13781	Amp	8	107786005	107787346	2.502083	0.129198
13782	Amp	8	107790515	107797140	2.379392	0.126685
13783	Amp	8	107800050	107813838	2.311329	0.125405
13784	Amp	8	107824222	107824222	2.356694	0.126255
13785	Amp	8	107828489	107854650	1.759227	0.11432
13786	Amp	8	107856021	107960070	1.908279	0.11736
13787	Amp	8	107960099	107960099	1.833307	0.115804
13788	Amp	8	107960417	107965546	1.769915	0.114635
13789	Amp	8	107969199	108026089	1.624655	0.111593
13790	Amp	8	108033704	108551923	1.456037	0.107703
13791	Amp	8	108551936	108584352	1.492063	0.108587
13792	Amp	8	108594766	108689708	1.604336	0.111156
13793	Amp	8	108691575	108780359	1.919241	0.117519
13794	Amp	8	108780542	108780542	2.52589	0.129454
13795	Amp	8	108944839	108950686	2.457251	0.128231
13796	Amp	8	108955413	108982015	2.52589	0.129438
13797	Amp	8	108988195	108998856	2.137445	0.121922
13798	Amp	8	109007083	109015362	2.502083	0.129177
13799	Amp	8	109018880	109033709	2.637742	0.131669
13800	Amp	8	109039415	109055505	2.256542	0.124415
13801	Amp	8	109061994	109085336	2.18114	0.122691
13802	Amp	8	109089182	109102059	2.537294	0.129794
13803	Amp	8	109102110	109103447	2.457251	0.128274
13804	Amp	8	109105281	109157356	2.997552	0.138338
13805	Amp	8	109159916	109182978	2.860424	0.135719
13806	Amp	8	109184400	109202309	3.057106	0.139596
13807	Amp	8	109203060	109220827	2.952409	0.137635
13808	Amp	8	109226315	109241637	2.825055	0.135142
13809	Amp	8	109241650	109320093	2.637742	0.131591
13810	Amp	8	109323156	109330370	2.684894	0.132602
13811	Amp	8	109334058	109406399	2.311329	0.125499
13812	Amp	8	109411513	109442776	2.661431	0.132223
13813	Amp	8	109443092	109508888	2.860424	0.135807
13814	Amp	8	109511176	109618496	2.571513	0.130494
13815	Amp	8	109618764	109626245	2.52589	0.129545
13816	Amp	8	109628004	109716568	2.345513	0.125976
13817	Amp	8	109716654	109856245	2.256542	0.124349
13818	Amp	8	109857515	109889924	2.137445	0.12182
13819	Amp	8	109890248	109939072	2.104978	0.121172
13820	Amp	8	109949884	109973853	1.769915	0.114448
13821	Amp	8	109977927	110039309	1.887378	0.11702
13822	Amp	8	110039660	110067651	2.016585	0.119589
13823	Amp	8	110069958	110100763	1.972776	0.118578
13824	Amp	8	110128298	110206628	1.407997	0.106642
13825	Amp	8	110207480	110283541	1.436692	0.107338
13826	Amp	8	110285305	110423458	1.456037	0.107825
13827	Amp	8	110424859	110438956	1.33853	0.105254
13828	Amp	8	110441974	110466933	1.358811	0.105725
13829	Amp	8	110469257	110472881	1.388256	0.106203
13830	Amp	8	110475032	110582820	1.407997	0.106667
13831	Amp	8	110584348	110655696	1.426786	0.107175
13832	Amp	8	110658040	110801090	1.522708	0.109399

13833	Amp	8	110812898	110857623	1.96212	0.11847
13834	Amp	8	110860578	110935884	2.038694	0.119914
13835	Amp	8	110937857	111099839	2.005947	0.119219
13836	Amp	8	111099993	111137851	2.027585	0.119724
13837	Amp	8	111143661	111156962	2.256542	0.124409
13838	Amp	8	111157443	111171830	2.137445	0.121839
13839	Amp	8	111173169	111196842	2.235168	0.123776
13840	Amp	8	111199256	111199256	2.137445	0.121839
13841	Amp	8	111206851	111213438	2.322689	0.125699
13842	Amp	8	111222410	111264705	2.368227	0.126376
13843	Amp	8	111269132	111352550	2.52589	0.129611
13844	Amp	8	111357724	111394699	2.571513	0.130408
13845	Amp	8	111401567	111602376	2.9061	0.136616
13846	Amp	8	111602681	111744762	2.952409	0.137476
13847	Amp	8	111745096	111745510	2.871582	0.136031
13848	Amp	8	111747526	111779837	2.390849	0.126961
13849	Amp	8	111789195	111976442	2.256542	0.124219
13850	Amp	8	111978594	112101169	2.094104	0.120984
13851	Amp	8	112102291	112167504	2.137445	0.121994
13852	Amp	8	112167635	112242933	2.094104	0.121134
13853	Amp	8	112249950	112256904	2.18114	0.122887
13854	Amp	8	112259619	112259619	2.390849	0.126867
13855	Amp	8	112259755	112265452	2.9061	0.13662
13856	Amp	8	112266589	112266589	2.765626	0.133995
13857	Amp	8	112269635	112279392	3.240397	0.142929
13858	Amp	8	112279703	112373296	3.323812	0.144588
13859	Amp	8	112376592	112401411	3.615896	0.149801
13860	Amp	8	112409685	112420916	3.435586	0.146536
13861	Amp	8	112424173	112428609	3.826187	0.153385
13862	Amp	8	112429267	112433111	3.985868	0.156243
13863	Amp	8	112436529	112521966	3.690482	0.15103
13864	Amp	8	112524815	112552558	4.090086	0.158033
13865	Amp	8	112557270	112557270	3.920287	0.155175
13866	Amp	8	112557770	112592833	3.701758	0.151298
13867	Amp	8	112600438	112684528	3.738665	0.151806
13868	Amp	8	112697461	112750614	3.985868	0.156277
13869	Amp	8	112753819	112807740	4.196711	0.159784
13870	Amp	8	112811817	112930015	3.9978	0.156458
13871	Amp	8	112941087	112949729	3.726055	0.151729
13872	Amp	8	112953656	112973203	4.010586	0.156683
13873	Amp	8	112976897	112982589	3.852343	0.153817
13874	Amp	8	112983983	112991796	3.933438	0.155343
13875	Amp	8	112992464	113036844	3.788687	0.15277
13876	Amp	8	113045051	113077812	4.209693	0.159917
13877	Amp	8	113078657	113081955	4.416011	0.163245
13878	Amp	8	113093468	113098428	4.47072	0.164061
13879	Amp	8	113100401	113120756	4.443348	0.163573
13880	Amp	8	113123415	113138206	4.223463	0.160066
13881	Amp	8	113142155	113142155	3.985868	0.156177
13882	Amp	8	113142361	113149041	4.116393	0.158454
13883	Amp	8	113149737	113167452	4.209693	0.159906
13884	Amp	8	113167697	113324231	4.429656	0.16337
13885	Amp	8	113326200	113424245	3.985868	0.156308

13886	Amp	8	113424495	113528294	3.959323	0.155802
13887	Amp	8	113529027	113549495	3.471122	0.147026
13888	Amp	8	113553196	113559094	3.387253	0.145574
13889	Amp	8	113560308	113567383	3.323812	0.144597
13890	Amp	8	113580472	113667023	3.387253	0.145652
13891	Amp	8	113671449	113777544	3.435586	0.146457
13892	Amp	8	113777995	113833755	3.458499	0.146988
13893	Amp	8	113837536	113849346	3.5415	0.148319
13894	Amp	8	113851117	113865231	3.898065	0.154693
13895	Amp	8	113868412	113913644	4.31834	0.161657
13896	Amp	8	113914457	113986561	4.183261	0.159461
13897	Amp	8	113989864	114000220	4.142958	0.158945
13898	Amp	8	114003711	114003711	4.024007	0.156963
13899	Amp	8	114006709	114063715	4.209693	0.15983
13900	Amp	8	114066845	114102043	3.887411	0.154573
13901	Amp	8	114104693	114135812	3.8021	0.153047
13902	Amp	8	114138483	114148881	3.640671	0.150181
13903	Amp	8	114150772	114150772	3.713605	0.151471
13904	Amp	8	114151333	114292762	3.483236	0.147375
13905	Amp	8	114293222	114381999	3.9978	0.156544
13906	Amp	8	114382832	114397340	4.169329	0.159295
13907	Amp	8	114400396	114403614	4.250544	0.160532
13908	Amp	8	114405631	114422482	4.223463	0.160045
13909	Amp	8	114426594	114438504	4.668674	0.167074
13910	Amp	8	114439115	114463058	4.584613	0.165783
13911	Amp	8	114465036	114475022	5.507674	0.179786
13912	Amp	8	114475633	114488582	4.924414	0.17096
13913	Amp	8	114493640	114527521	5.822221	0.184276
13914	Amp	8	114528281	114539586	5.732156	0.182873
13915	Amp	8	114543507	114611050	5.911851	0.185446
13916	Amp	8	114611629	114615356	6.304987	0.191227
13917	Amp	8	114661835	114683481	6.933875	0.200054
13918	Amp	8	114687413	114709075	7.295403	0.20507
13919	Amp	8	114713935	114717599	7.326935	0.205518
13920	Amp	8	114719658	114719658	7.042879	0.201602
13921	Amp	8	114719680	114719701	6.686496	0.196586
13922	Amp	8	114879397	114879820	5.702358	0.182605
13923	Amp	8	114879885	114897691	5.942149	0.186074
13924	Amp	8	114897769	114907650	6.122748	0.188509
13925	Amp	8	114908054	114926373	6.078164	0.188002
13926	Amp	8	114932651	115026808	6.686496	0.196633
13927	Amp	8	115029760	115063312	6.443221	0.193165
13928	Amp	8	115064188	115104379	6.564374	0.194777
13929	Amp	8	115104507	115104507	6.594741	0.195259
13930	Amp	8	115104783	115120693	6.65628	0.196105
13931	Amp	8	115123816	115166572	6.747966	0.197463
13932	Amp	8	115170368	115186542	6.443221	0.193087
13933	Amp	8	115198729	115202755	6.122748	0.188568
13934	Amp	8	115204637	115204637	6.062596	0.187635
13935	Amp	8	115205371	115210385	6.137384	0.188845
13936	Amp	8	115210613	115230290	5.987065	0.186688
13937	Amp	8	115230893	115292715	6.078164	0.187846
13938	Amp	8	115292995	115307295	6.503622	0.193945

13939	Amp	8	115307768	115317684	6.564374	0.194888
13940	Amp	8	115318550	115340340	6.032925	0.187386
13941	Amp	8	115340808	115340808	6.092521	0.188135
13942	Amp	8	115347355	115371906	6.549814	0.194712
13943	Amp	8	115375423	115378684	6.917796	0.199973
13944	Amp	8	115384365	115391630	8.088933	0.215909
13945	Amp	8	115391960	115397206	7.673484	0.210239
13946	Amp	8	115402196	115405335	7.6425	0.209791
13947	Amp	8	115405351	115427972	7.785234	0.211747
13948	Amp	8	115431664	115456588	8.073986	0.215648
13949	Amp	8	115458243	115508072	8.008326	0.214833
13950	Amp	8	115517846	115556437	8.378151	0.21988
13951	Amp	8	115561713	115585408	8.233714	0.217925
13952	Amp	8	115585471	115599675	8.20134	0.217447
13953	Amp	8	115599783	115599783	8.169654	0.216982
13954	Amp	8	115609517	115621472	8.121744	0.216304
13955	Amp	8	115621500	115651706	8.073986	0.215691
13956	Amp	8	115658120	115677027	8.409489	0.2202
13957	Amp	8	115679011	115687785	8.47374	0.221256
13958	Amp	8	115689971	115696131	8.671092	0.223869
13959	Amp	8	115696376	115714186	8.489568	0.221386
13960	Amp	8	115714866	115715368	8.60549	0.222813
13961	Amp	8	115718256	115722405	8.266115	0.218305
13962	Amp	8	115724598	115749434	8.409489	0.220203
13963	Amp	8	115749924	115761064	8.217372	0.21759
13964	Amp	8	115763645	115771843	7.531798	0.208378
13965	Amp	8	115774582	115800917	7.500789	0.207907
13966	Amp	8	115803992	115827639	7.437327	0.207101
13967	Amp	8	115829614	115906306	7.531798	0.208418
13968	Amp	8	115914475	115966032	7.516099	0.208168
13969	Amp	8	115968171	116008200	7.6425	0.20978
13970	Amp	8	116012792	116040994	8.489568	0.221424
13971	Amp	8	116041161	116041161	8.32882	0.219309
13972	Amp	8	116041726	116049897	8.057852	0.215407
13973	Amp	8	116056714	116097012	7.405471	0.206519
13974	Amp	8	116097420	116100141	7.167722	0.203292
13975	Amp	8	116107898	116115854	7.027404	0.201394
13976	Amp	8	116115877	116123304	7.058587	0.201918
13977	Amp	8	116136193	116136193	6.886863	0.199354
13978	Amp	8	116136939	116184247	6.763246	0.197768
13979	Amp	8	116184377	116202177	7.042879	0.201635
13980	Amp	8	116207537	116294872	6.686496	0.196711
13981	Amp	8	116296542	116403649	6.626113	0.195768
13982	Amp	8	116405284	116425393	6.33531	0.191706
13983	Amp	8	116426008	116444899	6.229291	0.190094
13984	Amp	8	116448233	116485410	6.686496	0.196599
13985	Amp	8	116489565	116513116	6.65628	0.19609
13986	Amp	8	116516930	116516930	6.488712	0.193727
13987	Amp	8	116520854	116530147	6.304987	0.191167
13988	Amp	8	116533161	116548290	5.882356	0.185125
13989	Amp	8	116551199	116552698	6.213438	0.189822
13990	Amp	8	116553724	116609461	6.443221	0.193233
13991	Amp	8	116610332	116612244	6.350097	0.191874

13992	Amp	8	116623338	116640003	5.717279	0.182754
13993	Amp	8	116640221	116661550	6.182794	0.189558
13994	Amp	8	116668080	116696582	6.33531	0.191555
13995	Amp	8	116697136	116738789	6.275802	0.190805
13996	Amp	8	116742583	116747788	5.911851	0.185544
13997	Amp	8	116751634	116756661	5.507674	0.179711
13998	Amp	8	116760110	116804981	5.184242	0.175015
13999	Amp	8	116805017	116834888	5.067861	0.173173
14000	Amp	8	116837823	116901778	4.626369	0.166369
14001	Amp	8	116901861	116951843	4.78228	0.168917
14002	Amp	8	116956373	116969105	4.924414	0.171009
14003	Amp	8	116972725	117027122	5.360902	0.177548
14004	Amp	8	117030623	117073151	5.492924	0.179557
14005	Amp	8	117074289	117098779	5.582276	0.180814
14006	Amp	8	117099325	117113327	5.957455	0.186267
14007	Amp	8	117122442	117122442	5.746988	0.183209
14008	Amp	8	117124964	117180682	5.582276	0.180773
14009	Amp	8	117180961	117200223	5.522349	0.179864
14010	Amp	8	117201377	117266463	5.687617	0.182206
14011	Amp	8	117268746	117273807	5.626868	0.181528
14012	Amp	8	117280207	117297592	4.570393	0.165592
14013	Amp	8	117297639	117309680	4.416011	0.16325
14014	Amp	8	117309848	117309848	4.129725	0.158577
14015	Amp	8	117310323	117328581	4.050096	0.157224
14016	Amp	8	117330381	117400951	4.264196	0.160864
14017	Amp	8	117401002	117402842	4.196711	0.159608
14018	Amp	8	117403523	117424857	4.290674	0.161194
14019	Amp	8	117428801	117428801	4.116393	0.158346
14020	Amp	8	117431286	117466731	3.738665	0.151808
14021	Amp	8	117469642	117534437	3.654159	0.15038
14022	Amp	8	117537137	117568145	3.505046	0.147832
14023	Amp	8	117571172	117590186	3.554363	0.148561
14024	Amp	8	117594737	117630734	3.505046	0.147832
14025	Amp	8	117630825	117742754	3.603523	0.149509
14026	Amp	8	117743156	117773221	3.578505	0.149022
14027	Amp	8	117778822	117897114	3.435586	0.146449
14028	Amp	8	117897155	118125766	3.240397	0.143037
14029	Amp	8	118125956	118143608	3.08058	0.139989
14030	Amp	8	118146524	118202745	3.009724	0.138751
14031	Amp	8	118208456	118226655	3.374864	0.145414
14032	Amp	8	118227477	118255257	3.411175	0.146103
14033	Amp	8	118256302	118291021	3.654159	0.150446
14034	Amp	8	118291218	118386737	3.566107	0.148871
14035	Amp	8	118388234	118452294	3.678399	0.150738
14036	Amp	8	118454051	118497438	3.8021	0.152875
14037	Amp	8	118498223	118519598	3.701758	0.151289
14038	Amp	8	118521578	118522090	3.590086	0.149279
14039	Amp	8	118523089	118545752	3.738665	0.151884
14040	Amp	8	118547616	118569966	3.972019	0.155978
14041	Amp	8	118572300	118672624	3.471122	0.147177
14042	Amp	8	118672942	118688913	3.446786	0.146676
14043	Amp	8	118690033	118725966	3.628913	0.149966
14044	Amp	8	118728292	118731947	3.516868	0.147971

14045 Amp	8	118737014	118743251	3.275143	0.143628
14046 Amp	8	118745361	118753330	3.021176	0.138812
14047 Amp	8	118756372	118836717	3.183474	0.141961
14048 Amp	8	118839780	118854460	3.566107	0.148834
14049 Amp	8	118858457	118858691	3.275143	0.14357
14050 Amp	8	118860759	118903022	2.986184	0.138117
14051 Amp	8	118914911	118939096	3.195626	0.142096
14052 Amp	8	118939606	118942040	3.264473	0.143434
14053 Amp	8	118942512	118963639	3.230405	0.142724
14054 Amp	8	118963905	118966898	3.349067	0.145004
14055 Amp	8	118971065	119008810	3.578505	0.149063
14056 Amp	8	119008925	119024503	3.628913	0.150042
14057 Amp	8	119028129	119041104	3.578505	0.149063
14058 Amp	8	119041157	119168212	3.814483	0.153207
14059 Amp	8	119174176	119247537	4.768128	0.168509
14060 Amp	8	119250018	119330183	4.236663	0.160327
14061 Amp	8	119330816	119363930	3.985868	0.156269
14062 Amp	8	119364314	119373212	4.156262	0.159064
14063 Amp	8	119373367	119373367	4.223463	0.160212
14064 Amp	8	119374067	119444830	4.050096	0.157416
14065 Amp	8	119450097	119517806	3.985868	0.15622
14066 Amp	8	119519341	119556645	4.250544	0.160629
14067 Amp	8	119556686	119566013	4.142958	0.158772
14068 Amp	8	119566594	119642848	3.628913	0.149968
14069 Amp	8	119642901	119694231	3.240397	0.143085
14070 Amp	8	119695360	119695360	2.788392	0.134615
14071 Amp	8	119697034	119697230	3.150497	0.141189
14072 Amp	8	119700371	119722554	3.115161	0.140633
14073 Amp	8	119722575	119726136	3.150497	0.141267
14074 Amp	8	119729945	119787091	3.374864	0.14533
14075 Amp	8	119790152	119801990	3.286256	0.143907
14076 Amp	8	119802326	119829083	3.361849	0.145107
14077 Amp	8	119829169	119867360	3.516868	0.147886
14078 Amp	8	119869935	119879141	3.654159	0.150285
14079 Amp	8	119879573	119916564	3.590086	0.14925
14080 Amp	8	119917092	119934865	3.458499	0.146851
14081 Amp	8	119935115	119970059	3.529607	0.148294
14082 Amp	8	119991712	119993504	3.690482	0.151038
14083 Amp	8	119997585	120007927	3.862695	0.154074
14084 Amp	8	120011915	120030274	3.690482	0.151038
14085 Amp	8	120032805	120034374	3.726055	0.151597
14086 Amp	8	120035559	120068914	3.471122	0.147188
14087 Amp	8	120069290	120073269	3.529607	0.148224
14088 Amp	8	120081156	120136538	3.361849	0.145202
14089 Amp	8	120139661	120175675	3.471122	0.147044
14090 Amp	8	120177986	120202683	3.640671	0.150146
14091 Amp	8	120204709	120257597	3.411175	0.145924
14092 Amp	8	120258116	120277636	3.374864	0.145364
14093 Amp	8	120280085	120297173	3.603523	0.149595
14094 Amp	8	120301031	120318446	3.713605	0.151446
14095 Amp	8	120323065	120408523	3.529607	0.148241
14096 Amp	8	120408861	120434676	3.492824	0.147592
14097 Amp	8	120435650	120486124	3.160681	0.14151

14098	Amp	8	120488040	120502870	3.5415	0.148402
14099	Amp	8	120502884	120563521	3.505046	0.147742
14100	Amp	8	120571043	120637114	3.399017	0.145892
14101	Amp	8	120637885	120685090	3.29895	0.14405
14102	Amp	8	120694165	120695316	2.594754	0.130734
14103	Amp	8	120695460	120763450	2.73206	0.13347
14104	Amp	8	120771719	120814582	2.594754	0.130734
14105	Amp	8	120815228	120939783	2.560422	0.130284
14106	Amp	8	120939985	121046809	2.605213	0.131095
14107	Amp	8	121047235	121105303	2.755154	0.133875
14108	Amp	8	121109053	121127312	3.195626	0.142086
14109	Amp	8	121128026	121226930	3.045223	0.139306
14110	Amp	8	121227849	121336061	3.09333	0.140059
14111	Amp	8	121337065	121367232	3.160681	0.141493
14112	Amp	8	121374850	121620519	3.127699	0.14074
14113	Amp	8	121620533	121702501	3.172599	0.141613
14114	Amp	8	121702731	121731672	3.264473	0.143457
14115	Amp	8	121732868	121803287	3.435586	0.1465
14116	Amp	8	121803634	121807369	3.195626	0.142087
14117	Amp	8	121807473	121851811	3.554363	0.148634
14118	Amp	8	121852813	121852813	3.411175	0.14603
14119	Amp	8	121853526	121876409	3.207115	0.142359
14120	Amp	8	121876665	121878655	3.138463	0.141021
14121	Amp	8	121879713	121944611	3.09333	0.140148
14122	Amp	8	121949609	122058644	3.183474	0.141958
14123	Amp	8	122058784	122326616	3.230405	0.142709
14124	Amp	8	122327011	122430008	2.882945	0.136162
14125	Amp	8	122432411	122445043	2.964305	0.137869
14126	Amp	8	122449049	122546011	2.882945	0.136162
14127	Amp	8	122546127	122555566	3.009724	0.138612
14128	Amp	8	122558271	122561831	3.08058	0.139988
14129	Amp	8	122562003	122578516	3.115161	0.140656
14130	Amp	8	122578743	122658757	2.986184	0.138168
14131	Amp	8	122664175	122684502	3.862695	0.154104
14132	Amp	8	122685632	122685632	4.142958	0.158779
14133	Amp	8	122691500	122742051	4.050096	0.157393
14134	Amp	8	122742105	122747877	3.839479	0.153543
14135	Amp	8	122750499	122788696	3.749777	0.152167
14136	Amp	8	122788717	122831723	3.615896	0.149784
14137	Amp	8	122836499	122957182	3.411175	0.146064
14138	Amp	8	122958841	122973468	3.067933	0.139705
14139	Amp	8	122975635	122992738	2.502083	0.129005
14140	Amp	8	122995838	123022571	2.605213	0.130982
14141	Amp	8	123023009	123105092	2.571513	0.130314
14142	Amp	8	123106286	123123082	2.514205	0.129375
14143	Amp	8	123125768	123142113	2.582422	0.130629
14144	Amp	8	123143646	123151322	2.626052	0.131562
14145	Amp	8	123151439	123252687	2.605213	0.131077
14146	Amp	8	123253348	123257584	2.848911	0.135547
14147	Amp	8	123258172	123339439	2.537294	0.129824
14148	Amp	8	123346048	123354907	3.286256	0.143879
14149	Amp	8	123367515	123367515	3.138463	0.141042
14150	Amp	8	123377368	123550789	3.492824	0.147634

14151 Amp	8	123552963	123553922	3.628913	0.149862
14152 Amp	8	123554116	123559837	3.275143	0.143581
14153 Amp	8	123560351	123560351	3.172599	0.141604
14154 Amp	8	123560416	123589421	3.471122	0.147077
14155 Amp	8	123597474	123603810	3.814483	0.153085
14156 Amp	8	123604556	123612330	4.076315	0.157791
14157 Amp	8	123614242	123682831	4.142958	0.158765
14158 Amp	8	123683508	123751361	4.090086	0.157968
14159 Amp	8	123755085	123866392	3.887411	0.154456
14160 Amp	8	123867758	123878311	3.814483	0.153201
14161 Amp	8	123883288	123955316	3.887411	0.154439
14162 Amp	8	123955753	124031492	3.933438	0.155381
14163 Amp	8	124033636	124155975	4.090086	0.15801
14164 Amp	8	124158853	124185526	3.959323	0.155782
14165 Amp	8	124186703	124237757	4.024007	0.156801
14166 Amp	8	124238164	124406614	3.946233	0.155608
14167 Amp	8	124407510	124409894	3.775276	0.152486
14168 Amp	8	124413527	124418519	3.887411	0.154442
14169 Amp	8	124420460	124445982	3.985868	0.156179
14170 Amp	8	124446564	124446564	3.240397	0.142902
14171 Amp	8	124447478	124469504	3.195626	0.142028
14172 Amp	8	124470716	124508933	3.045223	0.13935
14173 Amp	8	124512557	124541305	3.160681	0.141572
14174 Amp	8	124543795	124553737	2.755154	0.133853
14175 Amp	8	124556769	124604735	2.89426	0.136539
14176 Amp	8	124605876	124646661	2.755154	0.133853
14177 Amp	8	124647245	124656358	2.837591	0.135303
14178 Amp	8	124660126	124670284	2.986184	0.138154
14179 Amp	8	124670801	124680114	3.138463	0.14095
14180 Amp	8	124680914	124716527	3.8021	0.153065
14181 Amp	8	124719507	124758505	3.946233	0.155534
14182 Amp	8	124762736	124767839	3.8021	0.153065
14183 Amp	8	124767965	124861079	3.654159	0.150268
14184 Amp	8	124863064	124863064	3.985868	0.15613
14185 Amp	8	124863841	124913726	4.739548	0.168095
14186 Amp	8	124914482	125007875	4.78228	0.168724
14187 Amp	8	125010111	125010478	4.924414	0.170922
14188 Amp	8	125011810	125095313	4.966536	0.171603
14189 Amp	8	125096143	125176492	4.882872	0.170446
14190 Amp	8	125181503	125193485	4.69701	0.167596
14191 Amp	8	125196474	125200789	3.814483	0.153278
14192 Amp	8	125204346	125365325	3.738665	0.151828
14193 Amp	8	125365362	125438839	3.628913	0.149872
14194 Amp	8	125441036	125461199	3.5415	0.148413
14195 Amp	8	125462114	125779943	3.483236	0.147439
14196 Amp	8	125780460	125792744	3.446786	0.146758
14197 Amp	8	125793178	125833220	3.775276	0.152537
14198 Amp	8	125834240	125839338	3.690482	0.151103
14199 Amp	8	125843039	125957520	3.814483	0.15319
14200 Amp	8	125957896	125963688	3.946233	0.15554
14201 Amp	8	125966006	126023028	4.116393	0.158491
14202 Amp	8	126023682	126129689	4.223463	0.160039
14203 Amp	8	126133001	126231665	4.156262	0.159019

14204	Amp	8	126236134	126295912	3.887411	0.15447
14205	Amp	8	126298610	126321801	4.196711	0.159814
14206	Amp	8	126324250	126426783	5.241829	0.17575
14207	Amp	8	126427094	126516867	5.449463	0.178715
14208	Amp	8	126516916	126536542	5.360902	0.177551
14209	Amp	8	126539581	126569365	5.717279	0.182815
14210	Amp	8	126569582	126583939	5.882356	0.185112
14211	Amp	8	126585129	126602846	5.957455	0.186274
14212	Amp	8	126606181	126636644	6.1074	0.188374
14213	Amp	8	126638658	126639225	6.229291	0.190041
14214	Amp	8	126640668	126648882	6.078164	0.187954
14215	Amp	8	126649202	126685761	5.732156	0.183012
14216	Amp	8	126689082	126807278	5.777283	0.183597
14217	Amp	8	126809350	126852874	6.122748	0.18854
14218	Amp	8	126855918	126914776	5.957455	0.186243
14219	Amp	8	126915724	126974221	5.822221	0.184144
14220	Amp	8	126974238	127014165	5.927128	0.1858
14221	Amp	8	127016364	127040776	6.002088	0.18692
14222	Amp	8	127041601	127055192	6.229291	0.190148
14223	Amp	8	127062582	127094955	6.36593	0.191988
14224	Amp	8	127097285	127125487	6.291248	0.190959
14225	Amp	8	127128122	127154311	6.427493	0.193008
14226	Amp	8	127156415	127259011	6.579755	0.195206
14227	Amp	8	127259650	127334113	6.65628	0.196085
14228	Amp	8	127342067	127355489	7.120018	0.202704
14229	Amp	8	127359440	127377604	7.295403	0.205173
14230	Amp	8	127378415	127389527	7.452952	0.207214
14231	Amp	8	127390018	127392929	7.264196	0.204744
14232	Amp	8	127394754	127403489	7.689477	0.210523
14233	Amp	8	127404434	127412547	7.736725	0.211118
14234	Amp	8	127419449	127429290	7.689477	0.210491
14235	Amp	8	127429378	127446399	7.531798	0.208451
14236	Amp	8	127451968	127549994	8.154198	0.216747
14237	Amp	8	127551054	127588837	8.361498	0.219574
14238	Amp	8	127588957	127591361	7.801564	0.212076
14239	Amp	8	127591818	127630986	7.945028	0.213897
14240	Amp	8	127632710	127666536	8.217372	0.217602
14241	Amp	8	127666741	127721998	8.313762	0.218945
14242	Amp	8	127724453	127750916	8.088933	0.215914
14243	Amp	8	127753115	127780550	7.993387	0.214571
14244	Amp	8	127788055	127806155	6.825391	0.198635
14245	Amp	8	127806763	127841570	6.716522	0.196967
14246	Amp	8	127843886	127960475	6.886863	0.199496
14247	Amp	8	127961419	128081590	7.106137	0.202438
14248	Amp	8	128082343	128082343	7.183949	0.203673
14249	Amp	8	128082594	128098668	7.341956	0.205778
14250	Amp	8	128098699	128105409	7.673484	0.210362
14251	Amp	8	128105690	128105711	7.785234	0.211915
14252	Amp	8	128109092	128112456	8.105491	0.216258
14253	Amp	8	128112585	128112733	8.313762	0.218939
14254	Amp	8	128112902	128122355	8.47374	0.221255
14255	Amp	8	128123682	128178148	8.313762	0.219083
14256	Amp	8	128178298	128178713	8.073986	0.215747

14257	Amp	8	128181862	128182340	7.880674	0.213066
14258	Amp	8	128182604	128190180	6.79429	0.198179
14259	Amp	8	128190408	128211625	6.641348	0.196073
14260	Amp	8	128213622	128217832	6.380906	0.192368
14261	Amp	8	128220917	128222134	6.2443	0.19032
14262	Amp	8	128229364	128234169	5.942149	0.185977
14263	Amp	8	128236803	128308924	5.463735	0.179077
14264	Amp	8	128312669	128352539	5.746988	0.183136
14265	Amp	8	128356636	128406972	5.536915	0.180194
14266	Amp	8	128407204	128500745	5.081731	0.173288
14267	Amp	8	128501835	128520022	5.038261	0.172703
14268	Amp	8	128520424	128669495	4.924414	0.170927
14269	Amp	8	128669651	128670519	4.753798	0.168398
14270	Amp	8	128671188	128730029	4.725297	0.167861
14271	Amp	8	128771821	128786724	5.140232	0.174357
14272	Amp	8	128788524	128947215	4.966536	0.171728
14273	Amp	8	128948002	128952627	4.47072	0.164054
14274	Amp	8	128953334	128962079	4.388129	0.16283
14275	Amp	8	128965742	128970986	4.076315	0.157746
14276	Amp	8	128974886	128975973	3.374864	0.145404
14277	Amp	8	128989920	128990484	3.483236	0.14728
14278	Amp	8	128993912	129010623	3.505046	0.147717
14279	Amp	8	129010809	129028471	3.458499	0.146832
14280	Amp	8	129043735	129132280	3.387253	0.145598
14281	Amp	8	129134311	129139705	3.458499	0.146861
14282	Amp	8	129142087	129151609	3.590086	0.149238
14283	Amp	8	129158371	129226534	3.483236	0.147362
14284	Amp	8	129228986	129399660	3.898065	0.154649
14285	Amp	8	129400192	129745338	3.852343	0.153871
14286	Amp	8	129748892	129767448	3.920287	0.155074
14287	Amp	8	129772486	129980701	3.839479	0.153526
14288	Amp	8	129981572	130126329	3.862695	0.154036
14289	Amp	8	130127066	130311952	3.908795	0.15496
14290	Amp	8	130312781	130337843	4.062894	0.157587
14291	Amp	8	130344091	130380327	3.908795	0.15496
14292	Amp	8	130383058	130387722	3.946233	0.155474
14293	Amp	8	130388642	130515551	3.814483	0.153297
14294	Amp	8	130515584	130560769	3.933438	0.155289
14295	Amp	8	130564270	130654319	3.898065	0.154775
14296	Amp	8	130657175	130680276	4.024007	0.156985
14297	Amp	8	130682364	130746050	3.690482	0.150951
14298	Amp	8	130746080	130763242	3.654159	0.150441
14299	Amp	8	130766260	130831598	3.726055	0.151728
14300	Amp	8	130833706	130882655	3.275143	0.143535
14301	Amp	8	130890048	130890888	3.150497	0.141325
14302	Amp	8	130891033	131147959	3.264473	0.143491
14303	Amp	8	131148728	131155193	3.230405	0.142802
14304	Amp	8	131165838	131180946	4.129725	0.158635
14305	Amp	8	131182873	131222651	4.5989	0.166007
14306	Amp	8	131223169	131245720	4.264196	0.16078
14307	Amp	8	131249598	131249917	3.852343	0.153776
14308	Amp	8	131256037	131314167	3.713605	0.151499
14309	Amp	8	131315931	131326434	3.852343	0.153916

14310	Amp	8	131326565	131383467	3.887411	0.154551
14311	Amp	8	131384065	131392723	4.062894	0.157447
14312	Amp	8	131394713	131394713	3.908795	0.15503
14313	Amp	8	131396225	131483933	3.8021	0.153001
14314	Amp	8	131484902	131580378	3.678399	0.150856
14315	Amp	8	131580848	131623630	3.738665	0.151895
14316	Amp	8	131625731	131769821	3.887411	0.154433
14317	Amp	8	131770674	131832124	4.037384	0.157007
14318	Amp	8	131836046	131871099	3.887411	0.15447
14319	Amp	8	131873072	131900750	3.726055	0.151574
14320	Amp	8	131905496	131966767	4.129725	0.158681
14321	Amp	8	131968491	131968491	4.236663	0.160441
14322	Amp	8	131968707	131994679	4.169329	0.159226
14323	Amp	8	132004778	132010916	4.090086	0.157939
14324	Amp	8	132015153	132031275	4.062894	0.157503
14325	Amp	8	132031315	132051991	3.749777	0.152203
14326	Amp	8	132053860	132053860	3.628913	0.14998
14327	Amp	8	132055290	132056326	3.566107	0.148777
14328	Amp	8	132057207	132087427	3.862695	0.154077
14329	Amp	8	132088135	132121114	3.505046	0.147751
14330	Amp	8	132126847	132516898	3.150497	0.141259
14331	Amp	8	132517096	132517096	3.218362	0.142572
14332	Amp	8	132517961	132530968	3.127699	0.140752
14333	Amp	8	132537077	132642455	3.033246	0.139046
14334	Amp	8	132645714	132680208	3.435586	0.146498
14335	Amp	8	132680633	132684306	3.628913	0.149931
14336	Amp	8	132689257	132726286	3.738665	0.151929
14337	Amp	8	132728653	132734323	3.323812	0.144477
14338	Amp	8	132740109	132756211	3.045223	0.139275
14339	Amp	8	132763423	132918766	2.986184	0.138236
14340	Amp	8	132919503	132973721	3.057106	0.139501
14341	Amp	8	132977045	132990883	2.964305	0.137845
14342	Amp	8	132991509	133068951	3.057106	0.139557
14343	Amp	8	133069092	133072187	2.986184	0.138319
14344	Amp	8	133074093	133080308	3.160681	0.141392
14345	Amp	8	133083636	133120201	3.230405	0.142725
14346	Amp	8	133120568	133141329	3.335819	0.144636
14347	Amp	8	133142676	133148865	3.218362	0.142649
14348	Amp	8	133151196	133183793	2.707624	0.132891
14349	Amp	8	133184606	133204278	3.021176	0.138837
14350	Amp	8	133207034	133231905	3.160681	0.141501
14351	Amp	8	133234475	133259278	3.021176	0.138837
14352	Amp	8	133259964	133288123	2.860424	0.135765
14353	Amp	8	133289345	133293894	2.743935	0.133567
14354	Amp	8	133297844	133376239	2.673338	0.132373
14355	Amp	8	133376350	133461674	2.571513	0.130374
14356	Amp	8	133462350	133528207	2.871582	0.135933
14357	Amp	8	133529088	133582340	2.776997	0.134221
14358	Amp	8	133582395	133609421	2.860424	0.135894
14359	Amp	8	133609443	133728739	2.776997	0.134221
14360	Amp	8	133731984	133782580	2.468223	0.128486
14361	Amp	8	133787318	133856980	2.560422	0.130076
14362	Amp	8	133857471	133912035	2.468223	0.128361

14363	Amp	8	133913492	133922657	2.290039	0.124929
14364	Amp	8	133923143	134014229	2.202226	0.123322
14365	Amp	8	134014561	134068916	2.147988	0.122207
14366	Amp	8	134078334	134184883	2.127614	0.121724
14367	Amp	8	134185760	134193029	2.158817	0.122297
14368	Amp	8	134193264	134322305	2.235168	0.123948
14369	Amp	8	134324314	134333829	2.26699	0.124499
14370	Amp	8	134333870	134412571	2.235168	0.123948
14371	Amp	8	134413184	134437165	2.278531	0.124737
14372	Amp	8	134437327	134460725	1.502257	0.108801
14373	Amp	8	134462335	134487565	1.573014	0.110515
14374	Amp	8	134488589	134500943	1.645511	0.112033
14375	Amp	8	134501185	134549911	1.573014	0.110444
14376	Amp	8	134553025	134666561	1.456037	0.107869
14377	Amp	8	134685708	134785410	1.243254	0.103047
14378	Amp	8	134786334	134795604	1.388256	0.106189
14379	Amp	8	134796748	134968351	1.482378	0.108555
14380	Amp	8	134971982	135006204	1.407997	0.106756
14381	Amp	8	135011720	135029342	1.445847	0.107526
14382	Amp	8	135030341	135168213	1.407997	0.106756
14383	Amp	8	135174514	135190675	1.33853	0.105237
14384	Amp	8	135192443	135212129	1.369395	0.105873
14385	Amp	8	135212614	135255827	1.33853	0.105237
14386	Amp	8	135265154	135294158	1.984015	0.118853
14387	Amp	8	135296400	135334575	1.456037	0.107739
14388	Amp	8	135347669	135356272	0.950886	0.096151
14389	Amp	8	135356744	135375446	0.873678	0.094158
14390	Amp	8	135376049	135387846	0.98589	0.096859
14391	Amp	8	135397693	135436444	1.058188	0.098797
14392	Amp	8	135444207	135608540	1.041205	0.098167
14393	Amp	8	135611149	135629529	0.891602	0.094488
14394	Amp	8	135632012	135646248	0.765474	0.091208
14395	Amp	8	135646741	135654951	0.681869	0.088841
14396	Amp	8	135656286	135785508	0.751221	0.09091
14397	Amp	8	135791225	135844970	0.807647	0.092353
14398	Amp	8	135854934	135895247	0.704849	0.089652
14399	Amp	8	135898741	135914067	0.651644	0.088016
14400	Amp	8	135915212	136011353	0.667587	0.088535
14401	Amp	8	136011563	136011820	0.742723	0.090759
14402	Amp	8	136011842	136019440	0.765474	0.091269
14403	Amp	8	136021406	136023422	0.79909	0.092174
14404	Amp	8	136024010	136029187	0.781972	0.091655
14405	Amp	8	136029534	136142938	0.758088	0.091049
14406	Amp	8	136145332	136158454	0.431198	0.080848
14407	Amp	8	136163117	136266168	0.595248	0.086278
14408	Amp	8	136267575	136290349	0.689884	0.089089
14409	Amp	8	136290593	136303190	0.815228	0.092506
14410	Amp	8	136303422	136307544	0.602643	0.086468
14411	Amp	8	136310625	136319237	0.557122	0.085025
14412	Amp	8	136321954	136397207	0.636464	0.087549
14413	Amp	8	136397803	136458511	0.751221	0.090787
14414	Amp	8	136459431	136517270	0.815228	0.092653
14415	Amp	8	136521154	136583877	1.121584	0.100305

14416	Amp	8	136587476	136627456	1.049301	0.098428
14417	Amp	8	136632652	136634121	0.968522	0.096563
14418	Amp	8	136634167	136654485	0.941704	0.095928
14419	Amp	8	136655366	136655366	0.857296	0.093633
14420	Amp	8	136870980	136945309	0.758088	0.091108
14421	Amp	8	136949463	137003578	1.112463	0.10007
14422	Amp	8	137004627	137049636	0.758088	0.091108
14423	Amp	8	137049928	137058894	0.563096	0.085333
14424	Amp	8	137059602	137159236	0.622604	0.087074
14425	Amp	8	137159255	137166113	0.636464	0.087681
14426	Amp	8	137166199	137197130	0.660254	0.08836
14427	Amp	8	137202985	137219306	0.569771	0.085549
14428	Amp	8	137222653	137273932	0.355319	0.078302
14429	Amp	8	137273989	137333866	0.30524	0.076561
14430	Amp	8	137336423	137437447	0.322897	0.077286
14431	Amp	8	137441332	137907231	0.251218	0.07444
14432	Amp	8	137907458	137947004	0.271552	0.075263
14433	Amp	8	137947992	138003931	0.362016	0.078476
14434	Amp	8	138004170	138016848	0.417873	0.080346
14435	Amp	8	138024398	138025605	0.482379	0.082556
14436	Amp	8	138025730	138089978	0.494792	0.083
14437	Amp	8	138090024	138099915	0.515676	0.083733
14438	Amp	8	138099958	138132671	0.529451	0.084187
14439	Amp	8	138133509	138145481	0.557122	0.085108
14440	Amp	8	138146292	138272848	0.583752	0.085786
14441	Amp	8	138275874	138424416	0.557122	0.085108
14442	Amp	8	138447533	138465750	0.630438	0.087322
14443	Amp	8	138467932	138470417	0.644502	0.087767
14444	Amp	8	138471472	138575395	0.689884	0.089039
14445	Amp	8	138577928	138658741	0.86541	0.093851
14446	Amp	8	138660359	138677633	0.689884	0.089039
14447	Amp	8	138679373	138720819	0.882843	0.094425
14448	Amp	8	138723274	138770630	0.906385	0.094969
14449	Amp	8	138773401	139089711	0.72817	0.090206
14450	Amp	8	139093499	139149582	0.758088	0.090982
14451	Amp	8	139151208	139220692	0.933745	0.095575
14452	Amp	8	139223621	139289510	0.891602	0.09467
14453	Amp	8	139289520	139397104	0.924746	0.095496
14454	Amp	8	139397717	139414177	0.950886	0.09603
14455	Amp	8	139415142	139530027	0.977077	0.096803
14456	Amp	8	139530526	139559628	1.075952	0.099171
14457	Amp	8	139563107	139582115	1.093834	0.099662
14458	Amp	8	139584307	139603044	1.140417	0.100751
14459	Amp	8	139604312	139690865	1.225419	0.102675
14460	Amp	8	139690941	139690941	1.252022	0.103252
14461	Amp	8	139690970	139748767	1.290368	0.104021
14462	Amp	8	139753932	139765344	1.416817	0.106876
14463	Amp	8	139767382	139910045	1.463774	0.107968
14464	Amp	8	139919861	139989026	1.522708	0.10923
14465	Amp	8	139989179	140172623	1.492063	0.108785
14466	Amp	8	140174259	140178592	1.472629	0.108275
14467	Amp	8	140178814	140276484	1.780186	0.114854
14468	Amp	8	140276706	140286814	1.843664	0.116

14469	Amp	8	140290278	140294916	1.522708	0.109421
14470	Amp	8	140299948	140365372	1.445847	0.107497
14471	Amp	8	140365863	140381785	1.542638	0.109858
14472	Amp	8	140384297	140384297	1.492063	0.108712
14473	Amp	8	140384587	140395435	1.823153	0.115653
14474	Amp	8	140397511	140454006	1.68612	0.112799
14475	Amp	8	140455883	140479109	1.897704	0.117235
14476	Amp	8	140483350	140502973	1.833307	0.115901
14477	Amp	8	140504085	140543748	1.95165	0.118199
14478	Amp	8	140544364	140613553	1.727286	0.113763
14479	Amp	8	140619100	140620316	1.801583	0.115199
14480	Amp	8	141150299	141150299	1.994915	0.119095
14481	Amp	8	141153819	141154310	2.038694	0.120049
14482	Amp	8	141154347	141188497	1.95165	0.118191
14483	Amp	8	141188583	141247279	1.908279	0.117307
14484	Amp	8	141255160	141309970	2.016585	0.11963
14485	Amp	8	141310438	141310438	1.929887	0.11776
14486	Amp	8	141311437	141323524	1.823153	0.115562
14487	Amp	8	141323933	142423108	2.127614	0.12166
14488	Amp	8	142423309	142565427	2.202226	0.123119
14489	Amp	8	142570799	142582951	1.833307	0.115937
14490	Amp	8	142591627	142610477	1.727286	0.113576
14491	Amp	8	142616206	142903079	1.645511	0.11194
14492	Amp	8	142903464	142924657	1.759227	0.114271
14493	Amp	8	142927065	143029865	1.801583	0.115264
14494	Amp	8	143030864	143050947	1.940776	0.117954
14495	Amp	8	143051052	143064987	1.696256	0.112952
14496	Amp	8	143070953	143076925	1.759227	0.114362
14497	Amp	8	143081961	143137133	2.104978	0.121296
14498	Amp	8	143143176	143162209	2.049917	0.120256
14499	Amp	8	143162239	143228447	1.940776	0.117933
14500	Amp	8	143232207	143317866	1.96212	0.118478
14501	Amp	8	143325445	143413727	1.994915	0.119207
14502	Amp	8	143424267	143424267	1.929887	0.117807
14503	Amp	8	143445176	143462025	2.290039	0.125042
14504	Amp	8	143467990	143473146	2.614791	0.131347
14505	Amp	8	143484799	143563290	2.837591	0.135342
14506	Amp	8	143565794	143655392	2.479057	0.128657
14507	Amp	8	143658611	143667308	2.446878	0.127923
14508	Amp	8	143668378	143679240	2.127614	0.121618
14509	Amp	8	143680108	143680108	2.038694	0.120066
14510	Amp	8	143734812	143807002	1.843664	0.116072
14511	Amp	8	143822781	143830133	1.512549	0.10913
14512	Amp	8	143832522	143848452	1.604336	0.111009
14513	Amp	8	143856219	143876552	1.634701	0.111669
14514	Amp	8	143882296	143959566	1.780186	0.114844
14515	Amp	8	143963405	144011491	2.368227	0.126484
14516	Amp	8	144012083	144067190	2.300463	0.125274
14517	Amp	8	144071799	144080674	2.202226	0.123192
14518	Amp	8	144080783	144080783	2.104978	0.121312
14519	Amp	8	144081683	144084619	2.038694	0.119876
14520	Amp	8	144090023	144090784	1.833307	0.115757
14521	Amp	8	144091114	144100798	1.790459	0.115078

14522	Amp	8	144118018	144121574	1.502257	0.10887
14523	Amp	8	144121643	144123152	1.593878	0.110921
14524	Amp	8	144164776	144182542	1.290368	0.104105
14525	Amp	9	789794	802325	0	0.016095
14526	Amp	9	803158	834091	0	0.015116
14527	Amp	9	834510	872418	0	0.014634
14528	Amp	9	872798	1542744	0	0.014185
14529	Amp	9	1544557	1588442	0	0.013737
14530	Amp	9	1591055	1592138	0	0.013231
14531	Amp	9	1592353	2338786	0	0.012729
14532	Amp	9	2339647	2380595	0	0.020599
14533	Amp	9	2380734	2397564	0	0.020089
14534	Amp	9	2397918	2427182	0	0.021042
14535	Amp	9	2427960	2749297	0	0.020089
14536	Amp	9	2749911	3034259	0	0.018969
14537	Amp	9	3035385	3096930	0	0.019471
14538	Amp	9	3096934	3155550	0	0.020205
14539	Amp	9	3156066	3216078	0	0.019703
14540	Amp	9	3217054	3230477	0	0.018969
14541	Amp	9	3231375	3275784	0	0.011098
14542	Amp	9	3276396	3287307	0	0.007206
14543	Amp	9	3288470	3628349	0	0.010829
14544	Amp	9	3630088	3832812	0	0.011373
14545	Amp	9	3833449	4008653	0	0.013307
14546	Amp	9	4022111	4022111	0	0.013881
14547	Amp	9	4022162	4286898	0	0.011947
14548	Amp	9	4286932	4976751	0	0.011373
14549	Amp	9	4978643	5024452	0	0.010829
14550	Amp	9	5024480	5037453	0	0.025778
14551	Amp	9	5037454	5037462	0	0.029264
14552	Amp	9	5039341	5040333	0	0.014315
14553	Amp	9	5040386	6256586	0	0.010829
14554	Amp	9	7055236	7074970	0	0.008633
14555	Amp	9	7125060	7393274	0	0.010481
14556	Amp	9	7393426	7530883	0	0.008633
14557	Amp	9	7530885	7562043	0	0.009167
14558	Amp	9	7562072	7729573	0	0.008633
14559	Amp	9	7729692	8077872	0	0.007046
14560	Amp	9	8078449	8092620	0	0.007752
14561	Amp	9	8093062	8343617	0	0.007046
14562	Amp	9	8348417	8615045	0	0.007572
14563	Amp	9	8615659	8621368	0	0.010721
14564	Amp	9	8625545	8812385	0	0.014173
14565	Amp	9	8812415	8816665	0	0.016027
14566	Amp	9	8822267	9206006	0	0.031963
14567	Amp	9	9206144	9206144	0	0.028511
14568	Amp	9	9208635	9238929	0	0.025362
14569	Amp	9	9239154	9410073	0	0.028844
14570	Amp	9	9411107	9414766	0	0.030408
14571	Amp	9	9414834	9416962	0	0.027439
14572	Amp	9	9417120	9444744	0	0.025585
14573	Amp	9	9445231	9502693	0	0.020744
14574	Amp	9	9503477	9511247	0	0.017122

14575 Amp	9	9517017	9575298	0	0.020257
14576 Amp	9	9579104	9581692	0	0.012131
14577 Amp	9	9582128	9584526	0	0.01619
14578 Amp	9	9587003	9593858	0	0.012708
14579 Amp	9	9594429	9658042	0	0.009522
14580 Amp	9	9662343	9712549	0	0.008649
14581 Amp	9	9714838	9719384	0	0.009339
14582 Amp	9	9719887	9745020	0	0.010896
14583 Amp	9	9746909	9823820	0	0.01037
14584 Amp	9	9823870	9874677	0	0.008813
14585 Amp	9	9985419	10547481	0	0.007249
14586 Amp	9	10547511	10547923	0	0.048505
14587 Amp	9	10548235	11168641	0	0.007249
14588 Amp	9	11179675	11202702	0	0.005469
14589 Amp	9	11202763	11274289	0	0.007273
14590 Amp	9	11274798	11275208	0	0.006071
14591 Amp	9	11275227	12650198	0	0.007712
14592 Amp	9	12650750	12696148	0	0.006071
14593 Amp	9	12698363	13165967	0	0.00697
14594 Amp	9	13166140	13185150	0	0.003835
14595 Amp	9	13185984	13932847	0	0.002935
14596 Amp	9	13936771	13980585	0	0.006463
14597 Amp	9	13981419	14251918	0	0.006021
14598 Amp	9	14252209	14253180	0	0.006633
14599 Amp	9	14255427	14274726	0	0.003106
14600 Amp	9	14275240	14365308	0	0.007119
14601 Amp	9	14367591	14405159	0	0.003106
14602 Amp	9	14405556	14466059	0	0.002494
14603 Amp	9	14467619	14526765	0	0.005646
14604 Amp	9	14532723	14563606	0	0.002494
14605 Amp	9	14564103	14566408	0	0.001804
14606 Amp	9	14566931	14787708	0	0.002244
14607 Amp	9	14788409	15064518	0	0.001804
14608 Amp	9	15065869	15132512	0	0.003105
14609 Amp	9	15138602	15164722	0	0.019041
14610 Amp	9	15167379	15445221	0	0.01774
14611 Amp	9	15450519	15482357	0	0.019196
14612 Amp	9	15486267	16233087	0	0.019757
14613 Amp	9	16235373	16279541	0	0.020387
14614 Amp	9	16285779	16361582	0	0.022362
14615 Amp	9	16361682	16458660	0	0.021733
14616 Amp	9	16458843	16590841	0	0.021171
14617 Amp	9	16598108	16645896	0	0.019196
14618 Amp	9	16649934	16745594	0	0.023827
14619 Amp	9	16749756	16868693	0	0.019196
14620 Amp	9	16873467	17032246	0	0.003259
14621 Amp	9	17032793	17169491	0	0.00397
14622 Amp	9	17171371	17210041	0	0.005815
14623 Amp	9	17210515	17210516	0	0.006268
14624 Amp	9	17216959	17496116	0	0.005557
14625 Amp	9	17498758	17500441	0	0.007899
14626 Amp	9	17501079	17506496	0	0.006054
14627 Amp	9	17508776	17801557	0	0.003712

14628	Amp	9	17804529	17818648	0	0.004738
14629	Amp	9	17819383	17819832	0	0.003712
14630	Amp	9	17823395	18363379	0	0.003259
14631	Amp	9	18364340	18434649	0	0.004125
14632	Amp	9	18443062	18443062	0	0.005561
14633	Amp	9	18444713	18504310	0	0.007262
14634	Amp	9	18504831	18614151	0	0.009438
14635	Amp	9	18614353	18873379	0	0.007737
14636	Amp	9	18879084	19373257	0	0.005416
14637	Amp	9	19376410	19388175	0	0.005862
14638	Amp	9	19389772	19413866	0	0.008643
14639	Amp	9	19414904	19510858	0	0.020771
14640	Amp	9	19511098	19615356	0	0.024279
14641	Amp	9	19622270	19677545	0	0.008643
14642	Amp	9	19678271	19778773	0	0.006467
14643	Amp	9	19781412	19794692	0	0.008565
14644	Amp	9	19796863	19933000	0	0.01075
14645	Amp	9	19934209	19988977	0	0.012816
14646	Amp	9	19989586	20021049	0	0.022006
14647	Amp	9	20026340	20130229	0	0.01799
14648	Amp	9	20136173	20138516	0	0.019301
14649	Amp	9	20143594	20288153	0	0.022356
14650	Amp	9	20289075	20324372	0	0.026323
14651	Amp	9	20329677	20338714	0	0.028456
14652	Amp	9	20340661	20356743	0	0.03092
14653	Amp	9	20356838	20366034	0	0.028456
14654	Amp	9	20369577	20374247	0	0.034133
14655	Amp	9	20378538	20408622	0	0.036087
14656	Amp	9	20415620	20431058	0	0.03041
14657	Amp	9	20443349	20471662	0	0.032128
14658	Amp	9	20476132	20481820	0	0.034682
14659	Amp	9	20481906	20616256	0	0.037771
14660	Amp	9	20616619	20719868	0	0.039961
14661	Amp	9	20722831	20848719	0	0.037771
14662	Amp	9	20849578	20963340	0	0.036053
14663	Amp	9	20968660	20996452	0	0.033955
14664	Amp	9	20996710	21140673	0	0.0298
14665	Amp	9	21140928	21331198	0	0.025934
14666	Amp	9	21332348	21352378	0	0.029205
14667	Amp	9	21353872	21353872	0	0.017724
14668	Amp	9	21354181	21383194	0	0.026839
14669	Amp	9	21391232	21625102	0	0.011226
14670	Amp	9	21625579	21731100	0	0.00979
14671	Amp	9	21731367	21988897	0	0.007986
14672	Amp	9	21989073	22011657	0	0.010241
14673	Amp	9	22015435	22167975	0	0.007986
14674	Amp	9	22168129	22364627	0	0.00402
14675	Amp	9	22366804	22614986	0	0.005606
14676	Amp	9	22615800	22653272	0	0.00402
14677	Amp	9	22657615	22697536	0	0.001954
14678	Amp	9	22709362	23322377	0	0.003684
14679	Amp	9	23326891	23418574	0	0.00471
14680	Amp	9	23418807	23627726	0	0.006289

14681	Amp	9	23628588	24027325	0	0.00752
14682	Amp	9	24027683	24132560	0	0.023457
14683	Amp	9	24133240	24326492	0	0.00752
14684	Amp	9	24328052	25056827	0	0.008922
14685	Amp	9	25058339	25375429	0	0.009664
14686	Amp	9	25375724	25467901	0	0.01109
14687	Amp	9	25472083	25486471	0	0.025129
14688	Amp	9	25486863	25517944	0	0.024387
14689	Amp	9	25518346	25854606	0	0.022985
14690	Amp	9	25859416	26206192	0	0.02156
14691	Amp	9	26206846	26263020	0	0.022085
14692	Amp	9	26433574	26433574	0	0.028131
14693	Amp	9	26433919	26510949	0	0.0321
14694	Amp	9	26511002	26551355	0	0.02855
14695	Amp	9	26552715	26565915	0	0.029109
14696	Amp	9	26591875	26600980	0	0.025139
14697	Amp	9	26603476	26605500	0	0.028652
14698	Amp	9	26605988	26633843	0	0.031147
14699	Amp	9	26634030	26729881	0	0.030589
14700	Amp	9	26737217	26780890	0	0.032101
14701	Amp	9	26785715	26799084	0	0.02656
14702	Amp	9	26800361	26843962	0	0.025047
14703	Amp	9	26851151	26977028	0	0.027209
14704	Amp	9	26978990	27152211	0	0.028582
14705	Amp	9	27152294	27191013	0	0.044518
14706	Amp	9	27193505	27560676	0	0.028582
14707	Amp	9	27562916	27562916	0	0.026852
14708	Amp	9	27571460	27637031	0	0.023339
14709	Amp	9	27637181	27669414	0	0.021865
14710	Amp	9	27671296	27696703	0	0.023851
14711	Amp	9	27701894	27749955	0	0.021865
14712	Amp	9	27752808	27786811	0	0.0261
14713	Amp	9	27791974	27818943	0	0.021865
14714	Amp	9	27819542	27825456	0	0.023575
14715	Amp	9	27828908	27839435	0	0.023051
14716	Amp	9	27839532	27847128	0	0.024369
14717	Amp	9	27848033	27901737	0	0.023051
14718	Amp	9	27905144	28355727	0	0.024921
14719	Amp	9	28356133	28433572	0	0.022759
14720	Amp	9	28440396	28586158	0	0.020889
14721	Amp	9	28981483	28981483	0	0.025203
14722	Amp	9	28981528	29198673	0	0.028937
14723	Amp	9	29200388	29202018	0	0.026581
14724	Amp	9	29202338	29222705	0	0.027976
14725	Amp	9	29223063	29305921	0	0.029655
14726	Amp	9	29306967	29370344	0	0.036126
14727	Amp	9	29374156	29489192	0	0.029655
14728	Amp	9	29491886	29502778	0	0.027976
14729	Amp	9	29503207	29718538	0	0.022847
14730	Amp	9	29718733	29769928	0	0.025936
14731	Amp	9	29770178	30015538	0	0.024357
14732	Amp	9	30018158	30036940	0	0.025489
14733	Amp	9	30038862	30060382	0	0.022401

14734 Amp	9	30061026	30095438	0	0.025842
14735 Amp	9	30097790	30279615	0	0.022401
14736 Amp	9	30280022	30636430	0	0.025273
14737 Amp	9	30708218	30754279	0	0.02414
14738 Amp	9	30757463	30758418	0	0.02578
14739 Amp	9	30767293	30830159	0	0.016765
14740 Amp	9	30835548	30854786	0	0.015125
14741 Amp	9	30858988	30881815	0	0.015122
14742 Amp	9	30882216	31068072	0	0.014096
14743 Amp	9	31083833	31148010	0	0.014846
14744 Amp	9	31148520	31178231	0	0.013139
14745 Amp	9	31182136	31217393	0	0.014737
14746 Amp	9	31217536	31267195	0	0.021003
14747 Amp	9	31269874	31419131	0	0.022239
14748 Amp	9	31422433	31448153	0	0.019239
14749 Amp	9	31448966	31456863	0	0.018489
14750 Amp	9	31470200	31680707	0	0.017253
14751 Amp	9	31685373	31735437	0	0.010988
14752 Amp	9	31736474	31767554	0	0.009029
14753 Amp	9	31769890	31914329	0	0.00743
14754 Amp	9	31914689	31977452	0	0.008627
14755 Amp	9	31981000	32163279	0	0.010389
14756 Amp	9	32165675	32179568	0	0.009016
14757 Amp	9	32183269	32204660	0	0.011957
14758 Amp	9	32208637	32292143	0	0.013831
14759 Amp	9	32305836	32329380	0	0.012507
14760 Amp	9	32330517	32330517	0	0.013255
14761 Amp	9	32330739	32406163	0	0.016463
14762 Amp	9	32409924	32526148	0	0.014589
14763 Amp	9	32531273	32538242	0	0.011381
14764 Amp	9	32549604	32567824	0	0.01425
14765 Amp	9	32573149	32638448	0	0.01756
14766 Amp	9	32639351	32685509	0	0.017999
14767 Amp	9	32690445	32700102	0	0.02431
14768 Amp	9	32700821	32705364	0	0.026419
14769 Amp	9	32706143	32710129	0	0.028383
14770 Amp	9	32710251	32755030	0	0.022072
14771 Amp	9	32759619	32863971	0	0.023009
14772 Amp	9	32865571	32936384	0	0.022072
14773 Amp	9	32940751	33009415	0	0.038008
14774 Amp	9	33014580	33027218	0	0.022072
14775 Amp	9	33030887	33177184	0	0.033208
14776 Amp	9	33180815	33225649	0	0.040051
14777 Amp	9	33228191	33274457	0	0.03711
14778 Amp	9	33274476	33286158	0	0.040712
14779 Amp	9	33292804	33293407	0	0.033869
14780 Amp	9	33294093	33387453	0	0.030267
14781 Amp	9	33388291	33391217	0	0.032808
14782 Amp	9	33391590	33492001	0	0.034486
14783 Amp	9	33492017	33496883	0	0.033738
14784 Amp	9	33498249	33506303	0	0.03206
14785 Amp	9	33506672	33530769	0	0.03669
14786 Amp	9	33533623	33541555	0	0.038597

14787 Amp	9	33544975	33639740	0	0.044757
14788 Amp	9	33643263	33757000	0	0.042216
14789 Amp	9	33757011	33921935	0	0.037953
14790 Amp	9	33921979	33956634	0	0.036056
14791 Amp	9	33960004	33988708	0	0.034092
14792 Amp	9	33992631	34019604	0	0.036447
14793 Amp	9	34022404	34272092	0	0.034871
14794 Amp	9	34272260	34369383	0	0.032516
14795 Amp	9	34371790	34395252	0	0.034457
14796 Amp	9	34395818	34509748	0	0.036149
14797 Amp	9	34513625	34737705	0	0.043464
14798 Amp	9	34741755	34919433	0	0.045679
14799 Amp	9	34919519	34955697	0	0.044386
14800 Amp	9	34962080	34986902	0	0.052099
14801 Amp	9	34997572	35165180	0	0.048789
14802 Amp	9	35166766	35218644	0	0.051152
14803 Amp	9	35218696	35223317	0	0.056816
14804 Amp	9	35226707	35257731	0	0.054909
14805 Amp	9	35260920	35296669	0	0.049245
14806 Amp	9	35297428	35341112	0	0.043762
14807 Amp	9	35343530	35440437	0	0.03665
14808 Amp	9	35448365	35546738	0	0.041442
14809 Amp	9	35551636	35569636	0	0.039008
14810 Amp	9	35571633	35639124	0	0.034216
14811 Amp	9	35639632	35912174	0	0.034694
14812 Amp	9	35919471	35950498	0	0.032753
14813 Amp	9	35958336	35988453	0	0.032275
14814 Amp	9	35992700	36039070	0	0.02663
14815 Amp	9	36047660	36156971	0	0.019927
14816 Amp	9	36161436	36204915	0	0.020974
14817 Amp	9	36210414	36372712	0	0.023293
14818 Amp	9	36373332	36471215	0	0.021184
14819 Amp	9	36476956	36569097	0	0.023859
14820 Amp	9	36576196	36576196	0	0.020987
14821 Amp	9	36576345	36644314	0	0.022742
14822 Amp	9	36646725	36650845	0	0.020379
14823 Amp	9	36653687	36928929	0	0.018624
14824 Amp	9	36930539	36988854	0	0.015949
14825 Amp	9	36989026	37033162	0	0.015511
14826 Amp	9	37033364	37303492	0	0.015001
14827 Amp	9	37312957	37331608	0	0.023123
14828 Amp	9	37334733	37345152	0	0.021679
14829 Amp	9	37349074	37375129	0	0.01592
14830 Amp	9	37376125	37382102	0	0.01306
14831 Amp	9	37383641	37392090	0	0.021156
14832 Amp	9	37392146	37392869	0	0.016193
14833 Amp	9	37393051	37421240	0	0.021671
14834 Amp	9	37423235	37436108	0	0.018538
14835 Amp	9	37436928	37625872	0	0.013712
14836 Amp	9	37628283	38049777	0	0.014477
14837 Amp	9	38052132	38322404	0	0.017115
14838 Amp	9	38322901	38368853	0	0.019005
14839 Amp	9	38369462	38370268	0	0.016367

14840	Amp	9	38371437	38746909	0	0.014477
14841	Amp	9	38746940	38747691	0	0.012515
14842	Amp	9	68423153	68423153	0	0.021586
14843	Amp	9	68424128	68424128	0	0.021792
14844	Amp	9	68425024	68445781	0	0.019424
14845	Amp	9	68450451	68470315	0	0.003488
14846	Amp	9	68470687	69068713	0	0.019424
14847	Amp	9	69069453	69103291	0	0.023449
14848	Amp	9	69103543	69110518	0	0.019424
14849	Amp	9	69113463	69186476	0	0.020882
14850	Amp	9	69186778	69264895	0	0.022478
14851	Amp	9	69267940	69306772	0	0.021019
14852	Amp	9	69318241	69558428	0	0.01943
14853	Amp	9	69558813	69710146	0	0.017531
14854	Amp	9	69719671	69905354	0	0.019967
14855	Amp	9	69907785	70117832	0	0.01368
14856	Amp	9	70124533	70365865	0	0.011245
14857	Amp	9	70370001	70401258	0	0.001595
14858	Amp	9	70402137	70577009	0	0.003616
14859	Amp	9	70577238	70700346	0	0.001595
14860	Amp	9	70700651	71409229	0	0
14861	Amp	9	71413463	71414481	0	0.003102
14862	Amp	9	71421524	71466864	0	0.005582
14863	Amp	9	71467161	71603386	0	0.00248
14864	Amp	9	71617727	72068168	0	0
14865	Amp	9	72071722	72924729	0	0.00194
14866	Amp	9	72926762	73335763	0	0.002665
14867	Amp	9	73336290	73577710	0	0.003143
14868	Amp	9	73577946	73635212	0	0.004352
14869	Amp	9	73642196	73731019	0	0.003143
14870	Amp	9	73731076	73757637	0	0.002665
14871	Amp	9	73760027	73872016	0	0.00194
14872	Amp	9	73878421	74000516	0	0.002673
14873	Amp	9	74000746	74700069	0	0.000733
14874	Amp	9	74700100	74705081	0	0
14875	Amp	9	74707064	74836734	0	0.002368
14876	Amp	9	74838629	74882215	0	0.00495
14877	Amp	9	74883443	75114026	0	0.002368
14878	Amp	9	75114676	75752256	0	0
14879	Amp	9	75752430	75958223	0	0.000693
14880	Amp	9	75958785	75992407	0	0.001187
14881	Amp	9	75997137	76151035	0	0.004129
14882	Amp	9	76157543	76468556	0	0.003636
14883	Amp	9	76469992	76474586	0	0.00522
14884	Amp	9	76475649	76566363	0	0.006881
14885	Amp	9	76567499	76578068	0	0.00522
14886	Amp	9	76581816	76696274	0	0.002319
14887	Amp	9	76696935	76809252	0	0.002881
14888	Amp	9	76809929	76882316	0	0.004947
14889	Amp	9	76885238	77094908	0	0.002881
14890	Amp	9	77095342	77423639	0	0.002319
14891	Amp	9	77424742	77463822	0	0.004105
14892	Amp	9	77465519	77776566	0	0.007085

14893	Amp	9	77781556	77862861	0	0.005298
14894	Amp	9	77865068	77922704	0	0.010008
14895	Amp	9	77928885	78073279	0	0.011366
14896	Amp	9	78073425	78155814	0	0.006656
14897	Amp	9	78162597	78179590	0	0.010752
14898	Amp	9	78181172	78508776	0	0.009394
14899	Amp	9	78512055	78805904	0	0.010125
14900	Amp	9	78810199	78825092	0	0.012829
14901	Amp	9	78825547	78914291	0	0.010125
14902	Amp	9	78918628	78932215	0	0.007806
14903	Amp	9	78932910	79253109	0	0.007076
14904	Amp	9	79255267	79332887	0	0.008083
14905	Amp	9	79333470	79622470	0	0.009897
14906	Amp	9	79628786	79759994	0	0.008889
14907	Amp	9	79761669	79772315	0	0.012467
14908	Amp	9	79772350	80134738	0	0.008889
14909	Amp	9	80144393	80193698	0	0.007076
14910	Amp	9	80194288	80278792	0	0.002979
14911	Amp	9	80282365	80464012	0	0
14912	Amp	9	80464690	80547941	0	0.000536
14913	Amp	9	80548477	81339686	0	0
14914	Amp	9	81344496	81421990	0	0.000475
14915	Amp	9	81422146	81598880	0	0
14916	Amp	9	81599040	82082326	0	0.002858
14917	Amp	9	82082480	82191329	0	0.011847
14918	Amp	9	82191742	82247939	0	0.015936
14919	Amp	9	82248553	82267694	0	0.017692
14920	Amp	9	82267709	82341387	0	0.01888
14921	Amp	9	82343287	82347774	0	0.017692
14922	Amp	9	82348026	82383184	0	0.012215
14923	Amp	9	82384938	82400664	0	0.013475
14924	Amp	9	82405819	82572136	0	0.018953
14925	Amp	9	82575849	82637553	0	0.017197
14926	Amp	9	82641227	82647017	0	0.019864
14927	Amp	9	82656530	82691376	0	0.02042
14928	Amp	9	82691469	82975905	0	0.014015
14929	Amp	9	82976057	82986085	0	0.011907
14930	Amp	9	82986881	82999602	0	0.004483
14931	Amp	9	83002616	83039466	0	0.014933
14932	Amp	9	83044964	83107671	0	0.004483
14933	Amp	9	83107853	83173368	0	0.01363
14934	Amp	9	83174690	83360837	0	0.004483
14935	Amp	9	83361071	83388676	0	0.005281
14936	Amp	9	83388854	83478350	0	0.015274
14937	Amp	9	83478835	83494209	0	0.016112
14938	Amp	9	83495365	83576941	0	0.015274
14939	Amp	9	83583597	83751448	0	0.013028
14940	Amp	9	83757378	83808935	0	0.010361
14941	Amp	9	83811596	83996396	0	0.014723
14942	Amp	9	83997650	84132403	0	0.017793
14943	Amp	9	84135847	84154883	0	0.019772
14944	Amp	9	84161435	84331046	0	0.019216
14945	Amp	9	84336700	84363477	0	0.017955

14946 Amp	9	84363894	84412129	0	0.018712
14947 Amp	9	84412735	84602119	0	0.016733
14948 Amp	9	84602334	84696954	0	0.019181
14949 Amp	9	84702758	84809516	0	0.012664
14950 Amp	9	84810934	84852361	0	0.013337
14951 Amp	9	84852596	85106636	0	0.012664
14952 Amp	9	85107718	85270483	0	0.014234
14953 Amp	9	85273851	85310611	0	0.011786
14954 Amp	9	85311131	85651168	0	0.010217
14955 Amp	9	85657353	85762520	0	0.013436
14956 Amp	9	85769274	85906125	0	0.010217
14957 Amp	9	85906879	85970112	0	0.000797
14958 Amp	9	85970528	86198007	0	0.002139
14959 Amp	9	86202623	87713563	0	0.000797
14960 Amp	9	87713859	87899200	0	0.00185
14961 Amp	9	87907679	87973533	0	0.002408
14962 Amp	9	87974587	88075534	0	0.00185
14963 Amp	9	88075690	88433472	0	0.002711
14964 Amp	9	88449409	88488672	0	0.000797
14965 Amp	9	88489267	88497482	0	0.003227
14966 Amp	9	88500567	88583698	0	0.005963
14967 Amp	9	88584077	88614752	0	0.003533
14968 Amp	9	88614920	88648604	0	0.004504
14969 Amp	9	88653626	88667397	0	0.007223
14970 Amp	9	88668046	88974525	0	0.005533
14971 Amp	9	88976653	89095891	0	0.00685
14972 Amp	9	89099533	89120523	0	0.008746
14973 Amp	9	89122139	89175739	0	0.006026
14974 Amp	9	89180197	89201146	0	0.009459
14975 Amp	9	89203602	89242191	0	0.006981
14976 Amp	9	89244222	89247067	0	0.008478
14977 Amp	9	89248419	89425450	0	0.006726
14978 Amp	9	89429589	89482394	0	0.007499
14979 Amp	9	89483329	89568281	0	0.008304
14980 Amp	9	89573563	89608274	0	0.007531
14981 Amp	9	89609636	89717911	0	0.00904
14982 Amp	9	89718007	89817464	0	0.008069
14983 Amp	9	89817660	90155985	0	0.007264
14984 Amp	9	90167829	90267867	0	0.014544
14985 Amp	9	90270102	90564832	0	0.0232
14986 Amp	9	90565390	91102696	0	0.021235
14987 Amp	9	91102817	91140709	0	0.024849
14988 Amp	9	91143586	91174686	0	0.021235
14989 Amp	9	91183885	91194416	0	0.014727
14990 Amp	9	91197864	91241229	0	0.016941
14991 Amp	9	91249610	91465962	0	0.007513
14992 Amp	9	91466708	91552497	0	0.005299
14993 Amp	9	91553905	91575707	0	0.003802
14994 Amp	9	91583959	91618255	0	0.005205
14995 Amp	9	91618855	92278527	0	0.006688
14996 Amp	9	92285576	92316840	0	0.010358
14997 Amp	9	92598089	92605440	0	0.004792
14998 Amp	9	92608382	93598623	0	0.003931

14999 Amp	9	93598946	93860106	0	0.002448
15000 Amp	9	93860583	93934355	0	0.004071
15001 Amp	9	93934522	94026232	0	0.003026
15002 Amp	9	94027549	94233478	0	0.005004
15003 Amp	9	94236957	94269555	0	0.003381
15004 Amp	9	94281514	94996512	0	0.001403
15005 Amp	9	94997638	95494027	0	0.002772
15006 Amp	9	95496699	95846679	0	0.001403
15007 Amp	9	95848156	95906088	0	0.004556
15008 Amp	9	95910255	96085409	0	0.001403
15009 Amp	9	96085963	96122663	0	0.002151
15010 Amp	9	96122862	96310870	0	0.001403
15011 Amp	9	96310987	96352086	0	0.002789
15012 Amp	9	96352731	96500014	0	0.001403
15013 Amp	9	96503154	96789704	0	0.001993
15014 Amp	9	96790400	97278448	0	0.002986
15015 Amp	9	97278613	97292727	0	0.001993
15016 Amp	9	97294241	97347823	0	0.003522
15017 Amp	9	97347897	97655334	0	0.00431
15018 Amp	9	97673871	97852014	0	0.006467
15019 Amp	9	97861095	98052291	0	0.008193
15020 Amp	9	98059342	98072881	0	0.01485
15021 Amp	9	98073221	98082685	0	0.014063
15022 Amp	9	98083498	98111148	0	0.011905
15023 Amp	9	98117017	98133720	0	0.021184
15024 Amp	9	98133766	98276420	0	0.023268
15025 Amp	9	98277491	98454435	0	0.021184
15026 Amp	9	98455240	98674196	0	0.027491
15027 Amp	9	98674932	98727383	0	0.025765
15028 Amp	9	98730119	98955719	0	0.019458
15029 Amp	9	98959702	99000663	0	0.011064
15030 Amp	9	99017157	99017157	0	0.013335
15031 Amp	9	99019019	99144165	0	0.005794
15032 Amp	9	99145390	99225478	0	0.003522
15033 Amp	9	99228100	99540107	0	0.002932
15034 Amp	9	99543272	99597359	0	0.001529
15035 Amp	9	99597707	99953574	0	0
15036 Amp	9	99953684	100718895	0	0.001575
15037 Amp	9	100719413	101995103	0	0.003735
15038 Amp	9	102227112	102348786	0	0.001575
15039 Amp	9	102350109	102350109	0	0.006393
15040 Amp	9	102356339	102356339	0	0.014753
15041 Amp	9	102359752	102367712	0	0.019694
15042 Amp	9	102369109	102369109	0	0.006516
15043 Amp	9	102370801	102726354	0	0.001575
15044 Amp	9	102738445	102810714	0	0.009063
15045 Amp	9	102810755	102843335	0	0.009501
15046 Amp	9	102843736	103397066	0	0.002012
15047 Amp	9	103398137	103424946	0	0.005132
15048 Amp	9	103441838	105727944	0	0.002012
15049 Amp	9	105728489	105749741	0	0.00287
15050 Amp	9	105755553	106111716	0	0.004597
15051 Amp	9	106113439	106176817	0	0.004159

15052	Amp	9	106181854	106220261	0	0.006346
15053	Amp	9	106220584	106277338	0	0.005324
15054	Amp	9	106281604	106435107	0	0.004467
15055	Amp	9	106450496	107316535	0	0.00274
15056	Amp	9	107316905	108057520	0	0.003805
15057	Amp	9	108058035	109238461	0	0.001575
15058	Amp	9	109245396	109377228	0	0.003275
15059	Amp	9	109379628	109505345	0	0.001575
15060	Amp	9	109505698	110058510	0	0.002551
15061	Amp	9	110059903	110944308	0	0.006039
15062	Amp	9	110949956	111241665	0	0.005062
15063	Amp	9	111242324	111375286	0	0.00639
15064	Amp	9	111378202	111564225	0	0.009702
15065	Amp	9	111568495	111751059	0	0.008375
15066	Amp	9	111751935	111833761	0	0.010115
15067	Amp	9	111836680	111892876	0	0.010898
15068	Amp	9	111898926	111934987	0	0.013434
15069	Amp	9	111935123	111944606	0	0.011694
15070	Amp	9	111944693	112001101	0	0.012923
15071	Amp	9	112002715	112124134	0	0.013849
15072	Amp	9	112124469	112171834	0	0.01533
15073	Amp	9	112172760	112212328	0	0.014547
15074	Amp	9	112214205	112274710	0	0.012011
15075	Amp	9	112282060	112304298	0	0.010782
15076	Amp	9	112308648	112358465	0	0.009856
15077	Amp	9	112360045	112414448	0	0.011841
15078	Amp	9	112416736	112416736	0	0.01312
15079	Amp	9	112428431	112459764	0	0.013974
15080	Amp	9	112461018	112466868	0	0.018707
15081	Amp	9	112469885	112614181	0	0.013974
15082	Amp	9	112616014	112616014	0	0.010661
15083	Amp	9	112831449	112857747	0	0.010228
15084	Amp	9	112858779	112920927	0	0.011322
15085	Amp	9	112921580	112941850	0	0.012863
15086	Amp	9	112942342	113142540	0	0.016624
15087	Amp	9	113143557	113314969	0	0.015143
15088	Amp	9	113316991	113945033	0	0.01622
15089	Amp	9	113946543	113958718	0	0.016765
15090	Amp	9	113958866	113993052	0	0.017332
15091	Amp	9	113995190	114037646	0	0.013571
15092	Amp	9	114037812	114111033	0	0.017181
15093	Amp	9	114112075	114187442	0	0.01957
15094	Amp	9	114191471	114366329	0	0.017181
15095	Amp	9	114367105	114430926	0	0.016613
15096	Amp	9	114431336	114663036	0	0.016068
15097	Amp	9	114663715	114697870	0	0.014975
15098	Amp	9	114700560	114849011	0	0.01299
15099	Amp	9	114849633	114868396	0	0.010603
15100	Amp	9	114879737	114925924	0	0.01256
15101	Amp	9	114928990	115033044	0	0.015473
15102	Amp	9	115037441	115044069	0	0.01256
15103	Amp	9	115045801	115154911	0	0.01364
15104	Amp	9	115159142	115238588	0	0.014879

15105	Amp	9	115239391	115302561	0	0.012937
15106	Amp	9	115305687	115490127	0	0.014228
15107	Amp	9	115561337	115562133	0	0.022019
15108	Amp	9	115562425	115583714	0	0.020779
15109	Amp	9	115586397	115694572	0	0.022528
15110	Amp	9	115695699	115704392	0	0.020571
15111	Amp	9	115705094	116123909	0	0.021582
15112	Amp	9	116124398	116202814	0	0.022244
15113	Amp	9	116203470	116375693	0	0.021233
15114	Amp	9	116375849	116585378	0	0.019484
15115	Amp	9	116587017	117006043	0	0.017939
15116	Amp	9	117006293	117276522	0	0.014329
15117	Amp	9	117277280	117395807	0	0.01613
15118	Amp	9	117396305	117473895	0	0.017372
15119	Amp	9	117483823	117484594	0	0.018831
15120	Amp	9	117488114	117540682	0	0.020507
15121	Amp	9	117540773	117578886	0	0.021609
15122	Amp	9	117579236	117605733	0	0.023392
15123	Amp	9	117606870	117665041	0	0.021933
15124	Amp	9	117685120	117945332	0	0.020831
15125	Amp	9	117945347	118121065	0	0.019823
15126	Amp	9	118122378	118122378	0	0.01804
15127	Amp	9	118122993	118150636	0	0.009689
15128	Amp	9	118151090	118292525	0	0.012054
15129	Amp	9	118293620	118386175	0	0.010144
15130	Amp	9	118388816	118495060	0	0.011257
15131	Amp	9	118496278	118527551	0	0.01198
15132	Amp	9	118529487	118700932	0	0.010179
15133	Amp	9	118701218	118709564	0	0.011001
15134	Amp	9	118710322	118862437	0	0.010179
15135	Amp	9	118862680	118902630	0	0.009456
15136	Amp	9	118902635	118907071	0	0.011249
15137	Amp	9	118908258	119319766	0	0.011847
15138	Amp	9	119320014	119776801	0	0.010054
15139	Amp	9	119777167	119790955	0	0.011724
15140	Amp	9	119793098	119928980	0	0.010054
15141	Amp	9	119930116	120039844	0	0.012177
15142	Amp	9	120040551	120045324	0	0.012855
15143	Amp	9	120047692	120067519	0	0.014905
15144	Amp	9	120071190	120072752	0	0.014226
15145	Amp	9	120073775	120130325	0	0.012104
15146	Amp	9	120130699	120265682	0	0.01108
15147	Amp	9	120269954	120275798	0	0.009031
15148	Amp	9	120282917	120326419	0	0.012627
15149	Amp	9	120327741	120427103	0	0.010555
15150	Amp	9	120432858	120667614	0	0.009475
15151	Amp	9	120672220	120698491	0	0.010119
15152	Amp	9	120698896	120739918	0	0.01149
15153	Amp	9	120742260	120908312	0	0.013021
15154	Amp	9	120909242	120998149	0	0.015054
15155	Amp	9	121000728	121028585	0	0.018603
15156	Amp	9	121028854	121082311	0	0.017072
15157	Amp	9	121083586	121359505	0	0.01641

15158	Amp	9	121362985	121452105	0	0.018138
15159	Amp	9	121453779	121705531	0	0.014589
15160	Amp	9	121706464	121733560	0	0.018573
15161	Amp	9	121750775	121779750	0	0.014589
15162	Amp	9	121782266	121831177	0	0.016195
15163	Amp	9	121838096	121889511	0	0.019682
15164	Amp	9	121891832	121938886	0	0.016195
15165	Amp	9	121938990	121975408	0	0.024294
15166	Amp	9	121975888	122353124	0	0.015665
15167	Amp	9	122354451	122362786	0	0.016335
15168	Amp	9	122362922	122402657	0	0.016985
15169	Amp	9	122402946	122458945	0	0.016316
15170	Amp	9	122464190	122738481	0	0.027095
15171	Amp	9	122740508	122896382	0	0.029087
15172	Amp	9	122902724	122902724	0	0.027095
15173	Amp	9	122903379	122923375	0	0.032252
15174	Amp	9	122929676	122947044	0	0.035654
15175	Amp	9	122947292	122949230	0	0.022648
15176	Amp	9	122955270	123051708	0	0.019246
15177	Amp	9	123056323	123220885	0	0.022334
15178	Amp	9	123221705	123426263	0	0.019246
15179	Amp	9	123430569	123460067	0	0.022989
15180	Amp	9	123464413	123480934	0	0.02395
15181	Amp	9	123483384	123584007	0	0.025946
15182	Amp	9	123587947	123595397	0	0.022204
15183	Amp	9	123595958	123601512	0	0.02398
15184	Amp	9	123603411	123655238	0	0.02333
15185	Amp	9	123658624	123916152	0	0.020455
15186	Amp	9	123916346	123962172	0	0.024431
15187	Amp	9	123967192	124010337	0	0.022032
15188	Amp	9	124010791	124129667	0	0.020287
15189	Amp	9	124130794	124148309	0	0.021508
15190	Amp	9	124151078	124186800	0	0.026222
15191	Amp	9	124187007	124249540	0	0.029832
15192	Amp	9	124250189	124406564	0	0.028255
15193	Amp	9	124414088	124449429	0	0.033461
15194	Amp	9	124457854	124532633	0	0.031732
15195	Amp	9	124540341	124637503	0	0.036053
15196	Amp	9	124638799	124694155	0	0.034277
15197	Amp	9	124696437	124711602	0	0.029956
15198	Amp	9	124717883	124818725	0	0.019989
15199	Amp	9	124821170	124847016	0	0.021869
15200	Amp	9	124847597	124857862	0	0.023305
15201	Amp	9	124859365	124894316	0	0.030045
15202	Amp	9	124899366	124981955	0	0.034779
15203	Amp	9	124983261	124987610	0	0.044745
15204	Amp	9	124988130	125186414	0	0.047483
15205	Amp	9	125190546	125209909	0	0.053964
15206	Amp	9	125217716	125245860	0	0.047483
15207	Amp	9	125277127	125384160	0	0.045878
15208	Amp	9	125388855	125458029	0	0.047422
15209	Amp	9	125460061	125645084	0	0.040682
15210	Amp	9	125647117	125656488	0	0.041605

15211	Amp	9	125659895	125708737	0	0.042813
15212	Amp	9	125717810	125806857	0	0.031839
15213	Amp	9	125807811	125857976	0	0.026877
15214	Amp	9	125859031	125908316	0	0.025916
15215	Amp	9	125913592	126003914	0	0.041852
15216	Amp	9	126007265	126207186	0	0.049276
15217	Amp	9	126221236	126313635	0	0.058244
15218	Amp	9	126314001	126463259	0	0.061126
15219	Amp	9	126467917	126505786	0	0.063718
15220	Amp	9	126507054	126628886	0.153838	0.070397
15221	Amp	9	126632946	126672631	0.19964	0.072094
15222	Amp	9	126678371	126724813	0.311747	0.076697
15223	Amp	9	126733648	126788412	0.282828	0.075773
15224	Amp	9	126788727	126825278	0.128324	0.069293
15225	Amp	9	126829395	126907277	0	0.056666
15226	Amp	9	126907749	126952431	0	0.058317
15227	Amp	9	126952825	126954821	0	0.057055
15228	Amp	9	126958424	126961383	0	0.055202
15229	Amp	9	126963214	126998297	0	0.055954
15230	Amp	9	127003111	127154970	0	0.054498
15231	Amp	9	127159404	127275486	0	0.054008
15232	Amp	9	127278448	127281224	0	0.057214
15233	Amp	9	127281497	127305109	0	0.05867
15234	Amp	9	127308270	127406047	0	0.060482
15235	Amp	9	127407595	127457023	0	0.063247
15236	Amp	9	127457130	127457130	0	0.060958
15237	Amp	9	127457390	127466657	0	0.060206
15238	Amp	9	127468476	127468476	0	0.057706
15239	Amp	9	127472448	127494912	0	0.05601
15240	Amp	9	127495373	127673227	0	0.057162
15241	Amp	9	127673625	127827574	0	0.05601
15242	Amp	9	127833170	127953643	0	0.051508
15243	Amp	9	127954482	127990752	0	0.0503
15244	Amp	9	127991256	127993965	0	0.051986
15245	Amp	9	128002587	128028815	0	0.04081
15246	Amp	9	128029809	128039877	0	0.038998
15247	Amp	9	128040243	128045975	0	0.037562
15248	Amp	9	128048307	128097053	0	0.038895
15249	Amp	9	128098587	128164018	0	0.03721
15250	Amp	9	128171504	128172523	0	0.035688
15251	Amp	9	128172655	128388322	0	0.033809
15252	Amp	9	128388680	128473922	0	0.037219
15253	Amp	9	128475615	128481773	0	0.030989
15254	Amp	9	128483969	128483969	0	0.033473
15255	Amp	9	128488543	128530560	0	0.030267
15256	Amp	9	128536025	128558639	0	0.027501
15257	Amp	9	128563519	128592632	0	0.025013
15258	Amp	9	128592772	128644099	0	0.021416
15259	Amp	9	128644617	128765397	0	0.02301
15260	Amp	9	128768323	128792540	0	0.017485
15261	Amp	9	128794935	129077428	0	0.015001
15262	Amp	9	129082718	129281353	0	0.013407
15263	Amp	9	129286385	129478080	0	0.020991

15264	Amp	9	129493326	129500665	0	0.013407
15265	Amp	9	129501622	129730890	0	0.011931
15266	Amp	9	129731229	129798300	0	0.012593
15267	Amp	9	129806202	129957927	0	0.020093
15268	Amp	9	129958491	129965134	0	0.01943
15269	Amp	9	129965574	130047057	0	0.021704
15270	Amp	9	130056137	130088301	0	0.024099
15271	Amp	9	130093505	130397633	0	0.01943
15272	Amp	9	130400632	130483318	0	0.022927
15273	Amp	9	130485958	130565115	0	0.01943
15274	Amp	9	130565233	130607830	0	0.024352
15275	Amp	9	130608822	130837414	0	0.016852
15276	Amp	9	130837608	130890251	0	0.014772
15277	Amp	9	130890269	131241034	0	0.015325
15278	Amp	9	131241931	131755739	0	0.013869
15279	Amp	9	131757837	131834672	0	0.015578
15280	Amp	9	131836736	131872749	0	0.013869
15281	Amp	9	131873125	131973297	0	0.012734
15282	Amp	9	131980415	131980415	0	0.018661
15283	Amp	9	131980908	132074379	0	0.02674
15284	Amp	9	132076992	132130145	0	0.018661
15285	Amp	9	132133587	132140540	0	0.024869
15286	Amp	9	132141362	132176792	0	0.025567
15287	Amp	9	132178289	132198490	0	0.021628
15288	Amp	9	132198580	132199983	0	0.01964
15289	Amp	9	132200354	132569709	0	0.018943
15290	Amp	9	132587246	132687195	0	0.01839
15291	Amp	9	132691782	132920141	0	0.012181
15292	Amp	9	132920280	133090275	0	0.013737
15293	Amp	9	133092215	133134790	0	0.01594
15294	Amp	9	133137405	133141810	0	0.014384
15295	Amp	9	133141841	133250790	0	0.016318
15296	Amp	9	133253159	133258643	0	0.014384
15297	Amp	9	133259717	133277270	0	0.016158
15298	Amp	9	133278037	133345211	0	0.017975
15299	Amp	9	133345692	133356066	0	0.019718
15300	Amp	9	133361989	133497808	0	0.0179
15301	Amp	9	133498090	133538411	0	0.016127
15302	Amp	9	133542347	133543719	0	0.019199
15303	Amp	9	133543754	133705967	0	0.019828
15304	Amp	9	133706910	134241086	0	0.017625
15305	Amp	9	134244697	134400266	0	0.012846
15306	Amp	9	134413432	134432274	0	0.014373
15307	Amp	9	134447662	134488918	0	0.015051
15308	Amp	9	134495183	134634207	0	0.017364
15309	Amp	9	134646211	135021093	0	0.013524
15310	Amp	9	135023374	135248141	0	0.018006
15311	Amp	9	135418642	135519926	0	0.019364
15312	Amp	9	135520280	135539320	0	0.016852
15313	Amp	9	135539829	135929906	0	0.015494
15314	Amp	9	135930924	135949208	0	0.017401
15315	Amp	9	135968281	136063400	0	0.019876
15316	Amp	9	136065616	136081154	0	0.021861

15317	Amp	9	136083043	136099968	0	0.024011
15318	Amp	9	136104687	136129571	0	0.024711
15319	Amp	9	136130081	136138052	0	0.022097
15320	Amp	9	136138916	136160026	0	0.021397
15321	Amp	9	136161138	136161138	0	0.022096
15322	Amp	9	136169628	136456760	0	0.023486
15323	Amp	9	136462046	136465314	0	0.021491
15324	Amp	9	136470133	136509318	0	0.026898
15325	Amp	9	136512039	136514240	0	0.028089
15326	Amp	9	136514289	136563014	0	0.026698
15327	Amp	9	136573733	136963460	0	0.028756
15328	Amp	9	136966179	137074344	0	0.018723
15329	Amp	9	137075265	137124458	0	0.018024
15330	Amp	9	137128555	137257288	0	0.020477
15331	Amp	9	137294216	137333703	0	0.01857
15332	Amp	9	137396353	137414621	0	0.023387
15333	Amp	9	137415594	137463628	0	0.022196
15334	Amp	9	137472156	137489883	0	0.017379
15335	Amp	9	137496163	137656011	0	0.01541
15336	Amp	9	137656529	137843396	0	0.016979
15337	Amp	9	137843779	137857410	0	0.014526
15338	Amp	9	137909533	137957960	0	0.012957
15339	Amp	9	137959738	137983963	0	0.01531
15340	Amp	9	137984164	137989459	0	0.012957
15341	Amp	9	137996274	138005148	0	0.013855
15342	Amp	9	138005230	138016708	0	0.015051
15343	Amp	9	138017218	138044300	0	0.01616
15344	Amp	10	366509	381671	0	0.025499
15345	Amp	10	382136	546986	0	0.027177
15346	Amp	10	547465	985900	0	0.028032
15347	Amp	10	986231	1488703	0	0.026773
15348	Amp	10	1491806	1552960	0	0.027698
15349	Amp	10	1553721	1611765	0	0.030432
15350	Amp	10	1613560	1653099	0	0.027698
15351	Amp	10	1654741	1889282	0	0.027142
15352	Amp	10	1950452	1979566	0	0.032815
15353	Amp	10	1983495	2025329	0	0.032327
15354	Amp	10	2027763	2089169	0	0.034747
15355	Amp	10	2095417	2228618	0	0.033441
15356	Amp	10	2237769	2374908	0	0.031022
15357	Amp	10	2378658	2629073	0	0.032094
15358	Amp	10	2634107	3112917	0	0.032824
15359	Amp	10	3113025	3159873	0	0.034825
15360	Amp	10	3160490	3531093	0	0.033867
15361	Amp	10	3531452	3677226	0	0.034339
15362	Amp	10	3681417	3814101	0	0.033623
15363	Amp	10	3815413	4274884	0	0.030448
15364	Amp	10	4274890	4496280	0	0.03098
15365	Amp	10	4497427	4785032	0	0.031496
15366	Amp	10	4785110	4971324	0	0.031024
15367	Amp	10	4976632	4976632	0	0.062575
15368	Amp	10	4979676	4988189	0.342649	0.077741
15369	Amp	10	4991111	4991111	0	0.0624

15370	Amp	10	4997372	5125307	0	0.031475
15371	Amp	10	5125961	5266540	0	0.033563
15372	Amp	10	5313419	5346931	0	0.038669
15373	Amp	10	5347145	5595979	0	0.033563
15374	Amp	10	5596333	5596333	0	0.032152
15375	Amp	10	5678461	5678461	0	0.035763
15376	Amp	10	5678549	5694468	0	0.034189
15377	Amp	10	5695800	5724214	0	0.030577
15378	Amp	10	5727490	5878145	0	0.028594
15379	Amp	10	5884253	6275948	0	0.028143
15380	Amp	10	6276469	6553201	0	0.02887
15381	Amp	10	6554280	6631295	0	0.028353
15382	Amp	10	6803275	6964609	0	0.026009
15383	Amp	10	6964864	7050987	0	0.027276
15384	Amp	10	7053656	7056457	0	0.033298
15385	Amp	10	7063546	7097556	0	0.03203
15386	Amp	10	7097890	7143170	0	0.026009
15387	Amp	10	7150867	7788712	0	0.025282
15388	Amp	10	7789535	8015675	0	0.0304
15389	Amp	10	8016438	8048038	0	0.033293
15390	Amp	10	8059464	8059964	0	0.034065
15391	Amp	10	8062245	8062245	0	0.036681
15392	Amp	10	8068061	8073399	0	0.048283
15393	Amp	10	8074116	8075242	0	0.04539
15394	Amp	10	8077695	8089877	0	0.048778
15395	Amp	10	8090623	8092056	0	0.046162
15396	Amp	10	8096312	8096968	0	0.042774
15397	Amp	10	8100049	8180882	0	0.034852
15398	Amp	10	8181374	8199613	0	0.031172
15399	Amp	10	8199755	8235465	0	0.031685
15400	Amp	10	8236404	8441597	0	0.03255
15401	Amp	10	8442882	8493210	0	0.031477
15402	Amp	10	8494246	8702058	0	0.031919
15403	Amp	10	8702278	8722038	0	0.033266
15404	Amp	10	8722717	8853246	0	0.035
15405	Amp	10	8858053	8866608	0	0.037759
15406	Amp	10	8867961	8867961	0	0.039105
15407	Amp	10	8918380	8919651	0	0.044133
15408	Amp	10	8919808	8957327	0	0.044843
15409	Amp	10	8958878	8981662	0	0.047519
15410	Amp	10	8983075	8995310	0	0.049781
15411	Amp	10	8995529	9048262	0	0.052088
15412	Amp	10	9051887	9051887	0	0.049781
15413	Amp	10	9056421	9072408	0	0.04293
15414	Amp	10	9075594	9096447	0	0.043587
15415	Amp	10	9097301	9120963	0	0.041325
15416	Amp	10	9124382	9139845	0	0.040726
15417	Amp	10	9140785	9182131	0	0.039381
15418	Amp	10	9187263	9231669	0	0.036705
15419	Amp	10	9234417	9259729	0	0.033945
15420	Amp	10	9260662	9313704	0	0.033235
15421	Amp	10	9314004	9314668	0	0.030206
15422	Amp	10	9314766	9332605	0	0.031891

15423	Amp	10	9333192	9333192	0	0.033607
15424	Amp	10	9333577	9342681	0	0.03577
15425	Amp	10	9343123	9355857	0	0.034999
15426	Amp	10	9355980	9362212	0	0.033
15427	Amp	10	9364613	9387856	0	0.031315
15428	Amp	10	9390119	9519376	0	0.027436
15429	Amp	10	9533366	9535922	0	0.027982
15430	Amp	10	9536321	9736443	0	0.027325
15431	Amp	10	9739764	9804780	0	0.027837
15432	Amp	10	9804836	9869005	0	0.027395
15433	Amp	10	9878519	10152018	0	0.025556
15434	Amp	10	10155276	10196260	0	0.024701
15435	Amp	10	10196353	10335167	0	0.027936
15436	Amp	10	10336050	10454818	0	0.029797
15437	Amp	10	10454848	10551064	0	0.029048
15438	Amp	10	10551435	10832517	0	0.030598
15439	Amp	10	10832840	10835782	0	0.028649
15440	Amp	10	10836104	10975057	0	0.026727
15441	Amp	10	10981756	11005765	0	0.030254
15442	Amp	10	11006301	11046048	0	0.026727
15443	Amp	10	11046653	11100606	0	0.026214
15444	Amp	10	11100634	11127199	0	0.029216
15445	Amp	10	11131340	11191694	0	0.031751
15446	Amp	10	11194581	11473274	0	0.02989
15447	Amp	10	11478907	11483446	0	0.035944
15448	Amp	10	11487656	11490659	0	0.036545
15449	Amp	10	11499745	11553255	0	0.040926
15450	Amp	10	11555967	11623332	0	0.037924
15451	Amp	10	11628479	12039387	0	0.035389
15452	Amp	10	12049517	12159304	0	0.031498
15453	Amp	10	12165888	12183311	0	0.035368
15454	Amp	10	12191823	12370244	0	0.03488
15455	Amp	10	12372071	12481130	0	0.031009
15456	Amp	10	12485075	12718933	0	0.038336
15457	Amp	10	12719685	12767738	0	0.037471
15458	Amp	10	12768571	12836938	0	0.043571
15459	Amp	10	12851545	12886388	0	0.045885
15460	Amp	10	12887400	12911911	0	0.043571
15461	Amp	10	12915560	13255383	0	0.040741
15462	Amp	10	13255574	13342638	0	0.040228
15463	Amp	10	13343970	13368048	0	0.044725
15464	Amp	10	13369415	13372910	0	0.047638
15465	Amp	10	13373986	13385173	0	0.043141
15466	Amp	10	13385224	13625645	0	0.040228
15467	Amp	10	13625731	13778420	0	0.036975
15468	Amp	10	13778804	13801359	0	0.031548
15469	Amp	10	13801530	14033897	0	0.030624
15470	Amp	10	14036853	14139852	0	0.029822
15471	Amp	10	14141106	14311427	0	0.028272
15472	Amp	10	14311546	14336251	0	0.020271
15473	Amp	10	14336253	14347444	0	0.02648
15474	Amp	10	14347994	14887731	0	0.020271
15475	Amp	10	14888732	14933280	0	0.021296

15476 Amp	10	15028534	15108988	0	0.024579
15477 Amp	10	15113962	15169740	0	0.025627
15478 Amp	10	15173316	15175600	0	0.037023
15479 Amp	10	15181404	15265226	0	0.033739
15480 Amp	10	15283015	15634149	0	0.021296
15481 Amp	10	15637722	15646012	0	0.020694
15482 Amp	10	15651219	15675487	0	0.012827
15483 Amp	10	15676653	15712729	0	0.013354
15484 Amp	10	15719210	15760745	0	0.008985
15485 Amp	10	15760980	15844627	0	0.007713
15486 Amp	10	15848339	15938829	0	0.005794
15487 Amp	10	15941538	16924767	0	0.007161
15488 Amp	10	16927294	17037933	0	0.01097
15489 Amp	10	17043499	17204240	0	0.012126
15490 Amp	10	17204586	17245538	0	0.01097
15491 Amp	10	17245754	17283017	0	0.009603
15492 Amp	10	17286829	17395353	0	0.013925
15493 Amp	10	17396337	17540992	0	0.01213
15494 Amp	10	17541490	17565985	0	0.012683
15495 Amp	10	17568278	17581256	0	0.010548
15496 Amp	10	17586777	17696892	0	0.012715
15497 Amp	10	17698134	17711942	0	0.016234
15498 Amp	10	17712538	17735537	0	0.012715
15499 Amp	10	17953706	17958748	0	0.008393
15500 Amp	10	17959166	18060025	0	0.00784
15501 Amp	10	18068393	18578219	0	0.009552
15502 Amp	10	18578909	18706434	0	0.00784
15503 Amp	10	18707704	18735694	0	0.005673
15504 Amp	10	18737378	18744063	0	0.008293
15505 Amp	10	18744258	19477126	0	0.005673
15506 Amp	10	19478215	19506116	0	0.007254
15507 Amp	10	19506465	19563715	0	0.006708
15508 Amp	10	19563952	19632341	0	0.005126
15509 Amp	10	19636172	19696076	0	0.007439
15510 Amp	10	19697986	19769354	0	0.005126
15511 Amp	10	19773443	20060948	0	0.004102
15512 Amp	10	20061882	20237863	0	0.004572
15513 Amp	10	20247020	20789493	0	0.006775
15514 Amp	10	20790825	21103739	0	0.004761
15515 Amp	10	21112802	21139550	0	0.007016
15516 Amp	10	21141814	21181626	0	0.004761
15517 Amp	10	21182521	21328618	0	0.007975
15518 Amp	10	21330706	21400524	0	0.00962
15519 Amp	10	21404377	21404700	0	0.012915
15520 Amp	10	21406126	21435653	0	0.009204
15521 Amp	10	21439006	21485923	0	0.013226
15522 Amp	10	21486385	21611801	0	0.011582
15523 Amp	10	21613589	21759873	0	0.007559
15524 Amp	10	21760915	21857148	0	0.004264
15525 Amp	10	21857407	22041794	0	0.006222
15526 Amp	10	22042050	22174049	0	0.010249
15527 Amp	10	22176926	22200693	0	0.006222
15528 Amp	10	22200898	22226913	0	0.009037

15529 Amp	10	22244615	22324001	0	0.006222
15530 Amp	10	22330358	22454518	0	0.004134
15531 Amp	10	22454537	22541989	0	0.002177
15532 Amp	10	22544224	22593103	0	0.002789
15533 Amp	10	22593152	22670048	0	0.005532
15534 Amp	10	22677272	22744362	0	0.006211
15535 Amp	10	22751937	22881836	0	0.010796
15536 Amp	10	22894648	23066353	0	0.004505
15537 Amp	10	23069148	23111063	0	0.007117
15538 Amp	10	23113703	23241349	0	0.004505
15539 Amp	10	23243753	23650253	0	0.005364
15540 Amp	10	23650332	24243222	0	0.009548
15541 Amp	10	24245110	24423274	0	0.005364
15542 Amp	10	24423407	24469188	0	0.007557
15543 Amp	10	24470160	24490482	0	0.005638
15544 Amp	10	24490828	24784827	0	0.003445
15545 Amp	10	24785520	24978174	0	0.002975
15546 Amp	10	24988643	25037326	0	0.005759
15547 Amp	10	25039735	25318521	0	0.002975
15548 Amp	10	25324662	25337343	0	0.004852
15549 Amp	10	25361105	25444351	0	0.016867
15550 Amp	10	25446702	25495623	0	0.016418
15551 Amp	10	25496566	25735659	0	0.01709
15552 Amp	10	25736313	25852482	0	0.007256
15553 Amp	10	25887122	25972357	0	0.007749
15554 Amp	10	25975471	26003941	0	0.009185
15555 Amp	10	26003978	26014647	0	0.005337
15556 Amp	10	26015143	26111231	0	0.005918
15557 Amp	10	26112662	26150675	0	0.008886
15558 Amp	10	26164239	26175810	0	0.005918
15559 Amp	10	26176497	26196684	0	0.004482
15560 Amp	10	26197999	26568132	0	0.003901
15561 Amp	10	26569931	26582328	0	0.007052
15562 Amp	10	26584309	26667566	0	0.011416
15563 Amp	10	26670273	26689929	0	0.014291
15564 Amp	10	26693477	26761963	0	0.015743
15565 Amp	10	26764127	26844891	0	0.016522
15566 Amp	10	26847847	26909886	0	0.01585
15567 Amp	10	26912006	27089444	0	0.013973
15568 Amp	10	27089513	27534780	0	0.016871
15569 Amp	10	27535730	27560158	0	0.018168
15570 Amp	10	27561966	27766692	0	0.016275
15571 Amp	10	27768926	27822032	0	0.011911
15572 Amp	10	27831816	27939936	0	0.010613
15573 Amp	10	27942137	28014053	0	0.007378
15574 Amp	10	28017921	28529077	0	0.006081
15575 Amp	10	28530477	28536760	0	0.008723
15576 Amp	10	28543986	28599960	0	0.005571
15577 Amp	10	28600419	28617350	0	0.002929
15578 Amp	10	28620186	28816524	0	0.004573
15579 Amp	10	28816935	28856934	0	0.006427
15580 Amp	10	28857135	28975183	0	0.009422
15581 Amp	10	28977712	28977712	0	0.007568

15582	Amp	10	28977827	29014352	0	0.005925
15583	Amp	10	29014663	29018049	0	0.009148
15584	Amp	10	29018499	29028820	0	0.006153
15585	Amp	10	29029128	29130868	0	0.008367
15586	Amp	10	29131476	29161953	0	0.011072
15587	Amp	10	29162172	29220865	0	0.008367
15588	Amp	10	29222102	29253071	0	0.005144
15589	Amp	10	29253324	29332232	0	0.005677
15590	Amp	10	29339623	29472385	0	0.008064
15591	Amp	10	29474659	29596014	0	0.005677
15592	Amp	10	29596735	30302276	0	0.007529
15593	Amp	10	30302315	30395193	0	0.005677
15594	Amp	10	30397100	30427468	0	0.006285
15595	Amp	10	30442333	30462021	0	0.022221
15596	Amp	10	30462476	30586761	0	0.022727
15597	Amp	10	30590981	30611206	0	0.021149
15598	Amp	10	30613119	30669174	0	0.020644
15599	Amp	10	30671749	31297843	0	0.021194
15600	Amp	10	31305582	31328726	0	0.018979
15601	Amp	10	31332499	31479394	0	0.018446
15602	Amp	10	31483837	31554473	0	0.01962
15603	Amp	10	31560220	31563336	0	0.005087
15604	Amp	10	31567660	31620191	0	0.00687
15605	Amp	10	31622838	31792717	0	0.009047
15606	Amp	10	31793730	31823356	0	0.007265
15607	Amp	10	31828158	31877655	0	0.009413
15608	Amp	10	31879745	32014716	0	0.008863
15609	Amp	10	32017944	32168833	0	0.006686
15610	Amp	10	32169655	32232158	0	0.008282
15611	Amp	10	32232522	32391849	0	0.006686
15612	Amp	10	32392826	32404911	0	0.00996
15613	Amp	10	32404925	32452414	0	0.006686
15614	Amp	10	32456631	32545115	0	0.004537
15615	Amp	10	32545315	32584910	0	0.007284
15616	Amp	10	32589162	32738819	0	0.004537
15617	Amp	10	32742985	33125820	0	0.005331
15618	Amp	10	33129446	33276891	0	0.007779
15619	Amp	10	33277640	33323572	0	0.006986
15620	Amp	10	33323616	33435210	0	0.008338
15621	Amp	10	33438309	33929165	0	0.007164
15622	Amp	10	33933479	34018532	0	0.010385
15623	Amp	10	34021609	34377936	0	0.007164
15624	Amp	10	34381612	34518283	0	0.010177
15625	Amp	10	34522383	34878243	0	0.008774
15626	Amp	10	35038907	35050396	0	0.01499
15627	Amp	10	35060545	35218285	0	0.011977
15628	Amp	10	35318312	35682762	0	0.00576
15629	Amp	10	35684585	35824622	0	0.009802
15630	Amp	10	35825478	36040648	0	0.00576
15631	Amp	10	36044117	36148019	0	0.012614
15632	Amp	10	36152547	36198655	0	0.016797
15633	Amp	10	36205437	36206821	0	0.018691
15634	Amp	10	36208801	36346566	0	0.011837

15635	Amp	10	36348093	36362740	0	0.013067
15636	Amp	10	36365535	36412016	0	0.008885
15637	Amp	10	36412441	36417293	0	0.006991
15638	Amp	10	36420661	36453086	0	0.009875
15639	Amp	10	36457839	36473624	0	0.008645
15640	Amp	10	36476374	36504814	0	0.006658
15641	Amp	10	36504990	36508515	0	0.008193
15642	Amp	10	36512533	36512533	0	0.00929
15643	Amp	10	36513160	36574371	0	0.009851
15644	Amp	10	36574591	36604980	0	0.010697
15645	Amp	10	36606356	36611672	0	0.011364
15646	Amp	10	36612152	36612446	0	0.013042
15647	Amp	10	36612700	36740206	0	0.012183
15648	Amp	10	36741148	36749912	0	0.012713
15649	Amp	10	36755632	36807234	0	0.012046
15650	Amp	10	36808902	36863959	0	0.012743
15651	Amp	10	36866464	37026417	0	0.014473
15652	Amp	10	37028150	37044907	0	0.017018
15653	Amp	10	37053848	37054229	0	0.016172
15654	Amp	10	37054963	37087221	0	0.017357
15655	Amp	10	37087408	37091546	0	0.018326
15656	Amp	10	37095646	37159173	0	0.023474
15657	Amp	10	37165469	37165778	0	0.035092
15658	Amp	10	37193077	37193077	0	0.030817
15659	Amp	10	37195831	37238870	0	0.033648
15660	Amp	10	37242651	37242651	0	0.036205
15661	Amp	10	37252055	37273915	0	0.035674
15662	Amp	10	37276059	37290642	0	0.037213
15663	Amp	10	37293451	37326650	0	0.036028
15664	Amp	10	37326699	37377290	0	0.032027
15665	Amp	10	37377741	37380604	0	0.030488
15666	Amp	10	37380855	37381441	0	0.028811
15667	Amp	10	37390020	37408974	0	0.02598
15668	Amp	10	37412655	37417376	0	0.025283
15669	Amp	10	37425393	37429413	0	0.029917
15670	Amp	10	37429526	37446839	0	0.03255
15671	Amp	10	37453228	37500745	0	0.033832
15672	Amp	10	37502702	37588404	0	0.028052
15673	Amp	10	37590038	37744621	0	0.016434
15674	Amp	10	37747367	37777463	0	0.014445
15675	Amp	10	37783130	37798073	0	0.011997
15676	Amp	10	37798474	37803439	0	0.013125
15677	Amp	10	37804047	37942683	0	0.011666
15678	Amp	10	37944445	37985033	0	0.01249
15679	Amp	10	37989976	38092140	0	0.015292
15680	Amp	10	38094199	38347116	0	0.027224
15681	Amp	10	38347674	38388861	0	0.026089
15682	Amp	10	38388941	38397214	0	0.024807
15683	Amp	10	38407748	38467160	0	0.023678
15684	Amp	10	38619590	38702233	0	0.024163
15685	Amp	10	38706905	38706905	0	0.023194
15686	Amp	10	38706914	38752615	0	0.015468
15687	Amp	10	38752733	38770504	0	0.016496

15688	Amp	10	38770824	38770921	0	0.014961
15689	Amp	10	42450960	42457683	0	0.026716
15690	Amp	10	42459603	42459603	0	0.027447
15691	Amp	10	42460545	42462363	0	0.028282
15692	Amp	10	42462400	42493650	0	0.029997
15693	Amp	10	42494825	42579840	0	0.031928
15694	Amp	10	42583525	42672076	0	0.031359
15695	Amp	10	42678121	42701665	0	0.031853
15696	Amp	10	42704010	42780995	0	0.033399
15697	Amp	10	42785952	42935908	0	0.029692
15698	Amp	10	42936855	42944698	0	0.030668
15699	Amp	10	42946165	43007809	0	0.031322
15700	Amp	10	43010596	43013151	0	0.039903
15701	Amp	10	43016276	43055119	0	0.040565
15702	Amp	10	43057447	43062355	0	0.039903
15703	Amp	10	43066597	43092812	0	0.031322
15704	Amp	10	43092904	43093583	0	0.026587
15705	Amp	10	43093826	43153606	0	0.031506
15706	Amp	10	43154790	43162474	0	0.032956
15707	Amp	10	43162783	43298177	0	0.031928
15708	Amp	10	43305406	43309500	0	0.030831
15709	Amp	10	43310988	43354132	0	0.029614
15710	Amp	10	43354343	43412047	0	0.028638
15711	Amp	10	43413735	43445450	0	0.027188
15712	Amp	10	43445587	43469650	0	0.028143
15713	Amp	10	43471180	43545184	0	0.030399
15714	Amp	10	43550724	43582103	0	0.027285
15715	Amp	10	43583273	43674444	0	0.028608
15716	Amp	10	43676014	43680264	0	0.030737
15717	Amp	10	43685063	43801445	0	0.032042
15718	Amp	10	43803434	43854589	0	0.030568
15719	Amp	10	43857226	44122875	0	0.035481
15720	Amp	10	44125762	44251611	0	0.032679
15721	Amp	10	44252880	44256820	0	0.039859
15722	Amp	10	44256871	44256882	0	0.038121
15723	Amp	10	44257528	44274932	0	0.038626
15724	Amp	10	44278450	44373979	0	0.031446
15725	Amp	10	44374567	44421422	0	0.033367
15726	Amp	10	44422479	44455608	0	0.031446
15727	Amp	10	44457384	44477400	0	0.035151
15728	Amp	10	44479971	44516894	0	0.037875
15729	Amp	10	44517498	44520062	0	0.037324
15730	Amp	10	44522759	44544531	0	0.033619
15731	Amp	10	44544822	44698139	0	0.032456
15732	Amp	10	44698403	44872584	0	0.033884
15733	Amp	10	44874619	44887269	0	0.033379
15734	Amp	10	44888620	44899513	0	0.034677
15735	Amp	10	44900134	44900134	0	0.033853
15736	Amp	10	44906877	44951951	0	0.036138
15737	Amp	10	44955730	45057129	0	0.033853
15738	Amp	10	45060180	45100068	0	0.032425
15739	Amp	10	45100576	45368214	0	0.031127
15740	Amp	10	45369170	45494672	0	0.032129

15741	Amp	10	45502109	45574604	0	0.030786
15742	Amp	10	45602247	45622525	0	0.030178
15743	Amp	10	46013136	46013136	0	0.028138
15744	Amp	10	46015804	46015804	0	0.026834
15745	Amp	10	46016391	46041001	0	0.025832
15746	Amp	10	47084490	47117945	0	0.017466
15747	Amp	10	47119765	47250114	0	0.018919
15748	Amp	10	47250475	47326963	0	0.017562
15749	Amp	10	47327025	47327025	0	0.01552
15750	Amp	10	48188464	48188464	0	0.011969
15751	Amp	10	48189189	48569770	0	0.012726
15752	Amp	10	48570377	48599856	0	0.015202
15753	Amp	10	48600993	49023835	0	0.016946
15754	Amp	10	49025932	49499119	0	0.015594
15755	Amp	10	49500800	49579102	0	0.019733
15756	Amp	10	49582144	49604977	0	0.021864
15757	Amp	10	49605547	49663670	0	0.024363
15758	Amp	10	49664200	49724325	0	0.02654
15759	Amp	10	49727028	49779263	0	0.036071
15760	Amp	10	49780713	49849300	0	0.033895
15761	Amp	10	49863777	49868126	0	0.030446
15762	Amp	10	50716389	50808229	0	0.019587
15763	Amp	10	50818242	51038127	0	0.018829
15764	Amp	10	51038772	51038772	0	0.020207
15765	Amp	10	51045213	51245446	0	0.017708
15766	Amp	10	51246128	51271991	0	0.01633
15767	Amp	10	51272886	51457463	0	0.017323
15768	Amp	10	51457762	51971740	0	0.015192
15769	Amp	10	51971863	52204068	0	0.0142
15770	Amp	10	52206972	52210298	0	0.016092
15771	Amp	10	52210993	52338499	0	0.016803
15772	Amp	10	52338909	52619626	0	0.014911
15773	Amp	10	52619691	52851053	0	0.0142
15774	Amp	10	52851531	52917628	0	0.011222
15775	Amp	10	52918353	52920642	0	0.014267
15776	Amp	10	52920826	52925580	0	0.01494
15777	Amp	10	52926443	52934206	0	0.017174
15778	Amp	10	52936205	52973183	0	0.014129
15779	Amp	10	52977101	53369138	0	0.011895
15780	Amp	10	53369151	53563655	0	0.011321
15781	Amp	10	53569014	53841341	0	0.01225
15782	Amp	10	53841510	53898617	0	0.013408
15783	Amp	10	53904245	53975227	0	0.012478
15784	Amp	10	53975703	54101357	0	0.011321
15785	Amp	10	54106148	54111563	0	0.016182
15786	Amp	10	54112631	54377093	0	0.011321
15787	Amp	10	54377683	54487038	0	0.013216
15788	Amp	10	54490611	54573206	0	0.011321
15789	Amp	10	54575800	54849490	0	0.013405
15790	Amp	10	54936124	55041140	0	0.015441
15791	Amp	10	55042088	55060172	0	0.017713
15792	Amp	10	55063118	55148139	0	0.015629
15793	Amp	10	55579314	55670529	0	0.040591

15794 Amp	10	55670757	55670757	0	0.041398
15795 Amp	10	55971356	56115521	0	0.038141
15796 Amp	10	56117357	56219404	0	0.02224
15797 Amp	10	56219998	56231471	0	0.023449
15798 Amp	10	56235874	56329809	0	0.02224
15799 Amp	10	56330503	56398947	0	0.019969
15800 Amp	10	56402132	56479903	0	0.025948
15801 Amp	10	56494849	56606410	0	0.027991
15802 Amp	10	56607869	56903250	0	0.027183
15803 Amp	10	56904387	56930959	0	0.027936
15804 Amp	10	56935490	56972595	0	0.028734
15805 Amp	10	56972972	57178792	0	0.026691
15806 Amp	10	57180217	57206911	0	0.023696
15807 Amp	10	57210142	57335807	0	0.024746
15808 Amp	10	57337889	57679501	0	0.023696
15809 Amp	10	57679562	57681709	0	0.020972
15810 Amp	10	57685107	57883971	0	0.023934
15811 Amp	10	57887645	58054041	0	0.02095
15812 Amp	10	58056897	58392380	0	0.017987
15813 Amp	10	58396406	58504445	0	0.020163
15814 Amp	10	58504572	58526247	0	0.021491
15815 Amp	10	58526745	58815003	0	0.020163
15816 Amp	10	58816927	59005458	0	0.022153
15817 Amp	10	59006740	59037471	0	0.024163
15818 Amp	10	59038113	59051443	0	0.034799
15819 Amp	10	59052089	59156065	0	0.024163
15820 Amp	10	59159994	59398286	0	0.026513
15821 Amp	10	59402951	59421759	0	0.025716
15822 Amp	10	59421966	59436284	0	0.024963
15823 Amp	10	59437005	59503654	0	0.027054
15824 Amp	10	59505036	59582233	0	0.024963
15825 Amp	10	59586408	59689754	0	0.040899
15826 Amp	10	59689806	59705569	0	0.044346
15827 Amp	10	59711206	59786577	0	0.03999
15828 Amp	10	59786916	59794248	0	0.042837
15829 Amp	10	59795145	60021963	0	0.045815
15830 Amp	10	60023710	60077036	0	0.053783
15831 Amp	10	60080073	60081768	0	0.050805
15832 Amp	10	60087282	60200377	0	0.044511
15833 Amp	10	60200947	60476437	0	0.042035
15834 Amp	10	60479626	60523832	0	0.043897
15835 Amp	10	60525273	60556899	0	0.046206
15836 Amp	10	60564770	60620366	0	0.048393
15837 Amp	10	60622629	60660372	0	0.046403
15838 Amp	10	60662629	60747445	0	0.047605
15839 Amp	10	60750731	60798853	0	0.045742
15840 Amp	10	60800780	60805210	0	0.050098
15841 Amp	10	60805386	60813126	0	0.047789
15842 Amp	10	60814719	60831012	0	0.031853
15843 Amp	10	60831716	60840529	0	0.029666
15844 Amp	10	60843236	60860084	0	0.032713
15845 Amp	10	60864644	60883410	0	0.044574
15846 Amp	10	60886115	61006934	0	0.041527

15847	Amp	10	61010825	61013887	0	0.03992
15848	Amp	10	61013933	61386078	0	0.043995
15849	Amp	10	61386789	61427054	0	0.048525
15850	Amp	10	61431492	61706656	0	0.041985
15851	Amp	10	61706676	61728752	0	0.035267
15852	Amp	10	61728814	61754514	0	0.037248
15853	Amp	10	61759482	61816528	0	0.039527
15854	Amp	10	61817886	61844942	0	0.04069
15855	Amp	10	61845154	61906932	0	0.04137
15856	Amp	10	61910044	61928913	0	0.036627
15857	Amp	10	61928969	62019911	0	0.037366
15858	Amp	10	62020696	62055155	0	0.041061
15859	Amp	10	62057657	62060356	0	0.04247
15860	Amp	10	62064872	62102875	0	0.045422
15861	Amp	10	62107304	62109238	0	0.041728
15862	Amp	10	62115616	62119962	0	0.03251
15863	Amp	10	62124334	62129190	0	0.03177
15864	Amp	10	62129805	62246013	0	0.028818
15865	Amp	10	62254336	62263730	0	0.03453
15866	Amp	10	62274926	62368374	0	0.03731
15867	Amp	10	62377308	62422305	0	0.033367
15868	Amp	10	62422468	62440119	0	0.031412
15869	Amp	10	62441932	62494472	0	0.030731
15870	Amp	10	62497071	62557051	0	0.032527
15871	Amp	10	62557490	62583506	0	0.031239
15872	Amp	10	62589908	62771400	0	0.043431
15873	Amp	10	62772433	62918440	0	0.044036
15874	Amp	10	62918776	63060423	0	0.031843
15875	Amp	10	63064211	63451742	0	0.030433
15876	Amp	10	63452480	63454827	0	0.031856
15877	Amp	10	63454966	63454966	0	0.032847
15878	Amp	10	63458633	63588492	0	0.045291
15879	Amp	10	63591774	63836338	0	0.045766
15880	Amp	10	63846099	63846099	0	0.047211
15881	Amp	10	63846286	63846286	0	0.050682
15882	Amp	10	63846289	63953431	0	0.04926
15883	Amp	10	63953733	63976293	0	0.047815
15884	Amp	10	63976696	64027239	0	0.048301
15885	Amp	10	64032183	64061848	0	0.04483
15886	Amp	10	64066935	64151533	0	0.044344
15887	Amp	10	64152092	64190903	0	0.045211
15888	Amp	10	64192716	64673045	0	0.04323
15889	Amp	10	64673096	64693618	0	0.042363
15890	Amp	10	64693622	64749291	0	0.040512
15891	Amp	10	64749431	64759167	0	0.042011
15892	Amp	10	64761897	64792087	0	0.044357
15893	Amp	10	64795504	64816499	0	0.042011
15894	Amp	10	64818450	65081869	0	0.044313
15895	Amp	10	65082386	65084558	0	0.041259
15896	Amp	10	65092949	65160730	0	0.038909
15897	Amp	10	65161653	65194245	0	0.024669
15898	Amp	10	65197387	65211133	0	0.026766
15899	Amp	10	65213004	65278940	0	0.027803

15900	Amp	10	65283100	65337634	0	0.030366
15901	Amp	10	65346737	65388251	0	0.036204
15902	Amp	10	65394301	65416300	0	0.034107
15903	Amp	10	65416509	65458228	0	0.03793
15904	Amp	10	65458585	65500959	0	0.034107
15905	Amp	10	65580420	65657376	0	0.032795
15906	Amp	10	65658083	65741140	0	0.036183
15907	Amp	10	65741219	65790181	0	0.03793
15908	Amp	10	65790485	65807031	0	0.036892
15909	Amp	10	65807975	65822593	0	0.040463
15910	Amp	10	65824322	65871987	0	0.032214
15911	Amp	10	65874192	65881829	0	0.035922
15912	Amp	10	65884788	65902953	0	0.034175
15913	Amp	10	65903654	66022240	0	0.030788
15914	Amp	10	66039889	66074627	0	0.032218
15915	Amp	10	66081065	66126233	0	0.02994
15916	Amp	10	66132234	66147483	0	0.032292
15917	Amp	10	66150372	66176123	0	0.033985
15918	Amp	10	66177588	66199070	0	0.032555
15919	Amp	10	66204531	66238651	0	0.046795
15920	Amp	10	66239488	66289254	0	0.047623
15921	Amp	10	66289713	66289713	0	0.054621
15922	Amp	10	66789964	66792631	0.118001	0.068864
15923	Amp	10	66796599	66935610	0.164935	0.070831
15924	Amp	10	66939396	66963757	0.238246	0.073757
15925	Amp	10	66967071	66967086	0.271552	0.07525
15926	Amp	10	66967221	66967221	0.431198	0.08097
15927	Amp	10	67161806	67245437	0.47716	0.082298
15928	Amp	10	67254543	67377049	0.431198	0.080805
15929	Amp	10	67377336	67433843	0.362016	0.078453
15930	Amp	10	67450440	67524477	0	0.051157
15931	Amp	10	67526870	67576996	0	0.052343
15932	Amp	10	67586608	67627250	0	0.057028
15933	Amp	10	67629460	67707282	0.222041	0.072964
15934	Amp	10	67711515	67767188	0.251218	0.074346
15935	Amp	10	67769173	67825670	0.437198	0.08102
15936	Amp	10	67831473	67863513	0.522881	0.08397
15937	Amp	10	67864037	67882661	0.437198	0.081044
15938	Amp	10	67887271	67887526	0.282828	0.075781
15939	Amp	10	67890354	67905464	0.251218	0.074399
15940	Amp	10	67910008	67970108	0.210594	0.07258
15941	Amp	10	67970138	68136434	0.187449	0.071695
15942	Amp	10	68136579	68292198	0.259438	0.074845
15943	Amp	10	68293761	68304233	0.005328	0.064172
15944	Amp	10	68304248	68304341	0	0.061488
15945	Amp	10	68311184	68408543	0	0.05903
15946	Amp	10	68410900	68443452	0	0.043519
15947	Amp	10	68443477	68477885	0	0.047073
15948	Amp	10	68482708	68492961	0	0.04856
15949	Amp	10	68493137	68510208	0	0.045006
15950	Amp	10	68511518	68579504	0	0.031528
15951	Amp	10	68581456	68645782	0	0.032401
15952	Amp	10	68646098	68705225	0	0.02951

15953	Amp	10	68705373	68725744	0	0.025158
15954	Amp	10	68727506	68825821	0	0.029816
15955	Amp	10	68825943	68971047	0	0.030474
15956	Amp	10	68971776	69031503	0	0.029816
15957	Amp	10	69032585	69048275	0	0.030787
15958	Amp	10	69051229	69076358	0	0.029914
15959	Amp	10	69082267	69111374	0	0.034088
15960	Amp	10	69111842	69112425	0	0.04925
15961	Amp	10	69114060	69168218	0	0.048279
15962	Amp	10	69171718	69254657	0	0.048755
15963	Amp	10	69255633	69431264	0.015369	0.064691
15964	Amp	10	69434432	69757424	0.08435	0.067585
15965	Amp	10	69758170	69822027	0.040093	0.065693
15966	Amp	10	69822699	69858864	0	0.063412
15967	Amp	10	69858949	70087830	0.019169	0.064915
15968	Amp	10	70088678	70150506	0	0.062085
15969	Amp	10	70150797	70532718	0	0.046149
15970	Amp	10	70533812	70613462	0	0.052998
15971	Amp	10	70615502	70615892	0	0.051495
15972	Amp	10	70616045	70874210	0	0.051997
15973	Amp	10	70879328	70898281	0	0.06013
15974	Amp	10	70904392	70998972	0	0.062324
15975	Amp	10	71001176	71130546	0	0.063106
15976	Amp	10	71130618	71203358	0	0.060039
15977	Amp	10	71206851	71211981	0	0.056677
15978	Amp	10	71215705	71236288	0.210594	0.072613
15979	Amp	10	71236477	71251980	0	0.056677
15980	Amp	10	71254763	71313495	0	0.057181
15981	Amp	10	71320878	71494231	0	0.056399
15982	Amp	10	71497223	71504984	0	0.057453
15983	Amp	10	71505762	71572750	0	0.058016
15984	Amp	10	71573595	71577401	0	0.061737
15985	Amp	10	71577660	71733768	0	0.061261
15986	Amp	10	71745879	71745879	0	0.062711
15987	Amp	10	71746372	71895898	0.010261	0.064459
15988	Amp	10	71896723	72045641	0.060686	0.066478
15989	Amp	10	72046812	72048709	0	0.061716
15990	Amp	10	72050183	72052718	0	0.058966
15991	Amp	10	72055169	72073597	0	0.061902
15992	Amp	10	72081914	72106147	0	0.054351
15993	Amp	10	72106890	72131053	0	0.0529
15994	Amp	10	72132547	72142747	0	0.050881
15995	Amp	10	72151337	72196061	0	0.049138
15996	Amp	10	72196237	72227423	0	0.051447
15997	Amp	10	72233145	72267782	0	0.053601
15998	Amp	10	72268339	72349701	0	0.050665
15999	Amp	10	72352428	72373979	0.040093	0.065709
16000	Amp	10	72375477	72415453	0.089561	0.067783
16001	Amp	10	72415657	72458929	0.138391	0.06973
16002	Amp	10	72461447	72493718	0.19964	0.072117
16003	Amp	10	72494482	72494482	0.299433	0.076235
16004	Amp	10	72496272	72518615	0.602643	0.086557
16005	Amp	10	72519386	72575000	0.630438	0.087489

16006	Amp	10	72584262	72776400	0.689884	0.089167
16007	Amp	10	72782106	72845414	0.622604	0.087093
16008	Amp	10	72846503	72877255	0.4115	0.08031
16009	Amp	10	72883417	72960204	0.431198	0.080858
16010	Amp	10	72960240	73002790	0.470509	0.082259
16011	Amp	10	73003026	73021559	0.431198	0.080858
16012	Amp	10	73023728	73034011	0.4115	0.080297
16013	Amp	10	73034746	73081327	0.704849	0.089668
16014	Amp	10	73082831	73087876	0.815228	0.092657
16015	Amp	10	73091883	73110749	0.840289	0.093158
16016	Amp	10	73116129	73116129	0.924746	0.095384
16017	Amp	10	73118686	73128712	0.781972	0.091751
16018	Amp	10	73129080	73134388	0.636464	0.087709
16019	Amp	10	73134617	73211080	0.457501	0.081784
16020	Amp	10	73212576	73236069	0.329205	0.077349
16021	Amp	10	73242237	73268204	0.316877	0.076873
16022	Amp	10	73269718	73360301	0.299433	0.076371
16023	Amp	10	73376184	73447403	0.342649	0.077823
16024	Amp	10	73447729	73497399	0.329205	0.077323
16025	Amp	10	73509175	73518484	0.457501	0.081666
16026	Amp	10	73524213	73539577	0.417873	0.080371
16027	Amp	10	73541236	73562990	0.550755	0.084754
16028	Amp	10	73563400	73577099	0.487495	0.082806
16029	Amp	10	73578704	73578704	0.417873	0.080391
16030	Amp	10	73582710	73646383	0.47716	0.082308
16031	Amp	10	73650209	73658908	0.457501	0.081761
16032	Amp	10	73661450	73690961	0.368062	0.078771
16033	Amp	10	73693564	73724874	0.404718	0.08006
16034	Amp	10	73727740	73793821	0.470509	0.082097
16035	Amp	10	73794783	73794783	0.482379	0.082685
16036	Amp	10	73795544	73818085	0.557122	0.085062
16037	Amp	10	73828696	73859444	0.622604	0.087081
16038	Amp	10	73869057	73971837	0.557122	0.085044
16039	Amp	10	73974064	73974064	0.494792	0.083098
16040	Amp	10	73977369	73983860	0.47716	0.082494
16041	Amp	10	73986309	73990558	0.437198	0.081204
16042	Amp	10	73995773	74019047	0.576183	0.08556
16043	Amp	10	74019565	74023627	0.6737	0.088629
16044	Amp	10	74023741	74026637	0.711903	0.08974
16045	Amp	10	74027366	74071725	0.751221	0.090784
16046	Amp	10	74072830	74116251	0.704849	0.089673
16047	Amp	10	74117758	74190349	1.032525	0.098089
16048	Amp	10	74192912	74305647	0.704849	0.089673
16049	Amp	10	74308474	74369770	1.358811	0.105609
16050	Amp	10	74373521	74415218	1.270711	0.103692
16051	Amp	10	74415749	74476330	1.388256	0.106204
16052	Amp	10	74477740	74490027	1.33853	0.10516
16053	Amp	10	74492070	74507765	1.492063	0.108726
16054	Amp	10	74508701	74510808	1.542638	0.109779
16055	Amp	10	74512172	74640504	1.665486	0.112386
16056	Amp	10	74646693	74667886	1.759227	0.114374
16057	Amp	10	74669821	74678823	1.854421	0.116232
16058	Amp	10	74685304	74701261	2.213223	0.123398

16059	Amp	10	74704744	74721553	2.116108	0.121411
16060	Amp	10	74725407	74803400	2.18114	0.122832
16061	Amp	10	74805587	74840090	2.684894	0.132577
16062	Amp	10	74843724	74854104	2.490318	0.128883
16063	Amp	10	74854742	74873734	2.368227	0.126506
16064	Amp	10	74873906	74924027	2.468223	0.128398
16065	Amp	10	74924103	75027250	2.548433	0.129901
16066	Amp	10	75027370	75085874	2.696333	0.13273
16067	Amp	10	75087630	75125202	2.649655	0.131837
16068	Amp	10	75125210	75133854	2.479057	0.128579
16069	Amp	10	75134677	75150835	2.502083	0.129043
16070	Amp	10	75151291	75156942	2.661431	0.132129
16071	Amp	10	75156944	75217359	2.514205	0.129299
16072	Amp	10	75219460	75230150	2.256542	0.124265
16073	Amp	10	75231465	75232449	2.18114	0.122753
16074	Amp	10	75241575	75299911	2.027585	0.119777
16075	Amp	10	75301791	75347266	2.468223	0.128531
16076	Amp	10	75351137	75383422	2.548433	0.130043
16077	Amp	10	75383709	75439419	2.390849	0.126957
16078	Amp	10	75441350	75445926	2.322689	0.125536
16079	Amp	10	75447327	75457197	2.192012	0.12303
16080	Amp	10	75463055	75568909	1.463774	0.108026
16081	Amp	10	75571232	75587627	1.445847	0.107521
16082	Amp	10	75587916	75613776	1.398396	0.106402
16083	Amp	10	75614399	75637133	1.140417	0.100685
16084	Amp	10	75643310	75651024	0.557122	0.084966
16085	Amp	10	75651143	75654902	0.463534	0.081931
16086	Amp	10	75655127	75730195	0.543344	0.084498
16087	Amp	10	75733172	75789566	0.891602	0.094487
16088	Amp	10	75795733	75827208	0.815228	0.092595
16089	Amp	10	75836852	75854051	0.933745	0.095755
16090	Amp	10	75854824	75872974	0.660254	0.088178
16091	Amp	10	75875888	75875888	0.34892	0.077971
16092	Amp	10	75876292	75912523	0.4115	0.080258
16093	Amp	10	75913871	76002485	0.482379	0.082671
16094	Amp	10	76005478	76046041	0.667587	0.088413
16095	Amp	10	76051988	76111118	0.563096	0.085253
16096	Amp	10	76111853	76142091	0.14896	0.070209
16097	Amp	10	76149485	76170100	0.299433	0.076363
16098	Amp	10	76170249	76170249	0.329205	0.077499
16099	Amp	10	76171072	76269649	0.522881	0.083967
16100	Amp	10	76270234	76289603	0.193543	0.071946
16101	Amp	10	76293288	76298332	0.322897	0.077207
16102	Amp	10	76300621	76353248	0.233782	0.073554
16103	Amp	10	76354896	76452635	0.271552	0.075216
16104	Amp	10	76453246	76505371	0.355319	0.078228
16105	Amp	10	76508877	76533756	0.276783	0.075483
16106	Amp	10	76534094	76541858	0.233782	0.073401
16107	Amp	10	76542130	76598828	0.251218	0.074435
16108	Amp	10	76599818	76748859	0.233782	0.073503
16109	Amp	10	76756836	76779194	0.143787	0.069937
16110	Amp	10	76779688	76787483	0.08435	0.067651
16111	Amp	10	76788408	76793084	0.138391	0.06979

16112 Amp	10	76803061	77023994	0.08435	0.067597
16113 Amp	10	77029456	77081607	0	0.058953
16114 Amp	10	77084850	77236180	0	0.056426
16115 Amp	10	77238961	77305394	0	0.053859
16116 Amp	10	77307967	77492524	0	0.054913
16117 Amp	10	77492688	77559053	0	0.053584
16118 Amp	10	77562014	77564836	0	0.055623
16119 Amp	10	77574183	77779037	0	0.056446
16120 Amp	10	77782696	77957901	0.204831	0.072382
16121 Amp	10	77957957	77992218	0.215608	0.072827
16122 Amp	10	77993657	78002420	0.271552	0.075348
16123 Amp	10	78002686	78037772	0.259438	0.074831
16124 Amp	10	78039161	78111269	0.373599	0.078916
16125 Amp	10	78111758	78322403	0.704849	0.089622
16126 Amp	10	78328416	78347748	0.742723	0.090732
16127 Amp	10	78347864	78352815	0.6737	0.088725
16128 Amp	10	78352831	78449015	0.758088	0.091012
16129 Amp	10	78452705	78453082	0.6737	0.088634
16130 Amp	10	78453111	78520154	0.583752	0.085833
16131 Amp	10	78521979	78562220	0.251218	0.074358
16132 Amp	10	78563449	78579356	0.14896	0.070241
16133 Amp	10	78582918	78637113	0.583752	0.085886
16134 Amp	10	78639105	78676112	0.807647	0.092384
16135 Amp	10	78683111	78735845	1.329472	0.105075
16136 Amp	10	78736271	78929352	1.436692	0.107318
16137 Amp	10	78972382	78972656	2.368227	0.12657
16138 Amp	10	78980515	79010751	2.490318	0.128921
16139 Amp	10	79011141	79034112	2.605213	0.131005
16140 Amp	10	79034510	79125667	2.649655	0.131939
16141 Amp	10	79128316	79156851	2.005947	0.119247
16142 Amp	10	79158815	79162351	2.127614	0.121774
16143 Amp	10	79165198	79179757	2.005947	0.1193
16144 Amp	10	79180427	79271606	2.083076	0.120847
16145 Amp	10	79272798	79334945	2.060912	0.120408
16146 Amp	10	79337919	79344921	1.984015	0.118861
16147 Amp	10	79345099	79431500	1.95165	0.118273
16148 Amp	10	79437421	79440676	1.407997	0.106684
16149 Amp	10	79447919	79484181	1.823153	0.115669
16150 Amp	10	79484662	79484662	1.865019	0.116396
16151 Amp	10	79484771	79487437	2.038694	0.120075
16152 Amp	10	80248789	80248789	0	0.056387
16153 Amp	10	80252343	80282868	0	0.055612
16154 Amp	10	80283981	80359486	0	0.054389
16155 Amp	10	80367684	80505528	0	0.047998
16156 Amp	10	80508773	80521335	0	0.040577
16157 Amp	10	80523782	80537777	0	0.038014
16158 Amp	10	80540091	80547361	0	0.039034
16159 Amp	10	80553359	80570668	0	0.043985
16160 Amp	10	80571475	80646815	0	0.045618
16161 Amp	10	80648571	80757710	0	0.054315
16162 Amp	10	80758089	80758605	0	0.053588
16163 Amp	10	80758757	80982901	0	0.052189
16164 Amp	10	80988029	81006216	0	0.052785

16165	Amp	10	81007072	81030869	0	0.050615
16166	Amp	10	81034887	81055061	0	0.048562
16167	Amp	10	81056493	81165345	0.015369	0.064498
16168	Amp	10	81167894	81176270	0	0.048562
16169	Amp	10	81186077	81276654	0	0.046392
16170	Amp	10	81277235	81316627	0	0.045371
16171	Amp	10	81334591	81340817	0	0.048352
16172	Amp	10	81342095	81398821	0	0.046896
16173	Amp	10	81408966	81606815	0	0.045269
16174	Amp	10	81607248	81633472	0	0.048755
16175	Amp	10	81637800	81724759	0	0.044699
16176	Amp	10	81725247	81886309	0	0.044103
16177	Amp	10	81888990	81895974	0	0.035406
16178	Amp	10	81897657	82094036	0	0.037003
16179	Amp	10	82094116	82138381	0	0.040163
16180	Amp	10	82140463	82249370	0	0.038883
16181	Amp	10	82254297	82321944	0	0.034469
16182	Amp	10	82323236	82371089	0	0.033478
16183	Amp	10	82373084	82622843	0	0.039764
16184	Amp	10	82629425	82710254	0	0.041359
16185	Amp	10	82710617	82743625	0	0.041822
16186	Amp	10	82744435	82744435	0	0.050638
16187	Amp	10	82748495	82767755	0	0.053428
16188	Amp	10	82769985	82856266	0.1237	0.069204
16189	Amp	10	82856804	82908111	0.019169	0.064886
16190	Amp	10	82908144	83081580	0	0.059247
16191	Amp	10	83095675	83096055	0	0.061675
16192	Amp	10	83099687	83151083	0	0.061212
16193	Amp	10	83152768	83191596	0	0.063035
16194	Amp	10	83191748	83237631	0.015369	0.064501
16195	Amp	10	83238012	83248526	0	0.063035
16196	Amp	10	83255520	83397168	0	0.061151
16197	Amp	10	83398144	83535918	0	0.062959
16198	Amp	10	83540303	83544922	0	0.059978
16199	Amp	10	83547070	83566584	0	0.061058
16200	Amp	10	83569302	83602854	0	0.060126
16201	Amp	10	83607861	83700228	0	0.062206
16202	Amp	10	83700241	83702672	0	0.060126
16203	Amp	10	83706394	83796064	0	0.058532
16204	Amp	10	83797061	83826310	0	0.059817
16205	Amp	10	83826470	83882992	0	0.061642
16206	Amp	10	83884015	83887832	0.034113	0.065565
16207	Amp	10	83894550	83923813	0.089561	0.067827
16208	Amp	10	83925000	83999471	0.112543	0.068804
16209	Amp	10	84002158	84016981	0.019169	0.064881
16210	Amp	10	84042014	84101243	0.069848	0.06704
16211	Amp	10	84104597	84115055	0.019169	0.064881
16212	Amp	10	84116313	84175258	0.065715	0.066833
16213	Amp	10	84175809	84216250	0.019169	0.064881
16214	Amp	10	84217419	84391385	0	0.061148
16215	Amp	10	84393376	84401971	0	0.053779
16216	Amp	10	84402233	84526798	0	0.055864
16217	Amp	10	84531473	84584650	0	0.056975

16218	Amp	10	84584752	84650301	0	0.055254
16219	Amp	10	84650523	84846525	0	0.057076
16220	Amp	10	84853023	84943893	0	0.059326
16221	Amp	10	84944935	84946687	0	0.0575
16222	Amp	10	84946925	84983849	0	0.056389
16223	Amp	10	84985355	85413595	0	0.055204
16224	Amp	10	85417290	85423327	0	0.042805
16225	Amp	10	85423727	85471773	0	0.040786
16226	Amp	10	85472756	85487231	0	0.039708
16227	Amp	10	85488168	85562204	0	0.038628
16228	Amp	10	85566381	85609507	0	0.036532
16229	Amp	10	85612436	85702568	0	0.035787
16230	Amp	10	85703199	85737899	0	0.033795
16231	Amp	10	85741759	85883092	0	0.024163
16232	Amp	10	85884140	85935753	0	0.026386
16233	Amp	10	85935826	85944578	0	0.022661
16234	Amp	10	85944593	85954641	0	0.025839
16235	Amp	10	85954847	86004299	0	0.029682
16236	Amp	10	86004615	86074182	0	0.027528
16237	Amp	10	86081430	86087202	0	0.02435
16238	Amp	10	86097765	86116488	0	0.025058
16239	Amp	10	86120461	86128257	0	0.040681
16240	Amp	10	86128929	86246971	0	0.039705
16241	Amp	10	86248171	86298020	0	0.024082
16242	Amp	10	86303418	86336329	0	0.023338
16243	Amp	10	86341610	86460453	0	0.021088
16244	Amp	10	86461109	86593312	0	0.02038
16245	Amp	10	86596487	86622539	0	0.022314
16246	Amp	10	86622853	86646631	0	0.024461
16247	Amp	10	86646681	86677780	0	0.027038
16248	Amp	10	86693628	86729049	0	0.027582
16249	Amp	10	86732245	86983391	0	0.025434
16250	Amp	10	86985198	86985608	0	0.022354
16251	Amp	10	86996176	87116095	0	0.019961
16252	Amp	10	87406076	87406711	0	0.015039
16253	Amp	10	87407654	87423492	0	0.010972
16254	Amp	10	87426167	87639467	0	0.010294
16255	Amp	10	87640035	87727515	0	0.011196
16256	Amp	10	87732996	87748576	0	0.009113
16257	Amp	10	87750472	87852121	0	0.00821
16258	Amp	10	87852267	87872765	0	0.006166
16259	Amp	10	87881541	87902461	0	0.01345
16260	Amp	10	87905782	87981745	0	0.006166
16261	Amp	10	87982049	88044828	0	0.007676
16262	Amp	10	88046397	88119022	0	0.008194
16263	Amp	10	88123627	88333541	0	0.008819
16264	Amp	10	88337482	88662379	0	0.008194
16265	Amp	10	88669279	88728274	0	0.008677
16266	Amp	10	88728707	89037988	0	0.00816
16267	Amp	10	89047431	89138509	0	0.007676
16268	Amp	10	89140307	89178684	0	0.00835
16269	Amp	10	89191098	89243170	0	0.012576
16270	Amp	10	89245956	89317584	0	0.011903

16271	Amp	10	89322027	89346859	0	0.006631
16272	Amp	10	89346891	89422096	0	0.007575
16273	Amp	10	89422177	89518816	0	0.010977
16274	Amp	10	89520309	89531711	0	0.007575
16275	Amp	10	89531990	89576036	0	0.006631
16276	Amp	10	89578241	89777325	0	0.002405
16277	Amp	10	89783913	89829291	0	0.011955
16278	Amp	10	89829524	89995540	0	0.018389
16279	Amp	10	90000299	90096225	0	0.027793
16280	Amp	10	90100780	90401077	0	0.016511
16281	Amp	10	90407748	90592387	0	0.018729
16282	Amp	10	90595407	90654111	0	0.019301
16283	Amp	10	90657061	90667437	0	0.020112
16284	Amp	10	90668236	90805872	0	0.006469
16285	Amp	10	90811951	90993250	0	0.007573
16286	Amp	10	90996720	91015161	0	0.008608
16287	Amp	10	91016123	91050636	0	0.007573
16288	Amp	10	91051589	91079019	0	0.014217
16289	Amp	10	91082327	91269203	0	0.012377
16290	Amp	10	91270154	91414741	0	0.012842
16291	Amp	10	91416365	91421487	0	0.006934
16292	Amp	10	91423826	91453495	0	0.006469
16293	Amp	10	91459581	91593326	0	0.020112
16294	Amp	10	91599285	91784329	0	0.023155
16295	Amp	10	91786893	91845648	0	0.026771
16296	Amp	10	91856509	91962061	0	0.010933
16297	Amp	10	91962442	92025363	0	0.014853
16298	Amp	10	92028317	92049559	0	0.015909
16299	Amp	10	92051115	92098094	0	0.011989
16300	Amp	10	92098557	92296815	0	0.008947
16301	Amp	10	92298856	92717138	0	0.007891
16302	Amp	10	92717462	93058315	0	0.005673
16303	Amp	10	93066708	93099490	0	0.010014
16304	Amp	10	93104932	93127850	0	0.006398
16305	Amp	10	93130148	93244182	0	0.017895
16306	Amp	10	93246900	93257033	0	0.004952
16307	Amp	10	93258355	93292197	0	0.007058
16308	Amp	10	93292452	93318071	0	0.004422
16309	Amp	10	93318454	93465400	0	0.003748
16310	Amp	10	93465946	93473618	0	0.006379
16311	Amp	10	93473636	93485443	0	0.004014
16312	Amp	10	93488285	93612452	0	0.001384
16313	Amp	10	93613609	93684471	0	0.003586
16314	Amp	10	93688782	94012072	0	0.005784
16315	Amp	10	94012410	94023371	0	0.003581
16316	Amp	10	94023485	94149905	0	0.004062
16317	Amp	10	94150143	94300994	0	0.005317
16318	Amp	10	94302036	94377677	0	0.003581
16319	Amp	10	94381065	94535064	0	0.004589
16320	Amp	10	94535094	94736036	0	0.010147
16321	Amp	10	94736094	94736094	0	0.010786
16322	Amp	10	94799420	94890604	0	0.02113
16323	Amp	10	94894789	95069924	0	0.026252

16324	Amp	10	95070472	95105456	0	0.02113
16325	Amp	10	95105463	95143399	0	0.020297
16326	Amp	10	95144281	95284203	0	0.02113
16327	Amp	10	95284242	95420646	0	0.022135
16328	Amp	10	95422746	96360352	0	0.02113
16329	Amp	10	96360933	96501999	0	0.005229
16330	Amp	10	96502759	96594426	0	0.006412
16331	Amp	10	96595167	97095624	0	0.009402
16332	Amp	10	97095708	97350364	0	0.009913
16333	Amp	10	97351864	97424077	0	0.008729
16334	Amp	10	97424407	97483569	0	0.010948
16335	Amp	10	97488489	97540850	0	0.018614
16336	Amp	10	97546744	97546978	0	0.020162
16337	Amp	10	97547868	97609256	0	0.024585
16338	Amp	10	97615197	97652920	0	0.022119
16339	Amp	10	97657704	97693033	0	0.026804
16340	Amp	10	97696238	97836907	0	0.025256
16341	Amp	10	97836955	98073947	0	0.024745
16342	Amp	10	98085972	98338125	0	0.015638
16343	Amp	10	98339894	98972806	0	0.016766
16344	Amp	10	99075984	99285970	0	0.0091
16345	Amp	10	99287934	99306683	0	0.010323
16346	Amp	10	99310439	99386617	0	0.011368
16347	Amp	10	99390590	99564679	0	0.014373
16348	Amp	10	99565901	99848332	0	0.011368
16349	Amp	10	99850966	99874563	0	0.013415
16350	Amp	10	99878502	100295051	0	0.015322
16351	Amp	10	100343657	100417846	0	0.014798
16352	Amp	10	100420658	100492712	0	0.020316
16353	Amp	10	100496739	100558547	0	0.014798
16354	Amp	10	100558827	100591744	0	0.01275
16355	Amp	10	100593584	100904231	0	0.019654
16356	Amp	10	100905504	100985092	0	0.022061
16357	Amp	10	100985749	101128718	0	0.019654
16358	Amp	10	101135700	101166321	0	0.023178
16359	Amp	10	101167639	101526143	0	0.019654
16360	Amp	10	101528508	101563809	0	0.021673
16361	Amp	10	101571370	101700035	0	0.019654
16362	Amp	10	101707299	101756476	0	0.021126
16363	Amp	10	101765441	101853648	0	0.016952
16364	Amp	10	101862091	101912770	0	0.018787
16365	Amp	10	101915653	101999764	0	0.028212
16366	Amp	10	102005456	102333794	0	0.016952
16367	Amp	10	102334241	102344150	0	0.022716
16368	Amp	10	102348240	102367414	0	0.024331
16369	Amp	10	102372693	102401202	0	0.023203
16370	Amp	10	102407894	102555910	0	0.017438
16371	Amp	10	102556232	102978409	0	0.015824
16372	Amp	10	102981024	103135465	0	0.025228
16373	Amp	10	103138144	103146454	0	0.026454
16374	Amp	10	103150133	103388931	0	0.024256
16375	Amp	10	103464215	103621412	0	0.026328
16376	Amp	10	103624269	103658747	0	0.029205

16377	Amp	10	103659529	103784933	0	0.027134
16378	Amp	10	103785646	103797541	0	0.027602
16379	Amp	10	103802129	103841824	0	0.024724
16380	Amp	10	103844953	104527072	0	0.023913
16381	Amp	10	104528222	104607233	0	0.023445
16382	Amp	10	104609497	104852667	0	0.022219
16383	Amp	10	104853800	105005139	0	0.015076
16384	Amp	10	105006436	105106197	0	0.013604
16385	Amp	10	105112348	105182538	0	0.014842
16386	Amp	10	105183701	105197249	0	0.016801
16387	Amp	10	105202300	105318558	0	0.01911
16388	Amp	10	105324556	105421500	0	0.016801
16389	Amp	10	105422250	105735342	0	0.009897
16390	Amp	10	105741635	105745168	0	0.010825
16391	Amp	10	105746975	105771818	0	0.012494
16392	Amp	10	105771820	105854440	0	0.014971
16393	Amp	10	105928593	105930609	0	0.008273
16394	Amp	10	105930762	105939872	0	0.009367
16395	Amp	10	105940461	106027820	0	0.007698
16396	Amp	10	106029954	106040157	0	0.00677
16397	Amp	10	106047085	106529098	0	0.005677
16398	Amp	10	106534239	106567083	0	0.016708
16399	Amp	10	106567497	106576250	0	0.019894
16400	Amp	10	106576809	106740317	0	0.017675
16401	Amp	10	106740646	106857589	0	0.020646
16402	Amp	10	106859856	106888625	0	0.029686
16403	Amp	10	106892798	107104946	0	0.025007
16404	Amp	10	107104975	107783050	0	0.02257
16405	Amp	10	107783951	107810092	0	0.025414
16406	Amp	10	107810760	108285021	0	0.02257
16407	Amp	10	108285926	108300641	0	0.023612
16408	Amp	10	108301267	108370767	0	0.025852
16409	Amp	10	108370837	108450475	0	0.029311
16410	Amp	10	108451107	108456671	0	0.025852
16411	Amp	10	108456718	108666516	0	0.027956
16412	Amp	10	108667002	108699261	0	0.036542
16413	Amp	10	108699457	108886812	0	0.037226
16414	Amp	10	108886901	108939972	0	0.036542
16415	Amp	10	108942229	109146502	0	0.029775
16416	Amp	10	109148516	109470399	0	0.03427
16417	Amp	10	109474749	109646755	0	0.032166
16418	Amp	10	109649149	109682016	0	0.027671
16419	Amp	10	109687653	109718190	0	0.028853
16420	Amp	10	109721583	109725669	0	0.029695
16421	Amp	10	109727288	109833650	0	0.028853
16422	Amp	10	109834536	109853599	0	0.03511
16423	Amp	10	109854535	109972699	0	0.036143
16424	Amp	10	109973262	109976048	0	0.033127
16425	Amp	10	109976453	110024132	0	0.031945
16426	Amp	10	110038223	110108486	0	0.026603
16427	Amp	10	110112062	110159146	0	0.020388
16428	Amp	10	110162461	110295150	0	0.019251
16429	Amp	10	110295579	110352274	0	0.020234

16430	Amp	10	110353194	110527838	0	0.020703
16431	Amp	10	110533109	110548229	0	0.009138
16432	Amp	10	110549883	110554145	0	0.008155
16433	Amp	10	110554912	110620776	0	0.011652
16434	Amp	10	110623459	110650324	0	0.023353
16435	Amp	10	110655592	110655853	0	0.024581
16436	Amp	10	110659020	110665420	0	0.023942
16437	Amp	10	110667019	110682279	0	0.023473
16438	Amp	10	110686438	110730244	0	0.023933
16439	Amp	10	110730552	110850047	0	0.020436
16440	Amp	10	110850215	110880307	0	0.019209
16441	Amp	10	110883252	110931305	0	0.020008
16442	Amp	10	110931389	111183080	0	0.016821
16443	Amp	10	111185723	111214126	0	0.020178
16444	Amp	10	111222319	111398756	0	0.036114
16445	Amp	10	111400358	111433211	0	0.039365
16446	Amp	10	111433249	111514015	0	0.036114
16447	Amp	10	111518189	111651330	0	0.020178
16448	Amp	10	111652932	111747499	0	0.019717
16449	Amp	10	111754624	112137988	0	0.020334
16450	Amp	10	112141527	112289955	0	0.034969
16451	Amp	10	112291316	112451666	0	0.020334
16452	Amp	10	112451689	112516154	0	0.019535
16453	Amp	10	112516533	112689102	0	0.020824
16454	Amp	10	112690873	113582429	0	0.019535
16455	Amp	10	113583528	113638273	0	0.020025
16456	Amp	10	113639299	113650923	0	0.019535
16457	Amp	10	113651069	113830690	0	0.020737
16458	Amp	10	113832372	113910589	0	0.019535
16459	Amp	10	113915266	114029616	0	0.021615
16460	Amp	10	114032972	114032972	0	0.032539
16461	Amp	10	114053200	114089714	0	0.033807
16462	Amp	10	114090417	114141617	0	0.035237
16463	Amp	10	114145260	114212308	0	0.03188
16464	Amp	10	114214648	114809806	0	0.030612
16465	Amp	10	114810809	114843595	0	0.033036
16466	Amp	10	114847908	115069170	0	0.030957
16467	Amp	10	115070723	115162612	0	0.031636
16468	Amp	10	115164176	115211017	0	0.033386
16469	Amp	10	115212726	115247729	0	0.033886
16470	Amp	10	115252373	115264509	0	0.034392
16471	Amp	10	115268479	115551588	0	0.032642
16472	Amp	10	115553266	115559648	0	0.048579
16473	Amp	10	115563798	115601435	0	0.049623
16474	Amp	10	115603324	115753077	0	0.05291
16475	Amp	10	115753444	115808224	0	0.05223
16476	Amp	10	115808433	115869353	0	0.051185
16477	Amp	10	115878419	115961471	0	0.047899
16478	Amp	10	115967413	115974179	0	0.040223
16479	Amp	10	115977074	115984780	0	0.042721
16480	Amp	10	115986017	116134454	0	0.04063
16481	Amp	10	116134578	116140993	0	0.024694
16482	Amp	10	116146194	116207987	0	0.022196

16483	Amp	10	116208414	116220129	0	0.024259
16484	Amp	10	116220940	116236638	0	0.023753
16485	Amp	10	116237290	116241007	0	0.02133
16486	Amp	10	116242259	116251310	0	0.019266
16487	Amp	10	116252698	116276488	0	0.022654
16488	Amp	10	116281059	116427848	0	0.019266
16489	Amp	10	116427925	116569545	0	0.018766
16490	Amp	10	116573509	116882432	0	0.017336
16491	Amp	10	116882566	117312679	0	0.014472
16492	Amp	10	117313007	117338236	0	0.01605
16493	Amp	10	117338651	117781188	0	0.014472
16494	Amp	10	117786230	117972500	0	0.006412
16495	Amp	10	117973197	118204992	0	0.008139
16496	Amp	10	118215315	118590465	0	0.006204
16497	Amp	10	118590524	118843911	0	0.004477
16498	Amp	10	118847211	118889333	0	0.006471
16499	Amp	10	118889652	118906344	0	0.010179
16500	Amp	10	118906734	119294814	0	0.01185
16501	Amp	10	119294866	119599329	0	0.013128
16502	Amp	10	119601477	119601477	0	0.011575
16503	Amp	10	119603148	119622452	0	0.010297
16504	Amp	10	119622943	119657064	0	0.010841
16505	Amp	10	119659797	119674449	0	0.026591
16506	Amp	10	119677500	119740144	0	0.013205
16507	Amp	10	119743621	119846416	0	0.015572
16508	Amp	10	119848720	119858900	0	0.024349
16509	Amp	10	119859476	119968143	0	0.021982
16510	Amp	10	119972138	120437699	0	0.019618
16511	Amp	10	120438149	120557820	0	0.015994
16512	Amp	10	120561440	120562727	0	0.023181
16513	Amp	10	120571768	120587764	0	0.028095
16514	Amp	10	120590025	120616640	0	0.032513
16515	Amp	10	120621051	120668902	0	0.025326
16516	Amp	10	120669338	120802417	0	0.040917
16517	Amp	10	120802872	120829978	0	0.053029
16518	Amp	10	120831038	120853092	0	0.052484
16519	Amp	10	120857245	120885480	0	0.038269
16520	Amp	10	120887720	121001578	0	0.042326
16521	Amp	10	121030305	121030305	0	0.058163
16522	Amp	10	121030813	121052848	0	0.060153
16523	Amp	10	121055155	121098449	0	0.062308
16524	Amp	10	121101781	121155431	0	0.060153
16525	Amp	10	121155455	121163200	0	0.062039
16526	Amp	10	121163985	121211409	0.001323	0.064056
16527	Amp	10	121211940	121318482	0.024438	0.064983
16528	Amp	10	121321538	121329613	0.065715	0.066811
16529	Amp	10	121332690	121345160	0.251218	0.074451
16530	Amp	10	121345515	121428750	0.276783	0.075468
16531	Amp	10	121434324	121557671	0.386426	0.079292
16532	Amp	10	121560810	121564448	0.404718	0.079924
16533	Amp	10	121566024	121587820	0.386426	0.079292
16534	Amp	10	121588004	121736140	0.355319	0.078275
16535	Amp	10	121738707	121922574	0.399144	0.07977

16536	Amp	10	121924040	121982617	0.651644	0.088047
16537	Amp	10	121986932	122103401	0.583752	0.085853
16538	Amp	10	122105121	122120539	0.19964	0.0723
16539	Amp	10	122121308	122159420	0	0.058578
16540	Amp	10	122161269	122296407	0	0.058005
16541	Amp	10	122297031	122370279	0	0.053587
16542	Amp	10	122379070	122405700	0	0.037651
16543	Amp	10	122407488	122475543	0	0.045547
16544	Amp	10	122475780	122517501	0	0.044309
16545	Amp	10	122519178	122564250	0	0.044913
16546	Amp	10	122623497	122664273	0	0.037526
16547	Amp	10	122669246	122703177	0	0.044701
16548	Amp	10	122704084	122746052	0	0.043206
16549	Amp	10	122747469	122750316	0	0.045623
16550	Amp	10	122750764	122769497	0	0.043607
16551	Amp	10	122772902	122858633	0	0.041617
16552	Amp	10	122862338	122874713	0	0.042206
16553	Amp	10	122921307	123003769	0	0.028188
16554	Amp	10	123004200	123044303	0	0.030196
16555	Amp	10	123048002	123057364	0	0.027911
16556	Amp	10	123058191	123151549	0	0.029886
16557	Amp	10	123152482	123209932	0	0.032853
16558	Amp	10	123210405	123235791	0	0.037234
16559	Amp	10	123237190	123246883	0	0.03925
16560	Amp	10	123249711	123250965	0	0.038646
16561	Amp	10	123256190	123317318	0	0.040956
16562	Amp	10	123317644	123346678	0	0.036574
16563	Amp	10	123348987	123493721	0	0.038636
16564	Amp	10	123495557	123544768	0	0.039188
16565	Amp	10	123545035	123563813	0	0.037212
16566	Amp	10	123566863	123625550	0	0.039278
16567	Amp	10	123627060	123707399	0	0.037212
16568	Amp	10	123710096	123800328	0	0.035151
16569	Amp	10	123802456	123828639	0	0.037748
16570	Amp	10	123830670	123943507	0	0.038352
16571	Amp	10	123951019	123966293	0	0.039946
16572	Amp	10	123966512	123969265	0	0.040877
16573	Amp	10	123979493	124280459	0	0.037911
16574	Amp	10	124284440	124324747	0	0.039914
16575	Amp	10	124327313	124327313	0	0.039326
16576	Amp	10	124327871	124510720	0	0.0368
16577	Amp	10	124510733	124547850	0	0.038877
16578	Amp	10	124548032	124621325	0	0.036861
16579	Amp	10	124628830	124647589	0	0.034785
16580	Amp	10	124649524	124828775	0	0.037773
16581	Amp	10	124837549	124961310	0	0.035176
16582	Amp	10	124963492	124986801	0	0.037105
16583	Amp	10	124990352	125178422	0	0.038221
16584	Amp	10	125179065	125204035	0	0.030537
16585	Amp	10	125204903	125374505	0	0.029421
16586	Amp	10	125374932	125534083	0	0.028494
16587	Amp	10	125535228	125719022	0	0.026491
16588	Amp	10	125721713	125736294	0	0.023503

16589	Amp	10	125736490	125821686	0	0.019885
16590	Amp	10	125822154	125854619	0	0.018311
16591	Amp	10	125864450	125942231	0	0.017255
16592	Amp	10	125943916	126074802	0	0.019117
16593	Amp	10	126076388	126091181	0	0.018007
16594	Amp	10	126092242	126335835	0	0.021537
16595	Amp	10	126336777	126336777	0	0.03245
16596	Amp	10	126336815	126395995	0	0.033294
16597	Amp	10	126396369	126432586	0	0.034106
16598	Amp	10	126434342	126520387	0	0.032245
16599	Amp	10	126520571	126545998	0	0.03448
16600	Amp	10	126557793	126581593	0	0.032245
16601	Amp	10	126582130	126623277	0	0.034559
16602	Amp	10	126627355	126711810	0	0.03103
16603	Amp	10	126711940	126757105	0	0.030426
16604	Amp	10	126757899	127030354	0	0.031878
16605	Amp	10	127031073	127103267	0	0.036901
16606	Amp	10	127103345	127193365	0	0.038864
16607	Amp	10	127198387	127340866	0	0.037992
16608	Amp	10	127341867	127344907	0	0.031937
16609	Amp	10	127349027	127392346	0	0.029623
16610	Amp	10	127401278	127449216	0	0.032969
16611	Amp	10	127449780	127500828	0	0.033491
16612	Amp	10	127503996	127531291	0	0.035781
16613	Amp	10	127531793	127582669	0	0.0259
16614	Amp	10	127582749	127698843	0	0.022554
16615	Amp	10	127698959	127741639	0	0.024685
16616	Amp	10	127742789	127803405	0	0.022554
16617	Amp	10	127805707	127913710	0	0.022031
16618	Amp	10	127916621	127972938	0	0.025279
16619	Amp	10	127973639	128059164	0	0.022031
16620	Amp	10	128059796	128358621	0	0.035166
16621	Amp	10	128359605	128445820	0	0.039013
16622	Amp	10	128450036	128535360	0	0.041637
16623	Amp	10	128535685	128611070	0	0.037791
16624	Amp	10	128611389	129030109	0	0.035166
16625	Amp	10	129030679	129208706	0	0.037581
16626	Amp	10	129212507	129426731	0	0.053482
16627	Amp	10	129427452	129542001	0	0.059127
16628	Amp	10	129547208	129620735	0	0.045992
16629	Amp	10	129620829	129721364	0	0.055424
16630	Amp	10	129725400	129956096	0	0.045992
16631	Amp	10	129957407	130003196	0	0.054278
16632	Amp	10	130003567	130052762	0	0.053787
16633	Amp	10	130053515	130243835	0	0.054657
16634	Amp	10	130244350	130267577	0	0.053787
16635	Amp	10	130272917	130336278	0	0.049649
16636	Amp	10	130336693	130383003	0	0.050139
16637	Amp	10	131110205	131110205	0	0.057597
16638	Amp	10	131116646	131159440	0	0.059532
16639	Amp	10	131159632	131178688	0	0.054425
16640	Amp	10	131180564	131224972	0	0.05767
16641	Amp	10	131230003	131253190	0	0.061398

16642	Amp	10	131257865	131334780	0	0.05767
16643	Amp	10	131336720	131342778	0	0.058846
16644	Amp	10	131914143	131914230	0	0.050723
16645	Amp	10	131914424	131925938	0	0.049911
16646	Amp	10	131929960	132050299	0	0.051543
16647	Amp	10	132052643	132082131	0	0.054216
16648	Amp	10	132096453	132189263	0	0.054861
16649	Amp	10	132193685	132329993	0	0.056731
16650	Amp	10	132336493	132336493	0.100827	0.06839
16651	Amp	10	132385563	132473426	0.536238	0.084326
16652	Amp	10	132473842	132511579	0.550755	0.084816
16653	Amp	10	132525799	132536505	1.140417	0.100752
16654	Amp	10	132543713	132688515	0.550755	0.084816
16655	Amp	10	132690728	132731906	0.622604	0.087186
16656	Amp	10	132733532	132733532	0.550755	0.084816
16657	Amp	10	132734847	132747307	0.616549	0.086946
16658	Amp	10	132747703	132747703	0.470509	0.082119
16659	Amp	10	133038543	133055089	0.222041	0.073059
16660	Amp	10	133084562	133085133	0.271552	0.075253
16661	Amp	10	133097553	133190570	0.342649	0.07779
16662	Amp	10	133203815	133203815	0.431198	0.080986
16663	Amp	10	133242960	133249523	0	0.062513
16664	Amp	10	133255166	133364752	0	0.059358
16665	Amp	10	133364830	133365107	0	0.060472
16666	Amp	10	133366273	133366291	0	0.04966
16667	Amp	10	133370501	133377066	0	0.053072
16668	Amp	10	133381386	133411599	0	0.054512
16669	Amp	11	456120	516520	0	0.025538
16670	Amp	11	549959	573554	0	0.024515
16671	Amp	11	577454	577684	0	0.029911
16672	Amp	11	579753	620599	0	0.028937
16673	Amp	11	628753	894450	0	0.026912
16674	Amp	11	900485	942344	0	0.022695
16675	Amp	11	945039	1616914	0	0.030721
16676	Amp	11	1639207	1962212	0	0.020669
16677	Amp	11	2043393	2090301	0	0.026002
16678	Amp	11	2097889	2117533	0	0.028203
16679	Amp	11	2120356	2224667	0	0.022566
16680	Amp	11	2227332	2397429	0	0.020366
16681	Amp	11	2397803	2775412	0	0.018955
16682	Amp	11	2786292	2848935	0	0.016117
16683	Amp	11	2851644	3343128	0	0.013245
16684	Amp	11	3343287	3428208	0	0.01565
16685	Amp	11	3519839	3532742	0	0.014709
16686	Amp	11	3543276	3567829	0	0.015962
16687	Amp	11	3597187	3597187	0	0.015905
16688	Amp	11	3600405	3726982	0	0.020258
16689	Amp	11	3728965	3729930	0	0.023809
16690	Amp	11	3733823	3794630	0	0.019456
16691	Amp	11	3798037	3805057	0	0.022364
16692	Amp	11	3810408	3851233	0	0.021111
16693	Amp	11	3854181	3868153	0	0.022361
16694	Amp	11	3868867	4031054	0	0.025158

16695	Amp	11	4031586	4096479	0	0.022423
16696	Amp	11	4097873	4170231	0	0.018382
16697	Amp	11	4178175	4370299	0	0.011989
16698	Amp	11	4371081	4410445	0	0.010661
16699	Amp	11	4410517	4439356	0	0.015485
16700	Amp	11	4441676	5444005	0	0.010661
16701	Amp	11	5444167	5476399	0	0.013748
16702	Amp	11	5483631	5512884	0	0.010661
16703	Amp	11	5513760	5584086	0	0.011311
16704	Amp	11	5584775	5663545	0	0.014407
16705	Amp	11	5672509	5733822	0	0.011311
16706	Amp	11	5734375	5850293	0	0.010661
16707	Amp	11	5922814	6333786	0	0.012974
16708	Amp	11	6335493	6385943	0	0.017335
16709	Amp	11	6387863	6439942	0	0.020691
16710	Amp	11	6441858	6479079	0	0.024631
16711	Amp	11	6481550	6542533	0	0.029713
16712	Amp	11	6542816	6569927	0	0.025355
16713	Amp	11	6571879	6657901	0	0.019973
16714	Amp	11	6657951	6760295	0	0.024013
16715	Amp	11	6761173	6835946	0	0.029211
16716	Amp	11	6836570	6869010	0	0.03817
16717	Amp	11	6869670	7041618	0	0.029211
16718	Amp	11	7047235	7096538	0	0.030784
16719	Amp	11	7096780	7136931	0	0.02617
16720	Amp	11	7138066	7175311	0	0.024597
16721	Amp	11	7181990	7212661	0	0.028645
16722	Amp	11	7218677	7499074	0	0.03131
16723	Amp	11	7499302	7548891	0	0.023146
16724	Amp	11	7552510	7608159	0	0.025395
16725	Amp	11	7608209	7734206	0	0.021244
16726	Amp	11	7742174	7873000	0	0.018931
16727	Amp	11	7876154	8078366	0	0.019542
16728	Amp	11	8079531	8079626	0	0.020161
16729	Amp	11	8082263	8096706	0	0.01955
16730	Amp	11	8096925	8234785	0	0.025035
16731	Amp	11	8241376	8261239	0	0.022944
16732	Amp	11	8265068	8342460	0	0.024735
16733	Amp	11	8344601	8407192	0	0.024116
16734	Amp	11	8409391	8496034	0	0.038261
16735	Amp	11	8496179	8533002	0	0.031511
16736	Amp	11	8535722	8684217	0	0.029262
16737	Amp	11	8688004	8696060	0	0.027818
16738	Amp	11	8697629	8874708	0	0.02926
16739	Amp	11	8877853	9177466	0	0.014768
16740	Amp	11	9178465	9274337	0	0.030704
16741	Amp	11	9376691	9397074	0	0.032971
16742	Amp	11	9397659	9407007	0	0.031529
16743	Amp	11	9409973	10096530	0	0.015593
16744	Amp	11	10109483	10113683	0	0.017665
16745	Amp	11	10114302	10477925	0	0.018414
16746	Amp	11	10478306	10927161	0	0.017665
16747	Amp	11	10929426	11011441	0	0.020063

16748	Amp	11	11012292	11048858	0	0.018128
16749	Amp	11	11054470	11062253	0	0.015593
16750	Amp	11	11062454	11253944	0	0.01738
16751	Amp	11	11257587	11689856	0	0.021565
16752	Amp	11	11690395	11690482	0	0.026145
16753	Amp	11	11700094	11822969	0	0.025024
16754	Amp	11	11823161	11905484	0	0.026057
16755	Amp	11	11907574	11987361	0	0.021477
16756	Amp	11	11988022	12148937	0	0.019409
16757	Amp	11	12149070	12281860	0	0.020329
16758	Amp	11	12282453	12325863	0	0.019409
16759	Amp	11	12332380	12377064	0	0.020892
16760	Amp	11	12379114	12420944	0	0.019409
16761	Amp	11	12421676	12437158	0	0.02407
16762	Amp	11	12444429	12577832	0	0.025437
16763	Amp	11	12580387	12624152	0	0.020776
16764	Amp	11	12631421	12682014	0	0.021312
16765	Amp	11	12682678	12732040	0	0.019945
16766	Amp	11	12733101	12735476	0	0.021856
16767	Amp	11	12738025	12754920	0	0.019507
16768	Amp	11	12755919	12822331	0	0.023912
16769	Amp	11	12822388	12841648	0	0.026876
16770	Amp	11	12842503	12866450	0	0.030864
16771	Amp	11	12868811	12982791	0	0.03421
16772	Amp	11	12983914	13171159	0	0.036222
16773	Amp	11	13171955	13233713	0	0.0383
16774	Amp	11	13233943	13351042	0	0.036222
16775	Amp	11	13352217	13394725	0	0.032899
16776	Amp	11	13403891	13437198	0	0.034771
16777	Amp	11	13437824	13477813	0	0.035931
16778	Amp	11	13479231	13551161	0	0.033854
16779	Amp	11	13552007	13638386	0	0.032604
16780	Amp	11	13640901	13789080	0	0.030732
16781	Amp	11	13790455	14015005	0	0.033123
16782	Amp	11	14015881	14079255	0	0.034654
16783	Amp	11	14079328	14136634	0	0.037993
16784	Amp	11	14136891	14298843	0	0.034654
16785	Amp	11	14299552	14380918	0	0.032475
16786	Amp	11	14382619	14386821	0	0.028841
16787	Amp	11	14388837	14413087	0	0.041427
16788	Amp	11	14415400	14461728	0	0.031318
16789	Amp	11	14466390	14470383	0	0.033698
16790	Amp	11	14483380	14492097	0	0.027961
16791	Amp	11	14498191	14560216	0	0.025484
16792	Amp	11	14564451	14634675	0	0.028775
16793	Amp	11	14640900	14795415	0	0.030647
16794	Amp	11	14796292	14906721	0	0.028267
16795	Amp	11	14916629	14928586	0	0.027427
16796	Amp	11	14932332	14972944	0	0.031453
16797	Amp	11	14973318	15022109	0	0.0321
16798	Amp	11	15022381	15097880	0	0.034116
16799	Amp	11	15105643	15333700	0	0.036677
16800	Amp	11	15334943	15584872	0	0.038933

16801	Amp	11	15585176	15585176	0	0.035885
16802	Amp	11	15586790	15599040	0	0.037157
16803	Amp	11	15599068	15626383	0	0.040301
16804	Amp	11	15626481	15753812	0	0.037157
16805	Amp	11	15754796	15807992	0	0.034597
16806	Amp	11	15808305	15860064	0	0.033921
16807	Amp	11	15860153	16007548	0	0.037066
16808	Amp	11	16008071	16027906	0	0.039831
16809	Amp	11	16033367	16112463	0	0.037166
16810	Amp	11	16118139	16127357	0	0.037919
16811	Amp	11	16127839	16244350	0	0.036568
16812	Amp	11	16244871	16450572	0	0.034312
16813	Amp	11	16451436	16487515	0	0.040111
16814	Amp	11	16487674	16493887	0	0.039358
16815	Amp	11	16495751	16513745	0	0.041294
16816	Amp	11	16514410	16584046	0	0.039383
16817	Amp	11	16590218	16664517	0	0.037367
16818	Amp	11	16675232	16860502	0	0.032181
16819	Amp	11	16863195	16902023	0	0.035131
16820	Amp	11	16902777	16908324	0	0.042002
16821	Amp	11	16908411	16908940	0	0.038634
16822	Amp	11	16912602	17089725	0	0.039798
16823	Amp	11	17089788	17105795	0	0.037786
16824	Amp	11	17106451	17290213	0	0.03984
16825	Amp	11	17295120	17305040	0	0.041146
16826	Amp	11	17308294	17338569	0	0.038195
16827	Amp	11	17346848	17383105	0	0.038706
16828	Amp	11	17383786	17393410	0	0.034241
16829	Amp	11	17393422	17413056	0	0.035027
16830	Amp	11	17413667	17432687	0	0.03309
16831	Amp	11	17433178	17534003	0	0.031931
16832	Amp	11	17534484	17652683	0	0.029166
16833	Amp	11	17653533	17751907	0	0.030765
16834	Amp	11	17752926	17799301	0	0.032756
16835	Amp	11	17799397	18031998	0	0.031969
16836	Amp	11	18032391	18034678	0	0.030724
16837	Amp	11	18035833	18300987	0	0.033839
16838	Amp	11	18303584	18318694	0	0.033328
16839	Amp	11	18319655	18357250	0	0.034313
16840	Amp	11	18358376	18366505	0	0.039239
16841	Amp	11	18367171	18390604	0	0.039997
16842	Amp	11	18394140	18422657	0	0.041345
16843	Amp	11	18428589	18428803	0	0.03915
16844	Amp	11	18429549	18564176	0	0.041402
16845	Amp	11	18567737	18642015	0	0.036476
16846	Amp	11	18655622	18754784	0	0.034224
16847	Amp	11	18755335	18755624	0	0.035929
16848	Amp	11	18760584	18768273	0	0.040794
16849	Amp	11	18771189	18790910	0	0.046201
16850	Amp	11	18794455	18904545	0	0.040794
16851	Amp	11	18957582	19011540	0	0.038803
16852	Amp	11	19012977	19012977	0	0.041204
16853	Amp	11	19014764	19074752	0	0.0419

16854	Amp	11	19075752	19093000	0	0.04263
16855	Amp	11	19097811	19281495	0	0.044623
16856	Amp	11	19281520	19309579	0	0.043755
16857	Amp	11	19313005	19425158	0	0.04568
16858	Amp	11	19426000	19532959	0	0.048413
16859	Amp	11	19534114	19546807	0	0.047435
16860	Amp	11	19550646	19559395	0	0.046705
16861	Amp	11	19559569	19714466	0	0.045089
16862	Amp	11	19716764	19736413	0	0.042356
16863	Amp	11	19738364	19762974	0	0.040363
16864	Amp	11	19764406	20040907	0	0.038117
16865	Amp	11	20041158	20070373	0	0.039368
16866	Amp	11	20070531	20086946	0	0.038672
16867	Amp	11	20087933	20246150	0	0.04033
16868	Amp	11	20248709	20293960	0	0.036903
16869	Amp	11	20294647	20294647	0	0.033266
16870	Amp	11	20294669	20330028	0	0.030569
16871	Amp	11	20334898	20354553	0	0.030964
16872	Amp	11	20356353	20491714	0	0.033184
16873	Amp	11	20496179	20700097	0	0.02967
16874	Amp	11	20700903	20745006	0	0.030794
16875	Amp	11	20747555	20770223	0	0.033305
16876	Amp	11	20770736	20777453	0	0.030794
16877	Amp	11	20778734	20779303	0	0.031279
16878	Amp	11	20781207	20800077	0	0.030155
16879	Amp	11	20810054	20835175	0	0.031702
16880	Amp	11	20843285	20896184	0	0.036095
16881	Amp	11	20898311	20908305	0	0.040314
16882	Amp	11	20908838	20909992	0	0.041973
16883	Amp	11	20911973	20929114	0	0.03727
16884	Amp	11	20929315	21004889	0	0.035153
16885	Amp	11	21006639	21059731	0	0.03826
16886	Amp	11	21064544	21076923	0	0.036601
16887	Amp	11	21082474	21128086	0	0.034381
16888	Amp	11	21128589	21261706	0	0.036087
16889	Amp	11	21261981	21262812	0	0.037843
16890	Amp	11	21263499	21354000	0	0.035258
16891	Amp	11	21354754	21413862	0	0.033332
16892	Amp	11	21415348	21431877	0	0.033798
16893	Amp	11	21432932	21439942	0	0.031744
16894	Amp	11	21497985	21536945	0	0.028242
16895	Amp	11	21536968	21558508	0	0.030927
16896	Amp	11	21559952	21584869	0	0.030185
16897	Amp	11	21586435	21689838	0	0.032476
16898	Amp	11	21692916	21692916	0	0.034818
16899	Amp	11	21712549	21716622	0	0.039205
16900	Amp	11	21716804	21717273	0	0.045158
16901	Amp	11	21720690	21721221	0	0.042473
16902	Amp	11	21724212	21727380	0	0.038086
16903	Amp	11	21728917	21742497	0	0.035744
16904	Amp	11	21754732	21834424	0	0.03409
16905	Amp	11	21839632	21980439	0	0.039979
16906	Amp	11	21980563	21980563	0	0.038223

16907	Amp	11	21984495	21985482	0	0.033588
16908	Amp	11	21992668	22015932	0	0.032334
16909	Amp	11	22024286	22078651	0	0.031516
16910	Amp	11	22078903	22092537	0	0.047452
16911	Amp	11	22092547	22109479	0	0.049988
16912	Amp	11	22113529	22113860	0	0.051768
16913	Amp	11	22117077	22168463	0	0.047285
16914	Amp	11	22168535	22172847	0	0.045505
16915	Amp	11	22180939	22263978	0	0.049909
16916	Amp	11	22267830	22349323	0	0.045505
16917	Amp	11	22352884	22374993	0	0.04953
16918	Amp	11	22378461	22423574	0	0.052011
16919	Amp	11	22423768	22460644	0	0.053238
16920	Amp	11	22461094	22464850	0	0.061984
16921	Amp	11	22465068	22505068	0	0.063684
16922	Amp	11	22505204	22533076	0.089561	0.067907
16923	Amp	11	22533822	22578407	0.001323	0.063882
16924	Amp	11	22579375	22624928	0.040093	0.0656
16925	Amp	11	22625237	22629880	0	0.061377
16926	Amp	11	22634035	22668776	0	0.059033
16927	Amp	11	22671890	22710382	0	0.057225
16928	Amp	11	22718161	22738730	0.060686	0.06665
16929	Amp	11	22746977	22814014	0.024438	0.064932
16930	Amp	11	22816695	22844309	0	0.060751
16931	Amp	11	22850492	22878400	0	0.062987
16932	Amp	11	22882481	22956757	0	0.060696
16933	Amp	11	22958616	22991479	0	0.059004
16934	Amp	11	22991885	23084922	0	0.056467
16935	Amp	11	23085042	23125710	0	0.058022
16936	Amp	11	23127255	23196232	0	0.055786
16937	Amp	11	23197915	23247133	0	0.054549
16938	Amp	11	23247477	23296203	0	0.053063
16939	Amp	11	23305260	23348637	0	0.051357
16940	Amp	11	23348689	23408473	0	0.05298
16941	Amp	11	23413866	23462521	0	0.05516
16942	Amp	11	23465847	23511054	0	0.057261
16943	Amp	11	23511094	23531147	0	0.054464
16944	Amp	11	23533343	23560489	0	0.047198
16945	Amp	11	23560575	23562663	0	0.047684
16946	Amp	11	23562670	23682337	0	0.046231
16947	Amp	11	23684203	23721689	0	0.044677
16948	Amp	11	23722452	23801957	0	0.043054
16949	Amp	11	23803060	23803060	0	0.044226
16950	Amp	11	23808851	23847713	0	0.042125
16951	Amp	11	23848745	23862068	0	0.026297
16952	Amp	11	23863388	23897738	0	0.028496
16953	Amp	11	23897895	23910502	0	0.032496
16954	Amp	11	23911421	23929416	0	0.034457
16955	Amp	11	23932030	24167231	0	0.036626
16956	Amp	11	24176408	24176449	0	0.039065
16957	Amp	11	24243232	24260664	0	0.03578
16958	Amp	11	24271788	24420628	0	0.036251
16959	Amp	11	24433630	24433630	0	0.033812

16960	Amp	11	24436760	24578259	0	0.03264
16961	Amp	11	24578332	24656427	0	0.034455
16962	Amp	11	24659324	24829029	0	0.032287
16963	Amp	11	24829084	24841435	0	0.033329
16964	Amp	11	24846758	24847296	0	0.031678
16965	Amp	11	24849561	24941974	0	0.032314
16966	Amp	11	24943291	24949711	0	0.034749
16967	Amp	11	24956435	24988939	0	0.028714
16968	Amp	11	24989040	24993210	0	0.030827
16969	Amp	11	24994225	25012458	0	0.028392
16970	Amp	11	25013095	25026033	0	0.04351
16971	Amp	11	25028742	25029234	0	0.059338
16972	Amp	11	25033189	25102307	0	0.043583
16973	Amp	11	25102320	25102320	0	0.040292
16974	Amp	11	25112297	25112297	0	0.035399
16975	Amp	11	25274907	25274946	0	0.019572
16976	Amp	11	25274947	25275147	0	0.021684
16977	Amp	11	25275362	25275447	0	0.023597
16978	Amp	11	25284667	25288328	0	0.022382
16979	Amp	11	25294516	25392109	0	0.025458
16980	Amp	11	25397864	25404286	0	0.023497
16981	Amp	11	25404514	25493463	0	0.026173
16982	Amp	11	25495753	25584201	0	0.023497
16983	Amp	11	25584216	25706614	0	0.021584
16984	Amp	11	25706884	25752172	0	0.023399
16985	Amp	11	25755544	25837313	0	0.025498
16986	Amp	11	25839287	25844939	0	0.025027
16987	Amp	11	25847788	25961668	0	0.023212
16988	Amp	11	25962958	26048585	0	0.021013
16989	Amp	11	26054505	26153562	0	0.025459
16990	Amp	11	26154965	26176446	0	0.037574
16991	Amp	11	26184825	26244915	0	0.025945
16992	Amp	11	26246016	26289031	0	0.019993
16993	Amp	11	26292210	26303008	0	0.022656
16994	Amp	11	26305044	26389303	0	0.025317
16995	Amp	11	26389383	26391551	0	0.023219
16996	Amp	11	26397038	26404366	0	0.020556
16997	Amp	11	26407676	26500452	0	0.020069
16998	Amp	11	26507242	26622882	0	0.035897
16999	Amp	11	26629144	26704282	0	0.025298
17000	Amp	11	26714369	27032045	0	0.025857
17001	Amp	11	27037558	27161141	0	0.020629
17002	Amp	11	27161948	27170998	0	0.022523
17003	Amp	11	27172552	27253767	0	0.024756
17004	Amp	11	27257263	27356538	0	0.030538
17005	Amp	11	27358650	27396149	0	0.029373
17006	Amp	11	27400906	27447798	0	0.023592
17007	Amp	11	27448863	27545845	0	0.027951
17008	Amp	11	27547026	27590441	0	0.024308
17009	Amp	11	27592171	27639941	0	0.021359
17010	Amp	11	27641279	27721690	0	0.020292
17011	Amp	11	27725635	27937415	0	0.021499
17012	Amp	11	27945377	28104870	0	0.027638

17013	Amp	11	28111659	28127349	0	0.021499
17014	Amp	11	28127530	28156856	0	0.020292
17015	Amp	11	28157176	28445955	0	0.023912
17016	Amp	11	28448297	28462240	0	0.02532
17017	Amp	11	28462765	28529109	0	0.027129
17018	Amp	11	28530459	28540222	0	0.022615
17019	Amp	11	28543584	28761789	0	0.0217
17020	Amp	11	28787036	28799341	0	0.023946
17021	Amp	11	28809503	29030933	0	0.0217
17022	Amp	11	29032437	29429888	0	0.024111
17023	Amp	11	29430011	29437884	0	0.028618
17024	Amp	11	29440405	29488678	0	0.024111
17025	Amp	11	29490637	29526037	0	0.022217
17026	Amp	11	29527209	29580455	0	0.022689
17027	Amp	11	29580532	29626326	0	0.023284
17028	Amp	11	29627059	29674897	0	0.025283
17029	Amp	11	29677337	29679458	0	0.030419
17030	Amp	11	29680500	29753263	0	0.032026
17031	Amp	11	29754638	29773424	0	0.034134
17032	Amp	11	29773472	29786941	0	0.036073
17033	Amp	11	29787002	29842458	0	0.037925
17034	Amp	11	29849047	29900612	0	0.03279
17035	Amp	11	29904487	29967258	0	0.034645
17036	Amp	11	29971909	29983584	0	0.038891
17037	Amp	11	29986467	30004010	0	0.033816
17038	Amp	11	30004433	30090205	0	0.034966
17039	Amp	11	30094508	30143840	0	0.037702
17040	Amp	11	30159138	30159769	0	0.040088
17041	Amp	11	30159823	30191729	0	0.038149
17042	Amp	11	30191867	30211091	0	0.039665
17043	Amp	11	30212847	30254270	0	0.043146
17044	Amp	11	30254781	30290926	0	0.040518
17045	Amp	11	30291721	30304926	0	0.038893
17046	Amp	11	30305897	30357147	0	0.034373
17047	Amp	11	30362353	30478900	0	0.03997
17048	Amp	11	30485548	30523546	0	0.05493
17049	Amp	11	30527920	30601247	0	0.049333
17050	Amp	11	30603811	30616181	0	0.051924
17051	Amp	11	30617045	30641180	0	0.051451
17052	Amp	11	30643355	30670090	0	0.057416
17053	Amp	11	30680474	30684822	0	0.055029
17054	Amp	11	30685711	30685711	0	0.049065
17055	Amp	11	30686088	30728844	0	0.050805
17056	Amp	11	30730731	30756638	0	0.051319
17057	Amp	11	30759384	30791813	0	0.049579
17058	Amp	11	30797054	30797054	0	0.046475
17059	Amp	11	30797475	30811145	0	0.044623
17060	Amp	11	30811437	30882386	0	0.041143
17061	Amp	11	30883763	30888775	0	0.038689
17062	Amp	11	30889897	31134659	0	0.02373
17063	Amp	11	31136848	31145992	0	0.02258
17064	Amp	11	31151513	31293985	0	0.032447
17065	Amp	11	31303716	31363301	0	0.034765

17066	Amp	11	31364093	31418478	0	0.03417
17067	Amp	11	31418928	31543137	0	0.033634
17068	Amp	11	31552056	31581617	0	0.03613
17069	Amp	11	31588129	31621980	0	0.051415
17070	Amp	11	31627928	31665098	0	0.055311
17071	Amp	11	31665395	31674964	0	0.049926
17072	Amp	11	31675155	31742845	0	0.050391
17073	Amp	11	31750239	31760844	0	0.053209
17074	Amp	11	31761078	31768794	0	0.053876
17075	Amp	11	31771181	31793141	0	0.056507
17076	Amp	11	31801110	31829323	0	0.053876
17077	Amp	11	31831237	31846835	0	0.056327
17078	Amp	11	31847143	31887948	0	0.053734
17079	Amp	11	31889582	31915829	0	0.051239
17080	Amp	11	31916385	31919489	0.069848	0.067066
17081	Amp	11	31922322	31966532	0.494792	0.082987
17082	Amp	11	31967426	31997339	0.557122	0.085042
17083	Amp	11	31997822	32016342	0.494792	0.082987
17084	Amp	11	32018063	32055443	0.424557	0.080669
17085	Amp	11	32055926	32082148	0.482379	0.082515
17086	Amp	11	32082644	32100474	0.550755	0.084756
17087	Amp	11	32104309	32169206	0.482379	0.082515
17088	Amp	11	32175387	32188228	0.968522	0.096459
17089	Amp	11	32188764	32204323	1.023539	0.097809
17090	Amp	11	32204899	32221160	0.924746	0.095359
17091	Amp	11	32223975	32241797	0.72817	0.090312
17092	Amp	11	32244251	32262597	0.924746	0.095359
17093	Amp	11	32266135	32311449	1.032525	0.097964
17094	Amp	11	32311761	32328481	0.977077	0.096803
17095	Amp	11	32328710	32347198	1.041205	0.098151
17096	Amp	11	32347228	32360423	1.140417	0.100548
17097	Amp	11	32361674	32362763	1.093834	0.099617
17098	Amp	11	32362840	32386913	1.066493	0.098959
17099	Amp	11	32388771	32415177	1.049301	0.0984
17100	Amp	11	32415216	32425801	1.178324	0.101528
17101	Amp	11	32428253	32451004	1.407997	0.106771
17102	Amp	11	32453688	32471444	1.280185	0.103953
17103	Amp	11	32473416	32489906	1.426786	0.107215
17104	Amp	11	32491213	32496524	1.369395	0.105865
17105	Amp	11	32496545	32510376	1.531891	0.109473
17106	Amp	11	32510869	32571009	1.388256	0.106211
17107	Amp	11	32571218	32572408	1.416817	0.106955
17108	Amp	11	32574565	32587209	1.388256	0.106288
17109	Amp	11	32587708	32634097	1.358811	0.105677
17110	Amp	11	32637380	32780922	1.243254	0.103015
17111	Amp	11	32785707	32815103	1.084294	0.099407
17112	Amp	11	32816742	32830552	1.243254	0.103091
17113	Amp	11	32838592	32843364	1.112463	0.100016
17114	Amp	11	32849939	32869043	1.492063	0.108603
17115	Amp	11	32871566	32872231	1.604336	0.111119
17116	Amp	11	32872251	32877806	1.759227	0.11439
17117	Amp	11	32878395	32878395	1.940776	0.118111
17118	Amp	11	32878887	32904776	2.169666	0.122663

17119	Amp	11	32908670	32916597	1.416817	0.106835
17120	Amp	11	32917153	32941669	1.215958	0.102284
17121	Amp	11	32947712	32955374	1.290368	0.104023
17122	Amp	11	32965304	32979985	1.369395	0.105931
17123	Amp	11	32982442	33038612	1.358811	0.105625
17124	Amp	11	33040192	33139615	1.270711	0.103716
17125	Amp	11	33144516	33149277	1.066493	0.098888
17126	Amp	11	33152498	33165757	1.159209	0.10115
17127	Amp	11	33167470	33186124	1.041205	0.098254
17128	Amp	11	33200289	33244814	1.112463	0.099927
17129	Amp	11	33246908	33280928	1.178324	0.101602
17130	Amp	11	33463183	33471873	0.790614	0.09186
17131	Amp	11	33472426	33511221	0.891602	0.094539
17132	Amp	11	33515952	33516536	0.644502	0.087817
17133	Amp	11	33517807	33517807	0.667587	0.088584
17134	Amp	11	33519198	33541427	0.697374	0.089275
17135	Amp	11	33551621	33567965	0.906385	0.094955
17136	Amp	11	33569780	33581300	0.840289	0.093282
17137	Amp	11	33581367	33611977	0.86541	0.094017
17138	Amp	11	33613049	33637409	0.758088	0.091176
17139	Amp	11	33639873	33650095	0.790614	0.092022
17140	Amp	11	33651998	33704676	0.873678	0.094041
17141	Amp	11	33708715	33716721	0.711903	0.089733
17142	Amp	11	33720111	33776145	0.636464	0.087714
17143	Amp	11	33776268	33818361	0.494792	0.082978
17144	Amp	11	33819235	33826726	0.609133	0.086848
17145	Amp	11	33835537	33835537	0.494792	0.082978
17146	Amp	11	33835879	33942693	0.276783	0.075475
17147	Amp	11	33945148	33981675	0.069848	0.067057
17148	Amp	11	33984695	33989285	0.118001	0.068971
17149	Amp	11	33989537	34013363	0.073902	0.067297
17150	Amp	11	34014727	34045574	0.128324	0.069373
17151	Amp	11	34045943	34166603	0.143787	0.069938
17152	Amp	11	34168506	34206759	0.015369	0.064695
17153	Amp	11	34207297	34305538	0	0.06307
17154	Amp	11	34307230	34318985	0.010261	0.064479
17155	Amp	11	34321238	34321936	0.112543	0.068699
17156	Amp	11	34322103	34361451	0.065715	0.066768
17157	Amp	11	34364752	34489568	0	0.062548
17158	Amp	11	34491204	34598511	0.005328	0.064139
17159	Amp	11	34599457	34613112	0.040093	0.065587
17160	Amp	11	34613904	34617737	0.079652	0.067351
17161	Amp	11	34617953	34617953	0.040093	0.065761
17162	Amp	11	34767796	34767796	0.010261	0.064312
17163	Amp	11	34772477	34979436	0	0.062945
17164	Amp	11	34979551	34981275	0	0.057066
17165	Amp	11	34984355	35003774	0	0.058682
17166	Amp	11	35005049	35065512	0	0.060086
17167	Amp	11	35067974	35089745	0	0.052118
17168	Amp	11	35096707	35148200	0	0.054371
17169	Amp	11	35150575	35158104	0	0.056324
17170	Amp	11	35158142	35169898	0	0.056903
17171	Amp	11	35170667	35184842	0	0.059323

17172	Amp	11	35188651	35199334	0	0.059771
17173	Amp	11	35199350	35223900	0	0.058362
17174	Amp	11	35224360	35237644	0	0.05611
17175	Amp	11	35237860	35305323	0	0.054345
17176	Amp	11	35305494	35342338	0	0.055648
17177	Amp	11	35344627	35348193	0	0.053734
17178	Amp	11	35350573	35361512	0	0.05571
17179	Amp	11	35361668	35418269	0	0.054128
17180	Amp	11	35420845	35432752	0	0.058062
17181	Amp	11	35433575	35451264	0	0.058498
17182	Amp	11	35453542	35587878	0	0.052589
17183	Amp	11	35589270	35644007	0	0.060707
17184	Amp	11	35645315	35718554	0	0.061912
17185	Amp	11	35723682	35791434	0	0.059958
17186	Amp	11	35792371	35894057	0	0.057538
17187	Amp	11	35897669	35956639	0	0.054611
17188	Amp	11	35957266	35980758	0	0.05824
17189	Amp	11	35982301	35991418	0	0.058693
17190	Amp	11	35992406	36086947	0	0.056461
17191	Amp	11	36089241	36105521	0	0.058583
17192	Amp	11	36107345	36113364	0	0.056067
17193	Amp	11	36114999	36178566	0	0.055614
17194	Amp	11	36182775	36235629	0	0.053492
17195	Amp	11	36299004	36385110	0	0.049612
17196	Amp	11	36387645	36420888	0	0.048215
17197	Amp	11	36426791	36440897	0	0.04748
17198	Amp	11	36445607	36459292	0	0.050349
17199	Amp	11	36459436	36464328	0	0.04748
17200	Amp	11	36464514	36549958	0	0.049397
17201	Amp	11	36550227	36628913	0	0.058004
17202	Amp	11	36629167	36654147	0	0.057357
17203	Amp	11	36657198	36791360	0	0.055953
17204	Amp	11	36793245	36806299	0	0.047346
17205	Amp	11	36806319	36917979	0	0.045139
17206	Amp	11	36919576	36919576	0	0.045966
17207	Amp	11	36927497	36942243	0	0.045199
17208	Amp	11	36942293	36942730	0	0.047751
17209	Amp	11	37635347	37644100	0	0.048761
17210	Amp	11	37645912	37650304	0	0.051267
17211	Amp	11	37650611	37665349	0	0.052824
17212	Amp	11	37666990	37719212	0	0.054483
17213	Amp	11	37723900	37895534	0	0.056668
17214	Amp	11	37900222	37917817	0	0.059618
17215	Amp	11	37921805	37937033	0	0.06109
17216	Amp	11	37937425	37980351	0	0.054282
17217	Amp	11	37997391	38067370	0	0.055674
17218	Amp	11	38072843	38072843	0	0.062482
17219	Amp	11	38079641	38079836	0.010261	0.064478
17220	Amp	11	38081025	38081025	0.069848	0.067045
17221	Amp	11	38083230	38084414	0.044794	0.065871
17222	Amp	11	38084553	38101213	0.010261	0.064479
17223	Amp	11	38106128	38128885	0	0.063173
17224	Amp	11	38129683	38256724	0	0.062707

17225	Amp	11	38260390	38262757	0	0.061318
17226	Amp	11	38266909	38274416	0.001323	0.063863
17227	Amp	11	38274931	38281053	0	0.063426
17228	Amp	11	38281659	38303689	0.015369	0.064638
17229	Amp	11	38303809	38317441	0	0.062093
17230	Amp	11	38317678	38331776	0	0.047692
17231	Amp	11	38332845	38377407	0	0.045125
17232	Amp	11	38377594	38699877	0	0.043913
17233	Amp	11	38704876	38758315	0	0.046061
17234	Amp	11	38765693	38802626	0	0.048615
17235	Amp	11	38802947	38874264	0	0.04643
17236	Amp	11	38876216	38877031	0	0.048433
17237	Amp	11	38879645	38895917	0	0.050549
17238	Amp	11	38899001	38902810	0	0.041175
17239	Amp	11	38904531	38910212	0	0.04209
17240	Amp	11	38910398	38931527	0	0.040431
17241	Amp	11	38946110	39082816	0	0.041904
17242	Amp	11	39083172	39172911	0	0.039909
17243	Amp	11	39183221	39196753	0	0.041308
17244	Amp	11	39199711	39220558	0	0.043064
17245	Amp	11	39221088	39250878	0	0.040559
17246	Amp	11	39253998	39301111	0	0.038802
17247	Amp	11	39306975	39369157	0	0.036654
17248	Amp	11	39372501	39452742	0	0.038996
17249	Amp	11	39457353	39479828	0	0.03688
17250	Amp	11	39480592	39532991	0	0.042634
17251	Amp	11	39535580	39645667	0	0.044865
17252	Amp	11	39647900	39681056	0	0.042634
17253	Amp	11	39681759	39685849	0	0.04008
17254	Amp	11	39692379	39739744	0	0.040873
17255	Amp	11	39741081	39777328	0	0.042264
17256	Amp	11	39777391	39784020	0	0.044
17257	Amp	11	39784153	39821095	0	0.042609
17258	Amp	11	39825624	39837842	0	0.041138
17259	Amp	11	39841757	39907613	0	0.040345
17260	Amp	11	39908603	39972060	0	0.038946
17261	Amp	11	39975849	40048857	0	0.040207
17262	Amp	11	40049473	40131173	0	0.042453
17263	Amp	11	40133962	40135133	0	0.040182
17264	Amp	11	40137965	40142797	0	0.037936
17265	Amp	11	40143608	40197564	0	0.03565
17266	Amp	11	40198835	40200898	0	0.029896
17267	Amp	11	40204831	40231737	0	0.032627
17268	Amp	11	40233191	40236150	0	0.034043
17269	Amp	11	40238177	40326376	0	0.031311
17270	Amp	11	40328053	40384014	0	0.033983
17271	Amp	11	40394785	40485438	0	0.032511
17272	Amp	11	40486958	40515580	0	0.029645
17273	Amp	11	40518969	40535454	0	0.026957
17274	Amp	11	40535830	40539184	0	0.02622
17275	Amp	11	40545257	40549969	0	0.034522
17276	Amp	11	40550052	40565201	0	0.036103
17277	Amp	11	40568626	40582479	0	0.043781

17278	Amp	11	40593895	40620799	0	0.054098
17279	Amp	11	40621432	40639274	0	0.052518
17280	Amp	11	40641808	40688713	0	0.047133
17281	Amp	11	40689208	40689208	0	0.044878
17282	Amp	11	40689309	40755457	0	0.04648
17283	Amp	11	40758094	40785044	0	0.039723
17284	Amp	11	40786917	40984816	0	0.055659
17285	Amp	11	40985134	41050051	0	0.055159
17286	Amp	11	41050942	41086835	0	0.05714
17287	Amp	11	41089157	41102762	0	0.055537
17288	Amp	11	41106735	41180951	0	0.058142
17289	Amp	11	41187278	41204308	0	0.063836
17290	Amp	11	41204479	41209151	0	0.06242
17291	Amp	11	41210097	41419277	0	0.056727
17292	Amp	11	41423328	41423823	0	0.055386
17293	Amp	11	41430460	41555431	0	0.044891
17294	Amp	11	41556867	41831251	0	0.028955
17295	Amp	11	41832550	41993077	0	0.029516
17296	Amp	11	42031209	42065015	0	0.033283
17297	Amp	11	42069507	42117753	0	0.029516
17298	Amp	11	42120352	42150133	0	0.027536
17299	Amp	11	42154215	42170387	0	0.030755
17300	Amp	11	42172817	42175803	0	0.027157
17301	Amp	11	42175904	42236316	0	0.026595
17302	Amp	11	42243747	42252952	0	0.028207
17303	Amp	11	42253125	42318694	0	0.024988
17304	Amp	11	42319627	42397743	0	0.023727
17305	Amp	11	42399007	42652991	0	0.026452
17306	Amp	11	42654176	42872303	0	0.028632
17307	Amp	11	42876382	42881151	0	0.027116
17308	Amp	11	42881153	42905220	0	0.031924
17309	Amp	11	42911950	42937561	0	0.028371
17310	Amp	11	42955811	42957403	0	0.022999
17311	Amp	11	42959107	43477902	0	0.022172
17312	Amp	11	43477983	43507036	0	0.023053
17313	Amp	11	43508589	43645151	0	0.02469
17314	Amp	11	43647801	43653380	0	0.027413
17315	Amp	11	43661463	43739540	0	0.043349
17316	Amp	11	43745183	43857990	0	0.050044
17317	Amp	11	43858810	43919052	0	0.052404
17318	Amp	11	43919094	43921444	0	0.052866
17319	Amp	11	43921705	43940334	0	0.056275
17320	Amp	11	43945037	43995055	0	0.041672
17321	Amp	11	43997272	44004423	0	0.047836
17322	Amp	11	44006986	44059615	0	0.062439
17323	Amp	11	44062797	44063819	0	0.060827
17324	Amp	11	44064137	44084318	0	0.058173
17325	Amp	11	44089140	44120300	0	0.063199
17326	Amp	11	44120780	44216628	0.010261	0.064313
17327	Amp	11	44219215	44295369	0.089561	0.067796
17328	Amp	11	44297910	44341524	0.118001	0.068889
17329	Amp	11	44342050	44368007	0.138391	0.069874
17330	Amp	11	44368735	44400997	0	0.053938

17331	Amp	11	44401381	44551685	0	0.052585
17332	Amp	11	44560043	44563791	0	0.052241
17333	Amp	11	44569882	44575682	0	0.059034
17334	Amp	11	44576685	44608533	0	0.062038
17335	Amp	11	44610664	44685306	0	0.057788
17336	Amp	11	44685558	44761770	0	0.061958
17337	Amp	11	44762033	44899732	0.024438	0.065041
17338	Amp	11	44901353	45012196	0.056211	0.066322
17339	Amp	11	45016313	45083490	0.094967	0.068147
17340	Amp	11	45083704	45091415	0.065715	0.066866
17341	Amp	11	45093871	45110784	0.019169	0.064908
17342	Amp	11	45110836	45120149	0	0.06274
17343	Amp	11	45130481	45133802	0	0.059044
17344	Amp	11	45133860	45133860	0	0.058543
17345	Amp	11	45139909	45197443	0	0.057093
17346	Amp	11	45199449	45355265	0	0.059633
17347	Amp	11	45355783	45358999	0	0.061295
17348	Amp	11	45364863	45370005	0	0.057931
17349	Amp	11	45378268	45503599	0	0.058718
17350	Amp	11	45503856	45582935	0	0.057056
17351	Amp	11	45584877	45706009	0	0.058536
17352	Amp	11	45706905	45737393	0	0.057056
17353	Amp	11	45744220	45759034	0	0.050892
17354	Amp	11	45759637	45760721	0	0.050105
17355	Amp	11	45761888	45863816	0	0.049657
17356	Amp	11	45864549	45869693	0	0.049195
17357	Amp	11	45871621	45888300	0	0.051363
17358	Amp	11	45888622	45916266	0	0.047566
17359	Amp	11	45924220	46011553	0	0.043589
17360	Amp	11	46013915	46160058	0	0.037678
17361	Amp	11	46166032	46199995	0	0.042176
17362	Amp	11	46207714	46270504	0	0.048069
17363	Amp	11	46276080	46325615	0	0.04357
17364	Amp	11	46327078	46370750	0	0.046314
17365	Amp	11	46374929	46386919	0	0.050053
17366	Amp	11	46387761	46426112	0	0.049294
17367	Amp	11	46428103	46466436	0	0.052379
17368	Amp	11	46470199	46535915	0	0.04864
17369	Amp	11	46539273	46638067	0	0.045896
17370	Amp	11	46638660	46803915	0	0.037478
17371	Amp	11	46809469	46815996	0	0.040311
17372	Amp	11	46816341	46938254	0	0.037639
17373	Amp	11	46939028	46959626	0	0.043125
17374	Amp	11	46970823	46980583	0	0.0413
17375	Amp	11	46984082	47027229	0	0.039598
17376	Amp	11	47031075	47067830	0	0.036765
17377	Amp	11	47068783	47098658	0	0.038232
17378	Amp	11	47103590	47198243	0	0.040003
17379	Amp	11	47203692	47204445	0	0.032485
17380	Amp	11	47206346	47252021	0	0.029763
17381	Amp	11	47252880	47312973	0	0.032393
17382	Amp	11	47314769	47346060	0	0.038719
17383	Amp	11	47353024	47371752	0	0.034204

17384 Amp	11	47372087	47404084	0	0.039739
17385 Amp	11	47407014	47433211	0	0.041515
17386 Amp	11	47449829	47484022	0	0.038884
17387 Amp	11	47485878	47547272	0	0.035856
17388 Amp	11	47551582	47641229	0	0.03832
17389 Amp	11	47641361	47651755	0	0.040103
17390 Amp	11	47653930	47663473	0	0.042306
17391 Amp	11	47666492	47731223	0	0.047723
17392 Amp	11	47731533	47754857	0	0.045939
17393 Amp	11	47769739	47886253	0	0.047389
17394 Amp	11	47889271	47894233	0	0.043149
17395 Amp	11	47894980	48033911	0	0.041682
17396 Amp	11	48036761	48056324	0	0.040663
17397 Amp	11	48057845	48067931	0	0.039051
17398 Amp	11	48073217	48088815	0	0.037867
17399 Amp	11	48091680	48217170	0	0.038354
17400 Amp	11	48217945	48953076	0	0.032938
17401 Amp	11	48954804	48954804	0	0.027753
17402 Amp	11	48961561	48974607	0	0.025573
17403 Amp	11	48976261	48996010	0	0.028161
17404 Amp	11	48999554	49149366	0	0.032642
17405 Amp	11	49163141	49306978	0	0.028161
17406 Amp	11	49318863	49423807	0	0.030338
17407 Amp	11	49426977	49428175	0	0.028701
17408 Amp	11	49428344	49540287	0	0.030339
17409 Amp	11	49544412	49559138	0	0.030799
17410 Amp	11	49559504	49586087	0	0.031843
17411 Amp	11	49588210	49678015	0	0.032399
17412 Amp	11	49744609	49774918	0	0.033701
17413 Amp	11	49781039	49801915	0	0.033145
17414 Amp	11	49807576	49810079	0	0.031842
17415 Amp	11	49816812	49853162	0	0.030798
17416 Amp	11	49859841	49923730	0	0.039011
17417 Amp	11	49930993	49990649	0	0.044712
17418 Amp	11	49992133	49992218	0	0.047546
17419 Amp	11	50060063	50092125	0	0.051461
17420 Amp	11	50096159	50096159	0	0.040778
17421 Amp	11	50101778	50101778	0	0.04301
17422 Amp	11	56244856	56244856	0	0.02919
17423 Amp	11	56247303	56249180	0	0.017827
17424 Amp	11	56249220	56313324	0	0.01859
17425 Amp	11	56313348	56347137	0	0.017133
17426 Amp	11	56350479	56355718	0	0.013218
17427 Amp	11	56356411	56389319	0	0.011265
17428 Amp	11	56390232	56434217	0	0.012796
17429 Amp	11	56436845	56530047	0	0.011035
17430 Amp	11	56530489	56531635	0	0.013454
17431 Amp	11	56533408	56547032	0	0.014118
17432 Amp	11	56586901	56649785	0	0.017416
17433 Amp	11	56652157	56705570	0	0.014998
17434 Amp	11	56709849	56749665	0	0.015936
17435 Amp	11	56749717	56774503	0	0.028113
17436 Amp	11	56774520	56819930	0	0.030169

17437	Amp	11	56821670	56831151	0	0.03394
17438	Amp	11	56835129	56858299	0	0.03577
17439	Amp	11	56858541	56876235	0	0.034832
17440	Amp	11	56876738	56902914	0	0.037114
17441	Amp	11	56903141	56910970	0	0.040725
17442	Amp	11	56963025	56963025	0	0.046846
17443	Amp	11	56969083	57061764	0	0.055137
17444	Amp	11	57068624	57078463	0	0.051839
17445	Amp	11	57080194	57109185	0	0.050466
17446	Amp	11	57109774	57117667	0	0.047204
17447	Amp	11	57118403	57126113	0	0.048867
17448	Amp	11	57126126	57137168	0	0.047027
17449	Amp	11	57137492	57138975	0	0.049594
17450	Amp	11	57141384	57184291	0	0.04893
17451	Amp	11	57185158	57189042	0	0.040527
17452	Amp	11	57189944	57285799	0	0.038864
17453	Amp	11	57286177	57289378	0	0.036698
17454	Amp	11	57289556	57289600	0	0.036244
17455	Amp	11	57301448	57340304	0	0.038201
17456	Amp	11	57343577	57360843	0	0.036244
17457	Amp	11	57362999	57412165	0	0.037501
17458	Amp	11	57415477	57432517	0	0.044253
17459	Amp	11	57435069	57439974	0	0.045477
17460	Amp	11	57440995	57444173	0	0.038725
17461	Amp	11	57445588	57451616	0	0.036669
17462	Amp	11	57455067	57576686	0	0.038665
17463	Amp	11	57576803	57585089	0	0.040803
17464	Amp	11	57585556	57631599	0	0.03794
17465	Amp	11	57645499	57653730	0	0.036109
17466	Amp	11	57661063	57693265	0	0.045451
17467	Amp	11	57695470	57713546	0	0.046673
17468	Amp	11	57718115	57766617	0	0.049799
17469	Amp	11	57774038	57816074	0	0.055354
17470	Amp	11	57816196	57846140	0	0.058323
17471	Amp	11	57847431	57866721	0	0.054189
17472	Amp	11	57870695	57897076	0	0.050418
17473	Amp	11	57899139	57959612	0	0.041077
17474	Amp	11	57961298	57986318	0	0.038511
17475	Amp	11	58000816	58016835	0	0.041394
17476	Amp	11	58018545	58068777	0	0.039612
17477	Amp	11	58070484	58081219	0	0.051062
17478	Amp	11	58082979	58087259	0	0.039612
17479	Amp	11	58087296	58113573	0	0.041452
17480	Amp	11	58115676	58125955	0	0.045478
17481	Amp	11	58130134	58206178	0	0.047868
17482	Amp	11	58210726	58383323	0	0.044227
17483	Amp	11	58385060	58470409	0	0.043419
17484	Amp	11	58473988	58500617	0	0.05
17485	Amp	11	58502750	58519413	0	0.050947
17486	Amp	11	58524692	58550583	0	0.056314
17487	Amp	11	58555905	58586212	0	0.052288
17488	Amp	11	58587958	58587958	0	0.04692
17489	Amp	11	58594735	58599815	0	0.049044

17490	Amp	11	58603410	58653538	0	0.047462
17491	Amp	11	58657751	58678404	0	0.049269
17492	Amp	11	58685135	58690022	0	0.054362
17493	Amp	11	58693253	58741499	0	0.056363
17494	Amp	11	58743234	58745643	0	0.051269
17495	Amp	11	58747674	58777319	0	0.047355
17496	Amp	11	58780311	58781491	0	0.050952
17497	Amp	11	58786055	58834502	0	0.053806
17498	Amp	11	58835031	58849881	0	0.046965
17499	Amp	11	58850900	58880758	0	0.045588
17500	Amp	11	58881509	58887908	0	0.043781
17501	Amp	11	58890161	58904965	0	0.044585
17502	Amp	11	58907095	58907095	0	0.040988
17503	Amp	11	58910569	58913897	0	0.038134
17504	Amp	11	58917661	59011170	0	0.040035
17505	Amp	11	59023521	59038470	0	0.04674
17506	Amp	11	59039768	59097071	0	0.047876
17507	Amp	11	59101750	59117520	0	0.049292
17508	Amp	11	59122214	59126288	0	0.050765
17509	Amp	11	59126419	59168900	0	0.051388
17510	Amp	11	59170244	59181541	0	0.061667
17511	Amp	11	59181907	59181907	0.019169	0.064798
17512	Amp	11	59186138	59191556	0.222041	0.073118
17513	Amp	11	59193648	59257902	0.187449	0.071702
17514	Amp	11	59258842	59269220	0.024438	0.064997
17515	Amp	11	59272054	59322542	0	0.05119
17516	Amp	11	59323329	59328307	0	0.049061
17517	Amp	11	59331211	59348238	0	0.046092
17518	Amp	11	59355509	59389098	0	0.062028
17519	Amp	11	59389777	59401319	0	0.060892
17520	Amp	11	59403530	59476233	0	0.057761
17521	Amp	11	59479767	59497886	0.019169	0.064816
17522	Amp	11	59506197	59521112	0	0.04888
17523	Amp	11	59523572	59536627	0	0.046313
17524	Amp	11	59538674	59547844	0	0.044412
17525	Amp	11	59561366	59578204	0	0.041216
17526	Amp	11	59580907	59713720	0	0.043267
17527	Amp	11	59718386	59757855	0	0.049854
17528	Amp	11	59758268	59768706	0	0.040907
17529	Amp	11	59769227	59793994	0	0.04368
17530	Amp	11	59797583	59855478	0	0.044309
17531	Amp	11	59858538	59873601	0	0.04931
17532	Amp	11	59877137	59952943	0	0.0513
17533	Amp	11	59955604	59995615	0	0.055608
17534	Amp	11	59995798	59995798	0.079652	0.067314
17535	Amp	11	60022835	60053905	0.500841	0.08325
17536	Amp	11	60056738	60056738	0.630438	0.087377
17537	Amp	11	60057053	60072314	0.550755	0.084714
17538	Amp	11	60074525	60088594	0.399144	0.079713
17539	Amp	11	60088895	60107966	0.636464	0.087635
17540	Amp	11	60107979	60117647	0.72817	0.090213
17541	Amp	11	60120195	60130794	0.362016	0.078507
17542	Amp	11	60132633	60132633	0.711903	0.089849

17543	Amp	11	60133341	60151040	0.6737	0.08872
17544	Amp	11	60151865	60163882	0.933745	0.095714
17545	Amp	11	60163968	60183044	0.630438	0.087402
17546	Amp	11	60187804	60246384	0.758088	0.091141
17547	Amp	11	60248884	60270949	0.644502	0.087857
17548	Amp	11	60274624	60284677	0.404718	0.079978
17549	Amp	11	60286138	60340652	0.47716	0.082344
17550	Amp	11	60343649	60343649	0.431198	0.08087
17551	Amp	11	60343679	60347011	0.482379	0.082534
17552	Amp	11	60349653	60353081	0.536238	0.084333
17553	Amp	11	60353232	60394914	0.557122	0.084989
17554	Amp	11	60396162	60431573	0.758088	0.091042
17555	Amp	11	60441915	60464342	0.602643	0.086523
17556	Amp	11	60464513	60485468	0.463534	0.08187
17557	Amp	11	60485963	60496849	0.557122	0.085095
17558	Amp	11	60500297	60501767	0.379885	0.079043
17559	Amp	11	60511474	60572420	0.543344	0.084496
17560	Amp	11	60572718	60606374	0.450702	0.081569
17561	Amp	11	60609842	60611950	0.482379	0.08263
17562	Amp	11	60615994	60615994	0.616549	0.087021
17563	Amp	11	60617023	60628950	0.522881	0.084009
17564	Amp	11	60631682	60640801	0.34892	0.078128
17565	Amp	11	60646586	60684934	0.293269	0.076076
17566	Amp	11	60689426	60695482	0.373599	0.079018
17567	Amp	11	60697688	60705043	0.807647	0.092464
17568	Amp	11	60709688	60722181	0.924746	0.095501
17569	Amp	11	60727289	60736912	1.014678	0.097551
17570	Amp	11	60741425	60751314	0.848692	0.093424
17571	Amp	11	60753343	60772344	0.807647	0.092363
17572	Amp	11	60782493	60782697	0.933745	0.095679
17573	Amp	11	60788745	60797368	0.602643	0.086537
17574	Amp	11	60797771	60865388	0.522881	0.084024
17575	Amp	11	60865711	60865711	0.557122	0.085098
17576	Amp	11	60866749	60866749	0.6737	0.088672
17577	Amp	11	60868754	60892106	0.751221	0.090917
17578	Amp	11	60892996	60909523	0.857296	0.093594
17579	Amp	11	60910433	60971176	0.933745	0.095627
17580	Amp	11	60971360	61007120	1.014678	0.097575
17581	Amp	11	61014857	61051569	0.924746	0.095541
17582	Amp	11	61051949	61093561	0.941704	0.095915
17583	Amp	11	61094334	61094334	1.140417	0.100662
17584	Amp	11	61098329	61098329	0.995635	0.097088
17585	Amp	11	61098488	61113194	1.188121	0.101642
17586	Amp	11	61114006	61121776	1.290368	0.104067
17587	Amp	11	61122570	61122570	1.456037	0.107914
17588	Amp	11	61129817	61130533	1.665486	0.112264
17589	Amp	11	61131504	61143146	1.780186	0.114703
17590	Amp	11	61144690	61154570	1.843664	0.116111
17591	Amp	11	61157892	61158595	1.737623	0.113857
17592	Amp	11	61162404	61175244	1.769915	0.114446
17593	Amp	11	61175599	61197798	1.552619	0.109947
17594	Amp	11	61197828	61198004	1.812192	0.115372
17595	Amp	11	61252993	61255033	2.256542	0.12427

17596	Amp	11	61255079	61262511	2.322689	0.125575
17597	Amp	11	61265755	61311703	2.684894	0.132518
17598	Amp	11	61318915	61331857	2.649655	0.131929
17599	Amp	11	61344930	61344930	2.582422	0.130705
17600	Amp	11	61346817	61367060	2.435195	0.127763
17601	Amp	11	61369675	61377347	2.379392	0.12669
17602	Amp	11	61382892	61383879	2.345513	0.126061
17603	Amp	11	61385158	61390432	2.661431	0.132114
17604	Amp	11	61397808	61398269	2.594754	0.130794
17605	Amp	11	61402502	61403030	2.743935	0.133722
17606	Amp	11	61412256	61419362	3.08058	0.139865
17607	Amp	11	61424851	61430521	3.183474	0.141837
17608	Amp	11	61438761	61470748	3.045223	0.139323
17609	Amp	11	61473723	61495260	2.871582	0.135938
17610	Amp	11	61503723	61540785	3.09333	0.140091
17611	Amp	11	61549427	61556451	3.516868	0.148065
17612	Amp	11	61560494	61570773	2.860424	0.135868
17613	Amp	11	61573093	61573093	2.997552	0.138394
17614	Amp	11	61575224	61585305	2.929372	0.137019
17615	Amp	11	61593828	61604172	3.021176	0.138812
17616	Amp	11	61616211	61652472	2.356694	0.126197
17617	Amp	11	61655512	61660771	2.22459	0.123671
17618	Amp	11	61671062	61701467	1.919241	0.117618
17619	Amp	11	61702256	61731528	1.843664	0.11602
17620	Amp	11	61741917	61745944	1.696256	0.112934
17621	Amp	11	61749438	61766436	1.655056	0.112204
17622	Amp	11	61766538	61766538	1.706315	0.113191
17623	Amp	11	61770884	61785617	1.780186	0.11472
17624	Amp	11	61795125	61803311	1.696256	0.113057
17625	Amp	11	61803876	61834436	1.329472	0.104928
17626	Amp	11	61837310	61859449	1.445847	0.107664
17627	Amp	11	61860134	61884231	1.522708	0.109406
17628	Amp	11	61888057	61890235	1.416817	0.107034
17629	Amp	11	61890385	61890385	1.299876	0.104298
17630	Amp	11	61901041	61923429	1.23519	0.10277
17631	Amp	11	61923798	61945381	1.131695	0.100374
17632	Amp	11	61947644	61953405	1.014678	0.097697
17633	Amp	11	61956837	61959966	0.457501	0.081811
17634	Amp	11	61960319	61960319	0.386426	0.079444
17635	Amp	11	61960366	61987699	0.227787	0.073301
17636	Amp	11	61991100	61992218	0.368062	0.078631
17637	Amp	11	61998420	62002203	0.264858	0.074976
17638	Amp	11	62004442	62010997	0.133193	0.069646
17639	Amp	11	62012550	62043460	0.19964	0.072287
17640	Amp	11	62044623	62091577	0.187449	0.071727
17641	Amp	11	62096308	62148278	0.094967	0.067988
17642	Amp	11	62148351	62151786	0.143787	0.069925
17643	Amp	11	62154221	62209149	0.089561	0.067953
17644	Amp	11	62210120	62210120	0.170723	0.071115
17645	Amp	11	62211366	62237473	0.112543	0.068816
17646	Amp	11	62238650	62242411	0.040093	0.065653
17647	Amp	11	62243391	62243514	0	0.058861
17648	Amp	11	62243773	62297243	0	0.060933

17649	Amp	11	62300282	62301893	0	0.059134
17650	Amp	11	62312570	62321517	0	0.062448
17651	Amp	11	62389946	62433659	0	0.060082
17652	Amp	11	62433790	62438065	0	0.061115
17653	Amp	11	62442062	62453817	0	0.057801
17654	Amp	11	62456370	62484670	0	0.057589
17655	Amp	11	62487232	62492533	0	0.05573
17656	Amp	11	62493947	62536350	0	0.061598
17657	Amp	11	62547074	62560566	0	0.05573
17658	Amp	11	62561801	62573099	0	0.060384
17659	Amp	11	62583686	62599809	0	0.057805
17660	Amp	11	62603055	62655463	0	0.057183
17661	Amp	11	62658462	62681104	0	0.058052
17662	Amp	11	62688853	62746827	0	0.06181
17663	Amp	11	62759612	62798075	0	0.060611
17664	Amp	11	62799964	62810734	0	0.062661
17665	Amp	11	62813984	62847813	0	0.058903
17666	Amp	11	62849839	62852157	0	0.060581
17667	Amp	11	62852565	62877500	0	0.061311
17668	Amp	11	62885307	62928178	0	0.058606
17669	Amp	11	62928945	62941079	0	0.061177
17670	Amp	11	62944047	62944047	0	0.058536
17671	Amp	11	62947319	62966137	0	0.055966
17672	Amp	11	62977427	62977722	0	0.05265
17673	Amp	11	62982022	62984186	0	0.056239
17674	Amp	11	62986484	63020837	0	0.05027
17675	Amp	11	63022618	63033676	0	0.054599
17676	Amp	11	63035084	63035777	0	0.056765
17677	Amp	11	63036053	63046762	0	0.053176
17678	Amp	11	63048608	63070597	0	0.054582
17679	Amp	11	63070684	63105905	0	0.056844
17680	Amp	11	63108552	63113457	0	0.052573
17681	Amp	11	63125191	63182628	0	0.050991
17682	Amp	11	63192632	63220399	0	0.051944
17683	Amp	11	63221671	63228690	0	0.055479
17684	Amp	11	63230749	63320389	0	0.053517
17685	Amp	11	63320479	63320479	0	0.052467
17686	Amp	11	63321441	63350788	0	0.057559
17687	Amp	11	63351994	63380766	0	0.060816
17688	Amp	11	63384604	63395748	0	0.056162
17689	Amp	11	63397928	63418436	0	0.058589
17690	Amp	11	63441537	63441537	0	0.057182
17691	Amp	11	63442626	63443652	0	0.0612
17692	Amp	11	63445248	63461150	0	0.056933
17693	Amp	11	63461999	63489939	0	0.061499
17694	Amp	11	63493516	63527859	0	0.055054
17695	Amp	11	63530280	63530280	0	0.057912
17696	Amp	11	63532586	63533547	0	0.060576
17697	Amp	11	63539617	63546966	0	0.054115
17698	Amp	11	63548288	63564880	0	0.052323
17699	Amp	11	63566746	63575166	0	0.047758
17700	Amp	11	63575772	63593970	0	0.052449
17701	Amp	11	63599381	63610356	0	0.047758

17702	Amp	11	63611777	63628912	0	0.048854
17703	Amp	11	63629725	63647630	0	0.052974
17704	Amp	11	63648284	63664968	0	0.052244
17705	Amp	11	63673779	63706992	0	0.043795
17706	Amp	11	63707190	63757454	0	0.041131
17707	Amp	11	63758953	63758953	0	0.045306
17708	Amp	11	63770037	63792727	0	0.045764
17709	Amp	11	63796951	63848156	0	0.053139
17710	Amp	11	63855160	63855852	0	0.060093
17711	Amp	11	63858318	63874038	0	0.052718
17712	Amp	11	63876290	63899472	0	0.058583
17713	Amp	11	63901899	63924466	0	0.057487
17714	Amp	11	63925331	63928715	0	0.055437
17715	Amp	11	63935163	63942544	0	0.048483
17716	Amp	11	63949244	63949244	0	0.051099
17717	Amp	11	63987039	63987039	0	0.056141
17718	Amp	11	63987743	63990752	0	0.058154
17719	Amp	11	63991669	64062033	0	0.059142
17720	Amp	11	64064670	64107984	0.246818	0.074091
17721	Amp	11	64108510	64122070	0.227787	0.073265
17722	Amp	11	64126582	64177216	0.17597	0.071363
17723	Amp	11	64193264	64197185	0.241616	0.074026
17724	Amp	11	64205924	64220868	0.094967	0.068161
17725	Amp	11	64224329	64242772	0.08435	0.067715
17726	Amp	11	64258382	64294201	0.024438	0.065053
17727	Amp	11	64296608	64304772	0.170723	0.071174
17728	Amp	11	64309089	64324487	0.138391	0.069754
17729	Amp	11	64325257	64377053	0.065715	0.066754
17730	Amp	11	64378587	64391781	0.14896	0.07015
17731	Amp	11	64392796	64431315	0.17597	0.071433
17732	Amp	11	64432090	64464423	0.264858	0.075024
17733	Amp	11	64469561	64475263	0.386426	0.079423
17734	Amp	11	64482453	64521170	0.424557	0.080709
17735	Amp	11	64521519	64545819	0.444474	0.081403
17736	Amp	11	64547505	64557737	0.529451	0.084132
17737	Amp	11	64564446	64580182	0.34892	0.078011
17738	Amp	11	64584338	64593233	0	0.063495
17739	Amp	11	64613010	64617708	0.050473	0.066068
17740	Amp	11	64617966	64720582	0.005328	0.064078
17741	Amp	11	64724272	64743850	0	0.062329
17742	Amp	11	64747034	64748600	0	0.058793
17743	Amp	11	64756022	64772163	0	0.057841
17744	Amp	11	64772641	64775429	0	0.057049
17745	Amp	11	64782881	64817515	0	0.056591
17746	Amp	11	64817543	64817543	0	0.057919
17747	Amp	11	64825254	64825254	0	0.060676
17748	Amp	11	64825436	64825436	0	0.061235
17749	Amp	11	64828200	64828200	0	0.062987
17750	Amp	11	65902628	65902628	2.26699	0.124492
17751	Amp	11	65903039	65903039	3.067933	0.139747
17752	Amp	11	65906669	65922038	3.5415	0.148406
17753	Amp	11	65922184	65941956	3.286256	0.143803
17754	Amp	11	65943186	65945545	3.852343	0.153914

17755	Amp	11	65951146	65960837	4.102728	0.15808
17756	Amp	11	65961336	65961336	3.701758	0.151199
17757	Amp	11	65965818	65989869	3.726055	0.151674
17758	Amp	11	65996659	65996659	4.037384	0.157108
17759	Amp	11	65997636	65997636	3.150497	0.141172
17760	Amp	11	65998573	65998573	2.917729	0.137006
17761	Amp	11	65999682	66001182	3.150497	0.141348
17762	Amp	11	66003187	66008121	2.743935	0.133753
17763	Amp	11	66009114	66020333	3.009724	0.138729
17764	Amp	11	66020601	66035007	3.275143	0.14371
17765	Amp	11	66037497	66053219	3.458499	0.146809
17766	Amp	11	66055860	66074331	3.554363	0.148525
17767	Amp	11	66076244	66078166	3.701758	0.151268
17768	Amp	11	66081785	66081805	3.628913	0.149876
17769	Amp	11	66087670	66088674	3.654159	0.15038
17770	Amp	11	66097565	66097833	3.972019	0.156103
17771	Amp	11	66100180	66115267	4.076315	0.157792
17772	Amp	11	66116051	66124327	4.484961	0.164179
17773	Amp	11	66127182	66133306	4.443348	0.163675
17774	Amp	11	66138603	66139024	4.69701	0.167497
17775	Amp	11	66142658	66192940	4.753798	0.168351
17776	Amp	11	66196266	66197911	4.388129	0.162722
17777	Amp	11	66206009	66206009	4.979649	0.17191
17778	Amp	11	66210400	66220841	5.02373	0.172546
17779	Amp	11	66222287	66222287	5.140232	0.174317
17780	Amp	11	66224537	66238739	5.39004	0.177871
17781	Amp	11	66248302	66249476	5.942149	0.18594
17782	Amp	11	66262530	66270638	6.886863	0.199407
17783	Amp	11	66285772	66299522	6.291248	0.190904
17784	Amp	11	66300832	66300832	6.380906	0.192226
17785	Amp	11	66309662	66309662	6.840574	0.198775
17786	Amp	11	66309732	66310131	6.949342	0.200322
17787	Amp	11	66313740	66322561	7.75231	0.211447
17788	Amp	11	66324239	66328681	7.945028	0.213911
17789	Amp	11	66333189	66345103	8.34537	0.219379
17790	Amp	11	66346497	66377713	8.555266	0.222284
17791	Amp	11	66378833	66389360	9.054921	0.22865
17792	Amp	11	66399692	66403140	9.240499	0.231215
17793	Amp	11	66412397	66413597	9.592302	0.235814
17794	Amp	11	66416452	66417883	10.808758	0.251055
17795	Amp	11	66428834	66428834	11.662686	0.261762
17796	Amp	11	66433036	66444958	11.308217	0.257507
17797	Amp	11	66445748	66445748	11.590867	0.260956
17798	Amp	11	66450108	66475158	11.081157	0.25459
17799	Amp	11	66476983	66484325	12.094071	0.267008
17800	Amp	11	66486886	66492717	12.401399	0.270771
17801	Amp	11	66504766	66504766	12.383029	0.270455
17802	Amp	11	66505353	66505505	12.347637	0.269915
17803	Amp	11	66509105	66509105	12.383029	0.270509
17804	Amp	11	66513049	66520605	12.003259	0.265868
17805	Amp	11	66529338	66540908	11.464362	0.259401
17806	Amp	11	66558891	66558891	11.698383	0.262297
17807	Amp	11	66562261	66564938	11.716436	0.262328

17808	Amp	11	66565601	66565601	12.745398	0.274717
17809	Amp	11	66565728	66567837	12.964742	0.277343
17810	Amp	11	66577851	66578235	13.874487	0.288355
17811	Amp	11	66582642	66584269	13.729199	0.286507
17812	Amp	11	66595411	66595411	14.113089	0.291147
17813	Amp	11	66601587	66601587	14.725554	0.298327
17814	Amp	11	66610375	66623859	14.300473	0.293352
17815	Amp	11	66632210	66640689	14.577855	0.296553
17816	Amp	11	66647208	66658011	14.631516	0.297194
17817	Amp	11	66659167	66664759	14.968996	0.30114
17818	Amp	11	66666418	66684491	14.668813	0.297597
17819	Amp	11	66690573	66694936	14.169996	0.291622
17820	Amp	11	66695061	66695061	14.95044	0.300943
17821	Amp	11	66700237	66709684	14.725554	0.298351
17822	Amp	11	66713668	66734498	14.779959	0.298864
17823	Amp	11	66735528	66792844	15.137413	0.303088
17824	Amp	11	66794640	66801376	14.836401	0.299533
17825	Amp	11	66801472	66801472	14.447899	0.294955
17826	Amp	11	66803124	66803124	15.385452	0.305843
17827	Amp	11	66805802	66818733	15.672322	0.309121
17828	Amp	11	66827589	66827589	15.595327	0.30832
17829	Amp	11	66828026	66841520	15.442	0.306567
17830	Amp	11	66852333	66873601	15.977907	0.312475
17831	Amp	11	66876485	66877783	16.958374	0.323706
17832	Amp	11	66877907	66882589	17.038719	0.324612
17833	Amp	11	66889132	66899346	17.216433	0.326534
17834	Amp	11	66905608	66905721	16.224361	0.315302
17835	Amp	11	66906235	66906235	16.513277	0.318748
17836	Amp	11	66906778	66910537	16.340767	0.316798
17837	Amp	11	66912116	66912267	16.456552	0.318062
17838	Amp	11	66918174	66953557	16.224361	0.315436
17839	Amp	11	66956919	66956919	15.538779	0.307525
17840	Amp	11	66964195	66964195	15.940153	0.312117
17841	Amp	11	66965350	66972258	16.704765	0.320843
17842	Amp	11	66985824	66985824	16.397251	0.317447
17843	Amp	11	66987138	66990942	16.589822	0.319505
17844	Amp	11	66993461	66993992	16.070661	0.313587
17845	Amp	11	66999239	66999239	16.647433	0.320174
17846	Amp	11	66999340	67013013	16.938203	0.323532
17847	Amp	11	67016475	67025858	16.762041	0.321472
17848	Amp	11	67036566	67037277	16.126505	0.314308
17849	Amp	11	67046835	67046835	16.204394	0.315135
17850	Amp	11	67050897	67054048	17.392969	0.328437
17851	Amp	11	67057713	67064607	16.359113	0.316839
17852	Amp	11	67065041	67082396	16.958374	0.323658
17853	Amp	11	67084617	67091644	16.704765	0.320911
17854	Amp	11	67093470	67100374	17.038719	0.324455
17855	Amp	11	67103077	67112637	17.452845	0.329066
17856	Amp	11	67117271	67117271	18.307307	0.338438
17857	Amp	11	67122243	67122243	19.772229	0.354374
17858	Amp	11	67123185	67123185	19.47108	0.351017
17859	Amp	11	67126829	67128083	19.951408	0.356305
17860	Amp	11	67129317	67131021	20.195102	0.358797

17861	Amp	11	67134948	67137588	20.317454	0.360105
17862	Amp	11	67143355	67151807	21.727651	0.374918
17863	Amp	11	67155392	67156104	21.367762	0.371229
17864	Amp	11	67161007	67161007	21.557483	0.373228
17865	Amp	11	67162289	67162289	21.790241	0.375586
17866	Amp	11	67164973	67171237	22.8638	0.386695
17867	Amp	11	67171282	67192236	23.048513	0.388475
17868	Amp	11	67192934	67192934	23.282967	0.391064
17869	Amp	11	67199191	67208276	23.630134	0.394531
17870	Amp	11	67213037	67226807	24.436511	0.402538
17871	Amp	11	67230774	67235918	24.653584	0.404624
17872	Amp	11	67236827	67236827	25.765319	0.415778
17873	Amp	11	67248979	67253960	24.631658	0.40448
17874	Amp	11	67257903	67260072	24.96221	0.407752
17875	Amp	11	67264085	67264497	24.719836	0.405275
17876	Amp	11	67265037	67265037	25.938335	0.417475
17877	Amp	11	67268530	67273727	25.569265	0.413795
17878	Amp	11	67274739	67274739	25.827796	0.416287
17879	Amp	11	67286673	67286673	26.132798	0.419352
17880	Amp	11	67292163	67292163	25.006651	0.408055
17881	Amp	11	67292735	67292735	24.675589	0.40491
17882	Amp	11	67293901	67323722	24.415068	0.402351
17883	Amp	11	67324281	67324281	25.114202	0.409276
17884	Amp	11	67326389	67326389	25.006651	0.408197
17885	Amp	11	67331320	67337301	25.350724	0.411697
17886	Amp	11	67338511	67344325	25.178523	0.409871
17887	Amp	11	67346734	67348402	25.372008	0.411891
17888	Amp	11	67349148	67355846	25.634956	0.41434
17889	Amp	11	67357752	67357980	25.178523	0.409905
17890	Amp	11	67358493	67362651	25.28518	0.411028
17891	Amp	11	67370475	67370475	25.591369	0.414069
17892	Amp	11	67381553	67386141	26.112011	0.41928
17893	Amp	11	67386997	67386997	27.379331	0.431551
17894	Amp	11	67390233	67400259	27.516086	0.432953
17895	Amp	11	67402340	67403095	27.201197	0.429741
17896	Amp	11	67412338	67412554	27.447865	0.432178
17897	Amp	11	67413540	67413540	26.980394	0.427615
17898	Amp	11	67422921	67422921	27.180986	0.429633
17899	Amp	11	67425631	67429112	26.912741	0.427068
17900	Amp	11	67432612	67447734	27.025719	0.428053
17901	Amp	11	67457123	67460070	27.734305	0.435131
17902	Amp	11	67464418	67486981	26.155333	0.419527
17903	Amp	11	67487699	67489030	25.028351	0.408418
17904	Amp	11	67490085	67501654	25.350724	0.411608
17905	Amp	11	67508998	67514619	25.114202	0.40917
17906	Amp	11	67518604	67526837	25.006651	0.408013
17907	Amp	11	67529387	67529387	26.533906	0.42332
17908	Amp	11	68003122	68003122	24.501008	0.403204
17909	Amp	11	68003149	68003149	23.457147	0.392658
17910	Amp	11	68007631	68014099	21.07529	0.36806
17911	Amp	11	68018085	68020773	22.507409	0.382999
17912	Amp	11	68031956	68051786	22.904311	0.387114
17913	Amp	11	68052144	68052158	22.967626	0.387728

17914	Amp	11	68052335	68054155	22.761806	0.385438
17915	Amp	11	68058247	68059713	21.034527	0.367623
17916	Amp	11	68060617	68060617	20.296825	0.359804
17917	Amp	11	68066076	68075354	20.522128	0.362244
17918	Amp	11	68078103	68082569	20.399182	0.360936
17919	Amp	11	68087165	68087553	20.236975	0.359312
17920	Amp	11	68089043	68104771	20.440924	0.361399
17921	Amp	11	68105140	68115280	20.725611	0.364547
17922	Amp	11	68116898	68117036	20.440924	0.361389
17923	Amp	11	68123822	68126000	19.91297	0.355779
17924	Amp	11	68133959	68135293	20.032409	0.357027
17925	Amp	11	68139530	68140022	18.60467	0.341768
17926	Amp	11	68148226	68155201	19.368926	0.349915
17927	Amp	11	68160725	68163594	19.693709	0.353349
17928	Amp	11	68164294	68171381	19.852569	0.355103
17929	Amp	11	68171488	68192626	19.752676	0.354048
17930	Amp	11	68197993	68200664	20.032409	0.357139
17931	Amp	11	68202099	68202099	20.073356	0.35761
17932	Amp	11	68204943	68209809	19.91297	0.355858
17933	Amp	11	68210640	68212332	19.368926	0.349826
17934	Amp	11	68215524	68229339	19.12459	0.347368
17935	Amp	11	68232756	68233112	18.824364	0.344033
17936	Amp	11	68238256	68241286	19.185603	0.347907
17937	Amp	11	68245676	68250375	19.246606	0.34858
17938	Amp	11	68254538	68255794	19.792143	0.354583
17939	Amp	11	68256494	68260513	19.91297	0.355779
17940	Amp	11	68260636	68271224	19.633539	0.352736
17941	Amp	11	68276168	68292635	19.287215	0.349117
17942	Amp	11	68298566	68298566	19.892364	0.35555
17943	Amp	11	68307527	68307527	20.764613	0.364978
17944	Amp	11	68310060	68314225	21.876573	0.376349
17945	Amp	11	68314526	68314526	21.451458	0.372004
17946	Amp	11	68320527	68323797	21.706325	0.3746
17947	Amp	11	68326825	68353115	21.578837	0.373403
17948	Amp	11	68357571	68359582	21.472484	0.372264
17949	Amp	11	68368601	68369505	21.178346	0.369221
17950	Amp	11	68376815	68382244	21.727651	0.374819
17951	Amp	11	68385254	68399426	21.472484	0.372328
17952	Amp	11	68400760	68400760	21.157024	0.369109
17953	Amp	11	68407930	68408011	21.706325	0.374727
17954	Amp	11	68410240	68416451	22.380843	0.381625
17955	Amp	11	68432454	68432454	21.578837	0.373292
17956	Amp	11	68433534	68435112	21.790241	0.375591
17957	Amp	11	68438705	68439174	22.151048	0.379191
17958	Amp	11	68440465	68440465	22.507409	0.382961
17959	Amp	11	68444152	68444152	22.337402	0.381207
17960	Amp	11	68455977	68456574	20.011995	0.356964
17961	Amp	11	68458551	68469910	19.712397	0.353564
17962	Amp	11	68470511	68481999	19.892364	0.355537
17963	Amp	11	68484233	68484233	18.882836	0.344625
17964	Amp	11	68486860	68488842	19.084146	0.346842
17965	Amp	11	68489918	68489918	19.12459	0.347286
17966	Amp	11	68493759	68499317	19.451056	0.350775

17967	Amp	11	68514307	68520151	20.785556	0.365033
17968	Amp	11	68521772	68521772	20.99348	0.367246
17969	Amp	11	68526549	68530487	21.034527	0.367752
17970	Amp	11	68536161	68536161	21.199554	0.369535
17971	Amp	11	68539210	68539210	21.514974	0.372686
17972	Amp	11	68540882	68540882	21.367762	0.371269
17973	Amp	11	68541372	68541372	21.557483	0.373266
17974	Amp	11	68541640	68541640	22.151048	0.379189
17975	Amp	11	68543020	68545969	22.611544	0.383946
17976	Amp	11	68547096	68569706	22.027522	0.377976
17977	Amp	11	68573861	68578809	21.769764	0.375303
17978	Amp	11	68583446	68591156	23.630134	0.394383
17979	Amp	11	68592545	68599539	25.372008	0.411891
17980	Amp	11	68600235	68612528	25.157792	0.409674
17981	Amp	11	68617866	68620981	26.177122	0.419829
17982	Amp	11	68621099	68621120	25.765319	0.415636
17983	Amp	11	68621934	68621934	25.634956	0.414511
17984	Amp	11	68637532	68637532	27.003097	0.427779
17985	Amp	11	68640536	68646570	27.357173	0.431292
17986	Amp	11	68647188	68647188	28.132847	0.439001
17987	Amp	11	68647682	68647767	28.46833	0.442272
17988	Amp	11	68652187	68662219	30.141189	0.458208
17989	Amp	11	68663447	68678826	30.567717	0.462507
17990	Amp	11	68680729	68680823	31.630525	0.472503
17991	Amp	11	68694616	68704011	30.792422	0.464606
17992	Amp	11	68704576	68704576	30.902566	0.465576
17993	Amp	11	68707000	68713241	32.001606	0.476062
17994	Amp	11	68714022	68719040	32.504571	0.480774
17995	Amp	11	68721832	68727650	32.899565	0.48447
17996	Amp	11	68727713	68731999	32.946697	0.485065
17997	Amp	11	68732406	68734430	33.321384	0.488513
17998	Amp	11	68743669	68743669	34.394783	0.498174
17999	Amp	11	68745340	68745998	34.586301	0.500049
18000	Amp	11	68748153	68756486	34.873389	0.502648
18001	Amp	11	68760430	68760430	36.554634	0.517782
18002	Amp	11	68762106	68762850	38.158843	0.532167
18003	Amp	11	68764920	68769366	38.277845	0.533166
18004	Amp	11	68769371	68769544	38.372421	0.534039
18005	Amp	11	68771600	68772848	39.310455	0.542257
18006	Amp	11	68780102	68781000	42.033341	0.566131
18007	Amp	11	68782836	68782836	40.850623	0.555879
18008	Amp	11	68787127	68787127	40.701939	0.554557
18009	Amp	11	68788912	68791429	41.246772	0.5593
18010	Amp	11	68798089	68810356	41.022195	0.557243
18011	Amp	11	68814614	68814614	41.196513	0.558754
18012	Amp	11	68815088	68816203	40.899237	0.556297
18013	Amp	11	68818518	68819703	40.502975	0.552835
18014	Amp	11	68820358	68820358	40.353392	0.551507
18015	Amp	11	68823832	68831066	41.816506	0.564238
18016	Amp	11	68832636	68838202	42.58847	0.570896
18017	Amp	11	68838360	68859610	43.829855	0.581553
18018	Amp	11	68860067	68863772	44.787283	0.589973
18019	Amp	11	68864236	68866476	44.510779	0.587395

18020	Amp	11	68869202	68885097	44.787283	0.589794
18021	Amp	11	68887306	68887306	44.835982	0.590343
18022	Amp	11	68893329	68893329	46.035651	0.600469
18023	Amp	11	68894107	68905623	46.464557	0.604265
18024	Amp	11	68908532	68908532	47.938114	0.616736
18025	Amp	11	68911023	68914934	47.353489	0.611853
18026	Amp	11	68915027	68919965	48.189856	0.618933
18027	Amp	11	68924603	68925075	48.890926	0.62492
18028	Amp	11	68925568	68929237	49.958872	0.633711
18029	Amp	11	68932794	68932794	49.539433	0.630166
18030	Amp	11	68932956	68952067	50.037381	0.634441
18031	Amp	11	68952129	68952129	50.271116	0.636395
18032	Amp	11	68953903	68962196	51.491171	0.646554
18033	Amp	11	68964208	68964487	52.594816	0.655632
18034	Amp	11	68964557	68969593	52.798102	0.657374
18035	Amp	11	68976193	68977376	54.331533	0.6699
18036	Amp	11	68978214	68978430	54.699629	0.672941
18037	Amp	11	68981734	68983009	54.807035	0.673968
18038	Amp	11	68984439	68987575	53.726132	0.66489
18039	Amp	11	68996968	68996968	55.155084	0.676724
18040	Amp	11	68998066	68998296	55.741108	0.681414
18041	Amp	11	69001516	69008161	56.570063	0.688229
18042	Amp	11	69008843	69008843	56.949155	0.691352
18043	Amp	11	69012165	69012165	59.637963	0.712832
18044	Amp	11	69017247	69018317	58.847932	0.706485
18045	Amp	11	69018335	69018335	58.901215	0.706975
18046	Amp	11	69021240	69033149	59.941042	0.715134
18047	Amp	11	69037338	69037338	59.583176	0.7123
18048	Amp	11	69048055	69052961	61.093278	0.724266
18049	Amp	11	69053408	69053408	61.175778	0.72499
18050	Amp	11	69060158	69060249	61.997559	0.731338
18051	Amp	11	69065262	69065262	61.587607	0.72828
18052	Amp	11	69075023	69075127	63.604759	0.744262
18053	Amp	11	69079537	69079791	63.577543	0.744123
18054	Amp	11	69080705	69080705	64.190355	0.748915
18055	Amp	11	69081823	69081823	64.803766	0.753524
18056	Amp	11	69085565	69085565	64.915919	0.754408
18057	Amp	11	69088015	69088486	65.111392	0.755888
18058	Amp	11	69090568	69090681	66.546438	0.76724
18059	Amp	11	69093249	69093404	67.682508	0.776066
18060	Amp	11	69095162	69097201	69.758665	0.791965
18061	Amp	11	69098010	69109119	70.379593	0.796697
18062	Amp	11	69109694	69118988	69.758665	0.792008
18063	Amp	11	69119927	69119927	70.293955	0.796085
18064	Amp	11	69120669	69124816	69.868057	0.79299
18065	Amp	11	69125340	69125340	70.77795	0.79989
18066	Amp	11	69128364	69135233	72.470865	0.812694
18067	Amp	11	69136365	69156129	72.957898	0.816416
18068	Amp	11	69156525	69165764	73.205963	0.818191
18069	Amp	11	69167494	69168595	73.837693	0.823071
18070	Amp	11	69171448	69171448	76.197621	0.840589
18071	Amp	11	69172828	69179390	76.900086	0.845829
18072	Amp	11	69179860	69179860	77.073557	0.847278

18073	Amp	11	69180225	69180237	77.419666	0.849812
18074	Amp	11	69188379	69192736	77.830402	0.852754
18075	Amp	11	69192923	69192923	78.351904	0.856754
18076	Amp	11	69196009	69200189	79.140597	0.862596
18077	Amp	11	69205892	69217367	78.238397	0.855892
18078	Amp	11	69218030	69220641	78.557809	0.858258
18079	Amp	11	69222706	69222706	78.875245	0.860596
18080	Amp	11	69223643	69228976	80.73045	0.87433
18081	Amp	11	69229042	69229042	81.230018	0.878071
18082	Amp	11	69229256	69230205	81.496132	0.879932
18083	Amp	11	69233500	69233785	81.378225	0.878991
18084	Amp	11	69235483	69236833	81.17169	0.877542
18085	Amp	11	69241110	69243184	82.357235	0.886292
18086	Amp	11	69243588	69247794	82.090429	0.88427
18087	Amp	11	69248261	69257999	82.534031	0.887635
18088	Amp	11	69258086	69259237	82.884241	0.890149
18089	Amp	11	69272662	69274380	83.716455	0.896259
18090	Amp	11	69278747	69278747	86.267423	0.914767
18091	Amp	11	69280718	69291284	87.108525	0.92081
18092	Amp	11	69300416	69301278	89.832093	0.94044
18093	Amp	11	69305402	69307825	93.281762	0.964823
18094	Amp	11	69307945	69308810	92.669448	0.960505
18095	Amp	11	69328516	69332029	93.863541	0.969047
18096	Amp	11	69333346	69333346	94.256655	0.97175
18097	Amp	11	69335955	69336055	94.842686	0.976007
18098	Amp	11	69336243	69336243	95.119866	0.977864
18099	Amp	11	69339953	69340105	96.018221	0.984139
18100	Amp	11	69340624	69340624	96.234153	0.985612
18101	Amp	11	69351302	69354557	99.539133	1.008673
18102	Amp	11	69355815	69355815	100.430664	1.014961
18103	Amp	11	69357021	69363857	100.066375	1.012458
18104	Amp	11	69363863	69367952	100.55555	1.015835
18105	Amp	11	69370577	69370577	101.306432	1.021206
18106	Amp	11	69371512	69372653	100.711158	1.017088
18107	Amp	11	69380172	69380172	102.499676	1.029444
18108	Amp	11	69380218	69383521	102.65679	1.030447
18109	Amp	11	69383940	69383940	104.235125	1.041276
18110	Amp	11	69385925	69385925	104.611184	1.043924
18111	Amp	11	69387724	69387848	105.688535	1.051194
18112	Amp	11	69391976	69391976	106.545474	1.057204
18113	Amp	11	69399763	69399935	106.640367	1.057905
18114	Amp	11	69400081	69404387	107.111989	1.06115
18115	Amp	11	69404759	69417028	107.650405	1.064846
18116	Amp	11	69418029	69429308	108.909753	1.073615
18117	Amp	11	69431765	69446499	109.714521	1.079403
18118	Amp	11	69448736	69449854	110.177012	1.082511
18119	Amp	11	69452739	69452739	113.795094	1.108094
18120	Amp	11	69452952	69455897	113.325596	1.104826
18121	Amp	11	69460294	69460759	114.714688	1.11447
18122	Amp	11	69461097	69461097	115.019494	1.116743
18123	Amp	11	69462310	69467061	115.300106	1.118539
18124	Amp	11	69472725	69473878	117.915565	1.136453
18125	Amp	11	69477516	69479371	120.150179	1.151785

18126 Amp	11	69480882	69480882	120.918297	1.157176
18127 Amp	11	69489745	69492676	121.818303	1.164745
18128 Amp	11	69492927	69503323	122.267003	1.167904
18129 Amp	11	69503426	69503971	123.141687	1.174113
18130 Amp	11	69506105	69514906	123.419408	1.17617
18131 Amp	11	69517492	69534101	123.845229	1.179516
18132 Amp	11	69536953	69569215	121.788866	1.163693
18133 Amp	11	69570083	69584415	121.344657	1.160143
18134 Amp	11	69584881	69598492	121.639325	1.162411
18135 Amp	11	69616738	69619535	123.772328	1.179047
18136 Amp	11	69619812	69645337	123.874398	1.180552
18137 Amp	11	69645515	69661481	124.33382	1.184864
18138 Amp	11	69661498	69661511	124.407868	1.190758
18139 Amp	11	69666621	69669813	124.33382	1.187888
18140 Amp	11	69672780	69675970	124.33382	1.18561
18141 Amp	11	69677679	69678503	124.407868	1.190365
18142 Amp	11	69688161	69689724	123.068827	1.173571
18143 Amp	11	69697321	69708300	122.006702	1.16584
18144 Amp	11	69708781	69708781	121.639325	1.162362
18145 Amp	11	69715985	69716657	121.392272	1.160692
18146 Amp	11	69717013	69722153	123.066892	1.173244
18147 Amp	11	69724133	69724133	121.788866	1.1638
18148 Amp	11	69724944	69726222	121.484547	1.161352
18149 Amp	11	69728315	69735033	120.710738	1.155667
18150 Amp	11	69735836	69746737	120.408558	1.153481
18151 Amp	11	69749625	69753669	119.140234	1.145026
18152 Amp	11	69754456	69768209	118.892519	1.143441
18153 Amp	11	69771149	69772815	117.915565	1.136498
18154 Amp	11	69778046	69778046	118.536172	1.14096
18155 Amp	11	69778408	69778408	118.763589	1.142546
18156 Amp	11	69779237	69780246	119.266568	1.14586
18157 Amp	11	69781755	69781755	118.912718	1.143471
18158 Amp	11	69782674	69783019	116.75756	1.128574
18159 Amp	11	69785769	69795680	114.772981	1.114839
18160 Amp	11	69796026	69804942	115.180561	1.117758
18161 Amp	11	69808619	69808873	115.931793	1.122975
18162 Amp	11	69809331	69814168	117.635135	1.134485
18163 Amp	11	69814431	69814431	117.17876	1.131478
18164 Amp	11	69817131	69824617	116.285138	1.125436
18165 Amp	11	69827478	69832611	115.845189	1.122276
18166 Amp	11	69832982	69836687	116.569403	1.127282
18167 Amp	11	69843442	69843442	117.635135	1.134526
18168 Amp	11	69845437	69854915	117.998909	1.13713
18169 Amp	11	69854948	69854948	117.635135	1.134503
18170 Amp	11	69855949	69862486	118.119035	1.137921
18171 Amp	11	69866140	69884020	118.470925	1.140341
18172 Amp	11	69884534	69885178	117.915565	1.136305
18173 Amp	11	69885212	69885212	117.688252	1.134822
18174 Amp	11	69954050	69954050	110.177012	1.08246
18175 Amp	11	69959977	69960164	109.955886	1.08113
18176 Amp	11	69961088	69967139	109.365965	1.076913
18177 Amp	11	69967622	69968824	109.77195	1.079834
18178 Amp	11	69974461	69974461	110.203044	1.082746

18179	Amp	11	69981800	69984330	110.846085	1.087175
18180	Amp	11	69991152	69992893	111.388072	1.090725
18181	Amp	11	69993211	69995642	112.369637	1.097696
18182	Amp	11	69996418	70013719	112.496678	1.098702
18183	Amp	11	70016123	70023573	112.1503	1.096293
18184	Amp	11	70023976	70027827	112.768637	1.100842
18185	Amp	11	70027871	70056961	111.820789	1.093872
18186	Amp	11	70064734	70071927	112.649718	1.099954
18187	Amp	11	70074028	70075223	113.293255	1.104412
18188	Amp	11	70076466	70077193	113.50618	1.1063
18189	Amp	11	70086344	70086344	113.229833	1.103967
18190	Amp	11	70089341	70110559	112.798332	1.100966
18191	Amp	11	70113077	70120376	114.085788	1.110241
18192	Amp	11	70124531	70130087	114.303541	1.111648
18193	Amp	11	70132532	70159535	113.321388	1.104758
18194	Amp	11	70164035	70173336	112.502039	1.098763
18195	Amp	11	70173926	70181807	111.832891	1.094106
18196	Amp	11	70182038	70193720	111.763795	1.093487
18197	Amp	11	70195663	70195663	111.356851	1.090667
18198	Amp	11	70198918	70199230	110.146866	1.082284
18199	Amp	11	70202349	70230481	107.858786	1.066347
18200	Amp	11	70242901	70247076	108.824284	1.072996
18201	Amp	11	70248765	70250039	108.96289	1.074146
18202	Amp	11	70250479	70260564	109.33768	1.07659
18203	Amp	11	70265405	70281112	109.802117	1.080035
18204	Amp	11	70286674	70287433	109.492509	1.077722
18205	Amp	11	70288660	70288660	108.701307	1.07217
18206	Amp	11	70292144	70293559	107.268497	1.062188
18207	Amp	11	70294320	70309561	107.736615	1.065392
18208	Amp	11	70311538	70311538	108.138423	1.068262
18209	Amp	11	70313181	70319117	108.764553	1.072576
18210	Amp	11	70319399	70334487	109.31406	1.076452
18211	Amp	11	70335370	70346616	108.884873	1.073394
18212	Amp	11	70346954	70349872	108.424949	1.070259
18213	Amp	11	70351252	70354796	107.650405	1.064652
18214	Amp	11	70360478	70371239	108.424949	1.070172
18215	Amp	11	70374688	70374688	108.169591	1.068396
18216	Amp	11	70375611	70375611	107.827625	1.066144
18217	Amp	11	70377725	70379050	108.512484	1.070843
18218	Amp	11	70383065	70389762	106.924388	1.059822
18219	Amp	11	70390687	70394310	106.670701	1.05806
18220	Amp	11	70395829	70395998	105.53101	1.050149
18221	Amp	11	70402628	70402628	104.673891	1.044443
18222	Amp	11	70402794	70403081	104.29778	1.041733
18223	Amp	11	70405706	70412126	104.391397	1.042359
18224	Amp	11	70413092	70413092	103.79226	1.03824
18225	Amp	11	70414503	70417245	102.471851	1.029131
18226	Amp	11	70418584	70427820	100.184662	1.013195
18227	Amp	11	70428085	70428085	99.910271	1.011273
18228	Amp	11	70430712	70431560	99.539133	1.008665
18229	Amp	11	70437962	70437962	96.730689	0.989201
18230	Amp	11	70449792	70449792	94.47227	0.973265
18231	Amp	11	70453844	70469157	93.923211	0.969337

18232	Amp	11	70469880	70474991	92.76039	0.961183
18233	Amp	11	70480702	70481200	91.964035	0.955521
18234	Amp	11	70491272	70491578	90.470228	0.944865
18235	Amp	11	70501941	70516102	90.560635	0.945522
18236	Amp	11	70516150	70518005	90.197166	0.942963
18237	Amp	11	70518051	70520278	89.681501	0.939258
18238	Amp	11	70520939	70524002	89.499828	0.937851
18239	Amp	11	70524083	70524801	88.192601	0.928572
18240	Amp	11	70531373	70536949	86.780664	0.918446
18241	Amp	11	70542146	70542964	87.162692	0.921257
18242	Amp	11	70545611	70548831	84.284052	0.900421
18243	Amp	11	70550904	70550933	84.105164	0.899117
18244	Amp	11	70550964	70550964	83.65712	0.895908
18245	Amp	11	70553041	70553041	83.269543	0.893087
18246	Amp	11	70553754	70555049	82.765865	0.88939
18247	Amp	11	70557032	70557280	83.06249	0.891462
18248	Amp	11	70562750	70563977	83.50852	0.894705
18249	Amp	11	70564025	70564025	83.090576	0.891752
18250	Amp	11	70574714	70574714	82.587166	0.887929
18251	Amp	11	70574867	70575194	80.73045	0.87427
18252	Amp	11	70575601	70575692	80.58311	0.873234
18253	Amp	11	70578838	70579738	80.199069	0.870519
18254	Amp	11	70581179	70593048	79.640654	0.866373
18255	Amp	11	70593734	70606890	78.851316	0.860395
18256	Amp	11	70610584	70610584	77.155751	0.847833
18257	Amp	11	70610663	70624950	77.102388	0.847371
18258	Amp	11	70627703	70627703	76.284945	0.841398
18259	Amp	11	70627889	70629735	75.438329	0.834972
18260	Amp	11	70630858	70632696	73.6064	0.821314
18261	Amp	11	70632952	70633880	73.178522	0.818097
18262	Amp	11	70635859	70652205	72.13192	0.810149
18263	Amp	11	70655522	70655895	70.037192	0.794212
18264	Amp	11	70656615	70665519	69.561021	0.790536
18265	Amp	11	70666256	70666765	69.304421	0.78858
18266	Amp	11	70671399	70671399	68.962802	0.785976
18267	Amp	11	70671822	70672963	69.133794	0.787339
18268	Amp	11	70675286	70676684	67.570171	0.775258
18269	Amp	11	70676903	70703052	67.095598	0.771587
18270	Amp	11	70705518	70714204	65.111392	0.755929
18271	Amp	11	70718335	70718335	65.438973	0.758655
18272	Amp	11	70718971	70721589	65.764589	0.761298
18273	Amp	11	70725768	70726392	65.492062	0.759077
18274	Amp	11	70728820	70729255	67.069486	0.771413
18275	Amp	11	70733410	70741831	65.928486	0.762384
18276	Amp	11	70744277	70746128	65.084437	0.755848
18277	Amp	11	70749600	70750254	63.249531	0.741538
18278	Amp	11	70759623	70760395	62.048885	0.731832
18279	Amp	11	70763702	70766942	62.7927	0.737742
18280	Amp	11	70771257	70776238	63.097348	0.740121
18281	Amp	11	70780953	70793744	62.103469	0.732403
18282	Amp	11	70796715	70806899	63.553848	0.743821
18283	Amp	11	70817197	70820148	63.249531	0.741349
18284	Amp	11	70825755	70829798	61.507795	0.727633

18285	Amp	11	70829932	70837455	61.836004	0.730223
18286	Amp	11	70838600	70838998	61.587607	0.728152
18287	Amp	11	70839035	70849532	61.997559	0.731438
18288	Amp	11	70849761	70849761	61.039571	0.723752
18289	Amp	11	70850149	70850149	60.320513	0.718269
18290	Amp	11	70850732	70850752	61.561238	0.728019
18291	Amp	11	70851929	70851929	61.313803	0.725974
18292	Amp	11	70855163	70855163	60.513743	0.7198
18293	Amp	11	70858755	70869257	59.366525	0.710534
18294	Amp	11	70869413	70869413	59.22922	0.70942
18295	Amp	11	70874432	70875225	58.357834	0.70254
18296	Amp	11	70876988	70876988	58.166618	0.700994
18297	Amp	11	70880532	70880532	58.384593	0.702801
18298	Amp	11	70884341	70885875	58.061	0.700205
18299	Amp	11	70886863	70892216	58.46655	0.703375
18300	Amp	11	70892530	70893086	58.874405	0.706712
18301	Amp	11	70895783	70896697	59.093004	0.708474
18302	Amp	11	70896736	70903848	58.71192	0.705457
18303	Amp	11	70904188	70904188	58.657335	0.704863
18304	Amp	11	70906199	70908815	57.167018	0.693078
18305	Amp	11	70912479	70925859	57.790546	0.698102
18306	Amp	11	70927782	70936136	58.141533	0.700851
18307	Amp	11	70937447	70937447	57.167018	0.692911
18308	Amp	11	70937750	70937750	56.407139	0.686946
18309	Amp	11	70938094	70941018	55.794108	0.681902
18310	Amp	11	70941108	70943073	55.423607	0.678798
18311	Amp	11	70944747	70944747	55.020178	0.675725
18312	Amp	11	70945406	70947812	54.649243	0.672534
18313	Amp	11	70948969	70949160	54.675637	0.672871
18314	Amp	11	71096315	71096727	54.356714	0.670146
18315	Amp	11	71097018	71102882	54.517906	0.671455
18316	Amp	11	71109713	71110104	55.23503	0.677404
18317	Amp	11	71117422	71121768	55.767607	0.68164
18318	Amp	11	71125421	71131873	54.120423	0.668209
18319	Amp	11	71135358	71135358	53.380124	0.66226
18320	Amp	11	71135915	71139338	53.486074	0.663134
18321	Amp	11	71141810	71146153	53.988741	0.667204
18322	Amp	11	71148608	71160763	54.278226	0.669557
18323	Amp	11	71161413	71180500	56.220974	0.685494
18324	Amp	11	71180689	71183209	55.95567	0.683216
18325	Amp	11	71184267	71195027	55.979363	0.683357
18326	Amp	11	71196641	71204474	53.885452	0.666394
18327	Amp	11	71206597	71212773	52.750114	0.657003
18328	Amp	11	71213325	71216767	52.644753	0.656149
18329	Amp	11	71219287	71222582	53.805636	0.665539
18330	Amp	11	71222922	71225302	54.041406	0.667592
18331	Amp	11	71231578	71231578	52.249921	0.652769
18332	Amp	11	71232256	71232256	52.065816	0.651289
18333	Amp	11	71233341	71233432	51.699889	0.64837
18334	Amp	11	71235268	71236303	51.412684	0.645793
18335	Amp	11	71237563	71255951	52.014083	0.650965
18336	Amp	11	71258636	71261252	51.516465	0.646757
18337	Amp	11	71265255	71265414	51.386615	0.645766

18338	Amp	11	71266545	71271932	51.259917	0.644502
18339	Amp	11	71274371	71274752	50.978467	0.642302
18340	Amp	11	71274891	71291364	50.323772	0.636849
18341	Amp	11	71302169	71303384	47.912878	0.616597
18342	Amp	11	71304688	71323860	48.012914	0.617416
18343	Amp	11	71325140	71340250	47.733347	0.614997
18344	Amp	11	71340769	71340769	47.178485	0.610298
18345	Amp	11	71342252	71346418	46.6951	0.606241
18346	Amp	11	71346865	71352473	46.137615	0.601292
18347	Amp	11	71352709	71359921	45.806613	0.598598
18348	Amp	11	71360056	71378101	46.011588	0.600327
18349	Amp	11	71378164	71378188	46.644196	0.605787
18350	Amp	11	71381543	71382162	48.526752	0.621723
18351	Amp	11	71384345	71394165	47.606379	0.613991
18352	Amp	11	71395160	71399101	46.745804	0.606644
18353	Amp	11	71399260	71404400	46.362453	0.603357
18354	Amp	11	71406613	71406871	46.41329	0.603802
18355	Amp	11	71407522	71408187	46.311003	0.602918
18356	Amp	11	71412538	71413620	45.600645	0.596901
18357	Amp	11	71416865	71418477	45.3193	0.594345
18358	Amp	11	71419232	71432392	44.861515	0.590552
18359	Amp	11	71435645	71456403	44.081756	0.583796
18360	Amp	11	71456774	71480516	43.956711	0.582648
18361	Amp	11	71481435	71484951	44.306389	0.585752
18362	Amp	11	71486037	71486037	44.103948	0.583922
18363	Amp	11	71488566	71488620	42.967498	0.574145
18364	Amp	11	71494329	71494329	43.06679	0.575147
18365	Amp	11	71494362	71494362	42.537832	0.570519
18366	Amp	11	71496159	71499359	42.336344	0.568815
18367	Amp	11	71501961	71501961	41.863531	0.564576
18368	Amp	11	71510528	71510528	41.121064	0.558242
18369	Amp	11	71510559	71510559	41.070841	0.557638
18370	Amp	11	71517422	71519714	39.261591	0.541863
18371	Amp	11	71523250	71525717	38.694708	0.536945
18372	Amp	11	71526643	71527844	38.230723	0.532809
18373	Amp	11	71529966	71532526	35.637743	0.509501
18374	Amp	11	71535063	71542601	35.324942	0.506745
18375	Amp	11	71544576	71548456	34.969783	0.503398
18376	Amp	11	71549250	71549250	34.324464	0.497518
18377	Amp	11	71550088	71556405	34.205209	0.496516
18378	Amp	11	71556497	71556497	33.846472	0.493233
18379	Amp	11	71557473	71560963	33.110197	0.486575
18380	Amp	11	71566448	71566448	32.209205	0.478122
18381	Amp	11	71566780	71566780	31.862503	0.474695
18382	Amp	11	71568176	71568525	31.769293	0.473892
18383	Amp	11	71569220	71569220	32.922925	0.484662
18384	Amp	11	71570516	71572999	31.130801	0.467797
18385	Amp	11	71573179	71580444	31.062194	0.467123
18386	Amp	11	71582841	71582841	29.888511	0.455847
18387	Amp	11	71588129	71592950	27.80132	0.43575
18388	Amp	11	71615907	71623337	26.712236	0.425143
18389	Amp	11	71626328	71626328	26.308427	0.421088
18390	Amp	11	71629998	71641093	26.022514	0.418316

18391	Amp	11	71645745	71645745	21.5365	0.372975
18392	Amp	11	71649144	71649248	21.472484	0.372338
18393	Amp	11	71649989	71652965	19.83239	0.355014
18394	Amp	11	71653061	71653504	19.693709	0.353466
18395	Amp	11	71659485	71659754	19.59374	0.352421
18396	Amp	11	71713405	71713405	16.686433	0.320548
18397	Amp	11	71714949	71714949	16.841708	0.322251
18398	Amp	11	71716463	71719310	16.456552	0.317969
18399	Amp	11	71719657	71723459	15.842559	0.311155
18400	Amp	11	71729525	71748557	14.466246	0.295219
18401	Amp	11	71752517	71753030	13.510816	0.283865
18402	Amp	11	71802409	71802409	12.329307	0.269709
18403	Amp	11	71802551	71802551	11.39504	0.258576
18404	Amp	11	71812272	71816501	11.787092	0.263279
18405	Amp	11	71816650	71816665	12.510219	0.27198
18406	Amp	11	71859610	71900042	11.536709	0.260347
18407	Amp	11	71903690	71903690	11.290117	0.257267
18408	Amp	11	71908877	71908877	11.662686	0.261702
18409	Amp	11	71915043	71917562	10.378606	0.245766
18410	Amp	11	71917622	71929953	10.583831	0.24826
18411	Amp	11	71930047	71949346	10.808758	0.251193
18412	Amp	11	71952343	71967721	10.361022	0.245446
18413	Amp	11	71969544	71978553	10.600629	0.248508
18414	Amp	11	71986203	71994899	10.843331	0.251582
18415	Amp	11	71995791	71995791	10.499721	0.247165
18416	Amp	11	71998540	72008357	9.933591	0.240158
18417	Amp	11	72009894	72015166	9.812347	0.238455
18418	Amp	11	72017610	72037100	9.746461	0.237581
18419	Amp	11	72040559	72048041	9.289139	0.231754
18420	Amp	11	72054119	72054119	8.936662	0.227319
18421	Amp	11	72058862	72067776	9.373896	0.232928
18422	Amp	11	72068950	72068950	9.661346	0.236616
18423	Amp	11	72072197	72087933	9.916528	0.239758
18424	Amp	11	72089269	72089269	10.087806	0.241918
18425	Amp	11	72093072	72093467	10.000692	0.24086
18426	Amp	11	72096671	72104531	10.499721	0.247263
18427	Amp	11	72107417	72108294	10.583831	0.248237
18428	Amp	11	72112906	72119455	10.877729	0.252042
18429	Amp	11	72121089	72125232	10.618056	0.248723
18430	Amp	11	72125261	72127489	10.465282	0.246802
18431	Amp	11	72128352	72128610	10.703463	0.249891
18432	Amp	11	72130654	72132213	10.808758	0.251117
18433	Amp	11	72134352	72134495	10.346125	0.245345
18434	Amp	11	72183005	72183005	10.549344	0.247959
18435	Amp	11	72189920	72190626	11.627141	0.261333
18436	Amp	11	72196510	72196510	11.239373	0.25655
18437	Amp	11	72197463	72197463	11.063647	0.254378
18438	Amp	11	72197581	72230168	11.609398	0.261072
18439	Amp	11	72235896	72248149	12.401399	0.270687
18440	Amp	11	72251257	72251257	12.618951	0.273312
18441	Amp	11	72256571	72270928	12.364848	0.270198
18442	Amp	11	72275436	72280065	12.689522	0.274099
18443	Amp	11	72282158	72286451	12.873531	0.276298

18444	Amp	11	72287281	72292425	12.708276	0.274284
18445	Amp	11	72293791	72295350	12.383029	0.270383
18446	Amp	11	72297477	72298853	12.708276	0.274404
18447	Amp	11	72301530	72311356	12.456062	0.271308
18448	Amp	11	72311659	72311659	12.873531	0.276346
18449	Amp	11	72312784	72314141	12.383029	0.270509
18450	Amp	11	72315514	72315514	12.653484	0.273765
18451	Amp	11	72317158	72337363	12.983395	0.27763
18452	Amp	11	72337482	72348661	13.129509	0.279273
18453	Amp	11	72357853	72379492	13.600394	0.284985
18454	Amp	11	72380184	72380184	13.856199	0.287933
18455	Amp	11	72380337	72380337	13.800267	0.287283
18456	Amp	11	72381006	72390745	13.038387	0.278261
18457	Amp	11	72391325	72407153	12.689522	0.274149
18458	Amp	11	72407250	72413467	12.927625	0.276859
18459	Amp	11	72419907	72443485	14.243686	0.292612
18460	Amp	11	72443702	72444840	14.577855	0.296442
18461	Amp	11	72447531	72448220	14.393488	0.294283
18462	Amp	11	72454083	72487315	15.76669	0.310181
18463	Amp	11	72487383	72500231	15.690603	0.309355
18464	Amp	11	72505253	72505253	15.423788	0.30628
18465	Amp	11	72507808	72514775	15.76669	0.310279
18466	Amp	11	72514910	72514910	15.500252	0.307203
18467	Amp	11	72516805	72540347	15.709157	0.309522
18468	Amp	11	72541327	72552866	15.55852	0.307727
18469	Amp	11	72556445	72562841	15.595327	0.308277
18470	Amp	11	72567601	72572340	15.805309	0.310555
18471	Amp	11	72576306	72576306	16.053555	0.313369
18472	Amp	11	72577681	72580188	15.805309	0.310533
18473	Amp	11	72580883	72582998	15.940153	0.312218
18474	Amp	11	72594629	72594629	15.652786	0.308975
18475	Amp	11	72597527	72619167	15.461712	0.306698
18476	Amp	11	72619423	72619423	15.595327	0.308306
18477	Amp	11	72621171	72624866	15.823673	0.310791
18478	Amp	11	72625520	72625520	15.632849	0.308651
18479	Amp	11	72636272	72649380	16.107433	0.314073
18480	Amp	11	72653361	72659270	17.255967	0.326943
18481	Amp	11	72662452	72662452	16.762041	0.321522
18482	Amp	11	72662993	72666570	17.751967	0.332384
18483	Amp	11	72667293	72667293	18.049178	0.335695
18484	Amp	11	72668848	72674027	18.347493	0.338937
18485	Amp	11	72678606	72683401	18.14718	0.336617
18486	Amp	11	72690406	72693913	18.56575	0.341136
18487	Amp	11	72694229	72700619	18.347493	0.3388
18488	Amp	11	72706740	72718096	18.108258	0.336274
18489	Amp	11	72720634	72725638	18.167323	0.336896
18490	Amp	11	72728800	72749110	18.546429	0.340978
18491	Amp	11	72753480	72753480	18.367904	0.33896
18492	Amp	11	72754507	72754507	18.546429	0.340936
18493	Amp	11	72758742	72758742	17.038719	0.324467
18494	Amp	11	72760550	72794770	16.627883	0.319975
18495	Amp	11	72799316	72799619	15.9958	0.312801
18496	Amp	11	72805536	72813193	16.609099	0.319659

18497 Amp	11	72817928	72817928	16.841708	0.322254
18498 Amp	11	72819379	72825202	16.570587	0.319379
18499 Amp	11	72831222	72843515	15.900911	0.311708
18500 Amp	11	72846941	72846941	16.089694	0.313982
18501 Amp	11	72851263	72860230	15.977907	0.312504
18502 Amp	11	72872475	72872475	15.823673	0.310755
18503 Amp	11	72877685	72877685	15.366356	0.305701
18504 Amp	11	72878125	72884694	15.576957	0.308057
18505 Amp	11	72889434	72892319	14.430436	0.294709
18506 Amp	11	72895484	72896223	14.779959	0.298906
18507 Amp	11	72899463	72918901	15.55852	0.30781
18508 Amp	11	72921232	72929946	16.589822	0.3196
18509 Amp	11	72942246	72950824	16.416418	0.317624
18510 Amp	11	72954013	72955574	15.024711	0.301688
18511 Amp	11	72960411	72986032	14.818059	0.299414
18512 Amp	11	72988246	72988541	14.87478	0.29997
18513 Amp	11	72988559	73001757	14.650436	0.297347
18514 Amp	11	73005119	73009064	14.987459	0.301227
18515 Amp	11	73011396	73016420	14.912742	0.300464
18516 Amp	11	73017021	73032926	15.576957	0.308027
18517 Amp	11	73033471	73067417	15.823673	0.310793
18518 Amp	11	73068970	73089438	16.089694	0.313841
18519 Amp	11	73091219	73096717	17.492691	0.32947
18520 Amp	11	73100223	73117521	16.456552	0.318111
18521 Amp	11	73119438	73151797	15.100986	0.302482
18522 Amp	11	73154783	73169354	15.500252	0.307104
18523 Amp	11	73170643	73170643	15.348745	0.30542
18524 Amp	11	73171242	73183228	16.089694	0.313916
18525 Amp	11	73184153	73184797	16.053555	0.313366
18526 Amp	11	73184865	73195733	16.340767	0.316782
18527 Amp	11	73198827	73215965	16.070661	0.313687
18528 Amp	11	73216074	73223998	16.126505	0.314383
18529 Amp	11	73224780	73227375	16.282141	0.316133
18530 Amp	11	73232205	73247653	16.609099	0.319792
18531 Amp	11	73255409	73277421	17.831836	0.333104
18532 Amp	11	73283154	73310039	16.340767	0.316744
18533 Amp	11	73316804	73316804	15.40422	0.306109
18534 Amp	11	73319476	73325519	16.165422	0.314776
18535 Amp	11	73327936	73333306	15.747199	0.309869
18536 Amp	11	73337059	73349857	16.165422	0.314782
18537 Amp	11	73353739	73357724	15.881803	0.311547
18538 Amp	11	73359308	73359308	16.107433	0.314058
18539 Amp	11	73360218	73365359	15.900911	0.311768
18540 Amp	11	73365879	73368405	16.070661	0.313739
18541 Amp	11	73369360	73393596	15.920582	0.312002
18542 Amp	11	73396688	73423535	15.595327	0.308172
18543 Amp	11	73424841	73452440	14.931259	0.300593
18544 Amp	11	73458928	73458928	15.081766	0.302434
18545 Amp	11	73473325	73473325	16.493379	0.318371
18546 Amp	11	73475819	73483018	16.860248	0.322659
18547 Amp	11	73487042	73509339	16.533331	0.318972
18548 Amp	11	73513876	73519728	15.959834	0.312327
18549 Amp	11	73524376	73538374	15.348745	0.305468

18550 Amp	11	73540579	73565855	16.089694	0.313974
18551 Amp	11	73567991	73584783	16.609099	0.319694
18552 Amp	11	73590613	73620682	16.918195	0.323182
18553 Amp	11	73621018	73626884	16.762041	0.321572
18554 Amp	11	73629645	73629645	16.053555	0.313503
18555 Amp	11	73629842	73640259	15.977907	0.312583
18556 Amp	11	73646247	73651030	16.397251	0.317248
18557 Amp	11	73652165	73658536	16.551166	0.319002
18558 Amp	11	73661640	73710334	14.613267	0.296872
18559 Amp	11	73712700	73713224	14.058011	0.290441
18560 Amp	11	73715725	73722274	13.600394	0.285075
18561 Amp	11	73724251	73729914	13.510816	0.283966
18562 Amp	11	73733931	73741081	14.522375	0.295883
18563 Amp	11	73741896	73747829	14.243686	0.29263
18564 Amp	11	73750746	73750746	13.381152	0.282472
18565 Amp	11	73750890	73755594	13.165358	0.279872
18566 Amp	11	73756483	73756483	13.074762	0.278637
18567 Amp	11	73756502	73766000	13.293073	0.281221
18568 Amp	11	73781428	73788869	14.631516	0.297157
18569 Amp	11	73791715	73794186	13.856199	0.288001
18570 Amp	11	73797143	73797143	14.022374	0.289887
18571 Amp	11	73797880	73799600	14.281057	0.293063
18572 Amp	11	73800108	73832452	14.040669	0.290244
18573 Amp	11	73841485	73845148	12.873531	0.276393
18574 Amp	11	73851983	73854595	13.258073	0.280791
18575 Amp	11	73860838	73861282	12.855613	0.275996
18576 Amp	11	73861902	73870863	11.519317	0.26006
18577 Amp	11	73875484	73895353	11.098856	0.254818
18578 Amp	11	73896042	73896042	10.791286	0.250876
18579 Amp	11	73900081	73900630	10.720769	0.249948
18580 Amp	11	73902560	73908704	10.756361	0.250552
18581 Amp	11	73911118	73919292	10.51732	0.247377
18582 Amp	11	73919918	73954742	10.294658	0.244711
18583 Amp	11	73955168	73965382	10.843331	0.251538
18584 Amp	11	73965508	73968610	10.430994	0.246293
18585 Amp	11	73975718	73980729	10.720769	0.250133
18586 Amp	11	73983709	73990346	10.978295	0.253212
18587 Amp	11	73996963	73996963	11.115911	0.255116
18588 Amp	11	73999841	74009120	11.342933	0.257832
18589 Amp	11	74011576	74022230	11.698383	0.262099
18590 Amp	11	74023189	74029673	11.752146	0.262902
18591 Amp	11	74030309	74035210	10.961251	0.25315
18592 Amp	11	74041355	74043294	10.8261	0.251275
18593 Amp	11	74045426	74047664	10.634596	0.248939
18594 Amp	11	74047846	74048574	11.377246	0.25829
18595 Amp	11	74049462	74050282	11.239373	0.256448
18596 Amp	11	74057180	74059570	10.893171	0.252296
18597 Amp	11	74066502	74070669	10.893171	0.252224
18598 Amp	11	74070957	74077183	10.534461	0.247724
18599 Amp	11	74084589	74084589	10.669096	0.249491
18600 Amp	11	74085543	74092995	10.549344	0.247807
18601 Amp	11	74096656	74096656	10.860138	0.25175
18602 Amp	11	74104754	74127307	10.51732	0.247346

18603	Amp	11	74131564	74158290	10.8261	0.251325
18604	Amp	11	74160171	74175628	11.049458	0.254058
18605	Amp	11	74177106	74184615	10.9963	0.253502
18606	Amp	11	74187025	74230332	10.549344	0.247782
18607	Amp	11	74237754	74257791	10.9963	0.253506
18608	Amp	11	74260358	74296470	11.049458	0.254141
18609	Amp	11	74297483	74301042	10.000692	0.240887
18610	Amp	11	74303422	74322770	10.31211	0.244746
18611	Amp	11	74323428	74323428	10.534461	0.247654
18612	Amp	11	74327229	74342716	11.342933	0.257762
18613	Amp	11	74343378	74366113	10.961251	0.253096
18614	Amp	11	74367338	74370877	11.031992	0.253968
18615	Amp	11	74371566	74387450	10.651729	0.249144
18616	Amp	11	74387600	74387688	10.756361	0.250418
18617	Amp	11	74387738	74426506	10.893171	0.252224
18618	Amp	11	74430078	74435480	10.843331	0.251602
18619	Amp	11	74438045	74443641	10.534461	0.247623
18620	Amp	11	74444007	74450723	10.361022	0.245537
18621	Amp	11	74452791	74455275	11.644242	0.261473
18622	Amp	11	74456041	74457366	11.430328	0.259031
18623	Amp	11	74457546	74463919	10.877729	0.252072
18624	Amp	11	74464157	74466876	10.669096	0.249355
18625	Amp	11	74470871	74471903	10.361022	0.245512
18626	Amp	11	74475623	74487882	10.122701	0.242492
18627	Amp	11	74489378	74489694	10.277087	0.244311
18628	Amp	11	74493959	74502778	10.465282	0.246802
18629	Amp	11	74503963	74504850	9.442896	0.233674
18630	Amp	11	74505582	74517943	9.47615	0.234151
18631	Amp	11	74519247	74523493	8.919417	0.22696
18632	Amp	11	74524446	74546463	9.071495	0.229055
18633	Amp	11	74551859	74555027	8.953805	0.227447
18634	Amp	11	74557776	74559284	8.754268	0.224818
18635	Amp	11	74567131	74590147	8.818686	0.225748
18636	Amp	11	74590244	74594573	8.754268	0.224818
18637	Amp	11	74596569	74621217	8.654479	0.223535
18638	Amp	11	74621235	74627009	8.834661	0.226009
18639	Amp	11	74634537	74639568	9.763565	0.237971
18640	Amp	11	74646319	74653516	10.034845	0.241419
18641	Amp	11	74653521	74655710	9.373896	0.232791
18642	Amp	11	74662340	74690426	10.618056	0.248699
18643	Amp	11	74694303	74694303	10.9963	0.253434
18644	Amp	11	74695857	74725353	11.049458	0.254145
18645	Amp	11	74728081	74743202	10.877729	0.252051
18646	Amp	11	74744028	74744028	10.686888	0.249578
18647	Amp	11	74744377	74753753	10.105286	0.242138
18648	Amp	11	74754023	74754023	10.634596	0.249061
18649	Amp	11	74754531	74756593	10.756361	0.250494
18650	Amp	11	74756905	74767941	10.396417	0.245975
18651	Amp	11	74770273	74770658	11.500885	0.259804
18652	Amp	11	74770718	74777492	11.204236	0.25613
18653	Amp	11	74781731	74797564	11.984978	0.265636
18654	Amp	11	74799111	74804137	12.310616	0.26948
18655	Amp	11	74804438	74819974	12.021369	0.266147

18656 Amp	11	74822407	74824517	11.804955	0.263437
18657 Amp	11	74827360	74827360	13.129509	0.279373
18658 Amp	11	74828526	74830743	13.202322	0.28015
18659 Amp	11	74833619	74838562	13.258073	0.280973
18660 Amp	11	74841414	74847260	13.0564	0.278515
18661 Amp	11	74850203	74869006	12.891235	0.276433
18662 Amp	11	74870168	74871920	13.345022	0.28203
18663 Amp	11	74883610	74893700	13.856199	0.288
18664 Amp	11	74893873	74926590	13.764867	0.286974
18665 Amp	11	74935198	74940286	13.600394	0.284922
18666 Amp	11	74942710	74943747	13.398977	0.282537
18667 Amp	11	74944561	74967043	13.583613	0.284867
18668 Amp	11	74968999	74968999	14.243686	0.292526
18669 Amp	11	74969945	75001679	14.169996	0.291723
18670 Amp	11	75002963	75012922	13.800267	0.287384
18671 Amp	11	75016620	75016620	14.022374	0.289966
18672 Amp	11	75022293	75026872	13.837777	0.287754
18673 Amp	11	75027490	75028889	13.966925	0.289342
18674 Amp	11	75031830	75042447	13.673012	0.285826
18675 Amp	11	75046787	75066068	14.0045	0.2897
18676 Amp	11	75068604	75084108	13.528722	0.284155
18677 Amp	11	75085896	75089784	13.729199	0.28647
18678 Amp	11	75096248	75106002	14.762523	0.298743
18679 Amp	11	75109101	75116232	15.538779	0.307493
18680 Amp	11	75125557	75138174	15.977907	0.312661
18681 Amp	11	75142311	75167545	16.704765	0.320756
18682 Amp	11	75167841	75170211	17.512889	0.329759
18683 Amp	11	75170637	75194233	17.850895	0.333487
18684 Amp	11	75197271	75202711	17.711998	0.331934
18685 Amp	11	75205701	75205701	17.176717	0.325943
18686 Amp	11	75207903	75222593	16.821725	0.322099
18687 Amp	11	75226944	75230957	16.958374	0.323597
18688 Amp	11	75238358	75239273	16.743709	0.321171
18689 Amp	11	75251414	75258811	15.329	0.305262
18690 Amp	11	75260513	75279610	16.743709	0.321198
18691 Amp	11	75280652	75310966	17.532886	0.330025
18692 Amp	11	75312924	75318123	17.591497	0.330701
18693 Amp	11	75319903	75319903	17.452845	0.329165
18694 Amp	11	75322658	75348464	17.216433	0.32658
18695 Amp	11	75355359	75355445	18.683958	0.342517
18696 Amp	11	75359765	75363603	18.624086	0.34184
18697 Amp	11	75368056	75375626	18.56575	0.341205
18698 Amp	11	75375641	75385019	18.903702	0.345008
18699 Amp	11	75388224	75388339	17.452845	0.329072
18700 Amp	11	75388590	75392444	17.295317	0.327449
18701 Amp	11	75393168	75396292	17.691957	0.331583
18702 Amp	11	75401284	75416318	19.83239	0.354913
18703 Amp	11	75424402	75455823	20.175253	0.358716
18704 Amp	11	75460038	75463987	20.236975	0.359231
18705 Amp	11	75464848	75478137	20.317454	0.360072
18706 Amp	11	75480584	75480584	20.522128	0.362407
18707 Amp	11	75481750	75505548	20.236975	0.359188
18708 Amp	11	75509002	75523440	19.87244	0.355385

18709	Amp	11	75523590	75528858	19.792143	0.354567
18710	Amp	11	75529681	75530636	19.951408	0.356216
18711	Amp	11	75534013	75534261	19.83239	0.354979
18712	Amp	11	75534540	75558719	20.625864	0.363465
18713	Amp	11	75564590	75564590	20.974304	0.367101
18714	Amp	11	75565133	75565133	20.785556	0.36523
18715	Amp	11	75566712	75568402	20.704394	0.364324
18716	Amp	11	75571837	75571837	20.522128	0.362225
18717	Amp	11	75575146	75575146	20.974304	0.367011
18718	Amp	11	75576296	75576296	21.034527	0.367811
18719	Amp	11	75578172	75606128	20.686425	0.364008
18720	Amp	11	75606418	75633861	20.522128	0.362228
18721	Amp	11	75634045	75638472	20.785556	0.365073
18722	Amp	11	75638863	75638863	20.974304	0.367049
18723	Amp	11	75641235	75659511	20.255854	0.359411
18724	Amp	11	75664256	75713172	20.725611	0.364413
18725	Amp	11	75716567	75716748	21.221005	0.36971
18726	Amp	11	75719037	75720482	20.953203	0.366814
18727	Amp	11	75724134	75736394	21.115111	0.368549
18728	Amp	11	75740298	75748307	19.993383	0.356633
18729	Amp	11	75756791	75761034	20.134537	0.358185
18730	Amp	11	75761565	75764617	20.378428	0.360761
18731	Amp	11	75767569	75767569	20.093568	0.357673
18732	Amp	11	75769882	75773805	20.011995	0.356873
18733	Amp	11	75778424	75778930	19.633539	0.352695
18734	Amp	11	75781158	75789059	19.47108	0.350965
18735	Amp	11	75799139	75813751	18.724757	0.34287
18736	Amp	11	75820135	75829723	17.831836	0.333152
18737	Amp	11	75830262	75842144	17.11708	0.325282
18738	Amp	11	75843035	75848804	17.812399	0.332962
18739	Amp	11	75854213	75854213	18.327886	0.338725
18740	Amp	11	75854227	75854227	18.943031	0.345393
18741	Amp	11	75862533	75862533	19.246606	0.34871
18742	Amp	11	75865368	75876425	18.128001	0.336542
18743	Amp	11	75880859	75919374	17.452845	0.329065
18744	Amp	11	75924789	75984693	17.276105	0.327168
18745	Amp	11	75998789	76023116	17.812399	0.333027
18746	Amp	11	76024390	76024390	17.968693	0.334688
18747	Amp	11	76027868	76031374	18.683958	0.342536
18748	Amp	11	76035590	76035590	18.984039	0.345734
18749	Amp	11	76036506	76067965	18.84484	0.344263
18750	Amp	11	76070129	76073876	19.368926	0.349936
18751	Amp	11	76085775	76094803	17.948509	0.334505
18752	Amp	11	76094840	76121896	17.732001	0.33206
18753	Amp	11	76125569	76135655	17.651349	0.331283
18754	Amp	11	76141404	76152272	19.12459	0.347219
18755	Amp	11	76153533	76154787	18.96358	0.345503
18756	Amp	11	76155508	76185673	19.06482	0.34665
18757	Amp	11	76188877	76193093	18.745071	0.343202
18758	Amp	11	76193594	76211868	18.486439	0.340306
18759	Amp	11	76220976	76225898	18.18712	0.337107
18760	Amp	11	76226814	76238507	18.486439	0.340358
18761	Amp	11	76239007	76239007	18.267757	0.337971

18762	Amp	11	76244365	76247699	18.327886	0.338693
18763	Amp	11	76248711	76265470	18.467261	0.340255
18764	Amp	11	76266720	76269481	18.227868	0.337492
18765	Amp	11	76275110	76286566	18.60467	0.341577
18766	Amp	11	76286631	76299517	18.327886	0.338702
18767	Amp	11	76300130	76316892	18.546429	0.340957
18768	Amp	11	76317934	76345805	18.60467	0.341665
18769	Amp	11	76349240	76349240	19.61439	0.352474
18770	Amp	11	76349484	76371247	20.605119	0.363144
18771	Amp	11	76382975	76389153	20.053094	0.357219
18772	Amp	11	76393475	76398246	19.267324	0.348802
18773	Amp	11	76400875	76401446	19.044055	0.346473
18774	Amp	11	76404655	76410667	19.307452	0.34932
18775	Amp	11	76410865	76442824	20.806214	0.365256
18776	Amp	11	76452955	76452955	21.095587	0.368337
18777	Amp	11	76452968	76452968	21.408633	0.371545
18778	Amp	11	76456764	76456764	21.32672	0.370837
18779	Amp	11	76465995	76470587	20.501238	0.362166
18780	Amp	11	76479322	76479877	21.451458	0.372004
18781	Amp	11	76480593	76513024	21.347119	0.371045
18782	Amp	11	76518241	76520667	21.5365	0.372996
18783	Amp	11	76523282	76525460	21.472484	0.372393
18784	Amp	11	76527938	76548264	22.676769	0.384625
18785	Amp	11	76551872	76551872	23.090197	0.389019
18786	Amp	11	76552596	76553705	22.782563	0.385702
18787	Amp	11	76555128	76562304	23.028019	0.388422
18788	Amp	11	76562811	76564659	22.65511	0.384426
18789	Amp	11	76564690	76580581	22.782563	0.385758
18790	Amp	11	76590272	76590331	22.210585	0.379968
18791	Amp	11	76590970	76592641	22.444367	0.382338
18792	Amp	11	76595877	76611506	22.824016	0.386287
18793	Amp	11	76614416	76653682	22.151048	0.379204
18794	Amp	11	76653695	76657283	22.570418	0.383558
18795	Amp	11	76658960	76662995	23.391759	0.39214
18796	Amp	11	76663114	76681399	23.651501	0.394653
18797	Amp	11	76682126	76682899	23.413574	0.392283
18798	Amp	11	76683797	76685245	23.261742	0.390756
18799	Amp	11	76692497	76692497	22.782563	0.385709
18800	Amp	11	76694569	76708948	23.175552	0.389893
18801	Amp	11	76709140	76728739	22.967626	0.3877
18802	Amp	11	76729112	76733615	23.261742	0.390712
18803	Amp	11	76736415	76740553	23.19634	0.390112
18804	Amp	11	76740975	76747065	22.803189	0.385927
18805	Amp	11	76747662	76762691	23.111043	0.389263
18806	Amp	11	76764685	76777501	23.608558	0.394215
18807	Amp	11	76778424	76779672	23.499819	0.393068
18808	Amp	11	76785553	76787325	24.697822	0.405057
18809	Amp	11	76787394	76787394	25.850269	0.416586
18810	Amp	11	76798253	76798253	24.392895	0.402143
18811	Amp	11	76801387	76805624	24.940372	0.407555
18812	Amp	11	76807451	76807556	24.785232	0.405954
18813	Amp	11	76814386	76838168	24.284149	0.401002
18814	Amp	11	76839898	76842946	24.173763	0.3999

18815	Amp	11	76857858	76877253	23.847929	0.396565
18816	Amp	11	76881586	76881586	23.23982	0.39063
18817	Amp	11	76881790	76887784	23.326343	0.39149
18818	Amp	11	76899202	76927830	23.587837	0.393946
18819	Amp	11	76938387	76970944	26.112011	0.419116
18820	Amp	11	76971592	77001614	27.180986	0.429628
18821	Amp	11	77012740	77028687	27.538273	0.433003
18822	Amp	11	77030205	77030205	27.470572	0.432526
18823	Amp	11	77036346	77048993	28.242882	0.440136
18824	Amp	11	77051831	77059790	28.001632	0.43768
18825	Amp	11	77062342	77081385	28.491223	0.442428
18826	Amp	11	77081424	77103425	29.030889	0.447669
18827	Amp	11	77107967	77121673	28.896188	0.446241
18828	Amp	11	77124573	77126506	29.459956	0.451754
18829	Amp	11	77136867	77146216	30.141189	0.458381
18830	Amp	11	77152948	77216980	30.548921	0.462117
18831	Amp	11	77222248	77242985	30.365538	0.460349
18832	Amp	11	77245623	77245781	30.548921	0.462277
18833	Amp	11	77246788	77317797	31.449861	0.470832
18834	Amp	11	77320361	77357838	31.468722	0.47109
18835	Amp	11	77358701	77380529	32.575293	0.481415
18836	Amp	11	77382533	77384773	31.513925	0.471492
18837	Amp	11	77388772	77388772	31.382471	0.47016
18838	Amp	11	77392769	77392769	31.978878	0.475825
18839	Amp	11	77392953	77407671	31.885468	0.475055
18840	Amp	11	77409505	77432569	31.537213	0.471805
18841	Amp	11	77460846	77460846	31.468722	0.471093
18842	Amp	11	77461788	77478319	32.189017	0.477899
18843	Amp	11	77481346	77482787	31.931786	0.475382
18844	Amp	11	77483556	77510228	31.676767	0.473024
18845	Amp	11	77510880	77519503	31.978878	0.475829
18846	Amp	11	77532186	77543503	32.41139	0.479919
18847	Amp	11	77545812	77565417	32.575293	0.48156
18848	Amp	11	77565463	77565463	32.829371	0.483813
18849	Amp	11	77573003	77579425	33.038947	0.485901
18850	Amp	11	77585669	77588403	33.273719	0.488118
18851	Amp	11	77588943	77589003	33.110197	0.486457
18852	Amp	11	77598663	77602635	32.457841	0.480384
18853	Amp	11	77604186	77615323	33.512902	0.490141
18854	Amp	11	77623861	77628786	35.255407	0.506077
18855	Amp	11	77630083	77630083	36.848385	0.520322
18856	Amp	11	77633247	77633396	37.092271	0.522567
18857	Amp	11	77635840	77636829	37.288818	0.524365
18858	Amp	11	77639990	77681827	37.508261	0.526182
18859	Amp	11	77682495	77682546	37.699257	0.528005
18860	Amp	11	77684050	77709657	37.917023	0.52997
18861	Amp	11	77712507	77743031	38.182961	0.532251
18862	Amp	11	77743729	77773038	38.327083	0.533701
18863	Amp	11	77778696	77778845	37.917023	0.529884
18864	Amp	11	77781742	77814221	37.723632	0.52826
18865	Amp	11	77815603	77847087	38.013237	0.530869
18866	Amp	11	77850882	77857996	37.675355	0.527848
18867	Amp	11	77864229	77900821	39.483751	0.543784

18868	Amp	11	77902070	77904567	39.703842	0.545822
18869	Amp	11	77908887	77915917	39.483751	0.543755
18870	Amp	11	77916676	77917245	39.409721	0.543152
18871	Amp	11	77929801	77939083	36.12019	0.513797
18872	Amp	11	77945822	77955441	34.348468	0.497861
18873	Amp	11	77956119	77969353	35.135136	0.504914
18874	Amp	11	77970528	77993546	35.255407	0.506139
18875	Amp	11	77993738	77998340	35.06363	0.504211
18876	Amp	11	78010491	78013628	34.465843	0.498797
18877	Amp	11	78015218	78016877	34.205209	0.49658
18878	Amp	11	78019679	78020370	34.610062	0.500099
18879	Amp	11	78020709	78026070	35.759294	0.510661
18880	Amp	11	78028818	78033359	35.542113	0.508623
18881	Amp	11	78034631	78048753	34.969783	0.503338
18882	Amp	11	78050030	78050814	34.610062	0.500223
18883	Amp	11	78057815	78067996	32.876889	0.484412
18884	Amp	11	78069888	78071706	32.619783	0.481975
18885	Amp	11	78074750	78092585	32.29486	0.478978
18886	Amp	11	78094190	78128304	32.689482	0.48264
18887	Amp	11	78137903	78155234	33.017903	0.485694
18888	Amp	11	78157884	78170065	31.337869	0.469758
18889	Amp	11	78172432	78177224	33.017903	0.485569
18890	Amp	11	78180705	78181184	32.734247	0.483056
18891	Amp	11	78181598	78185117	33.086535	0.486287
18892	Amp	11	78190642	78192389	31.978878	0.475877
18893	Amp	11	78198414	78232897	31.405984	0.470509
18894	Amp	11	78236480	78236480	31.314367	0.469648
18895	Amp	11	78237049	78265833	30.792422	0.46449
18896	Amp	11	78266803	78285475	30.297306	0.459692
18897	Amp	11	78288474	78288474	30.567717	0.462295
18898	Amp	11	78289109	78319000	30.233001	0.459241
18899	Amp	11	78324098	78334374	29.636198	0.453576
18900	Amp	11	78346922	78352556	29.367408	0.450973
18901	Amp	11	78353037	78361981	29.074878	0.448168
18902	Amp	11	78367008	78387681	30.678399	0.463493
18903	Amp	11	78396562	78401582	29.030889	0.447556
18904	Amp	11	78404608	78405913	28.915681	0.446583
18905	Amp	11	78409225	78412785	28.60304	0.443493
18906	Amp	11	78413751	78428616	28.402074	0.441536
18907	Amp	11	78435047	78461069	26.198831	0.420076
18908	Amp	11	78461533	78471339	25.95936	0.417632
18909	Amp	11	78473595	78497892	26.264814	0.420612
18910	Amp	11	78498320	78519589	25.591369	0.413919
18911	Amp	11	78527697	78534527	25.372008	0.411771
18912	Amp	11	78537351	78537351	25.243712	0.410546
18913	Amp	11	78537794	78540439	25.415285	0.412308
18914	Amp	11	78543440	78551486	25.307231	0.411226
18915	Amp	11	78552712	78567746	25.263829	0.410717
18916	Amp	11	78568222	78568222	24.543628	0.403653
18917	Amp	11	78571374	78588744	23.672228	0.394895
18918	Amp	11	78589200	78633203	22.947264	0.387575
18919	Amp	11	78634295	78634295	22.904311	0.38707
18920	Amp	11	78637616	78642039	22.947264	0.387528

18921	Amp	11	78643824	78653468	22.676769	0.38462
18922	Amp	11	78655282	78672294	20.868849	0.365964
18923	Amp	11	78672987	78673503	19.972287	0.356515
18924	Amp	11	78673757	78677581	20.296825	0.359879
18925	Amp	11	78684144	78684994	19.93106	0.356048
18926	Amp	11	78689431	78707381	19.633539	0.35269
18927	Amp	11	78707877	78716277	17.968693	0.334683
18928	Amp	11	78717436	78724901	18.288413	0.338208
18929	Amp	11	78725033	78730368	18.028672	0.335342
18930	Amp	11	78730398	78730398	18.128001	0.336481
18931	Amp	11	78732190	78738076	17.831836	0.333106
18932	Amp	11	78738889	78760881	16.378681	0.31717
18933	Amp	11	78763749	78771544	16.533331	0.318896
18934	Amp	11	78773000	78781767	16.841708	0.322302
18935	Amp	11	78786476	78786476	16.551166	0.319132
18936	Amp	11	78790218	78794966	16.80163	0.321821
18937	Amp	11	78796516	78802063	16.686433	0.320681
18938	Amp	11	78802324	78820006	15.977907	0.312594
18939	Amp	11	78820481	78828608	15.576957	0.308063
18940	Amp	11	78829638	78840869	15.272026	0.304528
18941	Amp	11	78845233	78855620	14.95044	0.300866
18942	Amp	11	78857746	78897619	14.762523	0.298585
18943	Amp	11	78900932	78913201	15.100986	0.302598
18944	Amp	11	78914102	78957785	15.423788	0.306251
18945	Amp	11	78963242	78980499	15.348745	0.305363
18946	Amp	11	78981690	78988295	15.842559	0.311155
18947	Amp	11	78989604	79002145	16.570587	0.319287
18948	Amp	11	79003703	79016098	16.743709	0.321255
18949	Amp	11	79016503	79039174	16.589822	0.31953
18950	Amp	11	79039287	79060606	16.743709	0.321157
18951	Amp	11	79060718	79084175	16.378681	0.317143
18952	Amp	11	79085793	79114935	16.053555	0.313407
18953	Amp	11	79116804	79135093	14.818059	0.299251
18954	Amp	11	79136262	79179058	14.987459	0.301373
18955	Amp	11	79183811	79189924	13.510816	0.283987
18956	Amp	11	79199013	79199013	14.317432	0.293545
18957	Amp	11	79200244	79215696	14.113089	0.291015
18958	Amp	11	79215831	79234171	13.874487	0.288327
18959	Amp	11	79239437	79254543	14.169996	0.291649
18960	Amp	11	79254726	79259230	14.355711	0.293904
18961	Amp	11	79261047	79267468	14.262513	0.292803
18962	Amp	11	79270153	79270153	14.447899	0.294946
18963	Amp	11	79272928	79274928	14.243686	0.292691
18964	Amp	11	79275713	79276156	13.966925	0.289368
18965	Amp	11	79292881	79293290	14.169996	0.291673
18966	Amp	11	79293942	79311218	13.874487	0.288267
18967	Amp	11	79312576	79328743	14.169996	0.291806
18968	Amp	11	79330055	79335343	14.040669	0.290179
18969	Amp	11	79335372	79349848	14.169996	0.291745
18970	Amp	11	79351303	79357503	13.966925	0.289288
18971	Amp	11	79364331	79364331	13.710777	0.286325
18972	Amp	11	79364808	79403929	14.430436	0.294722
18973	Amp	11	79405411	79417191	13.710777	0.286325

18974	Amp	11	79417337	79458278	13.856199	0.287995
18975	Amp	11	79458419	79458419	13.729199	0.28654
18976	Amp	11	79458859	79463336	13.565854	0.28458
18977	Amp	11	79463733	79466621	13.381152	0.282325
18978	Amp	11	79468861	79476154	13.074762	0.278613
18979	Amp	11	79479813	79498762	12.964742	0.277329
18980	Amp	11	79498829	79517905	12.909694	0.276848
18981	Amp	11	79518387	79522059	12.003259	0.265948
18982	Amp	11	79522491	79522491	11.519317	0.260064
18983	Amp	11	79528352	79565468	10.430994	0.246296
18984	Amp	11	79571936	79581115	10.260441	0.244128
18985	Amp	11	79582041	79588028	10.465282	0.246838
18986	Amp	11	79591793	79606193	10.000692	0.24082
18987	Amp	11	79607344	79636086	10.346125	0.245198
18988	Amp	11	79637802	79686473	9.864718	0.239081
18989	Amp	11	79688563	79688563	9.678773	0.236827
18990	Amp	11	79688579	79688579	9.256952	0.23136
18991	Amp	11	79689364	79692127	9.575888	0.235421
18992	Amp	11	79693570	79706522	9.357173	0.232657
18993	Amp	11	79710319	79726523	8.169654	0.217027
18994	Amp	11	79727603	79734176	8.458247	0.220938
18995	Amp	11	79734217	79735522	8.572005	0.222516
18996	Amp	11	79735599	79755725	8.818686	0.225803
18997	Amp	11	79755768	79763222	8.720312	0.224507
18998	Amp	11	79763562	79788504	8.522585	0.221818
18999	Amp	11	79795054	79800830	8.041049	0.215301
19000	Amp	11	79802024	79802145	7.610655	0.209509
19001	Amp	11	79805388	79818806	7.736725	0.211163
19002	Amp	11	79821313	79837737	7.610655	0.209509
19003	Amp	11	79840342	79855878	7.500789	0.207839
19004	Amp	11	79859738	79890553	7.264196	0.204552
19005	Amp	11	79902560	79920825	7.042879	0.201528
19006	Amp	11	79929627	79938555	6.427493	0.192901
19007	Amp	11	79939735	79942755	6.36593	0.192159
19008	Amp	11	79947756	79956339	6.229291	0.190016
19009	Amp	11	79957690	79957834	6.36593	0.191996
19010	Amp	11	79959336	79986936	6.182794	0.189419
19011	Amp	11	79991116	80003927	6.810251	0.198297
19012	Amp	11	80004157	80055895	6.996189	0.200923
19013	Amp	11	80056046	80077791	6.763246	0.197742
19014	Amp	11	80078166	80078166	6.855677	0.199026
19015	Amp	11	80078585	80088505	6.610791	0.195501
19016	Amp	11	80089103	80093801	6.886863	0.199501
19017	Amp	11	80096256	80106067	7.027404	0.201391
19018	Amp	11	80106208	80118398	7.578613	0.209052
19019	Amp	11	80120079	80140252	7.248181	0.204438
19020	Amp	11	80143659	80152618	7.152075	0.20311
19021	Amp	11	80158251	80162487	7.011899	0.20113
19022	Amp	11	80162583	80175485	7.106137	0.202466
19023	Amp	11	80179243	80182714	7.295403	0.205158
19024	Amp	11	80185195	80195261	7.452952	0.207242
19025	Amp	11	80195458	80201362	8.249918	0.218199
19026	Amp	11	80201711	80238026	7.817451	0.212323

19027 Amp	11	80244506	80255788	7.673484	0.210312
19028 Amp	11	80274240	80278540	7.547251	0.208632
19029 Amp	11	80278923	80330289	7.485352	0.207773
19030 Amp	11	80331797	80351747	7.295403	0.20508
19031 Amp	11	80356160	80362587	7.135839	0.202996
19032 Amp	11	80363507	80415155	6.36593	0.192039
19033 Amp	11	80416796	80429389	6.04774	0.187451
19034 Amp	11	80431063	80446351	5.536915	0.180097
19035 Amp	11	80446442	80488915	5.762293	0.183358
19036 Amp	11	80490235	80500021	5.59628	0.180929
19037 Amp	11	80502054	80511510	5.626868	0.181447
19038 Amp	11	80512275	80534545	5.656875	0.181904
19039 Amp	11	80536401	80540490	5.536915	0.180014
19040 Amp	11	80542402	80546524	5.927128	0.18574
19041 Amp	11	80546925	80562651	6.078164	0.187851
19042 Amp	11	80565324	80565324	6.503622	0.19394
19043 Amp	11	80568482	80571320	6.122748	0.188686
19044 Amp	11	80581416	80599740	5.59628	0.180879
19045 Amp	11	80600022	80621068	4.979649	0.17184
19046 Amp	11	80621845	80621845	4.823878	0.169438
19047 Amp	11	80623948	80650989	4.654424	0.166812
19048 Amp	11	80651264	80666965	4.838749	0.169689
19049 Amp	11	80675177	80692014	4.979649	0.17186
19050 Amp	11	80693503	80752668	4.457399	0.163906
19051 Amp	11	80765032	80804102	4.897301	0.17064
19052 Amp	11	80815492	80830912	4.753798	0.168325
19053 Amp	11	80832563	80878875	4.484961	0.164353
19054 Amp	11	80882640	80889472	4.457399	0.163835
19055 Amp	11	80890376	80900126	4.345128	0.1621
19056 Amp	11	80900461	80909130	4.183261	0.159568
19057 Amp	11	80916380	80955416	4.91152	0.170868
19058 Amp	11	80955800	80955800	5.008935	0.172311
19059 Amp	11	80966053	80971331	5.052964	0.172904
19060 Amp	11	80976191	80991406	6.04774	0.187507
19061 Amp	11	80992924	80996717	5.86703	0.184862
19062 Amp	11	80997655	80998710	5.927128	0.185757
19063 Amp	11	81000348	81032186	5.184242	0.174835
19064 Amp	11	81033188	81053113	5.449463	0.178717
19065 Amp	11	81053987	81137646	6.549814	0.194653
19066 Amp	11	81138517	81159261	6.810251	0.198326
19067 Amp	11	81170083	81205587	5.626868	0.181429
19068 Amp	11	81205755	81217766	5.375101	0.177815
19069 Amp	11	81221699	81247363	5.241829	0.175694
19070 Amp	11	81253480	81360800	5.522349	0.179941
19071 Amp	11	81365643	81391574	5.434517	0.178575
19072 Amp	11	81392049	81396570	5.522349	0.179879
19073 Amp	11	81414542	81414542	5.626868	0.181443
19074 Amp	11	81415202	81415202	5.375101	0.17777
19075 Amp	11	81418555	81419208	5.067861	0.173148
19076 Amp	11	81419234	81500839	5.184242	0.174936
19077 Amp	11	81504890	81581467	4.416011	0.163215
19078 Amp	11	81585806	81608023	4.584613	0.165696
19079 Amp	11	81608036	81614957	4.47072	0.164134

19080	Amp	11	81622012	81622334	4.345128	0.162166
19081	Amp	11	81624190	81625615	4.526699	0.164841
19082	Amp	11	81627999	81653045	3.908795	0.155003
19083	Amp	11	81658001	81713589	3.713605	0.151457
19084	Amp	11	81713925	81736579	3.160681	0.141522
19085	Amp	11	81736763	81756369	3.240397	0.142977
19086	Amp	11	81756735	81864971	3.435586	0.146542
19087	Amp	11	81865927	81874808	3.483236	0.147301
19088	Amp	11	81874870	81900729	3.374864	0.145273
19089	Amp	11	81905181	81941302	3.471122	0.147073
19090	Amp	11	81941879	81979565	3.678399	0.150842
19091	Amp	11	81984747	82023878	4.196711	0.159775
19092	Amp	11	82028653	82063724	4.062894	0.157533
19093	Amp	11	82063911	82066026	3.933438	0.155361
19094	Amp	11	82073775	82089800	3.335819	0.144813
19095	Amp	11	82093347	82097556	3.009724	0.138588
19096	Amp	11	82098205	82098205	3.08058	0.139938
19097	Amp	11	82098269	82137251	3.009724	0.138653
19098	Amp	11	82138717	82138717	2.637742	0.131734
19099	Amp	11	82142180	82169547	2.800837	0.134664
19100	Amp	11	82173275	82195746	2.696333	0.132692
19101	Amp	11	82196062	82241656	2.765626	0.134084
19102	Amp	11	82242175	82273186	2.73206	0.133432
19103	Amp	11	82273917	82283567	3.195626	0.142072
19104	Amp	11	82284514	82317986	3.160681	0.141374
19105	Amp	11	82318056	82335835	2.673338	0.132441
19106	Amp	11	82342495	82365573	2.158817	0.122293
19107	Amp	11	82365793	82368968	2.278531	0.124811
19108	Amp	11	82370301	82392083	2.137445	0.121934
19109	Amp	11	82393083	82435727	2.005947	0.119275
19110	Amp	11	82440336	82447693	1.706315	0.113282
19111	Amp	11	82448109	82453597	1.624655	0.111545
19112	Amp	11	82457012	82489773	2.425182	0.127482
19113	Amp	11	82491085	82499227	2.626052	0.131449
19114	Amp	11	82503373	82503373	2.605213	0.130974
19115	Amp	11	82507169	82516510	2.560422	0.13008
19116	Amp	11	82521298	82521799	2.649655	0.13191
19117	Amp	11	82522003	82524136	2.871582	0.136055
19118	Amp	11	82648805	82648912	3.067933	0.139792
19119	Amp	11	82653388	82667323	3.103562	0.140357
19120	Amp	11	82672396	82705063	2.435195	0.127846
19121	Amp	11	82711859	82785054	2.300463	0.125171
19122	Amp	11	82787008	82791619	2.345513	0.126158
19123	Amp	11	82797945	82826235	2.468223	0.128495
19124	Amp	11	82827341	82835105	2.202226	0.123269
19125	Amp	11	82836889	82946179	2.18114	0.122704
19126	Amp	11	82946847	82969825	2.060912	0.120367
19127	Amp	11	82970226	83001576	2.882945	0.136304
19128	Amp	11	83005231	83017572	2.479057	0.128685
19129	Amp	11	83019480	83104768	1.68612	0.112749
19130	Amp	11	83108256	83156158	1.512549	0.109184
19131	Amp	11	83159435	83160219	1.299876	0.104392
19132	Amp	11	83164996	83164996	1.675319	0.112557

19133 Amp	11	83165061	83173679	1.727286	0.113738
19134 Amp	11	83177118	83184592	1.759227	0.11435
19135 Amp	11	83185938	83237910	1.093834	0.099562
19136 Amp	11	83249167	83277297	1.812192	0.115487
19137 Amp	11	83278087	83354888	1.093834	0.099562
19138 Amp	11	83354928	83389750	1.197658	0.10204
19139 Amp	11	83390438	83412004	1.075952	0.09911
19140 Amp	11	83412261	83432133	1.131695	0.100507
19141 Amp	11	83433568	83481312	1.225419	0.102668
19142 Amp	11	83482572	83528159	1.121584	0.10019
19143 Amp	11	83530738	83612277	0.774648	0.091564
19144 Amp	11	83613277	83613656	0.891602	0.094516
19145 Amp	11	83614071	83640230	0.807647	0.092478
19146 Amp	11	83646944	83660635	0.968522	0.096547
19147 Amp	11	83660648	83677098	0.79909	0.092198
19148 Amp	11	83683808	83707350	1.084294	0.099298
19149 Amp	11	83709512	83711238	0.79909	0.092198
19150 Amp	11	83712906	83745988	0.697374	0.089433
19151 Amp	11	83756716	83777708	0.569771	0.085364
19152 Amp	11	83778321	83808592	0.508568	0.083534
19153 Amp	11	83810246	83826039	0.482379	0.082547
19154 Amp	11	83827994	83874738	0.508568	0.083505
19155 Amp	11	83874896	83924027	0.857296	0.093737
19156 Amp	11	83924058	84136924	0.815228	0.092556
19157 Amp	11	84136962	84168801	0.781972	0.091733
19158 Amp	11	84170498	84200256	0.450702	0.081501
19159 Amp	11	84203653	84573165	0.204831	0.072424
19160 Amp	11	84574973	84593739	0.094967	0.068164
19161 Amp	11	84594395	84723195	0.079652	0.067354
19162 Amp	11	84726002	84726002	0.065715	0.066741
19163 Amp	11	84726514	84864223	0.128324	0.069424
19164 Amp	11	84864262	84864314	0.065715	0.066741
19165 Amp	11	84864329	84881181	0.073902	0.067221
19166 Amp	11	84881574	84932674	0.159312	0.070715
19167 Amp	11	84939606	84943541	0.019169	0.06492
19168 Amp	11	84948616	84951266	0.431198	0.080856
19169 Amp	11	84955791	84964051	0.355319	0.078313
19170 Amp	11	84964273	84990878	0.4115	0.080138
19171 Amp	11	84991055	85004928	0.47716	0.082426
19172 Amp	11	85007095	85054827	0.589605	0.086131
19173 Amp	11	85080090	85089556	0.857296	0.093775
19174 Amp	11	85100869	85133131	1.329472	0.105028
19175 Amp	11	85134881	85235421	1.624655	0.111535
19176 Amp	11	85235988	85257460	1.329472	0.105028
19177 Amp	11	85261128	85383614	0.959607	0.096197
19178 Amp	11	85384136	85445118	1.084294	0.099349
19179 Amp	11	85446006	85460777	1.023539	0.097786
19180 Amp	11	85461757	85466188	0.882843	0.094292
19181 Amp	11	85472602	85497911	1.093834	0.099511
19182 Amp	11	85502352	85503305	0.941704	0.095806
19183 Amp	11	85507545	85507545	0.563096	0.085189
19184 Amp	11	85508830	85525288	0.550755	0.084709
19185 Amp	11	85525718	85534728	0.704849	0.08962

19186	Amp	11	85536078	85556322	0.840289	0.093358
19187	Amp	11	85557609	85557609	0.299433	0.076244
19188	Amp	11	85571247	85579852	0.215608	0.072811
19189	Amp	11	85579868	85593111	0.316877	0.076999
19190	Amp	11	85593294	85595921	0.386426	0.079417
19191	Amp	11	85601792	85671825	0	0.063606
19192	Amp	11	85679362	85714813	0	0.061964
19193	Amp	11	85718031	85807542	0	0.060584
19194	Amp	11	85808579	85870235	0	0.058137
19195	Amp	11	85872772	85872772	0	0.055323
19196	Amp	11	85873026	85918173	0	0.05933
19197	Amp	11	85930588	85930588	0	0.060358
19198	Amp	11	85930912	86008242	0.19964	0.072156
19199	Amp	11	86020533	86027606	0	0.060358
19200	Amp	11	86033874	86152641	0	0.044422
19201	Amp	11	86154109	86206097	0	0.042179
19202	Amp	11	86207226	86227185	0	0.041152
19203	Amp	11	86228905	86325356	0	0.04515
19204	Amp	11	86326654	86352283	0	0.045727
19205	Amp	11	86355024	86355989	0	0.049886
19206	Amp	11	86359054	86500035	0	0.052076
19207	Amp	11	86500145	86559716	0	0.05484
19208	Amp	11	86562334	86642948	0	0.046946
19209	Amp	11	86656887	86669294	0	0.053966
19210	Amp	11	86674251	86679518	0	0.057678
19211	Amp	11	86681319	86720375	0.029031	0.065345
19212	Amp	11	86722028	86746881	0	0.060943
19213	Amp	11	86750923	86761863	0.133193	0.069534
19214	Amp	11	86762230	86771998	0.089561	0.067849
19215	Amp	11	86779538	86820076	0.001323	0.063993
19216	Amp	11	86833345	86844128	0.034113	0.065536
19217	Amp	11	86844147	86872418	0.153838	0.070398
19218	Amp	11	86872505	86882577	0.19964	0.072097
19219	Amp	11	86884701	86986618	0.379885	0.079168
19220	Amp	11	86988644	86988685	0.368062	0.078621
19221	Amp	11	86992291	86993054	0.316877	0.076921
19222	Amp	11	86997586	87044511	0.170723	0.071138
19223	Amp	11	87053883	87070001	0.19964	0.072239
19224	Amp	11	87075643	87093182	0.392611	0.079478
19225	Amp	11	87094747	87128494	0.17597	0.071321
19226	Amp	11	87129056	87129184	0.159312	0.070764
19227	Amp	11	87132332	87144648	0	0.063091
19228	Amp	11	87144996	87174530	0	0.06206
19229	Amp	11	87176550	87220993	0	0.063203
19230	Amp	11	87221561	87221574	0.029031	0.065224
19231	Amp	11	87224741	87236575	0.107017	0.068503
19232	Amp	11	87236646	87257867	0.089561	0.067887
19233	Amp	11	87262146	87304558	0.143787	0.070086
19234	Amp	11	87305959	87326523	0.089561	0.067926
19235	Amp	11	87326807	87339778	0.118001	0.06898
19236	Amp	11	87347675	87403908	0.001323	0.064012
19237	Amp	11	87405323	87420709	0.050473	0.066094
19238	Amp	11	87422711	87500207	0.128324	0.069287

19239	Amp	11	87501284	87584427	0.255918	0.074574
19240	Amp	11	87588315	87590884	0.595248	0.086363
19241	Amp	11	87595325	87660289	0.751221	0.090778
19242	Amp	11	87660310	87661249	0.765474	0.091283
19243	Amp	11	87661482	87718530	0.831947	0.092997
19244	Amp	11	87723886	87729467	0.891602	0.094485
19245	Amp	11	87742135	87757540	0.848692	0.093385
19246	Amp	11	87757650	87757650	0.758088	0.091186
19247	Amp	11	87758804	87932991	0.482379	0.082706
19248	Amp	11	87933148	87933866	0.529451	0.084114
19249	Amp	11	87934105	88034682	0.563096	0.085132
19250	Amp	11	88041502	88041616	0.595248	0.086365
19251	Amp	11	88046558	88050270	0.515676	0.083804
19252	Amp	11	88050677	88130250	0.636464	0.087708
19253	Amp	11	88132185	88347719	0.622604	0.087145
19254	Amp	11	88348575	88369784	0.711903	0.089846
19255	Amp	11	88375365	88519810	0.583752	0.085942
19256	Amp	11	88521916	88529578	0.463534	0.081935
19257	Amp	11	88531607	88714855	0.444474	0.081358
19258	Amp	11	88716227	88805047	0.404718	0.07995
19259	Amp	11	88810183	88810183	0.494792	0.082981
19260	Amp	11	88810547	88826927	0.522881	0.083828
19261	Amp	11	88826983	88877783	0.404718	0.080112
19262	Amp	11	88877796	88881048	0.373599	0.078879
19263	Amp	11	88881065	88891718	0.164935	0.070805
19264	Amp	11	88904573	88904855	0.089561	0.067774
19265	Amp	11	88905288	88918176	0.138391	0.069709
19266	Amp	11	88925975	89069560	0.153838	0.070551
19267	Amp	11	89072086	89125311	0.1237	0.069063
19268	Amp	11	89126263	89127571	0.029031	0.065324
19269	Amp	11	89135160	89137907	0	0.062637
19270	Amp	11	89147397	89161936	0	0.060448
19271	Amp	11	89163238	89163238	0	0.055132
19272	Amp	11	89163441	89189199	0	0.05429
19273	Amp	11	89194964	89417883	0	0.056436
19274	Amp	11	89421186	89647717	0.029031	0.06522
19275	Amp	11	89648409	89677295	0	0.061474
19276	Amp	11	89681629	89695811	0	0.044274
19277	Amp	11	90098873	90098873	0	0.05261
19278	Amp	11	90098900	90114103	0	0.050528
19279	Amp	11	90117678	90149750	0	0.053075
19280	Amp	11	90149939	90193519	0	0.055281
19281	Amp	11	90198942	90216867	0	0.052214
19282	Amp	11	90219297	90246910	0	0.050746
19283	Amp	11	90247014	90257741	0	0.047063
19284	Amp	11	90275625	90275779	0	0.049162
19285	Amp	11	90281816	90305263	0	0.046398
19286	Amp	11	90309083	90332357	0	0.045551
19287	Amp	11	90332362	90360480	0	0.041875
19288	Amp	11	90364061	90388720	0	0.044991
19289	Amp	11	90388828	90427385	0	0.046875
19290	Amp	11	90439581	90446998	0	0.048844
19291	Amp	11	90449673	90470766	0	0.05136

19292	Amp	11	90475226	90666128	0	0.049164
19293	Amp	11	90670513	90677449	0	0.047065
19294	Amp	11	90693209	90711236	0	0.050939
19295	Amp	11	90719871	90781453	0	0.045095
19296	Amp	11	90783806	90997894	0	0.0423
19297	Amp	11	90998085	91012636	0	0.04349
19298	Amp	11	91013331	91030944	0	0.043026
19299	Amp	11	91037080	91037080	0	0.045351
19300	Amp	11	91037923	91136560	0	0.05738
19301	Amp	11	91142032	91147990	0	0.059285
19302	Amp	11	91148871	91150131	0	0.054834
19303	Amp	11	91164099	91189609	0.159312	0.070761
19304	Amp	11	91190710	91226338	0.233782	0.073473
19305	Amp	11	91231986	91313966	0.170723	0.071148
19306	Amp	11	91316202	91327021	0.040093	0.065788
19307	Amp	11	91327062	91347143	0.069848	0.066949
19308	Amp	11	91347186	91442361	0.19964	0.072174
19309	Amp	11	91444625	91506829	0.463534	0.081936
19310	Amp	11	91513045	91524884	0.616549	0.086926
19311	Amp	11	91524898	91540629	0.630438	0.087466
19312	Amp	11	91542245	91543629	0.622604	0.087119
19313	Amp	11	91545724	91565649	0.264858	0.075091
19314	Amp	11	91571734	91578845	0.138391	0.069865
19315	Amp	11	91582810	91603816	0.069848	0.066926
19316	Amp	11	91604298	91613161	0.118001	0.069049
19317	Amp	11	91613584	91665925	0.065715	0.066669
19318	Amp	11	91673442	91713097	0.015369	0.064545
19319	Amp	11	91713258	91761933	0	0.057235
19320	Amp	11	91763018	91780760	0	0.059614
19321	Amp	11	91781603	91790361	0	0.058424
19322	Amp	11	91791316	91904209	0	0.060736
19323	Amp	11	91906946	91985122	0	0.061309
19324	Amp	11	91988816	91989896	0	0.058157
19325	Amp	11	91995828	92006987	0	0.045825
19326	Amp	11	92007541	92007567	0	0.043512
19327	Amp	11	92013563	92025187	0	0.041133
19328	Amp	11	92170971	92184288	0	0.045572
19329	Amp	11	92189350	92200763	0	0.04995
19330	Amp	11	92203825	92281213	0	0.048157
19331	Amp	11	92282773	92289319	0	0.052911
19332	Amp	11	92296034	92308687	0	0.050898
19333	Amp	11	92309668	92335271	0	0.047832
19334	Amp	11	92335487	92357360	0	0.04726
19335	Amp	11	92363669	92613212	0	0.035962
19336	Amp	11	92620823	92651404	0	0.033734
19337	Amp	11	92655767	92688661	0	0.034854
19338	Amp	11	92689383	92737422	0	0.043317
19339	Amp	11	92739095	92816366	0	0.029879
19340	Amp	11	92818062	92840745	0	0.039726
19341	Amp	11	92843300	92846969	0	0.045324
19342	Amp	11	92847647	93012353	0	0.035478
19343	Amp	11	93012411	93051037	0	0.032963
19344	Amp	11	93051762	93051762	0	0.032252

19345	Amp	11	93491739	93491739	0	0.033389
19346	Amp	11	93493618	93533428	0	0.03976
19347	Amp	11	93535287	93721241	0	0.032278
19348	Amp	11	93721358	93738837	0	0.028683
19349	Amp	11	93742626	93756811	0	0.034785
19350	Amp	11	93757356	93940379	0	0.033665
19351	Amp	11	93943027	94346117	0	0.03318
19352	Amp	11	94360122	94746262	0	0.032043
19353	Amp	11	94746930	94806766	0	0.030366
19354	Amp	11	94808569	94927986	0	0.028161
19355	Amp	11	94929512	95049556	0	0.027387
19356	Amp	11	95053692	95146052	0	0.028746
19357	Amp	11	95146154	95379693	0	0.027217
19358	Amp	11	95380806	95635290	0	0.028498
19359	Amp	11	95638188	95741898	0	0.030322
19360	Amp	11	95748585	95792953	0	0.029816
19361	Amp	11	95802056	95966423	0	0.024591
19362	Amp	11	95968431	96029801	0	0.026569
19363	Amp	11	96036176	96068960	0	0.027618
19364	Amp	11	96069088	96084035	0	0.025289
19365	Amp	11	96084215	96117744	0	0.027346
19366	Amp	11	96118113	96345177	0	0.030197
19367	Amp	11	96350785	96473562	0	0.028373
19368	Amp	11	96474442	96479510	0	0.026394
19369	Amp	11	96479838	96510025	0	0.031093
19370	Amp	11	96510173	96710502	0	0.027669
19371	Amp	11	96710715	96787136	0	0.026394
19372	Amp	11	96787692	97036109	0	0.022984
19373	Amp	11	97037425	97169308	0	0.027213
19374	Amp	11	97169910	97187949	0	0.028241
19375	Amp	11	97192595	97276384	0	0.036463
19376	Amp	11	97277352	97754084	0	0.033327
19377	Amp	11	97761649	97779928	0	0.035846
19378	Amp	11	97783504	98270972	0	0.048669
19379	Amp	11	98350106	98445211	0	0.045091
19380	Amp	11	98447659	98447971	0	0.046395
19381	Amp	11	98449684	98582366	0	0.048405
19382	Amp	11	98583664	98606545	0	0.047755
19383	Amp	11	98615330	98669417	0	0.042669
19384	Amp	11	98672488	98716984	0	0.043975
19385	Amp	11	98727812	98899472	0	0.047804
19386	Amp	11	98899582	99005163	0	0.046501
19387	Amp	11	99007289	99244808	0	0.042672
19388	Amp	11	99244928	99412940	0	0.038097
19389	Amp	11	99419669	99574153	0	0.034081
19390	Amp	11	99585885	99618643	0	0.040437
19391	Amp	11	99618748	99804289	0	0.042536
19392	Amp	11	99806957	99806996	0	0.03618
19393	Amp	11	99807947	99855766	0	0.034081
19394	Amp	11	99859176	99977201	0	0.033053
19395	Amp	11	99978232	100120232	0	0.035944
19396	Amp	11	100122909	100122909	0	0.037073
19397	Amp	11	100124550	100158966	0	0.05123

19398	Amp	11	100160299	100186013	0	0.055196
19399	Amp	11	100189162	100226060	0	0.05123
19400	Amp	11	100229587	100277899	0	0.038406
19401	Amp	11	100280530	100379779	0	0.035514
19402	Amp	11	100382774	100407914	0	0.034975
19403	Amp	11	100414978	100484361	0	0.032503
19404	Amp	11	100484831	100520319	0	0.020818
19405	Amp	11	100523353	100540738	0	0.026837
19406	Amp	11	100546214	100732652	0	0.022588
19407	Amp	11	100733500	100830762	0	0.022094
19408	Amp	11	100833218	100873847	0	0.026891
19409	Amp	11	100878298	100939612	0	0.024394
19410	Amp	11	100940098	100978942	0	0.02389
19411	Amp	11	100979342	101066576	0	0.02584
19412	Amp	11	101066914	101093760	0	0.02407
19413	Amp	11	101093764	101103372	0	0.025393
19414	Amp	11	101107231	101121437	0	0.03255
19415	Amp	11	101121453	101134955	0	0.028911
19416	Amp	11	101137771	101290317	0	0.025393
19417	Amp	11	101290732	101349305	0	0.02407
19418	Amp	11	101353925	101398247	0	0.030137
19419	Amp	11	101401695	101599814	0	0.02407
19420	Amp	11	101603215	101735389	0	0.026433
19421	Amp	11	101741911	101843429	0	0.01982
19422	Amp	11	101843762	101843762	0	0.020516
19423	Amp	11	101847972	102037910	0	0.018505
19424	Amp	11	102041840	102113897	0	0.027357
19425	Amp	11	102113987	102116371	0	0.026661
19426	Amp	11	102392217	102461995	0	0.022614
19427	Amp	11	102465687	102633740	0	0.026334
19428	Amp	11	102633856	102665683	0	0.02953
19429	Amp	11	102667105	102668221	0	0.031325
19430	Amp	11	102668571	102684818	0	0.028129
19431	Amp	11	102685878	102742459	0	0.028955
19432	Amp	11	102748760	102754183	0	0.032151
19433	Amp	11	102754867	102779693	0	0.032603
19434	Amp	11	102780073	102790368	0	0.029407
19435	Amp	11	102790864	103084362	0	0.026348
19436	Amp	11	103093410	103112894	0	0.024553
19437	Amp	11	103120500	103165062	0	0.015041
19438	Amp	11	103167558	103349218	0	0.016757
19439	Amp	11	103349550	103448231	0	0.017778
19440	Amp	11	103593639	103593639	0	0.019739
19441	Amp	11	103593797	103863593	0	0.020248
19442	Amp	11	103865759	104111225	0	0.021252
19443	Amp	11	104111982	104242848	0	0.023668
19444	Amp	11	104243934	104478004	0	0.021252
19445	Amp	11	104485170	104521144	0	0.024146
19446	Amp	11	104522303	104628581	0	0.021252
19447	Amp	11	104629450	104648316	0	0.022719
19448	Amp	11	104789903	105025596	0	0.016685
19449	Amp	11	105025790	105135186	0	0.025912
19450	Amp	11	105135209	105189064	0	0.024445

19451	Amp	11	105189456	105586474	0	0.014422
19452	Amp	11	105589253	106346445	0	0.013418
19453	Amp	11	106348964	106367355	0	0.004191
19454	Amp	11	106367981	106413079	0	0.003739
19455	Amp	11	106413648	107150265	0	0.007654
19456	Amp	11	107150372	107457818	0	0.005275
19457	Amp	11	107460607	107460608	0	0.017933
19458	Amp	11	107460612	107460612	0	0.027605
19459	Amp	11	107460616	107460977	0	0.043505
19460	Amp	11	107481240	107481240	0	0.021175
19461	Amp	11	107482587	107768035	0	0.005275
19462	Amp	11	107773570	107808194	0	0.001359
19463	Amp	11	107809102	108790310	0	0.003332
19464	Amp	11	108790891	109470062	0	0.001359
19465	Amp	11	109470863	110660283	0	0
19466	Amp	11	110665694	110720914	0	0.005234
19467	Amp	11	110724624	110861873	0	0.001353
19468	Amp	11	110863345	111538386	0	0
19469	Amp	11	111546855	111830322	0	0.001036
19470	Amp	11	111833311	111895389	0	0.003513
19471	Amp	11	111902445	111946958	0	0.006807
19472	Amp	11	111956023	112491739	0	0
19473	Amp	11	112503355	112915503	0	0.005677
19474	Amp	11	112915854	113079172	0	0.004053
19475	Amp	11	113080303	114610582	0	0
19476	Amp	11	114624276	114838533	0	0.000478
19477	Amp	11	114839776	116184810	0	0
19478	Amp	11	116187797	116332278	0	0.002167
19479	Amp	11	116337596	117027963	0	0
19480	Amp	11	117031392	117113733	0	0.002267
19481	Amp	11	117119148	117667052	0	0
19482	Amp	11	117673786	117723728	0	0.009318
19483	Amp	11	117731672	117742085	0	0.017008
19484	Amp	11	117743066	117925227	0	0.009318
19485	Amp	11	117927845	117942259	0	0.010594
19486	Amp	11	117946561	118069495	0	0.009318
19487	Amp	11	118070971	118435617	0	0.006479
19488	Amp	11	118444731	118485037	0	0
19489	Amp	11	118485066	118718449	0	0.001825
19490	Amp	11	118731998	118736969	0	0.008245
19491	Amp	11	118739920	118969999	0	0.00642
19492	Amp	11	118971542	119132859	0	0.003755
19493	Amp	11	119133976	119237402	0	0.004317
19494	Amp	11	119238138	119645470	0	0.000562
19495	Amp	11	119663625	119772568	0	0.003939
19496	Amp	11	119772947	120096567	0	0.006522
19497	Amp	11	120096696	120334263	0	0.003939
19498	Amp	11	120335454	121054039	0	0.000562
19499	Amp	11	121055731	121518624	0	0.001043
19500	Amp	11	121524065	121532520	0	0.003298
19501	Amp	11	121533842	121609320	0	0.001043
19502	Amp	11	121610698	121630082	0	0.000562
19503	Amp	11	121635262	121638807	0	0.002691

19504	Amp	11	121650615	122373276	0	0.004763
19505	Amp	11	122374405	122698666	0	0.004201
19506	Amp	11	122701924	123124371	0	0.005309
19507	Amp	11	123124571	123877297	0	0.006786
19508	Amp	11	123883497	123889804	0	0.007689
19509	Amp	11	123890751	123891564	0	0.013463
19510	Amp	11	123892534	124082913	0	0.012559
19511	Amp	11	124083173	124168662	0	0.017706
19512	Amp	11	124169100	124241904	0	0.016598
19513	Amp	11	124243021	124245439	0	0.059116
19514	Amp	11	124248114	124520488	0	0.016598
19515	Amp	11	124523888	125567803	0	0.01438
19516	Amp	11	125570843	125644096	0	0.017002
19517	Amp	11	125649982	125650953	0	0.032938
19518	Amp	11	125653708	125702804	0	0.027387
19519	Amp	11	125709161	125855911	0	0.02921
19520	Amp	11	125864487	125864487	0	0.034105
19521	Amp	11	125865669	125865669	0	0.035883
19522	Amp	11	125868723	125889332	0	0.03406
19523	Amp	11	125889780	125895010	0	0.029165
19524	Amp	11	125901710	126372802	0	0.032112
19525	Amp	11	126372862	126445129	0	0.033262
19526	Amp	11	126445832	126693096	0	0.032112
19527	Amp	11	126696691	126710445	0	0.021882
19528	Amp	11	126710921	126733062	0	0.023896
19529	Amp	11	126733168	126818110	0	0.021882
19530	Amp	11	126818457	126822302	0	0.023269
19531	Amp	11	126822915	126858460	0	0.021882
19532	Amp	11	126861772	126871988	0	0.015825
19533	Amp	11	126877441	126946394	0	0.013753
19534	Amp	11	126947045	127039261	0	0.026573
19535	Amp	11	127040685	127212651	0	0.036467
19536	Amp	11	127213160	127308868	0	0.038881
19537	Amp	11	127310636	127328155	0	0.036467
19538	Amp	11	127328327	127330648	0	0.02741
19539	Amp	11	127330669	127361529	0	0.036467
19540	Amp	11	127362993	127466440	0	0.038016
19541	Amp	11	127468655	127515702	0	0.022417
19542	Amp	11	127517599	128109078	0	0.009596
19543	Amp	11	128119717	128210622	0	0.022417
19544	Amp	11	128216099	128254949	0	0.010059
19545	Amp	11	128257837	128342823	0	0.016114
19546	Amp	11	128343044	128375436	0	0.018385
19547	Amp	11	128376136	128425536	0	0.019231
19548	Amp	11	128428889	128534223	0	0.012713
19549	Amp	11	128534550	128772493	0	0.010442
19550	Amp	11	128773658	128910802	0	0.008665
19551	Amp	11	128911444	129004130	0	0.021937
19552	Amp	11	129009303	129059271	0	0.024595
19553	Amp	11	129060150	129200689	0	0.025587
19554	Amp	11	129206274	129264747	0	0.027698
19555	Amp	11	129265217	129286853	0	0.025587
19556	Amp	11	129288276	130457304	0	0.026758

19557 Amp	11	130457987	130966328	0	0.025493
19558 Amp	11	130971084	131473433	0	0.023761
19559 Amp	11	131473623	131547657	0	0.024865
19560 Amp	11	131549994	131899560	0	0.019281
19561 Amp	11	131901501	132029179	0	0.024349
19562 Amp	11	132029513	132083280	0	0.02234
19563 Amp	11	132084253	132186822	0	0.024648
19564 Amp	11	132192492	132207023	0	0.019281
19565 Amp	11	132208038	132389839	0	0.013533
19566 Amp	11	132394119	132417434	0	0.015261
19567 Amp	11	132417566	132953650	0	0.013533
19568 Amp	11	132953663	133639648	0	0.015262
19569 Amp	11	133646952	133678166	0	0.017619
19570 Amp	11	133678332	133871397	0	0.015262
19571 Amp	11	133873927	134174115	0	0.028258
19572 Amp	11	134174205	134270258	0	0.027154
19573 Amp	12	780472	806855	0	0.059806
19574 Amp	12	813226	845898	0	0.054717
19575 Amp	12	855340	970002	0	0.052882
19576 Amp	12	971262	1041615	0	0.051246
19577 Amp	12	1042959	1070966	0	0.048777
19578 Amp	12	1071247	1132744	0	0.049366
19579 Amp	12	1135059	1197489	0	0.049977
19580 Amp	12	1752621	1755698	0	0.034041
19581 Amp	12	1757038	1757633	0	0.038866
19582 Amp	12	1758235	1770514	0	0.037504
19583 Amp	12	1771454	1815039	0	0.038075
19584 Amp	12	1819540	1877084	0	0.03325
19585 Amp	12	1878079	1891061	0	0.035915
19586 Amp	12	1893166	1915678	0	0.029894
19587 Amp	12	1916038	1984507	0	0.029448
19588 Amp	12	1986689	1995364	0	0.029911
19589 Amp	12	1997787	2000292	0	0.029448
19590 Amp	12	2011392	2018586	0	0.026782
19591 Amp	12	2019816	2031459	0	0.027311
19592 Amp	12	2032464	2095042	0	0.025719
19593 Amp	12	2096193	2110028	0	0.024334
19594 Amp	12	2113384	2295837	0	0.022525
19595 Amp	12	2300394	2305509	0	0.02086
19596 Amp	12	2306672	2575536	0	0.019917
19597 Amp	12	2576678	2646660	0	0.017759
19598 Amp	12	2649210	2773378	0	0.01617
19599 Amp	12	2774471	2840073	0	0.017556
19600 Amp	12	2842517	2872799	0	0.013121
19601 Amp	12	2875667	2879864	0	0.01251
19602 Amp	12	2880176	3133126	0	0.012
19603 Amp	12	3133626	3241417	0	0.013946
19604 Amp	12	3242186	3381322	0	0.012
19605 Amp	12	3381341	3528284	0	0.011428
19606 Amp	12	3532002	3546953	0	0.012696
19607 Amp	12	3548520	3626454	0	0.01156
19608 Amp	12	3627162	3659176	0	0.010975
19609 Amp	12	3659812	3663406	0	0.011508

19610	Amp	12	3664691	3670572	0	0.01024
19611	Amp	12	3670995	3676957	0	0.009651
19612	Amp	12	3677549	3726693	0	0.009122
19613	Amp	12	3759114	3810931	0	0.009711
19614	Amp	12	3816261	3817634	0	0.008669
19615	Amp	12	3821729	3880770	0	0.00808
19616	Amp	12	3882559	4083710	0	0.00728
19617	Amp	12	4085689	4203091	0	0.007852
19618	Amp	12	4203975	4346530	0	0.001892
19619	Amp	12	4353339	4388989	0	0.00132
19620	Amp	12	4389241	4685567	0	0.002894
19621	Amp	12	4685918	4688001	0	0.00338
19622	Amp	12	4694990	4766089	0	0.007466
19623	Amp	12	4766449	4831088	0	0.004604
19624	Amp	12	4831344	4868814	0	0.00338
19625	Amp	12	4869891	4897213	0	0.007859
19626	Amp	12	4898843	4930062	0	0.007265
19627	Amp	12	4930419	4993446	0	0.008072
19628	Amp	12	4994220	5009511	0	0.004186
19629	Amp	12	5009764	5219926	0	0.005771
19630	Amp	12	5220035	5420659	0	0.006237
19631	Amp	12	5422846	5477259	0	0.007851
19632	Amp	12	5477980	5540681	0	0.008444
19633	Amp	12	5547138	5667879	0	0.010426
19634	Amp	12	5670232	5692685	0	0.013854
19635	Amp	12	5693038	6020116	0	0.010426
19636	Amp	12	6023836	6099452	0	0.011608
19637	Amp	12	6104916	6278143	0	0.012813
19638	Amp	12	6281789	6353323	0	0.011608
19639	Amp	12	6353370	6361094	0	0.012971
19640	Amp	12	6379284	6414177	0	0.014406
19641	Amp	12	6417253	6417253	0	0.016091
19642	Amp	12	6418903	6456987	0	0.018045
19643	Amp	12	6460120	6489732	0	0.019105
19644	Amp	12	6490341	6491514	0	0.018619
19645	Amp	12	6494565	6510260	0	0.020073
19646	Amp	12	6512945	6536309	0	0.019541
19647	Amp	12	6536399	6562319	0	0.018221
19648	Amp	12	6572707	6723746	0	0.017162
19649	Amp	12	6723938	6725096	0	0.020717
19650	Amp	12	6732691	6780210	0	0.021422
19651	Amp	12	6784998	6826466	0	0.019808
19652	Amp	12	6833508	6860538	0	0.025908
19653	Amp	12	6866342	7014144	0	0.024474
19654	Amp	12	7019159	7063032	0	0.026046
19655	Amp	12	7067743	7165106	0	0.026703
19656	Amp	12	7165535	7179636	0	0.026046
19657	Amp	12	7182388	7183172	0	0.025581
19658	Amp	12	7194399	7194399	0	0.02034
19659	Amp	12	7203302	7212451	0	0.022963
19660	Amp	12	7214940	7215586	0	0.022314
19661	Amp	12	7215718	7269807	0	0.020739
19662	Amp	12	7274392	7485820	0	0.018117

19663 Amp	12	7489156	7630434	0	0.016663
19664 Amp	12	7632437	7669295	0	0.015078
19665 Amp	12	7672411	7750472	0	0.016752
19666 Amp	12	7750532	7805181	0	0.014797
19667 Amp	12	7805809	7807020	0	0.016996
19668 Amp	12	7810165	7810616	0	0.01629
19669 Amp	12	7982496	7987805	0	0.021304
19670 Amp	12	7989860	8044932	0	0.019631
19671 Amp	12	8048464	8144088	0	0.017432
19672 Amp	12	8147236	8157111	0	0.01507
19673 Amp	12	8443757	8443757	0	0.008969
19674 Amp	12	8444196	8447767	0	0.008376
19675 Amp	12	8449386	8481837	0	0.009871
19676 Amp	12	8482933	8785124	0	0.010578
19677 Amp	12	8793420	8851043	0	0.007926
19678 Amp	12	8855329	8996707	0	0.007219
19679 Amp	12	9000550	9045249	0	0.006412
19680 Amp	12	9046429	9243134	0	0.004918
19681 Amp	12	9243211	9638628	0	0.008217
19682 Amp	12	9642069	9686849	0	0.009657
19683 Amp	12	9687442	9871866	0	0.010798
19684 Amp	12	9876915	9894696	0	0.014399
19685 Amp	12	9895466	9905649	0	0.012959
19686 Amp	12	9908217	10046758	0	0.02306
19687 Amp	12	10052083	10097061	0	0.024507
19688 Amp	12	10099609	10112618	0	0.021208
19689 Amp	12	10116053	10402473	0	0.019761
19690 Amp	12	10407887	10524650	0	0.023124
19691 Amp	12	10564309	10667336	0	0.021983
19692 Amp	12	10671773	10722100	0	0.022466
19693 Amp	12	10723738	10728748	0	0.012365
19694 Amp	12	10732796	10762760	0	0.009002
19695 Amp	12	10772399	10863514	0	0.015248
19696 Amp	12	10868409	10999751	0	0.013675
19697 Amp	12	10999844	11000226	0	0.017989
19698 Amp	12	11200674	11243616	0	0.024887
19699 Amp	12	11246252	11507926	0	0.020573
19700 Amp	12	11508546	11639213	0	0.022688
19701 Amp	12	11643005	11681823	0	0.02009
19702 Amp	12	11683668	11825053	0	0.020757
19703 Amp	12	11825529	11894967	0	0.021354
19704 Amp	12	11895067	12003388	0	0.020757
19705 Amp	12	12003809	12075819	0	0.023496
19706 Amp	12	12076852	12154252	0	0.022132
19707 Amp	12	12155354	12244963	0	0.024453
19708 Amp	12	12252961	12334591	0	0.019393
19709 Amp	12	12338699	12535247	0	0.013147
19710 Amp	12	12536162	12721723	0	0.00625
19711 Amp	12	12726636	12890185	0	0.000666
19712 Amp	12	12892789	12892789	0	0.005524
19713 Amp	12	13183649	13602798	0	0.004858
19714 Amp	12	13603815	14172097	0	0
19715 Amp	12	14172743	14361479	0	0.0009

19716	Amp	12	14366661	14467256	0	0.002211
19717	Amp	12	14469068	14560557	0	0.003789
19718	Amp	12	14560859	14868147	0	0.00289
19719	Amp	12	14870471	14870623	0	0.005482
19720	Amp	12	14870892	14940519	0	0.006047
19721	Amp	12	14941411	15060262	0	0.003455
19722	Amp	12	15064900	15089204	0	0.004611
19723	Amp	12	15089717	15099206	0	0.006418
19724	Amp	12	15109143	15134542	0	0.009952
19725	Amp	12	15143876	15155848	0	0.013055
19726	Amp	12	15161398	15165268	0	0.009522
19727	Amp	12	15166300	15188673	0	0.006418
19728	Amp	12	15188979	15214078	0	0.007117
19729	Amp	12	15216806	15248862	0	0.006418
19730	Amp	12	15252465	15303903	0	0.009859
19731	Amp	12	15309093	15321694	0	0.008548
19732	Amp	12	15321889	15374620	0	0.006741
19733	Amp	12	15380035	15529355	0	0.005584
19734	Amp	12	15535089	15586386	0	0.008765
19735	Amp	12	15654433	15654433	0	0.005584
19736	Amp	12	15655744	15720087	0	0.007722
19737	Amp	12	15720911	15794500	0	0.004282
19738	Amp	12	15795422	15838792	0	0.003717
19739	Amp	12	15846909	15956970	0	0.008004
19740	Amp	12	15960098	15988877	0	0.003717
19741	Amp	12	15992148	16434407	0	0.004678
19742	Amp	12	16434835	16680526	0	0.003717
19743	Amp	12	16683048	16760700	0	0.002138
19744	Amp	12	16762571	16882753	0	0
19745	Amp	12	16883220	16898469	0	0.004588
19746	Amp	12	16898860	17213299	0	0
19747	Amp	12	17218520	17535595	0	0.000812
19748	Amp	12	17540482	17622469	0	0.00529
19749	Amp	12	17623759	17685902	0	0.006979
19750	Amp	12	17689902	17853215	0	0.008361
19751	Amp	12	17861685	18002617	0	0.010789
19752	Amp	12	18003275	18021269	0	0.009407
19753	Amp	12	18021851	18029474	0	0.006979
19754	Amp	12	18044873	18107563	0	0.012824
19755	Amp	12	18109877	18229867	0	0.008673
19756	Amp	12	18235011	18235793	0	0.041649
19757	Amp	12	18235993	18235993	0	0.035828
19758	Amp	12	18247102	18421893	0	0.008992
19759	Amp	12	18421963	18459800	0	0.011392
19760	Amp	12	18460395	18510910	0	0.009702
19761	Amp	12	18511397	18649873	0	0.007302
19762	Amp	12	18653414	18749246	0	0.009108
19763	Amp	12	18751180	18755843	0	0.011582
19764	Amp	12	18758221	18806409	0	0.009175
19765	Amp	12	18806749	18806749	0	0.011131
19766	Amp	12	18808113	18823678	0	0.013168
19767	Amp	12	18825257	19040840	0	0.013758
19768	Amp	12	19044016	19062867	0	0.016783

19769 Amp	12	19063109	19086855	0	0.013758
19770 Amp	12	19088459	19227111	0	0.013168
19771 Amp	12	19227825	19312553	0	0.012485
19772 Amp	12	19314022	19314022	0	0.014031
19773 Amp	12	19432224	19448983	0	0.015349
19774 Amp	12	19455504	19473881	0	0.020777
19775 Amp	12	19477263	19591245	0	0.015349
19776 Amp	12	19591337	19624063	0	0.012875
19777 Amp	12	19624557	19656321	0	0.016788
19778 Amp	12	19658522	19659534	0	0.012342
19779 Amp	12	19663428	19735611	0	0.008259
19780 Amp	12	19735702	20055967	0	0.009686
19781 Amp	12	20057002	20121998	0	0.01515
19782 Amp	12	20123579	20280706	0	0.009686
19783 Amp	12	20280996	20376412	0	0.008259
19784 Amp	12	20376704	20954357	0	0.00694
19785 Amp	12	20954515	21161748	0	0.003631
19786 Amp	12	21164200	21216257	0	0.011793
19787 Amp	12	21216392	21374189	0	0.010519
19788 Amp	12	21378094	21394047	0	0.01216
19789 Amp	12	21401766	21414671	0	0.015684
19790 Amp	12	21415628	21415628	0	0.018184
19791 Amp	12	21425337	21439472	0	0.01466
19792 Amp	12	21440754	21488759	0	0.01216
19793 Amp	12	21490289	21490289	0	0.006722
19794 Amp	12	21491733	21613949	0	0.008347
19795 Amp	12	21618969	21696414	0	0.010037
19796 Amp	12	21698535	21740315	0	0.010728
19797 Amp	12	21741608	22047803	0	0.012042
19798 Amp	12	22047891	22131985	0	0.015256
19799 Amp	12	22132368	22157931	0	0.012762
19800 Amp	12	22165521	22165521	0	0.011072
19801 Amp	12	22165615	22295729	0	0.012362
19802 Amp	12	22296759	22417433	0	0.013939
19803 Amp	12	22422812	22521613	0	0.010496
19804 Amp	12	22526960	22564006	0	0.008871
19805 Amp	12	22571137	22651201	0	0.011644
19806 Amp	12	22651341	22801281	0	0.013273
19807 Amp	12	22801998	22962095	0	0.020936
19808 Amp	12	22963031	23070086	0	0.022579
19809 Amp	12	23070178	23145021	0	0.014916
19810 Amp	12	23153548	23187649	0	0.015447
19811 Amp	12	23187669	23271185	0	0.017753
19812 Amp	12	23277142	23419263	0	0.018716
19813 Amp	12	23440132	23819768	0	0.017086
19814 Amp	12	23826812	23923213	0	0.022814
19815 Amp	12	23924414	24022705	0	0.024764
19816 Amp	12	24026788	24155667	0	0.032311
19817 Amp	12	24156729	24262002	0	0.024764
19818 Amp	12	24265608	24389650	0	0.022457
19819 Amp	12	24390908	24472460	0	0.021495
19820 Amp	12	24475499	24504255	0	0.022514
19821 Amp	12	24508190	24508190	0	0.0212

19822	Amp	12	24508427	24534199	0	0.022862
19823	Amp	12	24539449	24572718	0	0.024375
19824	Amp	12	24576201	24587403	0	0.018647
19825	Amp	12	24590281	24613769	0	0.016698
19826	Amp	12	24615467	24670904	0	0.01855
19827	Amp	12	24674547	24683712	0	0.020412
19828	Amp	12	24684323	24690644	0	0.021615
19829	Amp	12	24693588	24732783	0	0.024257
19830	Amp	12	24735028	24735028	0	0.02308
19831	Amp	12	25047631	25047631	0	0.028224
19832	Amp	12	25056830	25081712	0	0.026362
19833	Amp	12	25084104	25105523	0	0.042298
19834	Amp	12	25108334	25169730	0	0.054394
19835	Amp	12	25172979	25177517	0	0.056478
19836	Amp	12	25181420	25217841	0	0.056932
19837	Amp	12	25218528	25224758	0	0.058975
19838	Amp	12	25224775	25237555	0	0.055607
19839	Amp	12	25237846	25280057	0	0.057064
19840	Amp	12	25282529	25308105	0	0.058033
19841	Amp	12	25308585	25373314	0	0.057343
19842	Amp	12	25373763	25394406	0	0.059532
19843	Amp	12	25403770	25415235	0	0.048762
19844	Amp	12	25415289	25417466	0	0.048307
19845	Amp	12	25418742	25447712	0	0.046794
19846	Amp	12	25456779	25475251	0	0.048474
19847	Amp	12	25476365	25530562	0	0.046353
19848	Amp	12	25531013	25555886	0	0.04358
19849	Amp	12	25556598	25578967	0	0.041897
19850	Amp	12	25579129	25584887	0	0.04358
19851	Amp	12	25586225	25592183	0	0.045711
19852	Amp	12	25598155	25664531	0	0.049293
19853	Amp	12	25664945	25728670	0	0.047612
19854	Amp	12	25730171	25888770	0	0.048161
19855	Amp	12	25888994	25987471	0	0.048678
19856	Amp	12	25987716	26009842	0	0.050201
19857	Amp	12	26018643	26059513	0	0.048012
19858	Amp	12	26067794	26262262	0	0.04957
19859	Amp	12	26264479	26469165	0	0.050272
19860	Amp	12	26473455	26704293	0	0.055443
19861	Amp	12	26706086	26791785	0	0.057474
19862	Amp	12	26794882	26804563	0.187449	0.071852
19863	Amp	12	26813762	26892531	0	0.055916
19864	Amp	12	26903560	26957641	0	0.055104
19865	Amp	12	26962200	26981552	0	0.054402
19866	Amp	12	26981856	27028508	0	0.056112
19867	Amp	12	27031549	27074889	0	0.04283
19868	Amp	12	27077054	27196262	0	0.041325
19869	Amp	12	27196395	27267773	0	0.039241
19870	Amp	12	27269953	27293003	0	0.034097
19871	Amp	12	27298667	27309742	0	0.035965
19872	Amp	12	27309814	27346040	0	0.033834
19873	Amp	12	27350022	27435893	0	0.030954
19874	Amp	12	27438409	27465864	0	0.04689

19875	Amp	12	27465950	27465998	0	0.047565
19876	Amp	12	27468039	27541105	0	0.044514
19877	Amp	12	27545818	27565844	0	0.042646
19878	Amp	12	27565903	27700145	0	0.041069
19879	Amp	12	27701395	27823966	0	0.043407
19880	Amp	12	27824013	27841624	0	0.042732
19881	Amp	12	27842053	27846048	0	0.041275
19882	Amp	12	27846261	27909818	0	0.039566
19883	Amp	12	27915096	27941465	0	0.042891
19884	Amp	12	27941524	27967532	0	0.050671
19885	Amp	12	27974006	28067087	0	0.052037
19886	Amp	12	28069074	28229889	0	0.046865
19887	Amp	12	28233198	28317151	0	0.036432
19888	Amp	12	28320733	28481705	0	0.035066
19889	Amp	12	28481839	28538044	0	0.033862
19890	Amp	12	28539966	28680558	0	0.032218
19891	Amp	12	28681048	28867529	0	0.032802
19892	Amp	12	28869118	28999700	0	0.035345
19893	Amp	12	29001072	29030399	0	0.032479
19894	Amp	12	29030619	29180801	0	0.031895
19895	Amp	12	29180838	29181968	0.379885	0.079139
19896	Amp	12	29185506	29206411	0	0.031895
19897	Amp	12	29208088	29235839	0	0.030025
19898	Amp	12	29239070	29248064	0	0.035349
19899	Amp	12	29248941	29303157	0	0.038215
19900	Amp	12	29304070	29841251	0	0.03997
19901	Amp	12	29846366	29885929	0	0.042411
19902	Amp	12	29887784	29896097	0	0.041709
19903	Amp	12	29896148	29968652	0	0.039955
19904	Amp	12	29977858	30004659	0	0.036343
19905	Amp	12	30006753	30040759	0	0.037236
19906	Amp	12	30040824	30080397	0	0.039715
19907	Amp	12	30091773	30176221	0	0.037236
19908	Amp	12	30182019	30270581	0	0.015975
19909	Amp	12	30270664	30316270	0	0.016871
19910	Amp	12	30319316	30414359	0	0.016354
19911	Amp	12	30415241	30446149	0	0.019493
19912	Amp	12	30446483	30475317	0	0.017928
19913	Amp	12	30475429	30506305	0	0.014788
19914	Amp	12	30507880	30841582	0	0.01245
19915	Amp	12	30842552	30864547	0	0.015255
19916	Amp	12	30865177	31003566	0	0.01245
19917	Amp	12	31004579	31085371	0	0.014275
19918	Amp	12	31095328	31104761	0	0.014898
19919	Amp	12	31259292	31301084	0	0.020767
19920	Amp	12	31301628	31469807	0	0.019871
19921	Amp	12	31470914	31484492	0	0.020879
19922	Amp	12	31495892	31605333	0	0.021927
19923	Amp	12	31611757	31633811	0	0.019351
19924	Amp	12	31634267	31689709	0	0.018729
19925	Amp	12	31691810	31807781	0	0.019219
19926	Amp	12	31814156	31838572	0	0.018211
19927	Amp	12	31838672	31980592	0	0.020279

19928	Amp	12	31981027	32069712	0	0.017115
19929	Amp	12	32072014	32163685	0	0.01897
19930	Amp	12	32167896	32283563	0	0.018479
19931	Amp	12	32286812	32305347	0	0.016038
19932	Amp	12	32306136	32504342	0	0.010677
19933	Amp	12	32504865	32574668	0	0.012409
19934	Amp	12	32575185	32645858	0	0.011881
19935	Amp	12	32647193	32651833	0	0.010026
19936	Amp	12	32652119	32766739	0	0.009495
19937	Amp	12	32768632	32971116	0	0.008291
19938	Amp	12	32983041	33068557	0	0.010031
19939	Amp	12	33076742	33099244	0	0.015486
19940	Amp	12	33102734	33137723	0	0.012034
19941	Amp	12	33140973	33140973	0	0.013368
19942	Amp	12	33246810	33258407	0	0.015085
19943	Amp	12	33259943	33269603	0	0.018017
19944	Amp	12	33271972	33363499	0	0.015085
19945	Amp	12	33365705	33366515	0	0.013261
19946	Amp	12	33366590	33368216	0	0.011618
19947	Amp	12	38827290	38827290	0	0.018536
19948	Amp	12	38829764	38903226	0	0.020214
19949	Amp	12	38908445	39011660	0	0.022597
19950	Amp	12	39015104	39015104	0	0.020214
19951	Amp	12	39015221	39168609	0	0.02177
19952	Amp	12	39169360	39451288	0	0.020723
19953	Amp	12	39455158	39541392	0	0.026476
19954	Amp	12	39547873	39630276	0	0.024799
19955	Amp	12	39630447	39641895	0	0.025601
19956	Amp	12	39660451	40028427	0	0.019848
19957	Amp	12	40032854	40244652	0	0.021666
19958	Amp	12	40245375	40364850	0	0.024955
19959	Amp	12	40364981	40386092	0	0.021666
19960	Amp	12	40393583	40444549	0	0.02073
19961	Amp	12	40445527	40499786	0	0.023578
19962	Amp	12	40500186	40668503	0	0.025188
19963	Amp	12	40679662	40844726	0	0.02234
19964	Amp	12	40849946	40958628	0	0.025112
19965	Amp	12	40958875	41000770	0	0.023063
19966	Amp	12	41004577	41110024	0	0.025889
19967	Amp	12	41115611	41220939	0	0.028682
19968	Amp	12	41221686	41268090	0	0.026476
19969	Amp	12	41270712	41319017	0	0.028102
19970	Amp	12	41324736	41400118	0	0.030127
19971	Amp	12	41403584	41485311	0	0.027355
19972	Amp	12	41489723	41634677	0	0.028778
19973	Amp	12	41672741	41770287	0	0.027152
19974	Amp	12	41772002	41837710	0	0.029477
19975	Amp	12	41838533	41954393	0	0.026236
19976	Amp	12	41954551	41996037	0	0.024625
19977	Amp	12	41999325	42020959	0	0.022999
19978	Amp	12	42023162	42128625	0	0.025194
19979	Amp	12	42131099	42213169	0	0.022369
19980	Amp	12	42213315	42284595	0	0.02424

19981 Amp	12	42291855	42568097	0	0.032133
19982 Amp	12	42572506	42608993	0	0.034909
19983 Amp	12	42612192	42818897	0	0.032584
19984 Amp	12	43344680	43344680	0	0.031195
19985 Amp	12	43345446	43404470	0	0.029324
19986 Amp	12	43404973	43430746	0	0.031039
19987 Amp	12	43430911	43453458	0	0.029324
19988 Amp	12	43453779	43520500	0	0.027128
19989 Amp	12	43520975	43905616	0	0.024353
19990 Amp	12	43906254	44154877	0	0.016459
19991 Amp	12	44155632	44227111	0	0.017774
19992 Amp	12	44228698	44312244	0	0.016459
19993 Amp	12	44317772	44361411	0	0.013667
19994 Amp	12	44363222	44502746	0	0.014863
19995 Amp	12	44503748	44505429	0	0.015705
19996 Amp	12	44507960	44539639	0	0.017843
19997 Amp	12	44551274	44664827	0	0.016646
19998 Amp	12	44667624	44687841	0	0.01514
19999 Amp	12	44688325	44788477	0	0.017953
20000 Amp	12	44795569	44845619	0	0.019406
20001 Amp	12	44849226	44883384	0	0.018709
20002 Amp	12	44890302	44910798	0	0.02032
20003 Amp	12	44912411	45194187	0	0.019638
20004 Amp	12	45200317	45310402	0	0.022849
20005 Amp	12	45310756	45426739	0	0.024124
20006 Amp	12	45433724	45551822	0	0.022512
20007 Amp	12	45552519	45622565	0	0.024461
20008 Amp	12	45624901	45748004	0	0.023614
20009 Amp	12	45751717	45774445	0	0.026056
20010 Amp	12	45776425	45846405	0	0.027419
20011 Amp	12	45848874	45916640	0	0.028454
20012 Amp	12	45920014	46122026	0	0.029658
20013 Amp	12	46128417	46128417	0	0.030869
20014 Amp	12	46131678	46138093	0	0.043738
20015 Amp	12	46143340	46305119	0	0.045425
20016 Amp	12	46321298	46345926	0	0.044213
20017 Amp	12	46348952	46497792	0	0.047569
20018 Amp	12	46505815	46535224	0	0.046588
20019 Amp	12	46537037	46539477	0	0.045552
20020 Amp	12	46548124	46664263	0	0.043188
20021 Amp	12	46671940	46709472	0	0.047391
20022 Amp	12	46710074	46714880	0	0.034522
20023 Amp	12	46714881	46725590	0	0.039359
20024 Amp	12	46728283	46749878	0	0.030319
20025 Amp	12	46750488	46791001	0	0.02837
20026 Amp	12	46792477	47119014	0	0.026233
20027 Amp	12	47121075	47206649	0	0.022877
20028 Amp	12	47206724	47293929	0	0.023348
20029 Amp	12	47295469	47341617	0	0.018256
20030 Amp	12	47343490	47370650	0	0.020969
20031 Amp	12	47373177	47424180	0	0.025431
20032 Amp	12	47424706	47495557	0	0.022617
20033 Amp	12	47496051	47496203	0	0.022146

20034 Amp	12	47500191	47550395	0	0.024458
20035 Amp	12	47664492	47788304	0	0.027128
20036 Amp	12	47792398	47817999	0	0.035415
20037 Amp	12	47822342	47916896	0	0.033547
20038 Amp	12	47917148	47922390	0	0.03466
20039 Amp	12	47924049	48050705	0	0.03199
20040 Amp	12	48055665	48074766	0	0.028804
20041 Amp	12	48075779	48247620	0	0.032095
20042 Amp	12	48249731	48255160	0	0.038574
20043 Amp	12	48258851	48273911	0	0.041099
20044 Amp	12	48277081	48339301	0	0.03462
20045 Amp	12	48339668	48348741	0	0.032428
20046 Amp	12	48353466	48371797	0	0.029902
20047 Amp	12	48374991	48391302	0	0.026382
20048 Amp	12	48391706	48473872	0	0.023495
20049 Amp	12	48478712	48533758	0	0.025754
20050 Amp	12	48535778	48773477	0	0.025312
20051 Amp	12	48785775	48808707	0	0.022834
20052 Amp	12	48810638	48857472	0	0.031807
20053 Amp	12	48860765	48891851	0	0.03324
20054 Amp	12	48894974	48903586	0	0.033892
20055 Amp	12	48905412	49025894	0	0.036989
20056 Amp	12	49026295	49075195	0	0.038413
20057 Amp	12	49081737	49166790	0	0.030198
20058 Amp	12	49171740	49202828	0	0.028103
20059 Amp	12	49204057	49279721	0	0.025006
20060 Amp	12	49283708	49563900	0	0.023582
20061 Amp	12	49565818	49602372	0	0.025206
20062 Amp	12	49611507	49662339	0	0.024554
20063 Amp	12	49666611	49755564	0	0.023796
20064 Amp	12	49761222	49817550	0	0.025135
20065 Amp	12	49818381	49886933	0	0.028051
20066 Amp	12	49886941	49891779	0	0.026712
20067 Amp	12	49892339	50074438	0	0.028783
20068 Amp	12	50079049	50143277	0	0.030755
20069 Amp	12	50144032	50164060	0	0.027838
20070 Amp	12	50170323	50234683	0	0.030467
20071 Amp	12	50240571	50263301	0	0.031495
20072 Amp	12	50265864	50272264	0	0.032267
20073 Amp	12	50275752	50290679	0	0.034354
20074 Amp	12	50294896	50446988	0	0.03273
20075 Amp	12	50456157	50473336	0	0.030676
20076 Amp	12	50474929	50666391	0	0.031581
20077 Amp	12	50669284	50819650	0	0.030875
20078 Amp	12	50824331	50844033	0	0.030301
20079 Amp	12	50844665	50904979	0	0.031474
20080 Amp	12	50905562	50937381	0	0.033201
20081 Amp	12	50938796	51001244	0	0.033946
20082 Amp	12	51004251	51095903	0	0.032773
20083 Amp	12	51096625	51147044	0	0.030374
20084 Amp	12	51150075	51209045	0	0.028647
20085 Amp	12	51211036	51216581	0	0.026206
20086 Amp	12	51222798	51274402	0	0.02412

20087 Amp	12	51275501	51278127	0	0.027588
20088 Amp	12	51280717	51336980	0	0.026155
20089 Amp	12	51340167	51478646	0	0.028152
20090 Amp	12	51478795	51502098	0	0.027407
20091 Amp	12	51506144	51550878	0	0.029662
20092 Amp	12	51551561	51559427	0	0.03015
20093 Amp	12	51574298	51576458	0	0.029245
20094 Amp	12	51576690	51778851	0	0.028559
20095 Amp	12	51781520	51926892	0	0.029137
20096 Amp	12	51927942	51986070	0	0.029846
20097 Amp	12	51986206	52005470	0	0.030384
20098 Amp	12	52009326	52021848	0	0.030863
20099 Amp	12	52024880	52026175	0	0.028607
20100 Amp	12	52029395	52282619	0	0.027897
20101 Amp	12	52391992	52512802	0	0.030466
20102 Amp	12	52513456	52677598	0	0.031031
20103 Amp	12	52677776	52681126	0	0.033006
20104 Amp	12	52681433	52734506	0	0.033596
20105 Amp	12	52736501	52857625	0	0.033118
20106 Amp	12	52857700	52857700	0	0.031168
20107 Amp	12	52861260	52861383	0	0.03068
20108 Amp	12	52867084	52901610	0	0.031667
20109 Amp	12	52901767	52978521	0	0.031129
20110 Amp	12	52981050	53017933	0	0.030552
20111 Amp	12	53142366	53143439	0	0.031802
20112 Amp	12	53146581	53146581	0	0.026244
20113 Amp	12	53152261	53316141	0	0.027056
20114 Amp	12	53318810	53398204	0	0.025806
20115 Amp	12	53402538	53458437	0	0.022515
20116 Amp	12	53458940	53468055	0	0.021925
20117 Amp	12	53469537	53531587	0	0.019356
20118 Amp	12	53537494	53583284	0	0.020194
20119 Amp	12	53594979	53597916	0	0.021684
20120 Amp	12	53600166	53686771	0	0.02032
20121 Amp	12	53688748	53699330	0	0.022444
20122 Amp	12	53706972	53716196	0	0.026807
20123 Amp	12	53716413	53876485	0	0.027891
20124 Amp	12	53881341	53962129	0	0.026334
20125 Amp	12	53962144	54001298	0	0.028029
20126 Amp	12	54012999	54084046	0	0.026945
20127 Amp	12	54084834	54362766	0	0.02786
20128 Amp	12	54382766	54408922	0	0.029835
20129 Amp	12	54409088	54417924	0	0.030274
20130 Amp	12	54422444	54479154	0	0.029287
20131 Amp	12	54486407	54499243	0	0.031605
20132 Amp	12	54499821	54531890	0	0.035096
20133 Amp	12	54533464	54639902	0	0.031605
20134 Amp	12	54641124	54741881	0	0.030691
20135 Amp	12	54742620	54747950	0	0.032172
20136 Amp	12	54749063	54759867	0	0.033489
20137 Amp	12	54762698	54798905	0	0.038007
20138 Amp	12	54800172	54927230	0	0.035033
20139 Amp	12	54928374	54952650	0	0.032715

20140	Amp	12	54961449	54968139	0	0.03074
20141	Amp	12	54968218	55027816	0	0.029639
20142	Amp	12	55030855	55047154	0	0.031867
20143	Amp	12	55049255	55079984	0	0.03055
20144	Amp	12	55104752	55117348	0	0.031364
20145	Amp	12	55117654	55216199	0	0.035475
20146	Amp	12	55217397	55220652	0	0.030921
20147	Amp	12	55223260	55343619	0	0.032299
20148	Amp	12	55343681	55404021	0	0.035018
20149	Amp	12	55409338	55427421	0	0.034204
20150	Amp	12	55430210	55468046	0	0.030735
20151	Amp	12	55468316	55620370	0	0.028738
20152	Amp	12	55620985	55637920	0	0.030015
20153	Amp	12	55638053	55653923	0	0.028738
20154	Amp	12	55661007	55679928	0	0.028298
20155	Amp	12	55682904	55684405	0	0.02558
20156	Amp	12	55685681	55690850	0	0.024742
20157	Amp	12	55692872	55713907	0	0.023408
20158	Amp	12	55714036	55719701	0	0.02481
20159	Amp	12	55720985	55798869	0	0.025821
20160	Amp	12	55804287	55842534	0	0.025207
20161	Amp	12	55845472	56045130	0	0.024196
20162	Amp	12	56052259	56485565	0	0.021968
20163	Amp	12	56486888	56602931	0	0.022852
20164	Amp	12	56605946	56605946	0	0.02436
20165	Amp	12	56606647	56645345	0	0.025147
20166	Amp	12	56654812	56662444	0	0.028852
20167	Amp	12	56669714	56787096	0	0.027474
20168	Amp	12	56787478	56787478	0	0.028275
20169	Amp	12	56799657	56802199	0	0.026767
20170	Amp	12	56803447	57082440	0	0.025884
20171	Amp	12	57092864	57174358	0	0.023859
20172	Amp	12	57181450	57312070	0	0.032185
20173	Amp	12	57312298	57458585	0	0.031636
20174	Amp	12	57465648	57511802	0	0.02331
20175	Amp	12	57511904	57511904	0	0.022509
20176	Amp	12	57514929	57527405	0	0.027952
20177	Amp	12	57531918	57561513	0	0.043328
20178	Amp	12	57562596	57574955	0	0.047383
20179	Amp	12	57575135	57580207	0	0.04828
20180	Amp	12	57581787	57714636	0	0.048971
20181	Amp	12	57721488	57721488	0	0.061965
20182	Amp	12	57722795	57822054	0.001323	0.063871
20183	Amp	12	57828056	57842827	0.056211	0.066262
20184	Amp	12	57851618	57866874	0	0.050886
20185	Amp	12	57868156	57904378	0	0.048495
20186	Amp	12	57907364	57948969	0	0.047708
20187	Amp	12	57951074	58017027	0	0.045802
20188	Amp	12	58023291	58049492	0	0.053698
20189	Amp	12	58076366	58468328	0	0.056532
20190	Amp	12	58478775	58737311	0	0.053698
20191	Amp	12	58739698	58766697	0	0.055271
20192	Amp	12	58766832	58828192	0	0.0535

20193	Amp	12	58828198	58828282	0	0.041522
20194	Amp	12	58828295	58828516	0	0.034293
20195	Amp	12	58828619	58828619	0	0.035343
20196	Amp	12	58828895	58828895	0	0.041734
20197	Amp	12	58828938	58833794	0	0.05208
20198	Amp	12	58833809	58918606	0	0.055036
20199	Amp	12	58921427	59079020	0	0.050672
20200	Amp	12	59079191	59150422	0	0.051989
20201	Amp	12	59150650	59212440	0	0.052649
20202	Amp	12	59213677	59237495	0	0.049949
20203	Amp	12	59240273	59322596	0	0.052118
20204	Amp	12	59330185	59385263	0	0.050802
20205	Amp	12	59391262	59442105	0	0.052336
20206	Amp	12	59449692	59450080	0	0.039341
20207	Amp	12	59455896	59464192	0	0.040381
20208	Amp	12	59469977	59474198	0	0.038899
20209	Amp	12	59476713	59522849	0	0.034312
20210	Amp	12	59522986	59533789	0	0.033272
20211	Amp	12	59536269	59647696	0	0.032222
20212	Amp	12	59651082	59741431	0	0.034448
20213	Amp	12	59741723	59924388	0	0.032875
20214	Amp	12	59925984	59986789	0	0.031341
20215	Amp	12	59989288	60008236	0	0.033411
20216	Amp	12	60012592	60045245	0	0.028128
20217	Amp	12	60045375	60095741	0	0.028669
20218	Amp	12	60096559	60148143	0	0.031001
20219	Amp	12	60148151	60201426	0	0.031671
20220	Amp	12	60202083	60240427	0	0.035008
20221	Amp	12	60241561	60320973	0	0.044487
20222	Amp	12	60325899	60325899	0	0.045215
20223	Amp	12	60325953	60350916	0	0.042884
20224	Amp	12	60353482	60370482	0	0.052083
20225	Amp	12	60373300	60384427	0	0.05393
20226	Amp	12	60387057	60497616	0	0.056908
20227	Amp	12	60499255	60589056	0	0.056238
20228	Amp	12	60594247	60619541	0	0.042936
20229	Amp	12	60623994	60647496	0	0.044688
20230	Amp	12	60648581	60654381	0	0.04171
20231	Amp	12	60657688	60698951	0	0.040063
20232	Amp	12	60701485	60862246	0	0.041122
20233	Amp	12	60864548	60884983	0	0.032759
20234	Amp	12	60891939	60926558	0	0.034332
20235	Amp	12	60929107	61051091	0	0.029948
20236	Amp	12	61052848	61066054	0	0.029315
20237	Amp	12	61069122	61164142	0	0.027563
20238	Amp	12	61164935	61313830	0	0.026504
20239	Amp	12	61314146	61318825	0	0.027737
20240	Amp	12	61322691	61460220	0	0.029153
20241	Amp	12	61460951	61630585	0	0.028425
20242	Amp	12	61633370	61644590	0	0.027883
20243	Amp	12	61645015	61662138	0	0.021964
20244	Amp	12	61664096	61722593	0	0.019841
20245	Amp	12	61724176	61885690	0	0.026089

20246 Amp	12	61885804	61929187	0	0.019841
20247 Amp	12	61930881	62032249	0	0.022794
20248 Amp	12	62037256	62069155	0	0.024348
20249 Amp	12	62069658	62069768	0	0.036731
20250 Amp	12	62070057	62079355	0	0.033778
20251 Amp	12	62082243	62091462	0	0.021394
20252 Amp	12	62097283	62364426	0	0.023679
20253 Amp	12	62370623	62453005	0	0.036949
20254 Amp	12	62458998	62465095	0	0.037947
20255 Amp	12	62465640	62531419	0	0.039435
20256 Amp	12	62531490	62582317	0	0.042927
20257 Amp	12	62584826	62621682	0	0.044686
20258 Amp	12	62622258	62780671	0	0.042963
20259 Amp	12	62780955	62818336	0	0.044701
20260 Amp	12	62818395	62846892	0	0.043627
20261 Amp	12	62847091	62850778	0	0.044822
20262 Amp	12	62852848	62865478	0	0.045342
20263 Amp	12	62865901	62884687	0	0.051756
20264 Amp	12	62887666	62888562	0	0.042061
20265 Amp	12	62889959	62940375	0	0.044525
20266 Amp	12	62940419	63029212	0	0.046144
20267 Amp	12	63038793	63052637	0	0.047076
20268 Amp	12	63053745	63058633	0	0.048282
20269 Amp	12	63061301	63065585	0	0.047349
20270 Amp	12	63066952	63067647	0	0.0581
20271 Amp	12	63069290	63069777	0	0.056684
20272 Amp	12	63070281	63113506	0	0.059676
20273 Amp	12	63113540	63153868	0.024438	0.064929
20274 Amp	12	63154276	63188553	0	0.063097
20275 Amp	12	63195641	63250830	0	0.060329
20276 Amp	12	63250931	63401438	0	0.057246
20277 Amp	12	63406837	63422424	0	0.061072
20278 Amp	12	63425574	63472960	0	0.060577
20279 Amp	12	63473244	63487759	0	0.061129
20280 Amp	12	63487932	63526209	0	0.059934
20281 Amp	12	63751525	63790222	0	0.059329
20282 Amp	12	63790883	63808453	0	0.060556
20283 Amp	12	63812210	63833786	0	0.058271
20284 Amp	12	63838302	63873847	0	0.060148
20285 Amp	12	63876904	63884322	0	0.055598
20286 Amp	12	63884457	63957126	0	0.058037
20287 Amp	12	63957741	63957741	0	0.062272
20288 Amp	12	63958139	64031643	0	0.059834
20289 Amp	12	64037415	64056615	0	0.06061
20290 Amp	12	64060747	64108595	0.069848	0.066991
20291 Amp	12	64108774	64179579	0.128324	0.069469
20292 Amp	12	64185719	64255941	0.233782	0.073514
20293 Amp	12	64258762	64326446	0.204831	0.072432
20294 Amp	12	64326767	64337510	0.246818	0.074091
20295 Amp	12	64338514	64352418	0.259438	0.074908
20296 Amp	12	64357227	64359637	0.222041	0.073031
20297 Amp	12	64362956	64363765	0.08435	0.067732
20298 Amp	12	64368134	64368414	0.100827	0.068198

20299	Amp	12	64372042	64385236	0.112543	0.068635
20300	Amp	12	64388382	64434730	0.222041	0.073052
20301	Amp	12	64435462	64448593	0.681869	0.088852
20302	Amp	12	64450861	64450861	0.735486	0.09053
20303	Amp	12	64467289	64567904	0.823771	0.092856
20304	Amp	12	64576316	64612933	0.79909	0.092081
20305	Amp	12	64616360	64634618	0.689884	0.089232
20306	Amp	12	64634862	64709388	0.790614	0.092031
20307	Amp	12	64718319	64736643	0.950886	0.096047
20308	Amp	12	64736669	64747738	0.98589	0.096907
20309	Amp	12	64750355	64768663	1.102589	0.099785
20310	Amp	12	64783004	64876098	1.665486	0.112417
20311	Amp	12	64876130	64881988	1.780186	0.114773
20312	Amp	12	64884405	64906763	1.717671	0.113539
20313	Amp	12	64911156	64932716	1.573014	0.110371
20314	Amp	12	64933478	65018638	1.436692	0.107273
20315	Amp	12	65020594	65038834	1.369395	0.105821
20316	Amp	12	65039303	65069173	1.522708	0.109258
20317	Amp	12	65069235	65099126	1.604336	0.11104
20318	Amp	12	65099942	65125486	1.737623	0.113978
20319	Amp	12	65128330	65182536	1.604336	0.11104
20320	Amp	12	65190002	65335351	1.919241	0.117597
20321	Amp	12	65337484	65348759	2.169666	0.12262
20322	Amp	12	65353824	65378113	1.748606	0.114069
20323	Amp	12	65381327	65394149	2.169666	0.122657
20324	Amp	12	65410914	65410914	1.604336	0.111059
20325	Amp	12	65414227	65414227	1.675319	0.112668
20326	Amp	12	65414305	65443657	1.833307	0.115909
20327	Amp	12	65444749	65452348	1.972776	0.11872
20328	Amp	12	65456901	65468037	2.016585	0.119629
20329	Amp	12	65469804	65487315	2.049917	0.120187
20330	Amp	12	65490464	65664890	1.897704	0.117046
20331	Amp	12	65666177	65671164	2.038694	0.120042
20332	Amp	12	65671356	65671356	2.18114	0.12281
20333	Amp	12	65676520	65726528	2.104978	0.121191
20334	Amp	12	65734081	65734867	1.994915	0.119142
20335	Amp	12	65749877	65821512	1.823153	0.115726
20336	Amp	12	65822293	65885319	1.919241	0.117667
20337	Amp	12	65885592	65910481	1.843664	0.115989
20338	Amp	12	65914001	65915019	1.593878	0.11089
20339	Amp	12	65920051	66036089	1.717671	0.113432
20340	Amp	12	66037067	66071577	1.583409	0.110554
20341	Amp	12	66071605	66093279	1.502257	0.108979
20342	Amp	12	66093859	66093940	1.759227	0.114302
20343	Amp	12	66099271	66116869	1.645511	0.111942
20344	Amp	12	66119331	66132580	1.482378	0.108432
20345	Amp	12	66139595	66203921	1.542638	0.109685
20346	Amp	12	66205117	66206971	1.502257	0.108812
20347	Amp	12	66209554	66256386	1.876242	0.116679
20348	Amp	12	66260657	66359198	1.604336	0.111059
20349	Amp	12	66360399	66384149	1.563118	0.110216
20350	Amp	12	66387433	66442197	1.994915	0.119004
20351	Amp	12	66448260	66463150	1.96212	0.118451

20352	Amp	12	66463816	66491291	2.005947	0.119358
20353	Amp	12	66491357	66509497	1.96212	0.118451
20354	Amp	12	66511571	66511857	2.071483	0.120695
20355	Amp	12	66512030	66541340	1.984015	0.118912
20356	Amp	12	66542053	66687404	2.060912	0.120375
20357	Amp	12	66687454	66729503	1.833307	0.115762
20358	Amp	12	66734447	66805853	1.624655	0.111518
20359	Amp	12	66809410	66881867	1.456037	0.107807
20360	Amp	12	66882422	66905284	1.261401	0.103439
20361	Amp	12	66905393	66915175	1.329472	0.105006
20362	Amp	12	66915518	66917364	1.358811	0.105731
20363	Amp	12	66921166	66925403	1.593878	0.110808
20364	Amp	12	66926838	66956708	1.665486	0.112268
20365	Amp	12	66959691	66983867	1.696256	0.113067
20366	Amp	12	66984229	66988339	1.769915	0.114607
20367	Amp	12	66988678	67030294	1.887378	0.116929
20368	Amp	12	67033107	67101306	1.96212	0.1184
20369	Amp	12	67103215	67116639	1.897704	0.117173
20370	Amp	12	67116761	67164156	2.158817	0.122343
20371	Amp	12	67166303	67209204	2.22459	0.123648
20372	Amp	12	67216768	67223326	2.457251	0.128221
20373	Amp	12	67226203	67266436	2.425182	0.127495
20374	Amp	12	67271319	67342179	2.457251	0.128219
20375	Amp	12	67342716	67352063	2.582422	0.130591
20376	Amp	12	67352890	67374794	2.192012	0.1231
20377	Amp	12	67374979	67379963	1.994915	0.119068
20378	Amp	12	67380403	67381735	2.147988	0.122172
20379	Amp	12	67389328	67420002	2.192012	0.122904
20380	Amp	12	67420027	67425241	2.071483	0.120565
20381	Amp	12	67425596	67452951	2.027585	0.119832
20382	Amp	12	67453113	67453680	1.759227	0.114262
20383	Amp	12	67454521	67458687	1.780186	0.114831
20384	Amp	12	67459093	67498224	1.68612	0.112889
20385	Amp	12	67503464	67564920	1.96212	0.118387
20386	Amp	12	67565257	67569459	1.897704	0.117082
20387	Amp	12	67576149	67604927	2.060912	0.120394
20388	Amp	12	67605131	67621325	2.26699	0.124524
20389	Amp	12	67621467	67626760	2.73206	0.133369
20390	Amp	12	67628980	67714411	2.684894	0.132508
20391	Amp	12	67716043	67814715	2.929372	0.137101
20392	Amp	12	67821109	67831469	3.554363	0.148714
20393	Amp	12	67833900	67857323	3.374864	0.145401
20394	Amp	12	67861399	67899571	3.492824	0.147649
20395	Amp	12	67899694	67933182	3.067933	0.139727
20396	Amp	12	67937335	67950054	3.138463	0.141043
20397	Amp	12	67950432	67951812	3.387253	0.145663
20398	Amp	12	67956631	68084378	2.605213	0.130994
20399	Amp	12	68085433	68102642	2.425182	0.127486
20400	Amp	12	68102910	68177765	2.446878	0.12799
20401	Amp	12	68177886	68179952	2.290039	0.125062
20402	Amp	12	68183997	68366288	3.09333	0.140116
20403	Amp	12	68368234	68401323	3.505046	0.147782
20404	Amp	12	68401453	68474276	3.458499	0.146873

20405 Amp	12	68474456	68498192	3.483236	0.147324
20406 Amp	12	68500865	68565607	3.6657	0.150528
20407 Amp	12	68570882	68577445	3.701758	0.151266
20408 Amp	12	68580793	68591425	3.749777	0.152149
20409 Amp	12	68596338	68628940	3.422847	0.146224
20410 Amp	12	68629280	68644390	3.009724	0.138558
20411 Amp	12	68646635	68646635	3.471122	0.147165
20412 Amp	12	68649656	68721914	3.726055	0.151633
20413 Amp	12	68722360	68740622	4.037384	0.157166
20414 Amp	12	68742645	68768721	3.654159	0.15044
20415 Amp	12	68770866	68804332	3.701758	0.151332
20416 Amp	12	68805427	68842951	3.898065	0.154729
20417 Amp	12	68843839	68893965	3.920287	0.15517
20418 Amp	12	68896409	68909315	3.839479	0.15368
20419 Amp	12	68909484	68943030	4.156262	0.159133
20420 Amp	12	68945263	68958944	4.264196	0.160781
20421 Amp	12	68962247	69012335	4.010586	0.156647
20422 Amp	12	69013796	69018609	4.223463	0.160102
20423 Amp	12	69018849	69032051	4.31834	0.161732
20424 Amp	12	69042770	69063073	3.738665	0.151869
20425 Amp	12	69064377	69078911	3.788687	0.152693
20426 Amp	12	69079381	69131949	3.738665	0.1518
20427 Amp	12	69132107	69167939	3.933438	0.155278
20428 Amp	12	69172217	69197044	4.3738	0.162489
20429 Amp	12	69203865	69223896	4.753798	0.168436
20430 Amp	12	69224082	69279806	4.710978	0.167712
20431 Amp	12	69287633	69340795	4.264196	0.160767
20432 Amp	12	69344099	69356725	4.209693	0.160028
20433 Amp	12	69357059	69440421	3.862695	0.154081
20434 Amp	12	69452537	69473514	3.972019	0.155997
20435 Amp	12	69482397	69520738	3.387253	0.145604
20436 Amp	12	69523947	69525096	3.138463	0.141136
20437 Amp	12	69527388	69582420	3.286256	0.143848
20438 Amp	12	69584843	69606554	3.516868	0.148006
20439 Amp	12	69606738	69628658	3.640671	0.150179
20440 Amp	12	69630011	69648821	3.471122	0.147139
20441 Amp	12	69652902	69658668	3.399017	0.145883
20442 Amp	12	69660941	69697576	2.929372	0.137034
20443 Amp	12	69697652	69764010	2.825055	0.135118
20444 Amp	12	69769596	69769596	2.952409	0.137482
20445 Amp	12	69773384	69854689	2.800837	0.13477
20446 Amp	12	69865718	69887458	2.837591	0.135483
20447 Amp	12	69894548	69925053	2.975629	0.138002
20448 Amp	12	69933925	69949820	2.755154	0.133845
20449 Amp	12	69949890	69965173	2.986184	0.138147
20450 Amp	12	69965788	69977453	2.755154	0.133845
20451 Amp	12	69978175	69994224	3.045223	0.139217
20452 Amp	12	70003508	70009526	2.964305	0.137739
20453 Amp	12	70009561	70073832	3.115161	0.140558
20454 Amp	12	70074994	70094127	3.09333	0.140121
20455 Amp	12	70094690	70133044	3.218362	0.14252
20456 Amp	12	70139097	70141797	3.103562	0.140345
20457 Amp	12	70143731	70161712	3.264473	0.143524

20458	Amp	12	70161879	70216822	3.349067	0.144894
20459	Amp	12	70227036	70327971	3.240397	0.14288
20460	Amp	12	70331031	70339252	3.264473	0.143344
20461	Amp	12	70339549	70380598	3.399017	0.145818
20462	Amp	12	70381349	70386879	3.286256	0.143789
20463	Amp	12	70389074	70391266	3.458499	0.146892
20464	Amp	12	70391988	70427312	3.323812	0.144529
20465	Amp	12	70427564	70428153	3.183474	0.142009
20466	Amp	12	70433417	70444334	3.160681	0.141558
20467	Amp	12	70445626	70446657	3.323812	0.144405
20468	Amp	12	70447331	70470820	3.150497	0.141301
20469	Amp	12	70471798	70485879	3.554363	0.148671
20470	Amp	12	70486101	70517732	3.387253	0.145557
20471	Amp	12	70520769	70553759	3.492824	0.147523
20472	Amp	12	70554465	70570933	3.615896	0.149711
20473	Amp	12	70570970	70598937	3.323812	0.144541
20474	Amp	12	70599770	70605133	3.207115	0.142353
20475	Amp	12	70606251	70628230	3.311026	0.144254
20476	Amp	12	70633070	70647150	3.183474	0.141856
20477	Amp	12	70650496	70816898	3.578505	0.148987
20478	Amp	12	70817657	70895776	3.516868	0.147907
20479	Amp	12	70895899	70896544	3.578505	0.149158
20480	Amp	12	70907496	70918707	3.399017	0.145916
20481	Amp	12	70918847	70926994	3.361849	0.145092
20482	Amp	12	70931239	70954745	2.917729	0.136912
20483	Amp	12	70954795	70955380	3.045223	0.139327
20484	Amp	12	70960096	70986447	3.009724	0.138605
20485	Amp	12	70987240	71116475	2.848911	0.135507
20486	Amp	12	71127747	71140544	2.707624	0.133033
20487	Amp	12	71150894	71153950	2.104978	0.121226
20488	Amp	12	71164586	71165089	2.245479	0.124047
20489	Amp	12	71167104	71183321	2.311329	0.125394
20490	Amp	12	71183522	71194081	2.192012	0.122979
20491	Amp	12	71195441	71198339	2.049917	0.120158
20492	Amp	12	71198668	71235758	1.919241	0.117596
20493	Amp	12	71238289	71262767	2.137445	0.121863
20494	Amp	12	71280849	71321951	2.104978	0.121359
20495	Amp	12	71328749	71328749	1.897704	0.117231
20496	Amp	12	71330906	71333262	1.865019	0.11657
20497	Amp	12	71334960	71337024	2.137445	0.122029
20498	Amp	12	71341747	71341747	2.256542	0.124258
20499	Amp	12	71472182	71478099	2.192012	0.123053
20500	Amp	12	71484269	71572826	2.235168	0.123985
20501	Amp	12	71572916	71636719	2.290039	0.125038
20502	Amp	12	71639485	71666614	2.379392	0.126733
20503	Amp	12	71675093	71718752	2.027585	0.119819
20504	Amp	12	71718820	71720735	1.940776	0.117992
20505	Amp	12	71722979	71750874	1.887378	0.116824
20506	Amp	12	71765100	71778190	1.769915	0.114595
20507	Amp	12	71778991	71791295	1.531891	0.109622
20508	Amp	12	71796044	71814943	1.563118	0.110133
20509	Amp	12	71815045	71820366	1.492063	0.10858
20510	Amp	12	71830408	71830918	1.876242	0.11673

20511	Amp	12	71836689	71862326	2.605213	0.1311
20512	Amp	12	71862522	71872589	2.514205	0.129406
20513	Amp	12	71872961	71889499	2.290039	0.124911
20514	Amp	12	71894982	71933242	2.127614	0.121778
20515	Amp	12	71933264	71942014	2.290039	0.124911
20516	Amp	12	71942208	71970423	2.502083	0.129095
20517	Amp	12	71971324	71978821	2.457251	0.128248
20518	Amp	12	71979715	71979715	2.413643	0.127316
20519	Amp	12	71980750	72010169	2.33403	0.125804
20520	Amp	12	72010733	72020391	2.127614	0.12162
20521	Amp	12	72021254	72172295	2.594754	0.130828
20522	Amp	12	72176029	72213534	2.673338	0.13234
20523	Amp	12	72215588	72223717	2.479057	0.128655
20524	Amp	12	72223867	72279384	2.673338	0.13234
20525	Amp	12	72281897	72326232	2.571513	0.130439
20526	Amp	12	72326698	72361293	2.605213	0.131085
20527	Amp	12	72361695	72361695	2.696333	0.13276
20528	Amp	12	72368950	72386895	2.719563	0.133247
20529	Amp	12	72387999	72387999	2.322689	0.125635
20530	Amp	12	72397290	72460518	2.290039	0.124989
20531	Amp	12	72466718	72500891	1.984015	0.118919
20532	Amp	12	72505848	72581567	2.169666	0.122561
20533	Amp	12	72585289	72602868	2.083076	0.120758
20534	Amp	12	72604355	72621896	2.027585	0.119705
20535	Amp	12	72622465	72633607	2.049917	0.120156
20536	Amp	12	72634945	72646775	2.027585	0.119692
20537	Amp	12	72647009	72710268	2.094104	0.121001
20538	Amp	12	72714742	72722009	2.18114	0.122835
20539	Amp	12	72729816	72838560	2.300463	0.125273
20540	Amp	12	72977309	72992356	2.127614	0.121631
20541	Amp	12	72992372	72996304	1.759227	0.11442
20542	Amp	12	73008560	73008560	1.645511	0.111942
20543	Amp	12	73008595	73016504	2.071483	0.120601
20544	Amp	12	73016529	73050810	2.137445	0.121855
20545	Amp	12	73054484	73092716	2.256542	0.12436
20546	Amp	12	73095773	73147544	2.071483	0.120688
20547	Amp	12	73158632	73197766	1.522708	0.109379
20548	Amp	12	73202312	73209755	1.319393	0.104827
20549	Amp	12	73209978	73330726	1.270711	0.103721
20550	Amp	12	73332736	73344035	1.378521	0.106169
20551	Amp	12	73344625	73423989	1.329472	0.105049
20552	Amp	12	73424728	73435176	1.252022	0.103336
20553	Amp	12	73439501	73475170	1.23519	0.102768
20554	Amp	12	73476540	73498493	0.857296	0.093705
20555	Amp	12	73504571	73542933	0.915698	0.095186
20556	Amp	12	73544125	73544125	1.023539	0.09774
20557	Amp	12	73545738	73561419	1.058188	0.098768
20558	Amp	12	73568226	73698619	0.899693	0.094773
20559	Amp	12	73708367	73723468	0.807647	0.092326
20560	Amp	12	73723687	73804904	0.840289	0.093346
20561	Amp	12	73808328	73853135	0.781972	0.091841
20562	Amp	12	73855508	73906191	0.72817	0.090315
20563	Amp	12	73913012	74039612	0.899693	0.094738

20564	Amp	12	74044906	74068332	1.280185	0.103907
20565	Amp	12	74068622	74073685	1.149583	0.100757
20566	Amp	12	74080185	74105903	0.742723	0.090559
20567	Amp	12	74111037	74120739	0.758088	0.091033
20568	Amp	12	74121159	74121628	0.848692	0.093499
20569	Amp	12	74124048	74195039	0.807647	0.092485
20570	Amp	12	74196576	74302794	1.032525	0.097966
20571	Amp	12	74303049	74311705	1.014678	0.097514
20572	Amp	12	74318345	74368370	0.977077	0.09663
20573	Amp	12	74369890	74374477	0.790614	0.09201
20574	Amp	12	74376199	74388736	0.831947	0.092957
20575	Amp	12	74388897	74388897	0.790614	0.09201
20576	Amp	12	74389482	74444204	0.515676	0.083648
20577	Amp	12	74449133	74450916	0.34892	0.078168
20578	Amp	12	74454576	74537585	0.329205	0.077356
20579	Amp	12	74540504	74565059	0.210594	0.07274
20580	Amp	12	74565269	74607172	0.100827	0.068308
20581	Amp	12	74607335	74627338	0.170723	0.07108
20582	Amp	12	74628806	74629879	0.100827	0.068308
20583	Amp	12	74638365	74680600	0	0.063412
20584	Amp	12	74684960	74929251	0.044794	0.065857
20585	Amp	12	74931836	75041591	0.143787	0.069957
20586	Amp	12	75041989	75046096	0.204831	0.072454
20587	Amp	12	75048311	75070746	0.100827	0.068355
20588	Amp	12	75073519	75074382	0.287738	0.075892
20589	Amp	12	75074630	75179220	0.264858	0.07509
20590	Amp	12	75181528	75200285	0.19964	0.072171
20591	Amp	12	75201340	75242686	0.355319	0.078313
20592	Amp	12	75245650	75255576	0.215608	0.072756
20593	Amp	12	75256876	75306359	0.19964	0.072237
20594	Amp	12	75308950	75319512	0.282828	0.075778
20595	Amp	12	75319913	75394093	0.322897	0.077225
20596	Amp	12	75402941	75445290	0.153838	0.070477
20597	Amp	12	75450123	75469288	0	0.063741
20598	Amp	12	75471495	75545892	0	0.061612
20599	Amp	12	75546684	75546684	0.019169	0.064807
20600	Amp	12	75548607	75565078	0.065715	0.066665
20601	Amp	12	75568528	75766213	0.170723	0.071093
20602	Amp	12	75768455	75791728	0.255918	0.074667
20603	Amp	12	75800665	75800665	0.170723	0.071093
20604	Amp	12	75807080	75814030	0.181932	0.07163
20605	Amp	12	75814876	75821974	0.128324	0.069411
20606	Amp	12	75823187	75849612	0	0.060223
20607	Amp	12	75852392	75883290	0	0.059039
20608	Amp	12	75888067	75987834	0	0.060825
20609	Amp	12	75993702	76006849	0	0.060289
20610	Amp	12	76010729	76055288	0.001323	0.064007
20611	Amp	12	76060582	76143579	0	0.059579
20612	Amp	12	76143998	76154040	0	0.061238
20613	Amp	12	76154645	76156758	0	0.062634
20614	Amp	12	76157153	76392605	0	0.061703
20615	Amp	12	76393214	76418896	0	0.059177
20616	Amp	12	76428072	76546242	0	0.0569

20617 Amp	12	76554524	76554524	0	0.059727
20618 Amp	12	76561640	76641181	0	0.058357
20619 Amp	12	76648668	76736307	0	0.050614
20620 Amp	12	76740371	76799699	0	0.052479
20621 Amp	12	76800210	76948023	0	0.052928
20622 Amp	12	76948322	77058862	0	0.05676
20623 Amp	12	77060024	77132065	0	0.057539
20624 Amp	12	77134348	77192450	0	0.060834
20625 Amp	12	77192729	77233897	0	0.062734
20626 Amp	12	77237465	77256751	0	0.063333
20627 Amp	12	77257218	77335882	0	0.060039
20628 Amp	12	77343020	77378444	0	0.058139
20629 Amp	12	77378775	77458510	0	0.062568
20630 Amp	12	77461641	77479771	0.010261	0.064413
20631 Amp	12	77482862	77494669	0.001323	0.063898
20632 Amp	12	77495583	77504704	0.094967	0.06801
20633 Amp	12	77504761	77504781	0.029031	0.065183
20634 Amp	12	77505076	77601507	0	0.060465
20635 Amp	12	77602215	77675652	0	0.059225
20636 Amp	12	77676298	77774933	0	0.062423
20637 Amp	12	77776001	77798845	0	0.059836
20638 Amp	12	77802991	77831406	0.282828	0.075772
20639 Amp	12	77831594	77833716	0.246818	0.074167
20640 Amp	12	77834731	77972692	0.233782	0.073567
20641 Amp	12	77973210	78004072	0	0.063695
20642 Amp	12	78013170	78016778	0	0.061978
20643 Amp	12	78020999	78032058	0	0.056287
20644 Amp	12	78033884	78065900	0	0.058362
20645 Amp	12	78066160	78100922	0	0.056287
20646 Amp	12	78102935	78132115	0	0.057803
20647 Amp	12	78133822	78194662	0	0.059559
20648 Amp	12	78194907	78223102	0	0.056364
20649 Amp	12	78227292	78433021	0	0.054848
20650 Amp	12	78441238	78472064	0	0.05429
20651 Amp	12	78472408	78521118	0	0.056405
20652 Amp	12	78524839	78568116	0	0.055956
20653 Amp	12	78569248	78669996	0	0.052924
20654 Amp	12	78671051	78737240	0	0.054714
20655 Amp	12	78739867	78744848	0	0.052598
20656 Amp	12	78748237	78755612	0	0.054477
20657 Amp	12	78756224	78841836	0	0.057994
20658 Amp	12	78843899	78968439	0	0.059048
20659 Amp	12	78970800	78973443	0	0.057994
20660 Amp	12	78974137	79081679	0	0.056115
20661 Amp	12	79087062	79098188	0	0.057086
20662 Amp	12	79101413	79120636	0	0.056115
20663 Amp	12	79128578	79140428	0	0.052598
20664 Amp	12	79140569	79167184	0	0.058113
20665 Amp	12	79169548	79288874	0	0.056357
20666 Amp	12	79291429	79303507	0	0.054492
20667 Amp	12	79308216	79337729	0	0.057106
20668 Amp	12	79338987	79343075	0	0.054492
20669 Amp	12	79343835	79455488	0	0.056785

20670	Amp	12	79462323	79467683	0	0.052356
20671	Amp	12	79477222	79513776	0	0.044548
20672	Amp	12	79519465	79534962	0	0.046271
20673	Amp	12	79537290	79608169	0	0.045492
20674	Amp	12	79611503	79611503	0	0.043817
20675	Amp	12	79612324	79612913	0	0.045326
20676	Amp	12	79613428	79655450	0	0.043603
20677	Amp	12	79660974	79680288	0	0.054622
20678	Amp	12	79680980	79680980	0	0.043603
20679	Amp	12	79684759	79688972	0	0.045491
20680	Amp	12	79691924	79692487	0	0.050015
20681	Amp	12	79696195	79710535	0	0.050648
20682	Amp	12	79714636	79765300	0	0.051577
20683	Amp	12	79770335	79848636	0	0.050943
20684	Amp	12	79849550	80117932	0	0.04642
20685	Amp	12	80118397	80139954	0	0.050181
20686	Amp	12	80140161	80224813	0	0.052715
20687	Amp	12	80229288	80270139	0	0.037266
20688	Amp	12	80270855	80323774	0	0.033505
20689	Amp	12	80324085	80330363	0	0.032577
20690	Amp	12	80331247	80356254	0	0.034087
20691	Amp	12	80361187	80377555	0	0.032578
20692	Amp	12	80380231	80650391	0	0.031089
20693	Amp	12	80654120	80673879	0	0.034454
20694	Amp	12	80674215	80738476	0	0.032943
20695	Amp	12	80739586	80746325	0	0.029579
20696	Amp	12	80746505	80761800	0	0.028492
20697	Amp	12	80765362	80862933	0	0.032788
20698	Amp	12	80863409	80914564	0	0.033237
20699	Amp	12	80916873	81036185	0	0.032399
20700	Amp	12	81039135	81056738	0	0.03195
20701	Amp	12	81062225	81095058	0	0.034433
20702	Amp	12	81095935	81130538	0	0.039575
20703	Amp	12	81133362	81140671	0	0.04522
20704	Amp	12	81142665	81172328	0	0.040078
20705	Amp	12	81173805	81174191	0	0.0406
20706	Amp	12	81175657	81196313	0	0.036813
20707	Amp	12	81200266	81210382	0	0.03629
20708	Amp	12	81211661	81216918	0	0.041509
20709	Amp	12	81218124	81238261	0	0.045317
20710	Amp	12	81243871	81262593	0	0.042834
20711	Amp	12	81263086	81340395	0	0.044231
20712	Amp	12	81343555	81380587	0	0.040423
20713	Amp	12	81386677	81413944	0	0.041284
20714	Amp	12	81416664	81442825	0	0.040467
20715	Amp	12	81444780	81447201	0	0.041788
20716	Amp	12	81450714	81469054	0	0.05141
20717	Amp	12	81469221	81489481	0	0.041788
20718	Amp	12	81489901	81540169	0	0.043239
20719	Amp	12	81540492	81605617	0	0.044057
20720	Amp	12	81608871	81637721	0	0.042679
20721	Amp	12	81639709	81770264	0	0.044229
20722	Amp	12	81770894	81805963	0	0.042777

20723	Amp	12	81811685	81845365	0	0.035701
20724	Amp	12	81856341	81878852	0	0.038381
20725	Amp	12	81879533	81991593	0	0.041319
20726	Amp	12	81991680	82047753	0	0.039431
20727	Amp	12	82052193	82104633	0	0.036897
20728	Amp	12	82109453	82227601	0	0.0355
20729	Amp	12	82229330	82320802	0	0.033438
20730	Amp	12	82331399	82333080	0	0.035454
20731	Amp	12	82333981	82348496	0	0.040058
20732	Amp	12	82349930	82437197	0	0.037378
20733	Amp	12	82439858	82553409	0	0.039154
20734	Amp	12	82553902	82566148	0	0.043377
20735	Amp	12	82566162	82628929	0	0.042516
20736	Amp	12	82629664	82652373	0	0.0473
20737	Amp	12	82654168	82665514	0	0.042516
20738	Amp	12	82667642	82708042	0	0.040894
20739	Amp	12	82708257	82874098	0	0.042068
20740	Amp	12	82874829	82934685	0	0.042622
20741	Amp	12	82938571	83047755	0	0.038019
20742	Amp	12	83049470	83056246	0	0.033161
20743	Amp	12	83056790	83076665	0	0.028842
20744	Amp	12	83077095	83211796	0	0.030422
20745	Amp	12	83215619	83217377	0	0.022765
20746	Amp	12	83219150	83276157	0	0.020749
20747	Amp	12	83277947	83288612	0	0.020195
20748	Amp	12	83292210	83367768	0	0.018155
20749	Amp	12	83367891	83482151	0	0.016575
20750	Amp	12	83483135	83658148	0	0.018708
20751	Amp	12	83659067	83668999	0	0.020855
20752	Amp	12	83670246	83688418	0	0.019079
20753	Amp	12	83689692	83863686	0	0.013603
20754	Amp	12	83863779	83868201	0	0.014937
20755	Amp	12	83870574	83986145	0	0.013616
20756	Amp	12	83988466	84224087	0	0.012282
20757	Amp	12	84224734	84290743	0	0.013981
20758	Amp	12	84290973	84294268	0	0.017904
20759	Amp	12	84299095	84313262	0	0.016205
20760	Amp	12	84314417	84481925	0	0.012282
20761	Amp	12	84484702	84576320	0	0.017575
20762	Amp	12	84579939	84621898	0	0.015428
20763	Amp	12	84626953	84633717	0	0.010135
20764	Amp	12	84639877	84663495	0	0.012112
20765	Amp	12	84667503	84696325	0	0.010135
20766	Amp	12	84696756	84759392	0	0.013666
20767	Amp	12	84766804	84827124	0	0.01787
20768	Amp	12	84828360	84908915	0	0.02026
20769	Amp	12	84913310	84975474	0	0.019703
20770	Amp	12	84976764	85202555	0	0.017313
20771	Amp	12	85202612	85221177	0	0.013666
20772	Amp	12	85221633	85300709	0	0.018071
20773	Amp	12	85300831	85347832	0	0.019147
20774	Amp	12	85348156	85354129	0	0.019844
20775	Amp	12	85355535	85395583	0	0.015438

20776 Amp	12	85397031	85427829	0	0.017737
20777 Amp	12	85433563	85488139	0	0.014206
20778 Amp	12	85488545	85564853	0	0.018704
20779 Amp	12	85565230	85593242	0	0.034423
20780 Amp	12	85605437	85652876	0	0.050359
20781 Amp	12	85654843	85731586	0	0.051552
20782 Amp	12	85741914	85778744	0	0.053252
20783 Amp	12	85779479	85803080	0	0.052176
20784 Amp	12	85803930	85857117	0	0.03624
20785 Amp	12	85860072	85899690	0	0.020522
20786 Amp	12	85899938	85933224	0	0.019328
20787 Amp	12	85933726	86201147	0	0.023569
20788 Amp	12	86201268	86239588	0	0.021464
20789 Amp	12	86239808	86283134	0	0.023745
20790 Amp	12	86283352	86283352	0	0.021447
20791 Amp	12	86284878	86385535	0	0.019747
20792 Amp	12	86389894	86420736	0	0.022215
20793 Amp	12	86422928	86616525	0	0.017717
20794 Amp	12	86619205	86634576	0	0.019834
20795 Amp	12	86635204	86703066	0	0.017366
20796 Amp	12	86704696	86887807	0	0.030918
20797 Amp	12	86906212	87011146	0	0.035172
20798 Amp	12	87013255	87049992	0	0.032859
20799 Amp	12	87050544	87059408	0	0.03513
20800 Amp	12	87063596	87157990	0	0.035751
20801 Amp	12	87160965	87292729	0	0.034416
20802 Amp	12	87293424	87447333	0	0.032135
20803 Amp	12	87447979	87486957	0	0.018583
20804 Amp	12	87488497	87762315	0	0.022841
20805 Amp	12	87763660	87879305	0	0.024709
20806 Amp	12	87881119	87939816	0	0.023581
20807 Amp	12	87943697	88132220	0	0.020661
20808 Amp	12	88135742	88159588	0	0.018544
20809 Amp	12	88162939	88261323	0	0.014287
20810 Amp	12	88263803	88282684	0	0.012987
20811 Amp	12	88288090	88424737	0	0.016058
20812 Amp	12	88426526	88667060	0	0.020502
20813 Amp	12	88677956	88773088	0	0.024178
20814 Amp	12	88778448	88778448	0	0.020502
20815 Amp	12	88778935	88778935	0	0.023225
20816 Amp	12	88784356	89013922	0	0.022656
20817 Amp	12	89017405	89255899	0	0.019584
20818 Amp	12	89258988	89598412	0	0.02478
20819 Amp	12	89600270	89602991	0	0.032067
20820 Amp	12	89612978	89633621	0	0.030759
20821 Amp	12	89637910	89687627	0	0.027271
20822 Amp	12	89691594	89737103	0	0.022823
20823 Amp	12	89753657	90133970	0	0.01838
20824 Amp	12	90133971	90169816	0	0.02122
20825 Amp	12	90170268	90368363	0	0.022763
20826 Amp	12	90368710	90498540	0	0.02004
20827 Amp	12	90499151	90530634	0	0.024263
20828 Amp	12	90533944	90815520	0	0.023256

20829	Amp	12	90815795	90861258	0	0.025079
20830	Amp	12	90869773	90920851	0	0.024816
20831	Amp	12	90920878	90972820	0	0.020435
20832	Amp	12	90973533	91379597	0	0.018669
20833	Amp	12	91380415	91672037	0	0.016487
20834	Amp	12	91675066	91784131	0	0.014778
20835	Amp	12	91787503	91845844	0	0.017704
20836	Amp	12	91847317	91925873	0	0.014778
20837	Amp	12	91926569	91950254	0	0.020566
20838	Amp	12	91953588	92101099	0	0.014778
20839	Amp	12	92107879	92165968	0	0.030427
20840	Amp	12	92169366	92172899	0	0.033783
20841	Amp	12	92176960	92583688	0	0.02009
20842	Amp	12	92584259	92654954	0	0.023113
20843	Amp	12	92655056	92659430	0	0.020273
20844	Amp	12	92660944	92795281	0	0.027592
20845	Amp	12	92810173	92861886	0	0.039404
20846	Amp	12	92864439	92876757	0	0.040035
20847	Amp	12	92893434	92972207	0	0.039404
20848	Amp	12	92981260	93470237	0	0.035398
20849	Amp	12	93470514	93550181	0	0.032548
20850	Amp	12	93554264	93670479	0	0.033683
20851	Amp	12	93670497	93737535	0	0.036328
20852	Amp	12	93744303	93925452	0	0.038496
20853	Amp	12	93928823	93940715	0	0.035851
20854	Amp	12	93942675	93942675	0	0.032788
20855	Amp	12	93943571	93976380	0	0.033785
20856	Amp	12	93977119	94130222	0	0.029086
20857	Amp	12	94134707	94239729	0	0.026146
20858	Amp	12	94243080	94273499	0	0.023773
20859	Amp	12	94273682	94342772	0	0.01955
20860	Amp	12	94343411	94435138	0	0.022426
20861	Amp	12	94444054	94453775	0	0.01955
20862	Amp	12	94455345	94540566	0	0.011376
20863	Amp	12	94547192	94566536	0	0.011957
20864	Amp	12	94566619	94719637	0	0.012577
20865	Amp	12	94719730	94847006	0	0.011996
20866	Amp	12	94847481	95061965	0	0.009864
20867	Amp	12	95063364	95310266	0	0.010552
20868	Amp	12	95310807	95387946	0	0.017824
20869	Amp	12	95388761	95467994	0	0.015332
20870	Amp	12	95469729	95652949	0	0.019714
20871	Amp	12	95654303	95966572	0	0.022933
20872	Amp	12	95968361	96073789	0	0.019714
20873	Amp	12	96078153	96186633	0	0.021008
20874	Amp	12	96190265	96486063	0	0.02032
20875	Amp	12	96486464	96638079	0	0.023498
20876	Amp	12	96642211	96988768	0	0.019029
20877	Amp	12	96995181	97065062	0	0.014647
20878	Amp	12	97065607	97096572	0	0.019177
20879	Amp	12	97100179	97172398	0	0.023464
20880	Amp	12	97172684	97251645	0	0.025052
20881	Amp	12	97252734	97294893	0	0.025672

20882	Amp	12	97295410	97335504	0	0.023913
20883	Amp	12	97337476	97357416	0	0.026101
20884	Amp	12	97358519	97423912	0	0.02846
20885	Amp	12	97424651	97530334	0	0.026273
20886	Amp	12	97531339	97566283	0	0.024684
20887	Amp	12	97566828	97691949	0	0.021507
20888	Amp	12	97695509	97760590	0	0.024198
20889	Amp	12	97762049	98121587	0	0.021507
20890	Amp	12	98124176	98148802	0	0.01722
20891	Amp	12	98154580	98159796	0	0.017706
20892	Amp	12	98160253	98188011	0	0.019252
20893	Amp	12	98192170	98207879	0	0.018633
20894	Amp	12	98212867	98215508	0	0.01993
20895	Amp	12	98221392	98251095	0	0.022176
20896	Amp	12	98251317	98279977	0	0.024561
20897	Amp	12	98279992	98281416	0	0.024074
20898	Amp	12	98282309	98292655	0	0.024646
20899	Amp	12	98294158	98325993	0	0.023348
20900	Amp	12	98326819	98428696	0	0.022777
20901	Amp	12	98439574	98439574	0	0.026072
20902	Amp	12	98442658	98530736	0	0.023208
20903	Amp	12	98534868	98547445	0	0.019913
20904	Amp	12	98550670	98555734	0	0.015382
20905	Amp	12	98570351	98592314	0	0.01766
20906	Amp	12	98595455	98598127	0	0.015415
20907	Amp	12	98599535	98680994	0	0.016693
20908	Amp	12	98683563	98704283	0	0.017768
20909	Amp	12	98704609	98754889	0	0.019309
20910	Amp	12	98755922	98770836	0	0.016924
20911	Amp	12	98771841	98771841	0	0.015384
20912	Amp	12	98775898	98811935	0	0.017132
20913	Amp	12	98812962	98860378	0	0.016056
20914	Amp	12	98860602	98861442	0	0.015436
20915	Amp	12	98862962	98879812	0	0.01389
20916	Amp	12	98882234	98913442	0	0.00911
20917	Amp	12	98914130	98914504	0	0.007815
20918	Amp	12	98914992	99119060	0	0.009165
20919	Amp	12	99119907	99171796	0	0.012452
20920	Amp	12	99173756	99294280	0	0.009165
20921	Amp	12	99295329	99315614	0	0.009751
20922	Amp	12	99316424	99513851	0	0.008401
20923	Amp	12	99515517	99527513	0	0.006653
20924	Amp	12	99528663	99529126	0	0.011116
20925	Amp	12	99536040	99862165	0	0.008838
20926	Amp	12	99865217	99986420	0	0.013065
20927	Amp	12	99986624	100017297	0	0.014672
20928	Amp	12	100018651	100040174	0	0.014086
20929	Amp	12	100058771	100141745	0	0.017931
20930	Amp	12	100148505	100201669	0	0.018676
20931	Amp	12	100202579	100259552	0	0.019892
20932	Amp	12	100264734	100272419	0	0.015665
20933	Amp	12	100273182	100279463	0	0.013727
20934	Amp	12	100279854	100296936	0	0.015464

20935	Amp	12	100299272	100316386	0	0.013957
20936	Amp	12	100318702	100353471	0	0.015337
20937	Amp	12	100353900	100370719	0	0.017544
20938	Amp	12	100371212	100408483	0	0.0205
20939	Amp	12	100409077	100438440	0	0.015638
20940	Amp	12	100454578	100541532	0	0.020118
20941	Amp	12	100550730	100585734	0	0.016832
20942	Amp	12	100591143	100755639	0	0.015094
20943	Amp	12	100757206	100914934	0	0.012138
20944	Amp	12	100917595	101019148	0	0.007658
20945	Amp	12	101019897	101053863	0	0.008779
20946	Amp	12	101061669	101174340	0	0.006572
20947	Amp	12	101179441	101555328	0	0.004965
20948	Amp	12	101555493	101570303	0	0.007827
20949	Amp	12	101571223	101587376	0	0.004965
20950	Amp	12	101592954	101619398	0	0.007662
20951	Amp	12	101621001	101638127	0	0.00654
20952	Amp	12	101644413	101644413	0	0.003843
20953	Amp	12	101647979	101742429	0	0.003098
20954	Amp	12	101743022	101869410	0	0.001719
20955	Amp	12	101878881	101943532	0	0.002658
20956	Amp	12	101945179	101981128	0	0.003286
20957	Amp	12	101981279	102054572	0	0.005512
20958	Amp	12	102057949	102302275	0	0.010472
20959	Amp	12	102316906	102408461	0	0.007208
20960	Amp	12	102409079	102612289	0	0.006768
20961	Amp	12	102617381	102637418	0	0.012617
20962	Amp	12	102668819	102689461	0	0.006768
20963	Amp	12	102690111	102707382	0	0.009446
20964	Amp	12	102707634	102727441	0	0.010571
20965	Amp	12	102728164	103141156	0	0.007893
20966	Amp	12	103153828	103153828	0	0.006768
20967	Amp	12	103156660	103424133	0	0.0109
20968	Amp	12	103430591	103500649	0	0.006768
20969	Amp	12	103501036	103655414	0	0.007797
20970	Amp	12	103656163	103965812	0	0.006768
20971	Amp	12	103972539	104122436	0	0.010594
20972	Amp	12	104128279	104169243	0	0.013004
20973	Amp	12	104169609	104225405	0	0.011307
20974	Amp	12	104227764	104460233	0	0.008898
20975	Amp	12	104467716	105071479	0	0.011684
20976	Amp	12	105074560	105118097	0	0.015505
20977	Amp	12	105124998	105149888	0	0.015983
20978	Amp	12	105151205	105292853	0	0.012163
20979	Amp	12	105295659	105440796	0	0.023976
20980	Amp	12	105443192	105469946	0	0.019899
20981	Amp	12	105471808	105494564	0	0.023727
20982	Amp	12	105503950	106342240	0	0.019899
20983	Amp	12	106348214	106676271	0	0.013938
20984	Amp	12	106676797	106723904	0	0.014399
20985	Amp	12	106724315	107213920	0	0.012624
20986	Amp	12	107227988	107339644	0	0.014965
20987	Amp	12	107339753	107615889	0	0.012624

20988	Amp	12	107619180	107789168	0	0.01473
20989	Amp	12	107793600	107979973	0	0.01961
20990	Amp	12	107980247	107993922	0	0.020049
20991	Amp	12	107996496	108189673	0	0.01517
20992	Amp	12	108191242	108230501	0	0.016691
20993	Amp	12	108231830	108281409	0	0.016251
20994	Amp	12	108286303	108295500	0	0.017063
20995	Amp	12	108301810	108360795	0	0.016251
20996	Amp	12	108361457	108377872	0	0.015773
20997	Amp	12	108380772	108585414	0	0.014252
20998	Amp	12	108590967	108702242	0	0.01843
20999	Amp	12	108711110	108765495	0	0.016293
21000	Amp	12	108766597	109421295	0	0.014252
21001	Amp	12	109426794	109560173	0	0.013313
21002	Amp	12	109564334	109629564	0	0.009486
21003	Amp	12	109637055	109805740	0	0.010683
21004	Amp	12	109815750	110135538	0	0.013653
21005	Amp	12	110139402	110551081	0	0.010683
21006	Amp	12	110551241	110835532	0	0.008577
21007	Amp	12	110844147	110874286	0	0.011977
21008	Amp	12	110877553	110976567	0	0.008577
21009	Amp	12	110976657	111491366	0	0.009549
21010	Amp	12	111502241	111579614	0	0.013438
21011	Amp	12	111587721	111715482	0	0.014235
21012	Amp	12	111725842	111732572	0	0.010345
21013	Amp	12	111735404	111735404	0	0.0119
21014	Amp	12	111883947	112099934	0	0.010703
21015	Amp	12	112100501	112207597	0	0.009148
21016	Amp	12	112209218	112220474	0	0.008176
21017	Amp	12	112225137	112483678	0	0.010329
21018	Amp	12	112483694	112489468	0	0.009532
21019	Amp	12	112502132	112502519	0	0.057906
21020	Amp	12	112503767	112504183	0	0.042037
21021	Amp	12	112512962	112539973	0	0.010695
21022	Amp	12	112545202	112648311	0	0.011459
21023	Amp	12	112651536	112726720	0	0.010296
21024	Amp	12	112727097	112771380	0	0.008144
21025	Amp	12	112776023	112776023	0	0.009034
21026	Amp	12	112776983	112887439	0	0.010754
21027	Amp	12	112889213	112952609	0	0.014702
21028	Amp	12	112957379	112966220	0	0.012982
21029	Amp	12	112968820	113151935	0	0.009034
21030	Amp	12	113165195	113338339	0	0.008269
21031	Amp	12	113341370	113425125	0	0.007379
21032	Amp	12	113425138	113560797	0	0.010408
21033	Amp	12	113562166	113799824	0	0.010905
21034	Amp	12	113799869	114824045	0	0.007877
21035	Amp	12	114824978	115051589	0	0.009792
21036	Amp	12	115054027	115195535	0	0.014612
21037	Amp	12	115196095	115212355	0	0.017778
21038	Amp	12	115214192	115276724	0	0.014612
21039	Amp	12	115276770	115278842	0	0.009792
21040	Amp	12	115278851	115284157	0	0.014243

21041	Amp	12	115284197	115339528	0	0.01655
21042	Amp	12	115340364	115589933	0	0.018855
21043	Amp	12	115590962	115612976	0	0.01655
21044	Amp	12	115613277	115652459	0	0.019295
21045	Amp	12	115653489	115761914	0	0.01655
21046	Amp	12	115762355	115856322	0	0.017104
21047	Amp	12	115856762	115865494	0	0.01768
21048	Amp	12	115867102	115888459	0	0.017104
21049	Amp	12	115888484	115911666	0	0.020532
21050	Amp	12	115912975	116055361	0	0.017104
21051	Amp	12	116061474	116181805	0	0.012652
21052	Amp	12	116183354	116224214	0	0.015729
21053	Amp	12	116224782	116251296	0	0.017478
21054	Amp	12	116252303	116341254	0	0.01931
21055	Amp	12	116342740	116358546	0	0.017478
21056	Amp	12	116365302	116413315	0	0.021504
21057	Amp	12	116419357	116435675	0	0.019754
21058	Amp	12	116436871	116487021	0	0.016677
21059	Amp	12	116487041	116528120	0	0.018205
21060	Amp	12	116531060	116577591	0	0.020358
21061	Amp	12	116579707	116640866	0	0.018205
21062	Amp	12	116643636	116756395	0	0.016677
21063	Amp	12	116761830	116770436	0	0.018347
21064	Amp	12	116771860	117144344	0	0.020972
21065	Amp	12	117151051	117180220	0	0.022308
21066	Amp	12	117180517	117372495	0	0.024087
21067	Amp	12	117372677	117451964	0	0.024713
21068	Amp	12	117454942	117457329	0	0.023043
21069	Amp	12	117459076	117506266	0	0.027401
21070	Amp	12	117508040	117614989	0	0.025622
21071	Amp	12	117618435	117697063	0	0.027943
21072	Amp	12	117698091	117891188	0	0.025769
21073	Amp	12	117895724	117959794	0	0.027278
21074	Amp	12	117960334	118055062	0	0.029563
21075	Amp	12	118057582	118081611	0	0.028936
21076	Amp	12	118099050	118123493	0	0.026085
21077	Amp	12	118129701	118146312	0	0.023765
21078	Amp	12	118149854	118256331	0	0.022394
21079	Amp	12	118257784	118283986	0	0.024245
21080	Amp	12	118286713	118899154	0	0.022394
21081	Amp	12	118907086	118974082	0	0.02312
21082	Amp	12	118975200	119023205	0	0.021784
21083	Amp	12	119023846	119221248	0	0.021051
21084	Amp	12	119222052	119291833	0	0.023398
21085	Amp	12	119293345	119362728	0	0.02411
21086	Amp	12	119365722	119376927	0	0.024806
21087	Amp	12	119377844	119422160	0	0.026035
21088	Amp	12	119426276	119677137	0	0.030618
21089	Amp	12	119677604	119689249	0	0.031534
21090	Amp	12	119693726	119828458	0	0.030822
21091	Amp	12	119833296	119894128	0	0.033009
21092	Amp	12	119942498	120151048	0	0.03092
21093	Amp	12	120151356	120178189	0	0.030423

21094	Amp	12	120180660	120184883	0	0.029506
21095	Amp	12	120185378	120192213	0	0.026862
21096	Amp	12	120193703	120296902	0	0.024636
21097	Amp	12	120301472	120335247	0	0.026921
21098	Amp	12	120344697	120365980	0	0.025692
21099	Amp	12	120371388	120441870	0	0.023198
21100	Amp	12	120442294	120548388	0	0.023732
21101	Amp	12	120550901	120746031	0	0.022454
21102	Amp	12	120748746	120788498	0	0.024951
21103	Amp	12	120790607	120872048	0	0.022604
21104	Amp	12	120872259	121116375	0	0.025358
21105	Amp	12	121116620	121796285	0	0.018392
21106	Amp	12	121809376	121824293	0	0.021044
21107	Amp	12	121826917	121852934	0	0.024113
21108	Amp	12	121859371	122441604	0	0.021044
21109	Amp	12	122454411	122546241	0	0.017019
21110	Amp	12	122546636	122970879	0	0.016485
21111	Amp	12	122994180	123014268	0	0.032422
21112	Amp	12	123016762	123189405	0	0.033935
21113	Amp	12	123189994	123313035	0	0.032422
21114	Amp	12	123315427	123504344	0	0.034773
21115	Amp	12	123504703	123504914	0	0.018837
21116	Amp	12	123508110	123551547	0	0.019538
21117	Amp	12	123552865	123834712	0	0.018837
21118	Amp	12	123835317	123873574	0	0.019496
21119	Amp	12	123873962	124056984	0	0.023832
21120	Amp	12	124059983	124185150	0	0.02234
21121	Amp	12	124185247	124236825	0	0.023078
21122	Amp	12	124236873	124791998	0	0.023595
21123	Amp	12	124794574	124806460	0	0.020841
21124	Amp	12	124808949	124842793	0	0.025605
21125	Amp	12	124842806	124961356	0	0.024867
21126	Amp	12	124962876	124982692	0	0.026745
21127	Amp	12	124982919	125504487	0	0.027953
21128	Amp	12	125505078	125523512	0	0.029334
21129	Amp	12	125524623	125583135	0	0.030006
21130	Amp	12	125585215	125693450	0	0.029334
21131	Amp	12	125695581	125782846	0	0.02457
21132	Amp	12	125783071	126142510	0	0.020234
21133	Amp	12	126142778	126758411	0	0.019026
21134	Amp	12	126759332	126783528	0	0.016675
21135	Amp	12	126786701	126874646	0	0.021103
21136	Amp	12	126878286	126918181	0	0.021655
21137	Amp	12	126919857	127091148	0	0.019837
21138	Amp	12	127101309	127114409	0	0.022726
21139	Amp	12	127119263	127275736	0	0.018297
21140	Amp	12	127275904	127354465	0	0.016916
21141	Amp	12	127355234	127656971	0	0.016364
21142	Amp	12	127662810	128936743	0	0.020424
21143	Amp	12	128937187	129312264	0	0.022196
21144	Amp	12	129318981	129318981	0	0.02142
21145	Amp	12	130592328	130600810	0	0.019649
21146	Amp	12	130601378	130619369	0	0.02094

21147	Amp	12	130623843	130623843	0	0.030378
21148	Amp	12	130629188	130695706	0	0.030857
21149	Amp	12	130695842	130821733	0	0.032163
21150	Amp	12	130829072	130909552	0	0.035081
21151	Amp	12	130910883	130943539	0	0.038144
21152	Amp	12	130943720	131132872	0	0.038592
21153	Amp	12	131133090	131141761	0	0.039183
21154	Amp	12	131148153	131391438	0	0.026682
21155	Amp	12	131396733	131420574	0	0.029177
21156	Amp	12	131422427	131551883	0	0.026682
21157	Amp	12	131552315	131597785	0	0.027426
21158	Amp	12	131597972	131776139	0	0.026909
21159	Amp	12	131777564	131842636	0	0.027434
21160	Amp	12	131847692	131946017	0	0.026955
21161	Amp	12	131964088	132105630	0	0.026507
21162	Amp	12	132106128	132127961	0	0.025763
21163	Amp	12	132431226	132431226	0	0.026599
21164	Amp	12	132434972	132516535	0	0.026046
21165	Amp	12	132519299	132605822	0	0.02552
21166	Amp	13	18874255	18912909	0	0.032496
21167	Amp	13	18913643	18923307	0	0.028567
21168	Amp	13	18926606	18964312	0	0.027415
21169	Amp	13	18964746	19043758	0	0.025987
21170	Amp	13	19044000	19057017	0	0.029063
21171	Amp	13	19057328	19199374	0	0.025987
21172	Amp	13	19234783	19279168	0	0.023866
21173	Amp	13	19287677	19545013	0	0.021456
21174	Amp	13	19553745	19561562	0	0.024635
21175	Amp	13	19952621	20181120	0	0.027256
21176	Amp	13	20182160	20195611	0	0.030481
21177	Amp	13	20201429	20682654	0	0.027256
21178	Amp	13	20688592	20902177	0	0.03101
21179	Amp	13	20905785	21303323	0	0.030353
21180	Amp	13	21312851	21334385	0	0.033907
21181	Amp	13	21334571	21502690	0	0.035935
21182	Amp	13	21503850	21586197	0	0.037465
21183	Amp	13	21586722	21589728	0	0.033642
21184	Amp	13	21590247	21623771	0	0.032113
21185	Amp	13	21630533	21691425	0	0.031199
21186	Amp	13	21694257	21842035	0	0.028577
21187	Amp	13	21848321	22017116	0	0.032238
21188	Amp	13	22027596	22048282	0	0.028484
21189	Amp	13	22049390	22074930	0	0.024823
21190	Amp	13	22077105	22499658	0	0.027139
21191	Amp	13	22504933	22588688	0	0.037379
21192	Amp	13	22588942	22646065	0	0.033681
21193	Amp	13	22646509	22670541	0	0.041782
21194	Amp	13	22674769	22757318	0	0.046203
21195	Amp	13	22762170	22774798	0	0.043099
21196	Amp	13	22950543	23246906	0	0.034998
21197	Amp	13	23249749	23264486	0	0.030577
21198	Amp	13	23266349	23290899	0	0.028808
21199	Amp	13	23291572	23355916	0	0.041306

21200	Amp	13	23357594	23384178	0	0.045179
21201	Amp	13	23385875	23456954	0	0.04793
21202	Amp	13	23461797	23465604	0	0.050281
21203	Amp	13	23468215	23474969	0	0.049597
21204	Amp	13	23478904	23496486	0	0.044929
21205	Amp	13	23496931	23541608	0	0.042578
21206	Amp	13	23541791	23541791	0	0.043074
21207	Amp	13	24003455	24003455	0	0.046237
21208	Amp	13	24003893	24031661	0	0.047392
21209	Amp	13	24039867	24102888	0	0.04823
21210	Amp	13	24103168	24144009	0	0.048687
21211	Amp	13	24144241	24160017	0	0.047532
21212	Amp	13	24162953	24237484	0	0.060417
21213	Amp	13	24241602	24336058	0	0.054841
21214	Amp	13	24336509	24354315	0	0.046525
21215	Amp	13	24445886	24502485	0	0.048109
21216	Amp	13	24502892	24508492	0	0.046525
21217	Amp	13	24513626	24526883	0	0.048825
21218	Amp	13	24527202	24680060	0	0.045687
21219	Amp	13	24684706	24745608	0	0.04502
21220	Amp	13	24747734	24755539	0	0.044563
21221	Amp	13	24759567	24825715	0	0.049125
21222	Amp	13	24829225	24870991	0	0.047764
21223	Amp	13	24871588	24959630	0	0.048236
21224	Amp	13	24959996	25268650	0	0.043674
21225	Amp	13	25271722	25596916	0	0.047215
21226	Amp	13	25599516	25718816	0	0.048588
21227	Amp	13	25720065	25788018	0	0.052654
21228	Amp	13	25791240	25810442	0	0.055596
21229	Amp	13	25817337	25828302	0	0.051531
21230	Amp	13	25829057	25893949	0	0.047489
21231	Amp	13	25899746	25943671	0	0.054037
21232	Amp	13	25947461	26014336	0	0.059281
21233	Amp	13	26015725	26037756	0.001323	0.063998
21234	Amp	13	26039244	26040278	0.060686	0.066563
21235	Amp	13	26040307	26063458	0	0.063092
21236	Amp	13	26087895	26126626	0	0.061719
21237	Amp	13	26129548	26135322	0	0.062396
21238	Amp	13	26142828	26239588	0.246818	0.074144
21239	Amp	13	26242101	26395579	0.259438	0.074907
21240	Amp	13	26400425	26470043	0.246818	0.07423
21241	Amp	13	26470782	26473892	0.133193	0.069509
21242	Amp	13	26474805	26487379	0.056211	0.066259
21243	Amp	13	26489409	26736086	0	0.059877
21244	Amp	13	26740515	26780463	0.056211	0.066348
21245	Amp	13	26784561	26808189	0.100827	0.068238
21246	Amp	13	26808892	27005975	0.133193	0.069587
21247	Amp	13	27007397	27010165	0.065715	0.066682
21248	Amp	13	27011485	27037108	0.1237	0.069263
21249	Amp	13	27043407	27232124	0.079652	0.067373
21250	Amp	13	27232731	27244981	0.159312	0.070646
21251	Amp	13	27247325	27290656	0.170723	0.071086
21252	Amp	13	27293364	27369559	0.159312	0.070646

21253	Amp	13	27373838	27389754	0.050473	0.06612
21254	Amp	13	27391407	27443495	0	0.062323
21255	Amp	13	27447967	27475891	0	0.058451
21256	Amp	13	27476417	27490553	0	0.060339
21257	Amp	13	27490652	27513293	0	0.058564
21258	Amp	13	27515408	27677153	0	0.06198
21259	Amp	13	27677775	27818982	0	0.058564
21260	Amp	13	27827441	27844106	0	0.062413
21261	Amp	13	27851779	27913987	0.044794	0.065948
21262	Amp	13	27922634	28010607	0.222041	0.07315
21263	Amp	13	28012083	28048303	0.264858	0.075007
21264	Amp	13	28048329	28096426	0.238246	0.073828
21265	Amp	13	28096737	28174822	0.264858	0.075075
21266	Amp	13	28177043	28264975	0.107017	0.068411
21267	Amp	13	28266740	28276430	0.089561	0.06794
21268	Amp	13	28282151	28282151	0.238246	0.073748
21269	Amp	13	28284675	28629067	0.287738	0.075854
21270	Amp	13	28629821	28845641	0.241616	0.073966
21271	Amp	13	28849056	28856995	0.368062	0.078788
21272	Amp	13	28858725	28864836	0.246818	0.074243
21273	Amp	13	28865890	28932038	0.271552	0.075342
21274	Amp	13	28932139	29008983	0.210594	0.072685
21275	Amp	13	29010375	29036268	0.287738	0.075944
21276	Amp	13	29045799	29050503	0.233782	0.07356
21277	Amp	13	29052518	29090318	0.19964	0.07221
21278	Amp	13	29092195	29258809	0.316877	0.077083
21279	Amp	13	29267895	29287326	0.112543	0.068684
21280	Amp	13	29291541	29291541	0.153838	0.070396
21281	Amp	13	29291671	29307663	0.227787	0.073305
21282	Amp	13	29309370	29315946	0.362016	0.078461
21283	Amp	13	29318006	29324456	0.311747	0.076749
21284	Amp	13	29324631	29327713	0.233782	0.073567
21285	Amp	13	29328494	29444463	0.112543	0.068745
21286	Amp	13	29445440	29464654	0.060686	0.066661
21287	Amp	13	29465492	29582967	0.107017	0.068444
21288	Amp	13	29583115	29590222	0.060686	0.066661
21289	Amp	13	29590493	29673446	0.14896	0.070209
21290	Amp	13	29674362	29684735	0.19964	0.072182
21291	Amp	13	29691361	29700970	0.238246	0.073689
21292	Amp	13	29702682	29734693	0.259438	0.074702
21293	Amp	13	29741267	29750219	0.293269	0.076161
21294	Amp	13	29752458	29763194	0.329205	0.077303
21295	Amp	13	29763417	29781660	0.34892	0.077984
21296	Amp	13	29783018	29794730	0.392611	0.079648
21297	Amp	13	29799034	29821565	0.557122	0.084998
21298	Amp	13	29832947	29843693	0.704849	0.089488
21299	Amp	13	29852216	29862752	0.609133	0.086682
21300	Amp	13	29862831	29889947	0.711903	0.089767
21301	Amp	13	29894167	29894511	0.774648	0.091472
21302	Amp	13	29897152	29898121	0.704849	0.089499
21303	Amp	13	29901776	29941986	0.968522	0.096453
21304	Amp	13	29942031	30005318	0.857296	0.093651
21305	Amp	13	30005475	30071122	0.950886	0.096092

21306	Amp	13	30071167	30093712	1.014678	0.097643
21307	Amp	13	30093887	30101631	1.149583	0.100829
21308	Amp	13	30102839	30134117	1.23519	0.102772
21309	Amp	13	30134437	30199144	1.149583	0.100829
21310	Amp	13	30201400	30372796	1.23519	0.102722
21311	Amp	13	30374591	30393999	1.066493	0.098873
21312	Amp	13	30395873	30398654	0.98589	0.096979
21313	Amp	13	30400188	30508587	0.823771	0.092727
21314	Amp	13	30512203	30545589	0.550755	0.08476
21315	Amp	13	30550514	30584942	0.444474	0.081225
21316	Amp	13	30585774	30652044	0.368062	0.078783
21317	Amp	13	30653200	30669443	0.005328	0.064164
21318	Amp	13	30670043	30687963	0.024438	0.06503
21319	Amp	13	30689560	30692359	0	0.062046
21320	Amp	13	30692423	30730528	0	0.059138
21321	Amp	13	30736088	30753865	0	0.054648
21322	Amp	13	30756844	30804890	0	0.058313
21323	Amp	13	30809680	30865355	0	0.054783
21324	Amp	13	30865795	30909371	0	0.056588
21325	Amp	13	30909418	30916488	0	0.058952
21326	Amp	13	30918503	30960151	0	0.059609
21327	Amp	13	30960281	31021242	0	0.057737
21328	Amp	13	31023189	31049856	0	0.05915
21329	Amp	13	31049934	31064881	0	0.058283
21330	Amp	13	31065772	31065772	0	0.057626
21331	Amp	13	31068018	31095526	0	0.062419
21332	Amp	13	31097560	31108059	0	0.056804
21333	Amp	13	31108362	31150656	0.029031	0.065156
21334	Amp	13	31151237	31151237	0	0.058913
21335	Amp	13	31151480	31151480	0	0.057592
21336	Amp	13	31315918	31315918	0	0.049773
21337	Amp	13	31317024	31574428	0	0.046108
21338	Amp	13	31577955	31589152	0	0.045027
21339	Amp	13	31590112	31600958	0	0.043193
21340	Amp	13	31601087	31606534	0	0.042511
21341	Amp	13	31613629	31619896	0	0.041748
21342	Amp	13	31627026	31648562	0	0.047855
21343	Amp	13	31652262	31674357	0	0.046938
21344	Amp	13	31674622	31762600	0	0.045459
21345	Amp	13	31763057	31814729	0	0.046589
21346	Amp	13	31815195	31866535	0	0.053009
21347	Amp	13	31866648	31946446	0	0.046589
21348	Amp	13	31949791	31979540	0	0.043504
21349	Amp	13	31982427	31982427	0	0.048246
21350	Amp	13	31989157	32024772	0	0.036222
21351	Amp	13	32025164	32199422	0	0.038576
21352	Amp	13	32293372	32293530	0	0.03998
21353	Amp	13	32296306	32296306	0	0.043248
21354	Amp	13	32299016	32306377	0	0.037557
21355	Amp	13	32306489	32309770	0	0.033641
21356	Amp	13	32313101	32386822	0	0.039249
21357	Amp	13	32386985	32387065	0	0.03707
21358	Amp	13	32391593	32397255	0	0.030592

21359	Amp	13	32407005	32425814	0	0.043798
21360	Amp	13	32426384	32433211	0	0.040903
21361	Amp	13	32435137	32444087	0	0.027697
21362	Amp	13	32446976	32448850	0	0.030884
21363	Amp	13	32455438	32581972	0	0.044375
21364	Amp	13	32595560	32696277	0	0.046821
21365	Amp	13	32699829	32813361	0	0.05103
21366	Amp	13	32815113	32951301	0	0.035094
21367	Amp	13	32952260	33128732	0	0.027697
21368	Amp	13	33129191	33141960	0	0.028752
21369	Amp	13	33142864	33156138	0	0.028257
21370	Amp	13	33196616	33201882	0	0.026706
21371	Amp	13	33202253	33430900	0	0.02565
21372	Amp	13	33431097	33632789	0	0.030113
21373	Amp	13	33633563	33660890	0	0.027722
21374	Amp	13	33662305	33790581	0	0.024453
21375	Amp	13	33793808	33898216	0	0.023834
21376	Amp	13	33903867	34008773	0	0.022734
21377	Amp	13	34011596	34085603	0	0.024227
21378	Amp	13	34085753	34183788	0	0.026091
21379	Amp	13	34186300	34382991	0	0.022543
21380	Amp	13	34391274	34395031	0	0.025176
21381	Amp	13	34395094	34441348	0	0.030315
21382	Amp	13	34441944	34512456	0	0.028856
21383	Amp	13	34512623	34853032	0	0.027363
21384	Amp	13	34857252	35053720	0	0.027802
21385	Amp	13	35060028	35231915	0	0.025169
21386	Amp	13	35232541	35263344	0	0.0266
21387	Amp	13	35272338	35331530	0	0.028565
21388	Amp	13	35335727	35410813	0	0.023425
21389	Amp	13	35411974	35488006	0	0.023931
21390	Amp	13	35489387	35516543	0	0.026493
21391	Amp	13	35516909	35530810	0	0.027939
21392	Amp	13	35531263	35672750	0	0.02946
21393	Amp	13	35677373	35988034	0	0.029021
21394	Amp	13	35988873	36043936	0	0.032578
21395	Amp	13	36049309	36109370	0	0.035799
21396	Amp	13	36109372	36124078	0	0.035293
21397	Amp	13	36127788	36249097	0	0.032072
21398	Amp	13	36249594	36256633	0	0.029936
21399	Amp	13	36261875	36273031	0	0.03429
21400	Amp	13	36279892	36665091	0	0.032
21401	Amp	13	36668921	36678785	0	0.035593
21402	Amp	13	36679119	36778237	0	0.03182
21403	Amp	13	36780163	36896537	0	0.022606
21404	Amp	13	36899249	36939466	0	0.025379
21405	Amp	13	36942023	37093871	0	0.034606
21406	Amp	13	37095888	37129721	0	0.025379
21407	Amp	13	37129917	37353140	0	0.022606
21408	Amp	13	37353211	37355421	0	0.018417
21409	Amp	13	37357868	37416955	0	0.019759
21410	Amp	13	37422460	37485704	0	0.017794
21411	Amp	13	37486110	37493006	0	0.021745

21412	Amp	13	37493438	37555194	0	0.023192
21413	Amp	13	37555773	37636980	0	0.021671
21414	Amp	13	37642292	37675985	0	0.020224
21415	Amp	13	37676074	37706995	0	0.017643
21416	Amp	13	37712870	38072963	0	0.011717
21417	Amp	13	38079942	38102736	0	0.017956
21418	Amp	13	38104547	38315446	0	0.011717
21419	Amp	13	38318113	38345859	0	0.022406
21420	Amp	13	38350734	38380458	0	0.025034
21421	Amp	13	38385186	38418096	0	0.027116
21422	Amp	13	38423948	38606824	0	0.016427
21423	Amp	13	38614475	39044165	0	0.013865
21424	Amp	13	39054207	40080038	0	0.012158
21425	Amp	13	40083824	40303629	0	0.015209
21426	Amp	13	40304865	40445533	0	0.012158
21427	Amp	13	40447956	40510104	0	0.009531
21428	Amp	13	40510649	40614232	0	0.013012
21429	Amp	13	40617272	40775986	0	0.012084
21430	Amp	13	40776843	40917586	0	0.008602
21431	Amp	13	40918208	41186834	0	0.010366
21432	Amp	13	41187363	41930417	0	0.008284
21433	Amp	13	41934733	42219162	0	0.007428
21434	Amp	13	42222585	42389214	0	0.009982
21435	Amp	13	42390067	42742241	0	0.007428
21436	Amp	13	42745218	42752347	0	0.013321
21437	Amp	13	42757811	42852450	0	0.007428
21438	Amp	13	43216239	43216239	0	0.008215
21439	Amp	13	43216777	43825869	0	0.005938
21440	Amp	13	43827964	43858122	0	0.007302
21441	Amp	13	43859227	43964277	0	0.010669
21442	Amp	13	43966424	44134584	0	0.007302
21443	Amp	13	44136403	44411975	0	0.005938
21444	Amp	13	44422475	44619776	0	0.008693
21445	Amp	13	44624610	44878575	0	0.007906
21446	Amp	13	44878807	45398737	0	0.007077
21447	Amp	13	45399633	45729655	0	0.007742
21448	Amp	13	45729817	45806399	0	0.009223
21449	Amp	13	45806458	45870877	0	0.006468
21450	Amp	13	45870949	45888259	0	0.007416
21451	Amp	13	45889716	46307536	0	0.006468
21452	Amp	13	46307990	46503791	0	0.007169
21453	Amp	13	46504298	46592060	0	0.010248
21454	Amp	13	46593535	46635248	0	0.007169
21455	Amp	13	46635937	46841125	0	0.005688
21456	Amp	13	46842251	47366525	0	0.005024
21457	Amp	13	47369694	47504034	0	0.00662
21458	Amp	13	47505394	47506081	0	0.005024
21459	Amp	13	47507430	47667743	0	0.008689
21460	Amp	13	47667951	47754521	0	0.005024
21461	Amp	13	47754843	47763502	0	0.00559
21462	Amp	13	47765427	47774119	0	0.004186
21463	Amp	13	47776034	48062628	0	0.003485
21464	Amp	13	48062828	48639125	0	0.00404

21465	Amp	13	48642192	48923001	0	0.003485
21466	Amp	13	48925314	48937291	0	0.002918
21467	Amp	13	48937392	48950210	0	0.00448
21468	Amp	13	48989163	49456719	0	0.006098
21469	Amp	13	49457034	49768827	0	0.008197
21470	Amp	13	49769187	49860294	0	0.006098
21471	Amp	13	49864100	49917827	0	0.005588
21472	Amp	13	49920448	51788344	0	0.007135
21473	Amp	13	51790815	51922940	0	0.008157
21474	Amp	13	51927001	52034621	0	0.01013
21475	Amp	13	52035072	52091710	0	0.009021
21476	Amp	13	52092100	52208449	0	0.00746
21477	Amp	13	52214956	52633113	0	0.009468
21478	Amp	13	52635714	52657750	0	0.007496
21479	Amp	13	52661194	52690909	0	0.004466
21480	Amp	13	52691283	53054607	0	0.005386
21481	Amp	13	53054660	53722006	0	0.003839
21482	Amp	13	53722504	53789776	0	0.002918
21483	Amp	13	53793147	54299227	0	0.004334
21484	Amp	13	54299670	54384759	0	0.005243
21485	Amp	13	54385011	55077143	0	0.004334
21486	Amp	13	55079387	56452726	0	0.005337
21487	Amp	13	56452796	56678597	0	0.004334
21488	Amp	13	56679641	58056968	0	0.005716
21489	Amp	13	58057013	58749601	0	0.007012
21490	Amp	13	58756974	58779572	0	0.009203
21491	Amp	13	58780199	58828717	0	0.015014
21492	Amp	13	58828899	58885106	0	0.015718
21493	Amp	13	58885120	58914306	0	0.01423
21494	Amp	13	58917006	58920750	0	0.015397
21495	Amp	13	58921760	59143900	0	0.007395
21496	Amp	13	59144781	59223954	0	0.006099
21497	Amp	13	59223998	59289084	0	0.006814
21498	Amp	13	59289328	59347449	0	0.007278
21499	Amp	13	59348361	59391420	0	0.010459
21500	Amp	13	59394751	59527604	0	0.007278
21501	Amp	13	59530643	59530643	0	0.006112
21502	Amp	13	59538752	59604819	0	0.008108
21503	Amp	13	59615590	59656768	0	0.010771
21504	Amp	13	59665868	59713188	0	0.01252
21505	Amp	13	59716542	59953361	0	0.010771
21506	Amp	13	59954547	60437045	0	0.008108
21507	Amp	13	60441253	60724910	0	0.009783
21508	Amp	13	60725908	61028322	0	0.009319
21509	Amp	13	61028389	61073721	0	0.010217
21510	Amp	13	61077994	61408112	0	0.009319
21511	Amp	13	61416991	61494305	0	0.008604
21512	Amp	13	61497075	61709757	0	0.0079
21513	Amp	13	61713032	61870935	0	0.009518
21514	Amp	13	61876772	61883164	0	0.015505
21515	Amp	13	61883215	61894653	0	0.017324
21516	Amp	13	61902959	61968777	0	0.011337
21517	Amp	13	61973461	61993412	0	0.009518

21518	Amp	13	61995938	62223264	0	0.007842
21519	Amp	13	62224442	63321087	0	0.006224
21520	Amp	13	63327084	63681260	0	0.010373
21521	Amp	13	64372045	64372045	0	0.008754
21522	Amp	13	64372411	64475252	0	0.009384
21523	Amp	13	64476143	64593093	0	0.009989
21524	Amp	13	64599620	64661401	0	0.014342
21525	Amp	13	64661797	64732841	0	0.013737
21526	Amp	13	64734159	65104016	0	0.013107
21527	Amp	13	65104351	65418852	0	0.013816
21528	Amp	13	65418972	65591060	0	0.012401
21529	Amp	13	65591966	65702574	0	0.011692
21530	Amp	13	65704317	66100242	0	0.012779
21531	Amp	13	66103707	67805121	0	0.008426
21532	Amp	13	67809275	67940103	0	0.005896
21533	Amp	13	67940392	68527861	0	0.007356
21534	Amp	13	68529596	68851738	0	0.009903
21535	Amp	13	68853516	69083104	0	0.01042
21536	Amp	13	69087525	69252361	0	0.009333
21537	Amp	13	69253053	69391557	0	0.011272
21538	Amp	13	69404701	69426849	0	0.016322
21539	Amp	13	69429352	69499053	0	0.013775
21540	Amp	13	69500615	69891806	0	0.013258
21541	Amp	13	69892117	70067054	0	0.01499
21542	Amp	13	70067727	70081984	0	0.015567
21543	Amp	13	70083264	70098757	0	0.014786
21544	Amp	13	70100890	70160420	0	0.014209
21545	Amp	13	70201639	70431490	0	0.00722
21546	Amp	13	70437340	70443026	0	0.005838
21547	Amp	13	70446970	70516864	0	0.008575
21548	Amp	13	70517533	70675071	0	0.009984
21549	Amp	13	70675266	70788686	0	0.010485
21550	Amp	13	70789877	71043643	0	0.014773
21551	Amp	13	71043793	71172510	0	0.010485
21552	Amp	13	71172919	71704447	0	0.012225
21553	Amp	13	71706134	71863949	0	0.009488
21554	Amp	13	71864055	72056662	0	0.011554
21555	Amp	13	72058772	72133980	0	0.0135
21556	Amp	13	72136541	72190379	0	0.011554
21557	Amp	13	72190939	72625548	0	0.014088
21558	Amp	13	72631390	72763783	0	0.012348
21559	Amp	13	72767566	73003590	0	0.009814
21560	Amp	13	73013619	73571402	0	0.010437
21561	Amp	13	73571912	73675690	0	0.0123
21562	Amp	13	73676330	73903165	0	0.010437
21563	Amp	13	73903739	73950655	0	0.014089
21564	Amp	13	73950889	73967614	0	0.015575
21565	Amp	13	73968813	74322130	0	0.011923
21566	Amp	13	74322466	74491873	0	0.011301
21567	Amp	13	74506371	75090233	0	0.009235
21568	Amp	13	75090926	75159207	0	0.009932
21569	Amp	13	75161900	75270722	0	0.011066
21570	Amp	13	75272364	75645698	0	0.009932

21571	Amp	13	75645735	75892991	0	0.008446
21572	Amp	13	75893021	76246351	0	0.00896
21573	Amp	13	76251159	76313547	0	0.008446
21574	Amp	13	76313843	76964708	0	0.006796
21575	Amp	13	76966121	77185945	0	0.007861
21576	Amp	13	77186216	77441759	0	0.008505
21577	Amp	13	77442178	77757817	0	0.007441
21578	Amp	13	77758624	78512395	0	0.006796
21579	Amp	13	78522043	78534267	0	0.011544
21580	Amp	13	78534945	78540836	0	0.009087
21581	Amp	13	78558384	78715539	0	0.006796
21582	Amp	13	78715708	78973094	0	0.008416
21583	Amp	13	78973339	79044586	0	0.008896
21584	Amp	13	79053120	79369975	0	0.007466
21585	Amp	13	79374690	79768388	0	0.012216
21586	Amp	13	79775587	79800540	0	0.010596
21587	Amp	13	79802101	79902795	0	0.01192
21588	Amp	13	79902811	80030310	0	0.011387
21589	Amp	13	80030450	80073868	0	0.010063
21590	Amp	13	80076079	80246388	0	0.013396
21591	Amp	13	80253957	81012503	0	0.012916
21592	Amp	13	81015837	81570237	0	0.013429
21593	Amp	13	81570868	81601189	0	0.017171
21594	Amp	13	81601545	81873798	0	0.013429
21595	Amp	13	81873970	81906466	0	0.015175
21596	Amp	13	81906690	82013133	0	0.017844
21597	Amp	13	82018563	82116113	0	0.018417
21598	Amp	13	82120580	82834232	0	0.017844
21599	Amp	13	82840321	83441221	0	0.018326
21600	Amp	13	83441723	83647899	0	0.020637
21601	Amp	13	83656456	83875347	0	0.022177
21602	Amp	13	83883101	84699088	0	0.020637
21603	Amp	13	84701147	84875802	0	0.02554
21604	Amp	13	84883256	84907461	0	0.02245
21605	Amp	13	84908108	85349837	0	0.020637
21606	Amp	13	85351414	86282605	0	0.02246
21607	Amp	13	86282946	86563037	0	0.021978
21608	Amp	13	86571638	86603744	0	0.034499
21609	Amp	13	86603839	86612306	0	0.021978
21610	Amp	13	86616266	86632156	0	0.023159
21611	Amp	13	86632590	86645426	0	0.034613
21612	Amp	13	86652283	86668115	0	0.0395
21613	Amp	13	86668201	86840495	0	0.034613
21614	Amp	13	86842252	86858236	0	0.023159
21615	Amp	13	86859997	86915779	0	0.021978
21616	Amp	13	86922141	86954944	0	0.025039
21617	Amp	13	86960906	87280257	0	0.021978
21618	Amp	13	87288590	87301793	0	0.024648
21619	Amp	13	87309021	87338478	0	0.026913
21620	Amp	13	87340407	87730215	0	0.024648
21621	Amp	13	87730927	87892103	0	0.026518
21622	Amp	13	87892138	87963449	0	0.024648
21623	Amp	13	87965364	88563764	0	0.021079

21624	Amp	13	88568154	89210689	0	0.020566
21625	Amp	13	89212339	89265936	0	0.023567
21626	Amp	13	89266131	89283458	0	0.020566
21627	Amp	13	89285129	89339573	0	0.025281
21628	Amp	13	89339939	89355314	0	0.01788
21629	Amp	13	89364571	89825526	0	0.013165
21630	Amp	13	89826117	89865374	0	0.014535
21631	Amp	13	89865407	90199062	0	0.013438
21632	Amp	13	90202075	90217240	0	0.012068
21633	Amp	13	90218461	90543610	0	0.01343
21634	Amp	13	90545010	90701133	0	0.014905
21635	Amp	13	90702365	90864722	0	0.01343
21636	Amp	13	90870053	91042462	0	0.014285
21637	Amp	13	91047724	91410077	0	0.030221
21638	Amp	13	91455602	91813027	0	0.014285
21639	Amp	13	91815606	92158801	0	0.012923
21640	Amp	13	92159123	92396887	0	0.014275
21641	Amp	13	92399565	92446495	0	0.01769
21642	Amp	13	92449735	92449735	0	0.014275
21643	Amp	13	92450780	92693628	0	0.015777
21644	Amp	13	92695925	92707513	0	0.031765
21645	Amp	13	92711047	92715800	0	0.03922
21646	Amp	13	92722007	93284540	0	0.023232
21647	Amp	13	93286453	93540886	0	0.02173
21648	Amp	13	93545176	94004748	0	0.037667
21649	Amp	13	94005105	94316886	0	0.039651
21650	Amp	13	94317542	94318087	0	0.040121
21651	Amp	13	94318114	94389584	0	0.041068
21652	Amp	13	94389723	94889653	0	0.042276
21653	Amp	13	94891672	94965085	0	0.041645
21654	Amp	13	94965156	95051276	0	0.040791
21655	Amp	13	95056818	95570277	0	0.041499
21656	Amp	13	95570936	95747218	0	0.042797
21657	Amp	13	95749095	95966096	0	0.041499
21658	Amp	13	95968893	96585772	0	0.035218
21659	Amp	13	96593898	96949975	0	0.042491
21660	Amp	13	96957972	97173948	0	0.043266
21661	Amp	13	97176176	97481571	0	0.040955
21662	Amp	13	97484680	97484714	0	0.025019
21663	Amp	13	97490739	97490739	0	0.030657
21664	Amp	13	97494695	97522942	0	0.032623
21665	Amp	13	97522957	97525263	0	0.031675
21666	Amp	13	97525511	97764400	0	0.032582
21667	Amp	13	97775564	97824815	0	0.030617
21668	Amp	13	97826962	97939170	0	0.046553
21669	Amp	13	97955942	98095193	0	0.040915
21670	Amp	13	98095729	98317825	0	0.024979
21671	Amp	13	98318642	98359941	0	0.026687
21672	Amp	13	98360488	98557561	0	0.027313
21673	Amp	13	98558123	99078762	0	0.028563
21674	Amp	13	99081870	99502823	0	0.027937
21675	Amp	13	99508091	100088583	0	0.040115
21676	Amp	13	100093289	100157551	0	0.042408

21677	Amp	13	100158304	100175169	0	0.04474
21678	Amp	13	100176359	100245503	0	0.046811
21679	Amp	13	100246275	100338144	0	0.04474
21680	Amp	13	100340213	100355750	0	0.050945
21681	Amp	13	100357200	100485407	0	0.038766
21682	Amp	13	100486522	100514632	0	0.03658
21683	Amp	13	100514672	100653296	0	0.034287
21684	Amp	13	100663367	100811210	0	0.031956
21685	Amp	13	100813430	101210703	0	0.032776
21686	Amp	13	101214707	101235531	0	0.040588
21687	Amp	13	101235665	101292458	0	0.032776
21688	Amp	13	101293717	101328851	0	0.031869
21689	Amp	13	101333391	101419416	0	0.034339
21690	Amp	13	101420981	101654921	0	0.031869
21691	Amp	13	101655008	101658129	0	0.026782
21692	Amp	13	101667042	102009423	0	0.030322
21693	Amp	13	102009438	102484829	0	0.028862
21694	Amp	13	102484984	102924470	0	0.032375
21695	Amp	13	102924894	102972068	0	0.032832
21696	Amp	13	102973338	103926625	0	0.032375
21697	Amp	13	103927585	103972302	0	0.034716
21698	Amp	13	103972680	104482774	0	0.032375
21699	Amp	13	104487965	104546965	0	0.026913
21700	Amp	13	104547769	104737572	0	0.032804
21701	Amp	13	104738583	106637543	0	0.031631
21702	Amp	13	106637643	107238252	0	0.032134
21703	Amp	13	107242649	107436728	0	0.042645
21704	Amp	13	107440401	107469328	0	0.05162
21705	Amp	13	107470504	107645202	0	0.042645
21706	Amp	13	107648805	108204435	0	0.042142
21707	Amp	13	108204557	108220690	0	0.045372
21708	Amp	13	108221196	108257374	0	0.046625
21709	Amp	13	108260965	109214793	0	0.052126
21710	Amp	13	109261543	109702971	0	0.048895
21711	Amp	13	109703658	109735576	0	0.049508
21712	Amp	13	109741791	109939731	0	0.048955
21713	Amp	13	109940671	110213936	0	0.044007
21714	Amp	13	110214888	110291574	0	0.04668
21715	Amp	13	110293509	110639773	0	0.047549
21716	Amp	13	110642309	110731663	0	0.044876
21717	Amp	13	110732929	111608484	0	0.045433
21718	Amp	13	111608995	111668142	0	0.029701
21719	Amp	13	111851441	111977955	0	0.029749
21720	Amp	13	111981545	112137081	0	0.032082
21721	Amp	13	112137268	112404607	0	0.029363
21722	Amp	13	112418217	112497027	0	0.03132
21723	Amp	13	112497395	112522022	0	0.033641
21724	Amp	13	112522212	112535807	0	0.034854
21725	Amp	13	112542284	112860360	0	0.032533
21726	Amp	13	112862607	112862607	0	0.033978
21727	Amp	13	113081740	113181510	0	0.030505
21728	Amp	13	113184353	113508320	0	0.029059
21729	Amp	13	113508487	113563155	0	0.031134

21730	Amp	13	113567115	113582217	0	0.028402
21731	Amp	13	113587153	113885178	0	0.026328
21732	Amp	13	113887049	113934849	0	0.04206
21733	Amp	13	113936214	114221983	0	0.04124
21734	Amp	14	20033191	20151305	0	0.051781
21735	Amp	14	20154062	20164041	0	0.058654
21736	Amp	14	20167347	20184156	0	0.0498
21737	Amp	14	20186803	20205826	0	0.049006
21738	Amp	14	20205880	20209432	0	0.052057
21739	Amp	14	20218860	20230345	0	0.046508
21740	Amp	14	20243187	20390854	0	0.049153
21741	Amp	14	20394383	20479109	0	0.046076
21742	Amp	14	20482024	20482102	0	0.043764
21743	Amp	14	20536809	20541313	0	0.045242
21744	Amp	14	20541992	20555289	0	0.046866
21745	Amp	14	20556203	20566825	0	0.05223
21746	Amp	14	20568125	20590020	0	0.046866
21747	Amp	14	20590266	20676640	0	0.047472
21748	Amp	14	20676896	20681949	0	0.045847
21749	Amp	14	20720526	20779061	0	0.047226
21750	Amp	14	20781965	20996645	0	0.04787
21751	Amp	14	20997529	20998622	0	0.046392
21752	Amp	14	20999997	21045377	0	0.045014
21753	Amp	14	21052731	21052731	0	0.046158
21754	Amp	14	21057984	21121471	0	0.043514
21755	Amp	14	21121922	21177174	0	0.04287
21756	Amp	14	21178646	21178646	0	0.042108
21757	Amp	14	21200672	21200672	0	0.043614
21758	Amp	14	21201170	21250759	0	0.045018
21759	Amp	14	21253720	21267445	0	0.044387
21760	Amp	14	21268836	21570528	0	0.044993
21761	Amp	14	21573985	21634977	0	0.047181
21762	Amp	14	21635545	21635545	0	0.048132
21763	Amp	14	22664303	22664303	0	0.056723
21764	Amp	14	22664866	22904777	0	0.054158
21765	Amp	14	22905226	22908359	0	0.051987
21766	Amp	14	22920998	22996260	0	0.054728
21767	Amp	14	22999248	23043327	0	0.056712
21768	Amp	14	23047301	23055920	0	0.054978
21769	Amp	14	23063776	23113360	0	0.052237
21770	Amp	14	23114040	23275723	0	0.049187
21771	Amp	14	23287717	23310352	0	0.04479
21772	Amp	14	23319055	23372808	0	0.04199
21773	Amp	14	23373530	23393165	0	0.040586
21774	Amp	14	23395833	23413854	0	0.043461
21775	Amp	14	23416678	23463804	0	0.040586
21776	Amp	14	23466628	23487294	0	0.041409
21777	Amp	14	23489664	23501042	0	0.039442
21778	Amp	14	23503490	23630166	0	0.036807
21779	Amp	14	23631262	23667420	0	0.03399
21780	Amp	14	23668806	23682416	0	0.037642
21781	Amp	14	23682434	23689298	0	0.03399
21782	Amp	14	23693058	23693058	0	0.032345

21783	Amp	14	23694576	23729742	0	0.030252
21784	Amp	14	23729830	23816866	0	0.032113
21785	Amp	14	23820270	23926567	0	0.033765
21786	Amp	14	23932083	23938707	0	0.02809
21787	Amp	14	23939116	23955933	0	0.030326
21788	Amp	14	24024956	24024956	0	0.028096
21789	Amp	14	24025034	24031436	0	0.023243
21790	Amp	14	24031630	24031792	0	0.027873
21791	Amp	14	24041000	24044708	0	0.026221
21792	Amp	14	24049930	24203061	0	0.028226
21793	Amp	14	24203282	24241845	0	0.02961
21794	Amp	14	24247579	24321477	0	0.032682
21795	Amp	14	24321819	24341953	0	0.033342
21796	Amp	14	24342678	24440338	0	0.036601
21797	Amp	14	24533639	24533639	0	0.035397
21798	Amp	14	24533996	24550123	0	0.03604
21799	Amp	14	24557291	24796755	0	0.032934
21800	Amp	14	24796899	24814799	0	0.035643
21801	Amp	14	24819442	24835787	0	0.032934
21802	Amp	14	24839181	24872945	0	0.034544
21803	Amp	14	24875018	24909700	0	0.035097
21804	Amp	14	24914167	24969590	0	0.037293
21805	Amp	14	24970708	25035782	0	0.035909
21806	Amp	14	25037233	25228875	0	0.034837
21807	Amp	14	25230877	25259041	0	0.037037
21808	Amp	14	25266878	25288567	0	0.034837
21809	Amp	14	25288620	25307294	0	0.037117
21810	Amp	14	25307407	25384550	0	0.03154
21811	Amp	14	25393633	25426473	0	0.029679
21812	Amp	14	25427484	25500974	0	0.030813
21813	Amp	14	25502303	25537308	0	0.031569
21814	Amp	14	25537351	25587907	0	0.032104
21815	Amp	14	25595417	25676201	0	0.047872
21816	Amp	14	25683353	25797968	0	0.05691
21817	Amp	14	25800167	25800947	0	0.062862
21818	Amp	14	25801518	25903416	0.079652	0.067319
21819	Amp	14	25904973	25956908	0.094967	0.068134
21820	Amp	14	25959043	26160607	0.015369	0.064654
21821	Amp	14	26161794	26246343	0	0.063547
21822	Amp	14	26248678	26267195	0	0.062953
21823	Amp	14	26269085	26294421	0	0.047185
21824	Amp	14	26300516	26448809	0	0.046031
21825	Amp	14	26451287	26606643	0	0.045216
21826	Amp	14	26607271	26844770	0	0.044634
21827	Amp	14	26846757	26920155	0	0.046462
21828	Amp	14	26920652	27070175	0	0.044002
21829	Amp	14	27074590	27077330	0	0.048367
21830	Amp	14	27077590	27082212	0	0.050476
21831	Amp	14	27144154	27200592	0	0.046112
21832	Amp	14	27206387	27218655	0	0.053516
21833	Amp	14	27226764	27322990	0	0.046112
21834	Amp	14	27323518	27330971	0	0.04364
21835	Amp	14	27331228	27341574	0	0.041387

21836	Amp	14	27341657	27399274	0	0.040727
21837	Amp	14	27399888	27464323	0	0.043321
21838	Amp	14	27469909	27480630	0	0.040727
21839	Amp	14	27485979	27535932	0	0.035359
21840	Amp	14	27567831	27575532	0	0.0397
21841	Amp	14	27580835	27597143	0	0.037843
21842	Amp	14	27598020	27822531	0	0.035359
21843	Amp	14	27822899	27892565	0	0.036009
21844	Amp	14	27893464	27977913	0	0.034262
21845	Amp	14	28052155	28081034	0	0.031767
21846	Amp	14	28081802	28159219	0	0.032725
21847	Amp	14	28159286	28527942	0	0.033514
21848	Amp	14	28530059	28552722	0	0.036286
21849	Amp	14	28553659	28562426	0	0.038198
21850	Amp	14	28562693	28574871	0	0.037241
21851	Amp	14	28577496	28599209	0	0.03447
21852	Amp	14	28599454	28620187	0	0.037105
21853	Amp	14	28620527	28755627	0	0.039314
21854	Amp	14	28759695	28780495	0	0.040116
21855	Amp	14	28781096	28865245	0	0.041638
21856	Amp	14	28867311	28960918	0	0.039002
21857	Amp	14	28962231	29021395	0	0.041003
21858	Amp	14	29022569	29030549	0	0.038335
21859	Amp	14	29030900	29132147	0	0.04272
21860	Amp	14	29132806	29143825	0	0.052126
21861	Amp	14	29144304	29224828	0	0.057899
21862	Amp	14	29229542	29254523	0	0.058623
21863	Amp	14	29258648	29306925	0	0.058056
21864	Amp	14	29310904	29324550	0	0.059077
21865	Amp	14	29329010	29354925	0	0.052691
21866	Amp	14	29355467	29562433	0	0.053241
21867	Amp	14	29563241	29797635	0	0.055718
21868	Amp	14	29800020	29825365	0	0.054085
21869	Amp	14	29825680	29828154	0	0.052563
21870	Amp	14	29828885	29914087	0	0.054032
21871	Amp	14	29918314	30030717	0	0.052365
21872	Amp	14	30031037	30068720	0	0.057514
21873	Amp	14	30070056	30137993	0	0.058981
21874	Amp	14	30138756	30170205	0	0.061287
21875	Amp	14	30171877	30172242	0	0.062697
21876	Amp	14	30175856	30194492	0	0.063743
21877	Amp	14	30197191	30200557	0.100827	0.068284
21878	Amp	14	30204010	30232330	0.060686	0.066518
21879	Amp	14	30234136	30362315	0.153838	0.07057
21880	Amp	14	30369742	30394977	0.050473	0.066029
21881	Amp	14	30399897	30467211	0.015369	0.064619
21882	Amp	14	30482525	30525955	0.056211	0.066328
21883	Amp	14	30527568	30543645	0.094967	0.068021
21884	Amp	14	30551580	30612205	0.1237	0.069077
21885	Amp	14	30615882	30642654	0.14896	0.070348
21886	Amp	14	30643795	30654945	0.112543	0.068655
21887	Amp	14	30656765	30708320	0.181932	0.07155
21888	Amp	14	30721455	30801878	0.529451	0.084238

21889	Amp	14	30802146	30861076	0.557122	0.08495
21890	Amp	14	30863093	30985888	0.602643	0.086584
21891	Amp	14	30989923	30996925	0.293269	0.07615
21892	Amp	14	30998632	31051164	0.210594	0.072536
21893	Amp	14	31055293	31209008	0.282828	0.075597
21894	Amp	14	31211812	31304188	0.259438	0.074885
21895	Amp	14	31305791	31312369	0.34892	0.078149
21896	Amp	14	31315214	31610030	0.602643	0.086498
21897	Amp	14	31610457	31622846	0.644502	0.087726
21898	Amp	14	31622987	31660749	0.660254	0.088255
21899	Amp	14	31662768	31677306	0.831947	0.093026
21900	Amp	14	31684850	31703566	0.735486	0.090367
21901	Amp	14	31703900	31805431	0.751221	0.090804
21902	Amp	14	31805473	31813914	0.681869	0.08885
21903	Amp	14	31815671	31871472	0.933745	0.095728
21904	Amp	14	31872700	31884813	1.058188	0.098779
21905	Amp	14	31885781	32002883	0.857296	0.093615
21906	Amp	14	32003593	32032989	0.933745	0.0957
21907	Amp	14	32033702	32033702	0.915698	0.095263
21908	Amp	14	32035489	32074615	1.075952	0.099136
21909	Amp	14	32074903	32074903	1.121584	0.100308
21910	Amp	14	32083812	32085587	1.261401	0.103541
21911	Amp	14	32086024	32091003	1.102589	0.099668
21912	Amp	14	32091814	32198083	1.075952	0.099139
21913	Amp	14	32201900	32266547	1.252022	0.103299
21914	Amp	14	32278416	32298476	1.206641	0.102199
21915	Amp	14	32299135	32407729	1.159209	0.100971
21916	Amp	14	32408404	32458412	1.023539	0.097911
21917	Amp	14	32466262	32492211	1.131695	0.100488
21918	Amp	14	32492310	32563740	1.188121	0.101819
21919	Amp	14	32565969	32719642	1.131695	0.100517
21920	Amp	14	32722019	32892842	1.058188	0.098708
21921	Amp	14	32893724	32966328	1.131695	0.100427
21922	Amp	14	32969302	32996582	0.735486	0.09052
21923	Amp	14	32998585	33023204	0.899693	0.094838
21924	Amp	14	33023788	33048198	0.751221	0.090907
21925	Amp	14	33050729	33160815	0.660254	0.088193
21926	Amp	14	33161490	33352333	0.589605	0.08604
21927	Amp	14	33353629	33447410	0.667587	0.088518
21928	Amp	14	33447464	33455659	0.735486	0.090528
21929	Amp	14	33455926	33456636	0.720659	0.089978
21930	Amp	14	33456790	33478460	0.644502	0.087812
21931	Amp	14	33478799	33509453	0.616549	0.087045
21932	Amp	14	33510484	33512992	0.751221	0.090766
21933	Amp	14	33514909	33556024	0.616549	0.087045
21934	Amp	14	33559420	33608008	0.681869	0.08886
21935	Amp	14	33608839	33645761	0.774648	0.091515
21936	Amp	14	33648260	33653265	0.282828	0.075579
21937	Amp	14	33653286	33758926	0.251218	0.074316
21938	Amp	14	33759583	33763217	0.287738	0.075942
21939	Amp	14	33763505	33840083	0.246818	0.074127
21940	Amp	14	33843433	33902200	0.193543	0.071919
21941	Amp	14	33906940	33945516	0.222041	0.072978

21942	Amp	14	33951953	33985949	0.246818	0.074117
21943	Amp	14	33986802	34012578	0.293269	0.076086
21944	Amp	14	34017533	34017533	0.399144	0.079829
21945	Amp	14	34017580	34172875	0.457501	0.081686
21946	Amp	14	34183952	34185159	0.404718	0.079988
21947	Amp	14	34189214	34227523	0.457501	0.081802
21948	Amp	14	34228172	34297878	0.362016	0.078569
21949	Amp	14	34298099	34325733	0.251218	0.074408
21950	Amp	14	34329406	34359316	0.089561	0.067893
21951	Amp	14	34359532	34366177	0.1237	0.069107
21952	Amp	14	34366595	34444469	0.133193	0.069621
21953	Amp	14	34445630	34461721	0.282828	0.075687
21954	Amp	14	34467328	34475196	0.251218	0.074438
21955	Amp	14	34480606	34480606	0.227787	0.073266
21956	Amp	14	34482863	34496082	0.287738	0.075993
21957	Amp	14	34497034	34497730	0.264858	0.074937
21958	Amp	14	34500200	34502615	0.222041	0.073123
21959	Amp	14	34502916	34521568	0.153838	0.070396
21960	Amp	14	34521938	34521938	0.019169	0.064893
21961	Amp	14	34521995	34522419	0	0.06247
21962	Amp	14	34527119	34527508	0	0.058419
21963	Amp	14	34569707	34569707	0	0.062288
21964	Amp	14	34569888	34569888	0	0.05913
21965	Amp	14	34569954	34570001	0	0.061397
21966	Amp	14	34576753	34579436	0.019169	0.064795
21967	Amp	14	34579478	34579478	0.107017	0.068505
21968	Amp	14	34589610	34589610	0.118001	0.069027
21969	Amp	14	34591792	34601950	0.215608	0.072941
21970	Amp	14	34606747	34632388	0.264858	0.075071
21971	Amp	14	34634943	34634943	0.14896	0.070316
21972	Amp	14	34704418	34716383	0.293269	0.076158
21973	Amp	14	34719162	34749419	0.311747	0.07682
21974	Amp	14	34756768	34813037	0.222041	0.0731
21975	Amp	14	34815078	34859119	0.386426	0.079342
21976	Amp	14	34865115	34869281	0.522881	0.083969
21977	Amp	14	34869314	34875964	0.602643	0.086522
21978	Amp	14	34876049	34888144	0.543344	0.08458
21979	Amp	14	34891429	34929601	0.437198	0.081076
21980	Amp	14	34930654	34978092	0.362016	0.0786
21981	Amp	14	34982159	34988758	0.463534	0.082075
21982	Amp	14	34989539	35022070	0.386426	0.079283
21983	Amp	14	35022137	35043406	0.457501	0.081717
21984	Amp	14	35110721	35133750	0.529451	0.084102
21985	Amp	14	35156402	35157791	0.609133	0.08665
21986	Amp	14	35158151	35279476	0.557122	0.085011
21987	Amp	14	35284942	35284942	0.616549	0.086967
21988	Amp	14	35326248	35344491	0.576183	0.085752
21989	Amp	14	35349070	35349658	0.463534	0.082042
21990	Amp	14	35352486	35353059	0.522881	0.084022
21991	Amp	14	35353633	35353633	0.399144	0.079774
21992	Amp	14	35353778	35355819	0.431198	0.08084
21993	Amp	14	35356230	35376385	0.508568	0.083512
21994	Amp	14	35380264	35385840	0.450702	0.081557

21995 Amp	14	35387457	35401248	0.529451	0.084048
21996 Amp	14	35401264	35411536	0.508568	0.083481
21997 Amp	14	35411612	35489062	0.595248	0.086383
21998 Amp	14	35489787	35540565	0.522881	0.083892
21999 Amp	14	35546281	35558200	0.583752	0.085874
22000 Amp	14	35566472	35632735	0.742723	0.090755
22001 Amp	14	35634199	35644125	1.093834	0.099482
22002 Amp	14	35644667	35660855	1.159209	0.101076
22003 Amp	14	35666004	35705988	1.049301	0.098405
22004 Amp	14	35706295	35745289	0.977077	0.096664
22005 Amp	14	35747770	35813790	1.084294	0.099245
22006 Amp	14	35817390	35823145	0.924746	0.095332
22007 Amp	14	35823678	35867920	1.197658	0.102042
22008 Amp	14	35870975	35906824	1.243254	0.103024
22009 Amp	14	35909806	35912910	1.159209	0.101106
22010 Amp	14	35913400	35918478	1.058188	0.098608
22011 Amp	14	35918522	35970168	0.950886	0.096026
22012 Amp	14	35971119	36009797	0.630438	0.087505
22013 Amp	14	36010621	36141014	0.563096	0.08512
22014 Amp	14	36142915	36171395	0.482379	0.082686
22015 Amp	14	36172137	36195622	0.515676	0.083615
22016 Amp	14	36198343	36213619	0.482379	0.082556
22017 Amp	14	36214573	36267746	0.457501	0.081821
22018 Amp	14	36267934	36279480	0.543344	0.08447
22019 Amp	14	36280011	36288099	0.609133	0.086676
22020 Amp	14	36289628	36396634	0.6737	0.08869
22021 Amp	14	36396922	36412242	0.697374	0.089309
22022 Amp	14	36417628	36419185	0.636464	0.087715
22023 Amp	14	36419327	36419977	0.667587	0.088456
22024 Amp	14	36420484	36448257	0.72817	0.090174
22025 Amp	14	36452726	36484376	0.660254	0.088355
22026 Amp	14	36484883	36485971	0.751221	0.090906
22027 Amp	14	36486517	36489040	0.6737	0.08877
22028 Amp	14	36489085	36489085	0.602643	0.086465
22029 Amp	14	36492430	36531576	0.697374	0.089455
22030 Amp	14	36532704	36541621	0.622604	0.087129
22031 Amp	14	36547163	36555332	0.595248	0.086387
22032 Amp	14	36555706	36575957	0.651644	0.088082
22033 Amp	14	36577052	36631458	0.622604	0.087153
22034 Amp	14	36631488	36664403	0.774648	0.091443
22035 Amp	14	36665286	36665286	0.681869	0.088878
22036 Amp	14	36673349	36703660	0.774648	0.091419
22037 Amp	14	36704030	36789499	0.823771	0.092891
22038 Amp	14	36793042	36793042	0.86541	0.093814
22039 Amp	14	36793198	36803070	0.959607	0.096374
22040 Amp	14	36803799	36810742	0.933745	0.095712
22041 Amp	14	36810767	36884070	0.840289	0.093171
22042 Amp	14	36886485	36890426	0.774648	0.091454
22043 Amp	14	36891499	36954422	0.86541	0.094001
22044 Amp	14	36959266	36977845	0.711903	0.089711
22045 Amp	14	36984309	36984309	0.6737	0.088645
22046 Amp	14	36984426	37018291	0.86541	0.093868
22047 Amp	14	37018983	37080213	0.977077	0.096662

22048	Amp	14	37080305	37114054	1.041205	0.09836
22049	Amp	14	37114492	37153347	1.188121	0.101684
22050	Amp	14	37154384	37162122	1.066493	0.098889
22051	Amp	14	37164002	37174312	1.168655	0.10119
22052	Amp	14	37179175	37201854	1.492063	0.108729
22053	Amp	14	37205410	37205410	1.68612	0.112845
22054	Amp	14	37206715	37248588	1.583409	0.110715
22055	Amp	14	37257566	37267696	1.348715	0.105492
22056	Amp	14	37269222	37269314	1.270711	0.103723
22057	Amp	14	37274741	37275377	1.593878	0.110759
22058	Amp	14	37275469	37285368	1.737623	0.113953
22059	Amp	14	37286577	37290897	1.823153	0.115635
22060	Amp	14	37295543	37316496	1.624655	0.111519
22061	Amp	14	37316623	37432877	1.665486	0.112323
22062	Amp	14	37435326	37471855	1.512549	0.109129
22063	Amp	14	37473855	37479198	1.645511	0.111837
22064	Amp	14	37481700	37481778	1.748606	0.114163
22065	Amp	14	37485186	37495028	1.876242	0.116641
22066	Amp	14	37499431	37514045	2.26699	0.124525
22067	Amp	14	37514468	37557550	2.18114	0.122844
22068	Amp	14	37558382	37567944	2.116108	0.121402
22069	Amp	14	37576057	37576057	2.060912	0.12031
22070	Amp	14	37577553	37587218	1.95165	0.118296
22071	Amp	14	37593243	37593243	1.790459	0.114939
22072	Amp	14	37603948	37619267	1.706315	0.113212
22073	Amp	14	37626129	37644548	1.919241	0.11759
22074	Amp	14	37645618	37648779	1.972776	0.118665
22075	Amp	14	37650645	37650645	2.345513	0.126096
22076	Amp	14	37673871	37697892	2.049917	0.120295
22077	Amp	14	37705167	37705167	2.192012	0.123082
22078	Amp	14	37705253	37733358	2.083076	0.120756
22079	Amp	14	37734241	37751117	1.96212	0.118455
22080	Amp	14	37754497	37780005	1.823153	0.115668
22081	Amp	14	37780773	37809803	1.984015	0.118956
22082	Amp	14	37815552	37827139	1.854421	0.116248
22083	Amp	14	37827292	37869539	1.717671	0.113469
22084	Amp	14	37873212	37879783	1.675319	0.112525
22085	Amp	14	37882500	37930815	1.593878	0.110871
22086	Amp	14	37930889	37939792	1.696256	0.113003
22087	Amp	14	37943663	37972225	2.027585	0.119746
22088	Amp	14	37973496	37995538	1.706315	0.113239
22089	Amp	14	37996497	38009376	1.655056	0.112164
22090	Amp	14	38009764	38030587	1.737623	0.113818
22091	Amp	14	38030988	38052576	1.780186	0.114861
22092	Amp	14	38054369	38069148	1.748606	0.114138
22093	Amp	14	38069455	38077181	1.634701	0.111768
22094	Amp	14	38084786	38093067	1.329472	0.105025
22095	Amp	14	38096975	38233156	1.522708	0.109394
22096	Amp	14	38233467	38375683	1.833307	0.11592
22097	Amp	14	38376758	38397472	1.95165	0.11829
22098	Amp	14	38400801	38402727	1.833307	0.11592
22099	Amp	14	38404929	38462054	1.522708	0.109372
22100	Amp	14	38462128	38462128	1.388256	0.106299

22101 Amp	14	38462135	38462135	1.093834	0.099565
22102 Amp	14	38462150	38462150	0.98589	0.097018
22103 Amp	14	38462163	38462354	0.933745	0.095657
22104 Amp	14	38462461	38462461	1.014678	0.097527
22105 Amp	14	38462499	38462499	1.066493	0.098972
22106 Amp	14	38462510	38462510	1.206641	0.10222
22107 Amp	14	38462518	38481810	1.573014	0.110495
22108 Amp	14	38585376	38614208	1.319393	0.104777
22109 Amp	14	38615240	38673114	1.206641	0.102081
22110 Amp	14	38676533	38771504	1.159209	0.1011
22111 Amp	14	38777060	38823862	1.049301	0.098552
22112 Amp	14	38824390	39003697	1.032525	0.097933
22113 Amp	14	39003712	39058700	1.00505	0.09738
22114 Amp	14	39060167	39064301	0.959607	0.096337
22115 Amp	14	39065385	39217792	0.915698	0.095221
22116 Amp	14	39220574	39337363	0.968522	0.096622
22117 Amp	14	39340309	39357507	0.823771	0.092776
22118 Amp	14	39359606	39382691	0.873678	0.094023
22119 Amp	14	39384843	39401813	1.140417	0.100543
22120 Amp	14	39402962	39407832	0.72817	0.090214
22121 Amp	14	39409270	39423433	0.589605	0.086005
22122 Amp	14	39428009	39428558	0.470509	0.082239
22123 Amp	14	39428944	39434560	0.576183	0.085733
22124 Amp	14	39435162	39472384	0.508568	0.083423
22125 Amp	14	39473341	39545301	0.609133	0.086756
22126 Amp	14	39547384	39584427	0.644502	0.087853
22127 Amp	14	39591312	39622756	0.781972	0.091699
22128 Amp	14	39626653	39627734	0.735486	0.090451
22129 Amp	14	39627781	39763034	0.774648	0.091455
22130 Amp	14	39808104	39880158	0.735486	0.090424
22131 Amp	14	39880318	39939336	0.823771	0.092754
22132 Amp	14	39941162	40187113	0.774648	0.091483
22133 Amp	14	40187515	40211560	0.915698	0.095208
22134 Amp	14	40215121	40311747	1.058188	0.098645
22135 Amp	14	40311889	40366384	0.950886	0.096178
22136 Amp	14	40376359	40412601	0.915698	0.095132
22137 Amp	14	40440670	40639455	0.968522	0.096569
22138 Amp	14	40639857	40767119	0.873678	0.094239
22139 Amp	14	40775455	40787202	0.720659	0.090001
22140 Amp	14	40791035	40858889	0.840289	0.093349
22141 Amp	14	40863787	40907517	0.697374	0.089401
22142 Amp	14	40910057	41019628	0.583752	0.085907
22143 Amp	14	41019982	41020610	0.6737	0.088791
22144 Amp	14	41021353	41023225	0.765474	0.091345
22145 Amp	14	41027322	41035414	0.681869	0.088912
22146 Amp	14	41035905	41066013	0.287738	0.075961
22147 Amp	14	41066094	41137696	0.241616	0.073981
22148 Amp	14	41137910	41225810	0.210594	0.072581
22149 Amp	14	41226110	41229663	0.246818	0.074161
22150 Amp	14	41229722	41238007	0.193543	0.071982
22151 Amp	14	41242453	41276338	0.153838	0.070402
22152 Amp	14	41276583	41321114	0.1237	0.069233
22153 Amp	14	41321714	41360092	0.034113	0.065547

22154 Amp	14	41360444	41397386	0.015369	0.064671
22155 Amp	14	41398047	41417406	0.089561	0.067865
22156 Amp	14	41417740	41428887	0.293269	0.076114
22157 Amp	14	41435630	41438999	0.215608	0.072919
22158 Amp	14	41439105	41447855	0.015369	0.064671
22159 Amp	14	41452354	41553920	0.14896	0.070159
22160 Amp	14	41554339	41634591	0.241616	0.073841
22161 Amp	14	41635207	41676612	0.153838	0.07057
22162 Amp	14	41676645	41770229	0.215608	0.07295
22163 Amp	14	41770337	41840057	0.241616	0.073977
22164 Amp	14	41840400	41854699	0.282828	0.075604
22165 Amp	14	41855516	41868892	0.222041	0.073155
22166 Amp	14	41868908	41886568	0.282828	0.075647
22167 Amp	14	41893924	41906040	0.4115	0.080211
22168 Amp	14	41906267	41919942	0.335313	0.077592
22169 Amp	14	41926820	41928196	0.193543	0.072046
22170 Amp	14	41928293	41955942	0.170723	0.071018
22171 Amp	14	41960972	42000800	0	0.058957
22172 Amp	14	42001172	42022023	0	0.057519
22173 Amp	14	42022899	42034141	0	0.055821
22174 Amp	14	42045473	42045576	0	0.062336
22175 Amp	14	42046187	42084598	0	0.061814
22176 Amp	14	42089560	42143845	0	0.053557
22177 Amp	14	42147693	42164869	0	0.052847
22178 Amp	14	42175729	42181492	0	0.055706
22179 Amp	14	42186072	42205355	0	0.054063
22180 Amp	14	42251301	42259249	0	0.055113
22181 Amp	14	42260417	42260417	0	0.057945
22182 Amp	14	42260543	42276683	0	0.06032
22183 Amp	14	42276916	42322700	0	0.061722
22184 Amp	14	42326090	42496576	0	0.059596
22185 Amp	14	42496594	42496594	0	0.052369
22186 Amp	14	42622221	42631598	0	0.054681
22187 Amp	14	42637857	42668190	0	0.051439
22188 Amp	14	42671073	42730017	0	0.054989
22189 Amp	14	42730457	42808412	0	0.053471
22190 Amp	14	42808441	42867625	0	0.055243
22191 Amp	14	42867766	43018219	0	0.052869
22192 Amp	14	43023248	43054357	0	0.051693
22193 Amp	14	43054492	43088467	0	0.050832
22194 Amp	14	43090904	43098646	0	0.049829
22195 Amp	14	43100455	43136619	0	0.050995
22196 Amp	14	43140556	43145390	0	0.056085
22197 Amp	14	43149419	43161696	0	0.05843
22198 Amp	14	43161853	43204129	0	0.062066
22199 Amp	14	43206126	43236415	0.001323	0.063874
22200 Amp	14	43237809	43259827	0	0.062066
22201 Amp	14	43264666	43277628	0	0.05843
22202 Amp	14	43280768	43316291	0	0.060306
22203 Amp	14	43317347	43317347	0	0.057421
22204 Amp	14	43318881	43349029	0	0.058514
22205 Amp	14	43350333	43350333	0	0.057112
22206 Amp	14	43350726	43350726	0	0.0594

22207	Amp	14	43779976	43781302	0	0.05851
22208	Amp	14	43781620	43817338	0	0.055762
22209	Amp	14	43817933	43845670	0	0.057766
22210	Amp	14	43847089	43858346	0	0.05589
22211	Amp	14	43858621	43859381	0	0.06081
22212	Amp	14	44031914	44065957	0	0.060431
22213	Amp	14	44067092	44139380	0	0.058976
22214	Amp	14	44139597	44184986	0	0.061048
22215	Amp	14	44279793	44314815	0	0.057585
22216	Amp	14	44315448	44350506	0	0.057085
22217	Amp	14	44465304	44465399	0	0.056362
22218	Amp	14	44470676	44494498	0	0.059351
22219	Amp	14	44495083	44497550	0	0.060942
22220	Amp	14	44507909	44510812	0	0.056022
22221	Amp	14	44514220	44516152	0	0.053733
22222	Amp	14	44517428	44525094	0	0.050745
22223	Amp	14	44527346	44550595	0	0.054399
22224	Amp	14	44550791	44566748	0	0.052327
22225	Amp	14	44583115	44606751	0	0.049495
22226	Amp	14	44790006	44790006	0	0.047349
22227	Amp	14	44790792	44911269	0	0.045669
22228	Amp	14	44914555	45048037	0	0.048297
22229	Amp	14	45049006	45052380	0	0.050568
22230	Amp	14	45061885	45120222	0	0.042234
22231	Amp	14	45124870	45317636	0	0.044506
22232	Amp	14	45324279	45593536	0	0.040651
22233	Amp	14	45600770	45627788	0	0.049756
22234	Amp	14	45630702	45630880	0	0.053878
22235	Amp	14	45636715	45675938	0	0.044773
22236	Amp	14	45680126	45761842	0	0.049703
22237	Amp	14	45776348	45776348	0	0.047875
22238	Amp	14	45777220	45804580	0	0.045603
22239	Amp	14	45807340	45881899	0	0.061539
22240	Amp	14	45881943	45881943	0	0.059948
22241	Amp	14	45881968	45934348	0	0.055826
22242	Amp	14	45937167	46006727	0	0.059691
22243	Amp	14	46013196	46019290	0	0.042318
22244	Amp	14	46022625	46111930	0	0.04464
22245	Amp	14	46114465	46122974	0	0.042318
22246	Amp	14	46123697	46136401	0	0.045498
22247	Amp	14	46139772	46154521	0	0.050041
22248	Amp	14	46161083	46262865	0	0.046861
22249	Amp	14	46263557	46342732	0	0.052299
22250	Amp	14	46402212	46446828	0	0.055559
22251	Amp	14	46447605	46447605	0	0.056539
22252	Amp	14	46447844	46470761	0	0.059182
22253	Amp	14	46471110	46507940	0	0.058202
22254	Amp	14	46514775	46525368	0	0.052764
22255	Amp	14	46526648	46539309	0	0.053673
22256	Amp	14	46539576	46702084	0	0.057254
22257	Amp	14	46708629	46709246	0	0.061666
22258	Amp	14	46709716	46847301	0	0.059023
22259	Amp	14	46849325	46849487	0	0.060229

22260	Amp	14	46854924	46887549	0.024438	0.065041
22261	Amp	14	46889851	46901236	0.001323	0.063948
22262	Amp	14	46904911	46914493	0	0.063039
22263	Amp	14	46915237	46940760	0	0.063638
22264	Amp	14	46941371	47065050	0	0.059226
22265	Amp	14	47065103	47094343	0	0.061354
22266	Amp	14	47096279	47104079	0.138391	0.069905
22267	Amp	14	47112060	47112060	0.056211	0.066324
22268	Amp	14	47112418	47126775	0.079652	0.067356
22269	Amp	14	47126827	47145065	0.094967	0.068067
22270	Amp	14	47151013	47185331	0	0.063524
22271	Amp	14	47185658	47257148	0	0.062474
22272	Amp	14	47264041	47320887	0	0.06073
22273	Amp	14	47325301	47325301	0	0.063367
22274	Amp	14	47325910	47345098	0	0.062656
22275	Amp	14	47346916	47347555	0	0.060535
22276	Amp	14	47352831	47356933	0	0.061866
22277	Amp	14	47358246	47461054	0	0.057054
22278	Amp	14	47465804	47682044	0	0.05505
22279	Amp	14	47682230	47750079	0	0.055676
22280	Amp	14	47754840	47822883	0	0.062389
22281	Amp	14	47823251	47870315	0.005328	0.064159
22282	Amp	14	47870422	47882204	0.024438	0.065132
22283	Amp	14	47884689	47890127	0	0.061872
22284	Amp	14	47890271	47898614	0	0.059568
22285	Amp	14	47911033	47914042	0	0.062937
22286	Amp	14	47914844	47938579	0	0.060452
22287	Amp	14	47942211	47967616	0	0.057083
22288	Amp	14	47968862	48024368	0	0.061634
22289	Amp	14	48026716	48029561	0	0.059434
22290	Amp	14	48047980	48132385	0	0.057422
22291	Amp	14	48140580	48185766	0	0.054884
22292	Amp	14	48186243	48201968	0	0.044944
22293	Amp	14	48204107	48264531	0	0.045776
22294	Amp	14	48397525	48412499	0	0.043139
22295	Amp	14	48418430	48497000	0	0.046133
22296	Amp	14	48504373	48594067	0	0.048089
22297	Amp	14	48595200	48624861	0	0.053725
22298	Amp	14	48625515	48626565	0	0.05794
22299	Amp	14	48629869	48645526	0	0.055984
22300	Amp	14	48655929	48709107	0	0.060381
22301	Amp	14	48709796	48713407	0	0.062303
22302	Amp	14	48715250	48807595	0.128324	0.069468
22303	Amp	14	48808458	48894816	0.19964	0.072267
22304	Amp	14	48896805	48958114	0.060686	0.066632
22305	Amp	14	48958142	48973374	0.029031	0.065258
22306	Amp	14	48975846	49015406	0.14896	0.070226
22307	Amp	14	49016386	49040344	0.044794	0.066011
22308	Amp	14	49044893	49143575	0.08435	0.06769
22309	Amp	14	49143746	49155641	0.100827	0.068213
22310	Amp	14	49159547	49208233	0.060686	0.066534
22311	Amp	14	49210010	49226045	0.118001	0.068879
22312	Amp	14	49226102	49232910	0.222041	0.073029

22313 Amp	14	49237849	49304962	0.311747	0.076706
22314 Amp	14	49305537	49363029	0.431198	0.080934
22315 Amp	14	49364377	49367053	0.368062	0.078616
22316 Amp	14	49367381	49373033	0.392611	0.079634
22317 Amp	14	49373319	49478241	0.569771	0.085503
22318 Amp	14	49478632	49480810	0.589605	0.086062
22319 Amp	14	49483928	49487908	0.6737	0.088738
22320 Amp	14	49488297	49488297	0.660254	0.088246
22321 Amp	14	49488298	49494239	0.622604	0.087228
22322 Amp	14	49496675	49504812	0.711903	0.089882
22323 Amp	14	49505194	49512812	0.765474	0.091339
22324 Amp	14	49513716	49564310	0.667587	0.088545
22325 Amp	14	49570418	49612460	0.704849	0.089553
22326 Amp	14	49614278	49617083	0.622604	0.087178
22327 Amp	14	49621529	49621529	0.950886	0.09617
22328 Amp	14	49625610	49697734	1.075952	0.099158
22329 Amp	14	49700290	49704120	1.041205	0.098279
22330 Amp	14	49706188	49749281	0.950886	0.096008
22331 Amp	14	49754113	49795020	1.023539	0.097759
22332 Amp	14	49799603	49806565	1.140417	0.100695
22333 Amp	14	49807502	49809310	1.23519	0.102763
22334 Amp	14	49814018	49816068	1.290368	0.10404
22335 Amp	14	49825419	49825419	1.445847	0.10769
22336 Amp	14	49836875	49849939	1.876242	0.116635
22337 Amp	14	49850568	49850568	2.26699	0.124436
22338 Amp	14	49855436	49858631	2.435195	0.127793
22339 Amp	14	49861352	49920319	2.356694	0.126305
22340 Amp	14	49921175	49926002	2.468223	0.128504
22341 Amp	14	49928172	49958974	2.490318	0.12895
22342 Amp	14	49962461	49984408	2.379392	0.126751
22343 Amp	14	49984857	50008297	2.468223	0.128448
22344 Amp	14	50010478	50010478	2.594754	0.130809
22345 Amp	14	50013690	50039464	2.479057	0.128741
22346 Amp	14	50041519	50043254	2.137445	0.121831
22347 Amp	14	50047519	50059746	2.192012	0.122934
22348 Amp	14	50062595	50066449	2.402099	0.127037
22349 Amp	14	50066647	50066647	2.278531	0.124676
22350 Amp	14	50069314	50135178	2.22459	0.123573
22351 Amp	14	50137243	50191357	2.116108	0.121551
22352 Amp	14	50195638	50228690	1.940776	0.11803
22353 Amp	14	50232682	50257242	2.300463	0.125268
22354 Amp	14	50258805	50345465	2.22459	0.12357
22355 Amp	14	50354710	50381349	1.463774	0.107947
22356 Amp	14	50386301	50389731	1.261401	0.103565
22357 Amp	14	50389923	50499898	1.243254	0.103006
22358 Amp	14	50502239	50511252	1.112463	0.099989
22359 Amp	14	50519378	50536162	1.140417	0.100592
22360 Amp	14	50536996	50554305	0.933745	0.095624
22361 Amp	14	50564924	50705501	0.891602	0.094616
22362 Amp	14	50708469	50766381	0.848692	0.093466
22363 Amp	14	50770720	50773789	0.815228	0.092634
22364 Amp	14	50774771	50796695	0.848692	0.093581
22365 Amp	14	50808631	50828040	0.977077	0.09671

22366	Amp	14	50830010	50835739	1.33853	0.105154
22367	Amp	14	50839114	50870838	1.583409	0.11056
22368	Amp	14	50875135	50878004	1.445847	0.107566
22369	Amp	14	50879791	50914129	1.522708	0.109257
22370	Amp	14	50914568	50914568	1.445847	0.107524
22371	Amp	14	50920836	50966372	1.075952	0.09908
22372	Amp	14	50966436	51041407	1.197658	0.10198
22373	Amp	14	51046095	51106799	1.261401	0.103553
22374	Amp	14	51108547	51108547	1.388256	0.106215
22375	Amp	14	51111036	51220924	1.319393	0.104827
22376	Amp	14	51224807	51286575	1.378521	0.106097
22377	Amp	14	51286831	51304885	1.252022	0.103305
22378	Amp	14	51305863	51355651	1.049301	0.098479
22379	Amp	14	51356948	51405736	0.857296	0.093736
22380	Amp	14	51407259	51409306	0.977077	0.096813
22381	Amp	14	51411632	51440645	0.704849	0.089546
22382	Amp	14	51442444	51541338	1.023539	0.097739
22383	Amp	14	51541486	51544788	0.899693	0.094839
22384	Amp	14	51545773	51558548	0.995635	0.097153
22385	Amp	14	51564094	51614849	0.681869	0.08896
22386	Amp	14	51615143	51619877	0.891602	0.094478
22387	Amp	14	51619912	51668074	0.681869	0.08896
22388	Amp	14	51673111	51683243	0.595248	0.086298
22389	Amp	14	51685714	51704373	0.576183	0.085695
22390	Amp	14	51705314	51726404	1.178324	0.101526
22391	Amp	14	51727689	51737490	1.075952	0.099181
22392	Amp	14	51741299	51770668	0.98589	0.096868
22393	Amp	14	51778386	51808935	0.950886	0.096066
22394	Amp	14	51808979	51847141	1.014678	0.0975
22395	Amp	14	51849521	51855540	0.79909	0.092239
22396	Amp	14	51857135	51878599	0.735486	0.090489
22397	Amp	14	51883056	51905533	0.79909	0.092135
22398	Amp	14	51906635	51978945	0.689884	0.089147
22399	Amp	14	51979401	52077506	0.630438	0.087456
22400	Amp	14	52080394	52225867	0.660254	0.08832
22401	Amp	14	52227762	52274464	0.522881	0.083949
22402	Amp	14	52274928	52522617	0.589605	0.086172
22403	Amp	14	52525827	52688809	0.508568	0.083543
22404	Amp	14	52689898	52718977	0.602643	0.086525
22405	Amp	14	52723013	52763813	0.704849	0.089498
22406	Amp	14	52765587	52814591	0.781972	0.091699
22407	Amp	14	52817902	52873994	0.704849	0.089498
22408	Amp	14	52878660	52898083	0.536238	0.084305
22409	Amp	14	52899015	52913296	0.431198	0.080959
22410	Amp	14	52913373	52958790	0.342649	0.077917
22411	Amp	14	52961785	53082819	0.271552	0.075262
22412	Amp	14	53091029	53091455	0.19964	0.072289
22413	Amp	14	53100015	53101242	0.128324	0.069354
22414	Amp	14	53110094	53265215	0.227787	0.073316
22415	Amp	14	53303926	53303926	0.193543	0.072046
22416	Amp	14	53304015	53452591	0.1237	0.069064
22417	Amp	14	53454724	53514004	0.001323	0.063897
22418	Amp	14	53519000	53552168	0.056211	0.066292

22419	Amp	14	53553117	53576234	0.170723	0.071099
22420	Amp	14	53576265	53606762	0.073902	0.06729
22421	Amp	14	53614775	53626660	0.118001	0.068883
22422	Amp	14	53627058	53655187	0.222041	0.072966
22423	Amp	14	53655927	53671758	0.14896	0.070297
22424	Amp	14	53673006	53673006	0.210594	0.072657
22425	Amp	14	53675485	53709121	0.08435	0.067576
22426	Amp	14	53711764	53741331	0	0.060849
22427	Amp	14	53743125	53807316	0	0.06029
22428	Amp	14	53809506	53874830	0	0.044459
22429	Amp	14	53879079	54065005	0	0.0502
22430	Amp	14	54067825	54140627	0	0.048364
22431	Amp	14	54141044	54209518	0	0.047158
22432	Amp	14	54214950	54226750	0	0.050961
22433	Amp	14	54228014	54260517	0	0.049118
22434	Amp	14	54274562	54300931	0	0.051681
22435	Amp	14	54305715	54306475	0	0.047099
22436	Amp	14	54308216	54705088	0	0.04939
22437	Amp	14	54706891	54712634	0	0.053884
22438	Amp	14	54713625	54722365	0	0.050282
22439	Amp	14	54724785	54733130	0	0.04895
22440	Amp	14	54733196	54823654	0	0.046387
22441	Amp	14	54824977	54967240	0	0.048057
22442	Amp	14	54968074	55040395	0	0.050628
22443	Amp	14	55054205	55056071	0	0.045797
22444	Amp	14	55063402	55112795	0	0.055212
22445	Amp	14	55115502	55217522	0	0.057364
22446	Amp	14	55224984	55535704	0	0.054469
22447	Amp	14	55539215	55586643	0	0.055628
22448	Amp	14	55590332	55617359	0	0.05102
22449	Amp	14	55621138	55656283	0	0.055498
22450	Amp	14	55657022	55687034	0	0.054339
22451	Amp	14	55687269	55706969	0	0.051777
22452	Amp	14	55707387	55819990	0	0.053193
22453	Amp	14	55820993	55833711	0	0.048715
22454	Amp	14	55842433	55843513	0	0.040316
22455	Amp	14	55844240	55855729	0	0.036165
22456	Amp	14	55858405	55873273	0	0.038521
22457	Amp	14	55874271	55884904	0	0.03595
22458	Amp	14	55888216	55892226	0	0.041597
22459	Amp	14	55893356	56060387	0	0.03595
22460	Amp	14	56061402	56128027	0	0.037693
22461	Amp	14	56143814	56328430	0	0.04206
22462	Amp	14	56328948	56517117	0	0.039704
22463	Amp	14	56517528	56542364	0	0.04206
22464	Amp	14	56543220	56701785	0	0.039704
22465	Amp	14	56702243	56714781	0	0.038938
22466	Amp	14	56719234	56756748	0	0.036647
22467	Amp	14	56761197	56803107	0	0.038688
22468	Amp	14	56810965	56850295	0	0.041765
22469	Amp	14	56854094	56879139	0	0.045108
22470	Amp	14	56881874	56967581	0	0.043067
22471	Amp	14	56968100	56987591	0	0.045698

22472	Amp	14	56987721	57104583	0	0.048368
22473	Amp	14	57104636	57129912	0	0.051213
22474	Amp	14	57132842	57147088	0	0.048544
22475	Amp	14	57147841	57338290	0	0.045698
22476	Amp	14	57347605	57347605	0	0.043067
22477	Amp	14	57348066	57431060	0	0.045289
22478	Amp	14	57432710	57497770	0	0.047686
22479	Amp	14	57501799	57606176	0	0.044609
22480	Amp	14	57609610	57614830	0	0.041266
22481	Amp	14	57618073	57638536	0	0.04436
22482	Amp	14	57639002	57659928	0	0.046339
22483	Amp	14	57662452	57700342	0	0.048916
22484	Amp	14	57705974	57735657	0	0.04652
22485	Amp	14	57736005	57850806	0	0.042153
22486	Amp	14	57851332	57892280	0	0.046138
22487	Amp	14	57892741	57906710	0	0.049587
22488	Amp	14	57908490	57934656	0	0.048683
22489	Amp	14	57935816	57945253	0	0.050915
22490	Amp	14	57946281	57964067	0	0.04693
22491	Amp	14	57964369	57976449	0	0.048762
22492	Amp	14	57976653	58060797	0	0.047897
22493	Amp	14	58062362	58089063	0	0.04532
22494	Amp	14	58089716	58162172	0	0.041871
22495	Amp	14	58163072	58271010	0	0.03959
22496	Amp	14	58276223	58359332	0	0.041849
22497	Amp	14	58367588	58436290	0	0.043251
22498	Amp	14	58440701	58540086	0	0.041468
22499	Amp	14	58544028	58616299	0	0.038374
22500	Amp	14	58620743	58949420	0	0.042599
22501	Amp	14	58949890	59478128	0	0.040856
22502	Amp	14	59479741	59748292	0	0.044692
22503	Amp	14	59752771	59848095	0	0.040467
22504	Amp	14	59849042	60016003	0	0.039066
22505	Amp	14	60023392	60031304	0	0.036806
22506	Amp	14	60043738	60251558	0	0.029974
22507	Amp	14	60258339	60527726	0	0.034203
22508	Amp	14	60531064	60651301	0	0.036592
22509	Amp	14	60658222	60702455	0	0.039308
22510	Amp	14	60707300	61033331	0	0.041946
22511	Amp	14	61037395	61080670	0	0.041215
22512	Amp	14	61084802	61210679	0	0.043795
22513	Amp	14	61217620	61218874	0	0.041158
22514	Amp	14	61224982	61302808	0	0.0444
22515	Amp	14	61307628	61573593	0	0.04182
22516	Amp	14	61575830	61577576	0	0.042618
22517	Amp	14	61579917	61644488	0	0.040621
22518	Amp	14	61647174	61694969	0	0.042451
22519	Amp	14	61704251	61853694	0	0.040825
22520	Amp	14	61854706	62141151	0	0.042496
22521	Amp	14	62143264	62238419	0	0.043416
22522	Amp	14	62238887	62314807	0	0.041585
22523	Amp	14	62317715	62395353	0	0.038343
22524	Amp	14	62395987	62622605	0	0.039156

22525 Amp	14	62625523	62698735	0	0.041116
22526 Amp	14	62702996	62816578	0	0.042223
22527 Amp	14	62817062	62968806	0	0.040489
22528 Amp	14	62969239	62992112	0	0.039281
22529 Amp	14	62998746	63055944	0	0.035052
22530 Amp	14	63056017	63074756	0	0.033945
22531 Amp	14	63078670	63123203	0	0.03782
22532 Amp	14	63124371	63135152	0	0.037267
22533 Amp	14	63138827	63138827	0	0.036469
22534 Amp	14	63145478	63177396	0	0.033618
22535 Amp	14	63182087	63205286	0	0.029117
22536 Amp	14	63212204	63300126	0	0.025866
22537 Amp	14	63300268	63445499	0	0.027849
22538 Amp	14	63449056	63486734	0	0.031141
22539 Amp	14	63503815	63545037	0	0.033695
22540 Amp	14	63549300	63678680	0	0.031736
22541 Amp	14	63682171	64090713	0	0.031266
22542 Amp	14	64090912	64163328	0	0.033933
22543 Amp	14	64168445	64229656	0	0.031378
22544 Amp	14	64229671	64234466	0	0.028712
22545 Amp	14	64234738	64235606	0	0.031855
22546 Amp	14	64235663	64270143	0	0.028712
22547 Amp	14	64270290	64277954	0	0.030642
22548 Amp	14	64278162	64334312	0	0.028712
22549 Amp	14	64339114	64398643	0	0.022971
22550 Amp	14	64409915	64492594	0	0.019679
22551 Amp	14	64492835	64502750	0	0.020141
22552 Amp	14	64508121	64523371	0	0.02109
22553 Amp	14	64526559	64750193	0	0.023197
22554 Amp	14	64750506	64779905	0	0.025061
22555 Amp	14	64781655	64856375	0	0.022955
22556 Amp	14	64856894	64891858	0	0.02527
22557 Amp	14	64897939	64915046	0	0.023405
22558 Amp	14	64929078	64953234	0	0.029936
22559 Amp	14	64954556	64963610	0	0.024998
22560 Amp	14	64966177	64966613	0	0.027267
22561 Amp	14	64966960	64986345	0	0.029813
22562 Amp	14	64989807	64989807	0	0.025229
22563 Amp	14	64995503	65056025	0	0.018698
22564 Amp	14	65056619	65227202	0	0.016309
22565 Amp	14	65227517	65283067	0	0.012434
22566 Amp	14	65285606	65355775	0	0.009888
22567 Amp	14	65358893	65489320	0	0.012243
22568 Amp	14	65490179	65818956	0	0.012829
22569 Amp	14	65821256	65869635	0	0.015884
22570 Amp	14	65878651	65889605	0	0.025618
22571 Amp	14	65893576	65923546	0	0.03182
22572 Amp	14	65925680	65936880	0	0.035061
22573 Amp	14	65938712	65989316	0	0.029409
22574 Amp	14	65992675	66003971	0	0.026168
22575 Amp	14	66009998	66064386	0	0.015884
22576 Amp	14	66066743	66108173	0	0.018619
22577 Amp	14	66109938	66109938	0	0.018032

22578	Amp	14	66110503	66132588	0	0.016048
22579	Amp	14	66135923	66225392	0	0.013313
22580	Amp	14	66225596	66512775	0	0.012363
22581	Amp	14	66666062	66668441	0	0.009309
22582	Amp	14	66669952	66684733	0	0.014475
22583	Amp	14	66687882	66738028	0	0.009309
22584	Amp	14	66738569	66803405	0	0.010145
22585	Amp	14	66804385	67087403	0	0.013064
22586	Amp	14	67089810	67186915	0	0.015918
22587	Amp	14	67191396	67527666	0	0.012538
22588	Amp	14	67528911	67835727	0	0.012993
22589	Amp	14	67840070	67843012	0	0.014015
22590	Amp	14	67844914	68089714	0	0.01116
22591	Amp	14	68090227	68322266	0	0.009187
22592	Amp	14	68326837	68326987	0	0.012781
22593	Amp	14	68329475	68330213	0	0.011759
22594	Amp	14	68330226	68365970	0	0.014089
22595	Amp	14	68366097	68459751	0	0.014798
22596	Amp	14	68467973	68643813	0	0.012468
22597	Amp	14	68647648	68713254	0	0.011759
22598	Amp	14	68716080	68784198	0	0.008165
22599	Amp	14	68820874	68874888	0	0.010145
22600	Amp	14	68877453	69337662	0	0.008165
22601	Amp	14	69338207	71871942	0	0.00771
22602	Amp	14	71872504	72216599	0	0.009593
22603	Amp	14	72218104	72358023	0	0.008123
22604	Amp	14	72359459	72394148	0	0.008627
22605	Amp	14	72396443	72448413	0	0.010603
22606	Amp	14	72452928	72503794	0	0.010099
22607	Amp	14	72508738	72765547	0	0.008642
22608	Amp	14	72768742	72791736	0	0.009153
22609	Amp	14	72792053	73109015	0	0.008642
22610	Amp	14	73112892	73330961	0	0.011764
22611	Amp	14	73336954	73402929	0	0.009881
22612	Amp	14	73407146	73924660	0	0.006759
22613	Amp	14	73925724	74021285	0	0.012074
22614	Amp	14	74023561	74029765	0	0.010098
22615	Amp	14	74031630	74249800	0	0.009262
22616	Amp	14	74249972	74269097	0	0.016544
22617	Amp	14	74269638	74319930	0	0.009262
22618	Amp	14	74323634	74418103	0	0.003947
22619	Amp	14	74422808	74452422	0	0.004572
22620	Amp	14	74452905	74503325	0	0.020431
22621	Amp	14	74504671	74573635	0	0.019807
22622	Amp	14	74577327	74622392	0	0.003947
22623	Amp	14	74623033	74701003	0	0.006669
22624	Amp	14	74703092	74949406	0	0.003947
22625	Amp	14	74953428	75904187	0	0.001593
22626	Amp	14	75904424	76120078	0	0.002094
22627	Amp	14	76121191	76385056	0	0.002865
22628	Amp	14	76390182	77224697	0	0.004969
22629	Amp	14	77226368	77369569	0	0.007381
22630	Amp	14	77369693	77375705	0	0.00832

22631 Amp	14	77378918	77827045	0	0.006727
22632 Amp	14	77827751	78058228	0	0.005788
22633 Amp	14	78058615	78365884	0	0.004286
22634 Amp	14	78366348	78493912	0	0.003515
22635 Amp	14	78495739	78632935	0	0.004716
22636 Amp	14	78634788	78860929	0	0.005573
22637 Amp	14	78861581	79757711	0	0.003871
22638 Amp	14	79760880	79833404	0	0.002348
22639 Amp	14	79839392	80010388	0	0.004919
22640 Amp	14	80016189	80044934	0	0.004029
22641 Amp	14	80056767	80675347	0	0.001459
22642 Amp	14	80676964	81178673	0	0.000858
22643 Amp	14	81181534	81448415	0	0
22644 Amp	14	81449155	81603522	0	0.001813
22645 Amp	14	81607607	81683032	0	0.006216
22646 Amp	14	81686267	81714574	0	0.009274
22647 Amp	14	81721797	81744142	0	0.006216
22648 Amp	14	81746188	81951289	0	0.001813
22649 Amp	14	81955050	82175741	0	0.003196
22650 Amp	14	82179955	82634388	0	0.005243
22651 Amp	14	82634403	83055974	0	0.001383
22652 Amp	14	83058298	83088242	0	0.002618
22653 Amp	14	83094157	83185821	0	0.004767
22654 Amp	14	83196739	86339801	0	0.002618
22655 Amp	14	86347224	86403400	0	0.004718
22656 Amp	14	86406576	86551773	0	0.002618
22657 Amp	14	86555884	87067366	0	0.003774
22658 Amp	14	87073909	87314758	0	0.005432
22659 Amp	14	87318103	87420731	0	0.012403
22660 Amp	14	87420733	87919878	0	0.01415
22661 Amp	14	87920027	88196256	0	0.012994
22662 Amp	14	88198012	88270019	0	0.015572
22663 Amp	14	88272384	88318054	0	0.019117
22664 Amp	14	88320092	88443803	0	0.01654
22665 Amp	14	88455465	88527805	0	0.012994
22666 Amp	14	88533615	88674488	0	0.009755
22667 Amp	14	88676715	89488832	0	0.006023
22668 Amp	14	89489836	89563535	0	0.00668
22669 Amp	14	89567095	89634123	0	0.006023
22670 Amp	14	89637126	89657565	0	0.00814
22671 Amp	14	89659290	89686839	0	0.00678
22672 Amp	14	89692824	89710647	0	0.012952
22673 Amp	14	89711172	89827593	0	0.01473
22674 Amp	14	89828839	89929960	0	0.012952
22675 Amp	14	89930230	89935058	0	0.01437
22676 Amp	14	89935158	90122032	0	0.016763
22677 Amp	14	90129052	90156848	0	0.01976
22678 Amp	14	90159073	90192466	0	0.016763
22679 Amp	14	90192724	90295410	0	0.01437
22680 Amp	14	90300101	90848693	0	0.007442
22681 Amp	14	90849114	91246406	0	0.006023
22682 Amp	14	91247822	91396117	0	0.006742
22683 Amp	14	91397153	92075731	0	0.005083

22684	Amp	14	92075964	92830566	0	0.004365
22685	Amp	14	92833273	93849429	0	0.004846
22686	Amp	14	93852814	93864953	0	0.007595
22687	Amp	14	93867717	93955142	0	0.00576
22688	Amp	14	93955321	94110591	0	0.004846
22689	Amp	14	94113004	94365679	0	0.005343
22690	Amp	14	94372903	94512931	0	0.004846
22691	Amp	14	94512946	94727856	0	0.00313
22692	Amp	14	94734242	94763440	0	0.003814
22693	Amp	14	94776237	94842805	0	0.00313
22694	Amp	14	94845720	94891336	0	0.014666
22695	Amp	14	94893656	94936989	0	0.015982
22696	Amp	14	94939003	94951226	0	0.020383
22697	Amp	14	94953718	94996687	0	0.019067
22698	Amp	14	94999313	95251311	0	0.015147
22699	Amp	14	95254341	95255723	0	0.016276
22700	Amp	14	95264386	95304582	0	0.016856
22701	Amp	14	95308865	95518448	0	0.020361
22702	Amp	14	95520778	95536571	0	0.014682
22703	Amp	14	95538009	95566372	0	0.013299
22704	Amp	14	95567170	95567356	0	0.014209
22705	Amp	14	95568013	95746910	0	0.013629
22706	Amp	14	95748319	95857079	0	0.012719
22707	Amp	14	95859862	96524334	0	0.004005
22708	Amp	14	96535354	96574242	0	0.008488
22709	Amp	14	96574869	96725282	0	0.013688
22710	Amp	14	96725676	96808763	0	0.014779
22711	Amp	14	96808919	96812883	0	0.018534
22712	Amp	14	96821633	96821914	0	0.014051
22713	Amp	14	96825799	97028638	0	0.019251
22714	Amp	14	97029693	97055215	0	0.020723
22715	Amp	14	97055746	97232027	0	0.019251
22716	Amp	14	97233839	97287883	0	0.021116
22717	Amp	14	97290828	97564208	0	0.019251
22718	Amp	14	97568016	97624088	0	0.007721
22719	Amp	14	97625582	97789897	0	0.003967
22720	Amp	14	97790431	97934201	0	0.006953
22721	Amp	14	97936659	98707855	0	0.00784
22722	Amp	14	98728699	98783384	0	0.008337
22723	Amp	14	98783478	99102302	0	0.00745
22724	Amp	14	99115837	99341455	0	0.023386
22725	Amp	14	99343936	99500849	0	0.022295
22726	Amp	14	99501161	99576739	0	0.023496
22727	Amp	14	99578498	99765366	0	0.025146
22728	Amp	14	99765705	99856089	0	0.026319
22729	Amp	14	99858130	100022957	0	0.02736
22730	Amp	14	100027509	100027509	0	0.028472
22731	Amp	14	100030668	100065057	0	0.031243
22732	Amp	14	100065079	100230817	0	0.033636
22733	Amp	14	100236656	100386483	0	0.031243
22734	Amp	14	100387354	100444505	0	0.030071
22735	Amp	14	100451035	100485875	0	0.028958
22736	Amp	14	100487118	100532946	0	0.026187

22737	Amp	14	100547818	100632805	0	0.025146
22738	Amp	14	100633027	100669853	0	0.026218
22739	Amp	14	100673199	100728651	0	0.024568
22740	Amp	14	100731371	100814324	0	0.026895
22741	Amp	14	100815099	100998647	0	0.024568
22742	Amp	14	101000637	101048613	0	0.027419
22743	Amp	14	101049102	101063522	0	0.018566
22744	Amp	14	101063999	101088643	0	0.02248
22745	Amp	14	101088717	101131933	0	0.019503
22746	Amp	14	101132058	101247075	0	0.018302
22747	Amp	14	101250842	101380408	0	0.022958
22748	Amp	14	101384339	101480136	0	0.020107
22749	Amp	14	101480844	101537041	0	0.022838
22750	Amp	14	101543407	101624607	0	0.020107
22751	Amp	14	101630786	101687183	0	0.022112
22752	Amp	14	101753923	101943967	0	0.033934
22753	Amp	14	101945511	101996512	0	0.03447
22754	Amp	14	102002541	102035541	0	0.039116
22755	Amp	14	102036128	102040446	0	0.040576
22756	Amp	14	102042024	102114828	0	0.044062
22757	Amp	14	102117482	102397145	0	0.042576
22758	Amp	14	102398364	102405401	0	0.041504
22759	Amp	14	102413761	102465023	0	0.043066
22760	Amp	14	102466110	102703711	0	0.041504
22761	Amp	14	102703950	102730745	0	0.042484
22762	Amp	14	102736029	102799329	0	0.045086
22763	Amp	14	102806165	102884247	0	0.044106
22764	Amp	14	102885192	103200526	0	0.040946
22765	Amp	14	103201550	103252148	0	0.042382
22766	Amp	14	103252718	103403998	0	0.040946
22767	Amp	14	103407878	103462482	0	0.042929
22768	Amp	14	103466409	103471035	0	0.027001
22769	Amp	14	103473342	103496764	0	0.024399
22770	Amp	14	103498591	103590895	0	0.025861
22771	Amp	14	103590939	103626452	0	0.027174
22772	Amp	14	103627184	103796619	0	0.025191
22773	Amp	14	103800327	104126681	0	0.025685
22774	Amp	14	104128063	104313496	0	0.028228
22775	Amp	14	104314150	104314150	0	0.030546
22776	Amp	14	104480266	104481906	0	0.032533
22777	Amp	14	104484009	104559156	0	0.033224
22778	Amp	14	104568156	104673328	0	0.031236
22779	Amp	14	104673557	104674399	0	0.033732
22780	Amp	14	104683082	104749965	0	0.036061
22781	Amp	14	104750419	104818317	0	0.040146
22782	Amp	14	104819086	104903528	0	0.038141
22783	Amp	14	104907427	104957436	0	0.035812
22784	Amp	14	104958095	104994022	0	0.037334
22785	Amp	14	104994487	105034423	0	0.034413
22786	Amp	14	105037518	105081626	0	0.033878
22787	Amp	14	105095142	105099105	0	0.032355
22788	Amp	14	105101216	105341243	0	0.025952
22789	Amp	14	105354189	105360751	0	0.023457

22790	Amp	14	105362000	105437116	0	0.02485
22791	Amp	14	105446293	105521701	0	0.022307
22792	Amp	15	23442538	23890731	0	0.032618
22793	Amp	15	23891697	23965985	0	0.040695
22794	Amp	15	23973826	24076898	0	0.032618
22795	Amp	15	24077090	24652205	0	0.033084
22796	Amp	15	24655591	24713929	0	0.032319
22797	Amp	15	24724331	25207907	0	0.038753
22798	Amp	15	25208613	25241470	0	0.040594
22799	Amp	15	25243076	25403517	0	0.041922
22800	Amp	15	25403545	25936552	0	0.040594
22801	Amp	15	25936638	25938146	0	0.042966
22802	Amp	15	25940063	26016949	0	0.03859
22803	Amp	15	26017242	26068989	0	0.041563
22804	Amp	15	26070444	26076127	0	0.04054
22805	Amp	15	26078827	26145750	0	0.030292
22806	Amp	15	26146018	26224091	0	0.020071
22807	Amp	15	26224366	26279692	0	0.019605
22808	Amp	15	26280453	26388705	0	0.02079
22809	Amp	15	26388717	26437053	0	0.019971
22810	Amp	15	26437633	26661183	0	0.018786
22811	Amp	15	26661198	26794935	0	0.020089
22812	Amp	15	26801258	26945074	0	0.017043
22813	Amp	15	26945734	27060354	0	0.018372
22814	Amp	15	27062244	27098728	0	0.021821
22815	Amp	15	27104208	27456768	0	0.018372
22816	Amp	15	27458014	27621715	0	0.018937
22817	Amp	15	27623987	27709708	0	0.02091
22818	Amp	15	27709725	27853978	0	0.019581
22819	Amp	15	27855797	28213684	0	0.016167
22820	Amp	15	28217750	28315670	0	0.024806
22821	Amp	15	28315870	28315870	0	0.016167
22822	Amp	15	30839824	30855973	0	0.013253
22823	Amp	15	30856595	31114132	0	0.016978
22824	Amp	15	31116887	31307298	0	0.023192
22825	Amp	15	31308633	31364628	0	0.019466
22826	Amp	15	31367388	31500866	0	0.028772
22827	Amp	15	31502485	31522000	0	0.031604
22828	Amp	15	31522264	31626779	0	0.028772
22829	Amp	15	31634312	31664417	0	0.029414
22830	Amp	15	31664862	31664862	0	0.0288
22831	Amp	15	32636613	32702705	0	0.019696
22832	Amp	15	32705661	32870564	0	0.020758
22833	Amp	15	32871253	32914365	0	0.022163
22834	Amp	15	32914774	33368783	0	0.0211
22835	Amp	15	33368804	33955421	0	0.029689
22836	Amp	15	33955544	34128646	0	0.028967
22837	Amp	15	34133213	34366218	0	0.020378
22838	Amp	15	34692790	34697650	0	0.033875
22839	Amp	15	34698256	34714459	0	0.033031
22840	Amp	15	34714507	34720383	0	0.036652
22841	Amp	15	34723576	34734546	0	0.035741
22842	Amp	15	34736437	34740884	0	0.045166

22843	Amp	15	34745785	34814243	0	0.042026
22844	Amp	15	34814367	34853304	0	0.040224
22845	Amp	15	34855144	34916330	0	0.041208
22846	Amp	15	34916423	34916423	0	0.042933
22847	Amp	15	34922943	34979438	0	0.039312
22848	Amp	15	34985968	35023364	0	0.041014
22849	Amp	15	35023717	35262071	0	0.043138
22850	Amp	15	35265836	35338460	0	0.041435
22851	Amp	15	35338783	35920289	0	0.039312
22852	Amp	15	35922192	36457739	0	0.037418
22853	Amp	15	36459644	36462802	0	0.038703
22854	Amp	15	36468047	36514977	0	0.039903
22855	Amp	15	36518907	36576406	0	0.046896
22856	Amp	15	36576770	37090960	0	0.047694
22857	Amp	15	37111849	37262508	0	0.045969
22858	Amp	15	37262767	37372854	0	0.044769
22859	Amp	15	37372996	37554652	0	0.043633
22860	Amp	15	37556789	37557107	0	0.034208
22861	Amp	15	37557482	37686741	0	0.032922
22862	Amp	15	37687194	37810161	0	0.031518
22863	Amp	15	37812290	37826452	0	0.028358
22864	Amp	15	37830939	38003545	0	0.029718
22865	Amp	15	38034953	38433599	0	0.018486
22866	Amp	15	38441646	38448758	0	0.022519
22867	Amp	15	38450424	38547504	0	0.021159
22868	Amp	15	38550628	38737125	0	0.022192
22869	Amp	15	38737365	38808539	0	0.023545
22870	Amp	15	38809495	39013603	0	0.019511
22871	Amp	15	39021759	39370482	0	0.007675
22872	Amp	15	39377072	39394926	0	0.009407
22873	Amp	15	39396666	39595867	0	0.010157
22874	Amp	15	39598604	39608720	0	0.011432
22875	Amp	15	39611705	39637600	0	0.01286
22876	Amp	15	39637846	39668380	0	0.014474
22877	Amp	15	39668742	39717342	0	0.013676
22878	Amp	15	39717538	39746886	0	0.012401
22879	Amp	15	39756336	39777972	0	0.016169
22880	Amp	15	39778057	39778057	0	0.014741
22881	Amp	15	39787126	39973302	0	0.013991
22882	Amp	15	39977642	40142915	0	0.021771
22883	Amp	15	40144542	40468902	0	0.020418
22884	Amp	15	40472417	40580926	0	0.023664
22885	Amp	15	40585124	40715955	0	0.035068
22886	Amp	15	40717470	40856324	0	0.035753
22887	Amp	15	40861452	41343317	0	0.031515
22888	Amp	15	41343791	41537032	0	0.026716
22889	Amp	15	41537543	41623324	0	0.027215
22890	Amp	15	41625709	41645262	0	0.023969
22891	Amp	15	41657957	41745966	0	0.029635
22892	Amp	15	41749514	41833542	0	0.017835
22893	Amp	15	41833793	42095920	0	0.017336
22894	Amp	15	42100285	42457862	0	0.01999
22895	Amp	15	42461565	42533690	0	0.018376

22896	Amp	15	42538042	42570748	0	0.034312
22897	Amp	15	42594458	42765495	0	0.019387
22898	Amp	15	42770350	42944218	0	0.017076
22899	Amp	15	42944507	42986401	0	0.019387
22900	Amp	15	42990055	43063229	0	0.022431
22901	Amp	15	43065208	43159680	0	0.019387
22902	Amp	15	43164664	43351015	0	0.021633
22903	Amp	15	43355473	43381146	0	0.025604
22904	Amp	15	43384117	43437944	0	0.021633
22905	Amp	15	43444049	43553224	0	0.016952
22906	Amp	15	43553245	43553305	0	0.014706
22907	Amp	15	43722057	43859750	0	0.006128
22908	Amp	15	43860892	43883905	0	0.005164
22909	Amp	15	43885982	44116062	0	0.008822
22910	Amp	15	44122257	44210068	0	0.010875
22911	Amp	15	44215205	44474889	0	0.023153
22912	Amp	15	44478309	44542811	0	0.020535
22913	Amp	15	44546371	44568698	0	0.021485
22914	Amp	15	44570732	44634881	0	0.023348
22915	Amp	15	44637710	45177496	0	0.021295
22916	Amp	15	45179525	45521102	0	0.033835
22917	Amp	15	45525103	45795080	0	0.032802
22918	Amp	15	45801210	45933949	0	0.031088
22919	Amp	15	45939946	46789872	0	0.032602
22920	Amp	15	46797467	46883042	0	0.019283
22921	Amp	15	46883506	46960720	0	0.022301
22922	Amp	15	46963383	47072973	0	0.016863
22923	Amp	15	47075051	47109295	0	0.010409
22924	Amp	15	47114619	47175995	0	0.007391
22925	Amp	15	47178030	47214379	0	0.005597
22926	Amp	15	47216081	47251319	0	0.007392
22927	Amp	15	47252833	47282314	0	0.004881
22928	Amp	15	47282585	47419420	0	0.003931
22929	Amp	15	47420622	47572490	0	0.004475
22930	Amp	15	47573599	47854161	0	0.005856
22931	Amp	15	47857061	47882610	0	0.019785
22932	Amp	15	47883069	47900209	0	0.02332
22933	Amp	15	47913049	47937088	0	0.019785
22934	Amp	15	47943313	48432344	0	0.035326
22935	Amp	15	48432571	48648451	0	0.037333
22936	Amp	15	48652341	49050338	0	0.037833
22937	Amp	15	49053919	49067170	0	0.021897
22938	Amp	15	49067423	49149797	0	0.037833
22939	Amp	15	49151544	49178673	0	0.034953
22940	Amp	15	49185147	49418700	0	0.034453
22941	Amp	15	49422194	49804509	0	0.048971
22942	Amp	15	49809020	49832911	0	0.062943
22943	Amp	15	49839385	50017878	0.065715	0.06677
22944	Amp	15	50020831	50088543	0.112543	0.068697
22945	Amp	15	50090121	50192517	0.164935	0.071001
22946	Amp	15	50193286	50435102	0.073902	0.067174
22947	Amp	15	50438170	50545280	0.019169	0.06487
22948	Amp	15	50546548	50704424	0	0.062943

22949	Amp	15	50712499	50841358	0	0.06354
22950	Amp	15	50846387	51064427	0	0.062439
22951	Amp	15	51064531	51064531	0.040093	0.065641
22952	Amp	15	51071722	51090444	0.128324	0.069384
22953	Amp	15	51091997	51092193	0.050473	0.066181
22954	Amp	15	51093137	51215311	0	0.062439
22955	Amp	15	51220374	51276084	0	0.061057
22956	Amp	15	51281336	51329931	0	0.063646
22957	Amp	15	51334335	51368349	0	0.063001
22958	Amp	15	51369345	51488938	0.044794	0.065974
22959	Amp	15	51489215	51507342	0.069848	0.067075
22960	Amp	15	51511251	51609079	0	0.063319
22961	Amp	15	51609160	52121119	0	0.059684
22962	Amp	15	52122964	52224534	0.019169	0.064864
22963	Amp	15	52225416	52305314	0.112543	0.068624
22964	Amp	15	52310309	52340472	0.143787	0.070058
22965	Amp	15	52343186	52495854	0	0.057002
22966	Amp	15	52507612	52548669	0	0.055568
22967	Amp	15	52551319	52576902	0	0.057521
22968	Amp	15	52577384	52602818	0	0.059758
22969	Amp	15	52606318	52617128	0.034113	0.065506
22970	Amp	15	52620043	52630120	0	0.059758
22971	Amp	15	52630328	52657308	0	0.060265
22972	Amp	15	52657454	52668040	0	0.06177
22973	Amp	15	52673076	52681916	0	0.06101
22974	Amp	15	52684277	52686340	0	0.056742
22975	Amp	15	52689644	52706387	0	0.060373
22976	Amp	15	52711148	52865678	0	0.054505
22977	Amp	15	52868417	52869167	0	0.052773
22978	Amp	15	52869711	52880919	0	0.051268
22979	Amp	15	52881572	52895075	0	0.049315
22980	Amp	15	52895203	52959844	0	0.053042
22981	Amp	15	52960043	53281072	0	0.041282
22982	Amp	15	53281653	53580043	0	0.038308
22983	Amp	15	53586218	53683169	0	0.040151
22984	Amp	15	53683416	53783349	0	0.041862
22985	Amp	15	53790243	53900295	0	0.040018
22986	Amp	15	53902350	54097674	0	0.039525
22987	Amp	15	54097730	54119493	0	0.038308
22988	Amp	15	54123255	54131962	0	0.041394
22989	Amp	15	54133183	54144587	0	0.037666
22990	Amp	15	54145754	54518628	0	0.03707
22991	Amp	15	54521799	54562957	0	0.036057
22992	Amp	15	54563538	54642472	0	0.038925
22993	Amp	15	54646200	54811591	0	0.02542
22994	Amp	15	54813916	54859443	0	0.022552
22995	Amp	15	54862350	54977733	0	0.025295
22996	Amp	15	54978350	54980798	0	0.026053
22997	Amp	15	54983899	55059494	0	0.022967
22998	Amp	15	55061314	55114297	0	0.023497
22999	Amp	15	55114470	55151066	0	0.030016
23000	Amp	15	55151476	55329253	0	0.023497
23001	Amp	15	55330497	55452587	0	0.026028

23002 Amp	15	55455764	55638047	0	0.029637
23003 Amp	15	55638593	55675199	0	0.027106
23004 Amp	15	55676341	55798532	0	0.02768
23005 Amp	15	55799342	55936478	0	0.024532
23006 Amp	15	55954294	56134303	0	0.014834
23007 Amp	15	56141070	56186975	0	0.02869
23008 Amp	15	56187682	56219766	0	0.029333
23009 Amp	15	56234773	56244646	0	0.023781
23010 Amp	15	56248111	56335338	0	0.022522
23011 Amp	15	56341445	56416687	0	0.012655
23012 Amp	15	56417536	56736433	0	0.008667
23013 Amp	15	56740578	56755616	0	0.005971
23014 Amp	15	56756393	56817036	0	0.00643
23015 Amp	15	56819301	57235524	0	0.010779
23016 Amp	15	57235888	57279578	0	0.011269
23017 Amp	15	57284727	57291103	0	0.012511
23018 Amp	15	57296832	57311023	0	0.01202
23019 Amp	15	57311171	57550264	0	0.01252
23020 Amp	15	57551693	57588496	0	0.011279
23021 Amp	15	57590348	57662930	0	0.017069
23022 Amp	15	57663131	57840123	0	0.016569
23023 Amp	15	57841836	57899202	0	0.018017
23024 Amp	15	57904258	57915967	0	0.016569
23025 Amp	15	57917408	58133605	0	0.014283
23026 Amp	15	58134043	58254529	0	0.010779
23027 Amp	15	58255301	58543356	0	0.010021
23028 Amp	15	58547840	58949566	0	0.009477
23029 Amp	15	58953420	59073259	0	0.005128
23030 Amp	15	59077966	59166013	0	0.009787
23031 Amp	15	59166464	59242150	0	0.005128
23032 Amp	15	59243360	59374822	0	0.013956
23033 Amp	15	59374921	59670571	0	0.013312
23034 Amp	15	59670724	59682367	0	0.017538
23035 Amp	15	59684582	59819707	0	0.00871
23036 Amp	15	59820265	59836548	0	0.009417
23037 Amp	15	59840593	59848080	0	0.010422
23038 Amp	15	59848711	59933387	0	0.01106
23039 Amp	15	59933834	59975130	0	0.013981
23040 Amp	15	59975873	60018014	0	0.017936
23041 Amp	15	60019252	60109604	0	0.020741
23042 Amp	15	60119721	60291353	0	0.019736
23043 Amp	15	60294814	60301445	0	0.018983
23044 Amp	15	60304284	60436218	0	0.01624
23045 Amp	15	60439303	60488878	0	0.032177
23046 Amp	15	60489314	60920670	0	0.029372
23047 Amp	15	60920775	61025543	0	0.031194
23048 Amp	15	61026137	61047970	0	0.029372
23049 Amp	15	61050463	61116879	0	0.035806
23050 Amp	15	61118891	61152287	0	0.036434
23051 Amp	15	61154154	61186207	0	0.035806
23052 Amp	15	61190393	61192800	0	0.01987
23053 Amp	15	61196644	61312538	0	0.021219
23054 Amp	15	61316199	61418113	0	0.013039

23055	Amp	15	61426676	61461223	0	0.015484
23056	Amp	15	61462064	61550951	0	0.015026
23057	Amp	15	61554337	61612835	0	0.015501
23058	Amp	15	61613626	61671436	0	0.016272
23059	Amp	15	61673779	61683743	0	0.025091
23060	Amp	15	61683894	61756979	0	0.022645
23061	Amp	15	61757637	61886969	0	0.020285
23062	Amp	15	61892270	62047698	0	0.018936
23063	Amp	15	62047774	62498392	0	0.02428
23064	Amp	15	62498557	62558440	0	0.025313
23065	Amp	15	62563566	62586152	0	0.022392
23066	Amp	15	62587428	62650261	0	0.008229
23067	Amp	15	62654431	62706162	0	0.022515
23068	Amp	15	62708048	62807529	0	0.022039
23069	Amp	15	62807934	62842703	0	0.022791
23070	Amp	15	62843147	62953432	0	0.021758
23071	Amp	15	62956427	62978547	0	0.023077
23072	Amp	15	62980183	63030212	0	0.023605
23073	Amp	15	63030628	63068108	0	0.022287
23074	Amp	15	63068247	63158894	0	0.021535
23075	Amp	15	63168499	63236233	0	0.029192
23076	Amp	15	63236570	63367973	0	0.033082
23077	Amp	15	63368692	63414860	0	0.041665
23078	Amp	15	63418474	63487553	0	0.041136
23079	Amp	15	63499809	63565547	0	0.037247
23080	Amp	15	63574159	63576622	0	0.040481
23081	Amp	15	63580809	63664498	0	0.042425
23082	Amp	15	63667706	63690248	0	0.043962
23083	Amp	15	63692573	63753699	0	0.036305
23084	Amp	15	63754921	63761731	0	0.034534
23085	Amp	15	63763973	63903687	0	0.035867
23086	Amp	15	63906378	63986009	0	0.043032
23087	Amp	15	63994278	64025657	0	0.041088
23088	Amp	15	64027947	64167826	0	0.040382
23089	Amp	15	64168384	64210741	0	0.043144
23090	Amp	15	64213037	64237819	0	0.045363
23091	Amp	15	64238193	64405730	0	0.042129
23092	Amp	15	64413511	64414127	0	0.033546
23093	Amp	15	64414840	64434847	0	0.037035
23094	Amp	15	64438853	64454008	0	0.034075
23095	Amp	15	64458302	64550255	0	0.029757
23096	Amp	15	64551800	64669323	0	0.03059
23097	Amp	15	64670201	64678580	0	0.027735
23098	Amp	15	64678599	64695106	0	0.029215
23099	Amp	15	64696783	64867521	0	0.027149
23100	Amp	15	64868787	64928867	0	0.03098
23101	Amp	15	64929032	64987252	0	0.02655
23102	Amp	15	64991096	65265798	0	0.025779
23103	Amp	15	65270094	65345867	0	0.022931
23104	Amp	15	65348535	65638686	0	0.022099
23105	Amp	15	65641673	65930869	0	0.0252
23106	Amp	15	65933960	65940586	0	0.026297
23107	Amp	15	65946706	65973726	0	0.036782

23108	Amp	15	65973752	65983629	0	0.03833
23109	Amp	15	65984217	66001603	0	0.042564
23110	Amp	15	66005378	66224291	0	0.03833
23111	Amp	15	66225168	66331607	0	0.044487
23112	Amp	15	66332048	66445491	0	0.049913
23113	Amp	15	66449049	66686572	0	0.042552
23114	Amp	15	66687647	66865377	0	0.041218
23115	Amp	15	66866420	66917568	0	0.050646
23116	Amp	15	66919484	66981544	0	0.042212
23117	Amp	15	66983907	66993086	0	0.041573
23118	Amp	15	66995035	67026103	0	0.047517
23119	Amp	15	67026770	67029967	0	0.048136
23120	Amp	15	67032468	67109128	0	0.033851
23121	Amp	15	67109250	67257291	0	0.034677
23122	Amp	15	67257512	67350280	0	0.033129
23123	Amp	15	67350726	67374397	0	0.035321
23124	Amp	15	67379674	67448066	0	0.029376
23125	Amp	15	67455119	67523161	0	0.027184
23126	Amp	15	67523530	67754769	0	0.029168
23127	Amp	15	67758439	67931258	0	0.023742
23128	Amp	15	67934294	68107110	0	0.022645
23129	Amp	15	68109870	68144270	0	0.024951
23130	Amp	15	68145377	68214934	0	0.02681
23131	Amp	15	68216498	68225272	0	0.024504
23132	Amp	15	68231034	68547753	0	0.026047
23133	Amp	15	68552896	68628723	0	0.027135
23134	Amp	15	68629185	68654906	0	0.025151
23135	Amp	15	68655289	68717986	0	0.028858
23136	Amp	15	68718290	68930196	0	0.030207
23137	Amp	15	68931661	68953434	0	0.033254
23138	Amp	15	68956533	69069722	0	0.029547
23139	Amp	15	69075428	69111248	0	0.03176
23140	Amp	15	69114915	69269179	0	0.032207
23141	Amp	15	69275907	69499406	0	0.035889
23142	Amp	15	69501614	69608648	0	0.036536
23143	Amp	15	69610286	69705579	0	0.035187
23144	Amp	15	69712013	70046915	0	0.033643
23145	Amp	15	70051599	70063299	0	0.036452
23146	Amp	15	70065452	70084478	0	0.042802
23147	Amp	15	70084831	70105991	0	0.045761
23148	Amp	15	70109756	70290997	0	0.042952
23149	Amp	15	70291473	70373981	0	0.03927
23150	Amp	15	70378193	70459734	0	0.038823
23151	Amp	15	70461676	70540322	0	0.041512
23152	Amp	15	70541154	70606917	0	0.038823
23153	Amp	15	70609824	70712111	0	0.040332
23154	Amp	15	70712386	70736132	0	0.038887
23155	Amp	15	70738872	70741906	0	0.042897
23156	Amp	15	70741930	70741930	0	0.038887
23157	Amp	15	70741998	70815796	0	0.039693
23158	Amp	15	70827738	70859462	0	0.038184
23159	Amp	15	70860987	71012407	0	0.039339
23160	Amp	15	71014531	71021697	0	0.042769

23161	Amp	15	71026195	71026195	0	0.044628
23162	Amp	15	71034680	71085067	0	0.04672
23163	Amp	15	71089720	71130251	0	0.0462
23164	Amp	15	71131670	71144384	0	0.044341
23165	Amp	15	71145371	71162078	0	0.045839
23166	Amp	15	71166541	71324122	0	0.042409
23167	Amp	15	71347295	71398455	0	0.040837
23168	Amp	15	71398544	71400788	0	0.039682
23169	Amp	15	71401599	71423880	0	0.040126
23170	Amp	15	71426454	71509548	0	0.040575
23171	Amp	15	71509681	71601519	0	0.042073
23172	Amp	15	71602954	71627820	0	0.043376
23173	Amp	15	71628680	71632862	0	0.043868
23174	Amp	15	71643162	71643162	0	0.049212
23175	Amp	15	71645755	71650806	0	0.047714
23176	Amp	15	71651071	71653156	0	0.047265
23177	Amp	15	71653623	71681934	0	0.040915
23178	Amp	15	71683615	71737727	0	0.042912
23179	Amp	15	71738040	71885555	0	0.0458
23180	Amp	15	71901920	71901920	0	0.044995
23181	Amp	15	71906597	71927647	0	0.044551
23182	Amp	15	71929379	72011895	0	0.043249
23183	Amp	15	72013336	72016833	0	0.044687
23184	Amp	15	72018989	72037752	0	0.058623
23185	Amp	15	72039917	72078324	0	0.062152
23186	Amp	15	72080475	72233551	0	0.062598
23187	Amp	15	72236768	72255139	0	0.0606
23188	Amp	15	72255203	72414079	0.024438	0.064938
23189	Amp	15	72416883	72451117	0	0.059749
23190	Amp	15	72453092	72462978	0	0.063451
23191	Amp	15	72464303	72489956	0	0.059922
23192	Amp	15	72498526	72499815	0	0.063554
23193	Amp	15	72500479	72590887	0.107017	0.068479
23194	Amp	15	72595787	72685861	0.019169	0.064777
23195	Amp	15	72686275	72732039	0.050473	0.066118
23196	Amp	15	72732295	72766503	0.094967	0.068018
23197	Amp	15	72771088	72778941	0.153838	0.070459
23198	Amp	15	72784861	72874361	0	0.063775
23199	Amp	15	72875153	72912144	0	0.061738
23200	Amp	15	72913875	72934317	0	0.0603
23201	Amp	15	72934863	72992619	0	0.051189
23202	Amp	15	72993654	73066854	0	0.049288
23203	Amp	15	73071046	73081553	0	0.052142
23204	Amp	15	73081924	73117475	0	0.055269
23205	Amp	15	73121678	73194270	0	0.052415
23206	Amp	15	73196183	73311631	0	0.052887
23207	Amp	15	73317248	73355527	0	0.061484
23208	Amp	15	73364427	73426579	0	0.058437
23209	Amp	15	73427514	73518761	0	0.056621
23210	Amp	15	73526549	73531819	0	0.054408
23211	Amp	15	73536633	73795095	0	0.061793
23212	Amp	15	73795199	73802323	0	0.0638
23213	Amp	15	73806237	73806574	0.040093	0.065736

23214	Amp	15	73807791	73824208	0	0.060391
23215	Amp	15	73825171	74002789	0	0.058455
23216	Amp	15	74002854	74003049	0	0.055475
23217	Amp	15	74003063	74014065	0	0.055929
23218	Amp	15	74014082	74020453	0	0.050379
23219	Amp	15	74021632	74022119	0	0.025108
23220	Amp	15	74022307	74041072	0	0.056892
23221	Amp	15	74042396	74171842	0	0.055551
23222	Amp	15	74171859	74220887	0	0.053243
23223	Amp	15	74222965	74324289	0	0.049516
23224	Amp	15	74326035	74468392	0	0.045378
23225	Amp	15	74469916	74469916	0	0.047636
23226	Amp	15	74471540	74733647	0	0.0492
23227	Amp	15	74735539	74755085	0	0.055478
23228	Amp	15	74763486	74763486	0	0.0492
23229	Amp	15	74764017	75038405	0	0.050295
23230	Amp	15	75042019	75443118	0	0.049824
23231	Amp	15	75446325	75507150	0	0.053164
23232	Amp	15	75511181	75523890	0	0.059065
23233	Amp	15	75526393	75559588	0	0.056016
23234	Amp	15	75561490	75736946	0	0.056805
23235	Amp	15	75739839	75740986	0	0.054623
23236	Amp	15	75743585	75806710	0	0.06275
23237	Amp	15	75811388	75865169	0	0.052365
23238	Amp	15	75867552	75929595	0	0.045305
23239	Amp	15	75932604	75984088	0	0.047036
23240	Amp	15	75984318	76109822	0	0.046247
23241	Amp	15	76111446	76212128	0	0.05155
23242	Amp	15	76213565	76259787	0	0.054448
23243	Amp	15	76262881	76327772	0	0.058174
23244	Amp	15	76328231	76358361	0.040093	0.065579
23245	Amp	15	76367537	76409012	0.118001	0.068837
23246	Amp	15	76409454	76545880	0.073902	0.067106
23247	Amp	15	76549485	76723798	0.118001	0.068991
23248	Amp	15	76726952	76956020	0.293269	0.076047
23249	Amp	15	76965786	77127337	0.246818	0.074163
23250	Amp	15	77128639	77205573	0.299433	0.076235
23251	Amp	15	77214085	77255363	0.112543	0.068831
23252	Amp	15	77257092	77478931	0.08435	0.067735
23253	Amp	15	77479152	77515325	0.164935	0.070827
23254	Amp	15	77515595	77571364	0.069848	0.066962
23255	Amp	15	77571378	77652100	0.079652	0.067474
23256	Amp	15	77659818	77735999	0.044794	0.065944
23257	Amp	15	77737274	77801221	0	0.062685
23258	Amp	15	77801763	77865738	0.019169	0.064739
23259	Amp	15	77867048	77905451	0.222041	0.072965
23260	Amp	15	77908416	77947716	0.293269	0.076004
23261	Amp	15	77958923	77985922	0.404718	0.079973
23262	Amp	15	77989136	78004493	0.487495	0.082912
23263	Amp	15	78005137	78072617	0.47716	0.0824
23264	Amp	15	78078401	78085268	0.450702	0.081507
23265	Amp	15	78089318	78161677	0.667587	0.088552
23266	Amp	15	78162453	78345596	0.362016	0.078594

23267	Amp	15	78345891	78414707	0.431198	0.080806
23268	Amp	15	78419337	78510244	0.373599	0.078825
23269	Amp	15	78510527	78573551	0.342649	0.077867
23270	Amp	15	78573771	78714240	0.282828	0.075655
23271	Amp	15	78714427	78885397	0.342649	0.077881
23272	Amp	15	78888415	78910407	0.241616	0.073912
23273	Amp	15	78911232	78983388	0.215608	0.072752
23274	Amp	15	78985554	79001184	0.30524	0.076478
23275	Amp	15	79002705	79078071	0.287738	0.075936
23276	Amp	15	79080011	79321328	0.4115	0.080205
23277	Amp	15	79321864	79545479	0.431198	0.080895
23278	Amp	15	79546043	79602792	0.089561	0.067811
23279	Amp	15	79603250	79704322	0.210594	0.072675
23280	Amp	15	79704472	79785732	0.609133	0.086741
23281	Amp	15	79787205	79798192	0.47716	0.082337
23282	Amp	15	79801999	79802307	0.959607	0.096194
23283	Amp	15	79811676	79870680	1.058188	0.098715
23284	Amp	15	79874255	79882715	0.959607	0.096194
23285	Amp	15	79885941	79992774	1.149583	0.100881
23286	Amp	15	79994607	80112400	1.168655	0.101326
23287	Amp	15	80120830	80187879	0.630438	0.087468
23288	Amp	15	80191745	80282938	0.222041	0.073119
23289	Amp	15	80286498	80294338	0.569771	0.085496
23290	Amp	15	80299410	80323753	1.112463	0.099985
23291	Amp	15	80328156	80351284	1.261401	0.103371
23292	Amp	15	80351332	80384432	1.290368	0.104098
23293	Amp	15	80391133	80584550	1.014678	0.097645
23294	Amp	15	80591257	80631344	1.058188	0.09873
23295	Amp	15	80633809	80652064	1.675319	0.112598
23296	Amp	15	80657161	80676297	1.252022	0.103286
23297	Amp	15	80676775	80884653	1.041205	0.098271
23298	Amp	15	80885396	81114147	0.720659	0.090045
23299	Amp	15	81118764	81171054	0.79909	0.09214
23300	Amp	15	81171632	81234982	0.941704	0.095815
23301	Amp	15	81242479	81363621	0.751221	0.090952
23302	Amp	15	81366690	81423842	1.093834	0.099467
23303	Amp	15	81424717	81479994	1.215958	0.102285
23304	Amp	15	81480027	81480027	1.178324	0.101559
23305	Amp	15	81489579	81520668	1.225419	0.102621
23306	Amp	15	81526329	81626220	1.066493	0.098946
23307	Amp	15	81628453	81658614	0.924746	0.095341
23308	Amp	15	81807024	81820286	0.450702	0.081474
23309	Amp	15	81824409	82022443	0.417873	0.080412
23310	Amp	15	82023678	82088837	0.470509	0.082234
23311	Amp	15	82094117	82118710	0.386426	0.079336
23312	Amp	15	82119914	82240622	0.47716	0.082485
23313	Amp	15	82240659	82248157	0.322897	0.077168
23314	Amp	15	82251716	82289098	0.335313	0.07766
23315	Amp	15	82551194	82855505	0.697374	0.08932
23316	Amp	15	82855773	82868921	0.583752	0.085952
23317	Amp	15	82871501	82970206	0.329205	0.077436
23318	Amp	15	82970677	82981194	0.450702	0.08154
23319	Amp	15	82995889	83000399	0.899693	0.094828

23320 Amp	15	83000790	83029293	0.742723	0.090724
23321 Amp	15	83031360	83055560	0.857296	0.093687
23322 Amp	15	83056208	83099936	0.950886	0.09598
23323 Amp	15	83102944	83104159	1.032525	0.098069
23324 Amp	15	83109016	83133285	0.906385	0.094978
23325 Amp	15	83134794	83518114	0.550755	0.084781
23326 Amp	15	83518687	83604653	0.644502	0.087801
23327 Amp	15	83605251	83608398	0.742723	0.09075
23328 Amp	15	83609640	83665253	0.644502	0.08773
23329 Amp	15	83665822	83736822	0.899693	0.094742
23330 Amp	15	83737362	83806207	1.014678	0.097595
23331 Amp	15	83812327	84090586	0.899693	0.094742
23332 Amp	15	84095489	84111714	1.032525	0.097949
23333 Amp	15	84111812	84156533	0.915698	0.095187
23334 Amp	15	84158730	84158730	0.840289	0.093298
23335 Amp	15	84553803	84553803	0.720659	0.09009
23336 Amp	15	84554167	84581248	0.636464	0.08751
23337 Amp	15	84584141	84593584	0.6737	0.088713
23338 Amp	15	84597263	84648043	0.823771	0.092854
23339 Amp	15	84650802	84676218	0.807647	0.092361
23340 Amp	15	84680989	84744947	0.616549	0.086909
23341 Amp	15	84747382	84864649	0.891602	0.094579
23342 Amp	15	84868751	85001150	0.807647	0.092287
23343 Amp	15	85001223	85001898	0.758088	0.091084
23344 Amp	15	85006004	85101228	0.882843	0.094285
23345 Amp	15	85101690	85182267	0.968522	0.096556
23346 Amp	15	85183483	85183483	0.891602	0.094467
23347 Amp	15	85236347	85236347	1.041205	0.098199
23348 Amp	15	85236349	85236349	1.299876	0.104412
23349 Amp	15	85572645	85572645	1.33853	0.105115
23350 Amp	15	85575246	85580757	1.552619	0.109985
23351 Amp	15	85581324	85595629	1.215958	0.102314
23352 Amp	15	85597235	85705265	1.049301	0.098582
23353 Amp	15	85706353	85793403	1.075952	0.099178
23354 Amp	15	85796288	85859947	0.550755	0.084843
23355 Amp	15	85860499	85873583	0.508568	0.083582
23356 Amp	15	85876964	86069939	0.550755	0.084788
23357 Amp	15	86071436	86104574	0.644502	0.087836
23358 Amp	15	86108641	86112718	0.6737	0.088619
23359 Amp	15	86113754	86154769	1.243254	0.102948
23360 Amp	15	86158216	86166779	1.261401	0.103573
23361 Amp	15	86168094	86168094	1.33853	0.10517
23362 Amp	15	86172419	86233707	0.751221	0.090841
23363 Amp	15	86233762	86277508	0.807647	0.092305
23364 Amp	15	86277998	86507763	0.899693	0.094876
23365 Amp	15	86510328	86566977	0.941704	0.095943
23366 Amp	15	86571261	86612135	0.899693	0.094737
23367 Amp	15	86612325	86620426	1.041205	0.098289
23368 Amp	15	86623471	86625928	1.093834	0.099535
23369 Amp	15	86626040	86646028	1.426786	0.10715
23370 Amp	15	86646089	86756270	1.33853	0.105125
23371 Amp	15	86756890	86771849	1.369395	0.105781
23372 Amp	15	86773201	86828283	1.445847	0.107535

23373	Amp	15	86828667	86906653	1.369395	0.105781
23374	Amp	15	86909705	86997261	1.456037	0.107757
23375	Amp	15	86997644	87026654	1.727286	0.113731
23376	Amp	15	87026870	87070936	1.463774	0.107954
23377	Amp	15	87074296	87272407	1.206641	0.102168
23378	Amp	15	87274811	87274811	1.178324	0.101511
23379	Amp	15	87333928	87497038	1.416817	0.10688
23380	Amp	15	87497169	87507162	1.482378	0.108519
23381	Amp	15	87507832	87549553	1.252022	0.10315
23382	Amp	15	87607364	87613752	1.492063	0.108638
23383	Amp	15	87614230	87621043	1.552619	0.109977
23384	Amp	15	87625880	87830039	1.299876	0.104325
23385	Amp	15	87830355	87835563	1.252022	0.103205
23386	Amp	15	87840023	87885471	1.270711	0.10366
23387	Amp	15	87888102	87897911	1.112463	0.099889
23388	Amp	15	87901867	88049218	1.727286	0.113619
23389	Amp	15	88049241	88080107	1.769915	0.114641
23390	Amp	15	88081390	88159610	1.583409	0.110577
23391	Amp	15	88160709	88348266	1.369395	0.105917
23392	Amp	15	88355913	88403011	1.482378	0.108437
23393	Amp	15	88403321	88434327	1.645511	0.111886
23394	Amp	15	88436721	88437732	1.215958	0.102489
23395	Amp	15	88438248	88682543	1.472629	0.108304
23396	Amp	15	88693588	88761169	1.573014	0.110416
23397	Amp	15	88776705	88836619	1.522708	0.109311
23398	Amp	15	88836655	88846917	1.552619	0.109907
23399	Amp	15	88858820	88873742	2.147988	0.122181
23400	Amp	15	88875757	88929036	1.463774	0.108023
23401	Amp	15	88930574	88959758	1.542638	0.109726
23402	Amp	15	88960104	88998424	1.492063	0.108704
23403	Amp	15	89006295	89030246	1.552619	0.110059
23404	Amp	15	89032857	89058078	1.593878	0.110931
23405	Amp	15	89060497	89067856	1.522708	0.109228
23406	Amp	15	89067901	89084180	1.573014	0.110329
23407	Amp	15	89087028	89109321	1.472629	0.108217
23408	Amp	15	89109341	89172709	1.780186	0.114796
23409	Amp	15	89173187	89192666	1.854421	0.116369
23410	Amp	15	89192987	89205473	1.675319	0.112674
23411	Amp	15	89205622	89276830	1.624655	0.111573
23412	Amp	15	89285100	89365608	1.563118	0.110219
23413	Amp	15	89366095	89384958	1.426786	0.107092
23414	Amp	15	89388582	89533246	1.563118	0.110238
23415	Amp	15	89537001	89548145	2.33403	0.12592
23416	Amp	15	89549979	89624877	2.390849	0.126815
23417	Amp	15	89625462	89675716	2.468223	0.128508
23418	Amp	15	89683716	89733369	2.560422	0.130077
23419	Amp	15	89738690	89742946	2.514205	0.129307
23420	Amp	15	89744958	89756029	3.275143	0.143674
23421	Amp	15	89756283	89799539	3.150497	0.141326
23422	Amp	15	89800432	89953414	3.323812	0.144597
23423	Amp	15	89954611	90047456	3.29895	0.144039
23424	Amp	15	90049165	90049871	3.127699	0.140768
23425	Amp	15	90050715	90110279	3.230405	0.142862

23426	Amp	15	90115035	90115035	3.399017	0.145918
23427	Amp	15	90115098	90184008	3.874391	0.154373
23428	Amp	15	90187131	90209720	3.762283	0.152398
23429	Amp	15	90211234	90213060	3.862695	0.154165
23430	Amp	15	90216493	90233143	4.725297	0.167982
23431	Amp	15	90235099	90259484	3.749777	0.152045
23432	Amp	15	90260364	90261474	3.654159	0.150279
23433	Amp	15	90261581	90273107	3.492824	0.147635
23434	Amp	15	90277489	90298973	2.765626	0.134022
23435	Amp	15	90404326	90410416	3.713605	0.151542
23436	Amp	15	90411783	90426365	3.826187	0.153507
23437	Amp	15	90429209	90434118	3.057106	0.139449
23438	Amp	15	90434853	90456228	2.322689	0.125632
23439	Amp	15	90457213	90575782	2.290039	0.125008
23440	Amp	15	90578009	90626989	2.256542	0.124317
23441	Amp	15	90629366	90657927	2.083076	0.120855
23442	Amp	15	90659370	90672185	2.116108	0.121437
23443	Amp	15	90674815	90764180	2.213223	0.123433
23444	Amp	15	90765209	90799615	1.717671	0.113444
23445	Amp	15	90804401	90814996	1.624655	0.111448
23446	Amp	15	90815761	90828864	1.369395	0.105754
23447	Amp	15	90829468	90907294	1.522708	0.109336
23448	Amp	15	90908267	90911975	1.583409	0.110701
23449	Amp	15	90919176	90919176	1.299876	0.104418
23450	Amp	15	90920745	90941403	1.225419	0.102607
23451	Amp	15	90944035	90948530	1.093834	0.099569
23452	Amp	15	90949829	90953760	1.00505	0.097374
23453	Amp	15	90953847	90973533	0.704849	0.089499
23454	Amp	15	90974249	91000531	0.720659	0.089961
23455	Amp	15	91095131	91122455	1.225419	0.102669
23456	Amp	15	91122486	91129606	1.398396	0.106612
23457	Amp	15	91133330	91221758	1.482378	0.108534
23458	Amp	15	91223841	91253415	1.378521	0.106016
23459	Amp	15	91253663	91305942	1.512549	0.109078
23460	Amp	15	91314721	91331053	1.593878	0.110953
23461	Amp	15	91332997	91393360	1.696256	0.113009
23462	Amp	15	91393866	91403742	1.593878	0.110953
23463	Amp	15	91405098	91482986	1.748606	0.114169
23464	Amp	15	91484197	91528465	2.514205	0.129294
23465	Amp	15	91539671	91572550	2.537294	0.129757
23466	Amp	15	91574010	91625030	1.769915	0.114632
23467	Amp	15	91628670	91665538	2.537294	0.129726
23468	Amp	15	91665756	91710685	2.425182	0.127631
23469	Amp	15	91713973	91740785	2.560422	0.130279
23470	Amp	15	91741258	91779435	2.425182	0.127631
23471	Amp	15	91783034	91785036	2.33403	0.125757
23472	Amp	15	91786046	91799504	1.583409	0.110663
23473	Amp	15	91800166	91844938	1.68612	0.11287
23474	Amp	15	91854794	91889565	1.780186	0.114674
23475	Amp	15	91889853	91913532	1.665486	0.112406
23476	Amp	15	91913667	91943196	1.604336	0.111121
23477	Amp	15	91943920	91969962	1.655056	0.11224
23478	Amp	15	91970176	91983627	1.780186	0.114709

23479	Amp	15	91990684	91994615	1.655056	0.11224
23480	Amp	15	91995634	92019967	1.854421	0.116262
23481	Amp	15	92020855	92027220	1.897704	0.117064
23482	Amp	15	92032722	92039979	1.854421	0.116291
23483	Amp	15	92040462	92052969	1.897704	0.117064
23484	Amp	15	92052984	92063150	2.038694	0.119886
23485	Amp	15	92063216	92065393	2.060912	0.12044
23486	Amp	15	92067660	92152474	2.147988	0.122106
23487	Amp	15	92155347	92182708	2.060912	0.12044
23488	Amp	15	92183073	92234837	1.95165	0.118255
23489	Amp	15	92236688	92261512	1.823153	0.115735
23490	Amp	15	92261527	92267541	2.060912	0.120367
23491	Amp	15	92267889	92290259	2.213223	0.123451
23492	Amp	15	92291355	92327156	2.290039	0.125065
23493	Amp	15	92327365	92392217	2.137445	0.12198
23494	Amp	15	92395890	92437314	2.22459	0.123715
23495	Amp	15	92438604	92480269	2.137445	0.12198
23496	Amp	15	92480817	92496500	1.95165	0.118259
23497	Amp	15	92498834	92527512	1.854421	0.116173
23498	Amp	15	92527682	92549181	1.876242	0.116614
23499	Amp	15	92550919	92577885	1.994915	0.119103
23500	Amp	15	92579469	92613160	1.929887	0.117739
23501	Amp	15	92613580	92654567	2.379392	0.126715
23502	Amp	15	92656848	92656848	3.09333	0.140127
23503	Amp	15	92658632	92659015	3.435586	0.146505
23504	Amp	15	92662125	92673093	3.264473	0.143449
23505	Amp	15	92673227	92687776	3.150497	0.141169
23506	Amp	15	92688147	92729952	3.240397	0.143063
23507	Amp	15	92732011	92732579	3.505046	0.147684
23508	Amp	15	92737466	92750645	3.09333	0.140069
23509	Amp	15	92754769	92799030	2.986184	0.138175
23510	Amp	15	92801534	92801534	3.045223	0.139277
23511	Amp	15	92801862	92817550	3.160681	0.141404
23512	Amp	15	92817692	92842941	3.275143	0.143687
23513	Amp	15	92843533	92861590	3.458499	0.146996
23514	Amp	15	92869548	92869548	3.349067	0.144869
23515	Amp	15	92878014	92918945	3.160681	0.14156
23516	Amp	15	92921146	92950967	3.349067	0.144874
23517	Amp	15	92952227	93014380	3.483236	0.147318
23518	Amp	15	93018412	93036952	3.349067	0.144874
23519	Amp	15	93038458	93106210	3.009724	0.138738
23520	Amp	15	93106515	93107447	2.637742	0.131636
23521	Amp	15	93108090	93108090	2.626052	0.131545
23522	Amp	15	93466223	93486705	2.964305	0.137745
23523	Amp	15	93486971	93489370	2.837591	0.135473
23524	Amp	15	93728475	93728475	2.9061	0.136711
23525	Amp	15	93735122	93735122	2.765626	0.134024
23526	Amp	15	93735920	93735920	2.871582	0.13611
23527	Amp	15	93741940	93753197	2.776997	0.13439
23528	Amp	15	93756844	93826448	2.997552	0.138521
23529	Amp	15	93826903	93837508	2.89426	0.136468
23530	Amp	15	93839897	93910230	3.762283	0.152404
23531	Amp	15	93961485	94008378	4.024007	0.156782

23532	Amp	15	94008584	94090154	3.898065	0.154763
23533	Amp	15	94091012	94187469	3.654159	0.150386
23534	Amp	15	94195334	94199140	3.788687	0.152812
23535	Amp	15	94199669	94200141	3.738665	0.151917
23536	Amp	15	94202577	94233701	3.713605	0.151384
23537	Amp	15	94292369	94312684	2.837591	0.135448
23538	Amp	15	94315252	94321650	2.917729	0.13683
23539	Amp	15	94321990	94337909	3.057106	0.139483
23540	Amp	15	94338132	94358004	3.172599	0.141644
23541	Amp	15	94360592	94369307	3.103562	0.140384
23542	Amp	15	94474335	94498555	3.033246	0.139001
23543	Amp	15	94504423	94518825	3.6657	0.150672
23544	Amp	15	94529364	94564386	3.399017	0.145789
23545	Amp	15	94566317	94737983	3.218362	0.142588
23546	Amp	15	94738677	94771891	3.286256	0.143952
23547	Amp	15	94772486	94786479	3.264473	0.14349
23548	Amp	15	94789308	94982199	3.207115	0.142252
23549	Amp	15	94984218	95031550	3.127699	0.140888
23550	Amp	15	95032851	95118182	2.300463	0.125206
23551	Amp	15	95119012	95151966	2.202226	0.123125
23552	Amp	15	95152937	95162909	2.300463	0.125195
23553	Amp	15	95164617	95165104	2.094104	0.121064
23554	Amp	15	95167021	95196895	2.192012	0.122931
23555	Amp	15	95196958	95268143	2.083076	0.120861
23556	Amp	15	95270404	95276632	2.278531	0.124772
23557	Amp	15	95281199	95312953	2.390849	0.126922
23558	Amp	15	95313389	95315462	2.425182	0.12764
23559	Amp	15	95319847	95428751	2.917729	0.136804
23560	Amp	15	95429712	95459403	3.08058	0.139901
23561	Amp	15	95459741	95487297	2.917729	0.136804
23562	Amp	15	95488078	95543562	3.067933	0.139681
23563	Amp	15	95545223	95558405	2.964305	0.137814
23564	Amp	15	95560895	95608685	2.812598	0.134937
23565	Amp	15	95612302	95698057	2.626052	0.131568
23566	Amp	15	95698073	95709536	2.788392	0.134621
23567	Amp	15	95710014	95732805	2.848911	0.135557
23568	Amp	15	95733287	95743984	2.743935	0.13365
23569	Amp	15	95746501	95780223	2.390849	0.12689
23570	Amp	15	95780870	95788518	2.278531	0.124739
23571	Amp	15	95789303	95896376	2.446878	0.127986
23572	Amp	15	95898469	95968234	2.402099	0.12705
23573	Amp	15	95972674	96002576	2.614791	0.13117
23574	Amp	15	96006550	96040504	2.446878	0.127923
23575	Amp	15	96040589	96075616	2.425182	0.127481
23576	Amp	15	96076012	96126584	2.537294	0.12968
23577	Amp	15	96127379	96213426	2.425182	0.127481
23578	Amp	15	96214248	96218672	2.311329	0.125394
23579	Amp	15	96218929	96247065	2.390849	0.126818
23580	Amp	15	96247180	96247180	2.479057	0.128651
23581	Amp	15	96247377	96292052	2.696333	0.132878
23582	Amp	15	96295840	96427273	2.605213	0.131045
23583	Amp	15	96427728	96449583	2.73206	0.133489
23584	Amp	15	96449765	96453818	2.860424	0.135893

23585	Amp	15	96453868	96482897	2.986184	0.13811
23586	Amp	15	96483505	96483505	3.195626	0.142057
23587	Amp	15	96484257	96486527	3.240397	0.142901
23588	Amp	15	96487912	96564074	3.115161	0.140684
23589	Amp	15	96565470	96576681	3.874391	0.154216
23590	Amp	15	96578959	96603570	4.668674	0.167027
23591	Amp	15	96603613	96699212	4.264196	0.160708
23592	Amp	15	96699642	96716901	3.9978	0.156481
23593	Amp	15	96726551	96766524	3.887411	0.15458
23594	Amp	15	96767263	96851148	3.852343	0.153768
23595	Amp	15	96851386	96866185	3.908795	0.154946
23596	Amp	15	96869464	96884897	4.076315	0.157828
23597	Amp	15	96888838	96951177	4.037384	0.157111
23598	Amp	15	96951516	96951584	4.443348	0.163706
23599	Amp	15	96956075	96974212	4.513276	0.164646
23600	Amp	15	96974349	97005991	4.429656	0.163469
23601	Amp	15	97006387	97016555	4.882872	0.170265
23602	Amp	15	97017946	97078241	4.952273	0.171502
23603	Amp	15	97079551	97174531	5.067861	0.173273
23604	Amp	15	97174846	97189587	4.99404	0.172106
23605	Amp	15	97199791	97322373	5.271084	0.176151
23606	Amp	15	97326079	97386207	5.360902	0.177487
23607	Amp	15	97390145	97444347	5.300783	0.176547
23608	Amp	15	97447083	97497657	5.39004	0.177898
23609	Amp	15	97500892	97518580	5.59628	0.180974
23610	Amp	15	97518718	97536741	5.463735	0.17902
23611	Amp	15	97538471	97541159	6.291248	0.190911
23612	Amp	15	97542643	97564734	7.090354	0.202355
23613	Amp	15	97569110	97569827	7.961071	0.214232
23614	Amp	15	97573636	97577918	7.421526	0.206847
23615	Amp	15	97585807	97675673	6.291248	0.190911
23616	Amp	15	97676640	97689340	6.33531	0.191704
23617	Amp	15	97689787	97713917	6.488712	0.193732
23618	Amp	15	97714158	97736313	6.396376	0.192494
23619	Amp	15	97737685	97768686	6.473343	0.193586
23620	Amp	15	97770898	97874642	6.304987	0.19127
23621	Amp	15	97874742	97913890	6.167547	0.189242
23622	Amp	15	97918541	97965741	6.092521	0.188151
23623	Amp	15	97965977	97996702	6.229291	0.19008
23624	Amp	15	98012083	98030314	7.215959	0.204038
23625	Amp	15	98031318	98078219	7.833797	0.212544
23626	Amp	15	98080186	98118180	7.357785	0.206061
23627	Amp	15	98119562	98138085	7.547251	0.208551
23628	Amp	15	98138103	98142997	7.6425	0.209913
23629	Amp	15	98144718	98149196	7.801564	0.211991
23630	Amp	15	98150450	98243363	7.657996	0.210062
23631	Amp	15	98246145	98307263	7.500789	0.207984
23632	Amp	15	98311764	98334830	7.977624	0.214467
23633	Amp	15	98336596	98336596	8.088933	0.216024
23634	Amp	15	98337281	98363905	8.29898	0.218711
23635	Amp	15	98366955	98368802	9.240499	0.231145
23636	Amp	15	98369946	98371091	9.004172	0.228127
23637	Amp	15	98375054	98383127	8.802762	0.225439

23638	Amp	15	98386834	98399376	8.60549	0.222995
23639	Amp	15	98405824	98482370	8.704326	0.224153
23640	Amp	15	98493707	98494640	8.818686	0.225768
23641	Amp	15	98498578	98518981	9.122122	0.229547
23642	Amp	15	98519453	98521535	9.323109	0.232223
23643	Amp	15	98524312	98533887	9.20619	0.230666
23644	Amp	15	98534633	98576812	9.323109	0.232253
23645	Amp	15	98576923	98576923	9.39097	0.233193
23646	Amp	15	98578346	98589061	9.575888	0.235391
23647	Amp	15	98597342	98611432	9.39097	0.233193
23648	Amp	15	98611488	98627127	9.729466	0.237428
23649	Amp	15	98627267	98631771	10.000692	0.240893
23650	Amp	15	98631905	98660872	9.780264	0.23801
23651	Amp	15	98661796	98673765	9.847525	0.238951
23652	Amp	15	98675895	98690570	10.634596	0.248973
23653	Amp	15	98693273	98715787	10.877729	0.251991
23654	Amp	15	98716237	98754436	10.756361	0.250376
23655	Amp	15	98757533	98783431	10.482498	0.247097
23656	Amp	15	98787998	98821141	10.174276	0.243069
23657	Amp	15	98821622	98880797	10.448458	0.246459
23658	Amp	15	98881034	98944659	10.534461	0.247589
23659	Amp	15	98945499	98970002	10.448458	0.246648
23660	Amp	15	98978583	98996978	10.000692	0.240812
23661	Amp	15	98997029	99037386	10.174276	0.243073
23662	Amp	15	99039336	99056378	9.763565	0.23798
23663	Amp	15	99056730	99061812	9.47615	0.234201
23664	Amp	15	99062453	99127521	8.6219	0.223069
23665	Amp	15	99133735	99137986	7.626527	0.209749
23666	Amp	15	99139941	99146616	7.468873	0.207487
23667	Amp	15	99148672	99161466	8.233714	0.217826
23668	Amp	15	99162009	99166034	7.945028	0.21397
23669	Amp	15	99166428	99175147	7.865467	0.21284
23670	Amp	15	99177611	99179840	6.840574	0.198706
23671	Amp	15	99180057	99182650	6.626113	0.195828
23672	Amp	15	99186697	99202308	6.825391	0.19851
23673	Amp	15	99202358	99226657	7.027404	0.201429
23674	Amp	15	99226776	99233931	6.686496	0.196708
23675	Amp	15	99234337	99234337	6.213438	0.189826
23676	Amp	15	99235921	99235921	6.017563	0.187145
23677	Amp	15	99238337	99264236	5.911851	0.185558
23678	Amp	15	99267488	99267488	4.952273	0.171489
23679	Amp	15	99268352	99345453	4.640474	0.166585
23680	Amp	15	99346255	99363614	4.142958	0.158864
23681	Amp	15	99364066	99393794	4.037384	0.157126
23682	Amp	15	99397765	99514532	3.959323	0.155764
23683	Amp	15	99517721	99535267	4.076315	0.157857
23684	Amp	15	99542974	99572379	3.839479	0.153716
23685	Amp	15	99572652	99597529	3.690482	0.15104
23686	Amp	15	99598788	99619038	3.566107	0.148947
23687	Amp	15	99623543	99645216	3.471122	0.14708
23688	Amp	15	99653737	99674730	4.010586	0.156693
23689	Amp	15	99675053	99680173	4.169329	0.159213
23690	Amp	15	99681324	99707535	4.27644	0.160946

23691	Amp	15	99707989	99711969	3.701758	0.151211
23692	Amp	15	99715115	99759308	3.275143	0.143669
23693	Amp	15	99835668	99835668	3.207115	0.142377
23694	Amp	15	99836319	99837548	3.335819	0.144699
23695	Amp	15	99840164	99967042	3.566107	0.148936
23696	Amp	15	99968164	99968164	3.311026	0.144382
23697	Amp	15	100204464	100221475	4.250544	0.160557
23698	Amp	15	100221806	100243039	4.31834	0.161755
23699	Amp	15	100408652	100408652	3.985868	0.156238
23700	Amp	15	100415538	100422970	4.99404	0.172174
23701	Amp	15	100423550	100449634	5.052964	0.172971
23702	Amp	15	100541687	100542900	4.169329	0.159216
23703	Amp	15	100543482	100568082	4.076315	0.157806
23704	Amp	15	100569613	100595908	4.010586	0.156608
23705	Amp	15	100604708	100677761	3.862695	0.154119
23706	Amp	15	100682097	100689599	3.985868	0.156264
23707	Amp	15	100689668	100694975	4.129725	0.158635
23708	Amp	15	100695160	100709794	4.236663	0.160391
23709	Amp	15	100710686	100732193	4.102728	0.15821
23710	Amp	15	100735185	100792805	3.9978	0.156454
23711	Amp	15	100816310	100847281	4.156262	0.159079
23712	Amp	15	100847323	100875847	4.331178	0.161847
23713	Amp	15	100876537	100924710	4.183261	0.159525
23714	Amp	15	100927355	100996743	5.227494	0.175462
23715	Amp	15	100996951	101000996	5.081731	0.173473
23716	Amp	15	101003085	101014133	5.31534	0.176951
23717	Amp	15	101015509	101032805	5.360902	0.177406
23718	Amp	15	101033078	101066395	5.39004	0.177966
23719	Amp	15	101066942	101085446	5.125419	0.174048
23720	Amp	15	101085606	101175315	4.897301	0.17057
23721	Amp	15	101178495	101216255	4.640474	0.166681
23722	Amp	15	101220560	101241729	4.5989	0.166044
23723	Amp	15	101241884	101290039	4.196711	0.159713
23724	Amp	15	101293383	101293383	4.526699	0.164837
23725	Amp	15	101293916	101294724	4.47072	0.164057
23726	Amp	15	101304282	101309774	4.388129	0.162794
23727	Amp	15	101313378	101344124	4.5989	0.166061
23728	Amp	16	603333	618980	0	0.057908
23729	Amp	16	618985	618985	0	0.0587
23730	Amp	16	627854	627854	0	0.059483
23731	Amp	16	661905	722842	0	0.057598
23732	Amp	16	735279	738277	0	0.054384
23733	Amp	16	747786	749384	0	0.059541
23734	Amp	16	836376	930784	0	0.060634
23735	Amp	16	938889	942590	0.107017	0.068481
23736	Amp	16	944364	955666	0.187449	0.0717
23737	Amp	16	957268	1002943	0.170723	0.071183
23738	Amp	16	1039949	1084458	0.251218	0.074315
23739	Amp	16	1084683	1085845	0.233782	0.073495
23740	Amp	16	1087526	1184871	0.215608	0.072871
23741	Amp	16	1184989	1185314	0.362016	0.078455
23742	Amp	16	1201281	1238529	0.335313	0.077706
23743	Amp	16	1299186	1299186	0.4115	0.080196

23744	Amp	16	1299208	1303675	0.431198	0.080862
23745	Amp	16	1318009	1354829	0.508568	0.08346
23746	Amp	16	1364497	1509837	0.399144	0.079767
23747	Amp	16	1510885	1516381	0.417873	0.080462
23748	Amp	16	1517534	1542523	0.399144	0.079817
23749	Amp	16	1543271	1577467	0.379885	0.079181
23750	Amp	16	1581850	1615664	0.362016	0.078575
23751	Amp	16	1615722	1623884	0.238246	0.073678
23752	Amp	16	1629207	1634326	0.322897	0.077168
23753	Amp	16	1637704	1800445	0.311747	0.07672
23754	Amp	16	1801825	1812723	0.287738	0.075937
23755	Amp	16	1813078	1854505	0.276783	0.075406
23756	Amp	16	1854586	1938069	0.329205	0.077401
23757	Amp	16	1947613	2075833	0.392611	0.079487
23758	Amp	16	2095962	2128574	0.329205	0.077401
23759	Amp	16	2182793	2200206	0.34892	0.078114
23760	Amp	16	2204007	2317508	0.293269	0.07612
23761	Amp	16	2318012	2351818	0.271552	0.075316
23762	Amp	16	2357258	2365028	0.259438	0.074862
23763	Amp	16	2365690	2371235	0.355319	0.078268
23764	Amp	16	2379893	2518805	0.4115	0.080301
23765	Amp	16	2531358	2531461	0.238246	0.073684
23766	Amp	16	2537170	2741630	0.107017	0.068527
23767	Amp	16	2744306	2749691	0.089561	0.067891
23768	Amp	16	2754828	2860971	0.044794	0.065859
23769	Amp	16	2861319	2919696	0.024438	0.065007
23770	Amp	16	2919820	2949217	0.040093	0.065616
23771	Amp	16	2952823	2960509	0.094967	0.068149
23772	Amp	16	2962128	2985040	0.056211	0.066251
23773	Amp	16	2986550	3084264	0	0.063653
23774	Amp	16	3084753	3091937	0	0.061729
23775	Amp	16	3096027	3200228	0	0.061015
23776	Amp	16	3200250	3203161	0	0.057883
23777	Amp	16	3205707	3220459	0	0.05535
23778	Amp	16	3222363	3244475	0	0.058229
23779	Amp	16	3246230	3246230	0	0.061047
23780	Amp	16	3250044	3326686	0.170723	0.071143
23781	Amp	16	3326824	3357682	0.14896	0.070273
23782	Amp	16	3357842	3370814	0.193543	0.072068
23783	Amp	16	3375325	3609470	0.1237	0.069188
23784	Amp	16	3618935	3700619	0	0.062784
23785	Amp	16	3700974	3706847	0.019169	0.064762
23786	Amp	16	3711288	3767677	0.193543	0.071885
23787	Amp	16	3769780	3952319	0.368062	0.078659
23788	Amp	16	3954422	3960710	0.392611	0.079671
23789	Amp	16	3963466	4004689	0.368062	0.078643
23790	Amp	16	4004981	4073952	0.399144	0.07974
23791	Amp	16	4074299	4086417	0.34892	0.077968
23792	Amp	16	4087003	4162802	0.329205	0.077359
23793	Amp	16	4162913	4204915	0.34892	0.07798
23794	Amp	16	4208090	4301660	0.311747	0.076751
23795	Amp	16	4304040	4409948	0.322897	0.07721
23796	Amp	16	4410132	4501829	0.368062	0.078669

23797	Amp	16	4503191	4545747	0.386426	0.079365
23798	Amp	16	4548174	4723864	0.355319	0.078353
23799	Amp	16	4724236	4746145	0.379885	0.079251
23800	Amp	16	4748071	4769495	0.399144	0.07975
23801	Amp	16	4770190	4791379	0.373599	0.078957
23802	Amp	16	4791425	4814100	0.399144	0.079805
23803	Amp	16	4814178	4815632	0.417873	0.080399
23804	Amp	16	4819092	4837669	0.431198	0.080888
23805	Amp	16	4839070	4858955	0.450702	0.081475
23806	Amp	16	4859313	4869332	0.470509	0.082161
23807	Amp	16	4869595	4899945	0.482379	0.082626
23808	Amp	16	4902193	4907380	0.500841	0.08324
23809	Amp	16	4910938	5003397	0.444474	0.081263
23810	Amp	16	5004997	5006294	0.4115	0.080281
23811	Amp	16	5006935	5012080	0.373599	0.078904
23812	Amp	16	5012356	5057989	0.355319	0.078367
23813	Amp	16	5060799	5066160	0.342649	0.077882
23814	Amp	16	5066533	5198748	0.362016	0.078484
23815	Amp	16	5200004	5219252	0.379885	0.079161
23816	Amp	16	5220833	5223193	0.335313	0.077684
23817	Amp	16	5223715	5223715	0.322897	0.077169
23818	Amp	16	5225302	5320520	0.30524	0.076504
23819	Amp	16	5322534	5329093	0.362016	0.078493
23820	Amp	16	5330159	5334464	0.444474	0.081413
23821	Amp	16	5339353	5404822	0.431198	0.080967
23822	Amp	16	5406395	5418156	0.457501	0.081704
23823	Amp	16	5424695	5447266	0.4115	0.080244
23824	Amp	16	5448443	5503928	0.329205	0.077324
23825	Amp	16	5503985	5586182	0.355319	0.078218
23826	Amp	16	5586444	5587977	0.368062	0.078718
23827	Amp	16	5589771	5589797	0.379885	0.079209
23828	Amp	16	5591713	5696486	0.1237	0.069114
23829	Amp	16	5696732	5738914	0.133193	0.069585
23830	Amp	16	5739201	5782327	0.112543	0.06869
23831	Amp	16	5782945	5795199	0.073902	0.067135
23832	Amp	16	5799968	5815955	0.034113	0.06541
23833	Amp	16	5819616	6009980	0	0.06192
23834	Amp	16	6010571	6028140	0	0.061429
23835	Amp	16	6028180	6170315	0	0.059841
23836	Amp	16	6170983	6229305	0	0.060528
23837	Amp	16	6375599	6484368	0	0.050952
23838	Amp	16	6484601	6484772	0	0.050216
23839	Amp	16	6484935	6501088	0	0.049745
23840	Amp	16	6502164	6577458	0	0.047323
23841	Amp	16	6582487	6582887	0	0.0481
23842	Amp	16	6583259	6583259	0	0.047598
23843	Amp	16	7081613	7081613	0	0.044818
23844	Amp	16	7082182	7316368	0	0.044204
23845	Amp	16	7316940	7338839	0	0.045309
23846	Amp	16	7362448	7363354	0	0.050238
23847	Amp	16	7363392	7366995	0	0.051794
23848	Amp	16	7367418	7372479	0	0.051107
23849	Amp	16	7377024	7383337	0	0.048021

23850	Amp	16	7384140	7386686	0	0.057095
23851	Amp	16	7391343	7428256	0	0.049004
23852	Amp	16	7429066	7433032	0	0.048309
23853	Amp	16	7434220	7442976	0	0.04815
23854	Amp	16	7443923	7443986	0	0.049183
23855	Amp	16	7444364	7492751	0	0.048561
23856	Amp	16	7510978	7543573	0	0.043632
23857	Amp	16	7544956	7549510	0	0.042599
23858	Amp	16	7574054	7593245	0	0.046418
23859	Amp	16	7595394	7645604	0	0.042599
23860	Amp	16	7646603	7648812	0	0.044103
23861	Amp	16	7649335	7722378	0	0.043417
23862	Amp	16	7723248	7741217	0	0.046997
23863	Amp	16	7741983	7770883	0	0.048708
23864	Amp	16	7772705	7785760	0	0.045127
23865	Amp	16	7785989	7787451	0	0.045567
23866	Amp	16	7995410	8049534	0	0.047423
23867	Amp	16	8049696	8078056	0	0.048223
23868	Amp	16	8119039	8284709	0	0.047006
23869	Amp	16	8285157	8316863	0	0.046507
23870	Amp	16	8320623	8320623	0	0.049645
23871	Amp	16	8343360	8355534	0	0.052689
23872	Amp	16	8355848	8397265	0	0.050979
23873	Amp	16	8398483	8613610	0	0.052961
23874	Amp	16	8614341	8647408	0	0.054555
23875	Amp	16	8647931	8684294	0	0.056292
23876	Amp	16	8685828	8755599	0	0.058471
23877	Amp	16	8756503	8768439	0	0.058013
23878	Amp	16	8768787	8769133	0	0.05756
23879	Amp	16	8771962	8788110	0	0.056676
23880	Amp	16	8788795	8817217	0	0.053633
23881	Amp	16	8819257	8820661	0	0.052855
23882	Amp	16	8821001	8831869	0	0.050865
23883	Amp	16	8839164	9132117	0	0.0497
23884	Amp	16	9132513	9225050	0	0.049104
23885	Amp	16	9227141	9243583	0	0.047122
23886	Amp	16	9244856	9278048	0	0.047649
23887	Amp	16	9281443	9322833	0	0.045594
23888	Amp	16	9323431	9548647	0	0.045154
23889	Amp	16	9548763	9583646	0	0.042221
23890	Amp	16	9583804	9600803	0	0.046076
23891	Amp	16	9609060	9645347	0	0.042221
23892	Amp	16	9646683	9681469	0	0.040556
23893	Amp	16	9681807	9821365	0	0.041728
23894	Amp	16	9822521	9870064	0	0.039597
23895	Amp	16	9873143	9980747	0	0.049301
23896	Amp	16	9981321	10019377	0	0.050486
23897	Amp	16	10022810	10034098	0	0.051792
23898	Amp	16	10034319	10067369	0	0.051204
23899	Amp	16	10067460	10494687	0	0.052267
23900	Amp	16	10497393	10497393	0	0.048855
23901	Amp	16	10507035	10512947	0	0.047793
23902	Amp	16	10513977	10562464	0	0.048311

23903	Amp	16	10562779	10611055	0	0.05107
23904	Amp	16	10625744	10625744	0	0.041537
23905	Amp	16	10627117	10717830	0	0.039755
23906	Amp	16	10721616	10729265	0	0.035583
23907	Amp	16	10731376	10868495	0	0.034736
23908	Amp	16	10869276	10877446	0	0.033141
23909	Amp	16	10877613	10906991	0	0.032539
23910	Amp	16	10908769	10995532	0	0.03205
23911	Amp	16	10996554	10999538	0	0.033071
23912	Amp	16	11006476	11012850	0	0.033545
23913	Amp	16	11019987	11047464	0	0.031471
23914	Amp	16	11048131	11108087	0	0.034195
23915	Amp	16	11110872	11168926	0	0.030789
23916	Amp	16	11169699	11170755	0	0.030324
23917	Amp	16	11171463	11353501	0	0.029647
23918	Amp	16	11358641	11384291	0	0.030384
23919	Amp	16	11384698	11431263	0	0.030459
23920	Amp	16	11432324	11569041	0	0.029722
23921	Amp	16	11573305	11573421	0	0.033201
23922	Amp	16	11578272	11582162	0	0.036295
23923	Amp	16	11582604	11648171	0	0.037365
23924	Amp	16	11649476	11699242	0	0.036847
23925	Amp	16	11699374	11735390	0	0.039304
23926	Amp	16	11743826	11752839	0	0.036847
23927	Amp	16	11754677	11795203	0	0.03632
23928	Amp	16	11795742	11795742	0	0.039692
23929	Amp	16	11799663	11814939	0	0.042684
23930	Amp	16	11818418	11822505	0	0.041614
23931	Amp	16	11828862	11828862	0	0.028817
23932	Amp	16	11834061	11834061	0	0.025445
23933	Amp	16	11834124	11890323	0	0.022453
23934	Amp	16	11892328	12057580	0	0.023966
23935	Amp	16	12059517	12070828	0	0.026966
23936	Amp	16	12076666	12076716	0	0.027405
23937	Amp	16	12076905	12167767	0	0.024405
23938	Amp	16	12168577	12178426	0	0.021681
23939	Amp	16	12181751	12224199	0	0.024991
23940	Amp	16	12229420	12271282	0	0.022735
23941	Amp	16	12272877	12382803	0	0.023216
23942	Amp	16	12383049	12408900	0	0.025506
23943	Amp	16	12411612	12471050	0	0.027627
23944	Amp	16	12471094	12516588	0	0.026113
23945	Amp	16	12517040	12518221	0	0.024941
23946	Amp	16	12521945	12536662	0	0.023846
23947	Amp	16	12539061	12541756	0	0.026114
23948	Amp	16	12543125	12649466	0	0.02564
23949	Amp	16	12653250	13343141	0	0.026123
23950	Amp	16	13344838	13364963	0	0.029564
23951	Amp	16	13367773	13390608	0	0.030072
23952	Amp	16	13390882	13503769	0	0.026631
23953	Amp	16	13506445	13593934	0	0.02724
23954	Amp	16	13594055	13711737	0	0.028706
23955	Amp	16	13712527	13785839	0	0.031827

23956	Amp	16	13786011	13865244	0	0.032597
23957	Amp	16	13865509	13926095	0	0.032089
23958	Amp	16	13926525	13992570	0	0.031612
23959	Amp	16	13992902	14162693	0	0.03527
23960	Amp	16	14164821	14169143	0	0.034789
23961	Amp	16	14169702	14188625	0	0.031131
23962	Amp	16	14194829	14199179	0	0.035183
23963	Amp	16	14208151	14254244	0	0.034589
23964	Amp	16	14256675	14453424	0	0.031468
23965	Amp	16	14455252	14455922	0	0.027989
23966	Amp	16	14456451	14506538	0	0.027254
23967	Amp	16	14506728	14528120	0	0.023945
23968	Amp	16	14528312	14652868	0	0.023175
23969	Amp	16	14653245	14653245	0	0.026409
23970	Amp	16	14657199	14657199	0	0.027439
23971	Amp	16	18797068	18797068	0	0.019221
23972	Amp	16	18797081	18812259	0	0.015925
23973	Amp	16	18812285	18881779	0	0.01474
23974	Amp	16	18882258	18920752	0	0.014131
23975	Amp	16	18924799	18924799	0	0.01592
23976	Amp	16	18929351	18960312	0	0.017599
23977	Amp	16	18964091	19040554	0	0.018073
23978	Amp	16	19041278	19117416	0	0.016607
23979	Amp	16	19117453	19143368	0	0.017281
23980	Amp	16	19143599	19251022	0	0.016607
23981	Amp	16	19251712	19406858	0	0.018878
23982	Amp	16	19409435	19442234	0	0.021684
23983	Amp	16	19445593	19702560	0	0.018878
23984	Amp	16	19706049	19831413	0	0.020262
23985	Amp	16	19841492	19867938	0	0.024448
23986	Amp	16	19876531	20055957	0	0.021399
23987	Amp	16	20056775	20397020	0	0.017212
23988	Amp	16	20397055	20442299	0	0.018765
23989	Amp	16	20453198	20595007	0	0.01829
23990	Amp	16	20597488	20600480	0	0.020092
23991	Amp	16	20602420	20624663	0	0.019653
23992	Amp	16	20625234	20808396	0	0.019141
23993	Amp	16	20809248	20848533	0	0.020014
23994	Amp	16	20848704	20889754	0	0.022375
23995	Amp	16	20891485	21348993	0	0.021134
23996	Amp	16	22773239	22774621	0	0.017949
23997	Amp	16	22774844	22783418	0	0.016147
23998	Amp	16	22786531	22792283	0	0.014982
23999	Amp	16	22794988	22843045	0	0.014159
24000	Amp	16	22843236	22882636	0	0.013358
24001	Amp	16	22884141	22939551	0	0.013914
24002	Amp	16	22940532	23034533	0	0.015761
24003	Amp	16	23034622	23233879	0	0.013972
24004	Amp	16	23238103	23293622	0	0.016591
24005	Amp	16	23300447	23562289	0	0.019567
24006	Amp	16	23564368	23640264	0	0.021965
24007	Amp	16	23647007	23661948	0	0.019567
24008	Amp	16	23662821	23698153	0	0.0215

24009	Amp	16	23698370	23728667	0	0.023773
24010	Amp	16	23728681	23787285	0	0.025298
24011	Amp	16	23787611	24185334	0	0.023364
24012	Amp	16	24185861	24384139	0	0.026475
24013	Amp	16	24385257	24611396	0	0.027089
24014	Amp	16	24612525	24634475	0	0.028401
24015	Amp	16	24636150	24666013	0	0.027846
24016	Amp	16	24667997	24966205	0	0.025724
24017	Amp	16	24966837	25080089	0	0.024412
24018	Amp	16	25080798	25126705	0	0.021793
24019	Amp	16	25129508	25281997	0	0.029277
24020	Amp	16	25282046	25371354	0	0.026166
24021	Amp	16	25374319	25386000	0	0.029332
24022	Amp	16	25391983	25448730	0	0.029896
24023	Amp	16	25450801	25473948	0	0.02673
24024	Amp	16	25474434	25684412	0	0.03008
24025	Amp	16	25685097	25712971	0	0.032202
24026	Amp	16	25713600	26071575	0	0.03008
24027	Amp	16	26073253	26431920	0	0.02673
24028	Amp	16	26435253	26543566	0	0.025701
24029	Amp	16	26544782	26553842	0	0.028174
24030	Amp	16	26554566	26565981	0	0.030216
24031	Amp	16	26566889	26690437	0	0.028368
24032	Amp	16	26690910	26777244	0	0.029012
24033	Amp	16	26777381	26885637	0	0.029919
24034	Amp	16	26890308	26959049	0	0.029355
24035	Amp	16	26960772	26988272	0	0.028448
24036	Amp	16	26989569	26989569	0	0.030701
24037	Amp	16	26989829	26999386	0	0.032079
24038	Amp	16	26999441	27091364	0	0.032755
24039	Amp	16	27095311	27132575	0	0.030466
24040	Amp	16	27133778	27235617	0	0.029822
24041	Amp	16	27236007	27353485	0	0.028445
24042	Amp	16	27354147	27389186	0	0.029072
24043	Amp	16	27389554	27439242	0	0.02859
24044	Amp	16	27442767	27493974	0	0.025089
24045	Amp	16	27496874	27512905	0	0.027151
24046	Amp	16	27513641	27523861	0	0.03349
24047	Amp	16	27525875	27571496	0	0.035177
24048	Amp	16	27571819	27571819	0	0.034304
24049	Amp	16	27579385	27682048	0	0.034833
24050	Amp	16	27683864	27685692	0	0.040245
24051	Amp	16	27686237	27733012	0	0.04158
24052	Amp	16	27738792	27751789	0	0.035241
24053	Amp	16	27758573	27884827	0	0.033179
24054	Amp	16	27886004	27895885	0	0.030088
24055	Amp	16	27895931	28010511	0	0.031876
24056	Amp	16	28010660	28012531	0	0.032769
24057	Amp	16	28014319	28061349	0	0.048259
24058	Amp	16	28061519	28090564	0	0.048751
24059	Amp	16	28093903	28104930	0	0.047298
24060	Amp	16	28105080	28151434	0	0.046406
24061	Amp	16	28157736	28164890	0	0.044579

24062	Amp	16	28167116	28198404	0	0.0456
24063	Amp	16	28203452	28325415	0	0.047198
24064	Amp	16	28325856	29084019	0	0.038831
24065	Amp	16	29086168	29114764	0	0.036788
24066	Amp	16	29122775	29122775	0	0.035767
24067	Amp	16	29122783	29156685	0	0.038358
24068	Amp	16	29157733	29261283	0	0.039769
24069	Amp	16	29278656	29294233	0	0.045862
24070	Amp	16	29296764	29411088	0	0.048314
24071	Amp	16	29411192	29415091	0	0.050581
24072	Amp	16	29417218	29417218	0	0.054207
24073	Amp	16	29417224	30295647	0	0.051123
24074	Amp	16	30308694	30448897	0.019169	0.064781
24075	Amp	16	30452505	30600947	0.424557	0.08064
24076	Amp	16	30603876	30629842	0.392611	0.079516
24077	Amp	16	30630511	30716323	0.463534	0.081992
24078	Amp	16	30716844	30757607	0.47716	0.082507
24079	Amp	16	30758058	30774503	0.508568	0.083391
24080	Amp	16	30776976	30801240	0.463534	0.081866
24081	Amp	16	30811726	30883833	0.379885	0.07922
24082	Amp	16	30884224	30916950	0.470509	0.08225
24083	Amp	16	30917649	30933433	0.583752	0.085882
24084	Amp	16	30935185	30935185	0.681869	0.088824
24085	Amp	16	30940457	31036599	0.735486	0.090527
24086	Amp	16	31045967	31106199	0.522881	0.083858
24087	Amp	16	31112876	31152667	0.255918	0.074501
24088	Amp	16	31154707	31168037	0.322897	0.077264
24089	Amp	16	31170746	31189270	0.246818	0.074234
24090	Amp	16	31189916	31194793	0.293269	0.076148
24091	Amp	16	31197903	31273001	0.259438	0.074737
24092	Amp	16	31274953	31290803	0.215608	0.072823
24093	Amp	16	31291617	31299051	0.143787	0.070061
24094	Amp	16	31304356	31319403	0.112543	0.068677
24095	Amp	16	31319740	31336427	0.181932	0.07151
24096	Amp	16	31336667	31357495	0.622604	0.087199
24097	Amp	16	31358244	31392661	0.536238	0.084365
24098	Amp	16	31396631	31499980	0.550755	0.084834
24099	Amp	16	31501107	31525421	0.569771	0.085536
24100	Amp	16	31527592	31530950	0.651644	0.088068
24101	Amp	16	31539556	31554885	0.536238	0.084443
24102	Amp	16	31556213	31619182	0.500841	0.08328
24103	Amp	16	31620084	31689625	0.595248	0.086284
24104	Amp	16	31690173	31720735	0.482379	0.082699
24105	Amp	16	31734712	31741451	0.576183	0.085681
24106	Amp	16	31742618	31784206	0.681869	0.088994
24107	Amp	16	31786235	31817777	0.704849	0.089652
24108	Amp	16	31825824	31825824	0.622604	0.08729
24109	Amp	16	31830143	31834376	0.589605	0.086184
24110	Amp	16	31838978	31838978	0.644502	0.087875
24111	Amp	16	31841585	31841585	0.899693	0.094782
24112	Amp	16	46470502	46470502	0	0.029128
24113	Amp	16	46473753	46473753	0	0.024635
24114	Amp	16	46474548	46481615	0	0.021321

24115	Amp	16	46482583	46504043	0	0.020806
24116	Amp	16	46505480	46597673	0	0.023746
24117	Amp	16	46600539	46600539	0	0.032201
24118	Amp	16	46603868	46603868	0	0.04333
24119	Amp	16	46604997	46635420	0	0.045866
24120	Amp	16	46639499	46659538	0	0.048225
24121	Amp	16	46665305	46724689	0	0.049489
24122	Amp	16	46727329	46799785	0	0.058657
24123	Amp	16	46800699	46815946	0	0.050929
24124	Amp	16	46816493	46820330	0	0.048571
24125	Amp	16	46824225	46826622	0	0.042463
24126	Amp	16	46829898	46829898	0	0.043401
24127	Amp	16	46836939	46840785	0	0.046499
24128	Amp	16	46848146	46909793	0	0.047231
24129	Amp	16	46913266	46927790	0	0.048113
24130	Amp	16	46929995	46934815	0.001323	0.06405
24131	Amp	16	46936395	46979893	0.015369	0.064594
24132	Amp	16	46987928	47002208	0.029031	0.065204
24133	Amp	16	47002342	47046165	0.118001	0.068952
24134	Amp	16	47046973	47051077	0	0.063041
24135	Amp	16	47057927	47058872	0.128324	0.069312
24136	Amp	16	47060939	47074762	0.215608	0.07293
24137	Amp	16	47080410	47142547	0.311747	0.076776
24138	Amp	16	47155276	47177321	0.379885	0.079111
24139	Amp	16	47189154	47191256	0.335313	0.077662
24140	Amp	16	47195416	47202041	0.482379	0.082676
24141	Amp	16	47203574	47234295	0.543344	0.084601
24142	Amp	16	47234367	47304211	0.6737	0.088681
24143	Amp	16	47307936	47322243	0.636464	0.087651
24144	Amp	16	47328198	47346084	0.569771	0.085482
24145	Amp	16	47353265	47363930	0.6737	0.088668
24146	Amp	16	47364541	47435101	0.751221	0.090955
24147	Amp	16	47436756	47449942	0.873678	0.094162
24148	Amp	16	47450928	47450928	1.280185	0.103799
24149	Amp	16	47458152	47487872	1.178324	0.101513
24150	Amp	16	47552436	47612078	0.968522	0.096499
24151	Amp	16	47612513	47616316	1.665486	0.112435
24152	Amp	16	47619210	47638568	1.563118	0.110302
24153	Amp	16	47639403	47669488	1.542638	0.109691
24154	Amp	16	47671554	47720797	1.655056	0.112062
24155	Amp	16	47727571	47754351	1.563118	0.110187
24156	Amp	16	47760285	47824620	1.426786	0.10724
24157	Amp	16	47826255	47847926	1.737623	0.113961
24158	Amp	16	47849645	47875006	1.634701	0.111613
24159	Amp	16	47878853	47925263	1.897704	0.117103
24160	Amp	16	47935795	47951987	1.940776	0.117948
24161	Amp	16	47952246	47982692	1.843664	0.116024
24162	Amp	16	47984538	48012149	1.972776	0.118707
24163	Amp	16	48013930	48104361	1.23519	0.10277
24164	Amp	16	48105185	48129828	1.058188	0.098691
24165	Amp	16	48131667	48166449	0.995635	0.097143
24166	Amp	16	48168592	48229301	0.891602	0.09446
24167	Amp	16	48230182	48271793	1.102589	0.099709

24168	Amp	16	48272483	48299443	1.206641	0.102211
24169	Amp	16	48307297	48413461	1.280185	0.10391
24170	Amp	16	48421175	48425647	1.463774	0.108128
24171	Amp	16	48426224	48469683	1.168655	0.101229
24172	Amp	16	48479050	48479913	1.261401	0.103483
24173	Amp	16	48487509	48497417	1.583409	0.11055
24174	Amp	16	48497497	48515099	1.348715	0.105397
24175	Amp	16	48520338	48535326	1.416817	0.106986
24176	Amp	16	48535903	48547011	1.348715	0.105397
24177	Amp	16	48547699	48575684	1.23519	0.102861
24178	Amp	16	48578450	48596023	1.049301	0.098547
24179	Amp	16	48597977	48599917	0.924746	0.095432
24180	Amp	16	48602102	48628764	0.873678	0.09414
24181	Amp	16	48632589	48642611	0.781972	0.091638
24182	Amp	16	48646629	48687482	0.72817	0.090155
24183	Amp	16	48689715	48708350	0.681869	0.088902
24184	Amp	16	48714604	48814488	0.651644	0.088019
24185	Amp	16	48814938	48814938	0.697374	0.089253
24186	Amp	16	48814963	48941893	0.774648	0.091622
24187	Amp	16	48942558	48982618	0.840289	0.093214
24188	Amp	16	48983397	49003386	0.774648	0.091622
24189	Amp	16	49008480	49015061	0.735486	0.090388
24190	Amp	16	49015119	49033949	0.681869	0.088869
24191	Amp	16	49035029	49087952	0.831947	0.093043
24192	Amp	16	49088662	49127048	1.243254	0.103139
24193	Amp	16	49129211	49148285	1.319393	0.104841
24194	Amp	16	49149603	49181265	1.563118	0.110124
24195	Amp	16	49181517	49215983	1.426786	0.107128
24196	Amp	16	49216182	49216182	1.655056	0.112257
24197	Amp	16	49216708	49278762	0.959607	0.096321
24198	Amp	16	49286784	49332170	1.102589	0.099814
24199	Amp	16	49332795	49389016	1.058188	0.098636
24200	Amp	16	49391323	49459592	1.769915	0.114572
24201	Amp	16	49463054	49481517	1.95165	0.118229
24202	Amp	16	49481965	49506182	1.759227	0.114384
24203	Amp	16	49506702	49555484	1.843664	0.11608
24204	Amp	16	49556523	49556523	1.727286	0.113711
24205	Amp	16	49557874	49568800	1.482378	0.108427
24206	Amp	16	49570518	49583149	1.041205	0.098332
24207	Amp	16	49585031	49596412	1.121584	0.100182
24208	Amp	16	49598068	49598920	1.041205	0.098332
24209	Amp	16	49600729	49702417	0.977077	0.096635
24210	Amp	16	49703184	49829719	1.041205	0.098168
24211	Amp	16	49830880	49860153	1.093834	0.099579
24212	Amp	16	49862769	49880412	0.882843	0.094413
24213	Amp	16	49880470	49980056	0.742723	0.090756
24214	Amp	16	49984348	49986412	0.781972	0.091825
24215	Amp	16	49994588	49994588	0.287738	0.075917
24216	Amp	16	49994694	50030955	0.233782	0.073547
24217	Amp	16	50035317	50037204	0	0.062776
24218	Amp	16	50037319	50056455	0	0.061365
24219	Amp	16	50057424	50130510	0	0.057292
24220	Amp	16	50134625	50140772	0	0.055038

24221	Amp	16	50143067	50151652	0	0.053969
24222	Amp	16	50155593	50183698	0	0.052705
24223	Amp	16	50188029	50206121	0	0.054936
24224	Amp	16	50214108	50214108	0	0.056128
24225	Amp	16	50217646	50269602	0	0.040192
24226	Amp	16	50269902	50409496	0	0.042693
24227	Amp	16	50411020	50484125	0	0.050178
24228	Amp	16	50484724	50498317	0	0.05314
24229	Amp	16	50498531	50584825	0	0.05366
24230	Amp	16	50585960	50645452	0	0.051958
24231	Amp	16	50647175	50669787	0	0.054689
24232	Amp	16	50674941	50685661	0	0.051288
24233	Amp	16	50686455	50729878	0	0.053296
24234	Amp	16	50732159	50739429	0	0.054862
24235	Amp	16	50743306	50758750	0	0.057232
24236	Amp	16	50763072	50798821	0	0.055225
24237	Amp	16	50807884	50817665	0	0.052855
24238	Amp	16	50817918	50878008	0	0.052335
24239	Amp	16	50878035	51046303	0	0.054674
24240	Amp	16	51049180	51139648	0	0.057136
24241	Amp	16	51143251	51164165	0	0.047967
24242	Amp	16	51167140	51172148	0	0.04905
24243	Amp	16	51174782	51187637	0	0.040032
24244	Amp	16	51189027	51287649	0	0.042572
24245	Amp	16	51290803	51302658	0	0.039841
24246	Amp	16	51303699	51355117	0	0.03734
24247	Amp	16	51355438	51471712	0	0.042721
24248	Amp	16	51472649	51532457	0	0.040382
24249	Amp	16	51532898	51614396	0	0.03742
24250	Amp	16	51621168	51684794	0	0.040782
24251	Amp	16	51686386	51750462	0	0.038446
24252	Amp	16	51750575	51783599	0	0.037714
24253	Amp	16	51783831	51855787	0	0.036525
24254	Amp	16	51857470	51877019	0	0.037023
24255	Amp	16	51879936	52061199	0	0.036525
24256	Amp	16	52062389	52084541	0	0.038097
24257	Amp	16	52086456	52089697	0	0.044633
24258	Amp	16	52092564	52093102	0	0.058994
24259	Amp	16	52093213	52147329	0	0.056532
24260	Amp	16	52151403	52154763	0	0.058995
24261	Amp	16	52155664	52168117	0	0.061828
24262	Amp	16	52172748	52191483	0	0.058205
24263	Amp	16	52194833	52210446	0	0.059265
24264	Amp	16	52210530	52210723	0	0.061369
24265	Amp	16	52210854	52237915	0.001323	0.063969
24266	Amp	16	52244047	52267910	0	0.058479
24267	Amp	16	52276615	52333655	0	0.060599
24268	Amp	16	52343007	52365989	0.015369	0.064558
24269	Amp	16	52366493	52402297	0.040093	0.065624
24270	Amp	16	52403904	52413704	0	0.061665
24271	Amp	16	52416158	52445859	0	0.063807
24272	Amp	16	52446082	52489701	0.079652	0.0674
24273	Amp	16	52492937	52517703	0.450702	0.081623

24274	Amp	16	52519443	52519443	0.276783	0.075358
24275	Amp	16	52524155	52552429	0.164935	0.070966
24276	Amp	16	52552759	52686463	0.19964	0.072151
24277	Amp	16	52690336	52831764	0.644502	0.087783
24278	Amp	16	52836242	52964690	0.563096	0.085183
24279	Amp	16	52968466	52983885	0.494792	0.083062
24280	Amp	16	52984791	52988698	0.550755	0.08481
24281	Amp	16	52990967	53024946	0.622604	0.087183
24282	Amp	16	53027774	53034556	0.569771	0.085435
24283	Amp	16	53035647	53128113	0.437198	0.081052
24284	Amp	16	53143733	53147414	0.583752	0.085964
24285	Amp	16	53167051	53258004	0.660254	0.088244
24286	Amp	16	53259845	53315088	0.636464	0.087619
24287	Amp	16	53315779	53315779	0.569771	0.085339
24288	Amp	16	53318570	53319247	0.630438	0.087439
24289	Amp	16	53323525	53344701	0.758088	0.090991
24290	Amp	16	53353985	53373977	0.34892	0.078008
24291	Amp	16	53374550	53378950	0.417873	0.080538
24292	Amp	16	53379339	53405908	0.487495	0.082734
24293	Amp	16	53407820	53407820	0.4115	0.080243
24294	Amp	16	53421327	53421327	0.34892	0.078047
24295	Amp	16	53421612	53421751	0.386426	0.079425
24296	Amp	16	53421830	53439381	0.329205	0.077325
24297	Amp	16	53439515	53479087	0.287738	0.075947
24298	Amp	16	53479101	53487754	0.299433	0.07642
24299	Amp	16	53487769	53498663	0.329205	0.077456
24300	Amp	16	53499004	53502192	0.362016	0.078513
24301	Amp	16	53504245	53507566	0.335313	0.077571
24302	Amp	16	53511081	53511081	0.233782	0.073575
24303	Amp	16	53516230	53617929	0.299433	0.076225
24304	Amp	16	53619997	53681715	0.342649	0.07786
24305	Amp	16	53685342	53710639	0.424557	0.0806
24306	Amp	16	53713634	53778922	0.342649	0.077949
24307	Amp	16	53782363	53782363	0.299433	0.076314
24308	Amp	16	53786615	53852000	0.379885	0.079235
24309	Amp	16	53852707	53862347	0.404718	0.079974
24310	Amp	16	53864594	53874745	0.316877	0.077053
24311	Amp	16	53875243	53898590	0.362016	0.078602
24312	Amp	16	53903720	53915678	0.482379	0.082692
24313	Amp	16	53916004	53929727	0.576183	0.085714
24314	Amp	16	53930022	53949568	0.508568	0.083468
24315	Amp	16	53951361	53965018	0.463534	0.081877
24316	Amp	16	53965379	54001896	0.529451	0.08419
24317	Amp	16	54002331	54089505	0.444474	0.081365
24318	Amp	16	54089600	54099888	0.47716	0.082343
24319	Amp	16	54100890	54100890	0.543344	0.084651
24320	Amp	16	54101053	54117895	0.470509	0.082188
24321	Amp	16	54118708	54150090	0.508568	0.083478
24322	Amp	16	54152525	54204827	0.47716	0.082501
24323	Amp	16	54205789	54215144	0.322897	0.077116
24324	Amp	16	54217150	54253218	0.282828	0.075567
24325	Amp	16	54255193	54258063	0.210594	0.072545
24326	Amp	16	54273420	54294088	0.143787	0.070027

24327	Amp	16	54294415	54313509	0.417873	0.080413
24328	Amp	16	54319007	54319007	0.379885	0.079123
24329	Amp	16	54320568	54438871	0.311747	0.076814
24330	Amp	16	54439160	54498546	0.399144	0.079878
24331	Amp	16	54503696	54581124	0.494792	0.082958
24332	Amp	16	54582556	54752645	0.569771	0.085402
24333	Amp	16	54753332	54895684	0.494792	0.083089
24334	Amp	16	54897844	54913375	1.023539	0.097877
24335	Amp	16	54918120	54949465	1.058188	0.098626
24336	Amp	16	54953662	55125673	0.933745	0.095546
24337	Amp	16	55130541	55154049	1.066493	0.098877
24338	Amp	16	55155336	55184950	1.215958	0.102333
24339	Amp	16	55186942	55186942	1.178324	0.101606
24340	Amp	16	55187768	55235902	1.149583	0.100948
24341	Amp	16	55236081	55268943	1.206641	0.102086
24342	Amp	16	55271481	55315669	0.630438	0.087298
24343	Amp	16	55317195	55336042	0.550755	0.084694
24344	Amp	16	55344513	55344593	0.368062	0.07861
24345	Amp	16	55350098	55350098	0.210594	0.072641
24346	Amp	16	55356705	55360186	0.14896	0.070197
24347	Amp	16	55361830	55368304	0.073902	0.067134
24348	Amp	16	55370843	55391588	0.056211	0.066395
24349	Amp	16	55391821	55410526	0.029031	0.065257
24350	Amp	16	55411431	55422070	0	0.062727
24351	Amp	16	55424928	55460520	0.065715	0.06679
24352	Amp	16	55462716	55478410	0	0.063334
24353	Amp	16	55483250	55493510	0	0.061192
24354	Amp	16	55495499	55605542	0	0.062055
24355	Amp	16	55605628	55608392	0	0.058272
24356	Amp	16	55621512	55627099	0	0.061158
24357	Amp	16	55628920	55635790	0	0.063599
24358	Amp	16	55636401	55636401	0	0.062736
24359	Amp	16	55640316	55643134	0	0.057084
24360	Amp	16	55645425	55683165	0	0.059387
24361	Amp	16	55684571	55707544	0	0.056946
24362	Amp	16	55707589	55714586	0	0.053135
24363	Amp	16	55720390	55748372	0	0.050249
24364	Amp	16	55839497	55892329	0	0.04322
24365	Amp	16	55897884	55900247	0	0.040916
24366	Amp	16	55902162	55915075	0	0.047256
24367	Amp	16	55923749	55938070	0	0.043663
24368	Amp	16	55939115	55957653	0	0.035625
24369	Amp	16	55959410	56066292	0	0.034876
24370	Amp	16	56068846	56087397	0	0.024832
24371	Amp	16	56087946	56112627	0	0.023261
24372	Amp	16	56115802	56136025	0	0.022069
24373	Amp	16	56138416	56337609	0	0.019896
24374	Amp	16	56338549	56377856	0	0.014515
24375	Amp	16	56378614	56402447	0	0.013929
24376	Amp	16	56412700	56420510	0	0.016467
24377	Amp	16	56426369	56538461	0	0.019203
24378	Amp	16	56542758	56601840	0	0.020425
24379	Amp	16	56604736	56606667	0	0.019203

24380	Amp	16	56610353	56640199	0	0.016664
24381	Amp	16	56640946	56665589	0	0.018063
24382	Amp	16	56670002	56901208	0	0.019576
24383	Amp	16	56904332	56919784	0	0.016841
24384	Amp	16	56922555	56974816	0	0.015327
24385	Amp	16	56978467	57043593	0	0.019433
24386	Amp	16	57045441	57060102	0	0.017197
24387	Amp	16	57061863	57188830	0	0.012996
24388	Amp	16	57194923	57558341	0	0.010257
24389	Amp	16	57559178	57607431	0	0.014369
24390	Amp	16	57613483	57617174	0	0.006152
24391	Amp	16	57622607	58080236	0	0.005672
24392	Amp	16	58092256	58843597	0	0.004273
24393	Amp	16	58847365	58859929	0	0.008104
24394	Amp	16	58862370	59027965	0	0.004273
24395	Amp	16	59028818	59299170	0	0.005512
24396	Amp	16	59299692	59475138	0	0.006184
24397	Amp	16	59476259	59617725	0	0.007681
24398	Amp	16	59617899	59752653	0	0.008382
24399	Amp	16	59755485	59834769	0	0.011049
24400	Amp	16	59841209	59917465	0	0.010377
24401	Amp	16	59921210	59928263	0	0.008881
24402	Amp	16	59930164	59969265	0	0.006214
24403	Amp	16	59971136	60275871	0	0.008671
24404	Amp	16	60277210	60376786	0	0.007431
24405	Amp	16	60378427	60421165	0	0.008828
24406	Amp	16	60421645	60541170	0	0.011167
24407	Amp	16	60543296	60580110	0	0.008828
24408	Amp	16	60583425	60621744	0	0.011209
24409	Amp	16	60625263	60631754	0	0.024746
24410	Amp	16	60635372	60660460	0	0.026262
24411	Amp	16	60662747	60685496	0	0.023179
24412	Amp	16	60689674	60690720	0	0.020722
24413	Amp	16	60695152	60711531	0	0.021726
24414	Amp	16	60711945	60847803	0	0.019429
24415	Amp	16	60847931	60881236	0	0.018425
24416	Amp	16	60888601	60889183	0	0.016909
24417	Amp	16	60893421	61207457	0	0.017966
24418	Amp	16	61207694	61231476	0	0.019373
24419	Amp	16	61237511	61324884	0	0.022717
24420	Amp	16	61325472	61397601	0	0.02849
24421	Amp	16	61402527	61570744	0	0.025854
24422	Amp	16	61574457	61619484	0	0.027633
24423	Amp	16	61636887	61694668	0	0.025854
24424	Amp	16	61697079	61750266	0	0.011271
24425	Amp	16	61750281	61793708	0	0.013592
24426	Amp	16	61796498	61910678	0	0.010558
24427	Amp	16	61914841	61983509	0	0.004785
24428	Amp	16	61984930	62111566	0	0.003378
24429	Amp	16	62122069	62135569	0	0.0123
24430	Amp	16	62136825	62413208	0	0.009979
24431	Amp	16	62415331	63030492	0	0.001057
24432	Amp	16	63032203	63346980	0	0.010071

24433	Amp	16	63352513	63387467	0	0.012251
24434	Amp	16	63449136	63628775	0	0.010071
24435	Amp	16	63642322	63867498	0	0.011478
24436	Amp	16	63870790	64225485	0	0.002464
24437	Amp	16	64225597	64372707	0	0.004017
24438	Amp	16	64375760	64476705	0	0.002464
24439	Amp	16	64477138	64609679	0	0.004372
24440	Amp	16	64614673	64695840	0	0.003315
24441	Amp	16	64696886	65214124	0	0.001407
24442	Amp	16	65215749	65331332	0	0.003735
24443	Amp	16	65332099	65520667	0	0.001407
24444	Amp	16	65523046	65733313	0	0
24445	Amp	16	65733378	65819735	0	0.00201
24446	Amp	16	65822540	65895244	0	0
24447	Amp	16	65895540	65932526	0	0.001854
24448	Amp	16	65933189	65934713	0	0.003928
24449	Amp	16	65935948	65983492	0	0.002074
24450	Amp	16	65986155	66251134	0	0.00384
24451	Amp	16	66251383	66309138	0	0.001766
24452	Amp	16	66318492	67268448	0	0
24453	Amp	16	67276177	67324693	0	0.00073
24454	Amp	16	67324913	67878026	0	0
24455	Amp	16	67882458	68165925	0	0.001091
24456	Amp	16	68167392	68350136	0	0.014903
24457	Amp	16	68353593	68406229	0	0.009926
24458	Amp	16	68412244	68431132	0	0.021338
24459	Amp	16	68434755	68532884	0	0.009926
24460	Amp	16	68533649	68546880	0	0.005802
24461	Amp	16	68550914	68583328	0	0.00745
24462	Amp	16	68592681	68676734	0	0.004248
24463	Amp	16	68677781	68771881	0	0.0026
24464	Amp	16	68775842	69221116	0	0.005244
24465	Amp	16	69225412	69299974	0	0.007699
24466	Amp	16	69303500	69317365	0	0.008153
24467	Amp	16	69321603	69539184	0	0.005697
24468	Amp	16	69546812	70226836	0	0.006968
24469	Amp	16	70232872	70292705	0	0.00853
24470	Amp	16	70295122	70319988	0	0.006968
24471	Amp	16	70323731	70445443	0	0.007543
24472	Amp	16	70452696	70536137	0	0.008092
24473	Amp	16	70540076	70601992	0	0.005312
24474	Amp	16	70603713	71171291	0	0.004737
24475	Amp	16	71172005	71333018	0	0.002093
24476	Amp	16	71338580	71651403	0	0.011524
24477	Amp	16	71651499	71693719	0	0.012489
24478	Amp	16	71699044	71768078	0	0.014308
24479	Amp	16	71770011	71888253	0	0.012489
24480	Amp	16	71889278	72196908	0	0.011524
24481	Amp	16	72202780	72652451	0	0.002093
24482	Amp	16	72654751	72727835	0	0.004383
24483	Amp	16	72738145	72773514	0	0.006572
24484	Amp	16	72774057	72898072	0	0.004283
24485	Amp	16	72898561	72932592	0	0.01317

24486	Amp	16	72933180	72962594	0	0.012716
24487	Amp	16	72962914	73360716	0	0.010527
24488	Amp	16	73364750	73370052	0	0.012238
24489	Amp	16	73372403	73686965	0	0.014345
24490	Amp	16	73688535	73833382	0	0.014875
24491	Amp	16	73833948	73899017	0	0.013784
24492	Amp	16	73903506	73927761	0	0.015701
24493	Amp	16	73929412	74291454	0	0.013784
24494	Amp	16	74291845	74291845	0	0.016076
24495	Amp	16	74437522	74479886	0	0.017242
24496	Amp	16	74481534	74608495	0	0.01495
24497	Amp	16	74611744	74796077	0	0.016328
24498	Amp	16	74797393	74817014	0	0.026592
24499	Amp	16	74823389	75031599	0	0.016328
24500	Amp	16	75035832	75137129	0	0.014617
24501	Amp	16	75137291	75248066	0	0.016147
24502	Amp	16	75256214	75319794	0	0.017387
24503	Amp	16	75320180	75320180	0	0.02227
24504	Amp	16	75326198	75330143	0	0.026583
24505	Amp	16	75334999	75368905	0	0.025052
24506	Amp	16	75371014	75596381	0	0.015856
24507	Amp	16	75597420	75628985	0	0.01469
24508	Amp	16	75629522	75629522	0	0.018219
24509	Amp	16	75631439	75655646	0	0.022382
24510	Amp	16	75659066	75879545	0	0.01469
24511	Amp	16	75880598	76023657	0	0.016272
24512	Amp	16	76025327	76047621	0	0.02549
24513	Amp	16	76048709	76062826	0	0.023908
24514	Amp	16	76065561	76136983	0	0.01469
24515	Amp	16	76138376	76367899	0	0.015169
24516	Amp	16	76367972	76585125	0	0.01469
24517	Amp	16	76589264	76709260	0	0.012583
24518	Amp	16	76709732	76889773	0	0.011344
24519	Amp	16	76890165	77080602	0	0.016113
24520	Amp	16	77082190	77286708	0	0.007226
24521	Amp	16	77286805	77724161	0	0.010149
24522	Amp	16	77737755	77860690	0	0.011537
24523	Amp	16	77862124	77869183	0	0.012242
24524	Amp	16	77871557	77919728	0	0.022027
24525	Amp	16	77920799	78168923	0	0.012242
24526	Amp	16	78169325	78171169	0	0.007822
24527	Amp	16	78171279	78233200	0	0.007117
24528	Amp	16	78236767	78368552	0	0.005728
24529	Amp	16	78369156	78412159	0	0.006482
24530	Amp	16	78412705	78440559	0	0.007475
24531	Amp	16	78441511	78461464	0	0.009071
24532	Amp	16	78462927	78492530	0	0.007475
24533	Amp	16	78492850	78507458	0	0.006482
24534	Amp	16	78509102	78509102	0	0.022418
24535	Amp	16	78510145	78510825	0	0.019147
24536	Amp	16	78510958	78511451	0	0.020452
24537	Amp	16	78511787	78523872	0	0.025616
24538	Amp	16	78525837	78554674	0	0.024862

24539 Amp	16	78557987	78632255	0	0.018393
24540 Amp	16	78634311	78927209	0	0.002457
24541 Amp	16	78927286	79141514	0	0.003351
24542 Amp	16	79142440	79195837	0	0.002457
24543 Amp	16	79195926	79480258	0	0.005003
24544 Amp	16	79484512	79490425	0	0.004473
24545 Amp	16	79490803	79733055	0	0.005117
24546 Amp	16	79736167	79855630	0	0.003111
24547 Amp	16	79856062	79893248	0	0.003564
24548 Amp	16	79896441	79998634	0	0.003111
24549 Amp	16	80000306	80062935	0	0.005295
24550 Amp	16	80064352	80122932	0	0.003111
24551 Amp	16	80123127	80261573	0	0.002467
24552 Amp	16	80264908	80279946	0	0.004359
24553 Amp	16	80287120	80287120	0	0.010327
24554 Amp	16	80287775	80306835	0	0.01501
24555 Amp	16	80364824	80374364	0	0.004359
24556 Amp	16	80374842	81051432	0	0.002467
24557 Amp	16	81052637	81119313	0	0.004093
24558 Amp	16	81120843	82744486	0	0.002467
24559 Amp	16	82748061	82886321	0	0.003049
24560 Amp	16	82886856	83760358	0	0.002467
24561 Amp	16	83760390	83760629	0	0.011782
24562 Amp	16	83761711	83767158	0	0.002467
24563 Amp	16	83768033	83897876	0	0.002966
24564 Amp	16	83901550	84142954	0	0.003505
24565 Amp	16	84143137	84148194	0	0.005191
24566 Amp	16	84900412	84965159	0	0.006694
24567 Amp	16	84969689	85172303	0	0.004692
24568 Amp	16	85177856	85287521	0	0.003006
24569 Amp	16	85290292	85683032	0	0.00456
24570 Amp	16	85694905	85843796	0	0.005135
24571 Amp	16	85846553	85864282	0	0.00358
24572 Amp	16	85866717	86232426	0	0.003006
24573 Amp	16	86270638	86962213	0	0.007417
24574 Amp	16	86966284	86969540	0	0.004171
24575 Amp	16	86973376	87088821	0	0.003006
24576 Amp	16	87092216	87962783	0	0.0037
24577 Amp	16	87962849	88457407	0	0.003006
24578 Amp	16	88459366	89317317	0	0.004581
24579 Amp	17	1074619	1122457	0	0.027786
24580 Amp	17	1176906	1324207	0	0.023041
24581 Amp	17	1330321	1340647	0	0.02026
24582 Amp	17	1345098	1387273	0	0.022212
24583 Amp	17	1391320	1685248	0	0.014933
24584 Amp	17	1686489	1810639	0	0.014181
24585 Amp	17	1921252	1963598	0	0.012046
24586 Amp	17	1967221	2456983	0	0.009937
24587 Amp	17	2470590	2527447	0	0.011429
24588 Amp	17	2528316	2676032	0	0.014362
24589 Amp	17	2679222	2836935	0	0.0155
24590 Amp	17	2838482	2922179	0	0.014713
24591 Amp	17	2974709	2995998	0	0.01178

24592	Amp	17	3095770	3107682	0	0.019495
24593	Amp	17	3108252	3120573	0	0.020568
24594	Amp	17	3123598	3169761	0	0.021644
24595	Amp	17	3182610	3227419	0	0.029547
24596	Amp	17	3227584	3227584	0	0.03851
24597	Amp	17	3229256	3259013	0	0.030607
24598	Amp	17	3262595	3344132	0	0.028944
24599	Amp	17	3363511	3665125	0	0.021229
24600	Amp	17	3672253	3709496	0	0.02337
24601	Amp	17	3716844	3787636	0	0.021879
24602	Amp	17	3789918	3927390	0	0.026786
24603	Amp	17	3936391	4066233	0	0.029493
24604	Amp	17	4067684	4092611	0	0.026326
24605	Amp	17	4094737	4147148	0	0.024185
24606	Amp	17	4216699	4325708	0	0.010483
24607	Amp	17	4329976	4396818	0	0.011379
24608	Amp	17	4478453	4504417	0	0.015317
24609	Amp	17	4508210	4575541	0	0.014179
24610	Amp	17	4577608	4670758	0	0.015842
24611	Amp	17	4673817	5102453	0	0.014057
24612	Amp	17	5120631	5120631	0	0.01298
24613	Amp	17	5125207	5125207	0	0.011554
24614	Amp	17	5126298	5162952	0	0.013321
24615	Amp	17	5163344	5174348	0	0.015488
24616	Amp	17	5174992	5230512	0	0.021707
24617	Amp	17	5235218	5373236	0	0.013826
24618	Amp	17	5378044	5404158	0	0.011658
24619	Amp	17	5410551	5462410	0	0.009891
24620	Amp	17	5463330	5488052	0	0.006649
24621	Amp	17	5488370	5811149	0	0.008344
24622	Amp	17	5813675	5996610	0	0.006649
24623	Amp	17	5996960	6061538	0	0.009787
24624	Amp	17	6065938	6233230	0	0.006649
24625	Amp	17	6234404	6338112	0	0.005144
24626	Amp	17	6342702	6537887	0	0.001741
24627	Amp	17	6539924	6612134	0	0
24628	Amp	17	6615314	6661504	0	0.000576
24629	Amp	17	6662325	6823066	0	0
24630	Amp	17	6823472	7724147	0	0.000713
24631	Amp	17	7725392	7787395	0	0.006704
24632	Amp	17	7787792	7797886	0	0.01665
24633	Amp	17	7801089	7802092	0	0.026462
24634	Amp	17	7807637	7830791	0	0.01264
24635	Amp	17	7836346	7873896	0	0.010526
24636	Amp	17	7877743	7893365	0	0.004257
24637	Amp	17	7897764	8024546	0	0.010491
24638	Amp	17	8026459	8102587	0	0.01335
24639	Amp	17	8112695	8120732	0	0.01448
24640	Amp	17	8129964	8226373	0	0.010718
24641	Amp	17	8227549	8287899	0	0.011297
24642	Amp	17	8290260	8410924	0	0.008656
24643	Amp	17	8411801	8447713	0	0.008077
24644	Amp	17	8457600	8593740	0	0.011526

24645	Amp	17	8598243	8617501	0	0.013458
24646	Amp	17	8617849	8618923	0	0.01491
24647	Amp	17	8622935	8659776	0	0.012977
24648	Amp	17	8662038	8754569	0	0.011526
24649	Amp	17	8758708	8785416	0	0.013174
24650	Amp	17	8785947	8808490	0	0.009726
24651	Amp	17	8809017	8884178	0	0.003492
24652	Amp	17	8888776	8953275	0	0.019428
24653	Amp	17	8956249	9067912	0	0.003492
24654	Amp	17	9068870	9107428	0	0.006528
24655	Amp	17	9109384	9173220	0	0.003492
24656	Amp	17	9180060	9243942	0	0.001843
24657	Amp	17	9244743	9277162	0	0.009629
24658	Amp	17	9278497	9600094	0	0.001843
24659	Amp	17	9601611	9666348	0	0.004733
24660	Amp	17	9669305	9708011	0	0.003604
24661	Amp	17	9713388	9750535	0	0.00289
24662	Amp	17	9750747	11343188	0	0
24663	Amp	17	11343742	11484199	0	0.00102
24664	Amp	17	11484810	11916040	0	0
24665	Amp	17	11919133	11936714	0	0.001476
24666	Amp	17	11939643	12011145	0	0.003947
24667	Amp	17	12015324	12031709	0	0.002471
24668	Amp	17	12036193	12168800	0	0
24669	Amp	17	12172375	12195851	0	0.004046
24670	Amp	17	12198764	12322858	0	0
24671	Amp	17	12322873	12547373	0	0.003809
24672	Amp	17	12552765	12620950	0	0.009388
24673	Amp	17	12628368	12850067	0	0.003809
24674	Amp	17	12850248	13050478	0	0
24675	Amp	17	13056722	13097177	0	0.001962
24676	Amp	17	13097566	13720952	0	0
24677	Amp	17	13721185	13775518	0	0.000903
24678	Amp	17	13775742	13806480	0	0.001954
24679	Amp	17	13807593	13975996	0	0.002692
24680	Amp	17	13977100	14184629	0	0.004669
24681	Amp	17	14190945	14229461	0	0.005277
24682	Amp	17	14231324	14231324	0	0.004227
24683	Amp	17	15654216	15685870	0	0.010242
24684	Amp	17	15778279	15778279	0	0.013293
24685	Amp	17	15779264	16004943	0	0.012684
24686	Amp	17	16006972	16073196	0	0.015498
24687	Amp	17	16081727	16254177	0	0.031434
24688	Amp	17	16256248	16278539	0	0.037869
24689	Amp	17	16281007	16355784	0	0.04441
24690	Amp	17	16356234	16367946	0	0.045256
24691	Amp	17	16368410	16509028	0	0.048066
24692	Amp	17	16510295	16520784	0	0.04722
24693	Amp	17	16525187	16587259	0	0.048912
24694	Amp	17	16593088	16642735	0	0.04722
24695	Amp	17	16643163	16652195	0	0.043601
24696	Amp	17	16660167	16660167	0	0.045255
24697	Amp	17	16660241	16664225	0	0.061066

24698	Amp	17	16678690	16713872	0.015369	0.06463
24699	Amp	17	16718444	16880332	0.050473	0.066168
24700	Amp	17	16880973	16923551	0.015369	0.064514
24701	Amp	17	16924480	16952296	0.060686	0.066592
24702	Amp	17	16953103	16965558	0.118001	0.068868
24703	Amp	17	16969093	17028199	0.100827	0.068187
24704	Amp	17	17030669	17032880	0.044794	0.065897
24705	Amp	17	17035660	17078897	0	0.06382
24706	Amp	17	17079439	17124143	0.065715	0.066764
24707	Amp	17	17132967	17179623	0.138391	0.069814
24708	Amp	17	17180726	17233099	0.210594	0.07263
24709	Amp	17	17239882	17359473	0.133193	0.069685
24710	Amp	17	17365471	17438040	0	0.063308
24711	Amp	17	17438272	17517873	0	0.060876
24712	Amp	17	17519294	17525351	0.010261	0.064292
24713	Amp	17	17526860	17541802	0	0.060876
24714	Amp	17	17544705	17549011	0	0.063543
24715	Amp	17	17555721	17557360	0.019169	0.064795
24716	Amp	17	17569299	17572846	0	0.062128
24717	Amp	17	17574070	17581340	0.015369	0.064565
24718	Amp	17	17588174	17590593	0	0.048628
24719	Amp	17	17593146	17633889	0	0.045813
24720	Amp	17	17640098	17640098	0	0.047233
24721	Amp	17	17641390	17743462	0	0.031421
24722	Amp	17	17744082	17746147	0	0.033033
24723	Amp	17	17747688	17751779	0	0.035427
24724	Amp	17	17756543	17761440	0	0.019641
24725	Amp	17	17762138	17777157	0	0.021135
24726	Amp	17	17780470	17786776	0	0.018741
24727	Amp	17	17801230	17846424	0	0.017129
24728	Amp	17	17849495	17862746	0	0.019029
24729	Amp	17	17862982	17862982	0	0.021048
24730	Amp	17	17869852	17870375	0	0.019553
24731	Amp	17	17870975	17886152	0	0.029154
24732	Amp	17	17890577	17914439	0	0.030403
24733	Amp	17	17918079	18029839	0	0.029154
24734	Amp	17	18032563	18032563	0	0.027254
24735	Amp	17	18032959	18037661	0	0.029002
24736	Amp	17	18043309	18043309	0	0.027582
24737	Amp	17	18049400	18097424	0	0.030805
24738	Amp	17	18099122	18110489	0	0.03672
24739	Amp	17	18113786	18123666	0	0.033113
24740	Amp	17	18123679	18152742	0	0.033941
24741	Amp	17	18154717	18171905	0	0.035236
24742	Amp	17	18176959	18179790	0	0.041513
24743	Amp	17	18187705	18198577	0	0.043948
24744	Amp	17	18200986	18231613	0	0.045134
24745	Amp	17	18232087	18239426	0	0.048339
24746	Amp	17	18239614	18277301	0	0.047087
24747	Amp	17	18277375	18333236	0	0.047059
24748	Amp	17	18346132	18347747	0	0.031182
24749	Amp	17	18349125	18361950	0	0.029996
24750	Amp	17	18370635	18375453	0	0.024668

24751	Amp	17	18375514	18386413	0	0.028097
24752	Amp	17	18390113	18390113	0	0.0351
24753	Amp	17	18647546	18647546	0	0.039629
24754	Amp	17	18659407	18659407	0	0.050781
24755	Amp	17	18666219	18668032	0	0.057363
24756	Amp	17	18668351	18668351	0	0.060492
24757	Amp	17	18668662	18678032	0	0.057063
24758	Amp	17	18695470	18699163	0	0.044377
24759	Amp	17	18699219	18699219	0	0.046453
24760	Amp	17	18706323	18860764	0	0.04018
24761	Amp	17	18861511	18878377	0	0.041172
24762	Amp	17	18879175	18936521	0	0.056346
24763	Amp	17	18938277	18952298	0	0.041172
24764	Amp	17	18952494	18957997	0	0.038974
24765	Amp	17	19246050	19290029	0.159312	0.070759
24766	Amp	17	19290924	19320937	0.276783	0.075408
24767	Amp	17	19321506	19339154	0.293269	0.076067
24768	Amp	17	19341575	19390328	0.379885	0.079047
24769	Amp	17	19390414	19390414	0.316877	0.076899
24770	Amp	17	19393216	19457898	0.241616	0.074008
24771	Amp	17	19463329	19483500	0.170723	0.071029
24772	Amp	17	19483653	19512202	0.112543	0.068621
24773	Amp	17	19514415	19523662	0.187449	0.071803
24774	Amp	17	19527969	19694407	0.259438	0.074793
24775	Amp	17	19699059	19783133	0.373599	0.078929
24776	Amp	17	19786035	19791175	0.569771	0.085412
24777	Amp	17	19794102	19803988	0.742723	0.090588
24778	Amp	17	19804756	19817435	0.536238	0.084451
24779	Amp	17	19817525	19864454	0.424557	0.080725
24780	Amp	17	19865340	19883717	0.660254	0.08816
24781	Amp	17	19885914	19901828	0.417873	0.08037
24782	Amp	17	19902297	19924419	0.431198	0.08093
24783	Amp	17	19928098	19945938	0.355319	0.07833
24784	Amp	17	19956375	19969914	0.386426	0.079418
24785	Amp	17	19971349	20059930	0.355319	0.07833
24786	Amp	17	20064697	20065107	0.882843	0.094266
24787	Amp	17	20066084	20076946	0.977077	0.096822
24788	Amp	17	20078540	20079160	0.915698	0.095321
24789	Amp	17	20079592	20140497	1.197658	0.101881
24790	Amp	17	20150739	20163352	1.348715	0.105321
24791	Amp	17	20165258	20165415	1.032525	0.098102
24792	Amp	17	20171849	20171849	1.093834	0.099575
24793	Amp	17	20349166	20350100	2.18114	0.122688
24794	Amp	17	20367917	20387400	2.33403	0.125728
24795	Amp	17	20389632	20389632	2.368227	0.126541
24796	Amp	17	20403746	20426903	2.147988	0.12225
24797	Amp	17	20530272	20530556	1.994915	0.119199
24798	Amp	17	20554187	20559861	1.833307	0.115849
24799	Amp	17	20563168	20636913	1.940776	0.118007
24800	Amp	17	20644325	20652954	2.060912	0.120501
24801	Amp	17	20663490	20705970	1.994915	0.119028
24802	Amp	17	20716086	20716086	2.73206	0.133475
24803	Amp	17	20716473	20716473	2.837591	0.135416

24804	Amp	17	20717534	20721413	2.637742	0.131754
24805	Amp	17	20724896	20724896	2.825055	0.135223
24806	Amp	17	20724988	20766097	2.9061	0.136696
24807	Amp	17	20766551	20795034	2.800837	0.134755
24808	Amp	17	20805271	20835921	3.057106	0.139619
24809	Amp	17	20839307	20856240	2.929372	0.137124
24810	Amp	17	20859472	20879044	2.964305	0.137741
24811	Amp	17	20879173	20885792	3.067933	0.13968
24812	Amp	17	20889714	20898426	2.986184	0.138117
24813	Amp	17	20898523	20986798	2.929372	0.137074
24814	Amp	17	20987415	20987415	2.89426	0.136458
24815	Amp	17	20990259	20998260	2.696333	0.132714
24816	Amp	17	21000463	21005517	3.422847	0.146328
24817	Amp	17	21006331	21011322	3.505046	0.147765
24818	Amp	17	21014469	21032426	2.765626	0.134151
24819	Amp	17	21039255	21102632	2.975629	0.138039
24820	Amp	17	21109210	21129820	2.755154	0.1339
24821	Amp	17	21137758	21153986	2.571513	0.130298
24822	Amp	17	21161747	21230022	2.860424	0.135798
24823	Amp	17	21238301	21249216	2.571513	0.130298
24824	Amp	17	21255266	21257989	2.582422	0.130558
24825	Amp	17	21259349	21264981	1.780186	0.114674
24826	Amp	17	21268046	21277414	1.583409	0.110697
24827	Amp	17	21280472	21289118	1.463774	0.107948
24828	Amp	17	21347106	21353427	1.665486	0.11227
24829	Amp	17	21359367	21361139	1.542638	0.109688
24830	Amp	17	21361403	21370525	1.398396	0.106509
24831	Amp	17	21372211	21372211	1.023539	0.097733
24832	Amp	17	21372859	21391421	1.121584	0.100171
24833	Amp	17	21394488	21399126	0.995635	0.097152
24834	Amp	17	21399695	21399695	0.968522	0.096468
24835	Amp	17	26943491	26943491	2.311329	0.125304
24836	Amp	17	26943774	26943774	2.083076	0.120824
24837	Amp	17	26947350	26947350	2.290039	0.12498
24838	Amp	17	26949301	26949301	2.446878	0.127999
24839	Amp	17	26955079	26960035	2.755154	0.133951
24840	Amp	17	26970127	26975284	2.800837	0.134646
24841	Amp	17	26982437	26983019	3.628913	0.149995
24842	Amp	17	26986664	26999934	3.603523	0.149516
24843	Amp	17	27002448	27005190	3.678399	0.15075
24844	Amp	17	27006860	27006860	3.788687	0.152837
24845	Amp	17	27009727	27014516	3.701758	0.151276
24846	Amp	17	27015487	27036628	3.814483	0.153108
24847	Amp	17	27036713	27036773	3.590086	0.149278
24848	Amp	17	27049380	27049380	3.150497	0.141294
24849	Amp	17	27128159	27128159	3.207115	0.142241
24850	Amp	17	27128903	27128903	3.323812	0.144544
24851	Amp	17	27135382	27139975	4.457399	0.163836
24852	Amp	17	27141574	27148519	4.513276	0.164757
24853	Amp	17	27153627	27208042	4.443348	0.163563
24854	Amp	17	27209151	27217862	4.47072	0.164009
24855	Amp	17	27220417	27225574	4.584613	0.165798
24856	Amp	17	27226847	27230524	4.739548	0.168155

24857	Amp	17	27230970	27254773	4.809994	0.169155
24858	Amp	17	27257237	27266467	4.952273	0.171336
24859	Amp	17	27268251	27317024	5.169562	0.17465
24860	Amp	17	27317043	27331289	5.067861	0.17318
24861	Amp	17	27335248	27345634	5.852205	0.184686
24862	Amp	17	27346753	27347318	6.626113	0.195694
24863	Amp	17	27358026	27365327	6.825391	0.198672
24864	Amp	17	27365943	27365943	6.53507	0.19443
24865	Amp	17	27369128	27381934	7.074542	0.202036
24866	Amp	17	27382312	27387735	7.135839	0.202893
24867	Amp	17	27388573	27410050	7.357785	0.205869
24868	Amp	17	27410913	27434509	7.167722	0.203454
24869	Amp	17	27435891	27438955	7.248181	0.204475
24870	Amp	17	27441519	27445903	7.421526	0.206869
24871	Amp	17	27447869	27488287	7.594603	0.209228
24872	Amp	17	27488345	27496448	7.485352	0.207731
24873	Amp	17	27498646	27498938	7.310837	0.205337
24874	Amp	17	27499102	27505659	7.215959	0.204103
24875	Amp	17	27513213	27526624	6.671073	0.196497
24876	Amp	17	27531500	27532454	7.389113	0.206403
24877	Amp	17	27537790	27550676	7.594603	0.209252
24878	Amp	17	27558497	27587284	7.373439	0.206274
24879	Amp	17	27593982	27600902	7.531798	0.208305
24880	Amp	17	27601008	27611096	7.75231	0.211481
24881	Amp	17	27613792	27627330	7.421526	0.206769
24882	Amp	17	27627921	27647232	7.264196	0.204571
24883	Amp	17	27647591	27662327	7.437327	0.206948
24884	Amp	17	27662888	27688560	7.6425	0.209912
24885	Amp	17	27690219	27690219	7.689477	0.210413
24886	Amp	17	27690378	27700427	7.610655	0.209425
24887	Amp	17	27702197	27704599	7.074542	0.202024
24888	Amp	17	27706234	27709503	7.341956	0.205844
24889	Amp	17	27718804	27722899	7.215959	0.204055
24890	Amp	17	27723373	27729127	7.295403	0.205156
24891	Amp	17	27731367	27809669	8.073986	0.215754
24892	Amp	17	27811096	27822889	7.6425	0.209857
24893	Amp	17	27826999	27854839	8.249918	0.218154
24894	Amp	17	27858801	27858801	7.485352	0.207602
24895	Amp	17	27864760	27869146	7.578613	0.208904
24896	Amp	17	27870129	27876799	7.6425	0.209926
24897	Amp	17	27880781	27886628	8.137603	0.216638
24898	Amp	17	27889049	27894573	8.282609	0.218547
24899	Amp	17	27895459	27920670	7.896426	0.213302
24900	Amp	17	27920862	27937997	8.088933	0.216019
24901	Amp	17	27938662	27939811	8.555266	0.22225
24902	Amp	17	27940561	27955549	7.500789	0.208012
24903	Amp	17	27957894	27958499	7.817451	0.212336
24904	Amp	17	27959798	27980474	7.120018	0.202654
24905	Amp	17	27980692	27990702	7.279386	0.204935
24906	Amp	17	27994036	27994036	7.042879	0.201675
24907	Amp	17	28000866	28002165	5.942149	0.185921
24908	Amp	17	28002669	28010677	6.062596	0.187728
24909	Amp	17	28010866	28011680	6.137384	0.18883

24910	Amp	17	28014594	28028008	5.852205	0.184677
24911	Amp	17	28030073	28035263	5.762293	0.183374
24912	Amp	17	28039825	28057419	5.852205	0.184666
24913	Amp	17	28057983	28071104	5.927128	0.185813
24914	Amp	17	28071725	28077683	5.626868	0.181489
24915	Amp	17	28078782	28111827	5.732156	0.182923
24916	Amp	17	28113846	28119054	5.641615	0.181595
24917	Amp	17	28119955	28122663	5.687617	0.182195
24918	Amp	17	28123203	28127834	5.762293	0.183394
24919	Amp	17	28134797	28160781	5.641615	0.181586
24920	Amp	17	28166498	28168562	5.86703	0.184867
24921	Amp	17	28173585	28184050	5.434517	0.178613
24922	Amp	17	28185221	28185401	5.702358	0.182438
24923	Amp	17	28186884	28201049	5.611467	0.181291
24924	Amp	17	28204266	28205652	5.241829	0.175821
24925	Amp	17	28205828	28213604	5.345638	0.177379
24926	Amp	17	28217106	28217106	5.09526	0.173554
24927	Amp	17	28219464	28261333	6.032925	0.187367
24928	Amp	17	28262360	28262360	6.1074	0.188355
24929	Amp	17	28263287	28263287	6.032925	0.187255
24930	Amp	17	28277950	28282495	5.882356	0.185097
24931	Amp	17	28286650	28287993	6.380906	0.192291
24932	Amp	17	28293068	28308353	6.488712	0.193846
24933	Amp	17	28309717	28322841	7.199931	0.203744
24934	Amp	17	28331136	28333018	7.468873	0.207472
24935	Amp	17	28333963	28333963	6.473343	0.193528
24936	Amp	17	28338742	28343420	6.825391	0.198518
24937	Amp	17	28346290	28346290	6.949342	0.200233
24938	Amp	17	28348211	28348239	7.373439	0.206278
24939	Amp	17	28348367	28348367	7.75231	0.211466
24940	Amp	17	28350472	28383911	8.137603	0.21669
24941	Amp	17	28387191	28395910	8.361498	0.219732
24942	Amp	17	28406703	28423326	8.32882	0.219232
24943	Amp	17	28425576	28425576	8.458247	0.220918
24944	Amp	17	28426973	28441789	8.282609	0.218632
24945	Amp	17	28442114	28442114	8.393699	0.220166
24946	Amp	17	28446973	28464723	9.47615	0.234275
24947	Amp	17	28465889	28470564	9.796693	0.238361
24948	Amp	17	28479852	28479852	9.272857	0.231585
24949	Amp	17	28480716	28510504	9.18903	0.230585
24950	Amp	17	28510520	28551338	9.357173	0.232639
24951	Amp	17	28556295	28633740	9.712919	0.237125
24952	Amp	17	28649928	28666144	10.961251	0.253061
24953	Amp	17	28670493	28682841	10.808758	0.251153
24954	Amp	17	28687633	28694353	10.843331	0.251645
24955	Amp	17	28699905	28705809	12.021369	0.266204
24956	Amp	17	28713286	28728422	12.473924	0.27156
24957	Amp	17	28737334	28739945	12.564467	0.272625
24958	Amp	17	28746511	28750313	12.401399	0.270726
24959	Amp	17	28755692	28796471	12.27458	0.269172
24960	Amp	17	28796798	28805724	12.130806	0.267339
24961	Amp	17	28809674	28836344	11.894175	0.264673
24962	Amp	17	28843768	28855926	12.219704	0.268519

24963 Amp	17	28858809	28868899	12.600251	0.273092
24964 Amp	17	28873716	28885586	12.112615	0.267124
24965 Amp	17	28888388	28914928	12.347637	0.270107
24966 Amp	17	28919135	28929837	11.966547	0.265393
24967 Amp	17	28930108	28939125	12.148216	0.267572
24968 Amp	17	28940453	28940490	12.256206	0.269024
24969 Amp	17	28944821	28971983	10.961251	0.253088
24970 Amp	17	28978323	28978621	10.720769	0.250104
24971 Amp	17	28979365	29000723	10.877729	0.251931
24972 Amp	17	29000929	29006266	10.549344	0.247918
24973 Amp	17	29012187	29012187	11.049458	0.254177
24974 Amp	17	29012647	29055612	10.927313	0.252605
24975 Amp	17	29057130	29097298	11.049458	0.254224
24976 Amp	17	29097825	29116691	11.115911	0.255044
24977 Amp	17	29118978	29145225	10.961251	0.252991
24978 Amp	17	29146710	29152682	10.583831	0.248403
24979 Amp	17	29156274	29183658	10.465282	0.24687
24980 Amp	17	29184944	29201085	10.791286	0.250871
24981 Amp	17	29201365	29215463	10.893171	0.252261
24982 Amp	17	29216402	29238817	10.669096	0.249319
24983 Amp	17	29243604	29253219	10.346125	0.245317
24984 Amp	17	29254545	29258038	10.482498	0.247052
24985 Amp	17	29263936	29279685	10.8261	0.251272
24986 Amp	17	29283844	29323930	10.634596	0.248974
24987 Amp	17	29327586	29340150	10.51732	0.247344
24988 Amp	17	29341312	29353130	10.669096	0.249354
24989 Amp	17	29353857	29353857	9.644619	0.23625
24990 Amp	17	29357114	29370601	9.559998	0.235229
24991 Amp	17	29372277	29376331	9.644619	0.236382
24992 Amp	17	29379868	29404199	9.899054	0.239568
24993 Amp	17	29408699	29414859	9.780264	0.238178
24994 Amp	17	29418730	29425465	9.661346	0.236492
24995 Amp	17	29433125	29441030	9.54314	0.23504
24996 Amp	17	29445072	29445072	9.493034	0.234345
24997 Amp	17	29461239	29495783	9.155442	0.230125
24998 Amp	17	29500350	29510641	8.834661	0.225915
24999 Amp	17	29515260	29530680	9.02098	0.228243
25000 Amp	17	29532498	29537372	7.833797	0.212489
25001 Amp	17	29539043	29557587	7.058587	0.201817
25002 Amp	17	29557679	29628743	7.106137	0.202571
25003 Amp	17	29630244	29635375	7.279386	0.204941
25004 Amp	17	29640696	29640696	7.152075	0.203049
25005 Amp	17	29649718	29651676	7.279386	0.204888
25006 Amp	17	29656658	29659362	6.964631	0.200508
25007 Amp	17	29660223	29679262	6.886863	0.199401
25008 Amp	17	29682268	29685359	6.810251	0.198288
25009 Amp	17	29686309	29688372	6.182794	0.189385
25010 Amp	17	29689639	29699473	6.04774	0.187545
25011 Amp	17	29702842	29759404	4.966536	0.171609
25012 Amp	17	29763006	29789275	4.654424	0.166809
25013 Amp	17	29791417	29808434	4.924414	0.170913
25014 Amp	17	29820569	29838412	4.102728	0.158117
25015 Amp	17	29841555	29854557	4.290674	0.161153

25016 Amp	17	29855176	29855176	4.209693	0.159944
25017 Amp	17	29855667	29875832	4.809994	0.169244
25018 Amp	17	29876285	29894988	4.612121	0.166208
25019 Amp	17	29901674	29906544	4.169329	0.159175
25020 Amp	17	29907636	29920233	4.196711	0.159714
25021 Amp	17	29922284	29960909	4.062894	0.157637
25022 Amp	17	29965623	29998584	4.156262	0.159127
25023 Amp	17	30001801	30014862	3.972019	0.156026
25024 Amp	17	30021298	30022077	3.898065	0.154732
25025 Amp	17	30026375	30036495	3.839479	0.153667
25026 Amp	17	30037949	30037949	3.898065	0.154801
25027 Amp	17	30049540	30077563	4.102728	0.158185
25028 Amp	17	30079278	30079278	3.874391	0.154345
25029 Amp	17	30083754	30111845	3.775276	0.15261
25030 Amp	17	30117165	30131619	3.5415	0.14836
25031 Amp	17	30134181	30149440	3.690482	0.151015
25032 Amp	17	30149990	30167955	3.6657	0.150492
25033 Amp	17	30170180	30182310	3.323812	0.144488
25034 Amp	17	30188461	30196708	3.471122	0.147109
25035 Amp	17	30197396	30223885	3.8021	0.153003
25036 Amp	17	30225968	30264732	3.738665	0.151869
25037 Amp	17	30266065	30349613	3.603523	0.14951
25038 Amp	17	30352088	30358931	3.814483	0.153283
25039 Amp	17	30360178	30376863	3.554363	0.148658
25040 Amp	17	30377445	30388446	3.690482	0.151014
25041 Amp	17	30390515	30390515	3.578505	0.149124
25042 Amp	17	30390824	30421740	3.411175	0.145939
25043 Amp	17	30422394	30424353	2.825055	0.13515
25044 Amp	17	30430205	30463858	2.696333	0.132794
25045 Amp	17	30466299	30478928	2.235168	0.12391
25046 Amp	17	30480432	30538896	2.402099	0.127157
25047 Amp	17	30538951	30599410	2.425182	0.127612
25048 Amp	17	30601980	30606411	2.094104	0.121145
25049 Amp	17	30606938	30606938	2.049917	0.120153
25050 Amp	17	30609191	30609191	1.919241	0.117552
25051 Amp	17	30609785	30610646	1.876242	0.116782
25052 Amp	17	30625011	30625011	1.358811	0.105685
25053 Amp	17	30625268	30625268	1.299876	0.104251
25054 Amp	17	30848226	30848226	1.573014	0.110359
25055 Amp	17	30860091	30873571	1.270711	0.103746
25056 Amp	17	30882725	30887523	1.348715	0.105433
25057 Amp	17	30888624	30889227	0.602643	0.086425
25058 Amp	17	30891213	30898267	0.616549	0.08687
25059 Amp	17	30907487	30924755	0.431198	0.080919
25060 Amp	17	30928011	30956706	0.287738	0.075929
25061 Amp	17	30959567	30999717	0.362016	0.078509
25062 Amp	17	31000110	31000110	0.34892	0.078063
25063 Amp	17	31006498	31006498	0.276783	0.075484
25064 Amp	17	31009319	31018265	0.164935	0.070878
25065 Amp	17	31020014	31020014	0.079652	0.067361
25066 Amp	17	31020508	31022433	0.107017	0.068468
25067 Amp	17	31023751	31058215	0.069848	0.066997
25068 Amp	17	31063081	31099063	0	0.062853

25069	Amp	17	31101199	31105890	0.034113	0.065444
25070	Amp	17	31107288	31107288	0.056211	0.066255
25071	Amp	17	31108188	31125921	0	0.062678
25072	Amp	17	31130728	31149453	0.029031	0.065277
25073	Amp	17	31151564	31161822	0.005328	0.06417
25074	Amp	17	31165405	31222983	0.094967	0.068072
25075	Amp	17	31228136	31251342	0.14896	0.070273
25076	Amp	17	31251366	31270059	0.1237	0.069251
25077	Amp	17	31270475	31288983	0.227787	0.073234
25078	Amp	17	31294374	31295292	0.044794	0.06589
25079	Amp	17	31296270	31322150	0	0.062478
25080	Amp	17	31324462	31325438	0	0.059697
25081	Amp	17	31332183	31363075	0	0.061787
25082	Amp	17	31368557	31370980	0	0.059697
25083	Amp	17	31371736	31395601	0	0.061587
25084	Amp	17	31408634	31459383	0.001323	0.064022
25085	Amp	17	31467452	31469319	0	0.058551
25086	Amp	17	31473734	31490346	0	0.059315
25087	Amp	17	31491956	31491956	0	0.056881
25088	Amp	17	31496993	31502656	0	0.054991
25089	Amp	17	31506083	31524217	0	0.05417
25090	Amp	17	31525074	31545612	0	0.057465
25091	Amp	17	31547530	31547530	0	0.05417
25092	Amp	17	31552351	31576317	0	0.051548
25093	Amp	17	31577693	31588689	0	0.048086
25094	Amp	17	31593921	31596261	0	0.05217
25095	Amp	17	31599513	31615959	0	0.053776
25096	Amp	17	31621408	31624955	0	0.054565
25097	Amp	17	31626382	31635714	0	0.055792
25098	Amp	17	31638294	31715932	0	0.053591
25099	Amp	17	31716203	31748949	0	0.049608
25100	Amp	17	31751280	31758813	0	0.047737
25101	Amp	17	31782635	31790286	0	0.044886
25102	Amp	17	31790442	31793672	0	0.04328
25103	Amp	17	31794742	31809992	0	0.053141
25104	Amp	17	31812564	31930413	0	0.049057
25105	Amp	17	31932431	31967786	0	0.045743
25106	Amp	17	31971835	32017492	0	0.046251
25107	Amp	17	32020483	32035390	0	0.052987
25108	Amp	17	32036849	32085782	0	0.054232
25109	Amp	17	32086638	32086638	0	0.054968
25110	Amp	17	32086877	32098519	0	0.056032
25111	Amp	17	32102908	32109624	0	0.057558
25112	Amp	17	32109665	32123771	0	0.059807
25113	Amp	17	32128962	32148412	0	0.058562
25114	Amp	17	32153161	32171175	0	0.061028
25115	Amp	17	32174383	32176982	0	0.059964
25116	Amp	17	32182216	32182216	0	0.060411
25117	Amp	17	32184255	32184377	0.1237	0.069085
25118	Amp	17	32186096	32204078	0.079652	0.067388
25119	Amp	17	32206112	32252163	0	0.058715
25120	Amp	17	32253253	32261548	0.089561	0.067915
25121	Amp	17	32268820	32275119	0.019169	0.064844

25122	Amp	17	32275698	32319068	0.019169	0.06488
25123	Amp	17	32327167	32361741	0.118001	0.068869
25124	Amp	17	32365377	32368140	0.19964	0.072239
25125	Amp	17	32369841	32410813	0.17597	0.071247
25126	Amp	17	32411158	32421126	0.299433	0.076397
25127	Amp	17	32428471	32442634	0.689884	0.089103
25128	Amp	17	32445574	32445574	0.711903	0.089683
25129	Amp	17	32446459	32500014	0.774648	0.091484
25130	Amp	17	32502646	32511693	0.86541	0.093982
25131	Amp	17	32512101	32535046	0.765474	0.091281
25132	Amp	17	32537681	32537886	0.667587	0.088486
25133	Amp	17	32539263	32620465	0.583752	0.085987
25134	Amp	17	32621535	32632883	0.72817	0.090258
25135	Amp	17	32633427	32640250	0.704849	0.089494
25136	Amp	17	32640754	32647481	0.563096	0.085223
25137	Amp	17	32649504	32664479	0.128324	0.069287
25138	Amp	17	32665169	32678207	0.079652	0.067486
25139	Amp	17	32686070	32686070	0.508568	0.083422
25140	Amp	17	32689543	32703166	0.557122	0.084993
25141	Amp	17	32707856	32724758	0.508568	0.083422
25142	Amp	17	32727213	32730725	0.569771	0.085507
25143	Amp	17	32733510	32746830	0.417873	0.080357
25144	Amp	17	32748789	32748789	0.660254	0.088178
25145	Amp	17	32751204	32761392	0.882843	0.094314
25146	Amp	17	32764743	32768601	1.188121	0.101631
25147	Amp	17	32772311	32779179	0.924746	0.095495
25148	Amp	17	32779827	32806751	0.941704	0.09597
25149	Amp	17	32806792	32806792	0.857296	0.093727
25150	Amp	17	32807544	32821838	0.891602	0.094542
25151	Amp	17	32823280	32833441	0.941704	0.095908
25152	Amp	17	32834086	32834086	0.968522	0.096441
25153	Amp	17	32849022	32849022	0.98589	0.096897
25154	Amp	17	32851582	32853505	1.00505	0.09743
25155	Amp	17	32855516	32871458	1.472629	0.108205
25156	Amp	17	32878600	32895346	1.149583	0.100889
25157	Amp	17	32897445	32912697	1.309159	0.104502
25158	Amp	17	32914122	32923943	1.149583	0.100889
25159	Amp	17	32932023	32932023	0.720659	0.090114
25160	Amp	17	32934445	32934956	0.882843	0.094391
25161	Amp	17	32936555	32946860	0.86541	0.093935
25162	Amp	17	32948410	32957646	0.704849	0.089657
25163	Amp	17	32958726	32979750	1.112463	0.100004
25164	Amp	17	32983016	32983016	0.823771	0.092821
25165	Amp	17	32984659	32984659	0.848692	0.093495
25166	Amp	17	32985397	32993208	1.329472	0.105089
25167	Amp	17	32998518	33024103	0.689884	0.089153
25168	Amp	17	33027075	33028126	0.424557	0.080632
25169	Amp	17	33034989	33037988	0.689884	0.089183
25170	Amp	17	33038076	33045000	0.774648	0.091463
25171	Amp	17	33045170	33045291	1.436692	0.1074
25172	Amp	17	33045607	33045927	1.33853	0.105119
25173	Amp	17	33046256	33054800	1.261401	0.103515
25174	Amp	17	33055116	33057821	1.075952	0.099194

25175 Amp	17	33058725	33058989	1.225419	0.102545
25176 Amp	17	33065894	33068274	1.369395	0.105919
25177 Amp	17	33071828	33078063	1.522708	0.109321
25178 Amp	17	33080143	33122974	1.378521	0.105971
25179 Amp	17	33123646	33136391	1.319393	0.104715
25180 Amp	17	33136531	33146651	1.398396	0.106431
25181 Amp	17	33148479	33163863	0.704849	0.089622
25182 Amp	17	33164489	33167278	0.79909	0.092254
25183 Amp	17	33168568	33180367	0.924746	0.095527
25184 Amp	17	33180801	33180801	0.6737	0.088598
25185 Amp	17	33184168	33199906	0.616549	0.086881
25186 Amp	17	33201675	33201675	0.557122	0.085086
25187 Amp	17	33208279	33225531	0.735486	0.090472
25188 Amp	17	33229500	33229500	0.622604	0.087199
25189 Amp	17	33230867	33238250	0.609133	0.086691
25190 Amp	17	33239531	33266293	0.711903	0.089683
25191 Amp	17	33267149	33267149	0.765474	0.091326
25192 Amp	17	33273992	33283693	0.563096	0.085257
25193 Amp	17	33286245	33286245	0.667587	0.088508
25194 Amp	17	33286476	33299837	0.697374	0.089369
25195 Amp	17	33299894	33302953	0.790614	0.09186
25196 Amp	17	33305339	33322609	0.873678	0.094113
25197 Amp	17	33325304	33332489	0.774648	0.091423
25198 Amp	17	33332573	33342616	0.689884	0.089169
25199 Amp	17	33342829	33350142	0.494792	0.083012
25200 Amp	17	33351805	33376305	0.589605	0.086049
25201 Amp	17	33376335	33389964	0.536238	0.084442
25202 Amp	17	33391219	33401716	0.522881	0.083862
25203 Amp	17	33403363	33410137	0.681869	0.088821
25204 Amp	17	33413163	33420151	0.522881	0.083862
25205 Amp	17	33422327	33433074	0.431198	0.080849
25206 Amp	17	33433742	33447349	0.34892	0.078096
25207 Amp	17	33447681	33461505	0.222041	0.072969
25208 Amp	17	33462945	33470401	0.386426	0.079363
25209 Amp	17	33470685	33525930	0.222041	0.072969
25210 Amp	17	33525956	33532727	0.138391	0.069718
25211 Amp	17	33532953	33550232	0.069848	0.067087
25212 Amp	17	33551570	33551570	0.118001	0.068936
25213 Amp	17	33552571	33554867	0.044794	0.065898
25214 Amp	17	33560086	33569540	0.450702	0.081529
25215 Amp	17	33570078	33610568	0.424557	0.080668
25216 Amp	17	33612028	33617604	0.536238	0.084434
25217 Amp	17	33617879	33626719	0.765474	0.091229
25218 Amp	17	33626837	33630252	0.595248	0.086318
25219 Amp	17	33631162	33631162	0.543344	0.084469
25220 Amp	17	33631275	33631275	0.246818	0.074114
25221 Amp	17	34611015	34611015	0.065715	0.066831
25222 Amp	17	34616624	34616624	0.100827	0.068323
25223 Amp	17	34617146	34617146	0.170723	0.071024
25224 Amp	17	34620083	34624786	0.118001	0.06894
25225 Amp	17	34629490	34669731	0.044794	0.065954
25226 Amp	17	34669734	34670294	0.029031	0.065266
25227 Amp	17	34673067	34727870	0	0.062116

25228	Amp	17	34728921	34738582	0	0.061381
25229	Amp	17	34738975	34794151	0	0.060905
25230	Amp	17	34797565	34826300	0.001323	0.063843
25231	Amp	17	34826438	34831774	0.100827	0.068234
25232	Amp	17	34833762	34843009	0.08435	0.067701
25233	Amp	17	34850269	34850269	0.073902	0.067168
25234	Amp	17	34852943	34852943	0.029031	0.065307
25235	Amp	17	34854889	34867297	0.001323	0.063956
25236	Amp	17	34874599	34874599	0.08435	0.067629
25237	Amp	17	34877001	34883670	0.005328	0.064113
25238	Amp	17	34885348	34885847	0	0.063666
25239	Amp	17	34886914	34935363	0	0.048023
25240	Amp	17	34941290	34994291	0	0.050946
25241	Amp	17	34994480	35058954	0	0.047273
25242	Amp	17	35065066	35075145	0	0.04139
25243	Amp	17	35075433	35089986	0	0.045002
25244	Amp	17	35095139	35126223	0	0.045708
25245	Amp	17	35127211	35137845	0	0.047501
25246	Amp	17	35138854	35143830	0	0.046796
25247	Amp	17	35146648	35151934	0	0.047863
25248	Amp	17	35155270	35155270	0	0.054002
25249	Amp	17	35155873	35173107	0	0.055841
25250	Amp	17	35174117	35188145	0	0.055029
25251	Amp	17	35197079	35197079	0	0.046358
25252	Amp	17	35197656	35197656	0	0.041017
25253	Amp	17	35198080	35212918	0	0.047537
25254	Amp	17	35213457	35223003	0	0.041017
25255	Amp	17	35224278	35224278	0	0.04452
25256	Amp	17	35231256	35325827	0	0.046732
25257	Amp	17	35326451	35349487	0	0.051695
25258	Amp	17	35442187	35519398	0	0.054127
25259	Amp	17	35519689	35546331	0	0.056779
25260	Amp	17	35548950	35566359	0	0.055712
25261	Amp	17	35566490	35604029	0	0.058672
25262	Amp	17	35605189	35628224	0	0.055748
25263	Amp	17	35635990	35637180	0	0.053909
25264	Amp	17	35642388	35642388	0	0.057195
25265	Amp	17	35643663	35644202	0	0.061846
25266	Amp	17	35645470	35688078	0.015369	0.064608
25267	Amp	17	35695509	35710401	0	0.061219
25268	Amp	17	35717323	35717628	0	0.061658
25269	Amp	17	35717693	35717846	0.001323	0.064012
25270	Amp	17	35718179	35719905	0.019169	0.064864
25271	Amp	17	35727883	35728523	0.010261	0.064424
25272	Amp	17	35728624	35733982	0.034113	0.065483
25273	Amp	17	35739538	35745223	0.079652	0.067508
25274	Amp	17	35747690	35817153	0.056211	0.066246
25275	Amp	17	35821940	35821940	0	0.062426
25276	Amp	17	35822008	35849632	0.010261	0.064491
25277	Amp	17	35861288	35866084	0	0.058319
25278	Amp	17	35869551	35877803	0	0.061123
25279	Amp	17	35883693	35891322	0.005328	0.064069
25280	Amp	17	35900191	35923013	0	0.060784

25281	Amp	17	35925533	35925533	0.005328	0.064097
25282	Amp	17	35925775	35978820	0.170723	0.071135
25283	Amp	17	35979717	36002429	0.005328	0.064097
25284	Amp	17	36007429	36045781	0.14896	0.070196
25285	Amp	17	36046285	36067159	0.210594	0.072691
25286	Amp	17	36567187	36567187	0.299433	0.076343
25287	Amp	17	36567203	36567203	0.08435	0.067567
25288	Amp	17	36567250	36579005	0.019169	0.064915
25289	Amp	17	36586260	36586260	0.210594	0.072589
25290	Amp	17	36586276	36586320	0.227787	0.073185
25291	Amp	17	36586440	36590030	0.133193	0.06957
25292	Amp	17	36594718	36595100	0.164935	0.070832
25293	Amp	17	36601152	36604494	0.609133	0.086768
25294	Amp	17	36609563	36621378	0.379885	0.079094
25295	Amp	17	36624787	36661585	0.906385	0.09503
25296	Amp	17	36664701	36695244	1.270711	0.103597
25297	Amp	17	36695257	36695257	1.309159	0.104502
25298	Amp	17	36699270	36700930	1.131695	0.100451
25299	Amp	17	36703273	36725564	1.854421	0.116387
25300	Amp	17	36726659	36736537	1.456037	0.107821
25301	Amp	17	36737226	36766845	1.552619	0.110045
25302	Amp	17	36768968	36781105	1.68612	0.112847
25303	Amp	17	36790071	36790071	0.98589	0.096911
25304	Amp	17	36791018	36805589	1.206641	0.102163
25305	Amp	17	36811048	36811934	1.280185	0.104008
25306	Amp	17	36813767	36818067	1.084294	0.099289
25307	Amp	17	36819516	36827874	1.178324	0.101418
25308	Amp	17	36831827	36866720	1.093834	0.099573
25309	Amp	17	36869757	36869757	1.058188	0.098609
25310	Amp	17	36869832	36905815	0.891602	0.094546
25311	Amp	17	36906542	36917188	0.857296	0.09364
25312	Amp	17	36924242	37016003	1.023539	0.097928
25313	Amp	17	37018798	37031737	1.00505	0.097332
25314	Amp	17	37034170	37068298	1.131695	0.100424
25315	Amp	17	37070416	37071765	1.407997	0.106715
25316	Amp	17	37075428	37128755	0.98589	0.096883
25317	Amp	17	37130412	37130412	0.781972	0.091651
25318	Amp	17	37131744	37155331	0.79909	0.092151
25319	Amp	17	37156537	37156537	0.735486	0.090426
25320	Amp	17	37158767	37171564	0.848692	0.093546
25321	Amp	17	37173812	37202372	0.735486	0.090426
25322	Amp	17	37205334	37206006	0.840289	0.093304
25323	Amp	17	37206473	37260192	0.891602	0.0946
25324	Amp	17	37265128	37271209	0.704849	0.089487
25325	Amp	17	37276086	37312387	1.23519	0.102817
25326	Amp	17	37317803	37326084	1.178324	0.101521
25327	Amp	17	37327486	37350841	1.388256	0.106318
25328	Amp	17	37354230	37363057	1.68612	0.112781
25329	Amp	17	37364852	37387774	1.522708	0.109256
25330	Amp	17	37393422	37413676	1.33853	0.105182
25331	Amp	17	37413714	37413714	1.61378	0.111344
25332	Amp	17	37425380	37442797	1.823153	0.115556
25333	Amp	17	37444069	37453278	2.104978	0.121231

25334 Amp	17	37453526	37462222	2.345513	0.126111
25335 Amp	17	37464512	37468330	2.202226	0.123233
25336 Amp	17	37469812	37483896	1.897704	0.117072
25337 Amp	17	37488149	37488935	1.398396	0.106493
25338 Amp	17	37494525	37508035	1.215958	0.10228
25339 Amp	17	37512249	37525047	1.270711	0.103599
25340 Amp	17	37529428	37564796	1.655056	0.112065
25341 Amp	17	37566724	37569488	1.748606	0.114125
25342 Amp	17	37570871	37576496	2.169666	0.122496
25343 Amp	17	37578954	37587118	1.604336	0.110961
25344 Amp	17	37589863	37590352	1.014678	0.097631
25345 Amp	17	37592841	37602431	1.159209	0.101048
25346 Amp	17	37610983	37611838	1.102589	0.09985
25347 Amp	17	37617593	37620780	1.512549	0.109165
25348 Amp	17	37622983	37640578	2.147988	0.122185
25349 Amp	17	37642932	37644912	2.256542	0.12435
25350 Amp	17	37649948	37667147	1.727286	0.113782
25351 Amp	17	37668223	37684274	1.887378	0.116921
25352 Amp	17	37685804	37685804	1.96212	0.118484
25353 Amp	17	37686063	37690786	2.094104	0.121062
25354 Amp	17	37691774	37707951	1.655056	0.112088
25355 Amp	17	37708110	37739849	2.22459	0.123673
25356 Amp	17	37743574	37749832	2.743935	0.133707
25357 Amp	17	37752278	37758146	2.626052	0.131487
25358 Amp	17	37758363	37761579	2.661431	0.132083
25359 Amp	17	37762968	37762968	2.52589	0.129599
25360 Amp	17	37763542	37763542	2.776997	0.134214
25361 Amp	17	37763774	37763774	2.812598	0.134972
25362 Amp	17	38254377	38254377	5.227494	0.175465
25363 Amp	17	38254553	38254727	5.345638	0.177271
25364 Amp	17	38262539	38262539	6.04774	0.187489
25365 Amp	17	38268986	38291398	7.72044	0.21099
25366 Amp	17	38310064	38310064	7.011899	0.201206
25367 Amp	17	38312348	38312348	6.319529	0.19133
25368 Amp	17	38314737	38320537	6.641348	0.196026
25369 Amp	17	38320644	38339536	7.027404	0.20146
25370 Amp	17	38341114	38368782	7.074542	0.201946
25371 Amp	17	38373229	38375756	7.452952	0.207231
25372 Amp	17	38376410	38376410	6.840574	0.198686
25373 Amp	17	38380230	38443161	7.993387	0.214622
25374 Amp	17	38444036	38444036	7.610655	0.209456
25375 Amp	17	38445952	38475368	7.865467	0.212877
25376 Amp	17	38482665	38495020	7.610655	0.209456
25377 Amp	17	38495597	38542361	7.500789	0.207892
25378 Amp	17	38550545	38550545	8.008326	0.214818
25379 Amp	17	38550705	38551005	8.704326	0.224166
25380 Amp	17	38552041	38568104	8.802762	0.225446
25381 Amp	17	38573994	38573994	7.689477	0.210545
25382 Amp	17	38579549	38584355	7.849711	0.212614
25383 Amp	17	38588973	38600224	6.686496	0.196677
25384 Amp	17	38608238	38610045	6.062596	0.187636
25385 Amp	17	38611976	38621126	6.092521	0.188245
25386 Amp	17	38622776	38622776	7.232046	0.204181

25387	Amp	17	38625668	38629789	8.105491	0.216081
25388	Amp	17	38638315	38644236	7.849711	0.212732
25389	Amp	17	38648480	38654096	7.993387	0.214577
25390	Amp	17	38657093	38658037	7.120018	0.202715
25391	Amp	17	38662180	38689871	6.901838	0.199675
25392	Amp	17	38693724	38698373	7.279386	0.204803
25393	Amp	17	38699879	38719062	7.326935	0.205498
25394	Amp	17	38733306	38756275	7.547251	0.208618
25395	Amp	17	38764564	38773325	8.737278	0.224554
25396	Amp	17	38773603	38777871	9.592302	0.235636
25397	Amp	17	38778198	38785329	9.746461	0.237625
25398	Amp	17	38786378	38791091	8.505687	0.221689
25399	Amp	17	38791387	38825619	8.393699	0.220175
25400	Amp	17	38827709	38848196	8.18543	0.217256
25401	Amp	17	38852975	38863471	7.865467	0.212807
25402	Amp	17	38864727	38881877	8.073986	0.215812
25403	Amp	17	38886234	38907252	8.29898	0.218858
25404	Amp	17	38910197	38915939	8.885533	0.226641
25405	Amp	17	38916929	38928367	9.105383	0.229505
25406	Amp	17	38929872	38936035	9.256952	0.231464
25407	Amp	17	38937276	38959222	9.122122	0.229675
25408	Amp	17	38962432	38966861	9.256952	0.231278
25409	Amp	17	38975210	38991105	10.499721	0.247214
25410	Amp	17	38993320	39004193	10.791286	0.250875
25411	Amp	17	39004726	39018882	11.049458	0.254244
25412	Amp	17	39029593	39033888	11.627141	0.261301
25413	Amp	17	39035042	39047700	11.572965	0.260692
25414	Amp	17	39057832	39067205	11.948056	0.26514
25415	Amp	17	39067506	39074161	12.546325	0.272373
25416	Amp	17	39074882	39080579	13.874487	0.288309
25417	Amp	17	39087674	39096573	14.058011	0.290438
25418	Amp	17	39096811	39110808	13.800267	0.287393
25419	Amp	17	39113498	39139089	13.746952	0.286681
25420	Amp	17	39140069	39157452	13.528722	0.284037
25421	Amp	17	39164233	39176710	14.87478	0.299973
25422	Amp	17	39177143	39185296	14.560384	0.296312
25423	Amp	17	39186104	39208740	15.137413	0.303055
25424	Amp	17	39214442	39214442	15.690603	0.309304
25425	Amp	17	39229857	39263064	17.097145	0.32524
25426	Amp	17	39264512	39293136	17.373987	0.328159
25427	Amp	17	39293688	39309377	17.512889	0.329812
25428	Amp	17	39310284	39316067	17.671462	0.331366
25429	Amp	17	39316343	39322736	17.392969	0.328479
25430	Amp	17	39327459	39366443	17.870548	0.333697
25431	Amp	17	39370069	39383950	18.56575	0.341278
25432	Amp	17	39384117	39404487	18.78296	0.343673
25433	Amp	17	39406995	39430882	18.903702	0.344874
25434	Amp	17	39437234	39457761	20.378428	0.360696
25435	Amp	17	39463565	39469370	20.583986	0.362858
25436	Amp	17	39472646	39504749	21.769764	0.375353
25437	Amp	17	39506141	39506141	22.027522	0.378024
25438	Amp	17	39507733	39538569	22.169317	0.379492
25439	Amp	17	39539285	39547643	22.253967	0.380422

25440	Amp	17	39547964	39594767	20.725611	0.364486
25441	Amp	17	39602593	39608602	22.253967	0.380422
25442	Amp	17	39608922	39610186	22.761806	0.385483
25443	Amp	17	39623268	39633179	24.785232	0.40599
25444	Amp	17	39633909	39633909	25.006651	0.408158
25445	Amp	17	39639959	39648605	26.198831	0.42002
25446	Amp	17	39657603	39666058	26.846754	0.426429
25447	Amp	17	39669386	39677552	26.734801	0.425267
25448	Amp	17	39679601	39679601	28.11227	0.438684
25449	Amp	17	39680328	39720409	27.891609	0.436555
25450	Amp	17	39720514	39720514	27.645574	0.434101
25451	Amp	17	39720565	39720645	27.289486	0.430707
25452	Amp	17	39720681	39725020	27.113654	0.428945
25453	Amp	17	39725118	39734812	27.180986	0.429616
25454	Amp	17	39743435	39747958	27.688618	0.434587
25455	Amp	17	39749563	39749563	27.003097	0.427956
25456	Amp	17	39749917	39749917	27.357173	0.431315
25457	Amp	17	39753747	39777035	25.980631	0.417881
25458	Amp	17	39785909	39796188	25.872665	0.416805
25459	Amp	17	39799549	39814112	25.071082	0.408807
25460	Amp	17	39854562	39867188	22.169317	0.379454
25461	Amp	17	39869164	39877180	23.478835	0.392996
25462	Amp	17	39884459	39891218	21.941174	0.377174
25463	Amp	17	39896312	39896312	21.263085	0.370179
25464	Amp	17	39896881	39922978	21.095587	0.368333
25465	Amp	17	39926578	39929538	21.5365	0.372966
25466	Amp	17	39933291	39938921	20.032409	0.35703
25467	Amp	17	39939255	39950842	20.134537	0.358241
25468	Amp	17	39953914	39954556	20.053094	0.357311
25469	Amp	17	39960762	39972058	19.993383	0.356715
25470	Amp	17	39972395	39979157	20.338306	0.360392
25471	Amp	17	39984674	39990676	19.491404	0.351279
25472	Amp	17	39992088	40013359	19.430195	0.350585
25473	Amp	17	40013424	40013424	19.225845	0.348412
25474	Amp	17	40013441	40017100	18.923342	0.345053
25475	Amp	17	40020205	40041052	17.611387	0.330907
25476	Amp	17	40044698	40045777	17.392969	0.328384
25477	Amp	17	40059663	40109338	15.959834	0.312448
25478	Amp	17	40109696	40115933	16.456552	0.318009
25479	Amp	17	40116522	40116522	15.977907	0.312669
25480	Amp	17	40121215	40121215	15.461712	0.306614
25481	Amp	17	40126946	40130835	15.272026	0.304554
25482	Amp	17	40132808	40132888	14.169996	0.291694
25483	Amp	17	40134623	40167408	13.038387	0.278212
25484	Amp	17	40173757	40173757	11.698383	0.262276
25485	Amp	17	40176350	40185351	11.519317	0.26007
25486	Amp	17	40199581	40199581	10.260441	0.244134
25487	Amp	17	40200563	40200563	10.32955	0.24498
25488	Amp	17	40212548	40237596	9.071495	0.229043
25489	Amp	17	40241375	40252079	7.880674	0.213107
25490	Amp	17	40253812	40285259	7.801564	0.211932
25491	Amp	17	40291317	40291317	7.880674	0.213171
25492	Amp	17	40300899	40300899	8.008326	0.21477

25493	Amp	17	40310095	40324848	6.933875	0.200082
25494	Amp	17	40325043	40336376	6.411912	0.192768
25495	Amp	17	40349352	40349352	6.825391	0.198662
25496	Amp	17	40349368	40349368	6.901838	0.199709
25497	Amp	17	40349454	40349894	6.933875	0.200194
25498	Amp	17	40371518	40380731	6.53507	0.194376
25499	Amp	17	40387144	40398079	5.419518	0.17844
25500	Amp	17	40399270	40414212	5.792044	0.183731
25501	Amp	17	40431982	40434251	4.416011	0.16315
25502	Amp	17	40436311	40442978	4.612121	0.166319
25503	Amp	17	40452935	40454413	4.654424	0.166808
25504	Amp	17	40457926	40464707	4.484961	0.164295
25505	Amp	17	40465590	40470986	4.457399	0.163795
25506	Amp	17	40472963	40475061	4.250544	0.160625
25507	Amp	17	40477077	40477178	4.156262	0.158973
25508	Amp	17	40480767	40481999	4.290674	0.16117
25509	Amp	17	40484392	40484392	4.345128	0.162095
25510	Amp	17	40484492	40490424	4.499013	0.164406
25511	Amp	17	40493283	40509988	4.331178	0.161828
25512	Amp	17	40510293	40515092	4.196711	0.159631
25513	Amp	17	40515565	40534670	4.250544	0.160481
25514	Amp	17	40536505	40560309	3.887411	0.154462
25515	Amp	17	40574179	40616114	3.713605	0.151457
25516	Amp	17	40616408	40660914	3.603523	0.149572
25517	Amp	17	40672757	40672771	3.458499	0.146943
25518	Amp	17	40689379	40694727	3.275143	0.143602
25519	Amp	17	40694772	40701246	3.045223	0.139335
25520	Amp	17	40711435	40712315	3.207115	0.142438
25521	Amp	17	40713075	40714353	3.127699	0.140848
25522	Amp	17	40716967	40816808	3.275143	0.143606
25523	Amp	17	40817096	40817096	3.374864	0.145314
25524	Amp	17	40822818	40854292	2.571513	0.130299
25525	Amp	17	40854549	40854549	2.425182	0.127629
25526	Amp	17	40854610	40854610	2.356694	0.126202
25527	Amp	17	40867785	40869381	2.049917	0.120285
25528	Amp	17	40873495	40896269	1.675319	0.112481
25529	Amp	17	40897264	40927543	1.472629	0.108285
25530	Amp	17	40931731	40959087	1.243254	0.103099
25531	Amp	17	40959631	40967794	1.407997	0.106828
25532	Amp	17	40970410	40997990	1.140417	0.100563
25533	Amp	17	40998026	40998969	1.093834	0.099459
25534	Amp	17	40999972	41004769	0.508568	0.083523
25535	Amp	17	41004876	41010404	0.595248	0.086206
25536	Amp	17	41017956	41034936	0.47716	0.082491
25537	Amp	17	41035230	41035230	0.399144	0.079807
25538	Amp	17	41041508	41145460	0.287738	0.075997
25539	Amp	17	41145779	41194336	0.271552	0.075147
25540	Amp	17	41195952	41197963	0.299433	0.076251
25541	Amp	17	41199837	41224178	0.311747	0.076688
25542	Amp	17	41284704	41329907	0.010261	0.064342
25543	Amp	17	41331927	41346402	0.227787	0.073255
25544	Amp	17	41347661	41403800	0.010261	0.064342
25545	Amp	17	41404755	41415981	0	0.057372

25546	Amp	17	41416809	41445003	0	0.041458
25547	Amp	17	41445989	41462468	0	0.045412
25548	Amp	17	41464481	41564629	0	0.043492
25549	Amp	17	41570766	41643878	0	0.059429
25550	Amp	17	41650598	41650598	0	0.056182
25551	Amp	17	41650872	41653194	0	0.052742
25552	Amp	17	41660480	41663863	0.112543	0.068656
25553	Amp	17	41664532	41686993	0.08435	0.067731
25554	Amp	17	41692804	41701419	0.044794	0.065844
25555	Amp	17	41709504	41752766	0.069848	0.066896
25556	Amp	17	41752860	41782826	0	0.060643
25557	Amp	17	41783266	41803757	0	0.059175
25558	Amp	17	41806678	41853084	0	0.062335
25559	Amp	17	41853703	41861239	0.019169	0.064849
25560	Amp	17	41868030	41868030	0	0.063752
25561	Amp	17	41869011	41920849	0	0.051699
25562	Amp	17	41921764	41962637	0	0.051022
25563	Amp	17	41967616	41975519	0	0.035086
25564	Amp	17	41976083	42029815	0	0.033698
25565	Amp	17	42031620	42037468	0	0.043994
25566	Amp	17	42042257	42096735	0	0.036973
25567	Amp	17	42100949	42134336	0	0.034745
25568	Amp	17	42136994	42152491	0	0.042413
25569	Amp	17	42153740	42153740	0	0.044233
25570	Amp	17	42166716	42198957	0	0.041719
25571	Amp	17	42201821	42208625	0	0.034051
25572	Amp	17	42212883	42260218	0	0.029397
25573	Amp	17	42264082	42291348	0	0.035661
25574	Amp	17	42294404	42310047	0	0.039233
25575	Amp	17	42310602	42328363	0	0.037413
25576	Amp	17	42330786	42339998	0	0.039469
25577	Amp	17	42341988	42369957	0	0.041877
25578	Amp	17	42374643	42392660	0	0.031581
25579	Amp	17	42394249	42473264	0	0.030529
25580	Amp	17	42475008	42478822	0	0.027941
25581	Amp	17	42480709	42545129	0	0.023646
25582	Amp	17	42563724	42571083	0	0.021343
25583	Amp	17	42575091	42600646	0	0.017771
25584	Amp	17	42601977	42724247	0	0.015714
25585	Amp	17	42753891	42758668	0	0.013307
25586	Amp	17	42764500	42836360	0	0.015831
25587	Amp	17	42836533	42943614	0	0.014166
25588	Amp	17	42946737	43003423	0	0.017035
25589	Amp	17	43005523	43021318	0	0.020589
25590	Amp	17	43021544	43029040	0	0.017035
25591	Amp	17	43036325	43107667	0	0.018937
25592	Amp	17	43109546	43109546	0	0.02369
25593	Amp	17	43111997	43123845	0	0.027439
25594	Amp	17	43128962	43155084	0	0.031226
25595	Amp	17	43155380	43186470	0	0.027439
25596	Amp	17	43186631	43189750	0	0.02369
25597	Amp	17	43428081	43437078	0	0.023794
25598	Amp	17	43444725	43469427	0	0.02511

25599	Amp	17	43471615	43534811	0	0.024013
25600	Amp	17	43534982	43556748	0	0.031503
25601	Amp	17	43560907	43569361	0	0.028285
25602	Amp	17	43569798	43622595	0	0.020795
25603	Amp	17	43626186	43728330	0	0.013808
25604	Amp	17	43729994	43759814	0	0.018399
25605	Amp	17	43763434	43807620	0	0.023418
25606	Amp	17	43808403	43825754	0	0.02578
25607	Amp	17	43828940	43860817	0	0.022387
25608	Amp	17	43862070	43911568	0	0.01968
25609	Amp	17	43912708	43950431	0	0.018922
25610	Amp	17	43959505	43961919	0	0.015199
25611	Amp	17	43964243	44204705	0	0.011214
25612	Amp	17	44208669	44255219	0	0.01247
25613	Amp	17	44255435	44306946	0	0.014644
25614	Amp	17	44311540	44355399	0	0.013388
25615	Amp	17	44360386	44554648	0	0.015556
25616	Amp	17	44556040	44643012	0	0.01639
25617	Amp	17	44643548	44703780	0	0.017782
25618	Amp	17	44706392	44720147	0	0.016749
25619	Amp	17	44720380	44730047	0	0.018728
25620	Amp	17	44732726	44770829	0	0.021087
25621	Amp	17	44776840	44835984	0	0.019695
25622	Amp	17	44843739	44889927	0	0.021517
25623	Amp	17	44891052	44892360	0	0.026517
25624	Amp	17	44895089	44956911	0	0.024695
25625	Amp	17	44967312	45040055	0	0.025327
25626	Amp	17	45040445	45043940	0	0.026468
25627	Amp	17	45044122	45174877	0	0.025634
25628	Amp	17	45179669	45197029	0	0.028714
25629	Amp	17	45211105	45271153	0	0.02599
25630	Amp	17	45272186	45334403	0	0.02401
25631	Amp	17	45336085	45436394	0	0.02287
25632	Amp	17	45461373	45461373	0	0.02187
25633	Amp	17	46733528	46733732	0	0.036617
25634	Amp	17	46740644	46749321	0	0.031617
25635	Amp	17	46753695	46795576	0	0.038757
25636	Amp	17	46800902	46915478	0	0.044043
25637	Amp	17	46915752	46933458	0	0.046559
25638	Amp	17	46934055	46934721	0	0.048039
25639	Amp	17	46939061	46942762	0	0.045523
25640	Amp	17	46943889	46982458	0	0.046381
25641	Amp	17	46988859	46989235	0	0.062244
25642	Amp	17	46990645	46993322	0.050473	0.066059
25643	Amp	17	46993610	46995490	0.015369	0.064579
25644	Amp	17	46996597	47009371	0.034113	0.065465
25645	Amp	17	47013575	47074183	0.164935	0.071001
25646	Amp	17	47088789	47093070	0.227787	0.073367
25647	Amp	17	47097511	47101958	0.153838	0.070375
25648	Amp	17	47109832	47178750	0.010261	0.064354
25649	Amp	17	47186208	47190828	0.079652	0.067335
25650	Amp	17	47191993	47191993	0.107017	0.068448
25651	Amp	17	47194955	47194955	0	0.052585

25652	Amp	17	47201424	47230327	0	0.057857
25653	Amp	17	47230562	47237088	0	0.058748
25654	Amp	17	47238120	47239351	0	0.053462
25655	Amp	17	47239656	47293216	0	0.061418
25656	Amp	17	47293597	47351140	0	0.060365
25657	Amp	17	47364653	47364653	0.060686	0.066491
25658	Amp	17	47367502	47367539	0.112543	0.068806
25659	Amp	17	47370485	47402322	0.153838	0.070504
25660	Amp	17	47402367	47407680	0.187449	0.071871
25661	Amp	17	47412454	47425980	0.210594	0.07253
25662	Amp	17	47428062	47440041	0.271552	0.075312
25663	Amp	17	47440066	47441718	0.251218	0.074454
25664	Amp	17	47448848	47467907	0.100827	0.068236
25665	Amp	17	47469712	47472252	0.060686	0.066538
25666	Amp	17	47472901	47475547	0	0.05447
25667	Amp	17	47477081	47489525	0	0.060152
25668	Amp	17	47504596	47506009	0	0.06152
25669	Amp	17	47517165	47525646	0	0.056832
25670	Amp	17	47533708	47550228	0	0.062428
25671	Amp	17	47557019	47557019	0.040093	0.065595
25672	Amp	17	47566788	47567440	0.050473	0.066137
25673	Amp	17	47575998	47575998	0.187449	0.071769
25674	Amp	17	47592534	47592634	0.138391	0.069855
25675	Amp	17	47593698	47605413	0.204831	0.072496
25676	Amp	17	47605759	47617293	0.143787	0.070104
25677	Amp	17	47623967	47625700	0.335313	0.077632
25678	Amp	17	47628076	47644871	0.404718	0.079949
25679	Amp	17	47645887	47655408	0.444474	0.081346
25680	Amp	17	47658609	47665781	0.79909	0.092211
25681	Amp	17	47668592	47671388	0.616549	0.086978
25682	Amp	17	47671830	47671830	0.644502	0.08776
25683	Amp	17	47675763	47678180	0.915698	0.095296
25684	Amp	17	47685639	47727056	1.61378	0.111232
25685	Amp	17	47728550	47728550	1.492063	0.108612
25686	Amp	17	47732302	47763635	1.665486	0.112384
25687	Amp	17	47767264	47768577	1.369395	0.105838
25688	Amp	17	47769864	47784252	1.563118	0.110213
25689	Amp	17	47785975	47807039	2.038694	0.120015
25690	Amp	17	47807949	47826797	1.801583	0.115091
25691	Amp	17	47827299	47839581	1.876242	0.116721
25692	Amp	17	47839781	47876372	1.919241	0.117645
25693	Amp	17	47880097	47880097	1.876242	0.116656
25694	Amp	17	47880225	47888600	1.96212	0.118473
25695	Amp	17	47892239	47899199	1.843664	0.116157
25696	Amp	17	47905107	47931818	2.016585	0.119548
25697	Amp	17	47936242	47950779	2.094104	0.121066
25698	Amp	17	47956739	47962187	2.22459	0.123725
25699	Amp	17	47966137	47979629	2.137445	0.121908
25700	Amp	17	47979663	47999994	2.094104	0.121022
25701	Amp	17	48000516	48006366	2.614791	0.131291
25702	Amp	17	48008901	48022188	2.812598	0.13485
25703	Amp	17	48027053	48029268	3.678399	0.150786
25704	Amp	17	48032540	48033103	3.972019	0.155978

25705 Amp	17	48033806	48033806	4.264196	0.160744
25706 Amp	17	48036456	48071890	5.227494	0.175529
25707 Amp	17	48073537	48076385	5.375101	0.177749
25708 Amp	17	48080344	48080532	5.434517	0.178528
25709 Amp	17	48088338	48110701	5.536915	0.180162
25710 Amp	17	48113219	48117681	5.478024	0.179238
25711 Amp	17	48117973	48148639	5.626868	0.181407
25712 Amp	17	48149083	48149151	5.777283	0.183616
25713 Amp	17	48157173	48157173	5.86703	0.184955
25714 Amp	17	48160441	48171632	5.927128	0.185864
25715 Amp	17	48171769	48196677	6.092521	0.188217
25716 Amp	17	48199195	48207699	5.896595	0.185235
25717 Amp	17	48212887	48240813	6.275802	0.190744
25718 Amp	17	48242044	48262165	6.564374	0.194949
25719 Amp	17	48262250	48275101	6.732306	0.197206
25720 Amp	17	48279002	48289823	6.810251	0.198358
25721 Amp	17	48293797	48299306	6.871512	0.199199
25722 Amp	17	48302429	48328073	6.519288	0.194152
25723 Amp	17	48331110	48341227	6.949342	0.200406
25724 Amp	17	48342413	48367272	7.120018	0.202715
25725 Amp	17	48371235	48372952	6.933875	0.200015
25726 Amp	17	48381429	48397106	7.531798	0.208336
25727 Amp	17	48417237	48445177	7.485352	0.207757
25728 Amp	17	48446884	48447991	7.120018	0.202712
25729 Amp	17	48450356	48468096	7.326935	0.205586
25730 Amp	17	48471305	48471305	6.917796	0.199947
25731 Amp	17	48471328	48477663	6.626113	0.195674
25732 Amp	17	48478383	48480220	7.011899	0.201287
25733 Amp	17	48486395	48490793	7.421526	0.20683
25734 Amp	17	48491062	48501181	7.6425	0.209908
25735 Amp	17	48502550	48529290	7.279386	0.204773
25736 Amp	17	48529301	48529372	7.563028	0.208759
25737 Amp	17	48530197	48533835	7.75231	0.211286
25738 Amp	17	48534681	48538882	7.594603	0.209145
25739 Amp	17	48545758	48561246	7.72044	0.210949
25740 Amp	17	48561784	48563446	7.547251	0.208496
25741 Amp	17	48566799	48566799	7.610655	0.209363
25742 Amp	17	48589942	48590640	7.72044	0.210953
25743 Amp	17	48594430	48597308	7.896426	0.213375
25744 Amp	17	48600354	48603234	9.105383	0.229311
25745 Amp	17	48603334	48605268	9.004172	0.228098
25746 Amp	17	48606895	48606967	9.712919	0.23731
25747 Amp	17	48620396	48639345	10.9963	0.253545
25748 Amp	17	48647787	48652405	11.39504	0.258602
25749 Amp	17	48706805	48706805	11.644242	0.26147
25750 Amp	17	48707018	48712985	12.964742	0.277366
25751 Amp	17	48714439	48727378	12.20153	0.268369
25752 Amp	17	48727705	48729772	11.554491	0.260484
25753 Amp	17	48736169	48736169	12.021369	0.266146
25754 Amp	17	48750515	48751050	11.894175	0.264512
25755 Amp	17	48755936	48755936	11.752146	0.262833
25756 Amp	17	48756878	48771108	12.27458	0.269227
25757 Amp	17	48773120	48791348	12.745398	0.274759

25758	Amp	17	48791969	48796081	13.436139	0.283054
25759	Amp	17	48796910	48804386	13.293073	0.281209
25760	Amp	17	48805638	48806341	12.855613	0.276152
25761	Amp	17	48810445	48810939	13.436139	0.282967
25762	Amp	17	48820171	48846022	13.146748	0.279479
25763	Amp	17	48846102	48870856	13.038387	0.27835
25764	Amp	17	48871227	48880962	13.618338	0.285159
25765	Amp	17	48881245	48886265	13.417296	0.282848
25766	Amp	17	48886498	48911235	12.946363	0.277195
25767	Amp	17	48911792	48922889	13.202322	0.28029
25768	Amp	17	48924281	48929651	12.671608	0.273851
25769	Amp	17	48930135	48966303	12.456062	0.271234
25770	Amp	17	48967232	49006781	13.78167	0.287171
25771	Amp	17	49008953	49008953	13.985765	0.289539
25772	Amp	17	49012355	49012518	14.336365	0.293658
25773	Amp	17	49018134	49037120	14.779959	0.298945
25774	Amp	17	49037676	49048620	14.560384	0.296287
25775	Amp	17	49049358	49049358	14.743668	0.29845
25776	Amp	17	49050885	49056506	14.912742	0.300318
25777	Amp	17	49061511	49073685	14.631516	0.297263
25778	Amp	17	49081640	49108622	14.668813	0.297634
25779	Amp	17	49109694	49122706	14.541868	0.296004
25780	Amp	17	49130338	49134296	14.262513	0.292923
25781	Amp	17	49136891	49140544	14.560384	0.296336
25782	Amp	17	49141245	49144328	14.95044	0.300789
25783	Amp	17	49144970	49171554	15.272026	0.304591
25784	Amp	17	49172331	49179370	15.005353	0.301497
25785	Amp	17	49179760	49183403	15.747199	0.309882
25786	Amp	17	49186423	49188225	15.272026	0.304594
25787	Amp	17	49191157	49192747	15.480429	0.306989
25788	Amp	17	49194824	49199018	14.987459	0.301246
25789	Amp	17	49201127	49211095	16.647433	0.320128
25790	Amp	17	49212709	49216486	17.751967	0.332321
25791	Amp	17	49217555	49217555	18.128001	0.336552
25792	Amp	17	49228039	49246934	18.108258	0.336225
25793	Amp	17	49247040	49269167	18.167323	0.336992
25794	Amp	17	49274783	49278661	19.024158	0.346315
25795	Amp	17	49278738	49278738	19.368926	0.349903
25796	Amp	17	49294560	49294560	17.910589	0.333967
25797	Amp	17	49295311	49296468	17.77219	0.332504
25798	Amp	17	49297179	49307054	17.812399	0.333024
25799	Amp	17	49311576	49312652	18.14718	0.336574
25800	Amp	17	49312774	49320217	18.52593	0.34091
25801	Amp	17	49325114	49330119	18.704216	0.34274
25802	Amp	17	49330730	49336860	18.903702	0.344966
25803	Amp	17	49337793	49342487	19.185603	0.348062
25804	Amp	17	49342793	49354445	19.389328	0.350147
25805	Amp	17	49355102	49357687	19.633539	0.352814
25806	Amp	17	49359450	49360033	19.712397	0.353664
25807	Amp	17	49361262	49361262	20.399182	0.361085
25808	Amp	17	49362964	49385807	20.236975	0.359255
25809	Amp	17	49394190	49399784	19.852569	0.355182
25810	Amp	17	49400273	49408624	19.652987	0.352866

25811	Amp	17	49410388	49410388	18.167323	0.33693
25812	Amp	17	49412398	49412398	18.428857	0.339628
25813	Amp	17	49414221	49414221	17.792544	0.332688
25814	Amp	17	49415004	49416853	17.948509	0.33446
25815	Amp	17	49421653	49421653	17.611387	0.330842
25816	Amp	17	49431921	49431921	17.732001	0.332198
25817	Amp	17	49432124	49432244	16.743709	0.321264
25818	Amp	17	49432418	49436544	16.686433	0.320573
25819	Amp	17	49446966	49455841	16.627883	0.320052
25820	Amp	17	49456210	49483610	16.686433	0.320509
25821	Amp	17	49489049	49489224	16.107433	0.314151
25822	Amp	17	49492609	49494340	14.393488	0.294362
25823	Amp	17	49499986	49511453	13.710777	0.286236
25824	Amp	17	49514102	49514440	13.293073	0.281403
25825	Amp	17	49520934	49525277	13.038387	0.278262
25826	Amp	17	49539212	49541907	13.310331	0.281629
25827	Amp	17	49542151	49542151	13.001761	0.277727
25828	Amp	17	49544310	49580439	13.655032	0.285644
25829	Amp	17	49587519	49591468	13.258073	0.280909
25830	Amp	17	49593136	49595595	12.927625	0.276897
25831	Amp	17	49598108	49612185	13.111032	0.279087
25832	Amp	17	49612435	49621109	12.983395	0.277676
25833	Amp	17	49622020	49633917	13.310331	0.281574
25834	Amp	17	49634535	49649206	13.220925	0.280422
25835	Amp	17	49649532	49649532	13.093327	0.278941
25836	Amp	17	49654095	49699867	13.565854	0.284508
25837	Amp	17	49701813	49714142	13.363175	0.282153
25838	Amp	17	49718308	49718308	13.985765	0.289582
25839	Amp	17	49720034	49728156	14.206189	0.292139
25840	Amp	17	49729124	49738870	15.233242	0.304137
25841	Amp	17	49739594	49769863	15.727885	0.309672
25842	Amp	17	49775469	49781609	15.40422	0.30614
25843	Amp	17	49784383	49784383	16.107433	0.314003
25844	Amp	17	49795593	49796356	15.042671	0.302005
25845	Amp	17	49798502	49798502	15.194804	0.303612
25846	Amp	17	49801487	49836570	14.687681	0.297845
25847	Amp	17	49839435	49856979	14.743668	0.298462
25848	Amp	17	49865045	49869998	14.912742	0.300369
25849	Amp	17	49870140	49870643	15.100986	0.302669
25850	Amp	17	49872648	49875464	15.785635	0.310429
25851	Amp	17	49875497	49892023	15.920582	0.311854
25852	Amp	17	49893981	49899242	16.570587	0.319285
25853	Amp	17	49899569	49904253	16.666682	0.32047
25854	Amp	17	49909227	49943770	16.879456	0.32287
25855	Amp	17	49945718	49953292	17.512889	0.329639
25856	Amp	17	49953675	49974408	16.070661	0.313703
25857	Amp	17	49979692	50009186	15.385452	0.305942
25858	Amp	17	50011326	50029093	14.968996	0.301146
25859	Amp	17	50033146	50053573	15.9958	0.312779
25860	Amp	17	50058309	50072022	15.348745	0.305348
25861	Amp	17	50078407	50081837	16.551166	0.319116
25862	Amp	17	50082859	50093657	17.492691	0.329438
25863	Amp	17	50096523	50104193	18.467261	0.340185

25864	Amp	17	50107655	50123247	18.984039	0.345852
25865	Amp	17	50124424	50168801	18.765297	0.343452
25866	Amp	17	50173181	50179793	18.14718	0.336592
25867	Amp	17	50179887	50181423	18.624086	0.341977
25868	Amp	17	50181708	50181708	18.388632	0.339299
25869	Amp	17	50187356	50198467	18.584844	0.341467
25870	Amp	17	50198891	50221605	19.812781	0.354774
25871	Amp	17	50228607	50242754	20.053094	0.357403
25872	Amp	17	50248951	50251052	19.004359	0.346068
25873	Amp	17	50254633	50254633	19.06482	0.346759
25874	Amp	17	50261714	50261714	19.67284	0.353184
25875	Amp	17	50269226	50272268	19.12459	0.347369
25876	Amp	17	50275509	50284646	19.084146	0.346803
25877	Amp	17	50287058	50287058	18.923342	0.34513
25878	Amp	17	50288073	50300060	19.206111	0.348231
25879	Amp	17	50300517	50323254	19.772229	0.354237
25880	Amp	17	50326049	50330134	19.892364	0.355623
25881	Amp	17	50330504	50343333	19.267324	0.348855
25882	Amp	17	50348606	50362819	19.328184	0.349428
25883	Amp	17	50363098	50363098	18.644239	0.342042
25884	Amp	17	50366471	50366471	18.745071	0.343245
25885	Amp	17	50367578	50367578	18.227868	0.337645
25886	Amp	17	50367596	50384206	17.968693	0.334794
25887	Amp	17	50386302	50405727	16.533331	0.318857
25888	Amp	17	50406205	50448764	16.053555	0.313472
25889	Amp	17	50452256	50462251	16.359113	0.317015
25890	Amp	17	50477645	50480502	16.089694	0.313793
25891	Amp	17	50480742	50485928	16.436444	0.317718
25892	Amp	17	50488211	50488211	16.242583	0.315697
25893	Amp	17	50488316	50489341	16.475247	0.3182
25894	Amp	17	50492180	50492869	15.977907	0.312604
25895	Amp	17	50495454	50502588	15.823673	0.310754
25896	Amp	17	50502765	50524273	15.595327	0.308333
25897	Amp	17	50524785	50528952	15.76669	0.310109
25898	Amp	17	50528994	50528994	15.233242	0.304042
25899	Amp	17	50529176	50552885	15.11973	0.30285
25900	Amp	17	50554657	50565233	14.393488	0.294417
25901	Amp	17	50566354	50567176	14.779959	0.299002
25902	Amp	17	50571500	50582045	15.005353	0.301472
25903	Amp	17	50590373	50606130	14.836401	0.29954
25904	Amp	17	50608081	50615943	14.762523	0.298659
25905	Amp	17	50618396	50618396	14.447899	0.294886
25906	Amp	17	50625264	50628958	13.565854	0.284564
25907	Amp	17	50631561	50632580	13.239383	0.280576
25908	Amp	17	50642042	50658413	13.454936	0.283174
25909	Amp	17	50659042	50680056	12.855613	0.27618
25910	Amp	17	50681428	50688462	13.038387	0.278364
25911	Amp	17	50690307	50691968	13.363175	0.282242
25912	Amp	17	50696131	50765705	14.022374	0.290038
25913	Amp	17	50766035	50773733	13.856199	0.288144
25914	Amp	17	50773738	50785194	13.328506	0.281719
25915	Amp	17	50786818	50797667	13.729199	0.286485
25916	Amp	17	50805630	50805784	13.546866	0.284265

25917	Amp	17	50810445	50812944	12.964742	0.277354
25918	Amp	17	50819293	50819776	14.243686	0.292507
25919	Amp	17	50823514	50823836	14.631516	0.297243
25920	Amp	17	50824466	50859698	14.818059	0.299397
25921	Amp	17	50862166	50868277	14.07679	0.290621
25922	Amp	17	50869961	50869961	15.06204	0.302056
25923	Amp	17	50870593	50875309	14.762523	0.298751
25924	Amp	17	50875605	50879214	14.650436	0.29741
25925	Amp	17	50889442	50889442	14.931259	0.300641
25926	Amp	17	50891577	50904223	14.855744	0.29986
25927	Amp	17	50906414	50913996	14.430436	0.294852
25928	Amp	17	50925198	50950216	13.093327	0.278916
25929	Amp	17	50951829	50974490	12.745398	0.274824
25930	Amp	17	50974812	50990402	12.946363	0.277194
25931	Amp	17	50994809	51022303	13.398977	0.282544
25932	Amp	17	51028744	51039095	13.966925	0.289258
25933	Amp	17	51047012	51094053	13.328506	0.281728
25934	Amp	17	51096695	51097751	13.985765	0.28957
25935	Amp	17	51100270	51142283	13.019814	0.278135
25936	Amp	17	51149382	51150245	12.909694	0.276749
25937	Amp	17	51150921	51169113	12.726679	0.274483
25938	Amp	17	51174193	51174877	12.401399	0.270594
25939	Amp	17	51175576	51195889	12.437581	0.271066
25940	Amp	17	51198292	51204217	12.256206	0.268892
25941	Amp	17	51205855	51218628	12.130806	0.26746
25942	Amp	17	51228102	51242201	11.430328	0.258937
25943	Amp	17	51479558	51479558	8.555266	0.222332
25944	Amp	17	51487144	51487144	9.407903	0.233373
25945	Amp	17	51488186	51490411	9.559998	0.235276
25946	Amp	17	51491495	51491495	9.780264	0.238195
25947	Amp	17	51494146	51502364	10.087806	0.242039
25948	Amp	17	51504795	51505283	10.14039	0.242591
25949	Amp	17	51511901	51519955	10.190896	0.243249
25950	Amp	17	51526707	51526707	10.122701	0.242449
25951	Amp	17	51528569	51530058	10.51732	0.247365
25952	Amp	17	51530264	51533026	10.566416	0.248011
25953	Amp	17	51533864	51543510	10.860138	0.25168
25954	Amp	17	51544337	51544893	10.909195	0.252457
25955	Amp	17	51553571	51559784	10.32955	0.245071
25956	Amp	17	51560270	51586462	10.017642	0.241227
25957	Amp	17	51586637	51586637	10.190896	0.243223
25958	Amp	17	51587600	51629890	10.32955	0.244998
25959	Amp	17	51631080	51636860	10.843331	0.251505
25960	Amp	17	51638006	51638201	11.049458	0.254156
25961	Amp	17	51638862	51641064	11.081157	0.254661
25962	Amp	17	51641496	51643706	11.39504	0.258509
25963	Amp	17	51645527	51645527	11.446377	0.259161
25964	Amp	17	51646517	51653089	11.204236	0.256208
25965	Amp	17	51653387	51655343	11.1336	0.255317
25966	Amp	17	51656751	51656751	9.881657	0.239381
25967	Amp	17	51657113	51660832	8.654479	0.223445
25968	Amp	17	51661359	51667633	8.770924	0.225013
25969	Amp	17	51670198	51680479	8.393699	0.220098

25970 Amp	17	51683368	51684936	8.671092	0.223682
25971 Amp	17	51688309	51691363	8.572005	0.222562
25972 Amp	17	51692476	51715663	8.29898	0.218714
25973 Amp	17	51715686	51762533	8.008326	0.214836
25974 Amp	17	51762788	51766212	7.961071	0.214184
25975 Amp	17	51767153	51808410	7.993387	0.21464
25976 Amp	17	51808647	51817417	7.880674	0.213162
25977 Amp	17	51817563	51830663	7.673484	0.210294
25978 Amp	17	51831554	51841076	7.563028	0.208766
25979 Amp	17	51841611	51850430	7.801564	0.212071
25980 Amp	17	51851276	51875321	7.880674	0.213229
25981 Amp	17	51875498	51903412	7.945028	0.213942
25982 Amp	17	51905036	51905232	8.29898	0.218775
25983 Amp	17	51908776	51916137	8.32882	0.219216
25984 Amp	17	51916898	51922171	8.538535	0.221993
25985 Amp	17	51923836	51935917	8.073986	0.215753
25986 Amp	17	51936074	51952308	7.961071	0.214184
25987 Amp	17	51952902	51962819	8.266115	0.21824
25988 Amp	17	51966253	51985698	8.121744	0.216336
25989 Amp	17	51986703	51997302	8.057852	0.215508
25990 Amp	17	51999929	52010562	7.92856	0.213684
25991 Amp	17	52011327	52033480	7.704855	0.210718
25992 Amp	17	52036157	52060025	7.248181	0.2045
25993 Amp	17	52063544	52095891	6.122748	0.188563
25994 Amp	17	52098574	52133438	5.212889	0.175334
25995 Amp	17	52138338	52138521	5.404308	0.17812
25996 Amp	17	52141726	52156504	5.184242	0.174816
25997 Amp	17	52164889	52164889	4.853037	0.170021
25998 Amp	17	52169257	52175037	4.739548	0.168073
25999 Amp	17	52178040	52206808	4.401595	0.162864
26000 Amp	17	52207203	52210047	4.359416	0.162407
26001 Amp	17	52211685	52217038	4.196711	0.159631
26002 Amp	17	52217136	52232819	4.331178	0.161987
26003 Amp	17	52232885	52236807	4.823878	0.169377
26004 Amp	17	52240714	52249928	5.09526	0.173673
26005 Amp	17	52250818	52256195	4.169329	0.159233
26006 Amp	17	52262307	52266541	4.513276	0.164731
26007 Amp	17	52269896	52291365	4.484961	0.164259
26008 Amp	17	52296299	52309454	4.612121	0.166199
26009 Amp	17	52315150	52315150	4.264196	0.160702
26010 Amp	17	52317504	52349213	4.129725	0.158542
26011 Amp	17	52351582	52359089	3.678399	0.150833
26012 Amp	17	52361301	52367155	4.050096	0.157336
26013 Amp	17	52367371	52370441	4.443348	0.163513
26014 Amp	17	52370515	52393011	3.898065	0.154667
26015 Amp	17	52393798	52401191	3.788687	0.152742
26016 Amp	17	52401525	52438220	3.985868	0.156232
26017 Amp	17	52440035	52442911	3.862695	0.154112
26018 Amp	17	52443599	52482701	3.908795	0.154845
26019 Amp	17	52484234	52495030	4.062894	0.157626
26020 Amp	17	52499631	52500659	3.908795	0.154845
26021 Amp	17	52506223	52513168	4.290674	0.161227
26022 Amp	17	52513435	52522023	4.69701	0.167628

26023	Amp	17	52526380	52536352	4.290674	0.161227
26024	Amp	17	52536518	52536518	4.526699	0.164994
26025	Amp	17	52593672	52593672	3.422847	0.146287
26026	Amp	17	52601398	52605529	3.516868	0.147888
26027	Amp	17	52606287	52623793	3.435586	0.146382
26028	Amp	17	52626503	52640549	3.275143	0.143684
26029	Amp	17	52641857	52690812	3.399017	0.145731
26030	Amp	17	52691567	52701185	3.08058	0.140041
26031	Amp	17	52701331	52703983	3.972019	0.155977
26032	Amp	17	52705083	52706945	4.183261	0.159543
26033	Amp	17	52708931	52801248	4.388129	0.162855
26034	Amp	17	52801507	52801507	4.555821	0.165299
26035	Amp	17	52802289	52821706	4.416011	0.163205
26036	Amp	17	52821841	52845680	4.626369	0.166528
26037	Amp	17	52846150	52850890	4.823878	0.169493
26038	Amp	17	52851386	52861230	4.952273	0.171468
26039	Amp	17	52861306	52868536	4.924414	0.171027
26040	Amp	17	52870155	52871351	4.86791	0.170221
26041	Amp	17	52872660	52872660	5.271084	0.176286
26042	Amp	17	53239515	53239515	8.834661	0.225884
26043	Amp	17	53240500	53240500	8.885533	0.226502
26044	Amp	17	53240949	53253578	8.313762	0.219006
26045	Amp	17	53253994	53261886	8.47374	0.22109
26046	Amp	17	53262017	53265148	8.393699	0.219965
26047	Amp	17	53266655	53266655	8.654479	0.223605
26048	Amp	17	53266788	53270296	8.588477	0.222663
26049	Amp	17	53270517	53273042	8.47374	0.221117
26050	Amp	17	53274128	53285830	9.69597	0.237053
26051	Amp	17	53287307	53292454	9.933591	0.239943
26052	Amp	17	53293166	53300278	9.644619	0.236303
26053	Amp	17	53300769	53310931	9.407903	0.233413
26054	Amp	17	53318409	53329927	8.47374	0.221067
26055	Amp	17	53330649	53336373	8.637458	0.223344
26056	Amp	17	53336643	53385070	8.409489	0.220222
26057	Amp	17	53391351	53392420	8.953805	0.227496
26058	Amp	17	53394086	53417626	8.32882	0.219177
26059	Amp	17	53417889	53439917	7.295403	0.205035
26060	Amp	17	53442919	53444623	7.516099	0.208163
26061	Amp	17	53446942	53451636	7.373439	0.206263
26062	Amp	17	53457578	53459625	7.849711	0.212655
26063	Amp	17	53460224	53461675	8.073986	0.215766
26064	Amp	17	53463482	53468697	7.801564	0.212014
26065	Amp	17	53471415	53472963	7.704855	0.210667
26066	Amp	17	53475463	53487239	7.452952	0.207177
26067	Amp	17	53498610	53521438	6.964631	0.200618
26068	Amp	17	53529008	53529789	6.747966	0.197506
26069	Amp	17	53533205	53573664	6.933875	0.200155
26070	Amp	17	53573860	53577661	7.042879	0.201523
26071	Amp	17	53582957	53584155	7.610655	0.209428
26072	Amp	17	53592658	53593220	7.865467	0.21283
26073	Amp	17	53595451	53610020	7.610655	0.209517
26074	Amp	17	53610423	53614723	7.785234	0.211729
26075	Amp	17	53619928	53626257	7.689477	0.210472

26076 Amp	17	53633575	53637331	7.768843	0.211538
26077 Amp	17	53657402	53657402	8.02414	0.215169
26078 Amp	17	53657705	53667163	7.880674	0.213193
26079 Amp	17	53668073	53703935	7.736725	0.211094
26080 Amp	17	53704400	53704400	8.313762	0.21906
26081 Amp	17	53705026	53755935	8.409489	0.220315
26082 Amp	17	53756143	53771041	8.073986	0.215666
26083 Amp	17	53775497	53788567	7.865467	0.212892
26084 Amp	17	53790280	53807433	8.18543	0.217216
26085 Amp	17	53807637	53846486	8.378151	0.219795
26086 Amp	17	53851924	53852695	8.505687	0.221602
26087 Amp	17	53854139	53860020	8.555266	0.222136
26088 Amp	17	53861171	53868829	8.720312	0.224499
26089 Amp	17	53876889	53910988	8.885533	0.226611
26090 Amp	17	53911317	53934430	8.687806	0.224053
26091 Amp	17	53934968	53945906	8.770924	0.22502
26092 Amp	17	53948792	53948792	8.60549	0.222908
26093 Amp	17	53948887	53981787	8.786437	0.225221
26094 Amp	17	53983302	53983792	9.340097	0.232417
26095 Amp	17	53984764	53990523	9.054921	0.228855
26096 Amp	17	53992698	54026392	9.627477	0.236089
26097 Amp	17	54026848	54026848	9.47615	0.23413
26098 Amp	17	54029528	54032874	9.661346	0.23654
26099 Amp	17	54035367	54050134	8.572005	0.222373
26100 Amp	17	54052323	54052363	8.637458	0.223247
26101 Amp	17	54054996	54064452	8.505687	0.221665
26102 Amp	17	54066264	54066264	8.851521	0.226242
26103 Amp	17	54066791	54092406	8.953805	0.2274
26104 Amp	17	54096040	54104319	9.357173	0.232713
26105 Amp	17	54105205	54105205	9.038034	0.228627
26106 Amp	17	54107612	54127134	9.442896	0.23377
26107 Amp	17	54140320	54150435	9.746461	0.237597
26108 Amp	17	54150657	54196166	9.559998	0.235285
26109 Amp	17	54198042	54232164	9.442896	0.233768
26110 Amp	17	54233832	54233832	9.172152	0.230245
26111 Amp	17	54234163	54285187	9.105383	0.22937
26112 Amp	17	54291480	54295592	9.038034	0.228529
26113 Amp	17	54298663	54313667	9.272857	0.231678
26114 Amp	17	54329566	54335652	8.34537	0.219422
26115 Amp	17	54338603	54342701	9.559998	0.235358
26116 Amp	17	54353737	54353737	10.808758	0.251073
26117 Amp	17	54370002	54373849	11.221545	0.256308
26118 Amp	17	54376224	54379831	10.843331	0.251645
26119 Amp	17	54381514	54394330	11.049458	0.254066
26120 Amp	17	54395791	54434453	10.877729	0.251983
26121 Amp	17	54441533	54441533	10.583831	0.248227
26122 Amp	17	54441787	54448182	10.808758	0.251117
26123 Amp	17	54450457	54451871	11.204236	0.256121
26124 Amp	17	54464479	54473516	11.360465	0.258164
26125 Amp	17	54475390	54502491	12.039373	0.266425
26126 Amp	17	54518442	54527019	12.510219	0.271981
26127 Amp	17	54529698	54548568	12.364848	0.270307
26128 Amp	17	54551173	54554893	12.745398	0.274799

26129	Amp	17	54557365	54574022	11.430328	0.258863
26130	Amp	17	54574651	54582106	11.680554	0.26203
26131	Amp	17	54584863	54629674	11.204236	0.25614
26132	Amp	17	54634551	54634551	10.791286	0.250812
26133	Amp	17	54637990	54645896	10.583831	0.24828
26134	Amp	17	54648913	54662101	10.703463	0.249828
26135	Amp	17	54664292	54665427	10.346125	0.245325
26136	Amp	17	54665612	54723093	10.686888	0.249649
26137	Amp	17	54726371	54739886	10.843331	0.251578
26138	Amp	17	54742613	54746740	11.1336	0.255353
26139	Amp	17	54747158	54767989	11.031992	0.253864
26140	Amp	17	54769426	54771406	10.208075	0.243625
26141	Amp	17	54771612	54793153	10.260441	0.244174
26142	Amp	17	54793494	54836954	10.174276	0.243016
26143	Amp	17	54840219	54852704	9.780264	0.238042
26144	Amp	17	54854112	54859474	9.984309	0.240686
26145	Amp	17	54859747	54867813	9.526354	0.234931
26146	Amp	17	54868364	54869886	9.357173	0.232736
26147	Amp	17	54870061	54880462	9.830028	0.238706
26148	Amp	17	54882077	54882933	10.225188	0.24368
26149	Amp	17	54883390	54887365	10.260441	0.244164
26150	Amp	17	54887401	54897858	10.31211	0.24482
26151	Amp	17	54901313	54901313	10.105286	0.242176
26152	Amp	17	54904492	54908621	9.916528	0.23979
26153	Amp	17	54909385	54926331	9.780264	0.238071
26154	Amp	17	54928963	54933309	9.729466	0.237409
26155	Amp	17	54941793	54946879	9.172152	0.230174
26156	Amp	17	54947762	54950200	9.088236	0.229114
26157	Amp	17	54950494	54958241	9.54314	0.23496
26158	Amp	17	54967964	54986859	10.243045	0.243927
26159	Amp	17	54990360	55010682	10.686888	0.249586
26160	Amp	17	55016604	55020420	11.049458	0.254171
26161	Amp	17	55022807	55022807	11.168221	0.255771
26162	Amp	17	55022911	55026393	11.221545	0.256367
26163	Amp	17	55028085	55037889	11.822516	0.263663
26164	Amp	17	55038258	55065983	12.075847	0.266764
26165	Amp	17	55071788	55078063	12.347637	0.269983
26166	Amp	17	55089290	55101937	12.237724	0.26873
26167	Amp	17	55107370	55117224	12.671608	0.273984
26168	Amp	17	55117408	55131581	12.491673	0.271707
26169	Amp	17	55133749	55142771	12.708276	0.27428
26170	Amp	17	55142859	55142859	12.510219	0.272053
26171	Amp	17	55145938	55177984	12.184583	0.268154
26172	Amp	17	55178111	55178111	12.27458	0.269096
26173	Amp	17	55184650	55213116	11.840411	0.263849
26174	Amp	17	55215731	55232216	12.20153	0.268325
26175	Amp	17	55233789	55242430	11.464362	0.259358
26176	Amp	17	55242699	55259533	11.430328	0.258874
26177	Amp	17	55259910	55262850	11.377246	0.258322
26178	Amp	17	55263047	55274991	10.909195	0.252352
26179	Amp	17	55276729	55280172	9.864718	0.239217
26180	Amp	17	55280655	55288478	9.526354	0.234741
26181	Amp	17	55289087	55290427	9.172152	0.230235

26182	Amp	17	55293154	55309465	9.559998	0.235334
26183	Amp	17	55311036	55318378	10.566416	0.248086
26184	Amp	17	55320381	55322144	10.51732	0.24749
26185	Amp	17	55322749	55328290	10.686888	0.249692
26186	Amp	17	55328566	55332252	10.600629	0.248453
26187	Amp	17	55335233	55335233	9.712919	0.237292
26188	Amp	17	55337313	55362645	9.780264	0.238046
26189	Amp	17	55362963	55372802	9.712919	0.237292
26190	Amp	17	55374888	55378044	10.000692	0.241019
26191	Amp	17	55379223	55383991	10.260441	0.244094
26192	Amp	17	55397559	55401129	9.172152	0.230198
26193	Amp	17	55401533	55404494	9.223227	0.230888
26194	Amp	17	55406680	55411501	9.071495	0.22887
26195	Amp	17	55411640	55439186	8.834661	0.225888
26196	Amp	17	55441762	55443636	9.122122	0.229614
26197	Amp	17	55443772	55464869	8.851521	0.226129
26198	Amp	17	55465030	55465030	8.802762	0.225444
26199	Amp	17	55465230	55482664	8.60549	0.222871
26200	Amp	17	55485901	55495436	8.458247	0.220891
26201	Amp	17	55498112	55499259	8.409489	0.220326
26202	Amp	17	55499295	55510457	8.266115	0.218346
26203	Amp	17	55513023	55523807	7.768843	0.211619
26204	Amp	17	55524318	55524692	7.833797	0.212493
26205	Amp	17	55524829	55532165	7.563028	0.208738
26206	Amp	17	55533450	55538031	7.977624	0.214322
26207	Amp	17	55543066	55544131	7.279386	0.204959
26208	Amp	17	55548781	55550124	7.215959	0.204085
26209	Amp	17	55555543	55563081	7.027404	0.201435
26210	Amp	17	55564463	55577243	7.183949	0.203653
26211	Amp	17	55578549	55596627	6.964631	0.200579
26212	Amp	17	55604182	55609497	6.458214	0.193382
26213	Amp	17	55612076	55623406	6.304987	0.191164
26214	Amp	17	55625528	55638089	5.911851	0.185485
26215	Amp	17	55644816	55649615	6.04774	0.187456
26216	Amp	17	55650250	55657760	5.492924	0.179394
26217	Amp	17	55659984	55674041	5.942149	0.185945
26218	Amp	17	55674594	55707078	6.032925	0.187325
26219	Amp	17	55707135	55717495	5.777283	0.183641
26220	Amp	17	55721458	55744139	5.330541	0.17709
26221	Amp	17	55751949	55753803	6.275802	0.190804
26222	Amp	17	55757004	55757379	6.198231	0.189736
26223	Amp	17	55761141	55761141	6.04774	0.187534
26224	Amp	17	55761161	55770818	6.137384	0.188849
26225	Amp	17	55773484	55803918	6.04774	0.187519
26226	Amp	17	55806972	55807901	6.732306	0.19718
26227	Amp	17	55808679	55811383	6.855677	0.198971
26228	Amp	17	55812109	55817756	6.747966	0.197483
26229	Amp	17	55823882	55831728	6.503622	0.194006
26230	Amp	17	55833298	55882326	6.980586	0.20079
26231	Amp	17	55885594	55910309	5.882356	0.185075
26232	Amp	17	55910721	55933705	5.762293	0.183284
26233	Amp	17	55934822	55941082	5.987065	0.186737
26234	Amp	17	55945180	55960530	5.184242	0.174988

26235	Amp	17	55960880	55962210	5.39004	0.177876
26236	Amp	17	55963247	55963247	4.99404	0.172057
26237	Amp	17	55968960	55991682	5.081731	0.173373
26238	Amp	17	55995531	56010261	4.952273	0.171326
26239	Amp	17	56016143	56026456	4.979649	0.171827
26240	Amp	17	56029045	56080851	4.93817	0.171322
26241	Amp	17	56095906	56107118	5.081731	0.173403
26242	Amp	17	56107654	56111407	5.836931	0.184478
26243	Amp	17	56113025	56115643	6.350097	0.191925
26244	Amp	17	56118992	56124070	6.825391	0.198568
26245	Amp	17	56125999	56126113	6.549814	0.194592
26246	Amp	17	56127048	56138449	6.778132	0.197874
26247	Amp	17	56138944	56147091	6.901838	0.19964
26248	Amp	17	56149444	56167778	7.516099	0.208097
26249	Amp	17	56176957	56201269	8.29898	0.218716
26250	Amp	17	56206548	56218082	7.516099	0.208097
26251	Amp	17	56220293	56228989	7.452952	0.207272
26252	Amp	17	56233192	56248138	7.310837	0.205372
26253	Amp	17	56250699	56253835	6.871512	0.19929
26254	Amp	17	56256359	56275411	7.341956	0.205782
26255	Amp	17	56282927	56334747	7.626527	0.209664
26256	Amp	17	56334787	56352794	7.768843	0.211711
26257	Amp	17	56353598	56355318	7.817451	0.212251
26258	Amp	17	56356821	56367030	7.993387	0.214547
26259	Amp	17	56369600	56370372	8.088933	0.215968
26260	Amp	17	56371838	56389132	8.137603	0.216496
26261	Amp	17	56389307	56390503	7.977624	0.214513
26262	Amp	17	56391008	56391826	8.154198	0.216737
26263	Amp	17	56405869	56420249	8.233714	0.217864
26264	Amp	17	56422136	56422218	8.105491	0.216092
26265	Amp	17	56422406	56423371	7.993387	0.214617
26266	Amp	17	56424200	56437211	8.057852	0.215591
26267	Amp	17	56437444	56445022	8.105491	0.216154
26268	Amp	17	56445501	56445501	8.32882	0.219192
26269	Amp	17	56446225	56470647	8.802762	0.225515
26270	Amp	17	56470929	56473848	8.936662	0.227183
26271	Amp	17	56474848	56480729	8.737278	0.224558
26272	Amp	17	56481261	56486235	8.60549	0.222792
26273	Amp	17	56488342	56488342	8.754268	0.224891
26274	Amp	17	56489087	56489087	9.407903	0.233214
26275	Amp	17	56490453	56505747	9.272857	0.231656
26276	Amp	17	56505811	56524730	9.155442	0.229987
26277	Amp	17	56526616	56528012	9.340097	0.232549
26278	Amp	17	56528260	56538807	8.522585	0.221852
26279	Amp	17	56543245	56543245	8.802762	0.225591
26280	Amp	17	56546566	56568365	8.6219	0.223029
26281	Amp	17	56569991	56575049	8.538535	0.222055
26282	Amp	17	56587065	56602621	8.6219	0.223172
26283	Amp	17	56603264	56603264	7.833797	0.21251
26284	Amp	17	56604632	56604632	7.563028	0.208759
26285	Amp	17	56604955	56609063	7.516099	0.208046
26286	Amp	17	56615238	56617719	8.458247	0.220921
26287	Amp	17	56618871	56618871	8.282609	0.218596

26288	Amp	17	56619230	56626288	8.704326	0.224296
26289	Amp	17	56627468	56627828	8.754268	0.224772
26290	Amp	17	56627875	56628465	8.704326	0.224209
26291	Amp	17	56628959	56633114	8.936662	0.22733
26292	Amp	17	56637402	56637584	8.441738	0.220797
26293	Amp	17	56638206	56649650	8.121744	0.216379
26294	Amp	17	56650383	56657341	7.896426	0.21344
26295	Amp	17	56662018	56662882	7.357785	0.205997
26296	Amp	17	56666756	56670768	7.183949	0.203636
26297	Amp	17	56670981	56683950	7.295403	0.205072
26298	Amp	17	56684552	56701456	7.72044	0.210946
26299	Amp	17	56701543	56701903	7.405471	0.206703
26300	Amp	17	56702120	56709492	7.500789	0.207924
26301	Amp	17	56709856	56711017	7.468873	0.207447
26302	Amp	17	56711998	56732081	7.326935	0.205625
26303	Amp	17	56737481	56738203	8.505687	0.221561
26304	Amp	17	56738315	56745126	8.266115	0.21844
26305	Amp	17	56745735	56767756	8.121744	0.216338
26306	Amp	17	56768024	56772805	7.768843	0.211696
26307	Amp	17	56772968	56772968	7.673484	0.210376
26308	Amp	17	56776477	56791402	8.154198	0.216726
26309	Amp	17	56792112	56799503	7.610655	0.209345
26310	Amp	17	56799823	56817004	7.689477	0.210616
26311	Amp	17	56819126	56839839	7.880674	0.213168
26312	Amp	17	56841479	56854705	8.637458	0.223409
26313	Amp	17	56860640	56861535	9.18903	0.230441
26314	Amp	17	56862354	56863100	9.122122	0.229664
26315	Amp	17	56865575	56877361	9.256952	0.23137
26316	Amp	17	56884823	56895040	8.121744	0.216387
26317	Amp	17	56896721	56901719	8.008326	0.214797
26318	Amp	17	56902883	56969583	7.896426	0.213417
26319	Amp	17	56975809	57001580	7.993387	0.214638
26320	Amp	17	57002110	57011970	7.865467	0.212932
26321	Amp	17	57020703	57027535	8.313762	0.219
26322	Amp	17	57028431	57034747	8.409489	0.220273
26323	Amp	17	57034930	57034986	8.6219	0.223213
26324	Amp	17	57038368	57039914	8.588477	0.222737
26325	Amp	17	57039982	57047579	8.770924	0.225152
26326	Amp	17	57049617	57080337	8.671092	0.223813
26327	Amp	17	57081450	57087866	8.441738	0.220694
26328	Amp	17	57088071	57093435	8.572005	0.222436
26329	Amp	17	57096744	57100840	8.786437	0.225391
26330	Amp	17	57101967	57110834	8.987196	0.227786
26331	Amp	17	57111012	57122105	9.223227	0.231024
26332	Amp	17	57122969	57122969	9.729466	0.237376
26333	Amp	17	57124148	57164881	9.644619	0.236258
26334	Amp	17	57165424	57178658	10.105286	0.24231
26335	Amp	17	57185503	57185926	10.396417	0.245927
26336	Amp	17	57187472	57191349	10.566416	0.248118
26337	Amp	17	57191393	57202147	10.773589	0.25059
26338	Amp	17	57203381	57205971	10.9963	0.253618
26339	Amp	17	57210510	57213482	11.185948	0.255913
26340	Amp	17	57214139	57226567	11.27356	0.257042

26341	Amp	17	57226815	57226815	11.644242	0.26159
26342	Amp	17	57230281	57237228	11.519317	0.259965
26343	Amp	17	57237758	57242276	11.627141	0.261359
26344	Amp	17	57245236	57258794	11.377246	0.258321
26345	Amp	17	57258907	57269377	11.168221	0.255636
26346	Amp	17	57270272	57272776	11.221545	0.256305
26347	Amp	17	57272806	57278238	11.031992	0.253911
26348	Amp	17	57278826	57291439	10.277087	0.244409
26349	Amp	17	57293322	57306340	10.465282	0.246822
26350	Amp	17	57309277	57316749	10.378606	0.245697
26351	Amp	17	57317306	57317306	10.634596	0.248863
26352	Amp	17	57319492	57331951	10.978295	0.25321
26353	Amp	17	57336643	57349504	10.686888	0.249564
26354	Amp	17	57352083	57390615	10.549344	0.247822
26355	Amp	17	57390845	57400008	10.243045	0.244057
26356	Amp	17	57401131	57402717	9.916528	0.239858
26357	Amp	17	57404976	57445838	10.157784	0.2429
26358	Amp	17	57448476	57448476	10.31211	0.244882
26359	Amp	17	57448565	57448757	10.465282	0.246706
26360	Amp	17	57448852	57457150	10.566416	0.247982
26361	Amp	17	57462885	57491152	11.239373	0.256599
26362	Amp	17	57494944	57497302	11.063647	0.254304
26363	Amp	17	57498166	57499880	10.927313	0.25254
26364	Amp	17	57500528	57544180	11.049458	0.254066
26365	Amp	17	57544236	57556107	11.342933	0.257864
26366	Amp	17	57559763	57579654	11.221545	0.256338
26367	Amp	17	57581764	57585163	11.049458	0.254162
26368	Amp	17	57590361	57596237	11.1336	0.255304
26369	Amp	17	57598463	57601214	11.308217	0.257333
26370	Amp	17	57602868	57602868	10.843331	0.251459
26371	Amp	17	57603256	57623773	11.446377	0.259178
26372	Amp	17	57624085	57646974	10.720769	0.250024
26373	Amp	17	57651402	57676950	10.877729	0.25192
26374	Amp	17	57685888	57685910	11.168221	0.25566
26375	Amp	17	57686292	57701469	11.536709	0.260351
26376	Amp	17	57703909	57706214	11.698383	0.262277
26377	Amp	17	57709436	57715747	10.000692	0.240841
26378	Amp	17	57716583	57720574	10.877729	0.252098
26379	Amp	17	57721219	57726645	10.583831	0.2483
26380	Amp	17	57730026	57755232	10.943696	0.252903
26381	Amp	17	57757138	57766884	11.049458	0.25422
26382	Amp	17	57767828	57788969	9.967648	0.240396
26383	Amp	17	57792917	57802729	10.190896	0.243199
26384	Amp	17	57805250	57809828	10.51732	0.247348
26385	Amp	17	57810785	57823060	10.943696	0.252795
26386	Amp	17	57823656	57826133	10.600629	0.248589
26387	Amp	17	57826409	57835609	11.031992	0.253884
26388	Amp	17	57836665	57842482	11.27356	0.257041
26389	Amp	17	57844591	57848262	10.893171	0.252261
26390	Amp	17	57848719	57848719	11.063647	0.254314
26391	Amp	17	57851849	57851849	10.773589	0.250698
26392	Amp	17	57852014	57873527	10.893171	0.252192
26393	Amp	17	57873863	57876279	10.756361	0.250368

26394	Amp	17	57879064	57879064	9.746461	0.237624
26395	Amp	17	57879489	57895643	9.847525	0.239022
26396	Amp	17	57896360	57900875	9.812347	0.238482
26397	Amp	17	57903325	57903325	9.627477	0.236109
26398	Amp	17	57905581	57905581	9.508958	0.234711
26399	Amp	17	57905826	57931958	9.47615	0.234254
26400	Amp	17	57934649	57959034	9.967648	0.240528
26401	Amp	17	57963681	57963681	10.14039	0.242708
26402	Amp	17	57968210	58029222	10.32955	0.244961
26403	Amp	17	58031739	58031973	9.933591	0.240005
26404	Amp	17	58033296	58047913	10.31211	0.244886
26405	Amp	17	58054394	58058433	10.482498	0.246953
26406	Amp	17	58059698	58065050	10.346125	0.245158
26407	Amp	17	58068206	58071692	10.703463	0.249881
26408	Amp	17	58073107	58111225	10.860138	0.251683
26409	Amp	17	58111978	58123113	11.308217	0.257324
26410	Amp	17	58140037	58150377	11.734451	0.262615
26411	Amp	17	58150642	58151642	11.966547	0.265446
26412	Amp	17	58158965	58158965	12.600251	0.273071
26413	Amp	17	58164669	58172699	12.83688	0.275979
26414	Amp	17	58174244	58175345	13.892034	0.288416
26415	Amp	17	58180281	58187850	14.412004	0.294482
26416	Amp	17	58190575	58216203	14.725554	0.298341
26417	Amp	17	58220787	58226969	14.336365	0.293793
26418	Amp	17	58235367	58316700	14.595893	0.296781
26419	Amp	17	58317700	58321864	15.024711	0.301788
26420	Amp	17	58322155	58354092	15.348745	0.305483
26421	Amp	17	58354188	58354188	15.309262	0.305004
26422	Amp	17	58360147	58374760	14.95044	0.300938
26423	Amp	17	58388673	58394320	15.861935	0.311226
26424	Amp	17	58394758	58432504	15.805309	0.31057
26425	Amp	17	58432985	58433297	15.672322	0.309157
26426	Amp	17	58437025	58445050	15.727885	0.309688
26427	Amp	17	58445172	58472159	15.595327	0.308194
26428	Amp	17	58475337	58502133	14.447899	0.294928
26429	Amp	17	58506813	58516164	14.706125	0.298009
26430	Amp	17	58521630	58523678	14.779959	0.298814
26431	Amp	17	58524191	58524315	14.987459	0.301266
26432	Amp	17	58525591	58531040	14.631516	0.297117
26433	Amp	17	58531104	58545928	14.725554	0.298285
26434	Amp	17	58550586	58555279	14.300473	0.293232
26435	Amp	17	58556014	58565748	13.947841	0.289195
26436	Amp	17	58569501	58593607	13.892034	0.288379
26437	Amp	17	58594948	58621466	13.363175	0.282166
26438	Amp	17	58622046	58623311	14.706125	0.298102
26439	Amp	17	58634540	58634540	14.560384	0.2963
26440	Amp	17	58634557	58634845	14.224691	0.292475
26441	Amp	17	58637290	58637290	14.541868	0.296003
26442	Amp	17	58640946	58650977	15.881803	0.311409
26443	Amp	17	58660477	58662045	14.968996	0.301122
26444	Amp	17	58662634	58662634	14.466246	0.295124
26445	Amp	17	58663139	58675458	14.187091	0.292011
26446	Amp	17	58675701	58696921	14.48507	0.295453

26447	Amp	17	58697584	58710157	14.560384	0.296332
26448	Amp	17	58711925	58712843	14.412004	0.294482
26449	Amp	17	58716315	58716332	14.668813	0.29749
26450	Amp	17	58716439	58751807	15.233242	0.304084
26451	Amp	17	58753955	58764442	14.668813	0.29749
26452	Amp	17	58770639	58779900	14.743668	0.298374
26453	Amp	17	58780516	58794005	14.931259	0.300545
26454	Amp	17	58794827	58829019	15.272026	0.304475
26455	Amp	17	58832565	58837080	15.576957	0.308078
26456	Amp	17	58850431	58867279	16.145689	0.314519
26457	Amp	17	58876772	58880529	17.196473	0.326359
26458	Amp	17	58885777	58890487	16.513277	0.318734
26459	Amp	17	58892177	58907336	16.647433	0.32028
26460	Amp	17	58918962	58957666	17.433073	0.328952
26461	Amp	17	58958109	58962702	17.078925	0.324979
26462	Amp	17	58972170	58972297	17.373987	0.328249
26463	Amp	17	58976060	58976060	18.248294	0.337788
26464	Amp	17	59093350	59093350	18.704216	0.34278
26465	Amp	17	59093786	59100695	20.114426	0.357976
26466	Amp	17	59103294	59104385	20.82618	0.365612
26467	Amp	17	59107236	59134241	21.07529	0.368197
26468	Amp	17	59140533	59141926	21.32672	0.37077
26469	Amp	17	59141939	59186032	21.014881	0.367501
26470	Amp	17	59189022	59192836	21.242402	0.369972
26471	Amp	17	59193389	59202739	21.387318	0.371327
26472	Amp	17	59210890	59215465	21.769764	0.375401
26473	Amp	17	59216375	59216375	22.550798	0.383357
26474	Amp	17	59224035	59239368	23.069701	0.388719
26475	Amp	17	59239699	59254856	23.028019	0.388459
26476	Amp	17	59257426	59269345	23.478835	0.392938
26477	Amp	17	59290376	59290754	21.599523	0.373562
26478	Amp	17	59291909	59291909	21.748183	0.375006
26479	Amp	17	59295737	59305819	22.275029	0.380554
26480	Amp	17	59312945	59344874	22.486306	0.38266
26481	Amp	17	59346940	59360577	23.651501	0.394707
26482	Amp	17	59360804	59362048	23.891039	0.396959
26483	Amp	17	59362206	59377164	24.130107	0.399399
26484	Amp	17	59380182	59388543	25.678	0.414947
26485	Amp	17	59389143	59389712	25.524992	0.4134
26486	Amp	17	59390523	59414285	25.744542	0.415519
26487	Amp	17	59417086	59417086	27.357173	0.431307
26488	Amp	17	59417634	59438106	28.045209	0.438008
26489	Amp	17	59438273	59512032	27.581587	0.433501
26490	Amp	17	59514147	59519803	27.666235	0.434383
26491	Amp	17	59521767	59539052	27.538273	0.433191
26492	Amp	17	59539832	59540555	27.311632	0.430939
26493	Amp	17	59545150	59548899	28.17795	0.439408
26494	Amp	17	59549241	59564714	29.819844	0.455202
26495	Amp	17	59570579	59572197	29.979408	0.456783
26496	Amp	17	59576855	59621957	31.199674	0.468443
26497	Amp	17	59621970	59623792	31.337869	0.469879
26498	Amp	17	59631218	59635919	31.862503	0.474807
26499	Amp	17	59636260	59640778	32.048086	0.476518

26500	Amp	17	59643902	59665341	32.256002	0.478518
26501	Amp	17	59666290	59668173	32.048086	0.476413
26502	Amp	17	59675043	59677787	32.232731	0.478285
26503	Amp	17	59678652	59684873	32.48127	0.480581
26504	Amp	17	59685114	59687813	32.094452	0.477
26505	Amp	17	59689614	59705997	31.792747	0.474036
26506	Amp	17	59712845	59813811	32.256002	0.478476
26507	Amp	17	59814594	59820498	32.001606	0.47618
26508	Amp	17	59836174	59841009	32.189017	0.477776
26509	Amp	17	59842355	59843862	32.387839	0.4797
26510	Amp	17	59845081	59845081	32.619783	0.481869
26511	Amp	17	59847814	59847814	32.364292	0.479457
26512	Amp	17	59851871	59865666	32.273296	0.478664
26513	Amp	17	59866158	59881442	31.630525	0.472507
26514	Amp	17	59884331	59892474	31.427194	0.470685
26515	Amp	17	59899297	59899297	31.062194	0.467128
26516	Amp	17	59909426	59972128	30.971346	0.46622
26517	Amp	17	59972248	59977596	30.813876	0.464838
26518	Amp	17	59998150	59998150	30.902566	0.46567
26519	Amp	17	59998235	59998813	30.635708	0.463096
26520	Amp	17	60002609	60002609	31.062194	0.467183
26521	Amp	17	60003122	60003122	30.635708	0.463022
26522	Amp	17	60036209	60036209	30.991608	0.466465
26523	Amp	17	60040068	60040068	31.29255	0.469403
26524	Amp	17	60044092	60044092	30.410563	0.460935
26525	Amp	17	60044589	60055577	30.94943	0.466122
26526	Amp	17	60056228	60074524	30.233001	0.459117
26527	Amp	17	60076636	60077819	30.048152	0.457388
26528	Amp	17	60080572	60080696	29.796928	0.455036
26529	Amp	17	60084138	60085377	29.344359	0.450756
26530	Amp	17	60090124	60108275	29.18568	0.449161
26531	Amp	17	60110554	60111524	28.830451	0.445585
26532	Amp	17	60127486	60131885	28.132847	0.438968
26533	Amp	17	60132228	60136651	28.402074	0.441533
26534	Amp	17	60136763	60136763	28.310496	0.440626
26535	Amp	17	60144679	60164929	28.60304	0.443442
26536	Amp	17	60167902	60190276	28.739537	0.444797
26537	Amp	17	60198808	60209502	28.961732	0.446921
26538	Amp	17	60209859	60213345	29.321519	0.450478
26539	Amp	17	60218964	60257136	29.615095	0.453255
26540	Amp	17	60263583	60317687	29.459956	0.45187
26541	Amp	17	60318531	60318531	29.093942	0.448267
26542	Amp	17	60320697	60327323	28.896188	0.446394
26543	Amp	17	60332287	60356516	29.093942	0.448306
26544	Amp	17	60360805	60360805	28.938654	0.446871
26545	Amp	17	60361285	60377870	29.052181	0.447905
26546	Amp	17	60379655	60393506	28.830451	0.445676
26547	Amp	17	60395813	60395813	30.456814	0.461323
26548	Amp	17	60396954	60408376	30.025733	0.457292
26549	Amp	17	60411463	60416843	29.842234	0.455425
26550	Amp	17	60418954	60461768	28.17795	0.439489
26551	Amp	17	60465974	60475906	28.60304	0.443485
26552	Amp	17	60477012	60486757	28.045209	0.438084

26553	Amp	17	60489686	60496698	28.333456	0.440799
26554	Amp	17	60497098	60497098	28.536153	0.442755
26555	Amp	17	60497732	60502778	28.670637	0.444086
26556	Amp	17	60506585	60506585	28.58071	0.443206
26557	Amp	17	60515804	60519263	27.891609	0.43661
26558	Amp	17	60521897	60563025	28.11227	0.438735
26559	Amp	17	60563564	60564763	27.824091	0.43592
26560	Amp	17	60581112	60600504	29.18568	0.449243
26561	Amp	17	60603815	60651064	28.875293	0.446209
26562	Amp	17	60659066	60665805	30.342535	0.46029
26563	Amp	17	60665947	60665947	30.274095	0.459497
26564	Amp	17	60669619	60683322	30.723588	0.463983
26565	Amp	17	60686487	60694840	28.491223	0.442463
26566	Amp	17	60695416	60700417	28.132847	0.438938
26567	Amp	17	60709264	60709694	27.666235	0.434452
26568	Amp	17	60710151	60710151	26.846754	0.426285
26569	Amp	17	60712560	60713171	25.806057	0.416082
26570	Amp	17	60716833	60725498	25.372008	0.411814
26571	Amp	17	60725698	60725698	25.872665	0.416793
26572	Amp	17	60730462	60750927	25.95936	0.417601
26573	Amp	17	60751041	60818836	25.786817	0.41599
26574	Amp	17	60819584	60824355	26.001327	0.418087
26575	Amp	17	60827451	60840416	25.200555	0.410043
26576	Amp	17	60844600	60845710	25.938335	0.417372
26577	Amp	17	60851530	60853976	24.543628	0.403452
26578	Amp	17	60855371	60878315	24.719836	0.405187
26579	Amp	17	60880059	60921651	24.458179	0.402735
26580	Amp	17	60922010	60922010	24.96221	0.407601
26581	Amp	17	60923837	60923837	25.722092	0.415377
26582	Amp	17	60924070	60935692	25.916007	0.417158
26583	Amp	17	60939617	60945411	24.479113	0.402868
26584	Amp	17	60949327	60953064	22.984559	0.387987
26585	Amp	17	60957061	60969985	22.8638	0.386591
26586	Amp	17	60970027	60971716	23.006217	0.388082
26587	Amp	17	60977208	60980875	22.824016	0.386125
26588	Amp	17	60981224	60987773	22.925684	0.387297
26589	Amp	17	60992834	61012184	23.090197	0.389025
26590	Amp	17	61017836	61029363	23.435464	0.392549
26591	Amp	17	61033497	61034750	23.347862	0.391515
26592	Amp	17	61037180	61057572	22.380843	0.381686
26593	Amp	17	61058167	61074869	22.045129	0.378164
26594	Amp	17	61076197	61078859	22.444367	0.382294
26595	Amp	17	61082719	61094092	22.842906	0.386378
26596	Amp	17	61095204	61108785	23.153998	0.389701
26597	Amp	17	61116394	61141003	22.761806	0.385617
26598	Amp	17	61141594	61144501	22.984559	0.388016
26599	Amp	17	61146727	61169398	22.824016	0.386251
26600	Amp	17	61171185	61210997	22.8638	0.386724
26601	Amp	17	61212709	61245957	22.275029	0.380619
26602	Amp	17	61246902	61250476	21.876573	0.376489
26603	Amp	17	61252769	61272114	22.130946	0.379063
26604	Amp	17	61274260	61292104	22.444367	0.382251
26605	Amp	17	61294673	61307532	20.501238	0.362154

26606	Amp	17	61310878	61318925	20.911016	0.366491
26607	Amp	17	61319088	61351452	21.034527	0.367805
26608	Amp	17	61353863	61373778	20.974304	0.367055
26609	Amp	17	61376097	61376649	22.027522	0.377951
26610	Amp	17	61377861	61394011	22.88355	0.386845
26611	Amp	17	61395042	61395042	22.739997	0.385274
26612	Amp	17	61404012	61414305	21.408633	0.371675
26613	Amp	17	61414726	61415353	22.088184	0.378611
26614	Amp	17	61415647	61420889	22.189283	0.379684
26615	Amp	17	61422972	61423080	21.962553	0.377221
26616	Amp	17	61424092	61427361	21.095587	0.368328
26617	Amp	17	61427581	61432215	21.347119	0.370931
26618	Amp	17	61434087	61461302	21.095587	0.368457
26619	Amp	17	61466366	61468119	20.011995	0.356841
26620	Amp	17	61476192	61483324	19.693709	0.353333
26621	Amp	17	61484379	61489135	19.572994	0.35202
26622	Amp	17	61490398	61497237	19.12459	0.347394
26623	Amp	17	61500547	61502583	18.903702	0.344984
26624	Amp	17	61503927	61507204	18.486439	0.34029
26625	Amp	17	61508948	61544141	18.288413	0.338248
26626	Amp	17	61550516	61550516	17.928201	0.334251
26627	Amp	17	61555000	61560667	17.216433	0.32651
26628	Amp	17	61561830	61561830	17.157327	0.325855
26629	Amp	17	61562841	61568224	16.860248	0.322666
26630	Amp	17	61580759	61581390	18.207228	0.337251
26631	Amp	17	61584125	61592528	19.06482	0.346617
26632	Amp	17	61592928	61599741	18.288413	0.338267
26633	Amp	17	61599795	61613192	18.56575	0.341158
26634	Amp	17	61622225	61622225	18.428857	0.339725
26635	Amp	17	61622408	61627801	18.068998	0.335754
26636	Amp	17	61631060	61642448	18.128001	0.336451
26637	Amp	17	61642876	61647140	18.546429	0.341075
26638	Amp	17	61650878	61667650	18.288413	0.338184
26639	Amp	17	61674874	61711534	18.428857	0.33974
26640	Amp	17	61717851	61720720	18.108258	0.336171
26641	Amp	17	61722675	61743470	19.104535	0.347044
26642	Amp	17	61745674	61777920	19.491404	0.351165
26643	Amp	17	61778702	61786254	19.752676	0.35415
26644	Amp	17	61789186	61789186	19.91297	0.355841
26645	Amp	17	61789868	61790078	20.338306	0.360328
26646	Amp	17	61791950	61791950	19.712397	0.353607
26647	Amp	17	61793993	61800623	19.993383	0.356575
26648	Amp	17	61800636	61835013	20.114426	0.358046
26649	Amp	17	61835667	61848919	19.83239	0.354875
26650	Amp	17	61849946	61856717	19.451056	0.350715
26651	Amp	17	61858362	61858362	19.67284	0.353229
26652	Amp	17	61861375	61862437	20.563251	0.362647
26653	Amp	17	61862534	61874807	21.014881	0.367589
26654	Amp	17	61882025	61892224	20.296825	0.359994
26655	Amp	17	61894009	61897290	20.073356	0.357446
26656	Amp	17	61898011	61921072	20.563251	0.362669
26657	Amp	17	61926943	61938073	20.953203	0.366853
26658	Amp	17	61940527	61945627	20.667704	0.363792

26659	Amp	17	61949318	61951080	20.605119	0.363218
26660	Amp	17	61954132	61962968	20.482298	0.361887
26661	Amp	17	61964020	61993511	19.491404	0.351309
26662	Amp	17	61997119	62009641	20.255854	0.359578
26663	Amp	17	62024316	62028492	20.974304	0.366999
26664	Amp	17	62028881	62069101	21.32672	0.37079
26665	Amp	17	62070547	62093862	21.263085	0.370131
26666	Amp	17	62094589	62102146	21.014881	0.367501
26667	Amp	17	62102266	62102542	20.522128	0.362222
26668	Amp	17	62111248	62113269	19.61439	0.352425
26669	Amp	17	62115237	62132913	19.752676	0.354073
26670	Amp	17	62133360	62143514	20.053094	0.357243
26671	Amp	17	62147159	62147159	19.652987	0.352872
26672	Amp	17	62148059	62162178	19.451056	0.350701
26673	Amp	17	62164820	62190081	19.206111	0.348241
26674	Amp	17	62191558	62196680	19.004359	0.346071
26675	Amp	17	62197207	62200296	18.923342	0.345174
26676	Amp	17	62207746	62216059	19.307452	0.349288
26677	Amp	17	62216885	62229186	19.59374	0.352236
26678	Amp	17	62238581	62244793	20.093568	0.357678
26679	Amp	17	62245124	62245492	19.93106	0.356029
26680	Amp	17	62247491	62247861	20.296825	0.360014
26681	Amp	17	62278292	62278292	20.743272	0.364746
26682	Amp	17	62278668	62278668	21.034527	0.367666
26683	Amp	17	62285848	62286763	21.305424	0.370541
26684	Amp	17	62289615	62296315	21.727651	0.374947
26685	Amp	17	62300456	62369553	22.027522	0.377903
26686	Amp	17	62369759	62372954	21.451458	0.372126
26687	Amp	17	62374521	62374709	21.157024	0.368947
26688	Amp	17	62375289	62375289	20.686425	0.36398
26689	Amp	17	62378561	62391018	19.024158	0.346196
26690	Amp	17	62400384	62414205	18.60467	0.341565
26691	Amp	17	62414785	62446311	18.683958	0.342504
26692	Amp	17	62446641	62452523	19.752676	0.354041
26693	Amp	17	62457716	62490929	19.267324	0.348865
26694	Amp	17	62495585	62505681	18.745071	0.343249
26695	Amp	17	62511672	62535721	19.104535	0.347206
26696	Amp	17	62536174	62536174	19.732254	0.353854
26697	Amp	17	62540705	62554847	19.510188	0.35144
26698	Amp	17	62556470	62565781	20.275819	0.359783
26699	Amp	17	62566683	62566683	21.135588	0.368869
26700	Amp	17	62567316	62580664	21.727651	0.374946
26701	Amp	17	62584861	62618634	22.151048	0.379333
26702	Amp	17	62630037	62631216	21.621009	0.373788
26703	Amp	17	62631590	62637089	21.178346	0.36918
26704	Amp	17	62642398	62676543	20.338306	0.360441
26705	Amp	17	62678862	62707658	19.12459	0.347419
26706	Amp	17	62711871	62712044	18.78296	0.343521
26707	Amp	17	62713795	62714235	18.903702	0.344936
26708	Amp	17	62714421	62715169	19.084146	0.346821
26709	Amp	17	62715439	62745782	18.765297	0.343481
26710	Amp	17	62754882	62757401	18.96358	0.345559
26711	Amp	17	62765478	62786738	18.664489	0.342412

26712	Amp	17	62789386	62795399	18.408386	0.339457
26713	Amp	17	62800148	62816488	17.691957	0.331763
26714	Amp	17	62816718	62843564	17.571902	0.330348
26715	Amp	17	62844032	62850993	18.008387	0.335161
26716	Amp	17	62861823	62901582	18.388632	0.339211
26717	Amp	17	62905345	62943864	18.765297	0.34335
26718	Amp	17	62943908	62943908	18.52593	0.340708
26719	Amp	17	62946426	63014897	18.128001	0.336568
26720	Amp	17	63015392	63016547	18.248294	0.33766
26721	Amp	17	63017934	63039767	18.506432	0.340644
26722	Amp	17	63049434	63049875	19.185603	0.347869
26723	Amp	17	63057544	63058087	18.068998	0.335823
26724	Amp	17	63059957	63105337	17.078925	0.324939
26725	Amp	17	63111630	63111630	17.157327	0.325902
26726	Amp	17	63115499	63122345	17.353743	0.32803
26727	Amp	17	63129300	63129300	16.781541	0.321775
26728	Amp	17	63138544	63148216	16.978037	0.323898
26729	Amp	17	63150276	63168335	16.762041	0.321523
26730	Amp	17	63168386	63196446	16.533331	0.318828
26731	Amp	17	63198518	63243727	16.321318	0.316456
26732	Amp	17	63249203	63295450	17.968693	0.334703
26733	Amp	17	63299082	63305489	16.704765	0.320856
26734	Amp	17	63311129	63331117	16.860248	0.322593
26735	Amp	17	63334028	63334177	16.998219	0.324113
26736	Amp	17	63337539	63361254	16.533331	0.318913
26737	Amp	17	63361904	63374779	16.686433	0.320515
26738	Amp	17	63375307	63392508	16.898193	0.322948
26739	Amp	17	63395459	63429933	16.456552	0.317938
26740	Amp	17	63430677	63472877	16.224361	0.315505
26741	Amp	17	63478305	63484395	16.184688	0.314903
26742	Amp	17	63485810	63488974	15.519848	0.307433
26743	Amp	17	63490292	63490292	15.805309	0.310717
26744	Amp	17	63491783	63514291	16.126505	0.314336
26745	Amp	17	63515639	63545342	16.30158	0.31623
26746	Amp	17	63547188	63555561	17.018526	0.324206
26747	Amp	17	63560060	63562473	16.958374	0.323705
26748	Amp	17	63564739	63573104	16.475247	0.318195
26749	Amp	17	63579679	63584978	16.224361	0.315429
26750	Amp	17	63589129	63594463	16.456552	0.317937
26751	Amp	17	63599307	63610240	16.033601	0.313268
26752	Amp	17	63611468	63624488	15.9958	0.312831
26753	Amp	17	63625126	63640902	16.609099	0.319644
26754	Amp	17	63643836	63650606	17.890162	0.333886
26755	Amp	17	63651557	63651557	18.56575	0.341324
26756	Amp	17	63654250	63677646	18.664489	0.342263
26757	Amp	17	63678026	63685671	18.745071	0.343208
26758	Amp	17	63691324	63692121	18.546429	0.34108
26759	Amp	17	63705560	63710730	18.644239	0.342012
26760	Amp	17	63711499	63718429	18.408386	0.339455
26761	Amp	17	63718946	63718946	18.049178	0.335586
26762	Amp	17	63731243	63760899	17.910589	0.334066
26763	Amp	17	63761745	63781177	16.666682	0.320334
26764	Amp	17	63785824	63789514	16.533331	0.318931

26765	Amp	17	63791194	63852503	16.089694	0.313975
26766	Amp	17	63855553	63855553	16.165422	0.314723
26767	Amp	17	63859350	63860642	16.723558	0.321018
26768	Amp	17	63865580	63911447	16.647433	0.320086
26769	Amp	17	63911563	63911563	16.416418	0.317652
26770	Amp	17	63915777	63928647	17.05876	0.324672
26771	Amp	17	63929637	63933602	17.392969	0.328471
26772	Amp	17	63934867	63949113	17.176717	0.326012
26773	Amp	17	63951808	63959920	17.591497	0.330652
26774	Amp	17	63960810	63969956	17.850895	0.333339
26775	Amp	17	63971984	63995277	17.078925	0.32488
26776	Amp	17	63995929	63995929	17.078925	0.324858
26777	Amp	17	64000232	64088597	17.295317	0.327412
26778	Amp	17	64091885	64098143	17.552268	0.330107
26779	Amp	17	64100823	64100823	16.570587	0.319212
26780	Amp	17	64100919	64132496	16.397251	0.31728
26781	Amp	17	64137290	64151436	16.359113	0.316839
26782	Amp	17	64152084	64171026	16.224361	0.315356
26783	Amp	17	64171139	64171233	16.089694	0.313882
26784	Amp	17	64173798	64183409	15.290153	0.304676
26785	Amp	17	64183729	64198410	15.11973	0.302882
26786	Amp	17	64198641	64207007	14.968996	0.301039
26787	Amp	17	64215762	64221759	14.725554	0.298353
26788	Amp	17	64226560	64242323	14.968996	0.300968
26789	Amp	17	64243516	64259907	15.024711	0.301628
26790	Amp	17	64260114	64261974	15.272026	0.304649
26791	Amp	17	64266743	64282167	15.366356	0.305549
26792	Amp	17	64286156	64329884	15.672322	0.309055
26793	Amp	17	64332226	64333211	15.612951	0.30846
26794	Amp	17	64464879	64464879	13.454936	0.283234
26795	Amp	17	64468704	64472842	14.541868	0.296057
26796	Amp	17	64483156	64483156	14.987459	0.301201
26797	Amp	17	64484067	64484067	15.290153	0.304683
26798	Amp	17	64484994	64497225	14.762523	0.298617
26799	Amp	17	64497515	64504635	15.213793	0.303976
26800	Amp	17	64504888	64516353	15.423788	0.306213
26801	Amp	17	64520772	64548762	15.194804	0.303672
26802	Amp	17	64551697	64554178	15.366356	0.305544
26803	Amp	17	64556214	64562797	14.725554	0.298351
26804	Amp	17	64571715	64591736	14.48507	0.295383
26805	Amp	17	64594469	64603863	14.687681	0.297897
26806	Amp	17	64612193	64612193	15.881803	0.311474
26807	Amp	17	64615101	64615511	15.652786	0.308854
26808	Amp	17	64618111	64627915	15.747199	0.309993
26809	Amp	17	64636266	64654075	15.194804	0.30359
26810	Amp	17	64655971	64663439	14.987459	0.30123
26811	Amp	17	64663755	64666389	14.668813	0.297625
26812	Amp	17	64667139	64714646	14.317432	0.293379
26813	Amp	17	64714738	64720662	14.022374	0.289935
26814	Amp	17	64725497	64742314	12.873531	0.276357
26815	Amp	17	64744152	64751338	13.837777	0.287731
26816	Amp	17	64758554	64758554	13.655032	0.285557
26817	Amp	17	64759705	64766141	13.492157	0.283691

26818	Amp	17	64769796	64772394	13.436139	0.283083
26819	Amp	17	64787495	64792102	12.763765	0.275027
26820	Amp	17	64801333	64802452	12.039373	0.26629
26821	Amp	17	64802468	64802468	11.948056	0.265152
26822	Amp	17	64802530	64816408	11.734451	0.262678
26823	Amp	17	64843932	64843932	9.644619	0.23627
26824	Amp	17	64867438	64936226	8.555266	0.2223
26825	Amp	17	64936280	64936280	8.137603	0.216663
26826	Amp	17	64951737	64952455	8.424923	0.220527
26827	Amp	17	64952808	64959180	8.687806	0.223962
26828	Amp	17	64961745	64988259	8.538535	0.221919
26829	Amp	17	64989719	65036829	8.786437	0.225217
26830	Amp	17	65037416	65043397	8.834661	0.225854
26831	Amp	17	65045776	65049744	9.02098	0.228312
26832	Amp	17	65049958	65074741	8.770924	0.225015
26833	Amp	17	65077238	65077238	8.885533	0.226649
26834	Amp	17	65083792	65098606	9.272857	0.231489
26835	Amp	17	65099227	65120554	9.575888	0.235567
26836	Amp	17	65120581	65125511	9.881657	0.239402
26837	Amp	17	65134923	65135449	9.763565	0.237979
26838	Amp	17	65137910	65144842	10.017642	0.241171
26839	Amp	17	65149278	65150750	9.796693	0.238284
26840	Amp	17	65152202	65161004	10.190896	0.243411
26841	Amp	17	65161622	65175779	9.933591	0.239976
26842	Amp	17	65177638	65195540	9.746461	0.237659
26843	Amp	17	65195613	65198639	9.493034	0.234366
26844	Amp	17	65201802	65219013	9.678773	0.2367
26845	Amp	17	65219720	65220642	9.881657	0.239458
26846	Amp	17	65222350	65227844	9.933591	0.240069
26847	Amp	17	65228912	65249997	9.442896	0.233678
26848	Amp	17	65250160	65328308	9.493034	0.234411
26849	Amp	17	65328767	65342035	9.526354	0.234891
26850	Amp	17	65344691	65344691	9.780264	0.238167
26851	Amp	17	65345346	65355322	10.052391	0.241547
26852	Amp	17	65355511	65360770	9.916528	0.239913
26853	Amp	17	65366999	65373041	10.017642	0.241174
26854	Amp	17	65374057	65389443	9.881657	0.239379
26855	Amp	17	65389674	65416393	9.69597	0.237045
26856	Amp	17	65420712	65457354	9.984309	0.240605
26857	Amp	17	65461857	65473306	9.729466	0.23742
26858	Amp	17	65476910	65490062	9.442896	0.233739
26859	Amp	17	65490217	65525091	9.967648	0.240487
26860	Amp	17	65527485	65551029	10.034845	0.24146
26861	Amp	17	65562214	65563101	9.609886	0.235931
26862	Amp	17	65565474	65570281	10.791286	0.25095
26863	Amp	17	65572468	65598911	11.342933	0.257941
26864	Amp	17	65601456	65614201	11.572965	0.260601
26865	Amp	17	65614883	65617608	11.446377	0.259236
26866	Amp	17	65619344	65628829	11.168221	0.255746
26867	Amp	17	65635192	65639747	11.39504	0.258564
26868	Amp	17	65642009	65680032	11.1336	0.255318
26869	Amp	17	65681521	65723456	10.583831	0.248357
26870	Amp	17	65724408	65724408	10.909195	0.252342

26871	Amp	17	65726689	65726790	10.791286	0.251013
26872	Amp	17	65733408	65736855	10.51732	0.247468
26873	Amp	17	65736892	65736892	10.634596	0.24895
26874	Amp	17	65736973	65737611	10.9963	0.253491
26875	Amp	17	65737639	65755525	10.634596	0.248922
26876	Amp	17	65758742	65758742	11.185948	0.255883
26877	Amp	17	65763466	65795365	11.662686	0.261794
26878	Amp	17	65795429	65806353	11.446377	0.259068
26879	Amp	17	65807843	65807843	11.770282	0.263142
26880	Amp	17	65822038	65828251	10.843331	0.251638
26881	Amp	17	65834797	65834797	11.15007	0.255458
26882	Amp	17	65835699	65844352	11.25539	0.256828
26883	Amp	17	65857672	65857672	11.39504	0.258487
26884	Amp	17	65861859	65863026	12.364848	0.270251
26885	Amp	17	65867848	65909296	12.419384	0.270992
26886	Amp	17	65913131	65928669	12.112615	0.267172
26887	Amp	17	65930077	65964810	13.001761	0.277785
26888	Amp	17	65966482	66000369	12.637869	0.273566
26889	Amp	17	66000733	66022019	13.129509	0.279245
26890	Amp	17	66026581	66029232	13.239383	0.280555
26891	Amp	17	66035601	66041322	13.001761	0.277727
26892	Amp	17	66048922	66048922	12.637869	0.273462
26893	Amp	17	66051258	66052432	12.891235	0.276486
26894	Amp	17	66052552	66052592	12.473924	0.271497
26895	Amp	17	66052669	66087019	12.219704	0.2685
26896	Amp	17	66087570	66087570	12.347637	0.269946
26897	Amp	17	66094364	66104492	12.20153	0.268363
26898	Amp	17	66107967	66107967	11.412882	0.258687
26899	Amp	17	66109083	66118795	11.049458	0.254146
26900	Amp	17	66120648	66151880	11.168221	0.255755
26901	Amp	17	66153863	66169006	11.290117	0.257269
26902	Amp	17	66170297	66198386	11.360465	0.258116
26903	Amp	17	66201141	66229411	11.752146	0.262798
26904	Amp	17	66229742	66231556	12.094071	0.267075
26905	Amp	17	66232202	66233675	11.984978	0.265592
26906	Amp	17	66235413	66235413	11.734451	0.262691
26907	Amp	17	66235904	66238495	13.074762	0.278627
26908	Amp	17	66243628	66281938	13.764867	0.287058
26909	Amp	17	66285326	66285519	14.541868	0.296177
26910	Amp	17	66287030	66297853	14.300473	0.293358
26911	Amp	17	66298278	66299802	14.595893	0.296759
26912	Amp	17	66306132	66309043	14.836401	0.299655
26913	Amp	17	66310896	66341700	14.687681	0.297804
26914	Amp	17	66349341	66371929	14.87478	0.300035
26915	Amp	17	66372354	66391210	14.762523	0.298664
26916	Amp	17	66397724	66493013	15.024711	0.301697
26917	Amp	17	66493389	66499061	14.725554	0.298248
26918	Amp	17	66499129	66499129	14.779959	0.298901
26919	Amp	17	66503006	66503006	14.836401	0.29945
26920	Amp	17	66503190	66529767	15.024711	0.301705
26921	Amp	17	66530029	66534033	14.893571	0.300299
26922	Amp	17	66536939	66576598	14.650436	0.297281
26923	Amp	17	66578246	66586053	14.374364	0.294072

26924	Amp	17	66586700	66592543	14.503631	0.295739
26925	Amp	17	66593405	66605979	14.779959	0.298948
26926	Amp	17	66610018	66610606	14.040669	0.29016
26927	Amp	17	66611217	66615104	13.874487	0.288341
26928	Amp	17	66616644	66620262	13.929261	0.288839
26929	Amp	17	66620739	66630525	13.093327	0.278934
26930	Amp	17	66631194	66640654	12.781778	0.275155
26931	Amp	17	66647528	66655920	12.527954	0.272171
26932	Amp	17	66655994	66667167	12.383029	0.270503
26933	Amp	17	66670310	66672834	13.636576	0.285412
26934	Amp	17	66684904	66690136	13.764867	0.286974
26935	Amp	17	66691328	66691328	13.691717	0.286079
26936	Amp	17	66694140	66702263	12.437581	0.27117
26937	Amp	17	66705505	66707759	12.184583	0.268138
26938	Amp	17	66709082	66723831	12.546325	0.272325
26939	Amp	17	66725485	66726027	12.83688	0.275838
26940	Amp	17	66729772	66730423	13.276743	0.281165
26941	Amp	17	66734768	66734768	13.454936	0.283233
26942	Amp	17	66735060	66748195	13.565854	0.28457
26943	Amp	17	66749572	66753366	13.729199	0.286571
26944	Amp	17	66754027	66754027	13.528722	0.284154
26945	Amp	17	66759387	66763684	13.398977	0.282672
26946	Amp	17	66768840	66770961	13.454936	0.283252
26947	Amp	17	66772385	66788454	13.510816	0.283905
26948	Amp	17	66790923	66797215	14.040669	0.290193
26949	Amp	17	66801017	66813382	14.151192	0.291457
26950	Amp	17	66813717	66817735	14.187091	0.291955
26951	Amp	17	66823756	66831183	13.892034	0.288442
26952	Amp	17	66831435	66835539	14.058011	0.2905
26953	Amp	17	66838854	66872299	14.577855	0.296534
26954	Amp	17	66874731	66877810	14.743668	0.29842
26955	Amp	17	66885676	66885676	14.48507	0.295351
26956	Amp	17	66886575	66889334	14.300473	0.293154
26957	Amp	17	66890056	66893462	14.224691	0.292317
26958	Amp	17	66894870	66912892	14.040669	0.290259
26959	Amp	17	66914186	66917103	13.929261	0.288996
26960	Amp	17	66917582	66920528	14.0045	0.289788
26961	Amp	17	66920916	66922101	14.224691	0.292281
26962	Amp	17	66922873	66967197	14.560384	0.296307
26963	Amp	17	66967435	66967598	14.393488	0.294306
26964	Amp	17	66967626	66991553	14.541868	0.296178
26965	Amp	17	66994364	67013435	13.655032	0.285632
26966	Amp	17	67013883	67039119	13.729199	0.286506
26967	Amp	17	67039844	67045240	14.094981	0.290918
26968	Amp	17	67046385	67046500	14.243686	0.292498
26969	Amp	17	67046578	67068792	14.595893	0.296633
26970	Amp	17	67071829	67071829	14.48507	0.295461
26971	Amp	17	67072705	67112871	14.412004	0.294614
26972	Amp	17	67118973	67120375	14.169996	0.291704
26973	Amp	17	67120788	67121022	14.262513	0.292808
26974	Amp	17	67124575	67144308	14.355711	0.293854
26975	Amp	17	67144399	67149283	14.224691	0.292274
26976	Amp	17	67149885	67216690	14.0045	0.2898

26977	Amp	17	67217204	67234875	14.131967	0.291273
26978	Amp	17	67241073	67242635	14.0045	0.289711
26979	Amp	17	67252196	67264044	14.169996	0.291634
26980	Amp	17	67272728	67280785	13.636576	0.285394
26981	Amp	17	67281452	67287743	13.746952	0.286827
26982	Amp	17	67292922	67297018	14.022374	0.289938
26983	Amp	17	67299052	67311714	14.151192	0.291476
26984	Amp	17	67312582	67324214	14.058011	0.290454
26985	Amp	17	67325980	67327495	14.374364	0.294224
26986	Amp	17	67331266	67337270	14.560384	0.296202
26987	Amp	17	67344354	67354517	14.503631	0.295615
26988	Amp	17	67355392	67359619	14.07679	0.290681
26989	Amp	17	67360532	67372761	14.300473	0.293253
26990	Amp	17	67372783	67375850	14.613267	0.297002
26991	Amp	17	67390098	67390098	15.175853	0.303386
26992	Amp	17	67393189	67415812	15.519848	0.307326
26993	Amp	17	67416660	67426338	15.100986	0.302523
26994	Amp	17	67426884	67469039	15.175853	0.303354
26995	Amp	17	67479822	67503848	15.55852	0.307692
26996	Amp	17	67504229	67525963	15.709157	0.309524
26997	Amp	17	67530878	67532685	15.959834	0.312282
26998	Amp	17	67535543	67535543	15.442	0.306534
26999	Amp	17	67540499	67553854	15.348745	0.305469
27000	Amp	17	67553895	67562455	15.100986	0.30248
27001	Amp	17	67566770	67566924	15.137413	0.303113
27002	Amp	17	67569189	67583227	14.503631	0.29553
27003	Amp	17	67587135	67589383	15.213793	0.303797
27004	Amp	17	67593971	67598322	15.11973	0.302901
27005	Amp	17	67603380	67603380	13.985765	0.289603
27006	Amp	17	67603978	67641320	13.764867	0.287027
27007	Amp	17	67641603	67642898	13.129509	0.279281
27008	Amp	17	67646970	67659450	12.564467	0.272536
27009	Amp	17	67661684	67666804	12.437581	0.271015
27010	Amp	17	67667769	67671127	12.075847	0.266831
27011	Amp	17	67671617	67682372	12.237724	0.268711
27012	Amp	17	67682706	67682706	12.491673	0.271794
27013	Amp	17	67682741	67682826	12.419384	0.270971
27014	Amp	17	67687065	67692275	12.600251	0.273057
27015	Amp	17	67692709	67710082	12.855613	0.27609
27016	Amp	17	67710465	67710465	13.074762	0.278616
27017	Amp	17	67710632	67717680	12.81801	0.275583
27018	Amp	17	67720008	67729976	13.583613	0.284716
27019	Amp	17	67730562	67738712	13.454936	0.283178
27020	Amp	17	67740941	67751029	13.565854	0.284665
27021	Amp	17	67751269	67784234	13.800267	0.287427
27022	Amp	17	67784771	67797050	14.300473	0.293183
27023	Amp	17	67798370	67798390	14.577855	0.296489
27024	Amp	17	67798624	67801222	14.762523	0.298781
27025	Amp	17	67819788	67835374	15.11973	0.302799
27026	Amp	17	67836274	67876443	15.290153	0.304729
27027	Amp	17	67884837	67886965	14.893571	0.300122
27028	Amp	17	67891559	67891780	15.137413	0.302984
27029	Amp	17	67894773	67900436	15.785635	0.310411

27030 Amp	17	67901822	67910895	16.089694	0.313965
27031 Amp	17	67915279	67915279	16.204394	0.315249
27032 Amp	17	67921748	67976503	16.014682	0.312947
27033 Amp	17	67983025	67998511	15.842559	0.311116
27034 Amp	17	68000822	68000822	15.500252	0.307077
27035 Amp	17	68019847	68026544	16.145689	0.314487
27036 Amp	17	68029767	68029767	15.977907	0.312557
27037 Amp	17	68041227	68066200	15.940153	0.312213
27038 Amp	17	68178765	68178765	17.038719	0.324552
27039 Amp	17	68183009	68189163	16.781541	0.32169
27040 Amp	17	68209694	68220575	17.412814	0.328739
27041 Amp	17	68220670	68220670	17.333668	0.327815
27042 Amp	17	68221438	68229044	17.157327	0.325872
27043 Amp	17	68229216	68248848	17.157327	0.325738
27044 Amp	17	68249300	68257730	16.242583	0.315616
27045 Amp	17	68260815	68264868	15.309262	0.304877
27046 Amp	17	68265630	68281830	15.11973	0.302791
27047 Amp	17	68288654	68288973	14.541868	0.296134
27048 Amp	17	68292472	68300310	14.412004	0.294476
27049 Amp	17	68300703	68306301	14.631516	0.29724
27050 Amp	17	68316045	68319327	13.929261	0.288911
27051 Amp	17	68323759	68324304	14.281057	0.293079
27052 Amp	17	68324913	68328624	14.430436	0.294789
27053 Amp	17	68330444	68330444	14.094981	0.290905
27054 Amp	17	68338036	68348317	13.929261	0.289008
27055 Amp	17	68348454	68362782	14.262513	0.292924
27056 Amp	17	68364093	68371681	13.985765	0.289613
27057 Amp	17	68372043	68379852	13.800267	0.287318
27058 Amp	17	68380613	68391913	13.655032	0.285692
27059 Amp	17	68393046	68423256	13.220925	0.280459
27060 Amp	17	68424011	68424298	12.546325	0.272464
27061 Amp	17	68427870	68428196	12.510219	0.27187
27062 Amp	17	68428200	68459197	12.1666	0.26793
27063 Amp	17	68460522	68468405	12.130806	0.267318
27064 Amp	17	68468542	68483028	12.256206	0.269006
27065 Amp	17	68483075	68483075	12.184583	0.268104
27066 Amp	17	68490686	68507809	11.536709	0.260248
27067 Amp	17	68508878	68509715	11.239373	0.256619
27068 Amp	17	68513751	68541735	10.346125	0.245276
27069 Amp	17	68549204	68563037	9.172152	0.230228
27070 Amp	17	68566603	68602246	8.687806	0.223936
27071 Amp	17	68603904	68622278	8.885533	0.226505
27072 Amp	17	68622913	68626761	9.004172	0.228147
27073 Amp	17	68628613	68633040	8.409489	0.220251
27074 Amp	17	68634971	68648152	8.233714	0.21791
27075 Amp	17	68651699	68671422	8.105491	0.216269
27076 Amp	17	68671774	68671774	8.20134	0.217464
27077 Amp	17	68673972	68674431	7.768843	0.211627
27078 Amp	17	68675183	68694098	7.736725	0.211183
27079 Amp	17	68694445	68700570	8.137603	0.21667
27080 Amp	17	68706499	68708868	8.057852	0.21544
27081 Amp	17	68717640	68726525	8.008326	0.214807
27082 Amp	17	68730420	68738313	7.849711	0.212629

27083 Amp	17	68738359	68748641	7.689477	0.210504
27084 Amp	17	68748889	68753890	8.538535	0.222056
27085 Amp	17	68758594	68766174	8.18543	0.21728
27086 Amp	17	68770254	68778198	7.594603	0.20923
27087 Amp	17	68779830	68799894	7.264196	0.204636
27088 Amp	17	68803812	68847910	7.326935	0.205537
27089 Amp	17	68853445	68870247	7.531798	0.208453
27090 Amp	17	68873124	68875934	7.326935	0.205526
27091 Amp	17	68876592	68876661	7.421526	0.206853
27092 Amp	17	68879819	68882063	6.825391	0.198525
27093 Amp	17	68882237	68889385	7.011899	0.201186
27094 Amp	17	68890848	68895887	6.810251	0.198269
27095 Amp	17	68901399	68920603	7.232046	0.204236
27096 Amp	17	68922688	68944483	6.855677	0.199109
27097 Amp	17	68947071	68970346	6.380906	0.192328
27098 Amp	17	68974228	68996373	6.291248	0.190994
27099 Amp	17	68996474	69047810	6.198231	0.189667
27100 Amp	17	69048474	69048474	5.882356	0.185127
27101 Amp	17	69055254	69135746	6.53507	0.194425
27102 Amp	17	69142737	69149149	6.017563	0.187027
27103 Amp	17	69152586	69179842	5.806986	0.183988
27104 Amp	17	69185682	69192962	6.152783	0.188989
27105 Amp	17	69193354	69273360	5.972041	0.186329
27106 Amp	17	69275799	69315007	5.836931	0.184429
27107 Amp	17	69325814	69329129	5.672101	0.182064
27108 Amp	17	69329639	69333751	5.777283	0.183582
27109 Amp	17	69333885	69355424	5.987065	0.186632
27110 Amp	17	69363554	69416490	6.458214	0.193295
27111 Amp	17	69416825	69473307	6.488712	0.193738
27112 Amp	17	69473699	69475610	6.564374	0.194839
27113 Amp	17	69476771	69486173	7.183949	0.203601
27114 Amp	17	69486212	69539982	7.106137	0.202511
27115 Amp	17	69546606	69557000	6.901838	0.199648
27116 Amp	17	69557046	69566208	6.949342	0.200248
27117 Amp	17	69566222	69603596	7.264196	0.204655
27118 Amp	17	69607222	69623729	7.547251	0.208574
27119 Amp	17	69628414	69636998	7.074542	0.201977
27120 Amp	17	69637031	69648957	6.840574	0.19869
27121 Amp	17	69653183	69673103	6.092521	0.188069
27122 Amp	17	69673525	69681547	5.987065	0.186551
27123 Amp	17	69685026	69699637	6.396376	0.192546
27124 Amp	17	69702832	69725567	6.641348	0.195894
27125 Amp	17	69729514	69761363	6.488712	0.193747
27126 Amp	17	69761940	69778972	6.53507	0.194558
27127 Amp	17	69781272	69781272	6.732306	0.197329
27128 Amp	17	69786038	69798388	6.641348	0.196069
27129 Amp	17	69801482	69806039	6.411912	0.192721
27130 Amp	17	69807038	69847091	6.380906	0.192264
27131 Amp	17	69847559	69866940	6.062596	0.187674
27132 Amp	17	69867068	69887412	6.137384	0.188834
27133 Amp	17	69898906	69898906	5.972041	0.186437
27134 Amp	17	69899325	69916275	6.078164	0.187992
27135 Amp	17	69919557	69934533	6.443221	0.193051

27136	Amp	17	69934631	69945939	6.319529	0.191364
27137	Amp	17	69963203	69982604	6.229291	0.190204
27138	Amp	17	69982619	69986820	6.198231	0.189586
27139	Amp	17	69994712	70073088	6.152783	0.189076
27140	Amp	17	70073103	70109759	6.078164	0.187975
27141	Amp	17	70116392	70123096	6.122748	0.188504
27142	Amp	17	70124010	70124010	6.275802	0.190727
27143	Amp	17	70129831	70130786	6.137384	0.188867
27144	Amp	17	70137028	70147612	6.032925	0.187312
27145	Amp	17	70147955	70153588	5.746988	0.183262
27146	Amp	17	70157478	70170212	5.212889	0.175431
27147	Amp	17	70171366	70176185	5.375101	0.177652
27148	Amp	17	70176881	70180748	5.507674	0.179609
27149	Amp	17	70181624	70186718	5.702358	0.182441
27150	Amp	17	70190278	70193412	5.125419	0.174065
27151	Amp	17	70195243	70205020	4.979649	0.171842
27152	Amp	17	70205139	70205980	5.038261	0.172829
27153	Amp	17	70206372	70219296	4.93817	0.171315
27154	Amp	17	70219449	70221974	5.081731	0.173374
27155	Amp	17	70222267	70252017	5.02373	0.17253
27156	Amp	17	70254144	70258549	5.197738	0.175063
27157	Amp	17	70260154	70260154	5.038261	0.172842
27158	Amp	17	70261336	70262067	5.434517	0.178495
27159	Amp	17	70262210	70268476	5.360902	0.177508
27160	Amp	17	70268656	70268656	5.449463	0.17883
27161	Amp	17	70270365	70273294	5.582276	0.180829
27162	Amp	17	70273848	70273848	5.746988	0.183167
27163	Amp	17	70276213	70291615	6.198231	0.189755
27164	Amp	17	70292596	70320537	6.017563	0.186993
27165	Amp	17	70323392	70323392	5.59628	0.18101
27166	Amp	17	70323599	70328257	5.478024	0.179238
27167	Amp	17	70331792	70391079	5.882356	0.185137
27168	Amp	17	70391272	70412769	5.478024	0.179238
27169	Amp	17	70413598	70421144	5.067861	0.173243
27170	Amp	17	70422808	70433561	5.256006	0.176071
27171	Amp	17	70436455	70446360	4.345128	0.162134
27172	Amp	17	70446477	70525042	4.513276	0.164687
27173	Amp	17	70525720	70527421	4.345128	0.162187
27174	Amp	17	70530032	70538315	4.304307	0.161377
27175	Amp	17	70539207	70544611	4.5989	0.165952
27176	Amp	17	70544643	70560858	4.952273	0.171351
27177	Amp	17	70561062	70562192	4.853037	0.169996
27178	Amp	17	70562549	70572288	5.125419	0.173978
27179	Amp	17	70573184	70573184	4.91152	0.170707
27180	Amp	17	70573218	70576549	5.110288	0.173803
27181	Amp	17	70577737	70584869	4.91152	0.170835
27182	Amp	17	70585337	70599175	5.067861	0.17309
27183	Amp	17	70600491	70613091	5.300783	0.176729
27184	Amp	17	70613356	70613920	5.375101	0.177808
27185	Amp	17	70614674	70645335	4.897301	0.17048
27186	Amp	17	70647036	70663542	5.038261	0.17276
27187	Amp	17	70665151	70665739	5.300783	0.176637
27188	Amp	17	70666354	70667082	5.154391	0.174381

27189	Amp	17	70669389	70687363	4.91152	0.170742
27190	Amp	17	70692842	70708467	4.654424	0.166866
27191	Amp	17	70709882	70729064	4.541384	0.165232
27192	Amp	17	70729530	70799395	4.457399	0.163739
27193	Amp	17	70803930	70814060	4.31834	0.161741
27194	Amp	17	70814197	70824846	4.236663	0.160419
27195	Amp	17	70825548	70825548	4.062894	0.157454
27196	Amp	17	70826038	70844097	3.874391	0.154358
27197	Amp	17	70845900	70855603	3.814483	0.153163
27198	Amp	17	70856773	70902401	3.852343	0.153911
27199	Amp	17	70906067	70936650	4.037384	0.157059
27200	Amp	17	70938594	70946074	4.27644	0.160952
27201	Amp	17	70954121	70961852	4.076315	0.157803
27202	Amp	17	70964725	70977702	4.290674	0.161174
27203	Amp	17	70979552	70996927	4.555821	0.165258
27204	Amp	17	70997714	71016308	4.457399	0.163785
27205	Amp	17	71020884	71022852	4.196711	0.159702
27206	Amp	17	71023217	71075032	4.290674	0.161205
27207	Amp	17	71075065	71102842	4.31834	0.161688
27208	Amp	17	71105103	71180541	3.678399	0.150798
27209	Amp	17	71180738	71205313	3.762283	0.152288
27210	Amp	17	71205670	71219853	3.8021	0.152929
27211	Amp	17	71219999	71231471	3.240397	0.143096
27212	Amp	17	71234669	71236942	3.045223	0.139204
27213	Amp	17	71237929	71279888	3.251638	0.143161
27214	Amp	17	71285115	71291263	3.127699	0.140923
27215	Amp	17	71313622	71336308	3.033246	0.139134
27216	Amp	17	71340168	71347286	2.975629	0.1379
27217	Amp	17	71348539	71352163	3.09333	0.140241
27218	Amp	17	71368338	71417346	3.138463	0.141095
27219	Amp	17	71417678	71476022	3.009724	0.138646
27220	Amp	17	71478809	71509074	2.882945	0.136305
27221	Amp	17	71510616	71525589	3.021176	0.138929
27222	Amp	17	71534517	71534517	3.08058	0.139973
27223	Amp	17	71539060	71551702	3.762283	0.152428
27224	Amp	17	71552612	71556079	3.5415	0.148445
27225	Amp	17	71556441	71601302	3.127699	0.140914
27226	Amp	17	71601605	71601605	3.195626	0.142067
27227	Amp	17	71602076	71625533	2.637742	0.131721
27228	Amp	17	71625875	71650166	2.743935	0.133575
27229	Amp	17	71652624	71655550	2.560422	0.130184
27230	Amp	17	71657145	71658125	2.975629	0.138023
27231	Amp	17	71661204	71661429	3.009724	0.138742
27232	Amp	17	71662264	71676770	2.952409	0.137588
27233	Amp	17	71677315	71677315	3.251638	0.14323
27234	Amp	17	71679799	71681667	3.5415	0.148335
27235	Amp	17	71682170	71686235	3.615896	0.149736
27236	Amp	17	71687085	71689149	3.335819	0.14463
27237	Amp	17	71692968	71693719	3.183474	0.142007
27238	Amp	17	71693820	71693820	3.218362	0.142592
27239	Amp	17	71695284	71708543	3.150497	0.141191
27240	Amp	17	71709031	71713933	3.115161	0.140586
27241	Amp	17	71715537	71753567	2.975629	0.138054

27242	Amp	17	71753875	71753875	3.115161	0.140529
27243	Amp	17	71755005	71780465	3.275143	0.143594
27244	Amp	17	71782999	71782999	2.882945	0.136306
27245	Amp	17	71783541	71789148	2.825055	0.135132
27246	Amp	17	71789793	71811007	2.917729	0.136917
27247	Amp	17	71814316	71826146	3.275143	0.143711
27248	Amp	17	71826321	71865568	3.103562	0.140308
27249	Amp	17	71865999	71886931	2.964305	0.137796
27250	Amp	17	71888592	71897542	2.548433	0.129958
27251	Amp	17	71901793	71929225	2.696333	0.132812
27252	Amp	17	71929883	71939723	2.468223	0.128378
27253	Amp	17	71943725	71981398	2.571513	0.130348
27254	Amp	17	71981947	72009366	2.594754	0.130864
27255	Amp	17	72012780	72018020	2.435195	0.127799
27256	Amp	17	72018133	72018133	2.468223	0.128416
27257	Amp	17	72018339	72022322	2.490318	0.128895
27258	Amp	17	72022770	72046913	2.52589	0.129519
27259	Amp	17	72047277	72059593	2.548433	0.130015
27260	Amp	17	72064123	72108298	2.402099	0.127161
27261	Amp	17	72110815	72134404	2.490318	0.128882
27262	Amp	17	72134509	72186839	2.594754	0.130745
27263	Amp	17	72188419	72225603	2.468223	0.128454
27264	Amp	17	72226459	72251376	2.446878	0.127938
27265	Amp	17	72255028	72256844	2.345513	0.126075
27266	Amp	17	72257758	72300040	2.435195	0.127878
27267	Amp	17	72300124	72364187	2.413643	0.127395
27268	Amp	17	72366438	72396853	2.514205	0.129339
27269	Amp	17	72396939	72472895	2.707624	0.132925
27270	Amp	17	72475368	72475368	2.755154	0.133953
27271	Amp	17	72481409	72516180	2.719563	0.133234
27272	Amp	17	72520651	72574692	2.537294	0.129648
27273	Amp	17	72579749	72586407	2.502083	0.129119
27274	Amp	17	72587818	72654313	2.73206	0.133372
27275	Amp	17	72654873	72666749	2.825055	0.135183
27276	Amp	17	72675135	72680651	3.021176	0.138862
27277	Amp	17	72681384	72703205	3.057106	0.139581
27278	Amp	17	72703347	72703837	2.860424	0.135903
27279	Amp	17	72704969	72730105	3.045223	0.139221
27280	Amp	17	72731216	72743072	3.160681	0.141425
27281	Amp	17	72743222	72769836	3.275143	0.143728
27282	Amp	17	72770617	72772634	3.251638	0.143248
27283	Amp	17	72773952	72773952	3.138463	0.140945
27284	Amp	17	72774808	72788566	2.997552	0.138349
27285	Amp	17	72788602	72788602	2.848911	0.135508
27286	Amp	17	72789302	72798323	2.917729	0.136873
27287	Amp	17	72799796	72810256	2.860424	0.135845
27288	Amp	17	72814137	72817531	3.021176	0.138905
27289	Amp	17	72830013	72881640	3.852343	0.153785
27290	Amp	17	72882335	72908732	4.250544	0.16056
27291	Amp	17	72908956	72916676	3.852343	0.153785
27292	Amp	17	72917289	72920325	4.626369	0.166401
27293	Amp	17	72921232	72921232	3.422847	0.146214
27294	Amp	17	72922257	72928003	3.374864	0.145287

27295	Amp	17	72929081	72931546	3.5415	0.148428
27296	Amp	17	72936611	72936611	3.361849	0.145076
27297	Amp	17	72941476	72979972	3.505046	0.147841
27298	Amp	17	72984148	72990207	3.529607	0.148113
27299	Amp	17	72993742	72996932	3.887411	0.154488
27300	Amp	17	72998440	73010373	4.010586	0.156698
27301	Amp	17	73011293	73025540	3.959323	0.155754
27302	Amp	17	73030072	73033663	3.920287	0.155256
27303	Amp	17	73037627	73039474	3.726055	0.15171
27304	Amp	17	73045299	73054155	3.422847	0.14628
27305	Amp	17	73054470	73058174	3.5415	0.148326
27306	Amp	17	73061005	73061005	3.726055	0.151612
27307	Amp	17	73061414	73068799	3.826187	0.153389
27308	Amp	17	73069260	73076895	3.701758	0.151343
27309	Amp	17	73078803	73084939	3.578505	0.149132
27310	Amp	17	73085096	73134434	3.713605	0.151465
27311	Amp	17	73142268	73158377	4.050096	0.157363
27312	Amp	17	73159986	73169179	4.142958	0.158761
27313	Amp	17	73173244	73184288	4.457399	0.163786
27314	Amp	17	73187917	73221231	4.142958	0.158761
27315	Amp	17	73227828	73236548	3.701758	0.151227
27316	Amp	17	73237491	73242837	3.898065	0.154821
27317	Amp	17	73243459	73262247	4.102728	0.158273
27318	Amp	17	73267704	73274450	3.920287	0.155132
27319	Amp	17	73284074	73291021	4.102728	0.158084
27320	Amp	17	73295128	73302717	4.196711	0.159808
27321	Amp	17	73304051	73304051	4.739548	0.168243
27322	Amp	17	73308722	73308722	5.067861	0.173182
27323	Amp	17	73315500	73318138	5.39004	0.177959
27324	Amp	17	73319975	73390823	4.91152	0.170731
27325	Amp	17	73399034	73427123	4.683031	0.167279
27326	Amp	17	73432766	73450084	4.499013	0.164444
27327	Amp	17	73450098	73466156	4.69701	0.16752
27328	Amp	17	73466978	73504112	5.419518	0.178331
27329	Amp	17	73507780	73518434	5.806986	0.184133
27330	Amp	17	73518488	73548054	5.86703	0.184939
27331	Amp	17	73548167	73555579	5.822221	0.184188
27332	Amp	17	73555765	73583437	6.198231	0.189651
27333	Amp	17	73584084	73590256	6.2443	0.190361
27334	Amp	17	73591585	73607307	5.836931	0.184559
27335	Amp	17	73608084	73610137	5.536915	0.18002
27336	Amp	17	73612515	73615519	5.762293	0.183311
27337	Amp	17	73616799	73619496	5.626868	0.181368
27338	Amp	17	73619934	73646312	5.449463	0.178831
27339	Amp	17	73647371	73712491	5.582276	0.180754
27340	Amp	17	73714952	73718211	5.626868	0.181389
27341	Amp	17	73723234	73723234	5.536915	0.180168
27342	Amp	17	73724928	73763717	5.507674	0.179725
27343	Amp	17	73763821	73775199	5.285768	0.176439
27344	Amp	17	73775248	73798372	5.169562	0.174662
27345	Amp	17	73798511	73814343	4.78228	0.16874
27346	Amp	17	73814574	73814867	4.838749	0.169606
27347	Amp	17	73817489	73817489	4.076315	0.157805

27348	Amp	17	73817945	73820787	4.037384	0.157095
27349	Amp	17	73829384	73829384	3.678399	0.150859
27350	Amp	17	74695492	74695492	4.924414	0.170996
27351	Amp	17	74695789	74710490	3.762283	0.152253
27352	Amp	17	74711717	74732122	4.838749	0.169641
27353	Amp	17	74739490	74761382	4.725297	0.168042
27354	Amp	17	74769076	74771296	4.853037	0.169982
27355	Amp	17	74772662	74788510	4.809994	0.169175
27356	Amp	17	74793277	74797594	4.27644	0.160996
27357	Amp	17	74804288	74807755	3.775276	0.152443
27358	Amp	17	74812074	74838446	3.628913	0.149872
27359	Amp	17	74840873	74844490	3.590086	0.149254
27360	Amp	17	74845024	74848730	3.554363	0.148631
27361	Amp	17	74853558	74903688	3.603523	0.149561
27362	Amp	17	74904045	74942274	3.578505	0.149082
27363	Amp	17	74944196	74957542	3.788687	0.1527
27364	Amp	17	74959373	74965064	3.654159	0.15047
27365	Amp	17	74965959	74985341	3.933438	0.155433
27366	Amp	17	74985708	74985708	3.738665	0.151815
27367	Amp	17	74999282	75028701	3.628913	0.149893
27368	Amp	17	75029724	75036245	3.8021	0.15305
27369	Amp	17	75041973	75042250	4.129725	0.158556
27370	Amp	17	75042651	75091616	3.874391	0.154312
27371	Amp	17	75093757	75099857	3.726055	0.1516
27372	Amp	17	75100856	75117441	3.529607	0.148179
27373	Amp	17	75120947	75125314	3.654159	0.150322
27374	Amp	17	75126360	75143862	3.814483	0.153252
27375	Amp	17	75148189	75163986	3.920287	0.15516
27376	Amp	17	75167145	75168905	3.874391	0.154178
27377	Amp	17	75169821	75222082	4.037384	0.157022
27378	Amp	17	75224349	75246854	4.116393	0.158445
27379	Amp	17	75248953	75248953	3.985868	0.156303
27380	Amp	17	75256972	75274825	3.788687	0.152665
27381	Amp	17	75279427	75292850	3.908795	0.154974
27382	Amp	17	75293215	75337436	3.529607	0.148179
27383	Amp	17	75341034	75348774	3.374864	0.145335
27384	Amp	17	75349386	75412029	3.578505	0.149015
27385	Amp	17	75417368	75432295	3.323812	0.144491
27386	Amp	17	75433634	75445009	3.516868	0.147877
27387	Amp	17	75445658	75452850	3.590086	0.149391
27388	Amp	17	75454128	75470162	3.458499	0.146924
27389	Amp	17	75475238	75513460	3.749777	0.152028
27390	Amp	17	75518008	75594749	3.920287	0.155209
27391	Amp	17	75597346	75597493	3.852343	0.153815
27392	Amp	17	75598579	75600330	3.640671	0.150058
27393	Amp	17	75600348	75657542	3.446786	0.146672
27394	Amp	17	75659686	75712662	3.183474	0.141979
27395	Amp	17	75713473	75717916	2.986184	0.138299
27396	Amp	17	75718547	75761306	2.917729	0.136876
27397	Amp	17	75768556	75778162	2.548433	0.130037
27398	Amp	17	75785739	75785739	2.626052	0.131429
27399	Amp	17	75791316	75804098	2.743935	0.133736
27400	Amp	17	75806160	75853501	3.127699	0.140844

27401	Amp	17	75882548	75886726	2.594754	0.130842
27402	Amp	17	75903403	75943056	2.26699	0.124536
27403	Amp	17	75943480	75950561	2.390849	0.127005
27404	Amp	17	75950782	76096165	2.490318	0.128959
27405	Amp	17	76096987	76131329	2.278531	0.124745
27406	Amp	17	76142478	76158250	2.016585	0.119624
27407	Amp	17	76161455	76165397	1.96212	0.118353
27408	Amp	17	76165452	76209131	2.116108	0.121438
27409	Amp	17	76210259	76318692	2.192012	0.123009
27410	Amp	17	76319948	76344710	2.137445	0.122001
27411	Amp	17	76355062	76355062	2.18114	0.122819
27412	Amp	17	76361530	76361638	2.027585	0.119734
27413	Amp	17	76362462	76388178	1.897704	0.117145
27414	Amp	17	76389084	76429696	1.940776	0.117997
27415	Amp	17	76432281	76438390	1.865019	0.116536
27416	Amp	17	76438735	76511865	2.192012	0.123099
27417	Amp	17	76513298	76550857	2.158817	0.122409
27418	Amp	17	76553959	76583581	2.027585	0.119849
27419	Amp	17	76587008	76600320	2.413643	0.127458
27420	Amp	17	76601380	76644031	2.368227	0.126542
27421	Amp	17	76645768	76655610	2.684894	0.13259
27422	Amp	17	76656192	76673058	2.800837	0.13476
27423	Amp	17	76675126	76717489	3.29895	0.144127
27424	Amp	17	76721700	76732000	2.964305	0.137781
27425	Amp	17	76739260	76804917	2.837591	0.135287
27426	Amp	17	76835725	76857286	2.425182	0.127678
27427	Amp	17	76859323	76859736	2.560422	0.130145
27428	Amp	17	76867813	76909588	2.479057	0.128574
27429	Amp	17	76912983	76914310	2.169666	0.122526
27430	Amp	17	76942889	76979096	2.245479	0.124074
27431	Amp	17	76982798	77030770	2.127614	0.121607
27432	Amp	17	77032920	77092114	2.158817	0.122275
27433	Amp	17	77096200	77118793	2.235168	0.123873
27434	Amp	17	77119544	77126582	2.137445	0.121919
27435	Amp	17	77128872	77132113	2.368227	0.126556
27436	Amp	17	77134395	77267306	2.33403	0.125888
27437	Amp	17	77272257	77313269	2.425182	0.127627
27438	Amp	17	77315254	77318404	1.887378	0.116864
27439	Amp	17	77320744	77378026	1.801583	0.115256
27440	Amp	17	77385906	77401915	1.833307	0.115805
27441	Amp	17	77404018	77424154	1.96212	0.118378
27442	Amp	17	77426651	77452076	2.457251	0.128329
27443	Amp	17	77567552	77568225	2.560422	0.130158
27444	Amp	17	77568698	77635596	2.479057	0.128559
27445	Amp	17	77643885	77664981	2.614791	0.13135
27446	Amp	17	77669203	77672830	2.975629	0.138028
27447	Amp	17	77672834	77699327	2.89426	0.136513
27448	Amp	17	77699751	77755724	2.952409	0.137657
27449	Amp	17	77764505	77764921	3.021176	0.138904
27450	Amp	17	77765685	77772143	2.661431	0.132226
27451	Amp	17	77772301	77786654	2.696333	0.132881
27452	Amp	17	77786757	77789344	2.765626	0.13404
27453	Amp	17	77795095	77805714	2.684894	0.132537

27454	Amp	17	77808035	77826462	2.605213	0.131023
27455	Amp	17	77831289	77843798	2.707624	0.133004
27456	Amp	17	77847892	77921830	2.605213	0.131023
27457	Amp	17	77923722	77924902	2.743935	0.13369
27458	Amp	17	77926475	77938880	2.605213	0.131117
27459	Amp	17	77939804	78023274	2.548433	0.129957
27460	Amp	17	78025192	78033154	2.812598	0.134992
27461	Amp	17	78033188	78052893	2.871582	0.136097
27462	Amp	17	78057306	78077062	2.637742	0.131645
27463	Amp	17	78077984	78087937	2.964305	0.137747
27464	Amp	17	78089660	78106924	2.871582	0.136008
27465	Amp	17	78109650	78130123	3.021176	0.13896
27466	Amp	17	78133005	78203639	3.150497	0.141211
27467	Amp	17	78203704	78221902	3.264473	0.143483
27468	Amp	17	78223510	78272425	3.411175	0.145966
27469	Amp	17	78274006	78334238	3.615896	0.149611
27470	Amp	17	78339216	78347238	3.516868	0.148087
27471	Amp	17	78361731	78397291	3.505046	0.147769
27472	Amp	17	78397894	78412343	3.5415	0.148504
27473	Amp	17	78456702	78460053	3.726055	0.151624
27474	Amp	17	78463907	78463907	3.690482	0.150957
27475	Amp	17	78464649	78492202	3.566107	0.148772
27476	Amp	17	78493494	78511884	3.411175	0.145981
27477	Amp	17	78515861	78525593	3.374864	0.145397
27478	Amp	17	78529483	78563081	3.207115	0.142277
27479	Amp	17	78732558	78732558	3.045223	0.139308
27480	Amp	17	78732586	78733662	2.605213	0.131029
27481	Amp	17	78736256	78763483	2.356694	0.126249
27482	Amp	17	78766898	78808132	2.038694	0.119868
27483	Amp	17	78816093	78816758	1.929887	0.11779
27484	Amp	17	78817224	78835379	1.812192	0.115308
27485	Amp	17	78835701	78868723	1.645511	0.111904
27486	Amp	17	78870905	78901892	1.929887	0.117784
27487	Amp	17	78902983	78949566	2.094104	0.121132
27488	Amp	17	78949634	78961303	2.127614	0.121754
27489	Amp	17	78961717	78976253	2.158817	0.122377
27490	Amp	17	78980335	79030035	2.26699	0.124605
27491	Amp	17	79031928	79108711	2.468223	0.128448
27492	Amp	17	79111985	79124117	2.311329	0.125328
27493	Amp	17	79124495	79238441	2.245479	0.124184
27494	Amp	17	79238589	79238589	2.300463	0.125155
27495	Amp	17	79239524	79245328	2.345513	0.12603
27496	Amp	17	79245485	79254965	1.95165	0.118196
27497	Amp	17	79255108	79255178	2.094104	0.121081
27498	Amp	17	79256445	79270312	2.356694	0.126209
27499	Amp	17	79270927	79287148	2.379392	0.126771
27500	Amp	17	79289286	79353727	2.425182	0.127596
27501	Amp	17	79354260	79365779	2.468223	0.128533
27502	Amp	17	79406159	79408012	2.356694	0.126281
27503	Amp	17	79410326	79410326	2.425182	0.127473
27504	Amp	17	79411690	79430954	3.218362	0.142579
27505	Amp	17	79434511	79440229	3.411175	0.145943
27506	Amp	17	79444205	79493331	3.529607	0.148291

27507 Amp	17	79549025	79656995	3.483236	0.147416
27508 Amp	17	79667705	79669019	3.603523	0.149435
27509 Amp	17	79671823	79723538	3.933438	0.15542
27510 Amp	17	79724812	79724849	4.116393	0.158334
27511 Amp	17	79727803	79734466	4.345128	0.162049
27512 Amp	17	79735540	79782686	4.443348	0.163598
27513 Amp	17	79782961	79792183	4.209693	0.159949
27514 Amp	17	79796051	79807926	4.090086	0.157989
27515 Amp	17	79818238	79828320	4.169329	0.159195
27516 Amp	17	79846021	79866832	3.640671	0.150049
27517 Amp	17	79868380	79897012	3.5415	0.1485
27518 Amp	17	79898751	79910342	3.738665	0.151976
27519 Amp	17	79912261	79912261	3.554363	0.148613
27520 Amp	17	79917199	79935639	3.701758	0.151301
27521 Amp	17	79936215	79950106	3.483236	0.147407
27522 Amp	17	79950776	79970506	3.8021	0.153018
27523 Amp	17	79974124	79974124	3.775276	0.152455
27524 Amp	17	79976245	79997478	3.435586	0.146569
27525 Amp	17	80003498	80005417	3.251638	0.143221
27526 Amp	17	80030491	80033814	2.278531	0.12464
27527 Amp	17	80045981	80080445	2.235168	0.123774
27528 Amp	17	80080590	80151425	2.368227	0.126548
27529 Amp	17	80151658	80156511	2.479057	0.128686
27530 Amp	17	80161337	80180208	2.800837	0.134724
27531 Amp	17	80185561	80192498	2.917729	0.136887
27532 Amp	17	80193055	80198145	2.719563	0.133161
27533 Amp	17	80199168	80245008	2.514205	0.129318
27534 Amp	17	80352467	80352467	2.060912	0.120452
27535 Amp	17	80352501	80352501	1.95165	0.118289
27536 Amp	17	80357445	80396290	2.446878	0.128019
27537 Amp	17	80399457	80403430	2.368227	0.126453
27538 Amp	17	80412871	80420740	2.502083	0.12916
27539 Amp	17	80424332	80442111	2.537294	0.129699
27540 Amp	17	80453610	80500324	2.696333	0.132884
27541 Amp	17	80502268	80517808	2.560422	0.130282
27542 Amp	17	80519684	80588501	2.245479	0.124055
27543 Amp	17	80595422	80622528	2.104978	0.121349
27544 Amp	17	80641561	80798446	2.049917	0.120102
27545 Amp	17	80801771	80824774	2.049917	0.120134
27546 Amp	17	80829775	80848789	1.854421	0.116189
27547 Amp	17	80850614	80880339	1.573014	0.110352
27548 Amp	17	80880423	80978587	1.645511	0.111832
27549 Amp	17	80989081	80989145	1.897704	0.117217
27550 Amp	17	80993115	81008355	1.748606	0.114154
27551 Amp	17	81015311	81015311	1.624655	0.111488
27552 Amp	17	81019582	81170640	1.801583	0.115275
27553 Amp	17	81176185	81199384	1.759227	0.114424
27554 Amp	17	81204674	81217926	1.812192	0.115333
27555 Amp	17	81225322	81292757	1.769915	0.114508
27556 Amp	17	81293745	81310502	1.737623	0.113959
27557 Amp	17	81320655	81326387	1.706315	0.113337
27558 Amp	17	81331397	81377215	1.823153	0.115541
27559 Amp	17	81377990	81402211	1.727286	0.113689

27560	Amp	17	81415451	81415451	1.522708	0.109279
27561	Amp	17	81416257	81510690	1.378521	0.106077
27562	Amp	17	81510993	81607340	1.436692	0.107277
27563	Amp	17	81607688	81704905	1.492063	0.108733
27564	Amp	17	81708565	81726133	1.436692	0.107277
27565	Amp	17	81830747	81878759	1.522708	0.109434
27566	Amp	17	81886944	81886944	1.563118	0.11016
27567	Amp	17	81896908	81900044	1.492063	0.10868
27568	Amp	17	81916422	81951455	1.398396	0.106476
27569	Amp	17	81965842	81985599	1.737623	0.113851
27570	Amp	17	82018602	82041935	1.472629	0.108277
27571	Amp	17	82046065	82066440	2.094104	0.121028
27572	Amp	17	82066659	82121899	1.472629	0.108325
27573	Amp	17	82125680	82125680	1.426786	0.107124
27574	Amp	17	82256888	82256888	1.131695	0.100339
27575	Amp	17	82257710	82263309	1.197658	0.101969
27576	Amp	17	82267669	82268695	1.319393	0.104705
27577	Amp	17	82277019	82278184	1.790459	0.114979
27578	Amp	17	82285983	82337668	2.027585	0.119658
27579	Amp	17	82342874	82343577	1.790459	0.114979
27580	Amp	17	82358420	82382648	1.854421	0.116346
27581	Amp	17	82399121	82415624	1.780186	0.114715
27582	Amp	17	82416540	82417023	1.624655	0.1114
27583	Amp	17	82421456	82461316	2.104978	0.121203
27584	Amp	17	82462033	82464927	2.137445	0.121973
27585	Amp	17	82465310	82465521	2.027585	0.119676
27586	Amp	17	82470223	82506829	2.104978	0.121178
27587	Amp	17	82514951	82585274	2.038694	0.120019
27588	Amp	17	82597338	82622355	1.940776	0.117926
27589	Amp	17	82627157	82696259	2.158817	0.122359
27590	Amp	17	82696355	82696939	2.202226	0.12318
27591	Amp	17	82699889	82723196	2.235168	0.123862
27592	Amp	17	82726217	82745569	2.192012	0.122952
27593	Amp	17	82754905	82762370	2.116108	0.12145
27594	Amp	17	82768207	82823640	2.169666	0.122599
27595	Amp	17	82825133	82879867	1.727286	0.113651
27596	Amp	17	82887813	82890848	1.407997	0.106692
27597	Amp	17	82895012	82903330	1.436692	0.107336
27598	Amp	17	82905981	82905981	1.512549	0.109068
27599	Amp	17	82906378	82945581	1.593878	0.110934
27600	Amp	17	82946072	82959812	1.512549	0.109201
27601	Amp	18	326691	358695	0	0.017073
27602	Amp	18	359079	365815	0	0.01777
27603	Amp	18	365945	366043	0	0.014033
27604	Amp	18	367119	367725	0	0.014818
27605	Amp	18	891647	1159140	0	0.016903
27606	Amp	18	1162160	1176215	0	0.019398
27607	Amp	18	1181424	1359324	0	0.016903
27608	Amp	18	1359988	1391683	0	0.019331
27609	Amp	18	1398891	1419351	0	0.016903
27610	Amp	18	1421608	1446011	0	0.020871
27611	Amp	18	1458750	1483866	0	0.016903
27612	Amp	18	1485612	1491164	0	0.034041

27613	Amp	18	1495297	1601016	0	0.016903
27614	Amp	18	1601248	1624683	0	0.020297
27615	Amp	18	1687467	1713410	0	0.014513
27616	Amp	18	2079539	2089891	0	0.017267
27617	Amp	18	2090449	2092074	0	0.01657
27618	Amp	18	2094573	2268254	0	0.013817
27619	Amp	18	2270319	2289017	0	0.017163
27620	Amp	18	2292755	2293197	0	0.020088
27621	Amp	18	2298725	2304510	0	0.024333
27622	Amp	18	2305341	2475748	0	0.021408
27623	Amp	18	2478269	2490399	0	0.025027
27624	Amp	18	2490859	2493697	0	0.026515
27625	Amp	18	2496330	2498055	0	0.022896
27626	Amp	18	2498727	2501071	0	0.022112
27627	Amp	18	2502417	2548422	0	0.024068
27628	Amp	18	2783353	2786736	0	0.021662
27629	Amp	18	2787066	2809882	0	0.020174
27630	Amp	18	2811402	2898999	0	0.019412
27631	Amp	18	2900589	2935458	0	0.019897
27632	Amp	18	2941452	2941952	0	0.015652
27633	Amp	18	2943797	3031564	0	0.015167
27634	Amp	18	3039188	3608042	0	0.013211
27635	Amp	18	3610256	3619328	0	0.013992
27636	Amp	18	3620923	3641647	0	0.015492
27637	Amp	18	3644122	3740373	0	0.013406
27638	Amp	18	3742572	3750642	0	0.012483
27639	Amp	18	3753093	3959979	0	0.008633
27640	Amp	18	3964437	4122410	0	0.006193
27641	Amp	18	4126938	4246440	0	0.00864
27642	Amp	18	4249420	4943650	0	0.006193
27643	Amp	18	4943767	5055639	0	0.012064
27644	Amp	18	5057935	5073744	0	0.012777
27645	Amp	18	5081918	5216096	0	0.012064
27646	Amp	18	5218065	5364092	0	0.006193
27647	Amp	18	5371580	5452358	0	0.007614
27648	Amp	18	5455064	5527865	0	0.006193
27649	Amp	18	5530034	5548880	0	0.00865
27650	Amp	18	5549423	5572369	0	0.006193
27651	Amp	18	5575032	5602336	0	0.003039
27652	Amp	18	5603553	5676697	0	0.005225
27653	Amp	18	5678583	5705517	0	0.003039
27654	Amp	18	5706872	5733049	0	0.005219
27655	Amp	18	5733379	5959259	0	0.003039
27656	Amp	18	6602285	6668051	0	0.004483
27657	Amp	18	6674518	7009280	0	0.002065
27658	Amp	18	7014334	7037640	0	0.004535
27659	Amp	18	7042912	7050183	0	0.002065
27660	Amp	18	7054045	7346125	0	0.018001
27661	Amp	18	7346932	7382496	0	0.019548
27662	Amp	18	7382883	7396995	0	0.020062
27663	Amp	18	7403669	7528506	0	0.004125
27664	Amp	18	7528861	7699819	0	0.004603
27665	Amp	18	7701701	7816936	0	0.003057

27666	Amp	18	7818291	8093290	0	0.002436
27667	Amp	18	8096344	8134762	0	0.000992
27668	Amp	18	8137426	8162028	0	0.003491
27669	Amp	18	8162452	8162452	0	0.005224
27670	Amp	18	8163869	8187789	0	0.002725
27671	Amp	18	8187807	8226196	0	0.003465
27672	Amp	18	8226316	8284922	0	0.002951
27673	Amp	18	8287807	8307312	0	0.004878
27674	Amp	18	8312010	8373507	0	0.008533
27675	Amp	18	8375535	8395899	0	0.004878
27676	Amp	18	8397335	8507312	0	0.003144
27677	Amp	18	8509546	8607402	0	0.006488
27678	Amp	18	8610642	8634078	0	0.01274
27679	Amp	18	8638031	8643867	0	0.006488
27680	Amp	18	8652251	9108128	0	0.003144
27681	Amp	18	9110166	9233854	0	0.006515
27682	Amp	18	9237993	9395000	0	0.005775
27683	Amp	18	9395385	9511183	0	0.00848
27684	Amp	18	9518516	9533267	0	0.013072
27685	Amp	18	9533675	9568317	0	0.014391
27686	Amp	18	9570322	9604233	0	0.017571
27687	Amp	18	9609299	9774530	0	0.013595
27688	Amp	18	9775375	9775414	0	0.021058
27689	Amp	18	9775569	9949277	0	0.021528
27690	Amp	18	9949294	9960485	0	0.023767
27691	Amp	18	9960856	9967449	0	0.021528
27692	Amp	18	9970079	9990164	0	0.021058
27693	Amp	18	9995048	10096506	0	0.024194
27694	Amp	18	10097494	10104966	0	0.025635
27695	Amp	18	10112899	10121182	0	0.028814
27696	Amp	18	10121581	10133807	0	0.026887
27697	Amp	18	10145813	10240768	0	0.023751
27698	Amp	18	10245257	10250364	0	0.025663
27699	Amp	18	10251153	10269161	0	0.030365
27700	Amp	18	10270987	10287158	0	0.031773
27701	Amp	18	10287282	10313132	0	0.030365
27702	Amp	18	10313582	10341254	0	0.024442
27703	Amp	18	10341256	10424217	0	0.023079
27704	Amp	18	10425559	10442224	0	0.014875
27705	Amp	18	10445517	10458259	0	0.025641
27706	Amp	18	10458807	10468517	0	0.029757
27707	Amp	18	10471501	10525588	0	0.013821
27708	Amp	18	10535390	10668776	0	0.007793
27709	Amp	18	10669324	10674628	0	0.006353
27710	Amp	18	10674818	10687104	0	0.002982
27711	Amp	18	10687451	10695159	0	0.001166
27712	Amp	18	10695563	10797364	0	0.000688
27713	Amp	18	10798919	10928709	0	0.002421
27714	Amp	18	10929806	11020324	0	0.003249
27715	Amp	18	11020601	11036543	0	0.005151
27716	Amp	18	11038941	11292891	0	0.008368
27717	Amp	18	11295859	11372394	0	0.00754
27718	Amp	18	11376739	11380724	0	0.013623

27719	Amp	18	11381094	11382224	0	0.00754
27720	Amp	18	11384910	11408185	0	0.009872
27721	Amp	18	11408483	11489921	0	0.010416
27722	Amp	18	11490183	11504984	0	0.012127
27723	Amp	18	11505711	11648822	0	0.010394
27724	Amp	18	11649015	11658684	0	0.01513
27725	Amp	18	11658989	11670363	0	0.012798
27726	Amp	18	11670538	11694378	0	0.009966
27727	Amp	18	11695552	11847835	0	0.012192
27728	Amp	18	11848356	11854354	0	0.0139
27729	Amp	18	11857616	11877569	0	0.014972
27730	Amp	18	11878176	11907375	0	0.01136
27731	Amp	18	11908074	11949535	0	0.01267
27732	Amp	18	11949955	11976320	0	0.016063
27733	Amp	18	11976658	11999334	0	0.018874
27734	Amp	18	12001708	12004091	0	0.015482
27735	Amp	18	12006189	12192656	0	0.01267
27736	Amp	18	12238864	12409818	0	0.020526
27737	Amp	18	12409956	12450178	0	0.022423
27738	Amp	18	12454263	12493713	0	0.026088
27739	Amp	18	12499292	12569487	0	0.022423
27740	Amp	18	12570178	12621719	0	0.025661
27741	Amp	18	12623053	12812062	0	0.029042
27742	Amp	18	12812168	12966909	0	0.025804
27743	Amp	18	12972300	13052474	0	0.026403
27744	Amp	18	13052829	13255313	0	0.025331
27745	Amp	18	13257829	13295004	0	0.030181
27746	Amp	18	13295738	13387627	0	0.03073
27747	Amp	18	13387738	13387738	0	0.031565
27748	Amp	18	13389228	13404927	0	0.035231
27749	Amp	18	13406060	13412538	0	0.034683
27750	Amp	18	13419321	13424990	0	0.035911
27751	Amp	18	13425409	13481455	0	0.038931
27752	Amp	18	13483065	13483065	0	0.043796
27753	Amp	18	13486190	13605057	0	0.042486
27754	Amp	18	13606171	13702795	0	0.048644
27755	Amp	18	13703458	13725434	0	0.044977
27756	Amp	18	13727349	13785181	0	0.038819
27757	Amp	18	13788362	13822257	0	0.028409
27758	Amp	18	13824959	13849298	0	0.026468
27759	Amp	18	13852420	13855984	0	0.025624
27760	Amp	18	13856848	13872924	0	0.023783
27761	Amp	18	13879719	13894342	0	0.020764
27762	Amp	18	13895250	13895250	0	0.019896
27763	Amp	18	13908503	13916794	0	0.027782
27764	Amp	18	13925635	14062285	0	0.025556
27765	Amp	18	14071226	14071226	0	0.027815
27766	Amp	18	14355174	14355174	0	0.029417
27767	Amp	18	14362423	14362423	0	0.022598
27768	Amp	18	14363581	14363581	0	0.021313
27769	Amp	18	14366664	14467938	0	0.020037
27770	Amp	18	14469466	14602141	0	0.020916
27771	Amp	18	14603860	14604229	0	0.020372

27772	Amp	18	14620708	14632450	0	0.016991
27773	Amp	18	14641845	14648458	0	0.014733
27774	Amp	18	14653029	14660483	0	0.017754
27775	Amp	18	14661644	14661644	0	0.021384
27776	Amp	18	21109102	21114061	0	0.021626
27777	Amp	18	21117668	21143675	0	0.018888
27778	Amp	18	21147208	21147208	0	0.016578
27779	Amp	18	21147899	21371162	0	0.018278
27780	Amp	18	21385561	21427218	0	0.0174
27781	Amp	18	21427714	21557003	0	0.017898
27782	Amp	18	21558453	22070746	0	0.0174
27783	Amp	18	22076639	22099054	0	0.018803
27784	Amp	18	22099180	22226390	0	0.019265
27785	Amp	18	22226851	22573045	0	0.021092
27786	Amp	18	22574760	22583635	0	0.019098
27787	Amp	18	22583707	22713252	0	0.021219
27788	Amp	18	22714869	22736530	0	0.018516
27789	Amp	18	22742587	22803011	0	0.020337
27790	Amp	18	22804842	22918940	0	0.018216
27791	Amp	18	22923306	23029500	0	0.014586
27792	Amp	18	23031702	23044521	0	0.012766
27793	Amp	18	23046473	23525246	0	0.015216
27794	Amp	18	23529502	24125759	0	0.012766
27795	Amp	18	24129462	24141752	0	0.026646
27796	Amp	18	24143682	24329273	0	0.029416
27797	Amp	18	24331936	24545990	0	0.028013
27798	Amp	18	24547240	24732782	0	0.028516
27799	Amp	18	24734303	24785466	0	0.029611
27800	Amp	18	24788647	24880577	0	0.030465
27801	Amp	18	24881081	24915029	0	0.032745
27802	Amp	18	24921339	24959675	0	0.031892
27803	Amp	18	24962795	25255656	0	0.034605
27804	Amp	18	25258342	25280312	0	0.032325
27805	Amp	18	25280770	25290179	0	0.030105
27806	Amp	18	25293282	25367203	0	0.032466
27807	Amp	18	25367348	25829475	0	0.030105
27808	Amp	18	25831199	25841729	0	0.033287
27809	Amp	18	25842342	25921914	0	0.030105
27810	Amp	18	25922142	26086307	0	0.029602
27811	Amp	18	26092398	26264525	0	0.034955
27812	Amp	18	26265972	26339075	0	0.030603
27813	Amp	18	26345696	26574258	0	0.033734
27814	Amp	18	26576186	26584830	0	0.030603
27815	Amp	18	26584856	26928379	0	0.027889
27816	Amp	18	26928554	26973598	0	0.031491
27817	Amp	18	26973892	27017972	0	0.03371
27818	Amp	18	27019092	27250153	0	0.03094
27819	Amp	18	27254783	27518870	0	0.027918
27820	Amp	18	27521033	27537302	0	0.014038
27821	Amp	18	27543242	27562082	0	0.020002
27822	Amp	18	27563216	27816526	0	0.024621
27823	Amp	18	27817034	27889901	0	0.022922
27824	Amp	18	27890413	27977573	0	0.024717

27825	Amp	18	27977603	28036188	0	0.022922
27826	Amp	18	28036487	28531573	0	0.024651
27827	Amp	18	28535612	28589547	0	0.038531
27828	Amp	18	28591700	28593881	0	0.036704
27829	Amp	18	28601829	28662444	0	0.040604
27830	Amp	18	28662742	28755730	0	0.036704
27831	Amp	18	28822238	28959123	0	0.04053
27832	Amp	18	28961456	28987852	0	0.037621
27833	Amp	18	28991220	28991220	0	0.041023
27834	Amp	18	28993250	29026217	0	0.025086
27835	Amp	18	29041426	29745585	0	0.021685
27836	Amp	18	29747245	30117173	0	0.007805
27837	Amp	18	30118621	30368056	0	0.008851
27838	Amp	18	30371028	30378087	0	0.007805
27839	Amp	18	30390067	30450567	0	0.012042
27840	Amp	18	30451092	30966418	0	0.019176
27841	Amp	18	30966851	31162268	0	0.020837
27842	Amp	18	31162393	31203225	0	0.019741
27843	Amp	18	31203725	31240850	0	0.022725
27844	Amp	18	31241004	31268131	0	0.026068
27845	Amp	18	31268320	31341383	0	0.022725
27846	Amp	18	31346421	31384671	0	0.023177
27847	Amp	18	31384848	31606165	0	0.020193
27848	Amp	18	31610211	31672642	0	0.019741
27849	Amp	18	31673644	31955245	0	0.018081
27850	Amp	18	31957650	32394051	0	0.013811
27851	Amp	18	32397345	32404635	0	0.024341
27852	Amp	18	32419540	32661521	0	0.013811
27853	Amp	18	32666577	32941844	0	0.018629
27854	Amp	18	32942781	33731428	0	0.022233
27855	Amp	18	33731639	33797136	0	0.023706
27856	Amp	18	33797618	33969119	0	0.021977
27857	Amp	18	33971050	34103757	0	0.023837
27858	Amp	18	34106933	34277143	0	0.022364
27859	Amp	18	34283365	34590443	0	0.023697
27860	Amp	18	34590934	34806628	0	0.025084
27861	Amp	18	34809272	34942358	0	0.029726
27862	Amp	18	34945820	35019607	0	0.025084
27863	Amp	18	35022226	35163763	0	0.023751
27864	Amp	18	35164163	35202683	0	0.021634
27865	Amp	18	35202968	35427265	0	0.023536
27866	Amp	18	35430978	35452845	0	0.024089
27867	Amp	18	35466034	35627205	0	0.026404
27868	Amp	18	35633576	35635062	0	0.024916
27869	Amp	18	35749361	36072600	0	0.025873
27870	Amp	18	36073626	36085508	0	0.028928
27871	Amp	18	36085632	36134730	0	0.030794
27872	Amp	18	36138252	36207460	0	0.028928
27873	Amp	18	36210521	36316637	0	0.026613
27874	Amp	18	36325350	36675454	0	0.024753
27875	Amp	18	36675500	36797317	0	0.027409
27876	Amp	18	36801017	36843747	0	0.024753
27877	Amp	18	36844938	36929029	0	0.021698

27878	Amp	18	36929724	37252168	0	0.023088
27879	Amp	18	37256137	37401837	0	0.023728
27880	Amp	18	37403279	37692397	0	0.024368
27881	Amp	18	37692434	37694872	0	0.023728
27882	Amp	18	37697586	37774230	0	0.022341
27883	Amp	18	37775048	37826124	0	0.021701
27884	Amp	18	37836290	37854688	0	0.020745
27885	Amp	18	37864044	38015284	0	0.019731
27886	Amp	18	38015909	38085162	0	0.025429
27887	Amp	18	38086536	38458600	0	0.022885
27888	Amp	18	38459702	38542062	0	0.026344
27889	Amp	18	38542756	38652038	0	0.022885
27890	Amp	18	38656457	38942445	0	0.015682
27891	Amp	18	38946117	39023015	0	0.016756
27892	Amp	18	39024452	39046109	0	0.022271
27893	Amp	18	39046477	39057154	0	0.023714
27894	Amp	18	39057262	39103110	0	0.020113
27895	Amp	18	39105847	39679873	0	0.017373
27896	Amp	18	39680473	39713034	0	0.016299
27897	Amp	18	39716931	39723662	0	0.007222
27898	Amp	18	39724001	40489888	0	0.007971
27899	Amp	18	40493189	40519408	0	0.010539
27900	Amp	18	40523053	40551196	0	0.012127
27901	Amp	18	40555160	41680788	0	0.010539
27902	Amp	18	41683973	41711701	0	0.008637
27903	Amp	18	41711973	41732126	0	0.012248
27904	Amp	18	41733669	41900533	0	0.010768
27905	Amp	18	41902501	42242364	0	0.012605
27906	Amp	18	42242428	42659733	0	0.014201
27907	Amp	18	42670094	42706109	0	0.012364
27908	Amp	18	42707242	42760759	0	0.023037
27909	Amp	18	42760819	42778046	0	0.026143
27910	Amp	18	42784410	42933679	0	0.015469
27911	Amp	18	42935273	42991232	0	0.018483
27912	Amp	18	42994065	43610936	0	0.016747
27913	Amp	18	43611959	43741154	0	0.018722
27914	Amp	18	43744178	44007981	0	0.017805
27915	Amp	18	44017008	44044864	0	0.021867
27916	Amp	18	44046926	44431159	0	0.017805
27917	Amp	18	44434690	44671142	0	0.019559
27918	Amp	18	44671498	44954619	0	0.020924
27919	Amp	18	44955043	45195809	0	0.030616
27920	Amp	18	45196284	45584106	0	0.031577
27921	Amp	18	45590368	45612481	0	0.034498
27922	Amp	18	45616004	45657282	0	0.031577
27923	Amp	18	45659430	45710097	0	0.034427
27924	Amp	18	45715064	45801500	0	0.027048
27925	Amp	18	45801836	46156252	0	0.034345
27926	Amp	18	46163115	46222573	0	0.031495
27927	Amp	18	46224612	46232565	0	0.024198
27928	Amp	18	46238537	46544298	0	0.025763
27929	Amp	18	46544837	46565340	0	0.023142
27930	Amp	18	46566873	46808282	0	0.021578

27931	Amp	18	46819333	46866839	0	0.024167
27932	Amp	18	46871662	46915179	0	0.027095
27933	Amp	18	46915973	46947184	0	0.034718
27934	Amp	18	47037791	47068888	0	0.0292
27935	Amp	18	47069438	47075240	0	0.034104
27936	Amp	18	47078774	47078774	0	0.029384
27937	Amp	18	47084132	47390003	0	0.021762
27938	Amp	18	47392617	47414026	0	0.026472
27939	Amp	18	47416328	47554609	0	0.021762
27940	Amp	18	47555198	47599520	0	0.020192
27941	Amp	18	47602550	47643697	0	0.016857
27942	Amp	18	47646565	47692312	0	0.013843
27943	Amp	18	47696239	47832486	0	0.016226
27944	Amp	18	47833110	47949525	0	0.014684
27945	Amp	18	47950056	48230781	0	0.015886
27946	Amp	18	48233566	48842170	0	0.016989
27947	Amp	18	48847705	48999129	0	0.014607
27948	Amp	18	48999821	49055954	0	0.012632
27949	Amp	18	49057364	49258263	0	0.016731
27950	Amp	18	49260740	49385230	0	0.011205
27951	Amp	18	49385732	49463084	0	0.007105
27952	Amp	18	49464790	49670809	0	0.008424
27953	Amp	18	49673038	49842933	0	0.008906
27954	Amp	18	49843388	49865539	0	0.009423
27955	Amp	18	49867621	49998104	0	0.008104
27956	Amp	18	49998487	50040987	0	0.009215
27957	Amp	18	50044376	50092192	0	0.01134
27958	Amp	18	50093712	50097099	0	0.009215
27959	Amp	18	50098817	50212309	0	0.010306
27960	Amp	18	50213261	50637577	0	0.008104
27961	Amp	18	50638574	50700547	0	0.007622
27962	Amp	18	50700901	50742412	0	0.00237
27963	Amp	18	50744133	50758600	0	0.003077
27964	Amp	18	50759628	50856755	0	0.00237
27965	Amp	18	50858074	50916193	0	0.008449
27966	Amp	18	50918405	51467037	0	0.010475
27967	Amp	18	51473738	51875848	0	0.013445
27968	Amp	18	51876287	52008537	0	0.016345
27969	Amp	18	52010022	52024526	0	0.013375
27970	Amp	18	52026290	52145372	0	0.01514
27971	Amp	18	52146071	52167118	0	0.01224
27972	Amp	18	52171034	52516343	0	0.015033
27973	Amp	18	52517011	52601411	0	0.013032
27974	Amp	18	52637580	52642945	0	0.028354
27975	Amp	18	52644016	52662383	0	0.024675
27976	Amp	18	52665631	52825956	0	0.009353
27977	Amp	18	52826260	52972444	0	0.004135
27978	Amp	18	52973659	53379441	0	0.00802
27979	Amp	18	53379493	53468116	0	0.008475
27980	Amp	18	53470966	53635826	0	0.007958
27981	Amp	18	53639204	53723610	0	0.01513
27982	Amp	18	53726066	54026802	0	0.013268
27983	Amp	18	54027810	54115568	0	0.015721

27984	Amp	18	54118726	54118726	0	0.018009
27985	Amp	18	54128711	54285080	0	0.020424
27986	Amp	18	54288304	54438168	0	0.023028
27987	Amp	18	54445023	54629210	0	0.020614
27988	Amp	18	54632285	54914662	0	0.022838
27989	Amp	18	54916966	54964101	0	0.020386
27990	Amp	18	54967298	55222833	0	0.012471
27991	Amp	18	55225712	55234376	0	0.01563
27992	Amp	18	55235460	55280630	0	0.017957
27993	Amp	18	55285664	55380719	0	0.014797
27994	Amp	18	55381447	55457322	0	0.019948
27995	Amp	18	55459762	55613447	0	0.016063
27996	Amp	18	55614567	55666966	0	0.023173
27997	Amp	18	55666986	55761116	0	0.026622
27998	Amp	18	55762062	55822108	0	0.024857
27999	Amp	18	55822461	55997009	0	0.023753
28000	Amp	18	55997051	56217840	0	0.021648
28001	Amp	18	56219498	56364259	0	0.015374
28002	Amp	18	56365575	56628922	0	0.01862
28003	Amp	18	56630148	56821368	0	0.016199
28004	Amp	18	56823985	56913222	0	0.019089
28005	Amp	18	56914856	56952387	0	0.015374
28006	Amp	18	56955761	57077614	0	0.011691
28007	Amp	18	57078175	57143056	0	0.008579
28008	Amp	18	57145119	57334554	0	0.01145
28009	Amp	18	57334785	57367346	0	0.008579
28010	Amp	18	57368699	57491580	0	0.010585
28011	Amp	18	57493400	57533821	0	0.013188
28012	Amp	18	57534229	57569110	0	0.015956
28013	Amp	18	57569195	57833209	0	0.015501
28014	Amp	18	57836065	57880413	0	0.018177
28015	Amp	18	57882769	57988119	0	0.021854
28016	Amp	18	57988225	58006837	0	0.024311
28017	Amp	18	58006877	58067811	0	0.020634
28018	Amp	18	58071102	58183058	0	0.017866
28019	Amp	18	58188276	58454571	0	0.01971
28020	Amp	18	58455275	58547329	0	0.016486
28021	Amp	18	58549733	58600861	0	0.021557
28022	Amp	18	58603765	58917994	0	0.015533
28023	Amp	18	58918367	58940906	0	0.016429
28024	Amp	18	58944684	59060077	0	0.017571
28025	Amp	18	59060410	59072751	0	0.023386
28026	Amp	18	59074313	59107155	0	0.017571
28027	Amp	18	59108428	59204613	0	0.018671
28028	Amp	18	59207221	59216634	0	0.016637
28029	Amp	18	59218486	59290136	0	0.013268
28030	Amp	18	59294902	59301396	0	0.019071
28031	Amp	18	59302583	59312702	0	0.020212
28032	Amp	18	59316786	59448557	0	0.016819
28033	Amp	18	59451989	59467065	0	0.014812
28034	Amp	18	59467622	59515414	0	0.013712
28035	Amp	18	59515676	59515960	0	0.011303
28036	Amp	18	59516716	59517309	0	0.010162

28037	Amp	18	59523992	59541594	0	0.011264
28038	Amp	18	59544227	59669551	0	0.013631
28039	Amp	18	59669998	59682668	0	0.017928
28040	Amp	18	59685542	59785196	0	0.013631
28041	Amp	18	59787829	59807502	0	0.014209
28042	Amp	18	59812044	59823092	0	0.016789
28043	Amp	18	59824728	59896372	0	0.018384
28044	Amp	18	59897033	59962240	0	0.020594
28045	Amp	18	59962976	59972672	0	0.026128
28046	Amp	18	59981043	60117229	0	0.020594
28047	Amp	18	60120808	60220569	0	0.018384
28048	Amp	18	60220925	60245552	0	0.019414
28049	Amp	18	60245641	60275619	0	0.018346
28050	Amp	18	60280084	60300983	0	0.016122
28051	Amp	18	60301060	60376021	0	0.0227
28052	Amp	18	60376948	60378403	0	0.027794
28053	Amp	18	60378818	60409770	0	0.025547
28054	Amp	18	60411905	60520232	0	0.027706
28055	Amp	18	60521394	60571017	0	0.029686
28056	Amp	18	60574022	60658447	0	0.026498
28057	Amp	18	60662859	60685173	0	0.021404
28058	Amp	18	60688009	60797130	0	0.022475
28059	Amp	18	60797372	61009135	0	0.019833
28060	Amp	18	61009388	61034234	0	0.018237
28061	Amp	18	61034534	61059526	0	0.019119
28062	Amp	18	61062090	61091577	0	0.020626
28063	Amp	18	61096553	61139464	0	0.023355
28064	Amp	18	61140436	61146788	0	0.020626
28065	Amp	18	61148613	61156076	0	0.024225
28066	Amp	18	61156524	61271722	0	0.028896
28067	Amp	18	61277172	61388158	0	0.035896
28068	Amp	18	61389343	61535651	0	0.034388
28069	Amp	18	61535975	61603902	0	0.036112
28070	Amp	18	61607259	61621785	0	0.041464
28071	Amp	18	61624618	61707413	0	0.034465
28072	Amp	18	61710639	61753530	0	0.039309
28073	Amp	18	61754841	61757890	0	0.042413
28074	Amp	18	61768678	61793769	0	0.040432
28075	Amp	18	61794136	61809897	0	0.043513
28076	Amp	18	61842053	61876274	0	0.049714
28077	Amp	18	61882833	61882956	0	0.046769
28078	Amp	18	61888172	62016130	0	0.045341
28079	Amp	18	62016850	62055090	0	0.04751
28080	Amp	18	62058705	62161007	0	0.049771
28081	Amp	18	62161742	62298956	0	0.051241
28082	Amp	18	62299224	62459449	0	0.052738
28083	Amp	18	62470363	62480855	0	0.054702
28084	Amp	18	62481496	62577623	0	0.061699
28085	Amp	18	62580329	62586138	0	0.063601
28086	Amp	18	62586528	62609516	0	0.056604
28087	Amp	18	62614528	62686977	0.001323	0.063885
28088	Amp	18	62689370	62849059	0	0.062255
28089	Amp	18	62851190	62870392	0	0.055313

28090	Amp	18	62873627	62937660	0	0.053411
28091	Amp	18	62947164	63017832	0	0.058939
28092	Amp	18	63022691	63076247	0.040093	0.06571
28093	Amp	18	63078617	63245133	0.065715	0.066862
28094	Amp	18	63246710	63250228	0.034113	0.065364
28095	Amp	18	63251154	63335708	0.060686	0.066569
28096	Amp	18	63335900	63375575	0.005328	0.064147
28097	Amp	18	63376740	63459442	0.050473	0.066211
28098	Amp	18	63463156	63470628	0.010261	0.064408
28099	Amp	18	63470703	63585241	0	0.059838
28100	Amp	18	63586256	63641061	0	0.061049
28101	Amp	18	63646569	63646569	0	0.050169
28102	Amp	18	63646883	63758711	0	0.050667
28103	Amp	18	63762018	63764593	0	0.052434
28104	Amp	18	63766696	63800938	0	0.051937
28105	Amp	18	63802704	63934132	0	0.050726
28106	Amp	18	63937728	64108773	0	0.052419
28107	Amp	18	64117720	64236916	0	0.045504
28108	Amp	18	64237128	64294082	0	0.043583
28109	Amp	18	64295068	64441135	0	0.0469
28110	Amp	18	64441444	64607085	0	0.047586
28111	Amp	18	64608188	64612376	0	0.048567
28112	Amp	18	64613572	64681045	0	0.04732
28113	Amp	18	64689495	64739648	0	0.046634
28114	Amp	18	64740446	64740902	0	0.056273
28115	Amp	18	64751208	64767568	0	0.049366
28116	Amp	18	64768697	64782893	0	0.046333
28117	Amp	18	64785211	64826496	0	0.0436
28118	Amp	18	64826750	65083435	0	0.039656
28119	Amp	18	65083872	65106213	0	0.036645
28120	Amp	18	65109866	65110005	0	0.029364
28121	Amp	18	65110208	65195111	0	0.030752
28122	Amp	18	65195252	65278257	0	0.030306
28123	Amp	18	65278502	65278502	0	0.032819
28124	Amp	18	65282239	65335793	0	0.035647
28125	Amp	18	65335806	65515984	0	0.034667
28126	Amp	18	65516011	66372051	0	0.031377
28127	Amp	18	66372563	66554641	0	0.031994
28128	Amp	18	66562301	66850990	0	0.035893
28129	Amp	18	66851110	66855898	0	0.034448
28130	Amp	18	66856154	66878598	0	0.037786
28131	Amp	18	66881730	66936166	0	0.033831
28132	Amp	18	66937599	67317073	0	0.037213
28133	Amp	18	67317090	67336844	0	0.032063
28134	Amp	18	67337077	67624795	0	0.033793
28135	Amp	18	67651246	67710792	0	0.034373
28136	Amp	18	67711050	67723723	0	0.030163
28137	Amp	18	67728085	67855728	0	0.032676
28138	Amp	18	67857059	67871803	0	0.035062
28139	Amp	18	67871891	67884522	0	0.029171
28140	Amp	18	67886879	67889732	0	0.029942
28141	Amp	18	67889928	67936468	0	0.029362
28142	Amp	18	67936532	67938472	0	0.026222

28143 Amp	18	68118231	68124218	0	0.025452
28144 Amp	18	68124710	68178498	0	0.022938
28145 Amp	18	68178826	68228789	0	0.024633
28146 Amp	18	68228851	68273660	0	0.019789
28147 Amp	18	68275534	68316250	0	0.025357
28148 Amp	18	68316501	68346818	0	0.019789
28149 Amp	18	68563349	68563349	0	0.018319
28150 Amp	18	68563536	68721104	0	0.022054
28151 Amp	18	68724167	68872593	0	0.024869
28152 Amp	18	68873231	69021965	0	0.026617
28153 Amp	18	69022410	69071897	0	0.021187
28154 Amp	18	69100687	69282109	0	0.023108
28155 Amp	18	69288807	69338836	0	0.025205
28156 Amp	18	69341225	69370897	0	0.032122
28157 Amp	18	69371787	69434242	0	0.033334
28158 Amp	18	69436435	69476458	0	0.026417
28159 Amp	18	69476967	69521200	0	0.028667
28160 Amp	18	69521410	69654219	0	0.030267
28161 Amp	18	69661551	69700397	0	0.028346
28162 Amp	18	69706284	69950896	0	0.025531
28163 Amp	18	69953457	70027866	0	0.028369
28164 Amp	18	70030941	70137388	0	0.025531
28165 Amp	18	70137437	70202792	0	0.027311
28166 Amp	18	70207363	70470530	0	0.02947
28167 Amp	18	70471997	70475685	0	0.031103
28168 Amp	18	70479244	70479674	0	0.028943
28169 Amp	18	70484561	70551557	0	0.032433
28170 Amp	18	70555048	70571499	0	0.027798
28171 Amp	18	70573333	70653255	0	0.029598
28172 Amp	18	70653418	70682116	0	0.027998
28173 Amp	18	70683610	70824712	0	0.024508
28174 Amp	18	70826008	70854821	0	0.024994
28175 Amp	18	70859718	70975789	0	0.026377
28176 Amp	18	70979552	71089085	0	0.02589
28177 Amp	18	71089508	71119926	0	0.03356
28178 Amp	18	71120182	71183498	0	0.02589
28179 Amp	18	71186041	71277919	0	0.024685
28180 Amp	18	71278859	71342414	0	0.022885
28181 Amp	18	71343961	71381922	0	0.023329
28182 Amp	18	71382253	71382253	0	0.025712
28183 Amp	18	73699619	73699619	0	0.030386
28184 Amp	18	73699797	73699797	0	0.023096
28185 Amp	18	73701344	73702646	0	0.019684
28186 Amp	18	73707154	73943450	0	0.018302
28187 Amp	18	73943708	73943708	0	0.015919
28188 Amp	18	73945012	74191659	0	0.016398
28189 Amp	18	74194109	74244983	0	0.01807
28190 Amp	18	74247941	74709381	0	0.01759
28191 Amp	18	74710453	74770872	0	0.019363
28192 Amp	18	74771317	74932077	0	0.021239
28193 Amp	18	74932253	75126781	0	0.023595
28194 Amp	18	75132620	75153610	0	0.025653
28195 Amp	18	75153910	75182713	0	0.023297

28196	Amp	18	75183182	75202827	0	0.026004
28197	Amp	18	75210785	75210785	0	0.021239
28198	Amp	18	75212248	75573042	0	0.019467
28199	Amp	18	75575154	75619243	0	0.017719
28200	Amp	18	75621727	76209822	0	0.019562
28201	Amp	18	76211276	76776411	0	0.01789
28202	Amp	18	76779176	76984865	0	0.019543
28203	Amp	18	76989373	77008141	0	0.020322
28204	Amp	18	77008428	77168272	0	0.018971
28205	Amp	18	77168644	77193682	0	0.02184
28206	Amp	18	77200392	77478177	0	0.018971
28207	Amp	18	77478183	77530572	0	0.017169
28208	Amp	18	77530779	77784393	0	0.018892
28209	Amp	18	77790273	77989785	0	0.021195
28210	Amp	18	77990761	78124860	0	0.019319
28211	Amp	18	78171939	78189051	0	0.017016
28212	Amp	18	78189137	78239365	0	0.015292
28213	Amp	18	78242995	78655570	0	0.014207
28214	Amp	18	78656004	78943721	0	0.012558
28215	Amp	18	78944917	79051788	0	0.012113
28216	Amp	18	79051843	79098782	0	0.01582
28217	Amp	18	79102505	79191493	0	0.017788
28218	Amp	18	79191623	79349796	0	0.019582
28219	Amp	19	283868	305471	0	0.020901
28220	Amp	19	310174	335055	0	0.020388
28221	Amp	19	335323	335323	0	0.014999
28222	Amp	19	389805	415625	0	0.017488
28223	Amp	19	416231	464681	0	0.012937
28224	Amp	19	468402	547973	0	0.011518
28225	Amp	19	548446	970958	0	0.009154
28226	Amp	19	981661	987571	0	0.006665
28227	Amp	19	988135	1204475	0	0.006162
28228	Amp	19	1204594	1210607	0	0.004667
28229	Amp	19	1210939	1283189	0	0.003983
28230	Amp	19	1302966	1465199	0	0.005855
28231	Amp	19	1476534	1600598	0	0.008015
28232	Amp	19	1613848	1636590	0	0.010364
28233	Amp	19	1638920	1680988	0	0.008203
28234	Amp	19	1681481	1728502	0	0.009978
28235	Amp	19	1731832	1846112	0	0.012039
28236	Amp	19	1867913	1873721	0	0.01419
28237	Amp	19	1882763	2060090	0	0.016691
28238	Amp	19	2064620	2064620	0	0.020025
28239	Amp	19	2084161	2124622	0	0.017523
28240	Amp	19	2125087	2323963	0	0.019175
28241	Amp	19	2326989	2389786	0	0.020664
28242	Amp	19	2403566	2483299	0	0.033551
28243	Amp	19	2499529	2646942	0	0.031202
28244	Amp	19	2648184	2741142	0	0.033769
28245	Amp	19	2741606	2741606	0	0.031618
28246	Amp	19	2743904	2766093	0	0.022069
28247	Amp	19	2766386	2894153	0	0.023991
28248	Amp	19	2894880	2934284	0	0.025463

28249	Amp	19	2953089	2998070	0	0.020354
28250	Amp	19	2998719	3112080	0	0.021127
28251	Amp	19	3113905	3161259	0	0.021891
28252	Amp	19	3166717	3205977	0	0.023891
28253	Amp	19	3209485	3214116	0	0.02242
28254	Amp	19	3227104	3321679	0	0.024264
28255	Amp	19	3327945	3333279	0	0.026624
28256	Amp	19	3336541	3342441	0	0.030748
28257	Amp	19	3342948	3521410	0	0.033669
28258	Amp	19	3527112	3527112	0	0.030332
28259	Amp	19	3533408	3542985	0	0.038693
28260	Amp	19	3554785	3554785	0	0.037041
28261	Amp	19	3560636	3560636	0	0.035041
28262	Amp	19	3571448	3571448	0	0.033197
28263	Amp	19	3593690	3599136	0	0.030621
28264	Amp	19	3605541	3654183	0	0.027895
28265	Amp	19	3660621	3696305	0	0.031666
28266	Amp	19	3700861	3757411	0	0.031222
28267	Amp	19	3762121	3795705	0	0.032306
28268	Amp	19	3814394	3814435	0	0.029737
28269	Amp	19	3815537	3837071	0	0.035439
28270	Amp	19	3839182	3911771	0	0.027377
28271	Amp	19	3912905	3918049	0	0.030629
28272	Amp	19	3923223	3943269	0	0.032873
28273	Amp	19	3957923	3980369	0	0.035558
28274	Amp	19	3983993	4032531	0	0.034786
28275	Amp	19	4036396	4073730	0	0.035314
28276	Amp	19	4106113	4106786	0	0.038566
28277	Amp	19	4113314	4113314	0	0.037482
28278	Amp	19	4136330	4142635	0	0.034561
28279	Amp	19	4143214	4149863	0	0.031309
28280	Amp	19	4159104	4164804	0	0.033379
28281	Amp	19	4176088	4186146	0	0.029607
28282	Amp	19	4194093	4196017	0	0.026356
28283	Amp	19	4211184	4251036	0	0.024112
28284	Amp	19	4251072	4268405	0	0.027111
28285	Amp	19	4271625	4288558	0	0.027643
28286	Amp	19	4299133	4300624	0	0.025045
28287	Amp	19	4301848	4342356	0	0.022047
28288	Amp	19	4342850	4406163	0	0.024313
28289	Amp	19	4673369	4688832	0	0.029768
28290	Amp	19	4692342	4692342	0	0.031256
28291	Amp	19	4701120	4783686	0	0.02869
28292	Amp	19	4805732	4844140	0	0.028157
28293	Amp	19	4844621	4860658	0	0.030476
28294	Amp	19	4866427	4877715	0	0.034198
28295	Amp	19	4881494	4895650	0	0.035606
28296	Amp	19	4908081	5039124	0	0.038111
28297	Amp	19	5041167	5041167	0	0.039061
28298	Amp	19	5046059	5062521	0	0.04259
28299	Amp	19	5066038	5066038	0	0.0407
28300	Amp	19	5072291	5081470	0	0.04312
28301	Amp	19	5089784	5093009	0	0.04675

28302 Amp	19	5095978	5112250	0	0.046099
28303 Amp	19	5118606	5163852	0	0.043178
28304 Amp	19	5168009	5189778	0	0.040757
28305 Amp	19	5189956	5343923	0	0.037128
28306 Amp	19	5346471	5393737	0	0.04269
28307 Amp	19	5394754	5458580	0	0.043407
28308 Amp	19	5460194	5506446	0	0.04269
28309 Amp	19	5515782	5519433	0	0.038659
28310 Amp	19	5528958	5545333	0	0.042563
28311 Amp	19	5556902	5652689	0	0.044751
28312 Amp	19	5659854	5696951	0	0.04538
28313 Amp	19	5700464	5705064	0	0.043755
28314 Amp	19	5713224	5765858	0	0.046773
28315 Amp	19	5766079	5793693	0	0.05156
28316 Amp	19	5794706	5831096	0	0.052917
28317 Amp	19	5870270	5874092	0	0.054702
28318 Amp	19	5882858	5882858	0	0.04634
28319 Amp	19	5884662	5962751	0	0.050262
28320 Amp	19	5963589	6084981	0	0.046747
28321 Amp	19	6088140	6138016	0	0.046184
28322 Amp	19	6139058	6263484	0	0.044526
28323 Amp	19	6266767	6291991	0	0.040656
28324 Amp	19	6292969	6299337	0	0.036209
28325 Amp	19	6305990	6306960	0	0.031839
28326 Amp	19	6307101	6307491	0	0.031177
28327 Amp	19	6308498	6334651	0	0.02977
28328 Amp	19	6338359	6339586	0	0.029005
28329 Amp	19	6343831	6370186	0	0.025781
28330 Amp	19	6382140	6407729	0	0.022483
28331 Amp	19	6423884	6426360	0	0.025301
28332 Amp	19	6427353	6442329	0	0.023516
28333 Amp	19	6446640	6446640	0	0.022037
28334 Amp	19	6447208	6503297	0	0.020681
28335 Amp	19	6504754	6511955	0	0.022121
28336 Amp	19	6514299	6519458	0	0.023796
28337 Amp	19	6522783	6578029	0	0.035058
28338 Amp	19	6582438	6588490	0	0.03357
28339 Amp	19	6593591	6669125	0	0.030752
28340 Amp	19	6670899	6690999	0	0.031955
28341 Amp	19	6691620	6835657	0	0.034934
28342 Amp	19	6849895	6882419	0	0.035785
28343 Amp	19	6885312	6885312	0	0.034934
28344 Amp	19	7112112	7112112	0	0.036005
28345 Amp	19	7112165	7178867	0	0.034379
28346 Amp	19	7182742	7233930	0	0.036634
28347 Amp	19	7238249	7339450	0	0.037261
28348 Amp	19	7340749	7342337	0	0.039222
28349 Amp	19	7346095	7372463	0	0.02796
28350 Amp	19	7374024	7386990	0	0.027331
28351 Amp	19	7389136	7436730	0	0.02876
28352 Amp	19	7438859	7442056	0	0.027145
28353 Amp	19	7453326	7522426	0	0.02489
28354 Amp	19	7537535	7602987	0	0.021384

28355	Amp	19	7616626	7634461	0	0.032833
28356	Amp	19	7636372	7787451	0	0.030873
28357	Amp	19	7800602	7918601	0	0.028085
28358	Amp	19	7924444	8056555	0	0.030809
28359	Amp	19	8057853	8083355	0	0.034899
28360	Amp	19	8090962	8170129	0	0.030809
28361	Amp	19	8175529	8208395	0	0.031989
28362	Amp	19	8213676	8213676	0	0.029265
28363	Amp	19	8222932	8252908	0	0.032185
28364	Amp	19	8256678	8267912	0	0.033457
28365	Amp	19	8267916	8267916	0	0.033914
28366	Amp	19	8267922	8270055	0	0.032734
28367	Amp	19	8313986	8314410	0	0.03318
28368	Amp	19	8315317	8331596	0	0.036926
28369	Amp	19	8331919	8340107	0	0.035497
28370	Amp	19	8388925	8432398	0	0.036156
28371	Amp	19	8433215	8488438	0	0.032409
28372	Amp	19	8500286	8550384	0	0.033261
28373	Amp	19	8552805	8579617	0	0.036022
28374	Amp	19	8582114	8590905	0	0.033737
28375	Amp	19	8593226	8615429	0	0.035306
28376	Amp	19	8630575	8661521	0	0.042346
28377	Amp	19	8662168	8677192	0	0.043873
28378	Amp	19	8677305	8692885	0	0.044807
28379	Amp	19	8699081	8705006	0	0.043238
28380	Amp	19	8705645	8737202	0	0.042386
28381	Amp	19	8738496	8768031	0	0.040859
28382	Amp	19	8795410	8795425	0	0.045784
28383	Amp	19	8803416	8804153	0	0.049715
28384	Amp	19	8809491	8836223	0	0.045936
28385	Amp	19	8838919	8846888	0	0.042004
28386	Amp	19	8850216	8924180	0	0.045872
28387	Amp	19	8924638	8946265	0	0.042299
28388	Amp	19	8947011	8949132	0	0.04419
28389	Amp	19	8950136	8997889	0	0.041704
28390	Amp	19	9000303	9007827	0	0.043578
28391	Amp	19	9021939	9051570	0	0.047703
28392	Amp	19	9051706	9121131	0	0.045812
28393	Amp	19	9125992	9151266	0	0.040099
28394	Amp	19	9152860	9182271	0	0.042386
28395	Amp	19	9183329	9255816	0	0.044254
28396	Amp	19	9256072	9292588	0	0.049215
28397	Amp	19	9293630	9295994	0	0.045347
28398	Amp	19	9301539	9342203	0	0.046287
28399	Amp	19	9342478	9395237	0	0.048932
28400	Amp	19	9397416	9487076	0	0.050343
28401	Amp	19	9487911	9495432	0	0.048469
28402	Amp	19	9504478	9527369	0	0.050448
28403	Amp	19	9528829	9535965	0	0.051154
28404	Amp	19	9536186	9539095	0	0.050304
28405	Amp	19	9552462	9574461	0	0.048432
28406	Amp	19	9575143	9575143	0	0.046233
28407	Amp	19	9581881	9582234	0	0.045293

28408	Amp	19	9586249	9586249	0	0.050286
28409	Amp	19	9586287	9681224	0	0.04958
28410	Amp	19	9682675	9729549	0	0.050755
28411	Amp	19	9730551	9751958	0	0.04958
28412	Amp	19	9752177	9804077	0	0.048645
28413	Amp	19	9804504	9890249	0	0.040763
28414	Amp	19	9890824	9897799	0	0.035771
28415	Amp	19	9901518	9907519	0	0.033687
28416	Amp	19	9911402	9921069	0	0.032199
28417	Amp	19	9923070	9930431	0	0.033847
28418	Amp	19	9934449	9939508	0	0.037029
28419	Amp	19	9942635	9945333	0	0.035381
28420	Amp	19	9945453	10004943	0	0.037315
28421	Amp	19	10005400	10046528	0	0.034133
28422	Amp	19	10055256	10064792	0	0.037478
28423	Amp	19	10065948	10073330	0	0.035544
28424	Amp	19	10074203	10088431	0	0.040813
28425	Amp	19	10089132	10108400	0	0.042107
28426	Amp	19	10115006	10129990	0	0.040667
28427	Amp	19	10131268	10166137	0	0.039716
28428	Amp	19	10167123	10228000	0	0.041349
28429	Amp	19	10244839	10319307	0	0.046038
28430	Amp	19	10327700	10371675	0	0.048033
28431	Amp	19	10373876	10409388	0	0.049273
28432	Amp	19	10422127	10461930	0	0.046666
28433	Amp	19	10464035	10466964	0	0.048544
28434	Amp	19	10467167	10521916	0	0.05074
28435	Amp	19	10523308	10545128	0	0.043583
28436	Amp	19	10545766	10598160	0	0.044501
28437	Amp	19	10606674	10616046	0	0.046499
28438	Amp	19	10616338	10662539	0	0.049559
28439	Amp	19	10664411	10781332	0	0.047816
28440	Amp	19	10782968	10794212	0	0.048506
28441	Amp	19	10798095	10819583	0	0.04806
28442	Amp	19	10821497	10823506	0	0.049975
28443	Amp	19	10827588	10874897	0	0.050417
28444	Amp	19	10876902	10877410	0	0.042892
28445	Amp	19	10882455	10904326	0	0.041259
28446	Amp	19	10909121	10919955	0	0.043134
28447	Amp	19	10924170	10940676	0	0.040037
28448	Amp	19	10944048	10950750	0	0.042189
28449	Amp	19	10954492	11005590	0	0.048468
28450	Amp	19	11006760	11015412	0	0.045341
28451	Amp	19	11021404	11052925	0	0.043481
28452	Amp	19	11053013	11058376	0	0.041932
28453	Amp	19	11066732	11066732	0	0.040549
28454	Amp	19	11069603	11079146	0	0.04286
28455	Amp	19	11084147	11105898	0	0.045475
28456	Amp	19	11111879	11120755	0	0.048985
28457	Amp	19	11123101	11126329	0	0.04977
28458	Amp	19	11137314	11139463	0	0.054812
28459	Amp	19	11149384	11158677	0	0.048533
28460	Amp	19	11160191	11215416	0	0.046222

28461	Amp	19	11215873	11232027	0	0.044556
28462	Amp	19	11236683	11249046	0	0.040134
28463	Amp	19	11251903	11354453	0	0.037983
28464	Amp	19	11362739	11404757	0	0.035367
28465	Amp	19	11405457	11481388	0	0.037227
28466	Amp	19	11484705	11509803	0	0.036532
28467	Amp	19	11510579	11521099	0	0.033226
28468	Amp	19	11521394	11544195	0	0.032441
28469	Amp	19	11544412	11560950	0	0.034102
28470	Amp	19	11561979	11598669	0	0.031124
28471	Amp	19	11599494	11653681	0	0.036663
28472	Amp	19	11655490	11693055	0	0.039991
28473	Amp	19	11696225	11704071	0	0.038116
28474	Amp	19	11706450	11794528	0	0.039356
28475	Amp	19	11800973	11806016	0	0.035846
28476	Amp	19	11850377	11897388	0	0.031681
28477	Amp	19	11898361	11929468	0	0.032749
28478	Amp	19	11931930	11933296	0	0.03425
28479	Amp	19	11937820	11937820	0	0.03764
28480	Amp	19	11938087	11945938	0	0.043337
28481	Amp	19	11947135	11950569	0	0.041745
28482	Amp	19	11958031	11985758	0	0.038684
28483	Amp	19	11987887	12116025	0	0.041109
28484	Amp	19	12116420	12188375	0	0.044056
28485	Amp	19	12196905	12243840	0	0.047317
28486	Amp	19	12253499	12321749	0	0.044371
28487	Amp	19	12322481	12339584	0	0.042503
28488	Amp	19	12345468	12371342	0	0.046657
28489	Amp	19	12372756	12391400	0	0.048404
28490	Amp	19	12448899	12471337	0	0.051405
28491	Amp	19	12475571	12475770	0	0.052392
28492	Amp	19	12475890	12534067	0	0.054121
28493	Amp	19	12537272	12550332	0	0.05537
28494	Amp	19	12550364	12557409	0	0.056333
28495	Amp	19	12567173	12575480	0	0.052179
28496	Amp	19	12579493	12579493	0	0.048918
28497	Amp	19	12593981	12677447	0	0.053991
28498	Amp	19	12677959	12709441	0	0.055419
28499	Amp	19	12710182	12724406	0	0.057015
28500	Amp	19	12733247	12735709	0	0.05305
28501	Amp	19	12737870	12788107	0	0.055897
28502	Amp	19	12798558	12837124	0	0.050824
28503	Amp	19	12840049	12870716	0	0.050381
28504	Amp	19	12871984	12884608	0	0.051874
28505	Amp	19	12888564	12888564	0	0.050015
28506	Amp	19	12890683	12979159	0	0.051945
28507	Amp	19	12984871	12987321	0	0.058401
28508	Amp	19	12987605	13015157	0.060686	0.066541
28509	Amp	19	13015958	13067985	0.069848	0.067077
28510	Amp	19	13070658	13085866	0.107017	0.068458
28511	Amp	19	13090993	13107963	0.164935	0.070838
28512	Amp	19	13108899	13144465	0.19964	0.072308
28513	Amp	19	13148543	13148543	0.159312	0.070712

28514	Amp	19	13152524	13189880	0.029031	0.065256
28515	Amp	19	13194099	13213660	0	0.062409
28516	Amp	19	13214919	13245620	0.034113	0.0655
28517	Amp	19	13250736	13330527	0	0.062409
28518	Amp	19	13332956	13337386	0.034113	0.06546
28519	Amp	19	13337697	13387506	0.056211	0.066352
28520	Amp	19	13388161	13389263	0.015369	0.064606
28521	Amp	19	13389781	13410083	0	0.059976
28522	Amp	19	13410714	13413514	0	0.058047
28523	Amp	19	13415237	13421690	0	0.060147
28524	Amp	19	13423862	13451636	0	0.062031
28525	Amp	19	13458742	13462528	0	0.06065
28526	Amp	19	13463259	13565773	0	0.062126
28527	Amp	19	13567506	13675279	0.005328	0.064138
28528	Amp	19	13675842	13707138	0	0.062668
28529	Amp	19	13709442	13719828	0	0.063747
28530	Amp	19	13722850	13728862	0	0.061367
28531	Amp	19	13729552	13830024	0	0.063758
28532	Amp	19	13832321	13836478	0.112543	0.068688
28533	Amp	19	13843522	13860084	0.259438	0.074831
28534	Amp	19	13864543	13958756	0.227787	0.073338
28535	Amp	19	13969689	13999199	0.069848	0.066882
28536	Amp	19	14000913	14004906	0.044794	0.065803
28537	Amp	19	14012303	14035081	0.222041	0.073103
28538	Amp	19	14036451	14036451	0.181932	0.071456
28539	Amp	19	14036781	14078298	0.089561	0.067791
28540	Amp	19	14092798	14143226	0.100827	0.0682
28541	Amp	19	14143786	14143786	0.133193	0.06954
28542	Amp	19	14168665	14187045	0.08435	0.067552
28543	Amp	19	14187821	14203289	0.050473	0.06614
28544	Amp	19	14204575	14204575	0.215608	0.072792
28545	Amp	19	14216571	14216571	0.128324	0.069465
28546	Amp	19	14226547	14226547	0.060686	0.066463
28547	Amp	19	14229706	14246087	0.034113	0.065477
28548	Amp	19	14254625	14267329	0.056211	0.066402
28549	Amp	19	14269255	14274208	0	0.060259
28550	Amp	19	14274934	14295803	0	0.061144
28551	Amp	19	14305839	14306319	0	0.058078
28552	Amp	19	14315783	14390910	0	0.052852
28553	Amp	19	14396404	14439434	0	0.056833
28554	Amp	19	14439594	14445955	0	0.054969
28555	Amp	19	14446903	14456744	0	0.056691
28556	Amp	19	14462842	14513401	0	0.060044
28557	Amp	19	14513682	14522867	0	0.063024
28558	Amp	19	14542665	14542665	0.044794	0.06601
28559	Amp	19	14544181	14688689	0.065715	0.066839
28560	Amp	19	14691292	14728678	0.094967	0.068179
28561	Amp	19	14731430	14774029	0.112543	0.068755
28562	Amp	19	14777307	14794708	0.065715	0.066742
28563	Amp	19	14796868	14828368	0.222041	0.073096
28564	Amp	19	14829189	14901914	0.282828	0.075635
28565	Amp	19	14925918	14943167	0.379885	0.079104
28566	Amp	19	14943920	15004945	0.417873	0.080499

28567 Amp	19	15005325	15041494	0.470509	0.082235
28568 Amp	19	15043315	15105387	0.735486	0.090355
28569 Amp	19	15113369	15130220	0.576183	0.085699
28570 Amp	19	15130381	15135105	0.522881	0.083862
28571 Amp	19	15135425	15182134	0.450702	0.081436
28572 Amp	19	15182944	15183190	0.14896	0.070312
28573 Amp	19	15185496	15228293	0.417873	0.080401
28574 Amp	19	15238434	15252911	0.470509	0.082202
28575 Amp	19	15256263	15334032	0.543344	0.08452
28576 Amp	19	15335766	15356631	0.368062	0.07873
28577 Amp	19	15356698	15365356	0.444474	0.08132
28578 Amp	19	15372237	15402586	0.508568	0.083579
28579 Amp	19	15403849	15410956	0.470509	0.082133
28580 Amp	19	15415565	15421470	0.437198	0.08117
28581 Amp	19	15421851	15430479	0.404718	0.079921
28582 Amp	19	15442485	15466638	0.373599	0.078845
28583 Amp	19	15467115	15481053	0.287738	0.075824
28584 Amp	19	15481968	15521565	0.241616	0.073844
28585 Amp	19	15524124	15565939	0.342649	0.077853
28586 Amp	19	15590267	15590528	0.271552	0.075314
28587 Amp	19	15590942	15603506	0.316877	0.077016
28588 Amp	19	15609874	15626464	0.210594	0.072646
28589 Amp	19	15628505	15628787	0.276783	0.07543
28590 Amp	19	15630377	15725041	0.238246	0.073694
28591 Amp	19	15726410	15751726	0.17597	0.071435
28592 Amp	19	15752163	15923127	0.112543	0.068651
28593 Amp	19	15923205	15930219	0.069848	0.066985
28594 Amp	19	15931119	16013386	0.040093	0.065589
28595 Amp	19	16017078	16019794	0.079652	0.067336
28596 Amp	19	16021748	16063118	0.065715	0.06676
28597 Amp	19	16068520	16074300	0.128324	0.069378
28598 Amp	19	16078433	16082064	0	0.061395
28599 Amp	19	16082148	16082148	0	0.060748
28600 Amp	19	16083694	16084561	0	0.050603
28601 Amp	19	16087235	16130563	0	0.04727
28602 Amp	19	16134007	16179552	0	0.045315
28603 Amp	19	16180536	16203206	0	0.047488
28604 Amp	19	16203681	16225590	0	0.044019
28605 Amp	19	16228904	16238440	0	0.043133
28606 Amp	19	16239564	16260579	0	0.048153
28607 Amp	19	16261007	16284785	0	0.046814
28608 Amp	19	16287558	16343717	0	0.054613
28609 Amp	19	16347625	16384476	0	0.046935
28610 Amp	19	16389714	16553271	0	0.048949
28611 Amp	19	16554372	16554372	0	0.050712
28612 Amp	19	16559849	16566478	0	0.055088
28613 Amp	19	16625585	16702014	0	0.050401
28614 Amp	19	16702881	16749294	0	0.053763
28615 Amp	19	16758152	16758152	0	0.051748
28616 Amp	19	16758655	16760911	0	0.049865
28617 Amp	19	16765211	16766522	0	0.051856
28618 Amp	19	16767615	16773101	0	0.050952
28619 Amp	19	16786312	16794716	0	0.048779

28620	Amp	19	16795455	16901547	0	0.047862
28621	Amp	19	16903438	16984747	0	0.049202
28622	Amp	19	16985134	16985134	0	0.052281
28623	Amp	19	16987725	17000864	0	0.057139
28624	Amp	19	17000889	17023015	0	0.060787
28625	Amp	19	17023879	17024271	0	0.059958
28626	Amp	19	17033504	17049929	0	0.060915
28627	Amp	19	17052851	17069483	0.056211	0.066343
28628	Amp	19	17071092	17113862	0.073902	0.067163
28629	Amp	19	17119186	17135147	0	0.059364
28630	Amp	19	17135402	17171440	0	0.055716
28631	Amp	19	17172617	17196086	0	0.057088
28632	Amp	19	17196736	17250019	0	0.058762
28633	Amp	19	17250055	17267350	0	0.056772
28634	Amp	19	17271441	17315692	0	0.051914
28635	Amp	19	17315912	17351860	0	0.049721
28636	Amp	19	17351983	17353904	0	0.051337
28637	Amp	19	17356527	17452160	0	0.062611
28638	Amp	19	17459393	17483257	0	0.063444
28639	Amp	19	17488577	17495593	0.222041	0.073024
28640	Amp	19	17509997	17517543	0.17597	0.071408
28641	Amp	19	17522540	17549491	0.044794	0.06598
28642	Amp	19	17549792	17582703	0.107017	0.068608
28643	Amp	19	17585880	17729362	0.08435	0.067651
28644	Amp	19	17730160	17734133	0.193543	0.071987
28645	Amp	19	17737896	17775765	0.299433	0.076262
28646	Amp	19	17782565	17805721	0.143787	0.070001
28647	Amp	19	17809405	17817024	0.044794	0.065983
28648	Amp	19	17817859	17862349	0.005328	0.064253
28649	Amp	19	17862811	17871001	0	0.057887
28650	Amp	19	17878597	17887255	0	0.050954
28651	Amp	19	17888312	17931528	0	0.048325
28652	Amp	19	17932136	18015179	0	0.05096
28653	Amp	19	18015397	18017579	0	0.053489
28654	Amp	19	18017922	18062793	0	0.052079
28655	Amp	19	18065440	18065440	0	0.054175
28656	Amp	19	18069426	18090456	0	0.056124
28657	Amp	19	18092799	18154130	0	0.058841
28658	Amp	19	18156876	18184261	0	0.052165
28659	Amp	19	18184580	18216135	0	0.058737
28660	Amp	19	18216878	18221590	0	0.062145
28661	Amp	19	18224592	18270802	0	0.060634
28662	Amp	19	18273787	18404931	0	0.062036
28663	Amp	19	18413576	18414891	0	0.055464
28664	Amp	19	18422832	18505517	0	0.058877
28665	Amp	19	18505589	18542034	0	0.056242
28666	Amp	19	18544624	18553681	0	0.056771
28667	Amp	19	18562283	18563215	0	0.0581
28668	Amp	19	18566135	18590689	0	0.051983
28669	Amp	19	18596238	18612156	0	0.053115
28670	Amp	19	18625110	18625110	0	0.050663
28671	Amp	19	18625692	18625692	0	0.05288
28672	Amp	19	18634853	18638602	0	0.054577

28673 Amp	19	18639814	18662329	0	0.056501
28674 Amp	19	18664267	18741104	0	0.055738
28675 Amp	19	18744914	18865073	0	0.05404
28676 Amp	19	18871838	18911529	0	0.052908
28677 Amp	19	18912444	18913044	0	0.049829
28678 Amp	19	18914580	18935077	0	0.05158
28679 Amp	19	18948794	18965186	0	0.056448
28680 Amp	19	18968584	18983417	0	0.053919
28681 Amp	19	18986585	19004942	0	0.056298
28682 Amp	19	19005042	19066412	0	0.054624
28683 Amp	19	19069579	19069579	0	0.051109
28684 Amp	19	19072997	19157507	0	0.047271
28685 Amp	19	19157983	19186357	0	0.045348
28686 Amp	19	19186483	19204371	0	0.044252
28687 Amp	19	19207349	19219757	0	0.045865
28688 Amp	19	19222569	19266254	0	0.045044
28689 Amp	19	19266713	19287274	0	0.050499
28690 Amp	19	19288010	19390191	0	0.048997
28691 Amp	19	19390579	19395807	0	0.047247
28692 Amp	19	19398197	19421313	0	0.045142
28693 Amp	19	19421485	19595924	0	0.043529
28694 Amp	19	19598211	19603627	0	0.045956
28695 Amp	19	19606125	19632967	0	0.044627
28696 Amp	19	19639201	19652826	0	0.042411
28697 Amp	19	19652953	19666929	0	0.039984
28698 Amp	19	19667045	19669971	0	0.040667
28699 Amp	19	19670456	19698338	0	0.038769
28700 Amp	19	19701028	19776483	0	0.039295
28701 Amp	19	19776769	19827569	0	0.04027
28702 Amp	19	19827989	19835282	0	0.045265
28703 Amp	19	19836130	19836130	0	0.042189
28704 Amp	19	21064698	21064698	0	0.040998
28705 Amp	19	21065501	21066527	0	0.039658
28706 Amp	19	21066623	21088416	0	0.041103
28707 Amp	19	21089282	21116938	0	0.036828
28708 Amp	19	21120340	21120969	0	0.033031
28709 Amp	19	21123710	21135992	0	0.034058
28710 Amp	19	21136970	21352203	0	0.034559
28711 Amp	19	21357314	21362215	0	0.038272
28712 Amp	19	21364221	21404750	0	0.039835
28713 Amp	19	21404827	21413574	0	0.043524
28714 Amp	19	21414131	21451153	0	0.045912
28715 Amp	19	21452553	21456366	0	0.049923
28716 Amp	19	21458313	21521915	0	0.048479
28717 Amp	19	21522043	21540012	0	0.046379
28718 Amp	19	21543179	21565013	0	0.048152
28719 Amp	19	21584090	21612770	0	0.041287
28720 Amp	19	21613329	21615751	0	0.042463
28721 Amp	19	21619732	21771402	0	0.040075
28722 Amp	19	21773146	21781455	0	0.041176
28723 Amp	19	21781520	21803512	0	0.037367
28724 Amp	19	21806544	21814055	0	0.039655
28725 Amp	19	21819700	21898485	0	0.036294

28726 Amp	19	21900420	21906848	0	0.03441
28727 Amp	19	21907392	21932841	0	0.032426
28728 Amp	19	21933033	21938426	0	0.036615
28729 Amp	19	21938656	21955199	0	0.035514
28730 Amp	19	21957391	22005907	0	0.034678
28731 Amp	19	22009436	22059252	0	0.031622
28732 Amp	19	22059602	22059602	0	0.027434
28733 Amp	19	22318987	22338688	0	0.025633
28734 Amp	19	22339786	22345249	0	0.026489
28735 Amp	19	22449343	22531441	0	0.021186
28736 Amp	19	22531525	22626989	0	0.023025
28737 Amp	19	22628385	22637797	0	0.03018
28738 Amp	19	22639426	22748197	0	0.023025
28739 Amp	19	22748956	22883621	0	0.030398
28740 Amp	19	22885085	23017292	0	0.03844
28741 Amp	19	23017777	23062835	0	0.042268
28742 Amp	19	23063598	23274298	0	0.039034
28743 Amp	19	23278215	23331596	0	0.034503
28744 Amp	19	23333632	23538372	0	0.029817
28745 Amp	19	23540118	23577721	0	0.031928
28746 Amp	19	23582494	23664120	0	0.030535
28747 Amp	19	23675047	23817640	0	0.028423
28748 Amp	19	23818056	23831305	0	0.028955
28749 Amp	19	23834421	23868903	0	0.029438
28750 Amp	19	23870028	23870028	0	0.036154
28751 Amp	19	23870190	23870190	0	0.033867
28752 Amp	19	23872082	23872082	0	0.034378
28753 Amp	19	27749470	27749470	0	0.061253
28754 Amp	19	27749586	27749586	0	0.05754
28755 Amp	19	27749601	27749601	0	0.056454
28756 Amp	19	27752285	27758174	0	0.055479
28757 Amp	19	27765442	27765442	0	0.054303
28758 Amp	19	27780252	27784574	0	0.048264
28759 Amp	19	27786025	27787381	0	0.047407
28760 Amp	19	27790956	27797510	0	0.037016
28761 Amp	19	27798320	27845453	0	0.033602
28762 Amp	19	27849386	27956205	0	0.0322
28763 Amp	19	27958527	27974660	0	0.027957
28764 Amp	19	27974735	28091135	0	0.02842
28765 Amp	19	28093702	28124162	0	0.026791
28766 Amp	19	28125953	28137890	0	0.029049
28767 Amp	19	28139156	28156682	0	0.026913
28768 Amp	19	28158515	28211213	0	0.02947
28769 Amp	19	28213183	28322590	0	0.03855
28770 Amp	19	28323119	28356775	0	0.040107
28771 Amp	19	28357014	28369599	0	0.039423
28772 Amp	19	28372271	28404348	0	0.030342
28773 Amp	19	28407490	28438697	0	0.031391
28774 Amp	19	28439200	28443455	0	0.033892
28775 Amp	19	28446090	28455395	0	0.044318
28776 Amp	19	28459297	28599439	0	0.04125
28777 Amp	19	28599813	28639558	0	0.048105
28778 Amp	19	28644599	28679799	0	0.049712

28779	Amp	19	28682916	28733518	0	0.053575
28780	Amp	19	28736925	28794564	0	0.044043
28781	Amp	19	28794628	28818856	0	0.050276
28782	Amp	19	28824378	28861192	0	0.057588
28783	Amp	19	28861481	28919705	0	0.05865
28784	Amp	19	28923724	28923855	0	0.053036
28785	Amp	19	28925979	28958181	0	0.050562
28786	Amp	19	28962919	28974413	0	0.050107
28787	Amp	19	28979002	29022484	0	0.059453
28788	Amp	19	29024864	29091296	0	0.057103
28789	Amp	19	29091564	29164379	0	0.055496
28790	Amp	19	29169211	29267558	0	0.054434
28791	Amp	19	29267694	29271213	0	0.058235
28792	Amp	19	29271271	29276061	0	0.059018
28793	Amp	19	29279027	29319780	0	0.0625
28794	Amp	19	29321490	29374174	0	0.059669
28795	Amp	19	29374438	29385700	0	0.062807
28796	Amp	19	29385819	29389708	0.005328	0.06418
28797	Amp	19	29389800	29390024	0.065715	0.066689
28798	Amp	19	29395599	29437805	0.227787	0.073391
28799	Amp	19	29438154	29457118	0.100827	0.068263
28800	Amp	19	29465292	29489695	0.17597	0.071287
28801	Amp	19	29489902	29502949	0.138391	0.069913
28802	Amp	19	29503676	29523712	0.17597	0.071418
28803	Amp	19	29523902	29615852	0.14896	0.070197
28804	Amp	19	29621234	29640547	0.089561	0.067749
28805	Amp	19	29640655	29721196	0.118001	0.068869
28806	Amp	19	29721240	29765242	0.040093	0.065731
28807	Amp	19	29765571	29783716	0.100827	0.06832
28808	Amp	19	29783757	29852257	0.08435	0.067537
28809	Amp	19	29853014	29867165	0.14896	0.070228
28810	Amp	19	29867930	29961154	0.073902	0.067204
28811	Amp	19	29962116	29967456	0.100827	0.068274
28812	Amp	19	29967578	30100073	0	0.063657
28813	Amp	19	30100338	30138513	0.024438	0.064959
28814	Amp	19	30138662	30140646	0	0.06207
28815	Amp	19	30144566	30327006	0.029031	0.065333
28816	Amp	19	30327575	30357232	0	0.063759
28817	Amp	19	30357630	30382205	0	0.059814
28818	Amp	19	30383994	30389932	0	0.056787
28819	Amp	19	30391628	30434391	0	0.045183
28820	Amp	19	30440291	30440291	0	0.051198
28821	Amp	19	30440346	30477430	0	0.050696
28822	Amp	19	30477464	30535800	0	0.049192
28823	Amp	19	30537759	30552899	0	0.043177
28824	Amp	19	30555649	30636871	0	0.04412
28825	Amp	19	30643838	30785698	0	0.045477
28826	Amp	19	30786304	30852048	0	0.048043
28827	Amp	19	30852358	31120604	0	0.04998
28828	Amp	19	31120968	31193931	0	0.051902
28829	Amp	19	31196005	31275591	0	0.050959
28830	Amp	19	31275873	31511765	0	0.052766
28831	Amp	19	31512762	31595642	0	0.051403

28832	Amp	19	31596640	31607771	0	0.049509
28833	Amp	19	31612150	31678826	0	0.050384
28834	Amp	19	31679492	31768217	0	0.052737
28835	Amp	19	31769030	31884241	0	0.053961
28836	Amp	19	31887537	31897570	0	0.05049
28837	Amp	19	31897669	31968416	0	0.049267
28838	Amp	19	31972198	31974785	0	0.043194
28839	Amp	19	31978780	32060015	0	0.044419
28840	Amp	19	32061359	32081782	0	0.03051
28841	Amp	19	32082919	32130160	0	0.029636
28842	Amp	19	32130356	32146822	0	0.025997
28843	Amp	19	32149054	32170850	0	0.020531
28844	Amp	19	32174120	32200602	0	0.018968
28845	Amp	19	32201425	32211799	0	0.023908
28846	Amp	19	32213410	32373715	0	0.024711
28847	Amp	19	32375342	32443849	0	0.024248
28848	Amp	19	32447023	32541220	0	0.038036
28849	Amp	19	32542511	32574359	0	0.039681
28850	Amp	19	32575654	32605581	0	0.042378
28851	Amp	19	32607898	32663354	0	0.041021
28852	Amp	19	32663405	32687026	0	0.040231
28853	Amp	19	32689906	32725923	0	0.04253
28854	Amp	19	32730420	32771330	0	0.047955
28855	Amp	19	32771678	32919482	0	0.047443
28856	Amp	19	32919951	32951074	0	0.047909
28857	Amp	19	32953550	32991942	0	0.049725
28858	Amp	19	32997211	33190481	0	0.050662
28859	Amp	19	33190564	33192682	0	0.051815
28860	Amp	19	33197351	33214804	0	0.049999
28861	Amp	19	33217996	33378368	0	0.052898
28862	Amp	19	33379418	33379581	0	0.054103
28863	Amp	19	33384941	33422108	0	0.040315
28864	Amp	19	33422171	33450598	0	0.033856
28865	Amp	19	33450856	33491605	0	0.037769
28866	Amp	19	33492632	33674868	0	0.036564
28867	Amp	19	33676999	33686681	0	0.029343
28868	Amp	19	33686698	33716674	0	0.030471
28869	Amp	19	33719846	33728616	0	0.028172
28870	Amp	19	33730122	33899474	0	0.030094
28871	Amp	19	33899921	34001295	0	0.028172
28872	Amp	19	34001945	34002206	0	0.027019
28873	Amp	19	34004552	34064037	0	0.018221
28874	Amp	19	34065392	34100755	0	0.019568
28875	Amp	19	34100850	34153081	0	0.017334
28876	Amp	19	34155707	34239994	0	0.018604
28877	Amp	19	34246231	34246231	0	0.018078
28878	Amp	19	34246318	34361976	0	0.01878
28879	Amp	19	34367669	34450425	0	0.023612
28880	Amp	19	34453051	34467936	0	0.024316
28881	Amp	19	34467971	34517639	0	0.023188
28882	Amp	19	34527054	34533278	0	0.025565
28883	Amp	19	34536727	34562900	0	0.027173
28884	Amp	19	34566303	34581475	0	0.028741

28885	Amp	19	34583160	34605403	0	0.028038
28886	Amp	19	34612403	34751951	0	0.028988
28887	Amp	19	34752195	34757228	0	0.032357
28888	Amp	19	34758177	34842266	0	0.032849
28889	Amp	19	34842272	35003930	0	0.031281
28890	Amp	19	35006722	35043228	0	0.038826
28891	Amp	19	35043604	35063231	0	0.038334
28892	Amp	19	35063847	35105077	0	0.030788
28893	Amp	19	35113969	35118051	0	0.040652
28894	Amp	19	35121382	35159690	0	0.037283
28895	Amp	19	35161345	35195993	0	0.03817
28896	Amp	19	35202761	35221091	0	0.031219
28897	Amp	19	35221169	35259766	0	0.030753
28898	Amp	19	35259845	35288644	0	0.034768
28899	Amp	19	35294260	35327994	0	0.026555
28900	Amp	19	35329348	35518619	0	0.02813
28901	Amp	19	35519356	35678978	0	0.033217
28902	Amp	19	35683092	35683092	0	0.02813
28903	Amp	19	35686929	35773264	0	0.030857
28904	Amp	19	35777146	35896979	0	0.02997
28905	Amp	19	35897140	35982217	0	0.030736
28906	Amp	19	35986208	36047904	0	0.031187
28907	Amp	19	36051707	36262089	0	0.028459
28908	Amp	19	36265950	36265950	0	0.031828
28909	Amp	19	36356789	36356789	0	0.042242
28910	Amp	19	36356859	36390003	0	0.039902
28911	Amp	19	36390244	36412662	0	0.042697
28912	Amp	19	36415170	36451179	0	0.048451
28913	Amp	19	36457038	36469735	0	0.045656
28914	Amp	19	36472277	36497320	0	0.046493
28915	Amp	19	36509261	36509261	0	0.046041
28916	Amp	19	36510004	36510004	0	0.041532
28917	Amp	19	36514729	36609908	0	0.038163
28918	Amp	19	36622362	36632940	0	0.042441
28919	Amp	19	36633720	36633720	0	0.036687
28920	Amp	19	36634221	36654734	0	0.035311
28921	Amp	19	36671146	36756570	0	0.035229
28922	Amp	19	36758764	36758764	0	0.03756
28923	Amp	19	36764224	36794468	0	0.030298
28924	Amp	19	36807798	36827990	0	0.027214
28925	Amp	19	36829396	36843696	0	0.024837
28926	Amp	19	36847819	36848064	0	0.031191
28927	Amp	19	36862326	36870458	0	0.02985
28928	Amp	19	36873800	36932010	0	0.040939
28929	Amp	19	36932716	36950525	0	0.040333
28930	Amp	19	36952723	36967811	0	0.043397
28931	Amp	19	36970948	36979604	0	0.045149
28932	Amp	19	36981309	36990251	0	0.047813
28933	Amp	19	37003206	37048460	0	0.058067
28934	Amp	19	37050713	37070185	0	0.054198
28935	Amp	19	37089513	37244820	0	0.051135
28936	Amp	19	37244893	37263518	0	0.049005
28937	Amp	19	37264829	37264829	0	0.052036

28938	Amp	19	37303948	37303948	0	0.049888
28939	Amp	19	37303972	37355493	0	0.053757
28940	Amp	19	37361604	37382237	0	0.053088
28941	Amp	19	37384729	37387954	0.005328	0.064273
28942	Amp	19	37389847	37402398	0	0.057918
28943	Amp	19	37403973	37431893	0.233782	0.073598
28944	Amp	19	37432580	37444717	0.089561	0.067758
28945	Amp	19	37455923	37455923	0.14896	0.070196
28946	Amp	19	37457966	37526680	0.204831	0.072408
28947	Amp	19	37536888	37554129	0.355319	0.078248
28948	Amp	19	37555555	37780978	0.287738	0.075837
28949	Amp	19	37791518	37835294	0.457501	0.081752
28950	Amp	19	37844244	37880984	0.487495	0.08275
28951	Amp	19	37882838	37893316	0.667587	0.088394
28952	Amp	19	37893781	37900832	0.636464	0.087558
28953	Amp	19	37902572	37982655	0.72817	0.090195
28954	Amp	19	37983289	38040145	0.609133	0.086737
28955	Amp	19	38044368	38123072	0.536238	0.084405
28956	Amp	19	38129102	38157431	0.463534	0.081967
28957	Amp	19	38160800	38174235	0.609133	0.086719
28958	Amp	19	38176635	38185231	0.543344	0.084506
28959	Amp	19	38186704	38214502	0.431198	0.080981
28960	Amp	19	38216349	38230683	0.282828	0.07568
28961	Amp	19	38231413	38237317	0.259438	0.07473
28962	Amp	19	38241253	38262230	0.17597	0.071227
28963	Amp	19	38262985	38283781	0.210594	0.072732
28964	Amp	19	38289133	38318644	0.17597	0.071385
28965	Amp	19	38325898	38326637	0.089561	0.067824
28966	Amp	19	38326846	38359790	0.17597	0.071415
28967	Amp	19	38363459	38382219	0	0.059867
28968	Amp	19	38400218	38413090	0	0.055736
28969	Amp	19	38413444	38449635	0	0.05338
28970	Amp	19	38450959	38470925	0	0.054967
28971	Amp	19	38484339	38588311	0	0.041309
28972	Amp	19	38592259	38634237	0	0.036733
28973	Amp	19	38638766	38806984	0	0.038506
28974	Amp	19	38808543	38867726	0	0.035234
28975	Amp	19	38873223	38897323	0	0.04102
28976	Amp	19	38898068	38925769	0	0.044179
28977	Amp	19	38930748	38984962	0	0.040062
28978	Amp	19	38986163	38986163	0	0.040811
28979	Amp	19	38991476	39134150	0	0.042908
28980	Amp	19	39140011	39141240	0	0.045737
28981	Amp	19	39142173	39183394	0	0.044233
28982	Amp	19	39183502	39258399	0	0.043737
28983	Amp	19	39261137	39293422	0	0.051649
28984	Amp	19	39297802	39352962	0	0.053641
28985	Amp	19	39359388	39359566	0	0.0551
28986	Amp	19	39359841	39359841	0	0.062952
28987	Amp	19	39365606	39389023	0.08435	0.067656
28988	Amp	19	39395537	39398897	0	0.059803
28989	Amp	19	39401630	39430839	0	0.057707
28990	Amp	19	39440248	39440419	0	0.056622

28991 Amp	19	39449351	39475112	0	0.044127
28992 Amp	19	39475850	39523303	0	0.041181
28993 Amp	19	39533655	39583318	0	0.038283
28994 Amp	19	39583364	39701904	0	0.037005
28995 Amp	19	39705542	39725584	0	0.035546
28996 Amp	19	39726141	39758520	0	0.037727
28997 Amp	19	39759153	39774098	0	0.043931
28998 Amp	19	39775069	39802307	0	0.048765
28999 Amp	19	39804313	39845170	0	0.058907
29000 Amp	19	39847309	39867902	0	0.058376
29001 Amp	19	39868113	40028730	0	0.056383
29002 Amp	19	40031894	40051794	0	0.043967
29003 Amp	19	40085552	40085552	0	0.046558
29004 Amp	19	40090276	40105246	0	0.041855
29005 Amp	19	40110690	40132758	0	0.042922
29006 Amp	19	40144269	40205426	0	0.045673
29007 Amp	19	40207774	40304047	0	0.042922
29008 Amp	19	40305858	40357584	0	0.040331
29009 Amp	19	40357935	40448756	0	0.033658
29010 Amp	19	40449203	40463296	0	0.034614
29011 Amp	19	40468380	40481169	0	0.033529
29012 Amp	19	40485856	40498270	0	0.023387
29013 Amp	19	40499923	40546951	0	0.015945
29014 Amp	19	40550469	40646985	0	0.02194
29015 Amp	19	40653288	40653288	0	0.024869
29016 Amp	19	40654306	40734407	0	0.023282
29017 Amp	19	40739117	40757440	0	0.020353
29018 Amp	19	40760168	40798151	0	0.01926
29019 Amp	19	40798912	41011157	0	0.024344
29020 Amp	19	41011174	41068218	0	0.022737
29021 Amp	19	41073187	41161368	0	0.021934
29022 Amp	19	41164057	41209588	0	0.023009
29023 Amp	19	41211810	41221378	0	0.010123
29024 Amp	19	41224347	41346523	0	0.009047
29025 Amp	19	41346711	41363884	0	0.012147
29026 Amp	19	41366587	41383355	0	0.014959
29027 Amp	19	41387513	41388773	0	0.017807
29028 Amp	19	41398491	41413095	0	0.028551
29029 Amp	19	41414860	41433049	0	0.025739
29030 Amp	19	41435045	41531160	0	0.03402
29031 Amp	19	41531609	41609513	0	0.032933
29032 Amp	19	41613356	41649532	0	0.026262
29033 Amp	19	41663593	41724525	0	0.028324
29034 Amp	19	41731551	41741951	0	0.029718
29035 Amp	19	41742569	41901753	0	0.028324
29036 Amp	19	41904287	42015792	0	0.021428
29037 Amp	19	42022606	42056794	0	0.031742
29038 Amp	19	42063793	42063793	0	0.033632
29039 Amp	19	42064555	42130346	0	0.019244
29040 Amp	19	42131549	42131549	0	0.016397
29041 Amp	19	42140854	42181145	0	0.015441
29042 Amp	19	42181568	42416672	0	0.011387
29043 Amp	19	42418355	42545053	0	0.025868

29044	Amp	19	42545730	42628269	0	0.028157
29045	Amp	19	42632607	42635369	0	0.013677
29046	Amp	19	42636171	42683328	0	0.014213
29047	Amp	19	42683902	42697998	0	0.011924
29048	Amp	19	42699838	42707130	0	0.014721
29049	Amp	19	42707438	42712819	0	0.01539
29050	Amp	19	43458545	43510305	0	0.03067
29051	Amp	19	43514023	43541757	0	0.028409
29052	Amp	19	43544465	43551873	0	0.029791
29053	Amp	19	43553820	43564041	0	0.032024
29054	Amp	19	43567256	43570060	0	0.029254
29055	Amp	19	43571484	43743417	0	0.030204
29056	Amp	19	43743477	43827613	0	0.028947
29057	Amp	19	43828168	43975608	0	0.029668
29058	Amp	19	43978555	44383241	0	0.029132
29059	Amp	19	44383363	44505393	0	0.028195
29060	Amp	19	44505638	44596020	0	0.027473
29061	Amp	19	44601651	44613140	0	0.031895
29062	Amp	19	44615845	44615856	0	0.032999
29063	Amp	19	44623056	44867581	0	0.028578
29064	Amp	19	44869072	45114813	0	0.030156
29065	Amp	19	45115543	45136295	0	0.028578
29066	Amp	19	45148637	45259495	0	0.030364
29067	Amp	19	45262161	45262443	0	0.032448
29068	Amp	19	45264681	45479756	0	0.035814
29069	Amp	19	45482900	45490876	0	0.03773
29070	Amp	19	45502078	45556123	0	0.035959
29071	Amp	19	45561496	45651005	0	0.038296
29072	Amp	19	45651474	45799510	0	0.037345
29073	Amp	19	45801187	45804148	0	0.03942
29074	Amp	19	45810015	45879496	0	0.041743
29075	Amp	19	45898314	45898918	0	0.038565
29076	Amp	19	45900676	45938733	0	0.040884
29077	Amp	19	45943744	46010823	0	0.026719
29078	Amp	19	46011539	46034545	0	0.025337
29079	Amp	19	46035067	46037502	0	0.026384
29080	Amp	19	46043490	46159432	0	0.030579
29081	Amp	19	46159745	46236643	0	0.02826
29082	Amp	19	46237569	46257769	0	0.02874
29083	Amp	19	46261764	46279320	0	0.024545
29084	Amp	19	46279349	46309776	0	0.030421
29085	Amp	19	46316519	46476153	0	0.028084
29086	Amp	19	46477636	46550753	0	0.025344
29087	Amp	19	46563226	46626742	0	0.029147
29088	Amp	19	46627567	46683196	0	0.027361
29089	Amp	19	46684238	46740681	0	0.029161
29090	Amp	19	46741055	46837385	0	0.028114
29091	Amp	19	46840714	46918066	0	0.018011
29092	Amp	19	46918368	47035223	0	0.015434
29093	Amp	19	47038898	47076096	0	0.017548
29094	Amp	19	47079987	47180617	0	0.019678
29095	Amp	19	47180881	47313358	0	0.021415
29096	Amp	19	47313847	47661512	0	0.020771

29097 Amp	19	47668261	47748790	0	0.020291
29098 Amp	19	47751135	47821465	0	0.02303
29099 Amp	19	47823121	47881800	0	0.021157
29100 Amp	19	47883100	48088471	0	0.019304
29101 Amp	19	48088926	48124005	0	0.021406
29102 Amp	19	48128001	48161123	0	0.022852
29103 Amp	19	48162861	48177727	0	0.033425
29104 Amp	19	48185124	48217726	0	0.035483
29105 Amp	19	48217808	48289665	0	0.038334
29106 Amp	19	48294560	48332951	0	0.045744
29107 Amp	19	48337928	48519569	0	0.047513
29108 Amp	19	48520353	48537814	0	0.062184
29109 Amp	19	48538662	48572202	0	0.062697
29110 Amp	19	48572658	48572658	0.005328	0.06409
29111 Amp	19	48580236	48580939	0	0.062321
29112 Amp	19	48582438	48621546	0	0.063243
29113 Amp	19	48625353	48792339	0	0.05267
29114 Amp	19	48804204	48814982	0	0.051804
29115 Amp	19	48817584	48823935	0	0.053431
29116 Amp	19	48833980	48843103	0	0.056245
29117 Amp	19	48851265	48868987	0.193543	0.071952
29118 Amp	19	48870224	48889291	0.204831	0.072458
29119 Amp	19	48889473	48949205	0.386426	0.079275
29120 Amp	19	48951692	48958048	0.342649	0.077883
29121 Amp	19	48959720	48959874	0	0.058646
29122 Amp	19	48973910	48977742	0.164935	0.070868
29123 Amp	19	48985591	49035529	0.34892	0.077997
29124 Amp	19	49060867	49060867	0.500841	0.083338
29125 Amp	19	49067856	49068812	0.543344	0.084541
29126 Amp	19	49069290	49118098	0.622604	0.08722
29127 Amp	19	49121886	49123939	0.933745	0.095632
29128 Amp	19	49137037	49141336	0.667587	0.088503
29129 Amp	19	49145794	49163997	0.636464	0.08758
29130 Amp	19	49165499	49183346	0.255918	0.074691
29131 Amp	19	49185666	49185666	0.276783	0.075386
29132 Amp	19	49191132	49195256	0.1237	0.069147
29133 Amp	19	49195756	49230641	0.17597	0.071428
29134 Amp	19	49231805	49261491	0.457501	0.081773
29135 Amp	19	49268028	49268028	0.392611	0.079492
29136 Amp	19	49270742	49326568	0.529451	0.084228
29137 Amp	19	49335918	49348514	0.689884	0.089082
29138 Amp	19	49358063	49371962	0.735486	0.090463
29139 Amp	19	49372266	49372266	1.188121	0.101627
29140 Amp	19	49377132	49419739	1.348715	0.105407
29141 Amp	19	49436874	49440521	1.445847	0.107507
29142 Amp	19	49451759	49456107	1.512549	0.109064
29143 Amp	19	49457880	49457880	1.398396	0.106566
29144 Amp	19	49467046	49509788	2.158817	0.122431
29145 Amp	19	49526852	49563471	2.26699	0.124632
29146 Amp	19	49565435	49576993	2.356694	0.126172
29147 Amp	19	49577150	49613373	2.278531	0.124726
29148 Amp	19	49613779	49613844	2.060912	0.120428
29149 Amp	19	49628608	49685266	2.147988	0.122103

29150	Amp	19	49694149	49694858	2.060912	0.120425
29151	Amp	19	49699639	49719657	2.368227	0.126562
29152	Amp	19	49720562	49728398	1.940776	0.118069
29153	Amp	19	49728408	49756258	1.876242	0.116688
29154	Amp	19	49758827	49769443	2.005947	0.119338
29155	Amp	19	49774303	49781159	1.780186	0.114736
29156	Amp	19	49783736	49794202	1.593878	0.110884
29157	Amp	19	49796434	49797302	1.940776	0.117921
29158	Amp	19	49797418	49852402	1.717671	0.113373
29159	Amp	19	49856551	49856551	1.634701	0.111816
29160	Amp	19	49866809	49867421	1.542638	0.109703
29161	Amp	19	49871239	49874886	1.369395	0.105922
29162	Amp	19	50090736	50090736	1.188121	0.101743
29163	Amp	19	50135241	50135241	1.378521	0.106085
29164	Amp	19	50135318	50223062	1.655056	0.112109
29165	Amp	19	50224098	50319151	1.416817	0.107023
29166	Amp	19	50333798	50336657	0.933745	0.095577
29167	Amp	19	50338558	50356096	0.906385	0.095063
29168	Amp	19	50358476	50437426	0.790614	0.091927
29169	Amp	19	50438550	50457848	0.689884	0.089076
29170	Amp	19	50465559	50468051	0.47716	0.08237
29171	Amp	19	50469236	50555831	0.522881	0.083822
29172	Amp	19	50564172	50633484	0.437198	0.081143
29173	Amp	19	50633552	50642022	0.47716	0.082382
29174	Amp	19	50643541	50751116	0.543344	0.084595
29175	Amp	19	50752054	50779857	0.583752	0.085922
29176	Amp	19	50789795	50835951	0.494792	0.083108
29177	Amp	19	50910072	50922148	0.437198	0.081059
29178	Amp	19	50922619	50958056	0.765474	0.09126
29179	Amp	19	50959037	50988208	1.00505	0.097343
29180	Amp	19	50988504	50994388	0.831947	0.093043
29181	Amp	19	50997078	51022832	0.882843	0.094287
29182	Amp	19	51032701	51043385	0.79909	0.092074
29183	Amp	19	51045643	51190862	0.899693	0.094752
29184	Amp	19	51191332	51191332	1.00505	0.097435
29185	Amp	19	51193143	51233429	1.032525	0.098017
29186	Amp	19	51237068	51254199	1.149583	0.100937
29187	Amp	19	51254854	51269933	1.33853	0.10522
29188	Amp	19	51275476	51275698	1.131695	0.100495
29189	Amp	19	51277180	51277180	1.084294	0.099257
29190	Amp	19	51279200	51377066	0.959607	0.096336
29191	Amp	19	51377087	51379409	0.906385	0.095008
29192	Amp	19	51386932	51402739	0.941704	0.095777
29193	Amp	19	51403535	51414481	1.075952	0.099121
29194	Amp	19	51420109	51437983	1.159209	0.101156
29195	Amp	19	51439649	51450840	1.261401	0.103575
29196	Amp	19	51458781	51507163	1.593878	0.110945
29197	Amp	19	51507790	51546553	1.398396	0.106603
29198	Amp	19	51546646	51575676	1.634701	0.111761
29199	Amp	19	51575917	51605429	1.604336	0.110993
29200	Amp	19	51605677	51687306	1.645511	0.11199
29201	Amp	19	51692221	51754420	1.769915	0.114648
29202	Amp	19	51756318	51766834	1.940776	0.117961

29203 Amp	19	51767765	51767765	1.812192	0.115303
29204 Amp	19	52118391	52118391	2.137445	0.121831
29205 Amp	19	52121620	52125157	2.290039	0.125065
29206 Amp	19	52125358	52132078	2.502083	0.129169
29207 Amp	19	52133379	52135546	2.661431	0.13215
29208 Amp	19	52135581	52179313	2.368227	0.126467
29209 Amp	19	52182309	52189043	2.094104	0.120951
29210 Amp	19	52192727	52195617	2.137445	0.121966
29211 Amp	19	52198815	52256529	2.256542	0.124215
29212 Amp	19	52257778	52342211	2.192012	0.122971
29213 Amp	19	52344735	52355645	2.322689	0.125623
29214 Amp	19	52356667	52366799	1.897704	0.117241
29215 Amp	19	52469597	52473190	2.104978	0.121328
29216 Amp	19	52473427	52473521	2.027585	0.119691
29217 Amp	19	52475774	52537854	2.137445	0.121833
29218 Amp	19	52538742	52600839	2.213223	0.123498
29219 Amp	19	52605953	52618204	2.413643	0.12728
29220 Amp	19	52620336	52622219	2.038694	0.119985
29221 Amp	19	52624218	52624218	1.823153	0.115702
29222 Amp	19	52630781	52643509	1.876242	0.116678
29223 Amp	19	52644517	52695354	1.769915	0.114536
29224 Amp	19	52696373	52699649	1.675319	0.112553
29225 Amp	19	52702132	52702132	1.655056	0.112165
29226 Amp	19	52982662	52997053	1.624655	0.11161
29227 Amp	19	52997213	53005259	1.512549	0.109069
29228 Amp	19	53006207	53084117	1.908279	0.117267
29229 Amp	19	53084716	53085842	1.61378	0.111305
29230 Amp	19	53092614	53138328	1.823153	0.115594
29231 Amp	19	53166633	53176696	1.972776	0.118731
29232 Amp	19	53214367	53214367	1.790459	0.11496
29233 Amp	19	53215997	53217300	1.68612	0.112871
29234 Amp	19	53218048	53225122	1.769915	0.114648
29235 Amp	19	53247232	53259284	2.016585	0.119531
29236 Amp	19	53259332	53278637	2.446878	0.128032
29237 Amp	19	53284155	53293075	2.537294	0.12969
29238 Amp	19	53293145	53322825	2.446878	0.127913
29239 Amp	19	53322920	53324467	2.245479	0.124034
29240 Amp	19	53324587	53343525	2.490318	0.128768
29241 Amp	19	53344575	53349830	2.278531	0.124789
29242 Amp	19	53350496	53381940	2.537294	0.129849
29243 Amp	19	53382518	53392626	2.765626	0.133982
29244 Amp	19	53393380	53404859	2.626052	0.131526
29245 Amp	19	53514004	53519155	2.696333	0.132813
29246 Amp	19	53519945	53520489	2.964305	0.137855
29247 Amp	19	53520763	53529474	2.917729	0.136879
29248 Amp	19	53529475	53847931	2.755154	0.133898
29249 Amp	19	53849342	53875419	2.860424	0.135919
29250 Amp	19	53877979	53877979	2.964305	0.137873
29251 Amp	19	53885091	53885122	3.045223	0.139403
29252 Amp	19	53887334	53901222	3.654159	0.150362
29253 Amp	19	53901924	53937328	3.985868	0.1563
29254 Amp	19	53946019	53967099	4.331178	0.161862
29255 Amp	19	53967481	53994447	4.795971	0.169008

29256	Amp	19	53995322	54007771	5.463735	0.179021
29257	Amp	19	54015056	54022266	5.184242	0.174942
29258	Amp	19	54022502	54023454	4.979649	0.171834
29259	Amp	19	54030066	54066169	4.654424	0.166775
29260	Amp	19	54066194	54081592	4.683031	0.16737
29261	Amp	19	54081626	54097903	4.809994	0.169367
29262	Amp	19	54099351	54154140	5.687617	0.182193
29263	Amp	19	54154264	54155119	5.836931	0.184501
29264	Amp	19	54159784	54177886	5.125419	0.173998
29265	Amp	19	54178560	54182441	4.725297	0.167999
29266	Amp	19	54182540	54183205	4.626369	0.16646
29267	Amp	19	54193859	54214221	4.513276	0.164601
29268	Amp	19	54214239	54214239	4.331178	0.161923
29269	Amp	19	54215069	54215069	4.209693	0.159823
29270	Amp	19	54338128	54339958	3.349067	0.145017
29271	Amp	19	54340282	54362228	3.628913	0.150029
29272	Amp	19	54365045	54379018	3.749777	0.152026
29273	Amp	19	54380571	54390781	3.09333	0.140135
29274	Amp	19	54434666	54454023	3.590086	0.149332
29275	Amp	19	54454681	54476063	3.492824	0.147549
29276	Amp	19	54476886	54478884	4.010586	0.156645
29277	Amp	19	54481677	54507512	3.908795	0.154987
29278	Amp	19	54508081	54508081	4.116393	0.158422
29279	Amp	19	54515214	54520318	5.140232	0.174217
29280	Amp	19	54520336	54556551	4.612121	0.166148
29281	Amp	19	54558506	54641337	4.795971	0.169132
29282	Amp	19	54645888	54664840	5.008935	0.172292
29283	Amp	19	54670475	54697282	4.795971	0.169132
29284	Amp	19	54701426	54701727	4.331178	0.161983
29285	Amp	19	54702044	54703211	4.010586	0.156759
29286	Amp	19	54703251	54703854	4.102728	0.158191
29287	Amp	19	54713574	54715554	4.359416	0.162285
29288	Amp	19	54716380	54721301	4.809994	0.16917
29289	Amp	19	54871595	54905427	4.91152	0.170764
29290	Amp	19	54908679	54917123	5.463735	0.178947
29291	Amp	19	54917890	54924649	5.256006	0.176015
29292	Amp	19	54924934	54947448	4.710978	0.167832
29293	Amp	19	54947788	55013218	4.966536	0.171685
29294	Amp	19	55013384	55017474	5.125419	0.174055
29295	Amp	19	55018667	55037257	5.300783	0.176724
29296	Amp	19	55038018	55044464	5.419518	0.178484
29297	Amp	19	55054266	55066290	5.478024	0.179226
29298	Amp	19	55066305	55070143	5.806986	0.183999
29299	Amp	19	55070205	55079303	6.152783	0.189052
29300	Amp	19	55089368	55182643	6.33531	0.191654
29301	Amp	19	55189383	55189678	6.1074	0.188432
29302	Amp	19	55197107	55261085	6.458214	0.193385
29303	Amp	19	55262222	55296246	6.641348	0.196034
29304	Amp	19	55296263	55307406	6.411912	0.192761
29305	Amp	19	55313218	55349789	7.011899	0.201126
29306	Amp	19	55350268	55422178	6.810251	0.198262
29307	Amp	19	55423711	55425459	6.917796	0.199983
29308	Amp	19	55428418	55428418	7.326935	0.205451

29309	Amp	19	55428730	55472554	7.594603	0.209195
29310	Amp	19	55475270	55493064	7.027404	0.201319
29311	Amp	19	55496104	55514027	6.458214	0.193258
29312	Amp	19	55520796	55523708	6.182794	0.189515
29313	Amp	19	55525986	55533293	6.152783	0.189042
29314	Amp	19	55534101	55539477	6.350097	0.191777
29315	Amp	19	55551818	55561033	5.957455	0.186276
29316	Amp	19	55562094	55628996	6.53507	0.194546
29317	Amp	19	55632485	55639105	6.700554	0.196739
29318	Amp	19	55639133	55639133	6.732306	0.197194
29319	Amp	19	55640058	55655747	6.980586	0.200821
29320	Amp	19	55656614	55732301	7.152075	0.203085
29321	Amp	19	55737455	55744448	7.452952	0.207367
29322	Amp	19	55784873	55808050	6.732306	0.197239
29323	Amp	19	55808101	55819271	6.473343	0.193588
29324	Amp	19	55819627	55820038	6.917796	0.199821
29325	Amp	19	55820569	55900391	6.949342	0.200271
29326	Amp	19	55901361	55930080	7.106137	0.202547
29327	Amp	19	56013676	56023158	6.519288	0.19417
29328	Amp	19	56023300	56141223	6.610791	0.195442
29329	Amp	19	56142627	56168391	6.503622	0.193912
29330	Amp	19	56173153	56241947	6.291248	0.19089
29331	Amp	19	56243350	56256875	6.032925	0.187195
29332	Amp	19	56260073	56310987	5.896595	0.185271
29333	Amp	19	56311288	56329560	6.36593	0.192132
29334	Amp	19	56330177	56331093	6.2443	0.190372
29335	Amp	19	56333801	56333919	5.777283	0.183511
29336	Amp	19	56334187	56371260	6.411912	0.19282
29337	Amp	19	56376038	56386955	5.882356	0.185039
29338	Amp	19	56386973	56401667	5.717279	0.182762
29339	Amp	19	56405777	56414031	5.641615	0.181632
29340	Amp	19	56415548	56433732	5.212889	0.175399
29341	Amp	19	56437718	56437752	5.038261	0.172681
29342	Amp	19	56438315	56438598	5.212889	0.175244
29343	Amp	19	56438924	56439640	5.566851	0.180658
29344	Amp	19	56455429	56493382	5.702358	0.182411
29345	Amp	19	56494137	56494353	5.972041	0.186379
29346	Amp	19	56496622	56557711	5.852205	0.184752
29347	Amp	19	56558191	56558191	5.987065	0.186632
29348	Amp	19	56559317	56625670	4.91152	0.170696
29349	Amp	19	56631413	56651471	5.507674	0.17971
29350	Amp	19	56651772	56668317	5.300783	0.176725
29351	Amp	19	56668812	56690354	5.140232	0.174237
29352	Amp	19	56705460	56705460	5.732156	0.182959
29353	Amp	19	56706334	56800696	6.291248	0.190938
29354	Amp	19	56804628	56831125	6.152783	0.188984
29355	Amp	19	56831696	56871395	5.972041	0.186421
29356	Amp	19	56874691	56884477	5.86703	0.184989
29357	Amp	19	56886142	56919549	5.987065	0.186618
29358	Amp	19	56919767	56920463	5.762293	0.183312
29359	Amp	19	56922488	57003859	5.140232	0.174298
29360	Amp	19	57004702	57004702	5.285768	0.176472
29361	Amp	19	57006842	57012589	5.360902	0.177557

29362	Amp	19	57019563	57049654	5.792044	0.183817
29363	Amp	19	57050573	57055428	6.032925	0.18725
29364	Amp	19	57056605	57079489	6.549814	0.194695
29365	Amp	19	57079670	57088418	6.319529	0.19131
29366	Amp	19	57089823	57122284	5.792044	0.183865
29367	Amp	19	57122795	57131731	5.656875	0.181919
29368	Amp	19	57136792	57168248	5.39004	0.177846
29369	Amp	19	57172676	57185017	5.184242	0.174883
29370	Amp	19	57201137	57207496	5.732156	0.182937
29371	Amp	19	57213253	57223480	6.1074	0.188447
29372	Amp	19	57224448	57225759	7.011899	0.201125
29373	Amp	19	57226667	57243687	7.152075	0.203216
29374	Amp	19	57248079	57268099	6.549814	0.194636
29375	Amp	19	57268653	57289047	6.304987	0.191173
29376	Amp	19	57291691	57317528	6.04774	0.18753
29377	Amp	19	57318455	57318987	6.152783	0.189001
29378	Amp	19	57319128	57324544	6.33531	0.191554
29379	Amp	19	57326650	57326650	6.825391	0.198629
29380	Amp	19	57330625	57332552	7.011899	0.201263
29381	Amp	19	57336158	57361629	7.264196	0.204631
29382	Amp	19	57366165	57434452	6.840574	0.19883
29383	Amp	19	57437778	57441753	6.763246	0.197767
29384	Amp	19	57442213	57450244	6.641348	0.195963
29385	Amp	19	57451346	57455094	6.594741	0.195359
29386	Amp	19	57456329	57458786	7.042879	0.201551
29387	Amp	19	57458825	57538896	6.917796	0.199798
29388	Amp	19	57542760	57557293	6.840574	0.198784
29389	Amp	19	57557763	57560444	6.79429	0.198202
29390	Amp	19	57561172	57570500	6.871512	0.199292
29391	Amp	19	57570801	57570801	7.058587	0.201889
29392	Amp	19	57573562	57596849	6.641348	0.195942
29393	Amp	19	57601067	57608396	6.458214	0.193389
29394	Amp	19	57609841	57620208	6.53507	0.194356
29395	Amp	19	57647522	57647522	6.855677	0.198997
29396	Amp	19	57656083	57674132	7.042879	0.2017
29397	Amp	19	57674614	57698791	7.199931	0.203707
29398	Amp	19	57699121	57734808	6.855677	0.199045
29399	Amp	19	57738275	57768492	6.700554	0.196871
29400	Amp	19	57770441	57783618	6.825391	0.198519
29401	Amp	19	57840921	57847176	6.886863	0.199366
29402	Amp	19	57847211	57853532	6.732306	0.19736
29403	Amp	19	57857513	57884476	6.949342	0.200265
29404	Amp	19	57886303	57886303	6.763246	0.197617
29405	Amp	19	57887588	57890674	6.259932	0.190561
29406	Amp	19	57899088	57919256	6.564374	0.194877
29407	Amp	19	57921949	57944404	6.980586	0.200755
29408	Amp	19	57945948	57952706	7.437327	0.207087
29409	Amp	19	57954580	57954653	7.295403	0.204995
29410	Amp	19	57954945	57988055	7.357785	0.206
29411	Amp	19	57989309	58029574	7.152075	0.203109
29412	Amp	19	58030429	58030429	6.840574	0.198794
29413	Amp	19	58033742	58054375	6.996189	0.200949
29414	Amp	19	58061238	58076905	7.074542	0.202026

29415	Amp	19	58077482	58106196	6.747966	0.197495
29416	Amp	19	58107591	58141610	6.871512	0.199282
29417	Amp	19	58145736	58163638	6.810251	0.198277
29418	Amp	19	58164050	58171974	6.641348	0.195917
29419	Amp	19	58173152	58173152	6.519288	0.194322
29420	Amp	19	58173244	58175624	6.716522	0.197072
29421	Amp	19	58177063	58177063	7.264196	0.20458
29422	Amp	19	58191718	58191718	7.341956	0.205675
29423	Amp	19	58192137	58192137	7.833797	0.212383
29424	Amp	19	58192614	58214367	7.215959	0.203985
29425	Amp	19	58215050	58223917	6.763246	0.197653
29426	Amp	19	58229804	58240027	6.33531	0.191663
29427	Amp	19	58241829	58265102	5.717279	0.182712
29428	Amp	19	58265874	58265874	5.463735	0.179059
29429	Amp	19	58268723	58280610	5.582276	0.180795
29430	Amp	19	58280824	58288980	5.626868	0.181404
29431	Amp	19	58290418	58322560	5.507674	0.179601
29432	Amp	19	58328605	58370362	5.463735	0.179006
29433	Amp	20	475120	489160	0	0.050522
29434	Amp	20	493999	524027	0	0.044111
29435	Amp	20	524945	559857	0	0.041883
29436	Amp	20	560117	578519	0	0.040407
29437	Amp	20	583055	667787	0	0.039765
29438	Amp	20	668301	946105	0	0.0377
29439	Amp	20	946302	946814	0	0.038776
29440	Amp	20	951934	1213041	0	0.031059
29441	Amp	20	1214888	1360321	0	0.028825
29442	Amp	20	1361382	1405219	0	0.026739
29443	Amp	20	1405233	1428791	0	0.023071
29444	Amp	20	1429014	1507535	0	0.024158
29445	Amp	20	1508007	1635421	0	0.023325
29446	Amp	20	1635472	1673204	0	0.026087
29447	Amp	20	1673217	1734802	0	0.023509
29448	Amp	20	1736310	1759857	0	0.024284
29449	Amp	20	1766921	1784291	0	0.027439
29450	Amp	20	1785370	1790499	0	0.028012
29451	Amp	20	1790638	1800235	0	0.029609
29452	Amp	20	1801108	1811274	0	0.026453
29453	Amp	20	1814286	1876137	0	0.026923
29454	Amp	20	1876582	1881894	0	0.026148
29455	Amp	20	1882284	2033790	0	0.025072
29456	Amp	20	2039342	2144907	0	0.032825
29457	Amp	20	2145450	2165639	0	0.025072
29458	Amp	20	2169266	2192217	0	0.02634
29459	Amp	20	2196088	2212368	0	0.030081
29460	Amp	20	2215792	2320575	0	0.028993
29461	Amp	20	2322858	2398314	0	0.037045
29462	Amp	20	2398373	2458190	0	0.039754
29463	Amp	20	2460108	2468497	0	0.038329
29464	Amp	20	2469646	2474145	0	0.03311
29465	Amp	20	2477984	2520132	0	0.034495
29466	Amp	20	2520225	2551333	0	0.026444
29467	Amp	20	2557776	2663924	0	0.030668

29468	Amp	20	2664039	2700453	0	0.032023
29469	Amp	20	2700799	2713498	0	0.033727
29470	Amp	20	2718128	2729013	0	0.030965
29471	Amp	20	2731198	2820155	0	0.028257
29472	Amp	20	2820269	2860283	0	0.030966
29473	Amp	20	2862550	2879888	0	0.034579
29474	Amp	20	2880389	3024543	0	0.033194
29475	Amp	20	3024767	3075103	0	0.03149
29476	Amp	20	3081265	3095491	0	0.029533
29477	Amp	20	3100992	3123321	0	0.032093
29478	Amp	20	3127107	3199251	0	0.034899
29479	Amp	20	3204477	3344322	0	0.032093
29480	Amp	20	3348596	3381878	0	0.040145
29481	Amp	20	3385005	3393065	0	0.038877
29482	Amp	20	3393079	3393079	0	0.03722
29483	Amp	20	3402240	3449471	0	0.035019
29484	Amp	20	3452511	3574918	0	0.037323
29485	Amp	20	3579797	3704937	0	0.040449
29486	Amp	20	3705329	3782702	0	0.043256
29487	Amp	20	3791211	3805172	0	0.040131
29488	Amp	20	3810184	3815869	0	0.037323
29489	Amp	20	3819006	3822799	0	0.03995
29490	Amp	20	3823017	3823017	0	0.041732
29491	Amp	20	4123473	4127368	0	0.046819
29492	Amp	20	4129154	4254921	0	0.047713
29493	Amp	20	4254947	4308621	0	0.045153
29494	Amp	20	4317320	4328565	0	0.048856
29495	Amp	20	4328964	4329209	0	0.047713
29496	Amp	20	4331506	4376724	0	0.04401
29497	Amp	20	4379169	4490062	0	0.049408
29498	Amp	20	4490558	4706768	0	0.048938
29499	Amp	20	4708828	4708828	0	0.051891
29500	Amp	20	4709890	4721651	0	0.048724
29501	Amp	20	4721704	4731121	0	0.05335
29502	Amp	20	4732382	4750731	0	0.049448
29503	Amp	20	4750978	4784663	0	0.05149
29504	Amp	20	4791356	4814343	0	0.05505
29505	Amp	20	4815057	4907024	0	0.057815
29506	Amp	20	4907138	4983181	0	0.055357
29507	Amp	20	4984059	5005387	0	0.058052
29508	Amp	20	5007153	5025037	0	0.055748
29509	Amp	20	5026633	5040854	0	0.053053
29510	Amp	20	5041037	5136462	0	0.051699
29511	Amp	20	5138097	5183370	0	0.043868
29512	Amp	20	5188601	5205086	0	0.042183
29513	Amp	20	5209941	5248770	0	0.034131
29514	Amp	20	5256261	5259709	0	0.031502
29515	Amp	20	5259946	5300180	0	0.030929
29516	Amp	20	5303605	5373686	0	0.030035
29517	Amp	20	5377236	5417495	0	0.031753
29518	Amp	20	5417599	5417599	0	0.030035
29519	Amp	20	5737655	5769416	0	0.026081
29520	Amp	20	5774984	5818762	0	0.027305

29521	Amp	20	5822099	5888287	0	0.025708
29522	Amp	20	5892498	6068710	0	0.022483
29523	Amp	20	6068876	6086956	0	0.021969
29524	Amp	20	6089793	6219516	0	0.020525
29525	Amp	20	6226267	6243758	0	0.021721
29526	Amp	20	6244336	6336653	0	0.019012
29527	Amp	20	6337071	6421836	0	0.020686
29528	Amp	20	6422874	6738130	0	0.020201
29529	Amp	20	6738292	6884600	0	0.02184
29530	Amp	20	6888052	6920034	0	0.024957
29531	Amp	20	6922323	6955493	0	0.021397
29532	Amp	20	7136070	7177439	0	0.01828
29533	Amp	20	7178391	7193931	0	0.019187
29534	Amp	20	7196269	7219270	0	0.020404
29535	Amp	20	7226248	7253601	0	0.023072
29536	Amp	20	7254497	7312157	0	0.025486
29537	Amp	20	7312393	7399927	0	0.028399
29538	Amp	20	7400049	7503771	0	0.030006
29539	Amp	20	7503812	7516965	0	0.027338
29540	Amp	20	7517125	7528733	0	0.026121
29541	Amp	20	7529433	7535247	0	0.025541
29542	Amp	20	7535353	7540967	0	0.02454
29543	Amp	20	7544991	7603731	0	0.021482
29544	Amp	20	7606003	7682167	0	0.019808
29545	Amp	20	7686842	7751645	0	0.018169
29546	Amp	20	7751810	7780945	0	0.016969
29547	Amp	20	7781336	7821107	0	0.015211
29548	Amp	20	7824998	7831183	0	0.013429
29549	Amp	20	7841962	7883418	0	0.015197
29550	Amp	20	7886921	7960175	0	0.013429
29551	Amp	20	7961474	8104424	0	0.018079
29552	Amp	20	8104743	8104743	0	0.019107
29553	Amp	20	8605879	8605879	0	0.019213
29554	Amp	20	8606650	8619525	0	0.01165
29555	Amp	20	8619723	8631206	0	0.012618
29556	Amp	20	8631493	8667803	0	0.010895
29557	Amp	20	8668935	8785732	0	0.013597
29558	Amp	20	8785791	8794517	0	0.012629
29559	Amp	20	8794868	8832897	0	0.009927
29560	Amp	20	8833791	8841661	0	0.012655
29561	Amp	20	8841784	8842417	0	0.01379
29562	Amp	20	8843216	8909250	0	0.011062
29563	Amp	20	8910699	8994821	0	0.012524
29564	Amp	20	8995401	9063612	0	0.010423
29565	Amp	20	9063970	9156268	0	0.015866
29566	Amp	20	9158950	9290731	0	0.019577
29567	Amp	20	9291286	9297145	0	0.014134
29568	Amp	20	9297200	9498064	0	0.0156
29569	Amp	20	9500794	9501990	0	0.014601
29570	Amp	20	9508771	9515436	0	0.015468
29571	Amp	20	9515779	9570909	0	0.018201
29572	Amp	20	9572301	9694498	0	0.016219
29573	Amp	20	9695163	9716371	0	0.017683

29574 Amp	20	9721185	9827013	0	0.01495
29575 Amp	20	9827587	9834077	0	0.017522
29576 Amp	20	9834446	9928046	0	0.015095
29577 Amp	20	9928357	10042794	0	0.012523
29578 Amp	20	10044128	10104119	0	0.011495
29579 Amp	20	10104406	10104832	0	0.010228
29580 Amp	20	10106135	10123309	0	0.011901
29581 Amp	20	10127038	10307235	0	0.012955
29582 Amp	20	10309880	10312880	0	0.011901
29583 Amp	20	10313680	10377932	0	0.00819
29584 Amp	20	10378269	10380474	0	0.009545
29585 Amp	20	10388748	10428406	0	0.016933
29586 Amp	20	10429432	10612065	0	0.009545
29587 Amp	20	10617428	10754177	0	0.011258
29588 Amp	20	10756437	10846349	0	0.01282
29589 Amp	20	10847619	10918864	0	0.013436
29590 Amp	20	10922123	10975453	0	0.014619
29591 Amp	20	10976489	10997107	0	0.013436
29592 Amp	20	10999172	11113218	0	0.014559
29593 Amp	20	11124212	11164843	0	0.022545
29594 Amp	20	11165037	11413402	0	0.021422
29595 Amp	20	11413419	11520015	0	0.023548
29596 Amp	20	11520186	11520186	0	0.025245
29597 Amp	20	11520494	11542435	0	0.027605
29598 Amp	20	11542576	11593188	0	0.031489
29599 Amp	20	11601307	11707352	0	0.027605
29600 Amp	20	11708637	11778695	0	0.025932
29601 Amp	20	11780333	11908597	0	0.024235
29602 Amp	20	11914728	11953111	0	0.027192
29603 Amp	20	11956980	12006560	0	0.024235
29604 Amp	20	12006936	12140414	0	0.025125
29605 Amp	20	12145074	12159408	0	0.024235
29606 Amp	20	12159451	12170284	0	0.028231
29607 Amp	20	12174123	12284866	0	0.025871
29608 Amp	20	12287737	12314320	0	0.026398
29609 Amp	20	12315411	12574279	0	0.022403
29610 Amp	20	12575128	12600641	0	0.021875
29611 Amp	20	12600700	12660736	0	0.022667
29612 Amp	20	12668419	12779541	0	0.020954
29613 Amp	20	12780732	12793892	0	0.021789
29614 Amp	20	12800447	12818127	0	0.025187
29615 Amp	20	12911119	12911119	0	0.027074
29616 Amp	20	12912540	13125820	0	0.023676
29617 Amp	20	13125856	13184582	0	0.026601
29618 Amp	20	13184706	13206357	0	0.024521
29619 Amp	20	13208258	13232425	0	0.022634
29620 Amp	20	13235231	13349267	0	0.021072
29621 Amp	20	13351353	13420418	0	0.029475
29622 Amp	20	13422673	13451050	0	0.02721
29623 Amp	20	13451395	13550213	0	0.025431
29624 Amp	20	13552275	13795889	0	0.021665
29625 Amp	20	13797766	13808236	0	0.024243
29626 Amp	20	13812718	13950229	0	0.0137

29627	Amp	20	13956973	14099468	0	0.015637
29628	Amp	20	14104571	14127530	0	0.018982
29629	Amp	20	14130580	14156377	0	0.019522
29630	Amp	20	14159829	14167041	0	0.018299
29631	Amp	20	14167870	14191079	0	0.014826
29632	Amp	20	14191534	14214001	0	0.011481
29633	Amp	20	14221275	14234955	0	0.013245
29634	Amp	20	14235039	14243891	0	0.014883
29635	Amp	20	14247435	14279865	0	0.020301
29636	Amp	20	14280834	14282182	0	0.025473
29637	Amp	20	14284471	14314946	0	0.021742
29638	Amp	20	14317967	14326307	0	0.024386
29639	Amp	20	14332409	14338540	0	0.030773
29640	Amp	20	14338995	14378448	0	0.041138
29641	Amp	20	14385379	14408467	0	0.037269
29642	Amp	20	14410460	14436713	0	0.026904
29643	Amp	20	14473274	14473274	0	0.024795
29644	Amp	20	14474083	14576059	0	0.022858
29645	Amp	20	14580258	14628277	0	0.020816
29646	Amp	20	14628511	14628511	0	0.017973
29647	Amp	20	14629311	14705651	0	0.019839
29648	Amp	20	14705863	14711021	0	0.020618
29649	Amp	20	15182862	15182862	0	0.015803
29650	Amp	20	15184878	15186129	0	0.01668
29651	Amp	20	15188051	15212428	0	0.014994
29652	Amp	20	15217244	15318662	0	0.012323
29653	Amp	20	15318833	15327324	0	0.016081
29654	Amp	20	15341445	15424005	0	0.018487
29655	Amp	20	15424676	15446605	0	0.019194
29656	Amp	20	15447141	15466381	0	0.019736
29657	Amp	20	15466432	15513120	0	0.015978
29658	Amp	20	15513960	15574996	0	0.017875
29659	Amp	20	15575606	15578042	0	0.01916
29660	Amp	20	15579729	15599717	0	0.020912
29661	Amp	20	15602239	15629526	0	0.01916
29662	Amp	20	15629596	15663996	0	0.018453
29663	Amp	20	15664350	15692249	0	0.017911
29664	Amp	20	15700448	15710595	0	0.016014
29665	Amp	20	15711854	15713282	0	0.013888
29666	Amp	20	15713299	15743754	0	0.015204
29667	Amp	20	15748669	15790916	0	0.012798
29668	Amp	20	15791121	15821990	0	0.013973
29669	Amp	20	15822164	15894329	0	0.014935
29670	Amp	20	15894453	15905649	0	0.016675
29671	Amp	20	15905967	15917385	0	0.01539
29672	Amp	20	15918007	15918219	0	0.0163
29673	Amp	20	15918755	15931972	0	0.017418
29674	Amp	20	15933934	15935574	0	0.018836
29675	Amp	20	15936324	15958253	0	0.020305
29676	Amp	20	15962084	15971241	0	0.019513
29677	Amp	20	15972221	15979455	0	0.02009
29678	Amp	20	15979499	15988958	0	0.02236
29679	Amp	20	15989449	16035512	0	0.020494

29680	Amp	20	16035653	16055470	0	0.021259
29681	Amp	20	16055491	16082798	0	0.022419
29682	Amp	20	16083473	16149532	0	0.023528
29683	Amp	20	16155733	16179783	0	0.022352
29684	Amp	20	16181307	16188394	0	0.020714
29685	Amp	20	16189287	16191622	0	0.019297
29686	Amp	20	16193559	16206879	0	0.030166
29687	Amp	20	16206947	16257649	0	0.028696
29688	Amp	20	16264870	16305985	0	0.017828
29689	Amp	20	16306138	16308779	0	0.018767
29690	Amp	20	16309488	16360397	0	0.016497
29691	Amp	20	16364588	16403931	0	0.028753
29692	Amp	20	16404123	16489925	0	0.016497
29693	Amp	20	16490104	16555779	0	0.015718
29694	Amp	20	16556361	16575325	0	0.02175
29695	Amp	20	16577615	16577615	0	0.025132
29696	Amp	20	16578761	16584091	0	0.026358
29697	Amp	20	16584148	16606602	0	0.022975
29698	Amp	20	16606617	16653638	0	0.02175
29699	Amp	20	16654903	16736400	0	0.019118
29700	Amp	20	16742131	16748052	0	0.028278
29701	Amp	20	16748371	16823197	0	0.019118
29702	Amp	20	16823584	16832657	0	0.024925
29703	Amp	20	16833042	16846685	0	0.026365
29704	Amp	20	16846907	16921719	0	0.021639
29705	Amp	20	16922283	17021228	0	0.025634
29706	Amp	20	17021977	17149520	0	0.024319
29707	Amp	20	17150624	17204107	0	0.024825
29708	Amp	20	17204610	17243805	0	0.023717
29709	Amp	20	17245799	17246084	0	0.02609
29710	Amp	20	17247009	17284425	0	0.022094
29711	Amp	20	17286154	17309091	0	0.019721
29712	Amp	20	17316866	17323758	0	0.019145
29713	Amp	20	17324984	17483446	0	0.017704
29714	Amp	20	17484568	17499118	0	0.016939
29715	Amp	20	17499582	17536286	0	0.01492
29716	Amp	20	17537458	17547829	0	0.017595
29717	Amp	20	17548596	17588326	0	0.019255
29718	Amp	20	17597418	17599823	0	0.021339
29719	Amp	20	17600547	17600547	0	0.022065
29720	Amp	20	17601303	17620785	0	0.022857
29721	Amp	20	17623615	17643440	0	0.024638
29722	Amp	20	17645116	17665263	0	0.021713
29723	Amp	20	17666116	17679970	0	0.025383
29724	Amp	20	17685896	17688921	0	0.024473
29725	Amp	20	17689668	17716302	0	0.023533
29726	Amp	20	17719977	17726793	0	0.019863
29727	Amp	20	17729114	17786635	0	0.021375
29728	Amp	20	17788591	17848636	0	0.020498
29729	Amp	20	17850435	17861674	0	0.018838
29730	Amp	20	17863180	17882974	0	0.022179
29731	Amp	20	17887577	17903992	0	0.023211
29732	Amp	20	17913493	17914710	0	0.022179

29733	Amp	20	17914738	17917485	0	0.021387
29734	Amp	20	17918093	17945312	0	0.02266
29735	Amp	20	17949102	17964600	0	0.020152
29736	Amp	20	17964992	18063283	0	0.016811
29737	Amp	20	18069432	18085869	0	0.01758
29738	Amp	20	18085884	18331282	0	0.01704
29739	Amp	20	18338753	18348172	0	0.01802
29740	Amp	20	18354408	18371170	0	0.017153
29741	Amp	20	18372689	18438300	0	0.019105
29742	Amp	20	18441135	18482454	0	0.019639
29743	Amp	20	18491061	18507331	0	0.017555
29744	Amp	20	18520286	18567800	0	0.015069
29745	Amp	20	18573044	18621500	0	0.013556
29746	Amp	20	18625084	18639630	0	0.015227
29747	Amp	20	18641157	18665602	0	0.016722
29748	Amp	20	18666154	18709460	0	0.015051
29749	Amp	20	18710637	18753034	0	0.017579
29750	Amp	20	18754195	18810875	0	0.015051
29751	Amp	20	18811152	18819983	0	0.016432
29752	Amp	20	18820026	18910585	0	0.020678
29753	Amp	20	18911030	18974482	0	0.022955
29754	Amp	20	18974529	19156321	0	0.023567
29755	Amp	20	19160097	19177321	0	0.021417
29756	Amp	20	19177367	19239684	0	0.024594
29757	Amp	20	19239962	19319993	0	0.029098
29758	Amp	20	19320215	19435713	0	0.03307
29759	Amp	20	19435950	19471012	0	0.030395
29760	Amp	20	19472380	19521276	0	0.028118
29761	Amp	20	19522234	19542672	0	0.028667
29762	Amp	20	19545338	19602815	0	0.032098
29763	Amp	20	19605243	19652584	0	0.03155
29764	Amp	20	19653302	19661927	0	0.027578
29765	Amp	20	19662037	19703986	0	0.025827
29766	Amp	20	19704600	19731088	0	0.02913
29767	Amp	20	19733810	19772751	0	0.028517
29768	Amp	20	19772894	19776951	0	0.027022
29769	Amp	20	19777624	19871448	0	0.025023
29770	Amp	20	19871744	19991199	0	0.026311
29771	Amp	20	19992185	20019338	0	0.027475
29772	Amp	20	20022917	20064463	0	0.02827
29773	Amp	20	20065278	20089998	0	0.0301
29774	Amp	20	20090402	20320098	0	0.032177
29775	Amp	20	20321605	20338258	0	0.033173
29776	Amp	20	20341821	20454834	0	0.03362
29777	Amp	20	20458099	20464736	0	0.035261
29778	Amp	20	20467050	20526151	0	0.033522
29779	Amp	20	20527414	20666374	0	0.032486
29780	Amp	20	20669710	20707940	0	0.034385
29781	Amp	20	20708313	20708313	0	0.03303
29782	Amp	20	20726349	20752739	0	0.033685
29783	Amp	20	20756108	20772901	0	0.032043
29784	Amp	20	20773040	20792796	0	0.030189
29785	Amp	20	20795482	20803015	0	0.025944

29786 Amp	20	20804455	20838939	0	0.025289
29787 Amp	20	20839372	20991819	0	0.027246
29788 Amp	20	20994147	21046739	0	0.023814
29789 Amp	20	21047374	21112347	0	0.02137
29790 Amp	20	21117636	21117678	0	0.021988
29791 Amp	20	21119319	21127192	0	0.020828
29792 Amp	20	21130002	21215165	0	0.018871
29793 Amp	20	21215374	21265247	0	0.020883
29794 Amp	20	21265982	21267350	0	0.018871
29795 Amp	20	21270578	21302904	0	0.020956
29796 Amp	20	21310076	21338339	0	0.024151
29797 Amp	20	21341432	21463745	0	0.025425
29798 Amp	20	21470853	21471210	0	0.02223
29799 Amp	20	21471673	21594347	0	0.023671
29800 Amp	20	21594774	21602001	0	0.025807
29801 Amp	20	21603921	21615932	0	0.024845
29802 Amp	20	21616599	21662781	0	0.022945
29803 Amp	20	21663357	21794204	0	0.018408
29804 Amp	20	21795899	21810458	0	0.020679
29805 Amp	20	21811216	21895090	0	0.020232
29806 Amp	20	21897581	21934420	0	0.017962
29807 Amp	20	21937650	22008233	0	0.021294
29808 Amp	20	22008972	22094436	0	0.016913
29809 Amp	20	22099436	22126523	0	0.018557
29810 Amp	20	22129413	22129921	0	0.030229
29811 Amp	20	22135091	22238895	0	0.029435
29812 Amp	20	22244818	22279352	0	0.027994
29813 Amp	20	22285475	22341585	0	0.029576
29814 Amp	20	22341725	22358129	0	0.024568
29815 Amp	20	22358231	22364348	0	0.022986
29816 Amp	20	22364596	22370795	0	0.030104
29817 Amp	20	22372978	22373087	0	0.027969
29818 Amp	20	22374600	22459174	0	0.028587
29819 Amp	20	22459187	22482500	0	0.03771
29820 Amp	20	22483542	22510653	0	0.036005
29821 Amp	20	22510818	22590467	0	0.035498
29822 Amp	20	22594041	22631578	0	0.029543
29823 Amp	20	22633240	22688144	0	0.031878
29824 Amp	20	22688932	22797195	0	0.029543
29825 Amp	20	22800624	22938865	0	0.031645
29826 Amp	20	22939968	22940136	0	0.030604
29827 Amp	20	22944763	22974528	0	0.018148
29828 Amp	20	22974864	23009713	0	0.020913
29829 Amp	20	23010478	23040150	0	0.021819
29830 Amp	20	23040374	23060334	0	0.019055
29831 Amp	20	23062913	23135453	0	0.021765
29832 Amp	20	23136156	23139421	0	0.020859
29833 Amp	20	23140666	23167740	0	0.023161
29834 Amp	20	23167888	23217689	0	0.017512
29835 Amp	20	23223650	23268462	0	0.016238
29836 Amp	20	23269446	23412276	0	0.014593
29837 Amp	20	23416024	23423302	0	0.016682
29838 Amp	20	23596117	23596185	0	0.009647

29839	Amp	20	23596207	23887427	0	0.010087
29840	Amp	20	23888289	23910860	0	0.011365
29841	Amp	20	23911004	23938478	0	0.012258
29842	Amp	20	23942353	23975471	0	0.013332
29843	Amp	20	23976257	23998357	0	0.010979
29844	Amp	20	23998668	24018490	0	0.014687
29845	Amp	20	24018541	24107132	0	0.010979
29846	Amp	20	24110470	24899779	0	0.007047
29847	Amp	20	24950006	25161976	0	0.007617
29848	Amp	20	25163248	25375317	0	0.005528
29849	Amp	20	25385429	25495005	0	0.007269
29850	Amp	20	25501030	25673012	0	0.009455
29851	Amp	20	25673829	25774214	0	0.007735
29852	Amp	20	25847699	25938143	0	0.010847
29853	Amp	20	25948608	26096020	0	0.012508
29854	Amp	20	26098835	26125809	0	0.015683
29855	Amp	20	26127235	26184943	0	0.015216
29856	Amp	20	26186893	26196799	0	0.014598
29857	Amp	20	26197168	26215123	0	0.015037
29858	Amp	20	26215232	26215250	0	0.017309
29859	Amp	20	26215627	26217263	0	0.016417
29860	Amp	20	26217295	26217295	0	0.018581
29861	Amp	20	31247668	31256075	0	0.029439
29862	Amp	20	31256170	31258575	0	0.026506
29863	Amp	20	31259783	31263351	0	0.027088
29864	Amp	20	31267541	31267541	0	0.027808
29865	Amp	20	31272364	31297078	0	0.042541
29866	Amp	20	31299247	31311939	0	0.038218
29867	Amp	20	31312474	31329762	0	0.037636
29868	Amp	20	31330047	31413719	0	0.041883
29869	Amp	20	31414733	31423410	0	0.046133
29870	Amp	20	31428640	31457840	0	0.04726
29871	Amp	20	31460733	31581449	0	0.052786
29872	Amp	20	31584047	31633619	0	0.053265
29873	Amp	20	31640669	31641452	0	0.051412
29874	Amp	20	31644486	31720715	0	0.048888
29875	Amp	20	31727222	31791878	0	0.047761
29876	Amp	20	31794740	31798820	0	0.037411
29877	Amp	20	31803091	31845336	0	0.039903
29878	Amp	20	31852354	31868825	0	0.043388
29879	Amp	20	31877116	31895404	0	0.039903
29880	Amp	20	31901918	31911523	0	0.042898
29881	Amp	20	31918318	32070370	0	0.037394
29882	Amp	20	32073132	32085621	0	0.040335
29883	Amp	20	32086796	32107342	0	0.04268
29884	Amp	20	32116126	32199174	0	0.039685
29885	Amp	20	32199423	32227858	0	0.038966
29886	Amp	20	32232773	32299856	0	0.03952
29887	Amp	20	32304554	32334686	0	0.037974
29888	Amp	20	32334832	32410355	0	0.035034
29889	Amp	20	32413861	32417054	0	0.037507
29890	Amp	20	32417646	32417646	0	0.032428
29891	Amp	20	32420111	32469246	0	0.036224

29892 Amp	20	32470166	32496330	0	0.033858
29893 Amp	20	32510057	32516883	0	0.037015
29894 Amp	20	32517220	32736328	0	0.034543
29895 Amp	20	32737659	32745827	0	0.031385
29896 Amp	20	32748172	32826119	0	0.034055
29897 Amp	20	32832332	32847660	0	0.032093
29898 Amp	20	32851388	32944823	0	0.028734
29899 Amp	20	32945534	32953267	0	0.023998
29900 Amp	20	32958086	33000553	0	0.025127
29901 Amp	20	33001967	33092528	0	0.026202
29902 Amp	20	33092641	33104270	0	0.025073
29903 Amp	20	33105141	33143825	0	0.02258
29904 Amp	20	33146311	33238190	0	0.018784
29905 Amp	20	33241277	33318007	0	0.021056
29906 Amp	20	33319456	33351826	0	0.020617
29907 Amp	20	33352734	33352932	0	0.020063
29908 Amp	20	33354441	33357558	0	0.018988
29909 Amp	20	33359055	33394043	0	0.016976
29910 Amp	20	33395177	33415246	0	0.016537
29911 Amp	20	33416138	33463313	0	0.015879
29912 Amp	20	33467319	33479756	0	0.013128
29913 Amp	20	33499195	33506307	0	0.008877
29914 Amp	20	33508598	33660475	0	0.007634
29915 Amp	20	33665530	33822610	0	0.0049
29916 Amp	20	33829468	33829468	0	0.007365
29917 Amp	20	33832268	33860114	0	0.009034
29918 Amp	20	33864837	33883066	0	0.012673
29919 Amp	20	33886712	33886712	0	0.013247
29920 Amp	20	33890166	33902408	0	0.008528
29921 Amp	20	33907779	33943936	0	0.007939
29922 Amp	20	33947499	33962633	0	0.012601
29923 Amp	20	33964269	33997105	0	0.01602
29924 Amp	20	34006908	34020116	0	0.017632
29925 Amp	20	34020419	34020419	0	0.015167
29926 Amp	20	34025235	34052241	0	0.022751
29927 Amp	20	34056122	34057102	0	0.03202
29928 Amp	20	34057745	34151279	0	0.03624
29929 Amp	20	34159146	34186003	0	0.041684
29930 Amp	20	34188445	34284511	0	0.041169
29931 Amp	20	34291914	34293217	0	0.042987
29932 Amp	20	34300744	34365887	0	0.046086
29933 Amp	20	34367582	34388946	0	0.047704
29934 Amp	20	34391295	34431552	0	0.048859
29935 Amp	20	34432640	34447782	0	0.052363
29936 Amp	20	34448677	34458221	0	0.051171
29937 Amp	20	34467575	34489764	0	0.047667
29938 Amp	20	34489992	34490298	0	0.046049
29939 Amp	20	34494101	34494101	0	0.04976
29940 Amp	20	34494355	34522088	0	0.050818
29941 Amp	20	34522237	34537767	0	0.047774
29942 Amp	20	34544228	34587777	0	0.051
29943 Amp	20	34596157	34625665	0	0.052697
29944 Amp	20	34627491	34640868	0	0.058084

29945	Amp	20	34644819	34661394	0	0.056266
29946	Amp	20	34662522	34674495	0	0.055111
29947	Amp	20	34674901	34689049	0	0.055861
29948	Amp	20	34692049	34700707	0	0.056512
29949	Amp	20	34706549	34797150	0.005328	0.06418
29950	Amp	20	34797526	34835029	0	0.061908
29951	Amp	20	34836166	34844720	0	0.061257
29952	Amp	20	34846759	34912124	0	0.061753
29953	Amp	20	34913386	34927424	0.024438	0.064969
29954	Amp	20	34930910	34965861	0.276783	0.075464
29955	Amp	20	34966517	34967989	0.563096	0.085174
29956	Amp	20	34970361	35013449	1.066493	0.098879
29957	Amp	20	35019661	35100468	0.758088	0.091056
29958	Amp	20	35101505	35108244	0.807647	0.0924
29959	Amp	20	35111632	35132923	0.891602	0.094615
29960	Amp	20	35141967	35155909	1.140417	0.10055
29961	Amp	20	35156331	35158731	0.735486	0.090546
29962	Amp	20	35159394	35165498	0.765474	0.091298
29963	Amp	20	35166801	35167652	0.689884	0.089083
29964	Amp	20	35171451	35196485	0.276783	0.075372
29965	Amp	20	35200473	35201071	0.299433	0.076236
29966	Amp	20	35203478	35213480	0.322897	0.077114
29967	Amp	20	35216418	35232664	0.644502	0.087781
29968	Amp	20	35234186	35283858	0.735486	0.090437
29969	Amp	20	35283872	35294364	0.807647	0.092415
29970	Amp	20	35296856	35338437	0.924746	0.09546
29971	Amp	20	35340182	35417309	1.084294	0.099278
29972	Amp	20	35429495	35439830	1.280185	0.103838
29973	Amp	20	35443492	35452832	1.388256	0.106359
29974	Amp	20	35454649	35562506	1.329472	0.105015
29975	Amp	20	35566823	35583402	1.378521	0.10607
29976	Amp	20	35591132	35594802	1.472629	0.108188
29977	Amp	20	35597307	35603888	1.426786	0.107133
29978	Amp	20	35606132	35606889	1.843664	0.116076
29979	Amp	20	35609455	35645081	2.290039	0.124864
29980	Amp	20	35652984	35674089	2.356694	0.126163
29981	Amp	20	35675580	35679106	1.665486	0.112458
29982	Amp	20	35679821	35679821	1.492063	0.10864
29983	Amp	20	35686178	35751278	1.225419	0.102705
29984	Amp	20	35798377	35809199	0.781972	0.091824
29985	Amp	20	35810312	35818553	0.831947	0.093009
29986	Amp	20	35821638	35826617	0.595248	0.08631
29987	Amp	20	35827951	35853433	0.807647	0.0923
29988	Amp	20	35860223	35930240	0.644502	0.08774
29989	Amp	20	35930435	35930435	0.616549	0.086988
29990	Amp	20	35936925	35951466	0.899693	0.09486
29991	Amp	20	35956118	35956118	0.977077	0.096641
29992	Amp	20	35968044	35969697	1.407997	0.106767
29993	Amp	20	35974659	36060525	1.149583	0.100781
29994	Amp	20	36062024	36067022	0.720659	0.090104
29995	Amp	20	36073351	36089026	0.742723	0.090576
29996	Amp	20	36091633	36194824	0.790614	0.092007
29997	Amp	20	36204403	36207231	0.704849	0.089486

29998	Amp	20	36207450	36207450	0.651644	0.088055
29999	Amp	20	36209717	36226661	0.79909	0.092133
30000	Amp	20	36227615	36243733	0.644502	0.087774
30001	Amp	20	36249972	36359581	0.487495	0.082791
30002	Amp	20	36361587	36361587	0.241616	0.073849
30003	Amp	20	36367278	36407432	0.170723	0.071193
30004	Amp	20	36409462	36440382	0.259438	0.074874
30005	Amp	20	36444940	36470119	0.159312	0.070579
30006	Amp	20	36470685	36529004	0.138391	0.069715
30007	Amp	20	36530253	36615916	0.050473	0.066034
30008	Amp	20	36622147	36645320	0.107017	0.068598
30009	Amp	20	36646196	36667855	0.187449	0.07185
30010	Amp	20	36677699	36698304	0.112543	0.068719
30011	Amp	20	36700810	36712135	0	0.063332
30012	Amp	20	36715427	36738806	0	0.062454
30013	Amp	20	36749325	36763944	0.056211	0.066278
30014	Amp	20	36765221	36789224	0.089561	0.06796
30015	Amp	20	36789792	36821142	0.015369	0.064708
30016	Amp	20	36822262	36866682	0.050473	0.066098
30017	Amp	20	36867252	36871655	0.005328	0.064119
30018	Amp	20	36871748	36872447	0	0.063289
30019	Amp	20	36873837	36873837	0	0.055227
30020	Amp	20	36875752	36896408	0	0.053428
30021	Amp	20	36896959	36896959	0	0.050942
30022	Amp	20	36898585	36898585	0	0.04916
30023	Amp	20	36901371	36910534	0	0.047463
30024	Amp	20	36912739	36913988	0	0.049508
30025	Amp	20	36915209	36965075	0	0.047826
30026	Amp	20	36967009	36967009	0	0.046768
30027	Amp	20	36970612	37009834	0	0.049492
30028	Amp	20	37010371	37029348	0	0.051854
30029	Amp	20	37033542	37037384	0	0.051082
30030	Amp	20	37037435	37068156	0	0.04474
30031	Amp	20	37068159	37072159	0	0.043555
30032	Amp	20	37072242	37090567	0	0.041631
30033	Amp	20	37092246	37093618	0	0.042731
30034	Amp	20	37093952	37093952	0	0.044675
30035	Amp	20	37094427	37094427	0	0.046946
30036	Amp	20	37097999	37097999	0	0.062613
30037	Amp	20	37101547	37101547	0.001323	0.06404
30038	Amp	20	37101692	37101692	0.029031	0.06519
30039	Amp	20	37102518	37133091	0.143787	0.069963
30040	Amp	20	37134211	37148663	0.181932	0.071479
30041	Amp	20	37149272	37171650	0.19964	0.072249
30042	Amp	20	37172340	37179804	0.271552	0.07527
30043	Amp	20	37184911	37184911	0.215608	0.072955
30044	Amp	20	37190393	37216002	0.241616	0.073991
30045	Amp	20	37216768	37244193	0.30524	0.076449
30046	Amp	20	37244895	37336085	0.342649	0.077788
30047	Amp	20	37342875	37344024	0.450702	0.081619
30048	Amp	20	37344430	37381869	0.386426	0.079319
30049	Amp	20	37382727	37382727	0.355319	0.078272
30050	Amp	20	37386631	37426090	0.644502	0.087889

30051 Amp	20	37426319	37466037	0.515676	0.083673
30052 Amp	20	37466486	37485601	0.529451	0.08417
30053 Amp	20	37496355	37530910	0.470509	0.082241
30054 Amp	20	37531291	37577027	0.431198	0.080903
30055 Amp	20	37577356	37585513	0.316877	0.077037
30056 Amp	20	37589346	37596132	0.259438	0.074765
30057 Amp	20	37596422	37596422	0.170723	0.071173
30058 Amp	20	37596571	37606719	0.100827	0.068289
30059 Amp	20	37613969	37635591	0.164935	0.070902
30060 Amp	20	37635605	37637279	0.100827	0.068289
30061 Amp	20	37638319	37693197	0.089561	0.067793
30062 Amp	20	37695804	37697413	0.515676	0.083705
30063 Amp	20	37697445	37708780	0.583752	0.085899
30064 Amp	20	37710478	37751302	0.697374	0.089322
30065 Amp	20	37752179	37874942	0.616549	0.086865
30066 Amp	20	37876382	37927375	0.720659	0.090029
30067 Amp	20	37929137	37932909	0.98589	0.097005
30068 Amp	20	37935992	38029123	1.075952	0.099085
30069 Amp	20	38034663	38034663	0.720659	0.089911
30070 Amp	20	38037308	38043212	0.529451	0.084106
30071 Amp	20	38048406	38076200	0.4115	0.080274
30072 Amp	20	38078642	38088540	0.589605	0.086117
30073 Amp	20	38091105	38102181	0.4115	0.080339
30074 Amp	20	38108937	38110588	0.379885	0.079086
30075 Amp	20	38116769	38188749	0.251218	0.074424
30076 Amp	20	38189927	38220686	0.335313	0.077653
30077 Amp	20	38223266	38249939	0.159312	0.070716
30078 Amp	20	38250447	38277492	0.094967	0.068114
30079 Amp	20	38279596	38293108	0.065715	0.066686
30080 Amp	20	38293420	38334265	0.094967	0.068017
30081 Amp	20	38336198	38372987	0	0.059041
30082 Amp	20	38374215	38374656	0	0.061269
30083 Amp	20	38376710	38457717	0	0.062566
30084 Amp	20	38457897	38497214	0	0.05743
30085 Amp	20	38498397	38530078	0	0.056099
30086 Amp	20	38530549	38562225	0	0.054802
30087 Amp	20	38562835	38573743	0	0.056396
30088 Amp	20	38574319	38614593	0	0.056834
30089 Amp	20	38616914	38623282	0	0.049858
30090 Amp	20	38624671	38657011	0	0.04663
30091 Amp	20	38664856	38669133	0	0.040795
30092 Amp	20	38672659	38692638	0	0.041587
30093 Amp	20	38699225	38711446	0	0.039359
30094 Amp	20	38712224	38745573	0	0.03772
30095 Amp	20	38749720	38794083	0	0.041828
30096 Amp	20	38798659	38804267	0	0.043071
30097 Amp	20	38810716	38839350	0	0.037228
30098 Amp	20	38840613	38864778	0	0.037896
30099 Amp	20	38864933	38868696	0	0.036302
30100 Amp	20	38874539	38941011	0	0.034222
30101 Amp	20	38942082	38953346	0	0.050158
30102 Amp	20	38965645	38977034	0	0.034222
30103 Amp	20	38979610	38996372	0	0.029817

30104 Amp	20	38996725	39080825	0	0.031821
30105 Amp	20	39081792	39133685	0	0.03346
30106 Amp	20	39136736	39240858	0	0.037769
30107 Amp	20	39245170	39299832	0	0.045861
30108 Amp	20	39299856	39300658	0	0.044382
30109 Amp	20	39302563	39304531	0	0.050317
30110 Amp	20	39305066	39355117	0	0.049879
30111 Amp	20	39358762	39410926	0	0.051982
30112 Amp	20	39413403	39446558	0	0.058368
30113 Amp	20	39449471	39459293	0	0.056364
30114 Amp	20	39459604	39497469	0	0.058294
30115 Amp	20	39499781	39510927	0	0.055922
30116 Amp	20	39511457	39573752	0	0.058227
30117 Amp	20	39573955	39583544	0	0.059412
30118 Amp	20	39591138	39592113	0	0.06108
30119 Amp	20	39595705	39644662	0.010261	0.064295
30120 Amp	20	39647227	39663688	0	0.060952
30121 Amp	20	39663773	39736020	0	0.062118
30122 Amp	20	39737817	39740101	0.044794	0.065832
30123 Amp	20	39751621	39754278	0	0.059446
30124 Amp	20	39756465	39756465	0	0.055732
30125 Amp	20	39758982	39832853	0	0.060758
30126 Amp	20	39834088	39903905	0	0.062243
30127 Amp	20	39909900	39953156	0.001323	0.063968
30128 Amp	20	39954023	39976201	0	0.062931
30129 Amp	20	39976263	40135261	0.024438	0.065051
30130 Amp	20	40146008	40187447	0	0.060025
30131 Amp	20	40189230	40221002	0.17597	0.071237
30132 Amp	20	40221125	40227882	0.210594	0.07272
30133 Amp	20	40229271	40255529	0.316877	0.076998
30134 Amp	20	40256114	40278801	0.227787	0.073271
30135 Amp	20	40280951	40326628	0.079652	0.067337
30136 Amp	20	40329908	40332868	0.044794	0.065851
30137 Amp	20	40335371	40357346	0.015369	0.064666
30138 Amp	20	40358946	40399746	0.14896	0.070219
30139 Amp	20	40400387	40444435	0.005328	0.064089
30140 Amp	20	40446406	40446583	0	0.05981
30141 Amp	20	40446756	40474003	0	0.061074
30142 Amp	20	40476646	40530773	0.019169	0.064737
30143 Amp	20	40531522	40544088	0.060686	0.066493
30144 Amp	20	40545117	40591803	0.079652	0.067321
30145 Amp	20	40593304	40647722	0.040093	0.065754
30146 Amp	20	40647935	40759500	0.089561	0.067877
30147 Amp	20	40759574	40856872	0.322897	0.077193
30148 Amp	20	40860990	40897395	0.651644	0.088153
30149 Amp	20	40897857	40937391	0.595248	0.086276
30150 Amp	20	40939432	40974459	0.322897	0.077147
30151 Amp	20	40978476	41005726	0.660254	0.088226
30152 Amp	20	41005848	41102743	0.602643	0.086469
30153 Amp	20	41106241	41141335	0.276783	0.075509
30154 Amp	20	41144476	41146027	0.329205	0.07749
30155 Amp	20	41147406	41168825	0.386426	0.079397
30156 Amp	20	41168838	41173409	0.329205	0.07749

30157 Amp	20	41232176	41286875	0.094967	0.068174
30158 Amp	20	41287010	41291374	0.14896	0.070209
30159 Amp	20	41295124	41295124	0.210594	0.072598
30160 Amp	20	41301389	41332156	0	0.061519
30161 Amp	20	41332169	41351630	0	0.062504
30162 Amp	20	41352930	41373263	0	0.061021
30163 Amp	20	41374364	41398079	0	0.059513
30164 Amp	20	41401938	41430372	0	0.046821
30165 Amp	20	41434868	41449621	0	0.060858
30166 Amp	20	41452249	41452839	0	0.04952
30167 Amp	20	41453034	41464502	0	0.04847
30168 Amp	20	41465316	41474551	0	0.054589
30169 Amp	20	41475082	41476742	0	0.042701
30170 Amp	20	41478674	41486499	0	0.044349
30171 Amp	20	41489265	41501560	0	0.050606
30172 Amp	20	41501960	41526514	0	0.049267
30173 Amp	20	41528610	41547281	0	0.04999
30174 Amp	20	41552106	41612162	0	0.053214
30175 Amp	20	41615222	41625501	0	0.051179
30176 Amp	20	41627540	41629315	0	0.048206
30177 Amp	20	41632434	41678990	0	0.042764
30178 Amp	20	41680048	41709736	0	0.047559
30179 Amp	20	41712483	41726601	0	0.046731
30180 Amp	20	41728262	41730837	0	0.037644
30181 Amp	20	41730880	41731388	0	0.036308
30182 Amp	20	41732145	41732656	0	0.040935
30183 Amp	20	41732893	41732893	0	0.046198
30184 Amp	20	41732966	41798819	0	0.050733
30185 Amp	20	41801159	41813898	0.005328	0.064093
30186 Amp	20	41813912	41821287	0	0.062445
30187 Amp	20	41824887	41860398	0	0.05976
30188 Amp	20	41861676	41870268	0	0.056025
30189 Amp	20	41873763	41916769	0	0.059681
30190 Amp	20	41920710	41954196	0	0.058788
30191 Amp	20	41954515	41954914	0	0.053178
30192 Amp	20	41956852	41977481	0	0.053817
30193 Amp	20	41977841	42065024	0	0.051118
30194 Amp	20	42067802	42075267	0	0.052404
30195 Amp	20	42079297	42089519	0	0.063093
30196 Amp	20	42091201	42115569	0	0.061795
30197 Amp	20	42117251	42167521	0	0.060508
30198 Amp	20	42171905	42180780	0.005328	0.064202
30199 Amp	20	42180846	42194800	0	0.060508
30200 Amp	20	42199565	42246839	0.060686	0.06665
30201 Amp	20	42247195	42256036	0.133193	0.069489
30202 Amp	20	42257208	42260699	0.094967	0.067973
30203 Amp	20	42261750	42265680	0.069848	0.066988
30204 Amp	20	42270088	42270088	0.010261	0.064274
30205 Amp	20	42272906	42288732	0.094967	0.068004
30206 Amp	20	42290587	42311159	0.010261	0.064274
30207 Amp	20	42316999	42344620	0.073902	0.067121
30208 Amp	20	42348951	42390132	0.128324	0.069309
30209 Amp	20	42393209	42439810	0.181932	0.071509

30210 Amp	20	42443379	42453592	0.112543	0.06867
30211 Amp	20	42456244	42463331	0.044794	0.065824
30212 Amp	20	42465364	42529429	0.079652	0.067344
30213 Amp	20	42530165	42664160	0.044794	0.065824
30214 Amp	20	42666655	42671051	0	0.061499
30215 Amp	20	42672686	42787105	0	0.063243
30216 Amp	20	42789808	42816913	0	0.052554
30217 Amp	20	42820188	42842315	0	0.05081
30218 Amp	20	42857615	42886399	0	0.062573
30219 Amp	20	42888059	42993094	0.005328	0.064206
30220 Amp	20	42996464	43046280	0.040093	0.06577
30221 Amp	20	43049711	43062165	0.005328	0.064206
30222 Amp	20	43062610	43068763	0	0.052443
30223 Amp	20	43070719	43085925	0	0.054131
30224 Amp	20	43089283	43101823	0	0.052443
30225 Amp	20	43105283	43118070	0	0.054644
30226 Amp	20	43120691	43158162	0	0.052443
30227 Amp	20	43158524	43158524	0.100827	0.068219
30228 Amp	20	43168483	43185524	0	0.062631
30229 Amp	20	43194481	43251980	0	0.061227
30230 Amp	20	43255963	43265498	0	0.060789
30231 Amp	20	43267349	43319272	0	0.063054
30232 Amp	20	43319396	43337361	0.050473	0.066186
30233 Amp	20	43340879	43340879	0.024438	0.065021
30234 Amp	20	43341141	43372976	0	0.061122
30235 Amp	20	43373810	43373810	0	0.058933
30236 Amp	20	43374068	43399649	0	0.060548
30237 Amp	20	43400612	43449954	0	0.061885
30238 Amp	20	43451163	43528824	0	0.063433
30239 Amp	20	43529632	43541687	0.040093	0.065661
30240 Amp	20	43541777	43591191	0.342649	0.077746
30241 Amp	20	43594344	43647101	0.276783	0.075546
30242 Amp	20	43648004	43648076	0.19964	0.072195
30243 Amp	20	43655516	43655516	0.264858	0.075026
30244 Amp	20	43661508	43754861	0	0.062942
30245 Amp	20	43762967	43849579	0	0.060493
30246 Amp	20	43858274	43866762	0	0.063772
30247 Amp	20	43867832	43930679	0.118001	0.068969
30248 Amp	20	43931820	43936106	0.060686	0.06661
30249 Amp	20	43939434	43939512	0	0.063331
30250 Amp	20	43939924	43942380	0.128324	0.069316
30251 Amp	20	43944683	44062747	0.065715	0.066788
30252 Amp	20	44063607	44064041	0	0.061538
30253 Amp	20	44064443	44165444	0.029031	0.065353
30254 Amp	20	44166681	44220905	0.015369	0.06463
30255 Amp	20	44223497	44234153	0.056211	0.066238
30256 Amp	20	44238824	44277283	0.100827	0.068243
30257 Amp	20	44280325	44349608	0	0.063445
30258 Amp	20	44350543	44402226	0	0.059907
30259 Amp	20	44402316	44422573	0	0.060669
30260 Amp	20	44429180	44450564	0	0.061161
30261 Amp	20	44466857	44475549	0	0.05822
30262 Amp	20	44482257	44509328	0	0.062876

30263	Amp	20	44512534	44517232	0	0.061268
30264	Amp	20	44524579	44524579	0	0.05918
30265	Amp	20	44534968	44553756	0	0.057165
30266	Amp	20	44559205	44711771	0.14896	0.0702
30267	Amp	20	44713440	44757916	0.193543	0.072042
30268	Amp	20	44758720	44766939	0.342649	0.077855
30269	Amp	20	44770092	44814827	0.404718	0.079928
30270	Amp	20	44816719	44848232	0.342649	0.077855
30271	Amp	20	44848569	44868544	0.17597	0.071407
30272	Amp	20	44870675	44870675	0.241616	0.073986
30273	Amp	20	44871380	44962596	0.193543	0.071981
30274	Amp	20	44962647	44982033	0	0.057756
30275	Amp	20	44982589	45016653	0	0.060708
30276	Amp	20	45019613	45042167	0	0.057756
30277	Amp	20	45045156	45175881	0	0.063567
30278	Amp	20	45177653	45212530	0.251218	0.074419
30279	Amp	20	45214224	45251206	0.193543	0.071972
30280	Amp	20	45252085	45260361	0.255918	0.074493
30281	Amp	20	45261500	45264942	0.19964	0.072266
30282	Amp	20	45268950	45268950	0.368062	0.078713
30283	Amp	20	45269247	45269499	0.515676	0.083749
30284	Amp	20	45286303	45305054	0.550755	0.084742
30285	Amp	20	45307345	45346612	0.622604	0.08714
30286	Amp	20	45346811	45369281	0.569771	0.085421
30287	Amp	20	45377961	45507088	0.417873	0.080384
30288	Amp	20	45509271	45509271	0.543344	0.084674
30289	Amp	20	45511366	45511366	0.17597	0.0713
30290	Amp	20	45511629	45555736	0.316877	0.077065
30291	Amp	20	45558006	45573245	0.233782	0.073507
30292	Amp	20	45588272	45613926	0.287738	0.075905
30293	Amp	20	45615203	45648472	0.14896	0.07014
30294	Amp	20	45649864	45653538	0.1237	0.069148
30295	Amp	20	45660099	45708247	0.233782	0.073574
30296	Amp	20	45752208	45752208	0.342649	0.077952
30297	Amp	20	45752976	45824058	0.241616	0.073953
30298	Amp	20	45824936	45959540	0.181932	0.071555
30299	Amp	20	45959602	45990010	0.073902	0.067129
30300	Amp	20	45999282	46051095	0.17597	0.071264
30301	Amp	20	46051200	46075053	0.133193	0.069657
30302	Amp	20	46076490	46105732	0.246818	0.074199
30303	Amp	20	46107215	46107215	0.287738	0.075918
30304	Amp	20	46108465	46137314	0.368062	0.078691
30305	Amp	20	46137764	46161623	0.19964	0.072106
30306	Amp	20	46162247	46170283	0.373599	0.079033
30307	Amp	20	46170643	46188693	0.47716	0.082297
30308	Amp	20	46191517	46239656	0.392611	0.079525
30309	Amp	20	46242810	46275524	0.094967	0.068055
30310	Amp	20	46276516	46279888	0.056211	0.066324
30311	Amp	20	46285993	46294111	0.259438	0.074843
30312	Amp	20	46294270	46314580	0.181932	0.071579
30313	Amp	20	46314661	46334801	0.19964	0.072098
30314	Amp	20	46336653	46337136	0.457501	0.081751
30315	Amp	20	46340969	46371319	0.386426	0.079347

30316 Amp	20	46374411	46378744	0.322897	0.077264
30317 Amp	20	46378889	46469035	0.379885	0.07922
30318 Amp	20	46473627	46474611	0.457501	0.081817
30319 Amp	20	46476793	46482563	0.536238	0.084452
30320 Amp	20	46486462	46500194	0.557122	0.084929
30321 Amp	20	46504404	46517836	0.602643	0.086444
30322 Amp	20	46520419	46556118	0.522881	0.083847
30323 Amp	20	46556346	46558866	0.569771	0.085502
30324 Amp	20	46558974	46610230	0.704849	0.089519
30325 Amp	20	46611117	46654242	0.30524	0.076484
30326 Amp	20	46657435	46678289	0.193543	0.071886
30327 Amp	20	46679203	46694080	0.233782	0.073555
30328 Amp	20	46694448	46701208	0.159312	0.070754
30329 Amp	20	46715536	46715536	0.1237	0.069084
30330 Amp	20	46716503	46730769	0.193543	0.072077
30331 Amp	20	46731036	46845402	0.227787	0.073288
30332 Amp	20	46847935	46929885	0.282828	0.075591
30333 Amp	20	46929934	46930450	0.293269	0.076077
30334 Amp	20	46930695	46935803	0.576183	0.085555
30335 Amp	20	46938867	46959598	0.255918	0.074688
30336 Amp	20	46959708	47076288	0.233782	0.073477
30337 Amp	20	47077153	47107226	0.342649	0.077878
30338 Amp	20	47108129	47116878	0.4115	0.080252
30339 Amp	20	47122645	47133061	0.47716	0.082463
30340 Amp	20	47133318	47137690	0.417873	0.080373
30341 Amp	20	47139737	47139737	0.857296	0.093667
30342 Amp	20	47139766	47186894	1.112463	0.10007
30343 Amp	20	47187190	47215931	1.014678	0.097697
30344 Amp	20	47218932	47229476	1.084294	0.099395
30345 Amp	20	47229659	47238633	1.168655	0.101286
30346 Amp	20	47246748	47259866	0.98589	0.096884
30347 Amp	20	47262445	47278749	0.873678	0.094172
30348 Amp	20	47285041	47286306	0.915698	0.095184
30349 Amp	20	47292936	47325548	0.697374	0.089374
30350 Amp	20	47327640	47328322	0.636464	0.087718
30351 Amp	20	47329246	47369653	0.550755	0.084887
30352 Amp	20	47377405	47393812	0.355319	0.078296
30353 Amp	20	47397187	47399718	0.316877	0.077053
30354 Amp	20	47400638	47492766	0.246818	0.074198
30355 Amp	20	47493346	47499326	0.293269	0.07605
30356 Amp	20	47503054	47574746	0.015369	0.064595
30357 Amp	20	47577105	47601460	0.08435	0.067748
30358 Amp	20	47608081	47615095	0.015369	0.064595
30359 Amp	20	47616516	47624334	0.044794	0.065836
30360 Amp	20	47625101	47635430	0.019169	0.064824
30361 Amp	20	47640033	47653145	0.079652	0.067516
30362 Amp	20	47653975	47657495	0.094967	0.068058
30363 Amp	20	47659822	47692663	0.138391	0.069917
30364 Amp	20	47695480	47699141	0.282828	0.075757
30365 Amp	20	47699254	47700246	0.335313	0.077561
30366 Amp	20	47700323	47723423	0.431198	0.080862
30367 Amp	20	47726517	47739833	0.373599	0.079023
30368 Amp	20	47740370	47763481	0.444474	0.081329

30369	Amp	20	47764418	47792860	0.431198	0.080844
30370	Amp	20	47796832	47796832	0.500841	0.083295
30371	Amp	20	47801257	47811671	0.589605	0.086156
30372	Amp	20	47811691	47840772	0.644502	0.087792
30373	Amp	20	47841194	47849353	0.751221	0.090929
30374	Amp	20	47850819	47870585	0.644502	0.087792
30375	Amp	20	47875948	47884704	0.790614	0.091863
30376	Amp	20	47884769	47884769	0.765474	0.09132
30377	Amp	20	47887824	47888436	0.622604	0.087159
30378	Amp	20	47888592	47920315	0.774648	0.091577
30379	Amp	20	47922335	47930536	0.906385	0.094966
30380	Amp	20	47933424	47939462	0.924746	0.095512
30381	Amp	20	47940375	47943249	0.815228	0.092651
30382	Amp	20	47945039	47953827	0.840289	0.093214
30383	Amp	20	47960291	48004399	0.689884	0.089143
30384	Amp	20	48007784	48007784	0.742723	0.090572
30385	Amp	20	48009276	48076003	1.299876	0.104376
30386	Amp	20	48078923	48085305	1.215958	0.102485
30387	Amp	20	48090753	48140857	1.426786	0.107059
30388	Amp	20	48141468	48149831	1.502257	0.10887
30389	Amp	20	48151612	48157752	1.426786	0.10714
30390	Amp	20	48158473	48178118	1.280185	0.103839
30391	Amp	20	48181195	48229971	1.378521	0.105971
30392	Amp	20	48231239	48251954	1.319393	0.104731
30393	Amp	20	48257279	48309902	1.407997	0.106659
30394	Amp	20	48311345	48322660	1.378521	0.106113
30395	Amp	20	48322672	48353251	1.290368	0.104185
30396	Amp	20	48353334	48358560	1.369395	0.105823
30397	Amp	20	48366349	48407457	1.261401	0.103372
30398	Amp	20	48409427	48468767	1.329472	0.104981
30399	Amp	20	48468978	48469509	1.456037	0.107818
30400	Amp	20	48479209	48489530	1.833307	0.115893
30401	Amp	20	48519062	48519878	1.919241	0.117651
30402	Amp	20	48533224	48565908	2.071483	0.120667
30403	Amp	20	48568341	48574384	2.356694	0.126243
30404	Amp	20	48574634	48577468	2.514205	0.129243
30405	Amp	20	48577661	48602266	2.626052	0.131419
30406	Amp	20	48602950	48712604	2.479057	0.128582
30407	Amp	20	48714925	48748535	2.637742	0.131669
30408	Amp	20	48752478	48759648	2.571513	0.130304
30409	Amp	20	48765097	48805851	2.997552	0.138455
30410	Amp	20	48806412	48821544	2.917729	0.136944
30411	Amp	20	48825466	48844355	3.057106	0.139558
30412	Amp	20	48847175	48855288	2.975629	0.137889
30413	Amp	20	48859337	48868291	3.230405	0.142768
30414	Amp	20	48870147	48873360	3.067933	0.139681
30415	Amp	20	48875451	48882032	2.975629	0.137962
30416	Amp	20	48882633	48894445	2.882945	0.136324
30417	Amp	20	48896194	48901575	2.997552	0.138353
30418	Amp	20	48902661	48934285	2.964305	0.137801
30419	Amp	20	48934902	48950131	2.825055	0.135187
30420	Amp	20	48952962	48952962	3.021176	0.138919
30421	Amp	20	48953505	48967207	2.33403	0.125896

30422 Amp	20	48972031	48986704	2.986184	0.138234
30423 Amp	20	48987230	49025316	2.684894	0.132638
30424 Amp	20	49031125	49043365	2.060912	0.120299
30425 Amp	20	49044191	49076594	1.919241	0.117607
30426 Amp	20	49080383	49086793	1.426786	0.107267
30427 Amp	20	49087273	49098981	1.492063	0.108647
30428 Amp	20	49100535	49123277	1.583409	0.110597
30429 Amp	20	49128410	49239695	1.696256	0.112921
30430 Amp	20	49239772	49242385	1.655056	0.112052
30431 Amp	20	49242478	49251445	1.897704	0.117249
30432 Amp	20	49252654	49257468	1.398396	0.106402
30433 Amp	20	49258072	49260140	2.049917	0.120081
30434 Amp	20	49261407	49261407	2.637742	0.131671
30435 Amp	20	49269181	49270374	2.765626	0.133982
30436 Amp	20	49270392	49286239	2.684894	0.132659
30437 Amp	20	49286482	49286482	2.402099	0.127228
30438 Amp	20	49291099	49292084	2.571513	0.130339
30439 Amp	20	49294394	49294394	2.673338	0.132274
30440 Amp	20	49296489	49324218	3.172599	0.141708
30441 Amp	20	49325443	49333298	3.29895	0.144138
30442 Amp	20	49336930	49359651	3.160681	0.141434
30443 Amp	20	49363104	49406024	2.649655	0.132
30444 Amp	20	49406291	49419973	2.755154	0.133957
30445 Amp	20	49422179	49443609	2.860424	0.135821
30446 Amp	20	49444052	49469014	3.251638	0.143312
30447 Amp	20	49470871	49513450	3.150497	0.141355
30448 Amp	20	49513715	49524145	3.045223	0.139403
30449 Amp	20	49524207	49529692	3.399017	0.145789
30450 Amp	20	49622075	49630591	3.183474	0.14197
30451 Amp	20	49637438	49738173	4.570393	0.165468
30452 Amp	20	49742021	49743406	4.882872	0.170446
30453 Amp	20	49745031	49750087	5.038261	0.172754
30454 Amp	20	49820922	49820922	5.110288	0.173855
30455 Amp	20	49821295	49831743	5.492924	0.179414
30456 Amp	20	49836012	49859162	5.882356	0.18515
30457 Amp	20	49863023	49904753	5.551585	0.180418
30458 Amp	20	49908290	49911896	5.434517	0.17866
30459 Amp	20	49913224	49924646	6.017563	0.187033
30460 Amp	20	49928574	49928574	5.852205	0.184725
30461 Amp	20	49929073	49955136	5.285768	0.176352
30462 Amp	20	49999680	49999680	6.002088	0.18695
30463 Amp	20	50001086	50001086	5.732156	0.183015
30464 Amp	20	50001888	50031786	5.656875	0.181857
30465 Amp	20	50032011	50032011	5.942149	0.185967
30466 Amp	20	50041210	50057198	6.304987	0.19128
30467 Amp	20	50063398	50080728	6.458214	0.193454
30468 Amp	20	50083114	50083114	5.911851	0.185524
30469 Amp	20	50083590	50085225	5.822221	0.184238
30470 Amp	20	50095526	50095526	6.458214	0.193373
30471 Amp	20	50102403	50103142	6.503622	0.193941
30472 Amp	20	50106257	50108761	7.437327	0.207107
30473 Amp	20	50111064	50129652	8.378151	0.219769
30474 Amp	20	50131429	50140923	8.217372	0.217742

30475	Amp	20	50141531	50143009	8.378151	0.219901
30476	Amp	20	50146479	50147758	8.233714	0.217967
30477	Amp	20	50149751	50186081	9.272857	0.231519
30478	Amp	20	50186457	50193088	9.373896	0.232845
30479	Amp	20	50193365	50196372	8.919417	0.227006
30480	Amp	20	50202311	50208920	9.02098	0.228427
30481	Amp	20	50210630	50221324	8.687806	0.224059
30482	Amp	20	50224486	50231245	8.121744	0.216381
30483	Amp	20	50234655	50252422	7.704855	0.210805
30484	Amp	20	50255779	50264730	7.389113	0.206327
30485	Amp	20	50272298	50289979	7.817451	0.212325
30486	Amp	20	50291392	50344950	7.977624	0.214532
30487	Amp	20	50347482	50357711	8.34537	0.219527
30488	Amp	20	50357853	50371913	8.249918	0.218106
30489	Amp	20	50374046	50385804	8.073986	0.215704
30490	Amp	20	50386062	50400606	7.657996	0.21019
30491	Amp	20	50402897	50414686	7.896426	0.213423
30492	Amp	20	50414795	50416582	8.409489	0.220208
30493	Amp	20	50427668	50429252	8.266115	0.218328
30494	Amp	20	50431642	50448147	7.341956	0.205666
30495	Amp	20	50457109	50460373	6.980586	0.200717
30496	Amp	20	50461014	50471284	7.72044	0.21097
30497	Amp	20	50472450	50475660	7.279386	0.204972
30498	Amp	20	50477089	50481574	7.516099	0.208147
30499	Amp	20	50481748	50487822	7.437327	0.207103
30500	Amp	20	50489792	50526130	7.563028	0.208811
30501	Amp	20	50529513	50530318	7.421526	0.206799
30502	Amp	20	50530767	50532187	7.310837	0.20539
30503	Amp	20	50534089	50538017	6.579755	0.195137
30504	Amp	20	50538033	50538567	6.473343	0.193502
30505	Amp	20	50543407	50546005	7.135839	0.202955
30506	Amp	20	50546040	50550174	7.500789	0.207853
30507	Amp	20	50555207	50557587	6.810251	0.198399
30508	Amp	20	50557971	50558065	6.626113	0.195684
30509	Amp	20	50558183	50568538	6.65628	0.19622
30510	Amp	20	50569112	50571780	6.763246	0.197679
30511	Amp	20	50571854	50572631	6.79429	0.198231
30512	Amp	20	50574753	50580461	6.886863	0.199434
30513	Amp	20	50580639	50582634	6.980586	0.200834
30514	Amp	20	50582849	50583150	7.027404	0.201464
30515	Amp	20	50583425	50589607	6.871512	0.199288
30516	Amp	20	50593619	50622747	6.747966	0.197582
30517	Amp	20	50624830	50629046	6.626113	0.195832
30518	Amp	20	50632339	50645357	7.011899	0.201214
30519	Amp	20	50652059	50652059	7.961071	0.214108
30520	Amp	20	50652292	50670584	8.057852	0.215602
30521	Amp	20	50674452	50687933	8.249918	0.218121
30522	Amp	20	50688086	50708673	7.977624	0.214491
30523	Amp	20	50712951	50766474	6.825391	0.198671
30524	Amp	20	50768696	50772694	6.65628	0.196152
30525	Amp	20	50777407	50779344	6.062596	0.187638
30526	Amp	20	50783384	50797349	6.2443	0.190292
30527	Amp	20	50797655	50808921	6.36593	0.191982

30528 Amp	20	50817242	50834652	6.396376	0.192565
30529 Amp	20	50839494	50843836	6.671073	0.196513
30530 Amp	20	50844516	50849178	6.886863	0.199434
30531 Amp	20	50856205	50863226	7.152075	0.203159
30532 Amp	20	50865422	50875870	7.248181	0.204477
30533 Amp	20	50876391	50877208	7.341956	0.205643
30534 Amp	20	50878852	50885691	7.058587	0.201918
30535 Amp	20	50887672	50889658	7.689477	0.210415
30536 Amp	20	50890734	50903747	7.865467	0.212933
30537 Amp	20	50909163	50910481	7.6425	0.209849
30538 Amp	20	50914303	50918932	7.945028	0.21399
30539 Amp	20	50923437	50938116	8.18543	0.217151
30540 Amp	20	50940341	50949922	9.373896	0.232959
30541 Amp	20	50951163	50956703	9.881657	0.239299
30542 Amp	20	50957437	50957437	10.034845	0.241309
30543 Amp	20	50961420	50961420	9.933591	0.239981
30544 Amp	20	50962127	50962127	8.409489	0.220352
30545 Amp	20	50965112	50977881	9.289139	0.23189
30546 Amp	20	50977984	50977984	8.770924	0.225104
30547 Amp	20	50979189	50991175	8.47374	0.221205
30548 Amp	20	50991965	51006914	8.409489	0.220341
30549 Amp	20	51007963	51019576	8.770924	0.225064
30550 Amp	20	51027085	51027085	8.572005	0.222549
30551 Amp	20	51031065	51033173	8.671092	0.22387
30552 Amp	20	51035804	51035804	8.851521	0.226119
30553 Amp	20	51035878	51038035	9.122122	0.229671
30554 Amp	20	51045048	51051070	9.559998	0.235273
30555 Amp	20	51051196	51056294	9.644619	0.236254
30556 Amp	20	51057540	51069424	9.763565	0.237941
30557 Amp	20	51072189	51083137	9.712919	0.237149
30558 Amp	20	51084067	51096053	9.526354	0.234838
30559 Amp	20	51096789	51098572	9.575888	0.235389
30560 Amp	20	51098956	51102028	9.729466	0.237521
30561 Amp	20	51104606	51110819	8.737278	0.224627
30562 Amp	20	51110858	51129662	9.054921	0.22867
30563 Amp	20	51131020	51131020	8.936662	0.227311
30564 Amp	20	51132770	51132770	9.155442	0.230063
30565 Amp	20	51139689	51140869	9.02098	0.228227
30566 Amp	20	51143362	51147486	8.704326	0.224184
30567 Amp	20	51155972	51163062	9.575888	0.235561
30568 Amp	20	51165686	51172323	9.47615	0.234138
30569 Amp	20	51175764	51208337	9.950695	0.240162
30570 Amp	20	51216249	51239225	10.927313	0.252711
30571 Amp	20	51244897	51245897	10.122701	0.24247
30572 Amp	20	51248460	51268476	10.414382	0.246168
30573 Amp	20	51268832	51269770	10.773589	0.250797
30574 Amp	20	51272039	51278800	10.243045	0.243878
30575 Amp	20	51281567	51311883	10.893171	0.252108
30576 Amp	20	51313156	51323689	11.049458	0.254185
30577 Amp	20	51325176	51349708	10.618056	0.248716
30578 Amp	20	51353566	51355731	10.566416	0.248165
30579 Amp	20	51358442	51418416	10.534461	0.247704
30580 Amp	20	51419700	51477207	11.308217	0.257508

30581	Amp	20	51477390	51478896	11.536709	0.260175
30582	Amp	20	51484560	51487320	12.473924	0.271533
30583	Amp	20	51491244	51518144	13.093327	0.278957
30584	Amp	20	51519260	51530532	13.363175	0.282096
30585	Amp	20	51530861	51533921	13.310331	0.281619
30586	Amp	20	51534134	51538847	13.454936	0.28317
30587	Amp	20	51539420	51566701	12.873531	0.276398
30588	Amp	20	51566966	51574025	12.329307	0.26972
30589	Amp	20	51574317	51610753	11.804955	0.263559
30590	Amp	20	51611004	51637621	11.680554	0.261976
30591	Amp	20	51640589	51655623	11.308217	0.257348
30592	Amp	20	51656616	51657640	10.9963	0.253459
30593	Amp	20	51667081	51667081	11.857518	0.26419
30594	Amp	20	51667598	51667598	11.752146	0.262757
30595	Amp	20	51668909	51688027	11.787092	0.263249
30596	Amp	20	51688301	51693063	11.875821	0.264263
30597	Amp	20	51693461	51706078	11.911982	0.26474
30598	Amp	20	51706309	51791427	11.770282	0.263053
30599	Amp	20	51794628	51806016	11.822516	0.263723
30600	Amp	20	51806028	51806135	11.662686	0.261705
30601	Amp	20	51832686	51842991	10.877729	0.251901
30602	Amp	20	51843411	51846531	11.031992	0.25385
30603	Amp	20	51850873	51906271	11.239373	0.256606
30604	Amp	20	51907448	51908110	10.346125	0.245228
30605	Amp	20	51911233	51923515	10.566416	0.248024
30606	Amp	20	51924578	51934711	10.414382	0.246156
30607	Amp	20	51946940	51949289	11.081157	0.254518
30608	Amp	20	51949376	51949783	11.27356	0.257042
30609	Amp	20	51951043	51956695	10.961251	0.25302
30610	Amp	20	51958088	51990735	11.1336	0.255246
30611	Amp	20	51991484	52006649	11.572965	0.26071
30612	Amp	20	52011229	52027936	11.930548	0.26499
30613	Amp	20	52031054	52044359	12.130806	0.2674
30614	Amp	20	52044452	52046422	12.401399	0.270762
30615	Amp	20	52046539	52066076	12.057562	0.266625
30616	Amp	20	52067371	52072744	12.075847	0.266694
30617	Amp	20	52075535	52079310	13.398977	0.28263
30618	Amp	20	52082504	52112459	13.129509	0.279299
30619	Amp	20	52115232	52120996	13.345022	0.281865
30620	Amp	20	52123563	52123563	13.293073	0.281373
30621	Amp	20	52123750	52136218	13.746952	0.286834
30622	Amp	20	52139340	52145277	13.583613	0.284755
30623	Amp	20	52145709	52171196	12.983395	0.277525
30624	Amp	20	52171478	52183443	13.074762	0.278674
30625	Amp	20	52183980	52187221	13.454936	0.28327
30626	Amp	20	52195554	52211819	13.0564	0.278554
30627	Amp	20	52220647	52254398	13.129509	0.279312
30628	Amp	20	52257883	52277015	12.401399	0.270712
30629	Amp	20	52277319	52278796	12.364848	0.270235
30630	Amp	20	52278832	52295405	12.653484	0.273689
30631	Amp	20	52300383	52324232	11.787092	0.263258
30632	Amp	20	52325864	52335204	12.112615	0.267264
30633	Amp	20	52335842	52347428	11.840411	0.263903

30634 Amp	20	52347700	52352379	11.627141	0.26137
30635 Amp	20	52356417	52378513	11.430328	0.259008
30636 Amp	20	52380665	52417772	11.894175	0.264508
30637 Amp	20	52419025	52439907	12.003259	0.265813
30638 Amp	20	52449138	52449292	12.564467	0.272543
30639 Amp	20	52456081	52459734	13.019814	0.27812
30640 Amp	20	52461754	52477137	13.328506	0.28183
30641 Amp	20	52477334	52497433	13.381152	0.282413
30642 Amp	20	52501338	52505541	13.546866	0.284266
30643 Amp	20	52509510	52536024	13.636576	0.285479
30644 Amp	20	52537893	52558981	13.528722	0.284079
30645 Amp	20	52560557	52586947	13.764867	0.28686
30646 Amp	20	52588695	52602461	13.985765	0.289555
30647 Amp	20	52605707	52606521	14.577855	0.29657
30648 Amp	20	52606669	52623072	14.466246	0.295265
30649 Amp	20	52623284	52645136	14.613267	0.296887
30650 Amp	20	52645356	52664028	14.300473	0.293237
30651 Amp	20	52668633	52673780	14.95044	0.300851
30652 Amp	20	52675421	52676795	15.519848	0.307305
30653 Amp	20	52676818	52677905	15.40422	0.305987
30654 Amp	20	52678307	52686534	15.11973	0.302853
30655 Amp	20	52692805	52697981	14.743668	0.298461
30656 Amp	20	52698656	52702582	14.855744	0.299815
30657 Amp	20	52729148	52730370	14.595893	0.296776
30658 Amp	20	52732128	52744101	14.355711	0.293891
30659 Amp	20	52744518	52747657	14.893571	0.300247
30660 Amp	20	52748151	52753248	15.042671	0.30183
30661 Amp	20	52756999	52776235	14.206189	0.292063
30662 Amp	20	52776919	52779108	15.233242	0.304071
30663 Amp	20	52785193	52811800	15.595327	0.308298
30664 Amp	20	52813369	52818637	14.560384	0.296291
30665 Amp	20	52821018	52822771	14.87478	0.300029
30666 Amp	20	52823895	52830327	15.156258	0.303172
30667 Amp	20	52831495	52845818	15.366356	0.305604
30668 Amp	20	52898082	52898082	14.725554	0.298276
30669 Amp	20	52898333	52913853	14.206189	0.29226
30670 Amp	20	52914186	52934856	14.151192	0.291589
30671 Amp	20	52935062	52935062	14.022374	0.290034
30672 Amp	20	52937534	52946001	13.746952	0.286757
30673 Amp	20	52946443	52969158	14.447899	0.294955
30674 Amp	20	52973995	52973995	14.706125	0.297994
30675 Amp	20	52974738	52985949	14.855744	0.299847
30676 Amp	20	52989656	52996894	16.242583	0.315516
30677 Amp	20	53005383	53006573	16.014682	0.313084
30678 Amp	20	53006663	53017886	15.881803	0.311501
30679 Amp	20	53022971	53050295	15.959834	0.312317
30680 Amp	20	53052873	53074067	16.340767	0.316625
30681 Amp	20	53074391	53085791	16.475247	0.318278
30682 Amp	20	53086207	53092553	16.282141	0.316059
30683 Amp	20	53093423	53102136	16.242583	0.315622
30684 Amp	20	53102570	53108626	16.493379	0.318419
30685 Amp	20	53110370	53117584	16.879456	0.322845
30686 Amp	20	53117693	53117962	17.392969	0.328508

30687 Amp	20	53121481	53124525	17.910589	0.334166
30688 Amp	20	53127277	53153065	18.088995	0.336058
30689 Amp	20	53153231	53166147	18.584844	0.341401
30690 Amp	20	53167661	53167661	18.704216	0.342861
30691 Amp	20	53167766	53170211	18.923342	0.345143
30692 Amp	20	53171178	53184363	19.491404	0.351289
30693 Amp	20	53185049	53189390	19.552203	0.351826
30694 Amp	20	53189762	53192474	19.267324	0.348907
30695 Amp	20	53192999	53207395	19.409636	0.35046
30696 Amp	20	53210117	53213238	19.572994	0.352084
30697 Amp	20	53216237	53227195	19.93106	0.356104
30698 Amp	20	53231111	53231111	20.114426	0.358065
30699 Amp	20	53236495	53251317	20.440924	0.361354
30700 Amp	20	53251480	53270555	19.993383	0.35657
30701 Amp	20	53271118	53294248	19.812781	0.354619
30702 Amp	20	53299867	53308970	20.420161	0.361118
30703 Amp	20	53309296	53335552	20.338306	0.360361
30704 Amp	20	53336542	53383948	20.686425	0.364096
30705 Amp	20	53388544	53397906	21.242402	0.369903
30706 Amp	20	53398268	53434904	21.367762	0.371173
30707 Amp	20	53439069	53439410	21.557483	0.373184
30708 Amp	20	53441149	53441149	22.782563	0.38585
30709 Amp	20	53510024	53510504	25.157792	0.409657
30710 Amp	20	53510548	53520661	25.350724	0.411511
30711 Amp	20	53525664	53525664	26.935173	0.427332
30712 Amp	20	53527801	53527801	26.511296	0.423128
30713 Amp	20	53529335	53531956	26.308427	0.421058
30714 Amp	20	53534646	53557356	27.645574	0.434211
30715 Amp	20	53559068	53561351	26.957867	0.42737
30716 Amp	20	53562700	53572739	26.824594	0.426133
30717 Amp	20	53573412	53622571	27.069594	0.428563
30718 Amp	20	53624132	53624146	27.493371	0.432681
30719 Amp	20	53629166	53633236	27.75579	0.435246
30720 Amp	20	53634281	53656141	28.536153	0.442787
30721 Amp	20	53656198	53661719	28.739537	0.444737
30722 Amp	20	53680236	53692898	28.58071	0.443325
30723 Amp	20	53692913	53703011	28.896188	0.446235
30724 Amp	20	53703105	53735571	29.074878	0.448027
30725 Amp	20	53736099	53736651	29.750472	0.454685
30726 Amp	20	53736769	53743012	29.568915	0.452793
30727 Amp	20	53743038	53756553	29.276464	0.450072
30728 Amp	20	53757686	53810780	28.446018	0.441991
30729 Amp	20	53810937	53824302	28.001632	0.437647
30730 Amp	20	53825062	53834428	27.824091	0.435942
30731 Amp	20	53839906	53850438	27.223856	0.430094
30732 Amp	20	53852153	53864881	26.756945	0.425518
30733 Amp	20	53867869	53869880	27.357173	0.431446
30734 Amp	20	53871483	53879462	26.601595	0.423865
30735 Amp	20	53883654	53884966	26.177122	0.419846
30736 Amp	20	53885382	53885573	26.022514	0.418287
30737 Amp	20	53889922	53889922	25.850269	0.41663
30738 Amp	20	53891365	53898977	25.092641	0.408986
30739 Amp	20	53901219	53912688	24.56561	0.403844

30740	Amp	20	53915790	53919810	24.086469	0.399071
30741	Amp	20	53919886	53924067	24.284149	0.40097
30742	Amp	20	53926059	53929550	24.086469	0.399092
30743	Amp	20	53930271	53961068	23.956816	0.397632
30744	Amp	20	53963901	53969559	23.304899	0.391147
30745	Amp	20	53969580	53990857	23.069701	0.38877
30746	Amp	20	53991684	54021975	22.967626	0.387756
30747	Amp	20	54022745	54058649	22.294943	0.380825
30748	Amp	20	54059508	54061871	22.088184	0.378522
30749	Amp	20	54061925	54062598	22.401928	0.381811
30750	Amp	20	54062640	54064489	22.676769	0.384744
30751	Amp	20	54064568	54065071	22.842906	0.386497
30752	Amp	20	54070192	54071767	22.761806	0.385601
30753	Amp	20	54074127	54085397	22.803189	0.386052
30754	Amp	20	54085647	54085647	22.337402	0.381264
30755	Amp	20	54085787	54101190	22.401928	0.381929
30756	Amp	20	54104303	54130130	22.550798	0.383353
30757	Amp	20	54130897	54133520	22.130946	0.379045
30758	Amp	20	54134851	54150268	20.974304	0.367107
30759	Amp	20	54151823	54191523	20.667704	0.363782
30760	Amp	20	54192240	54211807	20.134537	0.358125
30761	Amp	20	54211845	54214923	20.255854	0.359585
30762	Amp	20	54214957	54238968	20.482298	0.361791
30763	Amp	20	54238973	54239779	19.93106	0.355914
30764	Amp	20	54248501	54248501	19.712397	0.353589
30765	Amp	20	54249296	54251574	18.624086	0.341803
30766	Amp	20	54251904	54256153	18.584844	0.341352
30767	Amp	20	54258540	54261209	18.049178	0.335559
30768	Amp	20	54261239	54263287	17.611387	0.330822
30769	Amp	20	54263980	54270492	17.831836	0.333201
30770	Amp	20	54272589	54286510	18.049178	0.335486
30771	Amp	20	54288100	54288738	17.591497	0.330607
30772	Amp	20	54289298	54295244	17.11708	0.325427
30773	Amp	20	54296158	54297826	15.785635	0.310358
30774	Amp	20	54298189	54315187	15.576957	0.307971
30775	Amp	20	54318950	54320253	16.126505	0.314218
30776	Amp	20	54320554	54331281	15.977907	0.312519
30777	Amp	20	54331578	54355312	16.475247	0.318203
30778	Amp	20	54355368	54360021	16.321318	0.316558
30779	Amp	20	54361264	54386155	15.385452	0.305894
30780	Amp	20	54388448	54397745	15.805309	0.31071
30781	Amp	20	54400111	54407275	16.184688	0.314957
30782	Amp	20	54407460	54449182	15.690603	0.309274
30783	Amp	20	54451874	54451874	15.519848	0.307375
30784	Amp	20	54458447	54458447	16.397251	0.317352
30785	Amp	20	54459698	54471905	16.879456	0.322788
30786	Amp	20	54476490	54481784	16.704765	0.320879
30787	Amp	20	54489521	54489814	16.378681	0.317229
30788	Amp	20	54491003	54515978	17.792544	0.33279
30789	Amp	20	54522046	54522046	17.412814	0.328739
30790	Amp	20	54524326	54525044	17.968693	0.334786
30791	Amp	20	54527706	54527706	16.570587	0.319224
30792	Amp	20	54529702	54529819	16.686433	0.320673

30793 Amp	20	54535319	54549073	15.805309	0.310696
30794 Amp	20	54551284	54552382	16.456552	0.31791
30795 Amp	20	54556389	54556618	16.089694	0.313837
30796 Amp	20	54560149	54579547	15.461712	0.306623
30797 Amp	20	54579999	54586887	15.024711	0.30169
30798 Amp	20	54586942	54588857	15.213793	0.303846
30799 Amp	20	54593876	54612815	14.912742	0.300422
30800 Amp	20	54617702	54618189	14.762523	0.298757
30801 Amp	20	54623891	54627659	13.381152	0.28229
30802 Amp	20	54630160	54643402	13.184	0.27991
30803 Amp	20	54643425	54651764	12.909694	0.276764
30804 Amp	20	54657841	54657841	13.276743	0.28102
30805 Amp	20	54659766	54659766	12.81801	0.275581
30806 Amp	20	54665002	54688332	12.582592	0.272799
30807 Amp	20	54688631	54696086	12.27458	0.269236
30808 Amp	20	54698444	54709776	11.930548	0.264979
30809 Amp	20	54711882	54774620	11.627141	0.261431
30810 Amp	20	54774837	54780358	11.734451	0.262615
30811 Amp	20	54780642	54780642	12.20153	0.268334
30812 Amp	20	54794480	54796361	12.456062	0.271263
30813 Amp	20	54797778	54798135	12.653484	0.27372
30814 Amp	20	54801180	54823202	12.873531	0.276223
30815 Amp	20	54824106	54826968	12.653484	0.273655
30816 Amp	20	54827024	54827341	12.745398	0.274788
30817 Amp	20	54828106	54839810	12.27458	0.269068
30818 Amp	20	54840382	54854044	12.329307	0.269786
30819 Amp	20	54856895	54888062	12.763765	0.274963
30820 Amp	20	54888352	54888352	13.184	0.280046
30821 Amp	20	54888910	54889360	13.146748	0.279477
30822 Amp	20	54894390	54942654	12.653484	0.273671
30823 Amp	20	54951470	54962062	11.360465	0.258002
30824 Amp	20	54963250	54992437	11.049458	0.254068
30825 Amp	20	54993426	55007820	11.239373	0.256467
30826 Amp	20	55007965	55017913	11.1336	0.255334
30827 Amp	20	55018221	55027828	11.39504	0.258566
30828 Amp	20	55028055	55033347	11.290117	0.257212
30829 Amp	20	55033520	55043960	10.860138	0.251787
30830 Amp	20	55051924	55071777	11.308217	0.257462
30831 Amp	20	55077492	55093626	11.609398	0.261029
30832 Amp	20	55096534	55098899	10.634596	0.24892
30833 Amp	20	55104286	55111516	10.225188	0.243836
30834 Amp	20	55111801	55128464	9.933591	0.24001
30835 Amp	20	55129442	55149209	9.559998	0.235219
30836 Amp	20	55149523	55211144	8.818686	0.225822
30837 Amp	20	55213862	55219234	9.240499	0.23106
30838 Amp	20	55220746	55253258	8.60549	0.222982
30839 Amp	20	55256275	55256879	8.47374	0.221128
30840 Amp	20	55257542	55257542	8.885533	0.22666
30841 Amp	20	55257762	55287727	8.770924	0.225038
30842 Amp	20	55290158	55330410	9.39097	0.233002
30843 Amp	20	55333515	55343594	9.575888	0.235497
30844 Amp	20	55346342	55356644	10.686888	0.249701
30845 Amp	20	55358115	55358115	10.634596	0.249036

30846 Amp	20	55358983	55386833	10.414382	0.246125
30847 Amp	20	55391043	55415015	9.984309	0.240769
30848 Amp	20	55415929	55420215	10.034845	0.241413
30849 Amp	20	55420440	55423515	9.847525	0.238918
30850 Amp	20	55425056	55433792	10.414382	0.246105
30851 Amp	20	55435983	55436527	10.8261	0.251248
30852 Amp	20	55437576	55438127	10.549344	0.247952
30853 Amp	20	55438182	55469504	10.703463	0.249859
30854 Amp	20	55469649	55469649	10.756361	0.250412
30855 Amp	20	55470458	55472039	10.720769	0.249955
30856 Amp	20	55472561	55475672	10.294658	0.244689
30857 Amp	20	55475904	55489802	10.499721	0.247174
30858 Amp	20	55492803	55506267	10.017642	0.241235
30859 Amp	20	55549400	55551096	8.868563	0.226421
30860 Amp	20	55552115	55575102	9.20619	0.230748
30861 Amp	20	55576130	55599040	8.770924	0.225021
30862 Amp	20	55599668	55613582	9.004172	0.228031
30863 Amp	20	55614116	55629681	8.754268	0.224799
30864 Amp	20	55630219	55636902	9.122122	0.229584
30865 Amp	20	55641936	55677152	8.953805	0.22756
30866 Amp	20	55677836	55682857	9.054921	0.228736
30867 Amp	20	55683132	55684281	9.442896	0.23384
30868 Amp	20	55700507	55748393	9.256952	0.231354
30869 Amp	20	55748850	55748850	8.987196	0.227787
30870 Amp	20	55753221	55757504	8.868563	0.22636
30871 Amp	20	55758269	55759633	8.720312	0.224349
30872 Amp	20	55761125	55763509	8.671092	0.223705
30873 Amp	20	55765025	55767093	8.555266	0.222256
30874 Amp	20	55768295	55778434	8.458247	0.22098
30875 Amp	20	55778772	55816265	7.704855	0.210813
30876 Amp	20	55820112	55824446	8.154198	0.216858
30877 Amp	20	55833038	55847583	7.704855	0.210813
30878 Amp	20	55848001	55869278	7.6425	0.209847
30879 Amp	20	55869980	55898430	7.865467	0.212811
30880 Amp	20	55899067	55899067	7.657996	0.210185
30881 Amp	20	55899252	55914860	7.389113	0.206394
30882 Amp	20	55914950	55914950	7.75231	0.211335
30883 Amp	20	55929540	55932757	8.073986	0.215713
30884 Amp	20	55933660	55936534	8.105491	0.216186
30885 Amp	20	55936625	55941282	7.673484	0.21023
30886 Amp	20	55942883	55943936	8.217372	0.21768
30887 Amp	20	55948107	55957176	8.6219	0.223167
30888 Amp	20	55957815	55957815	8.538535	0.221992
30889 Amp	20	55957834	55965272	8.105491	0.216115
30890 Amp	20	55965379	55993027	7.736725	0.211116
30891 Amp	20	55999488	56018994	6.825391	0.198567
30892 Amp	20	56021059	56022135	7.042879	0.201576
30893 Amp	20	56022887	56067168	7.011899	0.201087
30894 Amp	20	56069118	56069322	7.090354	0.202185
30895 Amp	20	56071855	56080250	7.437327	0.207097
30896 Amp	20	56087095	56090833	7.704855	0.210693
30897 Amp	20	56094366	56096562	7.75231	0.211361
30898 Amp	20	56097838	56131590	7.437327	0.207024

30899	Amp	20	56133331	56138027	7.833797	0.212434
30900	Amp	20	56139415	56140244	7.785234	0.211859
30901	Amp	20	56143868	56147076	7.578613	0.209006
30902	Amp	20	56147176	56175059	7.421526	0.206849
30903	Amp	20	56183811	56212516	7.865467	0.212915
30904	Amp	20	56217792	56225590	7.373439	0.206181
30905	Amp	20	56225778	56242106	7.801564	0.212037
30906	Amp	20	56242766	56267624	7.689477	0.210452
30907	Amp	20	56268969	56279355	7.594603	0.209268
30908	Amp	20	56282964	56287527	7.232046	0.204267
30909	Amp	20	56289433	56302418	7.310837	0.205344
30910	Amp	20	56302854	56334147	7.090354	0.202267
30911	Amp	20	56337844	56354810	6.871512	0.199162
30912	Amp	20	56356081	56376199	6.65628	0.196283
30913	Amp	20	56378228	56390845	7.058587	0.201872
30914	Amp	20	56390932	56391090	7.326935	0.205463
30915	Amp	20	56391777	56397240	7.610655	0.209381
30916	Amp	20	56397638	56408717	7.405471	0.206648
30917	Amp	20	56409479	56416484	7.232046	0.204146
30918	Amp	20	56420080	56425893	6.825391	0.19854
30919	Amp	20	56428601	56428601	6.549814	0.194729
30920	Amp	20	56429778	56435641	6.747966	0.197459
30921	Amp	20	56437262	56442244	6.79429	0.198176
30922	Amp	20	56444194	56454702	7.199931	0.203778
30923	Amp	20	56459548	56470769	6.996189	0.201048
30924	Amp	20	56476199	56476301	6.564374	0.194873
30925	Amp	20	56477864	56489602	6.488712	0.193873
30926	Amp	20	56491577	56499567	6.2443	0.190282
30927	Amp	20	56503517	56531604	6.380906	0.19231
30928	Amp	20	56536776	56539508	6.579755	0.195079
30929	Amp	20	56540778	56541941	6.503622	0.194002
30930	Amp	20	56558355	56567062	6.671073	0.196375
30931	Amp	20	56568278	56568278	7.833797	0.212441
30932	Amp	20	56573752	56573752	8.008326	0.214764
30933	Amp	20	56574229	56587388	8.378151	0.219765
30934	Amp	20	56589768	56619083	8.441738	0.220808
30935	Amp	20	56619487	56625734	8.770924	0.225086
30936	Amp	20	56635310	56635439	8.588477	0.22273
30937	Amp	20	56636166	56636650	9.155442	0.230133
30938	Amp	20	56643725	56679998	9.357173	0.232574
30939	Amp	20	56680231	56680418	9.678773	0.236806
30940	Amp	20	56680830	56695969	9.559998	0.235167
30941	Amp	20	56700846	56700846	9.272857	0.231515
30942	Amp	20	56702547	56717170	9.508958	0.234545
30943	Amp	20	56717423	56719483	9.442896	0.233709
30944	Amp	20	56781498	56781498	9.644619	0.23635
30945	Amp	20	56784762	56796525	9.256952	0.231262
30946	Amp	20	56796595	56796595	9.763565	0.237824
30947	Amp	20	56796859	56821601	9.54314	0.235114
30948	Amp	20	56821672	56824385	9.780264	0.238173
30949	Amp	20	56825399	56870221	9.240499	0.231129
30950	Amp	20	56870276	56901891	8.953805	0.22749
30951	Amp	20	56903129	56925381	9.272857	0.231593

30952 Amp	20	56930750	56933613	8.851521	0.226104
30953 Amp	20	56934774	56934774	9.627477	0.236243
30954 Amp	20	56934840	56946365	9.575888	0.235526
30955 Amp	20	56947460	56949733	9.442896	0.233672
30956 Amp	20	56952719	56966404	9.763565	0.23779
30957 Amp	20	56966818	56968690	9.442896	0.233672
30958 Amp	20	56970912	56979067	9.661346	0.23657
30959 Amp	20	56979995	56991302	9.004172	0.228125
30960 Amp	20	56991724	57022376	9.458858	0.233878
30961 Amp	20	57022772	57025820	9.54314	0.23502
30962 Amp	20	57027807	57032528	9.967648	0.24042
30963 Amp	20	57032728	57036756	9.54314	0.23501
30964 Amp	20	57038156	57052158	9.122122	0.22961
30965 Amp	20	57061636	57064806	9.712919	0.237333
30966 Amp	20	57064885	57069217	9.830028	0.238846
30967 Amp	20	57070652	57071392	11.081157	0.254666
30968 Amp	20	57071403	57102055	11.308217	0.257359
30969 Amp	20	57104129	57110029	12.021369	0.266043
30970 Amp	20	57110371	57140527	12.130806	0.267486
30971 Amp	20	57141188	57150087	12.473924	0.27157
30972 Amp	20	57150675	57155325	12.1666	0.267817
30973 Amp	20	57156588	57167080	11.875821	0.264387
30974 Amp	20	57167812	57225284	11.430328	0.25886
30975 Amp	20	57225330	57226609	11.464362	0.259349
30976 Amp	20	57229121	57255061	12.637869	0.27342
30977 Amp	20	57255095	57269326	12.946363	0.277173
30978 Amp	20	57273147	57283533	12.891235	0.276553
30979 Amp	20	57284157	57297833	12.855613	0.276004
30980 Amp	20	57299476	57337703	12.564467	0.272547
30981 Amp	20	57343636	57375984	13.310331	0.281513
30982 Amp	20	57380319	57386300	13.492157	0.283798
30983 Amp	20	57394641	57397861	13.019814	0.278141
30984 Amp	20	57400022	57400022	13.310331	0.281558
30985 Amp	20	57403942	57403942	12.021369	0.266204
30986 Amp	20	57404432	57412108	11.857518	0.264055
30987 Amp	20	57412753	57412753	10.8261	0.251245
30988 Amp	20	57414752	57416767	10.448458	0.246658
30989 Amp	20	57418499	57421839	10.651729	0.249091
30990 Amp	20	57422798	57436598	10.600629	0.248618
30991 Amp	20	57437029	57457686	11.840411	0.263972
30992 Amp	20	57461183	57467810	12.003259	0.265969
30993 Amp	20	57469047	57469047	11.098856	0.254809
30994 Amp	20	57473765	57513902	10.927313	0.252698
30995 Amp	20	57514360	57528700	10.686888	0.249572
30996 Amp	20	57529936	57537141	10.9963	0.253571
30997 Amp	20	57539439	57556761	10.8261	0.251397
30998 Amp	20	57557538	57558205	10.583831	0.248368
30999 Amp	20	57562751	57564911	10.843331	0.251582
31000 Amp	20	57567059	57580947	10.583831	0.248368
31001 Amp	20	57585237	57598413	9.712919	0.237136
31002 Amp	20	57599369	57608480	11.098856	0.254748
31003 Amp	20	57615324	57621387	11.27356	0.256998
31004 Amp	20	57624263	57651881	11.063647	0.254393

31005 Amp	20	57662385	57670055	10.686888	0.249608
31006 Amp	20	57673031	57676505	11.519317	0.260012
31007 Amp	20	57677866	57696661	11.609398	0.261075
31008 Amp	20	57700068	57734913	11.412882	0.258825
31009 Amp	20	57735763	57741316	10.600629	0.248421
31010 Amp	20	57744910	57762136	10.51732	0.247358
31011 Amp	20	57762559	57762559	10.791286	0.250839
31012 Amp	20	57762758	57762758	10.893171	0.252292
31013 Amp	20	57766887	57773534	11.081157	0.254493
31014 Amp	20	57775391	57775460	11.239373	0.256649
31015 Amp	20	57778074	57805985	11.430328	0.258943
31016 Amp	20	57806087	57819621	11.1336	0.255196
31017 Amp	20	57819649	57822525	11.049458	0.254227
31018 Amp	20	57822739	57838121	11.716436	0.262334
31019 Amp	20	57839073	57887337	11.360465	0.258152
31020 Amp	20	57888495	57896718	11.412882	0.25881
31021 Amp	20	57897078	57912333	11.770282	0.263061
31022 Amp	20	57912752	57917391	11.360465	0.258152
31023 Amp	20	57920385	57943989	11.115911	0.255082
31024 Amp	20	57944379	57965689	11.377246	0.258271
31025 Amp	20	57968753	57969007	10.738273	0.250165
31026 Amp	20	57970841	57976528	10.482498	0.246976
31027 Amp	20	57978819	57988845	10.346125	0.245301
31028 Amp	20	57989170	57993748	10.294658	0.244527
31029 Amp	20	57993938	58021469	9.916528	0.239936
31030 Amp	20	58021874	58027312	10.174276	0.243133
31031 Amp	20	58027928	58066548	10.534461	0.247561
31032 Amp	20	58069084	58076486	10.893171	0.252286
31033 Amp	20	58092489	58130231	10.534461	0.247561
31034 Amp	20	58130726	58134234	10.927313	0.252745
31035 Amp	20	58138813	58148814	11.15007	0.255375
31036 Amp	20	58150004	58152349	10.738273	0.250192
31037 Amp	20	58156334	58162583	10.961251	0.252991
31038 Amp	20	58163893	58165851	10.773589	0.250706
31039 Amp	20	58169067	58190296	11.1336	0.255219
31040 Amp	20	58191149	58194309	10.893171	0.252138
31041 Amp	20	58196113	58220978	11.098856	0.254795
31042 Amp	20	58221348	58222961	11.25539	0.256846
31043 Amp	20	58224405	58224456	11.482418	0.259696
31044 Amp	20	58227835	58241417	12.130806	0.267447
31045 Amp	20	58244502	58249118	12.527954	0.272294
31046 Amp	20	58249402	58249402	12.401399	0.270576
31047 Amp	20	58251612	58251827	12.637869	0.273565
31048 Amp	20	58260697	58264405	13.310331	0.28142
31049 Amp	20	58266366	58274343	13.746952	0.286681
31050 Amp	20	58275689	58278788	14.503631	0.295645
31051 Amp	20	58283953	58324445	14.094981	0.290798
31052 Amp	20	58325200	58366672	13.837777	0.287809
31053 Amp	20	58367119	58390286	14.040669	0.290108
31054 Amp	20	58393618	58397574	13.363175	0.282253
31055 Amp	20	58397877	58406609	13.239383	0.280741
31056 Amp	20	58407125	58407241	13.129509	0.279288
31057 Amp	20	58407979	58416027	13.0564	0.27854

31058	Amp	20	58417634	58481874	13.328506	0.281658
31059	Amp	20	58490397	58497384	13.184	0.279903
31060	Amp	20	58498269	58517278	13.510816	0.283878
31061	Amp	20	58517390	58528791	13.202322	0.280318
31062	Amp	20	58531853	58579539	13.276743	0.28119
31063	Amp	20	58579749	58600438	13.618338	0.285171
31064	Amp	20	58600456	58602133	13.856199	0.287933
31065	Amp	20	58602485	58604953	13.636576	0.285491
31066	Amp	20	58606993	58616274	14.577855	0.296453
31067	Amp	20	58616493	58633436	14.07679	0.290597
31068	Amp	20	58633667	58644400	13.892034	0.288495
31069	Amp	20	58645751	58655286	13.729199	0.286498
31070	Amp	20	58656859	58661774	14.094981	0.290913
31071	Amp	20	58664084	58678076	13.929261	0.288847
31072	Amp	20	58680514	58685234	13.746952	0.286691
31073	Amp	20	58685864	58740123	13.583613	0.284875
31074	Amp	20	58743065	58769837	13.276743	0.281123
31075	Amp	20	58770148	58777109	13.565854	0.284618
31076	Amp	20	58778527	58780493	13.111032	0.27907
31077	Amp	20	58780730	58790600	12.708276	0.274296
31078	Amp	20	58795974	58813105	12.473924	0.271455
31079	Amp	20	58814365	58908174	12.184583	0.26796
31080	Amp	20	58909030	58911925	12.347637	0.270089
31081	Amp	20	58912150	58938353	11.804955	0.263505
31082	Amp	20	58939371	58940048	12.237724	0.26867
31083	Amp	20	58943408	58965727	11.822516	0.263671
31084	Amp	20	58981914	58990083	12.364848	0.270255
31085	Amp	20	58991582	58991625	12.112615	0.267246
31086	Amp	20	58996357	59009835	13.345022	0.281863
31087	Amp	20	59012333	59014069	12.983395	0.2777
31088	Amp	20	59019375	59026064	12.347637	0.269989
31089	Amp	20	59027736	59085809	12.510219	0.271894
31090	Amp	20	59089533	59093234	12.130806	0.267483
31091	Amp	20	59093299	59126630	10.843331	0.251662
31092	Amp	20	59126927	59130952	11.698383	0.262129
31093	Amp	20	59131681	59136391	11.27356	0.256925
31094	Amp	20	59144236	59144236	11.098856	0.254907
31095	Amp	20	59148075	59187862	10.618056	0.248769
31096	Amp	20	59188362	59196555	10.361022	0.245371
31097	Amp	20	59197642	59203866	10.534461	0.247663
31098	Amp	20	59207073	59245339	10.346125	0.245364
31099	Amp	20	59249905	59259220	11.013813	0.253809
31100	Amp	20	59260334	59288966	11.239373	0.25648
31101	Amp	20	59290821	59323743	11.519317	0.259966
31102	Amp	20	59324068	59346585	11.734451	0.262559
31103	Amp	20	59349192	59350828	13.0564	0.278379
31104	Amp	20	59353611	59353611	14.374364	0.294088
31105	Amp	20	59354339	59359779	13.78167	0.287075
31106	Amp	20	59360011	59372917	14.300473	0.293229
31107	Amp	20	59374420	59391556	14.393488	0.294404
31108	Amp	20	59392620	59392851	13.856199	0.287955
31109	Amp	20	59393262	59397571	13.91041	0.288707
31110	Amp	20	59398069	59408822	13.710777	0.286385

31111	Amp	20	59409815	59417408	13.528722	0.284092
31112	Amp	20	59417701	59464827	13.293073	0.281237
31113	Amp	20	59465817	59474364	12.527954	0.272273
31114	Amp	20	59480731	59531631	12.800156	0.275523
31115	Amp	20	59545900	59559160	13.111032	0.279186
31116	Amp	20	59561322	59623253	12.637869	0.273393
31117	Amp	20	59623270	59631263	12.946363	0.277262
31118	Amp	20	59631654	59640217	13.381152	0.28236
31119	Amp	20	59640701	59645553	13.618338	0.285215
31120	Amp	20	59646521	59649639	13.837777	0.287812
31121	Amp	20	59658982	59663556	13.398977	0.282715
31122	Amp	20	59664810	59741136	13.583613	0.284775
31123	Amp	20	59741431	59754778	13.492157	0.28365
31124	Amp	20	59756228	59767213	13.417296	0.282898
31125	Amp	20	59769298	59780318	13.655032	0.285663
31126	Amp	20	59780736	59780736	13.691717	0.286139
31127	Amp	20	59781580	59781580	13.565854	0.284527
31128	Amp	20	59782884	59800947	13.91041	0.288692
31129	Amp	20	59803710	59832650	12.582592	0.272756
31130	Amp	20	59832827	59835526	11.984978	0.265669
31131	Amp	20	59835606	59862601	11.930548	0.265105
31132	Amp	20	59878803	59892221	12.148216	0.267636
31133	Amp	20	59897726	59897726	11.536709	0.260187
31134	Amp	20	59900135	59906790	10.843331	0.251649
31135	Amp	20	59907417	59927593	10.651729	0.249192
31136	Amp	20	59928686	59963202	10.346125	0.245174
31137	Amp	20	59964256	59968695	10.756361	0.250539
31138	Amp	20	59969692	59969692	11.500885	0.259812
31139	Amp	20	59972608	59994623	11.098856	0.254728
31140	Amp	20	59995534	60028562	10.877729	0.252014
31141	Amp	20	60031952	60031952	11.063647	0.254316
31142	Amp	20	60033355	60085206	11.27356	0.256894
31143	Amp	20	60086366	60094080	11.063647	0.254363
31144	Amp	20	60095894	60095894	10.260441	0.244087
31145	Amp	20	60096466	60131450	10.208075	0.243611
31146	Amp	20	60131496	60149835	10.618056	0.248695
31147	Amp	20	60149964	60153799	9.493034	0.234463
31148	Amp	20	60155915	60169641	9.172152	0.230382
31149	Amp	20	60170792	60175337	9.678773	0.236692
31150	Amp	20	60176202	60240455	8.458247	0.220872
31151	Amp	20	60240519	60273676	8.169654	0.217002
31152	Amp	20	60273745	60292330	8.424923	0.220571
31153	Amp	20	60293356	60294482	8.6219	0.223019
31154	Amp	20	60294878	60318057	9.038034	0.228514
31155	Amp	20	60319821	60347260	8.802762	0.225453
31156	Amp	20	60347607	60359081	8.47374	0.221068
31157	Amp	20	60361300	60367819	8.282609	0.21862
31158	Amp	20	60370986	60427838	8.489568	0.221328
31159	Amp	20	60427897	60445044	8.057852	0.215596
31160	Amp	20	60445635	60450006	8.32882	0.219261
31161	Amp	20	60453827	60466963	7.977624	0.214502
31162	Amp	20	60467146	60467358	7.578613	0.209049
31163	Amp	20	60469298	60469581	7.516099	0.208029

31164	Amp	20	60470415	60503016	7.578613	0.20894
31165	Amp	20	60506638	60515850	7.405471	0.206506
31166	Amp	20	60523338	60526857	7.485352	0.207677
31167	Amp	20	60532296	60567228	7.199931	0.20388
31168	Amp	20	60570516	60580782	6.747966	0.19757
31169	Amp	20	60581767	60581767	6.519288	0.19432
31170	Amp	20	60582076	60637921	6.671073	0.19649
31171	Amp	20	60640284	60679162	6.840574	0.198851
31172	Amp	20	60680797	60711690	6.763246	0.197617
31173	Amp	20	60712486	60712486	7.011899	0.201228
31174	Amp	20	60716689	60717098	8.169654	0.217049
31175	Amp	20	60720079	60758364	8.34537	0.21951
31176	Amp	20	60761032	60775666	8.393699	0.219968
31177	Amp	20	60776816	60776816	8.057852	0.215595
31178	Amp	20	60777120	60814571	7.833797	0.212507
31179	Amp	20	60815206	60815206	8.282609	0.218592
31180	Amp	20	60816933	60840114	8.654479	0.223624
31181	Amp	20	60843124	60851396	8.902195	0.226745
31182	Amp	20	60851524	60851524	8.34537	0.21947
31183	Amp	20	60856686	60883724	7.849711	0.212789
31184	Amp	20	60884322	60899371	8.770924	0.225028
31185	Amp	20	60900825	60904284	8.424923	0.220453
31186	Amp	20	60909227	60909227	8.637458	0.223427
31187	Amp	20	60910241	60915234	8.409489	0.220399
31188	Amp	20	60915639	60938762	8.282609	0.218635
31189	Amp	20	60941111	60962060	8.18543	0.217282
31190	Amp	20	60962469	60973708	8.572005	0.222392
31191	Amp	20	60974064	60989035	8.233714	0.217972
31192	Amp	20	61032969	61037483	8.409489	0.220398
31193	Amp	20	61041582	61055940	7.849711	0.212792
31194	Amp	20	61065674	61066911	7.689477	0.210591
31195	Amp	20	61067830	61068594	7.326935	0.205481
31196	Amp	20	61069423	61093750	7.373439	0.20615
31197	Amp	20	61098214	61135942	7.896426	0.213434
31198	Amp	20	61136255	61159029	7.373439	0.20615
31199	Amp	20	61159797	61159797	7.896426	0.213378
31200	Amp	20	61160447	61178031	8.34537	0.219369
31201	Amp	20	61178299	61198732	8.378151	0.219867
31202	Amp	20	61201699	61233602	8.20134	0.217441
31203	Amp	20	61234752	61278882	8.249918	0.218087
31204	Amp	20	61279114	61295317	8.441738	0.220775
31205	Amp	20	61295888	61296702	8.538535	0.222009
31206	Amp	20	61302306	61319137	7.880674	0.21323
31207	Amp	20	61319399	61322106	8.041049	0.215353
31208	Amp	20	61323223	61391791	8.29898	0.218802
31209	Amp	20	61392705	61408401	8.393699	0.22015
31210	Amp	20	61411413	61423835	8.20134	0.217462
31211	Amp	20	61424611	61424972	8.041049	0.215216
31212	Amp	20	61434025	61444691	7.92856	0.213686
31213	Amp	20	61444881	61512800	7.673484	0.210237
31214	Amp	20	61515326	61538219	7.896426	0.213408
31215	Amp	20	61539274	61589644	8.522585	0.22183
31216	Amp	20	61592662	61613628	8.572005	0.222425

31217 Amp	20	61616202	61621952	8.987196	0.227945
31218 Amp	20	61624035	61688728	9.796693	0.238316
31219 Amp	20	61690561	61707906	9.155442	0.230142
31220 Amp	20	61713053	61715436	9.088236	0.229241
31221 Amp	20	61715477	61719644	9.340097	0.232428
31222 Amp	20	61719737	61762696	9.256952	0.231257
31223 Amp	20	61762970	61783213	9.493034	0.234432
31224 Amp	20	61789636	61834745	8.802762	0.225601
31225 Amp	20	61835065	61835065	8.671092	0.223769
31226 Amp	20	61836400	61881335	9.240499	0.231084
31227 Amp	20	61883012	61915842	9.272857	0.231538
31228 Amp	20	61915858	61936132	9.442896	0.233747
31229 Amp	20	61940219	61970102	9.340097	0.232398
31230 Amp	20	61970501	62001936	9.39097	0.23307
31231 Amp	20	62002542	62007781	9.493034	0.234372
31232 Amp	20	62009016	62048212	9.950695	0.240282
31233 Amp	20	62050132	62061673	9.644619	0.236432
31234 Amp	20	62072127	62074878	9.054921	0.228765
31235 Amp	20	62075192	62079504	8.851521	0.226124
31236 Amp	20	62080339	62103556	8.754268	0.224858
31237 Amp	20	62105222	62135221	9.289139	0.231868
31238 Amp	20	62135463	62136161	9.609886	0.235911
31239 Amp	20	62137038	62149852	9.69597	0.237046
31240 Amp	20	62150481	62153711	9.933591	0.240095
31241 Amp	20	62161807	62176988	10.808758	0.251048
31242 Amp	20	62179401	62179401	10.31211	0.244884
31243 Amp	20	62184392	62188341	10.105286	0.242215
31244 Amp	20	62193129	62206338	10.773589	0.250758
31245 Amp	20	62214899	62246779	10.260441	0.244197
31246 Amp	20	62250539	62251711	9.763565	0.23782
31247 Amp	20	62256244	62267877	10.087806	0.242055
31248 Amp	20	62269313	62269313	9.847525	0.238885
31249 Amp	20	62274677	62280406	9.407903	0.233365
31250 Amp	20	62283456	62299134	9.729466	0.237367
31251 Amp	20	62300282	62300282	9.425321	0.233611
31252 Amp	20	62305147	62316232	8.834661	0.225932
31253 Amp	20	62331766	62332100	8.969911	0.22766
31254 Amp	20	62337061	62348076	8.47374	0.221181
31255 Amp	20	62350803	62386309	8.754268	0.224831
31256 Amp	20	62387981	62387981	8.720312	0.224342
31257 Amp	20	62389008	62392109	8.936662	0.227232
31258 Amp	20	62393838	62400808	9.038034	0.228462
31259 Amp	20	62400843	62405352	8.851521	0.226147
31260 Amp	20	62422274	62431702	8.6219	0.223191
31261 Amp	20	62436484	62443209	8.818686	0.225805
31262 Amp	20	62443319	62449442	8.987196	0.227873
31263 Amp	20	62449724	62490138	8.786437	0.225258
31264 Amp	20	62500776	62511210	9.02098	0.228355
31265 Amp	20	62511374	62611524	9.289139	0.231861
31266 Amp	20	62632769	62638388	9.138353	0.229793
31267 Amp	20	62643712	62647433	8.969911	0.227733
31268 Amp	20	62649310	62695865	9.796693	0.238397
31269 Amp	20	62696156	62712536	10.052391	0.241618

31270 Amp	20	62713232	62720438	9.967648	0.240387
31271 Amp	20	62722783	62810463	10.157784	0.242928
31272 Amp	20	62813960	62892960	10.346125	0.245226
31273 Amp	20	62893458	62927814	10.482498	0.247057
31274 Amp	20	62932854	62934086	9.763565	0.237784
31275 Amp	20	62939690	62943172	10.157784	0.242963
31276 Amp	20	62948159	62949530	10.105286	0.242293
31277 Amp	20	62964284	62971638	10.069962	0.241836
31278 Amp	20	62974417	63070677	10.190896	0.243258
31279 Amp	20	63074600	63077356	9.916528	0.239752
31280 Amp	20	63077653	63080185	9.526354	0.234827
31281 Amp	20	63141749	63148632	9.847525	0.23898
31282 Amp	20	63155226	63232289	9.984309	0.240807
31283 Amp	20	63252544	63269158	10.277087	0.244323
31284 Amp	20	63278747	63285965	10.087806	0.242071
31285 Amp	20	63289779	63424660	9.796693	0.238421
31286 Amp	20	63427378	63435440	9.559998	0.2352
31287 Amp	20	63439613	63510156	9.088236	0.2291
31288 Amp	20	63510707	63531914	8.868563	0.226392
31289 Amp	20	63539265	63556063	10.294658	0.244662
31290 Amp	20	63556289	63556493	10.378606	0.245666
31291 Amp	20	63556719	63556719	10.017642	0.241144
31292 Amp	20	63567905	63567905	10.346125	0.245341
31293 Amp	20	63578528	63588502	9.899054	0.239605
31294 Amp	21	13974127	14118973	0	0.024404
31295 Amp	21	14121040	14266612	0	0.024871
31296 Amp	21	14269275	14275110	0	0.024423
31297 Amp	21	14275833	14474591	0	0.023956
31298 Amp	21	14479756	14534899	0	0.00802
31299 Amp	21	14536321	14560310	0	0.010282
31300 Amp	21	14560725	14662917	0	0.00802
31301 Amp	21	14668621	14743691	0	0.012626
31302 Amp	21	14744071	14788030	0	0.01051
31303 Amp	21	14793610	14833427	0	0.005903
31304 Amp	21	14836016	14846888	0	0.007307
31305 Amp	21	14847177	14853155	0	0.010422
31306 Amp	21	14862361	15101699	0	0.008756
31307 Amp	21	15101858	15159913	0	0.009972
31308 Amp	21	15160479	15196375	0	0.014243
31309 Amp	21	15199803	15246144	0	0.015406
31310 Amp	21	15256273	15328642	0	0.01788
31311 Amp	21	15331229	15361620	0	0.023207
31312 Amp	21	15362390	15438791	0	0.021991
31313 Amp	21	15438816	15469641	0	0.023172
31314 Amp	21	15473474	15553866	0	0.021768
31315 Amp	21	15553905	15561971	0	0.026211
31316 Amp	21	15562245	15808783	0	0.021768
31317 Amp	21	15818090	16191078	0	0.016441
31318 Amp	21	16192390	16222084	0	0.012833
31319 Amp	21	16235162	16400372	0	0.01703
31320 Amp	21	16409783	16431256	0	0.020975
31321 Amp	21	16435942	16442124	0	0.022022
31322 Amp	21	16442441	16668982	0	0.017825

31323	Amp	21	16669000	16731467	0	0.019014
31324	Amp	21	16735940	16921337	0	0.012179
31325	Amp	21	16924846	17075001	0	0.014183
31326	Amp	21	17077728	17106237	0	0.017132
31327	Amp	21	17106480	17128402	0	0.016146
31328	Amp	21	17138363	17252388	0	0.021275
31329	Amp	21	17253037	17268739	0	0.018325
31330	Amp	21	17273919	17408537	0	0.012007
31331	Amp	21	17413338	17823949	0	0.012909
31332	Amp	21	17830623	17899674	0	0.015618
31333	Amp	21	17901984	17980778	0	0.012909
31334	Amp	21	17982041	18045155	0	0.011746
31335	Amp	21	18050622	18127037	0	0.009271
31336	Amp	21	18128703	18189038	0	0.007267
31337	Amp	21	18189321	18211293	0	0.004152
31338	Amp	21	18266878	18307643	0	0.006095
31339	Amp	21	18316186	18428344	0	0.005194
31340	Amp	21	18430010	18835717	0	0.008411
31341	Amp	21	18836413	18851640	0	0.009212
31342	Amp	21	18859658	18960335	0	0.012569
31343	Amp	21	18960365	19000479	0	0.009352
31344	Amp	21	19000702	19025379	0	0.009819
31345	Amp	21	19029086	19055807	0	0.012161
31346	Amp	21	19061122	19085723	0	0.013836
31347	Amp	21	19096334	19334522	0	0.018031
31348	Amp	21	19336769	19504174	0	0.013836
31349	Amp	21	19506194	19519366	0	0.013205
31350	Amp	21	19519645	19565839	0	0.015487
31351	Amp	21	19570894	19618307	0	0.020695
31352	Amp	21	19619906	19637047	0	0.024801
31353	Amp	21	19641117	19646580	0	0.022795
31354	Amp	21	19646769	19652132	0	0.021876
31355	Amp	21	19654930	19699244	0	0.017771
31356	Amp	21	19700456	19715478	0	0.017304
31357	Amp	21	19718304	19845180	0	0.015022
31358	Amp	21	19857307	19877950	0	0.014145
31359	Amp	21	19884265	19972255	0	0.013388
31360	Amp	21	19973465	20271880	0	0.011062
31361	Amp	21	20271965	20303606	0	0.015005
31362	Amp	21	20304049	20344687	0	0.017006
31363	Amp	21	20344735	20428325	0	0.020031
31364	Amp	21	20428340	20548597	0	0.018029
31365	Amp	21	20553560	20611579	0	0.022776
31366	Amp	21	20611618	20722402	0	0.026062
31367	Amp	21	20726399	20753519	0	0.027945
31368	Amp	21	20755797	20761782	0	0.03153
31369	Amp	21	20767142	20924579	0	0.027945
31370	Amp	21	20926535	20961981	0	0.02466
31371	Amp	21	20966364	21011073	0	0.022776
31372	Amp	21	21015249	21078651	0	0.031266
31373	Amp	21	21080805	21354580	0	0.026519
31374	Amp	21	21356333	21483571	0	0.02761
31375	Amp	21	21483725	21667437	0	0.033397

31376	Amp	21	21670526	21705637	0	0.031514
31377	Amp	21	21705997	21770521	0	0.032149
31378	Amp	21	21773595	21786905	0	0.024074
31379	Amp	21	21789030	21800075	0	0.02344
31380	Amp	21	21800155	21805185	0	0.026948
31381	Amp	21	21808571	21924184	0	0.030876
31382	Amp	21	21927614	21928444	0	0.03434
31383	Amp	21	21947803	22006863	0	0.030413
31384	Amp	21	22009608	22190055	0	0.026904
31385	Amp	21	22191262	22203758	0	0.030686
31386	Amp	21	22206095	22319471	0	0.028227
31387	Amp	21	22321112	22379557	0	0.024323
31388	Amp	21	22576499	22576499	0	0.020541
31389	Amp	21	22576819	22644664	0	0.023174
31390	Amp	21	22673071	22698424	0	0.028694
31391	Amp	21	22699761	22803187	0	0.029812
31392	Amp	21	22803376	23085532	0	0.024402
31393	Amp	21	23085610	23086045	0	0.02046
31394	Amp	21	23086170	23115151	0	0.025003
31395	Amp	21	23115164	23367100	0	0.028223
31396	Amp	21	23369733	23419770	0	0.024414
31397	Amp	21	23423044	23434596	0	0.017611
31398	Amp	21	23435175	23441087	0	0.021166
31399	Amp	21	23441414	23589106	0	0.022737
31400	Amp	21	23591588	23593938	0	0.024494
31401	Amp	21	23593970	23828573	0	0.022551
31402	Amp	21	23828963	23910875	0	0.016737
31403	Amp	21	23910980	23936508	0	0.02698
31404	Amp	21	23937372	23944944	0	0.02376
31405	Amp	21	23946434	24097978	0	0.026794
31406	Amp	21	24103984	24130715	0	0.024453
31407	Amp	21	24130814	24165980	0	0.023652
31408	Amp	21	24167086	24206371	0	0.013409
31409	Amp	21	24206423	24216026	0	0.012605
31410	Amp	21	24216643	24435880	0	0.013422
31411	Amp	21	24438262	24466404	0	0.013899
31412	Amp	21	24466960	24473523	0	0.01093
31413	Amp	21	24474590	24487963	0	0.016561
31414	Amp	21	24489243	24558224	0	0.01245
31415	Amp	21	24560641	24606468	0	0.011107
31416	Amp	21	24608841	24932515	0	0.012134
31417	Amp	21	24932980	24942019	0	0.019833
31418	Amp	21	24942056	25039364	0	0.022144
31419	Amp	21	25040343	25178847	0	0.024074
31420	Amp	21	25183619	25209639	0	0.02885
31421	Amp	21	25209827	25339531	0	0.033928
31422	Amp	21	25340671	25367833	0	0.032902
31423	Amp	21	25370038	25397616	0	0.025202
31424	Amp	21	25398908	25447979	0	0.017722
31425	Amp	21	25451900	25485577	0	0.017284
31426	Amp	21	25494500	25518538	0	0.014911
31427	Amp	21	25519021	25557001	0	0.015672
31428	Amp	21	25565010	25761479	0	0.025758

31429 Amp	21	25763494	25773517	0	0.024997
31430 Amp	21	25783228	25795572	0	0.021985
31431 Amp	21	25795682	25918019	0	0.020665
31432 Amp	21	25925065	25929026	0	0.02345
31433 Amp	21	25930936	26104770	0	0.025391
31434 Amp	21	26107249	26116450	0	0.029331
31435 Amp	21	26118280	26151670	0	0.03248
31436 Amp	21	26152388	26208964	0	0.029331
31437 Amp	21	26210519	26221221	0	0.026546
31438 Amp	21	26222068	26272271	0	0.031664
31439 Amp	21	26274499	26277417	0	0.029723
31440 Amp	21	26277931	26297111	0	0.027005
31441 Amp	21	26297440	26297440	0	0.029358
31442 Amp	21	26301758	26326743	0	0.030415
31443 Amp	21	26327490	26395366	0	0.028015
31444 Amp	21	26401036	26477733	0	0.026496
31445 Amp	21	26478652	26586409	0	0.025439
31446 Amp	21	26587055	26593387	0	0.026859
31447 Amp	21	26593511	26609408	0	0.025934
31448 Amp	21	26610437	26654193	0	0.025116
31449 Amp	21	26658381	26688292	0	0.022763
31450 Amp	21	26688704	26693648	0	0.025972
31451 Amp	21	26699466	26763965	0	0.029142
31452 Amp	21	26766649	26792220	0	0.025934
31453 Amp	21	26792711	26924070	0	0.024601
31454 Amp	21	26925535	26945816	0	0.033907
31455 Amp	21	26948973	27063575	0	0.024601
31456 Amp	21	27067489	27071455	0	0.020661
31457 Amp	21	27088258	27130885	0	0.021841
31458 Amp	21	27133009	27134618	0	0.02545
31459 Amp	21	27139787	27149167	0	0.022279
31460 Amp	21	27151602	27206837	0	0.018671
31461 Amp	21	27211481	27359138	0	0.026306
31462 Amp	21	27359658	27427105	0	0.024607
31463 Amp	21	27427598	27427645	0	0.034252
31464 Amp	21	27432355	27445140	0	0.040308
31465 Amp	21	27445442	27449574	0	0.030663
31466 Amp	21	27451836	27504973	0	0.024607
31467 Amp	21	27508885	27584825	0	0.026954
31468 Amp	21	27586362	27732169	0	0.028778
31469 Amp	21	27737682	27811877	0	0.026954
31470 Amp	21	27812209	27871070	0	0.029467
31471 Amp	21	27875712	27912117	0	0.026954
31472 Amp	21	27912651	28045883	0	0.025382
31473 Amp	21	28046581	28049437	0	0.01669
31474 Amp	21	28052608	28055779	0	0.026494
31475 Amp	21	28058653	28061247	0	0.030559
31476 Amp	21	28062278	28085833	0	0.039251
31477 Amp	21	28096016	28139595	0	0.045089
31478 Amp	21	28140568	28188489	0	0.043909
31479 Amp	21	28189200	28253603	0	0.041698
31480 Amp	21	28257844	28394475	0	0.03948
31481 Amp	21	28394559	28454414	0	0.042916

31482	Amp	21	28455643	28550068	0	0.045612
31483	Amp	21	28554425	28689209	0	0.042253
31484	Amp	21	28693785	28731476	0	0.03482
31485	Amp	21	28732078	28745928	0	0.037485
31486	Amp	21	28746004	28777296	0	0.034067
31487	Amp	21	28777632	28938374	0	0.03919
31488	Amp	21	28939398	29018966	0	0.045808
31489	Amp	21	29019272	29062544	0	0.044208
31490	Amp	21	29065418	29155923	0	0.044659
31491	Amp	21	29160465	29172802	0	0.039353
31492	Amp	21	29173679	29208686	0	0.04044
31493	Amp	21	29209574	29210706	0	0.042615
31494	Amp	21	29213186	29223524	0	0.048396
31495	Amp	21	29229195	29237253	0	0.047945
31496	Amp	21	29241145	29283290	0	0.03411
31497	Amp	21	29285512	29290693	0	0.031958
31498	Amp	21	29291400	29459232	0	0.033949
31499	Amp	21	29460151	29477295	0	0.037535
31500	Amp	21	29477375	29653505	0	0.049499
31501	Amp	21	29653876	29680575	0	0.053325
31502	Amp	21	29685984	29764010	0	0.049499
31503	Amp	21	29765463	29915838	0	0.043675
31504	Amp	21	29915891	29917613	0	0.044597
31505	Amp	21	29918000	30037472	0	0.048658
31506	Amp	21	30039057	30190949	0	0.046185
31507	Amp	21	30193050	30248833	0	0.039521
31508	Amp	21	30249408	30510388	0	0.035462
31509	Amp	21	30515309	30519317	0	0.03454
31510	Amp	21	30522334	30522334	0	0.025522
31511	Amp	21	30523442	30584989	0	0.027594
31512	Amp	21	30592942	30610148	0	0.011658
31513	Amp	21	30610669	30632027	0	0.012697
31514	Amp	21	30638389	30672491	0	0.013137
31515	Amp	21	30675446	30705096	0	0.014994
31516	Amp	21	30705213	30706741	0	0.012922
31517	Amp	21	30707396	30707396	0	0.011481
31518	Amp	21	30879355	30879355	0	0.020082
31519	Amp	21	30879818	30895443	0	0.019043
31520	Amp	21	30900583	30901666	0	0.012412
31521	Amp	21	30902227	30947145	0	0.010555
31522	Amp	21	30949531	31036916	0	0.009551
31523	Amp	21	31036972	31054913	0	0.007376
31524	Amp	21	31057340	31078427	0	0.013055
31525	Amp	21	31089136	31096296	0	0.007376
31526	Amp	21	31098803	31114402	0	0.010352
31527	Amp	21	31115405	31296972	0	0.006936
31528	Amp	21	31304045	31394434	0	0.008876
31529	Amp	21	31396244	31532983	0	0.011077
31530	Amp	21	31533041	31705232	0	0.012437
31531	Amp	21	31708000	31804218	0	0.010236
31532	Amp	21	31805620	31882519	0	0.014967
31533	Amp	21	31883133	31887197	0	0.020291
31534	Amp	21	31893461	31944897	0	0.022287

31535	Amp	21	31945396	31960965	0	0.023063
31536	Amp	21	31961019	32043201	0	0.01774
31537	Amp	21	32098850	32126289	0	0.020659
31538	Amp	21	32127137	32169067	0	0.021618
31539	Amp	21	32169304	32251133	0	0.02378
31540	Amp	21	32251135	32302884	0	0.021925
31541	Amp	21	32306444	32350724	0	0.023222
31542	Amp	21	32351468	32351468	0	0.023775
31543	Amp	21	32351961	32372940	0	0.027584
31544	Amp	21	32376629	32472129	0	0.025636
31545	Amp	21	32472696	32479879	0	0.023696
31546	Amp	21	32481241	32532545	0	0.021575
31547	Amp	21	32534135	32578508	0	0.017766
31548	Amp	21	32582753	32667961	0	0.013477
31549	Amp	21	32671260	32823170	0	0.014645
31550	Amp	21	32824665	32849719	0	0.014064
31551	Amp	21	32850940	32856308	0	0.015588
31552	Amp	21	32858923	32884320	0	0.013977
31553	Amp	21	32885039	32888171	0	0.019213
31554	Amp	21	32890924	32898969	0	0.020681
31555	Amp	21	32902120	32976302	0	0.019238
31556	Amp	21	32984264	33067430	0	0.021162
31557	Amp	21	33069258	33166506	0	0.024593
31558	Amp	21	33169998	33185006	0	0.022669
31559	Amp	21	33185797	33285501	0	0.023904
31560	Amp	21	33286061	33348155	0	0.026406
31561	Amp	21	33352013	33357071	0	0.029286
31562	Amp	21	33357841	33362204	0	0.027673
31563	Amp	21	33366168	33378054	0	0.030813
31564	Amp	21	33380121	33434808	0	0.022817
31565	Amp	21	33437399	33453484	0	0.02038
31566	Amp	21	33463622	33475967	0	0.023656
31567	Amp	21	33481572	33535099	0	0.020451
31568	Amp	21	33535527	33535527	0	0.021834
31569	Amp	21	33537340	33614556	0	0.018558
31570	Amp	21	33617321	33708734	0	0.016397
31571	Amp	21	33712616	33785207	0	0.018037
31572	Amp	21	33788871	33862756	0	0.016473
31573	Amp	21	33863243	33866783	0	0.015467
31574	Amp	21	33869576	33892070	0	0.012559
31575	Amp	21	33892093	33916942	0	0.014175
31576	Amp	21	33918172	34036485	0	0.015931
31577	Amp	21	34037590	34042166	0	0.016631
31578	Amp	21	34044606	34060035	0	0.021219
31579	Amp	21	34060564	34135322	0	0.016631
31580	Amp	21	34140258	34142372	0	0.015931
31581	Amp	21	34145483	34157537	0	0.012897
31582	Amp	21	34162166	34266581	0	0.011141
31583	Amp	21	34275480	34548330	0	0.015744
31584	Amp	21	34548792	34603840	0	0.016619
31585	Amp	21	34604537	34644536	0	0.019821
31586	Amp	21	34644923	34656826	0	0.018205
31587	Amp	21	34658693	34782523	0	0.013602

31588	Amp	21	34784252	34785133	0	0.015072
31589	Amp	21	34789049	34803153	0	0.020288
31590	Amp	21	34803780	34818303	0	0.021802
31591	Amp	21	34818526	34818526	0	0.023473
31592	Amp	21	34825643	34825854	0	0.026235
31593	Amp	21	34830143	34861903	0	0.030616
31594	Amp	21	34865359	34884011	0	0.026235
31595	Amp	21	34894945	34895793	0	0.023032
31596	Amp	21	34897211	34944654	0	0.021518
31597	Amp	21	34946441	35008307	0	0.018756
31598	Amp	21	35008780	35145859	0	0.027511
31599	Amp	21	35149358	35176351	0	0.031864
31600	Amp	21	35178732	35210535	0	0.029539
31601	Amp	21	35212295	35222220	0	0.027511
31602	Amp	21	35232611	35249206	0	0.030761
31603	Amp	21	35252251	35307967	0	0.027511
31604	Amp	21	35311760	35454053	0	0.030658
31605	Amp	21	35456072	35459294	0	0.033834
31606	Amp	21	35461598	35465938	0	0.02508
31607	Amp	21	35468097	35470663	0	0.026679
31608	Amp	21	35471620	35485190	0	0.025012
31609	Amp	21	35485319	35509133	0	0.026037
31610	Amp	21	35512695	35522358	0	0.031009
31611	Amp	21	35522721	35580869	0	0.032933
31612	Amp	21	35582455	35582741	0	0.035252
31613	Amp	21	35582915	35621049	0	0.034226
31614	Amp	21	35621512	35682027	0	0.035494
31615	Amp	21	35682098	35878537	0	0.035946
31616	Amp	21	35881801	35921154	0	0.033601
31617	Amp	21	35933528	35967591	0	0.028385
31618	Amp	21	35969239	35973859	0	0.02757
31619	Amp	21	35974466	36247984	0	0.037385
31620	Amp	21	36248238	36319058	0	0.036832
31621	Amp	21	36319881	36434775	0	0.03186
31622	Amp	21	36435020	36663420	0	0.033897
31623	Amp	21	36666161	36749236	0	0.037681
31624	Amp	21	36753814	36817106	0	0.040692
31625	Amp	21	36817419	36832208	0	0.045147
31626	Amp	21	36832294	36845538	0	0.04746
31627	Amp	21	36848082	36848756	0	0.04949
31628	Amp	21	36849033	36873978	0	0.045871
31629	Amp	21	36874186	36958320	0	0.048071
31630	Amp	21	36960468	36974603	0	0.046034
31631	Amp	21	36976837	36986332	0	0.045198
31632	Amp	21	36992685	36999027	0	0.043316
31633	Amp	21	37007925	37022022	0	0.041287
31634	Amp	21	37022678	37064058	0	0.040421
31635	Amp	21	37066873	37081421	0	0.038519
31636	Amp	21	37084532	37154999	0	0.028704
31637	Amp	21	37157748	37256802	0	0.032475
31638	Amp	21	37257296	37379387	0	0.034561
31639	Amp	21	37379562	37415115	0	0.033594
31640	Amp	21	37418854	37424426	0	0.040734

31641	Amp	21	37427735	37436925	0	0.044069
31642	Amp	21	37442476	37450773	0	0.036929
31643	Amp	21	37451269	37467535	0	0.038681
31644	Amp	21	37469470	37482177	0	0.041203
31645	Amp	21	37487876	37496779	0	0.038191
31646	Amp	21	37498060	37537361	0	0.036439
31647	Amp	21	37538265	37539183	0	0.042925
31648	Amp	21	37541990	37656195	0	0.03864
31649	Amp	21	37656229	37685205	0	0.040079
31650	Amp	21	37685319	37705799	0	0.03864
31651	Amp	21	37707907	37742921	0	0.043583
31652	Amp	21	37743146	37743146	0	0.0442
31653	Amp	21	37744670	37772636	0	0.039464
31654	Amp	21	37773242	37971395	0	0.041999
31655	Amp	21	37974030	37994506	0	0.040069
31656	Amp	21	37995141	38137632	0	0.04517
31657	Amp	21	38145227	38151798	0	0.043411
31658	Amp	21	38154337	38160065	0	0.040876
31659	Amp	21	38164700	38241621	0	0.041859
31660	Amp	21	38247971	38474017	0	0.040876
31661	Amp	21	38474054	38499585	0	0.046605
31662	Amp	21	38500772	38546100	0	0.045337
31663	Amp	21	38554421	38565161	0	0.049554
31664	Amp	21	38566173	38571099	0	0.046997
31665	Amp	21	38573467	38669145	0	0.04638
31666	Amp	21	38669371	38789148	0	0.048334
31667	Amp	21	38799529	38810253	0	0.045159
31668	Amp	21	38815826	38818612	0	0.042711
31669	Amp	21	38818682	38821021	0	0.043517
31670	Amp	21	38821285	38894014	0	0.045996
31671	Amp	21	38898773	38955771	0	0.041465
31672	Amp	21	38956395	38961311	0	0.041019
31673	Amp	21	38964626	38995174	0	0.039978
31674	Amp	21	38998237	39000237	0	0.041343
31675	Amp	21	39001837	39146070	0	0.043312
31676	Amp	21	39146853	39206620	0	0.04605
31677	Amp	21	39207758	39249643	0	0.054483
31678	Amp	21	39251092	39321697	0	0.063509
31679	Amp	21	39323849	39354553	0.044794	0.065927
31680	Amp	21	39354970	39369979	0.069848	0.067044
31681	Amp	21	39370990	39389619	0.210594	0.072566
31682	Amp	21	39389849	39425922	0.089561	0.067943
31683	Amp	21	39426237	39428269	0	0.063424
31684	Amp	21	39435131	39460937	0.128324	0.069407
31685	Amp	21	39468952	39554012	0.143787	0.070081
31686	Amp	21	39554726	39685597	0.005328	0.064097
31687	Amp	21	39686947	39731459	0.015369	0.064653
31688	Amp	21	39732421	39791619	0	0.060249
31689	Amp	21	39791790	39817797	0.010261	0.064478
31690	Amp	21	39820276	39874804	0	0.060249
31691	Amp	21	39874890	39883271	0.001323	0.063909
31692	Amp	21	39883545	39901656	0	0.063125
31693	Amp	21	39901996	39999124	0.024438	0.064942

31694	Amp	21	40000321	40074084	0	0.063125
31695	Amp	21	40075131	40098784	0	0.061091
31696	Amp	21	40100758	40116043	0	0.061657
31697	Amp	21	40116343	40121484	0	0.063111
31698	Amp	21	40122405	40129965	0	0.063642
31699	Amp	21	40134131	40174126	0	0.059982
31700	Amp	21	40174232	40367109	0	0.062092
31701	Amp	21	40367760	40398891	0	0.055522
31702	Amp	21	40399477	40422324	0	0.054848
31703	Amp	21	40423129	40469517	0	0.058215
31704	Amp	21	40469863	40476281	0	0.055032
31705	Amp	21	40479827	40498165	0	0.052713
31706	Amp	21	40499443	40579666	0	0.055528
31707	Amp	21	40589224	40594051	0	0.058846
31708	Amp	21	40594081	40594932	0	0.055528
31709	Amp	21	40608166	40615597	0	0.062751
31710	Amp	21	40618823	40649502	0	0.055528
31711	Amp	21	40650081	40652258	0	0.056963
31712	Amp	21	40653528	40668729	0	0.046764
31713	Amp	21	40676385	40700314	0	0.042073
31714	Amp	21	40703616	40738309	0	0.041507
31715	Amp	21	40740208	40771577	0	0.039538
31716	Amp	21	40772907	40789918	0	0.036172
31717	Amp	21	40794521	40849742	0	0.037365
31718	Amp	21	40852194	40857328	0	0.040603
31719	Amp	21	40857365	40881659	0	0.039168
31720	Amp	21	40882260	40914677	0	0.041041
31721	Amp	21	40918245	40934171	0	0.043432
31722	Amp	21	40936983	40972128	0	0.042876
31723	Amp	21	40972192	41021761	0	0.041682
31724	Amp	21	41024806	41146672	0	0.038445
31725	Amp	21	41147429	41275894	0	0.040946
31726	Amp	21	41277825	41354600	0	0.035244
31727	Amp	21	41355013	41479527	0	0.033134
31728	Amp	21	41484285	41484893	0.373599	0.078868
31729	Amp	21	41485167	41509923	0	0.033134
31730	Amp	21	41511244	41575353	0	0.033653
31731	Amp	21	41576526	41647085	0	0.036011
31732	Amp	21	41647779	41652916	0	0.036689
31733	Amp	21	41653447	41658722	0	0.036238
31734	Amp	21	41660551	41730694	0	0.036674
31735	Amp	21	41734793	41782175	0	0.039817
31736	Amp	21	41783802	41811036	0	0.037459
31737	Amp	21	41816240	41866553	0	0.034316
31738	Amp	21	41866956	41881672	0	0.036102
31739	Amp	21	41883012	41890848	0	0.049185
31740	Amp	21	41891334	41891334	0	0.052039
31741	Amp	21	41892839	42014492	0	0.049517
31742	Amp	21	42016191	42021503	0	0.051627
31743	Amp	21	42023389	42103862	0	0.050532
31744	Amp	21	42109159	42166548	0	0.054504
31745	Amp	21	42167275	42200375	0	0.058548
31746	Amp	21	42200888	42277878	0	0.056438

31747	Amp	21	42278705	42303403	0	0.0588
31748	Amp	21	42303761	42447273	0	0.056438
31749	Amp	21	42447677	42487718	0	0.059129
31750	Amp	21	42492837	42538805	0	0.057675
31751	Amp	21	42539939	42585053	0	0.0598
31752	Amp	21	42605776	42608841	0	0.063401
31753	Amp	21	42612602	42921874	0.001323	0.063869
31754	Amp	21	42923526	43033459	0	0.061647
31755	Amp	21	43034042	43034905	0.034113	0.065511
31756	Amp	21	43572694	43645080	0.282828	0.075735
31757	Amp	21	43645298	43651015	0.187449	0.07187
31758	Amp	21	43651860	43693748	0.094967	0.068016
31759	Amp	21	43695607	43700356	0	0.057126
31760	Amp	21	43703900	43762183	0	0.05497
31761	Amp	21	43766788	43837231	0	0.052944
31762	Amp	21	43837480	43896899	0	0.051071
31763	Amp	21	43900769	43935611	0	0.063741
31764	Amp	21	43936606	43939127	0.138391	0.069817
31765	Amp	21	43940575	43950155	0.08435	0.067588
31766	Amp	21	43950472	43977585	0	0.063525
31767	Amp	21	43977960	44016117	0	0.061651
31768	Amp	21	44021174	44026708	0	0.060134
31769	Amp	21	44028257	44458597	0	0.05041
31770	Amp	21	44459383	44573184	0	0.05178
31771	Amp	21	44573237	44622620	0	0.05041
31772	Amp	21	44623537	44667160	0	0.05124
31773	Amp	21	44667247	44753476	0	0.044553
31774	Amp	21	44755084	44761511	0	0.053862
31775	Amp	21	44761764	44858997	0	0.04525
31776	Amp	21	44859327	44909825	0	0.047859
31777	Amp	21	44910671	44921282	0	0.04525
31778	Amp	21	44921437	44935481	0	0.043209
31779	Amp	21	44937580	45088596	0	0.049896
31780	Amp	21	45090401	45136534	0	0.050512
31781	Amp	21	45136889	45227190	0	0.045644
31782	Amp	21	45227667	45227667	0	0.044814
31783	Amp	21	45670561	45670561	0	0.053827
31784	Amp	21	45671324	45676246	0	0.039784
31785	Amp	21	45682516	45722315	0	0.044913
31786	Amp	21	45725460	45735402	0	0.04628
31787	Amp	21	45735460	45822002	0	0.044486
31788	Amp	21	45822132	45822282	0	0.043877
31789	Amp	21	45822342	45833960	0	0.042209
31790	Amp	21	45834255	45888113	0	0.041123
31791	Amp	21	45889000	45894988	0	0.039756
31792	Amp	21	45897554	45925679	0	0.04033
31793	Amp	21	45930673	46104695	0	0.037064
31794	Amp	21	46106805	46106805	0	0.038698
31795	Amp	21	46107355	46116266	0	0.038261
31796	Amp	21	46122306	46178267	0	0.040535
31797	Amp	21	46182047	46220419	0	0.043116
31798	Amp	21	46221880	46262057	0	0.042597
31799	Amp	22	16934932	16952107	0	0.0577

31800	Amp	22	16952820	16982299	0	0.059401
31801	Amp	22	16983056	17097812	0	0.05736
31802	Amp	22	17101209	17203595	0	0.056065
31803	Amp	22	17207259	17259149	0	0.046036
31804	Amp	22	17261625	17265638	0	0.047609
31805	Amp	22	17266305	17318478	0	0.049099
31806	Amp	22	17318497	17377882	0	0.050509
31807	Amp	22	17380308	17380308	0	0.051334
31808	Amp	22	17413571	17433041	0	0.049765
31809	Amp	22	17433094	17434959	0	0.04745
31810	Amp	22	17436386	17462474	0	0.045653
31811	Amp	22	17469431	17500594	0	0.047663
31812	Amp	22	17506060	17520833	0	0.045653
31813	Amp	22	17523132	17612149	0	0.047281
31814	Amp	22	17612404	17694565	0	0.045871
31815	Amp	22	17696628	17731972	0	0.043187
31816	Amp	22	17732176	17780001	0	0.044787
31817	Amp	22	17782984	17782984	0	0.047541
31818	Amp	22	17792325	17812364	0	0.045914
31819	Amp	22	17815761	17823031	0	0.047281
31820	Amp	22	17824868	17840843	0	0.034696
31821	Amp	22	17841204	17841204	0	0.033123
31822	Amp	22	17841522	17928129	0	0.037315
31823	Amp	22	17931986	17965347	0	0.039032
31824	Amp	22	17965363	17975076	0	0.041977
31825	Amp	22	17980500	18100728	0	0.040611
31826	Amp	22	18101648	18119261	0	0.040054
31827	Amp	22	18119280	18128031	0	0.038337
31828	Amp	22	18128862	18130970	0	0.036885
31829	Amp	22	18131162	18131162	0	0.033164
31830	Amp	22	21741482	21741482	0	0.044879
31831	Amp	22	21742513	21751261	0	0.049379
31832	Amp	22	21751293	21753333	0	0.0488
31833	Amp	22	21753597	21753597	0	0.046346
31834	Amp	22	21755011	21835993	0	0.044206
31835	Amp	22	21836476	21859680	0	0.041703
31836	Amp	22	21861606	21875921	0	0.039036
31837	Amp	22	21881037	21903264	0	0.035792
31838	Amp	22	21903314	21920372	0	0.039514
31839	Amp	22	21921269	21936109	0	0.041662
31840	Amp	22	21939017	21949041	0	0.043096
31841	Amp	22	23474170	23475704	0	0.037071
31842	Amp	22	23479094	23535546	0	0.033582
31843	Amp	22	23538335	23546956	0	0.036096
31844	Amp	22	23547826	23553602	0	0.034291
31845	Amp	22	23641308	23659843	0	0.032143
31846	Amp	22	23661318	23690584	0	0.033063
31847	Amp	22	23691279	23754828	0	0.032143
31848	Amp	22	23760221	23833866	0	0.042638
31849	Amp	22	23836092	23856390	0	0.041584
31850	Amp	22	23861612	23899579	0	0.043553
31851	Amp	22	24015674	24015674	0	0.047494
31852	Amp	22	24020015	24028666	0	0.04301

31853	Amp	22	24029959	24034246	0	0.041187
31854	Amp	22	24036936	24054572	0	0.046174
31855	Amp	22	24063450	24069808	0	0.047797
31856	Amp	22	24072044	24121178	0	0.050179
31857	Amp	22	24122761	24177482	0	0.051014
31858	Amp	22	24186073	24186073	0	0.053059
31859	Amp	22	24186771	24186771	0	0.051436
31860	Amp	22	24195941	24225421	0	0.049054
31861	Amp	22	24225440	24243101	0	0.051052
31862	Amp	22	24262658	24291016	0	0.050471
31863	Amp	22	24299996	24320954	0	0.049796
31864	Amp	22	24321373	24336077	0	0.050334
31865	Amp	22	24336143	24336464	0	0.052044
31866	Amp	22	24339545	24339545	0	0.05358
31867	Amp	22	24489781	24501540	0	0.051535
31868	Amp	22	24502342	24569360	0	0.046706
31869	Amp	22	24579561	24591908	0	0.039936
31870	Amp	22	24594246	24643225	0	0.038613
31871	Amp	22	24663622	24678072	0	0.035264
31872	Amp	22	24714909	24904691	0	0.036329
31873	Amp	22	24905399	24954120	0	0.034619
31874	Amp	22	24957541	25005515	0	0.035965
31875	Amp	22	25005981	25094077	0	0.043299
31876	Amp	22	25095760	25245058	0	0.0402
31877	Amp	22	25245198	25245198	0	0.036642
31878	Amp	22	25608728	25608728	0	0.031381
31879	Amp	22	25612136	25623456	0	0.033042
31880	Amp	22	25623573	25646383	0	0.032504
31881	Amp	22	25646648	25731085	0	0.030359
31882	Amp	22	25738059	25816201	0	0.027059
31883	Amp	22	25818975	25865768	0	0.02479
31884	Amp	22	25866888	25882727	0	0.025664
31885	Amp	22	25883878	25884551	0	0.024319
31886	Amp	22	25888008	25954427	0	0.023445
31887	Amp	22	25959194	25980575	0	0.021762
31888	Amp	22	25982107	26102983	0	0.023365
31889	Amp	22	26104163	26138242	0	0.021806
31890	Amp	22	26141484	26147591	0	0.015773
31891	Amp	22	26147727	26240724	0	0.014111
31892	Amp	22	26241290	26282162	0	0.012508
31893	Amp	22	26285183	26398972	0	0.011551
31894	Amp	22	26400389	26530122	0	0.010332
31895	Amp	22	26531068	26533848	0	0.040996
31896	Amp	22	26534099	26641431	0	0.010332
31897	Amp	22	26649540	26830334	0	0.010832
31898	Amp	22	26831491	26845117	0	0.005534
31899	Amp	22	26849174	26871042	0	0.007094
31900	Amp	22	26875382	26884520	0	0.009323
31901	Amp	22	26884902	26954604	0	0.010201
31902	Amp	22	26959232	26974819	0	0.013975
31903	Amp	22	26975722	27039651	0	0.010201
31904	Amp	22	27040087	27044721	0	0.008642
31905	Amp	22	27047522	27493180	0	0.010197

31906	Amp	22	27498779	27560444	0	0.016801
31907	Amp	22	27560595	27647853	0	0.0202
31908	Amp	22	27647946	27653935	0	0.022621
31909	Amp	22	27654441	27836488	0	0.036127
31910	Amp	22	27847235	27857880	0	0.034571
31911	Amp	22	27858739	27865614	0	0.034071
31912	Amp	22	27865642	27977756	0	0.033193
31913	Amp	22	27983041	28012456	0	0.034699
31914	Amp	22	28014378	28042509	0	0.018772
31915	Amp	22	28045257	28059727	0	0.018105
31916	Amp	22	28060374	28092307	0	0.021496
31917	Amp	22	28092462	28141120	0	0.018105
31918	Amp	22	28142004	28147084	0	0.02233
31919	Amp	22	28152233	28191146	0	0.018931
31920	Amp	22	28215453	28222015	0	0.014707
31921	Amp	22	28224553	28230537	0	0.010339
31922	Amp	22	28232221	28259473	0	0.013805
31923	Amp	22	28259583	28299046	0	0.021714
31924	Amp	22	28308902	28440940	0	0.012881
31925	Amp	22	28440981	28584879	0	0.013521
31926	Amp	22	28585526	28747364	0	0.017873
31927	Amp	22	28749423	28749700	0	0.005613
31928	Amp	22	28750515	28807468	0	0.010909
31929	Amp	22	28807824	28925093	0	0.013272
31930	Amp	22	28925851	28979990	0	0.011766
31931	Amp	22	28982899	29079136	0	0.009404
31932	Amp	22	29084273	29297873	0	0.011596
31933	Amp	22	29299939	29336949	0	0.00813
31934	Amp	22	29337654	29559918	0	0.016974
31935	Amp	22	29560896	29566581	0	0.00813
31936	Amp	22	29568127	29693507	0	0.005937
31937	Amp	22	29703547	29837457	0	0.007256
31938	Amp	22	29840291	29859681	0	0.008038
31939	Amp	22	29860144	29903764	0	0.010101
31940	Amp	22	29909307	30004903	0	0.012452
31941	Amp	22	30005238	30141575	0	0.012911
31942	Amp	22	30141896	30155150	0	0.015278
31943	Amp	22	30156824	30216175	0	0.015857
31944	Amp	22	30222450	30237208	0	0.013793
31945	Amp	22	30253202	30269110	0	0.015769
31946	Amp	22	30273720	30335563	0	0.014451
31947	Amp	22	30339919	30346136	0	0.015876
31948	Amp	22	30367041	30492718	0	0.014516
31949	Amp	22	30498053	30706583	0	0.01375
31950	Amp	22	30709438	30899095	0	0.013291
31951	Amp	22	30899250	30909855	0	0.010925
31952	Amp	22	30914032	30938531	0	0.013981
31953	Amp	22	30942483	30982609	0	0.010925
31954	Amp	22	30984931	31421547	0	0.009714
31955	Amp	22	31434101	31450260	0	0.008288
31956	Amp	22	31451189	31517378	0	0.009607
31957	Amp	22	31519393	31715574	0	0.011682
31958	Amp	22	31717094	31724387	0	0.014592

31959	Amp	22	31724937	31818630	0	0.015519
31960	Amp	22	31820922	32041219	0	0.013445
31961	Amp	22	32047662	32131650	0	0.012518
31962	Amp	22	32134861	32166532	0	0.01488
31963	Amp	22	32171283	32188672	0	0.015329
31964	Amp	22	32188723	32259093	0	0.015768
31965	Amp	22	32260574	32303841	0	0.016389
31966	Amp	22	32306091	32449416	0	0.014026
31967	Amp	22	32450632	32486134	0	0.014648
31968	Amp	22	32486821	32529183	0	0.016134
31969	Amp	22	32534156	32612038	0	0.017401
31970	Amp	22	32617040	32677287	0	0.016761
31971	Amp	22	32685731	32733036	0	0.015494
31972	Amp	22	32740534	32984806	0	0.014007
31973	Amp	22	32987399	33043688	0	0.018804
31974	Amp	22	33044150	33088262	0	0.019586
31975	Amp	22	33092418	33134054	0	0.01479
31976	Amp	22	33135349	33249592	0	0.013022
31977	Amp	22	33256766	33288439	0	0.01005
31978	Amp	22	33289753	33344371	0	0.009267
31979	Amp	22	33345253	33444174	0	0.010901
31980	Amp	22	33446468	33630318	0	0.014107
31981	Amp	22	33632441	33651984	0	0.014587
31982	Amp	22	33657457	33870729	0	0.017174
31983	Amp	22	33872408	33874705	0	0.015541
31984	Amp	22	33875794	33929862	0	0.017003
31985	Amp	22	33931678	33991424	0	0.016523
31986	Amp	22	33995412	34024055	0	0.019356
31987	Amp	22	34024735	34136417	0	0.016523
31988	Amp	22	34141461	34187402	0	0.01554
31989	Amp	22	34192759	34199764	0	0.014078
31990	Amp	22	34199951	34321933	0	0.012474
31991	Amp	22	34327038	34408257	0	0.015235
31992	Amp	22	34410907	34778894	0	0.003821
31993	Amp	22	34781214	34852536	0	0.008592
31994	Amp	22	34860930	34893360	0	0.023415
31995	Amp	22	34896519	34899114	0	0.022794
31996	Amp	22	34899207	34951745	0	0.023908
31997	Amp	22	34952415	34999137	0	0.020269
31998	Amp	22	35002849	35143724	0	0.019136
31999	Amp	22	35144225	35199105	0	0.020507
32000	Amp	22	35199535	35304215	0	0.019136
32001	Amp	22	35315448	35316011	0	0.020563
32002	Amp	22	35316498	35356689	0	0.017393
32003	Amp	22	35356842	35367321	0	0.016954
32004	Amp	22	35368926	35375116	0	0.004188
32005	Amp	22	35382285	35473357	0	0.002761
32006	Amp	22	35479025	35523399	0	0.004491
32007	Amp	22	35523651	35568111	0	0.00897
32008	Amp	22	35712343	36007936	0	0.004491
32009	Amp	22	36010486	36042607	0	0.002761
32010	Amp	22	36087893	36108528	0	0.005899
32011	Amp	22	36108631	36218264	0	0.002761

32012 Amp	22	36320269	36356028	0	0.003938
32013 Amp	22	36356773	36422728	0	0.005629
32014 Amp	22	36428587	36437352	0	0.006431
32015 Amp	22	36440363	36460761	0	0.009247
32016 Amp	22	36462582	36495811	0	0.009857
32017 Amp	22	36496196	36613817	0	0.008166
32018 Amp	22	36614807	36665116	0	0.00535
32019 Amp	22	36670443	36731328	0	0.004173
32020 Amp	22	36732550	36866727	0	0.006612
32021 Amp	22	36869037	36893827	0	0.009011
32022 Amp	22	36915764	37037417	0	0.01449
32023 Amp	22	37039591	37043931	0	0.005156
32024 Amp	22	37046411	37085139	0	0.007438
32025 Amp	22	37095164	37155567	0	0.022392
32026 Amp	22	37158892	37356612	0	0.006456
32027 Amp	22	37356657	37401760	0	0.007235
32028 Amp	22	37412845	37574182	0	0.008667
32029 Amp	22	37574688	37594071	0	0.007888
32030 Amp	22	37597119	37619846	0	0.005605
32031 Amp	22	37632449	38049497	0	0.007688
32032 Amp	22	38053204	38195138	0	0.006256
32033 Amp	22	38195183	38244420	0	0.009314
32034 Amp	22	38244835	38287631	0	0.010073
32035 Amp	22	38287733	38345788	0	0.00799
32036 Amp	22	38356702	38464066	0	0.005229
32037 Amp	22	38475580	38486372	0	0.006852
32038 Amp	22	38488770	38538544	0	0.003795
32039 Amp	22	38538601	38704967	0	0.006304
32040 Amp	22	38708093	38774299	0	0.00468
32041 Amp	22	38785591	38939915	0	0.002172
32042 Amp	22	39007789	39198735	0	0.008151
32043 Amp	22	39205268	39205268	0	0.008782
32044 Amp	22	39228047	39303478	0	0.002803
32045 Amp	22	39304753	39328132	0	0.002044
32046 Amp	22	39329682	39360849	0	0.003464
32047 Amp	22	39360980	39439749	0	0.002833
32048 Amp	22	39443548	39492675	0	0.007178
32049 Amp	22	39492856	39584874	0	0.008101
32050 Amp	22	39585478	39751428	0	0.00579
32051 Amp	22	39757178	39911528	0	0.003755
32052 Amp	22	39912424	40094535	0	0.002334
32053 Amp	22	40095307	40146958	0	0.001532
32054 Amp	22	40156229	40184032	0	0.000922
32055 Amp	22	40184636	40225530	0	0.00263
32056 Amp	22	40230244	40239272	0	0.003989
32057 Amp	22	40240795	40313699	0	0.004782
32058 Amp	22	40321447	40376895	0	0.003074
32059 Amp	22	40377072	40455802	0	0.003848
32060 Amp	22	40475589	40480230	0	0.00703
32061 Amp	22	40493603	40706070	0	0.007883
32062 Amp	22	40711684	40821392	0	0.004702
32063 Amp	22	40823975	40926902	0	0.003344
32064 Amp	22	40933788	41251789	0	0.004012

32065 Amp	22	41252916	41331157	0	0.005034
32066 Amp	22	41332616	41359044	0	0.006229
32067 Amp	22	41363033	41544846	0	0.005306
32068 Amp	22	41548293	42020052	0	0.00309
32069 Amp	22	42021683	42108675	0	0.002421
32070 Amp	22	42109280	42238809	0	0.003966
32071 Amp	22	42244600	42337551	0	0.002421
32072 Amp	22	42337837	42385617	0	0.003384
32073 Amp	22	42586566	42601131	0	0.005042
32074 Amp	22	42601138	42677184	0	0.005925
32075 Amp	22	42682280	42745453	0	0.004267
32076 Amp	22	42749710	42776302	0	0.00588
32077 Amp	22	42780275	42900415	0	0.004267
32078 Amp	22	42901415	42946958	0	0.006209
32079 Amp	22	42947556	43040001	0	0.004267
32080 Amp	22	43046593	43053824	0	0.006128
32081 Amp	22	43054344	43081412	0	0.005245
32082 Amp	22	43085722	43108228	0	0.007774
32083 Amp	22	43114712	43230029	0	0.005912
32084 Amp	22	43233489	43317247	0	0.00823
32085 Amp	22	43318599	43322548	0	0.012842
32086 Amp	22	43323433	43354698	0	0.014603
32087 Amp	22	43355444	43420212	0	0.009992
32088 Amp	22	43420705	43445331	0	0.011058
32089 Amp	22	43445866	43551223	0	0.00874
32090 Amp	22	43551417	43643405	0	0.009204
32091 Amp	22	43645682	43711420	0	0.009897
32092 Amp	22	43713480	43769427	0	0.009204
32093 Amp	22	43769718	43861176	0	0.006675
32094 Amp	22	43863621	44022159	0	0.009079
32095 Amp	22	44025683	44060812	0	0.007318
32096 Amp	22	44063565	44273270	0	0.008892
32097 Amp	22	44277818	44288159	0	0.007318
32098 Amp	22	44293292	44592354	0	0.008884
32099 Amp	22	44593870	44720323	0	0.00811
32100 Amp	22	44721554	44754323	0	0.006543
32101 Amp	22	44760375	44764928	0	0.004139
32102 Amp	22	44765860	44852334	0	0.005998
32103 Amp	22	44865012	44904895	0	0.009677
32104 Amp	22	44905577	45006245	0	0.010801
32105 Amp	22	45011237	45083923	0	0.012502
32106 Amp	22	45084824	45087702	0	0.015003
32107 Amp	22	45087839	45087925	0	0.015443
32108 Amp	22	45088919	45100668	0	0.013742
32109 Amp	22	45101287	45171699	0	0.011884
32110 Amp	22	45172538	45175765	0	0.013818
32111 Amp	22	45177569	45192496	0	0.010138
32112 Amp	22	45193521	45255219	0	0.008204
32113 Amp	22	45261283	45319583	0	0.010335
32114 Amp	22	45321490	45382548	0	0.009211
32115 Amp	22	45383290	45390859	0	0.012223
32116 Amp	22	45391515	45467808	0	0.011782
32117 Amp	22	45469890	45483950	0	0.011318

32118	Amp	22	45484122	45564672	0	0.008818
32119	Amp	22	45565660	45614276	0	0.011008
32120	Amp	22	45615662	45883853	0	0.007996
32121	Amp	22	45887011	45956611	0	0.010834
32122	Amp	22	45970014	46037410	0	0.013239
32123	Amp	22	46038806	46067551	0	0.015595
32124	Amp	22	46075601	46082575	0	0.01915
32125	Amp	22	46082778	46117075	0	0.015801
32126	Amp	22	46129914	46288661	0	0.009841
32127	Amp	22	46288809	46372955	0	0.008162
32128	Amp	22	46373163	46434652	0	0.009813
32129	Amp	22	46446250	46549558	0	0.008162
32130	Amp	22	46552556	46582879	0	0.009975
32131	Amp	22	46583505	46626862	0	0.011981
32132	Amp	22	46627753	46679565	0	0.012425
32133	Amp	22	46682065	46692110	0	0.016926
32134	Amp	22	46693424	46704308	0	0.01492
32135	Amp	22	46705175	46718697	0	0.015382
32136	Amp	22	46718766	46788886	0	0.016634
32137	Amp	22	46794956	46807074	0	0.015382
32138	Amp	22	46809529	46839219	0	0.020588
32139	Amp	22	46839522	46874367	0	0.021024
32140	Amp	22	46875071	46920646	0	0.017517
32141	Amp	22	46921739	47004979	0	0.02133
32142	Amp	22	47005802	47010868	0	0.023472
32143	Amp	22	47011621	47037139	0	0.02166
32144	Amp	22	47042379	47144333	0	0.019961
32145	Amp	22	47151499	47389144	0	0.021435
32146	Amp	22	47390565	47426930	0	0.024824
32147	Amp	22	47429560	47483665	0	0.025296
32148	Amp	22	47484157	47557844	0	0.028276
32149	Amp	22	47561057	47734445	0	0.024887
32150	Amp	22	47736014	47813460	0	0.026941
32151	Amp	22	47815283	47820804	0	0.027553
32152	Amp	22	47821220	47860817	0	0.025197
32153	Amp	22	47861168	47877881	0	0.026814
32154	Amp	22	47877917	47887746	0	0.028887
32155	Amp	22	47887969	47904320	0	0.025907
32156	Amp	22	47904645	48006604	0	0.023853
32157	Amp	22	48006793	48007674	0	0.024833
32158	Amp	22	48013311	48095343	0	0.027374
32159	Amp	22	48096846	48134519	0	0.023684
32160	Amp	22	48134953	48136629	0	0.023072
32161	Amp	22	48140657	48306425	0	0.021598
32162	Amp	22	48308985	48377894	0	0.019455
32163	Amp	22	48378156	48521083	0	0.022007
32164	Amp	22	48525853	48561471	0	0.023828
32165	Amp	22	48564800	48619696	0	0.024697
32166	Amp	22	48621418	48624595	0	0.023828
32167	Amp	22	48626826	48632100	0	0.025566
32168	Amp	22	48643985	48780734	0	0.032091
32169	Amp	22	48780839	48825221	0	0.031618
32170	Amp	22	48829328	48885900	0	0.029797

32171	Amp	22	48891318	48913630	0	0.03628
32172	Amp	22	48913715	48935200	0	0.032164
32173	Del	1	3302046	3302046	19.097898	0.132386
32174	Del	1	3302359	3451567	18.810121	0.131264
32175	Del	1	3451579	3486887	16.894455	0.123785
32176	Del	1	3487663	3558286	16.153973	0.120793
32177	Del	1	3558637	3563585	16.481356	0.122102
32178	Del	1	3577515	3623098	16.153973	0.120793
32179	Del	1	3623660	3659269	15.473755	0.117988
32180	Del	1	3659365	3680479	15.202588	0.117053
32181	Del	1	3680520	3698012	15.461815	0.117988
32182	Del	1	3698042	3731704	15.552192	0.118362
32183	Del	1	3731781	3749303	15.656562	0.118736
32184	Del	1	3749737	3768876	15.678296	0.118923
32185	Del	1	3769754	3780042	15.774355	0.119297
32186	Del	1	3780075	3797469	15.656562	0.118736
32187	Del	1	3797918	3854839	15.868405	0.119671
32188	Del	1	3855795	3888691	16.258187	0.121167
32189	Del	1	3889073	3939532	16.04683	0.120419
32190	Del	1	3939577	4411673	15.937674	0.120045
32191	Del	1	4411966	4648927	15.098859	0.116492
32192	Del	1	4652424	4778181	14.624974	0.114623
32193	Del	1	4780903	4784763	14.58573	0.114436
32194	Del	1	4785052	5862018	16.340159	0.121541
32195	Del	1	5862221	5862761	15.879145	0.119671
32196	Del	1	5862774	5951845	15.333725	0.117427
32197	Del	1	5954503	6019642	15.480916	0.118175
32198	Del	1	6020227	6028304	15.623403	0.118736
32199	Del	1	6028611	6045049	15.675708	0.118923
32200	Del	1	6045070	6094386	15.764834	0.119297
32201	Del	1	6094681	6176837	15.856765	0.119671
32202	Del	1	6183275	6201763	16.106497	0.120606
32203	Del	1	6201811	6207865	16.157118	0.120793
32204	Del	1	6208050	6231954	16.056342	0.120419
32205	Del	1	6233415	6248849	16.157118	0.120793
32206	Del	1	6254762	6263905	16.067911	0.120419
32207	Del	1	6264330	6362615	15.644942	0.118736
32208	Del	1	6362699	6389578	15.578644	0.118549
32209	Del	1	6394399	6407783	15.568909	0.118362
32210	Del	1	6408568	6431233	15.020015	0.116305
32211	Del	1	6438103	6457443	14.908816	0.115744
32212	Del	1	6459544	6469763	15.044379	0.116305
32213	Del	1	6470129	6492439	14.985921	0.116118
32214	Del	1	6492498	6507189	14.941491	0.115931
32215	Del	1	6508890	6520190	14.84959	0.115557
32216	Del	1	6521223	6553130	14.803824	0.11537
32217	Del	1	6555047	6581698	14.97569	0.116118
32218	Del	1	6582434	6605539	14.952455	0.115931
32219	Del	1	6610335	6628798	15.044379	0.116305
32220	Del	1	6630080	6636818	14.997397	0.116118
32221	Del	1	6638914	6723993	14.548462	0.114436
32222	Del	1	6724532	6783774	14.446447	0.113875
32223	Del	1	6783868	6892266	14.196722	0.11294

32224	Del	1	6895273	7769373	10.905541	0.098729
32225	Del	1	7769713	7780842	13.814115	0.111257
32226	Del	1	7781489	7845814	14.913563	0.115744
32227	Del	1	7845948	7914015	14.781167	0.115183
32228	Del	1	7915729	7986078	14.699114	0.114997
32229	Del	1	7993075	8029121	14.814921	0.11537
32230	Del	1	8030168	8216849	14.838789	0.115557
32231	Del	1	8218752	8413753	15.310052	0.117427
32232	Del	1	8420102	8435345	15.520456	0.118175
32233	Del	1	8435459	8486854	14.746072	0.115183
32234	Del	1	8487820	8495974	15.60547	0.118549
32235	Del	1	8503397	8820214	14.746072	0.115183
32236	Del	1	8822128	8859635	15.816816	0.119484
32237	Del	1	8865053	8871090	15.879145	0.119671
32238	Del	1	8871304	8872100	15.816816	0.119484
32239	Del	1	8872295	8872295	15.780204	0.119297
32240	Del	1	8873183	8881093	15.879145	0.119671
32241	Del	1	8883906	8975955	15.960206	0.120045
32242	Del	1	8976555	9034472	16.031928	0.120419
32243	Del	1	9035435	9038363	15.902396	0.119858
32244	Del	1	9040939	9074131	15.948106	0.120045
32245	Del	1	9078408	9146408	16.044345	0.120419
32246	Del	1	9147248	9147248	15.585878	0.118549
32247	Del	1	9151464	9152724	15.785042	0.119297
32248	Del	1	9153118	9180199	15.585878	0.118549
32249	Del	1	9181688	9232223	15.70236	0.118923
32250	Del	1	9237613	9344634	15.713339	0.11911
32251	Del	1	9344921	9372103	15.493023	0.118175
32252	Del	1	9372331	9524383	15.785042	0.119297
32253	Del	1	9528050	9586562	15.656562	0.118736
32254	Del	1	9587064	9606629	15.690091	0.118923
32255	Del	1	9609028	9616944	15.644942	0.118736
32256	Del	1	9617009	9658873	15.774355	0.119297
32257	Del	1	9659607	9665467	15.493023	0.118175
32258	Del	1	9671279	9678728	15.70236	0.118923
32259	Del	1	9678821	9690399	15.774355	0.119297
32260	Del	1	9695116	9713660	15.493023	0.118175
32261	Del	1	9714660	9842347	15.978591	0.120045
32262	Del	1	9842842	9911730	15.631283	0.118736
32263	Del	1	9913870	9922525	15.884995	0.119671
32264	Del	1	9924821	9952956	15.921406	0.119858
32265	Del	1	9957375	9965785	15.754704	0.119297
32266	Del	1	9967328	9967482	15.968823	0.120045
32267	Del	1	9969855	9984433	15.754704	0.119297
32268	Del	1	9987812	9987812	15.743675	0.11911
32269	Del	1	9989119	10030667	15.874409	0.119671
32270	Del	1	10031603	10198748	15.575363	0.118549
32271	Del	1	10199842	10224178	15.685187	0.118923
32272	Del	1	10225186	10228739	16.296642	0.121354
32273	Del	1	10229443	10298108	15.685187	0.118923
32274	Del	1	10298533	10305205	16.296642	0.121354
32275	Del	1	10305228	10385876	15.685187	0.118923
32276	Del	1	10387735	10428257	17.665153	0.126777

32277	Del	1	10428498	10445075	17.625435	0.12659
32278	Del	1	10446134	10449254	17.536242	0.126216
32279	Del	1	10449646	10452095	17.651519	0.126777
32280	Del	1	10456970	10472924	17.638462	0.12659
32281	Del	1	10477033	10633983	16.689862	0.123037
32282	Del	1	10634068	10798689	16.593731	0.122663
32283	Del	1	10809488	11010551	17.182062	0.124907
32284	Del	1	11010638	11026077	16.603025	0.122663
32285	Del	1	11026275	11048652	16.584227	0.122476
32286	Del	1	11048996	11068018	16.329262	0.121541
32287	Del	1	11068597	11097442	16.146684	0.120793
32288	Del	1	11098048	11105344	16.329262	0.121541
32289	Del	1	11106118	11139159	15.422481	0.117801
32290	Del	1	11143046	11150047	16.293371	0.121354
32291	Del	1	11153818	11188058	15.422481	0.117801
32292	Del	1	11188180	11198979	16.329262	0.121541
32293	Del	1	11209739	11263560	15.422481	0.117801
32294	Del	1	11265464	11291667	16.267528	0.121354
32295	Del	1	11293314	11300136	16.207972	0.12098
32296	Del	1	11300900	11538613	16.406972	0.121915
32297	Del	1	11539717	11613733	16.615923	0.122663
32298	Del	1	11616244	11656237	16.667823	0.12285
32299	Del	1	11657243	11668882	16.594123	0.122663
32300	Del	1	11669565	11686443	16.641311	0.12285
32301	Del	1	11689851	11694530	16.615923	0.122663
32302	Del	1	11697946	11721916	16.441841	0.121915
32303	Del	1	11722711	11750787	16.584227	0.122476
32304	Del	1	11754574	11774397	17.923631	0.127712
32305	Del	1	11774997	11783110	18.106234	0.12846
32306	Del	1	11783141	11783141	17.923631	0.127712
32307	Del	1	11783840	11809839	18.030712	0.128273
32308	Del	1	11813694	11846011	17.768815	0.127151
32309	Del	1	11847591	11863925	17.464711	0.126029
32310	Del	1	11863970	11932127	18.009609	0.128086
32311	Del	1	11932953	11977700	17.820384	0.127338
32312	Del	1	11982204	12011376	17.705864	0.126964
32313	Del	1	12013007	12057844	17.981897	0.128086
32314	Del	1	12059512	12163425	17.923	0.127712
32315	Del	1	12165294	12170574	17.537192	0.126216
32316	Del	1	12171597	12190922	17.090816	0.124533
32317	Del	1	12190990	12196889	17.537192	0.126216
32318	Del	1	12197646	12228268	17.090816	0.124533
32319	Del	1	12229124	12504511	15.985269	0.120232
32320	Del	1	12505205	12508967	17.443732	0.125842
32321	Del	1	12513690	12513690	15.985269	0.120232
32322	Del	1	12514795	12577132	17.387359	0.125655
32323	Del	1	12578936	12579140	17.443732	0.125842
32324	Del	1	12588404	12623415	17.387359	0.125655
32325	Del	1	12624831	12708972	17.964708	0.127899
32326	Del	1	12709892	12739779	17.912125	0.127712
32327	Del	1	12739885	12764432	17.978168	0.128086
32328	Del	1	12765316	13471994	18.137269	0.128646
32329	Del	1	13473667	13580155	17.738373	0.127151

32330	Del	1	13582647	13618073	16.789543	0.123411
32331	Del	1	13619382	13704218	17.153896	0.12472
32332	Del	1	13704299	13744331	17.186744	0.124907
32333	Del	1	13745350	13789833	17.55488	0.126403
32334	Del	1	13790733	13828560	17.186744	0.124907
32335	Del	1	13830156	14920687	17.860659	0.127525
32336	Del	1	14921261	15071744	19.400502	0.134256
32337	Del	1	15073450	15105161	17.604329	0.12659
32338	Del	1	15108761	15155558	19.260096	0.133695
32339	Del	1	15157969	15157969	19.028658	0.132199
32340	Del	1	15158643	15173606	19.609231	0.135191
32341	Del	1	15173836	15243216	19.028658	0.132199
32342	Del	1	15245637	15399620	18.652472	0.130703
32343	Del	1	15400295	15433051	18.748319	0.131077
32344	Del	1	15436844	15476039	18.820826	0.131451
32345	Del	1	15479619	15494905	18.734197	0.131077
32346	Del	1	15495090	15527759	18.572129	0.130329
32347	Del	1	15528539	15569033	18.518535	0.130142
32348	Del	1	15569475	15592181	18.705414	0.13089
32349	Del	1	15592244	15659445	19.097898	0.132573
32350	Del	1	15659575	15666843	19.25632	0.133695
32351	Del	1	15666877	15761329	19.903491	0.1365
32352	Del	1	15761501	15761501	19.810844	0.135939
32353	Del	1	15761822	15778497	19.903491	0.1365
32354	Del	1	15779765	15788838	19.853909	0.136126
32355	Del	1	15788875	15850269	19.903491	0.1365
32356	Del	1	15854897	15868075	19.803329	0.135939
32357	Del	1	15870264	15907072	20.032434	0.137061
32358	Del	1	15907564	15933318	19.803329	0.135939
32359	Del	1	15940652	15976487	20.027734	0.137061
32360	Del	1	15977607	16006704	20.179795	0.137622
32361	Del	1	16007074	16018332	20.651381	0.139866
32362	Del	1	16018760	16123251	20.676581	0.140053
32363	Del	1	16123711	16157049	20.265044	0.137996
32364	Del	1	16157918	16213082	20.291824	0.138183
32365	Del	1	16213318	16237453	20.352702	0.138557
32366	Del	1	16268499	16398160	19.897368	0.136313
32367	Del	1	16398666	16435970	19.841337	0.136126
32368	Del	1	16438467	16459312	20.218824	0.137809
32369	Del	1	16459673	16470247	20.8784	0.141362
32370	Del	1	16471261	17016429	21.753871	0.147906
32371	Del	1	17018588	17065857	21.064186	0.143231
32372	Del	1	17066485	17122610	20.8784	0.141362
32373	Del	1	17129208	17245727	20.9581	0.142483
32374	Del	1	17247493	17265184	21.064186	0.143044
32375	Del	1	17266821	17296159	21.304804	0.144166
32376	Del	1	17296370	17364420	21.327297	0.144353
32377	Del	1	17365232	17406313	21.608184	0.145662
32378	Del	1	17407500	17534910	21.608184	0.145475
32379	Del	1	17536907	17577423	20.9581	0.142296
32380	Del	1	17578695	17611502	21.141199	0.143418
32381	Del	1	17613939	17699069	21.378331	0.14454
32382	Del	1	17699509	18061078	21.608184	0.146036

32383	Del	1	18061447	18105007	20.451968	0.138931
32384	Del	1	18105530	18378977	18.097232	0.12846
32385	Del	1	18379006	18625618	20.451968	0.138931
32386	Del	1	18629415	18738880	19.487735	0.13463
32387	Del	1	18739292	18751212	19.304206	0.133882
32388	Del	1	18751331	18868987	19.877971	0.136313
32389	Del	1	18869731	18878683	19.849616	0.136126
32390	Del	1	18882051	18886145	20.009353	0.136874
32391	Del	1	18886497	18895348	19.849616	0.136126
32392	Del	1	18896973	18899793	20.009353	0.136874
32393	Del	1	18903769	18903769	19.810252	0.135939
32394	Del	1	18905539	18958070	19.699544	0.135565
32395	Del	1	18958420	19199091	20.129853	0.137435
32396	Del	1	19199869	19230985	20.397614	0.138744
32397	Del	1	19231219	19243289	20.291509	0.138183
32398	Del	1	19243410	19247253	20.197663	0.137809
32399	Del	1	19250337	19256867	20.438295	0.138931
32400	Del	1	19257510	19296937	20.47639	0.139118
32401	Del	1	19300512	19314095	20.438295	0.138931
32402	Del	1	19314182	19332012	20.354831	0.138557
32403	Del	1	19332978	19334376	20.350237	0.13837
32404	Del	1	19336589	19372367	19.223282	0.133508
32405	Del	1	19372852	19490109	19.566247	0.135004
32406	Del	1	19490149	19599867	20.031753	0.137061
32407	Del	1	19601059	19605393	19.770611	0.135752
32408	Del	1	19606106	19609154	20.04316	0.137061
32409	Del	1	19612955	19629880	19.770611	0.135752
32410	Del	1	19632235	19637980	19.552585	0.135004
32411	Del	1	19638481	19647426	19.952238	0.136687
32412	Del	1	19647534	19662401	19.984552	0.136874
32413	Del	1	19668998	19682387	19.973125	0.136687
32414	Del	1	19682539	19802452	19.502942	0.13463
32415	Del	1	19804055	19878465	20.462085	0.139118
32416	Del	1	19878764	19914097	20.354831	0.138557
32417	Del	1	19915012	19969499	20.458536	0.139118
32418	Del	1	19969826	20069094	20.411827	0.138744
32419	Del	1	20069323	20093655	20.218824	0.137809
32420	Del	1	20094038	20122710	20.37526	0.138557
32421	Del	1	20125319	20154347	20.411827	0.138744
32422	Del	1	20156557	20182972	20.31925	0.13837
32423	Del	1	20183947	20187962	20.47639	0.139118
32424	Del	1	20190361	20289228	20.424028	0.138931
32425	Del	1	20289275	20356941	19.981175	0.136874
32426	Del	1	20357372	20431547	20.055774	0.137061
32427	Del	1	20431699	20496355	20.511182	0.139305
32428	Del	1	20498871	20509501	20.451968	0.138931
32429	Del	1	20510490	20585792	20.511182	0.139305
32430	Del	1	20587732	20619193	19.981405	0.136874
32431	Del	1	20625633	20635058	20.262162	0.137996
32432	Del	1	20636122	20641518	20.048679	0.137061
32433	Del	1	20642577	20662264	20.129853	0.137435
32434	Del	1	20664946	20715062	19.80041	0.135939
32435	Del	1	20717297	20740508	20.120489	0.137435

32436	Del	1	20741013	20805757	20.008822	0.136874
32437	Del	1	20806082	20940761	19.906247	0.1365
32438	Del	1	20940964	20987363	20.040107	0.137061
32439	Del	1	20987805	20990856	20.34032	0.13837
32440	Del	1	20990915	21168749	20.040107	0.137061
32441	Del	1	21169167	21188277	19.64192	0.135378
32442	Del	1	21189326	21280290	20.491479	0.139305
32443	Del	1	21281509	21281995	20.459394	0.139118
32444	Del	1	21287656	21314008	20.781847	0.140988
32445	Del	1	21314072	21349164	20.14935	0.137622
32446	Del	1	21349351	21503843	20.893376	0.141362
32447	Del	1	21508618	21581921	20.676581	0.140053
32448	Del	1	21590741	21654470	20.738525	0.140427
32449	Del	1	21655280	21677173	20.666214	0.140053
32450	Del	1	21677300	21722171	20.565586	0.139492
32451	Del	1	21722224	21804530	20.861641	0.141362
32452	Del	1	21811974	21837671	20.711825	0.140427
32453	Del	1	21838233	21938800	20.068402	0.137248
32454	Del	1	21939621	22116490	20.738525	0.140427
32455	Del	1	22117267	22146835	20.627736	0.139866
32456	Del	1	22146917	22449346	20.738525	0.140427
32457	Del	1	22450841	22533154	20.511351	0.139305
32458	Del	1	22534111	22590178	20.73163	0.140427
32459	Del	1	22591319	22606021	20.675835	0.140053
32460	Del	1	22608151	22635250	20.774395	0.140801
32461	Del	1	22636062	22639598	20.746851	0.140614
32462	Del	1	22643447	22650729	20.75842	0.140801
32463	Del	1	22651390	22704958	20.705975	0.14024
32464	Del	1	22705401	22717730	19.206405	0.133508
32465	Del	1	22719450	22721570	20.129853	0.137435
32466	Del	1	22722786	22860422	19.206405	0.133508
32467	Del	1	22862566	22865846	20.262162	0.137996
32468	Del	1	22866069	22925771	19.206405	0.133508
32469	Del	1	22926464	22966652	20.153462	0.137622
32470	Del	1	22967945	23016239	20.197663	0.137809
32471	Del	1	23019076	23036684	19.952238	0.136687
32472	Del	1	23044086	23049200	20.262162	0.137996
32473	Del	1	23049345	23051177	19.952238	0.136687
32474	Del	1	23055122	23059673	20.262162	0.137996
32475	Del	1	23059827	23084270	19.952238	0.136687
32476	Del	1	23084901	23171531	19.725828	0.135565
32477	Del	1	23174771	23279453	20.247617	0.137996
32478	Del	1	23281010	23302147	19.769109	0.135752
32479	Del	1	23304450	23348813	19.552585	0.135004
32480	Del	1	23349390	23365440	19.699544	0.135565
32481	Del	1	23367528	23376625	19.803578	0.135939
32482	Del	1	23380160	23486582	19.304396	0.133882
32483	Del	1	23486742	23555523	19.699544	0.135565
32484	Del	1	23556068	23623657	19.25632	0.133695
32485	Del	1	23624189	23686810	19.204597	0.133508
32486	Del	1	23689941	23736497	19.097898	0.132947
32487	Del	1	23738054	23774426	19.097898	0.13276
32488	Del	1	23778270	23787639	19.097898	0.132947

32489	Del	1	23790682	23805815	19.115727	0.133134
32490	Del	1	23808306	23833167	19.097898	0.132947
32491	Del	1	23833883	23870467	18.564243	0.130329
32492	Del	1	23871355	23914555	18.13931	0.128833
32493	Del	1	23920830	23930125	18.361623	0.129581
32494	Del	1	23961694	23962567	18.310077	0.129394
32495	Del	1	23964300	24055744	18.338694	0.129581
32496	Del	1	24055994	24107727	17.981096	0.128086
32497	Del	1	24118051	24148762	18.106234	0.128646
32498	Del	1	24149880	24191620	18.098649	0.12846
32499	Del	1	24191716	24213738	18.231454	0.12902
32500	Del	1	24214252	24320260	18.457435	0.129955
32501	Del	1	24320698	24357626	18.484026	0.130142
32502	Del	1	24359705	24368661	18.375378	0.129581
32503	Del	1	24368675	24417113	17.707647	0.126964
32504	Del	1	24417293	24468086	17.405173	0.125655
32505	Del	1	24468604	24477614	17.364062	0.125468
32506	Del	1	24483579	24507028	17.859734	0.127525
32507	Del	1	24507348	24507446	17.677055	0.126777
32508	Del	1	24507511	24511867	17.72523	0.126964
32509	Del	1	24512790	24537040	17.751897	0.127151
32510	Del	1	24537060	24550967	17.80465	0.127338
32511	Del	1	24552300	24640674	18.338694	0.129581
32512	Del	1	24641799	24741027	17.573636	0.126403
32513	Del	1	24742750	24846546	16.917185	0.123785
32514	Del	1	24846739	24912798	17.196276	0.124907
32515	Del	1	24913549	24924549	17.304906	0.125281
32516	Del	1	24924871	24934895	17.196276	0.124907
32517	Del	1	24935139	24965969	16.948417	0.123972
32518	Del	1	24966461	25027455	17.159064	0.12472
32519	Del	1	25030228	25423606	18.587509	0.130516
32520	Del	1	25427147	25446866	19.35259	0.134069
32521	Del	1	25447591	25613529	19.407097	0.134256
32522	Del	1	25615754	25786516	19.097898	0.13276
32523	Del	1	25789175	25818089	20.120489	0.137435
32524	Del	1	25818819	25828293	20.352702	0.138557
32525	Del	1	25828595	25850237	20.488907	0.139305
32526	Del	1	25855944	25882917	20.666214	0.140053
32527	Del	1	25883673	25898142	20.593827	0.139679
32528	Del	1	25900028	25957790	20.705975	0.14024
32529	Del	1	25957876	25999781	19.897368	0.136313
32530	Del	1	26001180	26037110	20.369677	0.138557
32531	Del	1	26038883	26049086	20.345747	0.13837
32532	Del	1	26052369	26071473	20.009353	0.136874
32533	Del	1	26072739	26125895	20.774395	0.140801
32534	Del	1	26127712	26174553	20.854762	0.141175
32535	Del	1	26178265	26191871	20.797862	0.140988
32536	Del	1	26193473	26212399	20.738525	0.140427
32537	Del	1	26212565	26223723	20.746851	0.140614
32538	Del	1	26233560	26262561	20.774395	0.140801
32539	Del	1	26268640	26274894	20.949928	0.141736
32540	Del	1	26277221	26289286	20.9581	0.142109
32541	Del	1	26290022	26312653	20.9581	0.141923

32542	Del	1	26313088	26315629	20.937103	0.141736
32543	Del	1	26316293	26321890	20.9581	0.142109
32544	Del	1	26322043	26358699	20.746851	0.140614
32545	Del	1	26358907	26429718	20.666214	0.140053
32546	Del	1	26429892	26457095	20.9581	0.14267
32547	Del	1	26460238	26467917	21.179708	0.143605
32548	Del	1	26468621	26477518	21.215052	0.143792
32549	Del	1	26477915	26546754	22.205123	0.150711
32550	Del	1	26549798	26555954	22.205123	0.151646
32551	Del	1	26556437	26576203	22.205123	0.150711
32552	Del	1	26576390	26699310	22.205123	0.151646
32553	Del	1	26705860	26793484	22.191782	0.15015
32554	Del	1	26801444	26823515	21.753871	0.147906
32555	Del	1	26824331	26861325	21.608184	0.145849
32556	Del	1	26861590	26870577	21.646941	0.146784
32557	Del	1	26876586	26884551	21.608184	0.146597
32558	Del	1	26885102	26904579	21.688855	0.147158
32559	Del	1	26905813	26915518	21.688855	0.147345
32560	Del	1	26919769	26946353	21.646941	0.146971
32561	Del	1	26946373	26986325	21.688855	0.147345
32562	Del	1	26986345	27000941	21.688855	0.147158
32563	Del	1	27001029	27097451	21.688855	0.147345
32564	Del	1	27098414	27229890	21.608184	0.146223
32565	Del	1	27229966	27331236	21.608184	0.145662
32566	Del	1	27333474	27336522	21.608184	0.145475
32567	Del	1	27336658	27354406	21.608184	0.145662
32568	Del	1	27357905	27363419	21.608184	0.145475
32569	Del	1	27363806	27401456	21.608184	0.145849
32570	Del	1	27401536	27525196	21.342499	0.144353
32571	Del	1	27530012	27609806	21.259511	0.143979
32572	Del	1	27610945	27628596	20.891255	0.141362
32573	Del	1	27628886	27659062	20.855691	0.141175
32574	Del	1	27665943	27723023	20.459394	0.139118
32575	Del	1	27725146	27767014	20.338233	0.13837
32576	Del	1	27767099	27829006	19.751604	0.135752
32577	Del	1	27829963	27833688	19.507618	0.13463
32578	Del	1	27833768	27842891	19.621244	0.135191
32579	Del	1	27846494	27856863	19.507618	0.13463
32580	Del	1	27861205	27889927	19.550488	0.134817
32581	Del	1	27890551	27918590	19.384151	0.134256
32582	Del	1	27919122	27948473	19.451133	0.134443
32583	Del	1	27958304	27968199	19.579089	0.135004
32584	Del	1	27969698	27972835	18.587202	0.130516
32585	Del	1	27975171	27987138	18.621906	0.130703
32586	Del	1	27987912	28098329	18.701833	0.13089
32587	Del	1	28098399	28186296	19.073742	0.132386
32588	Del	1	28188781	28194968	18.976873	0.132012
32589	Del	1	28200153	28229133	19.097898	0.132386
32590	Del	1	28234571	28252464	19.097898	0.13276
32591	Del	1	28255562	28320145	19.073742	0.132386
32592	Del	1	28328846	28366449	18.502983	0.130142
32593	Del	1	28369086	28403427	17.226096	0.125094
32594	Del	1	28408901	28502381	17.46663	0.126029

32595	Del	1	28502877	28513637	17.584206	0.126403
32596	Del	1	28513790	28541104	17.46663	0.126029
32597	Del	1	28546015	28576203	16.322999	0.121541
32598	Del	1	28576299	28581458	16.435396	0.121915
32599	Del	1	28586907	28586907	16.383111	0.121728
32600	Del	1	28587436	28600374	16.435396	0.121915
32601	Del	1	28604957	28665360	16.240561	0.121167
32602	Del	1	28665596	28733592	15.462475	0.117988
32603	Del	1	28734075	28808169	14.050128	0.112192
32604	Del	1	28808333	28873816	13.824902	0.111257
32605	Del	1	28882444	29145923	12.289158	0.105086
32606	Del	1	29146182	29235048	11.095153	0.099664
32607	Del	1	29235558	29327324	10.766296	0.098168
32608	Del	1	29329144	30006149	9.959163	0.094428
32609	Del	1	30006923	30666733	9.485191	0.092371
32610	Del	1	30667100	30867774	8.009368	0.085266
32611	Del	1	30868159	30930496	7.148724	0.081152
32612	Del	1	30930879	30935209	7.002215	0.080404
32613	Del	1	30935262	30938231	7.291587	0.081713
32614	Del	1	30940072	31175087	7.002215	0.080404
32615	Del	1	31177972	31258361	6.755327	0.079095
32616	Del	1	31258444	31301656	6.734224	0.078908
32617	Del	1	31301681	31368772	6.299196	0.076664
32618	Del	1	31369237	31408653	6.156682	0.075916
32619	Del	1	31408790	31411333	5.698102	0.073486
32620	Del	1	31412150	31493478	5.726692	0.073673
32621	Del	1	31495473	31572874	3.866942	0.062453
32622	Del	1	31576149	31616298	3.576005	0.060584
32623	Del	1	31616399	31628031	3.272945	0.058527
32624	Del	1	31628660	31651771	2.913475	0.056096
32625	Del	1	31652178	31725572	2.858971	0.055722
32626	Del	1	31726197	31787204	2.511969	0.053291
32627	Del	1	31787704	31903926	2.311559	0.051608
32628	Del	1	31905496	31919755	2.01335	0.049364
32629	Del	1	31920158	31945723	1.991089	0.049177
32630	Del	1	31948666	32068504	2.4303	0.052543
32631	Del	1	32070198	32079583	2.517254	0.053291
32632	Del	1	32079728	32087273	2.558655	0.053478
32633	Del	1	32088667	32103130	2.520411	0.053291
32634	Del	1	32104116	32175043	2.485898	0.052917
32635	Del	1	32175403	32199265	2.539127	0.053478
32636	Del	1	32209086	32232333	2.865731	0.055722
32637	Del	1	32233626	32245759	2.593901	0.053852
32638	Del	1	32246251	32255661	2.484632	0.052917
32639	Del	1	32260436	32293447	2.442131	0.05273
32640	Del	1	32300691	32329696	2.199102	0.05086
32641	Del	1	32332533	32365942	2.240394	0.051047
32642	Del	1	32366691	32397196	2.214575	0.05086
32643	Del	1	32397241	32490269	2.222962	0.051047
32644	Del	1	32490324	32606756	2.473189	0.052917
32645	Del	1	32607934	32653992	2.600654	0.053852
32646	Del	1	32658838	32737902	2.49092	0.053104
32647	Del	1	32740424	32783791	2.441756	0.05273

32648	Del	1	32783895	32859223	2.417084	0.052543
32649	Del	1	32860017	32905234	2.456337	0.05273
32650	Del	1	32906586	33004159	2.656754	0.054226
32651	Del	1	33006720	33009862	2.610403	0.053852
32652	Del	1	33010691	33080570	2.616643	0.054039
32653	Del	1	33080720	33102692	2.567669	0.053665
32654	Del	1	33104527	33143610	2.55314	0.053478
32655	Del	1	33144666	33177187	2.488033	0.053104
32656	Del	1	33177449	33252417	2.476094	0.052917
32657	Del	1	33252430	33302312	2.369427	0.052169
32658	Del	1	33303779	33323283	2.234207	0.051047
32659	Del	1	33324037	33330480	2.205353	0.05086
32660	Del	1	33330628	33334283	2.240394	0.051047
32661	Del	1	33334621	33337570	2.205353	0.05086
32662	Del	1	33347337	33364336	2.225659	0.051047
32663	Del	1	33365879	33380399	2.168227	0.050486
32664	Del	1	33380639	33510969	2.704243	0.0546
32665	Del	1	33512471	33854804	1.440378	0.044503
32666	Del	1	33857886	33865716	1.694754	0.046747
32667	Del	1	33865789	33866173	1.440378	0.044503
32668	Del	1	33866561	33886351	1.67059	0.04656
32669	Del	1	33889126	34156185	1.440378	0.044503
32670	Del	1	34156748	34173987	1.585794	0.045812
32671	Del	1	34175139	34668116	1.596151	0.045999
32672	Del	1	34668244	34862674	1.544387	0.045438
32673	Del	1	34862806	34974315	1.472391	0.044877
32674	Del	1	34974504	34985877	1.079865	0.041137
32675	Del	1	34985938	35122943	1.048335	0.04095
32676	Del	1	35123069	35195305	1.062823	0.041137
32677	Del	1	35202061	35554361	1.324901	0.043568
32678	Del	1	35555125	35632406	1.450147	0.04469
32679	Del	1	35633513	35710370	1.444497	0.044503
32680	Del	1	35710908	35725190	1.118303	0.041511
32681	Del	1	35731266	35794219	1.089403	0.041324
32682	Del	1	35798457	35865471	1.07635	0.041137
32683	Del	1	35869581	35929514	0.932176	0.039641
32684	Del	1	35929726	36075934	0.57598	0.035714
32685	Del	1	36081054	36159955	0.302066	0.031975
32686	Del	1	36161590	36300311	0.287875	0.031788
32687	Del	1	36300537	36324918	0.328213	0.032349
32688	Del	1	36327204	36389991	0.396677	0.033284
32689	Del	1	36390093	36412571	0.425428	0.033658
32690	Del	1	36417599	36451700	0.402094	0.033471
32691	Del	1	36455184	36464792	0.419498	0.033658
32692	Del	1	36467023	36791491	0.312465	0.032162
32693	Del	1	36795484	37158944	0.233506	0.03104
32694	Del	1	37159461	37167235	0.178336	0.029918
32695	Del	1	37169903	37487375	0.24576	0.031227
32696	Del	1	37489261	37497000	0.244722	0.03104
32697	Del	1	37498780	37501891	0.256185	0.031227
32698	Del	1	37503781	37569685	0.244722	0.03104
32699	Del	1	37569888	37600988	0.187626	0.030105
32700	Del	1	37603221	37638180	0.332289	0.032349

32701	Del	1	37641554	37691230	0.340864	0.032536
32702	Del	1	37694833	37711810	0.328213	0.032349
32703	Del	1	37712150	37757133	0.607148	0.036088
32704	Del	1	37758989	37792972	0.643563	0.036462
32705	Del	1	37793072	37810130	0.507255	0.034779
32706	Del	1	37814315	37863788	0.488653	0.034592
32707	Del	1	37864382	37956525	0.380987	0.033097
32708	Del	1	37956872	37990820	0.240579	0.03104
32709	Del	1	37999351	38008975	0.111428	0.028796
32710	Del	1	38016684	38089675	0.01015	0.026552
32711	Del	1	38089814	38837024	0.12338	0.028983
32712	Del	1	38837053	38877393	0	0.023186
32713	Del	1	38880948	38943463	0	0.025991
32714	Del	1	38944344	39077065	0.032844	0.027113
32715	Del	1	39078476	39487757	0.027227	0.026926
32716	Del	1	39488273	39505603	0	0.024121
32717	Del	1	39509470	39516785	0	0.024308
32718	Del	1	39517829	39559408	0	0.024121
32719	Del	1	39560301	39838328	0	0.025804
32720	Del	1	39838975	39891596	0	0.02356
32721	Del	1	39892380	39970534	0	0.021877
32722	Del	1	39971411	40068581	0	0.022251
32723	Del	1	40069170	40097686	0	0.025056
32724	Del	1	40101462	40244941	0.041309	0.0273
32725	Del	1	40245369	40260411	0	0.025243
32726	Del	1	40261444	40295010	0	0.025056
32727	Del	1	40296176	40371809	0	0.025243
32728	Del	1	40372964	40440176	0	0.025056
32729	Del	1	40441422	40620254	0	0.022438
32730	Del	1	40620552	40666503	0	0.022251
32731	Del	1	40668903	40692486	0	0.022438
32732	Del	1	40693557	40734483	0	0.022064
32733	Del	1	40734716	40780187	0	0.022251
32734	Del	1	40780629	40841771	0	0.019634
32735	Del	1	40843046	40974453	0	0.020008
32736	Del	1	40976444	40985939	0	0.019821
32737	Del	1	40987781	41046439	0	0.020008
32738	Del	1	41051967	41165158	0	0.01739
32739	Del	1	41169284	41246381	0	0.017016
32740	Del	1	41246988	41384214	0	0.017951
32741	Del	1	41384510	41505541	0	0.019073
32742	Del	1	41505556	41844611	0	0.017016
32743	Del	1	41846139	41920619	0	0.018512
32744	Del	1	41921259	42036809	0	0.017764
32745	Del	1	42037903	42166551	0	0.019073
32746	Del	1	42167508	42376391	0	0.020381
32747	Del	1	42378257	42451774	0	0.018886
32748	Del	1	42454655	42532730	0	0.019073
32749	Del	1	42534116	42655150	0	0.018512
32750	Del	1	42656131	42821170	0	0.019073
32751	Del	1	42821330	42843877	0	0.018886
32752	Del	1	42846421	42915394	0	0.019073
32753	Del	1	42921631	42985058	0	0.018886

32754	Del	1	42985601	43170047	0	0.01926
32755	Del	1	43170457	43212385	0	0.021316
32756	Del	1	43212652	43266448	0	0.021129
32757	Del	1	43267036	43390494	0	0.020195
32758	Del	1	43390962	43444575	0	0.019821
32759	Del	1	43447515	43521845	0	0.020195
32760	Del	1	43525964	43623961	0	0.019634
32761	Del	1	43624195	43693832	0	0.020008
32762	Del	1	43695142	43728081	0	0.020195
32763	Del	1	43728628	43833169	0	0.018886
32764	Del	1	43837594	43839092	0	0.020195
32765	Del	1	43839964	43931754	0	0.018886
32766	Del	1	43932531	44247017	0	0.020195
32767	Del	1	44247565	44247565	0	0.019447
32768	Del	1	44251149	44255119	0	0.020195
32769	Del	1	44258607	44358237	0	0.019447
32770	Del	1	44362473	44650765	0	0.020755
32771	Del	1	44650931	44678654	0	0.022438
32772	Del	1	44679178	44720421	0	0.022064
32773	Del	1	44721613	44722242	0	0.022438
32774	Del	1	44729408	44729408	0	0.022064
32775	Del	1	44730915	44735853	0	0.022438
32776	Del	1	44736778	44743488	0	0.022251
32777	Del	1	44746192	44816032	0	0.022438
32778	Del	1	44823546	44844138	0	0.022251
32779	Del	1	44847195	44870639	0	0.02169
32780	Del	1	44871422	44991606	0	0.021877
32781	Del	1	44991974	45009253	0	0.022438
32782	Del	1	45012003	45300520	0.036667	0.0273
32783	Del	1	45301759	45306599	0	0.02356
32784	Del	1	45307456	45336389	0	0.024495
32785	Del	1	45338378	45492413	0	0.025804
32786	Del	1	45493287	45575428	0	0.023373
32787	Del	1	45578370	45619440	0	0.023186
32788	Del	1	45619684	45621241	0	0.023373
32789	Del	1	45622290	45645368	0	0.02543
32790	Del	1	45645510	45647021	0	0.025617
32791	Del	1	45647432	45687413	0	0.02543
[truncated: 587,346 more chars]
